
JOURNAL INFORMATION
==============================
NLM Title Abbreviation: Intensive Care Med Exp
Journal ID: Intensive Care Med Exp
NLM Journal ID: 101645149

Intensive Care Medicine Experimental

EISSN: 2197-425X
Publisher: Springer

ARTICLE INFORMATION
==============================
PMCID: PMC5607052
PMID: 28933044
DOI: 10.1186/s40635-017-0151-4
Article ID: 151
Article version: 1
Subjects: Meeting Abstracts

ESICM LIVES 2017

30th ESICM Annual Congress. September 23-27, 2017

Electronic publication date: 2017 Sep 20
Publication date: 2017 Sep
Volume: 5
Issue: Suppl 2
Issue sponsor: Publication of this supplement has not been supported by sponsorship.
Electronic Location ID: 44
Copyright: © The Author(s). 2017
License: Open AccessThis article is distributed under the terms of the Creative Commons Attribution 4.0 International License (http://creativecommons.org/licenses/by/4.0/), which permits unrestricted use, distribution, and reproduction in any medium, provided you give appropriate credit to the original author(s) and the source, provide a link to the Creative Commons license, and indicate if changes were made.
License URL: https://creativecommons.org/licenses/by/4.0/

Conference name: 30th ESICM Annual Congress
Conference Acronym: ESICM 2017
Conference location: Vienna, Austria
Conference date: September 23-27, 2017
issue-copyright-statement: © The Author(s) 2017

==============================

### Oral Sessions Monday, 25 September 2017

#### Acute respiratory failure and MV experimental studies

##### 0001

**Comparison of three methods to measure lung compliance during a decremental PEEP trial in an experimental model of ARDS**

J.-C. Richard[1], B. Louis[2], C. Guérin[1,2]
[1]Hopital de la Croix Rousse, Lyon, France; [2]IMRB INSERM 955, Créteil, France
**Correspondence:** J.-C. Richard
*Intensive Care Medicine Experimental* 2017, **5(Suppl 2):**0001

**INTRODUCTION.** Optimal PEEP is still a matter of debate in ARDS patients. PEEP is by nature an expiratory setting aiming at maintaining lung recruitment reached during the breathing cycle and/or during a recruitment manoeuver. Decremental PEEP trial after a recruitment manoeuver is an attractive method to detect optimal PEEP. This latter can be defined from different variables, lung compliance being one of them.
**OBJECTIVES.** To compare three methods to measure lung compliance and to find out which is associated with the optimal PEEP (maximal compliance) during a decremental PEEP trial.
**METHODS.** Female piglets were anesthetized, paralyzed, tracheotomized and mechanically ventilated (Carestation, GE Healthcare) and acute lung injury was performed by saline lavage. Once PaO2 was lower than 100 mmHg under 100% FIO2, a recruitment manoeuver (sustained inflation to 40 cm H2O for 30 seconds) was performed followed by cycling mechanical ventilation in volume controlled mode, constant flow inflation, 100% FIO2, tidal volume 6 ml/kg body weight, respiratory rate 35 breaths/min. PEEP was initially set to 20 cmH2O then decreased by 2 $cmH_2O$-steps lasting 2 minutes each to 2 $cmH_2O$. At each PEEP step, airway pressure, esophageal pressure and airflow were acquired (Biopac 100), whole lung CT scan was performed during end-expiratory and end-inspiratory pause, and finally electrical impedance tomography (EIT) (Göttingen University) signal was acquired. Compliance was inferred using three methods: i) by fitting a R-C model on recorded pressure and flow signals using a least square method ($C_{lung}$). ii) by using CT data ($C_{scan}$) as ratio of tidal volume (sum of the difference between inspiration and expiration in volume of normally aerated, poorly aerated and overaerated lung compartments) to driving pressure (plateau pressure minus PEEP). iii) by computing ratio of change in electrical impedance to driving pressure ($C_{EIT}$). The relationships between $C_{lung}$, $C_{scan}$ and $C_{EIT}$ were performed by using the coefficients of determination over the 10 PEEP steps in each pig.
**RESULTS.** Thirteen pigs were analyzed. The table shows for each pig the values of coefficient of determination between each pair of compliance and the resulting optimal PEEP that maximized compliance.
**CONCLUSIONS.** Excellent correlation, except for pig B, was observed between $C_{lung}$ and $C_{scan}$ and the resulting optimal PEEP were in agreement with a difference generally less than 2 $cmH_2O$. Poor correlation ($r^2 \leq 0.8$) between $C_{EIT}$ and $C_{scan}$ was observed in 4 pigs. Nevertheless, even iIn these cases the difference between optimal PEEP computed from $C_{EIT}$ and the two other methods were small (≤2 $cmH_2O$).
**REFERENCE(S)**
Measurement of $C_{lung}$, which is easy to manage in adults, strongly correlated to $C_{scan}$ and should be used to titrate optimal PEEP in decremental PEEP trial. By contrast measurement of $C_{EIT}$, which is the only method possible in neonates, seems less correlated to Cscan.

**Table 1 (Abstract 0001)** See text for description

| | $r^2$ $C_{lung}$ vs $C_{EIT}$ | $r^2$ $C_{lung}$ vs $C_{scan}$ | $r^2$ $C_{scan}$ vs $C_{EIT}$ | Optimal PEEP $C_{lung}$ (cmH2O) | Optimal PEEP $C_{scan}$ (cmH2O) | Optimal PEEP $C_{EIT}$ ($cmH_2O$) |
|---|---|---|---|---|---|---|
| Pig A | 0.97 | 0.95 | 0.94 | 9.5 | 11.4 | 9.5 |
| Pig B | 0.55 | 0.50 | 0.99 | 14.7 | 10.7 | 10.7 |
| Pig C | 0.97 | 0.99 | 0.98 | 13.3 | 13.3 | 11.9 |
| Pig D | 0.80 | 1.00 | 0.80 | 11.3 | 7.8 | 7.8 |
| Pig E | 0.72 | 0.99 | 0.73 | 14.1 | 14.1 | 12.2 |
| Pig F | 0.99 | 0.99 | 0.98 | 9.9 | 9.9 | 11.8 |
| Pig G | 0.95 | 0.96 | 0.99 | 11.9 | 9.9 | 9.9 |
| Pig H | 0.96 | 0.98 | 0.97 | 7.9 | 7.9 | 7.9 |
| Pig I | 1.00 | 0.99 | 0.98 | 9.4 | 9.4 | 9.4 |
| Pig J | 0.10 | 0.94 | 0.06 | 8.7 | 10.7 | 12.8 |
| Pig K | 1.00 | 0.99 | 0.99 | 14.6 | 14.6 | 14.6 |
| Pig L | 1.00 | 0.99 | 0.99 | 10.3 | 10.3 | 10.3 |
| Pig M | 0.40 | 0.93 | 0.31 | 17.3 | 17.3 | 15.4 |

##### 0002

**Effect of external negative pressure versus positive end-expiratory pressure on respiratory mechanics during recruitment of experimentally induced lung injury**

A. Nestorowicz[1], A. Fijalkowska-Nestorowicz[2], J. Glapinski[3], J. Wosko[4]
[1]Medical University Lublin, Anaesthesiology and Intensive Therapy, Lublin, Poland; [2]Medical University Lublin, Anaesthesiological and Intensive Care Nursing, Lublin, Poland; [3]Wroclaw University of Science and Technology, Chair of Electronic and Photonic Metrology, Wroclaw, Poland; [4]University Hospital No 4, Anaesthesiology and Intensive Therapy, Lublin, Poland
**Correspondence:** A. Nestorowicz
*Intensive Care Medicine Experimental* 2017, **5(Suppl 2):**0002

**INTRODUCTION.** Positive end-expiratory pressure (PEEP) improves gas exchange and respiratory mechanics in patients suffering from acute lung injury. However, it itself may also induce the risk of lung overdistension and their damage. Some data suggest that the use of external negative pressure (eNP) in damaged lungs is less injurious compared to positive pressure ventilation [1].
**OBJECTIVES.** To compare the effect of eNP versus PEEP on lung mechanics in pigs with acute lung injury.
**METHODS.** Ten Large White pigs weighting 52 ± 5 kg were included in the study. Under general anaesthesia the animals were intubated and ventilation in a volume-controlled mode with $F_IO_2$ 1.0, VT 8–10 mL $kg^{-1}$ and I:E ratio 1:2. Respiratory rate was adjusted to maintain $PaCO_2$ wthin 35–45 mm Hg. A continuous infusion of Ringer´s lactate at the rate of 5–10 mL $kg^{-1}$ $h^{-1}$ was administered throughout the study.

The acute lung injury was produced by repeated bronchoalveolar lavage using warm 0.9% NaCl until $PaO_2/F_IO_2$ remainded stable below 100. Thereafter, each animal was secured in a whole body size-chamber and endotracheal PEEP followed by eNP were created, while lung ventilation mode was held unchanged. Peak airway pressure ($P_{aw}$peak), airway resistance (R) and dynamic compliance (Cdyn) were recorded before lung injury and after - at 0, 4, 8, 12, 16 cm $H_2O$ of positive and negative pressure, respectively.

**RESULTS.** There were no significant differences in Cdyn and R when either PEEP or eNP was applied. However, PEEP resulted in increase whereas eNP in decrease of $P_{aw}$peak and the obtained results differed significantly at the negative vs positive pressure of 12 and 16 cm $H_2O$. Both mode of lung recruitment resulted in a spectacular increase of oxygenation (Fig. 1).

**CONCLUSIONS.** This results demonstrate that both modes of lung recruitment constitutes similar profile of respiratory mechanics, but eNP decrease whereas PEEP increase $P_{aw}$peak.

**REFERENCE(S)**

1. Grasso F. et al.: Am J Respir Crit Care 2008; 177: 412–18.

**GRANT ACKNOWLEDGMENT**

NCN 2013/11/B/ST7/01173

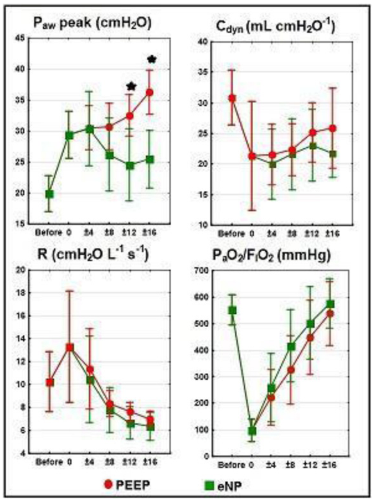

**Fig. 1 (abstract 0002)** Respiratory mechanics and oxygenation

### 0003

#### Dynamic changes on pulmonary artery flow caused by mechanical ventilation. Cyclic worsening of pulmonary artery function in acute respiratory distress syndrome

A. Santos[1,2], M.I. Monge-García[3], J.B. Borges[1], E. Gomez-Peñalver[4], J. Retamal[5], L. Lucchetta[6], G. Hedenstierna[7], A. Larsson[1], F. Suarez-Sipmann[1]

[1]Uppsala University, Hedenstierna Laboratory. Surgical Sciences Department, Uppsala, Sweden; [2]CIBER de Enfermedades Respiratorias (CIBERES), Madrid, Spain; [3]Hospital SAS Jerez, Intensive Care Unit, Jerez de la Frontera, Spain; [4]Hospital General de Villalba, Villalba, Spain; [5]Pontificia Universidad Católica de Chile, Departamento de Medicina Intensiva, Santiago, Chile; [6]San Mateo Hospital, Pavia, Italy; [7]Uppsala University, Department of Medical Sciences. Clinical Physiology, Uppsala, Sweden

**Correspondence:** A. Santos

*Intensive Care Medicine Experimental* 2017, **5(Suppl 2):**0003

**INTRODUCTION.** Mechanical ventilation (MV) induces cyclic changes in RV preload and afterload. However, evaluation of MV effects on RV and pulmonary circulation during the respiratory cycle and how these effects change after acute respiratory distress syndrome (ARDS) is not well known.

**OBJECTIVES.** To evaluate the cyclic effects of MV on RV and pulmonary artery function in a porcine model of ARDS.

**METHODS.** Continuous flow waveform analysis was applied beat by beat during a period of three minutes in 10 mechanically ventilated pigs before and after a model of ARDS. The model was created by means of lung saline lavages followed by two hours of injurious MV. Applied analysis was based on the described effect of MV on RV stroke volume (SV) (Fig. 2), mainly attributed to preload modification. However, also variables related with vascular function can be cyclically affected by MV (Fig. 3). We hypothesized that changes in RV and pulmonary artery function are also related with the SV changes and that this relationship is affected by ARDS. To test this hypothesis, slope and correlation ($r^2$) between variables related with vascular and ventricle function and SV were calculated along the respiratory cycle (Fig. 4). Studied variables were pulmonary artery acceleration time (PAAT, time to reach maximum flow normalized to the ejection time, which is inversely related with pulmonary vascular resistance), flow amplitude (maximum - minimum flow), and the maximum flow to the time to reach maximum flow ratio (Qmax/Qmaxt), which has been described as a contractility index. Flow signal was obtained from a sensor placed around the main pulmonary artery.

**RESULTS.** After creation of ARDS, mean SV decreased (39 ± 12 ml vs 30 ± 6 ml, p = 0.014) and its coefficient of variation increased (0.08 ± 0.03 vs 0.10 ± 0.03, p = 0.016), compared to baseline. Slopes between flow amplitude and SV (0.19 ± 0.05 vs 0.24 ± 0.09, p = 0.027), and between Qmax/Qmaxt and SV (1.6 ± 1.12 vs 2.9 ± 1.4, p = 0.008), as well as the correlation between PAAT and SV (0.21 ± 0.21 vs 0.54 ± 0.28, p = 0.012) and between Qmax/Qmaxt and SV (0.37 ± 0.21 vs 0.61 ± 0.26, p = 0.026) increased during ARDS. A non-significant trend to increase in the correlation between flow amplitude and SV (0.73 ± 0.30 vs 0.86 ± 0.14, p = 0.050) and to a decrease in the slope between PAAT and SV (−0.004 ± 0.005 vs −0.006 ± 0.004, p = 0.325) was observed during ARDS.

**CONCLUSIONS.** ARDS modified the hemodynamic effects of MV on RV and pulmonary vascular function: changes in SV during a respiratory cycle were more affected by pulmonary vascular function and RV contractility. These preliminary results could help to understand the tidal effects of MV on RV and pulmonary artery function.

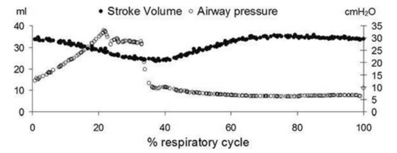

**Fig. 2 (abstract 0003)** See text for description

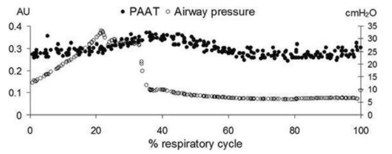

**Fig. 3 (abstract 0003)** See text for description

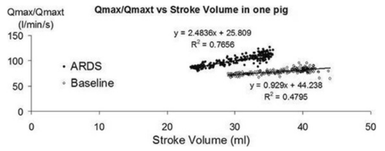

**Fig. 4 (abstract 0003)** See text for description

### 0004

#### Effects of prone positioning and ultra-low tidal volume on transpulmonary driving pressure in a porcine model of ards

F. Pino[1,2], G. Scaramuzzo[3], L. Ball[1], M. Pinol Ribas[4], J.B. Borges[2,5], G. Hedenstierna[6], A. Larsson[2], C. Guerin[7], P. Pelosi[1], G. Perchiazzi[2,8]
[1]University of Genoa, Dep. of Surgical Sciences and Integrated Diagnostics, Genova, Italy, [2]Hedenstierna Laboratory, Dep. of Surgical Science. Uppsala University, Uppsala, Sweden, [3]University of Ferrara, Ferrara, Italy, [4]Hospital La Fe, Valencia, Spain, [5]Hospital das Clínicas, Universidade de São Paulo, Sao Paulo, Brazil, [6]Hedenstierna Laboratory, Dep. of Medical Science. Uppsala University, Uppsala, Sweden, [7]Hopital de la Croix Rousse, Lyon, France, [8]Bari University, Bari, Italy
**Correspondence:** F. Pino
*Intensive Care Medicine Experimental* 2017, **5(Suppl 2):**0004

**INTRODUCTION.** In ARDS patients:

1) prone positioning can reduce mortality, and

2) respiratory system driving pressure is associated with outcome.

Therefore, a reduction in transpulmonary driving pressure ($DP_L$) could minimise ventilator-induced lung injury, yielding improved outcome in prone position.

**OBJECTIVES.** To test the effects of prone positioning on $DP_L$ during protective and ultra-low tidal volume ($V_T$) ventilation. We hypothesized that prone positioning can reduce $DP_L$ during mechanical ventilation at both $V_T$ sizes.

**METHODS.** We used a previously described two-hit porcine model of ARDS, comprising saline lavage followed by injurious mechanical ventilation. After lung injury, pigs were ventilated in volume-controlled mode, $V_T$ 6 mL/kg and positive end-expiratory pressure (PEEP) and $F_IO_2$ titrated according to the ARDSnet table ($PEEP_{ARDSnet}$). A decremental PEEP trial was performed from 20 $cmH_2O$ to 5 $cmH_2O$ in supine and prone position at $V_T$ of 6 mL/kg and 3 mL/kg, identifying the PEEP level with the lowest $DP_L$ ($PEEP_{BDP}$). Each animal was allocated, by random sequence and in crossover with a latin square design, to eight treatments: 6 and 3 mL/kg $V_T$ at constant minute ventilation, with $PEEP_{BDP}$ or $PEEP_{ARDSnet,}$ in prone and supine position. Each change in ventilation settings was preceded by a recruitment manoeuvre[GC1].The primary endpoint was $DP_L$, and secondary endpoints included gas-exchange and $PEEP_{BDP}$.

**RESULTS.** We analysed data from 8 pigs (32 ± 2 kg); after injury $PaO_2/F_IO_2$ ratio was 136 ± 22 mmHg. $DP_L$ was lower in prone than supine position at both 6 mL/kg (3.7 ± 1.8 vs. 5.2 ± 1.1 $cmH_2O$, $p = 0.006$) and 3 mL/kg (1.6 ± 0.9 vs. 2.6 ± 0.6 $cmH_2O$, $p = 0.002$) $V_T$. $PEEP_{BDP}$ was also lower in prone than supine position: 10.3 ± 1.0 vs. 13.3 ± 3.0 $cmH_2O$ ($p = 0.02$) at 6 mL/kg and 10.4 ± 1.4 vs. 12.8 ± 2.1 $cmH_2O$ ($p = 0.03$) at 3 mL/kg. In prone and supine positions, $PaO_2$ was 266 ± 63 vs. 234 ± 62 mmHg ($p = 0.17$) at 6 mL/kg and 219 ± 65 vs. 154 ± 39 $cmH_2O$ ($p = 0.06$) at 3 mL/kg, respectively; $PaCO_2$ was 68 ± 10 vs. 68 ± 17 $cmH_2O$ ($p = 0.99$) at 6 mL/kg and 90 ± 22 vs. 87 ± 24 $cmH_2O$ ($p = 0.66$) at 3 mL/kg, respectively.

**CONCLUSIONS.** In our ARDS animal model, prone positioning allowed a reduction of $DP_L$ and PEEP level with the lowest $DP_L$ during both protective and ultra-low $V_T$ ventilation.

### 0005

#### Abnormal diaphragm fibers in patients undergoing mechanical ventilation: a pilot study

J. Marin-Corral[1], I. Dot[1], M. Boguñà[1], C. Vilà[1], A. Castellví[1], S. Pascual-Guardia[2], M.P. Gracia[1], A. Zapatero[1], Y. Díaz[1], L. Cechini[3], J.R. Masclans[1,4]
[1]Hospital del Mar-Parc de Salut Mar, GREPAC (Grupo de Investigación en Patología Crítica), Institut Mar d'Investigaciones Médicas (IMIM), Critical Care Department, Barcelona, Spain; [2]Hospital del Mar-Parc de Salut Mar, Institut Mar d'Investigaciones Médicas (IMIM), Respiratory Department, Barcelona, Spain; [3]Hospital del Mar-Parc de Salut Mar, Urology Department, Barcelona, Spain; [4]Pompeu Fabra University (UPF), Barcelona, Spain
**Correspondence:** J. Marin-Corral
*Intensive Care Medicine Experimental* 2017, **5(Suppl 2):**0005

**INTRODUCTION.** Several studies are concordant with animal models of ventilator-induced diaphragm dysfunction (VIDD) in demonstrating ultrastructural injury and atrophy of diaphragmatic fibers, increased oxidative stress, activation of major proteolytic pathways and mitochondrial dysfunction, which are significantly correlated with mechanical ventilation (MV) duration. Area fractions of normal and abnormal diaphragm in patients undergoing MV have not been yet assessed.

**OBJECTIVES.** To assess and compare area fractions of normal and abnormal diaphragm and limb muscles of brain-dead organ donors (BD), type III Maastricht donors (MS), and stable patients who received mechanical ventilation during thoracic surgery (CT).

**METHODS.** Prospective and observational study in a polyvalent Spanish ICU. All organ donors admitted in the ICU during 6 months (BD or MS) were included. There was a control group with patients undergoing resection of a suspected early lung malignancy. Demographic and temporary variables, comorbidities, severity on admission and treatment were evaluated. Diaphragm biopsy specimens were obtained from the anterior costal diaphragm lateral to the insertion of the phrenic nerve and limb muscle from the vastus lateralis as internal control. Muscle samples were fixed and paraffin embedded and they were cut and stained with hematoxylin and eosin (H&E). Quantitative evaluation of normal and abnormal (internal nucleus, inflammatory cells, lipofuscin, abnormal viable and inflammed/necrotic) muscle fibers in sections were done following previous methodologies. Differences between groups were assessed using chi-square for categorical variables and Student's t-test or Mann–Whitney test for continuous variables. We considered $p < 0.05$ to be significant.

**RESULTS.** Fifteen patients were included of which 5(33%) were CT, 4(27%) were BD and 6(40%) MS. There were no differences between groups in age, sex, BMI, and comorbidities. BD patients presented a lower number of hours of mechanical ventilation with a higher proportion of controlled modalities than MS [63hrs(33–92) vs 216hrs(107–396);p = 0.010 and 33%(0–46) vs 5%(0–20);p = 0.128]. Diaphragm of BD presented higher percentage of abnormal muscle fibers than MS donors [19.1%(9.2) vs 2.4(2.3);p = 0.024] and CT subjects [19.1%(9.2) vs 4.5(1.1);p = 0.036]. Diaphragm of BD had lower percentage of internal nucleus [0%(0) vs 2.4%(1.0);p = 0.036] and MS diaphragms showed lower percentage of inflammatory cells [0%(0) vs 0.4%(0.1);p = 0.004] both compared to CT subjects. No differences were found in limb muscles evaluation of normal/abnormal fibers between different groups.

**CONCLUSIONS.** Diaphragm of BD donors presents an impaired number of abnormal fibers. Although MS donors are undergone mechanical ventilation during more hours their percentage of abnormal diaphragm fibers don't differ from CT subjects. It could be related with the ability of these patients to realise spontaneous breathing efforts.

### Insight into the pathophysiology of sepsis

#### 0006

**Characterization of metabolic signatures associated with early response to supportive therapy in patients with septic shock**

A. Cambiaghi[1], B. Bollen Pinto[2], L. Brunelli[3], F. Falcetta[3], A. Federico[1], K. Bendjelid[2], R. Pastorelli[3], M. Ferrario[1]
[1]Politecnico di Milano, Dipartimento di Elettronica, Informazione e Bioingegneria (DEIB), Milan, Italy; [2]Geneva University Hospitals, Anaesthesia, Pharmacology and Intensive Care, Geneva, Switzerland; [3]Istituto di Ricerche Farmacologiche Mario Negri, Milan, Italy
**Correspondence:** B. Bollen Pinto
*Intensive Care Medicine Experimental* 2017, **5(Suppl 2):**0006

**INTRODUCTION.** Elucidation of early metabolic signatures associated with the progression of septic shock and responsiveness to supportive therapy could be useful in the development of a new therapeutic strategies.

**OBJECTIVES.** The primary aim of this study was to verify whether a different response to therapy was associated with a particular trajectory in metabolite patterns.

**METHODS.** We examined the plasma metabolome of 21 septic shock patients (pts) enrolled in the Shockomics clinical trial (NCT02141607). Responsiveness to therapy was assessed as change in organ dysfunction assessed by SOFA score, measured at admission (T1, acute phase) and 48 hours after (T2, post-resuscitation). A patient was judged as non-responder if both $SOFA_{T2}$ was >8 and $\Delta = SOFA_{T1}$- $SOFA_{T2} < 5$ (NR, 7 pts); the remaining as responsive (R, 14 pts). We combined untargeted and targeted mass spectrometry-based metabolomics strategies to cover as much as possible the plasma metabolites repertoire. Firstly, a mass metabolic profiling, performed by direct flow injection-TOF-MS, was applied as untargeted screening. Afterwards, we performed a targeted analysis using a mass spectrometry-based quantitative approach with the Biocrates platform coupled to Triple-Quad 5500 LC-MS/MS system, which allowed to measure specific metabolic classes and the magnitude of their variation. We built classification models to predict non-responsiveness to therapy based on sets of metabolites. Explorative analyses by probabilistic graphical models - i.e. Markov Network (MN) - were also performed.

**RESULTS.** At ICU admission, plasma metabolome was similar between R and NR. In univariate analysis, NR presented less variation in metabolite concentration between T1 and T2. Different classification models using targeted metabolomics to predict NR status reproducibly reveled the presence of phosphatidylcholines (PCs) (e.g. lysoPC), alanine and Kynurenine (a metabolite of tryptophan). We combined untargeted metabolomics with those metabolites, and the set of features selected from the integrated models was consistent in showing the importance of PCs and alanine. All models had good performance (AUC > 0.9). The MNs showed that the two groups were characterized by different dependencies among metabolites, and the main differences were represented by PCs, kynurenine and alanine.

**CONCLUSIONS.** These findings support the emerging evidence that lipidome alteration plays an important role in response to infection. In addition, the identification of alanine as a consistent marker of NR to therapy could represent a possible shift in glucose-alanine cycle in the liver thus providing a more detailed characterization of liver dysfunction than clinically available tests. These results were strengthened by the explorative analyses performed by MNs suggesting that lipid species and alanine are important regulatory nodes.

**GRANT ACKNOWLEDGMENT**
This research is supported by the FP7 Health Programme (602706).

#### 0007

**Immune evasion mechanism of *E. coli* as a target for sepsis treatment**

M.D.T. Correia[1], F.P. da Silva[1], S.K. Ariga[1]
[1]Faculdade de Medicina da Universidade de São Paulo, Laboratory of Medical Investigation - LIM 51, São Paulo, Brazil
**Correspondence:** M.D.T. Correia
*Intensive Care Medicine Experimental* 2017, **5(Suppl 2):**0007

**INTRODUCTION.** Bacteria have evolved sophisticated strategies to evade immune defense mechanisms. Since our group discovered that *Escherichia coli* binds in an opsonin-independent manner to FcγRIII receptor (CD16) to escape immune system, we have sought to identify the protein sequence from such bacteria that is able to mimic IgG[1]. Using a Phage Display Technique, we recently identified and characterized a peptide of *E. coli* which is a ligand to CD16.

The peptide, called wzxe, is a translocase involved in the transbilayer movement of an trisaccharide-lipid (Lipid III) intermediate in the assembly of Enterobacterial Common Antigen (ECA). Peptide-receptor interactions induced CD16-mediated immunoreceptor ITAMi signaling, blocking the production of reactive species of oxygen(ROS) and bacterial killing. This CD16-mediated inhibitory signaling was abrogated in a wzxe$^{-/-}$ mutant of *E. coli* K12 restoring production of ROS and bacterial killing [2].

**OBJECTIVES.** To evaluate the role of *E. coli* peptide wzxe in a murine sepsis model of bacterial injection.

**METHODS.** Two group of 20 males mice C57Bl/6, at 10 weeks of age, were submitted to injection of 3 x $10^8$ *E. coli* K12 wild type or the same amount of *E coli* wzxe$^{-/-}$ mutant. Survival was observed during 96 hours then the mice were sacrificed for cytokines analysis. TNF alpha, IL-1beta, IL-6, MCP-1 and IL-10 were quantified by ELISA. In addition, control mice injected with saline were sacrificed in the 4$^{th}$ day for cytokine dosing.

**RESULTS.** A higher mortality and increased production of cytokines were observed in the group of mice injected with *E. coli* K12 wild type.

The Kaplan-Meier and log-rank methods were used to analyze mortality. Cytokine values were compared using the Mann–Whitney U-test. Differences were considered significant when P was < 0.05. All statistical analysis was performed using Prism 6.0 (GraphPad Software, USA).

**CONCLUSIONS.** In an era of growing resistance to antibiotics, preventing bacteria innate immune evasion could be a good approach to control infections. Regarding *E. coli*, peptide wzxe apparently plays an important role during sepsis, being an interesting therapeutic target to be explored.

**REFERENCE(S).**

1. Pinheiro da Silva F, Aloulou M, Skurnik D, Benhamou M, Andremont A, Velasco IT, et al. CD16 promotes Escherichia coli sepsis through an FcR gamma inhibitory pathway that prevents phagocytosis and facilitates inflammation. Nat Med. 2007;13(11):1368–74.
2. Beppler J, Mkaddem S Ben, Michaloski J, Honorato RV, Velasco IT, de Oliveira PSL, et al. Negative regulation of bacterial killing and inflammation by two novel CD16 ligands. Eur J Immunol. 2016;46(8):1926–35.

**GRANT ACKNOWLEDGMENT**
Financial Suport from FAPESP (São Paulo Research Foundation)

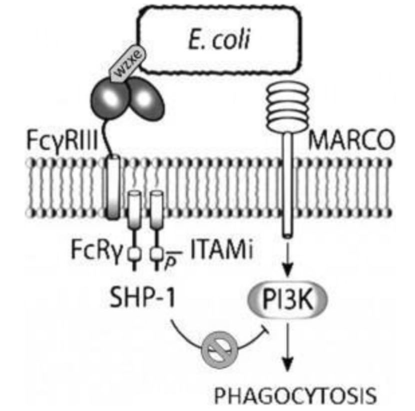

**Fig. 5 (abstract 0007).** E. coli immune evasion through ITAMi signaling

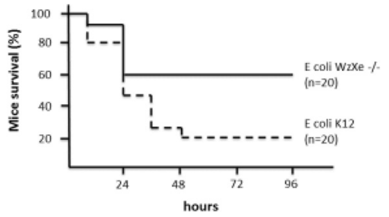

**Fig. 6 (abstract 0007).** Mice survival curves after bacterial injection

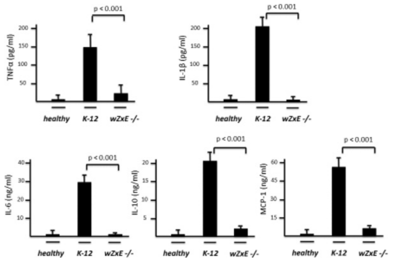

**Fig. 7 (abstract 0007).** Production of cytokines

### 0008

#### Oxidant/antioxidant status of adult and pediatric ICU patients: a key role for sepsis and mortality

M. Miliaraki[1], T. Tavladaki[2], A.-M. Spanaki[2], H. Dimitriou[3], S. Ilia[2], K. Vardas[4], E. Briassouli[5], E. Michalakakou[6], T. Karakonstantakis[6], E. Kondili[7], I. Papasotiriou[6], P. Papadopoulos[8], A. Pistiki[8], D. Georgopoulos[7], G. Briassoulis[2]

[1]University of Crete, Medical School, Pediatric Intensive Care Unit, Heraklion, Crete, Greece; [2]University of Crete, Medical School, Heraklion, Greece, Pediatric Intensive Care Unit, Heraklion, Crete, Greece; [3]University of Crete, Medical School, Heraklion, Greece, Pediatric Hamatology Oncology, Heraklion, Crete, Greece; [4]National and Kapodistrian University of Athens, First Critical Care Department, Athens, Greece; [5]National and Kapodistrian University of Athens, First Department of Internal Medicine - Propaedeutic, Athens, Greece; [6]National and Kapodistrian University of Athens, Biochemical Department, Children's Hospital 'Aghia Sophia', Athens, Greece; [7]University of Crete, Medical School, Heraklion, Greece, Adult Intensive Care Unit, Heraklion, Crete, Greece; [8]National and Kapodistrian University of Athens, 2nd ICU and 4th Internal Medicine Department, Athens, Greece

**Correspondence:** M. Miliaraki

*Intensive Care Medicine Experimental* 2017, **5(Suppl 2):**0008

**INTRODUCTION.** Sepsis is a major health problem and much emphasis has recently been placed on the role of redox imbalance, oxidative stress, chaperone heat shock proteins (HSP) 72, 90, and interleukin 27 (IL-27), in intensive care unit (ICU) patients' clinical course and outcome. Oxidative/antioxidative screening methods might assist the clinicians in discriminating critically ill septic patients, or at risk of death, from those with systemic inflammatory response syndrome (SIRS), and facilitate therapeutic decision-making upon admission of a patient.

**OBJECTIVES.** The present study seeks to ascertain whether the expected increased risk of mortality might be further determined by the presence of oxidant/antioxidant imbalance and altered IL-27 (a regulator T-cells having both pro and anti-inflammatory effects), Zn, glutamine, and HSP levels in septic ICU and pediatric ICU (PICU) patients, compared to SIRS and healthy controls.

**METHODS.** This prospective observational study was performed in a sample of critically ill adult (n = 180) and pediatric patients (n = 44) with sepsis or SIRS, compared to healthy controls (n = 116). Serum total oxidant status (TOS) and total antioxidant capacity (TAC) were measured on day 1 in ICU, using photometric test systems. Plasma glutamine (chromatography), serum zinc (Zn-Photometry), IL-27 (Elisa kits) and HSPs (Elisa and flow cytometry) were also analyzed. Clinical severity scores APACHE II and SOFA were calculated and ICU mortality was recorded.

**RESULTS.** A total of 180 adult ICU patients were included in the study. Mean APACHE II score was 22.5 ± 8 and ICU mortality rate 30.8% (PICU 4,3%). Septic patients had lower TAC, but higher TOS, glutamine, IL-6, IL-10, HSP72 and HSP90 levels, compared to SIRS and control individuals ($p < 0.05$). TOS was negatively related to TAS ($r = 0.5$, $p < 0.05$). Serum TOS correlated positively ($p < 0.05$) and TAS negatively ($p < 0.05$) with Zn, IL-27, HSP72/90 and clinical severity scores. For predicting sepsis among critically ill patients, TAC and TOS achieved a receiver operating characteristic curve (AUROC) >0.90 (95% CI 0,85-0,97, $p < 0.05$). In predicting mortality, TOS, IL-27, and Zn achieved an AUROC >0.68 (95% CI 0,50-0,85, $p < 0.05$). A pilot study of 44 pediatric patients showed the same trend of oxidant/antioxidant imbalance and lower Zn and glutamine levels in septic children ($p < 0.05$).

**CONCLUSIONS.** Oxidant/antioxidant status is significantly impaired in septic patients compared to SIRS. Decreased antioxidant defense and increased oxidative stress system, along with alterations in innate immune system integrity and IL-27, may play an important role in sepsis-related mortality.

**GRANT ACKNOWLEDGMENT**

This research has been co-financed by the European Union (European Social Fund- ESF) and Greek national funds through the Operational Program ´´Education and Lifelong Learning´´ of the National Strategic Reference Framework (NSRF)- Research Funding Program: THALES.

### 0009

#### Withdrawn

### 0010

#### Mitochondrial dysfunction in a tissue slice model of septic acute kidney injury is caused by oxidative stress

S.J. Pollen, M. Singer

UCL, Bloomsbury Institute of Intensive Care Medicine, London, United Kingdom

**Correspondence:** S.J. Pollen

*Intensive Care Medicine Experimental* 2017, **5(Suppl 2):**0010

**INTRODUCTION.** Mitochondrial modulation of cellular bioenergetics is increasingly viewed as an explanation for the apparent paradox between clinical/biochemical acute kidney injury (AKI) that occurs during sepsis, despite a relative lack of cell death, maintenance of adequate tissue oxygenation and recovery of organ function.[1] Previous *in vitro* studies from our lab show that exposing naïve kidney tissue to septic serum, thereby removing any haemodynamic influences, produces mitochondrial dysfunction.[2]

**OBJECTIVES.** To determine if the mitochondrial dysfunction that occurs following exposure to septic serum is driven by the observed increase in reactive oxygen species (ROS), and, if so, whether these changes can be inhibited and/or reversed using a targeted mitochondrial ROS scavenger.
**METHODS.** Live naïve kidney slices (200 µm thick) were exposed to serum from 24-hour sham operated or septic rats and imaged with a confocal microscope using fluorescent dyes to detect dynamic changes in mitochondrial function. Mitochondrial membrane potential (MMP), ROS generation and redox state (NADH) were all probed over a 90-minute time course. In separate studies, septic (and sham) serum exposed slices were co-incubated with the mitochondrial ROS scavenger MitoTEMPO, either at time zero or as a delayed (50 min) treatment.
**RESULTS.** The decreases in MMP and NADH, and the increase in ROS seen following exposure to septic serum were prevented by co-incubation with MitoTEMPO from time zero (Fig. 8).
Treating septic serum-exposed slices with MitoTEMPO at 50 min could partly reverse changes in mitochondrial function that had already begun, and prevent further progression (Fig. 9).
**CONCLUSIONS.** Our *in vitro* findings suggest that increased production of mitochondrial ROS is a direct cause of mitochondrial dysfunction in septic AKI, independent of circulatory changes, and this can be protected/reversed by ROS scavenging. Targeting mitochondrial ROS may provide therapeutic benefit.

**GRANT ACKNOWLEDGMENT**

UCL Medical School MBPhD Programme, Astor Foundation and Intensive Care Society

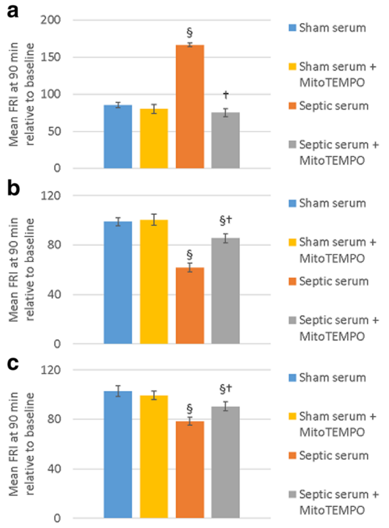

**Fig. 8 (abstract 0010).** See text for description

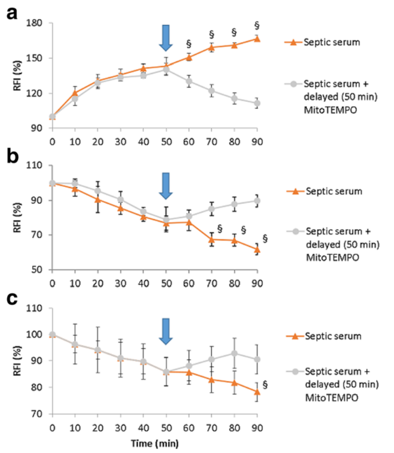

**Fig. 9 (abstract 0010).** See text for description

### Microcirculation and fluid responsiveness

#### 0011

#### Influence of the different fluid on the vascular endothelial barrier permeability and glycocalyx layer in a rats model of the acute normovolemic hemodilution

B. Ergin[1,2], P. Guerci[3], Z. Uz[1], Y. Ince[1], C. Ince[1,2]
[1]Academic Medical Center, University of Amsterdam, Department of Translational Physiology, Amsterdam, Netherlands; [2]Erasmus MC University Hospital, Department of Intensive Care, Rotterdam, Netherlands; [3]University Hospital of Nancy, Department of Anesthesiology and Critical Care Medicine, Nancy, France
**Correspondence:** B. Ergin
*Intensive Care Medicine Experimental* 2017, **5(Suppl 2):**0011

**INTRODUCTION.** Intravenous fluid administration is the most performed action in patients undergoing surgery. Hemodilution can be either intentional with the aim of limiting allogenic blood transfusions in elective surgery or used for correction of volume status of the patient. It has been suggested that colloidal solutions may preserve glycocalyx components, by maintaining the shear stress on endothelial due to an higher viscosity, osmotic pressure and also to the presence of macromolecules. We sought to evaluate the impact of hemodilution with either a balanced colloid, a balanced crystalloid or a normal saline solution on the glycocalyx components and the vascular barrier permeability.
**MATERIAL AND METHODS.** Experiments were conducted in 24 fully instrumented, mechanically ventilated and anesthetized rats. Acute normovolemic hemodilution (ANH) was induced by step-wise exchange of blood with the plasma expander according to the following ratio; 1:1 with balanced hydroxyethyl starch (HES 130/0.4), 1:3 with balanced crystalloid or normal saline 0.9%, untill targeted hematocrit (Hct) levels of Hct 30%, Hct 25%, Hct 20% and Hct 15% were achieved. An hand-held in vivo microscope, CytoCam was placed on the surface of the exposed biceps femoris. Plasma concentration-time curves of Dextran 40kD-Texas red, Albumin-Alexa 680 and Dextran 500kd-FITC were fitted for each experiment separately with a monoexponential function in order to assess both endothelial barrier function and plasma volume. At the end of the experiment, the heart, brain, kidney, lung and liver were harvested to determine their water content using the wet/dry weighing technique. Hyaluronan, syndecan-1, and heparan sulfate as indirect makers of glycocalyx degradation were measured by the ELISA in plasma samples.

**RESULTS.** MAP significantly decreased during the hemodilution process ($p < 0.05$) whereas CVP and femoral arterial blood flow (fABF) were maintained. At 15% of hematocrit, lactate levels increased significantly ($p < 0.01$), accompanied by changes in pH and bicarbonate levels for balanced crystalloid and normal saline groups ($p < 0.01$). Syndecan-1 levels were increased in all groups at the end of the experiment compared to baseline values respectively ($p < 0.0001$). Heparan sulfate increased only in saline group at the different hemodilution thresholds ($p < 0.01$) only in the normal saline group. Hyaluronan levels were increased in balanced crystalloid and normal saline groups compared to control ($p < 0.01$).

No significant alteration in power of the exponential decay-time of dyes were observed between the different fluid groups.

Neither was any alteration in the microcirculation nor the presence of tissue edema measured.

**CONCLUSION.** ANH per se may jeopardize the endothelial glycocalyx layer without inducing significant alterations in vascular barrier permeability regardless of the fluid composition. Normal saline appears to be the most unsuitable fluid for use for hemodilution.

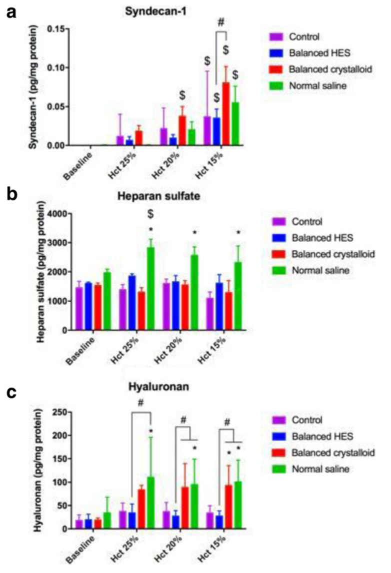

**Fig. 10 (abstract 0011).** No significant alteration in power of the exponential decay-time of dyes were observed between the different fluid groups.

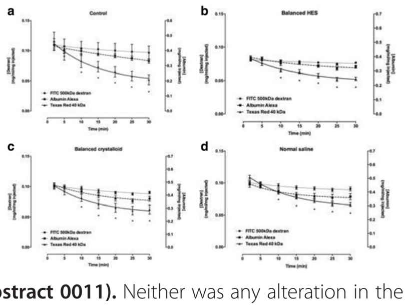

**Fig. 11 (abstract 0011).** Neither was any alteration in the microcirculation nor the presence of tissue edema measured.

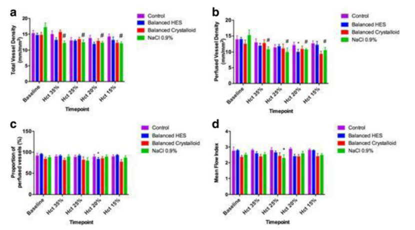

**Fig. 12 (abstract 0011).** See text for description

### 0012

#### Influence of fluid challenge administration rate on endothelial glycocalyx and major macrohemodynamic variables in surgical and septic patients

J. Pouska[1,2], V. Tegl[1,2], V. Cerny[3,4,5], J. Benes[1,2,6]

[1]Fakultní Nemocnice Plzeň, Klinika Anesteziologie, Resuscitace a Intenzivní Medicíny, Plzen, Czech Republic; [2]Lékařská Fakulta v Plzni, Univerzita Karlova, Klinika Anesteziologie, Resuscitace a Intenzivní Medicíny, Plzen, Czech Republic; [3]Fakultní Nemocnice Hradec Králové, Klinika Anesteziologie, Resuscitace a Intenzivní Medicíny, Hradec Kralove, Czech Republic; [4]Lékařská Fakulta v Hradci Králové, Univerzita Karlova v Praze, Hradec Kralove, Czech Republic; [5]Univerzita J. E. Purkyně, Masarykova Nemocnice Ústí Nad Labem, Klinika Anesteziologie, Perioperační a Intenzivní Medicíny, Usti nad Labem, Czech Republic; [6]Biomedicínské Centrum Lékařské Fakulty v Plzni, Univerzita Karlova, Plzen, Czech Republic

**Correspondence:** J. Pouska

*Intensive Care Medicine Experimental* 2017, **5(Suppl 2):**0012

**INTRODUCTION.** Fluid challenge (FC) is test of cardiac preload reserve, but how FC is performed varies widely. Slow FC may decrease the hemodynamic effect and contrary fast FC may lead to temporary hypervolemia. Hypervolemia harms endothelial glycocalyx (EG), and hence the rate of FC may significantly alter FC macro- and microhemodynamic effects.

**OBJECTIVES.** Major objectives of this study was to assess the influence of fast and slow FC administration on hemodynamic variables and EG.

**METHODS.** Randomized prospective study performed in a University hospital was approved by the local ethics committee. Patients undergoing major spinal surgery (group A) and critically ill with septic shock (group B) in whom a FC was indicated were randomly (1:1 randomization by predefined scheme) assigned to receive either fast (5-10min) or slow (20-30min) 500ml balanced crystalloid FC. In group A only one FC per patient was performed, in group B more FC were allowed if response to previous FC was positive. Intraoperatively preload reserve and volume effect was assessed using standard hemodynamic variables and pleth-variability index (PVI). In septic patients the PiCCO2 device was used for hemodynamic monitoring. Effect on EG was assessed using the intravital microscopy of sublingual circulation using the Glycocheck software (Glycocheck BV, Netherlands). Hemodynamic (PVI, stroke volume - SV) as well as EG variables (PBR - perfused boundary region and CD - capillary density) were monitored before (T0), immediately after (T1), and in 20 minutes intervals till the end of surgery (T2-4, group A) or after 1 and 2 hours (T2-3, group B). Microcirculatory parameters were assessed as absolute and relative change to baseline (%BL).

**RESULTS.** In 50 patients of group A 50 FC (25 fast and 25 slow) were performed; 26 FC in 15 patients (12 fast and 14 slow) of group B. All but 6 FC (12%) were positive in group A, whereas 13 (50%) were positive in group B equally distributed with 7 (54%) positive in fast and 6 (46%) in slow FC. In group A both fast and slow FC increased CD (T1 CD %BL 104 ± 5% and 109 ± 4%), but the effect vanished rapidly in the slow FC (T2 CD%BL 112 ± 16% vs. 96 ± 19%; $p = 0.02$). Similar effect was observed on PBR - post FC increment (T1 PBR %BL 104 ± 19% vs. 108 ± 16%) with significantly prolonged effect in the fast FC (T3 PBR%BL 109 ± 16% vs. 99 ± 15%; $p = 0.04$). In group B, rate of infusion have neither impact on PBR nor CD. Contrary, significantly higher PBR (1.9 ± 0.2 vs. 2.1 ± 0.3; p = 0.02) and lower CD (0.7 ± 0.1 vs. 0.6 ± 0.1; p = 0.02) values were observed in responders in T1.

**CONCLUSIONS.** In our study a fast 500ml balanced crystalloid FC had prolonged microcirculatory and hemodynamic effects in surgical patients than slow FC. In patients with septic shock no changes in terms of EG were observed.

**GRANT ACKNOWLEDGMENT**

Supported by Ministry of Health of the Czech Republic, grant nr. 15-31881A.

### 0013

#### Glycocalyx degradation is independent of vascular barrier dysfunction in non-traumatic hemorrhagic shock

P. Guerci[1,2,3], B. Ergin[1], Z. Uz[1], Y. Ince[1], M. Heger[4], C. Ince[1]
[1]Academic Medical Center, University of Amsterdam, Department of Translational Physiology, Amsterdam, Netherlands; [2]Centre Hospitalier Universitaire de Nancy, Anesthesiology and Intensive Care Medicine, Nancy, France; [3]University of Lorraine, Nancy, France; [4]Academic Medical Center, University of Amsterdam, Department of Experimental Surgery, Amsterdam, Netherlands
**Correspondence:** P. Guerci
*Intensive Care Medicine Experimental* 2017, **5(Suppl 2):**0013

**INTRODUCTION.** Glycocalyx shedding following traumatic hemorrhagic or septic shock has been linked to increased vascular barrier permeability (VBP) (1). Little is known regarding the VBP during non-traumatic hemorrhagic shock (NTHS). It is not yet elucidated whether or not there is positive correlation between the fluid resuscitation-mediated glycocalyx degradation and an increased VBP (2,3). It would be suggested that a colloidal solution would better preserve glycocalyx components, by maintaining the shear stress on endothelial due to an higher viscosity, osmotic pressure and also to the presence of macromolecules.

**OBJECTIVES.** The aim of this study was to determine the effects of different resuscitation fluids on the glycocalyx barrier and its relation with the VBP in short term of severe hemorrhage.

**METHODS.** Fully instrumented and hemodynamically monitored Wistar albino rats were subjected to a pressure-controlled NTHS for 60 min. Same spot of the microcirculatory unit of the biceps femoris muscle was monitored continuously with hand-held microscope. Rats were then fluid resuscitated with either Ringer's acetate or balanced starch solution or NaCl 0.9% till the MAP reaches 80 mmHg. Glycocalyx shed products (hyaluronan, syndecan-1, heparan) were determined at baseline and 60 min after fluid resuscitation by ELISA methods. VBP was assessed with 3 different techniques: plasma decay of 3 fluorescent dyes (Texas Red-40 KDa and FITC-500 kDa Dextrans and Alexa 680–70 kDa Albumin), tissue edema (wet/dry weight ratio) and intravital fluorescence microscopy.

**RESULTS.** NTHS and associated fluid resuscitation produced significant amount of glycocalyx shed products ($P < 0.05$) partially dampened by the use of balanced colloid. Despite glycocalyx degradation and microcirculatory functional density alterations ($P < 0.05$) we did not show an increase in VBP regardless of the technique used (plasma decay of fluorescent dyes,).

**CONCLUSIONS.** In NTHS, even though microcirculation was restored, fluid resuscitation was not associated with increased VBP despite the presence of glycocalyx degradation. This result challenges the role of the glycocalyx as a significant contributor to VBP. Each fluid used has a different impact on the glycocalyx.

**REFERENCE(S)**

1. Kottke MA. Shock 2016; 46:20–36
2. Torres Filho IP. Am J Physiol Heart Circ Physiol 2016; 310:H1468–78
3. Weinbaum S. Annu Rev Biomed Eng 2007; 9:121–67

**GRANT ACKNOWLEDGMENT**

Dr Guerci was supported by a grant from the French Society of Anesthesiology and Intensive Care Medicine (SFAR).

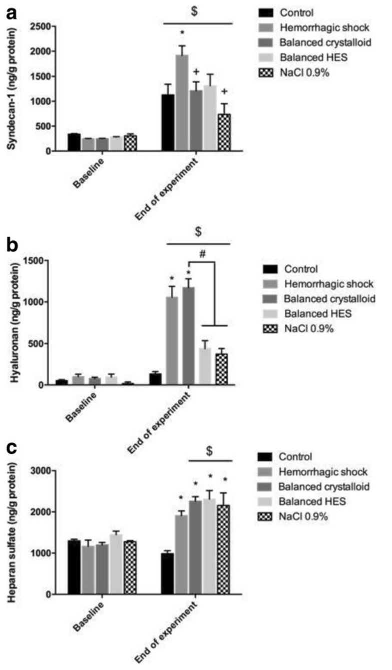

**Fig. 13 (abstract 0013).** See text for description

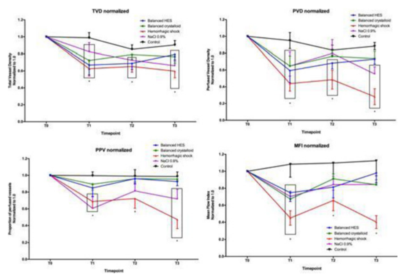

**Fig. 14 (abstract 0013).** See text for description

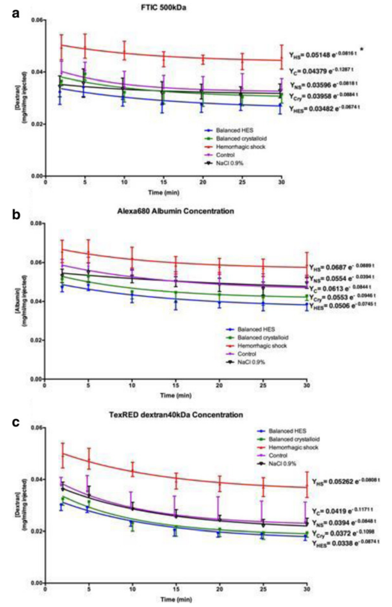

**Fig. 15 (abstract 0013).** See text for description

### 0014

#### Limitations of using grid-based point-of-care assessment for scoring microcirculatory alterations

Y. Ince[1,2], H. He[2,3], G. Guven[2,4], E. Boerma[1,5], C. Ince[1,2]
[1]Academic Medical Center, University of Amsterdam, Department of Translational Physiology, Amsterdam, Netherlands; [2]Erasmus MC-University Medical Center, Department of Intensive Care, Rotterdam, Netherlands; [3]Peking Union Medical College Hospital, Peking Union Medical College, Beijing, China; [4]Ankara University Faculty of Medicine, Department of Intensive Care, Internal Medicine, Ankara, Turkey; [5]Medical Centre Leeuwarden, Department of Intensive Care, Leeuwarden, Netherlands
**Correspondence:** Y. Ince
*Intensive Care Medicine Experimental* 2017, **5(Suppl 2):**0014

**INTRODUCTION.** The semi-quantitative score method introduced by Boerma and co-workers for measuring microcirculatory flow index (MFI) has been commonly used to evalute the microcirculatory alterations in critically ill patients using hand-held vital microscopes [1] and suggested as use for point-of-care assessment [2].MFI is based on scoring a representative vessel in one of four quadrants of the field of view (FOV) and calculating the mean value over the four quadrants. There remains uncertainty, however, about the reliability of the numerical value obtained from this grid-based scoring system.

**OBJECTIVES.** The aim of this study was to determine if this $MFI_{quadrants}$ would differ significantly from the MFI $_{per\ vessel}$ score based on the measurement of every vessel in the FOV.

**METHODS.** $MFI_{quadrants}$ is the mean flow score for the four quadrants of a representative vessel scored in each quadrant. $MFI_{per\ vessel}$ is the mean flow scores measured for each vessel in the entire FOV (Fig. 16).

Flow values are scored as 0 no flow, 1 (intermitent flow), 2 sluggish flow, 3 continuous flow [1]. The total vessel density (TVD), perfused vessel density (PVD), and percentage of perfused vessel (PVD) was also calculated and compared. 126 videos of skeletal muscle from 24 rats in shock and resuscitation were analyzed to measure the relationship between the $MFI_{quadrant}$ and $MFI_{per\ vessel}$.

**RESULTS.** The $MFI_{quadrants}$ was 2.29 + 0.74 (range from 0.5 to 3), and $MFI_{per\ vessel}$ was 2.13 + 0.74, range (0.17 to 3). A Bland-Altman plot showed the mean difference between $MFI_{quadrants}$ and $MFI_{per\ vessel}$ was − 0.16, and limits of agreement (bias ± 1.96SD) is from 0.95 to −0.64 (Fig. 17).

Moreover, the absolute value of variation between $MFI_{quadrants}$ and $MFI_{per\ vessel}$ was significantly correlated with PPV ($P < 0.001$), PVD ($P = 0.002$), $MFI_{per\ vessel}$ ($P < 0.0001$), but not with $MFI_{quadrants}$ ($P = 0.085$) and TVD ($P = 0.134$). The PPV (ROC area 0.81) was the best predictor of a 15% difference between $MFI_{quadrants}$ and $MFI_{per\ vessel}$, significantly better than PVD (ROC area 0.71), TVD(ROC area 0.6) (Fig. 18).

For predicting an 15% difference between $MFI_{quadrants}$ and $MFI_{per\ vessel}$, a PPV threshold of 71% was identified with a sensitivity of 42% and a specificity of 95%.

**CONCLUSIONS.** Quantative values of the MFI based on quadrants should be used with caution under conditions of low PPV (<71%). This finding is of importance if MFI values are to be used as thresholds for clinical decision making for point-of-care use at the bedside.

**REFERENCE(S)**

1. Boerma EC, Mathura KR, van der Voort PH, Spronk PE, Ince C: Quantifying bedside-derived imaging of microcirculatory abnormalities in septic patients: a prospective validation study. Crit Care 2005, 9: R601-R606.

2. De Backer D, Hollenberg S, Boerma C, Goedhart P, Büchele G, Ospina-Tascon G, Dobbe I, Ince C: How to evaluate the microcirculation: report of a round table conference. Crit Care 2007, 11:R101.

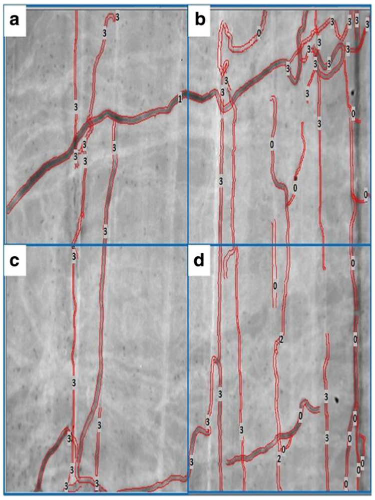

**Fig. 16 (abstract 0014).** See text for description

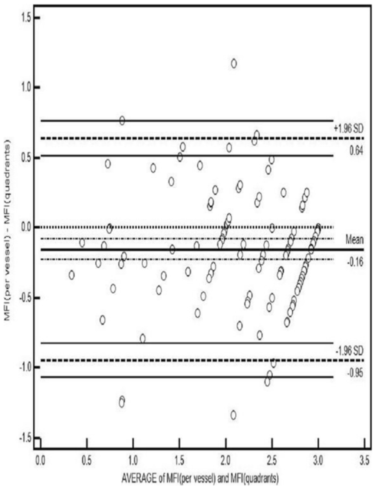

**Fig. 17 (abstract 0014).** See text for description

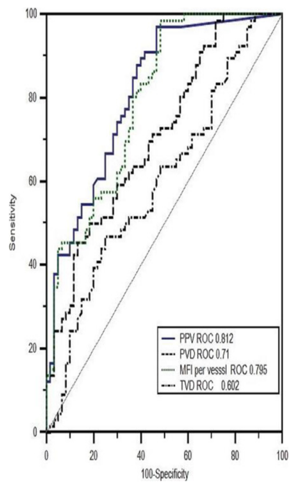

**Fig. 18 (abstract 0014).** See text for description

### 0015

#### The assessment of fluid responsiveness in undifferentiated shock using corrected flow time measured by Doppler ultrasound of carotid artery after passive leg raise maneuver

I. Barjaktarevic[1], S. Hu[2], S. Chang[1], E. Aquije Montoya[3], R. Buhr[1], W. Toppen[4], S. Ong[3], T. Wang[1], A. Chiem[3], D. Berlin[5], P. Paull[4], A. Maller[1], T. Rezayat[6]
[1]David Geffen School of Medicine at UCLA, Pulmonary and Critical Care Medicine, Los Angeles, United States; [2]David Geffen School of Medicine at UCLA, Division on Pulmonary and Critical Care Medicine, Los Angeles, United States; [3]David Geffen School of Medicine at UCLA, Los Angeles, United States; [4]David Geffen School of Medicine at UCLA, Department of Medicine, Los Angeles, United States; [5]Weil Cornell University, Pulmonary and Critical Care Medicine, New York, United States; [6]David Grant Medical Center, Pulmonary and Critical Care, Travis, United States
**Correspondence:** I. Barjaktarevic
*Intensive Care Medicine Experimental* 2017, **5(Suppl 2):**0015

**INTRODUCTION.** Adequate assessment of fluid responsiveness in undifferentiated shock has substantial impact on patient outcomes. Systolic flow time (FT) corrected for heart rate can be used to follow change in left ventricular preload. Recent data[1,2] suggests its possible value in predicting fluid responsiveness when combined with preload challenge maneuvers such as passive leg raise (PLR).

**OBJECTIVES.** Evaluation of PLR-induced change in carotid corrected FT (ccFT) as a predictor of fluid responsiveness in patients with undifferentiated shock.

**METHODS.** Prospective, non-interventional study included patients with newly diagnosed undifferentiated shock. Eligible subjects were adult patients with shock duration < 24 hours, resuscitated with >30cc/kg of intravenous fluids who had no history of significant cardiovascular disease or contraindications to perform PLR. Subjects were evaluated with point-of care (POC) Doppler ultrasound (Logiq e, GE Healthcare, Wauwatosa, WI) imaging of carotid artery and with stroke volume (SV) assessment via non-invasive cardiac output monitoring system (NICOM, Cheetah Medical) before and after PLR maneuver. ccFT was calculated based on Doppler waveform analysis, and ΔccFT was presented in milliseconds (msec) or percent-change ($_{\%}$ΔccFT), Fig. 19. Fluid responsiveness was defined as >10% ΔSV as per NICOM protocol.

**RESULTS.** Presented is preliminary analysis of the first 70 patients. All patients required vasopressor support, with 40 patients (57.1%) being on mechanical ventilation. Average length of stay prior to shock onset was 0.6 days, average age of patients 60.9 years with 51.4% of cohort being female, *Table* 2. Average cardiac index improved significantly with PLR from 3.75 to 4.24L/min/m2 with an average ΔSV 23.7 ± 23.0%, *Table* 3. Average ccFT at baseline was 314.0 ± 37.2msec and was increased after PLR to an average 324.0 ± 43.8msec with an average increase of 10.9 ± 23.0msec in ΔccFT or 3.6 ± 7.6% change in %ΔccFT. The majority of patients, 49 (70.0%), were fluid responders based on *ΔSV*. Fluid responders had higher average ΔccFT 17.7 ± 23.6 msec compared to non-responders, ΔccFT −4.7 ± 10.7msec (p = 0.000047). ROC curve analysis demonstrated slight improvement of ΔccFT (AUC = 0.873 [95% CI: 0.86-0.88]) in comparison to $_{\%}$ΔccFT (AUC = 0.860 [95% CI: 0.85-0.87]), Fig. 20. Using a ΔccFT cutoff of 9 msec, the test has positive predictive value of 95% with sensitivity 73% and specificity 90%, Table 4.

**CONCLUSIONS.** POC assessment of ccFT can be simple and broadly available test that, combined with PLR maneuver, may offer ability to predict fluid responsiveness in undifferentiated shock.

**REFERENCE(S)**

1) Blehar et al.Correlation of corrected FT in the carotid artery with changes in intravascular volume status. *J Crit Care*. 2014

2) Lafanechere et al.Changes in aortic blood flow induced by PLR predict fluid responsiveness in critically ill patients. *Crit Care*. 2006

**GRANT ACKNOWLEDGMENT**

This study has been financially supported by GE Healthcare

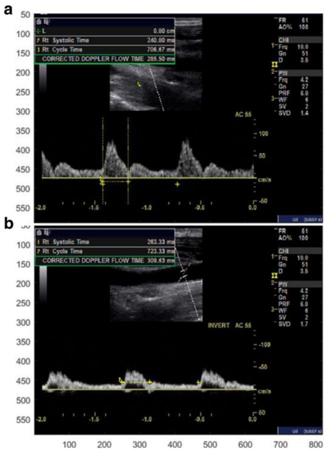

**Fig. 19 (abstract 0015).** See text for description

**Table 2 (Abstract 0015).** See text for description

| Parameter | Value |
|---|---|
| Number of patients enrolled, N | 70 |
| Sex (percent female subjects) | 51.4 |
| Age (years) | 60.9 |
| Heart rate, N (±SD) | 89.1 (±19.5) |
| Baseline mean arterial pressure, mmHg (±SD) | 70.6 (±11.4) |
| Vasopressor use, N (%) | 70 (100) |
| Mechanical ventilation, N (%) - Passively ventilated, N (%) - Positive end-expiratory pressure (PEEP) >5mmH2O, N (%) | 40 (57.1) - 19 (27.1) - 16 (22.9) |
| Lactate at enrollment, mg/dl | 34.2 |
| Hospitalization days prior to shock onset, N | 0.55 |

**Table 3 (Abstract 0015).** See text for description

| Parameter | Baseline Value | Post PLR challenge |
|---|---|---|
| NICOM cardiac index, L/min/m2 | 3.75 | 4.24 |
| NICOM SV, ml (±SD) | 62.1 (±24.5) | 74.9 (31.4) |
| NICOM ΔSV, % ±SD | | 23.7 (±23.0) |
| Fluid responsive, N (%) - NICOM ΔSV, % ±SD | | 49 (70) - 32.5 (21.8) |
| Fluid nonresponsive, N (%) - NICOM ΔSV, % ±SD | | 21 (30) - 3.1 (7.0) |
| Systolic time at baseline, msec (±SD) | 263.92 (42.84) | 272.70 (46.35) |
| Corrected FT at baseline, msec (±SD) | 314.03 (37.23) | 324.02 (43.78) |
| ΔccFT msec (±SD) and Percent ΔccFT, % (±SD) | | 10.94 (22.96) and 3.6 (7.6) |
| ΔccFT, fluid responsive vs. fluid non-responsive, msec (±SD) | | 17.65 (23.6) vs. -4.72 (10.68), p-value= 0.000047 |

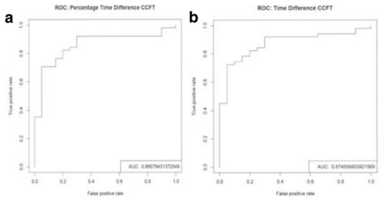

**Fig. 20 (abstract 0015).** See text for description

**Table 4 (Abstract 0015).** See text for description

| ΔccFT (msec) | Sensitivity | Specificity | Positive predictive value (PPV) | Negative predictive value (NPV) |
|---|---|---|---|---|
| 1 | 0.88 | 0.70 | 0.88 | 0.70 |
| 3 | 0.84 | 0.70 | 0.88 | 0.64 |
| 5 | 0.78 | 0.80 | 0.91 | 0.64 |
| 9 | 0.73 | 0.90 | 0.95 | 0.56 |
| 11 | 0.65 | 0.95 | 0.97 | 0.51 |
| 13 | 0.61 | 0.95 | 0.97 | 0.49 |
| 15 | 0.51 | 0.95 | 0.96 | 0.44 |
| 17 | 0.51 | 0.95 | 0.96 | 0.42 |
| 19 | 0.43 | 1.00 | 1.00 | 0.41 |

### Therapy and prognosis of acute brain injury

#### 0016

#### Acute magnetic resonance imaging predicts neurocognitive outcome after aneurysmal subarachnoid haemorrhage

M. J. Rowland[1,2], P. Garry[1,2], M. Ezra[1,2], R. Corkill[2], I. Baker[2], J. Westbrook[2], G. Douaud[1], K. T. Pattinson[1,2]

[1]University of Oxford, Nuffield Department of Clinical Neurosciences, Oxford, United Kingdom; [2]Oxford University Hospitals NHS Foundation Trust, Neurosciences Intensive Care Unit, Oxford, United Kingdom

**Correspondence:** M. J. Rowland

*Intensive Care Medicine Experimental* 2017, **5(Suppl 2):**0016

**INTRODUCTION.** Aneurysmal subarachnoid haemorrhage (SAH) is a devastating disease often leading to death or poor functional outcomes. Damage to the brain in the first 72 hours following aneurysm rupture ("early brain injury") is likely to play a key role in determining the incidence of morbidity and mortality after SAH. Acute MRI scanning may offer greater insight into early brain injury and help develop novel diagnostic and therapeutic biomarkers.

**OBJECTIVES.** To detect and quantify the impact of SAH on brain measurements of T1-structural and diffusion-weighted (DWI) MRI from the first 72 hours to 3 months post-SAH, and to correlate these with subsequent neurocognitive outcomes.

**METHODS.** 27 patients with "good grade” SAH (World Federation of Neurosurgeons Grade I and II) underwent T1-weighted structural and DWI MRI scanning at 3 time-points post-SAH: < 72 hours, 5–10 days and 3 months post-SAH. Patient MRI data was assessed longitudinally as well as being compared with data from age/gender-matched healthy controls. Patients underwent detailed assessment at 3 months post-SAH to quantify neurocognitive outcomes.

**RESULTS.** There was a significant global increase in grey matter (GM) volume measured from T1-weighted MRI in the first 72 hours post-SAH compared with healthy controls (Fig. 21). These changes were associated with restricted diffusion, suggesting widespread cytotoxic cerebral oedema resulting in this apparent increase of GM volume. By 3 months, structural changes had resolved, except for higher GM volume in patients compared with healthy controls in cognitive zones of the cerebellum (Crus I & II, and lobule VIIb – Fig. 22). This increase was negatively correlated with neurocognitive performance (r = 0.389, p = 0.006 Pearson's correlation). Retrospectively, patients with subsequent neurocognitive impairment at 3 months already showed higher GM volume at < 72 hours post-SAH in the same cognitive cerebellar regions compared with patients who did not develop cognitive impairment (Fig. 23). Notably, cerebellar GM measure during the acute phase made it possible to predict with 84% sensitivity and 86% specificity those patients who went on to develop poor neurocognitive outcomes post-SAH.

**CONCLUSIONS.** Structural and diffusion-weighted MRI are highly sensitive to the global as well as regional impact of cerebral oedema that occurs acutely following aneurysm rupture and is often undiagnosed. Results from this study also demonstrate the crucial role of cerebellar injury in modulating neurocognitive outcomes after “good grade” SAH. Using an automated approach, a simple T1-weighted structural MRI scan in the first 72 hours post-SAH can predict subsequent neurocognitive outcome with high accuracy. Damage to the cerebellum acutely post-SAH may therefore underlie neurocognitive impairment after SAH and act as a therapeutic and diagnostic marker of early brain injury.

**Grant Acknowledgement**

MRC (UK)

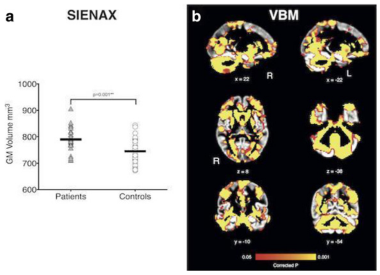

**Fig. 21 (abstract 0016).** <72 hours - Patients v Controls

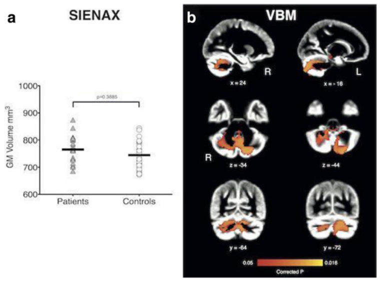

**Fig. 22 (abstract 0016).** 3 months - Patients v Controls

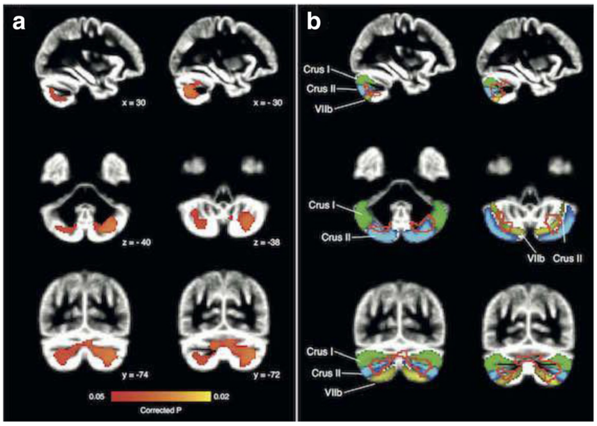

**Fig. 23 (abstract 0016).** <72 hours - Impaired vs Non-impaired

### 0017

#### Hypothermia to control intracranial pressure following traumatic brain injury is associated with significant differences in outcomes, management and physiology: results of additional data collection from the Eurotherm3235 trial

J. K. Rhodes[1], I. Puddu[2], F. S. Taccone[3], M. van der Jagt[4], A. Rodriguez[5], C. Battison[5], L. Sinclair[5], P. Andrews[1]
[1]University of Edinburgh/NHS Lothian, Edinburgh, United Kingdom, [2]University of Turin/S. Giovanni Battista-Molinette Hospital, Turnin, Italy, [3]Université Libre de Bruxelles/Hôpital Erasme, Brussels, Belgium, [4]Erasmus MC-University Medical Center, Rotterdam, Netherlands, [5]University of Edinburgh, Edinburgh, United Kingdom
**Correspondence:** J.K. Rhodes
*Intensive Care Medicine Experimental* 2017, **5(Suppl 2):**0017

**INTRODUCTION.** The Eurotherm3235 trial [1] was a high quality randomised controlled trial assessing the efficacy of therapeutic hypothermia, 32-35°C, (TH) to control intracranial pressure (ICP) following traumatic brain injury (TBI) verses standard care (Control (CG)). TH was associated with increased mortality at 6 months. To better understand this result study centres collected additional data retrospectively.

**METHODS.** Additional data, including the patients' background, management and physiology in the 7 days following randomisation, was collected from medical notes, 24 hour charts and hospital information systems between January and July 2016. This was collated into a secure database and analysed. Data are given as number (%) or mean daily values (standard deviation) for TH vs. CG.

**RESULTS.** 286 anonymised forms were received from 33 of 47 centres (74% of the trial population). 143 patients received TH and 143 were in the CG. Mortality at 6 months was significantly increased with TH (46 vs. 32 deaths, Hazard ratio 1.624 CI 1.025 - 2.573, p = 0.039). The cause of death may have differed between the groups, with an excess of TH patients dying with multi-organ failure, systemic inflammation or acute lung injury (15 (35.7%) vs. 5 (19.2%)). The numbers receiving Midazolam (96.0(12.8) vs. 78.7(16.0), p = 0.046), Alfentanil (37.1(4.7) vs. 27.7(5.2), p = 0.004, Atracurium (31.1(6.8) vs. 22.0(4.5), p = 0.012) were significantly greater with TH. TH was associated with increased numbers receiving Vasopressin (5.7(2.4) vs. 1.9(0.9), p = 0.002) and Dobutamine (7.3(3.1) vs. 2.3(1.0), p = 0.002). The number of patients with a metabolic acidosis (45(36.9%) vs. 27(22.1%), p = 0.017) and receiving bicarbonate (17(13.7%) vs. 5(4.2%), p = 0.018) was greater with TH. Serum lactate was also greater (1.20(0.10) vs. 1.06(0.11) mmol/l, p = 0.027). Furthermore, the number of patients receiving steroids was greater with TH (23(17.8%) vs. 9(6.7%), p = 0.01). Small but significant effects of TH on leucocytes (9.9(0.9) vs. 11.0(0.7) 10^9/l, p = 0.031), neutrophils (7.8(0.6) vs 9.0(0.7) 10^9/l, p = 0.005) and activated partial thromboplastin time (33.2(2.1) vs. 31.2(1.3) s, p = 0.046) were also found.

**CONCLUSIONS.** This post-hoc explorative study found significant between groups differences in outcome, management and physiology associated with TH. In light of Eurotherm3235´s principle finding that TH was associated with harm, these differences, which have previously been regarded as simple consequences of TH, could have pathological significance. Correcting for these differences in future studies of TH could be important.

**REFERENCE(S)**

1. Andrews, P.J., et al., *Hypothermia for Intracranial Hypertension after Traumatic Brain Injury.* N Engl J Med, 2015. 373(25): p. 2403–12.

**GRANT ACKNOWLEDGEMENT**

Supported by the National Institute for Health Research Health Technology Assessment program, which funded the main phase of the study. The European Society of Intensive Care Medicine funded the pilot phase.

### 0018

#### Decompressive craniectomy after traumatic brain injury and brain monitoring

A. V. Ferreira[1], J. Silva[2], E. Monteiro[3], A. Cerejo[4], C. Dias[3]
[1]Hospital Pedro Hispano, Matosinhos, Portugal; [2]Centro Hospitalar de Gaia, Neurosurgery, Gaia, Portugal; [3]Centro Hospitalar São João, Intensive Care, Porto, Portugal; [4]Centro Hospitalar São João, Neurosurgery, Porto, Portugal
**Correspondence:** A. V. Ferreira
*Intensive Care Medicine Experimental* 2017, **5(Suppl 2):**0018

**INTRODUCTION.** Decompressive craniectomy (DC) is largely used as treatment for refractory intracranial hypertension after traumatic brain injury (TBI). DC decision and its impact on clinical evolution has still unresolved controversies mainly regarding timing of intervention, patient selection and post-operative management.

**OBJECTIVES.** To characterize the before and after periods of DC using multimodal brain monitoring.

**METHODS.** We retrospectively studied 9 TBI patients, admitted to the Neurocritical Care Unit (NCCU) at Hospital São João, Porto that were submitted to primary or secondary DC and had brain monitoring during 12 hours before and 24 hours after surgery. At NCCU, TBI patients admitted are managed with an autoregulation-oriented CPP protocol. Variables analyzed were Intracranial Pressure (ICP), ICP dose over 20 mmHg (ICPd > 20), Cerebral Perfusion Pressure (CPP), optimal CPP (CPPopt), Arterial Blood Pressure (ABP), End-Tidal CO2 (ETCO2), cerebrovascular pressure reactivity based on ICP (PRx) and volume-pressure compensatory reserve (RAP).

**RESULTS.** Data analyzed included seven males and two females with mean age of 30.3 ± 11.1 years, with admission mean Glasgow Coma Scale (GCS) of 6.1 ± 2.1 and Simplified Acute Physiology Score II (SAPSII) of 42.2 ± 14.6. Marshall classification of the first CT scan was predominantly grade III and before DC was IV. The mean period from hospital admission to DC was 3.3 ± 2 days. DC led to significantly decrease in ICP maximum values ($p$ = 0.011) and lowered ICPd > 20 exposure (p = 0,038), in spite of non significant decrease of median ICP (Fig. 24). CPP before and after DC was significantly different ($p$ = 0.038) as it was CPPopt ($p$ = 0,021) (Fig. 25). Two hours before surgery PRx became positive indicating impairment of autoregulation (AR). The median values of PRx before and after DC show no statistically significant differences in spite of documented increase in its variance (Fig. 26). Furthermore in some patients after DC, PRx remained >0.25 (used cut-off for AR). Before DC, RAP index was positive and closer to 1 expressing poor volume-pressure reserve. After DC RAP decreased with a trend towards to zero ($p$ = 0.008) representing an improvement in brain compliance. Three months after hospital admission 67% of patients had Glasgow Outcome Scale (GOS) ≥3. Mortality rate was 17%.

**CONCLUSIONS.** ICP, CPP and compensatory reserve improved significantly as expected after DC. Sudden impairment of AR before DC may help to decide the timing of surgery. However, further studies are warranted to better understand DC influence on clinical decision and outcome.

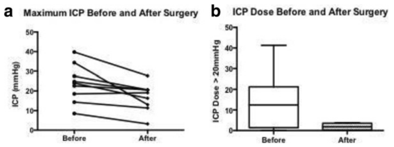

**Fig. 24 (abstract 0018).** ICP and ICP dose before and after DC

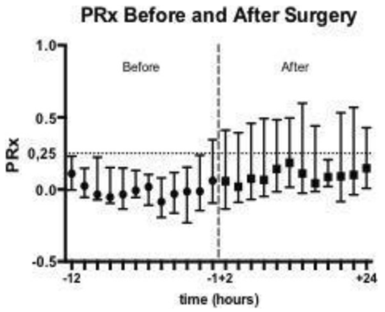

**Fig. 25 (abstract 0018).** PRx before and after surgery

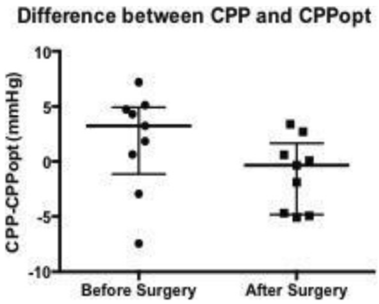

**Fig. 26 (abstract 0018).** CPP and CPPopt

### 0019

#### Transfusion Requirements after Head Trauma (TRAHT): a randomized clinical trial

A. Gobatto[1,2,3], M. L. Azevedo[1], D. J. F. Solla[1], W. S. Paiva[1], L. M. Malbouisson[1], E. B. Shu[1]

[1]Hospital da Clinicas - University of Sao Paulo Medical School, São Paulo, Brazil; [2]Hospital São Rafael, Salvador, Brazil; [3]Hospital da CIdade, Salvador, Brazil

**Correspondence:** A. Gobatto

*Intensive Care Medicine Experimental* 2017, **5(Suppl 2):**0019

**INTRODUCTION.** Anemia is frequent among traumatic brain injured (TBI) patients, and it is associated with an increased risk of poor outcomes. The optimal hemoglobin (Hb) level to trigger red blood cell (RBC) transfusions in TBI patients is yet to be defined.

**OBJECTIVE.** To evaluate the feasibility and safety of creating a hemoglobin gradient between TBI patients submitted to restrictive or liberal transfusion strategies in the ICU, as part of planning a larger randomized clinical trial.

**METHODS.** From June 2015 to June 2016, all adult patients with moderated or severe TBI were randomized either to a restrictive group, with an Hb transfusion threshold level of 7g/dL, or to a liberal group, with an Hb transfusion threshold level of 9g/dL. Transfusion strategies were maintained for up to 14 days or to ICU discharge. The primary outcome was the mean Hb difference between groups. Secondary outcomes included transfusion requirements, intracranial pressure (ICP) management resources use, transcranial doppler (TCD) parameters, length of stay and mortality. Patients were followed for 6 months after hospital discharge.

**RESULTS.** A total of 44 patients were randomized, 21 patients to the liberal group and 23 patients to the restrictive group. There was no baseline difference between the groups. The patients were predominantly male, 40 (90.9%) patients, mean age was 34.5 ± 13.4 years, and admission median Glasgow coma scale was 6 (quartiles 4–8). The mean CRASH score 14-day death risk was 40.7 ± 18.9% in the restrictive group and 39.3 ± 18.4% in liberal group ($p = 0.818$). The ICU admission Hb levels (g/dL) were 10.3 ± 1.6 in restrictive group and 10.1 ± 1.2 in liberal group ($p = 0.550$), and the average 14 days Hb level was 8.4 ± 1.0 and 9.3 ± 1.3 ($p < 0.001$), respectively. During ICU stay, RBC transfusion was administered to 13 (56.5%) patients in the restrictive group and to all 23 (100.0%) patients ($p = 0.001$) in the liberal group (a total of 35 and 66 RBC units, respectively; $p = 0.022$). There was an inverse correlation (−0.265) between Hb level and middle cerebral artery flow velocity ($p < 0.001$) at the side of trauma. This correlation was stronger on the liberal group (Hb range 7.1-12.6g/dL) when compared to the restrictive group (Hb 6.2-10.7g/dL). Hospital mortality was higher in the restrictive group, seven (30.4%), versus one (4.8%) patient ($p = 0.048$).

**CONCLUSION.** Creating a hemoglobin gradient between the groups was feasible. The restrictive group had lower hemoglobin levels and received less RBC transfusions. Mortality was higher in restrictive group.

### 0020

#### Cumulative intracranial pressure and cerebral perfusion pressure burdens as determinants of early mortality after severe traumatic brain injury

H. S. Mangat[1], X. Wu[2], L. M. Gerber[2], M. Fakhar[1], S. B. Murthy[1], P. E. Stieg[3], J. Ghajar[4], R. Härtl[3]

[1]Weil Cornell University, Neurology, New York, United States; [2]Weil Cornell University, Healthcare Policy & Research, New York, United States; [3]Weil Cornell University, Neurological Surgery, New York, United States; [4]Stanford University School of Medicine, Neurological Surgery, Palo Alto, United States

**Correspondence:** H.S. Mangat

*Intensive Care Medicine Experimental* 2017, **5(Suppl 2):**0020

**INTRODUCTION.** Increased Intracranial pressure (ICP) and low cerebral perfusion pressure (CPP) remain the two main cerebral hemodynamic goals to improve outcomes after severe traumatic brain injury (TBI). Yet debate remains over the actual thresholds, and whether they need to be individualised based on autoregulation status or other criteria.[1,2] Here, we used data from a large cohort to examine the association of cumulative ICP and CPP burden on outcome after severe TBI. Recent data from small cohorts has supported such an approach.[3]

**OBJECTIVES.** To determine association of cumulative burdens of intracranial pressure (ICP) and cerebral perfusion pressure (CPP) with two-week mortality after severe TBI.

**METHODS.** Retrospective analysis of prospectively collected data from the New York State TBI-trac® trauma database. Daily duration of ICP > 25mmHg ($ICP_{high}$) and CPP < 60mmHg ($CPP_{low}$) were recorded. Cumulative number of hours, averaged daily duration, and %total days with abnormal value were calculated. Outcome was recorded as 2-week mortality.

**RESULTS.** 1207 patients were included with 19.1% mortality. Mean cumulative $ICP_{high}$ burden was 8.85 hours, $ICP_{high}$ days were 25.1% of total days, averaged daily burden was 1.54 hours. Mean cumulative $CPP_{low}$ burden was 16.6 hours with $CPP_{low}$ days of 45.1% and daily average of 2.95 hours. In non-survivors compared to survivors, cumulative and averaged daily hours of $ICP_{high}$ (20.33 vs. 6.23 hours; 4.74 vs. 0.81 hours; p < 0.001) and $CPP_{low}$ (25.7 vs.14.46 hours; 5.83 vs.2.28 hours, p < 0.001) were significantly higher. Mortality increased dramatically with $ICP_{high}$ > 50% days (p < 0.001, c-statistic 0.71), or $CPP_{low}$ > 80% (p < 0.001, c-statistic 0.70). In multivariate analysis using age, GCS, hypotension, CT and pupillary abnormalities, and including $ICP_{high}$ > 40% and $CPP_{low}$ > 80%, model c-statistic was 0.834. Multivariate logistic regression demonstrated that the likelihood ratio statistic for cumulative $ICP_{high}$ (84.10) was higher than that for $CPP_{low}$ (45.97) in predicting mortality.

**CONCLUSIONS.** Cumulative $ICP_{high}$ and $CPP_{low}$ burdens have a high correlation with early mortality after severe TBI and warrant further evaluation in severe TBI patients with intracranial hypertension. $ICP_{high}$ burden appears to impact mortality more than $CPP_{low}$ burden, which has not been reported before.

**REFERENCE(S)**

1. Depreitere B, et al. J Neurosurg 2014;120:1451–7.
2. Lazaridis C, et al. J Neurosurg 2014; 120:893–900.
3. Guiza F, et al. Intensive Care Med 2015; 41:1067–76.

### Cardiac arrest management

#### 0021

#### Increase in bystander cardiopulmonary resuscitation is associated with increased rates of "chest compression only" resuscitation - a nationwide study

J. Hollenberg[1], G. Riva[2], M. Ringh[1], P. Nordberg[1], S. Rubertsson[3], J. Herlitz[4], L. Svensson[2]
[1]Karolinska Institutet, Center for Resuscitation Science, MICU, Dept of Cardiology, South Hospital Stockholm, Stockholm, Sweden; [2]Karolinska Institutet, Center for Resuscitation Science, Stockholm, Sweden; [3]Uppsala University, Uppsala, Sweden; [4]Sahlgrenska Akademin, Göteborgs Universitet, Gothenburg, Sweden
**Correspondence:** J. Hollenberg
*Intensive Care Medicine Experimental* 2017, **5(Suppl 2):**0021

**INTRODUCTION.** Bystander cardiopulmonary resuscitation (CPR) traditionally consists of both ventilations and chest compressions. The concept of Compression Only CPR (CO-CPR) has emerged since rescue breaths are difficult to perform, potentially delay the start of CPR, and interrupt chest compressions. Since 2010 guidelines suggest CO-CPR for telephone assisted CPR, for untrained bystanders and for trained bystanders unwilling or unable to perform rescue breaths.

**OBJECTIVES.** The aim was to describe changes in rates of bystander CPR, telephone assisted CPR, CO-CPR and survival during 3 different time-periods of CPR-guidelines on a national level. We hypothesized that dissemination of CO-CPR would be associated with increased CPR-rates and similar survival compared to standard CPR.

**METHODS.** A registry based cohort study including all bystander witnessed cases of out-of-hospital cardiac arrests reported to the Swedish Registry for Cardiopulmonary Resuscitation in 2000–2014. Exposure was categorized as bystander CPR or No-CPR. Bystander CPR was further categorized into Standard CPR (S-CPR) or CO-CPR. Primary outcome was 30-day survival.

**RESULTS.** In total 23 620 patients were included. Total rates of bystander CPR increased from 38% in 2000–2005 to 70% in 2011–2014. CO- CPR increased from 5% in 2000–2005 to 28% in 2011–2014. Any form of bystander CPR was associated with increased 30-day survival compared to no CPR in all time periods studied. Overall there was no significant difference in survival among patients receiving CO-CPR or S-CPR (13,6% vs. 12,9% p = 0,3).

**CONCLUSIONS.** The increase in bystander CPR during the last 15 years in Sweden is mainly attributed to an increase in CO-CPR. 30-day survival was not different when comparing CO-CPR to S-CPR.

#### 0022

#### Termination of resuscitation rule in pediatric patients with out-of-hospital cardiac arrest

T. Yoshida, K. Maekawa, K. Katabami, H. Sageshima, T. Wada, M. Hayakawa, A. Sawamura, S. Gando
Hokkaido University Hospital, Sapporo, Japan
**Correspondence:** T. Yoshida
*Intensive Care Medicine Experimental* 2017, **5(Suppl 2):**0022

**INTRODUCTION.** Although there is increasing acceptance of termination of resuscitation (TOR) in patients with out-of-hospital cardiac arrest (OHCA) following futile resuscitation, children are generally excluded from TOR protocols. Termination of pediatric resuscitation is a difficult topic for clinicians and patient's family, however, decision to withhold resuscitative efforts should be evidence based and free from emotion.

**OBJECTIVE.** We aimed to develop and validate a TOR rule in pediatric patients with OHCA, to guide emergency physicians in deciding whether to withhold further resuscitation efforts or terminate on-going resuscitation soon after patient arrival.

**METHODS.** We performed retrospective analysis using data from Japan's nationwide OHCA registry from January 2005 through December 2012, including pediatric OHCA patients < 18 yrs, transported to hospital by emergency medical services (EMS). We developed a new TOR rule predicting mortality at 1 month after cardiac arrest using multivariate logistic model. Internal validation was performed using split-sample methods. The derivation set comprised of 9702 patients registered in 2005–2010, and the validation set comprised 2875 patients registered in 2011–2012.

**RESULTS.** The overall mortality at 1 month after OHCA was 90.1%. We developed a new TOR rule including 5 criteria based on the results of multivariate logistic regression analysis: no prehospital return of spontaneous circulation (adjusted odds ratio 16.7; 95% confidence interval [CI] 13.4-20.8), no shockable initial cardiac rhythm (3.25; 2.50-4.21), time interval from EMS call to hospital arrival > 16 min (2.11; 1.69-2.65), no witness (1.83; 1.54-2.16), and no bystander CPR (1.48; 1.25-1.75). The new TOR rule had a specificity of 0.91 (95%CI 0.89-0.93) and a positive predictive value of 0.97 (0.97-0.98) in the derivation set, and a specificity of 0.91 (0.87-0.93) and a positive predictive value of 0.96 (0.95-0.97) in the validation set.

**CONCLUSIONS.** We developed and validated a new TOR rule in pediatric OHCA. This rule might help emergency physicians for counseling family and making medical decisions.

#### 0023

#### Impact of supplementation of selenium on oxidative stress in patient after cardiac arrest

M. Janotka, D. Vondráková, A. Krüger, P. Hladká, J. Pánková, P. Ošťádal
Hospital Na Homolce, Prague, Czech Republic
**Correspondence:** M. Janotka
*Intensive Care Medicine Experimental* 2017, **5(Suppl 2):**0023

**INTRODUCTION.** Prognosis of patients after cardiac arrest (CA) is still unfavourable despite improving prehospital and hospital care (10-20% survival to discharge).The anoxic phase of injury is followed by the reperfucion phase leading to postcardiac arrest syndrom.The oxidative stress is one of the main causes of post-cardiac arrest syndrome.There is a data showing that increasing of the antioxidative protection by the supplementation of selenium in patients in septic shock or severe sepsis might improve their prognosis.There is no clear data on the impact of this supplementation in patients after CA.

**OBJECTIVES.** We assumed that because there is a parallel between circulation impairment in sepsis and after CA and because the most prognosis limiting system is the brain that is full of lipids easilly targeted by oxidative stress, we could decrease the damage by selenium substitution in the same way as proven in septic shock.

**METHODS.** We randomized 26 patients in 2 groups. The first group was treated by standart therapy. The second group received selenium intravenously (sodium selenite) in addition to the standart therapy. 3mg of selenium was administered as initial bolus (the first day) in 30 minutes infusion followed by continuous infusion of 2mg of selenium per day for the next 5 days.We measured the products of reactive oxygen species reactions (ROM test and 8-hydroxy-guanosin as marker of the damage of RNA), serum antioxidative capacity (BAP test),markers of inflammation and tissue injury and clinical outcome (cerebral performance categories scale,CPC,incidence of infection).

**RESULTS.** Patients treated by selenium had less pronounced oxidative stress.The result of ROM test was significantly lower in the selenium treated group in the 3rd,4th and 5th day with greatest difference in the 4th day (340 vs.418 Carr units,p = 0,03).The result of BAP test was significantly higher in the selenium treated group in the 1st,2nd,3rd,4th and 5th day with greatest difference in the 3rd day (2337 vs.1938 μmol/l,p = 0,01). There was significantly lower concentration of 8-hydroxy-guanosin in selenium treated group in the 3rd day (1,2 vs. 2,3 ng/ml, p = 0,04). There was no difference in the level of the markers of renal,hepatal or cerebral (neuron specific enolase) injury and no difference in inflammation markers (leukocytes,CRP,-procalcitonin,incidence of infection). There was insignificantly better neurological outcome in the selenium treated group in 1st month follow up (80 vs. 50% of patients with CPC 1).

**CONCLUSIONS.** Preliminary results show that intravenously administered selenium in patients after CA is safe and might decrease the level of oxidative stress and increase the antioxidative capacity. Albeit the numerical trend to better neurological outcome does not mean necessarily positive effect on prognosis it shows that it does not cause any harm although the dosage of selenium exceeds the daily recommended dosage for healthy population.

### 0024

#### The optimum chest compression site with regard to heart failure demonstrated by a three-dimensional coordinate system imposed on computed tomography

K. Hwang, S.-B. Chon
CHA Bundang Medical Center, CHA University, Emergency Medicine, Seongnam, Korea, Republic of
**Correspondence:** S.-B. Chon
*Intensive Care Medicine Experimental* 2017, **5(Suppl 2):**0024

**INTRODUCTION.** Researchers suggested that the 'lowest' part of the sternum (vs. the 'lower half') should be compressed to maximize the stroke volume by compressing the maximum diameter of the left ventricle (LV) rather than the aorta.

**OBJECTIVES.** To determine the optimum chest compression site based on the presence/absence of heart failure (HF) by applying three-dimensional (3D) coordinates on computed tomography (CT).

**METHODS.** This retrospective, cross-sectional study involved adults who underwent echocardiography and CT within a day from 2007 to 2017. Patients who had incomplete CT or HF information, received cardiac medication between performing echocardiography and CT, or had thoracic abnormalities were excluded. Cases were classified as with or without HF through symptom/sign assessment, N-terminal pro-B type natriuretic peptide testing, and echocardiography. Whether the left ventricle (LV) was located just beneath the midpoint of lower half of the sternum (P_guide) was checked via CT. The 3D coordinates were drawn from the xiphisternal joint's midpoint (0, 0, 0). Leftward, upward, and into-the-thorax directions were designated as positive (Fig. 27). The 3D coordinates of the maximum LV diameter's midpoint (P_max) and the centre point of aortic annulus (P_ao) were identified (Fig. 28).

**RESULTS.** Finally, 148 patients were enrolled (mean age: 63.0 ± 15.1 years), of whom 87 were females and 76 had HF. HF cases had smaller proportion of LV located beneath the P_guide than normal cases (59.2% vs 77.8%). P_max of the former was also located more leftwards, lower, and deeper than the latter (5.69 ± 0.98, −1.51 ± 1.67, 5.76 ± 1.09 cm vs. 5.00 ± 0.83, −0.99 ± 1.36, 5.25 ± 0.71 cm, Table). The aorta (vs. LV) was compressed at 3 cm above the xiphisternal joint in 81.9% and 85.5% of normal and HF cases, respectively (Fig. 29).

**CONCLUSIONS.** The chest needs to be compressed at the lowest possible part just above the xiphisternal joint, especially for HF patients in cardiac arrest.

**REFERENCE(S)**

Shin J, Rhee JE, Kim K. Is the inter-nipple line the correct hand position for effective chest compression in adult cardiopulmonary resuscitation? Resuscitation 2007;75(2):305–10.
Hwang SO, Zhao PG, Choi HJ, et al. Compression of the left ventricular outflow tract during cardiopulmonary resuscitation. Acad Emerg Med 2009;16(10):928–33.

**Table 5 (Abstract 0024)**. Characteristics according to the absence/presence

| | Cases without HF (n=72) | Cases with HF (n=76) | p |
|---|---|---|---|
| LV (vs. aorta) below P_guide, n (%) | 56 (77.8) | 45 (59.2) | 0.025 |
| Coordinates, P_max (cm) | | | |
| x | 5.00±0.83 | 5.69±0.98 | <0.001 |
| y | −0.99±1.36 | −1.51±1.67 | 0.041 |
| z | 5.25±0.71 | 5.76±1.09 | 0.001 |
| Coordinates, P_ao (cm) | | | |
| x | 1.32±0.67 | 1.63±0.90 | 0.016 |
| y | 1.82±1.43 | 1.16±1.65 | 0.011 |
| z | 5.55±0.80 | 5.43±0.87 | 0.40 |

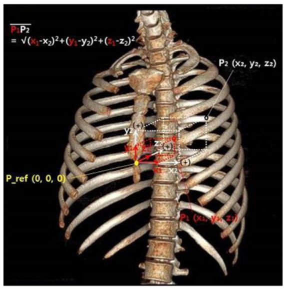

**Fig. 27 (abstract 0024).** A 3D coordinate system

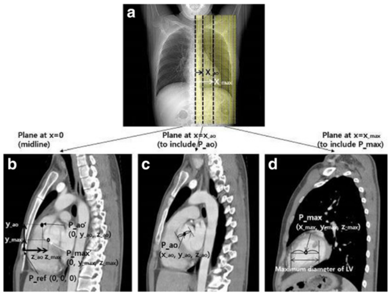

**Fig. 28 (abstract 0024).** 3D coordinates of (1) P_max and (2) P_ao

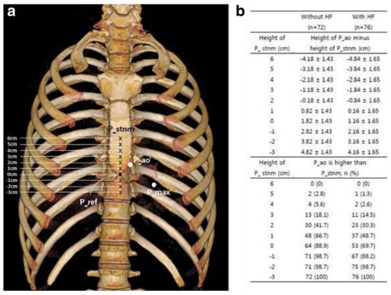

| | Without HF (n=72) | With HF (n=76) |
|---|---|---|
| Height of P_stnm (cm) | Height of P_ao minus height of P_stnm (cm) | |
| 6 | -4.18 ± 1.43 | -4.84 ± 1.65 |
| 5 | -3.18 ± 1.43 | -3.84 ± 1.65 |
| 4 | -2.18 ± 1.43 | -2.84 ± 1.65 |
| 3 | -1.18 ± 1.43 | -1.84 ± 1.65 |
| 2 | -0.18 ± 1.43 | -0.84 ± 1.65 |
| 1 | 0.82 ± 1.43 | 0.16 ± 1.65 |
| 0 | 1.82 ± 1.43 | 1.16 ± 1.65 |
| -1 | 2.82 ± 1.43 | 2.16 ± 1.65 |
| -2 | 3.82 ± 1.43 | 3.16 ± 1.65 |
| -3 | 4.82 ± 1.43 | 4.16 ± 1.65 |
| Height of P_stnm (cm) | P_ao is higher than P_stnm, n (%) | |
| 6 | 0 (0) | 0 (0) |
| 5 | 2 (2.8) | 1 (1.3) |
| 4 | 4 (5.6) | 2 (2.6) |
| 3 | 13 (18.1) | 11 (14.5) |
| 2 | 30 (41.7) | 23 (30.3) |
| 1 | 48 (66.7) | 37 (48.7) |
| 0 | 64 (88.9) | 53 (69.7) |
| -1 | 71 (98.7) | 67 (88.2) |
| -2 | 71 (98.7) | 75 (98.7) |
| -3 | 72 (100) | 76 (100) |

**Fig. 29 (abstract 0024).** Effect of hand position on the sternum

### 0025

#### Automated pupillometry for early prognostication in comatose cardiac arrest patients: preliminary results of a multicenter study

M. Oddo[1], A. Cariou[2], G. Citerio[3], H. Friberg[4], J. Horn[5], J.-F. Payen[6], C. Sandroni[7], P. Stammet[8], C. Storm[9], F.S. Taccone[10], from ESICM NeuroIntensive Care (NIC) and Trauma and Emergency Medicine (TEM) Sections
[1]CHUV and University of Lausanne, Department of Intensive Care Medicine, Lausanne, Switzerland; [2]Cochin University Hospital, Paris, France; [3]University Milano Bicocca and San Gerardo Hospital Monza, Monza, Italy; [4]Lund University, Lund, Sweden; [5]Academic Medical Center, University of Amsterdam, Amsterdam, Netherlands; [6]Grenoble University Hospital, Grenoble, France; [7]Catholic University of The Sacred Heart, Rome, Italy; [8]Luxembourg Medical Centre, Luxembourg, Luxembourg; [9]Charité University Hospital, Berlin, Germany; [10]Erasme University Hospital, Brussels, Belgium
**Correspondence:** M. Oddo
*Intensive Care Medicine Experimental* 2017, **5(Suppl 2):**0025

**INTRODUCTION.** Bilateral absence of pupillary reactivity 3 days after cardiac arrest (CA) predicts poor outcome, however prognostic accuracy of standard examination may be lower when performed at an earlier phase and is limited by lack of a quantitative tool.

**OBJECTIVES.** To examine the accuracy of quantitative pupillometry (NPi-200® automated infrared pupillometer, NeurOptics, Irvine, CA, USA) in predicting neurological recovery of post-CA coma and to compare its prognostic value to that of standard qualitative neurological examination.

**METHODS.** A prospective multicentre study was conducted amongst 10 European academic hospitals (clinicaltrials.gov NCT02607878). Blinded pupillometry tests (Neurological Pupil index [NPi] and % of Pupillary Light Reactivity [PLR]) were performed in parallel with standard neurological examination (motor response [GCS-M] and brainstem reflexes [BSR]) at day 1 and 2 after CA. Outcome was assessed at 3 months using the Cerebral Performance Categories (CPC) score; poor outcome was defined as CPC 4 (vegetative state) and 5 (death). Statistical analyses were performed by an independent statistician.

**RESULTS.** From March 2015 to October 2016, 371 consecutive patients were included in this ongoing study. On day 1, patients with poor outcome (n = 206; 56%) had lower NPi (3.6 ± 1.5 vs. 4.5 ± 0.3, p < 0.001) and lower PLR (13 [8, 19] vs. 20% [17, 26], p < 0.001) than patients with good outcome (n = 165; 44%). Similar results were found on day 2. Low NPi (<3) and PLR (<13%) had higher specificity and positive predictive value for predicting 3-month poor outcome than absent BSR and GCS-M, on both day 1 and day 2 after CA (Table 6).

**CONCLUSIONS.** These findings indicate that quantitative pupillometry had higher accuracy than standard qualitative neurological examination in predicting poor outcome in the early ICU phase after CA. Automated infrared pupillometry may be integrated in multimodal algorithms for coma prognostication following CA.

**ACKNOWLEDGEMENTS**
Automated infrared pupillometers were provided for the purpose of the study by NeurOptics.

**Table 6 (Abstract 0025).** Prognostic accuracy for poor outcome at 3 months

| Time after CA | Test | Specificity, % | Sensitivity, % | PP value, % | NP value, % |
|---|---|---|---|---|---|
| Day 1 | Quantitative PLR, % | 92.7 (87.3-96.3) | 50.5 (43.2-57.8) | 89.7 (82.3-94.8) | 59.7 (53.1-66) |
| | Quantitative NPi, % | 97.5 (93.6-99.3) | 18.7 (14.4-25.9) | 90.7 (77.9-97.4) | 49 (43.4-54.7) |
| | Absent brainstem reflexes | 65.4 (56.8-73.4) | 55.6 (46.8-64.1) | 61.5 (52.2-70.1) | 59.7 (51.4-67.7) |
| | Absent motor response | 17 (11.2-24.3) | 93.6 (88.6-96.9) | 55.7 (49.5-61.8) | 70.6 (52.5-84.9) |
| Day 2 | Quantitative PLR, % | 93.1 (86.4-97.2) | 39.3 (31.5-47.6) | 89.4 (79.4-95.6) | 51.1 (43.7-58.5) |
| | Quantitative NPi, % | 99.1 (95-100) | 23.6 (17.2-31) | 97.4 (86.2-99.9) | 47.6 (41-54.3) |
| | Absent brainstem reflexes | 86.7 (78.4-92.7) | 43.3 (34.3-52.7) | 80 (68.2-88.9) | 55.6 (47.3-63.6) |
| | Absent motor response | 63.8 (53.9-73) | 77.3 69.5-83.9) | 74.1 (66.3-81) | 67.7 (57.5-76.7) |

## New therapies for AKI

### 0026

#### Ciclosporin to protect renal function in cardiac surgery (CiPRICS). A double blind, randomized, placebo controlled, proof of concept study

P. Ederoth[1], A. Dardashti[1], E. Grins[1], B. Brondén[1], C. Metzsch[1], A. Erdling[1], S. Nozohoor[2], A. Mokhtari[2], M.J. Hansson[3], E. Elmér[3], L. Algotsson[1], S. Jovinge[4,5], H. Bjursten[6]
[1]Lund University, Department of Anesthesiology and Intensive Care, Clinical Sciences, Lund, Sweden; [2]Lund University, Department of Cardiothoracic Surgery, Clinical Sciences, Lund, Sweden; [3]Lund University, Department of Mitochondrial Medicine, Clinical Sciences, Lund, Sweden; [4]Van Andel Institute, Fredrik Meijer Heart and Vascular Institute Spectrum Health, Grand Rapids, United States; [5]Stanford University School of Medicine, Cardiovascular Institute, Stanford, United States; [6]Lund University, Department of of Cardiothoracic Surgery, Clinical Sciences, Lund, Sweden
**Correspondence:** P. Ederoth
*Intensive Care Medicine Experimental* 2017, **5(Suppl 2):**0026

**INTRODUCTION.** A possible mechanism for acute kidney injury (AKI) after cardiac surgery is ischemia-reperfusion injury caused by the Extracorporeal Circulation (ECC) [1], resulting in opening of the mitochondrial Permeability Transition Pore (mPTP) [2] in the kidneys, which in turn can led to cell injury or death [3]. Cyclophilin-D is a key regulator of the mPTP [4]. Animal studies have shown that ciclosporin may block the opening of mPTP by inhibition of cyclophilin-D [5] in the kidneys and decrease the renal injury [6] if administered before the ischemia-reperfusion event.

**OBJECTIVES.** We hypothesised that ciclosporin given as a single dose before start of ECC in coronary artery bypass grafting (CABG) surgery will decrease the degree of AKI.

**METHODS.** The CiPRICS study is an investigator-initiated, double-blind, randomized, placebo-controlled, single-centre study performed at Skane University Hospital (Lund, Sweden). The study was approved by the Regional Ethical Review Board and registered under NCT02397213 and EudraCT: 2014-004610-29. Before enrolment, oral and written informed consent was obtained. Between April 2015 and June 2016, 154 patients with estimated glomerular filtration rate of 15–90 mL/min/1.73m$^2$ planned for elective CABG with ECC were enrolled. Study patients were randomized in a 1:1 ratio to receive 2.5 mg/kg ciclosporin or placebo intravenously after anaesthesia induction but before surgery. Primary endpoint was relative plasma cystatin C changes from the preoperative day to postoperative day 3, tested with a linear mixed model in the mITT population. Secondary endpoints included biomarkers of kidney, heart, and brain injury. Safety data was collected from administration of study drug. An independent Data Safety Monitoring Board evaluated the study after 50 and 100 patients had received study drug and recommended the study to continue without changes in protocol.

**RESULTS.** The ciclosporin group showed a more pronounced relative increase from baseline plasma cystatin C to day 3 compared to placebo (1.36 ± 0.35 vs 1.16 ± 0.31, p < 0.001). The same pattern was observed for the other renal markers. There were no differences in clinical outcome or AE/SAE distribution between the groups.

**CONCLUSIONS.** Administration of ciclosporin 2.5 mg/kg before surgery did not protect CABG patients from AKI. Instead, ciclosporin caused a transient decrease in renal function compared to placebo.

**GRANT ACKNOWLEDGMENT**

Funding was received by the Swedish Heart-Lung foundation. NeuroVive Pharmaceutical AB, Lund, Sweden, provided the study drug together with unrestricted research grants covering costs for the research nurses and laboratory tests.

### 0027

#### Biomarker-guided intervention to prevent acute kidney injury after major surgery: the prospective randomized BigpAK study

I. Göcze[1], D. Jauch[2], M. Götz[1], P. Kennedy[1], B.M. Graf[1], B. Banas[1], T. Bein[1], H.J. Schlitt[1], T. Bergler[1]
[1]Universitätsklinikum Regensburg, Regensburg, Germany;
[2]Universitätsklinikum Freiburg, Freiburg, Germany
**Correspondence:** I. Göcze

*Intensive Care Medicine Experimental* 2017, **5(Suppl 2):**0027

**INTRODUCTION.** Early optimization of volume status, maintenance of adequate perfusion pressure and discontinuation of nephrotoxic medication before the occurrence of AKI may be the crucial step to reduce preventable AKI.

**OBJECTIVES.** To determine the impact of renal biomarker guided implementation of the Kidney Disease Improving Global Outcomes (KDIGO) guidelines on incidence of acute kidney injury (AKI) after major non-cardiac surgery in randomized clinical trial.

**METHODS.** Early administration of KDIGO bundle was compared to standard ICU care in 121 patients with increased AKI-Risk determined by elevated urinary [TIMP2 * IGFBP7] biomarkers. Incidence of overall AKI, severity of AKI; incidence of creatinine increase of >25%, length of stay, major kidney events (MAKE) at discharge and cost effectiveness between both groups were evaluated.

**RESULTS.** The incidence of overall AKI was not statistically different between both groups, but incidence of moderate and severe AKI (6.7% versus 19.7%; odds ratio [95%CI] 3.43 [1.0, 11.3], p = 0.04) and incidence of creatinine increase >25% of baseline value (40.0% versus 62.3%, OR 2.48 (1.19, 5.15), p = 0.01) were significantly reduced in the intervention group. Additionally, length of ICU and hospital stay decreased in the intervention group (p = 0.04) and the intervention significantly attenuated release of cellular stress biomarker (p = 0.03). There were no significant differences in the need for renal replacement therapy, in-hospital mortality or in MAKE by hospital discharge.

**CONCLUSIONS.** Among patients after major non-cardiac surgery early biomarker- based detection of AKI- risk followed by implementation of KDIGO bundle reduced AKI severity, postoperative creatinine increase, length of ICU and hospital stay.

### 0028

#### Resistin reversibly impairs intracellular bacterial killing in neutrophils by affecting reactive oxygen species generation

A. Bonavia[1], L. Miller[2], J. Kellum[3], K. Singbartl[4]
[1]Penn State Milton S. Hershey Medical Center, Anesthesiology and Perioperative Medicine, Hershey, United States; [2]Penn State Milton S. Hershey Medical Center, Hershey, United States; [3]University of Pittsburgh Medical Center, Pittsburgh, United States; [4]Mayo Clinic, Phoenix, United States
**Correspondence:** A. Bonavia

*Intensive Care Medicine Experimental* 2017, **5(Suppl 2):**0028

**INTRODUCTION.** Mounting evidence suggests that sepsis-induced morbidity and mortality are due to both immune activation and immunosuppression. Resistin is an inflammatory cytokine and uremic toxin. Septic hyperresistinemia (HR) has been associated with greater disease severity and worse outcome. Septic HR also impairs neutrophil (PMN) migration, a crucial first-line mechanism in host defense. Acute kidney injury (AKI) worsens septic HR. Preliminary studies indicate that conventional renal replacement therapies do not correct HR [1].

**OBJECTIVES.** Our central hypothesis is that HR causes PMN dysfunction and thereby potentially contributes to sepsis-induced immunosuppression. We have demonstrated that HR blocks PMN migration *in vitro*, likely due to impaired F-actin formation [1]. Here, we study the effects of resistin on intracellular bacterial killing and generation of reactive oxygen species (ROS). We also seek to examine the effects of hemoadsorption on HR and PMN dysfunction.

**METHODS.** An *in vivo* cohort of 13 patients with septic shock and 12 control ICU patients was analyzed for serum resistin levels and their effects on PMN migration. *In vitro*, following incubation with resistin-spiked serum samples, intracellular bacterial killing and ROS generation in PMNs was measured using a commercially available kit. *P. aeruginosa* was used to examine phagocytic and bacterial killing capacity of PMNs in a standard assay. *In vitro* hemoadsorption with both Amberchrome™ columns (AC) and CytoSorb™ cartridges (CC) were used to test correction of HR. We further tested AC for their effect on migration and ROS generation and CC for their effect on bacterial killing.

**RESULTS.** Patients with septic shock had higher serum resistin levels than control ICU patients and showed a strong correlation between HR and inhibition of PMN migration. *In vitro,* PMNs exposed to resistin exhibited lower intracellular bacterial killing rates compared to controls. Resistin impaired reactive oxygen species production in a dose-dependent manner. Hemoadsorption with AC reduced serum concentrations of resistin and restored PMN migration and generation of ROS to normal levels. Hemoadsorption with CC also corrected HR. Exposure of PMNs to resistin impaired intracellular *P. aeruginosa* killing compared to control. However, when this same resistin-spiked serum was subsequently treated with CC prior to exposure to PMNs, intracellular bacterial killing was restored to normal levels.

**CONCLUSIONS.** Septic HR significantly and reversibly impairs PMN intracellular bacterial killing and ROS generation. Hemoadsorption therapy with clinically approved CC may provide a therapeutic option to improve PMN function during septic HR and ultimately improve immunosuppression in these disease states.

**ACKNOWLEDGEMENTS**

CytoSorb cartridges provided by CytoSorbents Corporation.

### 0029

#### Impact analysis of an integrated electronic alert for those at risk or with acute kidney injury: effects on process measures

L. Hodgson[1], D. Hargreaves[2], G. Chater[2], J. de Carvalho[2,3], R. Venn[2], L. Forni[4,5]
[1]University of Southampton, Southampton, United Kingdom; [2]Western Sussex Hospitals Foundation NHS Trust, Worthing Hospital Department of Anaesthesia and Intensive Care Medicine, Worthing, United Kingdom; [3]Brighton and Sussex Medical School, Brighton, United Kingdom; [4]Royal Surrey County Hospital NHS Foundation Trust, Department of Intensive Care Medicine, Guildford, United Kingdom; [5]University of Surrey, Guildford, United Kingdom
**Correspondence:** L. Hodgson
*Intensive Care Medicine Experimental* 2017, **5(Suppl 2):**0029

**INTRODUCTION.** Acute Kidney Injury (AKI) is associated with high morbidity and mortality. Reports have suggested inadequate management of patients with hospital-acquired AKI (HA-AKI)(1), leading to subsequent recommended guidance.(2) One way of improving care for such patients is through electronic alerting and delivery of best practice care bundles.

**OBJECTIVES.** To investigate the impact on processes of care of a comprehensive electronic intervention: combining a prediction model for those at risk of AKI, an alert for those with new HA-AKI, alongside a care bundle.

**METHODS.** A dual-centre before and after with control design (2014–6) investigated the effects of the intervention on processes of care for medical ward patients. Methods included a review of electronic prescribing, coding of AKI patients and a retrospective case note review of a sample of patients with HA-AKI.

**RESULTS.** From 30,298 patient episodes there were 2,040 cases of HA-AKI over the study period. At the intervention site significantly more episodes of medications were stopped with AKI as the reason given vs the control site (n = 95 vs 36, P < 0 · 001, OR 2 · 1 [1 · 41-3 · 10]. Significantly more patients at the intervention site had ACE inhibitors or Angiotensin-receptor blockers stopped vs control site (308 vs 146, P < 0 · 001, OR 1 · 831 [1 · 493-2 · 247]). ICD-10 coding of AKI increased at both sites with the intervention site having a larger increase (+21% vs +17%). Coded AKI mortality at the intervention site significantly reduced (24% vs 20%, P = 0 · 0057) with no significant change at control site (20% vs 18% P = 0 · 44).

181 patients notes were reviewed with significant improvements at the intervention site found following the intervention including improved documentation (in the notes and on discharge), review of medications, monitoring of renal function and appropriate investigations (See Table 7 and Table 8)

**CONCLUSIONS.** The introduction of an electronic alert and care bundle significantly improved a number of process measures in this before-after with control site intervention study.

**REFERENCE(S)**

1. National Confidential Enquiry into Patient Outcome and Death. Adding insult to injury: a review of the care of patients who died in hospital with a primary diagnosis of acute kidney injury (acute renal failure) http://www.ncepod.org.uk/2009report1/Downloads/AKI_report.pdf. NCEPOD, 2009.

2. National Institute for Health and Care Excellence. Acute Kidney Injury: Prevention, Detection and Management Up to the Point of Renal Replacement Therapy. (Clinical guideline CG169). London: National Clinical Guideline Centre.; 2013 Aug.

**Table 7 (Abstract 0029)**. Pre-intervention analysis

| CARE BUNDLE ELEMENT | PRE INTERVENTION | | | |
|---|---|---|---|---|
| | Intervention | Control | P value | OR (95% CI) |
| Documented AKI | 9/43 | 22/47 | 0·014 | 0·30 (0·12-0·76) |
| Repeated U&Es | 29/43 | 33/40 | 0·076 | 0·36 (0·13-1·07) |
| Drugs checked | 22/43 | 8/32 | 0·032 | 3·14 (1·16-8·53) |
| Fluid Plan | 19/43 | 20/40 | 0·663 | 0·79 (0·33-1·88) |
| Search for cause | 17/43 | 22/40 | 0·190 | 0·54 (0·22-1·28) |
| Appropriate special tests | 14/41 | 14/37 | 0·815 | 0·85 (0·34-2·15) |
| AKI documented on discharge | 8/40 | 9/43 | 0·580 | 0·67 (0·22-1·98) |

**Table 8 (Abstract 0029)**. Post-intervention analysis

| CARE BUNDLE ELEMENT | POST INTERVENTION | | | |
|---|---|---|---|---|
| | Intervention | Control | P value | OR (95% CI) |
| Documented AKI | 25/37 | 13/44 | 0·033 | 2·71 (1·14-6·43) |
| Repeated U&Es | 39/44 | 31/43 | 0·062 | 3·02 (0·96-9·49) |
| Drugs checked | 24/44 | 10/43 | 0·004 | 3·96 (1·53-9·97) |
| Fluid Plan | 28/44 | 23/43 | 0·388 | 1·52 (0·65-3·59) |
| Search for cause | 20/44 | 9/43 | 0·023 | 3·15 (1·22-8·10) |
| Appropriate special tests | 16/43 | 7/43 | 0·050 | 3·05 (1·10-8·44) |
| AKI documented on discharge | 15/36 | 10/35 | 0·322 | 1·77 (0·67-4·80) |

### 0030

#### Comparison of two continuous renal replacement techniques associated with an increased absorption membrane: a randomized control trial

E.P. Plata-Menchaca[1], P. Cárdenas-Campos[2], G. Moreno-González[2], V. Corral-Velez[2], J.M. Vázquez-Rebellón[2], J.P. Pinseaun-Castillo[2], M. Pons-Serra[2], N. López-Suñé[2], M. Rojas-Lora[2], J. Sabater-Riera[2], J. Ordoñez-Llanos[3], N.D. Toapanta-Gaibor[2], J. Ballus-Noguera[2], M. Estruch-Alrich[3], A. Betbesé-Roig[4], J.C. Suárez-Garzón[4], P. López-Garzón[4], X. Perez-Fernandez[2]
[1]Instituto de Investigación Biomédica de Bellvitge, Research, L´Hospitalet de Llobregat, Spain; [2]Hospital Universitario de Bellvitge, Critical Care Medicine, L'Hospitalet de Llobregat, Spain; [3]Instituto de Investigación Biomédica Sant Pau, Barcelona, Spain; [4]Hospital Universitario de la Santa Creu i Sant Pau, Critical Care Medicine, Barcelona, Spain
**Correspondence:** E.P. Plata-Menchaca
*Intensive Care Medicine Experimental* 2017, **5(Suppl 2):**0030

**INTRODUCTION.** Acute kidney injury (AKI) represents a public health problem associated with high rates of morbidity and mortality among critically ill patients. Furthermore, it has been postulated that continuous venovenous hemofiltration (CVVH) might benefit critically ill patients with AKI by better clearing medium molecular weight inflammatory cytokines, whilst practice surveys have shown controversy in mode selection among countries and regions.
**OBJECTIVES.** To demonstrate in septic patients with sepsis-associated AKI (SA-AKI), a non-inferiority survival of continuous venovenous hemodialysis (CVVHD) compared to (CVVH), both techniques associated with an increased adsorption membrane (AN69-ST-150).
**METHODS.** A two-center, parallel-group and randomized single-blinded control trial was performed. Participating centers were two tertiary-level hospitals in Barcelona. We enrolled 106 critically ill adult patients with sepsis and AKI who met continuous renal replacement therapy (CRRT) initiation criteria were included, within the first 72 hours from intensive care unit (ICU) admission. One-hundred and six patients were randomly assigned to one of the two strategies [49 patients to CVVH + adsorption membrane (AN69-ST-150) and 57 patients to CVVHD + AN69-ST-150]. Patients were started on CRRT at a fixed dose of 30ml/kg/h during the first 72 hours. Clinical and laboratory data were collected. Follow-up was assessed at hospital discharge and 90 days.
**RESULTS.** Overall 90-day mortality was 57% in the CVVH group and 23% in the CVVHD group (p = 0.04). Mean age was 64 ± 13 years; 66% of patients were male. The most prevalent comorbidities were: hypertension, chronic heart failure and diabetes mellitus (in 58%, 35% and 26% of the patients, respectively). The main etiology of sepsis was intra-abdominal infection in 40% of patients. Mean SOFA score at ICU admission was 11 ± 3,8 and mean APACHE score was 25 ± 9. At CRRT initiation, 80% of patients met criteria for stage 3 AKI as stated by the Kidney Disease Improving Global Outcomes (KDIGO) guidelines. There were no differences between groups neither in terms of the number of extracorporeal circuits (EC) consumed (5.3 vs 4.5, p = 0.3105), nor in secondary outcomes such as ICU and hospital length-of-stay (LOS) [14 ± 13 vs 20 ± 49 (p = 0.89), and 25 ± 30 vs 30 ± 50 (p = 0.65), respectively].
**CONCLUSIONS.** In patients with SA-AKI, we demonstrated non-inferiority of CVVHD in terms of survival respect to CVVH in patients with SA-AKI. There were no differences respect to ICU and hospital LOS and the number of EC consumed during CRRT.
**REFERENCE(S)**
Friedrich JO, Wald R, Bagshaw SM, Burns KE, Adhikari NK. Hemofiltration compared to hemodialysis for acute kidney injury: systematic review and meta-analysis. Crit Care. 2012 6;16(4):R146.
**GRANT ACKNOWLEDGMENT**
Project PI12/01562, funded by Instituto de Salud Carlos III and co-funded by European Union (ERDF/ESF, "Investing in your future".

### Outcome prediction and scoring systems

#### 0031

#### Derivation and validation of the PEAR score to predict outcome in critically ill patients

L.E. Roeker[1], T. Moromizato[2], F. Gibbons[3], S.A. Quraishi[4], K. Christopher[5]
[1]Brigham and Women's Hospital, Department of Medicine, Boston, United States; [2]Okinawa Southern Medical Center and Children's Hospital, Renal and Rheumatology Division, Naha, Japan; [3]Massachusetts General Hospital, Pulmonary and Critical Care Division, Boston, United States; [4]Massachusetts General Hospital, Department of Anesthesia, Critical Care and Pain Medicine, Boston, United States; [5]Brigham and Women's Hospital, Renal Division, Boston, United States
**Correspondence:** K. Christopher
*Intensive Care Medicine Experimental* 2017, **5(Suppl 2):**0031

**INTRODUCTION.** Existing risk prediction models may not be practically applied in the clinical setting given their reliance upon a large number of physiologic or lab variables not uniformly collected in the course of clinical care.
**OBJECTIVES.** This study aims to assess the potential of a simple and practical score to function as an early predictor of mortality in critically ill patients. Our primary study objective was to derive and validate a simple risk prediction score based on demographics and blood laboratory values consistently measured at ICU admission.
**METHODS.** The PEAR (Platelet, Eosinophil, Age, Red cell distribution width) score, was constructed with demographics and laboratory measures immediately available at ICU admission. We included surrogates of chronic inflammation (red cell distribution width), acute inflammation (platelets) and extreme acute inflammation (eosinophil count). We performed a retrospective cohort study of 76,091 patients admitted to a medical or surgical ICU between 1997 and 2012, to 2 large Boston hospitals. All patients had platelet count, eosinophil count and red cell distribution width determined at ICU admission. The derivation cohort consisted of ICU patients from Brigham and Women´s Hospital (n = 27,706), and the validation cohort comprised of ICU patients from Massachusetts General Hospital (n = 48,385). A clinical prediction model was derived and validated based on logistic regression describing the risk of 90 day mortality as a function of the predictors at ICU admission: Red cell distribution width, platelet count, eosinophil count, age, gender and patient type (surgical vs. medical). Performance analysis of the PEAR score against APACHE II was completed in a subset of the validation cohort (n = 341).
**RESULTS.** The derivation cohort (n = 27,706) was 56% male, 53% surgical ICU with a mean age of 63 years and a 90-day mortality rate of 18.7%. The validation cohort (n = 48,385) was 59% male, 46% surgical ICU with mean age of 63 years and a 90-day mortality rate of 16.6%. The AUC for the prediction model was 0.740 in the derivation cohort and 0.743 in the validation cohort. For both the derivation and validation cohorts, the Hosmer-Lemeshow $\chi^2$ P values indicated good model fit. In the validation cohort, 90-day mortality was 5.5% in the low-risk group (0 to 20 risk score points), 9.9% in the intermediate-risk group (21 to 27 points), 17.6% in the high-risk group (28 to 36 points), and 34.9% in the very high-risk group (>36 points). In the validation cohort subset, differences in model discrimination between risk score and APACHE II were not significant ($\chi^2$ 1.73, P = 0.19).
**CONCLUSIONS.** Our PEAR score calculated from age, gender, patient type, red cell distribution width, platelet count and peripheral blood eosinophil count at ICU admission is a simple, quick and easily understandable score that may increase clinical utility for risk prediction in ICU patients.
**GRANT ACKNOWLEDGMENT**
NIH R01GM115774

#### 0032

#### Outcome prediction in ICU patients with a prolonged length of stay: how to use organ failure scores?

I.W. Soliman[1], D.W. De Lange[1], L.M. Peelen[1,2], M.C. Kerckhoffs[1], A.J.C. Slooter[1], O.L. Cremer[1], J.J.M. van Delden[3], D. van Dijk[1]
[1]University Medical Center Utrecht, Intensive Care Center, Utrecht, Netherlands; [2]Julius Center for Health Sciences and Primary Care University Medical Center Utrecht, Department of Epidemiology, Utrecht, Netherlands; [3]Julius Center for Health Sciences and Primary Care University Medical Center Utrecht, Department of Medical Humanities, Utrecht, Netherlands
**Correspondence:** I.W. Soliman
*Intensive Care Medicine Experimental* 2017, **5(Suppl 2):**0032

**INTRODUCTION.** In the process of prognostication and decision making about possible treatment limitations in patients with prolonged intensive care unit (ICU) admission, the extent, duration and temporal evolution of acute organ dysfunction is often taken into consideration. However, currently it is unclear way of using repeatedly measured SOFA scores is most helpful for predicting outcomes in these patients.
**OBJECTIVES.** To compare different ways of modelling daily SOFA score data observed during the first week of ICU stay with regard to the ability to predict poor long-term outcome.
**METHODS.** Patients admitted to a tertiary mixed ICU, staying at least 7 days, were included. APACHE IV predicted risk of mortality, daily SOFA scores, and outcome data were extracted from data routinely collected for national benchmarking and 1 year follow-up. First week SOFA scores were modelled in 35 different ways, with varying level of complexity (box 1). The main outcome studied was poor outcome at 1 year follow-up. This was defined as either death at 1 year follow-up or surviving with a EuroQoL-5D 3L health related quality of life index < 0.4. For each of the box 1 SOFA parameterizations, a logistic regression model was constructed with the APACHE IV predicted mortality and the SOFA parameterization as independent variables. Model fit, discrimination and calibration were assessed by Akaike's information Criterion (AIC), c-index, and the le Cessie-van Houwelingen global goodness of fit (CH) statistic, respectively.

**RESULTS.** The study population consisted of 1170 consecutive ICU patients with a prolonged stay (table 9). Mortality was 24% (274) and 43% (502) at day 30 and 1 year respectively. One year poor outcome occurred in 57% (n = 662) of patients. Figure 30 show the AIC, c-index and CH statistic for each of the SOFA parameterizations. Three consistently high performing SOFA representations were: A) the SOFA scores of day 1 and 7; B) the SOFA score on day 1, plus the change in SOFA score between day 1–4 and the change between day 4–7; and C) all individual SOFA scores of the first seven days. The SOFA representation using day 1, plus changes between day 1–4 and day 4–7 scored best when averaging performance over the three performance measures. In a sensitivity analysis where all analyses were repeated using 30 day or 1 year mortality as outcomes of interest, similar results were found, with the same top three performing SOFA parameterizations.

**CONCLUSIONS.** The way first week SOFA scores were combined with the APACHE IV model to predict 1 year poor outcome in patients admitted to the ICU for at least 7 days, greatly influenced predictive performance. Simple computations of organ failure scores consistently outperformed more complex ones.

**GRANT ACKNOWLEDGMENT**

This study was supported by the NutsOhra Foundation, project nr 1404–013.

**Box 1. SOFA parameterizations**

**SOFA score on a single day**
1. A single SOFA score, based on day 1
2. A single SOFA score, based on day 7

**SOFA scores on two days**
3. Two SOFA scores, based on day 1 and day 7
4. The difference in SOFA score between day 1 and day 4
5. The difference in SOFA score between day 4 and day 7

**SOFA scores on three days**
6. Both #4 and #5 entered as separate variables
7. #1, #4 and #5 entered all as separate variables
8. #1, #4 and #5 entered all as separate variables with interaction terms between #1 and #4, and between #1, #4 and #5

**SOFA score summary measures based on half a week (first half = day 1 to 3, second half = day 4 to 7)**
9. Maximum SOFA score during days 1 to 3
10. Minimum SOFA score during days 1 to 3
11. Mean SOFA score during days 1 to 3
12. Weighted SOFA score mean (day number is weight) during days 1 to 3
13. Sum of highest SOFA component scores during days 1 to 3 (SOFA totalmax)
14. Maximum SOFA score during days 4 to 7
15. Minimum SOFA score during days 4 to 7
16. Mean SOFA score during days 4 to 7
17. Weighted SOFA score mean (day number is weight) during days 4 to 7
18. Sum of highest SOFA component scores during days 4 to 7 (SOFA totalmax)

**SOFA score (summary measures) based on all 7 days**
19. Entering all seven SOFA scores as separate variables
20. Maximum SOFA score during days 1 to 7
21. Minimum SOFA score during days 1 to 7
22. Mean SOFA score during days 1 to 7
23. Weighted SOFA score mean (day number is weight) during days 1 to 7
24. Sum of highest SOFA component scores during days 1 to 7 (SOFA totalmax)
25. Delta SOFA score (difference between #25 and #1)
26. Entering both #25 and #1 as separate variables
27. Entering both #25 and #1 as separate variables, with an interaction term between #25 and #1
28. Entering both #9 and #14 as separate variables
29. Entering both #10 and #15 as separate variables
30. Entering both #11 and #16 as separate variables
31. Entering both #12 and #17 as separate variables
32. Entering both #13 and #18 as separate variables
33. Days with organ failure (SOFA component score >3) per SOFA component

**SOFA score trend or repeated measures analyses based on all 7 days**
34. Aligned trend analysis: predictors are multiple variables containing the presence/absence of a specific mutually exclusive prevalent trend (occurring in more than 5% of patients, without encompassing a shorter trend, and aligned to day 7)
35. Untrained cluster analysis group membership

[box01]

**Table 9 (Abstract 0032)**. Study population

| | |
|---|---|
| N | 1170 |
| Sex (female) | 410 (35%) |
| Age at ICU admission | 60 [49 - 70] |
| Admission type - | |
| Elective surgical | 163 (13.9%) |
| Urgent surgical | 352 (30.1%) |
| Medical | 655 (56%) |
| APACHE IV predicted mortality (%) | 27 [10 - 53] |
| SOFA Scores - | |
| Day 1 | 10 [7 - 12] |
| Day 2 | 10 [7 - 13] |
| Day 3 | 9 [6 - 12] |
| Day 4 | 9 [6 - 12] |
| Day 5 | 8 [5 - 11] |
| Day 6 | 7 [5 - 11] |
| Day 7 | 7 [4 - 10] |
| ICU length of stay (days) | 12.6 [9.1 - 20.2] |
| Mortality | |
| 30 day | 278 (24%) |
| 1 year | 502 (43%) |
| Poor outcome (death or low HRQoL*) | 662 (57%) |

Continuous variables are presented as median (interquartile range); categorical variables as n (percentage)
*ICU* Intensive Care Unit, *APACHE IV* Acute Physiology And Chronic Health Evaluation fourth edition, *SOFA* Sequential Organ Failure Assessment, *HRQoL* health related quality of life
* low HRQoL was defined as an EuroQoL 5D-3L index below 0.4

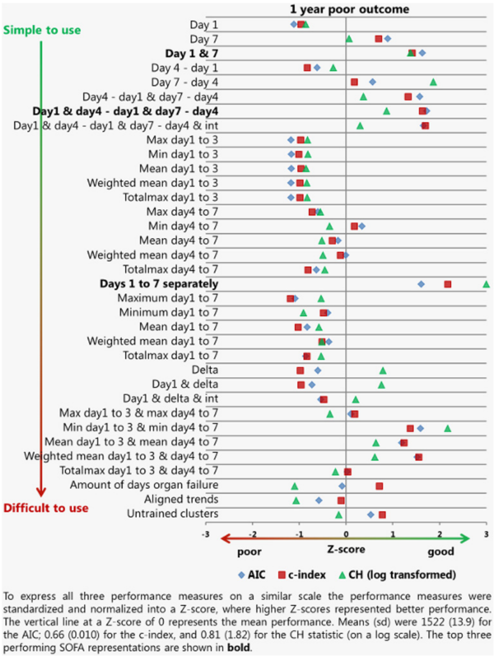

**Fig. 30 (abstract 0032)** Predictive performance for 1 year poor outcome

### 0033

#### Simplified prognostic model for critically ill patients in resource limited settings in South Asia

R. Haniffa[1,2,3], M. Mukaka[2], S. Munasinghe[1], P. De Silva[1,4], S. Jayasinghe[3], N. de Keizer[5], A. Dondorp[2]
[1]National Intensive Care Surveillance, Colombo, Sri Lanka; [2]Mahidol Oxford Tropical Medicine Research Unit, Bangkok, Thailand; [3]University of Colombo, Faculty of Medicine, Colombo, Sri Lanka; [4]Intensive Care National Audit & Research Centre, London, United Kingdom; [5]National Intensive Care Evaluation (NICE) Foundation, Amsterdam, Netherlands
**Correspondence:** R. Haniffa
*Intensive Care Medicine Experimental* 2017, **5(Suppl 2):**0033

**INTRODUCTION.** Current critical care prognostic models have been almost entirely developed in high income countries (HICs) and are usually based on hospital outcomes, which may not be feasible in LMIC ICUs due to the lack of electronic records or follow-up systems (1). Existing prognostic models cannot be imposed without validation in lower-and middle income countries (LMICs) as the diseases encountered in these settings (e.g. malaria, snake bite and poisoning), treatment modalities and available resources may be different (2).

**OBJECTIVE.** This study proposes a simplified critical care prognostic model for use at the time of Intensive Care Unit (ICU) admission.

**METHODS.** A cohort of ICU admissions were followed up until discharge in 21 ICUs in 4 countries: Bangladesh, India, Nepal and Sri Lanka. There were 3855 patients aged 18 and over admitted to these 21 ICUs.

Initial selection of candidate covariates for model development was based on their use in existing case-mix prediction models, perceived clinical importance and the feasibility of measurement in the settings of the participating ICUs. Multivariate logistic regression was used to develop three models - model 1 with clinical, laboratory and treatment variables, model 2 with clinical and laboratory variables and a purely clinical model 3 - for ICU mortality prediction.

Internal validation based on bootstrapping (1000 samples) was used to calculate discrimination (Area Under the Receiver Operating Characteristic, AUROC) and calibration (Hosmer-Lemeshow C-Statistic, HL C-S) (3). Comparison was made with the APACHE II model.

**RESULTS.** Model 1 retained respiratory rate, systolic blood pressure, GCS, blood urea, haemoglobin, mechanical ventilation and vasopressor use on ICU admission. Model 2, named TropICS (Tropical Intensive Care Score), included emergency surgery, respiratory rate, systolic blood pressure, GCS, blood urea and haemoglobin. Model 3 included respiratory rate, emergency surgery and GCS. AUC was 0.818 (95% CI 0.800-0.835) for model 1, 0.767 (95% CI 0.741-0.792) for TropICS and 0.725 (95% CI 0.688-0.762) for model 3, and. The H-L C- S p-values were less than 0.05 for models 1 and 3 and 0.18 for TropICS. In comparison, for APACHE II, AUC was 0.707 (95% CI- 0.688- 0.726) and H-L C-S was 124.84 ($p < 0.001$).

**CONCLUSION.** This paper proposes TropICS as the first multinational critical care prognostic model developed in a non-HIC setting.

**Table 10 (Abstract 0033)**. Multivariable logistic regression model of mortali

| Covariate | Model 1 Beta-coefficient | Model 1 95% CI | Model 2 Beta-coefficient | Model 2 95% CI | Model 3 Beta-coefficient | Model 3 95% CI |
|---|---|---|---|---|---|---|
| Emergency surgery | 0.437 | −0.136, 1.010 | 0.547 | 0.025, 1.069 | 0.730 | 0.439, 1.020 |
| Respiratory rate | 0.060 | 0.018, 0.102 | 0.005 | 0.002, 0.007 | 0.064 | 0.041, 0.087 |
| Systolic blood pressure | −0.019 | −0.031, -0.008 | 0.002 | 0.001, 0.004 | 0.003 | −0.002, 0.008 |
| Glasgow Coma Score | −0.099 | −0.139, -0.059 | −0.150 | −0.185, -0.115 | −0.128 | −0.151, -0.105 |
| Blood Urea | 0.006 | 0.003, 0.008 | 0.006 | 0.004, 0.009 | NA | - |
| Haemoglobin | −0.093 | −0.160, -0.027 | −0.098 | −0.161, -0.034 | NA | - |
| Vasoactive use | 1.057 | 0.5613, 1.5527 | NA | - | NA | - |
| Mechanical ventilation | 1.429 | 0.9919, 1.8661 | NA | - | NA | - |
| Constant | 6.164 | 4.374, 7.953 | 0.588 | −0.358, 1.534 | 0.229 | −0.061, 0.518 |

**Table 11 (Abstract 0033)**. Performance of the 3 models and APACHE II

| Performance item | Model 1 | Model 2 | Model 3 | APACHE II |
|---|---|---|---|---|
| Score, mean (SD) | - | - | - | 17.35 (6.16) |
| Probability, mean (SD) | 0.20 (0.20) | 0.20 (0.16) | 0.28 (0.14) | 0.26 (0.81) |
| Optimal cut-off probability | 0.18 | 0.18 | 0.26 | 0.20 |
| Sensitivity (at optimum cut-off) | 0.72 | 0.70 | 0.63 | 0.67 |
| Specificity (at optimum cut-off) | 0.77 | 0.69 | 0.67 | 0.71 |
| AUC (95% CI) | 0.812 (0.781-0.842) | 0.767 (0.734-0.800) | 0.689 (0.664-0.714) | 0.707 (0.688-0.727) |
| H/L C-statistic (p) | 16.91 (p=0.03) | 11.31 (p=0.19) | 15.94 (p=0.01) | 124.84 (P<0.01) |
| Brier score (95% CI) | 0.13 (0.11-0.14) | 0.14 (0.12-0.15) | 0.18 (0.18-0.19) | 0.18 (0.17-0.18) |

### 0034

#### Performance of critical care prognostic scoring systems in low and middle income countries - a systematic review

R. Haniffa[1,2,3], I. Isaam[4,5], A.P. De Silva[2,4], A.M. Dondorp[1,3], N.F. De Keizer[6]
[1]University of Oxford, Centre for Tropical Medicine and Global Health, Nuffield Department of Medicine, Oxford, United Kingdom; [2]National Intensive Care Surveillance, Ministry of Health, Colombo, Sri Lanka; [3]Mahidol Oxford Tropical Medicine Research Unit, Faculty of Tropical Medicine, Bangkok, Thailand; [4]Network for Improving Critical Care Systems and Training, Colombo, Sri Lanka; [5]AA (Ltd), London, United Kingdom; [6]Academic Medical Center, University of Amsterdam, Department of Medical Informatics, Amesterdam, Netherlands
**Correspondence:** R. Haniffa
*Intensive Care Medicine Experimental* 2017, **5(Suppl 2):**0034

**INTRODUCTION.** Prognostic models - used in critical care medicine for mortality predictions, benchmarking and for illness stratification in clinical trials- have been validated predominantly in high-income countries[1–3] These results may not be reproducible in low or middle income countries (LMICs) not only because of different case-mix characteristics but also because of missing predictor variables.

**OBJECTIVES.** To systematically review literature on the use of critical care scoring systems in LMICs and assess their ability to discriminate between survivors and non-survivors of ICU admissions, their calibration and accuracy, and the manner in which missing values are handled.

**METHODS.** The PubMed database was searched in March 2107 to identify research articles reporting the use and performance of prognostic models in the evaluation of mortality in ICUs in LMICs. Studies that were carried out in ICUs in high-income countries and/or paediatric ICUs, studies that evaluated disease specific scoring systems, were limited to a specific disease or single prognostic factor or studies published only as abstracts, editorials, letters and systematic and narrative reviews or in a language other than English were excluded. Quality assessment of the included studies was carried out in accordance with the critical appraisal for systematic reviews of prediction models (the CHARMS checklist[4]). Results were summarised using descriptive statistics.

**RESULT.** Fifty-four studies reporting 160 model validations met the review criteria and were selected for analysis. Three studies (4 evaluations) described the development of a new prediction model in a LMIC setting although no external validation was carried out.

The remaining 156 were external validations of APACHE, GCS, MPM, OSF, SAPS, SOFA and TISS or versions thereof; adaptations to existing models were attempted on seventeen occasions. Missing value handling was explicitly mentioned in only 26.9% of the studies. Where discrimination was reported as AUROC 20.6% (27 validations) and 65.7% (86 validations) reported excellent discrimination and very good/ good discrimination respectively. Only 102 (65.4%) evaluations reported calibration with 65.7% of these reporting a $p > 0.05$ for Hosmer-Lemeshow statistics. Thirteen evaluations that reported excellent discrimination also reported good calibration. Accuracy was reported in 45 evaluations (28.5%) and ranged from 55.20% to 98.5%.

**CONCLUSION.** Robust interpretations on the applicability of prognostic models are currently hampered by poor adherence to reporting guidelines, especially when reporting missing value handling. The performance of mortality risk prediction models for ICU patients-predominantly developed in and for HIC settings- in LMIC is at most moderate, especially with limitations in calibration. This justifies the need for continued efforts to develop and validate LMIC models with readily available prognostic variables, perhaps aided by medical registries.

### 0035

#### Long-term prognostication in the ICU: a systematic review into available prediction models

I.W. Soliman[1], L.M. Peelen[1,2], D.W. De Lange[1], A.J.C. Slooter[1], O.L. Cremer[1], J.J.M. van Delden[3], D. van Dijk[1]
[1]University Medical Center Utrecht, Intensive Care Center, Utrecht, Netherlands; [2]Julius Center for Health Sciences and Primary Care University Medical Center Utrecht, Department of Epidemiology, Utrecht, Netherlands; [3]Julius Center for Health Sciences and Primary Care University Medical Center Utrecht, Department of Medical Humanities, Utrecht, Netherlands
**Correspondence:** I.W. Soliman
*Intensive Care Medicine Experimental* 2017, **5(Suppl 2):**0035

**INTRODUCTION.** For the decision to start or withhold an intensive care unit (ICU) treatment, patient prognosis plays an important role. Most patients consider survival beyond hospital discharge with sufficient (health related) quality of life (HRQoL) as the desired result of their ICU treatment. Most current ICU prediction models, however, were designed to predict hospital survival. Although some of these models may also be used to provide insight into long-term HRQoL and survival, an overview of models available for this purpose is lacking.

**OBJECTIVES.** To provide an overview of prognostic models that can potentially be used for predicting the outcomes of ICU patients beyond hospital discharge.

**METHODS.** A search was set up using keywords and synonyms for prediction models, ICU patients, and survival or HRQoL, and was adapted for the MEDLINE, EMBASE, CINAHL and PsycINFO databases. The search was performed on the 6th of May 2015. Studies reporting the development of a prediction model in ICU patients for the outcome of survival, at least to hospital discharge, or HRQoL were eligible for inclusion. Screening and inclusion of search results was performed by two authors separately (IWS and LMP).

**RESULTS.** The search and selection led to the inclusion of 63 model development studies out of 8583 unique titles (see Fig. 31). Included studies were published between 1981 and 2015. Development datasets ranged from 50 to 216626 patients for individual models, with a median of 679 patients [IQR: 266–10738]. Most models targeted a general mixed ICU population (n = 48), but prediction models specifically designed for patients with non-surgical (medical) admission reasons (n = 8), acute neurological injuries (n = 2) or for the elderly ICU patient (n = 1) were also found. Half of the included prediction models (n = 32) were meant to be used for prognostication on day 1 of ICU admission. Almost all (n = 58) had in-hospital mortality as outcome of interest. Only 5 prediction models were developed with functional status or HRQoL incorporated into the outcome, but none of these were fitted in the general mixed ICU population (1 model for the elderly; 2 for patients with cardiac arrest; 2 for patients with traumatic brain injury). Ten out of the 63 studies used long-term outcomes (i.e. beyond 6 months). See Fig. 32 for more details on study characteristics. External validation was reported for 38 (60.3%) prediction models.

**CONCLUSIONS.** Prognostic research in ICU patients has traditionally been focused on predicting short-term mortality rather than long-term functional outcomes, such as HRQoL. Validation studies are required to determine whether available prediction models can also be used for long-term prognostication of ICU patients.

**GRANT ACKNOWLEDGMENT**
This study was supported by the NutsOhra Foundation, project nr 1404–013.

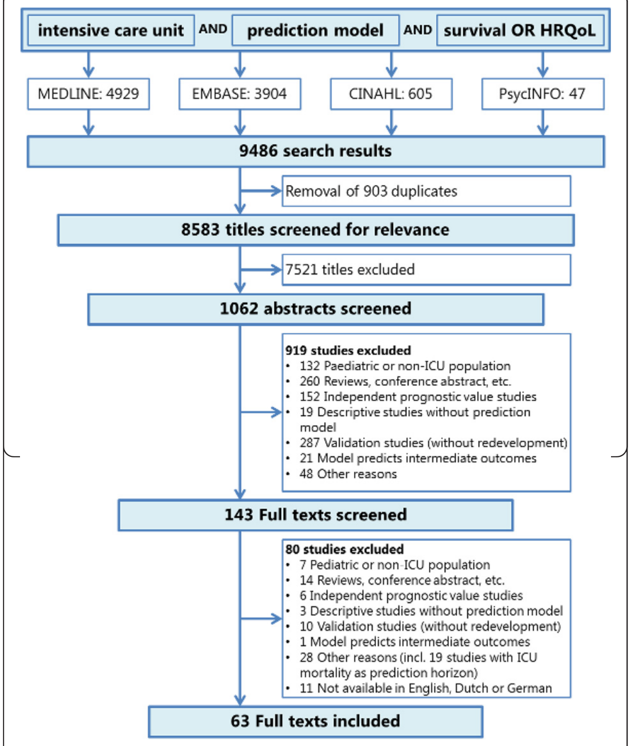

**Fig. 31 (abstract 0035).** Flowchart of included studies

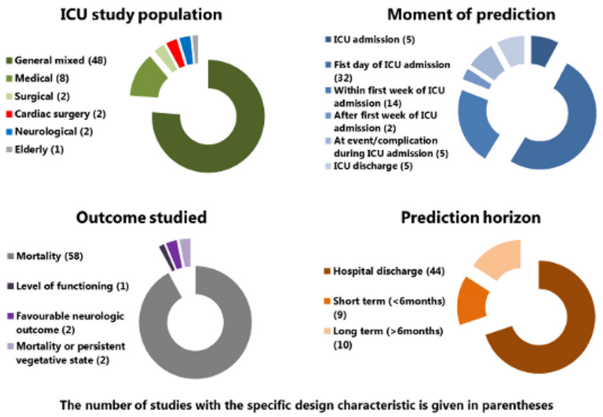

**Fig. 32 (abstract 0035).** Study characteristics

### Macro- & micronutrient supplementation

#### 0036

#### Iron deficiency diagnosed using hepcidin on ICU discharge is an independent risk factor for death and poor quality of life at one year: an observational study 1282 patients

S. Lasocki[1], T. Lefebvre[2], C. Mayeur[3], H. Puy[2], A. Mebazaa[3], E. Gayat[3], FROG-ICU
[1]LUNAM Université d'Angers, CHU d'Angers, Département Anesthésie Réanimation, Angers, France; [2]APHP, CHU Louis Mourier, Service de Biochimie, Colombes, France; [3]APHP, CHU Saint Louis - Lariboisiere, Service d'Anesthésie Réanimation, Paris, France
**Correspondence:** S. Lasocki
*Intensive Care Medicine Experimental* 2017, **5(Suppl 2):**0036

**INTRODUCTION.** Iron deficiency (ID) is responsible for anaemia but also for fatigue and muscle weakness. ID is frequent in critically ill patients on admission (around 20-40%) and probably on discharge (due to blood losses leading to iron losses), it may thus impair the post-ICU rehabilitation. Unfortunately, ID is difficult to diagnose using conventional markers (1). Hepcidin (Hepc) is the master regulator of iron metabolism and may indicate ID when it is low (2).

**OBJECTIVES.** To determine whether an Hepc dosage on ICU discharge is predictive of one year mortality and quality of life (QOL) in ICU patients.

**METHODS.** Ancillary study based on the prospective observational FROG-ICU study. Hep was measured using mass spectrometry on a discharge blood sample (3). We defined ID as an Hepc < 20 ng/l (<10 ng/l for severe ID). One year mortality as well as QOL using the SF-36 questionnaire was collected by phone interview and compared in ID and non-ID patients using univariate and multivariate analysis, adjusted for the principal cofounding factors (age, gender, diabetes, Liver disease, surgical/medical, septic shock and discharge Haemoglobin (Hb)).

**RESULTS.** Among the 1570 patients of the FROG-ICU cohort, alive on ICU discharge and for whom living status was available, 1282 patients (age, mean ± SD, 59 ± 17 yrs, 456(36%) Women, SAPS II 48 ± 18) had an Hepc dosage (median[Q1-Q3], 32.15[11.22-64.20] ng/l). The 448(35%) ID patients were more often women (41.5 vs 31.5%, p = 0.0017), had more liver disease (10.5 vs 3.9%, p < 0.0001), a lower discharge Hb (9.9[9.1-11.1] vs 10.2[9.1-11.6] g/dl, p = 0.02), but their renal function was better (eGFR 88.7[55.7-109.8] vs 80.7[39.9-105.5] ml/min, p = 0.00053). ID patients were less transfused during their ICU stay (38.6 vs 47.5%, p = 0.0029). At one year, ID patients had a higher mortality rate (100(23.1%) vs 130(16.9%) deaths, p = 0.012). The multivariate analysis adjusted for the main cofounding factors confirmed that ID is an independent predictor for one year mortality. A sensitivity analysis, using a lower threshold for ID found that the 284(22.8%) severe ID patients had a lower physical QOL (defined as a physical dimension of the SF-36 score < median) together with a higher one year mortality (Fig. 33).

**CONCLUSIONS.** ID (i.e. Hepc < 20 ng/l) is an independent risk factor for one-year mortality this is in accordance with data showing a poorer prognosis in ID cardiac patients. An interventional trial is ongoing to assess whether iron treatment of ID diagnosed using hepc (compared to treat ID using ferritin) on ICU discharge reduces post-ICU hospital length of stay, costs and improves Hb and QOL (Hepcidane trial NCT02276690).

**REFERENCE(S)**

1. Lasocki Crit Care. 2014;18(5):542
2. Lasocki Anesthesiology 2011;114(3):688–94
3. Lefebvre Ann Biol Clin 2017 ;75(1) :9–18

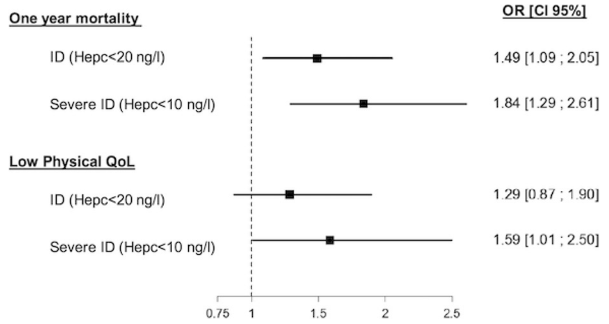

**Fig. 33 (abstract 0036).** Multivariate Analysis

### 0037

#### Omega-3 PUFAs in critically ill patients with acute respiratory distress syndrome: an updated systematic review and meta-analysis

P. Laferriere-Langlois[1], F. D'Aragon[2], W. Manzanares[3]
[1]Université de Sherbrooke, Sherbrooke, Canada; [2]Université de Sherbrooke, Anesthesiology, Sherbrooke, Canada; [3]Hospital de Clinicas, Intensive Care Unit, Montevideo, Uruguay
**Correspondence:** P. Laferriere-Langlois
*Intensive Care Medicine Experimental* 2017, **5(Suppl 2):**0037

**INTRODUCTION.** Acute respiratory distress syndrome (ARDS), a deadly complication of critical illness, is characterized by an acute inflammatory response in the lung parenchyma leading to hypoxemia. Due to its anti-inflammatory and immunomodulatory properties, omega-3 polyunsaturated fatty acids (n-3 PUFA) have been administered in ARDS patients, mostly by enteral route as diets enriched with eicosapentaenoic acid (EPA), γ-linolenic acid, and antioxidants. Clinical benefits of n-3 PUFA supplementation in ARDS patients remain unclear, as clinical trials have found conflicting results.

**OBJECTIVE.** Considering the most recent randomized controlled trials (RCT) and recent change in administration strategies, the aim of this updated systematic review and meta-analysis was to evaluate the benefits of n-3 PUFA administration on gas exchange, assessed by PaO2/FiO2 ratio, and clinical outcomes in critically ill patients with ARDS.

**METHODS.** We searched Medline, Embase, CINAHL and Cochrane database for RCT conducted in intensive care unit (ICU) patients with ARDS comparing the administration of n-3 PUFAs to placebo.

The outcomes assessed were PaO2/FiO2 ratio evaluated early (3–4 days) and late (7–8 days), mortality, ICU/hospital length of stay (LOS), length of mechanical ventilation (MV) and infectious complications. Two independent reviewers assessed eligibility, risk of bias and abstracted data. Authors of trials were contacted when required. Data was pooled using a random effect model to estimate the relative risk (RR) or weighted mean difference (WMD). Pre-defined subgroup analysis included high vs. low risk of bias and sensitivity analysis restricted to enteral administration and continuous enteral administration were conducted.

**RESULTS.** Twelve RCTs (n = 1280 patients) met our inclusion criteria. N-3 PUFA administration was associated with a significant improvement in early PaO2/FiO2 ratio (WMD = 49.33; 95% Confidence Interval [CI] 20.88-77.78; P = 0,0007; I2 = 69%), which persisted at days 7–8 (WMD = 27.87,; 95% CI 0.75-54.99; P = 0,04; I2 = 57%). With n-3 PUFA administration, there were trends towards improvement of ICU LOS (P = 0.08) and duration of MV (P = 0.06), while mortality, hospital LOS and infectious complications remained unchanged. Continuous enteral infusion was associated with improved mortality (P = 0.02) while analysis restricted to enteral administration either with or without bolus found improved early PaO2/FiO2 (P = 0.001) and MV duration (P = 0.03). Trials at higher risk of bias showed a significant reduction in mortality (P = 0.04), and improvement in late PaO2/FiO2 ratio (P = 0.003).

**CONCLUSIONS.** In critically ill patients with ARDS, n-3 PUFA administration was associated with an improvement in early and late PaO2/FiO2 ratio, while statistical trends exist for an improved ICU LOS and MV duration. In the face of these results, administering n-3 PUFA appears a reasonable course of action.

### 0038

#### A supplemental intravenous amino acid infusion sustains an improved protein balance for 24 hours in critically ill patients

M. Sundström Rehal[1,2], O. Rooyackers[1], F. Liebau[1,2], I. Tjäder[1,2], Å. Norberg[1,2], J. Wernerman[1,2], ICU Metabolism
[1]Karolinska Institutet, CLINTEC, Division of Anaesthesia and Intensive Care, Stockholm, Sweden; [2]Karolinska University Hospital, Department of Perioperative Medicine and Intensive Care, Stockholm, Sweden
**Correspondence:** M. Sundström Rehal
*Intensive Care Medicine Experimental* 2017, **5(Suppl 2):**0038

**INTRODUCTION.** Provision of protein during ICU stay may attenuate the well-known loss of lean body mass, but the effects of supplemental amino acids on protein synthesis and breakdown have been insufficiently described. It has previously been demonstrated that an intravenous infusion of balanced amino acids improves whole body net protein balance at three hours during the initial week in ICU [1].

It is unclear if this response can be sustained over a longer time period.

**OBJECTIVES.** The primary objective was to investigate if an intravenous amino acid infusion improves net protein balance for 24 hours in critically ill patients. The secondary objective was monitoring of safety aspects in terms of amino acid oxidation, urea and amino acid plasma concentrations.

**METHODS.** The study was conducted in the surgical-medical ICU of a tertiary referral hospital. All aspects of care were determined by the attending physician. Feeding rates were unaltered during the study period.

At T = 0 on day one, an infusion of [1-$^{13}$C]-phenylalanine was added to ongoing enteral nutrition to quantify the uptake of amino acids from the enteral nutrition. At T = 120 minutes, primed intravenous infusions of [ring-$^{2}H_5$]-phenylalanine and [3,3-$^{2}H_2$]-tyrosine were started. Baseline blood samples were drawn at expected isotopic equilibrium after T = 285–300 min. At T = 300 min, an intravenous amino acid infusion (Glavamin, Fresenius Kabi) was started at a rate corresponding to 1 g/kg/day and continued for 24 hours. Blood sampling was repeated at T = 465–480 minutes, after which the tracer infusions were discontinued.

On day two, enteral and parenteral tracers were administered in identical order and dose to day one, 24 hours after T = 0. A third set of blood samples were drawn between T = 285–300 min after the start of the enteral tracer on day two.

Whole body protein kinetics and protein oxidation were calculated using a single-pool model.

**RESULTS.** Eight patients were studied. The supplemental amino acid infusion resulted in an improved net protein balance over time, from −1.6 ± 7.9 μmol phe/kg/h at 0h to 6.0 ± 8.8 at 3h and 7.5 ± 5.1 at 24h (p = 0.002). Confirming earlier results protein balance increased between 0 and 3 h (p = 0.01), and remained unaltered between 3 and 24 h (p = 1.00). The sum of free amino acids in plasma increased from 3.1 ± 0.6 mmol/L at 0h to 3.2 ± 0.3 at 3h and 3.6 ± 0.5 at 24h (p = 0.038). Protein oxidation (p = 0.147) and plasma urea (p = 0.053) were not altered significantly.

**CONCLUSIONS.** We demonstrated that a supplemental intravenous amino acid infusion of 1 g/kg/24h improves whole body net protein balance for up to 24 hours in critically ill patients. The effects on lean body mass and on regional protein balance, as well as the implications upon autophagy remain to be established.

**REFERENCE(S)**

1. Liebau, F., et al. Crit Care, 2015. 19: p. 106.

**GRANT ACKNOWLEDGMENT**
Stockholm County Council (ALF)

### 0039

#### Current pharmaconutrition practices in the ICU: results of the International Nutrition Survey 2014–2015

W. Manzanares[1], P.L. Langlois[2], C. Szwec[3], I. Aramendi[1], D.K. Heyland[4]
[1]Universidad de la República, Critical Care, Montevideo, Uruguay; [2]Université de Sherbrooke, Anesthesiology, Shebrooke, Canada; [3]Hospital A. Posadas, Buenos Aires, Argentina; [4]Queen´s University, Clinical Evaluation Research Unit, Kingston, Canada
**Correspondence:** W. Manzanares
*Intensive Care Medicine Experimental* 2017, **5(Suppl 2):**0039

**INTRODUCTION.** Over the last few years several randomized controlled trials examining the role of pharmaconutrition in the critical care setting have shown controversial results. The purpose of this study was to describe current pharmaconutrition practices in intensive care units (ICUs) worldwide evaluating the compliance with the evidence-based Canadian Critical Care Nutrition Clinical Practice Guidelines (CPG).

**METHODS.** We conducted an international, prospective, observational, cohort study between September 2014-June 2015. Participating sites enrolled critically ill adult patients on mechanical ventilation within the first 48 hours of ICU admission and who remained for at least 72 hours in the ICU. Data on nutrition and pharmaconutrient practices were collected from admission to ICU discharge or a maximum of 12 days. Variables related to the CPG are reported as overall averages (or percentages) with the range of site averages.

**RESULTS.** 184 adult ICUs from 19 countries contributed 3926 patients to this analysis. Sixty-four percent were male, mean age was 59 (16–102) years old, average APACHE II score was 21.3 (1–55), and admission SOFA score was 6.2 (0–18).

5.4% of all patients (best site: 75.6%, and worst site: 0.0% of patients) received glutamine (GLN) by enteral, IV/parenteral or both routes. Enteral GLN was administered in 4.6%, and IV/parenteral GLN in 1.0% of patients. Moreover, 30% (0.0%-100%) of burn patients received enteral GLN, whereas this pharmaconutrient was provided in 4.4% of trauma patients (0.0%-87.5%). 6.1% of patients received selenium as single strategy or combined therapy. Most of ICUs avoid using arginine-enriched enteral formulas which were provided in 5.5% of patients (0.0%-94.4%). Fish oil (FO) enriched formula was administered in 14.7% of all patients, whereas in those with ARDS was provided in 22.4%. In patients on PN (or EN + PN) soybean oil (SO) based lipid emulsion (LE) was used in 25.3% of PN days. However, a SO sparing strategy was used in 35.6% of days, including an olive oil based LE: 13.0%, a mixture of SO, olive oil, medium chain triglycerides (MCT), and FO: 16.1%.

**Conclusions:** According to our recent data there is low utilization of enteral or parenteral pharmaconutrition in ICUs worldwide. The most frequently used pharmaconutrient is enteral GLN in the context of burn injury although we await the results of the ongoing Randomized Trial of Enteral Glutamine to Minimize Thermal Injury (RE-ENERGIZE) trial before making stronger recommendations for its use. Finally, in compliance with current guidelines in PN patients alternative lipid emulsions are more commonly used.

#### 0040

#### Leucine-enriched essential amino acid supplementation in mechanically ventilated trauma patients - a feasibility study

L. Wandrag[1], S. Brett[2], G. Frost[1], M. Hickson[3]
[1]Imperial College London, London, United Kingdom; [2]Imperial College Healthcare NHS Trust, London, United Kingdom; [3]University of Plymouth, Plymouth, United Kingdom
**Correspondence:** L. Wandrag
*Intensive Care Medicine Experimental* 2017, **5(Suppl 2):**0040

**INTRODUCTION.** Critically ill patients lose between 1-2% muscle mass per day (1). Efforts to ameliorate wasting may improve patient recovery. Leucine-enriched essential amino acid (L-EAA) has shown promise in enhancing muscle protein synthesis in older adults and in disuse atrophy (2).

**OBJECTIVES.** The aims of this study were to assess recruitment rate and feasibility of studying a mechanically ventilated trauma population given a leucine-enriched EAA supplement and to test which outcome measures could be used in a future randomised controlled trial to assess muscle mass, protein turnover and strength.

**METHODS.** This randomised study was performed over 6 months in mechanically ventilated trauma patients as part of a larger project. Standard enteral feeding was provided, with treatment group patients receiving a 5g L-EAA powder 5 times per day via the enteral feeding tube. CRP, albumin, IL-6, IL-10, urinary 3-MH, nitrogen balance, protein turnover ([1-$^{13}$C] Leucine stable isotopes) and muscle depth change on ultrasound was assessed on days 1, 3, 7 and 14 of study. The MRC Sum score was used upon awakening and at discharge from ICU as a measure of strength and to determine presence of ICU Acquired Weakness (ICU-AW).

**RESULTS.** Eight patients (9.5% of screened patients) were recruited over 6 months. L-EAA doses were provided on 91/124 (73%) occasions. Inflammatory and urinary marker data were collected; serial muscle depth measurements were lacking due to short length of stay of patients. Protein turnover studies were performed in four patients, protein balance ranged between −3.9 to −1.73g/24h. MRC-Sum score could not be performed as patients were not able to respond appropriately to the screening questions.

**CONCLUSIONS.** We found significant barriers to recruitment, delivery of the intervention and measurement of the chosen outcomes, this would need to be addressed in the study design of a future, large randomised controlled trial. Trauma patient ICU stay was shorter than anticipated, outcome measures were difficult to perform serially in this population. Although it was practical to provide L-EAA to patients, we were limited by short stay, frequent interruptions to testing and by complexity of protein turnover studies. One researcher conducted this project; a research assistant would facilitate data collection. The ICU patient population and outcome measures require careful consideration for future studies of amino acid or protein supplementation.

**REFERENCE(S)**

1. Reid CL et al. Muscle wasting and energy balance in critical illness. Clin Nutr 2004 Apr;23(2):273–80.
2. Borsheim E et al. Effect of amino acid supplementation on muscle mass, strength and physical function in elderly. Clin Nutr 2008 Apr;27(2):189–95.

**GRANTS**

LW was supported by a NIHR Clinical Doctoral Research Fellowship.

### Transfusion and haemostasis

#### 0041

#### Can arterio-venous-oxygen content difference be a target to guide transfusion in critically ill patients?

A. Fogagnolo[1], S. Spadaro[1], J. Creteur[2], E. Cavalcante[2], F.S. Taccone[2], C.A. Volta[1]
[1]Università Ferrara, Arcispedale Sant'Anna, Anestesia e Terapia Intensiva, Ferrara, Italy; [2]Hôpital Universitaire Erasme, Bruxelles, Belgium
**Correspondence:** A. Fogagnolo
*Intensive Care Medicine Experimental* 2017, **5(Suppl 2):**0041

**INTRODUCTION.** Red blood cells transfusion (RBCT) is a common intervention among intensive care (ICU) patients; however, RBCT are also associated with several adverse events. Few tools are available to target RBCT in this setting. The aim of this study was to evaluate the impact of the difference between arterial ($CaO_2$) and venous oxygen content ($CvO_{2)}$ (a-$vO_2$gap) on the outcome of ICU patients receiving RBCT.

**OBJECTIVES.** To investigate whether the adequacy of transfusion, defined on the a-$vO_2$gap, could be associated with ICU outcome.

**METHODS.** Prospective observational study (from January to December 2016) enrolling patients with hemoglobin (Hb) between 7 and 9 g/dL during the first 24hours after ICU admission. Patients were separated in two groups, according to the median value of the a-$vO_2$gap (=3.8 mL). The decision to transfuse on admission was taken by the treating physician unaware of the study protocol. "Appropriate" transfusion policy was defined when RBCT was given in patients with high a-$vO_2$gap or when patients with low a-$vO_2$gap were not transfused; otherwise, patients were categorized into the "non-appropriate" transfusion policy.

**RESULTS.** We enrolled 113 patients (23% with sepsis; Table 12); of those, 48 (42%) were transfused and 65 (58%) received non-appropriate transfusions policy; ICU mortality was 27%. Patients receiving non-appropriate transfusion policy showed a significantly higher 28-day mortality than others (23/65 vs. 8/48; p = 0.02 - HR 2.4 [95%CI: 1.1-4.6]) (Fig. 34). After the adjusted for several confounders, the odds ratio for mortality was 3.02 [95% CI: 1.2 - 6.2].

**CONCLUSIONS.** In an unselected population of ICU patients, the a-$vO_2$gap could be a valuable marker to identify patients who would benefit from RBCT (Fig. 34).

**Table 12 (Abstract 0041).** Demographic and clinical characteristics of patients at ICU admission. Data are expressed as Mean ± SD or median [IQ]

| Variables | Appropriate transfusion policy | | Non-appropriate transfusion policy | | P value |
|---|---|---|---|---|---|
| | *CaO2-CvO2<3.8mmHg* | *CaO2-CvO2>3.8mmHg* | *CaO2-CvO2<.8mmHg* | *CaO2-CvO2>3.8mmHg* | |
| | *Non-transfused* | *Transfused* | *Transfused* | *Non-Transfused* | |
| Age | 69±6 | 77 ± 7 | 69 ± 11 | 76 ± 9 | 0.19 |
| BMI | 25 ± 5 | 25 ± 4 | 30 ± 6 | 27 ± 4 | 0.88 |
| SAPS | 31 ± 8 | 39 ± 10 | 43 ± 7 | 41 ± 6 | 0.56 |
| SOFA at admission | 2 [1.5 – 4] | 5 [3 – 7] | 5 [3 – 6] | 2.5 [1 – 4] | 0.11 |
| Hemoglobin (g/dL) | 8.5 ± 0.5 | 8.4 ± 0.3 | 8.1 ± 0.5 | 8.9 ± 0.3 | 0.54 |
| Platelets ($10^9$/L) | 235 [170 – 262] | 130 [115- 225] | 175 [140 – 230] | 213 [180 – 255] | 0.39 |
| Lactate (mmol/L) | 1.3 ± 0.4 | 2.0 ± 0.7 | 1.9 ± 0.9 | 1.8 ± 1 | 0.31 |
| $O_2$ extraction rate (%) | 21 ± 8 | 38 ± 4 | 25 ± 7 | 37 ± 9 | <0.001 |

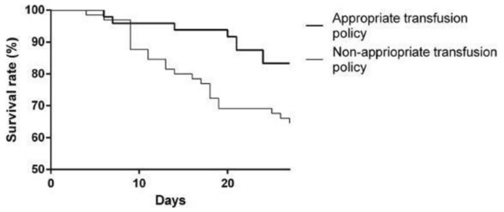

**Fig. 34 (abstract 0041).** See text for description

### 0042

#### High transfusion ratio of fresh frozen plasma (FFP): red blood cells (RBC) in severe trauma. Analysis of a French trauma registry (Traumabase)

F. Roquet[1], S. Hamada[2], J. Mantz[3], R. Pirracchio[1,3], Traumabase Group
[1]Saint-Louis Hospital, ECSTRA Team, Biostatistics and Clinical Epidemiology, Paris Diderot Sorbonne University, Paris, France; [2]Bicêtre University Hospital, Paris-South University Hospitals, Department of Anaesthesiology and Critical Care, Le Kremlin-Bicêtre, France; [3]Georges Pompidou European Hospital, Department of Anaesthesiology and Critical Care, Paris, France
**Correspondence:** F. Roquet
*Intensive Care Medicine Experimental* 2017, **5(Suppl 2):**0042

**INTRODUCTION.** Transfusion management is a major therapeutic issue in severe trauma patients. The benefit of a high FFP: RBC transfusion ratio remains uncertain. [1]

**OBJECTIVES.** This work aims at studying the effect on survival of a high FFP: RBC ratio (>0.75) during the first 24 hours in patients with haemorrhagic shock.

**METHODS.** Traumabase is a prospective French trauma registry of over 10,000 patients in 11 trauma centres. This public funded registry is approved by competent authorities for medical research and personal information protection. [2] Haemorrhagic shocks were identified on the following criteria:

(1) ≥ 4 RBCs over the first 6 hours;
(2) patients who died of haemorrhagic shock before receiving 4 RBCs. A 30-day survival analysis was performed using a Cox proportional hazards model on complete cases and then after multiple imputations (hazard ratio (HR) results [95% confidence interval]).

The choice of adjustment covariates was made according to

(1) their clinical relevance,
(2) previous knowledge and then
(3) by maximizing the Akaike Information criterion. A value of $p < 0.05$ was considered significant.

**RESULTS.** 836 observations were analyzed (table 13, 14, 15). A survival curve according to the transfusion ratio is presented in Fig. 35. After adjustment, the complete-case analysis (n = 543) revealed a protective effect of the high ratio: HR 0.69 [0.48-0.99] (p = 0.02). After multiple imputation (n = 836), this protective effect of the high transfusion ratio was confirmed: HR 0.73 [0.56-0.95] (p = 0.01). The area under the curve (AUC) of the final model was 0.84.

**CONCLUSIONS.** In trauma patients with massive transfusion, a high FFP: RGC ratio during the first 24 hours appears to be associated with a better 30-day survival.

**REFERENCE(S)**

[1] Holcomb JB et al. JAMA. 2015; 313(5):471–482
[2] National Commission on Informatics and Liberty (CNIL) authorisation № 911461

**Table 13 (Abstract 0042).** Demographics based on treatment group

| | Low ratio n=385 (46%) | High ratio n=401 (48%) | Univariate P (Wald) |
|---|---|---|---|
| Age (years) median [IQR] | 39 [26-55] | 35 [25-52] | *<0.001* |
| Male sex * n (%) | 276 (72%) | 285 (71%) | *0.59* |
| Anticoagulants | 10 (2.6%) | 9 (2.2%) | *0.004* |
| Antiplatelet drugs | 17 (4.4%) | 15 (3.7%) | *0.01* |
| Mechanism (% penetrating) | 56 (14%) | 57 (14%) | *0.06* |
| Intentionality | | | *0.02* |
| Unintentional | 218 (57%) | 233 (58%) | |
| Assault | 40 (10%) | 43 (11%) | |
| Self-inflicted | 104 (27%) | 91 (23%) | |

**Table 14 (Abstract 0042).** Outcome and prognostic markers by treatment group

| | Low ratio n=385 (46%) | High ratio n=401 (48%) | P (Wald) |
|---|---|---|---|
| Death n (%) | 131 (34%) | 126 (31%) | *0.18* |
| IGS II median [IQR] | 50 [36-68] | 51 [37-69] | *<0.001* |
| ISS | 34 [22-43] | 34 [18-45] | *<0.001* |
| Initial GCS <9 | 120 (31%) | 154 (38%) | *<0.001* |
| Initial prothrombin ratio (%) | 50 [36-64] | 47 [31-60] | *<0.001* |
| Initial lactate (mmol/L) | 4.6 [2.7-8.2] | 4.5 [2.7-8.1] | *<0.001* |

**Table 15 (Abstract 0042).** Surgery < 24h by treatment group

| | Low ratio n=385 (46%) | High ratio n=401 (48%) | P (Wald) |
|---|---|---|---|
| Orthopaedic | 163 (42%) | 168 (42%) | *<0.001* |
| Vascular -radio interventional * | 103 (27%) | 111 (28%) | *0.22* |
| Neurosurgery | 18 (4.7%) | 29 (7.2%) | *0.141* |
| Abdominal * | 82 (21%) | 96 (24%) | *0.27* |
| Thoracic * | 24 (6.2%) | 30 (7.5%) | *0.88* |

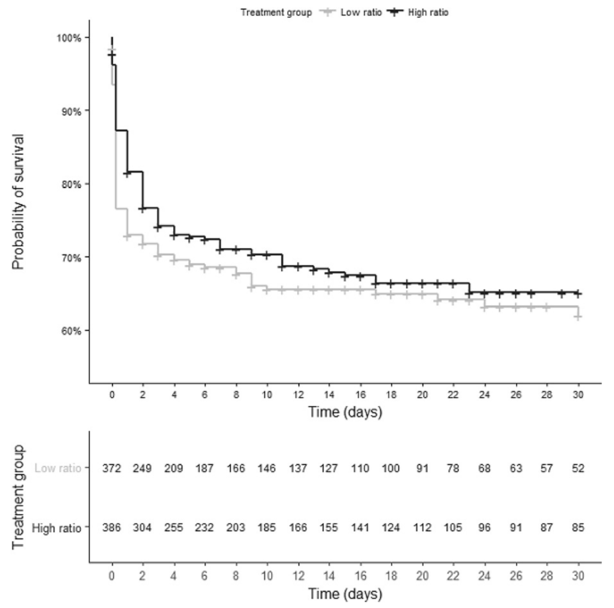

**Fig. 35 (abstract 0042).** KM survival curve according to transfusion ratio

### 0043

#### LOVE project - LMWHs prOphylaxis of venous thrombembolism in ICU patiEnts in the Czech Republic and Slovak Republic

R. Kula[1], M. Protuš[2]
[1]University Hospital Ostrava, Clinic of Anaesthesiology and Resuscitation, Ostrava, Czech Republic; [2]Institute for Clinical and Experimental Medicine, Anaesthesiology, Resuscitation and Intensive Care Department, Prague, Czech Republic
**Correspondence:** M. Protuš
*Intensive Care Medicine Experimental* 2017, **5(Suppl 2):**0043

**INTRODUCTION.** Deep venous thrombosis remains to be a frequent and serious complication in the critically ill patients. Therefore, effective thromboprophylaxis needs to be performed and subcutaneous (s.c.) low molecular weight heparins (LMWH) are the most commonly used drugs for this purpose. However, prophylactic s.c. LMWH dosing regimen used in standard wards, may not be sufficient for ICU patients, in which the availability of s.c. applied drugs is limited (positive fluid balance, vasopressor use, low cardiac output). Moreover, the critically ill tend to hypercoagulate (inflammatory response, artificial ventilation, immobilization).

**OBJECTIVES.** The aim of LOVE project was to test the real effectiveness of subcutaneously LMWH thromboprophylaxis in ICU patients in the Czech and Slovak Republic.

**METHODS.** A pilot, multicenter, prospective, observational study took place from 23.2. 2015 to 1.3. 2015 and investigated s.c. LMWH prophylaxis in ICU patients receiving s.c. LMWH for more than 3 days. The effectiveness of s.c. LMWH was evaluated according to the patient's anti-Xa serum levels, which were measured 3 hrs after LMWH application. Anti-Xa ≥0,2 kIU/l was considered to be sufficient for effective thromboprophylaxis. Before starting the study, only two centres monitored anti-Xa serum levels routinely.

**RESULTS.** In total, 101 patients, of average age 67 (34–83) and average weight 84 (54–120)kg were involved. 45 patients were admitted to ICU after surgery, 36 patients were admitted for internal diseases, 20 patients suffered trauma. Only 15 out of 101 patients had sepsis. Their average 72 hours fluid balance was 15 ml/kg (−17,1 - + 77,8), in 77 patients vasopressor circulatory support was used on the third day of ICU stay. The average SOFA score on the third day was 6 (0–11). LMWHs used in the study: Nadroparin in 76 patients, Enoxaparin in 20 patients, and Dalteparin in 5 patients. In 52 patients, s.c. LMWH was applied once a day, 49 patients received s.c. LMWH twice a day. The average LMWH dose was 68,7 UI/kg (31–175). Surprisingly, only 55% patients reached the effective anti-Xa serum level on the third day after ICU admission using standard LMWHs dosing regimen. The average LMWH dose of the patients whose antiXa < 0,2, was significantly different from the patients who reached antiXa ≥ 0,2 (58,3 UI/kg (21,3- 124,6) vs. 80,9 UI/kg (33,5- 181,8), p = 0,001), as well as their 72 hours fluid balance (25ml/kg (−17,1- + 92,7) vs. 12 ml/kg (−19,2 - + 50,7), p = 0,002).

**CONCLUSIONS.** We found, that in 45% patients involved in LOVE project, LMWH was underdosed and did not reach sufficient prophylactic serum level of antiXa ≥0,2. The optimal LMWH dose seems to be 75–100 UI/kg/24 hrs s.c.. Because in case of inadequate thromboprophylaxis the risk of deep venous thrombosis and thrombembolism rises substantially, the LMWH dosing regimens routinely used on ICUs should be reconsidered carefully, and guided by serum anti-Xa levels.

### 0044

#### Transfusion of fresh frozen plasma but not red blood cells transfusion increases the risk of pneumonia after cardiac surgery

A. González Pérez, J. Silva Guisasola
Hospital Universitario Central de Asturias, Oviedo, Spain
**Correspondence:** A. González Pérez
*Intensive Care Medicine Experimental* 2017, **5(Suppl 2):**0044

**INTRODUCTION.** Pneumonia is a known complication that may arise after cardiac surgery and increases the morbidity and mortality of these patients. On the other hand, it is known that blood compounds transfusion of increases the risk infections of postoperative cardiac surgery patients. Transfusion of fresh frozen plasma (FFP) units are at increased risk of acute lung injury. Red blood cells (RBC) transfusions increments the risk of pneumonia after cardiac surgery as described.

**OBJECTIVES.** The aim of this study is to analyze the relationship between intraoperative cardiac surgery FFP and RBC transfusions in the development of pneumonia during ICU stay.

**METHODS.** Retrospective observational study of a cohort of patients that underwent to cardiac surgery at our institution between 2006 and 2014. Demographic and clinical variables were collected from de Hospital Database. FFP and RBC units transfused during intraoperation procedure and the develop of nosocomial pneumonia were analysed to determinate their relationship. A multivariate logistic regression model was used to estimate the Odds Ratio of pneumonia associated with the FFP and RBC transfusion with confidence interval of 95%, p < 0.05. Values expressed as mean +/− SD or %.

**RESULTS.** Out of 3563 patients ; 64.8 males; 67.78+/− 11.13 years old; BMI 28.36 +/− 6.81 ; Logistic EuroScore 6.36 +/− 4.85; ICU stay 6.58 +/− 15.41 days ; postoperative bleeding at first 24 hours 560.74 +/− 447.95 cc; preoperative haemoglobin 13.58 +/− 5.72 gr/ dL. By pass time 104.31 +/− 231.75; cross clamp time 71.76 +/− 32.74 minutes. 265 patients were diagnostic of pneumonia during the ICU stay. These patients were most frequently males, older, less BMI, higher EuroScore and lower hematocrit and hemoglobin but no significant differences were found in these variables. Pneumonia group patients were transfused more FFP (1.31 +/− 1.85 vs 2.27 +/− 1.94 units p < 0.001) and presented a longer duration of CPB and clamp times (97.79 +/− 45.8 vs 126.19 +/− 3.52; p < 0.001 and 70.50 +/− 33.44 vs 98.88 +/− 24.09; p < 0.001 respectively. The relative risk of developing pneumonia was 1.50 for the group that received transfusion of blood components. In the multivariate analysis patients exposed to FFP transfusions had a significant OR 1.149 of pneumonia 95% CI (1.046 to 1.261) p < 0.001.No significant differences were found between RBC transfusion and the development of pneumonia.

**CONCLUSIONS.** Known deleterious effects that can cause transfusions of blood components clinical teams should explore strategies to prevent unnecessary transfusions. The transfusion of FFP demonstrated in this study that it could increase the morbidity after cardiac surgery. Further studies are needed to corroborate these results.

REFERENCE(S)
*Red Blood Cell Transfusions Impact Pneumonia Rates After Coronary Artery Bypass Grafting.* Likosky DS et all. Ann Thorac Surg. 2015 Sep;100(3):794–800.

### 0045

#### Platelet trajectories characterization and its association with mortal events

Y. Wei[1], J.G. Yabes[2], R.S. Parker[3], G. Clermont[4]
[1]University of Pittsburgh, Biostatistics, Pittsburgh, United States; [2]University of Pittsburgh, Medicine and Biostatistics, Pittsburgh, United States; [3]University of Pittsburgh, Chemical and Petroleum Engineering, Pittsburgh, United States; [4]University of Pittsburgh, Critical Care Medicine, Pittsburgh, United States
**Correspondence:** Y. Wei
*Intensive Care Medicine Experimental* 2017, **5(Suppl 2):**0045

**INTRODUCTION.** Platelets are involved in hemostasis and are a core component of the acute inflammatory response. No previous work has systematically examined the association between platelet trajectory, clinical evolution and outcome.

**OBJECTIVES.** To identify data-driven groups of platelet trajectories in acutely ill patients, predict group membership at baseline, and examine their association with 60-day mortality.

**METHODS.** From an observational dataset of 54,000 ICU admissions, we focused on patients surviving at least seven days with at least five platelet counts over that period. We applied latent class mixed models to identify groups of trajectories, modeled using third degree polynomials in time. Because of computational limitations, we split the data into 8 disjoint subsets and 5-group models were developed for each subset. Of the five groups, 2 were identified across all subsets (the "Large" set, N = 7,753). All other patients were pooled into the "Small" set (N = 3,565). Group assignment was reestimated within the Large and the Small sets. To study the association between platelet trajectory groups and 60-day mortality, logistic regression was used with age, sex, race, burden of chronic illness, baseline organ function (SOFA score), severity of disease (APACHE III), trauma status and surgery as additional covariates. To evaluate group membership prediction at baseline, multinomial logistic regression was used with platelet counts on the first and second day after admission, as well as baseline covariates.

**RESULTS.** 11,318 patients met study criteria. We identified 7 groups of platelet trajectories (Fig. 36). Trajectory groups were independently associated with 60-day mortality ($p < .001$). Compared to group 1, the odds of death within 60 days were significantly higher in group 2 (OR = 1.16 (1.04-1.30)), group 6 (OR = 2.62 (1.74-3.94)), and group 7 (OR = 1.29 (1.01-1.65)). AUC-ROC was 0.753. Platelet count on the first day predicts group membership after adjusting for baseline covariates ($p < .001$). A 50 increase in first day platelet count decreases the odds of being in group 2 (OR = 0.50 (0.48-0.52)) relative to group 1. In contrast, patients with higher first day platelet counts were more likely to be in group 3 (OR = 1.76 (1.52-2.03)), group 4 (OR = 1.81 (1.70-1.93)), group 5 (OR = 2.29 (2.09-2.51)), group 6 (OR = 2.17 (2.01-2.34)), and group 7 (OR = 1.27 (1.21-1.33)). The accuracy of a model that predicts group assignment improved from 66.2% to 72.6% when day-2 platelet count is added in the model.

**CONCLUSIONS.** Platelet trajectory group in the first week is independently associated with 60-day mortality, with the first and second day platelet counts predicting trajectory group membership with reasonable accuracy. Further analysis will focus on studying whether platelet trajectories are associated with morbidities such as late bleeding, embolism, and thrombosis.

**GRANT ACKNOWLEDGMENT**
Supported by National Institutes of Health (R21-HL-133891)

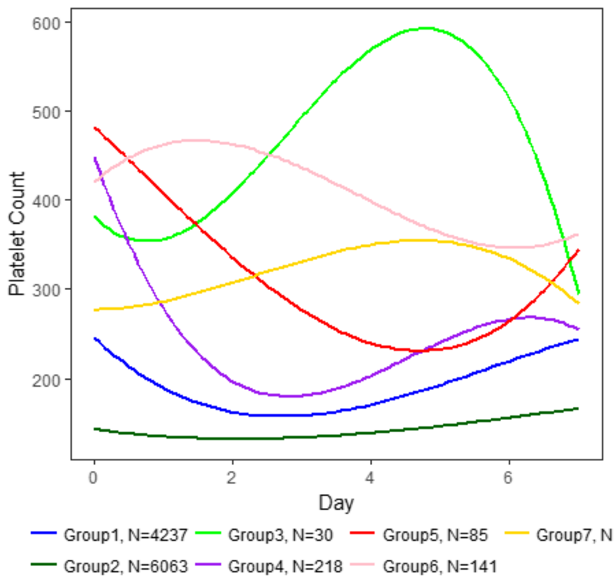

**Fig. 36 (abstract 0045).** Estimated mean trajectories of 7 groups

## Highlights of N&AHP research

### 0046

#### A sense of agency: an ethnographic exploration of being awake during mechanical ventilation in the intensive care unit

E. Laerkner[1], I. Egerod[2], F. Olesen[3], H.P. Hansen[4]
[1]Odense University Hospital, University of Southern Denmark, Odense C, Denmark; [2]University of Copenhagen, Rigshospitalet, Intensive Care Unit, Copenhagen, Denmark; [3]University of Aarhus, Department of Aesthetics and Communication - Information Studies, Aarhus, Denmark; [4]University of Southern Denmark, Odense, Denmark
**Correspondence:** E. Laerkner
*Intensive Care Medicine Experimental* 2017, **5(Suppl 2):**0046

**INTRODUCTION.** There is a current trend towards lighter or no sedation of mechanically ventilated patients in the intensive care unit (ICU). A study has shown advantages of no sedation such as shorter duration of mechanical ventilation and shorter ICU and hospital length of stay (1). Non-sedated patients are more awake during mechanical ventilation (2), but little is known about how this is experienced by the patient.

**OBJECTIVES.**
The aim was to explore patients' experiences of being awake during critical illness and mechanical ventilation in the ICU.

**METHODS.** Based on an ethnographic approach, 13 month of fieldwork following 28 awake, mechanically ventilated patients, in two ICU´s at a university hospital in Denmark where the no sedation strategy was implemented in practice. Participants were patients, who had been mechanically ventilated for at least 3 days and awake with a score of zero on the Richmond Agitation and Sedation Scale (RASS) for the majority of the time on mechanical ventilation. Twenty patients were interviewed during the week after ICU discharge, and 13 of these patients were interviewed again 2–4 month after ICU discharge. Interpretive description was used as an applied inductive approach, and data were analyzed using qualitative, thematic analysis.

**RESULTS.** Three themes were identified: "A sense of agency", "The familiar in the unfamiliar situation" and "Awareness of surrounding activities". Patients had the ability to interact from the first days of critical illness and sensed agency through initiating, directing and participating in communication and activities. Patients preferred competent and compassionate nurses who encountered them with respect. Patients appreciated nurses, who were attentive and involved them as individual persons, to feel encouraged and secure in the unfamiliar setting. Being critically ill and unable to speak with bodily weakness and in need of technological support, as well as being ignored by staff and involuntary witnessing other patients' suffering in the ICU, contributed to a feeling of powerlessness.

**CONCLUSIONS.** Being awake during mechanical ventilation entailed new opportunities and challenges for critically ill patients. Patients experienced being in an interface between agency and powerlessness as they had the ability to interact, yet were bound by bodily, relational and contextual factors. This knowledge is important to develop patient-centered nursing practice in the context of minimal sedation in the ICU.

**REFERENCE(S)**

1. Strøm, T et al. (2010) A protocol of no sedation for critically ill patients receiving mechanical ventilation: a randomised trial. Lancet (375) pp. 475–480.

2. Laerkner, E et al. (2016) No-sedation during mechanical ventilation: impact on patient's consciousness, nursing workload and costs. Nursing in Critical Care (21) pp. 28–35.

**GRANT ACKNOWLEDGMENT**

University of Southern Denmark, Odense University Hospital and Danish Nursing Research Society.

### 0047

### Nursing evaluation of comfort in acute hypoxemic respiratory failure patients with high flow nasal cannula

F. Binda[1], A. Galazzi[1], L. Masciopinto[1], I. Adamini[1], B. Cambiaghi[2], G. Grasselli[1], T. Mauri[1], A. Pesenti[1]

[1]Fondazione IRCCS Ca' Granda Ospedale Maggiore Policlinico, Università degli Studi di Milano, Milano, Italy; [2]ASST Monza, Università degli Studi Milano-Bicocca, Monza, Italy

**Correspondence:** F. Binda

*Intensive Care Medicine Experimental* 2017, **5(Suppl 2):**0047

**INTRODUCTION.** Comfort is a key feature for adherence to any type of oxygen therapy and is considered an outcome of nursing treatments[1]. In the last few years an increasing number of acute hypoxemic respiratory failure (AHRF) patients is undergoing oxygen therapy with high flow nasal cannula (HFNC)[2]. In the literature there aren't indications about flow and temperature best level to optimize the patient's comfort.

**OBJECTIVES.** To assess the comfort of patients, with PaO2/FiO2 < 300 mmHg, undergoing oxygen therapy with HFNC at different flows and temperatures.

**METHODS.** A prospective, randomized, cross-over study was conducted on 40 AHRF patients, admitted to intensive care unit (ICU) and receiving respiratory support by HFNC per clinical indication. Two flows (HFNC 60 l/min and 30 l/min) and 2 temperatures (31°C and 37°C) were randomly applied for 15 minutes (four steps per patient). We investigated during the last minutes of each step: comfort by numerical scale from 0 (extreme discomfort) to 5 (very comfortable), dyspnea by modified Borg scale form 0 (none) to 10 (unbearable), physiologic respiratory parameters. Clinical $FiO_2$ was left unchanged during all steps. Data of each step were compared using linear mixed-effects model analysis for repeated measures with Bonferroni or Tukey post-hoc test.

**RESULTS.** Forty patients (16 females), with age 57 ± 15 years-old, suffering from different types of AHRF (SAPS II 36.4 ± 9.4) were been studied. At enrolment patients had $PaO_2/FiO_2$ 218 ± 60 mmHg, $SpO_2$ was 97 ± 2% with $FiO_2$ 45 ± 10%. The main study results are reported in the table. The patient comfort level is higher at a lower temperature (31° C). The same temperature with the highest flow (60 L/min) improves both the $SpO_2$ and the RR, increasing the $SpO_2/FiO_2$ ratio.

**CONCLUSIONS.** Oxygen therapy with HFNC at lower temperature improves the patient's comfort. Titration of HFNC setting to obtain best comfort might be relevant to exploit the clinical efficacy of the device.

**Table 16 (Abstract 0047).** Study results

| | HFNC 30 L/min 31°C | HFNC 60 L/min 31°C | HFNC 30 L/min 37°C | HFNC 60 L/min 37°C | P-value flow | P-value temp. | P-value interaction |
|---|---|---|---|---|---|---|---|
| Comfort scale | 4.1 ± 1.0 | 3.7 ± 1.3 | 2.4 ± 1.3 | 2.4 ± 1.7 | 0.335 | <0.001 | 0.086 |
| Borg scale | 3.2 ± 2.2 | 3.0 ± 2.3 | 3.5 ± 2.2 | 3.2 ± 2.0 | 0.509 | 0.237 | 0.867 |
| SpO2 (%) | 96 ± 3 | 98 ± 2 | 96 ± 3 | 97 ± 2 | <0.001 | 0.529 | 0.158 |
| RR (bpm) | 24 ± 8 | 22 ± 6 | 24 ± 8 | 23 ± 6 | 0.022 | 0.130 | 0.713 |
| SpO2/FiO2 | 223 ± 44 | 227 ± 43 | 224 ± 45 | 227 ± 43 | <0.001 | 0.377 | 0.124 |

### 0048

### Evaluating risk factors that may predict pulmonary complications in patients with chest wall trauma

T.J. Lea, D. McWilliams

Queen Elizabeth Hospital NHS FT, Birmingham, United Kingdom

**Correspondence:** T.J. Lea

*Intensive Care Medicine Experimental* 2017, **5(Suppl 2):**0048

**INTRODUCTION.** The development of pneumonia is a significant risk factor for mortality in patients with blunt chest wall trauma[1]. Clinical symptoms are not considered an accurate predictor of outcome following blunt chest wall trauma[3] and there is a lack of research to identify risk factors for delayed deterioration[2].

**OBJECTIVES.** To identify potential risk factors for development of pulmonary complications for patients following chest wall trauma.

**METHODS.** Data was collected from all patients admitted to a large multi trauma centre in the west midlands with ≥ 3 rib fractures between November 2015 and August 2016. Development of pulmonary complications was assessed using the Brooks Brunn tool and analysed for association with a number of potential risk factors identified from previous literature. Data was analysed using the fisher exact test.

**RESULTS.** 71 patients were admitted with ≥ 3 rib fractures during the study period, of which 33 developed pulmonary complications. ISS on admission was significantly higher (23.6 vs 18.2, p = 0.0160) in the group that developed pulmonary complications (See Table 17) Those patients who developed pulmonary complications were more likely to be ventilated (Table 18) and had significantly longer duration of mechanical ventilation (12 v 7 days, p = 0.028) and incidence of tracheostomy (52% vs 13%, p = 0.002).

**CONCLUSIONS.** Patients with three or more rib fractures with a high ISS score are most at risk of developing pulmonary complications. When pulmonary complications develop, patients are more likely to require invasive ventilation or NIV and are more likely to have a tracheostomy. This may aid therapists in prioritising therapy for patients most at risk in the future.

**REFERENCE(S)**

1. Battle CE, Hutchings H, Evans P A. Risk factors that predict mortality in patients with blunt chest wall trauma: A systematic review and meta-analysis. *Injury. Int.J.Care Injured* 2012; 43; 8–17

2. Dubinsky I, Low A. Non-life -threatening blunt chest trauma: appropriate investigation and treatment.Am J Emerg Med 1997; 15: 240–3

3. Athanassiadi K, Gerazounis M,Theakos N.Management of 150 flail chest injuries:analysis of risk factors affecting outcomes. Eur J Cardiothoracic Surg 2004;26:373–6.

**Table 17 (Abstract 0048).** Patient Demographics

| | Pulmonary complications (n=33) | No pulmonary complications (n=38) | |
|---|---|---|---|
| Age | 60.4 | 56.2 | |
| Male n (%) | 27 (82%) | 28 (74%) | 0.5704 |
| APACHE II | 14.5 | 12.0 | 0.09435 |
| ISS | 23.6 | 18.2 | <0.05 |
| Respiratory History | 6 (18%) | 6 (16%) | 1.000 |

**Table 18 (Abstract 0048).** Outcomes

| | PPC's (n=33) | No PPC´s (n=38) | |
|---|---|---|---|
| NIV | 9 (27%) | 2 (5%) | 0.007 |
| Ventilated | 24 (73%) | 11 (29%) | 0.0001 |
| Tracheostomy | 17 (52%) | 5 (13%) | 0.0002 |
| Ventilator days | 12 (36%) | 7 (18%) | 0.2845 |
| Died | 4 (12%) | 0 (0%) | 0.031 |

### 0049

#### Feasibility of the Electrolarynx for Enabling Communication in the CHrOnically Critically Ill (The EECCHO study)

L. Rose[1], C. Dale[1], L. Istanboulian[2], S. Silencieux[2], B.H. Cuthbertson[3], O. Smith[4], A.C.K.B. Amaral[3], J. Grey[3]
[1]University of Toronto, Toronto, Canada; [2]Michael Garron Hospital, Toronto, Canada; [3]Sunnybrook Health Sciences Centre, Toronto, Canada; [4]St Michael's Hospital, Toronto, Canada
**Correspondence:** L. Rose
*Intensive Care Medicine Experimental* 2017, **5(Suppl 2):**0049

**INTRODUCTION.** Mechanically ventilated patients experience speech incapacity due to the need for an artificial airway. Speech incapacity has a particularly profound impact for chronically critically ill patients, who generally are conscious and receiving minimal or no sedation. The Electrolarynx has a vibrating head that serves as a sound source which when transmitted through soft tissues of the oropharynx adds sound to silent articulated speech. It has been shown to facilitate speech in case reports and feasibility studies, however there are limited data on speech intelligibility and comprehensibility.

**OBJECTIVES.** To assess the proportion of tracheostomized patients able to produce intelligible and comprehensible speech using the Electrolarynx. Secondary objectives were to measure ease of, and satisfaction with, communication before and after Electrolarynx training and anxiety.

**METHODS.** We included tracheostomized adults unable to tolerate cuff deflation who were able to follow commands, read English, and capable of mouthing words. We excluded patients with pre-existing hearing/speech impairment and with dementia. On study enrolment we measured baseline anxiety (Faces Anxiety Scale), Ease of Communication Scale and communication satisfaction (5-point Likert). The research team gave Electrolarynx instructions and daily practice for 5–15 minutes for 5 days. After training, 2 independent raters (1 facing, 1 facing away) assessed speech intelligibility using 50 words from the Assessment of Intelligibility of Dysarthric Speech (AIDS) tool. Intelligible speech was defined as ≥50% of words identified correctly. We used 5 X 5 word sentences from the AIDS and rated comprehensibility using a 9-point difficulty scale (9 = extreme difficulty). Testing was repeated on a subsequent day. We recorded the Electrolarynx Effectiveness Score (EES) and re-evaluated anxiety, ease of, and satisfaction with, communication.

**RESULTS.** From Jan 2015 to Dec 2016, we recruited 24 participants from a weaning centre and 2 ICUs. Most (63%) were male and admitted for medical reasons (58%); mean age was 62 years. Mean (SD) intelligibility score was 45% (17%) correct overall; 57% (21%) facing, 32% (19%) facing away. When rated facing, 68% of participants scored ≥50% correct. Mean (SD) comprehension difficulty was 6.4 (2.0) overall; 5.5 (2.5) facing, 7.3 (1.7) facing away. Mean (SD) EES was 2.9 (1.0) overall; 3.2 (1.2) facing, 2.9 (0.9) facing away (3 = improved lip-reading by producing recognizable sounds). Median anxiety score decreased from 3.8 to 2.0 (P = .007). Communication was rated easier (median score 15 to 12, P = .044) whereas satisfaction remained similar (median score 2 at both time-points; P = .059).

**CONCLUSIONS.** The Electrolarynx appears feasible for aiding intelligible speech in some patients when able to visualize the patient. Preliminary data suggest it may reduce anxiety and improve patient perception of communication ease.

**GRANT ACKNOWLEDGMENT**
Michael Garron Hospital/STTI

### 0050

#### Physiotherapist prediction of extubation outcome in the adult intensive care unit

G. Cork[1,2], L. Osman[2], H. Shannon[1]
[1]University College London, London, United Kingdom; [2]Guys and St Thomas' Hospital, London, United Kingdom
**Correspondence:** G. Cork
*Intensive Care Medicine Experimental* 2017, **5(Suppl 2):**0050

**INTRODUCTION.** Timing of extubation is critically important in the intensive care unit (ICU). Sub-optimal extubation timing may increase patient mortality, cost and ICU length of stay[1]. Factors such as cough strength, sputum abundance, respiratory muscle strength and work of breathing may contribute to the prediction of extubation outcome.[2] Physiotherapists play an important role in peri-extubation care so are well-placed to assess these factors. Accurate prediction of extubation outcome could help to inform both physiotherapy and medical management plans pre- and post-extubation.

**OBJECTIVES.** To report the accuracy of physiotherapists' prediction of extubation outcome in the adult ICU.

**METHODS.** Ethics approval was waived as the project was locally registered as a service evaluation.

A retrospective case note review was undertaken. All patients who were extubated during a three month period in the adult ICU of a large teaching hospital in the United Kingdom were included. Physiotherapy assessment including stratification of 'risk of extubation failure' as 'high', 'moderate' or 'low' was undertaken prior to extubation. Logistic regression analysis was performed to determine which pre-extubation factors were predictive of extubation outcome.

**RESULTS.** During the data collection period, 119 patients were extubated, 68(57%) following a physiotherapy assessment. Physiotherapy stratification as 'high risk' (OR 4 (95% CI 1.3 to 12); P = 0.009) and appropriate neurology (OR 0.3 (95% CI 0.09 to 0.96); P = 0.037) were the only pre-extubation factors significantly associated with extubation failure. Specialised physiotherapists predicted extubation failure with greater sensitivity but lower specificity compared with non-specialised physiotherapists and the logistic regression model (Table 19).

**CONCLUSIONS.** Patients classified as 'high risk' of extubation failure by a physiotherapist are significantly more likely to fail extubation. Specialised physiotherapists should be involved in the decision to extubate in the adult ICU. Further research is required to determine the optimal medical and physiotherapy management strategies for these high risk patients.

**Table 19 (Abstract 0050).** Prediction of Extubation Failure

| Predictor | Sensitivity | Specificity | Positive Predictive Value | Negative Predictive Value | Accuracy |
|---|---|---|---|---|---|
| All Physiotherapists | 40% (24-54) | 86% (79-92) | 56% (34-75) | 76% (70-82) | 72% (62-80) |
| Specialised Physiotherapists | 100% (57-100) | 68% (55-68) | 50% (28-50) | 100% (80-100) | 76% (55-76) |
| Non-specialised Physiotherapists | 22% (8-31) | 95% (88-99) | 67% (25-94) | 72% (67-75) | 71% (62-77) |
| Logistic Regression Model | 28% (15-34) | 96% (91-99) | 78% (42-96) | 75% (70-77) | 75% (67-79) |

Values are percentage (95% confidence interval)

## Poster Corner Sessions Monday, 25 September 2017

### Pneumonia: Basic and clinical research

#### 0051

#### Aerosol delivery of Aztreonam lysine (AZLI): *In vitro* comparison of two vibrating-mesh nebulizer during adult mechanical ventilation (MV)

A. Rodriguez[1], M. Cabrera[2], M. Bodi[1], S. Trefler[1], L. Canadell[3], L.F. Reyes[4,5], S. Ehrmann[2,6], I. Martín-Loeches[7], M.I. Restrepo[4,5], L. Vecellio[2]

[1]Hospital Universitario Joan XXIII/URV/IISPV/CIBERES, Critical Care, Tarragona, Spain; [2]Faculte de Medecine Bâtiment M, Centre d'Etude des Pathologies Respiratoires INSERM U 1100 Equipe 3. Aerosoltherapie et Biomedicaments a Visee Respiratoire, Tours Cedex, France; [3]Hospital Universitario Joan XXIII/URV/IISPV/CIBERES, Pharmacology, Tarragona, Spain; [4]University of Texas Health Science Center at San Antonio, Pulmonary Disease and Critical Care Medicine, San Antonio, United States; [5]South Texas Veterans Health Care System, Pulmonary Disease and Critical Care Medicine, San Antonio, United States; [6]Centre Hospitalier Regional et Universitaire de Tours, Medicine Intensive Reanimation, Tours Cedex, France; [7]St James'sUniversity Hospital - Trinity Centre for Health Sciences, MICRO- Anaesthesia and Critical Care, Dublin, Ireland

**Correspondence:** A. Rodriguez

*Intensive Care Medicine Experimental* 2017, **5(Suppl 2):**0051

**INTRODUCTION.** While aminoglycosides and colistin aerosolization might achieve better diffusion into the alveolar compartment than intravenous use, the information with other antibiotics is scarce.

**OBJECTIVES.** To evaluate *in vitro* performance of aerosol delivery of Aztreonam lysine (AZLI) during adult MV using two vibrating-mesh nebulizers: Aeroneb Solo® and M-neb® with and without Combihaler ® spacer.

**METHODS.** In the model (1) we used a test lung with a ventilator (Evita XL, Dräger) with following setting: a tidal volume of 450mL, a respiratory rate of 15/min, a PEEP of 5 $cmH_2O$ and, a flow rate of 40 L/min. The bench model was designed to reproduce real life clinical practice: a 7.5 mm endotracheal tube (ET) and a right-angle elbow adapter were inserted between the Y-piece and the test lung. The circuit was heated and humidified until the temperature was stable at 37°C. Four settings were tested when configuring the nebulizer in the circuit of the MV: a) Aeroneb solo® with a t-piece; b) Aeroneb solo® with the Combihaler® spacer; c) M-Neb® with a t-piece and d) M-Neb® with the Combihaler®. Three experiments were performed for each condition. Both nebulizers were placed before the Y-piece in the inspiratory limb and loaded with 150 mg of ALZI in 2 ml of diluents. The AZLI collected on the filter (inhalable mass) placed after the ET was quantified by spectrophotometry. The performance of nebulization was evaluated by: 1) Mass median aerodynamic diameter (MMAD); 2) Geometric standard deviation (GSD), 3) Fine particle dose (FPD), i.e. drug contained in particles smaller than 5 µm and considered to reach the lung, 3) Inhalable mass, i.e. fraction of total particles which is inhaled through the nebulizer, 4) Recovery rate (RR), i.e. percentage of AZLI mass placed in the nebulizer recovered on the filter.

**RESULTS.** In vitro aerosol delivery of AZLI during MV showed an excellent performance with both nebulizers tested, with a MMAD between 2.4-2.5 µm and 87% of particles smaller than 5 µm.Nebulizer aerosol delivery (assessed by FPD and inhalable mass) and RR was higher (50% and 70% respectively) for both nebulizers with the use of Combihaler® spacer (Table 20). *In vitro* aerosol delivery performance was similar for both nebulizers used.

**CONCLUSIONS.** Both Aeroneb Solo® as M-Neb® showed an excellent aerosol delivery profile for Aztreonam lysine during in vitro mechanical ventilation. A better aerosol delivery performance was obtained using the Combihaler ® spacer.

**REFERENCE(S)**

Boukhettala N, Poreé T, Diot P, Vecellio L. In Vitro Performance of Spacers for Aerosol Delivery during Adult Mechanical Ventilation. Journal of Aerosol Medicine and Pulmonary Drug Delivery 2015 ; 28: 130–136

**GRANT ACKNOWLEDGMENT**

Gilead Sciences

**Table 20 (Abstract 0051).** See text for description

| Variable | Aeroneb Solo® with a T-piece | Aeroneb Solo ® with the Combihaler ® | M-Neb® with a T-piece | M-Neb® with the Combihaler® |
|---|---|---|---|---|
| MMAD (µm)Mean (SD) | 2.4 (0.0) | 2.5 (0.1) | 2.4 (0.1) | 2.4 (0.2) |
| GSD, Mean (SD) | 1.9 (0.1) | 2.1 (0.3) | 2.0 (0.1) | 1.7 (0.1) |
| Inhalable mass (mg), Mean (SD) | 44.8 (20.2) | 68.6 (6.6) | 36.1 (17.4) | 60.7 (7.5) |
| FPD (<5% µm), Mean (SD) | 38.8 (17.8) | 59.8 (5.9) | 31.7 (14.8) | 52.6 (7.9) |
| Recovery rate (%), Mean (SD) | 30 (13) | 46 (4) | 24 (12) | 40 (5) |
| Duration (min), Maen (SD) | 4.59 (0.3) | 4.57 (0.3) | 4.22 (1.0) | 4.43 (1.1) |

#### 0052

#### The clinical characteristics and prognostic risk factors of healthcare-associated pneumonia in a Korean Tertiary Teaching Hospital

E.Y. Choi[1], J.H. Ahn[2]

[1]Yeungnam University Hospital, Department of Medical Science, Major in Internal Medicine, Daegu, Korea, Republic of; [2]Yeungnam University Hospital, Deagu, Korea, Republic of

**Correspondence:** E.Y. Choi

*Intensive Care Medicine Experimental* 2017, **5(Suppl 2):**0052

**Objectives.** Previous studies have shown that healthcare-associated pneumonia (HCAP) revealed worse clinical outcomes than community-acquired pneumonia (CAP). We investigated clinical features of patients with HCAP and identified the risk factors for HCAP mortality.

**Methods.** We conducted a retrospective, observational study of pneumonia patients who were hospitalized at a tertiary teaching hospital from March 2012 to February 2014. Identified pathogens that were not susceptible to β-lactams, macrolides and fluoroquinolones were defined as community-acquired pneumonia-drug resistant pathogens (CAP-DRPs). Primary endpoint was 28-day mortality.

**Results.** Of the 1046 patients, 399 were classified as HCAP and 647 as CAP. HCAP patients were older and had more comorbidities than CAP patients. Initial pneumonia severity index (PSI) was higher in patients with HCAP than with CAP. HCAP was associated with not only an increased rate of CAP-DRPs (HCAP, 57.5%; CAP, 22.6%; $P < 0.001$), but also increased rate of inappropriate initial antibiotic therapy (IIAT) (HCAP, 43.8%; CAP, 17.2%; $P < 0.001$). HCAP was also associated with increased 28-day mortality rate (HCAP, 14.5%; CAP, 6.3%; $P < 0.001$). In multivariable analysis, PSI was an independent risk factor of 28-day mortality in HCAP patients (OR 1.02, 95% CI 1.01-1.04). CAP-DRPs and IIAT were not associated with mortality.
**Conclusions.** Patients with HCAP revealed higher rate of CAP-DRPs, IIAT and mortality than patients with CAP. But, CAP-DRPs and IIAT were not associated with mortality. PSI was the main predicting factor for 28-day mortality in patients with HCAP.

### 0053

#### The association between implementation of "Bundles of Care" and possible ventilator associated pneumonia (pVAP) among mechanically ventilated patients in the intensive care unit: a quasi-experimental study

A.F.S. Abarquez[1], M.C. Orden[2], P.A. Estrella[1], M. Alejandria[3], E.V. Reside[2]
[1]The Medical City, Internal Medicine, Pasig, Philippines; [2]The Medical City, Pasig, Philippines; [3]The Medical City, Philippine General Hospital, Pasig, Philippines
**Correspondence:** A.F.S. Abarquez
*Intensive Care Medicine Experimental* 2017, **5(Suppl 2):**0053

**INTRODUCTION.** Ventilator Associated Pneumonia (VAP) is a serious medical condition causing significant morbidity and mortality among mechanically ventilated patients. The "VAP Bundles of Care" is a process improvement program designed to lessen the incidence of VAP.
**OBJECTIVES.** This study aims to determine the impact of implementation of "VAP Bundles of Care" program for reducing episodes of Possible Ventilator Associated Pneumonia (pVAP) in The Medical City Adult Intensive Care Unit (TMC-ICU) by comparing pVAP rates pre and post intervention. This study also aims to determine the association between the implementation of "Bundles of Care" with secondary outcomes of ventilator days, length of ICU stay, length of hospital stay among intubated adult patients at the TMC-ICU from January 2008-December 2015.
**METHODS.** A Quasi-experimental pretest/post-test research design with records review was done to compare pre-intervention VAP bundle pVAP rates with post intervention pVAP rates.
**RESULTS.** A total of 235 patients were included; 124 patients did not receive "VAP Bundles of Care" while 111 received "VAP Bundles of Care". The odds of acquiring pVAP among intubated patients is 0.23 lower when "Bundles of Care" was implemented. This translates to 4.35 higher pVAP rate without the "Bundles of Care" (n = 1/0.23). This association is significant with a p-value of < 0.001. There is no significant difference in secondary outcomes between the two groups.
**CONCLUSIONS.** This study showed that implementation of the "Bundles of Care" program for VAP reduced the episodes of pVAP in the TMC Intensive Care Unit. However it was noted that there was no significant difference between secondary outcomes of length of hospitalization, length of ICU stay, and duration of intubation before and after implementation of the "Bundles of Care".
**REFERENCE(S)**
1) American Thoracic Society Guidelines for the Management of Adults with Hospital-acquired, Ventilator-acquired and Healthcare-Associated Pneumonia, 2004
2) The Medical City- Hospital Infection Control and Epidemiology Center 2008–2015 Hospital Antibiogram
3) International Nosocomial Infection Control Consortium (INICC) Bundle to Prevent Ventilator-Associated Pneumonia (VAP) in ICU: An International Perspective 2013
4) Center for Disease Control. National Health Safety Network: Ventilator Associated Pneumonia Events. January 2016
5) Bird, et al. Adherence to Ventilator-Associated Pneumonia Bundle and Incidence of Ventilator-Associated Pneumonia in the Surgical Intensive Care Unit. Arch Surg. 2010;145(5):465–470.
6) International Nosocomial Infection Control Consortium (INICC) findings. Device-associated Infections rates in Adult, Pediatric and Neonatal Intensive Care Units of Hospitals in the Philippines. 2011.
7) The Medical City Hospital Infection Control and Epidemiology Center "Bundles of Care" Protocol

### 0054

#### Ventilator associated pneumonia rate in an anesthesiology and reanimation intensive care unit in Turkey: ten-year surveillance results

Ç.A. Hatipoğlu[1], Ç. Kaymak[1], F.Ş. Erdinç[1], N. Özcan[1], C. Bulut[2], A. Özcan[1], G.T. Ertem[1], H. Başar[1]
[1]University of Health Science, Ankara Training and Research Hospital, Ankara, Turkey; [2]University of Kırıkkale, Kırıkkale, Turkey
**Correspondence:** A. Özcan
*Intensive Care Medicine Experimental* 2017, **5(Suppl 2):**0054

**INTRODUCTION.** Ventilator associated pneumonia (VAP) is one of the most important device-associated hospital infections with high morbidity and mortality, prolonged length of stay and increased cost.
**OBJECTIVES.** The aim of this study was to evaluate the VAP rate, ventilator use rate and causative microorganisms isolated in VAP cases in a ten year study period.
**METHODS.** This study was conducted in the 12-bed anesthesiology and reanimation intensive care unit (AR-ICU) between 2007 and 2016. VAP rate per 1000 mechanical ventilator-days, device utilization ratio for mechanical ventilator and pathogens isolated in patients with VAP were evaluated retrospectively. Hospital infection definitions made according to the CDC criteria. Since 2011, we use a "Ventilator Bundle Checklist" including elevation of the head of the bed to 30–45 degrees, daily oral care with chlorhexidine, peptic ulcer prophylaxis, deep venous thrombosis prophylaxis and use of aseptic aspiration technique when suctioning endotracheal secretions in the AR-ICU to prevent ventilator associated pneumonia.
**RESULTS.** During the study period totally 4224 patients were followed in the AR-ICU for 35701 bed days. In 2007, the VAP rate was 23.1 per 1,000 mechanical ventilator days and the ventilator use rate was 0,54. Although the increased ventilator use, VAP rate was decreased significantly in recent years. The last VAP rate was 9,3 per 1,000 mechanical ventilator days and the ventilator use was 0,63, in 2016. *Acinetobacter baumannii* was the most commonly isolated microorganism as VAP pathogen at all years (rate: 42,9%-60,4%) except 2016. The most commonly pathogen of VAP was *Pseudomonas aeruginosa* in 2016 (52%) whereas the rate of *A. baumannii* was (25%).
**CONCLUSIONS.** In AR-ICU, VAP rate decreased in recent years although high ventilator usage. We think that the reason may be the adherence to VAP bundle and other infection control measures. Causative agent of VAP was *A. baumannii* except the last year.
**REFERENCES**
Magill SS., et al. Am J Crit Care 2013;22(6):469–73.
**GRANT ACKNOWLEDGMENT**
None

### 0055

#### Ventilator-associated pneumonia and ventilator-associated tracheobronchitis - what´s really the difference?

L. Sousa[1], A. Araújo[2], J. Pires[2], D. Carvalho[3], R. Araújo[3]
[1]Centro Hospitalar de Leiria, Leiria, Portugal; [2]Centro Hospitalar do Baixo Vouga, Aveiro, Portugal; [3]Hospital Pedro Hispano, Matosinhos, Portugal
**Correspondence:** L. Sousa
*Intensive Care Medicine Experimental* 2017, **5(Suppl 2):**0055

**INTRODUCTION.** Lower respiratory tract infections (RI) in intubated patients include ventilator-associated tracheobronchitis (VAT) and ventilator-associated pneumonia (VAP). The use of antibiotics is recommended in VAP, however the same is not truth in VAT. Studies demonstrated that antibiotic therapy for VAT may shorten the duration of mechanical ventilation, but it is uncertain whether it improves other clinical outcomes. The clinical distinction between these two entities is difficult.
**OBJECTIVES.** Our aim is to compare patients diagnosed with VAP and VAT in an intensive care unit (ICU) through a 4-years period; to access their differences and similarities as a way to conclude if there are any indicators that can support different approaches.

**METHODS.** Retrospective analysis using data from all patients diagnosed with RI from 2012 to 2015, in an ICU. A univariate analysis was performed.
**RESULTS.** 84 patients were included, with a mean age of 60,9 ± 14,9 years. Among these 58,3% were diagnosed with VAT. Patients with VAP had longer hospitalization (plus 51days) and ICU stay (plus 5.8days). Higher mortality rates (22.9%vs14.3%), an increased length of invasive mechanical ventilation (plus 6days) and antibiotic therapy (plus 3days), and lower paO2/FiO2 ratios were also observed in this group. In both groups, Pseudomonas aeruginosa was the most frequently isolated agent. All patients in both groups were treated with antibiotics. Similar results were found in both groups for the following variables: c-reactive protein levels, SAPS II and APACHE II scores, as well as SOFA score and temperature at diagnosis. It is relevant to state that only 26.5% of the patients in the VAT group had pneumonia excluded by computed tomography (CT), whether 48.6% of VAP patients had their diagnosis confirmed by CT. The only results with statistical difference ($p < 0.05$) were the hospitalization and antibiotic therapy lengths, and paO2/FiO2 ratios.
**CONCLUSIONS.** As expected our results showed worst outcomes in VAP, however there was no control group to establish if a different approach to VAT would lead to a different outcome and, likewise, most of the results were not statistically significant. Further studies are needed to ascertain the usefulness of different approaches in diagnosis, management and treatment of VAT and VAP; in addition, they should determine if the treatment of RI should be guided most by clinical features or by the differential diagnosis between VAT and VAP. A prospective study analysing 3 separate groups: VAP, VAT with antibiotics and VAT without antibiotics should be performed. It is recommended an independent analysis of several clinical variables that can influence the outcomes with a higher impact than the differential diagnosis of VAT/VAP. A bigger sample is needed to achieve significance in results. We further propose the use of CT in all patients diagnosed with VAT, if clinically possible, as to better prevent the presence of VAP in the VAT group.

### 0056

#### Ventilator associated pneumonia and ventilator associated tracheobronchitis. Are they different pathologies?

A. Ramírez Rodríguez, H. Rodríguez Pérez, L. Ugalde Jáuregui, A. Casamitjana Ortega, E. Hernández Medina, Á. Villanueva Ortiz
Hospital Universitario Insular de GC, Las Palmas de Gran Canaria, Spain
**Correspondence:** H. Rodríguez Pérez
*Intensive Care Medicine Experimental* 2017, **5(Suppl 2):**0056

**INTRODUCTION.** Ventilator associated pneumonia (VAP) and ventilador associated tracheobronchitis (VAT) are two common pathologies in the intensive care units (ICU). Their early diagnosis, differentiation between both entities and correct treatment helps reduce the morbidity and mortality of affected patients.
**OBJECTIVES.** To explore if in our ICU there are differentiating characteristics between VAP and VAT.
**METHODS.** It is a prospective, observational study during the year 2016, carried out in a 24 bed polyvalent ICU of a third level hospital. Patients with more than 24 hours of admission were included. We took into account the diagnosis of infection according to CDC criteria. The data was collected in the ENVIN-HELLICS database. A description of the main variables included in the study was made. For the qualitative variables, the relative frequency, absolute frequency and percentages were determined. For the quantitative variables, the mean, median, standard deviation and variance were calculated.
**RESULTS.** A total of 752 patients were studied, with mean age of 58 years, mostly male (65%), with an APACHE II score of 12. Most of them were patients with medical diseases (46,41%) followed by coronary heart disease (39,63%). The most frequent risk factors were urinary catheterization (48,94%), followed by the use of a central venous catheter (44%) and artifical airway path (37,63%). The mean length of stay was 9,57 days. Most of the patients included in our study came from their home (67%), followed by a hospitalized stay (28%). The most frequent infections were the VAP (39,73%) followed by the VAT (27,40%). The microorganism most frequently found was Klebsiella pneumoniae (29,23%) followed by Pseudomona aeruginosa (14,62%), both globally as for both type of respiratory infections. The rate for VAP was 5,97 VAPs for 1000 days of mechanical ventilation, as for 4,11 VATs for 1000 days of mechanical ventilation.
**CONCLUSIONS.** In our ICU we do not find differentiating characteristics between both pathologies. It is curious that patients with VAT have more days of mechanical ventilation until the development of the infection, and they do die more; but with no statistically significant differences possibly due to the few number of cases compared.
**REFERENCES**
1. Traqueobronquitis asociada a ventilación mecánica: incidencia, pronóstico y relación con la neumonía. Ramón Díaz-Alersi Hospital U. Puerto Real, Cádiz. REMI, http://medicina-intensiva.com. Octubre 2015.
2. Frequency, prevention, outcome and treatment of ventilator-associated tracheobronchitis: Systematic review and meta-analysis Michalis Agrafiotis a, Ilias I. Siempos a, Matthew E. Falagas. Respiratory Medicine (2010) 104, 325e336.

**Table 21 (Abstract 0056).** Results

| | VAP | VAT | |
|---|---|---|---|
| Cases | 29 | 20 | |
| Length of mechanical ventilation until infection (mean of days) | 12.62 | 16.45 | p=0.72 |
| Age (mean) | 59 | 64 | p=0.28 |
| APACHE II Score | 12.35 | 10.35 | p=0.40 |
| Mortality | 33.30% | 66.70% | p=0.21 |
| Male | 60.60% | 39.40% | p=0.63 |
| Immunosuppression | 85.70% | 14.30% | p=0.11 |

### 0057

#### Diagnostic and prognostic values of serum C-reactive protein, procalsitonin, soluble urokinase plasminogen activator receptor and neopterin levels in hospitalized patients in intensive care unit with ventilator associated pneumonia

R. Öztürk[1], H. Başar[2], V. Fidancı[2], S. Cesur[2], A. Özcan[2], N. Tülek[2], Ç. Kaymak[2], L. Şanal[3], E. Aktepe[2], A.P. Demiröz[2]
[1]University of Health Science, Keçiören Training and Research Hospital, Ankara, Turkey; [2]University of Health Science, Ankara Training and Research Hospital, Ankara, Turkey; [3]University of Health Science, High Specialization Training and Research Hospital, Ankara, Turkey
**Correspondence:** Ç. Kaymak
*Intensive Care Medicine Experimental* 2017, **5(Suppl 2):**0057

**INTRODUCTION.** Soluble urokinase plasminogen activator receptor (suPAR) and Neopterin are expressed systemic inflammation on peripheral blood mononuclear cells and neutrophils. In this study was evaluated to diagnostic parameters in patients with ventilator-associated pneumonia (VAP).
**OBJECTIVES.** The aim of this study was to investigate the diagnostic and prognostic values of serum C-Reactive Protein (CRP), Procalcitonin (PCT), suPAR, and Neopterin levels in the patients with VAP.
**METHODS.** Thirty eight patients diagnosed as VAP in intensive care unit and 40 patients without VAP in intensive care unit were enrolled the study. APACHE Acute Physiology and Chronic Health Evaluation (APACHE) II, Sequential Organ Failure Assessment Score (SOFA), Clinical Pulmonary Infection Scores (CPISm) of the patients were calculated at admission and daily PEEP account were measured. The serum levels of CRP, PCT, suPAR, and Neopterin were measured once in controls and were measured prior to antimicrobial treatment and on days 3, 5, and the end of treatment in VAP group. The results were compared between the groups. Subgroup analysis of the patients with VAP were performed, scores and levels of biomarkers were compared between the mortality and non-mortality group. Correlation between the levels of biomarkers and scores of the patients were investigated.

**RESULTS.** CRP, PCT, Neopterin, and suPAR levels were significantly higher at all timely measures (except suPAR values at the end of treatment) in VAP groups than those of controls (p < 0.005). While CRP and PCT values were significantly higher in mortality group on the day prior to treatment and days 3, 5 than those of non-mortality group, suPAR and Neopterin levels were higher on the days 3, 5. APACHE II and SOFA values were also higher in mortality group. CRP values > 14.5 ng/mL prior to the treatment and PCT levels > 1.23 ng/mL at day 5 were found as 100% specific for mortality in VAP group.
**CONCLUSIONS.** C-reactive protein, PCT, Neopterin, and suPAR values may be helpful for early diagnosis of VAP in intensive care patients. High CRP and PCT levels as well as high APACHE II and SOFA scores may have prognostic value for follow-up of the patients with VAP.
**REFERENCE**
Savva A., et al. J Infect. 2011; 63 (5) : 344–50.
**GRANT ACKNOWLEDGMENT**
This study supported fund from The Scientific And Technological Research Council Of Turkey (215S050 / 2015).

### 0058

#### Ventilator associated pneumonia: a cheshire & mersey critical care network pilot audit

D. Murray[1], A. Hall[1], K. Sim[2]
[1]Royal Liverpool University Hospital, Intensive Care Medicine, Liverpool, United Kingdom; [2]St Helen's & Knowsley Hospital, Intensive Care Medicine, Liverpool, United Kingdom
**Correspondence:** D. Murray
*Intensive Care Medicine Experimental* 2017, **5(Suppl 2):**0058

**INTRODUCTION.** Ventilator associated pneumonia (VAP) is an important Intensive Care Unit (ICU) associated infection. VAP is associated with increased mortality, length of stay & cost. ICUs should have standardised systems in place to monitor VAP rates[1]. Currently there is no unifying definition of VAP & many of the current definitions in use are complex. Finding an optimal definition remains controversial. Consequently, data collection between ICUs has been hampered by inconsistent definitions & methods of data collection.
**OBJECTIVES.**
1. Create a simple definition of VAP to standardize data collection
2. Design a daily data collection sheet
3. Carry out a pilot audit
**METHODS.** Our simplified VAP mnemonic based definition was based on the American Centre for Disease Control guidelines[2]. Inclusion criteria comprised a period of more than 48 hours of mechanical ventilation with stable or reducing $FiO_2$ & PEEP requirements. Exclusion criteria included non-infective causes for increased ventilator support &/or a current VAP.
A VAP was determined by:
Ventilator settings
An increase in $FiO_2$ OR PEEP to achieve designated targets for patient, sustained over a 24 hr period
***And***
*Associated features (**one** or more)*
· New WCC < 4.0 or >12.0 $x10^9$/L or increasing/decreasing from baseline
· New temperature change
· Positive microbiology consistent with VAP
***And***
*Pneumonia*
Evidence of new consolidation on CXR/CT (*Not required in **ARDS***)
We conducted a three month pilot audit at four ICUs across the Merseyside region (Whiston, Warrington, Leighton & Wirral). A data collection sheet was filled out each day. The data collection period was between 29/02/16 & 31/05/16 (93 days in total).
**RESULTS.** In total, we audited 1626 ventilator days, with 1314 ventilator days meeting the inclusion criteria.
**CONCLUSIONS.** In conclusion, we have standardised data collection for the diagnosis of VAP & have continued to use this tool across all ICUs in the Merseyside region. We found that our VAP rate was significantly lower that that quoted in the literature (20%)[3]. Although, the Scottish Intensive Care Society have recently quoted their VAP rate as 2.7/1000 invasive ventilator days[4]. This may be due to the patient population, an overestimation of VAP in the literature, improving clinical care or low sensitivity of our screening tool. However, if VAP rates are very low, this raises the questions as to whether measuring them is clinically beneficial. We are comparing our definition against other VAP scoring tools to assess validity.
**REFERENCE(S)**
1. https://www.ficm.ac.uk/standards-and-guidelines/gpics
2. https://www.cdc.gov/nhsn/pdfs/pscmanual/6pscvapcurrent.pdf
3. Chastre J, Fagon JY. Ventilator-associated pneumonia. *Am J Respir Crit Care Med*. 2002;165(7): 867–903
4. http://www.sicsag.scot.nhs.uk/docs/2016-08-09-SICSAG-report-web.pdf?25
**GRANT ACKNOWLEDGMENT**
Nil

**Table 22 (Abstract 0058).** Results

| ICU | VAP Rate |
|---|---|
| Warrington | 0.17% |
| Whiston | 0.76% |
| Wirral | 0.87% |
| Leighton | 0.94% |

### 0059

#### A study of ventilator associated pneumonia in a Tunisian medical ICU: incidence and risk factors

N. Fraj[1], M.A. Boujelbèn[1], N. Sma[1], K. Meddeb[1], J. Baroudi[1], E. Ennouri[1], W. Zarrougui[1], H. Kallel[1], A. Ben Abdelhafidh[1], I. Ben Saida[1], A. Azouzi[1], A. Khedher[1], J. Ayachi[1], I. Chouchene[1], M. Boussarsar[1,2], A. Ammar[3], A. Ben Cheikh[3], H. Ghali[3], O. Ezzi[3], M. Mahjoub[3], N. Bouafia[3], M. Njah[3], M. Marzouk[4], J. Boukadida[4]
[1]Farhat Hached University Hospital, Medical Intensive Care Unit, Sousse, Tunisia; [2]Ibn Al Jazzar Faculty of Medicine, Research Laboratory N° LR14ES05 Interactions of the Cardio-Pulmonary System, Sousse, Tunisia; [3]Farhat Hached University Hospital, Hospital Hygiene Departement, Sousse, Tunisia; [4]Farhat Hached University Hospital, Microbiology and Immunology Laboratory, Sousse, Tunisia
**Correspondence:** M. Boussarsar
*Intensive Care Medicine Experimental* 2017, **5(Suppl 2):**0059

**BACKGROUND.** Ventilator-associated pneumonia (VAP) is a serious concern for patients in medical ICU. The risks to patients are significant and the monetary costs are considerable. It remains a major cause of hospital morbidity and mortality despite recent advances in diagnosis and accuracy of management.
**OBJECTIVES.** To determine the rate and the risk factors of VAP in a Tunisian medical ICU.
**METHODS.** A prospective study was conducted in an 8-bed medical ICU during 18 months from September 15th, 2015 to march 15th,2017. All patients who remained in our ICU more than 48H were included. VAPs were diagnosed based on American Thoracic Society criteria. Risk factors were analyzed by conditional stepwise logistic regression. P values of less than 0.05 were regarded as statistically significant.
**RESULTS.** Among 258 included patients, 162(62.7%) were male with mean age 60 ± 18years. Acute respiratory failure was the main cause of admission 191(74%). The mean SAPS II score at admission was 31.7 ± 14. Mechanical ventilation was instituted in 223(87%) patients. 46(18%) patients developed VAP. VAP density incidence was at 14.5/1000. The most common microorganisms grown in the tracheal aspirates were: *Acinetobacter baumannii 22*(59.5%) and *Pseudomonas aeruginosa* 5(13.5%).
Univariate analysis showed that: NIV failure (p = 0.002), age (p = 0.009), Charlson comorbidities index (p = 0.033) and duration of sedation (p < 0.000) were associated to VAP. NIV as exclusive ventilatory support was considered a protective factor (p = 0.001).

Multivariate analysis identified the following independent risk factors for VAP : Duration of sedation (OR, 1.21 ; 95%CI, [1.06,1.37]; p = 0.003) and NIV failure (OR, 3.76 ; 95%CI, [1.07,13.26] ; p = 0.0039).
**CONCLUSION.** The present study demonstrated a high density-incidence of VAP in a Tunisian MICU. Duration of sedation and NIV failure were the only independently associated factors to VAP occurrence.

### 0060

#### Trends in ventilator associated pneumonia (VAP) rate in patients assisted with NIV for acute on chronic respiratory failure (AE/CRF) in a Tunisian medical ICU, 2000–2015

K. Meddeb[1], J. Ayachi[1], M.A. Boujelbèn[1], N. Fraj[1], J. Baroudi[1], H. Kallel[1], A. Ben Abdelhafidh[1], E. Ennouri[1], W. Zarrougui[1], N. Sma[1], A. Azouzi[1], I. Ben Saida[1], I. Chouchene[1], A. Khedher[1], M. Boussarsar[1,2]
[1]Farhat Hached University Hospital, Medical Intensive Care Unit, Sousse, Tunisia; [2]Ibn Al Jazzar Faculty of Medicine, Research Laboratory N° LR 14 ES 05 Interactions of the Cardio-Pulmonary System, Sousse, Tunisia
**Correspondence:** M. Boussarsar
*Intensive Care Medicine Experimental* 2017, **5(Suppl 2):**0060

**Background.** NIV is the first-line treatment for the management of (AE/CRF) in the intensive care unit. However, the impact of NIV use on nosocomial infections and mortality remains unclear.
**Aim.** To evaluate the effect of NIV use in patients admitted for AE/CRF on VAP and mortality.
**Methods.** Retrospective observational study conducted in an 8-bed Tunisian medical ICU during 16 years from January 2000 to December 2015. Were included all consecutive patients admitted for AE/CRF. Were assessed trends in density incidence of VAP and mortality rates.
**Result.** Among 4650 ICU admissions, 1245(26,7%) were admitted for AE/CRF. First line NIV was administered in 374(30%) patients. Figure 37 displays comparative VAP density incidence and mortality within 3 predefined periods.
**Conclusion.** The steady increase of NIV use did not have a significant impact on density incidence of VAP neither on mortality rate; showcasing the need for better management of NIV in the ICU where periods of organizational difficulties did not allow acquiring enough experience until the last five years.

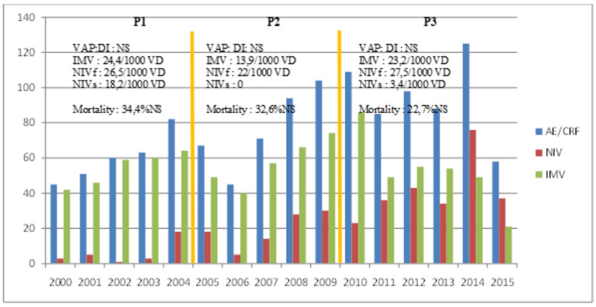

**Fig. 37 (abstract 0060).** Trends of VAP and mortality rates in patients having first line NIV. P1, period 1: 2000–2004; P2, period 2: 2005–2009; P3, period 3: 2010–2015; AE/CRF, acute exacerbation on chronic respiratory failure; VAP, ventilator associated pneumonia; NIV, non invasive ventilation; NIVs, NIV success; NIVf, NIV failure; IMV, Invasive Mechanical Ventilation; VD, Ventilator days

### 0061

#### Can we treat ICU-acquired respiratory infections like community-acquired infections?

M. Marques[1], C. Gonçalves[1], H. Castro[2], T. Cardoso[2], I. Aragão[2]
[1]Centro Hospitalar do Porto, Infectious Diseases, Porto, Portugal; [2]Centro Hospitalar do Porto, Intensive Care Unit (UCIP), Porto, Portugal
**Correspondence:** C. Gonçalves
*Intensive Care Medicine Experimental* 2017, **5(Suppl 2):**0061

**INTRODUCTION.** The unnecessary and/or prolonged use of broad-spectrum antibiotics is associated with the rapid emergence and dissemination of antibiotic-resistant microorganisms, therefore its use these should be restricted.
**AIM.** To analyze the microbiology profile of ICU-acquired respiratory infections in a mixed ICU and the possibility to use antibiotics recommended for community-acquired pneumonia (CAP) according to ICU length of stay.
**METHODS.** Retrospective study on all patients admitted from the community into a 12 bed mixed-ICU of a tertiary care university hospital for a minimum period of 48 hours, from February 2015 to July 2016 (18 months). Only microbiological documented ICU-acquired respiratory infection were included and categorized as sensitive to CAP antibiotic treatment recommendations or not. MDR was defined as a pathogen resistant to at least one antibiotic in three different classes to which it should be sensitive.
**RESULTS.** During the study period 286 patients were admitted to the ICU from the community for more than 48h. They had a mean ± SD age of 60 ± 16 years, 182 (64%) were male, 60% (n = 171) were medical admissions and 24% (n = 68) were trauma patients (medical or surgical admissions). Of these, 39 (14%) developed 46 episodes of ICU-acquired respiratory infections microbiological documented [8 tracheobronchitis (17%) and 38 pneumonias (83%)]. In this sample the rate of ventilator-associated infection was 12.1/1000 days of mechanical ventilation (MV) (10 pneumonias/1000 days of MV and 2.1 tracheobronchitis/1000 days of MV).
Up to three days of ICU admission there were 8 episodes of ICU-acquired (nosocomial) respiratory infection all sensitive to CAP guidelines (there was only one *Klebsiella pneumoniae* resistant to amoxicillin-clavulanate but sensitive to non-pseudomonal 3rd generation cephalosporins); the most frequent pathogen was *Haemophilus influenzae* (n = 4, 50%) and there was no infection by a MDR pathogen.
After day 4 there were 38 episodes of respiratory infection and the most frequent pathogens were: *Pseudomonas aeruginosa* (n = 11, 29%), *Klebsiella pneumoniae* (n = 6, 16%) and *Acinetobacter baumannii* (n = 4, 11%). In the period the proportion of adequate antibiotic therapy following CAP recommendations would fall to 50% or less. There were 11 (29%) caused by MDR pathogens.
**CONCLUSION.** In our unit ICU-acquired pneumonia can be treated as CAP up to 72 hours after ICU admission, in those patients directly admitted from the community setting, decreasing the use of unnecessary broad spectrum antibiotics.

### 0062

#### Ventilator-associated pneumonia (VAP) development in tracheostomized patients

E. Trujillo-Garcia[1], E. Curiel-Balsera[1], J.L. Muñoz-Muñoz[1], I. Bellido-Estévez[2], C.M. Gómez-Jiménez[1], M.J. Furones[1], M.C. Martínez-González[1], G. García-Quesada[1]
[1]H. R. U de Málaga, Intensive Care, Málaga, Spain; [2]Universidad de Málaga, Farmacología, Málaga, Spain
**Correspondence:** E. Trujillo-Garcia
*Intensive Care Medicine Experimental* 2017, **5(Suppl 2):**0062

**OBJECTIVES.** Analyzing VAP development in tracheostomized patients according to timing.
**MATERIAL AND METHODS.** Prospective and observational study where patients were followed up for 28 days or up to VAP development after being performed tracheostomy. Patients were divided into two groups: those who were performed tracheostomy within 7 days upon ICU admission (early group) and those performed tracheostomy afterwards (late group).
**RESULTS.** A total of 250 patients were analyzed. VAP global incidence was 23.4%. VAP incidence reached 13.6% and 30.3% in the early and late groups, respectively. No differences were observed regarding comorbidities, APACHE II-measured severity or the reason for admission between both groups. VAP direct impact was 9.8 and 14.54 in every 1000 days of MV in the early and late groups, respectively. Figure 38 shows differences between both groups in VAP development (Log Rank 0.003)

Multivariate Cox regression identified no other variables related to VAP development, except performance within the first 7 days: HR 0.42 IC95% (0.23-077), *p* = 0.005.

**CONCLUSIONS.** Early tracheostomy in critical patients is a protective factor against VAP development regardless of the patient´s reason for admission. It is since the third week after tracheostomy that VAP risks rocket in the late group relative to the early group. This is probably related to greater MV needs and more difficult weaning.

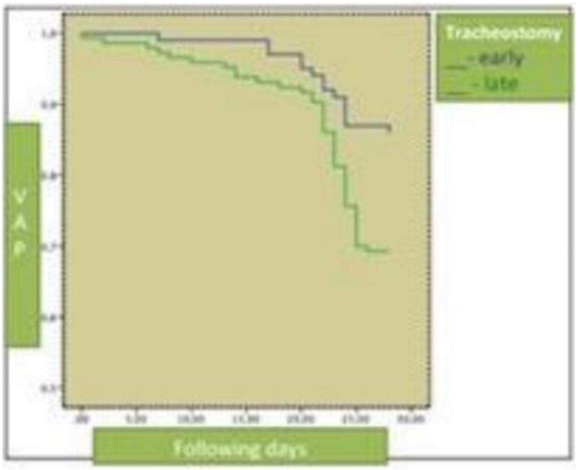

**Fig. 38 (abstract 0062).** VAP development

### 0063

#### Development of a clinical criteria for diagnosing and monitoring ventilator-associated pneumonia in a UK regional network

G. Lemma[1], D. Murray[2], K. Sim[3], V. Packham[2], A. Hall[2], I. Welters[2,4]
[1]University of Liverpool, School of Medicine, Liverpool, United Kingdom; [2]Royal Liverpool University Hospital, Intensive Care Unit, Liverpool, United Kingdom; [3]Whiston Hospital, Liverpool, United Kingdom; [4]University of Liverpool, Institute of Ageing and Chronic Disease, Liverpool, United Kingdom
**Correspondence:** G. Lemma
*Intensive Care Medicine Experimental* 2017, **5(Suppl 2):**0063

**INTRODUCTION.** It is estimated that ventilator-associated pneumonia (VAP) occurs in 10-20% of patients in intensive care[1]. Despite advances in prevention strategies, there remains a lack of consensus on standard criteria for clinical diagnosis of VAP. Although surveillance programmes are available, questions remain about their suitability as a clinical diagnostic tool[2]. In this regard, the Cheshire and Mersey Critical Care Network (CMCCN) have developed a regional audit tool in order to comply with recommendations published by the Faculty of Intensive Care Medicine[1].

**OBJECTIVES.** To complete and assess a new regional VAP audit tool provided by the CMCCN.

**METHODS.** Data were collected between August 2016 and January 2017 at the Royal Liverpool University Hospital Intensive Care Unit. Information was collated using clinical notes, observation charts and electronic investigation software (Sunquest ICE©).

Inclusion criteria were mechanical ventilation for greater than 48 hours with static or decreasing FiO2 or PEEP for more than 48 hours.

Exclusion criteria were current non-infected ventilator-associated event or current VAP without a 48 hour stability period.

Change in ventilator settings, white cell count, temperature, positive microbiology and evidence of new pulmonary infiltrates were also recorded.

**RESULTS.** 1343 ventilator days were identified, of which 1033/1343 were greater than 48 hours and 740/1033 met all other criteria.

The audit identified 8/740 (1.08%) positive cases of VAP.

**CONCLUSION.** We used this audit tool for an extended period at a large ICU within CMCCN. Our results identified a VAP frequency of 1.08%, which is much lower than expected. Limitations include variability in data collection, interpretation of clinical findings (e.g. radiology report), definition of mechanical ventilation, lack of data on microorganisms and antimicrobial treatment. The data collected were insufficient to calculate an accurate incidence rate.

Future research should focus on comparison against three commonly cited models: American CDC VAE Protocol (2017)[2], ECDC HAIICU Protocol (2015)[3] and Clinical Pulmonary Infection Score (CPIS)[4].

### 0064

#### Gram stain at 24 hours after antibiotic administration predicts effectiveness of antibiotics in ICU patients with respiratory tract infection?

E. Nakataki, T. Itagaki, M. Onodera, J. Oto, M. Nishimura
Tokushima University Hospital, Critical Care and Emergency Medicine, Tokushima, Japan
**Correspondence:** E. Nakataki
*Intensive Care Medicine Experimental* 2017, **5(Suppl 2):**0064

**INTRODUCTION.** Bacteria in Gram stain decrease after antibiotic administration, while few reports confirmed the relationship between change of Gram stain findings and antibiotic effectiveness.

**OBJECTIVES.** To examine if change of Gram stain findings at 24 hours after antibiotic administration predicts clinical effectiveness of antibiotics.

**METHODS.** This was a prospective observational study. We enrolled ICU patients who developed respiratory tract infections and were planned treatments. Attending physicians decided antibiotics by assessing tracheal aspirate Gram stain and clinical condition. Gram stain was performed again 24 hours after initial antibiotic administration. Three physicians independently assessed it, and when all of them evaluated bacteria decreased, we defined it positive. When patients were prescribed the same antibiotic at day 4 or de-escalated antibiotics, we defined the antibiotics were clinically effective. Main outcome was concordance ratio between change of Gram stain findings and clinical effectiveness.

**RESULTS.** Eighteen cases were enrolled. Fourteen cases showed a predominant morphotype while 4 cases showed a polymicrobial pattern. Thirteen were positive in Gram stain, whereas 5 were not. Among 12 of 13 cases, antibiotics were clinically effective and positive predictive value was 93%. Negative predictive value was 20%.

**CONCLUSIONS.** Gram stain at 24 hours after antibiotic administration was useful to predict clinical effectiveness of antibiotics.

**Table 23 (Abstract 0064).** Cross tabulation of Gram stain and effectiveness

| | | Antibiotic effectiveness | | |
|---|---|---|---|---|
| | | Yes | No | Total |
| Change of Gram stain findings | Positive | 12 | 1 | 13 |
| | Negative | 4 | 1 | 5 |
| | Total | 16 | 2 | 18 |

### 0065

#### Compared ventilator-associated pneumonia and mortality rates between NIV and IMV in a medical Tunisian ICU

K. Meddeb[1], J. Ayachi[1], H. Kallel[1], Y. Hamdaoui[1], J. Baroudi[1], M. Boujelbèn[1], N. Fraj[1], N. Sma[1], E. Ennouri[1], W. Zarrougui[1], A. Ben Abdelhafidh[1], I. Ben Saida[1], A. Azouzi[1], I. Chouchene[1], A. Khedher[1], M. Boussarsar[1,2]
[1]Farhat Hached University Hospital, Medical Intensive Care Unit, Sousse, Tunisia; [2]Ibn Al Jazzar Faculty of Medicine, Research Laboratory N° LR 14 ES 05 Interactions of the Cardio-Pulmonary System, Sousse, Tunisia
**Correspondence:** M. Boussarsar
*Intensive Care Medicine Experimental* 2017, **5(Suppl 2):**0065

**BACKGROUND.** (NIV) represents a first-line treatment for patients with acute hypercapnic respiratory failure. When possible this technique allows a more comfort for patients ; decreases intubation rate and IMV complications.

**AIM.** To assess VAP and mortality rates between NIV and IMV.

**METHODS.** Retrospective, observational, monocentric study conducted in an 8-bed medical Tunisian ICU from January, 2000 to December, 2015. Were included all consecutive patients admitted for acute on chronic respiratory failure for whom ventilatory support with either conventional mechanical ventilation or NIV initiated within the ICU as first line ventilatory support. Were assessed VAP and mortality rates between NIV and IMV.

**RESULTS.** Among the 4650 enrolled patients, 1245(26,7%) patients were admitted for AE/CRF. Firstline NIV use was in 374(30%). Overall NIV success rate was at 60,4%. In NIV failure group, mean delay to intubation was estimated at 2,3 ± 2,1days. VAP density incidence was higher in NIV failure group, compared to the NIV success group and the firstline IMV group, respectively, 29,3/1000 ventilator days vs 3,6/1000 ventilator days vs 26,9/1000 ventilator days (P = 0.0001). This resulted in a higher length of stay for the NIV failure group 14,7 ± 13,1days compared to NIV success group 7,4 ± 4,5days and the firstline IMV group 11,11 ± 10,9days (p = 0.0001). Similar results were found regarding mortality rate, which was higher in the NIV failure group compared to both other groups, 64,2% vs 1,3% vs 48,7% (p = 0.0001).

**CONCLUSIONS.** Compared to IMV, successful NIV management presents lower VAP density incidence and mortality rate, however delayed intubation after initial NIV seems to be a risk factor for VAP and mortality.

## The pathophysiology of sepsis

### 0066

#### Longitudinal changes of metabolic and bioenergetic patterns in critically ill children with sepsis (S) compared to those with SIRS

A. Spanaki[1], T. Tavladaki[1], H. Dimitriou[2], E. Kondili[3], A.V. Koslov[4], J.C. Duvigneau[5], E. Blevrakis[1], S. Dumitrescu[4], A. Weidiinger[4], D. Georgopoulos[3], G. Briassoulis[1]
[1]University Hospital of Heraklion/University of Crete, PICU, Heraklion, Greece; [2]University of Crete, Medical School, Paediatric Haematology Oncology, Heraklion, Greece; [3]University Hospital of Heraklion/University of Crete, ICU, Heraklion, Greece; [4]Ludwig Boltzmann Institute for Experimental and Clinical Traumatology, Vienna, Austria; [5]University of Veterinary Medicine, Vienna, Austria
**Correspondence:** A. Spanaki
*Intensive Care Medicine Experimental* 2017, **5(Suppl 2):**0066

**INTRODUCTION.** Systemic inflammatory response syndrome (SIRS) and sepsis (S) are associated with cell stress, inflammation, and metabolic derangements. Studies investigating the kinetics of metabolic and bioenergetic profiles in S or SIRS are limited.

**OBJECTIVES.** To compare acute cross-sectional and longitudinal changes of bioenergetics, inflammation and metabolism in adult and pediatric septic patients with those with trauma-related SIRS and healthy-controls (H).

**METHODS.** 68 children (S/18, SIRS/23, H/27) and 79 adults (S/23, SIRS/23, H/33) mechanically ventilated were included in the study. Blood samples were obtained within 24-hours upon admission and repeated on days 3 and 5. Mean Fluorescence Intensity (MFI) for HSP expression in monocytes (m) or neutrophils (n) was determined using 4-colour Flow Cytometry. Extracellular (e) HSP72 and 90 were measured using ELISA and energy-expenditure (EE) by the Gas Module E-COVX. ATP concentrations were measured by luminescence with a photometer. The levels of plasma amino acids and NO were determined by HPLC and Sievers NO-Analyzer, respectively; lipid peroxidation products (TBARS) by a colorimetric test.

**RESULTS.** On day 1, adult septic patients had lower VO2, VCO2, EE, metabolic pattern, albumin, mHSP72, and nHSP72 and higher TBARS, NO, nitrite, BVR, and lactate compared to controls and/or SIRS ($p < 0.05$). Among adult septic patients, VO2, VCO2, nitrite, arginine and metabolic pattern were increased and albumin and BVR decreased longitudinally ($p < 0.05$); in SIRS, NO and nitrite were increased while BVR and ATP decreased from day 1 to 3 and/or 5 ($p < 0.05$). Septic children also had lower ATP and mHSP72 compared to controls and/or SIRS longitudinally ($p < 0.05$). In pediatric sepsis, TBARS were decreased on day 3 and BVR on day 5, the day when ATP was increased ($p < 0.05$).

**CONCLUSIONS.** Metabolic patterns and bioenergetics differ between septic and non-septic critically ill adult and pediatric patients in acute stress and longitudinally. An acute metabolic and bioenergetics repression characterizes early-sepsis, following by inconsistent patterns of recovery by day 5.

**GRANT ACKNOWLEDGMENT**
This research has been co-financed by the European Union (European Social Fund (ESF)) and Greek national funds through the Operational Program "Education and Lifelong Learning" of the National Strategic Reference Framework (NSRF)-Research Funding Program: THALES.

**This abstract is now an oral abstract.**

### 0067

#### Kinetics of endothelial progenitor cells mobilization after neuromuscular electrical stimulation in septic ICU patients and the role of muscle contraction strength

E. Karatzanos[1], G. Mitsiou[1], C. Stefanou[1], K. Psarra[2], E. Angelopoulos[1], S. Dimopoulos[1], V. Gerovasili[1], C. Routsi[1], S. Nanas[1]
[1]1st Critical Care Department, Evangelismos General Hospital, School of Medicine, National & Kapodistrian University of Athens, Athens, Greece; [2]Immunology & Histocompatibility Department, Evangelismos General Hospital, Athens, Greece
**Correspondence:** S. Nanas
*Intensive Care Medicine Experimental* 2017, **5(Suppl 2):**0067

**INTRODUCTION.** Neuromuscular electric stimulation (NMES) has been recently shown to acutely mobilize endothelial progenitor cells (EPC), measures of the endothelial restoration potential, in septic ICU patients.

**OBJECTIVE.** The aim of the study was to explore the role of muscle contraction strength as well as the time course of EPCs mobilization.

**METHODS.** Twenty-two (age: 60 ± 14 yr) ICU septic patients were included in the analysis on the role of contraction strength. Eighteen patients (age: 58 ± 15 yr) were included in the analysis of the time course of EPCs changes. All patients were randomized to one of two 30-min NMES protocols of different characteristics applied in maximally tolerated intensity on vastus lateralis, vastus medialis and peroneus longus of both lower extremities. Muscle contraction of the quadriceps was evaluated with a scale from 0 (no contraction) to 4 (full knee extension) and the 22 patients were divided in two subgroups, using 2 (visible contraction) as a cut-off point. Blood was sampled before and immediately after NMES sessions. In relation to the 18 patients, blood samples were collected before ($t_{pre}$) immediately after ($t_{post}$) and 60 min following ($t_{post60}$) NMES sessions. EPC were quantified by cytometry markers CD34+/CD133+/CD45−, CD34+/CD133+/CD45−/VEGFR2+, and CD34+/CD45−/VEGFR2+. All values are mean ± SD. EPCs values are in cells / $10^6$ enucleated cells.

**RESULTS.** In relation to the role of contraction strength, CD34+/CD133+/CD45− and CD34+/CD45−/VEGFR2+ were overall increased ($p < 0.05$) with not any between-subgroup differences observed ($p > 0.05$). Regarding the time course of the changes, a significant time effect was found for all EPC populations ($p < 0.05$, table 24). Post-hoc analyses showed that CD34+/CD133+/CD45− and CD34+/CD45−/VEGFR2+ were significantly increased from $t_{pre}$ to $t_{post60}$ ($p < 0.05$), while a tendency to increase from $t_{pre}$ to $t_{post60}$ was observed for CD34+/CD133+/CD45−/VEGFR2+ (p = 0.10).

**CONCLUSION.** NMES muscle contraction strength did not found to affect EPC mobilization. NMES acutely increased EPC number and this effect was maintained for at least 60 min post sessions. The exact time course of the EPC increase after an NMES session and the clinical significance of these benefits need to be determined.

C. Stefanou, E. Karatzanos, G. Mitsiou, K. Psarra, E. Angelopoulos, S. Dimopoulos, E. Boviatsis, C. Routsi, S. Nanas. Neuromuscular electrical stimulation acutely mobilizes endothelial progenitor cells in critically ill patients with sepsis, Ann Intensive Care, 2016, 6(1), 21.

**GRANT ACKNOWLEDGEMENT**

This study was supported by the Special Account for Research Grants, National and Kapodistrian University of Athens, Greece.

**Table 24 (Abstract 0067).** Change of EPC populations over time

| | tpre | tpost | tpost60 |
|---|---|---|---|
| CD34+/CD133+/CD45− | 14.2 ± 10.9 | 22.1 ± 18.5 | 26.0 ± 19.5 * |
| CD34+/CD133+/CD45−/VEGFR2+ | 2.1 ± 2.5 | 5.0 ± 9.0 | 6.4 ± 9.0 |
| CD34+/CD45−/VEGFR2+ | 16.2 ± 11.6 | 25.5 ± 20.9 * | 38.9 ± 26.2 * |

* significant difference compared to tpre (p<0.05)

### 0068

#### Early increased levels of MR-proAdrenomedullin are associated with organ dysfunction and mortality in septic patients

J. Baldirà[1], J.C. Ruiz-Rodríguez[1,2], E. Papiol[3], A. Ruiz-Sanmartin[3], N. Varga[3], M. Acuña[3], L. Chiscano[3], P. Domingo[3], I. Benabou[2], V. Ribas[2], E. Gallart[3], J. Llaneras[4], E. Molas[4], L. Cruz[5], I. Comas[5], R. Ferrer-Costa[5], E. Casis[5], R. Ferrer[3]

[1]Hospital Universitari Vall d'Hebron, Intensive Care Department, Barcelona, Spain; [2]Vall d'Hebron Research Institute, Shock, Organ Dysfunction and Resuscitation Research Group (SODIR), Barcelona, Spain; [3]Hospital Universitari Vall d'Hebron, Critical Care Department, Barcelona, Spain; [4]Hospital Universitari Vall d'Hebron, Emergency Department, Barcelona, Spain; [5]Hospital Universitari Vall d'Hebron, Clinical Laboratories, Biochemistry Department, Barcelona, Spain

**Correspondence:** J. Baldirà

*Intensive Care Medicine Experimental* 2017, **5(Suppl 2):**0068

**INTRODUCTION.** MR-Proadrenomedullin (MR-proADM) is a novel biomarker of sepsis. Its levels are increased in septic patients. This biomarker can help in predicting mortality in this cohort of patients.

**OBJECTIVES.** To determine the role of MR-proADM as a predictor of mortality in septic patients at the moment of sepsis detection.

**METHODS.** Prospective observational study of 70 adult patients with activation of an in-hospital sepsis code (SC) in a tertiary center from 28-nov-2016 to 6-mar-2017. At the moment of sepsis detection, levels of procalcitonin (PCT), MR-ProADM, lactate and C reactive protein (CRP) were determined. Values of MR-ProADM were blinded to the investigators. Demographic data, SOFA score, laboratory data, physiological variables, need for ICU admision, source of infection, days of hospitalization and intrahospital mortality were recorded. This study was approved by Ethical Committee (PR(AG)333/2016). SEPSIS III definitions were used[(1)]. Quantitative variables with normal distribution have been expressed as mean ± standard deviation, non-normal distribution as median and interquartil range (25-75%) and categorical variables as a percentage. T-test and Chi-Square test was applied when necessary ($p < 0.05$). Statistical analysis was done with SPSS 18.0 (SPSS Inc., Chicago, IL, USA).

**RESULTS.** Of 70 patients, 6 were discarded because they had no infection. 42 (65.6%) were man with a mean age of 64 (±14.67) years. SOFA score was 6.94 (±3.48). Septic shock (SSH) was diagnosed in 42 (65.6%) patients, while 17 (26.6%) had sepsis (SS) and 5 (7.8%) had infection with no organ dysfunction. The source of the sepsis was intraabdominal in 19 (29.7%) patients, respiratory in 18 (28.1%), urinary tract 16 (25%). In 2 (3.1%), the source was not identified. At the moment of sepsis detection, lactate levels were 2.5 (1.6-5.65) mmol/dL, CRP 21.39 (±12.48) mg/dL, procalcitonin 8.03 (1.99-37.04) mg/dL and MR-proADM 3.91 (1.99-7.43)nmol/L. 41 (64.1%) patients were admitted to a critical care area. In-hospital mortality was 28.1% (18 patients). In SSH, MR-proADM levels were 4.6nmol/L (2.34-9.89) and in SS were 2.93 nmol/L (1.73-3.92). MR-proADM levels were higher in non survivors (4.9 vs 3.39; p = 0.035) while PCT (3.17 vs 10.53; p = NS) and PCR (22.71 vs 20.38; p = NS) did not show differences between survivors and non-survivors. In the subgroup of SSH patients, non survivors had higher levels (8.49 vs 4.26; p = 0.046), but not statistic differences were found in the subgroup of SS. Additionally, SOFA score was lower in survivors (6.28 vs 9.18; p = 0.002).

**CONCLUSIONS.** At the moment of sepsis detection, MR-proADM is the only biomarker able to predict mortality.

### 0069

#### advanced glycation end products (AGEs) in septic patients and the "two hit hypothesis" of inflammation

E. Rodriguez-Ruiz[1], R. Hernandez-Vaquero[1], I. Granja Gomez[1], J.L. Garcia Allut[1], A. Lopez Lago[1], A. Estany-Gestal[2], M.A. Garcia Gonzalez[3]

[1]Hospital Clínico Universitario, Intensive Care Unit, Santiago de Compostela, Spain; [2]Fundación Ramón Dominguez, Unidad de Epidemiología e Investigación Clínica, Santiago de Compostela, Spain; [3]Instituto de Investigaciones Sanitarias (IDIS), Grupo de Genética y Biología del Desarrollo de las Enfermedades Renales, Santiago de Compostela, Spain

**Correspondence:** E. Rodriguez-Ruiz

*Intensive Care Medicine Experimental* 2017, **5(Suppl 2):**0069

**INTRODUCTION.** Advanced glycation end products (AGEs) are stable products generated by non-enzymatic glycation of proteins due to the Maillard reaction, driven by oxidative stress; they appear in plasma and accumulate in tissues. High levels of AGEs have been reported associated to ageing process, smoking, diabetes mellitus, chronic kidney injury and cardiovascular diseases including heart failure, myocardial infarction and atrial fibrillation.

The "two hit hypothesis" of inflammation (Schmidt *et al.*) postulates that besides chronic upregulated interaction between AGEs and its receptors, superimposed critical illnesses with associated inflammation and oxidative stress, further elevates AGEs and its receptors, resulting in exaggerated and prolonged inflammation.

**OBJECTIVES.** Our aim was to determine the AGEs levels in the plasma of septic patients, and to study their association with chronic risk factors and medical history (age, smoking, diabetes mellitus, chronic renal failure and cardiovascular diseases). Also, we wanted to analyze the relationship between AGEs and inflammatory mediators (TNF-α, IL-1β, IL-6, IL-8, C-reactive protein and Procalcitonin).

**METHODS.** We included 89 consecutive patients admitted to our ICU with the diagnosis of sepsis, and checked in their medical history for age, smoking, diabetes mellitus, chronic kidney injury, and cardiovascular diseases including heart failure, myocardial infarction and atrial fibrillation. At day 1 of admission, plasma AGEs were measured by quantitative fluorescence spectroscopy. Simultaneously, we measured the next inflammatory mediators: TNF-α, IL-1β, IL-6, IL-8, CRP and Procalcitonin. Spearman correlation was performed to determine the relation between AGEs and age. Mann–Whitney test was applied to study the association between AGEs and risk factors, medical conditions and inflammatory mediators.

**RESULTS.** In our cohort of septic patients (mean age 68 ± 13), there were 72 survivors and 17 non-survivors. We found association between AGEs and the presence of chronic renal failure ($p < 0.001$), and between AGEs and atrial fibrillation (p = 0.013), but not with the other studied conditions. Besides, we could not find correlation between plasma AGEs and any of the acute phase inflammatory mediators.

**CONCLUSION.** Plasma AGEs at day 1 of the septic process are associated to the presence of chronic renal failure and to atrial fibrillation. AGEs could have a pathogenic role in the development of atrial fibrillation. We did not find correlation between AGEs and acute phase inflammatory products, which does not support the "two hit hypothesis" of inflammation previously exposed. Further studies are required to find out how AGEs and its receptors are involved in the inflammatory response of the septic process.

### 0070

#### Are gastrointestinal motility disorders a risk factor for the development of sepsis?

J. Schäper, C. Rundel, M. Quintel
University Medical Center Göttingen, Anaesthesiology, Göttingen, Germany
**Correspondence:** J. Schäper
*Intensive Care Medicine Experimental* 2017, **5(Suppl 2):**0070

**INTRODUCTION.** Gastrointestinal motility disorders are a common clinical sign during the ICU stay of critically ill patients. It is, however, unclear, whether gastrointestinal motility disorders (possibly associated with gut barrier dysfunction) are causative for the development of inflammatory complications (sepsis, death).

**OBJECTIVES.** This study retrospectively investigated in a patient cohort with spontaneous intracerebral parenchymatous hemorrhage the relation between gastrointestinal motility disorders and the development of sepsis and ICU mortality.

**METHODS.** Retrospective data analysis of clinical variables documented in IntelliSpace Critical Care & Anesthesia (ICCA™). The IT-based search of patients in the database (over the period 2010–2015) using 10 search items revealed a total of 597 patients,180 thereof were screened, 75 were evaluated. When applicable, clinic variables, such as time until 50% of nutritional needs were achieved, gastric reflux within 24 hours and time to first defecation were analysed on day 0, 1, 2, 3, 5, 7, 10, and 14. Statistics: multivariate analysis of prior evaluated variable-candidates for dichotomous outcome measures sepsis and ICU mortality. Comparison of variables of patients with gastrointestinal motility disorders with and without sepsis. Group comparison with Mann–Whitney U test. Significance level at $p < 0.05$.

**RESULTS.** Risk factors for sepsis were days in ICU ($p = 0.04$), chronic kidney failure ($p = 0.04$), and detection of gram-positive pathogens (0.04). Risk factors for ICU mortality were days on ICU ($p = 0.01$), sedation days ($p = 0.02$) and medication with ACE inhibitors ($p = 0.05$). Patients with sepsis showed a longer time to first defecation (137[100;186] vs. 100[68;130] h, median[quartile], $p = 0.012$), and time until 50% of nutritional needs were achieved (84 [63;120] vs. 71 [58;99] h, $p = 0.28$).

**CONCLUSIONS.** Variables of gastrointestinal motility disorders could not be determined as risk factors for sepsis or ICU mortality. Patients with sepsis, however, more often showed gastrointestinal motility disorders compared with critically ill patients without sepsis.

### 0071

#### Evaluation of RV function in patients with sepsis

M. Fawzy, M. Sayed, M. Afify, R. El-Sherif
Cairo University, Cairo, Egypt
**Correspondence:** M. Fawzy
*Intensive Care Medicine Experimental* 2017, **5(Suppl 2):**0071

**INTRODUCTION.** Myocardial depression is a well known phenomenon in sepsis. Limited studies assessed the right ventricuar (RV) function in sepsis.

**OBJECTIVES.** Evaluation of RV dimensions and function in patient with sepsis and its relation to outcome.

**METHODS.** Twenty patients with sepsis or severe sepsis were included. Patients with cardiomyopathies, known or suspected pulmonary HTN were excluded.

Within the first 24 hours of admission, the following echocardiographic parameters were obtained: RV dimensions (basal, mid and longitudinal), fractional area change (FAC), tricuspid annular plane excursion velocity (TAPSE), Pulmonary artery systolic pressure (PASP), peak systolic velocity of the RV free wall by tissue Doppler (Ś), the presence or absence of regional wall motion abnormalities (RWMAs) of the RV free wall, in addition to left ventricular (LV) internal dimensions and EF%.

APACHE II score on admission and its predicted mortality were assessed

**RESULTS.** The mean age of the study group was 49.9 ± 12.2 years with 15 females (75%). Sepsis with an identified pathogen (proved by microbiological culture) was documented in 19 patients (95%). The following were the sources of infection in order of frequency: Chest infection (50%), infected surgical wound (15%), intra-abdominal sepsis and urinary tract infection (10% each), infective endocarditis, perpural sepsis and suspicious for myocarditis with 2 positive categories (5% each).

63.1% of culture positive patients were of positive gram staining, while 36.9% were negative.

All patients showed normal RV internal dimensions, FAC, Ś velocity and TAPSE, in addition to normal LV internal dimensions and EF%. None of the studied patients had any evidence of RWMAs of the RV free wall.

Six patients (30%) had pulmonary HTN (PASP > 45 mmHg).

The mean APACHE II score was 16.8 ± 4.18 with average expected mortality 24% Average Length of ICU stay was 10.7 ± 6.2 days with no recorded deaths related to sepsis during hospital stay.

Since no in-hospital mortality due to sepsis or severe sepsis, correlations were done between the predicted mortality and the measured echocardiograophic parameters.

RV Longitudinal diameter and PASP showed significant positive correlations with expected mortality (r 0.483, P value 0.031 & r 0.644, P value 0.002 respectively). FAC and TAPSE showed significant negative correlations with expected mortality (r-0.496, P value 0.026 & r −0.498, P value 0.025 respectively).

**CONCLUSIONS.** In this small single centered study, patients with sepsis or severe sepsis did not have any evidence of RV or LV dilation and/or impaired systolic functions. PASP was elevated in one third of the study group.

### 0072

#### Myocardial cholesterol levels and adrenergic signalling in a septic rat model

A. Kleyman[1], W. Khaliq[1], D. Andreis[1,2], M. Singer[1]
[1]UCL, Bloomsbury Institute of Intensive Care Medicine, London, United Kingdom; [2]Università Politecnica delle Marche, Anesthesia and Intensive Care Unit, Milan, Italy
**Correspondence:** A. Kleyman
*Intensive Care Medicine Experimental* 2017, **5(Suppl 2):**0072

**INTRODUCTION.** Hypocholesterolaemia is both a hallmark of sepsis and a prognosticator. However, the reasons for this fall (decreased production/ingestion and/or increased utilization) are not known, neither is the effect of sepsis on tissue cholesterol levels. This may be highly relevant as cholesterol is a crucial component of the plasma membrane, including the organization of lipid rafts. Low membrane levels may impact on adrenergic receptor signalling as these receptors are located within the lipid rafts. To investigate this, we used our well-characterized rat model in which survivors and non-survivors can be identified with high sensitivity/specificity as early as 6h post-sepsis.

**OBJECTIVES.** To assess (i) the relationship between plasma and cardiomyocyte membrane cholesterol concentrations and adrenergic signalling in heart tissue taken at 24 hours post-sepsis.

**METHODS.** Awake, instrumented male Wistar rats (325 ± 15g) received an i.p. injection of faecal slurry with i.v. fluid resuscitation commenced from 2h. Control animals were treated identically except did not receive slurry. A heart rate cut-off of 460 bpm at 6h was used to classify animals into predicted survivors or non-survivors (1). At either 6 or 24h post-sepsis animals were killed and organs were immediately collected into liquid nitrogen. Cholesterol concentrations in membrane preps was measured by an Amplex® Red Cholesterol Assay (Invitrogen A12216). Alterations in adrenergic signalling at 24h were measured by serine23/24 phosphorylation of troponin I, a well-known target of adrenergic signalling. Phosphorylation of Erk1/2 was measured as a surrogate marker of desensitization of adrenergic receptors and b-arrestin signalling. Results are presented as mean ± SE, analysed using Student's t-test and considered statistically significant when $p < 0.05$.

**RESULTS.** A marked decrease in plasma cholesterol level was seen in septic animals as early as 6h post-sepsis, more so in non-survivors. At 24h the concentration of cholesterol in heart membrane preparations was significantly lower in septic animals, also greater in non-survivors. (Table 25). An increase in myocardial troponin I phosphorylation was seen at 24h in sepsis survivors only, whereas Erk1/2 phosphorylation at 24h was elevated in survivors and decreased in non-survivors.

**CONCLUSIONS.** Falls in plasma and heart membrane cholesterol occur in sepsis and are more exaggerated in non-survivors. These relates to changes in adrenergic signalling, as identified by alterations in phosphorylation of troponin I and Erk 1/2. The relevance of this finding warrants further investigation.

**REFERENCE(S)**

(1) Rudiger A. et al. Clin Sci 2013; 124:391–401

**Table 25 (Abstract 0072).** See text for description

| | Sham | Predicted survivor | Predicted non-survivor |
|---|---|---|---|
| Plasma cholesterol (mg/ml) at 6h | 2.34 ± 0.1 | 2.27 ± 0.05 | 1.95 ± 0,08 [ab] |
| Plasma cholesterol (mg/ml) at 24h | 2.5 ± 0.13 | 2.03 ± 0.03 [a] | 1.91 ± 0,03 [ab] |
| Heart membrane cholesterol at 24h (mg/mg protein) | 0.029 ± 0.003 | 0.023 ± 0.004 [a] | 0.018 ± 0.004 [ab] |
| Myocardial Troponin I phosphorylation at 24h | 100% | 204 ± 34.8% | 129±12.5% p=0.075 |
| Myocardial Erk 1/2 phosphorylation at 24h | 100% | 401 ± 93,6% | 59±11.2% [b] |

[a] *p< 0.05 vs control,* [b] *p< 0.05 vs survivors*

### 0073

#### Different expression profile in primary cells exposed to septic plasma of rats

J. Bringue[1,2,3], R. Guillamat-Prats[3,4], M. Camprubí[1,2,3], E. Torrents[5], M.L. Martinez[6], A. Artigas[1,2,3]

[1]CIBER de Enfermedades Respiratorias (CIBERES), Sabadell, Spain; [2]Institut d'Investigació i Innovació Parc Taulí, Sabadell, Spain; [3]Universitat Autónoma de Barcelona, Bellaterra, Spain; [4]Ludwig-Maximilians-University of Munich, Munich, Germany; [5]Corporació Sanitària i Universitària Parc Taulí, Critical Care Center, Sabadell, Spain; [6]Hospital General de Catalunya, Critical Care Center, Sant Cugat, Spain

**Correspondence:** J. Bringue

*Intensive Care Medicine Experimental* 2017, **5(Suppl 2):**0073

**INTRODUCTION.** The analysis of biological mediators in plasma of septic patients has provided us relevant information concerning systemic response of the body to septic process, although the molecular markers and the pathways that are activated in different cells and organs are not yet fully understood. A better understanding of the cellular response will lead to raise new effective therapeutic strategies.

**OBJECTIVES.** Determine the differences in molecular pathways activation in different primary cell exposed to septic plasma. Compare the expression profile of the primary cell to their septic organs.

**METHODS.** Plasma from septic rats was obtained after 6h and 24h of induced sepsis by a cecal ligation and puncture. 6 rats healthy male Sprague–Dawley rats for group weighting 250-300g were used for this purpose.

We isolated hepatocytes, type II alveolar cells, alveolar macrophages, blood neutrophils and myocytes from their tissues. For this purpose 10 healthy rats were used.

Isolated cells were incubated with media supplemented with 15% of plasma (septic 6h, septic 24h or control) and collected 6h and 10h after the incubation. We analyzed different molecular markers by RT-qPCR.

**RESULTS.** We used 6h plasma to mimic the first stages of a sepsis and 24h to mimic the established sepsis. We evaluated the response of the primary cells at two time points to determine the time course of the different molecular pathways.

Macrophages show increased pro-inflammatory markers TNFα, IL1β, IL6 and iNOS and decreased IL10 at 10h of incubation with both septic plasmas. No effects were observed in apoptosis. Neutrophils show an increase of TNFα, IL1β, iNOS and IL6 at 6h and 10h of incubation with plasma of the early sepsis. IL10 was increased with 24h plasma compared with 6h plasma. We observe increased apoptosis with the 6h septic plasma. Alveolar type II cells show an increase of TNFα and IL6 and decrease of IL10 after 10h of incubation with both septic plasmas. Caspase-3 was significant activated after 6h of incubation with both septic plasmas.

Hepatocytes show minor response to septic plasma; an increase of iNOS after 10h of incubation with 24h plasma and an increase of Bax and Smad2 at 6h incubation with plasma obtained in early.

**CONCLUSIONS.** Pro and anti-inflammatory markers present different regulation in different primary cells. Alveolar cells and inflammatory cells (neutrophils and macrophages) exhibit faster and highly activation in pro-inflammatory markers. Apoptosis is strongly activated in all the cells exposed to septic plasma, while the differences where in the extrinsic and intrinsic activation of apoptosis. Hepatocytes and myocytes present a less pro-inflammatory activation. Study in depth these pathways might help us to understand the MOF and prevent it with some specific treatments.

### 0074

#### Mechanisms of dysregulated host response in sepsis non-survivors of a 48 hours rat model of fecal peritonitis

V. Jeger[1,2], M. Arrigo[3], C.A. Schaer[1], G. Schoedon-Geiser[2], D.R. Spahn[1], D. Bettex[1], A. Rudiger[1]

[1]University Hospital Zurich, Institute of Anaesthesiology, Zürich, Switzerland; [2]University Hospital Zurich, Department of Internal Medicine, Zurich, Switzerland; [3]University Hospital Zurich, Department of Cardiology, University Heart Center, Zurich, Switzerland

**Correspondence:** V. Jeger

*Intensive Care Medicine Experimental* 2017, **5(Suppl 2):**0074

**INTRODUCTION.** Sepsis is a common and frequently fatal condition. We have shown that telemetry derived heart rate changes during early sepsis allow the prognostication of death with a good sensitivity and specificity (88% each) in a rat model of fecal peritonitis (mortality of 33%).

**OBJECTIVES.** To investigate mechanisms of disease in predicted survivors and predicted non-survivors of a 48 hours rat model of fecal peritonitis.

**METHODS.** 63 Male Wistar rats were instrumented with a venous line and an ECG telemetry transmitter. Sepsis was induced by i.p. injection of fecal slurry. Animals underwent arterial cannulation either at baseline (n = 9), or 4 (n = 16) or 24 (n = 6) hours after sepsis induction for hemodynamic measurements and terminal blood sampling. An increase in heart rate >50 bpm 4 hours after sepsis induction was used as a cut off to differentiate predicted survivors from predicted non-survivors. Changes in heart rate variability (HRV) in 24 septic rats and 8 sham rats were observed for up to 48 hours.

**RESULTS.** Septic animals were characterized by lethargy, fever, tachycardia and elevated cytokine (IL-6, TNF, CXCL-1) plasma levels at 4 hours of sepsis. At 24 hours, lactate and B-type natriuretic peptide levels were elevated. Despite tachycardia troponin levels were not increased at any time point. Mean arterial blood pressure and arterial blood gases were stable in all groups throughout the experiment. Hematocrit was higher in predicted non-survivors at 4 hours (45% ± 4, mean ± SD) compared to predicted survivors (34% ± 7; p = 0.011). The analysis of HRV demonstrated autonomic dysfunction during early sepsis in non-survivors: the ratio between low and high frequency (LF/HF) was decreased in non-survivors at 4 hours compared to sepsis-survivors (0.22 (±0.2) vs 1.07 (±0.69); p = 0.018).

**CONCLUSIONS.** Although MAP was maintained in predicted non-survivors, a decrease in intravascular volume was suggested by the increase of hematocrit. Respiratory failure or renal dysfunction were unlikely causes of death in septic animals considering the stability of the blood gas analysis. HRV indicated that autonomic dysfunction during early sepsis might be a mechanism of death in sepsis non-survivors.

**GRANT ACKNOWLEDGMENT**

This work was supported by a research grant from the *Privatdozenten-Stiftung* of the University of Zurich (2016) attributed to AR.

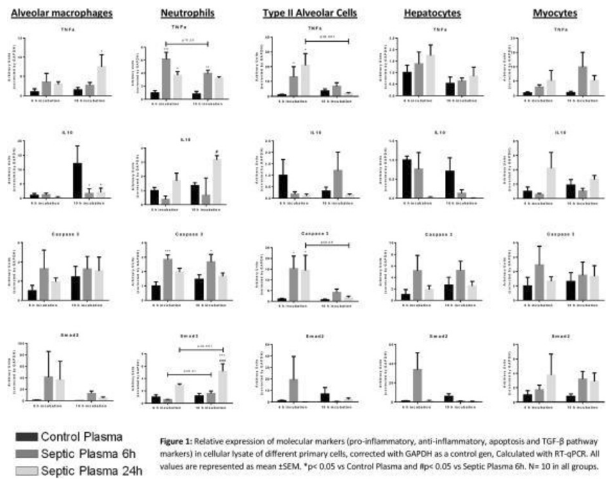

**Fig. 39 (abstract 0074).** See text for description

### 0075

#### Staphylococcus aureus sepsis elicit endothelial cells inflammation via activation of the TLR2/MAPK/NF-κB/COX2/PGE2 pathways

M.H. Tsai[1], J.-F. Hsu[2], I.-T. Lee[3]

[1]Yunlin Chang Gung Memorial Hospital, Pediatrics, Yunlin, Taiwan, Province of China; [2]Chang Gung Memorial Hospital, Linkou, Taiwan, Province of China; [3]China Medical University, Taichung, Taiwan, Province of China

**Correspondence:** M.H. Tsai

*Intensive Care Medicine Experimental* 2017, **5(Suppl 2):**0075

**INTRODUCTION.** The vascular endothelium is the first contact and important barrier during hematogenous spreading of *Staphylococcus aureus*, one of the most important pathogen of bacteremia and sepsis in the intensive care unit. The fundamental understanding of how *S. aureus* disrupts this barrier is still limited, and understanding of the pathogenesis may provide advanced insights toward new therapeutic strategies.

**OBJECTIVES.** To investigate the molecular mechanisms of how *S. aureus* specifically interacts with vascular endothelial cells and elicit inflammatory responses.

**METHODS.** Human aortic endothelial cells (HAECs) were infected with heat-killed *S. aureus* (HK-SA). Various inflammatory signaling pathways were surveyed. We also created an in vivo model of *S. aureus* sepsis to address this issue.

**RESULTS.** Our results indicated that HK-SA significantly induced COX-2 protein expression and mRNA levels, and increased promoter activity and $PGE_2$ secretion. In addition, HK-SA effectively promoted MMP-9 dependent cell migration of HAECs, which was inhibited by transfection with siRNA of COX-2, $PGE_2$, and IL-6. Furthermore, incubation with *S. aureus* also induced TLR2 but not TLR4 protein and mRNA expression. Transfection with siRNA of TLR2 significantly inhibited *S. aureus*-induced COX-2, IL-6 and MMP-9 expression, suggesting that TLR2 was a key receptor for *S. aureus* infection. Inhibition of MAPK (p38 and ERK1/2) and NF-κB by inhibitors and siRNA effectively attenuated *S. aureus*-induced COX-2, IL-6, MMP-9 expression and cell migration. Moreover, our *in vivo* study also showed that *S. aureus* infection induced COX-2 expression, C-reactive protein and IL-6 secretion and COX-2 mRNA expression was attenuated by pretreatment with inhibitors of p38, ERK1/2 and NF-κB.

**CONCLUSIONS.** These findings provide insight into how *S. aureus* sepsis may activate proinflammatory mechanisms of endothelial cells to elicit subsequent barrier function loss.

### 0076

#### Erythrophagocytosis and red blood cell complement regulatory proteins in critically ill patients

N. Selvais[1], K. Zouaoui Boudjeltia[2], A. Rousseau[2], P. Uzureau[2], P. Biston[1], M. Piagnerelli[1,2]

[1]CHU-Charleroi Marie Curie, Université Libre de Bruxelles, Dpt of Intensive Care, Charleroi, Belgium; [2]Experimental Medicine Laboratory, CHU Vésale, ULB 222 Unit, Montigny-le-Tilleul, Belgium

**Correspondence:** N. Selvais

*Intensive Care Medicine Experimental* 2017, **5(Suppl 2):**0076

**INTRODUCTION.** Red blood cell (RBC) rheology (deformability, aggregation and shape) is altered during inflammatory process, especially during sepsis. In patients with severe anemia due to malaria, RBCs are modified by the deposition of IgG and alterations in the complement regulatory proteins (CR1, CD 55 and CD 59) and these changes could contribute to the increased erythrophagocytosis (1). We have already showed an early decreased of RBC membrane sialic acid (SA) content- a carbohydrate of the RBC membrane-associated with a decreased RBC deformability in septic patients, as observed in senescence process (2). This SA decrease could also increase erythrophagocytosis..

**OBJECTIVES.** We studied the RBC surface proteins and erythrophagocytosis in ICU patients with and without sepsis.

**METHODS.** We, prospectively, studied RBCs from 14 ICU patients with (n = 9) or without sepsis (n = 5) compared to RBCs from 17 volunteers (V). By flow cytometry techniques, we studied the shape assessed by the moment (2) and the membrane content of IgG and in complement regulatory proteins (CR1, CD 55 and CD 59) in the 3 groups. Erythrophagocytosis was visualized directly by fluorescence microscopy and quantified by flow cytometry method. Values were expressed in median (25–75 th percentiles)and compared by the Kruskal Wallis one way of variance test and the Man-Whitney test.

A $p < 0.05$ was considered as significant.

**RESULTS.** RBCs from septic were more spherical than non- septic patients and V (Moment : V: −0.70 (−0.74 - - 0.68), NS: −0.53 (−0.56 - -0.39), S −0.31 (−0.48 - -0.24), $p < 0.001$).The complement regulatory proteins and Ig G were not statistically different between groups (for example: Ig G depot : V : 0.17 (0.16-0.17); NS: 0.17 (0.16-0.19); S: 0.17 (0.17-0.18), p = 0.53).With the cytometry, the percent of phagocytosis was higher for the septic patients than the healthy volunteers (6.3% vs 1.8%), in line with what was observed in microscopy.

**CONCLUSIONS.** RBCs from septic patients were more spherical and erythrophagocytosis process was enhanced but complement receptors at the membrane surface were not different between groups and thus not responsible of this increased phenomenon.This latter could be explained by the decreased SA membrane content as observed in senescence process (2).

**REFERENCE(S)**

1. Waitumbi JN et al. Blood 2000; 95: 1481–1486.
2. Piagnerelli M et al. Crit Care Med 2009 ; 37 : 1244–1250

**GRANT ACKNOWLEDGMENT**

CHU-Charleroi Scientific Commission

### 0077

#### Sublingual microcirculation assessment in one fixed area identify the presence of sepsis-induced plugging of capillary vessels

A. Lima[1], B. Ergin[2], T. van Rooij[3], M. Sorelli[4], Y. Ince[2], P. Specht[5], E. Mik[5], L. Bocchi[4], K. Kooiaman[6], N. de Jong[3], C. Ince[2]
[1]Erasmus MC University Medical Centre, Rotterdam, Netherlands; [2]Academic Medical Center, Department of Translational Physiology, Amsterdam, Netherlands; [3]Erasmus MC, Thorax Center, Department of Biomedical Engineering, Rotterdam, Netherlands; [4]University of Florence, Department of Information Engineering, Florence, Italy; [5]Erasmus MC, Laboratory of Experimental Anesthesiology, Department of Anesthesiology, Rotterdam, Netherlands; [6]Erasmus MC, Thorax Center, Department of Information Engineering, Rotterdam, Netherlands
**Correspondence:** A. Lima
*Intensive Care Medicine Experimental* 2017, **5(Suppl 2):**0077

**INTRODUCTION.** Microcirculatory slow flow combined with plugged capillaries is a hallmark of sepsis. We developed a new measurement technique using the Cytocam-Incident Dark Field (IDF) imaging to obtain recordings in a fixed sublingual area to better understand the dynamic changes of microcirculation during septic shock.

**OBJECTIVES.** To use Cytocam imaging to investigate capllary hemodynamic in a porcine model of sepsis in order to identify plugged capillary vessels.

**METHODS.** Experiments were conducted on fully instrumented and anesthetized 13 female pigs (BW 28.2 ± 1.9 kg). The animals were divided into 2 groups: with (LPS group n = 10) or without (control group, n = 3) infusion of LPS (2 $mg/kg^{-1}h^{-1}$). Measurements were performed at baseline (t0), during shock (t1), and one hour after starting fluids infusion (t2). Sublingual microcirculation was measured using the Cytocam (Braedius Medical, Naarden the Netherlands) supported by a mechanical arm to allow the operator to keep the microscope on the same sublingual area. Offline analysis (AVA 3.0 software; MicroVision Medical, Amsterdam, The Netherlands) involved the quantitative assessment of the functional capillary density and the velocity of the erythrocytes flowing in the perfused microvessels. Microcirculatory parameters were described as Perfused Vessel Density (PVD) and Erythrocyte Flow Velocity (EFV). EFV was assessed using the Space-Time Diagrams method in specific perfused capillaries sampled at baseline and identified and measured again at the successive time points.

**RESULTS.** Analysis of the Cytocam-IDF images in the fixed area allowed us to track specific capillaries during the entire experiment. We observed that normal perfused capillaries at the baseline became plugged or non-perfused in shock [arrow in Fig. 40]. More importantly, these plugged capillary vessels could not be reopened with fluid resuscitation despite correction of systemic hemodynamic variables.

**CONCLUSIONS.** Cytocam-IDF could identify the presence of sepsis-induced plugging of microcirculatory capillary vessels in one fixed sublingual area. In addition, our results demonstrated that microcirculatory alteration associated with sepsis persisted despite successfully normalized systemic hemodynamic variables.

**GRANT ACKNOWLEDGMENT**
This work is financially supported by Dutch Kidney Foundation Grant (14 OI 11)

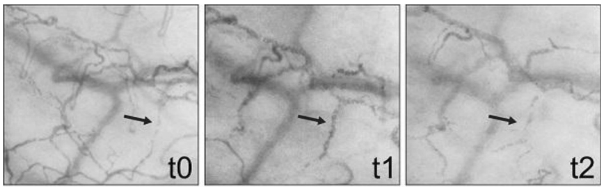

**Fig. 40 (abstract 0077).** See text for description

### 0078

#### Induction of human lymphoma cell death by adrenergic beta receptor stimulation

N. Matsuda, S. Xu, M. Higashi, M. Nishikimi, T. Yamamoto, A. Numaguchi, M. Kado, H. Tokuyama
Nagoya University Graduate School of Medicine, Emergency & Critical Care Medicine, Nagoya, Japan
**Correspondence:** N. Matsuda
*Intensive Care Medicine Experimental* 2017, **5(Suppl 2):**0078

**INTRODUCTION.** In systemic inflammation such as sepsis, adrenergic beta receptor stimulation can modify function of immunocompetent cells.

**OBJECTIVES.** This study aims to analyze beta receptor signaling in human lymphdma cell line and the differentiated neutrophils.

**METHODS.** Human Caucasian promyelocytic leukaemia cell line (HL-60) was differentiated into neutrophil-like cell by 1.25% dimethyl sulfoxide (DMSO). Free radical was detected inneutrophil-like cell by rapid and sensitive luminescent assay that measures the level of hydrogen peroxide (H2O2) ROS-Glo Assay. RT-PCR, immunohistochemistry and tunnel stain were used in the detection of molecular expression and apoptosis. For statistical analysis, t-test was used.

**RESULTS.** Neutrophil-like cells were differentiated from HL-60 by DMSO with Gr-1 and lobulated nucleus. It expressed all subtypes of adrenergic beta receptor. DOB (10-8 ~ 10-7 M) increased the production of reactive oxygen species and apoptosis by 40 times in a comparison with no adminstration of DOB by 24 hours. Randiorol and Beta1 receptor antagonist could inhibit the prpduction of reactive oxygen species and apoptosis reactive by 70%.

**CONCLUSIONS.** Beta receptor is expressed in neutrophils differentiated from HL-60. Beta receptor stimulation increases reactive oxygen species from the neutrophils and induces apoptosis in the neutrophils. Beta receptor antagonists can reduce production of reactive oxygen species.

### 0079

#### Central venous-to-arterial carbon dioxide difference combined with arterial-to-venous oxygen content difference ($P_{cva}CO_2/C_{av}O_2$) is associated with microcirculatory alterations in early septic shock

A. Pérez-Madrigal[1], C. Espinal[1], P. Saludes[1], L. Proença[2], E. Cortes[1], L. Enseñat[1], G. Gruartmoner[1], J. Mesquida[1]
[1]Hospital de Sabadell, Universitat Autònoma de Barcelona, Critical Care Department, Sabadell, Spain; [2]Hospital Prof. Dr. Fernando Fonseca, Serviço de Medicina I, Amadora, Portugal
**Correspondence:** A. Pérez-Madrigal
*Intensive Care Medicine Experimental* 2017, **5(Suppl 2):**0079

**INTRODUCTION.** Central venous-to-arterial carbon dioxide difference ($P_{cva}CO_2$) and the $P_{cva}CO_2$/arterial-venous oxygen content difference ratio ($P_{cva}CO_2/C_{av}O_2$) have been proposed as additional tools in the hemodynamic resuscitation process.

**OBJECTIVE.** To explore the relationship between global $CO_2$-derived parameters and microcirculatory oxygenation, evaluated by using near-infrared spectroscopy (NIRS).

**METHODS.** Observational study in a 30-bed mixed ICU. Fifty septic shock patients within the first 24 hours of ICU admission were studied. After restoration of mean arterial pressure, hemodynamic, metabolic and microcirculatory parameters were simultaneously evaluated. Local tissue oxygen saturation ($StO_2$), and local hemoglobin index (THI) were measured continuously on the thenar eminence by means of near-infrared spectroscopy (InSpectra[TM], Hutchinson Tech.). A transient vascular occlusion test was performed in order to obtain $StO_2$ deoxygenation rate ($DeO_2$), local oxygen consumption ($nirVO_2$), and reoxygenation rate ($ReO_2$). Statistical analysis: Three groups according to $P_{cva}CO_2$ were predefined: (1) < 6 mmHg, (2) 6–9.9 mmHg, and (3) ≥ 10 mmHg. Three groups were also predefined for $P_{cva}CO_2/C_{av}O_2$: (1) < 1, (2) 1–1.8, and (3) > 1.8. Differences among groups were assessed using the Kruskal-Wallis test, with a post hoc Mann–Whitney analysis.

**RESULTS.** At inclusion, $P_{cva}CO_2$ values inversely correlated with cardiac index, while $P_{cva}CO_2/C_{av}O_2$ correlated with lactate, and $ScvO_2$. Progressively lower $DeO_2$, $nirVO_2$, and $ReO_2$ were observed at higher $P_{cva}CO_2/C_{av}O_2$ values (Fig. 41). Low $P_{cva}CO_2$ values were associated with higher $StO_2$ and THI values, whereas no differences in $DeO_2$, $nirVO_2$ and $ReO_2$ were observed (Fig. 42).

**CONCLUSIONS.** In a population of early septic shock patients, increases in the $P_{cva}CO_2/CavO_2$ ratio were associated with alterations at the microcirculatory level. While $P_{cva}CO_2$ was related to global and local flow variables, the $P_{cva}CO_2/C_{av}O_2$ ratio reflected tissue dysoxia and impaired microvascular reactivity.

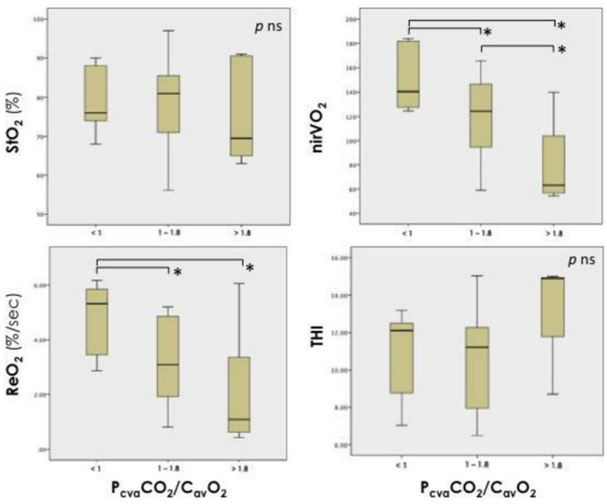

**Fig. 41 (abstract 0079).** NIRS-derived parameters for the three predefined $P_{cva}CO_2/C_{av}O_2$ groups

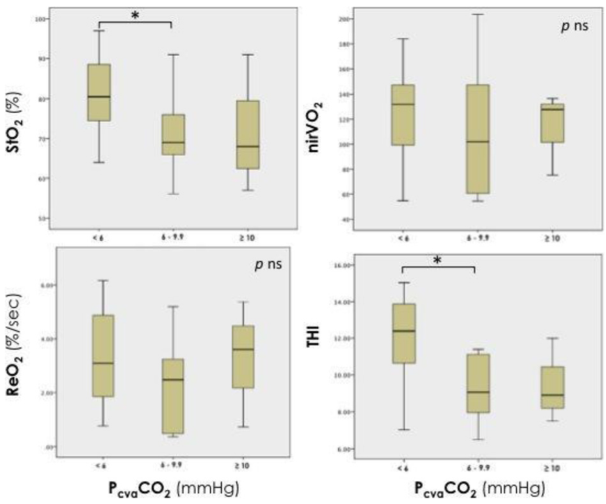

**Fig. 42 (abstract 0079).** NIRS-derived parameters for the three predefined $P_{cva}CO_2$ groups

### Fluid responsiveness

#### 0080

#### Transient or persistent fluid responders: toward a new definition of fluid responsiveness? The FC-Rev study

C. Roger[1], L. Zieleskiewicz[2], C. Demattei[1], K. Lakhal[3], G. Piton[4], J.-M. Constantin[5], R. Chabanne[6], J.S. Faure[6], Y. Mahjoub[7], I. Desmeulles[8], H. Quintard[9], J.-Y. Lefrant[1], AZUREA Group
[1]Nimes University Hospital, Nimes, France; [2]Hôpital Nord, Assistance Publique Hôpitaux de Marseille, Marseille, France; [3]Hôpital Laënnec, CHU Nantes, Nantes, France; [4]Réanimation Médicale, CHRU, Besançon, France; [5]Service de Réanimation Adulte, CHU Estaing, Clermont-Ferrand, France; [6]Hôpital Gabriel Montpied, CHU, Clermont-Ferrand, France; [7]Réanimation Chirurgicale, CHU, Amiens, France; [8]Service de Réanimation, CHU, Caen, France; [9]Réanimation Médico-Chirurgicale, CHU, Nice, France
**Correspondence:** C. Roger
*Intensive Care Medicine Experimental* 2017, **5(Suppl 2):**0080

**INTRODUCTION.** The goal of a fluid challenge (FC) is *in fine* to increase the stroke volume (SV) or the cardiac index (CI) when an episode of hypovolemia or a preload dependence status are observed.Fluid challenge is one of the most common practices in ICUs, however, the way to assess the response to FC is not standardized.

**OBJECTIVES.** The present study aimed to evaluate whether the trans-thoracic echocardiographic (TTE) assessment of the response to FC at the end of the infusion or 20 minutes later could affect the results of the FC.

**METHODS.** Prospective, observational, multicentre study including all ICU patients in septic shock requiring a FC. Were excluded patients with: arrhythmias, poor echogenicity and severe mitral or aortic regurgitation. The FC was performed administering 500 mL of crystalloids over 10 min. Fluid responsiveness was defined as a >15% increase in stroke volume (SV). Were collected MAP, HR and TTE variables at baseline ($T_0$), at the end of fluid challenge ($T_{10}$) and 10 ($T_{20}$) and 20 minutes ($T_{30}$) after the end of fluid challenge. The following echocardiographic parameters were recorded: E wave, A wave, E/A ratio, velocity-time integral (VTI), Ea wave and Sa wave. Quantitative data are expressed as mean and standard deviation (SD) or median and interquartile (IQR), according to their distribution. Qualitative data are expressed as absolute number and frequency (%). Statistical analysis was performed using R software version 3.0.2.

**RESULTS.** From May 20th 2014 to January 7th 2016, a total of 143 patients were enrolled in 11 French ICUs (mean age: 64 ± 14 years, median IGS II: 53 [43–63], median SOFA score: 10 [8–12]). Among the 76/143 (53%) patients responders to FC at T10, 37 patients were transient responders (TR), i.e. became non-responders at T30 (49%, 95%CI = [37–60]) and 39 (51%, 95%CI = [38–62])) patients were persistent responders (PR), i.e. remained responders at T30. Among the 67 non-responders (NR) at T10, 4 became responders at T30, (6%, 95%CI = [1.9-15.3]). In the subgroup analysis, no statistical difference in haemodynamic and echocardiographic parameters was found between non-responders, transient responders and persistent responders. The present study shows that, after a 15% VTI increase at the end of the FC, VTI returns to baseline at 30 minutes in half of the responders. Blood volume status before initiating the fluid infusion could explain the transient or persistent response to FC observed in septic patients.

**CONCLUSIONS.** This study describes, for the first time, three different responses to FC: non-responders, persistent responders, and transient responders. Our results suggest that the optimal timing to assess fluid responsiveness should be investigated in further studies.

**GRANT ACKNOWLEDGMENT**
Nimes University Hospital Academic grant

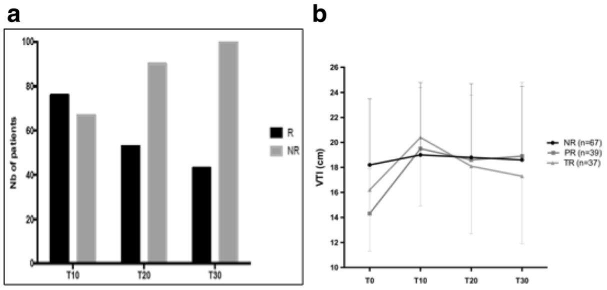

**Fig. 43 (abstract 0080). a** Proportion of R and NR at the end (T10), 10 min T(20), 20 min T(30) after the FC. **b** VTI time course during FC (T0 to T10) and during the 20 min after the FC (T10–T30). *R* responders, *NR* non-responders, *PR* persistent responders, *TR* transient responders

### 0081

#### Does echocardiographic finding of "hyperdynamic right ventricle unproportionally to low stroke volume" predict fluid responsiveness? An interim report

Y. Fujimoto[1], J. Kataoka[1], S. Fujitani[2], E. Hiraoka[1], Y. Norisue[1]
[1]Tokyo Bay Urayasu/Ichikawa Medical Center, Urayasu, Japan; [2]St Marianna University, Kawasaki, Japan
**Correspondence:** Y. Fujimoto
*Intensive Care Medicine Experimental* 2017, **5(Suppl 2):**0081

**INTRODUCTION.** In clinical practice, it is frequently experienced that hemodynamically unstable patients with hypovolemia show underfilled and hyperdynamic right ventricle to compensate inadequate preload on echocardiogram. Tricuspid annular plane systolic excursion (TAPSE) is a parameter easily measured by transthoracic echocardiogram (TTE) and indicates global RV function, which describes apex-to-base shortening[1]. On the other hand, left ventricular outflow tract velocity time integral (LVOT-VTI) directly reflects left ventricular stroke volume and is also easily measured by TTE. We hypothesized that the hemodynamically unstable patients who have fluid responsiveness show hyperdynamic right ventricle unproportionally to low stroke volume, thus, higher TAPSE/LVOT-VTI ratio compared to those without fluid responsiveness.

**OBJECTIVES.** The aim of this study is to evaluate the usefulness of TAPSE/LVOT-VTI ratio and to find the cutoff value of TAPSE/LVOT-VTI ratio to predict fluid responsiveness in critically ill patients.

**METHODS.** Patients with the clinical findings of tissue hypoperfusion for whom the attending physicians decided to conduct rapid fluid challenge test in the absence of contraindication for fluid infusion were include in this study. TTE was used to obtain VTI, LVOT diameter and TAPSE. After baseline hemodynamic measurements were obtained, an intravenous volume expansion consisting of 500 ml colloid solution was given over 10–20 minutes. The second hemodynamic measurements were performed within 5 minutes after volume expansion. A responder was defined as the patients with increase in stroke volume or cardiac output by 15% or more from baseline after fluid challenge measured with TTE.

**RESULTS.** A total of 32 critically ill patients were included so far in the intensive care unit at Tokyo Bay Urayasu/Ichikawa medical center. Of 32 patients, 17 patients were responders and 15 patients were non-responders. Receiver operating characteristic (ROC) curve was generated for TAPSE(mm)/LVOT-VTI(cm) with a cut off value of 0.95 which showed sensitivity of 88%, specificity of 75% and AUC of 0.84 for fluid responsiveness (95%CI 0.706-0.978).

**CONCLUSIONS.** TAPSE/LVOT-VTI ratio may be a useful parameter to predict fluid responsiveness in critically ill patients.

**GRANT ACKNOWLEDGMENT**
No grant

### 0082

#### Changes in Doppler E-wave Deceleration Time predicts non-fluid responsiveness in critically ill patients: a pilot study

E.D. Valenzuela Espinoza[1], C. Santis[1], M. Vera[1], L. Alegría[1], M. Silva[1], D. Carpio[1], S. Bravo[1], J. Retamal[1], G. Bugedo[1], A. Bruhn[1], R. Castro[1], G. Hernández[1], J. Bakker[1,2,3]
[1]Pontificia Universidad Católica de Chile, Facultad de Medicina, Medicina Intensiva, Santiago, Chile; [2]Columbia University Medical Center, Pulmonology, New York, United States; [3]Erasmus MC University Medical Centre Rotterdam, Intensive Care Adults, Rotterdam, Netherlands
**Correspondence:** E.D. Valenzuela Espinoza
*Intensive Care Medicine Experimental* 2017, **5(Suppl 2):**0082

**INTRODUCTION.** Changes in early relaxation strongly influence diastolic function. Doppler E-wave Deceleration Time (DT) is determined by ventricular stiffness and could thus be related to diastolic filling. Therefore, in patients with diastolic dysfunction, DT could be related to fluid responsiveness (FR+).

**OBJECTIVE.** To evaluate the performance of DT during a passive leg raising test (PLR).

**METHODS.** Patient with normal systolic function were enrolled to assess fluid responsiveness using passive leg raising. DT, left ventricular outflow tract velocity time integral (VTI), maximal E and A wave velocity where measured by a single operator trained in echocardiography. FR+ was defined as an increase in VTI > 10% following PLR. Paired - T test and Student´s *t* test were used for statistical analysis.

**RESULTS.** Twelve patients with a mean age of 68 ± 12 were included. Five patients were on mechanical ventilation. Three patients were fluid responsive. Baseline VTI and A-wave were lower in responders to PLR (table).

The change in DT during PLR was −54 ± 38 (p = 0.003) in the non responders and −22 ± 53 (p = 0.54) in responders.

**CONCLUSION.** DT decreases significantly in nonresponders following a PLR possibly reflecting a higher increase in ventricular filling pressure, even with a relatively small change in volume, compared to responders.

**Table 26 (Abstract 0082).** Clinical and Echocardiographic Variables

| Clinical and Echocardiographic Variables | All Baseline | PLR Responders n = 3 | | PLR Non Responders n = 9 | |
|---|---|---|---|---|---|
| | | Baseline | PLR | Baseline | PLR |
| HR, beats/min | 89 ± 22 | 114 ± 24 | 114 ± 20 | 83 ± 17 | 81 ± 18 |
| SBP, mmHg | 115 ± 18 | 110 ± 16 | 115 ± 8 | 116 ± 20 | 112 ± 21 |
| DBP, mmHg | 58 ± 10 | 64 ± 18 | 67 ± 10 | 57 ± 9 | 55 ± 9 |
| MAP, mmHg | 78 ± 12 | 83 ± 20 | 87 ± 16 | 77 ± 11 | 76 ± 13 |
| PP, mmHg | 57 ± 17 | 46 ± 4 | 47 ± 12 | 60 ± 18 | 57 ± 20 |
| LVOT VTI, cm | 20 ± 5 | 17 ± 1** | 20 ± 2* | 22 ± 5 | 21 ± 5 |
| E wave, m/sec | 0.73 ± 0.22 | 0.70 ± 0.11 | 0.78 ± 0.16 | 0.74 ± 0.25 | 0.80 ± 0.26 |
| E wave DT, ms | 214 ± 56 | 181 ± 69 | 159 ± 24 | 225 ± 50 | 171 ± 26* |
| A wave, m/sec | 0.65 ± 0.19 | 0.46 ± 0.14** | 0.47 ± 0.22 | 0.72 ± 0.17 | 0.72 ± 0.22 |

Values are expressed as mean ± SD. HR, heart rate; SBP, systolic blood pressure; DBP, diastolic blood pressure; MAP, mean arterial pressure; PP, pulse pressure; LVOT left ventricular outflow tract; VTI, velocity time integral; DT, deceleration time; PLR, passive leg raising; * (p<0.05) between baseline and PLR; ** (p<0.05) between fluid responders and non-fluid responders at baseline.

### 0083

#### Effects of end-expiratory occlusion test application during pressure support ventilation

A. Messina[1], D. Colombo[1], G. Cammarota[1], V. Rondi[1], F. Della Corte[1], P. Navalesi[2]
[1]AOU Maggiore della Carità, Novara, Italy; [2]Università della Magna Graecia, Catanzaro, Italy
**Correspondence:** A. Messina
*Intensive Care Medicine Experimental* 2017, **5(Suppl 2):**0083

**INTRODUCTION.** The end-expiratory occlusion test (EEO) is a functional hemodynamic test aiming to reveal preload dependency by increasing venous return after interrupting cyclic ventilation at end expiration (1). However, insofar only one study demonstrated that EEO is reliable also in critically ill patients with residual spontaneous breathing activity (1). The respiratory effect of interrupting the inspiratory support during EEO, when diaphragmatic activity is preserved has never been evaluated. In fact, inspiratory efforts may alter EEO reliability by reducing pleural pressure and, hence, increasing venous return to the right ventricle.

**OBJECTIVES.** Primary aim: to investigate the inspiratory efforts triggered by EEO application in critically ill patients undergoing pressure support ventilation (PSV). Secondary aim: to investigate EEO reliability in patients undergoing PSV.

**METHODS.** We enrolled 8 patients with a Richmond Agitation Sedation Scale ranging from −2 (light sedation) and 0 (calm) and requiring a fluid challenge (FC). The hemodynamic effects of EEO and FC were assessed by means of the MOSTCARE$^{TM}$ system and transthoracic echocardiography (TTE). Esophageal pressure (Pes) was measured using a latex balloon-tipped catheter system, connected to a pressure transducer, as previously described (2). Airway opening pressure, flow and Pes were displayed together with the arterial pressure waveforms by specific data acquisition software and a dedicated cable for interfacing the two systems (ICU-Lab System, KleisTEK; Bari, Italy). The dynamic occlusion test was performed to check the correct position of the balloon (2). The EEO was performed, as previously described (1), 3 minutes after the dynamic occlusion test ($EEO_1$) and then repeated again after 3 minutes ($EEO_2$). FC consisted of 500 ml infusion of crystalloids in 10 minutes. FR was defined as an increase in cardiac index ≥15% after FC administration, measured by means of both the MostCare and TTE. Receiving operator characteristic (ROC) was constructed for percent changes in cardiac index (CI) and pulse pressure (PP) induced by the EEO.

**RESULTS.** 33.3% of EEOs were interrupted because patient's triggering of ventilator or cough occurrence. The mean inspiratory pressure recorded was −21 ± 12 $cmH_2O$, ranging from −8 to −54 $cmH_2O$ (Fig. 44). Three patients were fluid responders (see Table 27). The ROC curves for CI and PP of the 13 EEO tests completed were not significant (p = 0.89 and p = 0.75, respectively).

**CONCLUSIONS.** In a selected population of critically ill patients undergoing PSV, the EEO application might determine significant variations in the inspiratory pressure, and hence, pleural pressure. These preliminary data also suggest that EEO reliability in lightly sedated critically ill patients undergoing PSV is still uncertain.

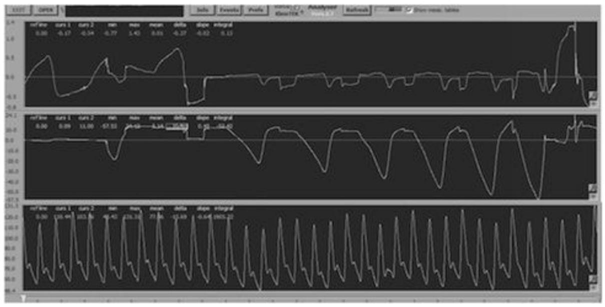

**Fig. 44 (abstract 0083).** See text for description

**Table 27 (Abstract 0083).** See text for description

| Patients | EEO1 | EEO1 | EEO1 | EEO2 | EEO2 | EEO2 | RASS | (%)ΔCI MOSTCARE post FC | (%)CIΔ TTE post FC |
|---|---|---|---|---|---|---|---|---|---|
| Responsiveness | Status | Δmax Inspiratory Pressure (cmH20) | ΔCI (%) | Status | Δmax Inspiratory Pressure (cmH20) | ΔCI (%) | | | |
| Non-responder | Completed | −21 | 13 | Completed | −18 | 4 | 0 | 8 | 6,7 |
| Responder | Completed | −27 | 4,8 | Completed | −12 | 11 | 0 | 16,8 | 18 |
| Non-responder | Interrupted | NA | NA | Completed | −18 | 0,73 | −1 | 3 | 5,5 |
| Non-responder | Interrupted | NA | NA | Completed | −29 | 14,4 | 0 | 4,8 | 4,2 |
| Responder | Completed | −14 | 8,67 | Completed | −8 | 8,4 | −2 | 19 | 18,4 |
| Responder | Completed | −54 | 0,66 | Interrupted | NA | NA | −2 | 17,5 | 16,8 |
| Non-responder | Completed | −24 | 5,6 | Interrupted | NA | NA | −1 | 3,2 | 4,2 |
| Non-responder | Interrupted | NA | NA | Completed | −13 | 3,8 | 0 | 6,8 | 7,4 |

### 0084

#### Assessing right ventricular volume responsiveness

M.F. Graessler[1], K.H. Wodack[1], H.O. Pinnschmidt[2], S. Nishimoto[1], C.R. Behem[1], D.A. Reuter[1], C.J.C. Trepte[1]
[1]University Medical Center Hamburg Eppendorf, Department of Anaesthesiology, Hamburg, Germany; [2]University Medical Center Hamburg Eppendorf, Department of Medical Biometry and Epidemiology, Hamburg, Germany
**Correspondence:** M.F. Graessler
*Intensive Care Medicine Experimental* 2017, **5(Suppl 2):**0084

**INTRODUCTION.** Correct assessment of a patient's volume responsiveness is crucial for guidance of fluid therapy. Dynamic indicators of preload were identified as most suitable parameters to predict volume responsiveness[1–2]. However, so far they only have been assessed systematically from systemic circulation, although particularly the right heart is sensitive to modifications of either preload or afterload. Thereby it potentially represents the weakest link in the chain when it comes to volume administration. Earlier publications showed that that during mechanical ventilation also changes in right ventricular stroke volume do occur[3–4].

**OBJECTIVES.** To date no dynamic right ventricular indicators have been established. Therefore, the aim of this study was to calculate dynamic indicators of right ventricular preload and assess their ability to properly predict right ventricular volume responsiveness in an experimental model.

**METHODS.** The study was designed as prospective trial in 20 domestic pigs. Animals were anaesthetized and mechanically ventilated. An ultrasonic flow probe was fit around the pulmonary artery to enable gold standard measurement of right ventricular stroke volume. Also a microtip catheter was directly placed in the pulmonary artery. Hypovolaemia was induced (withdrawal of blood 20ml/kg bodyweight) and thereafter three volume loading steps were performed re-transfusing the shed blood. ROC curves were calculated to assess the ability of dynamic right ventricular parameters to predict volume response ($SVV_{RV}$, $PPV_{PA}$, $SPV_{PA}$).An increase in stroke volume >15% was considered as a positive volume response.

**RESULTS.** ROC analysis revealed an area under the curve (AUC) of 0.82 (CI 95% 0.72-0.94; p < 0.001) for $SVV_{RV}$, an AUC of 0.74 (CI 95% 0.59-0.89; p < 0.01) for $PPV_{PA}$ and a AUC of 0.69 (CI 95% 0.53-0.84; p-value =0.03) for $SPV_{PA}$.

**CONCLUSIONS.** In our experimental animal model calculation of dynamic right ventricular indicators is possible and particularly $SVV_{RV}$ and $PPV_{PA}$ appear promising in predicting right ventricular volume responsiveness.

**REFERENCES(S)**

1. Zhang Z, Lu B, Sheng X, Jin N. Accuracy of stroke volume variation in predicting fluid responsiveness: a systematic review and meta-analysis. *J Anesth* 2011; **25**: 904–16

2. Yang X, Du B. Does pulse pressure variation predict fluid responsiveness in critically ill patients? A systematic review and meta-analysis. *Crit Care* 2014; **18**: 650
3. Vieillard-Baron A, Chergui K, Augarde R, et al. Cyclic changes in arterial pulse during respiratory support revisited by Doppler echocardiography. *Am J Respir Crit Care Med* 2003; **168**: 671–6
4. Vieillard-Baron A, Loubieres Y, Schmitt JM, Page B, Dubourg O, Jardin F. Cyclic changes in right ventricular output impedance during mechanical ventilation. *Journal of applied physiology* 1999; **87**: 1644–50

**GRANT ACKNOWLEDGMENT**
The study was supported by departmental funds of the Department of Anaesthesiology, University Medical Centre Hamburg-Eppendorf

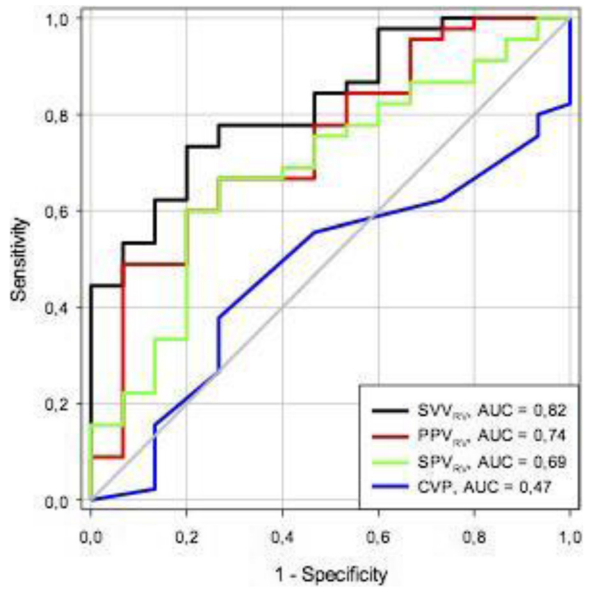

**Fig. 45 (abstract 0084).** ROC Curves

### 0085

#### Dynamic bedside evaluation of fluid responsiveness in hemodynamically unstable critically ill patients

U. La Brocca[1], S. Carelli[1], T. Taccheri[1], L. La Via[1], V. Piccoli[1], I. Cisterna[1], F. Torrini[1], A.M. Dell Anna[2], M. Antonelli[1]
[1]Fondazione Policlinico Universitario A. Gemelli, Roma, Italy; [2]Policlinico Universitario A. Gemelli, Roma, Italy
**Correspondence:** U. La Brocca
*Intensive Care Medicine Experimental* 2017, **5(Suppl 2):**0085

**INTRODUCTION.** Fluid administration is a common treatment in ICU patients affected by haemodynamic instability, in order to improve cardiac output and finally optimize tissue perfusion.

**OBJECTIVES.** This study aimed to assess whether dynamic haemodynamic parameters such as the variation in central venous pressure (D CVP) and D systemic perfusion pressure (D PP) after a fluid challenge are reliable tools to assess patients' fluid status.

**METHODS.** In this prospective, observational, monocentric trial we enrolled patients affected by haemodynamic instability, as suggested by common clinical parameters (HR > 120 bpm, MAP < 70 mmHg, diuresis < 0,5 ml/Kg/h and blood lactates >4 mmol/l). Exclusion criteria were age < 18, recent politrauma, moderate and severe left ventricular dysfunction, hemorrhagic shock and pregnancy. At baseline clinical characteristics were registered, as well as haemodynamic respiratory and metabolic parameter as SAP, DAP, MAP, HR, PVC, organ perfusion pressure (PP = MAP-PVC), data from arterial and venous blood gas analysis, urinary output; SVI, CI and derived parameters (SVRI, etc.) were also detected through transpulmonary thermodilution system EV1000 (Edwards Lifescience©TM). Fluid responsiveness was tested with the infusion of 500 ml of Ringer Lactate solution in 10 minutes. All measurements were repeated at the end of the fluid bolus and the test considered positive if followed by an increasing in CI > 12%, categorizing fluid responders (FR) and non fluid responders (NFR) patients.

**RESULTS.** We enrolled fourteen patients for a total of 19 measures. Baseline characteristics were not statistically different between FR and NFR. The rate of fluid responsiveness was 42%. After the fluid bolus, in the whole population MAP was significantly higher (p = 0.049) as well as CVP (9 [4–11] vs 12 [6–14] mmHg, p < 0.001), SVI (p = 0.014), CI (p = 0.013) and $DO_2I$ (p = 0.014) whilst $VO_2I$, $O_2ERI$, and DPP were not (p = ns); SVRI decreased significantly (p = 0.04). In FR MAP did not change significantly; otherwise, CVP increased significantly (8 [5–10] vs 11.5 [6.4-14], p = 0.015), while it did not change in NFR (9 [4,5-12,7] vs 12 [6–14], p = ns); SVRI decreased significantly only in FR (2208 vs 1937, p = 0.016).Nevertheless, DCVP was not significantly different between FR and NFR, as well as the DPP. In FR $DO_2I$ increased significantly after the fluid bolus (332 [235–369] vs 388 [304–488]ml/min/m2, p = 0.015) but $VO_2I$ and O2ERI did not.

**CONCLUSIONS.** CVP increased significantly after fluid challenge, particularly in FR. Moreover, both in the overall population and in FR the increasing in CI and $DO_2I$ was not associated with a significantly increase in $VO_2I$ and $O_2ERI$. Therefore, the question regarding the appropriateness of fluid expansion in critical patients remains still open, considering the possible damage due to fluid overload, and new prospective studies are warranted.

### 0086

#### Accuracy of the passing leg raising test in patients with intra-abdominal hypertension

A. Beurton[1,2], J.L. Teboul[1,2], V. Girotto[1,2], L. Galarza[1,2], C. Richard[1,2], X. Monnet[1,2]
[1]Hôpital Bicêtre, Hôpitaux Universitaires Paris-Sud, Assistance Publique - Hôpitaux de Paris, Le Kremlin-Bicêtre, France; [2]Inserm UMR S_999, Université Paris-Sud, Le Kremlin-Bicêtre, France
**Correspondence:** A. Beurton
*Intensive Care Medicine Experimental* 2017, **5(Suppl 2):**0086

**INTRODUCTION.** The passive leg raising (PLR) test is a reversible and repeatable volume challenge that is used to test preload responsiveness. During the PLR test, venous blood is transferred from the legs and the splanchnic territories toward the cardiac cavities, resulting in an increase in cardiac preload. Nevertheless, intra-abdominal hypertension (IAH) may impede the PLR-induced increase in cardiac preload by decreasing the venous return.

OBJECTIVE. We tested whether the reliability of the PLR test is reduced in case of IAH.

**PATIENTS AND METHODS.** We included patients with an intra-abdominal pressure (IAP) ≥12 mmHg. We measured the changes of cardiac output (CO, PiCCO2 device) before and during a PLR test, and before and after a subsequent volume expansion (500mL of saline). A patient was defined as fluid responder if cardiac output increased ≥ 15% during a volume expansion. IAP was measured through the bladder pressure.

**RESULTS.** We included 29 patients (IAP at baseline 20 ± 5 mmHg, 20 fluid responders, 9 fluid non-responders). During PLR, IAP significantly decreased in fluid responders (by 34 ± 13%) as well as in fluid non-responders (by 30 ± 16%). In fluid responders, cardiac output increased by 7 ± 9% during PLR and by 22 ± 6% during volume expansion. The PLR test was negative (PLR-induced increase in cardiac output < 10%) in 15 patients (false negatives) and positive in 5 patients (true positives). In fluid non-responders, the PLR test was negative in all patients. The sensitivity and specificity of the PLR test to detect fluid responsiveness were 25% (95% confidence interval: 60-98%) and 89 (57–98)%, respectively (area under the receiver operating characteristics curve: 0.58 ± 0.11).

**CONCLUSIONS.** Intra-abdominal hypertension is responsible for false negatives to the PLR test. The PLR test significantly reduces IAP.

### 0087

#### Can carotid and femoral Doppler assess the effects of passive leg raising?

V. Girotto[1], J.-L. Teboul[1], A. Beurton[1], L. Galarza[1], T. Guedj[2], C. Richard[1], X. Monnet[1]
[1]Service de Réanimation Médicale, Hôpital de Bicêtre, Hôpitaux Universitaires Paris-Sud, Assistance Publique - Hôpitaux de Paris, Le Kremlin-Bicêtre, France; [2]Service de Radiologie, Hôpital de Bicêtre, Hôpitaux Universitaires Paris-Sud, Assistance Publique - Hôpitaux de Paris, Le Kremlin-Bicêtre, France
**Correspondence:** V. Girotto
*Intensive Care Medicine Experimental* 2017, **5(Suppl 2):**0087

**INTRODUCTION.** A direct measurement of cardiac index is usually needed to assess the hemodynamic effects of passive leg raising (PLR) and fluid infusion. Nevertheless, changes in carotid and femoral blood flow may be proportional to changes in cardiac output.
**OBJECTIVES.** We tested if Doppler assessment of carotid and femoral blood flows and of their peak velocities could reflect the changes in cardiac output during a PLR and a fluid infusion.
**METHODS.** In 51 critically ill patients, we performed Doppler measurements of carotid and femoral blood flows and peak systolic velocities, as well as calibrated pulse contour cardiac index, before and during the PLR and before and after fluid infusion. Arterial diameter and velocity time integral or time average velocity were used to obtain Doppler blood flow values. If cardiac index increased ≥10% during PLR, the patient was considered as a "PLR responder". Fluid infusion (500 mL saline) was performed in PLR responders only (27 cases).
**RESULTS.** Considering all changes observed during PLR and fluid infusion (n = 120), cardiac index increased by 14 ± 15%, carotid blood flow by 15 ± 35% (n = 59) and femoral blood flow by 23 ± 36% (n = 14). No correlation was found between changes in cardiac index and changes in carotid and femoral blood flows (r = 0.07 and r = 0.28, respectively). In PLR responders, cardiac index increased by 19 ± 11% (n = 27), carotid blood flow by 13 ± 38% (n = 21) and femoral blood flow by 3 ± 12% (n = 3). We could not obtain a correct Doppler signal of the femoral artery in 38 patients. Neither the changes in carotid (area under the receiver operating characteristics curve (AUROC): 0.58 ± 0.10) and femoral blood flows nor the changes in their peak systolic velocities were able to detect a positive PLR test.
**CONCLUSIONS.** In our hands, Doppler assessment of carotid and femoral blood flows and of their peak velocities was not a reliable method to assess the changes in cardiac index during a PLR or fluid infusion.
**REFERENCES**
1. Marik PE, Levitov A, Young A, et al. The use of bioreactance and carotid Doppler to determine volume responsiveness and blood flow redistribution following passive leg raising in hemodynamically unstable patients. Chest. 2013;143:364–70
2. Préau S, Saulnier F, Dewavrin F, et al. Passive leg raising is predictive of fluid responsiveness in spontaneously breathing patients with severe sepsis or acute pancreatitis. Crit Care Med. 2010;38:819–25

### 0088

#### S wave variation to predict fluid responsiveness in icu patients undergoing controlled mechanical ventilation

A. Messina[1], F. Franchi[2], D. Colombo[1], F. Della Corte[1], P. Navalesi[3], S. Scolletta[2]
[1]AOU Maggiore della Carità, Novara, Italy; [2]Azienda Ospedaliera Universitaria Senese, Siena, Italy; [3]Università degli Studi Magna Græcia di Catanzaro, Catanzaro, Italy
**Correspondence:** A. Messina
*Intensive Care Medicine Experimental* 2017, **5(Suppl 2):**0088

**INTRODUCTION.** The role of echocardiography in predicting fluid responsiveness at bedside is still limited. The transesophageal echocardiography is a highly-skilled technique which was used to evaluate the aortic blood flow increase after volume expansion (VE)[1] or the respiratory changes in left ventricular outflow tract velocities (VTI)[2]. Moreover, also the use of the transthoracic echocardiography (TTE) to measure VTI is not always simply to obtain or reproduce in ICU patients[3]. TTE tissue Doppler imaging (TDI) has been proposed in different ICU setting to investigate diastolic dysfunction or false-positive pulse pressure variation. Considering that the systolic velocity wave (S wave) is correlated to the systolic ventricular function, we postulated that its variation during controlled mechanical ventilation (CMV) could be related to preload dependency.
**OBJECTIVES.** To assess the reliability of the dynamic variation of the systolic tissue Doppler wave (ΔSwave) to predict fluid responsiveness in ICU patients undergoing MCV.
**METHODS.** We studied 18 patients undergoing MCV requiring a VE (500 ml of crystalloids in 10 minutes). Hemodynamic measurements were obtained using MOSTCARE™ and TTE. All the predefined validity criteria of pulse pressure variation were respected, according to the literature[4]. Study protocol consisted of 3 steps:

1) at baseline the patients were ventilated with a tidal volume (Vt) of 6 ml/kg;
2) the Vt was increased up to at least 8 ml/kg for 5 minutes;
3) Vt was decreased to baseline values and a VE was administrated.

At each step, S waves were recorded for tricuspid annulus, septal and lateral mitral annulus. ΔSwave was calculated post-hoc as the percentage change between the two highest and two lowest values of 10 consecutive S waves recorded. ROC curves were constructed considering a patient showing a CI increase ≥ 15% after VE as fluid-responder.
**RESULTS.** 22 patients were enrolled but 4 were excluded because of technical limitations in obtaining reliable echo images after increasing Vt, either from tricuspid or mitral annulus. The AUCs of $PPV_{6\ ml/kg}$ and of $PPV_{8\ ml/kg}$ were 0.64 ($CI_{95}$ 0.36-0.91) and 0.84 ($CI_{95}$ 0.69-1.00), respectively. The AUC of ΔSwave of the tricuspid valve was 0.74 ($CI_{95}$ 0.50-0.97) and 0.91 ($CI_{95}$ 0.78-1.0) for a Vt of 6 and ≥ 8 ml/kg, respectively. The AUC of ΔSwave of the mitral septum annulus was 0.81 ($CI_{95}$ 0.61-1.00) and 0.86 ($CI_{95}$ 0.69-1.02) for a Vt of 6 and ≥ 8 ml/kg, respectively. The AUC of ΔSwave of the mitral lateral annulus was 0.78 ($CI_{95}$ 0.69-1.02) and 0.86 ($CI_{95}$ 0.69 to 1.02) for a Vt of 6 and ≥ 8 ml/kg, respectively (see also Table 28).
**CONCLUSIONS.** These preliminary data show that ΔSwave could be considered as a promising echographic measurement to predict fluid responsiveness in ICU patients.
**REFERENCES**
1) Monnet et al. ICM. 2005 Sep; 31(9):1195–201;
2) Marik CCM October 2016; 44 (10);
3) Feissel et al. CHEST 2001; 119:867–873;
4) Mahjoub et al. BJA 112 (4): 681–5 (2014).

**Table 28 (Abstract 0088).** See text for description

| | ΔSwave Tricuspid Vt ≥ 8 ml/kg | ΔSwave Mitral Septum Vt ≥ 8 ml/kg | ΔSwave Mitral Lateral Vt ≥ 8 ml/kg |
|---|---|---|---|
| Sensitivity | 80% | 60% | 70% |
| Specificity | 87.5% | 87.5% | 87.5% |
| Threshold | 17% | 14% | 14% |
| | ΔSwave Tricuspid Vt = 6 ml/kg | ΔSwave Mitral Septum Vt = 6 ml/kg | ΔSwave Mitral Lateral Vt = 6 ml/kg |
| Sensitivity | 40% | 50% | 50% |
| Specificity | 87.5% | 87.5% | 87.5% |
| Threshold | 10% | 11% | 11% |

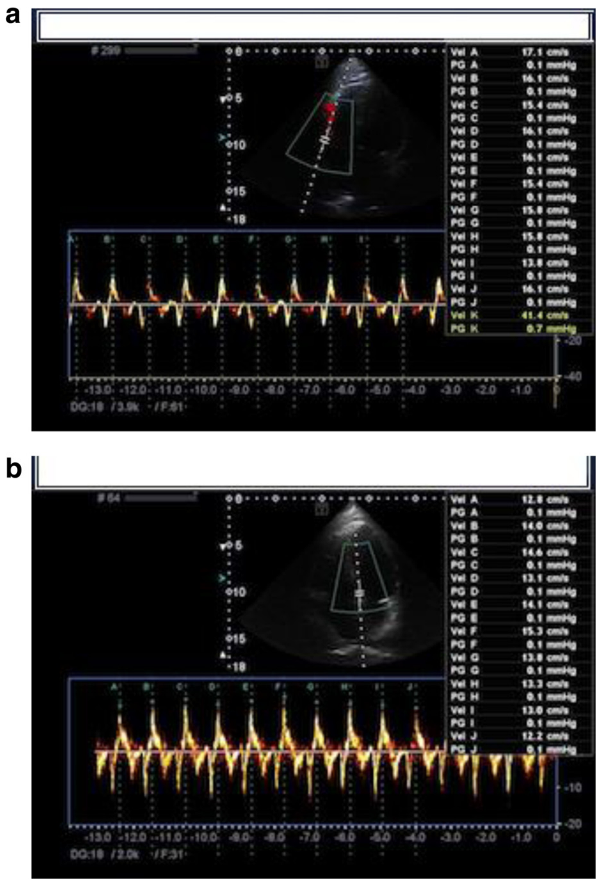

**Fig. 46 (abstract 0088).** See text for description

### 0089

#### Correlation of ultrasound measurement of IVC diameter with CVP and pulmonary artery pressures in mechanically ventilated patients

T. Melissopoulou, D. Xanthis, P. Vernikos, T. Kypraiou, M. Panoutsopoulou, K. Kolovou, N. Archontoulis, I. Floros
Laiko General Hospital, Intensive Care Unit, Athens, Greece
**Correspondence:** T. Melissopoulou
*Intensive Care Medicine Experimental* 2017, **5(Suppl 2):**0089

**INTRODUCTION.** Hemodynamic assessment of critically ill patients often involves the implementation of invasive techniques such as the placement of a central venous or a pulmonary artery catheter. Ultrasound measurement of IVC diameter may prove to be a useful, non-invasive alternative method of estimating volume status. Although in spontaneously breathing patients IVC dimensions seem to correlate well with CVP, data concerning mechanically ventilated patients are inconsistent.

**OBJECTIVES.** The aim of this study was to investigate whether a relationship between IVC diameter and collapsibility, CVP and pulmonary artery pressures exists in patients under mechanical ventilation.

**METHODS.**We performed a post-hoc analysis of prospectively collected data from 21 mechanically ventilated patients who were admitted to the ICU. A pulmonary artery catheter was inserted to all patients for hemodynamic monitoring and values of CVP, PAS, PAD, PAM were recorded. Ultrasound measurements of IVC diameter at end-expiration and at end-inspiration were made using the subxiphoid approach with the longitudinal plane for IVC imaging (GE LOGIC Q 500 ultrasound machine) and the IVC collapsibility index (IVCI) was calculated using the formula:

([IVCmax - IVCmin]/IVCmax)X100%

All measurements were made during controlled mechanical ventilation with Vt = 8ml/kg, R-R = 12/min and PEEP = 5cmH20.

**RESULTS.** Data from 21 measurements were statistically analyzed and tested for possible correlation using the Spearmans' Rho test. Significant correlations were found between IVCI and CVP (R = −0.870, p = 0.0001), PAS (R = −0.568, p = 0.007), PAD (R = −0.730, p = 0.0001) and PAM (R = −0.624, p = 0.0024). IVCe was correlated with CVP (R = +0.878, p = 0.0001), PAS (R = +0.579, p = 0.005), PAD (R = +0.650, p = 0.001) and PAM (R = +0.548, p = 0.01).

**CONCLUSIONS.** Respiratory variations of IVC diameter during mechanical ventilation seem to correlate with CVP as well as with pulmonary artery pressures. Ultrasound measurement of IVC dimensions and collapsibility could be a helpful tool in hemodynamic assessment in mechanically ventilated patients.

**REFERENCE(S)**

1. The efficacy of sonographic measurement of inferior vena cava diameter as an estimate of central venous pressure. Ciozda et al. Cardiovascular Ultrasound 2016;14:33

2. Correlation of IVC Diameter and Collapsibility Index With Central Venous Pressure in the Assessment of Intravascular Volume in Critically Ill Patients. Ilyas A. et al.(February 12, 2017). Cureus 9(2) e1025

### 0090

#### Prediction of fluid responsiveness in heterogeneous ICU-patients: a comparison of passive leg raising PLR, small volume challenge, CVC, $S_{cv}O_2$ and global enddiastolic volume index GEDVI with and without correction for femoral CVC

W. Huber, M. Hüttl, U. Mayr, R. Schmid
Klinikum rechts der Isar der TUM, Munich, Germany
**Correspondence:** W. Huber
*Intensive Care Medicine Experimental* 2017, **5(Suppl 2):**0090

**INTRODUCTION.** Appropriate fluid supply is crucial in critically ill patients. Parameters to predict fluid responsiveness (FR) include variabilities of the arterial pressure curve (e.g. stroke volume variation SVV), filling pressures (e.g. CVP) and volumes such as GEDVI. However, SVV is only applicable in case of sinus rhythm AND controlled Ventilation. The use of GEDVI may be limited due to an overestimation in case of femoral CVC, and filling pressures are confounded by intraabdominal and airway pressures. Therefore, − in case of doubt - a volume challenge VC is recommended as a gold-standard to measure FR. However, the infused volume might be harmful in non-responders. Therefore, passive leg raising PLR has been suggested as a reversible auto-transfusion. Furthermore, small VCs with a limited volume (1–3,5 mL/kg) might replace the standard VC (usually performed with 7mL/kg).

**OBJECTIVES.** Regarding a lack of studies comparing all these methods in one study, we compared the predictive capabilities of PLR (PLR vs. semi-recumbent-position) to those of CVP, GEDVI with and without correction for femoral CVC (1), $S_{cv}O_2$ and a small VC (SVC) with 3.5mL/kg to a conventional VC with 7mL/kg saline over 30min.

**METHODS.** 34 VCs with 7mL/kg in 27 patients (11f; 16m) were performed. APACHE-II 22 ± 6. Ventilation: spontaneous 16, assisted 16, controlled 2 patients. CVC jugular 2in 6, femoral in 8 patients. Transpulmonary thermodilution TPTD was performed before PLR, 5 min after PLR immediately before the VC (TPTD_2), after 15min with infusion of 3.5mL/kg (TPTD_3) and at the end of the VC (TPTD_4).

Pulse contour (PC) derived parameters such as CI_PC were documented at intervals of 15s during the PLR and of 5min during the VC.

**RESULTS.** FR defined as an increase in CI_TPTD_4 of 15% compared to CI_TPTD_1 (primary endpoint) was significantly predicted by a small volume challenge (ROC-AUC = 0.837; p = 0.009), GEDVI corrected for femoral CVC site (ROC-AUC = 0.905; p = 0.002) and $ScvO_2$ (ROC-AUC = 0.772; p = 0.034). By contrast, CVP (ROC-AUC = 0.673; p = 0.176) and percentage changes in CI-PC induced by a 120s PLR (ROC-AUC = 0.619; p = 0.353) were not predictive. Furthermore, GEDV not corrected for femoral CVC site slightly failed significance (ROC-AUC = 0,732; p = 0.057). Furthermore, different other read-outs of PLR such as maximum change in CI_PC during PLR (AUC = 0.551; p = 0.691) as well as changes in CI_PC after 30s (0.626; p = 0.326), 60s (0.667; p = 194) and 90s (0.578; p = 0.542) were not predictive for FR.

Finally, changes in CI_PC 10 minutes (AUC = 0.619; p = 0.353) and 15 minutes (AUC = 0.633; p = 0.301) after start of the small VC did not predict FR.

**CONCLUSIONS**.

1.) Small VC, $ScvO_2$ and GEDVI corrected for femoral CVC predicted FR, whereas PLR and CVP were not predictive.

2.) The predictive capacities of GEDVI were substantially improved by correction for femoral CVC.

3.) CI_TPTD_3 after 15min of a SVC was superior to CI_PC_15min.

**REFERENCE(S)**

Saugel, Huber et al., Crit Care 2010.

### 0091

#### Passive leg raising to predict fluid responsiveness after cardiac surgery

L. Camara[1], J.M. Jardim[1], R.M. Ianotti[1], A.A.C. Padula[2], G. Ferreira[2], L.A. Hajjar[2], F. Galas[1]

[1]Instituto do Coracao, Hospital das Clínicas, Universidade de São Paulo, Anesthesiology, Sao Paulo, Brazil; [2]Instituto do Coracao, Hospital das Clínicas, Universidade de São Paulo, Cardiology, Sao Paulo, Brazil

**Correspondence:** L. Camara

*Intensive Care Medicine Experimental* 2017, **5(Suppl 2):**0091

**INTRODUCTION.** Passive leg raising (PLR) represents a self-volume challenge that could predict fluid responsiveness with less limitation than other methods such as variation of stroke volume and pulse pressure variation.

**OBJECTIVE.** To assess the ability of PLR to predict fluid responsiveness after cardiac surgery.

**METHODS.** At the moment of intervention, all patients were intubated on mechanical ventilation (tidal volume 6 ml/kg) and were sedated with 50 mg fentanyl bolus and received 10 mg cisatracurium before the protocol started. Exclusion criteria were LVEF < 40%, significant arrhythmias, active bleeding, norepinephrine dose > 0,5 μg/kg/min, renal dysfunction and peripheral vascular disease involving femoral arteries. The decision to perform a fluid challenge was defined based on the presence of at least one sign of inadequate tissue perfusion: systolic blood pressure < 90 mmHg or need of vasopressors use, lactate ≥ 3mmol/L, cardiac index(CI) ≤ 2,5 L/min/$m^2$, $\Delta CO2 \geq$ 6mmHg. Four consecutive data sets were recorded: at baseline in the 45° semi-recumbent position; after 60s of PLR; at return to baseline in the 45° semi-recumbent position; and 10 min after fluid loading (Albumin 4% 500 ml over 10 min).The ventilator settings and vasoactive drugs remained unchanged during the study. The positive response was defined as an increase in CI of at least 10% after fluid challenge.

**RESULTS.** A total of 70 patients who underwent coronary artery bypass grafting were included. Fifty-five (75.6%) patients had an increase in CI ≥ 10% after fluid expansion and were classified as responders. Fifteen (21.4%) patients were non-responders. No difference was observed between both groups regarding baseline characteristics: mean age (63 ± 8 vs 66 ± 7 years, p = 0.184), BMI (kg/m2) (28 ± 4 vs 27 ± 4, p = 0.324), mean EuroSCORE [2 (1–3) vs 2 (1–3) p = 0,692] and mean LVEF [58% (49–63) vs 62% (54–66) p = 0.188]. Haemodynamic data at baseline were similar between the groups: mean arterial pressure (88 ± 15 vs 89 ± 21, p = 0.795), heart rate (100 ± 15 vs 106 ± 14, p = 0.234), stroke volume (50 ± 20 vs 51 ± 18, p = 0.922), stroke volume variation (18 ± 7 vs. 16 ± 7, p = 0.342) and central venous pressure [10 (7–17) vs 8 (5–13), p = 0.199]. Change in CI of 10% in response to PLR predicted volume responsiveness with a specificity of 89% (95% CI, 0.77 -0.95) and sensitivity of 27% (95% CI, 0.09 - 0.55).

**CONCLUSIONS**. The increase ≥ 10% in CI induced by PRL predicted fluid responsiveness with low sensitivity and high specificity after cardiac surgery. Further studies are needed to determine the better use of PRL as a noninvasive parameter to predict fluid responsiveness and guide therapy.

### 0092

#### Is bioreactance capable to assess the effects of passive leg raising and to track cardiac output variation in critically ill patients?

L. Galarza, P. Mercado, J.L. Teboul, V. Girotto, A. Beurton, C. Richard, X. Monnet

Service de Réanimation Médicale, Hôpital de Bicêtre, Hôpitaux Universitaires Paris-Sud, Assistance Publique - Hôpitaux de Paris, Le Kremlin-Bicêtre Inserm UMR S_999, Université Paris-Sud, Le Kremlin-Bicêtre, Paris, France

**Correspondence:** L. Galarza

*Intensive Care Medicine Experimental* 2017, **5(Suppl 2):**0092

**INTRODUCTION.** Bioreactance is a non-invasive method for cardiac output (CO) monitoring based on the measurement phase shift in voltage of an oscillating current that occurs when this crosses the thorax. We previously reported that the technique was inaccurate to assess the changes in CO during a passive leg raising (PLR) test (1).

**OBJECTIVES.** In the present study, we tested if a bioreactance device Starling® (Cheetah Medical, Portland, US) that had been modified to average CO on a shorter period could track the changes in CO during PLR and predict fluid responsiveness.

**METHODS.** In critically ill patients fitted with a PiCCO2 device, we set up a Starling device that had been modified for averaging cardiac index (CI) over 8 sec. only (noncommercial version). We recorded CI measured by PiCCO and by Starling devices before and during a PLR test and before and after a volume expansion (500mL of saline during 10 minutes).

**RESULTS.** We included 26 patients were included in the study in which 38 PLR tests and 14 volume expansions were performed. Shock was related to sepsis in 65% of the patients, 73% of patients received norepinephrine [0.17 (0.09-0.32) mcg/kg/min]. Ninety six percent of the patients were sedated and under mechanical ventilation. When considering all pairs of CI measurements performed by PiCCO with transpulmonary thermodilution and by Starling (n = 90), the bias was 0.08 L.min-1.m-2. The limits of agreement (LOA) were −1.86 and 2.01 L.min-1.m-2 (percentage error: 60%). When considering the changes in CI induced by PLR and volume expansion (n = 52), the polar plot had a mean polar angle of −14.4° and LOA of −48.8° to 20.1° (exclusion zone: 0.5 L/min). The concordance rate was 80% between 30° and −30°. The PLR test was positive (increase in CI ≥10%) in 14 "preload responsive" cases. The Starling device detected a positive PLR test with a sensitivity of 87 (60–98)% and a specificity of 74 (52–90)% (area under the receiver operating characteristics curve: 0.77 (0.60-0.94).

**CONCLUSIONS.** A version of the Starling device modified for averaging CO on 8 sec detected the effects of PLR with acceptable accuracy in ICU patients.

**REFERENCE(S)**

1. Kupersztych-Hagege E, Teboul JL, Artigas A, et al. Bioreactance is not reliable for estimating cardiac output and the effects of passive leg raising in critically ill patients. Br J Anaesth 2013;111:961–6.

### 0093

#### Predicting fluid responsiveness in paediatric septic shock: a Guytonian approach

P. Bertini[1,2], D. Colosimo[3], G. Mancino[1], G. Brizzi[1], L. Mirabile[3], F. Guarracino[1]

[1]Azienda Ospedaliero Universitaria Pisana, Anaesthesia and Intensive Care Medicine - Cardiothoracic and Vascular Anaesthesia and Critical Care, Pisa, Italy; [2]Royal Brompton & Harefield NHS Foundation Trust, Critical Care, London, United Kingdom; [3]Meyer Children Hospital, Intensive Care Unit, Florence, Italy

**Correspondence:** P. Bertini

*Intensive Care Medicine Experimental* 2017, **5(Suppl 2):**0093

**INTRODUCTION.** Assessing fluid balance and addressing hemodynamic restoration in the paediatric critically ill is challenging. Static and dynamic indexes of preload commonly used have been demonstrated not as accurate as in the adult population[1].

Recently, Guytonian approaches to the circulation have been proposed in order to better understand the response to fluid resuscitation in the critically ill and to better target volume expansion in septic shock patients according to guidelines [2].

**OBJECTIVES.** In order to provide a pathophysiological explanation of fluid responsiveness, we measured Pmsa, an analogue of the mean systemic filling pressure in 4 septic shock paediatric patients.

**METHODS.** Patients, (age 3+/− 4 years, height 97 +/− 39 cm, weight 20 +/− 19 kg) invasively ventilated in the intensive care unit, received 15 to 20 ml/kg as volume expansion (VE) in 30' and fluid responders (FR) were defined as the ones who increased CO by more than 20% as measured using MostCare®(PRAM).

SVV and PeakDeltaVTI as dynamic preload variables were also recorded.

Pvr, venous return driving pressure (Pvr = Pmsa-CVP) and heart performance (Eh = Pvr/Pmsa) were also calculated as previously reported [3].

**RESULTS.** As expected all patients increased Pmsa after VE increased both in FR and non FR, Pvr increased significantly in FR (50.6% +/− 7.9% vs 1.6% +/− 10.9% p = 0.001) and Eh decreased significantly in non FR (34.9% +/− 22.2%) while increased in FR (14.4% +/− 4.4%) p = 0.006.

Pvr, was considered a good predictor of FR as it was significantly less in FR (4.03 mmHg +/− 0.3mmHg) vs non FR (4.55 mmHg +/− 0.27 mmHg).

ROC analysis demonstrated a sensitivity and specificity of 100% in detecting FR for values of Pvr < 4.15 mmHg. PeakDelta VTI and SVV% were not able to predict FR vs not FR.

**CONCLUSIONS.** Guytonian approach to the circulation in paediatric septic shock is feasible and might provide better insights in order to address therapies able to restore hemodynamic derangement. Further studies are needed to confirm this preliminary results.

**REFERENCE(S)**

1. Desgranges FP, et al.: **Respiratory variation in aortic blood flow peak velocity to predict fluid responsiveness in mechanically ventilated children: a systematic review and meta-analysis**. *Paediatr Anaesth* 2016, **26**(1):37–47.

2. Rhodes A, *et al.*: **Surviving Sepsis Campaign: International Guidelines for Management of Sepsis and Septic Shock: 2016**. *Intensive Care Med* 2017, **43**(3):304–377.

3. Vos JJ, et al.: **The effect of fluid resuscitation on the effective circulating volume in patients undergoing liver surgery: a post-hoc analysis of a randomized controlled trial**. *J Clin Monit Comput* 2017.

### 0094

#### A comparison of echocardiography and the pressure recording analytical method (PRAM) for predicting fluid responsiveness after passive leg raising

A. Ozdemirkan, M. Aitakhanova, E. Gedik, P. Zeyneloglu, A. Pirat
Baskent University, Department of Critical Care, Ankara, Turkey
**Correspondence:** A. Ozdemirkan
*Intensive Care Medicine Experimental* 2017, **5(Suppl 2):**0094

**INTRODUCTION.** Pressure recording analytical method (PRAM) has been shown to estimate cardiac output (CO) with a good level of agreement with transthoracic echocardiography (TTE) under static conditions.

**OBJECTIVES.** This study aims to assess the agreement between the CO measured by PRAM and TTE before and after the passive leg raise (PLR) maneuver.

**METHOD.** This is a prospective observational study in critically ill patients. All patients were monitored with MostcareUp/PRAM (Vygon, Vytech, Padova, Italy). TTE was used to calculate CO before and after PLR. Cardiac index (CI) values and percent changes in CI values in response to PLR were recorded for the two methods. Compared with the baseline, patients who had an increase in CI that was greater than 10% were accepted as fluid responders (FR).

**RESULTS.** Data of a total of 25 patients were collected. The mean CI values that were calculated by TTE before and after PLR were 2.68 ± 0.91 L/min/m$^2$ and 3.05 ± 0.98 L/min/m$^2$, respectively. The mean CI values that were calculated by PRAM before and after PLR were 2.64 ± 0.81 L/min/m$^2$ and 2.83 ± 1.03 L/min/m$^2$, respectively. There was significant correlations between the measured CI values both by TTE and PRAM before and after PLR (r = 0.635, p = 0.001 and r = 0.610, p = 0.001, respectively).The mean percent changes in CI with TTE and PRAM were 0.18 ± 0.28 and 0.08 ± 0.21, respectively. Sixteen patients were determined as FR by TTE (64%) and 13 patients were determined as FR by PRAM (52%). The Kappa test showed that there was a moderate agreement between TTE and PRAM for predicting fluid responsiveness (k = 0.595; p = 0.002). The mean biases between the CI values measured by TTE and PRAM before and after PLR were 0.04 ± 0.77 L/min/m$^2$ and 0.22 ± 0.88 L/min/m$^2$, respectively.

**CONCLUSIONS.** This study showed a statistically significant correlation for CI values measured both by TTE and PRAM. For predicting fluid responsiveness there was agreement between the two methods after PLR. Further studies with larger numbers of patients are needed to assess the agreement of the two methods for predicting fluid responsiveness.

**REFERENCE(S)**

1) Romagnoli S, FloTrac/VigileoT M (Third Generation) and MostCares /PRAM Versus Echocardiography for Cardiac Output Estimation in Vascular Surgery. Journal of Cardiothoracic and Vascular Anesthesia, Vol 27, No 6 (December), 2013: pp 1114–1121.

2) Zangrillo A, Cardiac Index Validation Using the Pressure Recording Analytic Method in Unstable Patients. Journal of Cardiothoracic and Vascular Anesthesia, Vol 24, No 2 (April), 2010: pp 265–269

## Acute kidney injury: Biomarkers and outcome

### 0095

#### The use of creatinine and cystatin C to quantify renal dysfunction in AKI survivors

C. Stigare[1], B. Ravn[1], C.-R. Marling[1], M. Bottai[2], E. Löfberg[1], J. Mårtensson[1,3], M. Bell[1]
[1]Karolinska Institute, Department of Pharmacology and Physiology, Stockholm, Sweden; [2]Karolinska Institute, Institute of Environmental Medicine, Stockholm, Sweden; [3]Austin Hospital, Melbourne, Australia
**Correspondence:** C. Stigare
*Intensive Care Medicine Experimental* 2017, **5(Suppl 2):**0095

**INTRODUCTION.** The extent of renal dysfunction amongst acute kidney injury (AKI) survivors is unknown because structured follow-up is rarely performed. Serum creatinine decrease during prolonged ICU stay due to loss of muscle mass confounds it as a renal function marker[1]. Cystatin C may be a superior marker in critical illness. How long the catabolic state continues and whether creatinine can be used in the recovery period is unclear. Do creatinine and cystatin C give similar estimates of glomerular filtration rate (GFR) in the recovery period?

**OBJECTIVES.** To quantify the renal function of AKI survivors at 2–7 months using creatinine and cystatin C to estimate GFR. Identify ICU parameters which best predict AKI survivors renal function at 2-7months. To compare estimates of GFR using creatinine and cystatin C obtained a subgroup at 9 months when GFR was also measured using iohexal.

**METHODS.** Prospective cohort of AKI patients (RIFLE criteria) admitted to a mixed ICU during the September 2008 to May 2011. Patients were recruited at discharge and those under 18 and over 100 were excluded, as were subjects who died during the 3 months following discharge. Serum Creatinine and Cystatin C measurements were taken at 3 months. A sub-selection of patients with persisting renal dysfunction at first-follow-up were invited to return for a second 9-month visit, where, in addition to serum Creatinine and Cystatin C, GFR was measured using Iohexal clearance technique.

**RESULTS.** 358 AKI patients were included, of these 21 died before first follow-up and 44 were lost to follow-up; 293 remained. Only patients with first follow-up between 2 and 7 months (269) were included in analysis. Mean creatinine levels at follow up were (94.4 umol/L) and were linearly associated with maximum RIFLE grade in the ICU. Mean estimated GFR (eGFR) was 73.7 mL/min/1.73m$^2$ using the Lund Malmo formula. Close to a third, 32.4% of AKI patients, had creatinine eGFR under 60 mL/min/1.73m$^2$ at follow up. Multivariate analysis found that discharge creatinine, maximum ICU cystatin C and baseline creatinine were predictive of creatinine levels at first follow-up. Mean cystatin C level at first follow up were 1.55 (mg/l) and was linearly associated with maximum RIFLE during admission. Mean cystatin C eGFR was 54.1 mL/min/1.73m$^2$ ; 65.9% patients had cystatin C eGFR under 60 at follow up. Multivariate analysis identified discharge cystatin C, age and baseline creatinine as covariates associated with cystatin C at first follow-up. At second follow-up (N = 25) mean eGFR was 63.9, 47.4 for creatinine and cystatin C and 57.3 mL/min/1.73m$^2$ for iohexal *measured* GFR.

**CONCLUSIONS.** Fewer patients had an eGFR under 60 at follow-up using serum creatinine than with cystatin C at first follow up. In a small sub-group with an additional follow-up, cystatin C underestimated and creatinine overestimated GFR compared to iohexol measured GFR.

**GRANT ACKNOWLEDGEMENT**
Baxter Gambro

### 0096

#### Positive fluid balance is associated with increased time for correction of hypernatremia in critically ill patients

L.P. da Silva[1,2], K.O.B. Queiroz[1,2], B.B.R. Ramos[1,2], M.K.A. Barreto[1,2], R. Laruxa[1,2], A.M.S. Cerqueira Junior[1], F. Jones[3], N.M. Filgueiras Filho[1,2,4], P. Benigno[3], R. Passos[3], J.G.R. Ramos[3], A.L. Gobatto[1,3,5]
[1]Hospital da CIdade, Salvador, Brazil; [2]Universidade Salvador, Salvador, Brazil; [3]Hospital São Rafael, Salvador, Brazil; [4]Universidade do Estado da Bahia, Salvador, Brazil; [5]Hospital da Clinicas - University of Sao Paulo Medical School, São Paulo, Brazil
**Correspondence:** A.L. Gobatto
*Intensive Care Medicine Experimental* 2017, **5(Suppl 2):**0096

**INTRODUCTION.** Hypernatremia has been traditionally associated to fluid deficits and increased sodium load and treated by replacement of free water. However recent studies have challenged this assumption, putting more emphasis on the role of sodium excretion by the kidneys. Furthermore, a cumulative positive fluid balance has been associated to worse outcomes and may be a surrogate marker of increased water and electrolyte load and of the kidney capacity to effectively handle it. (1–3).

**OBJECTIVES.** Our aim was to analyze the association between fluid balance and hypernatremia correction in a population of hypernatremic critically ill patients.

**METHODS.** Unicentric, prospective, observational study of critically ill patients with hypernatremia (defined as serum sodium greater than 145 mEq/l). Patients with chronic kidney injury or in palliative care were excluded. Patients were categorized in two groups according to the fluid balance after the hypernatremia diagnosis: positive cumulative fluid balance (FB+) and negative cumulative fluid balance (FB-). The patients were followed up daily to sodium correction or ICU discharge. Clinical outcomes were evaluated at hospital discharge. The association between fluid balance and hypernatremia was evaluated by a Cox regression analysis.

**RESULTS.** During 7 months, 103 hypernatremic critically ill patients were evaluated. Median (IQR) cumulative fluid balance was 3929 (1527–6945) ml in FB+ patients and −2729 (−4094- -1580) in FB- patients. There was no difference between FB+ and FB- patients in mean age (69.5 vs 68.2 years), SAPS3 (56 vs 53) or median serum sodium before hypernatremia development (142 vs 142 mEq/l). Median (IQR) time for correction of hypernatremia was 3 (2–4) days for FB+ patients and 1 (1–2) days for FB- patients, p < 0.001. After adjustment for utilization of hypotonic fluids, acute kidney injury, utilization of thiazide diuretics and diuresis, a positive cumulative fluid balance was associated with a lower chance of correction of hypernatremia [HR (95%CI) = 0.38 (0.19-0.74), p = 0.005).

**CONCLUSIONS.** Positive fluid balance was associated with increased time for correction of hypernatremia in critically ill patients. The mechanisms of this association and whether therapies that target hypernatremia without increasing fluid balance are benefic should be evaluated in further studies.

### 0097

#### Proenkephalin, the new marker for kidney function on the Intensive care unit?

G. Leijte, R. Beunders, R. van Groenendael, E. Peters, M. Kox, P. Pickkers
Radboud University Medical Center, Intensive Care Department, Nijmegen, Netherlands
**Correspondence:** R. Beunders
*Intensive Care Medicine Experimental* 2017, **5(Suppl 2):**0097

**INTRODUCTION.** Deterioration of kidney function in critically ill patients is of important concern, as it is an early indicator of multi organ failure and is independently associated with adverse outcome. Diagnosis of acute kidney injury (AKI) is currently hampered by the lack of accurate markers to assess kidney function. Conventional creatinine-based methods to assess glomerular fitration rate (GFR) are known to be insensitive, late, and inaccurate. Plasma clearance of iohexol, an iodine contrast agent that is exclusively filtrated in the glomerulus, has been shown to be equally accurate in determining the GFR as inulin clearance, the current gold standard. Nevertheless, iohexol is not used in clinical practice as it is time-consuming and its determination is laboursome. A promising novel candidate to assess kidney function is plasma proenkephalin, a stable endogenous opioid precursor.

**OBJECTIVE.** To determine to what extent the plasma proenkephalin concentration reflects the GFR in sepsis patients.

**METHODS.** In this prospective pilot study, we included 24 septic patients admitted to our Intensive Care Unit. All patients received an intravenous bolus of 1 mL iohexol. During the subsequent 6-hour window, blood was sampled 4 times to determine the 'true GFR' using a mathematical reconstruction of the iohexol disappearance curve. The two most frequently used creatinine-based methods, the endogenous creatinine clearance (ECC) and the Modification of Diet in Renal Disease (MDRD), were assessed according clinical standards. Urine was collected cumulatively over the same 6-hour period and the plasma proenkephalin concentration was determined using a Sphincotec ELISA.

**RESULTS.** Patients had a median [IQR] iohexol GFR of 45 [15–75] ml/min. Both the MDRD and ECC overestimated this true GFR (bias ± SD) with 14% ± 40 and 30% ± 47, respectively. Using the MDRD or ECC would have resulted in misclassification of renal function in 6 (25%) and 8 (33%) patients, respectively. In contrary to these conventional methods, proenkephalin correlated strongly with the iohexol GFR ($r^2 = 0.91$; $p < 0.001$, Fig. 47).

**CONCLUSION.** Our study underlines the inaccuracy of conventional creatinine-based methods to assess GFR, which leads to frequent misclassification of renal function. The pilot results indicate that a single measurement of plasma proenkephalin strongly correlates with the true GFR in sepsis patients. Therefore, plasma proenkephalin may serve as a more feasible marker to assess kidney function in the critically ill. Results need to be confirmed in larger cohorts and other patient groups.

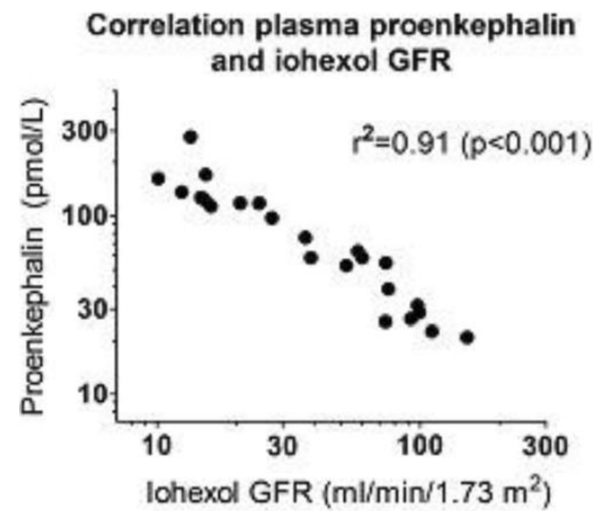

**Fig. 47 (abstract 0097).** See text for description

### 0098

#### Combined assessment of lactate and glucose early after ICU admission and its relation with acute kidney injury

P. Freire Jorge, N. Wieringa, E. de Felice, I.C. van der Horst, A. Oude Lansink, M.W. Nijsten
University Medical Center Groningen, Critical Care, Groningen, Netherlands
**Correspondence:** P. Freire Jorge
*Intensive Care Medicine Experimental* 2017, **5(Suppl 2):**0098

**INTRODUCTION.** In critically ill patients elevated lactate levels in the presence of an inappropriately low glucose levels can be a sign of impaired gluconeogenesis. The kidney is an important gluconeogenetic organ and therefore subtle derangements of lactate and glucose levels may anticipate and accompany the development of acute kidney injury (AKI).

**OBJECTIVES.** Examine the relation between early lactate and glucose levels after ICU admission and the development of AKI.

**METHODS.** We retrospectively analyzed all admissions to our 48-bed teaching hospital ICU from 2011 to 2014. Measurements of lactates and glucoses were regularly performed with point-of-care analyzers. We collected lactate and glucoses 6 hours before to 24 hours after ICU admission. Patients with ICU length of stay of less than 12 hours or with less than 4 lactate and glucose measurements were excluded. Mean lactate, glucose, and glucose squared were then grouped into quintiles. The outcome was the subsequent development of AKI grade 1 to 3 in the first 7 days after ICU admission.

**RESULTS.** We analyzed 92,000 blood samples from 9,074 patients with a mean age of 61 ± 15 years, 63% males, with an AKI incidence of 21% (AKI-grade 1, 2 and 3: respectively 10%, 4% 7%) and a hospital mortality of 11%. Lactate quintiles (≤1.0;1.0-1.3;1.3-1.7;1.7-2.3;>2.3 mmol/L) and glucose quintiles (≤7.0; 7.0-7.6; 7.6-8.2; 8.2-9.0;>9.0 mmol/L) were both univariately and positively related with AKI ($p < 0.001$). Glucose showed a U-shape relation with AKI and the combination of the highest lactate quintile with the lowest glucose quintile showed the highest AKI incidence. In multivariate regression analysis with APACHE-IV as fixed covariate lactate, glucose, and glucose squared were all significantly associated with AKI ($p < 0.001$).

**CONCLUSIONS.** The kidney is an important gluconeogenetic organ and the development of AKI may be preceded by derangements of gluconeogenesis. A 'normal' glucose in critically ill patients with elevated lactate should not be considered normal, as this points to an inability to (re)generate sufficient glucose under stress.

### 0099

#### Relationship between Body Mass Index and development of acute renal injury and mortality in severe acute pancreatitis

E.S. Cruz Montesinos, S.E. Zamora Gómez, M.A. Amezcua Gutierrez, J.O. Montoya Rojo, I.M. Lima Lucero, J. Garduño López, M.A. Toledo Rivera, M.A. Carrasco Flores, M. Hernández Romero, G.D. Hernández López
Hospital Juárez de México, Intensive Care Unit, Mexico, Mexico
**Correspondence:** E.S. Cruz Montesinos
*Intensive Care Medicine Experimental* 2017, **5(Suppl 2):**0099

**INTRODUCTION.** Obesity plays an important role in increasing the risks of cardiovascular diseases, kidney injury, metabolic diseases and death. There is persistent concern about the influence of the degree of obesity on mortality and morbidity in severe acute pancreatitis (SAP) patients. There is inconsistent correlation between BMI and outcome in SAP patients. BMI is the basic method used to determine obesity, and world health organization (WHO) classifies it as follows: Normal weight 18.5 to 24.9 Kg/m2, overweight 25 to 29.9 Kg/m2, obesity grade I 30 to 34.9 Kg/m2, obesity grade II of 35 to 39.9 Kg/m2 and obesity grade III >40 Kg/m2.

**OBJECTIVES.** Estimate the relationship between BMI and the development of acute renal injury (AKI), as well as on mortality in severe acute pancreatitis patients admitted to an tertiary level hospital intensive care unit (ICU) in Mexico City.

**METHODS.** Observational, retrospective study performed between the period of 2012 to 2015, involving SAP patients. Anthropometric characteristics were analyzed by BMI and its association with the development of acute kidney injury (AKI) and mortality. The obtained data were evaluated through a T-test analysis for independent samples.

**RESULTS.** 54 patients were analyzed, 36 males and 18 females. 16.67% with normal weight, 33.33% with overweight, 33.33% with degree I obesity, 12.96% with degree II obesity and 3.70% with degree III obesity. The analysis observed an overall mortality of 31.4% and presence of acute renal injury of 61.1%. The group analysis by Student´s t test showed a mean BMI of 29.52 kg / m2 in patients survivors and 31.73 kg / m2 in non survivors, with $p = 0.169$. On the other hand, the mean BMI that was associated with AKI were 30.15 kg / m2 and 30.33 kg / m2 in those without AKI, with $p = 0.908$. The analysis of variance of degrees of BMI and survival show the sum of the square = 11.64, 4 degrees of freedom intergroups and 49 intragroups, through F test we obtain $F = 1.2679$ and a critical value for $F = 2.5611$, and $p = 0.2952$, so there is no variation in the 5 degress of BMI in relation to survival.

**CONCLUSIONS.** In this study we did not found a significant association between body mass index and the development of acute renal injury nor prognostic information of mortality in severe acute pancreatitis patients. Obesity is now considered a risk factor for the development of multiple diseases, so additional studies, with a larger sample size, are necessary to evaluate other variables that influence the prognosis of severe acute pancreatitis.

**REFERENCE(S)**

1. Katuchova J, Bober J, Harbulak P, Hudak A, Gajdzik T, Kalanin R, Radonak J. Obesity as a risk factor for severe acute pancreatitis patients. Wien Klin Wochenschr. 2014;126(7–8):223–7.
2. Taguchi M, Kubo T, Yamamoto M et al. Body Mass Index Influences the Outcome of Acute Pancreatitis. Pancreas. 2014;863–66

### 0100

#### Urinary neutrophil gelatinase-associated lipocalin is associated with long-term renal outcomes in ICU survivors

K. Doi, R. Isshiki, M. Nangaku, N. Morimura, E. Noiri
University of Tokyo, Tokyo, Japan
**Correspondence:** K. Doi
*Intensive Care Medicine Experimental* 2017, **5(Suppl 2):**0100

**INTRODUCTION.** The concept of postintensive care syndrome has recently been suggested. Although several epidemiological studies suggested that acute kidney injury (AKI) in ICUs increases the risk of chronic kidney disease (CKD) development and progression, it is still unclear whether any AKI biomarker can predict long-term renal outcomes in ICU survivors.

**OBJECTIVES.** This study was undertaken to evaluate the possible role of urinary biomarkers for long-term renal outcome prediction after ICU discharge.

**METHODS.** We conducted a retrospective observational study examining 495 adult patients who had been admitted to the ICU of the University of Tokyo Hospital. Major adverse kidney events (MAKE): death, incident end-stage renal disease, and halving of estimated glomerular filtration rate (eGFR), at hospital discharge and long-term renal outcomes of 30% reduction of eGFR or incident end-stage renal disease were evaluated.

**RESULTS.** Among all the enrolled 495 patients, 393 patients discharged from the hospital without MAKE. Of them, data of eGFR up two years after ICU discharge was available in 173 patients and 63 patients (36.4%) were positive for long-term renal outcomes. A step-wise logistic regression analysis demonstrated that male sex and urinary neutrophil gelatinase-associated lipocalin (NGAL) measured at ICU admission showed significant associations with long-term renal outcomes. Receiver operating characteristic curve analysis showed the area under the curve of 0.66 (95% confidence interval 0.57-0.74) for prediction of long-term renal outcome by urinary NGAL.

**CONCLUSIONS.** Urinary NGAL measured at ICU admission was significantly associated with long-term renal outcomes after hospital discharge in MAKE-free ICU survivors. Measurement of urinary NGAL at ICU may be useful to identify a high risk population of kidney disease progression after intensive care.

**GRANT ACKNOWLEDGMENT**

This study was partly supported by grants from the Tokyo Society of Medical Sciences.

### 0101

#### Acute kidney injury (AKI) after lung transplantation (LT): perioperative associated factors

E. Atchade, S. Jean-Baptiste, A. Snauwaert, A. Tran-dinh, C. Genève, Y. Castier, O. Brugière, H. Mal, P. Montravers

Bichat-Claude Bernard Hospital, Paris, France

**Correspondence:** E. Atchade

*Intensive Care Medicine Experimental* 2017, **5(Suppl 2):**0101

**INTRODUCTION.** Acute kidney injury (AKI) is common after lung transplantation (LT) and is associated with increased short-term and long-term mortality, and increased long-term renal morbidity (1).

**OBJECTIVES.** Our primary goal was to assess the pre-operative, intraoperative and post-operative factors associated with AKI in the early post-operative period during ICU stay after LT. Secondary aims were to examine the incidence of post-operative AKI in our cohort and to evaluate its impact on the outcome.

**METHODS.** This was an observational, retrospective, monocentric study. AKI was defined as a ≥50% increase of serum creatinine from baseline or renal replacement therapy (RRT). AKI events were staged using the KDIGO criteria. Follow-up period was length of hospitalization in ICU. Results are expressed as medians, interquartile range, absolute numbers and percentages. Statistical analyses were performed using Chi squared and Fischer's exact tests ($p < 0.05$ as significance).

**RESULTS.** Between January 2016 and March 2017, 50 patients (pts) (60% men, median age 55 [49–49]) underwent LT (68% bilateral LT) in our institution. Most common diagnoses leading to LT were pulmonary fibrosis (34%) and COPD (30%). Median ICU length of stay was 14 days [9–23]. Intraoperative support by extra-corporeal membrane oxygenation (ECMO) was required for 42 pts (84%). Mortality rate at day 28 was 14%. AKI occurred during ICU stay in 19 pts (37%). According to the KDIGO criteria, Stage 1 AKI was observed in 3 pts (6%), Stage 2 in 3 pts (6%), and Stage 3 in 11 pts (22%) including 9 pts (18%) who required RRT. AKI occurred before day-5 after surgery in 15 pts (79%) or later in 4 pts (21%). There was no significant association between AKI and preoperative comorbidities. AKI was significantly associated with red blood cells and platelets transfusion during surgery ($p = 0.026$ and $p = 0.018$, respectively), stage 3 primary graft dysfunction (PGD) ($p = 0.043$), occurrence of a septic choc during ICU stay ($p = 0.001$), mechanical ventilation >7 days ($p = 0.045$), and extubation failure ($p = 0.026$). ICU mortality and at day-28 were significantly associated with AKI ($p = 0.001$ and $p = 0.009$, respectively). ICU mortality was significantly associated with the use of RRT ($p = 0.0004$).

**CONCLUSIONS.** Incidence of post-operative AKI is high after LT. Intraoperative blood and/or platelet transfusion, occurrence of a stage 3 PGD, or of a septic shock were significantly associated with AKI. Identification of patients at high risk of AKI in post-operative period could encourage physicians to adopt prevention strategies, such as avoiding most nephrotoxic immunosuppressive therapies.

**REFERENCE(S)**

(1) Wehbe, J Heart Lung Transplant 2012;31:244–51

**GRANT ACKNOWLEDGMENT**

None

**Table 29 (Abstract 101).** Association between preoperative factors and AKI

| | Patients with AKI, n=19 | Patients without AKI, n=31 | p |
|---|---|---|---|
| Age > 60, n (%) | 2 (11) | 10 (32) | 0.10 |
| High blood pressure, n (%) | 8(42) | 5 (16) | 0.05 |
| Diabete mellitus, n (%) | 2 (11) | 4 (13) | 1 |
| Left Ventricular Ejection Fraction < 60%, n (%) | 7 (37) | 11 (35) | 0.92 |
| Moderate to severe pulmonary hypertension, n (%) | 10 (53) | 16 (52) | 0.94 |
| Male gender, n (%) | 11 (58) | 19 (61) | 0.81 |
| BMI > 24.9 kg/m$^2$, n (%) | 11 (58) | 11 (35) | 0.12 |
| COPD, n (%) | 7 (37) | 8(26) | 0.41 |
| Pulmonary fibrosis, n (%) | 5 (26) | 12 (39) | 0.37 |

**Table 30 (Abstract 101).** Association between intraoperative factors and AKI

| | Patients with AKI, n=19 | Patients without AKI, n=31 | p |
|---|---|---|---|
| Single lung transplantation, n (%) | 4 (21) | 12(39) | 0.19 |
| ASA score > 3, n (%) | 10 (53) | 10 (32) | 0.15 |
| ECMO support, n (%) | 16 (84) | 26 (84) | 1 |
| Intraoperative catecholamines, n (%) | 14 (74) | 26 (84) | 0.47 |
| Norepinephrine > 2.5 mg/h or epinephrine, n (%) | 4 (21) | 10 (32) | 0.52 |
| Fluid administration > 30 mL/Kg, n (%) | 16 (84) | 26 (84) | 1 |
| Red blood cell transfusion, n (%) | 17 (89) | 18 (58) | 0.026 |
| Fresh frozen plasma transfusion, n (%) | 13 (68) | 13 (42) | 0.07 |
| Platelet transfusion, n(%) | 9 (47) | 4 (13) | 0.018 |

**Table 31 (Abstract 101).** Association between postoperative factors and AKI

| | Patients with AKI, n=19 | Patients without AKI, n=31 | p |
|---|---|---|---|
| Catecholamines > 3 days, n(%) | 11 (58) | 15 (48) | 0.51 |
| Stage 3 primary graft dysfunction, n (%) | 11 (58) | 9 (29) | 0.043 |
| Acute rejection, n (%) | 6 (32) | 7 (23) | 0.71 |
| Pneumonia in ICU, n (%) | 15 (79) | 24 (77) | 0.82 |
| Septic shock, n (%) | 9 (47) | 2 (6) | 0.001 |
| Extubation failure, n (%) | 3 (21) | 0 (0) | 0.026 |
| Mechanical ventilation > 7 days, n (%) | 8 (42) | 4 (13) | 0.045 |
| Mortality in ICU, n (%) | 8 (42) | 1 (3) | 0.001 |
| Mortality at day 28, n (%) | 6 (32) | 1 (3) | 0.009 |

### 0102

#### Cerebral oximetry alone can predict acute kidney injury after cardiac surgery

C. Balci
Health Sciences University, Derince Kocaeli, Intensive Care, Kocaeli, Turkey
*Intensive Care Medicine Experimental* 2017, **5(Suppl 2):**0102

**INTRODUCTION.** Acute kidney injury (AKI) is a common complication after cardiac surgery and is associated with significant morbidity and mortality. Near infrared technology (NIRS) is a non-invasive and real-time method to measure both cerebral and regional tissue oxygenation.
**OBJECTIVES.** The purpose of the present study was to evaluate a correlation between cerebral $SO_2$ measured intraoperatively and continued for at least 24 hours postoperatively and AKI in cardiac surgical patients.
**METHODS.** We retrospectively analysed the prospectively collected data of 45 adult patients who underwent isolated coronary artery bypass graft surgery (CABG) with normal renal function (baseline serum creatinine value < 1.4mg/dL) from January 2014 to May 2014. Kidney injury was interpreted according to Acute Kidney Injury Network (AKIN) criterias Cerebral oximetry was measured at different time points intraoperatively and the first 24 hours postoperatively.
**RESULTS.** Postoperative AKI occured in 12 (26%) patients. Baseline cerebral $SO_2$ values were significantly decreased 2 and 6 hours after cardiopulmonary bypass (CPB). Cerebral $SO_2$ values were significantly lower in AKI patients compaired to nonAKI patients.
**CONCLUSION.** This retrospective study shows that lower cerebral $SO_2$ values are correlated with AKI after CABG. We suggest that specific tissue NIRS measurements in addition to cerebral oximetry are not necessary and cerebral oximetry alone well predicts postoperative AKI.
**REFERENCE**
George Vretzakis, Stauroula Georgopoulou, Konstantinos Stamoulis, Georgia Stamatiou, Kosmas Tsakiridis, Paul Zarogoulidis, Nikolaos Katsikogianis, Ioanna Kougioumtzi, Nikolaos Machairiotis, Theodora Tsiouda, Andreas Mpakas, Thomas Beleveslis, Alexander Koletas, Stavros N. Siminelakis, Konstantinos Zarogoulidis. Cerebral oximetry in cardiac anesthesia..J Thorac Dis. 2014 Mar; 6(Suppl 1): S60-S69. doi: 10.3978.

### 0103

#### Hemofiltration veno-venous continouos high and very high volume, pulmonary (paO2/FiO2) function and mortality in refractory septic shock patients

F. Cota-Delgado, M.-V. de la Torre-Prados, E. Cámara-Sola, P. Nuevo-Ortega, T. Tsvetanova-Spasova, A. Fernández-Porcel, C. Rueda-Molina, L. Salido-Díaz, I. García- Gómez, M. Mateos-Rodríguez
Málaga/Ibima, Department of Intensive Care Medicine. Universitary H Virgen de la Victoria, Málaga, Spain
**Correspondence:** M.-V. de la Torre-Prados
*Intensive Care Medicine Experimental* 2017, **5(Suppl 2):**0103

**INTRODUCTION.** Refractory septic shock mortality is about 50%. In the early phase of septic shock, hemofiltration can attenuate the inflammatory cascade, alleviating cell and tissue damage in the lungs and reducing the mortality due to multiple-organ failure syndrome
**OBJECTIVES.** In patients with refractory septic shock Hemofiltration Veno-Venous Continuous High Volume (HFVVCHV, 35 ml / kgr / h) or Hemofiltration Veno-Venous Continuous Very High Volume (HFVVCVHV, 55 ml / kgr / h) improve pulmonary function (PaO2/FiO2) during the first 96 hours Intensive Care Unit (ICU) admission
**METHODS.** 102 refractory septic shock patients, with or without renal dysfunction and consecutively admitted to the ICU, were treated with early resuscitation and support bundles, according Surviving Sepsis Campaign (SSC) 2012, and 36 hours septic shock onset randomly assigned to group 1 (35 ml / kg / h) or group 2 (55 ml / kg / h). The variables studied were hemodynamic parameters, vasoactive drug parameters (dose and time of norepinephrine and hourly fluid intake), pulmonary (PaO2/FiO2), and renal function.
**RESULTS.** IIn both groups the paO2/FiO2 improve at 96 hours septic shock onset, higher in group 1 (100%) than group 1 (54%). The average of the respiratory quotient in both groups showed signs of Acute Distress Respiratory Syndrome (ADRS) with PaO2/FiO2 > 200. 100% of patients had needed Mechanical Ventilation to the admission in UCI. The evolution of quotient PAO2/FIO2 after 96 hours of the showed a progressive improvement. These differences between periods of time are significant at 48 hours, being superior in group 1 respect to group 2, as well as the duration in days of mechanical ventilation (P50). Tables 32 and 33.
Intensive care Unit (ICU) mortality in the series of patients globally was 19%, higher percentage in group 1 (23%) than in group 2 (16%). Hospital mortality showed an increase of 8 points.
**CONCLUSIONS.** In refractory septic shock patients, after the SSC measures correctly applied in time, an early horizontal treatment with HFVVCHV and HFVVCVHV improve the pulmonary function (paO2/FiO2) and prognosis.
**REFERENCE(S)**
Joannes-Boyau O, Honoré PM, Perez P, Bagshaw SM, Grand H, Canivet JL. High-volume versus standard-volume haemofiltration for septic shock

**Table 32 (Abstract 0103).** Respiratory dysfunction in septic shock

| | Group 1, 35ml/Kg/h, n=52 | Group 2, 55ml/Kg/h, n=50 |
|---|---|---|
| Variables | Media (IC95%) | Media (IC95%) |
| PaO2/FiO2>200 | 165 (140-190) | 153 (125-181) |
| Mechanical Ventilation | n=52, 100% | n=50, 100% |
| Group 1= HFVVCHV = Hemofiltration veno-venous continouos high volume | Group 2=HFVVCVHV= Hemofiltration veno-venous continouos very high volume | |

**Table 33 (Abstract 0103).** Respiratory quotient changes and HFVVC

| Variables | Group 1: 35 ml/Kg/h, N=52; P50 (P25-P75) | Group 2: 55 ml/Kg/h, N=50 | Total, N=102 |
|---|---|---|---|
| *PaO2/FIO2, ICU Admission | 154 (83-230) | 144 (77-187) | 146 (79-207) |
| PaO2/FIO2, 24 h | 187 (132-250) | 160 (108-244) | 180 (115-246) |
| *PaO2/FIO2, 48 h | 241 (187-298) | 151 (196-268) | 225 (167-282) |
| *PaO2/FIO2, 72 h | 271 (220-322) | 250 (180-289) | 265 (211-302) |
| *PaO2/FIO2, 96 h | 310 (258-283) | 276 (207-318) | 295 (240-350) |
| PaO2/FIO2 96 h- ICU Admission | 160 (81-234) | 120 (28-207) | 142 (59-220) |
| Mechanical Ventilation, days | 7 (4-12) | 9 (4.7-18) | 8 (4-16) |
| ICU: Intensive care Unit | Group 1: 35 ml/Kg/h. HFVVCHV: Hemofiltration veno-venous continouos high volume | Group 2: 55 ml/Kg/h HFVVCVHV: Hemofiltration veno-venous continouos very high volume | PaO2/FiO2: respiratory quotient |

*U de Mann-Whitney, p=<0,05

patients with acute kidney injury (IVOIRE study): a multicentre randomized controlled trial Intensive Care Med 2013; 39:1535–1546

### 0104

#### Dynamic contrast-enhanced ultrasound identifies microcirculatory alterations in sepsis-induced acute kidney injury

A. Lima[1], B. Ergin[2], T. van Rooijn[3], M. Sorelli[4], Y. Ince[2], P. Specht[5], E. Mik[5], L. Bocchi[4], K. Kooiman[3], N. de Jong[3], C. Ince[2]
[1]Erasmus MC, Department of Intensive Care Adults, Rotterdam, Netherlands; [2]Academic Medical Center, Department of Translational Physiology, Amsterdam, Netherlands; [3]Erasmus MC, Department of Biomedical Engineering, Thorax Center, Rotterdam, Netherlands; [4]University of Florence, Department of Information Engineering, Florence, Italy; [5]Erasmus MC, Laboratory of Experimental Anesthesiology, Department of Anesthesiology, Rotterdam, Netherlands
**Correspondence:** A. Lima
*Intensive Care Medicine Experimental* 2017, **5(Suppl 2):**0104

**INTRODUCTION.** Microcirculatory slow flow combined with plugged capillaries is a hallmark of sepsis-induced AKI. We developed a new measurement technique using dynamic contrast-enhanced ultrasound (CEUS) to identify these microcirculatory alterations in conjunction with laser speckle imaging (LSI).
**OBJECTIVES.** To use CEUS imaging to investigate microbubble kinetics in a porcine model of sepsis-induced AKI in order to identify plugged capillary vessels.
**METHODS.** Experiments were conducted on fully instrumented and anesthetized 13 female pigs (BW 28.2 ± 1.9 kg). The animals were divided into 2 groups: with (LPS group n = 10) or without (control group, n = 3) infusion of LPS (2 $mg/kg^{-1}h^{-1}$). While the auricular vein was used for anesthesia and fluid infusion, the left femoral artery was cannulated for monitoring arterial pressure and arterial blood samples, the swan-ganz catheter has been inserted in pulmonary artery through the right jugular vein used for microbubble injection and mix venous blood samples. The catheter in the right femoral vein was used for fluid resuscitation. After the retroperitoneal incision, transonic doppler flow probe was placed around the renal artery of exposed kidney. CEUS imaging was performed at baseline (t0), during shock (t1), and one hour after starting fluids infusion (t2). Cine loops of side-by-side B-mode and nonlinear contrast mode images were stored as lossless DICOM images for further offline analysis using MATLAB (The MathWorks, Natick, MA, USA). We calculated the final plateau intensity, the ratio of this plateau to the PE, and the difference in arrival time of the maximum contrast bolus in the artery and the microcirculation, i.e., the difference between the peak times, referred to as delta time of arrival (ΔT). Since the distance between the artery and the microcirculation is constant, the ΔT parameter was used to estimate intrarenal blood velocity.
**RESULTS.** CEUS effectively visualized renal microcirculatory hypoperfusion and allowed us to develop new analytical methods to measure dynamic variations in renal microvascular perfusion during shock and resuscitation. Plugged capillaries could be quantified by decreased peak enhancement and an increased ratio of the final plateau intensity to peak enhancement [Fig. 48]. Reduced intrarenal blood flow could be estimated by measuring the microbubble transit times between the interlobar arteries and capillary vessels in the renal cortex. LSI analysis confirmed that these CEUS parameters were related to cortical renal hypoperfusion.
**CONCLUSIONS.** These results confirmed the feasibility of dynamic CEUS imaging to visualize and quantify renal microcirculatory hypoperfusion by identifying the sepsis-induced plugging of microcirculatory capillary vessels.

**GRANT ACKNOWLEDGMENT**
This work is financially supported by Dutch Kidney Foundation Grant (14 OI 11)

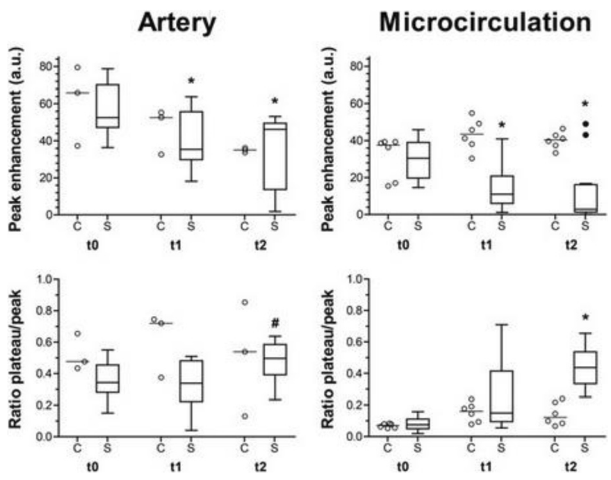

**Fig. 48 (abstract 0104).** Results of CEUS quantificationu

### 0105

#### The use of functional and structural biomarkers as predictors in the prognosis of acute kidney injury (AKI): preliminary report

A. Aisa Alvarez[1], M.E. Soto[2], G. Camarena Alejo[1], O. Torres Aguilar[1], J. Aguirre Sánchez[1], J. Franco Granillo[1]
[1]American British Cowdray Medical Center, Intensive Care Unit, Mexico City, Mexico; [2]American British Cowdray Medical Center, Department of Investigation, Mexico City, Mexico
**Correspondence:** A. Aisa Alvarez
*Intensive Care Medicine Experimental* 2017, **5(Suppl 2):**0105

**INTRODUCTION.** The 10th Consensus Conference of the Acute Dialysis Quality Initiative suggested that combining AKI biomarkers could have better precision when determining AKI outcome[1].
**OBJECTIVES.** To evaluate the prognostic utility of combining novel biomarkers of functional and structural renal injury in patients undergoing cardiac surgery.
**METHODS.** Prospective, observational and comparative cohort study. We included adults >18 years undergoing cardiac surgery without Chronic Kidney Disease who had not received contrast medium. We took baseline creatinine at the admission. After surgery, we took structural (urinary NGAL and cystatin, serum creatinine, albuminuria, and urinary sediment) and functional biomarkers (serum cystatin), and monitored patients with serum creatinine, urinary volume and fluid balance until their hospital discharge.
**RESULTS.** 31 patients were analyzed. Patients with AKI had higher levels of serum creatinine (1.12 ± 0.34 vs 0.81 ± 0.09; p = 0.002), serum cystatin [0.82 (0.56-1.65) vs 0.69 (0.40-1.12); p = 0.04] and urinary NGAL [48.3 (1.1-3103) vs 7.1 (1.2-71.3; p = 0.04]. Patients with persistent AKI had higher levels of serum cystatin [1.19 (0.78-1.65) VS 0.74 (0.56-1); p = 0.01] and albuminuria [123 (55–997) vs 25 (6.6-310); p = 0.01]. Patients with severe AKI (stages II and III) had higher levels of serum creatinine [1.6 (0.9-1.70) vs 0.95 (0.70 -1.5); p = 0.05] and serum cystatin [1.2 (1–1.65) vs 0.75 (0.56-0.97); p = 0.002]. The combination of a functional and structural biomarker was present in 20% of the AKI group, in 80% of the persistent group, and in 100% of the severe group. We found a low sensitivity (20%) and good specificity (100%) in the AKI group due to the low percentage of the biomarkers combination. In the persistent and severe form of AKI, this combination improves the sensibility (100% and 80%), specificity (80% and 80%), negative predictive value (100% and 92%) and accuracy (84% and 80%) when performing diagnostic test analysis.

**CONCLUSIONS.** In the persistent and severe form of AKI there is a higher frequency of functional and structural biomarker combinations with statistically significant difference. The presence of the combination improves the sensitivity, specificity, positive predictive value, negative predictive value and accuracy of the test for severe and persistent AKI.

**REFERENCE(S)**

1. McCullough PA, Bouchard J, Waikar SS, et al. Implementation of novel biomarkers in the diagnosis, prognosis, and management of acute kidneyinjury: executive summary from the Tenth Consensus Conference of the Acute Dialysis Quality Initiative (ADQI). Contrib Nephrol 2013;182:5–12.

### 0106

#### Short-term mortality after Continuous Renal Replacement Therapy (CRRT) in maintenance hemodialysis patients: a scoring system of short-term mortality risk after CRRT

T. Hamada, M. Sugiyama, N. Miyazawa, T. Morikawa, Y. Shikida, M. Mizobuchi, T. Shibata
Showa University, Division of Nephrology, Tokyo, Japan
**Correspondence:** T. Hamada
*Intensive Care Medicine Experimental* 2017, **5(Suppl 2):**0106

**INTRODUCTION.** Critically ill patients, suffering from serious diseases such as acute heart failure, severe acute pancreatitis, acute kidney injury, septic shock, and so on, often require continuous renal replacement therapy (CRRT) (1). It is well known that, maintenance hemodialysis (MHD) patients have many comorbidities such as hypertension, anemia, chronic kidney disease-mineral bone disorder (CKD-MBD), and are associated with high mortality.

Little is known about the outcome of CRRT in MHD patients, and what clinical parameters are risk factors of short-term mortality after CRRT.

**OBJECTIVES.** The aim of this study was to investigate whether MHD patients are at high risk of short term mortality after CRRT and to determine a scoring system relating to the mortality.

**METHODS.** In this study, 308 patients who required CRRT in our facility from April 2013 to March 2015 were retrospectively analyzed. Indication for CRRT and CRRT prescription were determined by nephrologists. We excluded patients who were indicated hemodialysis within 7 days before CRRT, transferred to other hospital, and lost to follow. Patients were stratified by two groups, MHD group and Non-MHD (control) group. Statistical analysis was performed using JMP pro for Windows, version 13. A p value < 0.05 was considered to be statistically significant.

**RESULTS.** 258 patients are included in our study. Sixty five % of them were male, mean age was 71 years old. Cumulative incidence of death for MHD group versus control group was 41.8% versus 26.9% at 7 days (p = 0.06), 60.4% versus 46.0% at 30 days (p = 0.09), respectively.

Kaplan-Meier analysis revealed that MHD group (log-rank test: p = 0.02), intubated patients (log-rank test: p < 0.0001), patients required catecholamine (log-rank test: p < 0.0001) had significant lower cumulative survival rate at 30-days after CRRT. Logistic regression analysis revealed that MHD patients were likely to die within 30-days after CRRT but not statistically significant (unadjusted odds ratio 1.79; 95% CI 0.92 - 3.54). After adjustment for elderly (age over 65 years), catecholamine administration, intubation, and MHD, MHD was an independent risk factor for 30-days mortality after CRRT (adjusted odds ratio 2.75; 95% CI 1.31 - 5.94; p = 0.0067).

Based on multivariate logistic regression analysis, we formulated a scoring system: MHD, Elderly, Intubation, Catecholamine (MEIC) score. The scoring system was derived as follows: (MHD × 5) + (Elderly (Age > 65 y) × 3) + (Intubation × 7) + (Catecholamine × 5). The area under the ROC curve was 0.73 for the MEIC score.

**CONCLUSIONS.** These results suggested that MHD, intubation, elderly, catecholamine administration were independent risk factors of 30-days mortality after CRRT. The MEIC score could be a useful scoring system for the mortality and further investigations are needed to confirm our results.

**REFERENCE(S)**

1 Joannidis M et al.Semin Dial 2009;22:160–164

**GRANT ACKNOWLEDGMENT**
No grants received

### 0107

#### Usefulness of SOFA score as a predictor of mortality in critical patients subjected to continuous renal replacement therapy

A. Cortina[1], M. Martínez[1], N. Varga[1], D. Oleas[2], N. Ramos[2], J. Baldirà[1], E. Papiol[1], R. Albertos[1], M. Pérez[1]
[1]Hospital Universitari Vall d'Hebron, Intensive Care, Barcelona, Spain; [2]Hospital Universitari Vall d'Hebron, Nephrology, Barcelona, Spain
**Correspondence:** A. Cortina
*Intensive Care Medicine Experimental* 2017, **5(Suppl 2):**0107

**INTRODUCTION.** Acute kidney injury is very common in critical patients. Many patients need renal replacement therapy, and the mortality of these patients is very high.

**OBJECTIVES.** To evaluate early prognostic factors in critical patients who require continuous renal replacement therapy (CRRT).

**METHODS.** Prospective observational study including patients admitted in an Intensive Care Unit requiring CRRT. Observational period was from March 2015 to March 2017. Chi-squared, Fisher Test, U of Mann–Whitney and logistic regression were used. Data expressed as frequency (%) and median (interquartile range).

**RESULTS.** Ninety-eight patients required CRRT during study period, men 67(68,4%), median age 63(53–70) years, APACHE II 29(24–35) and SOFA 11(8–14). Main etiology of AKI was multifactorial in 64(65.3%) patients, followed by sepsis in 26(26,5%) patients. In 73(74,5%) patients perfusion of furosemide was administered previously.

CRRT was initiated 1(0–4.3) days after ICU admission, and femoral access was used in 81(82,7%) patients. Treatment dose was protocolized. SOFA at the beginning of CRRT was 12.5(10–15), and after 48 hours of therapy, 12(9–15). Almost all the patients, 93(94,9%), received vasoactive support with norepinephrine and invasive mechanical ventilation, 87(88,8%).

Length of CRRT was 6(2–12,5) days, and length of stay in ICU was 13(7–33) days. Overall ICU mortality was 54,1%, and hospital mortality rate 61,2%.

According to Charlson Comorbidity Index, a greater mortality was presented by patients diagnosed with congestive heart failure (6(15%) vs. 1(1,9%); p = 0,04). All patients diagnosed with leukemia (acute or chronic) died (8(15,1%) (p = 0.009) vs. 0). According to the AKI etiology, there were no differences in mortality between groups.

A further analysis of the 83 patients still under CRRT after 48 hours of treatment was carried, showing that patients with favorable prognosis presented a SOFA score decrease of two or more points (OR:1,36 (CI 95%: 1,09-1,7);p = 0.006); being main cause the hemodynamic component.

**CONCLUSIONS.** Critical patients who required CRRT presented a high mortality, being leukemia and congestive heart failure factors of poor prognosis. A SOFA score decrease in the first 48 hours from therapy was presented as a factor of good prognosis.

### 0108

#### Long-term outcome in ICU patients treated with renal replacement therapy - a 2 yr follow up study in Ireland: mortality and dialysis dependency

G.J. Fitzpatrick[1], A.A. Mahmoud[1], D. Greaney[1], A. Magee[1], G. Rooney[1], H. Haslehurst[2], T. Lauren[2], M. Donnelly[1]
[1]Tallaght Hospital & Trinity College Dublin, Department of Anaesthesia & Critical Care, Dublin, Ireland; [2]Tallaght Hospital & Trinity College Dublin, Trinity College Medical School, Dublin, Ireland
**Correspondence:** A.A. Mahmoud
*Intensive Care Medicine Experimental* 2017, **5(Suppl 2):**0108

**INTRODUCTION.** ICU patients requiring CRRT (ICU/CRRT) have a high hospital mortality and reduced long term survival (1). A number of long term follow up studies have been carried out internationally but to date no such study has been carried out in Ireland.

**OBJECTIVES.** To examine the 2 year outcome of survivors of ICU/CRRT in an Irish population.

**METHODS.** We conducted a retrospective cohort study of all patients admitted to our ICU in 2013 and 2014 who required CRRT. Data was obtained from Clinical Information and laboratory systems and entered into a database for analysis. Parameters recorded included age, gender, APACHE 11 score, SOFA score and serum creatinine at initiation of CRRT and on hospital discharge.

Patients surviving to hospital discharge were followed up to 2 yrs to determine survival, dialysis dependency. For 69% of survivors, this information was obtainable from the hospital information and laboratory systems. For the remaining 31% GP's were contacted by written communication and follow up phone call. Categorical variables were compared using chi-square test and continuous variables were compared using the Mann Whitney U test. The study received ethical committee waiver.

Primary outcome was 2 year Mortality. Secondary outcomes included dialysis dependency and renal function at 2 years.

**RESULTS.** During the study period there were 686 admission to ICU and 201 patients (29.3%) were treated with CRRT. Following exclusion of 5 patients dialysed for reasons other than AKI and 15 patients with pre-exisiting ESRD, 181 were included for further analysis. 83 (46%) patients survived to hospital discharge The mean age of survivors was 60.7 yrs (range 18–86), 67% were male. The median APACHE 11 score was 20 (IQR 17–24), median SOFA score was 10 (IQR 8–12). 72 patients (87%) were dialysis independent (DI) at hospital discharge and 11 (13.3%) were dialysis dependent (DD). There was no significant difference between DD and DI patients with respect to age, gender, APACHE 11 score or SOFA score. The number of patients who required mechanical ventilation was significantly higher in DD group (63% v. 27% $p < 0.05$). The overall 2 year mortality was 29%. There was a trend towards greater mortality in the DD group (40% v 28%) not reaching statistical significance. All patients who were dialysis independent at hospital discharge and survived to 2 years remained dialysis independent. Two patients were lost to follow-up.

**CONCLUSIONS.** ICU CRRT is associated with a high hospital mortality and a significant mortality in the 2 years post discharge. The majority of survivors of ICU/CRRT were dialysis independent at hospital discharge and remained so after 2 years. There was a tendency towards improved long term survival in dialysis independent patients.

**REFERENCE(S)**

De Corte et al. Long Term outcome in ICU patients with acute kidney injury treated with renal replacement therapy: a prospective cohort study. Critical Care 2016 20:256

### Mechanical ventilation: Experimental studies

#### 0109

**Magnetic resonance imaging characterization of pulmonary artery dysfunction caused by acute lung injury in a porcine model**

A. Santos[1,2,3], E. Yazdanparast[3], I. Bilbao[3], I. Rodríguez[4], P. Cardinal-Fernández[5,6], R. Mota[3], F. Suarez-Sipmann[2], G. Peces-Barba[1,7], J. Ruiz-Cabello[1,4]

[1]CIBER de Enfermedades Respiratorias (CIBERES), Madrid, Spain; [2]Uppsala University, Hedenstierna Laboratory, Surgical Sciences Department, Uppsala, Sweden; [3]Centro Nacional de Investigaciones Cardiovasculares Carlos III (CINIC), Madrid, Spain; [4]Universidad Complutense de Madrid, Madrid, Spain; [5]Hospital Universitario HM Sanchinarro, HM Hospitales, Department of Emergency, Madrid, Spain; [6]HM Research Foundation, HM Hospitales, Madrid, Spain; [7]Fundación Jiménez Díaz, Department of Respiratory Medicine, Madrid, Spain

**Correspondence:** A. Santos

*Intensive Care Medicine Experimental* 2017, **5(Suppl 2):**0109

**INTRODUCTION.** Pulmonary Artery dysfunction and Right Ventricle (RV) failure worsen outcomes in Acute Respiratory Distress Syndrome (ARDS) patients. Due to the complexity of pulmonary circulation, a complete physiopathology description of pulmonary artery and RV disarrangements caused by ARDS is lacking. Regarding this, Magnetic Resonance Imaging (MRI) appears as a unique tool being considered as the gold standard for RV function evaluation.

**OBJECTIVES.** To evaluate RV and pulmonary artery dysfunction by means of MRI in a porcine model of ARDS.

**METHODS.** Seven anesthetized and mechanically ventilated pigs (≈35kgs) were submitted to lung saline lavages (until reaching a $PaO_2/O_2$ Inspired fraction below 200 mmHg) followed by 1.5 hours of injurious mechanical ventilation (PEEP 0 $cmH_2O$ and driving pressure of 30–35 $cmH_20$). A set of MRI datasets (Cine and Phase contrast) were acquired in baseline conditions and after the lung injury (with a delay or stabilization period of 30 minutes) in similar ventilation settings (PEEP 8 $cmH_2O$ and tidal volume 6–8 ml/kg in control volume ventilation). Acquired images were used for evaluation of RV function and flow-derived variables of vascular function estimation. Such variables included wave reflection phenomena evaluation coming from continuous flow and area analysis and forward and backward flow wave calculations.

**RESULTS.** Comparing to baseline conditions, ARDS demonstrated a non-significant trend to a lower ejection fraction (33 ± 8% vs 28 ± 5%, p = 0.195) and a higher end-systolic volume to stroke volume ratio (2.2 ± 0.7 vs 2.7 ± 0.7, p = 0.205), a MRI index of ventricular-arterial coupling. Additionally, time (normalized to the cardiac period) to backward wave arrival decreased from 0.19 ± 0.09 to 0.12 ± 0.0, p = 0.046 and reflection intensity (backward wave amplitude divided by backward and forward wave amplitude sum) increased from 0.24 ± 0.12 to 0.38 ± 0.05, p = 0.011. Mean pulmonary artery area increased after ARDS from 3.02 ± 0.31 $cm^2$ to 4.34 ± 0.52 $cm^2$, $p < 0.001$. Finally, a non-significant trend to increase in pulse wave velocity after ARDS was observed (2.2 ± 0.9 m/s vs 2.7 ± 1.4 m/s, p = 0.470).

**CONCLUSIONS.** In this porcine model, vascular function worsening caused by ARDS was non-invasively described by MRI. Of note, wave reflection phenomena increased after ARDS which can contribute to an increase in RV afterload. Interestingly, a significant decrease in the time to backward wave arrival was observed but the higher pulse wave velocity did not reach statistical significance. Maybe this can be related with the effect of other variables affecting the time to reflection arrival such as the distance to the reflection site. This distance is expected to decrease by the collapse effect that should reduce the effective longitudinal vascular length, although this interesting result would require further investigation. The potential use of MRI for pulmonary vascular function evaluation in ARDS is highlighted in this study.

#### 0110

**Effect of PEEP on recruitment/derecruitment during Neurally Adjusted Ventilatory Assist in experimental ARDS**

H. Lantz[1], M. Pellegrini[1], G. Hedenstierna[2], A. Larsson[1], G. Perchiazzi[1,3]

[1]Uppsala University, Hedenstierna Laboratory - Surgical Sciences, Uppsala, Sweden; [2]Uppsala University, Hedenstierna Laboratory - Medical Sciences, Uppsala, Sweden; [3]Bari University, Emergency and Organ Transplant, Bari, Italy

**Correspondence:** H. Lantz

*Intensive Care Medicine Experimental* 2017, **5(Suppl 2):**0110

**BACKGROUND.** It is questioned whether spontaneous breathing should be used in early acute respiratory distress syndrome (ARDS), as it may cause ventilator induced lung injury (VILI), by increasing the tidal strain/stress and tidal recruitment/derecruitment (R/D). However, spontaneous breathing has proven to offer beneficial effects, when used properly. We hypothesized that high positive end-expiratory pressure (PEEP) during spontaneous breathing would prevent derecruitment of lung regions, thereby reducing tidal R/D. We tested this hypothesis in experimental mild ARDS during continuous spontaneous breathing supported by Neurally Adjusted Ventilator Assist (NAVA).

**METHODS.** Mild experimental ARDS ($PaO_2/F_IO_2$-ratio of 200mmHg) was induced in tracheotomised and anesthetised pigs (n = 5), ventilated using optimally levelled (1) NAVA. PEEP was gradually increased in steps of 3 $cmH_2O$, from 0 to 15 $cmH_2O$ and thereafter decreased to 0 $cmH_2O$. Paradiaphragmatic dynamic computed tomography scans were acquired. Tidal volume, inspiratory time, peak flow and peak pressure were recorded continuously. Atelectatic tissue was defined as voxels with Hounsfield unit density of −100 to +100. R/D was calculated as the difference between end-expiratory and end-inspiratory atelectasis, in percentage of lung weight.

**RESULTS.** Tidal R/D decreased from 5.7 ± 7.4% to 2.3 ± 1.5% as PEEP was gradually increased from 0 $cmH_2O$ to 15 $cmH_2O$ (p < 0,01), and was increased to 5.5 ± 3.9% as PEEP was gradually decreased to 0 $cmH_2O$ (p < 0,01). Mean tidal volume increased from 83 ± 39 ml at PEEP 0 $cmH_2O$ to 386 ± 126 ml at PEEP 15 $cmH_2O$, while the respiratory rate decreased from 77 ± 20 bpm (PEEP 0) to 4.3 ± 1.9 (PEEP 15).

**CONCLUSIONS.** In this experimental early mild model of ARDS, tidal R/D occurred during spontaneous breathing using NAVA. However, the amount of tidal R/D was reduced by increasing PEEP. This indicates that PEEP higher than conventionally used clinically, in spontaneously breathing patients, might be applied in early ARDS in order to reduce R/D and, possibly, the risk of VILI.

(1) Brander et al. Chest 2009:135:695–703

### 0111

#### Effects of antithrombin and heparin for the treatment of acute lung injury in rats

N. Tantinyà[1], M. Camprubí-Rimblas[2,3], R. Guillamat-Prats[1], J. Bringué[1,2,3], M.N. Gómez[2], L. Blanch[1,2,4], A. Artigas[1,2,4]

[1]CIBER de Enfermedades Respiratorias (CIBERES), Sabadell, Spain; [2]Institut d'Investigació i Innovació Parc Taulí (I3PT), Sabadell, Spain; [3]Universitat Autònoma de Barcelona, Bellaterra, Spain; [4]Critical Care Center - Corporació Sanitària i Universitària Parc Taulí, Sabadell, Spain

**Correspondence:** N. Tantinyà

*Intensive Care Medicine Experimental* 2017, **5(Suppl 2):**0111

**INTRODUCTION.** Acute Lung Injury (ALI) is the result of an acute respiratory failure that develops from a variety of clinical disorders and is associated with a high mortality rate. In the first stages of ALI, proinflammatory mediators inhibit natural anticoagulant factors, which alter the normal balance between coagulation and fibrinolysis leading to a procoagulant state (1). Thus, patients with ALI may benefit from anticoagulant therapy. Recent studies revealed that local administration of anticoagulants could not only re-establish co-agulant activity in the lung but also prevent systemic bleedings (2).

**OBJECTIVES.** Evaluate the potential therapeutic effects of nebulized antithrombin (ATIII) and heparin in an ALI model.

**METHODS.** Sprague–Dawley rats (~300g) underwent intratracheal administration of HCL (1ml/g of HCL 0.1M, pH = 1.2). 2h later subjects underwent intratracheal administration of lipopolysaccharide (LPS 30mg/g body weight). Control groups received saline (0.9%) instead. ATIII (500IU/Kg body weight) alone or together with heparin (1000IU/Kg body weight) were nebulized through Aeroneb system (Philips Healthcare) 4h and 28h after HCL. An extra dose of nebulized heparin was administered 12h after HCL in heparin groups. Animals were sacrificed 48h after injury. Pro- and anti-inflammatory mediators as well as neutrophil and macrophages chemoattractant activity were evaluated in lung tissue using qRT-PCR. The permeability and the cellular infiltration were quantified in bronchoalveolar fluid and the integrity of the alveolar epithelium was evaluated by histology. Statistics: One-way ANOVA and Newman Keuls post-hoc test (p < 0.05).

**RESULTS.** A significant increase was observed in IL1beta, IL10, CXCL1, CCL2 and Caspase-3 in the lung tissue of the ALI group. AT-III and the combination of AT-III and heparin reduced the expression of all of them. A tendency was observed for plasminogen (Fig. 49).

**CONCLUSIONS.** These results indicate that nebulized anticoagulants are able to attenuate the lung injury through the decrease of neutrophils and monocytes recruitment and the reduction of inflammation.

**REFERENCE(S)**

1. Ware LB, Camerer E, Welty-Wolf K, Schultz MJ, Matthay MA. Bench to bedside:targeting coagulation and fibrinolysis in acute lung injury. Am J Physiol Lung Cell Mol Physiol 2006; 291(3): L307–11.

2. Hofstra JJ, Cornet AD, de Rooy BF, Vlaar AP, van der Poll T, Levi M, Zaat SA, Schultz MJ (2009) Nebulized antithrombin limits bacterial outgrowth and lung injury in Streptococcus pneumoniae pneumonia in rats. Crit Care. 2009;13(5):R145

**GRANT ACKNOWLEDGMENT**

This project has been supported by an award from Grifols, S.A.

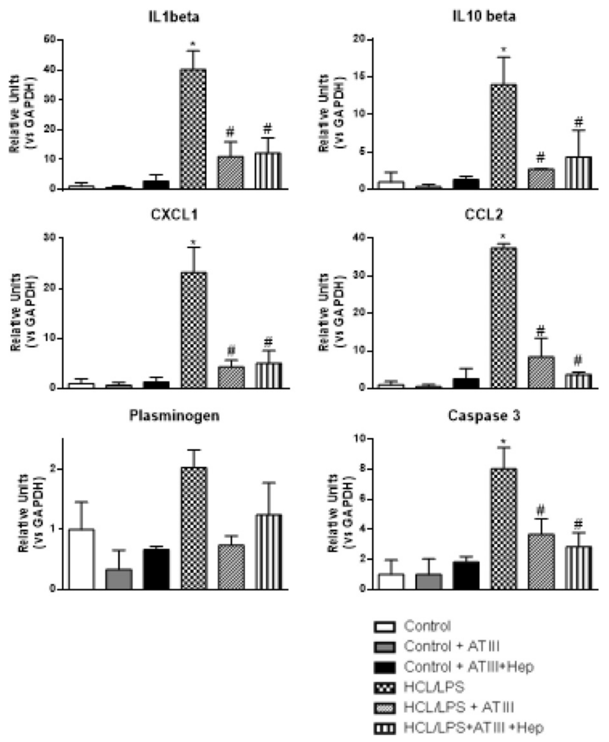

**Fig. 49 (abstract 0111).** Gene expression of pro- and anti-inflammatory markers, neutrophils and monocytes recruitment, coagulation and apoptosis. Groups: Control (n=8), control + ATIII (n=8), control+ATIII+Hep (n=8), HCL/LPS (n=8), HCL/LPS+ +ATIII (n=8), HCL/LPS+ +ATIII+HEP (n=8). *p≤0.05 vs Control: #p≤0.05 vs <HCL/LPS. Gene expression of inflammatory markers, coagulati

### 0112

#### The homing and protective effects of mesenchymal stem cells overexpressing CXCR7 in LPS-induced acute respiratory distress syndrome mice

J. Chen, L. Liu, L. Yang, X. Zhang, F. Guo, Y. Yang, H. Qiu

Zhongda Hospital, School of Medicine, Southeast University, Critical Care Medicine, Nanjing, China

**Correspondence:** J. Chen

*Intensive Care Medicine Experimental* 2017, **5(Suppl 2):**0112

**INTRODUCTION.** Mesenchymal stem cells (MSCs) have potential for re-epithelization and recovery in acute respiratory distress syndrome (ARDS). In a previous in vitro study, the results showed that the CXCL12/CXCR7 axis promoted the homing of MSCs, suggesting that the CXCL12/CXCR7 axis might be one of the key mechanisms underling the therapeutic effect of mouse MSCs in ARDS.

**METHODS.** mMSCs stable transfected with CXCR7 were transplanted intratracheally into the ARDS mice induced by lipopolysaccharide. Lung tissue injury and repair assessment were examined using haematoxylin and eosin staining, lung injury scoring. Homing of mMSCs were assayed by labelling and tracing MSCs using NIR815 dye and immunofluorescent staining.

**RESULTS.** mMSCs overexpressing CXCR7 engraftment led to more significant effects than the ZsGreen controls, including the homing of the mMSCs in the lung, improvement in endothelial permeability, and the pathologic impairment of the lung tissue.

**CONCLUSIONS.** These results suggest that overexpressing CXCR7 in mMSCs could further promote their homing to injured lung and improve the therapeutic effects of mouse MSCs in ARDS mice.

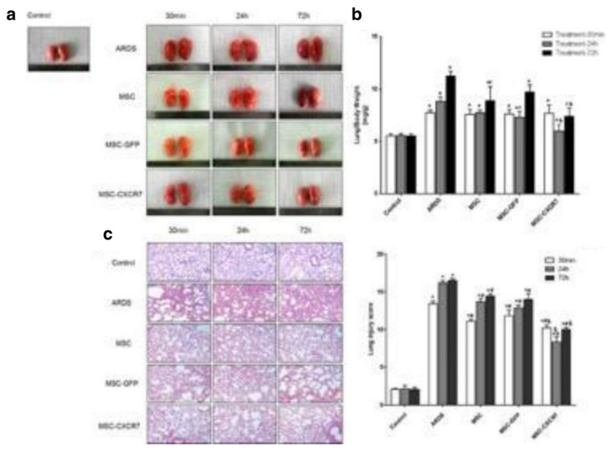

**Fig. 50 (abstract 0112).** See text for description

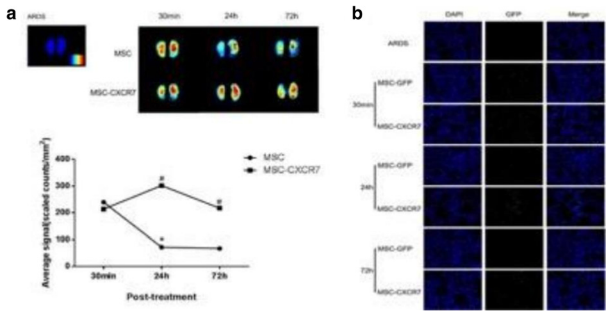

**Fig. 51 (abstract 0112).** See text for description

### 0113

#### Chemokine receptor 7 overexpression promotes mesenchymal stem cells homing via autocrine Chemokine ligand 12

L. Liu, J. Chen, X. Zhang, L. Yang, A. Liu, S. Hu, F. Guo, S. Liu, Y. Huang, Y. Yang, H. Qiu
Zhongda Hospital, School of Medicine, Southeast University, Critical Care Medicine, Nanjing, China
**Correspondence:** L. Liu
*Intensive Care Medicine Experimental* 2017, **5(Suppl 2):**0113

**INTRODUCTION.** Mesenchymal stem cells (MSCs) have several properties that make them attractive therapeutic candidates for treatment of acute disease, but in vivo homing, MSCs does not appear to be highly efficient. CXCL12/CXCR7 axis not only improves the motility of stem cells, but also regulates many essential biological processes. The aim of this study is to evaluate the effects of overexpressing or suppressing CXCR7 on proliferation and migration abilities of mice mesenchymal stem cells.

**METHODS.** The lentivirus vector overexpressing and suppressing the murine CXCR7 gene was transducted into mMSCs. The transfection efficiency of LV in passage 20 transduced-mMSCs was identified using fluorescence microscopy, and the percentage of ZsGreen positive cells was determined by flow cytometry analysis (FCM). CXCR7 mRNA expression in mMSCs was verified by quantitative real-time PCR, and CXCR7 protein expression was analyzed by FCM. The effect of CXCR7 on the migration of mMSCs was evaluated using the scratch assay and the transwell migration assay. In transwell assay, 50ng/ml CXCL12 was added in two groups to simulation inflammation microenvironments. The ELISA assay was used to detect the concentration of VCAM-1, CXCL12 the supernatant.

**RESULTS.** The efficiencies of the lentiviral vector transduction of MSC-OE-CXCR7 (overexpression of CXCR7), MSC-OENC-CXCR7 (normal control of overexpression of CXCR7), MSC-Sh-CXCR7 (suppression of CXCR7) and MSC-ShNC-CXCR7 (normal control of suppression of CXCR7) after 20 passages in mMSCs were 91.29%, 91.39%, 91.69% and 91.28% respectively. The CXCR7 mRNA and protein expression were significantly higher in the MSC-OE-CXCR7 cells than in the MSC-OENC-CXCR7 cells, and were significantly lower in the MSC-Sh-CXCR7 when compared with the MSC-ShNC-CXCR7.

Moreover, CXCR7 gene overexpression promoted mMSCs migration. In contrast, the suppression of CXCR7 inhibited mMSCs migration when compared with the MSC-ShNC-CXCR7 group.

Overexpression CXCR7 increased MSC-secreted CXCL12 and VCAM-1, which contributed to the improvement of mMSCs homing.

**CONCLUSION.** Overexpression CXCR7 improved the homing abilities of mMSCs, which might attribute mainly to increasement of CXCL12 and VCAM-1 secreted by mMSCs.

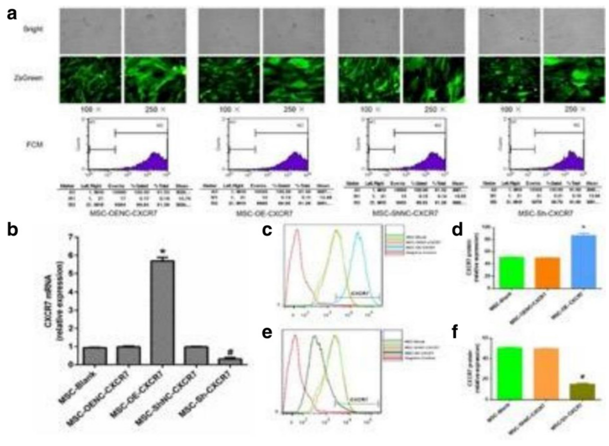

**Fig. 52 (abstract 0113).** See text for description

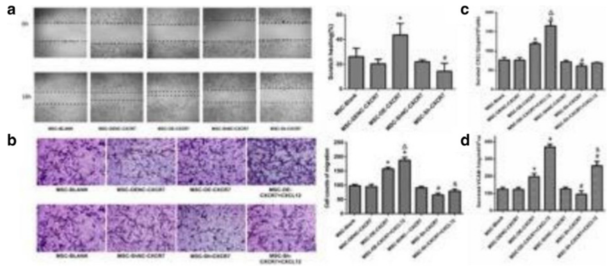

**Fig. 53 (abstract 0113).** See text for description

### 0114

#### Effects of mild-moderate hypothermia during lung protective ventilation

I. Blokhin[1], M. Pinol[2], F. Massaro[1,3], J.B. Borges[1], F. Freden[1], G. Göran Hedenstierna[1], G. Perchiazzi[1,3], A. Larsson[1], F.S. Sipmann[1,2]
[1]Uppsala University, Hedenstierna Laboratory, Surgical Sciences, Uppsala, Sweden; [2]Hospital Politècnic i Universitari La Fe, Unidad de Medicina Intensiva, Valencia, Spain; [3]University of Bari, Department of Emergency and Organ Transplant, Bari, Italy
**Correspondence:** I. Blokhin
*Intensive Care Medicine Experimental* 2017, **5(Suppl 2):**0114

**INTRODUCTION.** Therapeutic hypothermia has been extensively used in cardiac arrest but there are only a few reports on its effects on lung function and its use in the acute respiratory distress syndrome (ARDS). We hypothesized that mild hypothermia, while well tolerated, reduces the metabolic production of $CO_2$ ($VCO_2$) allowing decreasing the ventilatory demands and thereby enhancing the fulfillment of a lung protective ventilation strategy.

**OBJECTIVES.** To examine the effects of different levels of hypothermia applied during lung protective ventilation strategy on lung function, metabolism and gas exchange. In addition we tested the effects of a tidal volume (Vt) reduction at mild (34°C) and moderate (31°C) hypothermia.

**METHODS.** Hypothermia was sequentially induced by an intravascular temperature management system (Icy® IVTM,Zoll) in 4 healthy (30.1 ± 1.2kg) and 4 septic (endotoxin infusion) (30.4 ± 1.4) pigs ventilated under protective ventilation settings (Vt 6.5 ± 0.5ml/kg, PEEP 5cm$H_2O$, respiratory rate 30bpm, $FIO_2$ 0.4). T° was decreased from baseline (38.1 ± 0.5°C) to 36°C and then in steps of 1°C until 31°C each level maintained 30min. Two additional 30 min periods were performed at 34 and 31°C in order to evaluate the effects of Vt reduction. Stable Vt and cardiac output were maintained. Physiological measurements included lung mechanics, hemodynamics, gas exchange and metabolism.

**RESULTS.** Changes in physiological variables during T° reduction are depicted in Table 34, 35, 36. At stable ventilation and cardiac output (coefficient of variation of 0,17), $VCO_2$ decreased by 11.2 ± 1.6% and 5.8 ± 6.6 and VO2 by 5.8 ± 5 and 6.1 ± 8% per °C reduction in healthy and septic animals respectively. (Figure 54). At mild and moderate hypothermia Vt was reduced to 5 and 4 ml/kg in the healthy and to 5 ml/kg in the septic animals respectively. This resulted in decreased plateau and driving pressures of 30% in healthy and 15% in septic animals. At these settings $PaCO_2$ and pHa remained at acceptable clinical ranges (Table 36).

**CONCLUSIONS.** In this experimental model mild hypothermia (34°C) resulted in clinical relevant reductions in ventilatory demands in healthy and septic conditions. This may be an effective and promising early adjuvant intervention to enhance protective ventilation strategy.

**Table 34 (Abstract 0114).** Parameters during hypothermia experiment

| | Baseline | T36 | T35 | T34 | T33 | T32 | T31 | T34 low tidal volume | T31 low tidal volume |
|---|---|---|---|---|---|---|---|---|---|
| VCO2 healthy | 181±13 | 154±22 | 136±11 | 122±15 | 108 ±14 | 99±14 | 88±13 | 97±14 | 63±13 |
| VCO2 septic | 164±24 | 158±39 | 137±18 | 119±11 | 106 ±15 | 111 ±10 | 106 ±10 | 100±27 | 86±21 |
| VO2 healthy | 206±33 | 183±20 | 181±16 | 166±11 | 164 ±19 | 139 ±13 | 134 ±21 | 166±10 | 137±18 |
| VO2 septic | 237±34 | 212±26 | 176±52 | 187±56 | 165 ±29 | 161 ±26 | 152 ±28 | 187±38 | 139±48 |
| CO (L/min) healthy | 3,40 ± 0,3 | 3,79 ± 0,8 | 3,96 ± 0,7 | 3,64 ± 0,5 | 3,59 ±0,5 | 3,57 ±0,6 | 3,35 ±0,8 | 3,70 ±0,3 | 3,64 ± 0,8 |
| CO (L/min) septic | 3,91± 0,6 | 4,23± 0,6 | 4,19± 0,9 | 3,97± 0,77 | 3,87± 0,1 | 3,73± 0,2 | 3,79± 0,6 | 4,04± 0,5 | 3,71± 1,2 |

**Table 35 (Abstract 0114).** Lung mechanics during hypothermia experim

| | Baseline | T36 | T35 | T34 | T33 | T32 | T31 | T34 low tidal volume | T31 low tidal volume |
|---|---|---|---|---|---|---|---|---|---|
| VT (ml) healthy | 241±35 | 225 ±21 | 221 ±23 | 201 ±20 | 224 ±22 | 221 ±22 | 197 ±22 | 169±17 | 147,25 ±23 |
| VT (ml) septic | 217±29 | 218 ±22 | 219 ±21 | 219 ±23 | 228 ±14 | 227 ±16 | 227 ±16 | 159,75 ±22 | 167±16 |
| Pplat (cmH2O) healthy | 12±3,6 | 11,7 ±0,9 | 11,5 ±1,2 | 9,7 ±1,5 | 11,5 ±1,9 | 11,2 ±1,9 | 11,7 ±2,3 | 10±1,2 | 9,75±1,5 |
| Pplat (cmH2O) septic | 15,7 ±5.0 | 17,7±5 | 17,5 ±5,3 | 16,5 ±3,8 | 20±6,1 | 20,3 ±5,1 | 20,3 ±4,9 | 16±3,37 | 17±6,08 |
| Driving Pressure (cmH2O) healthy | 7±3.5 | 6,8 ±0,9 | 6,5 ±1,2 | 6,3 ±1,5 | 6,5 ±1,9 | 6,3 ±1,9 | 6,8 ±2,3 | 5±1,1 | 4,7±1,5 |
| Driving Pressure (cmH2O) septic | 13,5 ±3,9 | 13,5 ±4,4 | 13,3 ±4,7 | 13,8 ±4,6 | 15,4 ±5,7 | 16,4 ±4,1 | 15,6 ±4,6 | 11,2±3,2 | 12±3,8 |

**Table 36 (Abstract 0114).** Abg dring hypothermia experiments

| | Baseline | T36 | T35 | T34 | T33 | T32 | T31 | T34 low tidal volume | T31 low tidal volume |
|---|---|---|---|---|---|---|---|---|---|
| PaO2 (mmHg) healthy | 156±4 | 184 ±3 | 194 ±4 | 200 ±4 | 196 ±3 | 214 ±4 | 221 ±4 | 182±4 | 194±3 |
| PaO2 (mmHg) septic | 80±1 | 94±2 | 103 ±3 | 108 ±2 | 118 ±5 | 126 ±6 | 123 ±7 | 99±2 | 121±5 |
| PaCO2 (mmHg) healthy | 40±1 | 45±1 | 42±1 | 40±1 | 40±1 | 39±1 | 36±1 | 54±2 | 55±2 |
| PaCO2 (mmHg) septic | 56±0 | 54±1 | 53±1 | 54±1 | 52±1 | 51±1 | 50±1 | 73±1 | 64±1 |
| pHa healthy | 7,45 ±0,03 | 7,45 ±0,03 | 7,46 ±0,03 | 7,47 ±0,03 | 7,49 ±0,04 | 7,50 ±0,05 | 7,51 ±0,5 | 7,37 ±0,05 | 7,37 ±0,07 |
| pHa septic | 7,34 ±0,06 | 7,33 ±0,03 | 7,31 ±0,03 | 7,29 ±0,04 | 7,32 ±0,03 | 7,32 ±0,05 | 7,3 ±0,08 | 7,20 ±0,06 | 7,21 ±0,06 |

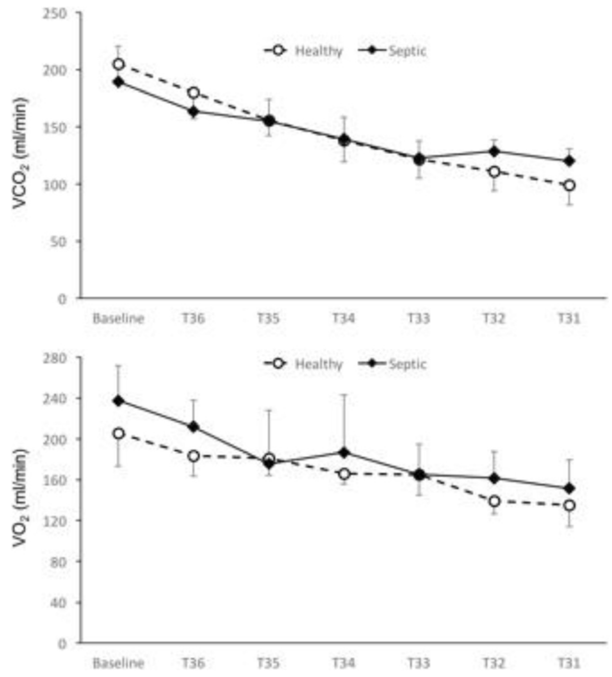

**Fig. 54 (abstract 0014).** VCO2 and VO2 dynamics related to hypothermia level

### 0115

#### Acceleration of lung-derived myofibroblast formation by dobutamine via adrenergic β receptor and CREB

N. Matsuda, S. Xu, M. Higashi, T. Yamamoto, A. Numaguchi, M. Kado, H. Tokuyama
Nagoya University Graduate School of Medicine, Emergency & Critical Care Medicine, Nagoya, Japan
**Correspondence:** N. Matsuda
*Intensive Care Medicine Experimental* 2017, **5(Suppl 2):**0115

**INTRODUCTION.** Catecholamines can proliferate fibroblasts and progress fibrosis via adrenergic beta receptors.

**OBJECTIVES.** This study aimed to analyze beta receptor action in human fibroblasts.

**METHODS.** The human fetal lung-derived fibroblast cell line IMR90 were used in this study. Expression of β receptor subtype was examined by RT-PCR and immunohistochem istry to evaluate fibroblast proliferation by dobutamine (DOB). A t test was used for the statistically analysis.

**RESULTS.** DOB accelerated the proliferation of IMR90 and the expression of α-smooth muscle actin(SMA)as myofibroblast in a concentration-dependent manner for 24 to 48 hours by $10^{-7}$ M. In RT-PCR analysis and immunohistochemical staining, IMR-90 expressed all subtypes of beta receptor. In the intracellular signaling analysis, the activity of cAMP and EPAC-1 was elevated, and it was suppressed by H89 and KT5720, whose main action was PKA inhibition. And also, transcription factor analysis showed that IMR-90 sustainably activated transcriptional factor cAMP response element binding protein (CREB) but not NF-κB and AP-1 after 15 min of dobutamine administration. The proliferative action of IMR-90 at 48 hours after DOB $10^{-7}$ M administration was significantly inhibited by CREB decoy oligonucleotides and selective β1 receptor agonist, CGP201712A and randiorol.

**CONCLUSIONS.** Fibroblasts could accelerate the proliferation and change the structural formation into myofibroblast by beta receptor stimulation.

GRANT ACKNOWLEDGMENT

Grants-in-Aid for Scientific Research of the Japan Society for the Promotion of Science.

### 0116

#### Cell therapies for the treatment of acute lung injury in an experimental model

M. Camprubí-Rimblas[1,2], R. Guillamat-Prats[3], N. Tantinyà[3], J. Bringué[1,2,3], M.N. Gómez[1], L. Blanch[1,3,4], A. Artigas[1,3,4]

[1]Institut d'Investigació i Innovació Parc Taulí (I3PT), Sabadell, Spain, [2]Universitat Autònoma de Barcelona, Bellaterra, Spain, [3]CIBER de Enfermedades Respiratorias (CIBERES), Sabadell, Spain, [4]Critical Care Center - Corporació Sanitària i Universitària Parc Taulí, Sabadell, Spain

**Correspondence:** M. Camprubí-Rimblas

*Intensive Care Medicine Experimental* 2017, **5(Suppl 2):**0116

**INTRODUCTION.** The lungs of patients with Acute Respiratory Distress Syndrome (ARDS) present widespread inflammation, coagulation and impaired fibrinolysis, producing the rupture of the endothelial and epithelial monolayer (1).

At present there is no effective treatment for ARDS. Cell therapy could be a promising therapeutic strategy for this disease (2,3). In our research laboratory, the transplantation of epithelial alveolar type II cells (ATII) in a model of Acute Lung Injury (ALI) has demonstrated promising results reducing lung injury. Potentiate immunomodulatory effect of ATII cells could improve their beneficial effects on ARDS.

**OBJECTIVE.** Evaluate the effect of genetically modified ATII cells focused in the control of the inflammatory process in ALI model.

**METHODS.** Sprague–Dawley rats (~250g) underwent intratracheal administration of 300 μl HCl (0.1M)and 2 h later of 500 μl Lipopolysaccharide (LPS 10μg/g body weight). Control animals received saline (0.9%). Genetically modified ATII cells with IL10, Il4 and IL13 were transplanted 11 h after HCl instillation. Animals were sacrificed 72 h after injury. The concentration of total proteins and the cell counts of macrophages, neutrophils and lymphocytes were assessed in the broncoalveolar lavage. Pro and anti-inflammatory pathways and markers of neutrophils and monocytes recruitment were evaluated in lung tissue by qRT-PCR. Data expressed as media ± SEM, relative to GAPDH and fold over saline group (n = 8 for all the study groups). Statistics: One-Way-ANOVA and Newman Keuls post-hoc test (Statistical significance: $p \leq 0.05$).

**RESULTS.** Higher levels of macrophages and neutrophils, lung weight and proteins were found in the HCl/LPS model and could not be reduced by transplanted modified ATII cells. Proinflammatory mediators were elevated in the ALI model. The transplantation of modified ATII cells increased pro and anti-inflammatory markers, mediators of recruitment and apoptosis.

**CONCLUSIONS.** Elucidate the pathways involved in the lung injury development and resolution is of interest to treat ARDS. Potentiate the immunomodulatory effect of ATII cells increased the recruitment of cells in to the lung and raised pro and anti-inflammatory pathways.

GRANT ACKNOWLEDGMENT

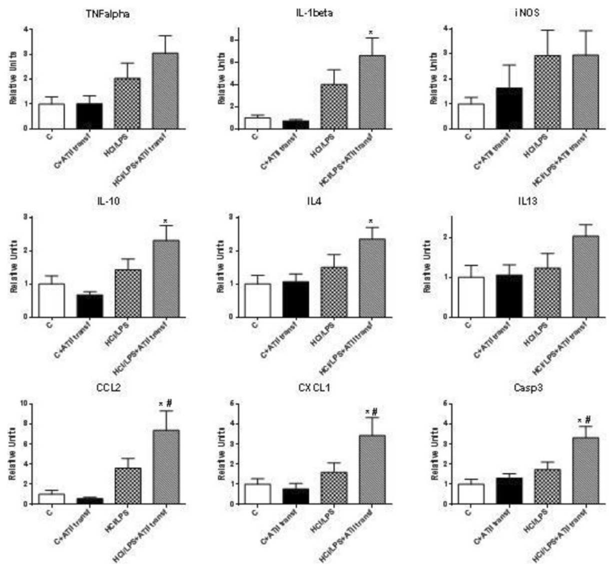

**Fig. 55 (abstract 0116).** Gene expression of pro and antiinflammatory markers, chemoattractant mediators and apoptosis in lung tissue. (n=8); *p≤0.05 vs C; #p≤0.05 vs HCL/LPS

### 0117

#### The protective effect of polydexyribonucleotide (PDRN) on acute lung injury (ALI)

J.J. Hwang[1], C.W. Choi[1], J.H. You[1], B.S. Chang[1], S.Y. Park[2], S.S. Paik[3], S.H. Park[1]

[1]Kyung Hee University Hospital at Gang-Dong, Department of Pulmonology and Critical Care Medicine, Seoul, Korea, Republic of; [2]Kyung Hee University Medical Center, Department of Pulmonology and Critical Care Medicine, Seoul, Korea, Republic of; [3]Dongnam Institute of Radiological & Medical Sciences, Pulmonology, Busan, Korea, Republic of

**Correspondence:** J.J. Hwang

*Intensive Care Medicine Experimental* 2017, **5(Suppl 2):**0117

**INTRODUCTION.** Acute lung injury (ALI) is an acute inflammatory syndrome that destroys the endothelium and epithelial barrier of the lungs due to various causes. PDRN, low molecular weight DNA complex, is a compound extracted from spermatozoa of salmon. PDRN has showed anti-inflammatory effect on very diverse inflammatory disease by A2 receptor stimulation. Recently, it has been reported that PDRN has ability to inhibit of cell death.

**OBJECTIVES.** We investigated whether PDRN could be used for ALI treatment.
**METHODS.** 36 male SD rats were classified into control group, ALI group, and ALI + PDRN treatment group. ALI was induced by intra-tracheal administration of 5 mg / kg Lipopolysaccharide (LPS).
The PDRN was injected into the abdominal cavity once at a dose of 0.3 ml (concentration of 8 mg/kg) 1 hour after LPS administration. The rats were sacrificed at 3 hours after PDRN administration.
RESULT. Compared with the control group, in lung tissue, inflammatory makers in ALI group were significant higher in lung tissue (TNF-a (1.00 ± 0.00 vs. 1.52 ± 0.08; P = 0.001) and IL-6 (**3h:** 1.00 ± 0.00 vs. 1.60 ± 0.14; P = 0.003)), and in the BALF (TNF-α (**3h:** 30.69 ± 3.07 vs. 3326.31 ± 162.06 pg/mL; P = 0.000) and IL-6 (**3h:** 35.37 ± 3.54 vs. 2495.50 ± 121.58 pg/mL; P = 0.000)). Between ALI group and ALI + PDRN treat group, ALI + PDRN treated group showed significant inflammation lowering effect in lung tissue (TNF-α (1.52 ± 0.08 vs. 1.01 ± 0.03) and IL-6 (1.60 ± 0.12 vs. 0.55 ± 0.02), all $P < 0.05$) and in BALF(TNF-α(3326.31 ± 162.06 pg/mL vs. 2112.16 ± 265.92 pg/mL), IL-6 (2495.50 ± 121.58 pg/mL vs. 1545.23 ± 194.54 pg/mL)and improving pathologic change(Lung injury score ALI 1.62 ± 0.18, ALI-PDRN 1.5 ± 0.16 $p < 0.02$).
**CONCLUSION.** The protective effect of PDRN on ALI may be associated with suppression of inflammatory responses. This demonstrate the possibility of PDRN as a therapeutic agent in acute lung injury.

### 0118

#### A new synthetic pulmonary surfactant for potential treatment of ARDS: preliminary results

J. Zebialowicz[1], F. Massaro[2,3], T. Curstedt[4], J. Johansson[1], A. Larsson[3], G. Perchiazzi[2,3], A. Rising[1]
[1]Department of Neurobiology, Care Sciences and Society, Karolinska Institutet, Stockholm, Sweden; [2]University of Bari, Department of Emergency and Organ Transplant, Bari, Italy; [3]Hedenstierna Laboratory, Dep. of Surgical Science. Uppsala University, Uppsala, Sweden; [4]Department of Molecular Medicine and Surgery, Karolinska University Hospital, Stockholm, Sweden
**Correspondence:** J. Zebialowicz
*Intensive Care Medicine Experimental* 2017, **5(Suppl 2):**0118

**INTRODUCTION.** Over the last decades, there have been many attempts to use exogenous pulmonary surfactant preparations in acute respiratory distress syndrome (ARDS) therapy. The success of this treatment has been restricted, probably due to several factors, one of which may be limited supply of pulmonary surfactant preparations.
**OBJECTIVES.** To test whether a new synthetic pulmonary surfactant, based on 2% (w/w) recombinant Surfactant Protein C (rSP-C33) in synthetic phospholipids, may be effective in a rabbit model of ARDS.
**METHODS.** Six adult rabbits were anesthetized, tracheotomized and ventilated in volume controlled mode. ARDS was induced by repeated lung lavages followed by injurious ventilation until pO2/FiO2 reached ~10kPa. We measured arterial pH, paO2, paCO2, HCO3-, mechanical ventilation and hemodynamic parameters at baseline, immediately after induction of ARDS, and then every half hour for 3 h. After the establishment of ARDS, rabbits were randomized to receive (n = 3) rSP-C33 surfactant (2,5 ml/kg of 80 mg phospholipids/ml) or an air bolus (n = 3), given at two time-points 30 minutes apart (intra-tracheal administration, half dose in the right lung, half dose in the left lung). The animals were sacrificed at the end of the experiment. Histological samples from lungs were taken.
**RESULTS.** rSP-C33 surfactant treatment gives improved pulmonary gas exchange (P/F Ratio), while it seems to have no influence on pulmonary dynamic compliance and Plateau Pressure. Immunohistochemical analysis of the samples is in progress.
**CONCLUSIONS.** The sample size is insufficient to allow detection of statistically significant differences, but the results show that the treated group has a better oxygenation compared with the non-treated group, suggesting that rSP-C33 surfactant is effective. We are awaiting the results from immunohistochemical analysis in order to analyse effects on lung histology. Further experiments are needed to confirm the results obtained, and to study additional parameters.

**REFERENCES.**
* Kronqvist, N., Sarr, M., Lindqvist, A., Nordling, K., Otikovs, M., Venturi, L., Pioselli, B., Purhonen, P., Landreh, M., Sjöberg, L., Robinson, C.V., Pelizzi, N., Jörnvall, H., Hebert, H., Jaudzems, K., Curstedt, T., Rising, A. & Johansson, J. (2017) Efficient protein production inspired by how spiders make silk. Nat. Commun. in press.
* Roger G. Spragg et al. Recombinant Surfactant Protein C-based Surfactant for Patients with Severe Direct Lung Injury, American Journal Of Respiratory and Critical Care Medicine, 2011,VOL 183,1055–1060;
* Valentin S. Rodriguez-Moya et al. Exogenous surfactant and alveolar recruitment in the treatment of the acute respiratory distress syndrome, the Clinical Respiratory Journal, 2016, ISSN 1752–6981;
* LI-NA ZHANG, Exogenous pulmonary surfactant for acute respiratory distress syndrome in adults: A systematic review and meta-analysis, Experimental and Therapeutic Medicine, 2013, 5:237–242;

### 0119

#### Haemodynamics during end-expiratory positive pressure and external negative pressure in a porcine model of lung injury

A. Fijalkowska-Nestorowicz[1], J. Glapinski[2], J. Wosko[3]
[1]Medical University Lublin, Department of Anaesthesiological and Intensive Care Nursing, Lublin, Poland; [2]Wroclaw University of Science and Technology, Chair of Electronic and Photonic Metrology, Wroclaw, Poland; [3]University Hospital No 4, Anaesthesiology and Intensive Therapy, Lublin, Poland
**Correspondence:** A. Fijalkowska-Nestorowicz
*Intensive Care Medicine Experimental* 2017, **5(Suppl 2):**0119

**INTRODUCTION.** Positive end-expiratory pressure (PEEP) may have a protective effect on lung aeration by enhance alveolar recruitment; however it may also induce lung hyperinflation and haemodynamic disturbances [1]. In contrast, external negative pressure (eNP) is known to improve oxygenation and to be less injurious compared to positive pressure ventilation.
**OBJECTIVES.** To assess the haemodynamic effects of eNP compared to PEEP during recruitment of experimentally induced lung injury.
**METHODS.** The study was conducted on ten adult Large White pigs (48–60 kg). Under continuous intravenous anaesthesia with propofol, the animals were put in a supine position, tracheotomized and intubated. Mechanical ventilation was administered in a volume-controlled mode with $F_IO_2$ -1.0, VT −8-10 mL $kg^{-1}$ and I:E ratio −1:2. Respiratory rate was adjusted to maintain $PaCO_2$ within 35–45 mm Hg. A continuous infusion of Ringer´s lactate at the rate of 5–10 mL $kg^{-1}$ $h^{-1}$ was administered throughout the study.
The acute lung injury was produced by repeated bronchoalveolar lavage using warm 0.9% NaCl until $PaO_2/F_IO_2$ remainded stable below 100. Thereafter, each animal was secured in a whole body size-chamber and endotracheal PEEP followed by eNP were created, while lung ventilation mode was held constant. Haemodynamic variables were recorded before and after the lung lavage - during recruitment manoeuvres. Heart rate (HR), mean arterial pressure (MAP), mean pulmonary artery (MPAP) and pulmonary wedge (PCWP) pressures, cardiac output (CO) and arterial oxygen tension ($PaO_2$) were compared when either PEEP or eNP were changed from 0 to 16 cm $H_2O$ by steps of 4 cm $H_2O$.
**RESULTS.** Bronchoalveolar lavage resulted in substantial deterioration of all measure variables. During lung aeration both mode of recruitment generated similar haemodynamic changes [Fig. 56], however a significant differences were recorded in MPAP, PCWP [Fig. 57] and CO [Fig. 58].
**CONCLUSIONS.** In a saline lavage lung injury eNP and PEEP generates similar haemodynamic changes. However, due to differences in MPAP, PCWP and CO, further studies are needed to confirm the haemodynamic safety of eNP during lung recruitment.

**REFERENCE**
Petitjenas F. et al.: Anaesthesiology Intensive Therapy 2016; 48: 314–38

**GRANT ACKNOWLEDGMENT**
NCN 2013/11/B/ST7/01173

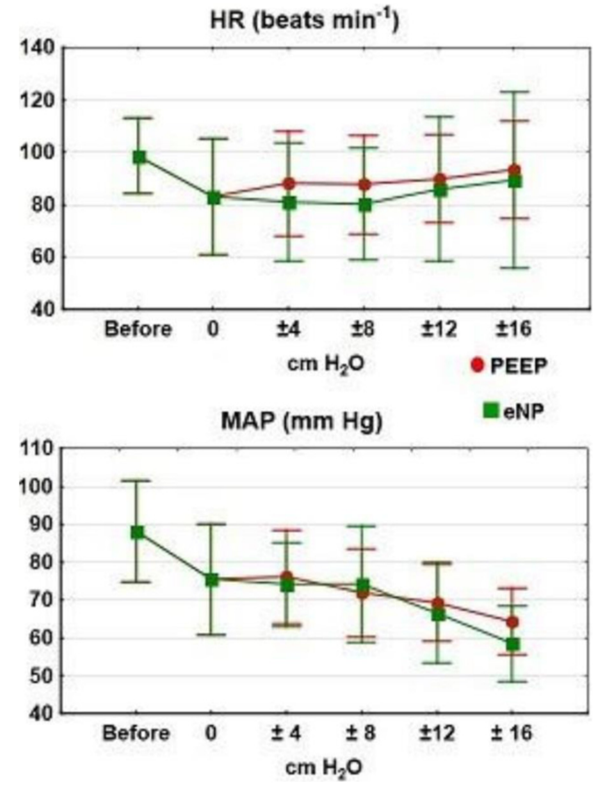

**Fig. 56 (abstract 0119).** HR and MAP during lung recruitment

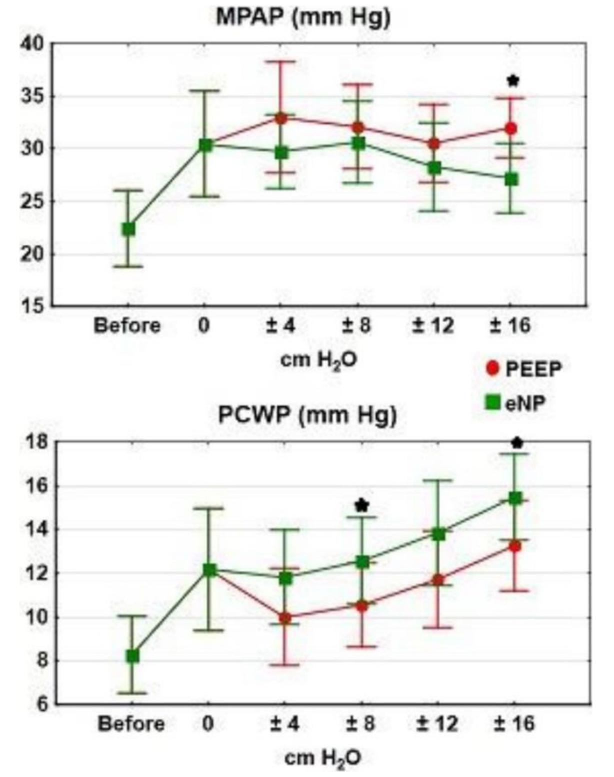

**Fig. 57 (abstract 0119).** MPAP and PCWP during lung recruitment

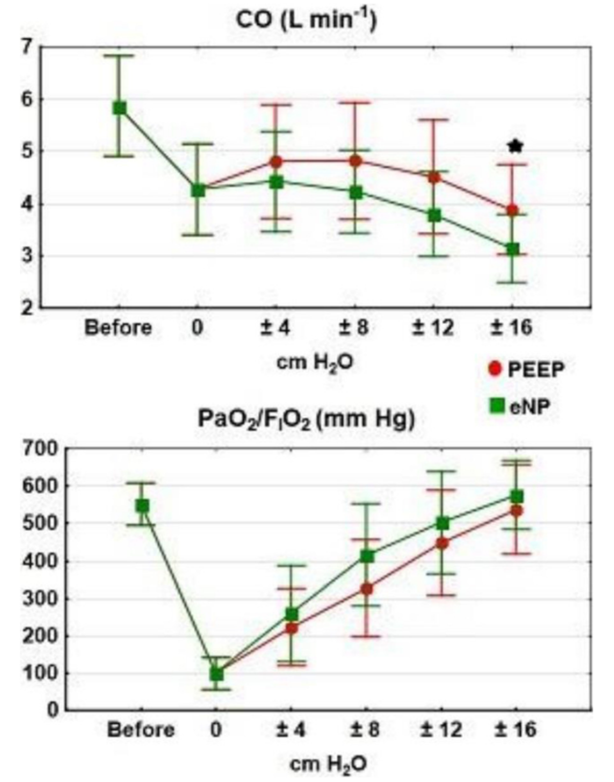

**Fig. 58 (abstract 0119).** CO and PaO2/FIO2 during lung recruitmen

### 0120

#### Molecular expression in ventilator-associated pneumonia (VAP)

L. Nogales-Martin[1], R. Almansa-Mora[2], M. Martín-Fernández[2], L. Rico-López[2], V. Iglesias[2], A. Ortega[2], D. Andaluz-Ojeda[1], L. Tamayo-Lomas[3], L. Socias[4], P. Ramírez[5], M. Balle[6], E. Villarreal[5], G. López-Campos[7], I. Martín-Loeches[8], J.F. Bermejo-Martín[2]
[1]Hospital Clínico Universitario de Valladolid, Intensive Care Unit, Valladolid, Spain; [2]Hospital Clínico Universitario de Valladolid, Laboratory of Biomedical Reseach in Sepsis (Bio.Sepsis), Valladolid, Spain; [3]Hospital Universitario Rio Hortega, Intensive Care Unit, Valladolid, Spain; [4]Hospital Son Llatzer, Intensive Care Unit, Palma Mallorca, Spain; [5]Hospital Politècnic i Universitari La Fe, Intensive Care Unit, Valencia, Spain; [6]Hospital Parc Tauli, Intensive Care Unit, Sabadell, Spain; [7]University of Melbourne, Health and Biomedical Informatics Centre (HABIC), Melbourne, Australia; [8]St James'sUniversity Hospital - Trinity Centre for Health Sciences, Intensive Care Unit, Dublin, Ireland
**Correspondence:** L. Nogales-Martin
*Intensive Care Medicine Experimental* 2017, **5(Suppl 2):**0120

**INTRODUCTION.** Ventilator-associated pneumonia (VAP) is a frequent intensive care unit (ICU)-acquired infection with worse consequences. Critically ill patients, especially septic patients, have depressed immune system. Transcriptomic analysis of circulating peripheral blood leucocytes has demonstrated to be a valuable approach to evaluate immunity in different infectious diseases and to identify new molecular markers for diagnosis proposes.

**OBJECTIVES.** The evaluation of the systemic immune dysfunction of VAP patients could be useful to understand the pathological events of this disease and to detect it early. In order to study these immune alterations in VAP we decided to use a molecular method, transcriptome microarrays.

**METHODS.** An observational prospective cohort study enrolling immune-competent adults with VAP, and no infection patients in seven medical ICU. A blood sample was collected in the first 24 hours following diagnosis of VAP by using PaxGene tubes. In the MV

control group, the sample was collected at 6th-7th day under MV (median day for diagnosis of infection in the VAP group). Clinical data also was collected; including medical history, physical examination and hematological, biochemical, radiological and microbiological data.

**RESULTS.** 41 mechanical ventilated (MV) patients (24 VAP and 17 control MV). ICU-admission reason, patient baseline characteristics are in Fig. 59. Both comparable in age, gender and comorbidities. The main reason for orotracheal intubation was low-consciousness level (72,5%). VAP group showed a lower PaO2/FiO2 ratio and higher CPIS score compared with non-infected patients. VAP patients stayed longer under mechanical ventilation (20 vs 12 days) and showed a longer ICU and hospital stay. Finally, no differences in terms of mortality were evidenced between both, control and pneumonia group.

1231 genes were identified whose expression levels were significantly different between the two groups. 680 genes were over-expressed in VAP patients and 551 genes showed lower transcript levels in this group. A large proportion of the implicated genes were down-regulated in patients with VAP. The molecules codified by these genes are related to immunological synapse, it`s suggested a depression of the antigen presentation in these patients.

**CONCLUSIONS.** These results support the idea of being able to be used the gene expression as biomarkers in the early diagnosis of ventilator adquired pneumonia.

**REFERENCE(S)**

Bermejo-Martin Jesús F, Andaluz-Ojeda David, Almansa Raquel, Gandía Francisco, Gómez-Herreras Jose Ignacio, Gomez-Sanchez Esther, et al. Defining immunological dysfunction in sepsis: A requisite tool for precision medicine. *J Infect* 2016;**72**(5):525–36.

**GRANT ACKNOWLEDGMENT**

It was granted by "Instituto de Salud Carlos III" and "Consejeria de Sanidad de Castilla y Leon" with a financial support, grant numbers [EMER 07/050] and [PI12/01815].

| | MVC (n=17, 42%) | VAP (n=24, 58%) | All patients (n=41) | p value (VAP vs MVC) |
|---|---|---|---|---|
| Age, years [Median ± IQR] | 68±26 | 54±23 | 55±25 | ns |
| Male, sex [n (%)] | 10 (58,8) | 17 (70,8) | 27 (65,9) | ns |
| APACHE II score [Median ± IQR] | 22±11 | 21±23 | 22±12 | ns |
| Comorbidities [n (%)] | | | | |
| Hypertension | 6 (35,3) | 1 (4,2) | 7 (17,1) | 0.01 |
| Diabetes mellitus | 5 (29,4) | 6 (25) | 11 (26,8) | ns |
| Chronic cardiac failure | 2 (11,8) | 4 (16,7) | 6 (14,6) | ns |
| Chronic renal failure | 1 (5,9) | 0 | 1 (2,4) | ns |
| Chronic lung disease/COPD | 2 (11,8) | 9 (37,5) | 11(26,8) | ns |
| Chronic hepatic failure | 3 (17,6) | 1 (4,2) | 4 (9,8) | ns |
| Reason for intubation [n (%)] | | | | |
| Respiratory failure | 0 | 5 (21,7) | 5 (12,5) | 0.04 |
| Cardiovascular failure | 2 (11,8) | 4 (17,4) | 6 (15) | ns |
| Coma (low-consciousness level) | 15 (88,2) | 14 (60,9) | 29 (72,5) | 0.05 |
| MV duration before diagnosis [Median ± IQR] | 0 | 8±6 | 7,5±3,75 | ns |
| At diagnosis | | | | |
| SOFA SCORE [Median ± IQR] | 5±5 | 7±3 | 6±3,5 | ns |
| CPIS score [Median ± IQR] | 3±2 | 7±1 | 6±4 | <0,01 |
| PaO2/FiO2 [Median ± IQR] | 235±62 | 160±90 | 214±102,5 | 0.03 |
| Temperature, ºC [Median ± IQR] | 37±1 | 38±0 | 37,6±1 | <0,01 |
| CRP,mg/L [Median ± IQR] | 150±126 | 164±162 | 164±171 | ns |
| PCT, ng/ml [Median ± IQR] | 0,4±1,55 | 0,1±0,85 | 0,19±1,07 | ns |
| Creatinine, mg/dL [Median ± IQR] | 0,71±0,29 | 0,78±0,45 | 0,72±0,38 | ns |
| Leukocytes, cells/mm3 [Median ± IQR] | 10590±2000 | 11630±5875 | 11110±4365 | ns |
| Lymphocytes, cells/mm3 [Median ± IQR] | 1160±683 | 1208±623 | 1180±721 | ns |
| Neutrophyles, cells/mm3 [Median ± IQR] | 8132±2161 | 9185±5312 | 8670±4434 | ns |
| Positive culture [n (%)] | 0 | 15 (62,5) | 15 (36,6) | <0,01 |
| ICU stay, days [Median ± IQR] | 17±13 | 25±30 | 20±16 | 0.01 |
| Hospital saty, days [Median ± IQR] | 28±49 | 32 ±32 | 31,5±40,25 | ns |
| Days of MV [Median ± IQR] | 12±4 | 20 ±21,5 | 15±14,25 | <0,01 |
| 28-day mortality | 3 (17,6) | 4 (16,7) | 7 (17,1) | ns |
| Hospital mortality | 4 (23,5) | 7 (29,2) | 11 (26,8) | ns |
| ICU mortality | 3 (17,6) | 7 (29,2) | 10 (24,4) | ns |

Results are expressed as median [interquartile range] for continuous variables and number (%) for categorical variables. MVC: Mechanical Ventilation Control, VAP: Ventilator-Associated pneumonia, APACHE II: Acute Physiology and Chronic Health Evaluation II, COPD: chronic obstructive pulmonary disease, SOFA: sepsis organ failure score, CPIS: Clinical Pulmonary Infection Score, CRP: C-reactive protein, PCT: procalcitonin, PaO2/FiO2: ratio of partial pressure of arterial oxygen to fraction of inspired oxygen, MV: mechanical ventilation. ICU: Intensive Care Unit.

**Fig. 59 (abstract 0120).** Clinical characteristics

| Gene (ng/μl) | MVC | VAP | *P*-value |
|---|---|---|---|
| CD40LG | 76.40 [64] | 37.60 [38.50] | 0.001 |
| CD28 | 118.40 [155.80] | 62.20 [76.50] | 0.003 |
| ICOS | 46 [45.40] | 24.80 [21.30] | 0.002 |
| IL2RA | 27.20 [27.20] | 19.40 [11.80] | 0.012 |
| CCR7 | 259.20 [327.80] | 146 [264.40] | 0.025 |
| CD1C | 41.20 [34.40] | 20.20 [17.10] | < 0.001 |
| CD3E | 274.80 [308.20] | 196.20 [233.70] | 0.009 |

**Fig. 60 (abstract 0120).** Gene expression

### 0121

### Isoplanar differences in healthy and injured lungs explored *in vivo* by synchrotron radiation computed tomography

G. Scaramuzzo[1], L. Broche[2,3], M. Pellegrini[2], L. Porra[4], S. Derosa[5], A.P. Tannoia[5], J.B. Borges[2], G. Hedenstierna[6], S. Bayat[7], A. Larsson[2], G. Perchiazzi[2,5]

[1]University of Ferrara, Dept. of Morphology, Surgery and Experimental Medicine, Section of Anesthesia and Intensive Care, Ferrara, Italy; [2]Uppsala University, Depts. of Medical and Surgical Sciences, Uppsala, Sweden; [3]European Synchrotron Radiation Facility, Grenoble, France; [4]University of Helsinki, Helsinki, Finland; [5]Università di Bari Aldo Moro, Dept. of Emergency and Organ Transplant, Bari, Italy; [6]Uppsala University, Hedenstierna Laboratory, Dept. of Medical Sciences, Clinical Physiology, Uppsala, Sweden; [7]Université de Picardie Jules Verne, Inserm U 1105 & Amiens University Hospital, Dept. of Pediatric Pulmonology, Amiens, France

**Correspondence:** G. Scaramuzzo

*Intensive Care Medicine Experimental* 2017, **5(Suppl 2):**0121

**INTRODUCTION.** The mechanisms and the microscopic distribution of alveolar volume changes induced by mechanical ventilation are still under debate for the difficulty of exploring them in vivo without altering the physiological properties of the respiratory system. Synchrotron Radiation Computed Tomography (SRCT) allows to image lung tissue at a higher resolution than conventional CT without modifying chest wall/lung interaction and to explore in vivo the microscopic effects of ventilatory parameters, like Positive End-Expiratory Pressure (PEEP).

**OBJECTIVES.** To test the effect of different PEEP on pulmonary microscopic structures characteristics in the core and in the peripheral areas of the lung.

**METHODS.** Seven anesthetized and paralyzed New Zealand rabbits underwent a decremental PEEP trial at 5 levels of PEEP (12, 9, 6, 3 and 0 $cmH_2O$); during the trial, end-expiratory SRCT scans (pixel size: 47.6 μm) were acquired. The PEEP sequence and image acquisition protocol was repeated after the induction of an experimental ARDS model (lung lavage + injurious ventilation) in 5 animals. Images were analyzed using MatLab (Mathworks, Natick, USA) Image Processing Toolbox after image contrast enhancement. Airspaces (AS) were automatically segmented based on X-ray density. The average airspace dimension (AsD), corresponding to the average number of pixels in each airspace, was evaluated in the peripheral lung (PL) and in the core lung (CL) for each level of PEEP and in both conditions. A two tails Mann–Whitney test ($\alpha = 0.05$) was used to evaluate any statistical difference between the AsD in the PL and CL for each level of PEEP.

**RESULTS.** In healthy lungs, the AsD was higher in the CL than in the PL at all PEEP levels. No statistically significant difference was found at PEEP 0 $cmH_2O$. In injured lungs, an opposite behavior was found, although without statistical significance. A high standard deviation was present in injured conditions, reduced at PEEP 12 $cmH_2O$ both for PL and CL.

**CONCLUSIONS.** The morphological characteristics of the microscopical structures of the lung are different between peripheral and core region both in healthy and in injured conditions. In healthy lungs, the AsD is bigger in the CL than in the PL at PEEP higher than 3 $cmH_2O$. In injured conditions, the AsD of the PL tended to be higher than in the CL. Moreover, a high inter-animal variability at low PEEP was present, that was reduced at PEEP 12 $cmH_2O$.

**REFERENCE(S)**

1. Hajari AJ, et al.: *J Appl Physiol* 2011;112 :937–943.
2. Perchiazzi G et al.: *Respir Physiol Neurobiol* 2014;201:60–70.
3. Dirocco JD, et al.: *Intensive Care Med* 2007;33:1204–1211.

**GRANT ACKNOWLEDGMENT**

The School of Anesthesia and the Master in Critical Care of Bari University; The Swedish Research Council; The Heart Lung Fund; The ESRF.

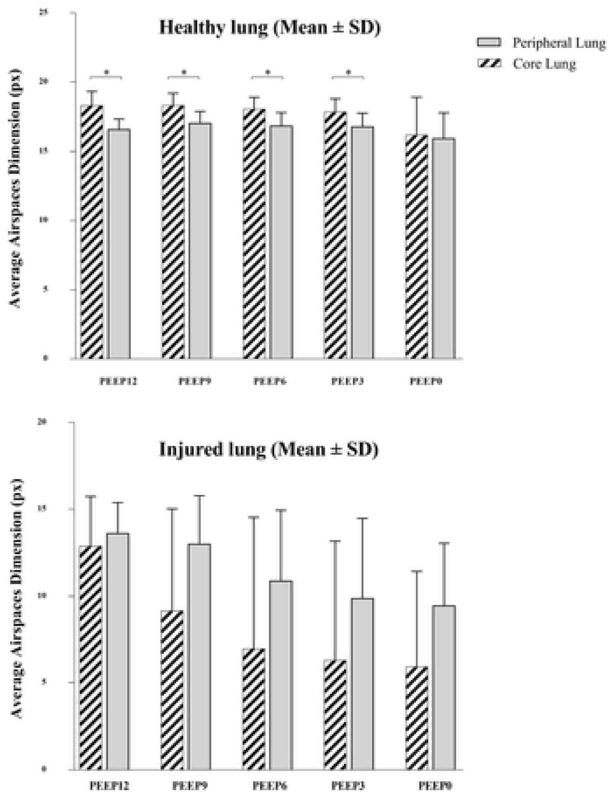

**Fig. 61 (abstract 0121).** AsD in Healthy and Injured lungs

### 0122

#### Multiple transient pendelluft phenomena occur during spontaneous breathing in experimental mild acute respiratory distress syndrome

G. Perchiazzi[1,4], M. Pellegrini[1], G. Hedenstierna[2], A. Roneus[1], M. Segelsjo[3], E.-L. Skogseid[1], A. Larsson[1]
[1]Uppsala University, Hedenstierna Laboratory, Dep. of Surgical Science, Uppsala, Sweden; [2]Uppsala University, Hedenstierna Laboratory, Dep. of Medical Science, Uppsala, Sweden; [3]Section of Radiology, Akademiska Sjukhuset., Uppsala, Sweden; [4]Bari University, Dep. of Emergency and Organ Transplant, Bari, Italy
**Correspondence:** M. Pellegrini
*Intensive Care Medicine Experimental* 2017, **5(Suppl 2):**0122

**INTRODUCTION.** Air redistribution inside the lung parenchyma - i.e. pendelluft phenomena - has been described as cause of occult overstretch in injured lungs during spontaneous breathing efforts [1].

**OBJECTIVES.** Objective of the current study was to test whether the pattern of air redistribution in injured lungs during spontaneous breathing is affected by the respiratory lung volume.

**METHODS.** Five anesthetized, tracheostomized spontaneous breathing pigs (25–30 kg) underwent lung lavages to achieve mild ARDS with a $PaO_2/FiO_2$ ratio of 250. High frequency (20 Hz) dynamic CT images were acquired at different lung volumes obtained by six continuous airway pressure levels (CPAP - from 15 to 0, in steps of 3 $cmH_2O$).The images acquired during the inspiratory phase were analyzed. Regions of interest (ROIs) outlining and image registration were performed to enable image comparisons. The registered ROIs were sequentially subtracted to obtain delta volumes (ΔV) both for complete breaths (total ΔV: end-inspiratory - end-expiratory images) and for consecutive inspiratory images (partial ΔV: volume $ROI_{(t+1)}$ - volume $ROI_{(t)}$).

The voxel ΔV distributions were classified in five histogram bins:

- highly negative ΔV (NN: from $-5*10^{-4}$ to $-2.5*10^{-4}$ mL),
- negative ΔV (N: from $-2.5*10^{-4}$ to 0 mL),
- zero ΔV (Z: 0 mL),
- positive ΔV (P: from 0 to $+2.5*10^{-4}$ mL),
- highly positive ΔV (PP: from $+2.5*10^{-4}$ to $+5*10^{-4}$ mL).

Student t-test was applied to determine differences in total ΔV distributions.

**RESULTS.** Multiple simultaneous transients of local air redistribution continuously occurred during the inspiratory phase. Negative regional ΔV indicated temporary decreases in regional lung volume during inspiration. The fugacious pendelluft phenomena were more represented at high lung volumes (high CPAP). By analyzing total ΔV distributions, CPAP lower than 6 $cmH_2O$ significantly changed the pattern of air redistribution, by reducing the pendelluft phenomena and increasing overinflation (Figure. *: statistical differences among different CPAP levels).

**CONCLUSIONS.** The current study demonstrates complex processes of air redistribution inside the lung parenchyma during spontaneous breathing.The present results show that pendelluft phenomena are not necessarily associated with detrimental ventilatory conditions. Our analysis introduces new understanding of lung aeration with pendelluft as a potential stabilizer of the lung.

**REFERENCE(S)**

1. Yoshida T, Torsani V, Gomes S, et al. (2013) Spontaneous effort causes occult pendelluft during mechanical ventilation. Am J Respir Crit Care Med 188:1420–7.

**GRANT ACKNOWLEDGMENT**

The Swedish Heart and Lung Foundation and the Swedish Research Council.

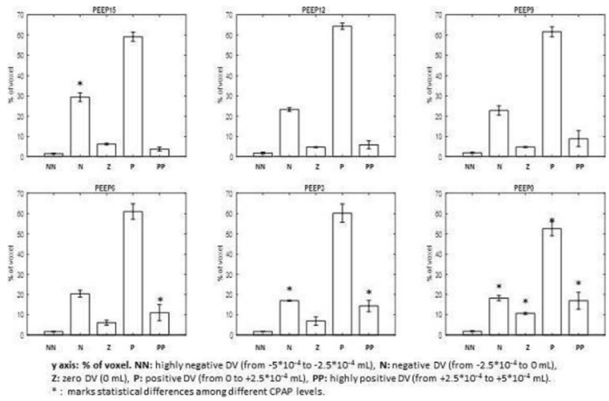

**Fig. 62 (abstract 0122).** Total delta volume distribution

### 0123

#### Long-term consequences of regional block of pulmonary perfusion in mechanically ventilated healthy pigs

T. Mauri[1], T. Langer[1], L. Di Girolamo[2], B. Cambiaghi[3], O. Biancolilli[1], M. Mirandola[2], V. Castagna[2], S. Caminada[2], G. Grasselli[1], A. Zanella[1], A. Pesenti[1]
[1]Fondazione IRCCS Ca' Granda Ospedale Maggiore Policlinico, Università degli Studi di Milano, Milan, Italy; [2]Università degli Studi di Milano, Milan, Italy; [3]Università degli Studi Milano Bicocca, San Gerardo Hospital, Monza, Italy
**Correspondence:** T. Mauri
*Intensive Care Medicine Experimental* 2017, **5(Suppl 2):**0123

**INTRODUCTION.** Previous studies showed that spontaneous ventilation coupled with complete block of pulmonary perfusion induces severe lung injury within 2–5 days [1–2]. In the present study, we aimed to assess the long-term effects of mechanical ventilation and partial pulmonary perfusion block.

**OBJECTIVE.** We investigated the effects on blood gases, respiratory mechanics and lung edema of prolonged controlled ventilation with tidal volume of 12 ml/kg and low driving transpulmonary pressure associated with constant inflation of a Swan-Ganz catheter balloon to partially occlude lung perfusion.

**MATERIAL AND METHODS.** We report preliminary data from 5 healthy female pigs (18 ± 5 Kg). After anesthesia and instrumentation (Baseline), controlled ventilation was started with 12 ml/Kg of tidal volume, PEEP 0 cmH2O, respiratory rate 20 bpm, I:E = 1:2 and FiO2 50% (T0), then the Swan-Ganz catheter balloon was filled with water to partially occlude left pulmonary vascular tree (T0). Ventilation and the Swan-Ganz catheter inflation and position were left unchanged until sacrifice. Animals were sacrificed at 48 hours or when diffuse bilateral infiltrates became evident at chest CT scan (Tend). At Baseline, T0 and Tend, we collected gas exchange, physiologic dead space (VdPhys), respiratory mechanics and we performed chest CT scans at end-expiration and end-inspiration for offline quantitative analysis (Maluna 3.17 software, Göttingen, Germany).

**RESULTS.** Two animals were sacrificed at 30 and 42 hours, having developed early diffuse lung injury, while 3 were sacrificed at 48 hours. Along the study, plateau pressure, driving airway pressure and driving transpulmonary pressure significantly increased because of steep bilateral decrease of respiratory system compliance, likely caused by worsened lung compliance (see Table 37). PaO2 declined, too, albeit non-significantly (Table 38). Quantitative analysis of CT scan showed decreased gas/tissue ratio and increased non-aerated lung parenchyma, especially in the non-occluded right lung (Table 39).

**CONCLUSIONS.** The present study generates the hypothesis that in healthy lungs mechanically ventilated with low transpulmonary pressure partial occlusion of the left pulmonary perfusion might induce edema and derecruitment within 48 hours.

**REFERENCES**
[1] Slutsky AS et al. N Engl J Med 2013;
[2] Kolobow T et al. Inter J Artif Organs 1981

**Table 37 (Abstract 0123).** See text for description

| | Baseline (n=5) | T0 (n=5) | Tend (n=5) | P-value RM-ANOVA One Way |
|---|---|---|---|---|
| Plateau pressure (cmH2O) | 10±1 | 11±2 | 21±11 | <0,05 |
| Driving airway Pressure (cmH2O) | 9±1 | 10±2 | 20±11 | <0,05 |
| Driving transpulmonary pressure (cmH2O) | 5±1 | 6±1 | 15±10 | <0,05 |
| Respiratory system compliance (ml/cmH2O) | 24±5 | 22±5 | 12±4 | <0,05 |
| Lung compliance (ml/cmH2O) | 48±13 | 39±7 | 22±16 | <0,05 |
| Chest wall compliance (ml/cmH2O) | 48±13 | 52±24 | 44±15 | 0,762 |
| Respiratory system compliance, right side (ml/cmH2O) | 11±2 | 12±3 | 7±2 | 0,01 |
| Respiratory system compliance, left side (ml/cmH2O) | 9±2 | 8±2 | 5±1 | <0,01 |
| Physiologic dead space | 0,28±0,09 | 0,34±0,08 | 0,48±0,15 | 0,120 |

**Table 38 (Abstract 0123).** See text for description

| | Baseline (n=5) | T0 (n=5) | Tend (n=5) | P-value RM-ANOVA One Way |
|---|---|---|---|---|
| PaO2 (mmHg) | 254±17 | 248±8 | 179±110 | 0,201 |
| PaCO2 (mmHg) | 38±3 | 41±4 | 43±16 | 0,766 |
| pH | 7,46±0,03 | 7,43±0,04 | 7,41±0,13 | 0,722 |
| PaO2/FiO2 | 508±34,5 | 496±16 | 346±238 | 0,196 |

**Table 39 (Abstract 0123).** See text for description

| | Baseline (n=5) | T0 (n=5) | Tend (n=5) | P-value RM-ANOVA One Way |
|---|---|---|---|---|
| Gas/tissue ratio, Right Lung | 43,2±10,2 | 40,8±8,9 | 30,0±16,8 | 0,04 |
| Gas/tissue ratio, Left Lung | 42,4±8,6 | 44,3±8,3 | 33,9±13,8 | 0,089 |
| Normally aerated tissue, Right Lung (%) | 32,9±24,3 | 29,6±19,5 | 20,4±22,2 | 0,071 |
| Normally aerated tissue, Left Lung (%) | 30,2±22,4 | 34,9±18,5 | 22,7±18,9 | 0,074 |
| Poorly aerated tissue, Right Lung (%) | 60,0±19,7 | 59,5±12,9 | 42,7±20,1 | 0,157 |
| Poorly aerated tissue, Left Lung (%) | 64,4±19,3 | 55,7±14,2 | 45,4±23,6 | 0,179 |
| Nonaerated tissue, Right Lung (%) | 6,9±6,1 | 10,9±6,7 | 36,9±30,8 | 0,05 |
| Nonaerated tissue, Left Lung (%) | 5,3±3,3 | 9,5±4,5 | 31,8±28,3 | 0,07 |

## Gastrointestinal dysfunction

### 0124

#### Thiazides for the treatment of hypernatremia in critically ill patients

R. Laruxa[1,2], M.K.A. Barreto[1,2], B.B.R. Ramos[1,2], L.P. da Silva[1,2], K.O.B. Queiroz[2,3], A.M.S. Cerqueira Junior[1], N.M. Filgueiras Filho[1,2,4], F. Jones[5], P. Benigno[5], R. Passos[5], J.G.R. Ramos[5], A.L. Gobatto[1,5,6]
[1]Hospital da Cidade, Salvador, Brazil; [2]Universidade Salvador, Salvador, Brazil; [3]Hospital da CIdade, Salvador, Brazil; [4]Universidade do Estado da Bahia, Salvador, Brazil; [5]Hospital São Rafael, Salvador, Brazil; [6]Hospital da Clinicas - University of Sao Paulo Medical School, São Paulo, Brazil
**Correspondence:** A.L. Gobatto
*Intensive Care Medicine Experimental* 2017, **5(Suppl 2):**0124

**INTRODUCTION.** Hypernatremia is associated with worse outcomes in critically ill patients and sodium renal excretion has been identified as one of its contributing factors. In addition to water administration, the treatment with thiazides has been suggested as an alternative to improve sodium renal excretion. However, evidences are scarce and available studies have been inconclusive. (1–3)

**OBJECTIVES.** Our aim was to analyze the kinetics of serum sodium in hypernatremic critically ill patients in whom thiazides were initiated for the treatment of hypernatremia.

**METHODS.** Unicentric, prospective, observational study of critically ill patients with hypernatremia (defined as serum sodium greater than 145 mEq/L) in whom thiazides were initiated for the treatment of hypernatremia. Patients with chronic kidney injury or in palliative care were excluded. The main outcome was the comparison of serum sodium variation before and after the thiazides were initiated. The patients were followed up to hospital discharge.

**RESULTS.** During 7 months, 103 hypernatremic critically ill patients were evaluated and a thiazide was initiated in 14 (13%). Twelve (86%) patients were male, and the most common admission diagnosis was respiratory failure with 7 (50%) patients. Mean (SD) age and SAPS 3 on admission were 67 (22) years and 60.3 (4.7), respectively and median (IQR) serum sodium and fluid balance before development of hypernatremia were 142 (141–144) mEq/L

and −17 (−332-790) ml, respectively. Median dose of thiazides was 58.5 (25–75) mg and maximum serum sodium was 150 (148–159) mEq/L. Thiazides initiation was associated with a decrease in serum sodium, with mean (SD) serum sodium variation before and after HCTZ of 3.3 (7.7) mEq/L and −5.6 (6.34) mEq/L, respectively, P = 0.02.

**CONCLUSIONS.** Initiation of thiazides for the treatment of hypernatremia was associated to a significant decrease in serum sodium. A causal association would be addressed by an adequately powered randomized clinical trial.

**REFERENCE(S)**

1. Pearson DC, McGain F. A survey of self-reported management of hypernatraemia acquired in Australasian intensive care units. Crit Care Resusc. 2014;16(2):140–2.

2. Bihari S, Holt AW, Prakash S, Bersten AD. Addition of indapamide to frusemide increases natriuresis and creatinine clearance, but not diuresis, in fluid overloaded ICU patients. J Crit Care. 2016;33:200–6.

3. van IMM, Buter H, Kingma WP, Koopmans M, Navis G, Boerma EC. Hydrochlorothiazide in intensive care unit-acquired hypernatremia: A randomized controlled trial. J Crit Care. 2017;38:225–30.

### 0125

#### Prognostic value of maximum acute gastrointestinal injury score, NUTRIC score and 1st-week cumulative caloric debt: a prospective, multicenter cohort study from the Sociedad Argentina de Terapia Intensiva (SATI)

C.I. Loudet[1,2], M.C. Marchena[1], L.I. Tumino[1], R. Gimbernat[3], M.L. Cabana[4], G. Capurro[5], P. Astegiano[6], M.A. Velásquez[7], M. Casanova[8], M.C. Roth[9], G. Roda[10], P. Okurzati[11], Y. Balmaceda[3], J. Rodríguez Bugueiro[8], M. Perman[12], R. Reina[1], E. Estenssoro[1], and other Investigators from the CALNUCI Group

[1]Hospital Interzonal General de Agudos San Martín de La Plata, Terapia Intensiva, La Plata, Argentina; [2]Facultad de Ciencias Médicas, Sección Terapia Intensiva, UNLP, La Plata, Argentina; [3]Centro de Cuidados Intensivos, San Juan, Argentina; [4]Hospital Pablo Soria, Jujuy, Argentina; [5]Hospital Interzonal General de Agudos Dr. Oscar Alende, Mar del Plata, Argentina; [6]Hospital José María Cullen, Santa Fe, Argentina; [7]Sanatorio Nuestra Señora del Rosario, Jujuy, Argentina; [8]Hospital El Cruce, Florencio Varela, Argentina; [9]Hospital San Juan de Dios, La Plata, Argentina; [10]Hospital Municipal Eva Perón, Merlo, Argentina; [11]Casa Hospital San Juan de Dios, Ramos Mejía, Argentina; [12]Asociación Argentina de Nutrición Enteral y Parenteral (AANEP), Buenos Aires, Argentina

**Correspondence:** C.I. Loudet

*Intensive Care Medicine Experimental* 2017, **5(Suppl 2):**0125

**INTRODUCTION.** Gastrointestinal dysfunction (GID) occurs frequently in the ICU and has been associated to increased mortality; yet it has not consistently been analyzed nor has been included in prognostic scores.

**OBJECTIVES.** To characterize GID and its impact on mortality in critically ill patients.

**METHODS.** Prospective multicenter cohort study launched by SATI. Between 1/1/12-6/1/14, patients admitted to the ICU on mechanical ventilation ≥48 hr were included. Epidemiological data, vasopressor use, $SOFA_{24hs}$, Charlson and NUTRIC scores, BMI, 1st-week cumulative caloric debt (1w-CC debt), early enteral nutrition and delivered/targeted kcal (efficacy) were collected. Acute gastrointestinal Injury (AGI) score was calculated until day 28; maximum AGI ($AGI_{max}$) was identified, and then patients were classified in 5 categories (0 to 4) according to AGI score[1]. GID was classified as gastrointestinal (primary) or secondary. High nutrition risk was considered as NUTRIC ≥5[2]. Hospital mortality was the main endpoint. Variables differing from survivors and nonsurvivors were entered in a logistic regression model. ROC curves were built to assess performance of $AGI_{max}$, NUTRIC, $SOFA_{24hs}$ and 1w-CC debt on mortality. A p value of 0.05 was considered significant for all comparisons. Adjustement for multiple comparisons was made if necessary.

**RESULTS.** This study was carried out in 10 ICUs from Argentina and 800 patients were included. Comparisons between epidemiological and nutritional variables for $AGI_{max}$ categories are shown in Tables 40 and 41. Figure 63 displays ROC curves for mortality with different scores. Logistic regression identified $AGI_{max}$(OR 1.97[1.51-2.59]), NUTRIC ≥5 (OR 5.28[3.43-8.13]), vasopressor use (OR 3.77[2.33-6.11]) age (OR 1.01[1.00-1.02]) and 1W-CC debt (OR 1.01[1.01-1.02]); all p < 0.01, as main predictors of mortality. Statistical analysis was performed with STATA 13 software.

**CONCLUSIONS.**

1) In this severely ill cohort of ICU mechanically-ventilated patients with frequent use of vasopressors, increasing severity of $AGI_{max}$ categories adequately predicts mortality, which highlights its prognostic accuracy. This association was also evident for NUTRIC score and 1w-CCdebt.

2) Discriminative power of $AGI_{max}$, NUTRIC and 1w-CC debt was excellent, and significantly better than $SOFA_{24hs}$.

3) This study underscores the prognostic validity of nutritional scores, thus supporting their inclusion in global severity scores like SOFA.

1. *Reintam et al., ESICM Working Group on Abdominal Problems, ICM 2012;38:384.*

2. *Heyland et al., Crit Care 2011; 15:R268.*

**GRANT ACKNOWLEDGMENT**

The present study was supported by the Argentinian Society of Critical Care (SATI)

**Table 40 (Abstract 0125).** Epidemiological characterstics

| | All N=800 | AGImax 0 N=138 | AGImax 1 N=194 | AGImax 2 N=217 | AGImax 3 N=197 | AGImax 4 N=54 | P (adjusted) |
|---|---|---|---|---|---|---|---|
| Age | 45±19 | 42±17 | 44±19 | 44±19 | 47±19 | 52±19 | 0.009 |
| Gender (male) | 529(66) | 94(68) | 126(65) | 143(66) | 127(64) | 39(72) | 0.83 |
| APACHE II | 17±6 | 14±6 | 17±6 | 17±6 | 19±6 | 19±7 | 0.0000 |
| SOFA24hs | 7[4-9] | 4[3-6] | 6[4-8] | 7[5-10] | 8[6-10] | 10[7-10] | 0.0001 |
| Charlson score | 0[0-1] | 0[0-1] | 0[0-1] | 0[0-1] | 1[0-2] | 2[1-2] | 0.0001 |
| Medical/ emergency surgery/ elective surgery admission (%) | 55/38/ 7 | 66/30/4 | 52/41/7 | 59/33/8 | 50/42/8 | 33/63/4 | 0.000 |
| Vasopressor use | 494(62) | 10(7) | 115(59) | 150(69) | 168(85) | 51(92) | 0.000 |
| ICU length of stay | 14[9-25] | 11[7-17] | 15[10-26] | 16[10-26] | 16[10-27] | 18[10-28] | 0.00001 |
| Hospital mortality | 318(40) | 7(5) | 31(16) | 90(41) | 143(73) | 47(87) | 0.000 |

**Table 41 (Abstract 0125).** Nutritional variables

| | All N=800 | AGImax 0 N=138 | AGImax 1 N=194 | AGImax 2 N=217 | AGImax 3 N=197 | AGImax 4 N=54 | P (adjusted) |
|---|---|---|---|---|---|---|---|
| Primary gastrointestinal failure | 137(17) | 2(1) | 10(5) | 20(9) | 64(32) | 41(76) | 0.000 |
| BMI | 23±8 | 22±7 | 22±8 | 23±8 | 23±8 | 20±12 | 0.58 |
| NUTRIC score | 4[2-5] | 2[1-4] | 3[2-5] | 4[3-5] | 5[4-6] | 5[4-6] | 0.0001 |
| NUTRIC score ≥5 (High nutrition risk) | 313(39) | 15(11) | 49(25) | 82(38) | 128(65) | 39(72) | 0.000 |
| Cumulative caloric debt (1st week) | −3300 [-6000 to -1200] | −560 [-800 to -320] | −2000 [-2700 to -1100] | −4000 [-5500 to -3000] | −7000 [-9000 to -5000] | −10000 [-12000 to -7335] | 0.0001 |
| Efficacy (Delivered/ targeted kcal, %) | 68[48-82] | 88[84-92] | 80[75-84] | 65[60-74] | 40[30-51] | 20[10-30] | 0.0001 |
| Early enteral nutrition (≤48 hr) | 497(63) | 127(92) | 136(70) | 146(67) | 81(42) | 7(11) | 0.000 |
| Supplemental Parenteral Nutrition | 65(8) | 0(0) | 3(2) | 4(2) | 28(14) | 30(57) | 0.000 |

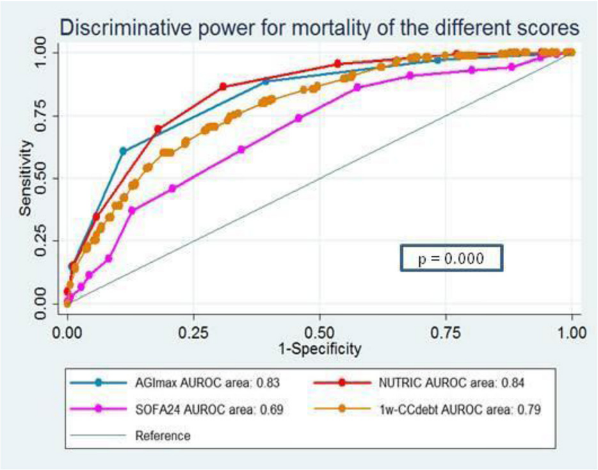

**Fig. 63 (abstract 0125).** Discriminative power for mortality

### 0126

#### Gastrointestinal failure affects outcome of intensive care

M. Padar[1], G. Uusvel[1], L. Starkopf[2], J. Starkopf[1,3], A. Reintam Blaser[3,4]
[1]Tartu University Hospital, Department of Anaesthesiology and Intensive Care, Tartu, Estonia; [2]University of Copenhagen, Faculty of Health and Medical Sciences, Section of Biostatistics, Institute of Public Health, Copenhagen, Denmark; [3]University of Tartu, Department of Anaesthesiology and Intensive Care, Tartu, Estonia; [4]Lucerne Cantonal Hospital, Department of Anaesthesiology, Intensive Care, Emergency and Pain Medicine, Lucerne, Switzerland
**Correspondence:** M. Padar
*Intensive Care Medicine Experimental* 2017, **5(Suppl 2):**0126

**INTRODUCTION.** Whereas a unified definition of gastrointestinal failure (GIF) is still lacking, number of concomitant GI symptoms has been suggested to identify GIF leading to impaired outcome [1].

**OBJECTIVES.** To describe the incidence and outcome of GIF in ICU patients and compare primary *versus* secondary GIF.

**METHODS.** We analysed a prospective database including all consecutive patients treated in a mixed ICU at Tartu University Hospital in years 2004–2015. Daily data were collected during patients' ICU stay and follow-up performed for 90-day survival using National Death Registry. Local Ethics Committee approved the study. GIF was considered present if a patient experienced ≥ 3 of the following 6 symptoms in one day: maximum gastric residual volume ≥ 500 mL; absent bowel sounds; vomiting or regurgitation; diarrhea; suspected or radiologically confirmed bowel distension; gastrointestinal bleeding [1]. Division into primary (gastrointestinal pathology leading to GIF) and secondary (GIF due to other conditions) GIF was made based on the origin of the syndrome.

**RESULTS.** GIF developed in 420 (10,4%) of the 4047 patients studied. Primary GIF was present in 251 (59,8%) and secondary GIF in 169 (40,2%) patients. Development of GIF was associated with longer mechanical ventilation (MV; median 9 (IQR 3–22) *vs* 2 (IQR 1–6) days), ICU stay (median 11 (IQR 5–25) *vs* 3 (IQR 1–8) days) and a higher ICU (34,8 *vs* 17,6%), 30-day (39,6 *vs* 26,4%) and 90-day mortality (49,6 *vs* 31,8%; Fig. 64), P < 0,001 for all.

Patients with primary origin of GIF experienced more gastrointestinal symptoms than those with secondary GIF (P < 0,05 on days 1–6, 10, 11). As opposed to those with secondary GIF, patients with primary GIF more frequently had sepsis (54,3 *vs* 40,7%; P = 0,007) and surgery on admission day (68,3 *vs* 46,7%; P < 0,001), whereas no differences were noted in MV days, ICU stay and ICU, 30- and 90-day mortality. All SOFA sub-scores on admission day independently predicted 90-day mortality, adding GIF to this model slightly improved mortality prediction (Table 42).

**CONCLUSIONS.** Gastrointestinal failure, independent of origin, is associated with impaired ICU outcome. Similarly to other organ failures, GIF independently predicts mortality.

**REFERENCE(S)**

1. Reintam Blaser A, Poeze M, Malbrain MLNG, et al. Gastrointestinal symptoms during the first week of intensive care are associated with poor outcome: a prospective multicentre study. *Intensive Care Medicine*. 2013;39(5):899–909. doi:10.1007/s00134-013-2831-1.

**GRANT ACKNOWLEDGMENT**
Ministry of Education and Research of Estonia (IUT34-24)

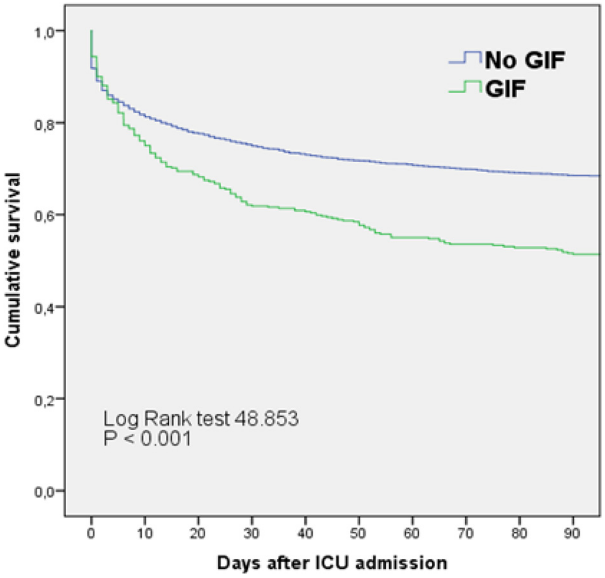

**Fig. 64 (abstract 0126).** Kaplan-Meier curve: GIF vs no GIF

**Table 42 (Abstract 0126).** SOFA and GIF predicting 90-day mortality

| Variable | OR | Lower CI 95 % | Upper CI 95 % | P value |
|---|---|---|---|---|
| SOFA respiratory | 1.097 | 1.018 | 1.182 | 0.015 |
| SOFA haematologic | 1.207 | 1.119 | 1.303 | <0.001 |
| SOFA hepatic | 1.172 | 1.071 | 1.283 | 0.001 |
| SOFA cardiovascular | 1.499 | 1.414 | 1.591 | < 0.001 |
| SOFA neurological | 1.445 | 1.377 | 1.515 | < 0.001 |
| SOFA renal | 1.342 | 1.276 | 1.410 | < 0.001 |
| GIF | 1.586 | 1.126 | 2.233 | 0.008 |

### 0127

#### Influence of Spanish society of intensive care (SEMICYUC) recommendations in the evolution of acute pancreatitis

R. Fernández Fernández[1], M.R. Ramírez Puerta[2], M.E. Yuste Ossorio[2]
[1]Hospital Santa Ana, Motril, Spain; [2]Complejo Hospitalario Granada, Granada, Spain
**Correspondence:** R. Fernández Fernández
*Intensive Care Medicine Experimental* 2017, **5(Suppl 2):**0127

**INTRODUCTION.** In 2012 the SEMICYUC published a consensus document with 82 recommendations for the management of acute pancreatitis in ICU. Of these we highlight the new classification of acute pancreatitis based on evolutionary determinants, new surgical techniques and nutritional recommendations. Based on these recommendations we have focused in the last 6 years the diagnosis and treatment of PA.

**OBJECTIVES.** Describe the epidemiology of patients with AP in the last 6 years in our ICU. Assess whether the adoption of the recommendations of SEMICYUC, has or not impact on the evolution of this disease.

**METHODS.** Retrospective descriptive study of 100 patients diagnosed with acute pancreatitis in the period from 01/01/2011 to 31/12/2016. We have studied the following variables: etiology, age, sex, APACHE II, SOFA, PIA, organ failure (respiratory, renal hemodynamics) severity rating, nutrition, need for mechanical ventilation (MV) or therapies extrarenal depuration (TDR) days of ICU stay and hospital, and mortality.

**RESULTS.**

Etiology: 32´35% alcohol; 38´23% bile; 11´76% post ERCP, 11.78% indeterminate; 5.88% hypertriglyceridemia.

Age: 58´87 ± 14´55,

APACHE II 15´58 ± 7´48,

SOFA 7´11 ± 4´3,

PIA 14´19 ± 3´74,

ICU stay ***2015*** 15´87 ± 27´26, ***2014*** 13´93 ± 17´65, ***2013*** 12´36 ± 14´43, ***2012*** 7´35 ± 10´43, ***2011*** 5´61 ± 15´83, respiratory disfunction ***no*** 49´04%, ***yes*** 50´96%, renal disfunction ***no*** 32´27%, ***yes*** 67´73%, hemodynamic dysfunction ***no*** 37´03%, ***yes*** 62,27%.

The most pancreatitis entered in 2011 and 2012 were serious (61´53% 54´16%) while in 2013 and 2014 the most frequent were moderate (55´66% and 50%).

Nutrition: Parenteral 43´96% 32´25% absolute, enteral 22´6%.

Need for mechanical ventilation: 56´86% and therapy renal clearance 33´74%.

Mortality. ***2011*** 35% ***2012*** 52% ***2013*** 28% ***2014*** 29´41%, ***2015*** 20´12%, ***2016*** 50%

**CONCLUSIONS.** In the past two years we have entered in ICU more moderate pancreatitis than severe. After the implementation of the SEMICYUC recommendations, has decreased overall mortality, but not ICU stay. Noted that we still use parenteral nutrition despite enteral via jejunal is a choice with strong recommendation grade and high-quality evidence.

**REFERENCE(S)**

http://www.medintensiva.org/es/semicyuc-2012-recomendaciones-el-manejo/articulo/S0210569113000119/

**GRANT ACKNOWLEDGMENT**

ENVIN, Dra. MªRosario Ramírez Puerta

### 0128

#### Pneumatosis intestinalis in critically ill patients: a retrospective observational study

S. Takeaki, M. Fujita, T. Irinoda, Y. Kawazoe, S. Akaishi, S. Kushimoto
Tohoku University Hospital, Emergency Center, Sendai-shi, Japan
**Correspondence:** S. Takeaki
*Intensive Care Medicine Experimental* 2017, **5(Suppl 2):**0128

**INTRODUCTION.** Although most of patients with pneumatosis intestinalis (PI) has been reported to be asymptomatic, bowel ischemia should be considered as a cause of PI in critically ill patients. However, there is a relative paucity of information on the characteristics of asymptomatic PI in critically ill patients.

**OBJECTIVES.** Purpose of this study was to clarify the characteristics of asymptomatic PI, and to assess the usefulness of clinical features for the differentiation between asymptomatic and bowel ischemia-related PI.

**METHODS.** This single-institutional retrospective observational study collected data from patients of PI between 2013 and 2015. PI was diagnosed with CT examination. Bowel ischemia/necrosis was diagnosed with operative findings or clinical course in non-operative patients. Clinical information on admission and at the time of diagnosis of PI was recorded.

**RESULTS.** Of 34 patients, 21 patients were asymptomatic and 13 were associated with bowel ischemia/necrosis. There was no difference in underlying diseases. In patients with bowel ischemia, APACHE-II and SOFA scores on admission (32 vs 21, p = 0.01; 11 vs 6, p = 0.01), C-reactive protein value at detection of PI (10.7 vs 1.5 mg/dL, p = 0.01, respectively) were higher, and onset of PI (5 vs 33 days, p = 0.01) was earlier as compared to those of asymptomatic patients. The rate of proceeding diarrhea and distribution of PI limited to colon was higher in asymptomatic patients with sensitivity and specificity: 92%, 71%, and 92%, 71%, respectively. On the other hand, ascites collection (sensitivity and specificity; 92%, 95%), PI in stomach and duodenum (83%, 71%), and in small intestine (92%, 71%) were pathognomonic for patients with bowel ischemia. Hospital mortality was 69% in patients with ischemia and 0% in asymptomatic.

**CONCLUSIONS.** PI may be relatively common condition in critically ill patient. Although PI located only in colon with proceeding diarrhea may be asymptomatic, those presented in upper gastrointestinal tract with ascites collection may be associated with bowel ischemia, with high mortality.

**GRANT ACKNOWLEDGMENT**

There was no grant.

### 0129

#### Epidemiology of severe acute pancreatitis in ICU

J.C. Moncayola Vicen, A.N. García Herrera, V. Mínguez Exposito, T. Caldito Ascargorta, E. Erice Azparren, M. García Parra, A. Orera Perez, E. Sanz Granado, M. García-Montesinos de la Peña, M. Barber Ansón[1]
Complejo Hospitalario de Navarra, Pamplona, Spain
**Correspondence:** A.N. García Herrera
*Intensive Care Medicine Experimental* 2017, **5(Suppl 2):**0129

**INTRODUCTION.** The incidence of acute pancreatitis (AP) ranges between 5 and 80 per 100,000 population. In Europe more patients tend to have biliary tract-related pancreatitis and less frequently alcohol, trauma or ERCP-related pancreatitis. About 25% of the patients with AP develop severe acute pancreatitis (SAP), which is strongly associated with organ failure and local complications such as pancreatic or peripancreatic necrosis and formation of pseudocyst. Overall mortality has decreased but still ranges 15%-25%.

**OBJECTIVES.** To analyze the epidemiological characteristics of patients admitted to the intensive care unit with diagnosis of SAP.

**METHODS.** Retrospective cohort study including patients admitted to the ICU of a referral hospital with the diagnosis of SAP between 2005 and 2015. Demographic variables, severity score (APACHE II), etiology, ICU and hospital mortality were collected from medical records. Quantitative variables were compared by Student´s T or Mann–Whitney test, as appropriate. The qualitative ones were compared by chi-square or Fisher´s test depending on data. Data were expressed as means and standard deviation (SD), odds ratio (OR) and 95% confidence intervals (95CI). p values < 0.05 were considered statistically significant.

**RESULTS.** 115 patients with SAP were admitted to the ICU, 54.8% males. 21.7% had a history of cholelithiasis, 7.8% had previous pancreatitis and 7.8% history of chronic alcoholism.

Among the etiology, biliary was the most frequent (39.1%) followed by indeterminate (21%), alcoholic (13.9%), ERCP-related (9.6%), hypertriglyceridemic (7%) and drug-induced (3.5%).

In terms of mortality according to etiology, higher mortality was observed in edematous pancreatitis (36.6%) compare to necrotizing pancreatitis (21.6%) without reaching statistical significance.

Higher mortality was observed in patients who received total parenteral nutrition (TPN) (60.7%) compared to those with enteral nutrition (OR: 8, 95CI: 3.1-20.8, p value: 0.00), without differences between nasogastric and nasojejunal feeding.

Pancreatic fluid collections were observed in 90.9% in ERCP-related pancreatitis without reaching statistical significance. 56.7% of patients with alcoholic related pancreatitis required percutaneous drainage of the fluid collections (OR, 3.4, 95CI: 1.16-10.11).

**CONCLUSIONS.** In the patients included in our study biliary tract-related pancreatitis is the most frequent etiology of SAP. We found no difference in mortality among the different etiologies.

Higher mortality was observed in edematous pancreatitis but without reaching statistical significance.TPN was associated with higher mortality compared to enteral nutrition.

### 0130

#### Measurement of salivary cortisol levels in critically ill patients

J. Lee, J.H. Kim, S.-M. Lee
Seoul National University Hospital, Seoul, Korea, Republic of
**Correspondence:** J. Lee
*Intensive Care Medicine Experimental* 2017, **5(Suppl 2):**0130

**INTRODUCTION.** Although the measurement of free cortisol remains ideal in assessment of hypothalamic pituitary adrenal function, it is not routinely measured. Cortisol in saliva correlates well with the biologically active free cortisol in the circulation.

**OBJECTIVES.** The aim of this study was to investigate the utility of morning basal and ACTH-stimulated salivary cortisol in critically ill patients and compare the results with non-critically ill patients.

**METHODS.** We prospectively enrolled 49 mechanically ventilated patients with various illnesses and 120 patients from the outpatient clinic. Serum and saliva samples were drawn between 8 am and 10 am. The salivary samples were inadequate in 14 critically ill patients (28.5%) and these patients were excluded in the final analysis. Salivary cortisol levels were measured using an enzyme immunoassay kit.

**RESULTS.** Critically ill patients (n = 34) were significantly older and had lower BMI, serum albumin and higher serum creatinine compared to the non-critically ill patients (n = 120). After adjustments for these parameters, both basal and stimulated salivary and serum cortisol levels were higher in critically ill patients. Increment of salivary cortisol, but not serum cortisol were higher in the critically ill patients. In critically ill patients, there was significant correlations among serum albumin, basal salivary cortisol, increment of serum cortisol and the patient's APACHE II scores. Increment of serum cortisol was the only significantly different variable between the APACHE II score ≥25 group and the APACHE II score < 25 group. Receiver-operating characteristic (ROC) analysis allowed the cutoff level of delta serum cortisol to be 8.2 mg/dL (sensitivity, 76.5%; specificity, 64.7%; AUC = 0.753).

**CONCLUSIONS.** Both basal and stimulated salivary and serum cortisol levels were higher in critically ill patients. Increment of serum cortisol was significantly higher in the APACHE II >25 group.

**GRANT ACKNOWLEDGMENT**
This work was supported by Research Resettlement Fund for the new faculty of SNU

### 0131

#### Clinical study of early enteral nutrition support in severe burn patients

Y. Wu, Y. Wu, J. Liu
Nanjing Medical University, Suzhou, China
**Correspondence:** Y. Wu
*Intensive Care Medicine Experimental* 2017, **5(Suppl 2):**0131

**OBJECTIVE.** To explore the value of early enteral nutrition in the treatment of severe burn patients.

**METHODS.** Choose 52 severe burn patients who are hospitalized in Suzhou ICU and fit inclusion criteria from August to September in 2014 to make retrospective analysis. According to starting time, they are divided into early enteral nutrition group (EEN group, n = 28) and non-early enteral nutrition group (NEEN group, n = 24). To compare the amount of intake energy to object energy, the amount of enteral and parenteral nutrient intake to total energy respectively when two group patients are burned from 1st to 7th day, at 14th day, 21st day and 28th day. And also, to compare scores of Acute physiology and chronic health evaluation (APACHE II), prealbumin, creatinine, urea nitrogen when two group patients are treated at 3rd day, 7th day, 14th day, 28th day, primary operation time in 28 days and operation numbers as clinical data.

**RESULTS.** Two group patients are both given enteral nutrition when hospitalized in 3 days. In 7 days, their total intake energy are both lower than object energy, and EEN group is much lower. At 14th day, 21st day and 28th day, their total intake energy are both meet object needed energy. And increase trend of EEN group is much stable. Their enteral nutrient intake is roughly equal to parenteral nutrient intake from 4th day, and take enteral way as first in 7 days. After treatment, scores of APACHE II are both improved, and scores of two group patients at 3rd day, 7th day, 14th day, and 28th day are both better than hospitalized in ICU(P < 0.05). Scores of APACHE II for EEN group at 7th day, 14th day, and 28th day are better than NEEN group. Prealbumin numbers for EEN group at 28th day are better than NEEN group(P < 0.05). And creatinine, urea nitrogen at 3rd day, 7th day, 14th day, 28th day, primary operation time in 28 days and total operation numbers for two group patients have no statistical differences. Also, the incidence of enteral nutrient adverse reaction for two group patients have no statistical differences.

**CONCLUSIONS.** Using early enteral nutrition in therapies for severe burn patients can make energy intake more stably, reduce disease severity of patients, improve plasma albumin numbers at 28th day, and will not increase the incidence of adverse reaction.

### 0132

#### The acute influence of acid suppression with esomeprazole on gastrointestinal microbiota and the gene expression profile of brain in a murine model of physiologic stress

R. Maclaren, D.N. Frank, R.A. Radcliffe
Univrsity of Colorado, Aurora, United States
**Correspondence:** R. Maclaren
*Intensive Care Medicine Experimental* 2017, **5(Suppl 2):**0132

**INTRODUCTION.** The central nervous system (CNS) and gastrointestinal tract (GIT) are linked through neuro-endocrine pathways and humoral mediators. Critically ill patients frequently receive acid suppressing agents to reduce the likelihood of GIT bleeding caused by physiologic stress; however, acid suppression during physiologic stress may alter GIT microbiota and CNS activity.

**OBJECTIVES.** To comparatively evaluate the GIT microbiota and the gene expression of neurocognitive mediators between esomeprazole and placebo in a murine model of physiologic stress.

**METHODS.** Twenty-four male C57BL/6J mice were randomly assigned to physiologic stress induced by hypothermic immobilization or control environment for three hours daily and either esomeprazole 2 mg/kg or saline by intraperitoneal injection daily. After three days, mice were sacrificed and stomach, ileum, colon, frontal lobe, and the hippocampus were harvested. Broad-range analysis of 16S rRNA genes of GIT samples was used to determine microbiome profiles and RNA-seq was performed to determine expression profiles of mRNA with enrichment analyses.

**RESULTS.** The mucosa of stressed mice showed mild gastritis. Both stress (p < 0.001) and esomeprazole (p = 0.006) had significant, independent effects on the stomach microbiota to alter the distribution of commensal organisms. Esomeprazole, but not stress, produced modest changes in the microbiota of the ileum (p = 0.056) and colon (p = 0.022). Stress induced differential expression of 5 genes in the frontal lobe, but no genes in the hippocampus. The addition of esomeprazole to stress induced differential expression of 124 genes in the hippocampus, the majority of which were down-regulated. The biological processes were most affected by these 124 genes included synaptic transmissions; locomotor behavior; associative learning; sensory perception to pain; feeding behavior; peristalsis; dopamine receptor signaling and synapse; response to hypoxia; and response to drugs such as ethanol, nicotine, amphetamine, and cocaine. Stress and esomeprazole had limited effect on the frontal lobe.

**CONCLUSIONS.** Acute physiologic stress has region-specific effects on the GIT microbiota. Distribution of commensal organisms is altered with stress and the effect is heightened with acid suppression. Acute stress alone has limited impact on CNS gene expression. The addition of acid suppression, however, has region-specific effects on CNS gene expression with the greatest impact in the hippocampus. Several key biological processes for neuro-cognition are affected. Further studies are needed to validate these results, determine associations between microbiota disturbances and altered gene expression, and assess associations between altered gene expression and biochemical aberrations and neuro-cognitive / behavioral outcomes.

**GRANT ACKNOWLEDGMENT**

Funding was provided by the Skaggs School of Pharmacy and Pharmaceutical Sciences Associated Dean for Research Grants Program.

### 0133

#### Potassium and phosphate excretion in the critically ill and their relation with sodium and chloride excretion

L. Hessels[1], A. Oude Lansink-Hartgring[1], M. Volbeda[1], M. Diemeer[2], C. Cordeiro[3], M.H. de Borst[4], M.W. Nijsten[1]
[1]University of Groningen, University Medical Center Groningen, Department of Critical Care, Groningen, Netherlands; [2]Sentron Europe B.V., Leek, Netherlands; [3]Brains On-Line B.V., Groningen, Netherlands; [4]University of Groningen, University Medical Center Groningen, Department of Internal Medicine, Division of Nephrology, Groningen, Netherlands
**Correspondence:** L. Hessels
*Intensive Care Medicine Experimental* 2017, **5(Suppl 2):**0133

**INTRODUCTION.** The stress response induced by critical illness leads to a catabolic state in ICU patients. The first observations that catabolism was accompanied by an increased excretion of intracellular ions, were made as early as the beginning of the 20th century. Recently, it was demonstrated that critically ill patients have a negative potassium balance, presumably resulting from the catabolic process as well, reflecting loss of lean body mass. However, there is not much information about the relation with other ions. Since both potassium and phosphate reside mainly intracellular, we hypothesized that potassium and phosphate excretion are related. We also explored the relationship of potassium and phosphate excretion with sodium and chloride excretion, both ions that reside mainly in the extracellular compartment.

**OBJECTIVE.** To compare potassium, sodium, chloride and phosphate excretions and identify which ions are most strongly related in critically ill patients.

**METHODS.** This study was a retrospective, observational study from September 2015 to April 2016, evaluating all patients with at least one adequate urinary measurement during the first three ICU days in our university teaching hospital. The first ICU admission of the last hospital admission was evaluated. Daily electrolyte excretions were measured in 24-h urine collections and mean electrolyte excretions were calculated for each patient. Patients who had AKI class 3 according to the KDIGO criteria were excluded.

**RESULTS.** A total of 1382 ICU patients were included with 4294 urine collections. Mean ± SD potassium, phosphate, sodium and chloride excretion were 57 ± 31, 18 ± 13, 92 ± 86, 103 ± 87 mmol/day respectively. Potassium excretion was most strongly associated with phosphate excretion ($R = +0.63$, $p < 0.001$). Sodium excretion was most strongly correlated with chloride excretion ($R = +0.95$, $p < 0.001$). Potassium excretion was also associated with sodium and chloride excretion ($R = +0.45$ and $R = +0.54$, $p < 0.001$). The correlation between phosphate and both sodium and chloride excretion was weaker but also significant ($R = +0.29$, $p < 0.001$).

**CONCLUSIONS.** Potassium and phosphate excretion were most closely related in critically ill patients, most likely due to their intracellular nature. Likewise the extracellular ions sodium and chloride were most strongly related. The relation of sodium and chloride with potassium excretion may be explained by the renal exchange of potassium and sodium and the administration of potassium chloride. Additional balance studies combined with a more detailed urine excretion analysis will most likely result in a better understanding of the association of potassium and phosphate excretion with catabolism and of sodium and chloride excretion with infusion-related extracellular volume expansion.

**GRANT ACKNOWLEDGMENT**
Partially funded by the IAG-4 grant from the Provincie Groningen.

### 0134

#### Nasogastric tube displacement and interruption to nutritional support and enteral drug administration

N. Ronan, R. Stoyanova, C. Clapham, I. Lazana
University College London Hospitals NHS Foundation Trust, London, United Kingdom
**Correspondence:** N. Ronan
*Intensive Care Medicine Experimental* 2017, **5(Suppl 2):**0134

**INTRODUCTION.** Appropriate delivery of nutritional support is fundamental to the care of the critically unwell patient. Regular interruptions to enteral feeding and medications due to Nasogastric Tube (NGT) displacement can be a frequent and avoidable occurrence in ICU patients.

**OBJECTIVES.** Evaluate the frequency and duration of enteral feeding interruptions and missed medications due to NGT displacement.

**METHODS.** Data were retrospectively collected from a tertiary ICU over a 3-month period (patients admitted to a single combined ICU/HDU between 1st of January and 1st of March 2016). Data collected included the number of enteral feed interruptions due to NGT displacement, specific reasons for tube dislodgement, duration of missed enteral feeding as well as the type and number of medications missed.

**RESULTS.** There were 519 patients admitted to the study ICU. Of these, 133 patients (26%) had a NGT in place for enteral nutrition. Overall there were 124 displaced NGTs over this time period; an average of 3 displaced tubes per patient. Forty-four patients (33%) had enteral feeding interrupted due to NGT displacement. The maximum number of dislodgements on one patient was 13 leading to a total 154 hours without nutrition which resulted in a deficit of 15,400kcal over a 73 day period (~210kcal/day, >10% estimated energy requirements). NGT pulled out by patient accounted for 49% of these displacements with migrated tubes accounting for 31%. Other reasons for dislodgement were NGTs removed due to blocking (13%), no aspirate (2%), not stated (4%) and 1 NGT was leaking. 64% of NGT displacements occurred in non-sedated patients. Total number of hours of missed enteral feeding was 1315 hours (an average 30 hours per patient). 264 medication doses were missed. Medications missed included beta-blockers, anti-hypertensives and lactulose (in an encephalopathic patient). The average length of stay the in ICU for patients who had at least one NGT displacement was 25 days.

**CONCLUSIONS.** NGT displacement is common amongst ICU patients, particularly those who are not sedated, and a frequent cause of interruption to nutritional support and medication administration in ICU/HDU patients.

### 0135

#### The value of monitoring gastric residual volume in the enterally fed critically ill patient

L. Hinder, E. Potter, K. Symes, J. Zekavica, J.F. Doyle
Royal Surrey County Hospital NHS Foundation Trust, Surrey Intensive Care Nutrition Collaborative (SIN-CO), Guildford, United Kingdom
**Correspondence:** J.F. Doyle
*Intensive Care Medicine Experimental* 2017, **5(Suppl 2):**0135

**INTRODUCTION.** Recent ESCIM[1] guidelines suggest measuring gastric residual volume (GRV) 6 hourly (Grade 2B) upon commencement of enteral nutrition (EN) and delaying this if aspirates are >500ml. ASPEN & SCCM[2,3] practice guidelines conclude that high GRV alone cannot be correlated with aspiration pneumonia, ICU mortality or ICU length of stay and only when considered with other markers of patient illness does the correlation with worsening patient outcome emerge. Despite this there remains some ambiguity with regards to the value of GRV once enteral feed is established with practice differing amongst ICUs.

**OBJECTIVES.** To assess incidence of high GRV amongst critically ill patients and their ICU outcome in a single centre and appraise current practice of response to high GRVs.

**METHODS.** All patients admitted to Royal Surrey County Hospital ICU in 2016 requiring mechanical ventilation for more than 24 hours and receiving EN were identified via IntelliVue Clinical Information Portfolio (ICIP). These patient's electronic records were reviewed for GRVs and if exceeding 500ml their subsequent management reviewed. ICU stay outcome was recorded as improved, worse or deceased.

**RESULTS.** 252 patients were identified during screening. 177 were analysed for rates of enteral nutrition. We identified a 52.5% EN rate via naso/oro-gastric tube. A GRV of greater than 500ml was found in 12% of these patients, increasing to 17.2% if a GRV of 400ml was to be deemed significant. Management of those patients with high GRV consisted of reduction or cessation of EN followed by a slow re-introduction. TPN was required in only one patient following recorded high GRVs. Prescription of single agent prokinetic was variable and at clinician discretion. ICU outcome for those with a GRV >500ml include a mortality rate of 45.4% with all other patients improved at discharge. This compares to a mortality rate of 24.2% for the unit for patients requiring mechanical ventilation for >24 hours.

**CONCLUSIONS.** Monitoring GRV is not a mandatory requirement in many ICUs however we found the practice of doing so directly affects the prescription of subsequent EN. Furthermore the presence of high GRVs may yet be shown to have value in such areas as patient prognosis, documented results such as this may be stimulus for further research.

### 0136

#### Enteral feeding in the critically ill patient on vasopressor support - what is safe? A review of the current literature, and UK Intensive care unit dietetic practice

Simon Andrew Hill, J. Zekavica, J.F. Doyle
Royal Surrey County Hospital NHS Foundation Trust, Surrey Intensive Care Nutrition Collaborative (SIN-CO), Guildford, United Kingdom
**Correspondence:** J. Zekavica
*Intensive Care Medicine Experimental* 2017, **5(Suppl 2):**0136

**INTRODUCTION.** Despite ESPEN[1], ASPEN[2] and recent ESCIM[3] guidelines, there remains some ambiguity as to the safety and tolerance in enteral feeding (EF) critically ill patients who are haemodynamically unstable requiring vasopressor support (VPS).

**OBJECTIVES.** To

(i) critically appraise current evidence on the safety and tolerance of EF in patients on VPS and

(ii) Review opinion of UK ICU dietitians and their clinical practice.

**METHODS.** EMBASE, CINAHL & MEDLINE databases were searched limited to 2007–2017, human, adult, English published studies, which reviewed VPS in critically unwell EF patients. A 9 question online survey of dietitians was conducted to determine opinion and practice in UK ICUs.

**RESULTS.**

(i) Literature search provided a total of 3 cohort studies[4–6], which were suitable for critical appraisal. All were retrospective analysis of American databases. All concluded that provision of EF is safe and tolerated in patients on VPS, although there may be a cut off dose at which EF is withheld or dose reduced.

(ii) Electronic survey results from 34 anonymous respondents found that 53% of respondents do assess VPS in EF critically ill patients. 6% going as far as to specify a VPS dose at which the rate of EF is reduced. 44% of respondents did not use VPS in isolation to advise a reduction in, or cessation of EF, instead respondents cited assessing alternative parameters of feed tolerance. 20% of respondents reduced and 14% withheld EF if patient requirements for VPS were “high” or “increasing”. 53% of practitioners expressed concern with patients on more than one VPS agent.

**CONCLUSIONS.** Although UK dietetic practice is varied, EF hemodynamically unstable patients on VPS is safe and tolerated. Further prospective research is required to result in consistent practice especially with regards to tailoring nutrition with vasopressor support in mind.

**GRANT**

No funding was received

### 0137

#### Is there any prognostic difference between severe and morbid obesity in cardiac surgery patients?

F. Ampatzidou, T. Tsermakian, C. Vardaxi, A. Madesis, T. Karaiskos, G. Drossos
G.Papanikolaou General Hospital, Cardiac Surgery Department, Thessaloniki, Greece
**Correspondence:** F. Ampatzidou
*Intensive Care Medicine Experimental* 2017, **5(Suppl 2):**0137

**INTRODUCTION.** Although many studies support the obesity paradox in cardiac surgery patients, an increased risk for postoperative complications in extremely obese patients has also been reported.

**OBJECTIVES.** To investigate whether staging obesity in categories II-severe and III- morbid (WHO classification) has any value in predicting early postoperative morbidity and mortality after cardiac surgery procedures.

**METHODS.** This study was based on our departments' prospectively collected data. From June 2012 to March 2017 a total of 2084 patients underwent cardiac surgery with the use of cardiopulmonary bypass. We categorized these patients in obesity stages according to WHO classification based on BMI. The following factors were compared between the severely obese and the morbidly obese patients: Sternal wound infections, septicemia, pneumonia, postoperative respiratory complications requiring non invasive ventilation (NIV), re-intubation, acute kidney injury based on RIFLE criteria, multiple organ dysfunction syndrome (MODS), atrial fibrillation, and mortality. We used $x^2$ test for statistical analysis.

**RESULTS.** A total of 704 (33.8%) consisted the obesity category (BMI > 30 kg/m$^2$). We studied 2 groups: 142(47 females) severely obese patients -Group A, mean age 63.5 ± 10.4, prognostic Euroscore II 2 ± 3.2 (BMI 35–39.9 kg/m$^2$) and 40(15 females) morbidly obese patients-Group B, mean age 61,8 ± 11.5, Euroscore II 1.65 ± 1.25 (BMI ≥40 kg/m$^2$)- group B. Results are shown in table 43.

**CONCLUSIONS.** Morbidly obese patients have higher incidence of pneumonia, acute kidney injury and higher mortality compared with severely obese cardiac surgery patients.

**REFERENCE(S)**

Ana P. Johnson et al.Body Mass Index, Outcomes, and Mortality Following Cardiac Surgeryin Ontario, Canada J Am Heart Assoc. 2015;4:e002140

**Table 43 (Abstract 0137)**. Results

| | Group A | Group B | x2, p value |
|---|---|---|---|
| Pneumonia,(n,%) | 3(2.1%) | 3(7.5%) | 4.12, p<0.05 |
| AKI | 15(10.6%) | 30(75%) | 4.42, p<0.05 |
| Deaths | 4(2.8%) | 3(7.5%) | 5.15, p<0.05 |

### Cardiac arrest 1

#### 0138

#### Cardiac arrest in intensive care units. A 8-year single center report

H. David, H. Ly, J.-Y. Bigeon, C. Halchini, P. Burtin, P. Courant
Clinique Millénaire, Montpellier, France
**Correspondence:** H. David

*Intensive Care Medicine Experimental* 2017, **5(Suppl 2):**0138

**INTRODUCTION.** Reports on cardiac arrests occurring in ICUs (ICUCA) are seldom. Current knowledge supports that ICUCA have a better hospital survival rate (HSR) due to continuous monitoring, presence of CA witness, shorter time to CPR and higher CPR skill of ICU staff (1–3).

No study investigates the performance of different ICUs inside the same institution. We conducted a retrospective observational study to assess the incidence and outcome of ICUCA in the 4 ICU and monitored areas of our institution in order to assess the actual survival rate and outcome factors of each unit.

**METHODS.** ICUCA was defined by chest compression and/or defibrillation performed in ICU. All ICUCA were treated by ICU staff and attending physicians. Datas recorded through a dedicated follow-up form were: demographic data, medical history, location, pre-CPR ventilation and vasopressors use, event time sequence, initial cardiac rhythm (VF, VT, PEA, Asystole), first treatment attempt, duration of CPR, number of EES, type of IV treatment, immediate survival rate (ISR); and for CPR-Survivors: SAPS II score, duration of post-arrest ventilation, duration of ICU stay, treatments applied and hospital survival rate (HSR). 4 groups were defined by location: CICU, ICU1 (Cardiac surgery, CS), ICU2 (Abdominal surgery AS) and PACU. Groups were compared using Chi2 and Kruskall-Wallis tests as indicated with $p < 0.05$ considered significant.

**RESULTS.** From 01/01/2009 to 12/31/2016, 226 ICUCA were treated. Overall ISR was 65.9% and HSR was 41.1%. Incidence density (n/1000) by location is described in Table 44. There was no differences between groups for age, gender, time to CPR. Myocardial ischemic disease was more frequent in CICU and Cancer disease was predominant in ICU2 and PACU.

Initial rhythm was predominantly shockable (VT+ VF) in CICU (53.6%) and ICU1 (46.9%) and non-shockable (PEA + Asystole) in ICU2 (53.1%) and PACU (71.1%) ($p < 0.04$). ISR was not different while HSR was significantly different between groups.

ISR and HSR varied according to the intensity of care prior to CA with the lowest survival rate for patients with ventilation and hemodynamic support.

CPR duration for patients who died after CPR was similar in the 4 groups.

**CONCLUSION.** Significant differences in HSR may be present between ICUs in the same institution independantly of patients age, comorbidities, time to CPR and night occurrence. For similar incidence density of ICUCA, AS patients have a worse prognosis than CS patients in our institution. Intensity of care (ventilation and hemodynamic support), and cancer disease portends poor outcome. CPR skills and staff commitment appear homogeneous over the 4 locations. The very low HSR of patients with invasive ventilation and hemodynamic support is an important information regarding post-ICUCA decision-making discussions.

**REFERENCE(S)**

1. Am J Respir Crit Care Med 2010; 182: 501–6
2. Resuscitation 2014: 81: 302–311
3. Resuscitation 2014: 85: 472–479

**Table 44 (Abstract 0138).** Demographic datas

| Groups | CICU n=112 | ICU1 n=64 | ICU2 n=38 | PACU n=12 | p value |
|---|---|---|---|---|---|
| Incidence density (n/1000) | 11.3 | 12.7 | 11.3 | 0.8 | NE |
| Night occurrence n (%) | 47 (42.7) | 21 (33.9) | 18 (47.4) | 4 (36.3) | 0.54 |
| Age mean (SD) | 73.1 (11.7) | 68.1 (12.0) | 71,8 (12.4) | 66.8 (17.2) | 0.07 |
| Male sexe n (%) | 80 (71.4) | 41 (65) | 23 (60.5) | 7 (58.3) | 0.53 |
| Myocardial ischemic disease n (%) | 91 (81.3) | 39 (61.9) | 21 (55.3) | 7 (58.3) | 0.004 |
| Respiratory insufficiency n (%) | 13 (11.8) | 13 (21.7) | 7 (19.9) | 2 (16.7) | 0.38 |
| Renal insufficiency n (%) | 34 (31.5) | 19 (31.7) | 10 (27) | 6 (54) | 0.39 |
| Cancer n (%) | 3 (2.8) | 4 (6.6) | 11 (29.9) | 3 (25) | 0.0001 |
| SAPS2 mean (SD) | 50.9 (8.3) | 56.1 (8.8) | 59.2 (9.8) | 50 (16.6) | 0.0007 |

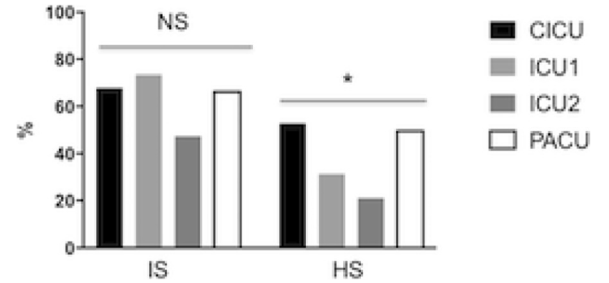

**Fig. 65 (abstract 0138).** See text for description

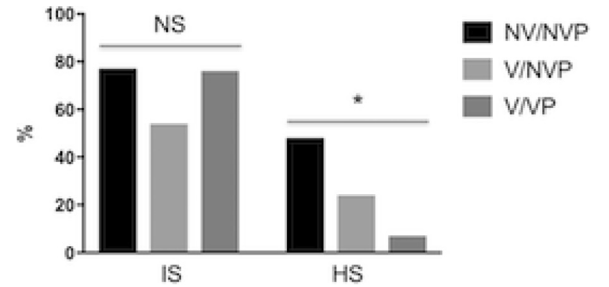

**Fig. 66 (abstract 0138).** See text for description

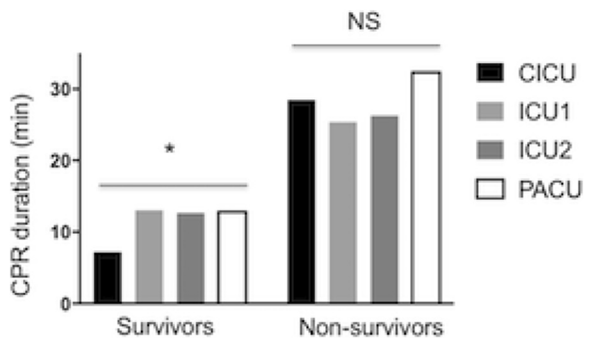

**Fig. 67 (abstract 0138).** See text for description

### 0139

#### Predicting intact survival after prolonged cardiopulmonary resuscitation for refractory out-of-hospital cardiac arrest - who benefit from novel therapies?

K. Maekawa, M. Hayakawa, T. Yoshida, Y. Itagaki, T. Tsuchida, K. Katabami, H. Sageshima, A. Sawamura, S. Gando
Hokkaido University Hospital, Sapporo, Japan
**Correspondence:** K. Maekawa
*Intensive Care Medicine Experimental* 2017, **5(Suppl 2):**0139

**INTRODUCTION.** Patients with out-of-hospital cardiac arrest (OHCA) who cannot achieve early return of spontaneous circulation (ROSC) are neurologically devastated. Alternative novel approaches including extracorporeal CPR can increase intact survival in patients refractory to conventional CPR, but also commit certain patients to "bridge to nowhere". Patients selection is crucial but we don't know who may benefit from novel therapies after prolonged CPR.

**OBJECTIVES.** To create a model for predicting intact survival after refractory OHCA to identify candidates for novel therapies.

**METHODS.** We performed retrospective analysis using data from Japan's nationwide OHCA registry from January 2005 through December 2012, which includes adult OHCA patients with time interval from EMS call to hospital arrival > 30 minutes and without prehospital ROSC. We created the prediction scores of intact survival at 1 month after prolonged CPR, using the β coefficients of prognostic factors from multivariable logistic models. Internal validation was performed using split-sample methods. The derivation cohort comprised of 260539 patients registered in 2005–2010, and the validation cohort comprised 111355 patients registered in 2011–2012.

**RESULTS.** The overall intact survival rate was 0.3% (n = 1162/ 371894). The characteristics of OHCA patients were similar between the derivation and validation cohort. The most accurate model was created based on multivariable logistic regression, including age < 71 yrs, first documented rhythm (VF, PEA, Asystole), delivered countershock > 1 time, witness and no-flow status (unwitnessed, no-flow interval < 2 min or > = 2min), interval from EMS call to EMS arrival < 11 min, with prediction score varying from 0 to 22 points. The C statistic for this model was 0.77 (95%CI, 0.75-0.79) in the derivation cohort and 0.83 (0.81-0.85) in the validation cohort. The calibration plots and goodness-of-fit test revealed good predictive accuracy of this model (p = 0.17). In patients with prediction score > 16 points, the negative predictive value for intact survival was 99.8%.

**CONCLUSIONS.** The new prediction model for refractory OHCA has been derived and validated. This model may help clinicians decide whether to apply novel therapies or terminate resuscitation efforts in OHCA patients refractory to conventional CPR.

**REFERENCE(S)**

Park SB, Yang JH, Park TK, et al. Developing a risk prediction model for survival to discharge in cardiac arrest patients who undergo extracorporeal membrane oxygenation. Int J Cardiol 2014. 177:1031–5.
Debaty G, Babaz V, Durand M, et al. Prognostic factors for extracorporeal cardiopulmonary resuscitation recipients following out-of-hospital refractory cardiac arrest. A systematic review and meta-analysis. Resuscitation. 2017. 112:1–10.

**GRANT ACKNOWLEDGMENT**
None

### 0140

#### Recurrent arrhythmia after resuscitation from ischemic ventricular fibrillation: analysis of the PROCAT registry

H. Bellut[1], L. Guillemet[1], F. Dumas[2], J. Charpentier[1], O. Ben Hadj Salem[1], J.-F. Llitjos[1], M. Paul[1], J.-D. Chiche[1], E. Marijon[3], F. Pène[1], O. Varenne[4], J.-P. Mira[1], A. Cariou[1]
[1]Cochin University Hospital (APHP) and Paris Descartes University, Medical ICU, Paris, France; [2]Cochin University Hospital (APHP) and Paris Descartes University, Emergency Department, Paris, France; [3]Georges Pompidou European Hospital (APHP) and Paris Descartes University, Cardiology, Paris, France; [4]Cochin University Hospital (APHP) and Paris Descartes University, Cardiology, Paris, France
**Correspondence:** H. Bellut
*Intensive Care Medicine Experimental* 2017, **5(Suppl 2):**0140

**INTRODUCTION.** In patients resuscitated from an ischemic ventricular fibrillation or tachycardia (VF/VT), the incidence of recurrent arrhythmia is unclear and the indication of prophylactic anti-arrhythmic (AA) treatments is debated during the post-resuscitation period.

**OBJECTIVES.** To evaluate the incidence of arrhythmia in patients resuscitated from a VF/VT caused by an acute coronary syndrome (ACS), according to the use of AA prophylactic treatments.

**METHODS.** The PROCAT registry captures all data from patients admitted in a tertiary hospital center after a resuscitated cardiac arrest (CA). We selected patients with an initial VF/VT caused by an ACS and who were treated with early percutaneous coronary intervention (PCI). The primary endpoint was the occurrence of arrhythmia between ICU admission and discharge. All arrhythmia resulting in CA recurrence, severe arterial hypotension or acute respiratory failure were classified as major, and all other arrhythmia as minor. We compared prophylactic AA recipients and non-recipients groups using exact Fisher or Kruskal-Wallis test. Multivariate logistic regression identified factors associated with the occurrence of major arrhythmia.

**RESULTS.** Between 01/2007 and 12/2016, 256 consecutive CA patients were included in the analysis. All patients underwent a successful PCI of the infarct-related artery on hospital arrival. A prophylactic AA treatment (amiodarone in 35 pts) was administered in 36 of them (14%). Characteristics and outcome of patients who did or did not receive a prophylactic AA treatment are described in the Table.

There was no significant difference in the incidence of major or minor arrhythmia between the 2 groups. In multivariate analysis, prophylactic AA treatment was not associated with recurrence of major arrhythmia (OR 0.55 (0.12-2.47), p = 0.44). Factors significantly associated with recurrence of severe arrhythmia were public location of CA (OR 0.12 [0.04-0.44]; p = 0.001) and male gender (OR 0.22 [0.08-0.57]; p = 0.02).

**CONCLUSIONS.** Despite early coronary reperfusion, more than 10% of patients experienced a recurrent severe arrhythmia during the post-resuscitation period, even in those treated with prophylactic anti-arrhythmic treatments. This incidence is much higher than what is reported in common ACS and further studies are needed to explore protective strategies.

**Table 45 (Abstract 0140).** Characteristics and outcome of patients

| | AA (n=36) | No AA (n=220) | p-value |
|---|---|---|---|
| Age (yrs), median(Q1-Q3) | 59 (50-66) | 59 (51-70) | 0.51 |
| Male gender, n(%) | 30 (83) | 175 (80) | 0.82 |
| Public location, n(%) | 16 (44) | 108 (49) | 0.72 |
| Bystander-CPR, n(%) | 26 (72) | 154 (72) | 1 |
| CA to ROSC, minutes median(Q1-Q3) | 27 (10-34) | 20 (12-31) | 0.49 |
| Induced hypothermia, n(%) | 32 (89) | 198 (90) | 0.77 |
| Major arrhythmia, n(%) | 4 (11) | 25 (11) | 1 |
| Minor arrhythmia, n(%) | 7 (19) | 43 (20) | 1 |
| In-hospital mortality rate, n(%) | 19 (53) | 95 (43) | 0.37 |

### 0141

#### Lower heart rate is associated with better outcome in out-of-hospital cardiac arrest patients

E. Wilkman, T. Oksanen, M. Skrifvars, V. Pettilä, the FINNRESUSCI Investigators
Helsinki University Central Hospital, ICU, Helsinki, Finland
**Correspondence:** E. Wilkman
*Intensive Care Medicine Experimental* 2017, **5(Suppl 2):**0141

**INTRODUCTION.** Optimal haemodynamic goals during intensive care after out-of-hospital cardiac arrest are not clear. Previous studies have reported associations between lower heart rate and good outcome1,2 during intensive care but possibly influenced by targeted temperature management (TTM), vasoactive medications and a greater degree of organ dysfunction.1

**OBJECTIVES.** To test the associations between heart rate and one-year neurologic outcome using a large prospective database, including patients treated with and without therapeutic hypothermia (TH).

**METHODS.** We analyzed heart rate and outcome data of 504 postresuscitation patients included between 1.3.2010 and 28.2.2011 in the prospective FINNRESUSCI study3. Continuous heart rate data of the first 72 hours in intensive care were converted into 10- or 15-minute medians and time-weighted mean heart rates were calculated. One-year neurologic outcome was dichotomised by the Cerebral Performance Category to good (1–2) or poor (3–5).

**RESULTS.** ICU mortality was 21.2% (107/504), and one-year mortality was 44.0% (222/504). Of 504, 202 (40.1%) had good neurologic outcome at one year. The lowest heart rate (HR) in patients with good and poor neurologic outcome was, 45 (IQR 39–52) beats per minute (bpm) and 50 (IQR 40–63) bpm respectively. The highest heart rate was 115 (IQR 102–131) bpm and 124 (IQR 108–142) bpm, and time-weighted mean heart rate (48 h) 69 (IQR 59–75) bpm and 77 (IQR 66–90) bpm; p < 0.001 for all. In multivariable regression analyses (five separate models) higher time-weighted HR for 0–48 h, higher time-weighted HR for 0–72 h, a higher percentage of heart rate recordings below 60 bpm, 80 bpm, and 100 bpm during the first 48 hours were all independently associated with poor neurological outcome at one year (p < 0.05 for all).

**CONCLUSIONS.** Lower heart rate during first 48 hours in ICU was associated with better one-year neurologic outcome in both TTM and non-TTM patients. Whether heart rate is more a prognostic indicator or a variable, which can be modulated by treatment strategies, needs future prospective trials.

**REFERENCE(S)**

1. Stær-Jensen H, Sunde K, Olasveengen TM, et al. Bradycardia During Therapeutic Hypothermia Is Associated With Good Neurologic Outcome in Comatose Survivors of Out-of-Hospital Cardiac Arrest*. Critical Care Medicine 2014;42(11):2401–8.
2. Thomsen JH, Hassager C, Bro-Jeppesen J, et al. Sinus bradycardia during hypothermia in comatose survivors of out-of-hospital cardiac arrest - a new early marker of favorable outcome? Resuscitation 2015;89:36–42.
3. Vaahersalo J, Hiltunen P, et al. Therapeutic hypothermia after out-of-hospital cardiac arrest in Finnish intensive care units: the FINNRESUSCI study. Intensive Care Med 2013;39(5):826–37.

### 0142

#### Differences in reasons of not initiating out-of-hospital CPR in nursing home patients in Munich - a retrospective analysis

S.J. Schaller[1], M. Fandler[2], J. Sattler[1], G. Schneider[1], K. Singler[2,3], M. Blobner[1]
[1]Klinikum rechts der Isar der TUM, Klinik für Anaesthesiologie, Munich, Germany; [2]Klinikum Nürnberg, Paracelsus Private Medical School, Nuremberg, Germany; [3]Friedrich-Alexander Universität Erlangen-Nürnberg, Institute of Biomedicine of Ageing, Nuremberg, Germany
**Correspondence:** S.J. Schaller
*Intensive Care Medicine Experimental* 2017, **5(Suppl 2):**0142

**INTRODUCTION.** Demographic shift poses challenges not only for in-hospital care, but also to prehospital emergency services. It is important to realize that older adults are very diverse and that baseline comorbidities and functional status are crucial factors for clinical outcomes (1).

**OBJECTIVES.** To investigate the reasons of not initiating and terminating out-of-hospital CPR in nursing homes residents vs. patients outside nursing homes ≥ 65 years old.

**METHODS.** After Ethics Committee approval, a retrospective analysis of the protocols of our prehospital physician staffed emergency service (EMS) location in Munich, Germany of 2014–2016. Statistical calculation was done using Mann–Whitney-U test and chi square test as appropriate using IBM SPSS Statistics 23.0 (SPSS, Chicago, IL, USA).

**RESULTS.** 8882 cases were assessed; after exclusion false alarms (n = 423), cases without documented age (n = 89) and cases with age < 65 (n = 4204), 4166 were analysed. Patients baseline characteristics were significantly different between patients in a nursing home vs. patients outside (table 46).

There were 162 circulatory arrests where CPR was not initiated without a difference between the groups (table 47). However, there was a significant difference in the reasons not initiating CPR: While non-nursing home CPRs were not initiated in 81% because the delay was too long, this only occurred in 33% of nursing home residents (RR 0.1 [0.0-0.3], p < 0.001). In nursing home residents, however, a patient´s provision or order resulted in omission of CPR attempts in 52% of cases vs. 12% of cases outside nursing homes (RR 8.0 [3.0-21.7], p < 0.001).

CPR was initiated in 208 cases, without differences between the groups. In 44% of cases outside nursing homes and 72% at nursing homes, resuscitation attempts were stopped and patient declared dead, a significant difference between groups (RR 1.0 [0.7-1.5], p = 0.004). Reasons for terminating CPR attempts were documented rarely; most likely stabilization of patients just could not be achieved.

**CONCLUSIONS.** Delay of CPR is still a major problem. Even if delays are less common in nursing homes, where supervision is present, the rate of 33% is still high. New technology (monitoring systems) might be able to reduce this factor in the future. Second, people living in nursing homes, are more likely to have a patient´s provision (or at least the provision is disclosed to the EMS personnel via the nursing home personnel more often). However, in the cases where CPR was initiated, the outcome of nursing home patients was worse. An advanced planning of strategy might be necessary to determine treatment strategies (e.g. comfort care) of nursing home patients to avoid futile care.

**REFERENCE(S)**

(1) Andrew, E., Z. Nehme, S. Bernard and K. Smith (2017). "The influence of comorbidity on survival and long-term outcomes after out-of-hospital cardiac arrest." Resuscitation 110: 42–47.

**Table 46 (Abstract 0142).** Baseline characteristics

| Table 1. Patient Characteristics | not nursing home n = 3586 | nursing home n = 580 | p-value | Relative Risk |
|---|---|---|---|---|
| Female, n (%) | 1881 (52%) | 386 (67%) | <0.001 | |
| Age, median (IQR) | 78 (72-84) | 85 (78-90) | <0.001 | |
| GCS initial, median (IQR) | 15 (14-15) | 14 (10.5-15) | <0.001 | |
| GCS end, median (IQR) | 15 (14-15) | 14 (11-15) | <0.001 | |
| Heart rate initial, median (IQR) | 85 (70-100) | 84 (71-100) | n.s. | |
| Blood pressure initial, median (IQR) | 147 (120-170) | 130 (110-160) | <0.001 | |
| NACA, median (IQR) | 4 (3-5) | 4 (3-5) | n.s. | |
| Disease Categories, n (%) | | | <0.001 | |
| Cardiovascular System | 1592 (44%) | 201 (35%) | <0.001 | 0.7 (0.6-0.8) |
| ... subgroup ACS cases | 503 (14%) | 34 (6%) | <0.001 | 0.4 (0.3-0.5) |
| Other | 541 (15%) | 98 (17%) | n.s. | 1.1 (0.9-1.4) |
| Traumatic | 453 (13%) | 59 (10%) | n.s. | 0.8 (0.6-1.0) |
| Central Nervous System | 359 (10%) | 86 (15%) | <0.001 | 1.6 (1.2-2.0) |
| Respiratory System | 321 (9%) | 78 (13%) | 0.001 | 1.6 (1.2-2.1) |
| Visceral System | 165 (5%) | 29 (5%) | n.s. | 1.1 (0.7-1.6) |
| Endocrinological System | 95 (3%) | 23 (4%) | n.s. | 1.5 (1.0-2.4) |
| Psychiatric | 59 (2%) | 5 (1%) | n.s. | 0.5 (0.2-1.3) |
| Paediatrical | (0%) | (0%) | n/a | n/a |
| Gynaecological/Obstetrical | (0%) | (0%) | n/a | n/a |

**Table 47 (Abstract 0142).** Reasons to withhold or stop CPR attempts

| Table 2. Reasons not initiating CPR and outcomes. | not nursing home n = 3586 | nursing home n = 580 | p-value | Relative Risk |
|---|---|---|---|---|
| No CRP attempts, n (%) | 141 (4%) | 21 (4%) | n.s. | 0.9 (0.6-1.4) |
| Too much delay | 114 (81%) | 7 (33%) | <0.001 | 0.1 (0.0-0.3) |
| Patients Provsion | 17 (12%) | 11 (52%) | <0.001 | 8.0 (3.0-21.7) |
| Palliative Care | 6 (4%) | 2 (10%) | n.s. | 2.4 (0.4-12.6) |
| Other | 4 (4%) | 1 (5%) | n.s. | 1.7 (0.2-16.1) |
| CPR started, n (%) | 179 (5%) | 29 (5%) | n.s. | 1.0 (0.7-1.5) |
| ROSC on scene, n (%) | 89 (50%) | 10 (34%) | n.s. | 0.5 (0.2-1.2) |
| CPR attempts terminated (not transported), n (%) | 78 (44%) | 21 (72%) | 0.004 | 3.4 (1.4-8.1) |
| No reason documented | 70 (90%) | 20 (95%) | n.s. | |
| Patients Provsion | 5 (6%) | 1 (5%) | n.s. | |
| Too much delay | 2 (3%) | 0 (0%) | n.s. | |
| Palliative Care | 1 (1%) | 0 (0%) | n.s. | |
| Hospital admission (after CPR), n (%) | 101 (56%) | 8 (38%) | 0.004 | 0.3 (0.1-0.7) |
| with ROSC | 70 (69%) | 6 (75%) | n.s. | |
| ongoing CPR | 31 (31%) | 2 (25%) | n.s. | |

### 0143

#### Early predictors of cardiac arrest in patients managed for convulsive status epilepticus

S. Legriel[1,2], E. Bresson[1], N. Deye[3], D. Grimaldi[4], B. Sauneuf[5], O. Lesieur[6], J.-B. Lascarrou[7], L. Argaud[8], J. Chelly[9], P. Beuret[10], D. Schnell[11], A.-L. Chateauneuf[1], M. Holleville[1], F. Perrier[1], V. Lemiale[12], C. Bruel[13], P. Cronier[14], N. Mongardon[15], N. De Prost[16], N. Pichon[17], F. Dumas[2,18], A. Cariou[2,19], CACTUS Study Group
[1]Versailles Hospital, Intensive Care Unit, Le Chesnay, France; [2]Paris Cardiovascular Research Center-INSERM, U970 (PARCC), Paris, France; [3]Centre Hospitalier Universitaire Lariboisière, Reanimation Medicale, Paris, France; [4]Université Libre de Bruxelles (ULB), Hopital Erasme, Department of Intensive Care, Brussels, Belgium; [5]Centre Hospitalier Public du Cotentin, Service de Réanimation Médicale Polyvalente, Cherbourg-en-Cotentin, France; [6]Centre Hospitalier Saint-Louis, Réanimation, La Rochelle, France; [7]District Hospital Center La Roche-sur-Yon, Medical-Surgical Intensive Care Unit, La Roche-sur-Yon, France; [8]Hospices Civils de Lyon, Edouard Herriot Academic Hospital, Medical Intensive Care Unit, Lyon, France; [9]Service de Médecine Intensive, Centre Hospitalier Marc Jacquet, Melun, France; [10]Centre Hospitalier de Roanne, Service de Réanimation et Soins Continus, Roanne, France; [11]Hôpital d'Angoulême, Intensive Care Unit, Angoulême, France; [12]Centre Hospitalier Universitaire Saint Louis, Medical Intensive Care Unit, Paris, France; [13]Groupe Hospitalier Saint Joseph, Medical-Surgical Intensive Care Unit, Paris, France; [14]Centre Hospitalier Sud-Francilien, Intensive Care Unit, Corbeil-Essonnes, France; [15]Centre Hospitalier Universitaire Henri Mondor, Department of Anesthesiology and Surgical Intensive Care Units, Creteil, France; [16]Centre Hospitalier Universitaire Henri Mondor, Medical Intensive Care Unit, Creteil, France; [17]Centre Hospitalier Universitaire de Limoges, Medical-Surgical Intensive Care Unit, Limoges, France; [18]Centre Hospitalier Universitaire Cochin Assistance Publique Hôpitaux de Paris, Emergency Departement, Paris, France; [19]Centre Hospitalier Universitaire Cochin Assistance Publique Hôpitaux de Paris, Medical Intensive Care Unit, Paris, France
**Correspondence:** S. Legriel
*Intensive Care Medicine Experimental* 2017, **5(Suppl 2):**0143

**INTRODUCTION.** Cardiac arrest (CA) is a dreadful event that may complicate the management of patients suffering from convulsive status epilepticus (CSE). Prevention of this complication would require an adequate identification of related risk factors.

**OBJECTIVES.** To describe clinical characteristics and outcome and to identify early predictors for CA during CSE management.

**METHODS.** We studied all consecutive patients admitted between 2000 and 2015 in17 French ICUs after successful resuscitation of a CA that occurred during the initial management of CSE. These cases (CSE with CA) were compared with controls coming from a single center registry of CSE patients with no cardiac arrest (CSE w/o CA). We collected baseline characteristics regarding medical history, management and etiology of CSE. We compared characteristics of these 2 groups according to occurrence of CA using univariate and multivariate logistic regression to identify potential risk factors for CA during CSE management.

**RESULTS.** Overall 284 CSE patients were studied: 49 CSE with CA and 235 CSE with CA. In overall population, patients were mainly male gender 154/284 (59%) with median age of 59 (IQR, 46–70) y.o. The median time from seizure to medical team arrival was 30 (IQR, 0–73) minutes with a total seizure duration median of 85 (IQR, 42–184) minutes. Benzodiazepines were given as first line therapy in 257/284 (93%) and 161/284 (57%) received another "classic" anticonvulsant as first or second line therapy.

Among the 49 CSE patients with CA, the arrest occurred in a median time of 25 (IQR, 5–85) minsafter medical team arrival. The first recorded rhythm was asystole in 25 (51%) patients, pulseless electrical activity in 13 (27%), ventricular fibrillation/tachycardia in 4 (8%), and other rhythm in 7 (14%). Survival at hospital discharge was observed in 216/235 (92%) in CSE w/o CA compared with19/49 (39%) in CSE with CA. One-year favorable outcome (CPC score 1 or 2) was observed in 13/49 (28%) CSE with CA.

Using multivariate analysis, independent positive predictors of CA occurrence were pulse oximetry < 97% at scene (OR, 2.66; 95%CI, 1.03-7.26, $p$ = 0.04), drug poisoning as the cause of CSE (OR, 4.13; 95%CI, 1.27-13.53, $p$ = 0.02), and complications occurring during early management (OR, 11.98; 95%CI, 4.67-34.69, $p$ < *0.0001*), whereas having at least one comorbidity among cardiac, respiratory and neurological (other than epilepsy) was a negative predictor of CA (OR, 0.28; 95%CI, 0.10-0.80, $p$ = 0.02).

**CONCLUSIONS.** In critically ill patients managed for convulsive status epilepticus, relative hypoxemia at scene, early management complications at scene and drug poisoning as the cause of CSE were strong early predictors of cardiac arrest occurrence, suggesting areas for improvement.

### 0144

#### Neurophysiology to aid prognostication following cardiac arrest in a UK tertiary referral centre, 2012–216

A. Skorko, M. Pachucki, N. Kane, T. Gould, M. Thomas
Bristol Royal Infirmary, Bristol, United Kingdom
**Correspondence:** A. Skorko
*Intensive Care Medicine Experimental* 2017, **5(Suppl 2):**0144

**INTRODUCTION.** Prognostication following cardiac arrest is a challenging and emotive topic. Clinicians must balance the risk of withdrawing care when there is a chance of recovery with that of treatment futility when there is none. Our neurophysiology department has provided neuroprognostication (NP) support with electroencephalogram (EEG) and Somatosensory Evoked Potentials (SSEPs) since 2012.

We present a retrospective analysis of 95 cases managed with the assistance of neurophysiology following cardiac arrest in a tertiary centre in the South West of the UK between 2012 and 2016.

**METHODS.** Every EEG and/or SSEP investigations carried out on ICU following cardiac arrest (in or out of hospital) was extracted and analysed. The system proposed by Synek to stratify EEG severity was used (Guerit et al.).

**RESULTS.** During the 4 year period approximately 630 patients were admitted following cardiac arrest and 95 had neurophysiology testing, of whom 91 underwent both EEG and SSEP.

The cardiac arrest population admitted to our unit have an ICU and hospital mortality of 49% and 55% respectively, an average age of 63 years, 74% are male. Those who underwent NP had an average age of 60 years, 80% were male. 92% were admitted post out of hospital arrest. All patients received therapeutic hypothermia. NP was undertaken following rewarming. Overall mortality in the NP group was 93%. Hospital mortality by NP finding is shown in Fig. 68.

Of the EEGs performed none were Synek grade 1 or 2, 13% were grade 3, 74% grade 4, and 13% grade 5. All patients with a poor EEG grade (4 or 5) and absent SSEPs died (Fig. 68).

Grade 4 or 5 EEG had a sensitivity of 89% at predicting mortality, a specificity of 50% and a false negative rate (FNR) of 0.1. The odds ratio (OR) of death with an EEG grade 4/5, adjusted for SSEPs, was 7.75 (CI 1.1-52, p = 0.21). AUROC for grade 4/5 EEG predicting death was 0.70.

49% of SSEPs demonstrated bilaterally present signal. 10% were non-diagnostic. Absent SSEPs had a sensitivity of 49% and specificity of 100% for predicting mortality. AUROC was 0.74 for absent SSEP predicting death. No patient with absent SSEPs survived. Hospital mortality despite present SSEPs was 85%.

**CONCLUSION.** In our case series, 12% of cardiac arrests underwent neuroprognostication. All survivors had bilaterally present SSEPs but 85% of patients with present SSEPs did not survive. Combing EEG and SSEPs findings identified those with the best survival.

The use of retrospective data, particularly the absence of SSEPs is problematic given the self-fulfilling prophecy of neuroprognostication. We propose to examine the notes of those with present SSEPs who died in order to understand which other factors impact outcome.

| | SSEPs present bilaterally | SSEPs absent bilaterally |
|---|---|---|
| Uncertain EEG (Synek grade 3) | 57.1% | 100% |
| Poor EEG (Synek grade 4 or 5) | 91.2% | 100% |

**Fig. 68 (abstract 0144).** Hospital mortality by NP finding

### 0145

#### Impact of intra-aortic balloon pump on extracorporeal cardiopulmonary resuscitation: a retrospective cohort study

W. Iwanaga[1], T. Kawashima[2], R. Sato[1], M. Nasu[1], T. Goto[1], T. Hozumi[1], K. Takahashi[1], Y. Kitahara[1], H. Fukui[1], T. Yonemori[1], M. Yagi[1]

[1]Urasoe General Hospital, Department of Emergency and Critical Care Medicine, Okinawa Urasoe, Japan; [2]Urasoe General Hospital, Department of Cardiology, Okinawa Urasoe, Japan

**Correspondence:** W. Iwanaga

*Intensive Care Medicine Experimental* 2017, **5(Suppl 2):**0145

**INTRODUCTION.** Although extracorporeal cardiopulmonary resuscitation (ECPR) for cardiac arrest has been reported to be more effective than conventional cardiopulmonary resuscitation recently, patients underwent ECPR are at high risk of refractory circulatory shock during the weaning of veno-arterial extracorporeal membrane oxygenation (ECMO) support.

**OBJECTIVES.** This study aimed to examine the impact of the intra-aortic balloon pump (IABP) on the survival rate of patients who underwent ECPR.

**METHODS.** In this retrospective cohort study, conducted in a Japanese tertiary care center, patients underwent ECPR between 2008 and 2016 were included. All ECMO cannulations were performed via femoral arteries and veins using an angiography. Patients were either performed ECPR only or ECPR plus IABP support. The primary outcome was the 30-day survival and the secondary outcome was the successful removal of ECMO. Log rank test and Fisher exact test were performed to compare these outcomes between two groups.

**RESULTS.** A total 116 consecutive adult patients were included in this study. The majority of study patients received bystander CPR (69% vs. 76%, n.s). There was no significant difference between the two groups in the median of circulatory collapse-to-ECMO time (50min [42–72] vs. 50min [39–69], *P* = 0.41). The 30-day survival rate was significantly higher in ECPR plus IABP support group than ECPR only group (37% vs. 10%; *P* = 0.0052). Successful rate of weaning from ECMO was also higher in ECPR plus IABP support group (50% vs. 17%, *P* = 0.0013).

**CONCLUSIONS.** In the present study, the use of IABP was associated with better survival and successful weaning from ECMO. Combination of ECPR and IABP support may lead to better outcome in patients with cardiac arrest.

**REFERENCE(S)**

Resuscitation. 2014 Jun;85(6):762–8.

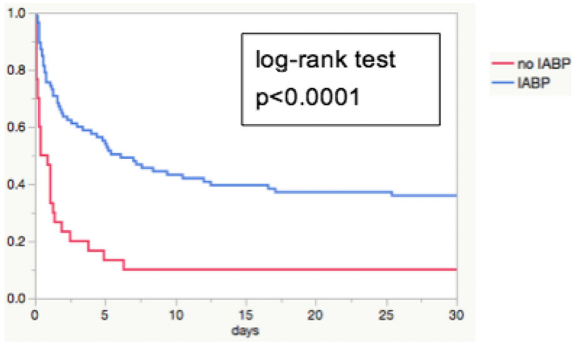

**Fig. 69 (abstract 0145).** Kaplan-Meier curves

### 0146

#### Severe metabolic acidosis after out-of-hospital cardiac arrest

M. Jamme[1], O. Ben Hadj Salem[1], L. Guillemet[1], P. Dupland[1], W. Bougouin[1,2], J. Charpentier[1], J.P. Mira[1], F. Pène[1], F. Dumas[2,3], A. Cariou[1,2], G. Geri[1,2]

[1]Cochin University Hospital, Réanimation Médicale, Paris, France; [2]INSERM U 970, Sudden Death Expertise Centre, Paris Cardiovascular Research Centre, Paris, France; [3]Cochin University Hospital, Services des Urgences, Paris, France

**Correspondence:** M. Jamme

*Intensive Care Medicine Experimental* 2017, **5(Suppl 2):**0146

**INTRODUCTION.** Metabolic acidosis is frequently observed as a consequence of global ischemia-reperfusion after out-of-hospital cardiac arrest (OHCA). However, the relationship between the level of this metabolic disturbance at hospital admission and the outcome of OHCA patients is still misunderstood and incompletely explored.

**OBJECTIVES.** We aimed to identify risk factors and assessing the impact of metabolic acidosis on outcome after OHCA.

**METHODS.** We included all consecutive OHCA patients admitted in our cardiac arrest center between 2007 and 2012. Metabolic acidosis was defined by a positive base deficit and was categorized by quartiles. Main outcome was survival at ICU discharge. Neurological recovery was assessed using the Cerebral Performance Categories (CPC) scale. Factors associated with acidosis severity were evaluated by linear regression. Factors associated with main outcome were assessed by logistic regression.

**RESULTS.** 826 patients (68.3% male, median age 61 years) were included in the analysis. Median base deficit was 8.8 [5.3,13.2] mEq/l. Male gender ($p = 0.002$), resuscitation duration ($p < 0.001$), initial shockable rhythm ($p < 0.001$) and post-resuscitation shock ($p < 0.001$) were associated with a deeper acidosis. Brain damage was the leading cause of death (n = 367, 67.6%) while multiple organ failure resulting from refractory shock was responsible for 173 (31.9%) deaths. ICU mortality rate increased across base deficit quartiles (39.1, 59.2, 76.3 and 88.3%, p for trend < 0.001) and base deficit was independently associated with ICU survival ($p < 0.001$). The proportion of CPC 1 patients among those discharged alive from ICU was similar across base deficit quartiles (72.8, 67.1, 70.5 and 62.5%, $p = 0.21$).

**CONCLUSIONS.** Severe metabolic acidosis is frequent in OHCA patients and is associated with poorer outcome, in particular due to refractory shock. However, we observed that about 10% of patients with a very severe metabolic acidosis survived to ICU discharge with a good neurological recovery.

**GRANT ACKNOWLEDGMENT**

GG was granted by the French Society of Intensive Care Medicine, the Assistance Publique Hôpitaux de Paris and by the Schueller-Bettencourt Foundation.

### 0147

#### Multimodal paradigm of short- and long-term prognostication in cardiac arrest survivors: results from a prospective registry

D. Vondrakova[1], P. Ostadal[2], A. Kruger[2], M. Janotka[2], P. Neuzil[2]

[1]Na Homolce Hospital, Cardiology, Prague, Czech Republic; [2]Na Homolce Hospital, Prague, Czech Republic

**Correspondence:** D. Vondrakova

*Intensive Care Medicine Experimental* 2017, **5(Suppl 2):**0147

**INTRODUCTION.** Accurate prediction of clinical outcomes is extremely important in cardiac arrest survivors and multimodal approach is recommeded for neuroprognostication by current international guidelines.

**OBJECTIVES.** The aim of our study was to evaluate prognostic importance of combining clinical and laboratory methods for short- and long-term outcomes using data from a prospective registry of out-of-hospital cardiac arrest survivors

**METHODS.** We analysed data from a single-center prospective registry of out-of-hospital cardiac arrest survivors admitted between 2010 and 2014 to the tertiary cardiovascular center. Study population was comprised from cardiac arrest survivors alive at least 24 hours after collapse. All subjects were treated with targeted temperature management using endovascular device (33°C for 24 hours). We evaluated the prognostic significance of age, gender, initial rhythm, time to return of spontaneous circulation (ROSC), initial levels of serum lactate, potassium, D-dimer and pH, serial measurements of neuron-specific enolase (NSE), C-reactive protein (CRP), procalcitonin, alanine aminotransferase (ALT), serum creatinine, somatosensory evoked potentials and electroencephalography (EEG) in this group. We investigated the association of these variables with neurological outcomes at 30 days and with one-year mortality. Good neurological outcome was defined according to the Cerebral Performance Category (CPC) as CPC 1–2.

**RESULTS.** One-hundred-and-fifty-three cardiac arrest survivors composed the study group (mean age 64.1 years; male 74.7%). Univariate analyses revealed that the initial rhythm, time to ROSC, the levels of NSE measured during the first four days after cardiac arrest, levels of ALT and serum creatinine 24 hours after arrest and the initial levels of serum lactate and pH are significantly associated with 30-days outcomes. Similar analyses showed that the same factors together with age were significantly associated also with-one year mortality. Multivariate analysis indicated that only the levels of NSE measured at day 3 or 4 and "burst-suppression" or "status epilepticus" pattern on EEG were independent predictors of 30-days outcomes; the same EEG patterns and age independently predicted one-year-mortality.

**CONCLUSIONS.** Our results indicate that among numerous available prognostic factors only NSE levels, EEG patterns and age were independently associated with short- and long-term outcomes in cardiac arrest survivors.

**GRANT ACKNOWLEDGMENT**

The study was supported by an Institutional grant MH CZ - DRO (Nemocnice Na Homolce - NNH, 00023884), IG150501

### 0148

#### The determination of neuron-specific enolase on day 3 and day 4 after cardiac arrest have the best predictive value. Results from a prospective study

D. Vondrakova, A. Kruger, M. Janotka, F. Malek, P. Neužil, V. Dudkova, P. Ostadal

Na Homolce Hospital, Prague, Czech Republic

**Correspondence:** D. Vondrakova

*Intensive Care Medicine Experimental* 2017, **5(Suppl 2):**0148

**INTRODUCTION.** The management of cardiac arrest survivors has improved during the past decades. The prognostication has become an integral part of post-resuscitation care and it is based on multimodal paradigm combining clinical examination and additional tools.

**OBJECTIVES.** The aim of our study was to compare the day-specific predictive values of neuron-specific enolase (NSE) collected during the first four days after cardiac arrest and to determine an optimal algorithm for neuroprognostication based on NSE

**METHODS.** Eligible patients were out-of-hospital cardiac arrest survivors treated with endovascular hypothermia (33°C for 24 h). Blood samples for NSE levels measurement were drawn on days 1, 2, 3 and 4 after hospital admission. Thirty-day neurological outcomes according to the Cerebral Performance Category (CPC) scale and 12-month mortality were evaluated as clinical end points.

**RESULTS.** A total of 153 cardiac arrest survivors (mean age 64.2 years) were enrolled in the present study. The NSE levels were significantly lower in the CPC 1–2 group in comparison with the CPC 3–5 group at each time point ($P < 0.05$). Using ROC analysis, optimal cut-off values of NSE for prediction of CPC 3–5 score on specific days were determined as: day 1 ≥ 20.4 mcg/L (sensitivity 63.3%; specificity 82.1%; $P = 0.002$); day 2 ≥ 29.0 mcg/L (72.5%; 94.4%; $P < 0.001$); and day 3 ≥ 20.7 mcg/L (94.4%; 86.7%; $P < 0.001$). The highest predictive value, however, was observed on day 4 ≥ 19.4 mcg/L (93.50%; 91%; $P < 0.001$); NSE value >50.2 mcg/L at day 4 was associated with poor outcome with 100% specificity and 42% sensitivity. Moreover, NSE levels measured on all individual days also predicted 12-month mortality ($P < 0.001$); the highest predictive value for death was observed on day 3 > 18.1 mcg/L (85.3%; 72.0%; $P < 0.001$). Significant association with prognosis was found also for changes in NSE at different time points. An NSE level on day 4 > 20.0 mcg/L, together with a change >0.0 mcg/L from day 3 to day 4, predicted poor outcome (CPC 3–5) with 100% specificity and 73% sensitivity.

**CONCLUSIONS.** Results of the present study suggest that NSE estimation is a useful additional tool for prediction of neurological outcome(s) and long-term mortality in out-of-hospital cardiac arrest survivors treated with targeted temperature management using endovascular device. The highest predictive values for NSE were observed on day 4 and day 3 after cardiac arrest. Using the NSE values from these days, poor prognosis can be predicted with 100% specificity and reasonable sensitivity.

**GRANT ACKNOWLEDGMENT**

The study was supported by an Institutional grant MH CZ - DRO (Nemocnice Na Homolce - NNH, 00023884), IG150501

### 0149

#### In-Hospital cardiac arrests after a new cardiac arrest code

M. López Carranza, A. Campanario García, F. Lucena Calderón, E. Palleja Gutiérrez, A. Loza Vázquez, J.A. Sánchez Román, A. Lesmes Serrano, RCP Andalucía Registry Investigators

Hospital Universitario Virgen de Valme, Sevilla, Spain

**Correspondence:** M. López Carranza

*Intensive Care Medicine Experimental* 2017, **5(Suppl 2):**0149

**OBJECTIVES.** To analyze the effect of a new Cardiac Arrest Code (CAC) for in-hospital Cardiac Arrest (CA): before and after overall mortality and neurological outcome, and describe the CA characteristics.

**METHODS.** Retrospective cohort study comparing mortality and neurological outcome for in-hospital CA before and after a new CAC (started on May 22, 2012).

Based on a national registry, we selected a cohort denominated "PRE" that included the CA's between January 1, 2009 and May 22, 2012 and another cohort called "POST" that included the CA's between May 23, 2012 and March 8, 2017.

Variables analyzed included: gender, age, characteristics of the CA, time of response, treatment, success resuscitation, admission to the ICU, neurological outcome and mortality.

A descriptive analysis was performed afterwards, expressing the qualitative variables in percentages and the quantitative variables in means ± standard deviation. A subsequent comparative study was made between the groups with T-student and Chi-squared tests.

**RESULTS.** A total of 267 patients were included, 52 in the period from January 1, 2009 to May 22, 2012, PRE implantation of the new CAC, and 215 patients in the POST CAC period, from May 23, 2012 to March 8, 2017.

Both groups were homogeneous, with no statistically significant differences in the variables of age, gender, site of CA, etiology and initial heart rhythm.

The time elapsed from the CPR to the warning to the Advence Life Support (ALS) team is 3.71 ± 4.09 min in the PRE period and 3.54 ± 4.64 min in the POST group (p = ns). A reduction in the response time of the ALS team of 4.96 ± 4.77 min in the PRE period to 3.41 ± 5.38 min was observed, although these differences did not reach statistical significance (p = 0.06). The duration of CPR in the PRE and POST groups was similar, being 20.74 ± 21.13 min vs 18.53 ± 15.03 min (p = ns) respectively.

Among patients with recovered CA, a statistically significant reduction in the use of therapeutic hypothermia from 26.1% in the PRE group to 9.5% in the POST group was observed (p = 0.030). The percentage of patients undergoing PCI increased from 26.1 in the PRE group to 30.5% in the POST group, although this difference is not statistically significant.

Neurologycal outcome was similar in both groups, but overall in-hospital mortality was significantly lower in the POST group, 78.8% compared to 63.3% with p = 0.045.

**CONCLUSIONS.** After the implementation of the CAC, there has been an improvement in the response time of the ALS team, and above all, a reduction in in-hospital mortality. Such reduction in mortality could be due to the improvement in post-resuscitation care with an increase in post-PCI.

We believe that periodically developing and reviewing a CAC is imperative to know what happens with the in-hospital CA and improve its outcome.

**GRANT ACKNOWLEDGMENT**

RCP - Andalucia Registry Investigators

**Table 48 (Abstract 0149).** Results

| | PRE (52) | POST (215) | p |
|---|---|---|---|
| Male/Female (n/%) | 41(78.8%)/ 11(21.2%) | 158(73.5%)/ 57(26.5%) | 0.0426 |
| Age(mean ± standard desviation) | 66.58 ± 14.77 | 69.77 ± 12.54 | 0.154 |
| Shockable rhythms/Non-Shockable rhythms (n/%) | 6(11.5%)/ 44(84.6%) | 40(18.6%)/ 167(77.7%) | 0.479 |
| Cardiac etiology/non cardiac etiology (n/%) | 25(48.1%)/ 27(51.9%) | 112(52.1)/ 103(48%) | 0.065 |
| Response time of ALS (mean ± standard desviation) | 4.96 ± 4.77 | 3.41 ± 5.38 | 0.062 |
| Initial outcome(Dead/Intensive care unit)(n/%) | 21(41.2%)/ 28(55%) | 78(40.2%)/ 112(57.7%) | 0.276 |
| In- hospital mortality (n/%) | 41(78.8%) | 136(63.3%) | 0.045 |

**Table 49 (Abstract 0149).** Treatment post PCR

| Treatment post PCR (n/%) | PRE (23) | POST (105) | p |
|---|---|---|---|
| Hypothermia | 6(26.1%) | 10(9.5%) | 0.03 |
| PCI | 6(26.1%) | 32(30.5%) | 0.679 |
| Fibrinolysis | 1(4.3%) | 6(5%) | 0.794 |

**Table 50 (Abstract 0149).** Neurologic outcome

| CPC Scale (n/%) (p= 0.497) | PRE | POST |
|---|---|---|
| 1. Good cerebral performance | 9(30%) | 49(42.2%) |
| 2. Moderate cerebral performance | 0(0%) | 2(1.7%) |
| 3. Severe cerebral performance | 0(0%) | 1(0.9%) |
| 4. Coma or vegetative state | 1(3.3%) | 1(0.9%) |
| 5. Brain death | 20(66.7%) | 63(54.3%) |

### 0150

#### Lower heart rate is associated with good neurologic outcome in postcardiac arrest patients regardless of therapeutic hypothermia

S.M. Shin, G.J. Suh, K.S. Kim, J.Y. Sun, J.-I. Ko, T. Kim

Seoul National University Hospital, Seoul, Korea, Republic of

**Correspondence:** S.M. Shin

*Intensive Care Medicine Experimental* 2017, **5(Suppl 2):**0150

**INTRODUCTION.** It has been known that bradycardia during targeted temperature management in out of hospital cardiac arrest patient have related to good neurological outcome in several studies recently(1,2).

**OBJECTIVES.** Purpose of this study is to evaluate the relationship between heart rate at 24-hour post-resuscitation and neurological outcome in cardiac arrest patients.

**METHODS.** Out of hospital cardiac arrest patients who visited 3 tertiary urban hospitals from March 2013 to April 2016 were enrolled. Patients were categorized therapeutic hypothermia (TH) group and non-HT group. Heart rate, body temperature at 0, 8, 24, and 72 hours were abstracted. Heart rate at 8-hour was divided into 4 quartiles and 28-days good cerebral performance criteria (CPC) rate was compared among those 4 quartiles.

**RESULTS.** Total 163 patients were enrolled and TH group was 90 (55.2%). Baseline characteristics were not different between two groups. 8-hr and 24-hr body temperature was significantly low in TH group (Fig. 70). 8-hr and 24-hr heart rate were significantly low in good CPC (Fig. 71). The good CPC ratio for the 8 hour HR quartile is higher at lower heart rates (Fig. 72).

**CONCLUSIONS.** Lower heart rate was associated with good neurological outcome in out of cardiac arrest patient during post cardiac arrest care. This result was found in both TH group and non-TH group.

**REFERENCE(S)**

(1) Thomsen JH, Hassager C, Bro-Jeppesen J, et al.: Sinus bradycardia during hypothermia in comatose survivors of out-of- hospital cardiac arrest—A new early marker of favorable outcome? Resuscitation; 89:36–42

(2) Stær-Jensen H, Sunde K, Olasveengen TM, et al.: Bradycardia during therapeutic hypothermia is associated with good neurologic outcome in comatose survivors of out-of- hospital cardiac arrest. Crit Care Med 2014; 42:2401–2408

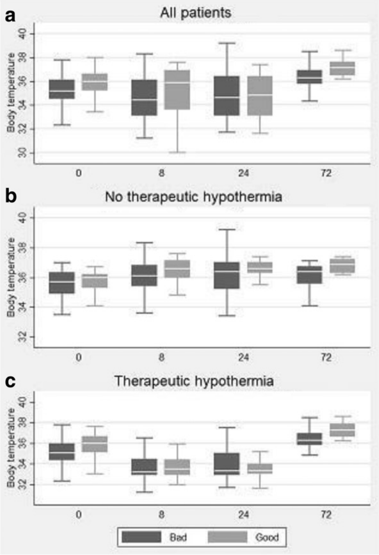

**Fig. 70 (abstract 0150).** See text for description

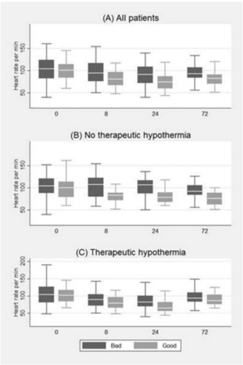

**Fig. 71 (abstract 0150).** See text for description

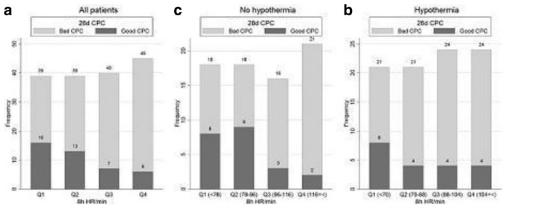

**Fig. 72 (abstract 0150).** See text for description

### 0151

#### Factors associated with survival of in-hopital recovered cardiac arrest. Results of the national registry in Spain

R. Algarte[1], B. Sánchez[1], L. Socias[2], L. Herrera[3], B. Vidal[4], J. Sánchez Ballesteros[5], D. Macias Guerrero[6], F. del Nogal Sáez[7], A. Loza[6], Grupo PCRR-HT of GTCIC-PNRCP of SEMICYUC

[1]Hospital Mutua de Terrassa, ICU, Terrassa, Spain; [2]Hospital Son LLatzer, Palma, Spain; [3]Complejo Hospitalario de Cartagena, Murcia, Spain; [4]Hospital General de Castellón, Castellón, Spain; [5]Hospital Universitario Rio Hortega, Valladolid, Spain; [6]Hospital Universitario de Valme, Sevilla, Spain; [7]Plan Nacional de RCP, Madrid, Spain

**Correspondence:** R. Algarte

*Intensive Care Medicine Experimental* 2017, **5(Suppl 2):**0151

**INTRODUCTION.** The cardiac arrest (CA) is defined as abrupt, unexpected and potentially reversible disruption of breathing and spontaneous circulation. It is essential to initiate manoeuvres to restore breathing and circulation, whose primary objective is to recover brain functions. The chance of successful recovery of a CA, depend directly on the precocity and correct performance of the resuscitation manoeuvres. Mild therapeutic hypothermia is recommended to improve neurological prognosis and survival in out-of-hospital cardiac arrest (OHCA) recovered with a shockable rhythm. In International CPR guidelines, this recommendation has been extended to other rhythms and in-hospital CA (IHCA) extrapolating information from OHCA to IHCA.

**OBJECTIVE.** To analyse the epidemiology, characteristics, survival and neuro-functional prognosis of the in-hospital recovered cardiac arrest (IHRCA) admitted to ICUs in Spain.
**METHODS.** We analysed "The national registry of attention to recovered cardiac arrest and use of hypothermia in the ICUs of Spain". An observational, prospective and multicenter study conducted between January 2014 and June 2016. We have selected only the IHRCA. Epidemiological, demographic, functional capacity (Barthel Index scale) and neurological prognosis (CPC scale) were analysed at hospital discharge and one-year survival. We make a descriptive analysis for factors associated with survival.
**RESULTS.** 46 ICUs, we included 310 patients, the most frequent place of the IHRCA was in general ward (32.8%). Table 51 shows the main features related to IHRCA.
Only in 44 patients were performed mild hypothermia. The use of controlled temperature techniques to perform mild hypothermia was applied in 20 (45.5%) of cases, in 18 (40.9%) of patients, the central temperature was higher 36°C before start hypothermia. The duration of hypothermia was lower of 24 hours in 23 (52.3%) of cases, and the rewarming was faster than 0.25°C/hour in 22 (50%) of cases.
**CONCLUSIONS.** The IHRCA is more frequent in general ward, at night and holidays. Although the percentage of witnesses IHRCA is high, it is not the performance of CPR manoeuvres or early defibrillation before arrive rapid response team. A high number of mild hypothermia techniques are performed by uncontrolled temperature systems. The cases of patients with a central temperature above 36°C prior the onset of mild hypothermia, the duration of the technique less than 24 hours as well as a rapid rewarming were high. The IHRCA is associated with a high mortality, but the neurological and functional prognosis of survivors is good. Survival is associated to shockable first rhythm, receive early defibrillation and cardiac etiology.

**Table 51 (Abstract 0151).** Describes the main features related to IHR

| | N=310 |
|---|---|
| Male. N (%) | 209 (67,4%) |
| Age. Years | 69 (SD 3,7) |
| Means hospital stay. Days | 27,6 (SD 29,4) |
| IHRCA at night or holidays. N (%) | 175 (56,5%) |
| Defibrillation before arrive rapid response team. N (%) | 21 (6,7%) |
| CPR manoeuvers > 20 min. N (%) | 96 (31%) |
| Start CPR manoeuvers before arrive rapid response team. N (%) | 127 (41%) |
| First rhythm shockable. N (%) | 89 (28,8%) |
| Cardiac etiologic. N (%) | 142 (45,8%) |

**Table 52 (Abstract 0151).** Shows the results of survivals, functional

| N=310 | Hospital discharge | One-year |
|---|---|---|
| Survivals. N (%) | 119 (38,4%) | 85 (27,4%) |
| CPC score 1-2. N (%) | 110 (35,5% of all IHRCA and 92,4% of all survivals) | 83 (26,7% of all IHRCA and 75,4% of all survivals) |
| Barthel Index score > 80. N (%) | 92 (29,7% of all IHRCA and 77,3% of all survivals) | 74 (23,8% of all IHRCA and 67,2% of all survivals) |

**Table 53 (Abstract 0151).** Shows the bivariate analysis of factors re

| N=310 | Alive | Dead | p |
|---|---|---|---|
| Age. Years (SD) | 65,4 (SD 16,3) | 70,7 (SD 12,6) | 0,009 |
| IHRCA at night or holidays. N (%) | 40 (47,1) | 126 (61,8) | 0,015 |
| First rhythm shockable. N (%) | 44 (51,8) | 35 (17,2) | <0,001 |
| Defibrillation before arrive rapid response team. N (%) | 14 (35) | 7 (8,5) | 0,001 |
| Cardiac etiology. N (%) | 52 (61,2) | 78 (38,2) | <0,001 |
| CPR manoeuvers < 20 min. N (%) | 66 (77,6) | 134 (65,7) | 0,03 |

### 0152

#### Use of extra-corporeal life support in severe poisoning: a retrospective study

L. Gaide-Chevronnay, A. Hebrard, G. Dessertaine, P.-H. Moury, M. Durand, J.-F. Payen, P. Albaladejo
CHU Grenoble, Grenoble, France
**Correspondence:** L. Gaide-Chevronnay
*Intensive Care Medicine Experimental* 2017, **5(Suppl 2):**0152

**INTRODUCTION.** The use of extra-corporeal life support (ECLS) in refractory cardiac arrest (RCA) is now well-known (1), and rules by French guidelines concerning indication on precise criterias, except for toxic causes (2). Indeed, datas about RCA in severe poisoning treated with ECLS are rare (3). Our study aims to describe survival in severe poisoning with ECLS.
**MATERIAL AND METHODS.** 20 patients treated with ECLS for severe poisoning were retrospectively included in an universitary hospital from 2007 to 2016. ECLS was indicated for RCA or for hemodynamic instability. Pregnant women and RCA from other cause were excluded.
**RESULTS.** Mean age was 49 ± 16 years. 60% of patients were women. 17 patients presented cardiac arrest (CA), 11 of them were RCA needing ECLS. 11 ECLS were started during cardiopulmonary resuscitation (CPR), and among these, 9 were out-of-hospital RCA(OH-RCA). Overall mortality was 14 patients, with 9 deaths among the 11 RCA and 8 among the 9 OH-RCA, and only 1 among the 2 patient presenting CA after hospital admission. Serum lactate and bicarbonate levels at admission were significantly associated to with mortality (respectively $p = 0.013$ and 0.017). Median low-flow was 70 min (4–250 min) for all patients, but 104 min (60–250 min) in OH-RCA. Medications and dosages were very heterogenous. All patients with CA had no No-Flow.
**CONCLUSION.** Overall mortality was high in severe poisoning treated with ECLS, particularly in OH-RCA. Other studies are needed to know if the use of ECLS in severe poisoning is justified regardless of the low-flow duration.

**REFERENCE(S)**

1. Intensive Care Med (2007) 33:758–764;
2. Annales Françaises d'Anesthésie et de Réanimation 28 (2009) 182–186;
3. Crit Care 2009, 13:R138 (doi:10.1186/cc8017)

**GRANT ACKNOWLEDGMENT**
None

### Acute brain injury 1

#### 0153

#### Factors associated with mortality and disability six months after discharge in patients with decompressive craniectomy in a neurotraumatic ICU

C. Sánchez Ramírez[1], C.F. Lübbe Vázquez[1], L.D.M. Díaz Suárez[1], C. Agüero Senovilla[1], R. Prada Osorio[1], L. Pastor Martín[2], S. Ruiz Santana[1]
[1]University Hospital of Gran Canaria Dr Negrín, Intensive Care Unit, Las Palmas de Gran Canaria, Spain; [2]University Hospital of Gran Canaria Dr Negrín, Neurosurgery Department, Las Palmas de Gran Canaria, Spain
**Correspondence:** C. Sánchez Ramírez
*Intensive Care Medicine Experimental* 2017, **5(Suppl 2):**0153

**OBJECTIVE.** To evaluate the factors associated with mortality and disability after discharge in patients with decompressive craniectomy (DC) in the ICU.

**METHODS.** Prospective study of patients admitted from January 1, 2013 to march 2017 who required DC. DC was performed by intracranial hypertension (ICH) refractory to medical treatment. They were collected and analyzed: main diagnosis at admission; demographic data; neurological data (clinical examination and Glasgow Coma Score: (GCS)); hypotension: at the site, at hospital admission and at ICU and at hospital discharge; type of craniectomy and DC complications; Rankin scale, and Glasgow outcome scale (GOS) at 30 days after UCI admission, at ICU discharge and 6 months after ICU discharge; preoperative serum lactate levels and number of hours with intracranial pressure (ICP) > 20, 25 or 30 mmHg before DC; hypo and hyperglycemia; application of mannitol or hypertonic saline solution before and after DC; dose of catecholamines needed; leukocytes and platelets before and after DC and other factors related to prognosis. Categorical variables were summarized as frequencies and percentages and the continuous variables as medians and interquartile ranges (IQR) or means and standard deviations. Univariate analysis of mortality and disability at ICU discharge and 6 months after ICU discharge were performed. Statistical significance was set at $p \leq 0.05$.

**RESULTS.** Twenty patients with DC were collected. Demographic data and types of admission are shown in Table 54.

Most were subarachnoid haemorrhages (SAH) (60%) and 70% of DC were hemicranial. The most frequent complications were reoperation due to complications (55%), hydrocephalus (50%) and extraaxial hematoma (40%). Five patients died at the ICU discharge (25%), 4 (80%) of them were SAH. Rankin mean at ICU discharge was 4.1 and GOS was 2.5. Rankin mean 6 months after ICU discharge was 3,16 and GOS was 3 (Table 55).

Mortality 6 months after discharge was significantly associated with the presence of focal contusion with edema and expansivity, bilateral pupillary reactivity prior DC (lower mortality), mean midline displacement (millimeters) on admission Computed Tomography (CT) as well as higher GCS at ICU discharge (lower motality) (Table 56).

We found no factors associated with mortality or disability at ICU discharge or disability 6 months of ICU discharge.We found no significant factors associated with mortality or disability at ICU discharge and disability six months after discharge in our study.

**CONCLUSIONS.** Preliminary data show low mortality (25%) of DC patients. Rankin and GOS reflect moderately severe disability in our DC patients on discharge and six months after discharge from the ICU. The presence of focal contusion with edema and expansivity, bilateral pupillary rectivity prior DC, mean midline displacement on admission CT and higher GCS at ICU discharge were significantly associated with mortality six months after discharge from the ICU.

**Table 54 (Abstract 0153).** See text for description

| Table 1. | n 20 |
|---|---|
| Age years, n (IQR) | 47 (36;55) |
| Male/Female, n (%) | 11/9 (55/45) |
| Diabetes, n, (%) | 2 (10) |
| Hypertension, n (%) | 2 (10) |
| APACHE-II, n ,(±SD) | 23 (4,23) |
| TBI on admission, n (%) | 6 (30) |
| Subarachnoid hemorrhage, n (%) | 12 (60) |
| Stroke Malignant middle cerebral artery, n (%) | 6 (30) |
| Tumor, n (%) | 3 (15) |
| Primary DC, n (%) | 12 (60) |
| Secondary DC, n (%) | 6 (30) |
| Bifrontal DC, n (%) | 2 (10) |
| Hemicraneal DC , n (%) | 14 (70) |
| Other, n (%) | 2 (10) |
| Mortality 6 months after discharge, n (%) | 8 (40) |
| ICU Mortality, n (%) | 5 (25) |
| Hospital mortality , n (%) | 2 (10) |
| Mortality after discharge from hospital, n (%) | 1 (5) |
| ICU stay days, m (IQR) | 47,9 (6;370) |
| Days with DC, m (IQR) | 72 (29;196) |
| Higher GCS in ICU, m (IQR) | 10,5 (7,5;15) |

ICU: Intensive Care unit; GCS: Glasgow Coma Score; GOS Glasgow Outcome Scale;n: number; DC: Decompressive Craniectomy;TBI: trauma brain injury; m: median;IQR: Intercuartile range.

**Table 55 (Abstract 0153).** See text for description

| | |
|---|---|
| GOS at ICU discharge , m (IQR) | 2,5 (1,5;3) |
| GOS 30 days after ICU admission, m (IQR) | 2,69 (1,5;4) |
| GOS 6 months after ICU discharge, m (IQR) | 3 (1:4,5) |
| Rankin at ICU discharge, m (IQR) | 4,1 (3,25:5) |
| Rankin 30 days after ICU admission, m (IQR) | 4,25 (3:5) |
| Rankin 6 months after ICU discharge, m (IQR) | 3,16 (1,25;5) |

ICU Intensive Care unit;, GOS Glasgow Outcome Scale; m: median;IQR: intercuartile range

**Table 56 (Abstract 0153)**. See text for description

| Table 3 | EXITUS Yes n 8 (%) | EXITUS No n 12 (%) | P |
|---|---|---|---|
| Female/Male | 6 (62,5)/ 4 (37,5) | 3 (33,3)/7 (66,7) | 0,362 |
| HBP | 1 (12,5) | 1 (8,3) | 1 |
| Dyslipemia | 2 (25) | 1 (8,3) | 0,537 |
| Tumor | 1,1 (14,3) | 2 (18,2) | 1 |
| SAH | 6 (71,4) | 7 (58,3) | 0,656 |
| Stroke Malignant middle cerebral artery | 2 (25) | 4,5 (37,5) | 0,642 |
| Acute subdural hematoma | 3,4 (42,9) | 6,5 (54,5) | 1 |
| Obliteration of the cisterns of the base | 2 (25) | 6,5 (54,5) | 0,352 |
| Focal contusion with edema and expansivity | 1,1 (14,3) | 7,6 (63,6) | 0,046 |
| Evacuated Injury | 3,4 (42,9) | 1 (9,1) | 0,245 |
| TBI | 2,2 (28,6) | 4 (33,3) | 1 |
| Pupillary reactivity prior DC ( Both) | 3 (37,5) | 11 (90,9) | 0,041 |
| Pupillary reactivity prior DC (None) | 1 (12,5) | 1 (8,3) | 1 |
| Prehospital endotracheal intubation | 2 (25) | 1 (8,3) | 0,537 |
| Emergency endotracheal intubation | 4 (50) | 4 (33,3) | 0,648 |
| Operating room endotracheal intubation | 2 (25) | 33,3 | 1 |
| Nº Platelets 24 hours after DC | 174000(105250;254500) | 221500(157250;386750) | 0,181 |
| Previous transfusion | 3,4 (42,9) | 1 (9,1) | 0,245 |
| Displacement midline on admission CT | 8,5 (1,;10) | 1(0,6) | 0,049 |
| GCS at admission | 7(6;14) | 15(10;15) | 0,069 |
| Higher GCS in ICU | 5(3;8) | 15(10;15) | 0,040 |
| Seizures prior DC | 3,4 (42,9) | 1 (9,1) | 0,245 |
| APACHE (median) | 25 | 23 | 0,171 |

Data are medians (IQR) and frequencies (%); n: number; IQR: interquartile range; ICU: Intensive Care unit; GCS: Glasgow Coma Score; TBI: trauma brain injury; HBP: High blood pressure; CT: computed tomography; SAH: subarachnoid hemorrhage; DC: Decompressive Craniectomy.

### 0154

#### Decompressive neurosurgery in traumatic brain injury. A systematic review and meta-analysis

A. Putzu, S. Clivio, T. Cassina
Fondazione Cardiocentro Ticino, Lugano, Switzerland
**Correspondence:** A. Putzu
*Intensive Care Medicine Experimental* 2017, **5(Suppl 2):**0154

**INTRODUCTION.** Traumatic brain injury (TBI) can lead to severe brain injury, major disability, and death. Current randomized literature presents contrasting results on the effect of neurosurgical decompression in this condition.

**OBJECTIVES.** The aim of this systematic review and meta-analysis of randomized trials was to study the effect of decompressive surgery on morbidity and mortality of TBI patients.

**METHODS.** PubMed, EMBASE, and the Cochrane Central Register of clinical trials were searched up to April 1st, 2017 for randomized controlled trials on the use of decompressive neurosurgery versus any control in patients with TBI. The primary outcomes were mortality, severe disability, and functional independence at longest follow-up available. Severe disability was defined as GOS-E (Extended Glasgow Outcome Scale) 2 to 4 and functional independence was defined as GOS-E 5 to 8. To analyse the outcomes we calculated risk ratios (RRs) and 95% confidence intervals (CIs).

**RESULTS.** Three studies for a total of 548 patients were included in the analysis [1–3]. Decompressive surgery was associated with significantly lower mortality compared to medical care (RR 0.64 [95% CI, 0.50, 0.81], p = 0.0002) (Fig. 73). After surgical decompression, no difference in functional independency was found (RR 0.96 [95% CI, 0.75, 1.22], p = 0.72), but severe disability was more frequent with surgery (RR 1.68 [95% Ci, 1.31, 2.16], p < 0.0001) (Fig. 73).

**CONCLUSIONS.** Decompressive craniectomy in patients with TBI resulted in lower mortality but higher rates of severe disability than medical care. The rate of functional independence was similar in the two groups. Future randomized trials should define the impact of this surgical strategy on patients´ outcomes and quality of life.

**REFERENCE(S)**

[1] Hutchinson PJ, Kolias AG, Timofeev IS, Corteen EA, Czosnyka M, Timothy J, et al. Trial of Decompressive Craniectomy for Traumatic Intracranial Hypertension. N Engl J Med 2016;375:1119–30.
[2] Cooper DJ, Rosenfeld J V., Murray L, Arabi YM, Davies AR, D'Urso P, et al. Decompressive Craniectomy in Diffuse Traumatic Brain Injury. N Engl J Med 2011;364:1493–502.
[3] Moein H, Sanati MA, Abbasi Fard S, Moein P, Hasheminasab SM. Outcome of Decompressive Craniectomy in Patients With Severe Head Injury. Neurosurg Q 2012;22:149–52.

**GRANT ACKNOWLEDGMENT**
The authors declare no conflicts of interests. The study had no funding.

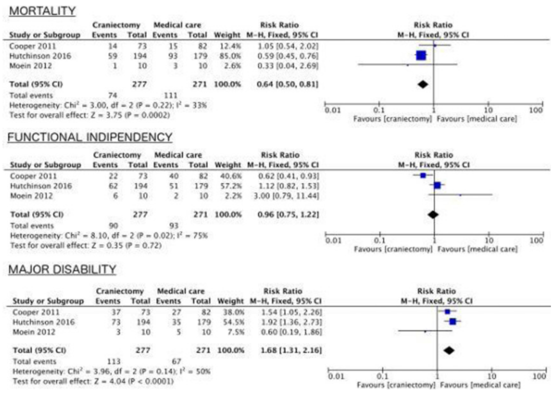

**Fig. 73 (abstract 0154)** See text for description

### 0155

#### Incidence of intracranial hypertension in spontaneous intracerebral hemorrhage patients

M. Carbonara[1], L. Zani[2], E. Frecchiami[2], F. Ortolano[1], N. Stocchetti[1,2]
[1]Fondazione IRCCS Ca' Granda Ospedale Maggiore Policlinico, Milano, Italy; [2]Università degli Studi di Milano, Milano, Italy
**Correspondence:** M. Carbonara
*Intensive Care Medicine Experimental* 2017, **5(Suppl 2):**0155

**INTRODUCTION.** Intracerebral hemorrhage (ICH), a non-traumatic bleeding in the brain parenchyma, is the second most common form of stroke and an important source of death and disability worldwide[1]. Treatment aims to support vital function and reverse possible causal factors, as arterial hypertension or antithrombotic therapy[2]. Surgical evacuation is still controversial, as indications for monitoring and treatment of intracranial pressure (ICP)[1,2].

**OBJECTIVES.** describe ICP and its treatment in a single-center consecutive case series of ICH

**METHODS.** Retrospective analysis of a prospectively collected database. Demographic characteristics, imaging of all ICH patients admitted to our intensive care unit (ICU) from January 2010 to December 2016 were collected. Mean (for every 12-hour period) and maximum (defined as higher value lasting more than 5 minutes in the same period) ICP were recorded. Intracranial hypertension (HICP) was defined as ICP maximum value higher than 20 mmHg Functional outcome (obey command) and mortality were evaluated at hospital discharge. Statistical analysis was performed using Prism. Significance was set at a p value ≤ 0.05.

**RESULTS.** During the 7-year period, we recruited 157 ICH patients (5% of all admissions), of which 53 had ICP monitoring. Baseline characteristics are shown in Table 57. ICP patients were younger (55

versus 63 years, $p < 0.05$) and had lower mortality (19% versus 61%, $p < 0.001$) than non-ICP. In the ICP group, hypertension was most likely cause in 60%, while ICH was secondary to coagulopathy (15%), vascular malformation (18%). ICP was monitored for 4 days (median, IQR 2–5.5), its median value was 15 mmHg (median, IQR 11–19). Brief episodes of HICP (9% of all monitored period) were found in 79% of patients. ICP was actively treated in 28% of patients, and 8 of them received extreme therapy (7 decompressive craniectomy and 1 barbiturates). There was no difference in mean ICP between evacuated versus non-evacuated patients. Mortality in the ICP group was low, with good short term outcome (62% of patients obeyed command at hospital discharge). Median hospital length of stay was 21 days (median, IQR 15–28.5).

**CONCLUSION.** In our institution, ICH surgical evacuation and ICP monitoring are preferred for patients with baseline predictors of good outcome. A relevant proportion of patients (79%) suffered episodes of HICP of rather short duration. Extreme therapy was necessary in 15% of population. Outcome was good compared to literature[1,2], supporting the idea that aggressive treatment in selected population can improve prognosis.

**GRANT ACKNOWLEDGMENT**

This research received no specific grant and it was supported by institutional funds.

**Table 57 (Abstract 0155)**. Baseline characteristics of population

| | |
|---|---|
| Age (median, IQR) | 55 (45-67) |
| Female (%) | 42% |
| Most likely cause (%) | |
| Arterial hypertension | 60% |
| Coagulopathy | 15% |
| Vascular anomaly | 18% |
| Other | 7% |
| Admission Glasgow Coma Scale score (median, IQR) | 8 (5-12) |
| Hematoma volume (median, IQR) | 58 (32-85) mL |
| Surgical evacuation | 72% |

### 0156

#### Association of Intensive care on patients with mild traumatic brain injury from the Japanese national neurotrauma registry

C. Tanaka, J. Kaneko, A. Takehara, S. Kudo, T. Tagami, K. Unemoto
Nippon Medical School Tamanagayama Hospital, Tokyo, Japan
**Correspondence:** C. Tanaka

*Intensive Care Medicine Experimental* 2017, **5(Suppl 2):**0156

**INTRODUCTION.** Brain injury is one of the major leading causes of disability and death following trauma. Previous studies suggested that among mild traumatic brain injuries (mTBI, as defined by a Glasgow Coma Scale score of 13 or greater on arrival), around 80% of patients had a favourable neurological recovery; however, some patients had residual severe neurological deficit. Although previous studies reported that patients with mTBI who required neurosurgery may have higher mortality rates and worse neurological outcomes, there were few studies that investigated an association between intensive care and mortality for patients with mTBI.

**OBJECTIVES.** We aimed to determine whether critical care management styles are connected with the neurological outcome in patients with mTBI who required neurosurgical intervention, using the Japan Neurotrauma Data Bank (JNTDB).

**METHODS.** We used the JNTDB, which is a national registry that includes patients with traumatic brain injury. Within the 2009–2011 records of JNTDB (n = 1092), we retrospectively identified patients with mTBI who needed neurosurgery. We used the Glasgow Outcome Scale as the main outcome measurement. We grouped these records into two groups: good outcome (good recovery and moderate disability) and poor outcome (severe disability, vegetative state, and death). We analysed neurological outcomes using a logistic regression analysis adjusted for within-hospital clustering and other factors that independently associated with neurological prognosis.

**RESULTS.** In total, 195 patients with mTBI under operative management were included in this study. Of these, the number of patients with a poor outcome was 70 (35.9%). Logistic regression analysis revealed that the use of osmotic agents resulted in poor outcomes (odds ratio: 5.32, 95% confidence interval: 1.76-16.11). However, following intensive treatments, these treatments had no significant association with a worse outcome: anticonvulsants, sedatives, and temperature management (odds ratio: 1.28, 95% confidence interval: 0.52-3.18; odds ratio: 1.59, 95% confidence interval: 0.28-9.05; and odds ratio: 0.71, 95% confidence interval: 0.23-2.19, respectively).

**CONCLUSIONS.** Our study using a Japanese nationwide brain trauma registry suggested that the requirement of osmotic agents was possibly associated with poor neurological outcome for patients with mTBI who required neurosurgical intervention, although anticonvulsants, sedatives, and temperature management was not significantly associated with outcome.

### 0157

#### Timing to emergency neurosurgery - are we doing enough?

S. Dias[1], S. Appleton[2], F. Pais Silva[1], A.M. Fernandes[1], R. Moreno[1,3]
[1]Hospital de São José, Centro Hospitalar de Lisboa Central, Unidade de Cuidados Intensivos Neurocríticos, Lisboa, Portugal; [2]Hospital de São José, Centro Hospitalar de Lisboa Central, Anaesthesia Department, Lisboa, Portugal; [3]Nova Medical School, New University of Lisbon, Lisboa, Portugal
**Correspondence:** S. Dias

*Intensive Care Medicine Experimental* 2017, **5(Suppl 2):**0157

**INTRODUCTION.** Contention of the primary damage and prevention of secondary damage is crucial in neurocritical care but the benefits of very early surgical treatment have not been clearly demonstrated. This leads to a lack of human and material resources specifically available for these patients in the emergency department and a dedicated track to the operating room.

PURPOSE. To understand the prognostic relevance of the hospital delay to the induction of anaesthesia in order to define priorities in patients admittance to the operating room.

**METHODS.** We collected data retrospectively about patients admitted after an emergency neurosurgery during one year in one neurocritical ICU (UCINC) of a central, tertiary hospital. 636 patients were admitted from 1st April 2016 to 31st March 2017. We selected the patients admitted after an emergency neurosurgical procedure (N = 93).

**RESULTS.** Our sample has 54% of men and an average age of 67,2 years (SD 15,9). Average SAPS II is 38,6, (SD 16,5), SAPS3 61,3 (SD 12,7), APACHE II 20,1 (SD 7,0).

Among those coming from the emergency department (77,4%), the median time between admission and induction of anaesthesia was 2,8 h. The median time from triage to neurosurgical observation was 0,5 h. The median time from neurosurgical formal operative indication to anaesthesia was 0,8 h. For patients already in the hospital this period was significantly longer - 1,8 h (Mann Whitney $p = 0.001$), without statistical significant difference in the preoperative Glasgow score (GCS). The median time between the end of anaesthesia and admittance to the ICU is 0,6 h.

There is a weak positive correlation between time to the operating room and GCS before the surgery, suggesting that patients with a lower GCS wait less for surgery ($R = 0{,}30$, $R^2 = 0{,}09\%$, $p = 0{,}003$).

No correlation was found between the waiting period for surgery or ICU admittance and hospital mortality, even after correcting for the preoperative GCS.

**CONCLUSIONS.** Neurocritical out-patients arriving to our emergency room are already treated with priority and we tend to treat first patients with more severe disease. More studies are needed to understand the prognostic relevance of a longer interval of time, including the determinants of outcome from those transferred from a secondary to a tertiary hospital. Also, patients already in the hospital that suffer neurological deterioration should be treated faster.

### 0158

#### Evaluation of intracerebral hemorrhage score and apache II scale in patients admitted in intensive care by non-traumatic brain hemorrhage

E. Aguilar-Alonso[1], M. Redondo-Orts[2], R. Vela-Colmenero[2], F. Guerrero-Lopez[3], M.D. Pola-Gallego de Guzman[2], L. Perez-Borrero[4], J. Mora-Ordoñez[5], P. Lara-Aguayo[1], M. Rojas-Amezcúa[1], J.F. Brea-Salvago[2], E. Castillo-Lorente[3]
[1]Intensive Care Unit - Hospital Infanta Margarita, Cabra, Spain; [2]Intensive Care Unit - Complejo Hospitalario, Jaen, Spain; [3]Intensive Care Unit - Hospital Universitario Virgen de las Nieves, Granada, Spain; [4]Intensive Care Unit - Hospital Serrania, Ronda, Spain; [5]Intensive Care Unit - Hospital Universitario Carlos Haya, Malaga, Spain
**Correspondence:** M. Redondo-Orts
*Intensive Care Medicine Experimental* 2017, **5(Suppl 2):**0158

**INTRODUCTION.** Intracerebral hemorrhage is a stroke subtype with high mortality and significant disability among survivors.
**OBJECTIVES.** To evaluate Intracerebral Hemorrhage (ICH) score and APACHE II scale in patients admitted to ICU with non-traumatic brain hemorrhage.
**METHODS.** Multicenter prospective observational study in three hospitals in Andalusia (Spain). We studied all patients with brain hemorrhage admitted to the Regional Hospital of Malaga (2006 to 2011), Neurotraumatology Hospital of Jaen (2010 to 2012) and Virgen de las Nieves Hospital of Granada (2006 to 2011).
Data were expressed as the mean and standard deviation for quantitative variables and percentages for qualitative variables. For the comparison of two means we used the Student's t-test and the chi- squared test was used to compare proportions. Area Under the ROC curve for analizing discrimination, Standardized Mortality Ratio (SMR) and Hosmer-Lemeshow Test for analizing calibration. Statistically significant differences $p < 0.05$.
**RESULTS.** N = 336 patients (263 supratentorial). Mean age 59.43 ± 14.75 years, Glasgow score (GCS) at admission 8 ± 4 points, APACHE-II 21.03 ± 7.6 points, intraventricular hemorrhage (IVH) was 58.6% of patients. 105 patients were treated by surgery. The hospital mortality was 54.17%.
Bilateral mydriasiswas observed in 37 cases (11.1%), with mortality at 30 days of 100% and predicted by the ICH score of 78.11%.
Patients who died in hospital were older 63.76 ± 12.21 vs 59,43 ± 14.75 ($p < 0.001$), lower GCS 6 ± 3. vs 10 4 ($p < 0.001$) and higher APACHE II 24.31 ± 6.37 vs 17.09 ± 7.08 ($p < 0.001$).
Mortality predicted by APACHE II scale (49.58%) compared with that observed (54.17%). The Hosmer-Lemeshow test was 12,28, so there not were statistically significant differences between the observed and predicted mortality by APACHE-II. Discrimination by the area under the ROC curve was 0.81 (0.76-0.85).
Mortality at 30 days predicted by ICH score (50,25%) compared with that observed (52,38%). Standardized Mortality Ratio (SMR): 1.04 (0.89-1.19)(not statistically significant). Mortality predicted by ICH score and observed: 0 point (N = 10) 0% vs. 10%, 1 point (N =52) 13% vs 21.15%, 2 points (N = 108) 26% vs 38.89%, 3 points (N = 109) 72% vs 66.97%, 4 points (N = 49) 97% vs 85.71%, 5 points (N = 7) 100% vs 85.71%, 6 points (N = 1) 100% vs 100%. Relation between mortality observed and predicted by ICH score, Hosmer-Lemeshow test (H) 45.01 ($p < 0.001$). Area under the ROC curve by ICH score 0.74 (0.69-0.79).
**CONCLUSIONS.** APACHE II scale has a correct discrimination and calibration. The ICH score has an acceptable discrimination, but not calibration. The differences between mortality observed and predicted by ICH score are low, although higher to the methodologically allowed. In spite of this we believe that ICH score is a useful instrument in ICU.

### 0159

#### A survey on fever monitoring and management in patients with acute brain injury (SUMMA)

E. Picetti[1], M. Oddo[2], L. Prisco[3], R. Helbok[4], F.S. Taccone[5]
[1]Parma University Hospital, Department of Anesthesia and Intensive Care, Parma, Italy; [2]CHUV-University Hospital, Department of Intensive Care Medicine, Lausanne, Switzerland; [3]University of Oxford and Oxford University Hospitals, NHS Foundation Trust, Oxford, United Kingdom; [4]Medical University of Innsbruck, Department of Neurology, Innsbruck, Austria; [5]Erasme Hospital, Department of Intensive Care, Brussels, Belgium
**Correspondence:** E. Picetti
*Intensive Care Medicine Experimental* 2017, **5(Suppl 2):**0159

**INTRODUCTION.** Fever occurs frequently in patients with acute brain injury (ABI) and may play a role in the development of secondary brain injury (1). The definition of fever and its clinical management across countries are different and heterogeneous.
**OBJECTIVES.** To survey practices in the management of fever in adult patients with ABI in Intensive Care Units (ICU). The main endpoints of the survey were the evaluation of: a) the definitions of fever, b) the thresholds and triggers to start intervention, c) and the methods to control temperature.
**METHODS.** A questionnaire including 26 items was available on the European Society of Intensive Care Medicine (ESICM) website (www.surveymonkey.com/r/SUMMAfever) between July 2016 and December 2016. The survey was endorsed and promoted by ESICM.
**RESULTS.** Respondents (RSP) were 231 [138 (60%) from Europe]; mainly intensivists [124 (54%)] and anesthesiologists [67 (29%)]. Body temperature (BT) was measured mostly with a bladder probe [93 (43%)]. Few RSP measured directly brain temperature [47 (22%)]. Fever was most often defined as a BT: > 38.3°C [71 (33%)], > 38 °C [56 (26%)] and > 37.5°C [38 (18%)]. The main thresholds for antipyretic therapy were 37.5°C [74 (34%)] and 38°C [86 (40%)]. However, the thresholds were lower in case of intracranial hypertension and cerebral ischemia. Principal methods for fever management are reported in Table 58. Fever was generally treated during the entire ICU stay [64 (33%)] or until intracranial hypertension and/or cerebral ischemia were resolved [61 (32%)]. Written protocols for fever and shivering were available respectively for 83 (43%) and 54 (28%) of RSP. Shivering was mainly managed with opiates [115 (60%)]. Rewarming was controlled (0.1-0.4 °C/h) to predefined temperature target for 75 (39%) of RSP and titrated to intracranial pressure for 55 (29%) of RSP.
**CONCLUSIONS.** A wide variability in practices on fever definition and management are identified in this survey. These findings may be helpful to define future investigations in this topic.

**ACKNOWLEDGEMENTS**
The Survey was endorsed and promoted by ESICM.

**Table 58 (Abstract 0159)**. Principal methods for fever management

| | DRUG | PHYSICAL METHOD | DEVICE |
|---|---|---|---|
| FIRST-LINE | Paracetamol [135 (70%)] | Ice Packs [90 (47%)] | External Non-Automated System [49 (25%)] |
| SECOND-LINE | Diclofenac [62 (32%)] | IV Cold Fluids [68 (35%)] | External Computerized Automated Systems [75 (39%)] |

### 0160

#### A comparison of non-invasive methods for estimation of intracranial pressure

S. Pozzebon, C. Castroviejo, F. Zama Cavicchi, B. Moro Salihovic, J. Creteur, J.-L. Vincent, F.S. Taccone
Erasme University Hospital, Intensive Care, Brussels, Belgium
**Correspondence:** S. Pozzebon
*Intensive Care Medicine Experimental* 2017, **5(Suppl 2):**0160

**INTRODUCTION.** Elevated intracranial pressure (ICP) is a well-known cause of secondary brain injury. The gold standard method of measuring ICP is using an intra-cerebral catheter. Several non-invasive techniques can be used to estimate ICP values, but they have never been compared.
**OBJECTIVE.** To compare three non-invasive methods of ICP assessment.
**METHODS.** Prospective observational study including ICU patients in whom invasive ICP monitoring was initiated. The following non-invasive methods were simultaneously used to estimate ICP: ocular ultrasound to measure the optic nerve sheath diameter (ONSD); transcranial Doppler to measure the pulsatility index (PI); and automated pupillometry (NeurOptics, Irvine, USA) to measure the neurological pupil index (NPi). The mean value from both eyes was calculated for all measurements and correlations assessed using a Pearson's or Spearman's test, as appropriate.
**RESULTS.** We studied 46 patients (traumatic brain injury = 12, subarachnoid hemorrhage = 22; others =12) with a median age of 51 years. Median Glasgow Coma Scale score on admission was 9 [5–12]. ICP assessment was performed 2 [1–3] days after ICU admission and median ICP values were 15 [11–25] mmHg. Median values from the different non-invasive techniques were: ONSD 5.2 [4.8-6.1] mm, PI 1.2 [0.9-1.7] and NPi 4.2 [3.6-4.7]. There was a significant, although weak, correlation between ONSD and PI ($r^2 = 0.11$; $p = 0.01$) and between PI and NPi ($r^2 = 0.37$; $p < 0.001$), but not between ONSD and NPi ($r^2 = 0.008$; $p = 0.53$).
**CONCLUSIONS.** Non-invasive techniques to estimate ICP values correlate poorly and cannot be considered as interchangeable.

### 0161

#### Who should place intraparenchymal pressure monitoring, neurosurgeons or intensivists? Comparative results of two centers

D.C. Robaglia, V.A. Hortigüela, A. Vidal, A.I. Tejero, H. Pérez, J.L. Pérez, L.M. Polanco, E. Rosas, C. Pérez
Fundación Jiménez Díaz, Intensive Care Medicine, Madrid, Spain
**Correspondence:** D.C. Robaglia
*Intensive Care Medicine Experimental* 2017, **5(Suppl 2):**0161

**INTRODUCTION.** There are common techniques to different medical specialties and we have to look for the best practice for patients.
**OBJECTIVES.** Evaluate the complications after placement of intraparenchymal pressure sensors in two different hospitals and compare the results.
**METHODS.** Descriptive retrospective observational study. We analyzed catheters placed consecutively by Intensivists at bedside at Hospital Virgen de la Salud in Toledo (Spain) during 2008 (GROUP I), and placed by neurosurgeons in the operating room at the Hospital Fundación Jiménez Díaz at Madrid (Spain) between the years 2012–2017 (GROUP II).
Data collected: sex, reason, location and implantation date time, complications (hemorrhage, malposition and infection) and functional evolution at ICU discharge according to Glasgow Coma Scale (GOS).
**RESULTS.**
* GROUP I: number 58; Sex (M/F) 22 (37.93%)/35 (60.34%); Reason: trauma brain injury (TBI) 46 (79.31%), spontaneous hemorrhage (SH) 11 (18.97%), Ischemia 1 (1.72%); Place (ipsilateral / contralateral: 39 (67.24%) / 19 (32.76%); Moment: at admission 36 (62.07%), < 24h 13 (22.41%), 2–3 day 8.62%), > 4 days 4 (6.90%); complications: hemorrhage 11 (18.96%), malposition 7 (12.07%).No infection, GOS at ICU discharge: GOS1 16 (27.59%), GOS2 6 (10.34%), GOS3 23 (39.66%), GOS4 6 (10.34%), GOS5 7 (12.07%).
* GROUP II: number 46; Sex (H/F) 12 (26.09%)/ 34 (73.90%); Reason: TBI 22 (47.82%), SH 18 (39.13%), Ischemia 1 (2.17%), Infection 4 (8.70%), cerebral hypoxia 1 (2.17%). Place (ipsilateral / contralateral: 39 (84.78%) / 7 (15.22%); Moment: at admission 37 (80.40%), < 24h 5 (10.87%), 2–3 day (24.39%), >4 days 2 (4.35%); complications: hemorrhage 10 (24.39%), malposition 10 (24.39%). No infection, GOS at ICU discharge: GOS1 17(36,96%), GOS2 4(8,70%), GOS3 12 (26.09%), GOS4 10 (21.74%), GOS5 2 (4.35%).
**CONCLUSIONS.** There were no differences between the moment of implantation and GOS to ICU discharge. In GROUP I there were more patients with TBI. In group II, more ipsilateral catheters were placed and more complications were observed. There are great differences in patients at each center and the time analyzed. Intensivists techniques are stricter compared to neurosurgeons, which in most cases end in craniotomy.
Intensivists are able to place intraparenchymal pressure catheters in cases that patient doesn´t need craniotomy, optimizing the initial management and the final result.
**REFERENCE(S)**
- Heck C1. **Invasive Neuromonitoring**. Crit Care Nurs Clin North Am. 2016 Mar;28(1):77–86.
- Forsyth RJ.**Routine intracranial pressure monitoring in acute coma.** J Trauma Acute Care Surg. 2015 Jul;79(1):85–90.
- Bonds BW. **Predicting secondary insults after severe traumatic brain injury.** Cochrane Database Syst Rev. 2015 Nov 2;(11):CD002043.
- Hawthorne C. **Monitoring of intracranial pressure in patients with traumatic brain injury.** Front Neurol. 2014 Jul 16;5:121.
**GRANT ACKNOWLEDGMENT**
None.

### 0162

#### Optimal cerebral perfusion pressure based on pressure-reactivity index in paediatric traumatic brain injury

H.K. Kanthimathinathan[1], J. Spry[1], R. Taylor-Smith[1], K. Morris[1], B. Scholefield[1,2]
[1]Birmingham Children's Hospital, Paediatric Intensive Care Unit, Birmingham, United Kingdom; [2]University of Birmingham, Birmingham, United Kingdom
**Correspondence:** H.K. Kanthimathinathan
*Intensive Care Medicine Experimental* 2017, **5(Suppl 2):**0162

**INTRODUCTION.** Optimal cerebral perfusion pressure(CPP) thresholds for use in the management of paediatric traumatic brain injury (TBI) are not well known. Brain Trauma Foundation guidelines do not recommend age-specific CPP targets other than a minimum of 40mmHg due to lack of robust evidence, despite widespread use of age-specific CPP targets(1). There has been recent interest in cerebrovascular autoregulation monitoring and individualised optimum CPP target derivation in children(2) after reports of the same in adult neurocritical care(3).
**OBJECTIVES.**
1. To assess feasibility of pressure-reactivity index (PRx) monitoring in paediatric TBI.
2. To analyse how often PRx monitoring results in an optimum CPP recommendation
3. To report differences between commonly used age-specific CPP thresholds (CPPopt_age) and optimum CPP (CPPopt_prx) generated by PRx monitoring.
**METHODS.** Prospective observational study of patients admitted to a paediatric intensive care unit (PICU) with severe TBI. Preliminary analysis of patients admitted between November 2016 and April 2017 is presented here. PRx monitoring was done using ICM+ (Cambridge Enterprises, UK). Clinicians were blinded to the data and retrospective analysis of prospectively collected data was performed. Intracranial pressure was monitored using an intraparenchymal pressure bolt (Camino). Details of PRx monitoring and the curve fitting procedure to generate optimum CPP has been described previously(4). Optimum CPP curves were plotted 4 hourly based on the distribution of PRx values in a series of 5 mmHg CPP bins.

**RESULTS.** Six patients (3 boys and 3 girls) had CPP monitored for 716 hours. As optimum CPP calculation was analysed in four hour windows, there were 179 four hour windows. The plot of PRx averaged within 5 mm Hg CPP bins showed a U-shape curve on 71% of the windows (127/179). An optimum CPP (CPPopt_prx) recommendation was available 65% (117) of the 179 windows. Among individual patients this varied from 40 to 75%. Where CPPopt_prx recommendation was available, it was higher than CPPopt_age on 75% of occasions (88/117). CPPopt_prx was equal to CPPopt_age on 3 (2%) occasions and was lower than CPPopt_age on 26 (22%) occasions. Mean (CPPopt_prx) MINUS (CPPopt_age) was +8 (range: −18 to +33). CPPopt_prx varied considerably with time even within individual patients

**CONCLUSIONS.** PRx monitoring and optimum CPP derivation are feasible in paediatric TBI patients. Optimum CPP recommendation was successfully obtained on 65% of occasions. Considerable differences exist between commonly reported age-specific CPP thresholds and optimum CPP recommended based on PRx.

**GRANT ACKNOWLEDGMENT**
Birmingham Children's Hospital Research Foundation Charity

### 0163

#### SEDLine™ System, a useful adjunt for assessment of brain death in traumatic brain injury patients

R. Badenes[1], C. Álvarez[1], R. Zaragoza[2], J. Galan[3], F.S. Taccone[4], F. Bilotta[5]
[1]Hospital Clinic Universitari de Valencia, Anesthesiology and Surgical-Trauma Intensive Care, Valencia, Spain; [2]Hospital Doctor Peset, Valencia, Spain; [3]Hospital Politècnic i Universitari La Fe, Valencia, Spain; [4]Hôpital Erasme, Université Libre de Bruxelles., Brussels, Belgium; [5]Sapienza University of Rome, Rome, Italy
**Correspondence:** R. Badenes
*Intensive Care Medicine Experimental* 2017, **5(Suppl 2):**0163

**INTRODUCTION.** Brain death (BD) is a catastrophic event associated with significant disturbances in the function of other organs. Even with maximum support, deterioration in cardiorespiratory function leading to asystole usually occurs. A continuous monitoring of brain and extra-cerebral organ function is needed in this setting.

**OBJECTIVES.** The goal of this study is to evaluate SEDLine™ System (Masimo Europe Limited, UK) values in traumatic brain injury (TBI) BD patients.

**METHODS.** Prospective observational study in a surgical and trauma tertiary intensive care of a universitary hospital including 18 consecutive TBI patients (2012–2016) diagnosed of BD during their ICU stay. The SEDLine™ System (Masimo Europe Limited, UK) was continuously recorded during the ICU stay but did not interfere with clinical decisions. Data from the 4-channel processed electroencephalograph (EEG) monitor, including EEG waveforms, Density Spectral Array (DSA) and the Patient State Index (PSI) numeric and trend plot, were collected.

**RESULTS.** In all patients (median age 47 [23–78] years), PSI values were < 5 at the moment BD was confirmed (clinical and electrophysiological tests, according to the Spanish legislation). PSI < 5 occurred 4.8 (±2.2) hours before BD could be confirmed by required testing.

**CONCLUSIONS.** Low PSI values on the SEDLine™ System could be a very useful method for early detection of brain death in acute brain injured patients. The impact of such early recognition on organ donors management and transplantion success need to be further evaluated.

### 0164

#### An audit of cerebral perfusion pressure monitoring in a level one trauma neurointensive care unit: target CPP and transducer positioning

S. Karimi[1], P. Candler[2]
[1]Queens Medical Centre, Anaesthetics, Nottingham, United Kingdom; [2]Queens Medical Centre, ICU, Nottingham, United Kingdom
**Correspondence:** S. Karimi
*Intensive Care Medicine Experimental* 2017, **5(Suppl 2):**0164

**INTRODUCTION.** The Brain Trauma Foundation recommends that cerebral perfusion pressure (CPP) is measured in all patients with severe traumatic brain injury. There is evidence that doing so decreases 2 week mortality from 22% to 13%.[1] The Brain Trauma Foundation recommends a target CPP of 60-70mmHg. Aggressive attempts to maintain CPP above 70mmHg poses a risk of adult respiratory failure and should be avoided.[2] To calculate CPP, the mean arterial pressure (MAP) used must be transduced at the level of the middle cranial fossa.[3] This means that the arterial line transducer should be positioned at the level of the tragus, an approximation for the middle cranial fossa.

**OBJECTIVES.** To assess compliance with the Brain Trauma Foundation guidelines for monitoring and targeting CPP.

**METHODS.** There were 48 data collection episodes from brain injured patients at Queens Medical Centre Intensive Care Unit between September and December 2016.

**RESULTS.** In 23% of cases the arterial line transducer was not correctly placed and therefore the CPP was measured incorrectly. 15% of measured CPPs were below the target of 60mmHg. This increased to 21% when the transducer was moved to the correct position (tragus).

27% of those patients whose transducers were incorrectly placed had CPPs measured that were within range, but their actual CPP was below target when correctly measured.

Also of note, 22% of patients had a CPP of above 80mmHg, well above the upper target of 70mmHg. Over 80% of these patients were on noradrenaline indicating that in some cases CPP is being over aggressively treated.

**CONCLUSIONS.** Greater awareness is needed that transducers should be placed at the level of the tragus when measuring CPP. Transducer position should be rechecked regularly, especially after patient repositioning. The cost effectiveness of a dual transducer system should also be investigated; this would allow simultaneous monitoring of the 'true' MAP (at heart level) and the CPP MAP (at tragus level). Finally care should be taken not to over titrate noradrenaline to avoid the risk of respiratory problems.

**REFERENCE(S)**

1. Gerber LM, Chiu YL, Carney N, Hartl R, Ghajar J. Marked reduction in mortality in patients with severe traumatic brain injury. J Neurosurg. Dec 2013;119(6):1583–1590. PMID: 24098983.
2. Brain trauma foundation, Guidelines for the Management of Severe Traumatic Brain Injury 4th Edition https://braintrauma.org/uploads/03/12/Guidelines_for_Management_of_Severe_TBI_4th_Edition.pdf
3. Joint position statement by the Councils of the Neuroanaesthesia Society of Great Britain and Ireland and the Society of British Neurological Surgeons with regards to the calculation of cerebral perfusion pressure in the management of traumatic brain injury. 2014.
4. Dr DIARMAID DILLON, calculation of cerebral perfusion pressure on icu, Belfast Health and Social care Trust, regional intensive care unit

### 0165

#### Optic nerve sheath diameter measurement by ultrasound to predict increase in intracranial pressure in traumatic brain injury

A. Al Tayar[1], A. Abouelela[2,3], K. Saleh[4], M. Khattab[4], A.A. Al Shaer[1], K. Swiefy[1], W. Gomaa[1], M. Hussain[1], A. Amin[1], M. Sohail[5], M. Diab[6]
[1]KFMMC, ICU, Dhahran, Saudi Arabia; [2]KFMMC, Dhahran, Saudi Arabia; [3]Alexandria University, Faculty of Medicine, Critical Care Medicine, Alexandria, Egypt; [4]KFMMC, Radiology, Dhahran, Saudi Arabia; [5]KFMMC, Neurosurgery, Dhahran, Saudi Arabia; [6]KFMMC, ER, Dhahran, Saudi Arabia
**Correspondence:** A. Al Tayar
*Intensive Care Medicine Experimental* 2017, **5(Suppl 2):**0165

**INTRODUCTION.** Prompt detection of raised intracranial pressure (ICP) in traumatic brain injury (TBI) is mandatory. Measurement of optic nerve sheath diameter (ONSD) by ultrasound (US) is emerging tool to predict high ICP and it is found to be comparable to computed tomography (CT) and invasive technique.

**OBJECTIVES.** To assess the accuracy of ONSD measurement by US to predict increased ICP in TBI compared to CT and invasive Ventriculostomy catheter technique as a gold standard, and to find cutoff value of ONSD to identify patients with high ICP.

**METHODS.** This a prospective study on 49 adult unconscious patients with TBI admitted to ICU at KFMMC in Dhahran,Saudi Arabia.ONSD was measured by portable US machine with high frequency probe by intensivist with previous training and experience in ocular US. Simultaneous CT measurement of both ONSD and signs of high ICP were determined by 2 different neuroradiologists. We compared these 2 techniques to invasive ventriculostomy catheter technique which was performed in 22 patients.

**RESULTS.** The study was done on 49 patients, invasive ICP monitor catheteres were inserted in 22 patients. ONSD by US mean value was 0.6 ± 0.08 mm versus 0.62 ± 0.08 by CT with no significant difference between the 2 modalities.17 patients with ICP > 20 mmHg have a mean ONSD by US of 0.7 ± 0.1 mm which was significantly higher than in 5 patients with ICP < 20 mmHg as the mean value was 0.5 ± 0.1 mm ($p < 0.001$). A cut off value of ONSD by US of 0.57 mm has been found to differentiate patients with high ICP from those with normal ICP with a sensitivity of 100% and specificity of 100%.There was no statistical significant difference in mortality and in neurologic outcome between those with ONSD above or below 0.57 mm.

**CONCLUSIONS.** ONSD by US is as accurate as CT measurements, it can be a good tool to predict the presence of increase in ICP with good sensitivity and specificity. However, further studies with higher number of patients are needed to validate these results.

**REFERENCE(S)**

1. Sekhon MS, et al.Optic nerve sheath diameter on computed tomography is correlated with simultaneously measured intracranial pressure in patients with severe traumatic brain injury. Intensive Care Med 2014;40:1267-74.

2. Komut E, et al. Bedside sonographic measurement of optic nerve sheath diameter as a predictor of intracranial pressure in ED. Am J Emerg Med 2016;34:963-7.

**GRANT ACKNOWLEDGMENT**

None

### 0166

#### Optic nerve ultrasound: a noninvasive tool for assessment of raised intracranial pressure (ICP)

R. Vara Arlanzón[1], O. Badallo Arévalo[1], C. Carbajales Pérez[1], M. Gero Escapa[1], A. Zabalegui Pérez[1], S. Calvo Simal[2], M. del Valle Ortiz[1], E. Martínez Barrio[1], D. Iglesias Posadilla[1], S. Ossa Echeverri[1], M.E. Perea Rodríguez[1], M. Montero Baladía[1], E. Pérez Cabo[1]

[1]Hospital Universitario de Burgos, Intensive Care Unit, Burgos, Spain; [2]Hospital Universitario de Burgos, Estatistics, Burgos, Spain

**Correspondence:** R. Vara Arlanzón

*Intensive Care Medicine Experimental* 2017, **5(Suppl 2):**0166

**INTRODUCTION.** Intracraneal hypertension is a common problem in the neurocritical care patients. Fast treatment of raised ICP prevent brain ischemia and brainstem herniation.

To measure ICP, invasive devices are the gold standard. In the last years, noninvasive ICP monitoring are been studied. Ultrasonography of optic nerve sheath diameter (ONSD) has been developed as a possible ICP Indicator.

**OBJECTIVES.**

To analise relationship between ICP and ONSD.

To study the ability of ultrasonography ONSD as a diagnostic tool for raised ICP.

**METHODS.** Prospective observational study. We included patients admitted in the ICU of Burgos Hospital from January to December 2015 with invasive ICP monitoring.

Ultrasound examination was made by trained investigators..

Measurements were performed every day and every ICP raised above 20 mmHg.

**RESULTS.**

We included 15 patients. The mean age was 61 years. Diagnoses were: traumatic brain injury 46,7%, subarachnoid hemorrhage 20%, intracerebral hemorrhage 33,3%. Mean GSC was 6,5 and mean ICP value was 13,83.

We made 113 ultrasound for each eye (total of 226 measurements). Only 34 ultrasound was performed with raised ICP above 20 mmHg. The median ONSD was 0,549 cm ±0,057.

There is a statistically significant relationship between ICP values and ONSD with Rho Spearman coefficient of 0,183 (p = 0,006).

We had 20 mmHg like raised ICP level, and made ROC curve. The area under the curve was 0,61 (95% IC 0,51-0,71) p = 0,043.

Optimal ONSD cutoff for the detection of raised ICP (>20 mmHg) was 0,58. Sensitivity 0,53, specificity 0,72.

**CONCLUSIONS.** Ultrasound ONSD measurement could guide intracraneal hypertension diagnosis but in our study this test didn´t have enough statistic power.

Ultrasound ONSD needs validation with another studies and more population.

- Dubourg J, Javouhey E, et al. Ultrasonography of optic nerve sheath diameter for detection of raised intracranial pressure: A systematic review and meta-analysis. Intensive Care Med. 2011;37(7):1059–68.
- Geeraerts T, Vigue B. Non-invasive assessment of intracranial pressure using ocular sonography in neurocritical care patients. 2008;2062–7.
- Rajajee V, Vanaman M, et al. Optic Nerve Ultrasound for the Detection of Raised Intracranial Pressure. 2011;506–15.
- Sekhon MS, Mcbeth P, et al. Association Between Optic Nerve Sheath Diameter and Mortality in Patients with Severe Traumatic Brain Injury. 2014;245–52.
- Dubourg J, Messerer M, et al. Individual patient data systematic review and meta-analysis of optic nerve sheath diameter ultrasonography for detecting raised intracranial pressure : protocol of the ONSD research group. 2013;
- Blaivas M, et al. Elevated Intracranial Pressure Detected by Bedside Emergency Ultrasonography of the Optic Nerve Sheath. :376–81.
- Frumin E, et al. Prospective Analysis of Single Operator Sonographic Optic Nerve Sheath Diameter Measurement for Diagnosis of Elevated Intracranial Pressure. 2014;XV(March):217–20.

## Trauma management

### 0167

#### Safety of the local magnesium infusion protocol

L. Lee-Warder[2], T. Ali[1], M. Hilary[2]

[1]Buckinghamshire Hospitals NHS Trust, Intensive Care Meidicine & Anaesthesia, Aylesbury, United Kingdom; [2]John Radcliffe Hospital, Neurosciences Intensive Care Unit, Oxford, United Kingdom

**Correspondence:** T. Ali

*Intensive Care Medicine Experimental* 2017, **5(Suppl 2):**0167

**INTRODUCTION.** Magnesium is amongst the most frequently used drug on critical care units for reasons including eclampsia[1], aneurysmal SAH[2] and correction of electrolyte imbalance.

**OBJECTIVES.** To assess the safety of the infusion protocol in Neurosciences Intensive Care Unit, John Radcliffe Hospital. As overshoots of plasma concentrations frequently occur, this trial was conducted to ascertain if patients suffer any complications.

The local protocol instructs users to infuse 20mmol of magnesium over 6 hours, diluted into 50ml of 0.9% Sodium Chloride.

**METHODS.** Retrospective analysis of 405 patients admitted to the Unit excluding aneurysmal SAH (alternative protocol). All data was collected using the electronic patient record system. The complications assessed were prolonged duration of ventilation, reduced consciousness and renal impairment (defined as reduction in eGFR).

**RESULTS.** Of the 405 patients 370 were excluded due to not having received magnesium or having received the aSAH protocol. Thirty five (8.6%) received magnesium infusions, of which 21 (60%) overshot plasma levels (Group A) and 14 (Group B) did not overshoot (40%). The highest plasma levels were 3.03 mmol/L with a mean rise of 1.27 mmol/L.
Regarding effects on ventilation in group A compared to B the total ventilator days were 173 (mean 8.2) compared to 130 (9.2). 7/21 (33.3%) were extubated compared to 4/14 (28.5%) and 2/21 (9.5%) did not require ventilation compared to 5/14 (35.7%).
Regarding the level of consciousness in Group A, 12/21 (57.1%) had reduced levels out of which 6 deceased, 3 were due to the primary brain injury and 3 were repatriated while under sedation. In Group B, 5/14 (35%) had a reduced consciousness out of which 1 patient deceased; 3 were due to the brain injury and 1 was repatriated.
The renal functions worsened in 6/21 (28%) patients in group A out of which 3 had pre-existing impairment and 1 demonstrated a fall in eGFR prior to magnesium administration. In group B, 1/14 (7%) demonstrated a reduction in eGFR.
**CONCLUSIONS.** Although the study size is small it demonstrates no significant differences in ventilator days between groups. Effects on level of consciousness cannot be accurately concluded as patients either had significant brain injury, deceased or were repatriated.
Reduction in eGFR occurred more frequently in Group A however 4/6 had a reduced eGFR on admission. This may imply that magnesium can worsen already impaired renal functions however the study numbers are too small to conclude this.
We recommend larger trials to obtain sufficient data for statistical analysis.

**REFERENCE(S)**

1. Magpie Trial Follow-Up Study Collaborative Group. The Magpie Trial: a randomised trial comparing magnesium sulphate with placebo for pre-eclampsia. Outcome for women at 2 years. *Bjog*.2007;114(3):300–309.
2. Wong GK et al. Intravenous magnesium sulphate for aneurysmal subarachnoid hemorrhage: an updated systemic review and meta-analysis. *Critical Care*. 2011;15(1).

### 0168

#### ICU admissions of self-harm patients - audit on demographics, mechanism of self-harm and outcomes

Y. Metodiev, C. Morris
Royal Derby Hospital, Anaesthesia and Intensive Care Medicine, Derby, United Kingdom
**Correspondence:** Y. Metodiev

*Intensive Care Medicine Experimental* 2017, **5(Suppl 2):**0168

**INTRODUCTION.** Self-harm and attempted suicide patients present a large proportion of emergency admissions and intensive care plays a significant role in managing patients with severe self-harm requiring organ support or invasive monitoring.
**OBJECTIVES.** The primary objective was to determine the outcome of patients who self-discharge from ICU and the rate of returns to hospital with another episode of self-harm.
**METHODS.** We looked at all ICU admissions of self-harm patients for the period between August 2014 and August 2016. We collected data from ICNARC database, electronic notes and case notes where needed. Data collected included patient demographics, type of self-harm, presence of mental conditions, ICU stay, outcome, destination at discharge and review by Mental Health Liaison Team.
**RESULTS.** There have been 142 admissions of self-harm patients for the period studied (5.2% of all admissions). One hundred and thirty-five patients presented after self-poisoning, alcohol and benzodiazepines being the most common agents used, the rest were mechanical self-harm. overall mortality was found to be 3.5%. Patients who self-discharge after being assessed by Mental Health Liaison Team were less likely to return to hospital with another self-harm episode.
**CONCLUSIONS.** The needs of mental health patients on ICU are not familiar to intensivists and managing challenging patients is an area that can be improved on by education of staff and interdisciplinary work. Creating joint pathways for assessment and referral at local and national levels could improve management of self-harm patients and prevent re-admissions.

**REFERENCE(S)**

1. Self-harm in over 8s: short-term management and prevention of recurrence. NICE clinical guideline CG16, 2004.
2. The National Confidential inquiry into Suicide and Homicide by People with Mental Illness. Making Mental Health Care Safer: Annual Report and 20-year Review. October 2016. University of Manchester.
3. Mental Capacity Act 2005. http://www.legislation.gov.uk/ukpga/2005/9/contents
4. Mental Health Act 2007. http://www.legislation.gov.uk/ukpga/2007/12/contents
5. Self-harm, Suicide and Risk: Helping People Who Self-harm. Royal College of Psychiatrists. College Report CR158. June 2010
6. Meehan, J et al. Suicide in Mental Health In-patients and within 3 Months of Discharge: National Clinical Survey. BJP 2006, 188: 129–134.
7. Powell, P et al. Suicide in Psychiatric Hospital In-patients. BJP 2000, 176: 266–272.

### 0169

#### Evaluation of a clasification tool for in-hospital triage of trauma patients

J. Sainz Cabrejas, C. García Fuentes, J. Barea Mendoza, M. Chico Fernández, J.C. Montejo González
H. U 12 de Octubre, Trauma and Emergency ICU, Madrid, Spain
**Correspondence:** J. Sainz Cabrejas

*Intensive Care Medicine Experimental* 2017, **5(Suppl 2):**0169

**INTRODUCTION.** In-hospital trauma care must be provided in different levels, according to patient's needs. Although classification of some patients with extremely serious or mild injuries may be simple, triage of others may be challenging. In several cases, *in-hospital partial trauma team activation* (HPTTA) is necessary because of the possibility of hidden life threatening injuries. Undertriage has been associated with an increase in mortality (OR 1.24). [1] Nevertheless, universal criteria for HPTTA are lacking.
In our institution, a classification tool based on *Field Triage Decision Scheme* of the *Centers for Disease Control and Prevention* (CDC) [1] is used in order to detect patients who may benefit from HPTTA.
**OBJECTIVES.** To evaluate the validity of our HPTTA criteria to detect patients in whom ICU admission is needed. To determine the frequency of undertriage and overtriage of each step of the triage process.
**METHODS.** This was a prospective cohort study including all patients requiring HPTTA at the moment of hospital arrival during a 56 month-period (May 1, 2012 through December 31, 2016). Patients arriving more than 30 minutes before activation were excluded. HPTTA criteria, data from physical examination, complementary tests and the need of ICU admission were recorded. There are four steps in triage process: step one: physiologic criteria, second: anatomic criteria, third: mechanism-of-injury criteria, and fourth: special considerations. For each criteria, sensitivity, specificity and under/overtriage rates (proportion of patients admitted to ICU who do not fulfill criteria and proportion of patients who fulfill criteria but ICU admission is not needed respectively) were calculated. The average number of patients who need to be evaluated to indicate and ICU admission (NND) was also determined. Analysis was performed using SPSS 16.0.
A total of 1548 patients were enrolled, 174 of which (11.24%) were admitted to ICU. 1170 patients were male (75.6%). The median age was 36 years old (IQR 27–47). HPTTA was indicated according to the first step in 97 patients, 164 to the second, 1101 to the third and 34 to the fourth. The proportion of patients admitted to ICU and the NND were respectively for each criteria 51.54% and 1.94 for the first; 21.34% and 4.68 for the second; 6.6% and 14.08 for the third and 8.82% and 11.69 for the fourth. Over/undertriage rates for each step of our triage process are summarized in Fig. 74.
**CONCLUSIONS.** *Field Triage Decision Scheme* implemented by CDC is a suitable tool to indicate HPTTA. First and second steps are associated with a high specificity while third and fourth ones present a high sensitivity, which is needed to decrease undertriage rate to acceptable values.

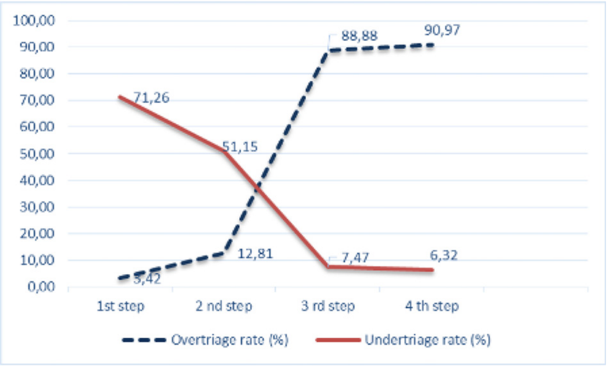

**Fig. 74 (abstract 0169)** Over/undertriage rates

**This abstract is now an oral abstract.**

### 0170

#### Tau protein as a diagnostic marker for diffuse axonal injury

K. Tomita, T.-A. Nakada, R. Abe, T. Motoshima, R. Kawaguchi, S. Oda
Chiba University, Emergency and Critical Care Medicine, Chiba, Japan
**Correspondence:** K. Tomita

*Intensive Care Medicine Experimental* 2017, **5(Suppl 2):**0170

**INTRODUCTION.** Diffuse axonal injury (DAI) is a severe traumatic brain injury (TBI), associated with poor outcome whose mortality exceeds 40% [(1)].However, diagnostics of DAI has not yet been established, especially in the early phase after injury. We hypothesized that serum level of tau protein, which was localized in neuronal axons, would be a useful biomarker to diagnose DAI.

**OBJECTIVES.** To determine the usefulness of serum tau protein measurement in early diagnosis of DAI in clinical setting and to elucidate relationship between severity of DAI and serum tau protein concentration, using an animal model.

**METHODS AND RESULTS.** We measured serum level of tau protein in patients suspected of DAI to evaluate its accuracy in the diagnosis of DAI and its prediction ability of neurological outcome at hospital discharge. DAI was diagnosed by clinical findings, CT scan and magnetic resonance imaging. Neurological outcome was evaluated using Glasgow Outcome Scale (GOS). Twenty-six patients were enrolled to the study. The serum tau levels were measured within 6 hours after TBI. The levels of serum tau in DAI group (n = 7) were significantly higher than those in non-DAI group (n = 19) (120.9 ± 207.9, 17.5 ± 57.2, respectively, P = 0.0007). A receiver-operating characteristic curve evaluating the diagnostic ability of serum tau level for DAI demonstrated an area under the curve of 0.853 with 89.5% for sensitivity and 85.7% for specificity. Concentration of serum tau was higher in unfavorable outcome group (GOS score = 1-3, n = 11) than in favorable outcome group (GOS score = 4-5, n = 15), but the difference was not statistically significant (P = 0.057). Next, using a rat model of DAI, we evaluated association between serum concentration of tau and severity of DAI. DAI was induced by dropping a weight in the head from a predetermined height (mild DAI group; 1 meter, severe DAI group; 2 meters), according to Marmarou's protocol[(2)]. Eighteen rats were divided into three groups, sham-operated (n = 4), mild DAI (n = 7) and severe DAI (n = 7). The serum levels of tau at 60 minutes after injury were statistically different among three groups (sham < mild < severe, sham vs. mild, p = 0.024; mild vs. severe, p = 0.018) .

**CONCLUSIONS.** Serum level of tau within 6 hours after TBI can be a useful biomarker for diagnosis of DAI. It may also be useful in predicting neurological outcome after TBI.

(1) Bruce DA, Alavi A, Bilaniuk L, et al.: Diffuse cerebral swelling following head injuries in children: the syndrome of "malignant brain edema". *J Neurosurg* 1981; 54: 170–178.
(2) Marmarou A, Foda MA, van den Brink W, et al.: A new model of diffuse brain injury in rats. Part I: Pathophysiology and biomechanics. *J Neurosurg* 1994; 80: 291–300

**GRANT**
Author has received a grant from Japan Society for the Promotion of Science(Grant Number JP15K20334) and The General Insurance Association of Japan(Grant Number 15-1-16).

### 0171

#### Intensive care unit with large trauma patient volume was associated to improved hospital mortality - a retrospective cohort study of the nationwide trauma database in Japan

A. Shiraishi[1], Y. Hayashi[2], Y. Otomo[3]
[1]Kameda Medical Center, Emergency and Trauma Center, Kamogawa, Japan; [2]Kameda Medical Center, Kamogawa, Japan; [3]Tokyo Medical and Dental University, Tokyo, Japan
**Correspondence:** A. Shiraishi

*Intensive Care Medicine Experimental* 2017, **5(Suppl 2):**0171

**INTRODUCTION.** Previous studies reported that increased patient volume in intensive care unit was associated to better patient survivability.

**OBJECTIVES.** To assess association of trauma patient volume in intensive care unit and hospital mortality.

**METHODS.** From the Japan Trauma Databank (JTDB, 244 hospitals) in Japan from the year of 2004 to 2014, this retrospective cohort study selected adult (≥16 y) hospitalized trauma patients in intensive care unit whose the injury severity score (ISS) ≥ 9. A logistic regression analysis after adjustment for age, mechanism of trauma, ISS assessed adjusted odds ratio of the quartile of patient volume in each intensive care unit (volume effect) for hospital mortality. Similarly, a generalized additive model after adjustment for the same baseline variables assessed adjusted odds ratio of patient volume in each intensive care unit as a continuous variable (Fig. 75).

**RESULTS.** Of a total of 183457 trauma subjects registered in JTDB, 74942 severely injured trauma patients hospitalized to the intensive care units and 9416 (12.6%) patients died during the hospitalizaton. Each the quartile of patient volume included 18356 (0-25th), 18489 (25-50th), 18583 (50-75th) and 19154 (75-100th) patients, respectively, and those hospital mortality were 30.4%, 25.2%, 21.0% and 23.4%, respectively. Adjusted odds ratio [95% confident interval] for hospital mortality in reference to the 25-50th quartile were 1.18 [1.11, 1.26] (0-25th quartile), 0.92 [0.86, 0.99] (50-75th quartile) and 0.96 [0.89, 1.03] (75-100th quartile), respectively.

**CONCLUSIONS.** Increased number of severe trauma patients volume negatively associated to risk of hospital mortality.

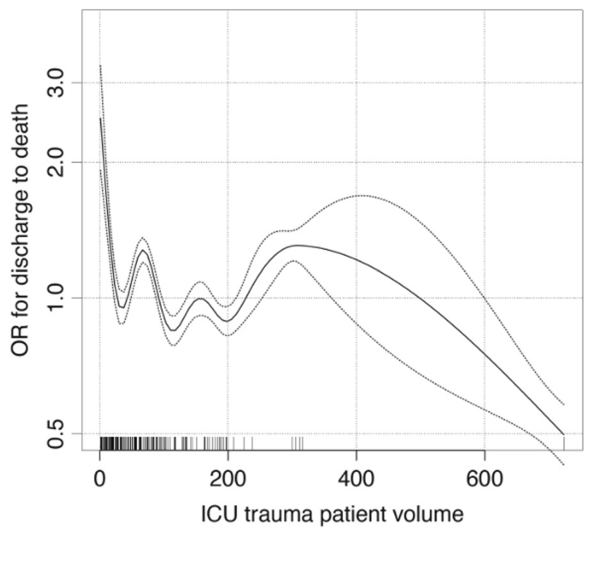

**Fig. 75 (abstract 0171)** Association of ICU trauma patients volume and mortality in generalized additive model

### 0172

#### What demand do major trauma patients put on the different surgical specialities?

P.A. Quinn[1], D. Lockey[2]
[1]University of Bristol, Medicine, Bristol, United Kingdom; [2]University of Bristol, Bristol, United Kingdom
**Correspondence:** P.A. Quinn
*Intensive Care Medicine Experimental* 2017, **5(Suppl 2):**0172

**INTRODUCTION.** The introduction of major trauma networks (MTN) into UK practice has led to a significant reduction in mortality (1). MTNs should have all surgical specialities on site and surgeons available to operate whenever required (2). In practice, some specialities aren´t always on the site of MTCs due to the development of specialist services in a region having many historical, political and geographical influences other than trauma care. The surgical demand from major trauma is poorly described and the lack of data makes it difficult to infer how often and how urgently surgeons are required. Therefore, it is difficult to fully assess whether the demand is met by the current supply of surgeons.

**OBJECTIVES.** This study aimed to quantify the major trauma load placed on different surgical specialities, focussing on urgent and non-urgent aspect of the interventions. It also discussed the implications of not having certain surgical specialities on site at the MTC.

**METHODS.** Anonymised datasets were retrieved from the Trauma Audit and Research Network spanning from 01/09/14 to 31/08/2016. Analysis of the registry provided a group of 1285 major trauma patients. Of these, 713 required at least one operation. To ensure quality of data, a random selection of 5% of the ISS > 15 who didn´t require an operation were analysed using patient records to certify that there were no misclassified cases.

**RESULTS.** The proportion of major trauma patients requiring surgery was lower than expected (55.49%). Neurosurgeons and orthopaedic surgeons were involved in most of the major trauma interventions (59.89% and 55.12% respectively). Due to the frequency and urgency of both specialities they must be on site. Cardiothoracic, general and maxillofacial surgeons were all required infrequently. However, general surgeons were needed urgently as 45.13% of their interventions were performed within 4 hours of arrival to the MTC. Two-thirds of the cardiothoracic interventions weren't urgent, but 31.43% of cardiothoracic-injured major trauma patients required thoracotomies- an urgent procedure. In this instance trained trauma team leaders performed the thoracotomies, negating the effect of not having cardiothoracics on site.

**CONCLUSIONS.** Having identified the urgency and frequency of the surgical requirement, some issues with training and exposure can be seen. By better understanding the surgical requirements of different health regions for major trauma, the organisation and delivery of major trauma care can be improved.

**REFERENCE(S)**

1. NHS England. NHS saves lives of hundreds more trauma victims just two years after changes to care - Independent audit NHS England; 2017 https://www.england.nhs.uk/2014/07/trauma-independent-audit/.
2. Surgeons CoT-ACo. Resources for optimal care of the injured patient. 6th ed. Chicago: American College of Surgeons; 2014. 215 p.

### 0173

#### Outcomes of trauma patients who receive a supra-massive red cell transfusion

C.G. Mason[1], C. Doughty[2], L. Green[2], C.J. Kirwan[1]
[1]Barts Health NHS Trust, Critical Care, London, United Kingdom; [2]Barts Health NHS Trust, Haematology, London, United Kingdom
**Correspondence:** C.G. Mason
*Intensive Care Medicine Experimental* 2017, **5(Suppl 2):**0173

**INTRODUCTION.** Patients suffering from traumatic haemorrhagic shock use substantial haematological resources. Features at presentation may be useful in helping influence clinicians' decision to continue resuscitation once certain transfusion thresholds have been reached.

**OBJECTIVES.** To identify factors associated with both a favourable and a poor outcome in trauma patients treated with a supra-massive blood transfusion.

**METHODS.** A retrospective analysis over 4.5 years (2012–2016) of patients receiving a supra-massive blood transfusion, defined as 15 units or more of red blood cells (RBC) in a 24 hour period, at the Royal London Hospital (largest trauma centre in UK). Electronic and paper patient notes were compared to data obtained from the transfusion laboratory.

**RESULTS.** We identified 49 trauma patients receiving a supra-massive transfusion; the median age was 29 (range 15–78) and 92% were male. 78% of patients died. Of the 11 who survived the median length of stay was 57 days (range 32–509). Survivors received a median 22 units RBC (range 15–30), non-survivors significantly more with a median 33 units RBC (range 15–74), p = 0.009. There were no survivors above 30 units RBC, as shown in graph.

10 patients had a co-existing traumatic brain injury; none of them survived. Survival of patients who had a thoracotomy was 11%, whilst those suffering a cardiac arrest had a survival of 8%. Isolated limb or pelvic injury was associated with a more favourable outcome with 70% of these patients surviving (See Table 59).

**CONCLUSIONS.** Traumatic brain injury, cardiac arrest and need for a thoracotomy are poor prognostic features when in combination with a supra-massive transfusion requirement. An isolated limb or pelvic injury has a much better chance of survival.

We propose that clinicians stop to review a patient's favourable and unfavourable features when resuscitation has reached 15units of RBC; ie at the point of a supra-massive transfusion. This could help decide whether or not to continue. Palliation should be considered once 30 units of RBC have been transfused in this group of patients.

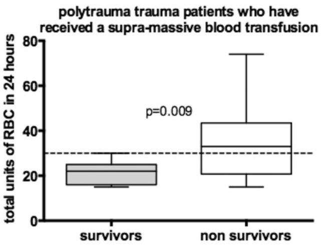

**Fig. 76 (abstract 0173)** Units Red Blood Cells; Survivors vs Non-survivors

**Table 59 (Abstract 0173)**. Comparison of survivors and non-survivors

| | Survivors | Non-survivors | Survival chance | |
|---|---|---|---|---|
| | | | With | Without |
| No. (%) | 11 (22) | 38 (78) | | |
| Age - median (range) | 32 (19-68) | 27 (15-78) | | |
| Intubated on arrival - No. (%) | 3 (27) | 30 (66) | 9% | 50% |
| Thoracotomy No. (%) | | | 11% | 30% |
| - pre-hospital | 0 (0) | 15 (40) | | |
| - in theatre | 2 (18) | 2 (5) | | |
| Cardiac Arrest No. (%) | | | 8% | 28% |
| - pre-hospital | 0 (0) | 10 (26) | | |
| - in theatre | 1 (9) | 2 (5) | | |
| Traumatic Brain Injury | | | 0% | 28% |
| - No. (%) | 0 (0) | 10 (26) | | |
| Isolated Limb/Pelvic Injury | | | 70% | 10% |
| - No. (%) | 7 (64) | 3 (8) | | |
| Received >30 units RBC | | | 0% | 38% |
| - No. (%) | 0 (0) | 20 (53) | | |

### 0174

#### Changes of para-, meta- and ortho-tyrosine over time in burned patients

P. Kovács[1], L. Szélig[1], S. Kun[2], C. Loibl[1], G.A. Molnár[2], I. Wittmann[2], L. Bogár[1], A. Miseta[3], C. Csontos[1]
[1]University of Pécs, Clincal Center, Department of Anaesthesiology and Intensive Care, Pécs, Hungary; [2]University of Pécs, Clincal Center, 2nd Department of Medicine and Nephrological Centre, Pécs, Hungary; [3]University of Pécs, Clincal Center, Department of Laboratory Medicine, Pécs, Hungary
**Correspondence:** P. Kovács
*Intensive Care Medicine Experimental* 2017, **5(Suppl 2):**0174

**INTRODUCTION.** Burn injury is associated with oxidative stress which can lead to production of abnormal amino acids (meta- and ortho-tyrosine).

**OBJECTIVES.** We aimed to assess the time course of tyrosine isoforms in blood and urine samples of burned patients and investigate their renal handling.

**METHODS.** Fifteen patients were involved in our study. Fifteen healthy individuals served as controls. Blood samples were taken on admission and on the next 4 consecutive days. Urine samples were collected for 24 hours. Tyrosine isoforms were determined using liquid chromatography with fluorescence detection.

**RESULTS.** Decreased serum para-tyrosine (normal isoform) levels were found in burned patients on days 2–5 ($p < 0.01$). Serum meta-tyrosine levels showed a significant elevation on days 2–4 ($p < 0.05$) similarly to ortho-tyrosine. Urinary para-tyrosine excretion tendency was increasing ($p < 0.01$), as well as its fractional excretion ($p = 0.001$). No significant tendency was found on fractional excretion of meta- and ortho-tyrosine. Urinary para-tyrosine excretion and fractional excretion were higher on days 2–5 ($p < 0.05$), urinary ortho-tyrosine fractional excretion was higher on days 1, 2 and 5 ($p < 0.05$) compared to controls. Significant positive correlations were found between serum meta-tyrosine and procalcitonin levels ($p < 0.05$) and between para-tyrosine and injury severity indexes ($p < 0.05$). Burned surface and fractional excretion of meta-, and ortho-tyrosine showed positive correlation ($p < 0.01$).

**CONCLUSIONS.** Increased serum meta- and ortho-tyrosine levels might be consequence of the oxidative stress similarly to the decreased serum para-tyrosine levels. Increased fractional excretion of ortho-tyrosine suggests synthesis of it in kidneys, whereas, increased fractional excretion of para-tyrosine might be sign of disturbed reabsorption.

**REFERENCE(S)**

Ritter C, Andrades M, Guerreiro M, et al. Plasma oxidative parameters and mortality in patients with severe burn injury. Intensive Care Med 2003 29:1380–3.
Molnár GA, Kun S, Sélley E et al. Role of Tyrosine Isomers in Acute and Chronic Diseases Leading to Oxidative Stress - A Review. Curr Med Chem. 2016 23(7):667–85.
Galano A, Cruz-Torres A. HO radical reactions with phenylalanine in free and peptide forms. Org Biomol Chem 2008 6:732–738.

**GRANT ACKNOWLEDGMENT**
SROP 4.2.4. A/2-11- 1-2012- 0001 'National Excellence Program'.

### 0175

#### Low-perfusion pressure-induced tissue hypoperfusion does not contribute to hyperfibrinolysis in disseminated intravascular coagulation associated with isolated traumatic brain injury

K. Katabami, T. Tsuchida, T. Wada, K. Maekawa, H. Sageshima, M. Hayakawa, A. Sawamura, S. Gando
Hokkaido University Hospital, Sapporo, Japan
**Correspondence:** K. Katabami
*Intensive Care Medicine Experimental* 2017, **5(Suppl 2):**0175

**INTRODUCTION.** Disseminated intravascular coagulation (DIC) during the early phase of isolated traumatic brain injury falls under the fibrinolytic phenotype.

**OBJECTIVE.** The aim of this study was to investigate the role of tissue hypoperfusion and brain damages on hyperfibrinolysis in DIC associated with isolated traumatic brain injury (iTBI), leading to poor outcome.

**METHODS.** iTBI was defined as an abbreviated injury scale of the head ≥3 and other body parts ≤ 2. Ninety-two patients with iTBI were retrospectively included in the present study. These patients were divided into DIC ($n = 45$) and non-DIC ($n = 47$) groups based on the Japanese Society for Acute Medicine DIC criteria on admission (day 0). The DIC patients were further subdivided into those with and without hyperfibrinolysis based on fibrin/fibrinogen degradation products (FDP) levels > 100 μg/mL. The FDP/D-dimer ratio was used as surrogate marker of fibrin(ogen)olysis. Systemic hypoperfusion was defined as a blood lactate level of ≥ 4 mmol/L and the severity of injury was assessed by the injury severity score. The sequential organ failure assessment (SOFA) and systemic inflammatory response syndrome (SIRS) scores were also evaluated. The main outcome measure was the all-cause hospital mortality.

**RESULTS.** All DIC patients developed SIRS. The SOFA scores of the DIC patients were significantly higher than those of the patients without DIC, leading to worse outcome. These changes were more prominent in DIC patients with hyperfibrinolysis. FDP/D-dimer ratios in DIC with hyperfibrinolysis were significantly higher than those of non-DIC patients. The mean blood pressures were identical between DIC and non-DIC patients and DIC with and without hyperfibrinolysis.

However, a significant difference was observed in the lactate levels between the DIC and non-DIC groups (5.3 [2.5] vs. 3.2 [1.6]), which was increased to a greater degree in DIC with hyperfibrinolysis. A logistic regression analysis revealed that hyperfibrinolysis is an independent predictor of death in iTBI patients, and a Kaplan-Meyer curve showed a significantly lower survival rate in DIC patients with hyperfibrinolysis than in those without hyperfibrinolysis. However, neither hypoperfusion nor brain damage was identified as an independent predictor of hyperfibrinolysis.

**CONCLUSION.** DIC with hyperfibrinolysis contributes to organ dysfunction and a poor outcome in patients with iTBI. DIC-induced low-flow-related hypoperfusion but not low-blood pressure-related hypoperfusion may be the cause of the increased lactate levels observed in this type of DIC.

### 0176

#### Should resuscitation in critical burn patients be started based on parkland formula?

E.M. Flores Cabeza, K.L. Nanwani Nanwani, N.A. Suarez Rovira, A. Saravia Moya, E. Herrero de Lucas, M. Sánchez Sánchez, A. Garcia de Lorenzo y Mateos
Hospital Universitario La Paz, Intensive Care Medicine, Madrid, Spain
**Correspondence:** E.M. Flores Cabeza
*Intensive Care Medicine Experimental* 2017, **5(Suppl 2):**0176

**INTRODUCTION.** The Parkland Formula has been used as gold standard for calculation of initial fluid resuscitation in severe burn patients (4 ml x TBSA burned x kg weight for 24 hours, half of that amount being given during the first 8 hours) adjusting the following requirements by urine output.

We studied the initial fluid administration needed when we used a resuscitation protocol guided by transpulmonary thermodilution and lactate levels and compared it with estimated fluid replacement with Parkland formula.

**METHODS.** We studied 84 severe burn patients resuscitated with Ringer lactate and after 12–18 hours we added colloids guided by a protocol based on transpulmonary thermodilution and lactate levels. We then measured fluid administration, hourly urinary output, and transpulmonary thermodilution parameters. Laboratory analyses were performed every 8 h.

**RESULTS.** The study included 84 patients with burns in 20% to 90% of the TBSA, aged between 18 and 85 years. The mean age was 45 ± 15 years, the mean percentage of TBSA was 36 ± 15% and the mean ABSI was of 8.2 ± 1.8. The mortality was 13%.

Fluid administration: The mean infusion rate per hour in the initial 8 hours after burn was 0,167 ± 0,117 ml /kg/hour per % of burned TBSA, lower than the Parkland formula estimation to be given in these first 8 hours (0,25 ml /kg/hour x % burn). During the period between 8 to 16 hours, and 16 to 24 hours after burn the mean infusion rate per hour was 0,165 ± 0,089 and 0,156 ± 0,095 respectively (Parkland formula estimates 0,125 ml /kg/hour per % of burned TBSA between 8 and 24 hours after the burn). During the 24–32 hours after burn the mean infusion rate per hour was 0.144 ± 0.076.

ITBV Index: in the initial 8 hours after burn; 8 to 16 h; 16–24 h and 24–32 h after burn, the results were 715 ± 256; 705 ± 225; 740 ± 262 and 754 ± 269 respectively.

EVLW Index: in the initial 8 hours after burn; 8–16 h; 16–24 h and 24–32 h after burn the results were 7.05 ± 2.6; 7.2 ± 2.7; 7.6 ± 3.6 and 8.5 ± 4.7 respectively

Cardiac Index: in the initial 8 hours after burn; 8–16 h; 16–24 h and 24–32 h after burn the results were: 2,74 ± 1; 2.72 ± 0.9, 2.9 ± 1.0 and 3,3 ± 1.1 respectively

Lactate levels: in the initial 8 hours after burn; 8–16 h; 16–24 h and 24–32 h after burn the results were: 2.8 ± 2; 2.5 ± 1.7; 2.5 ± 1.9 and 2.3 ± 1.7 respectively

Our patients required a relatively constant infusion of volume over the first 24 hours. There was no need for fluid addition during the first 8 hours as advised in the Parkland formula. This lower fluid intake did not deteriorate the preload (ITV 715 vs 705), maintaining an acceptable cardiac index as well as an adequate lactate clearance.

**CONCLUSIONS.** Starting with a higher fluid administration in the initial phase of resuscitation in severe burn patients can lead to an unnecessary supply of liquid when the phase of altered permeability has not yet occurred.

Resuscitation is a dynamic process and fluid administration should be guided according to the haemodynamic needs of each moment.

### 0177

#### Red cell distribution width at burn ICU admission and 5 year mortality in hospital survivors

C.V. Murphy[1], S.L. Nitzschke[2], A.C. Offodile[3], F.K. Gibbons[4], A. Salim[2], K.B. Christopher[5]
[1]Ohio State University Wexner Medical Center, Department of Pharmacy, Columbus, United States; [2]Brigham and Women's Hospital, Division of Trauma, Burn, and Surgical Critical Care, Boston, United States; [3]Johns Hopkins Bloomberg School of Public Health, Baltimore, United States; [4]Massachusetts General Hospital, Pulmonary and Critical Care Division, Boston, United States; [5]Brigham and Women's Hospital, Renal Division, Boston, United States
**Correspondence:** K.B. Christopher
*Intensive Care Medicine Experimental* 2017, **5(Suppl 2):**0177

**INTRODUCTION.** Little is known about long term survival risk factors in critically ill burn patients who survive hospitalization. Utilizing biomarkers elevated with chronic inflammation such as the red cell distribution width may inform risk prediction related to pre-existing disease states.

**OBJECTIVES.** We hypothesized that elevated red cell distribution width (RDW) at Burn ICU admission would be predictive of long term outcomes in hospital survivors.

**METHODS.** We performed a two center observational cohort study between 1998–2007 in 340 critically ill adult burn patients who survived to hospital discharge. The exposure of interest was RDW at Burn ICU admission and categorized a priori as ≤13.3% (normal), 13.3-14.0%, 14.0-14.7% and >14.7%. The primary outcome was all-cause 5 year mortality based on the US Social Security Administration Death Master File. Adjusted associations were estimated through fitting of multivariable logistic regression models. Time-to-event analysis was performed using Cox proportional hazard regression.

**RESULTS.** Of the cohort patients studied, 76% were male, 29% were non white, 14% were over 65, 41% had TBSA > 20%, and 44% had inhalational injury. The mean age was 45, 93% had partial thickness burns, 62% had full thickness burns, 28% received vasopressors, and 26% had sepsis. The mean TBSA was 20.5%. The mean Baux score was 65.1. Post hospital discharge 5 year mortality rate was 9.4%. The 30 day hospital readmission rate was 4%. Patients with RDW >14.7% had a significantly higher Baux score (74 vs 64), and higher development of inhalational injury and sepsis (all $P < 0.05$). In a logistic regression model adjusted for Charlson comorbidity index, gender, presence of full thickness burn, inhalational injury and the Baux score, patients with a RDW of 14.0-14.7% or RDW >14.7% at Burn ICU admission have an adjusted OR of 5-year post discharge mortality of 6.0 (95%CI, 1.4-25.5; P = 0.017) or 11.4 (95%CI, 3.2-40.7; P < 0.001) respectively, relative to patients with an RDW ≤13.3%. The adjusted model showed good discrimination (AUC 0.89) and calibration (Hosmer-Lemeshow $\chi 2$ P = 0.67). Cox proportional hazard multivariable regression modeling showed that Burn ICU admission RDW was predictive of mortality following hospital admission [RDW of 14.0-14.7% HR = 8.9 (95% CI 2.6-30.7; P = 0.001); RDW >14.7% HR = 11.1 (95% CI 3.6-34.4; P < 0.001)].

**CONCLUSIONS.** Burn patients with elevated RDW at ICU admission have increased 5 year mortality compared to those with normal RDW. The elevated RDW at the time of burn injury likely reflects a preexisting proinflammatory or oxidative stress state, or a combination thereof which may be reflective in long term recovery and outcomes.

### 0178

#### Clinical profile of intra-pelvic major arterial bleeding in patients with pelvic fracture

M. Kujo[1], N. Saitou[1], H. Kawamata[2], Y. Oda[1], H. Nakayama[1], K. Okada[1], T. Ootaguro[1], T. Motomura[1], T. Yagi[1], Y. Hara[1], H. Matsumoto[1], H. Yokota[3]
[1]Nippon Medical School Chiba Hokusoh Hospital, Emergency, Inzai Chiba, Japan; [2]Nippon Medical School Chiba Hokusoh Hospital, Radiology, Inzai Chiba, Japan; [3]Nippon Medical School Hospital Emergency and Critical Care Medicine, Emergency, Tokyo, Japan
**Correspondence:** M. Kujo
*Intensive Care Medicine Experimental* 2017, **5(Suppl 2):**0178

**INTRODUCTION.** In patients with pelvic fracture (PF), emergency angiography (AG) is a first process to treat intra-pelvic major arterial bleeding (IPMB). The typical indication of AG for these patients is unstable vital signs, but no clear consensus has been reached. We aimed to clarify the association between initial clinical profile and IPMB in patients with PF.

**METHODS.** A single-center retrospective study was conducted from January 2012 to December 2015. Among the 3,354 trauma patients admitted to a Japanese civilian trauma center, 390 had PF caused by a blunt mechanism during the study period. One hundred seven adult patients who underwent AG were included in this study. Patients who had no computed tomography (CT) scan or surgical intervention available before AG for PF were excluded. We classified patients into two groups according to the presence (E group, n = 70) or absence (NE group, n = 37) of contrast extravasation on AG as a diagnosis of IPMB and compared clinical profiles.

**RESULTS.** The median age of the patients was 66 years (interquartile range: 56–76 years), and their median injury severity score (ISS) was 34 (22–50). The percentage of the patients who had embolization was 76.6%. Univariate analysis indicated that age, the size of contrast leakage on enhanced CT scan and D-dimer on admission were higher in group E than in group NE. Admission vital signs were similar in both groups. The median vertical size of the contrast leakage (VSCL) was lager in the E group than in the NE group (E: 20 mm, NE: 0 mm, P = 0.021). Multivariate regression analysis revealed that the associated factors of IPMB were size of the VSCL (odds ratio [OR]: 0.316, 95% confidence interval [CI]: 0.119-0.838; P = 0.021) and D-dimer level (OR: 1.013, 95% CI: 1.003-1.023; P = 0.012). The area under the curve of the receiver-operating characteristics curve for arterial bleeding in VSCL was 0.68 (95% CI: 0.57-0.78). The cutoff value for VSCL from the sensitivity-specificity curve for IPMB was 10 mm (Sensitivity: 50%, specificity 75.4%).

**CONCLUSIONS.** VSCL on enhanced CT scans and D-dimer level on admission were associated with IPMB in patients with PF. These factors may be reasonable as additional information in selecting AG for PF.

### 0179

#### Quick sequential organ failure assessment score to predict in-hospital mortality among patients transported by physician-staffed helicopter

K. Miyamoto, N. Shibata, S. Kato
Wakayama Medical University, Wakayama, Japan
**Correspondence:** K. Miyamoto
*Intensive Care Medicine Experimental* 2017, **5(Suppl 2):**0179

**INTRODUCTION.** In the physician-staffed helicopter setting, on-the-scene prediction of patient mortality is important for the selection of an appropriate hospital. Recently, quick sequential organ failure assessment score (qSOFA) has been proposed for use as a simple prediction tool for mortality among patients with sepsis [1].

**OBJECTIVES.** This study evaluated the predictive ability of qSOFA for in-hospital mortality among patients who were transported by physician-staffed helicopters.

**METHODS.** We conducted a single center retrospective observational study using the physician-staffed helicopter registry data between 2003 and 2016. Out of 2453 adult patients transported to our hospital by helicopter, we excluded 604 patients because of missing information on patient outcomes and vital signs; hence, we included 1849 patients in our study. qSOFA score was evaluated for predicting in-hospital mortality in various diagnostic categories by the receiver operating characteristics curve analysis.

**RESULTS.** Median age of the patients was 66 (interquartile range 52–78) years; 1285 (69.5%) patients were men. Diagnostic categories included trauma (1038 patients, 56.1%), stroke (456 patients, 24.7%), cardiovascular disease (134 patients, 7.2%), and sepsis (50 patients, 2.7%). In-hospital mortality occurred in 169 patients (9.1%). In-hospital mortality rate among patients with qSOFA score 0, 1, 2 and 3 were 5 / 411 (1.2%), 69 / 797 (8.7%), 71 / 541 (13.1%), and 24 / 100 (24.0%), respectively. Area under the curve (AUC) was 0.67 (95% confidential interval [CI] [0.63, 0.71]) in all patients. AUC in patients with sepsis, trauma, stroke and cardiovascular disease were 0.66 (95% CI [0.43, 0.83]), 0.75 (95% CI [0.70, 0.80]), 0.58 (95% CI [0.52, 0.64]) and 0.66 (95% CI [0.38, 0.86]), respectively.

**CONCLUSION.** As the qSOFA score increased, the mortality rate gradually increased. In particular, patients with 0 score of qSOFA exhibited very low mortality rate. In-hospital mortality was well predicted in patients with trauma.

**GRANT ACKNOWLEDGMENT**

The present study was conducted without any support from funding agencies in the public, commercial or not-for-profit sectors.

### 0180

#### Serum glial fibrillary acidic protein is potential screening biomarker of CT positive mild to moderate traumatic brain injury in emergency department

T. Kaneko[1,2], H. Tanaka[1], T. Era[2], K. Karino[2], S. Yamada[2], M. Kitada[2], T. Sakurai[2], M. Harada[2], F. Kimura[2], T. Takahashi[2], S. Kasaoka[1]
[1]Kumamoto University Hospital, Emergency and General Medicine, Kumamoto, Japan; [2]Kumamoto Medical Center, Emergency and Critical Care Center, Kumamoto, Japan
**Correspondence:** T. Kaneko
*Intensive Care Medicine Experimental* 2017, **5(Suppl 2):**0180

**INTRODUCTION.** Glial fibrillary acidic protein (GFAP) is promised biomarker of traumatic brain injury (TBI) with positive head computed tomography (CT) findings (J Neurotrauma 2015;32:527–33, J Neurotrauma 2013;30:1490–7). On the other hand, traditional indication of head CT is decided by Glasgow coma scale (GCS).

**OBJECTIVES.** In the present study, in order to help avoid unnecessary head CT, serum GFAP and GCS were compared to judge which indication would predict positive CT findings in mild to moderate TBI patients of emergency department.

**METHODS.** All TBI cases were measured serum GFAP on admission, and all cases were performed head CT. Serum GFAP and GCS on admission were compared by receiver operating curve (ROC) analysis, univariate and multivariate analysis.

**RESULTS.** 57 cases of TBI cases were analyzed. Median age was 70 y.o. and 39% males. Median serum GFAP and GCS were 0.11 ng/mL and 15, respectively. 12 cases (21%) had positive head CT findings. ROC analysis showed that area under the curve (AUC) of GFAP v.s. GCS were 0.851 v.s. 0.756, respectively.

**CONCLUSIONS.** Serum GFAP showed the potential predictability of positive CT findings in TBI cases, which might be superior to GCS based on AUC data by ROC analysis.

**REFERENCE(S)**

McMahon PJ, et al.: Measurement of the glial fibrillary acidic protein and its breakdown products GFAP-BDP biomarker for the detection of traumatic brain injury compared to computed tomography and magnetic resonance imaging. J Neurotrauma 2015;32:527–33,
Okonkwo DO, et al.: GFAP-BDP as an acute diagnostic marker in traumatic brain injury: results from the prospective transforming research and clinical knowledge in traumatic brain injury study. J Neurotrauma 2013;30:1490–7

**GRANT ACKNOWLEDGMENT**

Grant by the Japanese Ministry of Health, Labor and Welfare (Grant no. 16K11409)

### Sepsis therapeutics 1

#### 0181

#### Norepinephrine (NE) requirements are associated with prognosis in sepsis-3 (S-3) septic shock patients independent of the blood pressure (BP) profile at ICU admission

D. Carpio, E.D. Valenzuela Espinoza, M. Silva, M. Vera, G. Ferri, L. Alegría, C. Santis, F. Araneda, R. Castro, J. Dreyse, S. Bravo, J. Bakker, G. Hernández
Pontificia Universidad Católica de Chile, Departamento de Medicina Intensiva, Facultad de Medicina., Santiago, Chile
**Correspondence:** D. Carpio
*Intensive Care Medicine Experimental* 2017, **5(Suppl 2):**0181

**INTRODUCTION.** S-3 consensus defined septic shock as NE requirements to maintain mean arterial pressure (MAP) > 65 mmHg and a serum lactate level >2 mmol/L despite fluid resuscitation, and claimed that these criteria will identify patients with a homogeneous high risk of death, allegedly in excess of 40% [1]. However, a recent study challenged this assumption demonstrating that the presence of a hypoperfusion context determined huge differences in mortality and morbidity among septic shock patients with similar lactate levels [2]. The purpose of this study was to explore if some hemodynamic parameters such as admission BP profile and NE requirements were independently associated to morbidity or mortality in S-3 septic shock patients.

**METHODS.** Retrospective analysis of a prospectively filled database of ninety S-3 septic shock patients treated at an academic ICU. Local IRB approved the study and waived the requirement of informed consent.

Patients were classified as follows according to admission BP profile: 1) Vasoplegic patients, diastolic blood pressure (DP) < 50 mmHg; 2) Patients with low pulse pressure (PP) < 50 mmHg and 3) mixed. These subgroups were compared according to demographic, hemodynamic, perfusion parameters, NE requirements. and outcome data. Statistical analysis, the distribution of continuous variables was explored using the Kolmogorov-Smirnov test. Student´s *t* test and Mann Whitney test were used to analyzing parametric and non-parametric continuous variables, respectively. Exact Fisher test was used for qualitative variables. Multivariate analysis was performed to determine the independent associations between NE requirements and comorbid variables. Statistical analyses were performed using IBM SPSS Statistics 20.

**RESULTS.** The main results are expressed in the Table 60

The BP profile at admission was not associated to prognosis. Multivariate analysis demonstrated that only NE doses > 0.1 ug/kg/min were associated to longer ICU stay and MV days after adjusting to lactate levels, p = 0.023 and p = 0.012, respectively.

Values are expressed as mean ± SD or median (interquartile range) or N (%); ICU, intensive care unit; LOS, length of stay; MV, mechanical ventilation; RRT, renal replacement therapy; * (p < 0.05) comparing group 1 and 2; **(p < 0.01).

**CONCLUSIONS.** S-3 septic shock patients with a vasoplegic profile require higher NE doses but do not present a worse prognosis as compared to patients with low pulse pressure at admission. NE requirements >0.1 ug/kg/min are associated with outcome, again challenging the homogeneity proclaimed by sepsis-3 consensus.

**Table 60 (Abstract 0181)**. See text for description

| | All | Group 1 (low DP) | Group 2 (low PP) | Group 3 (mixed) |
|---|---|---|---|---|
| n | 90 | 22 | 45 | 14 |
| Age (year) | 66 ± 16 | 70 ± 15 | 64 ± 16 | 67 ± 14 |
| APACHE II score/SOFA score | 21 ± 7/9 ± 4 | 23 ± 7/10 ± 3 | 20 ± 6/9 ± 3 | 21 ± 6/10 ± 3 |
| Lactate (mml/L) | 4.0 [2.9,5.8] | 3.5 [2.7,6.4] | 4.1 [3.0,4.9] | 3.9 [3.0,6.7] |
| NE dose (ug/kg/min) | 0.10 [0.02,0.29] | 0.16 [0.09,0.32]* | 0.13 [0.05,0.30] | 0.24 [0.12,0,44]** |
| MAP (mmHg) | 76 ± 19 | 58 ± 9 | 68 ± 17* | 55 ± 9** |
| ICU LOS (days)/MV (days) | 8 [6,14]/5 [3,9] | 8 [5,11]/4 [1,7] | 9 [7,12]/6 [3,9] | 8 [5,11]/4 [3,8] |
| RRT, (%) | 17 (19) | 4 (18) | 10 (22) | 4 (28) |
| Mortality, (%) | 12 (13) | 3 (14) | 4 (8) | 2 (14) |

#### 0182

#### Improved inflammation with dexmedetomidine in patients with sepsis required mechanical ventilation: a sub-analysis of the DESIRE Trial

Y. Ohta[1], Y. Kawazoe[2], K. Miyamoto[3], H. Yamamura[4], T. Morimoto[1], DESIRE Trial Investigators
[1]Hyogo College of Medicine, Nishinomiya, Hyogo, Japan; [2]Tohoku University Graduate School of Medicine, Sendai, Miyagi, Japan; [3]Wakayama Medical University, Wakayama, Wakayama, Japan; [4]Hirosaki University Graduate School of Medicine, Hirosaki, Aomori, Japan
**Correspondence:** Y. Ohta
*Intensive Care Medicine Experimental* 2017, **5(Suppl 2):**0182

**INTRODUCTION.** Dexmedetomidine was reported to improve immune system interactions such as cytokine and organ systems such as brain dysfunction, cardiovascular, lung injury, liver dysfunction and kidney injury in animal model of sepsis. There were limited data on the effects of dexmedetomidine on CRP or PCT as inflammation and Sequential Organ failure Assessment (SOFA) score as organ failure.

**OBJECTIVES.** To evaluate whether administration of dexmedetomidine could improve inflammation and organ failure in patients with sepsis requiring mechanical ventilation.

**METHODS.** DExmedetomidine for Sepsis in Intensive care unit Randomized Evaluation (DESIRE) Trial was randomized controlled trial to examine whether a sedation strategy with dexmedetomidine (DEX group) relative to without dexmedetomidine (non-DEX group) could improve clinical outcomes in patients with sepsis requiring mechanical ventilation. We compared CRP (days 1, 2, 4, 6, 8, 10, 12, 14); PCT (days 1, 4, 8, 14); and SOFA score (days 1, 2, 4, 6, 8, 10, 12, 14) during ICUs between groups in this sub-analysis. We assessed time to improvement of inflammation (CRP < 5 mg/dL and PCT < 0.5 ng/mL) with competing risk of death and censoring of discharge from ICUs. We used the Cox proportional hazards model to estimate the hazard ratio (HR) and 95%

confidence interval (CI) of DEX group against non-DEX group with the competing risk of death. We also used generalized linear models to estimate the overall differences in CRP and PCT between groups. We also used Wilcoxon rank sum test for SOFA scores at each day.

**RESULTS.** We includes 201 patients; the DEX group was 100 patients and the non-DEX group was the 101 patients. Mean age was 69 (standard deviation, 14) years and 63% were male. The number of abdominal infection was 74 (37%), thorax infection including pneumonia was 72 (36%), and other site infection was 55 (27%). Improvement of inflammation at 14 days was not significantly different (CRP < 5 mg/dL: 66% vs 54%, p = 0.15; PCT < 0.5 ng/mL: 51% vs 44%, p = 0.35). The HRs of DEX group were 1.23 (95% CI: 0.91-1.77) for CRP < 5 mg/dL and 1.16 (95% CI: 0.80-1.70) for PCT < 0.5 ng/mL. However, overall CRP and PCT during ICU stay were significantly lower in DEX group than non-DEX group (p = 0.03 and 0.04, respectively). Total SOFA score was not significantly different between DEX group and non-DEX group (day 1; 8 vs. 9, day 2; 9 vs. 9, day 4; 8 vs. 8, day 6; 8 vs. 7, day 8; 7 vs. 5, day 10; 6 vs. 6, day 12; 5 vs. 5.5, day 14; 4.5 vs. 4). The SOFA score for each organ system was not also significant different.

**CONCLUSIONS.** The sedation strategy with dexmedetomidine was associated with lower inflammation status during ICU stay than that without dexmedetomidine in patients with sepsis requiring mechanical ventilation. However, the time to improvement of inflammation and organ failure were not different between groups.

**GRANT ACKNOWLEDGMENT**

None declared

### 0183

#### Does dexmedetomidine increase lactate clearance in patients with septic shock? A sub-analysis of multicenter randomized controlled trial

T. Nakashima[1], K. Miyamoto[1], N. Shima[1], S. Katou[1], Y. Kawazoe[2], Y. Ohta[3], T. Morimoto[4], H. Yamamura[5]

[1]Wakayama Medical University, Department of Emergency and Critical Care Medicine, Wakayama, Japan; [2]Tohoku University Graduate School of Medicine, Division of Emergency and Critical Care Medicine, Sendai, Japan; [3]Hyogo College of Medicine, Division of General Medicine, Department of Internal Medicine, Nisinomiya, Japan; [4]Hyogo College of Medicine, Department of Clinical Epidemiology, Nishinomiya, Japan; [5]Hirosaki University Graduate School of Medicine, Department of Disaster and Critical Care Medicine, Hirosaki, Japan

**Correspondence:** T. Nakashima

*Intensive Care Medicine Experimental* 2017, **5(Suppl 2):**0183

**INTRODUCTION.** Lactate clearance is useful in resuscitating septic shock patients (1). Dexmedetomidine increased the lactate clearance in an experimental sheep model (2).

**OBJECTIVES.** To evaluate if dexmedetomidine increased lactate clearance in septic shock patients.

**METHODS.** DESIRE was a randomized controlled trial enrolling 201 mechanically ventilated adult patients with sepsis to test sedation strategy with and without dexmedetomidine in 8 ICUs in Japan (3). We included 111 septic shock patients (60 in the dexmedetomidine group and 51 in non-dexmedetomidine group) to evaluate the effect of dexmedetomidine on lactate clearance. A linear regression model, adjusted for baseline lactate value, was used to assess the effect of dexmedetomidine on lactate clearance at 6 h after randomization.

**RESULTS.** Mean age was 71.0 ± 13.4 years. Median Acute Physiology and Chronic Health Evaluation II score was 25 (interquartile range 19–31). The use group had a lower median serum lactate value at randomization than the non-use group (4.0 vs. 4.8 mmol/L). Lactate clearance at 6 h was higher in the use group, but was not significantly different (23.3 ± 29.8 vs. 11.1 ± 54.4; mean difference, 12.2; 95% confidence interval [CI], 4.4-28.8; *P* = 0.15). The multivariable model indicated lactate clearance at 6 h was significantly higher in the use group (adjusted mean difference, 18.5; 95% CI, 2.2-34.9; *P* = 0.03). There was no significant difference in 28-day mortality (13 [22%] in the use group and 18 [35%] in the non-use group, *P* = 0.11).

**CONCLUSIONS.** Compared with a sedation strategy without dexmedetomidine, a sedation strategy with dexmedetomidine increased lactate clearance in mechanically ventilated patients with septic shock. The increased lactate clearance effect of dexmedetomidine might be associated with the improved survival in sepsis patients.

**REFERENCE(S)**

1. Gu WJ, Zhang Z, Bakker J. *Intensive care medicine* 2015;41(10):1862–1863.

2. Hernandez G, Tapia P, Alegria L, et al. *Critical care* (London, England) 2016;20(1):234.

3. Kawazoe Y, Miyamoto K, Morimoto T, et al. *JAMA*. 2017;317(13):1321–1328.

**GRANT ACKNOWLEDGMENT**

The original study was partially supported by Hospira Japan that also provided a non-contractual research grant to Wakayama Medical University. However, the present study was conducted soley by the academic investigators without any support from pharmaceutical companies

### 0184

#### The effect of noradrenaline on resting energy expenditure in septic shock mechanically ventilated patients measured via indirect calorimetry, a prospective observational study

W. Hamimy, A. Zaghloul, S. Osman, Y. Albasha

Cairo University, Cairo, Egypt

**Correspondence:** A. Zaghloul

*Intensive Care Medicine Experimental* 2017, **5(Suppl 2):**0184

**INTRODUCTION.** Prediction of resting energy expenditure (REE) in septic shock patients is crucial to determine the optimal caloric requirement for patients on vasopressor therapy(1),(2). Many factors may affect the resting energy expenditure (REE) through manipulation of oxygen consumption (VO2) (3).

**OBJECTIVES.** To determine the effect of noradrenaline on REE and to compare the REE measured via indirect calorimetry and REE calculated by the Harris-Benedict equation.

**METHODS.** This prospective, observational study was conducted in the 24- bed trauma-surgical ICU at Cairo University Hospital. REE was measured and the corresponding vasopressor level was recorded on admission (or upon starting vasopressors in the ICU department), then after 24 hours and 48 hours thereafter. Measurements were taken with the patient lying supine and ventilator settings left unchanged for at least 60 minutes ahead of indirect calorimetry. The Harris Benedict equation for prediction of energy expenditure was also calculated(1).

**RESULTS.** A total of 40 patients fulfilling inclusion criteria were enrolled in our study, There was a negative correlation between noradrenaline dose and measured REE such that REE increased with decreasing dose of noradrenaline and vice versa at 24 and 48 hours {p value 0.002, 0.013(r = −0.469, −0.388)}. thirty one of the patients have REE measured by indirect calorimetriy greater than REE calculated by the equation.

**CONCLUSION.** There was negative association between resting energy expenditure and noradrenaline dose that may be used as a prognostic factor in septic shock patients. We found no agreement between REE measured by indirect calorimetry and REE calculated by Harris Benedict equation.

**GRANT ACKNOWLEDGMENT**

This research received no specific grant from any funding agency. There was no conflict of interest

### 0185

#### Selective LPS-adsorption in complex intensive therapy for children with gram-negative sepsis after heart surgery, preliminary report

M. Yaroustovsky, M. Abramyan, E. Komardina, A. Soldatkina, H. Nazarova, M. Plyushch, E. Rogalskaya
Bakulev Centre for Cardiovascular Surgery, Moscow, Russian Federation
**Correspondence:** M. Yaroustovsky
*Intensive Care Medicine Experimental* 2017, **5(Suppl 2):**0185

**INTRODUCTION.** Sepsis is still one of the most important problems of modern pediatric intensive care. As a component of the gram-negative cell wall, endotoxin plays a primary role in the pathogenesis of many cases of sepsis.

**OBJECTIVES.** To evaluate the effectiveness of selective LPS-sorbents: LPS-adsorber Toraymyxin - PMX-0.5R (Toray, Japan) in critically ill children with sepsis (S) secondary to gram-negative infection following cardiac surgery.

**METHODS.** During 2016 the study included 10 children aged 9–14 months and weighing 6.2 - 12.5 kg. The inclusion criteria were: clinical signs of sepsis with microbiologically confirmed focus of gram-negative infection or/and gram-negative BAL and blood culture, elevated blood procalcitonin (PCT) >2 ng/ml, LPS concentration according to the endotoxin activity assay (EAA) ≥0.6 and elevated levels of CRP. All children had thrombocytopenia, DIC-syndrome markers (D-dimers) and leukocytosis / leukopenia. Refractory hyperthermia (>38°C) was seen in all patients. Intensive therapy included inotropic and vasopressor support, mechanical ventilation and broad-spectrum antibiotics.

**RESULTS.** Each patient underwent 2 sessions of hemoperfusion with Polymyxin B-immobilized cartridge lasting a maximum of 180 minutes. Extracorporeal therapy was initiated within 24 hours after the diagnosis of sepsis was made. During the treatment the improvement of hemodynamic indices, oxygenation index, the normalization of leukocytosis and body temperature, positive dynamics on chest X-ray and the negative results of bacteriological tests were noted. After the procedures of LPS-adsorption we found the decrease of LPS concentrations according to the EAA, PCT, presepsin and levels of CRP. It was noticed that five out of six patients have survived. One child died from a surgical problem.

**CONCLUSIONS.** Our experience with clinical use of selective LPS-adsorption suggests that it is reasonable to include this method of extracorporeal therapy in complex intensive care treatment of sepsis in children after heart surgery.

### 0186

#### Role of CytoSorb ® in reduction of sepsis scores and better survival benefit in patients with multiorgan failure; when used early: a case series

R. Paul[1], D.B.K. Jha[2], D.V.K. Shetty[2]
[1]Apollo Hospital, Jubilee Hills, Internal Medicine and Intensive Care, Hyderabad, India; [2]Biocon Ltd, Bangalore, India, Immunotherapy and Critical Care, Bangalore, India
**Correspondence:** R. Paul
*Intensive Care Medicine Experimental* 2017, **5(Suppl 2):**0186

**INTRODUCTION.** Severe sepsis and multiorgan failure (MOF) are the commonest causes of ICU mortality. The extracorporeal cytokine adsorption column (ECAC; Cytosorb®, CytoSorbents Corporation, USA), a critical care therapeutic device, results in rapid reduction of cytokines and prevents organ failure. Use of ECAC in patients with sepsis is a new area of research. However, insufficient data are published till now. Studies published till dates have shown promising results. We report our clinical experience with ECAC in severe sepsis/septic shock/MOF patients.

**OBJECTIVES.** Objective of the study was to analyze survival benefit, selection of the eligible subgroup of patients, appropriate timing for initiation; number of device filters required per patient, and selective sepsis severity scores to identify rationales of ECAC therapy.

**METHODS.** A retrospective evaluation of ECAC in patients admitted to a tertiary care ICU in Apollo hospital, Hyderabad, from November 2015 to December 2016 was carried out. Patients were managed with standard of care therapy as per international sepsis guideline along with ECAC as adjuvant therapy. Vitals, APACHE II and SOFA scores were evaluated before and after ECAC therapy.

**RESULTS.** Twelve ICU patients (10 male, 2 female; mean age = 64.83 ± 12.1 [mean ± SD]; with baseline APACHE II 26.25 ± 0.84 [mean ± SD], SOFA score = 14.25 ± 0.41 [mean ± SD] and the majority having infection largely in the lung (n = 7; alone or with blood infection) followed by the abdomen (n = 4), and others (n = 1) were given ECAC (total ECAC = 23). Predicted mortality (PM) was >60% and 100% survived; all of them had were ECAC early (24 hours). APACHE II and SOFA scores after ECAC therapy were 11.66 ± 1.31 and 8.66 ± 1.39 respectively. Both SOFA and APACHE II score decreased >5 points in all patients after single application of ECAC.

**CONCLUSION.** All our patients had multiple organ failures and septic shock. Use of ECAC can be recommended as an adjuvant therapy in the treatment of severe sepsis/septic shock/MOF. Our patients had high predicted mortality and all could be saved with the use of ECAC when introduced early (≤24 hours).

If ECAC is introduced early (≤24 hr.) in septic shock/MOF patients' treatment, a better outcome in terms of mortality benefit can be expected. However, large prospective studies are required to understand the role of ECAC in patients with MOF/septic shock.

**Grant Acknowledgement**
In this clinical case series, we did not require any funding or grant.

### 0187

#### CytoSorb: what is the effect on the circulation?

S. Bok[1], A.F. Grootendorst[1], T.M. Kuijper[2], B.I. Cleffken[1]
[1]Maasstad Hospital, ICU, Rotterdam, Netherlands; [2]Maasstad Hospital, Science Department, Maasstad Academy, Rotterdam, Netherlands
**Correspondence:** S. Bok
*Intensive Care Medicine Experimental* 2017, **5(Suppl 2):**0187

**INTRODUCTION.** The removal of toxic cytokine in patients with septic multiple organ failure may be aided by the extracorporal cytokine adsorption filter(CytoSorb) as demonstrated in case studies and in-vitro experiments. The effect of Cytosorb on the circulation of the septic/septic shock patient however remains unknown.

**OBJECTIVES.** To examine the circulatory system in patients in the first twenty-four hours of CytoSorb therapy by means of PiCCO. In addition, to assess for possible decrease in norepinephrine-demand in respect of the thereby achieved Mean Arterial Pressure (MAP in mmHg) mentioned as Nor/MAP.

**METHODS.** Patients diagnosed with sepsis or septic shock were prospectively included in the period from January to August 2016 (Intervention group, N = 5) and retrospectively from January to December 2014 (Control group, N = 5)(Table 61). Patients in the intervention group received standard care and CRRT in combination with CytoSorb and hemodynamic monitoring with PiCCO. The control group was retrospectively assembled from Patiënt Data Management System, therapy included standard care and CRRT. The intervention group was assessed by measuring; Cardiac Index (CI), extravascular lung water index (EVLWI), systemic vascular resistance index (SVRI), Nor/MAP and fluid balance. Measurements were taken at 4 hour intervals in the initial 24-hour period. The demand for Norepinephrine and fluid balance was assessed in both groups.

**RESULTS.** Data (Table 61, 62) show a clear reduction in demand of Norepinephrine and the thereby achieved MAP (Nor/MAP) in the intervention group compared to the control group, at start 0.65 ± 0.50, after 24h 0.26 ± 0.13. The control group also shows a reduction, only less sharp 0.82 ± 0.41, after 24h 0.62 ± 0.42. At the conclusion of the study, the results showed a normal CI for 4

patients (3-5l/min/m2, N = 5). The initial values were 98 ± 1.53 and after 24h 3.6 ± 0.73. The patients with high CI at start of therapy showed a decline within 12h whilst values normalized for patients with a low CI within 12h. The 4 patients who showed a low SVRI at the beginning showed a sharp increase within the first 12h, however normal values (1700-2400dyn°sec°cm-5°m2) were not achieved after 24h, 1336.60 ± 486.90 and 1543 ± 333.84 at the end of the study.

**CONCLUSIONS.** In the cytosorb group, we demonstrated a trend towards stabilization of the CI, SVRI and Nor/MAP, especially in the first 12 hours. In addition, there was a reduction in norepinephrine requirement in this group. Our study was limited by small sample size, there was no significant statistical difference.

**Table 61 (Abstract 0187).** Baseline characteristics

| | Unit | Min | Max | Mean | SD |
|---|---|---|---|---|---|
| Age 1 | Years | 32 | 81 | 62 | 19 |
| Age 2 | Years | 68 | 85 | 76,6 | 7 |
| Weight 1 | Kg | 82 | 135 | 97,4 | 21 |
| Weight 2 | Kg | 75 | 100 | 86,8 | 10 |
| SOFA 1 | Score | 12 | 17 | 15 | 2 |
| SOFA 2 | Score | 13 | 20 | 15 | 3 |
| APACHE 1 | Score | 31 | 48 | 41 | 7 |
| APACHE 2 | Score | 34 | 47 | 41 | 5 |

1. Intervention 2. Control

**Table 62 (Abstract 0187).** Baseline versus end study

| | | Baseline | | | | End study 24H | | | |
|---|---|---|---|---|---|---|---|---|---|
| | Unit | Min | Max | Mean | SD | Min | Max | Mean | SD |
| Nor/MAP 1 | mcg/kg/hr mmHg | 0,15 | 1,4 | 0,65 | 0,50 | 0,11 | 0,45 | 0,26 | 0,13 |
| Nor/MAP 2 | mcg/kg/hr mmHg | 0,50 | 1,27 | 0,82 | 0,41 | 0,13 | 1,06 | 0,62 | 0,42 |
| CI 1 | l/min/m2 | 2,8 | 6,3 | 4 | 1,50 | 2,7 | 4,3 | 3,6 | 0,73 |
| SVRI 1 | dyn°sec°cm-5°m2 | 779 | 2074 | 1337 | 487 | 1282 | 2109 | 1543 | 334 |
| EVLWI 1 | ml/kg | 4 | 12 | 9 | 3,46 | 5 | 18 | 11 | 5,11 |
| Fluid Balance 1 | ml | | | | | −4546 | 9751 | 4783 | 5589 |
| Fluid Balance 2 | ml | | | | | 2358 | 10774 | 7120 | 3601 |

1. Intervention 2. Control

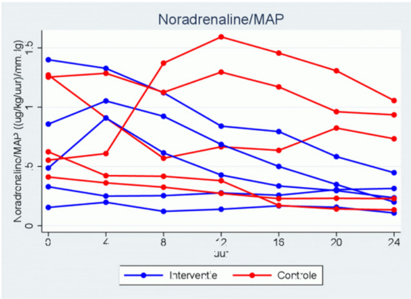

**Fig. 77 (abstract 0187)** See text for description

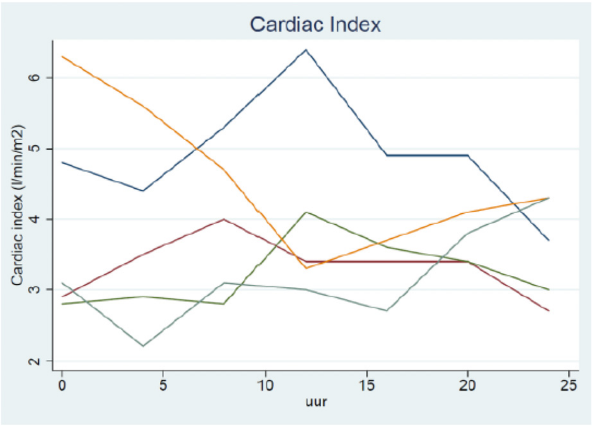

**Fig. 78 (abstract 0187)** See text for description

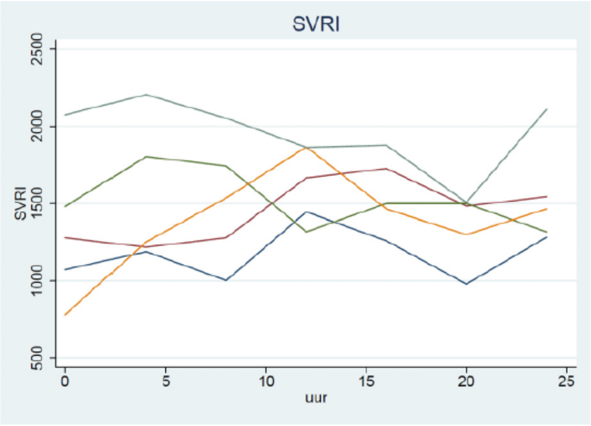

**Fig. 79 (abstract 0187)** See text for description

### 0188

#### Gelatin solutions versus albumin in septic shock resuscitation

S. Tongyoo, C. Chayakul, T. Viarasilpa, C. Permpikul
Mahidol University, Medicine, Bangkok, Thailand
**Correspondence:** S. Tongyoo

*Intensive Care Medicine Experimental* 2017, **5(Suppl 2):**0188

**INTRODUCTION.** Colloid solution was recommended for septic shock resuscitation, in the patient who didn't respond to crystalloid fluid[1]. Albumin is considered as the most suitable colloid[1], however; its use was limited by its price. Gelatin solution is an alternative colloid with lower price, but the efficacy had not been identified.

**OBJECTIVES.** This study aims to compare the outcome of gelatin versus albumin solution in septic shock resuscitation.

**METHODS.** A matched cohort study enrolled refractory septic shock patients, not respond to 30 ml/kg of crystalloid resuscitation and required either albumin or gelatin solution for fluid therapy. The primary outcome was 28-day mortality.

**RESULTS.** Overall 127 patients were recorded. After adjusted for patients' age, baseline mean arterial blood pressure, severity score and lactate level, 97 patients were included. 47 patients received albumin and 50 patients received gelatin for fluid therapy. There was no significant difference in patients' baseline characters. The 28-day mortality was 34% in albumin group, versus 42% in gelatin group, p = 0.28. The ICU and hospital mortality were not difference. Acute kidney injury, requiring renal replacement therapy occurred in 24% of patients in both groups. Among the low baseline serum albumin patients (<2.5g/dL), the 28-day mortality [4/21(19%) VS 13/25(52%), p = 0.03] and hospital mortality [4/21(19%) VS 13/25(52%), p = 0.03] were lower among albumin than gelatin group. The survival days in 28-day without organ support was significant longer in albumin group (16.9 ± 9.9 VS 7.7 ± 9.7, p = 0.002).

**CONCLUSIONS.** The outcome of septic shock resuscitated with albumin and gelatin solution was not different in overall population. Albumin resuscitation associated with more favorable outcome among the low baseline serum albumin septic shock patients.

**REFERENCE(S)**

1. Rhodes A, Evans LE, Alhazzani W, et al. Surviving Sepsis Campaign. International Guidelines for Management of Sepsis and Septic Shock: 2016. Intensive Care Med 2017;1–74.

**GRANT ACKNOWLEDGMENT**

Siriraj critical care research funding.

### 0189

#### Impact of fluid accumulation on the survival of patients with septic shock

J. Leache, I. Oliva, M. Cartanya, R. Carbonell, A. Rodríguez, M. Bodí, C. Villavicencio

Joan XXIII Tarragona University Hospital, Intensive Care Medicine, Tarragona, Spain

**Correspondence:** J. Leache

*Intensive Care Medicine Experimental* 2017, **5(Suppl 2):**0189

**OBJECTIVES.** To evaluate the fluid accumulation during the first week of septic shock and its impact on mortality.

**METHODS.** Prospective, observational study, conducted in a polyvalent ICU. 64 patients with diagnosis of septic shock were included in a period of 18 months. Demographic characteristics, comorbidities, APACHEII, SOFA, SAPSII, hemodynamic parameters, daily fluid balance (DFB), daily cumulative balance (DCB), percentage of fluid accumulation and the appearance of fluid overload were analysed during the first week of hospitalization. The impact in 28-day mortality was evaluated.

**DEFINITIONS.**

- DFB: Differences between volume income and daily diuresis.
- DCB: Addition of the DFB.
- Percentage of fluid accumulation: DCB divided by weight on admission x 100.
- Fluid overload: Percentage of fluid accumulation more than 10%.

**RESULTS.** We included 64 patients with mean age 65 years, 53% male, APACHE 28 ± 7, SAPS II 56 ± 20, SOFA on admission 8 ± 3, mechanical ventilation 76%, continuous renal replacement techniques 38%. The mean total volume administered during the first 7 days was 26 ± 8L with a mean DCB of 16 ± 8L and a mean fluid accumulation of 21% ± 13. Regarding Fluid accumulation: 17% have < 10%, 35% between 10-20% and 47,5% > 20%. 28th-day mortality and ICU mortality were 17% and 28% respectively. During the first week, the percentage of fluid accumulation was significantly higher in non-survivors than in survivors (28,5 ± 10,7 L vs. 18,7 ± 13,1 L, p 0.046) (Fig. 80). Cumulative survival was significantly lower (logRank = 6,05, p = 0,01) in patients with >20% of volume gain since the $6^{th}$ day (Fig. 81). >20% volume gain in the 6th day is a independently associated variable to mortality after adjusting by age, APACHE and hemodialysis (OR = 7,3; CI 95% 1,2-43,9; p = 0,02).

**CONCLUSIONS.** In septic shock patients, fluid overload more than 20% since the 6th day of evolution is associated with a higher 28-day mortality. Its early detection may influence the prognosis and survival.

**REFERENCE(S)**

1. Soroko SB, Chertow GM, Himmelfarb J, Ikizler TA. Fluid accumulation, survival and recovery of kidney function in critically ill patients with acute kidney injury. 2009:422–427. doi:10.1038/ki.2009.159.

2. Cordemans C, De I, Regenmortel N Van, Schoonheydt K, Dits H, Huber W. Fluid management in critically ill patients : the role of extravascular lung water, abdominal hypertension, capillary leak, and fluid balance. *Ann Intensive Care*. 2012;2(Suppl 1):S1. doi:10.1186/2110-5820-2-S1-S1.

3. Malbrain MLNG, Marik PE, Witters I, et al. 3, 4,. 2014;46(5):361–380. doi:10.5603/AIT.2014.0060.

4. Boyd JH, Frcp C, Forbes J, et al. Fluid resuscitation in septic shock: A positive fluid balance and elevated central venous pressure are associated with increased mortality*. 2011;39(2):259–265. doi:10.1097/CCM.0b013e3181feeb15.

**GRANT ACKNOWLEDGMENT**

None

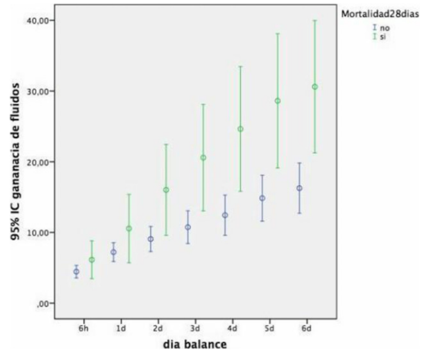

**Fig. 80 (abstract 0189)** Volume gain and mortality]

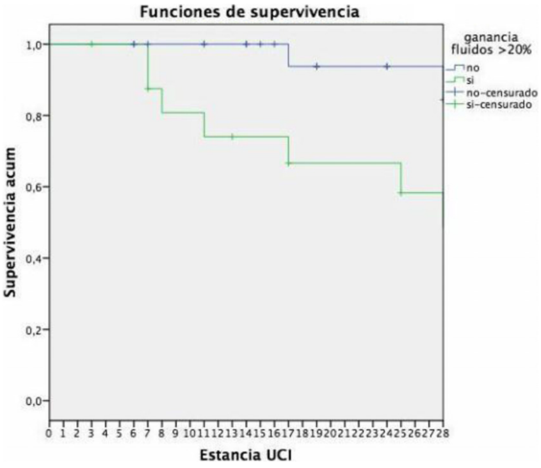

**Fig. 81 (abstract 0189)** Volume gain and 28th-day survival

### 0190

#### Polyclonal intravenous immune globuline treatment as additional strategy in sepsis

L. Mirabella[1], L. Tullo[1], A. Cotoia[1], L. Montrano[1], K. Mariano[1], A. Carretta[2], G. Cinnella[1]

[1]University of Foggia, Anaesthesia and Intensive Care, Foggia, Italy; [2]University of Foggia, Infectious Desease, Foggia, Italy

**Correspondence:** L. Mirabella

*Intensive Care Medicine Experimental* 2017, **5(Suppl 2):**0190

**INTRODUCTION.** Sepsis is defined as a life-threatening organ dysfunction caused by a dysregulated host response to infection associated with high mortality rate. The implementation of bundles for the treatment of sepsis has improved the outcome but it is not clear whether the success is tied to individual factors or to the synergy of therapies. Recently, several Meta-analyzes have evoked a potential role of (IG) as a treatment able to improve the outcome and reduce mortality after septic shock.

**OBJECTIVES.** Aim of the study was to evaluate efficacy of intravenous polyvalent immunoglobulin (IG) as additional therapy in sepsis treatment in reducing mortality and improve outcome.

**METHODS.** Patients with diagnosis of shock septic according with third International Consensus Definitions, received, as part of the standard treatment protocol and based on the discretion of the attending physician, IG at a dose of 5 mg /kg/day with slow infusion of 10 hours for 3 consecutive days. In the treated patients were assessed admission APACHE III score, Lactate, Procalcitonine and Proteine C Reactive (PCR) before and after treatment with IG, ICU length of stay, 28- day mortality and the occurrence of adverse events to the administration of IG.

**RESULTS.** Between March and December 2016, 9 patients (4F: 5M) were treated with IG, mean age 46 ± 10 years. The main diagnosis was pulmonary infection in 44% of cases, 2 patients with ARDS, 1 with thoraco-polmonary fistula, and 1 with diagnosis of VAP. In 33% of cases, the patients had Central Nervous System infection, 2 patients with bacterial meningoencephalitis and 1with autoimmune encephalitis complicated by sepsis. The remaining cases (23%), 1 patient with renal abscess and 1 with dental abscess complicated by mediastinitis. Two patients were concomitantly treated with polymyxin-B and 3 with renal replacement therapy. APACHE III score at admission was 65.5 ± 46, Lactate levels were 3.4 ± 0.9 mmol/L and 1.14 ± 0.2 respectively before and after treatment with IG, Procalcitonine was 46.1 ± 9 ng/mL and 2.18 ± 2.25 ng/mL, PCR was 425 ± 110 mg/L and 112.2 ± 90 mg/L. The duration of hospitalization in the ICU was 21 ± 15 days, no difference was observed with non-treated patients and the 28-day mortality was 33%, but 2 died patients; the death is due to the underlying disease and not to the sepsis. During treatment with IG no adverse events attributable to the administration of the drug was detected.

**CONCLUSIONS.** Currently the lack of randomized clinical trials and the small size of the sample treated do not supported sufficiently evident conclusions but treatment with IG polyclonal as adjunctive therapy in patients with septic shock diagnosis appears to improve survival when early administered.

**REFERENCE(S)**

Singer M, Deutschman CS, Seymour CW et al. The Third International Consensus Definitions for Sepsis and Septic Shock (Sepsis-3) JAMA. 2016; 315(8):801–810.

### 0191

#### Retrospective analysis of the use of polyclonal immunoglobulins as adjunctive therapy for septic shock

A. Wieczorek, A. Siebert, M. Lipińska-Gediga
4 WSK z Poliklinika we Wroclawiu, Wroclaw, Poland
**Correspondence:** A. Wieczorek
*Intensive Care Medicine Experimental* 2017, **5(Suppl 2):**0191

**INTRODUCTION.** International guidelines for management of sepsis and septic shock suggest against the use of IV immunoglobulins in patients with sepsis or septic shock. There are studies which show that the use of intravenous immunoglobulin therapy in adult septic patients may may be beneficial and seems to be associated with a reduce mortality. Immunoglobulins were used in our ICU during last year,but there were no protocol of administration or control the result of their use.

**OBJECTIVES.** The aim of the study was to assess agroup of patients with sepsis and septic shock which may improve after implementation of immunoglobulin.

**METHODS.** The retrospective chart review (April 2016–2017) of patients with septic shock treating with immunoglobulins.

**RESULTS.** 22 patients with septic shock received polyclonal IgG, IgM and IgA (IgGAM); Pentaglobin, 5ml/kg, 12 hours infusion repeated for the following 3 days. All of the patients were treated with abroad-spectrum antibiotic, in 20 cases adequate to the results of microbiological cultures. All of the patients needed renal replacement therapy, in most cases (20/22) the Oxiris (adsorb endotoxin and cytokines) filter was used. In all cases,hydrocortisone (4x50mg for 5–7 days) was administered. Patient with septic shock and without renal replacement therapywas not treated with Pentaglobin. 9 of 22 patients were surgical and died because of unsolved surgical condition (bowel ischemia, abdominal abscess, delayed surgical intervention). In blood, cultures form 5 of 22 patients was Acinetobacetbaumanii MDR, only Collistin sensitive, all of this patients died. After implementing immunoglobulin, start CRRT with Oxiris filter, in all cases was observed temporary improvement in patients condition- defined as adecrease of adose of catecholamines, FiO2 reduction. Survived 6 of 22 (26%) patients, in all cases, the origin of the infection was arespiratory system, in 2 cases Pentaglobin was a part ofsecondary infectionstreatment, all of them had lower SOFA score (less than 16) than so survivals in the moment of immunomodulatory treatment implementation.

**CONCLUSIONS.** Immunoglobulin was used as a part of animmunomodulatory bundle in patients with septic shock and renal replacement therapy and this group of patients may beneficial of such treatment. Based on this results we plan to prepare a protocol forimmunoglobulins implementation in our ICU.

**REFERENCE(S)**

1. Surviving Sepsis Campaign: International Guidelines for Management of Severe Sepsis and Septic Shock: 2016

2. Busani S, Damiani E, Cavazzuti I, Donati A, Girardis M. Intravenous immunoglobulin in septic shock: a review of the mechanisms of action and meta-analysis of the clinical effectiveness.MinervaAnestesiol. 2016 May;82(5):559–72.

3. Jesús F. Bermejo-Martina, Evangelos J. Giamarellos-Bourboulis. Endogenous immunoglobulins and sepsis: New perspectives for guiding replacement therapies.International Journal of Antimicrobial Agents 46(2015) S25–28.

### 0192

#### Percutaneous drainage (PCD) utility in critical patients of a tertiary hospital ICU

D.X. Cuenca Apolo[1], A. Díaz Martín[1], J. Pèrez Guerrero[1], S. Escalona-Rodríguez[2], N. Palomo-López[2], I. Palacios-García[2]
[1]Virgen del Rocío Universitary Hospital, Intensive Care Unit, Sevilla, Spain; [2]Hospital Universitario Virgen del Rocío, Intensive Care Unit, Sevilla, Spain
**Correspondence:** D.X. Cuenca Apolo
*Intensive Care Medicine Experimental* 2017, **5(Suppl 2):**0192

**INTRODUCTION.** Minimous invasive drainage of abscess is a tool that can decrease morbimortality in some ICU patients. The ecosonography is indispensable to carry out these procedures.

**OBJECTIVES.** To describe the characteristics of patients subjected to percutaneous drainage (PCD) during their stay in ICU and to analize if that implies fewer surgical interventions in certain high-risk patients.

**METHODS.** Retrospective study including patients undergoing percutaneous drainage in a tertiary hospital ICU during 2016. They are gathered by ICU admission reasons, clinical severity measured by APACHE II and SOFA scales, PCD date, duration and complications. We analyze PCD utility as a tool for preventable surgical intervention. Variables expressed as a mean or percentage (RIQ) as appropriate, and chi-squared when required.

**RESULTS.** A total of 52 patients undergoing PCD in our ICU in 2016 were included, with 62 years old mean (48–71), mean APACHE II 15 (10–19), SOFA 8 (6–12.75) and mean organ failure of 2 (1.25-3). Sepsis was the main admission, 33 cases (63.5%), in which 18

episodes (34,6%) was digestive septic focus, following 13 cases of post-surgical abdominal intervention (25%). Main reason of PCD was 11 cases of pulmonary empiema (21.%), following 9 cases of infected intraabdominal collections (17.35%) and pionefrosis in 8 cases (15.4%). In 46 cases (88%) the PCD was performed under ultrasound guidance. PCD was performed 1 day mean after ICU admission (0–5.75), it was maintained 4 days mean (2–13.75).

No complications related to PCD were observed in most episodes (44 cases, 85%). The most frequent complications were drainage obstruction in 5 cases (9.4%), and local bleeding in 2 episodes (3.4%), none of them related with decease.

Subsequent surgical intervention after PCD was required only in 8 cases (15.4% of patients). ICU mortality was 13.5% (7 cases) and in-hospital 19.2% (10 cases).

**CONCLUSIONS.** PCD can be a useful and safe tool for evacuation of collections in ICU patients. Prospective studies are needed to quantify surgical intervention reduction in these high-risk patients.

- Yu-Long Cai, Xian-Ze Xiong, Jiong Lu, Yao Cheng, Chen Yang, Yi-Xin Lin, Jie Zhang. Percutaneous needle aspiration versus catheter drainage in the management of liver abscess: a systematic review and meta-analysis. HPB (Oxford). 2015 Mar; 17(3): 195–201.

- Zerem E1, Omerovic S, Kunosic S. Sonographically guided percutaneous treatment of liver abscesses in critically Ill patients. J Clin Ultrasound. 2014 Nov-Dec;42(9):527–33.

### 0193

#### Early and sustained normalization of hyper-inflammatory response is associated with improved survival after a single dose administration of Reltecimod (AB103), a CD28 peptide antagonist, in a mice model of polymicrobial sepsis

A. Shirvan[1], R. Edgar[1], J. Palardy[2], M. Tarrio[3], G. Maislin[4], S. Opal[2]

[1]Atox Bio Ltd, Research & Development, Ness ziona, Israel; [2]Alpert Medical School, Brown University, Infectious Disease Division, Providence, United States; [3]Brown University, Molecular Microbiology and Immunology, Providence, United States; [4]Biomedical Statistical Consulting, Wynnewood, United States

*Intensive Care Medicine Experimental* 2017, **5(Suppl 2):**0193

**Correspondence:** A. Shirvan

**INTRODUCTION.** Co-stimulatory pathways provide crucial checkpoints for the host response to infection. Among them, CD28 receptor delivers signals essential for T cell proliferation, survival, cytokine production, and T-helper type-II development. Reltecimod, a CD28 antagonist short peptide, currently in phase 3 clinical study in NSTI, improves the host's ability to effectively fight the infection.

A single dose of Reltecimod confer higher survival rate compared to multiple doses.

**OBJECTIVES.** Compare the effect of a single dose vs. two doses on survival, circulating white blood cells profile and cytokine/chemokines levels, in animals undergoing sepsis induced by Cecal Ligation and Puncture (CLP).

**METHODS.** Sepsis was induced by CLP in BALB/c mice. PBS or Reltecimod (5 mg/kg) were administered intravenously, once or twice, at 2 hours or 2 and 24 hours post-CLP to randomized animal groups. Animals were euthanized at either 24 or 48 hours (h) after CLP. Blood was collected for measurements of various cytokines/chemokines (IL-6, IL-3, IFN-γ, IL-1a, IL-12, IL-17, IL-10, IL-5, MCP-1, MIP-1α, RANTES) and lymphocytes profile (T cells [CD4+; CD8+], B cells, neutrophils, macrophages/monocytes) determined using flow cytometry. Effect of a single dose was compared to control (PBS) or to two doses. Separate groups, treated similarly, were used for survival assessments.

**RESULTS.** Mice treated with a single dose of Reltecimod at 2h showed significant improvement in survival (90%) on day 6, compared to a control group (5%; $p < 0.002$) or to a group treated with two doses (at 2 and 24h) of Reltecimod (40%; $p < 0.002$). Average survival time was 2.9, 6.9 and 5.0 days, for control, a single dose or two doses of Reltecimod, respectively. In the control group at 24h post-CLP, levels of multiple pro-inflammatory cytokines/chemokines were elevated, increase in T cells subpopulation (CD4+; CD8+) and decrease in neutrophils were observed. Without treatment, these early changes normalized by 48h. Single dose of Reltecimod significantly reduced various cytokines/chemokines levels (mean and AUC) at 24h, which were positively correlated with (i) one another (ii) reduction in T cells subpopulations (number, %, AUC) and negatively correlated with neutrophils count (number, %), all compared to control. However, no further reduction at 48h post-CLP was detected in cytokine/chemokines or lymphocytes sub-populations when additional dose of Reltecimod was administered at 24h. These animals experienced longer exposure (>24 hours) to high cytokines/chemokines, without complete resolution of lymphocyte profile.

**CONCLUSIONS.** Improved mortality of mice following CLP and treatment with Reltecimod is associated with reduced exposure to cytokines during the early time period after infection and early and sustained normalization of lymphocytes profile. Both processes are enhanced when animals are treated with a single dose of Reltecimod, whereas a second dose has less favorable outcome.

### 0194

#### Intravenous beta-hydroxybutyrate has no impact in a long-term rat model of sepsis

B. Ficial[1,2], M. Singer[1]

[1]University College London, Bloomsbury Institute of Intensive Care Medicine, London, United Kingdom; [2]Università degli Studi di Verona, Scuola di Specializzazione in Anestesia, Rianimazione e Terapia Intensiva, Verona, Italy

**Correspondence:** B. Ficial

*Intensive Care Medicine Experimental* 2017, **5(Suppl 2):**0194

**INTRODUCTION.** A metabolic shift towards lipid use occurs during critical illness. This may represent an evolutionary conserved response to utilize body fat stores as a energy substrate. Ketone bodies, often regarded as a by-product of fatty acid beta-oxidation, are emerging as important metabolic regulators. Ketogenic diets are being used therapeutically in various diseases.

**OBJECTIVES.** To assess the impact of exogenous administration of the ketone, beta-hydroxybutyrate (BHB), on metabolism and cardiorespiratory function in a long-term rat model of fluid-resuscitated faecal peritonitis.

**METHODS.** Instrumented male Wistar rats had sepsis induced by intraperitoneal injection of faecal slurry. Sham-operated animals received no injection. Animals were then placed in metabolic cages for indirect calorimetric measurement of $O_2$ consumption and $CO_2$ production. They had free access to food and water. Fluid resuscitation with a balanced solution was started at 2h. At 6h animals were randomized to receive iv BHB infusion (180mg/kg/h) or an equivalent isocaloric volume of glucose solution. At 24h, blood and tissue samples were collected and animals euthanized.

**RESULTS.** At 24h BHB-treated animals had a metabolic alkalosis (7.53 ± 0.04 vs 7.49 ± 0.03 untreated; $p = 0.001$) with increased bicarbonate (33.4 ± 2.5 vs 28.2 ± 3.2 mEq/L; $p < 0.001$) and base excess (10.8 ± 3.2 vs 4.5 ± 3.9 mmol/L; $p < 0.001$), yet $PaCO_2$ levels were unchanged. BHB had no effect on respiratory exchange ratio (RER = $CO_2$ production/$O_2$ consumption) in sham-operated animals (0.85 ± 0.05 vs 0.89 ± 0.03 in placebo; $p = 0.169$). However, the septic animals, regardless of BHB treatment, had similarly reduced RER values at 24h (0.80 ± 0.03 vs 0.78 ± 0.01 untreated, p = ns; $p < 0.001$ compared to sham). $O_2$ consumption was unchanged at 24h, whereas $CO_2$ production was reduced in the septic groups (1245 ± 146 mL/kg/h vs 1441 ± 171 mL/kg/h sham; $p = 0.003$). Compared to placebo, BHB infusion in septic animals produced no significant changes in clinical severity score, temperature, blood pressure or cardiac function.

**CONCLUSIONS.** Sepsis produced a shift towards fat oxidation signified by a fall in RER. BHB infused over 18 hours resulted in a metabolic alkalosis but no effect on illness severity, haemodynamics or RER. No positive impact was seen with a ketogenic diet in this animal model of sepsis.

**REFERENCE(S)**
Robinson AM, Williamson DH. *Physiological roles of ketone bodies as substrates and signals in mammalian tissues*. Physiol Rev, 1980; **60**:143–87.
Newman JC, Verdin E. *Beta-hydroxybutyrate: much more than a metabolite*. Diabetes Res Clin Pract 2014; **106**:173–81.
**GRANT ACKNOWLEDGMENT**
Departmental funds

#### 0195

**Hemodynamic resuscitation with fluids bolus and norepinephrine increases severity of the lung damage in an experimental model of septic shock**

P. Guijo González[1], I. Monge García[2], M.A. Gracia Romero[1], B. Diez del Corral[1], A. Gil Cano[1]
[1]Hospital SAS Jerez, Unidad de Cuidados Intensivos, Jerez de la Frontera, Spain; [2]St George's University of London, Intensive Care Medicine, London, United Kingdom
**Correspondence:** P. Guijo González
*Intensive Care Medicine Experimental* 2017, **5(Suppl 2):**0195

**INTRODUCTION.** Hemodynamic resuscitation by means of fluid boluses and norepinephrine is currently considered as a cornerstone of the initial treatment of septic shock. However, there is growing concern about the undesirable effects of this treatment. Thus, previous studies have established a certain relationship between fluid administration and the appearance of ARDS, and there is increasing evidence of the detrimental effect that acute adrenergic overload seems to exert with direct organ damage and multiple "off-target" biological effects. Given the increased propensity of lung damage in the context of septic shock, it is possible to assume that resuscitation with fluids bolus and norepinephrine may increase odds of subsequent development of ARDS.

**OBJECTIVES.** To investigate, in an experimental septic shock model, if the hemodynamic resuscitation with fluids bolus and norepinephrine lead to lung injury development.

**METHODS.** Experimental study carried out on 18 randomly selected "New Zealand White" rabbits: 6 of them assigned to the control group (SHAM) and 12 of them assigned to the endotoxin shock group. These last ones received a Lipopolysaccharide (LPS strain O55:B5 of E. Coli) bolus of 1 mg/kg. After 3 hours, 6 of them received a 10 minutes infusion of 20 ml/kg of Ringer lactate. Afterwards, they received a norepinephrine infusion titrated up to achieve theirs initial arterial pressure (LPS-R). The other 6 animals did not receive additional treatment (LPS-NR). Minimally invasive monitoring with esophageal doppler probe and arterial catheter was performed, as well as airway pressure and flow monitoring. After 4 hours of experiment, animals were sacrificed. We analyzed the right lung pulmonary edema by the relation between lung wet and lung dry weight, and the left lung histopathological findings.

**RESULTS.** The animals of the SHAM group did not show any hemodynamic or respiratory changes. The administration of the LPS aimed at increasing cardiac output and arterial hypotension. In the LPS-NR group, animals remained hypotensive until the end of experiment. Conversely in animals from LPS-R group, the infusion of fluids increased cardiac output without changing arterial blood pressure, while the infusion of norepinephrine reversed arterial hypotension. Compared to the animals in the LPS-NR group, the animals in group LPS-R had more alveolar neutrophils and pneumocytes with atypical nuclei, thicker alveolar wall, more non-aerated pulmonary areas and less lymphocytes infiltrating the interstitial tissue. In addition, the airway pressure increased more in the group LPS-R, and the relationship between wet and dry lung weight, although slightly higher in the LPS-R, did not show significant differences.

**CONCLUSIONS.** In this model of experimental septic shock resuscitation with fluid bolus and norepinephrine increased cardiac output and normalized blood pressure but worsened lung damage.

### Prognostication 1

#### 0196

**The win ratio: a new tool to account for composite outcomes in clinical trials**

S. Bailly[1], S. Ruckly[1], W. Ibn Essaied[1], C. Dupuis[1], M. Wolff[2], C. Scwhebel[3], P.-E. Charles[4], E. Azoulay[5], J.-F. Timsit[1,2]
[1]INSERM UMR 1137, Paris, France; [2]Hôpital Bichât Claude Bernard, Paris, France; [3]Hôpital Albert Michallon, Grenoble, France; [4]Hôpital Dijon, Dijon, France; [5]Hôpital Saint Louis, Paris, France
**Correspondence**: S. Bailly
*Intensive Care Medicine Experimental* 2017, **5(Suppl 2):**0196

**INTRODUCTION.** Composite outcomes are often used as clinical endpoints in clinical trials. A composite outcome focuses only on the event that occurs first. Although there is some interest for the use of a composite outcome, it can lead to statistical biases mainly because each event defines the outcome, regardless clinical relevance and timing to occurrence. The win ratio (WR) is a new tool, based on risk-matched pairs of patients, which optimizes statistical power by considering hierarchical outcomes.

**OBJECTIVES.** Pot-hoc analysis of the EMPIRICUS trial using a modified WR that includes adverse events and antifungal therapy duration.

**METHODS.** The EMPIRICUS trial compared an empirical systemic antifungal treatment (micafungin) to a placebo for their impact on invasive fungal infection (IFI)-free survival at day 28 of mechanically ventilated patients with sepsis and fungal colonization. In this analysis, a patient died at day 7 is similar to a patient who presented an IFI at day 7 and ultimately survived. Micafugin and placebo patients were matched according to a propensity score of IFI-free survival computed by using a logistic regression with SOFA score and *Candida* score as adjustment variables. In each pair, patients of the micafugin arm were classified as winner or loser according to a hierarchical outcome (day-28 survival or death) (Fig. 82). Finally a win ratio (WR) was computed as the ratio of winners in the micafungin arm to winners in the placebo arm. A sub-group analysis was performed for patient with a baseline SOFA greater than 8.

**RESULTS.** Among the 251 patients included in the original analysis, 246 were successfully matched (123 from micafungin arm and 123 from placebo arm). For example, if a pair is constituted by two patients who died in each arm, at day 3 for placebo arm and day 25 for micafungin arm, the pair is considered as winner for micafungin. The original results using a multivariable Cox model with a composite outcome and results obtained using WR are presented in the following table. A WR > 1 is in favor of micafungin arm.

**CONCLUSION.** The win ratio is a simple tool to obtain a better estimation of a treatment effect by hierarchizing the outcomes in a RCT. This analysis showed great variation of the final effect of micafungin whether or not SAE and SAT duration are taken into account. Here, two improvements were made to the proposition of Pocock et al. by using a propensity score for matching and by considering more hierarchical levels for the outcome, by adapting the method proposed by Evans et al. This new approach should be extensively considered to replace composite outcomes in randomized clinical trials or observational studies.

**REFERENCE(S)**
Timsit JF et al. JAMA 2016 Oct;18;316(15):1555–64.
Pocock SJ et al. Eur Heart J. 2012 Jan;33(2):176–82.
Evans et al. CID 2015;61(5):800–6
**GRANTS**
Combacte statnet

**Table 63 (Abstract 0196)**. Results

| | | Number of events | | |
|---|---|---|---|---|
| | N total | Micafungin N (%) | Placebo N (%) | HR (95%CI) |
| IFI free survival Original result | 260 patients | 87 (68) | 74 (60) | 1.35 [0.87; 2.08] |
| IFI free survival (matched analysis) | 123 pairs | 85 (69) | 74 (60) | 1.33 [0.86; 2.05] |
| | | Winner micafungin | Winner placebo | WR [95% CI] |
| **Win ratio** IFI free survival without SAE | 246 patients 123 pairs | 52 | 43 | 1.13 [0.75; 1.70] |
| **Win ratio** IFI free survival without SAE and duration of SAT | | 53 | 70 | 0.76 [0.52; 1.08] |
| **SOFA >8** | | | | |
| **Win ratio** IFI free survival without SAE | 110 55 pairs | 25 | 23 | 1.09 [0.61; 1.96] |
| **Win ratio** IFI free survival without SAE and duration of SAT | | 25 | 30 | 0.83 [0.47; 1.42] |

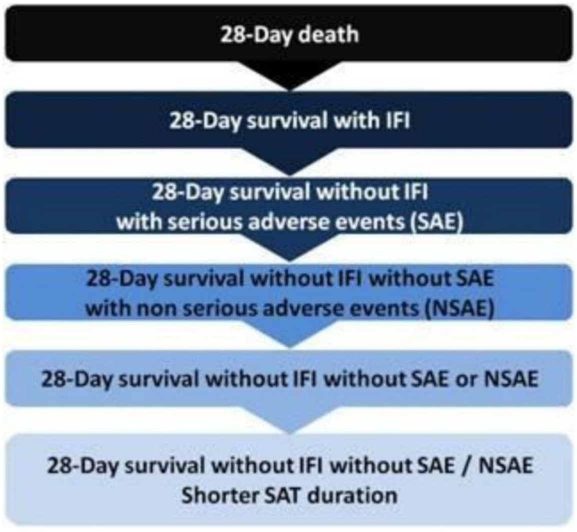

**Fig. 82 (abstract 0196)** Schema of the hierarchical outcome criteria

### 0198

#### Prognostic performance of qSOFA Score combined with Emergency Severity Index (ESI)

H. Kwak, G.J. Suh, Y.S. Jung, S.M. Shin, K.S. Kim, T. Kim, J.-I. Ko
Seoul National University Hospital, Seoul, Korea, Republic of
**Correspondence:** H. Kwak
*Intensive Care Medicine Experimental* 2017, **5(Suppl 2):**0198

**INTRODUCTION.** Emergency Severity Index (ESI) is both reliable and valid tool for triage. And qSOFA score, which was recently proposed, has good predictive validity for hospital outcome in patients outside of the ICU (1, 2).

**OBJECTIVES.** We conducted this study to investigate whether qSOFA score combined with Emergency Severity Index (ESI) predicts hospital outcome better than ESI does alone.

**METHODS.** We retrospectively reviewed data of adult patients (age ≥ 15 years) who visited an adult ED of a tertiary referral hospital from January 1st, 2015 to December 31st, 2015. We calculated and compared odds ratios, Akaike information criteria (AIC), and area under receiver-operating curve (AUROC) of ESI alone and qSOFA score combined with ESI for prespecified outcomes. The primary outcome was hospital mortality, and the secondary outcome was composite outcome of hospital mortality and ICU admission. Patients were divided into 4 subgroups according to ESI levels; we also calculated hospital mortality rates by positivity of qSOFA score in each subgroup.

**RESULTS.** 44367 patients visited the adult ED during study period; 43748 among them were included for analysis. The AICs of the logistic regression models with qSOFA combined with ESI and the model with ESI alone for hospital mortality were 4358 and 4417.2, respectively, and the AICs of the logistic regression models for composite outcome were 12295 and 12342, respectively. The AUROCs were as follows: 0.786 vs 0.777 ($p < 0.001$) for hospital mortality; 0.778 vs 0.774 ($p < 0.001$) for composite outcome. Hospital mortalities were higher for groups with positive qSOFA score compared with those for groups with negative qSOFA score in every prespecified subgroup with any hospital mortality (20.4% vs 14.7% in ESI level 1 subgroup; 11.3% vs 2.7% in ESI level 2 subgroup; 2.3% vs 0.4% in ESI level 3 subgroup; 0.0% vs 0.0% in ESI level 4 & 5 subgroup).

**CONCLUSIONS.** The prognostic performance of qSOFA score combined with ESI for mortality did not show significant difference from that of ESI alone. But within every ESI groups, positivity of qSOFA score were related with higher hospital mortalities.

**GRANT ACKNOWLEDGMENT**
None.

### 0199

#### scores are superior to biomarkers to predict mortality in septic patients

I. Granja Gomez[1], E. Rodriguez-Ruiz[1], R. Hernandez-Vaquero[1], A. Lopez Lago[1], J.L. Garcia Allut[1], A. Estany-Gestal[2], M.A. Garcia Gonzalez[3]
[1]Hospital Clínico Universitario, Intensive Care Unit, Santiago de Compostela, Spain; [2]Fundación Ramón Dominguez, Unidad de Epidemiología e Investigación Clínica, Santiago de Compostela, Spain; [3]Instituto de Investigaciones Sanitarias (IDIS), Grupo de Genética y Biología del Desarrollo de las Enfermedades Renales, Santiago de Compostela, Spain
**Correspondence:** I. Granja Gomez
*Intensive Care Medicine Experimental* 2017, **5(Suppl 2):**0199

**INTRODUCTION.** In sepsis, due to the lack of diagnostic and prognostic precision of different scoring systems, multiple biomarkers have been evaluated to date. After initial enthusiasm for some of these biomarkers, well reviewed in the literature and reported as useful for predicting sepsis survival, they can become frustrating in the daily practice, and only a few have a realistic application in sepsis.

A more recent biomarker, advanced glycation end products (AGEs), is increasingly described as playing a role in many diseases. AGEs accumulation in different tissues including skin, and elevated circulating levels of AGEs in plasma have been reported under inflammatory conditions.

**OBJECTIVES.** Our aim was to study different biomarkers (TNF-α, IL-1β, IL-6, IL-8, C-reactive protein, Procalcitonin), including AGEs in skin and plasma, and well known ICU scores (APACHE II and SOFA), and to analyze their ability to predict mortality.

**METHODS.** We assessed 89 consecutive septic patients admitted to our medical-surgical ICU. TNF-α, IL-1β, IL-6, IL-8, C-reactive protein, Procalcitonin, and AGEs in skin and plasma were determined on day 1. AGEs in skin were measured detecting skin auto-fluorescence with the AGE Reader mu (DiagnOptics, Groningen, The Netherlands). Plasma AGEs were measured by quantitative fluorescence spectroscopy. APACHE II was calculated on day 1, and daily SOFA score was calculated for 5 consecutive days from admission. Chi-squared and Mann–Whitney tests were performed to study the association of these variables with 28-day mortality, and ROC curves were carried out to verify their predictive power.

**RESULTS.** There were 72 survivors and 17 non-survivors. We did not find association between the values of any biomarker on day 1: TNF-α, IL-1β, IL-6, IL-8, C-reactive protein, Procalcitonin, AGEs in skin and plasma, and the mortality on day 28. However, when analyzing scoring systems, APACHE II showed association with 28-day mortality (p = 0.009), and SOFA scores on days 1 to 5, were also associated with 28-day mortality: SOFA 1 (p < 0.033), SOFA 2 (p = 0.005), SOFA 3 (p = 0.002), SOFA 4 (p < 0.001), and SOFA 5 (p < 0.001). The ROC curve analysis showed the best prediction ability for the values of SOFA 4 (AUC 0.798, p < 0.001), and SOFA 5 (AUC 0.819, p < 0.001).

**CONCLUSION.** In our cohort of septic patients, we found correlation between APACHE II (day 1) and SOFA scores (day 1 to 5), and the 28d-mortality, being values for SOFA at days 4 and 5, good predictors of mortality. Meanwhile, none of the studied biomarkers showed this correlation. Our results support that, if we want accurate prediction models, an approach of simultaneous evaluation of multiple targets and their serial measurements should be favored over the single target and single time-point approach.

### 0200

#### The prognostic value of N-terminal pro-B-type natriuretic peptide in patients admitted to the intensive care unit

S. Snouda[1], M.F. Abbes[2], F. Hebaieb[1], M. Kaddour[1], H. Ben Ghezala[1]
[1]Regional Hospital of Zaghouan, Emergency and Intensive Care, Zaghouan, Tunisia; [2]Regional Hospital of Zaghouan, Cardiology Department, Zaghouan, Tunisia

*Intensive Care Medicine Experimental* 2017, **5(Suppl 2):**0200

**Correspondence:** S. Snouda

*Intensive Care Medicine Experimental* 2017, **5(Suppl 2):**0200

**INTRODUCTION.** N terminal probrain natriuretic peptide (Nt-proBNP) is secreted by the heart in response to ventricular wall stress. Increased Nt-proBNP plasma levels are also found in noncardiac diseases, such as sepsis, renal failure, pulmonary embolism and chronic obstructive pulmonary disease, which are common in intensive care unit patients. We suggested that Nt-proBNP could predict intensive care unit outcome in this setting.

**OBJECTIVES.**

1. To evaluate the relationship between Nt-proBNP and hospital mortality.
2. To investigate the relationship between Nt-proBNP and severity of disease (SAPS 3).

**METHODS.** This was a prospective study performed in the emergency and intensive care department in the regional hospital of Zaghouan. The patients were recruited between June 2015 and December 2015. All adult patients admitted to the intensive care unit (ICU) were included. Nt-proBNP plasma levels were measured at admission. Illness severity was assessed using the SAPS 3 score. Hospital length of stay, and mortality rate were recorded in all patients.

**RESULTS.** During the study period, 108 patients were included. Patients discharged alive from ICU (n = 83) had significantly lower Nt-Pro-BNP levels (860 pg/ml [range, 25–4120 pg/ml]) than ICU non survivors (n = 25) (2485 pg/ml [range, 80–5180 pg/ml]); p = 0.004. Likewise, hospital survivors (n = 76) were characterized by significantly lower Nt-Pro-BNP levels than hospital non survivors (n = 32) (862.5 pg/ml [range, 25–4120 pg/ml] vs. 1950 pg/ml [range, 60–5180 pg/ml], respectively; p = 0.004). The area under the ROC curve was 0.76 (95% confidence interval [CI], 0.66-0.86; p < 0.001) for Nt-Pro-BNP and 0.83 for SAPS 3 (95% confidence interval [CI], 0.75-0.92; p < 0.001). The difference between the curves did not reach statistical significance (p = 0.125). The difference in mortality rates becomes evident after 6 days from admission. Survival distribution was significantly different in the two groups (P < 0.001). Median survival was 12 days (95% [CI], 3–20 days) in the group Nt-Pro-BNP ≥ 1300 pg/ml vs. 32 days (95% [CI], 22–41 days) in the group Nt-Pro-BNP < 1300 pg/ml, p < 0.001. Multi logistic regression analysis revealed that SAPS 3 score (odds ratio [OR], 9.6; 95% [CI], 3.7 - 25.22; p < 0.001) and an Nt-Pro-BNP level more than 1300 pg/ml (OR, 4.38; 95% [CI], 1.78 - 10.78; p < 0.001) and sepsis
(OR, 9.42; 95% [CI], 2.7 - 32.69; p = 0.018) were significant and independent outcome predictor.

**CONCLUSIONS.** In this study, elevated admission Nt-proBNP levels is an independent predictor of mortality in critically ill patients.

### 0201

#### Psychological impact in post-ICU survivors

J. B. Costa[1], S. Taba[1], J. Ricardi[2], L. Lucchesi[2], I.R. Porto[3], P.A.D. Duarte[3]
[1]Hospital Universitário do Oeste do Paraná, Dept of Psychology, Cascavel, Brazil; [2]Hospital Universitário do Oeste do Paraná, Cascavel, Brazil; [3]Hospital Universitário do Oeste do Paraná, General ICU, Cascavel, Brazil
**Correspondence:** I.R. Porto

*Intensive Care Medicine Experimental* 2017, **5(Suppl 2):**0201

**INTRODUCTION.** Survivors from ICU admissions are more likely to develop anxiety, posttraumatic stress disorder, and depression, culminating in a poorer quality of life both physically and mentally. The objective of this study was to evaluate the incidence of psychological disorders of adult patients, 2 months after discharge from the ICU.

**METHODS.** Retrospective cohort study, with all adult patients post-ICU survivors (at General ICU in a teaching hospital) attended in the post-ICU Outpatient Office in the same institution. Patients with previously diagnosed mental illness, terminal illness, admitted for attempted suicide, unable to talk, and with neurological damage after ICU discharge were excluded. It was made psychological evaluation by specific instruments:

(1) Intensive Care Unit Memory/ICUM (for evaluate recollections),
(2) Hospital Anxiety and Depression Scale/HADS, and
(3) Impact of Event Scale-Revised (IES-R), for evaluating Post-Traumattic Stress Disorder (PTSD) symptoms.

**RESULTS.** During the study period (2008–2014), XXX patients were discharged alive from the ICU, of which 629 were attended by the psychology team at the post-ICU clinic (62% male, age 42.7 y; the most common causes of admission to the ICU were trauma [42%] and medical [23%]). The incidence of psychological disorders was 45.0%, with 30.4% with anxiety, 13.8% depression and 19.4% with PTSD symptoms. The distribution of incidences is shown in Fig. 83.
The main factors related to the presence of psychological disorders were age, type / cause of ICU admission (with elective surgery patients having a lower incidence of psychological disturbances, and neurological (trauma or not) patients with a trend to more psychological disorders), and use/time of MV during the ICU.

**CONCLUSION.** The incidence of psychological disturbances 2 months after discharge from the ICU is high, especially in elderly patients, those with acute neurological diseases and with longer MV use.

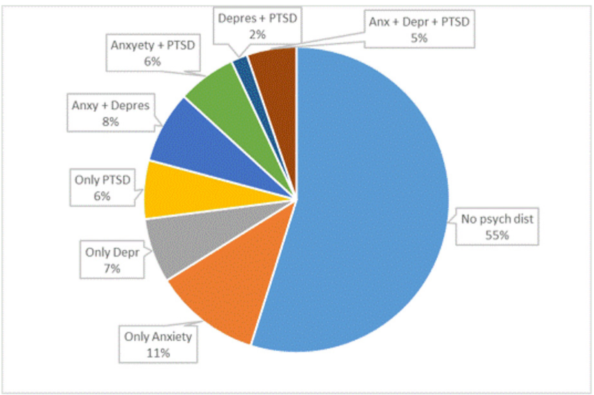

**Fig. 83 (abstract 201)** Distribution of psychological disorders in post-ICU patients. n = 629.

### 0202

#### Assessment of presence and dynamic evolution of organ failures during early intensive management by MERCIC model can guide inappropriate 'Futile Care' in the first week in critically ill cirrhotics

R. Maiwall[1], P. Singh[2], P. Jain[3], G. Kumar[1], S. Sarin[1]
[1]ILBS, Hepatology, New Delhi, India; [2]Jaypee Hospital, Hematology, New Delhi, India; [3]ILBS, Biostatistics, New Delhi, India
**Correspondence:** R. Maiwall
*Intensive Care Medicine Experimental* 2017, **5(Suppl 2):**0202

**BACKGROUND AND AIMS.** Admission of patients with cirrhosis with organ failures to the intensive care unit (ICU) is associated with high mortality despite aggressive management and involves a complex resource usage, and considerations for withdrawing or withholding life sustaining treatments. We aimed to develop and validate a dynamic model, the **M**odel for **E**arly **R**ecovery for **C**ritically **I**ll **C**irrhotics (MERCIC)to predict intensive care futility in a prospective cohort of critically ill cirrhotics.

**METHODS.** Consequtive[A1] 349 patients with cirrhosis admitted to dedicated Liver ICU, were included, 145 in the derivation and 204 in validation cohort.Binary logistic regression models were used to identify score parameters and the derived estimated coefficients were used as relative weights to compute the score which was also tested for sequential use with repeated measures Generalised Estimating Equations (GEE). The predictive ability of the score was compared to MELD, CTP and SOFA scores.

**RESULTS.** The mean age of the cohort was 48(±11) years, 87% males, with mean MELD of 30 (±8.3) and SOFA score of 11.6 (±4.5). Of these only 53% were alive at 1-month. At day 7, 45% patients recovered and were shifted out from the ICU. Of all patients with non-recovery at day 7 only 3% were alive at 28 days.Presence of circulatory shock (none versus one versus two vasopressors), serum bilirubin (gm/L) (<5 versus 5–15 versus >15), PaO2/FiO2 ratio(<200, versus 200–350 versus ≥ 350) and urine output (<0.5ml/kg/hr versus ≥0.5ml/kg/hr) at day 0 were identified as significant predictors of non-recovery at day 7 and as components of the MERCIC. Further, each unit change in score was significantly associated with recovery at day 7 ($p < 0.001$,OR 0.74, 95% CI 0.68-0.81) on sequential evaluation from day 0 to day7. Patients who recovered at day 7 showed decline ($p < 0.05$) in bilirubin (OR,95% CI) [(0.91, 0.87-0.95), (0.95,0.91-0.99)] and improvement in mean arterial pressure [(1.04,1.02-1.06; (1.03,1.01-1.05)], PaO2/Fio2 ratio [(1.27,1.02-1.59);1.17, 1.01-1.4) and urine output [(1.02,1.003-1.03); 1.02,1.005-1.04)] in both the derivation and validation cohorts respectively.

The AUROC of model was 0.85 and 0.74 in the derivation and the validation cohorts which was better than the MELD (0.57, 0.50), CTP (0.50,0.52) and SOFA scores (0. 66,0.61) respectively ($p < 0.05$).
The model also predicted 28-day mortality with fair accuracy ($p < 0.001$, HR 1.18, 95% CI 1.11-1.25).

**CONCLUSIONS.** The MERCIC model can be used as an accurate tool to guide intensive care futility in the first week in critically ill cirrhotics.
[A1] Check if correct

### 0203

#### Predictors of morbidity and mortality among adult Filipino post-coronary artery bypass graft patients at the Makati Medical Center from January 2011 to March 2016

L. A. R. Labrador[1], J.M.B. Bernardo[2]
[1]Makati Medical Center, Medicine, Parañaque, Philippines; [2]Makati Medical Center, Cardiology and Vascular Medicine, Makati, Philippines
**Correspondence:** L. A. R. Labrador
*Intensive Care Medicine Experimental* 2017, **5(Suppl 2):**0203

**INTRODUCTION.** Coronary artery bypass graft is indicated for revascularization therapy in severe and unstable coronary artery disease and risk assessment has been a major concern in cardiac surgery.

**OBJECTIVES.** To determine whether patient demographics, EuroSCORE II factors, creatinine and ALT are predictive in the morbidity and mortality of post-coronary artery bypass graft patients.

**METHODS.** Patient dermographics, EuroSCORE II factors, ALT, serum creatinine, postoperative outcomes, length of ICU and hospital stay from in-hospital charts were collected using retrospective cohort analysis of 169 patients who underwent coronary artery bypass graft (CABG) surgery from January 2011 to March 2016.

**RESULTS.** Presence of extra-cardiac arteriopathy (OR 4.37, 1.29-14.79 CI) is predictive in determining outcomes of patients after CABG surgery.Inotrope use (75.1%) and blood transfusion (100%) were common complications after CABG surgery.

**CONCLUSIONS.** It is important to determine presence of extracardiac arteriopathy in predicting outcomes of post-CABG patients.

**REFERENCE(S)**

1. Kasper, DL, Hauser, SL, Jameson, JL, Fauci, AS, Longo, DL, Loscalzo, J. Harrison's Principles of Internal Medicine. (19th ed). McGrawHill USA, 2015; Volume II
2. Passamani E, Davis KB, Gillespie MJ, Killip T. A randomized trial of coronary PHA Clinical Practice Guidelines for the Management of Coronary Artery Disease 59 artery bypass surgery: survival of patients with a low ejection fraction. N Engl J Med. 1985; 312: 1665–1671.
3. The VA Coronary Artery Bypass Surgery Cooperative Study Group. Eighteenyear follow-up in the Veterans Affairs Cooperative Study of Coronary Artery Bypass Surgery for stable angina. Circulation. 1992; 86: 121–130.
4. Varnauskas E. Twelve-year follow-up of survival in the randomized European Coronary Surgery Study. N Engl J Med. 1988; 319: 332–337
5. Sipahi, I., Akay, H., Dagdelen, S., Blitz, A., Alhan, C. Coronary Artery Bypass Grafting vs Percutaneous Coronary Intervention and Long-term Mortality and Morbidity in Multivessel Disease: Meta-analysis of Randomized Clinical Trials of the Arterial Grafting and Stenting Era. JAMA Intern Med 2014; 174(2): 223–230
6. Al-Alao BS, Parissis H, McGovern E, et al. Gender influence in isolated coronary artery bypass graft surgery: a propensity match score analysis of early outcomes. Gen Thorac Cardiovasc Surg 2012;60:417–24.
7. Saxena A, Dinh D, Smith JA, et al. Sex differences in outcomes following isolated coronary artery bypass graft surgery in Australian patients: analysis of the Australasian Society of Cardiac and Thoracic Surgeons cardiac surgery database. Eur J Cardiothorac Surg 2012;41:755–62.

ACKNOWLEGDMENT
The researcher would like to thank Dr. Jasmin Melissa Bernardo and Dr. Tarcela Gler for their guidance and insight in making this research an achievement.

**Table 64 (Abstract 0203)**. Multivariate regression analysis

| Significant Variables in the equation | OR (95% CI) | p-value |
|---|---|---|
| Extracardiac Arteriopathy (n=20) | 4.37(1.29-14.79) | 0.018 |
| Pulmonary Hypertension | | |
| Normal | 0 | 0.003 |
| Moderate (n=49) | 0.88(0.07-11.57) | 0.921 |
| Severe (n=3) | 3.60(0.27-47.64) | 0.331 |

### 0204

#### Blood glucose variation concentration as a predictor of mortality in critically patients in ICU Dr. Hasan Sadikin Hospital

T. T. Maskoen, S. L. Novita, S. Suwarman
Faculty of Medicine University of Padjadjaran, Anesthesiology and Intensive Therapy, Bandung, Indonesia
**Correspondence:** T. T. Maskoen
*Intensive Care Medicine Experimental* 2017, **5(Suppl 2):**0204

**INTRODUCTION.** Hyperglycaemia stress is common during critical illness and become one of important targets in intensive care. In severe hyperglycemia, there are changes in metabolism and cytokine response as well as anti-regulatory hormones release that cause cell damage due to accumulation of reactive oxygen species. Blood sugar level regulation within a narrow range (80–110 mg/dl) is recommended as an effort to reduce mortality in critically ill patients in the ICU.

Previous studies show an association between a wide variations of blood sugar levels with increasing mortality rate. APACHE II is an assessment system generally used to estimate mortality in ICU patients that involves a few examinations such as, body temperature, MAP, heart rate, respiratory rate, Oxygen fraction, pH, Natrium serum, kalium serum, creatinine, haematocrit, lecocyte and Glasgow coma score.

**OBJECTIVES.** This study aims to determine the usefulness of variations in blood sugar levels as a simplier predictor of mortality of critically ill patients in the ICU of Dr. Hasan Sadikin Hospital.

**METHODS.** Analytic Observational Retrospective Study. The patients blood sugar level were recorded as the object of this study and were divided into 2 groups based on the high standard deviation values reprensenting variations in blood sugar levels with a high range (SD > 20.61) and a lower standard deviation (SD < 20.61) representing variations in blood sugar levels with narrow range. Reassessment survival rate performed on day 28 for each group to assess whether any of these patients died.

**RESULTS.** The results showed that variations in blood sugar levels had a sensitivity of 71.43, specificity of 86.36, positive predictive value of 86.96, negative predictive value of 70.37, positive likelyhood ratio variations in blood sugar levels of 5.23, and likelyhood ratio of negative variations in blood sugar levels of 0.33.

**CONCLUSIONS.** Conclusion of this study is that variations in blood sugar levels can be used to predict mortality critically ill patients admitted to the ICU RSHS Bandung.

**REFERENCE(S)**

1. Hermanides J, Vriesendorp TM, Bosman RJ, Zandstra DF, Hoekstra JB, Devries JH. Glucose variability is associated with intensive care unit mortality. Crit Care Med 2010;38(3):834–42.
2. Todi S, Bhattacharya M. Glycemic variability and outcome in critically ill. Indian J Crit Care Med 2014;18(5):285–90.
3. Egi M, Bellomo R, Stachowski E, French CJ, Hart G. Variability of blood glucose concentration and short-term mortality in critically ill patients. Anesthesiology 2006;105(2):244–52.
4. Falciglia M, Freyberg RW, Almenoff PL, D´Alessio DA, Render ML. Hyperglycemia-related mortality in critically ill patients varies with admission diagnosis. Crit Care Med 2009;37(12):3001–9.

### 0205

#### The Simplified Mortality Score for the Intensive Care Unit (SMS-ICU): development and internal validation of a simple score to predict 90-day mortality

A. Granholm[1], A. Perner[1,2], M. Krag[1], P.B. Hjortrup[1], N. Haase[1], L.B. Holst[1], S. Marker[1], M.O. Collet[1], A.K.G. Jensen[3], M.H. Møller[1]
[1]Copenhagen University Hospital - Rigshospitalet, Department of Intensive Care 4131, Copenhagen, Denmark; [2]Centre for Research in Intensive Care, Copenhagen, Denmark; [3]University of Copenhagen, Section of Biostatistics, Copenhagen, Denmark
**Correspondence:** A. Granholm
*Intensive Care Medicine Experimental* 2017, **5(Suppl 2):**0205

**INTRODUCTION.** Existing intensive care unit (ICU) mortality prediction scores suffer from complexity and limited accuracy of predictions. An updated, simple score could prove a valuable tool in both research and clinical practice.

**OBJECTIVES.** To develop and internally validate a new and simple score that predicts 90-day mortality upon acute admission to the ICU.

**METHODS.** We developed a simple score as recently recommended [1] and according to a predefined, published protocol and statistical analysis plan [2]. We used binary logistic regression and multinational datasets including 2139 acutely admitted general ICU patients and 1947 with severe sepsis/septic shock (the AID-ICU and SUP-ICU inception cohorts and the 6S, TRISS and CLASSIC trials). We selected variables through backward elimination, multiply imputed missing data and assessed discrimination, overall performance, calibration, and internal validity.

**RESULTS.** The Simplified Mortality Score for the ICU (SMS-ICU) comprises seven variables readily available following ICU admission (Fig. 84): age, hematologic malignancy or metastatic cancer, acute surgical admission, lowest systolic blood pressure, and use of vasopressors/inotropes, respiratory support and renal replacement therapy.

Discrimination (area under the receiver operating characteristic curve) was 0.72 (95% CI: 0.71-0.74), overall performance (Nagelkerke´s $R^2$) was 0.19, and calibration (intercept and slope) was 0.00 and 0.99, respectively. Optimism-corrected performance (internal validity using bootstrapping) was similar to apparent performance.

**CONCLUSIONS.** The SMS-ICU predicted 90-day mortality upon ICU admission with reasonable and very stable performance. The score will be externally validated and compared to existing scores. If performance remains adequate, it could prove a valuable tool for ICU clinicians and researchers because of its simplicity and expected low number of missing values.

**REFERENCE(S)**

1. Labarère J, Bertrand R, Fine MJ (2014). How to derive and validate clinical prediction models for use in intensive care medicine. Intensive Care Med 40:513–527. doi: 10.1007/s00134-014-3227-6
2. Granholm A, Perner A, Krag M, Hjortrup PB, Haase N, Holst LB, Marker S, Collet MO, Jensen AKG, Møller MH (2017). Simplified Mortality Score for the Intensive Care Unit (SMS-ICU): protocol for the development and validation of a bedside clinical prediction rule. BMJ Open 7:e015339. doi:10.1136/bmjopen-2016-015339

**GRANT ACKNOWLEDGMENT**
The present work was supported by a grant from Rigshospitalet's Research Council.

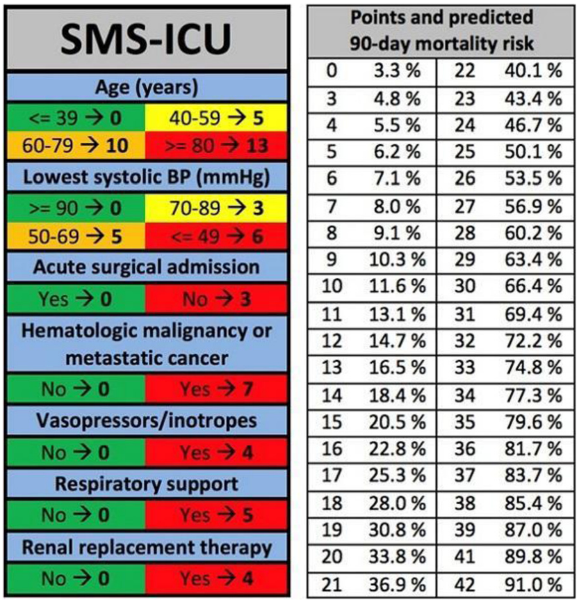

| SMS-ICU | |
|---|---|
| Age (years) | |
| <= 39 → 0 | 40-59 → 5 |
| 60-79 → 10 | >= 80 → 13 |
| Lowest systolic BP (mmHg) | |
| >= 90 → 0 | 70-89 → 3 |
| 50-69 → 5 | <= 49 → 6 |
| Acute surgical admission | |
| Yes → 0 | No → 3 |
| Hematologic malignancy or metastatic cancer | |
| No → 0 | Yes → 7 |
| Vasopressors/inotropes | |
| No → 0 | Yes → 4 |
| Respiratory support | |
| No → 0 | Yes → 5 |
| Renal replacement therapy | |
| No → 0 | Yes → 4 |

| Points and predicted 90-day mortality risk | | | |
|---|---|---|---|
| 0 | 3.3 % | 22 | 40.1 % |
| 3 | 4.8 % | 23 | 43.4 % |
| 4 | 5.5 % | 24 | 46.7 % |
| 5 | 6.2 % | 25 | 50.1 % |
| 6 | 7.1 % | 26 | 53.5 % |
| 7 | 8.0 % | 27 | 56.9 % |
| 8 | 9.1 % | 28 | 60.2 % |
| 9 | 10.3 % | 29 | 63.4 % |
| 10 | 11.6 % | 30 | 66.4 % |
| 11 | 13.1 % | 31 | 69.4 % |
| 12 | 14.7 % | 32 | 72.2 % |
| 13 | 16.5 % | 33 | 74.8 % |
| 14 | 18.4 % | 34 | 77.3 % |
| 15 | 20.5 % | 35 | 79.6 % |
| 16 | 22.8 % | 36 | 81.7 % |
| 17 | 25.3 % | 37 | 83.7 % |
| 18 | 28.0 % | 38 | 85.4 % |
| 19 | 30.8 % | 39 | 87.0 % |
| 20 | 33.8 % | 41 | 89.8 % |
| 21 | 36.9 % | 42 | 91.0 % |

**Fig. 84 (abstract 0205)** See text for description

### 0206

#### Severity of illness scores may misclassify critically ill obese patients

R. O. Deliberato[1,2], S. Ko[2,3], L.A. Celi[2,4], D.J. Stone[2,5]
[1]Hospital Israelita Albert Einstein, Critical Care, São Paulo, Brazil; [2]Massachusetts Institute of Technology, Harvard-MIT Health Sciences and Technology, Cambridge, United States; [3]Harvard Medical School, Harvard T.H. Chan School of Public Health, Boston, United States; [4]Beth Israel Deaconess Medical Center, Department of Medicine, Boston, United States; [5]University of Virginia School of Medicine, Anesthesiology and Neurosurgery, Charlottesville, United States
**Correspondence:** R. O. Deliberato
*Intensive Care Medicine Experimental* 2017, **5(Suppl 2):**0206

**INTRODUCTION.** Obesity is currently a global pandemic, responsible for 3–4 million deaths per year [1].Obesity is over-represented in the intensive care unit (ICU), comprising approximately one third of patients, compared to the 20% prevalence of being overweight or obese worldwide. ICUs commonly use severity of illness scores, such as APACHE, SAPS-II or SOFA to predict mortality, but none of these scoring systems incorporates obesity into their risk adjustment parameters.

Although obese and normal weight patients may present to the ICU with a similar physiological "snapshot or phenotype" as reflected by the same severity of illness scores, these identical scores may actually represent inherently different levels of deviation from the prior baseline state. This may inadvertently result in misclassification, leading to potential errors in mortality prediction and severity adjustment.

**OBJECTIVES.** We analyzed a large ICU database (which included baseline laboratory results prior to hospital admission) to compare the deviation of laboratory tests utilized in scoring systems from baseline to ICU admission in both obese and normal weight patients.

**METHODS.** Retrospective cohort study performed in an intensive care unit (ICU) database.We included obese and normal weight patients with age greater ≥ 16 years old who were admitted to the ICU between 2001 to 2012, and had laboratory results documented between 3 days to 1 year prior to hospital admission. Deviation on ICU admission from baseline in the laboratory tests used in calculating SAPS-II and SOFA score was compared between obese and normal weight patients.

**RESULTS.** 769 normal weight patients were compared to 1,258 obese patients. Adjusting for SAPS-II score, age, comorbidity index, baseline result and ICU type, the deviation in white blood cell count (WBC) was 0.80 (95%CI 0.27-1.33) x$10^9$/L higher in obese patients than normal weight patients (p = 0.003); and the deviation in blood urea nitrogen (BUN) was 1.50 (95%CI 0.28-2.71) mg/dl higher in obese patients than normal weight patients (p = 0.016). Adjusting for SOFA score, age, comorbidity index, baseline result and ICU type, the deviation in log(creatinine) was 0.03 (95%CI 0.02-0.05) higher in obese patients than normal weight patients (p < 0.001).

**CONCLUSION.** Among patients with the same severity of illness score, deviations in WBC, creatinine and BUN from baseline were significantly higher in obese compared to normal weight patients. Accounting for the extent to which critically ill patients deviate from their own baseline may improve the objectivity, precision, and generalizability of ICU mortality prediction and severity adjustment models.

**REFERENCE(S)**

1. Ng M, Fleming T, Robinson M, et al. Global, regional and national prevalence of overweight and obesity in children and adults during 1980–2013: a systematic analysis for the Global Burden of Disease Study 2013. Lancet. 2014; 384 (9945): 766.

**Table 65 (Abstract 0206)**. Demographic Characteristics

| | Normal weight (BMI = 18.5 - 24.9) N = 769 | Obese (BMI >30) N = 1,258 | p-value |
|---|---|---|---|
| Age (years), (median, IQR) | 66.9 (53 -78.5) | 64.3 (55.5 - 72.7) | 0.01 |
| Male, n (%) | 431 (56%) | 741 (59%) | 0.22 |
| Ethnicity (white), n (%): | 583 (76%) | 583 (76%) | <0.001 |
| Comorbidity index (median,IQR) | 5 (0 - 10) | 2 (0- 7) | <0.001 |
| Smoker (yes), n(%): | 379 (49%) | 649 (52%) | 0.20 |
| Primary ICD-9 diagnosis (cardiovascular disease),n (%) | 357 (46%) | 528 (42%) | 0.01 |
| mechanical ventilation (1st 24h), n(%) | 453 (59%) | 937 (74%) | <0.001 |
| SAPS-II score (median, IQR) | 35 (27 - 45) | 32 (25.2 - 40) | <0.001 |
| SOFA score (median, IQR) | 4 (2 - 6) | 4 (2 - 6) | 0.02 |

**Table 66 (Abstract 0206)**. Unadjusted laboratory deviation (ICU-baseline)

| | Normal weight | Obese | P-value |
|---|---|---|---|
| WBC x109/L, mean ± SD | 5 ± 5.9 | 6.4 ± 5.9 | <0.001 |
| Sodium, mmol/L, mean ± SD | −2.6 ± 4.3 | −3.2 ± 3.6 | 0.003 |
| Potassium, mmol/L, mean ± SD | 0.8 ± 1.1 | 1 ± 0.9 | 0.001 |
| BUN, mg/dl, mean ± SD | 3.1 ± 15.2 | 1.9 ± 14.2 | 0.10 |
| BIC, mg/dL, mean ± SD | −4 ± 4.1 | −4 ± 3.6 | 0.8 |
| Creatinine, mg/dL, mean ± SD | 0.1 ± 0.8 | 0.2 ±0.8 | 0.05 |
| Platelets x109/L, mean ± SD | −75 ± 118.8 | −70.6 ± 79 | 0.37 |

**Table 67 (Abstract 0206)**. Adjusted laboratory deviation (ICU -baseline)

| | Adjusted difference in deviation (Δ (ICU - baseline) between obese and normal weight individuals (95%CI) | P-value |
|---|---|---|
| Δ WBC, x109/L | 0.80 (0.27 -1.33) | 0.003 |
| Δ Sodium, mmol/L | −0.06 (-0.40 - 0.28) | 0.712 |
| Δ Potassium, mmol/L | 0.01 (-0.07 - 0.09) | 0.857 |
| Δ BUN, mg/dL | 1.50 (0.28 - 2.71) | 0.016 |
| Δ BIC, mg/dL | −0.19 (-0.50 - 0.13) | 0.254 |
| Δ log (Creatinine, mg/dL) | 0.03 (0.02-0.05) | <0.001 |
| Δ Platelets, x109/L | 4.94 (-2.48 - 12.36) | 0.192 |

### 0207

#### Evaluation of APACHE II scale in patients admitted in intensive care by non-traumatic brain hemorrhage

M. Redondo-Orts[1], E. Aguilar-Alonso[2], M.D. Pola-Gallego de Guzman[1], E. Castillo-Lorente[3], C. De La Fuente-Martos[2], E. Moran-Fernandez[2], R. Vela-Colmenero[1], A.M. Castillo-Rivera[1], C. Lopez-Caler[4], J.M. Garcia-Alvarez[5], F. Guerrero-Lopez[3]

[1]Intensive Care Unit - Complejo Hospitalario, Jaen, Spain; [2]Intensive Care Unit - Hospital Infanta Margarita, Cabra, Spain; [3]Intensive Care Unit - Hospital Universitario Virgen de las Nieves, Granada, Spain; [4]Intensive Care Unit - Hospital Universitario Carlos Haya, Malaga, Spain; [5]Intensive Care Unit - Hospital Serrania, Ronda, Spain

**Correspondence:** M. Redondo-Orts

*Intensive Care Medicine Experimental* 2017, **5(Suppl 2):**0207

**INTRODUCTION.** Intracerebral hemorrhage is a stroke subtype with high mortality and significant disability among survivors.

**OBJECTIVES.** To evaluate the APACHE II scale in patients admitted to ICU with non-traumatic brain hemorrhage.

**METHODS.** Multicenter prospective observational study in three hospitals in Andalusia (Spain). We studied all patients with brain hemorrhage admitted to the Regional Hospital of Malaga (between 2006 to 2011), Neurotraumatology Hospital of Jaen (between 2010 to 2012) and Virgen de las Nieves Hospital of Granada (between 2006 to 2011).

Data were expressed as the mean and standard deviation for quantitative variables and percentages for qualitative variables. For the comparison of two means we used the Student's t-test and the Chi-squared test was used to compare proportions. Area under the ROC curve for analizing discrimination, Standardized Mortality Ratio (SMR) and Hosmer-Lemeshow Test for analizing calibration. Statistically significant differences: $p < 0.05$.

**RESULTS.** N = 336 patients (263 supratentorial). Mean age 59.43 ± 14.75 years, Glasgow score (GCS) at admission 8 ± 4 points, APACHE-II 21.03 ± 7.6 points, intraventricular hemorrhage (IVH) was 58.6% of patients. 105 patients were treated by surgery. The hospital mortality was 54.17%. Patients who died in hospital were older 63.76 ± 12.21 vs 59,43 ± 14.75 ($p < 0.001$), lower GCS 6 ± 3. vs 10 ± 4 ($p < 0.001$) and higher APACHE II 24.31 ± 6.37 vs 17.09 ± 7.08 ($p < 0.001$).

Hospital mortality predicted by APACHE II scale was 49.58% and observed hospital mortality (54.17%), SMR: 1.09 (0.94-1.24) (not statistically significant differences). The Hosmer-Lemeshow test was 12,28, so there not were statistically significant differences between the observed and predicted mortality by APACHE-II. Discrimination by the area under the ROC curve was 0.81 (0.76-0.85).

**CONCLUSIONS.** Patients admitted to the ICU with non-traumatic brain hemorrhage have a high mortality. APACHE II scale has a good discrimination and calibration.

**Table 68 (Abstract 0207)**. Analysis of APACHE II model calibration by the Hos

| Probability of death | N.° patients | Observed deaths | Predicted deaths | Observed survivors | Predicted survivors |
|---|---|---|---|---|---|
| <0.10 | 9 | 0 | 0.80 | 9 | 8.20 |
| 0.10-0.20 | 29 | 3 | 4.37 | 26 | 24.63 |
| 0.20-0.30 | 52 | 12 | 12.77 | 40 | 39.23 |
| 0.30-0.40 | 44 | 17 | 15.36 | 27 | 28.64 |
| 0.40-0.50 | 41 | 24 | 18.83 | 17 | 22.17 |
| 0.50-0.60 | 32 | 21 | 17.73 | 11 | 14.27 |
| 0.60-0.70 | 44 | 34 | 28.26 | 10 | 15.74 |
| 0.70-0.80 | 42 | 33 | 31.39 | 9 | 10.61 |
| 0.80-0.90/>0.90 | 32/11 | 29/9 | 26.89/10.21 | 3/2 | 5.11/0.79 |

### 0208

#### Validation of SAPS 3 and Charlson comorbidity index in the prognosis of cardiac patients admitted to Brazilian intensive care unit

N. M. Filgueiras Filho[1,2,3], R.L. Silva[2,4], E.F. Ricaldi[2,4], R.C. Menezes[3], I.B. Ferreira[3,5], A.L. Silva[2,3], C. S. Ballalai[2,4], C.V. De Lucia[2,3], J.C. Oliveira[3], C.S. Silva[2,3,4], M.E. Garrido[2,3], G.P. Araponga[3,5], L.N. Veiga[3,5], A.L. Gobatto[6], GEMINI

[1]Universidade do Estado da Bahia, Medicina, Salvador, Brazil; [2]Universidade Salvador, Medicina, Salvador, Brazil; [3]Núcleo de Ensino e Pesquisa - Hospital da Cidade, Medicina Intensiva, Salvador, Brazil; [4]Núcleo de Pesquisa Clínica - UNIFACS, Medicina, Salvador, Brazil; [5]Universidade do Estado da Bahia, Medicina Intensiva, Salvador, Brazil; [6]Hospital da CIdade, Medicina Intensiva, Salvador, Brazil

**Correspondence:** N. M. Filgueiras Filho

*Intensive Care Medicine Experimental* 2017, **5(Suppl 2):**0208

**INTRODUCTION.** The scores have been widely used in the Intensive Care and Cardiac Units. The prognosis system SAPS 3 (Simplified Acute Physiology Score 3) aims to establish a predictive mortality rate for patients admitted to Intensive Care Units (ICU). The Charlson Comorbidity Index (CCI) is a method of predicting mortality by classifying or weighting comorbid conditions. Acute coronary syndromes (ACS) are a common cause ICU admission. Specific prognostic scores have been developed and validated for ACS patients, like Thrombolysis in Myocardial Infarction (TIMI) and Global Registry of Acute Coronary Events (GRACE) scores.

**OBJECTIVES.** To evaluate the accuracy of the SAPS 3 and CCI scores in the prognosis of cardiac patients admitted to the ICU of a general hospital in northeast region of Brazil.

**METHODS.** Prospective cohort study placed in a general ICU. In the study were included 234 patients with cardiac disease of 1138 admitted patients to general ICU, from August 2015 and March 2017. All the patients were assessed using the SAPS 3 (Global Equation and Central & South American Equation) and CCI scoring systems. Logistic regressions analysis was used to calculate the sensitivity, specificity and accuracy as well as the OR probability of mortality. The ability to predict mortality was performed with ROC curves. The differences between observed-to- predicted mortality were analyzed with the Hosmer-Lemeshow test.

**RESULTS.** The study population had the following general characteristics: age 67 ± 16.15 years, men 59.8%, with a median time of 4 days of hospitalization (IQR = 2.0-7.0). In the subgroup of patients with ASC, the mean age was 65.89 ± 14.75 years, 48.6% female, length stay of 4 days (IQR = 1.0-5.0), with diagnosis at admission, ST-elevation myocardial infarction - STEMI (20.3%), non-

ST-elevation myocardial infarction - NSTEMI (64.9%) and unstable angina - UA (14.9%) and there were only three deaths. The median TIMI and Grace score were 3 (IQR = 2.00-4.00) and 112 (IQR = 86.8-142.8), respectively. The median (in points) in SAPS 3 score was 44 (CI = 38.0-50.0) and CCI was 1 (IQR = 0–2.0) and the area under the ROC curve was 0.74 (CI 95% = 0.85-1.00) and 0.52 (CI 95% = 0.89-1.00), respectively. Calibration was confirmed by the Hosmer-Lemeshow test for SAPS 3-$X^2$ = 6.029 df: 8 (p > 0.644) and CCI was $X^2$ = 3.08 df:8 (p > 0.214).

**CONCLUSIONS.** The results showed that SAPS 3 score can be effective in the prognostic assessment of cardiac patients admitted to a general ICU, with good calibration and adequate discrimination value. However, the CCI score doesn't have good discrimination in this population.

### 0209

#### Do prehospital risk assessment tools predict ICU admission within 48 hours?

M. Hoikka[1,2], T. Silfvast[3,4], T.I. Ala-Kokko[1,2]
[1]Oulu University Hospital, Anesthesiology, Division of Intensive Care, Oulu, Finland; [2]University of Oulu, Medical Research Centre, Research Unit of Surgery, Anaesthesia and Intensive Care, Oulu, Finland; [3]Helsinki University Central Hospital, Emergency Medicine, Helsinki, Finland; [4]University of Helsinki, Helsinki, Finland
**Correspondence:** M. Hoikka
*Intensive Care Medicine Experimental* 2017, **5(Suppl 2):**0209

**INTRODUCTION.** Critical components of an efficient emergency medical service (EMS) include correct risk assessment and emergency patient identification (1). Under-triage may delay patients' appropriate medical care and negatively impact patient survival (2). Knowledge about prehospital risk assessments´ predictability to ICU admissions is scarce (3).

**OBJECTIVES.** To compare risk assessments made by dispatchers and on the scene by EMS providers with ICU admission within 48 hours.

**METHODS.** All EMS patients over 15 years of age in two hospital districts (Kainuu and Länsi-Pohja) in Northern Finland during 1.1.2014-30.6.2014 were prospectively studied. The risk assessments according to the national criteria based dispatch protocol and prehospital National Early Warning Score (NEWS) were coupled with the ICU database to calculate admission and risk rates.

**RESULTS.** 204 out of 12.728 (1.6%) EMS patients were admitted to the ICU. The highest priority A and high-risk NEWS groups were associated with the highest risk ratio for ICU admission (RR 11.1; RR 17.6) (Table 69). There were 92 (45.1%) cases where either of the risk assessment tools classified the patient as a high-risk. In 36 (17.6%) patients admitted to the ICU both risk assessment tools classified the patients as low-risk in the prehospital phase.

**CONCLUSIONS.** Both the criteria based dispatch protocol and the prehospital NEWS indicated applicability to predict ICU admission in an undifferentiated EMS population. However, approximately one-sixth of ICU patients were classified as low-risk during the prehospital phase. Better tools are needed to identify critically ill patients in the early phase of the chain of medical care.

(1) Ornato JP. Science of emergency medical dispatch. Circulation. 2009; 119:2023–5.
(2) Jacobs IG. Emergency medical dispatch - more than merely sending the ambulance! Resuscitation 2011;82:1473–4.
(3) Williams TA, et al. The ability of early warning scores (EWS) to detect critical illness in the prehospital setting: A systematic review. Resuscitation 2016;102:35–43.

**GRANT ACKNOWLEDGMENT**

The study was funded by Oulu University EVO-grant.

**Table 69 (Abstract 0209).** ICU admission rate within 48 hours from EMS encounter classified according to either dispatch priorities or NEWS

| | Dispatch priorities | | | | NEWS class | | |
|---|---|---|---|---|---|---|---|
| | A | B | C | D | High | Medium | Low |
| N total | 616 | 3192 | 5637 | 3283 | 736 | 1871 | 10121 |
| ICU admission rate % (n) | 7.5 (46) | 2.4 (78) | 1.0 (58) | 0.7 (22) | 10.6 (78) | 3.5 (65) | 0.6 (61) |
| RR (95%CI) | 11.1 (6.8-17.7) | 3.6 (2.3-5.7) | 1.5 (0.94-2.5) | 1.0 (ref) | 17.6 (12.9-23.8) | 5.8 (4.1-8.1) | 1.0 (ref) |
| p | <0.001 | <0.001 | 0.085 | | <0.001 | <0.001 | |

### 0210

#### Can nT-proBNP level predict prognosis in acute exacerbations of COPD in intensive care unit?

O. Ediboglu, S. Ataman, F. Tuksavul, C. Kirakli
Dr. Suat Seren Chest Diseases and Surgery Training and Research Hospital, Intensive Care Unit, Izmir, Turkey
**Correspondence:** O. Ediboglu
*Intensive Care Medicine Experimental* 2017, **5(Suppl 2):**0210

**INTRODUCTION.** The prognostic value of nT-proBNP levels in COPD patients due to acute exacerbations in the intensive care unit (ICU) is unknown.

**OBJECTIVES.** In this study, we aimed to evaluate the relationship between nT-proBNP level, NIV success, weaning and mortality in patients who underwent invasive or noninvasive mechanical ventilation (IMV / NIV) support.

**METHODS.** In this prospective cohort study, demographics, comorbidity, APACHE II score, NIV success, arterial blood gases, intensive care unit and hospital stay, weaning and mortality rates of patients with acute exacerbation of COPD followed in the ICU between December 2015 and December 2016 were recorded. The relationship between these dataand nT-proBNP levels and trends were evaluated.

**RESULTS.** 110 patients (75 males) were included in the study. Median age was 69 (61–76) years and APACHE II was 19 (15–23) years. The highest median nT-proBNP level was found to be lower in cases with NIV success than in those with failure (p: 0.053). In addition, the highest median nT-proBNP level was significantly higher (4740 pg / ml vs. 3004 pg / ml, p: 0.001) in patients who underwent IMV than the ones who did not. During hospitalization, mortality was significantly higher in patients with a trend of elevated nT-proBNP levels (59% vs. 23%, p: 0.015).

**CONCLUSION.** In patients with acute exacerbations of COPD requiring mechanical ventilation, nT-proBNP measurement and trend monitoring may be valuable in predicting prognosis.

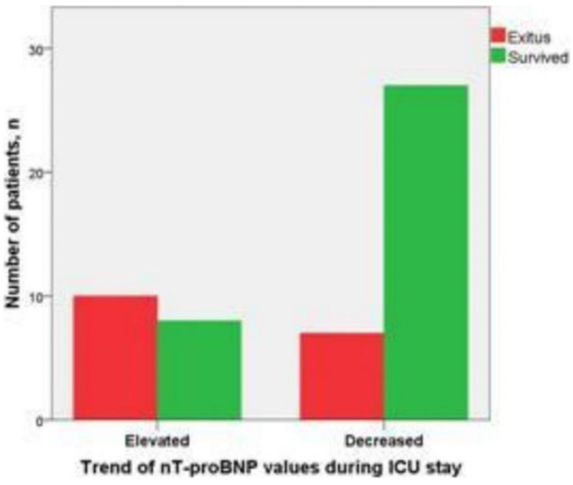

**Fig. 85 (abstract 0210)** nT-proBNP mortality

### Cardiovascular monitoring

#### 0211

#### Relationship between mean systemic filling pressure and mortality in critically ill patients

V. A. Bennett[1,2], H. D. Aya[1,2], J. Mellinghoff[1,2], B. Philips[1,2], N. Fletcher[1,2], A. Rhodes[1,2], M. Cecconi[1,2]
[1]St George's University Hospital NHS Foundation Trust, London, United Kingdom; [2]St George's University of London, London, United Kingdom
**Correspondence:** V. A. Bennett
*Intensive Care Medicine Experimental* 2017, **5(Suppl 2):**0211

**INTRODUCTION.** Optimisation of cardiac function and fluid management are well recognised as key components in the care of the critically ill patient. A cumulative positive fluid balance is associated with both increased mortality and morbidity.[1,2] Mean systemic filling pressure (Pmsf) is the pressure in the vascular system when the heart is stopped and there is no blood flow. It depends on the intravascular volume and mean vascular capacitance and it is a quantitative measurement of intravascular filling.[3]

**OBJECTIVES.** To determine if higher values of Pmsf are associated with increased 28, 60 and 90-day mortality in critically ill patients.

**METHODS.** Retrospective analysis of data collected in two different clinical trials at the ICU in a tertiary centre. 160 patients admitted to ICU were included for the analysis. 80 patients were admitted following cardiac surgery, and 80 because of septic shock. Pmsf-arm was measured at time of randomisation (recently admitted to ICU), using a stop-flow transient occlusion method. Linear regression analysis was performed to find the differences in Pmsf-arm between survivors and non-survivors at 28, 60 and 90 days post randomisation, adjusted by sepsis status (septics, non-septics) severity (APACHE II score) and diabetes mellitus status. Area under the receiver-operator curve (ROC) was calculated. Statistical analysis was performed with SPSS v24.

**RESULTS.** Mortality at 28, 60 and 90 days were 16.3%, 18.1% and 18.1% respectively. The mean Pmsf-arm for the whole sample is 21.12 mmHg (95% CI 20.09, 22.15). The estimated difference in Pmsf-arm between survivors and non-survivors at 28, 60 and 90 days adjusted by sepsis status and APACHE score is 0.38 mmHg (95% CI −2.71, 3.47, $p$ = .81), 1.01 mmHg (95%CI −1.91, 3.94, $p$ = .49) and 0.95 mmHg (95%CI −1.93, 3.83, $p$ = .52). Area under the ROC for mortality at 28, 60 and 90 days against Pmsf-arm values were 0.62 (95%CI .50, .74), 0.59 (95%CI .47, .70) and 0.59 (95%CI .47, .71) respectively.

**CONCLUSIONS.** High values of Pmsf-arm are not related to increased mortality in critically ill patients.

**REFERENCE(S)**

1. Vaara ST, Korhonen AM, Kaukonen KM, Nisula S, Inkinen O, Hoppu S et al. Fluid overload is associated with an increased risk for 90-day mortality in critically ill patients with renal replacement therapy: data from the prospective FINNAKI study 2012; 16(5):R197
2. Silversides JA, Major E, Ferguson AJ, Mann EE, McAuley DF, Marshall JC et al. Conservative fluid management or deresuscitation for patients with sepsis or acute respiratory distress syndrome following the resuscitation phase of critical illness: a systematic review and meta-analysis. Intensive Care Med 2017; 43(2):155–170
3. Aya HD, Rhodes A, Ster IC, Fletcher N, Grounds RM, Cecconi M. Hemodynamic effect of different doses of fluid for a fluid challenge: A quasi-randomized controlled study. Critical Care Medicine 2017;45(2):e161–e168

**GRANT ACKNOWLEDGMENT**
None

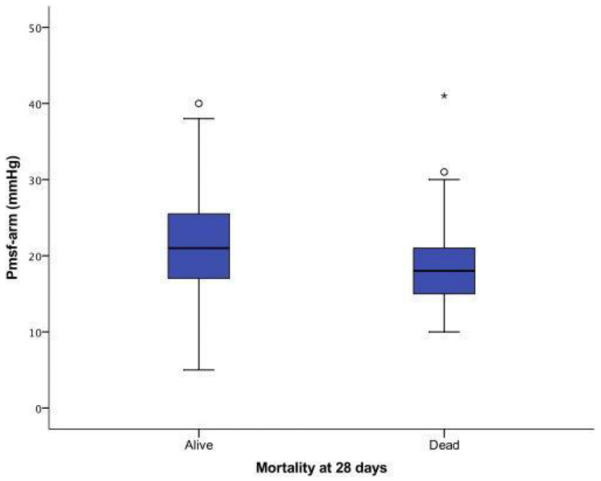

**Fig. 86 (abstract 0211)** Pmsf-arm values and 28 day mortality

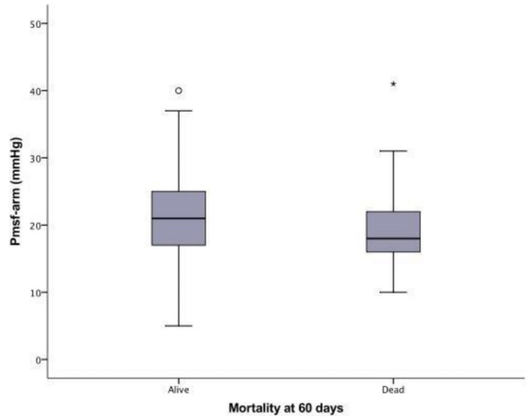

**Fig. 87 (abstract 0211)** Pmsf-arm values and 60 day mortality

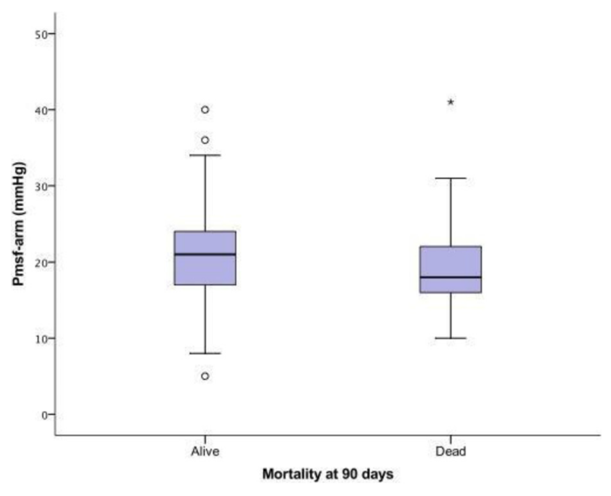

**Fig. 88 (abstract 0211)** Pmsf-arm values and 90 day mortality

### 0212

#### Estimation of thermodilution-derived Cardiac Index: a comparison of body surface temperatures and biometric data vs. the uncalibrated pulse contour analysis device Clearsight®

W. Huber, T. Lahmer, A. Herner, U. Mayr, S. Rasch, G. Batres-Baires, R. Schmid, M. Heilmaier
Klinikum rechts der Isar der TUM, Munich, Germany
**Correspondence:** W. Huber
*Intensive Care Medicine Experimental* 2017, **5(Suppl 2):**0212

**INTRODUCTION.** Cardiac Index CI is a key target of haemodynamic monitoring. Initial clinical assessment of shock patients is based on body surface temperature BST. Accurate measurement of BST with an infrared thermometer (Thermofocus, Tecnimed) combined with biometric data might provide a complete non-invasive estimate of CI (CI_CNI) with a precision and accuracy comparable to a non-invasive pulse-contour-analysis-estimate of CI (CI_CS; ClearSight, Edwards; USA).

**OBJECTIVES.** We compared BSTs alone and combined with biometrics ("CI_CNI") to CI_CS and to a gold-standard CI derived from transpulmonary thermodilution (TPTD; CI_TD; PiCCO; Pulsion; Germany).

**METHODS.** In 30 pats. (17m; 13f; APACHE-II 20 ± 9) 240 datasets were recorded (8 datasets per patient within 24h). Immediately before TPTD we measured BST on the forehead, forearm, finger and great toe and recorded un-calibrated CI_CS. These data were compared to TPTD-derived CI-TD (PiCCO). Statistics: SPSS 23.

**RESULTS.** CI-TD was univariately ($p < 0.001$ except as indicated) associated to the BSTs of forearm ($r = 0.507$; $p = 0.001$), forehead ($r = 0.433$), finger ($r = 0.250$) and great toe ($r = 0.228$) as well as to age ($r = -0.192$) and male gender ($r = 0.236$; $p = 0.012$) and to CI_CS ($r = 0.578$).

Multiple regression analysis including the BSTs demonstrated that BST_forehead ($p < 0.001$), BST_forearm ($p < 0.001$) and BST_finger ($p = 0.005$) were independently associated to CI_TD. This regression resulted in an CI-estimate based on BSTs (CI_BST; $R^2 = 0.333$).

A combined regression (CI_CNI; $R^2 = 0.428$) demonstrated independent association of BSTs on forehead, forearm and finger as well as male gender, age and height to CI_TD.

In a next step, we analysed, if these data could improve the estimation of CI by CI_CS. Several regression analyses regarding CI_TD demonstrated independent association of CI_CS in addition to CI_BST ($R^2 = 0.504$) or CI_CNI ($R^2 = 0.541$).

The final model (CI_BST_CS_biometry; $R^2 = 0.541$) included CI_BST, CI_CS, age, gender and height. The bias (L/min/m$^2$) and percentage error values for CI_BST (0.023; 40%), CI_CNI (−0.108; 38%), CI_CS (−0.347; 51%), CI_BST_CS (0.001; 35%) and CI_BST_CS_Biometry (0.057; 33%) demonstrated at least comparable accuracy and precision for CI_BST and CI_CNI compared to CI_CS. The combination of CI_CS with CI_BST or CI_CNI substantially improved its predictive capabilities.

This is supported by the ROC-AUCs regarding CI ≥ 5L/min/m$^2$ (all $p < 0.001$) for CI_BST (AUC = 0.779), CI_CS (0.835), CI_CNI (0.856), CI_BST_CS (0.869) and CI_BST_PA_Biometry (0.853) as well as regarding CI ≤ 3.25L/min/m$^2$ for CI_BST (AUC = 0.731; $p < 0.001$), CI_CS (0.717; $p = 0.001$), CI_CNI (0.652; $p = 0.016$), CI_BST_CS (0.773; $p < 0.001$) and CI_BST_A_Biometry (0.791; $p < 0.001$).

**CONCLUSIONS.** BSTs alone or combined with biometrics provide an estimate of CI with predictive capabilities at least comparable with those of CI_CS. Combination of CI_CS with CI_BST and biometrics substantially improves its prediction of CI_TD.

### 0213

#### Prediction of hypotension in surgical patients

T.W. Scheeren
University Medical Center Groningen, Anesthesiology, Groningen, Netherlands
*Intensive Care Medicine Experimental* 2017, **5(Suppl 2):**0213

**INTRODUCTION.** Patients in the perioperative setting are often at risk of developing arterial hypotension, which can lead to poor outcomes such as acute kidney and myocardial injury [1]. The objective of this observational study is to assess if the hypotension probability indicator (HPI) recently developed by Edwards Lifesciences (Irvine, USA) can predict hypotensive events before they occur more reliably than changes of mean arterial pressure (MAP) can.

**METHODS.** We studied 34 patients that were mainly undergoing off-pump Coronary Artery Bypass Grafting (OPCABG) surgery. The patient demographics are listed in Table 70. Radial arterial pressure waveforms were monitored with FloTrac IQ™ sensors (Edwards Lifesciences, Irvine, USA) and saved for offline analysis. A hypotensive event was defined as MAP < 65 mmHg for at least one minute. The ROC analysis was performed to assess AUC of HPI and ΔMAP to identify and predict a hypotensive event: four different ΔMAP were analyzed: they are calculated from two MAPs that are 20 seconds, 1 minute, 3 and 5 minutes apart, respectively.

**RESULTS.** A total of 389 hypotensive events were registered, 11 events on average per patient, and the cumulative time (mean (SD)) a patient spent in hypotension was 63 (±81) minutes. The area under the curve, sensitivity and specificity of HPI and ΔMAP in predicting hypotension 5 minutes prior to the actual occurrence of hypotension are listed in Table 71.

**CONSLUSION.** This study shows that the change of MAP cannot be used to predict hypotension, while HPI predicts the occurrence of hypotension with an acceptable accuracy (sensitivity and specificity >80%).

**Table 70 (Abstract 0213)**. Patient demographics

| | |
|---|---|
| Age, years, mean (SD) | 65 (13) |
| Weight, kg, mean (SD) | 81 (16) |
| Height, cm, mean (SD) | 173 (9) |
| Gender, n | 34 (10 F, 24 M) |

**Table 71 (Abstract 0213)**. Performance of HPI and ΔMAP in predicting

| Variable | AUC | Sensitivity | Specificity |
|---|---|---|---|
| HPI | 0.914 | 83.7% | 82.6% |
| ΔMAP 20sec | 0.644 | 56.5% | 65.2% |
| ΔMAP 1min | 0.625 | 51.9% | 52.2% |
| ΔMAP 3min | 0.564 | 47.4% | 47.8% |
| ΔMAP 5min | 0.615 | 57.0% | 56.5% |

### 0214

#### Norepinephrine increases arterial load and impairs left ventricular efficiency in septic shock

M. I. M. Monge García[1], A. Santos[2,3,4], B. Diez del Corral Fernández[1], P. Guijo González[1], M. Gracia Romero[1], A. Gil Cano[1], M. Cecconi[5]

[1]Hospital SAS Jerez, Intensive Care Unit, Jerez de la Frontera, Spain; [2]Hedenstierna Laboratory, Dep. of Surgical Science. Uppsala University, Upsala, Sweden; [3]Centro Nacional de Investigaciones Cardiovasculares Carlos III (CINIC), Madrid, Spain; [4]CIBER de Enfermedades Respiratorias (CIBERES), Madrid, Spain; [5]St George's University Hospital NHS Foundation Trust, London, United Kingdom

**Correspondence:** M. I. M. Monge García

*Intensive Care Medicine Experimental* 2017, **5(Suppl 2):**0214

**INTRODUCTION.** Persistent and profound vasoplegia is a common phenomenon associated with septic shock. The use of vasopressors for increasing arterial pressure is necessary to restore an adequate perfusion pressure. However, the impact of vasopressor therapy could potentially be detrimental if cardiac afterload is increased, especially if systolic function is already impaired.

**OBJECTIVES.** We aimed to test whether vasopressors alter the arterial pressure propagation and reflection phenomena in septic shock patients, and how this could affect left ventricular (LV) efficiency.

**METHODS.** 17 septic shock patients were monitored with an oesophageal Doppler and a radial arterial line. Pulse wave velocity (PWV) was calculated using the carotid-to-femoral PWV (Sphygmocor). Aortic pressure waveform was estimated from the carotid arterial pressure obtained by applanation tonometry. Arterial waveform analysis was performed separating the aortic pressure waveform in its forward (Pfw) and backward (Pbw) components. The ratio between the systolic area of Pbw and Pfw ($Pbw_{area}/Pfw_{area}$%), and Pbw and measured pressure ($Pbw_{area}/Pmeasured_{area}$%), augmentation index (the ratio of augmentation pressure to aortic pulse pressure), and reflection index (Pbw/Pbw + Pfw), were used as measures of LV load because of arterial reflections. Total, steady and oscillatory LV power, subendocardial viability ratio (SEVR, an index of myocardial perfusion relative to LV workload), and energy transmission ratio (the ratio between the hydraulic power in measured pressure and Pfw, expressing the effects of wave reflections on reducing LV hydraulic power) were calculated from the analysis of the central pressure and flow waveforms. Measurements were obtained before (Fig. 89) and after introduction or increasing norepinephrine dosage (Fig. 90).

**RESULTS.** Changing norepinephrine dosage from 0.05 (0 to 0.10) to 0.15 (0.08 to 0.27) $\mu g^{-1}Kg^{-1}min^{-1}$ increased mean arterial pressure (from 83 ± 13 to 98 ± 10 mmHg; p < 0.001); PWV (from 8.3 ± 2.2 to 10.3 ± 3 m/s; p < 0.001); $Pbw_{area}/Pfw_{area}$ (from 16.9 ± 9 to 20.7 ± 10.5%; p < 0.001); $Pbw_{area}/Pmeasured_{area}$ (from 13.9 ± 6.6 to 16.5 ± 7.2; p < 0.001); augmentation index (from −3 ± 25 to 11 ± 23%; p < 0.001), and reflection index (from 0.26 ± 0.05 to 0.28 ± 0.06; p < 0.001). Cardiac output and stroke volume did not change (5.86 ± 2.15 to 5.83 ± 2.3 L/min; p = 0.885; 66 ± 18 to 68 ± 18 mL; p = 0.201, respectively). Total, steady and oscillatory LV power increased (from 0.97 ± 0.48 to 1.14 ± 0.51 W; from 0.76 ± 0.36 to 0.87 ± 0.39 W; and from 0.21 ± 0.14 to 0.26 ± 0.15 W; p < 0.001, respectively), without increasing LV efficiency (ETR from 88 ± 7.5 to 85.1 ± 8.8%; p < 0.001, and SEVR from 154 ± 61 to 153 ± 62%; p = 0.871).

**CONCLUSIONS.** Vasopressor support modifies the arterial pressure propagation and reflection phenomena, increasing LV afterload and impairing LV efficiency.

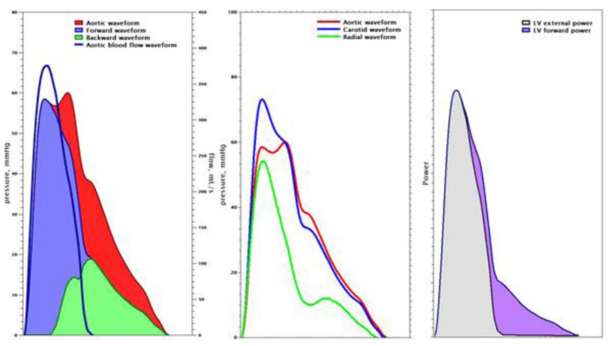

**Fig. 89 (abstract 0214)** See text for description

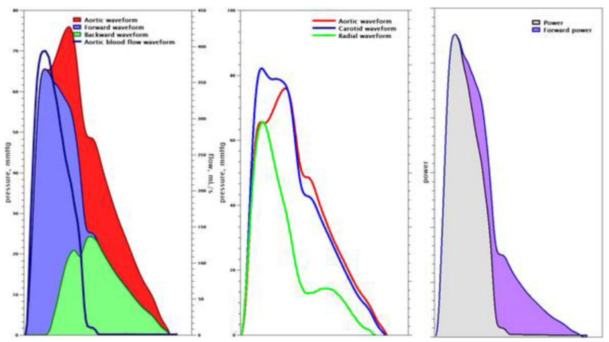

**Fig. 90 (abstract 0214)** See text for description

### 0215

#### Ventriculo-arterial coupling in patients with shock

K. El Sayed, S. El-Hadidy, Y. Nassar, R. El-Sherif

Cairo University, Cairo, Egypt

**Correspondence:** R. El-Sherif

*Intensive Care Medicine Experimental* 2017, **5(Suppl 2):**0215

**INTRODUCTION.** Shock is a state of acute circulatory failure. Understating the hemodynamic alterations in shocked patient is important for therapeutic planning.

**OBJECTIVES.** Assessment of change in ventricuo-arterial (V-A) coupling in patients with shock and its effect on short-term outcome.

**METHODS.** 40 patients with shock were included. At the time of preparing initial fluid resuscitation, the following transthoracic echocardiographic views were obtained (parasternal long axis, apical 4 & 5 chambers) for offline analysis and calculation of Venticulo-arterial coupling (VA coupling). VA coupling was measured using the following equation:

Ea/Ees = (ESP/SV)/(ESP/ESV)

Where Ea is arterial elastance, Ees is ventricular elastance, ESP is end systolic pressure (0.9 x systolic blood pressure (SBP)), SV is stroke volume and ESV is end-systolic volume.

Values more or less than 1 ± 0.36 were considered decoupled.
APACHE II score & its predicted mortality were calculated on admission and were compared with the in-hospital mortality.

**RESULTS.** 40 patients with mean age 60.25 ± 15.62 years and 60% males. They included 24 patients with cardiogenic shock, 15 with septic shock and one patient with hypovolemic shock. VA decoupling was detected in 36 patients (90%), 100% of septic shock, 83.3% of cardiogenic shock, and the patient with hypovolemic shock. In septic shock, decoupling was due to decreased Ea in 80% & decreased Ees in 40%.

In cardiogenic shock, decoupling was mainly due to decreased Ees (95.8%), increased Ea (62.5%), and decreased Ea (12.5%). The patient with hypovolemic shock had increased Ea and normal Ees.

The mean APACHE II scoring was 43.3 ± 11.5 with mean predicted mortality 68.4% ± 8. In-hospital mortality was 85%.

Predicted mortality by APACHE II showed good -ve correlation with Ea /Ees (r = −0.7) and poor correlation with SBP on admission (r = 0.16). Significant association was found between Ea/Ees and DM, predicted and in-hospital mortality, all with p < 0.001. No association was found between mortality and SBP on admission (p 0.5).

**CONCLUSIONS.** The incidence of VA decoupling was very high in patients with shock and appeared to be correlated with worse outcome. SBP on admission was not a good predictor of prognosis in shocked patients.

### 0216

#### Comparison of cardiac function index CFI derived from *jugular* and *femoral* indicator injection using the PiCCO-2 device: a prospective observational study

A. Herner, R. Schmid, W. Huber
Klinikum rechts der Isar der TUM, Munich, Germany
**Correspondence:** W. Huber
*Intensive Care Medicine Experimental* 2017, **5(Suppl 2):**0216

**INTRODUCTION.** Cardiac function index (CFI) is a transpulmonary thermodilution (TPTD)-derived index of systolic function. CFI is defined as the ratio of cardiac output CO divided by global enddiastolic volume GEDV: CFI = CO/GEDV. The validity of CFI relies on an accurate measurement of global end-diastolic volume GEDV. Several studies demonstrated that the use of *femoral* venous access results in a marked overestimation of GEDV. This also falsely reduces CFI. One of these studies suggested a correction formula for *femoral* venous access that markedly reduced the bias for GEDVI. Consequently, the last PiCCO-algorithm requires information about the CVC-position, and correction of GEDVI for *femoral* access has been shown. However, a recent study suggests inconsistencies of the last PiCCO algorithm using uncorrected GEDV to calculate CFI despite an obvious correction of GEDVI. Nevertheless, the study was based on mathematical analyses of the data displayed in a total of 15 patients equipped with only a *femoral*, but not with a *jugular* CVC.

**OBJECTIVES.** Therefore, this study compared CFI_fem derived from *femoral* TPTD to values derived from *jugular* indicator injection in 29 patients with both jugular and femoral CVCs.

**METHODS.** 29 ICU-patients with PiCCO-monitoring were included. Each dataset consisted of three triplicate TPTDs using the jugular venous access as the gold standard CFI_jug, and the femoral access with (CFI_fem_cor) and without (CFI_fem_uncor) information about the femoral indicator injection to evaluate, if correction for femoral GEDV pertains to CFI_fem.

**RESULTS.** 29 patients (14f; 15m; 26/29 mechanically ventilated; 20/29 under vasopressors). CFI_fem_uncor was significantly lower than CFI_jug (4.34 ± 1.70 vs. 5.29 ± 1.90 $min^{-1}$; p < 0.001). Similarly, CFI_fem_cor was significantly lower than CFI_jug (4.31 ± 1.6 vs. 5.29 ± 1.90 $min^{-1}$; p < 0.001). This is explained by the finding that CFI_fem_uncor was not different to CFI_fem_cor (4.34 ± 1.70 vs. 4.31 ± 1.6 $min^{-1}$; p = 0.884). This clearly suggests that correction for femoral CVC does not pertain to CFI. Calculatory correction of CFI_fem_uncor was performed by multiplying CFI_fem_uncor by the ratio GEDVI_fem_uncor/GEDVI_jug. This resulted in CFI_fem_formula which was not significantly different from CFI_jug (5.56 ± 1.94 vs. 5.29 ± 1.90 $min^{-1}$; p = 0.136). The agreement of measurements classified in the same category of CFI (decreased (<4.5), normal (4.5-6.5) and increased (>6.5 $min^{-1}$)) as CFI_jug was high for CFI_fem_formula (identical categories in 25 of 27 comparisons), whereas the agreement with CFI_jug was significantly lower for CFI_fem_cor (13 out of 27; p < 0.001) and CFI_fem_uncor (14 out of 27; p = 0.001).

**CONCLUSIONS.** Femoral indicator injection for TPTD results in significantly lower values for CFI. While the last PiCCO algorithm obviously corrects GEDV(I), this correction is not applied to CFI. Therefore, CFI-values are substantially underestimated in case of femoral CVC.

### 0217

#### The cardiovascular reserve index (CVRI) - a potential indicator of hemodynamic deterioration

U. Gabbay[1,2], R. Durst[3], E. Glassberg[4], M. J. Segel[5,6], K. Yoram[7,8]
[1]Rabin Medical Center, Quality Unit, Petach TiQwa, Israel; [2]Tel Aviv University, Epidemiology and Preventive Medicine, School of Public Health, Sackler Faculty of Medicine, Tel Aviv, Israel; [3]Haddasah Hebrew University, Cardiology, Jersusalem, Israel; [4]Israeli Defence Force, Medical Corps, Surgeon General Headquarter, Ramat Gan, Israel; [5]Sheba Medical Center, Lung Insitute, Ramat Gan, Israel; [6]Tel Aviv University, Sackler Faculty of Medicine, Tel Aviv, Israel; [7]Rambam Medical Center, General Surgery, Haifa, Israel; [8]Technion - Israeli Institute Technology, Rappaport Faculty of Medicine, Haifa, Israel
**Correspondence:** U. Gabbay
*Intensive Care Medicine Experimental* 2017, **5(Suppl 2):**0217

**INTRODUCTION.** The cardiovascular reserve hypothesis focuses on the remaining momentary cardiovascular reserve (the potential to gain cardiac output to meet increasing metabolic needs) rather than momentary cardiovascular performance per-se. It proposes a comprehensive explanation to aerobic exhaustion (the reserve decreased below exhaustion threshold), heart failure (low reserve which reaches exhaustion threshold even in daily activity) and shock (cardiovascular reserve decline to the sustainability limit regardless if the cardiac output is low or high) [1].The cardiovascular reserve index (CVRI) was proposed as an estimate of the assumed cardiovascular reserve based on the principles of control theory [CVRI = f(BP,CVP,HR,RR,BSA) where BP = blood pressure, CVP = central vein pressure, HR = heart rate, RR = respiratory rate, BSA = body surface area]. We review the entire results as for today.

**OBJECTIVES.** Empirical validity of CVRI was conducted by three researches: 1) Validity of CVRI in diverse hemodynamic morbidities and conditions. 2) Validity of CVRI during hemorrhage related hemodynamic deteriorations and 3) Validity of CVRI during exercise

**METHODS.** Validity of CVRI in diverse hemodynamic morbidities was conducted in ptients of 3 existing databases were evaluated (1. patients with diverse exercise capacity, 2. heart failure and 3. pending shock). Each database was stratified by severity to 3–4 subgroups. CVRI was computed for each patient. Validity of CVRI during hemorrhage was evaluated in 17 swine experiment in the surgeon general headquarter IDF. Validity of CVRI during exercise in patients who underwent cardio-pulmonary exercise testing (CPX) in Sheba Medical Center.

**RESULTS.** In diverse conditions highest mean CVRI was in the normal exercise capacity group (around 1.0) and the lower at shock (0.2) with intuitive ordinal order in-between [2]. During hemorrhage CVRI demonstrated nearly linear decrease slop from pre-hemorrhage (around 1.0) through increase in cumulative blood loss toward shock (0.2). Interestingly, CO exhibits considerable detectability delay [3]. During exercise CVRI demonstrated highest CVRI at rest and decrease with exercise intensity reaching a nadir at peak exercise (0.35) and regain with recovery [4].

**CONCLUSIONS.** all three researches comply with the assumed cardiovascular reserve. The results show that CVRI may indicate a rough hemodynamic state in single measurement. CVRI may indicate hemodynamic deterioration through monitoring or repeated measurements of an individual patient. Prospective studies are planned to validate CVRI predictive in diverse clinical settings.

**REFERENCE(S)**
1. Medical Hypotheses. 2014;82:694–699
2. Clinical Trials and Regulatory Science in Cardiology 2015;12:1–5
3. Annals of Medicine and Surgery.2017;14:1–7
4. Int J Cardiol 2017;234:33–37.
**GRANT ACKNOWLEDGMENT**
Studies 1–2 were supported by Cardio-Scale LTD who has a patent on CVRI in the US.

### 0218

#### Estimation of thermodilution-derived Cardiac Index: a comparison of body surface temperatures and biometric data vs. the uncalibrated pulse contour analysis device ProAqt ®

W. Huber, B. Brandt, T. Lahmer, U. Mayr, S. Rasch, R. Schmid, M. Heilmaier
Klinikum rechts der Isar der TUM, Munich, Germany
**Correspondence:** W. Huber
*Intensive Care Medicine Experimental* 2017, **5(Suppl 2):**0218

**INTRODUCTION.** Cardiac Index CI is a major target of haemodynamic monitoring. Initial clinical assessment is based on body surface temperature BST ("cold shock vs. warm shock"). We hypothesized that accurate measurement of BST with an infrared thermometer (Thermofocus, Tecnimed) combined with biometric data might provide a complete non-invasive estimate of CI (CI_CNI) with precision and accuracy comparable to a pulse contour analysis estimate of CI (CI_PA; ProAqt, Pulsion, Germany).

**OBJECTIVES.** We compared BSTs alone and in combination with biometric data (CI_CNI) to CI_PA and to a gold-standard CI derived from transpulmonary thermodilution (TPTD; CI_TD; PiCCO; Pulsion).

**METHODS.** In 29 patients (20m; 9f; APACHE-II 16 ± 5) 232 datasets were recorded (8 datasets per patient within 24h). Immediately before TPTD we measured BST on the forehead, forearm, finger and great toe and recorded CI_PA. Statistics: SPSS 23.

**RESULTS.** CI-TD was *univariately* ($p < 0.001$) associated to BST on the forehead ($r = 0.601$), forearm ($r = 0.473$), finger ($r = 0.392$) and great toe ($r = 0.391$) as well as to age ($r = -0.408$) and CI_PA ($r = 0.365$).

*In multiple regression analysis* BST_forehead ($p < 0.001$) and BST_forearm ($p < 0.001$) were independently associated to CI_TD. This regression resulted in an CI-estimate based on BSTs (CI_BST; $R^2 = 0.358$). Furthermore, age ($p = 0.001$), gender ($p = 0.047$) and height ($p = 0.053$) were independently associated to CI_TD. A combined regression (CI_CNI; $R^2 = 0.465$) demonstrated independent association of BSTs on forehead, forearm and toe as well as male gender, young age and height to CI_TD.

In a next step, we analysed, if these data could improve the estimation of CI by CI_PA. CI_TD was independently associated with CI_BST($R^2 = 0.434$) and/or CI_CNI in addition to CI_PA. The final model (CI_BST_PA_biometry; $R^2 = 0.485$) included CI_BST, CI_PA, age, gender and height as independent predictors of CI_TD. The bias (L/min/$m^2$) and percentage error values for CI_BST (0.010; 47%), CI_CNI (−0.077; 44%), CI_PA (0.117; 56%), CI_BST_PA (0.001; 43%) and CI_BST_PA_Biometry (−0.001; 41%) demonstrated at least comparable accuracy and precision for CI_BST and CI_CNI compared to CI_PA. The combination of CI_PA with CI_BST or CI_CNI substantially improved the predictive capabilities of CI_PA.

This is supported by the ROC-AUCs regarding CI ≥ 5L/min/$m^2$ (all $p < 0.001$) for _BST (AUC = 0.794), CI_PA (0.724), CI_CNI (0.819), CI_BST_PA (0.842) and CI_BST_PA_Biometry (0.847) as well as regarding CI ≤ 2.5L/min/$m^2$ for CI_BST (AUC = 0.853), CI_PA (0.684), CI_CNI (0.878), CI_BST_PA (0.858) and CI_BST_A_Biometry (0.888).

**CONCLUSIONS.**
1.) BSTs alone or combined with biometrics provided an estimate of CI with predictive capabilities at least comparable to those of CI_PA.
2.) Combination of CI_PA with CI_BST and biometrics substantially improved the predictive capabilities of CI_PA.
3.) CI_BST and CI_CNI could be used alone or as an external calibration to improve accuracy and precision of CI_PA

### 0219

#### Hemodynamic monitoring during spontaneous breathing trials

M. Rauseo, L. Mirabella, S. Gadaleta, A. Z. Di Fiore, R. Terenzio, D. G. Marzocca, L. Tullo, G. Cinnella
University Hospital of Foggia, Anesthesia and Intensive Care Unit, Foggia, Italy
**Correspondence:** M. Rauseo
*Intensive Care Medicine Experimental* 2017, **5(Suppl 2):**0219

**INTRODUCTION.** Failure to wean from controlled mechanical ventilation is associated with significant morbidity and mortality in the intensive care unit[1]and this commonly occurs in patients with limited cardiac reserve. The process of weaning leads to changes in the hemodynamic and autonomic nervous systems. Spontaneous breathing trial (SBT) assesses the patient´s ability to breathe while receiving minimal or no ventilator support.[2] It has been showed that reduced heart rate variability during SBTs was significantly associated with extubation failure.[3] Based on strong evidence, the duration of SBT should be at least 30 minutes. The initial few minutes of the SBT should be monitored closely before judgment is made to continue the SBT.

**OBJECTIVES.** The aim of this study is to evaluate whether hemodynamic parameters measured during a 30-minute SBT using a CPAP level of 5 $cmH_2O$ are able to detect weaning failure from weaning success.

**METHODS.** This was a prospective observational study, which included intubated ICU patients with an expected duration of mechanical ventilation of >24hrs.

Recordings of hemodynamic parameters were obtained using the Vigileo™ monitor (Edwards Life Sciences LLC, Irwine, CA, USA, software version 01.10) at three time points (at 5, 15 and 30 minutes, respectively), during the weaning trial.

**RESULTS.** Fifteen patients were enrolled, M/F: 10/5, with a mean age of 69 (SD 14). The main causes of ICU admission were shock (30%), pneumonia and cardiac arrest (20%).

Data collected showed a significant difference in mean arterial pressure (MAP), 76.1 ± 16.2 mmHg vs 99.7 ± 14.3 mmHg ($p < 0.01$) and in heart rate (HR), 94.7 ± 14.9 bpm vs 120.83 ± 21.5 bpm ($p < 0.009$), between the success and failure SBT groups, respectively, with no difference of the other hemodynamic parameters (Table 72.)

**CONCLUSIONS.** We observed a significantly difference of MAP and HR between groups of patients who succeeded or failed a SBT. Preload optimization may have influenced other hemodynamic parameters in both groups.

**REFERENCE(S)**
1. MacIntyre NR, Cook DJ, Ely EW, Jr., Epstein SK, Fink JB, Heffner JE, et al. Evidence-based guidelines for weaning and discontinuing ventilatory support: a collective task force facilitated by the American College of Chest Physicians; the American Association for Respiratory Care; and the American College of Critical Care Medicine. Chest. 2001;120(6 Suppl):375s–95s.
2. Boles JM, Bion J, Connors A, Herridge M, Marsh B, Melot C, et al. Weaning from mechanical ventilation. The European respiratory journal. 2007;29(5):1033–56.
3. Huang CT, Tsai YJ, Lin JW, Ruan SY, Wu HD, Yu CJ. Application of heart-rate variability in patients undergoing weaning from mechanical ventilation. Critical care (London, England). 2014;18(1):R21.

**Table 72 (Abstract 0219)**. See text for description

| | SBT success | SBT failure | P value |
|---|---|---|---|
| MAP (mmHg) | 76.1±16.2 | 99.7±14.3 | 0.01 |
| HR (bpm) | 94.7±14.9 | 120.83±21.5 | 0.009 |
| SVV (%) | 9.9±4.1 | 12.2±5.8 | 0.36 |
| SVI (ml/beat/m2) | 47±14.4 | 40±17.4 | 0.44 |
| CO (L/min) | 6.8±2.4 | 8±2 | 0.33 |
| CI (L/min/m2) | 4±1.7 | 4.7±1.2 | 0.38 |

Haemodynamic parameters during 30 minutes of SBT with CPAP 5 cmH2O. Data are expressed as mean±SD of the three time points (5-15-30 minutes). P value from paired T-test.
*HR* heart rate, *MAP* mean arterial blood pressure, *CI* cardiac index, *SVV* stroke volume variation, *CO* cardiac output, *SVI* stroke volume index

### 0220

#### Internal yugular vein colapsability index as a predictor for hemodynamic optimization in prone position ARDS patients

J. O. Montoya Rojo[1], S. E. Zamora Gómez[1], L. A. Gorordo Delsol[1], M. A. Amezcua Gutierrez[1], I. M. Lima Lucero[1], E. S. Cruz Montesinos[1], J. Garduño López[1], B. Bolado Morales[2], M. A. Carrasco Flores[1], M. Hernández Romero[1], M. A. Toledo Rivera[1], G. D. Hernández López[1], J. A. Castañon González[1]
[1]Hospital Juárez de México, Intensive Care Unit, Mexico, Mexico; [2]Hospital General del Estado de Sonora, Emergency Deparment, Hermosillo, Mexico
**Correspondence:** J. O. Montoya Rojo
*Intensive Care Medicine Experimental* 2017, **5(Suppl 2):**0220

**INTRODUCTION.** The goal of fluid resuscitation is an increase in cardiac output as result of increase in venous return carrying an improvement of regional and global microcirculatory perfusion (1). Hemodynamic optimization can be achieved by using vasopressors, preload optimization or inotropic in an individual way of each patient. Bedside ultrasound (US) has proven to be useful for determinate volume response; superior or inferior cava vein (collapsibility and distensibillity index) are used to decide if a fluid bolus is beneficial, with controversial results. Internal jugular vein collapsibility index (IJV-CI) ≥18% has proven to be an equivalent of inferior cava vein measurements with a good predictive value of volume responsiveness (2–4).

**OBJECTIVES.** Determine if IJV-CI is a good predictor for hemodynamic optimization in prone position (PP) ARDS patients

**METHODS.** A retrospective analysis was made during the first 3 months of 2017 in a tertiary hospital ICU at Mexico City, including ARDS patients who were in PP and requiring vasopressor; a bedside US was used to measure IJV-CI and analyze the behavior of vasopressor dose after an infusion of 250 ml of crystalloid.

**RESULTS.** Fifteen patients were analyzed, 28 measures were done. A bedside US was performed to measure IJV-CI with a linear probe, in B and M-mode, calculating the IJV-CI with this formula:.When IJV-CI ≥18% and a 250 ml of crystalloid fluid challenge was infused we observed vasopressor withdraw in 26 of 28 measures (92.8%). Normal distribution was proven by Shapiro Wilks, hence a Student t-test for related samples was made, resulting in a significant difference ($p = 0.0008$, CI 95%) for vasopressor withdraw in patients who received a fluid challenge. Fisher's test was calculated ($F = 0.0421$, $p < 0.05$, CI 95%) when the IJV-CI was ≥18%, with sensibility 92%, specificity 67%, positive predictive value 96% and negative predictive value 50% for vasopressor withdraw. The correlation between IJV-CI value and amount vasopressor withdraw by the Pearson $r = 0.41$.

**CONCLUSIONS.** IJV-CI ≥18% appears to be a good predictor for vasopressor withdraw after fluid challenge in prone position ARDS patients, a prospective comparative study is needed to elucidate the validity of this index as an hemodynamic optimization predictor in this population.

**REFERENCE(S)**

1. Vincent JL, Annual Update in Intensive Care and Emergency Medicine 2017, Springer I, Brussels, Belgium 2017
2. Guaraccino F et al. Jugular vein distensibility predicts fluid responsiveness in septic patients, Critical Care 2014 18:647
3. Kent A, et al. Jugular and femoral vein versus IVC collapsibility, Annals of Thoracic Medicine 2015;10(1):1
4. Thudium M, et al. Is internal jugular vein extensibility associated with indices of fluid responsiveness in ventilated patients, Acta Anaesthesiologica Scandinavica 2016;60:723–33

**Table 73 (Abstract 0220).** Patient's characteristics

| Variable (n = 15) | Mean |
|---|---|
| Age | 44 years |
| Body mass index (BMI) | 32.35 |
| PBW Tidal Volume | 6.55 ml/kg |
| PaO2/FiO2 | 75.73 |
| PEEP | 11.84 cmH2O |
| Driving Pressure | 14 cmH2O |
| Compliance (Cstat) | 28.39 ml/cmH2O |
| APACHE II | 17 points |
| SOFA | 9.5 points |

### 0221

#### Effects of fluid resuscitation on mean systemic filling pressure in cardio-surgical spontaneous breathing patients

P. Bertini[1,2], G. Brizzi[1], G. Mancino[1], L. Doroni[1], R. Baldassarri[1], F. Guarracino[1]
[1]Azienda Ospedaliero Universitaria Pisana, Anaesthesia and Intensive Care Medicine - Cardiothoracic and Vascular Anaesthesia and Critical Care, Pisa, Italy; [2]Royal Brompton & Harefield NHS Foundation Trust, Critical Care, London, United Kingdom
*Intensive Care Medicine Experimental* 2017, **5(Suppl 2):**0221

**Correspondence:** P. Bertini
*Intensive Care Medicine Experimental* 2017, **5(Suppl 2):**0221

**INTRODUCTION.** In the ICU the assessment of haemodynamic variables is critical in unstable patients. We usually focus on cardiac output and on fluid responsiveness, which is assessed using dynamic indices such as systolic pressure variation and pulse pressure variation induced by positive pressure ventilation. Although we can easily predict fluid responsiveness at the bedside using these approaches, none of these parameters can explain why a patient is or is not volume "requiring", and they also require mechanical ventilation and a sinus rhythm. For this reason we have studied the venous side of the circulation as a determinant of cardiac output, and in particular the mean systemic filling pressure.

**OBJECTIVES.** To evaluate the mean systemic filling pressure and its analogous value derived by a mathematical model (Pmsa), the venous return gradient and the heart efficiency as predictors of fluid responsiveness in post-surgical cardiac patients.

**METHODS.** We performed our study in a post-surgical cardiac ICU. In spontaneously ventilating patients, monitored with a central venous catheter and invasive arterial blood pressure, we evaluated the Pmsa and the venous return as previously described[1], using the formula[2]: $Pmsa = aRAP + bMAP + cCO$

Knowing the heart rate, CO was measured with echocardiography measuring the LVOT diameter and LVOT VTI. We performed a passive leg raising maneuver as described in literature[3] and we considered an increase of stroke volume or cardiac output greater than 10% as a positive response.

We then measured the venous return gradient (the difference between mean systemic filling and central venous pressure) and the heart efficiency ($Eh = (Pmsa - CVP)/Pmsa$) and its change after fluid challenge (ΔEh).

**RESULTS.** A total of 8 patients were studied. In 6 patients SV and CO increased more than 10% after PLR. Pmsa increased in the 8 patients (Pmsa 15 ± 4 mmHg before PLR, and 18 ± 4 mmHg after PLR). The venous return gradient (dVR) increased in the "responder group", but not in the "non-responder group". Heart performance did not change significantly in either group.

**CONCLUSIONS.** In this small preliminary study we have calculated bedside the changes in Pmsa, dVR and Eh, and tried to correlate them with fluid responsiveness. It seems that an increase of dVR correlates with fluid responsiveness. Further studies are needed to confirm this correlation and to assess its usefulness in clinical practice.

**REFERENCE(S)**

1. Maas JJ, Pinsky MR et al. Estimation of mean systemic filling pressure in postoperative cardiac surgery patients with three methods. Intensive Care Medicine. 2012;38(9):1452–1460. doi:10.1007/s00134-012-2586-0.

2. Parkin WG, Leaning MS (2008) Therapeutic control of the circulation. J Clin Comput Monit 22:391–400

3. Monnet X, Teboul J-L. Passive leg raising: five rules, not a drop of fluid! Critical Care. 2015;19(1):18. doi:10.1186/s13054-014-0708-5.

### 0222

#### Extra vascular lung water for predicting mortality in ARDS and septic shock patients: a systematic review and meta-analysis

P. Mercado, V. Girotto, J. L. Teboul, M. Jozwiak, C. Richard, X. Monnet
Service de Réanimation Médicale, Hôpital de Bicêtre, Hôpitaux Universitaires Paris-Sud, Assistance Publique - Hôpitaux de Paris, Le Kremlin-Bicêtre, France
**Correspondence:** P. Mercado
*Intensive Care Medicine Experimental* 2017, **5(Suppl 2):**0222

**INTRODUCTION.** The prognostic value of extravascular lung water index (EVLWI) has been investigated in various settings by studies that were often of small size.

**OBJECTIVES.** With this systematic review and meta-analysis, we wanted to assess the ability of EVLWI to predict mortality in the specific population of adult patients with septic shock and/or acute respiratory distress syndrome (ARDS).

**METHODS.** We searched for all relevant observational studies evaluating the ability of EVLWI to predict mortality in adult patients with sepsis or septic shock and/or ARDS, from 1966 to March 2017 on MEDLINE database and the Cochrane Database of Systematic Reviews.

**RESULTS.** We included 14 studies (1338 patients). EVLWI was significantly higher in non-survivors than in survivors (mean difference: 5.1 mL/kg). The analysis of the receiver operating characteristic (ROC) curve was available in 10 studies (1017 patients). EVLWI predicted mortality with a pooled sensitivity of 0.68 (confidence interval: 0.64-0.72) and a pooled specificity of 0.72 (0.67-0.76). The positive likelihood ratio was 3.04 (2–4.6) and the negative likelihood ratio was 0.40 (0.31-0.54). The thresholds reported to discriminate between survivors and non-survivors ranged from 9 to 21 mL/kg. None of the covariates were found to be a significant source of heterogeneity. In particular, the fact that EVLWI was indexed to predicted or actual body weight and the fact that studies included ARDS patients or not did not influence the predictive accuracy of EVLWI. The pooled area under the receiver ROC curve was 0.76 ± 0.45 ($p < 0.05$ vs. 0.50).

**CONCLUSIONS.** EVLWI seems to be a good predictor of mortality in patients with ARDS and/or septic shock. This suggests that EVLWI has its own pathophysiological meaning and that the estimation of EVLWI by thermodilution is reliable.

### 0223

#### Comparison of two mini-invasive technique to CO and CO variations monitoring during different types of shock

V. Piccoli[1], G. De Pascale[1], M. S. Vallecoccia[1], S. Carelli[1], T. Taccheri[1], L. La Via[1], U. La Brocca[1], A. M. Dell Anna[2], M. Antonelli[1]
[1]Fondazione Policlinico Universitario A. Gemelli, Roma, Italy; [2]Policlinico Universitario A. Gemelli, Roma, Italy
**Correspondence:** V. Piccoli
*Intensive Care Medicine Experimental* 2017, **5(Suppl 2):**0223

**INTRODUCTION.** The assessment of cardiac output (CO) is a cornerstone in haemodynamic management of shock patients. Many minimally invasive systems have been introduced to provide CO changes beat-to-beat and to allow a more widespread use of CO monitoring bedside, replacing more invasive methods (i.e. pulmonary artery catheter).

**OBJECTIVES.** The aim of this study was to evaluate if the estimation of CO with two mini-invasive monitoring systems, MostCare (Vygon©TM) and CardioQ (Deltex MedicalTM) was reliable and able to follow CO changes in shock patients with profound hemodynamic instability.

**METHODS.** In this prospective, observational, monocentric study, we enrolled patients with diagnosis of shock, in particular septic and hemorrhagic shock, as suggested by common clinical parameters (HR > 120 bpm, MAP < 70 mmHg, urinary output < 0,5 ml/Kg/h and blood lactates >4 mmol/l, bleeding >1000 ml). Exclusion criteria were: age < 18, pregnancy, survival expectancy < 24h and need for urgent surgery. All patients might receive different intervention according to clinical needs: volume expansion with 500 ml of Ringer's lactate in 10 minutes, increase or decrease of norepinephrine or increase or decrease of dobutamine. CardioQ and Mostcare measurements were compared with transpulmonary thermodilution (TPTD-EV1000) as reference method. A patient was characterized as fluid responder (FR) if CO increase was > 12% after fluid bolus. For each stage, we detected haemodynamic, respiratory and metabolic parameters as SAP, DAP, MAP, HR, CVP, SVi, Ci, SVRi, Ea, Cla, blood gas analysis and urinary output.

**RESULTS.** We obtained 23 measures on six patients, with an average age of 65 ± 18 (30% male). At baseline a significant difference between CO measured by CardioQ and TPTD emerged by Bland-Altman analysis (mean −1.1, CI −7.2-5 L/min/m$^2$) as well as between MostCare and TPTD (0.5 CI −1.9-3 L/min/m$^2$) but with lower differences. Mountain Plot showed that MostCare overestimates CI but with a smaller error compared to CardioQ (50 percentile 0.5 vs 0 L/min/m$^2$). When we compared trending ability of the two systems, by plotting the difference in CO before and after intervention, MostCare had a smaller error compared to CardioQ (mean 0.21 CI −0.89-1.31 vs mean 0.5 CI −2.3-3.2 L/min/m$^2$, respectively).

**CONCLUSIONS.** Despite the major accuracy of MostCare to detect CO values and acute changes than CardioQ, these mini-invasive monitoring systems tend to be less accurate than TPTD also in following changes in cardiac output more than their absolute value. The small number of patients enrolled hampers to draw definitive conclusion on such devices and further studies are therefore needed.

### 0224

#### Estimation of Cardiac Index: a comparison of body surface temperatures and biometric data vs. The uncalibrated pulse-contour-analysis device Flotrac®

W. Huber, C. Zachoval, T. Lahmer, A. Herner, U. Mayr, S. Rasch, G. Batres-Baires, M. Heilmaier, R. Schmid
Klinikum rechts der Isar der TUM, Munich, Germany
**Correspondence:** W. Huber
*Intensive Care Medicine Experimental* 2017, **5(Suppl 2):**0224

**INTRODUCTION.** Cardiac Index CI is a key target of haemodynamic monitoring. Initial clinical assessment of shock patients is based on the assessment of body surface temperature BST. We hypothesized that accurate measurement of BST with an infrared thermometer (Thermofocus, Tecnimed) combined with biometric data provides a completely non-invasive estimate of CI (CI_CNI) with a precision and accuracy comparable to a pulse-contour-analysis estimate of CI (CI_FT; FloTrac, Edwards Lifesciences; USA).

**OBJECTIVES.** We compared BSTs alone and in a model (CI_CNI) combined with biometric data and pulse pressure PP to CI_FT and to a gold-standard CI derived from transpulmonary thermodilution (TPTD; CI_TD; PiCCO; Pulsion).

**METHODS.** 240 datasets (eight per patient) in 30 patients (24m; 6f; APACHE-II 20 ± 6) were recorded within 24h. Each dataset included pre-TPTD BSTs of great toe, finger, forearm and forehead, CI_FT and thermodilution-derived CI_TD.

Statistics: SPSS 23. Preliminary data not including CI_CNI and other combined models to predict CI_TD were presented at the SCCM-meeting 2013.

**RESULTS.** CI-TD was univariately ($p < 0.001$ except as indicated) associated to surface temperatures of forehead (r = 0.441), great toe (r = 0.423) and distal forearm (r = 0.209; p = 0.001) as well as to age (r = −0.756), height (r = 0.256), gender (r = 0.162; p = 0.012), PP (r = 0.322) and CI-FT (r = 0.830). Multiple regression including BSTs and biometrics demonstrated that BST_forehead (p = 0.001), BST_toe (p = 0.012), age and male gender were independently associated to CI_TD. This regression formula resulted in CI_BB ("Biometry and BST"; $R^2$ = 0.541). Inclusion of PP resulted in a further improved model CI_CNI ($R^2$ = 0.646). In this model BST_forehead (p = 0.001), BST_toe (p = 0.004), age ($p < 0.001$), height (p = 0.002), weight (p = 0.033) and PP ($p < 0.001$) were independently associated to CI_TD.

In a next step, we analysed, if these models could improve the estimation of CI by CI_FT. CI_BB and CI_FT were independently ($p < 0.001$) associated with CI_TD. This model was termed CI_BB_FT ($R^2$ = 0.683). Similarly, CI_CNI and CI_FT were independently associated with CI_TD. The resulting formula CI_CNI_FT provided the largest $R^2$-value (0.706). In Bland-Altman-analyses the bias (L/min/$m^2$) and percentage error values (%) compared to CI_TD were −0.123 and 44% for CI_FT, −0.001 and 37% for CI_CNI_FT, −0.049 and 47% for CI_BB, 0.031 and 41% for CI_CNI, and −0.003 and 39% for CI_BB_FT.

The ROC-AUCs regarding CI ≤ 2.5 and CI ≥ 5L/min/$m^2$ were comparable for CI_FT (0.936; 0.883), CI_BB (0.930; 0.856) and CI_CNI (0.953; 0.888). They could be further improved by combination of the non-invasive techniques with CI_FT for CI_BB_FT (0.957; 0.897) and for CI_CNI_FT(0.962; 909; $p < 0.001$ for all ROCs).

**CONCLUSIONS.**

1.) BSTs combined with PP and biometrics provide an estimate of CI with similar predictive capabilities as for the CI-FT.

2.) CI_CNI could be used alone or as an external calibration of CI_FT.

### 0225

#### Comparison of pocket-sized and high end ultrasound devices for inferor vena cava diameter measurements in critically ill adults

K. Inci[1], G. Gursel[2]

[1]Gazi University School of Medicine, Department of Internal Medicine, Division of Critical Care, Ankara, Turkey; [2]Gazi University School of Medicine, Department of Pulmonary Critical Care Medicine, Ankara, Turkey

**Correspondence:** K. Inci

*Intensive Care Medicine Experimental* 2017, **5(Suppl 2):**0225

**INTRODUCTION.** Determination of a patient´s volume status in intensive care units (ICU) is still a challenging issue. Point of care USG (POCUS) to measure inferior vena cava diameter (IVCD) and variability with respiration have been shown to correlate with central venous pressure, stroke volume and pulse pressure variation as a fluid status determination method in critically ill adults. On the other hand, during a routine ICU day, intensivist has to do ultrasonographic evaluation of heart, lung and IVC several times for planning fluid therapy. For this reason, a practical, reliable, faster and affordable ultrasound device may be more convenient. Previous studies reported pocket-sized (PS) devices can be used for POCUS in Cardiology, Emergency Departments and ICU's effectively.

**OBJECTIVES.** We aimed to determine the diagnostic accuracy of (PS) ultrasound in comparison with a high end(HE) device for measurement of IVCD and variability with respiration.

**METHODS.** Patients were examined as a randomized order with the PS (GE V-scan) and HE(GE Vivid Q) devices. Examinations were carried out according to a standardized protocol by two intensivists with sector probes of the devices. All images were saved as cineloop records and measurements were performed on this records according to recent guideline recommendations. Paired t test, and Bland-Altman analyses were used for statistical analysis.

**RESULTS.** 27 patients were included in this study. There was no statistically significant difference in inspiratory (PS;1,34 ± 0,61cm, HE; 1,38 ± 0,64cm) and expiratory (PS;1,95 ± 0,45cm, HE;2,04 ± 0,49cm) diameter of IVC among PSand HE devices(p > 0,05). There was also no significant difference between the calculated respirational variability rates with PS (35 ± 20%) and HE (34,6 ± 20%) devices (p > 0,05). The Bland-Altman analysis revealed that the width of the 95% limits of agreement were similar for both devices(Figs. 91, 92 and 93).

**CONCLUSIONS.** There was good inter-device agreement among PS and HE ultrasound devices for measurements of IVC in both respiration phases and there was no significant difference in the respirational IVC variability between the measurements with both devices.

**REFERENCE(S)**

1. Marik PE, Monnet X, Teboul J-L. Hemodynamic parameters to guide fluid therapy. Annals of Intensive Care,2011

2. Guidelines for the Appropriate Use of Bedside Cardiac Usg. in the Evaluation of Critically Ill Patients. Levitov,Alexander MD, et al. Critical Care Medicine,2016

**GRANT ACKNOWLEDGMENT**

Study was supported by Gazi University Scientific Reserch Projects Foundation(Project No: 01/2015-48).

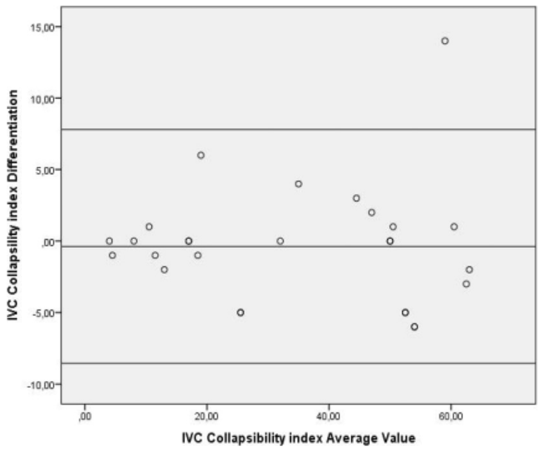

**Fig. 91 (abstract 0225)** See text for description

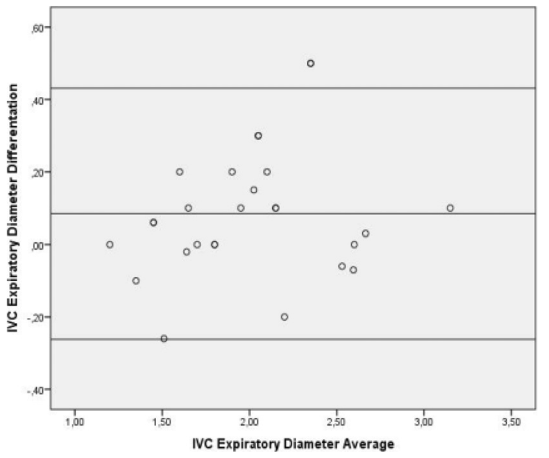

**Fig. 92 (abstract 0225)** See text for description

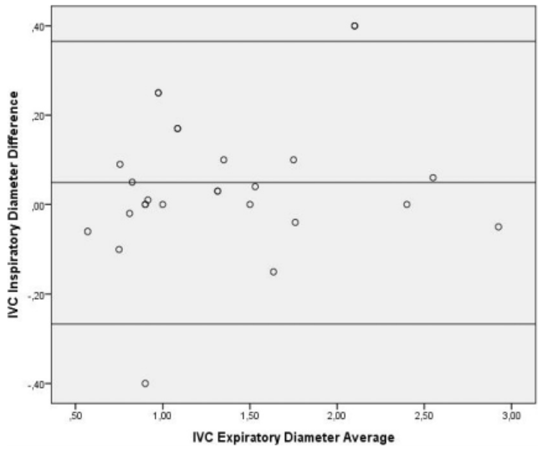

**Fig. 93 (abstract 0225)** See text for description

### Acute respiratory failure: Clinical studies

#### 0226

**Diagnostic and prognostic value of heparin binding protein in patients with acute respiratory distress syndrome**

S. El Maraghi[1], M. Ali[1], M. Sayed[1], A. Samir[2]
[1]Beni-Suef University, Critical Care Department, Cairo, Egypt; [2]Cairo University, Critical Care Department, Cairo, Egypt
**Correspondence:** S. El Maraghi
*Intensive Care Medicine Experimental* 2017, **5(Suppl 2):**0226

**INTRODUCTION.** The acute respiratory distress syndrome (ARDS) is a common clinical disorder characterised by injury to the alveolar epithelial and endothelial barriers of the lung and acute respiratory failure [1]. Serum biomarkers released from inflammatory cells are of a great value in the diagnosis and follow up of such condition [2].HBP is one of these biomarkers that is released from azurophilic granules of activated neutrophils and carries a great value in the diagnosis and follow up of ARDS [3].

**OBJECTIVE.** To validate the diagnostic and prognostic role of HBP in patients with ARDS and to correlate it with other prognostic markers and scoring systems.

**METHODS.** Forty patients presented to the critical care department at Beni-Suef & Cairo University and diagnosed as having ARDS according to the New Berlin Definition [4] during the period from March to November 2015 in addition to 10 non ARDS patients who served as controls. Plasma HBP level was measured within 24 hours from admission [HBP 1] & on day 5 [HBP 5] and only once for controls by ELISA technique & correlated it with CRP & APACHE II, SOFA & Lung Injury scores [LIS].

**RESULTS.** HBP1 was significantly higher in patients than in controls with a p- value 0.001. HBP 5 was significantly higher in non survivors than survivors with a p-value 0.006. HBP showed an insignificant correlation with APACHE II score with a p-value 0.65 & 0.35 while it showed a moderate correlation with SOFA score with a p-value 0.04 & 0.05. Also a negative insignificant correlation was found between HBP and LIS with a p-value 0.107 & 0.742. HBP showed moderate significant correlation with CRP with a p-value 0.026 & 0.025. HBP was significantly higher in septic than in non-septic patients with a p-value 0.025 & 0.026. The cut-off point of HBP for diagnosis of ARDS was 16.2 ng/ml with a sensitivity of 97.5% & specificity of 100% with a p-value of 0.001 while 52 ng/ml was the cut-off point to predict mortality with a sensitivity of 89.7% and a specificity of 72.7% with a P value of 0.001.

**CONCLUSION.** Heparin Binding Protein can be used as an early diagnostic and prognostic biomarker for ARDS with a high sensitivity and specificity.

#### 0227

**Timing of ICU admission and clinical outcome in patients with sepsis requiring invasive mechanical ventilation**

Y. Shen, Y. Dong, O. Gajic, P. R. Bauer
Mayo Clinic, Rochester, United States
**Correspondence:** Y. Shen
*Intensive Care Medicine Experimental* 2017, **5(Suppl 2):**0227

**INTRODUCTION.** Sepsis has initial hyper-inflammatory and subsequent immune-suppressive phase with different clinical outcomes. Thus, the timing of sepsis and critical care support may influence outcome in septic patients.

**OBJECTIVES.** To determine the impact of the timing of intensive care unit (ICU) admission on the outcomes of patients with sepsis and respiratory failure who originated from the emergency department (ED).

**METHODS.** Retrospective study of prospectively collected electronic data from ICU patients with sepsis 2.0, identified by a sepsis alert system and who required invasive mechanical ventilation between January 2006 to December 2014 in 3 ICUs from a tertiary medical center. Variables identified by univariate analysis were included in multivariate logistic regression analysis to analyze independent risk factors associated with hospital mortality, using SPSS 18.0 for Windows.The study was approved by the local Institutional Review Board (#14-008754).

**RESULTS.** From a total of 637 patients initially evaluated in the ED, 313 patients were directly admitted to the ICU and 324 patients were first admitted to the floor and subsequently transferred to ICU. Median time from ED to ICU admission was 3 hours [2–4] for direct admits and 48 hours [16–138] for transfers from the floor ($p < 0.001$). All patients developed sepsis and were intubated after ICU admission. Patients transferred from the floor to ICU had longer ICU (5.3 [2.8-9.4] vs. 4.6 [2.7-8.4] days, $p = 0.017$) and hospital (14.6 [8.4-25] vs. 9.8 [4.9-15.7], p 0.002) length of stay, higher ICU (33 vs. 21%, $p < 0.001$) and hospital (44 vs. 31%, $p < 0.001$) mortality than those admitted directly. Indications for intubation varied according to the admission route: patients coming from the floor were less often intubated for airway protection

(30 vs. 41%, p 0.003) and more often for respiratory failure (74 vs. 66%, $p = 0.029$). When compared with survivors, the non-survivors were older (69 [59–79] vs. 63 [52–74] year-old, $p < 0.001$), more predominantly males (63 vs. 54%, $p = 0.024$), with higher APACHE III (96 [79–119] vs. 83 [70–100], $p < 0.001$), SOFA

(9 [7–13] vs. 8 [6–11], $p < 0.001$) and Charlson (3 [1–5] vs. 2 [1–5], $p = 0.002$) scores, lower PaO2/FiO2 ratio (153 [97–206] vs. 185 [119–246], $p < 0.001$), and delayed intubation time (12.1 [3.1-290] vs. 6 [2.2-20.3] hours, $p = 0.014$). By multivariate analysis independent predictors of hospital mortality included initial floor status (OR 1.746, 95% CI 1.227-2.486, $p = 0.002$), APACHE III score (OR 1.017, 95% CI 1.006-1.028, $p = 0.002$), lower PaO2/FiO2 ratio (OR 0.997, 95% CI 0.995-0.999, $p = 0.004$) and timing of intubation

(OR 1.011, 95%CI 1.004-1.017, $p = 0.001$).

**CONCLUSIONS.** Patients originated from the ED, who were admitted to ICU with sepsis requiring invasive mechanical ventilation, had a poorer outcome if they were transferred from the floor, with hypoxemic respiratory failure and delayed intubation in ICU, even after adjustment for APACHE III score.

### 0229

#### Determinants of one-year mortality in lung transplant recipients readmitted to the ICU

M. Martínez[1], A. Cortina[1], N. Varga[1], M. García[1], C. Laborda[1], L. Romero[2], C. Berastegui[3], R. Ferrer[1,4], J. Sacanell[1], O. Roca[1,4]
[1]Vall d´Hebron University Hospital and Vall d´Hebron Research Institute, Critical Care, Barcelona, Spain; [2]Vall d´Hebron University Hospital, Thoracic Surgery, Barcelona, Spain; [3]Vall d´Hebron University Hospital, Pneumology, Barcelona, Spain; [4]Instituto de Salud Carlos III, Ciber Enfermedades Respiratorias (Ciberes), Madrid, Spain
**Correspondence:** M. Martínez
*Intensive Care Medicine Experimental* 2017, **5(Suppl 2):**0229

**INTRODUCTION.** Few data are available on determinants of long-term outcomes among lung transplant (LTx) patients who require re-admission to the ICU.
**OBJECTIVES.** Our aim was to describe the variables associated with one-year mortality in LTx recipients who require readmission to the ICU beyond the postoperative period.
**METHODS.** Single-center, observational, retrospective analysis of a prospectively assessed cohort of LTx patients who were readmitted to the ICU over a 6-year period (2011–2016). LTx patients who required readmission beyond 30 days after transplantation were included. Patients were followed up until one year after ICU re-admission. Demographic data, transplantation-related aspects, and ICU-related variables were collected. Data were expressed as means (standard deviation), medians (interquartile range) or frequencies (percentage). Differences between categorical variables were assessed by the chi-square or Fisher exact test when necessary. Continuous variables were compared using the Student t test or Mann–Whitney test, as appropriate. To determine which variables were independently associated with one-year mortality, a backward stepwise logistic regression analysis was performed. Variables with $p < 0.1$ in the univariate analysis were introduced into the multi-variate model. Continuous variables were categorized for this analysis using the observed mean in the overall cohort. A two-sided $p$ value of 0.05 or less was considered statistically significant.
**RESULTS.** During the study period, 342 LTx were performed and 84 LTx recipients required ICU readmission, with a total of 100 episodes. Mean age was 53 (12) years and the mean APACHE II score was 16 (6). The main reason for ICU readmission was respiratory failure (70%) and the main aetiology was lower respiratory tract infection (LRTI) (59.6% of respiratory failure episodes). Fifty-four (64.3%) patients died during the follow-up period. APACHE II, SOFA at ICU re-admission, need for MV during ICU stay, previous LRTI, presence of chronic lung allograft dysfunction (CLAD) and reported FEV1(%) value prior to ICU readmission were included in the multivariate logistic regression analysis. The model showed an association between previous LRTI (OR 17.71 [95%CI 3.52 - 89.15]; $p < 0.001$), presence of CLAD (OR 5.46 [95%CI 1.34 - 22.25]; $p = 0.018$), and need for MV during ICU stay (OR 17.97 [95%CI 4.85 - 66.64]; $p < 0.001$). Thus, patients diagnosed with LTRI and CLAD prior to ICU readmission and who needed MV during their ICU stay had a predicted probability of one-year mortality of 97%.
**CONCLUSIONS.** LTx patients who require ICU readmission present a high risk of death during the following year. Previous LTRI and CLAD as well as the need for MV during ICU stay are independent predictors of one-year mortality.

### 0230

#### Prospective observational study on the association between serum mannose-binding lectin levels and severe outcome in critically ill patients with pandemic influenza type A (H1N1) infection

E. Zogheib[1,2], R. Nyga[3], M. Sellier[1], B. Sendid[4], J. Monconduit[5], V. Jounieaux[5], J. Maizel[6], C. Segard[7], T. Chouaki[3], H. Dupont[1,2]
[1]Cardiothoracic and Vascular Intensive Care Unit, Amiens University Hospital, Amiens, France; [2]INSERM U 1088, Jules Verne University of Picardy, Amiens, France; [3]Medical Parasitology and Mycology Department, Amiens, France; [4]Medical Parasitology and Mycology Department, Lille, France; [5]Respiratory Intensive Care Unit, Amiens, France; [6]Medical Intensive Care Unit, Amiens, France; [7]Medical Virology Department, Amiens, France
**Correspondence:** E. Zogheib
*Intensive Care Medicine Experimental* 2017, **5(Suppl 2):**0230

**INTRODUCTION.** Mannose-Binding Lectin (MBL) plays an important role in the innate immune response. It can also contribute to a deleterious inflammatory response in mice infected with influenza A (H1N1) virus (pdmH1N1) infection.
**OBJECTIVES.** Our aim was to determine whether baseline serum levels of MBL at admission to ICU could predict mortality in ICU patients with pdmH1N1 infection.
**METHODS.** Prospective observational study performed in ICU patients with ARDS due to pdmH1N1 virus. Demographic characteristics and severity indices were recorded at ICU admission. MBL was assayed from blood drawn at H1N1 diagnosis. Outcomes were compared according to MBL levels. Results are expressed as median and interquartile range.
**RESULTS.** MBL levels were studied in 27 patients (median age: 56 [29] years) with severe pdmH1N1 infection. Median admission SAPS II and SOFA scores were 49 [26] and 12 [5], respectively. Thirty-day mortality rate was 37% (n = 10/27). MBL was significantly higher in non-survivors (3741ng/ml [2336]) vs survivors (215 ng/ml [1307]), p = 0.005. MBL cut-off >1870ng/ml had a sensitivity of 80% and a specificity of 88.2% for mortality (AUC = 0.82 (95%CI = 0.63-0.94)). Kaplan-Meier analysis demonstrated a strong association between MBL levels and mortality (logrank 7.8, p = 0.005). MBL > 1870ng/ml was the only factor independently associated with mortality (adjusted HR = 8.7, 95%CI = 1.2-29.1, p = 0.007).
**CONCLUSIONS.** This study shows that baseline MBL > 1870ng/ml is associated with higher mortality in ICU patients with severe pdmH1N1 infection.

**REFERENCE(S)**

Mannose-binding lectin contributes to deleterious inflammatory response in pandemic H1N1 and avian H9N2 infection. J Infect Dis. 2012;205:44–53.

### 0231

#### Does the response in urine output to a small dose of furosemide predict organ failure after achievement of negative fluid balance in acute respiratory failure? The interim analysis

J. Kataoka[1], Y. Fujimoto[1], Y. Norisue[1], S. Fujitani[2]
[1]Tokyo Bay Urayasu Ichikawa Medical Center, Department of Critical Care Medicine, Urayasu, Japan; [2]St Marianna University, Kawasaki, Japan
**Correspondence:** J. Kataoka
*Intensive Care Medicine Experimental* 2017, **5(Suppl 2):**0231

**INTRODUCTION.** In hemodynamically stable patients with acute respiratory distress syndrome (ARDS), a conservative fluid management strategy has been shown to improve lung function and shorten the duration of mechanical ventilation (1). However premature initiation of diuresis can increase non-pulmonary organ failure such as acute kidney injury and there is no proven parameter or test to determine when or who to start diuresis. We hypothesized that a patient who is "ready for diuresis" has a good response in urine output to a small dose of furosemide.
**OBJECTIVES.** We investigated whether the urine output after a small dose of furosemide predicts organ tolerability to negative fluid balance (NFB) in acute respiratory failure.
**METHODS.** This study was a prospective observational study. All mechanically ventilated and hemodynamically stable patients with evaluations of hypervolemia for whom diuresis were planned were included. A small amount of furosemide was administered (10mg for eGFR > 50, 20mg for eGFR 30–50), and the urine output was examined for 4 hours (a furosemide stress test; FST), which is a part of the traditional practice in our hospital to predict tolerability to NFB although no study has validated the practice. After a FST, the patients were diuresed to achieve an NFB of more than 1000ml in 24 hours with additional doses of furosemide as needed. The primary outcome of this study was the tolerance to NFB. Tolerance to NFB was defined as absence of hypotension, acute kidney injury, or need for fluid expansion.

**RESULTS.** A total of 12 mechanically ventilated patients were included in the intensive care unit at Tokyo Bay Urayasu/Ichikawa medical center. 9 patients (75%) had a tolerance of NFB and 3(25%) patients did not have. The urine output following FST in patients who did not have tolerance of NFB showed significantly lower urine output than that of patients with tolerance to NFB in the first 1 hour (p = 0.018). The area under the receiver operator characteristic curves for the urine output in the first 1 hour following FST to predict tolerance to NFB was 0.96 (p = 0.021). The cutoff point of 120ml has the sensitivity of 0.89, and the specificity of 1.00.

**CONCLUSIONS.** The furosemide stress test is a potential predictor of tolerance to negative balance. Larger sample size is needed to further determine the usefulness of the test.

**REFERENCE(S)**

(1) Wiedemann HP, et al. Comparison of two fluid-management strategies in acute lung injury. N Engl J Med. 2006 Jun 15;354(24):2564–75.

**GRANT ACKNOWLEDGMENT**

None

### 0232

#### Correlation between Selenium level, body mass index and anti-oxidant capacity respiratory critical patients

C. Cobilinschi[1], R.C. Tincu[2,3], I.F. Tincu[4], Z. Ghiorghiu[2], R.A. Macovei[2,3]
[1]Clinical Emergency Hospital Bucharest, Anesthesiology and Intensive Care, Bucharest, Romania; [2]Clinical Emergency Hospital Bucharest, Anesthesiology and Intensive Care II Toxicology, Bucharest, Romania; [3]University of Medicine and Pharmacy Carol Davila, Clinical Pharmacology, Toxicology and Psychopharmacology, Bucharest, Romania; [4]Clinical Emergency Hospital for Children Grigore Alexandrescu, Pediatrics, Bucharest, Romania
**Correspondence:** C. Cobilinschi
*Intensive Care Medicine Experimental* 2017, **5(Suppl 2):**0232

**INTRODUCTION.** Selenium is an important micronutrient implicated in anti-oxidants enzymes modulation, incorporated in selenoproteins and essential in oxidative defense. Glutathione peroxidases is a crucial agent against oxidative stress associated with critical care condition. Inadequate Selenium intakes give rise to o wide spectrum of disease and abnormal anti-oxidant activity.

**OBJECTIVES.** The hypothesis tested if abnormal nutritional status and poor anti-oxidant capacity are implicated in bronchopneumonia clinical evolution (considering acute respiratory distress syndrome (ARDS) according to Berlin criteria).

**METHODS.** We enrolled 32 patients aged over 65 years old, with bronchopneumonia and acute respiratory failure evaluated in terms of body mass index (BMI), Selenium and glutathione peroxidase levels.

**RESULTS.** Mean Selenium levels were different according to BMI (patients having BMI under 20 had 48.50 ± 4.94 μg/L compared to 65.25 ± 15.46 μg/L Selenium level for normal BMI subjects). Glutathione peroxidase level depends on BMI in a model adjusted for Selenium level (p < 0.005). Subjects having low Selenium and glutathione peroxidase levels had 2.5 higher risk for developing ARDS (95% confidence interval [CI], 1.09 to 3.59; $p < 0.001$).

**CONCLUSIONS.** Selenium level might be a protective microelement in critical care patients with bronchopneumonia. Abnormal nutritional status is correlated with low anti-oxidant glutathione peroxidase activity.

### 0233

#### Efficacy and safety of acetazolamide for hypercapnic respiratory failure associated with metabolic alkalosis in patients with chronic obstructive pulmonary disease: a meta-analysis of randomized controlled trials

C. E. D. Barbon, A. Z. Ortal, J. E. D. Duya, N. Maghuyop
Philippine General Hospital, Department of Medicine, Manila, Philippines
**Correspondence:** C. E. D. Barbon
*Intensive Care Medicine Experimental* 2017, **5(Suppl 2):**0233

**BACKGROUND.** COPD patients can develop respiratory acidosis and metabolic alkalosis, the latter causing impaired ventilation. Acetazolamide, a carbonic anhydrase inhibitor, can theoretically stimulate breathing by inducing mild metabolic acidosis. This study aims to determine safety and effectiveness of acetazolamide in reducing the duration of mechanical ventilation, length of ICU stay and mortality among COPD patients with hypercapnic respiratory failure.

**METHODS.** Various research databases were searched using predefined MeSH terms. Unpublished data were obtained by correspondence with authors. Studies selected are randomized clinical trials of COPD patients with hypercapnic respiratory failure comparing acetazolamide versus placebo measuring duration of mechanical ventilation, length of intensive care unit stay and mortality, and arterial blood gas parameters including change in pH, $PaCO_2$, $PaO_2$, and bicarbonate.

**DATA ANALYSIS.** Data were extracted and analyzed by two independent reviewers. Continuous outcomes reported as mean differences ± standard deviation and dichotomous outcomes as relative risks using 95% confidence intervals.

**RESULTS.** Three randomized placebo-controlled trials were included in the analysis, with a total of 499 patients (246 - acetazolamide; 251 - placebo). All trials have low risk of bias. A trend towards benefit with acetazolamide was seen in the duration of mechanical ventilation, length of ICU stay, and all-cause mortality, but was not statistical significant. There was no significant changes in pH and $PaCO_2$, but produced a mild improvement in $PaO_2$ (MD = 0.48kPa; 95%CI 0.15,0.82; P = 0.004). There was no statistically significant serious adverse effects.

**CONCLUSIONS.** A decrease in duration on mechanical ventilation, days in the ICU, and all-cause mortality is seen with acetazolamide use in COPD patients with ventilatory failure, but the small sample sizes limited its statistical significance. Acetazolamide is not shown to improve blood gas parameters apart from a small improvement in oxygenation. Its use in such patients cannot be recommended yet based on currently available data.

### 0234

#### Sevoflurane as an add on therapy to treat severe asthma

R. Knafelj, M. Noc
University Medical Centre Ljubljana, MICU, Ljubljana, Slovenia
**Correspondence:** R. Knafelj
*Intensive Care Medicine Experimental* 2017, **5(Suppl 2):**0234

**INTRODUCTION.** Severe asthma episodes leading to need for mechanical ventilation are rarely seen in Slovenia due to efficient national prevention strategies. Typically asthma worsening is seen during winter months usually due to viral infections, especially Respiratory Syncytial Virus (RSV). Muscular fatigue, hypercapnia and severe hyperinflation are hallmarks of severe asthma. Mainstream therapy consists of sedation, inhalation bronchodilator, steroids, fluid management and bronchial lavage. Ventilator settings should include moderately low respiratory rate (12–14 / min), low PEEP (3 cm H2O), high inspiratory flow (!00 L/min) in order to relieve hyperinflation.

**OBJECTIVES.** We compared bronchodilatatory add on effects of sevoflurane and propofll to fenoterol/ipratropium (F/I) in patients ventilated because of severe asthma.

**METHODS.** We retrospectively analyzed 20 consecutive patients admitted to our ICU with respiratory failure due to severe asthma, 10 sedated with propofol or sevoflurane (Anaconda, Sedana Medical). Inspiratory and expiratory hold was performed while on volume control ventilation with square flow pattern (90 L/min) and values for Resistance (R), compliance (C) and intrinsic PEEP (PEEPi) obtained. All patients received inhaled bronchodilator, steroids and antihistamines in emergency department.

**RESULTS.** Admission values for C, R and PEEPi in sevoflurane group were 35 ± 9, 25 ± 7 and 11 ± 4 respectively and in propofol group 42 ± 9, 27 ± 3 and 11 ± 4 respectively (p > 0.1). With F/I only R and PEEPi decreased in both groups (20 ± 4 vs 21 ± 3, p = 0.04 and 6 ± 2 vs 8 ± 2, p = 0.02 respectively). In sevoflurane group C increased (46 ± 8, p = 0.01), R and PEEPi decreased (13 ± 3, p = 0.003 and 6 ± 2, p = 0.02). Deep sedation (RAAS −5) was achieved with 3.3 ± 1 mg/kg/h propofol drip and 1.2 ± 0.3 vol% sevoflurane.

**CONCLUSIONS.** When used in combination with fenoterol/ipratorpium, sevoflurane ensures sufficient sedation level while decreasing PEEPi and R in severe asthma. Propofol as a sedation agent does not add to bronchodilation beyond fenoterol/ipratorpium effect. Increase in C was seen in sevoflurane group only, probably due to reduced lung hyperinflation.

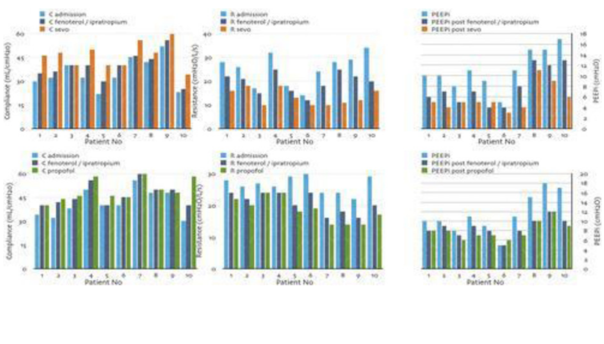

**Fig. 94 (abstract 0234)** Measurements

### 0235

#### Acute respiratory failure in cardiosurgery. Update in the first decade of the new century

I. Kasherininov, A. Bautin, V. Mazurok, V. Rubinchik, D. Laletin, A. Marichev, M. Gordeev

Almazov North-West Federal Medical Research Centre, St. Petersburg, Russian Federation

**Correspondence:** I. Kasherininov

*Intensive Care Medicine Experimental* 2017, **5(Suppl 2):**0235

**INTRODUCTION.** Cardiosurgical interventions are associated with variable complications, affecting the main organs, including the respiratory system [1]. Different pathophysiological changes lead to variable clinical forms of acute respiratory failure (ARF) [2]. The main reasons of ARF after open-heart cardiac surgery, are: acute respiratory distress-syndrome (ARDS), cardiogenic pulmonary edema (CPE), exacerbation of chronic obstructive pulmonary disease (COPD), pneumonia, pneumothorax and atelectasis [3].

**OBJECTIVES.** To evaluate ARF prevalence in cardiac surgery and observe its main causes.

**MATERIALS AND METHODS.** A retrospective study included 8859 patients, who underwent open-heart cardiosurgical interventions in Almazov North-West Federal Medical Research Centre from 2008 to 2012. The main inclusion criteria were the hypoxemia with $PaO_2/FiO_2 < 300$ mmHg and prolonged mechanical ventilation after surgery (>24 hours).

**RESULTS.** ARF developed in 377 cases (4.2%). The most common clinical variant was ARDS - 159 patients (1.8% of all and 42.2% of ARF). CPE occurred in 95 cases (1.1% of all patients and 25.2% of ARF). Severe COPD exacerbations were registered in 43 patients (0.49% of all population and 11.4% of ARF). 26 patients developed pneumonia (0.3% of all and 6.9% of ARF). Atelectasis and pneumothorax appeared both in 22 cases (0.25% of all and 5.8% of ARF). 9 patients with ARF died (2.4%) and ARDS was the sole cause of mortality.

We studied the severity of ARDS after cardiac surgery in accordance with the Berlin definition (2012). The majority of ARDS cases were mild - 107 (67.2% of ARDS population), moderate ARDS occured in 35 cases (22.0%) and severe ARDS developed in 17 patients (10.8%). All-cases mortality in ARDS was 5.7%. Mortality rate was associated with the severity of ARDS. In mild ARDS population mortality was 2%, in moderate cases - 13%, and it was maximal in severe forms - 27.3%. There were no lethal cases, caused by uncontrolled hypoxemia. All mortality cases were associated with multi-organ failure.

**CONCLUSIONS.** This study confirmed that ARDS is the main cause of ARF in cardiosurgery. Severe ARDS continues to be associated with high mortality.

**GRANT**

Not applicable

**ACKNOWLEDGMENT**

The support of Almazov North-West Medical Research Centre is gratefully acknowledged.

### 0236

#### The importance of chest drain position after drainage for pneumothorax in critically ill

C. M. Mokotedi[1], L. Lambert[2], L. Simakova[2], M. Balik[1]

[1]1st Medical Faculty, Charles University, Anaesthesia and Intensive Care, Prague, Czech Republic; [2]1st Medical Faculty, Charles University, Radiology, Prague, Czech Republic

**Correspondence:** M. Balik

*Intensive Care Medicine Experimental* 2017, **5(Suppl 2):**0236

**INTRODUCTION.** Malposition of percutaneously inserted chest drains (CD) is underestimated with a reported prevalence of 30% (1). As the lung cross-section tapers in the cranial direction, a CD inserted for pneumothorax drainage may turn cranially, laterally and ultimately fall dorsally. As thus, an occult ventral pneumothorax may persist particularly on mechanical ventilation.

**OBJECTIVES.** Retrospective study to assess several simple X-ray (CR) parameters in diagnosing chest tube (CD) malposition inserted for pneumothorax in non-trauma mechanically ventilated patients.

**METHODS.** Patients with a CD inserted to the pleural space from the safe triangle with a subsequent CR and computerized tomography (CT) scan performed for various reasons up to 24 hours apart were assessed for CD foreshortening, angle of inclination of the CD and CD tortuosity.

**RESULTS.** 28 patients were included in the study. The median time between the CR and CT examinations was 5.4 hours (IQR3.8-6.9). The angle of inclination above the horizontal line at pleural space entry was the best marker of a misplaced CD with AUC 0.97(95%CI 0.83-1.0, $p < 0.0001$), 100% sensitivity and 96% specificity for a cut-off value of 50 degrees. CD tortuosity on single plane CR imaging was higher in misplaced drains. Three patients with drains misplaced laterally beyond axillary line had an occult ventral pneumothorax.

**CONCLUSIONS.** The steep angle of inclination of the CD above the horizontal at chest entry measured on a CR should raise suspicion of CD misplacement. Foreshortening of the CD requires additional information like how much of the CD is actually inserted in the chest. These CR signs herald further investigation by chest ultrasound to rule out an occult pneumothorax not detected on a CR.

1.) Remerand F, Luce V, Badachi Y, Lu Q, Bouhemad B, Rouby JL. Incidence of Chest Tube Malposition in the Critically Ill. A Prospective Computed Tomography Study. Anesthesiology 2007; 106:1112–9

### 0237

#### Single centre experience of clinical manifestation and laboratory parameters evaluation of scrub typhus patients with and without ARDS

A. Taggu[1], N. Darang[2], M.H. Ismail[3]
[1]Mahatma Gandhi Medical College and Reseach Institute, Critical Care Medicine, Pondicherry, India; [2]Sribalaji Vidhyapeeth University, Pondicherry, India; [3]St.Johns Medical College, Bangalore, India
**Correspondence:** A. Taggu
*Intensive Care Medicine Experimental* 2017, **5(Suppl 2):**0237

**OBJECTIVES.** To evaluate clinical features, lab values, and outcome in patients with scrub typhus and comparison in patients with or without ARDS.

**METHODS.** Prospective observational study of 210 adult patients with acute febrile illness admitted in MICU of tertiary hospital in South India. Total of 198 patients suspected to have scrub typhus were tested positive with Immune-chromatography test and higher titres of weil felix test.Group A :110 out of 198 were patients having either Immune-chromatography test/ Weil felix test positive. whereas group B (88 out of 198) were negative for both Immune-chromatography and Weil felix test were excluded. Clinical manifestations, laboratory parameters, and outcome were evaluated in all group A patients with scrub typhus. Statistical analysis done using SPSSS version 10.0.

**RESULTS.** Among 110 group A patients who were included 65 patients had no ARDS and 45 patients had ARDS as per the Berlin definition. Clinical presentations like shortness of breath, cough, hypotension (MAP < 65 mmHg), and laboratory parameters like Hemoglobin, Hematocrit(HCT),, worsening serum creatinine, serum total bilirubin, serum Albumin(admission value), AST, ALT, LDH, CPK, and serum lactate were statistically significant (P < 0.0001) in group A patients. Group B patients recovered completely. Amongst group A patients, 36 out of 45 recovered, 6 patients died and 3 were discharged against medical advice.

**CONCLUSIONS.** Scrub typhus associated with ARDS has high morbidity and mortality. Early diagnosis and treatment with doxycycline is recommended.

### 0238

#### Discrepancies in pharmacological treatment of stable COPD patients' in reference to the 2011 GOLD recommendations, in a Tunisian medical ICU, 2011–2016

H. Kallel[1], E. Ennouri[1], J. Ayachi[1], R. Ben Jazia[2], K. Meddeb[1], A. Khedher[1], N. Fraj[1], M.A. Boujelbèn[1], W. Zarrougui[1], A. Ben Abdelhafidh[1], I. Ben Said[1], A. Azouzi[1], I. Chouchene[1], M. Boussarsar[1,3]
[1]Farhat Hached University Hospital, Medical Intensive Care Unit, Sousse, Tunisia; [2]Ibn El Jazzar Hospital, Department of Pulmonology, Kairouan, Tunisia; [3]Ibn Al Jazzar Faculty of Medicine, Research Laboratory N° LR14ES05 Interactions of the Cardio-pulmonary System, Sousse, Tunisia
**Correspondence:** M. Boussarsar
*Intensive Care Medicine Experimental* 2017, **5(Suppl 2):**0238

**INTRODUCTION.** COPD represents an important public health challenge that is both preventable and treatable. Its treatment success may be largely influenced by the proper implementation of recommendations, such as Global Strategy for Diagnosis, Management, and Prevention of COPD (GOLD 2011).

**OBJECTIVES.** To assess the frequency and the severity of discrepancies, in reference to the 2011 GOLD recommendations, in pharmacological treatment of stable COPD patients recruited upon ICU admission for an acute exacerbation.

**METHODS.** A retrospective observational study conducted in an 8-bed Tunisian medical ICU over a 6-years´ period (January 2011 to December 2016). Were included all consecutive patients admitted for AE/COPD. Were described, patients´ past history characteristics and at ICU admission and instrumental and pharmacological treatments. GOLD 2011 stage, frequency and severity of discrepancies were assessed using consensual two experts´ (JA, RB) definitions and opinions.

**RESULTS.** 314 patients were admitted for AE/COPD over the 6-years´ study period. They were 68.59 ± 10.8years aged, mainly men 281(89.5%). They presented severe clinical status attested by an mMRC ≥ 2 in 268(85.4%) ; GOLD D 270(86%), frequent exacerbations (≥3) in the last year 101(32.2%) with mean exacerbations episodes at 3.6 ± 3.6. Long term oxygen therapy and home mechanical ventilation were respectively used in 56(18.5%) and 18(5.7%).
138(43.9%) patients were admitted in the ICU with a severe hypercapnic encephalopathy. Mean pH, $PaCO_2$ and bicarbonates were respectively 7.31 ± 0.1 ; 59.8 ± 18.5mmHg and 31.4 ± 8.4mmol/L. Mean SAPSII score was 32.5 ± 12. Firstline invasive mechanical ventilation (IMV) was registered in 165(52.5%) patients. Mean length of stay (LOS) was 10.9 ± 10.7days with mortality rate at 132(42%).
Pharmacological treatment including : Short acting beta agonists (SABA), long acting beta agonists (LABA), inhaled corticosteroids (ICS), long acting muscarinic agonists (LAMA), Theophyllin and LABA/ICS combination were prescribed respectively in : 117(37.3%) patients ; 16(5.1%) ; 68(21.7%) ; 15(4.8%) ; 33(10.5%) and 42(13.4%). Only one patient benefited from rehabilitation. Discrepancies was objectified in 261(83.1%) and were classified as severe in 165(52.5%) patients.

**CONCLUSIONS.** In developing countries with low income, the present study highlights the poor access of severe COPD patients to health care system demonstrated by the huge discrepancies either in frequency and in severity of recommended medication and over-utilization of ICU´s facilities.

### 0239

#### NTproBNT as prognostic marker of mortality in the patient with inhalation injury

L. Cachafeiro Fuciños, E. Herrero de Lucas, A. Agrifoglio Rotaeche, M. Sanchez Sanchez, M.J. Ansensio Martin, B. Estebanez Montiel, A. Garcia de Lorenzo
Hospital Universitario la Paz, Madrid, Spain
**Correspondence:** L. Cachafeiro Fuciños
*Intensive Care Medicine Experimental* 2017, **5(Suppl 2):**0239

**INTRODUCTION.** Elevation of NTproBNP in pathologies other than heart disease, such as COPD, is related to prognosis. The elevation of NTproBNP in these cases is due to the production of NTproBNP in the right ventricle by the vasocontriction produced by hypoxia

**OBJECTIVES.** The aim of our study is to evaluate the relationship between NTproBNP and the inhalation injury of burn patients.

**METHODS.** We conducted a three-year prospective cohort study of 84 consecutive critically burned patients with inhalation injury admitted to a critical burn unit considered as a national burn reference center.We used in all patients admitted to our unit with inhalation injury, a resuscitation protocol guided by the parameters obtained with transpulmonary thermodilution and tissue perfusion dataDemographic data, TBSA, severity scores, mechanical ventilation, length of stay and mortality were collected, in addition to the data provided by thermodilution and lactate value, troponin and NTproBNP.

**RESULTS.** During this period, 84 consecutive patients were admitted with inhalation injuries, and the majority were men (75.6) with a mean age (52.5.2 ± 17.5). The severity scores obtained were: ABSI 8.2 ± 2.8, Baux 80.5 ± 27.8, APACHE II 13.3 ± 6.8. 89.3% required VM. The avarage stay was 25.5 ± 25.1 days and the mortality was 28.6%
With thermodilution we observed that patients who died had higher cardiac dysfunction (with higher troponin and NTproBNP), higher tissue hypoperfusion (elevated lactic) and a greater inflammatory response with more altered permeability (elevations of ELWI).
Having a logistic regression with these values, the only factor that was correlated with mortality was NTproBNP. Using as cutoff point > 400 pg/ml, we found significant differences between both groups (p 0.046). 71.4% of patients with inhalation injury who died had a mean NTproBNP above this value.

**CONCLUSIONS.** In our study, NTproBNP is related to the mortality of patients inhalation injury, with the optimum cutoff found at 400 pg / dl. Possibly this is due to the cardiac dysfunction that is produced by the initial aggression and hypoxia secondary to inhalation.

**REFERENCE(S)**
M. Sánchez-Marteles, A. Cecilio-Irazola et a. NT-proBNP in chronic obstructive pulmonary disease patients. An. Sist. Sanit. Navar. 2009, 32(2):235–41
Pavasini R, Tavazzi G, Biscaglia S, et al. Amino terminal pro brain natriuretic peptide predicts all-cause mortality in patients with chronic obstructive pulmonary disease: Systematic review and meta-analysis. Chronic Respiratory Disease 2017, Vol. 14(2) 1–10

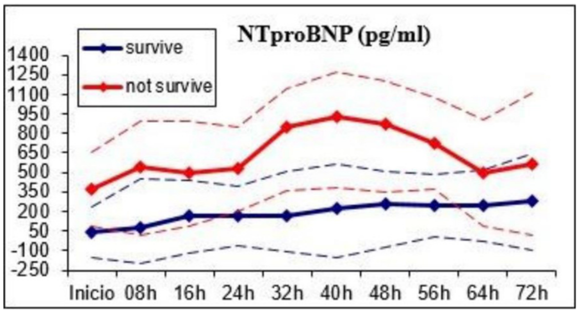

**Fig. 95 (abstract 0239)** NTproBNP

### 0240
### Acute respiratory events: incidence on general wards in England

R. Weissbrod[2], M. Cecconi[1], C. Leonard[3], M. Hamilton[1]
[1]St George's University Hospitals NHS Foundation Trust, London, United Kingdom; [2]Medtronic, Jerusalem, Israel; [3]Medtronic, Watford, United Kingdom
**Correspondence:** M. Cecconi
*Intensive Care Medicine Experimental* 2017, **5(Suppl 2):**0240

**INTRODUCTION.** Respiratory Compromise is a state in which there is a high likelihood of decompensation into respiratory insufficiency, respiratory failure, unplanned admission to intensive care or death, but in which early intervention might prevent decompensation.

**OBJECTIVES.** Our goal was to estimate the incidence and in-hospital mortality of acute respiratory events for elective patients admitted onto general wards in England.

**METHODS.** This is a retrospective analysis of the HES (Hospital Episode Statistics) records of 1,162,807 elective admissions between April 2015 and May 2016 to NHS hospitals in England. Descriptive statistics were used to characterize incidence and distribution of acute respiratory events. Acute events were pre-defined using existing ICD-10 and OPCS codes for respiratory events. Statistical techniques used were Welch t-test, Mann–Whitney test, ANOVA, Chi-Squared test.

**RESULTS.** There were 25852 acute events and 3791 deaths reported, representing 2.22% and 0.33% of the entire cohort. Adverse events were significantly more common in those over 71 years of age; (Welch t-test; $p < 0.05$). Opioid related events were reported in 1% of all acute events.

Medicine and thoracic medicine had the highest number of acute events at 4191 which accounted for 25% of the events in that therapy area.

On surgical wards, 1656 events were reported for general surgery, 1482 for trauma and orthopedics accounting for over 60% of the events in that therapy area.

OPCS Chapter K (Heart) had a significant higher number of events per 100,000 procedures than all other OPCS Chapters (Mann–Whitney; $p < 0.05$).

Comorbidities relating to codes used for resistant infection and severe acute respiratory syndrome have the highest number of acute events per 100,000 (n = 934) followed by diseases for respiratory system (n = 676).

Patient characteristics most strongly associated with adverse respiratory events were age, primary hypertension, respiratory failure, other diseases and ischemic heart failure.

**CONCLUSIONS.** Acute respiratory events on general wards in England are relatively common and the incidence is similar to that reported for US retrospective database analyses[1]. These events are related to age, with those in their senior years more likely to suffer an event. Multiple factors are associated with in-hospital events and mortality and may suggest a need for better patient monitoring solutions to detect respiratory compromise earlier.

**REFERENCE(S)**
Development Of Postoperative Pulmonary Complications After Admission To General Care Floor In Elective Surgery Cases- Kelley, Scott; Agarwal, Santosh; Parikh, Niraj; Erslon, Mary; Morris, Peter /Critical Care Medicine Volume 40(12) Supplement 1, December 2012
**GRANT ACKNOWLEDGMENT**
Study funded by Medtronic

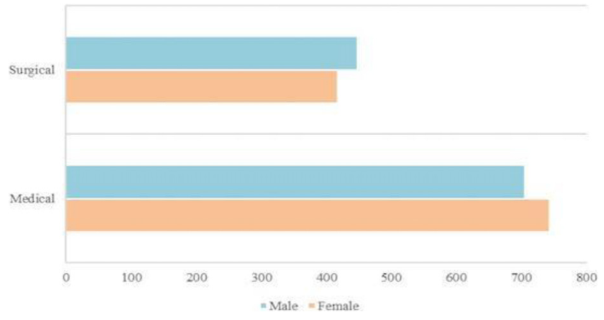

**Fig. 96 (abstract 0240)** Respiratory events

## Imaging to assess patients' needs

### 0241
### Competency of medicine residents in using ultrasound to detect lung abnormalities in cadaveric models

N. Kongpolprom
King Chulalongkorn Memorial Hospital, Thai Red Cross, Chulalongkorn University, Bangkok, Thailand
*Intensive Care Medicine Experimental* 2017, **5(Suppl 2):**0241

**INTRODUCTION.** Chest ultrasonography is a noninvasive and useful tool for rapid diagnosis of lung abnormalities at bedside, especially in emergency situations. For this reason, our institute set a 3-hour lung ultrasound session for second and third year medical residents, which was composed of 1-hour lecture and 2-hour hands on workshop. However, this learning program might not improve their competency in using ultrasound in real clinical practice.

**OBJECTIVES.** We aimed to evaluate the competency of medicine residents in using ultrasound to detect lung abnormalities at 6 months after they attended the lung ultrasound session and surveyed the number of their experience cases.

**METHODS.** Four cadaveric models, including pneumothorax, atelectasis, consolidation and pleural effusion were created. All cadavers were assisted with mechanical ventilation. The medicine residents had 10 minutes per the model to perform ultrasound, describe lung abnormalities and make final diagnoses. Additionally, the numbers of residents' experience cases were surveyed.

**RESULTS.** Fifty-six residents were enrolled to our study. The median number of lung ultrasound examination was 2[1,4] cases per month. The majority (66-82%) of them had never performed ultrasound to detect pneumothorax, atelectasis or consolidation while 60% of them had experience in using ultrasound to detect pleural effusion for at least 5 cases. Approximately, 84% of the residents correctly detected pneumothorax, however 45% of them used nonspecific signs to make diagnosis. Around 27% and 34% of the residents accurately diagnosed atelectasis and consolidation, nevertheless only 7% and 21% described correct signs of atelectasis and consolidation, respectively. Most of the residents made diagnosis of pneumothorax, atelectasis and consolidation based on inadequate information. Besides, only 50% of the residents detected small and loculated pleural effusion. Due to variable and small numbers of experience cases, competency in using lung ultrasound to detect the abnormalities was not associated with the residents' experience.

**CONCLUSIONS.** The medicine residents had the highest skill to detect pneumothorax by ultrasound, compared with the diagnosis of atelectasis, consolidation and pleural effusion. However, due to the fact that a large number of them made the diagnosis by non-pathognomonic signs and inadequate information, competency in using ultrasound to detect lung abnormalities might be lower than these findings. Thus, we need to provide more effective strategies to improve this competency.

### 0242

#### Using a blended learning environment as a key to the development of innovative medical education in critical ultrasound point of care teaching

J. Ortega Salas[1], E. Urias[2]

[1]Instituto Mexicano Del Seguro Social, Culiacan, Sinaloa, Mexico; [2]Centro de Investigacion y Docencia en Ciencias de la Salud, Culiacan, Mexico

**Correspondence:** J. Ortega Salas

*Intensive Care Medicine Experimental* 2017, **5(Suppl 2):**0242

**INTRODUCTION.** The most efficient learning process consists of supporting traditional teaching with e-learning methods. Classes conducted in traditional educational institutions are complemented with virtual labs. Teachers provide a variety of materials, guidelines and instructions using distance learning techniques. This method works well for those who do not fully understand a particular part of the material. By learning in blended mode, a learner is able to revise and consolidate individual issues.

**OBJECTIVES.** Show a blended learning enviroment as an innovative medical education technique for critical ultrasound point of care teaching.

**METHODS.** Observative, prospective, transversal study of three months in a blended learning environment.

**RESULTS.** 120 students; 96% from Mexico, 1.01% from Peru, 1.01% from Ecuador and 1.01% from Guatemala. There were 13 modules on line with different formative evaluation skills. Forums (40% of final evaluation), integrated activities (30% of final evaluation), questionnaires (20% of final evaluation) and a final test (10% of the final grade) The sumative evaluation was made of these percentages plus the assistance of the students to the six presencial modules at the Hospital. There were a 10% of students who gave up the course, the final grades for forums were 39.52%, these also included a space where students could put their scanning ultrasound images and the professor could grade them and give their own opinion. The final grades for integrated activities were 28.4%, the final grade for questionnaires was 19.09% and the total final grades of the entire course was of 99.52%. Satisfaction of the students was measured with a grading score showing that 93% of the students were satisfied with the course and the results in ther education. We also measured if students continued to use ultrasound after ending the course. 37% continued to use ultrasound in ther units and 20% bought and ultrasound equipment.

**CONCLUSIONS.** The blended environment permited evaluation of the students in different areas as forums, integrative activities and skill evaluations. Basing on the satisfaction of the students we conclude that the use of this environment allows students to learn properly ultrasound point of care techniques. However only a small percentage continue to use ultrasound perhaps of the lack of interest of hospital directions; other students bought ultrasounds so they could continue performing.

**REFERENCE(S)**

Liu, Q., Peng, W., Zhang, F., Hu, R., Li, Y., & Yan, W. (2016). The Effectiveness of Blended Learning in Health Professions: Systematic Review and Meta-Analysis. Journal of Medical Internet Research, 18(1), e2. http://doi.org/10.2196/jmir.4807

Cook DA1, Hatala R, Brydges R, Zendejas B, Szostek JH, Wang AT, Erwin PJ, Hamstra SJ.(2011) Technology-enhanced simulation for health professions education: a systematic review and meta-analysis. 306(9):978–88. doi: 10.1001/jama.2011.1234.

**GRANT ACKNOWLEDGMENT**

None

### 0243

#### Regional pulmonary perfusion in experimental regional ARDS - a comparison of EIT and CT

G. Elke[1], M. Kircher[2], O. Dössel[2], M.K. Fuld[3], A.E. Halaweish[3], E.A. Hoffman[3], N. Weiler[1], I. Frerichs[1]

[1]University Medical Center Schleswig-Holstein, Campus Kiel, Department of Anesthesiology and Intensive Care Medicine, Kiel, Germany; [2]Karlsruhe Institute of Technology (KIT), Institute of Biomedical Engineering, Karlsruhe, Germany; [3]University of Iowa Carver College of Medicine, Department of Radiology, Division of Physiologic Imaging, Iowa City, United States

**Correspondence:** G. Elke

*Intensive Care Medicine Experimental* 2017, **5(Suppl 2):**0243

**INTRODUCTION.** Electrical impedance tomography (EIT) can trace ventilation and perfusion related changes in electrical properties of lung tissue. So far, the ability of EIT to assess lung perfusion has been examined experimentally by electron-beam and single-photon-emission computerized tomography (CT) in healthy or globally injured lungs [1,2].

**OBJECTIVES.** To validate EIT measurements of regional perfusion by dynamic CT in two animal models of regional sublobar ARDS.

**METHODS.** The study was approved by the University of Iowa Committee for Animal Care. Regional lung injury was induced in two adjacent sublobar segments of the right lung by repetitive saline lavage (N = 3) or endotoxin injury (N = 5) in anesthetized mechanically ventilated pigs (33 ± 2 kg). EIT measurements (Goe-MF II, CareFusion, Höchberg, Germany) were performed at 25 scans/s during a breath-hold and subsequent bolus injection of 0.45 ml/kg 5.8% saline via a right ventricle catheter. EIT scans were generated using a FEM based linearized Newton–Raphson reconstruction algorithm. To determine pulmonary blood flow (PBF) via CT, axial (4 cm z-axis) ECG-gated dynamic CT (80 kV, 150 mAs, 0.75 mm section thickness, 0.5 mm increments, 0.28s rotation time, 25 time points, ~4.2 mSv) was performed during breath-hold after right ventricular injection of iodinated contrast agent (0.5 mL/kg over 2 s). PBF was estimated both in CT and EIT data based on the indicator dilution theory [3] and compared by Spearman correlation and Bland-Altman analysis. For CT calculations, the necessary arterial input function (AIF) was determined within the automatically segmented left and right pulmonary artery. AIF for EIT perfusion estimation was chosen within the right ventricle. The influence of the right cardiac region on regional PBF was removed by image post-processing.

**RESULTS.** PBF estimated by dynamic CT was compared to PBF obtained by EIT. For CT, PBF within the lung volume was projected onto a cross-section to enable comparison with the 2D EIT image. Further, regional distribution of PBF was compared within four quadrants respecting the centroid of the segmented lungs. In each of the quadrants relative perfusion with respect to overall PBF was used for comparison. The estimated regional perfusion distributions from CT

and EIT showed good overall agreement in healthy and locally injured lung regions ($r^2$ = 0.88). EIT slightly overestimated PBF within the ventral quadrants likely caused by incomplete heart region removal during calculation.

**CONCLUSIONS.** EIT is able to monitor regional pulmonary perfusion using a saline bolus. The perfusion estimation algorithm will be extended in future research to better account for the right cardiac phase of the bolus.

**GRANT ACKNOWLEDGMENT**

Restricted research grant: CareFusion, Höchberg, Germany.

### 0244

#### Accuracy of an ultra sound protocol of muscle thickness compared to CT-scan in a neuro ICU population

C. Tourel[1], P. Croisille[2], J. Morel[1]
[1]CHU Saint Etienne, Anesthésie-Réanimation, Saint Etienne, France; [2]CHU Saint Etienne, Radiologie, Saint Etienne, France
**Correspondence:** C. Tourel
*Intensive Care Medicine Experimental* 2017, **5(Suppl 2):**0244

**INTRODUCTION.** Muscular atrophy is frequent in ICU patients and is associated with increased mortality and long term muscle weakness. Bedside ultrasound has been proposed to assess muscle atrophy. This tool has not been validated in ICU patients yet.

**OBJECTIVES.** The objective of this prospective observational study was to compare the agreement between 2D ultrasound and 2D CT-scan for the measurement of thigh quadriceps muscle thickness.

**METHODS.** Consecutive patients admitted in our neuro ICU were included if they presented a Glasgow score ≤ 8 at admission, required mechanical ventilation and were scheduled for repeated follow-up CT scans of the brain. A written informed consent was obtained from patient next-of-kin before study enrollment.

Muscle thickness was measured 15cm above the upper edge of the patella perpendicular to the patella- anterior superior iliac spine axis. A landmark was drawn on the skin with a permanent marker. Iterative brain CT scans were associated with a quadriceps-centered acquisition sequence. Concomitantly, an ultrasound of the quadriceps was performed. The position of the leg was standardized for both technics. A custom-made system was developed to enable a precise and ergonomic positioning of the US transducer. (Fig. 97).

A maximum of three cerebral-CTs coupled with quadriceps imaging were performed between ICU admission and the tenth day of hospitalization. The thickness of the quadriceps was measured by an independent operator on the CT and ultrasound sections. We calculated that 38 patients were necessary to show a different between both technics.

**RESULTS.** Patients were included from August 2015 to December 2016, and 86 measurements of US and CT quadriceps thickness were compared. The agreement between these two techniques was very good as shown by the correlation coefficient ($R^2$ = 0.86) and the Intra-Class Correlation index of 0.96 (95% CI: 0.93-0.97). We also observed a very good intra-operator reproducibility and inter-operator variability.

**CONCLUSIONS.** We show for the first time in an ICU population that the US set up used here for quadriceps thickness measurement is reliable and reproducible.

• Tillquist M, and al. *Bedside ultrasound is a practical and reliable measurement tool for assessing quadriceps muscle layer thickness.* JPEN; 2014 Sep; 38(7):886–90
• Parry SM and al. *Ultrasonography in the intensive care setting can be used to detect changes in the quality and quantity of muscle related to muscle strength and function.* J Crit Care; 2015 Oct; 30(5):1151.e9–1151.e14

**GRANT**

None

**ACKNOWLEDGMENT**

None

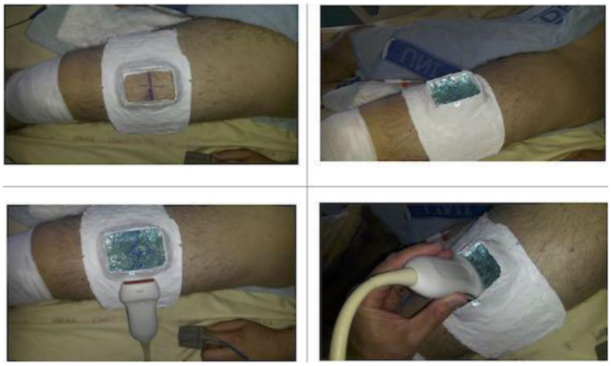

**Fig. 97 (abstract 0244)** Custom-made system

### 0245

#### Lung CT scan in acute respiratory distress syndrome (ARDS) and in cardiogenic pulmonary edema: a quantitative analysis

G. Vergani[1], M. Cressoni[1], F. Crimella[2], C. L'Acqua[3], E. Sisillo[3], M. Gurgitano[4], A. Liguori[4], A. Annoni[5], G. Carrafiello[6], D. Chiumello[7]
[1]Università degli Studi di Milano, Dipartimento di Scienze della Salute, Milan, Italy; [2]Università degli Studi di Brescia, Spedali Civili di Brescia., Scuola di Specialità di Anestesia, Rianimazione, Terapia Intensiva e del Dolore., Brescia, Italy; [3]IRCCS Centro Cardiologico Monzino, Department of Anesthesia and Intensive Care, Milan, Italy; [4]Università degli Studi di Milano, Postgraduation School in Radiodiagnostics, Milan, Italy; [5]IRCCS Centro Cardiologico Monzino, Department of Cardiovascular Imaging, Milan, Italy; [6]ASST Santi Paolo e Carlo, Università degli Studi di Milano, Radiologia Diagnostica e Interventistica. Dipartimento di Scienze della Salute, Milan, Italy; [7]ASST Santi Paolo e Carlo. Università degli Studi di Milano, SC Anestesia e Rianimazione. Dipartimento di Scienze della Salute, Milan, Italy
**Correspondence:** G. Vergani
*Intensive Care Medicine Experimental* 2017, **5(Suppl 2):**0245

**INTRODUCTION.** The cardiogenic pulmonary edema (CPE) and the acute respiratory distress syndrome (ARDS) are both characterized by an increase in extravascular lung water. Computed tomography (CT) provides not only a morphological but also a quantitative description of the lung [1].

**OBJECTIVES.** Aim of this study was to evaluate the quantitative CT findings in CPE and ARDS patients during the early phase.

**METHODS.** 60 ARDS (mild, moderate and severe) and 20 CPE patients were enrolled. The amount of not inflated tissue, well inflated tissue and edema were computed with a dedicated software along the sterno-vertebral axis [2]. The edema was estimated as the difference of lung weight measured with CT scan and the one predicted from the patient height [3].

**RESULTS.** Considering both CPE and ARDS groups moving from the non-dependent to the dependent lung regions, the not inflated lung tissue significantly increased while the well inflated lung tissue decreased. Significant differences were found between CPE and ARDS mostly in dependent regions. In CPE, the estimated edema was significantly lower than in severe ARDS (532 ± 637 vs 757 ± 740g) but higher compared to moderate and mild ARDS (385 ± 673 and 447 ± 525g).

**CONCLUSIONS.** Although CPE has a lower and higher amount of not inflated and well inflated tissue compared to severe ARDS, the overall regional distribution of these tissues is similar within the lung.

**REFERENCE(S)**

[1] M. Zompatori, F. Ciccarese, e L. Fasano, «Overview of current lung imaging in acute respiratory distress syndrome», *Eur. Respir. Rev. Off. J. Eur. Respir. Soc.*, vol. 23, n. 134, pagg. 519–530, dic. 2014.

[2] L. Gattinoni, A. Pesenti, L. Avalli, F. Rossi, e M. Bombino, «Pressure-volume curve of total respiratory system in acute respiratory failure. Computed tomographic scan study», *Am. Rev. Respir. Dis.*, vol. 136, n. 3, pagg. 730–736, set. 1987.

[3] M. Cressoni *et al.*, «Limits of normality of quantitative thoracic CT analysis», *Crit. Care Lond. Engl.*, vol. 17, n. 3, pag. R93, mag. 2013.

**GRANT ACKNOWLEDGMENT**

None

**Table 74 (Abstract 0245)**. Lung aeration computed by CT in ARDS and CPE

| | ARDS Mild (n=20) | ARDS Moderate (n=20) | ARDS Severe (n=20) | CPE (n=20) | *p* |
|---|---|---|---|---|---|
| Not inflated lung tissue (%) | 37±13 | 41±17$^{c}$ | 53±14$^{b,c}$ | 33 ±20$^{a,b,c}$ | <0.0001 |
| • Non- dependent regions | 7±11 | 8±10 | 13±13$^{b,c}$ | 10±14 | <0.001 |
| • Intermediate regions | 16±21 | 24±22$^{c}$ | 40±25$^{b,c}$ | 22±21$^{a}$ | <0.0001 |
| • Dependent regions | 63±31 | 63±31 | 78±22$^{b,c}$ | 49 ±34$^{a,b,c}$ | <0.0001 |
| Well inflated lung tissue (%) | 32±12 | 23±12$^{c}$ | 14±8$^{b,c}$ | 37 ±21$^{a,b,c}$ | <0.0001 |
| • Non- dependent | 64±19 | 49±22$^{c}$ | 42±21$^{b,c}$ | 58 ±24$^{a,b}$ | <0.0001 |
| • Intermediate regions | 47±25 | 34±25$^{c}$ | 17±16$^{b,c}$ | 45 ±25$^{a,b}$ | <0.0001 |
| • Dependent regions | 13±18 | 8±12 | 4±6$^{c}$ | 24 ±28$^{a,b,c}$ | <0.0001 |

**Table 75 (Abstract 0245)**. Estimated edema computed by CT in CPE and ARDS

| | ARDS Mild (n=20) | ARDS Moderate (n=20) | ARDS Severe (n=20) | CPE (n=20) | *p* |
|---|---|---|---|---|---|
| Estimated edema (g) | 447±525 | 385±673 | 757±740$^{b,c}$ | 532 ±637$^{a,b}$ | <0.0001 |
| • Non-dependent regions | 8±6 | 18±19$^{c}$ | 25±20$^{b,c}$ | 13±15$^{a}$ | <0.0001 |
| • Intermediate regions | 17±12 | 36±33$^{c}$ | 55±45$^{b,c}$ | 31±35$^{a,c}$ | <0.0001 |
| • Dependent regions | 23±15 | 45±41$^{c}$ | 65±47$^{b,c}$ | 40±52$^{a,c}$ | <0.0001 |

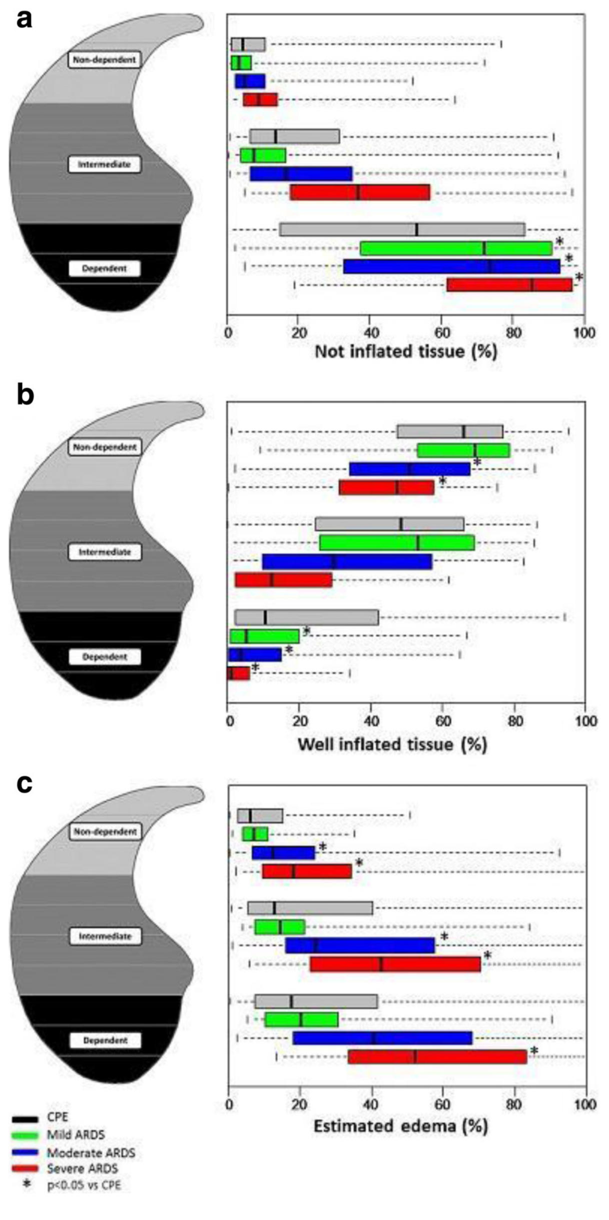

**Fig. 98 (abstract 0245)** Regional distribution of aeration and edema

### 0246

#### Speckle tracking echocardiography in evaluation of the left ventricular function during severe sepsis or septic shock

M. Daiki[1], H. Mahfoudhi[2], S. Koubaji[1], A. Rafrafi[1], K. Taamallah[2], C. Bouguerra[3], I. Labbène[1], M. Ferjani[1]

[1]university Tunis Elmanar, critical care, Tunis, Tunisia; [2]Tunis Elmanar, cardiology, Tunis, Tunisia; [3]university Tunis Elmanar, Department of statistics and preventive medecine, Tunis, Tunisia

**Correspondence:** M. Daiki

*Intensive Care Medicine Experimental* 2017, **5(Suppl 2):**0246

**INTRODUCTION.** Sepsis is a clinical syndrome caused by severe infection. It is characterized by a systemic inflammatory reaction with varying degrees of organ dysfunction. Myocardial dysfunction has a cumulative effect on sepsis-related mortality[1]. Hence, echocardiography-Doppler remains essential for the assessment of myocardial function in severe sepsis and septic shock. More recently, a new imaging modality is coming into clinical focus called speckle tracking imaging (STE)[2].

**OBJECTIVES.** The aim of our study is to investigate left ventricular dysfunction in patients with severe sepsis or septic shock by comparing standard echocardiography-based indices and STE as well as their prognostic implications.

**METHODS.** we prospectively included 30 consecutive adult patients (age > =18 years old) admitted to the intensive care unit of military hospital of Tunis within 48 hours of diagnosis of severe sepsis or septic shock. Standard trans-thoracic echocardiography and left ventricular STE imaging were performed within 48 hours of sepsis onset(D1). They were reassessed after 48 hours (D3) and 7 days (D7)in survival patients. Troponion T high sensitive (ThT-hs) measurement was performed on D1 of sepsis onset and reassessed on D3 and D7 in survival patients. All hypertensive patients, those with known cardiovascular disease and post operative cardiac surgery patients were not included in the study.

**RESULTS.** There was no difference in age, heart rate, systolic and diastolic blood pressure, vasoactive medication, ThT-hs levels and left ventricular ejection fraction between survival group and non survival group. However, survivor patients dilated their left ventricule in the early phase of sepsis (survival group vs non survival group respectively on D3 and D7 of sepsis onset: D3: 47.63 ± 4.17vs 44 ± 3.84, p = 0.031/ D7: 49.6 ± 4.4 vs44.15 ± 4.56, p = 0.009). Left ventricular filling pressure assessed by E/e' ratio were high in the two groups of patients in D1 of sepsis onset. However, E/e' ratio decline in survival group and remains high in non survival group with a statistically significant result on D7 of sepsis onset (survival group vs non survival group: D1: E/e': 10.42 ± 5.48 vs 9.97 ± 3.71 p = 0.8/ D7 E/e': 8.27 ± 3.84 vs12.06 ± 2.55 p = 0.018). The global longitudinal peak systolic strain GLPSS in survival group was higher in D3 and D7 of sepsis onset (survival group vs non survival group GLPSS D3: −18.44% ± 3.37 vs −14.68% ±4.03, p = 0,013/ GLPSS D7: −17.74% ± 4.53 vs −11.75% ± 4.34,p = 0,005).

**CONCLUSIONS.** Speckle tracking echocardiography could be an important parameter for evaluating LV function that would detect cardiac dysfunction at an early stage of the disease in septic patients.

**REFERENCE(S)**

1. Wenzel RP. **Treating sepsis**. N Engl J Med 2002;347:966–7

2. Blessberger H, Binder T. **Two dimensional speckle tracking echocardiography: basic principles**. Heart 2010;96:716–722

**GRANT ACKNOWLEDGMENT**

No grant was received for this work.

### 0247

#### Bedside ultrasound is a practical measurement tool for assessing quadriceps muscle: a pilot study

R. L. Cordioli, D. Toledo, D. C. de Lima e Silva, J. M. Silva, S. M. F. Piovacari, E.J.A. Figureiredo, I.C. de Paula, D. M. dos Santos, R. Dib
Hospital Israelita Albert Einstein, Adult Intensive Care, São Paulo, Brazil
**Correspondence:** R. L. Cordioli

*Intensive Care Medicine Experimental* 2017, **5(Suppl 2):**0247

**INTRODUCTION.** Bedside ultrasound measurement has previously been used to quantify muscle layer thickness at the quadriceps muscle. Tillquist et al.[1] showed that the technique had excellent intra and inter-reliability when used to assess healthy volunteers.

**OBJECTIVES.** To evaluate the intra and inter-reliability of measuring QMLT using bedside ultrasound.

**METHODS.** The study was approved by the ethics committee of Hospital Israelita Albert Einstein. We conducted a prospective observational study of measuring quadriceps muscle layer thickness (QMLT) in healthy volunteers. The thickness of the quadriceps musculature was quantified with a portable B-mode ultrasound device with volunteer in the lying supine position with knees extended and relaxed, 2 landmarks on each quadriceps were identified and marked. QMLT was calculated by measuring at the border between the lower third and upper two-thirds between the anterior superior iliac spine (ASIS) and the upper pole of the patella, as well as the measurement of the midpoint between the ASIS and the upper pole of the patella (Fig. 99). The muscle thickness was quantified as the distance between the upper margin of the femoral bone and the lower boundary of the deep fascia of the femoral rectus muscle (Fig. 100). To standardize measurements, a procedural manual and an accompanying training with practical lessons were held with load time of 6 hours for the team's training. Trainer were 1 physician with advanced training in bedside ultrasound. Trainees were comprised of 3 dietitians, 2 physician, 1 physiotherapists the majority with no prior ultrasound experience. To validate the image collection by the quadriceps muscle ultrasound measurements were performed in two healthy volunteers of different gender comparing between trainer and trainees and between trainees.

**RESULTS.** Total 16 samples were examinations by trainer in the procedure and compared the same exams for trainees. The Person's correlation was found relation between trainer and trainees with $r^2 > 0.90$ (Fig. 101). The better association was between trainer and dietitians with $r^2$ of 0.99 P < 0.001 and the relation between trainer and physicians was $r^2$ to 0.92 P < 0.001. In comparison to Bland Altman, evaluating the tests made by the trainer and the trainees, the highest percentage of error was 5.12% (IC 95% 3.64 -12.37) and the lowest was 1.01% (CI 95% 0.72 -2.58), on the other hand *the highest* bias of the values described was −0,12 ± 0,19 and smallest was −0,01 ± 0,04.

**CONCLUSION.** The data analyzed showed an excellent correlation of measures between trainer and trainees. Therefore, this analysis shows that the exam is feasible and easy application for all professionals involved in the treatment of patients may present nutritional deficits and loss of muscle mass.

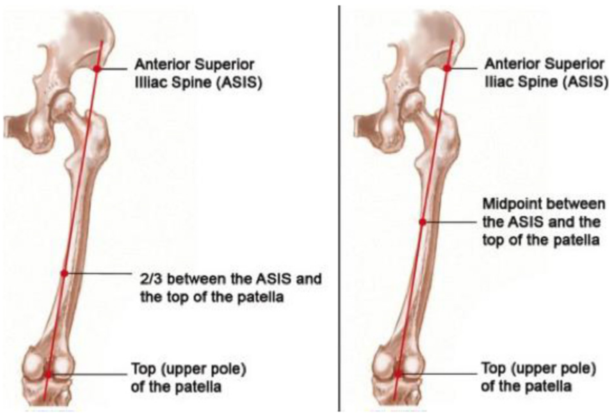

**Fig. 99 (abstract 0247).** See text for description

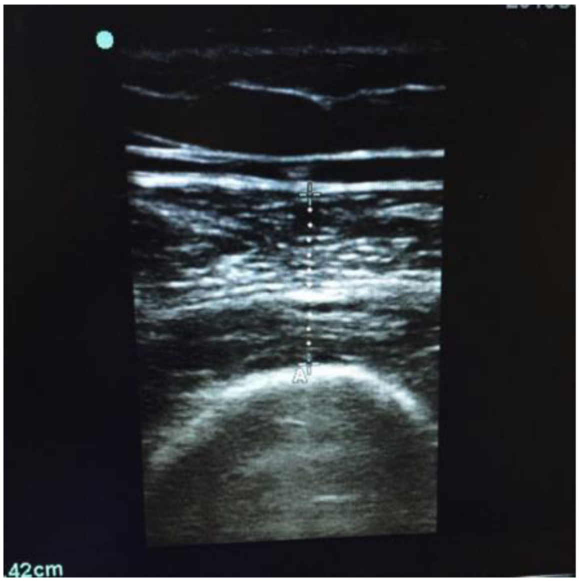

**Fig. 100 (abstract 0247)** See text for description

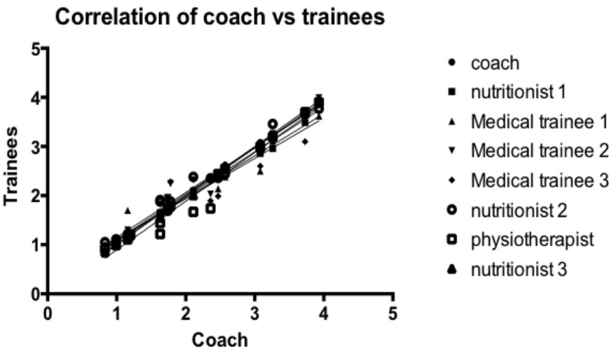

**Fig. 101 (abstract 0247)** See text for description

### 0248

#### Use of ultrasound imaging for diagnosis of intestinal structural changes and correction of treatment of postoperative gastrointestinal failure after major abdominal surgeries

M. V. Petrova[1], A. V. Butrov[1], M. V. Vatsik[2], M. F. I. Nakade[1], R. Mohan[1], M. N. Storchai[1], G. R. Mahmutova[1]
[1]RUDN University, Anesthesiology and Intensive Care, Moscow, Russian Federation; [2]City Hospital 64, Anesthesiology and Intensive Care, Moscow, Russian Federation
**Correspondence:** M. V. Petrova
*Intensive Care Medicine Experimental* 2017, **5(Suppl 2):**0248

**INTRODUCTION.** Gastrointestinal Failure (GIF) is frequent early postoperative(PO) period complication after abdominal surgery, characterized by impaired motor, secretory, absorptive, barrier function of gastrointestinal tract (GIT), usually develops as result of sudden disturbance in abdomen leading to severe water-electrolyte imbalance, if untreated leads to Abdominal Compartment Syndrome, ultimately multi-organ failure. Common diagnostic method is barium-contrast X-ray but unfortunately is less informative & doesn´t allow monitoring of intestinal condition to observe changes in real time. We tried to adopt Ultrasound Imaging (USI) as diagnostics method allowing better evaluation of gut condition.

**OBJECTIVES.** Observing intestinal wall structural changes, nature of intestinal peristalsis after abdominal surgery using X-ray & USI.

**METHODS.** 70 PO patients after major abdominal surgeries. All patients were examined with X-ray & USI to observe intestinal lumen diameter, wall thickness, nature of peristalsis, amount of contents in GIT, free fluid in abdomen, intestinal microcirculation.

**RESULTS.** Comparison of both diagnostic methods, we observed:

(1) 26 patients couldn't undergo x-ray examination due to unstable condition, unfit for transportation to X-ray room,
(2) All 70 patients underwent USI,
(3) 44 patients underwent x-ray exam & USI. It was observed that USI was more informative,
(4) 24 patients had pneumatised intestines in x-ray films, 20 patients had air-fluid levels showing insufficient information for deciding further treatment tactics.
(5) Using X-ray exam except for air-fluid levels, any other details weren't possible to detect. Whereas USI 38(54%) patients lumen diameter < 3cm with unchanged peristalsis proving 1$^{st}$ stage GIF, 22(31%) patients lumen diameter < 4cm, slightly edematous intestinal wall, decelerated peristalsis shows 2$^{nd}$stage GIF, 10(14%) patients with lumen diameter > 4 cm, intestinal wall > 4 mm with large amount of gastric-stasis shows 3$^{rd}$stage GIF

**CONCLUSIONS.** Detailed USI information helps recognize GIF stage & treatment options which is difficult using x-ray exam due to lack of information. X-ray is difficult in early PO since patients need to assume vertical position, consume oral contrast unlike in USI, special positioning isn't required & is bed-side technique which doesn´t require transportation of unstable patients allowing evaluation of intestinal wall condition, motor activity in real-time & thus, it's advisable to be used as first-line diagnostic method of IF

**REFERENCE(S)**

Berzakova I.V., Kalanov R.I. Ultrasound Exam during acute intestinal obstruction, with complicated peritonitis// All Russian conference of Surgeons. Volgograd, 2000. P.152.

**GRANT ACKNOWLEDGMENT**
Not applicable

**Table 76 (Abstract 0248)**. Comparison between X-ray & USI exam of GIT

| | Barium-contrast X-ray | USI |
|---|---|---|
| Intestinal lumen diameter | - | +++ |
| Intestinal wall thickness | — | +++ |
| Nature of peristalsis (Active, decelerated, absent) | 0 | +++ |
| Contents in GIT | - | +++ |
| Presence of free fluid in abdomen | 0 | +++ |
| Intestinal microcirculation | 0 | +++ |
| | Used to just state presence/absence of IF | Used to evaluate stage of IF,condition of GIT for further Intensive Therapy & Enteral Feeding correction |

Well observed: +++, Poorly observed: -, Very poorly observed: —, Cannot be observed: 0

### 0249

#### Precision of measurements with transthoracic echocardiography in critically ill patients

M. Jozwiak[1,2], J. Gimenez[1,2], J.-L. Teboul[1,2], P. Mercado[1,2], F. Depret[1,2], C. Richard[1,2], X. Monnet[1,2]
[1]Service de Réanimation Médicale, Hôpital de Bicêtre, Hôpitaux Universitaires Paris-Sud, Assistance Publique - Hôpitaux de Paris, Le Kremlin Bicêtre, France; [2]Inserm UMR S_999, Univ Paris-Sud, Le Kremlin Bicêtre, France
**Correspondence:** M. Jozwiak
*Intensive Care Medicine Experimental* 2017, **5(Suppl 2):**0249

**INTRODUCTION.** So far, no study has specifically investigated the precision of transthoracic echocardiography (TTE) in the intensive care unit, *i.e.* how close repeated measurements are to each other. We had two objectives: (1) to determine the least significant change (LSC) of various measurements when the TTE probe is kept in the same position. This allowed us to determine how many measurements should be averaged within a TTE examination, (2) to determine the LSC of these measurements between two examinations. This allowed us to determine the least change that can be considered as significant between two TTE examinations performed at different times by the same operator.

**METHODS.** Two successive TTE examinations were performed by a board certified operator in 100 hemodynamically stable patients (age 67 ± 16 y.o., 54% under invasive mechanical ventilation, 16% with atrial fibrillation). Within each TTE examination, all measurements were performed at end-expiration, the probe being kept in the same position. We investigated the velocity time-integral of the subaortic flow (VTI), the ratio of the early peak velocity of transmitral flow over the early diastolic peak velocity of the lateral mitral annulus (E/e' ratio), the left ventricular ejection fraction (LVEF), the ratio of the end-diastolic right over left ventricular areas (RVEDA/LVEDA) and the tricuspid annular plane systolic excursion (TAPSE).

**RESULTS.** When the probe was kept in the same position, i.e. within one TTE examination, the LSC of VTI was 9[25-75% interquartile: 5–15]% when only one single measurement was performed. It dropped to 5[3–9]% if three measurements were averaged. In these conditions, the LSC was close to 10% for all others measurements. The LSC of VTI was higher in patients with atrial fibrillation (12[4–17]%) than in the other ones (5[3–8]%, $p = 0.007$). There was no significant difference in the LSC of VTI between patients with and without invasive mechanical ventilation. Similar results were found for TAPSE. No difference was observed for the other measurements between subgroups of patients. Between the two TTE examinations, the LSC of VTI was 11[5–18]%, 8[4–15]% for LVEF and close to 20% for the other studied variables. Whatever the measurements, the LSC was similar between subgroups of patients with and without atrial fibrillation and between subgroups of patients with and without invasive mechanical ventilation.

**CONCLUSIONS.** Averaging three measurements within one TTE examination is enough for obtaining an acceptable LSC for TTE measurements. Between two examinations, one can rely on changes larger for 11% for VTI, 8% for LVEF and 20% for other usual variables.

### 0250

#### Lung ultrasound and blood gas based classification of critically ill patients with dyspnea: a pathophysiologic approach

S. Saigal[1], R. Joshi[2], J.P. Sharma[3]
[1]Aiims Bhopal, Trauma & Emergency, Bhopal, India; [2]Aiims Bhopal, Medicine, Bhopal, India; [3]Aiims Bhopal, Anaesthesia, Bhopal, India
**Correspondence:** S. Saigal
*Intensive Care Medicine Experimental* 2017, **5(Suppl 2):**0250

**INTRODUCTION.** Tachypnea is single most indicator of critical illness. The causes of tachypnea vary from lung parenchyma disorders, airway disorders, pulmonary vascular disorders, cardiac disorders and metabolic disorders. Each and every tachypnoeic patient doesn't need intubation. Furthermore an ABG analysis is immediately done in ICU, by which we assess respiratory & metabolic parameters of the patient. But ABG doesn't localize the cause of tachypnea, hence doesn't define definite treatment. We combined this parameter with LUS which is readily available as bedside tool in ICU, to localize the cause of tachypnea. Once the cause of tachypnea is localized, definite treatment could be initiated early.

**OBJECTIVES.** The objective of this study was to classify tachypnoeic patient and to evaluate outcome variables on the basis of LUS and arterial blood gas (ABG) findings.

**METHODS.** It was a retrospective chart based review in which all tachypnoeic patients admitted in ICU were enrolled. On the basis of LUS (presence of A- lines/ B-Lines) and ABG (Hypoxia/Hypercarbia) patients were classified into six groups:

a) Airway disorder
b) Perfusion disorder
c) Metabolic disorder
d) Airway and Alveolar disorder
e) Pulmonary edema
f) Pneumonia.

The patient's demographic data, SOFA score, need for intubation, vasopressor and central venous catheter, form of mechanical ventilation, ICU outcome and length of stay were noted.

**RESULTS.** There were 8 patients with airway disorder, 4 with perfusion, 83 with metabolic, 16 with airway & alveolar disorder, 46 with pulmonary edema and 87 with pneumonia. The median age was 55 (6–93) years in our study. The median SOFA score was 7(1–18). 12.5% patients with airway disorder, 50% with perfusion, 14.5% with metabolic, 56.3% with airway and alveolar, 21.7% with pulmonary edema and 60.9% patients with pneumonia were intubated ($p < 0.0001$). Non-invasive ventilation (NIV) was required in 87.5% of patients with airway disorder, 3.6% with metabolic, 37.5% with ventilation & alveolar, 43.5% with cardiac, 9.2% with pneumonia($p < 0.0001$). We had maximum mortality in patients with pneumonias (21.7%) followed by metabolic (10.8%), pulmonary edema (8.7%) and in patients with airway disorder it was nil ($p < 0.0001$).

**CONCLUSIONS.** This classification gives an organized approach in managing patients with tachypnea. It predicts the need for intubation/ mechanical ventilation early on. It also predicts that patients with pneumonia are most sick of all the six groups and needs immediate intervention.

**REFERENCE(S)**

Xirouchaki N[1], Kondili E, Prinianakis G, Malliotakis P, Georgopoulos D. Impact of lung ultrasound on clinical decision making in critically ill patients. Intensive Care Med. 2014 Jan;40(1):57–65. doi: 10.1007/s00134-013-3133-3. Epub 2013 Oct 25.

### 0251

#### Lung ultrasound for the diagnosis of pneumonia in intensive care unit

Z. Bitar[1], O. Maadarani[2], A. Elshalaby[3], M. Alajmi[4]
[1]Ahmadi Hospital, Kuwait Oil Company, Intensive Care Medicine, Fahahil, Kuwait; [2]Ahamdi Hospital, Cardiology, Ahamdi, Kuwait; [3]Ahmadi Hospital, Kuwait Oil Company, Ahamdi, Kuwait; [4]Ahmadi Hospital, Kuwait Oil Company, Head of Respiratory Unit, Ahmadi, Kuwait
**Correspondence:** Z. Bitar
*Intensive Care Medicine Experimental* 2017, **5(Suppl 2):**0251

**BACKGROUND.** Despite Chest radiography (CXR) is the preferred tool for lung imaging and a cornerstone for the diagnosis of pneumonia, the diagnostic accuracy is 65% when compared with CT scan (1). CT scan is the diagnostic gold standard. Because of high radiation dose and high cost, it is not used routinely as diagnostic procedure of patients with suspected pneumonia in ICU (2).

**AIM.** To assess the role of bedside lung ultrasound examination by the critical care physician in the diagnosis of acute pneumonia in ICU, in comparison with CXR and CT chest.

**MATERIAL AND METHODS.** This is an observational, prospective and single-center study conducted between October 2015 and October 2016 in the intensive care unit of Ahamdi general hospital. The Lung ultrasound examination (LUS) was performed by trained critical care physicians, and a chest radiograph was interpreted by another critical care physician blinded to LUS result. CT scans were obtained when considered clinically indicated by the senior physician in charge of the patient. The final diagnosis of pneumonia was made by physicians in charge of the patients, on the basis of the clinical presentations, radiological examinations, clinical evolution, markers of inflammation and microbiology (3).

**RESULTS.** We studied 92 patients and 73 confirmed pneumonia (79.3%): 34 (36.6%) males with a mean age of 68.3 years (SD, 13.56). Eleven (15%) patients were community acquired pneumonia 64 (85%) were hospital acquired pneumonia. In the group of confirmed pneumonia, we had 72 (98.6%) positive LUS and 40 (54.8%) positive

CXR for consolidation (Table 1). Based on the diagnosis of pneumonia the specificity and sensitivity of CXR were 63% and 54.7%, while in LUS 84% and 9806% respectively (Table 2). A chest CT scan was performed in 38 of the 93 enrolled patients and was diagnostic for pneumonia in 32 cases. LUS was positive in 31 of 32 patients with CT confirmed pneumonia and CXR was positive in 5 of 32 patients with CT confirmed pneumonia. Sensitivity and specificity of LUS were 96.9% and 83%, while in CXR were 15.6 and 16.6% in relation to CT chest (Table 3).

We could perform a complete LUS examination (scanning anterior, lateral and posterior chest wall) in all the patients.

**CONCLUSION.** Bedside lung ultrasound is a reliable and accurate tool for diagnosing pneumonia in the ICU setting, and superior to CXR. It allows a faster, non-invasive and without radiation tool for the diagnosis of pneumonia in the ICU.

### 0252

#### Can point of care diaphragm usg reliably be performed with pocket-sized ultrasound devıces in ICU

K. Inci[1], G. Gursel[2]

[1]Gazi University School of Medicine, Department of Internal Medicine, Division of Critical Care, Ankara, Turkey; [2]Gazi University School of Medicine, Department of Pulmonary Critical Care Medicine, Ankara, Turkey

**Correspondence:** G. Gursel

*Intensive Care Medicine Experimental* 2017, **5(Suppl 2):**0252

**INTRODUCTION.** Current literature suggests that diaphragm ultrasound (DUS) could be a useful and accurate tool to detect diaphragmatic dysfunction in critically ill patients to predict extubation success, monitoring, respiratory workload and assessing atrophy in mechanically ventilated patients. On the other hand, there is an increasing body of evidence comparing the diagnostic accuracy of the pocket-sized ultrasonography devices (PSUD) with standard, high quality ones suggesting that they may be safely used to enhance the diagnostic accuracy of cardiovascular examination and proposed their use in various clinical settings such as emergency department, intensive care units and outpatient clinics.

**OBJECTIVES.** Aim of this study is to compare measurement accuracy of (PSUD) with high end ultrasound device (HEUD) in the measurement of diaphragm thickness(DT) and excurtion(DE) in ICU patients.

**METHODS.** Ultrasonographic evaluation was performed using Vivid-Q and V-Scan (GE, Fairfield, CT) devices with linear and sector probes. Diaphragmatic thickness was measured in B mode in two devices and excurtion was measured in M-Mode(MM) in HEUD. Because PSUD does not have MM DE's were measured using the scale on the right side of the screen on cineloop records. Blant - Altman statistical method was used for bias and agrement determination.

**RESULTS.** Twenty-six patients were included in the study and all of them had respiratory failure. Seven (27%) of them were intubated and 12(46%) of them were receiving nononinvasive mechanical ventilation therapy. In all patients diaphragm images were acquired with both devices and there was a paradox movement in 2 patients. The Bland-Altman analysis revealed that the width of the 95% limits of agreement were similar in both devices.

**CONCLUSIONS.** Pocket sized ultrasound devices may be very reliable in the evaluation of diaphragm thickness and excurtion in the ICU.

**REFERENCE(S)**

1- Massimo Zambon et al. Mechanical Ventilation and Diaphragmatic Atrophy in Critically Ill Patients: An Ultrasound Study Crit Care Med 2016; 44:1347–1352

2- Bruce J Kimura,et al. Point-of-care cardiac ultrasound techniques in the physical examination: better at the bedside Heart 2017;0:1–8. doi:10.1136/heartjnl-2016-309915

**GRANT ACKNOWLEDGMENT**

Study was supported by Gazi University Scientific Reserch Projects Foundation(Project No: 01/2015-48).

**Table 77 (Abstract 0252)**. Results

| Measurements | Mean±SD | Mean diff±SD | 95%CI of the differences | p |
|---|---|---|---|---|
| Expiratory diaphragm thickness(DT), cm | | 0,0046±0,031 | -0,016-0,0075 | P>0,05 |
| HEUD | 0,30±0,11 | | | |
| PSUD | 0,30±0,12 | | | |
| Tidal inspiratory DT,cm | | 0,0047±0,031 | -0,007- 0,017 | P>0,05 |
| HEUD | 0,40±0,18 | | | |
| PSUD | 0,40±0,18 | | | |
| Maximal inspiratory DT,cm | | 0,0046±0,031 | -0,034-0,013 | P>0,05 |
| HEUD | 0,49±0,17 | | | |
| PSUD | 0,50±0,19 | | | |
| Tidal thickness ratio(%) | | 2,93±7,3 | -6,08-6,20 | P>0,05 |
| HEUD | 32,1±13,1 | | | |
| PSUD | 30,4±13,5 | | | |
| Maximal thickness ratio(%) | | 0,062±15,5 | -0,11-0,072 | P>0,05 |
| HEUD | 57,2±30,4 | | | |
| PSUD | 58,9±29,3 | | | |
| Tidal excurtion,cm | | 0,017±0,22 | 0,089-5,76 | P>0,05 |
| HEUD | 1,54±0,73 | | | |
| PSUD | 1,52±0,75 | | | |
| Maximal excurtion,cm | | 0,11±0,26 | -0,22- -0,03 | P>0,05 |
| HEUD | 2,50±1,22 | | | |
| PSUD | 2,40±1,10 | | | |

### 0253

#### Verification of correct central venous catheter placement using ultrasound with two acoustic windows

G. Zick[1], T. Becher[1], J. Renner[1], L. Balke[2], D. Schädler[1], G. Elke[1], I. Frerichs[1], N. Weiler[1]

[1]University Medical Centre Schleswig-Holstein, Campus Kiel, Department of Anaesthesiology and Intensive Care Medicine, Kiel, Germany; [2]University Medical Centre Schleswig-Holstein, Campus Kiel, Department of Internal Medicine I, Kiel, Germany

**Correspondence:** G. Zick

*Intensive Care Medicine Experimental* 2017, **5(Suppl 2):**0253

**INTRODUCTION.** After insertion of a central venous catheter, its correct placement has to be confirmed and complications ruled out. The proper placement in the vena cava is usually assumed with correct projection in the chest radiograph. To reduce unnecessary radiation exposure, an alternative ECG-based approach has been suggested. Since this method is only reliable with sinus rhythm, it cannot be applied in patients with atrial fibrillation. We present here a novel approach for confirmation of proper catheter placement using ultrasound offering a safe alternative method even in this specific group of patients.

**OBJECTIVES.** The aim of this study was to visualize the j-wire in the vena cava using ultrasound with two acoustic windows. Additionally, an estimation of the correct length of insertion was examined.

**METHODS.** 100 critically ill patients (29 women, 65 men; mean age 66 ± 14yr) requiring a central venous catheter were examined. The vena cava was visualized from the right body side through the liver or the subcostal window displaying the heart with the connection of the vena cava to the right atrium. In the last 48 patients of our study population, the disappearance of the j-wire from the acoustic window was used to estimate the appropriate inserted length of the catheter. The patients´ examinations were performed using the vivid S6 with a sector scanner (M4S-RS, 1.5 to 3.6 MHz; GE Healthcare, Munich, Germany). They were carried out during the insertion of the j-wire before dilatation and insertion of the catheter.

**RESULTS.** After excluding duplicate examinations, 94 patients could be analysed. The j-wire was correctly identified in the vena cava or the right atrium in 91 patients. In one patient, where no wire was seen, the catheter was inserted in the wrong way. In two patients, the j-wire could not be identified although the catheter was later shown to be placed correctly according to the X-ray. The examination of the inserted length of the catheter in 48 patients revealed the following: in 42 patients, the catheter was correctly placed in the vena cava superior, in 4 patients, in the vena anonyma and in 2 patients, the disappearance of the j-wire could not be identified. No catheter was advanced into the right ventricle in our study patients.

**CONCLUSIONS.** Identification of the j-wire in the vena cava or the right atrium with ultrasound verifies the proper intravascular placement and direction of catheter insertion as confirmed in nearly all our patients. The appropriate length of the inserted catheter was also correctly estimated. Since complications like pneumothorax and haemothorax can also be ruled out with ultrasound [1], radiological X-ray examinations would only be needed in the very few cases where the ultrasound approach fails.

**REFERENCE(S)**

1. Lichtenstein et al. Anaesthesiol Intensive Ther 2014, 46:323–35

#### 0254

#### Diafragmatic ultrasound in intensive care units: feasible and reproducible

O. Moreno Romero, M. Muñoz Garach, A. Carranza Pinel, M. E. Yuste Ossorio
Complejo Hospitalario de Granada, Granada, Spain
**Correspondence**: O. Moreno Romero
*Intensive Care Medicine Experimental* 2017, **5(Suppl 2):**0254

**INTRODUCTION.** The use of ultrasounds in the intensive care units has increased in the last decades due to an easy learn & use, great accessibility, low cost and especially non invasive technique. Nowadays, has become relevant the use of diaphragmatic ultrasound to evaluate the patients response under invasive mechanical ventilation and to guide the weaning process and safe artificial airway removal avoiding re-intubation and thus, morbimortality.

**OBJECTIVES.** To describe the intraclass correlation index of diaphragmatic movement in healthy volunteers measured by ultrasound and between two intensive care physicians randomly selected in a university intensive care unit hospital.

**METHODS.** Prospective study in which we registered 19 volunteers from in 2 months (August and September 2016). An ultrasound video of the right diaphragm was made in everyone of them by a third professional not involved in the final measures. Afterwards, two ICU professionals were randomly selected to measure diaphragmatic movement as described in the literature. At the end, an intraclass correlation index of the results was analyzed.

**RESULTS.** From the 19 volunteers none was withdrown. None of the ICU physicians were prior trained in diaphragmatic ultrasound and the measures were made blind and independently in two different days. From all measures, the intraclass correlation index was 0.923 with a 95% CI (0,83 - 0.96).

**CONCLUSIONS.** Measurement of the diaphragmatic kinetics in the critical care units is a feasible and reproducible technique that might be part of the dairy practice in intensive care units.

### Perioperative intensive care: General

#### 0255

#### Early detection of lung congestion in pre-eclampsia using lung ultrasound and electrical cardiometry

Y. Hammad, A. Omran, A. Refaie, A. El-Sakka, A. Lotfy, A. Hasanin, A. Kamal, H. Mostafa
Kasralainy Faculty of Medicine, Cairo University, Cairo, Egypt
**Correspondence:** Y. Hammad
*Intensive Care Medicine Experimental* 2017, **5(Suppl 2):**0255

**INTRODUCTION.** Pre-eclampsia is a common pregnancy syndrome associated with poor maternal outcomes. Acute pulmonary edema is a common complication of pre-eclampsia associated with serious sequelae. Evaluation of lung water would be a helpful tool for early detection of patients with pending acute pulmonary edema.

**OBJECTIVES.** To evaluate the ability of extra-vascular lung water (EVLW) measured by lung ultrasound and total thoracic fluid content (TFC) measured by electrical cardiometry in early detection of pulmonary edema. We also correlated between EVLW and TFC using spearman correlation coefficient.

**METHODS.** A prospective observational study including pre-eclamptic parturients scheduled for delivery in Cairo university Hospitals. Lung ultrasound was performed on patient admission, and lung score (LUS) was calculated. Electrical cardiometry was used for measurement of TFC. Patients were divided into two groups: Congested group (defined as patients with clinical congestion requiring diuretics) and non-congested group. Area under receiver operating characteristic (AUROC) curve was calculated for both LUS and TFC for early detection of lung congestion. Spearman correlation coefficient was calculated for correlation between LUS and TFC.

**RESULTS.** Sixty parturients were available for final analysis. Eleven patients (18.3%) developed clinically manifested congestion. Clinically congested patients had a higher median lung ultrasound score 17(15–19) and thoracic fluid content 46(40–48) compared to non-congested patients 2(2–3) and 28(26–30) respectively (P value < 0.001). LUS showed excellent predictive properties for clinical congestion [AUROC = 0.964(0.881-0.995), sensitivity = 100%, and specificity = 93.8% at cutoff value of >4]. TFC also showed excellent predictive value [AUROC = 0.968(0.886-0.996), sensitivity = 100%, specificity = 89% at cutoff value of >37]. There was a good correlation between LUS and TFC with a spearman correlation coefficient (r) of 0.77.

**CONCLUSIONS.** Both LUS and TFC have excellent predictive properties for early detection of clinical congestion in parturients with severe pre-eclampsia. The presence of good correlation between TFC and LUS makes electrical cardiometry a promising alternative to ultrasound for assessment of EVLW.

#### 0256

#### Monitoring of gas exchange and patient state index: can we predict postoperative cognitive dysfunction?

M. Sokolova, L. Rodionova, V. Kuzkov, N. Izotova, M. Kirov
Northern State Medical University, Anesthesiology and Intensive Care Medicine, Arkhangelsk, Russian Federation
**Correspondence:** M. Sokolova
*Intensive Care Medicine Experimental* 2017, **5(Suppl 2):**0256

**INTRODUCTION.** General anaesthesia and surgery can be complicated by postoperative cognitive dysfunction. Among different factors, the impairment of mental functions and cerebral blood flow can be associated with disturbances of gas exchange. However, it is still unsettled whether the deterioration of cognitive function and gas exchange is related with depth of anaesthesia. One of the novel parameters for assessment of anaesthesia depth is a patient state index (PSI).

**OBJECTIVES.** Thus, the aim of this study was to explore the relationship of cognitive function with gas exchange and PSI in laparoscopic cholecystectomy.

**METHODS.** Totally 170 patients scheduled for laparoscopic cholecystectomy were enrolled into a prospective study and randomized into four groups:

1) normoxia-normocapnia ($nO_2$-$nCO_2$),
2) hyperoxia-normocapnia ($hO_2$-$nCO_2$),
3) normoxia-hypocapnia ($nO_2$-$lCO_2$),
4) hyperoxia-hypocapnia ($hO_2$-$lCO_2$).

Normoxia was defined as $PaO_2$ within 70–150 mm Hg, hyperoxia — within 150–300 mm Hg. Normocapnia was referred to $PaCO_2$ of 35–48 mm Hg, and hypocapnia — to 25–35 mm Hg. The intervention was conducted under total intravenous anaesthesia (propofol/fentanyl). All patients were tested for cognitive function using Montreal Cognitive Assessment Score (MoCA), monitoring the depth of anaesthesia was assessed by PSI (Masimo Root, USA).

**RESULTS.** Totally 109 patients strictly met the inclusion criteria and targeted gas exchange. We observed the decrease in MoCA at 6 hours after surgery in the hO2-nCO2 and the hO2-lCO2 groups (p < 0.03). Compared with the nO2-nCO2 group, arterial lactate and pH increased significantly at the end of surgery in the hO2-lCO2 and the nO2- lCO2 groups (p < 0.001). The PSI before induction of anesthesia correlated with age (rho = −0.41, p = 0.01) and baseline MoCA value (rho = 0.80, p < 0.001). In addition, we observed a positive correlation between PaCO2 and PSI at the end of intervention (rho = 0.32, p = 0.046).

**CONCLUSIONS.** The short-term combination of hyperoxia and hypocapnia during laparoscopic cholecystectomy leads to transient postoperative decline in cognitive function. The PSI correlates with age, baseline cognitive function and PaCO2 at the end of the operation, thus the monitoring of anaesthesia depth can be used for prediction of postoperative cognitive dysfunction.

### 0257

#### Adults sedation during MRI: dexmedetomidine versus propofol

K. Bielka, I. Kuchyn
Postgraduate Institute of Bogomolets National Medical University, Anesthesiology and Intensive Care, Kyiv, Ukraine
**Correspondence:** I. Kuchyn
*Intensive Care Medicine Experimental* 2017, **5(Suppl 2):**0257

**INTRODUCTION.** Sedation is often required during MRI for adult uncommunicative patients or those with different psychiatric disorders (phobia, anxiety etc.). Although it can be challenging to obtain the deep sedation level required to prevent the patient's movement while maintaining respiratory and hemodynamic stability. Limited access to the patient may pose a safety risk during MRI. Dexmedetomidine can be assosiated with better sedation quality and lower risk of breathing complications during MRI (1).
**OBJECTIVES.** To compare efficacy and safety of dexmedetomidine sedation versus propofol during MRI in adults.
**METHODS.** Prospective randomised study was conducted in the private clinic "Boris" at department of anesthesiology and intensive care of Bogomolets medical university (Kyiv, Ukraine) during 2015–2016. Uncommunicative conscious patients with acute ischemic stroke were included in the study and randomly allocated to 2 groups - dexmedetomidine (D) and propofol (P). The sedation goal was the same in the both group (RASS 0 to −2). Patients in group D receive dexmedetomidine infusion in dose 0,2-1.4 mcg/kg/h, in group P - propofol 1–4 mg/kg/h. Data are presented as median and 25–75 quartiles. Nonparametric criteria were used for data analysis (Fisher test, Mann–Whitney test).
**RESULTS.** 84 patients (42 in each group) with median age 64 [56–75] years were included in the study. There were no statistical differences between groups by demographics, severity of stroke, comorbidity. The goal level of sedation was achieved during 81 [75–90]% of total sedation time in group D, and in 65 [50–75]% of time in group P ($p < 0.05$). The incidence of complications varied between groups: arterial hypotension occurred in 6/42 (14%) patients in group D and in 2/42 (5%) patients in group P ($p > 0.05$), bradycardia in 5/42 (12%) and 3/42 (7%) ($p > 0.05$), desaturation in 2/42 (5%) and 10/42 (24%) (p = 0.26, OR 6 95%CI 1.2 to 61), bradypnoe in 0/42 and 2/42 (5%) ($p > 0.05$).
**CONCLUSIONS.** In this prospective randomized study dexmedetomidine comparing to propofol was associated with higher sedation quality and lower incidence of complication (desaturation) during acute ischemic stroke patients sedation for MRI. Dexmedetomidine could be an effective and safe alternative to propofol for procedure sedation during MRI.
**REFERENCE(S)**
1) Siddappa R, Riggins J, Kariyanna S. et al. High-dose dexmedetomidine sedation for pediatric MRI.Paediatr Anaesth. 2011 Feb;21(2):153–8.
**GRANT ACKNOWLEDGMENT**
None

### 0258

#### Analgesia Nociception Index-based analgesia protocol reduces pain during nursing in critically ill patients: a before/after study

T. Bernard, T. Gaillard, S. Gergaud, S. Lasocki
Angers University Hospital, Angers, France
**Correspondence:** T. Bernard
*Intensive Care Medicine Experimental* 2017, **5(Suppl 2):**0258

**INTRODUCTION.** Nursing care is a frequent procedure in the critically ill, repeated several times a day, but it is often painful. The Analgesia Nociception Index (ANI) evaluates analgesia/nociception balance, by studying the heart rate variability. It has been validated in anesthesia (1), but not in the critical care.
**OBJECTIVES.** The objective of this study was to assess whether an ANI based analgesia protocol may reduce the incidence of pain during nursing care.
**METHODS.** We performed a prospective, monocentric (surgical ICU), before/after study, assessing the incidence of pain during nursing care without and then with an ANI-based analgesia protocol. Pain during nursing was assessed using a as a Numerical Scale ($NS \geq 4$) in patient able to communicate (communicating patient (CP)) or using the Behavior Pain Scale ($BPS \geq 5$) if not (non-communicating patient (NCP)). During the first phase (5 months), we measured the resting ANI, before nursing. We used Receiver-operating characteristic (ROC) curves to defined the best resting ANI thresholds able to predict a painful nursing. During the second phase (4 months), nurses gave an analgesia bolus according to the resting ANI before the nursing. We compared the incidence of painful nursing during the two periods, in both CP and NCP.
**RESULTS.** We evaluated 137 nursing in 30 patients (age 57 ± 15 YO, 12(40%) women, IGSII 47 ± 15) and 146 in 32 patients (age 52 ± 18 YO, 11(34%) women, IGSII 46 ± 17) during the two successive phases. An ANI ≤71 predicts the occurrence of pain during the nursing procedures in CP (AUC = 0.85, Sensitivity 71%, Specificity 100%), and an ANI ≤ 63 in NCP (AUC = 0.63, Sensitivity 63%, Specificity 66[SI1] %). Administration of a bolus before nursing was more frequent during the second phase: 51(35%) vs 25(18%), p = 0.002. The incidence of resting pain was not different between the 2 phases (p = 0.118), but the incidence of pain during the nursing was lower with the use of ANI (27% with vs 43%, p = 0.004) (Fig. 102). Regarding the nursing procedures without bolus, 47 (42%) were painful without ANI, vs 15 (16%) with ANI (p < 0,001).
**CONCLUSIONS.** Pain during nursing care is common in critically ill patients. The use of an ANI-based analgesia protocol allows a significant reduction of this incidence, by almost 40%.
**REFERENCE(S)**
(1) Boselli E, Daniela-Ionescu M, Begou G, Bouvet L, Dabouz R, Magnin C, et al. Prospective observational study of the non-invasive assessment of immediate postoperative pain using the analgesia/nociception index (ANI). Br J Anaesth. 2013 Sep 1;111(3):453–9

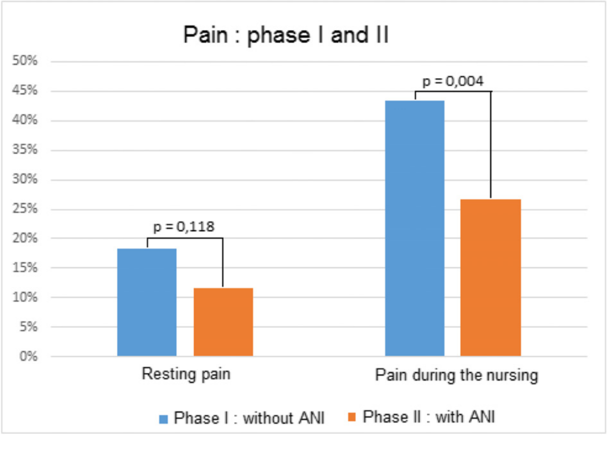

**Fig. 102 (abstract 0258).** See text for description

### 0259

#### Sleep patterns in nonsedated critically ill patients with severe sepsis and COPD

Y. Boyko[1], P. Jennum[2], H. Oerding[3], J.T. Lauridsen[4], M. Nikolic[5], P. Toft[1]
[1]Odense University Hospital, University of Southern Denmark, Anesthesia and Intensive Care Medicine, Odense C, Denmark; [2]Rigshospitalet, University of Copenhagen, Danish Center for Sleep Medicine, Department of Clinical Neurophysiology, Copenhagen, Denmark; [3]Vejle Hospital, University of Southern Denmark, Anesthesia and Intensive Care Medicine, Vejle, Denmark; [4]University of Southern Denmark, Department of Business and Economics, Odense M, Denmark; [5]Rigshospitalet, Danish Center for Sleep Medicine, Department of Clinical Neurophysiology, Copenhagen, Denmark
**Correspondence:** Y. Boyko
*Intensive Care Medicine Experimental* 2017, **5(Suppl 2):**0259

**INTRODUCTION.** Abnormal sleep in critically ill patients may be associated with delirium, prolonged stay in intensive care unit and increased mortality. Polysomnography, the standard criterion method of sleep assessment, is complicated in ICU. Standard sleep scoring classification (American Academy of Sleep Medicine, AASM) is not suitable in critically ill patients due to abnormal electrophysiological patterns. Modified classification for critically ill patients has been suggested by Watson et al. This classification has not been tested in the homogeneous groups of critically ill patients and has not yet been validated.

**OBJECTIVES.**

1. To describe sleep patterns in 2 homogeneous groups of unsedated critically ill patients on mechanical ventilation: a severe sepsis group, and a Chronic Obstructive Pulmonary Disease (COPD) group;
2. To determine the association of sleep patterns with the severity of critical illness scores, Systemic Inflammatory Response Syndrome criteria and delirium; 3.to compare PSG findings to subjective sleep evaluation performed by nurses in the ICU.

**METHODS.** PSG was performed in 2 homogeneous groups of nonsedated conscious critically ill patients on mechanical ventilation: a severe sepsis group (n = 16) and a group with COPD (n = 17). AASM classification was used for sleep scoring in presence of wake-sleep characteristics. Watson's classification was used if the characteristics of normal sleep were absent. Subjective sleep assessment by nurses was done with 15 min intervals.

**RESULTS.** We found significantly different distribution of sleep stages in the severe sepsis and the COPD groups. COPD, higher APACHE II and SOFA scores, and delirium were significantly associated with the higher risk of atypical sleep. The correlation between the nurse sleep assessment and PSG was only observed in the presence of normal sleep characteristics.

**CONCLUSIONS.** COPD diagnosis, critical illness scores and presence of delirium were associated with risk of atypical sleep. There was no correlation between subjective sleep assessment by nurses and objective PSG sleep recording in case of atypical sleep.

**REFERENCE(S)**

Watson PL, Pandharipande P, Gehlbach BK, Thompson JL, Shintani AK, Dittus BS, Bernard GR, Malow BA, Ely EW: Atypical sleep in ventilated patients: empirical electroencephalography findings and the path toward revised ICU sleep scoring criteria. Crit Care Med 2013; 41:1958–67

**GRANT ACKNOWLEDGMENT**

Thanks for the financial support to University of Southern Denmark, the Region of Southern Denmark, Department of Anesthesia and Intensive Care, Odense University Hospital, Department of Anesthesia and Intensive Care, Vejle Hospital, and the following foundations: The Danish Society of Anesthesiology and Intensive Care Medicine, Aage and Viola Holm-Madsens Research Foundation, A.P. Moellers Foundation, The exchange pool Rigshospitalet/Odense University Hospital.

### 0260

#### Patient sleep in the intensive care unit remains elusive: an observational study using portable EEG monitoring

J. L. Darbyshire[1], J. D. Young[2], Kadoorie Centre for Critical Care Research & Education
[1]University of Oxford, Nuffield Department of Clinical Neurosciences, Headington, Oxford, United Kingdom; [2]University of Oxford, Nuffield Department of Clinical Neurosciences, Oxford, United Kingdom
**Correspondence:** J. L. Darbyshire
*Intensive Care Medicine Experimental* 2017, **5(Suppl 2):**0260

**INTRODUCTION.** Sleep for patients in Intensive Care Units (ICU) is known to be poor and is difficult to measure. There does not seem to be a simple, robust alternative to full EEG monitoring which is expensive and inappropriate for most patients in the ICU.

**OBJECTIVES.** To assess quality and quantity of sleep in patients admitted to the intensive care unit using a small portable EEG monitor designed for home use.

**METHODS.** An observational study was performed in a general adult intensive care unit in a large teaching hospital in the UK. Patients aged ≥16 years were recruited to wear a portable EEG monitor (Sleep Profiler, Advanced Brain Monitoring Inc, Carlsbad, CA, USA) for up to 48 hours. Sleep stage was determined using the validated auto-staging algorithm supplied with the EEG monitor. Total sleep time, percentage spent in sleep stages, and duration of unbroken sleep periods were measured. Times are reported as hh:mm:ss.

**RESULTS.** (Recruitment is ongoing, data will be updated prior to ESICM) 44 patients recruited (26 male), between August 2014 - April 2017; mean age 60.4 (SD16.4), median APACHE II 24 (range 10–31), median length of stay in ICU 4.5 days (range 1–36 days). Total recording time 610:54:00 (per patient median 14:42:30). During their recording time, each patient slept for a median duration of 02:05:45 (range 00:00:30–12:38:30). Of this, 00:07:00 (5.6%) was spent in REM; 00:12:45 (10.2%) in slow wave sleep, and 01:26:30 (69.2%) in NREM1 and 2. 17% of sleep was unable to be characterised and 142mins (16%) per patient recording was classified 'invalid'. Patients experienced a median of 3.5 sleep periods per hour (maximum 13.6), each period being a median of 00:01:00 in duration (maximum single sleep period duration 05:26:00).

**CONCLUSIONS.** The total amount of sleep experienced by patients admitted to ICU is considerably lower than the eight hours recommended by the WHO[1]. Sleep was also highly fragmented with up to 13.6 awakenings every hour. Patients also had reduced REM sleep and increased non-REM sleep relative to normal sleep patterns[1].

**REFERENCE(S)**

1 World Health Organisation, Night noise guidelines for Europe, 2009.

**GRANT ACKNOWLEDGMENT**

National Institute for Health Research, Research for Patient Benefit (ref: PB-PG-0613-31034).

### 0261

#### Performance of new AnacondaS in clinical setting

R. Knafelj, M. Noc
University Medical Centre Ljubljana, Ljubljana, Slovenia
**Correspondence:** R. Knafelj
*Intensive Care Medicine Experimental* 2017, **5(Suppl 2):**0261

Sedation strategies using volatiles via classical Anaconda (Anaconda-C) is gaining popularity over intravenous sedation with propofol and midazolam. It's been demonstrated that sevoflurane exerts organoprotective properties and shortens time to extubation. In our ICU volatila sedation is mostly used mode of sedation and sevoflurane only is used.

**OBJECTIVES.** Currently used Anaconda-C adds 100 mL of additional death space in breathing circuit which can be challenging during ultraprotective ventialtion under ECMO. In such circumstances, Anaconda C can be inserted into inspiratory limb in which case conserving effect of the device is abolished. Newly developed AnacondaS being half the size and volume (50 mL) should address these issues, while ensuring adequate sedation level. There is no published data on volatile anesthetic consumption with Anaconda S.

**METHODS.** Different infusion rates of sevoflurane using AnacondaS (Sedana, Sweden) were used in 20 consecutive patients were ventilated with controled mechanical ventilation with constant minute volumes (Elisa 800 EIT, Heinen + Löwenstein, Germany) aiming to achieve sevoflurane concentration of 0.5 and 1.0 volume% respectively. Data was obtained, analyzed, nomograms constructed using statistical program Numbers (Apple, USA) and compared to nomogram for currently marketed Anaconda-C.

**RESULTS.** To achieve 0.5 vol% of sevoflurane, almost twice the infusion rate of sevoflurane is needed at MV up to 10 L/min when using AnacondaS compared to Anaconda-C. In order to achieve 1 vol% of sevoflurane, difference in infusion rate is less pronounced. Infusion rate nomogram curves converge as the MV increases, but never intersecting.

**CONCLUSIONS.** In order to achieve 0.5 vol% sevoflurane concentration higher infusion rates of sevofluran are needed compared to Anaconda-C. At 1 vol% and MV ≥ 9 L sevoflurane consumption is comparablewith Anaconda C and Anaconda S.

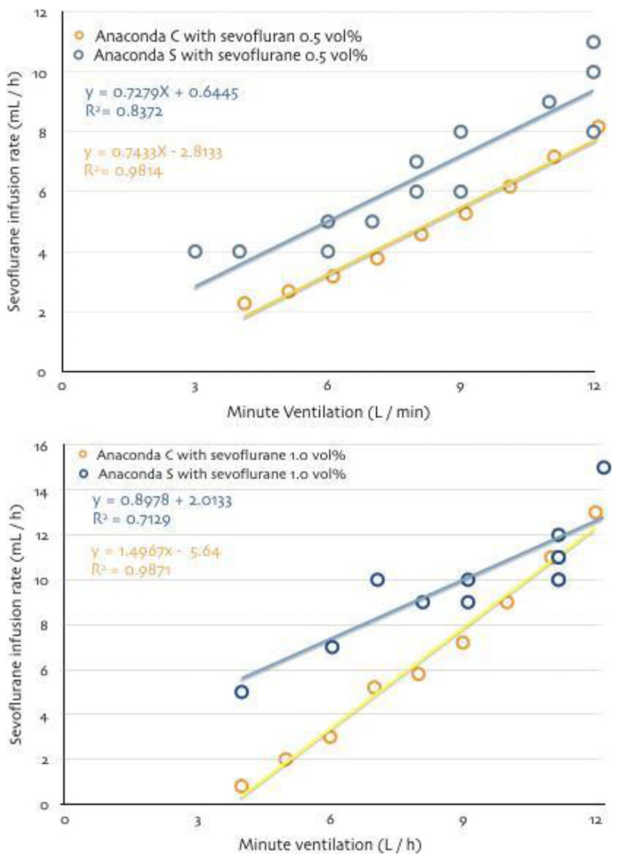

**Fig. 103 (abstract 0261).** Performance comparison

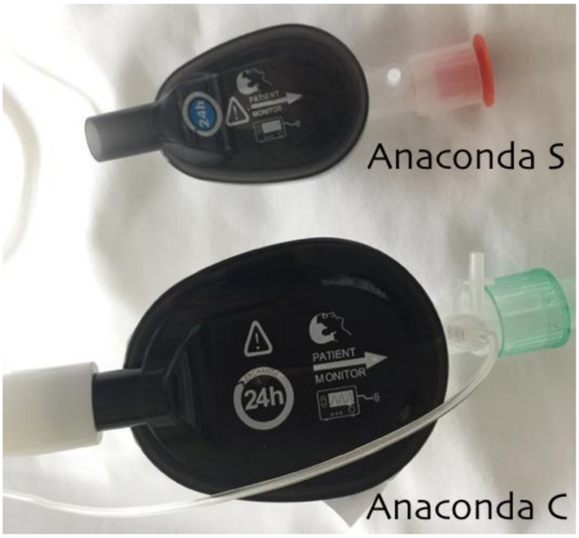

**Fig. 104 (abstract 0261).** Anaconda S (upper) and Anaconda C (lower)

### 0262

#### Disease severity impacts negative effects on the sleep status of non-sedated critically ill patients

S. Park[1], S. Kim[1], Y.-J. Cho[2]
[1]Sheikh Khalifa Specialty Hospital, Department of Intensive Care Medicine, Rak, United Arab Emirates; [2]Seoul National University Bundang Hospital, Seongnam, Korea, Republic of
**Correspondence:** S. Park
*Intensive Care Medicine Experimental* 2017, **5(Suppl 2):**0262

**OBJECTIVES.** Many critically ill patients treated in the intensive care unit (ICU) experience sleep disruption. This study was undertaken to identify the sleep status of non-sedated critically ill patients in Korea medical ICU.

**METHODS.** This is a prospective study. Polysomnography recording was performed over 24 hour to assess the quantity and quality of sleep.

**RESULTS.** Total 20 patients were enrolled. Median total sleep time was 03:43 (hh:mm, IQR: 00:49–06:10). The majority of sleep was stage 1 (median 03:02 [00:47–04:34]) with scant stage 2 (median 00:00 [00:00–00:46]), REM (median 00:00 [00:00–00:15]) and absent stage 3. The number of waking episodes in 1 hour was a median of 14.0 (7.7 - 29.1). The APACHE II score showed a significantly negative correlation with total sleep time (r = −0.49, P = 0.028). Patients who stayed more than 5 days in the ICU showed similar total sleep times. However, they showed significant reduction in night sleep time compared to patients who stayed less than 5 days (00:42 ± 0:46 vs 2:04 ± 1:25, P = 0.012).

**CONCLUSIONS.** The quantity and quality of sleep in critically ill patients were poor. More severe disease influenced negative effects on sleep. Long duration of ICU stay disrupted circadian rhythm in critically ill patients.

### 0263

#### EARLY-PRE-DELIRIC model as a predictor of delirium in a mixed ICU

H. Torrado, G. Moreno- González, J.C. López- Delgado, E. Farrero, S. Contreras-Medina, R. Ávila, E. Casanova, R. Soley
Hospital Universitario de Bellvitge, Intensive Care Department, L'Hospitalet de Llobregat, Spain
**Correspondence:** G. Moreno- González
*Intensive Care Medicine Experimental* 2017, **5(Suppl 2):**0263

**INTRODUCTION.** Delirium is highly prevalent in critically ill patients and is related to poor outcomes. Identifying at admission those patients at higher risk for developing delirium would allow apply early preventive interventions.

**OBJECTIVE.** To assess the EARLY-PRE-DELIRIC model as a predictor of delirium in a mixed medical- surgical ICU in a tertiary hospital in Barcelona Spain.

**METHODS.** A prospective observational cohort study of all patients consecutively admitted to a mixed ICU, in a tertiary Hospital in Barcelona Spain, between April and June 2016. Demographic data, prognostic scores, and the E-PRE-DELIRIC score were collected at ICU admission.

The presence of delirium was determined using the CAM-ICU scale or by the clinical criterion of the physician in charge.

We performed a descriptive analysis of the demographic data and prognostic scores. With a multivariate analysis we determined the factors associated with the presence of delirium.

The sensitivity, specificity, and positive and negative likelihood ratios were assessed by ROC curves. The statistical analysis was performed with SPSS 18.0.

**RESULTS.** During the study period, 110 patients were admitted. Fifteen patients were excluded because they were unable to be assessed for delirium at any time for persistent coma or death during their ICU stay. Of the 95 patients evaluated 66.3% were men; mean age 63.3 ± 13.2, APACHE II 14.3 ± 5.1, SOFA at admission 5.8 ± 2.6, SAPS II 27 ± 9.4, SAPS III 47.6 ± 12. The overall incidence of delirium was 23,15%. The E-PREDELIRIC stratify the risk for developing delirium during ICU stay in 4 subgroups:

a) Very low risk (0 - 10%),
b) Low risk (10-20%),
c) Moderate risk (20-35%) and
d) High risk (>35%).

In our study we had 5,1% in the group 1, 25% in the group 2, 41,2% in the group 3 and 42,1% in the group 4. In our study the power of the E-PRE-DELIRIC was moderate with a sensitivity of 72% and a specificity of 69,5% with a cut-off of 20%, and was correlated with the published in the literature.

**CONCLUSIONS.** The E-PRE-DELIRIC score is simple to obtain and was a predictor of delirium in our ICU. The identification of patients at greater risk for developing delirium at admission would allow the implementation of intensive early preventive strategies mainly in moderate and high-risk patients.

### 0264

#### Comparative analysis of different methods of pain management for critical cancer patients in the surgical intensive care unit

B. W.-Y. Huang[1,2], C.-K. Fang[2], Y.-L. Lai[2,3,4]
[1]Mackey Memorial Hospital, Critical Care Medicine, Taipei, Taiwan, Province of China; [2]Mackey Memorial Hospital, Hospice and Palliative Care Center, Taipei, Taiwan, Province of China; [3]MacKay Memorial Hospital, Department of Radiation Oncology, Taipei, Taiwan, Province of China; [4]MacKay Medical College, Taipei, Taiwan, Province of China
**Correspondence:** B. W.-Y. Huang
*Intensive Care Medicine Experimental* 2017, **5(Suppl 2):**0264

**INTRODUCTION.** With technological advancements, more critical cancer patients are undergoing surgery and need surgical intensive care unit (SICU) care[1]. Post -operation optimal pain management can not only release cancer and surgical pain but also provide a more stable hemodynamic status which shorten length of hospital stay as well as *reduced medical cost*[2].

**OBJECTIVES.** We aimed to determine the pain management method that provides better acute pain control in critical SICU cancer patients.

**METHODS.** This retrospective cohort study included a chart review of patients treated in a 26-bed surgical intensive care unit from April 2011 through September 2012. Cancer patients who were unconscious, uncooperative, post-brain surgery, or had an American Society of Anesthesiologists classification < III, were excluded. The primary aim was to compare visual analogue scale (0–100) scores between three different methods of pain management. pethidine/NSAIDs, intravenous patient-controlled analgesia (PCA), and patient-controlled epidural analgesia (PCEA), under different conditions (rest, movement, and coughing). The secondary endpoints were patient satisfaction.

**RESULTS.** We chart reviewed 1872 patients and 1593were excluded.VAS results presented as mean ± SD. Data analysis using ANOVA with Scheffe post hoc test. The average age was 67 years, and 64% were male. At rest, the PCEA group exhibited significantly lower pain scores (11.92 ± 10.23) compared with the other two groups. During movement, the PCEA group also has significantly lower pain scores (32.96 ± 12.65) than PCA and pethidine/NSAIDs groups. While coughing, the PCEA and pethidine/NSAIDs showed no difference; both exhibited lower pain scores than the PCA group (59.72 ± 14.73) The PCEA group showed highest patient satisfaction (4.27 ± 0.51).

**CONCLUSIONS.** PCEA appears to be a better choice for pain management for critical SICU cancer patients.

**REFERENCE(S)**

1.Ward CW. Procedure-specific postoperative pain management. *Medsurg Nurs*.2014;23:107–110.
2.Momeni M, et al. Patient-controlled analgesia in the management of postoperative pain. *Drugs*. 2006;66:2321–2337.

**GRANT ACKNOWLEDGMENT**
The authors certify that they have NO affiliations with or involvement in any organization or entity with any financial interest, or non-financial interest in the subject matter or materials discussed in this abstract.

**Table 78 (Abstract 0264)**. VAS of 3 conditions with 3 pain management methods

| | Pethidine/NSAIDs (n =135) | PCA (n = 71) | PCEA (n = 73) | p-value |
|---|---|---|---|---|
| VAS—Rest | 23.41±14.26[ξ] | 22.11±8.44[ξ] | 11.92±10.23 | <0.001 |
| VAS—Movement | 40.60±18.96[ξ] | 42.96±12.58[ξ] | 32.96±12.65 | <0.001 |
| VAS—Coughing | 47.24±23.02 | 59.72±14.73[ξ] | 50.27±14.34 | <0.001 |
| Patient satisfaction | 4.01±0.24[ξ] | 4.03±0.29[ξ] | 4.21±0.52 | <0.001 |

[ξ]Significant difference (p< 0.05) compared to PCEA

### 0265

#### Isoflurane and sevoflurane consumption with the AnaConDa S™ anaesthetic conserving device versus the conventional AnaConDa™ using an artificial lung model

L. Procopiuc, I. Georgevici, D. Drees, V. Vinnikov, T. P. Weber, M. Bellgardt
St. Josefs University Hospital, Ruhr-University Bochum, Anaesthesiology and Intensive Care, Bochum, Germany
**Correspondence:** L. Procopiuc
*Intensive Care Medicine Experimental* 2017, **5(Suppl 2):**0265

**INTRODUCTION.** Volatile anaesthetics (VA) are increasingly finding a place in intensive care sedation, and their benefits on patient outcome are beginning to gather (1). The AnaConDa™ (ACD) incorporates a reflector between the breathing circuit and the patient which captures exhaled VA and resupplies it during the next inspiration, obviating the need for a circle system to administer VA. Concerns have been voiced that this device increases dead space ventilation and arterial $PaCO_2$ values, limiting its use in low tidal volume ventilation strategies like ARDS (2) or in children. The AnaConDa S™ (ACD S) features a smaller reflector which decreases dead space from 100 to 50 ml.

**OBJECTIVES.** We aimed to test the reflection ability of the ACD S by comparing its VA consumption to that of the original ACD system in an artificial lung model.

**METHODS.** We assembled an artificial lung model of 2x2L volume with an additional reservoir of 3.9 L simulating the functional residual capacity. This was connected to the ACD S or to the ACD system, a Puritan Bennett™ 840 (Covidien, Mansfield, MA, USA) ventilator and a Vamos monitor (Dräger, Lübeck, Germany) to measure end tidal $CO_2$ and expired VA concentration. Measurements were each made continuously for 30 min after achieving steady state at end tidal concentrations of 0.5, 1.0, 1.5, 2.0 and 2.5 Vol% for Isoflurane (ISO) and Sevoflurane (SEVO) respectively. Every measurement was repeated 3 times. The breathing parameters were held constant (MV 5L/min, RR 10/min, TV 0.5 L). The groups were compared using the Mann Whitney U test (SPSS® v. 24.0).

**RESULTS.** ACD S utilises an equivalent amount of ISO as ACD to maintain end tidal concentrations in the range between 0,5% (0,73 ml/h vs 0,62 ml/h) and 1,5% (3,7 ml/h vs 2,6 ml/h). Beginning at 1.5%, the consumption of ISO rises steadily for the ACD S, surpassing that of the ACD (3,7 ml/h vs 2,6 ml/h). In the case of SEVO, the consumption of the ACD S remains equivalent with ACD only in the end tidal concentration domain between 0.5% (0,73 ml/h vs 1,1 ml/h) and 1% (1,63 ml/h vs 1,7 ml/h). Beginning at 1%, the efficiency of the ACD S falls rather unexpectedly until 1.5%, surpassing the ACD (4,3 ml/h vs 2,8 ml/h). At 2,5% the consumption is one and a half times that of the classic ACD (10,7 ml/h vs 6,9 ml/h). For end-tidal concentrations of 2.0 and 2.5 Vol% SEVO and 1.5, 2.0 and 2.5 Vol% ISO ACD S utilizes significantly more VA than ACD ($p < 0.01$).

**CONCLUSION.** The new ACD S has a similar efficiency to the original ACD in the concentration domain between end tidal VA 0.5-1.0 Vol%, corresponding to ICU sedation targets (MAC 0.5), with the additional benefit of reducing dead-space.

### 0266

#### Volume of AnaConDa® cut in half: evaluation of a new small volume anesthetic reflector in a test lung model

F. Meiser[1], P. Daume[1], H. Bomberg[1], M. Bellgardt[2], T. Volk[1], H. V. Groesdonk[1], A. Meiser[1]
[1]University Medical Centre, University of Saarland, Department of Anaesthesiology, Intensive Care Medicine and Pain Medicine, Homburg/Saar, Germany; [2]St. Josef Hospital, Katholisches Klinikum Bochum, University Hospital, Ruhr-University of Bochum, Department of Anaesthesiology and Intensive Care Medicine, Bochum, Germany
**Correspondence:** P. Daume
*Intensive Care Medicine Experimental* 2017, **5(Suppl 2):**0266

**INTRODUCTION.** Inhalation sedation with the anesthetic conserving device (ACD, AnaConDa®, Sedana Medical, Uppsala, Sweden) is increasingly used to sedate critically ill patients in the ICU. Because of the high volumetric dead space of 100 ml and the partial reflection of carbon dioxide ($CO_2$), concerns about $CO_2$ retention have been raised when using the AnaConDa®-100ml (ACD-100). Since recently, a smaller reflector, AnaConDa®-50ml (ACD-50), with volumetric dead space reduced to the half is available.

**OBJECTIVES.** We wanted to compare $CO_2$ elimination and isoflurane reflection efficiency of both reflectors on the bench.

**METHODS.** A test lung was ventilated with 500 mL, 10 breaths per minute while constantly insufflating $CO_2$. At the beginning of each experiment a heat moisture exchanger (HME, 35ml) was connected while adjusting $CO_2$ flow to reach an end-tidal $CO_2$ (et-$CO_2$) of 5.3 ± 0.1 kPa. Successively connecting ACD-100 and ACD-50, et-$CO_2$ was measured under different conditions: under ambient temperature pressure (ATP), body temperature pressure saturated conditions (BTPS), and BTPS with 0.4 or 1.2 Vol% isoflurane (ISO-0.4/ISO-1.2). In a second experiment ACD-100 and ACD-50 were again successively connected. For each reflector tidal volume was stepwise adjusted to maintain normocapnia.

To determine reflection efficiency of both ACDs isoflurane was administered into the test lung via a syringe pump. Isoflurane concentrations were measured in steady state at varying isoflurane infusion rates (0.5-20 ml/hour) under ATP and BTPS + $CO_2$. Reflection efficiency was defined as:

$$\text{Efficieny}[\%] = 100 \times (\text{Isoflurane}_{\text{reflected}} \div \text{Isoflurane}_{\text{exhaled}})$$

**RESULTS.** Under all conditions ACD-100 caused higher et-$CO_2$ than ACD-50. $CO_2$ reflection was highest under ATP, less under BTPS, smallest with ISO-0.4 and ISO-1.2. Beside the enlarged volumetric dead space compared to a normal HME there is an additional *reflective dead space* of ACD-100 and ACD-50 which could be quantified as 40ml or 25ml under BTPS with isoflurane, respectively. Regarding the reflection efficiency of both ACDs Isoflurane reflection was highest under ATP. Under BTPS + $CO_2$ reflection efficiency was above 80% up to 4.2 Vol% with ACD-100 and up to 0.4 Vol% when using the ACD-50.

**CONCLUSIONS.** While isoflurane reflection is still sufficient with ACD-50 at lower concentrations, $CO_2$ elimination is much less impaired compared to ACD-100. Thus, it should be possible to use ACD-50 with tidal volumes as low as 200ml for lung protective ventilation even of small patients.

**GRANT ACKNOWLEDGMENT**
AnaConDa-50mL were kindly provided by the manufacturer. AM and TV have received honoraria from Sedana Medical (Uppsala, Sweden).

### 0267

#### Impact on kidney delayed graft function of two different retrieval techniques in controlled circulatory death donors

M. L. Pérez Pérez, I. Fernandez Simón, A. Pérez Lucendo, A. P. Rodriguez Villamizar, A. Naharro Abellán, P. Matía Almudevar, J. Veganzones Ramos, S. Alcántara Carmona, M. Pérez Redondo
Hospital Universitario Puerta de Hierro, Intensive Care, Majadahonda, Spain
**Correspondence:** M. L. Pérez Pérez
*Intensive Care Medicine Experimental* 2017, **5(Suppl 2):**0267

**OBJECTIVES.** To describe the general characteristics of kidney donors after controlled circulatory death (CCD) and to evaluate the incidence of delayed graft function (DGF) after normothermic regional perfusion with extracorporeal membrane oxygenation (NRP) versus *in situ* preservation (ISP).

**METHODS.** Descriptive and retrospective study (January 2012-October 2016) performed at Hospital Universitario Puerta de Hierro Majadahonda (Madrid, Spain). Variables analyzed included donors age, sex, ICU length of stay, cause of death, body mass index (BMI) and previous history of hypertension (HT) or diabetes mellitus (DM). Age and sex of the recipients, development of DGF, preservation methods and warm and cold ischemia times (WIT, CIT) were also registered. We considerer WIT as the period between systolic arterial pressure below 60 mmHg to initiation of NRP or preservation solutions (ISP), and CIT to the period between preservation solution infusion and implant.

**RESULTS.** In the period studied, 30 patients were CCD donors, which accounted for 60 transplanted kidneys. Forty-seven of them were implanted in our center and are the subject of this study. Sixteen donors were men (46%). Mean age was 53.4 ± 13.4 years and mean ICU days of stay were 9.7 ± 8.4. The main causes of death were: anoxic encephalopathy [14 patients (47%)] and intracranial hemorrhage or thrombosis [9 patients (29.9%)]. ISP was the technique of choice in 38 patients (80.8%) vs. NRP in 9 of the cases (19%). Median WIT was 20 minutes (8–41) and CIT was 6 hours (4–11).

In the recipient group (47), there were 34 (72.3%) men and mean age was 54.5 ± 12.4 years. Twenty-two of them (40%) suffered a DGF.

There was no statistical association between DGF and age, sex, BMI, HT, DM or cause of death of the donors. There were 20 patients who developed DGF after ISP vs. 2 patients after NRP (90.9% vs. 9%; $p = 0.1$). Median time of CIT in the group of patients who developed DGF after ISP vs. NRP was 6.75 hours (4–12.5) vs. 5.5 hours (4–8) respectively; p 0.43.

**CONCLUSIONS.** CCD has contributed to increase the number of kidney donors in our centre in the recent years.
The incidence DGF after CCD is high, but NRP seems to reduce the risk of this complication.
Larger series are needed in order to further asses the roll of NRP as a preservation technique for CCD donors.

### 0268

#### Kidney transplantation results comparing donors after brain death vs donors after circulatory death

J. M. Manciño, S. Canelles, B. Catalan, S. Martínez Vega, H. Pérez Moltó, M. Izura, E. Argudo, P. Ricart
Hospital Universitari Germans Trias i Pujol, Intensive Care Medicine, Badalona, Spain
**Correspondence:** S. Canelles
*Intensive Care Medicine Experimental* 2017, **5(Suppl 2):**0268

**INTRODUCTION.** Kidney grafts' hypoperfusion due to longer warm ischemia time is thought for a donor after circulatory death (DCD), a suboptimal type of organ donor.[1]
**OBJECTIVES.** To compare the primary kidney transplantation results in brain-death donors (DBD) vs DCD.
**METHODS.** Observational prospective study in one year period of kidney grafts transplantation from DBD and controlled-DCD patients in a tertiary care hospital (551 total beds which 88 belongs to critical area). Analysis of data was performed with $X^2$ and T Student.
**RESULTS.** 14 controlled-DCD and 13 DBD donors were included in this period, in which a total number of 50 kidneys were obtained (a non valid donor in each group of patients). Among DCD, 4 kidneys were excluded from transplantation because of tissue biopsy score or vascular anomalies. 35 kidney transplantation were performed in our hospital; 4 kidneys were transferred to other hospitals and 7 (all from DBD) were included in the Hyperimmunized Spanish Program. Mean age was 61,46 years old in DCD and 60,53 years old in DBD. Urea and Creatinine values in the day of organ removal were 54,02 mg/dL and 0,70 mg/dL respectively for DCD group and 31,33 mg/dL and 0,77 mg/dL for the DBD group. Delayed Graft Function (DGF) was established in 8 recipients from the DCD group (40%), whereas in the DBD group were only 4 (26,6%). Mean cold ischemic time for DCD and DBD group were 4,3 hours and 20,65 hours respectively. Mortality among the recipient group was 1 patient for each type of donation.
**CONCLUSIONS.** Controlled-DCD's recipients tend to have a higher amount of DGF, although there is a lower cold ischemia time comparing to DBD donors. However, there is no significance in the results obtained. Comparing both DCD and DBD groups there is an homogeneity of patients' characteristics included in each group (age, renal function).
**REFERENCE**
1. Donación en Asistolia en España: situación actual y recomendaciones. Consenso Nacional de Donación, año 2012. *Organización Nacional de Transplantes*.

**Table 79 (Abstract 0268)**. Donors' characteristics

| | DBD (n=13) | DCD (n=14) | p |
|---|---|---|---|
| Mean age | 60,53 (44-71) | 61,46 (45-75) | ns |
| Male sex (%) | 7 (53,84%) | 10 (71,42%) | ns |
| Blood type | | | ns |
| *O* | 5 (38,40%) | 5 (35,70%) | |
| *A* | 6 (46,20%) | 9 (64,30%) | |
| *B* | 1 (7,70%) | 0 | |
| *AB* | 1 (7,70%) | 0 | |
| Mean Creatinine (mg/dL) | 0,77 | 0,70 | ns |
| Mean Urea (mg/dL) | 31,33 | 54,02 | ns |

**Table 80 (Abstract 0268)**. Recipients' characteristics

| | DBD recipients (n=15) | DCD recipients (n=20) | p |
|---|---|---|---|
| *Mean age* | 61,66 (43-70) | 60 (36-73) | ns |
| *Male sex (%)* | 10 (66,6%) | 16 (80%) | ns |
| *Cold ischemia time (mean hours)* | 20,65 | 5,4 | <0,001 |
| *Delayed graft function (%)* | 4 (26,6%) | 8 (40%) | ns |
| *Exitus (%)* | 1 (6,6%) | 1 (5,4%) | ns |

### 0269

#### Postoperative in the ICU of the renal transplantation of living donor success; guarantees?

N. Palomo-Lopez, A. Roldan-Reina, D. Cuenca-Apolo, M. Adriaensens, M. Porras-López, L. Martin-Villen, Z. Ruiz de Azua-López, J. J. Egea-Guerrero
Hospital Universitario Virgen del Rocío, Sevilla, Spain
**Correspondence:** N. Palomo-Lopez
*Intensive Care Medicine Experimental* 2017, **5(Suppl 2):**0269

**INTRODUCTION.** Living donation process has an important risk for the donor. For that reason, an optimal postoperative care must be achieved.
**OBJECTIVES.** To analyze the differences between living and cadaveric donors after renal transplantation, at the ICU admission.
**METHODS.** Observational retrospective study peformed from January 2013 to December 2016. We collected demographic data,comorbidity, type of donation, analytical values at ICU admission, results of radioisotope tests, complications (both in ICU and during follow-up during hospitalization) days of ICU stay, hospital and mortality. Descriptive statistical analysis was performed. The Xi square test was used for qualitative variables and the Student´s T or Mann Whitney U test for the quantitative variables depending on their distribution. We considered significance statistical results $p < 0.005$.
**RESULTS.** We studied a total of 387 kidney transplants. The general characteristics of the patients; the analytical values and the rest of the variables, are shown in Table 81. 13% of patients received an organ from a living donor, with a significant decrease in the development of complications during admission (it is defined as the need of dialysis an blood transfusion) compared to other donors. They also had a shorter hospital stay and there was no exitus in the live group.
**CONCLUSIONS.** That patients who receive an organ from living donor have a more optimal postoperative outcome than those from a cadaver donation. Our results suggest that transplanting a living donor provides guarantees of success, thanks also to the careful selection process and donor compatibility.
**REFERENCE(S)**
1. Nicholson ML, Metcalfe MS, White SA, et al. A comparison of the results of renal transplantation from non-heart-beating, conventional cadaveric, and living donors. Kidney Int. 2000; 58 : :2585–91
2. Summers DM, Watson CJ, Pettigrew GJ, et al. Kidney donation after circulatory death (DCD): state of the art. Kidney International. 2015; 88: 241–9.
3. Unek T, Egeli T, Özbilgin M, Çelik A, Atilla K, Ağalar C, Arslan NÇ, Karademir S, Bora S, Gülay H, Derici ZS, Astarcıoğlu I.Long-term results od Living Donors in Simultaneous Kidney and LIver transplantaion. Transplant Proc. 2017 Apr;49(3):403–406
**GRANT ACKNOWLEDGMENT**
We gratefully appreciate the invaluable of the ICU staff

**Table 81 (Abstract 0269)**. General characteristics of the 387 transplants acc

| DONOR | LIVING | CADAVER | P |
|---|---|---|---|
| n (%) | 50 (13) | 337 (87) | - |
| Age, media (DE) | 44,0 (13,3) | 52,2 (12,6) | 0,000 |
| Male Sex, n (%) | 31 (62,0) | 213 (63,2) | 0,869 |
| creatinine median ICU admission (IR) | 5,49 (4,23 - 6,66) | 5,98 (4,55 - 7,59) | 0,052 |
| Pathological MAG-3, n (%) | 5 (10,0) | 49 (14,5) | 0,387 |
| Hospital, complications n (%) | 22 (44,0) | 217 (64,4) | 0,060 |
| ICU complications, n (%) | 4 (8,0) | 87 (22,8) | 0,060 |
| Length of stay, Hospital, median (IR) | 13 (9,75 - 17,00) | 15 (12,00 - 20,00) | 0,010 |
| Exitus, n (%) | 0 (0,0) | 2 (0,5) | 0,585 |

### Stroke and intracranial haemorrhage

#### 0270

**Speckle-tracking evaluation of left ventricular longitudinal systolic function in patients with moderate to severe traumatic brain injury**

R. Cinotti[1], T. Le Tourneau[2], M. Le Courtois du Manoir[3], A. Roquilly[3], K. Asehnoune[3]
[1]Chu - Hôtel Dieu, Nantes, France; [2]Hôtel-Dieu, Explorations Fonctionnelles, Nantes, France; [3]Hôtel-Dieu, Nantes, France
**Correspondence:** R. Cinotti
*Intensive Care Medicine Experimental* 2017, **5(Suppl 2):**0270

**INTRODUCTION.** Stress cardiomyopathy has been extensively described after aneurysmal subarachnoid haemorrhage, but there is little evidence after traumatic brain injury (TBI). Speckle-tracking echocardiography (STE) analyses longitudinal left ventricular systolic function, and is more sensitive than left ventricular ejection fraction (LVEF) to detect early systolic dysfunctions.

**OBJECTIVES.** Incidence of infra-clinical stress cardiomyopathy after TBI by using STE.

**METHODS.** Mono-centric longitudinal study in one university hospital, in patients undergoing TBI with a Coma Glasgow Score ≤ 13 before ICU admission. STE was performed in the first 24 hours after admission, at day 3 and day 7 after admission. Patients were excluded in the setting of thoracic trauma, out-of-hospital rescuscitated cardiac arrest, history of major heart surgery or ischemic cardiomyopathy, clinical signs of brain death at admission, withdrawal of life-sustaining therapies in the first 24 hours. Hyper-sensitive Troponin was dosed in the first 24 hours after admission. The primary outcome was the incidence Global Longitudinal Strain (GLS) > −16% which defines significant longitudinal systolic function impairment.

**RESULTS.** We included 102 patients with TBI from March 2014 to March 2017, 78(75%) male and 26(25%) female. Mean age was 43(±20). Glasgow Coma Score at admission was 7(±3). 77(75%) patients received norepinephrine at day 1. 85 patients were kept in final STE analysis because of appropriate echogenicity. At day 1, LVEF was preserved 66(±21)% as well as GLS −20.3(±3.6)%. Troponin hs at admission was 61(±156) pg/mL, but there was no correlation with GLS. Only 4(4.7%) patients displayed a severe GLS impairment (> − 16%) at day 1. There was a significant improvement in GLS at day 3 (−22.4(±3)% (p < 0.05), which did not sustain at day 7 (−20.7(±4)%). There was no link between GLS and in-ICU mortality and day-90 neurological outcome evaluated with Glasgow Outcome Scale.

**CONCLUSIONS.** STE could detect up to 36% of stress cardiomyopathies in patients with aneurysmal subarachnoid haemorrhage (1). In patients undergoing TBI, stress cardiomyopathy evaluated assessed with STE is very infrequent. Although it has been witnessed through case reports, our data suggest that stress cardiomyopathy will not be a relevant clinical issue in TBI patients.

**REFERENCE(S)**

(1) Cinotti et al. Speckle tracking analysis allows sensitive detection of stress cardiomyopathy in severe aneurysmal subarachnoid haemorrhage. *Intensive Care Med* 2016

**GRANT ACKNOWLEDGMENT**
None

#### 0271

**Troponin as a marker of severity in cerebral hemorrhage**

R. Rodríguez García, I. Astola Hidalgo, L. Forcelledo Espina, L. Marqués Álvarez, J.A. Gonzalo Guerra, R. Yano Escudero, E. Garcia Prieto, L. López Amor, L. Viña Soria, D. Escudero Augusto, L. Martín Iglesias, G. Muñíz Albaiceta
Hospital Universitario Central de Asturias, Oviedo, Spain
**Correspondence:** R. Rodríguez García
*Intensive Care Medicine Experimental* 2017, **5(Suppl 2):**0271

**INTRODUCTION.** Despite a large body of evidence testifying to the development of myocardial injury in subarachnoid hemorrhage the true incidence in this population remains unknown. Troponin (cTn) release is accepted as the most specific marker of myocardial necrosis. There is evidence on the use of cTnT as a predictive factor in patients with ischemic stroke and subarachnoid hemorrhage. However, there are few reviews that have studied the relationship between elevated levels of cTnT and mortality of patients with intracerebral hemorrhage (ICH).

**OBJECTIVES.** The aim of this study was to define the incidence of cardiac injury in patients with ICH as determined by cTnT release. To evaluate the relationship between elevated cardiac troponin T (cTnT > 14 ng/L) and the outcome from ICH.

**METHODS.** We made a retrospective and observational study of patients admitted to the ICU during one year and who have identified an ICH. On admission, we recorded medical examinations, including CT and cardiac troponin T (cTnT) measurements. Cerebral hemorrhages secondary to TBI, vascular malformation, cerebral aneurysm, or ischemic hemorrhagic transformation were excluded. CT scans were reviewed to determine the hematoma size, location, presence of intraventricular or subarachnoid hemorrhage, hydrocephalus, and midline shift.

**RESULTS.** 44 patients were identified. The mean age was 63.98 ± 9.47 years and 54.5% were women. On admission the mean value of APACHE II and Glasgow Coma Score were 17.04 ± 6.3 and 10.2 ± 4.15, respectively. The most common site of hemorrhage were basal ganglia (54.6%) and the cortical region (22.7%). 31.8% had previous heart disease, 18 patients (40.9%) were received some type of antiplatelet or anticoagulant treatment. 17 patients (38.6%) had elevated cTnT values (>14 ng / L) with a mean of 37.52 ± 78.78 ng/L. Factors related to increase cTnT values were a previous history of cardiac disease (71.4%) and need of vasopressors after admission (72.7%). The average stay in ICU was 19.95 ± 22.19 days. Significantly higher in patients with elevated TnT (28 vs 15 days p = 0.018). The most frecuent cause of death was neurological complications (40%). Mortality was higher in the group of patients with enzymatic mobilization, 52.9% compared to 22.2%. Troponin T values > 14 ng/L was an independent predictor of ICU mortality (OR 3.94, 95% CI 1.06-14.67, p 0.041).

**CONCLUSIONS.** Elevated cardiac troponin T (cTnT) values occur frequently in ICH and are independently associated with higher in-hospital mortality.

#### 0272

**Coagulation disorders after aneurysmatic subarachnoid haemorrhage**

A. Vahtera[1], E. Junttila[1], L. Pyysalo[2], H. Huhtala[3], P. Helen[2], A. Kuitunen[1]
[1]Tampere University Hospital, Critical Care Medicine Research Group, Tampere, Finland; [2]Tampere University Hospital, Department of Neurosurgery, Tampere, Finland; [3]Tampere University, Faculty of Social Sciences, Tampere, Finland
**Correspondence:** A. Vahtera
*Intensive Care Medicine Experimental* 2017, **5(Suppl 2):**0272

**INTRODUCTION.** Aneurysmatic subarachnoid haemorrhage (aSAH) patients exhibit hypercoagulability state from six hours after the initial bleed[1] predisposing these patients to thromboembolic complications. Rotational thromboelastometry (ROTEM) is a point-of-care test providing a targeted and dynamic analysis of coagulation cascade[2] and it can identify hypercoagulability by increased level of maximum clot firmness (MCF)[3].

**OBJECTIVES.** To evaluate the on-going coagulation process after aSAH by ROTEM analyses and compare the results to ROTEM analyses of elective neurosurgery patients undergoing craniotomy.

**METHODS.** This prospective, observational study was conducted in the Tampere University Hospital, Finland between September 2015 and June 2016. The ROTEM analyses were done at 12, 24, 48 and 72 hours from the onset of aSAH symptoms. In the control group the ROTEM analyses were done prior the surgery (i.e. baseline) and at 24 and 48 hours. To screen an asymptomatic deep venous thrombosis (DVT), a bilateral compression ultrasound was performed to all patients on day 3–5. The primary endpoint was MCF of EXTEM test. It was calculated that 16 patients were needed to achieve 80% power to detect a clinically meaningful increase (from 65mm to 70 mm, SD 5 mm) in MCF with *p*-level of 0.05.

**RESULTS.** 17 aSAH (age 49, IQR 40–60 years, male 35.5%) and 16 control patients (age 62, IQR 56–67 years, male 43.8%) were enrolled. At 72 hours, the MCF of EXTEM was significantly higher in aSAH patients compared to the baseline value of the control group: 68 (IQR 66–71) vs. 65 (IQR 60–67), p = 0.024 (Fig. 1). The same comparison done in MCF of FIBTEM analysis resulted in similar results: 23 (IQR 19–25) vs. 15 (IQR 13–18), p = 0.001. No difference was detected when compared EXTEM MCF results at 24 h 68 (IQR 63–70) vs. 65 (IQR 61–67), p = 0.37 or at 48 h 66 (IQR 66–69) vs 67 (IQR 64–70), p = 0.79. Two DVTs was detected in the study group and both patients developed also a pulmonary embolism. In two patients with DVT + PE after aSAH MCF values were not higher compared with other aSAH patients at 72 hours (62 vs. 69, p = 0.076). No thromboembolic complications were observed in the control group.

**CONCLUSIONS.** It seems that after 72 hours from the aneurysmatic subarachnoid haemorrhage, the patients develop a hypercoagulability state, that may be detected by ROTEM EXTEM-analysis.

**REFERENCE(S)**

1. Ji Y, Meng QH, Wang ZG. Changes in the coagulation and fibrinolytic system of patients with subarachnoid hemorrhage. *Neurol Med Chir (Tokyo)*. 2014;54(6):457–464.
2. Theusinger OM, Nurnberg J, Asmis LM, Seifert B, Spahn DR. Rotation thromboelastometry (ROTEM) stability and
3. Akay OM, Ustuner Z, Canturk Z, Mutlu FS, Gulbas Z. Laboratory investigation of hypercoagulability in cancer patients using rotation thrombelastography. *Med Oncol*. 2009;26(3):358–364

**GRANT ACKNOWLEDGMENT**

The Finnish Society of Intensive care and CSL Behring

### 0273

#### Prevalence and outcome of polyuria in patients with subarachnoid haemorrhage

L. Vandenbulcke, E. Hoste, D. Benoit, K. Colpaert

Ghent University Hospital, Intensive Care, Ghent, Belgium

**Correspondence:** L. Vandenbulcke

*Intensive Care Medicine Experimental* 2017, **5(Suppl 2):**0273

**BACKGROUND - OBJECTIVES.** Polyuria and disturbances in electrolytes are common complications after subarachnoid haemorrhage (SAH) and can be potentially harmful. Previous studies have demonstrated that patients with polyuria have an increased risk of symptomatic cerebral vasospasm or delayed cerebral ischemia.[1] The exact cause of this polyuria is not always very obvious.[2] In this study the prevalence and possible causes of polyuria and the association of polyuria with length of stay (LOS) and long term outcome in critically ill patients with spontaneous SAH were examined.

**METHODS.** In this retrospective study data about demographics, diuresis, LOS and outcome of patients with spontaneous SAH were collected during a period of 7 years (January 2010 - December 2016). Exclusion criteria were: traumatic SAH, secondary transport ≥24 hours after first admission and elective coiling. Polyuria was defined as a diuresis of >80 ml/kg/day. Statistical analysis was performed using Mann–Whitney U and Chi-Square tests with SPSS.

**RESULTS.** 317 patients were identified with SAH of which 95 experienced polyuria (30.0%). The median duration of polyuria was 2.0 (1.0-5.0) days. There was no significant difference in demographics or Hunt and Hess scale at admission between both groups. In 184 patients (58.0%), SAH was caused by a ruptured aneurysm; of these, 67 (36.4%) had polyuria. Diabetes insipidus was diagnosed in 90 patients (28.4%) and cerebral salt wasting in 134 (42,3%). Hyponatremia (sodium < 135mEq/L) was more frequent in polyuria versus no-polyuria patients (82.1% vs 65.9%, p < 0.001). High urinary sodium concentration (>150mEq/L) occurred in 89 (93.7%) patients in the polyuria group vs 140 (63.1%) patients in the no-polyuria group (p < 0.001). Patients with polyuria experienced significantly more vasospasm compared to those without (39.4% vs 12.9%, p < 0.001). Polyuria patients in comparison to no-polyuria patients had a longer LOS at both ICU (8.7 (6.0-16.0) vs 3.0 (2.0-9.3), p < 0.001) and the hospital (26 (14.5-49.5) vs 14.5 (9.0-30.0), p < 0.001). ICU mortality in polyuria patients was similar compared to those without polyuria (7.4% vs 14.4%, p = 0.080); hospital mortality was significantly lower in patients with polyuria (7.4% vs 17.6%, p = 0.018). No significant difference could be demonstrated in Glasgow Outcome Scale after circa six months in the polyuria versus no-polyuria group (GOS 1–3: 36.6% vs 32.8%, GOS 4–5: 63.4% vs 67.2%, p = 0.546).

**CONCLUSION.** Polyuria is a frequent complication in patients with SAH during ICU stay. Cerebral salt wasting and diabetes insipidus were common causes of polyuria, but other causes have to be taken into account. Patients with polyuria were more likely to have vasospasms and had a longer ICU and hospital LOS. There was no significant difference in long term outcome, except hospital mortality was lower in polyuria patients.

### 0274

#### Variability of cerebral glucose concentration and its relationship with three levels of Cerebral Blood Flow (a microdialysis and hemedex study)

D. C. Papadopoulos[1], V. Zisopoulou[1], S. Karagiannis[1], A. S. Filippidis[2], P. Papamichalis[1], T. Bekiari[3], A. Komnos[1], G. Vretzakis[4], K. N. Paterakis[5], K. N. Fountas[5]

[1]General Hospital of Larisa, Intensive Care Unit, Larisa, Greece; [2]Harvard Medical School. Beth Israel Deaconess Medical Center, Neurosurgery, Boston, United States; [3]Prefectural Administration of Thessaly, Department of Public Health Services, Larisa, Greece; [4]University of Thessaly. School of Medicine, Anaesthesiology, Larisa, Greece; [5]University of Thessaly. School of Medicine, Neurosurgery, Larisa, Greece

**Correspondence:** D. C. Papadopoulos

*Intensive Care Medicine Experimental* 2017, **5(Suppl 2):**0274

**INTRODUCTION.** Cerebral microdialysis (MD) monitoring enables assessment of regional metabolic physiology and provides biomarkers for clinical correlation in critical conditions, such as subarachnoid hemorrhage (SAH).

**OBJECTIVES.** The aim of our current study was to investigate the correlation between regional cerebral blood flow (CBF) and (MD) parameters (Glucose (Glu), Lactate (Lac), Glycerol (Gly), Pyruvate (Pyr) concentrations and Lactate/Pyruvate metabolic ratio- L/P) in patients with SAH.

**METHODS.** 21 patients with SAH were enrolled in this prospective study. A minimally invasive, multimodal, neuromonitoring system was placed in the first 8 hours after admission to the ICU. A frontal burr hole was placed at Kocher's point under aseptic conditions and a bolt kit was used for insertion of:

(a) an intracranial pressure (ICP) measurement catheter (Codman microsensor kit, Codman, Johnson & Johnson, Raynham. MA USA),
(b) a brain tissue-oxygen monitoring catheter ($P_{br}O_2$ measuring catheter, Licox, Integra NeuroSciences. Plainsboro. NJ. USA) and
(c) an MD catheter (CMA 70 Brain Microdialysis Catheter, 10 mm membrane length, 20 kDa cut off, CMA. Stockholm, Sweden).

A second burr hole for inserting a CBF catheter (QFlow500, Hemedex, Bowman, Boston, MA, USA) was drilled adjacently to the first one. CBF based on thermal diffusion methodology, the thermal coefficient K, and MD biochemical markers were recorded. All patients were treated using a cerebral perfusion pressure (CPP) guided protocol. The duration of the brain monitoring was 10 days.

**RESULTS.** Regarding the correlations of the MD parameters and CBF of the sample, Glu correlated positively with CBF, cerebral Temperature (T), Lac, Pyr, and negatively to the L/P, Gly and K coefficient of thermal conductivity of the brain (K). Pyr was positively correlated, while Gly and Lac correlated negatively with CBF. K correlated positively with the CBF and T. When the CBF was bisected in the median flow rate (25ml/100gr/min), Glu was correlated inversely with T, the L/P ratio and the CBF, when the CBF exceeded the limit value. However, when, CBF was below the limit value, K correlated negatively with CBF and T and positively with all other variables. Glu was positively correlated with the L/P and negatively with the T. At even lower CBF (corresponding to ischemia), very strong negative correlations between the L/P and CBF emerge. Glu continues to positively correlate with the L/P ratio as was previously described for CBF less than 25ml/100gr/min. However at this state there is a positive Glu and CBF correlation. Gly shows a negative correlation to the CBF.

**CONCLUSIONS.** Regarding the correlations of MD parameters, with CBF, K, and T, and also between them, the correlations vary depending on the level of CBF. In summary, as the CBF drops, the statistically significant relationships, are strengthened

**REFERENCE(S)**

Bauernfeind A.L. et al.2014. Rostami E. et al.2014. Bouzat P. et al.2015

### 0275

#### The role of sepsis in the outcome of subarachnoid hemorrhage patients: an exploratory analysis

B. Gonçalves, R. Turon, D. de Barros, N. Melo, P. Lacerda, M. A. dos Prazeres, F. Miranda, P. Kurtz, F. Bozza, C. Righy
Paulo Niemeyer State Brain Institute, Rio de Janeiro, Brazil
**Correspondence:** B. Gonçalves
*Intensive Care Medicine Experimental* 2017, **5(Suppl 2):**0275

**INTRODUCTION.** Subarachnoid hemorrhage (SAH) following aneurysmal rupture is a catastrophic cerebrovascular event with high mortality and morbidity. Intense systemic inflammatory response (SIRS) is one of the main characteristics of the disease occurring in up to 83% of patients. In this study, we evaluated the prevalence of SIRS and sepsis and its association with outcomes in this population.

**OBJECTIVES.** Our goal was to describe the incidence of SIRS and sepsis and its relation to the outcomes in patients with SAH, as part of a large ongoing prospective study.

**METHODS.** From April 2016 to March 2017, all consecutive patients admitted with aneurysmal SAH to the Neuro-ICU of a reference center in Rio de Janeiro were enrolled in our cohort. Demographic, clinical characteristics and laboratory variables related to sepsis were collected during the first 14 days of hospital stay. The primary endpoint was mortality and dichotomized functional outcome (poor outcome defined as Modified Rankin Scale 4–6) at hospital discharge. Numeric variables were expressed as median and interquartile interval, and categorical variables were summarized as frequency and percentage. We used the Chi-square test to analyze categorical variables.

**RESULTS.** A total of 55 patients were enrolled in the study. Median age was 51 years (22–79), and 41 patients were female (75%). 10 patients (18%) had poor-grade SAH (World Federation of Neurologic Surgeons score of 4 and 5).

From our cohort, 73% patients developed SIRS criteria during hospital stay. Using the current definitions (Sepsis-3, defined as an acute change in total SOFA score ≥2 points consequent to the infection), 12 patients (22%) had the diagnosis of sepsis during the first 14 days of hospital stay.

The most common site of infection was pulmonary (6 patients - 50%), followed by urinary tract (2 patients), upper airway (2), pressure ulcer (1) and central nervous system (1). Median time to infection (from bleeding) was ten days (4–24 days). When stratified by severity, on the septic group, four patients (33%) had poor-grade SAH (WFNS 4 or 5), against six patients in the non-septic group (14%).

On the studied outcomes, among septic patients, 6 had vasospasm (50%), 2 had DCI (17%), two died (17%), and 9 had poor outcome (75%).

In the non-septic group, 16 (37%) had vasospasm (p = 0.42), 10 (23%) had DCI (p = 0.62), 4 (9%) died (p = 0.47) and 11 (25%) patients had poor outcome (p = 0.001).

**CONCLUSIONS.** Our data is in tandem with the hypothesis that SIRS is highly prevalent in SAH patients. However, about a quarter of them develop sepsis, and, in this cohort of patients with SAH, sepsis was associated with poor outcome, but not with either mortality or DCI.

### 0276

#### Role of serum biomarkers in the diagnosis of sepsis in aneurysmal subarachnoid hemorrhage patients

B. Gonçalves, R. Turon, D. de Barros, N. Melo, P. Lacerda, M. A. dos Prazeres, F. Miranda, P. Kurtz, F. Bozza, C. Righy
Paulo Niemeyer State Brain Institute, Rio de Janeiro, Brazil
**Correspondence:** B. Gonçalves
*Intensive Care Medicine Experimental* 2017, **5(Suppl 2):**0276

**INTRODUCTION.** Subarachnoid hemorrhage (SAH) following aneurysmal rupture is a catastrophic cerebrovascular event with high mortality and morbidity. The intense systemic inflammatory response is one of the main characteristics of the disease occurring in up to 83% of patients. Differential diagnostic between infection, sepsis and systemic inflammatory response (SIRS) associated with acute brain injury is a challenge in this particular group.

**OBJECTIVES.** Our goal was to describe the role of biomarkers for the diagnosis of infection and sepsis in this particular cohort, as part of a large ongoing prospective study.

**METHODS.** From April 2016 to March 2017, all consecutive patients admitted with aneurysmal SAH to the Neuro- ICU of a reference center in Rio de Janeiro were enrolled in our cohort. Demographic and clinical characteristics were collected during the first 14 days of hospital stay. Procalcitonin and C-reactive protein levels were measured in the first and third days of hospital stay. The primary endpoint was sepsis (using the current definitions - Sepsis-3 - defined as an acute change in total SOFA score ≥2 points consequent to the infection). Numeric variables were expressed as median and interquartile interval and were analyzed using Mann–Whitney U-test.

**RESULTS.** A total of 55 patients were enrolled in the study. Median age was 51 years (22–79), and 41 were female (75%). 10 patients (18%) had poor-grade SAH (World Federation of Neurologic Surgeons score of 4 and 5).

Twelve patients (22%) had the diagnosis of sepsis during the first 14 days of hospital stay.

Median Procalcitonin levels on the first day on septic patients was 0.085 ng/ml (interquartile range 0.28, 0.05 - 0.33), and 0.05 ng/ml (IQR 0.04, 0.04 - 0.08) on non-septic (p = 0.14). On the third day, median procalcitonin concentration was 0.11 ng/ml (IQR 0.21, 0.05 - 0.26) in septic patients, and 0.05 ng/ml (IQR 0.06, 0.03 - 0.09), in non-septic patients (p = 0.2).

On the first day, median C-reactive protein level was 93 mg/L (IQR 165.6, 45.5 - 211) on septic patients, and 29.3 mg/L (IQR 55.3, 9.8 - 65.1) on non-septic patients (p = 0.0008). On the third day, median C-reactive protein concentration was 137.5 mg/L (IQR 99.7, 80.3 - 180) on septic patients and 37.8 mg/L (IQR 58.7, 13.7 - 72.4) on non-septic patients (p = 0.0001).

**CONCLUSIONS.** In this small cohort, procalcitonin did not differ between the septic and non-septic groups, but CRP was higher in patients with sepsis, which could aid the differential diagnosis and prompt early treatment of patients at high risk of sepsis.

### 0277

#### Systemic glucose variability and functional outcome after subarachnoid hemorrhage

B. Gonçalves, L. E. Novis, N. M. Costa, D. F. De Barros, M. A. Prazeres, F. Miranda, C. Righy, P. Kurtz
Paulo Niemeyer State Brain Institute, Rio de Janeiro, Brazil
**Correspondence:** P. Kurtz
*Intensive Care Medicine Experimental* 2017, **5(Suppl 2):**0277

**INTRODUCTION.** Hyperglycemia has been associated with poor outcome in patients with subarachnoid hemorrhage, and increased systemic glucose variability was associated with cerebral metabolic distress and increased hospital mortality in these patients in a retrospective cohort. The impact of glycemic control in neurocritical patients remains controversial.

**OBJECTIVE.** The objective of this study was to assess whether glucose variability is associated with functional outcome after subarachnoid hemorrhage.

**METHODS.** From July 2015 to December 2016, all consecutive patients admitted with aneurysmal SAH to the Neuro-ICU of a reference center in Rio de Janeiro were enrolled in our cohort. Demographic, clinical characteristics and serum glycemic values were collected on the first 7 days after admission. The primary endpoint was mortality and dichotomized functional outcome (poor outcome defined as Modified Rankin Scale 4–6) at hospital discharge.

**RESULTS.** A total of 72 patients and 2865 measurements of serum glucose were analyzed, along with arterial pressure, temperature and Glagow coma scale. Glucose variability was expressed as the standard deviation (SD) of all serum glucose measurements. Fifty six patients (78%) were female, 18 (25%) were poor-grade SAH with WFNS 3 to 5 and 40 (56%) had modified Fisher III or IV. Seven patients (10%) rebled, 21 (29%) developed cerebral infarcts and only 1 patient died during hospital admission. At hospital discharge, 34 (47%) patients had poor outcome and systemic glucose SD was higher in this group as compared to those with good outcome (median [IQR] 30.6 [23.6 - 37.6] vs 26.4 [20.4 - 32.4] P = 0.09). Multivariate analysis will be undertaken to adjust to other clinically relevant variables.

**CONCLUSIONS.** In this retrospective analysis of a prospective cohort of SAH patients, increased systemic glucose variability during ICU admission was associated with poor outcome at hospital discharge.

### 0278

#### Influence of infectious complications in the outcome of critically ill patients with acute stroke

A. Estella
Hospital SAS Jerez, Unidad de Gestión Clínica de Medicina Intensiva Hospital del SAS de Jerez, Jerez, Spain
*Intensive Care Medicine Experimental* 2017, **5(Suppl 2):**0278

**INTRODUCTION.** Infections occurs frequently after stroke, pneumonia is a dreaded complication in stroke patients.

**OBJECTIVES.** The aim of the present study is to describe the incidence of pneumonia in patients admitted in ICU with a stroke. To analyze the prognosis of stroke patients who develop pneumonia as complication during the acute phase of stroke.

**METHODS.** Prospective observational study performed in a community hospital. Inclusion criteria were patients admitted in ICU with an acute stroke. Time of study recruitment was 12 months. Variables analyzed were: age, gender, type of stroke: ischemic or hemorrhagic, NIHSS at admission, vascular risk factors, mechanical ventilation requirements, vasoactive drugs use, ICU lenght of stay and mortality. Two groups of patients were classified according development of pneumonia. Statistic analysis was performed by SPSS v18 program, chi square test was applied to compare qualitative variables and t Student for the quantitative variables.

**RESULTS.** During the study period, 62 stroke patients were admitted to the ICU. Pneumonia occurred in 25.8%.

**CONCLUSIONS.** In our serie of patients development of pneumonia was related with the presence of vascular risk factors.

Pneumonia in stroke patients increases ICU lenght of stay, support therapies requirements and mortality.

**Table 82 (Abstract 0278)**. Shows clinical characteristics and outcome according development of pneumonia

| | Stroke without pneumonia (n = 46) | Stroke with pneumonia (n = 16) |
|---|---|---|
| Age (years) mean±sd | 60,4±14 | 61±11 |
| Gender %(Male/Female) | 60,9/39,1 | 56,3/43,7 |
| NIHSS at admission (mean±sd) | 15,2±7 | 17,1±8 |
| ICU lenght of stay (days) mean±sd* | 3,35±2,5 | 9,8±5,1 |
| Vascular risk factors Hypertension/ Diabetes/Dyslipidemia/Atrial fibrillation | 67,4%/54,3%/ 19,6%/28,3%/15,2% | 87,5%/81,3%/ 37,5%/25%/37,5% |
| Mechanical ventilation requirements (%)* | 45,7 | 93,8 |
| Vasoactive drugs (%)* | 34,8 | 75 |
| Stroke: Ischemic/hemorrhagic (%) | 54,3/45,7 | 43,8/56,2 |
| Mortality (%)* | 26,1% | 81,3% |

* $P < 0.05$

### 0279

#### DDimer as perioperative predictor of hemorrhage after brain tumor surgery

E. Val Jordan[1], M. D. Vicente Gordo[1], J. Casado Pellejero[2], N. Fernandez Monsteirin[3], A. C. Nebra Puertas[1], B. Virgos Señor[1]
[1]Miguel Servet University Hospital, Trauma ICU, Zaragoza, Spain; [2]Miguel Servet University Hospital, Neurosurgery, Zaragoza, Spain; [3]Miguel Servet University Hospital, Hematology, Zaragoza, Spain
**Correspondence:** E. Val Jordan
*Intensive Care Medicine Experimental* 2017, **5(Suppl 2):**0279

**INTRODUCTION.** Unexplained cerebral hemorrhage could appear after brain tumor surgery because of some hemostatic abnormalities not routinely studied. Perioperative D-Dimer levels could predict the appearance of that cerebral hemorrhage.

**OBJECTIVES.** The aim of this study was to analyse the association between perioperative levels of DDimer and cerebral hemorrhage after brain tumor neurosurgery.

**METHODS.** We prospectively performed, during 18 months at Miguel Servet University Hospital in Spain, 3 DDimer testings (A presurgery, B postsurgery, C 24 hours after surgery) in patients with brain tumor. Ethical approval from a Committee and Informed Consent were required. We accepted 0-200ng/ml as normal values. A Head CT scan done the day after surgery to evaluate surgical complications. Cerebral hemorrhage was defined by intracranial hypertension due to blood volumen or mass effect.

**RESULTS.** We included 109 patients, 69 male and 40 female. A total of 67 patients in A (61,4%), 82 in B (75,22%) and 91 in C (83,4%) had DDimer elevated. A total of 67 patients in A, 82 in B and 91 in C had DDimer elevated. The average was abnormally elevated in 3 testings (A 930,04ng/ml, B 728,51ng/ml, C 1105,55ng/ml, 95% confidence interval 599.536 to 1260.464, 590.504 to 865.496, 915.825 to 1294.175 respectively). 39 of 109 patients included in the study (35,78%) suffered from cerebral hemorrhage. The Wilcoxon-Mann–Whitney test stadistic showed significance between cerebral hemorrhage and DDimer levels in A and C testings (p 0,039 and 0,042 respectively).
**CONCLUSIONS.** The average of perioperative DDimer was elevated in patients with brain tumor. DDimer abnormalities in, presurgery and the day after neurosurgery, testings were associated with cerebral hemorrhage. So, we can conclude that high levels of DDimer could be considered as risk marker for cerebral hemorrhage after brain tumor surgery.

**REFERENCE(S)**

1. Barber M, Langhorne P, Rumley A, Lowe GD, Stott DJ. Hemostatic function and progressing ischemic stroke: D-dimer predicts early clinical progression. Stroke 2004 (35):1421–5
2. Hoffmann M.E, Ma O.J, Gaddis G. Utility of an initial D-dimer assay in screening for traumatic or spontaneous intracranial hemorrhage. Acad Emerg Med.2001;(8):859–65
3. Juvela S, Siironen J. D-Dimer as an Independent Predictor for Poor OutcomeAfter Aneurysmal Subarachnoid Hemorrhage. Stroke.2006;37:1451–6
4. Delgado P, Álvarez-Sabín J, Abilleira S, Santamarina E, Purroy F, Arenillas J.F, Molina C.A, Fernández-Cadenas I, Rosell A, Montaner J. Plasma d-dimer predicts poor outcome after acute intracerebral hemorrhage. Neurology.2006; 67 (1): 94–8
5. Komarov A et al. D-dimer and platelet aggregability are related to thrombotic events in patients with peripheral arterial occlusive disease. Eur Heart J. 2002;23(16):1309–16

**GRANT ACKNOWLEDGMENT**

This scientific work was supported by a grant from Miguel Servet University Hospital

### 0280

#### Lung mechanics in critically ill patients in acute period of intracranial hemorrhages

A. Solodov, V. Krylov, S. Petrikov, E. Mekhia Mekhia, S. Kosenkov, A. Yushkova, S. Kalashnikova
Sklifosovsky Research Institute, Moscow, Russian Federation
**Correspondence:** A. Solodov
*Intensive Care Medicine Experimental* 2017, **5(Suppl 2):**0280

**INTRODUCTION.** Primary brain injury leads to a cerebral and systemic inflammatory reactions, a massive release of catecholamines, which can effects of the respiratory system state.
**OBJECTIVES.** To assess the lung mechanics during mechanical ventilation (MV) in patients in acute period of intracranial hemorrhages (ICH).
**METHODS.** 58 patients with ICH requiring ventilation for more than 24 hours enrolled in the study (age - 48,9 ± 10,5 years, male/female - 37/21). 18 patients had severe traumatic brain injury, 40 - cerebral aneurism rupture. We used lung protective ventilation in all pts. In first day after the beginning MV we analyzed every hour the heart rate (n = 1068), mean blood pressure (BPmean) (n = 1062), saturation ($SpO_2$) (n = 985), end-tidal CO2 ($EtCO_2$) (n = 985), peak airway pressures (Ppeak) (n = 1076) and mean airway pressures (Pmean) (n = 1048), dynamic respiratory compliance (n = 1010), expiratory resistance (n = 914), minute ventilation (MV) (n = 1022) and respiratory *quotient* (RQ) (n = 914).
**RESULTS.** Parameters of hemodynamics and gas exchange were stable: heart rate - 90 ± 21 beats per minute, BPM - 107 ± 19 mmHg, $SpO_2$ - 99 ± 1%. We identified a tendency to hyperventilation in pts in acute period of ICH: $EtCO_2$ - 32 ± 12 mm Hg, MV - 10.6 ± 3.5 l/min, RQ - 0.78 ± 0.15. The airway pressures: Ppeak - 23.7 ± 9.6 mmHg, Pmean - 12.7 ± 4.5 mmHg. The dynamic compliance of the respiratory system was 62 ± 26.5 ml/ $cmH_20$, expiratory resistance - 9.2 ± 2.8 $cmH_20$/l/s.
**CONCLUSIONS.** Already in the first day after start of MV in patients in acute period of ICH lung mechanics there are changes, due to a decrease in the respiratory compliance and an increase expiratory resistance.

### 0281

#### Heart rate and central venous oxygen content are key determinants of brain tissue oxygenation in critically ill patients

M. D. Wood[1], D. M. Maslove[2], J. Muscedere[2], J. G. Boyd[2], Cerebral Oxygenation and Neurological Outcomes Following Critical Illness-CONFOCAL Study group, and the Canadian Critical Care Trials Group
[1]Queen's University School of Medicine, Centre for Neuroscience Studies, Kingston, Canada; [2]Queen's University School of Medicine, Medicine and Critical Care, Kingston, Canada
**Correspondence:** J. G. Boyd
*Intensive Care Medicine Experimental* 2017, **5(Suppl 2):**0281

**INTRODUCTION.** Low levels of near-infrared spectroscopy derived brain tissue oxygenation ($BtO_2$) are associated with poor neurological outcomes, such as delirium, in critically ill patients.[1]
**OBJECTIVE.** To characterize the physiological determinants of BtO2 in critically ill patients.
**METHODS.** This cohort was part of the CONFOCAL study (NCT02344043 clinicaltrials.gov). Participants (n = 103) with respiratory failure and/or shock were enrolled within 24h of admission. $BtO_2$ was measured with the FORESIGHT cerebral oximeter (Casmed, Caster Medical). Simultaneous multiple linear regression analyzed which hemodynamic/physiological parameters predict $BtO_2$. A multivariable model was then selected using stepwise backwards selection. Spearman's correlation coefficients were calculated for each individual patients' hemodynamics and BtO2. Hierarchical cluster was applied to these correlations in order to identify unique physiological phenotypes.
**RESULTS.** The full regression model included: mean arterial pressure (MAP), heart rate (HR), peripheral oxygen saturation ($SpO_2$), temperature, pH, central venous $pO_2$ (CV-$pO_2$), venous $pCO_2$ ($vPCO_2$), and hemoglobin (Hb). This model accounted for a significant proportion of the variance in $BtO_2$, $R^2$ = 0.405, p = 0.012. Backwards model selection indicated that the best fitting model included: HR, $SpO_2$, CV-$pO_2$, $vPCO_2$, and Hb. This final model also accounted for a significant proportion of the variance in $BtO_2$, $R^2$ = 0.379, p = 0.002. The percentage of $BtO_2$ increased significantly as HR and CV-$pO_2$ increased. However, $SpO_2$, $vPCO_2$, and Hb were not significant predictors of $BtO_2$. Correlational analysis indicated that the per patient $BtO_2$ correlations with hemodynamic parameters were highly variable. For example, some clusters of patients had significant positive correlations between $BtO_2$ and MAP, whereas others had inverse correlations.
**CONCLUSIONS.** HR and central venous $pO_2$ contribute significantly to $BtO_2$. The significant inter-individual variability may limit the prospects of a universal algorithm to optimize $BtO_2$, and suggest the need for precision approaches based on individual patient physiology.

**GRANT ACKNOWLEDGMENT**

Physician Services Incorporated (to JGB).

### 0282

#### EEG in patients undergoing ECMO

S. Rechichi[1], F. Franchi[2], S. Pozzebon[1], S. Scolletta[2], A. Brasseur[1], N. Gaspard[3], J.-L. Vincent[1], J. Creteur[1], F. S. Taccone[1]
[1]Hopital Erasme, Intensive Care, Brussels, Belgium; [2]UOC Rianimazione e Medicina Critica, Azienda Ospedaliera Universitaria Senese, Department of Biotechnologies, Siena, Italy; [3]Hopital Erasme, Neurology, Brussels, Belgium
**Correspondence:** S. Rechichi
*Intensive Care Medicine Experimental* 2017, **5(Suppl 2):**0282

**INTRODUCTION.** Neurologic injury remains a significant complication and one of the most frequent causes of death in patients undergoing extracorporeal membrane oxygenation (ECMO). As ECMO patients usually require sedation, their neurological examination is not reliable, but neuromonitoring can help. However, the use of electroencephalogram (EEG) in adult ECMO patients has not yet been analyzed.

**OBJECTIVES.** To assess the occurrence of EEG abnormalities and their relationship to outcome in patients treated by ECMO.

**METHODS.** We reviewed data all patients undergoing venous-venous (VV) or venous-arterial (VA) ECMO with a contemporary (either intermittently or continuous) EEG monitoring (April 2009 - June 2016). EEG findings of interest were: a) "mild-moderate encephalopathy" (i.e. diffuse slowing with reactivity/variability) vs. "severe encephalopathy" (i.e. diffuse slowing without reactivity/variability); b) "burst suppression" or flat; c) epileptiform activity (i.e. ictal EEG pattern, sporadic epileptiform discharges or periodic discharges); d) EEG reactivity. EEG findings were analyzed according to the primary diagnosis (presence of cardiac arrest (CA) or not), the use of VA vs. VV ECMO and ICU mortality.

**RESULTS.** We studied 94 sedated patients (50 [16–83] years; 30 (32%) male gender) out of 434 treated with ECMO). ICU mortality was 60%. There were more unreactive EEGs among VA-ECMO (n = 72) patients than in VV-ECMO (n = 22; 57% vs. 23%; p = 0.006). As expected patients after cardiac arrest (n = 64) more frequently had a burst suppression or flat EEG than the others (n = 30; 27% vs. 0%; p = 0.001). Also the non-survivors (n = 57) more frequently had a severe encephalopathy (56% vs. 27%; p = 0.006), burst suppression or flat EEG (28% vs. 3%; p = 0.002) and unreactive EEG (60% vs. 32%; p = 0.01) than the survivors. The presence of these three EEG findings was significantly associated with ICU mortality (OR 14.05 [1.77-111.25]; p = 0.01), even after adjustment for confounders.

**CONCLUSIONS.** EEG monitoring can be very helpful in sedated patients during ECMO.

### 0283

#### Comparison of the influence of clonidine and dexmedetomidine on the basic hemodynamic parameters during removal of the posterior fossa tumors

M. Rumiantceva, R. Nazarov, A. Kondratyev
Russian Polenov Neurosurgical Institute, Saint Petersburg, Russian Federation
**Correspondence:** M. Rumiantceva
*Intensive Care Medicine Experimental* 2017, **5(Suppl 2):**0283

**INTRODUCTION.** From 1989 in RNSI of Prof. A.L.Polenov we have been successfully using the method of anesthesia that includs a combined effect on opioid (fentanyl) and adrenergic (clonidine) antinociceptive system. The use of more selective ALPHA2-adrenoagonists (dexmedetomidine) can be considered as an option for application in the structure of anesthesia during removal of subtentorial brain tumors.

The aim of the study was to compare clonidine and dexmedetomidine´s effect on the major hemodynamic parameters during the removal of posterior fossa tumors.

**MATERIALS AND METHODS.** The study included 89 patients (average age of 52,3 ± 13), underwent the elective surgery in RNSI for subtentorial brain tumors. In all patients induction of anesthesia included: muscle relaxants (nondepolarizing muscle relaxant), hypnotic (propofol 2mg/kg), an opioid analgetic (fentanyl 4,8 ± 0,6μg/kg) + ALPHA2-adrenoagonists (clonidine or dexmedetomidine). Maintenance of anesthesia: hypnotics (propofol and 5,2 ± 1,6mg/kg/h), opioid analgetic (fentanyl and 1,2μg /kg/h) + ALPHA2-adrenoagonists (clonidine or dexmedetomidine). All patients were divided into three groups: in group I (21 patients) induction of anesthesia: Clonidine 1,5 ± 0,4μg/kg; maintenance of anesthesia: Clonidine 0,4 ± 0,2μg/kg/h. In group II (25 patients) induction of anesthesia: Dexmedetomidine 0,7 ± 0,1μg/kg; maintenance of anesthesia: Dexmedetomidine 0,18 ± 0,07μg/kg/h. In group III (43 patients) induction of anesthesia: Dexmedetomidine and 1,4 ± 0,3μg/kg; maintenance of anesthesia: Dexmedetomidine 0,4 ± 0,2μg/kg/h.

**RESULTS.** In group I, the initial value of the mean BP was (mean ± standard deviation) 108 ± 14 mm Hg., HR 77 ± 14 beats per min. After induction of anesthesia mean BP 76 ± 19mm Hg, HR 53 ± 8 per min. 20 minutes after induction of anesthesia mean BP 71 ± 14mm Hg, HR 55 ± 7per min. By the time of full closure of the wound mean BP 83 ± 11mm Hg, HR 59 ± 13 per min.

In group II the initial value of the mean BP 110 ± 18mm Hg, HR 76 ± 15 per min. After induction of anesthesia mean BP 110 ± 21mm Hg, HR 42 ± 7 per min. 20 min. after induction of anesthesia mean BP 83 ± 17mmHg, HR 52 ± 6 per min. By the time of full closure of the wound mean BP 84 ± 10mm Hg, HR 51 ± 7 per min.

In group III, the initial value of mean BP 101 ± 13mm Hg, HR 72 ± 11 per min. After induction of anesthesia mean BP 110 ± 17mmHg, HR 43 ± 8 per min. 20 min. after induction of anesthesia mean BP 93 ± 16mmHg, HR 51 ± 10 per min. By the time of full closure of the wound mean BP 90 ± 13mm Hg, HR 55 ± 9 per min.

**CONCLUSION.** Thus, in the I and II groups after induction of anesthesia central simpatolytic effect dominated. Such changes of the hemodynamic parameters were preserved during the entire operation.

And in group III after induction of anesthesia dominated the peripheral vasoconstrictor effect.Such changes of hemodynamic parameters were observed for a short period of time and later during operations also dominated central simpatolytic effect.

## Sedation, analgesia and delirium

### 0284

#### Pharmacological interventions for delirium in Intensive Care Unit (AID-ICU): an international inception cohort study

M.O. Collet[1,2], H. Wøien[3], A. Schandl[4], J. Hästbacka[5], M. van den Boogaard[6], M. Hänggi[7], P. Nydahl[8], G. Citerio[9], R. Sonneville[10], F.A. Bozza[11], K. Colpaert[12], J. Caballero[13], L. Rose[14], M. Barbateskovic[2,15], T. Lange[16], A. Jensen[2,16], M.B. Krog[17], I. Egerod[1,2], H.L. Nibro[2,17], J. Wetterslev[2,18], A. Perner[1,2]
[1]University Hospital Copenhagen, Intensive Care Unit 4131, Copenhagen, Denmark; [2]Centre for Research in Intensive Care, Copenhagen, Denmark; [3]Oslo University Hospital, Division of Emergencies and Intensive Care, Oslo, Norway; [4]Karolinska University Hospital, Department of Perioperative Medicine and Intensive care, Stockholm, Sweden; [5]Helsinki University Hospital, Intensive Care Units, Helsinki, Finland; [6]Radboud University Nijmegen Medical Center, Department of Intensive Care Medicine, Nijmegen, Netherlands; [7]University Hospital, Department of Intensive Care Medicine, Bern, Switzerland; [8]University Hospital of Schleswig-Holstein, Nursing Research, Kiel, Germany; [9]H San Gerardo Monza University Milano Bicocca, Neuroanaesthesia and Neurointensive care, Milano, Italy; [10]Hôpital Bichat, Intensive Care Medicine, Paris, France; [11]Ministry of Health, National Institute of Infectious Disease, Rio de Janeiro, Brazil; [12]University Hospital Gent, Department of Intensive Care, Gent, Belgium; [13]Vall d'Hebron University Hospital and Vall d'Hebron Institut de Recerca, Barcelona, Spain; [14]Lawrence S. Bloomberg Faculty of Nursing, Toronto, Canada; [15]Copenhagen University Hospital, Copenhagen Trial Unit, Copenhagen, Denmark; [16]Section of Biostatistics, University of Copenhagen, Department of Public Health, Copenhagen, Denmark; [17]Aarhus University Hospital, Department of Intensive Care, Aahus, Denmark; [18]Copenhagen University Hospital, Copenhagen Trial Unit, Copenhagen OE, Denmark
**Correspondence:** M.O. Collet
*Intensive Care Medicine Experimental* 2017, **5(Suppl 2):**0284

**INTRODUCTION.** Delirium occurs frequently in Intensive Care Unit (ICU) patients and is associated with poor outcome. In spite of this, there is limited evidence to support any specific pharmacological interventions, and recent Clinical Practice Guidelines make no strong recommendations.[1] However, widespread use of haloperidol for ICU-delirium is likely.

**OBJECTIVES.** To describe current use of haloperidol and other pharmacological interventions for delirium in critically ill patients admitted to the ICU and to explore associations to outcomes.

**METHODS.** We conducted the international AID-ICU cohort study with a 14-day inception period (March - August 2016) in Denmark, Norway, Sweden, Finland, the Netherlands, Switzerland, Germany, Belgium, Spain, Italy, Canada, Brazil and France. We included all patients aged 18 years or older who were acutely admitted to a participating ICU not meeting an exclusion criterion (e.g. mental illness, dementia). Follow-up was until 90 days after ICU admission. We estimated that at least 1000 patients was needed to obtain 95% CI's of 11% - 15% around an estimated rate of the primary outcome (the number of patients with delirium intervened with haloperidol) of 13%. Patient consent was obtained according requirements in each country.

**RESULTS.** (analysis in progress)

A total of 99 ICUs screened 1922 patients and excluded 648; 8 patients withdrew consent and 6 patients had incomplete data leaving 1260 patients for the analyses. We will report the number of ICU patients with delirium intervened with haloperidol (the primary outcome) including dosages and types of administration, and the number of ICU patients with delirium intervened with other agents for delirium (other antipsychotics than haloperidol, dexmedetomidine and benzodiazepines); the number of patients with delirium; mortality at 90 days; days alive in ICU without coma or delirium; days alive without mechanical ventilation, and days alive out of hospital within the 90-day period. In the explorative analyses we will assess any associations between baseline risk factors and use of haloperidol and mortality.

**CONCLUSIONS.** With the results of the AID-ICU cohort study, we will provide important data on the use of haloperidol for delirium in a large number of ICUs Europe, Brazil and Canada. This will inform the design of a large-scale, international, randomized, placebo-controlled trial of ICU patients with delirium.

**REFERENCE(S)**

1. Barr J, Fraser GL, Puntillo K, et al. Clinical practice guidelines for the management of pain, agitation, and delirium in adult patients in the intensive care unit. *Crit Care Med*. 2013;41(1):263–306. doi:10.1097/CCM.0b013e3182783b72.

**GRANT ACKNOWLEDGMENT**

The AID-ICU study is funded by the Innovation Foundation Denmark.

### 0285

#### Postoperative delirium in the ICU: the impact of early brief delirium and persistent delirium

S. M. Yang[1], J. Bae[2], H. G. Ryu[2], H. Lee[2]
[1]Seoul National University Hospital, Anesthesia and Pain Medicine, Seoul, Korea, Republic of; [2]Seoul National University Hospital, Seoul, Korea, Republic of
**Correspondence:** J. Bae
*Intensive Care Medicine Experimental* 2017, **5(Suppl 2):**0285

**INTRODUCTION.** Immediate postoperative patients are under the influence of anesthetics, which might affect the results of CAM-ICU. And the consequence of immediate postoperative brief delirium might be different from that of persistent delirium.

**OBJECTIVES.** The aims of our study were to compare early brief delirium, late brief delirium and persistent delirium in patients with an ICU LOS of more than 24 hours after surgery and to evaluate the risk factors of persistent delirium in postoperative surgical ICU patients.

**METHODS.** We performed a retrospective study of development of delirium from November 1, 2012, to September 30, 2014. A total of 581 patients admitted to SICU for more than 24 h were enrolled. Patients were evaluated for development of delirium using the Confusion Assessment Method for ICU Patients (CAM-ICU). Patients were categorized into 4 groups according to the onset and duration of delirium: no delirium, delirium for less than 1 day on postoperative day 0 and abated within first 24 hours postoperatively (early brief delirium), delirium for less than 1 day after postoperative day 0 (late brief delirium), delirium for more than 1 day (persistent delirium).

**RESULTS.** The overall incidence of postoperative delirium was 23.1% (134/581). ICU and hospital length of stay and hospital and 1-year mortality were not different between early brief delirium and no delirium groups. Patients with persistent delirium showed longer length of stay (ICU and hospital) and higher mortality rate (ICU, hospital, 1-year) compared to patients with no delirium. After adjusting APACHE II score, persistent delirium group was associated with hospital mortality (hazard ratio (HR) 3.26, 95% CI [1.30-8.1], $p = 0.012$) and 1-year mortality (HR 2.82, 95% CI [1.43-5.58], $p = 0.003$) in time varying multivariable cox regression analyses. Multivariable analysis revealed age (OR 1.05, 95%CI [1.03-1.08], $p < 0.001$), higher APACHE II score (OR 1.08, 95%CI [1.03-1.12], $p < 0.001$), emergency operation (OR 2.29, 95%CI [1.31-4.02], $p = 0.004$), and alcoholism (OR 6.38, 95%CI [1.24-32.99], $p = 0.03$) as significant predictors of persistent delirium.

**CONCLUSIONS.** In conclusion, we found that early brief delirium group had similar outcome to no delirium group and significant difference with persistent delirium group in ICU stay and mechanical ventilation duration. And persistent postoperative delirium was associated with increased ICU and hospital stay and increased hospital and 1-year mortality.

**REFERENCE(S)**

1. Ely EW, Shintani A, Truman B, Speroff T, Gordon SM, Harrell FEJ, Inouye SK, Bernard GR, Dittus RS Delirium as a predictor of mortality in mechanically ventilated patients in the intensive care unit. JAMA. 2004;291:1753–62
2. Shehabi Y, Riker RR, Bokesch PM, Wisemandle W, Shintani A, Ely EW Delirium duration and mortality in lightly sedated, mechanically ventilated intensive care patients. Crit Care Med. 2010;38:2311–8

**GRANT ACKNOWLEDGMENT**

No

### 0286

#### Analysis of the management of sedation, analgesia and delirium on ICU

M. Barber Ansón, A. N. García Herrera, A. Martiarena Orce, T. Caldito Ascargorta, J. Barado Hualde, L. Macaya Redín, M. García Parra, E. Erice Azparren, S. Ajona Martínez Polo, S. Barace De Carlos
Complejo Hospitalario de Navarra, Pamplona, Spain
**Correspondence:** A. N. García Herrera
*Intensive Care Medicine Experimental* 2017, **5(Suppl 2):**0286

**INTRODUCTION.** Sedoanalgesia is a key component in the treatment of many critical patients and providing the optimal level of sedation in each patient is crucial. It is essential to use adequate doses of sedatives and analgesics, as well as targeting and monitoring constantly the level of sedation.

OBJECTIVE. Analyze the sedoanalgesia and delirium management in the ICU of a referral hospital.

**METHODS.** Prospective observational study, masked for non-participating professionals. Studied variables included type of admission (scheduled surgery, urgent surgery, medical and trauma), age, gender, APACHE II, underlying disease, median length of stay and ICU mortality. Therapeutic management related to sedation, analgesia and delirium data were collected daily for 15 consecutive days in all patients. Sedoanalgesic management in the ICU was analyzed during the first, fifth and ninth days of admission. Values were expressed as mean ± SD and frequency.

**RESULTS.** 46 patients were included, mean age was 63.6 years (SD 16.3). 68.1% were male. 45.7% were admitted after scheduled surgery. Hypertension (45.7%), heart disease (41.3%), and smoking (28.3%) were the three most common underlying conditions. APACHE II of 13.2 (SD 6.9). 73.9% required mechanical ventilation and 54.3% vasopressors. Median length of stay was 4.3 days (SD 5.0) with a mortality of 16.3%. Delirium was not monitored in 97.5%, pain in 91.3% and sedation in 81.1% of patients respectively. The management of sedatives and analgesics with average doses (D) and average hours (H) with minimum and maximum values in both D and H are shown in Table 83. Results of sedation and agitation scales obtained by the researches are shown in Table 84.

**CONCLUSIONS.** The results revealed a lack of monitoring of pain, sedation and delirium in our ICU as well as the use of medium-high doses of sedatives but lower doses of analgesics reaching degrees of moderate-deep sedation.

**Table 83 (Abstract 0286)**. Characteristics of sedatives and analgesics

| Variables | Day 1 | Day 5 | Day 9 |
|---|---|---|---|
| Propofol (mg/kg/h) | D:2,5(0,01-4,59) H:8,9(1-23) | D:2,7(0,95-4,18) H:23,6(23-24) | D:2,5(1,46-3,51) H:19,8(4-24) |
| Remifentanyl (mcg/kg/min) | D:0,07(0,03-0,11) H:8,9(3-23) | D:0,07(0,03-0,11) H:23,6(23-24) | D:0,07(0,05-0,08) H:19(4-24) |
| Midazolam (mg/kg/h) | D:0,21 H:17 | D:0,17(0,06-0,29) H:17,5(12-23) | D:0,08(0,05-0,12) H:24 |
| Fentanyl (mcg/kg/h) | D:3,2 H:17 | D:1,59(1-2,19) H:17,5(12-23) | D:1,37(0,09-1,76) H:24 |
| Morphine (mg/kg/h) | D:0,03(0,02-0,04) H:5,25(4-6,5) | - | - |
| Dexmedetomidine (mcg/kg/h) | - | - | D:0,81(0,66-0,99) H:24 |

**Table 84 (Abstract 0286)**. Sedation and agitation scales' results

| Variables | Day 1 | Day 5 | Day 9 |
|---|---|---|---|
| ESCID 0 | 82,4% | 92,3% | 80,5% |
| EVN < o = 4 | 76,9% | 100% | 92,6% |
| RASS < o = -3 | 38,6% | 67,6% | 45,5% |
| CAM-ICU (+) | 3,8% | 15,4% | 16,7% |

### 0287

#### Early detection of ICU-acquired encephalopathy with the use of automated quantitative pupillometry

P. Morelli, D. Solari, J.-P. Miroz, P. Eckert, M. Oddo
CHUV and University of Lausanne, Lausanne, Switzerland
**Correspondence:** D. Solari
*Intensive Care Medicine Experimental* 2017, **5(Suppl 2):**0287

**INTRODUCTION.** ICU-acquired encephalopathy is frequent and is associated with increased morbidity and length of stay (LOS), yet there are no recognized monitoring tools for early prediction of delirium in this setting.

**OBJECTIVES.** To examine the value of automated infrared pupillometry (Neurolight-Algiscan®, ID-Med, France) to predict delirium.

**METHODS.** Cohort analysis of consecutive patients admitted to a mixed medical/surgical ICU (December 2016-March 2017), who underwent sedation and mechanical ventilation for > 48 hours and had no acute/previous history of neurological dysfunction. Standard neurological examination (GCS-motor response [GCS-M] and the FOUR score) and quantitative pupillary light reflex (qPLR, expressed as % pupillary response to a calibrated light stimulus) were performed in parallel (5 [4–7] days following ICU admission). Delirium was defined as a Richmond Agitation Sedation Score (RASS) > 1 requiring antipsychotic medication.

**RESULTS.** 24 patients were included (median age 65 [IQR 54–72] yrs; APACHE II score 24 [19–29]; SOFA score 12 [9–13]). All subjects had sepsis, and 12 (50%) of them developed delirium. Delirium was associated with longer ICU LOS (22 ± 18 vs. 8 ± 4 days in non-delirium patients, p = 0.01). Quantitative pupillometry - performed a median of 4 [1–8] days previous to delirium diagnosis - revealed significantly lower qPLR in patients with delirium than in those without delirium (20 [16–29] vs. 30 [25–39] %, p = 0.03). All - except one - subjects with impaired qPLR < 20% developed delirium (92% specificity; 86% positive predictive value). In contrast, simultaneous GCS-M and FOUR score did not differ significantly between delirium and non-delirium patients (p > 0.2). Similarly, no significant differences were found between the two subgroups for age, APACHE II score, SOFA score, and the cumulative dose of sedatives/analgesics/vasopressors.

**CONCLUSIONS.** These preliminary data suggest that impaired quantitative PLR may be used for early prediction of ICU-acquired encephalopathy in critically ill non-neurological mechanically ventilated patients.

### 0288

#### Pain, agitation and delirium (PAD) in a community ICU - audit and survey of nurses

F. Miller[1], C. Marshall[1], P. Yung[1], M. Camargo[2], K. Ross[2], D. Fleming[2], M. Law[3], J. L. Tsang[1,4,5]
[1]McMaster University, Niagara Regional Campus, Michael G. DeGroote School of Medicine, St. Catharines, Canada; [2]Niagara Health, St. Catharines, Canada; [3]Brock University, St. Catharines, Canada; [4]McMaster University, Medicine, Hamilton, Canada; [5]Niagara Health, Medicine/Critical Care, St. Catharines, Canada
**Correspondence:** J. L. Tsang
*Intensive Care Medicine Experimental* 2017, **5(Suppl 2):**0288

**INTRODUCTION.** Delirium is a common manifestation of acute brain dysfunction in critically ill patients with a prevalence of 80% in intensive care unit (ICU) patients[1]. It is associated with multiple complications[1]. A revised version of the ICU pain, agitation, and delirium (PAD) guidelines was published in 2013[2]. Yet, its dissemination in a community ICU is unclear.

**OBJECTIVES.** As the initial phase of a single center multifaceted and multidisciplinary intervention with an overall aim to improve PAD management in a community ICU, this study aimed to:

1. Review PAD management practice.
2. Examine nurses' comfort in, perception and satisfaction of PAD management.
3. Explore potential barriers and improvement strategies of PAD management.

**METHODS.** Daily collection of process and outcome measures was conducted on all patients admitted to the ICU for >24 hours over a 20-week period (from April to August 2016). An anonymous 23-question paper-based survey was administered to ICU nurses to understand their comfort in, perception and satisfaction of PAD management.

**RESULTS.** Pain using Numeric Report System, agitation using Richmond Agitation Sedation Scale (RASS) and delirium using Confusion Assessment Method for the ICU were assessed on a median of 53, 73 and 24% of patients per day, respectively. Benzodiazepines were administered to a median of 24% of patients per day. A median of 20 and 36% of patients per day were found to be in pain and over-sedated (RASS < −1), respectively.

For the survey, there were 81 responses (98% response rate) with a median ICU experience of 6 years. >85% of nurses were comfortable with pain and agitation assessment. Only 41% of nurses were comfortable with delirium screening. 94, 70 and 57% of nurses were comfortable with pain, agitation and delirium treatment, respectively. 70% of nurses would calm down an agitated patient first. 54% of nurses performed sedation vacation or used non-pharmacological therapy. 47 and 43% of nurses were satisfied with PAD management by nurses & physicians, respectively. Multiple barriers to optimal PAD management included inconsistencies in PAD management among physicians and nurses, inadequate education and staff shortage. Nurses also identified education, PAD protocol, consistency of patient care among physicians and tailored non-pharmacological therapy as potential improvement strategies.

**CONCLUSION.** Our audit data suggests a gap in PAD management. Information elicited from our survey helps to explain this gap and will guide subsequent nurse focused interventions as part of our multifaceted interventional study.

**GRANT ACKNOWLEDGMENT**
Jennifer LY Tsang holds the McMaster University Internal Career Research Award.

### 0289

#### Clonidine for sedation in mechanically ventilated, critically ill adults: a retrospective chart review

L. Burry[1], E. Purivatra[2], T.H.T. Tran[2], B. Coleman[1], M. Guenette[1]
Mount Sinai Hospital, Toronto, Canada; [2]University of Toronto, Toronto, Canada
**Correspondence:** L. Burry
*Intensive Care Medicine Experimental* 2017, **5(Suppl 2):**0289

**INTRODUCTION.** Intensive care unit (ICU) patients often require analgesia and sedation, especially to ensure safety and comfort in the context of mechanical ventilation (MV). To this end, intravenous sedatives (e.g. midazolam) and opioids (e.g., morphine) are routinely administered. These drugs however, do have adverse effects (e.g. respiratory depression, delirium), do not permit rapid neurological assessment, and are associated with prolonged MV and ICU length of stay (LOS). Using the lowest effective sedative and analgesic doses while maintaining desirable pain control and sedation has been shown to improve clinical outcomes. Clonidine is an alpha-2 agonist traditionally used for hypertension, but is also gaining popularity in the ICU setting, where it is used for its sedating and analgesic properties. To date, however, little research has been published on clonidine for sedation in adult ICU patients.

**OBJECTIVES.**

1) describe clonidine dosing regimens,
2) report incidence of adverse events (hypotension, bradycardia), and
3) investigate possible sparing effects on traditional drugs used for pain, sedation and agitation (e.g., opioids, sedatives, antipsychotics).

**METHODS.** Single-centre retrospective chart review. Inclusion: adults (≥18 years) admitted to the ICU during a 5-year period and receiving ≥1 dose of enteral clonidine. Exclusion: patients on clonidine prior to ICU admission. Data were extracted from pharmacy records and medical charts. Demographic and outcome data: age, sex, MODS, admission type, adverse events (e.g., hypotension), duration of MV, ICU and hospital LOS, and ICU mortality. Drug data: duration of clonidine use, dosing regimen, and method of discontinuation. Subjects were divided into those ever receiving 0.4 mg/day or more (high dose) versus those never receiving 0.4 mg/day (low dose). Adverse drug events were collected for the three days before and after clonidine initiation.

**RESULTS.** Preliminary results are available for 95/195 patients meeting inclusion criteria. Median age 56 years (36% female) and median MODS 6.7. Fifty-six percent (53/95) of patients had clonidine titrated beyond an initial dose (high dose) (Table 85). Median duration of clonidine use was 5 days. High dose patients had a longer duration of clonidine use (6 vs. 2.5 days, p = 0.002) and MV (14 vs. 9 days, p = 0.037). Within 24 hours of starting clonidine, 21% of all patients experienced SBP < 90 mmHg, 53% experienced MAP < 65, and 7.4% had ≥1 episode of HR < 60 (Table 86). There was no difference in adverse events between groups. No differences were found in terms of reduction of overall opioid and sedative exposure (Table 87); however, the high dose group had greater antipsychotic usage.

**CONCLUSIONS.** Preliminary review shows only ~50% of patients have their clonidine dose titrated beyond an initial dose. No differences were found between groups in the incidence of hypotension or bradycardia, nor in opioid and sedative exposure after clonidine initiation.

**Table 85 (Abstract 0289).** Clonidine Prescription Information

| | | Total daily dose of clonidine (mg) | | | |
|---|---|---|---|---|---|
| | | All (n = 95) | ≤ 0.4 (n = 53) | > 0.4 (n = 42) | P-value (≤ 0.4 vs > 0.4) |
| Duration of use, days | median (IQR) | 5 (2,8) | 3.5 (2,7) | 6 (4,12) | 0.002 |
| Transferred out of the ICU on clonidine | n (%) | 26 (27) | 16 (30) | 10 (24) | 0.57 |
| Tapered (while in ICU) | n (%) | 5 (5.3) | NA (cannot taper low dose) | 5 (12) | 0.015 |

**Table 86 (Abstract 0289).** Adverse Events

| Timeframe [hours since clonidine initiation] | Hypotension/ [total available for estimate] | | | MAP<65/[total available for estimate] | | | HR<60/[total available for estimate] | | |
|---|---|---|---|---|---|---|---|---|---|
| | Total daily dose of clonidine (mg) | | | Total daily dose of clonidine (mg) | | | Total daily dose of clonidine (mg) | | |
| | All | ≤0.4 | >0.4 | All | ≤0.4 | >0.4 | All | ≤0.4 | >0.4 |
| 24 | 20/95 (21%) | 12/53 (23%) | 8/42 (19%) | 50/95 (53%) | 30/53 (57%) | 20/42 (48%) | 7/95 (7%) | 2/53 (4%) | 5/42 (12%) |
| 48 | 16/76 (21%) | 9/36 (25%) | 7/40 (18%) | 29/76 (38%) | 16/36 (44%) | 13/40 (33%) | 8/76 (11%) | 1/36 (3%) | 7/40 (18%) |
| 72 | 11/58 (19%) | 4/25 (16%) | 7/33 (21%) | 31/58 (53%) | 12/25 (48%) | 19/33 (58%) | 12/58 (21%) | 4/25 (16%) | 8/33 (24%) |

**Table 87 (Abstract 0289).** Utilization of opioids, sedatives and anti

| | | Mean daily difference (72h before - 72h after clonidine start) | | | |
|---|---|---|---|---|---|
| | | Total dose of clonidine (mg) | | | |
| | | All | ≤0.4 | >0.4 | p value |
| Opioids (fentanyl equivalents, mcg) | median (IQR) | 98.3 (-318.8, 881.7) | 83.3 (-225, 608.3) | 124.2 (-325, 1016.7) | 0.716 |
| Benzodiazepines (midazolam equivalents, mg) | median (IQR) | 0 (-0.7, 16.2) | 0 (-0.33, 9.33) | 0.58 (-4.67, 21.0) | 0.848 |
| Antipsychotics (olanzapine equivalents, mg) | median (IQR) | −0.25 (-20.38, 0) | 0 (-9.2, 0.76) | −4.8 (-32.6, 0) | 0.039 |
| Propofol (mcg) | median (IQR) | 0 (-43.4, 113.3) | 0 (0, 20.0) | 0 (-580, 318.3) | 0.945 |

### 0290

#### Analgesia, sedation and delirium management - the unwalked path

C. Paulino[1,2], I. J. Pereira[3,4,5], P. Póvoa[1,6], C. Granja[3,4,5], GASD (Analgesia, Sedation and Delirium Study Group - Task Force of SPCI/SPA Sociedade Portuguesa de Cuidados Intensivos/Sociedade Portuguesa de Anestesiologia)
[1]Hospital de São Francisco Xavier, CHLO, Polyvalent Intensive Care Unit, Lisbon, Portugal; [2]Nova Medical School, New University of Lisbon, Lisbon, Portugal; [3]Algarve Hospital Center, Intensive Care Unit, Faro, Portugal; [4]University of Algarve, Department of Biomedical Sciences and Medicine (DCBM), Faro, Portugal; [5]Faculty of Medicine, University of Porto, Center for Health Technology and Services Research (CINTESIS), Porto, Portugal; [6]Nova Medical School, CEDOC/ New University of Lisbon, Lisbon, Portugal
**Correspondence:** C. Paulino
*Intensive Care Medicine Experimental* 2017, **5(Suppl 2):**0290

**INTRODUCTION.** Critically ill patients frequently need analgesics and sedatives namely to improve patient ventilator synchrony, to relieve anxiety and improve comfort. Eventually, some of those will develop delirium known to increase morbidity and mortality.

**OBJECTIVES.** The aim of this study was to characterize the practices of Portuguese Intensive Care Units (ICU) physicians towards analgesia, sedation and delirium management (ASD).

**METHODS.** Between September 2016 and April 2017, an on-line survey was distributed to ICU physicians in Portugal. The database was built from the Portuguese Intensive Care Society database.

**RESULTS.** A total of 117 physicians answered the survey (28% response rate) and the majority of respondents (88%) work mainly in ICU. The existence of protocols for ASD is considered useful by 94% of physicians, but less than 50% refer having such protocol in their ICU.

Pain is considered a frequent problem in ICU (95%), but only 86% monitor pain daily and only 67% use standardized scales. Opioids are the most frequently used analgesic in ICU (94%), followed by acetaminophen (77%).

Concerning sedation, the most frequently used medication is propofol (91%), followed by opioids, both natural and synthetic (79%), midazolam (68%) and dexmedetomidine (50%). Richmond Agitated Sedation Scale is the most commonly used scale for sedation assessment. Forty-four physicians consider that patients are over sedated most of the time. Physicians are aware of delirium as an important cause of mortality and morbidity but only 65% assess it daily. Confusion Assessment Method for Intensive Care Unit (CAM-ICU) is the most frequently applied delirium scale (55 physicians). Some physicians report diagnosing delirium on clinical grounds, without the use of scales (42 physicians). Almost half of the respondents consider delirium diagnostic scales difficult to apply (49%). Quetiapine (72%) and haloperidol (92%) are the most commonly used drugs in hyperactive delirium.

**CONCLUSION.** Despite the recent advances in knowledge regarding ASD, some recommendations are still not translated into clinical practice. Concerning sedation, the regular use of sedation scales and sedation assessment lacks effect as clinicians consider there is still over-sedation. Even thought the recommendations towards the use of sedation strategies with non-benzodiazepine sedatives, it is still largely used in ICU. Pain is routinely monitored in almost every patient, and preemptive analgesia over baseline analgesia is becoming a frequent approach. Delirium is considered a serious problem in ICU, but only a small percentage of physicians use specifics screening tool. The authors consider that there is still a long path to walk concerning ASD in Portuguese ICU. Recently a group of physicians created a national task force to address issues related with ASD practices and education, hoping for better practices in the near future.

**Acknowledgment**
Eng Jorge Gomes (CINTESIS)

### 0291

#### A prospective, observational, longitudinal cohort study of sedation practices in SGH intensive care units

Y. L. Lee[1], S.Y. Ng[1], G. Kalyanasundaram[2], L. K. Ti[3]
[1]Singapore General Hospital, Department of Anaesthesiology, Singapore, Singapore, [2]Singapore General Hospital, Department of Respiratory and Critical Care Medicine, Singapore, Singapore, [3]National University Hospital, Department of Anaesthesiology, Singapore, Singapore
**Correspondence:** Y. L. Lee
*Intensive Care Medicine Experimental* 2017, **5(Suppl 2):**0291

**INTRODUCTION.** Critically ill patients who are admitted to the intensive care unit (ICU) are often sedated as agitation may lead to ventilator dysynchrony and the inadvertent dislodgement of vital equipment and medications. Despite the rampant use of sedation, to date there is no consensus on the best sedation practices. In a landmark trial by Shehabi [1], he found that patients who were lightly sedated in the early admission period were more likely to be extubated earlier and to have increase survival rates.

**OBJECTIVES.** We attempt to investigate our sedation practices, and to determine if there are any association between depth of sedation and outcomes.

**METHODS.** This was a prospective, observation cohort study in patients admitted to SGH MICU/SICU who were ventilated and sedated for >24 hours. Baseline demographics were obtained, and patients followed up for 28 days or up to ICU discharge. Details on type and dose of sedative agents, ventilation duration, hospital/ICU length of stay/mortality, delirium (by CAM-ICU), and sedation depth (by RASS) were collected.

**RESULTS.** 58 patients were recruited from April-July 2012 (mean age 63.1+/−14.3 years; mean APACHE II, 20.2+/−8.5). Hospital mortality rates were 32.8%.

Patients were followed up for 387 ICU patient-days. In the early (first 48h) period, the most popular sedative used was propofol(74.1% patient-days), followed by morphine(29.3%), midazolam(13.8%), dexmedetomidine(7.8%), no sedation(6%) and fentanyl(2.6%). In the subsequent period, most patients were not sedated(47.6%), morphine became the most popular sedative(32.5%) followed by propofol(31%), midazolam(3.7%), dexmedetomidine(3%) and fentanyl(0.4%).

1994 RASS assessments were performed with a prescribed target in 11.1%. Amongst them, 86% met their targets.

3 patients were excluded from outcome analysis as they were ventilated for less than 48 hours. Lightly sedated(−2 > =RASS > = 1) patients were less likely to be delirious (0.9% vs 7.7%, p = 0.0095), with a reduction in ICU mortality (0% vs 21.1%, p = 0.048). There was a trend towards a reduction in vasopressor use (41.2% vs 76.3%, p = 0.057). There was no difference in rates of renal replacement therapy, hospital/ICU length of stay and hospital mortality.

**CONCLUSIONS.** Propofol and morphine are the most common ICU sedatives. Routine sedation goals should be prescribed as lighter sedation leads to improved survival and delirium rates.

**REFERENCE(S)**

[1] Shehabi Y, Bellomo R, Reade MC, Bailey M, Bass F, Howe B, McArthur C, Seppelt IM, Webb S, Weisbrodt L. Early intensive care sedation predicts long - term mortality in ventilated critically ill patients. Am J Respir Crit Care Med 2012; 186: 724–731

**GRANT ACKNOWLEDGMENT**
Not applicable

### 0292

#### Inadequate sedation: influence on length of stay and mortality in ICU

A. N. García Herrera, M. Barber Ansón, A. Martiarena Orce, E. Erice Azparren, J. C. Moncayola Vicen, V. Mínguez Exposito, T. Caldito Ascargorta, E. Sanz Granado, A. Orera Perez, J. Barado Hualde
Complejo Hospitalario de Navarra, Pamplona, Spain
**Correspondence:** A. N. García Herrera
*Intensive Care Medicine Experimental* 2017, **5(Suppl 2):**0292

**INTRODUCTION.** Sedation and pain control are crucial in the management of ICU patients. Deep sedation, defined as a Richmond Agitation-Sedation Scale (RASS) of −4 or −5 is indicated in intracranial hypertension, epileptic status, therapeutic hypothermia, complex respiratory situations, superficial sedation failure and terminal sedation. Precise control of the depth of sedation is often not well managed; patients are frequently oversedated with an accompanying increase in morbidity and mortality.

**OBJECTIVES.** Analysis of the repercussion of inadequate sedation on the length of stay and mortality in ICU patients.

**METHODS.** Observational and prospective study, masked for non-participating professionals. The study was conducted in the 24-bed capacity ICU of a referral hospital and was carried out over a period of 15 days. All patients admitted to the ICU were included. Demographic data, severity scales and characteristic of sedoanalgesia were collected daily for the whole period of the study. Inadequate sedation was defined as a RASS < −3 (deep sedation) without indication (without ARDS, intracranial hypertension, epileptic status, terminal sedation or superficial sedation failure). Univariable analysis was performed to explore association with days of inadequate sedation and ICU lenght of stay and mortality. Those variables associated with mortality (p-value ≤0.10) and those associated with length of stay (p-value ≤0.05) were included in a multivariate model to quantify the adjusted weight of days of inadequate sedation with mortality and length of stay. The quantitative variables were analyzed by Student´s t or Mann–Whitney U. The qualitative ones were analyzed by Chi-square or Fisher´s test and correlation coefficients of Pearson or Spearman as appropriate. The multivariate model was performed by logistic regression for mortality and multiple linear regression for the length of stay.

**RESULTS.** 46 patients were included with a total of 207 days analyzed with a median of 4.7 days analyzed by patient (range 1–15). 68.1% were male, mean age 63.6 years (SD 16.3 years). 16.3% mortality. Inadequate sedation in 19 patients, with an average of 3.6 days (SD 2.6, range 1–9). In the multivariate analysis, (B) value for days of inadequate sedation was 2.20 (95%CI 0.04 - 4.25) and p-value 0.047. For mortality 1.22 (95%CI 0.75 - 2.00) for each day of inadequate sedation, with p-value 0.419.

**CONCLUSIONS.** There was a high prevalence of inadequate sedation in our study. Each day of inadequate sedation was associated with an increase in the length of stay of 2.20 days and an increase in mortality by 1.22 for each day of inadequate sedation - although this last value does not reach statistical significance - probably due to the shortness of the study.

### 0293

#### Initial experience of a dexmedetomidine sedation protocol in critically ill patients admitted to an intensive care unit (ICU) in a secondary-level university hospital

J. L. Martinez Melgar[1], E. Moreno Lopez[2], A. Ortega Montes[1], E. Sanmartin Mantiñan[1], J. Bravo Doviso[1], I. Cenoz Osinaga[1], T. Sanchez de Dios[1], A. Pais Almozara[1]

[1]Complexo Hospitalario Universitario de Pontevedra, Intensive Care Unit, Pontevedra, Spain; [2]Complexo Hospitalario Universitario de Ferrol, Anesthesiology and Postoperative Care, Ferrol, Spain

**Correspondence:** E. Moreno Lopez

*Intensive Care Medicine Experimental* 2017, **5(Suppl 2):**0293

**INTRODUCTION.** To describe our experience (in terms of safety and efficacy) of a sedoanalgesia protocol using dexmedetomidine (Dx) in critically ill patients, under mechanical ventilation (MV) and spontaneous breathing (SB), admitted to our Unit (medical ICU).

**METHODS.** Descriptive and prospective study carried out during a 18-month period, according to dexmedetomidine indications for mechanical ventilation: sequential sedation, failure- tolerance to sedation, agitation- withdrawal syndrome or hyperactive delirium; and in SB: agitation-withdrawal syndrome, hyperactive delirium or adaptation to non-invasive mechanical ventilation. The following parameters were analyzed in both groups: age, gender, APACHE II score, reason for admission, length of ICU stay, past psychiatric history and toxic habits, time to achieve RASS goal (between −2 and 0): before 12 hours, between 12–24 hours or more than 24 hours, mean dosage and maximum dosage, adverse effects, non controllable patients (failure of Dx strategy), time spent under sedoanalgesia and time of de-escalation with Dx (hours). Statistical analysis: quantitative variables are expressed as the mean and standard deviation (SD), and qualitative variables as a percentage. The difference between qualitative variables are expressed using chi-square test and quantitative using an ANOVA analysis.

**Results:** 57 patients were included (35 male), mean age: 60 ± 22 years old, APACHE II score: 17,4 ± 9, reason for ICU admission: trauma 9, respiratory 18, neuro 8, cardio 3, infectious 10, intoxications 8, other 1. Past psychiatric history 12 and toxic habits 9. Indication for Dx according to protocol (patients under MV/SB: 37/20): sequential sedation 8/0, failure-tolerance 15/0, agitation-withdrawal syndrome 10/7, hyperactive delirium 4/5 and adaptation to non-invasive MV 0/8 (post-extubation 5). Time to achieve RASS goal (between −2 and 0), in the MV group: < 12 hours:7, between 12–24 hours:21 and >24 hours: 9 patients; in the SB group: < 12 hours: 4, between 12–24 hours: 10 and >24 hours: 6 patients. Mean dosage (Dx): 0,83 ± 0,25μgr/kg/h (MV) and 0,4 ± 0,24μgr/kg/h (SB) and maximum dosage: 1,2 ± 0,16μgr/kg/h (MV) and 0,7 ± 0,12μgr/kg/h (SB). Failure of Dx: 7 patients. Adverse effects: bradycardia 4, hypotension 8. Time spent under Dx: 39 ± 18 hours(MV) and 28 ± 14 hours (SB)

**CONCLUSIONS.** According to our protocol of sedoanalgesia with dexmedetomidine, the RASS goal was achieved in 70% and 75% of the patients under MV or SB in less than 24 hours respectively. Failure of the strategy of sedation with Dx: 12% in our series. The most common adverse effect was hypotension (14%).

### 0294

#### Dexmedetomidine - does it really make a difference to ICU length of stay?

C. Remmington, A. Rosenberg, S. Kaul

Harefield Hospital, London, United Kingdom

**Correspondence:** C. Remmington

*Intensive Care Medicine Experimental* 2017, **5(Suppl 2):**0294

**INTRODUCTION.** The incidence of ICU delirium remains high[1] particularly in mechanically ventilated patients[2] and is associated with substantial morbidity, mortality and ICU length of stay (ICU-LOS)[3]. Dexmedetomidine was introduced on our Cardiothoracic ICU (CT ICU) in April 2015 primarily for patients with ICU delirium and this case series provides a report on our experience of using the drug.

**OBJECTIVES.**

- To analyse our use of dexmedetomidine in a clinical setting
- To report the mean duration of mechanical ventilation (MV) and ICU-LOS in all patients admitted to the ICU before and after the introduction of dexmedetomidine, as secondary outcome measures.

**METHODS.** Data were retrieved retrospectively from an electronic prescribing system over an 18 month period for all patients treated with dexmedetomidine on the CT ICU. The mean MV and ICU-LOS for all patients admitted pre (2014) and post-introduction of dexmedetomidine (2016) was also calculated using a database retrieval system.

**RESULTS.** A total of 72 patients were included in the case series during April 2015 to October 2016. The mean age was 58 years old. The mean initial and maintenance doses were 0.54 and 0.95micrograms/kg/hour respectively. The mean duration of treatment was 2.43 days and the mean MV and ICU-LOS were 5.66 days and 9.07 days respectively. Adverse effects were found in 72% of patients, the most common were hypotension (61%) and bradycardia (10%).

For all patients admitted to the CT ICU in 2016 the mean MV (days) was 4.7 (n = 634) compared to 5.12 (n = 598) in 2014 (P-value 0.4056, unpaired t-test). The mean ICU-LOS (days) for all patient admitted to the unit in 2016 was 7.07 (n = 770) compared to 7.97 (n = 702) in 2014 (P-value 0.1424, unpaired t-test).

**CONCLUSIONS.** Dexmedetomidine has become a useful adjunct to our sedation armamentarium in a specialist Cardiothoracic and Transplant Centre. Our secondary outcome measure demonstrated a reduction in mean MV and LOS-ICU when comparing 2016 to 2014 for all patients admitted to the unit, but this did not reach statistical significance. This case series also illustrated that patients treated with dexmedetomidine for delirium receive mechanical ventilation for longer and spend more time in the ICU.

**Table 88 (Abstract 0294)**. MV_ICU_LOS

| | 2014 | 2016 | | 2014 | 2016 |
|---|---|---|---|---|---|
| Mean MV (days) | 5.12 | 4.7 | Mean ICU-LOS (days) | 7.97 | 7.07 |
| Number of patients | 598 | 634 | Number of patients | 702 | 770 |
| Standard deviation | 9.9 | 7.93 | Standard deviation | 13.23 | 10.29 |
| **P value = 0.4056 (Unpaired T-Test)** | | | **P value = 0.1424 (Unpaired T-Test)** | | |

### 0295

#### Promote the accuracy of using the confusion assessment method for the intensive care unit in critically ill patients by a two-stage clinical teaching program

A. Chao[1], S. Chu[2], C. Yang[3], Y. Yeh[1]
[1]National Taiwan University Hospital, Department of Anesthesiology, Taipei, Taiwan, Province of China; [2]National Taiwan University Hospital, Department of Nursing, Taipei, Taiwan, Province of China; [3]National Taiwan University Hospital, Department of Emergency Medicine, Taipei, Taiwan, Province of China
**Correspondence:** A. Chao
*Intensive Care Medicine Experimental* 2017, **5(Suppl 2):**0295

**INTRODUCTION.** Despite the occurrence of delirium is common in intensive care unit (ICU) and there is growing evidence demonstrates that delirium is harbinger of worse patient outcome, many doctors and nurses show little awareness towards delirium. This highlights routine screening of all ICU patients for the presence of delirium is important to its successful and early management. Nurses are on the frontline to look after patients they can detect, manage and even prevent delirium. Therefore we decided to launch a two-stage teaching program to make sure nurses were competent to implement the Confusion Assessment Method of the Intensive Care Unit (CAM-ICU) correctly in our SICU.

**OBJECTIVES.** The present study is to use the Direct Observation of Procedural Skills (DOPS) to assess the effectiveness of the two-stage teaching program of implementing CAM-ICU to screen delirium in a surgical ICU.

**METHODS.** 32 SICU nurses were included in this study. Stage 1 consisted of self-study and a lecture of CAM-ICU. References and websites of delirium and CAM-ICU were provided to the nurses before the lecture. All participants took a test before and after the lecture. Stage 2 was hands-on clinical practice of CAM-ICU on SICU patients. First DOPS took place one month after the clinical screening of delirium, another DOPS were repeated six months later.

**RESULTS.** The effect of self-studying of acquiring the knowledge of delirium and learning of how to perform CAM-ICU was inadequate (Table 89).

After the lecture as shown by the results of first DOPS, the nurses performed CAM-ICU with awkwardness. Second DOPS showed that nurses were more adept at performing CAM-ICU (Table 90).

**CONCLUSIONS.** Mere self-studying and lecture related to screening delirium with CAM-ICU were inadequate. ICU culture and clinical hands-on practicing CAM-ICU were keys to implement delirium screening in the ICU.

**REFERENCE(S)**

1. Pandharipande P, Cotton B, Shintani A, et al. Prevalence and risk factors for development of delirium in surgical and trauma intensive care unit patients. J Trauma. 2008;65:34–41.
2. Bellelli G, Morandi A, Zanetti E, Bozzini M, Lucchi E, Terrasi M, Trabucchi M. Recognition and management of delirium among doctors, nurses, physiotherapists, and psychologists: an Italian survey. Int Psychogeriatr. 2014; 26:2093–2102.
3. Selim AA, Ely EW. Delirium the under-recognised syndrome: survey of healthcare professionals' awareness and practice in the intensive care units. J Clin Nurs. 2016. Doi: 10.1111/jocn.13517

**GRANT ACKNOWLEDGMENT**
The study was supported, in part, by the National Taiwan University Hospital (NTUH 105–29).

**Table 89 (Abstract 0295)**. Results of pre- and post-lecture tests of

| Question | Number of participants answered correctly (n=32) | |
|---|---|---|
| | Pre-lecture | Post-lecture |
| Delirium is a chronic onset of consciousness disturbance. | 9 | 31 |
| There are three subtypes of delirium in ICU: hyperactive, hypoactive and mixed. Hyperactive delirium tends to occur more in elderly patients and has a worse outcome. | 7 | 31 |
| Haldol is the drug of choice to manage delirium. | 8 | 31 |
| CAM-ICU is to assess the severity of delirium | 5 | 30 |
| Opiates and benzodiazepines are an independent risk factor for delirium. | 31 | 27 |
| The four features of CAM-ICU are: (I) Acute change or fluctuating course of mental status, (II) Inattention, (III) Altered level of consciousness, and (IV) Disorganized thinking | 13 | 30 |
| Delirium is presence when any 2 features of CAM-ICU are positive. | 19 | 31 |
| Feature III was absent if RASS is -2. | 17 | 30 |

**Table 90 (Abstract 0295)**. Results of first and second DOPS

| Assessment (1 point to 6 points) | First DOPS (n=28) | Second DOPS (n=25) |
|---|---|---|
| Explains indication of assessment | 3.5 | 4.7 |
| Demonstrates appropriate preparation | 3.9 | 4.8 |
| Explains assessment to patient | 4.0 | 4.8 |
| Performs assessment | 3.7 | 4.2 |
| Provides consideration for patient | 4.0 | 5.5 |
| Communication skills | 4.0 | 5.3 |
| Overall clinical competence | 3.9 | 4.9 |

### 0296

#### Postoperative delirium in critically ill surgical patients: perioperative risk factors and outcomes

O. Chaiwat
Siriraj Hospital, Mahidol University, Anesthesiology, Bangkok, Thailand
*Intensive Care Medicine Experimental* 2017, **5(Suppl 2):**0296

**INTRODUCTION.** Incidence of postoperative delirium ranges from 9 to 73% with high incidence found in critically ill surgical patients. Delirium is associated with increase mortality and cost of treatment. Factors associated with delirium vary between the study population. The intraoperative and postoperative factors which can be modified or prevented are less studied and the results are inconclusive.

**OBJECTIVE.** To identify risk factors associated with postoperative delirium in patients admitted to surgical ICUs and to compare outcomes of patients who did and did not develop delirium.

**METHOD.** This was a prospective cohort study in patients admitted to general surgical ICUs at Siriraj hospital, Mahidol University, Thailand. Adults patients (>18 years) who expected to stay in SICUs for more than 24 hours and who were undergone the operations within 7 days were included. Demographic data, intraoperative and postoperative data were recorded. CAM-ICU was used as an assessment for delirium.

**RESULTS.** A total of 251 patients were enrolled. Delirium occurred in 61 patients (24.6%). Hypoactive type was found in 44 patients (72%) and delirium frequently occurred on day 1 after SICU admission (72%). Age, diabetes mellitus, severity of disease (SOFA score), perioperative used of benzodiazepine and the use of mechanical ventilation were independent risk factors for delirium in multivariate analysis. The predictive score (Age + 4*SOFA + 19*DM + 14*BZP +

18*Vent) was created. The cut point of 110 demonstrated a sensitivity of 74% and a specificity of 72%, respectively. Delirious patients showed significantly higher hospital mortality than non-delirious patients (25% vs. 6%, p < 0.01, respectively).
The incidence of physical restraint, sleep deprivation and propofol used were significantly higher in delirious patients.(p < 0.01, all).
**CONCLUSION.** The incidence of delirium was approximately one-fourth of surgical patients admitted to SICUs and the mortality was much higher than non-delirious patients. The identification of high risk patients together with the prevention strategy will be the most important role in clinical practice.

### 0297

#### Effect of lormetazepam administration for prolonged sedation on incidence of delirium in critically ill patients

K. Pilarczyk[1], F. Wiens[1], H. Jakob[2], B. Huschens[2], E. Demircioglu[2], F. Dusse[3]
[1]imland Klinik Rendsburg (managed by Sana AG), Department of Intensive Care Medicine, Rendsburg, Germany; [2]West German Heart and Vascular Center, University Hospital Essen, Department for Thoracic and Cardiovascular Surgery, Essen, Germany; [3]University Witten/Herdecke, Medical Centre Cologne-Merheim, Department of Anaesthesiology and Intensive Care Medicine, Köln, Germany
**Correspondence:** K. Pilarczyk
*Intensive Care Medicine Experimental* 2017, **5(Suppl 2):**0297

**INTRODUCTION.** Long-term sedation with commonly used agents, e.g. midazolam or propofol in intensive care units (ICUs) has serious adverse effects. The intravenous benzodiazepine lormetazepam is approved for anxiolysis and sedation in critically ill patients in Germany and might offer some advantages over other sedatives. However, the literature lacks reports about the safety and efficacy of lormetazepam as well as its effect on delirium in critically ill patients.
**OBJECTIVE.** It was the aim of the present study to evaluate the effect of intravenous lormetazepam administration for prolonged sedation after cardiac surgery on incidence and severity of delirium.
**METHODS.** 50 adult patients on the cardiothoracic ICU with the need for sedation for more than 24 hours were included in this prospective observational study. Intravenous lormetazepam was administered with an initial bolus followed by a continuous infusion as clinically indicated for a maximum of five days. Sedation level and delirium were assessed using the Richmond Agitation-Sedation Scale (RASS) and Intensive Care Delirium Screening Checklist (ICDSC) every 8 hours. Primary endpoint was the ICDSC score 72 h after initiation of lormetazepam sedation.
**RESULTS.** 50 patients with a mean age of 60.8 ± 18.0 years (18 female) were included in this study. Mean duration of lormetazepam administration was 77.7 ± 43.8 h with a mean dosage of 14.6 ± 11.9 mg per day. Total ICDSC scores significantly improved after lormetazepam administration (2.68 ± 2.32 vs. 3.8 ± 2.23, p = 0.045) as well as several subscores e.g. inappropriate speech or mood (0.05 ± 0.19 vs. 0.34 ± 0.43, p = 0.01) or altered level of consciousness (0.38 ± 0.47 vs. 0.69 ± 0.50 vs. p = 0.03) accompanied by a significant reduction of other sedatives. A mild bradypnoe was observed in one patient possibly related to the administration of lormetazepam in combination with opioids without need for further intervention.
**CONCLUSION.** Our data suggest that lormetazepam represents a safe and efficient agent for prolonged sedation in cardiothoracic patients with beneficial effects on incidence and severity of delirium. However, randomized controlled trials are needed to investigate whether administration of lormetazepam compared to other sedatives might be favorable for the ICU patient.

### 0298

#### Dexmedetomidine and traditional sedation: a surgical intensive care experience

N. Shaikh, S. Tahseen, A. G. M. Tharayli, S. H. Sheik, A. Ganaw, A. H. Chanda, F. M. Malmstrom
Hamad Medical Corporation, Doha, Qatar
**Correspondence:** N. Shaikh
*Intensive Care Medicine Experimental* 2017, **5(Suppl 2):**0298

**INTRODUCTION.** Dexmedetomidine (Dex) is a sedative agent with analgesic activity and a unique mechanism of action than the traditional sedation by benzodiazepine and propofol. This traditional sedative agent acts on Gamma aminobutyric acid (GABA) receptors1. Dex causes sedation and anaxiolysis by acting on Locus ceruleus in midbrain whereas analgesic effect is by acting on presynaptic receptor in the spinal card. Its analgesic effect is through its effect on alpha 2-adrenargeic receptors in the dorsal horn of spinal cord and modulating the release of substance P2.
**OBJECTIVES.** Our study was to know the efficacy, safety and analgesic sparing effect of dexemedetomidine (Dex) in critically ill surgical patients.
**METHODS.** All patients receiving dexemedetomidine (Dex) in the surgical intensive unit of a tertiary health care facility were included in the study. Patients´ demographic data, diagnosis, surgical interventions, traditional sedation, Dex dosage and days, post extubation Dex use, adverse effects, adverse effects in lower and higher Dex dose, analgesic requirement, and rescue sedation requirement were prospectively recorded. Data entered in SPSS program, required test were used for comparison and P < 0.05 was considered as statistically significant.
**RESULTS.** A total of 428 patients were enrolled in the study. Majority of patients were males (73.3%). Most common diagnosis was acute abdomen and frequently performed surgery was laparotomy (28.9%). Duration of Dexemedetomidine was ranging from 2 to 28 days; common dose was 0.5 to 1.4 μg/kg/hours. 78% patients required Dex in the post-extubation period in a dose of 0.2 μg/kg/hours. There was significant reduction in the analgesia requirement in the post Dex period (P < 0.001). Adverse effects were bradycardia 6.1%, hypotension 1.6% and hypertension (4%) and there was no significant difference in adverse effects between lower and higher dose of Dex (P < 0.82). Patients in higher dose of Dex required significantly higher rescue traditional sedation (P < 0.01).
**CONCLUSIONS.** Dexmedetomidine is a safe sedative agent in variable dosage in surgical critically ill patients. It has a significant analgesic sparing effect. Increasing dosage of Dex will not increase the sedation efficacy but requires more rescue traditional sedation.

**REFERENCE(S)**

1. RR Riker, Shehabi Y, Bokesch PM,Ceraso D, Wisemandle W, Koura F et al. Dexmedetomidine Vs Midazolam for sedation of critically ill patients. *JAMA2009*; 301:489–99

**GRANT ACKNOWLEDGMENT**
None

## Severe infections in the ICU

### 0299

#### Impact of empiric antibiotic therapy on mortality in intra-abdominal infections with *Enterococcus* acquired in the ICU

A.-C. Morvan[1], B. Hengy[1], M. Garrouste-Orgeas[2], C. Schwebel[3], S. Ruckly[4], G. Marcotte[1], B. Mourvillier[5], J.-F. Timsit[5], OUTCOMEREA Study Group
[1]Edouard Herriot University Hospital, Department of Anesthesiology and Critical Care Medicine, Lyon, France; [2]St Joseph Hospital, Polyvalent ICU, Paris, France; [3]Albert Michallon Hospital, Grenoble 1 University, Medical ICU, Grenoble, France; [4]Bichat-Claude Bernard Hospital, UMR 1137 - IAME Team 5 - DeSCID, Decision SCiences in Infectious Diseases, Control and Care INSERM, Paris Diderot - Sorbonne Paris Cité University, Paris, France; [5]Bichat-Claude Bernard Hospital, Medical and Infectious Diseases ICU, Paris, France
**Correspondence:** A.-C. Morvan
*Intensive Care Medicine Experimental* 2017, **5(Suppl 2):**0299

**INTRODUCTION.** Pathogenicity of *Enterococcus* in intra-abdominal infections in the ICU is debated (1–3). The treatment of intra-abdominal infections is first surgical and the antibiotic therapy is an adjunctive treatment. An inadequate antibiotic therapy is often associated with a poor prognosis.
**OBJECTIVE.** To determine if an empiric antibiotic therapy active against *Enterococcus spp.* in ICU patients who underwent surgery for an intra-abdominal infection helps to reduce 30-day mortality.

**METHODS.** Retrospective study of the OUTCOMEREA database which includes 22 French ICU from 1997 to 2016. All patients hospitalized in an ICU with intra-abdominal infection growing with *Enterococcus spp*. who had undergone surgery were selected. The patient inclusion was confirmed after reading the hospitalization records. Demographics, severity scores, microbiology, surgical complications and 30-day outcome after surgery were collected. Chi2 and Mann Whitney tests were used. Results are shown in median (interquartile) or numbers (%). Stepwise multivariate logistic regression was used to identify independent factors associated with mortality.

**RESULTS.** Ninety-three patients were included but 66 were analyzed. The empiric antibiotic therapy was active against *Enterococcus spp.* in 40 (70%) patients and inactive in 26 (30%) patients.

Inactive empiric antibiotic therapies included one penicillin inactive against *E. faecium* in 21 cases (81%) and one cephalosporin inactive against *Enterococcus spp*. in 5 cases (19%). Thirty-day mortality was lower in patients with *E. faecalis* than with other types of *Enterococcus*: 4 (14.8) vs 16 (41) p = 0.023. In the multivariate analysis, SAPS score (HR = 1.038 per point 95% CI (1.006 - 1.070) p = 0.0185) and an empiric antibiotic therapy inactive against *Enterococcus spp.* (HR = 3.391 95% CI (1.337 - 8.599) p = 0.0101) were independently associated with 30-day mortality.

**CONCLUSION.** In our small cohort of ICU patients surgically treated for an intra-abdominal infection, an empiric antibiotic therapy active against *Enterococcus spp.* is associated with a reduced 30-day mortality when an *Enterococcus* is isolated in the peritoneal fluid.

**GRANT ACKNOWLEDGMENT**

the OUTCOMEREA study group

**Table 91 (Abstract 0299)**. Comparison active vs inactive antibiotic therapy

| Variables | Empiric antibiotic therapy active against Enterococcus (n = 40) | Empiric antibiotic therapy inactive against Enterococcus (n = 26) | p |
|---|---|---|---|
| SOFA | 7.5 (4.6) | 9.2 (3.4) | 0.046 |
| Number of E. faecium isolated in peritoneal fluid | 13 (35.1) | 16 (66.7) | 0.016 |
| 30-day mortality | 7 (17.5) | 13 (50) | 0.005 |

### 0300

#### The incidence and mortality rates of necrotising soft tissue infections in Denmark in 2005–2013

M. B. Madsen[1], L. B. Madsen[2], O. Hyldegaard[2]

[1]Rigshospitalet, Copenhagen University Hospital, Department of Intensive Care, Copenhagen, Denmark; [2]Rigshospitalet, Copenhagen University Hospital, Department of Anaesthesia, Centre of Head and Orthopaedics, Copenhagen, Denmark

**Correspondence:** M. B. Madsen

*Intensive Care Medicine Experimental* 2017, **5(Suppl 2):**0300

**INTRODUCTION.** Necrotising soft tissue infection (NSTI) is a fast progressing bacterial infection involving any layers of the soft tissue and is associated with high rates of morbidity and mortality [1]. The incidence and mortality rates vary widely between cohorts.

**OBJECTIVES.** The objective of the study was to determine the incidence and mortality rate of NSTI in Denmark in the period 2005–2013.

**METHODS.** Data were obtained from the Danish National Patient Register (DNPR), which collects data from all Danish hospitals, and the Civil Registration System, which includes data on date of birth and death [2]. Data can be linked at an individual level by the social security number, a unique number assigned to every person living in Denmark. The National Registry of Death collects data on causes of death from death certificates. Data on population levels are available on-line at Statistics Denmark [3].

Data from 2005 to 2013 were obtained. The following codes from the Danish diagnosis system were used: DA480, gasgangrene; DM425A, necrotising fasciitis (before 2012); DM726, necrotising fasciitis (from 2012) and DN498C, Fournier's gangrene. The DNPR was searched for all patients with any contact to a hospital with one of the above codes registered. Contacts registered as outpatient visits were excluded.

Incidence rates were calculated from the yearly number of patients with NSTI divided by the population registered midyear.

The study was approved by the Danish Health and Medicines Authority and the National Data Protection Agency.

**RESULTS.** A total of 2668 contacts by 945 unique patients were registered, leaving 918 when patients with only outpatient visits were removed. The median age was 62 (IQR 49–71), 65% were male. The average incidence of NSTI was 1.84 per 100,000 inhabitants/year (Fig. 105). Of the 918 patients 737 (80%) were registered by the diagnosis necrotising fasciitis, 86 (9%) by the diagnosis gas gangrene and 283 (31%) by the diagnosis Fournier's gangrene. 17% were registered with more than one of the diagnoses. Mortality rates are seen in Table 92. NSTI was reported as the underlying cause in 60 (22%) of the deaths. One-year follow-up was achieved for 99.7% of the patients.

**CONCLUSIONS.** The average incidence of NSTI in Denmark during the study period was 1.84 per 100,000 inhabitants/year, and 90-day mortality was 25% (95% CI: 23%-28%).

**GRANT ACKNOWLEDGMENT**

The study is part of the INFECT project (NCT01790698), supported by the European Union's Seventh Framework Programme.

**Table 92 (Abstract 0300).** All-cause mortality rates for patients with NSTI

| | Number of patients/total number in group | Percentage (95% CI) |
|---|---|---|
| 30-day mortality | 178/917 | 19% (17%-22%) |
| 90-day mortality | 232/916 | 25% (23%-28%) |
| 1-year mortality | 274/915 | 30% (27%-33%) |

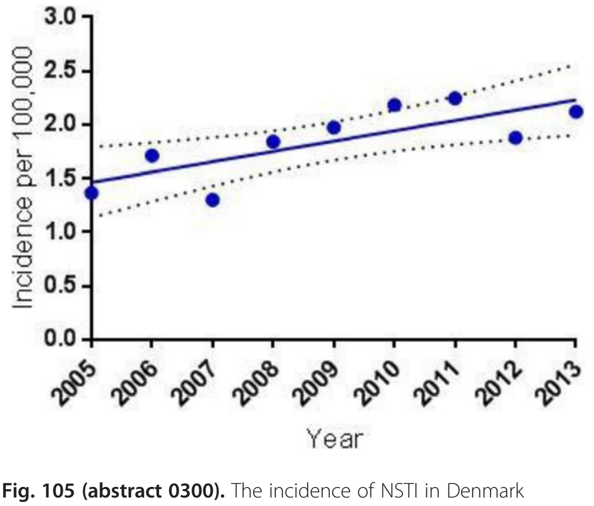

**Fig. 105 (abstract 0300).** The incidence of NSTI in Denmark in 2005–2013

### 0301

#### Tracheo-bronchial mucosal lesions during flexible bronchoscopy in ICU patients to diagnose invasive pulmonary aspergillosis

A. Alexandrescu[1], M. Barile[2], M. Nollevaux[2], G. Horlait[1], J. Petit[1], P. Evrard[1], I. Michaux[1], A. Dive[1], P. Bulpa[1]
[1]UCL Namur Godinne Hospital, Intensive Care, Yvoir, Belgium; [2]UCL Namur Godinne Hospital, Pathology, Yvoir, Belgium
**Correspondence:** A. Alexandrescu
*Intensive Care Medicine Experimental* 2017, **5(Suppl 2):**0301

**INTRODUCTION.** Mortality remains high in the ICU patients (pts) with invasive pulmonary aspergillosis (IPA), but early diagnosis could improve survival. However, definitive diagnosis is still difficult even with extensive diagnostic procedure.

**OBJECTIVES.** To analyze the interest of thorough inspection of the tracheobronchial tree associated with biopsies of the mucosal lesions to confirm IPA diagnosis.

**METHODS.** Between 01/1997 and 03/2017,174pts presented positive *Aspergillus* cultures in the airways during their ICU stay. Among them 169 underwent flexible bronchoscopy to exclude IPA. Suspicion of IPA was also based on positive *Aspergillus* culture from the airways with consistent clinical and radiological findings.

**RESULTS.** During bronchoscopy, tracheal and/or bronchial lesions were observed in 57/169pts(34%).These consisted of areas of erythema of the mucosa, associated with either patchy necrosis or non-removable false membranes. In each case, biopsy of the lesions revealed the presence of *Aspergillus* hyphae. In addition to these 57 patients, IPA was also proven in 16pts after autopsy, and finally, it was probable in 21 others. Consequently, IPA was finally diagnosed in 94 patients, thus bronchoscopy confirmed IPA in 61%(57/94pts). In the remaining 75pts, *Aspergillus* was considered as a contaminant; in these, although some mucosal lesions were encountered during endoscopy, patchy necrosis or non-removable false membranes were never found and none developed IPA.

**CONCLUSIONS.** Thorough endoscopic inspection of the tracheobronchial tree associated with guided biopsies appears to be minimally invasive and useful to diagnose invasive pulmonary aspergillosis in ICU pts.

### 0302

#### Predictors of mortality in adult severe Chikungunya patients from 2016 epidemic in India: comorbid conditions and organ failure not stable hemodynamic status on presentation, defines outcome

N. Tyagi, R. Kumar, V. K. Singh, S. Mittal, S. Taneja, S. C. Sharma, D. Dhar, B. K. Rao
Sir Gangaram Hospital, Critical Care & Emergency Medicine, Delhi, India
**Correspondence:** N. Tyagi
*Intensive Care Medicine Experimental* 2017, **5(Suppl 2):**0302

**INTRODUCTION.** Chikungunya fever(CHIK V) is considered a self-limiting disease with relapsing arthralgias. In the outbreak of Chikungunya in the Reunion Island epidemic, there were 237 deaths with case fatality rate of 1/1000 cases. However, it was not sure whether the mortality was directly associated with Chikungunya or coexisting morbidities worsened with Chikungunya infections.

**OBJECTIVES.** This study was conducted to assess the clinical manifestations and to find out the prognostic factors of critical ill patients with severe chikungunya.

**METHODS.** A single center retrospective observational study conducted at the intensive care unit of a tertiary care teaching hospital in North India.

Study period : 31st September 2016 to 31st December 2016.

Adult patients (>18 years) admitted to the intensive care unit with history of an acute febrile illness and found to have lab confirmed CHIKV infection by a positive RT-PCR test or detection of anti-CHIKV IgM by ELISA, were enrolled.

Severe CHIKV infection was defined by the presence of a new organ dysfunction or worsening of pre-existing organ dysfunction.

The medical records of all the recruited patients were retrospectively reviewed and the following information was collected: age, gender, clinical manifestations, disease severity scores, underlying conditions, laboratory examinations, and outcome.

The primary endpoint was to find the predictors of in-hospital mortality.

**RESULTS.** During study period, a total of 410 patients with chikungunya infections were admitted to hospital. 76(18.5%) patients with severe chikungunya infection required ICU admission.

Among these 76 critically ill patients, mean age was 69.7 years. (Male: Female = 55:21)

Respiratory failure was the most common organ dysfunction on presentation (n = 16, 21.0%) followed by acute kidney injury (n = 11,14.4%).

Overall, a total of 40 patients died, and 28 day mortality was 39.4% (n = 30).

For these 30,Mean SOFA on day 1 was 11.6 while only 9 had Cardiovascular SOFA 2+ or above.

In multivariate analysis, we found that in-hospital mortality was significantly high in age above 60 years(n = 22/50 44%) and in those with co existence of diabetes mellitus & hypertension (n = 8/11 72.7%).

Requirement of positive pressure ventilation(n = 23/54, 42.6%) and renal replacement therapy (n = 19/32, 59.4%) were also associated with high mortality.

**CONCLUSIONS.** Severe chikungunya requiring ICU admission is associated with high mortality. Mortality is likely to be more in those aged 60 years and above, with co morbid conditions particularly hypertension and diabetes mellitus if present together.

Worsening of respiratory failure and acute kidney injury was associated with highest mortality in our study. These organ failures were present despite not so poor hemodynamic status.

**REFERENCE(S)**

1. Lemant J, et al. Serious acute chikungunya virus infection requiring intensive care during the Reunion island outbreak in 2005–2006. *Crit Care Med 2008;36:2536–41*

### 0303

#### Nosocomial infections in an intensive care unit: predictive mortality factors

A. Jamoussi[1], K. Ben Ismail[1], S. Ayed[1], T. Merhebene[1], E. Mhiri[2], L. Slim[2], J. Ben Khelil[1], M. Besbes[1]
[1]Faculty of Medicine of Tunis, Medical ICU, Ariana, Tunisia; [2]Faculty of Medicine of Tunis, Microbiology Departement, Ariana, Tunisia
**Correspondence:** T. Merhebene
*Intensive Care Medicine Experimental* 2017, **5(Suppl 2):**0303

**INTRODUCTION.** Nosocomial infection (NI) is a public health problem due to its frequency, severity and costs, especially in intensive care unit (ICU).

**OBJECTIVES.** We aimed to investigate mortality's rate and potential death risk factors related to NI.

**METHODS.** This was a retrospective study, from January 1, 2016 to December 31, 2016; carried out within a 22-bed medical ICU of Abderrahmane Mami's pneumology hospital in Tunis. Demographic, microbiological and outcome data were recorded and multivariate regression analysis was conducted to identify potential death's risk factors. The endpoint of this study is to identify independent predictive mortality's factors in ICU patients having nosocomial infections.

**RESULTS.** During the study period (12 months), 590 patients were hospitalized; 59 patients (10%) presented at least one nosocomial infection. We recorded 91 episodes of nosocomial infections (59 patients) divided into pneumonia (n = 45, 49.5%), bacteremia (n = 26, 28.6%), catheter related infections (n = 13, 14.3%) and urinary infections (n = 7, 7.6%). *Acinetobacter Baumanii* Resistant to Imipenem (ABRI) was the most frequent pathogen responsible of pneumonia (n = 27, 60%) and bacteremia (n = 9, 34%).

Mortality in patients with nosocomial infections (n = 59) was of 55.9% while mortality in all patients (n = 590) was of 32.88%. Multivariate regression analysis identified 5 independent mortality risk factors which are detailed in Table 93.

**CONCLUSIONS.** In ICU patients with nosocomial infections, mortality is high. Pulmonary location of nosocomial infections is a major factor leading to pejorative outcome, especially when it is an ABRI. More efforts in hygiene precautions should be deployed to limit this fatal scourge.

**Table 93 (Abstract 0303)**. Mortality multivariate regression analysis

| | Surviving (n=26) | Dead (n=33) | OR | CI 95% | p |
|---|---|---|---|---|---|
| SAPS II | 21.5 ± 17.3 | 73 ± 13.5 | 1.199 | 1.043 - 1.377 | 0.011 |
| APACHE II | 16.7 ± 7.4 | 28.5 ± 6.3 | 1.457 | 1.009 - 2.105 | 0.045 |
| Nosocomial pneumonia | N=15 | N=30 | 6.206 | 1.351 - 28.510 | 0.019 |
| Days with vasopressive drugs | 4.4 ± 7.18 | 13 ± 8 | 1.151 | 1.055 - 1.256 | 0.001 |
| Pneumonia caused by ABRI | N=5 | N=22 | 5.500 | 1.434 - 21.096 | 0.013 |

### 0304

#### Epidemiology and outcomes of severe acute respiratory viral infections

S. M. A. Lobo[1], L.H.S. Covello[1], J.P. Nepomuceno[1], R. Ferrari[1], L. Godoy[1], M.L.M. Salomão[2], N.A. Furlan[3], A.S. Watanabe[3], J.V. Gandolfi[1], M.L. Nogueira[3]
[1]Faculdade de Medicina de São José do Rio Preto - FAMERP, Intensive Care Division, São José do Rio Preto, Brazil; [2]Faculdade de Medicina de São José do Rio Preto - FAMERP, Epidemiology Department, São José do Rio Preto, Brazil; [3]Faculdade de Medicina de São José do Rio Preto - FAMERP, Virology Laboratory - FAMERP, São José do Rio Preto, Brazil
**Correspondence:** S. M. A. Lobo
*Intensive Care Medicine Experimental* 2017, **5(Suppl 2):**0304

**INTRODUCTION.** Severe acute respiratory viral infections are among the main causes of morbimortality around the world.
**OBJECTIVES.** To evaluate the epidemiology and outcomes of patients admitted to the emergency department with signs of Severe Acute Respiratory Syndrome (SARS) due to viral infections
**METHODS.** Clinical, epidemiological and laboratory data were collected from patients older than 12 years old admitted to the emergency department from January to May 2016. Multiplex qPCR was used for viral detection
**RESULTS.** From January to November 2016, 159 patients were admitted in the emergency department with cough, fever, throat sore and respiratory symptoms (tachypnea or hypoxemia). A total of 78 patients were positive for Influenza, being 75 influenza A (all H1N1pmd2009) and 3 Influenza B, with mean age of 47 ± 16 years. Another 10 adult patients (Media age: 62 years) were negative for Influenza A H1N1, and positive for Human Respiratory Syncytial Virus (HRSV), of whom one patient aged 25 years old died (20%). A total of 28 Influenza A H1N1 and 3 Influenza B patients were admitted in the ICU with ARDS and mortality rate of 57%. Non survivors had a longer period between first symptom and first dose of oseltamivir (6,5 vs. 4 dias) or hospital admission (7 vs. 3,5 dias) in comparison to survivors. In H1N1 patients, C-reactive protein on admission was higher in non survivors (NS) than in Survivors (S) (31,3 vs. 18,3 mg/dL, p = 0,035 e 11,8 vs. 4,0 mg/dL, p = 0,035). NS had also higher serum lactate levels, leucocytes and SOFA scores in the first days of admission. Coinfection with bacterial pneumonia was suspected in 14 patients of whom, 9 (Early: 3, late:6) microbiologically confirmed: pseudomonas aeruginosa (n = 3), *staphylococcus aureus* (n = 3), *acinetobacter baumannii* (n = 2), *klebsiella pneumoniae* (n = 1). Patients with bacterial coinfection had more severe hypoxemia (PaO2 / FiO2 at ICU admission; 56 vs 184, p = 0.043), more organ dysfunction (SOFA day 3: 10 vs 3.5, p = 0.023), as well as longer period of time in the ventilator and on hospital.
**CONCLUSIONS.** Acute Respiratory Syndrome due to viral infections are associated with significant morbimortality even among adult immunocompetent patients.
**REFERENCE(S)**
World Health Organization - Global Burden Diseases, 2004. In http://www.who.int/healthinfo/global_burden_disease/GBD_report_2004update_full.pdf
**GRANT ACKNOWLEDGMENT**
CNPQ scholarship for medical student..

### 0305

#### Clinical characterization of fungal infections in the intensive care units at the Fundación Santa Fe de Bogotá, Colombia

Y. R. Cardenas[1], D. P. Diaz[1], P. Calvahi[1], A. M. Cardona[1], J. F. Devia[1], E. Celis[1], J. L. Nates[2]
[1]Fundación Santa Fe de Bógota, Bogota D.C, Colombia; [2]University of Texas M. D. Anderson Cancer Center, Texas, United States
**Correspondence:** Y. R. Cardenas
*Intensive Care Medicine Experimental* 2017, **5(Suppl 2):**0305

**INTRODUCTION.** Fungal infections continue to be a major cause of mortality in Intensive Care Units (ICUs), commonly associated with immunocompromised patients. Lately, there has been an increase in immunocompetent patients, being *Candida spp* the most prevalent fungal infection. With the introduction of new antifungals, it has been observed an increase resistance to them.
**OBJECTIVES.** To describe the incidence of candida infections, related risk factors, comorbidities, resistance to antifungals, use of vasopressors (VP), mechanical ventilation (MV) and mortality rate in patients admitted into the ICUs at Fundación Santa Fe de Bógota, Colombia in a five-year period.
**METHODS.** We conducted a retrospective observational study between Jan 2012 and Dec 2016. Adult patients from FUINC database with evidence of fungal isolation were reviewed. We describe the frequency of candida infections, resistance patterns, risk factors, use of VP, MV; outcomes as mortality and length of stay (LOS) in general wards ICUs. The analysis was performed with SPSS Statistic 23.0.
**RESULTS.** 159 patients were included in the study (male 63.52%). The mean age was 59 years old. The most frequent diagnoses were sepsis (n = 63, 39.62%) and respiratory pathologies (n = 29, 18.24%). *C. Albicans* was the main isolated fungus (n = 72, 45.28%), followed by *C.Tropicalis* (n = 29, 18.24%) and *C.Parapsilosis* (n = 26, 16.35%). Bronchoalveolar lavage (n = 65,40.88%) and hemorrhage (n = 35,22.01%) were the most frequent sites of isolation. 99.37% of the patients presented at least one of the risk factors (sepsis 37.74%, antibiotic management prior to infection 15.72%, and prolonged hospitalization 13.27%). The use of VP and MV were required in 135 patients (84.28%). The overall mortality was higher in our population (n = 83, 52%), compared with literature; the average of LOS at the ICU was 27 days and 42 days for general wards. Clinical resistance to antifungal treatment azoles (AZ), fluconazole (FZ), voriconazole (VZ) echinocandins (EC) and polyenes (PY) were measured per years. In 2012 (n = 29), the percentage of resistance to medical treatment was 3% for AZ, FZ, and VZ; reported mortality was 41%. In 2013 (n = 36), resistance to AZ and FZ was 3%, and mortality was 56%. In 2014 (n = 35), the resistance for AZ and FZ was (9%), EC (3%); and mortality was 49%. In 2015 (n = 31), no patients with resistance and mortality were 58%. Finally, by 2016 (n = 28), resistance to AZ, FZ and PY was 3.5% and mortality was 57%.
**CONCLUSIONS.** The incidence of candida infection in our population is relatively low. The mortality rate is slightly higher compared to the literature. The diagnosis of sepsis is present in the great majority of our patients. Other risk factors were antibiotic management prior to infection and prolonged hospitalization. In general, resistance rates to antifungals were very low.
**REFERENCE**
Michael A. Pfaller. Antifungal Drug Resistance: Mechanisms, Epidemiology and Consequences for Treatment. Am J Med. 2012;125.S3–S13

### 0306

#### Epidemiology and prognosis of early antibiotic therapy during acute pancreatitis

P. Montravers[1], M. Jabaudon[2], S. Bulyez[2], N. Belhadj-Tahar[3], T. Rimmele[4], O. Joannes-Boyau[5], J.Y. Lefrant[6], P. Seguin[7], M. Leone[8], C. Paugam[9], B. Veber[10], P. Guerci[11], T. Kerforne[12], R. Cinotti[13], L. Jacob[14], P. Verdier[15], T. Dugernier[16], J.M. Constantin[2], H. Dupont[17]
[1]CHU Bichat Claude Bernard, DAR, Paris, France; [2]CHU Clermont-Ferrand, DAR, Clermont-Ferrand, France; [3]CHU St Antoine, DAR, Paris, France; [4]CHU Edouard Herriot Hospices Civils de Lyon, DAR, Lyon, France; [5]CHU Bordeaux, DAR, Bordeaux, France; [6]CHU Nimes, DAR, Nimes, France; [7]CHU Rennes, DAR, Rennes, France; [8]CHU Marseille, DAR, Marseille, France; [9]CHU Beaujon, DAR, Clichy, France; [10]CHU Rouen, DAR, Rouen, France; [11]CHU Nancy, DAR, Nancy, France; [12]CHU Poitiers, DAR, Poitiers, France; [13]CHU Nantes, DAR, Nantes, France; [14]CHU Saint Louis, DAR, Paris, France; [15]CH Montluçon, Montlucon, France; [16]Clinique St Pierre, Ottignies, Belgium; [17]CH Amiens, DAR, Amiens, France
**Correspondence:** P. Montravers
*Intensive Care Medicine Experimental* 2017, **5(Suppl 2):**0306

**INTRODUCTION.** Antibiotic therapy (AB) of acute pancreatitis (AP) at the time of ICU admission has been poorly investigated. It is usually recommended to delay AB to decrease selection pressure and to avoid the emergence of multidrug resistant (MDR) bacteria.

**OBJECTIVES.** The frequency of early AB and their consequence on the emergence of MDR bacteria and outcome were assessed in a retrospective multicentre analysis.

**METHODS.** From 06/2009 to 03/2014, all patients (pts) admitted with a diagnosis of AP in 17 ICUs were included in a database. Demographic characteristics, underlying diseases, severity criteria on ICU admission (Day0), AB and their reasons, emergence of MDR bacteria in clinical samples during ICU stay and outcome at Day 30 were collected. Results are presented in medians (IQR) or proportions. The pts receiving an early AB (AB+ started at Day0) were compared to the AB-free pts using Fisher's exact test and Wilcoxon test ($p < 0.05$ significant).

**RESULTS.** Among 860 collected cases, 355 (41.3%) pts received AB from the admission in the ICU. Compared with AB-free pts, AB+ pts were older (60 [49–73] versus 56 [43–71], $p = 0.004$), with a higher SAPS II score (42 [32–63] vs 36 [25–50], $p < 0.001$). They had more frequent mechanical ventilation (37% vs 19%, $p < 0.001$), renal replacement therapy (12% vs 7%, $p = 0.02$) and vasoactive agents (46% vs 16%, $p < 0.001$). The two groups did not differ in terms of underlying disease or frequency of morbid obesity (BMI >30: 28%). In the AB+ group, a septic shock was observed in 108 (30%) pts or sepsis in 200 (57%) pts. Empiric AB was administered in 284 pts and targeted AB in 54 pts. The most frequently used AB were betalactams (n = 271 (85% of AB), including carbapenems n = 81 (23%)), aminoglycosides (n = 120 (35%)), anti-Gram-positive AB (n = 50 (14%)). Antifungal therapy was prescribed in 53 (15%) pts, mainly azoles (n = 46) and empiric (n = 40)). The main reasons for AB prescription were: intra-abdominal infection (n = 209 (60%)), pneumonia (n = 46 (13%)), bacteremia (n = 45 (13%)), catheter related infection (n = 9 (3%)) or urinary tract infection (n = 9 (3%)). During their ICU stay, 34/132 (26%) pts in the AB+ group had a clinical isolate yielding MDR bacteria compared to 25/122 (20%) in the AB-free pts (NS). The most frequent MDR strains were ESBL-producing *Enterobacteriaceae* (n = 37 including 19 (56% of MDR strains) in AB+ pts), methicillin-resistant *Staphylococcus aureus* (n = 13, 6 (18%) in AB+ pts) and *Pseudomonas aeruginosa* (n = 16, 9 (26%) in AB+ pts). No link was evidenced between MDR bacteria during the ICU stay and AB at Day0. Overall, 197/860 (23%) pts died. The two groups did not differ for the delay of death in ICU (5 [1–27] days) or death rate at Day30 99/505 (20%) vs 78/355 (22%).

**CONCLUSIONS.** Initial AB is frequently reported in ICU pts admitted for AP. Compared to AB-free pts, early AB has no significant impact neither in terms of emergence of MDR bacteria nor mortality.

### 0307

#### Decompressive craniectomy in acute onset infectious diseases: a systematic review of the literature

A. C. Kastoris[1], M. Michaelides[1], G. Fragkiadakis[1], T. Kyprianou[1,2]
[1]Nicosia General Hospital, Intensive Care Department, Nicosia, Cyprus; [2]University of Nicosia Medical School, Nicosia, Cyprus
**Correspondence:** A. C. Kastoris
*Intensive Care Medicine Experimental* 2017, **5(Suppl 2):**0307

**INTRODUCTION.** The role of decompressive craniectomy in infectious diseases has been sporadically documented in the literature in patients with increased intracranial pressure and risk of brain herniation. Evidence is lacking regarding the benefits or detriments of neurosurgical intervention.

**OBJECTIVES.** The aim of this study is to systematically review the literature regarding the role of decompressive craniectomy in cases of infectious diseases.

**METHODS.** Data from the available medical literature were systematically reviewed regarding decompressive craniectomy in cases of central nervous system infection. Relevant studies published in any language using the key terms "encephalitis", "meningitis", "meningoencephalitis", "decompression," "decompressive craniectomy" in the PubMed and Scopus databases. Bibliographies of the potentially relevant articles were also hand searched. Our systematic review included case reports and small case series reporting decompressive craniectomy in acute course viral or bacterial meningitis, meningoencephalitis or encephalitis including adults and children. Data was extracted regarding the characteristics of the included studies such as author and date of publication, study design, the number of patients reported in the study.

**RESULTS.** 43 studies were included in the systematic review reporting on a total of 57 patients, with a mean age of 27 years (range 1 to 66 years), including 27 females, 29 males and 4 cases where sex was not reported. Six studies did not report the pathogen involved. Viral pathogens were reported in 29 of 51 patients, whereas bacterial pathogens in 22 of the 51 patients.

The initial Glasgow Coma Scale on admission to the hospital was reported in 33 patients with a mean of 12 (range 3 to 15), pre-ICU admission GCS was reported in 32 patients with a mean of 6 (range 3 to 14).

In 11 patients, decision for decompressive craniectomy was taken by direct measurement of intracranial pressure after conservative measures failed. In 34 patients CT scan findings, compatible with herniation in combination with clinical signs of herniation led to decision for decompressive craniectomy, whereas in 12 patients, decision for decompressive craniectomy was guided by clinical signs of herniation alone. Time from symptom onset to the first CT brain scan was reported in 35 patients with a mean of 4.7 days (range of 0 to 17 days). Time after symptom onset until initiation of antibiotic treatment was reported in 29 of the cases with a mean of 6.7 days (range of 0 to 24 days).

In the ICU, neurological deficit was reported in 42 patients (Table 94). Long term outcome was reported according to the Glasgow outcome score more than 6 months after craniectomy in 43 of the 57 patients (Table 95).

**CONCLUSIONS.** Decompressive craniectomy may play a role in infectious diseases in cases of impending herniation or in increased intracranial pressure resistant to medication but further, higher quality research is necessary.

**Table 94 (Abstract 0307).** Neurological Deficit in the ICU

| No or Mild Neurological Deficit | Moderate Neurological Deficit | Hemiparesis | Paraplegia | Epilepsy | GCS < 4 | Death |
|---|---|---|---|---|---|---|
| 20 | 8 | 5 | 1 | 1 | 1 | 6 |

**Table 95 (Abstract 0307).** Long term outcome (Glasgow Outcome Scale)

| GOS 5 | GOS 4 | GOS 3 | GOS 2 | GOS 1 |
|---|---|---|---|---|
| 22 Patients | 9 Patients | 4 Patients | 2 Patients | 6 Patients |

### 0308

#### Severe influenza cases in a public pediatric intensive care unit in Greece during the epidemic seasons 2015 to 2017

C. Dimolitsa, K. Tziouvas, M. Mahaira, P. Bonos, F. Nikolaou, J. Papadatos
P. & A. Kyriakou Children's Hospital of Athens, Pediatric Intensive Care Unit, Athens, Greece
**Correspondence:** C. Dimolitsa
*Intensive Care Medicine Experimental* 2017, **5(Suppl 2):**0308

**INTRODUCTION.** Influenza is one of the most common vaccine-preventable viral diseases, with the highest morbidity reported for children and elderly patients. Data on complications in children with seasonal influenza virus infection are limited.

**OBJECTIVES.** We initiated a 2-year surveillance of children who were admitted to our pediatric intensive care unit (PICU) with severe seasonal influenza.

**METHODS.** Active surveillance from October 2015 through April 2017 was performed among all children admitted to our PICU. Cases to be reported were all hospitalized children < 16 years of age with PCR-confirmed influenza treated in our PICU due to severe complications.

**RESULTS.** Eight severe influenza-associated cases were reported from our PICU during two influenza seasons (2015–2017), all of them treated with oral oseltamivir. Among these children there were 4 males and 4 females and their age ranged from 45 days to 14 years. None had received vaccination against influenza. In 6 (75%) patients, the infection had been caused by influenza A and in 2 (25%) by influenza B. Patients spent a median of 10,5 days (IQR 1–20) in the PICU; 5 (62,5%) needed mechanical ventilation and one of them required HFOV. Most frequent diagnoses were influenza-associated encephalitis/encephalopathy (50%), myocarditis/syncope (25%), secondary bacteremia (12,5%) and ARDS (12,5%). Three (37,5%) children had chronic underlying medical conditions; one of our patients who had history of muscle weakness and ataxia was diagnosed with NARP (Neuropathy, Ataxia, and Renitis Pigmentosa) after severe neurological sequelae caused by influenza B infection. All three patients with encephalitis/encephalopathy had possible permanent sequelae. One influenza-associated death was reported due to ARDS.

**CONCLUSIONS.** Influenza viruses may be associated with significant childhood morbidity and PICU admissions. Many of the severe cases might have been prevented by following the recommendations for vaccination of risk groups.

### 0309

#### A tertiary centre prospective study of patients with dengue fever with encephalitis and use of laboratory parameters for early identification and management

A. Taggu[1,2], N. Darang[2], M.H. Ismail[3]
[1]Mahatma Gandhi Medical College and Reseach Institute, Critical Care Medicine, Pondicherry, India; [2]Sribalaji Vidhyapeeth University, Pondicherry, India; [3]St.Johns Medical College, Bangalore, India
**Correspondence:** A. Taggu
*Intensive Care Medicine Experimental* 2017, **5(Suppl 2):**0309

**OBJECTIVES.** To compare laboratory parameters, clinical manifestations and outcome in dengue infection patients presenting with or without encephalitis.

**METHODS.** Prospective observational study of 166 adult patients with acute febrile illness and IgM-anti-DENV ELISA proven Dengue fever admitted to MICU were included in the study. Baseline laboratory parameters, clinical spectrum and outcome were compared in patients with or without encephalitis.

**RESULTS.** Out of 200 patients admitted during the 9 months period, only 166 were positive for IgM-anti-DENV ELISA. Total of 78.3% patients had features of Dengue fever with no encephalitis (groupA) while 21.6% patients presented with encephalitis (groupB). Altered sensorium with fever dominated the second group (P-value < 0.05). No significant baseline parameters differences between the two groups. Notably Serum Ig-M, Ig-G antibodies, APTT, SGOT and SGPT were statistically significant in encephalitis group (P-value < 0.05). Group A patients recovered completely without any complications. Among group-B patients with encephalitis, 30 patients recovered completely, 4 patients died of multi-organ failure and 2 patients went discharged against medical advice.

**CONCLUSIONS.** Combination of laboratory parameters along with high index of clinical suspicion may help in identification of Dengue fever patients with possible encephalitis.

### 0310

#### Epidemiology of Clostridium difficile infections in intensive care unit of a General Hospital

A. Margaritis[1], N. Zotos[2], M. Gerasimou[2], M. Bolosi[3], E. Tatsina[2], O. Ygropoulou[1], D. Rizos[1], G. Papadopoulos[3]
[1]General Hospital of Ioannina 'G Hatzikosta', ICU, Ioannina, Greece; [2]General Hospital of Ioannina 'G Hatzikosta', Microbiology, Ioannina, Greece; [3]Ioannina University Hospital, Anesthesiology, Ioannina, Greece
**Correspondence:** A. Margaritis
*Intensive Care Medicine Experimental* 2017, **5(Suppl 2):**0310

**INTRODUCTION.** Clostridium Difficile is the most frequent etiology for nosocomial diarrhea. The organism may asymptomatically colonize the gut or cause illness extending from watery diarrhea through pseudomembranous colitis, toxic megacolon, and even death.

**OBJECTIVES.** The aim of this research was to study the epidemiology of Clostridium difficile infections in Intensive Care Unit of our Hospital.

**METHODS.** All patients admitted to general intensive care unit with a planned hospital stay > 3 days were investigated (baseline measurement, carriers control) and then, they were checked again per week or during symptoms, such as intense or prolonged diarrhea episodes. Trinity Biotech Uni Gold was used which requires small amount of feces with results in 20 minutes. It is a rapid immunochromatography membrane test for immediate determination of glutamate dehydrogenase antigen and toxins A and B in fecal samples.

**RESULTS.** In 10 months 31 middle-aged patients (66,4 ± 1,9 years old) with a planned hospital stay > 3 days and ability to check fecal samples within 24 hours after hospital admittion for carriers control, were investigated.

APACHE II was 20,6 ± 1,0, SOFA was 9,4 ± 0,7 and mean duration of hospitalization was 26,3 ± 4,3 days.

43 measurements were performed (baseline και recheck).

5 patients (16,1%) were positive for antigen by entering ICU but only one (3,2%) patient was positive for toxins A and B. Between these 5 patients, 2 were rechecked because they developed diarroic syndrome and both they were positive for antigen and negative for toxins A and B.

Of the remaining 23 patients, 9 were rechecked because they developed diarroic syndrome and all of them were negative for antigen and negative for toxins A and B.

**CONCLUSIONS.** Clostridium difficile carriers in ICU of general hospital are frequent enough (≈16%), although the disease appears to be not so frequent.

### 0311

#### Characteristics and Outcomes of patients requiring valve surgery during active infective endocarditis

H. Jouini[1], I. Nefzi[1], B. Jeribi[1], A.G. Ammous[1], N. Hamrouni[1], Y. Mannai[1], S. Mlayhi[2], A. Ammous[1], R. Denguir[2], A. Cherif[1]
[1]La Rabta Hospital, Anesthesiology & Intensive Care, Tunis, Tunisia; [2]La Rabta Hospital, Cardiac Surgery, Tunis, Tunisia
**Correspondence:** I. Nefzi
*Intensive Care Medicine Experimental* 2017, **5(Suppl 2):**0311

**INTRODUCTION.** Infective endocarditis is a serious disease with high morbidity and mortality.

**OBJECTIVES.** The Purpose of our study was to analyze the characteristics and outcomes of patients requiring valve surgery during active infective endocarditis.

**METHODS.** Retrospective review of data related to surgically treated patients in our cardiovascular surgery department from January 2010 to December 2016 with a diagnosis of infective endocarditis requiring surgical management.

**RESULTS.** Forty one patients data were analyzed. Mean age was 45 (17) years, with majority of males (72.2%). Native valves were involved in 29 patients (70.73%). Infective endocarditis mainly involved the aortic (24), mitral (17), and tricuspid valve (2). The most common causative pathogens were streptococci (9), staphylococci (7), and enterococci (3) (Table 96). The antibiotic treatment was adapted to bacteriological results in 8 cases (22.22%). Perioperative mortality was 41.7%. Timing for surgical procedure decision was 6.86 (9) days. Reasons for indicating surgery in 36 patients included heart failure (36.2%), vegetation (27.8%), no control of infection (11.1%) and unstable prosthesis (8.4%).

In our study, the biological profile of infectious endocarditis is marked by the preponderance of streptococcus and staphylococcus according to the international cohort [1] taken here as a reference with a larger percentage of negative cultures. This would have affected the low percentage of treatment adaptation (22,22%) and subsequently the high rate of perioperative mortality (41,7%) vs 18% in the reference study.

**CONCLUSIONS.** Endocarditis remains a serious problem both in terms of diagnosis and management. Current trends are for earlier surgery favoring valve repair (AHA and ESC guidelines). A better knowledge of microbiological characteristics would improve the quality of management.

**REFERENCE(S)**

[1]: Clinical presentation, etiology, and outcome of infective endocarditis in the 21st century: the International Collaboration on Endocarditis-Prospective Cohort Study, Murdoch, DR; et al., Arch Intern Med. 2009 Mar 9;169(5):463–73.

**Table 96 (Abstract 0311).** Common pathogens in active infective endocarditis

| | n= 41 (%) |
|---|---|
| Streptococci | 9 (22) |
| Staphylococci | 7 (17) |
| Enterococci | 3 (7) |
| Other pathogens | 9 (22) |
| Negative Culture | 13 (32) |

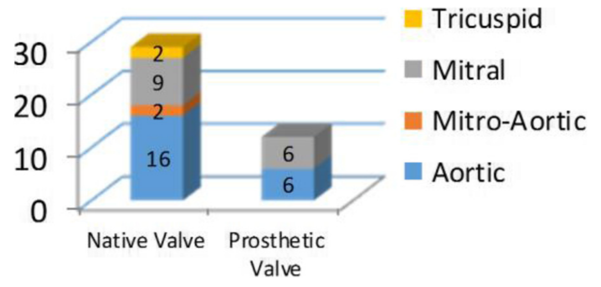

**Fig. 106 (abstract 0311).** Common pathogens in active infective endocarditis

### 0312

### ICU acquired Gram-negative bacteremia (ICU-GNB) in a tertiary hospital ICU

P. Rodríguez Villamizar, A. Pérez Lucendo, B. Balandín Moreno, S. Alcántara Carmona, B. Lobo Valbuena, N. Martínez Sanz, J. Palamidessi, R. Fernández Rivas, L. Pérez Pérez, P. Matía Almudevar, A. Naharro Abellán, J. Veganzones Ramos

Hospital Puerta de Hierro, Intensive Care Medicine, Madrid, Spain

**Correspondence:** P. Rodríguez Villamizar

*Intensive Care Medicine Experimental* 2017, **5(Suppl 2):**0312

**OBJECTIVE.** To describe the epidemiology, clinical characteristics, evolution and mortality related factors of ICU acquired Gram-negative bacteremia (ICU-GNB) in a tertiary hospital ICU.

**METHODS.** Descriptive study (2009–2016) of critically ill patients with infection by ICU acquired Gram negative bacteria, defined by at least one positive blood culture obtained ≥ 48 h after ICU admission. Demographic data, comorbidities, invasive devices, source and severity of infection, causative microorganism, antimicrobial susceptibility and therapy were recorded. APACHE II, SOFA and Pitt bacteremia scores, and the outcome were analyzed.

**RESULTS.** During the study period 42 ICU-GNB were diagnosed. Twenty six were men and the mean age was 59 years (44–73). Most patients came from emergency department (54%) and 23.8% patients were severely immunodepressed. The main reasons for ICU admission were sepsis (29%), neurocritical (19.6%) and trauma (16.6%). Time from admission to ICU-GNB was 13.5 days (9–43). Median days of central venous line exposure were 16 (10.5-45); arterial line 11 (7–24) and of mechanical ventilation 17 (12–33).

Almost all patients (95%) had received antibiotics prior ICU-GNB. Fifteen patients (35%) were on septic shock. Sources of bacteremia were the catheter-related (45.4%), respiratory (16.7%) and urinary (11%). Microorganisms isolated are shown in Fig. 107.

After receiving the antibiogram, 56% of patients received monotherapy with carbapenems (45%), the remaining 44% received a combination and the association between meropenem and tigecycline was the most frequent (33%). Resistance to carbapenems was documented in 58% of nonfermenting-GNB and 28% in *Enterobacteriaceae*. Twenty-two percent of patients were colonized by Gram negative bacteria, 7% of infections were caused by the colonizing pathogen.

The median ICU and hospital lengths of stay were 36 (26–39) and 73 (44–141) days. In-hospital mortality was 38% and mortality attributable to bacteremia was 9 (21.6%). APACHE II at ICU admission was 16.5 (11–23). Median SOFA and Pitt's scores on the day of bacteremia in survivors and non-survivors were 7 (5–10) versus 10 (8–12) and 3 (2–6) versus 6 (5–8) ($p < 0.05$), respectively. The incidence of ICU-GNB decreased significantly along the study period ($p < 0.04$).

**CONCLUSIONS.** ICU-GNB occur in critically ill patients with common risk factors for nosocomial infections such as antibiotic exposure and invasive procedures. Patients with ICU-GNB have a high in-hospital mortality. Higher SOFA a Pitt's scores are associated with a poor outcome. In our study, ICU-GNB experienced a steady decrease over the last years.

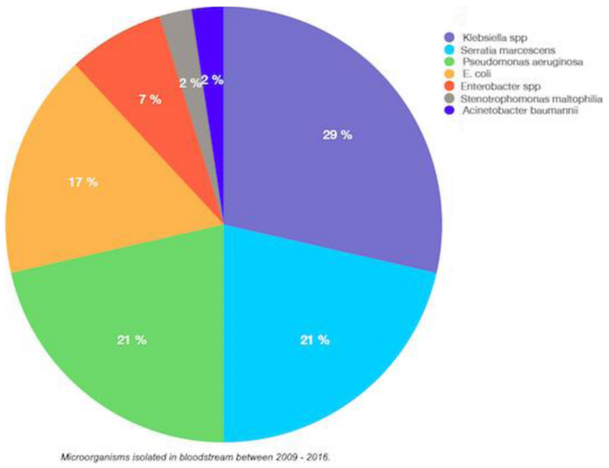

**Fig. 107 (abstract 0312).** See text for description

### 0313

#### Characteristics of patients with influenza (H1N1) admitted to the ICU

C. Duman, H. Sungurtekin, S. Serin, V. Yarar
Pamukkale University, Department of Anesthesiology and Reanimation, Denizli, Turkey
**Correspondence:** H. Sungurtekin
*Intensive Care Medicine Experimental* 2017, **5(Suppl 2):**0313

**AIMS.** Analysis of demographics characteristics, clinics and outcomes of patients with influenza A (H1N1) admitted to our intensive care units.

**METHODS.** All patients with Influenza Virus A (H1N1) confirmed with PCR were included in this study between January 2016- March 2016. Ventilatory strategy, demographics, hemodynamic data and outcomes were recorded.

**RESULTS.** Six patients included in this study with H1N1, two of those was man, four of those was women. H1N1 was diagnosed when two of the patients was arrived. Test results of other patients have been concluded in our clinic. Two patients admitted from emergency clinics, the other from wards in our hospital. The average age was 61.6 years. Four patient have comorbidities. The most common symptoms were respiratory failure (100%), fever (66.6%; mean temperature, 38.9°C) and cough (66.6%). The other symptoms were sputum extraction (50%) and sore throat (33.3%). Five patients have type 1 hypoxemic respiratory failure, one patient has combined respiratory failure (type 1 and 2). Two patients have high leucocyte count whereas three patients have high leucocyte count. All patients have high plasma C reactive protein. Two of patients admitted with hyponatremia, one of those has thrombocytopenia. Patients were present with lung infiltrates on chest X-ray. Patients were given two times a day 75 mg Oseltamivir. Five patients admitted to the ICU with acute respiratory distress syndrome whereas four of those were died during hospital stay.

**CONCLUSION.** Mortality is very high with pneumonia and ARDS in patients with H1N1 influenza virus infection. Survival is possible with early and effective treatment.

## Acute respiratory failure monitoring

### 0314

#### Effects of esophageal pressure-guided PEEP in ARDS patients on the amount of set PEEP and respiratory mechanics in the supine and the prone positions

M. Mezidi[1,2], H. Yonis[1,2], A. Louf-Durier[1], R. Tapponnier[1], F. Lissonde[1], M. Aublanc[1,2], Z. Riad[2], L. Baboi[1], B. Moro[1], J.-C. Richard[1,2], J. Mancebo[3], C. Guérin[1,2,4]
[1]Hôpital de la Croix Rousse, Réanimation Médicale, Lyon, France; [2]Université de Lyon, Lyon, France; [3]Sant Pau Hospital, Medical ICU, Barcelona, Spain; [4]INSERM 955, Créteil, France
**Correspondence:** M. Mezidi
*Intensive Care Medicine Experimental* 2017, **5(Suppl 2):**0314

**INTRODUCTION.** The use of esophageal pressure (Pes) to set PEEP in ARDS patients has been shown to result in a trend towards lower mortality as compared to a PEEP/FIO2 table [1]. Prone positioning (PP) for long sessions in association with use of low tidal volume and cisatracurium infusion has been shown survival benefit in ARDS patients. PP, due to the removal of the weight of the mediastinum might allow a more reliable measurement of Pes.

We hypothesized that Pes values are lower in PP than in supine position (SP) and, hence, PEEP level required to reach a 3 ± 2 cmH2O PEEPtot,L target should be lower in PP.

**OBJECTIVES.** The main objective of the study was to assess the amount of external PEEP set to reach positive PEEPtot,L in SP and in early and late PP.

**METHODS.** A prospective interventional physiologic study was performed in patients with moderate to severe ARDS (PaO2/FIO2 < 150 mmHg), intubated, sedated and paralyzed, requiring PP. Patients were assessed in SP at 30° and in PP at 0°or 15° from the horizontal plane. The baseline PEEP was set according to PEEP/FIO2 table [2] in SP 30° and applied for 60 minutes. Respiratory mechanics and blood gases were assessed. Then, PEEP was titrated to reach 3 ± 2 cmH2O PEEPtot,L and applied for one hour and measurements were done again. The baseline PEEP was resumed and patient was turned to PP with 0° or 15° inclinaison. Measurements were done after one hour and PEEP titration performed as above. For the rest of the PP session PEEP was randomly allocated between baseline PEEP or Pes-guided PEEP and measurements done at the end of it (16h).

**RESULTS.** We enrolled 32 consecutive ARDS patients (31 with complete data) during 15 months.

Pes-guided PEEP did not lead to significant variation of the amount of PEEP, except for a trend to higher PEEP in PP 0° (+3 [0–5.8] cmH2O, p = 0.1). Pes-guided PEEP strategy led to large (>5cmH20) changes in 5/31 patients, in SP, and in 7/31 in PP.

Respiratory mechanics were not altered by the Pes-guided PEEP strategy (Table).

PaO2/FiO2 ratio did not vary with Pes-guided PEEP in both SP (p = 0.31) and PP (p = 0.12), but improved with PP itself : +19 [2.5-50]mmHg, p < 0.003 at H + 1 and then +36 [16–66] mmHg (p < 0.001) at H + 16.

Respiratory mechanics did not change after 16 hours in PP.

**CONCLUSIONS.** PEEP required to reach a positive PEEPtot,L was similar in PP and in SP. Pes-guided PEEP strategy did not significantly alter respiratory mechanics in either SP or PP.

**REFERENCE(S)**

1. Talmor D, Sarge T, Malhotra A, O´Donnell CR, Ritz R, Lisbon A, Novack V, Loring SH, (2008) Mechanical ventilation guided by esophageal pressure in acute lung injury. N Engl J Med 359: 2095–2104
2. ARDSnet, (2000) Ventilation with lower tidal volumes as compared with traditional tidal volumes for acute lung injury and the acute respiratory distress syndrome. The Acute Respiratory Distress Syndrome Network. N Engl J Med 342: 1301–1308

**GRANT**

This work was partially funded by Bernhard Dräger Award ESICM Award.

**Table 97 (Abstract 0314).** See text for description

| | PEEP/FIO2 table-guided PEEP | | | Pes-guided PEEP | | | P value for Pes-guided PEEP in | | |
|---|---|---|---|---|---|---|---|---|---|
| | SP 30° N=31 | PP 0° N=15 | PP 15° N=16 | SP 30° N=31 | PP 0° N=15 | PP 15° N=16 | SP 30° | PP 0° | PP15° |
| PEEPapplied (cmH2O) | 10 [8–10] | 10 [8–11] | 10 [9.5–10] | 10 [9–13] | 14 [10.5–16.5] | 9.5 [7.8–10.2] | 1 | 0.10 | 1 |
| PEEPtot,es (cmH2O) | 8.7 [7–11.1] | 11 [8.2–12.6] | 7.4 [6.2–9.6] | 9.6 [6.7–13] | 12.9 [8.7–15] | 7.5 [5.4–9.8] | 0.4 | 0.07 | 0.52 |
| PEEPtot,L (cmH2O) | 2.4 [0.2–3.8] | 1.3 [-1.3–2.4] | 3.2 [2.6–4.6] | 2.8 [1.6–3.2] | 2.6 [1.8–2.8] | 2.5 [1.4–3.2] | 1 | 0.12 | 0.56 |
| Pplat,rs (cmH2O) | 22.1 [19,4–26,5] | 20.9 [18–23,2] | 23.3 [20,7–26] | 24.6 [19,6–27,8] | 23 [20,9–27,5] | 21.6 [18,7–24,2] | 0.9 | 0.2 | 0.59 |
| Pplat,L (cmH2O) | 9.3 [6,7–12,8] | 6.8 [3,7–8,1] | 10.4 [7,9–13,6] | 9.6 [7,7–12,5] | 8.1 [6,4–9,5] | 8.9 [7–11.2] | 1 | 0.17 | 0.6 |
| DP,rs (cmH2O) | 11.1 [8.8–14.8] | 8.6 [7.6–11.1] | 11.4 [9,7–13] | 12.5 [8.4–14.2] | 8.6 [7.6–10.4] | 11.4 [9,9–13,2] | 1 | .73 | 0.84 |
| DP,L (cmH2O) | 7 [5,7–10,3] | 5.4 [3,9–7] | 7.5 [5,7–9,3] | 6.6 [5,7–10,1] | 5.5 [3,4–6,5] | 6.8 [4,4–8,3] | 0.30 | .62 | 0.37 |
| Est,rs (cmH2O/L) | 29.6 [23,8–41,6] | 26.5 [22,2–29,1] | 30.1 [24,4–39,4] | 31.1 [23,2–43,8] | 25.9 [22,7–32,5] | 32.7 [25,3–41,6] | 0.81 | 1 | 1 |
| Est,L (cmH2O/L) | 18.8 [14,8–28,2] | 14.8 [10,3–20,2] | 19.3 [15,4–25] | 18.5 [12,8–25,1] | 17.8 [10–19,4] | 19.9 [12,3–26,3] | 0.76 | 0.91 | 0.87 |

### 0315

#### The effect of different flow rates of high-flow nasal cannula on end-expiratory lung volume in patients with acute lung injury

A. Shono[1], T. Kotani[2], T. Mihara[1], T. Nikai[1], Y. Saito[1]
[1]Shimane University Hospital, Anesthesiology, Izumo, Japan; [2]Showa University, Department of Anesthesiology and Critical Care Medicine, Tokyo, Japan
**Correspondence:** A. Shono
*Intensive Care Medicine Experimental* 2017, **5(Suppl 2):**0315

**INTRODUCTION.** A previous research using electrical impedance tomography (EIT) has demonstrated that in healthy volunteers, rising flow rate of HFNC resulted in increase in pharyngeal pressure and end-expiratory lung impedance (EELI), suggesting an increase in end-expiratory lung volume (EELV) in a flow-dependent manner. However, it is still unknown whether this effect is promising in patients with acute lung injury (ALI).
**OBJECTIVES.** In this study we investigated the effect of flow rate during HFNC on EELV and regional ventilation in ALI patients.
**METHODS.** 5 different flow rates of 10, 20, 30, 40, 50 L/min of HFNC were applied with random order in ALI patients. Each flow rate was maintained for 10 min, and EIT measurements were performed and recorded for 2 min at the end of each step. Respiratory rates and P/F ratio were measured simultaneously and oropharyngeal pressures were also monitored. EELI (impedance value at the end of expiration) and TIV (tidal impedance variation of a respiratory cycle), corresponding to EELV and tidal volume, respectively, were analyzed by EIT software. Changes in EELI and TIV from at 10L/min of HFNC were calculated and expressed as a percentage of TIV at 10 L/min. The patients were not asked to breathe with their mouths closed during the measurement period. Data was analyzed by one-way ANOVA with repeated measures and Turkey test as post-hoc analysis.
**RESULTS.** We enrolled seven ALI patients with severe acute pancreatitis, pneumonia, pregnancy induced hypertension. Mean P/F ratio before HFNC was 244 mmHg. Mean changes in EELI between at 10 L/min and at 20, 30, 40, 50 L/min were 24, −6, −19, 11%, respectively, indicating that flow rate was not associated with EELI. In addition, neither respiratory rate nor P/F ratio changed significantly with increased flow rate. On the other hand, mean oropharyngeal pressure increased from 2.4 $cmH_2O$ at 10 L/min to 5.2 $cmH_2O$ at 50 L/min.
**CONCLUSIONS.** Increase in flow rate during HFNC does not necessarily augment EELV in patients with ALI, despite low positive airway pressure generated in the oral cavity.
**REFERENCE(S)**
Respir Care. 2015; 60 :1397–1403.
**GRANT ACKNOWLEDGMENT**
None

### 0316

#### A high flow nasal cannula during sleep reduces inspiratory effort but does not reduce respiratory rate

Y. Onodera[1], R. Akimoto[1], T. Kobayashi[2], H. Suzuki[1], M. Nakane[2], K. Kawamae[1]
[1]Yamagata University Faculty of Medicine, Department of Anesthesiology, Yamagata, Japan; [2]Yamagata University Faculty of Medicine, Department of Emergency and Critical Care Medicine, Yamagata, Japan
**Correspondence:** Y. Onodera
*Intensive Care Medicine Experimental* 2017, **5(Suppl 2):**0316

**INTRODUCTION.** The use of high flow nasal cannula (HFNC) is increasing. Clinical studies show that HFNC reduce respiratory rate (RR), but tidal volume and minute ventilation vary, and thus the respiratory effect of HFNC is not fully understood. The majority of these studies were performed on patients while awake and thus the influence on respiratory patterns caused by conscious (comfort, discomfort) rather than the respiratory physiological effect itself cannot be excluded. We thus conducted a study using a HFNC in sleeping patients and recorded respiratory parameters to determine respiratory physiological effects that excluded the influence of conscious effects.
**OBJECTIVES.** To evaluate respiratory physiological effects of HFNC excluding the influence of conscious effects.
**METHODS.** The study was approved by our institutional ethical committee. Eight patients who underwent cardiac surgery and provided informed consent were included. A nasogastric tube equipped with electrodes to measure electrical activity of the diaphragm (EAdi) was inserted after extubated. Each patient received dexmedetomidine to achieve RASS < −1. At midnight, after recording the EAdi for 10 min with a venturi-mask (VM) to reach a $SpO_2$ of 95 ~ 98%, a HFNC was applied using a flow of 20 and 40 L/min for 10 min each while recording EAdi. The $F_IO_2$ of the HFNC was adjusted to reach the same $SpO_2$ with the VM in each patient. The RR and mean value of peak EAdi for each breath (p-EAdi) were determined from stable 1-min data from each phase. Blood gas analysis was done at the end of each phase. Data of RR, p-EAdi, $PaCO_2$, and $PaO_2$ for HFNC at 20 and 40 L/min were expressed as median (IQR) and compared with the VM data using the Wilcoxon signed-rank test. A $p$ value < 0.05 was considered statistically significant.
**RESULTS.** RR (VM; 17/min (12–20) at HFNC 20 L/min; 16 (11–19) at HFNC 40 L/min; 17(12–20)) $PaCO_2$ (38 mmHg (37–40); 40 (37–41); 39 (34–40)) $PaO_2$ (88 mmHg (78–96); 89 (86–92); 92 (87–103)) were not affected by HFNC. With the HFNC at a flow of 20 L/min, p-EAdi was reduced significantly, but showed no difference at a flow of 40 L/min (8.4 uV (6.4-12.5), 6.7 (5.1-10.4), and 6.3 (4.9-15.4).
P-EAdi is correlated closely with inspiratory effort (1). As shown in previous studies, a reduction in p-EAdi using a HFNC at a flow of 20 L/min reduced inspiratory effort without increasing $PaCO_2$. Increasing HFNC flow to 40 L/min may have caused increased inspiratory effort. This change is not fully understood and more bench and clinical studies are required.
Although many studies have reported a reduction in RR with HFNC our study did not show this effect. This result indicated that a reduction in RR using a HFNC while awake may be caused by the conscious rather than respiratory physiological effect.
**CONCLUSION.** Using a HFNC during sleep reduces inspiratory effort but does not reduce RR.
**GRANT ACKNOWLEDGMENT**
None
**REFERENCE**
1. Bellani G, et al. Crit Care Med 2013;41:1483–1491.

### 0317

#### A comparison of optimal positive end-expiratory pressure determination guided by electrical impedance tomography and using pressure-volume curve guided by EVITA 4 in moderate and severe acute respiratory distress syndrome patients

C. Tsai -Fen[1], C. Mei Yun[2], C. Hou.-Tai[3]
[1]Taipei Medical University, Chest, New Taipei City, Taiwan, Province of China; [2]Far Eastern Memorial Hospital, Chest, New Taipei City, Taiwan, Province of China; [3]Far Eastern Memorial Hospital, Banciao Dist, New Taipei City, Taiwan, Province of China
**Correspondence:** C. Tsai -Fen
*Intensive Care Medicine Experimental* 2017, **5(Suppl 2):**0317

**INTRODUCTION.** Acute respiratory distress syndrome (ARDS) is characterized by acute diffuse inflammatory lung injury presents with rapid onset of respiratory failure, dishomogeneity of the lung parenchymal[1], and significantly non-aeration region in the dependent parts. It is largely accepted that the benefit of applying of lung protective mechanical ventilation with low tidal volume and high PEEP strategy to maintain the alveolar pressure above atmosphere pressure to prevent ventilator associated lung injury in ARDS[2,3].
**OBJECTIVES.** Clinical studies indicate that chest EIT is able to monitor ventilation distribution at the bedside and may help to develop lung protective ventilation strategies. EIT-guided PEEP titration for ECMO-treated ARDS patients showed promising results, The aim of this study was to test To compare the treatment outcome in moderate and severe acute respiratory distress syndrome patients by using pressure-volume curve guided and electrical impedance tomography guided positive end-expiratory pressure setting.

**METHODS.** Patients in both groups were ventilated with EVITA 4 (Dräger Medical, Lübeck, Germany) with low tidal volume (6 ml/kg predicted body weight). In EIT group, the EIT data were recorded at 20 Hz and reconstructed with EIT Data Analysis Tool 6.3 (Dräger Medical, Lübeck, Germany). Offline analysis of regional compliance was achieved using a customized software compiled with MATLAB (MathWorks, Natick, MA, USA).

**RESULTS.** A total of 68 ARDS patients were included,records of 34 patients titrated with pressure-volume curve were retrospectively analyzed.survival rate, weaning successful rate, APACHEII24 hurs, $PaO_2/FiO_2$ in EIT group were significantly higher than the control group($p < 0.05$).

**CONCLUSIONS.** The preliminary results show that our protocol is feasible and the PEEP titration guided by EIT might lead to a better clinical outcome in ARDS patients.

**REFERENCE(S)**

1. Chiumello D, Froio S, Bouhemad B, Camporota L, Coppola S. Clinical review: Lung imaging in acute respiratory distress syndrome patients–an update. Critical care. 2013;17(6):243.

2. Briel M, Meade M, Mercat A, et al. Higher vs lower positive end-expiratory pressure in patients with acute lung injury and acute respiratory distress syndrome: systematic review and meta-analysis. Jama. Mar 3 2010;303(9):865–873.

3. Caironi P, Cressoni M, Chiumello D, et al. Lung opening and closing during ventilation of acute respiratory distress syndrome. American journal of respiratory and critical care medicine. Mar 15 2010;181(6):578–586.

**GRANT ACKNOWLEDGMENT**

This work was financially supported by project Far Eastern Memorial Hospital (FEMH-2017-C-055). The authors would like to thank Zhanqi Zhao for his continuous, enthusiastic, and smart support for this article.

**CONTRIBUTIONS OF AUTHORS.** Tsai-Fen Chen, Mei Yun Chang: contributes equally in this study.

### 0318

#### Impact of body inclination in the supine and prone positions on esophageal pressure in ARDS patients

M. Mezidi[1,2], H. Yonis[1,2], A. Louf-Durier[1], R. Tapponnier[1], F. Lissonde[1], M. Aublanc[1,2], Z. Riad[1,2], L. Baboi[1], B. Moro[1], J.-C. Richard[1,2], J. Mancebo[3], C. Guérin[1,2,4]

[1]Hôpital de la Croix Rousse, Réanimation Médicale, Lyon, France; [2]Université de Lyon, Lyon, France; [3]Sant Pau Hospital, Medical ICU, Barcelona, Spain; [4]INSERM 955, Créteil, France

**Correspondence:** M. Mezidi

*Intensive Care Medicine Experimental* 2017, **5(Suppl 2):**0318

**INTRODUCTION.** Esophageal pressure (Pes), as a reflect of pleural pressure, has been proposed to help clinicians to set PEEP by computing transpulmonary pressure (Ptp = airway pressure minus Pes). In the supine position (SP), due to the compression of esophagus by the mediastinum weight and by the displacement of abdominal content toward the thorax due to inclination of the thorax from the horizontal plane, the absolute value of Pes may be misleading. PP should be less affected by both effects, and hence Pes measurement more reliable.

**OBJECTIVES.** The objective of the study was to evaluate the variation of end-expiratory esophageal pressure at zero flow ($PEEPtot_{es}$) in PP and in SP, each at two body inclinations.

**METHODS.** A prospective interventional physiologic study was performed in patients with moderate to severe ARDS (PaO2/FIO2 < 150 mmHg) requiring PP. Thorax angulation was 30 ° and 0 ° in SP and 0 ° and 15 ° in PP. Data are expressed as medians [interquartile range] and compared through Friedman test and pairwise comparison with Holm correction.

**RESULTS.** Thirty-two consecutive ARDS patients were included. $PEEPtot_{es}$ did not vary significantly between SP (30 °) and PP (0 ° or 15 °). However, opposite variations were found according to thorax angulation in PP: a rise of 2.1 [0.7—3.1] cmH2O in PP (0 °), p =0.01 and a drop in PP (15 °) of 1.6 [0—2.6] cmH2O, p = 0.21. PP was associated with lower $\Delta P_L$ [−1 cmH20, P = < 0.001].

In a complementary analysis of 24 patients, $PEEPtot_{es}$ was studied in four positions [SP (30 °), SP (0 °), PP (0 °) and PP (15 °)] and followed an inverted U-shape pattern with median values of 9.7 [7.4—12.1], 12.8 [11.2—16], 11 [8.9—12.9] and 8.8 [6.7—11.1] cmH2O, respectively (Fig. 108).

These differences were statistically significant (Holm adjusted p value for multiple comparisons < 0.001) except between SP (30 °) and PP (0 ° or 15 °).

As a consequence, $PEEPtot_L$ rose of 2.8 cmH2O between SP (0 °) and PP (0 °), p < 0.001. With postural variations, EELV was significantly altered. Hence, $PEEPtot_L$ at PP (0 °) was computed at the EELV in SP (0 °), allowing to eliminate the impact of the change of EELV between postures: 83% of $PEEPtot_L$ changes were due to change of posture per se

[+3.5 cmH2O, p < 0.001]. Between SP (30 °) and PP (0 °), posture effect on $PEEPtot_L$ variation was +2 cmH2O, p < 0.001. A linear mixed model disclosed that rise of $PEEPtot_L$ was explained by PP for an amount of 2.8 (SD 0.4) cmH2O and by thorax angulation for 0.10 (SD 0.02) cmH2O per degree ($p < 10^{-7}$).

**CONCLUSIONS.** In PP (0 °) Pes increased as compared to SP (30 °) and decreased as compared to SP (0 °). However, when analyzing PEEPtotL variation due only to posture change (and not EELV change), a rise close to 3 cmH2O of PEEPtotL was seen in PP (0 °) compared to SP (0 °) or SP (30 °). Clinicians might therefore consider lowering PEEP level in PP.

**GRANT**

This work was partially funded by Bernhard Dräger Award ESICM Award.

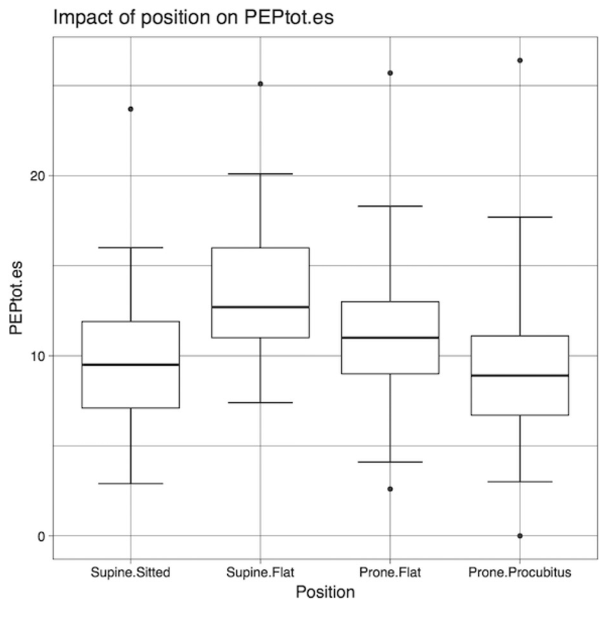

**Fig. 108 (abstract 0318).** See text for description

### 0319

#### Comparison of electrical impedance tomography images in critically ill patients with and without pleural effusion

T. Becher, M. Bußmeyer, I. Lautenschläger, D. Schädler, N. Weiler, I. Frerichs
Universitätsklinikum Schleswig-Holstein, Campus Kiel, Anästhesiologie und Operative Intensivmedizin, Kiel, Germany
**Correspondence:** T. Becher
*Intensive Care Medicine Experimental* 2017, **5(Suppl 2):**0319

**INTRODUCTION.** Electrical impedance tomography (EIT) is a non-invasive and radiation-free imaging modality that is increasingly used for monitoring of ventilation distribution within the lungs in patients with or at risk for respiratory failure. Clinical observations in patients with pleural effusion (PLE) and previous studies [1, 2] suggest that areas of low impedance, like fluid accumulations, in close proximity to areas of high impedance, like the ventilated lungs, cause out-of-phase impedance changes that can easily be detected in EIT images.

**OBJECTIVES.** To investigate whether out-of-phase impedance changes in the dorsal lung quadrants are a typical finding in patients with PLE.

**METHODS.** We conducted a prospective observational study in 30 intensive care unit patients with and without PLE. EIT data were recorded with PulmoVista 500 (Drägerwerk AG, Lübeck, Germany). In patients with PLE, EIT data were recorded before, during and after unilateral thoracocentesis. In patients without PLE, EIT data were recorded without any intervention. EIT images were separated into four quadrants of equal size. The sum of out-of-phase impedance changes in the dorsal lung quadrants was compared between patients without and with PLE before drainage by the Mann–Whitney test and receiver operating characteristics (ROC) curve. The comparison between patients with PLE before and after drainage was performed by the Wilcoxon matched pairs test.

**RESULTS.** We included 20 patients with PLE (PLE group) and 10 patients without PLE (control group). There were no between-group differences in age, height, weight, simplified acute physiology score and gas exchange. The amount of drained fluid in the PLE group was 525 ± 214 (mean ± SD) ml. The sum of out-of-phase impedance changes was 70, 49–119 (median, $25^{th}$-$75^{th}$ percentile) arbitrary units (a.u.) in the PLE group before drainage, 25, 12–46 a.u. after drainage ($p < 0.0001$) and 11, 6–17 a.u. in the control group ($p < 0.0001$ vs PLE group before drainage). The area under the ROC curve was 0.96 (0.91-1.01) for comparison between PLE group before drainage and control group.

**CONCLUSIONS.** In patients with PLE, out-of-phase impedance changes in the dorsal lung quadrants are a typical EIT finding and might be useful for detection and monitoring of PLE.

**GRANT ACKNOWLEDGMENT**
Not applicable

### 0320

#### Positive end expiratory pressure levels evaluated by two different methods using electrical impedance tomography had different effects on oxygenation after cardiac surgery

T. Kotani[1], K. Bito[1], S. Kimura[1], A. Shono[2]
[1]Showa University, Anesthesiology and Critical Care Medicine, Tokyo, Japan; [2]Shimane University, Anesthesiology, Izumo, Japan
**Correspondence:** T. Kotani
*Intensive Care Medicine Experimental* 2017, **5(Suppl 2):**0320

**INTRODUCTION.** Positive end expiratory pressure (PEEP) is one of the important factors to provide better global and regional ventilation distribution that leads to better oxygenation and to prevent ventilator-induced lung injury. However, the method to set PEEP safely and effectively is still not established. It is reported that electrical impedance tomography (EIT) can provide valuable information to determine PEEP level in an independent patient.

**OBJECTIVES.** In this study we assessed PEEP to obtain even distribution of global and regional ventilation using two different methods of EIT in patients after elective cardiac surgery, and investigated the clinical efficacy of each EIT-evaluated PEEP.

**METHODS.** A decremental PEEP trial (from 20 to 4 $cmH_2O$ of PEEP, 8 steps of every 2 $cmH_2O$) was performed in 22 consecutive post-cardiac surgery patients in the intensive care unit. At each PEEP step, EIT measurements were performed and recorded. Using EIT machine at bedside (online PEEP) and software later (offline PEEP), the lowest PEEP at which dorsal ventilation was preserved was determined in each way. The surgeon who had no knowledge of the experiment could change the PEEP one hour after determining PEEP. Time course of oxygenation and ventilation distribution were investigated and compared between the patients ventilated with EIT-evaluated PEEP until weaning and the patients ventilated with reduced PEEP.
Wilcoxon rank sum test and chi-square test were used for statistical analysis as appropriate.

**RESULTS.** Online and offline PEEP were 10 (6–15) and 10 (8–14) $cmH_2O$, respectively (median (interquartile range), p = 0.96). After 1 hour PEEP was decreased in 9 cases and in 6 of them dorsal ventilation decreased later. In contrast, PEEP was maintained at EIT-evaluated level in 13 cases and dorsal ventilation decreased in 3 of them (p = 0.04). In 8 cases EIT-evaluated PEEP was maintained until weaning process was started and their oxygenation was preserved by standard oxygen therapy, whereas in 6 of 14 cases PEEP was decreased from EIT-evaluated level and the patients required non-invasive positive pressure ventilation or high flow nasal cannula oxygen therapy (p = 0.03).

**CONCLUSIONS.** Offline EIT-evaluated PEEP provided better dorsal ventilation distribution and might have an impact on oxygenation and oxygen therapy after extubation.

**GRANT**
Departmental.

### 0321

#### Assessing respiratory mechanics using a new method in mechanically ventilated patient with respiratory failure

J.A. Benítez Lozano[1], P. Carmona Sánchez[2], H. Molina Díaz[1], M. Delgado Amaya[1], F. Ruíz Ferrón[3], J. M. Serrano Simón[2]
[1]Hospital Quirón, Intensive Care Unit, Málaga, Spain; [2]Hospital Universitario Reina Sofía, Intensive Care Unit, Córdoba, Spain; [3]Complejo Hospitalario de Jaén, Intensive Care Unit, Jaén, Spain
**Correspondence:** J. M. Serrano Simón
*Intensive Care Medicine Experimental* 2017, **5(Suppl 2):**0321

**INTRODUCTION.** Different methods of mechanics respiratory analysis have been used, as Multiple linear regression (MLR[1]), Time constant (TC[2]), Fourier analysis (fft[3]), and Occlusion techniques (Occ[4]). Some of these methods are intrusive and complex to apply in clinical practice. Futher, there are wide agreement limits between them.

**OBJETIVES:** To evaluate a new method for measurement of mechanics respiratory in ventilated subjects. Our method is a variant of isovolume technique[5], simple and easy to apply by any ventilator.

**METHODS.** We studied a group of mechanically ventilated patients during volume controlled mode with constant flow, due to acute respiratory failure of different diseases. Airway, esophageal pressure, and airway flow were recorded, sampling 561 Hz. The flow-resistance was calculated by choosing the points in both the inspiratory and expiratory phases of a respiratory cycle when the lung volume were identical and flow rate were about maximal. The ratio of this pressure change to the corresponding change in flow between these points was calculated as the average flow-resistance for inspiration and expiration (Rs). The elastance (Ers) and total PEEP was obtained from linear regression between component elastic of pressure and volume, between 10%-90% of each respiratory phase. The results from this method (iso*) were compared with those obtained from MLR, TC, Occ and fft techniques. Data are expressed as mean (SD) in absolute values, and as a percentage. The comparison of the data was made using one-way ANOVA, and Bonferroni post-hoc. The Bland-Altman analysis and linear regression was applied to assess the agreement.
**RESULTS.** 30 patients were studied. For all data: Ers: Iso* 27.41 ± 1.43 cmH2O/L; MLR 27.49 ± 1.42 cmH2O/L; TC 24.5 ± 1.34 cmH2O/L; Occ 23.13 ± 1.15 cmH2O/L; fft 30.14 ± 1.52 cmH2O/L. Rrs: Iso* 17.19 ± 3.58cmH2O/L/sec; MLR 16.41 ± 3.36 cmH2O/L/sec; TC 19.15 ± 3.61 cmH2O/L/sec; Occ 18.53 ± 4.21cmH2O/L/sec; fft 16.67 ± 3.54 cmH2O/L/sec. The mean comparison of the Ers and Rrs between Iso* and other techniques did not show statistical differences. Solely for Ers, Occ vs fft p = 0.018. The Tables 98 and 99 show the concordance analysis and correlation ($R^2$) for Ers and Rrs, respectively.
**CONCLUSIONS.** The new method is equivalent to other techniques established to compute the respiratory mechanics. The best fit is shown with the most dynamic methods (MLR and fft), and less with Occ and TC, perhaps because they are required to maintain a linear relationship between flow and volume not always real in patients with severe respiratory failure.

**Table 98 (Abstract 0321).** The Bland-Altman analysis and correlation for Ers

| Ers Iso* vs: | Means differences (SD), (cmH2O/L) | 95% Limits of agreement | Percentage Error | R2 |
|---|---|---|---|---|
| MLR | 0.09 (0.09) | −0.09 to 0.027 | 0.33 | 0.99 |
| TC | −2.96 (4,30) | −11.34 to 5.53 | 17.86 | 0.71 |
| Occ | −4.27 (3.94) | −12.15 to 3.64 | 15.59 | 0.75 |
| fft | 2.74 (3.21) | −3.68 to 9.16 | 11.16 | 0.85 |

**Table 99 (Abstract 0321).** The Bland-Altman analysis and correlation for Rrs

| Rrs Iso* vs: | Means differences (SD), (cmH2O/L/sec) | 95% Limits of agreement | Percentage Error | R2 |
|---|---|---|---|---|
| MLR | −0.79 (0.76) | −2.32 to 0.73 | 4.54 | 0.96 |
| TC | 1.95 (2.59) | −3.23 to 7.13 | 14.25 | 0.55 |
| Occ | 1.33 (1.73) | −2.12 to 4.78 | 9.68 | 0.84 |
| fft | −0.53 (0.7) | −1.92 to 0.87 | 4.32 | 0.96 |

### 0322

#### Improving the ability to monitor respiratory effort and work with esophageal pressure

J.A. Benítez Lozano[1], P. Carmona Sánchez[2], H. Molina Díaz[1], M. Delgado Amaya[1], J. M. Serrano Simón[2]
[1]Hospital Quirón, Intensive Care Unit, Málaga, Spain, [2]Hospital Universitario Reina Sofía, Intensive Care Unit, Córdoba, Spain
**Correspondence:** J. M. Serrano Simón
*Intensive Care Medicine Experimental* 2017, **5(Suppl 2):**0322

**INTRODUCTION.** Instability of pleural pressure measured with esophageal balloon by mobilizations, swallowing, changes with patient movement, etc. make constant and reliable monitoring of muscle pressure difficult with this technique. This has made its introduction into the clinic scarce, and its use is usually relegated to research.
**OBJETIVES:** We have designed a system that improves the ability to monitor pleural pressure, respiratory effort (PTP), and work (WOB) by replacing distending pressure (DP) by the equation of motion (EM) with the parameters resulting from a multiple linear regression (MLR) of 3–5 selected cycles between DP, Flow and volume.
**METHODS.** We studied a group of mechanically ventilated patients under pressure-support ventilation. Airway, esophageal (Paw, Pes) and airway flow signals were recorded, sampling 561Hz for more than 500 cycles. The volume was obtained from the flow integration. Previously we are measured on passive ventilation the chest wall elastance (Etw) as function of pleural pressure change and volume. On a visual inspection of each record, 3 to 10 successive cycles were selected, close to a Baydur test in which the Pes signal was regular, there was no swallowing, significant displacement of the baseline, or the maximum displacement of each cycle. The Pes of these cycles was transformed into muscle pressure (Pmusc) by subtracting of esophageal pressure, the product from the instant volume by Etw. The Paw - Pmusc + PEEP of each cycle was used to generate the DP. MLR of this pressure was performed with flow and volume to determine Ers, Rrs and total PEEP. The correlation ($R^2$) between EM and DP in the chosen cycles were > 0.96. PTP, WOB were obtained simultaneously from DP and EM, and it´s was extended to 60 cycles which included the previous selected records; of these were excluded five with the lowest and the highest frequency. Data were expressed as mean ± SD, the comparison was made with Student's t-test and agreement with concordance analysis.
**RESULTS.** Comparison in the 1500 cycles. WOB: DP vs EM: 2.25 ± 0.77J/L vs 2.39 ± 0.66 J/ L, p < 0.001. PTP: DP vs EM: 443.56 ± 175.02 cmH2O/s · min vs 477.876 ± 152.99 cmH2O/s · min, p < 0.001. The Table 100 show concordance analysis. Figures 109 and 110 show two examples of our approach. Figure 109a: The transformation contributes little to direct measurement but serves to demonstrate that it can be substituted. Figure 109b: In this other case the transformation contributes and much, to the direct measurement, otherwise it becomes impossible. Figure 110: Improvement of Pes by filtering effect and stabilization of signal.
**CONCLUSIONS.** Although equation of motion (EM) vs distending pressure (DP) show wide dispersion, however EM can be used for the monitoring of muscular effort and provides optimization of esophageal pressure by filtered and calibration with respect to Paw.

**Table 100 (Abstract 0322).** The Bland-Altman analysis and correlation coeffici

| Distending Pressure vs Equation Motion | Means differences (SD) | 95% Limits of agreement | % Error | R2 |
|---|---|---|---|---|
| PTP(cmH2O/s.min) | 34.32 (129.32) | −224.31 to 292.94 | 28.07 | 0,48 |
| WOB(J/L) | 0.13 (0.61) | −1.07 to 1.35 | 26.26 | 0,43 |

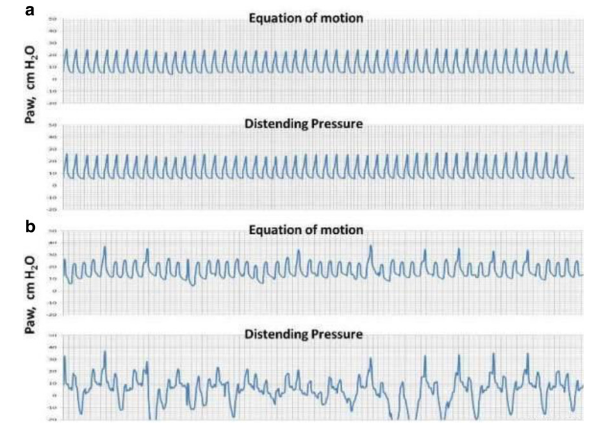

**Fig. 109 (abstract 0322).** Distending Pressure vs Eq Motion

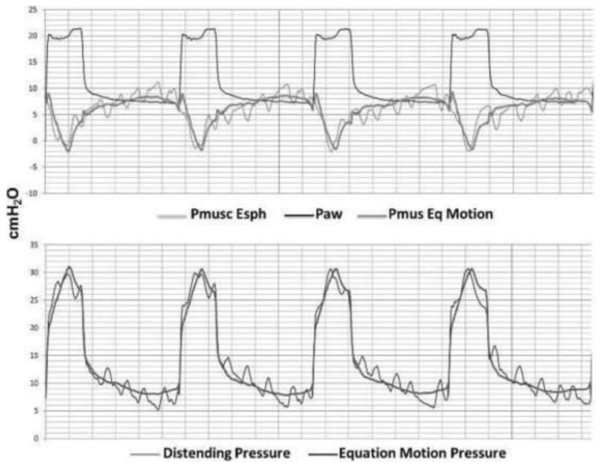

**Fig. 110 (abstract 0322).** Signals

### 0323

#### Alveolar collapse and overdistension in patients with ARDS versus post cardiac thoracic surgery patients assessed by electrical impedance tomography

S. Heines, U. Strauch, M. C. van de Poll, P. M. Roekaerts, D. C. Bergmans
Maastricht University Medical Centre+, Intensive Care, Maastricht, Netherlands
**Correspondence:** S. Heines
*Intensive Care Medicine Experimental* 2017, **5(Suppl 2):**0323

**INTRODUCTION.** Lung recruitment decreases alveolar collapse (CL) but may induce regional overdistension (OD). "Optimal PEEP" can be defined as a PEEP level where CL and OD are balanced. However, the effect of incremental or decremental PEEP steps on OD and CL between patients post cardiac thoracic surgery (CTS) and with ARDS is different. Electrical impedance tomography (EIT) enables visualization of CL and OD[1].

**OBJECTIVES.** To compare the effect of PEEP on CL and OD in ARDS and post-CTS.

**METHODS.** Twenty-nine ARDS patients (mean $PaO_2/FiO_2$-ratio 141, SD 55) and 17 CTS patients (mean $PaO_2/FiO_2$-ratio 339, SD 73) were retrospectively analysed. Overdistension and CL were measured by EIT during an incremental and decremental PEEP trial of 4 steps, each with a delta pressure of 2 $cmH_2O$. The balance between overdistended and collapsed alveoli is calculated by adding CL to OD (ODCL). An ODCL of 0% indicates an optimal balance between CL and OD. Data are expressed as mean (SEM). Changes in ODCL between ARDS and CTC were tested using 2-way ANOVA.

**RESULTS.** At baseline ODCL in CTS patients was 22% (1,5) and 14% (1,2) in patients with ARDS. During the increment in PEEP, ODCL significantly increased to 30% (1,7) in CTS and 32% (1,3) in ARDS ($p < 0.001$). The increase and decrease was significantly different in both groups ($p < 0.0001$, Fig. 111). The changes in ODCL are more gradual in the CTS group.

**CONCLUSIONS.** Alveolar OD and CL occur simultaneously and add up to the ODCL. The ODCL can be influenced by increasing PEEP, making the OD more pronounced than CL. During the incremental PEEP trial the increase of OD is larger in ARDS patients than in post-CTS patients, which is probably due to a lower respiratory system compliance. At the beginning of the decremental PEEP trial the ODCL is more optimal in the post-CTC patients, which could be explained by a better alveolar recruitability. When determining optimal PEEP settings, variations in the balance between OD and CL should be taken into account. To this end visualization of local ventilation by EIT appears to be a useful tool.

**REFERENCE(S)**

1. Costa E, et al., Intensive Care Med 2009;**35**(6):1132–7

**GRANT ACKNOWLEDGMENT**
No grant was received for this study.

Changes in percentages of ODCL in patients with ARDS (blue triangle) and post CTS patients (red dot) during an incremental and decremental PEEP trial.

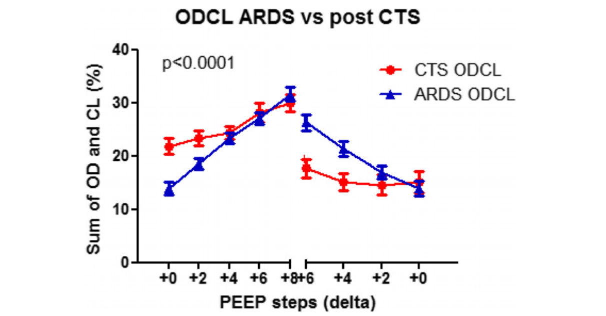

**Fig. 111 (abstract 0323).** See text for description

### 0324

#### Regional lung perfusion measured by electrical impedance tomography and single photon emission computed tomography

T. Muders[1], B. Hentze[1,2], S. Leonhardt[2], G. Hedenstierna[3], H. Wrigge[4], C. Putensen[1]
[1]University of Bonn, Department of Anaesthesiology and Intensive Care Medicine, Bonn, Germany; [2]RWTH Aachen University, Philips Chair for Medical Information Technology (MedIT), Aachen, Germany; [3]Uppsala University, Department of Medical Sciences, Clinical Physiology, Uppsala, Sweden; [4]University of Leipzig, Department of Anaesthesiology and Intensive Care Medicine, Leipzig, Germany
**Correspondence:** T. Muders
*Intensive Care Medicine Experimental* 2017, **5(Suppl 2):**0324

**INTRODUCTION.** Pulmonary gas exchange results from the coupling between alveolar ventilation (V) and perfusion (Q). In order to individually optimize ventilator settings, combined measurement of regional V and Q might be helpful (1). Electrical Impedance Tomography (EIT) is a noninvasive imaging technique to monitor regional V at bedside (2). We recently developed an EIT-based approach to measure regional Q (3).

**OBJECTIVES.** To validate an EIT-based approach to measure regional lung perfusion against Single Photon Emission Computed Tomography (SPECT) in a porcine animal trial.

**METHODS.** After admission of the local ethics committee (Uppsala University Hospital, Uppsala, Sweden) experimental lung injury was induced in 4 pigs by repeated recruitment and derecruitment. Regional Q was measured using EIT and SPECT at PEEP levels of 0 and 15 cm $H_2O$, respectively. Injections of a conductive contrast agent (NaCl 10%, 10 ml) into a central venous catheter were performed under temporary apnea. EIT measurements (EEK 2, Draeger Medical GmbH, Luebeck, Germany) were acquired at a frame rate of 40 Hz and images of conductivity change were reconstructed from the measurements using individual finite element models (FEM). Additionally, 99mTc-labeled albumin was injected and spatial Q distribution was acquired using a dual-head gamma camera. EIT- and SPECT-derived images of relative regional Q distributions (16x16 matrix) were compared in ventral-to-dorsal and right-to-left direction, respectively, using linear correlations and Bland-Altman plots.

**RESULTS.**

**CONCLUSIONS.** EIT- and SPECT-based perfusion measurements showed good to excellent correlation and agreement. Regional lung perfusion can reliably be measured by EIT using conductive contrast agent injection during temporary apnea.

**REFERENCE(S)**

1) Pelosi, P., Abreu, M.G. de, 2015. Acute respiratory distress syndrome: we can't miss regional lung perfusion! BMC Anesthesiology 15, 35

2) Muders, T., Luepschen, H., Putensen, C., 2010. Impedance tomography as a new monitoring technique. Curr Opin Crit Care 16, 269–275.

3) Luepschen H et al. 2010, Electrical Impedance Tomography for the Assessment of Pulmonary Perfusion. Poceedings of Biosignal 2010, July 14–16

**GRANT ACKNOWLEDGMENT**

This study was supported by the German Research Council, DFG (PU 219/2-1; WR47-1-1)

**Table 101 (Abstract 0324).** See text for description

| Direction | R/L | R/L | V/D | V/D |
|---|---|---|---|---|
| PEEP (cm H2O) | 0 | 15 | 0 | 15 |
| $r^2$ | 0.69 | 0.83 | 0.96 | 0.94 |
| p | <0.001 | <0.001 | <0.001 | <0.001 |
| CoV (%) | 27 | 18 | 9.7 | 13 |

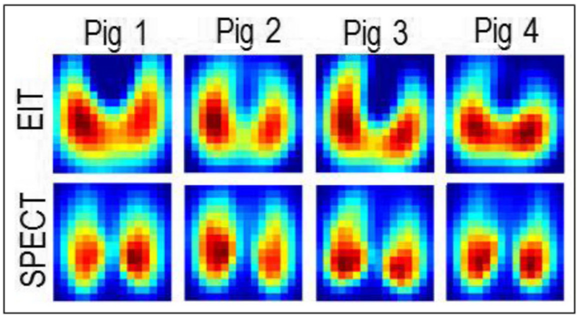

**Fig. 112 (abstract 0324).** Regional perfusion images measured by EIT and SPECT at a PEEP of 15 cm $H_2O$

### 0325

#### Effect of PEEP and esophageal catheter calibration filling volume and balloon size on the occurrence of negative transpulmonary pressure

F. Magni[1], A. Asta[1], V. Punzi[1], S. Spina[1], C. Valsecchi[1], A. Zanella[2], A. Pesenti[2], N. Patroniti[3]

[1]San Gerardo Hospital, Anesthesiology and Critical Care, Monza, Italy; [2]Università degli Studi di Milano, Department of Pathophysiology and Transplantation, Milano, Italy; [3]Università degli Studi Milano Bicocca, School of Medicine and Surgery, Milano, Italy

**Correspondence:** F. Magni

*Intensive Care Medicine Experimental* 2017, **5(Suppl 2):**0325

**INTRODUCTION.** The use of esophageal balloon catheter to estimate pleural pressure has gained renewed popularity in recent years. Indeed, measurement of transpulmonary pressure ($P_L$, computed from the difference between airway pressure and esophageal pressure) may allow a more pathophysiological-based approach to ventilator strategy in ARDS patients. The occurrence of a negative $P_L$ at end expiration ($P_L$ee) has been interpreted by some author has a tendency of the lung to collapse. A recent study has shown that setting of the PEEP level to reverse a negative $P_L$ee in acute respiratory distress syndrome (ARDS). However, other authors refused the possibility of a negative $P_L$ and interpreting it as measurement artefacts.

**OBJECTIVES.** To assess the effect of PEEP, and calibration filling volume (fv) and balloon size of the esophageal catheter on measurement PL,ee.

**METHODS.** Prospective experimental study conduct on 16 (13 ARDS, 3 non ARDS) controlled mechanically ventilated. Esophageal pressure was monitored with two different esophageal catheter: Cooper Surgical (2 ml maximum fv) in 9 patients and Nutrivent (5 ml maximum fv) in 7 patients.

Three PEEP levels were randomly applied: $low_{PEEP}$ (8 and 4 $cmH_2O$ respectively in ARDS and non ARDS patients), $medium_{PEEP}$ (12 and 8 $cmH_2O$) and $high_{PEEP}$ (16 and 12 $cmH_2O$).

At each PEEP level we inflated the esophageal balloon with increasing amount of air (from 0.2 to 2 ml and from 1 to 10 ml, respectively in Cooper and in Nutrivent). For each fv, we performed an end-inspiratory occlusion, an end-expiratory occlusion maneuver during which we applied two manual chest compression to check calibration (ΔPaw/ΔPes ratio between 0.8 and 1.2). At each fv we computed the PL,ee. Effects of PEEP, fv (expressed as percentage of the maximum volume), and catheter type on PL,ee were analyzed with a three ways ANOVA.

**RESULTS.** Acceptable calibrations were obtained only in the range of fv between 30 and 70% of maximum balloon volume and analysis was restricted to fv in the range of 30 and 70%.

PL,ee decrease with increasing fv (Fig. 113). Occurrence of negative PL,ee was higher at higher filling volume.

The effect of fv on PL,ee was significantly associated with the catheter type: catheter with bigger balloon showed a faster decrease in PL,ee; PL,ee was more negative with Cooper compared to Nutrivent catheter at fv of 60 and 70%.

PL,ee increase with increasing PEEP independently of fv and catheter type.

**CONCLUSIONS.** Even if well calibrated, the calibration filling volume and catheter type have a great effect on transpulmonary pressure measurement. Though effects of PEEP on PL,ee are not affected by calibration filling volume or catheter type, lower filling volumes and smaller balloon catheters would result in setting lower PEEP levels.

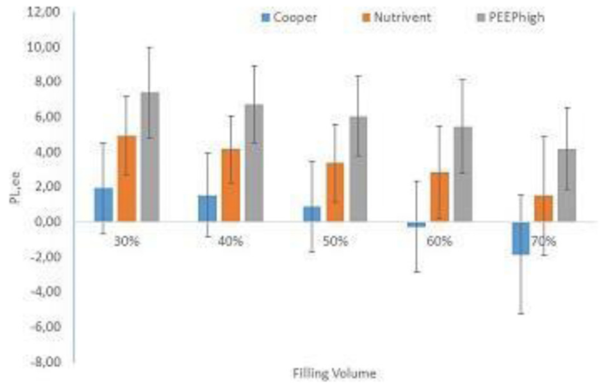

**Fig. 113 (abstract 0325).** See text for description

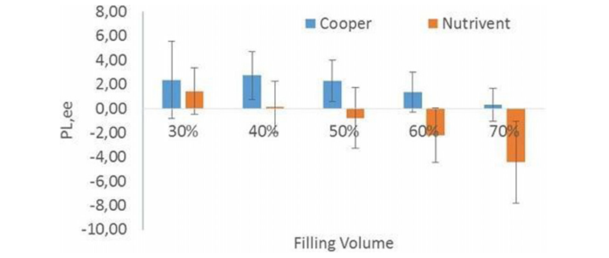

**Fig. 114 (abstract 0325).** See text for description

### 0326

#### Model-based regional lung ventilation and perfusion by electrical impedance tomography

B. Hentze[1,2], T. Muders[2], H. Luepschen[2], S. Leonhardt[1], C. Putensen[2], M. Walter[1]
[1]RWTH Aachen University, Philips Chair for Medical Information Technology (MedIT), Aachen, Germany; [2]University of Bonn, Department of Anaesthesiology and Intensive Care Medicine, Bonn, Germany
**Correspondence:** B. Hentze
*Intensive Care Medicine Experimental* 2017, **5(Suppl 2):**0326

**INTRODUCTION.** Electrical impedance tomography (EIT) is a noninvasive imaging technique that can be used to monitor regional lung ventilation (V) of mechanically ventilated patients in intensive care units (ICU) at bedside (1, 2). In order to achieve an improved adjustment of ventilation therapy, additional measures of regional lung perfusion (Q) and regional lung ventilation to perfusion ratio (V/Q) would be highly beneficial.

**OBJECTIVES.** To develop an approach to extract functional images of V, Q and V/Q from EIT measurements and validate the method in a porcine animal trial.

**METHODS.** After admission of the local ethics committee (Uppsala University Hospital, Uppsala, Sweden) a healthy pig was anesthetized and mechanically ventilated in supine position (3). Seven injections of a conductive contrast agent (NaCl 10%, 10 ml) into a central venous catheter were performed under temporary apnea at different PEEP levels (0 to 20 $cmH_2O$). During the injections, EIT measurements (EEK 2, Draeger Medical GmbH, Luebeck, Germany) were acquired at a frame rate of 40 Hz. EIT images of conductivity change were reconstructed from the measurements based on a cylindrical finite element model (FEM). Functional images of V were obtained by Principal Component Analysis (PCA). A gamma-variate model (4) was combined with a semi-negative matrix factorization to extract functional images of Q from the EIT images.

**RESULTS.**

Functional images of V (see Fig. 115, line 1) and Q (line 2) at different PEEP levels were obtained. A regional V/Q ratio (line 3) and histograms of distribution (line 4) were calculated from the images. At PEEP 0 $cmH_2O$ an inhomogeneous V/Q distribution is observed. Modification of PEEP from 0 to 20 $cmH_2O$ yields a more homogeneous V/Q which might indicate reduction in both dead space ventilation and shunt perfusion. Gradually stepping down to PEEP 0 $cmH_2O$ results in a more inhomogeneous V/Q as observed before.

**CONCLUSIONS.** The presented approach allows for reliable extraction of regional lung perfusion (Q) from EIT images.

It was possible to trace changes of regional V/Q after modification of PEEP in a porcine animal trial. The described approach might thus be a step into the direction of providing measures for improved adjustment of ventilation therapy.

**REFERENCE(S)**

(1) T. Muders et al. "Impedance tomography as a new monitoring technique", Current Opinion in Critical Care, vol. 16, no. 3, pp. 269–275, 2010.

(2) S. Leonhardt et al. "Electrical impedance tomography: the holy grail of ventilation and perfusion monitoring?" Intensive Care Medicine, vol. 38, no. 12, pp. 1917–1929, 2012.

(3) H. Luepschen et al. "Bestimmung der Lungenperfusion mittels elektrischer Impedanztomographie", 44. Jahrestagung der DGBMT, Rostock, Germany 2010.

(4) H. K. Thompson et al. "Indicator Transit Time Considered as a Gamma Variate", Circulation Research, vol. 14, no. 6, pp. 502–515, 1964.

**GRANT ACKNOWLEDGMENT**

This research is supported by a DFG grant (PU 219/2-1).

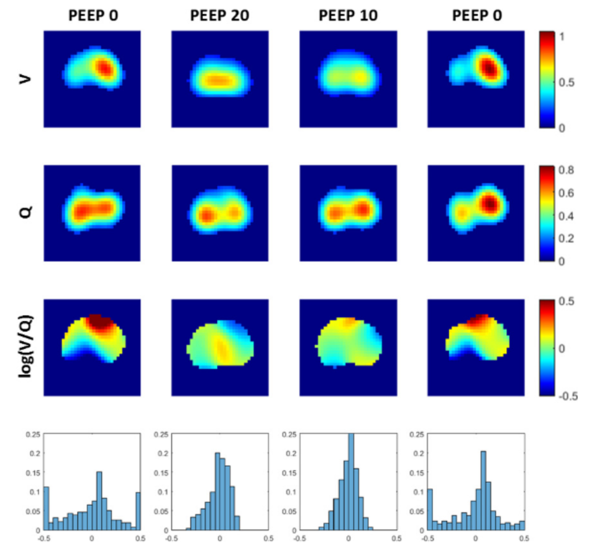

**Fig. 115 (abstract 0326).** See text for description

### 0327

#### Pleural, esophageal and transpulmonary pressure: a validation study

D. L. Grieco[1,2], E. Charbonney[3,4], S. Delisle[5], P. Ouellett[6], M. Rigollot[7], B. Badat[7], G. Bronchti[8], A. Drouet[7], D. Savary[7], J. Mancebo Cortes[5], T. Yoshida[9], B. Kavanagh[9], M.B. Amato[10], A. Mercat[11], J.C.M. Richard[7,12], L. Brochard[1], CAVIAR Group of Researchers
[1]St. Michael's Hospital/University of Toronto, Keenan Centre for Biomedical Research, Li Ka Shing Knowledge Institute/Interdepartmental Division of Critical Care Medicine, Toronto, Canada; [2]Catholic University of The Sacred Heart, Anesthesiology and Intensive Care Medicine, Rome, Italy; [3]Université de Montréal, Hôpital du Sacré-Coeur de Montréal, Montreal, Canada; [4]Université du Quebec à Trois-Rivières, Trois-Rivieres, Canada; [5]Centre de Recherche du Centre Hospitalier Universitaire de Montréal (CHUM), Montreal, Canada; [6]Vitalité Health Network, Zone 4, Edmundston, Canada; [7]General Hospital, SAMU 74, Emergency and ICU Department, Annecy, France; [8]Université du Quebec à Trois-Rivières, Anatomy Department, Trois-Rivieres, Canada; [9]Hospital for Sick Children, Physiology and Experimental Medicine, Departments of Critical Care Medicine and Anesthesia, Toronto, Canada; [10]Heart Institute (Incor) Hospital das Clínicas da Faculdade de Medicina da Universidade de São Paulo, Laboratório de Pneumologia LIM-09, Disciplina de Pneumologia, Sao Paulo, Brazil; [11]Angers University Hospital, Critical Care Department, Angers, France; [12]INSERM UMR 955, Creteil, France
**Correspondence:** D. L. Grieco
*Intensive Care Medicine Experimental* 2017, **5(Suppl 2):**0327

**INTRODUCTION.** The measurement of Esophageal pressure (PES) permits to estimate pleural pressure (PPL) and to compute transpulmonary pressure (PL), which is the amount of pressure actually dissipated across the lung independently from the effects of the chest wall. PL is increasingly used in ARDS patients for monitoring and, possibly, to tailor interventions regarding ventilator settings. Nonetheless, it is still unclear how reliable is PES in estimating PPL.

**OBJECTIVES.** We conducted a study on Thiel cadavers (an embalming method that maintains the elastic and dynamic properties of the corpse) to assess the relationship between PES and

PPL directly measured in sternal (non-dependent) and vertebral (dependent) regions (PPLs and PPLv). We previously showed that Thiel cadavers' respiratory mechanics are similar to ARDS patients [1].

**METHODS.** Three cadavers from a donation program of the Université du Quebec à Trois-Rivieres were studied in the supine position. To standardize lung volume history, each cadaver was intubated, recruited and mechanically ventilated for 30–60 minutes with Vt 6 = ml/Kg PBW and PEEP = 20cmH2O.

An oesophageal balloon was inserted to monitor PES. PPLs and PPLv were measured through dedicated sensors (wafer-type, flat balloon) surgically implanted in the pleural space and inflated with 'non-stress' volume.Chest x-ray and ultrasound excluded the presence of pneumothorax. A 4-step decremental PEEP trial (20 to 5 cmH2O) was then performed, with each step kept for 15 minutes.

Airway pressure (PAW), flow, PES, PPLs, and PPLv were recorded and analysed during the last minutes of each step.

We evaluated the relationship between PES, PPLs, PPLv and the corresponding PL at end-expiration and end-inspiration: end-expiratory and end-expiratory PL were computed as the absolute difference between PAW and PES, PPL,and PPLv.in static conditions. End-inspiratory PL was also calculated according the Elastance-derived method.

**RESULTS.** At all PEEP levels, end-expiratory and end-inspiratory absolute PES values were intermediate between PPLv and PPLs. Consistently, end-inspiratory and end-expiratory PL computed as PAW-PES were at a mid-value between PL measured using PPL in the sternal and vertebral regions (Fig. 116a-b). Elastance-derived PL at end-inspiration was close to the directly measured PL in non-dependent regions (Fig. 116b).

**CONCLUSIONS.** The difference between the PAW and PES values accurately reflects transpulmonary pressure at a mid-chest between sternal and vertebral regions. In the supine position, end-inspiratory PL derived from lung-elastance allows to estimate the distending pressure in sternal, non-dependent lung regions. More data in specific groups of patients (obese etc.) are needed to fully validate this concept.

**REFERENCE(S)**

1. Delisle S, Charbonney E, Gosselin P, et al. (2016). Study Of Ventilation During Cardio Pulmonary Resuscitation (cpr): A New Model Using Thiel Cadavers. Am J Respir Crit Care Med 193;2016:A7030

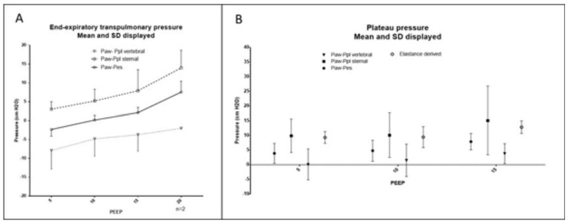

**Fig. 116 (abstract 0327).** Transpulmonary pressure

### 0328

#### Use of esophageal pressure in the ICU patients with multiple mechanical abnormalities And chest wall problems

I. Kara[1], N. Boyacı[2], K. Inci[2], B.B. Aydogan[3], G. Gursel[3]
[1]Gazi University School of Medicine, Ankara, Turkey; [2]Gazi University School of Medicine, Department of Internal Medicine, Division of Critical Care, Ankara, Turkey; [3]Gazi University School of Medicine, Department of Pulmonary Critical Care Medicine, Ankara, Turkey
**Correspondence:** G. Gursel
*Intensive Care Medicine Experimental* 2017, **5(Suppl 2):**0328

**INTRODUCTION.** The respiratory changes in esophageal pressure (Pes) are representative of changes in pleural pressure. The difference between plato airway pressure (Ppl) and Pes is a valid estimate of transpulmonary pressure(TPP). Recently it has been shown that setting respiratory parameters according to inspiratory and expiratory TPP may allow more accurate tidal volume and PEEP settings and improve oxygenation.

**OBJECTIVES.** In this study we wanted to evaluate the use of esophageal pressure measurements in mechanically ventilated ICU patients who has multiple respiratory system mechanical abnormalities including chest wall problems.

**METHODS.** All measurements were performed (AVEA ventilator, CareFusion) while patients sedated, paralyzed and ventilated with volume control mode. Peak, plato, esophageal pressures were measured and inspirtory, expiratory transpulmonary pressures(TPPi, TPPe) driving pressure, transpulmonary driving pressure and total respiratory system, lung and chest wall elastances were calculated. Patients were classified according to mechanical abnormalities of the respiratory system as airway, paranchimal and chest wall abnormalities(CWA).

**RESULTS.** 28 patients were included in the study. Mean age was 67 and mean APACHE II scores was 28.Sixty-one percent(17) of the patients had airway, 50%(14) patients had chest wall abnormalities(obesity, massive pleural effusion, pleural thickening, kyphoscoliosis) and all patients had paranchimal problems(like edema, pneumonia, fibrosis). Mean Pplat was 24 ± 3 in CWA positive group(CWAPG)and 20 ± 5 cmH2Oin CWA negative group)CWANG(p:0.001). Mean driving pressure was 21 ± 2.2 in CWAPG and 14 ± 5 cmH2O in CWANG(0.0001). Mean transpulmonary driving pressure was 15.5 ± 5.4 in the CWAPG and 11.6 ± 4.7 cmH2O in the CWANG, p:0.51. Mean TPPi was 16 ± 10 in CWAPG and 13 ± 7 in CWANG p:0.311. In 10 patients TPPe was negative(mean −8 cm H2O). While mean Pplat and total resp system elastance were significantly higher in CWAPG than the CWANG, TPPi and transpulmonary driving pressures were similar across the groups.

**CONCLUSIONS.** Monitoring Pes might be more useful than Pplat in ICU patients with CWAs to better understand underlying mechanical problems and safer ventilatory settings.

**REFERENCE(S)**

1. Talmor D, Sarge T, Malhotra A et al. (2008) Mechanical ventilation guided by esophageal pressure in acute lung injury. N Engl J Med 359:2095–2104.

2. Mauri T,et al. (2016) Esophageal and transpulmonary pressure in the clinical setting: meaning, usefulness and perspectives. Intensive Care Med 42:1360–1373

**GRANT ACKNOWLEDGMENT**

Study was supported by Gazi University Scientific Reserch Projects Foundation(Project No: 01/2015-48).

## Quality issues in ICU

### 0329

#### Using indicators to improve the quality of care: an example of unplanned extubation in patients with mechanical ventilation in the surgical ICU

K. S. Chan[1], M. I. Sung[2]
[1]Chi Mei Medical Center, Department of Intensive Care Medicine, Tainan, Taiwan, Province of China; [2]Chi Mei Medical Center, Tainan, Taiwan, Province of China
**Correspondence:** K. S. Chan
*Intensive Care Medicine Experimental* 2017, **5(Suppl 2):**0329

**INTRODUCTION.** Unplanned extubation (UE) is an unprecedented happening in intensive care unit (ICU); which may lead to severe complications in patients. It has been considered as an important quality indicator of care and recognized as the most common airway adverse event in the ICU.

**OBJECTIVES.** The objective of this study was to investigate that if using indicators could improve the care quality by reducing the incidence rate of unplanned extubation in the ICU.

**METHODS.** This study was conducted in a medical center with a total capacity of 1276 beds with inclusive of 38 surgical ICU beds. International quality indicator project was firstly introduced in the year 1999, improvement initiatives were implemented since year 2001. The improvement initiatives, for examples, standardization of procedures, improvement of communication skills, revision of sedation and weaning protocols, changing strategy for physical restraints, establishment of task force for identification and management of high-risk patients, implementation of quality improvement models including Breakthrough Series (BTS) and Team Resource Management(TRM) as well as new fixation method of endotracheal tube, were launched during different periods to reduce the incidence rate of unplanned extubation in mechanically ventilated patients.

**RESULTS.** The overall incidence rate of unplanned extubation decreased from 6.4% in the year 2001 to 0.8% in the year 2016 without prolonged days of mechanical ventilation. In addition, rate of failed UE was increasing over the years from 42.7% in the year 2001 to 88.9% in the year 2016 and it represented that the low possibility of delayed extubation among these patient.

**CONCLUSIONS.** In conclusion, using indicators as the reference of the implementation of serial improvement initiatives could reduce the incidence of unplanned extubation in the ICU without prolonged length of mechanical ventilation.

**REFERENCE(S)**

1. da Silva PS, Fonseca MC. Unplanned endotracheal extubations in the intensive care unit: systematic review, critical appraisal, and evidence-based recommendations. Anesth Analg 2012: 114:1003–1014.

2. Jarac hovic M, Mason M, Kerber K, McNett M. The role of standardized protocols in unplanned extubations in a medcal intensive care unit. Am J Crit Care 2011;20:304–11.

**GRANT ACKNOWLEDGMENT**

None

### 0330

#### Cost and health outcomes associated with mechanical ventilation: a cost-effectiveness evaluation in the United Kingdom

R. Saunders[1], D. Georgopoulos[2]

[1]Coreva Scientific GmbH & Co KG, Freiburg, Germany, [2]University of Crete, University Hospital of Heraklion, Heraklion, Greece

**Correspondence:** R. Saunders

*Intensive Care Medicine Experimental* 2017, **5(Suppl 2):**0330

**INTRODUCTION.** Mechanical ventilation is a lifesaving and integral part of intensive care unit (ICU) medicine. It is, however, an expensive intervention and is not problem free. Optimizing use and outcomes of mechanical ventilation (MV) could result in substantial cost savings and improved patient outcomes.

**OBJECTIVES.** Evaluate the cost-effectiveness of proportional assist ventilation (PAV + TM ventilation [PAV + TMV]) compared with pressure support ventilation (PSV).

**METHODS.** A cohort-level, in-silico, clinical model was built using data from recent clinical studies and randomized, controlled trials (RCTs). The model estimates the incidence of patient-ventilator asynchrony >10%, tracheostomy, ventilator-associated pneumonia (VAP), other nosocomial infections, spontaneous breathing trial (SBT) success, hypoxemia, and death. Patients, mean 55.4 years and 37.7% female,[1] are on MV until a successful SBT or until they leave the ICU. Time on MV is influenced by asynchrony >10% and VAP. MV is either PSV or PAV + TMV, with differences between these interventions taken from RCTs.[2,3] PAV + TMV was associated with lower asynchrony, shorter time on MV and in the ICU, but longer time in hospital. Mortality was estimated from rates in peer-reviewed literature up to 3 years and with UK life tables thereafter. Quality-adjusted life expectancy (QALE) used EQ5D utilities. All event costs are taken from peer-reviewed literature and expressed in 2015 £. The time horizon extends from 1 to 40 years. Probabilistic sensitivity analysis provides estimates of outcome significance via the credible interval (CrI).

**RESULTS.** Over 40 years, the mean cost of care per patient was £56,508 with PSV and £59,403 with PAV + TMV, difference GBP 2,895. PAV + TMV increased life expectancy by 2.06 years (13.82 vs. 15.87) and QALE by 0.97 quality-adjusted life years (QALYs, 6.47 vs. 7.44). The cost per life year and QALY gained with PAV + TMmode was £1,407 and £2,987, respectively. PAV + TMV reduced mean days on MV from 6.8 to 5.3 and days with VAP from 7.2% to 5.4%. In 2,000 simulations using sampled input parameters, PAV + TMV reduced costs (95% CrI −7,743 to +19,078) in 29% of simulations and increase QALE (95% CrI −0.5 to +2.5) in 91%. At a willingness to pay threshold of GBP 30,000 per QALY gained, PAV + TMV was cost-effective in 89% of simulations. If patients' NHS costs after discharge were excluded then PAV + TMV would reduce costs in 62% of simulations. With a time horizon of 1 and 5 years, PAV + TMV was more likely than PSV to be cost saving and maintained its increase in QALE in 91% of simulations.

**CONCLUSIONS.** PAV + TMV is likely to be considered a cost-effective alternative to PSV in the UK. In the short term, PAV + TMV increased patient life expectancy and decreased healthcare costs.

**REFERENCE(S)**

1. Marti. *J. Intensive Care* **4,** 56 (2016).

2. Bosma. *Crit. Care Med.* **44,** 1–11 (2016).

3. Xirouchaki. *Intensive Care Med.* **34,** 2026–2034 (2008).

**GRANT ACKNOWLEDGMENT**

Medtronic Inc. (Boulder, CO)

### 0331

#### Value of arterial blood gas measurements in intensive care unit

S. Thienchairoj

Ramathibodi Hospital, Department 270, Bangkok, Thailand

*Intensive Care Medicine Experimental* 2017, **5(Suppl 2):**0331

**INTRODUCTION.** Arterial blood gas (ABG) ability to be performed at the patient's bedside, and its rapid analysis make it an important tool used by physicians to direct and redirect the treatment of their patients, especially in patients who are critically ill.

**OBJECTIVES.** We aim to assess the value of the ABG is necessary and appropriate in Intensive care unit (ICU) situation.

**METHODS.** This prospective observational study was conducted in adult patients who admitted in ICU. Residents, fellows or staff who were intimately involved in the care of the patient at the time of the ABG test responded to the ABG survey such as for reason, interesting parameters and change in management that response to ABG test results.

**RESULTS.** There were 274 patients had been enrolled in this study which 155 patients from MICU and 119 patients from SICU. We collected 837 ABG tests performed in the laboratory. The most common reasons for requesting an ABG test were changes in ventilator settings 30.8% (258/837). Of the clinicians, 14.5% (121/837) selected routine as their reason for ordering an ABG test in the morning before round ward. The most interesting parameters in the ABG test results was $PaO_2$ that showed 34.5% (289/837) tests then pH and PaCO2, respectively. A change in patient management occurred in 438 times (52.3%). There was a change in ventilator settings in 91.6% (401/438) of all management. The changes in ventilator settings were 401 times (93.2%) of all management, 22.4% (98/438) consisted of a change in $FiO_2$.

**CONCLUSIONS.** Sometime though testing may not be indicated or change management that maybe unnecessary testing eg. routine or change in $FiO_2$. If we reduce in unnecessary testing that help to decrease risk from blood exposure, workload in healthcare providers, blood loss and incremental cost saving.

**REFERENCE(S)**

1. Wang TJ, Mort EA, Nordberg P, et al. A utilization management intervention to reduce unnecessary testing in the coronary care unit. *Arch Intern Med.* 2002;162:1885–1890.

2. Brzezinski M, Luisetti T, London MJ. Radial artery cannulation: a comprehensive review of recent anatomic and physiologic investigations. *Anesth Analg*. 2009 Dec. 109(6):1763–81.

3. Pawson SR, DePriest JL. Are blood gases necessary inmechanically ventilated patients who have successfully completed a spontaneous breathing trial? *Respir Care.* 2004;49:1316–1319.

### 0332

#### A look into quality standards for management and documentation of interhospital transfers from a district general hospital. Is what we are doing adequate?

G. Wong, S. Cole, S. Pearson, H. Agostini
Colchester Hospital University Foundation NHS Trust, Critical Care, Colchester, United Kingdom
**Correspondence:** G. Wong
*Intensive Care Medicine Experimental* 2017, **5(Suppl 2):**0332

**INTRODUCTION.** Good communication is crucial when transferring critically ill patients between hospitals as they are exposed to potential physiological compromise. “The magnificent seven” are evidence-based quality aspirations, agreed by all critical care units in the East of England. One standard is all interhospital transfers are conducted by adequately trained personnel with adequate equipment and monitoring[1]. Our 14-bedded District General ICU employs a regional transfer form in in order to ensure standardisation and document this process.

**OBJECTIVES.** Assessing all interhospital transfers initiated from our hospital over a 12-month period, we aimed to determine whether those clinicians involved in critical care transfer had adequate transfer training; and to evaluate adequacy of documentation and handover.

**METHODS.** A retrospective audit was conducted of all interhospital transfer forms completed in 2016. Seniority of clinician and reason for transfer were analysed. The target standard was that all areas of the transfer form should have been completed, especially relevant times and observations during transfer including pupils for neurosurgical cases.

**RESULTS.** We reviewed 23 interhospital transfer forms, yet were aware of at least 6 transfers for which the forms were never completed. The majority were for clinical reasons (96%) with 52% for neurosurgical intervention. There was no documentation regarding critical incidents in 57% of cases, though subjectively in all 23 cases the observations recorded suggested that the patient had been stable during transfer. Documentation of medications administered was poor and observations recorded during transfer were inadequate in 52% of cases missing at least 1 vital component for the duration of the transfer for example a lack of pupillary responses for neurosurgical cases. Level 3 transfers were all managed by anaesthetists, 88% had at least 2 years experience. There was one anaesthetist escort who had not attended a transfer course and 5 where it was unknown whether they had attended a course however all other anaesthetists had been on a transfer course in the prior 4 years.

**CONCLUSIONS.** Transfer documents were not used for all cases and when used, documentation was almost universally inadequate. We have highlighted the availability and location of transfer forms. We have raised awareness amongst nursing staff to help avoid cases being missed where locum anaesthetists are involved. Attendance of the local transfer course is now mandatory for novice anaesthetic trainees prior to starting indirectly supervised on calls. The department discussed that evaluation of available personnel would occur on an individual case basis within the on call anaesthetic and ICU teams. A re-audit will occur after changes have been implemented.

**GRANT ACKNOWLEDGMENT**
Nil

### 0333

#### Understanding quality measurement in intensive care units: healthcare professionals' knowledge of quality indicators and attitudes to quality assessment

A. Breen
Leeds Teaching Hospitals NHS Trust, Department of Adult Critical Care, Leeds, United Kingdom
*Intensive Care Medicine Experimental* 2017, **5(Suppl 2):**0333

**INTRODUCTIONS.** Quality measurement in healthcare is based on a construct devised over 50 years ago, but as healthcare has become more complex, so too has the measurement of quality in healthcare. The delivery of quality improvement might be improved by knowledge of how well healthcare professionals understand quality measurement.

**OBJECTIVES.** This study examines the perspectives of healthcare professionals working in intensive care units on the methodology and use of quality indicators, and the scientific basis on which they are chosen. The study also aims to ascertain attitudes to quality measurement amongst healthcare professionals, and the potential benefits of improving their understanding of these quality indicators.

**METHODS.** A cross-sectional inquiry was performed amongst doctors, nurses, allied health professionals and managers working in intensive care units. A questionnaire was designed to elicit the level of understanding and views on quality measurement in intensive care units, and this was conducted using an online survey platform.

**RESULTS.** 273 healthcare professionals responded. They demonstrated a good understanding of the quality indicators in use at a national and local level within intensive care medicine, but there were important differences in how these indicators are perceived between doctors and nurses. In particular, doctors appear to place less emphasis on patient safety indicators than nursing staff do. There was good support for adherence to care-bundle approaches to improving quality. Patient safety was found to be more highly valued than other dimensions of healthcare quality. Respondents indicated that quality of care may be enhanced with improved education on quality measurement methodology, and improved feedback of performance against a range of quality indicators.

**CONCLUSIONS.** It is recommended that quality measurement and quality improvement methodology be integrated at an early stage into educational programmes for nursing and medical staff working in intensive care medicine, and that feedback to staff should be broadened to include clinical effectiveness measures as well as patient safety measures.

### 0334

#### Exploring a quality improvement collaborative for enhancing antimicrobial stewardship programs in intensive care units: insights from clinicians

L. Jeffs[1], M. Law[2], M. Zahradnik[3], M. Steinberg[4], M. Maione[3], A. Morris[4], C. Bell[4], Y. Nakamachi[4]
[1]St Michael's Hospital in Toronto, Li Ka Shing Knowledge Institute, Keenan Research Centre, Toronto, Canada; [2]Brock University, St Catharines, Canada; [3]St Michael's Hospital, Toronto, Canada; [4]Sinai Health System, Toronto, Canada
**Correspondence:** L. Jeffs
*Intensive Care Medicine Experimental* 2017, **5(Suppl 2):**0334

**INTRODUCTION.** Antimicrobial Stewardship Programs (ASPs) are being employed to mitigate increasing antimicrobial resistance rates and complications associated with inappropriate and unnecessary prescriptions. Evidence exists on the benefits of ASPs however, less is known about what program factors and conditions influence the sustainability of ASPs in the intensive care unit (ICU) environment.

**OBJECTIVES.** To explore clinician's experiences associated with an integrated Quality Improvement (QI) and Knowledge Translation (KT) strategy aimed at improving ASPs in ICUs in four large academic hospitals in Ontario, Canada.

**METHODS.** The QI-KT strategy focused on QI methods and sustainability of change and involved five learning modules delivered in an interactive Communities of Practice model with an assigned QI mentor. Each team implemented an AS project that focused on improving antimicrobial use in the ICU setting. Projects included the reduction of

1) unnecessary use of antimicrobials in patients with possible ventilator-associated pneumonia,
2) duration of empiric therapy, and
3) vancomycin prescription; and an
4) increase in appropriate use and days of therapy.

A qualitative research design was employed including focus groups with clinicians followed by a constant comparative data analysis to examine emergent themes.

**RESULTS.** A total of 25 individuals participated in the six focus groups conducted in January 2016 (n = 15) and December 2016/ January 2017 (n = 10) with participating ICU clinicians. Key themes regarding their experiences included:

1) the QI-KT Collaborative helped to keep projects on track and aided in scoping projects to manageable change ideas;
2) having leadership and local level stakeholder engagement enhanced the uptake of the QI activities;
3) embedding changes in daily work flow through their focused project work aided in sustaining optimal antimicrobial use;
4) learning from other project teams aided in the development of individual projects; and
5) their engagement in the collaborative provided confidence for scaling the ASP activities to other areas.

**CONCLUSIONS.** Participants valued the QI-KT learning strategy and its usefulness to sustain appropriate antimicrobial prescriptive practices in the ICUs and to spread to other units in their respective hospitals. Findings from the larger study are informing the development of a toolkit that can be used by other ICUs in their efforts to improve and sustain their ASPs.

**GRANT ACKNOWLEDGMENT**
The authors would like to acknowledge the Canadian Institutes of Health Research for the funding for this project.

### 0335

#### Description of our protocol to detect the needs of patients with mechanical ventilation

R. Fernandez, R. Sánchez Rico, P. Morales Laborías, M. Á. Díaz Castellanos
Hospital Santa Ana, Motril, Spain
**Correspondence:** R. Fernandez
*Intensive Care Medicine Experimental* 2017, **5(Suppl 2):**0335

**INTRODUCTION.** Patients with mechanical ventilation (MV) in ICU is a challenge for professionals because of the complexity of the care and the multiple potential complications.

**OBJECTIVES.**
- Improve the quality of care that we provide to our patients.
- Describe the tools needed to detect the needs of those patients
- Describe the development of the individualized our care plan

**METHODS.**
- Descriptive study. Start 01-01-16.
- 8-bed ICU. Hospital district of sanitary area of about 160,000 people.
- We followed the model of Virginia Henderson.
- It included all staff of ICU, a total of 31 professionals.
- We used international taxonomy NANDA, NOC, NIC and clinimetric tables of
valuation.
- The nurses´ interventions were put in common in the clinical sessions.
- Started from the medical profile of our patients, we elaborated a profile of the patient in MV. We detected the altered needs of these patients admitted using the Virginia Henderson model.

**RESULTS.** Nurses´ diagnoses were associated with the indicators and with a specific intervention for each patient, we organized the activities to carry out.

The results of the assessments unit following the model of Virginia Henderson, are

*AUTONOMY PROBLEMS:* food and hydratation, urinary and fecal elimination, mobilization, cleanliness, body temperature maintenance.

*CARE PLAN: Nursing Diagnostics* 00146 anxiety, 00148 fear, 00051 deterioration of verbal communication, 00034 dysfunctional response to weaning, 00095 deterioration of the sleep pattern, 00128 acute confusión.

*COLLABORATION ISSUES*: *Ineffective ventilatory function*: NIC 6680 Monitoring vital signs, NIC 3120 Intubation and stabilization of airways, NIC 3300 Mechanical ventilation, NIC 3180 Management of artificial airways, NIC 1910 Acid–base management, NIC 2300 Medication administration, NIC 7780 Management of technology.

*DISCONNECTION OF MECHANICAL VENTILATION (MV):* NIC 6880 Monitoring vital signs, NIC 3310 Disconnection from mechanical ventilation, NIC 3270 Tracheal extubation

*PROBLEMS ASSOCIATED WITH MV* : pneumonia, atelectasia, retention of secrections, barotrauma-neumotorax, renal, hemodynamic, gastrointestinal, neurological complications

**CONCLUSIONS.** The establishment of care plans allows the logical, rational and systematic delivery of care through the scientific method, supporting, supplying and helping the patient to cover their needs individually.

It also allows the detection of deficiencies in plans already initiated, being also an important tool in the orientation and help for the staff.

During the implementation of the care plan, we needed the family help in order to fully and comprehensively evaluate our patients, since they are sometimes sedated or with serious communication problems. In this way we have also detected in the main caregivers problems of independence that can be treated by nursing, which has given us the opportunity to develop a specific care plan for the family.

### 0336

#### An audit of unit acquired infections in the blood: report from a tertiary referral adult intensive care unit in the United Kingdom

J. Pakpoor[1], A. Ward[1], A. Wong[1], R. Jayaram[2]
[1]Oxford University Hospitals NHS Foundation Trust, Adult Intensive Care Unit, Oxford, United Kingdom; [2]University of Oxford, Nuffield Department of Clinical Neurosciences, Oxford, United Kingdom
**Correspondence:** R. Jayaram
*Intensive Care Medicine Experimental* 2017, **5(Suppl 2):**0336

**INTRODUCTION.** The Case Mix Programme (CMP) of Intensive Care National Audit and Research Centre (ICNARC), United Kingdom (UK)[1] is an audit of patient outcomes and selected quality measures from patient-level data submitted by 227 intensive care units (ICU). Standards for the provision of adult critical care services in the UK were published in 2015 as the 'General Provision of Intensive Care Services' (GPICS). ICNARC's annual quality report describes metrics pertaining to patient outcomes, process and sepsis and provides a high-level 'dashboard' of the hospital's performance against GPICS standards. Review of the ICNARC quality report - 2016 to our hospital suggested that sepsis-related metrics are a cause for concern due to high rates of "unit-acquired infection" in the blood defined as the presence of an infection in any blood sample taken for microbiological culture 48hrs or more after admission to the ICU. If more than one organism is isolated, the primary organism isolated in two rather than one blood culture bottle is given priority (except for MRSA, which takes precedence). Potential contaminants such as Staphylococcus epidermidis are excluded.

**OBJECTIVES.** Audit of the specific quality measure of "Unit acquired infections in blood" flagged up as an area of concern as compared to national benchmarks.

**METHODS.** Two investigators independently verified, the accuracy of the database of patients coded as having unit acquired infection in the blood, by checking corresponding electronic case records. True positive results were analysed further by referring to the notes by the Microbiologist and Infectious Diseases specialist. For confirmed unit acquired infections in the blood, source of bacteraemia ascertained by analysis of electronic patient records.

**RESULTS.** Out of twenty patients flagged positive, thirteen patients did not meet the criteria for unit-acquired infections in blood and five patients had ICU-acquired catheter-related blood stream infections. Reasons for false positive results included reporting non-blood specimens such as bronchoalveolar lavage or ascites fluid, Staphylococcus epidermidis flagging incorrectly as Staphylococcus aureus, blood cultures with an organism that initially flagged within the first 48 hours and should, therefore, have been excluded and blood cultures with an organism that initially flagged pre-ICU admission and should therefore have been excluded. The risk-adjusted hospital mortality and selected quality measures did not give rise to any significant concerns when compared to 227 UK Critical Care Units.

**CONCLUSIONS.** A detailed audit of unit-acquired infection in blood demonstrates systematic errors in reporting rather than a major clinical concern in an adult ICU acting as a tertiary referral centre for critical care, regional vascular and major trauma services, and a large transplant department in the UK.

**REFERENCE(S)**

1) *Crit Care*. 2004, 8: R99–R111. 10.1186/cc2834

### 0337

#### Evaluation of implementing a pressure ulcers prevention bundle in a mexican intensive care unit

S. E. Zamora Gómez[1], J.O. Montoya Rojo[1], E. Lozano Cruz[2], M.A. Amezcua Gutierrez[1], J. Garduño López[1], E.S. Cruz Montesinos[1], B. Bolado Morales[3], M.A. Carrasco Flores[1], M. Hernández Romero[1], M.A. Toledo Rivera[1], G.D. Hernández López[1]
[1]Hospital Juárez de México, Intensive Care Unit, Mexico, Mexico; [2]Hospital Juárez de México, Quality Division, Mexico, Mexico; [3]Hospital General del Estado de Sonora, Emergency Deparment, Hermosillo, Mexico
**Correspondence:** S. E. Zamora Gómez
*Intensive Care Medicine Experimental* 2017, **5(Suppl 2):**0337

**INTRODUCTION.** The incidence of hospital acquired pressure ulcers (HAPUs) in intensive care unit (ICU) is high and numerous strategies have been implemented as a comprehensive management of critical ill patients. Patients with pressure ulcers (PUs) experience significantly increased morbidity, mortality and financial burden; confirming that PUs prevention is an essential component of patient care; immobility is a significant PUs risk factor[1], being prone position (PP) a major cause of it in severe ARDS patients. In this study we compare the incidence of PUs before and after an implementation of evidence based care bundle[2].

**OBJECTIVES.** Evaluate the impact in the incidence of PUs before and after the implementation of a prevention care bundle.

**METHODS.** Retrospective-prospective observational study in a tertiary level intensive care unit including patients < 65 years who developed PUs during ICU stay. In a retrospective way we included patients at ICU from January 2016 to September 2016, evaluating the incidence and risk factors with a conventional care; the prospective observational phase was from October 2016 to March 2017 after the implementation of a PUs prevention bundle. PUs incidence was analyzed in both groups. The care bundle is shown on Table 102.

**RESULTS.** In retrospective phase 212 patients were admitted in a 6 months period, 25 (12%) patients developed PUs including 11 patients in PP, 17 (68%) males and 8 (32%) females, the mean BMI was 30.84 m2/kg, mean age was 40.32, mean APACHE II score at admission was 20.16, mean ICU stay of 13.12 days, and average albumin at admission of 2.7 g/dl and 19 (76%) patients has high (<12) Brader score.In prospective phase of the study, 163 patients were admitted in a second 6 month period, 31 (19%) patients developed PUs, 24 were in PP, 24 males (77.42%) and 7 females (22.58%), the mean BMI was 28.89 m2/kg, mean age 35.58 years, mean APACHE II of 19.45, mean ICU stay 14 days, mean albumin at admission 2.55 g/lL and 26 (83.8%) patients has high Brader score. In a sub analysis of the data excluding the prone position patients the incidence was of 7% before and 5% after care bundle implementation.

**CONCLUSIONS.** PUs is a worrying condition in intensive care unit, the implemented bundle care has promising results; PP is a high risk factor for develop PUs and this can bias the results of this study. The persistent application of the prevention care bundle and its benefit will be evaluated again in prospective way, looking for more homogeneous population.

**REFERENCE(S)**

1. Xiao-Lin Z, Fan-Jie M, A care bundle for pressure ulcer treatment in intensive care units, International Journal of Nursing Sciencies, 2015, 340–347

2. Tayyib N, Coyer F, Lewis P, Implementing a pressure ulcer prevention bundle in an adult intensive care, Intensive and Critical Care Nursing 2016, Elsevier

**Table 102 (Abstract 0337).** Pressure Ulcers (PUs) prevention care bundle

| ISSUE | INTERVENTION |
|---|---|
| Risk assesment | Brader score calculation |
| Skin care | Bed bath once daily using clorhexidine, and application of mixture of solid hydrocarbons and heavy mineral oils |
| Mobility | Patients repositioned using a three hourly turning schedule using a 'turn clock'. Foot of the bed elevated by 20 degrees if clinically permitted Where clinically possibly patients are mobilized daily to sit out of bed on chair |
| Support surfaces | All ICU patients were managed on air mattresses |

### 0338

#### A retrospective audit: diagnostic blood tests in the critical care setting

H. Agostini, J. Crorkin
Colchester Hospital University Foundation NHS Trust, Colchester, United Kingdom
**Correspondence:** J. Crorkin
*Intensive Care Medicine Experimental* 2017, **5(Suppl 2):**0338

**INTRODUCTION.** Routine blood tests have been shown to increase anaemia in the critically ill patient leading to an increase number of patients needing a blood transfusion[1,3]. They can also lead to irrelevant clinical findings and do have a cost implication for the department. Literature searches reveal a number of methods aimed at reducing the number of blood tests. These included introduction of specialist software (which resulted in a 12% reduction)[2] another introduced written guidelines in the critical care setting[3]. Furthermore, literature suggests that changing the method for which samples are obtained resulted in an 80% reduction in phlebotomy associated blood loss[4].

**OBJECTIVES.** To evaluate the number of patients in the Critical Care Unit(CCU) at Colchester Hospital having routine regular blood tests and assess if these were appropriate with a view to suggest potential methods of reducing recurrence.

**METHODS.** A retrospective audit was carried out identifying the last 50 patients that were discharged from CCU (six were excluded due to being transferred or had died). Our pathology database was then used to see the number of tests that each patient had and excessive/erroneous samples counted.

**RESULTS.** Of the 44 patients 75% were emergency admissions, 18% elective admissions and 7% repatriations. Average number of samples per patient was 33, with an approximate cost of £3,156. Forty-one Group and Saves were sent with only 29% of patients receiving a transfusion. We identified that 33% of coagulation samples were excessive, followed by Group and Saves(17%), Magnesium(17%), Full Blood Count(13%), Liver Function Tests(12%) and Urea and electrolytes(12%).

**CONCLUSIONS.** Blood tests are being ordered excessively. Each time a sample is taken from a patient there is always a risk of acquiring an infection. In today's climate we should also be financially savvy. Suggestions to improve this practice included disseminating the already published evidence and the results from this audit to all staff in the department. We also suggest that blood tests should be performed on clinical basis and documenting the need for such tests and frequency for each patient.

**REFERENCE(S)**

1. Howard L. Corwin; Kathy C. Parsonnet; Andrew Gettinger RBC Transfusion in the ICU: Is there a reason? Chest. 1995; 108(3);767–771
2. May TA. Clancy M Critchfield J, et al. Reducing Unnecessary Inpatient Laboratory Testing in a Teaching Hospital Am J Clin Pathol 2006;126(2):200–206
3 Flabouris A. Bishop G. Williams L, Cunningham M Routine Blood Test Ordering for Patient in Intensive Care, Anaesth Intensive Care 2000 28(5): 562–565
4 Harber C R, Sosnowski K J, Hegde R M. Highly conservative Phlembotomy in Adult Intensive Care- A prospective Randomized Controlled Trial Anaesth Intensive Care 2006 34(4): 434–437

**GRANT ACKNOWLEDGMENT**
Dr Helen Agostini
Dr Pete Bishop

### 0339

#### Critical care medicine quality in Taiwan from 1997 to 2013 under national health insurance

K.-C. Cheng[1], C.-C. Lai[2], C.-H. Ho[3]
[1]Chi Mei Medical Center, Internal Medicine, Yangkang Dist, Tainan City, Taiwan, Province of China; [2]Chi Mei Medical CenterMei Medical Center, Liouying, Intensive Care Medicine, Tainan, Taiwan, Province of China; [3]Chi Mei Medical Center, Medical Research, Tainan, Taiwan, Province of China
**Correspondence:** K.-C. Cheng
*Intensive Care Medicine Experimental* 2017, **5(Suppl 2):**0339

**INTRODUCTION.** Continued monitoring of trends in the utility of the ICU and the outcomes of ICU patients is important for estimating the full future impact of ICU utilization.

**OBJECTIVE.** To investigate critical care medicine quality in Taiwan between 1997 and 2013.

**METHODS.** Patients > 18 years and < 105 years old with first-time Intensive Care Unit (ICU) admission from January 1997 through December 2013 recorded in the Taiwan National Health Insurance Database were enrolled. Age, gender, Charlson comorbidity index (CCI), age-adjusted Charlson comorbidity index (ACCI), invasive mechanical ventilator (IMV), noninvasive ventilation (NIV), continuous renal replacement therapy (CRRT), extracorporeal membrane oxygenation (ECMO), and intra-aortic balloon pump (IABP) data were collected. ICU mortality and ICU length of stay (LOS) were the main outcomes.

**RESULTS.** We identified 3,451,157 eligible patients (mean age: 65.4 years; mean ICU LOS: 5.9 ± 9.0 days; overall ICU-mortality rate: 19.8%). Annual ICU admissions increased from 115,754 in 1997 (incidence: 734/100,000 population) to 244,820 in 2013 (incidence: 1266/100,000 population) ($P < 0.0001$). The admission rate was highest for patients > 75 years old. The uses of NIV, CRRT, and ECMO increased from 4.3%, 1.1%, and 0.01% in 1997 to 11.5%, 2.0% and 0.4% in 2013,respectively. ICU LOS remained stable, but the annual mortality rate significantly decreased from 23.0% in 1997 to 16.3% in 2013.

**CONCLUSIONS.** ICU admissions significantly increased between 1997 and 2013, especially for patients [3]75 years old, but the ICU mortality rate for adult patients significantly declined.

**Acknowledgment**
Financial support by Chi Mei Medical Center

### 0340

#### Which multicenter randomized controlled trials have shown reduced mortality in critical care medicine? A systematic review

C. A. Santacruz Herrera[1], A.J. Pereira[2], E. Celis[1], J.L. Vincent[3]
[1]Hospital Universitario Fundación Santa Fe de Bogota, Intensive Care, Bogota, Colombia; [2]Hospital Israelita Albert Einstein, Sao Paolo, Sao Paolo, Brazil; [3]Erasme University Hospital, Intensive Care, Brussels, Belgium
**Correspondence:** C. A. Santacruz Herrera
*Intensive Care Medicine Experimental* 2017, **5(Suppl 2):**0340

**INTRODUCTION.** Multicenter prospective randomized controlled trials (RCTs) are an important method of obtaining unbiased estimates of treatment effect, but are difficult to conduct in the heterogeneous intensive care unit (ICU) population.
**OBJECTIVES.** To determine which multicenter RCTs in critically ill patients have reported that an intervention reduced mortality rates.
**METHODS.** A systematic review of the literature was performed using the MEDLINE database to identify multicenter, randomized, placebo-controlled trials evaluating any pharmacological, extracorporeal membrane oxygenation or ventilatory strategy in critically ill patients and which reported mortality as a primary or secondary outcome. We included only trials written in English and no date limit was applied. The trials were classified as reporting reduced, increased or no effect on mortality. RCT methodology was assessed using the Jadad scale.
**RESULTS.** A total of 159 trials were identified. Eighteen of the trials reported a significant reduction in mortality, including 5 trials that evaluated interventions to limit ventilator-induced lung injury in patients with acute respiratory distress syndrome (ARDS), such as prone position, neuromuscular blockers, and low tidal volume mechanical ventilation. The other 13 trials evaluated anti-inflammatory therapies in sepsis, ultrafiltration strategies in patients with cardiac arrest, invasive management of ventilator-associated pneumonia and hemodynamic optimization using gastric tonometry. Eleven trials reported an increase in mortality because of iatrogenic effects of anti-inflammatory therapies in sepsis, colloids for resuscitation in patients with hemodynamic shock, high frequency oscillatory ventilation in patients with ARDS and non-invasive positive pressure ventilation for respiratory failure after extubation. In 130 trials, there were no significant differences in mortality between the two study arms. The Jadad scores showed poor mean methodological quality.
**CONCLUSIONS.** Eighteen of the 159 RCTs identified in our literature search reported a reduction in mortality with the intervention under evaluation; 11 trials showed an increase in mortality and 130 reported no effect. Reducing the risk of iatrogenic interventions was associated with improved survival and may serve as a more attainable outcome than mortality. This review raises questions about the standard approach of targeting mortality as an endpoint in RCTs in critical care medicine.

### 0341

#### Maintaining compliance with the UK National ICU Rehabilitation Guidelines (CG83)

L. M. Enoch[1], J. Hyde[1], J. Mcadam[1], S. Rees-pedlar[2], S. Law[3]
[1]Addenbrookes NHS Trusts Hospital, Critical Care, Cambridge, United Kingdom; [2]Addenbrookes NHS Trusts Hospital, Major Trauma, Cambridge, United Kingdom; [3]Addenbrookes Hospital, Critical Care, Cambridge, United Kingdom
**Correspondence:** L. M. Enoch
*Intensive Care Medicine Experimental* 2017, **5(Suppl 2):**0341

**BACKGROUND.** Survivors of Critical Care illness are faced with many long term adverse sequelae both cognitive and physical which can decrease their quality of life post hospital (Jones 2007). Recovery can be prolonged and often incomplete.
Prior studies have successfully demonstrated the benefits of early rehabilitation in Critical Care. UK national ICU rehabilitation guidelines (CG83) (NICE 2009) were introduced to improve patient care and outcome by providing an integrated approach with a continuum to recovery. Critics of CG83 have commented that it is extremely time-consuming and a tick box exercise.
**OBJECTIVES.** We sought to demonstrate compliance and challenges with CG83.
**METHOD.** Addenbrooke's Hospital is a 1200 bed tertiary referral centre with two adult Critical Care Units (41 beds open at the time). Data was prospectively collected and analysed using Excel for the 3 months from October to December 2016. Approval obtained from the Trust audit department. CG83 Assessments were performed by Critical Care Specialist nurses (1.8 whole time equivalent). Qualitative data was also obtained by interviewing patients and relatives in a focus group.
**RESULTS.** There were 399 admissions across the two Critical Care Units. The results are summarised in Table 103. The majority (365; 91.5%) were assessed on admission. 170 further assessments had to be performed prior to ICU discharge, and a further 166 assessments were performed on the wards, making a total of 701 assessments in total over the 3 month period.
Quotes from the focus groups:
Question: Do you think it was a valuable service?

- The advice about rehab and to re-establish a routine to help regulate my days
- Very much so - I was referred for counselling to help with PTSD
- very motivating

**CONCLUSION.** A large number (>700) of assessments were required over the 3 months to comply with CG83; this volume of assessments can be labour intensive. CG83 has not been fully implemented outside of Critical Care and there is a need for cohesive working with Multidisciplinary teams to facilitate ongoing rehabilitation.
Qualitative data suggested that the most valuable assessments were prior to discharge from ICU and on the ward - preparing for a step down in care. Assessments provide time for planning individualised care, setting goals, referrals, and information giving opportunities. It is an inclusive process accounting for relatives needs and addressing their levels of life altering experiences.
Areas for future evaluation:
To collect data measuring the interventions and referrals made as a result of the assessments completed.
To document and calculate the length of time for each assessment.
To implement a more robust method for collecting valuable qualitative data.

**REFERENCES**
NICE, 2009. Rehabilitation after Critical Illness (CG83), London.
Jones C, 2007. Aftermath of intensive care: the scale of the problem. British Journal of Hospital Medicine. Vol.68. No 9.

**Table 103 (Abstract 0341).** Assessment Audit

| | Admission Assessment | Pre discharge from ICU | Ward based Assessment | Pre hospital discharge | Total |
|---|---|---|---|---|---|
| Completed | 365 | 170 | 117 | 49 | 701 |
| Does not meet criteria | 4 | 101 | 126 | 130 | 361 |
| Not completed | 10 | 3 | 4 | 22 | 39 |
| Clinical transfer | 8 | 5 | 5 | 6 | 24 |
| Died during hospital stay | 4 | 61 | 66 | 73 | 204 |
| Non clinical transfers | 8 | 0 | 0 | 0 | 0 |
| Continue on other pathway | | 42 | 63 | 63 | 168 |
| Repatriation to own trust | | 17 | 18 | 49 | 84 |
| No data | | | | 7 | 7 |

### 0342

#### Automatic quality improvement through intensive care unit data management (ICU-DAMA) tool. Quality in information enhances quality in critical patient care

M. Bodí[1], J. Gómez[2], G. Moreno[2], F. Esteban[2], M. Olona[2], A. Rodríguez[1], J.J. Guardiola[3], G. Sirgo[2]
[1]Hospital Universitario Joan XXIII/URV/IISPV/CIBERES, Intensive Care Medicine, Tarragona, Spain; [2]Hospital Universitario Joan XXIII/IISPV, Tarragona, Spain; [3]University of Louisville, Department of Pulmonary, Critical Care and Sleep Medicine, Louisville, United States
**Correspondence:** M. Bodí
*Intensive Care Medicine Experimental* 2017, **5(Suppl 2):**0342

**INTRODUCTION.** It is a priority in modern Intensive Care Medicine to obtain quality indicators that are measurable, reliable and reproducible from the data contained in the clinical information systems (CIS).

**OBJECTIVES.**

1) To determine the feasibility of the automatic generation of health care quality metrics from the data of the CIS, using the ICU-Dama ® tool, and compare them with the gold standard (manual measurement).

2) Describe and classify the differences between both methods.

**METHODS.** Descriptive study. University Hospital. Period of study: two months. Selection of 25 variables to measure the quality of care. Automatic method: using the ICU-DaMa ® tool based on Business Discovery (designed to analyse data generated in the CIS (visualization, generation of indicators, ad hoc studies of patient subgroups) through a previous process of extraction, transformation and loading of data from the CIS in a warehouse. Manual method: construction of a database created from the CIS by two trained professionals. The differences between the two methods were classified as plausibility, completeness or conformance errors.

**RESULTS.** All variables could be captured automatically. There were no significant differences between the automatic method and the manual method. Errors of plausibility (sex), completeness (destination for ICU discharge and days of use of continuous renal replacement techniques) and conformance (reason for isolation and brain death) were detected.

| METRIC | OVERALL (n = 149) | Type of error | Accuracy |
|---|---|---|---|
| Sex | 1 (0.7%) | Plausibility | 99.3% |
| Reason for isolation | 2 (1.3%) | Conformance | 98.7% |
| Destination for ICU discharge | 2 (1.3%) | Completeness | 98.7% |
| Brain death | 1 (0.7%) | Conformance | 99.3% |
| days of CRRT | 1 (0.7%) | Completeness | 99.3% |

*[Data Quality Errors. ICU DaMa vs Gold Standard]*

**CONCLUSIONS.** The automatic generation of quality metrics using ICU-DaMa ® is feasible, and it allows optimizing the time of professionals. Discrepancies regarding the standard gold could be identified and potentially correctable. The training of professionals in the culture of data quality is fundamental.

**GRANTS**

## ICU discharge and re-admission

### 0343

#### Relationship between ICU discharge during weekend and evolution of patients

A. Lopez Martinez, A. Carrillo Alcaraz, L. Casado Mansilla, G. Quintanilla, M. C. Lorente, M. N. Alcazar, L. Lopez
Hospital JM Morales Meseguer, ICU, Murcia, Spain
**Correspondence:** A. Lopez Martinez
*Intensive Care Medicine Experimental* 2017, **5(Suppl 2):**0343

**INTRODUCTION.** It has been suggested that patients admitted to the ICU and discharged to the hospital ward during the weekend may have a worse outcome because they are attended the ward staff on call in the hospitals and not by the corresponding specialists.

**OBJECTIVES.** To analyze the clinical outcome of patients admitted in the ICU and discharged to the ward during the weekend, comparing them with discharged patients in working days.

**METHODS.** Retrospective study of all patients admitted in the ICU, consecutively, discharged alive to conventional ward in a university hospital during a period of 20 years. Patients were divided in two groups, Group I: discharge day at the weekend (saturday or sunday) and Group II: discharge, the rest of days. Sociodemographic, clinical and evolutionary variables were analyzed. Quantitative variables were expressed as mean ± standard deviation or median (first, third quartile), and qualitative as absolute and relative frequency. Comparisons between variables were performed using Pearson´s Ji2, Student´s T or Mann Whitney tests.

**RESULTS.** During the study period, 18740 patients were discharged alive to the ward, 3837 (20.5%), during the weekend. Neither age, Group I: 63.6 ± 16.7 and Group II: 63.7 ± 17.3 ($p = 0.779$) or gender (64.5% and 63.3% males, respectively; $p = 0.157$) differ in the two groups. Charlson´s comorbidity index was similar in both groups, median: 2 (1.3) [$p = 0.896$]. The severity at admission as measured by the SAPS II index was higher in Group I: 34.1 ± 12.2 vs Group II: 35.1 ± 13.7 ($p < 0.001$), but the maximum SOFA index was lower, 2.6 ± 3.3 and 3.1 ± 3.5 respectively ($p < 0.001$). Patients with do not intubate order were 216 (5.6%) in Group I and 933 (6.3%) in Group II ($p = 0.146$), and 76 (2%) and 359 (2.4%) respectively presented no readmission order ($p = 0.116$). Hospital stay was lower in patients in Group I (16 ± 17 days) than group II (18 ± 20 days) [$p < 0.001$.] The readmissions were slightly lower in Group I: 197 patients (6.3%) compared to 859 (7.3%) in group II ($p = 0.051$), and hospital mortality was 5.1% (202 patients) and 5.9% (890 patients) respectively ($p = 0.082$).

**CONCLUSIONS.** In our cohort, the fact of being discharged during the weekend does not result in an increased risk for patients, Since there is no greater number of re-admissions nor a higher mortality rate.

### 0344

#### To return or not to return: how good are Intensivists at formulating treatment escalation plans for patients on discharge from ICU?

S. Arasaretnam, K. Dombrowsky, J. Gross
Northwick Park & St Mark's Hospitals, London, United Kingdom
**Correspondence:** S. Arasaretnam
*Intensive Care Medicine Experimental* 2017, **5(Suppl 2):**0344

**INTRODUCTION.** Intensive Care can be associated with unpleasant and distressing experiences for patients and in the longer term may result in significant physical and psychosocial morbidity, poor quality of life and excess mortality[1,2]. Whilst published guidelines relating to ICU discharge stipulate the need for clear plans for ongoing treatment[3], there is no specific reference to treatment escalation planning (including ICU readmission status) at the time of ICU discharge in the event of recurring critical illness.

**OBJECTIVES.**

1. To investigate the proportion of patients who have a documented treatment escalation plan (TEP) on discharge from ICU (including "Do Not Attempt Cardiopulmonary Resuscitation" - DNACPR orders).

2. For patients who do have TEPs, to determine the proportion that were discussed with patients.

**METHODS.** Retrospective analysis at our institution of all patients who survived an ICU admission over a 6-month period between 1st September 2016 and 29th February 2017. Discharge summaries for all patients were analysed to ascertain ceilings of care (including

DNACPR orders), readmission status and whether these decisions were discussed with the patient. Subgroup analysis was performed on those who were intubated and had an ICU stay of 4 days or more.

**RESULTS.** Of the 283 patients admitted to ICU during the data collection period, 215 survived to ICU discharge. Five patients were excluded due to a lack of available discharge summary. Of the 210 patients remaining, 134 (63.8%) were male, with a mean age of 60.1 years (SD 18.1). Median ICU stay was 3.8 days (IQR 1.71-7.13). On ICU discharge, 133 (63.3%) patients had no TEP, 148 (70.5%) had no documented DNACPR status and 181 (86.2%) patients were not consulted about treatment re-escalation and potential future ICU readmission.

For patients who had been intubated with a length of stay (LOS) of 4 days or more (n = 73), the median LOS was 7.8 days (IQR 5.7-14.29). In this subgroup, 40 (54.8%) had no TEP, 50 (68.5%) did not have a documented DNACPR status and 59 (80.8%) patients were not consulted about treatment re-escalation and potential future ICU readmission.

**CONCLUSIONS.** Our study shows that treatment escalation planning on ICU discharge is inconsistent and patients are rarely consulted about treatment re-escalation and ICU readmission at the point of ICU discharge. It is recommended that treatment escalation plans (that takes into account potential benefits, risks and patient's wishes) should be routinely considered and documented on discharge from the ICU.

*2.* Cuthbertson BH et al. Quality of life in the five years after intensive care: a cohort study. *Critical Care. 2010;14(1):R6*

### 0345

#### An observational study on early mobilization and pain -agitation/ sedation -delirium as quality indicators in two intensive care units

A. Petosic[1], H. Wøien[1,2], H. Flaatten[3], K. Olafsen[1], K. Sunde[1,4]

[1]Oslo University Hospital, Division of Emergencies and Critical Care, Oslo, Norway; [2]University of Oslo, Institute of Health and Society, Oslo, Norway; [3]Haukeland University Hospital and University of Bergen, Dep. of Clinical Medicine, Bergen, Norway; [4]University of Oslo, Institute of Clinical Medicine, Oslo, Norway

**Correspondence:** A. Petosic

*Intensive Care Medicine Experimental* 2017, **5(Suppl 2):**0345

**INTRODUCTION.** The use of quality indicators (QIs) to improve care in the intensive care unit (ICU) is increasing. In Norway, process-QIs for Early Mobilization (EM) or Pain-Agitation/sedation-Delirium (PAD) assessment are infrequently used.

**OBJECTIVES.** The aim of this pilot study was to evaluate the frequency of these two processes measured by specific QIs in two ICUs at the Oslo University Hospital Ullevål (OUHU).

**METHODS.** In this prospective observational study, all patients admitted at two different ICUs at OUHU were included during a predefined period in autumn 2015. Data for determining EM and PAD-assessment were retrieved from the daily electronic patient record (MetaVision). EM was defined as mobilization within 72 hours from ICU admittance to either edge of bed, standing position, walking or sitting in a chair. PAD was defined as documentation of pain- (P), agitation/sedation- (A/S) and delirium- (D) scores during each nursing shift. Data analysis was performed using SPSS version 21. Descriptive statistics are presented as frequencies (percentages) for categorical variables, and as median with interquartile range (IQR) for continuous variables.

**RESULTS.** We included 143 adult ICU patients (73% male, median age 59 years (37, 71)), 92% being surgical patients. Among those, 69% were trauma patients and 20% surgical emergency patients. Median SAPS II score was 28 (21, 41), median duration of mechanical ventilation (MV) was 1 day (0, 6) and median ICU length of stay was 4 days (2, 10). Altogether 97 patients (68%) were mobilized in the ICU, and EM was performed in 60 patients (42%). In patients treated with MV (n = 107), EM was performed in 36 patients (34%). The 143 patients were followed in 1192 ICU days corresponding to 3147 nursing shifts, and P, A/S and D were assessed in 22%, 52% and 0,004% of the shifts, respectively.

**CONCLUSIONS.** Guideline adherence for both EM and PAD-assessments at OUHU is suboptimal, although we have to take into account the large number of polytraumatic patients. Systematic monitoring and focus on QIs like EM and PAD is important to improve quality of care in an ICU.

**GRANT ACKNOWLEDGMENT**

Oslo University Hospital, Division of Emergencies and Critical Care, Department of Postoperative and intensive care.

### 0346

#### Magnificent Seven: quality standards for sedation in critical care

C. Bird[1], P. Bishop[1], G. Wong[2]

[1]Colchester Hospital University Foundation NHS Trust, Anaesthetics Department, Colchester, United Kingdom; [2]Colchester Hospital University NHS Foundation Trust, Anaesthetics department, Colchester, United Kingdom

**Correspondence:** G. Wong

*Intensive Care Medicine Experimental* 2017, **5(Suppl 2):**0346

**INTRODUCTION.** Sedation in critical care is a balancing act. Whilst patient comfort is paramount, depth of sedation also affects haemodynamic stability, ventilatory requirement and neurological assessment. Current guidelines advocate a light sedation technique using tools such as the Richmond Agitation-Sedation scale (RASS) to record and titrate depth[1]. Analgesia-first sedation and daily interruption are recommended strategies to manage pain, agitation and delirium in critical care[1]. These recommendations form one of the "Magnificent Seven" principles agreed by the East of England Critical Care Operational Delivery Network for delivering evidence-based quality care in the areas of diagnostic tests, red cell transfusion, parenteral nutrition, sedation, ceiling of care, antibiotic use and inter-hospital transfers[2]. This audit focuses on current sedation practices in Colchester General Hospital, a 560-bed district general hospital serving a population of 370,000 in North Essex.

OBJECTIVESI To audit current practice in Colchester General Hospital Critical Care Unit against the recommendation from the East of England Network and local guidelines. To highlight key areas for improvement in sedation practice.

**METHODS.** Data were collected prospectively from all mechanically ventilated critical care patients for one month. Patients were excluded on day of intubation. Bedside notes were reviewed for documentation of depth of sedation, use of analgesic, sedation hold within previous 24 hours and reasons for deviation from above.

**RESULTS.** During the 45 ventilated patient days depth of sedation was recorded daily in all patients using the RASS score. All patients had multimodal sedation comprising opiate infusion with propofol, dexmedetomidine or midazolam. Sedation holds were performed on 29 out of 45 ventilator days. Nine of the 16 reasons for omission were consistent with local guidelines, including PEEP >12, FiO2 > 60%, ongoing neuromuscular block and raised intracranial pressure.

**CONCLUSIONS.** The audit revealed good nursing documentation of depth of sedation but limited medical documentation of rationale for sedation strategy. There was variable practice with sedation holds, not always consistent with local guideline. Recommendations include discussion of sedation on daily ward rounds involving assessment of, and plan for weaning and withdrawal of sedation. When sedation hold is not appropriate the reason should be documented. These changes to practice will ensure judicious use of sedation in order to optimise ventilatory wean and minimise side effects of over sedation.

2. East of England Critical Care Operational Delivery Network. The Magnificent Seven: Evidence-based quality principles agreed by all critical care units in the East of England. www.eoeccn.org.uk/documents

### 0347

#### Validation of the French 18-item questionnaire IPREA for quantitative assessment of perceived discomforts in critically ill patients

P. Kalfon[1,2], M. Boucekine[2], P. Estagnasie[3], M.-A. Geantot[4], A. Berric[5], G. Simon[6], B. Floccard[7], A. Sossou[8], M. Venot[9], T. Signouret[10], M. Fromentin[11], R. Robert[12], A. Follin[13], J. Audibert[1], A. Ben Salah[1], A. Renault[14], J. Amour[15], O. Mimoz[16], N. Revel[17], E. Azoulay[9], T. Sharshar[18], M. Garrouste-Orgeas[19], A. Loundou[2], K. Baumstarck[2], P. Auquier[2], the IPREA AQVAR Study Group

[1]CH Chartres, Réanimation Polyvalente, Chartres, France; [2]Aix Marseille Université, Centre d'Etudes et de Recherche sur les Services de Santé et la Qualité de Vie - CEReSS, Marseille, France; [3]Clinique Ambroise Paré, Réanimation, Neuilly/Seine, France; [4]CHU Dijon Bourgogne, Dijon, France; [5]CHI Toulon/La Seyne sur Mer, Réanimation Polyvalente, Toulon, France; [6]CH Troyes, Réanimation, Troyes, France; [7]CHU Edouard Herriot Hospices Civils de Lyon, Réanimation Polyvalente, Lyon, France; [8]CH Emile Roux, Réanimation Chirurgicale, Le Puy-en-Velay, France; [9]CHU Saint Louis Assistance Publique Hôpitaux de Paris, Réanimation Médicale, Paris, France; [10]Hôpital Européen de Marseille, Réanimation, Marseille, France; [11]CHU Cochin Assistance Publique Hôpitaux de Paris, Réanimation Chirurgicale, Paris, France; [12]CHU Poitiers, Réanimation Médicale, Poitiers, France; [13]Hôpital Européen Georges Pompidou Assistance Publique Hôpitaux de Paris, Réanimation Chirurgicale, Paris, France; [14]CHU Brest, Réanimation Médicale, Brest, France; [15]CHU Pitié-Salpêtrière, Assistance Publique Hôpitaux de Paris, Anesthésie Réanimation, Paris, France, [16]CHU Poitiers, Poitiers, France; [17]Hôpital Louis Pasteur, CHU Nice, Réanimation Polyvalente, Nice, France; [18]CHU Raymond Poincaré, Assistance Publique Hôpitaux de Paris, Réanimation, Garches, France; [19]Groupe Hospitalier Paris Saint Joseph, Réanimation, Paris, France

**Correspondence:** P. Kalfon

*Intensive Care Medicine Experimental* 2017, **5(Suppl 2):**0347

**INTRODUCTION.** Critically ill patients in intensive care units (ICUs) are exposed to stressful conditions and experience discomfort from multiple sources. To assess the level of overall discomfort perceived by the ICU patient, we developed the 16-item IPREA questionnaire[1]. However, based on the high level of respiratory symptoms reported by ICU patients as well as depressive symptoms that may persist after ICU discharge, the content validity of IPREA can be increased by introducing 2 items, shortness of breath and depression.

**OBJECTIVES.** The objective of this study was to assess the validity of the overall discomfort score derived from the 18-item version in comparison with that derived from the original 16-item version of IPREA.

**METHODS.** We conducted the IPREA3 study, a multicenter, cluster-randomized, two-period crossover trial, controlled study involving 34 French adult ICUs and 2130 ICU survivors to assess the efficacy of a multicomponent tailored program to reduce discomfort in the ICU[2]. On the day of ICU discharge, the bedside nurse asked patients to rate the severity of each discomfort item from 0 to 10 (minimal to maximal) using the 18-item version of IPREA. The primary outcome of the IPREA3 study was the overall discomfort score derived from the already validated 16-item version from 0 to 100 (minimal to maximal). The validation process of the 18-item version was based on a confirmatory factor analysis (CFA) applied to patients not impacted by the program. CFA consisted of calculating the root mean square error of approximation (RMSEA), the comparative fit index (CFI), the Tucker Lewis Index (TLI) and the standardized root mean square residual (SRMR).

**RESULTS.** Before implementation of the program, 994 ICU survivors were included (age 62.4 ± 15.6, SAPS2 35.9 ± 16.5). The overall discomfort scores were resp. 21.99 ± 13.88 and 21.92 ± 13.73 for the 16-item and 18-item versions. The scores for shortness of breath and depression were resp. 2.84 ± 3.09 and 1.44 ± 2.57. CFA is presented in Table 104.

**CONCLUSIONS.** The 18-item version of IPREA including the items shortness of breath and depression presents the same satisfactory psychometric properties as the 16-item version. Its use can be recommended to monitor discomfort in unselected adult ICU patients.

**REFERENCE(S)**

1. Kalfon P, Mimoz O, Auquier P, Loundou A, Gauzit R, Lepape A, et al. Development and validation of a questionnaire for quantitative assessment of perceived discomforts in critically ill patients. Intensive Care Med. 2010; 36(10): 1751–8.

2. Kalfon P, Mimoz O, Loundou A et al.: Reduction of self-perceived discomforts in critically ill patients in French intensive care units: study protocol for a cluster-randomized controlled trial. *Trials* 2016, 16; 17:87.

**GRANT ACKNOWLEDGMENT**

French Ministry of Health

**Table 104 (Abstract 0347).** See text for description

| | 16-item IPREA, n = 994 | 18-item IPREA, n = 994 |
|---|---|---|
| RMSEA (<0.05 good) | 0.039 (0.03 - 0.05) | 0. 042 (0.04 - 0.05) |
| CFI (>0.9 good) | 0.932 | 0.918 |
| TLI (>0.9 good) | 0.916 | 0.901 |
| SRMR (<0.08 good) | 0.034 | 0.04 |

### 0348

#### Pain: a retrospective look at hospital pain levels and the effect on clinical outcomes

E. Doty[1], R. Gollub[2], N. McCague[3], L. A. Celi[4,5]

[1]Harvard Medical School, Department of Biomedical Informatics, Boston, United States; [2]Massachusetts General Hospital, Boston, United States; [3]Boston University Graduate School of Public Health, Boston, United States; [4]Beth Israel Deaconess Medical Center, Boston, United States; [5]Massachusetts Institute of Technology, Cambridge, United States

**Correspondence:** E. Doty

*Intensive Care Medicine Experimental* 2017, **5(Suppl 2):**0348

**INTRODUCTION.** The effect of pain experienced by post-cardiac surgical patients on clinical outcomes has not been well studied.

**OBJECTIVES.** Determine the association between patient-reported pain levels after coronary artery bypass graft (CABG) and hospital length of stay, 30-day mortality, and 1-year mortality.

**METHODS.** Using the Medical Information Mart for Intensive Care (MIMIC-III) database, a cohort of 844 post CABG patients, extubated within 24 hours, was identified. Pain levels after extubation, reported on a scale of 1 to 10, were represented as mean, median, and maximum values during their intensive care unit (ICU) stay, and then categorized into no (0/10), mild (1–3), moderate (4–6), and severe pain (7–10). Regression analysis was used to study the relationship between the pain scores, mortality, and hospital length of stay (LOS). Falsification hypothesis testing using nausea, a symptom with no known effect on the outcomes of interest, was performed on the patient cohort.

**RESULTS.** Adjusting for age, sex, Oxford Acute Severity of Illness Score (OASIS), the Elixhauser comorbidity index, and use of extracorporeal circulation during surgery, increased levels of pain were found to be significantly associated with reduced length of stay and reduced mortality at 30 days and 1 year after among the patient cohort. One point increase in mean pain level was found to be associated with a 0.92 (95%CI 0.67-1.16, p < 0.001) day decrease in hospital LOS, and a reduction in the odds of 30-day and 1-year mortality by a factor of 0.44 (95%CI 0.28-0.65, p < 0.001) and 0.70 (95%CI 0.55-0.86, p < 0.01) respectively. Compared to patients who reported no pain after extubation in the ICU, those with mild pain had hospital LOS decreased by 3.8 (95%CI 2.56-5.34, p < 0.001), those with moderate pain had a reduction of 5.19 (95%CI 3.60-6.79, p < 0.001)

and those with severe pain had a reduction of 6.57 (95%CI 3.68-9.46, p < 0.001). Falsification testing showed nausea was not related to hospital length of stay or 30-day mortality in this patient cohort.
**CONCLUSIONS.** Some level of pain post-CABG may be related to improved patient outcomes. This finding has not been reported and is counterintuitive, and needs to be validated in other databases and settings.

### 0349

#### Time to ICU readmission and outcomes: a retrospective cohort study

T. D. Corrêa, C. R. Ponzoni, A. Serpa Neto, R. R. Filho, M. S. Assunção, A. Pardini, G. P. Schettino
Hospital Israelita Albert Einstein, Intensive Care Unit, São Paulo, Brazil
**Correspondence:** T. D. Corrêa
*Intensive Care Medicine Experimental* 2017, **5(Suppl 2):**0349

**INTRODUCTION.** Readmission to intensive care unit (ICU) is associated with poor clinical outcomes and increased costs [1]. Moreover, it has been demonstrated that ICU readmission after 72 hrs from ICU discharge is associated with increased hospital mortality in developed countries [1].
**OBJECTIVES.** To evaluate the effect of the time interval between ICU discharge and readmission on resource use and outcomes in a developing country.
**METHODS.** This retrospective single center cohort study was conducted in a forty bed, medical-surgical, open model ICU of a private, tertiary care hospital in São Paulo, Brazil. The Local Ethics Committee approved the study protocol, and the need for informed consent was waived. All consecutive adult (≥18 years) patients admitted to the ICU between June 1, 2013 and July 1, 2015 were enrolled in this study. Comparisons were made between patients readmitted within 72 hrs or less (Early) and after 72 hrs (Late) after ICU discharge.
**RESULTS.** In total, 5,779 patients were included in this analysis. The incidence of ICU readmission during the same hospitalization was 10% (576/5,779 patients). Among the readmitted patients, 169 (29.3%) were readmitted to the ICU within 72 hrs or less and 407 (70.7%) were readmitted after 72 hrs. Patients readmitted after 72 hrs of ICU discharge were slightly older [68 (56–81) vs. 65 (53–76) years, respectively for Late and Early; p = 0.028)], more frequently male [258/407 (63.4%) vs. 91/169 (53.8%); p = 0.039] and had a higher severity of illness [Simplified Acute Physiology (SAPS) III score] on index ICU admission [52 (42–63) vs. 47 (38–56); p = 0.001)] than those readmitted within 72 hrs or less. The need for vasopressors, invasive mechanical ventilation, and renal replacement therapy did not differ between Late and Early ICU readmissions. Late ICU readmission was associated with higher ICU [3 (2–8) vs. 2 (1–5) days, respectively for Late and Early; p = 0.001)] and hospital [43 (26–70) vs. 20 (12–42) days; p < 0.001)] length of stay and with a higher hospital mortality [149/407 (36.6%) vs. 47/169 (27.8%); p = 0.043] than Early ICU readmission. Nevertheless, after adjusting for confounders in a multivariable logistic regression analysis, time to ICU readmission was no longer associated with increased hospital mortality.
**CONCLUSIONS.** Late ICU readmissions were frequent and associated to higher ICU and hospital lengths of stay. The impact of late ICU readmission on hospital mortality and the degree at which late ICU readmissions are preventable in developing countries need to be further determined.
**REFERENCE.** [1] Kramer AA, et. al. Intensive Care Unit Readmissions in U.S. Hospitals: Patient Characteristics, Risk Factors, and Outcomes. Crit Care Med 2012;40:3–10.

### 0350

#### Characteristics of the deaths of patients readmitted to the ICU compared to the deaths of those who have not been readmitted in the ICU, in a tertiary-level hospital in Brazil

B. B. Campos[1,2], D. Deheinzelin[2]
[1]State University of Campinas, CAISM - Critical Care, Campinas, Brazil; [2]Instituto Sírio-Libanês de Ensino e Pesquisa, Critical Care, Sao Paulo, Brazil
**Correspondence:** B. B. Campos
*Intensive Care Medicine Experimental* 2017, **5(Suppl 2):**0350

**INTRODUCTION.** ICU readmission has been related to poor outcomes and greater risk of death. By studying the specific group of deceased patients and the death process, we believe that better characterizing this subgroup would provide relevant information in order to improve the quality of care and the use of ICU.
**OBJECTIVES.** To compare the characteristics of the deaths after the first ICU stay with the characteristics of the deaths after readmission to the ICU, and to identify behaviors associated with withholding/withdrawing of life support therapies.
**METHODS.** A retrospective case-case study of all patients admitted consecutive to the Intensive Care Unit of the Sírio-Libanês Hospital in São Paulo, Brazil, with 370 total beds and 32 ICU beds, attending highly complex clinical and surgical patients. A total of 2098 patients were admitted, with analysis of 152 deaths in the single admission group and 24 in the readmitted group. Readmission was defined as follows: any patient transferred back to the ICU prior to death or hospital discharge. Demographic, physiological variables, presence of situations or orders of withholding or withdrawing life support therapies were collected. Life Support Therapies were considered defibrillation for ventricular fibrillation, cardiopulmonary resuscitation, treatment of acute cardiac arrhythmias, treatment with vasopressors and / or inotropes, tracheal intubation and mechanical ventilation, surgeries, transfusion of blood products, antimicrobial treatment and renal replacement therapy. Statistical analysis has been performed using the statistical program Stata® (version 11.2).
**RESULTS.** Readmission rate was 8.4%, mortality rate was 8.5% (95% CI: 7.3% to 9.9%) among not readmitted patients and 14.1% (95% CI: 9.3% to 20, 1%) among those who were readmitted. The groups were similar according to the physiological parameters studied, gender, age range or marital status, except for length of stay. No significant associations were found between an existence of cancer diagnosis or causes of admission to ICUs with a number of passages through the unit. Limitations of Therapeutic Efforts (LTE) were adopted for 116 (65.9%) patients. The commonest LTE decisions were: "Do not resuscitate" (DNR) and do not start renal replacement therapy.
**CONCLUSION.** The similar rates of LTE in both groups may indicate that they had the same intensity of care and died similarly, regardless ICU readmission. There should be some perception that the outcome will be adverse, regardless of the readmission event, and this causes the high LTE rate.
**REFERENCES.** Al-Jaghbeer MJ, Tekwani SS et al. Incidence and Etiology of Potentially Preventable ICU Readmissions. Critical Care Medicine. 2016;44(9):1704–9. Phua J, Joynt GM, Nishimura M et al. Withholding and withdrawal of life-sustaining treatments in low-middle-income versus high-income Asian countries and regions. Intensive Care Med. 2016;42(7):1118–27.
**GRANT ACKNOWLEDGMENT**
None

### 0351

#### In-hospital mortality after ICU discharge

G. Seller-Perez[1], D. Arias-Verdu[1], M. Delgado-Amaya[1], P. Benitez-Moreno[1], I. De.Dios-Chacon[2], M.E. Herrera-Gutierrez[1]
[1]Regional University Hospital Málaga, ICU, Malaga, Spain; [2]Hospital Son Llatzer, Palma de Mallorca, Spain
**Correspondence:** D. Arias-Verdu
*Intensive Care Medicine Experimental* 2017, **5(Suppl 2):**0351

**OBJECTIVE.** To evaluate possible predicting variables related to mortality after ICU discharge.
**METHODS.** Prospective observational cohorts study. All patients (n = 1128) admitted to our unit for one year, excluding cardiac patients and with < 24 hours stay, followed-up until hospital discharge. We defined two groups: early [EM] (<48h) and late [LM] after discharge mortality. A logistic regression analysis was performed. With approval by the hospital Ethics Committee.

**RESULTS.** Population: 35% women, 59.4(15.9) years, SOFA at admission 4.1(3.1), APACHE II 16.6(7.9), expected mortality 29.2(21.5)%. From elective surgery 23.9%, urgent surgery 14.5%, emergency area 34.5% and medical wards the rest. 18.4% developed infection during the admission, 39.8% AKI and 7.2% required CRRT.

Overall mortality 21.8% (5.9% for elective surgery, 22.5% in the rest). 56 out of 939 patients (5.9%) died after ICU: 10 in EM group and 46 in the LM group. In EM group, 7 patients were readmitted and died in the Unit and 3 died unexpectedly in the hospital (hidden mortality). (Fig. 117)

Age, longer hospital stay before ICU, from medical ward, AKI and Sabadell Score were related to after discharge mortality. The Sabadell Score did not discriminate between the EM and LM groups (Fig. 118).

**CONCLUSIONS.** The distribution of mortality in our series is similar to that published elsewhere. Even when the Sabadell Score detected fairly well those patients with highest probability of death after ICU discharge, regarding hidden mortality (an infrequent but serious problem considering that are those patients not expected to die) was not of aid for their detection.

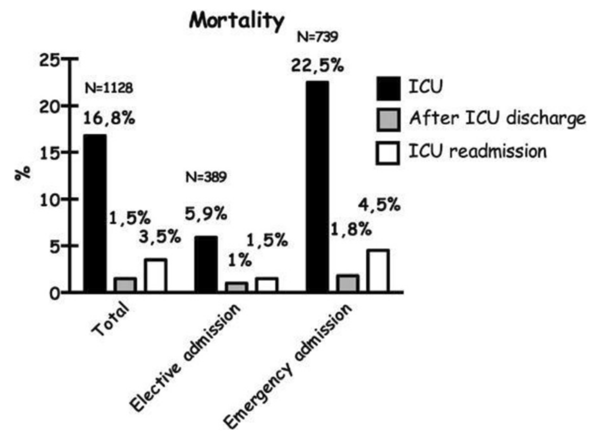

**Fig. 117 (abstract 0351).** See text for description

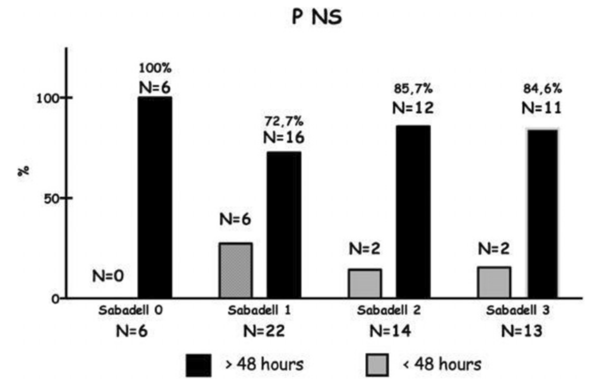

**Fig. 118 (abstract 0351).** See text for description

### 0352

### Risk factors of readmission after ICU discharge. One year survey

J. Baroudi[1], M.A. Boujelbèn[1], N. Fraj[1], K. Meddeb[1], N. Sma[1], E. Ennouri[1], H. Kallel[1], W. Zarrougui[1], A. Ben Abdelhafidh[1], I. Chouchene[1], A. Azouzi[1], A. Khedher[1], I. Ben Saida[1], J. Ayachi[1], M. Boussarsar[1,2]
[1]Farhat Hached University Hospital, Medical Intensive Care Unit, Sousse, Tunisia; [2]Ibn Al Jazzar Faculty of Medicine, Research Laboratory N° LR14ES05 Interactions of the Cardio-pulmonary System, Sousse, Tunisia
**Correspondence:** M. Boussarsar
*Intensive Care Medicine Experimental* 2017, **5(Suppl 2):**0352

**INTRODUCTION.** Surviving intensive care stay important, but readmission after ICU discharge is associated with increased risk of mortality and poor prognosis. The epidemiology of ICU readmission remains unknown in Tunisia. To focus on patients at risk of readmission, could help health caregivers to improve their ICU discharge.

**OBJECTIVES.** To identify the frequency and factors associated with hospital readmission after ICU discharge.

**METHODS.** An observational prospective cohort study was performed in a Tunisian medical ICU from January 2014 to December 2015 involving survivors after ICU stay. An analysis of collected data from all patients readmitted to the hospital wards or ICU during 12-month period after their ICU stay was conducted. A between group-comparison of patients who were readmitted and not readmitted to ICU was performed. Significant factors with a p value of < 0.2 were entered into a backward conditional binary logistic regression to determine independent factors leading to hospital readmission.

**RESULTS.** In this prospective study, 71(33%) patients were readmitted. The first hospital readmission occurred within a median of 89[1–365] days. 37(56%) were readmitted within the first month. 120(56%) were readmitted in the ICU and 23 (11%) in the pulmonology department. The most common reason for readmission was respiratory disorders for 47(66%) patients. 7(10%) persons were readmitted for sepsis and a further 12(17%) were readmitted for a cardiovascular event.

Multivariate analysis, identified significant independent factors for readmission were found to be coronary heart disease (OR, 2.7 ; 95%CI, [0.96-8.02] ; p = 0.05) and physiological reserve (OR, 2.6 ; 95%CI, [1.5-4.6] ; p = 0.001) as the two independent associated factors with post ICU readmission.

**CONCLUSIONS.** The present study revealed a high risk of post ICU readmission in a Tunisian medical ICU. Physiological reserve and coronary heart disease were identified as independent associated risk factors.

### 0353

### The incidence and risk factors of delirium in patients who admitted to the ICU of Dr. Hasan Sadikin Hospital Bandung Indonesia

T. T. Maskoen[1,2], R. Adiwinata[2], E. Oktaliansah[1]
[1]Faculty of Medicine University of Padjadjaran, Anesthesiology and Intensive Therapy, Bandung, Indonesia; [2]Cicendo Hospital, Anesthesiology, Bandung, Indonesia
**Correspondence:** T. T. Maskoen
*Intensive Care Medicine Experimental* 2017, **5(Suppl 2):**0353

**INTRODUCTION.** Delirium is an acute and fluctuative changes of mental status and level of consciousness. Delirium will prolong length of stay in ICU, higher cost during hospitalization, increase morbidity and mortality and also will decrease the functional recovery of ICU patients.

**OBJECTIVES.** To measure the incidence and find out the risk factors of delirium in patients who admitted to the ICU Dr. Hasan Sadikin Hospital during 3 months from January to March, 2015.

**METHODS.** Descriptive observational cohort study using Confusing Agitation Method-Intensive Care Unit (CAM-ICU) and Richmond Agitation Sedation Score (RASS).

**RESULTS.** From 105 patients who admitted to the ICU, 22 patients were excluded. Delirium was found in 37.3% (31/83) in ICU patients. The risk factors in patients with delirium included: geriatric patient 48.4%, using mechanical ventilator 38.7%, morphine 29%, sepsis and other infection 29%, cardiac disfunction 25.8%, High APACHE SCORE II 25.8%, renal disfunction 22.6%, laboratory abnormality 22.6%, sedation midazolam 19.4%, endocrine disfunction 16.1%, fentanyl 6.5% and stroke 3.2%

**CONCLUSIONS.**

We made a concussion that the incidence of delirium in ICU patients of Dr. Hasan Sadikin Hospital was 37.3% and the most common risk factor was geriatric patients

**REFERENCE(S)**

1. Steiner LA. Postoperative delirium Part 1: Pathophysiology and risk factor. Eur J Anesthesiol 2011;28(9):628–36

2. Steiner LA. Postoperative delirium. Part 2: detection, prevention, and treatment. Eur J Anestheiol 2011;28(10):723–32

3. American Psychiatric Association Diagnostic and Statistical Manual of Mental Disorder (DSM-IV). 4th ed. APA, 2006.

4. Connor D, Williams E in critical care. Intensive care medicine. Royal cornwall Hospital UK. 2011;18:232

5. Boogard VM, Pickkers P, van der Hoeven H, Roodbol G, Achterberg VT, Schoonhoven L. Development and validation prediction of delirium in ICU patients. BMJ. 2012;344;420

6. Ouimet S, Kavanagh BP, Gottfried SB, Skrobik Y. Incidence, risk factors and consequences of ICU Delirium. Intensive Care Med. 2007;33:66–73

### 0354

#### Machine learning techniques for improving prediction of unplanned intensive care readmission

A. Ercole[1], T. Desautels[2], R. Das[2], J. Calvert[2], M. Trivedi[3], C. Summers[4], D. Wales[5]

[1]University of Cambridge, Division of Anaesthesia, Cambridge, United Kingdom; [2]Dascena Inc, Hayward, United States; [3]Cambridge University Hospitals NHS Foundation Trust, Cambridge, United Kingdom; [4]University of Cambridge, Cambridge, United Kingdom; [5]University of Cambridge, Department of Chemistry, Cambridge, United Kingdom

**Correspondence:** A. Ercole

*Intensive Care Medicine Experimental* 2017, **5(Suppl 2):**0354

**INTRODUCTION.** Unplanned readmission to intensive care is highly undesirable in that it contributes to increased variance in care, disruption, difficulty in resource allocation and may increase length of stay and mortality particularly if subject to delays. Unlike the ICU admission from the ward, readmission prediction has received relatively little attention, perhaps in part because at the point of ICU discharge, full physiological information is systematically available to the clinician and so it is expected that readmission should be largely due to unpredictable factors. However it may be that there are multidimensional trends that are difficult for the clinician to perceive that may nevertheless be predictive of readmission.

**OBJECTIVES.** We investigated whether machine learning (ML) techniques could be used to improve on the simple published SWIFT score [1] for the prediction of unplanned readmission to ICU within 48 hours.

**METHODS.** We extracted systolic BP, pulse pressure, heart and respiration rate, temperature, $SpO_2$, bilirubin, creatinine, INR, lactate, white cell count, platelet count, pH, $FiO_2$, and total Glasgow Coma Score from ICU stays of over 2000 adult patients from our hospital electronic patient record system. We trained our own custom multidimensional / time-sensitive algorithmic ML system to predict failed discharges defined as either readmission or unexpected death within 48 hours of discharge. We used 10-fold cross validation to assess performance. We also assessed the effect of augmenting our system by transfer learning (TL) with 44,000 additional cases from the MIMIC III database.

**RESULTS.** The SWIFT score performed relatively poorly with an AUROC of around 0.6 which our ML system trained on local data was also able to match. However when augmented with an additional dataset by TL, the AUROC for the ML system improved statistically and clinically significantly to over 0.7.

**CONCLUSIONS.** Machine learning is able to improve on predictors based on simple multiple logistic regression. Thus there is likely to be information in the trends and in combinations of variables. A disadvantage with this technique is that ML approaches require large amounts of data for training. However, ML approaches can be improved by TL. Basing prediction models on locally derived data augmented by TL is a potentially novel approach to generating tools that customised to the institution yet can exploit the potential power of ML algorithms.

**Grant Acknowledgement**

This work was internally funded.

## Perioperative intensive care: Abdominal surgery

### 0355

#### Epidural analgesia prolongs ICU stay in patients after major abdominal surgery

D. Vriens, J. van Bommel

Erasmus MC, Intensive Care, Rotterdam, Netherlands

**Correspondence:** D. Vriens

*Intensive Care Medicine Experimental* 2017, **5(Suppl 2):**0355

**INTRODUCTION.** Epidural analgesia (EDA) for major abdominal surgery is usually associated with a beneficial effect on postoperative outcome. It is thought to reduce pulmonary complications, cardiac dysrhythmias and to accelerate the return of gastrointestinal transit.It is associated with a shorter length of ICU and hospital stay after HIPEC and esophagectomy procedures.[1] Yet in our center we see a longer ICU stay in patients with EDA because of hypotension and vasopressor use.

**OBJECTIVES.** To investigate the association between epidural analgesia and vasopressor use after elective major abdominal surgery.

**METHODS.** Medical records were reviewed from all consecutive patients admitted to the ICU after elective major abdominal surgery from June 2013 until November 2015. Prolonged duration of stay was defined as more than two days. The association between EDA and vasopressor use was quantified with a Mann–Whitney U test.

**RESULTS.** 195 patients were included, 46.2% after thoracoabdominal esophagectomy (TAE), 25.6% after HIPEC, 15.4% after abdominal aorta repair and 12.8% after liver surgery. 82% of patients received EDA. Of all patients with EDA, 61 patients (38%) required additional intravenous analgesia. TAE patients were most at risk for prolonged ICU stay.

Of patients with a prolonged duration of stay (36 patients), 72% had EDA and mean duration of vasopressors was twice compared to patients who stayed 2 days or less. We found a significant association between EDA and vasopressor use, especially in the TAE patients ($p = 0.009$). A trend towards significance was observed for HIPEC patients ($p = 0.06$).

**CONCLUSIONS.** These results confirm our perception that EDA is associated with a longer stay in patients after major abdominal surgery. This prolonged stay is mainly explained by hypotension and need for vasopressors.

In patients receiving EDA, sympathic outflow is blocked resulting in loss of vasomotor tone. Combined with a strong surgical trauma induced systemic inflammatory response, this can result in significant hypotension. This is detrimental for anastomotic healing and interferes with mobilization due to orthostasis. Vasopressor use, prolonged hypotension and fluid overload are all associated with an increase in gastro anastomotic leaks. [2]

We conclude that the possible benefits of EDA have to be considered carefully in patients undergoing major abdominal surgery when side effects lead to additional treatment and prolonged ICU stay, especially when ICU capacity is scarce and costly.

**REFERENCE(S)**

1. Heinrich, S., et al., *Short- and long term effects of epidural analgesia on morbidity and mortality of esophageal cancer surgery*. Langenbecks Arch Surg, 2015. **400**(1): p. 19–26.

2. Zakrison, T., et al., *Perioperative vasopressors are associated with an increased risk of gastrointestinal anastomotic leakage*. World J Surg, 2007. **31**(8): p. 1627–34.

**GRANT ACKNOWLEDGMENT**

No grands received.

### 0356

#### Factors relates to medium-term survival after liver transplantation (LT)

M. D. Arias-Verdú[1], M. Delgado-Amaya[1], J. Barrueco-Francioni[1], I. De Dios-Chacón[2], M.E. Herrera-Gutiérrez[1], G. Seller-Pérez[1]

Hospital Regional Universitario Carlos Haya de Málaga, Intensive Care Unit, Málaga, Spain; [2]Hospital Son Llatzer, Intensive Care Unit, Mallorca, Spain

**Correspondence:** M. D. Arias-Verdú

*Intensive Care Medicine Experimental* 2017, **5(Suppl 2):**0356

**OBJECTIVE.** To analyze the medium-term survival after a LT and to determine which factors are related with survival.

**METHODS.** Prospective cohort. Patients after LT over the period 2009–2014, follow up until 2016. We analyzed variables related to the long-term prognosis. Data: mean,median, Hazard-Ratio [H-R] Statistic: non-parametric tests, Chi-square, Kaplan-Meier, Log-Rank, Cox regression.

**RESULTS.** N = 253; 76.3% male; Mean age 54.7 ± 0.62 years Ethanol etiology 45.8%, mean MELD 16.36 ± (0.43, SOFA on admission 6.53 ± 0.19. Median days in ICU: 3 and hospital 8. ICU Mortality 4.7%, on ward 2.8% and overall 22.1%.

We performed a follow-up of 1174 days (632–1820). The complications were: no liver problems 78.7%, vascular problems 5.9%, hemorrhagic 3.6%, rejection 3.2%, recurrence of basal 3.2% and bile problems 3.2%. Mortality during follow-up was 14.6% and the cause of mortality at discharge from the hospital was recurrence of the underlying disease 21.6%, vascular problems 13.5%, bile 10.8%, rejection 5.4% and other causes 48.6%.

Univariate analysis: we found a relationship between survival and the history of renal failure, viral etiology, previous LT, SOFA at admission, plasma and noradrenaline supply after surgery, AKIN 3 in the immediate postoperative period and graft dysfunction, Multivariate analysis only found relationship between the survival and the viral etiology [HR 2.72 (1.54-4.81) (p0.001), previous LT [HR 3.63 (1.75-7, 53) (p 0.001)], SOFA at admission [HR 1.15 (1.06-1.25) (p 0.001)] and plasma transfusion [HR 2.14 (1.16-3.95) p 0.015].

**CONCLUSION.** Although the prognosis of LT patients is good, more than a quarter will present graft-related problems and mortality in the medium term remains high, close to 15%.

### 0357

#### Elective surgical admissions to Torbay Hospital ICU

S. Brown, T. Clark

Torbay Hospital, Intensive Care, Devon, United Kingdom

**Correspondence:** S. Brown

*Intensive Care Medicine Experimental* 2017, **5(Suppl 2):**0357

**INTRODUCTION.** Elective admission to the Intensive Care Unit (ICU) following major surgery comprises a significant proportion of all admissions. Historically this was reserved for high-risk cases, however there is increasing appreciation that lower risk patients may also benefit by preventing excess morbidity. Recent evidence shows that elective admission of patients post colorectal resection that are predicted to have a post-operative mortality of between 1 and 3% reduces complications, length of stay and costs[1].

At our District General Hospital we see all elective major surgical cases in a 'Shared Decision Making' pre-assessment clinic where we categorise patients depending on their predicted 30 day post-operative mortality (category (Cat) A if greater than 6%, Cat B if between 3 and 6%, Cat C if between 1 and 3% and Cat D if less than 1%). All patients with a predicted mortality of greater than 1% are considered for High Dependency Unit (HDU) care. Elective surgery currently accounts for 21% of all our ICU / HDU admissions, in comparison with emergency/urgent surgery that accounts for 23%. For the year 2015–16, 128 out of 613 patients admitted were elective surgical patients.

**OBJECTIVES.** To gain a better understanding of why patients with a 1-3% predicted post-operative risk benefit from HDU level care in the immediate post-operative period.

**METHODS.** All elective surgical admissions that were admitted to ICU/HDU between the months of August and November 2016 and who had a length of stay greater than 8 hours were considered for inclusion.

We collected baseline data on age, comorbidities, gender and pre-operative risk status. Outcome data included mean arterial blood pressure, vasopressor requirements, Sequential Organ Failure Assessment (SOFA) scores, development of complications and other levels of support.

**RESULTS.** Forty-six patients were included in data analysis.

| | |
|---|---|
| Categorisation (predicted mortality A > 6%, B 3 to 6%, C 1 to 3%, D < 1%) | Cat A n = 4 (95), Cat B n = 9 (20%), Cat C n = 24 (52%) Cat D n = 1 (2%) Uncategorised n = 8 (17%) |
| Surgical case split | General surgical n = 37 (80%) Urology n = 11 (23%) Vascular n = 2 (4%) |
| Mean age (years) | 72 (range 28 to 88) |
| Mean SOFA score | 4 (range 2 to 9) |
| Mean length of stay (days) | 1 (range 0 to 4) |
| Vasopressor use | 19 patients (41%) |
| Median vasopressor duration (hours) | 27 (range 2 to 50) |
| Vasopressor start time post-op (hours) | Immediate n = 7 (37%) <5 hours n = 8 (42%) 5 to 10 hours n = 1 (5%) 10 to 15 hours n = 3 (16%) |

[Table of results]

| | |
|---|---|
| Incidence Acute Kidney Injury | 8 (17%) |
| Incidence other complications | 2 (4%) |
| Incidence readmission post discharge | 2 (4%) |
| Incidence return to theatre | 1 (2%) |

[Incidence of complications]

Of the patients who were supported with vasopressors, 3 were Cat A, 4 were Cat B, 9 were Cat C (38% of all 24 cat C patients) and 1 was Cat D. 2 patients were uncategorised. Of the patients who developed acute kidney injury (AKI) 1 was Cat A, 2 were Cat B, 2 were Cat C and 3 were uncategorised.

**CONCLUSIONS.** Lower risk patients admitted to HDU / ICU after elective surgery have a significant rate (38%) of vasopressor use to maintain adequate mean arterial pressure. A service that provides this support may reduce post-operative morbidity by detecting and treating relative hypotension otherwise underreported on the ward.

**GRANT ACKNOWLEDGMENT**

Not applicable.

### 0358

#### The change of tissue oxygen saturation measured by dynamic near infrared spectroscopy following general anesthesia in healthy population: observation on the effect of age

A.-R. Cho[1], H.-J. Lee[1], J.-Y. Yoon[2], E.-J. Kim[2]

[1]Pusan National University Hospital, Anesthesia and Pain Medicine, Busan, Korea, Republic of; [2]Pusan National University Dental Hospital, Dental Anesthesia and Pain Medicine, Yangsan, Korea, Republic of

**Correspondence:** A.-R. Cho

*Intensive Care Medicine Experimental* 2017, **5(Suppl 2):**0358

**INTRODUCTION.** The key function of microcirculation is to deliver nutrients and oxygen to tissues to maintain cellular and organ function. The mirocirculatory dysfunction leads to regional hypoxia and organ failure. Vascular occlusion test (VOT) using near infrared spectroscopy (NIRS) is a method of observing the change in tissue oxygen saturation ($StO_2$) after applying a brief ischemic challenge. The reduction rate of $StO_2$ (occlusion slope) in the ischemic period reflects oxygen extraction. The rate of $StO_2$ increase (recovery slope) in the reperfusion period is known to reflect microvascular reactivity. The effects on microcirculation of general anesthetics have begun to be studied recently, and the results are still inconsistent. We assume that the inconsistent results may be due to the different states of microvasculature in the subjects. In order to understand the effects of general anesthesia on microcirculation, it is essential to study the effects on healthy patients first.

**OBJECTIVES.** The main purpose of this study is to investigate how general anesthesia affects microcirculation in healthy patients using VOT derived parameters with NIRS. We also examined the effect of age on microcirculation by analyzing young and elderly patients without accompanying diseases.

**METHODS.** This prospective observational study was performed on 57 patients without comorbidities who had undergone elective surgery under general anesthesia with inhalational agent. We measured $StO_2$ during VOT before (T0) and after general anesthesia (T1). We divided the patients into two subgroups at the age of 50 and compared VOT derived parameters.

**RESULTS.** General anesthesia increased $StO_2$ values and the recovery slope (2.91 ± 1.06% $sec^{-1}$ at T0 and 3.56 ± 1.70% $sec^{-1}$ at T1, p = 0.006) and reduced the occlusion slope (0.21 ± 0.08% $sec^{-1}$ at T0 and 0.15 ± 0.05% $sec^{-1}$ at T1, p < 0.001) (Fig. 119).

Similar changes were observed in young patients. On the contrary, in elderly patients, the recovery slope did not increase after general anesthesia (2.86 ± 0.98% $sec^{-1}$ at T0 and 3.16 ± 1.47% $sec^{-1}$ at T1, p = 0.227) (Fig. 120).

**CONCLUSIONS.** General anesthesia increases microcirculatory flow through vasodilation with improved microvascular reactivity, but these beneficial effects were not seen in elderly patients.

**REFERENCE(S)**

1. Butler E, Chin M, Aneman A. Peripheral Near-Infrared Spectroscopy: Methodologic Aspects and a Systematic Review in Post-Cardiac Surgical Patients. J Cardiothorac Vasc Anesth 2016 Jul 28.

2. Bernet C, Desebbe O, Bordon S, Lacroix C, Rosamel P, Farhat F, et al. The impact of induction of general anesthesia and a vascular occlusion test on tissue oxygen saturation derived parameters in high-risk surgical patients. J Clin Monit Comput 2011;25(4):237–44.

3. Kim TK, Cho YJ, Min JJ, Murkin JM, Bahk JH, Hong DM, et al. Tissue microcirculation measured by vascular occlusion test during anesthesia induction. J Clin Monit Comput 2016;30(1):41–50.

**GRANT ACKNOWLEDGMENT**

None.

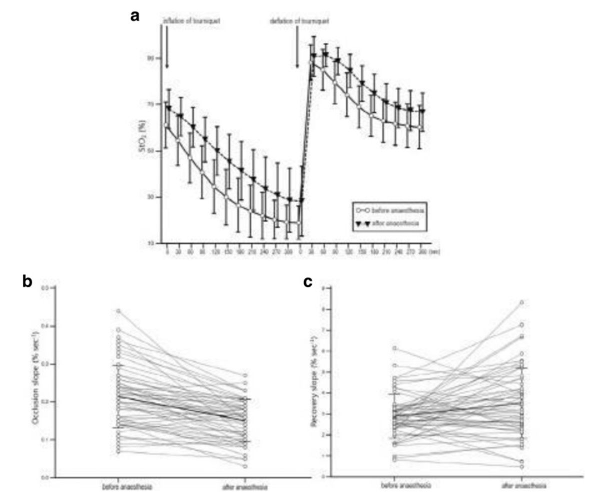

**Fig. 119 (abstract 0358).** See text for description

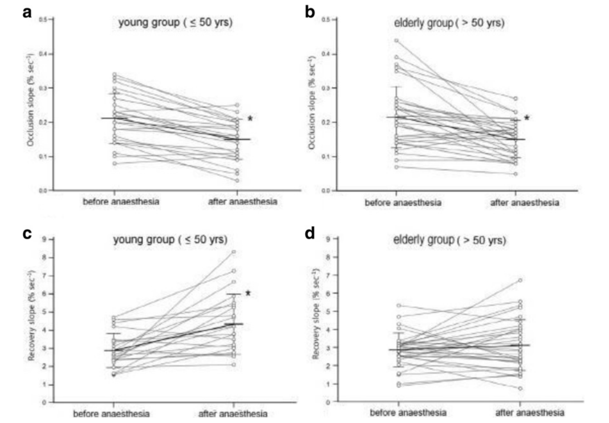

**Fig. 120 (abstract 0358).** See text for description

### 0359

#### Time course of C-reactive protein in patients with and without infection after liver resection as a function of functional liver remnant

J. Ijzerman[1], R. Steenstra[2], H. Banierink[1], M. W. Nijsten[1], M. Hoekstra[1]

[1]University Medical Center Groningen, Intensive Care, Groningen, Netherlands; [2]University Medical Center Groningen, Anesthesiology, Groningen, Netherlands

**Correspondence:** J. Ijzerman

*Intensive Care Medicine Experimental* 2017, **5(Suppl 2):**0359

**INTRODUCTION.** Post-operative infection is a common complication after liver resection. C-Reactive Protein (CRP) is an acute-phase protein that is frequently used as a marker of infection. As it is produced by the liver, major resection of the liver can influence the postoperative time course of CRP.

OBJECTIVE. In this retrospective study we determined the time course of CRP after liver resection as a function of functional liver remnant (FLR) in patients with and without infection.

**METHODS.** All consecutive patients (2008–2014) who were postoperatively admitted to the intensive care unit (ICU) after liver resection were included. Patients were classified based on a FLR of ≥60% (small resection), 35-60% (medium resection) and < 35% (large resection). Postoperative infection was defined as any infection during the first 10 postoperative days which resulted in antibiotic treatment. Numbers are expressed as mean ± standard deviation.

**RESULTS.** A total of 382 patients admitted to the ICU after liver resections were included (33% after small resection, 48% after medium resection and 19% after large resection). The main indication was colorectal liver metastasis (63%). A total of 120 patients (31%) developed a postoperative infection. In patients with a small liver resection there were significantly higher CRP levels during the first postoperative week compared to the larger resections (peak CRP 162 ± 61 mg/L versus 98 ± 47 mg/L, $P < 0.001$). Also in patients with a small liver resection the time course of CRP is markedly different in those who develop a postoperative infection ($P < 0.005$), with a more gradual decline after the peak at day 2. However in patients with a medium or large liver resection, there is no difference in the time course of CRP in patients with and without infection.

**CONCLUSIONS.** In patients after medium and major liver resection, there is no difference in the time course of postoperative CRP in patients with and without infection. Therefore CRP is not a useful marker of inflammation in this patient group.

### 0360

#### Intraoperative oxygen administration in patients undergoing orthotopic liver transplantation

W. J. Liew[1], C. M. Morkane[1,2], A. Strachan[2], D. S. Martin[1,2]
University College London, London, United Kingdom; [2]Royal Free Hospital NHS Foundation Trust, London, United Kingdom
**Correspondence:** C. M. Morkane
*Intensive Care Medicine Experimental* 2017, **5(Suppl 2):**0360

**INTRODUCTION.** Supplemental oxygen is the most commonly administered drug during general anaesthesia. Hyperoxaemia ($PaO_2 > 13.3$ kPa) is associated with adverse changes to the respiratory and cardiovascular systems (1,2). This has particular relevance during liver transplantation where ischaemia-reperfusion injury (IRI) post liver reperfusion is potentiated by oxygen-induced production of reactive oxygen species and immune cell activation (3). Avoidance of excessive oxygen levels during general anaesthesia could therefore lead to improved perioperative outcomes.

**OBJECTIVES.** This service evaluation characterised current anaesthetic practice as regards intraoperative oxygenation during liver transplantation in a single centre and looked at postoperative markers of IRI and outcome.

**METHODS.** This retrospective service evaluation looked at intraoperative data for sequential liver transplants performed over a period of 26 months (November 2013 to January 2016) at the Royal Free Hospital. Data were retrieved from anaesthetic charts, arterial blood gases (ABGs), laboratory reports and hospital records.

We used ABG data to generate a graphical intraoperative oxygenation profile for each patient and calculate the area under the curve (AUC) of $PaO_{2;}$ allowing us to determine cumulative intraoperative $PaO_{2.}$

**RESULTS.** 201 patients were included; 72.8% were male (n = 150). The median age of all patients was 51.5 years. The median number of intraoperative ABGs per patient was 9 (range 4 to 23). Table 105 demonstrates oxygenation and haemoglobin values according to operative stage for all patients:

The mean cumulative oxygen dose during liver transplantation was 10,872 kPa with a mean duration of surgery of 433 minutes. The relationship between cumulative oxygen dose and a number of postoperative outcomes and markers of IRI was explored (Table 106):

**CONCLUSIONS.** We have demonstrated the consistent presence of intraoperative hyperoxaemia throughout liver transplantation surgery in our centre and there appears to be no adjustment of $FiO_2$ in response to this. No significant associations were found between oxygen dose and markers of ischaemia-reperfusion injury. Debate persists around the area of perioperative oxygenation however and the question of 'how much is too much' remains unanswered in the field of liver transplant surgery. Further research is required to explore this question in other transplant centres.

**REFERENCE(S)**
1. Milone SD, Newton GE, Parker JD. Hemodynamic and biochemical effects of 100% oxygen breathing in humans. Can J Physiol Pharmacol. 1999;77:124–130.
2. Farquhar H, Weatherall M, Wijesinghe M et al. Systematic review of studies of the effect of hyperoxia on coronary blood flow. Am Heart J. 2009;158:371–377.
3. Zangl Q, Martignoni A et al. Postoperative hyperoxia (60%) worsens hepatic injury in mice. Anaesthesiology. 2014; 121:1217–25

**Table 105 (Abstract 0360).** Oxygenation values according to operative stage

| Operative stage (number of samples) | Median FiO2 (IQR) | Median PaO2 in kPa (IQR) | Median P:F ratio in kPa (IQR) | Median Haemoglobin g/l (IQR) |
|---|---|---|---|---|
| Stage 1: dissection (583) | 0.48 (0.09) | 24.82 (11.10) | 54.96 (20.84) | 92 (25) |
| Stage 2: anhepatic (305) | 0.49 (0.07) | 26.60 (8.79) | 56.50 (19.01) | 90 (23) |
| Stage 3: reperfusion (641) | 0.47 (0.11) | 24.60 (10.61) | 54.69 (19.48) | 85 (17) |

**Table 106 (Abstract 0360).** Postop outcomes & relationship to cumulative O2

| | Median (IQR) | p value |
|---|---|---|
| Peak AST (U/l) | 1493 (2085) | 0.98 |
| Day 3 AST (U/l) | 154 (180) | 0.58 |
| 24 hr creatinine (umol/l) | 110 (93) | 0.40 |
| ICU LOS (nights) | 4 (4) | 0.14 |

### 0361

#### Addition of abnormal preoperative coronary CT angiographic findings improves predictive performance of revised cardiac risk index in liver transplant surgery

Y.-S. Park, Y.-J. Moon, I.-G. Jun, J.-G. Song, G.-S. Hwang
University of Ulsan College of Medicine, Asan Medical Center, Department of Anesthesiology and Pain Medicine, Seoul, Korea, Republic of
**Correspondence:** Y.-S. Park
*Intensive Care Medicine Experimental* 2017, **5(Suppl 2):**0361

**INTRODUCTION.** With improved surgical techniques of liver transplant (LT), perioperative major cardiac events (PMCE) have been emerged as one of leading cause of death in the perioperative period. The revised cardiac risk index (RCRI) is well-known risk stratification index. We investigated the predictive value of preoperative coronary CT angiography (CTA) in patients undergoing LT.

**OBJECTIVES.** Identify the value of coronary CTA in predicting perioperative cardiac risk in liver transplant recipients.

**METHODS.** Of patients undergoing living-donor LT surgery from April 2010 to October 2015, we retrospectively analyzed 1512 patients (mean age, 53 years; male sex, 77%; mean MELD score, 16) who underwent coronary CTA for screening of coronary artery disease (CAD) within a year before the surgery. PMCE were defined as non-fatal myocardial infarction (MI), death, or combined events within 30 days after LT surgery. The severity of CAD was classified as normal (no atherosclerosis), minimal (<25%), mild (25-50%), moderate (50-70%), or severe (>75% narrowing) stenosis. Non-obstructive CAD was defined as stenosis degree of no more than minimal or mild in all coronary artery and obstructive CAD was defined as moderate or severe stenosis. The RCRI score was calculated as the total number of known independent predictors.[1]

**RESULTS.** Non-obstructive and obstructive CAD were found in 33.9% (512/1512 patients) and 7.7% (117/1512 patients), respectively. PMCE developed in 2.9% (44/1512 patients). The incidence of PMCE was significantly increased with increasing severity of CAD [2.4% (21/883) vs 2.7% (14/512) vs 7.7% (9/117) for patients with normal coronary vessel, non-obstructive, and obstructive CAD, respectively, P = 0.001]. The RCRI score 1 identified 1, 240 (82.0%) patients, score 2 identified 251 (16.6%) patients, and score 3 identified 21 (1.4%) patients, and each score group showed event rate of 1.3% (16/1240 patients), 9.6% (24/251 patients), and 19.0% (4/21 patients), respectively (P < 0.001). In receiver operating characteristic (ROC) curve analysis, predictive performance of RCRI alone (model 1) showed significant better C-statistic compared with predictive model of RCRI plus MELD score (model II) and RCRI plus MELD score plus severity of CAD (model III) (C-statistic, 0.739; 0.800; 0.817; P < 0.01, respectively, Fig. 121).
**CONCLUSIONS.** Addition of abnormal coronary CTA to MELD score and RCRI improved perioperative risk stratification, suggesting that preoperative severe CAD deserves to be identified using coronary CTA to predict the development of PMCE in patients undergoing LT surgery.
**REFERENCE(S)**
1. Fleisher LA, Fleischmann KE, Auerbach AD, et al. 2014 ACC/AHA guideline on perioperative cardiovascular evaluation and management of patients undergoing noncardiac surgery: a report of the American College of Cardiology/American Heart Association Task Force on practice guidelines. *J Am Coll Cardiol*. 2014;64:e77-137.

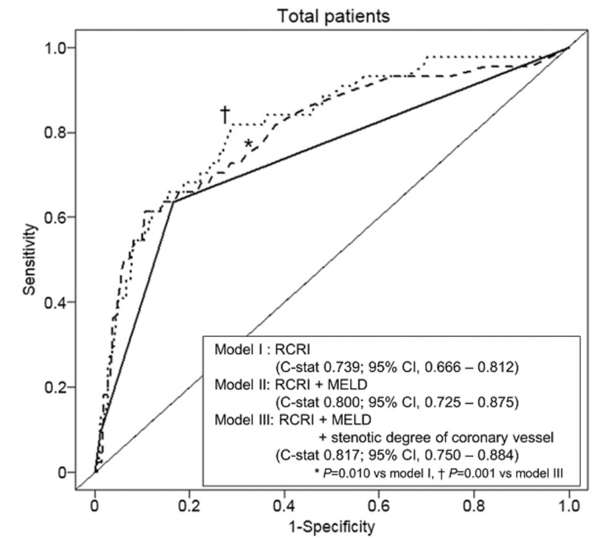

**Fig. 121 (Abstract 0361).** See text for description

### 0362
### Predictive factors of anastomotic leakage in the Intensive Care Unit at Punta de Europa Regional Hospital
A. Ubeda[1], I. Fernández[1], R. Torcuato[1], L. Alonso[2], A. Fregosi[1], J. Garcia[1]
[1]Hospital Punta de Europa, Intensive Care Unit, Algeciras, Spain;
[2]Pediatric La Lobilla, Estepona, Spain
**Correspondence:** A. Ubeda
*Intensive Care Medicine Experimental* 2017, **5(Suppl 2):**0362

**INTRODUCTION.** Anastomotic leakage (AL) is one of the most serious complications of gastrointestinal tract surgery. Several studies have evaluated a variety of risk factors of AL; however there is no universal agreement regarding the associated risk factors.
**OBJECTIVES.** To analyze postoperative patients admitted to an Intensive Care Unit (ICU) and to determine predictive factors of AL in ICU.
**METHODS.** Retrospective cohort study of all the adult patients admitted to a 12-bed Intensive Care Unit after gastrointestinal tract surgery between 1 January 2011 and 31 December 2015. Statistical analysis: categorical variables (frequencies and percentages) and numerical variables (mean and standard deviation or medians and interquartile range). Comparisons: X2 test (percentages), Student´s test (means) and Kruskal-Wallis test (medians). Statistical significance p < 0.05. Multivariate logistic regression models were calculated. The analysis was performed with the software SPSS v21.
**RESULTS.** We identified 376 patients; mean age 66.6 ± 14.6, 63% were male. Mean APACHE II 11.3 ± 4.9, SOFA 5.2 ± 3.8. Comorbidities: solid tumor (61.4%), DM (34.3%), liver disease (26.3%), COPD (24.5%), chronic renal failure (12.5%), obesity (14.3%). Emergency surgery 41.8%, elective 58.2%. Procedures: partial colectomy (n = 74), hepatectomy (n = 49), small bowell resection (n = 35), colostomy/ileostomy (n = 29), pancreaticoduodenectomy (n = 17), total colectomy (n = 15). We found 64 (17%) anastomotic leakage. ICU mortality 20.5%, hospital mortality 24.8%. Blood transfusion during surgery 20.2%. Clinical situation at ICU admission: sepsis (15.7%), severe sepsis/septic shock (37.2%). Vasoactive agents (first 24 hours in ICU): noradrenaline (NAD) 43.1%, dobutamine 9.8%, dopamine 2.1%. Patients with AL: higher APACHEII (12.7 ± 4.9 vs. 11.1 ± 4.9, p = .012), hospital lenght of stay (24.1 ± 22.2 vs. 18 ± 18.1 days, p = .033), COPD (39.1% vs. 21.5%, p = .003), elective surgery (70.3% vs. 55.8%, p = .032), clinical situation severe sepsis/septic shock (46.8% vs. 35.2%, p = .006), vasoactive drugs (NAD 53.1% vs. 41%) (p = .001), mechanical ventilation (64.1% vs. 47.1%, p = .014), corticosteroids (14.1% vs. 3.8%, p = .004), haemoglobin at admission (9.8 ± 1.6 vs. 10.5 ± 2.0 g/dL, p = .007). Multivariate logistic regression model: elective surgery and use of corticosteroids were independent predictors of AL in ICU.
**CONCLUSIONS.** Elective surgery and use of corticosteroids were predictors of AL in adult patients admitted to ICU after gastrointestinal surgery. There were no relation between AL and mortality.
**REFERENCE(S)**
None.
**GRANT ACKNOWLEDGMENT**
None.

### 0363
### Postoperative intensive care course of patients undergoing first and second stage of Associating Liver Partition and Portal Vein Ligation for Staged hepatectomy (ALPPS)
A.C. Kastoris[1], G. Fragkiadakis[1], M. Michaelides[1], C. Charalambous[2], A. Petrou[2], T. Kyprianou[1,3]
[1]Nicosia General Hospital, Intensive Care Department, Nicosia, Cyprus;
[2]Nicosia General Hospital, Surgical Department, Nicosia, Cyprus;
[3]University of Nicosia Medical School, Nicosia, Cyprus
**Correspondence:** A.C. Kastoris
*Intensive Care Medicine Experimental* 2017, **5(Suppl 2):**0363

**INTRODUCTION.** Associating liver partition and portal vein ligation for staged hepatectomy (ALPPS) first performed in Germany in 2007 may minimize the risk of post-hepatectomy liver failure by producing rapid marginal future liver remnant hypertrophy decreasing the time between the first surgery and hepatectomy. Postoperative intensive care course of patients undergoing ALPPS has not been previously described as published series are still few.
**OBJECTIVES.** The aim of this study is to document the postoperative intensive care course, hemodynamic and metabolic management and ICU outcome of patients undergoing the ALPPS procedure.
**METHODS. Inclusion criteria:** All patients undergoing ALPPS in Nicosia General Hospital since 2015.
Tertiary, referral, University affiliated Hospital, multidisciplinary ICU/HDU reporting.
Prospective, observational study, data collection from Hospital EMR / ICU CIS. Patients' demographics, type and staging of cancer, comorbidities, and time elapsed between the 2 stages are reported as well as intra-operative data. Postoperative Intensive Care course is described in detail in terms of hemodynamic management including fluids and vasopressors, acid–base balance, kinetics of lactate, blood sugar, clotting profile, and liver function tests. ICU outcome is documented in terms of morbidity, length of ICU stay and ICU mortality.
**RESULTS.** Four patients had undergone the ALPPS procedure between January 2015 and April 2017. Patient demographics are presented in Table 107.
Three patients had metastatic cancer, 2 with metastatic colon cancer and 1 patient with ovarian cancer, all of whom had undergone several cycles of chemiotherapy. Only one patient presented a Klatskin tumour. Cancer staging in all patients was Stage IV cancer. All four patients underwent an extended right hepatectomy.
The mean time for stage 1 of the operation was 3.5 hours (3–4 hours). For stage 2, the mean operation time was 5.25 hours (4.5-6 hours).
Depicted in Figs. 122 and 123 are fluid management and lactate kinetics.
During the first part of the ICU stay the mean daily fluid infusions were 4.5 litres, whilst during the second part of the ICU stay fluids were infused with a daily average of 3.8 Litres.
ICU morbidity, mortality and length of stay are presented in Table 108.
ICU length of stay for both parts of the procedures ranged between 3 and 12 days with a mean of 7.5 days. The mean length of stay for the second procedure was 4 days with a range between 1 and 9 days. The patient with the longest stay in the ICU died less than 24 hours after the completion of the second part of the procedure and required renal replacement therapy during his stay. No infections were recorded during the ICU stay in any of the patients.
**CONCLUSIONS.** We present the post-operative course in the ICU of four patients that underwent the ALPPS procedure. Further research and evaluation in larger scale studies is necessary in order to optimise the post-operative care of such patients.

**Table 107 (Abstract 0363)**. Patient Demographics

| Patient Number | Age, Sex | Type of Cancer | Cancer Staging | Comoridities | Time elapsed between two stages in days |
|---|---|---|---|---|---|
| 1 | 67, M | Metastatic Colon Cancer | Stage IV | Moderate COPD | 12 |
| 2 | 79, M | Klatskin Tumor | Stage IV | None | 12 |
| 3 | 78, F | Metastatic Ovarian Cancer | Stage IV | Hypertension, Atrial Fibrillation | 9 |
| 4 | 72, M | Metastatic Colon Cancer | Stage IV | Deep vein thrombosis, pulmonary embolism, pleural effusions | 6 |

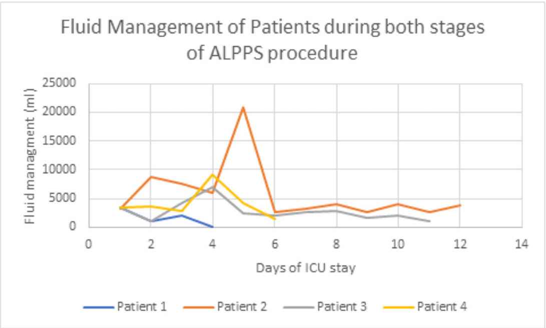

**Fig. 122 (Abstract 0363).** Fluid management in the ICU

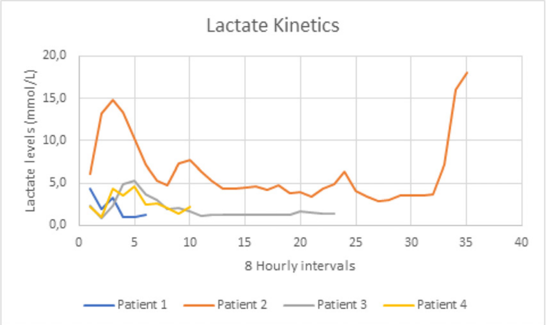

**Fig. 123 (Abstract 0363).** Lactate Kinetics

**Table 108 (Abstract 0363).** ICU morbidity, mortality, length of stay

| Patient Number | ICU Morbidity | ICU length of stay in days - 1st procedure | ICU length of stay in days - 2nd procedure | ICU Mortality - Glasgow outcome scale |
|---|---|---|---|---|
| 1 | None | 1 | 2 | 5 |
| 2 | Renal replacement therapy (5 of the 12 days spent in ICU), Hepatic insufficiency | 11 | 1 | 1 |
| 3 | Atrial Fibrillation | 1 | 9 | 5 |
| 4 | None | 1 | 4 | 5 |

### 0364

#### Effect of perioperative low-dose dexmedetomidine on postoperative delirium in the intensive care unit after liver transplantation - a preliminary report

S.M. Yang, H. Lee, J. Bae, S. Yoon, C.-W. Jung, H.G. Ryu
Seoul National University Hospital, Department of Anesthesiology and Pain Medicine, Seoul, Korea, Republic of
**Correspondence:** S.M. Yang
*Intensive Care Medicine Experimental* 2017, **5(Suppl 2):**0364

**INTRODUCTION.** Delirium after liver transplantation is one of the major challenges in postoperative care because it is associated with increased hospital length of stay and higher morbidity and mortality. Dexmedetomidine has been reported to be effective in lowering the incidence of delirium when used as a sedative agent in the intensive care unit (ICU). A randomized controlled clinical trial was performed to investigate whether perioperative low dose dexmedetomidine infusion decreases the incidence of delirium after liver transplantation.
**METHODS.** In this randomized controlled trial we enrolled patients scheduled for living donor liver transplantation between August 11, 2014 and March 27, 2017, with informed consent. The liver recipients were randomized to either the control group or the dexmedetomidine group. Dexmedetomidine was continuously infused during anesthesia and was continued until 72 hours postoperatively in the ICU in the dexmedetomidine group while 0.9% normal saline was infused in the same manner in the control group. The incidence of delirium after liver transplantation was compared between the two groups. Factors that can affect the occurrence of delirium such as patient age, Acute Physiology and Chronic Health Evaluation score, preoperative and postoperative ammonia level, the difference in pre and postoperative ammonia, surgical time, and preoperative hepatoencephalopathy were also analyzed. ICU length of stay (LOS) and hospital LOS were also compared.

**RESULTS.** A total of 158 patients were enrolled and a total of 144 patients were used in the analysis. From the 67 patients in the dexmedetomidine group and 77 patients in the control group, delirium occurred in a total of 12 patients. There was no significant difference in the incidence of delirium in both groups (7% [5/67] in the dexmedetomidine group vs 9% [7/77] in the control group p = 0.752, and the results showed no significance in multivariate analysis (OR 1.448, 95% CI 0.268 - 7.818, p = 0.667) as well. Only the Model for End-Stage Liver Disease (MELD) score showed to be significant in its relation with delirium. MELD score greater than 17 showed to have an OR 19.074 with 95% CI 2.599 - 139.988. However, subgroup analysis of the MELD score did not show any significance. There was no significant difference in the ICU LOS and hospital LOS in the two groups as well.

**CONCLUSION.** Perioperative low dose dexmedetomidine infusion did not reduce the incidence of delirium in living donor liver transplantation recipients in the ICU. Because of the low incidence of delirium, however, this study could have been underpowered, and further studies are needed. MELD score greater than 17 was the only significant variable in the incidence of delirium in the ICU in this study.

### 0365

#### Evaluation of the influence of sufentanil and reminfentanil on colon motility via kappa receptors in vitro

M. Schörghuber[1], E. Tatzl[1], P. Holzer[2], W. Toller[1], S. Fruhwald[1]
[1]Medical University of Graz, Department for Anesthesiology and Intensive Care Medicine, Graz, Austria; [2]Medical University of Graz, Graz, Austria
**Correspondence:** M. Schörghuber
*Intensive Care Medicine Experimental* 2017, **5(Suppl 2):**0365

**INTRODUCTION.** ICU patients frequently develop gastrointestinal (GI) motility disorders. Opioids, mandatory for pain management, play a major role in the development of GI motility disorders. Their influence on the GI tract is reduced by the administration of opioid receptor antagonists (ORA) like Naloxon and Methylnaltrexon (MNTX). Naloxon, a pan-ORA with a high antagonistic potency, crosses the blood brain barrier and by this may alter analgesia while the peripheral ORA MNTX has no effects on analgesia but restores or improves bowel motility in only about 50% treated patients. This may be explained by its low affinity to κ receptors.

**OBJECTIVES.** We used an experimental setting to evaluate if and to which extent Remifentanil and Sufentanil inhibit colonic motility via their interaction with κ receptors.

**METHODS.** Guinea pig's colonic segments were fixed on a polyacrylic tray in a tissue bath. The speed of an intraluminally placed pellet along these segments was measured before ($V_0$) and after administration ($V_1$) of the test substances. The influence of the substances on the transit speed was calculated as ratio $V_1/V_0$. A $V_1/V_0$ ratio of 1 means no influence on peristalsis, while $V_1/V_0 > 1$ means stimulation and a $V_1/V_0 < 1$ demonstrates an inhibition of peristalsis. In a first step Sufentanil 1nM and Remifentanil 30nM were tested alone to quantify their inhibitory effect on colon transit. In a second step we evaluated a combination of each opioid with the antagonists Naloxon (0.03µM) or MNTX (1µM). Finally we tested a combination of the highly selective κ ORA Norbinaltorphimine (nor-BNI 0.03µM) and Remifentanil or Sufentanil to quantify the κ receptor associated effect.

**RESULTS.** (mean ratio ± SEM): Sufentanil (0.2 ± 0.08) and Remifentanil (0.02 ± 0.01) had a pronounced inhibitory effect on colon motility. Naloxon (1.0 ± 0.1, p < 0.001) or MNTX (0.9 ± 0.14, p < 0.001) plus Sufentanil had a high potency to restore colonic motility. Although not significant, Naloxon's antagonistic effect seemed more pronounced compared to MNTX. Nor-BNI (0.59 ± 0.14, p = 0.026) also had a distinct antagonistic effect.

In combination with Remifentanil Naloxon (0.85 ± 0.08, p < 0.001) had a greater antagonistic effect by trend than MNTX (0.73 ± 0.2, p < 0.001). Again nor-BNI (0.3 ± 0.1, p = 0.02) also markedly reduced the inhibitory effect of Remifentanil.

**CONCLUSIONS.** Despite the 30 times higher concentration MNTX had a lower potency to restore the inhibitory opioid effects compared to Naloxon. This may be explained by the high affinity of Naloxon to all opioid receptors in contrast to the peripheral antagonist MNTX. Furthermore we were able to demonstrate a distinct potency of nor-BNI to restore opioid induced colonic motility disorders in this experimental setting which shows a notably effect of opioids on colon peristalsis via κ receptors.

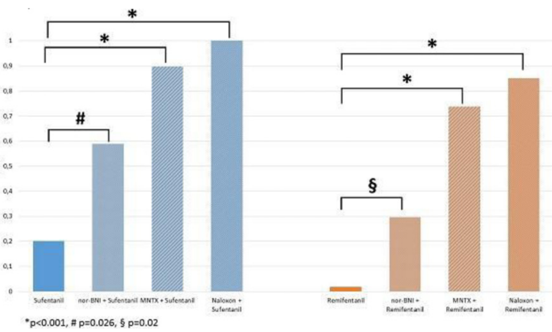

**Fig. 124 (Abstract 0365).** graphs

### 0366

#### Recombinant thrombomodulin attenuates remote liver injury induced by extracellular histones in intestinal ischemia-reperfusion injury

N. Hayase[1], T. Hiruma[1], K. Mayumi[2], T. Yamashita[2], Y. Hamasaki[2], E. Noiri[2], M. Nangaku[2], N. Morimura[1], K. Doi[1]
[1]University of Tokyo, Department of Acute Medicine, Tokyo, Japan; [2]University of Tokyo, Department of Nephrology and Endocrinology, Tokyo, Japan
**Correspondence:** N. Hayase
*Intensive Care Medicine Experimental* 2017, **5(Suppl 2):**0366

**INTRODUCTION.** Intestinal ischemia is a life-threating abdominal emergency encountered in a variety of clinical settings. Not only ischemia but reperfusion (I/R injury) triggers an intense inflammatory injury locally and subsequently in remote organs[1]. Recently, extracellular histones have been demonstrated as mediators of distant organ damage in acute kidney injury and severe trauma[2, 3]. Recombinant thrombomodulin (rTM) has been shown to possess anti-inflammatory roles by inhibition of circulating histones[4].

**OBJECTIVES.** We aimed to elucidate the effect of rTM on intestinal I/R injury and the underlying mechanism of I/R injury-induced remote organ damage.

**METHODS.** Intestinal ischemia was induced in male C57BL/6J mice by clamping superior mesenteric artery for 30 minutes or 45 minutes. Intraperitoneal administration of 10 mg/kg rTM was conducted at the initiation of reperfusion. For mice with intestinal I/R injury (ischemia for 30 minutes and reperfusion for 180 minutes), the levels of tissue damage, expression of inflammatory genes, and extracellular histones were assessed in the jejunum, liver, lung, and kidney. Neutrophil extracellular traps (NETs) formation were also evaluated by immunostaining.

**RESULTS.** Treatment with rTM markedly improved survival of intestinal I/R injured mice (hazard ratio 0.31; p = 0.014, n = 11 per group). We detected significantly decreased expression of tumor necrosis factor alpha, interleukin-6, and keratinocyte-derived chemokine (n = 7 per group, p < 0.05), and degree of tissue damage in the liver, but not in the intestine compared with untreated I/R mice. Moreover, intense accumulation of histones and NETs formation were found in the liver among remote organs (liver 0.049 ± 0.011 AU/μg wet tissue, lung 0.014 ± 0.008 AU/μg wet tissue, and kidney 0.006 ± 0.008 AU/μg wet tissue, n = 4–5 per group, p < 0.05 vs. lung and kidney), and rTM significantly decreased the level of histones in the liver (n = 8 per group, p < 0.05).

**CONCLUSIONS.** Our finding suggests that NETs and extracellular histones cause liver injury through release of inflammatory cytokines following intestinal I/R injury. Recombinant thrombomodulin may have significant impact on blocking histones and decreasing expression of inflammatory cytokines in the liver and contribute to improvement of survival in mice with intestinal I/R injury.

**GRANT ACKNOWLEDGMENT**

This study was supported by KAKEN-HI 16K09603 (to KD), MEXT, Japan.

### 0367

#### Microcirculation in healthy volunteers, dialysis patients and kidney transplant recipients

Y.-C. Yeh[1], A. Chao[1], C.T. Lee[1], M.-K. Tsai[2]

[1]National Taiwan University Hospital, Department of Anesthesiology, Taipei, Taiwan, Province of China; [2]National Taiwan University Hospital, Department of Surgery, Taipei, Taiwan, Province of China

**Correspondence:** Y.-C. Yeh

*Intensive Care Medicine Experimental* 2017, **5(Suppl 2):**0367

**INTRODUCTION.** Microcirculatory dysfunction are associated with acute and chronic kidney diseases [1]. Only few studies tried to compare microcirculation between healthy volunteers and patients with chronic kidney disease. In addition, we found only one study demonstrating that systemic microcirculatory dysfunction in type I diabetes mellitus patients was improved in the first year after simultaneous transplantation of the pancreas and kidney [2].

**OBJECTIVES.** The study aimed to compare the difference in microcirculation among healthy volunteers, dialysis patients, and kidney transplant recipients with a latest generation of hand-held video microscope.

**METHODS.** This observational study was approved by the National Taiwan University Hospital Research Ethics Committee, which is registered on the ClinicalTrials.gov Protocol registration system (ID: NCT02412839 and NCT02940275). Sublingual microcirculation was examined using incident dark field imaging and was compared among healthy volunteers, dialysis patients, and kidney transplant recipients.

Age, height, and body weight were recorded. Heart rate and blood pressure were measured. Data were analyzed using the statistical software program SPSS 20 (IBM SPSS, Chicago, IL, USA). Numerical data are expressed as mean (standard deviation) and were compared using one-way analysis of variance, followed by Tukey multiple comparison tests.

**RESULTS.** Heart rates, systolic blood pressure, diastolic blood pressure, and mean arterial pressure were higher in the dialysis patients and kidney transplant recipients than in the healthy volunteers.

| Group | Healthy volunteers | Dialysis patients | Kindey transplant recipients | P value |
|---|---|---|---|---|
| Female/Male (n/n) | 40/30 | 2/8 | 7/8 | 0.084 |
| Age (years) | 43.7 (13.5) | 46.1 (11.6) | 49.5 (13.2) | 0.299 |
| Height (cm) | 165 (8) | 166 (5) | 162 (9) | 0.445 |
| Weight (kg) | 61.8 (10.0) | 65.6 (8.2) | 62.4 (12.0) | 0.546 |
| Heart rate (bpm) | 71 (9) | 86 (14)* | 79 (14)* | <0.001 |
| Systolic blood pressure (mm Hg) | 119 (11) | 141 (28)* | 134 (19)* | <0.001 |
| Diastolic blood pressure (mm Hg) | 71 (9) | 88 (13)* | 79 (11)* | <0.001 |
| Mean arterial pressure (mm Hg) | 87 (9) | 106 (18)* | 97 (12)* | <0.001 |

*[Basic characteristics]*

Perfused vessel density and proportion of perfused vessels were lower in the dialysis patients than in the healthy volunteers.

| Group | Healthy volunteers | Dialysis patients | Kidney transplant recipients | P value |
|---|---|---|---|---|
| n | 70 | 10 | 15 | |
| TVD (mm/mm$^2$) | 27.3 (2.1) | 26.0 (1.8) | 26.9 (2.2) | 0.149 |
| PVD (mm/mm$^2$) | 26.8 (2.1) | 25.0 (2.2)* | 26.2 (2.4) | 0.046 |
| PPV (%) | 98 (2) | 96 (4)* | 98 (2) | 0.042 |
| TSVD (mm/mm$^2$) | 25.7 (2.2) | 24.9 (2.3) | 26.1 (2.5) | 0.372 |
| PSVD (mm/mm$^2$) | 25.2 (2.3) | 23.9 (2.8) | 25.5 (2.6) | 0.218 |
| PPSV (%) | 98 (2) | 96 (4)* | 98 (2) | 0.048 |
| Microvascular flow index | 2.9 (0.1) | 2.8 (0.2) | 2.9 (0.1) | 0.094 |
| Heterogeneity index | 0.08 (0.10) | 0.13 (0.12) | 0.11 (0.14) | 0.212 |

*[Microcirculation parameters]*

Perfused vessel density and proportion of perfused vessel did not differ significantly between kidney transplant recipients and healthy volunteers.

**CONCLUSIONS.** Perfused vessel density was lower in the dialysis patients than in the healthy volunteers, and it did not differ significantly between kidney transplant recipients and healthy volunteers.

**REFERENCE(S)**

1. Zafrani L, Ince C. Microcirculation in Acute and Chronic Kidney Diseases. Am J Kidney Dis 2015;66:1083–1094.

2. Khairoun M, de Koning EJ, van den Berg BM, Lievers E, de Boer HC, Schaapherder AF, et al. Microvascular damage in type 1 diabetic patients is reversed in the first year after simultaneous pancreas-kidney transplantation. American Journal of Transplantation2013;13:1272–1281.

**GRANT ACKNOWLEDGMENT**

This study was supported, in part, by grants from the National Taiwan University Hospital (NTUH 105-A125).

#### 0368

**Donor's age impact on cumulative survival rates of live graft and recipient of liver transplantation in 20 years of experience in Santiago de Compostela (Spain)**

J.R. Fernandez Villanueva[1], R. Fernandez Garda[1], R. Hernandez Vaquero[1], E. Rodriguez Ruiz[1], A.M. Lopez Lago[1], C. Galban Rodriguez[1], E. Varo Perez[2]

[1]Hospital Clínico Universitario de Santiago de Compostela, Intensive Care Unit, Santiago de Compostela, Spain; [2]Hospital Clínico Universitario de Santiago de Compostela, Abdominal Transplant Unit, Santiago de Compostela, Spain

**Correspondence:** R. Hernandez Vaquero

*Intensive Care Medicine Experimental* 2017, **5(Suppl 2):**0368

809 Liver Transplants (LT) were made at Hospital Clínico Universitario of Santiago de Compostela (Spain) in the period 1994–2014, accounting 4.25% of all LT performed in Spain. Review of the impact of the Donor´s Age on the Survival of Graft and Recipient in 20 years of experience in our center.

**MATERIALS AND METHODS.** Retrospective and descriptive review of 809 cases of liver transplantation performed in the 1994–2014 period at the Hospital Clínico Universitario of Santiago de Compostela (Spain) acording to the Local Registry and the Spanish Registry of Liver Transplantation (RETH).

**RESULTS.** 809 cases, 12 cases were Hepato-renal transplantation, 3 requiered Re- transplant. Media of Donors: 33 cases/year. 79.35% Men and 20.64% Women with Recipeint Mean Age: 51 years old. Predomintaly Blood group A (49%). Most frequent LT indication: Alcoholic Cirrhosis (43,%), Idiopathic (43.01%) and Fulminant Liver Failure (6.18%). Cumulative Survival at 20 years in our serie: 47% higher than the comparison with the global data provided by RETH (37% applying Kaplan-Meier curve p <0.01) and in the last 5 years (2008–2013): 77% compared to RETH data for the same period (70% with p <0.05). The Mean Age of the Donor in Brain Death: 53.5 years, increasing exponentially from the beginning of the program with a Median of 35 years (1994) to 68 years (2014).

Impact of Survival Rate of the Receiver based on the Donor's Age divided into Age gropus (p 0.07): <50 years: 70%, 50–59 years: 59%, 60–69 years: 55% and >70 years: 53%. Impact on Cumulative Survival Rate of the Graft (p 0.06): <50 years: 65%, 50–59 years: 58%, 60–69 years: 50%, >70 years: 50%. Segregated by Indication of LT (p <0.05): Cirrhosis non HCV and non-Hepatocarcinoma: <50 years: 58%, 50–59 years: 50%, 60–69 years: 49%, >70 years: 49% and the group of Cirrhosis with Hepatocarcinoma: <50 years: 45%, 50–59 years: 40%, 60–69 years: 32% > 70 years: 35%.

**CONCLUSIONS.** An exponential increase in Donor's Age in Brain Death in our serie has non-statistically significant impact on Graft and Recipient Cumulative Survival Rates, although it was statistically significant in the Segregated group of Cirrhosis with Hepatocarcinoma with low Cumulative Survival Rates in all Age groups and even lower in those recipients with grafts coming from Elderly Donors. Cumulative Survival Rate globally is similar to the ones found out in other Transplantation Programs.

#### 0369

**Eotaxin-1 (CCL11) is detectable in donated blood products and increases with the donor`s age**

J. Hoefer[1], M. Luger[2], C. Dal-Pont[3], Z. Culig[4], H. Schennach[3], S. Jochberger[5]

[1]Medical University Astana, University of Innsbruck, Urology, Innsbruck, Austria; [2]Medical University of Innsbruck, Innsbruck, Austria; [3]Medical University of Innsbruck, Central Institute for Blood Transfusion & Immunological Department, Innsbruck, Austri0; [4]Medical University of Innsbruck, Urology, Innsbruck, Austria; [5]Anaestesiology and Critical Care Medicine, Innsbruck, Austria

**Correspondence:** S. Jochberger

*Intensive Care Medicine Experimental* 2017, **5(Suppl 2):**0369

**INTRODUCTION.** Eotaxin-1 (CCL11) is a chemokine involved in eosinophil-dependent immunologic reactions, however, it has also been implicated in further biological processes such as neurogenesis and ageing [1]. Therefore, Eotaxin-1 is hypothesized as critical factor which might influence a recipient`s cognitive functions and ageing after transfusion of blood, a phenomenon which has recently been described in mice [1]. However, until now it is unknown whether Eotaxin-1 is detectable in transfusion blood products.

**OBJECTIVES.** The present study addresses the question if Eotaxin-1 is detectable in different blood products processed for transfusions and if yes, its level depends on demographic factors or storage time of the blood product.

**METHODS.** Eotaxin-1 was measured in fresh frozen plasma (FFP; n = 168), erythrocyte concentrates (EC; n = 160) and platelet concentrates (PC; n = 8) ready-to-use for transfusions using a Q-Plex immunoassay for detection of Eotaxin-1 and quantified using Q-view software. For statistical analyses, Mann–Whitney as well as Kruskal-Wallis tests were employed. Correlations have been assessed with Pearson correlation analysis. Age groups of donors were defined as follows: Group 1: 18–25 years, group 2: 26–55 years, and group 3: >55 years.

**RESULTS.** Eotaxin-1 was consistently detected in FFP (median 69.4 pg/ml) and EC (median 42.6 pg/ml) but was only detectable in 3 of 8 PC (median 10.38 pg/ml). Eotaxin-1 levels did not differ between male and female donors but increased with rising age in both, FFP (median Eotaxin-1 group 1: 52.4 pg/ml; group 2: 72.4 pg/ml; group 3: 80.1 pg/ml) and EC (group 1: 36.1 pg/ml; group 2: 47.6 pg/ml; group 3: 48.7 pg/ml). A statistically significant increase in Eotaxin-1 was detected between age groups 1 and 2 as well as groups 1 and 3 in FFP and EC, respectively ($p$ < 0.001 for all comparisons). Consequently, Eotaxin-1 correlated with age in both blood products (Pearsons correlation coefficient 0.35 [p = $3.8E^{-5}$] and 0.44 [p = $6.8E^{-9}$], respectively. Importantly, no significant correlation of Eotaxin-1 and storage time in neither blood product could be identified. Finally, we demonstrated that Eotaxin-1 is subject to only minor fluctuations within one donor over a longer period of time.

**CONCLUSIONS.** Eotaxin-1 is detectable and stable over time in FFP and ECs and increases significantly with the donor`s age. Considering the presumed involvement in ageing and cognitive impairment in mice, differences in donor- and recipient Eotaxin-1 levels may affect ageing and cognitive function after transfusion of blood products. Further studies should decipher the exact role of Eotaxin-1 in cognitive processes and assess the impact of high Eotaxin-1 levels in blood products on recipient's cognitive function.

**REFERENCE(S).**

Villeda, S.A., et al., The ageing systemic milieu negatively regulates neurogenesis and cognitive function. Nature, 2011. 477(7362): p. 90–4.

**GRANT ACKNOWLEDGMENT**

FWF grant T 738-BBL.

### Ethical issues: What do we know?

#### 0370

**Huge variation in obtaining ethical permission for a non-intervention observational study in Europe**

D.W. de Lange[1], B. Guidet[2,3,4], R. Moreno[5], S. Christensen[6], H. Flaatten[7], on behalf of the VIP1 Study Group

[1]University Medical Center Utrecht, Intensive Care, Utrecht, Netherlands; [2]Assistance Publique, Hôpitaux de Paris, Hôpital Saint-Antoine, Service de Réanimation Médicale, Paris, France; [3]Sorbonne Universités, Institut Pierre Louis d'Epidémiologie et de Santé Publique, Paris, France; [4]INSERM, Institut Pierre Louis d'Epidémiologie et de Santé Publique, Paris, France; [5]Hospital de São José, Unidade de Cuidados Intensivos Neurocríticos, Lisbon, Portugal; [6]University Hospital Aarhus, Department of Anaesthesia and Intensive Care Medicine, Aarhus, Denmark; [7]Haukeland University Hospital, University of Bergen, Department of Clinical Medicine, ICU, Bergen, Norway

**Correspondence:** D.W. de Lange

*Intensive Care Medicine Experimental* 2017, **5(Suppl 2):**0370

**INTRODUCTION.** Although clinical research is vital for the improvement of the quality of care there will always be a potential disparity between the interests of the researcher and the patient. Therefore medical ethical approval has to be obtained before research can be started. The European Union (EU) has harmonized the process for ethical approval for high-risk studies, like interventional trials in their clinical trials directive(s) (EU-CTD1/2). [1] However, these directives do not focus on non-interventional observational studies.

**OBJECTIVES.** To analyze the differences in ethical approval for an anonymous, non-interventional, observational study between various European countries using our experiences from a recent European multicenter study in the very old ICU patients.

**METHODS.** European national coordinators (NCs) of that study (www.vip1study.com) were asked to fill out an questionnaire on their experience to get ethical approval.

**RESULTS.** 16 out of 17 NCs responded. N = 8/16 of the NCs could apply at one central ethical committee (EC), while the others had to apply to various ECs, individual hospital institutional research boards (IRBs) or a combination thereof. The time between applying for ethical approval and the final decision varied between 7 days and 300 days. In n = 9/16, informed consent from the patient was not deemed necessary, and required from patients or relatives in the rest (n = 7/16). One country accepted retrospective consent in survivors. The upload of anonymous data to a central database required ethical approval in n = 14/16. One country had to apply for this from a separate EC. In n = 4/16 the NCs had to ask ethical approval to keep a subject identification code list to de-anonymize the patients if questions would occur. Only n = 2/16 of the NCs agreed that informed consent was necessary for such an observational study. Overall, n = 6/16 of the NCs were satisfied with the entire process of ethical approval, n = 8/16 were (very) unsatisfied and n = 2/16 were neutral (time consuming but necessary). N = 11/16 would welcome a European central EC that would judge observational studies for all European countries, while n = 5/16 were "not sure" fearing "more bureaucracy" in the ethical approval process.

**CONCLUSIONS.** There are huge variations between European countries in the process and the time needed to get ethical approval for low-risk, anonymized, observational studies (e.g. quality registries, research studies or "big data" analyses). Such variations hamper inclusion of patients as well as countries and might have a negative influence the external validity of such observational studies. Further harmonization of ethical approval across European countries is also welcomed for low-risk observational studies.

**GRANT ACKNOWLEDGEMENT**

The VIP1-study was endorsed by the ESICM.

### 0371

#### One third of patients reviewed by ICU's rapid response team are over 75 years old - should an alternative intervention be considered?

J. Tirkkonen[1,2], P. Setälä[1], S. Hoppu[1]
[1]Tampere University Hospital, Department of Intensive Care Medicine, Tampere, Finland; [2]Seinäjoki Central Hospital, Department of Anaesthesia and Intensive Care Medicine, Seinäjoki, Finland
**Correspondence:** J. Tirkkonen
*Intensive Care Medicine Experimental* 2017, **5(Suppl 2):**0371

**INTRODUCTION.** Rapid response teams (RRTs) form the efferent limb of rapid response systems (RRSs) intended to improve outcome of deteriorating ward patients.[1] As both the general and hospital populations age, RRTs probably review geriatric patients with comorbidities daily. This aspect sets new challenges to the 'ICU without walls'[2], but has not been studied.

**OBJECTIVES.** To investigate the prevalence and outcome of geriatric RRT population and to evaluate, whether previously identified risk factors for mortality among the general RRT population predict tenuous outcome among the geriatric RRT patients.

**METHODS.** Data on patients reviewed by ICU's RRT in Tampere university hospital, Finland, between 1 May 2012 and 30 April 2015, were prospectively collected. Independent risk factors for 30-day mortality were identified from the whole study population using multivariate logistic regression (MLR), and accumulation of these factors were investigated among the geriatric sub population without treatment limitations (patients ≥ 75 years). The local Ethics Committee approved the study protocol (Approval no: R10111).

**RESULTS.** A total of 1372 patients were reviewed 1722 times. Geriatric patients (n = 449, 33% of the study population) had more comorbidities, received new limitations of medical treatment more frequently and were more rarely admitted to the ICU as compared to patients < 75 years old (n = 923, 67%). Geriatric patients had substantially higher 30-day and one-year mortality as compared to non-geriatric population (33% vs. 21%, $p < 0.001$ and 54% vs. 35%, $p < 0.001$). In a MLR model, seven independent risk factors for 30-day mortality among the whole RRT population were identified. Of these, five factors (positive RRT criteria measured during the review, Charlson comorbidity score ≥ 5, non-elective hospital admission, medical reason for admission, afferent limb failure) were cumulatively applied to the geriatric sub population without preceding treatment limitations (n = 411) as data on these factors are feasibly obtainable for both the ward staff and/or RRT. The risk for 30-day mortality increased as these risk factors accumulated, $p < 0.001$ (Fig. 125).

**CONCLUSIONS.** One third of patients reviewed by RRT were over 75 years old. To no surprise, these patients have higher cumulative comorbidity and poorer prognosis than younger RRT patients. ICU's RRT staff are often faced with the dilemma of escalating or de-escalating the care. Our results suggest, that the cumulative burden of independent risk factors for 30-day mortality among the general RRT population could provide some assistance in these situations, and even trigger discussions on treatment goals rather than RRT activations. Our results, however, must be validated in future studies.

**REFERENCE(S)**

1. Jones D, DeVita M, Bellomo R. Rapid-response teams. N Engl J Med 2011;365:139–46.
2. Hillman K. Critical care without walls. Curr Opin Crit Care 2002;8:594–9.

**GRANT ACKNOWLEDGMENT**

None.

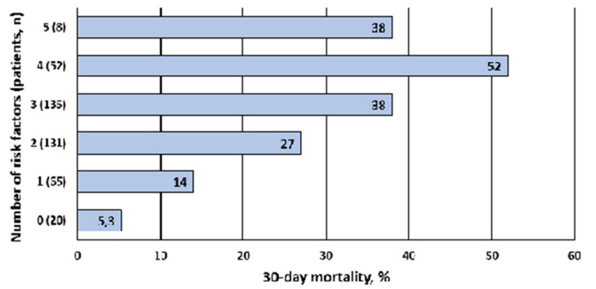

**Fig. 125 (Abstract 0371).** See text for description

### 0372

#### Treatment Escalation Plans (TEPs): how important are they and can we improve?

M. Dhankhar, K. Dombrowsky, J. Gross
Northwick Park & St Mark's Hospitals, London, United Kingdom
**Correspondence:** J. Gross
*Intensive Care Medicine Experimental* 2017, **5(Suppl 2):**0372

**INTRODUCTION.** Deciding whether or not to admit to the Intensive Care Unit (ICU) requires careful consideration as to whether the likelihood of successful outcome (which includes not only survival but also a quality of life that is acceptable to the patient) outweighs risks of harm that may result in physical and psychosocial morbidity and low quality adjusted life years[1]. Pre-existing frailty and comorbidity may sway the balance against ICU admission. ICU admission should be regarded as a treatment and as such patient's wishes should be ascertained where possible.

**OBJECTIVES.** To look at the quality of referrals for ICU admission in relation to

- consideration of treatment escalation plans by referring teams prior to or at the time of referral
- patient/family involvement in escalation plans (including ICU admission).

**METHODS.** Prospective observational analysis at our institution between July-October 2016 for all new ICU referrals and cardiac arrest calls attended. Primary outcome included the % of patients who had treatment escalation plans (TEPs) (+/− "Do Not Attempt Cardiopulmonary Resuscitation" - DNACPR orders) made before or at the time of referral. The nature of these TEPs and DNACPR orders was further analysed according to who made the TEP (referring or ICU team) and whether or not this was discussed with the patient or family. Subgroup analysis included patients with limited functional status and severe comorbidity at baseline (as defined by ICNARCs CMP) and in-hospital cardiac arrests during data collection period.

**RESULTS.** Out of 147 patients referred to ICU, only 24 patients (16%) had a documented TEP. For the patients who did have a TEP, 12/24 (50%) were made by the ICU consultant and 11/24 (46%) were made before ICU referral. Only 3/24 (13%) referrals/TEPs involved a discussion with the patient (which represents 2% of all referrals made) and 13/24 (54%) involved a discussion with the patient's family. Similar trends were observed for the DNACPR orders. The uptake of TEPs was poor and inconsistent even in those with limited functional status and severe comorbidity at baseline (Table 109).

Eleven referrals were made following an in-hospital cardiac arrest. Seven of these patients (64% of in-hospital cardiac arrests) either had significant comorbidity and/or limited functional status but none of them had a prior TEP considered.

**CONCLUSIONS.** The uptake of TEPs and decisions relating to CPR was low, even for patients with limited functional status and severe comorbidity at baseline. The nature as to how these are implemented are inconsistent and patient/family involvement in the decision making process is inadequate. There is an urgent need to address these issues which should look into ways of increasing the earlier uptake of earlier TEPs and involving patient and their surrogates in these decisions.

**Table 109 (Abstract 0372).** TEPs according to functional status & comorbidity

| TEP documented? | Independent/ No comorbidity | Independent/ 1 or more comorbidity | Limited functional status/No comorbidity | Limited functional status/ 1 or more comorbidity |
|---|---|---|---|---|
| Yes | 8 (11%) | 5 (24%) | 5 (18%) | 6 (24%) |
| No | 65 (89%) | 16 (76%) | 23 (82%) | 19 (76%) |

### 0373

#### Perceptions, practices and level of knowledge of intensive care physicians about the application of palliative care in intensive care units (ICUs)

M.C. Calle[1], S.L. Pareja[1], M.M. Villa[2], M. Lemos[2], S. Navarro[3], J.P. Román-Calderón[2], A. Krikorian[4]

[1]Universidad CES, Medicine, Medellin, Colombia; [2]Universidad EAFIT, Psychology, Medellin, Colombia; [3]Clinica Universitaria Bolivariana - Universidad Pontificia Bolivariana - Universidad CES, Intensive Care - Bioethics, Medellin, Colombia; [4]Universidad Pontificia Bolivariana, Grupo de Dolor y Cuidado Paliativo, Medellin, Colombia

**Correspondence:** S. Navarro

*Intensive Care Medicine Experimental* 2017, **5(Suppl 2):**0373

**INTRODUCTION.** The benefits of a complementary palliative care (PC) approach in gravely ill patients in the Intensive Care Unit (ICU) are well known. However, barriers limiting the interaction between the specialties persist, more probably in contexts were PC is developing. Factors influencing this interaction have been poorly explored.

**OBJECTIVES.** To explore the perceptions, level of knowledge, practices, and perceived barriers that intensive care physicians have about the integration of a PC approach in ICUs.

**METHODS.** A descriptive-correlational design was used. An online survey exploring the above-mentioned variables was developed *ad hoc*. Members of the Colombian Association of Critical Care and Intensive Medicine were invited to participate. Descriptive and correlational statistics were obtained and a Partial Least Squares Structural Equation Modelling analysis to identify predictors of interaction practices.

**RESULTS.** 101 physicians working in ICUs completed the questionnaire (82,2% intensive care specialists). 31 participants (30,7%) referred having some training in PC (average hours of training was 61,24; SD: 177,08). Significant positive correlations were found between perceptions and knowledge (r = 0.86, p < 0.01), perceptions and practices (r = 0.33, p < 0.01), knowledge and practices (r = 0.41, p < 0.01), and hours of training and practices (r = 0.34, p < 0.01).

A negative and significant relation was found between perceptions and barriers (r = −0,24, p < 0.5). The structural model analyzed showed that hours of training had a positive significant effect on perception about PC ($b$ = .70, $t$ = (95) 11.74, $p$ < .01), which in turn exerted a positive significant influence on CP practices ($b$ = .58, $t$ = (95) 6.03, $p$ < .01), and a negative effect on barriers ($b$ = −.31, $t$ = (95) 5.12, $p$ < .01). Finally, perceived barriers exerted a negative and significant effect on practices ($b$ = −.14, $t$ = (95) 2.03, $p$ < .05). The model explained a significant proportion of variance in perception about PC ($R^2$ = .49, $p$ < 01), barriers to PC ($R^2$ = .09, $p$ < 05), and PC practices ($R^2$ = .39, $p$ < 01).

**CONCLUSIONS.** Training in PC impacts on its positive perception, while diminishing perceived barriers; in turn, all the above variables favor interaction practices. A more formal training in PC in intensive care educational programs will probably result in an increased use of PC in ICUs.

**REFERENCE(S)**

D.B. White, J.M. Luce. Palliative care in the intensive care unit: barriers, advances, and unmet needs. Crit Care Clin 20 (2004) 329–343

Baker et al. Integration of Palliative Care Services in the Intensive Care Unit. A Roadmap for Overcoming Barriers. Clin Chest Med 36 (2015) 441–448

M.P. Aulisio et al. Ethics and palliative care consultation in the intensive care unit. Crit Care Clin 20 (2004) 505–523

**GRANT ACKNOWLEDGMENT**

None.

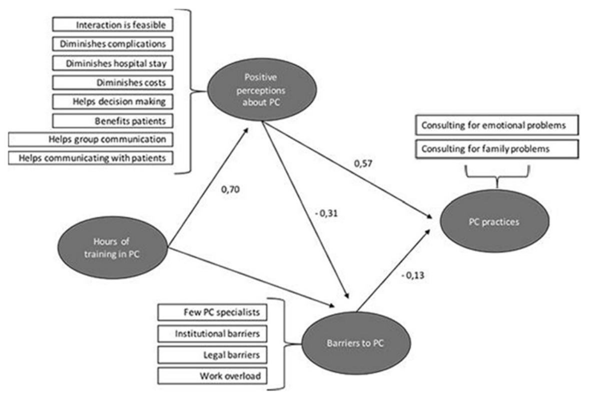

**Fig. 126 (Abstract 0373).** MODEL

### 0374

#### ICU nurses' grief and coping after patients dying

T. van Galen[1], L. Duijn[1], N. Rouw[1], H.J. de Grooth[2], A.R.J. Girbes[1]
[1]VU University Medical Center, Department of Intensive Care, Amsterdam, Netherlands; [2]VU University Medical Center, Department of Anesthesiology, Amsterdam, Netherlands
**Correspondence:** T. van Galen
*Intensive Care Medicine Experimental* 2017, **5(Suppl 2):**0374

**INTRODUCTION.** ICU nurses are confronted frequently with patients dying. This being a significant stressor due to the emotional implications and irreversibility [1,2]. Frequent exposure to patients dying is related to an increased risk of stress and the potential development of mental and physical symptoms, compassion fatigue or burnout syndrome (BOS). This is increased when emotions accumulate if not handled adequately or being ignored [3,4]. With an 11% mortality rate and patients dying every other day, it was decided to explore feelings of grief among our ICU nursing staff.

**OBJECTIVES.**

- Identify the emotional response.
- Inquire the prevalence.
- Investigate possible demographic deviations.
- Examine why emotions occur and what provoking factors are.
- Determine if peer support is effective.

**METHODS.** A quantitative study was performed using a peer validated questionnaire that was distributed to the ICU nursing staff (N = 124). The questionnaire was returned by 78% (N = 96). For qualitative and explorative purposes peer group discussions were organized with randomly selected ICU nurses (N = 50), divided into multiple groups.

**RESULTS.** Nurses' emotions after patients dying were common and usually ending after a 3 day period. Minimal differences based on gender, age or experience were found.

| | Yes | No |
|---|---|---|
| Disbelief | 66% (N = 63) | 34% (N = 33) |
| Sadness | 66% (N = 63) | 34% (N = 33) |
| Anger | 48% (N = 46) | 52% (N = 50) |
| Shame | 20% (N = 19) | 80% (N = 77) |

*[Different emotions after a patient dying.]*

| | Yes | No |
|---|---|---|
| Up to 3 days | 65% (N = 62) | 35% (N = 34) |
| Up to 1 week | 21% (N = 20) | 79% (N = 76) |
| Up to 1 month | 3% (N = 3) | 97% (N = 93) |
| For more than 1 month | 3% (N = 3) | 97% (N = 93) |

*[Duration of emotions after a patient dying.]*

| | Yes | No |
|---|---|---|
| Are there differences in experiencing grief to how a patient has died? | 67% (N = 64) | 33% (N = 32) |
| Confrontation with a dying patient is an extra burden to the job. | 35% (N = 34) | 65% (N = 62) |
| I have a need for support/counseling after a patient's death. | 86% (N = 83) | 14% (N = 13) |
| ICU peer support/counseling is sufficient. | 50% (N = 48) | 50% (N = 48) |
| Informal and small scale peer support/counseling is more effective. | 86% (N = 83) | 14% (N = 13) |
| I have sufficient support in my private life/network. | 92% (N = 88) | 8% (N = 8) |
| I am satisfied with the nursing role related to the process of EOL decision making. | 55% (N = 53) | 45% (N = 43) |
| I am satisfied with the nursing and intensivist communication related to EOL Care. | 73% (N = 70) | 27% (N = 26) |
| I am satisfied with the nursing role related to EOL Care execution. | 70% (N = 67) | 30% (N = 29) |

*[ICU nurses needs and experiences.]*

Although peer support was considered ineffective by 50%, most nurses did not experience long term emotions due to their own coping mechanisms or supportive network. Frequently grief was provoked or amplified by EOL care imperfections related to doctor-nurse cooperation and communication. Nurses were dissatisfied by lack of EOL care information and an unclear role in the EOL process, however this was evaluated differently between younger and older nurses.

Cross examining data we found a small group (8%) prone for developing problems with peer support being ineffective (p = 0,003) and having an insufficient supportive network (p = 0,014). These nurses needed more time for effective coping (p = 0,000).

**CONCLUSIONS.** Confrontation and dealing with patients dying is unavoidable and emotions with a short duration are normal for almost all our ICU nurses. Peer support is not to be considered effective for all team members. Combined with the absence of an effective supportive network it makes a small group prone for developing problems. Ward management should focus on identifying these vulnerable team members besides offering sufficient and continuous support to all ICU employees. Other provocative factors were found related to an ineffective nursing role in EOL process. These can be solved by (re)defining the nursing role in the EOL care process.

**GRANT ACKNOWLEDGMENT**
None.

### 0375

#### A survey of doctors attitudes towards the patient with devastating brain injury

C.T. Kaye, S. Stott
NHS Grampian, Intensive Care Unit, Aberdeen, United Kingdom
**Correspondence:** C.T. Kaye
*Intensive Care Medicine Experimental* 2017, **5(Suppl 2):**0375

**INTRODUCTION.** The patient with a devastating brain injury (DBI) may present to a wide range of specialities and the decision to refer or admit these patients to the Intensive Care Unit (ICU) can be difficult for both parties. Recent work has suggested that the ability to predict outcomes of these patients at first presentation is debatable and highlighted several potential benefits for admitting these patients to the ICU, despite the high likelihood of non-reversible pathology (1).

**OBJECTIVES.** To Identify:

1. Referring physician's & intensivist's attitudes towards the management of the patient with DBI.
2. Perceived barriers to providing ICU care for patients with DBI.

**METHODS.** An online survey was sent to all medical staff in ICU and the specialities who refer patients with DBI to the ICU in a large, Scottish teaching hospital. The survey presented two cases of DBI, differentiated by the necessity for endotracheal intubation prior to referral. Follow on questions examined whether the doctor would refer the patient and enquired about the rationale surrounding their decision.

**RESULTS.** A total of 83 respondents completed the survey with a good representation of specialties (58% Anaesthesia, 14% Emergency Medicine, 8% ICM, 16% Medicine and 4% Neurosurgery). The majority of responses (54%) were from Consultants.

Doctors were less likely to refer the non-intubated patient (54% compared to 83%). For both cases, the most frequent reason to refer the patient was for potential organ donation. Looking at reasons against referral, for both cases the most frequent reasons were perceived futility and the ability to provide end of life care outside of the ICU. A number of respondents in each case would not refer to the ICU based purely on the CT diagnosis of DBI.

Optimisation for organ donation and providing time for families were the most commonly identified benefits of ICU referral; whereas bed/capacity issues and the attitudes of the duty ICU Consultant were identified as the leading barriers to referral.

**CONCLUSIONS.** In our survey, doctors were less likely to refer to the ICU if there was no support to withdraw, or no requirement for ICU-level interventions at the time of referral. Furthermore, several respondents made their decision based on the CT imaging and neurosurgical diagnosis. Further work should be done to see if these beliefs are similar to those of staff in other hospitals who care for these patients.

**REFERENCE(S)**

1. Manara AR, Thomas I, Harding R. A case for stopping the early withdrawal of life sustaining therapies in patients with devastating brain injuries. Journal of the Intensive Care Society 2016, May 5;17(4):295–301.

**GRANT**

None

**ACKNOWLEDGMENT**

None

### 0376

#### End-of-life care for patients dying in an intensive care unit - how do patients die in ICU?

J.M. Ribeiro, R. Costa, D. Silva, I. Moniz, Z. Costa e Silva

University Hospital of Santa Maria, CHLN, Intensive Care Department, Lisbon, Portugal

**Correspondence:** R. Costa

*Intensive Care Medicine Experimental* 2017, **5(Suppl 2):**0376

**INTRODUCTION.** Dignity and compassion should head the care for patients dying in hospitals. Environment in the intensive care unit (ICU) can be very impersonal and lead to additional suffering. Recently, a report from the World Federation of Societies of Intensive and Critical Care Medicine identified regional differences in the processes of care for dying persons. A statement that will apply to all regions and cultures of the world might be intangible, and so, triggering local initiatives will be crucial to identify keystone interventions to improve end-of-life care in the ICU.

**OBJECTIVES.** Characterization of the process of care for dying patients in a 22-bed ICU in a university-affiliated hospital.

**METHODS.** Family integration in the care of patients admitted to our ICU has been previously outlined and implemented. Relatives are allowed to stay with the patient for long periods of time. Routine care for patients include daily multidisciplinary rounds. In this study, we did a protocol-driven retrospective review of prospectively registered data regarding the care for patients dying in our ICU (from January 2016 till March 2017).

**RESULTS.** In the study period, a total of 696 patients were admitted to our ICU. SAPS II was 40.7 (mean score). A total of 100 patients died (ICU mortality rate: 14.3%). Only two patients died from unexpected cause. Regarding this population of patients who died, main causes of death were sepsis and pure cardiogenic shock. SAPS II score was 61.1 ± 16.4 and SOFA score at admission was 10.3 ± 3.9. Eighty nine patients had respiratory failure and needed invasive ventilation, 85 had shock and needed vasopressor therapy, 73 had acute kidney injury and 38 needed CRRT. Regarding the dying process, a collegial decision has been registered in 77% of the cases. This consisted in withdrawal of therapy in 81% of patients and definition of limits for intervention in 84% of patients. Vasopressors were withdrawn in 26 and an upper dose limit defined for additional 36 patients, renal replacement therapy was stopped in 31 patients and ventilation was withdrawn or withhold in 12 and 22 patients, respectively. Time from decision to death averaged 9.1 hours (minimum zero; maximum 144). Pharmacological intervention to treat pain, anxiety, xerostomy and breathlessness included opioid (72 patients) and propofol (54 patients). Only one patient referred persistent pain that required additional analgesia. Family has been present at time of death in 27 patients.

**CONCLUSIONS.** Characterization of the dying process in the ICU is important to encourage local initiatives and to develop recommendations that boost excellence in end-of-life care. Decision process registration need some improvement. Withdrawal and withhold respect ethical and legal precepts in our country, and are perceived by relatives and professionals as adequate ways to dignify the process of death in ICU.

### 0377

#### Retrospective observational study of the documentation of end of life care in a Hong Kong ICU

K.H. Lam[1], W.T. Wong[2], G. Joynt[2]

[1]Prince of Wales Hospital, Anaesthesia and Intensive Care, Shatin, Hong Kong, China; [2]The Chinese University of Hong Kong, Anaesthesia and Intensive Care, Shatin, Hong Kong, China

**Correspondence:** W.T. Wong

*Intensive Care Medicine Experimental* 2017, **5(Suppl 2):**0377

**INTRODUCTION.** Documentation in end of life (EOL) care in Intensive Care Unit (ICU) is an effective means of communication and also a prerequisite for audit and research in quality of care.

**OBJECTIVES.** This retrospective study is to investigate the documentation of key components of EOL care in an university hospital ICU in Hong Kong.

**METHOD.** Key components in clarifying the precondition before EOL care decision, making the EOL care decision and implementing the order were identified before reviewing the medical records. Clear documentation was defined as explicitly written notes by the ICU doctors in the medical record.

**RESULTS.** There were 65 adult cardiac deaths with full medical record at ICU from 1st January to 31st December 2013 and 52 adult patients with EOL care implemented were included in the study.

**Clarifying the precondition of EOL care in ICU**

The trigger of EOL discussion was documented in 43 patients' medical records (82.7%) out of 52 recruited patients. The initiator of the EOL care decision was documented in 46 records (89%). Only two patients' medical records (4%) included documentation of checking EOL decision making capacity. Consensus among ICU physicians and consensus with primary care team clinicians were documented in 29.6% and 17.3% of the records respectively.

**Documentation of EOL care decision making process**

Documentation of checking patients´ expressed value was found in 19% and clarification of consensus among patient's family in 15% of the records. Documentation of explaining shared decision making and substituted decision concept was found in 1 patient's record (2%) and 2 patients' records (4%) respectively.

**Documentation of life support therapy (LST) being withheld (WH) or withdrawn (WD)**

"Do-Not-Resuscitate" (DNR) order was implemented in all 52 patients, 48% of the records documented implementation of the order and 42% documented explanation to the family. All patients whose ventilator support was WH/WD had the explanation in the family meeting and implementation of the order clearly documented. In those 38 patients whose vasopressor support was WH/WD, 30 (78.9%) patients' record documented the implementation order while 27 (71.1%) patients' record documented the explanation to family. There are 12 patients whose renal replacement therapy was WH/WD. All except one (91.7%) records documented the implementation order and 9 records (75%) documented the explanation to the family.

**CONCLUSIONS.** In this study, we found inadequate documentation in key components in EOL care in ICU. In clarifying the precondition, documentation of clarification of patient's decision making capacity and consensus among health care workers are frequently lacking. In the decision making process, documentation of reaching consensus among family members, shared decision making and substituted decision making concept are commonly missed out. The documentation of implementing DNR order and explaining the order to family is less common than limiting ventilator support.

### 0378

#### The use of neural monitoring to assist decision-making in terminal extubation or terminal weaning as part of end of life care in a critical care setting

D. Hadfield[1], B. Penhaligon[2], C. Bell[2], H. Noble[2], L. Thompson[2], E. Clarey[2], J. Smith[2], S. Birch[2], C. Harris[2], P.A. Hopkins[1], ACET Research Group
[1]Kings Health Partners, King's Critical Care, London, United Kingdom; [2]King's College Hospital, King's Critical Care, London, United Kingdom
**Correspondence:** P.A. Hopkins
*Intensive Care Medicine Experimental* 2017, **5(Suppl 2):**0378

**INTRODUCTION.** The management of airway, oxygenation and ventilatory support in patients who are dying on the critical care unit remains a challenging area, particularly in relation to treatment limitation (1). Specifically, the relative merits of reductions in ventilatory support ('terminal weaning') versus removal of the endotracheal tube ('terminal extubation') have not been explored objectively. Further, concerns in relation to potential discomfort from dyspnea or even airway obstruction may lead to overuse of analgesic or hypnotic agents (2). Here we describe some preliminary data from patients with pre-existing neural ventilatory drive monitoring where these observations were extended to guide treatment withdrawal and palliative care.

**OBJECTIVES.** To test the feasibility, acceptibility and utility of EADi neural drive monitoring (NAVA, Getinge) in terminal weaning and terminal extubation of patients receiving treatment limitation as part of end-of-life care.

**METHODS.** Research and patient-public approvals were gained and all data anonymised prior to publication. Retrospective data were taken over a three-year period from 50 patients who underwent either terminal weaning without extubation or terminal extubation. 25 patients had neural catheters already in place at the time of the decision to target palliative goals of care and 25 matched only by date and decision. Staff were surveyed to record their perceptions and use of EADi monitoring.

**RESULTS.** 15 patients underwent neural monitoring during terminal extubation and 10 patients who had terminal ventilatory weaning without extubation, Electrical activity of the diaphragm (EAdi) data was obtained in all 25 patients throughout either palliative strategy. Patients with neural monitoring received less hypnotic and analgesic drugs and had fewer increases in these therapies. There were no differences in time to death or time with nasogastric tubes or use of enteral nutrition between groups. Critical care staff reported high levels of satisfaction with neural monitoring during end-of-life care and perceived it to be safe and useful.

**CONCLUSIONS.** To our knowledge neural demand monitoring has not been used previously in the context of terminal weaning or extubation. Neural monitoring appeared to be feasible and acceptable to staff; provide clinically useful information and did not appear to lead to extended use of nasogastric tubes when compared to standard care. We believe that further research is needed, and that dying patients can benefit from new technologies.

**REFERENCE(S)**

1. Cottereau A et al. ICU physicians´ and nurses´ perceptions of terminal extubation and terminal weaning: a self-questionnaire study. Intensive Care Med. 2016 42: 1248–57
2 Billings JA. Humane terminal extubation reconsidered: the role for preemptive analgesia and sedation.. Crit Care Med. 2012 40: 625–30.

**GRANT ACKNOWLEDGMENT**
NIHR Fellowship (DH) and the research program is supported by the CRN critical care specialty.

### 0379

#### Treatment limitations before and during ICU admission affect hospital mortality: a pilot study

N. Kusadasi[1], K. Kaasjager[2], M. van Dam[1]
[1]University Medical Center Utrecht, Intensive Care Center, Utrecht, Netherlands; [2]University Medical Center Utrecht, Internal Medicine, Utrecht, Netherlands
**Correspondence:** M. van Dam
*Intensive Care Medicine Experimental* 2017, **5(Suppl 2):**0379

**INTRODUCTION.** Most patients admitted to the Intensive Care Unit (ICU) are offered treatment without any limitations. Withholding resuscitation or mechanical ventilation could affect patients' survival. This pilot study examines the effect of treatment limitations before and during ICU admission on hospital mortality of ICU patiients.

**OBJECTIVES.** To examine the presence of treatment limitations set before and during ICU admission and the hospital mortality of this category of patients.

**METHODS.** In this single center, retrospective observational pilot study in an academic 30-bed ICU all consecutive patients admitted from February 1$^{st}$ until March 1$^{st}$ 2017 with a length of stay (LOS) of more than 24 hours were included. Data collected were age, gender, treatment limitations before and during admission to ICU, referring specialty, vasoactive medication, invasive mechanical ventilation, ICU LOS and hospital mortality. Follow-up was obtained through information from the electronic patient file in the hospital. Statistic analyses: P- values are determined by Chi-square and Fisher tests (OpenEpi, version 3.01, www. openepi.com). A waiver was obtained from the Medical Research and Ethics Committee.

**RESULTS.** Overall, 174 patients were admitted to the ICU in Februari 2017; 76 patients were included in this pilot study. Limitations of treatment were determined for 13 patients (17%) before or during ICU admittance. Patients characteristics are shown in Table 110. Patients with treatment limitations were older (61 ± 18 yrs) compared to patients without treatment limitations (56 ± 17 yrs). Gender distribution was equal (male: female in the treatment limitation group 62: 38% and 65:35% in the full treatment group. The patients with treatment limitations were more often referred from medical specialties, such as internal medicine, cardiology, cardiovascular, respiratory, neurology. APACHE II and IV scores were higher for patients with treatment limitations 21 ± 6 vs 17 ± 6 and 75 ± 24 vs 62 ± 22, respectively. Less patients with treatment limitations were treated with mechanical ventilation 62% vs 81% and vasopressors 62% vs 75%. Strikingly, the length of stay for both patients groups was almost equal 6 ± 5 days for the treatment limitations group versus 6 ± 7 days for the full treatment group. Hospital mortality was 69% in the cohort with treatment limitations compared to 11% in patients full treatment. This is significantly higher P = 0,00008 as determined by Fisher exact test using two by two table.

**CONCLUSIONS.** Patients with treatment limitations before and during ICU admittance have a significantly higher hospital mortality in this study compared to patients with full treatment.

**Table 110 (Abstract 0379)**. See text for description

| Characteristics | Treatment limitations N=13 | Full treatment N=63 |
|---|---|---|
| Age (years) | 61 ± 18 | 56 ± 17 |
| Gender Male (%) Female (%) | 62/38 | 65/35 |
| Referring specialisms Medical (%)/Surgical (%)(Cardiothoracic/Gastroenterloogic/Vascular/Urogenital/Trauma/Neuro/) | 69/8/15/-/-/1/-/ | 29/44/5/3/6/8/5/ |
| APACHE II* | 21 ± 6 | 17 ± 6 |
| APACHE IV** | 75 ± 24 | 62 ± 22 |
| Mechanical ventilation (%) | 62 | 81 |
| Vasopressors (%) | 62 | 75 |
| LOS (days) | 6 ± 5 | 6 ± 7 |
| Mortality (%)*** | 69 | 11 |

*n=8; **n=46; ***P=0,00008 as determined by Fisher

### 0380

#### Decision-making process and limitation of therapeutic effort in a Mexican ICU: a pilot study

L.A. Gorordo-Delsol[1], M.D. Delgado-Ochoa[2], G.D. Hernández-López[1], J.A. Castañón-González[1]
[1]Hospital Juárez de México, Intensive Care Unit, México City, Mexico; [2]Hospital Juárez de México, Bioethics Committee, México City, Mexico
**Correspondence:** L.A. Gorordo-Delsol
*Intensive Care Medicine Experimental* 2017, **5(Suppl 2):**0380

**INTRODUCTION.** In clinical practice physicians, patients and their families are involved in decision-making process (DMP). This process is of paramount importance in patients with critical, chronic and/or terminal illnesses, where bioethical dilemmas increase. DMP must respect human rights of patient and physicians. Mexico City has a "Law of advance directives" and recently legislated in favor of passive euthanasia [1].

**OBJECTIVES.** To describe the DMP of medical staff regarding the limitation of the therapeutic effort (LTE) in the ICU of Hospital Juárez de México (HJM)

**METHODS.** Research was qualitative and descriptive. Authorization was obtained from the Research Committee, Jenssen et al. survey [2] was translated, adapted and applied to ICU´s medical staff (n = 11).

**RESULTS.** DMP included an item related if it is a difference between withhold or withdraw treatment, 7/11 responded that there is a difference. Cause to consid...

We also asked about who should participate in DMP:

a. Patient = 11/11
b. Family or surrogate = 7/11 (when patient is unable to decide)
c. Physician = 4/11 (when patient and family are unable to decide)

Other item asked was if nurses should participate in DMP, 4 responded "always", 1 "frequently", 1 "sometimes", 1 "rarely" and 4 "never", but in practice only 2 of them consider consult nurses on a regular basis.

Although all physicians agree to write down DMP´s including functional prognosis and quality of life scales; in the medical record, only 4 actually did it. Record in files

Tanatology, Algology and Palliative Care departments, are rarely consulted by the ICU physicians.

**CONCLUSIONS.** This pilot study give us the opportunity to expand our research to pediatric and obstetric patients.

Less than half of physicians write down in medical record DMP´s about LTE.

Patients are not sufficiently involved because of their conditions limits their capacity for DMP, that´s what we strongly support advance directives; the opinion of family members and nurses seems to be underestimated.

The paternalistic model of doctor - patient relationship prevails in our ICU.

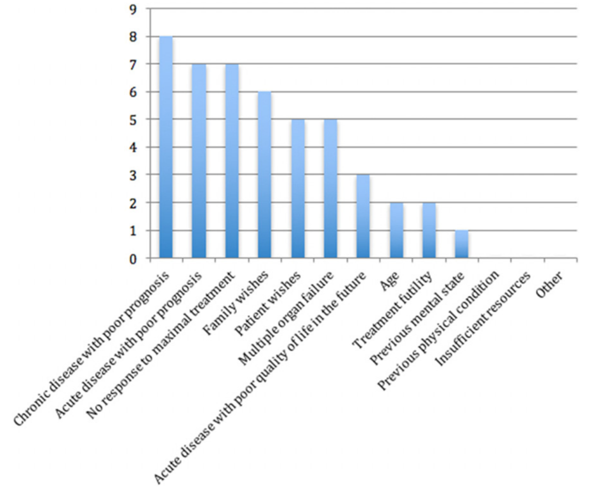

**Fig. 127 (Abstract 0380).** Cause to consider LTE

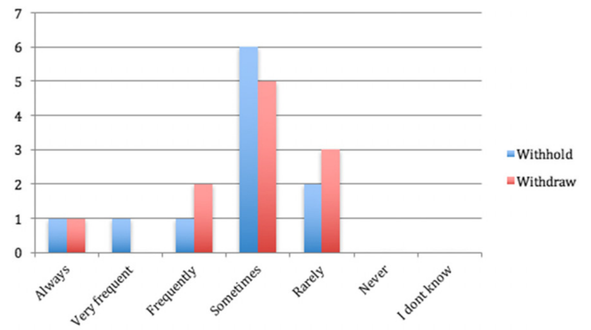

**Fig. 128 (Abstract 0380).** Record in files

### 0381

#### Have the workers of the differents intensive care units the same opinion and knowledge about the limitation of therapeutic effort?

M.R. Diaz Contreras, F.C. Santiago Ruiz, C.M. Rodríguez Mejías, M. Duro Lombardo, J. Castaño Pérez, E. Aguayo de Hoyos
Complejo Hospitalario de Granada, Medicina Intensiva, Granada, Spain
**Correspondence:** F.C. Santiago Ruiz
*Intensive Care Medicine Experimental* 2017, **5(Suppl 2):**0381

**OBJECTIVES.** Nowadays there are several subtypes of intensive care units in our hospital: cardiologic, neurotraumatologic, and general-postsurgical. Our objective is to know if all the professionals have a similar view about the limitation of the therapeutic effort (LTE) or it varies according to the subunit in which they work.

**MATERIAL:** Surveys were carried out in an anonymous and voluntary way to the intensive care workers (physicians, nurses, nursing assistants, and caretakers). Our intensive care unit consists of the following subunits: cardiological (C), neurotraumatology (NT), and general-postsurgical (G-PS).

**THE SURVEY:**

A - Sex

B - Age

C - Professional category

D - Years of work activity: 0–3 / 3–6 / 6–9 / More than 9.1

E - Do you know what LTE means? Yes / No

F - Do you know what LTE is? Active Euthanasia / - Passive Euthanasia / Non-establishment of treatment is an active measure and a withdrawal is an active measure / None of the above.

G - Are you in favor of the LTE application? Yes / No

H - Do you think it is the same not to start a treatment as to withdraw a treatment already in place? Yes / No

I - Do you consider that palliative or terminal sedation is similar to euthanasia? Yes / No

J - Should we take into account the document of anticipated willingness of the patient when implementing LTE?

K - Do you feel ready to make a decision about LTE? Yes / No

L - Do you think that sometimes you fall, even with the best intentions, in the therapeutic cruelty, prolonging uselessly situations of Suffering for both the patient and his / her family?

**RESULTS.** A total of 148 surveys were answered (92G-PQ, 36NT and 20C), 77.3% women, 61.5% > 45 years and 82.5% > 9 years of experience. 18.5%, Nurses 54%, Nurses assistants 25.5% and 2% caretakers. In favor of LTE: 99% G-PQ, 100% NT and 99% C. They think it is the same to withdraw a treatment that not start it 20% G-PQ, 17% NT and 20% C. Are LTE and passive euthanasia the same? 13% G-PQ, 11% NT and 20% C.No instauration is a passive measure and withdrawal is an active one; 20% G-PQ, 11.1% NT and 10% C. Is Terminal sedation equal to euthanasia: 5.4% G-PQ, 11.1% NT and 15% C. Do you feel capable of making a LTE decision? 46% G-PQ, 44% NT and 50% C. Almost 100% in all units believe that sometimes we fall into therapeutic cruelty, even with the best intentions.

**CONCLUSIONS.** Despite working in different units, the feeling is similar and most of the workers are in favor of LTE application. However, many of them are not clear on what LTE is. A large part of them believe that in too many cases, we fall into therapeutic cruelty.

### 0382

#### Evaluation of refusals to donate organs in case of marginal donors

I. Camerzan[1], C. Gutu-Bahov[1,2], R. Avadanii[1], V. Garbuz[1], T. Dumitras[3], S. Matcovschi[3]
[1]Clinical Municipal Hospital 'Sfanta Treime', Intensive Care Unit, Chisinau, Republic of Moldova; [2]'Nicolae Testemitan' State University of Medicine and Pharmacy, Chair of Anaesthesiology and Reanimatology Nr.2, Chisinau, Republic of Moldova; [3]'Nicolae Testemitanu' State University of Medicine and Pharmacy, Department of Internal Medicine, Chisinau, Republic of Moldova
**Correspondence:** I. Camerzan
*Intensive Care Medicine Experimental* 2017, **5(Suppl 2):**0382

**INTRODUCTION.** Currently, there are 250 patients requiring kidney transplantion and 300 patients suffering from liver failure in the waiting list of the National Transplantation Agency of Republic of Moldova. Due to the organs shortage individual waiting time and the number of deaths is increasing. Because of the organs shortage all over the world, the criteria of donation have been revised. As a result, organ transplantation based on expanded criteria has been accepted in case of the so called marginal donors. The donation agreement in these cases plays an important role.

**OBJECTIVES.** To assess the frequency and causes of refusal to donate organs (kidneys or liver) in case of marginal donors.

**METHODS.** During the years 2014–2016, a total number of 47 brain dead donors (18 males and 29 females) were maintained in Intensive Care Unit of Chisinau Municipal Hospital “Sfanta Treime”, of them 43 corresponded to the criteria of marginal donors. The study represents a retrospective analysis based on the data from informational system “SIA Transplant” and donors` files.

**RESULTS.** According to our data, the mean age of those 43 marginal donors was 62.33 ± 11.36 years. The duration of treatment in Intensive Care Unit untill the declaration of brain death was less than 48 hours in 55.8% (24/43) cases, 48–72 hours - in 39.5% (17/43) cases and more than 72 hours - in 4.7% (2/43) cases. The cause of brain death included intracerebral hemorrhage with eruption in ventricles in 58.1% (25/43) cases and massive ischaemic stroke in 41.9% (18/43) cases. The duration of maintenance of donors in brain death was less than 12 hours in 25.6% (11/43) cases, 12–24 hours - in 20.9% (9/43) cases and more than 24 hours - in 53.5% (23/43) cases.The refusals from family members to donate organs were registered in 10 (23.3%) marginal donors explained by the fear of negative opinions from close family friends and in 2 (4.7%) cases the acceptance from forensic medicine was not obtained due to the presence of posttraumatic subcutaneous hematoma. The transplantologists` team was present soon after the positive decision of the family, thus minimizing the donor maintaining period. As a result, 31 organs (14 livers and 17 kidneys) were taken and no deaths of organs` recipients were registered in posttransplantation period.

**CONCLUSIONS.** In our study the frequency of refusals of organ donation in case of marginal donors during the years 2014–2016 was 27.9%, of which the majority of cases were due to donors` family negative decision.

### 0383

#### Analysis of the process of organ and tissue donation between the Spanish population and foreigners

H. Rodríguez Pérez, J. Blanco López, B. Del Amo Nolasco, L. Ugalde Jáuregui, A. Casamitjana Ortega, R. Vega Rodríguez
Hospital Universitario Insular de GC, Las Palmas de Gran Canaria, Spain
**Correspondence:** H. Rodríguez Pérez
*Intensive Care Medicine Experimental* 2017, **5(Suppl 2):**0383

**INTRODUCTION.** Transplantation is possible thanks to a life cycle, enabling a society which donates to benefit from the process. In order to keep this cycle working, it is essential to involve society, which depend on cultural, religious, ethical, and educational factors, among others.

**OBJECTIVES.** To analyze the differences of the process of organ and tissue donation between Spanish population and foreigners.

**METHODS.** It is a descriptive, retrospective study performed in a third level hospital accredited for organ and tissue extraction and renal implant, between years 2010 and 2016 inclusive. Two comparison groups were performed: patients with Spanish nationality (group 1) versus patients with foreign nationality (group 2). We studied epidemiological variables, cause of death, nationality and place of residence, judicial cases, organs donated and reasons for non-donation. Our sample included all patients who arrived at a diagnosis of brain death in our study period, who were assessed by the transplant coordinator of our hospital. Different statistical tests were used, including ANOVA and Chi square test.

**RESULTS.** During the study period, 143 patients were diagnosed of brain death in our hospital. The mean age of all patients was 55.78 years and 65.7% were men. The cause of the death in 68.5% of the cases was cerebral stroke, in 18.2% of the cases was recovered cardiorrespiratory arrest and finally in 13.3% the TBI. When analyzed by study groups, we found differences in the cause of death, the most frequent cause being TBI in the group of patients with foreign nationality (p = 0.049, 95% CI: 0.14-1.01). Of the patients studied, 69.9% were effective donors, being the family negative the most frequent reason to dismiss the donation process with 21% in the total population studied. When analyzing by groups, although the most frequent reason remained the family negative, there were significant differences between both groups (p = 0.0025, 95% CI: 1.11-5.73).

**CONCLUSIONS.** Although our sample is small, there are some differences in the process of organ and tissue donation between Spanish and foreign patients. Probably attributable, among other things, to cultural motives.

**REFERENCE(S)**

"El modelo español de coordinación y trasplantes" R. Matesanz 2008. "Transplant Coordination Manual", 3rd Edition 2014. Curso avanzado "El proceso de donación y trasplante de órganos y tejidos" XXV Edición internacional, Alicante, 2017.

**GRANT ACKNOWLEDGMENT**
None

### 0384

#### Evolution of renal transplantation in controlled and uncontrolled DCD during admission to the ICU

N. Palomo-Lopez, A. Roldan-Reina, D. Cuenca-Apolo, M. Adriaensens, M. Porras-Lopez, L. Martin-Villen, Z. Ruiz de Azua-Lopez, J.J. Egea-Guerrero
Hospital Universitario Virgen del Rocío, Sevilla, Spain
**Correspondence:** N. Palomo-Lopez
*Intensive Care Medicine Experimental* 2017, **5(Suppl 2):**0384

**INTRODUCTION.** Insteed of the delay in creatinine evolution in those who receive an organ afer CDC tipe II, survivial seems similar.

**OBJECTIVES.** To study the renal transplant evolution from controlled and uncontrolled Donation after Circulatoy Death (DCD) (type II and type III in Maastricht clasification) (super-rapid tecnique) in patients who were admitted in ICU.

**METHODS.** Observational retrospective study conducted from January 2013 to December 2016. We collected demographic data, comorbidity, type of donation, analytical values at ICU admission, creatinine values at admission in ICU, at the time of discharge from ICU and from hospital, results of radioisotope tests, days in ICU stay, hospital stay and mortality. Statistical descriptive analysis was performed; Xi square test was used for qualitative variables and the Student´s T or Mann Whitney U test for the quantitative variables depending on their distribution. We consider significance statistical results p < 0.005.

**RESULTS.** A total of 87 kidney transplants were studied. The general characteristics of the patients, the analytical values and other variables are shown in Table 111. The 42,5% of grafts came from donation type II DCD, comparing his creatinine levels with donation type III we can a see a significant delay in the decrease in creatinine levels in the first group (Fig. 129). The average time of ICU stay was 1 day (IR 1–2). Survival rate during ICU stay was 100%.

**CONCLUSIONS.** Renal transplant patients admitted to the ICU after a donation protocol in type II DCD have a slower recovery of kidney function than those who receive an organ after DCD type III.

**REFERENCE(S)**

1. Steinbrook R. Organ donation after cardiac death. N Engl J Med. 2007; 357: 209–13
2. Summers DM, Watson CJ, Pettigrew GJ, et al. Kidney donation after circulatory death (DCD): state of the art.Kidney International. 2015; 88: 241–9.
3. Salmeron-Rodriguez MD, Navarro-Cabello MD, Agüera_morles ML, López-Andreu M, Rodriguez-Benot, A et al. Short-Term Evolution of Renal Transplant With Grafts From Donation After Cardiac Death: Type III Maastricht Category. Transplant Proc. 2015; 47, 23–26
4. Tomita Y, Tojimbara T, Iwadoh K, Nakajima I, Fuchinoue S.Long-Term Outcomes in Kidney Transplantation From Expanded-Criteria Donors After Circulatory Death.Transplant Proc. 2017 Jan - Feb;49(1):45–48

**GRANT ACKNOWLEDGMENT**
We gratefully appreciate the invaluable of ICU staff.

**Table 111 (Abstract 0384).** General characteristics of the 87 transplant

| DONOR | TYPE II | TYPE III | P |
|---|---|---|---|
| n (%) | 37 (42,5) | 50 (57,5) | - |
| Male sex, n (%) | 30 (48,4) | 32(51,6) | 0.09 |
| creatinine at ICU admission median (RI) | 6,96 (5,26 - 8,87) | 5,71 (4,47 - 7,67) | 0,081 |
| Creatinine at Hospital ward, median(RI) | 7,84 (5,41 - 9,30) | 5,60 (4,50 - 7,73) | 0,004 |
| Hospital discharge creatinine, median (RI) | 4,37 (2,07 - 6,18) | 2,56 (1,93 - 3,98) | 0,036 |
| Exitus, n (%) | 0 | 0 | - |
| Lenght of stay at ICU, median (RI) | 1 (1 - 2) | 1 (1 - 1) | 0,016 |
| Lenght of stay at Hospital median (RI) | 18 (15 - 22) | 15 (12,72 - 20,25) | 0,035 |
| Age, media (DE) | 47,97 (11,33) | 51,98 (12,23) | 0,123 |

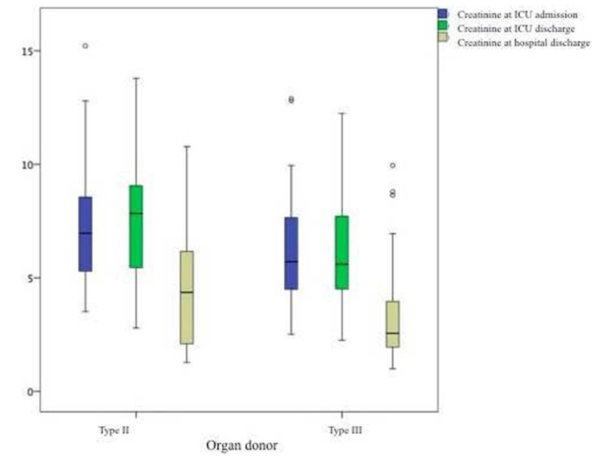

**Fig. 129 (Abstract 0384).** Evolution of creatinine levels

## Outcome 1

### 0385

#### Clinical impact of a post-ICU follow-up program

I. Dot Jordana[1], A. Vázquez Sánchez[1,2], Y. Díaz Buendía[1,3], M.P. Gracia Arnillas[1,3], A. Zapatero Ferrandiz[1,2], P.-T. Purificación[1,2], S. Vallés Angulo[1], J. Marín-Corral[1], M. Gas Amat[1], F. Alvarez-Lerma[1,3], J.R. Masclans Enviz[1,2], J. Nolla Salas[1,2]
[1]Hospital del Mar, Critical Care Department, IMIM, Barcelona, Spain; [2]Universitat Pompeu Fabra, Barcelona, Spain; [3]Universitat Autónoma de Barcelona, Barcelona, Spain
**Correspondence:** I. Dot Jordana
*Intensive Care Medicine Experimental* 2017, **5(Suppl 2):**0385

**INTRODUCTION.** After Intensive Care Unit (UCI) discharge some patients have a high readmission risk, long-lasting impairments in physical functioning, as well as, risk of complications and death.

**OBJECTIVES.** Post-discharge programs are supposed to reduce adverse outcomes in high-risk patients. The objective of this study is to investigate the clinical impact on readmission and mortality of ICU patients after the application of a post discharge ICU follow-up program.

**METHODS.** In a longitudinal prospective study performed at the ICU of the Hospital del Mar de Barcelona, patients were included from February 2013 to September 2015. Ethical Medical Committee of the hospital approved the study. We studied all adult patients discharged alive from the ICU who did not meet the exclusion criteria were. Exclusion criteria were the transfer to other ICU/other hospital/home and the admission for a scheduled neuroangiography. All the included patients should had undergone at least one follow-up visit, allowing the communication between ICU and general ward professionals. The reasons for not performing follow-up were related to physician criteria. Demographic collected data included age, sex, ICU length of stay, admission type (medical, scheduled surgery) and origin (emergency room, general ward). APACHEII, SOFA, clinical data, type of discharge (emergent or planned) and evolution were also analysed. Quantitative characteristics of patients were described using their mean and median values. The qualitative characteristics were compared using the Chi square test.

**RESULTS.** We included 1048 patients in the study. 63% (n = 661) men, median age of 63 years (49–74), median APACHE II 15 (9–22), median SOFA at admission 4 (1–6) and median SOFA at discharge 1 (0–3). ICU admission was due a medical diagnosis in 66.7%, and after emergency room admission in 60.4%.

Followed-up patients were 768 (73%), who required a lower need for readmission compared with those who were non-followed, 7% (n = 54) vs 11.4% (n = 32) respectively ($p < 0.05$). ICU global readmission rate was 8.2% (2.5% < 48h), with a significant decrease in patient readmission from 2013 (11.8%) to 2015 (4.8%), $p < 0.05$. This outcome translates into a decrease in the global mortality after ICU discharge (IntraUCI mortality after readmission plus occult mortality) over the years, from 11.4% (n = 40) in 2013 to 6.6% (n = 22) in 2015. Intra ICU mortality of readmitted patients had a reduction from 3.7% (n = 13) in 2013 to 0.3% (n = 1) in 2015.

**CONCLUSIONS.** The implementation of follow-up programs after ICU discharge, in collaboration with general ward doctors, can lead to improve communication and outcomes, with less readmissions and a decrease in readmitted mortality.

**GRANT ACKNOWLEDGMENT**

2012 Hospital del Mar Star project

### 0386

#### Outcomes of ventilated patients with sepsis who undergo inter-hospital transfer: a nationwide linked analysis

B.A. Rush[1,2], P.D. Tyler[3], D.J. Stone[4], B.P. Geisler[5], K.R. Walley[1,2], L.A. Celi[6,7]
[1]University of British Columbia, Division of Critical Care Medicine, St. Paul's Hospital, Vancouver, Canada; [2]University of British Columbia, Centre for Heart Lung Innovation (HLI), Vancouver, Canada; [3]Beth Israel Deaconess Medical Center, Department of Internal Medicine, Boston, United States; [4]University of Virginia School of Medicine, Departments of Anesthesiology and Neurosurgery, Charlottesville, United States; [5]Massachusetts General Hospital, Hospital Medicine Group, Boston, United States; [6]Beth Israel Deaconess Medical Center, Division of Pulmonary, Critical Care, and Sleep Medicine, Boston, United States; [7]Massachusetts Institute of Technology, Cambridge, United States
**Correspondence:** P.D. Tyler
*Intensive Care Medicine Experimental* 2017, **5(Suppl 2):**0386

**INTRODUCTION.** The outcomes of critically ill patients who undergo inter-hospital transfer (IHT) are not well understood. Physicians assume that patients who undergo IHT will receive more advanced care that may translate into decreased morbidity or mortality relative to a similar patient who is not transferred [1]. However, there is little empirical evidence to support this assumption.

**OBJECTIVES.** To understand whether, in mechanically ventilated (MV) patients with sepsis, IHT is associated with a mortality benefit.

**METHODS.** This is a retrospective cohort study using the Nationwide Readmissions Database (NRD), which contains information about hospital admissions from 22 States, accounting for roughly half of U.S. hospitalizations [2]. The database contains linkage numbers so that admissions and transfers for the same patient can be linked. From the 2013 NRD Sample, 14,325,172 hospital admissions were analyzed. There were 61,493 patients with sepsis and on MV. Of these, 1630 (2.7%) patients were transferred during their hospitalization. A propensity-matched cohort of 1630 patients who did not undergo IHT was identified. The propensity score included age, gender, insurance coverage, do not resuscitate (DNR) status, use of hemodialysis, presence of shock and Elixhauser co-morbidities index. The exposure of interest was inter- hospital transfer to an acute care facility. Primary outcome was hospital mortality. Secondary outcome was hospital length of stay (LOS).

**RESULTS.** After propensity matching, IHT was not associated with a difference in in-hospital mortality (12.3% IHT vs 12.7% non-IHT, $p = 0.74$). IHT was associated with a longer total hospital LOS (12.8 days IQR 7.7-21.6 for IHT vs 9.1 days IQR 5.1-17.0 for non-IHT, $p < 0.01$).

**CONCLUSIONS.** Patients with sepsis requiring MV who underwent IHT did not have improved outcomes compared to those who were not transferred. The study raises questions about the risk-benefit profile of IHT as an intervention.

**REFERENCE(S)**

1. Iwashyna TJ (2012) The incomplete infrastructure for interhospital patient transfer. Crit Care Med 40:2470–2478. doi: 10.1097/CCM.0b013e318254516f

2. Nationwide Readmissions Database Overview. Healthcare Cost and Utilization Project (HCUP). Agency for Healthcare Research and Quality, Rockville, MD. www.hcup-us.ahrq.gov/nrdoverview.jsp.

### 0387

#### High red-cell distribution width (RDW) is a marker of ward mortality after ICU discharge: a cohort study

R. Fernandez[1,2,3], J.-M. Alcoverro[1], I. Catalan[1], O. Rubio[1,2], C. Subira[1], J. Masclans[1], G. Rognoni[1], L. Ventura[1], C. Macharete[1], S. Cano[1]
[1]Hospital Sant Joan de Deu / Fundacio Althaia, Manresa, Spain; [2]Universitat Internacional de Catalunya, Barcelona, Spain; [3]CIBERES, Group 33, Sabadell, Spain
**Correspondence:** R. Fernandez
*Intensive Care Medicine Experimental* 2017, **5(Suppl 2):**0387

**INTRODUCTION.** High red-cell distribution width (RDW) has been associated with worse outcome in a variety of clinical conditions, including critically ill patients. Ward survival after ICU discharge is a challenge, but Sabadell score is one of the better predictors.

**OBJECTIVES.** To define the association of RDW with survival after ICU discharge, and whether RDW can improve accuracy of Sabadell score.

**DESIGN:** Observational cohort study.

**SETTING:** General ICU in a university-affiliated hospital.

**PATIENTS:** All patients discharged to the ward from the ICU from January2010 to October 2016.

**VARIABLES:** Demographics on ICU admission (age, comorbidities, severity score), during ICU stay (treatments, complications, and length of stay), and at ICU discharge (Sabadell score and RDW). Primary outcome was hospital mortality. Statistical analyses included multiple logistic regression models and ROC analyses.

**RESULTS.** We studied 3.366 patients with a ward length of stay of 7 [4–13] days and ward mortality of 5.2%. Mean RDW at ICU discharge was 15.4 ± 2.5%, and each quartile of RDW was associated with higher mortality (0.7%, 2.9%, 7.5%, 10.3%) and AUROC of 0.81. The most accurate logistic regression model for predicting ward mortality included age, SAPS3 and Sabadell score (SS) with AUROC of 0.90, but the inclusion of RDW in the model did not improve accuracy. A recursive partitioning analysis demonstrated higher mortality of patients with high RDW at each level of Sabadell Score (1.6% vs. 0.3% in SS0, 9.7% vs. 1.1% in SS1, 21.9% vs. 9.7% in SS2, see Fig. 130).

**CONCLUSIONS.** After ICU discharge, high RDW is a marker of severity and improves the accuracy of Sabadell score for predicting ward mortality.

**GRANT ACKNOWLEDGMENT**

None.

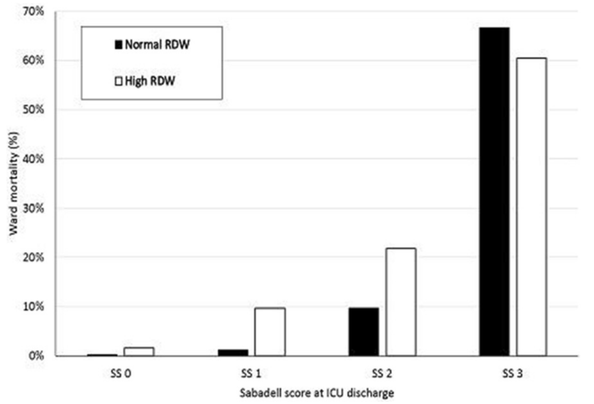

**Fig. 130 (Abstract 0387).** See text for description

### 0388

#### Early screening of intensive care unit survivors for psychological follow-up - a prospective cohort study

A. Milton[1], A. Schandl[1], M. Bottai[2], P.V. Sackey[1], Karolinska Intensive Care Outcomes Study Group

[1]Karolinska Institutet, Physiology and Pharmacology, Stockholm, Sweden; [2]Karolinska Institutet, Environmental Medicine, Stockholm, Sweden

**Correspondence:** P.V. Sackey

*Intensive Care Medicine Experimental* 2017, **5(Suppl 2):**0388

**INTRODUCTION.** A majority of patients survive their episode of critical illness but up to 30% of patients suffer from psychological problems such as post-traumatic stress, anxiety and depression in the year after intensive care unit (ICU) stay(1,2). A method to identify discharged patients at risk for adverse psychological outcome would be helpful in the triage for ICU follow up and enable early intervention(3).

**OBJECTIVES.** The objective of this study was to evaluate if early screening with validated questionnaires after ICU discharge can identify patients at risk for symptoms of post-traumatic stress, anxiety and depression three months after ICU stay.

**METHODS.** We performed a prospective observational cohort study at the general ICU at the Karolinska University Hospital Solna, Stockholm, Sweden. All adult patients surviving $^{3}$24 hours in the ICU in a 9-month period were eligible for inclusion. Patients with mental disability, serious auditory and visual disorder, aphasia or who were unable to understand Swedish were excluded. One hundred thirty-two patients were included and visited by a follow-up nurse within a week after ICU discharge. The Hospital Anxiety and Depression Scale (HADS) and Post-Traumatic Stress Symptoms Checklist-10 (PTSS-10) were administered. Three months after ICU discharge patients received the same questionnaires by postal mail.

**RESULTS.** Eighty-two patients [Office3] returned the follow-up questionnaires. We found correlation between early and late scores and reasonable predictive precision regarding three-month outcomes, with an area under the receiver operating characteristic curve (AUROC) of 0.9 for PTSS-10 part B, 0.8 for HADS anxiety subscale and 0.75 for HADS depression subscale.

**CONCLUSIONS.** Symptoms of post-traumatic stress, anxiety and depression assessed one week after ICU stay correlate with three-month psychological outcome. HADS and PTSS-10 may be useful aids to identify ICU survivors at high-risk for clinically significant symptoms of post-traumatic stress, anxiety and depression three months post-ICU.

**GRANT ACKNOWLEDGEMENTS**

Funding supported by grants provided by the Stockholm County Council (ALF), the Olle Byggmästare Foundation and the Department of Perioperative Medicine and Intensive Care, Karolinska University Hospital Stockholm, Sweden.

### 0389

#### Experiences of ICU survivors in a low middle income country - a multicentre study

L. Pieris[1,2], P.C. Sigera[1], A.P. De Silva[3], S. Munasinghe[1], A. Rashan[1], P.L. Athapattu[4], K.A.S. Jayasinghe[5], K. Samarasinghe[6], A.M. Dondorp[7], R. Haniffa[1]

[1]Network for Improving Critical Care Systems and Training, Colombo, Sri Lanka; [2]Sri Lanka Nursing Council, Colombo, Sri Lanka; [3]National Intensive Care Surveillance, Colombo, Sri Lanka; [4]Ministry of Health, Colombo, Sri Lanka; [5]Faculty of Medicine, University of Colombo, Colombo, Sri Lanka; [6]Kristianstad University, Kristianstad, Sweden; [7]Mahidol Oxford Tropical Medicine Research Unit, Bangkok, Thailand

**Correspondence:** L. Pieris

*Intensive Care Medicine Experimental* 2017, **5(Suppl 2):**0389

**INTRODUCTION.** Patients' experiences during the Intensive Care Unit (ICU) stay is associated with dissatisfaction (Hunziker et al. 2012).

**OBJECTIVES.** To describe the recall of ICU survivors' experiences, stressors and their satisfaction with ICU services in a Low Middle Income Country (LMIC).

**METHODS.** This follow-up study was carried out in 32 state ICUs in Sri Lanka between July and December 2015. ICU experiences, stress and satisfaction were gathered using an interviewer-administered questionnaire adapted from two previous studies (Granja et al. 2005; Wright et al. 2015).

**RESULTS.** There were 1665 eligible ICU survivors and 818 (49.1%) patients were not contactable and 389 (23.3%) patients died after discharge from the ICU. Twenty (4.4%) patients were excluded due to being unable to communicate (12) or not consenting (8), 438 successfully contacted ICU survivors were included in this study. Although 78.1% (n = 349) of patients remembered their admission to the hospital, only 42.3% (n = 189) could recall their admission into the ICU. Only 3 patients (0.7%) of patients said they had no recall of any of their ICU stay.

| Memory of ICU stay | Number | Precentage |
|---|---|---|
| Prefer not to remember | 3 | 0.67% |
| Don't remember anything | 3 | 0.67% |
| Don´t mind to remember | 396 | 88.59% |
| Want to remember everything | 34 | 7.61% |
| None of them | 11 | 2.46% |

*[Memory of your ICU stay]*

| Experiences | Number | Precentage |
|---|---|---|
| Dreams during ICU stay | 59 | 13.20% |
| Nightmares during ICU stay | 54 | 12.08% |

*[Experiences during ICU stay]*

The three commonest experiences patients were unable to recall were dependence on ventilator, disconnection from the ventilator and music being played in the ICU. The commonest stressful experiences were being limited to bed, pain and general discomfort, daily need punctures, family worries, fear of dying or uncertainty in the future.

Overall, the patients found the level of health care received in the ICU to be "very satisfactory" (91.5%, n = 409) with none stating they were either "dissatisfied" or "very dissatisfied".

**CONCLUSIONS.** In common with HIC, ICU survivors were very satisfied with the critical care they received but in contrast, they are frequently do not appear to recall specific ICU experiences. Stressful experiences, in common with HIC, are commonly over the future, dependency, family, and economic concerns. This follow up study highlights two concepts which are central to the delivery and evaluation of critical care services in LMIC settings; the feasibility and importance of determining outcomes beyond ICU endpoints; the necessity of engaging with ICU survivors in order to identify discriminators aiding quality improvement efforts.

The authors wish to acknowledge the support received from participating ICUs and the Ministry of Health, Sri Lanka.

**REFERENCE(S)**

Granja, C. et al., 2005. Patients' recollections of experiences in the intensive care unit may affect their quality of life. *Critical care (London, England)*, 9(2), pp.R96-109.

Hunziker, S. et al., 2012. Predictors and correlates of dissatisfaction with intensive care. *Critical care medicine*, 40(5), pp.1554-61.

Wright, S.E. et al., 2015. *Family-Reported Experiences Evaluation (FREE) study: a mixed-methods study to evaluate families' satisfaction with adult critical care services in the NHS.*

**GRANT ACKNOWLEDGMENT**

None.

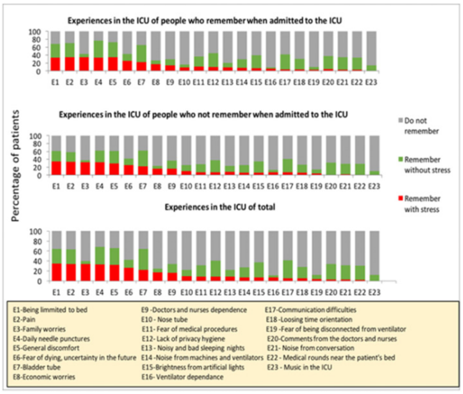

**Fig. 131 (Abstract 0389).** Recollection of stressful experiences in the ICU

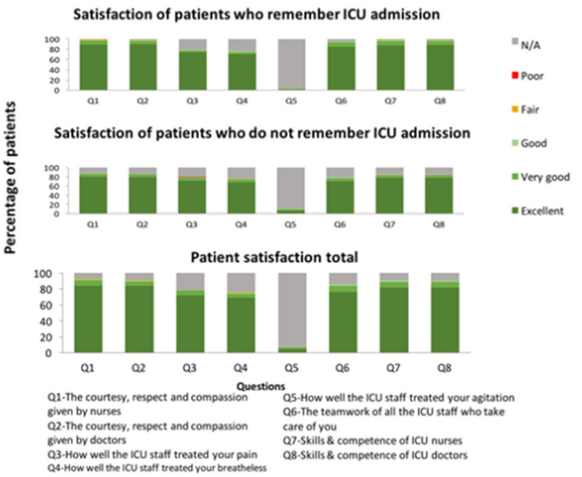

**Fig. 132 (Abstract 0389).** Patients' perception on care received

### 0390

#### Incidence and characteristics of the post intensive care syndrome

C. Leggieri[1], A. Battaini[2], L. Dezza[2], B. Oltolini[2], S. Villa[2], B. Noto[2], D. Manara[2], L. Cabrini[1], R. Lembo[1], A. Zangrillo[1,2], G. Landoni[1,2]
[1]IRCCS San Raffaele Hospital, Anesthesia and Intensive Care, Milan, Italy; [2]Vita-Salute San Raffaele University, Milan, Italy
**Correspondence:** L. Cabrini
*Intensive Care Medicine Experimental* 2017, **5(Suppl 2):**0390

**INTRODUCTION.** Post Intensive Care Syndrome includes physical, psychological and cognitive deficits that arise and may persist after critical disease. Patient's quality of life and long-term health perception are usually the most compromised items. The awareness of this syndrome and of the possibility to prevent or at least to minimize it should modify the primary goal of critical care, extending it beyond patient survival.

**OBJECTIVES.** Evaluation of physical, psychological and cognitive sequelae, including the quality of life (QoL) of patients and their perception of health in ICU survivors up to 180 days after discharge.

**METHODS.** The study was performed in the general ICU of a large University Hospital (San Raffaele of Milan, Italy), submitting two questionnaires to all patients discharged over a 6-month period after discharge for intensive treatment. The questionnaires were SF-36, EQ-ED-5L, to be completed at ICU discharge, and then at 30, 90 and 180 days after discharge. The quality of life and the perception of health were estimated by the synthetic physical index (ISF) and mental index (ISM) from the SF-36 and by the subjective percentage expressed by patients from 0 to 100 in the EQ-5D-5L questionnaire, where 50 and 100 are respectively the best imaginable perception of health from the ISF/ISM and from the EQ-5D-5L.

**RESULTS.** During the study period 173 patients were discharged, 90 (52%) being admitted for intensive treatment and 83 (48%) for postoperative monitoring (questionnaires not submitted).

The average ICU length of stay was 6,7 ± 6,7 days. The mean ISF was 31,5 ± 9,5 at ICU discharge, 36,7 ± 10,7 at 30 days, 44,2 ± 11,6 at 90 days, 47,6 ± 11,5 at 180 days. The ISM was an average 43,2 ± 11,1 at ICU discharge, 40,2 ± 11,6 at 30 days, 45,7 ± 11 at 90 days, 53,1 ± 11 at 180 days. The mean reported percentage about the perception of health in the EQ-5D-5L was 50/100 (±23) at ICU discharge, 55/100 (±22) at 30 days, 65/100 (±23) at 90 days and 73/100 (±19) at 180 days; the positive trends reached a statistical significance for ISF at 90 days (p = 0.0006) and at 180 days (p = 0.0009) and for ISM at 180 days (p = 0.018).

**CONCLUSIONS.** We noticed a positive trend of the reported perception of health, but six months after discharge from ICU the values were almost 30 points below the best value, suggesting a marked and persistent reduction in the quality of life. Moreover, ISF

and ISM at 180 days are below the standard reference population average score (50), confirming the impact of ICU stay on patients'physical and mental abilities, when they return to their daily lives. It is more important work on mental/cognitive problems. Our findings confirm that patients discharged from an ICU report a relevant worsening of the perceived quality of life. After ICU (and hospital) discharge, patients should be followed long-term to identify and possibly treat the post ICU sequelae.

**REFERENCE(S)**

D. Needham et al. CCM 2012
M.A. Harvey CCM 2016

### 0391

#### The Barthelindex is not a suitable method for follow-up of functional independence in discharged intensive care unit patients

J.J. van Arkel, E.C. Boerma, C.M. de Jager, N. Bruins
Medisch Centrum Leeuwarden, Intensive Care Unit, Leeuwarden, Netherlands
**Correspondence:** J.J. van Arkel
*Intensive Care Medicine Experimental* 2017, **5(Suppl 2):**0391

**INTRODUCTION.** In 2013 the ICU in Medical Center Leeuwarden, initiated interventions aimed at reducing post intensive care syndrome (PICS)[1] related morbidity. In the following years, this lead to increased functional independence, measured via the Barthelindex (BI), at ICU discharge[2].

**OBJECTIVES.** This paper attempts to determine whether the observed improvements in functional independence are maintained over the medium-term.

**METHODS.** The primary outcome of this research is the BI, measure of functional independence. Over the period of 2012–2016, 289 patients were selected for having been admitted to the ICU for >48h as well as having been seen at the outpatient department of the ICU-MCL.

**RESULTS.** Patient group composition remained consistent, length of stay and the duration of mechanical ventilation were significantly reduced, post-intervention. The BI, at ICU discharge, rose from 8[5–12] in 2012 to 13[9–18] in 2016.

At follow-up, performance on the 6 minute walking test (6mwt), and MFI-20 score for fatigue improved significantly, whilst other scores describing functional independence remained constant. At follow-up we encountered a ceiling effect of the BI, all patient groups gained a maximum 20[20–20] BI score. A BI of < 16 at ICU discharge is a significant predictor for not reaching the ceiling effect thus scoring sub-optimally on the BI.

When analyzing scores for physical abilities, a lower BI at discharge predicts poor performance on both the 6mwt test and the physical section of the Short Form 36. Poor performance on the ability to climb stairs at ICU discharge, significantly predisposes to depression with an AUC of 0,78.

**CONCLUSIONS.** The BI is not suitable for the follow-up of ICU patients post discharge. The BI is validated as adequate for the monitoring patient functional abilities during hospital admission. This research shows however that the BI is not sensitive for determining subtle but debilitating problems that ex-ICU patients face[3].

Secondary parameters show that the implemented interventions have subtle but positive effects on PICS related morbidity.

**GRANT ACKNOWLEDGMENT**

This research did not receive any external funding.

### 0392

#### Virtual Reality Exposure Therapy is a preferred intervention among survivors of critical illness suffering from posttraumatic stress disorder: a patient's perspective

M.E. van Genderen[1,2], N. Poublon[3], E.J. Wils[3], A. Brouwers[3], A. Schut[4], M. Verkade[5], L. Mandigers[5], E. Berger[3], J. van Bommel[2]
[1]Franciscus Gasthuis, Internal Medicine, Rotterdam, Netherlands; [2]Erasmus MC University Hospital, Intensive Care, Rotterdam, Netherlands; [3]Franciscus Gasthuis, Intensive Care, Rotterdam, Netherlands; [4]Ikazia Hospital, Intensive Care, Rotterdam, Netherlands; [5]IJsselland Hospital, Intensive Care, Rotterdam, Netherlands
**Correspondence:** M.E. van Genderen
*Intensive Care Medicine Experimental* 2017, **5(Suppl 2):**0392

**INTRODUCTION.** Survivors of critical illness suffer frequently from loss of quality of life after discharge from the ICU. In recent years, follow-up studies pointed at psychiatric morbidity as an important contributing factor, especially symptoms of posttraumatic stress disorder (PTSD) were demonstrated in ICU survivors. Interventions like ICU diaries, early in-ICU psychological assessment and ICU follow-up clinics appear ineffective to improve psychological recovery.

Recently, Virtual Reality Exposure Therapy (VRET) was shown to be effective in treating patients with combat-related posttraumatic stress disorder, pain after burn injury and mental health disorders. The need and possible effect of VRET in ICU survivors is not yet known.

**OBJECTIVES.** To examine the prevalence of PTSD symptoms in a representative sample of Dutch adult ICU survivors and assess their preferred method of intervention, including VRET.

**METHODS.** A retrospective cohort of patients was recruited from 4 Dutch ICU's. Due to the retrospective nature of the study we set the start of the study at October 1 2016. Patients, respectively 1 month (4-8weeks), 6 months (26–30 weeks), 12 months (52–56 weeks), 2 years (104–108 weeks), and after 2,5 years (130–134 weeks) after ICU discharge were eligible for inclusion. Eligible patient's had to be mechanically ventilated for more than 2 days. Patients were sent a postal survey containing a self-report questionnaire to assess: symptoms of anxiety, depression, and post-traumatic stress (Impact of Event Scale (IES)) and quality of life (EuroQol health classification system (EQ-5D)). They were also asked regarding different methods that could improve their recovery and well-being the best, including an information brochure and VRET.

**RESULTS.** A total of 67 patients received questionnaires of which 46 (69%) responded. Of these patients 22% were included in the 1 month cohort, 22% after 6 months, 19% after 12 months, 21% after 24 months, and 16% after 30 months. PTSD-related symptoms were reported in 61% of the patients, with a maximum prevalence at 6 months. The presence of PTSD symptoms was associated with more problems on all EQ-5D domains (mean 0.17 (SD 0.17) versus 0.49 (SD 0.36); $P < 0.01$)). Patients with PTSD reported significantly more anxiety/depression (60% versus 40%; $P < 0.01$) indicating decreased Quality Of Life. Especially patients with PTSD more frequently expected a possible effect from VRET to cope with their problems, compared to patients without PTSD ((62% versus 38%; $P = 0.047$)).

Moreover, of the patients with PTSD, 65% favored VRET compared to 38% an information brochure; P < 0.01.

**CONCLUSIONS.** PTSD related symptoms persist up to 30 months after recovery from critical illness and are associated with a considerable decrease in Quality Of Life. Patients with PTSD appear motivated to undergo Virtual Reality Exposure Therapy in the aftermath of critical illness to finally improve quality of life.

### 0393

#### Outcome and quality of life following implantation of left ventricle assist devices (LVAD) in cardiogenic shock patients with multiorgan failure (MOF)

A. Radjou[1], R. Sonneville[2], L. Bouadma[2], M. Neuville[2], E. Mariotte[3], S. Ruckly[2], R. Smonig[2], E. Magalhaes[3], J. Lebut[3], O. Andremont[2], M. Lermuzeaux[2], C. Dupuis[2], L. Lepage[4], R. Dorent[4], P. Nataf[5], M. Wolff[2], B. Mourvillier[2], J.F. Timsit[2]

[1]Bichat-Claude Bernard Hospital, Réanimation Médicale et Infectieuse, Paris, France; [2]Bichat-Claude Bernard Hospital, Paris, France; [3]Hopital Bichat, Paris, France; [4]Bichat-Claude Bernard Hospital, Cardiology, Paris, France; [5]Bichat-Claude Bernard Hospital, Cardiac Surgery, Paris, France

**Correspondence:** A. Radjou

*Intensive Care Medicine Experimental* 2017, **5(Suppl 2):**0393

**INTRODUCTION.** Heart transplantation (HT) is considered the "gold standard" therapy for patients with end stage heart failure. However, only few patients can benefit from HT in the emergency setting, essentially because of the lack of heart donors and/or associated organ failure(s). LVAD implantation is a valid alternative in the absence of multiorgan failure, but data on the potential interest of this procedure in cardiogenic shock patients with MOF are scarce.

**OBJECTIVES.** We aimed to determine the prognosis and quality of life of patients who underwent LVAD implantation with preoperative cardiogenic shock and multiorgan failure.

**METHODS.** We performed a retrospective chart review of 37 cardiogenic shock patients with MOF who underwent LVAD (n = 36 Heart Mate II® and n = 1 Heartware® devices) implantation, between October 2011 and September 2016. The primary end-point was all-cause mortality one year following LVAD implantation. We also analyzed quality of life by phone interviews between February 2017 and April 2017, using the EUROQOL and the Short-Form General Health Survey (SF-36) scales (scores range from 0 to 100, with higher scores indicating a better health care status). Results are presented as n (%) or median [interquartile range].

**RESULTS.** Thirty-seven patients (male gender, n = 33 (89%); age, 60 [45; 66.5] years; SAPS II, 48 [34; 62]; SOFA 8 [6–13]) were analyzed. The main cause of heart failure was ischemic cardiomyopathy (n = 18 (49%) patients). All patients were mechanically ventilated, 34 (92%) patients received vasopressor/inotropic support, 21 (57%) patients received renal replacement therapy, and 22 patients (59%) received venoarterial ECMO support before LVAD implantation. LVAD implantation was performed after 10.5 [5.5; 16] days of ICU stay. Overall, twenty-one (57%) patients were alive at ICU discharge. Duration of mechanical ventilation and duration of ICU stay were 9 [3.5; 21] and 32 [22; 58] days, respectively. Hospital survival and 1-year survival were 57% (21/37) and 54% (20/37), respectively. Seventeen (46%) patients underwent HT 264 [51; 342] days after LVAD implantation, 2 (5%) patients were still waiting for HT at 1 year, and 2 (5%) patients received LVAD implantation as a destination therapy. The score on the EUROQOL scale was 67.5 [57.5; 76.3]. Among the SF-36 items, the lower score was observed on the role physical (RP) item (12.5 [0; 50]), whereas higher scores were observed for other items (i.e., Physical Functioning (PF) item: 80 [64; 95] and Social Functioning (SF) item: 75 [60.1; 87.5]). The main qualitative complaint in survivors was the incapacity to take showers or bathes.

**CONCLUSIONS.** The implantation of a LVAD in cardiogenic shock patients with MOF is associated with a 54% survival rate at 1 year, with an acceptable quality of life in most of survivors.

**REFERENCE(S)**

Dembitsky WP. Ann Thorac Surg. 1999 Mar;67

Rogers et al. JACC Vol. 55, 2010

Lynne Warner Stevenson Circulation. 2004

### 0394

#### Functional status after one year of post ICU discharge

J. Baroudi[1], N. Fraj[1], J. Ayachi[1], K. Meddeb[1], E. Ennouri[1], W. Zarrougui[1], N. Sma[1], M.A. Boujelbèn[1], A. Ben Abdelhafidh[1], H. Kallel[1], I. Chouchene[1], A. Azouzi[1], A. Khedher[1], I. Ben Saida[1], M. Boussarsar[1,2]

[1]Farhat Hached University Hospital, Medical Intensive Care Unit, Sousse, Tunisia; [2]Ibn Al Jazzar Faculty of Medicine, Research Laboratory N° LR14ES05 Interactions of the Cardio-pulmonary System, Sousse, Tunisia

**Correspondence:** M. Boussarsar

*Intensive Care Medicine Experimental* 2017, **5(Suppl 2):**0394

**INTRODUCTION.** Independence in activities of daily living was absolutely decreased after the ICU stay and quality of life is altered. The major challenge for medical staff is to decrease mortality and to improve functional status for critically ill patients after discharge. The effects of an ICU stay in patients with severe illness and comorbidity on autonomy and functional capacity is certainly important.

**OBJECTIVES.** To evaluate functional autonomy and to quantify the physical and mental activity in daily life in long term ICU survivors.

**METHODS.** A prospective cohort study was conducted with a follow-up from hospital discharge to 12 months after for ICU survivors during the period from January 2014 to December 2015. Functional status was assessed by evaluation of autonomy for all survivors using Activities of daily living (ADL)(1) and Instrumental activities of daily living (IADL)(2) scales in telephone interviews with patient or their proxies.

**RESULTS.** During the predetermined period of data collection, the follow up concerned 280 patients. Only 215(77%) patients responded. Among a total of 215 patients, 152(71%) patients survived to 12 months. At the time of interview, 180(84%) patients survived independently without specialized care. The median values of ADL/IADL scales were respectively 4 and 6. The proportion of those reporting combined difficulties in ADL (<3) or IADL (<5) were respectively 16% and 8.6% with more patients reporting difficulty with ADL than IADL. There were significant differences between patients with or without difficulties; in age (45,5 ± 22,5 vs 54,3 ± 21,1 ; p = 0.02) ; comorbidities of cancer (4(11,1%) vs 5 (2,9%) ; p = 0.02) ; clinical diagnosis (neurological diagnosis at ICU admission for 13 patients (36,1%) vs 16 patients (9,1%) ; p = 0.000), invasive therapy such as tracheostomy (6(16,7%) vs 8(4,6%) ; p = 0.0008) and nosocomial infection for (13(36,1%) vs 31 (17,8%) ; p = 0.01).

**CONCLUSIONS.** Partial and moderate reduction in physical activity, exercise capacity was detected in critically ill patients, but persisting even 12 months after hospital discharge. Neurological critical illness is the strongest factor associated with difficulties in daily living activity.

**REFERENCE(S)**

1. White DK, Wilson JC, Keysor JJ. Measures of adult general functional status: SF-36 Physical Functioning Subscale (PF-10), Health Assessment Questionnaire (HAQ), Modified Health Assessment Questionnaire (MHAQ), Katz Index of Independence in activities of daily living, Functional Independence Measure (FIM), and Osteoarthritis-Function-Computer Adaptive Test (OA-Function-CAT). Arthritis Care Res. nov 2011;63 Suppl 11:S297-307.

2. Barberger-Gateau P, Commenges D, Gagnon M, Letenneur L, Sauvel C, Dartigues JF. Instrumental activities of daily living as a screening tool for cognitive impairment and dementia in elderly community dwellers. J Am Geriatr Soc. nov 1992;40(11):1129-34.

### 0395

#### Incidence and outcomes of accidental hypothermia upon admission to the intensive care unit: a single center observational study

A. Hussain[1,2], O. Gural[1], J. Zhurova[1], N. Izotova[1,2], L. Bjertnaes[3], T. Tveita[3], M. Kirov[1,2,3]
[1]Northern State Medical University, Anesthesiology and Intenisve Care Medicine, Arkhangelsk, Russian Federation; [2]City Hospital #1, Arkhangelsk, Russian Federation; [3]University of Tromsø, Tromsø, Norway
**Correspondence:** A. Hussain
*Intensive Care Medicine Experimental* 2017, **5(Suppl 2):**0395

**INTRODUCTION.** Accidental hypothermia (AH) is defined as an unintended decrease in core temperature < 35 °C. In addition to acting as a risk factor in itself, AH occurs in different categories of patients and thus worsen underlying diseases and their clinical courses.
**OBJECTIVES.** The aim of our study was to estimate the incidence and outcomes of AH in patients upon admission to the intensive care unit (ICU).
**METHODS.** We performed a retrospective analysis of the ICU charts of all patients admitted to the 52-bed adult ICU of the City hospital #1 of Arkhangelsk in year 2016. The hospital has 1050 beds and serves as a main emergency hospital in the region of North Russia. We included for analysis all patients who had axillary temperature < 35.0 °C upon admission to ICU and recorded demographic data, diagnosis, type of surgery, duration of ICU stay and mortality.
After checking data distribution, we used the Student´s t-test and the chi-square test to compare the data according to their type. The results are presented as mean ± standard deviation.
**RESULTS.** Totally, 3299 ICU charts were examined. Upon admission to ICU, AH was observed in 172 patients (5.2%). The mean age of patients was 63.5 ± 13 years. The mean body temperature was 34.86 ± 0.42°C. AH was observed more frequently in cardiac ICU than in general ICU [104 (60.5%) vs. 68 (39.5%), $p = 0.008$] patients. The postoperative patients had higher incidence of AH [160 (93%) vs. 12 (7%), $p = 0.0001$]; the majority (65%) of postoperative patients were after cardiac surgery. In general ICU, the main causes of AH included abdominal surgery (50%), sepsis (17.6%), poisoning (10.3%), burns (4.4%), freezing injury (2.9%). The axillary temperature was lower in general ICU: 34.6 ± 0.6°C vs. 34.9 ± 0.1°C in cardiac patients ($p = 0.001$). The mean values of plasma lactate and INR in patients with AH on admission to ICU were 3.28 ± 3.7 mmol/L and 1.29 ± 0.56, respectively. The mean ICU stay of patients with AH was 3.6 days and overall mortality rate was 12.8%. Mortality rate was higher in general ICU patients with AH: 22.6% vs. 6.8% in cardiac ICU patients (p = 0.007).
**CONCLUSIONS.** The incidence of AH on admission to ICU is 5.2% and occurs most frequently in postoperative patients, mostly after cardiac and abdominal surgery. AH is accompanied by an overall mortality rate of 12.8% and requires correction of metabolic and coagulation abnormalities.

### 0396

#### Outcomes for haematology patients in the intensive care unit: a brighter perspective but ongoing challenges

M. Shea, M. Rowland, A. Wong
Oxford University Hospitals NHS Trust, Oxford, United Kingdom
**Correspondence:** M. Shea
*Intensive Care Medicine Experimental* 2017, **5(Suppl 2):**0396

**INTRODUCTION.** Haematology patients have historically had high mortality rates in the Intensive Care Unit (ICU) (1). Despite an improving trend in recent years, the perception remains among many intensivists that haematology patients have a poor prognosis in ICU (2).
**OBJECTIVES.** To analyse characteristics of all haematology and oncology patients admitted to ICU over a 3 year period, with the aim of analysing mortality rates and identifying predictors of in ICU mortality.
**METHODS.** Data from electronic patient records for a 3-year period (2014–2016) was used to generate descriptive statistics for patients admitted from haematology and oncology to ICU. The primary outcome was survival to discharge from ICU vs death in ICU. Independent variables were: age, sex, referring service (haematology vs oncology), APACHE II score, number of days requiring level 2 or 3 care, duration of stay, percentage of patients discharged within 1 day, primary diagnosis, and primary reason for ICU admission. Continuous variables were initially analysed using Student's t test and categorical variables using Chi square test. A logistic regression model was then applied.
**RESULTS.** 95 admissions to ICU from haematology and oncology services were documented from 2014–2016 (66 from haematology, 29 from oncology). Survival to discharge from ICU, at 1 month post discharge, and at 6 months post discharge was 69.5%, 51.6%, and 33.7% for the combined haematology and oncology patients, 65.7%, 48.4%, and 31.8% for haematology patients, and 78.6%, 55.2%, and 35.8% for the oncology patients. In univariate analysis, significant differences between patients surviving to discharge and those that died in ICU were observed for the following variables: Length of stay in ICU ($p < 0.05$), number of days requiring level 2 support ($p < 0.01$) or level 3 support ($p < 0.01$), maximum organ support required ($p < 0.01$), and Apache II score ($p < 0.01$). The referring specialty was not significantly associated with in ICU mortality. Logistic regression confirmed that APACHE II score is a predictor of mortality in a multivariate analysis.
**CONCLUSIONS.** Contrary to expectations, this study did not show a significant excess of mortality for haematology patients over oncology patients. However, the high mortality rates at 6 months and the length of stay for patients who died in ICU (mean stay 8.9 days, but 24% died within 24 hours of ICU admission) raise two questions. First, is patient selection for ICU adequately managed, and second, have the patients and their families been sufficiently counselled on their prognosis and the likely interventions while in ICU. In line with previous studies, we find that a higher Apache II score is associated with a higher mortality rate. Unfortunately, a robust set of criteria for selecting haematology and oncology patients for ICU treatment is still awaited.

**REFERENCE(S)**
1) Crit Care Med. 2003 Jan;31(1):104-1204-12,
2) Br J Anaesth. 2012 Mar;108(3):452–9
**GRANT ACKNOWLEDGMENT**
None

### 0397

#### Incidence of Post Intensive Care Syndrome (PICS) in patients with Subarachnoid Hemorrhage (SAH): results of an ICU aftercare program

D. van Rijswijk, A. Rutten, G. van der Nat, N. van der Lely, D. Ramnarain
Elisabeth-TweeSteden Hospital Tilburg, Intensive Care, Tilburg, Netherlands
**Correspondence:** D. van Rijswijk
*Intensive Care Medicine Experimental* 2017, **5(Suppl 2):**0397

**INTRODUCTION.** Spontaneous Subarachnoid Hemorrhage (SAH) is a neurological disease with high morbidity and mortality[1]. ICU survivors of SAH often experience negative outcomes after ICU discharge, including physical complaints, cognitive decline and mental health problems. Recognition of ICU related risk factors and early treatment of probable negative outcomes in these patients is of great importance.
**OBJECTIVES.** The aim of the study was to examine the incidence of somatic complaints, cognitive dysfunction, and negative emotional outcomes, including depression, anxiety and post-traumatic stress disorder (PTSD), in patients with SAH treated in our ICU. Also, we studied the association of ICU related factors with somatic complaints, cognitive dysfunction, anxiety, depression and PTSD.

**METHODS.** Patients with SAH treated in our ICU between January 2012 and December 2016 for a minimum of 4 days were invited to the ICU aftercare clinic. Six weeks after hospital discharge, patients received an invitation letter along with three questionnaires: a health-related questionnaire for identifying somatic and cognitive complaints, the Hospital Anxiety and Depression Scale (HADS) and the Impact of Event Scale - Revised (IES-R). Patient characteristics and ICU admission characteristics were obtained from electronic patient records.

**RESULTS.** A total of 110 SAH patients visited our aftercare program (mean age = 58, SD = 10.40), of which 31.8% was male. Median Apache II and Apache IV were 11 and 36, respectively. Median ICU LOS and hospital LOS were 6 and 15 days, respectively. Of all patients, 31.8% was mechanically ventilated with a median length of 4 days. Delirium during ICU treatment was observed in 24.5% of the patients. Most frequently reported somatic complaints after discharge were muscle weakness (41.8%), fatigue (73.6%), weight loss (30.9%) and pain (36.4%). Reported cognitive complaints were problems with concentration (63.6%), memory problems (45.8%) and reduced thinking capacity (60.9%). The prevalence of clinically significant anxiety and depression was 24.3% and 19.6%, respectively. Additionally, 11.4% of the patients showed possible PTSD. Length of mechanical ventilation was associated with PTSD ($p < 0.05$) Clinical factors including ICU LOS, hospital LOS, APACHE scores and drugs administered during ICU treatment were not associated with somatic complaints, cognitive dysfunction or negative emotional outcomes.

**CONCLUSIONS.** Incidence of PICS related symptoms, including somatic complaints, cognitive dysfunction, anxiety, depression and PTSD, was high in patients with SAH surviving the ICU. Patients with longer duration of mechanical ventilation showed more PTSD symptoms. No other ICU related risk factors could be identified.

**REFERENCE(S)**

1. De Oliveira Manoel AL et al. Functional outcome after poor-grade subarachnoid hemorrhage: A single center study and systematic literature review. Neurocrit. Care 2016, 25(3):338–350.

### 0398

#### Outcomes of hypoxic/ ischaemic brain injury following out of hospital cardiac arrest

I. Fale, V. Newey
Barts Health NHS Trust, Physiotherapy, London, United Kingdom
**Correspondence:** I. Fale
*Intensive Care Medicine Experimental* 2017, **5(Suppl 2):**0398

**INTRODUCTION.** Hypoxic brain injury (HBI) is a common complication of out of hospital cardiac arrest (OOHCA). With improved pre-hospital treatment and acute care interventions the prevalence of patients with HBI is increasing. Research in this area is limited and largely focused on prognostication with limited evidence on long-term outcome and on-going rehabilitation needs (1).

**OBJECTIVES.** Physiotherapy plays a key role in rehabilitation of HBI patients. The aim of this study was to evaluate clinical characteristics, prognostic factors and outcomes following OOHCA.

**METHODS.** This study was a retrospective case note review of all HBI patients admitted to our critical care unit over three months. Targeted temperature management was used in all patients. The CT and EEG findings were classified by a consultant neurologist and neurophysiologist respectively as mild, moderate or severe. Outcome measures were the Glasgow Outcome Scale (GOS) and the Cerebral Performance Categories scale (CPC). The GOS and CPC were recorded 1 month after admission and 1 month after discharge.

**RESULTS.** Twenty two patients were admitted during this time. 14% of patients were discharged home with no or only minor deficits within one month; 32% remained in either acute care or specialist neurological rehabilitation and 54% of patients died. At one month post discharge from acute care 18% of patients were at home with no or only minor deficits. Patient demographics are shown in Table 112.

The average time to neurological investigation (CT and EEG) was 3 days; these investigations were performed in 91% of patients. Neurological investigations and clinical examination were performed after sedation hold when patients did not wake appropriately and/or had adverse neurological signs e.g. seizure activity. Clinical seizure activity was noted in 59% of patients. Of those who experienced seizure activity the CT scan was classified as severe in 69% whilst in 77% the EEG was classified as severe. In 62% the CPC score was 4 or 5, indicating severe disability or vegetative state. All patients with both severe EEG and CT findings had a poor CPC outcome (Tables 113 and 114). Of note some patients made a good functional recovery and were discharged home despite adverse prognostic criteria.

**CONCLUSIONS.** Severe abnormalities seen on both EEG and CT are strongly associated with a poor outcome or death in this patient population. This small sample demonstrates that certain patients with initially poor prognostic features can achieve a good functional outcome at two months. Seizure activity does not always predict a poor outcome. We will continue to prospectively enrol patients to this study to achieve a larger sample size and a longer period of follow-up.

**Table 112 (Abstract 0398).** Patient Demographics

| Variable | Data (n=22) |
|---|---|
| Age (years) | 61 +/- 11 |
| Male sex | 21 |
| Estimated downtime (mins) | 26.6 +/- 16.5 |
| Independent function pre admission | 22 |

**Table 113 (Abstract 0398).** Relationship between CT, EEG and CPC of 5

| | CT : Mild | CT : Moderate | CT : Severe |
|---|---|---|---|
| EEG: Mild | 0/2 (0%) | | 0/1(0%) |
| EEG: Moderate | 1/3 (33.3%) | 0/1 (0%) | |
| EEG: Severe | 1/2 (50%) | 0/1 (0%) | 9/9 (100%) |

Number/total number (%)

**Table 114 (Abstract 0398).** Relationship between CT, EEG and CPC of 1 or 2

| | CT : Mild | CT: Moderate | CT: Severe |
|---|---|---|---|
| EEG: Mild | 2/2 (100%) | | 1/1(100%) |
| EEG: Moderate | 1/3 (33.3%) | 1/1 (100%) | |
| EEG: Severe | 1/2 (50%) | 1/1 (100%) | 0/9 (0%) |

Number/total number (%)

### 0399

#### Incremental utility of laboratory results over demographics and vital signs in predicting future cardiorespiratory instability

L. Chen[1], A. Dubrawski[1], T. Pellathy[2], G. Clermont[3], M. Pinsky[3], M. Hravnak[2]
[1]Carnegie Mellon University, Auton Lab, Robotics Institute, Pittsburgh, United States; [2]University of Pittsburgh, Acute and Tertiary Care, Pittsburgh, United States; [3]University of Pittsburgh, Critical Care Medicine, Pittsburgh, United States
**Correspondence:** M. Hravnak
*Intensive Care Medicine Experimental* 2017, **5(Suppl 2):**0399

**INTRODUCTION.** Correctly identifying patients likely to develop future cardiorespiratory instability (CRI) has implications for patient triage, allocation of surveillance resources and preemptive care.

**OBJECTIVES.** To determine if laboratory test values available within the first 4 hr of unit admission improves forecasting of CRI compared to models that include only patient demographics and vital sign (VS) features.

**METHODS.** We collected continuous noninvasive VS monitoring data (heart rate, respiratory rate, blood pressure, SpO2) from 1092 stepdown unit (SDU) patients and identified 584 patients with CRI episodes (positive) defined as VS deviation beyond stability thresholds, and 508 patients never displaying CRI (negative). This dataset was augmented with demographics (age, gender, race, Charlson Comorbidity Index) and 13 serum laboratory results (blood chemistries, hematology, liver function). We extracted last lab test values and changes of lab test values from the second-last test within a 24-hour window up to 4 hours after SDU admission. For VS, we computed mean and interquartile range during the first 4 hours of SDU stay. After filtering out insignificant features using two-sample t-tests, the final dataset included 9 lab test values features, 6 VS features and 4 demographic features. We fit three random forest models: model 1 includes only demographics, model 2 adds VS features, and model 3 further adds lab test value features. We report model performance based on 10-fold cross validation.

**RESULTS.** The Area Under Receiver Operating Curve (ROC) scores are 0.52, 0.60 and 0.65 for models 1, 2 and 3 respectively. To better illustrate the models' ability to identify true positives and negatives, Fig. 133. shows a sub-part of the ROC diagram with true and false positive and negative rates plotted on log scale. The left panel shows that at a low level of false positive rate (<0.01) there is no significant difference in true positive rate between the models. However, the right panel shows that at a low level of false negative rate, model 3 exhibits a significantly higher true negative rate than models 1 and 2. For example, at a false negative rate of 1 in 1000, the true negative rate is 0.08 for model 3 (95% CI [0.07, 0.09]), compared to 0.04 for model 2 (95% CI [0.04, 0.03]) and 0.02 for model 1 (95% CI [0.02, 0.03]).

**CONCLUSIONS.** Adding laboratory test values to models forecasting CRI carries extra predictive utility compared to models relying solely on demographics and the initial 4 hours of VS after admission, and in particular is advantageous in identifying those patients who will never develop CRI at a very low miss rate. This insight may provide guidance in developing a clinically relevant predictive triage model to identify very early in their hospital unit admission patients who need closer surveillance and those who do not using continuously and automatically acquired clinical data.

**GRANT ACKNOWLEDGMENT**
NIH NINR R01NR013912

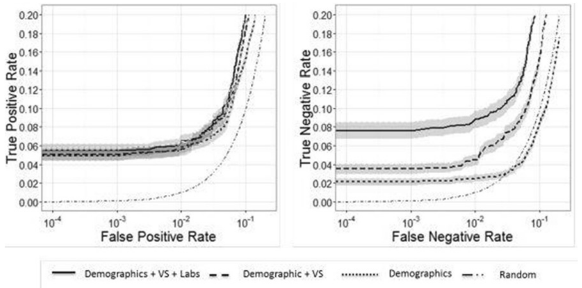

**Fig. 133 (Abstract 0399).** Portion of ROC diagram comparing 3 models

## Oral Sessions Tuesday, 26 September 2017

### Abstract award winning session

#### 0400

#### The effects of normal saline versus balanced crystalloid solution as a resuscitation fluid on acute kidney injury in shock patients: a randomized opened label controlled trial

R. Ratanarat, P. Sanguanwit, A. Chitsomkasem
Mahidol University, Division of Critical Care, Dept. of Medicine, Faculty of Medicine Siriraj Hospital, Bangkok, Thailand
**Correspondence:** R. Ratanarat
*Intensive Care Medicine Experimental* 2017, **5(Suppl 2):**0400

**INTRODUCTION.** Isotonic saline (0.9% sodium chloride) is the most commonly used intravenous fluid for resuscitation. Conflicting data regarding its association with greater incidence of AKI in comparison to buffered crystalloids.

**OBJECTIVES.** To assess the association of 0.9% saline versus a physiologically balanced crystalline (Sterofundin®) as a resuscitation fluid with AKI incidence in shock patients.

**METHODS.** We randomly assigned shock patients diagnosed within 1 hour to receive either a balanced crystalloid or 0.9% saline (NSS) for intravascular-fluid resuscitation during the first 72 hours. The primary outcome was an incident of AKI according to KDIGO classification during the 7-day period after randomization. Secondary outcomes included the resolution from peak to baseline creatinine level in the ICU, need for renal replacement therapy (RRT), length of stay in ICU and hospital, mortality, and metabolic derangements.

**RESULTS.** Of 181 patients enrolled, 88 received a balanced crystalline and 93 received saline. There were no significant differences in baseline demographic and metabolic data between two groups at enrollment. The median volume of fluid therapy in the first 3-day were 11,158 ml and 11,189 ml in a balanced crystalloid, and saline groups, respectively ($p$ = 0.9). 64/88 (72%) patients in the balanced crystalloid group and 69/93 (74%) patients in the saline group developed AKI during 7 day of admission ($p$ = 0.82). At 7 days after randomization, the number of cases who still had AKI decreased to 18/88 (20%) in the balanced crystalloid group, and 19/93 (20%) in the saline group ($p$ = 0.85). In the buffered crystalloid group, RRT was used in 4 of 88 patients (4.6%) compared with 12 of 93 patients (12.2%) in the saline group ($p$ = 0.06). There were no difference in 28-d, ICU, or hospital mortality, hospital or ICU length of stay, or ventilator-free and vasopressure-free days.

**CONCLUSIONS.** The use of a balanced crystalloid as the resuscitation fluid in patients with shock did not reduce the incident of AKI compared to saline.

#### 0401

#### A prospective international observational one-day prevalence study on prone positioning in ARDS. The APRONET study

C. Guérin[1,2], P. Beuret[3], J.-M. Constantin[4], G. Bellani[5,6], L. Baboi[7], A. Mercat[8,9], J.-M. Chrétien[10,11], G. François[12], APRONET group
[1]Hopital de la Croix Rousse, Réanimation Médicale, Lyon, France; [2]IMRB INSERM 955, Créteil, France; [3]Centre Hospitalier de Roanne, Roanne, France; [4]CHU Estaing, Clermont-Ferrand, France; [5]A.O. San Girardo, Dipartimento di Emergenza e Urgenza, Monza, Italy; [6]Università degli Studi Milano Bicocca, Dipartimento di Medicina e Chirurgia, Monza, Italy; [7]Hopital de la Croix Rousse, Lyon, France; [8]CHU Angers, Réanimation Médicale, Angers, France; [9]Université d'Angers, Angers, France; [10]CHU Angers, DRCi - Cellule Gestion des Données et Evaluation, Angers, France; [11]CIL, Angers, France; [12]ESICM, Bruxelles, France
**Correspondence:** C. Guérin
*Intensive Care Medicine Experimental* 2017, **5(Suppl 2):**0401

**INTRODUCTION.** Whereas prone positioning (PP) has been shown to improve patient survival in moderate to severe ARDS patients, its rate of use has been found as low as 16% in the Lung Safe study. However, this latter did not specifically focus on PP.

**OBJECTIVES.** To determine prevalence of use of PP, physiologic effects and reasons for not using PP in ARDS patients.

**METHODS.** The APRONET study was a prospective international one-day prevalence study. Data collection was performed 4 times in April, July, October 2016 and January 2017 in ICUs recruited through ESICM website. Each ICU had to select one day per period among 4 proposals. At each study day, investigators in each ICU had to screen every patient staying in the ICU from 0 to 24 hours and to fill a specific electronic CRF. For patients with ARDS oxygenation, ventilator settings and plateau pressure (Pplat) were recorded. For those receiving PP these variables were recorded before and at the end of PP session. Reasons for not proning were also collected. ARDS was defined according to the Berlin definition from the worst PaO2/FIO2. Values are presented as median ($1^{st}$-$3^{rd}$ quartiles). Prevalence rate of ARDS was compared by using chi-square for trend and groups with nonparametric tests.

**RESULTS.** Overall 6,614 patients were screened in 138 ICUs from 19 countries, and 743 had ARDS. ARDS prevalence was overall 11.2%, and 13.7, 9.0, 10.2 and 11.6% in periods 1 to 4, respectively (P = 0.113).

One hundred and one patients received at least one PP session (13.6%). This rate was 13.9, 12.9, 15.0 and 14.1% in periods 1 to 4, respectively (P = 0.826). It was 6.5, 11.3 and 37.0% in mild, moderate and severe ARDS stages, respectively (P < 0.001), and 26.0% in those with PaO2/FIO2 ratio < 150 mmHg.

First PP session lasted 18 [15–23] hours. Before and after first PP session, PaO2/FIO2 was 101 [76–136] vs.171 [118–220]mmHg (P = 0.0001), driving pressure 14 [11–17] vs.13 [10–16]cmH2O (P = 0.001), FIO2 77 [60–100] vs. 60 [40–75]% (P = 0.0001), and PEEP (12 cmH2O), tidal volume (6 ml/kg predicted body weight) and Pplat were similar.

Main reason for not proning was not severe hypoxemia (60.7%). PaO2/FIO2 at the time of inclusion was 164 [122–211] vs 102 [80–142] mmHg in not proned vs proned, respectively (P = 0.0001). Other reasons were: low mean arterial pressure (5.4%), tracheotomy (2.8%), elevated intracranial pressure (1.6%), pneumothorax (1.2%), unstable bone dislocation (1.1%), sternotomy (1.1%), excessive workload (1.1%), facial trauma (0.7%), hemoptysis (0.4%), other (21.7%).

**CONCLUSIONS.** This study confirms a 11% prevalence rate of ARDS with no significant seasonal variation. Rate of PP use is overall similar as but higher in severe ARDS than in the Lung Safe study, which could reflect change in practice or ICU selection bias. Significant oxygenation improvement is confirmed together with decrease in driving pressure. The main reason for not proning is lack of severe hypoxemia.

### 0402

#### ICU survivors have a substantial higher risk of developing chronic conditions compared to a population based control group

I. van Beusekom[1,2], F. Bakhshi-Raiez[1,2], N.F. de Keizer[1,2], M. van der Schaaf[3,4], W.B. Busschers[1,2], D.A. Dongelmans[2,5]
[1]Academic Medical Center, Medical Informatics, Amsterdam, Netherlands; [2]National Intensive Care Evaluation (NICE) Foundation, Amsterdam, Netherlands; [3]Academic Medical Center, Rehabilitation, Amsterdam, Netherlands; [4]University of Applied Sciences, Amsterdam School of Health Professions, ACHIEVE, Amsterdam, Netherlands; [5]Academic Medical Center, Intensive Care Medicine, Amsterdam, Netherlands
**Correspondence:** I. van Beusekom
*Intensive Care Medicine Experimental* 2017, **5(Suppl 2):**0402

**INTRODUCTION.** Intensive Care Unit (ICU) patients consume significant healthcare resources during their ICU admission and studies have shown that factors present before ICU admission, such as comorbidities, are strong predictors of hospital resource use [1]. However, little is known about the chronic conditions patients suffer before their ICU admission and the development of chronic conditions over time. Moreover, it is unclear whether there is a difference in developing chronic conditions between ICU patients and the general population. Identifying groups of ICU patients with a high risk of developing chronic conditions after ICU admission is important for prevention and targeted care in order to reduce healthcare expenditure.

**OBJECTIVE.** To describe the differences between an ICU population and a population based control group with respect to existing chronic conditions during the year before ICU admission and the risk of developing chronic conditions during the year after ICU discharge.

**METHODS.** A retrospective cohort study combining data of the Dutch National Intensive Care Evaluation (NICE) registry [2], with data of the insurance claims database of Vektis [3]. A population based control group was created by Vektis, based on the ICU population and weighed on age, gender and socioeconomic status (SES).

**RESULTS.** The study population consisted of 56,760 ICU patients and 75,232 persons in the population based control group. The ICU population had more chronic conditions during the year before ICU admission compared to the control population (p < 0.05). The ICU population showed an increase of 24.7% (CI 23.8%; 25.6%) in number of chronic conditions in the year after ICU admission compared to the year before admission. For the control population the increase was 6.5% (CI 5.8%; 7.2%). After adjusting for gender, age and SES, ICU patients had a higher odds on developing high cholesterol, heart diseases, COPD, DM type II, DM type I and depression, ranging from 2.0 (CI 1.8; 2.2) to 7.9 (CI 7.2; 8.6), compared to the control group.

**CONCLUSIONS.** ICU patients have more chronic conditions during the year before ICU admission and over time ICU patients develop almost four times more chronic conditions compared to the control group. After adjusting for gender, age and SES, differences in risk of developing specified chronic conditions remained elevated for ICU patients. We believe that the differences between the two populations were not completely explained by demographics and propose that factors related to the ICU admission may play an important role in the development of chronic conditions in ICU patients.

**REFERENCE(S)**

1. Lone NI et al. Five Year Mortality and Hospital Costs Associated With Surviving Intensive Care. Am J Respir Crit Care Med. 2016;194(2):198–208.
2. Dutch National Intensive Care Evaluation (NICE) registry. http://www.stichting-nice.nl
3. Vektis. http://www.vektis.nl

### 0403

#### Effect of protocol-based physiotherapy and muscle activating measures on muscle synthesis and degradation balance in intensive care unit acquired weakness

J. Grunow[1,2], T. Wollersheim[1,3], N.M. Carbon[1], M. Kny[2], M. Giesecke[4], C. Birchmeier[3,5], J. Fielitz[2,6,7], S. Weber-Carstens[1,3]
[1]Charité Universitätsmedizin Berlin, Department for Anesthesiology and Intensive Care Medicine, Berlin, Germany; [2]Charité Universitätsmedizin Berlin, Experimental and Clinical Research Center (ECRC), Berlin, Germany; [3]Berlin Institute of Health (BIH), Berlin, Germany; [4]Charité Universitätsmedizin Berlin, Center for Musculoskeletal Surgery (CMSC), Berlin, Germany; [5]Max-Delbrück-Center for Molecular Medicine (MDC), Berlin, Germany; [6]German Centre for Cardiovascular Research (DZHK), Partner Site Greifswald, Greifswald, Germany; [7]Heart Center Brandenburg and Medical University Brandenburg (MHB), Department of Cardiology, Bernau, Germany
**Correspondence:** J. Grunow
*Intensive Care Medicine Experimental* 2017, **5(Suppl 2):**0403

**INTRODUCTION.** Intensive Care Unit acquired weakness (ICUAW) is characterized by muscle weakness, wasting and a misbalance between myosin synthesis and degradation. Recently we reported that protocol-based physiotherapy (pPT) alone or with additional measures (adMeas), such as electrical muscle stimulation, whole body vibration and the combination of both, maintains larger myocyte cross-sectional areas when compared to standard physiotherapy (sPT) in muscle biopsies of ICUAW patients[1].

**OBJECTIVE.** To assess the effect of pPT and pPT + adMeas on muscular protein synthesis and degradation.

**METHODS.** Within this prospective randomized controlled trial 37 patients at high risk for ICUAW (SOFA ≥ 9) were randomized to receive either pPT (n = 11) or pPT + adMeas (n = 26). Reference patients receiving sPT (n = 22) were available from our previous trial[2]. Muscle biopsies obtained on day 15 from the *vastus lateralis* muscle were assessed by qPCR for gene expression and by Western Blot for protein abundance. Biopsy specimens from six healthy patients undergoing elective orthopedic surgery were included for baseline values (Charité EA 2/041/10).

**RESULTS.** Myosin expression increased significantly with pPT + adMeas as opposed to sPT for *MYH1/2/4* and for *MYH1/4* in comparison to controls. In sPT patients, no differences were observed for *MYH1* expression while *MYH2* showed a decreased and *MYH4* an increased expression (Fig. 134). *FBXO32* and *TRIM62* expression was increased in all critically ill patients with no differences between the interventions. *TRIM63* expression presented baseline values for sPT and an increase for pPT and pPT + adMeas as opposed to controls or sPT (Fig. 135). The increase in myosin expression was accompanied by a significantly increased myosin total/fast/slow protein content for pPT + adMeas as opposed to sPT. In line with the expression data pPT showed an increased and sPT a decreased myosin protein abundance (Fig. 136). MuRF-1 protein was increased with pPT and pPT + adMeas, which was coherent with its gene expression. Atrogin-1 showed an increased protein content in pPT + adMeas patients which was inconsistent with its gene expression.

**CONCLUSION.** In muscle of ICUAW patients, pPT + adMeas increases myosin synthesis as well as muscular protein degrading systems. Our data suggest that in pPT + adMeas treated patients protein synthesis outbalances its degradation resulting in higher myosin protein contents preventing muscle atrophy.

**GRANT ACKNOWLEDGMENT**

Twinning research grant (TRG-3) was given to SWC, CB and JF by the BIH. TW is a participant, in the clinical scientist program of Charité and BIH.

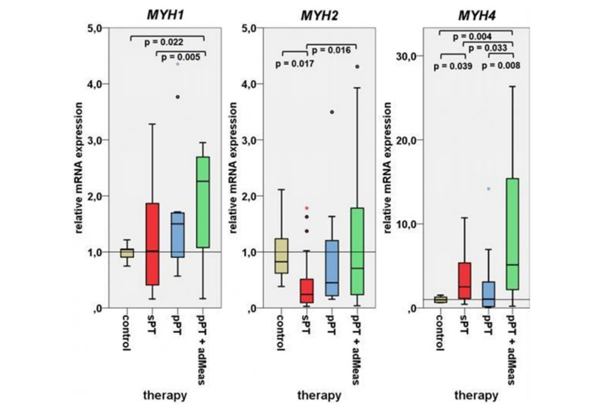

**Fig. 134 (Abstract 0403).** Relative gene expression MY1/MYH2/MYH4

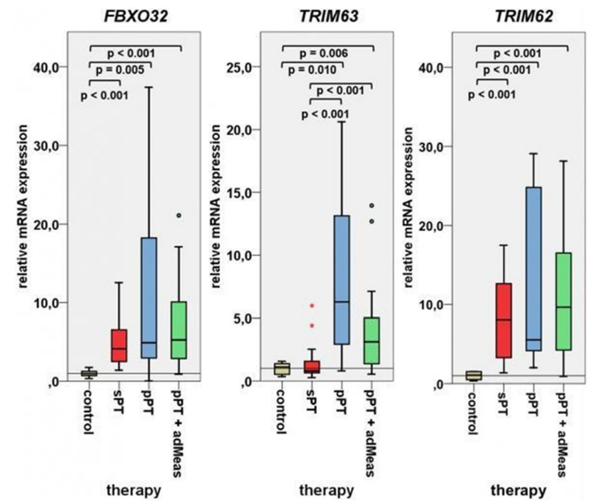

**Fig. 135 (Abstract 0403).** Relative gene expression FBXO32/TRIM63/TRIM

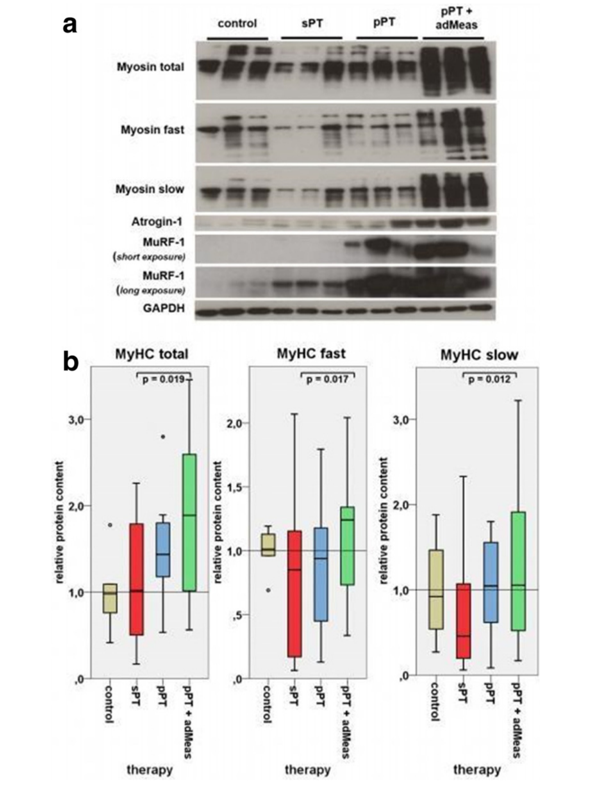

**Fig. 136 (Abstract 0403).** Protein content Myosin/Atrogin-1/MuRF-1

### 0404

#### Delirium prediction in the intensive care unit: head to head comparison of two delirium prediction models

A. Wassenaar[1], M. van den Boogaard[1], L. Schoonhoven[2,3], R. Donders[4], P. Pickkers[1,5], DECISION Study Group
[1]Radboud University Medical Center, Department of Intensive Care Medicine, Nijmegen, Netherlands; [2]University of Southampton, Faculty of Health Sciences, Southampton, United Kingdom; [3]Radboud University Medical Center, Scientific Institute for Quality of Healthcare, Nijmegen, Netherlands; [4]Radboud University Medical Center, Department for Health Evidence, Nijmegen, Netherlands; [5]Radboud University Medical Center, Radboud Center for Infectious Diseases, Nijmegen, Netherlands
**Correspondence:** A. Wassenaar
*Intensive Care Medicine Experimental* 2017, **5(Suppl 2):**0404

**INTRODUCTION.** Delirium occurs frequently in Intensive Care Unit (ICU) patients and is associated with poor outcome. Identification of high risk patients is important in the facilitation of delirium prevention. Currently, two ICU delirium prediction models are available, that both predict delirium for the complete ICU length of stay. The PRE-DELIRIC model reliably predicts ICU patients' risk for delirium using ten predictors obtained within 24 hours after ICU admission and the E-PRE-DELIRIC model uses nine predictors obtained at ICU admission. The use of a delirium prediction model is not yet implemented in daily clinical practice, as it is unknown which of the two available models can best be used to predict ICU delirium.

**OBJECTIVES.** To compare the predictive and clinical performance of the PRE-DELIRIC model and the E-PRE-DELIRIC model.

**METHODS.** A multinational prospective cohort study in eleven ICUs from seven countries. All ICU patients aged ≥18 years from the participating centers were included within three consecutive months, up to a maximum of 300 patients per participating ICU. Patients were excluded if: they were already delirious at ICU admission; had an expected ICU stay shorter than six hours; or were unable to be reliably assessed for delirium. Delirium was assessed using the Confusion Assessment Method-ICU or the Intensive Care Delirium Screening Checklist. The predictive performance of both prediction models was determined using the area under the receiver operating characteristic curve (AUROC) and calibration was assessed graphically. The predictive performance of both models was compared using the Hanley & McNeil method. Clinical performance was determined by means of a questionnaire for physicians about the user convenience of both prediction models.

**RESULTS.** A total of 2192 patients were included. 620 patients were excluded, of whom N = 162 were delirious at ICU admission; N = 76 had an expected ICU stay shorter than six hours; in N = 272 delirium could not reliably be assessed and N = 110 were excluded for other reasons. Patient characteristics are depicted in Table 115. The AUROC of the E-PRE-DELIRIC model was 0.68 (95%CI 0.65-0.71) and the AUROC of the PRE-DELIRIC model 0.73 (95%CI 0.71-0.76) (Fig. 137). Both models were well calibrated (Fig. 138). The predictive performance of the PRE-DELIRIC model was significantly better compared to the E-PRE-DELIRIC model with a Z-score of −2.64, p < 0.01. The evaluation of the clinical performance is ongoing.

**CONCLUSIONS.** This study shows that both ICU delirium prediction models statistically perform well. While the predictive accuracy of the PRE-DELIRIC is somewhat better, the clinical relevance of this significant difference is limited. Moreover, this model needs data obtained during 24 hours, while the E-PRE-DELIRIC can be obtained at ICU admission, allowing direct preventive measures in high risk patients. Which model warrants use as instrument of choice also depends on the ongoing clinical evaluation.

**Table 115 (Abstract 0404).** Patient characteristics

| Variable | Patients (N=2192) |
|---|---|
| Age in years, Mean (SD) | 62.1 (15.2) |
| Male, N (%) | 1330 (60.7) |
| Admission category, N (%) Surgery - Medical - Trauma - Neurology/neurosurgery | 1081 (49.3) - 859 (39.2) - 89 (4.1) - 163 (7.4) |
| Urgent admission, N (%) | 1357 (61.9) |
| APACHE-II score, Mean (SD) | 17.4 (7.1) |
| Delirium, N (%) | 470 (21.4) |
| E-PRE-DELIRIC score, Median (Q1-Q3, min/max) | 16.9 (9-32, 2/100) |
| PRE-DELIRIC score, Median (Q1-Q3, min/max) | 18.6 (12-31, 3/100) |

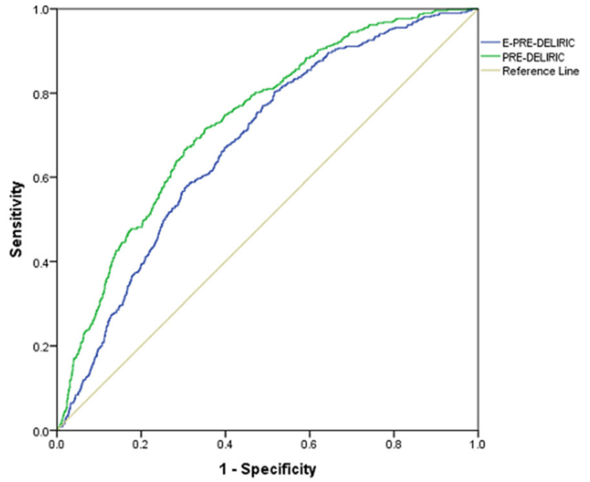

**Fig. 137 (Abstract 0404).** AUROC (E-)PRE-DELIRIC

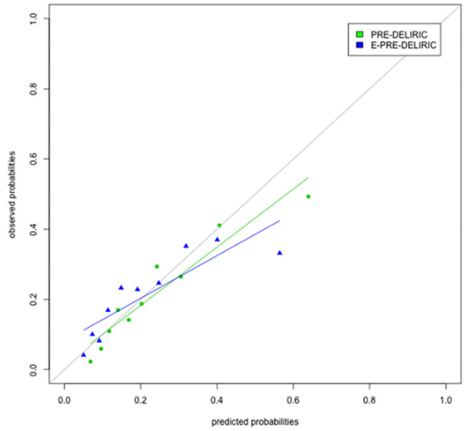

**Fig. 138 (Abstract 0404).** Calibration plot (E-)PRE-DELIRIC

### Acute respiratory failure clinical studies

#### 0405

**Circulating 1,25(OH)$_2$-vitamin D concentration is associated with increased mortality and predicts responsiveness to statins in patients with the acute respiratory distress syndrome**

M. Fitzgerald[1], R. Verghis[1], K. Amrein[2], B. Obermayer-Pietsch[2], J. McNamee[1,3], D. Thickett[4], D. McAuley[1,3], C. O'Kane[1]
[1]Queen's University Belfast, Centre for Experimental Medicine, Belfast, United Kingdom; [2]Medical University of Graz, Department of Internal Medicine, Graz, Austria; [3]Royal Victoria Hospital, Intensive Care Unit, Belfast, United Kingdom; [4]University of Birmingham, Centre for Translational Inflammation Research, Birmingham, United Kingdom
**Correspondence:** M. Fitzgerald
*Intensive Care Medicine Experimental* 2017, **5(Suppl 2):**0405

**INTRODUCTION.** The acute respiratory distress syndrome (ARDS) is characterised by a dysregulated alveolar inflammatory response. Vitamin D is a pleiotropic steroid hormone, increasingly implicated in the regulation of the immune response of critically ill patients. Vitamin D therapy may improve outcome in the critically ill (1). HMG-CoA-reductase inhibitors (statins) may influence vitamin D metabolism.

**OBJECTIVES.** To assess the relationship between vitamin D deficiency, simvastatin and outcome in patients with ARDS.

**METHODS.** All participants in a multicentre randomised controlled trial of simvastatin vs. placebo (2) were included in a post hoc analysis. Patients received once-daily simvastatin at a dose of 80 mg or placebo enterally for up to 28 days. Plasma concentrations of 25(OH)D, a biomarker of vitamin D status, and the active form of vitamin D, 1,25(OH)$_2$D, were analysed by automated enzyme immunoassay. 25(OH)D concentrations of < 12ng/ml and 1,25(OH)$_2$D < 26pg/ml were considered deficient. Outcomes were 28-day mortality and ventilator free days (VFD) to 28 days. Data were analysed using Fisher's exact test.

A $p < 0.05$ was considered significant.

**RESULTS.** Of 506 patients, 246 had received the intervention drug simvastatin, and 260 received placebo. The majority of patients, 330/506 (65%), were 25(OH)D deficient at baseline. No association between 25(OH)D and mortality or VFD to 28 days was seen. 12% of patients were 1,25(OH)$_2$D deficient at baseline. 28 day mortality was significantly higher in those patients who were 1,25(OH)$_2$D deficient vs. sufficient at baseline (38 vs. 23%, $p = 0.02$, Fig. 139a). This mortality rate was reduced in those patients who received simvastatin compared with control (25% vs. 52%, $p = 0.04$, Fig. 139b). No association between 1,25(OH)$_2$D and VFD to 28 days was seen.

**CONCLUSIONS.** Vitamin D deficiency was common in ARDS patients in this RCT. The 28 day mortality was higher in those with 1,25(OH)$_2$D deficiency, which was reduced by simvastatin. Further investigation of how simvastatin mediates a protective effect in 1,25(OH)$_2$ D deficient patients with ARDS is warranted.

**GRANT ACKNOWLEDGMENT**
Supported by a grant from the Intensive Care Society of Ireland.

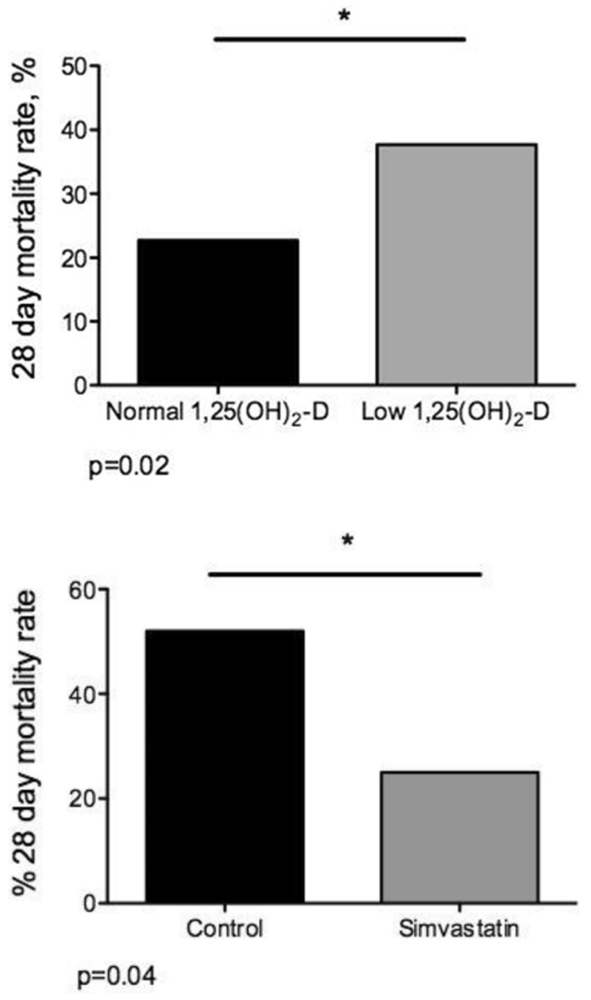

**Fig. 139 (Abstract 0405).** a (upper image) and b (lower image)

#### 0406

**Do patients with unilateral opacities have a similar outcome than patients with ARDS? An ancillary analysis of the LUNG SAFE study**

T. Pham[1,2], J.G. Laffey[3,4], G. Bellani[5,6], E. Fan[1,7], G. Bugedo[8], J.A. Lorente[9], F. Rios[10], A. Bruhn[8], L. Brochard[1,2], G.D. Rubenfeld[1,11], on behalf of the LUNG SAFE Investigators and the ESICM Study Group
[1]University of Toronto, Interdepartmental division of Critical Care Medicine, Toronto, Canada; [2]Keenan Research Centre for Biomedical ScienceKeenan Research Centre, Li Ka Shing Knowledge Institute, St Michael's Hospital, Toronto, Canada; [3]University of Toronto, Departments of Anesthesia, Physiology and Interdepartmental division of Critical Care Medicine, Toronto, Canada; [4]Keenan Research Centre for Biomedical Science, St Michael's Hospital, Toronto, Canada; [5]San Gerardo Hospital, Department of Emergency and Intensive Care, Monza, Italy; [6]University of Milan-Bicocca, School of Medicine and Surgery, Monza, Italy; [7]University Health Network and Mount Sinai Hospital, Department of Medicine, Toronto, Canada; [8]Facultad de Medicina, Pontificia Universidad Católica de Chile, Departamento de Medicina Intensiva, Santiago, Chile; [9]Universidad Europea, Hospital Universitario de Getafe, CIBER de Enfermedades Respiratorias, Madrid, Spain; [10]Hospital Nacional Alejandro Posadas, Intensive Care Unit, Buenos Aires, Argentina; [11]Sunnybrook Health Sciences Centre, Program in Trauma, Emergency and Critical Care Organization, Toronto, Canada
**Correspondence:** T. Pham
*Intensive Care Medicine Experimental* 2017, **5(Suppl 2):**0406

**INTRODUCTION.** According to the BERLIN definition, chest imaging must show bilateral opacities to fulfil ARDS criteria. Chest X-ray interpretation is challenging in the ICU with poor inter-rater reliability. There is no data in the literature comparing patients with ARDS to hypoxemic patients having unilateral air-space disease.

**OBJECTIVES.** We aimed to compare the outcome of patients with ARDS to patients of similar severity having only unilateral opacity/ opacities on the chest X-ray.

**METHODS.** We enrolled all intubated patients included in the LUNG SAFE study with hypoxemia (PF ratio < 300mmHg) and compared patients fulfilling the ARDS definition to patients who fulfilled the same other criteria but whose chest imaging only showed unilateral opacities involving 1 or 2 quadrant(s) (Unilateral Acute Hypoxemic Respiratory Failure « u AHRF »). The number of quadrants involved was available for all patients. In order to determine in an adjusted analysis the factors associated with outcome, severity variables associated with hospital mortality with a p-value < 0.20 in a bivariable analysis were selected as candidate variables for multivariable logistic regressions.

**RESULTS.** A total of 3146 patients were included in the present analysis comprising 2377 patients with ARDS and 769 patients with uAHRF. Patients with uAHRF were less severe than ARDS and their main characteristics and outcome are presented in Table 116.

Among patients with uAHRF, 451 (71%) had 1 quadrant involved, 188 (29%) had 2 as compared to 978 (42%), 535 (23%) and 830 (35%) patients with ARDS who had respectively 2, 3 or 4 quadrants involved on the chest imaging.

In a multivariable analysis adjusting on baseline severity (age, weight, pH, non-pulmonary SOFA score, PaO2/FiO2 ratio, respiratory rate, peak inspiratory pressure, medical cause of admission, chronic liver failure, immunodeficiency, pancreatitis, the present of concomitant cardiac failure), bilateral opacities were associated with increased hospital mortality (OR = 1.35, $_{95\%}$CI = 1.09-1.67, p < 0.01). In the same model using number of quadrants instead of bilateral status, having 3 quadrants (OR = 1.42, $_{95\%}$CI =1.05-1.93 p = 0.02) or 4 quadrants (OR = 1.54, $_{95\%}$CI =1.16-2.06, p < 0.01) involved were associated with higher mortality.

We then restricted the analysis to the 1166 patients with 2 quadrants involved. There was no difference of mortality between the 188 uAHRF and 978 ARDS in bivariate (33% vs 36%, p = 0.51) or multivariable analysis: OR = 1.17, $_{95\%}$CI =0.82-1.68, p = 0.39.

**CONCLUSIONS.** Patients with uAHRF have a high mortality comparable to ARDS with 2 quadrants involved. The total number of quadrants involved is as important as the bilateral characteristic of the parenchymal injury. Although, the ability of physicians to reliably count the number of involved quadrants was not tested, 3 or 4 quadrants of radiographic involvement are associated with independent risk of death.

**Table 116 (Abstract 0406).** See text for description

| | u AHRF (n=769) | ARDS (n=2377) | p-value |
|---|---|---|---|
| Pneumonia | 371 (48%) | 1385 (58%) | <0.01 |
| Non pulmonary SOFA score | 6.3±3.9 | 6.8±4.0 | <0.01 |
| PaO2/FiO2, mmHg | 190±64 | 160±68 | <0.01 |
| Tidal Volume, mL/kg PBW | 7.9±2.0 | 7.6±1.9 | <0.01 |
| PEEP, cmH2O | 6 [5;8] | 8 [5;10] | <0.01 |
| Ventilator-Free Days, days | 18 [0;24] | 10 [0;22] | <0.01 |
| ICU length of stay, days | 9 [5;17] | 10 [5;20] | <0.01 |
| Hospital length of stay, days | 17 [9;31] | 17 [8;33] | 0.95 |
| Hospital mortality | 244 (32%) | 952 (40%) | <0.01 |

### 0407

#### The efficacy of the Whispherflow CPAP system versus high flow nasal cannula in patients at high risk for postextubation failure

P. Theerawit, N. Natpobsuk, Y. Sutherasan
Ramathibodi Hospital, Bangkok, Thailand
**Correspondence:** P. Theerawit
*Intensive Care Medicine Experimental* 2017, **5(Suppl 2):**0407

**INTRODUCTION.** Either high-flow nasal cannula (HFNC)or noninvasive mechanical ventilation (NIV) may reduce need for reintubation in patients at risk for post-extubation failure. The CPAP generated by WhisperFlow system, a type of NIV, principally works like the HFNC. However, there are no established data to compare the effectiveness of HFNC and WhisperFlow CPAP system in patients ready to wean from mechanical ventilation.

**OBJECTIVES.** We conducted a randomized controlled study to compare the efficacy of HFNC and WhisperFlow CPAP System regarding the rate of reintubation in high risk patients. The secondary outcomes were post-extubation acute respiratory failure requiring any type of assisted ventilation, mortality and effect on the physiologic variables.

**METHODS.** The randomized controlled open-label trial was conducted at the medical ICU of Ramathibodi hospital. The inclusion criteria were age over 18 years with respiratory failure. All subjects must be ready to wean from mechanical ventilation and had at least one of the following high-risk criteria for post-extubation failure:

1) chronic heart failure;
2) an ineffective cough and excessive tracheobronchial secretions;
3) Acute Physiology and Chronic Health Evaluation (APACHE) II score more than 12 points on day of extubation; and
4) upper airway obstruction without immediately required intubation.

All subjects were randomly assigned to CPAP or HFNC. The duration of intervention was 48 hours in both arms. The outcome was determined during the intervention period.

**RESULTS.** A total of 116 subjects were recruited. 28 subjects were excluded according to exclusion criteria. All 88 subjects were eligible for intention to treat analysis. Forty-five subjects were randomly assigned to CPAP group, and forty-three subjects were randomly assigned to HFNC group. The baseline characteristics between groups were not significantly different, except the APACHE II score was higher in CPAP group than HFNC group. The reintubation rate was similar [3(7%) in HFNC vs 5 (11.1%) in CPAP; P = 0.71]. Postextubation respiratory failure was not significantly different between groups [6 (14%) in HFNC vs 5 (11.1%) in CPAP; P = 0.27]. Effects on the physiologic variables and mortality were also similar in both groups.

**CONCLUSIONS.** HFNC provided the similar results to WhisperFlow CPAP system for preventing reintubation and post-extubation respiratory failure in high risk patients.

### 0408

#### Epidemiology and predictors of outcome in patients with acute respiratory distress syndrome: the Calgary experience

K. ParharK. Parhar, K. Zjadewicz, A. Soo, A. Sutton, M. Zjadewicz, L. Doig, A. Ferland, C. Lam, H.T. Stelfox, C.F. Doig
University of Calgary, Department of Critical Care Medicine, Calgary, Canada
**Correspondence:** K. Parhar
*Intensive Care Medicine Experimental* 2017, **5(Suppl 2):**0408

**INTRODUCTION.** Acute respiratory distress syndrome (ARDS) in an inflammatory syndrome of the lungs resulting in increased vascular permeability and a clinical syndrome of hypoxemia and reduced lung compliance with associated lung infiltrates. ARDS is associated with significant morbidity and mortality. Limited data on the epidemiology of this condition within Canadian centers exists.

**OBJECTIVES.**

(1) To define the baseline incidence and prevalence of hypoxemic respiratory failure and ARDS (Berlin Definition) within 4 Intensive Care Units in Calgary, Alberta, Canada.

(2) To determine patient demographics for patients diagnosed with ARDS

(3) To describe the practice patterns of patients treated for ARDS

(4) To determine predictors of patient mortality associated with ARDS

**METHODS.** As a quality improvement measure a prospective standardized screening program for ARDS was set up at 4 intensive care units (ICUs) within Calgary Alberta from 2010 to 2012. All patients ventilated for greater than 24 hours were screened with a routine nightly ABG performed on a standard FiO2 of 1.0, and patients with a PaO2:FiO2 (PF) ratio of less than 300 were considered to have hypoxemic respiratory failure. A manual review of all chest X-Ray images by two independent clinicians was performed to determine if criteria for ARDS (Berlin definition) were met. Patient demographics, details of ICU course, therapeutic interventions, as well as ICU and hospital outcomes were analysed by univariate and multivariable linear regression analysis.

**RESULTS.** During the study period 7944 patients were eligible for screening. 986 patients (12.4%) upon screening were determined to have hypoxemic respiratory failure (PF < 300), of which 731 patients (9.2%) met criteria for ARDS. Overall ICU and hospital mortality for the ARDS cohort were 31.6% and 37.9% respectively. Patients with sustained ARDS had a hospital of mortality of 26.5% for mild, 31.8% for moderate, and 60% for severe ARDS. Factors that were associated with increased ICU mortality in patients with ARDS included older age, higher admission SOFA score, higher cumulative fluid balance, higher median peak and plateau pressure, as well as a higher driving pressure. While on controlled ventilation, patients received tidal volumes of less than 8ml/kg 56.5% of the time. Advanced intervention use was variable with 35.9% receiving recruitment manoeuvres, 36.2% requiring neuromuscular blockade, 2.7% of patients required prone positioning, and 0.6% of patients requiring extracorporeal life support.

**CONCLUSIONS.** Among 4 Calgary ICUs, ARDS was common and associated with significant mortality, in a severity dependant fashion. There was variability in the application of evidence based treatments highlighting the potential for further study to determine barriers to the use of evidence based therapy.

**GRANT ACKNOWLEDGMENT**

This project was funded by the Chief Medical Office/Calgary Zone Medical Affairs - Alberta Health Services.

### 0409

#### Feasibility and organizational implications of interhospital ECMO rescue programs

J.M. Ribeiro, C. Candeias, J. Gouveia, A.C. Santos, A. Farias, M.C. Rocha, Z. Costa e Silva
University Hospital of Santa Maria, CHLN, Intensive Care Department, Lisbon, Portugal
**Correspondence:** J.M. Ribeiro
*Intensive Care Medicine Experimental* 2017, **5(Suppl 2):**0409

**INTRODUCTION.** Extracorporeal membrane oxygenation (ECMO) is expensive and demands high volume clinical experience and proficiency to assure good clinical outcomes. Acknowledgement that every hospital should have an ECMO program seems unrealistic in terms of its financial and clinical implications, which obviously imposes that ECMO centers develop and implement rescue competence in their files.

**OBJECTIVES.** Primary objective was evaluation of an ECMO rescue program in terms of clinical outcomes; secondary objectives were evaluation of performance, efficacy, and complications. Additionally, we would like to contribute to the development of benchmark recommendations for interhospital transport of patients under ECMO.

**METHODS.** Retrospective review of prospectively collected data from our protocol-driven ECMO program. Characterization of the population of patients referred from peripheral hospitals and transported to our center under venovenous ECMO. Comparison of rescued (RP) versus non-rescued population (NRP) by case-match control and identification of learning points to adequate triage of patients.

**RESULTS.** Sixty-six adult patients have been submitted to venovenous ECMO in our center, of which 36 have been rescued from hospitals without ECMO programs. Rescue team consisted of two ECMO physicians, two nurses and a perfusionist. Total distance was 5831 kilometres (*min = 0.2; max = 1447km*) and total time spent on transport was 2405 minutes (*min = 10; max = 220min*). Most frequent cause of respiratory failure was $H_1N_1$ infection (17 patients) and acute bacterial pneumonia (13 patients). When comparing the rescued population with patients primarily treated at our center, we found significant differences in ECMO run duration (RP = 11.3 ± 8.2 vs NRP = 16.5 ± 12.4 days; $p < 0.05$) and ventilation free days (RP = 8.6 ± 7.6 vs NRP = 4.2 ± 6.1 days; $p < 0.05$). Six-month mortality rate was also significantly inferior in rescued patients (RP = 22.2% vs NRP = 46.6%; $p < 0.05$). There was no statistical difference in many other parameters, including age (RP = 48.1 ± 13.0 vs NRP = 47.7 ± 11.6), Murray score (RP = 3.3 ± 0.3 vs NRP = 3.2 ± 0.4), SAPS II (RP = 42.2 ± 17.0 vs NRP = 38.9 ± 12.9), $PaO_2$:$FiO_2$ ratio (RP = 67.4 ± 14.8 vs NRP = 76.7 ± 41.8) and $PaCO_2$ (RP = 60.9 ± 22.1 vs NRP = 70.8 ± 23.4) before cannulation, and hospital mortality rate (RP = 20% vs NRP = 29%; $p = 0.393$). Days of disease before ECMO tend to be inferior in rescued patients (RP = 5.2 ± 3.5 vs NRP = 10.8 ± 19.6; $p = 0.103$). Four transports were done by air. There were no major complications during transport of patients to our ECMO center.

**CONCLUSIONS.** Well prepared ECMO rescue programs, with early referral and early implementation of ECMO, are safe and are associated with good clinical outcomes. We favour the organizational concept of ECMO centers and privilege an ECMO rescue team including physicians, nurses and a perfusionist.

## Mechanisms and prognosis of acute brain injury

### 0410

#### Added value of video oculography in ocular responses to caloric stimulation in ICU patients

C. Marois[1], M. Quirins[1], M. Valente[2], B. Rohaut[1,2], F. Faugeras[2], S. Demeret[1], M. Seasseau[2,3], A. Demoule[4], L. Naccache[1,2], N. Weiss[1,5]
[1]Assistance Publique-Hopitaux de Paris, Hôpital Universitaire Pitié Salpétrière, Réanimation Neurologique, Paris, France; [2]Institut du Cerveau et de la Moelle Épinère - ICM, Paris, France; [3]Société Eye-Brain, Paris, France; [4]Assistance Publique-Hopitaux de Paris, Hôpital Universitaire Pitié

Salpêtrière, Réanimation Médicale et Pneumologie, Paris, France; [5]Institut de Neurosciences Translationnelles, Institut-Hospitalo-Universitaire-A-Institut du Cerveau et de la Moelle (IHU-A-ICM), Paris, France
**Correspondence:** C. Marois
*Intensive Care Medicine Experimental* 2017, **5(Suppl 2):**0410

**INTRODUCTION.** The early identification of patients with poor neurological outcome is becoming a major challenge for intensivists. The absence of any ocular movement after caloric stimulation on comatose patients has been proposed as a major factor for poor prognosis. We also showed that the fast component of nystagmus after caloric stimulation could predict consciousness recovery in vegetative patients. Video oculography (VOG) could provide a more objective measurement of ocular responses to caloric stimulation than naked eye.

**OBJECTIVES.** The main objective was to evaluate the added diagnostic value of VOG in the neurological assessment of ocular response to caloric stimulation in ICU patients. The secondary objective was to assess its prognostic value.

**METHODS.** We conducted a prospective study between 2013 and 2015 in two medical ICUs in La Pitié-Salpêtrière hospital. We included all patients that presented disorders of consciousness, were intubated, ventilated and free from sedation for more than 48 hours. Patients with history of ORL or oculomotricity disease were excluded. Baseline characteristics were noted. Bedside neurological examination was performed by neurointensivists that scored Glasgow coma scale, Full Outline of UnResponsiveness (FOUR) and Coma Recovery Scale-revised (CRS-R). Caloric stimulation was elicited by the injection of 60cc of cold water in the outer auditory canal. Clinical characteristics of ocular responses were noted. VOG was performed with the EBT mobile (Eyebrain, Paris, France) that enables the quantitative evaluation of frequency, number of jerks and latency of nystagmus. One-year survival and sequelae were retrieved. Fifteen healthy controls were evaluated as control group. Statistical analysis was performed with JMP 9.0 (SAS Institute) using Khi2, Wilcoxon and Spearman correlation tests. Patients or their relative and healthy controls give their written consent. This study was approved by local IRB.

**RESULTS.** 35 patients and 15 healthy control subjects were included. Patients were 53 years-old, 57% were men. Their median GCS was 8 [6–9], their FOUR 10 [7–12] and their CRS-R 8 [5–10]. VOG detected a fast component of nystagmus in 82% of the subjects versus 70% with the naked eye (p = 0,06). The fast component of the fast component of nystagmus had a higher frequency in controls vs patients (0,44 Hz vs 0,05 Hz, p < 0,0001) and more jerks were detected (63 vs 6, p < 0,0001). These parameters were correlated with the Glasgow score, the FOUR score and the CRS-R (p < 0,05). Fast component of nystagmus latency was longer (59,9 vs 24,9 seconds, p = 0,018) for deceased patients than for survivors at one-year follow-up.

**CONCLUSION.** The VOG tend to detect higher number of fast component of nystagmus than the naked eye. Higher frequency and number of fast component of nystagmus jerks are correlated with higher level of consciousness. Longer latency of the fast component of nystagmus is correlated with poorer 1-year prognosis.

### 0411

#### Clinical Value of Blood Biomarkers in Acute Stroke

F.N. Backes, A. Souza, A.N. Backes, S.R.R. Vieira, M.M. Bianchin
Hospital de Clínicas de Porto Alegre, Porto Alegre, Brazil
**Correspondence:** F.N. Backes
*Intensive Care Medicine Experimental* 2017, **5(Suppl 2):**0411

**BACKGROUND AND PURPOSE.** Identifying a panel of blood biomarkers that correlates with stroke severity and prognostic might be useful for treatment of patients with acute stroke. The objective of this study is to investigate the variability of common blood serum biomarkers of neural injury according with stroke severity and evaluate if these biomarkers could be used as prognostic tools in acute stroke and if they could be predictors of hospital stay after stroke.

**DESIGN AND METHODS.** We investigated 60 consecutive patients with acute stroke who were admitted within 24 h of event onset in our hospital. We evaluate blood levels of neuron-specific enolase (NSE), S100ß protein (S100ß), interleukin-6 (IL-6), C-reactive protein (CRP) and brain-derived neurotrophic factor (BDNF) within 24 h of the acute event, on the third day and on the fifth day after the stroke. Neurological stroke severity and global disability were determined with the National Institutes of Health Stroke Scale (NIHSS) and modified Rankin Scale (mRS) at the same three times of blood collection and at the time of hospital discharge.

**RESULTS.** Levels of S100ß, IL-6 and CRP, but not NSE or BDNF, showed significant positive correlations with stroke severity and prognosis of patients. When patients were subdivided into two groups according to the NIHSS and mRS scores, both neurologic scores for worse outcome (NIHSS > 6 and mRS > 3) at hospital discharge were significantly related to the S100ß protein and IL-6 levels at all of the measured time points. Moreover, the length os hospital stay was longer in patients with high levels of CRP within 24 h and on the third day of stroke.

**CONCLUSION.** We conclude that S100ß protein, IL-6 and CRP may be useful in acute stroke scenario associated with stroke scales to predict stroke severity, prognosis of patients and length of hospital stay after acute stroke.

### 0412

#### Modest elevation in troponin predicts functional myocardial impairment by echocardiogram after aneurysmal subarachnoid hemorrhage

K. M. Yousef[1,2], N. Kagiyami[3], E. Crago[2], T. Lagattuta[2], K. Mahmoud[2], R. M. Friedlander[4], J. Gorcsan[3], M. Hravnak[2]
[1]University of Jordan, Amman, Jordan; [2]University of Pittsburgh, Acute and Tertiary Care, Pittsburgh, United States; [3]University of Pittsburgh, Department of Cardiology, Pittsburgh, United States; [4]University of Pittsburgh, Department of Neurological Surgery, Pittsburgh, United States
**Correspondence:** M. Hravnak
*Intensive Care Medicine Experimental* 2017, **5(Suppl 2):**0412

**INTRODUCTION.** Patients with aneurysmal subarachnoid hemorrhage (aSAH) frequently display elevated cardiac troponin I (cTnI) levels, but the association between cTnI and aSAH associated myocardial injury (SAHMI) as displayed by functional myocardial impairment on echocardiography is not well established.

**OBJECTIVES.** We aimed to determine the ability of peak cTnI levels to predict functional myocardial impairment as quantified by early transthoracic echocardiography (echo; days 1–5) findings of depressed cardiac index (CI), stroke volume index (SVI), and left ventricular ejection fraction (LVEF).

**METHODS.** Longitudinal prospective analysis of 50 patients with aSAH. Inclusion criteria: age 21–75 years, spontaneous aneurysm rupture, Fisher grade ≥ 2 and/or Hunt and Hess grade ≥ 3, and with cTnI levels and echocardiogram. Exclusion: traumatic SAH, and recent myocardial infarction. cTnI was obtained daily, and the peak level from days 0–3 for each patient was used in the analyses, and dichotomized into cTnI ≥ 0.3ng/ml and < 0.3ng/ml. Body surface area was derived by the Mosteller calculation. From the echocardiogram, Doppler-derived SVI

and CI, and LVEF by biplane Simpson method were obtained. SVI was dichotomized as ≥ 35 ml/beat/m$^2$ and < 35, CI was dichotomized as ≥ 2.1 L/min/m$^2$ and < 2.1, and LVEF was dichotomized as ≥ 60% and < 60. Patients were then further dichotomized as a composite variable for myocardial impairment positive by echo if they had any of SVI < 35, or CI < 2.1, or LVEF < 60. Logistic regression was performed to assess the ability of cTnI ≥0.3ng/ml to predict myocardial impairment positive, with cTnI < 0.3ng/ml as the referent group in the model.

**RESULTS.** Of the 50 patients in the sample, only 46 had both cTnI levels plus echo data available. Of these 46 patients, 83% were female, with a mean age of 53.2 years ±11. The sample had a mean cTnI of 0.96 ng/ml ± 2.3, a mean SVI of 58 ± 9, a mean CI of 3.0 ± 0.9 and a mean LVEF of 64 ± 7. For dichotomized variables, 35% had cTnI >0.3 ng/ml, 39% had SVI < 35, 22% had CI < 2.1, and 17% had LVEF < 60%. The composite variable of SAHMI positive by echo held 54% of the sample. In logistic regression, patients with a peak cTnI ≥0.3 ng/ml had 4 times the odds of being positive for myocardial impairment by echo (OR = 3.98, 95% CI 1.025-15.01, p = 0.046).

**CONCLUSIONS.** Patients with even modest elevations of cTnI level >0.3ng/ml after aSAH have 4 to 1 odds of experiencing functional myocardial impairment by CI, SVI or LVEF when compared to patients with cTnI < 0.03ng/ml. Clinicians should closely observe patients with cTnI above this threshold for perfusion abnormalities that may be a consequence of such impairment.

**GRANT ACKNOWLEDGMENT**
NIH NINR R01NR014221

### 0413

### FOUR score predicting outcomes in subarachnoid hemorrhage: multicenter study

D. Iglesias Posadilla[1], M. Gero Escapa[1], A. Domínguez Berrot[2], M. González Vaquero[2], Á. García Miguel[3], A.I. Diego Calvo[3], L. Nogales Martín[4], Á. Bueno Sacristán[4], A.M. Olmos Linares[5], Á. González Salamanca[5], O. Badallo Arévalo[1], A. Zabalegui Pérez[1]
[1]Hospital Universitario de Burgos, Intensive Care Department, Burgos, Spain; [2]Complejo Asistencial Universitario de León, Intensive Care Department, León, Spain; [3]Complejo Asistencial Universitario de Salamanca, Intensive Care Department, Salamanca, Spain; [4]Hospital Clínico Universitario de Valladolid, Intensive Care Department, Valladolid, Spain; [5]Hospital Universitario Río Hortega, Intensive Care Department, Valladolid, Spain
**Correspondence:** D. Iglesias Posadilla
*Intensive Care Medicine Experimental* 2017, **5(Suppl 2):**0413

**INTRODUCTION.** Full Outline of UnResponsiveness (FOUR) score was developed by J Widjicks in 2005 (1), and includes additional information not assessed by the Glasgow Coma Scale (GCS) such as brainstem reflexes, visual tracking, breathing patterns, and respiratory drive.

**OBJECTIVES.** The aim of the study was to determine whether the Full Outline of UnResponsiveness (FOUR) score, which includes eyes opening (E), motor function (M), brainstem reflex (B), and respiratory pattern (R), can be used as an alternate method in predicting intensive care unit (ICU) mortality in subarachnoid hemorrhage (SAH) patients.

**METHODS.** We performed an observational prospective multicenter-study in 5 spanish neurocritical ICUs in Castilla y León (Spain), covering a 2 years' period (from January 1th, 2015 to December 31, 2016). The study was approved by the Ethics Committee of Burgos. All adult patients admitted in ICU were recruited. The GCS, FOUR score, Hunt-Hess, Fisher, modified-Fisher and WFNS was registered at ICU admittance. APACHE-II was calculated 24 h after admission. GOS was registered at hospitalization discharge and after 6 months.

**RESULTS.** 169 patients were recruited. 53,85% female. 58,33 (SD 15,22) years old. APACHE-II 14,26 (SD 7,58). Cause of SAH: 66,27% aneurysmatic, 4,14% arteriovenous malformation (AVM), 15,38% perimesencephalic and 14,20% others.

The median with interquartile range in the FOUR score and GCS was 16 (9–16) and 14 (8–15), respectively.

Global mortality was 20,24%.

Figure 140 shows the plot of the ROC curves to compare the different scores used in SAH prognosis predicting mortality.

The area under the curve for the receiver operator characteristics (AUC ROC) predicting ICU mortality was 78,28% for the FOUR score, 83,58% for the GCS, 81,26% for the Hunt-Hess scale, 81,03% for the WFNS, 71,49% for the Fisher modified scale and 67,63% for the Fisher scale. The difference between GCS and FOUR results statistically significant (p = 0,039).

**CONCLUSIONS.** FOUR score at admission has a fair predictive power of mortality at the ICU in our observational study. FOUR score was higher than 14 in almost 2/3 of the sample. That suppose an important bias. FOUR score should be tested as mortality predictor in a larger population that includes more low score punctuation, to be able to get a true understanding of this score as predicting tool.

**REFERENCE(S)**

1. Wijdicks EF, Bamlet WR, Maramattom BV, Manno EM, McClelland RL. Validation of a new coma scale: The FOUR score. Annals of neurology. 2005;58(4):585–93.

**GRANT.** This study did not receive any grant from any funding agency.

**ACKNOWLEDGMENT**
The entire staff of the Neurocritical ICUs in Castilla y León.

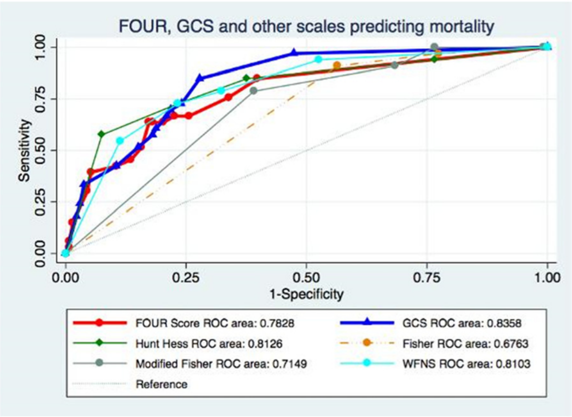

**Fig. 140 (Abstract 0413).** FOUR ROC

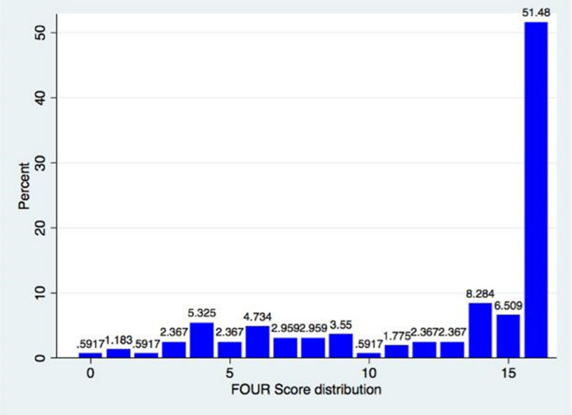

**Fig. 141 (Abstract 0413).** FOUR distribution

### 0414

#### The effect of moderate hyperoxaemia on neurological injury, inflammation and oxidative stress - a randomised controlled pilot trial (BRAINOXY)

M.S. Lång[1], M.B. Skrifvars[2], J. Siironen[3], P. Tanskanen[4], M. Ala-Peijari[5], S. Bendel[1]
[1]Kuopio University Hospital, Intensive Care Medicine, Kuopio, Finland; [2]Helsinki University Central Hospital, Intensive Care Medicine, Helsinki, Finland; [3]Helsinki University Central Hospital, Neurosurgery, Helsinki, Finland; [4]Helsinki University Central Hospital, Anesthesia and Intensive Care, Helsinki, Finland; [5]Tampere University Hospital, Intensive Care Medicine, Tampere, Finland
**Correspondence:** M.S. Lång
*Intensive Care Medicine Experimental* 2017, **5(Suppl 2):**0414

**INTRODUCTION.** In patients with traumatic brain injury (TBI) regional ischemia is a common finding and normobaric hyperoxia is one possible intervention used to alleviate secondary brain ischemia. Moderate hyperoxemia increases cerebral aerobic metabolism and has been suggested to improve outcome after TBI but thus far clinical evidence is limited. Hyperoxemia introduced with high fractions of inspired oxygen (FiO2) may cause formation of lung atelectasis and some studies even suggest an increase in mortality in general ICU patients.

**OBJECTIVES.** To compare daily blood concentrations of reactive oxygen spieces (ROS), interleukin-6 (IL-6) and neuron spesific enolase (NSE) over time and as well as the incidence of pulmonary complications between different oxygen groups.

**METHODS.** An open label randomized controlled study to compare the hyperoxemia (FiO2 70%) to control (Fi02 40%) and its effect on markers of oxidative stress, neurological injury, inflammation and pulmonary complications after severe TBI.

**RESULTS.** We enrolled 27 patients in the control group and 38 in the hyperoxemia group. The groups were well balanced on randomisation, apart from a difference in hemoglobin consentration (13.1 ± 2 g/dl in the 40% group and 12.4 g/dl in the 70% group, $p < 0.05$) and a difference in TBI severity according to the The International Mission for Prognosis and Analysis of Clinical Trials in TBI [1] (IMPACT) risk of poor outcome (0,45 ± 0,21 in the 40% group and 0.56 ± 0.20 in the 70% group, $p < 0.05$).

After randomisation there were no differences in ROS, IL-6 and NSE over time:ROS concentrations in the control group and hyperoxemia group were at admission 64.8 nM (22.6-102.1) and 64.9 nM (26.8-96.3), p = 0.80., at day one 24.2 nM (20.6-33.5) and 29.2 nM (22.7-69.2), p = 0.10, and at day two 25.4 nM (21.7-37.4) and 47.3 nM (34.4-126.1), p = 0.95, respectively. IL-6 concentrations in the control group and hyperoxemia group at admission were 92.4 pg/ml (52.9-171.6) and 94.3pg/ml (54.8-133.1), p = 0.52, at day one 112.7 pg/ml (65.9-168.9) and 83.9 pg/ml (51.8-144.3), p = 0.41 and at day 3 55.0 pg/ml (34.2-115.6) and 49.3 pg/ml (34.4-126.1), p = 0.95, respectively. NSE concentration in control group and hyperoxemia group were at admission 21.04 ug/l (14.0-30.7) and 17.8 ug/l (14.1-23.9), p =0.35., at day one 15.9 ug/l (9.0-24.3) and 15.3 ug/l (12.2-26.3), p = 0.62, respectively. There were no differences in the indidence of pulmonary complications.

**CONCLUSIONS.** In this pilot trial the use of a higher fraction of inspired oxygen did not result in increased blood concentrations of markers of oxidative stress, inflammation or neurological injury in patients with severe TBI.

**REFERENCE(S)**

1. Marmarou A, Lu J, Butcher I, et al. IMPACT database of traumatic brain injury: design and description. J Neurotrauma 2007;24:239–50.

**GRANT ACKNOWLEDGMENT**

None.

## Severe infections in the critically ill

### 0415

#### Prevention of nosocomial infections in critically ill patients with Lactoferrin (PREVAIL Study)

J. Muscedere[1], N. O'Callaghan[2], D. Maslove[2], J.G. Boyd[2], X. Jiang[2], S. Reynolds[3], M. Albert[4], R. Hall[5], A. Day[2], G. Jones[6], F. Lamontagne[7]
[1]Queen's University, Critical Care, Kingston, Canada; [2]Queen's University, Kingston, Canada; [3]University of British Columbia, Vancouver, Canada; [4]Universite de Montreal, Montreal, Canada; [5]Dalhousie University, Halifax, Canada; [6]University of Ottawa, Ottawa, Canada; [7]University of Sherbrooke, Sherbrooke, Canada
**Correspondence:** J. Muscedere
*Intensive Care Medicine Experimental* 2017, **5(Suppl 2):**0415

**INTRODUCTION.** Nosocomial infections (NIs) are a cause of morbidity, mortality, and increased health care costs in critically ill patients. Lactoferrin (LF), a protein component of the innate immune system, has antibacterial, probiotic, and immunomodulatory properties, making it of interest for the prevention of NIs in the critically ill.

**OBJECTIVES.** To inform on the potential utility of LF for the prevention of NIs and the conduct of a phase 3 study powered on clinically important outcomes.

**METHODS.** A phase 2 randomized, multi-center, double-blind study investigating LF for the prevention of NIs in mechanically ventilated (MV) adult patients. Patients expected to be MV > 72 hours were randomized to placebo or LF delivered both enterally and through an oropharyngeal swab in a blinded fashion (using opaque syringes and containers). The primary outcome was antibiotic free days, defined as number of days alive and free of antibiotics 28 days post randomization. The study was powered to detect a 25% increase in antibiotic free days. Secondary outcomes included: occurrence of NIs, hospital length of stay (LOS), hospital and 90-day mortality and impact on biomarkers including cytokines. Data to inform the conduct of a larger definitive trial, including recruitment rates and protocol adherence was also collected.

**RESULTS.** Of the 214 patients randomized, 212 receiving the study intervention were analyzed. See Table for baseline patient characteristics. Patients in the LF group had a higher severity of illness and need for vasopressors. Antibiotic free days and NIs per patient between placebo and LF were (mean ± SD): 18.5 ± 7.1 vs. 17.3 ± 9.0, p = 0.91 and 0.4 ± 0.6 vs. 0.3 ± 0.7, p = 0.48, respectively. Clinical outcomes for placebo and LF were: ICU length of stay (15.0 ± 37.3 vs 14.5 ± 18.0 days, p = 0.82), hospital length of stay (28.1 ± 44.6 vs. 25.0 ± 25.9 days, p = 0.57), hospital mortality [32 (30.5%) vs. 44 (41.1%), p =0.11], 90 day mortality [34 (32.4%) vs. 48 (44.9%), p =0.06], respectively. There was no difference between the groups in the biomarkers collected (data not shown). Protocol adherence was high (87.5%) and no serious adverse events were reported.

| | Placebo (n = 105) | Lactoferrin (n = 107) |
|---|---|---|
| Age: mean + SD | 62.5 ± 16.2 | 66.3 ± 13.5 |
| Male: (%) | 54 (51.4%) | 59 (55.1%) |
| APACHE II: mean (SD) | 23.5 ± 7.9 | 26.8 ± 7.8 |
| Vasopressor Use: n(%) | 74 (70.5%) | 85 (79.4%) |
| Baseline Infection: n(%) | 63 ((60%) | 75 (70%) |
| Sepsis on Admission: n(%) | 51 (48.6%) | 62 (57.9%) |
| Intervention duration (days): mean (SD) | 8.8 ± 6.2 | 10.0 ± 8.6 |

*[Baseline Patient Characteristics]*

**CONCLUSIONS.** We did not find an effect of LF on the primary outcome of antibiotic free days or secondary clinical outcomes in critically ill adult patients. Our data does not support the conduct of a future, large-scale phase 3 randomized controlled trial powered on clinically important outcomes.

**REFERENCE(S)**

Muscedere J et al. Prevention of nosocomial infections in critically ill patients with lactoferrin (PREVAIL study): study protocol for a randomized controlled trial. Trials 2016;17:474

**GRANT ACKNOWLEDGMENT**

Lotte and John Hecht Foundation, SEAMO Innovation Grant.

### 0416

#### Impact of bronchial colonization with fungus on risk of bacterial Ventilatory-acquired pneumonia in ICU. The FUNGIBACT prospective cohort study

J.-F. Timsit[1,2,3], C. Schwebel[3,4], L. Styfalova[5], M. Cornet[6], P. Poirier[7], C. Forrestier[8], S. Ruckly[3,5], R. Veyrat-Masson[9], M.-J. Stasia[10], B. Souweine[3,11], FUNGIBACT

[1]IAME Inserm Université Paris Diderot, DescID, Paris, France; [2]APHP Bichat, Medical and Infectious Diseases ICU, Paris, France; [3]OUTCOMEREA, Paris, France; [4]CHU Grenoble, Medial ICU, Grenoble, France; [5]ICU Research SA, Statistics, Paris, France; [6]CHU Grenoble, Mycology-Paasitology, Grenoble, France; [7]CHU Clermont-Ferrand, Mycology-Paasitology, Clermont-Ferrand, France; [8]CNRS, Clermont-Ferrand, France; [9]CHU Clermont-Ferrand, Immunology, Clermont-Ferrand, France; [10]CHU Grenoble, Immunology, Grenoble, France; [11]CHU Clermont-Ferrand, Medical ICU, Clermont-Ferrand, France

**Correspondence:** J.-F. Timsit

*Intensive Care Medicine Experimental* 2017, **5(Suppl 2):**0416

Controversies exist about the potential of bronchial colonization (COL) with *Candida* (C) to promote bacterial VAP. This association has been identified for P. aeruginosa (PA) VAP. In vitro, PA exploits filamentous fungi resulting in fungal killing and limitation of C growth in the host. *C*, *PA* and *S. aureus* (SA) are the most common pathogens retrieved from endotracheal tube co-biofilms and secretions in patients with VAP. Finally, it has been demonstrated that previous bronchial *C. albicans* COL enhanced the incidence of PA pneumonia in a murine model and epidemiological studies reversed by antifungals. This pathophysiologic scheme and clinical association did not firmly demonstrate causal association since confounding factors such as mutiple-organ failure (MOF) associated immunoparalysis have not been taken into account.

**OBJECTIVES.** To study prospectively the relationship between candidal COL and bacterial VAP on mechanically ventilated (MV) patients with MOF.

**INCLUSION:** MV Patients with MOF ventilated for more than 4 days were included. At inclusion (D0) and every 4 days until extubation bronchial COL with *C* was determined. All the episodes of VAP using quantitative proximal and bronchial cultures were recorded. HLA-DR and PMN/lymphocytes ratio were used to monitor immunoparalysis at D0 and D7. PA, SA and *C* recovered simultaneously from VAP episodes were stored for subsequent analysis of virulence pattern and co-biofilm formation capability. Cause-specific models for repeated events with adjustment on time-dependent confounders and immune factors were used to model the relationship between C COL and VAP.

**RESULTS.** Between Feb 2012 and Dec 2015, 213 patients (median age 64, SAPS II 55, SOFA 10, medical 197(92%)), could be enrolled in 2 ICUs. The median ICU stay was 24 days and death occurred in 69 (32%) cases. HLA-DR was 5916 Ab bound per cell [3863 ; 8934], lower than 8000 in 68% of the cases. Median lymphocyte count was 0.9 Giga/L [0.6 ; 1.3] and PMN/lymphocytes ratio was 10.9% [6.5 ; 19.7].bronchial colonization with *C* was observed in 146 cases (*C albicans* n = 103). The first episode of VAP occurred in 62 (29.1%) cases (13 *SA*, 15 PA), 5.5 days in median after D0 the second episode in 12 cases (1 SA, 2 PA), 15.5 days in median after DO.

After adjustment on risk factors of VAP, HLA-DR and PMN/lymphocytes ratio, bronchial colonization with C was not associated with VAP (adjusted Cause specific model CSHR = 0.98 [0.59 - 1.65] with p = 0.950 significance level for the case specific adjusted model). In subgroup analyses, similar results were observed for SA (CSHR = 0.54 [0.18 - 1.67], p = 0.286) and PA (CSHR = 0.90 [0.32 - 2.52], p = 0.841).

**CONCLUSIONS.** Bronchial colonization with Candida was not associated with VAP even after adjustment on immune function in MV patients for more than 4 days with MOF.

**REFERENCE(S)**

1. Azoulay E et al. Chest 2006 Jan;129(1):110–7.

2. Ader F et al. Path Biol 2008.

**GRANT ACKNOWLEDGMENT**

PHRC 11-PHR-01; NCT01770015.

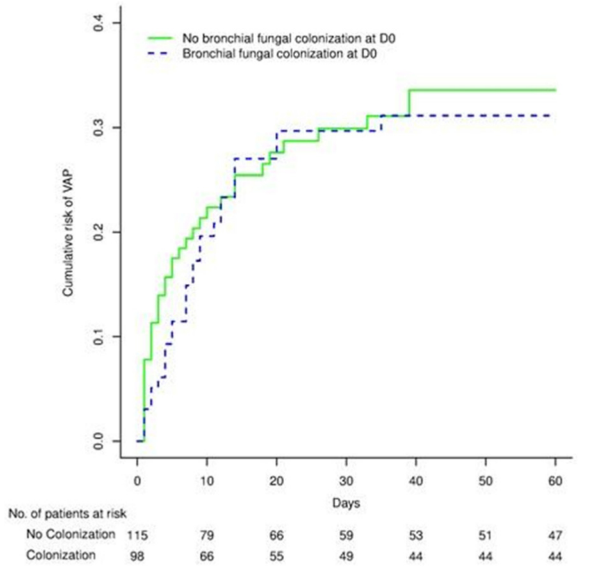

**Fig. 142 (Abstract 0416).** Cumulative incidence if VAP

### 0417

#### Temporal trends of unit-risk-adjusted bacterial resistance in bloodstream infections, from a nationwide surveillance network in the intensive care unit, 2005–2015: a contrasted picture

S. Bailly[1], J.-C. Lucet[1,2], A. Lepape[3], F. L'Hériteau[4], M. Aupée[5], C. Bervas[6], S. Boussat[7], A. Berger-Carbonne[8], A. Machut[3], A. Savey[9], J.-F. Timsit[1,2], REARAISIN Network

[1]INSERM UMR 1137, Paris, France; [2]Hôpital Bichât Claude Bernard, Paris, France; [3]Hospices Civils de Lyon, Lyon, France; [4]CH Aix En Provence, Aix En Provence, France; [5]CHU Rennes, Rennes, France; [6]CHU Bordeaux, Bordeaux, France; [7]CHU Nancy, Nancy, France; [8]Santé Publique, Paris, France; [9]CCLIN Sud Est, Lyon, France

**Correspondence:** S. Bailly

*Intensive Care Medicine Experimental* 2017, **5(Suppl 2):**0417

**INTRODUCTION.** Healthcare-associated infections (HAI) affect 20 to 25% of patients admitted in the ICU, and are associated with considerable morbidity, mortality and costs. Antimicrobial resistance (AMR) is high in ICU, with the need of epidemiological studies assessing trends.

**OBJECTIVES.** To assess the ICU-adjusted temporal trends of ICU-acquired bloodstream infection (BSI) in France, with a particular emphasis to those due to AMR over 11 years.

**METHODS.** Data from the REARAISIN French national ICU surveillance network, including ICUs participating for at least 5 successive years from 2005 to 2015. Data at center unit level, patient level and infection level were recorded. The annual incidence was computed as the ratio of all BSI during one year to the number of patient-days during this year. The patients with a BSI were censored at the time of the first bacteraemia. Univariate analyses of the temporal trends of bacteraemia were performed using an autoregressive model.

**RESULTS.** During the period study, 150 ICUs participated for at least 5 years, corresponding to 241,510 patients, for a total of 2,589,678 ICU patient.days; 6,305 patients developed 7915 ICU-acquired bacteraemia. The yearly incidence of ICU-acquired BSI had a non significant increase from 122 in 2005 to 183.9 BSIs per 100,000 patient.days in 2015 (p = 0.77) (Fig. 143). The incidence of ICU-acquired *S. aureus* was stable, with a sharp decrease of MRSA over time, from 63% to 15% (p = 0.001). Regarding *Enterobacteriacae*, the incidence of BSI due to *E. coli* remained stable with a resistance rate to third generation cephalosporin (3GC) at about 20% (Fig. 144). The incidence of BSI due to *Klebsiella* spp. non-significantly increased, associated with a significant increasing incidence of C3G-R isolates, from about 33% to 51% (p = 0.004). The incidence of *E. faecalis* and *E. faecium* increased over time, the former not significantly, whereas the increasing incidence of *E. faecium* was significant (p = 0.008), mainly due to *E. faecium* ampicillin R (p = 0.001). Finally the incidence of BSI due *to P. aeruginosa* and *A. baumannii* remained stable over time, at 20–30 and 2–4 per 100,000 patient.days, without increasing proportion of resistance of *P. aeruginosa* (~20%) or *A. baumannii* (~60%) to ceftazidime. Nearly no VRE or CRE were identified.

**CONCLUSIONS.** Analysis of data from the REARAISIN network during an 11-year period showed no significant trends of ICU-acquired BSI, except for *E. faecium*. Major changes were observed: decrease in *S. aureus* meticillin resistant and increase in *Enterobacteriacae* 3GC resistant.

**GRANT ACKNOWLEDGMENT**

The RAISIN project is supported by the French Ministry of Health and an annual grant from French National Public Health agency (Santé Publique France).

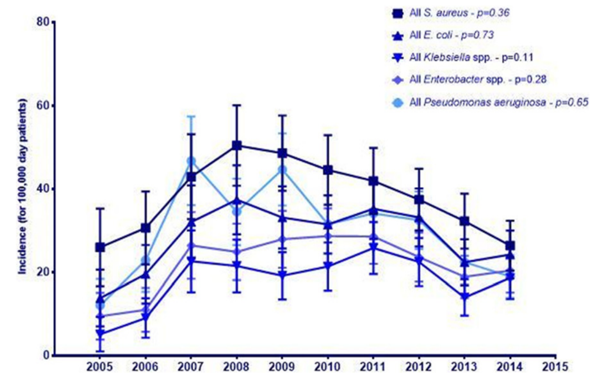

**Fig. 143 (Abstract 0417).** Evolution of incidence of nosocomial BSI

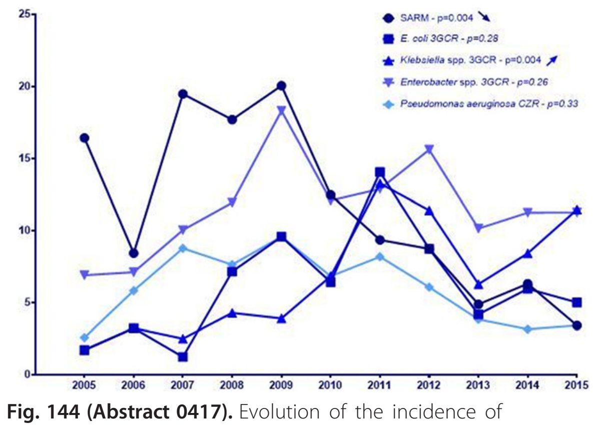

**Fig. 144 (Abstract 0417).** Evolution of the incidence of resistant strains

### 0418

#### Burden of Pneumococcal pneumonia requiring ICU admission in France (2014): 1-year prognosis, resource use and costs

J.-F. Timsit[1,2], J. Gaillat[3], A. Saighi[4], S. Fievez[4], G. Chaize[5], A. Vaintchock[5]
[1]APHP Bichat, Medical and Infectious Diseases ICU, Paris, France; [2]ICU Research SA, Paris, France; [3]Centre Hospitalier d'Annecy-Genevois, Service des Maladies Infectieuses, Annecy, France; [4]Pfizer, Paris, France; [5]Heva, Lyon, France
**Correspondence:** J.-F. Timsit
*Intensive Care Medicine Experimental* 2017, **5(Suppl 2):**0418

**INTRODUCTION.** Pneumonia remains a main cause of ICU admission. The cost in the acute care setting with pneumonia has already been reported but little is known about 1-year prognosis, resource use and costs. We focused on pneumococal pneumonia, accessible to prevention by vaccine therapy.

**METHODS.** We used the "Programme de médicalisation des systèmes d'information" PMSI national administrative database. This administrative database contains ICD-10 codes of all the hospitalization in the acute care beds in France. It also includes many individual demographic data, SAPS II, resource and procedure used during the ICU stay. Individual patients can be tracked across multiple hospitalisations through a unique anonymous patient identifier. It allows the follow-up of all the hospital admissions in acute care bed after the index stay. We include all the patients with a diagnosis of pneumococcal pneumonia (ICD-10 codes :J13 "Pneumonia due to Streptococcus pneumoniae" and B953 "Streptococcus Pneumoniae as the cause of diseases classified elsewhere") who required ICU admission. Valuation of the stays was done through the French official tariffs (T2A, Tarification À l'Activité) from the healthcare system perspective. All costs are presented in 2016 euros.

**RESULTS.** Of the 182858 patients hospitalized for pneumonia, 10587 were due to *S pneumonia. T*he number of rehospitalization was 5816 during the first year. Comorbidities were as follows cardiac 74%, respiratory 52%, liver 16%, renal 14%, cancer/hemopathy 30%, organ transplant 16%, diabetes 25%. An history of smoking (35%) an alcohol abuse (26%) was frequent. 1665 (aged 65 ± 15 years old; 58% men, SAPS II 48 ± 19) required ICU admission. ICU admission was more frequent for male, patients with chronic liver, cardiac and respiratory diseases, and for alcohol and tobacco users. The mean duration of ICU and hospital stay (index hospitalization) were respectively 13 and 23 days. KM estimated of 1-year survival was 68%.The index hospitalization and the 1-year median (25$^{th}$-75$^{th}$ percentiles) costs were €4446 (2349–6698) and €7173 (3502–16011) for all the cohort of P-PAC. It increased up to €14244 (9900–22752) and €21004 (12623–32953) in case of ICU admission. Finally 1-year median costs for ICU survivors were €22042 (14148–35613).

Limitations: Out-of-hospital death and costs are not recorded.

**CONCLUSIONS.** Severe pneumococcal pneumonia is associated with poor hospital and 1-year prognosis, important costs and resource use. These results emphasized the need for preventive strategies.

**GRANT ACKNOWLEDGMENT**

Supported by Pfizer.

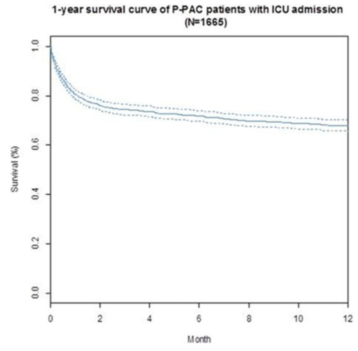

**Fig. 145 (Abstract 0418).** See text for description

### 0419

#### Cessation of screening for intestinal carriage of extended-spectrum beta-lactamase-producing *Enterobacteriaceae* (ESBLE) in a low-prevalence intensive care unit (ICU) with universal contact precautions

W. Jalalzai[1], M. Boutrot[1], J. Guinard[2], A. Guigon[2], L. Bret[2], D.-M. Poisson[2], T. Boulain[1], F. Barbier[1]
[1]La Source Hospital - CHR Orléans, Medical ICU, Orléans, France; [2]La Source Hospital - CHR Orléans, Department of Microbiology, Orléans, France
**Correspondence:** W. Jalalzai
*Intensive Care Medicine Experimental* 2017, **5(Suppl 2):**0419

**INTRODUCTION.** It remains unclear whether a policy of systematic screening for intestinal carriage of ESBLE may reduce the incidence of healthcare-associated infections due to these pathogens in ICU with otherwise optimized prevention measures.
**OBJECTIVES.** To investigate the impact of ceasing the screening for ESBLE carriage on the incidence of ICU-acquired ESBLE infections, patient outcome and carbapenem consumption in a low-prevalence ICU with single-patient rooms and universal contact precautions (CP).
**METHODS.** A single-center study based on prospectively collected data and including all patients admitted for a first ICU stay ≥ 3 days during two consecutive one-year periods : a first period with routine screening for ESBLE carriage, and a second period without screening.
**RESULTS.** A total of 1,069 patients were enrolled [male, 61.5%, age, 64 [53–75] years, SAPS II at admission, 45 [33–59] points, recruitment from the Emergency department, 84.1%], including 524 and 545 patients during the first period and the second periods, respectively. Average consumptions of alcohol-based hand rub (149 mL per patient-day), single-use gloves (51 pairs per patient-day) and single-use gowns (22 gowns per patient-day) remained stable throughout the study. During the first period, 28 EBLSE carriers (5.3%) were identified (imported carriage, 3.2%, acquisition rate, 2.4 events per 1,000 patient-days). Only 6 patients from the first period (1.1%) and 8 patients (1.5%) from the second period developed ≥ 1 ICU-acquired ESBLE infection ($P = 0.64$), with crude incidence densities of 1.2 and 1.4 episodes per 1,000 patient-days, respectively ($P = 0.80$). The cumulative incidence of ICU-acquired ESBLE infections did not differ between inclusion periods after adjustment on the competing risk of death or ICU discharge [standardized hazard ratio (SHR), 2.32, 95% confidence interval (CI), 0.80-6.73, $P = 0.12$], while an admission during the second period had no independent impact on the hazard of ICU-acquired ESBLE infections after adjustment on potential confounders (stepwise logistic regression, odds ratio, 1.16, 95% CI, 0.38-3.50, $P = 0.79$). Empirical antimicrobial regimens were adequate in all cases. Crude ICU mortality rates (overall, 20.6%) and lengths of stay (overall, 6 [4–11] days) did not vary between inclusion periods, with no period-dependent effect on the competing hazards of death (SHR, 1.22, 95% CI, 0.93-1.59, $P = 0.15$) and ICU discharge (SHR, 0.89, 95% CI, 0.79-1.01, $P = 0.08$). Carbapenem exposure in patients without ESBLE infection decreased between the first and the second periods (75 versus 61 carbapenem-days per 1,000 patient-days, $P = 0.01$).
**CONCLUSIONS.** In a low-prevalence ICU with universal CP, stopping the screening for ESBLE carriage had no deleterious effect on the incidence of ICU-acquired ESBLE infections and patient outcomes, and was associated with a slight decrease in empirical carbapenem consumption.
**REFERENCE**
None
**GRANT ACKNOWLEDGMENT**
None.

## Mechanical ventilation monitoring

### 0420

#### Are ‘ventilator associated events´ relevant for the detection of ventilator associated lower respiratory tract infections?

O. Pouly[1], A. Rouze[1], B. Voisin[1], F. Wallet[1], S. Nseir[2]
[1]University Hospital of Lille, Critical Care Center, Lille, France; [2]University of Lille, School of Medicine, Lille, France
**Correspondence:** O. Pouly
*Intensive Care Medicine Experimental* 2017, **5(Suppl 2):**0420

**INTRODUCTION.** In 2013, The Centers for Disease Control and Prevention (CDC) introduced a new surveillance paradigm for ventilated patients. Ventilator-associated events (VAE), reflecting worsening oxygenation, are defined as a persistent and significant increase in FiO2 or PEEP level after a period of stability on the ventilator. VAE definition includes ventilator-associated conditions (VAC), infection-related ventilator-associated complications (IVAC) and probable ventilator-associated pneumonia (PVAP). The relevance of VAE for ventilator-associated pneumonia (VAP) is low. However, the correlation between the three VAC, IVAC, and PVAP, and the onset of ventilator-associated low respiratory tract infection (VALRTI), including ventilator-associated tracheobronchitis (VAT) and pneumonia (VAP), has never been studied yet.
**OBJECTIVES.** To investigate the concordance between the onset of three VAE tiers and VALRTI, and their impact on outcomes.
**METHODS.** We performed a retrospective analysis of prospectively collected data from patients requiring mechanical ventilation for more than 5 days in a 50-bed mixed ICU of a tertiary university teaching hospital, between January 1 and December 31, 2016. VAT and VAP episodes were assessed by prospective surveillance of nosocomial infections, according to the American Thoracic Society criteria. VAE were identified retrospectively, according to current CDC definitions. The agreement between VAC, IVAC, PVAP and VALRTI was assessed by k statistic. The impact of VAE and VALRTI on duration of mechanical ventilation, ICU and hospital length of stay and mortality was also assessed for the first episode of VAT and VAP.
**RESULTS.** We included 545 patients (7927 ventilator days). 126 VAP (15.9 per 1000 ventilator-days), 80 VAT (10.1 per 1000 ventilator-days) and 115 VAE (14.5 per 1000 ventilator-days) were diagnosed. There was no agreement between VAT and VAE and the agreement was poor between VAP and VAC (k = 0.12, 95%CI 0.03-0.20), VAP and IVAC(k = 0.23, 95%CI 0.14-0.32) or VAP and PVAP (k = 0.30, 95%CI 0.22-0.40). Patients who developed VAT, VAP or VAE had significantly longer duration of mechanical ventilation, ICU and hospital length of stay, compared to patients who did not, with similar mortality rates.
**CONCLUSIONS.** VAE are not relevant for VAT diagnosis and have low agreement with VAP, despite their negative impact on ventilation duration, ICU and hospital length of stay.
**REFERENCES**
1) Muscedere J, Sinuff T, Heyland DK, Dodek PM, Klompas M. et al. The clinical impact and preventability of ventilator-associated conditions in critically ill patients who are mechanically ventilated. Chest. 2013;144:1453–60.
2) Fan Y, Gao F, Wu Y, Zhang J, Zhu M, Xiong L. Does ventilator-associated event surveillance detect ventilator-associated pneumonia in intensive care units? A systematic review and meta-analysis. Critical Care. 2016;20:338.

### 0421

#### Skeletal troponin I in serum and diaphragmatic ultrasound in mechanically ventilated ICU patients: a pilot study

S. Spadaro[1,2], A. Trentini[3], F. Dalla Corte[1], V. Rosta[3], F. Pavan[1], A. Fogagnolo[1], L. Mirabella[2], G. Cinnella[2], C. Rizzuto[1], T. Bellini[3], C.A. Volta[1]
[1]University of Ferrara, Morphology, Surgery and Experimental Medicine, Ferrara, Italy; [2]University of Foggia, Foggia, Italy; [3]University of Ferrara, Department of Biomedical and Specialist Surgical Sciences, Ferrara, Italy
**Correspondence:** S. Spadaro
*Intensive Care Medicine Experimental* 2017, **5(Suppl 2):**0421

**INTRODUCTION.** Prolonged mechanical ventilation (MV) can cause muscle atrophy and adversely affect diaphragmatic force-generating capacity, a condition referred to as ventilator-induced diaphragmatic dysfunction (VIDD) [1]. Diaphragmatic ultrasound has been proposed as a bedside daily monitoring of diaphragm function [2]; however, to date there are no specific serum biomarkers for muscular damage and dysfunction. Foster et al. [3] suggested that skeletal troponin I (sTnI) could be a sensitive marker to detect early signs of muscle injury.
**OBJECTIVES.** Firstly, to study the trend of a novel biomarker (sTnI) in mechanically ventilated ICU patients; secondly, to determine whether this trend was associated with the development of VIDD as assessed with diaphragmatic ultrasound.
**METHODS.** Serial serum samples were obtained from 24 mechanically ventilated ICU patients at 24 (T0), 48 (T1) and 72 (T3) hours after admission. Patients were not considered for inclusion if they had a history of neuromuscular disease or a previously documented diaphragm paralysis. Specimens were analysed and specific isoforms for sTnI (slow (ssTnI) and fast (fsTnI)) were assayed by commercially available ELISA kits (Human TNNI ELISA kit, Mybiosource). Simultaneously, in 16 patients diaphragmatic displacement (DD), thickness at end expiration ($T_{EE}$) and thickening fraction (TF) were measured with ultrasound. Values were compared using Friedman´s analysis for repeated measures and Wilcoxon test for paired samples; $p < 0.05$ was considered statistically significant.
**RESULTS.** Patients' clinical charateristics are reported in Table 117.
Ventilatory variables did not change over time (Table 118).
The two sTnI isoforms had a different behaviour over time (Table 119). ssTnI levels remained unchanged during the course of MV (p = 0.957), while sfTnI significantly decreased over time (p < 0.003), with the lowest level after 72 hours from admission (T0 vs T2, p = 0.004) (Fig. 146).
Both DD (p = 0.004) and TF (<0.0001) decreased over time (Fig. 147). while $T_{EE}$ remained stable (Table 119).
We found a statistically significant correlation between the percent change from baseline of sfTnI and TF at 72 hours from admission (r = 0.661, p = 0.007).
**CONCLUSIONS.** Our results seem to demonstrate that both sTnI and ultrasound describe adequately the decrease in diaphragmatic function over time in mechanically ventilated ICU patients. Moreover, fsTnI seems to be better correlated with echographical signs of diaphragmatic dysfunction.
**REFERENCE(S)**
[1] Vassilakopoulos T et al. Am J Respir Crit Care Med 2004;169:336–341.
[2] Goligher EC et al. Am J Respir Crit Care Med 2015;192:1080–1088.
[3] Foster G et al. Eur J Appl Physiolg 2012;112:3547–3558.
**GRANT ACKNOWLEDGMENT**
This study was supported by a research grant from the Italian Ministry of Health.
(Bando Ricerca Finalizzata 2013 Progetto "GR-2013- 02355391 "Diaphragmatic dysfunction in critically patients undergoing mechanical ventilation" - P.I. Dr. Savino Spadaro).

**Table 117 (Abstract 0421).** See text for description

| | Total population n = 24 |
|---|---|
| Age, years | 70 ± 11 |
| Male, n (%) | 15 (63) |
| BMI, kg/m$^2$ | 27.4 ± 4.1 |
| SAPS II score on admission | 45 [36 - 58] |
| SOFA score on admission | 7.50 [5.75 - 9.00] |
| ICU LOS, days | 15 [7 - 23] |
| Hospital LOS, days | 40 [19 - 53] |
| ICU mortality, n (%) | 4 (17) |

**Table 118 (Abstract 0421)**. See text for description

| | T0 | T1 | T2 | p-value |
|---|---|---|---|---|
| Vt, ml | 508 [412 - 654] | 548 [438 - 620] | 554 [500 - 673] | 0.496 |
| RR, bpm | 16 [13 - 19] | 15 [14 - 18] | 15 [12 - 20] | 0.636 |
| PS, cmH2O | 8 [7 - 10] | 8 [8 - 12] | 8 [6 - 10] | 0.217 |
| PEEP, cmH$_2$O | 8 [6 - 10] | 8 [5 - 10] | 8 [6 - 10] | 0.844 |
| P0.1, cmH$_2$O | 1.5 [0.9 - 2.1] | 0.95 [0.65 -1.60] | 1.20 [0.58 - 2.18] | 0.199 |

**Table 119 (Abstract 0421)**. See text for description

| | T0 | T1 | T2 | p-value |
|---|---|---|---|---|
| slTnI, pg/ml | 176.3 [119.8 - 685.9] | 363.5 [121.2 - 623.1] | 277.8 [99.7 -902.1] | 0.957 |
| sfTnI, pg/mL | 104.8 [38.0- 313.0 | 67.7 [18.3 - 431.4] | 58.7 [9.0 -140.6] | 0.003 |
| DD, mm | 18.5 [12.8 - 20.1] | 14.0 [10.5 - 17.5] | 11.0 [8.0 - 15.5] | 0.004 |
| TF, % | 45 [39 - 49] | 39 [29 - 43] | 30 [26 - 39] | < 0.0001 |
| $T_{EE}$, cm | 0.30 [0.26 - 0.39] | 0.34 [0.32 - 0.37] | 0.36 [0.31 - 0.39] | 1.000 |

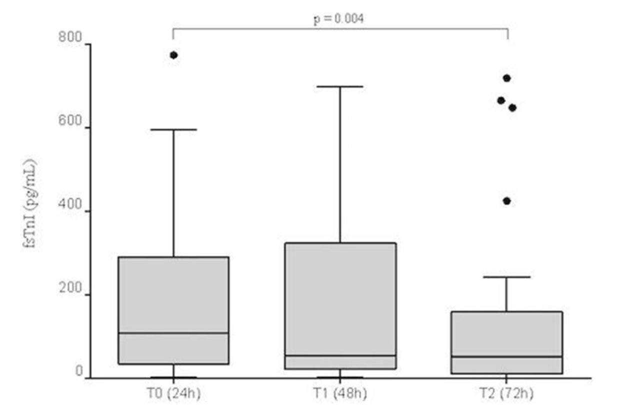

**Fig. 146 (Abstract 0421).** See text for description

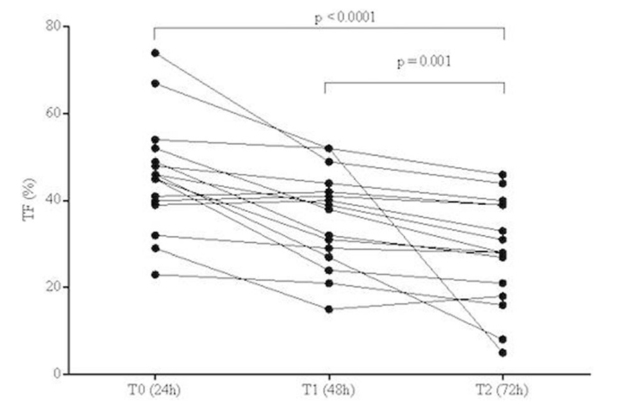

**Fig. 147 (Abstract 0421).** See text for description

### 0422
## Minute ventilation to carbon dioxide production ratio ($VE/VCO_2$) is an accurate and non-invasive index of ventilatory inefficiency in mechanically ventilated patients

R. López[1], I. Caviedes[2], J. Graf[1]
[1]Clínica Alemana de Santiago, Departamento de Paciente Crítico, Santiago, Chile; [2]Clinica Alemana de Santiago, Unidad de Enfermedades Respiratorias, Santiago, Chile
**Correspondence:** R. López
*Intensive Care Medicine Experimental* 2017, **5(Suppl 2):**0422

**INTRODUCTION.** Pulmonary dead-space fraction ($V_D/V_T$) has been associated to outcome in acute respiratory distress syndrome[1]. Even though Bohr-Enghoff's dead-space ($V_{Dphys}/V_T$) overestimates true dead-space[2], it is the best available index of overall ventilatory inefficiency (VI). Surrogate indices of VI available for critical ill patients are: Arterial to end-tidal $CO_2$ pressure difference ($PaCO_2$-$EtCO_2$), ($PaCO_2$-$EtCO_2$)/$PaCO_2$, minute ventilation (VE) corrected for ideal $PaCO_2$($VE_{CORR}$) and the ratio of the product of actual $PaCO_2$ and VE to the product of their ideal counterparts, termed the ventilatory ratio (VR)[3]. In cardiopulmonary exercise testing VI is usually assessed with the minute ventilation to carbon dioxide production ratio (VE/$VCO_2$), with prognostic value in chronic cardiac and pulmonary diseases[4].

**OBJECTIVE.** The aim of this study was to test the correlation of VE/$VCO_2$ to $V_{Dphys}/V_T$ as compared to other indices of VI in critically ill patients.

**METHODS.** 43 mechanically ventilated patients monitored with volumetric capnography (VCap) were included. Simultaneously collected arterial blood gases and VCap parameters were recorded. Surrogate VI indices were compared to $V_{Dphys}/V_T$ using Pearson correlation. Significance was set at $p < 0.01$.

**RESULTS.** 162 measurements were recorded from 43 patients, mean ± SEM $PaO_2/FiO_2$ ratio 253 ± 6, $PaCO_2$ 40 ± 4 mmHg, VE 9.0 ± 0.2 L/min. The following correlations to $V_{Dphys}/V_T$ were obtained: $PaCO_2$-$EtCO_2$, r = 0.63; ($PaCO_2$-$EtCO_2$)/$PaCO_2$, r = 0.60; r = 0.18; $VE_{CORR}$, r = 0.62; VR, r = 0.45 and VE/$VCO_2$,r = 0.88 (Fig. 148). When only patients with $PaO_2/FiO_2$ < 200 (n = 17 patients, 47 measurements) or $PaCO_2$ > 45 mmHg (n = 12 patients, 27 measurements) were considered all correlations improved, but VE/$VCO_2$ still performed best (r = 0.91 for $PaO_2/FiO_2$ < 200 and r = 0.96 for $PaCO_2$ > 45 mmHg).

**CONCLUSIONS.** VE/$VCO_2$ is an accurate, continuous and non-invasive index of VI in mechanically ventilated patients. It correlates better to $V_{Dphys}/V_T$ than other indices that use $PaCO_2$. Its performance seems to improve in severe respiratory failure.

**REFERENCE(S)**

1. Nuckton TJ, Alonso JA, Kallet RH, Daniel BM, Pillet JF, Eisner MD, Matthay MA (2002) Pulmonary dead-space fraction as a risk factor for death in the acute respiratory distress syndrome. N Engl J Med 346:1281–1286.
2. Kuwabara S, Duncalf D (1969) Effect of anatomic shunt on physiologic dead space-to-tidal volume ratio–a new equation. Anesthesiology 31:575–577.
3. Sinha P, Fauvel NJ, Pradeep S, Soni N (2013) Analysis of ventilatory ratio method to monitor ventilatory adequacy at the bedside. Crit Care 17:R34.
4. Tumminello G, Guazzi M, Lancellotti P, Piérard LA (2007) Exercise ventilation inefficiency in heart failure: pathophysiological and clinical significance. Eur Heart J 28:673–678.

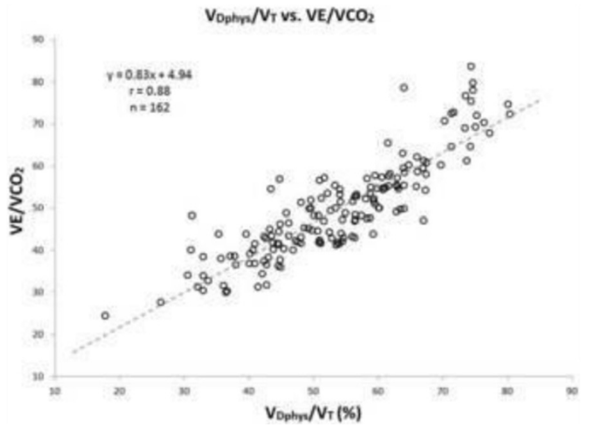

**Fig. 148 (Abstract 0422).** VDphys/VT vs. VE/VCO2

### 0423

#### Expiratory muscle function during weaning from mechanical ventilation

D. Jansen[1], J. Doorduin[1], L. Roesthuis[1], J.G. van der Hoeven[1], H.W.H. van Hees[1], L.M.A. Heunks[2]

[1]Radboud University Medical Center, Nijmegen, Netherlands; [2]VU University Medical Center, Amsterdam, Netherlands

**Correspondence:** D. Jansen

*Intensive Care Medicine Experimental* 2017, **5(Suppl 2):**0423

**INTRODUCTION.** Respiratory muscle dysfunction is an important cause for difficult weaning from mechanical ventilation. Several studies have evaluated inspiratory muscle function in weaning patients. Although the expiratory muscles play an important role in patients with inspiratory muscle weakness or high breathing effort, the function of the expiratory muscles during weaning has not been studied in detail.

**OBJECTIVES.** To compare the recruitment of the inspiratory muscles and expiratory muscles during weaning.

**METHODS.** Twenty-one adult patients receiving pressure support ventilation underwent a spontaneous breathing trial (T-tube with supplemental oxygen) of maximal 1 hour. During the trial flow, gastric pressure, esophageal pressure and diaphragm electrical activity were recorded continuously. Transdiaphragmatic pressure, neuromechanical efficiency of the diaphragm, pressure–time product of the inspiratory muscles and expiratory muscles were calculated.

**RESULTS.** Ten patients failed the spontaneous breathing trial. In the failure group the contribution of the expiratory muscles to total respiratory muscle effort increased up to 26 ± 4% during spontaneous breathing compared to 10 ± 3% in the weaning success group ($p$ < 0.05). In the weaning failure group diaphragm electrical activity was higher (71%; $p$ < 0.05) and neuro-mechanical efficiency of the diaphragm was lower (41%; $p$ < 0.05).

**CONCLUSIONS.** The expiratory muscles significantly contribute to respiratory muscle effort in patients who fail a trial of spontaneous breathing. In addition, our findings confirm that impaired pressure-generating capacity of the diaphragm, regardless of its origin, plays a central role in failure to wean from mechanical ventilation.

### 0424

#### Early recognition of acute respiratory distress syndrome: mechanical ventilation might not be mandatory for the diagnosis

R. Coudroy[1,2], J.-P. Frat[1,2], F. Boissier[1,2], D. Contou[3,4], R. Robert[1,2], A.W. Thille[1,2]

[1]CHU de Poitiers, Service de Réanimation Médicale, Poitiers, France; [2]INSERM CIC 1402, Poitiers, France; [3]Centre Hospitalier Victor Dupouy, Service de Réanimation, Argenteuil, France, [4]Hôpital Henri Mondor, Service de Réanimation Médicale, Créteil, France

**Correspondence:** R. Coudroy

*Intensive Care Medicine Experimental* 2017, **5(Suppl 2):**0424

**INTRODUCTION.** According to the current Berlin definition, mechanical ventilation is needed to identify patients with acute respiratory distress syndrome (ARDS). Therefore the use of noninvasive positive pressure ventilation in patients breathing spontaneously allows categorizing them as ARDS. However it has recently been suggested that NIV may be deleterious in ARDS, especially in the most severe patients.

**OBJECTIVES.** We aimed to assess whether patients breathing spontaneously under standard oxygen could be recognized early as ARDS patients.

**METHODS.** In a post-hoc analysis from two prospective studies (1, 2), all ICU patients admitted for acute hypoxemic respiratory failure and treated with noninvasive ventilation (NIV) were analyzed. Patients with cardiogenic pulmonary edema, acute exacerbation of chronic obstructive pulmonary disease or hypercapnia were excluded. The $PaO_2/FiO_2$ ratio was estimated at admission under standard oxygen using the following formula: oxygen flow in liters per minute x

0.04 + 0.21, then measured after 1 hour of NIV, and repeated within the first 24 hours according to respiratory status. Severity of hypoxemia was considered as mild when $PaO_2/FiO_2$ ranged from 201 to 300, moderate from 101 to 200 and severe when ≤ 100 mmHg.

**RESULTS.** Among the 219 patients admitted for acute hypoxemic respiratory failure, 180 (82%) had bilateral infiltrates and 172 (79%) fulfilled ARDS criteria under NIV within the first 24h following ICU admission. Among all patients with bilateral infiltrates on chest radiograph and a $PaO_2/FiO_2 \leq 300$ mm Hg under standard oxygen, 96% (154 of 161 patients) had ARDS within the first 24 hours. Their mortality (29%) was very similar to that of intubated patients in the current Berlin definition of ARDS (30%).

**CONCLUSIONS.** Almost all patients with bilateral pulmonary infiltrates and a $PaO_2/FiO_2 \leq 300$ mm Hg under standard oxygen fulfilled ARDS criteria under NIV within the first 24 hours after ICU admission. Their mortality rate was not different from that reported in the Berlin definition of ARDS. Therefore, patients with ARDS under NIV could be diagnosed early while breathing spontaneously without positive pressure ventilation.

**REFERENCE(S)**

1. Frat JP, Thille AW, Mercat A, Girault C, Ragot S, Perbet S, et al. High-Flow Oxygen Through Nasal Cannula in Acute Hypoxemic Respiratory Failure. N Engl J Med. 2015;372(23):2185–96.
2. Thille AW, Contou D, Fragnoli C, Cordoba-Izquierdo A, Boissier F, Brun-Buisson C. Non-invasive ventilation for acute hypoxemic respiratory failure: intubation rate and risk factors. Crit Care. 2013 Nov 11;17(6):R269.

### Sepsis therapy, any advances?

#### 0425

#### Effect of PMX-DHP longer than 2 hours on mortality in patients with septic shock: a sub-analysis of multicenter randomized controlled trial

Y. Kawazoe[1], T. Sato[2], N. Miyagawa[2], Y. Yokokawa[2], S. Kushimoto[1], K. Miyamoto[3], Y. Ohta[4], T. Morimoto[4], H. Yamamura[5], DESIRE Trail Group
[1]Tohoku University Graduate School of Medicine, Sendai, Japan; [2]Tohoku University Hospital, Sendai, Japan; [3]Wakayama Medical University, Wakayama, Japan; [4]Hyogo College of Medicine, Nishinomiya, Japan; [5]Hirosaki University Graduate School of Medicine, Hirosaki, Japan
**Correspondence:** Y. Kawazoe
*Intensive Care Medicine Experimental* 2017, **5(Suppl 2):**0425

**INTRODUCTION.** The polymyxin-B direct hemoperfusion therapy (PMX-DHP) of 2 hours in patients with septic shock has been failed to demonstrate the consistent effect on mortality.[1)] Prolonged duration of PMX-DHP has been suggested to be beneficial for shock reversal in those patients.

**OBJECTIVES.** To determine whether PMX-DHP longer than 2 hours improve outcomes in patients with septic shock.

**METHODS.** We conducted DESIRE trial, a randomized controlled trial with sedation strategy with or without dexmedetomidine in 201 adult patients with sepsis.[2)] Patients treated with polymyxin-B direct hemoperfusion (PMX group) and patients treated without it (non-PMX group) were compared. Then, we compared those with PMX-DHP of 2 hours (conventional PMX group) and PMX-DHP longer than 2 hours (prolonged PMX group). A primary outcome was 28-day mortality and compared by Kaplan-Meier estimate and log rank test. The secondary outcome was vasopressor-free days within 7 days and compared by Wilcoxon rank sum test. We developed Cox proportional hazard model and linear model for these outcomes adjusting for the sedation strategy with dexmedetomidine, age, the APACHE II score, infectious sites was lung, emergency surgery.

**RESULTS.** The mean age was 70.6 (standard deviation (SD) 13.8) and male was 61.6%. The median APACHE II score was 25.0 (interquartile range (IQR) 19.3, 31.0). Among 112 patients with septic shock, PMX-DHP was introduced in 36 patients. The 28 day mortality was 19.4%% and 31.6% in PMX group and non-PMX group, respectively (P = 0.18). The adjusted hazard ratio (HR) of PMX group was 0.85 (95% confidence interval: 0.32-2.10). Among the patients treated with PMX-DHP, 22 patients received PMX-DHP for 2 hours and the rest 14 patients received it longer than 2 hours. The time of PMX-DHP was 5.5 (IQR 3.75, 12) hours in prolonged group at the first hemoperfusion. Patients characteristics did not differ between the 2 groups. The 28 day mortality was 7 (31.8%) in conventional PMX group and 0 (0%) in prolonged PMX group (p = 0.029). The HR was < 0.001 (95%CI 0, 0.36) (P = 0.0036). The 7 day vasopressor-free days were 2.5 (SD 2.2) in conventional PMX group and 3.1 (SD 2.1) in prolonged PMX group (p = 0.25).

**CONCLUSIONS.** PMX-DHP was not shown effective for patients with septic shock when we compared those with PMX-DHP and without, that was the same result as the previous study. However, PMX-DHP longer than 2 hours was more effective than PMX-DHP of 2 hours. Our result indicated that the prolonged duration-PMX-DHP therapy may contribute better clinical outcome for the patient with septic shock than conventional duration-PMX-DHP therapy.

**REFERENCE(S)**

1. Intensive Care Med. 2015 Jun;41(6):975–84.
2. JAMA. 2017 Mar 21.

**GRANT ACKNOWLEDGMENT**
This post hoc analysis was conducted by the academic investigators without support from any pharmaceutical companies.

#### 0426

#### Early norepinephrine administration vs. standard treatment during severe sepsis/septic shock resuscitation: a randomized control trial

C. Permpikul, S. Tongyoo, T. Viarasilpa, T. Trainarongsakul, K. Noppakaorattanamanee
Mahidol University, Medicine, Bangkok, Thailand
**Correspondence:** C. Permpikul
*Intensive Care Medicine Experimental* 2017, **5(Suppl 2):**0426

**INTRODUCTION.** Norepinephrine has been recommended as the first choice vasopressor for septic shock resuscitation.[1] However, the appropriate timing for administration has not been clarified. Whether the medication is given after adequate volume replacement or it should be initiated along with fluid resuscitation is ongoing debated.

**OBJECTIVES.** To compare the efficacy of early norepinephrine administration together with fluid resuscitation during septic shock resuscitation with the use of this medication after adequate volume replacement.

**METHODS.** In this double-blind, single-center (Siriraj Hospital, Mahidol University, Bangkok, Thailand) randomized, placebo-controlled trial, we enrolled adult septic shock patients within one hour after the diagnosis. Patients were randomly assigned, in 1:1 ratio, to early norepinephrine administration (intravenous norepinephrine 0.05 mcg/kg/min together with fluid resuscitation) or standard treatment (placebo norepinephrine together with fluid resuscitation). Open-label norepinephrine was added if the target mean arterial pressure (MAP) of 65 mmHg was not reached after adequate volume. Other managements were given according to Surviving Sepsis Campaign 2012.[1] The primary outcome was the achievement of MAP goal and the tissue perfusion target, as judged by urine output of more than 0.5 mL/kg/hour or the decrement of serum lactate more than 10% from baseline, at 6 hours after resuscitation. This study was approved by the Siriraj Hospital Ethics Committee and was registered in ClinicalTrials.gov (NCT 01945983).

**RESULTS.** From September 2013 to March 2017, 320 patients were included. Of these, 162 patients were assigned to “early group” and 158 patients to “standard group”. All were included in the intention-to-treat analysis. Patients' mean age was 64.4 ± 15.9 years and mean baseline APACHE II score was 21.8 ± 7.4. The average MAP was 54.8 ± 7.5 mmHg and mean lactate level 4.5 ± 6.9 mmol/L. The "early group" had higher rate of primary outcome achievement (76.2% vs 43.3%; odds ratio 1.96; 95% CI, 1.52 to 2.58; $p < 0.001$). The amount of intravenous fluid given at 6 and 24 hours were similar (2,718.2 ± 1,000.4 vs. 2,834.6 ± 1,097.3 mL; $p = 0.39$ and 5,125.1 ± 1,640.1 vs. 5,084.4 ± 1,686.2 mL; $p = 0.85$). Open label norepinephrine was given in 69.7% of early group and 80% in control group ($p = 0.08$). Also, there were non-significant lower rate of adverse events, namely cardiogenic pulmonary edema (25.6% vs 30.8%; $p = 0.39$) and new onset arrhythmia (12.4% vs. 20.8%; $p = 0.09$).

**CONCLUSIONS.** Early norepinephrine administration during septic shock resuscitation, when compared with standard therapy, resulted in higher rate of shock reversal at 6 hours.

**REFERENCE(S)**

1. Dellinger RP, Levy MM, Rhodes A, Annane D, Gerlach H, Opal SM, et al. Surviving Sepsis Campaign: international guidelines for management of severe sepsis and septic shock, 2012. Intensive Care Med. 2013;39(2):165–228.

**GRANT ACKNOWLEDGMENT**

Siriraj critical care research funding.

### 0427

#### Safety, tolerability and pharmacokinetics/-dynamics of the anti-adrenomedullin antibody Adrecizumab: a first in man study

C.B.C.A.G. Geven[1,2], M. Kox[1,2], P. Scigalla[3], A. Bergmann[3], E. Peters[1,2], P. Pickkers[1,2]

[1]Radboud University Medical Center, Department of Intensive Care Medicine, Nijmegen, Netherlands; [2]Radboud University Medical Center, Radboud Center for Infectious Diseases (RCI), Nijmegen, Netherlands; [3]Adrenomed AG, Hennigsdorf, Germany

**Correspondence:** C.B.C.A.G. Geven

*Intensive Care Medicine Experimental* 2017, **5(Suppl 2):**0427

**INTRODUCTION.** Adrenomedullin (ADM) is an important regulator of endothelial barrier function and vascular tone during sepsis and septic shock; it therefore may represent a potential novel treatment target. Adrecizumab is a humanized IgG high-affinity antibody against the N-terminus of ADM, which only partially inhibits ADM signalling. Administration of Adrecizumab in septic animals reduced catecholamine requirements and vascular leakage, while improving blood pressure, renal function, and survival.

**OBJECTIVES.** The aim of this study was to investigate the safety, tolerability and pharmacokinetics/-dynamics of Adrecizumab in a *first-in-man* study.

**METHODS.** 24 healthy male volunteers (18 to 35 years), were recruited for this randomized, double-blind, placebo-controlled phase I study. Subjects were randomized into four groups (n = 6 each): 0.5, 2, or 8 mg/kg Adrecizumab, or placebo. The study drug was administrated intravenously over 1 hour. Due to the expected long half-life of Adrecizumab, subjects were followed for 90 days. Blood was sampled at various points on the study drug administration day and during the follow-up period. Several clinical safety parameters were monitored, including local tolerability, vital signs and electrocardiographic, biochemical, and haematological parameters. Additionally, pharmacokinetic and -dynamic analyses were performed, including plasma levels of Adrecizumab, ADM and MR-proADM (fragment of the ADM precursor).

**RESULTS.** Adrecizumab was well tolerated and showed an excellent safety profile. No severe adverse events occurred. Study drug administration did not result in relevant changes in vital signs and electrocardiographic evaluations. Apart from transient laboratory abnormalities, which were deemed not clinically significant, 37 adverse events (AEs) (22 possibly related, 15 unrelated) were reported of which the most common were headaches (n = 9) and symptoms of common cold (n = 11). AEs were evenly distributed across all groups (11 out of 37 AEs were reported in the placebo group), implying no relation to the study drug. All AEs occurred transiently and did not require intervention. PK of Adrecizumab showed proportional increases of the maximum observed plasma concentration (Cmax; 9.7 ± 0.9, 44.1 ± 4.5 and 179 ± 21.1 μg/ml for 0.5, 2 and 8 mg/kg Adrecizumab, respectively), a small volume of distribution (~100 ml/kg), a low clearance rate (~0.2 ml/h/kg) and a terminal $T_{1/2}$ of approx. 14 days. Adrecizumab administration elicited a pronounced increase of plasma ADM levels, while levels of MR-proADM remained unchanged (Fig. 149), indicating that de novo synthesis of ADM is not influenced.

**CONCLUSIONS.** Administration of Adrecizumab is safe and well-tolerated in humans. These findings pave the way for further investigation of Adrecizumab during systemic inflammation using the human endotoxemia model, and in an upcoming phase II clinical trial in sepsis patients.

**GRANT ACKNOWLEDGMENT**

This study was supported by Adrenomed AG.

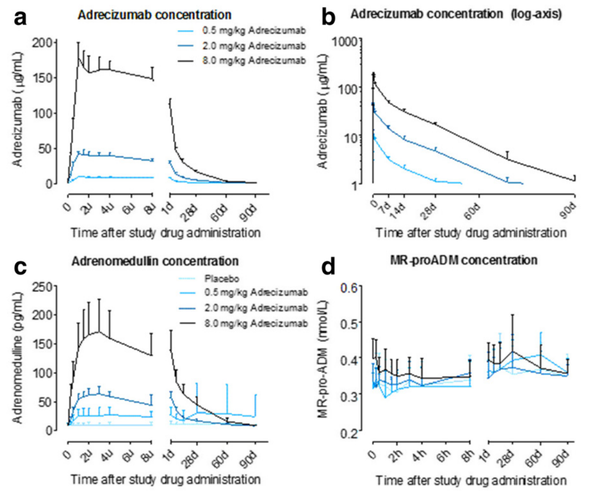

**Fig. 149 (Abstract 0427).** Plasma concentrations of Adrecizumab (A,B), Adrenomedullin (C) and MR- proADM (D) after sinlge i.v. administration of placabo oe Adrecizumab (0.5, 2 or 8 mg/kg) in healty male volunteers (n = 6 per group). Data are expressed as mean + SD.

### 0428

#### Acetylsalicylic acid partially reverses *in vivo* endotoxin tolerance in humans

G. Leijte[1,2], D. Kiers[1,2], A. Jansen[1,2], V. Boerrigter[1,2], M. Kox[1,2], P. Pickkers[1,2]

[1]Radboud University Medical Center, Intensive Care Department, Nijmegen, Netherlands; [2]Radboud University Medical Center, Radboud Center for Infectious Diseases (RCI), Nijmegen, Netherlands

**Correspondence:** G. Leijte

*Intensive Care Medicine Experimental* 2017, **5(Suppl 2):**0428

**INTRODUCTION.** Sepsis is a major health care burden with increasing incidence and high mortality rates. A severely suppressed state of the immune system called immunoparalysis is increasingly recognized as the overriding immune dysfunction in septic patients. Experimental studies have demonstrated that ASA exerts pro-inflammatory effects and epidemiologic data show that prehospital use of low dose acetylsalicylic acid (ASA) is associated with improved outcome of patients with sepsis. However, it remains to be determined whether ASA can reverse immunoparalysis.

**OBJECTIVE.** We investigated whether ASA prophylaxis or treatment prevents or reverses *in vivo* endotoxin tolerance induced by experimental human endotoxemia (a model for sepsis-induced immunoparalysis).

**METHODS.** We performed a double-blind placebo-controlled randomized study in 30 healthy male volunteers (age 18–35 years) who were intravenously challenged with bacterial endotoxin (LPS) twice (each challenge consisting of a bolus of 1 ng/kg followed by continuous administration of 1 ng/kg/hr during 3 hours). The two LPS

challenges were separated by a week. Subjects were randomized into three groups, a prophylaxis group (80mg ASA daily for a 14-day period starting 7 days before the first LPS challenge), a treatment group (80mg ASA daily for the 7-day period in-between both LPS challenges), and a placebo group that was challenged with LPS twice but received no ASA. We measured plasma cytokine levels (TNFα, IL-6, IL-8, IL-10, MIP-1α, MIP-1β and MCP-1) and changes in vital parameters such as temperature and heart rate.

**RESULTS.** The first LPS challenge resulted in a profound increase of plasma cytokine levels ($p < 0.0001$ for all cytokines), temperature (+2.9°C, $p < 0.001$) and heart rate (+68%, $p < 0.001$) in all groups. Prophylactic use of ASA enhanced plasma concentrations of TNFα by 50% compared with the placebo group ($p < 0.001$, Fig. 150a). The development of endotoxin tolerance was illustrated by a severely blunted plasma cytokine response upon the second LPS challenge in the placebo group (reduction of TNFα: 58%, $p < 0.001$; IL-6: 73%, $p = 0.004$; IL-8: 65%, $p = 0.003$; IL-10: 56%, $p = 0.003$; MIP-1α: 42%, $p = 0.002$; MIP-1β: 55%, $p = 0.01$; MCP-1: 38%, $p < 0.01$). ASA prophylaxis did not result in different cytokine levels during the second LPS challenge compared with the placebo group. ASA treatment resulted in enhanced plasma levels of TNFα (+53%, $p = 0.02$, Fig. 150b), IL-6 (+91%, $p = 0.03$) and IL-8 (+42%, $p = 0.02$) upon the second LPS challenge compared with the placebo group, whereas the plasma level of the key anti-inflammatory cytokine IL-10 was lower compared with the placebo group (−40%, $p = 0.003$).

**CONCLUSION.** Low dose ASA partially reverses the development of *in vivo* endotoxin tolerance in humans. These findings may partly explain the beneficial effect of ASA in sepsis patients observed in epidemiological studies and might provide rationale for use of ASA in this group of patients.

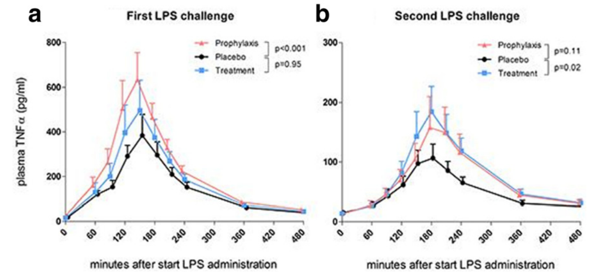

**Fig. 150 (Abstract 0428).** Plasma TNFα concentration during the first LPS challenge (A) and the second LPS challenge (B)

### 0429

#### Effects of the humanized anti-Adrenomedullin antibody Adrecizumab on vascular barrier function and survival during systemic inflammation and sepsis

C.B.C.A.G. Geven[1,2], E. Peters[1,2], J. Struck[3], A. Bergmann[3], O. McCook[4], P. Radermacher[4], M. Kox[1,2], P. Pickkers[1,2]
[1]Radboud University Medical Center, Department of Intensive Care Medicine, Nijmegen, Netherlands; [2]Radboud University Medical Center, Radboud Center for Infectious Diseases (RCI), Nijmegen, Netherlands; [3]Adrenomed AG, Hennigsdorf, Germany; [4]Ulm University Hospital, Institute of Anesthesiological Pathophysiology and Process Engineering, Ulm, Germany
**Correspondence:** C.B.C.A.G. Geven
*Intensive Care Medicine Experimental* 2017, **5(Suppl 2):**0429

**INTRODUCTION.** Adrenomedullin (ADM) is an important regulator of endothelial barrier function during sepsis and septic shock. Previously, administration of a murine antibody targeted against the N-terminus of ADM resulted in improved outcome in models of murine sepsis.

**OBJECTIVES.** To determine the effects of Adrecizumab on vascular barrier dysfunction and survival in rodent models of systemic inflammation and sepsis.

**METHODS.** Male Wistar rats (n = 48) received Adrecizumab (0.02, 0.1, 0.5 or 2.5 mg/kg) or placebo, directly followed by lipopolysaccharide (LPS; 5 mg/kg). Evans Blue dye was administered 24 hours later and vascular leakage was assessed in kidney tissue. In subsequent experiments, C57BL/6 mice (n = 24) received Adrecizumab or placebo, immediately followed by CLP-surgery. Eighteen hours later, kidneys were harvested for immunohistochemical analysis of albumin, vascular endothelial growth factor (VEGF) and angiopoietin-1 (Ang-1). Finally, survival was assessed in a CLP model using C57BL/6 mice (n = 60) that received single (2 mg/kg) or repeated dosages (4 mg/kg followed by 2 mg/kg after 24 and 48 hours) of Adrecizumab or placebo.

**RESULTS.** In rats, LPS administration resulted in a 3.5-fold increase in renal albumin leakage compared with saline-treated controls, which was significantly attenuated by Adrecizumab at dosages of 0.1 and 2.5 mg/kg (71% and 40% attenuation, respectively), whereas a trend towards decreased renal albumin leakage was observed for the 0.5 mg/kg dose (33% attenuation). Adrecizumab administration resulted in significantly attenuated albumin (79%, 75% and 78% attenuation for 0.1, 2.0 and 20 mg/kg Adrecizumab, respectively) and VEGF concentrations (55%, 45% and 59% attenuation) in the kidneys of septic mice, whereas concentrations of the protective protein Ang-1 were augmented (387%, 474% and 379% augmentation, respectively). Both single and repeated administration of Adrecizumab resulted in improved survival during murine sepsis (single dose: from 10% to 50%; repeated dose: from 0% to 40%; Fig. 151).

**CONCLUSIONS.** Pre-treatment with the humanized anti-ADM antibody Adrecizumab improves vascular barrier function and survival in rodent models of systemic inflammation and sepsis. These results pave the way for clinical development of this antibody. A phase I study is in its final stages and a phase II study in septic patients is planned.

**GRANT ACKNOWLEDGEMENT**
This study was supported by Adrenomed AG.

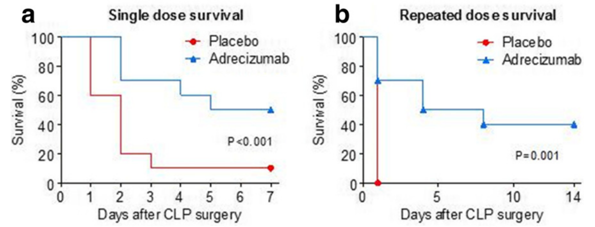

**Fig. 151 (abstract 0429).** Survival after CLP-surgery in mice, comparing sinlge (A) or repeated do ses (B) of either placebo or Adrecizumab (n = 10 per group). Kaplan-Meier curves are depicted and p-values were calculated using log-rank tests

### Non-mortality outcomes after critical illness

### 0430

#### Psychiatric symptoms in unselected ICU survivors one year after the ICU discharge

P. Kalfon[1,2], S. Renoult[3], M.-A. Geantot[4], S. Deparis-Dusautois[5], M. Boucekine[2], O. Mimoz[6], B. Mauchien[1], C. Jourdain[1], A. Ben Salah[1], J. Audibert[1], J. Amour[7], N. Revel[8], E. Azoulay[9], T. Sharshar[10], M. Leone[11], M. Garrouste-Orgeas[12], A. Loundou[2], K. Baumstarck[2], P. Auquier[2], The IPREA AQVAR Study Group
[1]CH Chartres, Réanimation Polyvalente, Chartres, France; [2]Aix Marseille Université, Centre d'Etudes et de Recherche sur les Services de Santé et la Qualité de Vie - CEReSS, Marseille, France; [3]Clinique Ambroise Paré, Neuilly/Seine, France; [4]CHU Dijon Bourgogne, Dijon, France; [5]CH Troyes, Réanimation, Troyes, France; [6]CHU de Poitiers, Poitiers, France; [7]CHU Pitié-Salpêtrière, Assistance Publique Hôpitaux de Paris, Paris, France; [8]Hôpital Louis Pasteur, CHU Nice, Réanimation Polyvalente, Nice, France; [9]CHU Saint Louis Assistance Publique Hôpitaux de Paris, Réanimation Médicale, Paris, France; [10]CHU Raymond Poincaré, Assistance Publique Hôpitaux de Paris, Garches, France; [11]Hôpital Nord, Assistance Publique

Hôpitaux de Marseille, Anesthésie Réanimation, Marseille, France; [12]Groupe Hospitalier Paris Saint Joseph, Réanimation, Paris, France
**Correspondence:** P. Kalfon
*Intensive Care Medicine Experimental* 2017, **5(Suppl 2):**0430

**INTRODUCTION.** Being discharged from an ICU is often just the beginning of a long recovery process ahead that may be impacted by anxiety and depressive symptoms or post traumatic stress disorder (PTSD). This psychiatric morbidity is associated with increased use of psychiatric medications or other mental health services[1]. Available studies exploring this issue have numerous limitations, including a low number of patients coming from single sites, the focus on specific pathologies, and the multiplicity of the methods used to recognise these symptoms.

**OBJECTIVES.** To evaluate the prevalence of psychiatric symptoms one year after ICU discharge in a large cohort of unselected adult ICU survivors.

**METHODS.** After carrying out the IPREA3 study (a multicenter, cluster-randomized, controlled study involving 34 French adult ICUs to assess the efficacy of a multicomponent tailored intervention to reduce discomfort in the ICU[2]), we conducted a 1-year follow-up of the patients included in IPREA3 in 30 of 34 ICUs. We assessed anxiety and depressive symptoms and PTSD using resp. the Hospital and Anxiety Depression Scale (HADS) and the Impact of Event Scale-Revised (IESR). The HADS anxiety and depression subscales consist of 7 items, each rated on a 4-point scale from 0 to 4 leading to potential scores for each subscale from 0 to 21. Patients with score ≥ 8 are considered symptomatic for anxiety or depression. The IESR consists of 22 items, each rated on a 4-point scale leading to a mean total score ranging from 0 to 4. We used the threshold of 1.6 for which the sensitivity and the specificity for detecting full DSM-IV PTSD were resp. 100% and 85%. HADS and IESR were administered either by telephone or returned by patients after mailing.

**RESULTS.** Of the 1893 enrolled patients, 297 (16%) died before 1-year follow-up and 590 could not be interviewed for the following reasons: lost to follow-up (302), declined participation (93), no questionnaires return (117), and disability (78). Data exploring psychiatric morbidity were available for 1006 patients (response rate: 63%). One year after ICU discharge, resp. 31%, 26%, and 8% of survivors had anxiety, depressive, and PTSD symptoms; 42% of them presented symptoms in at least one domain.

**CONCLUSIONS.** Psychiatric comorbidity affects nearly one in two survivors 1 year after ICU discharge. This high prevalence should raise awareness of the need for a health policy applied to unselected adult ICU survivors.

**REFERENCE(S)**

1. Davydow DS, Hough CL, Zatzick D, Katon WJ: Psychiatric symptoms and acute care service utilization over the course of the year following medical-surgical ICU admission: a longitudinal investigation. *Crit Care Med* 2014, 42:2473–81.
2. Kalfon P, Mimoz O, Loundou A et al.: Reduction of self-perceived discomforts in critically ill patients in French intensive care units: study protocol for a cluster-randomized controlled trial. *Trials* 2016, 16; 17:87.

**GRANT ACKNOWLEDGMENT**
French Ministry of Health

### 0431

#### The impact of frailty on intensive care unit outcomes: a systematic review and meta-analysis

B. Waters[1], A. Varambally[2], S.M. Bagshaw[3], J.G. Boyd[4], D. Maslove[4], S. Sibley[4], K. Rockwood[5], J. Muscedere[4]
[1]McMaster University, Critical Care Medicine, Hamilton, Canada; [2]Midwestern University, School of Medicine, Glendale, United States; [3]University of Alberta, Critical Care Medicine, Edmonton, Canada; [4]Queen's University, Critical Care Medicine, Kingston, Canada; [5]Dalhousie University, Geriatrics, Halifax, Canada
**Correspondence:** B. Waters
*Intensive Care Medicine Experimental* 2017, **5(Suppl 2):**0431

**INTRODUCTION.** Frailty is a multiply determined at-risk state, typically accompanied by decreased mobility, weakness, poor muscle mass, poor nutritional status, diminished cognitive function and characterized by reduced functional reserve (1). This renders frail individuals more susceptible to extrinsic stressors. Assessing baseline frailty in critically ill patients may provide important prognostic and diagnostic information.

**OBJECTIVE.** To determine the impact of frailty on critical care outcomes through the conduct of a systematic review and meta-analysis.

**METHODS.** We searched the Cochrane Central Register of Controlled Trials, MEDLINE, EMBASE, Pubmed, and Clinicaltrials.gov. All study types except for narrative reviews, case reports and editorials were included. Studies must have assessed frailty in critically ill patients > 18 years of age and compared frail and non-frail patients. Two reviewers independently applied eligibility criteria, assessed quality, and extracted data. The primary outcome was hospital mortality and long-term mortality (≥6 Months). Secondary outcomes included the prevalence of frailty, health-related quality-of-life (HRQL) among survivors and health service utilization. Quality was assessed using the Newcastle-Ottawa Scale (NOS) for observational trials.

**RESULTS.** Ten observational studies enrolling a total of 3030 patients (927 frail and 2103 non-frail patients) were included. The overall methodologic quality on the NOS was moderate with 5 high, 5 moderate, and no low quality studies. Frailty was assessed with the Clinical Frailty Scale (7 studies), Frailty Index (4 studies) and Frailty Phenotype (2 studies). The pooled prevalence of frailty was 30% (95% CI 29 - 32%). Frailty was associated with higher hospital mortality [Relative Risk (RR), 1.71; 95% CI, 1.43, 2.05; $p$ < 0.00001; $I^2$ = 32%] and long term mortality (RR, 1.53; 95% CI, 1.40, 1.68; $p$ < 0.00001; $I^2$ = 0%). Frail patients remained in hospital for 3.39 days longer than non-frail patients, although this was not statistically significant (mean difference; 95% CI, −0.33, 7.10, p = 0.07, $I^2$ = 77%). Frail patients were also less likely to be discharged home than non-frail patients (RR, 0.59; 95% CI, 0.49, 0.71; $p$ < 0.00001; $I^2$ = 12%). Reduced HRQL was found in the only two studies assessing this.

**CONCLUSIONS.** Frailty is common among ICU patients and is associated with increased hospital and long-term mortality. Frail patient are less likely to be discharged to home than non-frail patients. Routine screening for clinical frailty in critically ill patients may provide important diagnostic and prognostic information to clinicians, patients and their families. Further studies to reduce mortality and disability in frail patients should be conducted.

**GRANT ACKNOWLEDGEMENT**
None

### 0432

#### Hopelessness: independent associations with health-related quality of life and short-term mortality after critical illness

L. Orwelius[1], M. Kristenson[2], M. Fredrikson[3], F. Sjoberg[4], S. Walther[2]
[1]Linköping University, Departments of Intensive Care, Clinical and Experimental Medicine, Linkoping, Sweden; [2]Linköping University, Department of Medicine and Health Sciences, Linkoping, Sweden; [3]Linköping University, Department of Clinical and Experimental Medicine, Linkoping, Sweden; [4]Linköping University, Department of Clinical and Experimental Medicine, Burns, Hand and Plastic Surgery, Linkoping, Sweden
**Correspondence:** L. Orwelius
*Intensive Care Medicine Experimental* 2017, **5(Suppl 2):**0432

**INTRODUCTION.** It is well known that survivors after critical illness perceive poorer health-related quality of life (HRQoL) compared with an age and sex adjusted control group. In addition to the effects of medical treatments, individual psychological factors may also influence HRQoL. These include psychological resources such as ability to cope, sense of coherence and perceived control, together with psychological risk factors such as depression, exhaustion, and hopelessness. The ability to cope with life is central for health and shown to be associated with better HRQoL in patients with chronic disease. If the individual cannot cope, this can lead to feeling of hopelessness.

**OBJECTIVES.** To assess the independent associations between the psychological factors ability to cope and hopelessness with measures of HRQoL and their effects on mortality up to 3 years after discharge in patients who have been treated in an ICU.

**METHODS.** A prospective, cross-sectional, multicenter study of 980 adult patients (mean age 58 yrs.,42% women). Ability to cope, hopelessness, and HRQoL were evaluated using validated instruments: the Mastery Scale questionnaire, Eversons's 2-item scale and Short Form-36. Linear mixed models and multiple linear and logistic regressions adjusted for confounding variables (i.e. age, sex, marital status, level of education, APACHE II score, comorbidity, and duration of ICU and hospital stay) were used.

Questionnaires were sent to patients 6, 12, 24, and 36 months after discharge from ICU.

**RESULTS.** Low scores for ability to cope and high scores for hopelessness were both related to poorer HRQoL for all SF-36 subscales (except for coping with bodily pain). Effects were in the same range as coexisting disease for physical subscales, and stronger for social and mental subscales. High scores for hopelessness also predicted mortality up to 3 years after discharge from ICU ($p < 0.001$).

**CONCLUSIONS.** Ability to cope and hopelessness showed significant and independent effects on HRQoL after ICU care, and this effect was stronger than the effect of coexisting disease. Hopelessness also predicted mortality after critical illness. Awareness of the psychological state of patients after critical illness is important to identify those with increased risk.

### 0433

#### Association between tracheostomy and functional, neuropsychological and healthcare utilization outcomes in the RECOVER cohort

A. Brondani[1,2], G. Tomlinson[3], L. Chu[3], P. Robles[3,4], A. Matte[3], S. Burns[3], C. Thomas[3], F. Lamontagne[5], N. Adhikari[1,6], N. Ferguson[1,3], J. Freidrich[1,7], J. Rudkowski[5,8], Y. Skrobik[9], H. Meggison[10], J. Cameron[3,11,12], M. Herridge[1,3], S. Mehta[13], RECOVER Program Investigators and Canadian Critical Care Trials Group

[1]University of Toronto, Interdepartmental Division of Critical Care, Toronto, Canada; [2]Azienda Sanitaria Universitaria Integrata di Udine, Department of Anesthesia and Critical Care, Udine, Italy; [3]University of Toronto, Department of Medicine, Interdepartmental Division of Critical Care Medicine, Toronto General Research Institute, Institute of Medical Science, University Health Network, Toronto, Canada; [4]University of Toronto, Graduate Department of Rehabilitation Science, Toronto, Canada; [5]Université de Sherbrooke, Centre de Recherche du CHU de Sherbrooke, Sherbrooke, Canada; [6]Sunnybrook Health Sciences Centre, Department of Critical Care Medicine, Toronto, Canada; [7]University of Toronto, Departments of Medicine and Critical Care Medicine, St. Michael's Hospital, Toronto, Canada; [8]McMaster University, Departments of General Internal Medicine and Critical Care, St. Joseph's Healthcare, Hamilton, Canada; [9]McGill University, Department of Medicine, Montreal, Canada; [10]University of Ottawa, Department of Critical Care Medicine, Ottawa, Canada; [11]University of Toronto, Department of Occupational Science and Occupational Therapy, Toronto, Canada; [12]University of Toronto, Toronto Rehabilitation Institute, Toronto, Canada; [13]University of Toronto, Department of Medicine, Interdepartmental Division of Critical Care Medicine, Sinai Health System, Toronto, Canada

**Correspondence:** A. Brondani

*Intensive Care Medicine Experimental* 2017, **5(Suppl 2):**0433

**INTRODUCTION.** Towards RECOVER (Phase I: RECOVER Program) stratified survivors of ≥1 week of mechanical ventilation (MV) into 4 disability groups (Grs) based on age and ICU length of stay (LOS) that determine 1-year recovery trajectories and healthcare use independent of admitting diagnosis or illness severity (1). Tracheostomy (T) was performed in 49% of patients (pts). In this study we evaluated the impact of T on pt outcomes in the 'Older' ('Older Long LOS', age 46–66 years; ICU LOS ≥ 2 weeks) and 'Oldest' ('Oldest Long LOS', age > 66 years; ICU LOS ≥ 2 weeks) risk Grs.

**METHODS.** There were 237 pts in the 'Older' (N = 144) and 'Oldest' (N = 93) Grs. We compared baseline and outcome variables in T versus non-tracheostomy (NT) pts within each Gr, assessed severity of illness using Acute Physiology and Chronic Health Evaluation II (APACHE II), Multiple Organ Dysfunction Score (MODS), Elixhauser (Elixsum), Charlson score. At 1 week, 3-6-12 months post ICU discharge we compared: survival, Functional Independence Measure (FIM) total, motor and cognitive subscales, distance walked in 6 minutes, Short Form 36 (SF36), Medical Research Council Scale (MRC), Impact of Event Scale (IES), Beck Depression Inventory-II (BDI-II); disposition at hospital discharge, ICU readmission, number of specialist visits post ICU discharge.

**RESULTS.** Of the 237 pts, 144 (60.8%) had T performed on median ICU day 13. Baseline severity of illness scores were not significantly different between T and NT pts. Total FIM, FIM motor subscale were significantly lower in T pts at day 90 ($p = 0.02$), FIM cognitive subscale was never significantly different. Duration of MV, ICU-hospital LOS were significantly longer in T pts. T was not associated with higher mortality in the disability Grs at any time point. T pts had higher ICU readmission rates ($p = 0.09$). No interactions between Gr and T in any other outcome variables, number of specialist visits, hospital readmission. Total lung capacity, vital capacity, forced expiratory volume 1 second, and carbon monoxide diffusion capacity, were significantly lower in the T Gr ($p = 0.04, 0.01, 0.01, 0.02$ respectively). The effects of T on the disposition at hospital discharge were not the same for the 2 RECOVER Grs, a single effect of T could not be estimated. Disposition at 1 year was not significantly different between the 2 Grs.

**CONCLUSIONS.** In this population of pts older than 46 years, MV ≥ 1 week, ICU stay ≥ 2 weeks, T was not independently associated with mortality. Pts who received a T had worse pulmonary outcomes, longer duration of MV, ICU, hospital LOS. T after 2 weeks of MV was not independently associated with functional or cognitive outcomes. The daily burden of care required by the patient, expressed by FIM, was not significantly influenced by T.

### 0434

#### Determinants of depressive symptoms at one year after Intensive Care Unit discharge in survivors of ≥ 7 days of mechanical ventilation: results from the RECOVER Program

M. Hamilton[1], G. Tomlinson[2], L. Chu[2], P. Robles[2,3], A. Matte[2], S. Burns[2], C. Thomas[2], F. Lamontagne[4], N. Adhikari[1,5], N. Ferguson[1,2], J. Friedrich[1,6], J. Rudkowski[4,7], Y. Skrobik[8], H. Meggison[9], J. Cameron[3,10,11], M. Herridge[1,2], RECOVER Program Investigators and the Canadian Critical Care Trials Group

[1]University of Toronto, Interdepartmental Division of Critical Care, Toronto, Canada; [2]Toronto General Research Institute, Institute of Medical Science, Department of Medicine, Interdepartmental Division of Critical Care Medicine, University of Toronto, Toronto, Canada; [3]University of Toronto, Graduate Department of Rehabilitation Science, Toronto, Canada; [4]Universite de Sherbrooke, Centre de Recherche du CHU de Sherbrooke, Sherbrooke, Canada; [5]Sunnybrook Health Sciences Centre, Department of Critical Care Medicine, Toronto, Canada; [6]St Michael's Hospital, Departments of Medicine and Critical Care Medicine, Toronto, Canada; [7]McMaster University, Departments of General Internal Medicine and Critical Care, St. Joseph's Healthcare, Hamilton, Canada; [8]University of Montreal, Department of Medicine Division of Critical Care, Maisonneuve Rosemont Hospital, Montreal, Canada; [9]University of Ottawa, Department of Critical Care, Ottawa, Canada; [10]University of Toronto, Department of Occupational Science and Occupational Therapy, Toronto, Canada; [11]Toronto Rehabilitation Institute, University of Toronto, Toronto, Canada

**Correspondence:** M. Hamilton

*Intensive Care Medicine Experimental* 2017, **5(Suppl 2):**0434

**BACKGROUND.** Moderate to severe depressive symptoms occur in 20-33% of survivors at one year after Intensive Care Unit (ICU) discharge and may have a negative impact on functional outcomes. The extent to which patient and caregiver factors impact the development of depressive symptoms remains unclear.

**OBJECTIVES.** We sought to evaluate patient and caregiver factors associated with the development of post-ICU depressive symptoms.

**METHODS.** Using the RECOVER Program cohort of 391 medical/surgical ICU patients, we investigated factors associated with depressive symptoms captured by the Beck Depression Inventory-II (BDI-II) score. We determined the association between BDI-II and baseline characteristics (age, sex, socioeconomic status, Charlson Comorbidity Index (CCI) score and ICU length of stay (LOS)), Functional Independence Measure (FIM) motor subscale score and caregiver characteristics (Caregiver Assistance Scale, Center for Epidemiologic Studies Depression Scale and caregiver BDI-II) using linear regression and mixed models at the 3, 6 and 12-month time points. All analyses were preplanned.

**RESULTS.** BDI-II data was available for 246 patients. Median age at ICU admission was 54 years, 58% were male (n = 143) and median LOS was 19 days. Mean BDI-II scores at 3, 6 and 12 months were 12.3, 11.4 and 10.7. Across all time points 17% of patients had a BDI-II score ≥21, indicating moderate-severe depressive symptoms. At each time point, a lower FIM motor subscale score was significantly associated with more severe depressive symptoms. At 6 and 12 months, age had a curvilinear relationship to BDI-II, with the highest depressive symptoms associated with ages 45–50. Over the 12 months, mixed models found more severe depressive symptoms in patients with lower FIM motor subscale scores, those who had not completed secondary education, and the same curvilinear relationship to age. No statistically significant associations were found between patient BDI-II and patient CCI, sex, LOS, income or caregiver variables.

**CONCLUSIONS.** Severity of post-ICU depressive symptoms is associated with less education, greater functional dependency, and has a curvilinear relationship with age. No associations between caregiver variables and patient outcome were identified. Knowledge of risk factors for clinically important depressive symptoms may inform surveillance and targeted mental health follow-up. Rehabilitation programs aiming to improve the FIM may serve to modify mood disorders.

**REFERENCE(S)**

Herridge MS, Chu LM, Matte A, Tomlinson GT et al. The RECOVER Program: Disability Risk Groups & One Year Outcome after ≥ 7 Days of Mechanical Ventilation. Am J Respir Crit Care Med. 2016 Oct 1;194(7):831–844

Cameron JI, Chu LM, Matte A, Tomlinson G et al. One year outcomes in caregivers of critically ill patients. N Engl J Med. 2016 May 12;374(19):1831–41

Supported by CIHR, MOH-AFP, John and Gail MacNaughton Family fund, Don and Jane Luck Family fund, Jason Tham and Andrea Chan Family fund.

### Sedation, analgesia and delirium

#### 0435

#### Conscious sedation versus general anesthesia for thrombectomy in acute ischemic stroke. A customized election

S. Alcántara Carmona, N. Martínez Sanz, A. Pérez Lucendo, L. Pérez Pérez, P. Matia Almudevar, A. Naharro Abellán, J. Veganzones Ramos, M. Pérez Redondo

Hospital Universitario Puerta de Hierro Majadahonda, Intensive Care, Majadahonda, Spain

**Correspondence:** S. Alcántara Carmona

*Intensive Care Medicine Experimental* 2017, **5(Suppl 2):**0435

**OBJECTIVE.** Whether patients undergoing thrombectomy (TRB) in acute ischemic stroke (AIS) should be managed with conscious sedation (CS) or general anesthesia (GA) remains controversial. Our objective is to compare both strategies and study the differences in mortality, neurological improvement and those related with TRB.

**METHODS.** Retrospective study (October 2013 - July 2016). AIS patients treated with TRB admitted to our ICU. Data collected are shown in Tables 120 and 121. We considerer neurological improvement as a difference ≥ 4 points between the initial and before discharge NIHSS scores, and reperfusion as angiographic flow recovery. ICU and hospital length of stays, in-hospital and 6-month overall mortalities together with modified Rankin scores at 6-months where also registered. Statistical analysis included $X^2$ and Mann–Whitney tests together with logistic regression.

**RESULTS.** During the period studied, 183 patients were admitted to our ICU with a diagnosis of AIS. Of them, 108 (59%) underwent TRB. Fifty-four were managed with CS and 54 with GA. Only APACHE II and NIHSS scores were higher in the GA group (Table 120). Twenty-one patients (38.8%) in the CS group and 32 (59.2%) in the GA had a NIHSS ≥ 18. Reasons for GA were: elective (37), diminished level of consciousness (8), agitation (7) and respiratory impairment (2). There were no crossovers between the CS and GA groups.

GA was associated with longer arrival to puncture times and a more frequent usage of vasoactive drugs, but onset to reperfusion times were similar between groups and GA was associated with better reperfusion rates. Although neurological improvement (NIHSS ≥ 4) was higher in the GA group, modified Rankin scores at 6 months were better for the CS group (Table 121). Median time until extubation was 13h (5–31.2). Patients undergoing GA had longer ICU stays [1 day (1–2) vs. 2 days (2–5);p < 0.005) but hospital length of stay was similar between groups [14 days (9–23) vs. 11 days (8–26.5);p = 0.5]. The GA group had higher in-hospital and 6-month overall mortality rates [3 (5.6%) vs. 13 (24.5%);p = 0.07 and 6 (11.5%) vs. 15 (29.5%);p = 0.02) but, after adjusting by APACHE II, initial NIHSS, reperfusion rate, vasoactive drug usage and ICU length of stay we found no differences in in-hospital [OR 3.47 (0.51-23.38)] or overall 6-month mortalities [OR 3,19 (1,12-9,05)]. Initial NIHSS for deceased patients was 18 (15–20).

**CONCLUSION.** Even though GA was associated with better reperfusion rates and larger NIHSS improvements, patients under CS had a better neurological recovery, which could be related with lower initial NIHSS scores.

In our series, GA was used in patients with higher APACHE II and initial NIHSS scores without causing significant delays in treatment times.

GA patients had higher in-hospital and overall six-month mortalities, however, after adjusting by confounding factors we found no differences in mortality rates between groups.

**Table 120 (Abstract 0435).** See text for description

| | CS (n = 54) | GA (n = 54) |
|---|---|---|
| Age (years) | 65 (58 – 76) | 71 (59 – 78) |
| Sex (male) | 35 (64.8) | 31 (57.4) |
| Nº comorbidities | 0: 5 (9.2)<br>1: 15 (27.8)<br>2: 11 (20.4)<br>3: 11 (20.4)<br>4: 10 (18.5)<br>5: 2 (3.7) | 0: 4 (7.4)<br>1: 11 (20.4)<br>2: 22 (40.7)<br>3: 11 (20.4)<br>4: 6 (11.1)<br>5: 0 (0) |
| APACHE II | 10 (7 – 13) | 12.5 (10 – 16)* |
| Prior Modified Rankin Scale | 0 (0 – 0) | 0 (0 – 0) |
| Previous ischemic stroke (YES) | 4 (7.4) | 6 (11.1) |
| Previous hemorrhagic stroke (YES) | 1 (1.85) | 0 (0.0) |
| Initial NIHSS | 16 (12 – 18) | 19.5 (16 – 21)* |
| Vascular territory involved | LMCA: 25 (46.3)<br>RMCA: 25 (46.3)<br>Basilar: 1 (1.85)<br>ICA: 2 (3.7)<br>ACA: 1 (1.85) | LMCA: 29 (53.7)<br>RMCA: 20 (37.05)<br>Basilar: 4 (7.4)<br>ICA: 1 (1.85)<br>ACA: 0 (0) |
| ASPECTS | 8 (7 – 10) | 8 (7 -10) |

Data are shown as absolute values (%) or as median (q25 – q75). Modified Rankin Scale score range: 0 to 6 (0 = symptom free to 6 = dead). NIHSS: National Institute of Health Stroke Scale. LMCA: left middle cerebral artery; RMCA: right middle cerebral artery; ICA: internal carotid artery; ACA: anterior cerebral artery. ASPECTS: Alberta Stroke Program Early CT Score. * p < 0.01

**Table 121 (Abstract 0435)**. See text for description

| | CS (n = 54) | GA (n = 54) |
|---|---|---|
| Reason for thrombectomy | Thrombolysis failure: 34 (63.0)<br>Thrombolysis contraindication: 14 (25.9)<br>Symptoms for > 4.5 h: 6 (11.1) | Thrombolysis failure: 27 (50)<br>Thrombolysis contraindication: 18 (33.4)<br>Symptoms for > 4.5 h: 9 (16.6) |
| Symptoms to thrombolysis (min) | 122.5 (100 – 145) | 107 (80 – 134) |
| Onset to arrival to TRB center (min) | 200 (129 – 280) | 190 (97 – 231) |
| Arrival to puncture (min) | 55 (40 – 99) | 73 (50 – 128)* |
| Puncture to reperfusion (min) | 67 (47 – 104) | 53 (40 – 75) |
| Onset to reperfusion (min) | 349 (260 – 390) | 309 (250 – 360) |
| Type of endovascular treatment | Stent retriever: 19 (52.8)<br>Direct aspiration: 4 (11.1)<br>Retriever+ aspiration: 13 (36.1) | Stent retriever: 21 (52.5)<br>Direct aspiration: 7 (17.5)<br>Retriever+ aspiration: 12 (30.0) |
| Reperfusion (YES) | 37 (75.5) | 45 (93.7)* |
| Thrombectomy complications (YES) | 2 (3.7) | 5 (9.6) |
| Vasoactive drugs (YES) | 3 (5.6) | 13 (24.4)* |
| Hypotensive drugs (YES) | 9 (16.7) | 10 (18.5) |
| Hemorrhagic transformation (YES) | 11 (20.4) | 12 (22.2) |
| Change in NIHSS score | < 4: 24 (45.3)<br>≥ 4: 29 (54.7) | < 4: 10 (23.3)*<br>≥ 4: 33 (76.7)* |
| Destination upon discharge | Home: 30 (62.5)<br>Rehabilitation facility: 18 (37.5) | Home: 26 (66.7)<br>Rehabilitation facility: 13 (33.3) |
| Modified Rankin Scale at 6 months | 0 -2: 32 (66.7)<br>3 – 6: 16 (33.3) | 0 -2: 20 (40.0)*<br>3 -6: 30 (60.0)* |

Data are shown as absolute values (%) or as median (q25 – q75). Modified Rankin Scale score range: 0 to 6 (0 = symptom free to 6 = dead). NIHSS: National Institute of Health Stroke Scale * $p < 0.05$

### 0436

#### Pain, Agitation and Delirium (PAD) in a community ICU - nurse focus group to inform PAD Quality Improvement (QI) program development

M. Law[1], P. Yung[2], F. Miller[2], C. Marshall[2], K. Ross[3], M. Camargo[1,3], D. Fleming[3], J.L. Tsang[2,4,5]
[1]Brock University, St. Catharines, Canada; [2]McMaster University, Niagara Regional Campus, Michael G. DeGroote School of Medicine, St. Catharines, Canada; [3]Niagara Health, St. Catharines, Canada; [4]McMaster University, Medicine, Hamilton, Canada; [5]Niagara Health, Medicine/ Critical Care, St. Catharines, Canada
**Correspondence:** J.L. Tsang
*Intensive Care Medicine Experimental* 2017, **5(Suppl 2):**0436

**INTRODUCTION.** Delirium is a common manifestation of acute brain dysfunction in critically ill patients with a prevalence of 80% in intensive care unit (ICU) patients[1]. It is associated with multiple complications[1]. In 2013, a revised version of the ICU pain, agitation, and delirium (PAD) guidelines was published[2]. Yet, its dissemination in a community hospital is unclear. More importantly, nurses play an integral part in PAD management and their perspectives should inform the development of a multifaceted and multidisciplinary quality improvement (QI) program to improve PAD management.

**OBJECTIVES.** This qualitative study explored the views of ICU nurses regarding PAD management in order to inform the subsequent development of a multifaceted and multidisciplinary QI program with the aim to improve PAD management in a community ICU.

**METHODS.** Focus groups (1-hour) with nurses were conducted using a structured interview guide containing six questions designed to explore themes related to nursing practice, professional preferences and improvement strategies around PAD management. Transcripts were analyzed using a constant comparative approach to identify themes that emerged from the data. Responses for each question were analyzed individually and then compared across sessions to examine similarities or differences across the responses.

**RESULTS.** Forty-five out of sixty-eight nurses attended 1 of 5 focus group sessions. Themes that developed from the analysis outlined differences in nurse perceptions of optimal sedation and physical restraint use, but there was alignment in their perception regarding the impact of environmental factors on delirium development. Differences were found related to physical restraint use as some nurses felt that they contribute to delirium while some felt that they are necessary for safety. Some nurses preferred deep sedation to avoid awareness of traumatic experience, whereas others preferred wakefulness facilitating participation in their medical care. Most nurses agreed that noise, lights, medications, disjointed care and physical restraint use contribute to delirium development. They agreed that consistencies among healthcare professionals in the form of an algorithm, individual patient-tailored non-pharmacological therapy involving input from family members and education for staff and family members would aid in PAD management.

**CONCLUSIONS.** The results of this study will guide subsequent interventions as part of our multifaceted and multidisciplinary QI program. Core to this project is the nurse engagement in the development of these interventions which in turn will impact improvement for PAD in the ICU.

**REFERENCE(S)**

1. *Intensive Care Med* **33,** 66–73 (2007)
2. *Critical Care Med* **41,** 263–306 (2013)

**GRANT ACKNOWLEDGMENT**

Jennifer Tsang holds the McMaster University, Department of Medicine, Internal Career
Research Award. This work was supported by the Ontario Nurses' Association Superior
Education Fund.

### 0437

#### Nicotine replacement therapy in the intensive care unit: a randomized controlled pilot study

A. Schuppers[1], B. de Jong[2,3], H. van den Oever[1], M. Arbouw[4], A. Kruisdijk-Gerritsen[1], A. van Zanten[2]
[1]Deventer Hospital, Intensive Care, Deventer, Netherlands; [2]Gelderse Vallei Hospital, Intensive Care, Ede, Netherlands; [3]Radboud University Medical Center, Intensive Care, Nijmegen, Netherlands; [4]Deventer Hospital, Clinical Pharmacy, Deventer, Netherlands
**Correspondence:** A. Schuppers
*Intensive Care Medicine Experimental* 2017, **5(Suppl 2):**0437

**INTRODUCTION.** Delirium and agitation are common in mechanically ventilated patients admitted to the intensive care unit (ICU). Retrospective data have shown that smoking, or the acute abstinence of smoking, is an additional risk factor for agitation. A safe and effective method to treat nicotine withdrawal symptoms in outpatient and hospitalized smoking adults is transdermal nicotine replacement therapy (NRT). Previous studies on the use of transdermal NRT in critically ill smokers admitted to the ICU have produced conflicting results.The main goal of this pilot study was to investigate the feasibility of a phase III trial, by conducting a test run of a randomized controlled trial. We aimed to assess the safety and efficacy of transdermal NRT in mechanically ventilated smokers admitted to the ICU.

**METHODS.** In this two-centre, randomized, placebo-controlled pilot study, mechanically ventilated smokers admitted to the ICU were included. Subjects received either a nicotine patch (14 or 21 mg/day) or a placebo patch daily until ICU discharge or for a maximum of 30 days. The primary outcome was 30-day mortality. Secondary outcomes included 90-day mortality and number of (serious) adverse events. In a post hoc analysis, we defined a composite outcome parameter as time spent alive without sedation and delirium in the first 30 days.

**RESULTS.** The study was stopped after the inclusion of 47 patients. The two groups were comparable with respect to baseline characteristics. No differences were found in 30-day mortality and 90-day mortality between NRT and control group (2/21 vs. 2/26; p = 0.843 and 3/21 vs. 5/26; p = 0.665). There was no difference in the number of adverse and serious adverse events, between NRT and control patients (102 vs. 177; p = 0.096, and 5 vs. 11; p = 0.251). Patients in the NRT group had spent more time alive without sedation and delirium during the first 10 days (160 hours (96–216) vs. 88 hours (20–210); p = 0.043) and during the first 20 days (400 hours (316–448) vs. 304 hours (110–432); p = 0.033) of treatment.

**CONCLUSION.** Transdermal NRT in mechanically ventilated smokers had no effect on 30- and 90-day mortality, although our study was underpowered to establish such differences.
Furthermore, the number of (serious) adverse events between the two groups was comparable.
Finally, patients with NRT spent more time alive without sedation and delirium during the first 10 and 20 days.
We suggest that future trials should focus on a possible effect of NRT on predefined delirium endpoints, particularly in the second and third weeks of ICU stay.
The results of this pilot study showed that, with some modifications of the trial design, a randomized controlled phase III study to assess the safety and efficacy of transdermal NRT in mechanically ventilated smokers admitted to the ICU is feasible.

### 0438

#### Guidelines and consensus statements on delirium-, analgesia- and sedation- management in the ICU - a systematic literature search and critical quality appraisal with AGREE II

M. Rosenthal, B. Weiss, J. Grunow, R. Moergeli, F. Balzer, C. Spies
Charité Universitätsmedizin Berlin, Department of Anesthesiology and Intensive Care Medicine CC7, Berlin, Germany
**Correspondence:** M. Rosenthal
*Intensive Care Medicine Experimental* 2017, **5(Suppl 2):**0438

**INTRODUCTION.** The management of delirium, analgesia and sedation (DAS) is an integral part of critical care. Various guidelines and recommendations are available to assist clinicians in their everyday decision-making. However, the benefit of these guidelines and their actual potential of bringing evidence into clinical practice is highly dependent on their quality, which is largely unknown.
**OBJECTIVES.** The purpose of the study was a literature search and quality assessment of DAS guidelines to identify
(1) high quality guidelines and
(2) possible domains to improve guideline-development in the future.
**METHODS.** We conducted a systematic literature search of DAS-guidelines in the critical care context published between 2007 and 2017. Guidelines were systematically identified by searching six electronic bibliographic databases. Critical appraisal was performed by three independent expert reviewers using the Appraisal of Guidelines Research and Evaluation (AGREE II).
**RESULTS.** 2The search algorithm revealed 625 manuscripts that were manually screened for inclusion and exclusion criteria. We identified five guidelines and one consensus statement meeting the criteria. Three guidelines (50%) were predominantly developed on a regional/national level, the others were international/continental (other specifications s.f. Table 122).
AGREE II domain-scores varied considerably among the six guidelines (Fig. 152). Two guidelines exceed the quality threshold of 60% in all domains, although the full version of one of these guidelines is exclusively available in German language, which limits applicability outside German-speaking countries.
From the methodological perspective, the highest AGREE II-scores were achieved in the domains "scope&purpose" (median 80.6% [min 25.9%; max 90.7%]) and "editorial independence" (median 70.8% [min 0%; max 94.4%]). In contrast, the domains "stakeholder involvement" (median 54.6% [min 9.3%; max 81.5%]) and "applicability" (median 44.4% [min 0%; max 84.7%]) scored lowest (Table 123). These quality gaps in particular evolved from a lack of consideration of patient's treatment preferences and the concrete development of implementation strategies.
**CONCLUSION.** In the field of DAS-management, there are three guidelines of highest quality available. However, our analyses revealed a quality deficiency in stakeholder involvement and applicability of current guidelines. These are vital domains for implementation and acceptance of guidelines and should be increasingly considered in future guideline development.

**Table 122 (Abstract 0438)**. See text for description

| Title | Organization | Year of publication | No. of statements / recommendations | Scope | Appraisal tool | Endorsement | Documents used for appraisal |
|---|---|---|---|---|---|---|---|
| DAS-Guideline | DAS-Task Force (DGAI, DIVI) | 2015 | 145 | for all ICU professionals and all critically ill patient populations. | Oxford criteria | multiprofessional and interdisciplinary endorsement | Long Version (Langfassung), Short version |
| PAD Guideline | SCCM | 2013 | 54 | adult ICU patients | GRADE | multiprofessional and interdisciplinary endorsement | Published version and Supplemental Digital Content 1-3 |
| NICE Guideline | NICE | 2013, reviewed in 2015 | 27 | Adults (18 years and older) in hospital; Adults (18 years and older) in long-term residential care | GRADE | NICE | Full Guideline and online supplements |
| FEPIMCTI Guideline | FEPIMCTI | 2013 | 138 | adult ICU patients, with or without tracheal intubation (nasal or orotracheal) and ventilatory support, and/or with certain conditions or diseases. | GRADE | Colombian Association of Critical Medicine and Intensive Care (AMCI) | Published Guideline, technical report was requested but not available at the time of analysis |
| eCASH | | 2016 | 11 | ICU patients | n/a | n/a | Published Statement |
| DSIT Guideline | DSIT, DASAIM | 2015 | 8 | ICU patients | n/a | DSIT, DASAIM | Published Guideline |

**Table 123 (Abstract 0438)**. See text for description

| Domain | 1 | 2 | 3 | 4 | 5 | 6 |
|---|---|---|---|---|---|---|
| DAS | 90,7 | 81,5 | 84,9 | 81,8 | 77,8 | 94,4 |
| PAD | 85,2 | 61,1 | 88,5 | 90,7 | 61,1 | 94,4 |
| NICE | 87 | 79,6 | 78,4 | 57,4 | 84,7 | 61,1 |
| FEPIMCTI | 75,9 | 48,2 | 56,8 | 57,4 | 27,8 | 13,9 |
| eCASH | 44,4 | 27,8 | 14,4 | 44,4 | 22,2 | 80,6 |
| DSIT | 25,9 | 9,3 | 6,5 | 20,4 | 0 | 0 |

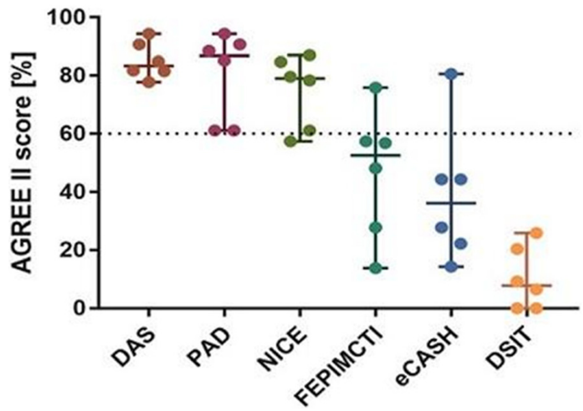

**Fig. 152 (Abstract 0437)** Comparison of guideline Domain Scores. DAS = DAS Guideline, PAD = PAD Guideline, NICE = NICE Guideline, FEPIMCTI Guideline, eCASH = eCASH statement, DSIT = DSIT Guideline. The dotted line represents the cut-off for a high quality score (60%)

### 0439

#### The relationship of the timing and dose of dexmedetomdine administration with the incidence and duration of postoperative delirium in the elderly patients after major non-cardiac surgery

C. Lee, S. Kim
Wonkwang University School of Medicine Hospital, Anesthesiology and Pain Medicine, Iksan, Korea, Republic of
**Correspondence:** C. Lee
*Intensive Care Medicine Experimental* 2017, **5(Suppl 2):**0439

**INTRODUCTION.** Postoperative delirium in elderly patients with major surgery including cardiac surgery is a relatively frequent complication. Although dexmedetomidine has been known be effective to prevent delirium, its use is still controversial. The present study was aimed to investigate the effect of the timing and dose of dexmedetomidine on postoperative delirium.

**MATERIALS AND METHODS.** Postoperative delirium was evaluated for 5days after surgery in 318 patients older than 65 years undergoing non-cardiac surgery under general anesthesia. Patients received either dexmedetomidine (1 μg/kg bolus followed by 0.2 to 0.7 μg/kg/ h infusion from induction of anesthesia to the end of surgery (Group D1), or 1 μg/kg bolus at 15 minutes before the end of surgery (Group D2) or saline (Group S).The primary endpoint was incidence of delirium. Secondary outcomes were duration of delirium, visual analog scale (VAS) for pain, patient controlled analgesia (PCA) volume for 24 h after surgery, and haloperidol use in delirium patients). As secondary analysis, cytokines (TNF-a, IL-1beta, IL-2, IL-6, IL-8, and IL-10), C-reactive protein (CRP), and cortisol were measured on 1st hour and 1st day after surgery.

**RESULTS.** Group D1 reduced incidence and duration of delirium. However group D2 didn't decrease incidence of delirium but its duration in patients with delirium. VAS and PCA volume consumed for 24 h after surgery in group D1was significantly lower than that of other two groups. Haloperidol dose used in patients with delirium was lower in group D1 and D2 than in group S. IL-6 in group D1 was significantly lower at 1 h and 24h after surgery than in group S and lower at 24 h after surgery than in group D2. IL-6 in group D2 was significantly lower at only 1 h after surgery than in group S. However, Il-6 in group D2 with delirium was significantly lower at 1h and 24 h after surgery than in group S. Cortisol at 1 h after surgery was significantly lower in group D1 and D2 than group S.

**CONCLUSIONS.** In present study, dose and timing of dexmedetomidine may be important in preventing delirium. The reduced incidence and duration of delirium by dexmemdetomidine is associated with reduced IL-6 level at 1st day after surgery.

**REFERENCE(S)**

1.Orena EF, King AB, Hughes CG.The role of anesthesia in the prevention of **postoperative delirium**: a systematic **review**.Minerva Anestesiol. 2016 ;82:669–83.

2. Vasunilashorn SM, Ngo L, Inouye SK, Libermann TA, Jones RN, Alsop DC, Guess J, Jastrzebski S, McElhaney JE, Kuchel GA, Marcantonio ER.Cytokines and Postoperative **Delirium** in Older Patients Undergoing Major Elective Surgery. J Gerontol A Biol Sci Med Sci. 2015;70:1289–95

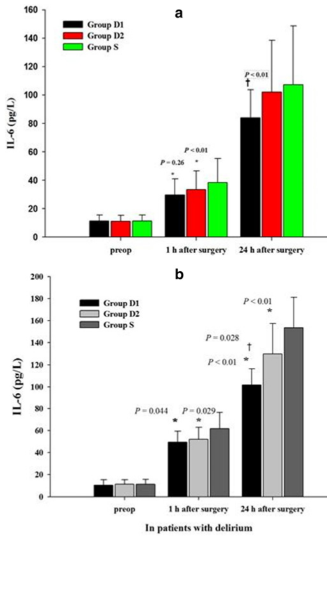

**Fig. 153 (Abstract 0439)** IL-6 concentration in three groups (A) and in patients with delirium (B). *P vs Group S, †P vs other two groups.

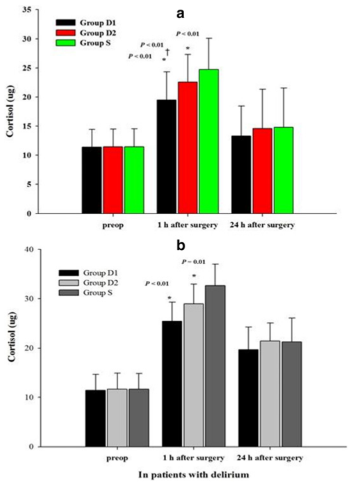

**Fig. 154 (Abstract 0439)** Cortisol concentration in three groups (A) and in patients with delirium (B). *P vs Group S, †P vs other two groups

### Outcome research in sepsis

#### 0440

#### Causes of long-term mortality in severe sepsis and septic shock

S.B. Wilhelms, S.M. Walther, F. Sjöberg, L. De Geer
Linköping University, Linköping, Sweden
**Correspondence:** S.B. Wilhelms
*Intensive Care Medicine Experimental* 2017, **5(Suppl 2):**0440

**INTRODUCTION.** Severe sepsis and septic shock are conditions with high acute mortality [1]. It may also contribute to long-term mortality [2], but the causes of long term mortality in severe sepsis patients are poorly described.

**OBJECTIVES.** To describe the causes of death ≥ one year after being treated in an ICU due to severe sepsis or septic shock.

**METHODS.** The study cohort consisted of 8760 patients with severe sepsis or septic shock registered in the Swedish Intensive Care Register (year 2008–2013, American College of Chest Physicians/ Society for Critical Care Medicine definition). These patients were compared with a control group consisting of 8760 patients treated in the intensive care unit (ICU), but not suffering from severe sepsis or septic shock (matched by gender, age, SAPS3 score and ICU length of stay). Death causes (primary and secondary diagnoses as International Classification of Diseases codes) were obtained from the Swedish Cause of Death Register (year 2008–2014).

**RESULTS.** A total of 903 patients with severe sepsis died > = 365 days after their initial intensive care unit stay, but within the study period (year 2008–2014). In the control group, 884 patients died > =365 days after the initial event. The most common causes of death in patients surviving more than 1 year were heart diseases (50,2% in the severe sepsis and septic shock patients, 48,6% in the non-sepsis patients) and cancer (33,7% and 31,7%, respectively). Infectious diseases were more common as a cause of death in the septic cohort than in the control group, even in those patients surviving more than 1 year after the initial ICU episode (24,3% and 19,6% respectively, $p < 0.05$).

**CONCLUSIONS.** The most common cause of death after severe sepsis and septic shock is heart disease, which is similar to a matched non-septic ICU cohort. However, septic patients have a significantly higher incidence of infectious diseases as a cause of death than non-sepsis patients, even one year after the initial septic episode. The reasons for this suggests an immune related difference but more studies regarding this are needed.

**REFERENCE(S)**
[1] Fleischmann C et al. Assessment of Global Incidence and Mortality of Hospital-treated Sepsis. Current Estimates and Limitations. Am J Respir Crit Care Med. 2016;193(3):259–72.
[2] Shankar-Hari M et al. Evidence for a casual link between sepsis and long-term mortality: a systematic review of epidemiologic studies. Crit care 2016;20:101. DOI 10.1186/s13054-016-1276-7.

**GRANT ACKNOWLEDGMENT**
This study was supported by grants from Region Östergötland and Linköping University, Sweden.

### 0441
### Persistent immune defects are detectable in survivors of a septic insult

S.-J. Forsyth[1], J. Borgel[1], T. Ashken[1,2], H.D.T. Torrance[1,2], E.R. Longbottom[1,2], C.J. Hinds[1,2], R.M. Pearse[1,2], M.J. O'Dwyer[1,2]
[1]William Harvey Research Institute, Translational Medicine and Therapeutics, London, United Kingdom; [2]Royal London Hospital, Barts Health NHS Trust, Adult Critical Care Unit, London, United Kingdom
**Correspondence:** S.-J. Forsyth
*Intensive Care Medicine Experimental* 2017, **5(Suppl 2):**0441

**BACKGROUND & OBJECTIVE.** Patients surviving a septic illness experience significant reductions in long term survival that persists for up to 8 years after hospital discharge (1). Recurrent infectious illnesses in previously healthy patients is a major contributor to this increased long-term morbidity (2). Our hypothesis was that epigenetic alterations induced by a septic episode imparts a persistent, newly acquired immunological defect.
**METHODS.** Patients with positive blood cultures underwent blood sampling within 24 hours of blood culture result, 4–7 days later and 6–12 months following hospital discharge. Healthy volunteers served as a comparator group. Gene expression was determined using quantitative polymerase chain reaction. Peripheral blood mononuclear cells (PBMCs) were cultured in 10% serum with and without lipopolysaccharide (LPS) and PMA/ionomycin (Sigma) for 24 hours. Cytokine concentration in the supernatants were quantified using Cytometric Bead Array (Biolegend).
**RESULTS.** Clinical and demographic features are outlined in Table 124. Median age of the volunteer group was 41 (IQR 36.6-49.5) and 22 (60%) were male. Patients with acute infection produced less IL-10, IL-6, IFNγ and TNFα than healthy volunteers following stimulation. This persisted in the convalescent samples (Fig. 155). In comparison to healthy volunteers, gene expression of histone modification enzymes EHMT2 and KDM6B were downregulated and upregulated, respectively. This persisted in the convalescent samples (Fig. 156).
Table 124 Demographic and clinical features of the patient cohort. Numbers refer to median with IQR in parenthesis or absolute count and percentages.
**CONCLUSIONS.** Patients recovered from an infectious insult display features of immune suppression which could be attributable to infection induced histone modifications. Further Chromatin Immunoprecipitation assays are required to inform causation.

**GRANTS**
*This project is funded by the British Journal of Anaesthesia and the Royal College of Anaesthetists and Barts Charity.*

**REFERENCES**
1. Czaja AS *et al*. Readmission and late mortality after paediatric severe sepsis. *Paediatrics*. 2009;123:8492.
2. Mira JC *et al*. Sepsis Pathophysiology, Chronic Critical Illness, and Persistent Inflammation-Immunosuppression and Catabolism Syndrome. *Critical Care Medicine*. 2017; 45:2

**Table 124 (Abstract 0441)**. Patient clinical and demographic features

| n=69 | |
|---|---|
| Age | 59(51-68.5) |
| Male sex | 41 (59%) |
| Patient location: | Intensive care: 23 (33.3%) |
| | General ward: 46 (66.6%) |
| SOFA score day 0 (ICU cohort) | 6.4 (2-13) |
| Gram -ve infection | 37 (53.6%) |
| Gram +ve infection | 31 (44.9%) |
| Fungal infection | 1 (1.4%) |

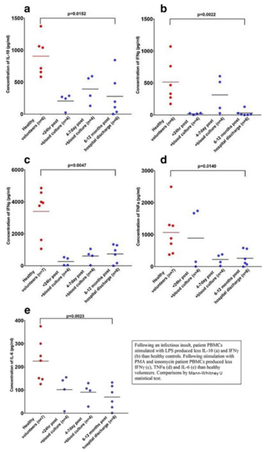

**Fig. 155 (Abstract 0441)** Cytokine production (pg/ml) of PBMCs stimulated with LPS (a. IL-10, b. IFNγ) and PMA (c. IFNγ, d. TNFα, e. IL-6)

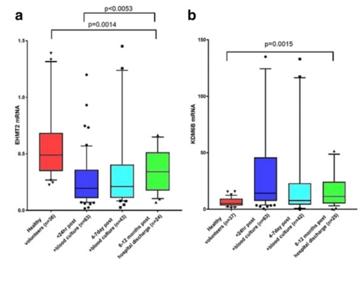

**Fig. 156 (Abstract 0441)** Gene expression levels of a) EHMT2 and b)KDM6B

### 0442

#### Basophils and mortality in septic shock

M. Martín-Fernández[1], R. Almansa[1], E. Tamayo[2], D. Andaluz-Ojeda[3], J. Blanco[4], M. Heredia-Rodríguez[2], A. Muriel-Bombín[4], E. Gómez-Sánchez[2], R. Herrán-Monge[4], J.F. Bermejo-Martín[1]
[1]Hospital Clínico Universitario de Valladolid, SACYL/IECSCYL, Group for Biomedical Research in Sepsis (Bio·Sepsis), Valladolid, Spain; [2]Hospital Clínico Universitario de Valladolid, SACYL, Anesthesiology and Reanimation Service, Valladolid, Spain; [3]Hospital Clínico Universitario de Valladolid, SACYL, Intensive Care Medicine Service, Valladolid, Spain; [4]Hospital Universitario Río Hortega, Intensive Care Medicine Service, Valladolid, Spain
**Correspondence:** M. Martín-Fernández
*Intensive Care Medicine Experimental* 2017, **5(Suppl 2):**0442

**INTRODUCTION.** Septic shock is defined as a subset of sepsis in which underlying circulatory, cellular, and metabolic abnormalities are associated with a greater risk of mortality than sepsis alone[1]. Septic syndromes deeply perturb immune homeostasis and impair innate and adaptive immunity. In this sense, leukocytes and its subpopulations are crucial components of the innate immune response and the first line of defense against infection[2].

**OBJECTIVES.** The aim of this study is to evaluate the role of basophil in septic shock and its ability to predict mortality.

**METHODS.** We conducted a two-stage multicenter, retrospective study, aimed at evaluating the prognostic value of leukocyte subpopulations in patients with septic shock and no immunosuppression. Thus, we developed a Derivation cohort formed by 238 patients and a Validation cohort of 186 patients. Statistical analyses were performed to evaluate the impact of basophil count on mortality at 28 days.

**RESULTS.** When patients of the Derivation cohort were split based on deciles for basophils count at 28-days mortality prediction, those with basophils > 110 cells/mm3 died earlier (Fig. 157). Thus, multivariate COX regression analysis was performed based on statistically significant variables and it demonstrated that 110 basophils cut-off was statistically significant ($p$ = 0.048 and HR = 2.32) (Table 125).

The cut-off identified in the Derivation cohort was tested in the Validation Cohort. Kaplan-Meier survival analysis (Fig. 158) and COX regression multivariate analysis based on statistically significant variables confirmed the previous results obtained in the Derivation cohort ($p$ = 0.012 and HR = 2.288) (Table 126).

**CONCLUSIONS.** In conclusion, monitoring basophil count represents a promising prognostic tool in septic shock patients.

**GRANT ACKNOWLEDGMENT**

This study was supported by Proyectos de Investigación en Biomedicina, Consejería de Sanidad, JCYL (BOCYL-D-26072010). This work was also supported by Fondo de Investigaciones Sanitarias, Instituto de Salud Carlos III, Spain (grant number PI13/02110).

**Table 125 (Abstract 0442)**.COX regression analysis in Validation Cohort

| | HR | 95% CI | p-value |
|---|---|---|---|
| Age | 1.014 | [0.99-1.04] | 0.240 |
| APACHE II | 1.067 | [1.03-1.10] | 0.000 |
| COPD | 1.460 | [0.74-2.89] | 0.278 |
| Mechanical Ventilation | 2.768 | [1.22-6.28] | 0.015 |
| Gram + | 0.554 | [0.27-1.15] | 0.111 |
| Fungi | 2.553 | [0.98-6.66] | 0.055 |
| Basophils cut-off (> 110 cells/mm3) | 2.320 | [0.98-6.66] | 0.048 |

**Table 126 (Abstract 0442)**. COX regression analysis in Derivation Cohort

| | HR | 95% CI | p-value |
|---|---|---|---|
| Age | 1.029 | [1.00-1.06] | 0.046 |
| APACHE II | 1.045 | [1.00-1.09] | 0.043 |
| Diabetes | 0.536 | [0.24-1.18] | 0.121 |
| Gram + | 0.564 | [0.32-0.99] | 0.045 |
| Basophils cut-off (> 110 cells/mm3) | 2.288 | [1.20-4.37] | 0.012 |

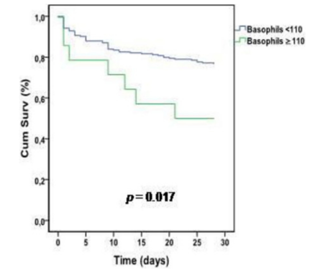

**Fig. 157 (Abstract 0442)**Kaplan-Meier analysis in Derivation cohort

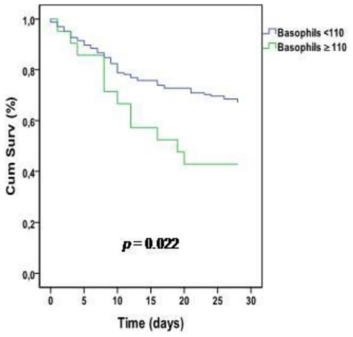

**Fig. 158 (Abstract 0442)**Kaplan-Meier analysis in Validation cohort

### 0443

#### Hypothermia and fever in sepsis - different responses associated with differences in mortality

D. Thomas-Rüddel[1,2], K. Reinhart[1,2], F. Bloos[1,2], for the MEDUSA Study Group
[1]Jena University Hospital, Anesthesiology and Intensive Care, Jena, Germany; [2]Jena University Hospital, Center for Sepsis Control and Care (CSCC), Jena, Germany
**Correspondence:** D. Thomas-Rüddel
*Intensive Care Medicine Experimental* 2017, **5(Suppl 2):**0443

**INTRODUCTION.** Fever has recently been reported to be associated with lower mortality in septic patients in the emergency department (1,2) and is still widely believed to be strongly associated with bacteremia (3).

**OBJECTIVES.** To assess the association of fever and hypothermia with bacteremia, different foci of infection and mortality in a large, mixed population of hospital and community acquired sepsis.

**METHODS.** We present a post hoc analysis from a 4 year multicenter quality improvement study where ICU patients with sepsis (all with organ dysfunction) were documented prospectively. The most pathological central body temperature within 24 hours after sepsis onset was recorded resulting in 7599 patients available for analysis.
**RESULTS.** Body temperature showed a bimodal distribution in patients with and without bacteremia. Lower body temperatures were associated with higher mortality (p < 0.001 in binary logistic regression). Fever was more frequent in pulmonary and urogenital sepsis (p < 0.001).
In a binary logistic regression analysis female sex, older age, negative blood cultures, lower procalcitonin values and abdominal or soft tissue and bones infections were independently associated with a hypothermic response, but only a small portion of variance (Nagelkerke R square 0.06) was explained by those factors.
**CONCLUSIONS.** While normothermia is rare, a hypothermic and a hyperthermic response can be observed in a mixed population with severe sepsis and septic shock. Fever is more frequent in bacteremic patients but this association is not as absolute as commonly believed. Further research should analyze which underlying factors trigger the possibly maladaptive hypothermic response in sepsis.

**REFERENCES**
1. Sundén-Cullberg et al., Crit Care Med 2017
2. Henning et al., Crit Care Med 2017
3. Linsenmeyer et al., J Hosp Med 2016

**GRANT ACKNOWLEDGMENT**
Financial support by the Federal Ministry of Education and Research (BMBF) via the Integrated Research and Treatment Center, Center for Sepsis Control and Care (CSCC, FKZ 01EO1002).

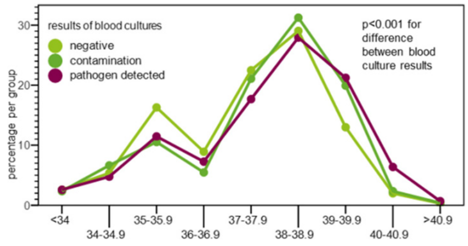

**Fig. 159 (Abstract 0443)** Temperature distribution by blood culture results

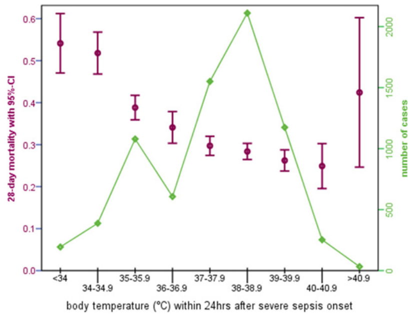

**Fig. 160 (Abstract 0443)** Frequency and associated mortality

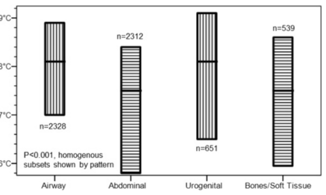

**Fig. 161 (Abstract 0443)**Temperature boxplot and focus of infection

### 0444

#### Derivation of a predictive model for in-hospital mortality in a UK emergency department cohort identified as potentially septic: the LUCA$^2$ score

D. Hargreaves[1], J. de Carvalho[1,2], L. Hodgson[3], R. Venn[1]
[1]Western Sussex Hospitals Foundation NHS Trust, Worthing Department of Anaesthesia and Intensive Care Medicine, Worthing, United Kingdom; [2]Brighton and Sussex Medical School, Brighton, United Kingdom; [3]University of Southampton, Southampton, United Kingdom
**Correspondence:** D. Hargreaves
*Intensive Care Medicine Experimental* 2017, **5(Suppl 2):**0444

**INTRODUCTION.** The qSOFA score has been proposed to assist identification of potentially septic patients at high risk of mortality presenting outside the ICU, but in external validation has been found to lack discrimination early on in the presentation of sepsis [1]. UK guidance for the emergency departments (EDs) recommends risk stratification of potentially septic patients using the National Early Warning Score (NEWS) and additional parameters [2] which can be time consuming in the context of a busy ED.
**OBJECTIVES.** To derive a parsimonious, readily calculated clinical prediction model (CPM) with practical utility in the ED to assist with rapid identification of those potentially septic patients at high risk of mortality.
**METHODS.** A dual centre ED study was performed using a prospective cohort of 1,552 patients (2014–16) identified at triage as potentially septic. Univariate analysis identified 15 variables associated with 30-day mortality (p < 0.01). Multiple imputation was used to adjust for missing values. Multivariate logistic regression in a stepwise backwards elimination method in 11 steps was performed until 6 variables remained. Area under receiver operator curves (AUROC) were constructed for the new predictive model and were compared to those for qSOFA and NEWS at the point of decision to admit to hospital (leaving the ED).
**RESULTS.** Six factors independently associated with mortality were weighted to generate a CPM: LUCA$^2$. Odds ratios and 95% confidence intervals were calculated (Fig. 162).
AUROC for LUCA$^2$ (95% CI) was 0.79 (0.72-0.81). Hosmer-Lemeshow demonstrated good fit (p = 0.291). This compared with an AUROC of 0.67 (0.65-0.69) for NEWS and 0.63 (0.61-0.65) for qSOFA (Fig. 163).
A cut-off of 4 points had the best sensitivity of 97% but poor specificity of 23%. At a cut-off of 5 points, sensitivity was 80%, sensitivity 68%, Positive predictive value 30% and Negative Predictive Value 95%. This compared with NEWS and qSOFA as follows (Fig. 164).
Though not included in the model, *absence* of chills was identified as a significant univariate predictor of mortality (p < 0.001) but because of negative association was excluded from the final score for simplicity of use.

**CONCLUSIONS.** We describe a new CPM that could be made automatable and assist the ED team in risk stratifying patients with signs of infection early in their presentation. Further internal and external studies are required to validate this score for clinical use.

**REFERENCE(S)**

1. Churpek M, Snyder A, Han X, Sokol S, Pettit N, Howell MD et al. Quick Sepsis-related Organ Failure Assessment, Systemic Inflammatory Response Syndrome, and Early Warning Scores for Detecting Clinical Deterioration in Infected Patients outside the Intensive Care Unit. *Am J Respir Crit Care Med* 2017 Apr 1;195(7):906–911.

2. National Institute for Health and Care Excellence. *Sepsis; early recognition, diagnosis and management.* (National guideline NG51). London: National Clinical Guideline Centre.; 2016 Jul.

| LUCA² Score Parameter | Points weighting | Odds Ratio | 95% Confidence Interval |
|---|---|---|---|
| **L** actate > 4 mmol/L | +3 | 2.83 | 1.58 - 5.07 |
| **U** rea > 10 mmol /L | +3 | 3.20 | 2.13 - 4.79 |
| **C** ongestive Cardiac Failure PMHx | +2 | 2.17 | 1.40 - 3.38 |
| **C** old <36 °C | +6 | 5.86 | 2.95 - 11.65 |
| **A** ge ≥ 65 years | +4 | 3.63 | 1.61 - 8.16 |
| **A** VPU less than alert | +3 | 2.94 | 1.19 - 7.30 |
| *TOTAL* | *21* | | |

**Fig. 162 (Abstract 0444)**: LUCA2 Score Odds Ratios

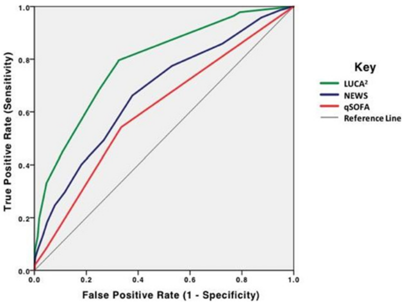

**Fig. 163 (Abstract 0444)**: AUROC Curve for LUCA2 Score

| SCORE & Cut-off | Sensitivity (95% CI) | Specificity (95% CI) | PPV (95% CI) | NPV (95% CI) |
|---|---|---|---|---|
| LUCA² ≥ 4 | 97% (92-99) | 23% (20-26) | 18% (17-19) | 97% (94-99) |
| LUCA² ≥ 5 | 80% | 68% | 30% | 95% |
| NEWS ≥ 5 | 69% (62-74) | 62% (59-65) | 27 (25-30) | 91% (89-92) |
| qSOFA ≥ 2 | 14% (10-19) | 96% (95-97) | 42% (32-52) | 85% (84-86) |

**Fig. 164 (Abstract 0444)**: LUCA2 Score Cut Offs

### Muscle mass preservation

#### 0445

#### Effects of protocol based physiotherapy and added physiotherapeutic measures on insulin sensitivity in critically ill patients with multiple organ failure

N.M. Carbon[1,2], T. Wollersheim[1,2], M. Krebs[1], K. Haas[1], R. Mörgeli[1], J. Spranger[2,3], S. Weber-Carstens[1,2]
[1]Charité Universitätsmedizin Berlin, Anesthesiology and Intensive Care Medicine, Berlin, Germany; [2]Berlin Institute of Health (BIH), Berlin, Germany; [3]Charité Universitätsmedizin Berlin, Endocrinology, Berlin, Germany
**Correspondence:** N.M. Carbon
*Intensive Care Medicine Experimental* 2017, **5(Suppl 2):**0445

**INTRODUCTION.** Intensive care-unit acquired weakness (ICUAW) is frequent (incidence as high as 80% [1]) in survivors of multi organ failure. Bioenergetic failure has been discussed as one of the possible mechanisms. Previous analyses indicate improved metabolic function in critically ill patients after protocol based physiotherapy [2].

**AIM.** To investigate the impact of different types of physiotherapy on glucose and lipid metabolism in skeletal muscle.

**METHODS.** We compare the effects between three groups of ICU patients (SOFA-Score ≥9 within 72h of ICU admission): standard physiotherapy (sPT), protocol based physiotherapy (pPT) and protocol based physiotherapy with added measures (pPT+). In the context of a randomized interventional trial the effects of pPT with and without additional measures (whole body vibration and electrical muscle stimulation) were compared to results of a previous trial with sPT alone in ICU patients and 4 healthy controls. Hyperinsulinemic euglycemic clamp (HE-clamp) was used to measure insulin sensitivity index (ISI). Before and during the maximal insulin stimulus of the HE-clamp baseline and flexibility of muscular metabolism was assessed as lactate, pyruvate and glycerol concentrations in *musculus vastus lateralis*

obtained by microdialysis (CMA 600, Mdialysis). MRC scoring on the first day of being adequately awake. Data shown as Median (25th/75th percentile). Ethic vote (Charité EA 2/041/10).

**RESULTS.** HE-clamp was performed in 50 severely critically ill patients with ICUAW (SAPS-II: 51(39/63), SOFA at admission: 13(10/14), MRC: 3.0(2.1/3.5)) in the groups sPT (n = 22), pPT (n = 8) and pPT+ (n = 20) on median day 17 of critical illness. First, ICU patients show a significant reduction in ISI compared to healthy controls without effect of the type of intervention (Fig. 165). Second, while healthy controls show a massive induction of the glycolytic pathway during insulin stimulus and glucose supply, ICU patients do not and remain metabolically inflexible (Fig. 166). Third, healthy controls and ICU patients show a significant suppression of lipolysis due to insulin stimulus measured by glycerol concentration (Fig. 167).

**CONCLUSIONS.** In our severely critically ill patients the type of physiotherapeutic intervention does not have any impact on systemic insulin sensitivity which is strongly reduced in ICU patients. This finding goes in line with a local metabolic inflexibility of the glycolytic pathway despite maximal insulin stimulus and glucose supply. Lipolytic energy delivery may play a role in skeletal muscle of ICUAW-patients. Lipolysis seems to be active and its regulation is sensitive to insulin in our cohort.

[1]Latronico N.,F1000Res,2014

[2]Patel et al. Chest 2014

**GRANT ACKNOWLEDGMENT**

BIH Twinning Research Grant 1.2.3; TW is BIH/Charité Clinical Scientist

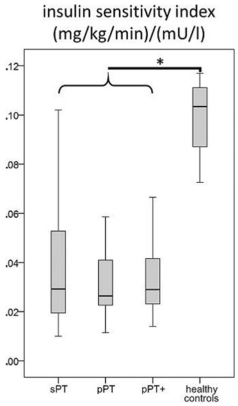

**Fig. 165 (Abstract 0445).** insulin sensitivity index, intervention groups: standard, physiotheraphy (sPT), protocol based physiotheraphy (pPT), protocol based physiotheraphy and added measures (pPT+). healthy controls shown fro comparison. *indicates p<0.05.

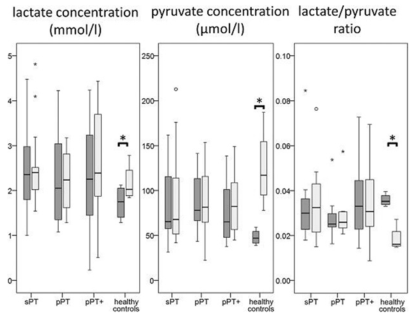

**Fig. 166 (Abstract 0445).** lactate and pyruvate concentration, lactate/pyruvate ratio in dialysate obtained by microdialysis of *m. vastus lateralis* before (dark grey) and during (light grey) insulin stimulus: there is no significant increase in muscle metabolism measured by metabolites and no effect of interventions standard physiotheraphy(sPT). protocol based physiotheraphy(pPT), protocol based physotheraphy and added measures (pPT+) healthy controls shown for compariosn. *indicates p<0.05

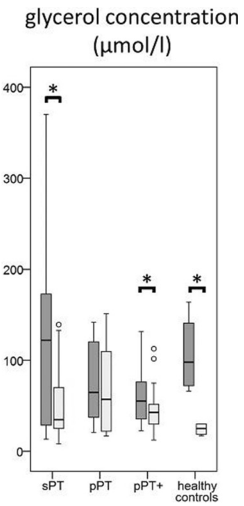

**Fig. 167 (Abstract 0445).** glycerol concentration in dialysate obtained by microdialysis of *m. vastus lateralis* before (dark grey) and during (light grey) insulin stimulus: there is a significant decrease in glycerol levels in healthy controls, standard physiotheraphy(sPT) and protocols based physiotheraphy and added measures (pPT+). *indicates p<0.05

### 0446

#### Association of body mass index and outcome in chronic hemodialysis patients requiring intensive care therapy

W. Druml[1], W. Winnicki[1], P. Metnitz[2], P. Zajic[2], T. Fellinger[3], B. Metnitz[3]
[1]Medical University of Vienna, Department of Medicine III, Division of Nephrology, Wien, Austria; [2]Medical University of Graz, Department of Anaesthesiology and Intensive Care Medicine, Division of General Anaesthesiology, Emergency- and Intensive Care Medicine, Graz, Austria; [3]Austrian Centre for Documentation and Quality Assurance in Intensive Care, Wien, Austria
**Correspondence:** W. Druml
*Intensive Care Medicine Experimental* 2017, **5(Suppl 2):**0446

**INTRODUCTION.** In patients with end-stage renal disease (ESRD) a positive association of body mass index (BMI) and outcome, the "obesity paradox" has been described. It remains unknown whether this association extends also to ESRD patients treated at an ICU.

**OBJECTIVES.** In a large group of intensive care patients we assessed whether a potential beneficial effect of a high body mass is seen in ESRD patients with critical illness.Methods and Participants: In a retrospective analysis of a prospectively collected data base of 82,323 patients from 98 Austrian intensive care units (ICUs) in whom BMI was available, in 9,869 patients with ESRD the association of 6 groups of BMI and outcome was assessed. Results were adjusted for severity of disease, age, sex and other acute and chronic comorbidities.

**MAIN RESULTS.** The 9,869 patients with ESRD were older, sicker, had a longer ICU stay and a higher ICU and hospital mortality. Within the group of ESRD patients a high BMI (>25) was associated with an improved survival in a multivariate analysis (BMI 25–30: OR 0.88, CI 0.77-0.99; 30–35: OR 0.8; CI 0.68-0.93; 35–40, OR 0.74, CI 0.6-0.92) but this was not seen in morbidly obese patients with a BMI > 40. The association was significant in patients with the highest disease severity (SAPS-3 Score > 56) but remarkably, also in those patients without systemic inflammatory response syndrome (SIRS) and those not requiring mechanical ventilation.

**CONCLUSIONS.** Also in ESRD patients who have acquired an acute intermittent disease and are admitted to an ICU an increased BMI is associated with an improved outcome. This association however, is not seen in morbidly obese patients with a BMI > 40. This improved tolerance to acute disease processes may in part explain the "obesity paradox" observed in ESRD patients.

### 0447

#### Does hypoxia play a role in the development of sarcopenia in humans? Mechanistic insights from the Caudwell Xtreme Everest expedition

L. Wandrag[1], M. Siervo[2], H.L. Riley[3], M. Khosravi[4], B.O. Fernandez[3], C.A. Leckstrom[3], D.S. Martin[4], K. Mitchell[5], L. Denny[6], H. Montgomery[4], M. Mythen[4], M. Stroud[7], M.P. Grocott[4], M. Feelisch[5], Caudwell Extreme Everest Research Group
[1]Imperial College London, London, United Kingdom; [2]University of Newcastle, Newcastle, United Kingdom; [3]University of Warwick, Coventry, United Kingdom; [4]University College London, London, United Kingdom; [5]University of Southampton, Southampton, United Kingdom; [6]Southampton NIHR Respiratory Biomedical Research Unit, Southampton, United Kingdom; [7]University Hospital Southampton NHS TrustS, Southampton, United Kingdom
**Correspondence:** L. Wandrag
*Intensive Care Medicine Experimental* 2017, **5(Suppl 2):**0447

**INTRODUCTION.** Sarcopenia refers to the involuntary loss of skeletal muscle and is a predictor of physical disability and mortality. Its pathogenesis is poorly understood, although roles for altered hypoxic signalling, oxidative stress, adipokines and inflammatory mediators have been suggested. Sarcopenia also occurs upon exposure to the hypoxia of high altitude.

**OBJECTIVES.** Using data from the Caudwell Xtreme Everest expedition (1) we therefore sought to analyse the extent of hypoxia-induced body composition changes and identify putative pathways associated with fat-free mass (FFM) and fat mass (FM) loss.

**METHODS.** After baseline testing in London (75m), 24 investigators ascended from Kathmandu (1,300m) to Everest base camp (EBC 5,300m) over 13 days. Fourteen investigators climbed above EBC, eight of whom reached the summit (8,848m). Assessments were conducted at baseline, during ascent and after one, six and eight week(s) of arrival at EBC. Changes in body composition (FM, FFM, total body water, intra- and extra-cellular water) were measured by bioelectrical impedance. Biomarkers of nitric oxide and oxidative stress were measured together with adipokines, inflammatory, metabolic and vascular markers.

**RESULTS.** Participants lost a substantial, but variable, amount of body weight (7.3 ± 4.9 kg by expedition end; p < 0.001). A progressive loss of both FM and FFM was observed, and after eight weeks, the proportion of FFM loss was 48% greater than FM loss (p < 0.008). Changes in protein carbonyls (p < 0.001) were associated with a decline in FM whereas 4- hydroxynonenal (p < 0.001) and IL-6 (p < 0.001) correlated with FFM loss. GLP-1 (r = −0.45, p < 0.001) and nitrite (r = −0.29, p < 0.001) concentration changes were associated with FFM loss. In a multivariate model, GLP-1, insulin and nitrite were significant predictors of FFM loss while protein carbonyls were predicted FM loss.

**CONCLUSIONS.** The putative role of GLP-1 and nitrite as mediators of the effects of hypoxia on FFM is an intriguing finding. If confirmed, nutritional and pharmacological interventions targeting these pathways may offer new avenues for prevention/treatment of loss of skeletal muscle at high altitude and in susceptible patient groups including older patients, critically ill patients, or in those susceptible to hypoxia as a result of cardiovascular or pulmonary disease.

GRANTS
Xtreme Everest funding see http://archive.xtreme- everest.co.uk/ for details. MF acknowledges ongoing support by the UK Medical Research Council (G1001536).

### 0448

#### Body hydration as a confounder of muscle mass as assessed by bioelectrical impedance analysis in critically ill patients: a prospective cohort study

N. Denneman, M.C. de Waard, S.N. Stapel, H.M. Oudemans-van Straaten
VU University Medical Center, Intensive Care, Amsterdam, Netherlands
**Correspondence:** N. Denneman
*Intensive Care Medicine Experimental* 2017, **5(Suppl 2):**0448

**INTRODUCTION.** Assessment of muscle mass in intensive care (IC) patients remains a challenge. Bioelectrical impedance analysis (BIA) is a validated method to assess muscle mass in people with fluid homeostasis. However, IC patients often have an abnormal fluid balance.

**OBJECTIVES.** To determine the confounding effect of fluid balance and body hydration on the change in BIA-derived muscle mass (ΔMM) during IC admission.

**METHODS.** Prospective observational study in 46 adult IC patients, with BMI ≤ 35 kg/m$^2$. Cum fluid balance, total body water (TBW = (height(cm)$^2$/resistance)*0.713)[1,2], and BIA-derived phase angle and muscle mass (BIA 101 Anniversary, Akern®, Italy) were measured at day 1, 3 and 7. Analysis of variance (ANOVA), univariate regression analysis and multivariate regression analysis using the stepwise backward method were performed.

**RESULTS.** Mean age 63 ± 16 years, APACHE II score 23 ± 8, SOFA score$_{admission}$ 9 ± 3. Median cum fluid balance (L) was significantly different between day 1, 3 and 7. Mean phase angle (°), muscle mass (kg) and total body water (L) were not different between day 1, 3 and 7 (Table).

ΔMM day 1–3 was positively associated with ΔTBW ($R^2$ = 0.63; p < 0.001) but not related to Δcum fluid balance ($R^2$ = 0.03; p = 0.28). ΔMM day 1–7 showed positive associations with both ΔTBW ($R^2$ = 0.43; p < 0.001) and Δcum fluid balance ($R^2$ = 0.40; p < 0.001). ΔMM day 1–3 and ΔMM day 1–7 were not significantly associated with SOFA (day 1–3 p = 0.51; day 1–7 p = 0.38) or APACHE II score (day 1–3 p = 0.91; day 1–7 p = 0.19). ΔPhase angle (odds ratio (OR) = 2.41-3.68; $p < 0.001$) and ΔTBW (OR = 0.31-0.51; $p < 0.001$) appeared as independent explanatory variables of ΔMM day 1–3 ($R^2 = 0.93$), and day 1–7 ($R^2 = 0.87$; Δphase angle OR = 2.17-3.34; $p < 0.001$; ΔTBW OR = 0.18-0.33; $p < 0.001$) while Δcum fluid balance did not contribute.

**CONCLUSIONS.** Although mean BIA-derived muscle mass did not significantly change over the first week, individual changes were great. The change in muscle mass was explained by changes in total body water by more than two thirds, supporting the confounding effect of changes in body hydration on BIA-derived muscle mass. Future studies should focus on the validation of a muscle mass equation taking changes in body hydration into account.

**REFERENCE(S)**

[1]Biasioli S, ASAIO J 1993,39:596–601.

[2]Nyboer J, NRC on Aviation Medicine 1943, Report No 149.

**GRANT ACKNOWLEDGMENT**

No grants received.

**Table (Abstract 0448).** Results of BIA, hydration and fluid balance

| | Day 1 | Day 3 | Day 7 | P-value (ANOVA) |
|---|---|---|---|---|
| Phase angle (°) | 4.7 ± 1.6 | 4.2 ± 1.5* | 4.1 ± 1.8 | 0.10 |
| ΔPhase angle | | -0.45 ± 1.33 | -0.49 ± 1.48 | |
| MM (kg) | 34.7 ± 8.7 | 34.7 ± 8.7 | 33.4 ± 9.2 | 0.46 |
| ΔMM | | 0.10 ± 6.93 | -0.23 ± 6.27 | |
| TBW (L) | 51.8 ± 13.6 | 56.3 ± 14.5* | 53.3 ± 17.7 | 0.09 |
| ΔTBW | | 4.41 ± 10.15 | 2.93 ± 16.82 | |
| Cum fluid balance (L) | 2.03[0.63-4.18] | 5.78[3.98-8.72]* | 6.21[1.71-11.26]* | <0.01 |
| ΔCum fluid balance | | 3.65 ± 4.02 | 3.19 ± 6.04 | |

Data as mean ± SD or median [IQR]. Δ = change day 1–3 or day 1–7 respectively, MM = muscle mass, TBW = total body water. *p < 0.05 vs. day 1.

### 0449

#### Perioperatively acquired weakness

G. Lachmann[1], N.M. Carbon[1], S. Kuenz[1], S. Weber-Carstens[1,2], C. Spies[1], B. Study Group[1], T. Wollersheim[1,2]

[1]Charité Universitätsmedizin Berlin, Berlin, Germany; [2]Berlin Institute of Health (BIH), Berlin, Germany

**Correspondence:** T. Wollersheim

*Intensive Care Medicine Experimental* 2017, **5(Suppl 2):**0449

**INTRODUCTION.** Intensive Care Unit Acquired Weakness (ICUAW) is a common complication in critically ill patients and develops early during ICU stay (1). Even 2 h of mechanical ventilation in healthy patients leads to weakness and muscle protein degradation in respiratory muscle (2). Hyperglycemia is a major risk factor for ICUAW and is frequently seen perioperatively.

**OBJECTIVES.** Our aim is to investigate whether perioperatively acquired weakness occurs and the impact of glucose metabolism.

**METHODS.** We analyzed a subgroup of 19 patients enrolled in a prospective, ethically approved (EA2/092/14), observational study undergoing elective non-thoracic surgery of at least 60 min. Lung function respectively hand grip strength (dominant hand) were assessed preoperatively, as well as the day after surgery by Spirometry (*Pneumotrac, Vitalograph)* respectively Dynamometer *(SH5002, SAEHAN)*. Data of vital capacity (VC), forced expiratory volume in 1 second (FEV1) and hand grip strength are shown as percentage of patients individually expected standard values. Blood glucose was measured every 20 min throughout the procedure via arterial blood gas analyses (*ABLflex 800, Radiometer*). Maximum, minimum, mean and variability by standard deviation (SD) were calculated for intraoperative blood glucose. Non-parametric Wilcoxon tests were performed. Data shown as median [25th/75th perc].

**RESULTS.** VC (p = 0.003) and FEV1 ($p < 0.001$) decreased significantly from preoperative to postoperative (median postoperative day 1 [1/3])

The FEV1/VC ratio remains unchanged (preoperative median 76% [68/87], postoperative 76% [69/82]; p = 0.286). Hand grip strength significantly decreased after surgery by 13.3% (Fig. 168). Surgical time was significantly positively correlated with FEV1 decrease (p = 0.012) and hand grip strength decrease (p = 0.028) whereas no influence was seen on decrease of VC (p = 0.085). Furthermore, perioperative glucose metabolism as characterized by maximum (144 mg/dL [128/195]), minimum (112 mg/dL [93/132]), and mean blood glucose concentration (128 mg/dL [114 /156]), as well as blood glucose variability (SD 11.9 mg/dL [7.4/20.4]) showed no impact on lung function and hand grip strength by correlation in our data.

**CONCLUSION.** Decreased lung function test by about 13% for VC and by about 18% for FEV1 may be explained in all probability by perioperative acquired respiratory muscle weakness. Unchanged FEV1/VC ratio makes obstructive reasons implausible. Furthermore, hand strength decreased by about 13% in our cohort. We could not find an impact of intraoperative blood glucose levels on the development of perioperative acquired reduced lung function or hand grip strength.

**ACKNOWLEDGMENT**

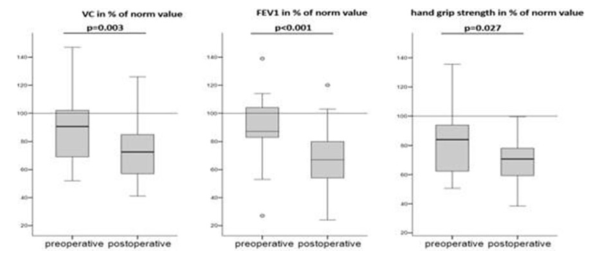

**Fig. 168 (Abstract 0449).** VC (vital capacity), FEV1 (forced expiratory volume in 1 second) and hand grip strength pre- and postoperatively for n = 19 patients

## Poster Corner Sessions Tuesday, 26 September 2017

### Elderly: Triage and outcome

### 0450

#### Factors influencing triage decisions in patients referred for ICU admission

M.L. Hariz, I. Chinna, J. AL Khan

BDF Hospital, Anaesthesia-ICU, Manama, Bahrain

**Correspondence:** M.L. Hariz

*Intensive Care Medicine Experimental* 2017, **5(Suppl 2):**0450

**INTRODUCTION.** Healthcare resource allocation refers to the distribution of healthcare resources among individuals and populations and encompasses rationing and triage. With more complex medical procedures, increasing patient age and expectations, and the increased severity of diseases, there is a greater demand for all medical services. Decisions on whether to admit a critically ill patient to ICU or not are complex, since they need to balance the potential risks and benefits for the individual patient with the limited bed availability and thus the implication for future patients. Unfortunately, the indications for admission to ICU remain poorly defined, and the identification of patients who can benefit from intensive care is extremely difficult.

**OBJECTIVES.** The objective of this study is to evaluate factors influencing triage decisions among patients referred for ICU admission and to assess its impact in outcome.

**METHODS.** A single-center, prospective, observational study of 252 consecutive triage evaluations was conducted in patients referred for ICU admission that were either accepted, or refused and treated on the medical or surgical wards.

**RESULTS.** One hundred ninety-eight patients (78.6%) were accepted for ICU admission. The majority of referrals were of medical patients (62.3%), followed by surgical (22.2%), trauma (10.7%) and obstetrics and gynaecology (4.7%). The chief indications for referral were the need for cardiovascular and respiratory support. Mean Acute Physiology and Chronic Health Evaluation (APACHE)-II score was 19.5 (0–52) and 19.8 (4–50) for accepted and refused patients, respectively. Younger age, chronic heart failure, and higher APACHE-II score at the time of evaluation were associated with acceptance of ICU admission with OR of 6.59, 12.01 and 6.69 respectively. Thirty-six (18.2%) died in ICU, while the in-hospital mortality for refused patients was 31.5%.

**CONCLUSION.** The majority of referred patient was admitted to ICU. Refusal of ICU admission was associated with higher mortality. Further efforts are needed to define which patients are most likely to benefit from ICU admission. Triage protocols or guidelines to promote efficient critical care beds use are warranted.

1.Guidelines for intensive care unit admission, discharge, and triage. Task Force of the American College of Critical Care Medicine, Society of Critical Care Medicine. Crit Care Med. 1999;27(3):633–638.

### 0451

### Comparative analysis: elderly patient in the intensive care unit. (75–79) vs (80–90) years

J. Higuera[1], M. Aroca[2], G. Narváez[2], D. Cabestrero[2], S. Saéz[2], R. De Pablo[2]
[1]Hospital Ramón y Cajal, Intensive Care Medicine, Madrid, Spain;
[2]Hospital Ramón y Cajal, Intensive Care, Madrid, Spain
**Correspondence:** J. Higuera
*Intensive Care Medicine Experimental* 2017, **5(Suppl 2):**0451

**INTRODUCTION.** Historically, intensivists have took age as a determinant factor in a patient´s admission to intensive care units. However, the increase in life expectancy, the development in chronic treatments and the parallel improvement of ventilation techniques, renal replacement therapy, and drugs with lower adverse effects... have made us think that elderly patients may not present greater comorbidities only in relation to his age.

**METHODS.** Our goal is to perform a comparative retrospective analysis of patients aged 75 years and above. We performed a comparative study between patients with 75–80 years old and 80–90 years between April 2013 - August 2016. We describe the incidence of mechanical ventilation(MV), renal replacement therapy (RRT), vasopressors drugs (VD) and mortality in both groups and then, we compare them to each other.

**RESULTS.** A total of 157 patients older than 75 years (75–90) were included. 45.9% Women 54.1%Men. Age 79.87 ± 3.6 (75–90) SOFA 8.21 ± 8.12 (1–80) APACHE II20.84 ± 8.7 (4–47) SAPS II54.7 ± 18.47(2–100). Tabulated diagnostic: cardiovascular (26/157) Gastrointestinal (15/157) Infectious (23/157) Metabolic (6/157) Neurological (29/157) Surgical (15/157) Respiratory (40/157) Traumatology (3/157).

7% of patients required RRT (11/157), 63%MV (99/157), and 62%VD (98/157). The mortality rate was 33% (53/157) with limitation of the therapeutic effort (LTE) in 12% of the cases (19/157).

Performing non-parametric tests, higher mortality was observed in elderly patients requiring MV (P < 0.001)and in those requiring VD (P < 0.02)

In the75-79years group, a total of 81 patients were collected.43.2%-Women. Age 76.98, SOFA 8.81 APACHE II 21.95 SAPS II 54.32.

9.8% of the patients in this group required RRT (8/81), 65.3%MV (53/81) and 64.2%VD (52/81). The mortality was 33.3% (27/81) with LTE in 12.3% (10/81).

Performing non-parametric tests, higher mortality was observed in elderly patients whose age ranged from 75–79 years and required MV. P < 0.0001.

In the group of 80–90 years. 76 patients have been studied. 48.7% Women. Age 83; SOFA7.56; APACHE II19.65; SAPS II55;

1.3% of the patient's required RRT (1/76), 60.5% MV (46/76), and 60.5% VD (46/76). Mortality rate was 34% (26/76) with LTE in 11.8%(9/76)

Performing non-parametric tests, a higher mortality was observed in elderly patients aged 80–90 years who require MV P < 0.0001 and in those who require VD. P < 0.03.

Performing a comparative statistical analysis between both groups, we found differences in mean age in both groups 76.98vs83. (P < 0.0001). Performing chi-square analysis, we did not observe statistically significant differences in the use of MV, VD and RRT in both groups. There were also no significant differences in mortality in any of the groups.

**CONCLUSIONS.** The comparative analysis between patients aged 75–79 years vs 80–90 years does not show significant differences in mortality, use of MV, VD and RRT.

The entry of older patients 80–90 years does not equate to a worse prognosis compared to the elderly 75–80 group.

### 0452

### Chronic critically ill patients admitted to the ICU of two different social level Hospitals in São Paulo, Brazil: an overview of their socio-economic status, emotional disorders and quality-of-life

R.R.L. Fumis[1], I. de Castro[2], A.B. Ferraz[3], H.S.B. de Oliveira[1], M. Moock[3], J.M. Vieira Junior[1]
[1]Hospital Sírio-Libanês, Intensive Care Unit, São Paulo, Brazil; [2]Hospital Sírio-Libanês, São Paulo, Brazil, [3]Hospital Geral do Grajaú, Intensive Care Unit, São Paulo, Brazil
**Correspondence:** R.R.L. Fumis
*Intensive Care Medicine Experimental* 2017, **5(Suppl 2):**0452

**INTRODUCTION.** The chronic critically ill (CCI) comprise a growing population of patients who have survived acute critical illness. There are few studies about their characteristics as well as their emotional, social status and quality-of-life.

**OBJECTIVES.** To assess the characteristics of CCI patients and their family members admitted to the ICU of two different social level hospitals in São Paulo, Brazil and to identify their socio-economic status, emotional disorders and quality-of-life.

**METHODS.** A prospective study was conducted in two hospitals of different social levels. Patients were defined as CCI after a stay of at least 8 days in the ICU plus one of the six eligible clinical conditions: mechanical ventilation for at least 96 hrs; tracheotomy; sepsis; severe wounds; stroke; traumatic brain injury [1]. Family members filled out the Hospital Anxiety and Depression Scale (HADS) and the WHOQOL-BREF. Regarding patients, family members also responded the EQ-5D and KATZ index of patients at the ICU, 30-and 90-days after first assessment.

**RESULTS.** Between May 2015 and March 2017, 100 patients from a hospital in the central region and 86 from a hospital in the suburbs, fulfilled the CCI criteria and participated in this study. Patients from the suburban hospital were younger (59 vs 69 years old; p < 0.001), had a similar median SAPS3 (55 vs 54; p = 0.689) and Charlson index (1.0 vs 2.0; p = 0.059). The difference was found in the ICU mean length of stay (21 vs 16; p = 0.006) and in the median days on mechanical ventilation (13 vs 9; p = 0.026).Furthermore, patients were more often submitted to tracheostomy in the suburban hospital (42% vs 16%; p < 0.0001). The ICU mortality was higher in the suburban no hospital (32.6% vs 17.2%, p = 0.026) but with no difference in hospital mortality (38.1% vs 35.1%, p = 0.837). No major difference was found in EQ-5D (p = 0.651). Regarding family members, in the suburban hospital there were more protestants than catholic (p < 0.001), their monthly family income was less than $1,000 compared to that of the central region (>$ 2,400; p < 0.001), 32.6% were unemployed vs 1.0%, p < 0.001. Presented worse scores in all domains of WHOQOL-BREF [Psychological (70 vs 75, p = 0.032), Social Relationships (75 vs 83, p < 0.001), Environment (62 vs 87, p < 0.001) and Physical Health (71 vs 78, p = 0.011)] and had a higher HADS score (p = 0.003). The difference in Physical Health remains similar in 30-and 90-days after first assessment.

**CONCLUSIONS.** It was observed that the CCI patients' outcomes are the same, regardless of socio-economic differences. Their characteristics are similar, that is, they had similar comorbidities and severity. However, family members with a lower socio- economic level had high HADS scores and worse quality of life.

**GRANT ACKNOWLEDGMENT**

FAPESP Support Process 15/17687-4

### 0453

#### Outcomes and prognostic factors in old and very old patients admitted to Intensive Care Unit

M.C. Rebelo[1], J. Louro[2], A.G. Almeida[1], T. Lamas[1], I. Gaspar[1], I. Simões[1], E. Carmo[1]

[1]Centro Hospitalar de Lisboa Ocidental- Hospital Egas Moniz, Unidade de Cuidados Intensivos Polivalente, Lisbon, Portugal; [2]Hospital de Vila Franca de Xira, Vila Franca de Xira, Portugal

**Correspondence:** M.C. Rebelo

*Intensive Care Medicine Experimental* 2017, **5(Suppl 2):**0453

**INTRODUCTION.** Long-held assumptions of poor prognoses for elderly patients have meant that physicians have been reluctant to admit them to the Intensive Care Unit (ICU). Heterogeneity of this elderly population need a broader geriatric assessment and adequate evaluation of the burden of comorbidities and critically ill disease on prognosis.

**OBJECTIVES.** The objective of the present study was to analyze the characteristics of old and very old patients admitted to an ICU, identify predictors for ICU mortality and assess the relative effects of age on ICU outcome.

**METHODS.** A single-center, retrospective cohort study was carried out among geriatric patients, aged 75 years or older, admitted to an ICU of a Tertiary Hospital between 1 January 2008 and 31 December 2016. Patients who underwent uncomplicated elective surgery were excluded from the study. The study population was divided in two groups: old patients (≥74 to 80) and very old patients (≥80 years old). Data acquired included: patient characteristics, reason for ICU admission, Hospital length of stay prior to admission (HLOS), comorbidities, SAPSII, modified SAPSII (without age) and APACHE II. Laboratory values, organ failures, and level of organ support were recorded on ICU admission. Predictors for ICU mortality were evaluated using uni- and multivariate analysis.

**RESULTS.** A total of 667 elderly patients (old patients: 294, very old: 373) were admitted to ICU during the study period. The mean APACHE II (p = 0.023), SAPSII (p < 0001) and modified SAPSII (p = 0.012) were significantly higher in the very old group. The mean HLOS prior to admission was 14 ± 35 days, the mean ICU length of stay was 13.6 ± 19.8 and there were no significant differences between the two groups. ICU mortality was higher in the very old group (33.9% vs 41.6%, p = 0.039). When comparing patients who survived with those who didn't survive, there were significant differences in APACHE, SAPSII and modified SAPSII (all p < 0.001), age > 80 (p = 0.039), Chronic Liver disease (CLD) (p = 0.003), renal replacement therapy (p = 0.002), Organ dysfunction and Malignancy (both p < 0.001). By multivariate analysis, factors identified as associated with ICU mortality were modified SAPS II (p < 0.001, OR 1,069, IC95%: 1.056-10.82), CLD (p < 0.007, OR 17,88, IC95%: 1.918-166.5); Hematologic disease (p = 0.009, OR 0,363; IC95%: 0.170-0.774), Malignancy (p = 0.03, OR: 2.330; IC95%: 1,338-4,057) and organ dysfunction prior to admission (p = 0.015, OR: 1.676; IC95%: 1,106-2,542).

**CONCLUSIONS.** Our findings suggest that old age per se does not markedly influence ICU mortality, while other factors, such as disease severity and comorbidities (CLD, Hematologic disease and Malignancy) appear to be responsible for the poorer prognosis. More appropriate admissions decisions based on shared decision-making and improved prediction models that include comorbidities and functional status are needed for this particular patient population.

### 0454

#### 4th age at ICU: a reality

D. Iglesias Posadilla, O. Badallo Arévalo, R. Vara Arlanzón, C. Carbajales Pérez, E.M. Pérez Cabo, P. De La Torre Vélez, M.E. Perea Rodríguez, M.E. Martinez Barrio, M. Del Valle Ortíz, S.A. Ossa Echeverri, M. Montero Baladia, M. Gero Escapa, L. Fisac Cuadrado, M. Martínez Barrios, F. Callejo Torre, M. Arroyo Díez, A. Zabalegui Pérez, J.A. Fernández Ratero

Hospital Universitario de Burgos, Intensive Care Department, Burgos, Spain

**Correspondence:** D. Iglesias Posadilla

*Intensive Care Medicine Experimental* 2017, **5(Suppl 2):**0454

**INTRODUCTION.** Very old patients´ admission at ICU are growing in the last years due to the aging of the population.

**OBJECTIVES.** To study the clinical mortality and ICU supports of the very old aged patients (>80 years old - 4th age-) in comparison to middle old aged (65–80 years old -3rd age-) or youngers, admitted to the Intensive Care Unit of the Burgos University Hospital during 2015 and 2016.

**METHODS.** We performed a descriptive observational study that was approved by the Ethics Committee of Burgos. The main interesting variables: demographic data and admission diagnoses, APACHE II, procedures, ICU length of stay and ICU and hospital´s mortality. The continuous variables are described as mean (SD), using T-Student Test to compare them, or medians (IQR); the qualitative ones are written as rates, and were compared using Chi-Square Test.

**RESULTS.** During the period 01/2015 to 12/2016, 2587 patients were admitted to ICU. 961 (37,15%) of those belong to 3rd age group and 480 (18,55%) to the 4th age group, being male 68,54% and 57,50% respectively. Within the 4th age, 167 (34,8%) were extremely old (>85 years old).

The 4th age patients were admitted due to coronary heart disease more than the 3rd age (34,17% vs 25,70% p =0,001).

The severity measured by APACHE II scale was 18,07 (SD 8,74) for the 3rd age and 19,15 (SD 9,21) for the 4th age, presenting no significant difference when APACHE was corrected by age (p = 0,43).

The mean stay was 5,41 days (SD 8,75) in < 65 years old, 6,61 (SD 11,73) for the 3rd age and 4,96 (SD 9,28) for the 4th age, appearing significant difference in this last two (p = 0,007). The extremely old had less stay (3,87) tan the very old ones (5,54), nearly touching the statistical significance (p = 0,06).

Invasive mechanical ventilation (IMV) was needed by 38,18% of the 4th age against to 47,37% in the 3rd age, difference that turned out statistically significant (p < 0,001). About the IMV length there were no statistically significant differences (2,94 vs 3,85) (p = 0,11), even though < 65 years. The use of non-invasive ventilation (NIVM) was similar in both groups 20,60% (3rd age) vs 25,18%(4th age) (p = 0,07). Both groups had a highest use of NIMV (22,10%) compared with the youngers (11,88%) (p < 0,001).

The ICU mortality was 14,46% for the 3rd age, meanwhile for the 4th age was 17,71% (15,97% very old and 20,35% extremely old) without statistical difference (p = 0,11). However, hospital mortality was 21,23% for the 3rd age and 30% for the 4th age. By causal disease, in the 4th age patients were found greater mortality in those after trauma (with or without TBI) (100% vs 16,67% p = 0,003; 50% vs 9,09% p = 0,048) and after surgery (17,72% vs 7,14% p = 0,006).

**CONCLUSIONS.** According to our experience, the correct selection at admission to ICU of very old age patients could involve acceptable survival rates for that ages, without using more resources and longer ICU´s stays.

**GRANT**
This study did not receive any grant from any funding agency.

**Table (Abstract 0454)**. Results

| | 3rd age | 4th age | p |
|---|---|---|---|
| Coronary heart disease | 25,70% | 34,17% | 0,001 |
| APACHE II | 18,07 (SD 8,74) | 19,15 (SD 9,21) | 0,033 |
| APACHE II corrected (– age) | 12,75 (SD 8,70) | 13,15 (SD 9,21) | 0,432 |
| LOS | 6,61 (SD 11,73) | 4,96 (SD 9,28) | 0,007 |
| IMV (%) | 47,37% | 38,18% | <0,001 |
| IMV length | 3,85 (SD 9,04) | 2,94 (SD 8,37) | 0,11 |
| NIMV (%) | 20,60% | 25,18% | 0,001 |
| ICU Mortality | 14,2% | 17,5% | 0,11 |
| Hospital Mortality | 21,23% | 30% | <0,001 |

### 0455

#### Elderly patients undergoing whipple operation: resource utilization and postoperative complications in intensive care unit

C.-T. Lee, Y.-C. Yeh, A. Chao
National Taiwan University Hospital, Taipei, Taiwan, Province of China
**Correspondence:** C.-T. Lee

*Intensive Care Medicine Experimental* 2017, **5(Suppl 2):**0455

**INTRODUCTION.** The risk of developing pancreatic cancer increases with age and the Whipple operation remains the most effective treatment for patients with a localized periampullary neoplasm. With the advance of medicine and aging population, more elderly patients of pancreatic malignancy are undergoing the Whipple operation, even it is still one of the most complex gastrointestinal surgeries. Although several studies showed that Whipple operation could be carried out safely in elderly patients with acceptable results, elderly patients may still present challenges in their postoperative period [1].

**OBJECTIVES.** The purpose of this study is to investigate intensive care unit (ICU) resource utilization in elderly patients undergoing the Whipple operation.

**METHODS.** This is a retrospective study reviewing 167 patients undergoing Whipple operation consecutively between January 2011 and December 2013. Enrolled patients were categorized into two groups: the “Young group” (<75 year-old) and the “Elderly group” (≥80 year-old). Patients demographic, comorbidity, preoperative, intraoperative and postoperative data were collected from the medical records of the patients. The main outcomes were postoperative complications, ICU length of stay, hospital length of stay, and mechanical ventilator hours.

**RESULTS.** There were 133 patients in the Young group (mean age 62.0 year-old) and 34 patients in the Elder group (mean age 78.8 years). Basic characteristics were similar in the two groups, despite higher American Society of Anesthesiologists.classification in the Elder group ($p = 0.002$).

Intra-operative outcomes were all similar in the two groups.

Although postoperative complications were more frequently occurred in the elder group, especially the incidence of delayed gastric emptying ($p = 0.001$) and the incidence of pneumonia/ARDS ($p = 0.029$), only the mechanical ventilation hours ($p = 0.004$) was higher in the elder group($p = 0.055$). The rest of ICU resource utilization were not statistically significant higher than the young group (Table 129).

**CONCLUSIONS.** The present study demonstrated that patients aged 75 years or elder undergoing the Whipple operation required increased medical resources in treating airway and pulmonary complications even they have similar intra-operative outcomes compared with the younger counterpart. Careful preoperative evaluation and meticulous perioperative managements, especially focus on respiratory system, are mandatory to decrease morbidity and ICU resource utilization.

**REFERENCE(S)**

1. Beltrame V, Gruppo M, Pastorelli D, Pedrazzoli S, Merigliano S, Sperti C. Outcome of pancreaticoduodenectomy in octogenarians: Single institution´s experience and review of the literature. J Visc Surg. 2015;152(5):279–284.

**GRANT ACKNOWLEDGMENT**
This study was supported, in part, by grants from the National Taiwan University Hospital (NTUH 104-S2808).

**Table 127 (Abstract 0455)**. Patient demographics and preoperative data

| | Young group (N=133) | Elder group (N=34) | P-value |
|---|---|---|---|
| Age (years, mean(SD)) | 57.7(12.3) | 78.8(3.81) | <0.001 |
| Gender (male/female) | 69/64 | 18/16 | 0.912 |
| ASA (I and II/III and IV) | 58/75 | 5/29 | 0.002 |
| Underlying CAD (n,%) | 8(6) | 5(15) | 0.142 |
| Underlying COPD (n,%) | 6(5) | 2(6) | 1.0000 |
| Underlying renal insufficiency (n,%) | 4(3) | 0(0) | 0.583 |
| Underlying cirrhosis (n,%) | 6(5) | 2(6) | 0.666 |
| Baseline hemoglobin (g/dl, mean(SD)) | 12.7(2.0) | 12.0(1.9) | 0.089 |
| Baseline albumin(g/dL, mean(SD)) | 4.2(0.5) | 3.8(0.6) | 0.001 |

**Table 128 (Abstract 0455)**. Intra-operative outcomes

| | Young group (N=133) | Elder group (N=34) | P-value |
|---|---|---|---|
| Operation duration (mins, mean(SD)) | 291(63) | 284(46) | 0.540 |
| Blood loss (ml, mean(SD)) | 488(337) | 397(249) | 0.145 |
| Packed RBC transfusion (ml, mean(SD)) | 103(197) | 141(184) | 0.305 |
| FFP transfusion (ml, mean(SD)) | 390(370) | 498(321) | 0.093 |
| Platelet transfusion (ml, mean(SD)) | 158(1043) | 0(0) | 0.380 |
| Total fluid intake (ml, mean(SD)) | 2701(948) | 2462(726) | 0.173 |
| Fluid balance (ml, mean(SD)) | 1511(783) | 1507(703) | 0.980 |
| Urinary output (ml, mean(SD)) | 703(658) | 558(388) | 0.221 |
| Intraoperative lactate (mmol/L, mean(SD)) | 1.4(1.1) | 1.2(0.8) | 0.642 |

**Table 129 (Abstract 0455)**. Post-operative outcomes in SICU

| | Young group (N=133) | Elder group (N=34) | P-value |
|---|---|---|---|
| ICU admission APACHE-II (mean(SD)) | 6.9(3.7) | 10.3(3.0) | <0.001 |
| Mechanical ventilation (hours, mean(SD)) | 8.8(37.2) | 30.8(92.4) | 0.004 |
| length of ICU stay (day, mean(SD)) | 3.3(6.4) | 6.2(10.2) | 0.127 |
| Length of hospital stay (day, mean(SD)) | 26.8(16.4) | 30.9(14.5) | 0.178 |
| Hospital mortality (n, %) | 2(1.5) | 1(3.0) | 0.497 |
| Delayed gastric (n, %)emptying | 17(13) | 13(38) | 0.001 |
| Pneumonia/ARDS (n, %) | 9(7) | 7(21) | 0.029 |
| Bacteremia (n, %) | 9(7) | 2(6) | 1.000 |
| Bowel ischemia (n, %) | 1(1) | 0(0) | 1.000 |

### 0456

#### Intensive care of elderly patients with solid tumors: a retrospective observational study

L.U. Taniguchi[1,2], P.V. Mendes[1,2], E.M. Pires[1], J.M. Vieira Jr[1,3]
[1]Hospital Sírio-Libanês, Education and Research Institute, Sao Paulo, Brazil; [2]Hospital das Clínicas, Universidade de São Paulo, Emergency Medicine Discipline, Sao Paulo, Brazil; [3]Hospital Sírio-Libanês, Intensive Care Unit, Sao Paulo, Brazil
**Correspondence:** L.U. Taniguchi
*Intensive Care Medicine Experimental* 2017, **5(Suppl 2):**0456

**INTRODUCTION.** There is an increase demand for intensive care unit (ICU) admission of elderly oncological patients, but the outcome and factors related to hospital mortality in this subgroup of critically ill patients are still understudied. Our objective was to assess risk factors for hospital death in a cohort of elderly patients with solid cancer admitted to the ICU.

**METHODS.** This is a retrospective cohort study conducted at a private tertiary oncological hospital (Hospital Sírio-Libanês) at São Paulo, Brazil. We extracted relevant information from the adult ICU database (sistema Epimed™). All consecutive patients ≥ 65 years old with a diagnosis of active solid cancer were evaluated. We used univariate and multivariate analysis to identify risk factors associated with hospital mortality.

**RESULTS.** Between July 2012 to June 2016 we studied 1562 patients ≥ 65 years old who had cancer (hospital mortality of 17.1%). Patients who died were more severely ill compared to patients those who survived (SAPS 3 of 59 [52–68] versus 42 [36–53] respectively; $p < 0.001$), were older, more frequently came from the ward with longer hospital length of stay before ICU admission, had more comorbidities and worse performance status, higher prevalence of metastatic disease, more frequently required mechanical ventilation and vasoactive drugs as well as dialysis, and had a different pattern of primary tumor site. Independent risk factors for hospital mortality in our multivariate logistic model were higher SOFA score, admission source other than the operating room, longer length of stay before ICU transfer, invasive life support procedures, metastatic disease, primary tumor site and worse physiological parameters at ICU admission.

**CONCLUSION.** Most of risk factor for hospital death in our cohort were similar to non-oncological patients and reflect the severity of critical illness.

### 0457

#### Short-and long-term outcomes of very old patients admitted to intensive care unit

E. Marques Mendes, J.M. Pereira, C. Sousa Dias, T. Honrado
Centro Hospitalar São João, Serviço de Medicina Intensiva, Porto, Portugal
**Correspondence:** E. Marques Mendes
*Intensive Care Medicine Experimental* 2017, **5(Suppl 2):**0457

**INTRODUCTION.** As average life expectancy increases, the expectations of care delivered to the elderly population also rise.However, very old patients (≥80years) intensive care unite (ICU) admissions are often questioned by the presence of multiple comorbidities and previous impaired functional status.

**OBJECTIVES.** To describe the epidemiology and outcome of patients ≥80years admitted to an ICU.

**METHODS.** Retrospective analysis of very old patients consecutively admitted to the ICU in a portuguese university hospital between 01/01/2015 and 30/06/2016.

**RESULTS.** A total of 228 patients were admitted (~12% of total admissions), mostly male(53%) with a mean age of 84(±3,4) years. The majority of patients(92%) had ≥ 2 comorbidities, mainly congestive heart failure (17%) and chronic renal disease (17%) and were considered severe in 23% of them. ICU causes of admission were: medical (41%), urgent (39%) and elective surgery (10%) and trauma (10%). At admission, mean SAPS II score was 53,2(±16,5) with an expected hospital mortality of 50%.Mean SOFA score was 7,1(±3,6) and the most prevalent organ dysfunctions were respiratory(70%) and neurologic(70%). ICU and hospital mortality were 25% and 36% respectively.Mean ICU length of stay was 6,7(±6,7) days.After 30 days post-ICU discharge, 28% of patients regain their previous functional status.At 6 months, mortality was 53% and hospital readmission rate was 33%. In univariate analysis, ICU mortality was associated with total SOFA score at admission (9,3 vs 6,7;p < 0,01), cardiovascular SOFA ≥ 3 (35,7% vs 17,3%;p = 0,003), neurologic SOFA ≥ 3 (41,8% vs 16,4%;p < 0,001) and SAPS II (66 vs 51;p < 0,001) while 6 months mortality was associated with total SOFA (8,0 vs 6,6;p = 0,003), neurologic SOFA ≥ 3 (66,7% vs 48,3%;p = 0,011), SAPSII scores (59,6 vs 48,9;p < 0,001) and presence of severe comorbidities (71,4% vs 51,0%;p = 0,018). Multivariate analysis showed that SAPSII (OR 1,06; 95%CI 1,03-1,09) and SOFA (OR 1,13; 95%CI 1,01-1,27) scores and admission type (OR 1,74; 95%CI 1,00-3,04) were independently associated with ICU mortality. In addition, age(OR 1,13; 95%CI 1,02-1,24), SAPSII (OR 1,05; 95%CI 1,02-1,07), presence of severe comorbidities(OR 2,66; 95%CI 1,12-6,32) and frailty index (OR 0,86; 95%CI 0,75-0,99) were independently associated with 6-months. mortality.

**CONCLUSION.** Although a small percentage (25%) of very old patients died in ICU and hospital mortality was lower than predicted, a significant number of patients succumb or are readmitted to a hospital within a 6 months period.Nevertheless,28% regain their previous functional status 30 days after ICU discharge. SAPS II, SOFA score and admission type were associated with ICU mortality. Variables associated with 6 months-mortality were age, SAPSII, the presence of severe comorbidities and frailty index.

### 0458

#### Comparative analysis of mortality between elderly and non elderly sepsis and septic shock patients

A. Pérez González, D. Almudi Ceinos, S. Martin Alfonso, O. Lopez del Moral Lopez, J. Rico Feijoo, C. Aldecoa Alvarez- Santullano
Hospital Universitario Río Hortega, Valladolid, Spain
**Correspondence:** A. Pérez González
*Intensive Care Medicine Experimental* 2017, **5(Suppl 2):**0458

**INTRODUCTION.** Sepsis in a prevalent medical condition in critically ill patients with high mortality and significant consumption of health-care resources (1).

**OBJECTIVES.** To compare outcomes between elderly (≥65 years old) and non-elderly (<65 years old) patients with sepsis and septic shock in our ICU and determine short and long term mortality in both groups.

**METHODS.** Retrospective cohort study including 261 sepsis and septic shock patients admitted to the ICU of the the University Hospital of Río Hortega de Valladolid, for more than 48 hours between January 2011 to January 2017. Our study consisted of 261 patients, of whom 55 belonged to the group of < 65 years and 206 over 65 years old. We determined the following variables: age, divided into groups, sex, diagnosis of sepsis, severe sepsis or septic shock, SOFA scale at 24 h of admission and during the first 7 days of recovery, APACHE II scale at 24 h of admission, presence of comorbidities (two groups: < 2 comorbidities and > 2), acute renal failure, need for renal replacement therapy, need for mechanical ventilation, stay in resuscitation unit, mortality in ICU unit, at 28 days, 90 days and 1 year before.

**RESULTS.** Non-elderly patients accounted for 21,1% (55/261) and elderly patients for 78,9% (206/261). Elderly patients had a higher APACHE II score [18 (13–22,25) versus 14 (10–17); $p < 0.001$], and SOFA score at 24 H [7 (5–9), versus 6 (5–9) $p < 0.05$] compared to non-elderly patients, although the number of organ dysfunctions was not stadistically significant between both groups neither the number of days with organ dysfunction. Significant differences were found in 28-day (14,5% versus 32,5%), in-hospital mortality (18,2% Vs 35,4%),

90-day (20% Vs 37,9%) and 1 year mortality (27,3% Vs 47,6%) rates between non-elderly and elderly patients respectively.
Predictors of death among elderly patients included the number of organ dysfunctions, presence of acute kidney failure, heart disease, non hematological cancer and liver dysfunction.
**CONCLUSIONS.** Studies published so far of elderly critically ill patients show a death rate higher than 65%, reporting a mortality "greater than 92% at 6 months and 97% at 12 months in patients older than 85 years" according to P Biston (2). In our study, mortality was increased according to age, but also with some comorbidities wich may be an independent factor of mortality, and not only age as reported in other studies until the date. Elderly population will increase in the forthcoming years so new prospective studies for long-term impact on functional status and quality of life are necessary.
**REFERENCE(S).**
1. Martin S., Pérez A., Aldecoa C. Sepsis and immunosenescence in the elderly Patient: A Review, Front. Med. 4:20. doi: 10.3389/fmed.2017.00020
2. Biston P, Aldecoa C, Devriendt J, Madl C, Chochrad D, Vincent JL, et al. Outcome of elderly patients with circulatory failure. Intensive care medicine. 2014;40(1):50–6.

### 0459
### When the critic becomes chronic - what shall we do?
A. Clara, R. Bernardo, T. Gago, J. Moreno, S. Castro, C. Granja
Algarve Hospital Center, Faro, Portugal
**Correspondence:** A. Clara
*Intensive Care Medicine Experimental* 2017, **5(Suppl 2):**0459

**INTRODUCTION.** Up to 10% of critical ill patients may present a slow recovery developing chronic critical illness (CCI)[1]. Although there's no consensus in it's definition, it's known that CCI is a complex syndrome that involves metabolic, neuroendocrine, neuropsychic and immunological mechanisms[2]. This group of patients contribute to an increase in personal, family, financial and institutional burden and costs.
**OBJECTIVES.** To identify the main dysfunctions that are related to the increase of ICU length of stay and mortality.
**METHODS.** Observational retrospective study, with clinical information from and ongoing database. The study included patients admitted to the ICU between 1/1/2015 31/12/2015 and with a n ICU lenght of stay ≥ 14 days. Criteria for organ dysfunction was according to clinical criteria SAPS II e APACHE scores.
**RESULTS.** Of the 474 patients admitted to the ICU during that period, 72 patients (15.2%) were eligible for the study, 62.5% were females, with a mean age of 62.5 years old, with mean APACHE scores of 28 and SAPS II of 60. ICU mortality was 26%. Reason for admission: 44% medical, 39% non-scheduled surgery, 14% trauma and 3% elective surgery. The mean ICU length of stay was 24 days, with a mean time of invasive mechanical ventilation (IMV) of 18 days; 22 patients were submitted to tracheostomy between the 12th and 49th days of ICU stay; 48 patients underwent exclusive enteral feeding, 13 patients received exclusive parenteral nutrition and 10 patients underwent both. The identified dysfunctions that justified an increase in the ICU length of stay were respiratory (cRd) - 93%, neurological (cNd) - 44%, cardiovascular (cCd) - 29%, renal (cR'd) 18%, with patients presenting more than one dysfunction. Despite the large number of patients with cRd, patients with cNd presented a greater tendency to the need of tracheostomy ($p < 0.007$). The dysfunctions that best correlated with mortality, at ICU discharge or hospital discharge, were cCd ($p < 0.000$ and $p < 0.000$, respectively) and cR'd ($p < 0.013$ and $p < 0.009$, respectively).
**CONCLUSIONS.** The respiratory and neurological dysfunctions significantly increased the mean ICU length of stay. The cardiovascular and renal dysfunctions were significantly associated with mortality.
The need for traqueostomy was related with neurological dysfunction. Early identification of these organ dysfunctions might help us to identify CCI patients.
**REFERENCES**
1) Leung, D; *in* American Journal of Hospice and Palliative Medicine; May 2016;
2) Marchioni, A; in European Journal of Clinical Investigation; 2015

### 0460
### A retrospective study reviewing the mortality of emergency laparotomy patients admitted to the intensive care unit at heart of England foundation trust
A.S. Blaggan, J. Hares, N. Arora
Heart of England Foundation Trust, Birmingham, United Kingdom
**Correspondence:** A.S. Blaggan
*Intensive Care Medicine Experimental* 2017, **5(Suppl 2):**0460

**INTRODUCTION.** The National Emergency Laparotomy Audit (NELA) is a set of national clinical audit projects designed to provide trusts with reports on their compliance and performance against evidence based standards for patients undergoing emergency laparotomies[1]. It was introduced in 2012 to improve patient care following a high incidence of death following emergency laparotomy. This quality improvement project interrogates data from the Heart of England Foundation Trust (HEFT) and looks at mortality and the characteristics of patients who have underwent emergency laparotomy requiring intensive care in order to identify areas to improve patient care.
**OBJECTIVES.**
1) Number of emergency laparotomies requiring admission to the Intensive Care Unit (ICU) at HEFT
2) Length of stay in ICU
3) Number of laparotomy admissions resulting in death
4) Age of patients who died following surgery and ICU admission.
**METHODS.** Data (n = 316) consisted of all patients admitted to ICU following emergency laparotomy between the years 2015 and 2016 from two sites across HEFT. All information on patient age, type of surgery, admission time, discharge time and mortality were collected from patient notes and compiled onto a spreadsheet. Analyses were conducted using descriptive statistics.
**RESULTS.** During 2015 and 2016 there were 316 emergency laparotomies requiring ICU admission. Small bowel and large bowel pathology accounted for 58% (n = 173) of cases. Average length of stay was 88.6 hours but lower for large (72.7 hours) and small bowel (85.4 hours) conditions compared to non-bowel pathology (100.4 hours). Overall ICU mortality was 11.0% (n = 33) but had increased from 8.2% in 2015 to 13.3% in 2016. Small bowel laparotomy resulted in the greatest mortality (14.1%) followed by non-bowel (11.1%) and large bowel procedures (6.8). 60% of those who died were admitted out of hours. 63% of all patients who died were above 71 years of age.
**CONCLUSIONS.** Patients aged over 70 years have an increased risk of mortality. This may be because elderly patients are more likely to have significant comorbidity and required increased cardiovascular and respiratory support. Other considerations for increased mortality include the surgical procedure and admission time.
**REFERENCE**
1. NELA - National Emergency Laparotomy Audit [Internet]. Nela.org.uk. 2017 [cited 12 April 2017]. Available from: http://www.nela.org.uk/NELA_home

### 0461
### Hospital mortality in elderly patients admitted to surgical intensive care units at a tertiary referral hospital in Thailand
A. Piriyapatsom, O. Chaiwat, S. Kongsayreepong
Faculty of Medicine Siriraj Hospital, Mahidol University, Anesthesiology, Bangkoknoi, Thailand
**Correpondence:** A. Piriyapatsom
*Intensive Care Medicine Experimental* 2017, **5(Suppl 2):**0461

**INTRODUCTION.** The number of elderly patients admitted to intensive care unit (ICU) has been increased worldwide. Elderly patients are subjected to various physiologic changes related to aging process and it has been recognized that aging is an important predictor for mortality.

**OBJECTIVES.** The aim of this present study is to determine the hospital mortality in elderly patients admitted to general surgical ICUs at a tertiary referral hospital in Thailand.
**METHODS.** This was a retrospective study of the prospective observational cohort study conducted in two general surgical ICUs at Siriraj Hospital between April 2011 and November 2012. Elderly patients were defined as patients whose age of more than 70 years old. Demographic data and clinical outcomes were collected. The adjusted logistic regression analysis was performed to identify independent risk factors for hospital mortality.
**RESULTS.** There were 377 elderly patients admitted to general surgical ICU, which was accounted for 41.7% (95% CI, 38.5-45.0%) of all admission and their mortality was 12.5% (95% CI, 9.4-16.3%). Age of more than 70 years old was an independent risk factor for hospital mortality in patients admitted to ICU (adjusted OR, 1.85; 95% CI, 1.07-3.21). Three independent risk factors for hospital mortality in elderly patients admitted to ICU included APACHE II score of more than 15 (adjusted OR, 7.06; 95% CI, 3.29-15.16) and presence of coagulation and cardiovascular dysfunction at ICU admission (adjusted OR, 3.39; 95% CI, 1.38-8.31 and adjusted OR, 2.66; 95% CI, 1.21-5.84; respectively).
**CONCLUSIONS.** Hospital mortality in elderly patients admitted to general surgical ICU was 12.5%. Age of more than 70 years old was a risk factor for mortality in patients admitted to general surgical ICU. Among elderly patients, severity of acute illness was an important risk factor for hospital mortality.
**REFERENCE(S)**
1. Pisani MA. Considerations in caring for the critically ill older patient. J Intensive Care Med. 2009;24(2):83–95.
2. de Rooij SE et al. Factors that predict outcome of intensive care treatment in very elderly patients: a review. Crit Care. 2005;9(4):R307-14.
3. Fuchs L et al. ICU admission characteristics and mortality rates among elderly and very elderly patients. Intensive Care Med. 2012;38(10):1654–61.
4. Shum HP et al. Outcome of elderly patients who receive intensive care at a regional hospital in Hong Kong. Hong Kong Med J. 2015;21(6):490–8.

### 0462

#### Usefulness of modified early warning score on ederly patient with dyspnea in emergency department as a predictor for ICU admission

T.H. Kim[1], H.B. Kim[1], Y.D. Jang[2], K.Y. Seo[3]
[1]College of Medicine, Inje University, Department of Emergency Medicine, Haeundae Baik Hospital, Busan, Korea, Republic of; [2]College of Medicine, Inje University, Department of Emergency Medicine, Busan Baik Hospital, Busan, Korea, Republic of; [3]Seoul Bumin Hospital, Department of Emergency Medecine, Seoul, Korea, Republic of
**Correspondence:** T.H. Kim
*Intensive Care Medicine Experimental* 2017, **5(Suppl 2):**0462

**INTRODUCTION.** Early warning scores (EWS) are recommended as part of tools in the early recognition and response to patient who get worse breathing 65 years or more than older patients.
**OBJECTIVES.** The objective of this research is to evaluate the predictability of Modified Early Warning Scores (MEWS) for the elderly patients with respiratory difficulties in emergency department.
**METHODS.** This retrospective study was conducted demographic data, laboratory data, and Framingham criteria influencing MESW using medical records older than 65 years who had visited tertiary emergency department from January 2015 to December 2016. The usefulness of MEWS as a predictor for the patient's need for ICU care was analyzed using IBM SPSS Statistics for Windows.
**RESULTS.** Of the 213 patients, 142 patients (84 males) were enrolled in the analysis. Pulmonary disease was diagnosed in 74 patients (52.2%) and 49 patients (34.5%) in cardiogenic disease. We found the following factors influencing MEWS; arterial carbon dioxide ($\beta = .203$, $p < 0.001$) and body temperature ($\beta = .176$, $p < 0.006$), heat beat rate ($\beta = .247$, $p < 0.001$), systolic blood pressure ($\beta = .329$, $p < 0.001$), and lactate ($\beta = .282$, $p < 0.001$). The receiver operator characteristic (ROC) curve shows that MEWS has higher sensitivity and specificity than lactate and temperature.
**CONCLUSIONS.** MEWS is good predictor for the patients aged 65 or older with dyspnea in emergency department with higher sensitivity and specificity. MEWS can be used to predict for ICU care need and clinical deterioration.
**REFERENCE(S)**
1. Gary B.Smith, David R. Prytherch, Paul Meredith et al. The ability of the National Early Warning Score (NEWS) to discriminate patients at risk of early cardiac arrest, unanticipated intensive care unit admission, and death. Resuscitation 2013;84 465–70.
2. Jeroen Ludikhuize, Susanne M. Smorenburg, Sophia E. de Rooij et al. Identification of deteriorating patients on general wards; measurement of vital parameters and potential effectiveness of the Modified Early Warning Score. Crit Care Med 2012;4(27) 424e7-424e13.

### 0463

#### Very elderly donors as a potential source for liver transplant. The next frontier?

M.A. Pérez Lucendo[1], B. Lobo Valbuena[2], S. Alcántara Carmona[1], M. Pérez Redondo[1], P. Matía Almudévar[1], A. Naharro Abellán[1], P. Rodríguez Villamízar[1], J. Veganzones Ramos[1], L. Pérez Pérez[1], J. Palamidessi Domínguez[1], F. Somoza Sáez[3], I. Lipperheide Valhonrat[1], N. Martínez Sanz[1], I. Fernández Simón[1], R. Fernández Rivas[1], J.J. Rubio Muñoz[1]
[1]Hospital Universitario Puerta de Hierro Majadahonda, Madrid, Spain; [2]Hospital Universitario del Henares, Madrid, Spain; [3]Hospital Universitario de Getafe, Madrid, Spain
**Correspondence:** M.A. Pérez Lucendo
*Intensive Care Medicine Experimental* 2017, **5(Suppl 2):**0463

**INTRODUCTION.** Donor shortage together with the increase in organ demands have led to the development of different strategies aimed at raising organ retrieval rates. Amongst these strategies, the evaluation of expanded criteria donors has aroused as an alternative.
**OBJECTIVES.** To assess the viability of livers harvested from very elderly donors (≥80 years), and to study the evolution of both the graft and the recipient during a minimum follow-up period of six months.
**METHODS.** Retrospective study (January 2011 - December 2016), including very elderly liver donors, which were offered by the National Transplant Organization to the Transplant Coordination Team of Puerta de Hierro Majadahonda University Hospital. We compared livers discarded exclusively because of donor's age to those who were implanted. The following variables were studied: age, sex, body mass index, cardiovascular risk factors, toxic habits, comorbidities, natraemia, use of vasoactive drugs, development of diabetes insipidus, use of antibiotics (prophylactic or therapeutic) and length of ICU stay. We also conducted a six-month minimum follow-up of recipients, assessing graft and patient survival.
**RESULTS.** During the period studied a total of 66 livers were offered (Fig. 169). Of the 49 (74.2%) livers rejected, in 26 (39.4%) the reason stated for rejection was age exclusively. Seventeen (25.8%) livers ≥ 80 years of age were implanted, of which two needed retransplantation. There were no statistically significant differences between livers rejected and implanted in the variables detailed above, except for the number of comorbidities, which were lower in the rejected group. During the follow-up period four deaths were registered.
**CONCLUSIONS.** In our series, liver rejection due exclusively to age was frequent. Recipients of livers that came from very elderly donors had an evolution that is similar to what has been previously described for younger livers.
Very elderly patients should be evaluated carefully as liver donors before discarding them based exclusively on age criteria.

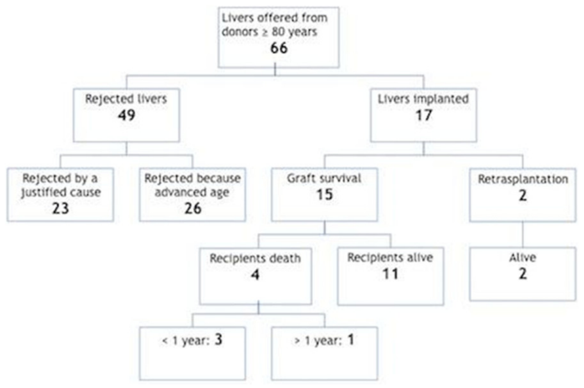

**Fig. 169 (Abstract 0463)** See text for description

### Sepsis epidemiology and early identification

#### 0464

#### The risk-stratification of emergency department sepsis (REDS) score, a new score combining the MISSED score, qSOFA, refractory hypotension and hyperlactaemia, compared with the change in SOFA score to risk-stratify emergency department patients admitted with an infection, for in-hospital mortality

N. Sivayoham[1], L. Newgrosh[1], S.E. Tharimoopantavida[1], M. Cecconi[2,3]
[1]St George's University Hospitals NHS FT, Emergency Department, London, United Kingdom; [2]St George's University of London, London, United Kingdom; [3]St George's University Hospitals NHS FT, London, United Kingdom
**Correspondence:** N. Sivayoham
*Intensive Care Medicine Experimental* 2017, **5(Suppl 2):**0464

**INTRODUCTION.** The objective criteria defining sepsis is a ≥2 point increase in the Sequential Organ Failure Assessment (SOFA) score. This is associated with a minimum 10% mortality rate, thus risk stratifying patients for mortality. But accurate calculation of the SOFA score in a busy emergency department (ED) is difficult and inaccurate as some criteria are not met or measured in the ED.

The quick SOFA (qSOFA) is a rule-in score [1] and cannot be used alone. The Mortality In Severe Sepsis in the Emergency Department (MISSED) score together with refractory hypotension and hyperlactaemia would identify the majority but not all deaths [2]. Both the qSOFA and MISSED (Age ≥65y, albumin ≤27g/l, INR ≥ 1.3) scores have three variables each and range from 0–3 and a score ≥2 is deemed high-risk.

**OBJECTIVES.** To create a scoring system combining the validated MISSED and qSOFA scores with refractory hypotension and hyperlactaemia, to risk-stratify ED patients admitted with an infection for in-hospital mortality and compare its performance to that of the change in SOFA (ΔSOFA) score to do the same.

**METHODS.** Patients aged ≥18y admitted from the ED who received intravenous antibiotics for an infection and met at least one Red-Flag or two SIRS criteria on presentation, were included. Each patient was scored 0–3 for MISSED and qSOFA scores. Refractory hypotension and lactate were allocated scores ranging from 0 to 3. Refractory hypotension with lactate ≤2 and >2mmol/l were scored 2 and 3, respectively, a score of 1 was not allocated. Lactates ≤2, 2.1-3.9 and ≥4mmol/l were scored 0, 1 and 3, respectively. The cumulative score of all four scoring systems, the Risk-stratification of ED Sepsis (REDS) score, ranged from 0–12. A score ≥2 in any one of the fours constituent scoring systems placed the patient in a high-risk category as did a cumulative score of ≥3. Patients with a ΔSOFA score of ≥2 were classed as high-risk. Missing values were assumed to be normal.

**RESULTS.** Population: 942 patients including 120 deaths. The area under the receiver operator characteristic (AUROC) curve for the REDS score 0.774 [95%CI 0.745-0.800) was greater than the AUROC curve for the ΔSOFA score 0.707 [95%CI 0.677-0.736]; p = 0.0036. Odds ratio for mortality, between high-risk and low-risk categories in the REDS score was 6.01[95%CI 3.57-10.10]; p < 0.0001, and that for the ΔSOFA score was 3.97[95%CI 2.56-6.16]; p < 0.0001.

**CONCLUSIONS.** The REDS score is equivalent to ΔSOFA to risk stratify ED patients with an infection, for mortality.

**REFERENCE(S)**

1. Churpek MM, Snyder A, Han X *et al*. qSOFA, SIRS, and Early Warning scores for detecting clinical deterioration in infected patients outside the ICU. Am J Resp and Crit Care Med Published online 20th September 2016 http://dx.doi.org/10.1164/rccm.201604-0854OC

2. Sivayoham N, Holmes P, Cecconi M, Rhodes A. (2015) Internal emergency department validation of the simplified MISSED score. Eur J Emerg Med;22:321–326

**GRANT ACKNOWLEDGMENT**

None

**Table (Abstract 0464).** Test charactertistic of high and low risk groups

| Risk | Sensitivity (95% Confidence Interval) | Specificity (95% CI) | Positive Predictive Value (95% CI) | Negative Predictive Value (95% CI) |
|---|---|---|---|---|
| REDS score- Low | 15.00 (9.14-22.67) | 48.54 (45.07-52.02) | 4.08 (2.69- 6.15) | 79.64 (77.92-81.26) |
| REDS score-High | 85.00 (77.33-90.86) | 51.46 (47.98-90.86) | 20.36 (18.74-22.08) | 95.92 (93.85-97.31) |
| ΔSOFA-Low | 24.17 (16.28-32.83) | 44.16 (40.73-47.63) | 5.94 (4.38-8.02) | 79.96 (77.84-81.91) |
| ΔSOFA-High | 75.83 (67.17-83.18) | 55.84 (52.37-59.27) | 20.04 (18.09-22.16) | 94.06 (91.98-95.62) |

#### 0465

#### Rapid pathogen identification using matrix-assisted laser desorption/ionization time-of-flight improves appropriateness of antimicrobial therapy in patients with sepsis

W.-Y. Kim, E.S. Jeong, I. Kim, K. Lee
Pusan National University School of Medicine, Department of Internal Medicine, Busan, Korea, Republic of
**Correspondence:** W.-Y. Kim
*Intensive Care Medicine Experimental* 2017, **5(Suppl 2):**0465

**INTRODUCTION.** In the presence of sepsis or septic shock, delay in administration of appropriate antimicrobials is associated with an increase in mortality [1]. Matrix-assisted laser desorption/ionization time-of-flight (MALDI-TOF) has been found to decrease time to microorganism identification by 1.2-1.5 days compared to conventional methods [2]. However, there are limited data focusing the use of MALDI-TOF on patients with sepsis.

**OBJECTIVES.** To evaluate the impact of MALDI-TOF on clinical and antimicrobial therapy-related outcomes in patients with sepsis.

**METHODS.** A total of 98 adult patients with bacteremia who met ≥2 quick SOFA criteria and were admitted to the intensive care unit (ICU) were reviewed. Patients for whom microorganisms were identified via MALDI-TOF (between March 2014 and February 2016; n = 58) were compared with patients for whom microorganisms were identified using conventional methods (between March 2011 and February 2013; n = 40).

**RESULTS.** The mean time from blood culture drawn to microorganism identification and the availability of antimicrobial susceptibility results was significantly shorter in the MDLDI-TOF group compared with the conventional group (90.2 ± 32.1 h vs. 108.7 ± 43.1 h; $P = 0.02$). In the MALDI-TOF group, the proportion of patients with appropriate antimicrobial therapy before susceptibility results were significantly higher than those in the conventional group (77% vs. 47%; $P = 0.005$). The 28-day mortality after ICU admission was 40% in the MALDI-TOF group and 70% in the conventional group ($P = 0.003$). In survivors, there were no differences between the groups with regard to duration of mechanical ventilation and ICU

and hospital length of stay. Univariate analysis indicated that microorganism identification via MALDI-TOF associated significantly with mortality (OR, 0.28; 95% CI, 0.12-0.66) along with malignancy (OR, 8.83; 95% CI, 2.76-28.27) and baseline SOFA score (OR, 1.31; 95% CI, 1.14-1.50).

**CONCLUSIONS.** Our results suggest that rapid identification of microorganisms in blood via MALDI-TOF was associated with appropriate antimicrobial therapy and decreased mortality in patients with sepsis.

**REFERENCE(S)**

1. Kumar A, Roberts D, Wood KE, Light B, Parrillo JE, Sharma S et al. (2006) Duration of hypotension before initiation of effective antimicrobial therapy is the critical determinant of survival in human septic shock. Crit Care Med 34:1589–1596

2. Huang AM, Newton D, Kunapuli A, Gandhi TN, Washer LL, Isip J et al. (2013) Impact of rapid organism identification via matrix-assisted laser desorption/ionization time-of-flight combined with antimicrobial stewardship team intervention in adult patients with bacteremia and candidemia. Clin Infect Dis 57:1237–1245

**GRANT ACKNOWLEDGMENT**

This work was supported by a National Research Foundation of Korea grant funded by the Korean government (Ministry of Science, ICT and Future Planning) (2016R1C1B1008529).

### 0466

#### Epidemiology of sepsis and validation of the Sepsis-3 definitions in a high-middle income country: a prospective cohort study from the Sociedad Argentina de Terapia Intensiva (SATI) in 809 patients

E. Estenssoro[1], V. Edul[2], C. Loudet[1], J. Osatnik[3], F. Ríos[4], A. Dubin[5], D. Vazquez[6], M. Pozo[7], B. Lattanzio[8], G. Tuhay[9], A. García[10], G. Zakalik[11], P. Rubatto[5], A. Santamaría[12], and other Investigators of the SATISEPSIS Group

[1]Hospital Interzonal General de Agudos San Martín de La Plata, La Plata, Argentina; [2]Hospital Fernández, Buenos Aires, Argentina; [3]Hospital Alemán, Buenos Aires, Argentina; [4]Hospital A. Posadas, Haedo, Argentina; [5]Sanatorio Otamendi, Buenos Aires, Argentina; [6]Sanatorio Anchorena, Buenos Aires, Argentina; [7]Clinica Bazterrica, Buenos Aires, Argentina; [8]Clínica Santa Isabel, Buenos Aires, Argentina; [9]Fundación Favaloro, Buenos Aires, Argentina; [10]Hospital Misericordia, Córdoba, Argentina; [11]Hospital Lagomaggiore, Córdoba, Argentina; [12]Sanatorio Trinidad Mitre, Buenos Aires, Argentina

**Correspondence:** E. Estenssoro

*Intensive Care Medicine Experimental* 2017, **5(Suppl 2):**0466

**INTRODUCTION.** The Sepsis-3 definition recently released claimed for validation in low and middle income economies. Besides, there was no data regarding sepsis characteristics and outcomes in Argentina. Hence the SATI and the National Ministry of Health launched a prospective cohort study to address these relevant issues.

**OBJECTIVES.** To analyze epidemiological features and validate the Sepsis-3 definitions.

**METHODS.** Prospective national cohort study. SATI sent invitations, and a website-based database was designed to collect data. Between 7/1-9/1/16,patients admitted to the ICU with suspected infection that triggered blood cultures and antibiotic administration were included as these categories: 1. Infection; 2.Sepsis (infection + SOFA ≥ 2); 3.Septic shock (inotropics + lactate ≥2 mmol/L); 4.Cardiovascular dysfunction (lactate ≥2 without inotropics); 5. Inotropics without lactate measurement. Epidemiological, self-perceived health status (E-QoL VAS),Sepsis-3, infectological and management data were recorded. Hospital mortality was the main endpoint. Kaplan-Meier curves were constructed for each category and compared (logrank). Variables differing from survivors and nonsurvivors were entered in a logistic regression model. ROC curves were built to assess SIRS, qSOFA and SOFA performance. A p value of 0.05 was considered significant. Adjustement for multiple comparisons was made if necessary.

**RESULTS.** 809 patients from 47 UCIs were included; 44% had respiratory infections, 19% UTI, 9% intrabdominal, 6%soft tissue and 3% catheter-related. 28% of cultures were negative. Different variables and outcomes for the entire population, comparisons between categories, and Kaplan Meier curves are shown in Table 130 and Fig. 170.
General mortality was 38%, and by categories 13%, 20%, 38%, 51% and 39% respectively; independent predictors are displayed on Table 131.
Crude AUROC values for SIRS, qSOFA and SOFA were 0.51[0.47-0.55], 0.52[0.48-0.55] and 0.73[0.79-0.77] respectively (P = 0.000).

**CONCLUSIONS.**

1) Increasing severity of Sepsis-3 categories adequately tracks mortality.
2) Lactate measurement was not an impediment for diagnosis of septic shock (available in 97%of patients).
3) As recently published, SOFA shows acceptable predictive validity for ICU patients, whilst SIRS and qSOFA exhibited low discrimination.
4) The findings of this study, the first prospectively carried out in a LMIC, support the usefulness of the Sepsis-3 definition recently issued.

**GRANT ACKNOWLEDGMENT**

SATI, Ministerio de Salud de la Nación, Argentina

**Table 130 (Abstract 0466).** See text for description

| | All patients N=809 | 1. Infection 62/809 (8) | 2. Sepsis 225/809 (28) | 3. Septic Shock 324/809 (40) | 4. CVD with normal lactate 164/809 (20) | 5. CVD without lactate measurement 28/809 (3) | P corrected |
|---|---|---|---|---|---|---|---|
| Age | 60±20 | 48±21 | 57±18 | 63±39 | 62±18 | 57±17 | 0.000 |
| Female gender | 354/809 (44) | 35/62 (56) | 91/225 (40) | 135/324 (42) | 69/164 (42) | 14/28 (50) | 0.154 |
| Charlson score | 2[0-3] | 0[0-2] | 1[0-3] | 2[1-4] | 2[0-4] | 0.5[0-2] | 0.001 |
| Preexisting health status (0-100 points, EQ-VAS) | 69±23 | 83±22 | 72±22 | 66±23 | 65±29 | 70±27 | 0.0002 |
| Origin: community/ Intrahospital/ICU/ other (%) | 55/32/10/3 | 38/35/25/2 | 63/26/11/3 | 57/33/8/3 | 47/37/9/7 | 56/30/11/0 | 0.000 |
| APACHE II | 19±8 | 15±8 | 15±6 | 22±8 | 20±7 | 18±8 | 0.000 |
| Lactate (mmol/L) | 2.1[1.3-3.4] | 1.2[0.9-2.1] | 1.8[1.2-2.8] | 3.2[2.5-4.7] | 1.4[1.1-1.7] | 0[0-0] | 0.000 |
| $SOFA_{24hs}$ score | 7[4-10] | 1[0-1] | 4[3-6] | 9[7-11] | 8[6-10] | 8[6-11] | 0.000 |
| Mortality | 303/809 (38) | 8/62 (13) | 44/225 (20) | 166/324 (51) | 63/164 (38) | 11/28 (39) | 0.000 |

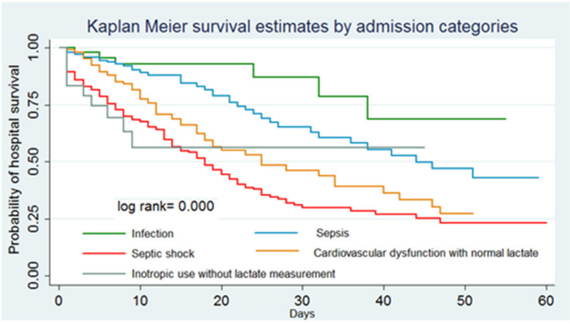

**Fig. 170 (Abstract 0466).** See text for description

**Table 131 (Abstract 0466).** See text for description

| | OR | [95% CI] | P |
|---|---|---|---|
| SOFA | 1.13 | [1.07-1.20] | 0.000 |
| Lactate | 1.22 | [1.12-1.34] | 0.000 |
| Charlson score | 1.19 | [1.10-1.30] | 0.000 |
| Previous health state [0-100 points, EQ-VAS] | .99 | [.98-.99] | 0.001 |
| Extremely-resistant microorganisms. | 1.80 | [1.15-2.82] | 0.011 |
| Mechanical ventilation | 8.96 | [5.33-15.05] | 0.000 |
| AUROC 0.8377 [0.80646-0.86896] | | | |

### 0467

#### Incorporating IRIDICA PCR / ESI-MS technology for the rapid identification of bloodstream microorganisms in severe sepsis and code sepsis activation (CS) at the Vall d´Hebron University Hospital

E. Papiol[1,2], J. Baldirà[1,2], A. Fàbrega[3,4], J.J. González[3,4], A. Mir[3,4], N. Larrosa[3,4], L. Chiscano[1,2], N. Varga[1,2], M. Acuña[1,2], S. Ramírez[1,2], P. Domingo[1,2], A. Ruiz[1,2], J. Llaneras[5], E. Molas[5], R. Alcaraz[1,2], X. Nuvials[1,2], J. Caballero[1,2], R. Albertos[1,2], M. Pérez[1,2], J. Serra[1], M. de Nadal[6], A. Ricart[5], T. Pumarola[3,4], J.C. Ruiz[1,2], R. Ferrer[1,2]
[1]Hospital Universitari Vall d'Hebron, Critical Care Department, Barcelona, Spain; [2]Vall d'Hebron Research Institute, Shock, Resuscitation and Organ Dysfunction Research Group, Barcelona, Spain; [3]Hospital Universitari Vall d'Hebron, Microbiology Department, Barcelona, Spain; [4]Vall d'Hebron Research Institute, Barcelona, Spain; [5]Hospital Universitari Vall d'Hebron, Emergency Department, Barcelona, Spain; [6]Hospital Universitari Vall d'Hebron, Anestesiology Department, Barcelona, Spain
**Correspondence:** E. Papiol
*Intensive Care Medicine Experimental* 2017, **5(Suppl 2):**0467

**INTRODUCTION.** The early identification of the microorganism causing infection in a patient with sepsis could have an influence in the initial treatment and an impact on prognosis. Regular cultures don't usually provide a rapid diagnosis. IRIDICA PCR / ESI-MS (Abbott) is a blood qualitative diagnostic technique that identifies bacteria and Candida sp nucleic acids in septic patients in 7 hours. It also identifies genes that encode resistance to antibiotics (*$bla_{KPC}$, mecA, vanA, vanB*). DNA is amplified by PCR and analyzed by mass spectrometry ionization electrospy (PCR / ESI-MS).
**OBJECTIVES.** Evaluation of PCR / ESI-MS ability to detect bloodstream microorganisms in septic patients.
**METHODS.** We included all septic patients with CS activation admitted to the hospital between May and December 2016.We analyzed 1 blood sample with PCR / ESI-MS and two blood cultures taken at least 1 hour apart. We were on continuous communication with the Department of Microbiology to perform the technique and read the results. We compared the results of blood cultures and PCR / ESI-MS. The quantitative variables are expressed as average (SD) and categorical variables as percentage. The Ethics Committee approved the study IRIDICA and CS: PR (AG) 162/2016, PR (AG) 88/2016 and PR (AG) 336/2016.
**RESULTS.** We obtained 252 samples, 4.3% were rejected for technical error and 19.44% for polymicrobial detection and confusing interpretation. Of the remaining 190 samples, 62.1% were septic shock infection and 37.9% with sepsis. Age of the patients were 60.1 (SD 16.4), men 63.7%, SOFA score 6.6 (SD 3.4). The sources of infection were: abdominal 32.1%, respiratory 26.8%, urinary 22.1% and other 19%. Required drainage of the source of infection 22.1% and Intensive Care Unit (ICU) admission 52.6%. Required norepinephrine 46.3%, mechanical ventilation 21%, high flow nasal cannules 19.4% and renal replacement therapy 12.1%. The hospital mortality was 25.8% and the hospital mortality in ICU patients was 38.9%.Taking as gold standard all clinical and available cultures, the obtained values for sensitivity, specificity, positive predictive and negative predictive were 91.2%, 86.9%, 89.2% 90 2% for IRIDICA® and 61.6%, 89.9%, 85.5%, 70.8% for blood cultures. The results of PCR / ESI-MS were obtained in the following 7 hours whereas positivity of blood cultures ranged from 10 hours to several days.
**CONCLUSIONS.** PCR / ESI-MS provides a fast and reliable identification of microorganisms responsible for a blood infection in patients with sepsis. The high sensitivity and specificity make technical PCR / ESI-MS a valuable tool for diagnosing septic patients and could improve their optimal management and prognosis.

**REFERENCE(S)**

Metzgar D, Frinder MW, Rothman RE, Peterson S, Carroll KC, Zhang SX, et al. (2016) The IRIDICA BAC BSI Assay: Rapid, Sensitive and Culture-Independent Identification of Bacteria and Candida in Blood. PLoS ONE 11(7): e0158186. doi:10.1371/journal.pone.0158186

### 0468

#### The mortality attributable to sepsis in adult patients in intensive care

M. Schout[1,2], N. Hammond[1,3,4], F. Bass[1,4], A. Delaney[1,3,4], S. Finfer[1,3,4]
[1]Royal North Shore Hospital, Intensive Care Unit, St. Leonards, Australia; [2]Radboud University Nijmegen, Nijmegen, Netherlands; [3]University of Sydney, Sydney, Australia; [4]The George Institute of Global Health, Sydney, Australia
**Correspondence:** M. Schout
*Intensive Care Medicine Experimental* 2017, **5(Suppl 2):**0468

**INTRODUCTION.** Chronic comorbid conditions are present in the majority of sepsis patients and these patients have a significantly worse outcome than patients without comorbidities. It is thought that sepsis occurs as a terminal event in a proportion of sepsis patients dying from a disease process other than sepsis.
OBJECTIVE. To determine the attributable mortality of sepsis in critically ill adult patients in intensive care.
**METHODS.** We conducted an inception cohort study including all patients admitted to the ICU at a tertiary hospital in Sydney, Australia between October 1st 2016 and December 31st 2016. In all patients diagnosed with sepsis using clinical criteria we used an adjudication system where clinicians estimated the pre-sepsis life expectancy of patients who died in-hospital within 60 days. Patients who had an estimated life expectancy of less than 90 days were adjudicated to have died with sepsis rather than from sepsis.
**RESULTS.** From 864 patients admitted, 146 (16.9%) patients were prospectively diagnosed with sepsis and included. Comorbidities were present in 117/146 (80.1%) of patients. 39/146 (26.7%) died in-hospital within 60 days. 30/39 (76.9%) of deceased patients were adjudicated not to be in a terminal phase of an underlying disease process before onset of sepsis. These deaths were therefore adjudicated to be directly attributable to sepsis.
**CONCLUSIONS.** Despite the presence of comorbidities in the majority of patients, most patients who died were not in a terminal stage of an underlying disease process when sepsis occurred. Therefore, sepsis did result in an appreciable loss of life years. Further research in this area is warranted.

**REFERENCE(S)**

1. Martin GS. Sepsis, severe sepsis and septic shock: changes in incidence, pathogens and outcomes. Expert Rev Anti Infect Ther. 2012;10(6):701–706.
2. Angus DC, Linde-Zwirble WT, Lidicker J, Clermont G, Carcillo J, Pinsky MR. Epidemiology of severe sepsis in the United States: analysis of incidence, outcome, and associated costs of care. Crit Care Med. 2001;29(7):1303–1310.
3. Kaukonen KM, Bailey M, Suzuki S, Pilcher D, Bellomo R. Mortality related to severe sepsis and septic shock among critically ill patients in Australia and New Zealand, 2000–2012. JAMA. 2014;311(13):1308–1316.
4. Kadri SS, Rhee C, Strich JR, et al. Estimating Ten-Year Trends in Septic Shock Incidence and Mortality in United States Academic Medical Centers Using Clinical Data. Chest. 2016.
5. Martin GS, Mannino DM, Eaton S, Moss M. The epidemiology of sepsis in the United States from 1979 through 2000. N Engl J Med. 2003;348(16):1546–1554.
6. Fleischmann C, Scherag A, Adhikari NK, et al. Assessment of Global Incidence and Mortality of Hospital-treated Sepsis. Current Estimates and Limitations. Am J Respir Crit Care Med. 2016;193(3):259–272.
7. Martin GS, Mannino DM, Moss M. The effect of age on the development and outcome of adult sepsis. Crit Care Med. 2006;34(1):15–21.

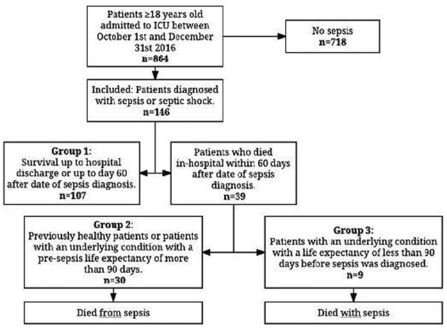

**Fig. 171 (Abstract 0468).** Inclusion and adjudication process

### 0469

**Withdrawn**

### 0470

#### Early recognition and treatment for sepsis and septic shock in a tertiary hospital in Spain

V.D. Gumucio-Sanguino[1], P. Sastre-Pérez[1], H. Alanez-Saavedra[1], L. Anguela-Calvet[1], C.J. Barreda[2], J. Sabater-Riera[1], X.L. Perez-Fernandez[1], SIRAKI Group
[1]Hospital Universitari de Bellvitge, Critical Care Medicine, L`Hospitalet de Llobregat, Spain; [2]Clinica Universitaria Reina Fabiola, Critical Care Medicine, Córdoba, Argentina
**Correspondence:** V.D. Gumucio-Sanguino
*Intensive Care Medicine Experimental* 2017, **5(Suppl 2):**0470

**INTRODUCTION.** According to changes for definitions in sepsis and septic shock during the last year and the latest recommendations published by the Surviving Sepsis Campaign there is an important request for improving our system of detection and management of septic patients.

**OBJECTIVES.** Evaluate the usefulness of sepsis bundles accomplishment at the emergency department and intensive care unit in septic patients admitted in a tertiary hospital.

**METHODS.** A prospective observational study was performed in a tertiary hospital in Spain. Data was collected from patients with criteria for sepsis at all emergency levels, and from all the patients admitted with septic shock at the intensive care unit between the period from December 2015 to March 2017. We evaluated the accomplishment of the initial measures of resuscitation, control of infection source and adjuvant treatment in patients with sepsis and septic shock.

**RESULTS.** 171 patients were enrolled. 60,8% of patients were men, and mean age was 62,2 +/− 14 years. Sepsis was identified at emergency department (72%), at other hospital (11%) and at ward (10%). Sepsis protocol was electronically activated in only 20% of these patients. 38% of these patients developed septic shock. The most frequent etiologies of sepsis were pneumonia 32,7% and urinary infection with 32,2%. 43% of the blood cultures were positive. Mean Systolic blood pressure (SBP) was 96.4mmHg, mean leucocytes were 16800 +/− 14000 mm3, mean creatinine was 137+/− 81 umol/l, mean central venous oxygen saturation was 76,5%. Mechanical ventilation was required in 16,3% of patients, and non invasive ventilation in 12,9%. Steroids were used in 23,7% of patients and 7,7% required renal replacement therapy. Accomplishment of bundles in patients with electronically protocol activation was better specially concerning initial resuscitation measures. Global mortality was 20,7%.

**CONCLUSIONS.** Electronically sepsis protocol activation can be a useful tool in septic patients but our actual utilization is still low.

**REFERENCE(S)**

Rhodes A et al. Surviving Sepsis Campaign: International Guidelines for Management of Sepsis and Septic Shock: 2016. Intensive Care Med. 2017;43:304–377.

### 0471

#### MALDI-TOF MS is not superior to standard blood-cultures in patients with infection who require mechanical ventilation or noradrenalin

J. Golub[1], A. Markota[1], S. Lorenčič-Robnik[2], A. Sinkovič[3]
[1]University Medical Centre Maribor, Medical Intensive Care Unit, Maribor, Slovenia; [2]National Laboratory of Health, Environment and Food, Centre for Microbiology and Imunology, Maribor, Slovenia; [3]Faculty of Medicine University of Maribor, Maribor, Slovenia
**Correspondence:** A. Sinkovič
*Intensive Care Medicine Experimental* 2017, **5(Suppl 2):**0471

**INTRODUCTION.** Identifying microorganisms from blood in septic patients is paramount in sepsis(1). Matrix assisted laser desorption ionisation-time mass spectroscopy (MALDI-TOF MS) is one of new methodes for rapid microbal idetification that has proven to have many advantages in the past studies (2).

**OBJECTIVES.** The aim of this study was to compare blood culture (BC) technique and MALDI-TOF MS.

**METHODS.** We performed a retrospective study on critically ill patientes who were hospitalised between January and March 2015 in a medical intensive care unit. We included patients with infection who required mechanical ventilation or noradrenalin. Standard BC were used as the gold standard. Multiple sets of BC (2 aerobic and 2 anaerobic bottles, 40 ml of blood in total) were allowed for each patient. One pair of BC was considered as one study sample. MALDI-TOF MS assay was performed from positive BC bottles in an automated system (BacT/ALERT®,Biomérieux, USA). Medical documentation for 81 patients was examined for basic demographic data, BC results, MALDI-TOF MS results, time to results and time to change of antimicrobial therapy.

**RESULTS.** We included 81 patients, 51 (63%) were men. The total number of samples was 147. Mean age was 64 ± 14 years. Survival at discharge from intensive care unit was 65,4%, and survival at discharge from hospital was 53,0%. Concordance between BC and MALDI-TOF MS was: +/+ 16 (10,8%), −/− 114 (77,5%), +/− 17 (11,6%) and −/+ 0 (0%). Sensitivity was 48,5%, specificity was 100% and negative predictive value was 87%. Positive BC bottles are the starting point for MALDI-TOF MS assay, which needs to be considered when interpreting concordance with BC. In cases where MALDI-TOF MS was positive and BC negative other microbiological samples (e.g. urine, tracheal aspirates, wound swabs…) were examined for concordance with MALDI-TOF MS, and none were discovered. Time to results was 31,1 ± 15,2h for BC, and 31,4 ± 17,3h for MALDI-TOF MS, p = 0.6.

**CONCLUSIONS.** According to our study MALDI-TOF MS is not superior to standard BC in identifying pathogens in patients with infection who required mechanical ventilation or noradrenalin. Further studies are required to evaluate the role of MALDI-TOF MS assay in critically ill.

**GRANT ACKNOWLEDGMENT**
The authors have nothing to declare.

### 0472

#### Sensitivity of the SOFA score, quick SOFA score and SIRS criteria used in a tertiary critical care emergency center: a single-center observational study

M. Todani[1], T. Yagi[1], T. Nakahara[1], Y. Kawamura[1], M. Fujita[1], K. Kaneda[1], Y. Oda[1], R. Tsuruta[1]
Yamaguchi University Hospital, Advanced Medical Emergency and Critical Care Cente, Yamaguchi, Japan
**Correspondence:** M. Todani
*Intensive Care Medicine Experimental* 2017, **5(Suppl 2):**0472

**INTRODUCTION.** The Third International Consensus Definition Task Force released clinical criteria for sepsis and septic shock (Sepsis-3) in 2016[1)]. Although the Task Force encouraged clinicians to apply the Sequential Organ Failure Assessment (SOFA) score in intensive care units (ICUs) and the quickSOFA (qSOFA) score outside the ICU, there is no evidence to suggest which scoring system should be used in tertiary critical care emergency centers, which primarily treat referred and/or complicated patients.

**OBJECTIVES.** To compare the prognostic value of the SOFA score, qSOFA score, and systemic inflammatory response syndrome (SIRS) criteria for patients with suspected infection admitted to a tertiary critical care emergency center.

**METHODS.** In this retrospective observational study, we reviewed the medical records of patients admitted to our emergency department between September 2014 and August 2016 who underwent bacteriological culture and treatment with antibiotics within 2 days of a primary diagnosis of infection. The patients' clinical data were retrieved from electronic medical records. The SOFA score, qSOFA score, and number of SIRS components on admission were retrospectively determined using these data. We evaluated the prognostic value of the SOFA and qSOFA scores and the SIRS criteria for the following outcomes: in-hospital mortality, ICU-free days, and length of stay.

**RESULTS.** Overall, 174 patients were included in this study. Their mean age was 69 years (standard deviation: 16 years), 160 patients (92%) were treated in the ICU, and 29 patients (17%) died in hospital. The hospital mortality rates were significantly greater in patients with SOFA scores ≥ 2 vs < 2 (22% vs 2%, $P < 0.01$) and qSOFA scores ≥ 2 vs < 2 (27% vs 8%, $P < 0.01$) but not between ≥ 2 vs < 2 SIRS components (16% vs 19%, $P = 0.73$). A SOFA score of ≥ 2 on admission was a better predictor of in-hospital mortality compared with the qSOFA score and SIRS criteria (sensitivity: 0.97, 0.76, and 0.21, respectively). Furthermore, in-hospital mortality was significantly associated with SOFA (odds ratio [OR] 1.36, 95% confidence interval [CI] 1.20-1.54, $P < 0.01$) and qSOFA (OR 1.93, 95% CI 1.20-3.10, $P = 0.01$) but not with SIRS criteria (OR 0.82, 95% CI 0.55-1.24, $P = 0.35$).

**CONCLUSION.** A SOFA score of ≥ 2 on admission showed greater sensitivity and prognostic utility for detecting patients with severe infection compared with a qSOFA score of ≥ 2 or the presence of ≥ 2 SIRS components among patients admitted to a tertiary critical care emergency center.

**GRANT ACKNOWLEDGMENT**
No grants received.

### 0473

#### Sepsis code activation in a high complexity hospital

J. Baldirà[1], E. Papiol[1], A. Ruiz-Sanmartin[1,2], M. Acuña[1], L. Chiscano[1], N. Varga[1], N. Larrosa[3], J.J. González[3], R. Bartolomé[3], E. Molas[4], J. Llaneras[4], T. Pumarola[3], R. Ferrer[1,2], J.C. Ruiz-Rodríguez[1,2]
[1]Hospital Universitari Vall d'Hebron, Critical Care Department, Barcelona, Spain; [2]Vall d'Hebron Research Institute, Shock, Organ Dysfunction and Resuscitation Research Group (SODIR), Barcelona, Spain; [3]Hospital Universitari Vall d'Hebron, Microbiology Department, Barcelona, Spain; [4]Hospital Universitari Vall d'Hebron, Emergency Department, Barcelona, Spain
**Correspondence:** J. Baldirà
*Intensive Care Medicine Experimental* 2017, **5(Suppl 2):**0473

**INTRODUCTION.** An in-hospital sepsis code can help in the standardization of management of sepsis/septic shock and improve morbimortality.

**OBJECTIVES.** To analyze the activations of an in-hospital sepsis code (SC) and to analyze the demographic and diagnostic characteristics of sepsis/septic shock patients.

**METHODS.** Prospective observational study of SC patients during 1 year (15-oct-2015 - 15-oct-2016) in a third level hospital. Demographic data, APACHE II score, laboratory data, physiological variables, Intensive care unit (ICU) and intrahospital mortality were recorded in patients with suspected sepsis or septic shock. SEPSIS III definitions were used. This study was approved by Ethical Committee (PR(AG)336/2016). Quantitative variables with normal distribution have been expressed as mean ± standard deviation, non-normal distribution as median and interquartile range (25-75%) and categorical variables as a percentage. Statistical analysis was done with SPSS 18.0 (SPSS Inc., Chicago, IL, USA).

**RESULTS.** 517 patients (55.3% men) were included with a mean age 63.7 (±16.6) years and APACHE II 23.5 (±8.9). 44.9% of the activations were made by the Emergency Department, 18.6% by the Intensive Care Unit and 5.8% by the General Surgery Department. Of all SC activations, 28.8% were by sepsis; 51.93% septic shock; 11.6% for infection without sepsis and 7.11% were not infection. The most frequent sepsis focuses were abdominal (32.17%) followed by respiratory (27.8) and urinary (23.8%). Blood cultures were performed in 93.8% of the cases (42.9% positive). The most frequent microorganisms were *E. coli* (30.9%), *K. pneumoniae* (7.9%) and *P. aeruginosa* (7.9%). The control of the septic focus was performed in 39.5% of the cases. 64.8% of patients required vasoactive drugs and 31.8% mechanical ventilation, 11.7% HFNC and 11.7% renal support techniques. 180 patients were admitted to the ICU (49.4%), 47 died (26.1%). The in-hospital mortality of patients admitted to the ICU was 54 (29.4%) and those not admitted to the ICU 39 (21.1%) with an overall mortality of 96 (26.5%).

**CONCLUSIONS.** Sepsis code is mainly activated in the emergency room and the wards. Most activations are on septic shock patients. The knowledge of the most frequents focus of infection and associated pathogens can help with treatment.

### 0474

#### Prevalence and short-term prognosis of sepsis and septic shock defined according to Sepsis-3 definitions in a single tertiary care ICU in Turkey

A. Topeli, N.D. Eyupoglu, E. Ortac Ersoy, K. Rollas, K. Ozmen Suner, M.N. Gullu, P. Hanci, O. Kilic, B. Halacli, S. Ocal
Hacettepe University Faculty of Medicine, Medical Intensive Care Unit, Ankara, Turkey
**Correspondence:** A. Topeli
*Intensive Care Medicine Experimental* 2017, **5(Suppl 2):**0474

**INTRODUCTION.** As a major worldwide public health problem, sepsis carries a high disability and mortality rate. Epidemiologic data about sepsis are still scarce and are mainly from developed countries.

**OBJECTIVES.** To determine prevalence of sepsis and septic shock at admission to an ICU and to determine factors affecting 28th-day mortality.

**METHODS.** Patients admitted to a 9-bed medical-ICU of a university hospital between 1.1.2007-31.12.2014 were analyzed retrospectively. Patients were classified as no-sepsis, sepsis and septic shock groups at admission according to new Sepsis-3 definitions. Comparisons were done between 3 groups and between survivors and non-survivors. Kaplan-Meier survival analysis was performed and logistic regression analysis was done to determine independent predictors of 28th-day mortality.

**RESULTS.** Data of 1404 patients were analyzed. Sepsis was the reason for admission in 536 (38.3%) patients, 164 (11.7% of all) of whom had septic shock. When no-sepsis, sepsis and septic shock groups were compared, APACHE-II, SOFA and Charlson co-morbidity scores were highest in septic shock group (p < 0.001 for all). 28th-day mortality was 16.5%, 31.2%, 49.4% and hospital mortality was

26.3%, 50.3% and 70.1% in no-sepsis, sepsis and septic shock groups, respectively (p < 0.001 for both). Kaplan-Meier survival analysis showed similar results in terms of 28th-day and hospital mortality, revealing that the survival was worst in septic shock group (log-rank test, p < 0.001, for both). When 8 year period was divided into quartiles, frequency of sepsis increased from 1st to 4th quartile (35.3%, 35.0%, 40.6% and 41.8%; respectively; p = 0.002). However, there was no significant difference in 28th-day (24.7%, 24.5%, 25.5%, 22.4%; respectively; p = 0.80) and hospital mortality (35.0%, 35.3%, 43.9%, 37.2%; respectively; p = 0.06) between quartiles. In logistic regression analysis, presence of sepsis at admission was an independent risk factor for 28th-day mortality as compared to no-sepsis group (OR (95% CI): 1.51 (1.06-2.16); p = 0.02). Other factors affecting mortality were a high admission APACHE-II, SOFA, Charlson co-morbidity scores; admission lactate level >2mmol/L and administration of fresh frozen plasma.

**CONCLUSIONS.** According to new sepsis definitions, sepsis is responsible from 38.3% of admissions during an 8 year period in a medical-ICU with high 28th-day and hospital mortality rates. Although frequency of sepsis increased from 2007 to 2014, mortality rates did not increase significantly. Presence of sepsis at admission was an independent risk factor for 28th-day mortality.

**REFERENCE(S)**

1. Vincent *et al*. Assessment of the worldwide burden of critical illness: the Intensive Care Over Nations (ICON) audit. Lancet Respir Med 2014;2:380.

2. Fleischmann *et al*. Assessment of Global Incidence and Mortality of Hospital-treated Sepsis Current Estimates and Limitations. Am J Respir Crit Care Med Vol 2016;193:259.

### 0475

#### Analysis of the bacteremias admitted to the Intensive Care Unit of a regional hospital

A. Ubeda[1], I. Fernández[1], R. Torcuato[1], J. Garcia[1], A. Fregosi[1], L. Alonso[2]
Hospital Punta de Europa, Intensive Care Unit, Algeciras, Spain; [2]Pediatric La Lobilla, Estepona, Spain
**Correspondence:** A. Ubeda
*Intensive Care Medicine Experimental* 2017, **5(Suppl 2):**0475

**INTRODUCTION.** Blood cultures are routinely taken at the time of admission to the intensive care unit (ICU) for patients suspected to have infection. We undertook this study to determine the incidence of bacteremia in our area and to assess its impact on the outcome of our patients.

**OBJECTIVES.** To analyze the characteristics of the bacteremias admitted in the ICU of Punta de Europa Hospital: community-acquired (CB) and nosocomial-acquired (NB).

**METHODS.** Descriptive retrospective analysis performed in a 12-bed ICU between 2015 and 2016. BC was considered to be the one with positive blood culture at < 48 hours of admission, and BN if blood culture was positive after 48 hours of admission. Demographic variables, comorbidities, risk factors, clinical situation, severity scores, ICU and hospital length of stay (LOS) were recorded.

Statistical analysis: categorical variables (frequencies and percentages) and numerical variables (mean and standard deviation or medians and interquartile range). Comparisons: X2 test (percentages), Student´s test (means) and Kruskal-Wallis test (medians). Statistical significance p < 0.05.

**RESULTS.** 72 bacteremias were included: 33 (45,8%) CB and 39 (54,1%) NB. Significant differences were found in patient type (medical: CB 84.8% vs NB 38.5%, p < 0.01), diabetes (CB 42.4% vs NB 69.2%, p = .022), previous hospitalization (CB 3% vs NB 30.8%, p = .002), previous isolation of multiresistant bacteria (CB 3% vs NB 23.1%, p = .017), bladder catheter (CB 54.5% vs NB 89.7%, p = .001), central catheter (CB 69.7% vs NB 92.3%, p = .013), postoperative wound (CB 15.2% vs NB 64.1%, p < .001), SOFA (CB 2.8 [1.5] vs NB 3.6 [1.5], p = .029), use of catecholamines (CB 57.6% vs NB 92.3%, p = .001), LOS in ICU (CB 5 [3; 12] vs NB 14 [6; 27], p = .009), hospitalization (10 [7; 17.5] vs 32 [16; 69], p < .001). Site of infection (p = .025): CB (urinary 30.3%, respiratory 24.2%, abdominal 15.2%), NB (abdominal 33.3%, CRB 25.6%, unknown 15.6%). Most frequent microorganisms isolated: CB (*E. coli* 33.3%, *S. epidermidis* 18.2%), NB (*S. epidermidis* 25.6%, *E. coli* 17.9%). Empiric antibiotic (ATB): CB (without ATB 42.4%, ceftriaxone 21.2%), NB (without ATB 30.8%, piperacillin/tazobactam 25.6%). Directed ATB: CB (ceftriaxone 18.2%, piperacillin/tazobactam 15.2%, meropenem 12.1%), NB (piperacillin/tazobactam 23.1%, meropenem + vancomycin 23.1%, imipenem 12.8%).

**CONCLUSIONS.** NB were related to increased use of central catheters, bladder catheter, surgical procedures, days of hospitalization and previous isolation of multiresistant bacteria. The mortality of bacteremia is high, although there were no differences between CB and NB. The most frequent site of infection were urinary (CB) and abdominal (NB).

### 0476

#### Mortality in septic patients admitted to the Intensive Care Unit (ICU) of a regional hospital

A. Ubeda[1], I. Fernández[1], R. Torcuato[1], A. Fregosi[1], J. Garcia[1], L. Alonso[2]
[1]Hospital Punta de Europa, Intensive Care Unit, Algeciras, Spain;
[2]Pediatrics La Lobilla, Estepona, Spain
**Correspondence:** A. Ubeda
*Intensive Care Medicine Experimental* 2017, **5(Suppl 2):**0476

**INTRODUCTION.** Septic patients frequently require ICU admission as well as agressive treatments such as intravenous antibiotics, mechanical ventilation and use of corticosteroids. Delay in time to start the treatment may lead to severe complications or death.

**OBJECTIVES.** To analyze the factors related to the mortality of septic patients admitted to the ICU of a regional hospital during 2016.

**METHODS.** Descriptive retrospective analysis performed in a 12-bed ICU during the year 2016. Demographic variables, comorbidities, risk factors, mortality predictors (APACHE II and SOFA), antibiotic therapy (ATB), ICU and hospital length of stay (LOS) and mortality. Statistical analysis: categorical variables (frequencies and percentages) and numerical variables (mean and standard deviation or medians and interquartile range). Comparisons: X2 test (percentages), Student test (means) and Kruskal-Wallis test (medians). Statistical significance with p < 0.05.

**RESULTS.** 94 patients were included: 58.5% male, medical patients 56.4%, surgical patients 40.4%, trauma patients 3.2%. Mean age 61.6 ± 14.3. APACHE II 17.6 ± 5.9. SOFA 7.4 ± 3.4. 48.9% came from the Emergency area and 35.1% came from a surgical area. The most common site of infection was abdominal (44.7%), followed by respiratory (26.6%) and urinary (13.8%). Global mortality: 36.2%. There were no statistically significant diferences between gender, age, type of patient, comorbidities, site of infection, ICU and hospital LOS. Patients who died had more days of mechanical ventilation (76.5% vs 43.4%, p = .002), corticoids (20.6% vs 5.0%, p = .033), higher APACHE II score (20.7 [5.2] vs 15.9 [5.6], p < .001), and SOFA score (9.5 [3.2] vs 6.2 [2.9], p < .001) at ICU admission. Most used antibiotics: piperacillin/tazobactam (17.6%), meropenem (17.6%) and ceftriaxone + levofloxacion (17.6%). Appropriate antimicrobial treatment: deceased 29.4% vs survivors 43.4%, p = .182. There was no difference in the de-escalation percentage.

**CONCLUSIONS.** The use of mechanical ventilation and corticosteroids were associated with higher mortality in septic patients admitted to the ICU, as well as a higher APACHE II and SOFA score at admission. The choice of the initial antimicrobial had no influence on mortality. The inadequate antimicrobial treatment was not significantly associated with higher mortality.

### 0477

#### Pre-hospital identification of septic patients: a comparison of process measures between patients identified pre-hospital and those identified in the emergency department

J. de Carvalho[1,2], D. Hargreaves[2], L. Hodgson[3], R. Venn[2]
[1]Brighton and Sussex Medical School, Brighton, United Kingdom;
[2]Western Sussex Hospitals Foundation NHS Trust, Worthing Department of Anaesthesia and Intensive Care Medicine, Worthing, United Kingdom;
[3]University Of Southampton, Southampton, United Kingdom
**Correspondence:** J. de Carvalho
*Intensive Care Medicine Experimental* 2017, **5(Suppl 2):**0477

**INTRODUCTION.** UK national guidelines advocate completion of Surviving Sepsis Campaign (SSC) resuscitation bundle within 1 hour of a patient being identified septic.[1] Bundle implementation has been associated with improved mortality outcomes.[2] Furthermore, pre-hospital identification of sepsis has been associated with more timely delivery of resuscitation measures [3].

**OBJECTIVES.** To compare the impact on measures of process and outcome in potentially septic patients flagged pre-hospital or in the emergency department (ED).

**METHODS.** A single-centre, prospective cohort (2014–16) of ED patients (n = 672), identified as potentially septic, was analysed for measures of process (time to antibiotics, to completion of SSC resuscitation bundle) and outcome (30 day mortality, admission to intensive care, length of hospital stay, final coded diagnoses of sepsis and/or infection). Those identified pre-hospital were compared with those identified on ED arrival.

**RESULTS.** 307 of the cohort were identified by ambulance crew pre-hospital and a further 365 were identified in the ED. Age, gender, previous medical history, lactate level and acute kidney injury incidence were not significantly different between the groups. Incidence of final coded diagnosis of sepsis was not significantly different between groups. However, when infection was added as final diagnosis code, significantly more pre-hospital patients had a final diagnosis of ´sepsis and/or infection´ (89% vs 82% $p < 0.05$).

There was a significant association with earlier delivery of processes of care in patients identified pre-hospital including antibiotics < 1 hour (72% vs 53%, $p < 0.0001$) and completion of a resuscitation bundle < 1 hr (32% vs 23%, $p < 0.01$) [Fig. 172a & b].

There was no significant difference in any outcome measures between either group. Additionally, for the whole cohort analysis of mortality against time to antibiotics and time to resuscitation bundle completion showed no significant association between variables ($p = 0.183$ and $p = 0.491$, respectively).

**CONCLUSIONS.** Pre-hospital use of a sepsis identification tool significantly improved time to antibiotics and completion of the SSC resuscitation bundle, but did not affect outcomes.

**REFERENCE(S)**

1. National Institute for Health and Care Excellence. Sepsis: Recognition, Diagnosis and early management London; NG51, National Clinical Guideline Centre July 2016.

2. D'Amore J.A., Abberbock S., Ward M., Masick K.D., Kabcenell A., Parmentier D., et al. The ESP (Early Sepsis Prophylaxis) study: Can implementation of a basic sepsis bundle reduce mortality? A three-year sepsis research registry experience. In: American College of Emergency Physicians 2013 Research Forum. Seattle, Washington: *Ann Emerg Med*; 2013: S3-4.

3. Lane D, Ichelson RI, Drennon IR, Scales DC. Prehospital management and identification of sepsis by emergency services: a systematic review. *Emerg Med J* 2016; 33: 408–13

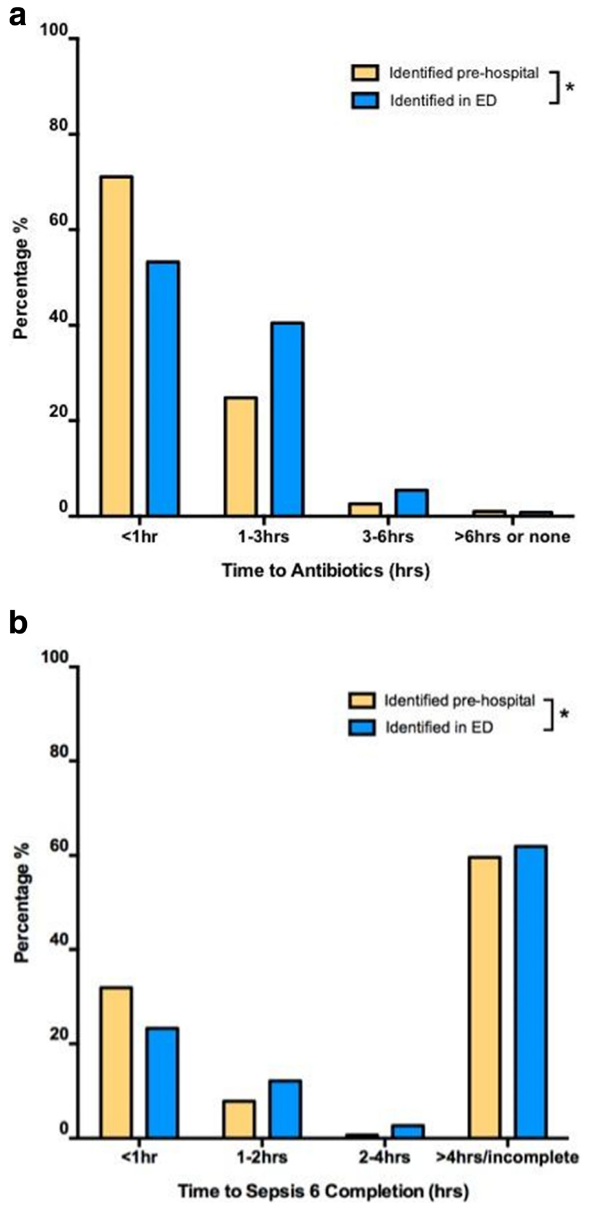

**Fig. 172 (Abstract 0477). a** Time to Antibiotics. **b** Time to Sepsis 6

#### 0478

**The incidence of sepsis in an Australian ICU: a prospective clinical diagnosis versus a retrospective database diagnosis**

M. Heldens[1,2,3], N. Hammond[2,3,4], F. Bass[2,3,4], A. Delaney[2,3,4], S. Finfer[2,3,4]
[1]Radboud University Nijmegen, Nijmegen, Netherlands; [2]Royal North Shore Hospital, Sydney, Australia; [3]University of Sydney, Sydney, Australia; [4]The George Institute of Global Health, Sydney, Australia
**Correspondence:** M. Heldens
*Intensive Care Medicine Experimental* 2017, **5(Suppl 2):**0478

**INTRODUCTION.** Estimates of the incidence of sepsis are often based on retrospective database or coding studies and the accuracy of these estimates in comparison with prospective cohort studies is not clear.

**OBJECTIVES.** To compare the incidence of sepsis derived from a prospective inception cohort study (clinically diagnosed sepsis) and that derived from retrospective database (ANZICS CORE) coding in ICU patients.

**METHODS.** This study is an inception cohort study including all patients admitted between the 1st of October and the 31st of December 2016 at the ICU of The Royal North Shore Hospital, St Leonards, Australia.

MAIN OUTCOME MEASURES: Incidence of sepsis and septic shock using a clinical and a database definition.

**RESULTS.** A total of 864 patients were admitted during the study period, of these 146 patients (16.9%) met the clinical definition of sepsis, of which 49/146 (33.6%) met the clinical definition for septic shock. A total of 98/864 patients (11.3%) met the database definition of sepsis, with 83/98 (84.7%) diagnosed with septic shock. Hospital mortality was higher in patients who met the clinical definition of sepsis (39/146, 26.7%), compared to patients that met the database definition of sepsis (17/98, 17.3%).

**CONCLUSIONS.** Our study showed that the incidence of sepsis using the ANZICS CORE database definition is lower and the incidence of septic shock higher when compared with a prospective clinical definition. The database definition also provides a falsely low estimate of sepsis mortality.

**GRANT ACKNOWLEDGMENT**
Radboud University Programme ´Beyond the Frontiers´

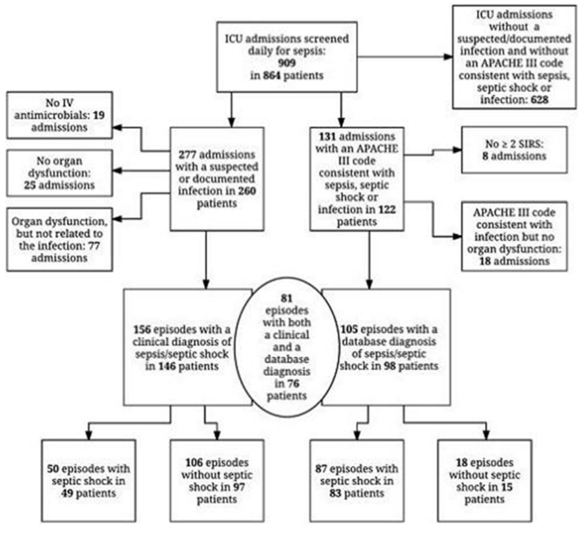

**Fig. 173 (Abstract 0478).** Outcome of 909 ICU admissions

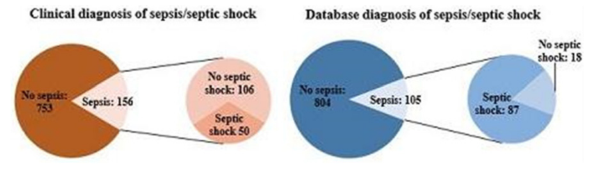

**Fig. 174 (Abstract 0478).** Incidence of episodes with sepsis and septic shock

### Trauma and burns management

#### 0479

**Differential cytokine expression between antigen responders and non-responders in critically ill burn and neurosurgical patients**

S.W. Mueller[1], E.T. Van Matre[1], R. Powell[2], K. Doenges[2], R. Maclaren[1], T.H. Kiser[1], D.N. Fish[1], R. Reisdorph[2], N. Reisdorph[2]
[1]University of Colorado Skaggs School of Pharmacy and Pharmaceutical Sciences, Department of Clinical Pharmacy, Aurora, United States; [2]University of Colorado Skaggs School of Pharmacy and Pharmaceutical Sciences, Department of Pharmaceutical Sciences, Aurora, United States
**Correspondence:** S.W. Mueller
*Intensive Care Medicine Experimental* 2017, **5(Suppl 2):**0479

**INTRODUCTION.** Previously we have shown divergent humoral responses to 23-valent pneumococcal polysaccharide vaccine (PPSV23) when administered to critically ill burn and neurosurgical (NS) patients. Burn patients generated a robust response to PPSV23 compared to a blunted response in NS patients.

**OBJECTIVES.** Utilizing PPSV23 responsiveness as a marker of antigen challenge, we assessed differences in biomarker concentrations at time of vaccination (D0), 14 days after vaccination (D14) and changes in concentration over 14 days; correlating values with successful antigen challenge in two critically ill populations.

**METHODS.** Following consent, patients were administered PPSV23 within six days of injury. Blood samples were obtained at D0 and D14. Samples from 32 (10 burn and 22 NS) patients were analyzed.

Of those, 16 were male with a mean age of 51 ± 16, APACHE II score of 16 ± 8, 22 required mechanical ventilation, 20 required vasopressors, 6 died prior to discharge, and 17 responded to PPSV23 antigen challenge. An electrochemiluminescence immunoassay was used to measure 30 cytokines (V-plex, MesoScaleDiscovery). Associations between antigen responsiveness and cytokine differences at D0, D14 and the change over time was assessed by non-parametric methods using a significance value of $p < 0.05$. Further, we compared cytokine expression between burn and NS patients.

**RESULTS.** At D0, antigen responders had significantly higher levels of G-CSF, IL-12p70, IL-15, IL-4, and IL-6; and significantly lower Eotaxin and MCP-4. No significant associations were observed between cytokine level and antigen responsiveness at D14. Increases in IL-16, MIP-1α, and TNFα, and significant decreases in G-CSF and IL-12p70 over 14 days were significantly associated with antigen response. Burn patients had significantly higher levels of G-CSF, IL-10, IL-12p70, IL-1β, IL-2, IL-4, IL-6 and IL-8, as well as lower levels Eotaxin compared to NS patients at D0. At D14, burn patients had higher Eotaxin-3, G-CSF, IL-1β, and VEGF levels compared to NS patients. Those with burn injuries had significant decreases in G-CSF, IL-10, IL-12p70, IL-1β, IL-2, IL-6, and IL-8 levels; whereas, TARC and VEGF significantly increased over 14 days compared to NS patients.Further cytokine analysis and models of antigen responsiveness will be constructed.

**CONCLUSIONS.** Our findings suggest that immunologic responsiveness by PPSV23 antigen challenge during critical illness is related to measurable differences in cytokines at the time of challenge and changes over time. Although exploratory, we've identified differences in biomarkers that relate to a T-cell independent immune response. Burn patients have higher levels of many measured cytokines at D0 and a greater decrease by D14 which, coupled with our previous observations of burn patients being more likely to respond to PPSV23, supports an immune responsiveness phenotype. Further research is needed to identify derangements in immune non-responsive phenotypes.

### 0480

#### Mortality study of burn patients in a critical burn unit

L. Cachafeiro Fuciños[1], A. Agrifoglio Rotaeche[2], E. Herrero de Lucas[2], M. Sanchez Sanchez[2], P. Millan Estañ[2], E. Flores Cabezas[2], A. Garcia de Lorenzo[2]
[1]Hospital Universitario La Paz, Critical Care, Madrid, Spain, [2]Hospital Universitario La Paz, Madrid, Spain
**Correspondence:** L. Cachafeiro Fuciños
*Intensive Care Medicine Experimental* 2017, **5(Suppl 2):**0480

**INTRODUCTION.** Total burned surface area, age and inhalation injury are the main factors related with mortality of burn patients.
**OBJECTIVES.** The aim of our study is to evaluate the mortality in a critical burn unit.
**METHODS.** A prospective, observational and descriptive study was conducted over a period of two years in burn patients admitted to a critical burn unit considered as a national burn reference center. We considered critical burn patient when total burn surface area (TBSA) > 20% or while having a minor TBSA the patient has associated at least one of the following issues: trauma, deep affectation of the face and / or neck, advanced age (greater than 75 years), previous illness, inhalation injury, electrical or chemical mechanism. Demographic data, TBSA, ABSI, Baux score, APACHE II, SOFA, mechanical ventilation, complications, length of stay, hospital course and mortality data were collected. Data are presented as number and percentage or as median and interquartile range and they were analyzed with Fisher exact test and Mann–Whitney test.
**RESULTS.** During this period 362 burns patients were admitted in our unit. Nearly 50% (180 patients) were considered as critical ill patients. 84 patients had inhalation injury. Demographic characteristics and severity scores are shown in the Table 132.
34.3% of burn patients required mechanical ventilation, 59.4% of critical burn patients and 88.1% of those who had inhalation injury. Critical burn patients had more respiratory, renal, infectious complications and more need of vasoactive support than those who were not considered critical, but there were no significant differences with patients who also had inhalation injury. There was no statistically significant difference in volume used during initial resuscitation in the different groups, nor in the average stay.
Mortality was 9% for burn patients, 18.8% for critical burn patients and 28.6% for inhalation injury. The multivariate analysis shows that the age TBSA and inhalation injury are independent factors of mortality in our population. However, when we analyzed mechanical ventilation as a mortality factor, we observed that mechanical ventilation is also an independent factor of mortality. Mechanical ventilation and TBSA are the most influence mortality's factors.
**CONCLUSIONS.** In our study, patients with inhalation injury had the highest severity scores, with a greater necessity for mechanical ventilation and higher mortality.
We found that TBSA, age, inhalation injury and mechanical ventilation were independent risk factors for mortality in burn patients. However, mechanical ventilation and TBSA are the most related factors to mortality in our population.

**REFERENCE(S)**
Sheridan RL.Fire-related inhalation injury. N Engl J Med (2016);375:464–9
Mackie DP.Inhalation injury or mechanical ventilation: which is the true killer in burn patients? Burns. 2013;39:1329–30

**Table 132 (Abstract 0480)**. See text for description

| | Burn patients | Critical burn patients | Inhalation injury |
|---|---|---|---|
| Patients | 362 | 96 | 84 |
| Male (%) | 76,2 | 72.9 | 75,6 |
| Age | 45.2 ±19.1 | 44.9±18.5 | 52.5.2 ±17.5 |
| TBSA (%) | 18.7 ± 16.8 | 34,8±17,5 | 28.1 ± 25.7 |
| ABSI | 5.9 ± 2.2 | 7,6±2,3 | 8.2 ± 2.8 |
| Baux | 64,1± 26,8 | 79,82±28.90 | 80,5± 27,8 |
| APACHE II | 7.4 ± 6.9 | 12.2±6.8 | 13.3 ± 6.8 |
| SOFA | 1.7 ± 2.2 | 2.3 ± 2.7 | 4.7 ± 2.8 |

### 0481

#### Microbiologic study of pad used for packing in damage control laparotomy

Y. Park, J. Kim, W. Kang, Y. Jo
Chonnam National University Hospital, Gwangju, Korea, Republic of
**Correspondence:** J. Kim
*Intensive Care Medicine Experimental* 2017, **5(Suppl 2):**0481

**INTRODUCTION.** Damage control laparotomy (DCL) is a technique utilized to control the massively injured abdominal trauma patients. This operation is well known as a life saving procedure, especially in coagulopathy, nevertheless, prolonged open abdomen and pad packing is likely associated with increased morbidity and mortality.
**OBJECTIVES.** We conducted microbiologic analysis of the pad used for packing in DCL and studied its association with morbidity and mortality.
**METHODS.** This is a retrospective review of all patients undergoing immediate laparotomy at Chonnam National University Hospital Trauma Center between 2011 and 2015. DCL was defined as temporary abdominal closure at the initial surgery. 18 consecutive patients undergoing DCL were analyzed. Microbiologic samples from pad used in DCL were collected and analyzed.
**RESULTS.** 15 microorganisms were cultured. Samples from 12 (66.7%) patients were positive by microbiologic culture and six (33.3%) patients were negative. Morbidity rate (91.7% vs. 66.7%) and mortality rate (41.7% vs. 16.7%) were higher in patients with positive culture than patients with negative culture. Infection rates such as surgical site infection (75.0% vs. 33.3%) and sepsis (41.7% vs. 16.7%) were higher in the culture-positive patients. Four patients underwent two or three take back surgeries and all samples from these patients were positive for microorganism.

**CONCLUSIONS.** There was a high infectious complication rate in patients with positive culture of the pad. And two or more frequent take back surgery seems to increase risk of infection in DCL.

**REFERENCE(S)**

ABIKHALED, John A., et al. Prolonged abdominal packing for trauma is associated with increased morbidity and mortality. *The American surgeon*, 1997, 63.12: 1109.

**GRANT ACKNOWLEDGMENT**

None

### 0482

#### Traumatic brain injury (TBI) outcomes in an LMIC tertiary care centre and performance of trauma scores

S. Samanmalee[1], P.C. Sigera[2], A.P. De Silva[3], K. Thilakasiri[2], A. Rashan[2], S. Wadanambi[4], P.L. Athapattu[1], K.A.S. Jayasinghe[5], A.M. Dondorp[6], R. Haniffa[2]

[1]Ministry of Health, Colombo, Sri Lanka; [2]Network for Improving Critical Care Systems and Training, Colombo, Sri Lanka; [3]National Intensive Care Surveillance, Colombo, Sri Lanka; [4]National Hospital of Sri Lanka, Colombo, Sri Lanka; [5]Faculty of Medicine, University of Colombo, Colombo, Sri Lanka; [6]Mahidol Oxford Tropical Medicine Research Unit, Bangkok, Thailand

**Correspondence:** R. Haniffa

*Intensive Care Medicine Experimental* 2017, **5(Suppl 2):**0482

**INTRODUCTION.** The burden of TBI is greatest in Low and Middle Income Countries (LMIC), where 85% of the world´s population live (De Silva et al., 2008).

**OBJECTIVES.** This study evaluates post-ICU outcomes of patients admitted with moderate and severe TBI to a tertiary neurocritical care unit in an LMIC and the performance of RTS, ASCOT, ISS and TRISS in this setting.

**METHODS.** Consecutive adult patients directly admitted to the Neuro Trauma Intensive Care Unit (NTICU) I, II and Emergency Treatment Unit (ETU) of the National Hospital of Sri Lanka (NHSL) between 21st July 2014 and 1st October 2014 with moderate or severe TBI were recruited for this study. Socio-demographic and injury related data were gathered from the patient or the next of kin during their stay in the setting. Medical data was extracted from case notes.

A telephone administered questionnaire based on the GOSE scale was used to assess functional outcome of patients at 3 and 6 months after injury. The economic impact of the injury was assessed before injury, and at 3 and 6 months after injury based on an adapted tool from Griffiths et al.(Bouamra et al. 2015).

**RESULTS.** 101 patients with TBI were included in the study. Majority of patients had severe TBI (n = 63) and others had moderate TBI (n = 38). Survival at ICU discharge, 3 months and 6 months was 68.3%, 49.5% and 45.5% respectively.

Out of survivors at 3 months after ICU/ETU discharge, 43 (86%) were living at home while others were in rehabilitation centres or in hospital. Respectively, only 19 and 20 patients had a good recovery (as defined by GOSE 7 and 8) at 3 and 6 months after ICU/ETU discharge. Three months and six months after ICU/ETU discharge, respectively 25 patients and 14 patents had become "economically dependent". Similarly, 17 families who were "economically independent" prior to the event became dependent and 10 families were still dependent 6 months after discharge.

ISS, RTS, ASCOT and TRISS had poor discriminatory ability in predicting mortality.

**CONCLUSIONS.** This observational study of patients sustaining moderate or severe TBI in Sri Lanka reveals only 46% of patients were alive at 6 months after ICU discharge and only 20% overall having a good recovery. The social and economic consequences of TBI were long lasting in this setting. ISS, RTS, ASCOT and TRISS all performed poorly in predicting mortality in this setting and illustrate the need for setting adapted tools.

The authors acknowledge the support given by staff at NTICU I, II and ETU of the NHSL, Sri Lanka.

**REFERENCE(S)**

Bouamra, O. et al., 2015. Prediction modelling for trauma using comorbidity and "true" 30-day outcome. Emergency medicine journal : EMJ, 32(12), pp.933-938.

De Silva, MJ. et al., 2008. Patient outcome after traumatic brain injury in high, middle and low income countries analysis of data on 8927 patients in 46 countries. International Journal of Epidemiology, 38(2Pp), pp.452-458.

**GRANT ACKNOWLEDGMENT**

None

**Table (Abstract 0482).** Functional outcome at 3 months after discharge

| | Moderate injury (n = 38) | Severe injury (n = 63) | Total (n = 101) | RTA (n = 68) | Fall (n = 24) |
|---|---|---|---|---|---|
| Dead/Vegetative (GOSE 1,2) | 16(42) | 33(52) | 49(49) | 36(53) | 9(38) |
| Disability (GOSE 3,4,5,6) | 10(26) | 16(25) | 26(26) | 17(25) | 7(29) |
| Good Recovery (GOSE 7,8) | 9(24) | 10(16) | 19(19) | 10(15) | 6(25) |

**Table (Abstract 0482).** Trauma scores predicting 30 days mortality

| Trauma severity scores | ROC AUC (upper 95% CI, lower 95% CI) | Hosmer Lemeshow C-statistic | HL p-value |
|---|---|---|---|
| ASCOT | 0.62 (0.51,0.73) | 7.49 | 0.4853 |
| TRISS | 0.67 (0.56,0.78) | 15.83 | 0.0449 |
| RTS score | 0.53 (0.41,0.65) | | |
| ISS score | 0.46 (0.36,0.56) | | |

### 0483

#### Complications and mortality in severe trauma inpatient after intensive care unit discharge

L. Marqués Álvarez[1], R. Rodríguez García[1], L. Villota Ferreiro[2], L. Pérez Belmonte[1], D. Escudero Augusto[1], L.A. Cofiño Castañeda[1], R. Yano Escudero[1], L. Álvarez García[1], C. Palomo Antequera[1], M. Martínez Revuelta[1], B. García Arias[1]

[1]Hospital Universitario Central de Asturias, Oviedo, Spain; [2]Universidad de Oviedo, Oviedo, Spain

**Correspondence:** R. Rodríguez García

*Intensive Care Medicine Experimental* 2017, **5(Suppl 2):**0483

**OBJECTIVES.** Trauma is a major health problem in the world whose complications are associated with increased morbidity, length of stay, and mortality and are also responsible for a increase of the overhead costs. Identification of the epidemiology, clinical parameters, and causes of complications following trauma may provide useful information for improving treatment strategies. The aims of our study were to evaluate the incidence and type of complications after trauma among patients admitted to the ICU.

**METHODS.** We made a retrospective and observational study of polytrauma patients admitted to the ICU from January 2015 to December 2015. Information was collected on the severity of illness. Demographic and clinical data, including age, sex, and mechanism of injury, procedures, hospital length of stay, complications, and inhospital mortality were obtained.

**RESULTS.** This study included 190 patients, 18 years of age or older, presenting with trauma and admitted to ICU. The mean age was 57 + 18 years and 70% were men. On admission the mean value of APACHE II and GCS was 19 and 12 points respectively. Most frequent medical history was alcoholism (35%), smoking (29%) and psychiatric history (29%). 55% came from other hospital, 41% from emergency department and 3% from operating room. Overall falls (53%) were the most frequent cause of trauma, followed by traffic accidents (33%) and autolytic attempts (7%). Autolytic attemps were the most frequent in < 50 years. The most common type of trauma was cranioencephalic (45%) followed by thoracic (36%). The complications developed in the hospitalization ward, from highest to lowest frequency, were: 39.5% cardiovascular (AHT, arrhythmias, pulmonary embolism and coronary artery disease), 30.5% infectious (skin and soft tissue, urinary with/without bacteremia), 25.8% hematological (anemia and coagulopathy), 24% metabolic (hyponatremia), 19.5% abdominal (paralytic ileus and acute cholecystitis) and 14% neurological (acute confusional syndrome and epileptic seizures). Cardiocirculatory diseases (82%) were more frequent in patients older than 50 years and neurological (40%) in patients younger than 50 years. Overall, the acute confusional syndrome was the most frequent complication in the hospital ward (34.2%). Most frequent indications of returned to the ICU were infections and decreased level of consciousness. The average stay in ICU and in the hospital ward was of 9 and 18 days respectively. Hospital ward mortality was 29.2%.

**CONCLUSIONS.** Critical polytrauma patients have been hospitalized for a long time. In general, most frequent complications were cardiocirculatory, followed by infectious, hematological and metabolic, although the most common was acute confusional syndrome. Inpatient mortality is low and usually occurs within the first ten days.

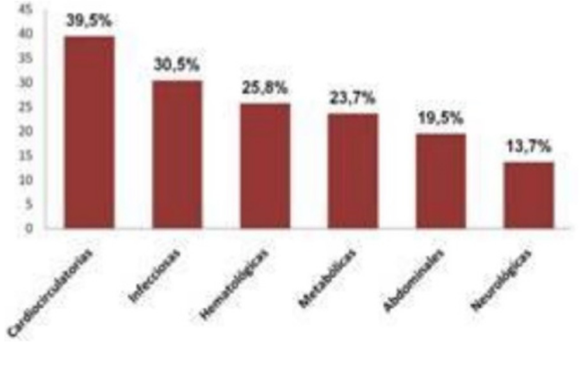

**Fig. 175 (Abstract 0483).** Global complications

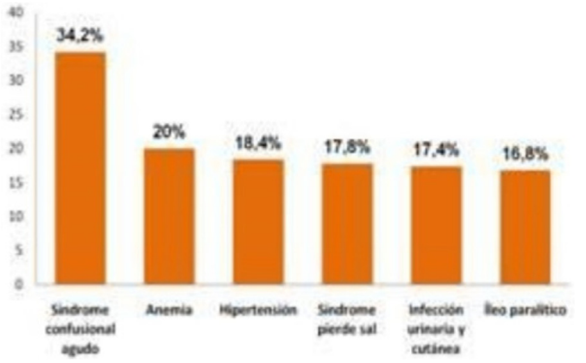

**Fig. 176 (Abstract 0483).** Specific complications

### 0484

#### The impact of cumulative fluid balance on mortality and late multiple organ failure of trauma patients with prolonged ICU stay

K. Okada[1], N. Saito[1], M. Kujo[1], T. Yagi[1], H. Matsumoto[1], H. Yokota[2]
[1]Nippon Medical School Chiba Hokusoh Hospital, Shock and Trauma Center, Inzai, Japan; [2]Nippon Medical School, Emergency and Critical Care Medicine, Tokyo, Japan
**Correspondence:** K. Okada
*Intensive Care Medicine Experimental* 2017, **5(Suppl 2):**0484

**INTRODUCTION.** The increased attention has been paid to detrimental effects of excessive volume resuscitation in acute phase of trauma, including ARDS, coagulopathy and abdominal compartment syndrome[1,2]. However, the optimal long-term fluid management remains unclear in trauma patients.

**OBJECTIVES.** To clarify the effects of cumulative fluid balance on the outcome of trauma patients with prolonged stay in ICU.

**METHODS.** We retrospectively reviewed trauma patients who had stayed in ICU for more than 10 days. Patients were classified into high (≥12L) or low (<12L) fluid balance according to cumulative balance for 10 days in ICU. Multivariate logistic regression was conducted to assess the associated factors of 28-day mortality rate and developing late multiple organ failure.

**RESULTS.** 81 patients were included in this analysis. 28 patients (34.6%) had the high cumulative fluid balance (≥12L). The high fluid balance group was associated with higher 28-day mortality rate compared to low balance group (17.9% vs 1.9%, $P = 0.017$). The maximum SOFA score during the period of 10 days in ICU of both groups were 12.6 ± 3.0 and 9.5 ± 3.0, respectively ($P < 0.001$). Multivariate analysis revealed that high cumulative fluid balance was independently associated factors of 28-day mortality (odds ratio [OR] 15.0, 95% confidence interval [CI] 1.3 to 174.7, $P < 0.03$) and developing SOFA score greater than 10 during the period of ICU stay (odds ratio [OR] 6.8, 95% confidence interval [CI] 2.0 to 23.3, $P = 0.002$).

**CONCLUSIONS.** High cumulative balance for 10 days in ICU is significantly associated with risk of mortality and late multiple organ failure.

**REFERENCE(S)**

1. Kasotakis G, Sideris A, Yang Y, et al. Aggressive early crystalloid resuscitation adversely affects outcomes in adult blunt trauma patients: an analysis of the Glue Grant database. J Trauma Acute Care Surg 2013;74:1215–21; discussion 21–2.

2. Barmparas G, Ko A, Harada MY, et al. Decreasing maintenance fluids in normotensive trauma patients may reduce intensive care unit stay and ventilator days. J Crit Care 2016;31:201–5.

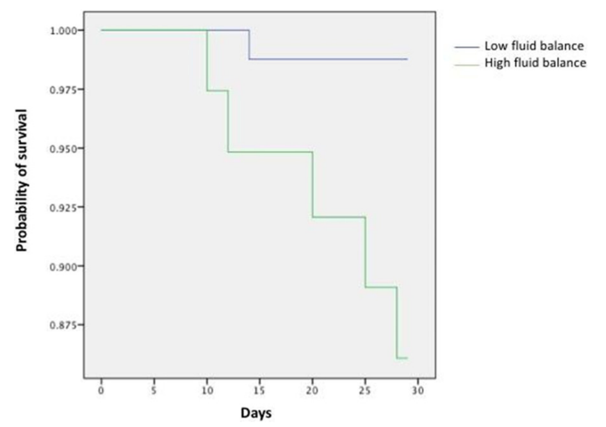

**Fig. 177 (Abstract 0484).** Comparison of probability of survival

### 0485

#### Prognostic analysis of pulmonary contusion in severe head trauma patients

S. Snouda[1], F. Hebaieb[1], M.F. Abbes[2], M. Kaddour[1], H. Ben Ghezala[1]
[1]Regional Hospital of Zaghouan, Emergency and Intensive Care, Zaghouan, Tunisia; [2]Regional Hospital of Zaghouan, Cardiology Department, Zaghouan, Tunisia
**Correspondence:** S. Snouda
*Intensive Care Medicine Experimental* 2017, **5(Suppl 2):**0485

**INTRODUCTION.** Severe head trauma is a common reason for patient's admission to the intensive care unit and it is the leading cause of death in young adults. The outcome of these patients depends on many factors such as cerebral hypoxia. Pulmonary contusion can cause an acute respiratory failure that aggravates head trauma.

**OBJECTIVES.** To evaluate the impact on morbidity and mortality of pulmonary contusion in patients admitted for severe head trauma.

**METHODS.** This was a retrospective study performed in the emergency and intensive care department in the regional hospital of Zaghouan. The patients were recruited during 3 years, from January 2013 to December 2015. All adult patients admitted for severe head trauma (Glasgow coma score (GCS) ≤ 8) were included and evaluated by quantified injury severity scores including APACHE2, GCS, ISS, TISS. The diagnosis of pulmonary contusion was based on the history of blunt chest trauma and the appearance of four types of lesions on chest CT scans. Hospital length of stay, mortality rate, incidence of adverse events including pneumonia, ARDS and shock were recorder and compared into the tow groups (with or without pulmonary contusion).

**RESULTS.** During the study period, 104 patients were included. The diagnosis of pulmonary contusion was retained in 34 patients (32.7%). The two groups were compared for age, genre, APACHE 2 and GCS. There was a significant difference in the ISS and TISS ($p < 0.001$). Patients with pulmonary contusion had significantly lower $PaO_2/FiO_2$ ratio (240 [range, 160–380]) vs. 430 [range, 210–590]; p = 0.009); and significantly higher $PaCO_2$ levels (43.5 mmHg [range, 38–52 mmHg] vs. 38 mmHg [range, 32–40 mmHg], $p < 0.001$). The number of cases of pneumothorax and hemothorax were significantly higher in the group of pulmonary contusion (p = 0.003, p = 0.021; respectively). Likewise, The occurrence of pneumonia and ARDS was significantly higher in the group of pulmonary contusion (p = 0.031, p = 0.023; respectively) with a shorter length of time between ICU admission and the occurrence of such complications; (6 days [range, 6–10 days]) vs. 13 days [range, 8–16 days]; p = 0.024) and (6 days [range, 2–8 days]) vs. 10 days [range, 8–10 days]; p = 0.016), respectively. No significant difference was observed with regard to the number of cases of shock (p = 0.174). The length of hospital stay was significantly higher in the group of pulmonary contusion (13.44 ± 6 days vs. 12.44 ± 4 days; $p < 0.001$). The overall mortality rate was 11.5% in pulmonary contusion group and 7.7% in the other group (Odds ratio, 4.22; 95% confidence interval, 1.527 to 11.703; p = 0.004).

**CONCLUSIONS.** In this study, pulmonary contusion alters gas exchange and appears to increase the morbidity and mortality in patients with severe head trauma.

### 0486

#### Risk factors for delirium in patients with abdominal trauma

J. Kim, Y. Park, W. Kang, Y. Jo
Chonnam National University Hospital/Chonnam National University Medical School, Department of Surgery, Gwangju, Korea, Republic of
**Correspondence:** Y. Park
*Intensive Care Medicine Experimental* 2017, **5(Suppl 2):**0486

**INTRODUCTION.** The occurrence of delirium is well known risk factors associated with poor prognosis in ICU patients.

**OBJECTIVES.** The purpose of this study is to identify risk factors for delirium after abdominal trauma and to predict the development of delirium.

**METHODS.** Data was collected retrospectively from August 2015 to December 2016 at a regional truma center on consecutive trauma patients. Head trauma patients and patients under 18 years were excluded. A multivariate logistic regression was performed to identify risk factors for delirium.

**RESULTS.** Of the 264 patients who met criteria, 32 (12.1%) were diagnosed with delirium. The mean age of the patients was 52 ± 19 years, 15.7 for Injury Severity Score(ISS), 14.4 for Glasgow Coma Scale(GCS) and 3.6 days for Intensive Care Unite(ICU) stay. In addition, chest Abbreviated Injury Score(AIS) was 1.2 ± 0.9, abdomen AIS was 2.9 ± 0.1 and extremity AIS was 1.0 ± 0.9. Among these factors, Age(odds ratio[OR], 1.05; 95% confidence interval [CI], 1.02-1.08; p = 0.01), ICU day(odds ratio[OR], 1.14; 95% confidence interval [CI], 1.04-1.24; p = 0.04), abdominal operation(odds ratio[OR], 2.54; 95% confidence interval [CI], 1.10-5.85; p = 0.03) were correlated with delirium.

**CONCLUSIONS.** Abdominal operation is strongly asscociated with delirium in patients with traumatic injury. Careful observation of changes in consciousness after operation is necessary.

**REFERENCE(S)**

PANDHARIPANDE, Pratik, et al. Prevalence and risk factors for development of delirium in surgical and trauma ICU patients. *The Journal of trauma*, 2008, 65.1: 34.

**GRANT ACKNOWLEDGMENT**
None

### 0487

#### Disseminated intravascular coagulation during the early stage of isolated traumatic brain injury

K. Katabami, Y. Itagaki, T. Wada, K. Maekawa, H. Sageshima, M. Hayakawa, A. Sawamura, S. Gando
Hokkaido University Hospital, Sapporo, Japan
**Correspondence:** K. Katabami
*Intensive Care Medicine Experimental* 2017, **5(Suppl 2):**0487

**INTRODUCTION.** There is ample evidence demonstrating that disseminated intravascular coagulation (DIC) occurs in patients with traumatic brain injury.

**OBJECTIVE.** The aim of this study was to test the hypothesis that DIC during the early stage of isolated traumatic brain injury (iTBI) contributes to systemic hypoperfusion and organ dysfunction, which affects the outcome of these patients.

**METHODS.** We conducted a retrospective cohort study of 335 trauma patients with brain injury. iTBI was defined as an abbreviated injury scale of the head ≥3 and other body parts ≤ 2. Out of 335 patients, 92 patients with iTBI were included in the present study. These patients were divided into DIC (n = 45) and non-DIC (n = 47) groups based on the Japanese Society for Acute Medicine DIC criteria on admission (day 0). Systemic hypoperfusion was defined as a blood lactate level of ≥ 4 mmol/L and the severity of injury was assessed by the injury severity score (ISS). The sequential organ failure assessment (SOFA) and systemic inflammatory response syndrome (SIRS) scores were also evaluated. The main outcome measure was the all-cause hospital mortality.

**RESULTS.** There were no significant differences in the background data, including the ISS of the two groups. The mean (SD) DIC scores of the DIC and non-DIC patients were 5.1 (1.4) and 1.5 (0.8), respectively. The prevalence of systemic hypoperfusion among the DIC patients (64.4%) was significantly higher than that among the non-DIC patients (23.4%), and a significant difference was observed in the lactate levels of the two groups (5.3 [2.5] vs. 3.2 [1.6]). All DIC patients developed SIRS. The SOFA scores of the DIC patients were significantly higher than those of the patients without DIC. DIC patients required a larger volume of transfusion than the non-DIC patients. The mortality rate of the DIC patients (28.9%) was more than twice that of the non-DIC patients (10.6%) ($p = 0.025$).

A stepwise logistic regression analysis and the area under receiver operating characteristic curves confirmed that DIC is an independent predictor of death. The Kaplan-Meier curves showed that DIC significantly reduced the survival rate of patients with iTBI.

**CONCLUSION.** DIC accompanied by systemic hypoperfusion and organ dysfunction contributes to a poor outcome in patients with iTBI.

### 0488

#### Differential time course of creatinine and urea after major trauma and their association with duration of hospitalisation

R. Haines[1,2], D. Lin[1,2], C.J. Kirwan[1,2], J.R. Prowle[1,2]
[1]Queen Mary University of London, William Harvey Research Institute, London, United Kingdom; [2]Barts Health NHS Trust, Adult Critical Care Unit, The Royal London Hospital, London, United Kingdom
**Correspondence:** R. Haines
*Intensive Care Medicine Experimental* 2017, **5(Suppl 2):**0488

**INTRODUCTION.** During critical illness assessment of renal function using serum creatinine may be confounded by decrease in its generation [1,2]. Major trauma patients are at particular risk of acute muscle wasting resulting in reduced creatinine production while the time course of changes in urea after major trauma has not been well-described.

**OBJECTIVES.** To examine the time course of changes in creatinine, urea and urea:creatinine ratio in major trauma cases admitted to ICU.

**METHODS.** Single centre retrospective analysis of major-trauma ICU admissions hospitalised for ≥7 days, excluding those who received renal replacement therapy and deaths before d7.

**RESULTS.** In 1021 patients, median age was 43 (IQR: 27–58), Injury Severity Score 27 (17–33), Hospital length of stay 24d (17–33), ICU length of stay 8d (4–15); 8.7% died in hospital and 15% experienced non-RRT-requiring acute kidney injury. After admission median creatinine fell rapidly over the first 7 days while median urea *rose* progressively after d4 (Figs. 178 and 179). In 647 patients with measurements on d7, creatinine had fallen from median 81μmol/L (66–102) at admission to 56μmol/L (47–71) by d7 ($p < 0.0001$), over the same period urea rose from median 4.9 (3.9-6.4) to 6.6mmol/L (5.0-8.9) ($p < 0.0001$) and median Urea:Creatinine ratio almost doubled from 62 (45–77) to 116mmol/mmol (89–147) ($p < 0.0001$). Median Ure:Cre ratio at d7 was significantly higher in patients who had either died or were still in hospital ten days later (at d17) compared to those who were discharged in this interval: 122 (96–154) vs 85mmol/mmol (63–108) $p < 0.0001$, Fig. 180. However, in isolation creatinine at d7 did not predict longer hospitalisation, falling similarly to day 7 values of 56 vs. 57μmol/L respectively in those discharged by d17 and those dying or remaining in hospital ($p = 0.65$).

**CONCLUSIONS.** After major trauma creatinine progressively fell while urea rose, potentially reflecting differential changes in their production or clearance in response to major injury, muscle wasting and tissue catabolism. In support of this hypothesis, longer hospitalisation or death was associated with an acute and sustained rise in Ure:Cre ratio in the first seven days after injury. Higher Ure:Cre ratios have been previously associated with adverse outcomes in critical illness [3] however this study is novel in documenting dynamic changes in Ure:Cre ratio and outcome. Studies examining both production and clearance of creatinine and urea are required to understand the changes in these markers of renal function after major trauma and their association with patient outcomes.

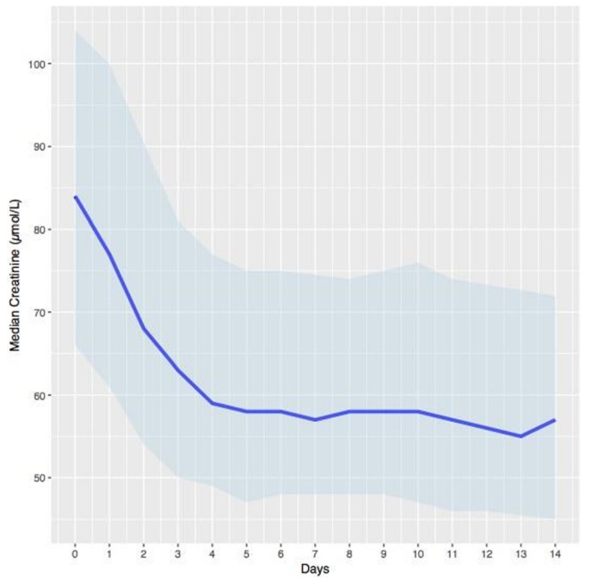

**Fig. 178 (Abstract 0488).** Time-course of Creatinine

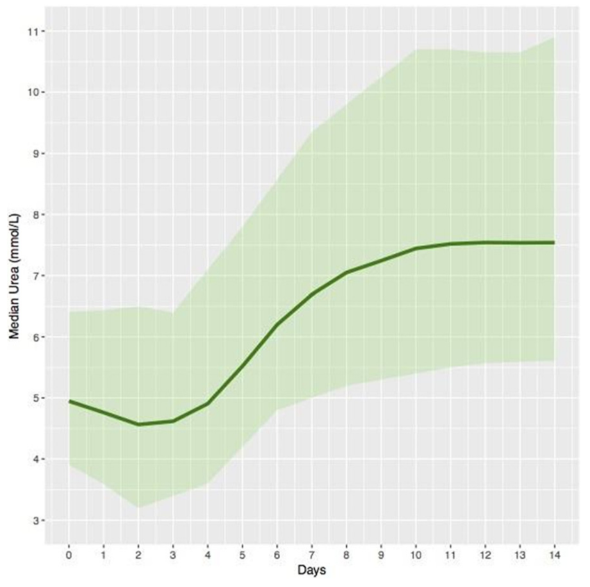

**Fig. 179 (Abstract 0488).** Time-course of Urea

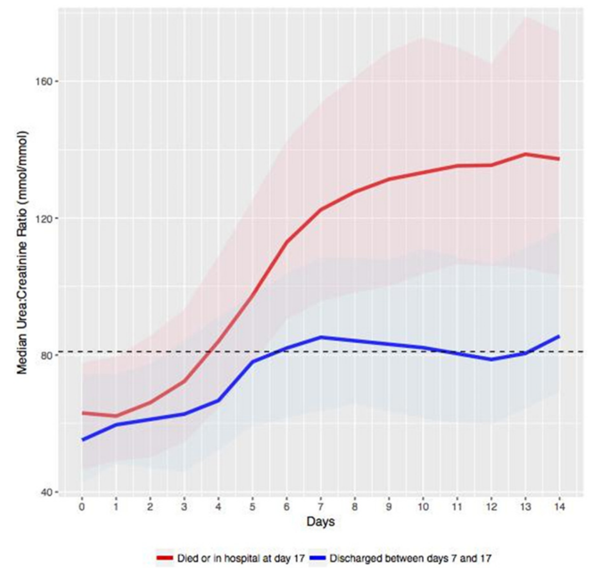

**Fig. 180 (Abstract 0488).** Time course of Ure:Cre ratio by outcome

### 0489

#### Hemostatic effect and complications of preperitoneal pelvic packing in patients with hemodynamic instability due to pelvic fracture

G.S. Ha[1], H. Shim[2], H.Y. Kwon[2], H.-J. Chung[3], P.Y. Jung[2], K.S. Bae[2], S. Kim[2], J.Y. Jang[2]
[1]Wonju Severance Christian Hospital, Department of Surgery, Wonju, Korea, Republic of; [2]Yonsei University Wonju College of Medicine, Department of Surgery, Wonju, Korea, Republic of; [3]Yonsei University Wonju College of Medicine, Department of Orthopedic Surgery, Wonju, Korea, Republic of
**Correspondence:** G.S. Ha
*Intensive Care Medicine Experimental* 2017, **5(Suppl 2):**0489

**INTRODUCTION.** Despite using a multidisciplinary treatment approach, the mortality with hemodynamic instability due to severe pelvic fracture is still 40-60%. Recently, several studies have showed that preperitoneal pelvic packing (PPP) was useful hemostatic technique for these patients in the acute phase. However, there are few studies about complications after the procedure.

**OBJECTIVES.** The purpose of present study was to evaluate the efficacy and complications of PPP in these patients.

**METHODS.** We retrospectively reviewed the medical charts of 53 patients with hemorrhagic shock due to pelvic fracture between March 2011 and February 2017.

**RESULTS.** Twenty-eight patients in the PPP group had significantly lower hemorrhagic mortality rate than 25 patients in the non-PPP group (17.9% vs 48.0%, p = 0.019), although both groups had similar patient characteristics(age, Injury severity score, and initial serum lactate). In 28 patients of PPP group, the mean age and injury severity score were 61.6 years and 39.3. Pelvic external fixation (EF) was performed concurrently with PPP in 7 patients (25%). Complications occurred in 11 of 23 patients except 5 patients who died of bleeding in the PPP group. The most common complication was pneumonia (72.7%), followed by surgical site infection (36.4%), and meningitis (18.2%). Patients with complication underwent more frequently pelvic EF with PPP, compared with patients without complication
(26.1% vs 0%, p = 0.005).

**CONCLUSIONS.** Although PPP is an effective method of hemostasis, complications after the procedure should be considered.

**REFERENCE(S)**

Jang JY et al. Preperitoneal pelvic packing in patients with hemodynamic instability due to severe pelvic fracture: early experience in a Korean trauma center. Scand J Trauma Resus. 2016;24.

Burlew CC et al. Preperitoneal Pelvic Packing/External Fixation with Secondary Angioembolization: Optimal Care for Life-Threatening Hemorrhage from Unstable Pelvic Fractures. J Am Coll Surgeons. 2011;212(4):628–35

Burlew CC et al. Preperitoneal pelvic packing reduces mortality in patients with life-threatening hemorrhage due to unstable pelvic fractures. Journal of Trauma and Acute Care Surgery. 2016.

**GRANT ACKNOWLEDGMENT**

None

**Table (Abstract 0489).** Comparison according to infectious complication

| | No complication (12) | Complication (11) | P-value |
|---|---|---|---|
| Age | 57.4 ± 15.3 | 64.6 ± 9.6 | 0.195 |
| Injury severity score | 40.4 ± 9.8 | 37.6 ± 5.7 | 0.405 |
| Initial serum lactate | 4.27 ± 2.37 | 5.44 ± 3.44 | 0.348 |
| Pelvic fracture type (B & Y) | | | 0.680* |
| Pelvic binder use | 1 (8.3%) | 6 (54.5%) | 0.027* |
| Concurrent pelvic external fixation | 0 | 6 (26.1%) | 0.005* |
| Duration of surgical pad maintenance (hours) | 58.8 ± 27.4 | 51.3 ± 19.1 | 0.465 |
| Total requirement of packed RBC | 14.3 ± 5.1 | 16.3 ± 11.3 | 0.597 |
| Mortality | 4 (33.3%) | 4 (36.4%) | 1.000* |

### 0490

#### Predictors of mortality and profile of thoracic injury patients requiring mechanical ventilation: a retrospective study

K. D. Soni, R. Agarwal, M. Uniyal, A. Gupta, S. Kumar, S. Sagar
J.P.N Apex Trauma Centre, AIIMS, New Delhi, Delhi, India
**Correspondence:** K.D. Soni
*Intensive Care Medicine Experimental* 2017, **5(Suppl 2):**0490

**INTRODUCTION.** Previous studies have demonstrated numerous factors which predict the mortality in blunt chest injuries but most include patients who were not ventilated thereby increasing the case mix and heterogeneity.Factors determining survival or mortality are different in middle income(MIC) or low-income countries with limited resources.The present study was conducted to know the clinical profile of patients with chest injuries requiring mechanical ventilation and to know the factors predicting mortality in the particular cohort presenting to trauma centre of MIC.

**OBJECTIVES.**

1. To study the demographic, clinical and biochemical parameters of patients with chest trauma requiring mechanical ventilation.
2. To compare demographic, clinical and biochemical parameters between the survivors and non-survivors.
3. To assess the clinical and biochemical variables which are independent predictors of mortality.

**METHODS.** Patients (n = 2058) presented over a period of four years (Jan 2012 to Dec 2015)were selected.Out of these,746 (36.24%) required mechanical ventilation. Patients were identified from the Trauma registry after ethical clearance. These 746 patients were further divided in to two groups -Group A (survivors) and Group B (non-survivors). Demographic, clinical and biochemical parameters were compared between these two groups. Independent predictors of mortality were assessed using multivariate logistics regression model.

**RESULTS.** 746 (36.24%) patients with thoracic trauma required mechanical ventilation. Majority, 479 (64.2%) survived inhospital while 267 (35.8%) died during the stay. Age was comparable in both survivors and non-survivors. Tachycardia was a feature of non-survivor group. Both Systolic (110 mmHg) as well as diastolic blood pressures (69 mmHg) were significantly low in non-survivor arm. (p value < .001) Glasgow coma scale (GCS) score was significantly lower in the non-survivor group (median 4) as compared to the survivor group. (median 10).

Systolic blood pressure < 90mmHg, Random Blood Sugar (<60 and >400mg/dl), HCO3 (<23 and >29 mmol/L), Serum Lactate (>6 mmol/L), GCS score (<8), Injury by physical assault and no Intercostal drainage were significant predictors of mortality in this group of patients.

**CONCLUSIONS.** Shock,hyper or hypoglycemia,associated severe head injury, hyperlactatemia, and without intercostal drainage at presentation are significantly associated with worse outcome in patients put on mechanical ventilation following chest injures.

**REFERENCE(S)**

1. Battle C,Hutchings H,Lovett S,et al.Predicting outcomes after blunt chest wall trauma: development and external validation of a new prognostic model. Crit Care. BioMed Central; 2014 May 14;18(3):R98

2. Battle C,Hutchings,H,Evans P.Risk factors that predict mortality in patients with blunt chest wall trauma: a systematic review and meta-analysis. 2012 Jan;43(1):8–17

**GRANT ACKNOWLEDGMENT**

None

### 0491

#### Safety evatualion of passive verticalization for patients with polytrauma in intensive care unit

V. Movsisyan, Z. Marutyan, S. Petrikov, I. Aliev, A. Evdokimov
N.V. Sklifosovsky Research Institute of Emergency Medicine, Healthcare Department, Moscow, Russian Federation
**Correspondence:** Z. Marutyan
*Intensive Care Medicine Experimental* 2017, **5(Suppl 2):**0491

**INTRODUCTION.** Passive verticalization is one of the most essential part of early rehabilitation in Intensive care unit (ICU). However, there is no information provided about verticalization in intensive care unit for patients with polytrauma.

**OBJECTIVES.** Adaptation of the passive verticalization (PV) protocol for patients with polytrauma in ICU.

**METHODS.** In the Intensive care unit of N.V. Sklifosovsky Research Institute of Emergency Medicine were held 34 episodes of PV for 15 patients with polytrauma, of these, 5 women and 10 men. Verticalization was provided by the medical tilt table. In 11 (32%) cases we verticalizated patients on artificial lung ventilation (ALV), in 23 (68%) cases on spontaneous breathing. Verticalization was provided according to Russian clinical recommendations for passive verticalization of 2015 y. Before every episode of PV assessment of patient's volume status was held. Also for every patient was defined a comfortable orthostatic position.

Verticalization was provided up to 15° with the exposition on each level during 15 minutes.

Maximum duration of the episode was 2 hours, maximum angle of tilt able - 60°. In case of aggravation (arterial hypotension, tachycardia, subjective complaints of the patient), the angle of the tilt table was returned to the previous level, with the duration of the exposure up to 15 minutes. The episode was stopped if aggravations lasted for more than 15 minutes and continued if the regression of aggravation has been completed during this time.

During an episode was held dynamic monitoring of the level of consciousness, hemodynamic parameters, saturation of peripheral blood and acid–base status of arterial blood.

**RESULTS.** Patients on ALV successfully reached the target value during 9 (82%) episodes. Most of the episodes (n = 6 (67%)) were performed without aggravation; in 3 cases (33%), were noted some insignificant aggravation were noted. Two of the episodes (18%), had to be stopped because of the orthostatic failure (arterial hypotension, tachycardia).

Patients on spontaneous breathing reached a target angle in 10 episodes (43%), four of them (40%) had no aggravation, 6 (60%), had insignificant aggravation. Thirteen episodes (57%) had to be interrupted because of the aggravation.

The most frequent aggravations during the verticalization are tachycardia (n = 9 (37,5%)), arterial hypotension (n = 11 (46%)) and complaints on subjective discomfort (n = 4 (16,5%)).

**CONCLUSIONS.** Passive verticalization in intensive care unit for patients with polytrauma is safety to use for both groups of patients: on ALV and on spontaneous breathing.

Most usual aggravation are arterial hypotension and tachycardia.

### 0492

#### Traumatic brain injury and quality indicators: how is our compliance?

A. Clara, P. Cavaleiro, A. Coutinho, R. Bernardo, J. Moreno, C. Granja
Algarve Hospital Center, Faro, Portugal
**Correspondence:** A. Clara
*Intensive Care Medicine Experimental* 2017, **5(Suppl 2):**0492

**INTRODUCTION.** Traumatic brain injury (TBI) is one of the main causes of morbidity and mortality, especially among young individuals[1]. According to the Glasgow Coma Scale (GCS) it can be classified as mild if GCS 15–13, moderate if GCS 12–9 and severe if GCS < 8, which has important implications in its approach[1]. The approach to the patient has improved in the last two decades with the implementation in our country of a protocol-based acute management of TBI patients and quality indicators for moderate and severe injuries have been defined: GCS < 13 - head-computerized tomography (CT) in the first 4 hours after injury; GCS < 9 - prompt endotracheal intubation (ETI), surgery in the first 4 hours when indicated and intracranial pressure (ICP) monitoring[2].

**OBJECTIVES.** To verify the adherence to quality indicators in patients with TBI admitted to the ICU and its morbidity by applying GOSE (Extended Glasgow Outcome Score) after 3 months.

**METHODS.** Observational retrospective study, with data collected of an ongoing database. The study included patients diagnosed with moderate and severe TBI requiring admission to the ICU from 1/11/2013 to 31/12/2016.

**RESULTS.** Seventy-five patients were included in the study with a mean age of 51 [18–88] years, 72% were male. The sample was similar over the years: 29 patients in 2014, 21 patients in 2015 and 24 patients in 2016. The ICU mortality was 14.7% with a length of stay of 12,32 [1–68] days. Of the 85.3% survivors, 73.8% remained alive at 28 days. Clinical records of GCS in pre-hospital setting were found in 95.2% of the patients, either on the pre-hospital setting or in the emergency department; 39% presented severe TBI with ETI in 100% of patients and 71.7% had ICP monitoring. The median time for cranial CT-scan was 0h40min[±3h20min47s]. When craniotomy was indicated, it was performed in < 4 hours in 38.7% patients. Only 29.4% met all the quality indicators. We were able to evaluate the GOSE at 3 months in 66,6% of the patients; 21 patients had a GOSE < 6 and 18 patients had a GOSE ≥ 6. The lower GOSE score was related with longer ICU length of stay (95% CI 0,902 to 15,271;$p$ = 0.028) and longer overall hospitalization (95% CI 3,562 to 66,528;$p$ = 0.03), as well as GCS at UCI discharge

(95% CI −3,491 to −0,728;$p$ = 0.006).

**CONCLUSION.** Regarding the adherence to quality indicators ICP monitoring and craniotomy performance are the areas that need more improvement. Patients with a GOSE score < 6 were significantly associated to lower GCS at ICU discharge, longer ICU length of stay, and longer overall hospitalization. Due to the small sample, we weren't able to establish a association between the adherence to quality indicators and 3 months TBI outcome.

#### 0493

**Shedding of glycocalyx-derived heparan sulfate is not associated with auto-heparinization**

J. Zipperle[1], J. Hoefer[1,2], G. Iapichino[1], C. Keibl[1], N. Hofmann[1], M. Ashmwe[1], A. Bahrami[1], J. Jilge[1], M. Jafarmadar[1], S. Bahrami[1], H. Schöchl[1,2]
[1]Ludwig Boltzmann Institute for Experimental and Clinical Traumatology, AUVA Trauma Research Centre, Vienna, Austria; [2]AUVA Trauma Centre Salzburg, Department of Anaesthesiology and Intensive Care, Salzburg, Austria
**Correspondence:** J. Zipperle
*Intensive Care Medicine Experimental* 2017, **5(Suppl 2):**0493

**INTRODUCTION.** Heparan sulfate (HS) represents an integral component of the endothelial glycocalyx, a thin anticoagulant layer on the surface of the undisrupted vasculature. Hypoperfusion and sympathoadrenal activation following major trauma result in endotheliopathy and the shedding of HS, which is suspected to promote the allosteric activation of plasmatic antithrombin. This endogenous mechanism of heparinization might represent a contributor to trauma-induced coagulopathy.

**OBJECTIVES.** We investigated whether the trauma-induced shedding of HS could exert heparin-like effects of anticoagulation.

**METHODS.** ROTEM was performed on whole blood of trauma patients, using an intrinsically-activated assay (INTEM) in the presence or absence of Heparinase (HEPTEM) upon emergency room admission. To assess the course and magnitude of HS shedding following injury, rats were subjected to a well-characterized model of hemorrhage, trauma and resuscitation (HTR). INTEM/HEPTEM were performed at time points with peaking HS levels. Clinically relevant doses of two different HS preparations were added to whole blood from healthy donors and measured in an INTEM assay with heparin as a positive control.

**RESULTS.** Severe injury was not associated with a prolongation of INTEM clotting time (CT) in trauma patients which was unaltered in the presence of Heparinase (HEPTEM). In our rat model of HTR, shock and resuscitation resulted in the shedding of endothelial HS without a prolongation of INTEM CT or an effect of Heparinase. When compared with Heparin, clinically relevant concentrations of HS did not exert anticoagulant effects.

**CONCLUSIONS.** Our data suggest that severe injury results in the shedding of HS from the endothelium. Although structurally similar, the amount of shed HS does not appear to exert an anticoagulant effect comparable to that of heparin. Our data do not substantiate the concept of clinical autoheparinization.

### Microcirculation and tissue oxygenation

#### 0494

**Hemodynamic and functional parameter (Co2 gap and Scvo2) as targets for resuscitation during septic shock**

L. La Via, T. Taccheri, S. Carelli, U. La Brocca, V. Piccoli, M. Cicetti, A. M. Dell Anna, M. Antonelli
Fondazione Policlinico Universitario A. Gemelli, Roma, Italy
**Correspondence:** L. La Via
*Intensive Care Medicine Experimental* 2017, **5(Suppl 2):**0494

**INTRODUCTION.** Septic shock remains one of the most common causes of mortality in intensive care unit (ICU). In order to ensure an adequate cellular oxygen utilization, macro haemodynamic optimization is a main issue. Validated markers of organ hypoperfusion are serum lactates, ScvO2 and veno-arterial CO2 difference (CO2gap).

**OBJECTIVES.** This study aimed at exploring the relationship between these markers mortality at 28 days.

**METHODS.** This prospective, observational, monocentric study enrolled all consecutive patients (pts) with septic shock, diagnosed according to SSC guidelines of 2012, within 8h from ICU admission. Exclusion criteria were age < 18 years, a life expectancy < 28 days, pregnancy and severe immunodeficiency.
At admission SOFA, SAPSII, Charlson's comorbidity scores, haemodynamic and metabolic parameter as SAP, DAP, MAP, HR, CVP, CI, organ perfusion pressure (MAP-CVP), venous and arterial blood gas analysis, $DO_2$, $VO_2$, $O_2ER$, urinary output were recorded. All updatable parameters were then detected at 6h, 12h, 24h, 36h, 48h, 60h and 72h after admission and daily up to a week. Haemodynamic assessment was performed through transpulmonary thermodilution EV1000 (Edwards Lifescience©TM).

**RESULTS.** This study included 25 pts, with an average age of 64 ± 15 (52% male). Overall mortality at 28 days was 56%. At admission, haemodynamic parameters showed no significant differences between the survivors (S) and non survivors (NS) at 28 days. Lactate were higher in NS [5.2 vs 1.7 $p < 0.01$]; Charlson's, SOFA and SAPSII showed significantly higher values in NS ($p = 0.025$, $p = 0.005$, $p = 0.01$ respectively). MAP, CI and MAP-CVP were also significantly higher in S [$p < 0.001$], with similar CVP.
The CO2gap over time was significantly higher in S ($p = 0.045$) as well as blood lactate ($p < 0.01$). CO2 gap was also significantly related with MAP-CVP ($p < 0.001$, $r = 0.71$), CI ($p = 0.038$, $r = -0.42$) and DO2 ($p < 0.001$, $r = 0.67$). VO2 and DO2 were significantly higher in S ($p < 0.001$ and $p = 0.014$, respectively); similarly, $O_2ER$, particularly in the first 48h ($p = 0.003$). Conversely, no significant differences were detected in terms of ScvO2 and $pCO_2$ gap/$CaO_2$-$CvO_2$ between the two groups. Regarding to the positive predictive value of 28 days mortality of the analyzed variables, lactates at admission showed the highest AUC (0.76, $p = 0.014$) with a specificity of 91% and a sensitivity of 64% for lactate >2.9 mmol/l. S had a longer ICU stay than NS (16 vs 6 days, $p = 0.01$), as well as more mechanical ventilators and less vasopressors free days (8 vs 1, $p = 0.01$ and 9 vs 2, $p = 0.01$ respectively).

**CONCLUSIONS.** This work showed that parameters associated with tissue hypoperfusion were closely related to septic shock patients' outcome. In particular, lower values of $CO_2$gap in the first hours of hospitalization seemed to be associated to poor outcome because mirrored tissue inability to employ oxygen supply.

#### 0495

**Alterations in peripheral microcirculatory in patients with Acute Respiratory Distress Syndrome (ARDS)**

W. Mongkolpun, D. O. Cortés, J.-L. Vincent, J. Creteur
Erasme University Hospital, Université Libre de Bruxelles, Department of Intensive Care, Brussels, Belgium
**Correspondence:** W. Mongkolpun
*Intensive Care Medicine Experimental* 2017, **5(Suppl 2):**0495

**INTRODUCTION.** Peripheral perfusion may be altered in patients with ARDS and the degree of alteration may be associated with disease severity.

**OBJECTIVE.** To compare skin microvascular perfusion in patients with ARDS with and without shock and survivors and non-survivors.

**METHODS.** We studied 26 healthy volunteers and 27 patients within 24 hours after a diagnosis of ARDS (using the Berlin criteria). All patients were receiving mechanical ventilation. Using skin laser Doppler (Periflux, Perimed5000), we evaluated, at baseline (T0) and 6 (T6), 24 (T24) and 48 (T48) hours, skin blood flow before and after a thermal challenge test (TCT) performed by increasing the skin probe temperature to 37°C for 3 minutes. SOFA and APACHE II scores were obtained at baseline. We compared healthy volunteers with ARDS patients, ARDS patients with circulatory shock (defined as need for vasopressors to maintain mean arterial pressure [MAP] ≥65 mmHg with >1 sign of poor tissue perfusion [altered consciousness, oliguria, skin hypoperfusion, lactate ≥2 mmol/L]) and those without shock, and survivors with non-survivors. Results were analyzed using STATA 14.0 and $p < 0.05$ was considered statistically significant.

**RESULTS.** Baseline clinical characteristics are shown in Table 133. The causes of ARDS were sepsis (19), pancreatitis (3), near drowning (2), inhalation (2), chest contusion (1); overall mortality was 55%. Twenty patients had circulatory shock. At baseline (T0) the SBF and the relative change in SBF after TCT (ΔSBF) were lower in ARDS patients than in volunteers; the ΔSBF was significantly lower in ARDS patients with than in those without shock; and the SBF and ΔSBF were lower in the non-survivors than in the survivors (Table 134). At 24 and 48 hours, the SBF was lower in ARDS patients with than in those without shock. The SBF was lower in the non-survivors than in the survivors at 48 hours and the ΔSBF was lower in the non-survivors than in the survivors at 24 and 48 hours (Table 134). The SBF increased from 48(18–64) at T6 to 101(39–157) at T24 (p = 0.03) in the survivors but did not change significantly in the non-survivors.

**CONCLUSION.** Cutaneous microvascular perfusion is altered in ARDS and these changes are related to disease severity.

**Table 133 (Abstract 0495)**. See text for description

| | Volunteers (N=26) | Total Patients (N=27) | p | ARDS without shock (N=7) | ARDS with shock (N=20) | p | Survivors (N=12) | Non survivors (N=15) | p |
|---|---|---|---|---|---|---|---|---|---|
| Age | 30(29-31) | 62(42-75) | <0.01 | 41(26-74) | 63 (54-72) | 0.7 | 63(52-77) | 59(26-75) | 0.3 |
| SOFA score | - | 15(13-16) | - | 15(11-16) | 15(13-16) | 0.4 | 14(12-16) | 15(13-16) | 0.2 |
| APACHE II score | - | 30(24-33) | - | 30(24-32) | 31(27-35) | 0.6 | 28(22-32) | 31(27-33) | 0.3 |
| Mean arterial pressure (mmHg) | - | 69(66-79) | - | 68(65-79) | 75(70-79) | 0.2 | 73(68-81) | 68(65-78) | 0.1 |
| Cardiac index (L/min/m2) | - | 2.1(1.5-2.4) | - | 2(2-2.1) | 2(1.1-3) | 0.6 | 3.1(1.5-3.8) | 1.8(1.5-2.1) | 0.1 |
| Norepinephrine dose(μg/kg/min) (No. of patients) | - | 0.3(0.1-0.8) (20) | - | - | 0.3(0.1-0.8) | <0.01 | 0.3(0.1-0.5) (9) | 0.5(0.2-0.8) (11) | 0.2 |
| Lactate (mmol/L) | - | 3(2-7) | - | 2(2-4) | 4(2-7) | 0.1 | 3(2-7) | 3(2-7) | 0.3 |
| PaO2/FiO2 | - | 92(70-150) | - | 132(74-216) | 112(66-154) | 0.6 | 122(73-231) | 85(67-111) | 0.1 |
| Venovenous extracorporeal membrane oxygenation (N%) | - | 9 (33%) | - | 1 | 8 (40%) | 0.2 | 3(25%) | 6(40%) | 0.4 |

**Table 134 (Abstract 0495)**. See text for description

| | Volunteers (N=26) | Total (N=27) | p | ARDS without shock(N=7) | ARDS with shock (N=20) | p | Survivors (N=12) | Non Survivors (N=15) | p |
|---|---|---|---|---|---|---|---|---|---|
| SBF at T0 (PU) | 226(171-254) | 24(9-74) | 0.01 | 86(24-132) | 16(6-35) | 0.07 | 60(26-120) | 16(6-31) | 0.03 |
| Relative change at T0(%) | 76(61-85) | 34(13-102) | 0.01 | 56(18-125) | 13(10-16) | 0.03 | 112(28-295) | 15(11-78) | 0.01 |
| SBF at T6 (PU) | - | 47(13-75) | - | 31(7-76) | 47(15-75) | 0.5 | 48(18-64) | 47(9-97) | 0.8 |
| Relative change at T6 (%) | - | 62(18-114) | - | 42(18-111) | 45(19-70) | 0.7 | 66(21-137) | 42(14-96) | 0.4 |
| SBF at T24 (PU) | - | 47(18-146) | - | 140(88-179) | 21(10-41) | 0.01 | 101(39-157) | 27(13-95) | 0.05 |
| Relative change at T24 (%) | - | 57(24-133) | - | 42(14-73) | 58(40-133) | 0.2 | 120(55-284) | 40(18-79) | 0.02 |
| SBF at T48 (PU) | - | 33(17-157) | - | 118(51-151) | 29(21-83) | 0.03 | 156(92-180) | 21(12-28) | 0.01 |
| Relative change at T48 (%) | - | 26(17-64) | - | 65(40-96) | 38(29-75) | 0.3 | 48(25-99) | 20(12-45) | 0.02 |

### 0496

#### Hyperoxia does not alter oxygen delivery in healthy volunteers

B. Smit[1], Y. M. Smulders[2], H. J. S. de Grooth[1], H. P. M. M. Gelissen[1], A. R. J. Girbes[1], H. H. M. Schotman[3], P. G. Scheffer[3], H. M. Oudemans - van Straaten[1], A. M. E. Spoelstra - de Man[1]
[1]VU University Medical Center, Intensive Care, Amsterdam, Netherlands; [2]VU University Medical Center, Internal Medicine, Amsterdam, Netherlands; [3]VU University Medical Center, Clinical Chemistry, Amsterdam, Netherlands
**Correspondence:** B. Smit
*Intensive Care Medicine Experimental* 2017, **5(Suppl 2):**0496

**INTRODUCTION.** Administration of supplementary oxygen may lead to hyperoxia, which can have acute cardiovascular effects, such as vasoconstriction and a reduction in cardiac output, which may even result in a paradoxical reduction in oxygen delivery. Most hemodynamic effects have been studied in healthy volunteers inhaling either air or oxygen. Due to the lack of blood gas analysis in healthy volunteers, very few data exist on the effect of hyperoxia on oxygen delivery. Because of this common strategy, the direct relation between $P_aO_2$ and oxygen delivery remains ambiguous. Here we describe, in a $P_aO_2$ guided fashion, the relation between several levels of hyperoxia and hemodynamic effects and oxygen delivery in healthy volunteers.

**OBJECTIVES.** To study the dose–response relationship between $P_aO_2$, systemic hemodynamics and resulting oxygen delivery, as well as sublingual microcirculatory perfusion.

**METHODS.** Ten healthy volunteers received five incremental $F_iO_2$s for 10 minutes each via a non-invasive ventilation mask, without mechanical ventilatory support, to reach $P_aO_2$s of baseline (air), 20, 40, 60 and max kPa (oxygen). An arterial line was placed for blood gas sampling. During each period, hemodynamics (Heart Rate, Mean Arterial Pressure, Cardiac Index, Stroke Volume and Systemic Vascular Resistance Index) were measured continuously by the volume-clamp method (Nexfin(r)). At the end of each period, the sublingual microcirculation was recorded by sidestream darkfield imaging.

**RESULTS.** Seven males and three females with a mean age of 28 years (SD ± 7), without cardiac or respiratory illness completed the protocol. $P_aO_2$s during the five sequential periods were 14 (SD ± 1.5), 21(±1.5), 39(±3.0), 56(±5.6) and 74 (±2.4) kPa. Cardiac Index decreased linearly (linear regression, P = 0.0003) with increasing $P_aO_2$, with a maximum decrease of 0.33 L/min/m$^2$ (SD ± 0.22) when breathing oxygen (−8% vs baseline, P < 0.0001; paired t-test, Fig. 181a). The reduction in CI was explained by a decrease in Heart Rate (7 bpm, −10% vs baseline, P < 0.0001) and not Stroke Volume. Systemic Vascular Resistance Index rose slightly (linear regression, P = 0.043) with a maximum of 110 dynes · sec/cm$^5$/m$^2$ (SD ± 101, +5% vs baseline, P = 0.007, Fig. 181b). MAP was not affected. Oxygen delivery remained stable at an average of 77 ml $O_2$/min/m$^2$ at each $P_aO_2$ (Fig. 181c). Sublingual vessel density and perfused vessel density were decreased by 16% (P = 0.06) and 17% (P = 0.04, Fig. 181d), respectively.

**CONCLUSIONS.** In healthy volunteers, oxygen has hemodynamic effects over the entire range of 14–74 kPa, evident by a stepwise decrease of cardiac index (through a reduction in heart rate), and a small increase in systemic vascular resistance index. In these subjects, the increased arterial oxygen content is sufficient to maintain oxygen delivery despite a decrease in blood flow. However, whether the reduction in (sublingual) perfusion affects oxygenation at the tissue level in vital organs remains to be determined.

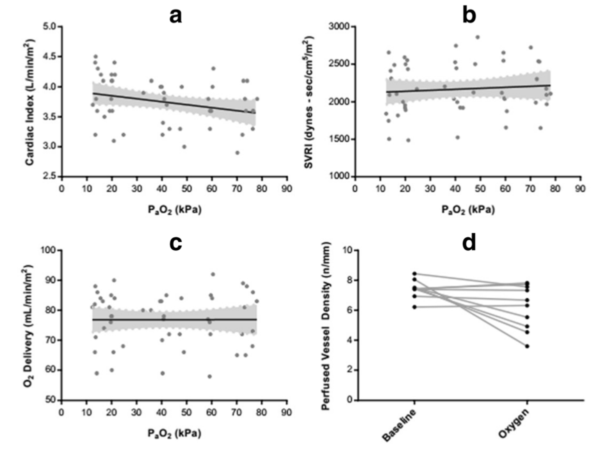

**Fig. 181 (Abstract 0496).** See text for description

### 0497

#### Mitochondrial DNA predicts mortality in acute but not in chronic heart failure

K. Krychtiuk[1], R. Wurm[1], S. Ruhittel[1], M. Lenz[1], K. Huber[2], J. Wojta[1], G. Heinz[1], M. Hülsmann[1], W.S. Speidl[1]
[1]Medical University of Vienna, Department of Internal Medicine II, Vienna, Austria; [2]Wilhelminenhospital Vienna, 3rd Department of Medicine, Vienna, Austria
**Correspondence:** K. Krychtiuk
*Intensive Care Medicine Experimental* 2017, **5(Suppl 2):**0497

**INTRODUCTION.** Severe acute heart failure (AHF) is associated with a poor short-term outcome, while patients with chronic heart failure (CHF) exhibit a poor long-term prognosis. Tissue hypoxia may lead to cellular damage and the release of intracellular mitochondrial DNA (mtDNA), which may activate the immune system due to its resemblance to bacterial DNA.
**OBJECTIVES.** The aim of this study was to analyze circulating levels of mtDNA as a possible predictor of outcome in patients with AHF and CHF.
**METHODS.** Plasma levels of circulating mtDNA were measured in 90 consecutive patients with AHF admitted to our ICU and in 109 consecutive patients with CHF at our HF outpatient department by real-time PCR.
**RESULTS.** Patients in the ICU group were 64.7 (49.4-74.3) years old and median NT-proBNP levels were 4986 (1525–23842) pg/mL. 30-day survival was 64.4%. In the CHF group, median age was 63 (IQR 52–72) years. 49.5% of patients had ischemic and 50.5% had a non-ischemic etiology of CHF. 38.5% were in NYHA class III/IV, and patients had a median NT-proBNP level of 1025 (IQR 450–3480) pg/mL.
Patients with AHF showed significantly higher circulating levels of mtDNA as compared to patients with CHF (27.0 IQR 8.2 - 52.2 ng/mL vs. 14.5 IQR 8.5 - 25.4 ng/mL, $p < 0.005$). In CHF patients, mtDNA levels were associated with NYHA functional class but did not differ according to HF etiology and outcome. On the contrary, in patients with severe AHF, mtDNA levels were significantly higher in patients that died within 30 days after ICU admission (30.6 IQR 13.0 - 90.1 ng/mL vs. 22.8 IQR 6.4 - 41.6 ng/mL, $p < 0.05$); patients with plasma levels of mtDNA in the highest quartile (mtDNA > 50.9 ng/mL) had a 3.1-fold risk ($p = 0.002$) of dying.
**CONCLUSIONS.** Circulating levels of mtDNA predict mortality in patients with severe AHF but are not associated with outcome in patients with CHF. Reduced tissue perfusion with release of mtDNA may play a role within the pathophysiology of AHF and severe CHF.

### 0498

#### Hyperlactatemia at 24 h and acute kidney injury predict mortality in cardiogenic shock patients requiring intra-aortic balloon pump in setting of acute coronary syndromes

M. Stefan, A. Stan, A. Cristian, E. Oleineac, M. Luchian, I. Marinica, A. Paunescu, D. Filipescu
[1]Emergency Institute for Cardiovascular Diseases 'Prof Dr CC Iliescu', ICU, Bucharest, Romania
**Correspondence:** M. Stefan
*Intensive Care Medicine Experimental* 2017, **5(Suppl 2):**0498

**INTRODUCTION.** Acute heart failure syndromes (AHFS) after acute coronary syndromes (ACS) still pose a significant clinical challenge for the intensivist. Despite recent progress in extracorporeal assist device therapy, little evidence suggests outcome benefit. Intra-aortic balloon pump (IABP) remains a viable option for improving coronary perfusion and ventricular unloading in this population.
**OBJECTIVES.** To identify clinical predictors of mortality in patients with CS requiring IABP support in the setting of ACS.
**METHODS.** We performed a cross-sectional study, retrospectively analysing patients with AHFS after ACS, admitted to our ICU between 1 January 2014 and 31 December 2015. Patients presenting with CS and requiring IABP support were further analysed. Independent predictors of mortality were identified using a multivariate logistics model.
**RESULTS.** 241 patients were identified. Of these, 43 (17%) presented with CS and received IABP support.
Baseline characteristics (Table 135) show that they were predominantly male (74,4%), with an average age of 61,9 (±10,4) years. 82,2% received primary PCI, with a median time to PCI of 314 min (IQR 150–460). IABP was placed after PCI in all patients. Median left ventricular ejection fraction was 30% (IQR 20–35).
46,5% of patients died. We found significant differences between survivors and non-survivors (Table 136) in admission APACHE II scores (14,8 ± 2,8 vs 22 ± 4,1, p = 0,01), incidence of acute kidney injury (AKI - 69,6% vs 95%, p = 0,033), peak creatinine (1,80 ± 0,41 vs 2,72 ± 0,59 mg/dl, p = 0,035) and serum lactate levels at 24 hours after hospitalization (2,07 ± 0,67 vs 7,12 ± 3,5 mmol/L, p = 0,003). We also noted a trend toward increased renal replacement therapy usage in survivors (35% vs 13%, p = 0,089). There were no differences in sepsis incidence and admission lactate between the groups.
Using multiple logistic regression analysis, we identified 2 independent predictors for in-hospital mortality - lactate levels > 2,5 mmol/l at 24 hours (odds ratio [OR] 1,64, CI 95% 1,072-2,52) and worsening of renal function defined as elevation of creatinine levels by >0,3 mg/dl (OR 2,6, CI 95% 1,12-6,022).
**CONCLUSIONS.** Normalization of serum lactate at 24 hours after admission in patients with CS requiring IABP after ACS, as well as avoiding AKI could be clinical objectives worth following. Further studies to prove outcome improvement are warranted.

**Table 135 (Abstract 0498)**. Baseline characteristics

| | |
|---|---|
| Age (years) | 61,9 ± 10,4 |
| Male | 74,4% (32/43) |
| PCI | 82,2% (37/43) |
| Time to PCI (min) | 314 (150-460) |
| Time to IABP | 325 (150-660) |
| Admission lactate (mmol/L) | 6,7 ± 1,37 |
| Admission NT-proBNP (pg/ml) | 2862 (790-7671) |
| Admission LVEF | 30% (20-35) |

**Table 136 (Abstract 0498)**. Results

| Results | Total | Survivors | Non-survivors | Significance |
|---|---|---|---|---|
| Death | 46.5% (20/43) | | | |
| Admission APACHE II | 18,1±2,5 | 14,8±2,8 | 22 ±4,1 | p= 0,01 |
| AKI | 81.4% (35/43) | 69,6% | 95% | p= 0,033 |
| Peak creatinine | 2,23±0,36 | 1,80 ±0,41 | 2,72±0,59 | p= 0,035 |
| RRT | 23,3% (10/43) | 35% | 13% | p= 0,089 |
| Sepsis | 30.2% (13/30) | 30% | 30,4% | p= 0,975 |
| Admission lactate (mmol/L) | 6,7±1,37 | 5,5 ±1,2 | 8,1 ±2,6 | p= 0,38 |
| 24 h lactate | 4,4±1,7 | 2,07 ±0,67 | 7,12 ±3,5 | p= 0,003 |

### 0499

#### Hyperlactatemia after cardiac surgery: association with outcomes

J. M. Jardim[1], F. Galas[1], D. H. P. Souza[2], I. A. A. Souza[2], L. Camara[1], G. Ferreira[2], R. Kalil Filho[2], L. A. Hajjar[2]
[1]Hospital das Clínicas, Universidade de São Paulo, Serviço de Anestesia, São Paulo, Brazil; [2]Hospital das Clínicas, Universidade de São Paulo, Cardiologia, São Paulo, Brazil
**Correspondence:** J. M. Jardim
*Intensive Care Medicine Experimental* 2017, **5(Suppl 2):**0499

**INTRODUCTION.** Causes of hyperlactatemia include tissue hypoxia as well as nonhypoxic causes such as drug therapy, cardioplegia, hypothermia, and cardiopulmonary bypass (CPB), but, although hyperlactatemia is common after cardiac surgery, its value as a prognostic marker remains controversial.

**OBJECTIVES.** To identify the intraoperative factors causing early high lactate levels (lactate >3mmol/L) at intensive care unit (ICU) admission in patients undergoing cardiac surgery and to evaluate the association between early high blood lactate levels and postoperative outcomes.

**METHODS.** A retrospective observational study including 102 patients who underwent cardiac surgeries in a tertiary care center from April to August 2016. The patients were divided into 2 groups based on serum lactate levels at ICU admission; those with serum lactate levels greater than or equal to 3 mmol/L considered as hyperlactatemia and those with serum lactate levels less than 3 mmol/L. Preoperative and intraoperative clinical data and postoperative outcomes were evaluated in both groups.

**RESULTS.** Hyperlactatemia was present in 46 patients (45%) at ICU admission. There was no significantly association between lactate levels and preoperative clinical data. Age (P = 0.619), body index mass (P = 0.154), EuroSCORE (P = 0.954) and left ventricular ejection fraction (P = 0.132) were similar in both groups. Hyperlactatemia group received significantly higher intraoperative blood transfusion (50% *vs* 28,6%; P = 0.027). Other intraoperative factors such as type of surgery, duration of cardiopulmonary bypass, cross clamp and fluid balance were not associated with hyperlactatemia. There were also no significant differences in the postoperative outcomes, except for low cardiac output syndrome which occurred more frequently in the hyperlactatemia group (37% *vs* 10.7%, P = 0.002). There were no differences in the surgical complications: 30-day mortality (6.5% *vs* 1.8%, P = 0.325), cardiogenic shock (6.5% *vs* 3.6%, P = 0.656), bradyarrhythmias (6.5% *vs* 1.8%, P = 0.325), tachyarrhythmias (34.8% *vs* 41.1%, P = 0.516), acute renal failure (54.3% *vs* 41.1%, P = 0.181), stroke (0% *vs* 3.6%, P = 0.500) or infection (52.2% *vs* 50%, P = 0.827). Patients from the hyperlactatemia group had more frequent low output syndrome in the postoperative period (37% *vs* 10.7%, P = 0.002).The length of ICU stay (5,57 days *vs* 5,38 days, P = 0,655), duration of mechanical ventilation (2348 min *vs* 1349 min, P = 0,863) and hospital stay (22,87 days *vs* 22,27 days, P = 0,828) were similar in both groups.

**CONCLUSIONS.** Patients with hyperlactatemia at the ICU admission received more blood transfusion at the surgery and had a higher incidence of low cardiac output in the postoperative period. On the other hand, the occurrence of hyperlactatemia had no significant association with other postoperative complications.

**REFERENCE(S)**

Hajjar LA et al. High lactate levels are predictors of major complications after cardiac surgery. J Thorac Cardiovasc Surg. 2013;146(2):455–60.

### 0500

#### Mortality prediction in septic shock patients evaluated by Cardiac Index and Dv-aCO2/Da-vO2 index

I. M. Lima Lucero, S. E. Zamora Gómez, M. A. Amezcua Gutierrez, J. O. Montoya Rojo, E. S. Cruz Montesinos, J. Garduño López, M. Hernández Romero, M. A. Toledo Rivera, M. A. Carrasco Flores, G. D. Hernández López[1]

Hospital Juárez de México, Intensive Care Unit, Mexico, Mexico

**Correspondence:** I. M. Lima Lucero

*Intensive Care Medicine Experimental* 2017, **5(Suppl 2):**0500

**INTRODUCTION.** Mortality associated to septic shock is related with initial macrohemodynamic disturbances and ongoing hypoperfusion, ending in irreversible mitocondrial disfunction. Early recognition of mortality predictive variables in these patients encourage therapeutic strategies directed to hemodynamic optimization in macro and microcirculatory level. Cardiac Index (CI) is the prefered macrohemodynamic variable and recently Dv-aCO2/Da-vO2 index appeared as the most integral noninvasive way to evaluate global tissue perfusion.

**OBJECTIVES.** Estimate mortality risk in septic shock patients, evaluated by CI and Dv-aCO2/Da-vO2 index behaviour.

**METHODS.** Retrospective, observational study, in a tertiary level intensive care unit during march 2013 to july 2016 including patients with diagnostic of septic shock, with hemodynamic monitorization by continuos cardiac index by wave pulse contour analysis, in which Dv-aCO2/Da-vO2 index was calculated. Mortality was evaluated in this group of patients.

**RESULTS.** 77 patients were analyzed (46.8% males and 53.2% females), with a mean age of 48.01 years, mean APACHE II score of 23.7 points and SOFA score of 11.4 points at entry. Abdominal infection was the main cause of septic shock (48.1%). The macrohemodynamic analysis was obtained of CI at entry, with a median of 3 L/min/m2, becoming this the cut-off point. 44 patients (57%) had an CI >3 and 33 patients (43%) had an CI < 3. Global tissue perfusion was analized with Dv-aCO2/Da-vO2 index at entry, with a cut-off point of 1.4; 54 patients (71%) had an index >1.4 and 23 patients (29%) < 1.4. Global mortality was of 31% (24 patients), 31.8% (14 patients) in the group of CI >3 (OR = 1.05, IC 95%(0.535 a 2.06), p = 0.02) and 30.3% (10 patients) in the group of CI < 3; Mortality according to Dv-aCO2/Da-vO2 index was of 24.67% in the group >1.4 (OR = 1.79, IC 95% 0.178 - 1.745 p = 0.557) and 6.49% in the group < 1.4.

**CONCLUSIONS.** In this group of septic shock patients, macrohemodynamic alteration characterized by an CI > 3 L/min/m2 was associated with an increase in mortality risk of 1.05 folds, with stadistic significance. Conversely, in the same group there was a trend in increase mortality risk when Dv-aCO2/Da-vO2 index >1.4 was present, but without stadistic significance. We suggest further prospective studies to elucidate the validity of this study.

**REFERENCE(S)**

1. Ospina- Tascón G, Hernández Glenn, Cecconi Maurizio. Understanding the venous- arterial CO2 to arterial- venous O2 content difference ratio. Intensive Care Med 2016; 42(11):1801–1804

2. Cecconi M,et al., A Consensus on circulatory shock and hemodynamic monitoring. Task force of the European Society of Intensive Care Medicine. Intensive Care Med 2014; 40:1795–1815

3. De Backer D, Ortiz JA, Salgado D. Coupling microcirculation to systemic hemodynamics. *Curr Opin Crit Care* 2010; 16:250–254.

### 0501

#### Cardiac CO2gap and perfusion coronary pressure during cardiopulmonary by-pass: a new hemodynamic and functional parameter

T. Taccheri, S. Carelli, M. Calabrese, G. Arlotta, L. Montini, F. Cavaliere, A. M. Dell'Anna, M. Antonelli

Catholic University of the Sacred Heart - Pol. A. Gemelli, Anesthesiology and Intensive Care Medicine, Rome, Italy

**Correspondence:** T. Taccheri

*Intensive Care Medicine Experimental* 2017, **5(Suppl 2):**0501

**INTRODUCTION.** During coronary artery by-pass graft (CABG) venous to arterial (v-a) carbon dioxide content difference (CCO2gap) is a useful marker of tissue hypoperfusion. Coronary sinus (CS) CCO2 can also be explored during cardiac surgery and its behavior may be a possible marker of myocardium hypoperfusion. This work aimed at comparing CS pCO2 and CCO2 gap with central venous (CV) and mixed venous (MV) blood before and during cardiopulmonary bypass (CPB) and at exploring the behavior of myocardial CO2 production.

**METHODS.** This prospective, observational, monocentric study enrolled all consecutive patients undergoing CABG. Exclusion criteria were age < 18 years, combined surgery, pregnancy. At baseline clinical characteristics were registered, as well as SOFA, SAPSII, Euroscore and Charlson's comorbidity scores. Haemodynamic and metabolic parameters as SAP, DAP, MAP, systolic, diastolic and mean coronary sinus pressure (SCSP, DCSP, MCSP respectively), SPAP, DPAP, MPAP, CO, CI, coronary perfusion pressure [CPP] (MAP-mean coronary sinus pressure [MCSP]), MV, CV, CS and arterial blood gas analysis, $DO_2$, $VO_2$ and $O_2ER$ were recorded before and in later CPB. All clinical and hemodynamic parameters, HS troponin, fluid balance and urinary output were collected at 6h and 24h after ICU admission. Haemodynamic assessment was performed with Swan-Ganz catheter (Edwards Lifescience©™).

**RESULTS.** We enrolled 5 male patients with a mean age of 69 ± 17 years, SAPS II at 24 h of 32 ± 3, Euroscore of 1 ± 0.3, NYHA of 3, BMI of 26 ± 4; TnI at 6h and 24h was 7 ± 11 and 5 ± 12 mcg/l, respectively. $CCO_2$ gap was significantly higher in CS compared to MV and CV before CPB (10 [7–11] vs. 4.4 [2.2-7.7] vs. 3 [1.9-4.2], p = 0.04-Fig. 1). $O_2$ consumption was also higher for CS before CBP (31 [27–33] vs. 24 [22–27] vs. 22 [19–27] p = 0.015). $pCO_2$ gap was similar among groups, as well as the ratio between $CCO_2$ gap and arterial-venous (a-v) difference in $O_2$ content (Respiratory quotient - RQ). RQ was not different among groups, however a clear trend towards higher RQ in CS was evident (1.9 [1.3-2] vs. 0.9 [0.5-1.5] vs. 0.8 [0.5-1.2] p = ns). During CPB $O_2ER$ from CS was not significantly lower (14 [7–23] vs. 31[26–34], p = 0.06), as well as $CCO_2$ gap (1.5 [0.9-18] vs. 10 [4.7 - 11.6] p = ns). A decrease in CPP below 45 mmHg caused an abrupt decrease in $CCO_2$ gap (r = 0.84, p = 0.07) and, similarly, a sudden decrease in RQ below 1.9 (r = 0.81, p = 0.09-Fig. 2).

**CONCLUSIONS.** We showed that myocardial CO2 production is not negligible and contributes to differences observed in CO2 and O2 content observed between central venous and mixed venous blood samples, meaning that in certain situation CV samples may not be considered interchangeable with MV samples. Moreover, relationship between myocardial perfusion pressure and CCO2 gap or RQ suggests that myocardial CO2 production decreases linearly below a critical value of blood flow. Other studies are warranted to confirm such preliminary results.

### 0502

#### Dynamic parameters of preload predict impairments of intestinal microcirculation in a porcine model of ischemia-reperfusion

C. R. Behem[1], M. F. Graessler[1], T. Friedheim[1], R. Kluttig[1], A. Duprée[2], D. Manzoni[3], S. H. Wipper[3], E. S. Debus[3], D. A. Reuter[1], C. J. C. Trepte[1]
[1]University Medical Center Hamburg-Eppendorf, Center for Anesthesiology and Intensive Care Medicine, Hamburg, Germany; [2]University Medical Center Hamburg-Eppendorf, Department of General, Visceral and Thoracic Surgery, Hamburg, Germany; [3]University Medical Center Hamburg-Eppendorf, Department of Vascular Medicine, Hamburg, Germany
**Correspondence:** C. R. Behem
*Intensive Care Medicine Experimental* 2017, **5(Suppl 2):**0502

**INTRODUCTION.** Dynamic preload parameters such as stroke volume variation (SVV) and pulse pressure variation(PPV) have been widely recommended for evaluation of volume responsiveness. However, when guiding volume therapy, either hypovolaemia as well as volume-overload have to be avoided. Over-infusion results in impairments of microcirculation, which are associated with decreased outcome[1]. Endothelial dysfunction - that can be caused by ischemia/reperfusion - aggravates this problem and promotes interstitial swelling leading to further deterioration of microcirculation[2]. To date no effort has been made to evaluate whether SVV and PPV potentially can help to avoid decreases of microcirculation due to volume overload.

**OBJECTIVES.** The aim of this study was to evaluate, whether low values of PPV and SVV do have the potential to predict an impairment of intestinal microcirculation caused by volume-overload in experimental ischemia/reperfusion.

**METHODS.** In 8 pigs ischemia/reperfusion was induced during experimental aortic bypass surgery. Mean microcirculatory blood flow (mFlux) of the ileum was measured using direct laser-speckle-contrast-imaging. PPV and SVV were measured using aortic flow-probes and femoral microtip-catheters. 6 hours after ischemia/reperfusion injury measurements were performed during 4 consecutive volume-loading steps (VLS). We performed ROC Analysis to determine ability and potential thresholds of SVV and PPV to predict a microcirculatory decrease due to volume loading. A reduction of ≥10% mFlux was considered a relevant decrease.

**RESULTS.** I In total 28 VLS were analysed. Haemodynamic changes throughout the protocol are shown in Table 137. In ROC-analysis SVV presented with an AUC of 0,76 (CI 95% 0,57-0,96; p = 0,034) and PPV with an AUC of 0,84 (CI 95% 0,68-0,99; p = 0,003). ROC curves are presented in Fig. 182. Youden Index was calculated to determine ideal cut-off values predicting microcirculatory decrease. The following cut-off values can be proposed: SVV < 12,69% (Youden Index 0,57; sensitivity 77,78%, specificity 73,33%), PPV < 8,02 (Youden Index 0.64; sensitivity 72,73%; specificity 87,50%).

**CONCLUSION.** The results of our study show that low values of SVV and PPV do have the potential to predict decreases of intestinal microcirculatory blood flow caused by volume-overload in experimental ischemia/reperfusion. Therefore, dynamic preload parameters may not only be used to detect volume responsiveness but might also help to prevent microcirculatory impairments.

**GRANT ACKNOWLEDGMENT**

This study was supported by departmental funds.

**Table 137 (Abstract 0502)**. See text for description

| Table 1 | Before I/R | After I/R | VLS 1 | VLS 2 | VLS 3 | VLS 4 |
|---|---|---|---|---|---|---|
| CO (l/min) | 3,22±0,39 | 1,67±0,41* | 2,46±0,47* | 3,03±0,66 | 2,92±1,14 | 2,83±1,28 |
| SV (ml) | 49,63±6,50 | 19,02±7,29* | 28,86±5,55* | 35,87±5,84* | 36,53±8,22 | 36,16±8,21 |
| MAP (mmHg) | 84,11±21,94 | 47,71±13,65* | 59,38±13,76* | 60,86±16,82 | 61,06±18,65 | 54,69±26,10 |
| SVV (%) | 10,66±5,83 | 26,05±4,98 | 14,73±2,60 | 12,12±1,35 | 8,52±1,82 | 8,65±1,83 |
| PPV (%) | 4,37±1,75 | 29,41±16,67* | 14,18±10,97* | 8,49±3,04 | 7,36±1,83 | 8,06±3,51 |
| mFlux | 742,72±238,41 | 434,80±172,89 | 450,88±191,42 | 449,12±204,35 | 390,62±181,70* | 338,83±162,53* |

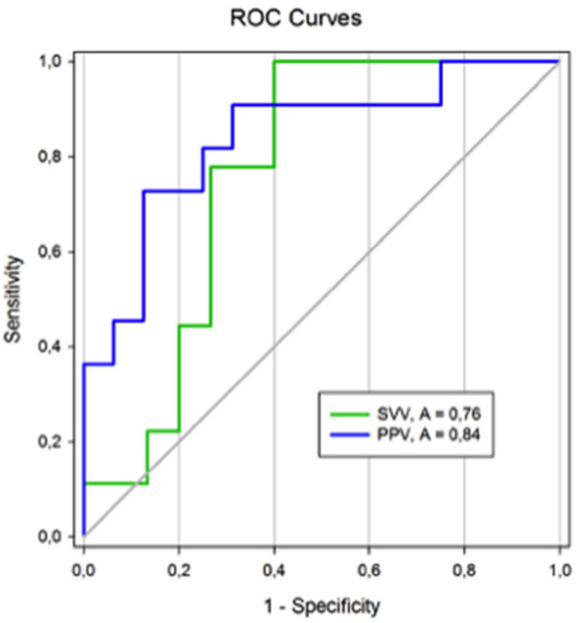

**Fig. 182 (Abstract 0502).** Receiver operating characteristic (ROC) for stroke volume variation (SVV) and pulse pressure variation (PPV) for prediction of a decline of 10% mean microcirculatory bloos flow (mFlux) of the ileum due to volume administration. Areas under the curves (AUC) are given beside the curves.

### 0503

#### Accuracy of mixed venous oxygen saturation (SvO2) and mixed venous-to-arterial carbon dioxide difference (pCO2 gap) in predicting adverse outcomes in adult patients undergoing cardiac surgery: the Philippine Heart Center Experience

J. B. T. Gemarino[1], C. C. Permejo[2], L. M. I. Habana[3]
[1]Philippine Heart Center, Department of Adult Cardiology Critical Care Medicine, Quezon City, Philippines; [2]Philippine Heart Center, Department of Adult Cardiology, Division of Critical Care Cardiology, Quezon City, Philippines; [3]Philippine Heart Center, Department of Ambulatory, Emergency and Critical Care Medicine, Quezon City, Philippines
**Correspondence:** J. B. T. Gemarino
*Intensive Care Medicine Experimental* 2017, **5(Suppl 2):**0503

**INTRODUCTION.** SvO2 and pCO2 gap are indices of tissue perfusion to detect early organ dysfunction. Many studies have validated them as predictors of organ failure, prolonged ventilatory support and hospital stay, and mortality. In the Philippines, the use of SvO2 and pCO2 gap as tools for monitoring cardiac surgical patients is still underutilized and there is a paucity of evidence regarding their use.
**OBJECTIVES.** To determine the accuracy of SvO2, pCO2 gap, and their combination in predicting adverse outcomes in adult patients undergoing cardiac and aortic aneurysm surgeries.
**METHODS.** A prospective cohort study approved by the Technical Review Committee (TRC) and the Institutional Ethics Review Board (IERB) which included 108 adult patients who underwent cardiac and aortic aneurysm surgeries from October to December 2016 in the Philippine Heart Center (PHC). PHC is the Center of Excellence in Cardiovascular Care in the Philippines which operates a semi-open cardiac surgical intensive care unit (SICU) where attending cardiologists can opt to refer or not to refer to intensivists. SvO2 and pCO2 gap of subjects were taken on admission to the SICU and were analyzed as to their accuracy in predicting adverse outcomes namely, organ dysfunction, in-hospital, and 30-day mortality.
**RESULTS.** To predict organ dysfunction, SvO2 < 60% and in combination with pCO2 gap ≥6 mmHg showed high specificity of 90% (SvO2 < 60% PPV = 82.35%; pCO2 gap ≥6mmHg PPV = 81.36%). To predict in-hospital mortality, they showed good specificity of 82.93% and 85.37%, respectively (SvO2 < 60% NPV = 82.93%; pCO2 gap ≥6mmHg NPV = 82.98%; combined parameters NPV = 82.35%). To predict 30-day mortality, they likewise showed good specificity of 84.44% and 86.67%, respectively (SvO2 < 60% NPV = 83.52%; pCO2 gap ≥6mmHg NPV = 83.67%; combined parameters NPV = 82.98%). The receiver operating characteristic (ROC) curves of SvO2 and pCO2 gap to predict organ dysfunction, in-hospital, and 30-day mortality were 0.43 and 0.64, 0.52 and 0.47, and 0.49 and 0.47, respectively. This can be explained by factors like absence of standard protocol for post-op care in our institution for hemodynamic optimization and goal-directed therapy and varying hemoglobin levels as transfusion trigger.
**CONCLUSIONS.** SvO2 < 60% and its combination with pCO2 gap ≥6mmHg revealed high specificity and good PPVs in predicting organ dysfunction. They also revealed good specificity and good NPVs in predicting in-hospital and 30-day mortality.

**REFERENCE(S)**

1. Robin E, Futier E., Pires O, Fleyfel M, Tavernier B, Lebuffe G, Vallet B, Central venous-to-arterial carbon dioxide difference as a prognostic tool in high-risk surgical patients, Critical Care (2015) 19:227
2. Holm J, Hakanson E, Vanky F, Svedjeholm, R. Mixed venous oxygen saturation predicts short- and long-term outcome after coronary artery bypass grafting surgery: a retrospective cohort analysis, British Journal of Anaesthesia 2011; 107 (3): 344–50

**GRANT ACKNOWLEDGMENT**
None

### 0504

#### Intra observer variability of endothelial glycocalyx measurements in ICU patients using sidestream dark field imaging

M. Bol[1], M. Suverein[1], T. Delnoij[1], R. Driessen[1], S. Heines[1], T. Delhaas[2], M. van de Poll[1], J. W. Sels[1]
[1]Maastricht University Medical Centre+, ICU, Maastricht, Netherlands; [2]Maastricht University, Maastricht, Netherlands
**Correspondence:** M. Bol
*Intensive Care Medicine Experimental* 2017, **5(Suppl 2):**0504

**INTRODUCTION.** There is a growing interest in the state of the microcirculation during critical illness [1]. Sidestream Dark Field Imaging (SDFI) is widely used to study microcirculation in vivo. One such SDFI device is the Glycocheck®, which estimates the thickness of the endothelial glycocalyx by measuring the thickness of the Perfused Boundary Region (PBR). Though single measurements are current clinical practice, intra observer variability is unknown.
**OBJECTIVES.** This study aims to determine the intra observer variability of the PBR as measured with the Glycocheck®.
**METHODS.** Microcirculation was assessed in a pilot study of 49 mechanically ventilated mixed ICU patients using SDFI (Glycocheck®). Each patient was assessed three times consecutively within 30 minutes by the same experienced operator. The operator was unaware of the results of the measurements until all three measurements were completed. The Glycocheck® calculated the PBR for each of the measurements. The intra-observer agreement was calculated using an intra-class correlation coefficient (ICC) using SPSS.
**RESULTS.** The mean (SD) PBR was 2,02 (0,26) μm. The ICC for single measures was 0,362, indicating a poor intraobserver agreement for single measures. ICC for average measures was 0,630 indicating reasonable reliability of the averaged value of 3 consecutive measurements.
**CONCLUSIONS.** Intraobserver variability in this pilot study was higher than expected. Averaging the results of three consecutive measurements improved data quality. Whether intraobserver agreement was limited by patient factors such as hemodynamic instability or whether it is inherent to the method itself remains unclear. Reliability of measurement of PBR by SDFI should be increased by averaging (at least three) multiple consecutive measurements. A single measurement is insufficient to yield reliable data on PBR diameter assessed by glycocheck.

**REFERENCE(S)**

[1] Bezemer R, Bartels SA, Bakker J, Ince C., Critical Care. 2012 Jun 19;16(3):224

### 0505

#### Microcirculation and slightly increased lactate levels: is there a bedside relationship? A cross-sectional pilot study

E. ter Bals[1], W. W. J. van Strien[2], N. Papaloukas[2], S. Rijkenberg[2], P. H. J. van der Voort[2]
[1]Onze Lieve Vrouwe Gasthuis, Intensive Care, Amsterdam, Netherlands; [2]Onze Lieve Vrouwe Gasthuis, Amsterdam, Netherlands
**Correspondence:** E. ter Bals
*Intensive Care Medicine Experimental* 2017, **5(Suppl 2):**0505

**INTRODUCTION.** Microcirculatory dysfunction cannot be predicted by systemic hemodynamic parameters.[1] A serum lactate above 4 mmol/l was previously considered abnormal but this upper level has been lowered to 2 mmol/l.[2] This is considered to indicate inadequate oxygen delivery.[3]
**OBJECTIVES.** To determine whether a lactate level above 2 mmol/L is associated with microvascular dysfunction compared to normal lactate.
**METHODS.** We conducted a cross-sectional observational pilot study. In each intensive care patient sublingual microcirculation was assessed with the Cytocam-IDF (incident dark field illumination) device simultaneously with the collection of an arterial blood gas for lactate measurement. Microcirculatory images were analysed automatically with Cytocam analysis software. We assessed the direct bedside relationship between total vessel density (TVD), perfused vessel density (PVD), proportion of perfused vessels (PPV) and the lactate level of blood collected at the same time. Descriptive analyses and Pearson's correlation coefficient were used. Mann–Whitney U tests were used for comparison between the lactate groups ≤ 2 mmol/L and > 2 mmol/L.

**RESULTS.** 32 patients were included, 21 of them after heart surgery, 8 with sepsis. Lactate level was below 2.0 mmol/L in 14 and in 17 the lactate level was above 2.0 mmol/L. Mean age was 65 years (SD 8.1), median APACHE IV predicted mortality 4.9% (IQR 23), mean SOFA score 6.4 (SD 2.8). Lactate levels were median 2.4 mmol/L (IQR 1.7) and varied between 0,5 mmol/L and 6.7 mmol/L.

TVD, PVD and PPV were non-significantly different between groups (all $p > 0.3$). All correlation coefficients between lactate and TVD, PVD and PPV were below 0.2 and non-significantly different from zero.

**CONCLUSIONS.** No significant correlation was found between sublingual IDF microcirculation parameters and lactate levels. Groups with normal and slightly elevated lactate were comparable too. Higher lactate ranges need further research.

**REFERENCE(S)**

1. G. Hernandez, et al. Severe abnormalities in microvascular perfused vessel density are associated to organ dysfunction and mortality and can be predicted by hyperlactemia and norepinephrine requirements in septic shock patients. J Crit Care 2013;28(4):538.e9-14.

2. L.W. Andersen, et al. Etiology and therapeutic approach to elevetad lactate level. Mayo Clin Proc 2013;88(10):1127–40.

3. C. Jung, M. Kelm. Evaluation of the microcirculation in critically ill patients. Clin Hemorheol Microcirc 2015;61:213–24

**GRANT ACKNOWLEDGMENT**

None.

### 0506

#### The characteristics of venous-to-arterial $CO_2$ difference/arterial-central venous $O_2$ difference ratio in clinical classification based on P(v-a)$CO_2$ and Scv$O_2$

H. He[1,2], D. Liu[1], C. Ince[2,3], Y. Long[1], B. Tang[1]

[1]Peking Union Medical College Hospital, Peking Union Medical College, Beijing, China; [2]Erasmus MC University Hospital, Rotterdam, Netherlands; [3]Amsterdam University Medical Center, Amsterdam, Netherlands

**Correspondence:** H. He

*Intensive Care Medicine Experimental* 2017, **5(Suppl 2):**0506

**INTRODUCTION.** Studies had shown that venous-to-arterial $CO_2$ difference/arterial-central venous $O_2$ difference(P(v-a)$CO_2$/C(a-v)$O_2$)ratio is related to lactate levels, lactate clearance and the response of $VO_2$to an acuteincrease of $DO_2$. The P(v-a)$CO_2$/C(a-v)$O_2$ is calculated from a well-known formula[1], using the values of central venous saturation(Scv$O_2$) and thevenous-to-arterial $CO_2$ difference (P(v-a)$CO_2$). To our knowledge, thedependency of the calculation of P(v-a)$CO_2$/C(a-v)$O_2$ ratio to Scv$O_2$ and P(v-a)$CO_2$has not been explored sufficiently in clinical practice.

**OBJECTIVES.** Determine the relationship between Scv$O_2$, hemoglobin, Sa$O_2$, venous-to-arterial $CO_2$ difference and P(v-a)$CO_2$/C(a-v)$O_2$ ratio, and define the corresponding factors which identify give a high P(v-a)$CO_2$/C(a-v)$O_2$ ratio in critically ill patients.

**METHODS.** A total of 1294 pairs of simultaneous arterial and central venous blood gas measurements were retrospectively studied in 354critically ill patients. A cutoff of P(v-a)$CO_2$/C(a-v)$O_2$ ratio > 1.68 was defined as an abnormally high ratio based on published literature[1]. The measurements were divided into four categories based on cutoff values of Scv$O_2$ (70%) and P(v-a)$CO_2$ (6mmHg): group I (P(v-a)$CO_2$ ≤ 6mmHg on Scv$O_2$ < 70%), group II (P(v-a)$CO_2$ ≤ 6mmHg on Scv$O_2$ ≥ 70%), group III (P(v-a)$CO_2$ > 6mmHg on Scv$O_2$ < 70%), and group IV (P(v-a)$CO_2$ > 6mmHg on Scv$O_2$ ≥ 70%).

**RESULTS.** P(v-a)$CO_2$ show a significantly stronger correlation with P(v-a)$CO_2$/C(a-v)$O_2$(r =0.692, $P < 0.05$) than Scv$O_2$ (r = 0.104, $P < 0.05$) and hemoglobin(r = −0.159, $P < 0.05$). The group I measurements had the lowest P(v-a)$CO_2$/C(a-v)$O_2$ ratio value (1 ± 0.46) and percentage of high P(v-a)$CO_2$/C(a-v)$O_2$ ratio(5.8%(8/136)). Moreover, the group IV measurements had the highest P(v-a)$CO_2$/C(a-v)$O_2$ ratio value (2.32(1.9-3.4)) and percentage of high P(v-a)$CO_2$/C(a-v)$O_2$ ratio(84%(176/209)). (Figure 183) However, there was a significantly lower Sa$O_2$, hemoglobin and higher lactate in the group I. The P(v-a)$CO_2$(ROC area 0.793) was with the best ability to predict the high ratio and was significantly better than Scv$O_2$ and hemoglobin. (Figure 184). For predicting the high P(v-a)$CO_2$/C(a-v)$O_2$ ratio, a P(v-a)$CO_2$ threshold of 7mmHg was associated with a sensitivity of 41.77% and a specificity of 90.62%.

**CONCLUSIONS.** P(v-a)$CO_2$ is predictive of high P(v-a)$CO_2$/C(a-v)$O_2$ ratio, and P(v-a)$CO_2$ is more relevant to P(v-a)$CO_2$/C(a-v)$O_2$ ratio than Scv$O_2$. There might be a "pseudo-normalization" of P(v-a)$CO_2$/C(a-v)$O_2$ ratio in the group I with low P(v-a)$CO_2$(≤6mmHg) and low Scv$O_2$(<70%).

**REFERENCE(S)**

1. Mallat J, et al. Ratios of central venous-to-arterial carbon dioxide content or tension to arteriovenous oxygen content are better markers of global anaerobic metabolism than lactate in septic shock patients. Ann Intensive Care.Dec; 2016.6(1):10.

**GRANT ACKNOWLEDGMENT**

This study was supported by China Scholarship Council (NO. 201608110082) and the Organization Department of Beijing Municipal Committee (No. 2015000020124G072).

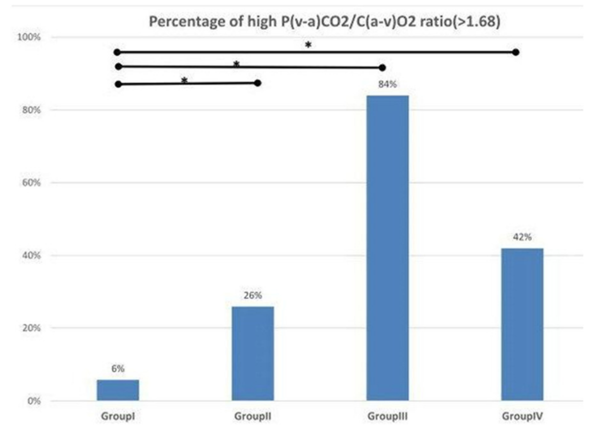

**Fig. 183 (Abstract 0506).** The percentages of high ratio in the four groups

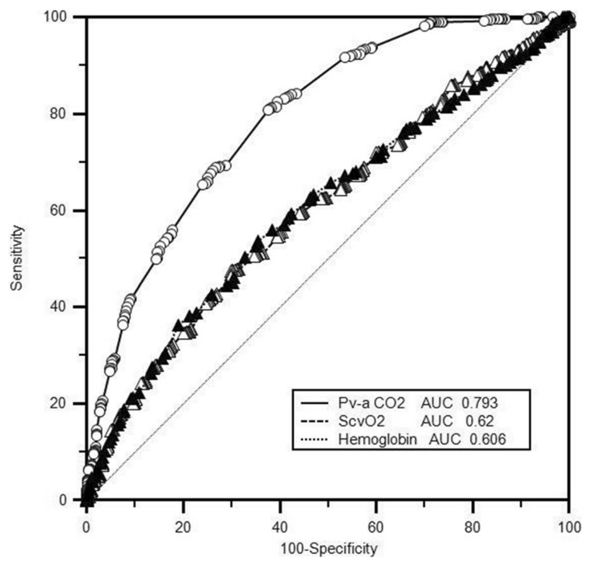

**Fig. 184 (Abstract 0506).** ROC curves for predicting an high P(v-a)CO2/C(a-v)

### 0507

#### CO2 variables after cardiac surgery and its association with renal failure. Preliminary results

T. F. Fariña González[1], J. Latorre[2], J. M. Gil Perdomo[1], F. Martinez Sagasti[1], A. García de Lorenzo y Mateos[3]
[1]Hospital Clinico San Carlos, Intensive Care Department, Madrid, Spain; [2]Hospital Universitario la Paz, Anaesthesiology Department, Madrid, Spain; [3]Hospital Universitario la Paz, Intensive Care Department, Madrid, Spain
**Correspondence:** T. F. Fariña González
*Intensive Care Medicine Experimental* 2017, **5(Suppl 2):**0507

**INTRODUCTION.** Renal function impairment is common after cardiac surgery,. The early mortality rate in patients with AKI is around 5% but raises up to 50% when renal replacement therapy (RRT) is needed. Various factors related to CPB have been implicated as possible determinants of AKI but few studies analyze the C02 and 02 metabolism. Venous-arterial CO2 derivatives (ΔpCO2, ΔpCO2/ΔO2content, ΔCO2content/ΔCO2) are intensively studied in sepsis, but there are lack of studies concerning cardiac surgery.

**OBJECTIVES.** To analyze the relationship between CO2-derived variables measured during the first 12 hs after an elective cardiac surgery and renal failure in the following 28 days. The calculated sample is 150 patients. These are preliminary results.

**METHODS.** prospective, observational study; following a cohort of adults who underwent cardiac surgery and were admitted in a 16-bed cardiac ICU. Informed consent was obtained from the next-of-keen or reconsent was signed by the patient after extubation. The study was approved by the Hospital Ethics Committee.

Previous history of renal and hepatic disease was recorded as well as type of surgery and surgical times. Central venous and arterial gases were analyzed at ICU admission, 6 hs and 12 hs later, as well as, hemoglobin, creatinine, lactate, and hemodynamic variables. Fluid balance was also collected. In the following 28 days, ICU mortality, hospital and ICU stay and acute renal failure incidence, according AKIN and RIFLE classification were analyzed. All normally distributed data were expressed as the mean ± standard deviation (SD) unless otherwise specified. Quantitative variables were compared using non-parametric tests. Qualitative variables were compared with chi square or Fisher's tests. $p < 0,05$ was considered significant.

**RESULTS.** 22 patients were analyzed. 13 were male (59,1%). Mean age was 66,68 years (SD ± 12,77). Four patients had previous renal disease (18,2%) but none of them had hepatic disease. Type of surgery: valvular (11), CABG (5), CABG + valvular (4) and aortic root (2). EuroScore II was 1,63 (IQR 0,92-2,44) and admission CASUS was 3 (1,75-5). CPB was used in 18 surgeries (81,8%): CPB time was 101 min (SD ± 51,64) and cross clamp time 78 min (SD ± 36,31). Fluid balance at 12 hs was 578 ml (IQR −55-928). Four patients (18,2%) were ventilated and 8 (36,4%) treated with vasoactive drugs >12 hs. Perioperative ARF was diagnosed in 7 patients (31,8%) but none of them received RRT. CPB was not associated with renal failure ($p = 0,19$) No patients died. Hospital stay was 11 days (SD ± 6,63) and ICU 1,81 days (SD ± 1,25). delta-12hs Svc02, Lac, CO2 variables and fluid balance were not associated either with renal failure, mortality or lengths of stays ($p > 0,05$).

**CONCLUSION.** These preliminary results show that in the first 12 hs, CO2 derivates are not related either with postoperative renal failure or mortality.

### 0508

#### Central venous-arterial pCO2 difference as a tool to guide postoperative management of cardiac surgery patients

I. Ben Naoui[1], I. Nefzi[1], B. Jeribi[1], K. Ben Mahmoud[1], H. Jouini[1], S. Mlayhi[2], A. Ammous[1], R. Denguir[2], A. Cherif[1]
[1]La Rabta Hospital, Anesthesiology & Intensive Care, Tunis, Tunisia; [2]La Rabta Hospital, Cardiac Surgery, Tunis, Tunisia
**Correspondence:** I. Nefzi
*Intensive Care Medicine Experimental* 2017, **5(Suppl 2):**0508

**INTRODUCTION.** The arteriovenous difference in $CO_2$ ($\Delta PCO_2$) is an indicator of macro and microcirculatory perfusion during shock states. In septic shock, an arteriovenous pressure gradient of $CO_2 < 6$ mmHg (in addition to venous saturation of oxygen [$SvO_2$] > 70%) demonstrates a restored central hemodynamics and constitutes a therapeutic objective [1].

**OBJECTIVES.** The purpose of this study was to evaluate the clinical relevance of high values of $\Delta PCO_2$ and to assess the association between both $\Delta PCO_2$ and arterial lactate levels with cardiac surgery outcomes.

**METHODS.** Between January and March 2017, all patients scheduled to undergo open heart surgery using cardiopulmonary bypass were included in this prospective single-center observational study. A standard technique for anesthesia was followed. Demographic and biological data, $PCO_2$ gap, central venous oxygen saturation, lactate levels, vasopressor/inotropic support and postoperative complications were recorded for all patients at ICU admission, then 1 hour (H1), 2 hours (H2), 3 hours (H3), 6 hours (H6) and 10 hours (H10) after admission.

**RESULTS.** Thirty-three consecutive patients were included. Mean age was 48 (9) years. Nine patients (27%) required mitral valve replacement. Eight (24%) required aortic valve replacement. Another eight (24%) required coronary bypass surgery, and eight (24%) underwent Bentall procedure. The mean left ventricular ejection fraction (LVEF) was 50 (2)%. Correlation between $\Delta PCO_2$ and lactate levels was significant at all time points. We found a significant correlation between mortality rate and $\Delta PCO_2$ up to H2 (57%, $p = 0.001$) but no correlation with length of ventilation, and hospital length of stay.

**CONCLUSIONS.** The arteriovenous difference in $CO_2$ might be a useful tool to detect global and microcirculatory hypoperfusion in postoperative cardiac surgical patients.

**REFERENCE(S)**

[1] The role of central venous oxygen saturation, blood lactate, and central venous-to-arterial carbon dioxide partial pressure difference as a goal and prognosis of sepsis treatment, J Crit Care, 2016 Dec; 36:223–229

**Table (Abstract 0508).** Correlation between lactate levels and ΔPCO2

| | H0 | H1 | H2 | H3 | H6 | H10 |
|---|---|---|---|---|---|---|
| Correlation between Lactate levels and ΔPCO2 (%) | 59 | 50 | 49 | 57 | 59 | 53 |
| p | 0.001 | 0.03 | 0.04 | 0.001 | 0.001 | 0.002 |

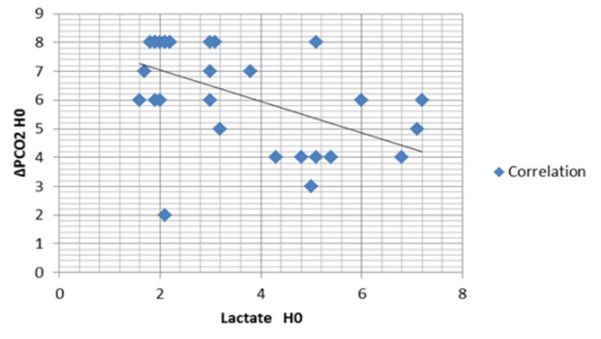

**Fig. 185 (Abstract 0508).** Correlation between ΔPCO2 and Lactate levels

### Mechanical ventilation: Clinical studies

#### 0509

**The effect of evacuation of massive pleural effusions on the distribution of tidal volume and lung mechanics in mechanically ventilated patients**

C. Nouris, K. Marmanidou, M. Vasiliou, K. Dimitroulakis, E. Soilemezi, D. Matamis
Papageorgiou General Hospital, ICU, Thessaloniki, Greece
**Correspondence:** C. Nouris
*Intensive Care Medicine Experimental* 2017, **5(Suppl 2):**0509

**INTRODUCTION.** Pleural effusions are common in ICU patients, making therapeutic thoracocentesis one of the most commonly performed medical procedures. However, the effect of pleural effusions drainage on lung mechanics and on the distribution of tidal volume between the thoracic and abdominal compartments has not been elucidated.

**OBJECTIVES.** To investigate the distribution of tidal volume among the thoracic and abdominal compartments before and after drainage of pleural effusions in mechanically ventilated patients and the effect of positive end expiratory pressure (PEEP) on this distribution.

**METHODS.** Six sedated and mechanically ventilated ICU patients with bilateral pleural effusions were enrolled in the study. A central venous catheter was inserted in each pleural cavity for fluid drainage and direct pleural pressure measurement. The following variables were measured: Rib cage (RC) and abdominal (AB) compartment contribution to tidal volume (Vt) using Respiratory Inductive Plethysmography, Functional Residual Capacity (FRC) using the dilutional nitrogen wash in and wash out method (Carescape R860 ventilator), static compliance (Cstat), diaphragmatic displacement using ultrasonography, $PaO_2/FiO_2$ ratio and transpulmonary pressure. Our measurements were recorded during four different steps: i: before drainage of pleural fluid, ii: after drainage with zero peep (ZEEP), iii: after drainage and application of 14 $cmH_2O$ of PEEP for 20 minutes and iv: after release of PEEP. ANOVA with post hoc analysis was performed using IBM statistics SPSS 23.

**RESULTS.** The mean volume of pleural fluid drained was 1700ml. The distribution of Vt in RC and AB did not change significantly after the evacuation of pleural fluid. On the contrary the application of 14 cm $H_2O$ of PEEP increased the distribution of Vt in the RC compartment by 13% (p = 0,047). The application of PEEP also induced an increase in FRC by 1lt (p = 0,01) and $PaO_2/FiO_2$ ratio by 120 mmHg (p = 0,078). Additionally, transpulmonary pressure also increased by 123% (p = 0,046), whereas Cstat remained constant. All the effects of PEEP on lung mechanics were abolished after the release of PEEP except for the effect on oxygenation. Moreover, an increase in diaphragmatic displacement was observed with ultrasonography in all patients after the release of PEEP (p = 0,04) probably due to reopening of basal atelectasis.

**CONCLUSIONS.** Pleural fluid drainage itself does not accomplish sufficient lung recruitment. Only high levels of PEEP are associated with a significant improvement in FRC; however, the improvement is temporal and is abolished after the release of PEEP.

#### 0510

**An international study to assess the effect of high altitude over ARDS and mortality rate in invasive mechanically ventilated patients: a preliminary report**

M. Jibaja[1,2], G. Ortiz[3,4], M. F. Garcia Aguilera[1], M. Garay[3], F. D. J. Montelongo[5], J. Martinez[6], J. A. Viruez[7], O. Baez-Pravia[8], S. Salazar[9], F. Villacorta[10], F. Morales[11], A. Tinoco[12], P. Cardinal-Fernandez[13,14], RELAMI - Red Latinoamericana de Investigadores de Medicina Intensiva en la Altura
[1]Hospital Eugenio Espejo, Intensive Care Unit, Quito, Ecuador; [2]Universidad Internacional del Ecuador, Escuela de Medicina, Quito, Ecuador; [3]Universidad del Bosque, Internal Medicine and Pulmonology, Bogota, Colombia; [4]Universidad de Barcelona, Barcelona, Spain; [5]Hospital General de Ecatepec 'Las Americas', Intensive Care Unit, Ecatepec, Mexico; [6]Hospital 'San Pedro', Intensive Care Unit, Pasto, Colombia; [7]Hospital de El Alto, Intensive Care Unit, La Paz, Plurinational State of Bolivia; [8]Hospital Universitario HM Sanchinarro, HM Hospitales, Intensive Care Unit, Madrid, Spain; [9]Hospital Carlos Andrade Marin, Intensive Care Unit, Quito, Ecuador; [10]Hospital Provincial General Docente de Riobamba, Intensive Care Unit, Riobamba, Ecuador; [11]Hospital SOLCA, Intensive Care Unit, Portoviejo, Ecuador; [12]Hospital Nacional Ramiro Priale Priale, Intensive Care Unit, Huancayo, Peru; [13]Hospital Universitario HM Sanchinarro, HM Hospitales, Department of Emergency, Madrid, Spain; [14]Hospital Universitario HM Sanchinarro, HM Hospitales, HM Research Foundation, Madrid, Spain
**Correspondence:** P. Cardinal-Fernandez
*Intensive Care Medicine Experimental* 2017, **5(Suppl 2):**0510

**INTRODUCTION.** It is estimated that more than 140 million of people lives in areas situated higher than 2500 mts above sea level. The effect of low airway pressure induced by high altitude has never been studied in invasive mechanically ventilated (IMV) patients.

OBJECTIVE. To define the effect of high-altitude over ARDS and mortality rate in IMV patients.

**METHODS.** This international, prospective, cohort and observational study of patients (> = 16 years-old) undergoing IMV was conducted during 4 consecutive weeks (August 1st to 31st) of 2016 in a convenience sample of 27 ICUs from Bolivia, Brazil, Colombia, Ecuador, México, Peru and Spain. Exclusion criteria: change the altitude over sea level during the 40 days before ICU admission. Demographic data, comorbid diseases, and clinical and laboratory data were collected. Patients were followed up until death, hospital discharge or for 28 days, Day 0: pa02/Fio02 < =300mmHh.ARDS was defined according to Berlin. Results are shown as median (IQR). Categorical and continuous variables were compared with $\chi 2$ and Mann–Whitney or kruskal-wallis, respectively. Ethical committee approved this study.

**RESULTS.** From 178 patients that were recruited, n = 24 belong to the sea level group (≤600mt); n = 75 to the mild altitude group (>600mts & ≤2600mts); n = 69 to the high altitude group (>2600mts & ≤ 3600mts) and n = 10 to the extreme high altitude group (>3600mts). Apache II scores were 18 (12; 26); 16 (12; 21); 19 (11; 26) and 17 (15; 18) respectively (p = 0.50). Age (years-old) were 65 (56; 71); 60 (44; 74); 58 (39; 71) and 40 (36; 56) respectively (p = 0.01). Mortality rates were 33%, 23%, 26% and 30%, respectively (p = 0.76). ARDS rates were 42%, 14%, 21% and 60%, respectively (p = 0.01).

**CONCLUSIONS.** In this pioneer study the increase in the altitude above sea level seems to be influence the risk of ARDS but not the mortality of patients undergoing IMV.

**TRIAL REGISTRATION.** Clinicaltrials.gov Identifier: NCT02871063 .

**GRANT**
Escuela de Medicina de la Universidad Internacional del Ecuador

#### 0511

**Evidence supporting the clinical uses of proportional assist ventilation: a systematic review and meta-analysis**

B. K. Tirupakuzhi Vijayaraghavan[1,2], S. Hamed[1], A. Jain[1], T. Chimunda[1], I. Telias[1], J. O. Friedrich[1,3], K. E. A. Burns[1,4]
[1]University of Toronto, Critical Care Medicine, Toronto, Canada; [2]Sunnybrook Health Sciences Centre, Critical Care Medicine, Toronto, Canada; [3]St. Michael's Hospital/University of Toronto, Critical Care Medicine, Toronto, Canada; [4]St. Michael's Hospital/University of Toronto, Critical Care Medicine, Toronto, Canada
**Correspondence:** B. K. Tirupakuzhi Vijayaraghavan
*Intensive Care Medicine Experimental* 2017, **5(Suppl 2):**0511

**INTRODUCTION.** Proportional Assist Ventilation (PAV) generates pressure in proportion to instantaneous effort by amplifying inspiratory effort without a preselected target in spontaneously breathing patients. PAV plus measures compliance and resistance, calculates work of breathing and elastance, and adjusts the level of support provided according to a preset level of assistance. Although PAV and PAV+ are expected to augment patient comfort and synchrony, whether they confer clinical benefits remains unknown.

**OBJECTIVES.** To summarize randomized controlled trials (RCTs) comparing invasive and noninvasive PAV and PAV+ in critically ill adults and children reporting clinical outcomes.

**METHODS.** We searched Medline (1946-Sept 2015), EMBASE (1947-Sept 2015) and CENTRAL (August 2015) to identify potentially eligible trials using database-specific search strategies. Five authors hand-searched conference proceedings from 5 meetings (Society of Critical Care Medicine, European Society of Intensive Care Medicine, International Symposium of Intensive Care and Emergency Medicine, American College of Chest Physicians, and the American Thoracic Society) from 2005–2015. The search was not limited by language or publication status.

We selected parallel group and crossover RCTs that enrolled critically ill adults or children receiving invasive or noninvasive PAV or PAV+, compared them to an alternate mode, and reported at least one clinically important outcome.

**RESULTS.** We identified 13 RCTs (10 parallel-group, 3 crossover) involving 881 patients. Eight trials evaluated PAV and 5 trials evaluated PAV+. Of these, 4 parallel-group RCTs evaluated noninvasive PAV in patients with acute respiratory failure (ARF). Two parallel-group trials evaluated invasive PAV as a weaning strategy. Four trials evaluated PAV+ as an initial support strategy (n = 1), weaning strategy (n = 2), and as an SBT technique (n = 1). Three cross-over trials [2 invasive PAV and 1 invasive PAV+] assessed sleep quality. Trials were of low to moderate quality.

Compared to noninvasive Pressure Support (PS), noninvasive PAV showed a nonsignificant reduction in intubation rate [RR 0.92 (0.59,1.43); 2 trials, n = 161; $I^2 = 0\%$] and no effect on ICU or hospital mortality or length of stay. Compared to invasive PS, we found no effect of invasive PAV on the proportion of rapid eye movement sleep [MD −2.93 (−14.20, 8.34) (percentage); 2 trials, n = 50; $I^2 = 43\%$]. Compared to invasive PS, invasive PAV+ showed a nonsignificant increase in weaning time [MD 0.46 (−0.71,1.63); (hours)2 trials, n = 74; $I^2 = 0\%$].

**CONCLUSIONS.** Available RCT data do not support use of invasive or noninvasive PAV or invasive PAV+ for various indications in critically ill adults. No trial evaluated PAV or PAV+ in children. Promising areas for future investigation include evaluation of (i) noninvasive PAV as initial support for patients with ARF, (ii) invasive PAV on sleep quality, and (iii) invasive PAV+ in weaning.

**GRANT ACKNOWLEDGMENT**

Nil

### 0512

#### Impact of the introduction of a standardised ventilator associated pneumonia (VAP) prevention bundle and endotracheal tubes (ETTs) with subglottic suction ports on VAP rates in a large tertiary critical care centre

T. Moorthy[1], A. Prichard[1], E. Prower[1], A. Forbes[2], A. Fife[1], S. Patel[1]
[1]King's College Hospital, London, United Kingdom; [2]King's College Hospital, Department of Critical Care, London, United Kingdom
**Correspondence:** A. Forbes
*Intensive Care Medicine Experimental* 2017, **5(Suppl 2):**0512

**INTRODUCTION.** VAP is the most common healthcare-associated infection in critical care units, occurring in up to 30% of patients requiring mechanical ventilation. Attributable mortality is estimated at 9–13.5% and the additional financial cost of each VAP episode ranges from £7000-£25,000. VAP rates are a quality indicator, and a reduction in VAP rates has been demonstrated to result in reduced length of stay, more efficient use of critical care beds, and financial savings.[1,2]

**OBJECTIVES.** To demonstrate a reduction in VAP rates through two separate interventions:

a) implementation of a standardised VAP prevention bundle for every intubated patient and
b) introduction of subglottic suction port ETTs.

**METHODS.** Prospective study conducted in a large tertiary critical care centre with two phases: Phase 1 over 20 working days (July-August 2016) followed by phase 2 on working days over 8 weeks (February-April 2017). All patients ventilated >48 hours across four specialist critical care units were audited (total 68 beds). Patients were assessed using the HELICS criteria, and the impact of two consecutive interventions on VAP rates:

a) implementation of a standardised VAP prevention bundle for every intubated patient (between phases 1 and 2) and
b) introduction of subglottic suction port ETTs (during phase 2). VAP prevention bundle compliance was audited simultaneously.

**RESULTS.** VAP rates during phase 1 were 38 per 1000 ventilator days. VAP rates reduced to 29 per 1000 ventilator days following the introduction of the VAP prevention bundle (phase 2a). Compliance with the VAP bundle was considered sub-optimal at 84%. During phase 2b VAP rates were further reduced in those patients with subglottic suction port ETTs (14.7 per 1000 ventilator days) compared to those with standard ETTs (26.6 per 1000 ventilator days). VAP prevention bundle adherence was similar during both phases.

**CONCLUSIONS.** Overall compliance with the VAP prevention bundle was considered sub-optimal, however despite this, a trend towards improvement in VAP rates was seen with this intervention. Introduction of ETTs with subglottic suction ports in addition to the VAP prevention bundle showed a marked improvement in VAP rates. Based on our data, optimisation of VAP prevention bundle adherence and routine use of subglottic suction port ETTs is recommended. There is likely to be a significant cost saving associated with a reduction in VAP rates following these interventions.

**REFERENCE(S)**

1. Ventilator associated pneumonia in the ICU. Kalanuria et al. Critical Care 2014, 18:208

2. Number needed to treat and cost effectiveness in the prevention of ventilator-associated pneumonia. Wyncoll and Camporota. Critical Care 2012, 16:430

### 0513

#### Dynamic stress may not be modified by conventional lung protective strategies in spontaneous ventilation compared to controlled ventilation modality

J. I. Gomez Ramirez, E. Monares Zepeda, G. Camarena Alejo, J. S. Aguirre Sanchez, J. Franco Granillo
American British Cowdray Medical Center, Critical Care Medicine, Ciudad de Mexico, Mexico
**Correspondence:** J. I. Gomez Ramirez
*Intensive Care Medicine Experimental* 2017, **5(Suppl 2):**0513

**INTRODUCTION.** Calculated dynamic stress measured as mechanical power in respiratory system ($Power_{rs}$) is a recently described tool for assessment of lung injury[1]. However information related to spontaneous ventilation (SV) is limited, moreover whether the $Power_{rs}$ is modified by conventional protective lung strategies (CLPS) is unknown in SV.

**OBJECTIVES.** To measure and to compare $Power_{rs}$ in patients with SV and controls with controlled ventilation (CV) modality and to stratify according to CLPS.

**METHODS.** We conducted a case–control study with critical patients from a single center in an intensive care unit (ICU) in Mexico. Study subjects included subsequent cases of patients with spontaneous ventilation and 1:1 age-and sex-matched-controls with CV modality. Demographic, and Clinical variables were collected from clinical files. We included the following Ventilator parameters: tidal volume (TV), predictive body weight (PBW), compliance ($C_{RS}$), respiratory rate (RR), total positive end expiration pressure (tPEEP), peak pressure (PeakP), plateau pressure (PlatP) and $Power_{rs}$ calculated according to an equation described according to the work published on intensive care med in 2016 a cutoff > = 13 J/min was defined as high dynamic stress. We tested the following CLPS:

1) TV < 6ml/Kg/PBW,
2) Platp < 30 mm H2O,
3) Driving pressure < 15 cm H2O.

**RESULTS.** We included data from 30 patients, age 65 (SD ± 15) years and 63% males with 30 matched controls. Overall, 23% of patients had full CPLS. PBW of 62 (SD ± 10.5 kg, mean RR of 18 (SD ± 5.5)min-1, mean TV of 0.46 (SD ± 0.1) liters, mean $C_{RS}$ of 55 (SD ± 22) mL/cm H2O, tPEEP of 7.6 (SD ± 3.3) cm H20, PeakP 20.4 (SD ± 6.9) cm H2O, PlatP of 17.05 (SD ± 5.8) cm H2O. Global mean $Power_{rs}$ 12.7 (SD 8.25) J/min. $Power_{rs}$ in SV was similar for both protected and unprotected patients 7.25 (SD ±1.59) vs. 6.09 (SD ±1.8) J/min (p = 0.11), and lower in controls with 12.3 vs 20.35 J/min (p = 0.02) when in CPLS. Case patients had lower overall risk for high dynamic stress OR = 0.26 (95% CI 0.14 - 0.48, p < 0.01) and was not modified by any of the CLPS.

**CONCLUSIONS.** Patients in SV modality showed lower estimated $Power_{rs}$ vs. control group. CPLS did not modify the risk for with high dynamic stress since any of subjects in this group reached > = 13 J/min. Lower cutoff points for dynamic stress might not be clinically relevant because the risk for lung injury at lower cutoff values is improbable and additional CPLS possibly should not be further required.

**REFERENCE(S)**

1. John J. Marini and Samir Jaber, Dynamic predictors of VILI risk: beyond the driving pressure Intensive Care Med (2016) 42:1597–1600.

### 0514

#### A randomized controlled trial comparing full closed loop controlled ventilation (IntelliVent-ASV™) with conventional ventilation in intubated COPD patients (interim analysis results)

C. Kirakli[1], I. Naz[2], O. Ediboglu[1], S. Ataman[1]

[1]Dr. Suat Seren Chest Diseases and Surgery Training and Research Hospital, Intensive Care Unit, Izmir, Turkey; [2]Katip Celebi University, Faculty oh Health Sciences, Physiotherapy and Rehabilitation, Izmir, Turkey

**Correspondence:** C. Kirakli

*Intensive Care Medicine Experimental* 2017, **5(Suppl 2):**0514

**INTRODUCTION.** There are some studies suggesting that adaptive support ventilation (ASV), a closed loop ventilation mode may shorten the duration of intubation and weaning duration in some patient groups (Kirakli et al. 2015; Domingo et al. 2010). IntelliVent-ASV™ (IV-ASV) is a full closed-loop ventilation mode that automatically adjusts ventilation (according to end tidal CO2) and oxygenation (according to pulse oximetry) parameters in both passive and active patients with promising results (Arnal et al. 2013; Bialais et al. 2013).

**OBJECTIVES.** We aimed to investigate the effect of IV-ASV on the duration of intubation and weaning, workload regarding the setting of the ventilator and other ventilation parameters in COPD patients when compared to conventional modes.

**METHODS.** COPD patients who were intubated longer than 24 h were randomized into IV-ASV or pressure assisted controlled ventilation (P-ACV). Weaning was performed with the "Quickwean" function and automatic spontaneous breathing trial (SBT) in IV-ASV and pressure support ventilation and SBT with a t-tube trial in P-ACV. In both groups ventilation data were recorded with dedicated software connected to the ventilator. Duration of intubation and weaning, number of manual and automatic settings of the ventilator and other clinical endpoints were compared between the two groups.

**RESULTS.** Data are expressed as median (25th-75th percentiles). Eighty one patients out of 188 (study sample size) until now were enrolled. Median duration of intubation from randomization and weaning duration seemed insignificantly shorter with IV-ASV. Number of manual adjustments per hour for setting the ventilator was significantly lower in the IV-ASV group. IV-ASV performed median 93 (76–136) automatic adjustments per hour for setting the ventilator parameters.

**CONCLUSIONS.** IV-ASV may decrease the total duration of mechanical ventilation and weaning and significantly staff's workload when performed from intubation until extubation.

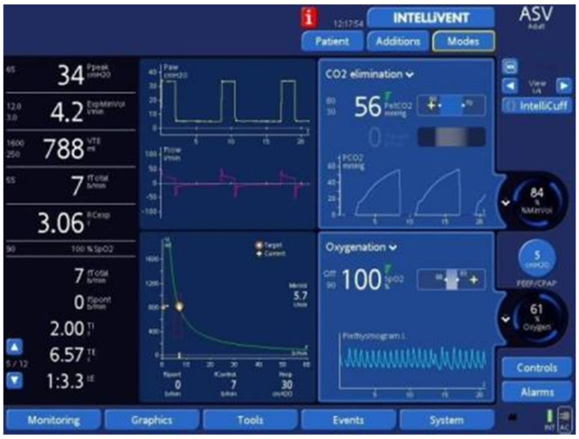

**Fig. 186 (Abstract 0514).** Representative screenshot of a patient on IV-ASV

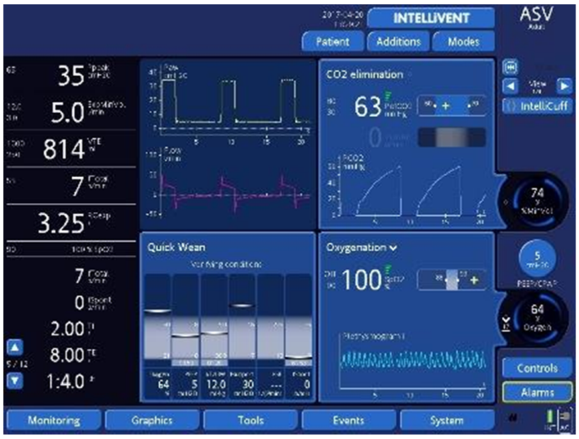

**Fig. 187 (Abstract 0514).** Automatic weaning function of IV-ASV

**Table (Abstract 0514).** Characteristics of the patients at randomization

| | IV-ASV | P-ACV | p |
|---|---|---|---|
| Age, years | 70 (62–77) | 67 (59–73) | 0.19 |
| SAPS II | 41 (37–45) | 43 (35–48) | 0.86 |
| pH | 7.20 (7.16-7.27) | 7.20 (7.16-7.26) | 0.54 |
| PaCO2, mmHg | 99 (82–116) | 101 (87–115) | 0.95 |
| PaO2/FiO2 | 152 (115–195) | 200 (155–260) | **0.018** |

**Table (Abstract 0514).** Comparison of the two ventilation modes

| | IV-ASV | P-ACV | p |
|---|---|---|---|
| Weaning duration, h | 35 (18–70) | 41 (18–69) | 0.91 |
| Duration of intubation, h | 63 (23–91) | 71 (45–101) | 0.34 |
| Number of manual adjustments per hour, n | 0.05 (0.01-0.07) | 0.28 (0.19-0.34) | **<0.001** |
| Proportion of the ventilation time patient is actively triggering the ventilator, % | 94 (72–100) | 64 (59–88) | **0.002** |
| Tidal volume/IBW, ml/kg | 8.6 (8.1-9.7) | 8.1 (6.4-8.4) | 0.07 |
| ABG number/day, n | 0.94 (0.56-1.44) | 2.18 (1.75-3) | **<0.001** |
| Weaning success, n (%) | 37 (93) | 33 (97) | 0.37 |

### 0515

#### Partition of respiratory mechanics during the first day after lung transplantation. Influence on prognostic

P. Carmona Sánchez[1], J. Rodríguez Gómez[1], J. C. Robles Arista[1], J. C. Pozo Laderas[1], J. Redel Montero[2], J. M. Serrano Simón[1]
[1]Hospital Universitario Reina Sofía, Intensive Care Unit, Córdoba, Spain; [2]Hospital Universitario Reina Sofía, Pneumology Service, Córdoba, Spain
**Correspondence:** P. Carmona Sánchez
*Intensive Care Medicine Experimental* 2017, **5(Suppl 2):**0515

**INTRODUCTION.** Different factors may to modify the mechanics properties of respiratory system following lung transplantation (LT). Changes in the airway resistance, lung elasticity and chest wall, could be provide detailed information allow some intervention in order to improve the outcome (1,2).

**OBJETIVES:** To assess the mechanics of lung and chest wall after of postoperative LT and its usefulness to discriminate the prognosis.

**METHODS.** Prospectively collected data from 27 patients undergoing LT between 2016–2017. During volume controlled mechanical ventilation with constant flow at the first day(d) after LT. Airway and Esophageal pressure, Flow signals were recorded. Multiple linear regression was used to compute elastance and resistance of respiratory system (Ers, Rrs) and his components. Demographic data, complications, prolonged mechanical ventilation ≥ 21d(PMV), stay ICU, survival 90d, were evaluated. Data were analyzed by the Fisher exact test, Mann–Whitney test or t-test as appropriate.

**RESULTS.** Males 21(77.8%); 51.18 ± 11.33y. Fibrosis 13(48,14%), Emphysema 12(44.4%), fibro-emphysema 1(3.7%), Idiopathic Pulmonary Hypertension 1(3.7%). Double LT 8(29.6%). Death 5(18,5%) 3 in double. Preservation 345,8 ± 68,4min. **Respiratory system mechanics:** All Data: Rrs 14.23 ± 5.94 cmH2O.s/L, Ers 33.48 ± 11.02cmH2O/L, EL 23.73 ± 10.94 cmH2O/L, Rcw 0.92 ± 0.93 cmH2O.s/L, Ecw 7.1 ± 2.9cmH2O/L, PEEP 5.8 ± 1.2cmH2O, V' 0.69 ± 0.14 L/s, Vt 0.5 ± 0.09L, RR 19.7 ± 2.6/min. **Single vs double LT:** Rrs 15.78 ± 6.32 vs 10.56 ± 2.56cmH2O.s/L, p = 0.034; EL 24.58 ± 11.41 vs 21.7 ± 10.15cmH2O/L, p = 0.54. Ecw 7.05 ± 3.14 vs 7.28 ± 2.48cmH2O/L, p = 0.86. **Emphysema vs fibrosis:** Rrs 14.30 ± 6.36 vs 12.45 ± 2.91cmH2O.s/L, p = 0.38; EL 19.08 ± 6.14 vs 27.44 ± 13.10cmH2O/L, p = 0.048; Ecw 7.42 ± 3.04 vs 7.01 ± 2.98cmH2O.s/L, p = 0.74. **Survival:** Rrs 12.2 ± 3.1 vs 19.5 ± 9.9cmH2O.s/L, p = 0.007; EL 20.01 ± 6.8 vs 32.5 ± 9.3cmH2O/L, p = < 0.002; Ecw 7.02 ± 2.9 vs 7.49 ± 2.9cmH2O/L, p = 0.76. **Stay ICU** 9(7–25)d < />: Rrs 13.47 ± 5.8 vs 15.75 ± 6.3cmH2O.s/L, p = 0.36; EL 19.25 ± 6.77 vs 26.55 ± 9.48cmH2O/L, p = 0.04; Ecw 6.99 ± 3.1 vs 7.36 ± 2.8cmH2O/L, p = 0.78. **PMV** < />: Rrs 13.48 ± 5.54 vs 18.54 ± 7.22cmH2O.s/L, p = 0.12; EL 18.81 ± 5.88 vs 31.54 ± 10.67cmH2O/L, p = 0.003; Ecw 5.9 ± 1.8 vs 7.2 ± 3.2cmH2O/L, p = 0.34. **Reperfusion injury:** Rrs 10.44 vs 17.18 ± 8.23cmH2O.s/L, p < 0.03; EL 19.32 ± 6.19 vs 27.02 ± 7.75cmH2O/L, p = 0.03; Ecw 7.2 ± 6.9 vs 6.9 ± 3.6cmH2O/L, p = 0.84. **Rejection or pneumoniae:** Rrs 14.3 ± 6.5 vs 14.2 ± 5.4cmH2O.s/L, p = 0.97; EL 18.75 ± 6.8 vs 29.95 ± 12.2cmH2O/L,p = 0.006; Ecw 6.83 ± 3.2 vs 7.51 ± 2.6cmH2O/L,p = 0.58.

**CONCLUSIONS.** The mechanics respiratory following LT tend to be worse in the patients with worse results. Particularly, EL could discriminate morbidity and mortality. The surgery modality can influence in the respiratory mechanics. We could speculate that early assessment of respiratory mechanics after LT can provide better understanding of patient's conditions and therapeutic approach.

### 0516

#### Comparison and concordance of three different ventilator associated pneumonia (VAP) criteria versus clinician diagnosis in a large tertiary critical care centre

A. Forbes[1], E. Prower[1], A. Prichard[2], T. Moorthy[1], A. Fife[1], S. Patel[1]
[1]King's College Hospital, London, United Kingdom; [2]King's College Hospital, Department of Critical Care, London, United Kingdom
**Correspondence:** A. Prichard
*Intensive Care Medicine Experimental* 2017, **5(Suppl 2):**0516

**INTRODUCTION.** The clinical diagnosis of VAP is notoriously difficult and there is often significant discordance between units and individual clinicians. There is currently no gold standard for diagnosis with significant variability between existing diagnostic criteria.[1]

**OBJECTIVES.** To assess VAP rates using three different diagnostic criteria (HELICS, CPIS, CDC NHSN 2008) versus clinician diagnosis, followed by an assessment of the criteria for concordance. The objective was to choose a diagnostic tool for long term use based on ease of use and concordance with clinician diagnosis.

**METHODS.** Prospective study conducted in a large tertiary critical care centre on working days over 8 weeks (February-April 2017). All patients ventilated >48 hours across four specialist critical care units were audited (total 68 beds). Patients were assessed using HELICS, CPIS and CDC NHSN 2008 criteria for identification of VAP. This was compared to patients with a documented clinician diagnosis of VAP. These data were analysed for concordance between clinician diagnosis and the three VAP diagnostic criteria and additionally between the VAP criteria themselves.

**RESULTS.** VAP rate according to clinician diagnosis was 31 per 1000 ventilator days. VAP rates were 24 per 1000 ventilator days, 36 per 1000 ventilator days and 15 per 1000 ventilator days according to HELICS, CPIS and CDC NHSN 2008 diagnostic criteria, respectively. Concordance with clinician diagnosis was highest for HELICS (28.2%) followed by CPIS (23.9%) and CDC NHSN 2008 (18.2%). Concordance between criteria was greatest for HELICS and CDC (88.2%).

**CONCLUSIONS.** CPIS and HELICS appear to have similar VAP detection rates compared with clinician diagnosis. CDC NHSN 2008 appears to underestimate VAP rates compared to the other criteria. However, all three criteria have poor concordance with clinician diagnosis. Of the three, HELICS had the best concordance with clinician diagnosis. Based on this data we have adopted HELICS as our tool for VAP diagnosis.

**REFERENCE(S)**

1. Ventilator associated pneumonia in the ICU. Kalanuria et al. Critical Care 2014, 18:208

### 0517

#### A comparison of response to reverse triggering between two ventilation modes: biphasic positive airway pressure ventilation and pressure controlled assist-control ventilation

T. Yamashita, A. Uchiyama, Y. Fujino
Osaka University Hospital, Anesthesiology and Intensive Care, Suita, Japan
**Correspondence:** T. Yamashita
*Intensive Care Medicine Experimental* 2017, **5(Suppl 2):**0517

**INTRODUCTION.** Reverse triggering is a form of patient-ventilator dyssynchrony referred to as patient's breathing effort induced by mandatory ventilation. Reverse triggering is often seen in deeply sedated critically ill patients. Reverse triggering causes double breathing accompanied by large tidal volume ($V_T$) increased transpulmonary pressure ($P_L$ = Airway pressure - pleural pressure) and can exacerbate the lung injury. There has been little knowledge about appropriate ventilation modes for reverse triggering phenomenon.

**OBJECTIVES.** Responses to induced spontaneous breathing effort in reverse triggering are different between Pressure Controlled Assist-Control ventilation (PC-AC) mode and biphasic positive airway pressure ventilation (Bilevel) mode. We hypothesized that Bileve mode would limit $V_T$ and reduce $P_L$ in comparison with PC-AC mode.

**METHODS.** We simulated reverse triggering phenomenon by using a twin bellow-type lung model, which can produce spontaneous breathing effort. The lung model consisted of two bellows implanted into one closed box. One bellow represents lung and the other bellow make activity of inspiratory muscles, which can be controlled by a personal computer. We can know pleural pressure by measuring the pressure inside the box. Spontaneous breathing of the lung model was set at 500 mL of tidal volume, 1.5 or 2.0 s of inspiratory time, and sine-wave inspiratory flow pattern. The lung model was connected to the Puritan-Bennett$^{TM}$ 840 ventilator (Nellcor Puritan Bennet, Carlsbad, CA). The mandatory ventilation was performed by Bilevel mode or PC-AC mode with 20 $cmH_2O$ of inspiratory airway pressure, 5 $cmH_2O$ of end-expiratory pressure, 1.0 s of inspiratory time, and 10 /min of ventilatory rate. Reverse triggering phenomenon was simulated by making spontaneous inspiratory efforts following the mandatory ventilation with short, moderate, or long delay (0.3, 0.6, and 1.0 s after the beginning of mandatory inspiration, respectively). Respiratory flow, airway pressure, and pleural pressure were measured. $V_T$ and maximum $P_L$ were calculated as an average of 10 breaths at each condition.

**RESULTS.** Airway pressure rose at all the entrained inspiratory efforts in PC-AC mode. Airway pressure did not rise in response to the entrained triggering efforts in Bilevel mode except the condition of 1.5 s of inspiratory time with short delay. $V_T$ and $_{max}P_L$ during BILEVEL mode were significantly smaller than those during PC-AC.

**CONCLUSIONS.** Biphasic positive airway pressure ventilation mode might be advantageous to suppressing the excessive tidal volume and transpulmonary pressure in reverse triggering phenomenon.

**Table (Abstract 0517).** The VT and PL at each condition

| | | VT (mL) | | maxPL (cmH2O) | |
|---|---|---|---|---|---|
| inspiratory time | delay | A/C | Bilevel | A/C | Bilevel |
| 1.5 sec | short | 650 ± 7 | 650 ± 13 | 17.8 ± 0.2 | 18.4 ± 0.2 |
| | moderate | 947 ± 5 | 629 ± 14 | 18.6 ± 0.2 | 16.9 ± 0.3 |
| | long | 968 ± 8 | 641 ± 4 | 18.7 ± 0.1 | 13.5 ± 0.1 |
| 2.0 sec | short | 912 ± 14 | 614 ± 18 | 17.6 ± 0.1 | 12.4 ± 7.4 |
| | moderate | 982 ± 12 | 619 ± 22 | 18.2 ± 0.4 | 15.9 ± 0.3 |
| | long | 936 ± 10 | 596 ± 22 | 18.6 ± 0 | 13.6 ± 0.1 |

### 0518

#### Home mechanical ventilation

M. Muñoz-Garach, R. Ramirez-Puerta, S. Narbona-Galdó
Complejo Hospitalario de Granada, Granada, Spain
**Correspondence:** M. Muñoz-Garach
*Intensive Care Medicine Experimental* 2017, **5(Suppl 2):**0518

**INTRODUCTION.** Patients with chronic dependence on mechanical ventilation should not stay all the time in intensive care unit. Ideally, if their personal situation allows, it is follow at home, for this it is necessary the support of a Home Mechanical Ventilation Program. Currently, in Granada, Spain, there is a program, that started more than 20 years ago and created with the objective of improving the quality of life of these patients and facilitating their family reintegration. It has an infrastructure of eleven beds, a office and an intensivist care physician who plans, coordinates and evaluates all the process, providing integral care due to its multidisciplinary training.

**OBJECTIVES.** Show the sociohealth care utility of Home Mechanical Ventilation Program in Granada led by intensive care physicians.

**METHODS.** Observational, descriptive retrospective study of patients treated from 1/10/95 to 31/3/17, by management of an anonymous database, regarding patients included in the program and referred from intensive care unit, follow up visit at the medical office, telephone attention, sex, age, pathologies and therapies.

**RESULTS.** Patients included in program: 405. Average of patients/year: 90–130. Medical office/year:> 300, telephone support: 3–5 calls/day. Beds availability for acute admission, respiratory therapies training, family rest and palliative care.

Patients derived from ICU 110, Men 66 (60%), mean age at admission: 51.3 ± 17.9 years; Pathologies: amyotrophic lateral sclerosis 33 (30%); Myopathies 24 (21.8%); Thoracogens 15 (13.6%); Multifactorial 18 (16.4%), others 20 (18.2%). Invasive ventilation 80 (72.7%), noninvasive 25 (22.7%), non-ventilated tracheotomy 5 (4.6%).

**CONCLUSIONS.** The program leads by intensive care physcians is fully justified because:

- Achieves an integral approach to the patients
- Allows the transfer from the ICU to the home through a structured care circuit

**REFERENCE(S)**

Cox C, Carson S. Medical and econonic implications of prolonged mechanical ventilation and expedited post-acute care. Semin Respir Crit Care Med 2012, 33 (04): 357–361.
Baines RJ, de Bruijne MC, Langelaan M. What are the safety risks for patients undergoing treatment by multiple specialties: a retrospective patient record review study. Health Serv Res 2013; 13(1): 497.
Garner DJ, Berlowitz DJ, Douglas J et al. Home mechanical ventilation in Australia and New Zealand. Eur Respir J 2013 Jan; 41 (1):39–45.
Divo MJ, Murray S, Cortopassi F et al. Prolonged mechanical ventilation in Massachusett: the 2006 prevalence survey. Respir Care 2010 Dec; 55 (12):1693–1698.
Dellweg D, Gerhard F, Hoehn E et al. Survey of nursing services with regard to mechanical ventilation at home. Pneumologie. 2011 Nov; 65(11): 685–691.
Fernández R, Rubinos G, Cabrera C et al. Ventilación mecánica domiciliaria: dependencia y carga de cuidados en domicilio. Arch Bronconeumolog 2009 45 (8): 383–386.

### 0519

#### Characteristics, mortality and outcomes in patients treated with airway pressure release ventilation (APRV) for severe acute respiratory distress syndrome (ARDS)

M. S. Echeverria León, G. E. Kaminsky Lara, A. Alonso Ovies, A. Escribá Bárcena, R. Del Olmo Monge, V. E. Gálvez Villarrubia, B. Nieto Martino, W. A. Ramírez Lajones, M. E. Fuentes Salazar, C. Campos Moreno, C. I. Vaquerizo Alonso, J. Álvarez Rodríguez
Hospital Universitario de Fuenlabrada, Intensive Care Unit, Fuenlabrada, Madrid, Spain
**Correspondence:** M. S. Echeverria León
*Intensive Care Medicine Experimental* 2017, **5(Suppl 2):**0519

**OBJECTIVE.** To describe the clinical characteristics and physiological effects of APRV (Airway Pressure Release Ventilation) in patients with ARDS (Acute Respiratory Distress Syndrome) at the Intensive Care Unit (ICU).

**METHODS.** Retrospective descriptive observational study in a polyvalent 10 - bed adult intensive care unit in an University Hospital in Madrid, Spain. Patients with ARDS (based on Berlin criteria) were included from October 2013 to December 2016. Patients receiving APRV for less than 24 hours were excluded.

**RESULTS.** 17 patients fulfilled the eligibility criteria. A total of 21 episodes on APRV were included (because 4 of these patients had 2 APRV episodes each one). 86% were men; median age was 60 years (interquartile range (IQR) 41–66). Initial APACHE II and SAPS 3 median were 25 (11–29) and 58 (53–79), respectively. SAPS 3 associated predicted mortality was 28% (18–71). The majority were medical patients. Prior to APRV initiation, median Murray Lung Injury Score was 2,5 (IQR 2,25 - 3,25) and median PaO2/FiO2 was 117 (80,5 - 145). At the beginning of APRV, most of patients had a −4 in the RASS sedation scale. PaO2/FiO2 significantly improved within 24 hours post - APRV initiation (ANOVA F (2) = 8, 07; $n_P^2$ = 0,288; p < 0,05).There were no significant differences related to partial pressure of carbon dioxide on APRV. 4 of the 17 patients were place in prone position. Only one patient had a pneumothorax associated to barotrauma and required intercostal catheter insertion during APRV ventilation. The half of these patients were changed of ventilation mode for improving, and the other half were changed because of respiratory deterioration. Only one patient was extubated in APRV. ICU mortality rate was 33.3%. **(Tables showing median and interquartile range (IQR), mean and standard deviation (SD).**

**CONCLUSIONS.** In patients with ARDS and severe hypoxemic respiratory failure, initiation of APRV was associated with an early and sustained improvement in oxygenation and low incidence of clinically significant complications. The safety and efficacy of APRV as a rescue strategy for patients with ARDS and severe hypoxemic respiratory failure requires further consideration.

**Table (Abstract 0519).** Baseline characteristics of patients (n = 21)

| | |
|---|---|
| Age | 60 (41–66) |
| Gender, n (%) | Male 18 (85,7); Female 3 (14,3) |
| Admission type, n (%) | Medical 14 (66,7); Surgical 7 (33,3) |
| Lung injury mechanism, n (%) | Pulmonary 17 (81); Extrapulmonary 4 (19) |
| APACHE II score | 25 (11–29) |
| SAPS 3 score | 58 (53–78,5) |
| Predicted mortality based on SAPS 3 score | 28 (18–71) |
| ARDS severity (Berlin criteria), n (%) | Mild 2 (9,5); Moderate 7 (33,3); Severe 12 (57,1) |
| Murray criteria, n | 2,5 (2,25 - 3,25) |
| Mode of ventilation prior to APRV (%) | Pressure control 14 (66,7), volume control 2 (9,5), pressure support 5 (23,8) |

**Table (Abstract 0519).** Baseline characteristics of patients 2

| | |
|---|---|
| Hours from intubation to APRV, mean (SD) | 201,95 (1–1100) |
| Noradrenaline requirement on initiation of APRV, n (%) | With noradrenaline: 11 (52,4%); mean 0,26 (0,26 ug/kg/min); without noradrenaline / vasopresors: 10 (47,6) |
| PaO2/FiO2 ratio prior to APRV (mmHg) | 126,14 (68–250) |
| RASS scale at the beginning of APRV, n (%) | −4: 11 (52,4), −3: 4 (19), −2: 3 (14,3), −1: 1 (4,8), 0: 1 (4,8) |
| Tracheostomy (%) | 11 (52,4) |
| Duration on APRV (hours) | 82 (IQR 11–228) |
| Mechanical ventilation duration (days) | 21,5 (IQR 1–55) |
| Mode of ventilation after APRV, n (%) | Pressure support 9 (42,9); pressure control 11 (52,45); extubation 1 (4,8) |
| ICU LOS (days), mean (SD) | 46,33 |
| Mortality, n (%) | ICU Mortality 7 (33,3%); Hospital Mortality 7 (33,3%) |

**Table (Abstract 0519).** Ventilatory, gasometric and hemodynamics outcomes

| Ventilatory parameter, gasometric and hemodynamics outcomes. Mean (SD) | On initiation of APRV | 12 h post initiation of APRV | 24 h post initiation of APRV |
|---|---|---|---|
| High pressure ($cmH_2O$); Low pressure ($cmH_2O$) | 24,76 (3,83); 0,14 (0,47) | 24,66 (3,81); 0,14 (0,47) | 24,28 (4,05); 0,14 (0,47) |
| Peak airway pressure ($cmH_2O$); Mean airway pressure ($cmH_2O$) | 25,95 (3,85); 21,80 (3,57) | 25,52 (3,86); 22,04 (3,77) | 25,80 (3,41); 21,57 (3,21) |
| Minute volumen (ml); spontaneous breathing frequency | 10,92 (2,95); 15,19 (6,88) | 10,54 (3,18); 12,36 (5,56) | 10,00 (3,51); 10,71 (6,47) |
| High time (sec); Low time (sec) | 4,13 (0,80); 0,47 (0,10) | 4,13 (0,80); 0,47 (0,10) | 4,06 (0,67); 0,52 (0,10) |
| pO2 (mmHg) | 85,04 (22,12) | 113,28 (34,54) | 108,94 (54,19) |
| FiO2; pO2/FiO2 ratio (mmHg) | 0,68 (0,16); 130,36 (48,48) | 0,61 (0,15); 198,24 (81,72) | 0,56 (0,16); 206,93 (110,48) |
| PH; pCO2 (mmHg) | 7,35 (0,04); 54,05 (13,59) | 7,38 (0,05); 54,57 (15,08) | 7,38 (0,05); 52,08 (18,74) |
| Heart rate (beats/min); Mean arterial pressure (mmHg) | 91,57 (15,41); 84,09 (12,30) | 87,38 (13,80); 78,09 (12,54) | 93,04 (16,88) 87,71 (15,25) |

### 0520

#### Clinical characteristics of patients with late onset pompe's disease requiring mechanical ventilation in the long term

I. Ben Saida[1], W. Zarrougui[1], A. Khedher[1], J. Baroudi[1], N. Fraj[1], M. A. Boujelbèn[1], N. Sma[1], E. Ennouri[1], H. Kallel[1], A. Ben Abdelhafidh[1], A. Azouzi[1], I. Chouchene[1], J. Ayachi[1], D. Orlikowski[2], M. Boussarsar[1,3]
[1]Farhat Hached University Hospital, Medical Intensive Care Unit, Sousse, Tunisia; [2]Raymond Poincaré Teaching Hospital, Medical Intensive Care Unit, Garches, France; [3]Ibn Al Jazzar Faculty of Medicine, Research Laboratory N° LR 14 ES 05 Interactions of the Cardio-Pulmonary System, Sousse, Tunisia
**Correspondence:** M. Boussarsar
*Intensive Care Medicine Experimental* 2017, **5(Suppl 2):**0520

**INTRODUCTION.** Pompe´s disease (Acid Maltase deficiency) is a rare metabolic myopathy. Unlike other neuromuscular diseases in which respiratory problems occur after loss of ambulation, respiratory failure in pompe disease progress more rapidly than motor impairment and hypoventilation during sleep may occur even if the patient is still fully mobile.

**OBJECTIVES.** To study correlations between clinical features and physiological respiratory parameters to better predict respiratory disorders onset.

**METHODS.** It´s a retrospective observational study. All patients with late onset Pompe´s disease followed at the Raymond Poincaré teaching hospital (Garches, France) until November 2013 were included. Data recorded were: demographic characteristics, clinical features, ABG´s parameters, ventilatory functional tests assessed at diagnosis and at the time of ventilation. Patients were divided in two groups patients who needed mechanical ventilation (group 1) and patients who didn't require home ventilation until November 2013 (group2). The two groups were compared using an unpaired t test. Correlations between respiratory parameters and clinical features were studied with Wilcoxon rank sum test with continuity correction.

**RESULTS.** A total of 33 patients (48,5% male; 51,5% female) were included. The mean age at the first motor symptoms and at diagnosis of pompe´s disease was respectively 38,3 ± 14,6 and 46,4 ± 14,5 years. 80,6% of patients could walk without aids, and

9,7% were confined to wheelchair. The average score of Walton was at 3,16 ± 1,5 while 54% of patients required home ventilation. 72,2% of patients complained of dyspnea and 61,1% had clinical signs of hypercapnia. The comparison of the two groups showed no difference in age at diagnosis, age at onset of motor symptoms and diagnostic delay.

However, Walton score was higher in group 1 (4,1 ± 1,9 vs 2,6 ± 1; p = 0,04). Vital capacity (VC), Maximal inspiratory pressure (Pimax) assessed at diagnosis was significantly lower in group 1: VC in sitting position (1,1 ± 0,4 vs 1,8 ± 0,8; p = 0,01), VC in supine position (1,8 ± 0,5 vs 2,4 ± 0,9; p = 0,03) and Pimax (24,3 ± 8,1 Vs 43,8 ± 29; p = 0,03).

No significant correlations were found between clinical symptoms and ventilator capacity. However, significant correlation was found between excessive daytime sleepiness and VC in sitting position (W = 63.5; p = 0.026).

**CONCLUSIONS.** An early onset of respiratory disorders preceding muscular weakness distinguishes Acid maltase deficiency from other neuromuscular diseases. These patients may warrant home ventilation early in the natural course of motor disorders. A sharp follow up using ABG's, ventilatory functional tests and Walton Score may add a valuable discriminative properties to the clinical monitoring.

### 0521

#### Compliance and outcomes of home ventilated chronic hypercapnic respiratory failure: a prospective observational study

N. Sma[1], A. Khedher[1], N. Fraj[1], K. Meddeb[1], M. A. Boujelbèn[1], J. Ayachi[1], H. Kallel[1], E. Ennouri[1], W. Zarrougui[1], A. Ben Abdelhafidh[1], A. Azouzi[1], I. Chouchene[1], M. Boussarsar[1,2]

[1]Farhat Hached University Hospital, Medical Intensive Care Unit, Sousse, Tunisia; [2]Ibn Al Jazzar Faculty of Medicine, Research Laboratory N° LR14ES05 Interactions of the Cardio-pulmonary System, Sousse, Tunisia

**Correspondence:** M. Boussarsar

*Intensive Care Medicine Experimental* 2017, **5(Suppl 2):**0521

**INTRODUCTION.** Invasive positive-pressure home ventilation via Tracheostomy (TPPV) and home non invasive ventilation (NIV) are being increasingly used to treat chronic hypercapnic respiratory failure after ICU discharge. However, there are little data evaluating compliance with ventilation prescriptions and long term outcome remains uncertain.

**OBJECTIVES.** To assess the compliance with home ventilation (TPPV and NIV) and outcomes of patients ventilated at home for chronic hypercapnic respiratory failure after ICU discharge.

**METHODS.** Prospective observational study conducted in an 8-bed Tunisian medical ICU over a two year period (January 2015 to December 2016). Were included all consecutive ICU survivors discharged with home TPPV or home NIV. Were collected patients´ characteristics, clinical presentation on admission and at discharge, SAPSII, type of home ventilation at ICU discharge. Compliance reports, readmissions and outcomes were assessed.

**RESULTS.** A total of 23 patients were collected. The Median age was 65 years, 25-75CI [17–76]. Sixteen (70%) patients were male. Twelve (54%) were admitted for acute exacerbation of COPD. Five (22%) and 6(24%) were respectively admitted for acute exacerbation of restrictive and mixed chronic respiratory failure. Among all the admitted patients, 4(17%) were already under home NIV. Median SAPSII was 28, 25-75CI [22–36]. Overall, firstline NIV use was registered in 15(65%) patients and 6(40%) failed. Thirteen(56%) patients required invasive mechanical ventilation (IMV) during ICU stay. The median duration of IMV was 5 days (range 4–14). At ICU discharge, median pH was 7.43, 25-75CI [7.36-7.46] and median PaCO2 was 49mmHg, 25–75 CI [41–66]. Eleven(56%) were discharged with tracheostomy all performed for weaning difficulties. Twelve (52%) patients were discharged with home NIV. Respectively, median daily use of home NIV and TPPV was 4h 25-75CI [3.25-7.75] and 6.5 h 25-75CI [5–6.5]. The median NIV use (≥4h per day) was respectively 48% for NIV and 98% for TPPV. Three(25%) of Home NIV and 1(9%) of TPPV patients were readmitted. Survival rates at 6 months for the home NIV and home TPPV patients were respectively 90% and 70%.

**CONCLUSION.** This study shows a low adherence to home NIV as well as TPPV ventilation for ICU survivors of acute exacrbation of chronic hypercapnic failure. Otherwise, this therapy seems to provide survival for long periods of time.

### 0522

#### Effectiveness and safety of respiratory support with adaptive support ventilation in patients with severe traumatic brain injury

Z. Marutyan, A. Evdokymov, S. Petrikov, I. Aliev, A. Shakotko, V. Movsisyan, A. Kinishemova

N.V. Sklifosovsky Research Institute of Emergency Medicine of the Moscow Healthcare Department, Moscow, Russian Federation

**Correspondence:** Z. Marutyan

*Intensive Care Medicine Experimental* 2017, **5(Suppl 2):**0522

**INTRODUCTION.** Maintenance of a normal level of carbon dioxide in arterial blood (PaCO2) is an important goal of intensive care patients with severe traumatic brain injury (TBI). However, methods of providing stable PaCO2 in this category of patients are not completely defined.

***Goal of research is:*** To assess the safety and efficacy of implementing the "Intellivent-ASV" in patients with dire TBI.

**METHODS.** We surveyed 15 patients with TBI and oppression of level of the wakefulness to 8 and less points according to GCS. In 11 (73%) cases there was a concomitant injury with the prevalence of TBI, and 4 (27%) patients had isolated TBI. The victim's mean age was 27 ± 6 years, the proportion between male/female was 13/2. The victims were carried out on ALV - adjustable volume (Volume Control Synchronized Intermittent Mandatory Ventilation), and then shifted into intellectual mode "Intellivent-ASV" ("HAMILTON-G5", SWITZERLAND). Duration of respiratory support in each mode was 2 hours.

The mean airway pressure (Reap) was designated to all the patients, the partial pressure of oxygen in arterial blood (PaO2), PaCO2, PaO2 relation to the fraction of oxygen in the inspired air (FiO2) in an hour and at 2 hours after the start of mechanical ventilation in different modes.

**RESULTS.** Data analysis revealed no differences between the average values PaO2 in patients ventilated by volume monitored modes, and in intelligent way: PaO2 183,9 ± 18.8 mm.Hg.(n = 30) vs 180,3 ± 24.4 mm.Hg.(n = 30) relationship PaO2/ FiO2 368 ± 53 (n = 30) vs 360 ± 67 (n = 30), respectively. Noted a trend towards a more sustainable retention level PaCO2 when using the "Intellivent-ASV" 34,5 ± 1,7 mm.Hg.(n = 30) against 35,3 ± 2,6 mm.Hg. (n = 30) in volume monitored modes. The patients who underwent volume monitored modes, the minimum value PaCO2 was 29.4 mm.Hg. vs 30.3 mm.Hg. in patients who underwent respiratory support in intelligent mode, the maximum value PaCO2 - 41 mm.Hg. against 39,5 mm.Hg. respectively.

The fluctuation PaCO2 was 12.5 mm.Hg. in patients ventilated in controlled-volume vs 9.5 mm.Hg. in patients who underwent respiratory support in an intelligent way. The average amplitude of fluctuations of PaCO2 during the observation period were −0.2 (−1,5;1,1) mm.Hg. at patients who underwent volume monitored modes, vs. 1.7 (0;3) mm.Hg. in patients ventilated in an intelligent way, the maximum change in level PaCO2 during the observation period was 8.2 mm.Hg. vs 5.9 mm.Hg., respectively.

The mean airway pressure was comparable during respiratory support in both ventilation modes:11,7 ± 1.5 cm.wg. at volume monitored modes, compared with the 8.5 ± 1.4 cm.wg. in patients ventilated in an intelligent way.

**CONCLUSIONS.** The intelligent ventilation mode "Intellivent-ASV" is effective and safety to use for patients with TBI, providing a high level PaO2 and adequate level PaCO2.

#### 0523

**Chronic obstructive pulmonary disease patients admitted in ICU for acute exacerbation requiring mechanical ventilation: the role of limitation of therapeutic effort on outcomes**

D. García Huertas[1], F. Manzano[1], A. Fuentes Calatayud[1], A. López Fernández[1], S. Ramiro González[1], F. Villagrán Ramírez[1], M. Colmenero[2]
[1]Hospital Universitario Virgen de las Nieves, Granada, Spain; [2]Hospital Clinico San Cecilio, Granada, Spain
**Correspondence:** D. García Huertas
*Intensive Care Medicine Experimental* 2017, **5(Suppl 2):**0523

**INTRODUCTION.** Acute exacerbation of patients with chronic obstructive pulmonary disease (COPD) has been a classical reason for admission in ICU. The advances in mechanical ventilation (noninvasive modality) and changes in patient decision-making (preferences for treatment) have substantially modify the pattern of ICU utilization for this type of patients.

**OBJECTIVE.** The objective was to study the epidemiology and outcomes of patients admitted in ICU for acute exacerbation of COPD patients requiring mechanical ventilation and to determine the impact of patient characteristics on decisions to limit therapeutic effort.

**METHODS.** Retrospective cohort study from January 2014 to December 2016 in the ICU of a University Hospital. We described the epidemiology, treatment and outcome of COPD patients admitted in ICU requiring mechanical ventilation. Variables related to comorbidities, functional status (number of visits to the Emergency department in previous year), acute physiology derangement (APACHE II) of the episode and type of mechanical ventilation (invasive vs noninvasive) were recorded. A multivariate analyses (logistic regression) was made to relate this factors with the decision to limit therapeutic efforts. Results are expressed as adjusted OR and 95% confidence intervals.

**RESULTS.** 44 patients were included. The mean age was 68 ± 10 years and sex ratio (male/female): 4.1. APACHE II score was 19.9 ± 4.8 points. 22.7% of patients had chronic right heart failure. The use of invasive mechanical ventilation was required in 40 patients (90%). Of these, 7 of 40 patients requiring invasive mechanical ventilation due to failure of non-invasive mechanical ventilation. ICU and overall hospital mortality were 20.5% and 25%, respectively. Primary cause for mortality was limitation of therapeutic effort for advanced respiratory disease (9 of 11 patients, 82%).The APACHE II score (22 ± 6 points vs 19 ± 4, respectively) and emergency department visits (3.8 ± 4.9 vs 1.1 ± 1.6, p > 0.001) were significantly higher in non-survivors. Patients with diabetes mellitus (40% versus 10.3%, p = 0.02) and with right heart failure had a higher mortality (57.1% versus 13.5%, p < < 0.009). In the adjusted multivariate model, emergency department visits was a significant independent predictor of limitation of therapeutic effort (adjusted OR, 1.39; 95% confidence interval, 1.00-1.97; *P* = 0.05).

**CONCLUSION.** Limitation of therapeutic effort represents the most common reason of mortality in exacerbation of COPD. Emergency department visits is an independent risk factor for the decision to limit therapies for life support in acute exacerbation of COPD.

### Which nutritional practices in ICU ?

#### 0524

**Albumin mass balance and kinetics in liver transplantation**

M. Amouzandeh[1], G. Nowak[2], A. Januszkiewicz[1], J. Wernerman[1,3], O. Rooyackers[1,3], Å. Norberg[1,3]
[1]Karolinska University Hospital, Anesthesiology and Intensive Care, Stockholm, Sweden; [2]Karolinska University Hospital, Transplant Surgery, Stockholm, Sweden; [3]Karolinska Institute, CLINTEC, Stockholm, Sweden
**Correspondence:** M. Amouzandeh
*Intensive Care Medicine Experimental* 2017, **5(Suppl 2):**0524

**INTRODUCTION.** Large amounts of albumin are used during and after liver transplantation, mainly to keep up plasma albumin concentration (P-alb). However, the fate of albumin beyond the initial volume effect is poorly understood. Likewise, the recovery rate of synthesis capacity for albumin in the transplanted liver is unknown.

**OBJECTIVES.** To study the cumulative perioperative loss of albumin from plasma up to the end of surgery and until post-operative day 3 (POD 3) as well as albumin synthesis rate and transcapillary escape rate (TER) on POD 3 after liver transplantation.

**METHODS.** Patients (n = 16) were studied during liver transplantation, and 11 of these had complete data until POD 3. Albumin net loss was assessed by mass balance of albumin and hemoglobin obtained from measures of P-alb, blood hemoglobin and hematocrit, and the sum of albumin given and lost. Synthesis rate of albumin was estimated by the flooding technique using deuterium labeled phenylalanine. TER was assessed by radio iodinated ($^{125}$I) human serum albumin.

**RESULTS.** P-alb was 31 ± 8 g/L at the start of surgery, 30 ± 4 g/L at the end of surgery, and decreased marginally to 26 ± 4 g/L on POD 3 (p = 0.049). At the end of surgery, 91 ± 37 g of exogenous albumin had been administered, and 33 ± 22 g (p = 0.0014) of albumin was lost from the circulation. Until POD 3 another 47 ± 35 g of exogenous albumin was administered, and at that time a total of 44 ± 38 g (p = 0.0085 vs baseline, p = 0.458 vs end of surgery) had left plasma since start of surgery. On POD 3 albumin absolute synthesis rate was 239 ± 84 mg/kg/day, TER was 5.9 ± 1.9% per hour, and the corresponding plasma volume was 4.8 ± 1.0 L.

**CONCLUSIONS.** Albumin net leakage from plasma progressed until the end of surgery, and was maintained until POD 3. This is in contrast with the absence of albumin leakage found at day 3 after major abdominal surgery, when P-alb was low and only small amounts of exogenous albumin was administered.[1] On POD 3 albumin synthesis rate was high compared to reference values.

**REFERENCE(S)**

1) Norberg Å, Rooyackers O, Segersvärd R and Wernerman J: Leakage of albumin in major abdominal surgery, Critical Care (2016) 20:113

**GRANT ACKNOWLEDGMENT**
Swedish Medical Research Council, and the County Council of Stockholm

#### 0525

**Frailty syndrome among critically ill patients undergoing nutrition support therapy in a Brazilian tertiary hospital**

I. A. O. Souza[1,2], T. S. Vieira[3], P. C. Ribeiro[4], L. U. Taniguchi[1,2,5]
[1]Hospital Sírio-Libanês, Intensive Care Unit, Sao Paulo, Brazil; [2]Hospital Sírio-Libanês, Education and Research Institute, Sao Paulo, Brazil; [3]Hospital Beneficência Portuguesa Mirante, Intensive Care Unit, Sao Paulo, Brazil; [4]Hospital Sírio-Libanês, Nutrition Support Team, Sao Paulo, Brazil; [5]Hospital das Clínicas, Universidade de São Paulo, Emergency Medicine Discipline, Sao Paulo, Brazil
**Correspondence:** L. U. Taniguchi
*Intensive Care Medicine Experimental* 2017, **5(Suppl 2):**0525

**INTRODUCTION.** Frailty is a multidimensional syndrome characterized by loss of physiologic and cognitive reserves that confers vulnerability to adverse outcomes during hospitalization, with higher morbidity and mortality rates.

**OBJECTIVES.** To describe the prevalence and outcomes associated with frailty among critically ill patients who required nutrition support therapy in a Brazilian tertiary hospital.

**METHODS.** We conducted a prospective single-center cohort study of patients admitted to the Intensive Care Unit (ICU) of Hospital Sírio-Libanês (São Paulo, Brazil), between October 2015 and March 2017. Frailty syndrome was classified using the Canadian Study on Health and Aging Clinical Frailty Scale (CFS). Patients were considered to be frail if they had a score > 4[1]. Multivariable analyses were used to evaluate the independent association between frailty and hospital mortality.

**RESULTS.** We studied 548 patients during the study period (mean age was 71.7 years, 66.6% were admitted for non-surgical reasons, 47.3% had cancer, 58.8% were frail and 27.6% died during hospitalization). 436 (79.6%) patients undergone enteral nutrition and 173 (31.6%) parenteral nutrition. Patients who died were more severely ill compared to patients those who survived (SAPS 3 of 53 [40–63] versus 42 [34–53.5] respectively; p < 0.001), were older, more frequently came from the ward due to non-surgical reasons, had more comorbidities, more frequently required mechanical ventilation and vasoactive drugs, had longer ICU and hospital length of stay, and more frequently were frail (75.5% vs 52.4% respectively, $p < 0.001$). After controlling for baseline differences, adjusted odds ratio for hospital death associated with frailty was 2.00 (95% CI 1.15 - 3.50; $p = 0.014$).

**CONCLUSIONS.** Frailty is very common among our population of critically ill patients undergoing nutrition support therapy, and it was associated with higher adjusted odds ratio for hospital mortality.

**REFERENCE(S)**

1. Bagshaw SM, Stelfox HT, McDermid RC, et al. Association between frailty and short- and long-term outcomes among critically ill patients: a multicentre prospective cohort study. CMAJ 2014; 186(2): E95-102.

### 0526

#### Current status of nutritional practice in Japan: a multicenter observational study

T. Yatabe[1], M. Egi[2], A. Nishigaki[1], M. Nishimura[3], ICUPRO Study Investigators
[1]Kochi Medical School, Department of Anesthesiology and Intensive Care Medicine, Nankoku; Japan, [2]Kobe University, Department of Anesthesiology, Kobe, Japan; [3]Tokushima University, Department of Emergency and Critical Care Medicine, Tokusima, Japan
**Correspondence:** T. Yatabe
*Intensive Care Medicine Experimental* 2017, **5(Suppl 2):**0526

**INTRODUCTION.** Although guidelines recommend a protein intake of 1.2-2.0 g/kg/day, no randomized trials have investigated the relationship between protein intake and outcomes. Additionally, the mean body mass index (BMI) in most nutritional studies has generally been greater than 25 kg/m$^2$. Therefore, the optimal practice for populations with low BMI, such as the Japanese remains unclear.

**OBJECTIVES.** We conducted a multicenter observational study to assess the current status of nutritional practices in Japanese ICUs and the relationship between amino acid and protein intake, and outcomes in critically ill patients.

**METHODS.** The study population consisted of patients who were on mechanical ventilation for at least 24 hours and in the ICU for longer than 72 hours between April 2015 and March 2016. The reasons for ICU admission, APACHE II and SOFA scores, type and amount of nutrition received in the ICU on days 3 and 7 and at ICU discharge, ICU-free and ventilator-free days during the first 28 days following ICU discharge, 28-day mortality rate, and rehabilitation status (bed rest, sitting, end sitting, sitting on wheelchair, or walking) at ICU discharge were recorded. The primary outcomes were the protein and amino acid intake in the ICU on days 3 and 7 and at ICU discharge; the secondary outcome was the relationship between the intake of protein and amino acids and patient rehabilitation status at ICU discharge. We defined a good rehabilitation status as more than end sitting and poor rehabilitation status as bed rest and sitting. Data are expressed as the median (interquartile range).

Values of $p < 0.05$ were considered statistically significant.

**RESULTS.** The data collected from 403 patients (34% women) from 13 hospitals were analyzed. The age and BMI were 70 (60–78) years and 22.3 (19.7-25.2) kg/m$^2$, respectively. The APACHE II and SOFA scores were 19 (14–26) and 11 (9–13), respectively. The protein and amino acid intake on days 3 and 7 in the ICU and at ICU discharge were 0.2 (0–0.5), 0.5 (0.3-0.9), and 0.6 (0.2-1.0) g/kg/day, respectively. Although the caloric intake in the ICU on day 7 was significantly lower in patients with a good rehabilitation status than in those with a poor rehabilitation status (13 [9–22] vs. 18 [12–27] kcal/kg/day, $P = 0.01$), the protein and amino acid intake in both groups did not significantly differ. Oral intake in the ICU on day 7 and at ICU discharge was achieved by a significantly higher proportion of patients with a good rehabilitation status than by those with a poor rehabilitation (12 vs. 2%, $P < 0.01$ on day 7 and 35 vs. 7%, $P < 0.01$ at ICU discharge). The ICU-free and ventilator free days were 16 (0–21) and 19 (0–24), respectively. The 28-day mortality rate was 15%.

**CONCLUSIONS.** Our study showed that the protein and amino acid intake during the acute phase was 0.6 g/kg/day in Japanese ICU patients. Our findings indicated that high early caloric intake might be associated with poor ICU rehabilitation.

### 0527

#### Facilitated glucose control in critically Ill patients utilizing a very high protein low carbohydrate formula

T. W. Rice[1], D. C. Files[2], P. Morris[3], A. Bernard[4], T. Ziegler[5], J. Drover[6], J. Kress[7], K. R. Ham[8], D. Grathwohl[9], M. Huhmann[10], J. B. Ochoa[11,12]
[1]Vanderbilt University, Medicine, Nashville, United States; [2]Wake forest School of Medicine, Winston-Salem, United States; [3]University of Kentucky Healthcare, Pulmonary, Lexington, United States; [4]University of Kentucky Healthcare, Surgery, Lexington, United States; [5]Emory University, Medicine, Atlanta, United States; [6]Queen's University School of Medicine, Surgery, Kingston, Canada; [7]University of Chicago, Pulmonary critical care, Chicago, United States; [8]Regions Hospital, Saint Paul, United States; [9]Nestle Research Center, Ver Chez le blanc, Switzerland; [10]Nestle Health Science, Clinical Sciences, Florham Park, United States; [11]Nestle Health Science, Livingston, United States; [12]Geisinger Medical Center, Critical Care Medicine, Danville, United States
**Correspondence:** T. W. Rice
*Intensive Care Medicine Experimental* 2017, **5(Suppl 2):**0527

**INTRODUCTION.** Hyperglycemia in critically ill patients is associated with increased morbidity and mortality. Control of hyperglycemia has concentrated on the use of insulin but there are significant side effects.

**OBJECTIVES.** Determine whether blood glucose control can be facilitated by using an enteral nutrition formula containing low carbohydrates, very high level of hydrolyzed whey protein, and medium chain triglycerides ensuring optimal protein delivery.

**METHODS.** Multi-center, randomized, open label clinical trial in mechanically ventilated critically ill, overweight and obese subjects requiring enteral nutrition assigned to an experimental - very high protein (37%) low carbohydrate (29%) group (Peptamen® Intense VHP) or control - high protein (25%), high carbohydrate (45%) group (Replete®). Both groups were prescribed 1.5 g protein/kg ideal body weight. Primary endpoint was glucose variability and was measured as incidence of glycemic blood glucose levels outside the range of 6.1 - 8.3 mmol/L.

**RESULTS.** 105 patients were randomized (52 experimental, 53 control) and 102 were included in the intention to treat population. Forty nine patients (mean ages 63 ± 12, 61 ± 15, ns) completed at least 5 days on protocol. Data presented are n, mean (sd). Both groups received similar amounts of protein
(47, 0.95 (0.32) g/kg/d vs. 49, 0.86 (0.38) g/kg/d, $p = 0.25$) with lower total energy (47, 10.19 (3.47) vs. 49, 13.77 (6.04) kcals/kg/d kcals/kg/d $p < 0.0001$) and lower carbohydrate load (47, 0.79 (0.27) g/kg/d vs. 49, 1.54 (0.68) g/kg/d $p < 0.0001$) in experimental vs. control, respectively. There was no significant difference in the incidence of glucose levels outside the range between 6.1 mmol/L and 8.3 mmol/L (primary endpoint): 52, 0.59 (0.25) vs. 50, 0.62 (0.24) $p = 0.54$. In the experimental group there was a lower incidence of glucose levels above 8.3 mmol/L (delta = −13%, $p = 0.015$) and higher incidence of levels between 4.4 and 6.1 mmol/L (delta = +14% $p = 0.0007$). Mean glucose level was lower in the experimental group than the control: 7 mmol/L [5.5, 8.9] mg/dl vs. 7.7 [6, 9.8] $p = 0.0039$. Glycemic excursion (standard deviation) was 10% lower in the experimental group ($p = 0.0015$). There were no significant differences in adverse events. Insulin use was lower in the experimental group ($p = 0.048$). Twenty three patients experienced glucose below 4.4 mmol/L with no differences between groups; use of rescue dextrose for hypoglycemia did not differ between groups. One patient died in the experimental group while 6 patients died in the control group during the study.

**CONCLUSIONS.** A very high hydrolyzed whey protein and low carbohydrate formula facilitates blood glucose control in critically ill overweight patients and is associated with a lower incidence of hyperglycemia, lower glycemic excursion and a decrease in insulin use without increased adverse events or mortality.

**REFERENCE(S)**

Finfer,S. NEJM 2009;360(13):1283–97

**GRANT ACKNOWLEDGMENT**

Nestle Health Science

### 0528

#### A single mega dose supplementation of liquid cholecalciferol in 22 young adults with major burns from a large starch-based powder explosion: a retrospective data analysis

Y. -Y. Han, Y. -C. Hsu, S.-R. Lai, H.-C. Tai
National Taiwan University Hospital, Taipei, Taiwan, Province of China
**Correspondence:** Y. -Y. Han
*Intensive Care Medicine Experimental* 2017, **5(Suppl 2):**0528

**INTRODUCTION.** Burn patients are at risk of vitamin D (VD) deficiency. It has been recommended stoss therapy of cholecalciferol (VD3) benefit critically ill patients through its pleiotropic effects. However, little is known specifically in adult burn setting with a large VD3 bolus.

**OBJECTIVES.** Aim of this study was to assessed the effect as well as safety of a single mega-dose VD3 supplementation in young major burn patients.

**METHODS.** This is a retrospective data analysis. There was a major explosion from flammable starch-based powder at a party at the Coast Water Park, New Taipei, Taiwan, on June 27, 2015. Out of 499 young people injured, 34 patients were transferred to our hospital including 28 major burns. 22 patients received a single mega dose of 576000 IU of liquid VD3 supplementation enterally at the $4^{th}$ week of intensive care unit (ICU) stay. The main outcome of interest was trajectory of serum 25(OH)D level before and after supplementation. Blood samples were collected before (D0), 7 days, 14 days and 21 days after VD3 bolus to measure 25(OH)D, intact-parathyroid hormone (iPTH), TNF-α and CRP.

**RESULTS.** 25 major burns who remained in ICU at the $4^{th}$ weeks after injury were included. The mean age and BSA were, respectively, 21.8 ± 4.3 years and 49.4 ± 19.6%. At D0, the serum 25(OH)D averaged 13.7 ± 3.9ng/ml with 8% of patients presenting a VD insufficiency (20-30ng/ml) and 92.0% a deficiency (≤19.9 ng/ml). Furthermore, severe deficiency (<13ng/ml) was disclosed in 48% of patients. The baseline 25(OH)D deficiency was associated with BSA, regardless of age, sex, inhalation injury, TNF-α, CRP, and number of surgery. Hypocalcemia and elevated iPTH were noted. After VD3 supplementation in 22 of them, 25(OH)D levels increased quickly, peaking on day 3 at 28.8 ± 12.6ng/ml. iPTH and TNF-α were inversely associated. 25(OH)D levels dropped below 30 ng/mL soon during the follow-up.

**CONCLUSIONS.** This is the first study providing valuable data of stoss therapy of VD3 on burn patients, particularly young adult burned en masse during a large explosion event. High prevalence of profound VD deficiency were shown. A single mega-dose VD3 (576000 IU) increased serum 25(OH) levels, however, even insufficient to ensure restoring body reserve above 30ng/ml. A followed rapid drop implicates high consumption which mainly comes from complex immuno-inflammatory disarrangement, a specific problem of burn due to critical illness, multiple procedures and frequent infection. No hypercalcemia or hypercalciuria was noted after supplementation. Our findings suggested a single high loading dose of cholecalciferol is safe and mandatory to correct the potential severe VD deficiency in acute major burn and maintenance therapy should start earlier to meet the greatly elevated metabolic demand.

**REFERENCE(S)**

1. Rousseau AF et al. Burns. 2015 Aug;41(5):1028–34.
2. Braun AB et al. Crit Care Med. 2012 Jan;40(1):63–72.
3. Amrein K et al. JAMA. 2014 Oct 15;312(15):1520–30.

**GRANT ACKNOWLEDGMENT**

None

### 0529

#### In-Hospital mortality among different caloric target in critically Ill patients

C. Y. Wang[1,2], C.-H. Tseng[1,3]
[1]Taichung Veterans General Hospital, Department of Critical Care Medicine, Taichung, Taiwan, Province of China; [2]Chung Shan Medical University, Department of Nutrition, Taichung, Taiwan, Province of China; [3]National Taiwan University Hospital, Institute of Epidemiology and Preventive Medicine, College of Public Health, Taipei, Taiwan, Province of China
**Correspondence:** C. Y. Wang
*Intensive Care Medicine Experimental* 2017, **5(Suppl 2):**0529

**INTRODUCTION.** Adequate energy supplement has been shown to reduce mortality and the nosocomial infection. The 2016 ASPEN/SCCM nutrition guideline also recommend rating nutrition risk with NUTRIC score based on expert consensus. Rahman et al. removed IL-6 level and reported a modified-NUTRIC score which is easier to be applied in clinical practice. However, the optimal caloric target have not yet been widely reported according to different levels of nutrition risk. This study aims to provide evidence in optimal caloric target among critically ill patients.

**OBJECTIVES.** In the present study, we reviewed the information in the hospital database from September 2015 to August 2016. To be eligible for the study, patients were required to admitted to medical and surgical intensive care unit (ICU) for more than 48 hours and older than 20 years old.

**METHODS.** Clinical data for analysis included basic demographic data, body mass index and total calories intake, including parenteral nutrition and enteral nutrition. The nutrition supplement targets were separated into three groups, less than 50%, 50-79% and more than 80% of calculated goal calories intake. Clinical outcomes included in-hospital mortality, and ventilator dependent days. High nutrition risk was defined by modified NUTRIC score≥5; low nutrition risk was defined by modified NUTRIC score < 5. Adjusted hazard ratios (aHRs) and the associated 95% confidence intervals (CIs) were estimated using the Cox proportional hazards model with APACHEII score as covariate.

**RESULTS.** Of 1649 patients enrolled in the study, 1035 (62.8%) patients were at high nutrition risk. In-hospital mortality rate among patients at high nutrition risk was 33.6%, and compared to those target less than 50% of calculated goal calories (46.9%), that was lowest in who targeted more than 80% (24.3%, aHR: 0.31 [95% CI, 0.24-0.41], p < 0.001), and then who targeted 50-79% (29%, aHR: 0.34 [95% CI, 0.26-0.44], p < 0.001). On the other hand, the mortality rate in patients at low nutrition risk was 11.2%, compared to lowest nutrition target (9.8%), that was best in who targeted 50-79% (8.8%, aHR: 0.32 [95% CI, 0.16-0.69], p = 0.003), and then who targeted more than 80% (14.8%, aHR: 0.49 [95% CI, 0.28-0.87], p = 0.015).

**CONCLUSIONS.** The optimal nutrition supplement goal could be more than 80% of calculated target among patients at high nutrition risk, and 50-79% of calculated target among patients at low nutrition risk.

**REFERENCE(S)**

1. Rahman A, et al. Identifying critically-ill patients who will benefit most from nutritional therapy: Further validation of the "modified NUTRIC" nutritional risk assessment tool. ClinNutr. 2016;35(1):158–62
2. McClave SA, et al. Guidelines for the Provision and Assessment of Nutrition Support Therapy in the Adult Critically Ill Patient: Society of Critical Care Medicine (SCCM) and American Society for Parenteral and Enteral Nutrition (A.S.P.E.N.). JPEN J Parenter Enteral Nutr. 2016;40(2):159–211.

### 0530

#### Metabolic effects of a very high protein low carbohydrate enteral formula in critically ill medical patients

T. Rice[1], D.C. Files[2], P. Morris[3], A. Bernard[4], T.R. Ziegler[5], J. Drover[6], J. Kress[7], K.R. Ham[8], D. Grathwohl[9], M. Huhmann[10], J.B. Ochoa[11,12]
[1]Vanderbilt University, Critical Care Medicine, Nashville, United States; [2]Wake Forest School of Medicine, Pulmonary and Critical Care Medicine, Winston Salem, United States; [3]University of Kentucky Healthcare, Pulmonary and Critical Care Medicine, Lexington, United States; [4]University of Kentucky Healthcare, Surgery, Lexington, United States; [5]Emory University, Medicine, Atlanta, United States; [6]Queen's University School of Medicine, Surgery, Kingston, Canada; [7]University of Chicago, Pulmonary and Critical Care Medicine, Chicago, United States; [8]Regions Hospital, Saint Paul, United States; [9]Nestle Research Center, Ver Chez le Blanc, Switzerland; [10]Nestle Health Science, Medical Unit, Florham Park, United States; [11]Nestle Health Science, Medical Unit, Livingston, United States; [12]Geisinger Medical Center, Critical Care Medicine, Danville, United States
**Correspondence:** T. Rice
*Intensive Care Medicine Experimental* 2017, **5(Suppl 2):**0530

**INTRODUCTION.** The metabolic effects of an enteral formula containing High protein in the form of hydrolyzed whey and low carbohydrates needs are unknown.

**OBJECTIVES.** Describe the metabolic effects of the use of such a formula in critically ill patients.

**METHODS.** Multi-center, randomized, open label, clinical trial, in subjects requiring enteral nutrition for at least 5 days. Medical critically ill ventilator dependent patients with a BMI of > 26 receiving a very high protein (37%) low carbohydrate (29%) formula (Experimental formula - Peptamen® Intense VHP) or control protocol with a high protein (25%), high carbohydrate (45%) formula (Replete®) infused to provide 1.5 g protein/Kg.

**RESULTS.** 105 patients were randomized (52 experimental (E), 53 control (C)) with 102 providing data according to the intention to treat principle. Groups were comparable with respect to age, BMI and sex. Most frequent diagnoses on admission were acute respiratory failure, pneumonia and sepsis. Data presented are n, mean (sd) for patients completing 7 days on protocol (n = 36). Both groups received similar amounts of protein (19, 1.15 (0.43) g/Kg/d vs. 17, 1.41 (0.37) g/Kg, p = 0.11) with lower total energy (19, 18.36 (6.86) vs. 17, 15.11 (4.00) p < 0.03) in the experimental group.

Carbon dioxide ($CO_2$) accumulation (as measured by blood levels) was lower in the experimental group and was maintained across time, along with lower bicarbonate levels (Table - data presented as the difference between means (delta-D), significance per day (p=). Alkaline phosphatase (AP) progressively increased in the controls with no change in the experimental group. No differences were observed in C- peptide, C-reactive protein or albumin levels. In the experimental group at day 7 a modest decrease in total protein and an increase in the number of patients with detectable ketones in blood and urine were observed. There were no differences in renal function (plasma creatinine & eGFR). White blood cell counts were lower in the experimental group by day 7.

**CONCLUSIONS.** A very high protein, low carbohydrate whey based formula is associated with metabolic changes that could potentially be of clinical significance. Lower $CO_2$ and bicarbonate may translate to a potential clinical benefit in patients with acute respiratory failure and renal dysfunction. Increasing AP (suggesting cholestasis) in the control group was avoided with the experimental product. Higher ketones observed with lower caloric loads may benefit obese patients. The significance of lower circulating total protein with no difference in albumin between groups needs to be defined.

**REFERENCE(S)**

Martindale,R. Nutr 2011;35(5 suppl): 80S-87S

**GRANT ACKNOWLEDGMENT**

Nestle Health Science

**Table (Abstract 0530).** Timeline

| Day | 0 | 1 | 2 | 3 | 4 | 5 | 6 | 7 |
|---|---|---|---|---|---|---|---|---|
| AP D % | 1 | −8 | −8.2 | −15.8 | −21.5 | −27.2 | −20.9 | −28.7 |
| AP p= | ns | 0.3 | 0.3 | 0.7 | 0.02 | 0.005 | 0.04 | 0.02 |
| CO2 D % | 1.1 | −3.8 | −7.5 | −7.1 | −10.1 | −8.9 | −10.5 | −14.8 |
| CO2 p= | ns | 0.3 | 0.05 | 0.06 | 0.01 | 0.04 | 0.02 | 0.004 |
| Bicarb D mmol/L | −0.9 | 0.112 | 0.0007 | −0.055 | −0.123 | −0.169 | −0.125 | −0.191 |
| Bicarb p= | ns | 0.06 | 0.9 | 0.4 | 0.09 | 0.03 | 0.1 | 0.03 |

### 0531

#### Vitamin D levels in liver transplantation recipients. prospective observational study, preliminary data

G. Martucci[1], R. Volpes[2], D. Bellavia[3], P. Asaro[1], D. Di Carlo[4], F. Lullo[1], M. Farbo[1], G. Chiaramonte[1], C. Ricotta[5], G. Panarello[1], G. Burgio[1], S. Gruttadauria[5], G. Vizzini[6], K. Amrein[7], A. Arcadipane[1]
[1]Ismett, Department of Anesthesia and Intensive Care, Palermo, Italy; [2]Ismett, Hepatology Unit, Palermo, Italy; [3]Ismett, Cardiology Unit, Palermo, Italy; [4]Ismett, Department of Laboratory Medicine, Palermo, Italy; [5]Ismett, Organ Transplantation Unit, Palermo, Italy; [6]Ismett, Department for the Treatment and Study of Abdominal Diseases and Abdominal Transplantation, Palermo, Italy; [7]Medical University of Graz, Department of Internal Medicine, Division of Endocrinology, Graz, Austria
**Correspondence:** G. Martucci
*Intensive Care Medicine Experimental* 2017, **5(Suppl 2):**0531

**INTRODUCTION.** Vitamin D deficiency is common in chronic liver disease and it has been correlated with a worse outcome after orthotopic liver transplantation (OLT).[1]

**OBJECTIVES.** We evaluated the change of the level of vitamin D, Parathyroid hormone (PTH), calcium and phosphate in OLT recipients 28 days post-OLT as compared to the pre-OLT asset and we correlated their possible association, with the rate of infections, surgical complications and death after OLT.

**METHODS.** Single center prospective observational study. All consecutive recipients of cadaveric OLT were included (Sep 2016 - Jan 2017). As descriptive statistics mean ± SD, or median (IQR) were used. Comparisons between biochemical assets pre- and 28 days post-OLT were assessed using Wilcoxon Rank Sum Test.

**RESULTS.** Twenty-nine patients were enrolled. Clinical characteristics of the patients were Age 57 ± 7, BMI 27 ± 5, Meld score 16.6 ± 8.3, Child Pugh score 6.5 ± 1.3; overimposed hepatocarcinoma was found in 20 patients (69%). Table 138

All the recipients but one, were deficient in vitamin D (<20 ng/dl) pre-LT: median 8.1 (IQR 0.9 - 9.1), and all of them remained deficient at day 28 after OLT: median 7.25 (IQR 4.6 - 8.6), with a slight reduction that was not statistically significant (p = 0.79). We found a trend in reduction of PTH and Phosphate with a significant increase of total calcium levels (p < 0.001).

The length of stay was 9 ± 12 days in ICU and 27 ± 24 days in hospital; pts were mechanically ventilated for 4 ± 10 days and received vasopressor for 2.9 ± 7.3 days. 14 pts (48%) experienced at least one infection, 10 (34%) had surgical complication and 3 patients deceased. Table 140

The incidence of early post-OLT surgical complications were not correlate with change in vitamin D, PTH, calcium and phosphate whereas total calcium level increased more in the patients alive (p < 0.01) than in those deceased (p = 0.18) arising the hypothesis that an improvement in calcium metabolism may be a positive marker after OLT. Infections were not correlated to changes in vitamin D, PTH, calcium and phosphate. Moreover we found a lower pre-OLT vitamin D level in those patients who developed an infection during the first 28 days after OLT (mean value 7 vs 15).

**CONCLUSIONS.** Liver transplantation, with its consequent metabolic storm, might have a strong impact in further reducing vitamin D blood levels, and this in turn could have a significant impact on clinical outcomes.
Our preliminary results show a high prevalence of vitamin D deficiency pre-OLT (97%), and that it was correlated with an increased risk of infection in the early post-OLT period. Although a larger study is mandatory to confirm such results, we believe that a protocol with vitamin D supplementation should be used in all patients both in the pre- and post- OLT period.

**REFERENCES**
1. Reese PP, et al. Changes in vitamin D binding protein and vitamin D concentrations associated with liver transplantation. Liver Int 2012;32(2):287–296

**Table 138 (Abstract 0532).** See text for description

| | |
|---|---|
| Age, years | 57 ± 7 |
| Body Mass Index | 27 ± 5 |
| MELD | 16.6 ± 8.3 |
| Charlson Comorbidity Index | 6.5 ± 1.3 |
| Child Pugh Score | 8.6 ± 2.2 |
| Hepatocarcinoma N (%) | 20 (69) |
| Creatinine mg/dl | 0.89 (0.4 - 2.96) |
| MDRD | 86 (22 - 139) |
| Donor Age, years | 58 ± 22 |
| Cold ischemia Time, minutes | 435 ± 102 min |

**Table 139 (Abstract 0532).**See text for description

| Variable | Pre OLT | 28 days post OLT | p value |
|---|---|---|---|
| Vitamin D, ng/ml | 8.1 (0.9-9.1) | 7.3 (4.6-8.6) | 0.79 |
| Parathyroid Hormone, pg/ml | 55.8 (15.7-160) | 49.7 (21.4-142.7) | 0.61 |
| Total Calcium, mg/dl | 8.3 (7.1-9.5) | 9.2 (8.0-10.0) | <0.001 |
| Phosphate, mg/dl | 3.6 (2.2-5.5) | 3.5 (1.2-4.5) | 0.52 |

**Table 140 (Abstract 0532).** See text for description

| Clinical Outcomes | |
|---|---|
| ICU length of stay, days | 9 ±12 |
| Mechanical Ventilation, days | 4 ± 10 |
| Vasopressor, days | 2.9 ± 7.3 |
| Hospital length of stay, days | 27 ± 24 |
| Bilirubin Day28, mg/dl | 1.24 (0.8 - 24.6) |
| INR Day28 | 1.06 (0.9 - 2.1) |
| Infection, N(%) | 14 (48) |
| Surgical Complication, N(%) | 10 (34%) |
| Death, N(%) | 3 (10%) |

### 0532

### Inconsistent fasting practice before procedures: a national survey of United Kingdom intensive care units

E. Segaran[1], T. Lovejoy[2], C. Proctor[3], W. Bispham[4], R. Jordan[4], B. Jenkins[5], E. O'Neill[6], S. Harkess[7], M. Terblanche[8]
[1]Imperial College Healthcare NHS Trust, Nutrition and Dietetics, London, United Kingdom; [2]Nottingham University Hospitals NHS Trust, Nutrition and Dietetics, Nottingham, United Kingdom; [3]Northwick Park & St Mark's Hospitals, Nutrition and Dietetics, London, United Kingdom; [4]Lewisham and Greenwich NHS Trust, Nutrition and Dietetics, London, United Kingdom; [5]University Hospital Southampton NHS Trust, Department of Nutrition and Dietetics, Southampton, United Kingdom; [6]City Hospitals Sunderland NHS Foundation Trust., Department of Nutrition and Dietetics, Sunderland, United Kingdom; [7]County Durham and Darlington NHS Foundation Trust, Department of Nutrition and Dietetics, Darlington, United Kingdom; [8]King's College London, Division of Health and Social Care Research, School of Medicine & Life Sciences, London, United Kingdom
**Correspondence:** E. Segaran
*Intensive Care Medicine Experimental* 2017, **5(Suppl 2):**0532

**INTRODUCTION.** Intensive care unit (ICU) patients' nutritional requirements are frequently unmet. On average patients receive 60% of nutritional requirements (1, 2). Enteral nutrition (EN) delivery is frequently interrupted for surgical and airway procedures to avoid aspiration of stomach contents. Recurrent fasting leads to under delivery of EN and is associated with worse outcomes (3, 4). International fasting recommendations do not provide guidance for intubated patients receiving EN. This may be due to a dearth of research on fasting times for stopping EN in this patient population. Consequently, clinical practice varies considerably and extended fasting times is commonplace. Currently, no data are available on practice in the United Kingdom.
**OBJECTIVES.** This study aimed to gain a detailed perspective of UK critical care fasting practices.
**METHODS.** A web-based survey was sent to 232 UK ICUs via critical care dietitians, consisting of questions relating to local ICU fasting practices, presence of guidelines, average fasting times for common procedures and dietitian time per ICU bed. Qualitative analysis was undertaken to assess any common themes within the free text comments.
**RESULTS.** 176 ICU responded (72% of all ICUs in the UK). Only 20% of units had a fasting guideline and these were not consistently adhered to (mean compliance was 66%). Units with greater dietetic involvement were more likely to have guidelines. Fasting times were shorter for abdominal surgery (p = 0.002), non-abdominal surgery (p = 0.016) and radiology (p = 0.015) if a guideline was present. Fasting for extubation and tracheostomy was predominately 4–6 hours irrespective of the presence of a guideline. Considerable variation in fasting times was reported, usually due to inconsistencies in clinical decision-making.
**CONCLUSIONS.** This comprehensive survey of national practice, demonstrates that fasting times are varied and inconsistent, leading to long fasting times and under-delivery of EN. Where guidelines were present, fasting was significantly shorter for surgery and radiology. However, for procedures perceived to be at higher risk (such as extubation and tracheostomy), fasting times were similar between all units irrespective of the presence of a guideline. More dietetic input was associated with increased likelihood of a fasting guideline.

**REFERENCE(S)**
1. Elke G, et al. *Critical Care* 2014;18:1.
2. Nicolo M, et al. *Journal of Parenteral and Enteral Nutrition* 2015:0148607115583675.
3. Singer P et al. The Tight Calorie Control Study (Ticacos): *Intensive Care Medicine* 2011;37:601–609.
4. Villet S et al. *Clinical Nutrition* 2005;24:502–509.

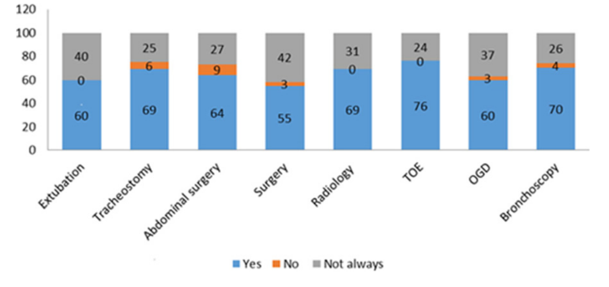

**Fig. 188 (Abstract 0532).** Adherence to fasting guidelines

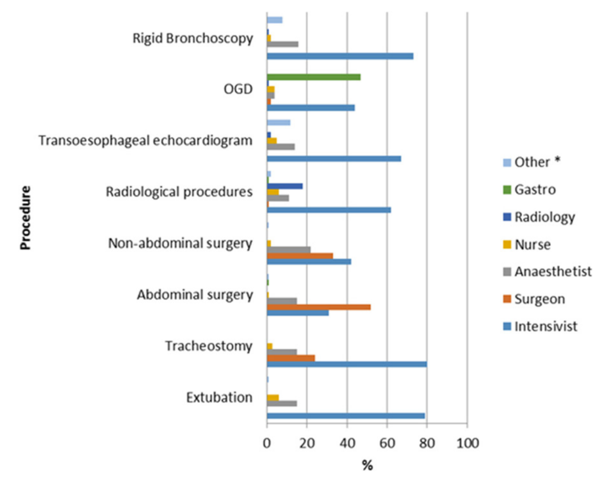

**Fig. 189 (Abstract 0532).** Decision makers without guideline

### 0533

#### A retrospective analysis of the diagnosis and management of acute colonic pseudo-obstruction in a 60-bedded ICU

R. Barelli[1], T. Gilbey[2], K. Lane[2], S. Assadullah[3], R. Wan[4], A. Langan[5], K. Dingli[2]
[1]Barts Health NHS Trust, Intensive Care Medicine, London, United Kingdom; [2]Guy's and St Thomas' Hospital, Intensive Care Medicine, London, United Kingdom; [3]King's College London, Pharmacy, London, United Kingdom; [4]Guy's and St Thomas' Hospital, Pharmacy, London, United Kingdom; [5]Guy's and St Thomas' Hospital, Nutrition and Dietetics, United Kingdom
**Correspondence:** T. Gilbey
*Intensive Care Medicine Experimental* 2017, **5(Suppl 2):**0533

**INTRODUCTION.** Acute colonic pseudo-obstruction (“ACPO”) is characterised by dilatation of the colon in the absence of an anatomical lesion that obstructs the flow of intestinal contents and is also known as Ogilvie's syndrome. A clinical diagnosis of ileus is commonly made in critically unwell patients but the prevalence of ACPO underlying this is unknown. ACPO can result in colonic ischaemia, perforation and death (1).

**OBJECTIVES.** We aimed to retrospectively identify cases of ACPO over the course of one year. We reviewed the diagnosis and management of ACPO, including complications arising. We assessed our practice against a recently developed institutional guideline to examine if there were further needs of the critical care population.

**METHODS.** A search of our electronic patient records for the terms “ileus”, “distension” and “obstruction” yielded 273 patients. Full clinical notes and imaging were examined by two independent clinicians to confirm the clinical features of ileus, associated medication and nutritional parameters, and treatment. Abdominal computed tomography scans were examined to find evidence of colonic dilatation without a structural obstructing lesion.

**RESULTS.** Sixty-eight cases of clinical ileus representing 5.2% of critical care admissions were identified. Twenty-two of 68 patients (32%) patients had abdominal imaging consistent with ACPO, but most did not have the colonic diameter documented on the report. Only 63% had cross-sectional imaging.

82% of cases were prescribed opioids, and alpha agonists were prescribed in 32% of cases. The most common interventions were a reduction in the opioid dose and the use of prokinetics.

Eight patients had enteral nutrition formally prescribed and a further 5 had parenteral nutrition in addition to this. Of these 13, only 3 attained >80% of their target energy intake.

Five patients were treated with neostigmine and 6 were re-imaged. 77% received surgical review but only 6 were treated with a decompression tube and 5 had endoscopy. Three patients (14%) experienced a bowel ischaemia and/or perforation, and two of these required laparotomy. There were 3 deaths amongst the 22 cases, 2 of which were related to ACPO.

CONCLUSIONS.

1. Clinical ileus is common; a subset of patients will develop ACPO and there is a significant risk of complications.
2. Many of our patients were not imaged. Of those that were, plain films are sub-optimal for diagnosing ACPO and CT reports did not contain sufficient detail.
3. Opioid and alpha-agonist use was common in this cohort and may be difficult to avoid.
4. Patients with ACPO did not attain their target nutritional intake.
5. Neostigmine and endoscopic decompression, recommended by local protocols, were used in fewer than 25% of cases, probably on an individual risk/benefit basis.

**Table (Abstract 0533).** Patient Demographics (Median[Range])

| | |
|---|---|
| Age (y) | 63 |
| Sex | 18 Male, 4 Female |
| APACHE | 16 [8–26] |
| Length of stay (d) | 22 [2–229] |
| Days of mechanical ventilation | 17 [6–229] |
| Days of RRT | 0 [0–56] |
| Days until diagnosis ACPO | 5.5 [1–35] |

### 0534

#### Early goal-directed enteral nutrition in NAGOYA University Emergency ICU

M. Naoyuki, M. Higashi, T. Yamamoto, M. Nishikimi, A. Numaguchi, M. Kado, H. Tokuyama
Nagoya University Graduate School of Medicine, Emergency & Critical Care Medicine, Nagoya, Japan
**Correspondence:** M. Naoyuki
*Intensive Care Medicine Experimental* 2017, **5(Suppl 2):**0534

**INTRODUCTION.** We created early goal-directed enteral nutrition protocol (EGDN NAGOYA) in 2010.

**OBJECTIVES.** The purpose of this study is to evaluate the relation between nutrition management methods and the outcome in emergency and medical ICU.

**METHODS.** We investigated retrospectively the patients hospitalized for 48 hours or more, in the ICU of in Nagoya University hospital in Japan, from May 2011 to june 2015. According to the first nutrition method, the patients were classified into the following three groups: peroral intake group (PO), enteral nutrition group, and total parenteral nutrition group (TPN). Enteral nutrition group was classified into the following four groups, according to the time to initiate enteral feeding: within 6 hours(EN6), 6 hours or more, and less than 24 hours(EN6-24), 24 hours or more, and less than 48 hours(EN24-48), and 48 hours or more(EN48). We evaluated APACHEII score and mortality in ICU of each group. A paired t-test was used for statistical analysis.

**RESULTS.** There were 745 cases in the criteria of this study. Enteral nutrition according to EGDN NAGOYA were 377 cases (50.6%) were classified into enteral group: EN6 63 (8.5%), EN6-24 166 (22.3%), EN24-48 110 (14.8.4%) and EN48 38 (5.1%). The mean of the APACHE II score was 29.4, 29.9, 29.2 and 29.3, and the ICU mortality was 1.6%,7.2%,8.2%, 10.5%, respectively. On the other side, in TPN, the APACHE II score was 29.4 and its ICU mortality was 20.3%. EN according to EGDN NAGOYA showed the significantly better outcome than PN in spite of the similar severity.

**CONCLUSIONS.** This study showed that early beginning enteral nutrition with a protocol could lead to good prognosis of critically ill patients.

### 0535

#### Effects of enteral nutrition on stress ulcer hemorrhage in critically ill patients: multicenter randomized controlled trial

K. Gundogan[1,2], E. Karakoc[3], T. Teke[4], A. Zerman[5], A. Coruh[1], M. Sungur[1]
[1]Erciyes University, School of Medicine, Intensive Care Unit, Kayseri, Turkey; [2]Erciyes University Hospital, Hospital Enteral and Parenteral Nutrition Unit, Kayseri, Turkey; [3]Cukurova University School of Medicine, Intensive Care Unit, Adana, Turkey; [4]Necmettin Erbakan University, Pulmoner Critical Care Unit, Konya, Turkey; [5]Adana Numune Research and Training Hospital, Intensive Care Unit, Adana, Turkey
**Correspondence:** K. Gundogan
*Intensive Care Medicine Experimental* 2017, **5(Suppl 2):**0535

**INTRODUCTION.** Mucosal erosions can occur on luminal surface of stomach in approximately 75-100% patients during the first 24 hours of intensive care unit admission. These erosions often cause bleeding with penetrating superficial capillaries. Enteral nutrition (EN) may have protective effects against stress ulcer bleeding by neutralizing the acidic pH in the stomach lumen, providing a structural and functional integrity of the mucosal surface and trophic effect on the GI mucosa. There is no sufficient data showing the relation between enteral nutrition and stress ulcer hemorrhage.

**OBJECTIVES.** To determine effect of enteral nutrition on stress ulcer bleeding in critically ill patients.

**METHODS.** This study was performed as prospective randomized controlled multicenter trial in critically ill patients. Patients randomized into two groups; Group 1 received only oral/enteral nutrition, group 2 received oral/enteral nutrition and pantoprazole.

**RESULTS.** We enrolled 158 (group 1: 78, group 2: 80) patients. The mean age was 61.3 ± 17.6 years. The most common cause of admission to ICU was respiratory failure in 48 patients (30%), followed by renal failure 18 (11%) and sepsis/septic shock in 14 patients (9%). Mean APACHE II score was 19.8 ± 6.0, first day median SOFA score was 4 (range: 0–12), first day mean GCS was 12.9 ± 3.2 and mean NUTRIC score was 4.0 ± 1.8. Time to starting nutrition was 8.1 ± 5.7 hours. Length of nutrition time was 3 (1–36) days and length of ICU stay was 3 (1–130) days (Table 1). Overall, 119 (75%) patients were fed by oral route, 37 (24%) patients fed by enteral tube and 2 (1%) patients were fed by oral + entral tube. There was no any overt GI bleeding in any patients in both groups. ICU mortality rate was 16%.

**CONCLUSIONS.** This study showed that stress ulcer related GI bleeding was not observed in critically ill patients who were fed via oral/enteral route in both groups. Stress ulcer prophylaxis may not be needed in critically ill patients receiving oral/enteral nutrition.

**GRANT ACKNOWLEDGMENT**
This study has been supported by NESTLE
Note: This study will be terminated in in August 15, 2017 and approximately 400 patients will be recruited.
**ClinicalTrials.gov Identifier:** NCT03098537

### 0536

#### Olanzapine associated to glucose disturbances in intensive care unit

H. Pena Carrillo
Hospital Independencia/Benemérita Universidad Autónoma de Puebla, Critical Care Unit, Tehuacán, Mexico
*Intensive Care Medicine Experimental* 2017, **5(Suppl 2):**0536

**INTRODUCTION.** In intensive care unit (ICU) we have the combination of several problems, thats the reason why sometimes the cases in ICU are a puzzle to manage. We have patients in sepsis, with glucose, psychotics abnormalities and shock. So to find the clue or the perfect phamacology combination is complex, though the evidence supports them. Psychosis is a mayor problem in critical care patient in any stage, now the evidence points to atypical antipsychotic use, the most studied in critical areas is olanzapine with its accute use.

**OBJECTIVES.** The purpose of this study is to evaluate the hyperglycemia in ICU patients receiving newly prescribed olanzapine.

**METHODS.** From January 2015 to february 2017 we accepted in our ICU 186 patiets charts reviewed, from this example 51 patients were included in this study. We find that 46% of patients at least report 1 hyperglycemic episode (blood glucose >160 mg/dL) after the we start dose olanzapine in the ICU in front of any kind of pshycotic episode. But the patients at least with more than 5 hyperglycemic episodes (64.3%) that experienced it was associeted to the septic state with multiple organic faillures. 19 (38%) patients with severe neumonias discharged from the ICU with olanzapine, 3 (6%) patients died in the ICU and all the patients with glucose disturbance, 31 (62%) patients were treated with fast insulin scale. Logistic regression analysis showed that patients in ICU with a higher APACHE II score were significantly more likely to have multiple hyperglycemic episodes.

**CONCLUSIONS.** Psychosis is a commom mayor problem in the ICU as well as infections and hyperglycemia, so olanzapine as an antipsychotic drug is a fast and extraordinary option, but base don our results, we recommend to be careful with its use in ICU for the risk to develop hyperglycemia of other glucose-disturbance. We need to investigate the link between the use of atypical antipsychotics and acute hyperglycemia and its clinical significance.

**REFERENCE(S)**

Girard TD, Pandharipande PP, Carson SS,.Feasibility, efficacy, and safety of antipsychotics for intensive care unit delirium: the MIND randomized, placebo-controlled trial. Crit Care Med. 2010; 38(2):428–437.

Barr J, Fraser GL, Puntillo K,.Clinical practice guidelines for the management of pain, agitation, and delirium in adult patients in the intensive care unit. Crit Care Med. 2013;41(1):263–306.

Devlin JW, Roberts RJ, Fong JJ,.Efficacy and safety of quetiapine in critically ill patients with delirium: a prospective, multicenter, randomized, double-blind, placebo-controlled pilot study. Crit Care Med. 2010;38(2):1–9.

American Diabetes Association: Standards of medical care in diabetes - 2014. Diabetes Care. 2014;37(suppl 1):S14-S80. Google Scholar Medline

Liebzeit KA, Markowitz JS, Caley CF. New onset diabetes and atypical antipsychotics. Euro Neuropsychopharmacol. 2001;11(1):25–32.

**GRANT ACKNOWLEDGMENT**
No financial disclosure; No Product/Research disclosure information.

### 0537

#### Trace element repletion following severe burn injury: a 16-year retrospective study

O. Pantet[1], P. Stoecklin[2], M. Charrière[1], A. Vernay[3], M.M. Berger[1]
[1]CHUV, Intensive Care & Burn Center, Lausanne, Switzerland; [2]Inselspital, Bern University Hospital, Intensive Care Medicine, Bern, Switzerland; [3]CHUV, Computer Sciences, Lausanne, Switzerland
**Correspondence:** O. Pantet
*Intensive Care Medicine Experimental* 2017, **5(Suppl 2):**0537

**INTRODUCTION.** Trace elements (TE) repletion has been shown to be beneficial in burn patients who suffer from large exudative losses of Copper (Cu), Selenium (Se) and Zinc (Zn), in terms of infectious complication reduction and improved wound healing.

**OBJECTIVES.** The study aimed at checking if our repletion protocols were appropriate to normalize TE plasmatic levels of our burn patients during the first 3 weeks after injury over a period of 16 years.

**METHODS.** Retrospective analysis of a prospective cohort of patients admitted to the ICU between 1999 and 2015. Inclusion criteria: burn injury requiring an ICU stay > 7 days. The cohort was divided into 4 groups according to the period defined by changes in our management protocol. Period 1 (P1): 1999–2000, P2: 2001–2005, P3: 2006–2010, P4: 2011–2015. Changes were mainly increasing TE repletion doses and better compliance with the repletion protocol. Demographic data, daily TE intakes and weekly TE plasma levels were retrieved for the first 21 days. Data is expressed as median and (IQR). Study periods were compared using the $\chi 2$ test, Fisher´s exact test or one-way-ANOVA when applicable.

**RESULTS.** 253 patients aged 43 (32) years, burned on 25 (24)% body surface area were included (no difference between periods). Daily Cu, Se and Zn intakes increased significantly between 1999 and 2015, allowing normalization of plasma Cu (19mg/l) and Zn (12mg/l) levels during P4. Median plasma Se levels were elevated during P4, flirting with the maximal range (1404μg/l).

**CONCLUSIONS.** The study shows that our supplementation protocol normalizes Cu and Zinc levels, but Se doses seem too high, suggesting a reduction of the dose.

**REFERENCE(S)**

Berger MM, Shenkin A. Trace element requirements in critically ill burned patients. *J Trace El Med Biol :* 2007; 21 (suppl 1):44–48.

**GRANT ACKNOWLEDGMENT**

None.

**Table (Abstract 0537).** Results

| Variable | All periods | Period 1 (99–01) | Period 2 (02–05) | Period 3 (06–10) | Period 4 (11–15) | P-value |
|---|---|---|---|---|---|---|
| Number of patients | 253 | 32 | 57 | 85 | 79 | |
| Daily Cu intake (mg) | 2.5 (3.4) | 2.7 (3.4) | 2.0* (3.0) | 2.2 (3.0) | 3.3* (4.2) | <0.001 |
| Cu level (mg/l) (12.5-23.6) | 14.2 (11.7) | 8.3* (5.2) | 11.7 (16.4) | 17.6 (9.3) | 19.3* (8.7) | <0.001 |
| Daily Se intake (μg) | 334 (484) | 300* (374) | 323 (397) | 392* (540) | 292 (647) | <0.001 |
| Se level (μg/l) (750–1500) | 1233 (769) | 530* (430) | 1053 (540) | 1461* (683) | 1404 (538) | <0.001 |
| Daily Zn intake (mg) | 33 (48) | 27* (35) | 30 (37) | 45 (50) | 40* (61) | <0.001 |
| Zn level (mg/l) (10.1-17) | 12.3 (5.8) | 6.7* (3.9) | 13 (7) | 13.2* (5.6) | 11.5 (4.2) | <0.001 |

### 0538

#### Bedside ultrasound of muscle layer thickness of the quadriceps in the critically ill patient

R. L. Cordioli[1], D. C. Lima e Silva[1], J. M. da Silva[1], S.M. Fraga Piocavari[1], E. J. Figueiredo[1], I. C. de Paula[1], D.M. dos Santos[1], R. Dib[1], D. Toledo[1]
[1]Hospital Israelita Albert Einstein, Adult Intensive Care, São Paulo, Brazil
**Correspondence:** R. L. Cordioli

*Intensive Care Medicine Experimental* 2017, **5(Suppl 2):**0538

**INTRODUCTION.** Survivors of critical illness experience significant skeletal muscle wasting that may predict clinical outcome. Ultrasound is a noninvasive method that can measure muscle quadriceps muscle layer thickness (QMLT) at the bedside. [1,2]

**OBJECTIVE.** To evaluate the measuring quadriceps muscle layer thickness using bedside ultrasound in critically ill patient.

**METHODS.** This was a prospective, single-center study, conducted in a tertiary care hospital. The study was approved by the ethics committee of Hospital Israelita Albert Einstein and written informed consent obtained from each study participant. Demographic data, anthropometric data, prognostic index (SAPS 3), nutrition risk screening (NRS) and image of US QMLT were collected for analysis. The thickness of the quadriceps musculature was quantified with a portable B-mode ultrasound device. With the patient lying supine, knees extended and relaxed, 2 landmarks on each quadriceps were identified. The underlying tissues were then maximally compressed by the ultrasound probe, and the screen image was frozen. The muscle thickness was quantified by the use of onscreen calipers and taken as the distance between the upper margin of the femoral bone and the lower boundary of the deep fascia of the rectus femoris. Each landmark was imaged and averaged across each leg and then between legs. Measurements of the first (D1), third (D3) and seventh (D7) days were performed and the percentage of QMLT was compared by comparing the left and right legs.

**RESULTS.** It was enrolled 20 patients underwent to 40 QMLT using US in each leg. The mean age was 57.0 ± 20.2 years, 75% male, BMI 24.6 ± 3.0 kg/m$^2$, SAPS 3 was 51.3 ± 17.4 and NRS 3.2 ± 1.0. Overall, 8.4% (22.0% to −8.9%) of muscle wasting in the right leg (Fig. 190) and 5.2% (22.6% to −11.0%) in the left leg (Fig. 191) assessed by the US occurred from the first to the seventh day. In the right leg the median values evaluated by the US were 1.16 (0.92 to 1.75) D1, 1.13 (0.92 to 1.60) D3, 1.02 (0.81 to 1.41) D7; P = 0.005. In the left leg, the median values evaluated by the US were 1.23 (0.93 to 1.43) D1, 1.16 (0.92 to 1.34) D3, 1.13 (0.89 to 1.31) D7; P = 0.017.

**CONCLUSION.** The measuring quadriceps muscle layer thickness using ultrasound demonstrated that critical ill patients present muscle wasting daily, and this procedure can be a great differential to identify patients most likely to benefit from enhanced nutritional and rehabilitation support.

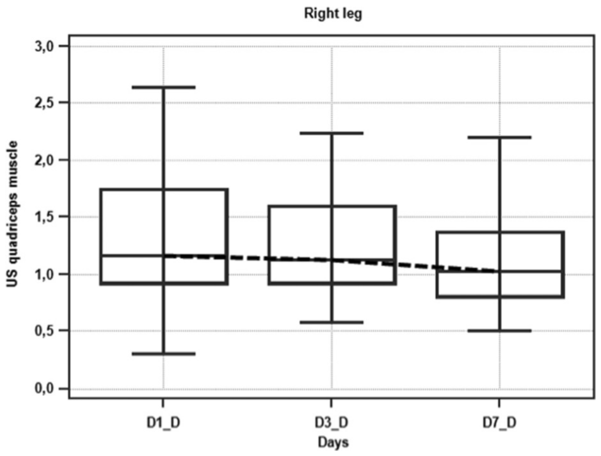

**Fig. 190 (Abstract 0538).** See text for description

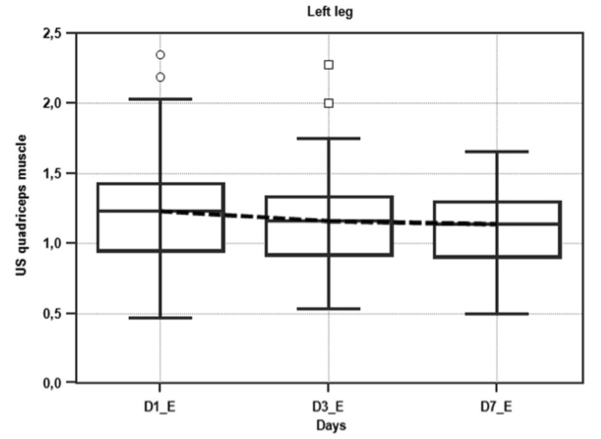

**Fig. 191 (Abstract 0538).** See text for description

### Cardiac arrest 2

#### 0539

#### Outcomes and eligibility criteria of extracorporeal cardiopulmonary resuscitation in refractory cardiac arrest

S. Luzon-Chetrit[1], V. Chhor[1], C. Ursat[2], C. Lebard[1], A. Follin[1], B. Champigneulle[1], J. Chatelon[1], G. Fave[1], A. Neuschwander[1], T. Loeb[2], R. Pirracchio[1]
[1]Hopital Europeen Georges Pompidou, Paris, France; [2]SAMU 92, Garches, France
**Correspondence:** S. Luzon-Chetrit
*Intensive Care Medicine Experimental* 2017, **5(Suppl 2):**0539

**INTRODUCTION.** Extracorporeal life support (ECLS) is a rescue technique for refractory cardiac arrest (CA). However, eligibility criteria and benefit on survival or neurological outcome are still controversial. The goal of our study was to assess the effect of extracorporeal cardiopulmonary resuscitation (e-CPR) on mortality during refractory CA.

**OBJECTIVES.** The primary goal was to study the effect of e-CPR on survival in refractory CA (low flow > 20 minutes). Secondary goals were, i) to describe the characteristics of patients proposed for e-CPR, ii) to identify the factors associated with survival after refractory CA.

**METHODS.** Two observational monocentric prospective cohorts were concatenated and studied: the first one (RéAC) included all out-of-hospital refractory CA patients managed between March 2012 and June 2016 by a local ambulance service. The second cohort included all patients admitted for e-CPR at our center between May 2004 and April 2016 for refractory cardiac arrest.

**RESULTS.** Six hundred and thirty-five patients were included, 240 (38%) were proposed for e-CPR whereas 395 (62%) were not. One hundred fifty six patients (25%) actually underwent e-CPR. Patients proposed for e-CPR were younger, had more witnessed CA and initial shockable rhythm. 30-day survival rate was 2.5% for patients who actually benefited of e-CPR.

Survival at 30 days was not different among patients proposed or not for e-CPR (7 (3.0%) vs 6 (1.5%) respectively, p = 0.23). In a multivariate analysis, the only factor associated with survival after refractory CA was initial shockable rhythm (OR 0.26, 95%CI = 0.09-0.81, p =0.02) whereas treatment with e-CPR was not.

**CONCLUSIONS.** e-CPR did not improve survival in patients treated for refractory CA. Reducing the duration of low-flow might improve the results of e-CPR for refractory CA.

#### 0540

#### Outcome of patients with refractory cardiac arrrest treated with extracorporeal cardiopulmonary resuscitation (ECPR)

A. Krüger[1], P. Ošťádal[1], M. Janotka[1], D. Vondráková[1], Š. Černý[2], I. Skalský[2]
[1]Na Homolce Hospital, Cardiology, Prague, Czech Republic; [2]Na Homolce Hospital, Cardiac Surgery, Prague, Czech Republic
**Correspondence:** A. Krüger
*Intensive Care Medicine Experimental* 2017, **5(Suppl 2):**0540

**INTRODUCTION.** Substantial proportion of patients who suffered cardiac arrest do not respond to conventional cardiopulmonary resuscitation. Recently, extracorporeal cardiopulmonary resuscitation (ECPR) has been introduced as a potentially life-saving procedure in refractory cardiac arrest.

**METHODS.** Eligible patients for this analysis had to undergo ECPR after unsuccessful cardiopulmonary resuscitation with a minimum of three defibrillation attempts. For extracorporeal life support (ECLS) we used Cardiohelp system (Maquet-Cardiopulmonary-AG, Hirrlingen, Germany) or Levitronix CentriMag blood pump (Levitronix LLC, Waltham, MA, USA). LUCAS II (Physio-Control, Lund, Sweden) system was used for chest compressions during ECPR insertion and cannulas were placed with percutaneous puncture under fluoroscopy or ultrasound control. The relations of blood lactate and pH levels, measured before ECPR insertion and after 24 hours as well as comorbities (diabetes, hypertension, BMI) to the clinical outcomes at 3, 6 and 12 months were evalueated.

**RESULTS.** We analyzed data from 29 patients treated with ECPR for refractory cardiac arrest. The mean age of our patients was 58 years (31–81). Out-of-hospital cardiac arrest (OHCA) occurred in 16 patients, 13 patients suffered from in-hospital arrest (IHCA). Baseline value of lactate was 11.52 ± 3.97 mmol/l, initial pH 6.98 ± 0.21. In comparison with survivors, patients who died had significantly higher initial lactate levels (15.05 ± 1.56 vs. 10.01 ± 1.03; P < 0.05) and lower baseline pH (6.87 ± 0.06 vs 7.04 ± 0.04; P < 0.05). Moreover, survivors had significantly lower lactate levels after 24 hours and lower BMI (26.7 vs 33.1; P < 0.05). Diabetes or hypertension in our group have no influence on the mortality. The difference of mortality in the group of OHCA or IHCA was also not significant. With good neurological outcome (CPC 1–2) survive 34% three months, 28% six months and one year 24% of the patients.

**CONCLUSIONS.** ECPR represents virtually the last chance to survive refractory cardiac arrest. The levels of blood lactate, pH are significantly associated with clinical outcomes of ECPR.

#### 0541

#### Lactate values after out-of-hospital cardiac arrest: associations with one-year neurologic outcome

J. Laurikkala[1,2], M. B. Skrifvars[1,3], V. Pettilä[2,4], M. Tiainen[2,5], S. Bendel[6], T. Varpula[1,2], E. Wilkman[1,2], The FINNRESUSCI study group
[1]Helsinki University Central Hospital, Department of Anesthesiology, Intensive Care and Pain Medicine, Helsinki, Finland; [2]University of Helsinki, Helsinki, Finland; [3]Monash University, Melbourne, Australia; [4]Helsinki University Central Hospital, Helsinki, Finland; [5]Helsinki University Central Hospital, Department of Neurology, Helsinki, Finland; [6]Kuopio University Hospital, Kuopio, Finland
**Correspondence:** J. Laurikkala
*Intensive Care Medicine Experimental* 2017, **5(Suppl 2):**0541

**INTRODUCTION.** Out-of hospital cardiac arrest (OHCA) associates with high mortality. Current ERC guidelines recommend targeting normal or decreasing lactate levels during post-resuscitation care. Hyperlactatemia is a well-known marker of tissue hypoperfusion and hypoxia in critically ill patients. Recent data have suggested that early lactate reduction over the first six hours during ICU care is associated with better survival but data on associations with long term outcome are lacking.

**OBJECTIVES.**

To determine the associations between lactate values during the first 72 h after OHCA and one-year neurologic outcome. We hypothesized that high early lactate values after out-of-hospital cardiac arrest are associated with poor long- term neurologic outcome.

**METHODS.** We included 458 OHCA patients with data on lactate values, treated in 19 ICUs from the FINNRESUSCI study between 2010 and 2011. Using separate multivariate backward regression models along with factors at resuscitation, during ICU treatment and SAPS scores, we tested associations with long-term neurologic outcome, dichotomized to good (cerebral performance category (CPC 1–2) and poor (CPC 3–5).

**RESULTS.** Of 458 OHCA patients, 185 (40%) patients had good and 273 (60%) patients had poor one-year outcome. We analyzed 10299 lactate values, a median of 16 (7–35) values per patient. Admission lactate (p < 0.001), the lowest measured lactate (p < 0.001), the highest measured lactate (p < 0.001) and the last measured lactate (p < 0.001) were all significantly higher in patients with poor one-year neurological outcome. Areas under receiver operating characteristic (AUCs) regarding poor one-year outcome were: admission lactate (AUC 0.638, 95% CI 0.588-0.689), time-weighted lactate for ICU stay (AUC 0.654, 95% CI 0.604-0.703), and last measured lactate (AUC 0.719, 95% CI 0.673-0.765). In multivariate backward regression analyses lactate at 72 h (OR 0.80, 95% CI 0.64-0.99, p < 0.01), time-weighted total lactate (OR 0.68, 95% CI 0.52-0.90, p < 0.01) and last measured lactate (OR 0.48, 95% CI 0.27-0.79, p < 0.01) were independent predictors of good one-year outcome.

**CONCLUSIONS.** In this prospective multicenter study, we found that higher lactate at 72 h, higher time-weighted lactate during ICU stay, and higher last measured lactate were independent predictors of poor one-year outcome.

**REFERENCE(S)**

1) Nolan, J. P. *et al.* European Resuscitation Council and European Society of Intensive Care Medicin Guidelinesfor Post-resuscitation Care 2015: Section 5 of the European Resuscitation Council Guidelines forResuscitation 2015. *Resuscitation* 95, 202–222 (2015).

2) Vaahersalo, J. et al. Therapeutic hypothermia after out-of-hospital cardiac arrest in Finnish intensive care unitthe FINNRESUSCI study. Intensive Care Med. 39, 826–837 (2013).

**GRANT ACKNOWLEDGMENT**

HUS funding (TYH2014221), FLS, Medicinska Understödsföreningen Liv och Hälsa, Stiftelsen Dorothea Olivia, Karl Walter och Jarl Walter Perkléns minne

### 0542

#### Prediction of outcomes in cardiac arrest survivors remaining comatose more than four days after collapse: results from a prospective registry

P. Ostadal, D. Vondrakova, A. Kruger, M. Janotka, J. Naar, D. Vondrackova, Z. Vojtech, V. Dudkova, P. Neuzil
Na Homolce Hospital, Prague, Czech Republic
**Correspondence:** P. Ostadal
*Intensive Care Medicine Experimental* 2017, **5(Suppl 2):**0542

**INTRODUCTION.** Current options for outcome prediction in cardiac arrest survivors remain significantly limited. Prognosis assessment is extremely important especially in patients remaining comatose even after the end of temperature intervention and withdrawal of sedation.

**OBJECTIVES.** The aim of our study was to evaluate the prognostic value of individual clinical characteristics and recommended examinations in specific subpopulation of cardiac arrest survivors remaining comatose more than four days after collapse.

**METHODS.** We analyzed data from a single-center prospective registry of out-of-hospital cardiac arrest survivors admitted between 2010 and 2014 to the tertiary cardiovascular center. All subjects were treated with endovascular hypothermia (33°C for 24 hours). Studied population comprises individuals remaining comatose on artificial ventilation more than four days after arrest. We evaluated the prognostic significance of age, gender, initial rhythm, time to return of spontaneous circulation (ROSC), initial levels of serum lactate, potassium, D-dimer and pH, serial measurements of neuron-specific enolase (NSE), C-reactive protein (CRP), procalcitonin, alanine aminotransferase, serum creatinine, and electroencephalography (EEG) in this group. Clinical outcomes were assessed according to the Cerebral Performance Category (CPC) at 30 days (good neurologic outcome was defined as CPC 1–2).

**RESULTS.** A total of 99 patients remaining comatose four days after arrest were included in the study (mean age 65 years, males 64%). Univariate analysis revealed that age, EEG, and values of NSE, initial serum lactate and D-dimer were significantly associated with clinical outcomes. On the other hand, several well-known prognostic factors (e.g. initial rhythm, pH or time to ROSC) were not associated with prognosis in this specific population. Multivariate analysis found that only EEG (“burst-suppression” pattern, P = 0.0007), initial level of D-dimer (P = 0.0009) and NSE value at 72 hours (P = 0.0008) were independent predictors of prognosis in these patients. Receiver operating characteristic analysis revealed that especially EEG pattern and NSE levels had high value for accurate prediction of poor prognosis.

**CONCLUSIONS.** Prognosis prediction is extremely important especially in those cardiac arrest survivors remaining comatose even after withdrawal of sedation. Our results indicate that EEG pattern, levels of D-dimer and NSE are independent predictors of prognosis in this subpopulation of cardiac arrest survivors.

**GRANT ACKNOWLEDGMENT**

The study was supported by an Institutional grant MH CZ - DRO (Nemocnice Na Homolce - NNH, 00023884), IG150501

### 0543

#### Comparing to predicting mortality of scoring systems after CPR

M. Azizoglu, H. Birbicer
Mersin University, Anesthesia and Reanimation, Mersin, Turkey
**Correspondence:** H. Birbicer
*Intensive Care Medicine Experimental* 2017, **5(Suppl 2):**0543

**INTRODUCTION.** Scoring systems have been used for evaluating severity of disseases and predicting mortality. Some of scoring systems also have been developed for predicting mortality and good neurologic outcome after CPR. It has been estimated 192.000 in hospital and 395.000 out of hospital cardiac arrests in the US annually[1,2].

**OBJECTIVES.** We evaluated to success of predicting mortality of ICU and CPR scoring systems after CPR.

**METHODS.** We calculated of 105 patient's GOFAR, PAM, MODS, LODS, SOFA, APACHE II, SAPS II scores after succesful CPR retrospectively after approval ethics committee. We used on the day of resuscitation parameters when calculating scores. Student t test was used to compare mean scores of patients in which survive or death. Receiver operating characteristic curve (ROC) was used to evaluate of scoring systems success on predicting mortality.

**RESULTS.** Characteristics of patients showed in Table 141.

Survived and died patient had statistically significant mean GCS, GOFAR, PAM and SAPS II scores (Table 142) ($p < 0.05$).

Predicting in first 3 day mortality succes of GOFAR, PAM and SAPS II were calculated statistically significant. (AUC values; GOFAR:0.629, PAM:0.691, SAPSII:0.653 respectively, $p < 0,05$). There was no difference in terms of success of predicting mortality in three scoring systems (Fig. 192).

**CONCLUSIONS.** We found SAPS II could be an helpful instrument to evaluate mortality after CPR near to GO-FAR and PAM. More studies needed to evaluate scoring systems and mortality rate to prevent poor neurologic outcome after resuscitation.

**REFERENCE(S)**

1. Merchant RM, Yang L, Becker LB, Berg RA et al. Incidence of treated cardiac arrest in hospitalized patients in the United States. Crit Care Med. 2011;39(11):2401–6.

2. Daya MR, Schmicker RH, Zive DM, Rea TD et al. Out-of-hospital cardiac arrest survival improving over time: Results from the Resuscitation Outcomes Consortium (ROC). Resuscitation. 2015;91:108–115.

**Table 141 (Abstract 0543).** Patients characteristics

| | |
|---|---|
| Age(year) | 59.65±20.46 |
| Gender (M/F) | 69/36 |
| Hospital discharge (day) | 14.66±16.50 |
| First documented rhythm(n:105) Asystole and PEA VF and pulseless VT Unknown | 74 (70%) 12 (12%) 19 (18%) |
| Survive in 3 days (n:105) | 83 (79%) |
| Survive in 7 days (n:105) | 70 (66%) |
| Outcome (n:105) Discharge with neurologically disability Discharge without neurologically disability Death | 7(7%) 17(16%) 81 (77%) |
| Location of arrest (n:105) In hospital Out of hospital | 54 (53%) 49 (47%) |
| Duration of Resuscitation (min) | 18.75±11.71 |

**Table 142 (Abstract 0543)**. Mean scores of survived and death patients

| | Survived | Death |
|---|---|---|
| Age (year) | 54.50±4.03 | 61.18±2.78 |
| GCS | 3.81±0.35α | 3.08±0.04 |
| GOFAR | 1.41±2.38α | 6.29±1.29 |
| PAM | 1.7±0.84α | 4.83±0.58 |
| APACHE II | 28.79±1.23 | 30.59±0.84 |
| LODS | 7.83±0.41 | 8.71±0.31 |
| SOFA | 11.33±0.61 | 12.43±0.37 |
| SAPS II | 59.13±2.16α | 68.72±1.88 |
| MODS | 7.08±0.57 | 7.66±0.22 |

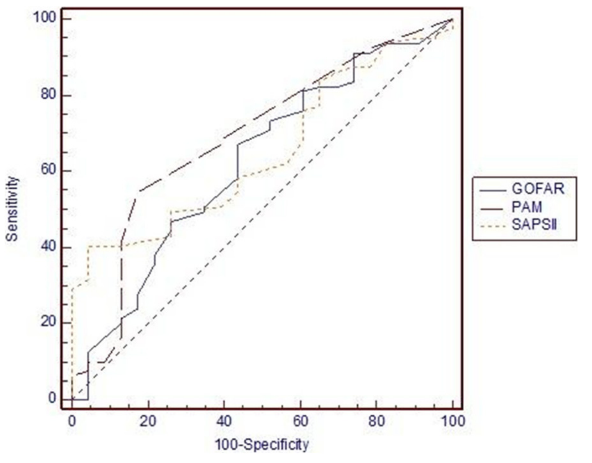

**Fig. 192 (Abstract 0543).** ROC curve of GOFAR, PAM and SAPS II

### 0544

#### Air ambulance and out-of-hospital cardiac arrest: ´highflying´ outcomes?

H. Garland[1], R. Green[2], T. Gauss[2], A. Downes[2], A. Wilson[2], W. Davies[3], A. Vuylsteke[1,3]
[1]University of Cambridge, Cambridge, United Kingdom; [2]East Anglian Air Ambulance, Cambridge, United Kingdom, [3]Papworth Hospital NHS Foundation Trust, Cambridge, United Kingdom
**Correspondence:** H. Garland
*Intensive Care Medicine Experimental* 2017, **5(Suppl 2):**0544

**INTRODUCTION.** Out-of-hospital cardiac arrest (OOHCA) remains associated with significant mortality. Guidelines suggest that all non-traumatic OOHCA patients should be cared for at a centre with ICU and primary percutaneous coronary intervention (PPCI) services. Air ambulance transfer can facilitate this. We analysed data from all OOHCA patients who were treated by advanced practitioners from the air ambulance service before being transferred to a tertiary regional specialist cardiac centre.

**OBJECTIVES.** Determine current rates of survival to discharge and sought to identify novel prognostic indicators for survival to discharge post cardiac arrest.

**METHODS.** A retrospective analysis of all consecutive hospital records extracted from our regional Air Ambulance service and tertiary regional angioplasty centre databases. Data presented as mean ± SEM.

**RESULTS.** Out of all OOHCA patients studied, 15/27 (55.6%) had ROSC before and 5/27 (18.5%) after arrival of the air ambulance and 1/27 (3.7%) achieved ROSC during PPCI. All patients achieved ROSC for a period, although this was short in some cases, however the location of ROSC was not recorded in 6/27 (22.2%) cases. 25/27 (92.6%) underwent coronary angiography and 13/25 (52%) went on to have stenting performed. Patients who were discharged alive spent an average of 8.5 ± 1.7 days in hospital including an average of 5.9 ± 1.4 days in ICU. All patients who died did so in ICU following an average length of stay of 2.7 ± 0.7 days. None of the patients died more than 6 days after admission. Overall, 18/27 (66.7%) of patients survived. Multivariate analysis confirmed that suffering a witnessed arrest and receiving bystander CPR are both independently associated with higher likelihood of achieving pre-hospital ROSC.

**CONCLUSIONS.** Current survival figures are comparable to previously published data in relation to out of hospital cardiac arrests attended by air ambulance services (1). Only around half the patients had stenting performed but we were unable to identify on-the-scene variables predicting requirement for the need of coronary stenting or for survival. This is in keeping with other previous studies that have shown that ECG on ROSC is a poor predictor of STEMI (2). Pre-hospital evaluation by echocardiography or use of advanced electrocardiographic techniques might be considered.

**REFERENCE(S)**

1. Chesters A, Harris T, Hodgetts TJ, Keefe N. Survival to discharge after cardiac arrest attended by a doctor-paramedic helicopter emergency medical service: An Utstein-style multiservice review of 1085 activations. Journal of Emergency Medicine. 2015;49(4):439–47.

2. Salam I, Hassager C, Thomsen JH, Langkjaer S, Soholm H, Bro-Jeppesen J, et al. Editor´s Choice-Is the pre-hospital ECG after out-of-hospital cardiac arrest accurate for the diagnosis of ST-elevation myocardial infarction? Eur Heart J Acute Cardiovasc Care. 2016;5(4):317–26.

**GRANT ACKNOWLEDGMENT**
None

### 0545

#### Multivariate analysis of time-course of veno-arterial extracorporeal membrane oxygenation used in patient with cardiac arrest

D. F. Ottolina, A. Corona, T. Fossali, A. Perotti, R. Colombo, R. Rech, P. Bergomi, B. Borghi, E. Ballone, A. Castelli, E. Catena
ASST Fatebenefratelli Sacco, Anestesia Rianimazione i Ospedale Sacco, Milano, Italy
**Correspondence:** D. F. Ottolina
*Intensive Care Medicine Experimental* 2017, **5(Suppl 2):**0545

**INTRODUCTION.** In recent years veno-arterial extracorporeal membrane oxygenation (VA-ECMO) has been more frequently used for treatment of refractory cardiac arrest (CA) as the last step of cardiopulmonary resuscitation (ECPR), with different results between studies regarding outcomes and prognostic factors (1).

**OBJECTIVES.** To evaluate possible predictors of mortality in ECPR along the whole chain of survival, from the event to ECMO, in Table 143 a metropolitan Italian city.

**METHODS.** Among all VA-ECMO performed in a ECMO-tertary single-center hospital from 2014 to April 2017, we have considered those used for ECPR, in a setting of CA. VA-ECMO was performed in a standardized protocol, maintaining 36°C of internal body temperature, positioning intra-aortic balloon pump and inotropic support with epinephrine. An electronic-dedicated database was created to prospectively collect clinical and pathophysiological variables.

**RESULTS.** We analyzed data from 30 ECPR, two of these were excluded for impossibility to ECMO implantation. 26 patients had Out-of-Hospital cardiac arrest (OHCA), 2 patients In-Hospital cardiac arrest (IHCA). The overall hospital-survival and within 24-hours were respectively 4% and 36%. When we have analysed the time of the various steps of the survival chain up to ECPR, we have registered a time-to first shock of 7 [5–12] minutes, a time-to ACLS of 16 [12–20] minutes and a time-to hospital of 55 [45–59] minutes (median, 25th-75th perc). The time-to-ECMO for OHCA is averagely the same of literature reports (2–3), with an incannulation time of 30 [23–44] minutes. At the end no-flow time was 5 [0–10] minutes, low-flow time 80 [69–88] minutes. When performed a multivariate analysis, the main factors predicted 24-hours survival was low-flow (Cox's Regression-Table 143).

**CONCLUSIONS.** As reported in this work the key variable to reduce unfavourable 24-hour outcome was the reduction of pre-hospital timing, in particular low-flow time, emphasizing, at the same time, that witnessed-CA, with an immediate RCP leading to zero no-flow time, and the presence of automatic devices are essential preconditions to increase the survival rate. We consider that protocols with targeted timing should be used in order to obtain better outcomes including the possibility to position VA-ECMO circuit on the place of the event.

**REFERENCE(S)**

[1] Extracorporeal life support during cardiac arrest and cardiogenic shock: a systematic review and meta-analysis. Ouweneel DM, Schotborgh JV, Limpens J, et al. Intensive Care Med. 2016 Dec;42(12):1922–1934.

[2] *Extracorporeal life support following out-of-hospital refractory cardiac arrest.* Le Guen M, Nicolas-Robin A, Carreira S, et al. Crit Care. 2011;15(1):R29.

[3] *Favourable survival of in-hospital compared to out-of-hospital refractory cardiac arrest patients treated with extracorporeal membrane oxygenation: an Italian tertiary care centre experience.* Avalli L, Maggioni E, Formica F, et al. Resuscitation. 2012 May;83(5):579–83

**Table 143 (Abstract 0545)**. See text for description

| Variables | P | HR | 95% CI for HR | |
|---|---|---|---|---|
| RCPby-stander | 0.706 | 0.71 | 0.11 | 4.3 |
| Automatic RCP devices | 0.239 | 0.22 | 0.12 | 2.8 |
| Low Flow | 0.001 | 1.22 | 1.1 | 1.4 |
| Age | 0.40 | 1.06 | 1.003 | 1.128 |

### 0546

#### Well controlled peripheral and core temperature reduces shivering of therapeutic hypothermia for post cardiac arrest syndrome

H. Arimoto, H. Rinka, T. Miyaichi, A. Fuke, J. Ishikawa, T. Morooka, K. Shigemitsu, A. Tsuruoka, T. Morimoto, K. Suzuki, T. Yamashita, H. Tanaka
Osaka City General Hospital, Emergency and Critical Care Medical Center, Osaka, Japan
**Correspondence:** H. Arimoto

*Intensive Care Medicine Experimental* 2017, **5(Suppl 2):**0546

**INTRODUCTION.** Mild therapeutic hypothermia has been integrated into management strategies for improving the neurological outcome of out-of-hospital cardiac arrest. However, the shivering leads difficult body control during therapeutic hypothermia (TH).

**OBJECTIVES.** We monitored peripheral and core temperature to achieve adequate peripheral perfusion. This study aims to evaluate the dissociation between peripheral and core temperature affects temperature control during TH for post cardiac arrest syndrome (PCAS) in ICU.

**METHODS.** From January 2014 to March 2017, 23 patients were treated with mild hypothermia (34 degrees centigrade, for 24 hours). Each patients were inducted with Arctic-sun®, Thermogard XP®, or ECMO for unconscious out-of-hospital-cardiac arrest patients of PCAS. All patient were measured both peripheral and bladder temperature as core temperature. Analgo-sedation were administered during TH, however, neuro-muscular blocking agent was not given routinely. We divided 23 patients into Group C (well controlled peripheral temperature) and Group U (uncontrolled peripheral temperature). Ventilator associated pneumonia, shivering and neurological outcomes were evaluated. Shivering was evaluated with bedside shivering assessment scale. A favorable outcome was defined as a Cerebral Performance Category (CPC) of 1–2.

**RESULTS.** As compared with group C (N = 10), group U (N = 13) had higher rates of shivering (20% vs. 62%, $p < 0.05$). Skin counter warming was used higher rate in Group C (60% vs. 15%, $p < 0.05$). There were no significant differences between the two groups about pneumonia and favorable outcomes.

**CONCLUSIONS.** Though we cannot found the advantage of control peripheral temperature, the dissociation between peripheral and core temperature increases the rate of shivering as results. In addition, skin counter warming is useful method to achieve good peripheral perfusion.

### 0547

#### Predictors of ischemic brain injury in survivors of out-of-hospital cardiac arrest

A. Sinkovic[1,2], A. Markota[1]
[1]University Clinical Centre Maribor, Department of Medical ICU, Maribor, Slovenia; [2]Medical Faculty of University of Maribor, Maribor, Slovenia
**Correspondence:** A. Sinkovic

*Intensive Care Medicine Experimental* 2017, **5(Suppl 2):**0547

**INTRODUCTION.** Ischemic brain injury is one of the most important postresuscitation syndrome in patients after out-of-hospital cardiac arrest (OHCA) and associated with increased mortality.

**OBJECTIVES.** To evaluate independent predictors of ischemic brain injury in successfully resuscitated OHCA patients, admitted to medical ICU.

**METHODS.** We retrospectively included 119 successfully resuscitated OHCA patients, admitted in 2011–2013 (73.1% men, mean age 64 ± 13,5 years). Therapeutic hypothermia within the first 24 hours, early treatment of acute coronary syndromes, prevention of recurrent cardiac arrests, maintainance of adequate ventilation and circulation were among measures in the ICU to improve the outcome. Neurological outcome was classified by cerebral performance category (CPC) scale 1–5. Significant ischemic brain injury was defined by CPC scale 3–5 and included patients with brain death, vegetative state or other severe impairment of cerebral function. Good neurological recovery was defined by CPC scale 1–2 and included minor to intermediate brain injury with the ability for independent life. Predictors of brain injury were tested by logistic regression.

**RESULTS.** Among successfully resuscitated OHCA patients, admitted to ICU good neurological recovery was observed in 35.3% and significant ischemic brain injury in 64.7%. Patients with significant brain injury in comparison to those with good neurological outcome were significantly older (66 ± 12.9 years vs 60.2 ± 13.9 years, $p < 0.05$), resuscitated longer (8.3 ± 5 min vs 4.3 ± 4.8 min, $p < 0.05$), less likely due to ventricular fibrillation or tachicardia (VF/VT) (45.5% vs 71.4%, $p < 0.05$), but more likely due to asystole (53.2% vs 21.4%, $p < 0.05$), with significantly increased mean admission lactate level (7.5 ± 4.3 mmol/l vs 4.2 ± 2.9 mmol/l, $p < 0.05$), less likely performed PCI (32.5% vs 54.8%, $p < 0.05$), more likely observed heart failure (96,1% vs 83.3%, $p < 0.05$) and with increased in-hospital (75.3% vs 7.1%, $p < 0.05$) and 6-month mortality (77.9% vs 7.1%, $p < 0.05$). There were nonsignificant differences in comorbidities, achieved hypothermia < 34°C and hypothermia < 36°C. Independent predictors of ischemic brain injury were age (OR 1.044, 95% CI 1.000 to 1.090, p = 0.048), admission lactate (OR 1.241, 95% CI 1.007 to 1.528, p = 0.042) and resuscitation time (OR 1,106; 95% CI 1.015 to 1,205, p = 0.022).

**CONCLUSIONS.** Among several predictors only older age, increased admission serum lactate and resuscitation time independently predicted ischemic brain injury in our OHCA patients.

**REFERENCE(S).**
1. Nolan JP, Soar J, Cariou A, et al. European Resuscitation Council and European Society of IntensiveCare Medicine Guidelines for Post-resuscitation Care 2015. Section 5 of the European Resuscitation Council Guidelines for Resuscitation 2015. Resuscitation 2015; 95: 202–22.

### 0548

#### Prognostic value of early brain computed tomography after successful cardio-pulmonary resuscitation

A. Margaritis[1], M. Bolosi[1], E. Kostami[2], E. Kosma[1], D. Lepida[1], O. Ygropoulou[1], D. Theodorou[2], G. Papadopoulos[3]
[1]General Hospital of Ioannina 'G Hatzikosta', ICU, Ioannina, Greece; [2]General Hospital of Ioannina 'G Hatzikosta', CT-MRI, Ioannina, Greece; [3]Ioannina University Hospital, Anesthesiology, Ioannina, Greece
**Correspondence:** A. Margaritis
*Intensive Care Medicine Experimental* 2017, **5(Suppl 2):**0548

**INTRODUCTION.** Cardiac arrest is a disastrous event and is related with high mortality. Some of the patients will survive and will be admitted in ICU. The most important cause for a poor prognosis in these patients, who will survive initially, remains the post-cardiac arrest syndrome. This is a pathophysiological situation characterized by cerebral damage, myocardial disfunction and systemic ischemia. The cerebral damage remains the most common cause of death in these patients.

**OBJECTIVES.** We designed this clinical research to study the prognostic value of early brain CT scan in patients' outcome after cardiac arrest.

**METHODS.** Our study included 19 patients. These patients developed spontaneous circulation (SC) after successful CPR and received a brain CT scan within 1 hour after the return of SC. Then, they were admitted in ICU of our hospital. After that, we recorded if the CT was positive or negative for brain damage, and the final outcome of these patients. We hypothesized as positive CT the loss of gray-white matter differentiation or/and basilar cistern or/and sulcal effacement (Figs. 193 and 194), and negative without any recently findings.

**RESULTS.** 19 patients were included in our study with min age 69 ± 16 years old (min 20, max 87). The duration of their hospitalization in ICU was 12 ± 10 days.

In 7 (37%) patients brain damage in cerebral CT was observed. In the rest 12 (63%) patients the cerebral CT was negative for brain damage. All the 7 patients with positive CT did not survive. From the 12 patients with negative CT only 3 survived and left ICU in good mental status.

**CONCLUSIONS.** From the above mentioned results, we can conclude that all patients with positive CT did not survive. It is obvious that there is a correlation between brain damage and mortality, so we can say that in our study the cerebral CT has a positive prognostic value of 100%. From the patients with negative CT survived only 25%. That means that the examination has poor negative prognostic value (25%), due to the fact that most patients with negative CT for brain damage had the worst outcome. To sum up, the cerebral CT in the first hour after successful CPR in patients after cardiac arrest may be a useful tool for the prognosis of these patients only when findings of brain damage appear.

**REFERENCE(S)**
1. SooHyun Kim, et al.: Early brain computed tomography findings are associated with outcome in patients treated with therapeutic hypothermia after out-of-hospital cardiac arrest. Scand J Trauma Resusc Emerg Med. 2013;21:57
2.Taccone F, et al.: How to assess prognosis after cardiac arrest and therapeutic hypothermia. Crit Care, 2014;18(1):202

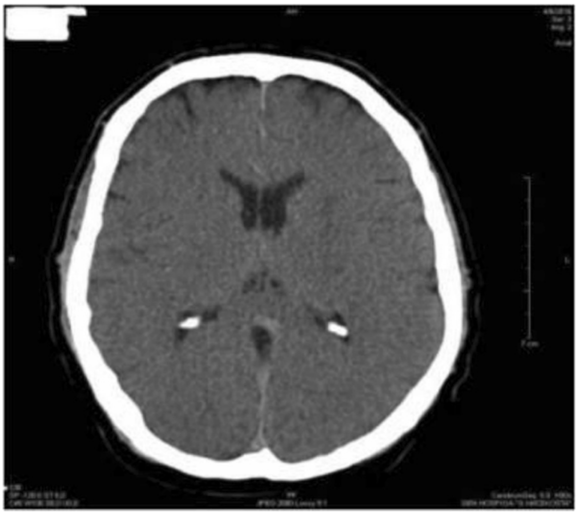

**Fig. 193 (Abstract 0548).** See text for description

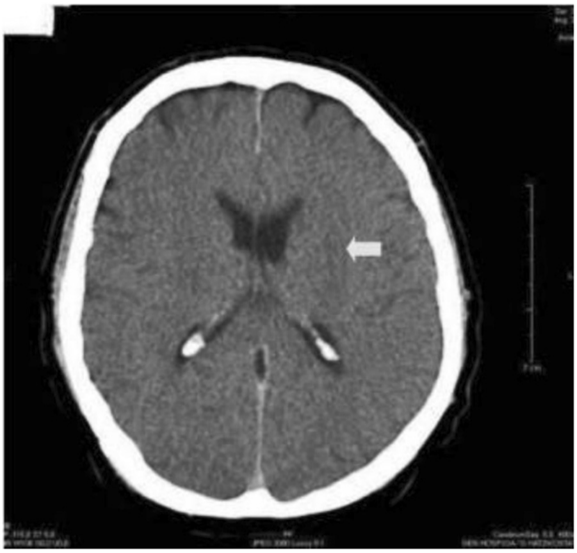

**Fig. 194 (Abstract 0548).** See text for description

### 0549

#### Hippocampal slice cultures as an *in vitro* model for ischemic brain damage induced by cardiac arrest

P. Meyer[1,2,3], D. Grandgirard[1], M. Haenggi[2], S.M. Jakob[2], J. Takala[2], S.L. Leib[1]
[1]University of Bern, Institute for Infectious Diseases, Neuroinfection Laboratory, Bern, Switzerland; [2]University of Bern, Bern University Hospital, Departement of Intensive Care Medicine, Bern, Switzerland; [3]University of Bern, Graduate School for Cellular and Biomedical Sciences, Bern, Switzerland
**Correspondence:** P. Meyer
*Intensive Care Medicine Experimental* 2017, **5(Suppl 2):**0549

**INTRODUCTION.** Sudden cardiac arrest (CA) is the most important cause of global cerebral ischemia. Due to a lack of effective therapies to treat the subsequent brain damage, most patients are left with incomplete neurological recovery. Several brain regions, including the cornu ammonis field 1 (CA1) of the hippocampus are particularly affected by a transient hypoxic-ischemic insult. This "selective vulnerability" is observed in rodent models as well as in humans.

**OBJECTIVES.** To develop an *in vitro* surrogate for the *in vivo* rat model of cardiac arrest/resuscitation, using organotypic hippocampal slices, and test it for regenerative therapy by neuronal progenitor cells (NPCs) grafting.

**METHODS.** Hippocampi from rats pups are cut into slices, cultivated for one week, and then subjected to oxygen-glucose deprivation (OGD) to reproduce *in vitro* the hypoxic-ischemic injury observed after CA. Duration of OGD is optimized to reproduce the extent and pattern of damage observed *in vivo*. Neuronal damage is quantified by Fluoro-Jade B (FJ) staining, specific for degenerating neurons. For transplantation experiments, NPCs are isolated from hippocampi of newborn rats, expanded as neurospheres for one week and grafted into injured cultures. Their survival, differentiation and migration are assessed by immunohistochemistry (IHC).

**RESULTS.** Organotypic cultures submitted to 33 minutes of OGD developed hippocampal damage to a similar extent as observed in the *in vivo* model. A significantly higher amount of FJ-positive cells was found after OGD in the CA1 segment compared to the normoxic control (582 ± 244 FJ+ cells/mm$^2$, n = 9 versus 155 ± 84 FJ+ cells/mm$^2$, n =5; P = 0.0034). The cellular composition of the neurospheres, as tested by IHC, showed the presence of numerous nestin- and doublecortin-positive cells, indicating NPCs. After NPCs transplantations into OGD-injured hippocampal cultures, viable cells were found at different time points after grafting, confirming the overall feasibility of the procedure.

**CONCLUSIONS.** We could successfully reproduce damage to CA1 neurons of the hippocampus *in vitro* after OGD, as observed *in vivo* after CA. Neurospheres were mostly composed of neuronal progenitors, as confirmed by IHC. The survival, migration and differentiation of grafted cells are currently assessed using NPCs isolated from Green Fluorescent Protein transgenic rats, therefore facilitating the identification of the grafted cells. This project represents a first step in the development of a cell-based regenerative therapy after global cerebral ischemia consecutive to CA.

**GRANT ACKNOWLEDGMENT**

This project is mainly funded by the Department of Intensive Care Medicine, Inselspital, Bern University Hospital. Additional funding is provided by the Swiss National Science Foundation grant nr. 310030–162583.

### 0550

#### The EFFECCT (Electronic feedback for effective chest compressions) trial - a pilot study

W.Y. Lim[1], H.L. Tan[1], V.K. Ho[2]

[1]Singapore General Hospital, Singapore, Singapore; [2]Singapore General Hospital, Anaesthesia and Intensive Care, Singapore, Singapore

**Correspondence:** W.Y. Lim

*Intensive Care Medicine Experimental* 2017, **5(Suppl 2):**0550

**INTRODUCTION.** Cardiac arrest survival is determined by the 4 links within the chain of survival: early access, early CPR, early defibrillation and post-resuscitation care. Studies have shown that early cardiopulmonary resuscitation (CPR) with minimal interruption is crucial for improving cardiac arrest survival rates. One of the determinants for successful resuscitation is effective CPR with adequate depth (5-6cm) and rate (at least 100 per min) while allowing complete chest recoil. Current AHA guidelines recommend that rescuers switch every 2 minutes, while allowing assessment of ECG rhythm, despite the harmful effect of interrupting chest compressions. This also allows recognition of fatigue and replacement of the rescuer to maintain optimal chest compressions. A variety of feedback devices have been designed to improve the quality of chest compressions.

**METHODS.** We evaluated the quality of chest compressions using an electronic feedback device in a simulated resuscitation during a 1-week period. The monitoring device was a ZOLL 'R' series defibrillator with an accelerometer, providing real-time audio-visual feedback prompts. 40 participants were grouped in pairs, resulting in 20 pairs. Each pair performed two rounds of chest compressions and defibrillation according to ACLS protocol on a manikin in 10-minute resuscitation periods.

**RESULTS.** In the pairs with no feedback first (NFF), overall CPR performance was poor. However, with the use of feedback, the quality of chest compressions improved from 11.8% to 58.1%. In the pairs with feedback first (FF), percentage of quality chest compressions improved from from 24.7% to 47.1%. The results emphasized the variability of CPR quality between clinicians and the need for CPR coaching, which may translate to improved patient outcomes in the clinical setting. Our results showing an improvement in the quality of CCs induced by CPR feedback, were consistent with those previously reported. To our knowledge, our study is the first to date to evaluate the effect of chest compression quality in a continuous 10-minutes x2 simulation (with a short 5-minute interval), mirroring clinical scenarios where resuscitations often last longer than 2–5 minutes in previously published studies.

**CONCLUSIONS.** Chest compressions are the cornerstone to successful cardiopulmonary resuscitation. Good quality chest compression is vital for patient survival and improved neurological outcomes following cardiopulmonary arrest. Our study showed significant variation in chest compression quality among clinicians with room for improvement. The ability to improve clinicians' performance of chest compressions with technological advancements (CPR feedback device) and training may translate to better survival outcomes in real patients in the future. Good quality CPR continues to be a focus of quality assurance and improvement programs.

### 0551

#### Factors associated with successful on-site return of spontaneous circulation by automated external defibrillator

C.-C. Chao[1,2], W.-F. Kao[1,2], T.-H. Wang[3,4]

[1]Taipei Medical University, Taipei, Taiwan, Province of China; [2]Taipei Medical University Hospital, Taipei, Taiwan, Province of China; [3]National Taiwan University, Taipei, Taiwan, Province of China; [4]Ministry of Health and Welfare, Department of Medical Affairs, Taipei, Taiwan, Province of China

**Correspondence:** C.-C. Chao

*Intensive Care Medicine Experimental* 2017, **5(Suppl 2):**0551

**INTRODUCTION.** Many countries have legislation for encouraging the use of automated external defibrillators (AEDs) to reduce the mortality of out-of-hospital cardiac arrest (OHCA) patients. Taiwan Legislature passed amendments to the Emergency Medical Services Act, thereby requiring the installation of AEDs in mandatory areas in 2013.

**OBJECTIVES.** Our study aims to analyze the efficacy of the policy regarding AED installation.

**METHODS.** 215 cases received AED treatment was reviewed between July 11, 2013 and July 31, 2015. The data was reported by lay persons or supervisors of public facilities; however, only 124 of all cases had complete data. The analysis of data was performed using SPSS 14.0.

**RESULTS.** Trained responders rescued 139 (64.7%) cases. Approximately 55% cases were male. In mandatory areas, schools, mass gathering places, and special institutions are places with most usage of AEDs, accounting 33 cases (15.3%). Long-term care facilities had the most cases among the non-mandatory areas, 32 cases (14.9%). The odds for long distance transport were lower than those for commuting stations, 0.481 (95% CI, 0.24 ~ 0.962). The odds ratio for commuting stations and schools, large -scale gathering places, and special institutions was 4.474 (95% CI, 2.497 ~ 8.015). Non-mandatory areas had a higher frequency of AED use, 4.259 (95% CI, 2.223 ~ 8.159).

| District | Value | 95% CI |
|---|---|---|
| Non-mandatory area + V.S. Mandatory areas | 2.302 | 1.407-3.768 |
| Mandatory areas: compared with commuting station | | |
| Long distance transport | 0.481 | 0.24-0.962 |
| Sightseeing area | 1.978 | 0.906-4.318 |
| School, mass gathering place, and special institution | 4.474 | 2.497-8.015 |
| Large entertainment facility | 1.546 | 0.648-3.688 |
| Large shopping mall | 1.847 | 0.733-4.656 |
| Hotel | 1.324 | 0.525-3.337 |
| Hot spring area | 0.696 | 0.196-2.476 |

*[Odds ratio of AED usage]*

**CONCLUSIONS.** The policy regarding AEDs improved the prevalence. However, more AEDs are required in schools, mass gathering places, and long-term care facilities. Furthermore, mandatory areas should include long-term facilities.

**REFERENCE(S)**

1. McNally B, Robb R, Mehta M, et al. Out-of-hospital cardiac arrest surveillance — Cardiac Arrest Registry to Enhance Survival (CARES), United States, October 1, 2005–December 31, 2010. MMWR Surveill Summ. 2011 Jul 29;60(8):1–19.GRANT

### 0552

#### Extracorporeal life support in polytrauma patients with acute respiratory failure

R. Avila[1], N. Carrizo[1], A. Fernadez[1], M. Filippi[1], C. Casabella[2], F. Palizas[2]
[1]Hospital Cullen, Santa Fe, Argentina; [2]Clinica Bazterrica, Buenos Aires, Argentina
**Correspondence:** R. Avila
*Intensive Care Medicine Experimental* 2017, **5(Suppl 2):**0552

**INTRODUCTION.** On average 20 people per day die in traffic accidents, and major trauma is the leading cause of death of young adults in Argentina. New approaches in trauma care and advanced treatments are needed to improve the actual therapeutic strategy and treatment protocols. Extracorporeal life support (ECLS) has proven to be effective in cases of shock (in venous-arterial modality, VA-ECLS) and pulmonary failure (in Venous-Venous modality, VV-ECLS or ECMO), when standard therapies have failed. However, the need for anticoagulation has historically limited the use of ECLS in polytrauma patients because of the increased risk of bleeding. We report our initial experience with ECLS,which would be the first cases reported in our country as a rescue therapy in severely polytraumatized patients in a refractory clinical setting.

**OBJECTIVES.** To evaluate the feasibility of performing ECMO in severe trauma patients with acute respiratory failure to whom the optimal ventilator strategies and adjunctive measures had failed. Also describe the outcome of patients and hemorrhagic complications during the procedure.

**METHODS.** We performed a retrospective analysis of ECMO patients who had severe polytrauma with acute respiratory failure at a tertiary-level referral trauma center. Demographic, severity of illness and outcome data were recorded, and we also described ECMO configuration used and hemorrhagic complications.

**RESULTS.** We performed 10-VV ECMO during a 2-year period. The mean age of the survivors was 21 years vs. 29 years for non-survivors and the mean time of ECLS treatment was 9 days in survivors vs. 3 days in non-survivors.The ISS and APACHE II were 38, 17.25 in survivors vs 42, 19.4 in non-survivors. Out of 10 patients, 5 patients (50%) were discharged from the hospital. All patients were treated with intravenous heparin with a mean onset of 12.5 h from the beginning of ECMO, and the mean dose of heparin was 18.25 u/kg. Two patients required transient anticoagulation discontinuation due to episodes of major bleeding, which was subsequently restarted. In patients efficiently supported by ECLS, the cardiac index, mean arterial pressure, blood lactate concentration, arterial oxygen tension, arterial carbon dioxide tension, and pH all showed significant improvement within the first 6 hours.

**CONCLUSIONS.** In our review, we conclude that the use of ECMO in polytrauma patients who develop refractory respiratory distress or high-debit pleural fistulas is a feasible therapy if it is performed by a properly trained team and with close monitoring of anticoagulation.

**REFERENCE(S)**

1. Advanced extracorporeal therapy in trauma. Curr Opin Crit Care 2016, 22:578–583
2. Extracorporeal life support in patients with severe trauma: An advanced treatment strategy for refractory clinical settings. J Thorac Cardiovasc Surg 2013;145:1617–26

### 0553

#### Laparotomy following cardiopulmonary resuscitation after traumatic cardiac arrest: is it futile?

J. Kim, Y. Park, W. Kang, Y. Jo
[1]Chonnam National University Hospital/Chonnam National University Medical School, Department of Surgery, Gwangju, Korea, Republic of
**Correspondence:** Y. Park
*Intensive Care Medicine Experimental* 2017, **5(Suppl 2):**0553

**INTRODUCTION.** Survival of cardiopulmonary resuscitation (CPR) for traumatic cardiac arrest patients is very poor. Furthermore, some consider laparotomy for abdominal trauma after CPR futile.

**OBJECTIVES.** This study aimed to describe the outcomes for victims of trauma who is pulseless and received CPR with laparotomy.

**METHODS.** We conducted a retrospective review of medical records of our hospital from January 2009 to March 2016. Patient's demographics, injury severity score, CPR time, operative data and mortality data were collected and analysis.

**RESULTS.** 385 patients underwent laparotomy due to trauma. Sixty two (16.1%) patients died after laparotomy. Sixteen patients underwent CPR at emergency room and after successful CPR, these patients underwent laparotomy for treatment of abdominal injuries. Among these patients, fifteen patients (93.7%) died after laparotomy, and only one patient (6.2%) survived who suffered by iliac artery injury. Five (5/15, 33.3%) patients had a length of stay of < 1 day, Three patients (3/15, 20.0%) had a length of stay of >7 days. Mean time from injury to admission was 172 minute. All patients were victims of blunt injury.

**CONCLUSIONS.** Survival rate of laparotomy following CPR after traumatic cardiac arrest was very poor. But this modality is not always futile. And more improvement of emergency transport system to trauma center seems to be needed.

**REFERENCE(S)**

WILLIS, Cameron D., et al. Cardiopulmonary resuscitation after traumatic cardiac arrest is not always futile. *Injury*, 2006, 37.5: 448–454.

**GRANT ACKNOWLEDGMENT**
None.

## Intensive care after cardiothoracic surgery

### 0554

#### Early post-operative lung ultrasound to predict respiratory distress

E. Roldi[1], O. Dransart-Raye[2], S. Mongodi[1], F. Mojoli[1], B. Bouhemad[2]
[1]Fondazione IRCCS Policlinico S. Matteo, Anesthesia and Intensive Care, University of Pavia, Pavia, Italy; [2]CHU Dijon Bourgogne, Anesthesia and Intensive Care, CHU Dijon, Dijon, France
**Correspondence:** E. Roldi
*Intensive Care Medicine Experimental* 2017, **5(Suppl 2):**0554

**INTRODUCTION.** Acute respiratory Distress (ARD) is a condition rarely present before an elective surgical procedure and should be a preventable postoperative complication. Extravascular lung water

(EVLW) is an early marker of lung edema and ARD[1,2]. Lung ultrasound (LUS) has proven to be a reliable tool in assessing EVLW and may help in early identification of postoperative ARD (PARD)[3]

**OBJECTIVES.** Evaluating the predictive value of EVLW assessed by LUS as an early marker of PARD in patients admitted to ICU after major surgery.

**METHODS.** In adult patients admitted to ICU after major surgery, LUS was performed at admission: a LUS score ranging from 0 to 36 was computed on the basis of percentage of occupied pleura [4] in the 12 standard thoracic areas. PARD was defined as length of mechanical ventilation >6h or need for non-invasive ventilation within 48 hours after extubation.

**RESULTS.** We prospectively enrolled 139 patients (age 65 ± 13; BMI 27 ± 6; male 59%, ASA score 3 [2–3]): 32 patients (23%) presented PARD. LUS score was significantly higher in patients who developed PARD compared to the non-PARD (11 ± 4 vs. 9 ± 4; p = 0.001). A LUS score >6 was associated with a prolonged mechanical ventilation and a longer ICU stay (Figs. 195 and 196). Fluid resuscitation during surgery didn't differ between the 2 groups; on the contrary, transfusions were significantly more frequent in the PARD group (59% of patients vs. 32%, p = 0.005)(Table 144).

**CONCLUSIONS.** EVLW assessed by LUS seems to be an early marker of PARD in patients admitted to ICU after major surgery

**REFERENCE(S)**

1. Kor et al., Crit Care Med 2015;
2. Chew et al., Crit Care 2012;
3. Zhao et al., BMC Pulm Med 2015;
4. Mongodi et al., Eur J Ultrasound 2017

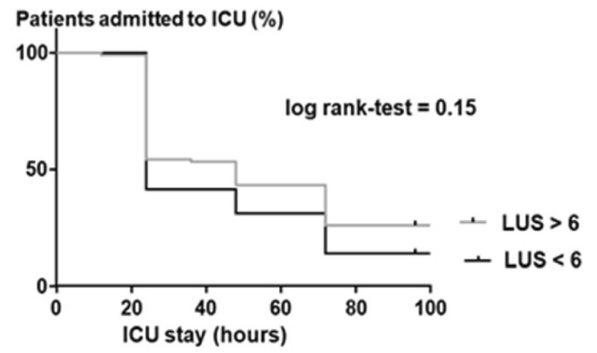

**Fig. 195 (Abstract 0554).** ICU stay in function of LUS score

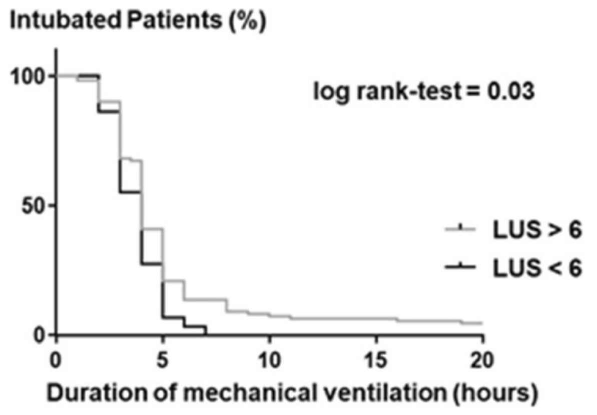

**Fig. 196 (Abstract 0554).** Duration of MV in function of LUS score

**Table 144 (Abstract 0554).** Differences between NON-PARD and PARD group

| | | Non-PARD(N = 107) | PARD (N = 32) | p |
|---|---|---|---|---|
| Fluid Input | mean ± SD | 3665 ± 1857 | 3801 ± 2475 | 0.73 |
| Transfusions | n (%) | 34(32) | 19(59) | 0.005 |
| LUS score | mean ± SD | 9 ± 4 | 11 ± 4 | 0.001 |
| ICU stay (hours) | median (IQR) | 24(24–72) | 95(72–102) | <0.0001 |
| Mortality at day 8 | n (%) | 0(0) | 0(0) | n.s. |
| Mortality at day 28 | n (%) | 0(0) | 1(3) | n.s. |

### 0555

#### Mechanical power in obese patients during general anesthesia: how much energy?

D. Chiumello[1], M. Guanziroli[1], M. Gotti[2], A. Marino[1], G. Casotto[1], M. Cressoni[1], L. Gattinoni[3]
[1]Università degli Studi di Milano, Milan, Italy; [2]ASST Santi Paolo e Carlo, Milan, Italy; [3]University of Algarve Gottingen, Gottingen, Germany
**Correspondence:** D. Chiumello
*Intensive Care Medicine Experimental* 2017, **5(Suppl 2):**0555

**INTRODUCTION.** The Mechanical Power (MP) measures the energy applied by the ventilator to the respiratory system of the patient during mechanical ventilation[1]. An excessive energy load applied could lead to ventilator-induced lung injury (VILI) development [2].

**OBJECTIVES.** We quantified MP and investigated MP's components distribution in obese patients without pulmonary disease and in ARDS patients at 2 PEEP levels.

**METHODS.** Flow and airway pressure (Paw) were recorded during volume controlled ventilation in 19 obese and 50 ARDS patients at different tidal volumes (6 to 12 ml/kg) at 2 levels of PEEP (5 and 15 $cmH_2O$), and respiratory rate (RR) 13 ± 4 breaths/min. MP was computed as the area between the inspiratory limb of Paw-Volume curve and the Volume axis times RR. Elastic recoil-, gas motion- and PEEP-related components were obtained [1]. MP and its 3 components were then normalized by the functional residual capacity (FRC).

**RESULTS.** MP/FRC was significantly higher in obese than ARDS patients at both PEEP levels (25 ± 12 vs 14 ± 8 and 31 ± 16 vs 21 ± 12 mJ/min/ml, respectively at 5 and 15 $cmH_2O$; p < 0.001 for both). As shown in Fig. 197, MP's PEEP-related component/FRC was higher in obese than in ARDS patients at both PEEP levels (6 ± 2 vs 4 ± 2 and 18 ± 9 vs 12 ± 6 mJ/min/ml at 5 and 15 $cmH_2O$, respectively; p < 0.001 for both).

Interestingly, in obese patients the MP's elastic recoil-related component/FRC was lower at higher PEEP (9 ± 5 vs 7 ± 4mJ/min/ml, p < 0.001).

**CONCLUSIONS.** Mechanical ventilation in obese patients was associated with higher MP than in ARDS patients. The PEEP-related component could play a central role in modulating the total MP applied.

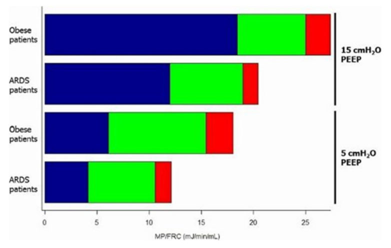

**Fig. 197 (Abstract 0555).** MP's components-FRC ration in obese and ARDS patients at two PEEP levels. The meam values of PEEP-, elastic recoil- and gas motion-related components are represented in blue, green and red, respectively

### 0556

#### Evaluation of perioperative protective lung mechanical ventilation

D. Casserly[1], G. Tuan[1], C. B. Groba[2]
[1]Brighton and Sussex Medical School, Brighton, United Kingdom; [2]Brighton and Sussex University Hospitals, Critical Care, Brighton, United Kingdom
**Correspondence:** C. B. Groba
*Intensive Care Medicine Experimental* 2017, **5(Suppl 2):**0556

**INTRODUCTION.** Mechanical ventilation is often a necessary supportive intervention during general anaesthesia. Over the past 20 years, the pro-inflammatory potential of mechanical ventilation has been recognised. Volutrauma, barotrauma, atelectrauma, biotrauma and hyperoxia are all mechanisms implicated in ventilator induced lung injury leading to potentially serious pulmonary and systemic complications. Historically, high tidal volumes (10-15ml/kg) were used to prevent atelectasis and ensure adequate oxygenation during surgery. Since the ARDSnet trial in 2000, the role of protective lung ventilation using tidal volumes 6-8ml/kg (predicted body weight), application of Positive End Expiratory Pressure (PEEP), recruitment manoeuvres and judicious titration of $FiO_2$ has been established as a gold standard of care for patients in the critical care setting. There is recent evidence that the translation of this standard of care to those with healthy lungs in the perioperative setting may be of benefit to patients.

**OBJECTIVES.** The aim is to evaluate that current mechanical ventilation settings in the operating theatre comply with current proposed lung ventilation strategy.

**METHODS.** Intraoperative ventilation data were voluntary collected by anaesthetists prospectively using a proforma data collection form on patients undergoing elective or emergency surgery. Those on mandatory, supported or combined modes of ventilation were included. Missing variables were obtained by retrospective review of hospital notes. Data was initially recorded in Microsoft® Excel® 2011 and analysed using IBM® SPSS® Statistics Version 22.

**RESULTS.** Compliance with protective low tidal volume ventilation (≤8ml/kg PBW) was 66.4% (n = 79). Less than 10ml/kg PBW was achieved in 101 cases (84.9%). Interestingly, those who were ventilated with more than 10ml/kg (n = 18; 15.1%) were all female with height < 167 cm and 47.6% had a BMI ≥ 30kg/m$^2$. Overall, females received larger Vt (9ml/kg PBW) compared to men (7ml/kg PBW). PEEP 2–8 cmH$_2$O was applied in 73.6% of cases (n = 92) with neurosurgical patients receiving the least amount of PEEP (39.4%). Peak pressure < 30 cmH$_2$O was achieved in 94.4% of cases (n = 118). $FiO_2 \geq 0.5$ was used intraoperatively 61.4% of the time (n = 62) despite median $SpO_2 \geq 95\%$.

**CONCLUSIONS.** Protective lung ventilation (PLV) is being employed in theatre, however the delivery of higher tidal volumes to females is a concern, as it is the used of low PEEP. A consistent use of high concentration of administered O2 ($FiO_2 \geq 0.5$ despite $SpO_2 \geq 95\%$) was also identified. A default setting on the ventilator of 500 ml and a PEEP of zero might have been partly responsible. To encourage compliance with the PLV we have proposed to changed the default setting to 400 ml $V_T$ and PEEP 4, as well as more sensible use of O2.

**REFERENCE(S)**

1. Sutherasan Y et al. Crit Care. 2014;18:211.
2. Guay J, Ochroch EA. The Cochrane Database Syst Rev. 2015;2:CD011151.

### 0557

#### Is left ventricular global longitudinal strain (AvgGLS) impairment after cardiopulmonary bypass related to anesthesia technique? A single center pilot study

G. Faivre, P. Burtin, C. Leclercq, R. Berthezene, A. Roussiaux, C. Halchini
Clinique Millénaire, Montpellier, France
**Correspondence:** G. Faivre
*Intensive Care Medicine Experimental* 2017, **5(Suppl 2):**0557

**INTRODUCTION.** Strain Analysis by 2D TEE is a new technology allowing a semiobjective and quantitative measure of regional myocardial function (1) which is less preload dependant than left ventricular ejection fraction (LVEF). Applications of Speckle-tracking Strain imaging in the perioperative period of cardiac surgery are still being evaluated (2). The only study available with a before/after CPB design demonstrated a LV improvement and a RV impairment after aortic valve replacement (3).

**OBJECTIVES.** We conducted a pilot observational study to assess LV strain modification before/after CPB and investigate the role of anesthesia technique.

**METHODS.** All scheduled cardiac surgery patients with a complete perioperative TEE examination (10/01/2016 to 01/31/2017) were included. TEE was performed with a GE Vivid S70 device, before sternal incision and after sternal closure. Speckle-tracking Strain imaging calculations were made on site using the vendor software (AFI) using 3 incidences (APLAX, 4C, 2C). Patients were divided in two groups depending on the anesthesia technique with volatile anesthetics (HALOG) or with total intravenous anesthesia (TIVA). Normothermic blood cardioplegia is used in all patients. Groups were compared using Student t and Chi2 tests as indicated ($p < 0.05$ significant).

**RESULTS.** 63 patients were included : 25 AVR, 36 CABG, 1 MVR, 1 AVR + CABG. 25 patients are in group TIVA, 38 in group HALOG. Some characteristics are shown in Table 145.

Overall, a significant decrease is observed for AvgGLS before and after CPB (−13,60% +/− 4,44 (IC95% : [− 12,48%;-14,72%]) vs −10,60% +/− 5,70 (IC95% : [−9,17%:-12,02%]), p < 0,05). There is no difference between TIVA and HALOG groups for the characteristics studied (Table 146).

This is the first study investigating the role of anesthesia technique on LV strain modification in cardiac surgery. We observed an overall impairment of LV strain in our study, and this result is not consistent with the only study available to date (3). While mean decrease of AvgGLS was larger in the HALOG group, there is no statistically significant role of volatile anesthetics on LV strain variation. These findings should be investigated through a randomized controlled trial in order to assess the factors accounting for LV strain impairment in the perioperative period of cardiac surgery. Further studies are also needed to assess the role of GLS impairment on patients outcome. Speckle-tracking Strain imaging could be used as a unique clinical tool to investigate the pharmacologic effect on the myocardium of anesthesia and cardioplegia techniques.

**CONCLUSIONS.** TEE measurement of the global longitudinal strain didn't reveal any significant difference on the alteration of LV function and patient's outcome according to the type of anesthesia in cardiac surgery with CPB.

**REFERENCE(S)**

1-Anesth Analg 2014;118:525–44
2-JCVA 2014;28:128–40
3-Anesth Analg 2017;124:406–18

**Table 145 (Abstract 0557).** Characteristics

| | OVERALL | TIVA | HALOG | p |
|---|---|---|---|---|
| PATIENTS | 63 | 25 | 38 | |
| AGE | 65.67 +/- 9.18 | 66.72 +/- 8.02 | 64.97 +/- 9.93 | 0.22 |
| SEX M | 48 | 16 | 32 | 0.06 |
| BMI | 26.59 +/- 3.76 | 26.32 +/- 3.46 | 26.76 +/- 3.98 | 0.65 |
| CPB time (min) | 76.07 +/- 28.25 | 79.6 +/- 30.05 | 73.82 +/- 27.1 | 0.42 |
| Clamping (min) | 62.00 +/- 26.53 | 67.24 +/- 31.71 | 58.64 +/- 22.41 | 0.2 |
| HCT (%) | 29.13 +/- 4.30 | 28.66 +/- 4.83 | 29.46 +/- 3.94 | 0.52 |
| LV mass | 214.80 +/- 62.70 | 198.76 +/- 61.13 | 223,31 +/- 62.8 | 0.19 |

**Table 146 (Abstract 0557).** Results

| | OVERALL | TIVA | HALOG | p |
|---|---|---|---|---|
| LVEF (%) BEFORE | 59.45 +/- 9.61 | 62.18 +/- 7.37 | 57.87 +/- 10.46 | 0.06 |
| LVEF (%) AFTER | 58.69 +/- 11.83 | 62.65 +/- 8.7 | 56.52 +/- 12.84 | 0.056 |
| GLOBAL LONGITUDINAL STRAIN BEFORE(%) | −13.60 +/- 4.44 | −12.88 +/- 4.48 | −14.07 +/- 4.41 | 0.3 |
| GLOBAL LONGITUDINAL STRAIN AFTER (%) | −10.60 +/- 5.70 | −9.58 +/- 6.4 | −11.25 +/- 5.18 | 0.25 |
| DELTA STRAIN (%) | 16.32 +/- 0.4 | 12.98 +/- 0.5 | 18.52 +/- 0.29 | 0.58 |
| TROPO (ng/l) | 428.46 +/- 642.11 | 458.36 +/- 570.23 | 409.31 +/- 690.81 | 0.76 |
| VENTIL (h) | 4.94 +/- 2.51 | 5.32 +/- 3.39 | 4.69 +/- 1.73 | 0.33 |
| LOS ICU (d) | 3.66 +/- 1.55 | 3.52 +/- 1.85 | 3.74 +/- 1.53 | 0.6 |
| MAJOR EVENT (n) | 16 | 6 | 10 | 0.55 |

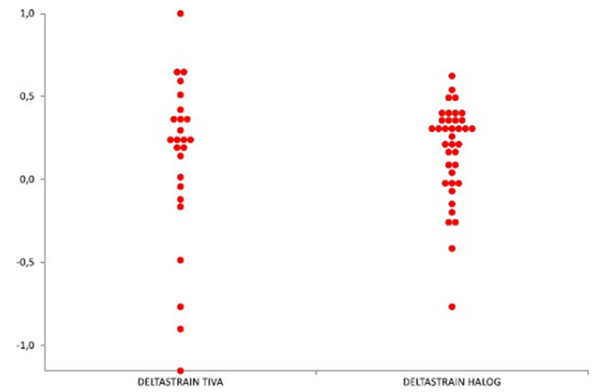

**Fig. 198 (Abstract 0557).** Spread plot of the ration of variable of AvgGLS between before and after CPB (Deltastrain) in patients in group TIVA (n = 25) and group HALOG (n = 38). Y axis values from 0 to +1 feature an AvgGLS impairment. Y axis values from 0 to −1 feature an AvgGLS improvement. p = 0.58

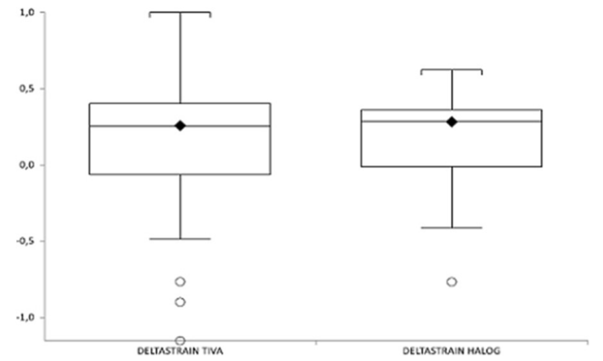

**Fig. 199 (Abstract 0557).** box & whisker plot for deltastrain TIVA & deltastrain HALOG. Y axis values from 0 to +1 feature an AvgGLS impairment, values from 0 to −1 feature an AvgGLS improvement

### 0558

#### Evaluation of Direct Electrical Cardioversion applied to the heart during weaning from cardiopulmonary bypss on Troponin I levels and outcomes after cardiac surgery

O. Valles-Fructuoso[1], L. Costa-Capella[1], J. C. Lopez Delgado[1], M. Alaidroos[2], D. Berbel-Franco[1], H. Torrado-Santos[1], E. Farrero[1], M. L. Carriò[1], G. Landoni[2]
[1]Hospital Universitari de Bellvitge, Intensive Care, Hospitalet de Llobregat, Spain, [2]San Raffaele Scientific Institute/ Vita-Salute San Raffaele University, Department of Anesthesia and Intensive Care, Milano, Italy
**Correspondence:** J. C. Lopez Delgado
*Intensive Care Medicine Experimental* 2017, **5(Suppl 2):**0558

**INTRODUCTION.** Direct electrical cardioversion (DEC) may be needed during weaning from cardiopulmonary bypass (CPB), which may lead to a higher cardiac troponin I (cTnI) peak. Mild elevations in cTnI may be associated with lower survival after cardiac surgery.

**OBJECTIVES.** Evaluate factors associated with the need of major number of DEC, the influence of DEC over cTnI levels and if there is any association between DEC needs and outcomes.

**METHODS.** Prospective, observational study in our Surgical ICU in a tertiary-level university hospital. cTnI was measured at 0h, 6h, 12h, 24h and 48h following CS. We divided our population in 3 subgroups: those needing ≤1 DEC (64.6%;1731), 2 DECs (17.8%;478) and ≥3 DECs (17.6%;472).

**RESULTS.** 2681 patients were included. Mean age was 64.4 ± 11.7 years; Mean APACHE II: 12.2 ± 4.5; Body Mass Index: 27.9 ± 4.2Kg · $m^{-2}$; 64.3% ($n$ = 1725) were male.

We showed that valvular (OR: 2.008; 95% IC:1.077-3.744; $P = 0.028$) or combined procedures(valvular plus bypass graft) (OR: 2.400; 95% IC:1.107-5.204; $P = 0.027$) were associated with the need of ≥3 DECs, whereas hypertrophic myocardiopathy in the preoperative echocardiography (OR: 0.632; 95% IC:0.469-0.851; $P = 0.003$) was protector.

Despite pattern of levels of cTnI was similar between subgroups, the area under the curve of different levels of cTnI was different between those needing ≤1 DEC and ≥3 DECs when calculated during 24h (186 ± 478 vs. 248 ± 388ng·$mL^{-1}$·$h^{-1}$; $P = 0.021$) and 48h (379 ± 651 vs.510 ± 803ng·$mL^{-1}$·$h^{-1}$; $P = 0.001$). This was also confirmed when we only analyzed patients who underwent valvular surgery. As well, higher levels of cTnI 12h were showed in the subgroup of patients with ≥3 DECs (OR: 1.009; 95% IC:1.003-1.015; $P = 0.004$), which was also confirmed when we only analyzed patients who underwent coronary artery bypass graft surgery. We did not find any association between DEC needs and outcomes.

**CONCLUSIONS.** DEC may lead to a modest increase in cardiac troponin levels in our population, which did not have any influence over outcomes. Type of surgery and hypertrophic myocardiopathy may be associated with the need of DEC during weaning from CPB.

**REFERENCE(S)**

Lurati Buse GA. The prognostic value of troponin release after adult cardiac surgery - a meta-analysis. Eur J Cardiothorac Surg. 2010; 37(2):399–406.

Zangrillo A. A 20-joule electrical cardioversion applied directly to the heart elevates troponin I by at least 1.5 ng.mL-1. Can J Anaesth. 2005; 52:336–7.

### 0559

#### Tracheal ultrasound combined with clinical characteristics to select adequate left double lumen tubes

E. Roldi[1], P. Inghilleri[2], S. Mongodi[1], F. Mojoli[1], B. Bouhemad[2]
[1]Fondazione IRCCS Policlinico S. Matteo, Anesthesia and Intensive Care, University of Pavia, Pavie, Italy, [2]CHU Dijon Bourgogne, Anesthesia and Intensive Care, CHU Dijon, Dijon, France
**Correspondence:** E. Roldi
*Intensive Care Medicine Experimental* 2017, **5(Suppl 2):**0559

**INTRODUCTION.** Left Double-Lumen Tube (LDLT) incorrect choice is frequent and may lead to tracheal damage, mainly if oversized [1]. Correlation between left bronchial diameter (LBD) and patients' characteristics (height, gender) or tracheal diameter (TD) alone is poor [2]. Thus patients' characteristics or TD alone are inaccurate for predicting LBD. Ultrasound (US) may help in improving LDLT choice adding TD to standard parameters.

**OBJECTIVES.** Comparison of standard LDLT choice approach to a new approach, integrating TD measured by US to standard parameters.

**METHODS.** Patients requiring LDLT were prospectively enrolled. Step 1: LDLT was chosen by anaesthesiologists based on patients' characteristics. LDLT size was deemed appropriate if volume inflated into bronchial-cuff to achieve isolation was 0,5 to 2,5ml. LDLT requiring bronchial-cuff volume < 0.5ml or >2,5ml were considered oversized or undersized respectively [3]. TD was measured above the sterno-clavicular junction to identify cut-off values[2]. Step 2: LDLT was chosen according to TD cut-off and height outlined in step 1. Men whose height was 165-175cm had 37F if TD < 17mm or 39F if TD >17mm; when taller than 175cm they had 39F if TD < 18mm or 41F if TD >18mm. Women whose height was 150-165cm had 35F if TD < 14mm or 37F if TD >14mm; when taller than 165cm they had 35F if TD < 15mm or 37F if TD >15mm.

**RESULTS.** Step 1 and Step 2 enrolled 102 and 50 adult thoracic surgery patients respectively. Step 1: conventional approach is associated with high rate of incorrect tubes. Compared to step 1, in step 2 we had statistically significant lower inappropriate tubes (61% vs. 14%; p < 0.01)(Table 147). In particular, the US integrated approach significantly reduced the number of oversized tube (38.2% vs. 6.0%, p < 0.001).

**CONCLUSIONS.** Ultrasound measurement of TD matched with patients' characteristics significantly increased the rate of adequate LDLT and reduced oversized and potentially harmful tube. Further studies are needed to confirm that US measurement of TD may guide LDLT choice.

**REFERENCE(S)**

1.Sustic, J Clin Anesth (2008); 2.Olivier, Chest (2006); 3.Brodsky, J Cardiothor Vasc Anest (2003).

**Table 147 (Abstract 0559).** Accuracy of 2 approaches (Step 1 vs Step 2)

| Gender | Used Tube | Step 1 (n= 102)<br>Small / Appropriate / Big<br>Small + Appropriate / Big | Step 2 (n= 50)<br>Small / Appropriate / Big<br>Small + Appropriate / Big | P value |
|---|---|---|---|---|
| Women | 35 F | 1 / 4 / 3 (n= 8)<br>5 / 3 | 1 / 2 / 0 (n= 3)<br>3 / 0 | 0.41<br>0.21 |
| | 37 F | 2 / 10 / 11 (n= 23)<br>12/ 11 | 1 / 15 / 1 (n= 17)<br>16 / 1 | 0.01<br>0.004 |
| | 39 F | 1 / 0 / 4 (n= 5) | | |
| | 41 F | 0 / 1 / 0 (n= 1) | | |
| Men | 37 F | | 0 / 1 / 0 (n= 1) | |
| | 39 F | 5 / 6 / 6 (n= 17)<br>11 / 6 | 2 / 15 / 1 (n= 18)<br>17 / 1 | 0.01<br>0.03 |
| | 41 F | 14 / 19 / 15 (n= 48)<br>33 / 15 | 0 / 10 / 1 (n= 11)<br>10 / 1 | 0.008<br>0.09 |

### 0560

### Analysis of the impact of two doses of methylprednisolone on the development of acute renal injury after cardiac surgery

A. González Pérez, J. Silva Guisasola
Hospital Universitario Central de Asturias, Oviedo, Spain
**Correspondence:** A. González Pérez

*Intensive Care Medicine Experimental* 2017, **5(Suppl 2):**0560

**INTRODUCTION.** Cardiac surgery with cardiopulmonary bypass (CPB) triggers a systemic inflammatory response syndrome (SIRS) that is associated with postoperative morbidity and mortality. Steroids may attenuate this response but there is currently controversy over its use.

**OBJECTIVES.** We aimed to assess the effects of two dosage of steroids in CPB in AKI development during ICU stay.

**METHODS.** Retrospective observational study of a cohort of patients that underwent to cardiac surgery at our institution between 2006 and 2014. Two groups were compared, one group administers 1 gr of methylprednisolone (MTPN) during cardiopulmonary bypass and another group administers 2 gr. AKI development during ICU stay was defined as impaired renal function with elevation to twice baseline creatinine or need of renal replacement therapy. Demographic data, clinical and surgical variables were collected from de Hospital database. Values expressed as mean +/− SD or %. Significant variables in the univariate analysis were entered into a multivariate logistic regression model to calculate the odds ratio with confidence interval 95%.

**RESULTS.** Out of 4573 patients; 64.8 males; 67.78+/− 11.13 years old; BMI 28.36 +/− 6.81; Logistic EuroScore 6.36 +/− 4.85; ICU stay 6.58 +/− 15.41 days; postoperative bleeding at first 24 hours 560.74 +/− 447.95 cc; preoperative haemoglobin 13.58 +/− 5.72 gr/ dL; preoperative urea and creatinine 51.39 +/− 23.76; 1.13 +/− 0.57 mg / dl respectively. By pass time 104.31 +/− 231.75; cross clamp time 71.76 +/− 32.74 minutes. Two groups were compared: group 1 : MPNL (1 gr) ;group 2 with 2 gr of MPNL during CPB and analyzed its relation with the development of AKI during ICU stay. Patients who received higher doses of steroids had a lower risk of developing AKI: Relative Risk 0.891, 95% CI (0.817 to 0.972) p = 0.009. Multivariate analysis showed that a dose of 2 grams of MTPN reduced the incidence of AKI in the postoperative period of cardiac surgery OR 1.44 95% CI (1.09 to 1.91) p = 0.012.

**CONCLUSIONS.** While new circuits of extracorporeal circulation have been shown to decrease the incidence of SIRS in the postoperative period of cardiac surgery and there is controversy about its use, in this study a higher dose of steroids was shown to reduce the incidence of AKI after cardiac surgery.

**REFERENCE(S)**

1. Ho KM et all. Benefits and risks of corticosteroid prophylaxis in adult cardiac surgery: a dose–response meta-analysis.Circulation. 2009 Apr 14;119(14).

2. Whitlock RP et all. Methylprednisolone in patients undergoing cardiopulmonary bypass (SIRS): a randomised, double-blind, placebo-controlled trial.Lancet. 2015 Sep 26.

### 0561

### Incidence of respiratory complications after thymectomy in myasthenia gravis patients

A. Suphathamwit, S. Lertkowit, S. Suksompong
Faculty of Medicine Siriraj Hospital, Mahidol University, Anesthesiology, Bangkok, Thailand
**Correspondence:** A. Suphathamwit

*Intensive Care Medicine Experimental* 2017, **5(Suppl 2):**0561

**INTRODUCTION.** In myasthenia gravis (MG),an autoimmune disease, the number of postsynaptic acetylcholine receptors decrease, resulting in weakness and fatigability. Therefore, performing thymectomy is challenging because of the increased risk for postoperative respiratory complications (PORCs).

**OBJECTIVES.** To determine the incidence and risk factors for PORCs in MG patients.

**METHODS.** Patients who underwent thymectomy at Siriraj Hospital between 2010 and 2014 were retrospectively analyzed. PORCs were defined as delayed extubation or need for reintubation. Patients' characteristics and perioperative data were recorded and analysed.

**RESULTS.** Among 134 MG patients, 9.7% developed PORCs. Univariate analysis identified preoperative pyridostigmine alone[odds ratio (OR) = 3.82, 95% confidence interval (CI) = 1.12-13.07, $p$ = 0.033], preoperative plasmapheresis (OR = 10.82, 95% CI = 1.39-84.46, $p$ = 0.023),and prolonged operation
(OR = 1.02, 95% CI = 1.00-1.03, $p$ = 0.033) as risk factors for PORCs. Multivariate analysis identified preoperative pyridostigmine alone (OR = 6.37, 95% CI = 1.32-30.88, $p$ = 0.021) and Osserman stage 3 (OR = 5.44, 95% CI = 1.02-24.63, $p$ = 0.028) as predictors of PORCs.
**CONCLUSIONS.** The incidence of PORCs in myasthenic patients who underwent thymectomy in this study was 9.7%. Muscle function monitoring by TOF before extubation did not warrant safety. Preoperative use of pyridostigmine and Osserman stage 3 were the significant risk factors for PORCs.

### 0562

#### Use of brain protein "Cellex" for prevention of postoperative cognitive dysfunction after cardiac surgery

M. Kirov[1], M. Putanov[2], M. Sokolova[1], P. Lenkin[1], V. Slastilin[2], I. Baskakova[2], A. Kiriluk[1], K. Chetskaja[1], T. Isakova[1], M. Rumjantseva[1,2]
[1]Northern State Medical University, Arkhangelsk, Russian Federation; [2]City Hospital #1 of Arkhangelsk, Arkhangelsk, Russian Federation
**Correspondence:** M. Kirov
*Intensive Care Medicine Experimental* 2017, **5(Suppl 2):**0562

**INTRODUCTION.** Cardiac surgery can be accompanied by cognitive dysfunction and complicated postoperative period. Recently, a novel brain protein "Cellex" has been introduced into clinical practice. However, despite several promising pilot studies after stroke, its' role for attenuation of postoperative cognitive dysfunction is still unsettled.
**OBJECTIVES.** The aim of our study was to evaluate the efficacy of brain protein "Cellex" for prevention of postoperative cognitive dysfunction after cardiac surgery.
**METHODS.** Our study included 60 patients undergoing elective cardiosurgical operations who were randomized into two groups. In the “Cellex” group, the patients received 1,0 ml of "Cellex" subcutaneously daily during 8 days, beginning from the preoperative day; the control group received a saline treatment as a placebo. The cognitive function was assessed using Montreal cognitive assessment (MoCA) test. The plasma concentration of S 100b protein was measured before the surgery, at Days 3 and 7. The patients received monitoring of gas exchange, hemodynamics and cerebral oxygenation. In addition, the efficacy of "Cellex" and the severity of cognitive dysfunction were evaluated in operations with cardiopulmonary bypass (CPB).
**RESULTS.** There was no difference between groups in age and preoperative MoCA score. The duration of surgery and mechanical ventilation, as well as hemodynamics and cerebral oxymetry data also did not differ significantly. There was a transient decrease of cognitive functions at Day 3 after surgery in both groups ($p < 0.01$). At Day 7, the MoCA score was still decreased in the control group ($p < 0.003$) but returned to baseline in the "Cellex" group; these effects were more evident after surgery with CPB. The plasma concentrations of S100b protein at Day 7 after surgery correlated with baseline MoCA score and $PaCO_2$ during early postoperative period; intraoperative $PaCO_2$ was also associated with cerebral oxygenation.
**CONCLUSIONS.** The perioperative use of "Cellex" can attenuate cognitive dysfunction after cardiac surgery, especially after interventions with CPB.

**GRANT ACKNOWLEDGMENT**
The study was partially funded by the grant of FarmSintez company.

### 0563

#### Adherence and feasibility of an active decision support system based on a goal directed therapy protocol during high-risk vascular surgery: a prospective trial

A. Fischer, J. Menger, M. Mouhieddine, M. Seidel, M. Stanger, M. Bevilacqua, M. Hiesmayr, M. Dworschak
Medical University of Vienna, Vienna, Austria
**Correspondence:** A. Fischer
*Intensive Care Medicine Experimental* 2017, **5(Suppl 2):**0563

**INTRODUCTION.** Most GDT (goal directed therapy) protocols[1] are passive decision support systems where physicians apply a GDT protocol as seen fit. In passive protocols, reasons for non-adherence are frequently not immediately obvious. In an active decision-support system (ADSS) where digital alerts or another person actively give GDT-based advice to the treating physician, reasons for non-adherence may be recognised more easily.
OBJECTIVE. The objective of this study was to evaluate the adherence and feasibility of an active decision-support system that is based on a GDT protocol.
**METHODS.** In this prospective trial, 31 patients were included who underwent major elective vascular surgery except carotid artery surgery. A study anaesthesiologist was solely dedicated to actively give GDT-based advice to the anaesthesiologist in charge. The latter could reject the advice by indicating a clinically justified reason. The GDT protocol determined the need for fluid, vasoactive drugs and inotropes in series based on individualized optimal stroke volume variation (SVV), mean arterial pressure (MAP), and cardiac output (CO). SVV and CO were measured with a LiDCO rapid® device.
**RESULTS.** Median (IQR) duration of use of the ADSS given as percentage of length of surgery was 100% (94-100%). During ADSS the anaesthesiologist in charge followed the ADSS advice in 634 out of 743 (85%) interventions. Reasons for non-adherence were either inadequate time to assess the action of given drugs or artificially increased SVV (1%), foreseen or unexpected surgical conditions (2%), orders of a senior physician (2%), dobutamine-induced ST-segment changes (2%), or unstable haemodynamics (6%). Adaptation of either too low or too high individualized goals for MAP and CO were necessary in five patients prior to ADSS use.
**CONCLUSIONS.** The majority of interventions occurred according to the ADSS. Surgical conditions, haemodynamic changes and inherent limitations of SVV were reasons for non-conformity.

**REFERENCE(S)**

[1] Hamilton et al. A systematic review and meta-analysis on the use of preemptive hemodynamic intervention to improve postoperative outcomes in moderate and high-risk surgical patients. Anesth Analg 2011;112:1392–402.

### 0564

#### Prolonged ICU length of stay after cardiac surgery: risk factors and outcomes

J. C. Lopez Delgado[1], H. Marmol-Alonso[2], G. Moreno-Gonzalez[1], M. Alaidroos[3], D. Berbel-Franco[1], F. Esteve-Urbano[1], H. Torrado-Santos[1], D. Rodriguez-Castro[1], G. Landoni[3]
[1]Hospital Universitari de Bellvitge, Intensive Care, Hospitalet de Llobregat, Spain, [2]Hospital Universitari Vall d'Hebron, Intensive Care, Barcelona, Spain, [3]San Raffaele Scientific Institute/ Vita-Salute San Raffaele University, Department of Anesthesia and Intensive Care, Milano, Italy
**Correspondence:** J. C. Lopez Delgado
*Intensive Care Medicine Experimental* 2017, **5(Suppl 2):**0564

**INTRODUCTION.** Nowadays, the proportion of patients with underlying comorbidities who underwent cardiac surgery (CS) is higher. Those suffer more frequently from prolonged ICU length of stay after CS (PICULOS), which leads to more complications and worst outcomes.

**OBJECTIVES.** To evaluate the risk factors associated with PICULOS and outcomes of this group of patients who underwent CS.

**METHODS.** Prospective, observational study in our Surgical ICU in a tertiary-level university hospital. PICULOS was defined the need of >72h of ICU stay following CS. A complete follow-up during 4.6 ± 2.4 years was performed in 2840 patients.

**RESULTS.** 2939 patients were included. Mean age was 64.6 ± 11.6 years; 67% ($n$ = 1880) were male; Body Mass Index: 28 ± 4.3Kg · $m^{-2}$. 66.3% ($n$ = 1950) experienced PICULOS.

In our population, we showed that a high chest drain loss during the first 12h after procedure (OR: 1.002; 95% IC:1.001-1.003; $P = 0.001$), a low preoperative hemoglobin (OR: 0.762; 95% IC:0.659-0.881; $P < 0.001$), and a lower arterial partial pressure of $O_2$ and fraction of inspired oxygen ratio ($PaO_2/FiO_2$) 24h after CS (OR: 0.996; 95% IC:0.992-0.999; $P = 0.02$) were variables associated with PICULOS.

Regarding outcomes, we showed higher mean hospital stay (OR: 1.025; 95% IC:1.010-1.039; $P = 0.001$), higher mechanical ventilation needs (OR: 1.054; 95% IC:1.037-1.072; P < 0.001), higher pacemaker needs (OR: 7.698; 95% IC:1.315-45.056; $P < 0.001$) and higher mortality (OR: 16.846; 95% IC:4.593-61.787; P < 0.001).

Subgroups of patients with PICULOS showed worst survival during hospital stay (93.4% vs. 95.2%; $P = 0.035$) and from the long-term mortality scenario (80.3% vs. 87.7%; $P < 0.001$).

**CONCLUSIONS.** Patients who experienced PICULOS suffer from higher mortality in our population, even in the long-term scenario. Lower levels of preoperative hemoglobin and $PaO_2/FiO_2$ 24h after CS, and higher chest drain losses were variables associated with PICULOS.

**REFERENCE(S)**

Yu PJ, et al. Outcomes of Patients with Prolonged Intensive Care Unit Length of Stay After Cardiac Surgery. J Cardiothorac Vasc Anesth. 2016; 30:1550–54.

Kapadohos T, et al. Determinants of prolonged intensive care unit stay in patients after cardiac surgery: a prospective observational study. J Thorac Dis. 2017; 9:70–79.

### 0565

#### CO2 variables after cardiac surgery as markers of adequate perfusion. Preliminary results

T. F. Fariña González[1], J. Latorre[2], I. García González[1], F. Martinez Sagasti[1], A. García de Lorenzo y Mateos[3]
[1]Hospital Clinico San Carlos, Intensive Care Department, Madrid, Spain; [2]Hospital Universitario La Paz, Anaesthesiology Department, Madrid, Spain; [3]Hospital Universitario La Paz, Intensive Care Department, Madrid, Spain
**Correspondence:** T. F. Fariña González
*Intensive Care Medicine Experimental* 2017, **5(Suppl 2):**0565

**INTRODUCTION.** Classic disoxia markers, such as central venous saturation (Svc02) and lactate (Lac) have been questioned throughout in the last years because they might not be adequate to predict tissue disoxia in certain situations. Venous-arterial CO2 derivatives (ΔpCO2, ΔpCO2/ΔO2content) have been proposed as global markers of the adequacy of perfusion and O2 utilization in shock. In lastest studies, Haldane effect has been taken in account, so the ratio between CO2 and O2 contents is proposed as more accurate. These CO2 derivatives are intensively studied in sepsis, but there are lack of studies concerning cardiac surgery.

**OBJECTIVE.** Compare the adequacy of CO2-derived variables with Svc02 and Lac during the first 12 hs after an elective cardiac surgery as markers of perfusion. The calculated sample is 150 patients. These are preliminary results.

**METHODS.** Prospective, observational study; following a cohort of adults who underwent cardiac surgery and were admitted in a 16-bed cardiac ICU. Informed consent was obtained from the next-of-keen or by the patient after extubation.

Previous history of renal and hepatic disease were recorded as well as type or surgery and surgical times. Central venous and arterial gases were analyzed at ICU admission, 6 hs and 12 hs later, as well as, hemoglobin, creatinine, lactate, total bilirrubin and hemodynamic variables. Fluid balance was also collected. Normally distributed data were expressed as the mean ± standard deviation (SD) unless otherwise specifed. Quantitative variables were compared using non-parametric tests. Qualitative variables were compared with chi square or Fisher's tests. p < 0,05 was considered significant.

**RESULTS.** 22 patients were analyzed. 13 were male (59,1%). Mean age was 66,7 years (SD ± 12,77). Four patients had previous renal disease (18,2%) but none of them had hepatic disease. Type of surgery: valvular (11), CABG (5), CABG + valvular (4) and aortic root (2). EuroScore II was 1,63 (IQR 0,92-2,44) and admission CASUS 3 (1,75-5). CPB was used in 18 surgeries (81,8%): CPB time was 101 min (SD ± 51,64) and cross clamp time 78 min (SD ± 36,31). Fluid balance at 12 hs was 578 ml (IQR −55-928).

4 patients (18,2%) were ventilated and 8 (36,4%) treated with vasoactive drugs >12 hs. ΔSvc02 0–12 hs is not statistically correlated with CO2 measured variables. Changes in Lac are not correlated with changes in Scv (correlation −0,274, p 0,21) and ΔpCO2 (−0,36, p 0,093) ; they are inversely correlated when O2 content is analyzed: ΔCCO2/ΔCO2 (−0,618, p 0,004) and ΔpCO2/ΔC02 (−0,55, p 0,007). 12hs-fluid balance was correlated with ΔCCO2/ΔCO2 (0,598, p 0,005) and ΔpCO2/ΔC02 (0,482 p 0,023) but not to ΔpCO2 (0,318, p 0,15), ΔLac (−0,514, p 0,14) and ΔSvc (0,162, p 0,47).

**CONCLUSION.** These preliminary results show that, after cardiac surgery, CO2 derivatives that analyzed CO2 consumption are related with more fluid requirements although they might not correlate with disoxia (using lactate as a standard marker).

### 0566

#### The use of fluoroscopy may contribute to safe pulmonary artery catheter insertion without prolonging general anesthesia induction time

K. Katsuta, H. Komura, Y. Hara, N. Kuriyama, T. Nakamura, C. Yamashita, J. Shibata, O. Nishida
Fujita Health University, Anesthesiology and Critical Care Medicine, Toyoake, Japan
**Correspondence:** K. Katsuta
*Intensive Care Medicine Experimental* 2017, **5(Suppl 2):**0566

**INTRODUCTION.** There are many institutions in which the pulmonary artery catheter (PAC) is blindly inserted with reference to the pressure waveform. However, occasionally, position abnormality or aberration may be caused, possibly requiring an unexpectedly long time for insertion and causing iatrogenic complications such as severe arrhythmia or pulmonary artery injury. In our hospital, in order to insert the PAC safely and surely, from June 28th, 2016, in principle, insertions are all performed under X-ray fluoroscopy. The fluoroscopic device is installed and operated by a radiological technician.

**OBJECTIVES.** We examined the influence and safety of PAC insertion under X-ray fluoroscopy on the time of general anesthesia induction (from the start of anesthesia to the start of surgery) retrospectively.

**METHODS.** Adult cardiovascular surgical cases in which PAC was inserted at the time of induction of general anesthesia were investigated at our hospital between January 1st and October 31st, 2016. We divided the cases into the former group and the latter group before and after the principle of fluoroscopic use (June 28th, 2016). We analyzed electronic records and anesthesia records retrospectively, and extracted the anesthesia induction time and complications. As they influence anesthesia induction time, cases of one-lung ventilation, multiple arterial pressure measurement, and intra-aortic balloon pumping insertion during the induction of anesthesia were excluded.

**RESULTS.** There were 98 cases in the former group and 56 cases in the latter group. The number of cases used for comparing the anesthesia induction time was 64 cases in the former group and 41 cases in the latter group. The anesthesia induction time [minutes] [median (interquartile range)] was 80 (72–91) in the former group and 86 (78–93) in the latter group (Mann–Whitney U test, p = 0.26). Complications in the former group were observed in 2 cases (loop formation 1 case and pulmonary artery injury 1 case), but none were observed in the latter group.

**DISCUSSION.** Position confirmation of PAC by transesophageal echocardiography and echo operation are difficult for an anesthesiologist to perform alone. Although X-ray fluoroscopy takes time to set up and cooperation with a radiological technician is necessary, it is possible to check the position of the endotracheal tube and central venous catheter, as well as whether there is pneumothorax or hemothorax at the same time. Furthermore cardiologists generally insert PAC under fluoroscopy during percutaneous coronary intervention. Hybrid operating rooms are more common and fluoroscopy can be used easily by anesthesiologists and the use of that can contribute to the safety.

**CONCLUSIONS.** We suggest that PAC insertion under fluoroscopy can be performed safely without affecting the anesthesia introduction time.

D. C. Evans et al. Scandinavian Journal of Surgery 98: 199–208, 2009

**GRANT ACKNOWLEDGMENT**

None.

### 0567

#### The frequency of postoperative BNP measurement and intervention threshold of BNP concentration in pediatric cardiac intensive care unit: multicenter study in Japan

T. Kanazawa[1], K. Ohe[2], S. Fujii[3], Y. Otsuka[4], M. Yamasaki[5], H. Mori[6], N. Taga[4], T. Iwasaki[1], H. Morimatsu[1]

[1]Okayama University Hospital, Anesthesia and Intensive Care Unit, Okayama, Japan; [2]Showa University Northern Yokohama Hospital, Anesthesia, Yokohama, Japan; [3]Ehime University Graduate School of Medicine, Department of Anesthesia and Perioperative Medicine, Toon, Japan; [4]Jichi Children's Medical Center Tochigi, Jichi Medical University, Department of Pediatric Intensive Care and Anesthesia, Shimono, Japan; [5]Kyoto Prefectural University of Med, Department of Anesthesiology and Intensive Care, Kyoto, Japan; [6]Shimane University, Faculty of Medicine, Department of Anesthesiology, Izumo, Japan

**Correspondence:** T. Kanazawa

*Intensive Care Medicine Experimental* 2017, **5(Suppl 2):**0567

**INTRODUCTION.** In pediatric cardiac patients, various setting of congenital heart disease is well studied to show the relationship between BNP and heart failure. However, we still need discuss the utility of BNP in children during postoperative period because we do not have strong evidence.

OBJECTIVE. We conducted a prospective multicenter observational study to investigate the recent consideration of Japanese pediatric cardiac intensivists how to use BNP for postoperative management in pediatric cardiac intensive care unit.

**METHODS.** Study period: From August 21st 2015 to July 31st 2016.

Study site: Six tertiary teaching hospitals in Japan.

Study population: Children under 15 years old who underwent pediatric cardiac surgery.

BNP measurement: All BNP measurement for postoperative patients was prospectively collected in PICU. We checked how often did we measure BNP concentration postoperatively. Then we checked whether each BNP measurement was used for the decision-making of intervention or not. The intervention for patients on the strength of BNP was defined as follows: 1 inotropic support, 2 ventilator setting, 3 respiratory support device (NIPPV, HFNC), 4 others. To investigate the frequency of postoperative BNP measurement and intervention threshold of BNP we divided each BNP measurement into 4 groups: A 0–299 pg/ml (reference), B 300–999 pg/ml, C 1000–1999 pg/ml, D >2000 pg/ml.

Statistical analysis: Logistic regression analysis was performed to compare the intervention ratio between A (reference) and B, C, D. We also did multiple comparison analysis to compare intervention ratio in each group.

**RESULTS.** 246 BNP measurements for 353 patients were included in this study. Thirty six (15.9%) measurements were used as a criterion to decide to intervene children. The number of BNP measurements in each group is as follows: A 113 (45.9%), B 81 (32.9%), C 45 (18.3%), D 7 (2.8%). The intervention ratio in each group was 4.4% (A, 5/113), 8.6% (B, 7/81), 44.4% (C, 20/45), and 71.4% (D, 5/7). The intervention ratio of C and D were significantly higher than A (reference): (Odds ratio (95%CI) 12.1(4.8-33.9),p < 0.0001, 25.2(5.2-146.2),p < 0.0001). The relationship of the intervention ratio in each group from the result of multiple comparison is similar to logistic regression analysis; the intervention ratio of C and D compared to A (p < 0.0001).

**CONCLUSIONS.** BNP was not measured regularly for postoperative pediatric cardiac patients. High BNP concentration measured in PICU especially more than 1000pg/ml were frequently considered as a good sign for intervening children compared to that of less than 1000pg/ml.Further study should be conducted to investigate whether BNP guide therapy for postoperative pediatric cardiac patients may improve patient's prognosis or not.

### 0568

#### Which anatomical measurements affect the misplacement of central catheters at the postoperative period in patients undergoing thoracotomy?

S. Sen[1], O. Tuncyurek[2], S. Cokpinar[3], S. Guler[1], S. Sen[3], I. Kurt Omurlu[4]

[1]Adnan Menderes University Medical Faculty, Anesthesiology and Reanimation Department, Aydin, Turkey; [2]Adnan Menderes University Medical Faculty, Radiology Department, Aydin, Turkey; [3]Adnan Menderes University Medical Faculty, Thoracic Surgery, Aydin, Turkey; [4]Adnan Menderes University Medical Faculty, Statistics, Aydin, Turkey

**Correspondence:** S. Sen

*Intensive Care Medicine Experimental* 2017, **5(Suppl 2):**0568

**INTRODUCTION.** Central venous catheters (CVCs) are required in much major thoracic operation. The use of ultrasound guidance during CVC placement can use to improve patient safety. However, serious complications may also occur due to catheter tip malposition. Our study aims to assess CVC placement and the malposition ratios in cases that underwent pulmonary resection.

**METHOD.** CVC placement was retrospectively examined in the patients who underwent pulmonary resection between 2012 and 2016. The 2nd intercostal space-carina (C2-carina), carina-catheter tip (carina-tip), clavicular notch-carina (notch-carina) and clavicular notch-catheter tip (notch-tip) distances were measured in the postoperative lung graphs. Zone A, upper right atrium and lower superior vena cava (SVC) and Zone B, upper SVC and junction of the right innominate vein(1). The tip of catheter positioned below the carina was evaluated as Zone C (in the right atrium), whereas the positioning of a catheter outside the superior vena cava was considered a malposition.

**RESULTS.** Of 234 patients, 103 had the catheter tip on Zone A, 85 on Zone B, and 27 on Zone C, whereas the malposition number was 19 (8.11%). When the patients were divided into two groups as < 165

cm (short, n = 109) and >165 cm (tall, n = 125) by their height, the carina-tip and notch-tip distances were greater, but the carina-notch distance was smaller in short patients. The Zone 0 patients and malposition were also higher in short patients (15 vs 4). The most placement of malposition was right subclavian vein.

**CONCLUSION.** The malposition ratio is 7% in the literature and may be related to vascular structural deformity, obesity, malignity and postural changes (2). Larger diameters at the brachiocephalic vein due to malignancy, besides mediastinal displacement because of lateral position, the short carina-notch distance may also cause the misplacement of the right internal jugular CVC in patients with lung resection.

### Acute kidney injury: Epidemiology & outcome

#### 0569

**Renal outcome and mortality of ICU survivors up to 7 years after hospital discharge**

S. Ayis[1], J. Powell-Tuck[2], H. Leonard[2], R. Haines[2], J. Peacock[1], M. Ostermann[2]
[1]King's College London, London, United Kingdom; [2]Guy's & St Thomas Hospital, London, United Kingdom
**Correspondence:** S. Ayis
*Intensive Care Medicine Experimental* 2017, **5(Suppl 2):**0569

**OBJECTIVE.** Worsening of estimated glomerular filtration rate (eGFR) is associated with a higher risk of end stage renal disease (ESRD) and increased mortality. However, little is known about the long-term patterns of change in eGFR, particularly in critically ill patients with acute kidney injury (AKI). In a recent study, we elucidated the heterogeneity of these patients and identified four distinct trajectories of eGFR. In this study, we extended our analysis to explore dialysis free survival rates among these four groups.

**METHODS.** We studied 1,223 patients (60% males) who were admitted to a multi-disciplinary ICU in a University Hospital between 2004–2008 and discharged from hospital alive, and collected follow-up data up to June 2016. The eGFR was obtained at 6 months, then annually up to 7 years after hospital discharge with 1223 patients recording at least 2 eGFR measuremnts anda subgroup of 427 patients with at least four. Trajectories of eGFR were identified using group based trajectory modelling (GBTM).[1] The rate of dialysis free survival was examined using Kaplan-Meier estimates, and hazards ratios (HRs) were calculated from multivariable Cox regression models, using Stata (14.0).

**RESULTS.** Four patterns of eGFR were identified: Persistently low (PL) 11.3% of patients; Rapidly increasing (RI) 2.2%; Persistently moderate (PM), 81.7% and Rapidly dropping (RD) 4.8%. (Fig. 200)

The 4 groups differed in clinical and demographic characteristics. The risk of death or dialysis was significantly higher in the PL group, used as a reference. The HRs within 7 years of hospital discharge, adjusting for age, gender and pre-existing chronic conditions, were: 0.28 (95% CI: 0.12 to 0.65); 0.35 (95% CI: 0.27 to 0.46) and 0.61 (95% CI: 0.40 to 0.95), and P values were: 0.003, < 0.001, and 0.029, for the 3 trajectories RI, PM and RD respectively, compared to the reference (HR = 1.0). Results in the subgroup of 427 patients were consistent with those in the full sample, with slight differences in magnitudes but not in significance. The median eGFRs over 7 years of hospital discharge were higher for patients with no AKI, and lowest for those with maximum AKI stage III (Fig. 201)

**CONCLUSIONS.** Recognition of the heterogeneity of patterns of eGFR, and the mortality risk associated with each, is useful for the application of cost effective personalized long term management of AKI survivors after ICU discharge.

**Acknowledgements**
Dr Salma Ayis was supported by the National Institute for Health Research (NIHR) Collaboration for Leadership in Applied Health Research and Care South London at King´s College Hospital NHS Foundation Trust.

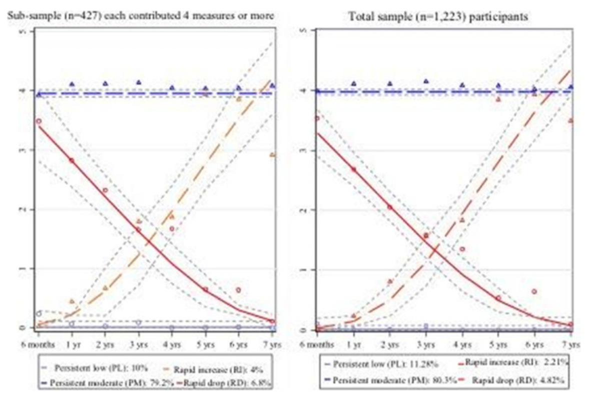

**Fig. 200 (Abstract 0569).** Trajectories of eGFR

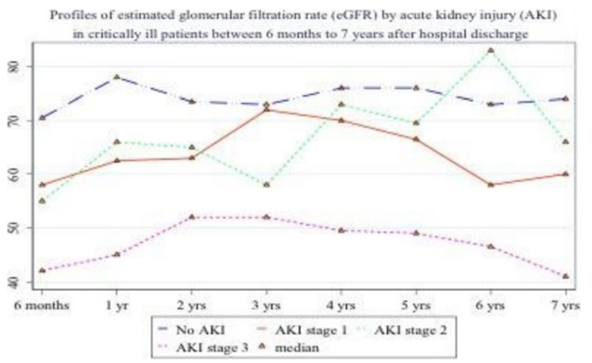

**Fig. 201 (Abstract 0569).** Long-term renal function in AKI patients

#### 0570

**Long-term outcomes after acute kidney injury: a prospective observational cohort**

E. P. Plata-Menchaca[1], G. Moreno-González[2], L. Anguela-Calvet[2], G. Muñóz-del Río[2], A. Salinas-Cánovas[2], X. L. Perez-Fernandez[2], P. Rebelo-Esquibel[2], K. Maisterra-Santos[2], A. Farré-Estebe[2], I. Romera-Peregrina[2], M. González-Romero[2], J. Pérez-Sánchez[2], A. Martínez-Pascual[2], V. Fuentes-Mila[2], R. Abellan-Lencina[2], J. Ballus-Noguera[2], J. Sabater-Riera[2], SIRAKI Group
[1]Instituto de Investigación Biomédica de Bellvitge, Research, L'Hospitalet de Llobregat, Spain; [2]Hospital Universitario de Bellvitge, Critical Care Medicine, L'Hospitalet de Llobregat, Spain
**Correspondence:** E. P. Plata-Menchaca
*Intensive Care Medicine Experimental* 2017, **5(Suppl 2):**0570

**INTRODUCTION.** Acute kidney injury (AKI) represents a public health problem associated with high rates of morbidity and mortality among critically ill patients, along with considerable short- and long-term clinical consequences. AKI survivors are at increased risk of developing acute kidney disease (AKD), chronic kidney disease (CKD) and end-stage renal disease (ESRD).

**OBJECTIVES.** To describe the clinical characteristics, complications, short- and long-term outcomes (up to 5 years of follow-up) of critically ill patients with AKI.

**METHODS.** A prospective observational cohort study from January 2011 to January 2016 was performed, which included 370 patients with AKI at or during admission in the intensive care unit (ICU) of Bellvitge University Hospital, in Barcelona, Spain. Clinical data, comorbidities and long-term outcomes [incidence of CKD, as stated by Kidney Disease Improving Global Outcomes (KDIGO) guideline criteria, requirement of renal replacement therapy (RRT) and mortality] were assessed at 6 months, 2 years and 5 years after a single AKI episode. We performed a binary logistic regression model with an adjusted analysis of risk factors for long-term RRT dependence. 11 patients were lost during the follow-up period.

**RESULTS.** Mean age was 58 ± 16 years; 65% of patients were men. One-hundred and twenty-three patients (34.6%) did not require RRT during ICU stay. Among survivors (174 patients), 39.1% (68 patients) presented CKD stage 3 or more according to KDIGO criteria and 22 patients (12.6%) required RRT at 5 years of follow-up. Independent risk factors associated with long-term RRT requirement were a decreased renal function (measured in terms of serum creatinine) at baseline and at hospital discharge. Overall 5-year mortality was 34.8% after hospital discharge.

**CONCLUSIONS.** Long-term morbidity and mortality of patients presenting with AKI are high. Risk factors associated with long-term RRT dependence were an elevated baseline serum creatinine and elevated creatinine at hospital discharge.

**REFERENCE(S)**

Chawla LS, Bellomo R, Bihorac A, Goldstein SL, Siew ED, Bagshaw SM, et al. Acute Disease Quality Initiative Workgroup 16. Acute kidney disease and renal recovery: consensus report of the Acute Disease Quality Initiative (ADQI) 16 Workgroup. *Nat Rev Nephrol* 2017 Apr;13(4):241–257.

### 0571

#### Preoperative albuminuria as a predictive marker of perioperative acute kidney injury (AKI) in non-cardiac major surgery

D. Marouli[1], E. Papadakis[2], P. Malliotakis[1], E. Pediaditis[1], E. Kondili[1], A. Papaioannou[2], D. Georgopoulos[1], H. Askitopoulou[2]
[1]University Hospital of Heraklion, Intensive Care Unit, Heraklion, Greece; [2]University Hospital of Heraklion, Anesthesiology Department, Heraklion, Greece
**Correspondence:** D. Marouli
*Intensive Care Medicine Experimental* 2017, **5(Suppl 2):**0571

**INTRODUCTION.** Perioperative AKI is associated with significant morbidity and mortality[1]. Albuminuria has recently been recognized as a significant risk factor for AKI, even when estimated Glomerular Filtration Rate (eGFR) is >90 mL/min/1.73 $m^2$ [2]. Moreover, only one clinical AKI prediction model, the AKI Risk Index, has been developed for the general surgery population[3]. While several predictive markers for AKI have been proposed in the non-cardiac surgery population[4], preoperative albuminuria has not been studied in this context.

**OBJECTIVES.** The aim of this observational study was the evaluation of the AKI Risk Index, as well as preoperative albuminuria as AKI predictors in a cohort of non-cardiac surgery patients.

**METHODS.** 61 major surgery patients without CKD stage IV or V were studied. AKI was defined according to AKIN criteria within 48 hrs after surgery[5]. The AKI Risk Index (which includes nine risk factors: age ≥ 56yrs, male sex, congestive heart failure, hypertension, diabetes, ascites, serum creatinine > 1.2mg/dl, emergency and intraperitoneal surgery) was calculated for each patient, with one point being assigned to each risk factor. At pre-defined time points (preoperatively, recovery room and on postoperative days 1 to 7) the following markers were measured: Serum creatinine, cystatine C, urine albumin and creatinine, whereas eGFR (using the CKD-EPI Creatinine Equation)[6] and urine albumin to creatinine ratio (UACR) were calculated. An eGFR < 90ml/min/$m^2$ and a UACR value ≥ 30mg/g were considered abnormal.

**RESULTS.** 11 patients (18%) developed AKI. While there was no difference in preoperative eGFR between AKI and non-AKI patients (75.3 ± 16 vs 83.9 ± 15.2 ml/min/$m^2$, p = 0.09), recovery room eGFR was already lower in AKI patients (mean: 69.5 ± 18.7 vs 85.7 ± 15.6 ml/min/$m^2$, p = 0.001) and remained significantly lower until day 7. A significantly higher mean AKI Risk Index score was observed in AKI versus non-AKI patients (4.36 vs 3.48, p = 0.02). On multivariate regression analysis each point in AKI Risk Index score increase was associated with a higher OR (2.01, 95% CI: 1.07-3.77, p = 0.03) for AKI, while an abnormal pre-op UACR was also strongly associated with AKI development (OR = 5.47, 95% CI:1.36-21.92, p = 0.019).

**CONCLUSIONS.** In a general surgery population the AKI Risk Index proved to be a simple yet powerful predictive tool for perioperative renal dysfunction. Furthermore, an abnormal preoperative UACR was strongly associated with postoperative AKI development, regardless of baseline eGFR and other comorbidities. Being based on readily available data, the AKI Risk Index and the UACR should be considered part of a routine preoperative patient evaluation.

**REFERENCE(S)**

1. Hobson C et al.: Ann Surg 2015; 261:1207
2. James MT et al.: Lancet 2010; 376 (9758): 2096,
3. Kheterpal S et al.: Anesthesiology 2007;107: 892
4. Schley G et al.: PLoS One 2015;10(12): e0145042
5. AKI Work Group: Kidney Int..2012; 2 :1
6. Inker LA et al.: NEJM 2012; 367: 20

**GRANT ACKNOWLEDGMENT**
None

### 0572

#### Organ failures associated with acute kidney injury in critically ill cirrhotics have a major influence on disease progression and outcomes: a prospective ICU based study

R. Maiwall[1], R. Moreau[2], P. Jain[3], G. Kumar[1], S. Sarin[1]
[1]ILBS, Hepatology, New Delhi, India; [2]Inserm, Hepatology, Paris, France; [3]ILBS, Biostatistics, New Delhi, India
**Correspondence:** R. Maiwall
*Intensive Care Medicine Experimental* 2017, **5(Suppl 2):**0572

**INTRODUCTION AND OBJECTIVES.** Acute kidney injury (AKI) is a known detrimental complication in patients with cirrhosis. Currently, there are no studies evaluating AKI and the impact of extra-renal organ failures (E-OF) on the course of AKI and outcome in critically ill cirrhotics.We addressed this question in a large prospective cohort of cirrhotics admitted to Liver intensive-care.

**METHODS.** Patients were prospectively followed from admission until death, recovery or liver transplant for prevalence, development and progression of AKI and it's association with extra-renal organ failures (E-OF) (i.e. cerebral failure, circulatory failure and respiratory failure). Binary logistic regression models using multivariate repeated measures Generalised Estimating Equations (GEE) were used to identify significant variables associated with AKI progression at day 7. Cox-regression analysis was done to identify predictors of 28-day mortality.

**RESULTS.** A total of 291 patients with cirrhosis, aged 48. ± 11years, 87% males, with mean MELD of 30.1 ± 8.4; SOFA score of 11.6 ± 4.5 were included of which only 145(49.8%) were alive at 1 month. AKI at admission was present in 194(67%), (Stage I:II:III in 21%:29%:50% respectively); 244 patients (84%) had at least one E-OF. At day 7, 49% had AKI progression with peak AKIN stage III:II:I in 66%: 25%:9% respectively. Presence of any E-OF was strongly associated with progression of AKI (p = 0.002, OR 3.99, 95%CI 1.6-9.8). This risk further increased with increase in the number of E-OFs (p < 0.001,OR 1.8, 95%CI

1.4-2.4). Further, AKI at admission alone was not associated with mortality, but predicted mortality together with any E-OF (p = 0.02, OR 1.9, 95%CI 1.12-3.4). On multivariate analysis using GEE model, number of extrarenal organ failures (OR 1.67, 95% CI 1.32-2.14), increasing serum bilirubin (OR 1.05, 95% CI 1.02-1.08), declining urine output (OR 0.98, 95% CI 0.97-0.99) and increase in the components of SIRS (OR 2.5, 95% CI 1.2-4.8) predicted AKI progression at day 7.

**CONCLUSION.** Extra-renal organ failures are commonly associated with AKI in critically ill patients with cirrhosis which determine progression of AKI as well as mortality. Presence of AKI alone is not associated with worse outcome in critically ill cirrhotics.

### 0573

#### Impact of acute kidney injury requiring renal replacement therapy on prognosis of cancer patients treated at the ICU

W. Druml[1], P. Schellongowski[2], P. Metnitz[3], P. Zajic[3], T. Fellinger[4], B. Metnitz[4]

[1]Medical University of Vienna, Department of Medicine III / Division of Nephrology, Wien, Austria; [2]Medical University of Vienna, Department of Medicine III / Intensive Care Unit, Wien, Austria; [3]Medical University of Graz, Department of Anaesthesiology and Intensive Care Medicine, Division of General Anaesthesiology, Emergency- and Intensive Care Medicine, Graz, Austria; [4]Austrian Centre for Documentation and Quality Assurance in Intensive Care, Wien, Austria

**Correspondence:** W. Druml

*Intensive Care Medicine Experimental* 2017, **5(Suppl 2):**0573

**INTRODUCTION.** Solid cancer in many instances has become a chronic disease process. When cancer patients are admitted to the ICU because of reversible complications the prognosis is not necessarily worse than in other patients groups.

**OBJECTIVE.** In a large group of ICU patients we assessed how a complicating acute kidney injury (AKI) requiring renal replacement therapy (RRT) affects outcome in this especially vulnerable patient group.

**METHODS.** In a retrospective analysis of a prospectively collected data base of 165,848 patients from 151 Austrian intensive care units (ICUs) 20,689 patients with solid cancer were identified of whom 996 patients had AKI requiring RRT (AKI-3). Impact of AKI on prognosis was assessed and results were adjusted for severity of disease, age, sex and other acute and chronic comorbidities.

**MAIN RESULTS.** ICU patients with solid cancer were older but had a comparable severity of disease, need of mechanical ventilation, length of ICU stay and ICU/ hospital mortality (10.28/ 16.81 vs. 9.99/ 14.53%). Cancer patients with AKI versus patients without AKI had a much higher severity of disease, requirement of mechanical ventilation and an increased ICU/ hospital mortality (49.05/ 59.24% vs. 8.32/ 14.67%, p < 0.001). Moreover, mortality was much higher in cancer than in non-cancer ICU patients with AKI (49.05/ 59.24% vs. 36.60/ 45.52%, p < 0.001).

**CONCLUSIONS.** Cancer patients in the ICU have a comparable survival as other patients groups but have much worse prognosis when developing AKI requiring RRT during their ICU stay. Survival of these patients is much lower than in other ICU patients with AKI. Nevertheless, diagnosis of cancer per se does not justify withholding RRT in these patients, a decision which must be based on a thorough individual assessment.

### 0574

#### Predictive modelling for acute kidney injury and renal replacement therapy after major trauma

R. Haines[1,2], D. Lin[1,2], C.J. Kirwan[1,2], J.R. Prowle[1,2]

[1]Queen Mary University of London, William Harvey Research Institute, London, United Kingdom; [2]Barts Health NHS Trust, Adult Critical Care Unit, The Royal London Hospital, London, United Kingdom

**Correspondence:** R. Haines

*Intensive Care Medicine Experimental* 2017, **5(Suppl 2):**0574

**BACKGROUND.** Acute Kidney Injury (AKI) complicating major trauma is associated with mortality and prolonged hospitalization. Trauma-associated AKI has specific risk factors and predictable timing of injury making diagnostic prediction of AKI for targeted intervention attractive.

**OBJECTIVES.** To develop and test risk prediction models for AKI after major trauma based on data close to ICU admission.

**METHODS.** In a single centre, retrospective observational study we studied major trauma ICU admissions who survived for at least 24h after ICU admission excluding patients with advanced renal impairment at hospital admission (creatinine > 356μmol/L). We defined a development cohort admitted from February 2012 to Oct 2014 and a validation cohort admitted Nov 2014 to May 2016. AKI was defined by KDIGO creatinine criteria in the first 7 days or need for Renal replacement therapy (RRT). For predictive modelling, we considered demographics, injury severity, observations, blood transfusion, first blood results and ICD-10 comorbidities. Missing values were handled by simultaneous transformation and single imputation. To avoid over-fitting we use hierarchical clustering and principal component analysis for data-reduction. We then used backward variable selection to develop final logistic regression models. Model performance was assessed by the c-statistic in the development and validation datasets.

**RESULTS.** We identified 830 patients for development and 564 for validation, AKI occurred in 19.6% and 17.4% respectively, with ~5% receiving RRT (Table 148). Models predicting need for RRT and all AKI were developed and validated (Table 149). First serum creatinine, first serum phosphate, units of packed reds cells transfused in the first 24h after injury, age and Charlson comorbidity score were found to be predictive of AKI. A model predicting need for RRT had a c-statistic of 0.92 (95%CI 0.88-0.96) in the development cohort and 0.91 in the validation group (Fig. 202). However even with apparently excellent discrimination, positive predictive value for RRT at the optimal cutoff was only 22% in both groups, as need for RRT was rare overall. A model predicting all AKI performed less well, c-statistic 0.77 (0.72-0.81), and was worse in validation (c-stat: 0.70, 0.64-0.77).

**CONCLUSIONS.** Risk prediction modelling has demonstrated that age, first values of serum creatinine and phosphate, and need for major blood transfusion are associated with development of AKI and need for RRT after trauma. Interestingly, variables such as injury severity and serum creatine kinase did not appear in our final models, potentially because other variables such as transfusion and serum phosphate were better measures of underlying severity of injury and muscle damage. However, even with apparently excellent model performance, ability to predict need for a rare intervention such as RRT was limited.

**Table 148 (Abstract 0574).** Baseline characteristics

| | Development | | | | Validation | | | |
|---|---|---|---|---|---|---|---|---|
| | All Patients | No AKI | AKI 1-3 | p AKI vs No AKI | All Patients | No AKI | AKI 1-3 | p AKI vs No AKI |
| Number | 830 (100%) | 667 (80.4%) | 163 (19.6%) | - | 564 (100%) | 466 (82.6%) | 98 (17.4%) | - |
| Age (y) | 42 (24-50) | 40 (27-54) | 50 (32-66) | 0.4 | 41 (27-58) | 40 (25-56) | 52 (33-71) | <0.001 |
| Male Sex | 676 (81.4%) | 539 (80.8%) | 137 (84.0%) | <0.001 | 437 (77.5%) | 361 (77.5%) | 76 (77.6%) | 1.0 |
| New Injury Severity Score | 34 (24-50) | 34 (22-50) | 34 (25-50) | 0.66 | 34 (22-50) | 34 (21-50) | 43 (29-57) | <0.001 |
| Hospital LOS (d) | 19 (8-39) | 18 (8-38) | 21 (8-45) | 0.32 | 15 (7-32) | 15 (7-31) | 19 (7-37) | 0.44 |
| Mortality | 156 (18.8%) | 103 (15.4%) | 53 (32.5%) | <0.001 | 103 (18.3%) | 63 (13.5%) | 40 (40.8%) | <0.001 |
| RRT | 42 (5.1%) | - | 42 (25.8%) | - | 25 (4.4%) | - | 25 (25.5%) | - |

Median (IQR) or Number (%). Wilcoxon or Fisher's Tests

**Table 149 (Abstract 0574).** Predictive Models for AKI and RRT

| | Model for RRT | | Model for all AKI | |
|---|---|---|---|---|
| | Odds Ratio | p | Odds Ratio | p |
| Age (y) | 1.031 | 0.0007 | 1.020 | 0.0001 |
| First Phosphate (mmol/L) | 4.56 | <0.0001 | 2.19 | 0.0002 |
| First Creatinine (μmol/L) | 1.009 | 0.027 | 1.008 | 0.0017 |
| Packed cells in first 24h (Units) | 1.121 | 0.0007 | 1.085 | <0.0001 |
| Charleston Index 1-2 (Ref 0) | Not retained in model | | 2.36 | 0.0002 |
| Charleston Index ≥3 (Ref 0) | Not retained in model | | 2.92 | 0.0066 |
| Development c-statistic | 0.92 (0.88-0.96) | <0.0001 | 0.77 (0.72-0.81) | <0.0001 |
| Validation c-statistic | 0.91 (0.86-0.97) | <0.0001 | 0.70 (0.64-0.77) | <0.0001 |

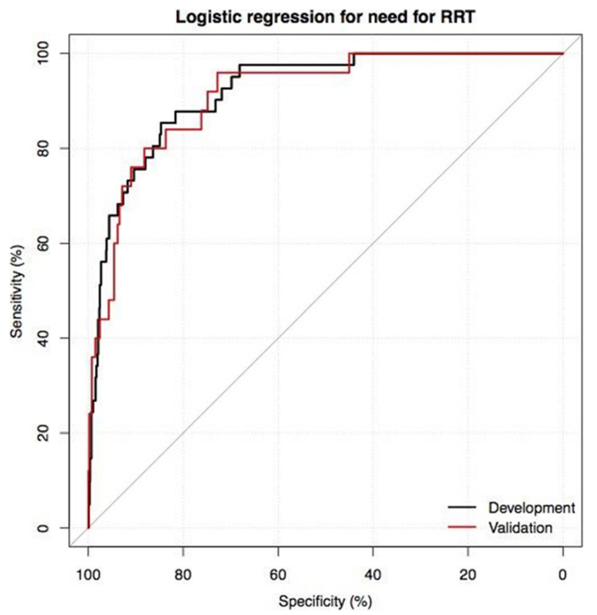

**Fig. 202 (Abstract 0574).** ROC curve analysis for RRT prediction model

### 0575

#### Acute kidney injury and pneumococcal infection in critically ill patients

M. Cecchia[1], C. A. Belluardo[2], M. Giovini[1], S. Gandolfi[3], M. Nolli[2], S. Orlando[1], E. Antonucci[1]
[1]Intermediate Care Unit - Guglielmo da Saliceto Hospital, Piacenza, Italy; [2]Intensive Care Unit - Guglielmo da Saliceto Hospital, Piacenza, Italy; [3]Local Health Unit, Piacenza, Italy
**Correspondence:** M. Cecchia
*Intensive Care Medicine Experimental* 2017, **5(Suppl 2):**0575

**INTRODUCTION.** Acute kidney injury (AKI) is a recurring finding in critically ill patients and is linked to some short and long-term adverse outcomes.Contextually, Streptococcus pneumoniae (SP) infections can be associated with an increased risk of AKI[1]even if, as far as we know, this relationship has been not extensively analysed in critically ill patients yet.

**OBJECTIVES.** We hypothesized that SP infections could be significantly associated with an high incidence of AKI in critically ill patients. We analysed mortality rates, Intensive Care Unit (ICU)/ Intermediate Care Unit (IMCU) stays and the different factors that could have facilitated AKI onset during SP infections.

**METHODS.** We reviewed all patients admitted in our ICU and/or IMCU from June 2014 to December 2016 (21 months). The inclusion criteria were age ≥ 18 y.o; positive microbiological samples (urinary antigen; endotracheal aspirate with a bacterial count ≥ $10^6$ CFU/mL; broncho-alveolar lavage with a bacterial count ≥ $10^4$ CFU/mL; blood culture; pleural culture or cerebro-spinal fluid culture).The exclusion criteria were: patients with bronchitis, aspiration pneumonia or with non-pneumococcal infections. KDIGO criteria were used to classify AKI. We also analysed two subgroups: patients with AKI and patients without AKI.

**RESULTS.** 55 patients respected inclusion criteria. Demographic characteristics and comorbidities are shown in Tables 150 and 151. The main causes of admission were : acute respiratory failure in 53/55 patients (96,4%) and circulatory failure in 19/55 patients (34,6%).We collected different kinds of positive microbiological samples: 27 urinary antigen, 24 endotracheal aspirates, 4 broncho-alveolar lavage, 1 pleural fluid culture, and 1 cerebro-spinal fluid culture. 11/55 patients (20.0%) had SP bacteraemia. AKI occurred in 22/55 patients (40%): stage 1 in 10/22 patients (45,5%) ; stage 2 in 8/22 patients (36,4%); stage 3 in 4 patients (18,2%). No patient needed any renal replacement therapy. A compromised renal function persisted at discharge in 2 patients (3,6%). All of AKI patients showed mortality rates (see Fig. 203) and ICU/IMCU stays (17 days [1–112] vs 7.7 [1–37], p value: 0.02) significantly higher compared to no AKI patients. Chronic kidney disease (CKD) and circulatory failure at admission were significantly related to AKI onset (see Table 152). No difference was found in two groups about SAPS 2, the use of iodinated contrast media, aminoglycosides, glycopeptides, ACEi/ sartans, colistin.

**CONCLUSIONS.** AKI is a recurring complication in critically ill patients with SP infection. Patients with AKI show higher mortality rates and more prolonged ICU/IMCU stay compared to no AKI patients. CKD and circulatory failure are significantly related to AKI onset during SP infections.

**REFERENCE(S)**

1) Mongardon N et al. Epidemiology and outcome of severe pneumococcal pneumonia admitted to intensive care unit: a multicenter study. Critical Care 2012;16(4):R155.

**Table 150 (Abstract 0575).** Demographic characteristics

| | |
|---|---|
| Male gender, n (%) | 34/55 (61,8%) |
| Median age (years) | 73 [39-93] |
| Median BMI (kg/m^2) | 26,1 [17,3-52,2] |
| ICU/IMCU stay (days) | 12 [1-112] |
| Total duration of hospitalization (days) | 16 [1-112] |
| Median SAPS 2 score at admission | 45 [13-97] |
| Median SOFA score at admission | 7 [2-15] |

**Table 151 (Abstract 0575).** Comorbidities

| | |
|---|---|
| Cardiovascular, n (%) | 39/55 (70,9%) |
| Diabetes, n (%) | 14/55 (25,5%) |
| COPD-Asthma, n (%) | 28/55 (50,9%) |
| Tumors, n (%) | 8/55 (14,55%) |
| CKD, n (%) | 12/55 (21,8%) |
| Tobacco, n (%) | 20/55 (36,4%) |
| Cirrhosis, n (%) | 11/55 (20%) |

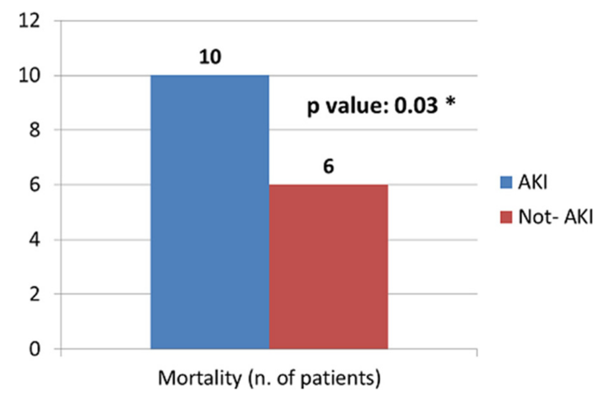

**Fig. 203 (Abstract 0575).** Mortality rate

**Table 152 (Abstract 0575).** Factors associated to AKI onset

| | Patients with AKI | Patients without AKI | p value |
|---|---|---|---|
| CKD, n. of patients | 8/22 | 4/33 | 0,03* |
| Circulatory failure, n. of patients | 12/22 | 7/33 | 0,01* |

### 0576

#### Acute kidney injury after out-of-hospital cardiac arrest

L. Colino Gomez, J. L. Perez, A. Vidal, H. Pérez, P. Turrión, J. M. Milicua, A. Tejero, V. Hortigüela, N. Arias, M. Á. Alcalá, C. Pérez Calvo
Hospital Fundación Jiménez Díaz, Madrid, Spain
**Correspondence:** L. Colino Gomez
*Intensive Care Medicine Experimental* 2017, **5(Suppl 2):**0576

**INTRODUCTION.** Mortality and morbidity from cardiac arrest are high. Subsequent shock and tissue ischemia effects are associated with worse outcome. Acute kidney injury (AKI) has been identified like predictor of increased mortality.
**OBJECTIVES.** Our aim was to evaluate the prevalence of acute kidney injury (AKI) and identifying risk factors and clinical course in out-of-hospital cardiac arrest.
**METHODS.** We conducted a retrospective, observational and descriptive study analyzing all out-of-hospital cardiac arrest patients admitted our unit, from Jan 2012 to March 2016. AKI was defined as KDIGO criteria. Demographics, comorbidities, cardiac arrest conditions and ICU interventions were collected. The results are expressed as mean, median or percentage.
**RESULTS.** 95 patients were included, 64,2% were male, mean age 65,3 years old, average admission APACHE II score was 32. An initial shockable rhythm was in 55,8% of patients and hypothermia is achieved in 57 (60%). 25 patients (26,3%) developed AKI. 8 of whom (32%) presented AKI stage 3, requiring renal replacement therapy 3 cases. 6 patients suffered chronic renal disease previously. Between AKI group and non-AKI group there are not significant differences in comorbidities as COPD, cardiopathy, oncologic diseases or drugs abuse. Longer duration of cardiac arrest, initial higher lactate, initial decreased Ph, lower bicarbonate and increased transaminases were independent predictors of AKI development as well as norepinephrine and dobutamine postarrest administration. History of pre-arrest renal insufficiency was significantly more common in AKI vs. non-AKI patients (1,5% vs 25%; $p < 0,05$). However, no independent association was between AKI and diagnostic or therapeutic coronary catheterization and hypothermia. ICU stay (9,4 vs 5,9 days), and hospital mortality were significantly higher in AKI group ($p < 0,05$).
**CONCLUSIONS.** Post-cardiac arrest AKI occurs in more than 25% of patients. AKI is not influenced by the initial rhythm. These patients have higher severity of shock and more serious hypoperfusion markers and therefore are associated with increased acute renal failure and ICU and hospital mortality.

### 0577

#### Acute kidney injury in critical trauma patients: incidence and clinical outcomes

R. Avila, N. Carrizo, M. Filippi, A. Fernandez, V. Reichert
Hospital Cullen, Santa Fe, Argentina
**Correspondence:** R. Avila
*Intensive Care Medicine Experimental* 2017, **5(Suppl 2):**0577

**INTRODUCTION.** Many factors are involved in the prognosis of trauma patients. Acute kidney injury (AKI) is one of the most threatening complications because of its high association with bad outcomes, increasing morbid mortality, length of stay in ICU (LOS) and higher treatment costs.These patients are exposed to multiple injuries such as shock, rhabdomyolysis, abdominal hypertension and administration of IV contrast, which make them vulnerable to developing AKI. In this study we describe the incidence and outcomes of patients with trauma related AKI who were managed in the Trauma Intensive Care Unit.
**OBJECTIVES.** To identify the incidence and outcomes of patients with trauma related acute kidney injury (AKI), as defined by KDIGO criteria, at a single level I trauma center in Santa Fe, Argentina.
**METHODS.** We performed a retrospective study of 611 trauma patients admitted to the Hospital Cullen intensive care unit from January 2014 to January 2016. The trauma unit database (Hardinero Q) was used to retrieve patient data for this analysis. We collected patient demographics: age, sex and injury severity score (ISS).
We conducted multivariable logistic regression to identity independent predictors for AKI and mortality. In the mortality prediction model we evaluated the following risk factors: age, mechanism of injury (blunt vs. penetrating) or traumatic brain injury (TBI), BMI, hematocrit on admission and AKI.
**RESULTS.** The overall incidence of AKI was 19.5% (n = 119), ICU LOS in non AKI 14 days vs 21 days in AKI. Mean ISS was 23 and mean age was 37 (SD 15.2) in the AKI group. Overall mortality in the AKI group was 71% (n = 85) but was significantly lower in the non AKI group (71% versus 27% $P < 0.00001$). AKI was an independent predictor of mortality (OR 11,6 95% CI 4.51-29.82). Hematocrit on admission was an independent risk factor for AKI. Forty-one patients (36%) required renal replacement therapy.
**CONCLUSIONS.** Our findings are similar to other studies where AKI in trauma patients was an independent risk factor for mortality and LOS ICU. Renal replacement therapy treatment is high in this group and represents a significant health care cost burden.
**REFERENCE(S)**
Acute kidney injury following severe trauma: Risk factors and long-term outcomeJ Trauma Acute Care Surg. 2015 Sep;79(3):407–12.
The incidence and outcomes of acute kidney injury amongst patients admitted to a level I trauma unit.Injury, Int. J. Care Injured 45 (2014) 259–264

### 0578

#### Higher scores in the dynamic prediction for renal replacement model may be associated with mortality and fewer CRRT-free days

C. Gomez Moctezuma[1], E. Monares Zepeda[1], J. Franco Granillo[1], J. Aguirre Sanchez[1], G. Camarena Alejo[2], A. Sanchez Rodriguez[3], L. Cid Mena[3]
[1]American British Cowdray Medical Center, Critical Care, Mexico City, Mexico, [2]American British Cowdray Medical Center, Mexico City, Mexico, [3]American British Cowdray Medical Center, Internal Medicine, Mexico City, Mexico
**Correspondence:** C. Gomez Moctezuma
*Intensive Care Medicine Experimental* 2017, **5(Suppl 2):**0578

**INTRODUCTION.** The dynamic prediction score (DPS) for renal replacement therapy (RRT) model is a recently validated tool to predict RRT in acute kidney injury (AKI), however its possible utility for sepsis, mortality, and need for CRRT are unknown.
**OBJECTIVES.** To assess the capability of a new model to predict mortality, CRRT-free days and the need for CRRT in septic patients in an intensive care unit (UCI).

**METHODS.** We conducted an observational cohort with critical patients admitted with diagnosis of sepsis and AKI from august 2014 through august 2016 in a single center in Mexico. Demographic, clinical, and laboratory data was collected for all patients. We used the model described by Erdfelder1 et al. for all patients. Primary outcomes included mortality, median free - CRRT days, and higher levosimendan-vasoactive-inotropic score (L-VIS)2,3. Statistical analyses were done in STATA SE 11.0.

**RESULTS.** Preliminary data from 26 patients were included, mean age of 68 (SD ± 13) years, 61.54% male. Overall mortality of 42.31% and need for CRRT in 50% with a global median of CRRT-free days of 10 (IQR 3–14) days. The DPS model was found to be accurate (= > 4 points) for discriminate mortality [AUC = 0.65 (0.43 - 0.86)], fewer CRRT-free days with a median of 3(IQR2-9) vs.12 (IQR 8–18) days, p =0.013, the need for CRRT [AUC = 0.70 (0.49 - 0.91)] and risk for CRRT with HR = 6.46 (95% CI 1.23 -33.9, p = 0.027). Additionally L-VIS (> = 33 points) was found to be able to discriminate the need for CRRT [AUC = 0.72 (0.54 - 0.91)] and HR = 8.8 [(95% CI = 1.7 - 46, (p = 0.009)].

**CONCLUSIONS.** DPS may be accurate to predict renal outcomes related to CRRT including need to initiate, survival free days and mortality in septic patients with AKI, additionally a higher L-VIS predicted the need for CRRT in this clinical setting.

**REFERENCE(S)**

1. Erdfelder F, Grigutsch D, Hoeft A, Reider E, Matot I, Zenker S. Dynamic prediction of the need for renal replacement therapy in intensive care unit patients using a simple and robust model.J Clin Monit Comput. 2017 Feb;31(1):195–204.

2. Gaies MG, Gurney JG, Yen AH, Napoli ML, Gajarski RJ, Ohye RG, et al. Vasoactive-inotropic score as a predictor of morbidity and mortality in infants after cardiopulmonary bypass. Pediatr Crit Care Med. 2010 Mar;11(2):234–8.

3. The Vasoactive-Inotropic Score and Levosimendan: Time for LVIS?. Favia, Isabella et al. Journal of Cardiothoracic and Vascular Anesthesia, Volume 27, Issue 2, e15 - e16

### 0579

#### Effect of AKI in ICU on ICU outcomes

M. A. Pachucki[1], E. Ghosh[2], T. Gould[1], M. Thomas[1], L. Eshelman[2], K. Palanisamy[2], C. Bourdeaux[1]

[1]University Hospitals Bristol NHS Foundation Trust, Department of Anaesthesia, Bristol, United Kingdom; [2]Acute Care Solutions Philips Research North America 2 Canal Park, Cambridge, United States

**Correspondence:** M. A. Pachucki

*Intensive Care Medicine Experimental* 2017, **5(Suppl 2):**0579

**INTRODUCTION.** Acute Kidney Injury (AKI) is estimated to occur in 7-18% patients in hospital and approximately in 50% patients admitted to Intensive Care[1]. AKI is associated with worse clinical outcomes, increased length of stay (LOS), increased morbidity and mortality that extend beyond ICU stay[1].

**OBJECTIVES.** In this retrospective analysis we quantify the effect of developing AKI in ICU on clinically relevant outcomes such as mortality and ICU and hospital LOS.

**METHODS.** Using routinely collected data from integrated electronic health records (EHR - Phillips IntelliVue Clinical Information Portfolio - ICIP) incorporating laboratory results and hourly observations, we developed a novel method of interrogating time series of serial (hourly) weight adjusted urine output measurements and daily serum creatinine levels to describe the incidence and progression of acute kidney injury (according to AKIN stage definition[2]) in our cohort. Electronic Health Records data from 4000 encounters was selected. This equates to 3781 patients admitted to ICU for more than 24 hours during 18 month period in 2015/16. Outcomes including mortality, length of stay, ICU outcome were collected as part of routine national audit and the odds risk of outcomes from AKI were calculated.

**RESULTS.** Developing AKI in ICU increases the risk of mortality by 5 times (Fig. 204: Mortality by AKI Stage). Moreover, patients who do not develop AKI in ICU are 5 times more likely to have an improved ICU outcome (Fig. 205: ICU Outcome by AKI Stage). Median ICU length of stay is 3 days for patients with no AKI vs 8 days for patients with AKI stage 3.

(Figure 206: Median LOS by AKI Stage).

**CONCLUSIONS.** To our knowledge this is the first study utilizing the evaluation of time series of hourly weight adjusted urine output measurements and daily serum creatinine levels to define AKI score and progression during ICU stay. Developing AKI in ICU correlates with adverse ICU outcomes, including a longer ICU and hospital length of stay, higher risk of mortality and lower chance of improved outcome upon ICU discharge.

**REFERENCE(S)**

1. Hoste EAJ, Bagshaw SM, Bellomo R et al. (2015) Epidemiology of acute kidney injury in critically ill patients: the multinational AKI-EPI study. Intensive Care Med. doi:10.1007/s00134-015-3934-7

2. Mehta RL et al. Acute Kidney Injury Network: report of an initiative to improve outcomes in acute kidney injury, Crit Care, 2007, vol. 11 pg. R31

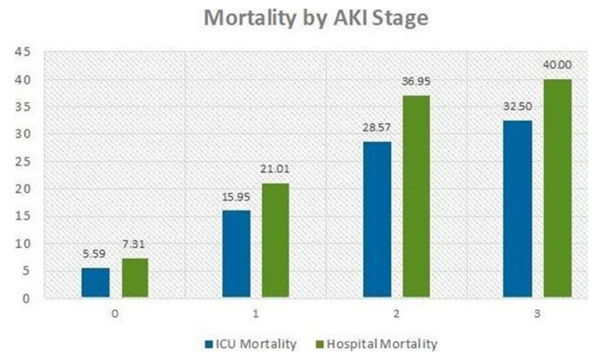

**Fig. 204 (Abstract 0579).** See text for description

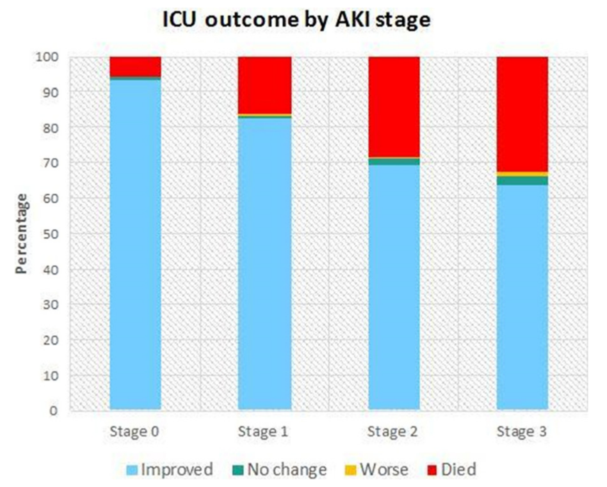

**Fig. 205 (Abstract 0579).** See text for description

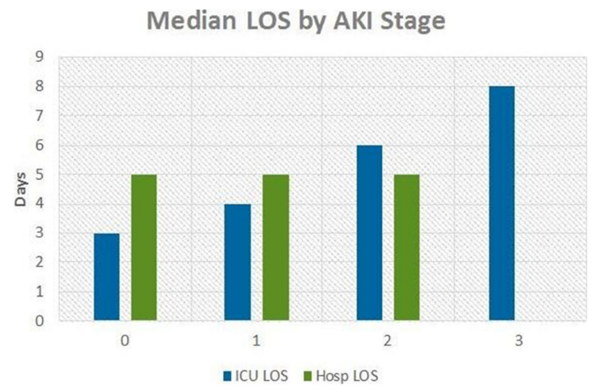

**Fig. 206 (Abstract 0579).** See text for description

### 0580

#### Acute kidney injury incidence in septic patients

A. García-de la Torre[1], M.-V. de la Torre-Prados[2], T. Tsvetanova-Spasova[2], E. Cámara-Sola[3], P. Nuevo-Ortega[2], A. Fernández-Porcel[2], C. Rueda-Molina[2], L. Salido-Díaz[2], M. Mateos-Rodríguez[2], I. García-Gómez[2]
[1]Málaga/Ibima, Clinical Chemistry Department. Universitary H Virgen de la Victoria, Málaga, Spain; [2]Málaga/Ibima, Department of Intensive Care Medicine. Universitary H Virgen de la Victoria, Málaga, Spain; [3]Málaga/Ibima, Department of Intensive Care Medicine, Málaga, Spain
**Correspondence:** M.-V. de la Torre-Prados
*Intensive Care Medicine Experimental* 2017, **5(Suppl 2):**0580

**INTRODUCTION.** Acute kidney injury (AKI) is a common and potentially fatal complication of sepsis. The aim of this study was to describe the incidence of and its prognostic value within the first 24 hours after the diagnosis of severe sepsis (SS) or septic shock (SSh), Surviving Sepsis Campaign 2008.

**METHODS.** Prospective study in a cohort of consecutive septic adults, hospitalized, from August 2008 to July 2015, in a polyvalent Intensive Care Unit (ICU). Demographic variables, severity score, mortality, and renal dysfunction parameters were recorded within the first 24 hours. Statistical analysis was performed using SPSS 18.0 for Windows (SPSS Inc. Chicago, IL, USA).

**RESULTS.** We analyzed 741 consecutive septic patients episodes admitted in the ICU. The median age of the study sample was 64 (inter-quartile range, 51–73) years old; 61.4% were male and 85.5% had SSh. Chronic renal failure was presented in 7.3% of our serie and 23.1% had diabetes. The main sources of infection were: respiratory tract 36.2% and intra-abdomen 33.2%. The median length of stay was 6 [3–14] days, with APACHE II severity score of 24 [20–29] and SOFA score of 9 [7–11] and mortality was 27.9% (n = 207).

Of our serie, 88.3% presented oliguria and 58.4% creatinine increased. In the group of patients with SS, 80.3% had oliguria and 52.3% presented pathologic creatinine values vs. 89.7% and 60.1%, respectively in SSh group, $p < 0.05$.

The difference in oliguria among those who survived was not significant 87.4% vs. 90.3%; however, it was in the case of organic dysfunction according to creatinine values 52.1% vs. 68.3%, $p < 0.05$. Non-survivors had significantly higher creatinine values 68.3% vs. 52.1%, however they had no significant higher oliguria 90.3% vs. 87.4%. The following table shows the creatinine values and mortality, chi square = 18.91 $p < 0.05$.

| Values Creatinine* | Survivors (%) n = 534 | Non-survivors (%) n = 207 | TOTAL (%) |
|---|---|---|---|
| <0.6 mg/dl | 4.3 | 0.5 | 3.2 |
| 0.6-1.1 mg/dl | 22.3 | 14.1 | 19.6 |
| 1.1-1.4 mg/dl | 14.6 | 15.1 | 13.5 |
| 1.5-1.9 mg/dl | 20.2 | 20 | 20 |
| 2-3.4 mg/dl | 27.5 | 32.2 | 28.8 |
| 3.5-4.9 mg/dl | 8.1 | 11.7 | 9.1 |
| >4.9 mg/dl | 2.8 | 6 | 5.4 |
| *Chi square = 18.91 p <0.05 | | | |

*[AKI and Mortality in Sepsis]*

**CONCLUSIONS.** The incidence of AKI is very high in septic patients, being significantly higher in patients with SSh. Higher values of creatinine show significantly worse prognosis.

### 0581

#### Acute kidney injury incidence & progression: a single center analysis

M. A. Pachucki[1], E. Ghosh[2], T. Gould[1], M. Thomas[1], L. Eshelman[2], K. Palanisamy[2], C. Bourdeaux[1]
[1]University Hospitals Bristol NHS Foundation Trust, Department of Anaesthesia, Bristol, United Kingdom; [2]Acute Care Solutions Philips Research North America 2 Canal Park, Cambridge, United States
**Correspondence:** M. A. Pachucki
*Intensive Care Medicine Experimental* 2017, **5(Suppl 2):**0581

**INTRODUCTION.** Acute kidney injury (AKI) is a common condition occurring in more than half of Intensive Care Unit (ICU) patients worldwide and is associated with increased mortality and morbidity that extends beyond ICU stay[1]. It is a condition clinicians may fail to diagnose early.

**OBJECTIVES.** In this study, we use Electronic Health Records (EHR - Phillips IntelliVue Clinical Information Portfolio -ICIP) to retrospectively evaluate the incidence and progression of AKI in a general and cardiac ICU at a large teaching hospital in UK.

**METHODS.** Using routinely collected data from an integrated EHR incorporating laboratory results and hourly observations we developed a novel method of interrogating time series of serial (hourly) weight adjusted urine output measurements and daily serum creatinine levels to describe the incidence and progression of acute kidney injury according to AKI stage using AKIN criteria [2] in our cohort. EHR data from 4000 encounters was selected. This equates to 3781 patients admitted to ICU for more than 24 hours during 18 month period in 2015/16. The prevalence of AKI at ICU admission, improvement or deterioration in AKI stage during ICU stay and on ICU discharge were evaluated.

**RESULTS.** 10% of the population had AKI at ICU admission and 14% had AKI at discharge. 32.6% developed AKI in ICU. Of these patients, 25% were discharged with AKI. AKI risk progression is shown in Fig. 207a. The largest increase in AKI risk occurs between Day 1 and Day 2 of ICU admission. The proportion of patients with AKI Stage 3 increases from Day 1 through Day 5. Analysis of AKI stage improvement and deterioration (Fig. 207b) reveals that most deteriorations occur between Day 1 and Day 2. From Day 3 to Day 5 the proportion of patients improving is more than deteriorating.

**CONCLUSIONS.** To our knowledge this is the first study utilizing the evaluation of time series of hourly weight adjusted urine output measurements and daily serum creatinine levels to define AKI stage and progression during ICU stay. A significant proportion of ICU patients develop AKI, although most are discharged with no risk of AKI. AKI typically manifests on Day 2 of ICU admission and the largest proportion of patients develop low risk of AKI (Stage 1 AKIN criteria). This risk is typ ically reduced over the course of ICU stay.

**REFERENCE(S)**

1. Hoste EAJ, Bagshaw SM, Bellomo R et al. (2015) Epidemiology of acute kidney injury in critically ill patients: the multinational AKI-EPI study. Intensive Care Med. doi:10.1007/s00134-015-3934-7

2. Mehta RL et al. Acute Kidney Injury Network: report of an initiative to improve outcomes in acute kidney injury, Crit Care, 2007, vol. 11 pg. R31

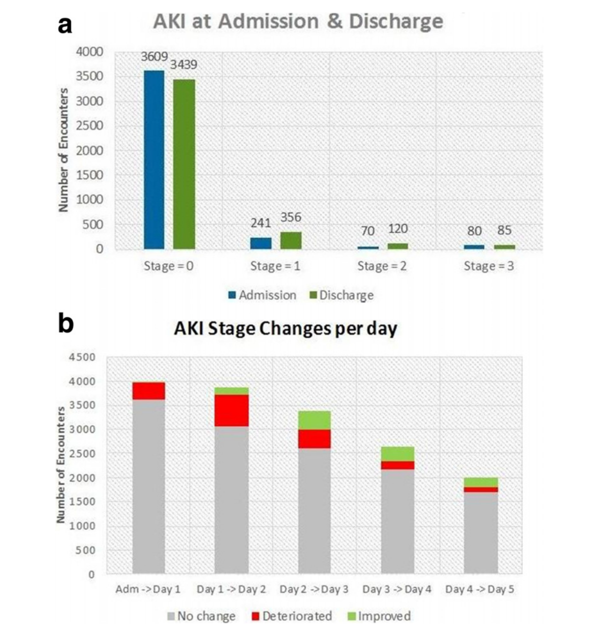

**Fig. 207 (Abstract 0581).** See text for description

### 0582

#### Incorporating Acute Kidney Injury Network (AKIN) criteria with National Early Warning Scores (NEWS) to predict patient outcomes in ICU admissions

P. Parulekar[2], C. E. Perrett[1]

[1]Brighton and Sussex University Hospitals, ICU, BN BE, United Kingdom; [2]Brighton and Sussex University Hospitals, Brighton, United Kingdom

**Correspondence:** C. E. Perrett

*Intensive Care Medicine Experimental* 2017, **5(Suppl 2):**0582

**INTRODUCTION.** The Royal College of Physicians developed the use of NEWS across the UK to help to provide enhanced clinical review of patients at risk of acute deterioration (1). The six parameters used in scoring system do not incorporate urine output or other measure of renal function.

The Acute Kidney Injury Network developed a score designed to observe changes in creatinine and urine output over a 48 hour period to aid the identification of acute kidney injury.(3) It is known that even patients with a mild creatinine elevation have an increased mortality. (2) The AKIN score is sensitive to smaller changes in creatinine and therefore able to pick up those early changes which are still associated with a poorer outcome.

**OBJECTIVES.** The aim of this retrospective study was to assess whether adding the AKIN score to the NEWS in patients subsequently admitted to ICU could have helped to predict the need for renal replacement therapy (RRT), organ support and 28 day mortality.

**METHODS.** Data was collected from ICU admissions at the Royal Sussex County Hospital, UK. The last NEWS score and creatinine for each patient prior to ICU admission were obtained. Due to limited documentation of urine output a decision was made not to include this. Need for renal replacement therapy, other organ support, and 28 day mortality were recorded. Outcomes of NEWS versus NEWS + AKIN score were then compared to see whether the combined score was a better prediction tool for outcomes.

**RESULTS.** Interestingly 20% of patients with a NEWS score of 0–5 went onto require RRT suggesting NEWS alone is a poor indicator for need for renal replacement therapy. However, the NEWS + AKIN score showed a difference of 5% between the lower score group (score 0–6) compared to the higher score group (score >6). With regards to 28-day mortality the NEWS score versus the NEWS + AKIN score showed similar increase between the lower and higher score groups (increase of 13% in the NEWS group versus 8% in NEWS + AKIN).

**CONCLUSIONS.** This study has shown that NEWS + AKIN score may be a better predictor for need for RRT compared to NEWS alone. With increasing national focus on identifying and managing AKI as early as possible, this study highlights the need to regularly review renal parameters combined with clinical assessment and haemodynamic markers in the assessment of all acutely unwell patients. We aim to repeat this study prospectively adding urine output and creatinine to the NEWS to assess if this is a more accurate predictor of outcome.

1.) Royal College of Physicians. National Early Warning Score (NEWS): Standardising the assessment of acute illness severity in the NHS.

2.) Chertow GM, B et al.: Acute kidney injury, mortality, length of stay, and costs in hospitalized patients. *J Am Soc Nephrol* 2005,

3.) Mehta R, et al. AKINetwork: Acute Kidney Injury Network: Report of an Initiative to Improve Outcomes in Acute Kidney Injury *Critical Care 2007*

### 0583

#### Renal function recovery and long term mortality in critical care patients with acute kidney injury subjected to continuous renal replacement therapy

N. Varga[1], A. Cortina[1], M. Martinez[1], J. Jaramillo[2], K. Arredondo[2], D. García[2], R. Albertos[1], E. Papiol[1], J. Baldirà[1], N. Ramos[2], M. Pérez[1]

[1]Vall d´Hebron University Hospital, Critical Care, Barcelona, Spain; [2]Vall d´ Hebron University Hospital, Nephrology, Barcelona, Spain

**Correspondence:** N. Varga

*Intensive Care Medicine Experimental* 2017, **5(Suppl 2):**0583

**INTRODUCTION.** Critically ill patients with acute kidney injury that require continuous renal replacement therapy present a high mortality rate. The recovery of renal function is unknown in the long term in the survivors, as well as its relation with the mortality.

**OBJECTIVES.** To determine the differences in terms of renal function recovery, depending on the cause, in patients with acute kidney injury (AKI) that required continuous renal replacement therapy (CRRT) and evaluate mortality according to renal function recovery.

**METHODS.** Prospective observational study including AKI patients that required CRRT, of a tertiary care hospital between january 2013 and december 2014 with at least 2 year follow-up. Chi-Square, Fisher´s Test, T-Student, U of Mann Whitney and Kaplan Meier curves were used. Data expressed as frequency (%) and median (interquartile range).

**RESULTS.** One hundred sixty seven patients were included, median age 66 (55–74) years, men 103 (61.7%), Charlson comorbidity index 3 (1–6) and APACHE II 23 (16–28).

AKI etiology was multifactorial in 61 (37.2%) patients, septic in 60 (36.6%), hemodynamic in 32 (19.5%), toxic in 4 (2.4%) and miscellany in 7 (4.3%). There were no differences between groups and the CRRT protocol was similar.

Baseline creatinine was 0.98 (0.75-1.37) mg/dL, on admission 2 (1–3) mg/dL and at CRRT initiation 2.57 (1.82-3.58) mg/dL. Length of CRRT was 4 (2–8) days, initiated mostly before the 4th day of admission. Twenty-eight patients (23.9%) required one or more subsequent intermittent hemodialysis (IHD) sessions. Overall ICU mortality was 60.5% and mortality rate at 2 years 71.9%.

Nineteen patients (30.2%) died during follow-up period, only 4 (21.1%) patients had presented data compatible with recovery of baseline renal function (BRF). Patients who recovered BRF during follow-up had a greater survival (87.1% vs. 60.9%, p = 0.03).

Forty-four (60.8%) patients survived at least 2 years. BRF was recovered by 27 (65.9%) patients, only 3 (6.8%) needed chronic IHD. According to AKI etiology, BRF was recovered by 11 (77.8%) patients of multifactorial group, 11(73.3%) septic group and 6 (54.5%) hemodynamic group; (p = 0.56).

**CONCLUSIONS.** The etiology of AKI in critically ill patients submitted to CRRT could guide us through the recovery of basal renal function, which influences long-term survival.

### Sepsis therapeutics 2

#### 0584

#### Impact of the use of biomarkers on early discontinuation of empirical antifungal therapy in critically ill patients: a randomised controlled study

A. Rouzé[1], S. Loridant[2], J. Poissy[1], B. Sendid[2], S. Nseir[1], STAFE Study Group

[1]CHU de Lille, ICU, Lille, France; [2]CHU de Lille, Mycology, Lille, France

**Correspondence:** A. Rouzé

*Intensive Care Medicine Experimental* 2017, **5(Suppl 2):**0584

**INTRODUCTION.** Empirical antifungal treatment (EAT) is frequently prescribed to septic critically ill patients with risk factors for invasive *Candida* infections (ICI). However, antifungal discontinuation is rarely performed in patients with no proven ICI, resulting in unnecessary overuse of antifungals.

**OBJECTIVES.** The primary objective of this study was to determine the impact of a biomarker-based strategy on early discontinuation of EAT.

**METHODS.** Prospective randomized controlled unblinded study performed in a single mixed ICU. 110 patients receiving EAT for the first time, with an expected length of stay of at least 6 days, were randomly assigned to one of the two following decision-making strategies regarding EAT duration:

(1) a strategy in which EAT duration was determined by β-D-1,3-glucan, mannan, and anti-mannan serum assays, performed at day 0 (day of EAT initiation) and day 4 (biomarker strategy); or

(2) a routine care strategy, based on international guidelines, which recommend 14 days of treatment (routine strategy). Primary outcome was the percentage of patients receiving early discontinuation of EAT, defined as a discontinuation strictly before day 7. Secondary endpoints included duration of EAT, renewal of any antifungal therapy after EAT discontinuation, proven ICI after EAT discontinuation, intensity of *Candida* colonisation during ICU stay, antifungal-free days at day 28, ventilator-free days at day 28, ICU-free days at day 28, ICU mortality and day 28-mortality. This trial was registered at ClinicalTrials.gov, number NCT02154178.

**RESULTS.** 109 patients were analyzed (one patient withdraw consent), including 54, and 55 in intervention and routine strategy, respectively. Patient characteristics were similar in the two study groups. EAT was early discontinued in 29 patients in biomarker strategy, compared with 1 patient in routine strategy (54% *vs* 2%, p < 0.001, OR (95% CI) 62.6 (8.1-486)). Duration of EAT was significantly shorter in biomarker strategy compared with routine strategy (median (IQR): 6 (4–13) *vs* 13 (12–14) days, p < 0.001). After EAT discontinuation, antifungal treatment was restarted in 6 patients (5 in the biomarker-strategy group, and 1 in the routine-strategy group, p = 0.113). The percentage of patients with proven recurrent ICI was similar in the two groups (4% versus 2% in intervention and control group, respectively, p = 0.54). Biomarker strategy significantly increased antifungal-free days, compared with routine strategy (3.5 (0–17) *vs* 0 (0–7) days, p = 0.004). Ventilator-free days, ICU-free days, ICU mortality and day 28-mortality were similar in the two study groups.

**CONCLUSIONS.** The use of a biomarker-based strategy increased the percentage of early discontinuation of EAT among critically ill patients with suspected ICI, without negative impact on outcomes.

**GRANT ACKNOWLEDGMENT**

Supported in part by a Grant from MSD.

#### 0585

#### Empirical therapy with an echinocandin reduces mortality in critically ill patients with candidaemia: a propensity score-adjusted analysis of a multicenter study. A propensity score-adjusted analysis of a multicenter study

J. Rodriguez-Gomez[1], J. L. Garcia Garmendia[2], A. Díaz-Martin[3], L. Cantón[4], E. Villarreal Tello[5], R. Sierra[6], A. Loza[7], D. Arias-Verdú[8], M. Rodriguez-Delgado[9], J. Ruiz-Ramos[5], J. Garnacho-Montero[4]

[1]Hospital Universitario Reina Sofía, Cordoba, Spain; [2]Hospital San Juan de Dios De Ajaraje, Bormujos, Spain; [3]Hospital Universitario Virgen del Rocío, Sevilla, Spain; [4]Hospital Universitario Virgen Macarena, Sevilla, Spain; [5]Hospital La Fe, Valencia, Spain; [6]Puerta del Mar University Hospital, Cádiz, Spain; [7]Hospital Universitario Virgen de Valme, Sevilla, Spain; [8]Hospital Regional Universitario de Málaga, Malaga, Spain; [9]Hospital Universitario Reina Sofía, Córdoba, Spain

**Correspondence:** J. Rodriguez-Gomez

*Intensive Care Medicine Experimental* 2017, **5(Suppl 2):**0585

**INTRODUCTION.** Clinical, guidelines advocate recommend empirical therapy with an echinocandin in critically ill patients with candidaemia.[1] However, recent observational studies have questioned the superiority of echinocandins over fluconazole for empirical treatment of candidaemia in critically ill patients.[2]

**OBJECTIVES.** To determine if, compared with fluconazole and after adjustment for confounders, the empirical therapy with an echinocandin is associated with a lower mortality rate (at 30 and 90-day) in patients with proven candidaemia admitted to intensive care units (ICUs) at disease onset. The microbiological isolations of these proven candidaemias were described.

**METHODS.** It is a Retrospective multicenter study carried in nine Spanish medical-surgical ICU. Adult patients (≥18 yr) with an episode of *Candida* bloodstream infection during admission to the ICU from January 2011 to April 2016 were included. All patients were followed up for 90 days after the episode of candidaemia. There were reviewed patients characteristics, severity of illness, infection-related variables, severity of illness the day of candidaemia, microbiological data, therapeutic interventions and metastatic complications. Univariante statistical analysis (SPSS v18) was performed using chi-square o exact Fisher test for qualitative variables and t-Student or U Mann–Whitney test for quantitative variables when appropriate. After that a propensity score (PS) adjusted -multivariable analysis was performed to identify the risk factors significantly associated with 30-day and 90-day mortality.

**RESULTS.** 294 patients were included. Candida albicans was the most common species isolated (46.6%) followed by C. parapsilosis (21.8%), C. glabrata (17.3%), C. tropicalis (9.9%) and others (2.0%). 60 were excluded (other antifungals in the primary therapy or died without empirical antifungal therapy). The study group is comprised by 115 patients that received fluconazole (30-day mortality 37.4%) and 119 patients treated empirically with an echinocandin (30-day mortality 31.9%). The use of an echinocandin in the empirical therapy was a protective factor of 30-day (OR 0.32; 95%CI 0.16-0.66; p = 0.002) and

90-day mortality (OR 0.50; 95%CI 0.27-0.93; p = 0.014) in the propensity score adjusted-multivariable analysis.

**CONCLUSIONS.** In critically ill patients with documented candidaemia, empirical use of an echinocandin significantly reduces mortality at 30 and 90 days.

**REFERENCE(S)**

1. Cornely OA et al. ESCMID Fungal Infection Study Group. ESCMID* guideline for the diagnosis and management of Candida diseases 2012: non-neutropenic adult patients. Clin Microbiol Infect. 2012 Dec;18 Suppl 7:19–37.

2. Murri R et al. Initial antifungal strategy does not correlate with mortality in patients with candidemia. Eur J Clin Microbiol Infect Dis 2016; 35:187–93.

### 0586

#### Monotherapy vs. combination therapy for bacteriemia treatment in adult intensive care patients. A retrospective cohort study

A. Naharro Abellán[1], M. Ripa[2], C. De La Calle[3], L. Morata[3], A. Soriano[3], S. Alcantara[1], P. Matía almudevar[1], J.A. Martínez[3]

[1]Hospital Universitario Puerta de Hierro Majadahonda, Medicina Intensiva, Majadahonda, Spain; [2]Hospital San Raffaele, Milano, Italy; [3]Hospital Clinic, Malalties Infeccioses, Barcelona, Spain

**Correspondence:** A. Naharro Abellán

*Intensive Care Medicine Experimental* 2017, **5(Suppl 2):**0586

**INTRODUCTION.** Combination therapy refers to the use of two different classes of antibiotics for the treatment of a single pathogen that is sensitive to both. Nowadays no firm evidence exists regarding the benefit or harm of combination therapy[1] and current guidelines only recommend it for the treatment of septic shock[2].

**OBJECTIVES.** To assess the benefits of empirical monotherapy versus combination therapy in bacteriemia developed in adult patients admitted the Intensive Care Unit (ICU) of a Spanish tertiary hospital (Hospital Clínic of Barcelona). The primary outcome was all cause mortality.

**METHODS.** Retrospective study (2010–2016). We selected all mono-pathogen positive blood cultures of patients admitted to the ICU for more than 24 hours. We collected demographic, clinical, microbiological data (including antibiogram) and antibiotic treatment. Empiric antibiotics were evaluated separately in order to quantify the number of correct antibiotics and three group categories were created: incorrect empirical therapy (not analyzed in this study), monotherapy and combination therapy. Statistical analysis with Chi square, Student´s t-test and forward logistic regression using SPSS IBM software.

**RESULTS.** During the period studied, 443 mono-pathogen positive blood cultures were identified. A total of 269 patients received a correct antibiotic therapy and of those 204 (76%) were treated with monotherapy and 65 (24%) with combination therapy.

In the monotherpy group, the most frequent isolated microorganisms were Gram-positive cocci (42.9 vs 20.3%;p = 0.001) while in the combination therapy group there was a higher rate of Gram negative bacilli (45.8 vs 76.6%; p < 0.001). In the statistical analysis we found that the monotherapy group had shorter hospital lengths of stay (mean of 22 vs 33 days; p = 0.001) and less solid organ transplant recipients (10.3 vs 20.3%; p = 0.04).

Crude analysis of all-cause mortality between monotherapy and combination therapy showed no differences (29.9% vs 30.8%;p = 0.89) and this result was sustained (p = 0.79) after logistic regression analysis adjusted by shock (HR 6.43;p < 0.001) and prognosis (using McCabe classification;p < 0.001). We didn´t find differences in related mortality (21.6% vs 16.9%;p = 0.42) nor in the subgroup of patients with shock.

**CONCLUSIONS.** In our study we found no differences between mortality in patients treated with a correct monotherapy vs. combination therapy.

**REFERENCE(S)**

1. Sjövall, F., Perner, A. & Hylander Møller, M. Empirical mono- versus combination antibiotic therapy in adult intensive care patients with severe sepsis - A systematic review with meta-analysis and trial sequential analysis. *J. Infect.* **74,** 331–344 (2017).

2. Rhodes, A. *et al. Surviving Sepsis Campaign: International Guidelines for Management of Sepsis and Septic Shock: 2016. Intensive Care Medicine* **43,** (Springer Berlin Heidelberg, 2017).

**Table 153 (Abstract 0586).** Demographic and comorbidities

| | Motherapy | | | Combined Therapy | | | |
|---|---|---|---|---|---|---|---|
| | Count | % | Mean | Count | % | Mean | p |
| Sex (women) | 66 | 32,35% | | 29 | 44,62% | | 0,072 |
| Age | | | 60 | | | 59 | 0,422 |
| Comorbidities | | | | | | | |
| Enolism | 19 | 9,3% | | 6 | 9,2% | | 0,984 |
| Diabetes | 33 | 16,2% | | 14 | 21,5% | | 0,321 |
| Cirrhosis | 21 | 10,3% | | 5 | 7,7% | | 0,536 |
| Chronic renal failure | 34 | 16,7% | | 12 | 18,5% | | 0,738 |
| COPD | 28 | 13,7% | | 6 | 9,2% | | 0,342 |
| Uropathy | 1 | ,5% | | 1 | | | 0,572 |
| HIV | 6 | 2,9% | | 1 | 1,5% | | 0,536 |
| Bone marrow transplant | 2 | 1,0% | | 2 | 3,1% | | 0,224 |
| Neoplasm | 49 | 24,0% | | 9 | 13,8% | | 0,082 |
| **Solid Organ Transplantation** | **21** | **10,3%** | | **13** | **20,0%** | | **0,040*** |
| No comorbidities | 4 | 2,0% | | 1 | | | 0,255 |
| Focus | | | | | | | |
| Pulmonary | 13 | 6,4% | | 7 | 10,8% | | 0,239 |
| Urinary | 16 | 7,9% | | 4 | 6,3% | | 0,665 |
| Unknown | 52 | 25,5% | | 16 | 24,6% | | 0,888 |
| Catheter | 93 | 45,6% | | 33 | 50,8% | | 0,466 |

**Table 154 (Abstract 0586).** Data at time of positive blood culture

| | | Motherapy | | | Combined Therapy | | | |
|---|---|---|---|---|---|---|---|---|
| | | Count | % | Mean | Count | % | Mean | p |
| **Days of previous admission** | | | | **23** | | | **33** | **0,001*** |
| Surgery | | 80 | 39,8% | | 27 | 42,2% | | 0,735 |
| Invasive manipulation | | 42 | 29,4% | | 10 | 21,7% | | 0,313 |
| Mechanical ventilation | | 96 | 48,5% | | 31 | 47,7% | | 0,912 |
| Urinary catheter | | 173 | 87,4% | | 56 | 87,5% | | 0,979 |
| Fever | | 166 | 82,6% | | 52 | 86,7% | | 0,455 |
| Shock | | 54 | 26,6% | | 23 | 37,1% | | 0,111 |
| CID | | 5 | 2,5% | | 2 | 3,3% | | 0,713 |
| ARDS | | 12 | 5,9% | | 2 | 3,3% | | 0,425 |
| Granulopenia | | 14 | 7,1% | | 3 | 4,7% | | 0,496 |
| Corticoids | | 64 | 33,3% | | 28 | 43,8% | | 0,133 |
| Prognosis | Non-Fatal | 129 | 64,8% | | 39 | 63,9% | | 0,73 |
| | Ultimately fatal | 59 | 29,6% | | 20 | 32,8% | | |
| | Rapidly fatal | 11 | 5,5% | | 2 | 3,3% | | |
| **Gram-positive cocci** | | **87** | **42,6%** | | **13** | **20,0%** | | **0,001*** |
| **Gram negative bacilli** | | **93** | **45,6%** | | **50** | **76,9%** | | **0,000*** |
| White blood cell count | | | | 12773 | | | 13389 | 0,507 |
| PCR | | | | 13,82 | | | 12,93 | 0,08 |
| Lactic | | | | 15,63 | | | 13,66 | 0,36 |

### 0587

#### Abnormal capillary refill time after 6 hours of resuscitation predicts the requirement of high-volume hemofiltration (HVHF) as a rescue therapy in septic shock patients

M. Silva[1], E. D. Valenzuela Espinoza[1], J. Dreyse[1], M. Vera[1], L. Alegría[1], G. Ferri[1], C. Santis[1], D. Carpio[1], R. Castro[1], S. Bravo[1], J. Bakker[1,2,3], G. Hernández[1]

[1]Hospital Clínico Universidad Católica, Departamento Medicina Intensiva, Santiago, Chile; [2]Columbia University Medical Center, Dept. Pulmonology & Critical Care, New York, United States; [3]New York University Medical Center, Dept. Pulmonology & Critical Care, New York, United States

**Correspondence:** M. Silva

*Intensive Care Medicine Experimental* 2017, **5(Suppl 2):**0587

**INTRODUCTION.** The presence of a hypoperfusion context determines huge differences in mortality and morbidity among septic shock patients. The purpose of this study was to explore if the normalization of capillary refill time (CRT) after 6 hours of resuscitation is associated to decrease in morbidity (HVHF, Mechanical Ventilation, ICU and Hospital length of stay) or mortality in S-3 septic shock patients.

**METHODS.** Retrospective analysis of a prospectively filled database of ninety S-3 septic shock patients treated at an academic ICU. Patients were classified according to the presence of persistent abnormal CRT (>3 sec) vs. normal CRT after 6 hours of intense treatment. These subgroups were compared according to demographic, hemodynamic, perfusion parameters, NE requirements.

**RESULTS.** 90 patients with septic shock were enrolled; the main demographic and clinical variables are shown in the table. Persistent abnormal CRT after 6 hours of resuscitation is a predictor of HVHF requirement (OR: 3.37 95%CI 1.14-10.24, p = 0.032).

**CONCLUSIONS.** Persistent abnormal peripheral perfusion predicts requirement of HVHF as a rescue therapy in septic shock patients.

**Table 155 (Abstract 0587).** See text for description

| | All | 6 hours CRT > 3 sec | 6 hours CRT ≤ 3 sec |
|---|---|---|---|
| n | 90 | 23 | 67 |
| Age (year) | 66 ± 16 | 68 ± 16 | 65 ± 16 |
| APACHE II score | 21 ± 7 | 24 ± 8* | 20 ± 6 |
| SOFA score | 9 ± 4 | 10 ± 4 | 9 ± 4 |
| Lactate (mmol/L) | 4.0 [2.9,5.8] | 3.5 (2,7.4)* | 2 (1.4,2.6) |
| NE dose (ug/kg/min) | 0.10 [0.02,0.29] | 0.24 (0.11,0.43)* | 0.06(0.02,0.16) |
| MAP (mmHg) | 76 ± 19 | 72 ± 7* | 78 ± 11 |
| ICU LOS (days) | 8 [6,14] | 9 (7,12) | 8 (6,14) |
| Hospital LOS (days) | 25 ± 19 | 27 ± 20 | 24 ± 19 |
| MV (days) | 5 [3,9] | 7 (4,10) | 4 (3,8) |
| HVHF (%) | 17 (19) | 8 (35)* | 9 (13) |
| Mortality (%) | 12 (13) | 6 (26)* | 6 (9) |

Values are expressed as mean ± SD or median (interquartile range) or N (%). APACHE, Acute Physiology and Chronic Health Evaluation; SOFA, Sequential Organ Failure Assessment; MAP, mean arterial pressure; NE, norepinephrine; ICU, intensive care unit; LOS, length of stay; MV, mechanical ventilation; HVHF, high-volume hemofiltration; *(p<0.05) between groups.

### 0588

#### De-escalation in a mixed intensive care unit with selective digestive decontamination significantly decreases ICU mortality

C. Sánchez Ramírez[1], L. Caipe Balcázar[1], M. Cabrera Santana[1], S. Hípola Escalada[1], M.A. Hernández Viera[1], R.E. Morales Sirgado[1], C.F. Lübbe Vázquez[1], F. Artiles Campelo[2], P. Saavedra Santana[3], S. Ruiz Santana[1]
[1]University Hospital of Gran Canaria Dr Negrín, Intensive Care Unit, Las Palmas de Gran Canaria, Spain; [2]University Hospital,of Gran Canaria Dr Negrín, Pharmacy Department, Las Palmas de Gran Canaria, Spain; [3]University of Las Palmas de Gran Canaria, Mathematics and Informatics Department, Las Palmas de Gran Canaria, Spain
**Correspondence:** C. Sánchez Ramírez
*Intensive Care Medicine Experimental* 2017, **5(Suppl 2):**0588

**OBJECTIVES.** To assess the appropriate use of antibiotics and their de-escalation (DE) to treat nosocomial infections along 5 years in an ICU with Selective Digestive Decontamination (SDD).

**METHODS.** In a 30 bed mixed ICU from October 1, 2011 to September 30, 2016 nosocomial infections (pneumonia, urinary tract infections, catheter-related bacteremia (CRB) and bacteremia of unknown origin and secondary nosocomial bacteremia) were prospectively collected. ENVIN-HELICS diagnostic criteria were applied. Etiology, inflammatory response to infection, antibiotic treatment (ATB T) and treatment modifications according to culture results, were analyzed. SDD was applied to all admitted patients requiring endotracheal intubation over 48 hours. For each groups categorical variables were summarized as frequencies and percentages and number in means and standard deviations (SD) or median with interquartile ranges (IQR). Percentages were compared, as appropriate, with the Fisher´s exact test.or $X^2$ test and medians with the Wilcoxon test for independent samples. For those variables that were associated with DE in the univariate analysis were entered into a logistic multidimensional analysis. The model obtained was expressed by p-values and odd-ratios, which were estimated by confidence intervals at 95%. A hypothesis test was considered statistically significant when p-value was less than .05.

**RESULTS.** DE was done in 105 out of 339 patients. There were no significant differences in demographics or type of admission (Table 156). Mortality was lower in patients receiving DE antibiotic (ATB) (27.6% vs. 43.2%, p: 0.006). In the multivariate study, ICU mortality, CRB, severe sepsis and septic shock were statistically significant (Table 157).

Fifty-four patients with CRB (51.4%) and 44 with nosocomial pneumonia (41.9%) had DE. The ATB T was inadequate in 58 infections (15%).Targeted therapy was performed in 101 infections (21%). In 85 patients DE was applied once, 17 twice and 3 three times and 76 had septic shock. The number of antibiotics used was 845 and in 128 ATB DE were performed. The most DE ATB were levofloxacin, meropenem and cefepime. Frequency of use and DE are shown in Table 158.

**CONCLUSIONS.** ICU patients who received, compared to those that did not received DE, had a significantly lower mortality. Factors independently associated to DE were in addition to ICU mortality, severe sepsis, septic shock and CRB infection. Inadequate ATB T was applied to 15% of nosocomial infections. Finally, the most commonly DE ATB were levofloxacin, meropenem and cefepime.

**Table 156 (Abstract 0588).** See text for description

| | Total N = 339 | De-escalation No N = 234 | De-escalation Yes N = 105 | P |
|---|---|---|---|---|
| ***Age**, years* | 61.2 ± 15.4 | 61.8 ± 15.5 | 60.0 ± 15.4 | .327 |
| ***Sex*** | | | | .097 |
| Male | 217 (64.0) | 143 (61.1) | 74 (70.5) | |
| Female | 122 (36.0) | 91 (38.9) | 31 (29.5) | |
| ***Apache-II*** | 22.0 ± 7.5 | 22.4 ± 7.4 | 21.4 ± 7.7 | .256 |
| ***Admission*** | | | | .362 |
| Medical | 237 (69.9) | 166 (70.9) | 71 (67.6) | |
| Program surgical | 51 (15.0) | 37 (15.8) | 14 (13.3) | |
| Urgent surgical | 51 (15.0) | 31 (13.2) | 20 (19.0) | |
| ***Inflammatory response*** | | | | .007 |
| Non Sepsis | 19 (5.6) | 18 (7.7) | 1 (1.0) | |
| Sepsis | 116 (34.2) | 88 (37.6) | 28 (26.7) | |
| Severe sepsis | 34 (10.0) | 22 (9.4) | 12 (11.4) | |
| Septic shock | 170 (50.1) | 106 (45.3) | 64 (61.0) | |
| ***Traumatic patients*** | 38 (11.2) | 25 (10.7) | 13 (12.4) | .657 |
| ***Catheter related bacteremia*** | 140 (41.3) | 86 (36.8) | 54 (51.4) | .011 |
| ***Secondary bacteremia*** | 68 (20.1) | 48 (20.5) | 20 (19.1) | .755 |
| ***Nosocomial pneumonia*** | 124 (36.6) | 80 (34.2) | 44 (41.9) | .375 |
| ***Diabetes mellitus*** | 107 (31.6) | 72 (30.8) | 35 (33.3) | .639 |
| ***Cirrhosis*** | 20 (5.9) | 17 (7.3) | 3 (2.9) | .111 |
| ***COPD*** | 53 (15.6) | 38 (16.2) | 15 (14.3) | .647 |
| ***Urinary infection*** | 93 (27.4) | 74 (31.6) | 19 (18.1) | .010 |
| ***Chronic renal failure*** | 69 (20.4) | 49 (20.9) | 20 (19.1) | .689 |
| ***Neoplasms*** | 35 (10.3) | 26 (11.1) | 9 (8.6) | .477 |
| ***Coronary patient*** | 74 (21.8) | 49 (20.9) | 25 (23.8) | .554 |
| ***RRT*** | 127 (37.5) | 89 (38.0) | 38 (36.2) | .746 |
| ***Total parenteral nutrition*** | 125 (36.9) | 94 (40.2) | 31 (29.5) | .060 |
| ***Neutropenic patients*** | 12 (3.5) | 10 (4.3) | 2 (1.9) | .355 |
| ***Death*** | 128 (38.3) | 99 (43.2) | 29 (27.6) | .006 |
| ***ICU days*** | 30 (17 ; 48) | 29 (16.5 ; 48) | 36 (19 ; 49) | .364 |

Data are means ± SD, frequencies (%) and medians (IQR) COPD: Chronic Obstructive Pulmonary Disease; RRT: Renal Replacement Therapy

**Table 157 (Abstract 0588).** See text for description

| | P | OR (95% CI) |
|---|---|---|
| *Catheter related bacteremia* | .009 | 1.914 (1.174 ; 3.121) |
| *Death* | < .001 | 0.370 (0.215 ; 0.637) |
| *Severe sepsis or septic shock* | < .001 | 3.015 (1.748 ; 5.203) |

**Table 158 (Abstract 0588).** See text for description

| *Antibiotic* | | *Frequency of use* | *Frequency of de-escalation* |
|---|---|---|---|
| Acyclovir | 1 | 1 | 0 |
| Fusidic acid | 2 | 1 | 0 |
| Amikacin | 6 | 26 | 5 |
| Ampicilin | 8 | 5 | 0 |
| Amoxicillin-clavulanate | 11 | 1 | 0 |
| Cefotaxime | 30 | 19 | 1 |
| Ceftazidime | 34 | 41 | 7 |
| Ceftriaxon | 35 | 4 | 0 |
| Cefuroxime | 37 | 2 | 0 |
| Ciprofloxacin | 40 | 23 | 1 |
| Clindamycin | 42 | 2 | 1 |
| Cloxacilina | 44 | 15 | 0 |
| Colistin | 45 | 18 | 1 |
| Cotrimoxazole | 46 | 7 | 1 |
| Doxycycline | 52 | 1 | 0 |
| Erythromycin | 56 | 1 | 0 |
| Fluconazole | 61 | 37 | 1 |
| 5- fluorocytosine | 62 | 1 | 0 |
| Fidoxamicina | 63 | 1 | 1 |
| Fosfomycin | 65 | 16 | 0 |
| Gentamycin | 67 | 24 | 3 |
| Imipenem | 71 | 35 | 1 |
| Metronidazole | 85 | 1 | 0 |
| Penicillin | 105 | 1 | 0 |
| Piperacilin-Tazobactam | 107 | 95 | 5 |
| Ampicilin-Sulbactam | 119 | 2 | 0 |
| Teicoplanin | 123 | 28 | 4 |
| Tobramicyn | 129 | 10 | 1 |
| Vancomycin | 131 | 39 | 2 |
| Other antifungal | 134 | 1 | 0 |
| Cefepime | 151 | 89 | 20 |
| Meropenem | 161 | 105 | 20 |
| Levofloxacin | 204 | 103 | 29 |
| Grepafloxacin | 206 | 1 | 0 |
| Linezolid | 210 | 68 | 17 |
| Voriconazole | 211 | 4 | 0 |
| Caspofungin | 212 | 9 | 3 |
| Tigecycline | 215 | 2 | 0 |
| Daptomycin | 216 | 1 | 0 |
| Anidulafiugin | 218 | 5 | 1 |

### 0589

#### Immunomodulation of therapeutic normothermia in febrile septic shock patients: a randomized controlled trial

J. Saoraya[1], K. Musikatavorn[2], P. Puttaphaisan[1], A. Komindr[1], N. Srisawat[3]
[1]King Chulalongkorn Memorial Hospital, The Thai Red Cross Society, Emergency Department, Bangkok, Thailand; [2]Emergency Medicine Unit, Faculty of Medicine, Chulalongkorn University and King Chulalongkorn Memorial Hospital, The Thai Red Cross Society, Department of Medicine, Bangkok, Thailand; [3]Chulalongkorn University, Excellent Center for Critical Care Nephrology, Division of Nephrology, Department of Medicine, Bangkok, Thailand
**Correspondence:** J. Saoraya
*Intensive Care Medicine Experimental* 2017, **5(Suppl 2):**0589

**INTRODUCTION.** Therapeutic normothermia was previously found to decrease early mortality in patients with septic shock. However, the pathophysiologic effect of therapeutic normothermia in sepsis is still unclear. Recently, immunomodulation effect has been proposed as one of the key mechanism on sepsis outcome.

**OBJECTIVES.** To study immunomodulation of the febrile septic shock patients undergoing therapeutic normothermia when compared with standard fever control using CD11b, neutrophil chemotaxis, IL-6, IL-10, CRP and mHLA-DR levels.

**METHODS.** In a single-center, randomized, open-label trial, febrile patients with septic shock who presented to the emergency department were enrolled. The patients were randomly assigned to either standard fever control or therapeutic normothermia, which was aggressive fever control to maintain body temperature around 36–37 degree Celsius for the first 24 hours. Primary outcome was immunomodulation as measured by CD11b, neutrophil chemotaxis, IL-6, IL-10, CRP and mHLA-DR. Secondary outcomes were various clinical outcomes.

**RESULTS.** Fifteen patients, consisting of 7 patients in the therapeutic normothermia group, were enrolled and analyzed. The changes on day 0, 1 and 7 of CD11b, neutrophil chemotaxis, IL-6, IL-10, CRP and mHLA-DR were not statistically significantly different between groups ($p > 0.05$). Both groups demonstrated the increase in neutrophil chemotaxis and mHLA-DR, and the decrease in CD11b, CRP, IL-6 and IL-10 over time. The standard fever control group showed statistically significant changes from day 0 to day 7 of CD11b (−10.9 ± 7.5%, $p = 0.016$), neutrophil chemotaxis (+9.5 ± 3.2%, $p = 0.001$), and mHLA-DR (+16.7 ± 8.7%, $p = 0.005$). The therapeutic normothermia group showed statistically significant changes from day 0 to day 7 of IL-6 (−1135.7 ± 908.9pg/ml, $p = 0.049$). There were no statistically significant differences between groups regarding to secondary outcomes. There was more shivering in the therapeutic normothermia group ($p = 0.007$), which led to protocol violation in two patients.

**CONCLUSIONS.** In this study, therapeutic normothermia when compared to standard fever control did not result in change in immunomodulation, though more significant shivering ensued.

**REFERENCE(S)**

1. Schortgen F, Clabault K, Katsahian S, Devaquet J, Mercat A, Deye N, et al. Fever control using external cooling in septic shock: a randomized controlled trial. Am J Respir Crit Care Med. 2012;185(10):1088-95
2. Hotchkiss RS, Monneret G, Payen D. Sepsis-induced immunosuppression: from cellular dysfunctions to immunotherapy. Nat Rev Immunol. 2013;13(12):862–74

**GRANT ACKNOWLEDGMENT**

This research has been supported by the Ratchadaphiseksomphot Endowment Fund of Chulalongkorn University (CU-59-001-HR) and the Ratchadapiseksompotch Fund, Faculty of Medicine, Chulalongkorn University (RA59/040).

### 0590

#### Histone deacetylases inhibition reduces mortality in a murine model of secondary pneumonia

F. Alby-Laurent[1,2], C. Rousseau[2], A. Gavaud[2], J.-F. Llitjos[1,2], H. Merdji[2], J.-P. Mira[1,2], F. Pène[1,2], J. Toubiana[2,3], J.-D. Chiche[1,2]
[1]Cochin University Hospital (APHP), Medical Intensive Care Unit, Paris Cedex 14, France, [2]Institut Cochin, Infection, Immunity & Inflammation, Paris, France, [3]Hôpital Necker (APHP), Pédiatrie Générale, Paris, France
**Correspondence:** F. Alby-Laurent
*Intensive Care Medicine Experimental* 2017, **5(Suppl 2):**0590

**INTRODUCTION.** With an increasing incidence and high mortality rates, sepsis is a public health issue. There is growing evidence that sepsis induces long lasting alterations of transcriptional programs through epigenetic mechanisms that may lead to protracted inflammation, organ failure, sepsis-induced immune suppression (SIIS), secondary infections and death. We hypothesized that epigenetic changes contribute to the pathophysiology of SIIS. To test this hypothesis, we studied the effects of histone deacetylases (HDAC) inhibition with trichostatin A (TSA) in a murine model of SIIS and secondary pneumonia.

**METHODS.** C57BL/6 mice were treated with TSA (2 mg/kg ip) or saline serum (CTL) 30 min before induction of sepsis by cecal ligation puncture (CLP). Surviving mice underwent intratracheal instillation of $1.5x10^6$ CFU of *P. aeruginosa* 8 days after CLP. We evaluated the effect of TSA on survival and cellular responses to the primary and secondary infections. Cellular responses in the blood, spleen and BAL were assessed by flow cytometry at different time points after CLP (Days 1,3&8) and after pneumonia (4 & 12h). We also studied lymphocyte apoptosis and dendritic cells (DC) expression of CD40,

CD86, and CMHII. Bacterial clearance was assessed in the BAL and in the blood 4 and 12 h after pneumonia. Continuous variables represented as means ± SD were compared using Student t test. Kaplan-Meier curves were compared by the log rank test. $P < 0.05$ indicated statistically significant differences.

**RESULTS.** Whereas treatment with TSA did not change survival after CLP, TSA significantly improved survival after tracheal instillation of *P. aeruginosa* (P = 0.009, Fig. 208).

Treatment with TSA partially reversed features of SIIS. TSA-treated mice had significantly higher absolute dendritic cells, T-lymphocytes counts with reduced lymphocyte apoptosis after CLP and reduced dendritic cells deactivation. Four hours after secondary pneumonia, TSA-treated mice improved bacterial clearance in the BAL, with reduced systemic dissemination of *P. aeruginosa*

**CONCLUSIONS.** In murine model of secondary pneumonia, HDAC inhibition with TSA improves survival, improve bacterial clearance and attenuate cellular features of SIIS. These results suggest that sepsis-induced epigenetic changes contribute to the advent of SIIS.

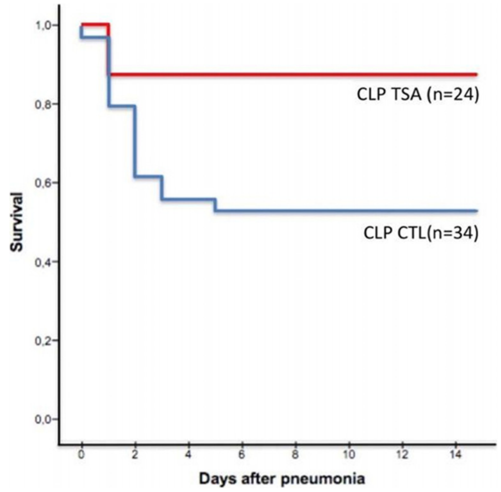

**Fig. 208 (Abstract 0590).** See text for description

### 0591

#### Computer-controlled closed-loop drug infusion system for automated hemodynamic resuscitation in septic shock

K. Uemura, T. Kawada, C. Zheng, M. Li, M. Sugimachi
National Cerebral and Cardiovascular Center, Department of Cardiovascular Dynamics, Suita, Japan
**Correspondence:** K. Uemura
*Intensive Care Medicine Experimental* 2017, **5(Suppl 2):**0591

**INTRODUCTION.** Hemodynamic resuscitation in septic shock requires aggressive fluid replacements and appropriate use of vasopressors to optimize mean arterial pressure (AP) and peripheral perfusion, i.e. cardiac output (CO). Because responses to these drugs vary between patients and within patient over time, strict monitoring of patient condition and repetitive adjustment of drug dose are required. This is time and labor consuming task, which has been associated with poor adherence to resuscitation guidelines of Surviving Sepsis Campaign. One potential way to ease these tasks is to automate them via the use of closed-loop control schemes.

**OBJECTIVES.** The aim of this study was to develop a closed-loop drug infusion system for automated hemodynamic resuscitation in septic shock by extending our previous system [1], and to validate performance of the system in a canine model of endotoxin shock.

**METHODS.** Our system continuously monitors AP, CO and central venous pressure (CVP), and computes arterial resistance (R), stressed blood volume (V) and Frank-Starling slope of left ventricle (S). Our system controls R with noradrenaline (NA), and V with Ringer's acetate solution (RiA), thereby controlling AP and CO. In 4 dogs, CO was measured invasively with aortic flow probe. In 4 dogs, CO was measured less-invasively using transthoracic Doppler aortic velocimetry and peripheral AP contour to further develop the system for clinical application. In all the 8 dogs, we created endotoxin shock by injections of bacterial lipopolysaccharide, which significantly decreased AP from 101 ± 20 to 42 ± 5mmHg, CO from 123 ± 27 to 60 ± 17 ml/min/kg, and increased blood lactate level from 1.7 ± 0.4 to 3.0 ± 0.8 mmol/L. The system was then connected to the dogs and activated.

**RESULTS.** Our system immediately started NA and RiA. Within 40 min, RiA increased V to its target, and NA maintained R to its target while S was concomitantly increased. These resulted in restoration of AP to 70 ± 2 mmHg and CO to 130 ± 10 ml/min/kg. Steady state deviations from target values were small for AP (−1 ± 4 mmHg) and CO (−3 ± 10 ml/min/kg). AP and CO were controlled stably over a period of 4 h even when CO was measured less invasively. Blood lactate level at 4 h of hemodynamic resuscitation (3.3 ± 1.5 mmol/L) was not significantly different from that observed before resuscitation.

**CONCLUSIONS.** In a canine model of endotoxin shock, our system automatically improved and stably maintained the hemodynamics over the period of 4 h. In clinical settings, the period may be extended without difficulty until the infection causing septic shock can be eradicated. Our system could be an attractive clinical tool for rescuing patients with septic shock.

**GRANT ACKNOWLEDGMENT**

This work was supported by JSPS KAKENHI Grant Number 15K01307.

### 0592

#### APJ receptor uncoupling from the Gαi/Adenylyl Cyclase/cAMP signaling pathway enhances the inotropic response to apelin-13 (APL-13) during experimental sepsis: impact of biased ligands

D. Coquerel[1], L. Dumont[2], J. Coté[3], X. Sainsily[3], F. Chagnon[1], R. Dumaine[3], P. Sarret[3], E. Marsault[3], D. Salvail[4], M. Auger-Messier[5], O. Lesur[1]
[1]University of Sherbrooke, ICU, Dept of Medicine, Sherbrooke, Canada; [2]University of Sherbrooke, Sherbrooke, Canada; [3]University of Sherbrooke, Pharmacology/Physiology, Sherbrooke, Canada; [4]IPS Therapeutique, Sherbrooke, Canada; [5]University of Sherbrooke, Cardiology/Medicine, Sherbrooke, Canada
**Correspondence:** O. Lesur
*Intensive Care Medicine Experimental* 2017, **5(Suppl 2):**0592

**INTRODUCTION.** APL-13, the dominant isoform of APLs in human heart with strong inotropic effect, is a promising non-catecholaminergic drug to improve cardiac performance during sepsis. APL-13 binds to the apelin receptor (APJ), a GPCR highly expressed in the cardiovascular system. APJ activation by APL-13 engages either G-protein or β-arrestin stimulating downstream intracellular pathways and triggers physiological responses. Although efficient and cardioprotective, the myocardial APJ signaling is still poorly understood during sepsis. Engineered C-terminal modifications of APL-13 by unnatural amino acids can change ligand-receptor interactions and downstream signaling. These biased ligands (*BLs*) are new pharmacological tools to investigate the apelinergic system modulation and protection of cardiac functions.

**OBJECTIVES.** To develop and trial BLs triggering the Gai pathway and better understand how the apelinergic system influences cardiomyocyte's function and signals in healthy and septic conditions.

**METHODS.** Signal transduction was first screened in HEK293 cells and cardio-specific pathways were assessed by Western blots in rat neonatal ventricular cardiomyocytes (RNVC) with or without human APJ over-expression. Sepsis-related myocardial dysfunction was induced by Cecal Ligature and Puncture (CLP) in rats. Myocardial function was assessed by Langendorff assay *ex-vivo* and by echocardiography *in vivo*.

**RESULTS.** Inotropic impact of several APJ ligands was inversely correlated with their potency to decrease cAMP content in forskolin-stimulated HEK293 (Spearman r = 0.61; p = 0.023). *BLs* inducing high cAMP inhibition (cAMP EC50: APL-13 1.4 ± 0.1 nM; *BL1* 0.04 ± 0.1 p < 0.05 vs. APL-13; BL2 0.07 ± 0.1 p < 0.05 vs. APL-13) were more efficient than APL-13 to inhibit the inotropic response induced by forskolin (Fsk) in isolated-perfused hearts (increased contractility from baseline; Fsk (1μM) 340 ± 81%; Fsk + APL-13 (0.1nM) 266 ± 33; Fsk + *BL1* 142 ± 40.34 p < 0.05 vs. Fsk) as well as to reduce phospholamban Ser-16 and Threo-17-phosphorylations in Fsk stimulated RNVC. After CLP induction, these later responses were ineffective (Fsk 279 ± 70%; Fsk + APL-13 304 ± 44%; *BL1* 269 ± 68%) and BLs became highly cardiotonic *ex vivo* as well as *in vivo* (Cardiac index in continuously infused rats; CLP (6h) 11.2 ± 0.8 mL/min/100g, CLP + APL-13 15.9 ± 1.4 vs. CLP; CLP + *BL1* 15.8 ± 1.2 and CLP + *BL2* 15.7 ± 1.1, p < 0.05 vs. CLP) comparatively to their effects in healthy rats (CI; Sham 20.8 ± 0.9 mL/min/100g, APL-13 23.7 ± 0.7; *BL1* 20.7 ± 1.3; *BL2* 20.5 ± 0.5).

**CONCLUSIONS.** Sepsis uncouples APJ from the Gαi/Adenylyl Cyclase/cAMP pathwayand acts as a negative feedback for the inotropic response of APJ agonists. This standpoint of knowledge is important for the future pharmacological development of the apelinergic pathway in the context of sepsis-related myocardial dysfunction.

**REFERENCE(S)**

PMID:27571457; Intensive Care Med. Exp. 2016, 4 (Suppl 1) :27; PIMD26986036

**GRANT ACKNOWLEDGMENT**

Merck-Dhome FMSS, CRSNG-SEP, MITACS

### 0593

#### Fluid and norepinephrine resuscitation fail to correct renal failure in endotoxic shock

G. Ferrara[1], V. S. Kanoore Edul[1], J. F. Gonzalez Eguillor[1], M. G. Buscetti[1], H. S. Canales[1], B. Lattanzio[1], L. Gatti[1], C. Ince[2], A. Dubin[1]
[1]Facultad de Ciencias Médicas, UNLP, Cátedra de Farmacología Aplicada, La Plata, Argentina; [2]Academic Medical Center, University of Amsterdam, Translational Physiology, Amsterdam, Netherlands
**Correspondence:** A. Dubin
*Intensive Care Medicine Experimental* 2017, **5(Suppl 2):**0593

**INTRODUCTION.** The pathophysiology of renal failure in sepsis is complex. In addition, the role of hypoperfusion is controversial.

**OBJECTIVES.** To characterize kidney perfusion, oxygenation, and function in shock and resuscitation. Our hypothesis was that the normalization of systemic oxygen transport fails to correct renal dysfunction occurring in endotoxic shock.

**METHODS.** We studied 24 anesthetized and mechanically ventilated sheep. We measured systemic hemodynamics, and oxygen transport and consumption ($DO_2$ and $VO_2$), renal blood flow, $DO_2$, and $VO_2$, cortical microcirculation, and creatinine clearance. Endotoxic shock (n = 12) was induced by intravenous injection of E. Coli endotoxin (5 mg/kg endotoxin, followed by 2.5 mg/kg/hr for 180'). After 60' of shock, resuscitation was performed by fluid infusion (30 mL/kg of saline solution) and norepinephrine, which was titrated to reach a mean arterial pressure (MAP) of 70 mm Hg. We also included a sham group (n = 12). Data are shown as median (25–75 IQR).

**RESULTS.** As shown in Table 159, endotoxic shock decreased MAP, cardiac index, and systemic $DO_2$ and $VO_2$, which returned to basal values after resuscitation. Renal blood flow, $DO_2$ and $VO_2$, cortical perfused vascular density, and creatinine clearance (Fig. 209) were reduced in endotoxic shock. Resuscitation only partially improved some of them. All these variables were unchanged in sham group.

**CONCLUSIONS.** Endotoxic renal failure was characterized by reductions in renal blood flow, $DO_2$ and $VO_2$, and cortical microvascular perfusion. The administration of fluids and norepinephrine improved systemic hemodynamics but was unable to normalize kidney perfusion and oxygenation. Nevertheless, the magnitude of these alterations might not be enough to explain the persistent and severe reduction in creatinine clearance. Consequently, other mechanisms might be also involved.

**Table 159 (Abstract 0593)**. See text for description

| | Mean blood pressure (mm Hg) | Cardiac index (mL/min/kg) | Systemic $DO_2$ (mL/min/kg) | Systemic $VO_2$ (mL/min/kg) | Systemic $O_2$ extraction ratio | Left renal blood flow (mL/min/100 g) | Renal $DO_2$ (mL/min/100 g) | Renal $VO_2$ (mL/min/kg) | Renal $O_2$ extraction ratio |
|---|---|---|---|---|---|---|---|---|---|
| Sham 0 min | 80 (74-94) | 131 (112-149) | 15.9 (14.4-18.1) | 6.1 (5.2-7.8) | 0.40 (0.36-0.45) | 198 (150-443) | 24.5 (17.1-62.2) | 5.1 (3.4-9.1) | 0.18 (0.16-0.23) |
| Endotoxic shock 0 min | 83 (71-98) | 112 (92-128) | 14.1 (12.2-16.6) | 5.3 (5.0-6.0) | 0.40 (0.29-0.48) | 205 (157-293) | 28.4 (19.0-38.2) | 5.3 (4.0-8.8) | 0.21 (0.15-0.24) |
| Sham 60 min | 87 (78-99) | 121 (106-145) | 15.2 (13.0-17.7) | 5.8 (4.9-6.9) | 0.40 (0.33-047) | 199 (157-394) | 25.4 (18.9-51.8) | 5.4 (4.6-9.4) | 0.22 (0.18-0.26) |
| Endotoxic shock 60 min | 34 (31-40)*† | 71 (53-82)*† | 8.4 (7.4-10.2)*† | 4.4 (3.8-5.4)*† | 0.54 (0.46-0.66)*† | 131 (99-185)*† | 15.8 (13.5-23.2)*† | 3.7 (3.3-4.5)*† | 0.26 (0.18-0.36) |
| Sham 180 min | 93 (74-107) | 130 (114-170) | 17.2 (14.9-19.2) | 6.6 (4.7-7.2) | 0.39 (0.32-0.45) | 221 (170-279) | 25.8 (19.2-35.7) | 5.1 (4.5-7.1) | 0.21 (0.18-0.26) |
| Endotoxic shock 180 min | 72 (70-73)† | 123 (97-147) | 14.3 (9.6-16.0) | 5.1 (4.3-5.9) | 0.36 0.30-0.51) | 174 (92-186)† | 20.5 (10.8-22.7)* | 3.8 (1.9-4.8)*† | 0.21 (0.16-32) |

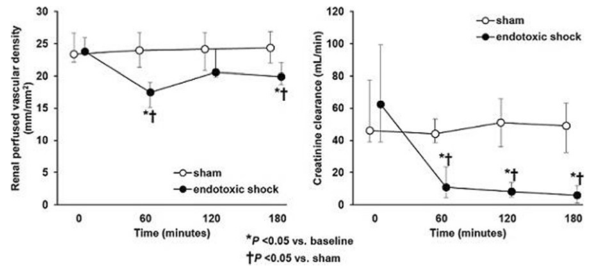

**Fig. 209 (Abstract 0593).** See text for description

### 0594

#### Defibrotide prevents sepsis induced endothelial cell activation

S. Fernández[1], M. Palomo[2], J. Aibar[1], M. Díaz-Ricart[2], J. M. Nicolás[1], P. Castro[1]
[1]Hospital Clínic, Medical Intensive Care Unit, Barcelona, Spain; [2]Hospital Clínic, Hemotherapy-Hemostasis Department, Barcelona, Spain
**Correspondence:** S. Fernández
*Intensive Care Medicine Experimental* 2017, **5(Suppl 2):**0594

**INTRODUCTION.** Endothelial dysfunction plays a key role in sepsis physiopathology, leading to multiorgan failure and death. Defibrotide (DF) is a product effective in the treatment of veno-occlusive disease (VOD), a complication of hematopoietic stem cell transplanted patients where endothelial activation is also cornerstone. Although the mechanism of action of DF is uncertain, it seems to modulate endothelial cells activation. Therefore, DF may have a role as potential treatment for sepsis complications.

**OBJECTIVES.** Characterize in an in-vitro model of sepsis the activation and damage of endothelial cells in different sepsis syndrome and determine the influence of DF in these alterations.

**METHODS.** Human umbilical vein endothelial cells (HUVEC) were grown in the presence of 20% pooled sera collected from patients with septic syndrome (sepsis, severe sepsis and septic shock) or systemic inflammatory response syndrome (SIRS) within the first 24 hours of symptoms onset. Sera from healthy volunteers was used as control. We assessed changes in the expression of endothelial cell receptors at the surface (intercellular adhesion molecule-1 (ICAM-1) and vascular cell adhesion molecule-1 (VCAM-1)), the presence of extracellular adhesive proteins such a Von Willebrand factor (VWF) and the thrombogenicity of the extracellular matrix (ECM) generated by these cells. To explore the effect of DF, cells were incubated with DF (100 μg /mL) for 24 hours before being exposed to the sera and every 24 hours after the exposition.

**RESULTS.** Endothelial cell activation, determined by an increase in expression of ICAM-1 and VCAM-1 and an increase in expression of

VWF and platelet adhesion on the ECM, was higher in septic syndrome compared with SIRS and control group ($p < 0.001$) with a progressive increase correlated with sepsis severity (sepsis < severe sepsis ≤ septic shock) ($p < 0.05$ for VCAM-1, ICAM-1 and VWF; $p < 0.001$ for platelet adhesion). Previous exposure and continuous incubation of endothelial cells with DF modified the endothelial response by significantly reducing VWF, ICAM-1, VCAM-1 and platelet adhesion induced by sera from patients with septic syndrome in all gropus ($p < 0.001$).

**CONCLUSIONS.** DF prevents activation and endothelial damage induced in septic syndrome in an ex-vivo model of sepsis.

**REFERENCE(S)**

Palomo M, Díaz-Ricart M, Rovira M et al. Defibrotide Prevents the Activation of Macrovascular and Microvascular Endothelia Caused by Soluble Factors Released to Blood by Autologous Hematopoietic Stem Cell Transplantation. Biol Blood Marrow Transplant 2011; 17:497–506.

**GRANT ACKNOWLEDGMENT**

FIS PI12/00832, Instituto de Salud Carlos II y cofinanciado con Fondo Europeo de Desarrollo Regional (FEDER), Unión Europea, Una manera de hacer Europa.

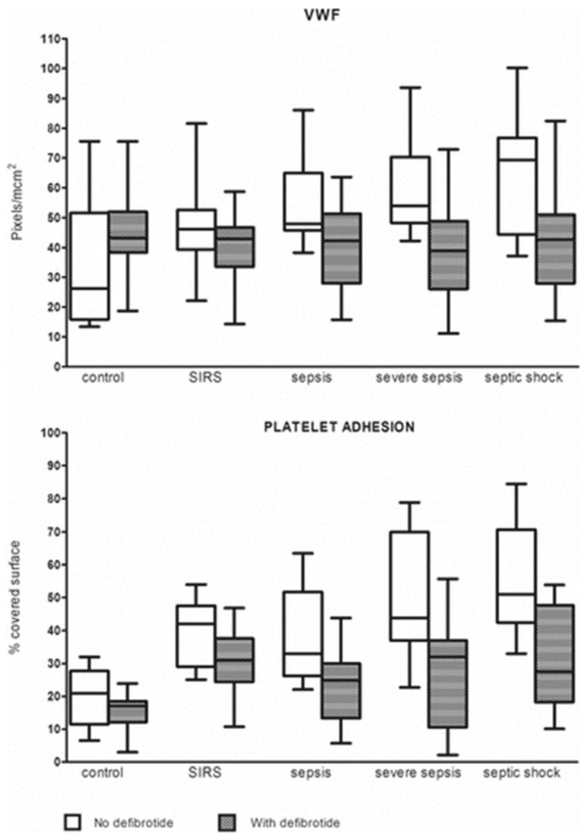

**Fig. 210 (Abstract 0594).** VWF expression and platelet adhesion

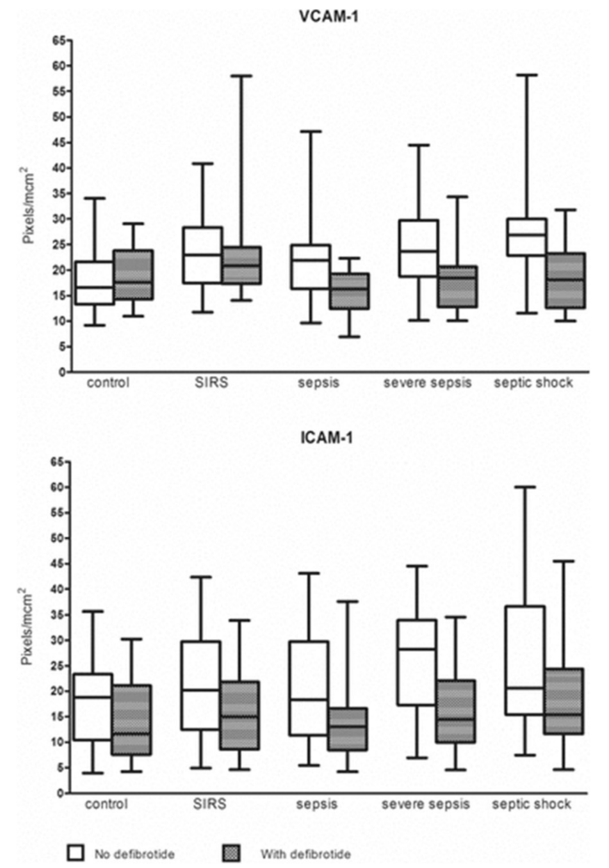

**Fig. 211 (Abstract 0594).** Endothelial cell receptors

### 0595

#### IgM enriched immunoglobulins as adjunctive treatment to antimicrobial therapy for patients in septic shock

A. Corona[1], A. Gandola[2], F. Stretti[2], A. Veronese[2], C. Soru[2], C. Fulvio[2], R. Colombo[2], E. Catena[2]

[1]PO Luigi Sacco, Emergency, Milano, Italy; [2]PO Luigi Sacco, Milano, Italy

**Correspondence:** A. Corona

*Intensive Care Medicine Experimental* 2017, **5(Suppl 2):**0595

**INTRODUCTION.** Sepsis is responsible of both an immune hyperactivity damage from inflammation and an immune suppression and paralysis causing vulnerability to infection. Literature provides few retrospective studies supporting the role of IgM enriched immunoglobulins (IgM-e-IG) as adjunctive of antimicrobial treatment (1).

**OBJECTIVES.** To evaluate the likely roleof IgM enriched immunoglobulins (IgM-e-IG) as adjunctive of antimicrobial treatment.

**METHODS.** Since December 2016 to the end of March 2017, patients experiencing a septic shock and admitted with a first 24h SAPS II > 25, associated with a SOFA-score > 4 - were considered for the treatment with IgM-e-IG, given for three days at the total dosage of 500 mg/kg. Response to the therapy was based on clinical, microbiological and rheological data.

**RESULTS.** A total of 9 patients (3 pneumococcal meningitis, 2 pneumococcal pneumonia, 2 E. coli peritonitis, 1 MRSA thigh necrotising fasciitis and bacteraemia), median (IQR): age: 48 (28–60), 1st 24 hrs. SAPS II: 55 (43–68), SOFA-score: 12 (7–14) were treated with IgM-e-IG in association with median (IQR) adequate antimicrobial treatment of 7 (8–14) days. Clinical response to therapy occurred by 72 hrs, whereas median (IQR) duration of mechanical ventilation and vasopressor infusion were respectively 3 (2–7) and 3 (2–3). We recorded a median (IQR) 80% decreasing procalcitonin [47(7–209)ng/ml vs.7.1 (0.3-9.1)] and CRP [333(225–407)mg/ml vs. 27 (10–70)] respectively on day 3 and 5, whereas of SOFA-score on day 6. All patient were alive on 30th day, even if one of them (pneumococcal pneumonia) died after 2 months for a KP-cp bacteraemia.

**CONCLUSIONS.** Despite the exiguous sample size it seems that early adjunctive treatment with IgM may be associated with a survival benefit. A larger patient sample and a control group will be warranted to corroborate this preliminary results.

**REFERENCE(S)**

Cavazzuti I, Serafini G, Busani S, Rinaldi L, Biagioni E, Buoncristiano M,Girardis M. Early therapy with IgM-enriched polyclonal immunoglobulin in patients with septic shock. Intensive Care Med. 2014 Dec;40(12):1888–96.

**GRANT ACKNOWLEDGMENT**

NONE.

### 0596

#### Stem cells mobilization in surgical patients with septic shock

A. Cotoia[1], S. Altamura[1], R. Villani[1], G. Palumbo[2], F. Cutillo[2], L. Tullo[1], L. Mirabella[1], S. Capalbo[2], G. Cinnella[1]

[1]University Hospital of Foggia, Critical Care Medicine, Foggia, Italy; [2]University Hospital of Foggia, Hematology, Foggia, Italy

**Correspondence:** A. Cotoia

*Intensive Care Medicine Experimental* 2017, **5(Suppl 2):**0596

**INTRODUCTION.** Complicated intra-abdominal infection is a frequent cause of sepsis and septic shock in surgical patients. Several efforts have been made to study the stem cells role in septic patients, but we currently lack of informations regarding septic surgical patients.

**OBJECTIVES.** The aim of the study is to evaluate the time course level circulating endothelial stem cells in septic shock patients undergoing major abdominal surgery.

**METHODS.** In the prospective observational study were enrolled consecutive patients undergoing major abdominal surgery at University Hospital, Foggia. After written informed consent, blood samples were collected on admission of the post surgical patients in intensive care unit (ICU) or in surgery ward at 24 hours (T1), and 3 (T3), and 7 (T7) days postoperatively. Quantitative analysis of endothelial progenitor stem cells (CD34) was performed. At any time were also collected the clinical parameters, the laboratory values of interleukins, procalcitonin, C-reactive protein and endotoxin, white blood cells, red blood cells, hemoglobin and platelets. The outcome was also recorded. The data are presented as median ± SE.

**RESULTS.** 33 patients undergoing major abdominal surgery were analysed. 20 patients with septic shock required ICU admission following surgery and 13 patients (controls) were admitted in post-surgical ward. 13 of 20 patients with septic shock deceased within 15 days postoperatively. At T1, CD34 cells were 0.23 ± 1.5/mcl in control group, 0.06 ± 0.09/mcl in septic shock survivors and 0.32 ± 2.2/mcl in septic shock non survivors (NS). At T3, the circulating stem cells slightly increased in septic shock survivors and control while they reduced, whereas non significantly, in non survivors patients (0.2 ± 0.9/mcl, 0.27 ± 1.1/mcl, 0.16 ± 2.5 respectively). At T7 the CD34 stem cells were slightly higher in septic shock survivors as compared to control and non survivors (0.36 ± 0.1/mcl, 0.21 ± 0.06/mcl and 0.25 ± 0.4/mcl, respectively).

**CONCLUSIONS.** The presented preliminary findings indicate a increased level of the circulating CD34 stem cells in the blood of surgical patients likely due to an adaptive response to surgical stress. Moreover it appears that patients capable of increasing the production of CD34 stem cells exhibit a better outcome following major abdominal surgery. This pilot study encourages the continuation of the research project on a larger sample size of patients to better understand if and how CD34 stem cells are involved in the surgical patients with septic shock.

**GRANT ACKNOWLEDGMENT**

Intervento cofinanziato dal Fondo di Sviluppo e Coesione 2007–2013 - APQ Ricerca Regione Puglia “Programma regionale a sostegno della specializzazione intelligente e della sostenibilità sociale ed ambientale - FutureInResearch”.

### 0597

#### Is it possible to predict the hemodynamic profile of ventilated patients with septic shock using front-line hemodynamic parameters?

B. Evrard[1], A.-L. Fedou[1], X. Repessé[2], A. Aubry[2], C. Jacob[3], G. Prat[3], K. Bouferrache[4], J. Maizel[5], M. Slama[5], P. Delour[6], L. Chimot[6], F. Vargas[7], J. Jabot[8], D. Vandroux[8], B. Riu[9], S. Silva[9], A. Vieillard-Baron[2], P. Vignon[1]

[1]CHU de Limoges, Limoges, France; [2]Hôpital Ambroise Paré, Assistance Publique Hôpitaux de Paris, Boulogne-Billancourt, France; [3]CHU Brest, Brest, France; [4]Hôpital Foch, Paris, France; [5]CHU Amiens, Amiens, France; [6]CH Périgueux, Périgueux, France; [7]CHU Bordeaux, Bordeaux, France; [8]Hôpital Félix Guyon - CHU La Réunion, Saint-Denis, France; [9]CHU Toulouse, Toulouse, France

**Correspondence:** B. Evrard

*Intensive Care Medicine Experimental* 2017, **5(Suppl 2):**0597

**INTRODUCTION.** The Surviving Sepsis Campaign 2016 recommends that serial assessments of hemodynamic status be performed after initial fluid loading. Front-line hemodynamic parameters include blood pressure, heart rate, central venous pressure, which are interpreted in light of ScvO2 and lactate levels.

**OBJECTIVES.** To describe the hemodynamic profile of a cohort of ventilated patients assessed within twelve hours of ICU admission for septic shock using transesophageal echocardiography (TEE) and evaluate the diagnostic value of front-line hemodynamic parameters.

**METHODS.** This is an ancillary study of two prospective, observational, multicenter, French cohorts of ventilated patients who were hemodynamically assessed for a septic shock using TEE (HEMOSEPSIS and HEMOPRED studies). In each patient, cardiac index (CI), left ventricular ejection fraction (LVEF), respiratory variations of the superior vena cava (ΔSVC) and of the subaortic Doppler velocities (ΔVmaxAo), and the ratio of end-diastolic areas of both the right and left ventricle in the long axis view of the heart (RVEDA/LVEDA) were measured. A LVEF < 40% defined LV systolic dysfunction, a CI < 3 l/min/m$^2$ defined low cardiac output, and a RVEDA/LVEDA ratio > 0.6 (± associated with a paradoxical septal motion in the short axis of the heart) defined RV dysfunction (± acute cor pulmonale). The preload-dependence was evaluated using ΔSVC or ΔVmaxAo. Front-line hemodynamic parameters were noted at the time of TEE assessment.

**RESULTS.** LVEF and CI could be simultaneously measured in 388 of 410 patients who were hemodynamically assessed (95%). 74 patients (19%) had a low CI related to LV systolic dysfunction (lactate: 4.36 ± 3.49 mmol/l), 141 patients (36%) had a low CI and a preserved LVEF related to a RV dysfunction or to a sustained preload-dependence (lactate: 3.58 ± 3.28 mmol/l), 146 patients (38%) had preserved CI and LVEF (lactate: 3.38 ± 3.32 mmol/l) including only 23 patients (6%) with a hyperkinetic profile (high CI and LVEF > 70%), and 27 patients (7%) has preserved CI but altered LVEF (lactate: 3.49 ± 2.95 mmol/l) due to a marked tachycardia. None of the frontline hemodynamic parameters was discriminatory to identify the circulatory profile identified by TEE assessment (Table 160).
**CONCLUSIONS.** Front-line hemodynamic parameters fail to discriminate between the distinct hemodynamic profiles identified during TEE assessment of ventilated patients with septic shock.

**Table 160 (Abstract 0597).** See text for description

| | | EF < 40% | EF ≥ 40% |
|---|---|---|---|
| CI ≥ 3 l/min/m[2] | n (%) | 27 (7%) | 146 (38%) |
| | mBP (mmHg)/HR (bpm) | 82 ± 15/119 ± 26 | 76 ± 16/114 ± 20 |
| | CVP (mmHg)/ScvO2 (%) | 10 ± 4/76 ± 13 | 10 ± 4/80 ± 10 |
| CI < 3 l/min/m[2] | n (%) | 74 (19%) | 141 (36%) |
| | mBP (mmHg)/HR (bpm) | 78 ± 16/108 ± 26 | 77 ± 17/96 ± 23 |
| | CVP (mmHg)/ScvO2 (%) | 11 ± 5/76 ± 11 | 10 ± 5/76 ± 11 |

### 0598

#### Audit of adherence to the magnificent seven care bundle on antimicrobial usage in Colchester intensive care

P. Cooke, P. Bishop
Colchester General Hospital, Essex, UK, Intensive Care Unit, Colchester, United Kingdom
**Correspondence:** P. Cooke
*Intensive Care Medicine Experimental* 2017, **5(Suppl 2):**0598

**INTRODUCTION.** Antimicrobial resistance threatens the prevention and treatment of an ever-increasing range of infections and leads to increased morbidity and costs of care. 30-40% of antibiotics prescribed in ICU´s are unnecessary, inappropriate or suboptimal.
**OBJECTIVES.** We aimed to examine whether the ICU was meeting recommendations for antibiotic prescribing, according to the Magnificent Seven standards. In doing so, we aimed to look for ways of improving practice and patient outcomes, and to find practical ways of avoiding unnecessary antibiotic usage.
**METHODS.** A retrospective audit was performed on notes of all patients commenced on antibiotics on ITU within a 3 week period. Interrogation looked for documented evidence of infection, an indication for antibiotics, evidence that blood cultures were taken prior to antibiotic doses, a planned duration for treatment, and a review of antibiotics at 48 hours.
**RESULTS.** 21 patients were included, among which 20 errors were made. 18 errors (90%) occurred in 8 particular patients. All patients had documented evidence of infection but this was felt to be subjective in some cases. Only 1 patient did not have antibiotics reviewed, 4 (19%) had no cultures taken and 8 (38%) had no planned duration for treatment. 7 (33%) had no documented indication for the antibiotics.
**CONCLUSIONS.** Amongst staff, there was a lack of awareness of the Magnificent Seven standards. Ambiguity around which member of the team carries the ultimate responsibility for antibiotic stewardship creates the potential for gaps in care. In some instances, a lack of regular review meant antibiotics were continued for longer than necessary, or despite negative microbiology results. Recommendations to the department included assigning a junior doctor to microbiology rounds to ensure adequate documentation, and training on the Magnificent Seven at staff inductions. The electronic medical records system, CareVue, is due to be expanded on ITU. The authors recommended a specific microbiology section be created, containing prompts for each of the standards. We also recommended using serum procalcitonin tests to provide evidence of infection and to guide treatment. Once these changes are implemented, we hope to perform a PDSA re-audit to assess their impact on the standards.

**REFERENCE(S)**
1. Jong et al. Efficacy and Safety of Procacitonin Guidance in Reducing the Duration of Antibiotic Treatment in Critically Ill Patients: A Randomised, Controlled, Open-Label Trial. Lancet Infect. Dis. 2016 Jul;16(7):819–27.
2. WHO Antimicrobial Resistance Factsheet, updated 2016.
3. Harbarth et al. Diagnostic Value of Procalcitonin, Interleukin-6 and Interleukin-8 in Critically Ill patients Admitted with Suspected Sepsis. American Journal of Respiratory and Critical Care Medicine 2001, Aug;164(3).
4. Public Health England. Start Smart Then Focus. Antimicrobial Stewardship Toolbox Kit for English Hospitals. Updated March 2015.

## Transfusion and blood management

### 0599

#### D-Dimer testing in patiens with concomitant renal insufficiency in critical ilness: usefuleness and new perspectives

C. A. Pfortmueller[1], G. Lindner[2], G.-C. Funk[3], A. B. Leichtle[4], G. M. Fiedler[4], C. Schwarz[5], A. K. Exadaktylos[6]
[1]Inselspital, Bern University Hospital, Department of Intensive Care, Bern, Switzerland; [2]Hirslanden Klinik Im Park, Department of General Internal Medicine & Emergency Medicine, Zurich, Switzerland; [3]Otto Wagner Hospital and Ludwig-Boltzmann Institute for COPD and Respiratory Epidemiology, Department of Respiratory and Critical Care Medicine, Vienna, Austria; [4]Inselspital, University Hospital Bern, Center for Laboratory Medicine, Bern, Switzerland; [5]Landeskrankenhaus Steyr, Department of Internal Medicine and Nephrology, Steyr, Austria; [6]Inselspital, Bern University Hospital, Department of Emergency Medicine, Bern, Switzerland
**Correspondence:** C. A. Pfortmueller
*Intensive Care Medicine Experimental* 2017, **5(Suppl 2):**0599

**BACKGROUND.** D-Dimer was found to be elevated in renal insufficiency. We tried to A.) evaluate whether D-Dimer shows a relation to renal function in critically ill patients with suspected VTE; B.) identify renal function adjusted D-Dimer cut-offs.
**METHODS.** In this retrospective analysis, all patients presenting to an emergency department between 01 January 2009 and 01 November 2013 with suspected VTE were included. Linear regression analysis was used to identify factors related to D-Dimer.
**RESULTS.** 9,716 patients (55% male) were included. D-Dimer was 583 μg/L (297 to 1,274) and it was >500 μg/L in 5,372 (55%) patients. 7,439 (77%) had an eGFR > 60 ml/min, 1,759 (18%) an eGFR of 30–60 ml/min and 518 (5%) an eGFR < 30 ml/min. Prevalence of negative D-Dimer decreased with declining eGFR. There was a significant inverse correlation between eGFR and D-Dimer. While sensitivity of D-Dimer was unchanged by declining eGFR, its specificity for VTE decreased substantially. After adjustment for renal function D-Dimer cut-off values yielded a post-test probability for VTE of 1%.
**CONCLUSIONS.** Our findings confirm the relationship between renal function and D-Dimer. Use of renal function adjusted D-Dimer cut-offs in combination with clinical prediction rules could result in less need for further investigations to rule out VTE.

### 0600

#### Downward bias of conductivity based point-of-care hemoglobin measurement compared with optical methods

J. H. Kim, M. H. Heo
Inje University / Ilsan Paik Hospital, Anesthesiology and Pain Medicine, Goyang, Korea, Republic of
**Correspondence:** M. H. Heo
*Intensive Care Medicine Experimental* 2017, **5(Suppl 2):**0600

**INTRODUCTION.** Point-of-care (POC) arterial blood gas analysis (ABGA) is widely used for checking hemoglobin (Hb) level. However, there is the tendency of downward bias of conductivity-based POC ABGA Hb measurement compared with optical methods.

**OBJECTIVES.** Authors tried to correct that bias by linear regression equation.

**METHODS.** We retrospectively collected a total of 86 Hb result pairs during surgeries. Hb measured by the Sysmex XE-2100 in the laboratory was set as the gold standard and was compared with that measured by the GEM Premier 3500. Data were compared using the Bland-Altman analysis, the reliability of trasfusion decision was assessed using three-zone error gird. The linear regression analysis was performed to find out the relation between the Hb results of POC ABGA and those of laboratory based test.

**RESULTS.** The bias of the Hb measured between Sysmex XE-2100 and GEM Premier 3500 was - 0.85 g/dl ($p < 0.0001$).

The percentage error was 16.4%. According to error grid methodology, zone A, B and C encompassed 89.5%, 10.5% and 0% of data pairs.

After adjusting the POC ABGA Hb values, the bias of the Hb measured by two methods was 0 g/dl ($p = 0.99$). The percentage error was 18.2%. The zone A, B and C encompassed 91.9%, 8.1% and 0% of data pairs.

**CONCLUSIONS.** Hb measurements obtained with reference to conductivity via a POC ABGA were significantly lower than those obtained via optical methods. Correcting the bias by linear regression formula reduced data pairs in zone B and C, but increased percentage error. This bias may deserve attention of anesthesiologists when POC ABGA Hb level is used as a transfusion guideline.

**REFERENCE(S)**

[1] Wu P, Morey TE, Harris NS, Gravenstein N, Rice MJ. Intravenous fluids cause systemic bias in a conductivity-based point-of-care hematocrit meter. Anesth Analg. 2012;114:314–21.

[2] Steinfelder-Visscher J, Weerwind PW, Teerenstra S, Pop GAM, Brouwer RMHJ. Conductivity-based hematocrit measurement during cardiopulmonary bypass. J Clin Monit Comput. 2007;21:7–12.

**GRANT ACKNOWLEDGMENT**

None.

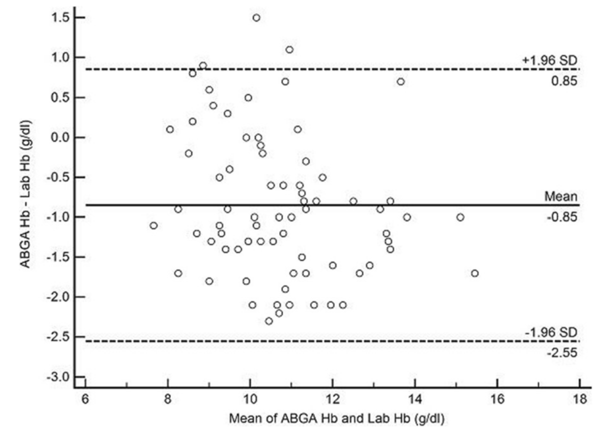

**Fig. 212 (Abstract 0600).** Bland-Altman plot of difference before adjustment

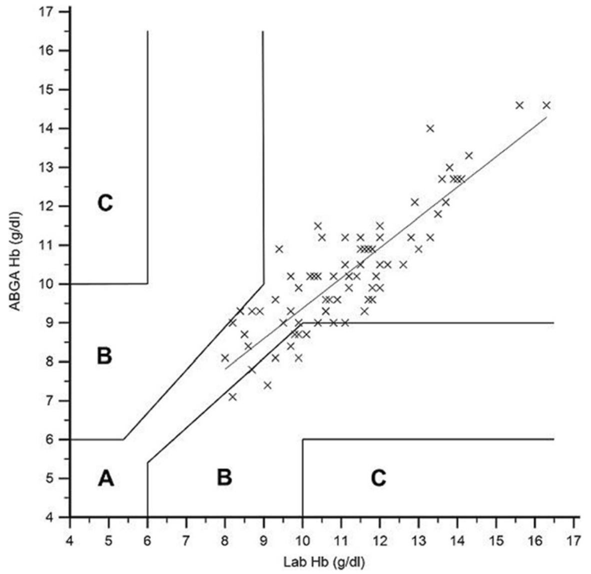

**Fig. 213 (Abstract 0600).** Three zones error grid plot before adjustment

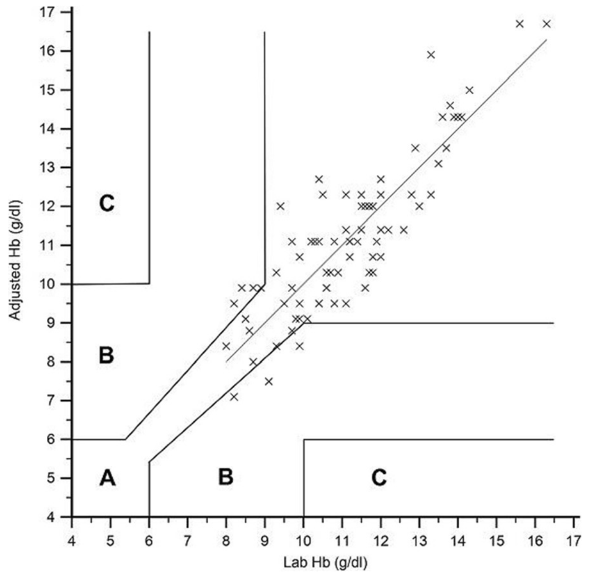

**Fig. 214 (Abstract 0600).** Three zones error grid plot after adjustment

### 0601

#### Use of recombinant factor seven for severe haemorrhage including patients who have had major trauma

C. G. Mason[1], C. Doughty[2], L. Green[2], C. J. Kirwan[1]

[1]Barts Health NHS Trust, Critical Care, London, United Kingdom; [2]Barts Health NHS Trust, Haematology, London, United Kingdom
**Correspondence:** C. G. Mason
*Intensive Care Medicine Experimental* 2017, **5(Suppl 2):**0601

**INTRODUCTION.** Recombinant Factor VIIa (rFVIIa) is an expensive but effective therapy for promoting haemostasis in certain haematological conditions. Although its efficacy is questionable for use in haemorrhage secondary to major trauma[1] it is often used as a 'last ditch attempt'. Local policy allows for rFVIIa use in certain haematology patients, those with post partum haemorrhage but not recommended in major haemorrhage due to trauma.

**OBJECTIVES.** To review guideline compliance for the use of rFVIIa and compare patient outcomes and amount of blood transfused.

**METHODS.** Audit of adult patients prescribed rFVIIa over a 3 year period at the Royal London Hospital (largest trauma centre in UK). Electronic pharmacy records of rFVIIa prescription were reviewed along with patient paper and electronic notes. Data on red blood cell transfusion was obtained from the transfusion laboratory.

**RESULTS.** rFVIIa use is rare; 16 patients were prescribed rFVIIa of whom 13 went on to receive it; 7 according to the guidelines and 6 off label.

Of the 7 cases compliant with the guidelines 3 received rFVIIa due to a clotting abnormality or extreme intolerance to blood transfusion (Glanzmann's thrombasthenia, Bernald-Soulier syndrome, sickle cell anaemia with numerous red cell antibodies); the remaining 4 as part of an obstetric major haemorrhage. 6 patients were given rFVIIa off label after a supra-massive blood transfusion due to a traumatic reason (2 stabbings, 2 road traffic collisions, 1 pancreatic surgery, 1 for hepatic drain).

All the patients with a haematological condition were treated for minor bleeding that was halted with the use of rFVIIa and platelet transfusion; none received any red blood cells (RBC). The obstetric haemorrhage patients received median 15 units RBC (range 7–29) whilst the trauma patients received significantly more blood products, median 41 units RBC (range 24–55), p = 0.03, see Table 161. All patients given rFVIIa for a traumatic reason received it after at least 15 units RBC and numerous other blood products.

All of the patients with a haematological abnormality and obstetric related haemorrhage survived whereas all of the trauma patients died.

**CONCLUSIONS.** Almost half the cases of rFVIIa use are not compliant with current hospital guidelines. Our findings do not support its use in patients who have suffered major haemorrhage secondary to trauma.

This audit should serve as further evidence that rFVIIa has limited value in the treatment of patients who have already received a supra-massive blood transfusion.

**Table 161 (Abstract 0601)**. Comparison of groups treated with rFVIIa

| | Haematological | Obstetric | Traumatic |
|---|---|---|---|
| Number | 3 | 4 | 6 |
| Packed red cells - median, (range) | 0 | 15 (7-29) | 41 (21-55) |
| Length of Hospital Stay Post Bleed - median (range) | 6 (3-31) | 17 (9-26) | 1 (0-7) |
| Survived to Hospital Discharge - No. (%) | 3 (100) | 4 (100) | 0 (0) |
| Cost of rFVIIa Per Patient - median | £4963 | £3309 | £5239 |

### 0602

#### Efficacy of prophylactic anticoagulation with enoxaparin in ICU patients measured by antiXa activity: a retrospective study

I. Zykova, B. Paldusova, J. Ferech, D. Morman
Regional Hospital Liberec, Department of Anesthesiology and Intensive Care, Liberec, Czech Republic
**Correspondence:** I. Zykova
*Intensive Care Medicine Experimental* 2017, **5(Suppl 2):**0602

**INTRODUCTION.** Standard dose of 40 mg of enoxaparin subcutaneously once a day is so far recommended for prophylactic anticoagulation in all patients regardless their weight and physical status although it has been shown in ICU patients in numerous studies to be unreliable and not effective enough according to antiXa activity. Evidence suggests decreased bioavailability of enoxaparin in ICU population.We have started testing antiXa activity routinely in our ICU patients after every enoxaparin given for prophylactic anticoagulation in 2013. The dosing of enoxaparin is adjusted according to antiXa activity in each patient. The initial dose used in our ICU is 40 mg in most patients, higher initial dose is used mostly in obese patients, but the initial dosage is left to the ICU physician, we do not have a set protocol.

**OBJECTIVES.** Evaluation of the efficacy of prophylactic anticoagulation with dosing of enoxaparin guided by routine testing of antiXa activity.

**METHODS.** Data was retrospectively collected from patients admitted to our 9 bed mixed ICU over 3 months period (1.4.-30.6.2016). Included were all patients on prophylactic anticoagulation by subcutaneously administered enoxaparin in whom antiXa activity was measured. AntiXa activity is measured 3 hours after the subcutaneous application of enoxaparin in our institution, the recommended prophylactic range of antiXa activity is 0,2-0,5 U/ml.

**RESULTS.** 49 patients who met the set criteria were admitted during the 3 month study period to our ICU. 284 antiXa measurements were taken, 190 (66,9%) were within the prophylactic range (0,2-0,5 U/ml), 80 (28,2%) were under the prophylactic range and 14 were over the prophylactic range.

40 mg of enoxaparin was used 118 times (41,5%), 60 mg 106x (37,3%), 80 mg 45x (15,8%),100 mg 5x (1,8%) and 20 mg 10x (3,5%).

The initial dose of enoxaparin was 40 mg in 40 patients, in 19 antiXa was within prophylactic range, in 19 was antiXa activity too low and in 2 patients was antiXa activity too high. The initial dose was 60 mg in 8 patients, in 5 patients antiXa activity was within the recommended range and in 3 patients it was too low. The initial dose was 80 mg in one patient with antiXa activity within prophylactic range. In summary the initial dose was correct in 25 patients, it was too low in 22 patients and too high in 2 patients.

| initial dose of enoxaparin | No. of patients | adequate antiXa range (0,2-0,5 U/ml) | low antiXa activity (<0,2 U/ml) | high antiXa activity (>0,5 U/ml) |
|---|---|---|---|---|
| 40 mg | 40 patients (81,6%) | 19 patients (47,5%) | 19 patients (47,5%) | 2 patients (5%) |
| 60 mg | 8 patients (16,3%) | 5 patients (62,5%) | 3 patients (37,5%) | 0 |
| 80 mg | 1 patient (2%) | 1 patient | 0 | 0 |
| all initial doses | 49 patients (100%) | 25 patients (51%) | 22 patients (44,9%) | 2 patients (4,1%) |

*[Initial dose of enoxaparin]*

**CONCLUSIONS.** Prophylactic anticoagulation in critically ill patients is essential but poses many problems. The standard recommended dosage is often not sufficient, in our study standard dosing was used only in 41,5%. Even though we used higher dosage than recommended in 54,9%, the antiXa activity was adequate only in 66,9%. The bioavailability of enoxaparin changes in a patient during his critical illness. The initial dose of enoxaparin should also be individualized. Monitoring of antiXa activity seems to be essential but even routine monitoring is not the only answer. More studies need to be done.

**REFERENCE(S)**

On poster

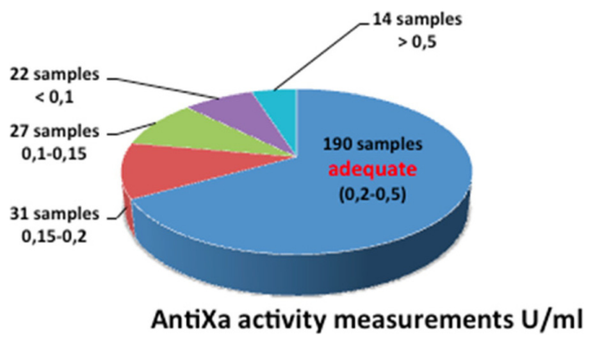

**Fig. 215 (Abstract 0602).** AntiXa activity measurements

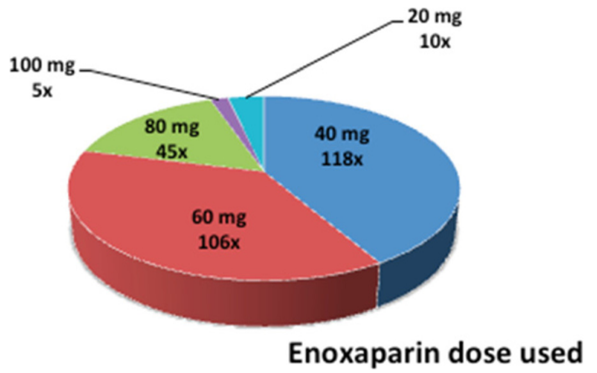

**Fig. 216 (Abstract 0602).** Enoxaparin dose used

### 0603

#### Venous thromboembolism in the Intensive care unit: a prospective observational study of occurrence, risk factors and outcome

S. Beitland[1], T. Lorentsen[1], H. Wimmer[2], M. Kjelås[2], C. Hotvedt[2], T. Drægni[2], N.E. Kløw[2,3], P.M. Sandset[2,3], K. Sunde[1,3]
[1]Oslo University Hospital, Department of Anaesthesiology, Oslo, Norway; [2]Oslo University Hospital, Oslo, Norway; [3]University of Oslo, Institute of Clinical Medicine, Oslo, Norway
**Correspondence:** S. Beitland
*Intensive Care Medicine Experimental* 2017, **5(Suppl 2):**0603

**INTRODUCTION.** Intensive care unit (ICU) patients may develop deep vein thrombosis (DVT) or pulmonary embolism (PE).

**OBJECTIVES.** The aim of the study was to explore occurrence, risk factors and outcome of venous thromboembolism (VTE) in ICU patients.

**METHODS.** Prospective observational study of adult medical and surgical patients at Oslo University Hospital in Norway from 2012 to 2016. Patients with ICU length of stay above 48 hours were included according to predefined inclusion and exclusion criteria. Doppler Ultrasound (DUS) was used for screening of the neck and the upper and lower extremity veins to elbow and knee level. The DUS examination was performed at inclusion, and thereafter twice a week until discharge from the ICU or up to 30 days. Computed tomography angiography was used when clinically indicated for any medical reason.

**RESULTS.** Seventy patients were included (mean age 62 years, 79% males), half with normal kidney function and half with acute kidney injury undergoing renal replacement therapy. All included patients received thromboprophylaxis with dalteparin, 44 (63%) additionally used graduated compression stockings. VTE was found in 19 (27%) patients, DVT in 15 (21%) and PE in four (6%). Among those with VTE, 11 (58%) events were present within the first 48 hours after admission, and nine (47%) were related to central vein catheters. The VTE was clinically suspected in five (26%) patients, three with PE and two with DVT. Treatment was therapeutic anticoagulation in 15 (79%) patients, and removal of the central vein catheter related to the DVT in seven. Risk factors for VTE were abdominal surgery (53% vs. 28%, p = 0.05), malignant disease (32% vs.10%, p = 0.03) and central vein catheter in the internal jugular vein (100 vs. 67%, p = 0.04), but not acute kidney injury treated with renal replacement therapy (47% vs. 51%, p = 0.79). Patients with and without VTE had comparable ICU LOS (13 days vs. 11 days, p = 0.79) and mortality (16% vs. 20%, p = 0.72).

**CONCLUSIONS.** In adult ICU patients, DVT was observed in 21% and PE in 6% of the patients. Most of the VTE events were present within the first 48 hours after admission, but not clinically suspected. Abdominal surgery, malignant disease and central vein catheters in the internal jugular vein were risk factors for VTE. Presence of VTE did not impact on ICU length of stay or mortality.

1.Minet C, Potton L, Bonadona A, Hamidfar-Roy R, Somohano CA, et al. Venous thromboembolism in the ICU: main characteristics, diagnosis and thromboprophylaxis. Crit Care. 2015;19:287.
2.Boonyawat K, Crowther MA. Venous thromboembolism prophylaxis in critically ill patients.Semin Thromb Hemost. 2015 Feb;41:68–74.

**GRANT ACKNOWLEDGMENT**
The authors declare that they have no competing interest. Financial support was provided solely from institutional sources.

### 0604

#### ROTEM EXTEM clotting time *versus* thrombin generation in experimentally reconstituted whole blood

G. E. Iapichino[1,2], M. Ponschab[1], J. Zipperle[1], C. J. Schlimp[1], H. Schoechl[1]
[1]Ludwig Boltzmann Institute for Experimental and Clinical Traumatology, Department of Intensive Care, Vienna, Austria; [2]Università degli Studi di Milano, Scuola di Specializzazione in Anestesia, Rianimazione e Terapia Intensiva, Milano, Italy
**Correspondence:** G. E. Iapichino
*Intensive Care Medicine Experimental* 2017, **5(Suppl 2):**0604

**INTRODUCTION.** EXTEM clotting time (CT) is considered representative of the activation of the coagulation cascade and therefore of the thrombin generation potential. However CT is defined as the time needed to reach 2 mm amplitude on the viscoelastic tracing and it is thus also dependent on fibrinogen concentration.

**OBJECTIVES.** To test the correlation between EXTEM CT and parameters of thrombin generation in the setting of reconstituted whole blood (RWB) with increasing plasmatic dilution and fibrinogen supplementation.

**METHODS.** Whole blood (WB) was collected from six healthy donors and divided into red blood cells, platelet-rich and platelet-poor plasma (PPP) by consecutive centrifugation steps. These components were then re-mixed to produce aliquots of reconstituted whole blood (RWB) with increasing plasma dilutions (20, 40, 60, 80% by addition of saline) but constant hematocrit and platelet count. WB and RWB aliquots were tested with EXTEM after the addition of either 10 μL of saline (EXTEM) or 10 μL (600 μg) of fibrinogen concentrate (EXTEM + Fib) to the cup. PPP of these aliquots was then tested with CAT assay (thrombin generation).

**RESULTS.** Blood cell count and plasma dilution are displayed in Table 162.

Dilution of the plasmatic component was associated with an increase (not significant) of thrombin generation area under the curve (TG AUC) that was maintained from 20 to 80%. EXTEM CT was shortest at 20% dilution and then significantly increased with increasing dilution. Addition of fibrinogen to the test (EXTEM + Fib) significantly reduced CT (Fig. 217). Linear regression of EXTEM CT and AUC and Lag Time parameters of TG are displayed in Fig. 218.

**CONCLUSIONS.** No correlation between EXTEM CT and thrombin generation parameters was found. Our experiments also showed that EXTEM CT is significantly dependent on fibrinogen, a substrate of the activated thrombin enzyme.

**GRANT ACKNOWLEDGMENT**

This study was supported with a grant of the AUVA Böhler Fonds.

**Table 162 (Abstract 0604).** Blood cell count and plasma dilution

| | WB | 20% diluted RWB | 40% diluted RWB | 60% diluted RWB | 80% diluted RWB |
|---|---|---|---|---|---|
| Hematocrit (%) | 41 ± 6 | 24 ± 1 | 25 ± 1 | 24 ± 2 | 25 ± 1 |
| Platelet count (*1000/μL) | 227 ± 47 | 90 ± 8 | 89 ± 10 | 92 ± 7 | 89 ± 7 |
| Plasma dilution (%) | 0 ± 0 | 20 ± 0 | 40 ± 0 | 60 ± 0 | 74 ± 6 |

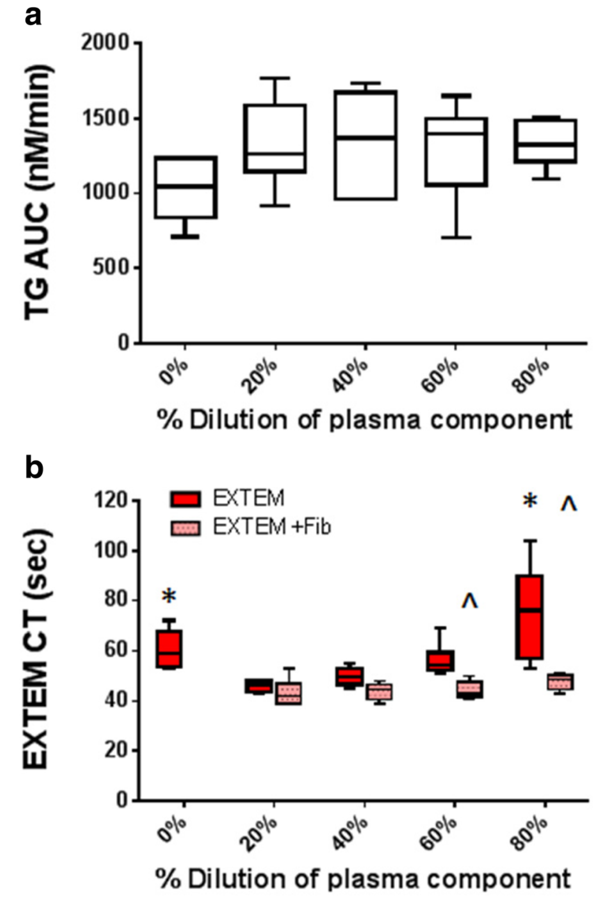

**Fig. 217 (Abstract 0604).** See text for description

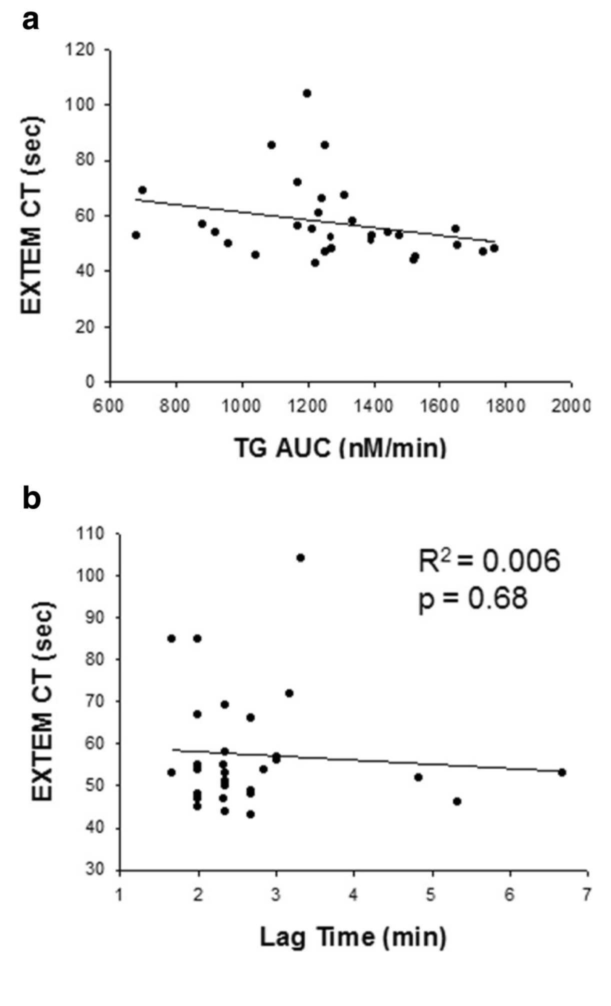

**Fig. 218 (Abstract 0604).** See text for description

### 0605

#### Rotational thromboelastometry to detect hyperfibrinolysis with tissue plasminogen activator for catheter thrombosis in critically ill patients

A. Mittel, A. F. Yen, R. Ittoop, G. Wagener, M. Patel, O. Panzer
Columbia University Medical Center, Anesthesiology, New York, United States
**Correspondence:** A. Mittel
*Intensive Care Medicine Experimental* 2017, **5(Suppl 2):**0605

**INTRODUCTION.** Central venous catheter (CVC) thrombosis is a common problem in the ICU. Tissue plasminogen activator (tPA) may be required to restore lumen patency, but carries a risk of bleeding. Studies supporting this practice have not evaluated for the possible complication of systemic hyperfibrinolysis.[1]

**OBJECTIVES.** We sought to determine if tPA given for CVC declotting is associated with hyperfibrinolysis, as directly measured using rotational thromboelastometry (ROTEM), the clinical gold standard for hyperfibrinolysis detection.[2]

**METHODS.** We conducted an observational study of patients who required tPA administration for CVC thrombosis. At our center, 2 mg of tPA is dissolved into 2 mL of sterile water, administered into the occluded CVC lumen for 90 minutes, and then removed. We tested for evidence of hyperfibrinolysis before (pre-tPA), 5–10 min after tPA was inserted (mid-dwell), and after tPA (post-tPA) using EXTEM and INTEM ROTEM assays. The primary outcome was the difference in mean maximum lysis index (ML) between each time point above.

**RESULTS.** 20 patients were enrolled. Of these, 8 patients were excluded from analysis due to technical errors. In the remaining 12 patients, the mean EXTEM ML pre-tPA, at mid-dwell, and post-tPA were as follows (standard deviations in parentheses): 2.5% (2.5%), 10.9% (28.2%), and 3.3% (2.5%), respectively. The mean INTEM ML pre-tPA, at mid-dwell, and post-tPA were as follows: 1.5% (1.6%), 10.1% (28.4%), and 2.8% (1.7%), respectively. In one patient, INTEM ML pre-tPA was 0%, was 100% at mid-dwell, and was 1% post-tPA. This patient's EXTEM ML post-tPA assay was inadvertently not performed, but EXTEM ML pre-tPA ML was 0% and mid-dwell ML was 100%. When analyzing the other 11 patients, the mean EXTEM ML at pre-tPA, mid-dwell, and post-tPA were as follows: 2.5% (2.4%), 2.8% (3.4%), and 3.3% (2.5%), respectively. Similarly, in these same 11 patients, the mean INTEM ML at pre-tPA, mid-dwell, and post-tPA were as follows: 1.6% (1.6%), 1.9% (2.0%), and 2.9% (1.7%).

When analyzing all 12 patients ML values at each time point via matched pairs analysis, there was a significant difference between INTEM ML at pre-tPA and post-tPA ($p = 0.045$). When including only those 11 patients that did not have ML elevations at any time point, the INTEM ML between pre-tPA and post-tPA was not significant ($p = 0.062$). There were no significant differences between ML's at any other time points.

**CONCLUSIONS.** Low dose tPA for CVC declotting can be associated with systemic hyperfibrinolysis. However, our results were likely influenced by one outlier who did become hyperfibrinolytic. Importantly, post hoc review determined that this was the only patient that had blood samples drawn from a CVC lumen directly adjacent to the lumen in which tPA was administered, thus the clinical implications of this are unclear.

**SUPPORTING GRANT**
None.

### 0606

#### Recombinant activated VII factor at massive bleeding in case of invasive placenta

E. Shifman[1], A. Kulikov[2], A. Zhilin[3], A. Matkovski[3], S. Mamayev[3]
[1]M.F. Vladimirskiy Moscow Regional Research Clinical Institute, Moscow, Russian Federation; [2]Ural State Medical University, Yekaterinburg, Russian Federation; [3]Regional Perinatal Center, Yekaterinburg, Russian Federation
**Correspondence:** E. Shifman
*Intensive Care Medicine Experimental* 2017, **5(Suppl 2):**0606

**INTRODUCTION.** In obstetrics, the massive blood loss (MBL) occupies a leading position in the structure of maternal mortality. The application of VII factor at massive bleeding in obstetrics is debatable and requires further investigations.

**OBJECTIVES.** The aim of the study was to evaluate the efficiency of the recombinant activated VII factor (FVIIa) for coagulopathy correction at massive bleeding against the background of invasive placenta.

**METHODS.** A prospective study included 60 patients with MBL with the invasive placenta. In 30 patients, the correction of acute coagulopathy in the background of MC with a volume of 2939.7 ± 248.2 ml was performed with freshly frozen plasma (FFP) and in 30 patients, with the MBL volume of 3146.1 ± 640.2 ml - fVIIa (90 μg / kg) + FFP. The state of haemostasis was assessed by thromboelastography (TEG) with the TEG 5000 apparatus (Haemonetics); the indices of platelets, fibrinogen, MNO and APTV have been analyzed. The time of elimination of coagulopathy from the moment of registration (min) and the volume of injected FFP (ml) have been recorded.

**RESULTS.** At the peak of blood loss, in all patients, the structural and chronometric hypocagulation was recorded by the TEG method, which was eliminated in the SLE group after 43.4 ± 2.1 min after registration of coagulopathy, and in the fVIIa + FFP group - after 5.4 ± 1.8 min ($p < 0.0001$). The first group required 1297 ± 67.5 ml FFP, while the group with fVIIa - 766.0 ± 242.0 ml ($p < 0.05$). Complications of fVIIa have not been registered.

**CONCLUSIONS.** The application of fVIIa at the MBL with invasive placenta reliably reduces the correction time of coagulopathy compared to the isolated use of FFP and is accompanied by a significant decrease in the required volume of FFP to achieve the desired effect. In case of the MBL in obstetrics it is necessary to apply FVIIa immediately after the registration of coagulopathy.

**REFERENCE(S)**

Collins P, Abdul-Kadir R, Thachil J; Subcommittees on Women´ s Health Issues in Thrombosis and Haemostasis and on Disseminated Intravascular Coagulation. Management of coagulopathy associated with postpartum hemorrhage: guidance from the SSC of the ISTH. J Thromb Haemost. 2016 Jan;14(1):205–10

### 0607

#### Agreement between activated partial thromboplastin time (aPTT) and anti-Xa activity (antiXa) in critically ill patients under therapeutic anticoagulation

D. Ratano[1], L. Alberio[2], F. Delodder[1], M. M. Berger[1]
[1]Lausanne University Hospital (CHUV), Service of Intensive Care Medicine, Lausanne, Switzerland; [2]Lausanne University Hospital (CHUV), University of Lausanne, Division of Hematology and Central Hematology Laboratory, Lausanne, Switzerland
**Correspondence:** D. Ratano
*Intensive Care Medicine Experimental* 2017, **5(Suppl 2):**0607

**INTRODUCTION.** Anticoagulation with unfractionated heparin (UFH) is a mainstay in critical care. Monitoring of this therapy is based either on aPTT or antiXa. There is no clear recommendation on which method should be used in critical care: studies addressing this issue are a few and include small cohorts of patients. In clinical setting this often leads to the simultaneous determination of both parameters, increasing costs with no obvious advantage. Indeed the two measures may disagree with regard to their therapeutic range leading to confusion.

**OBJECTIVES.** This study aimed at analysing the agreement between aPTT and antiXa in a large population of therapeutically anticoagulated critically ill patients. Patients treated with extracorporeal devices such as continous renal replacement therapy (CRRT) and circulatory assist devices (CAD) were predefined subgroups.

**METHODS.** Retrospective study of prospectively collected data of a 35 beds mixed-ICU population between years 2006 and 2016 in a University teaching hospital. Inclusion criteria were the delivery of a UFH dose superior to 15'000U/24h during at least 1 day with a concomitant antiXa determination. Analysed data: demographic variables, aPTT, antiXa, extracorporeal devices (CAD, CRRT). Couples of simultaneously dosed antiXa and aPTT were analysed on the basis of their agreement to the subtherapeutic, therapeutic (aPTT 50-80", antiXa 0.3-0.7 U/ml) or supratherapeutic range. Data as mean and percentage.

**RESULTS.** Out of 23'334 admissions, 2'254 were analysed: 2'123 patients, age 63.7 yrs, SAPSII 46.9 pts, median ICU stay 8.2, ICU mortality 13%. In 2006, antiXa was used in 9% of the patients increasing to >96% after 2012. Altogether 40'795 antiXa and 80'696 aPTT were analysed, resulting in 40'578 couples of values. Only 217 antiXa (0.53%) were determined without simultaneous aPTT. The overall agreement between antiXa and aPTT was 59.8% (disagreement 40.2% with aPTT > antiXa 30.5% and aPTT < antiXa 9.7%). Agreement was lower in patients with extracorporeal devices (CAD 57.9% / CRRT 57.3%), being lowest with both devices (agreement 54.1%, disagreement 45.9% with aPTT > antiXa 39% and aPTT < antiXa 6.9%).

**CONCLUSIONS.** The agreement between antiXa and aPTT is < 60% and lower in patients with CAD and/or CRRT. The analysis of the proportions in the disagreement group suggests a factitious prolongation of aPTT in presence of extracorporeal devices. The very large proportion of simultaneous aPTT/antiXa determination probably reflects a lack of confidence in usual UFH monitoring methods and causes unnecessary costs. Establishing the factors influencing the antiXa/aPTT agreement may assist the clinician choosing the optimal monitoring for UFH in ICU patients.

Accuracy of aPTT monitoring in critically ill patients treated with unfractionated heparin. Van Roessel S.,Neth J Med 2014;72:305

**GRANT ACKNOWLEDGMENT**

None

### 0608

#### The correlation between conventional coagulation tests and thromboelastography in each phase of liver transplantation

J.-H. Cheon[1], J.-U. Yoon[1], S.-W. Baik[1], J.-Y. Yoon[2], E.-J. Kim[2], Jiuk Yoon
[1]Pusan National University Yangsan Hospital, Yangsan, Korea, Republic of; [2]Pusan National University Dental Hospital, Yangsan, Korea, Republic of
**Correspondence:** J.-H. Cheon
*Intensive Care Medicine Experimental* 2017, **5(Suppl 2):**0608

**INTRODUCTION.** Recently, thromboelastography (TEG) has been used in liver transplantation (LT) as well as conventional coagulation tests (CCTs) such as prothrombin time (PT), activated partial thromboplastin time (aPTT), antithorombin III (ATIII), platelet count and fibrinogen concentration.

**OBJECTIVES.** The purpose of this study was to investigate the correlation between the TEG and CCT values during each phase of LT and to evaluate the clinical utility of TEG in LT.

**METHODS.** Patients who underwent deceased donor liver transplantation (DDLT) at Pusan National University Yangsan Hospital, Yangsan, Korea, between October 2010 and July 2015, were retrospectively evaluated. Samples were obtained from all patients at 1 hour after initiation of the operation (pre-anhepatic phase), 30 minutes after total hepatectomy of the recipient (anhepatic phase), and 1 hour after reperfusion (neo-hepatic phase). A portion of the sample was used for TEG and the rest was subjected to CCTs.

**RESULTS.** The Spearman correlation coefficient between TEG and CCT was obtained. At the pre-anhepatic phase (Table 1), the reaction time (R), PT, and aPTT did not show a high correlation with each other, but rather showed a negative correlation with the number of platelets. Clot formation time (K) showed a similar correlation with R, but a negative correlation with fibrinogen. The maximal amplitude (MA) and α-angle (α) were positively correlated with the number of platelets and fibrinogen, and inversely correlated with aPTT. During the anhepatic phase (Table 2), MA was significantly correlated with PLT, and inversely correlated with aPTT; other parameters had no significant correlation. During the neo-hepatic phase (Table 3), R and K were significantly correlated with aPTT, and inversely correlated with the number of platelets and fibrinogen, similar to the pre-anhepatic phase. A correlation of MA and α with PLT, aPTT, and fibrinogen was also reported. It was revealed in this study that clot lysis at 30 minutes (LY30; percentage) and estimated percent lysis (EPL) were inversely correlated with levels of ATIII and fibrinogen. PT was not correlated with TEG during all of these phases.

**CONCLUSIONS.** This study demonstrated that conventional coagulation tests and TEG tests show particularly poor agreement during the anhepatic period of liver transplantation. TEG can be more reliable in the anhepatic phase, which is more complicated and during which dynamic hemostatic changes occur.

**REFERENCE(S)**

Kang YG, Martin DJ, Marquez J, Lewis JH, Bontempo FA, Shaw BW Jr, et al. Intraoperative changes in blood coagulation and thrombelastographic monitoring in liver transplantation. Anesth Analg 1985; 64: 888–896.

Tripodi A, Caldwell SH, Hoffman M, Trotter JF, Sanyal AJ. Review article: the prothrombin time test as a measure of bleeding risk and prognosis in liver disease. Aliment Pharmacol Ther 2007; 26:141–148.

### 0609

#### Assessment of venous thromboprophylaxis in various adult intensive care units regarding caprini score

M. Masjedi[1,2], R. Chegini[3], F. Zand[1,2], B. Maghsoudi[1,2], G. Sabetian[1,4], M. Savaie[3,5]
[1]Shiraz University of Medical Sciences, Anesthesiology and Critical Care, Shiraz, Iran, Islamic Republic of; [2]Shiraz University of Medical Sciences, Anesthesiology and Critical Care Research Center, Shiraz, Iran, Islamic Republic of; [3]Shiraz University of Medical Sciences, Shiraz, Iran, Islamic Republic of; [4]Trauma Research Center, Shahid Rajaee (Emtiaz) Trauma Hospital, Shiraz, Iran, Islamic Republic of; [5]Ahwaz University of Medical Sciences, Ahwaz, Iran, Islamic Republic of
**Correspondence:** M. Masjedi
*Intensive Care Medicine Experimental* 2017, **5(Suppl 2):**0609

**INTRODUCTION.** Venous thromboembolism is a common complication in intensive care units, which increases mortality morbidity. According to different risk assesment methods, without use of thromboprophylaxis, the risk of deep venous thrombosis may reach up to 81% and in case of its usage,can be reduced to 44% or even lower based on predisposing factors.

**OBJECTIVES.** We were to assess the appropriate use and extent of adherence to venous thromboprophylaxis in different intensive care units (ICUs) according to Caprini scoring system.

**METHODS.** In this study, 296 patients admitted in five different types of ICUs in two referral, university affiliated hospitals were studied during a 6 month period in year, 2016 . Data were collected in a predefined questionnaire based on caprini risk assesment tool and appropriatness of thromboprophylaxis consumption was assessed.

**RESULTS.** Among the studied population, 87 (29.4%) cases were female and 209 (70.6%) were male. Distribution of patients among different ICUs was, medical 27%, trauma 32.4%, neurosurgical 25%, surgical 11.5% and neurological 4.1%. Distribution of patients according to risk level was as follows ; very low 2.7%, low 507%, moderate 17.2% and severe 74.3%. Overall, most of the patients who required prophylaxis received it but appropriateness and sufficiencies, varied.From 296 studied cases, only 62 patients (20.9%) received appropriate and sufficient prophylaxis while 64 patients (21.6%) did not received it at all. 78 patients (26.4%) had contraindication to administeration of drug prophylaxis, among them, 67 cases need compression device but it was applied in only 19 cases (28.3%). Although according to Caprini score, all patients admitted in neurological ICU were at severe risk, but none of them received sufficient prophylaxis.

**CONCLUSIONS.** There is a gap between real life VTE prophylaxis and what is suggested by the Caprini scoring recommendations in our ICUs.Improved prescription of extended thromboprophylaxis is warranted to ensure adherence to standard guidelines.

**REFERENCE(S)**

1- Cunningham R, Murray A, Byrne JS, Hammond L, Barry M, Mehigan D, Sheehan S. Venous thromboembolism prophylaxis guideline compliance: a pilot study of augmented medication charts.Ir J Med Sci. 2015 Jun;184(2):469–74.
2- Vallano A, Arnau JM, Miralda GP, Pérez-Bartolí J. Use of venous thromboprophylaxis and adherence to guideline recommendations: a cross-sectional study.Thromb J. 2004 Apr 1;2(1):3.
3- Panju M, Raso D, Patel A, Panju A, Ginsberg J. Evaluation of the use of venous thromboembolism prophylaxis in hospitalised medical patients.J R Coll Physicians Edinb. 2011 Dec;41(4):304–8.
**GRANT ACKNOWLEDGMENT**
This study was financially supported by the Vice chancellery for research affairs of Shiraz University of Medical Sciences, Shiraz, Iran

### 0610

#### Survival and associated predictor in the critically ill patient after transfusion therapy

L. Marqués Álvarez[1], R. Rodríguez García[1], D. Escudero Augusto[1], R. Victoreo Fernández[2], E. Martínez Revuelta[1], R. Yano Escudero[1], S. De Cima Iglesias[1], M.J. Espina Angulo[1], C. Palomo Antequera[1]
[1]Hospital Universitario Central de Asturias, Oviedo, Spain; [2]Universidad de Oviedo, Oviedo, Spain
**Correspondence:** R. Rodríguez García
*Intensive Care Medicine Experimental* 2017, **5(Suppl 2):**0610

**INTRODUCTION.** Blood transfusions are an usually practice at the ICU. There is an increasing evidence of potential harm and sometimes worsen patient outcomes so, nowadays, we suggest that blood and component transfusions carry significant though poorly quantified risks. It has been estimated that greater than 40% of patients receive one or more red blood cell transfusions (RBC) while in the ICU.
**OBJECTIVES.** The aim of our study was to determinate short term survival and associated predictors obtained on admission and during staying at ICU.
**METHODS.** We made a retrospective and observational study of patients admitted to the ICU during six months and who have received at least one blood component. Patients admitted with cardiac diseases were excluded. On admission, we recorded clinical and laboratory parameters. Survival of patients at the end of the study was investigated. Kaplan-Meier survival curves were calculated and compared by Long Rank test. Factors associated with survival were obtained using multivariate Cox regression analysis.
**RESULTS.** 137 patients were identified. The mean age was 61.6 ± 15 years and 65% were men. On admission the mean value of APACHE II was 22 ± 7. 43% came from the emergency department, 37% from the hospital ward and 20% from another hospital. 61% required invasive ventilation, 48% received vassopresors and 19% CVVHDF. Among of 1144 units were transfused, 602 (53%) were red blood cells, 305 (27%) platelets, 234 (20%) fresh frozen plasma (FFP) and 3 (<1%) cryoprecipitates. Most patients received RBC (79%). The average pretransfusion hemoglobin concentration was 7.9 ± 2 g/dl and the ratio RBC/patient was 5.6 ± 6.2. The common indication for transfusion was low hemoglobin (39% had Hb < 7 g/dl, 42% between 7–9 g/dl). Overall, 67 (49%) and 31 (23%) of the patients were transfused platelets and FFP, respectively. The indications for transfusion were hemorrhage, low platelet counts, prolonged prothrombin time or to provide cover for invasive interventions. Most platelet transfusions were given at values in the order of > 50.000/ mcl (53.7%). Total survival achieved 63.5%. The accumulated survival decreased below 50% at the month. Immunosuppression, APACHE II, ventilation, CVVHDF, vasopressors, number of RBC transfused, hemoglobin and platelet pretransfusion values were statistically significant variables related to survival analysis ($p < 0.05$). The number of transfused RBCs was the only independent predictor of survival ($p = 0.035$). The average stay in ICU and in the hospital ward was of 14 ± 15 and 21 ± 28 days respectively. ICU mortality was 29.2%. Most patients died within the first week.
**CONCLUSIONS.** Transfusion practice in ICU is consistent with the clinical practice guidelines. The most frequent indication is the hemorrhage. We have a restrictive therapy. ICU mortality of transfused patients is high. Up to 1/3 dies during the first week. The number of transfused RBCs was the only predictor of survival at the ICU.

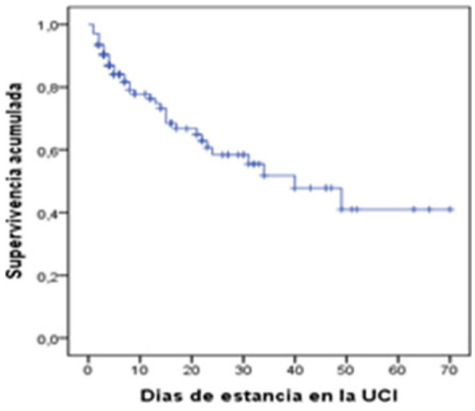

**Fig. 219 (abstract 0610).** ICU Survival

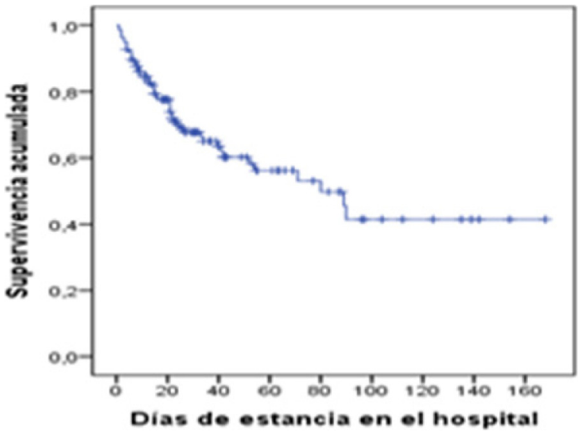

**Fig. 220 (abstract 0610).** Hospital Ward Survival

### 0611

#### EXTEM ROTEM profile in a cohort of patients with non recovered cardiac arrest

J. A. Barea-Mendoza, L. Terceros-Almanza, C. García-Fuentes, C. Mudarra-Reche, S. Bermejo-Aznárez, I. Prieto-Portillo, J. A. Cantalapiedra, J. C. Montejo-González, M. Chico-Fernández, Trauma and Emergency Research Group (GINTEM_12O)
[1]12 de Octubre Hospital, Trauma and Emergency ICU (UCITE), Critical Care Department, Madrid, Spain
**Correspondence:** J. A. Barea-Mendoza
*Intensive Care Medicine Experimental* 2017, **5(Suppl 2):**0611

**INTRODUCTION.** Traditionally, coagulopathy has been diagnosed with the usual laboratory tests. However, these determinations are not sensitive and do not allow us to detect the mechanisms responsible. ROTEM has been developed to enable early point of care in ICU. Hypoperfusion has been described as a coagulopathy trigger. Several studies have assessed the incidence of coagulopathy during and after cardiac arrest. Cardiac arrest (CA) is the most extreme hypoperfusion example in the human specie. The study of those process during CA could offer a better understanding about the role of hypoperfusion in the coagulation during critical illness.[1]
**OBJECTIVES.** To describe the ROTEM (Rotational Thromboelastometry) pattern in patients with non shockable cardiac arrest.
**METHODS.** Prospective, unicentric study was developed according with prespecified protocol. We include all patients admitted in our center with out-of-hospital cardiac arrest in non-shockable rhythm from February to December 2015. No exclusion criteria were established. We collected initial heart rhythm, time between arrest and admission, and characteristics of cardiopulmonary resuscitation (CPR). Blood sampling was performed at admission. Coagulation tests were performed within 30 min after blood collection. The routine coagulation tests performed: prothrombin activity (PT), partial thromboplastin time (aPTT) and fibrinogen determination. We analyzed the ROTEM EXTEM assay in all samples. The included parameters were: clot time (CT), clot formation time (CFT), maximum clot firmness (MCF), maximum lysis (ML). Quantitative data are reported as median (Interquantile Range; 25–75). Categorical ones are shown in N (%). All analyses are performed with STATA 12.

**RESULTS.** The study included 14 patients. The median age was 48 years (42.7-54.31), 12 were men (82.2%). The time from arrest until hospital admission was 83 min (62.3-105.4). All patients stayed in cardiac arrest during blood sampling. Initial rhythm was ventricular fibrillation in four patients, asystole in seven patients and unknown for three patients. At admission the hemoglobin levels were 13.6 g/dl (12–15.3) and the platelet count was $137.6 \times 10^9$/L (115.2-160). The results of the routine coagulation tests were: AP 75% (66.4-83.7); TTPA 64 (52–75.5) and fibrinogen levels 235 mg/dl (114–355). For the ROTEM EXTEM assay the results expressed as median (IQR) were: CT 125 (96–153), CFT 287 (179–396), MCF 34 (25–43), ML 80 (63–96). ML > 15% for 13 patients.

**CONCLUSIONS.** In our sample, patients after non reversed cardiac arrest presented prolonged TTPA. The EXTEM assays presented a pattern suggestive of alterations in the extrinsic pathway and fibrinolysis. The study of the coagulation disturbances after CA could be an interesting model of hipoxia-couagulation relationships.

### 0612

#### Causes of hypofibrinogenemia in the ICU

D. Koutrafouri[1], E. Boutzouka[1], E. Tsigou[1], K. Fragoulopoulou[2], T. Katsoulas[1], G. Spanou[2], G. Fildissis[1]
[1]ICU, Faculty of Nursing, National & Kapodistrian University of Athens, Agioi Anargiroi Hospital, Athens, Greece; [2]Hematology Laboratory, Agioi Anargiroi Hospital, Athens, Greece
**Correspondence:** D. Koutrafouri
*Intensive Care Medicine Experimental* 2017, **5(Suppl 2):**0612

**INTRODUCTION.** Hypofibrinogenemia is defined as fibrinogen levels < 150 mg/dL, and is distinguished in acquired and congenital, the latter being very rare. The acquired disorder is usually attributed to reduced hepatic production, as it happens in hepatic failure or when consumption is increased in cases of DIC. Rarer causes include medicines that inhibit synthesis in the liver, and primary fibrinolysis.

**OBJECTIVES.** To evaluate the incidence, causes and significance of hypofibrinogenemia in the ICU.

**METHODS.** All pts admitted to an 8-bed general ICU during 2015 were retrospectively assessed. Demographics, cause of admission, APACHE II and SOFA scores, ICU LOS and outcome were recorded in all pts that developed hypofibrinogenemia during their ICU stay. Data are presented as means ± SD or as median (min, max), for normal and skewed distributions, respectively. Graph Pad Prism 5.0 was used for the descriptive statistics.

**RESULTS.** A total of 173 pts were admitted during 2015. The number of pts who developed hypofibrinogenemia was 12 (6.94%, 8 females) with a mean age of 66 ± 19 years, APACHE II 26 ± 7.20 and SOFA 10 ± 2.90. One pt presented 2 episodes of hypofibrinogenemia during ICU stay, and 8 pts were septic (66.67%). ICU LOS and mortality rate was 39 ± 42 days and 75%, respectively.

Hypofibrinogenemia occurred in the $11^{th}$ day of stay ($1^{st}$ to 63th) and lasted 5.40 ± 3.70 days. Median levels of fibrinogen were 110 mg/dL (unspecified-150). Seven pts had DIC and 2 hepatic failure, 8 had received tigecycline (for ≥1 reasons in 6 cases) and 2 had an unknown cause.

Hemorrhagic complications occurred in 2 cases (15.38%), while FFP transfusion or dry fibrinogen substitution was undertaken in 3 cases (23.08%).

**CONCLUSIONS.** Hypofibrinogenemia was observed in only a small percentage of critically ill pts who usually were septic, more seriously ill and had a worse outcome. Hypofibrinogenemia was more frequently associated with the use of tigecycline, a finding that has been referred in small case reports up to now. Physicians should be aware of this complication and monitor fibrinogen levels during tigecycline administration.

**REFERENCE(S)**

1. Mosesson MW. Fibrinogen and fibrin structure and functions. J Thromb Haemost 2005; 3:1894–1904.
2. Besser MW, MacDonald SG. Acquired hypofibrinogenemia: current perspectives. J Blood Med 2016; 7:217–22.

## Artificial airways and complications

### 0613

#### Ultrasound-guided percutaneous dilational tracheostomy: a systematic review of randomized controlled trials and meta-analysis

A. Gobatto[1,2,3], B.A.M.P. Besen[1], M. Cestari[4], L.M. Malbouisson[1], P. Pelosi[5]
[1]São Paulo University, São Paulo, Brazil; [2]Hospital São Rafael, Salvador, Brazil; [3]Hospital da CIdade, Salvador, Brazil; [4]Hospital Santa Catarina, São Paulo, Brazil; [5]University of Genoa, Genoa, Italy
**Correspondence:** A. Gobatto
*Intensive Care Medicine Experimental* 2017, **5(Suppl 2):**0613

**INTRODUCTION.** Percutaneuos dilational tracheostomy (PDT) is a common and increasingly used procedure in the intensive care unit (ICU), usually performed with bronchoscopy guidance. Ultrasound has emerged as a useful tool to assist PDT, potentially improving its success rate and reducing procedure-related complications.

**OBJECTIVE.** To investigate whether ultrasound-guided PDT is equivalent or even superior to bronchoscopy-guided and anatomical landmark-guided PDT with regards to the procedure-related and clinical complications.

**METHODS.** We conducted a systematic review of randomized clinical trials comparing ultrasound-guided PDT to control groups (either bronchoscopy- or landmark-guided PDT) in patients undergoing PDT in the Intensive Care Unit (ICU). Our primary outcome was major complications rate and our secondary outcome was minor complications rate. A random-effects meta-analysis was used to pool the results.

**RESULTS.** Four studies fulfilled inclusion criteria and were analyzed, comprising 588 subjects. There was no difference in the major complications rate between patients assigned to ultrasound-guided PDT compared to control groups (pooled RR 0.48; 95% confidence interval [CI] 0.13-1.71, I2 = 0%). Minor complications rate were not different between groups, with high heterogeneity (pooled RR 0.49; 95% confidence interval [CI] 0.16-1.50, I2 = 85%). Sensitivity analysis including only RCTs using landmarks-guided PDT as the control group showed lower rates of minor complications in the ultrasound-guided PDT group (pooled RR 0.55; 95% CI 0.31-0.98, I2 = 0%).

**CONCLUSION.** Ultrasound-guided PDT seems feasible and comparable to bronchoscopy-guided PDT regarding major and minor complications, while it seems to reduce minor complications when compared to landmark-guided PDT.

### 0614

#### Predictors of an airway complication during intubation among the critically Ill: a nested case–control study of airway management - part I

N. Smischney[1], M. O. Seisa[1], O. Demirci[1], D. R. Schroeder[1], D. Diedrich[1], Hemodynamic and Airway Management Group (HEMAIR)
[1]Mayo Clinic, Rochester, MN, United States
**Correspondence:** O. Demirci
*Intensive Care Medicine Experimental* 2017, **5(Suppl 2):**0614

**INTRODUCTION.** Endotracheal intubation (ETI) is one of the most commonly performed procedures in the intensive care unit (ICU)[1]. ETI can be life-saving for patients presenting with acute respiratory failure. However, if the clinician is not vigilant during the procedure, complications can arise that may impact the overall outcome of patients. In addition, ETI carried out in the ICU, as compared to other environments, can be associated with increased complications, likely resulting from the lack of time for optimization before ETI as well as patient-related factors [1].

**OBJECTIVES.** Our primary aim was to identify predictors of an immediate airway complication during the peri-intubation period and to assess outcomes of those who developed an airway complication. Secondary aims were to report short-term outcomes of those who developed an airway complication compared to those who did not.

**METHODS.** We conducted a nested case control study of adult critically ill patients admitted to a medical and/or surgical ICU requiring emergent and non-emergent ETI during two year study period. For the primary aim, data on airway management was collected during the peri-intubation period (60 minutes pre- and post- intubation). We defined an immediate airway complication as hypoxemia (pulse oximetry reading < 90%) occurring at any time 30 minutes following ETI. Once identified within the cohort, patients were then classified as cases (those who experienced hypoxia) and controls (those who did not experience hypoxia).

**RESULTS.** 74 out of 420 patients experienced an airway complication (incidence of 18%). Patients who experienced an immediate airway complication were of lower age, male gender, and higher weight. These patients also had significantly higher illness severity APACHE 3 score at 24 hours of ICU admission. A history of obstructive lung disease did not lead to increased odds of experiencing an airway complication. However, intubating patients with acute respiratory failure was associated with experiencing an airway complication. On the contrary, intubating for airway protection and/or for a procedure resulted in decreased odds of experiencing an airway complication.

| | AIRWAY complication (N = 74) | No AIRWAY Complication (N = 346) | p-value |
|---|---|---|---|
| Age (years), mean ± SD | 62.1 ± 17.2 | 63.0 ± 16.1 | .655 |
| Male, n (%) | 45 (60.8) | 199 (57.5) | .602 |
| Weight (kg), mean ± SD | 86.2 ± 33.1 | 84.5 ± 28.6 | .639 |
| APACHE III 24hr score, mean ± SD | 89.6 ± 33.8 | 82.6 ± 25.0 | .041 |
| Reasons for intubation, n (%) | | | |
| Airway protection | 20 (27.0) | 153 (44.2) | .006 |
| Acute respiratory failure (dyspnea and/or Sa02 < 90%) | 61 (82.4) | 221 (63.9) | .002 |
| Neurologic (stroke/altered mental status) | 18 (24.3) | 115 (33.2) | .135 |
| Procedure-related (endoscopy/ bronchoscopy) | 9 (12.2) | 94 (27.2) | .007 |

*[Patient characteristics]*

| | AIRWAY complication (N = 74) | No AIRWAY Complication (N = 346) | p-value |
|---|---|---|---|
| Sa02(30 mins pre-), mean ± SD | 89.1 ± 8.1 | 94.6 ± 6.2 | <.001 |
| Sa02(30 mins post-), mean ± SD | 88.6 ± 6.3 | 96.9 ± 2.7 | <.001 |
| MAP (mmHg), mean ± SD | 84.5 ± 22.0 | 83.7 ± 18.5 | .740 |
| Emergency, n (%) | 61 (82.4) | 252 (72.8) | .085 |
| Operator level, n (%) | | | .050 |
| ICU attending | 9 (12.2) | 22 (6.4) | |
| ICU Fellow | 60 (81.1) | 314 (90.8) | |
| Resident | 5 (6.8) | 10 (2.9) | |

*[Variables obtained]*

| | AIRWAY complication (N = 74) | No AIRWAY Complication (N = 346) | p-value |
|---|---|---|---|
| Invasive ventilation (days), median [25th, 75th] | 1.8 [0.6, 6.5] | 1.9 [0.6, 4.6] | .524 |
| Non-invasive ventilation (days), median [25th, 75th] | 0.0 [0.0, 0.9] | 0.0 [0.0, 0.5] | .300 |
| Tracheostomy during ICU, n (%) | 10 (13.5) | 25 (7.2) | .076 |
| Tracheostomy during hospital, n (%) | 13 (17.6) | 36 (10.4) | .082 |
| ICU LOS (days), median [25th, 75th] | 4.6 [1.8, 9.1] | 4.0 [1.9, 7.2] | .480 |
| Hospital LOS (days), median [25th, 75th] | 9.5 [4.4, 21.4] | 11.9 [6.2, 21.0] | .139 |
| ICU death, n (%) | 22 (29.7) | 58 (16.8) | .010 |
| Hospital death, n (%) | 35 (47.3) | 85 (24.6) | <.001 |

*[Outcomes]*

**CONCLUSIONS.** Known risk factors associated with a difficult airway and hence hypoxia did not result in increased risk of experiencing an airway complication. Rather, patients not identified earlier in their clinical deterioration are more likely to decompensate (intubation for acute respiratory distress and pre-existing hypoxia). Furthermore, in-experienced intubating providers are more likely to have an airway complication as compared to experienced intubating providers. This study sheds light on the fact that early recognition is paramount to preventing an immediate airway complications,

**REFERENCE(S)**

[1] Simpson GD et al.. Tracheal intubation in the critically ill: a multi-centre national study of practice and complications. *Br J Anaesth* 2012;108(5):792–799.

### 0615

#### Optimum timing

E. Trujillo-Garcia[1], E. Curiel-Balsera[1], C. M. Gómez-Jiménez[1], I. Bellido-Estévez[2], J. M. Mora-Ordóñez[1], C. Joya-Montosa[1], G. Gómez-Gallego[1], G. Quesada-García[1]
[1]H. R. U de Málaga, Intensive Care, Málaga, Spain; [2]Universidad de Málaga, Farmacología, Málaga, Spain
**Correspondence:** E. Trujillo-Garcia
*Intensive Care Medicine Experimental* 2017, **5(Suppl 2):**0615

**OBJECTIVES.** Analyzing the impact of early tracheostomyon the impact of ventilator-associated pneumonia (VAP), as well as analyzing whether early tracheostomy reduces VM, sedation, ICU and hospital stay duration, and whether it has any impact on death rate.

**MATERIAL AND METHODS.** Observational, prospective and comparative study between patients who had been performed elective early tracheostomy (within the first 7 days of MV) and patients who were performed tracheostomy after 7 days of MV while they were admitted in ICU from March 2012 to June 2015. Alpha error was 5%. Multivariate logistic regression was applied to identify factors which may predict interesting variables, thus controlling confusion factors and potentially-related interactions.

**RESULTS.** A total number of 250 tracheostomies were performed in that period. The profile of the tracheostomized patient is a middle aged (58 years old) man who is admitted upon ICU usually after a mainly neurological problem, followed by non-surgical reasons with average APACHE II and SAPS 3 upon admission. Tracheostomy was performed to 3.4% of the patients admitted upon this ICU in this period, most of them being percutaneous (86.8%).

A total number of 104 early and 146 late tracheostomies were performed. No significant differences are observed between both groups regarding age, gender, comorbidities or severity on admission scales. Early tracheostomized patients generally presented VAP upon 18.4 ± 6.2days, while late tracheostomized patients showed VAP upon 20 ± 6.6. However, VAP impact was significantly lower in the group of early tracheostomized patients (14.4 vs. 30.1%; $p < 0.05$). VAP was diagnosed according to SEMICYUC and ATS/IDSA criteria. Multivariate analysis shows that early tracheostomy is a protective factor (OR 0.359, IC 95% 0.184-0.699) against VAP when stratified according to severity on admission (valued by means of the APACHE II and SAPS 3 scales) and patient type (neurological subgroup). Early tracheostomized patients presented fewer days of MV (13.63 ± 11.69 days) relative to their late tracheostomized counterparts (20.72 ± 11.84 days); $p$ = 0.0001. Likewise, the first group showed fewer days of sedation ($p$ = 0.0001) and shorter ICU stays ($p$ = 0.0001) but not shorter hospital stays ($p$ = 0,08). Early tracheostomy shows no protective effects against mortality (OR 1.44, IC 95% 0.75-2.75).

**CONCLUSIONS.** Early tracheostomized patients show significantly lower VAP incidence, shorter VM and sedation (which involves numerous related problems) duration, and shorter hospital stay relative to late tracheostomized patients. Early tracheostomy shows no impact on either ICU death rates or hospital stay.

### 0616

#### Airway management in critical care - an audit of practice & complication rate following implementation of an intubation bundle of care

S. Sethi, C. Lockie, S. Tricklebank, P. Sherren
Guys and St Thomas' Hospital, ICU, London, United Kingdom
**Correspondence:** S. Sethi
*Intensive Care Medicine Experimental* 2017, **5(Suppl 2):**0616

**INTRODUCTION.** Rapid sequence induction of critically unwell patients outside of the operating theatre is high risk and associated with significant morbidity (1). It is often performed by inexperienced trainees with lack of expert assistance or advanced equipment, in an unwell cohort of patients. The Fourth National Audit Project (NAP4) highlighted this, and also the higher frequency of adverse airway events (2). Following a local audit, which revealed a high complication rate (39%), an Intubation Bundle was introduced to standardise airway management in Critical Care. This consisted of a standardised checklist and equipment preparation sheet, difficult airway algorithms with regular in situ MDT training & an Airway Rescue in Critical Care Course (ARCC).

**OBJECTIVES.** To undertake a snapshot audit of Airway Management in the ICU. To evaluate the efficacy of an "Intubation Bundle".

**METHODS.** Performed in a 67-bedded intensive care department in a central London teaching hospital. Data was collected from August to October 2016. Proforma completed by staff performing the RSI.

**RESULTS.** Data was collected for 24 intubations. Majority performed for respiratory failure (n = 8, 33.3%). First pass intubation performed in 92% (n = 22) of RSIs and waveform capnography and an intubation checklist were used in 96% (n = 23). All intubations performed by doctors with more than 2 years of airway experience, with 2 doctors present in 33% and a consultant present in 29% of cases. 8 patients (33%) suffered one or more adverse events & included significant hypoxaemia (21%), significant hypotension (17%), bradycardia and 2 multiple intubation attempts (3 attempts in both cases). In total, there were 12 adverse events affecting 8 patients compared with 15 adverse events affecting 7 (39%) patients prior to the introduction of the intubation bundle. This was not statistically significant (p = 0.7).

**CONCLUSIONS.** We have demonstrated a snapshot of RSI practice in a busy, tertiary ICU. A high first-pass success rate was shown with good use of the Intubation Bundle. A non-significant reduction in complications is reported, which may be related to the Intubation Bundle and its safety features. This bundle of care has heightened airway awareness across critical care and is now being introduced throughout the the hospital. Airway documentation in the ICU has been updated, with electronic proforma which will enable ongoing analysis. Education with MDT training, including simulation, combined with audit will hopefully improve airway management in this challenging environment.

(1) Lapinsky SE, Endotracheal intubation in the ICU Crit Care. 2015; 19(1): 258.

(2) Cook TM, Woodall N, Harper J, Benger J: Major complications of airway management in the UK: results of the fourth national audit project of the royal college of anaesthetists and the difficult airway society: part 2: intensive care and emergency departments. Br J Anaesth. 2011, 106 (5): 632–642. 10.1093/bja/aer059. Epub 2011 Mar 29

### 0617

#### The utility of the C-MAC as a training tool for direct laryngoscopy in the emergent endotracheal intubation

S. W. Jo, S. Y. Hwang, I. J. Jo, S.U. Lee, T. R. Lee, H. Yoon, W. C. Cha, M. S. Sim, T. G. Shin
Samsung Medical Center, Emergency Medicine, Seoul, Korea, Republic of
**Correspondence:** S. W. Jo
*Intensive Care Medicine Experimental* 2017, **5(Suppl 2):**0617

**INTRODUCTION.** Direct laryngoscope (DL) has long been used as a standard device to facilitate endotracheal intubation (ETI), however, it is very difficult to be skilled in the technique.

**OBJECTIVES.** The goal of this study was to determine the utility of the C-MAC (Karl Storz, Tuttlingen, Germany) as a training tool for DL in the emergent ETI.

**METHODS.** This was a single-center, retrospective propensity-matched study of continuous quality-improvement data from April 1, 2014 to October 30, 2016. All ETI for adult patients (18 years of age or older) performed by emergency medicine residents with conventional Macintosh DL or C-MAC as DL were included for the analysis. When C-MAC was used as DL, operator was not allowed to see the monitor and identified anatomy through the mouth of the patient. Supervising physician saw the monitor and gave instructions in real time to find glottis. The primary outcome was the first pass success rate (FPS). The secondary outcome was multiple attempts and intubation-related complications. Patients were matched based on propensity scores determined by variables including indication of intubation, presence of difficult airway characteristics, level of residency (junior vs senior) and intubation experiences.

**RESULTS.** A total of 939 intubations took place over the study period, and 744 were included for the analysis. Of the eligible patients, 163 were performed using C-MAC as DL, and 581 using conventional DL. From these, 163 propensity score matched pairs were generated (1-to-n matching: C-MAC as DL group, 163 vs. conventional DL group, 428). Before matching, the overall FPS rate of eligible patients was 72% (539/744). For the propensity-matched groups, overall FPS rate was 69% (409/591), 79% (129/163) for the C-MAC as DL group and 65% (129/428) for the conventional DL group. Overall multiple attempts were 8% (44/591), 4% (6/163) for the C-MAC as DL group and 9% (38/428) for the conventional DL group. Overall intubation related complication rate was 11% (65/591), 4% (7/163) for the C-MAC as DL group and 14% (58/428) for the conventional DL group.

**CONCLUSIONS.** The C-MAC (Karl Storz, Tuttlingen, Germany) is useful as a training tool for DL indicated by increased FPS and decreased multiple attempts and intubation-related complications compared to conventional DL.

**GRANT ACKNOWLEDGMENT**

There is nothing to declare.

### 0618

#### Tracheostomy teaching for newly qualified doctors

E. Ford[1], L. Ruff[2]

[1]Oxford University Hospitals NHS Foundation Trust, Anaesthetics, Oxford, United Kingdom; [2]Aintree University Hospital NHS Foundation Trust, Intensive Care, Liverpool, United Kingdom

**Correspondence:** E. Ford

*Intensive Care Medicine Experimental* 2017, **5(Suppl 2):**0618

**INTRODUCTION.** Tracheostomies are performed within Intensive Care. With increased throughput, patients are discharged to wards prior to decannulation. NCEPOD in 2014 stated members of staff on wards should be 'competent in recognising and managing common airway complications including tube obstruction or displacements' [1]. In a review of NPSA reports it was shown that 453 tracheostomy incidents over 2 years were largely due to blocked or displaced tubes and were associated with patient harm [2]. Newly qualified (foundation) doctors haven't got experience with managing tracheostomies but they may be called to manage emergencies when on call, without seniors immediately accessible. Therefore a session involving lectures and scenario training was held for foundation doctors at a District General Hospital (DGH).

**OBJECTIVES.**

1. Assess Foundation Doctors' knowledge of tracheostomies and management of a blocked tracheostomy emergency scenario
2. Teach the above
3. Assess success of teaching sessions
   a. Knowledge
   b. Feedback

**METHODS.** A pre-course survey was sent to all foundation doctors at a DGH to assess knowledge and experience. Each cohort received 1 teaching session of:

1 Lecture- tracheostomies, laryngectomies, types of tracheostomy tubes and management of blocked tracheostomies.

2 Practical session- different tubes and associated equipment.

3 Scenario teaching session running through a blocked tracheostomy scenario.

Post-course questionnaires were circulated.

**RESULTS.** There were 43 pre and 39 post course responses. 72% hadn't received tracheostomy teaching previously; 88% had come into contact with tracheostomy patients.

Confidence improved managing blocked tracheostomies, the majority of trainees said they would score their confidence as 3-4/5 post course. (1- not confident, 5- very confident)

There was improvement in knowledge comparing pre and post course questionnaires of managing blocked tracheostomies; application of oxygen via mouth and tracheostomy, passing a suction catheter and removal of the inner tube.

**CONCLUSION.** We identified requirement for tracheostomy training for Foundation Doctors. Using multimodal sessions we saw an improvement in knowledge and confidence managing blocked tracheotomies. This is a short, cheap, repeatable intervention which could be adapted for multi disciplinary use. Increasing tracheostomy knowledge and familiarity should translate to improved patient safety post discharge [3].

**GRANT ACKNOWLEDGMENT**

None.

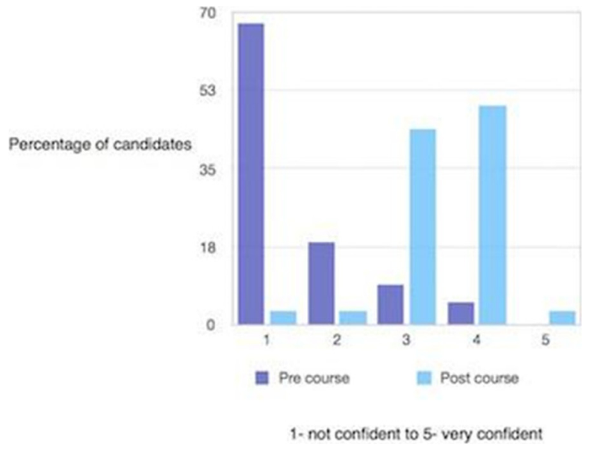

**Fig. 221 (Abstract 0618).** Comparison of Pre and post course confide

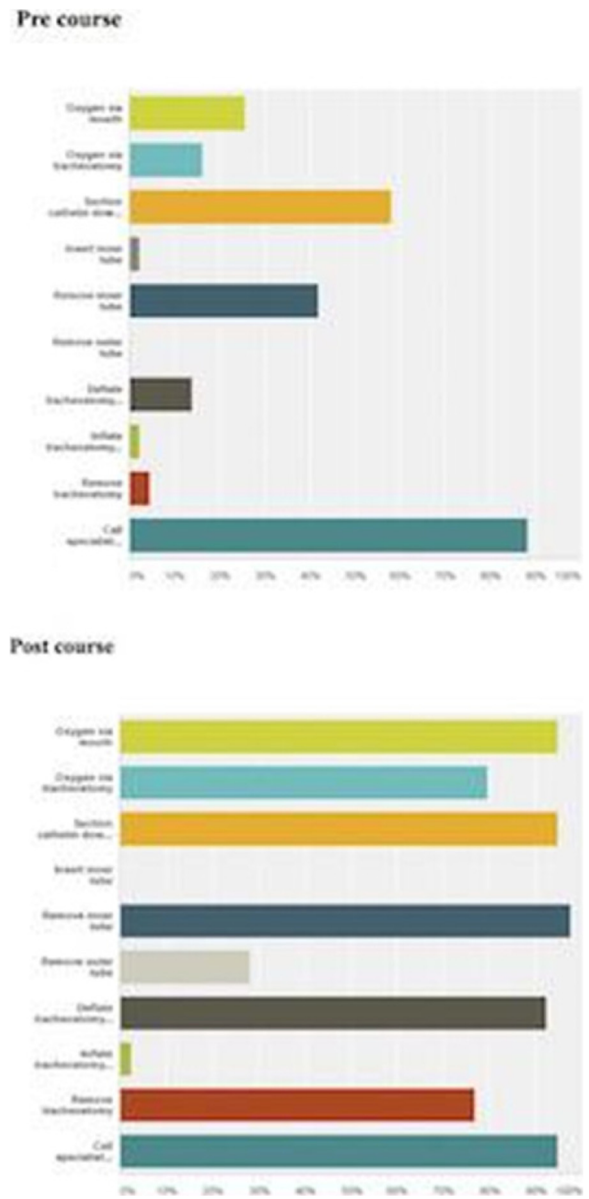

**Fig. 222 (Abstract 0618).** Steps involved in emergency blocked trach

### 0619

#### ICU-acquired swallowing disorders after prolonged intubation in the elderly: a fiberoptic endoscopic study

M. Nakanishi[1], H. Okamoto[2], T. Fujiwara[3], T. Fukuoka[1]
[1]Kurashiki Central Hospital, Intensive Care Medicine, Kurashiki City, Japan; [2]St.Luke's International Hospital Center, Emergency Medicine, Chuoku, Japan; [3]Kurashiki Central Hospital, Otolaryngology, Kurashikishi, Japan
**Correspondence:** M. Nakanishi
*Intensive Care Medicine Experimental* 2017, **5(Suppl 2):**0619

**INTRODUCTION.** Critically ill elderly patients requiring prolonged intubations often develop ICU-acquired swallowing disorders (ICU-ASD), which related to the worse outcomes. However, few research has examined the incidence of ICU-ASD using fiberoptic endoscopy.

**OBJECTIVES.** The aim of our study is to describe the incidence and clinical characteristics of ICU-ASD in the elderly patients using fiberoptic endoscopy.

**METHODS.** A cross-sectional study was conducted in our medical-surgical ICU from May 2016 to March 2017. The inclusion criteria were all consecutive patients aged ≥65 years old who required prolonged intubation (endotracheal intubation ≥48 hours). Exclusion criteria were patients who had preexisting dysphasia, tracheostomy, and altered mental status (GCS < 13). All included patients were examined for ICU-ASD using fiberoptic endoscopy within 48 hours after extubations. The severity of ICU-ASD was evaluated using the Hyodo-Komagane score which consists of four categories (0: normal, 1: mild, 2: moderate, 3: severe) and assessed four findings (salivary pooling in the vallecular and piriform sinuses, response of the glottal closure reflex, swallowing reflex initiation, pharyngeal clearance after swallowing of test water). The primary outcome was incidence of ICU-ASD. The secondary outcomes were incidence and characteristics of vocal code injury (edema, ulceration, granulation, and immobility), and the length of ICU stay.

**RESULTS.** 59 patients were included in our study. Median age was 79.0 (73.5-83.0) years old and 59% were male. The median intubated days were 6.0 (5.0-9.0) days. Indications of intubations were sepsis (42.3%), respiratory failure (22.0%), trauma (16.9%), and others (18.8%). The median APACHE II score was 21 (17–13). The incidence of ICU-ASD was 16 patients (27.1%). The severity of ICU-ASD was normal 22 patients (37.3%), mild 12 patients (20.3%). And observed abnormal findings were as follows: 16 patients had aspiration, 3 patients had moderate to severe salivary pooling degree, 6 patients had that reduction of the glottal closure reflex, 11 patients had delayed time of the swallowing reflex, and 6 patients had reduction of pharyngeal clearance. The vocal cord injuries were seen in 14 patients (edema 5 patients, ulceration 3 patients, granulation 6 patients, and immobility 6 patients, respectively). The length of ICU stay was 8.0(6.0-10.0) days.

**CONCLUSIONS.** The incidence of ICU-ASD was 27.1% in the elderly patients. Further studies are warranted to investigate the risk factors of ICU-ADS in elderly patients.

### 0620

#### Percutaneous vs surgical tracheostomy outcomes in critical patients

J. F. Martin-Lazaro[1], M. Michael[1], R. Despiney[1], M. Brys[1], D. Mateo[1], A. Thomas[2]
[1]Newham University Hospital, Intensive Care Unit, London, United Kingdom; [2]Barts Health NHS Trust, Intensive Care Unit, London, United Kingdom
**Correspondence:** J. F. Martin-Lazaro
*Intensive Care Medicine Experimental* 2017, **5(Suppl 2):**0620

**INTRODUCTION.** Surgical tracheostomy (SgT) is a time-established procedure. Percutaneous tracheostomy (PcT) has been said to have many advantages over SgT: a smaller skin incision and less dissection and tissue trauma, and therefore fewer wound complications such as hemorrhage, infection, tracheal stenosis or scar problems, and less cost of the intervention. Studies comparing PcT and SgT show that advantages of PcT are less obvious. However, there are not many large studies comparing outcomes.

**OBJECTIVES.** To analyse quality indicators and compare the outcome in patients that underwent for a SgT or PcT.

**METHODS.** A retrospective study was undertaken to compare outcome, in critical care patients admitted in Barts Health Trust (Barts Hospital, Royal London Hospital, Newham University Hospital and Whipps Cross Hospital) during 2016, PcT technique performed at the bedside vs SgT technique performed in the operating room. Criteria for elective SgT remained on the decision of the intensivist, based on: anatomy, morbid obesity or possible major complications (i.e. bleeding). Indicators used were: time to insertion, time to decannulation, decannulation success, complications and mortality. Statistical analysis for description of the sample and averages comparison were performed by Excel and SPSS.

**RESULTS.** Our population consisted on 274 patients (n = 274): 190 males (69%) and 84 females (31%) with an average age of 52 ± 17 years. SgT was performed in 56% (n = 154) of our patients while PcT

was inserted in 44% (n = 120). We found statistical differences ($p < 0.005$) for the time to insertion of Tracheostomy: 12.77 days for SgT and 10.60 days for PcT; as well as in relation to SgT and PcT mortality: 9% (n = 26) and 4% (n = 11) respectively. Successful decannulation occurred in 97% of our patients but all the patients that failed (n = 4) had a SgT and was related to secretion load. Our results show no significant differences between the two techniques of tracheostomy, in terms of time to decannulation: 23.15 days for SgT and 23.82 days for PcT or complications (5% SgT; 2% PcT). No major complications were observed with any of the techniques.

**CONCLUSIONS.** SgT and PcT techniques were both safe when conducted by experienced, skilled practitioners, with no difference in major complications or time to decannulation, but SgT showed higher rate of failure at the time of decannulation and carried, in our population, double rate of mortality.

**REFERENCE(S)**

- Percutaneous and surgical tracheostomy in critically ill adult patients: a meta-analysis. Christian Putensen, Nils Theuerkauf, Ulf Guenther, Maria Vargas, Paolo Pelosi. Crit Care. 2014; 18(6): 544.

- An overview of complications associated with open and percutaneous tracheostomy procedures. Anthony Cipriano, Melissa L Mao, Heidi H Hon, Daniel Vazquez, Stanislaw P Stawicki, Richard P Sharpe, David C Evans

### 0621

#### Pilot study on the safety of mechanical ‚Insufflation-Exsufflation' in patients with artificial airway

T. Fariña[1], Á. Del Pino[1], P. Santos[2], G. Rodríguez[3], F. Martínez[1], M. Sanchez Garcia[1,4]

[1]Hospital Clinico San Carlos, Critical Care, Madrid, Spain; [2]Hospital Clinico San Carlos, Medicina Física y Rehabilitación, Madrid, Spain; [3]Hospital Clinico San Carlos, Pulmonology, Madrid, Spain; [4]Universidad Complutense de Madrid, Medicina, Madrid, Spain

**Correspondence:** T. Fariña

*Intensive Care Medicine Experimental* 2017, **5(Suppl 2):**0621

**INTRODUCTION.** Catheter suctioning of respiratory secretions in intubated patients is limited to the proximal airway and associated with complications like traumatic lesions to the mucosa and poor tolerance. “Mechanical insufflation-exsufflation” (MIE) with “airway clearance devices” exerts positive pressure, followed by an abrupt drop to negative pressure. Potential advantages of MIE are aspiration of distal airway secretions, avoiding trauma and improving tolerance.

**OBJECTIVES.** We evaluated the safety of MIE (50 cmH2O, 3 seconds; −45 cmH2O, 4 seconds), with high frequency oscillation on both pressure plateaus.

**METHODS.** Clinical, laboratory, respiratory and ventilator parameters were recorded at baseline and compared to values collected at 5 and 60 minutes after MIE. Patients were reconnected to baseline ventilator parameters immediately after MIE. Quantitative variables were compared by Student's t test, analysis of variance for repeated measurements, or non-parametric tests, as appropriate. Qualitative variables were compared with chi square or Fisher's tests. Patients were followed during their ICU stay until discharge or death.

**RESULTS.** We studied 7 male and 6 female patients requiring suctioning who were connected to the MIE device a total of 26 times on median day 12 (IQR 6.25; 28.25) after ICU admission and 11.5 (IQR 6.25; 25.75) after endotracheal intubation. Per session, a median of 2 (IQR 1; 2) cycles were performed. Mean tidal volume during mechanical insufflation was 1043.6 ± 649.9 ml. The device was connected 16 times to a tracheostomy tube and 10 times to an endotracheal tube. All, except 1, MIE cycles were productive and well tolerated. No statistically significant differences were identified between baseline and post-MIE time points, except for an increase in PaO2 (Table 163). Barotrauma, desaturation, atelectasis, hemoptysis or hemodynamic complications were not detected after MIE.

**CONCLUSIONS.** Mechanical insufflation-exsufflation seems safe and effective in this pilot study. Safety and efficacy need to be confirmed in larger studies with different patient populations.

**Table 163 (Abstract 0621).** Baseline and follow-up parameters

| | Baseline | 5´ | 60´ | p |
|---|---|---|---|---|
| Heart rate | 97.7 ± 14.4 | 97.1 ± 15.1 | 97.3 ± 14.2 | 0.82 |
| Mean blood pressure | 87 ± 11.9 | 87.6 ± 14.7 | 83.6 ± 14.7 | 0.57 |
| PaO2 | 105.5 ± 23.9 | 124 ± 55.5 | 143 ± 42.3 | 0.031 |
| PaCO2 | 37.5 ± 12.6 | 40.5 ± 13.3 | 35.2 ± 8.2 | 0.058 |
| PaO2/FiO2 | 239.8 ± 96.8 | 285.5 ± 140 | 328.7 ± 104.7 | 0.14 |
| Minute Volume | 10.6 ± 3.6 | 11.0 ± 3.2 | 9.6 ± 2.6 | 0.13 |
| Static Compliance | 18.7 ± 8.8 | 17.2 ± 9.1 | 19.1 ± 9.3 | 0.33 |
| Dynamic compliance | 17.7 ± 9.5 | 16.3 ± 9.4 | 17.6 ± 9.6 | 0.4 |

### 0622

#### Cumulative results from 10 years of tracheostomy data collection in a large tertiary university hospital in Ireland

S. Healy, R. Plant, CUH Tracheostomy MDT

Cork University Hospital, Cork, Ireland

**Correspondence:** S. Healy

*Intensive Care Medicine Experimental* 2017, **5(Suppl 2):**0622

**INTRODUCTION.** A multidisciplinary tracheostomy team (MDT) was set up in Cork University Hospital (CUH) in Ireland in 2005. This team comprised physiotherapists, speech and language therapists, nurses and intensivists. The purpose of this quality improvement initiative was to enhance the care provided to adult patients with tracheostomies. Developments included various forms of education, weekly ward rounds (including review of clinical practices of tracheostomy care), support for staff caring for tracheostomy patients and an annual database report is sent to all hospital consultants. Data were collected prospectively over 10 years to monitor changing trends after introducing the MDT.

**Objectives:**

- Examine numbers and outcome of patients with tracheostomies
- Identify changing trends
- Explore differences across subspecialties

**Methods:** Analysis of prospective data collected over 10 years during weekly hospital wide MDT tracheostomy ward rounds. Data were analysed using Microsoft excel descriptive statistics.

**Results:** There were 368 new patients (258 (70%) male) with tracheostomies in CUH between Jan 2007 and December 2016. The decannulation rate improved from 48% in 2007 to 63.5% in 2016 (Fig. 223).

Hospital mortality rate fell from 36% in 2007 to 12% in 2016 (Fig. 224) with an average of 23% for the 10 years. The mortality rate by subspecialty can be seen in Table 164.

Overall 1 year mortality rates are currently being collated via national register. There was no tracheostomy-related mortality during the 10 years.

Average time to decannulation dropped from 48 days in 2007 to 30 days in 2016 showing a slight downward trend over the 10 years.

**Conclusions:** Cumulative results over 10 years of MDT input in tracheostomy care show improving outcomes in terms of decannulation rate and mortality with a trend towards shorter times to decannulation. Subgroup analysis shows that cardiothoracic, respiratory and renal patients have higher than average mortality rates, while neurosurgery patients have a much lower mortality rate. These results are now being used as one aspect of informing decision making on tracheotomy insertion in CUH. The database facilitates local review and audit of the service provided to this patient group. Overall the development of a tracheostomy multi-disciplinary team has been a very welcome development whose success is supported by these encouraging data.

**Grant Acknowledgement**

None.

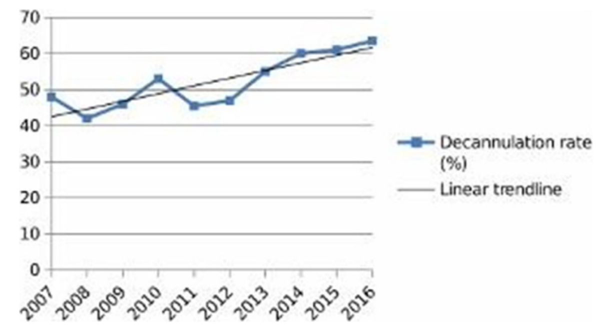

**Fig. 223 (Abstract 0622).** Decannulation rate (%)

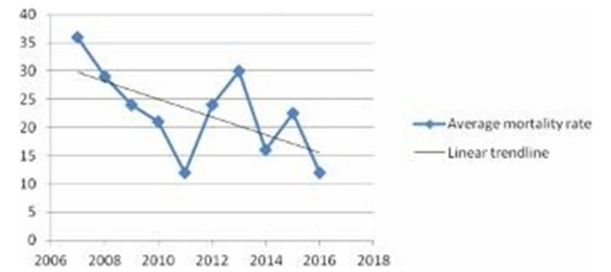

**Fig. 224 (Abstract 0622).** In-hospital mortality rates (%)

**Table 164 (Abstract 0622).** Average mortality rate of subspecialties over 10 years

| Discipline | Number of patients | Average mortality rate |
|---|---|---|
| Neurosurgery | 95 | 9% |
| Cardiothoracics | 76 | 33% |
| Respiratory | 43 | 33% |
| Vascular/General surgery | 37 | 27% |
| Neurology | 32 | 25% |
| Renal | 22 | 36% |
| Others | 63 | 21% |

Average mortality of subspecialties over 10 years

### 0623

#### SLT Management of tracheostomised patients: a comparative audit

N. Maistry[1], J. Storah[2]
[1]Royal Brompton and Harefield NHS Trust, London, United Kingdom; [2]Royal Brompton and Harefield NHS Trust, Rehabilitation and Therapies, London, United Kingdom
**Correspondence:** N. Maistry
*Intensive Care Medicine Experimental* 2017, **5(Suppl 2):**0623

**INTRODUCTION.** Standardised care provided by a specialised multidisciplinary tracheostomy team is associated with decreased time to decannulation and fewer tracheostomy related complications (Yu 2014 & de Mestral 2011) Patients with tracheostomies can therefore benefit from a co-ordinated, multidisciplinary approach to care. Speech and Language Therapy (SLT) specific expertise in assessing and managing communication and swallowing needs is a vital part of this process. (McGrath 2014) This SLT role includes assessment and management of swallowing, voice and communication function (Freeman - Sanderson 2011) Standardized care provided by a specialised MDT tracheostomy team was associated with fewer tracheostomy-related complications and an increase in the use of a speaking valve.

**OBJECTIVES.** To audit (SLT) intervention with patients with a Tracheostomy in a Cardio-Respiratory ICU in London as compared to a clinical audit carried out in an Australian Tertiary hospital ICU in 2011.

**METHODS.** Data was retrospectively analysed of all patients referred to SLT, with a tracheostomy in situ in ICU, for 5 month period, evaluating average time taken to commence verbal communication, average time taken to commence oral intake from date of tracheostomy insertion and length of tracheostomy cannulation. Patient demographic data and reason for intensive care admission were also collected. These results were compared to the audit data presented in the Australian study.

**RESULTS.** A total of 25 patients were tracheostomised over the 5 month period. There were 9 males and the median age was 60.2.

The average time taken to commence phonation was 7 days post tracheostomy insertion and the average time to commence oral intake was 12 days post insertion (as compared to 16 days for phonation and 15 days for oral intake in the Australian audit)

17/25 patients were initially assessed for speaking valve usage, to allow phonation, whilst still on mechanical ventilation (Pressure support).

**CONCLUSIONS.** Significantly improved length of time in commencing phonation in patients with a tracheostomy in situ and comparable times in facilitating oral intake, were demonstrated as compared to the Australian audit. It is surmised that speaking valve trials to facilitate verbal communication are commenced earlier in our cardiothoracic intensive care unit, whilst most patients are still on mechanical ventilation.

### 0624

#### Does unplanned extubation have an impact on ICU outcome?

O. Ediboglu[1], P. Cimen[2], C. Anar[2], D. Tatar[2], C. Kirakli[1]
[1]Dr. Suat Seren Chest Diseases and Surgery Training and Research Hospital, Intensive Care Unit, Izmir, Turkey; [2]Dr. Suat Seren Chest Diseases and Surgery Training and Research Hospital, Pulmonary Division, Izmir, Turkey
**Correspondence:** C. Kirakli
*Intensive Care Medicine Experimental* 2017, **5(Suppl 2):**0624

**INTRODUCTION.** When unplanned extubation (UE) occurs in intensive care patients, mechanical ventilation therapy is affected negatively(Epstein SK 2000, Kapadia F 2001).

**OBJECTIVES.** In this study, we aimed to investigate the characteristics of UE patients, the duration of extubation and the risk factors for re-intubation.

**METHODS.** In this retrospective cohort study of patients with UE between May 1, 2010 and December 31, 2012; re-intubated patients were defined as Group 1 (n = 12) and patients who did not need re-intubation were defined as Group 2 (n = 12). Demographic features, mechanical ventilation mode, UE source (patient - staff), APACHE II score, presence of ventilator associated pneumonia, extubation time, sedation, noninvasive mechanical ventilation (NIV) requirement after extubation and reintubation requirement were recorded.. Group 1 and 2 were compared in terms of risk factors for reentubation.

**RESULTS.** Twenty-four patients (23 male) were included in the study. Median age was 75 years (56–81). In group 1, APACHE II score was significantly higher than group 2 (27 vs 23, p = 0.03); The pH was significantly lower (7.26 vs 7.35, p = 0.008). Weaning was significantly lower and mortality was significantly higher in group 1 (1/12 vs 8/12, p = 0.003) and (11/12 vs 1/12, p = 0.0001) respectively. In group 1, the number of patients with ventilator-associated pneumonia was higher (7/12 vs 2/12, p = 0.035). NIV support was applied to all cases except 1 case.

**CONCLUSIONS.** Unplanned extubation is an undesirable condition in intensive care. Patients with advanced age and high APACHE II scores may be at higher risk of re-entubation and mortality.

**REFERENCE(S)**

Epstein SK, Nevins ML, Chung J: Effect of unplanned extubation on outcome of mechanical ventilation. Am J Respir Crit Care Med 2000, 161:1912–1916.

20. Kapadia F. Effect of unplanned extubation on outcome of mechanical ventilation. Am J Respir Crit Care Med 2001; 163: 1755–6.

### 0625

#### Tracheostomy complications. Checklist

E. Trujillo-Garcia, E. Curiel-Balsera, C. M. Gómez-Jiménez, C. Joya-Montosa, C. M. Martínez-González, H. Molina-Díaz
H. R. U de Málaga, Intensive Care, Málaga, Spain
**Correspondence:** E. Trujillo-Garcia
*Intensive Care Medicine Experimental* 2017, **5(Suppl 2):**0625

**OBJECTIVE.** Analyzing complications both during and up to 1 year after the procedure of electively tracheostomized patients upon ICU admission. Elaborating a checklist which shall allow us to prevent some of the aforementioned procedure-related complications, thus improving patient safety.

**MATERIAL AND METHODS.** Observational, prospective and comparative study with patients who had been tracheostomized during their ICU stay. Tracheostomies performed in UCI-admitted patients from March 2012 to June 2015 were analyzed, as well as complications.

**RESULTS.** 250 tracheostomies were performed in that period. Tracheostomy was performed to 3.4% of the patients admitted upon this ICU in this period, most of them being percutaneous, with no procedure-related lethal complications. Tracheostomies were performed by widely experienced personnel.

Complications registered during the procedure represent 12% (no = 23) of the analyzed population. The most frequent complication, by far, was bleeding demanding cauterization, bonding or surgical revision; followed by cannula misplacement (2.8%, no = 7), pneumothorax (1.9%, no = 3) and, finally, tracheal rupture (0.8%, no = 2). Late complications represent 7.6% (no = 19), the most frequent one being tracheal stenosis (3.2%, no = 8) diagnosed after ENT revision, followed by stoma bleeding and infection (both 2.8%, no = 7). Of all tracheostomies performed in this period, 86.8% were performed percutaneously by intesivists. The remaining tracheostomies were performed in the operating theater by ENT specialist. No significant differences are observed regarding immediate complications between both techniques. More late complications are generally observed with the surgical technique (*p* = 0.001) (24.2 vs. 5.1%) in each of the three considered complications. Ward monitoring was performed by ENT personnel by means of interconsultation forms. We design our check list in order to reduce procedure complications.

**CONCLUSIONS.** Tracheostomy is not exempt from risks and complications, although they are not usually lethal. Even though, comparing complications between the different existing studies is a complex process due to the different definitions followed for each complication. On the other hand, most complications depend on the patient´s anatomy or basal features and personnel´s experience.

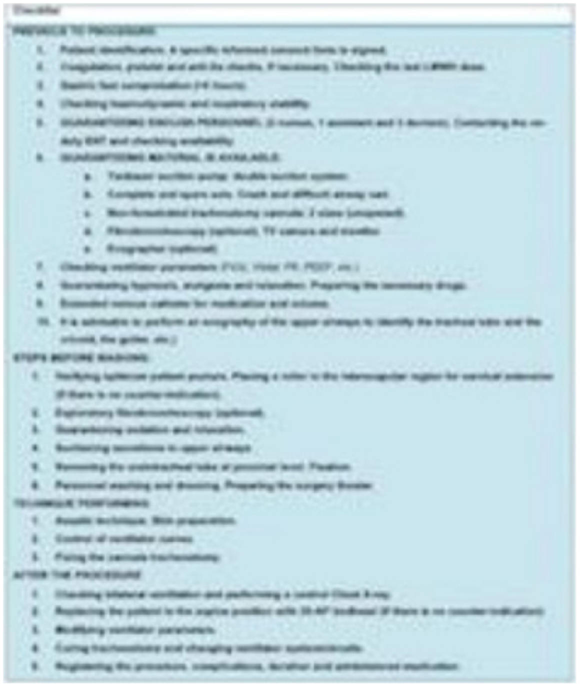

**Fig. 225 (Abstract 0625).** Checklist

### 0626

#### Respiratory management in children with tracheostomy airway prosthesis - clinical experience

A. Oglinda[1], G. Ciobanu[2], C. Oglinda[2], M. Casian[2]
[1]Institute Mother and Child, Intensive Care, Chisinau, Republic of Moldova; [2]SUMP "Nicolae Testemiţanu", Chisinau, Republic of Moldova
**Correspondence:** A. Oglinda
*Intensive Care Medicine Experimental* 2017, **5(Suppl 2):**0626

**INTRODUCTION.** The common denominator of the reanimation wards is to avoid their overcrowding with patients who need long-term mechanical ventilation (LTMV) support.

**OBJECTIVES.** The necessity to be applied an advanced respiratory management in children with tracheostomy airway prosthesis.

**METHODS.** The study is formed from 81 of patients from pediatric reanimation ward of the IM&C and who have been under LTMV during the last 9 years. Children were divided in 2 groups - the Ist contained 46 children who were under LTMV without the application of tracheostomy and the IInd which contained 35 children whose ventilation management was with tracheostomy airway prosthesis.

**RESULTS.** This retrospective trial has identified the following facts: children who were under artificial prolonged ventilation spent on average 12 ± 2,9 days in bed. The causes for which the children were under APV were the following diseases: Multiple developmental abnormalities 28; Cerebral diseases and muscle diseases 25 children; Congenital diseases of the bronchopulmonary apparatus and cardiovascular system 14; Genetic diseases 14 children, inclusively 4 children with Down syndrome. The age of children was 1 month - 17 years. All the children have benefited from a special care for a critic child who is under APV and an approach of the respiratory management according to the protocol. At the same time the respiration support by the artificial respiration through the cannula tracheostomy in Volume SIMV or Pressure SIMV needed Vt 5 ml/kg and smaller pressure in comparison with IET children under APV. Simultaneously with the amelioration of general condition, resumption of spontaneous breathing it was possible to re-initiate oral feeding in 9 children of the second group beginning with the second day after the installation of the tracheostomy. It should be mentioned the significant decrease of the duration of being under artificial respiration which was identified in the second group of children 18 ± 1,9 days, and in the first group it constituted 37 ± 1,7 days. Because of the severity of the main disease in the first group died 14 children and in the second group died 8 children, thus we have founded high mortality in both groups, but it was succeeded to transfer the children from the second group to palliative care.

**CONCLUSIONS.**

1. If comparing with IET children the advanced respiratory management in children with tracheostomy airway prosthesis need ventilator support with small volumes and pressure.
2. besides the increase of the survival rate in the second group, we have founded a decrease of the duration of stay in intensive care, improving the transfer of these children, thus decreasing the prime cost of medical care.

**REFERENCE(S)**

1. A.De Jong, B.Jung, et all. Intubation in the ICU: We Could Improve our practice. Edited by J.-L.Vincent. Annual Update in Intensive care and Emergency Medicine 2014. pag 107–117.

**GRANT ACKNOWLEDGMENT**

Conflicts of interest: none.

### 0627

#### Effect of low tidal volume on absolute humidity delivered by heated humidifiers in ICU ventilators - a bench study

L. Baboi[1], B. Moro[1], H. Yonis[1], Z. Riad[1], M. Aublanc[1], A. Louf-Durier[1], F. Lissonde[1], J.-C. Richard[1], C. Guérin[1,2]
[1]Hopital de la Croix Rousse, Lyon, France; [2]INSERM 955, Créteil, France
**Correspondence:** C. Guérin
*Intensive Care Medicine Experimental* 2017, **5(Suppl 2):**0627

**INTRODUCTION.** In ARDS patients under ECMO common ventilator strategy aims at resting the lung by lowering tidal volume (VT). We were wondering whether such low VT can impact the efficacy of heated humidifiers (HH) in ICU ventilators to achieve the recommended target of 30 mg/L or more absolute humidity (HA).

**OBJECTIVES.** To assess on the bench HA over a range of low VT in ICU ventilators.

**METHODS.** A pneumatic test lung (TTL, Michigan inc. Grand Rapids, USA) was set at 20 ml/cmH2O compliance and 20 cmH2O/L/s resistance and attached to either 3 ICU ventilators (V 500 (Drager), Carescape R 860 (GE Healthcare), Servo U (Maquet)) equipped with Fisher-Paykel MR 850 HH and double wired limb ventilator circuit. PEEP was set to 12 cmH2O and FIO2 0.21. HA (mg/L) was assessed by using the psychrometric method. Two probes measured the inspiratory gas temperature into a double circuit inserted between Y piece and lung model. HH was set in manual mode and targeted 37°C into the chamber and 40°C at the Y piece. HH was run during 45 minutes at VT 500 ml then 100 ml, each at respiratory rate 30 breaths/min. Temperature in both probes was continuously monitored (Biopac M150) during the whole experiment. Once both temperatures leveled off (change of 1% or less) at VT 100 ml, 10 cycles were counted then 120 ml VT was delivered til next plateau temperature and so on by 20 ml-increment in VT up to 280 ml. Then VT was lowered to 100 ml and respiratory rate to 15 breaths/min. At the time of plateau temperature same VT increment as above was performed. The experiment was performed by using adult (RT 380 EVAQUA Fisher Paykel) and repeated with neonate (RT 266 EVAQUA Fisher-Paykel) ventilator circuit. The primary end-point was the impact of VT on HA. Data were analyzed by using linear mixed model.

**RESULTS.** Figure shows the box and whisker plots of HA across VT for each ventilator, at 15 (upper panels) and 30 (lower panels) breaths/min with adult (left panels) and neonate (right panels) circuits.

Over all the ventilators (taken as the factor with random effect in the model), there was a significant effect of VT on HA: the mean value of HA intercept of the reference (adult circuit and respiratory rate 15/min) (25.75 mg/L) increased significantly by 0.02 mg/L per 1 ml VT increase (P = 0.0001). It was significantly reduced by 4.17 mg/L with the neonate circuit (P = 0.0011) and significantly increased by 0.19 mg/L with the 30 breaths/min respiratory rate (P = 0.0001). There was no significant interaction between circuit and respiratory rate.

The table summarizes the effect of VT on HA in each panel of the figure

**CONCLUSIONS.** A safe HA can be achieved at any low VT by using adult circuit and respiratory rate 30 breaths/min with any ICU ventilator presently investigated.

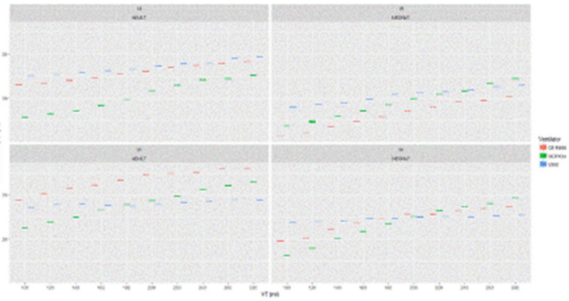

**Fig. 226 (Abstract 0627).** See text for description

**Table 165 (Abstract 0627).** See text for description

| circuit/ respiratory rate | HA intercept of the reference GE ventilator (mg/L) | effect of VT on HA per ml VT | effect of Servo U on HA | effect of V 500 on HA |
|---|---|---|---|---|
| adult/15 | 29.44 | +0.02* | −2.57* | +0.65* |
| neonate/ 15 | 23.63 | +0.02* | +1.42* | +2.22* |
| adult/30 | 33.18 | +0.02* | −2.61* | −2.62 |
| neonate/ 30 | 28.13 | +0.02* | −0.32* | +0.21 |

## Organisation issues

### 0628

#### The science and the art of practicing intensive care medicine in Latin America: the human factor

L. Alegria[1], N. Nin Vaeza[2], F. Rios[3], E. Estensoro[4], G. Murias[5], G. Friedman[6], M. Jibaja[7], G. Ospina-Tascon[8], F. Machado[9], A. Biasi Cavalcanti[10], A. Dubin[11], L. Azevedo[12], M. Cecconi[13], J. Bakker[14], J. Hurtado[15], M. C. Marin[16], G. Hernández[1], R. Castro[17]
[1]Pontificia Universidad Católica de Chile, Santiago, Chile; [2]Universidad de la República, Montevideo, Uruguay; [3]Hospital A. Posadas, Buenos Aires, Argentina; [4]Hospital Interzonal San Marin La Plata, Buenos Aires, Argentina; [5]Clinica Bazterrica, Buenos Aires, Argentina; [6]Universidade do Estado da Bahia Universidade Federal do Rio Grande do Sul, Porto Alegre, Brazil; [7]Unidad de Cuidados Intensivos. Hospital Eugenio Espejo, Quito, Ecuador; [8]Fundación Valle del Lili - Universidad ICESI, Cali, Colombia; [9]Universidade Federal de São Paulo, Sao Paulo, Brazil; [10]Research Institute HCor, Hospital do Coração, Sao Paulo, Brazil; [11]Universidad Nacional de La Plata, Buenos Aires, Argentina; [12]Federal University of São Paulo, Sao Paulo, Brazil; [13]St George's University Hospitals NHS Foundation Trust, London, United Kingdom; [14]Columbia University Medical Center, New York, United States; [15]Hospital Español de Montevideo, Montevideo, Uruguay; [16]Hospital Regional 1 de Octubre,

ISSSTE, Ciudad de Mexico, Mexico; [17]Pontificia Universidad Católica de Chile, Satiago, Chile
**Correspondence:** L. Alegria
*Intensive Care Medicine Experimental* 2017, **5(Suppl 2):**0628

**INTRODUCTION.** Intensive care medicine (ICM) is a relatively young discipline that has rapidly grown into a full-fledged specialty. Intensivists are responsible for managing an ever-increasing number of patients with complex, life-threatening diseases. Several factors may influence their performance, including age, training, experience, workload, and their socioeconomic contexts.
**OBJECTIVES.** To gain insight on individual and behavioral aspects of the intensivist workforce in Latin America (LA).
**METHODS.** Cross-sectional study through a web-based electronic survey, in public and private ICUs from LA countries, academic and non-academic. Study population were intensivists from Argentina, Bolivia, Brazil, Chile, Colombia, Ecuador, Guatemala, Mexico, Paraguay, Peru, and Uruguay, currently working in the ICU.
The survey included questions about training, workload, competencies, continuous education, research activities, and experiential aspects. Additionally, considering septic shock as a key ICU condition that integrates diverse facets of clinical functioning, we incorporated specific question regarding its treatment. Intercountry comparisons were performed using the Gross National Income indicator from the World Bank. The survey was submitted by email to a list of LA intensivists obtained by national scientific societies and personal contacts, with weekly reminders from June through September 2016 to non-respondents.
**RESULTS.** 735 surveys (45% return) were responded from Brazil (29%), Argentina (19%), Chile (17%), Uruguay (12%), Ecuador (9%), Mexico (7%), Colombia (5%); Bolivia, Peru, Guatemala and Paraguay (2%). LA intensivists' workforce is predominantly male (68%) and young (42.0 ± 9.2 yr-old). ICU formal training among countries is between 2–4 years. ICU workload runs from 39–60 hours/week. Academic rounds are more frequent in academic units, mainly from high-income countries (55 vs 46%, p = .014). Satisfaction with conditions for appropriate septic shock management were reported as adequate by most participants (85%). Unsatisfactory conditions were attributed to insufficient technology (11%), laboratory support (5%), imaging resources (5%), and limited drug availability (5%). Satisfaction with septic shock management was significantly associated with country income (p =0.0148). A 70% of intensivists participate in research, and a 54% read scientific papers regularly (1–3 times a week). On the other hand, 32% of them reads no more than one scientific paper a month. Intensivists are mostly unsatisfied with their income (81%). However, a minority of them (27%) report having considered quitting their job as intensivists.
**CONCLUSIONS.** LA intensivist workforce faces resources and time constraints. New challenges arise in terms of learning new competencies, while balancing clinical, academic and personal areas. National income seems to be an important determinant for the quality of care in LA ICUs.

### 0629

#### Evaluation of a peer support training program in a university medical center

M. van Dam[1,2], B. de Vries[3], K. de Lange[4], M. Heringa[3]
[1]University Medical Center Utrecht, Intensive Care Center, Utrecht, Netherlands; [2]University Medical Center Utrecht, Educational Center, Utrecht, Netherlands; [3]University Medical Center Utrecht, Quality and Patient Safety, Utrecht, Netherlands; [4]Kim de Lange Trainingen, Zwolle, Netherlands
**Correspondence:** M. van Dam
*Intensive Care Medicine Experimental* 2017, **5(Suppl 2):**0629

**INTRODUCTION.** Adverse clinical events occur to all clinicians at some point in their careers. These events can lead to a variety of strong negative emotional consequences, e.g. isolation, self-doubt, depression, rumination, and anxiety, that may result in reduced professional performance and prolonged psychological stress. It is important that clinicians don't feel left alone and are supported under these circumstances. Recently, a Peer Support (PS) program was implemented in the hospital to address this need. Physicians, nurses and paramedics from a wide variety of departments and levels of experience were trained to provide PS during this difficult time for their colleagues (peers).
**OBJECTIVES.** To evaluate the training program for physicians, nurses and paramedics to offer PS with enough self confidence to professional victims of an adverse event.
**METHODS.** The PS training program is developed after models used in the Harvard teaching hospitals[i] and the OLVG, Amsterdam. The one-day course covers an introduction to urgency of PS, the organization in the hospital, an inventory of personal communicative styles and a half day of interactive training of true PS conversations and interactions of all participants with training actors. The training is completed with a anonymous written evaluation.To become a Peer supporter we asked all clinical co-workers in the hospital, over 8400 people, through e-mail to propose a colleague suitable for PS. This lead to 195 suggestions from all kind of backgrounds: nurses, medical doctors, physicians assistants, OR nurses, both experienced and in training, young and old, covering almost all specialties in the hospital. Colleagues introducing themselves or who were not clinically involved were excluded. Few colleagues declined.
**RESULTS.** 68 participants (15 nurses, 25 medical specialists, 11 residents, 14 paramedics e.g. OR nurses,physician assistants and midwives) were trained to perform PS. 66 completed the evaluation. The training program was considered a positive educational experience (n = 65) with a mean score of 4.3/scale 1–5 (SD 0.5)The training met their expectations (n = 63) mean 4.2/5 (SD 0.7). 64 participants appraised the training offering guidance for performing PS themselves in clinical practice, mean score 4.3/5 (SD 0.6).During verbal feedback the trainees mentioned repeatedly that for many clinicians the first impulse is to act when suffering is observed while true PS is essentially listening and supporting the colleague with the attention they need. The practical training made them more aware of this mechanism. Overall satisfaction was scored 8.2 on a10 point scale (mean, SD 0.8).
**CONCLUSIONS.** These results suggest that a practical training program to strengthen clinicians´ confidence to offer PS may help to actually to give that support, and so enhance professional performance and learning of adverse events.
**REFERENCE(S)**
[i] *Yue-Yung Hu etal. Arch Surg. 2012;147(3):212–217*

### 0630

#### Prognostic factors and strategies of flow management in sepis cases

H. H. G. Pires[1], F. F. Neves[2], A. Pazin - Filho[3]
[1]Federal University of Triangulo Mineiro, Internal Medicine, Uberaba, Brazil; [2]Federal University of São Carlos, Internal Medicine, São Carlos, Brazil; [3]Ribeirão Preto School of Medicine of the University of São Paulo, Internal Medicine, Ribeirão Preto, Brazil
**Correspondence:** H. H. G. Pires
*Intensive Care Medicine Experimental* 2017, **5(Suppl 2):**0630

**INTRODUCTION.** Sepsis is a clinical condition of disseminated and uncontrolled inflammation associated with an infectious outbreak. The organization of patient flow through the health system, ensuring ICU beds is critical.
**OBJECTIVES.** Evaluate the association of the prioritization of Vacancies in ICU with the mortality, morbidity and hospital stay time of the patients;the strategy of prioritizing vacancies in the access of patients in severe sepsis or septic shock to ICU beds; the strategy of prioritization of vacancies in the delay to access of patients in severe sepsis or septic shock to ICU beds and the strategy of prioritization of vacancies in the mortality of patients in severe sepsis or septic shock to ICU beds. The Quick SOFA prognostic index in patients with severe sepsis or septic shock admitted to U.E.-HCFMRP-USP.
**METHODOLOGY.** This is a retrospective cohort based on administrative data obtained from January 01,2010 to December 31,

2016. We used Student t tests, Analysis of Variance or non-parametric equivalents, chi-square or Fisher´s exact test and Receiver Operating Curves for univariate analysis.Multivariate logistic regression with binary or categorical outcome and multivariate Poisson regression as appropriate for the multivariate analysis. A p < 0.05 or the exclusion of the unit from the confidence interval signaled statistical significance.

**RESULTS.** Patients who received higher priority for ICU access(priority 1–5826; 62,5%) were younger (55;12-100-p < 0,01), had less comorbidities(Charlson 0,3583; 61,5%, p < 0,01) and less severity (Quick SOFA 0,2170; 37,2%-p < 0,01;SOFA < 10%-1782; 0,5%-p < 0,01). They were admitted in greater proportion (2097; 35,9%-p < 0,01) and had faster access to ICU (1081;52,5%-p < 0,01),presenting lower mortality (1853;31,8%- p < 0,01).The odds ratio to receive priority 1, higher value of the Charlson class OR 0,53; 0,49-0,57, the Quick SOFA (Severity)-OR 0,45;0,43-0,48 and the presence of Sepsis condition-OR 0,20-0,17;0,23 were independently associated with a lower chance of being classified as priority 1.Sepsis was more associated to lower chance of receiving priority 1(0,2; IC 95%-0,17;0,23). It persisted as an independent factor for total in-hospital mortality(2,7; IC 95%- 2,32;3,17) and for the mortality of patients admitted to the ICU (2,38;IC 95%-1,82;3,11). The prioritization of vacancies facilitated the access of the septic patients to the ICU. There was no delay in ICU admission for septic patients who received priority 1 (0,43; IC 95%-0,35;0,53). The Quick SOFA prognostic index had low accuracy in patients with severe sepsis or septic shock admitted to U.E.-HCFMRP-USP (AUROC = 0,5646, IC95%-0,52991; 0,59930-p < 0,001).

**CONCLUSIONS.** Sepsis presented a high mortality even when admission to the ICU was guaranteed. Flow management strategies were effective in securing access in ICU.

**REFERENCE(S)**

Neves FF, Pazin-Filho A.Management of resources in intensive care:application Of an information system for row organization.2016;7(2):730–41

### 0631

#### Improving referrals to a general adult intensive care unit at a UK tertiary centre

D. Day, M. Rowland, A. Wong
Oxford University Hospitals NHS Trust, Adult Intensive Care Unit, Oxford, United Kingdom
**Correspondence:** A. Wong
*Intensive Care Medicine Experimental* 2017, **5(Suppl 2):**0631

**INTRODUCTION.** Intensive Care services are under increasing pressure, with higher than recommended bed occupancy (1), an ageing population and burden of chronic disease. This results in an increased workload for ICU teams. It is recommended that senior clinicians should be involved in unplanned admissions to ICU, both in the referring and reviewing teams. Prompt admission to critical care is associated with reduced morbidity and mortality (2).

There is no international standard for the referral process. In our unit, there are no formal data for referral numbers and details, and no formal system for documentation of telephone advice or follow-up of patients who do not need immediate admission but are at risk of deterioration.

**OBJECTIVES.** This project aimed to quantify the workload generated by referrals to a tertiary centre ICU, understand who is making them and look at the outcomes of these referrals to assess whether an electronic system is required.

**METHODS.** A purpose designed referral proforma booklet was provided to doctors receiving referrals. Details for referrals were collected continuously over a 3 week period. Various matrix of the referral process was recorded including time of referral, patient demographics, prior ceilings of treatment, seniority of referrer and whether the Consultant was involved was noted.

**RESULTS.** 117 referrals were recorded over 3 weeks. 18/117 (15.4%) were from a Consultant and 25/117 (21.4%) were from a doctor below registrar level. In 47/117 (40.2%) the patient's Consultant was unaware of the referral. 7/117 (6.0%) had a DNACPR order in place. 26/117 (22.2%) referrals were given telephone advice only with no documentation being made by the ICU team. 93/117 (79.5%) of referrals were not discussed with the ICU Consultant at the time of referral. 23/117 (19.7%) were admitted to ICU or another specialist critical care unit on site. 50/117 (42.7%) were felt to not require ICU and 22/117 (18.8%) were felt to be inappropriate to admit to ICU. Of referrals from doctors below registrar grade, 22/25 (88%) did not require ICU admission. Documentation of timing of referral, review and admission was poor, with all 3 only recorded in 8 cases.

**CONCLUSIONS.** Recording data for referrals to ICU yields useful information, which impacts on teams throughout the hospital. An electronic system for referrals is needed. This will allow effects of interventions to be measured. It will also allow the ICU team to document advice given over the phone and discussions with consultants about referrals.

**REFERENCE(S)**

(1) Guidelines for the prevision of Intensive Care Services. (2017) http://www.ics.ac.uk/ICS/guidelines-and-standards.aspx
(2) Harris, S. et al. Delay to admission to critical care and mortality among deteriorating ward patients in UK hospitals: a multicentre, prospective, observational cohort study. Lancet. 2015 Feb 26;385 Suppl 1:S40

**GRANT ACKNOWLEDGMENT**
Nil.

### 0632

#### Improving referrals to a general adult intensive care unit at a university teaching hospital

D. Day, A. Wong, M. Rowland
Oxford University Hospitals NHS Foundation Trust, Adult Intensive Care Department, Oxford, United Kingdom
**Correspondence:** D. Day
*Intensive Care Medicine Experimental* 2017, **5(Suppl 2):**0632

**INTRODUCTION.** Prompt admission to critical care is associated with reduced morbidity and mortality, and it is recommended that senior clinicians should be involved in unplanned admissions. Recommendations for target timescales of under 4 hours (UK ICS) and under 6 hours (SCCM) to admission have been made but there is no gold standard referral process (1) (2).

In our unit, there are no data for referral numbers and details, and no formal system for documentation of telephone advice or follow-up of patients referred but not admitted. Times from referral to admission are not recorded. This project aimed to quantify the workload generated by referrals to a tertiary centre adult intensive care unit (AICU). It also looked at who is making them and at the outcomes, to see whether an electronic referrals system is needed.

**OBJECTIVES.** To record prospective data for referrals to the AICU at a university teaching hospital made over a 3 week period.

**METHODS.** A purpose designed paper referral proforma booklet was carried by registrar doctors receiving referrals to AICU at a university teaching hospital. Details of the patient, condition, referring team, actions taken and outcomes were collected continuously over a 3 week period.

**RESULTS.** 117 referrals were recorded. 18/117 (15.4%) were from a consultant and 25/117 (21.4%) were from a doctor below registrar level. In 47/117 (40.2%) the patient's consultant was unaware of the referral. 7/117 (6.0%) had a not-for-CPR order in place. 26/117 (22.2%) referrals were given telephone advice only with no documentation being made by the ICU team. 93/117 (79.5%) of referrals were not discussed with the ICU consultant at the time of referral. 23/117 (19.7%) were admitted to ICU or another specialist critical care unit on site. 50/117 (42.7%) were felt to not require ICU and 22/117 (18.8%) were felt to be inappropriate to admit to ICU. Of referrals from doctors below registrar grade, 22/25 (88%) did not require ICU admission. Documentation of timing of referral, review and admission was poor, with all 3 only recorded in 8 cases.

**CONCLUSIONS.** Recording data for referrals to ICU yields surprising and useful information, which impacts on teams throughout the hospital as well as in ICU. An electronic system for ICU referrals is required, to allow measurement of interventions to increase referring doctor seniority and home consultant involvement. It will also allow documentation of telephone advice given and ICU consultant opinion. Finally it will allow measurement of the timescale of admissions to ICU.

(1) Guidelines for the prevision of Intensive Care Services. (2017) [online] Available at: http://www.ics.ac.uk/ICS/guidelines-and-standards.aspx

(2) Nates, J.L., et al. ICU Admission, Discharge, and Triage Guidelines: A Framework to Enhance Clinical Operations, Development of Institutional Policies, and Further Research. (2016) Critical Care Medicine: 44:8, pp 1553–1602

### 0633

#### Blood glucose control in critically ill adult patients: results of a practice survey in French intensive care units

G. Besch[1,2], L. Carteron[1,2], C. Schneider[1], J. Paillot[1], E. Samain[1,2], S. Pili-Floury[1,2]

[1]Centre Hospitalier Universitaire de Besancon, Anesthesiology and Intensive Care Medicine, Besancon, France; [2]Université de Bourgogne - Franche-Comté, EA 3920 Marqueurs Pronostiques et Facteurs de Régulation des Pathologies Cardiaques et Vasculaires, Besancon, France

**Correspondence:** L. Carteron

*Intensive Care Medicine Experimental* 2017, **5(Suppl 2):**0633

**INTRODUCTION.** Stress hyperglycemia is associated to an increase in morbidity and in mortality in intensive care unit (ICU) patients. Intravenous (iv) insulin infusion is the gold standard treatment of stress hyperglycemia in the ICU. The modalities of blood glucose (BG) control remain a matter of debate as contradictory results have been reported with the various iv insulin therapy protocols that have been published. If everyone agrees that hyperglycemia has to be corrected to improve the outcome of ICU patients, no consensual protocol has emerged. To date, the daily practice in BG control in French ICU patients has never been studied.

**OBJECTIVES.** To assess how BG control is managed in French ICUs.

**METHODS.** An on-line questionnaire (SurveyMonkey®) was sent by email to all heads-of-department of French ICUs that were accredited for post graduate teaching of French residents from January to December 2016. The questionnaire has been build, tested and validated by 4 attending physicians from our surgical intensive care unit prior to on-line submission. The 14 first questions were used to describe the characteristics of the ICU and to ask if a standardized BG management was protocolized. If the answer was "yes", 29 more questions were added to precise the modalities of BG control and of the iv insulin therapy protocol. Data presented are median [interquartile range] and number of ICUs (percentage).

**RESULTS.** Among the 385 ICUs interviewed, 187 (49%) answers have been analyzed. An iv insulin therapy protocol was used in 154 (82%) ICUs. 116 (75%) ICUs have declared iv versus subcutaneous infusion of insulin in BG control at the acute phase. The insulin infusion rate was adjusted in 110 (71%) ICUs on the current BG value, in 50 (32%) ICUs on the previous BG value, in 66 (39%) ICUs on the current insulin infusion rate, and in 47 (31%) ICUs on the caloric intake. The median values of the upper and of the lower limits of the BG target range were respectively 150 [140–180] and 90 [80–110] $mg.dl^{-1}$. The safety and the efficacy of the iv insulin therapy protocol have never been assessed in respectively 121 (78%) and 125 (81%) ICUs.

**CONCLUSIONS.** A practice survey conducted in French ICUs suggests a great heterogeneity in the modalities of BG control management at the acute phase of care in critically ill adult patients. A global strategy based on actualized guidelines appears desirable to improve the quality of these practices.

### 0634

#### Evaluation of the practice of proning in patients with ARDS across 22 Intensive Care Units in London, United Kingdom

T. Samee, S. C. Robert

Homerton University Hospital, Intensive Care Unit, London, United Kingdom

**Correspondence:** T. Samee

*Intensive Care Medicine Experimental* 2017, **5(Suppl 2):**0634

**INTRODUCTION.** Despite lung-protective ventilation and other measures inpatient mortality for acute respiratory distress syndrome (ARDS) remains high at over 40%[1]. Proning has long been suggested to improve oxygenation in mechanically ventilated patients with ARDS.

Recent research demonstrated that proning longer than 16 hours within 36 hours of starting mechanical ventilation significantly reduces 28-day mortality in patients with severe ARDS[2]. There are further meta-analyses which also recommend proning[1,3].

However, the United Kingdom currently has no national guidelines on proning. The European Society for Intensive Care Medicine has not published any either so the management of ARDS patients is currently entirely dictated by local practice and individual preferences.

**OBJECTIVES.** The primary aim of our project was to establish how many hospitals in London had a pre-set formal local or regional proning guideline for patients with ARDS. We also sought to evaluate their specific triggers for proning and the duration. Our hypothesis was that there was likely to be wide variations in practice.

**METHODS.** A total of 23 intensive care units in London were contacted via telephone and/or email. Senior staff members were asked to complete eight questions about their local proning practice. 22 intensive care units were very forthcoming and provided insight into their local practice.

**RESULTS.** Of the 22 hospitals, only 18% *(n = 4)* had set guidelines on proning.

64% *(n = 14)*of the ITUs routinely considered it as a way of treating patients with ARDS whereas 36% *(n = 8)* did not.

67% *(n = 14)* favoured proning only in severe ARDS compared to 33% *(n = 7)* which used proning in moderate ARDS. The mean maximum duration of proning was 18.3 hours over a 24 hour period, ranging from 4–24 hours. The triggers for proning varied greatly between the different units.

**CONCLUSIONS.** We found considerable variations in practice in the 22 units evaluated. Given that current evidence supports proning as a beneficial measure in managing ARDS patients, there is an urgent need for national guidelines to streamline the management of this particular patient group.

[3] Lee JM, Bae W, Lee YJ, Cho YJ. The efficacy and safety of prone positional ventilation in acute respiratory distress syndrome: updated study-level meta-analysis of 11 randomized controlled trials. Critical Care Medicine. 2014 May 1;42(5):1252–62.

**GRANT ACKNOWLEDGMENT**
Not applicable.

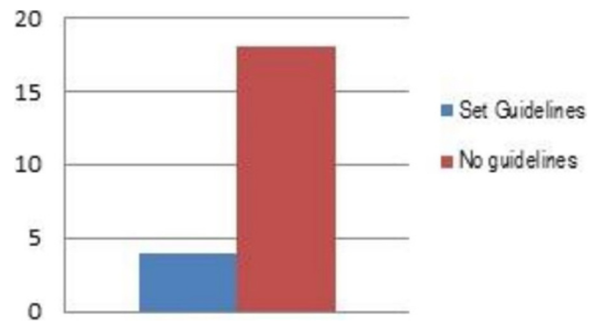

**Fig. 227 (Abstract 0634).** Hospitals with guidelines versus no guidelines

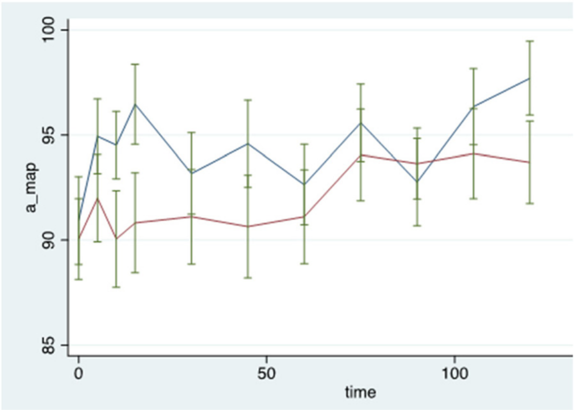

**Fig. 228 (Abstract 0634).** Regular proning of ARDS patients

### 0635

#### Epidemiological characteristics of patients accepted to the Medical Intensive Care service in a tertiary hospital in Qatar: a retrospective review of critical care registry from January 2015 to December 2015

A. S. Ibrahim, I. Hassan, T. Raza, A. Abdussalam, H. Jaouni, J. Ibrahim, A. Akbar, I. Alatoum
Hamad Medical Corporation, Medicine Critical Care Divsision, Doha, Qatar
**Correspondence:** A. Abdussalam
*Intensive Care Medicine Experimental* 2017, **5(Suppl 2):**0635

**INTRODUCTION.** Data registry in critical care is a valuable tool to understand patients' demographics, disease characteristics, resource utilization, quality measures, and service performance. Yet, only limited objective information is available to describe the specific epidemiological characteristics of critically ill patients in Qatar, where the critical care services are overwhelmed by the substantial growth of the population over the last few years.

**OBJECTIVE.** To study the demographic and clinical characteristics of patients admitted to Medical Intensive Care Unit (MICU) in Hamad General Hospital (HGH), the main tertiary hospital in the State of Qatar.

**METHOD.** A retrospective descriptive study of the data registry for adult patients above the age of 14 years, who were admitted to MICU in year 2015. Descriptive statistics were given as numbers and percentages for categorical variables, or as the mean ± standard deviation for continuous variables. Calendar days were used to calculate patients' days.

**RESULTS.** in 2015, a total of 1472 out of 1679 referrals were accepted for admission to MICU. From all accepted referrals, 682 (46.3%) patients were admitted to MICU, and 790 (53.7%) patients were managed by the outreach team outside the ICU. Of those managed outside the ICU, 657 (83.2%) patients did not get access to MICU, while 61 (7.7%) patients died, and 72 (9.1%) patients were transferred to other hospitals. Planned extubation occurred in 32 patients, and a total of 1879 patients-days in the whole year were managed outside the ICU. Of the 682 patients physically admitted to MICU, 62% came from the emergency department, 25.8% were from inpatient wards, and 12.3% were from other sources. Males formed 65.5%, while females were 34.5%. Mean age was 55.8 ± 19 years. Qatari patients formed 39.1% of all admissions. The most common reasons for admission were respiratory failure in 28.7%, septic shock in 27.8%, and central nervous system pathology in 24.2%. The Crude Mortality was 26.1%. The mean disease adjusted APACHEII score of 20.8 ± 9.4 predicated a mean mortality of 38.5 ± 27.0%. The mean length of stay observed was 10.1 ± 11.5 days, including 429 wait days to get floor beds after ICU discharge decision. Out of the 70.8% intubated; planned extubation was performed in 47.3%, re-intubation occurred in 9.2%, and tracheostomy was performed in 22.2%.

**CONCLUSION.** This Epidemiological registry demonstrates the variation in critical care practice, when the service is overwhelmed to a breaking-point. More than half of the patients who did not get access to critical beds were assessed, triaged, and managed by ICU physicians outside the ICU. The difference in their outcome requires further research that links it to a validated severity scoring system.

### 0636

#### Mechanical ventilation: use and weaning practice in Thailand

N. Kongpolprom[1], N. Rittayamai[2], V. Tangsujaritvijit[3], A. Lertamornpong[4], K. Kawkitinarong[1], A. Wongsa[4], Assembly of Critical Care, The Thoracic Society of Thailand
[1]King Chulalongkorn Memorial Hospital, Chulalongkorn University, the Thai Red Cross Society, Division of Pulmonary and Critical Care Medicine, Department of Medicine, Faculty of Medicine, Bangkok, Thailand; [2]Siriraj Hospital, Mahidol University, Division of Respiratory Diseases and Tuberculosis, Department of Medicine, Faculty of Medicine, Bangkok, Thailand; [3]Ramathibodi Hospital, Mahidol University, Division of Pulmonary and Critical Care Medicine, Department of Medicine, Faculty of Medicine, Bangkok, Thailand; [4]Phramongkutklao Hospital, Division of Pulmonary and Critical Care Medicine, Department of Internal Medicine, Bangkok, Thailand
**Correspondence:** N. Kongpolprom
*Intensive Care Medicine Experimental* 2017, **5(Suppl 2):**0636

**INTRODUCTION.** International survey demonstrated variable practice and responsibilities in mechanical ventilation and weaning in different countries. In addition, a number of mechanically-ventilated patients are managed outside the intensive care unit in limited-resource countries that may affect clinical outcomes.

**OBJECTIVES.** This study aimed to evaluate current practice in starting and stopping of mechanical ventilation among Thai physicians, and professional responsibilities of mechanical ventilation in Thailand.

**METHODS.** A survey questionnaire was sent to new first-year medical residents in 3 university hospitals before medical residency training was started. The questionnaire was composed of 4 parts including

1) personal demographics,
2) professional responsibilities of ventilator,
3) mechanical ventilation practice and
4) weaning practice.

We analyzed data as numbers, percentages and proportions.

**RESULTS.** One hundred and seven residents responded to the questionnaire. Almost all (99%) worked in public hospitals before the training. Approximately, 65% of them had experience in taking care of mechanically-ventilated patients in general wards. The median number of ventilated patients was 5[3, 8.5] cases per day in 1 ward. Interestingly, nurses had authorities to set and adjust mechanical ventilation in 26% of the hospitals. Moreover, in the physicians' view, nurses had the important role in adjustment of ventilator with the median score of 7/10 (0 = no role and 10 = the most important role). Nurses also played role in evaluation of the readiness for weaning in 18.7% of the hospitals, while in determination of weaning failure and termination of weaning in 35% of the hospitals. Besides, nurses decided to extubate the patients in 12% of the hospitals. For the weaning practice, 56% of the responders used weaning protocols, and T-piece was the most common method used for weaning. Rapid shallow breathing index was the most frequently used parameter for prediction of weaning outcomes. Additionally, 67% of the responders evaluated cuff leak test before extubation but only 37% of them routinely assessed the test. Furthermore, 43% of the responders provided noninvasive ventilation after extubation, however only 22.4% of them applied it early as the prophylaxis for extubation failure.

**CONCLUSIONS.** Due to limited ICU resources, Thai physicians had to provide ventilatory care in general wards. Additionally, Thai nurses had the important role in ventilator management and weaning.

### 0637

#### Reliability of a standardized protocol for bedside quadriceps strength measurement in critically ill patients

A.-F. Rousseau[1], G. Freycenon[2], I. Kellens[1], N. Dardenne[3], O. Bruyère[4], P. Damas[1], J.-L. Croisier[5]

[1]University Hospital of Liège, General Intensive Care Unit and Burn Centre, Liège, Belgium; [2]University of Liège, Liège, Belgium; [3]University of Liège, Support Unit in Epidemiology and Biostatistics, Liège, Belgium; [4]University of Liège, Department of Public Health, Epidemiology and Health Economics, Liège, Belgium; [5]University of Liège, Department of Sports Sciences and Rehabilitation, Liège, Belgium

**Correspondence:** A.-F. Rousseau

*Intensive Care Medicine Experimental* 2017, **5(Suppl 2):**0637

**INTRODUCTION.** Muscle weakness is a common complication in critically ill (CI) patients. Objective quantification of muscle strength is now considered essential when assessing functional status of CI survivors. To date, published data are very heterogeneous in terms of device, patient positioning and measurement standardization.

**OBJECTIVES.** To assess feasibility and intra-observer reliability of a standardized bedside protocol of quadriceps strength measurement performed in supine position in collaborative CI patients.

**METHODS.** Maximal isometric quadriceps strength was assessed using a handheld dynamometer (MicroFet2). Standardized patient positioning was ensured through an adjustable system of vertical and horizontal traction plain bars and clamp bars, aiming to get a 45° hip flexion and 40° knee flexion. The operator was positioned in front of the patient, at foot of the bed, withstanding the subject's movement (raising the leg). The MicroFet2 was localized at the anterior face of the ankle, two fingers above external malleolus level. The same trained operator performed measurements at 3 different sessions (T0, after 1h and after 24h). He provided strictly standardized instructions and encouragements during testing. RASS score was used to identify collaborative patients. Vital parameters were recorded during T0 session. Interclass correlation coefficients (ICC) and minimal detectable changes (MDC%) were computed to assess respectively relative reliability and absolute reliability of the tests. Paired data were analyzed using Friedman test ($p < 0.05$: significant).

**RESULTS.** Thirty consecutive CI adults were enrolled (68 [54–76] years, 77% men). Quadriceps strength was highly variable among patients. Median strength was 192.5 [113.4 - 266.1] N at H0, 206.5 [111.8 - 273.5] N at H1 and 181.5 [122.7 - 281] at H24. Peak forces measured during the three sessions were not statistically different (p = 0.69), showing a good stability over time in the absence of intercurrent acute event. The relative intra-observer reliability was very high: ICC of the tests were > 0.9. MDC% ranged from 17.13 to 27.33, which are acceptable values when considering the potential of muscle strength improvement in CI patients. Procedures were highly feasible without any clinically significant changes in vital parameters.

**CONCLUSIONS.** Very high relative and absolute intra-observer reliabilities were observed for quadriceps strength measurement using the MicroFet2 device. The present extremely standardized protocol was well tolerated. Reproduced with strict observance, such assessment, feasible at bedside, could be used in clinical practice and future studies. Handheld dynamometry may be viewed as a key step in weakness assessment, from diagnosis and identification of patients most likely to benefit from rehabilitation, to follow-up and assessment of post-rehabilitation progresses.

### 0638

#### Associated factors with frequent doctors´ visits in ICU survivors. One year survey

J. Baroudi[1], N. Fraj[1], J. Ayachi[1], K. Meddeb[1], E. Ennouri[1], W. Zarrougui[1], N. Sma[1], M. A. Boujelbèn[1], A. Ben Abdelhafidh[1], I. Chouchene[1], A. Azouzi[1], A. Khedher[1], I. Ben Saida[1], M. Boussarsar[1,2]

[1]Farhat Hached University Hospital, Medical Intensive Care Unit, Sousse, Tunisia; [2]Farhat Hached University Hospital, Research Laboratory N° LR14ES05 Interactions of the Cardio-pulmonary System, Sousse, Tunisia

**Correspondence:** M. Boussarsar

*Intensive Care Medicine Experimental* 2017, **5(Suppl 2):**0638

**INTRODUCTION.** Health service use following ICU discharge is generally increased. Studies showed that doctors´ visits are very important for the follow up. The study was performed to determine the frequency of medical visits over one year follow up after an ICU stay and to identify factors associated with a higher frequency of medical visits.

**METHODS.** A prospective cohort study was conducted in a tertiary Tunisian medical ICU. Adult survivors after an ICU stay between January 2014 and December 2015 were included. ICU survivors were interviewed through regular phone calls from 3 months after discharge until 1 year. Data recorded were medical visits frequency, causes of visits concluded by the investigating physician on the basis of non-standardized questions. 2 groups of patients were compared regarding frequency of visits (≤3 and >3 visits per year). Significant factors with a p value of < 0.2 were entered into a backward conditional binary logistic regression to determine independent factors for frequent visits.

**RESULTS.** 325 patients were included. 215 among them were able to follow up with phone interviews over 1 year. ICU survivors were mainly transferred to the hospital ward 174(62%) and only 106(38%) were discharged home. 157(73%) patients consulted at least once during the year following the hospital discharge, however 58(27%) patients had no medical visit after their discharge. 141(90%) patients saw general practitioner or specialist physician in the first three months. The mean frequency of visits during the year was 3. Pulmonologist, cardiologist and ED practitioner were the frequent physicians visited respectively by 42, 23, and 17 patients. Reasons for doctor visits were classified into 3 categories: systematic control for (108 patients), exacerbation of a chronic disease for (43patients) and a new disease for (6 patients). Multivariate analysis showed that chronic respiratory failure (OR, 1.77 ; 95%CI, [1–3.1] ; P = 0.04), and the patients with ventilatory device at discharge (OR, 3.22 ; 95%CI, [1.4-7] ; P = 0.002) were the independent factors for frequent visits after ICU discharge.

**CONCLUSION.** After ICU discharge, the doctors´ visits seem to be rather frequent in survivors. Chronic respiratory failure and a home ventilatory device at discharge are the independent factors for these frequent visits.

### 0639

#### Influence of increasing intensive care units beds on ICU admission decisions, mortality and hospital length of stay

S. Paydar[1], G. Sabetian[2], A. Ahmadi[3], E. Paktinat[4], H. Khalili[2], M. Masjedi[5], F. Zand[5]

[1]Trauma Research Center,Shiraz University of Medical Sciences, Shiraz, Iran, Islamic Republic of; [2]Trauma Research Center, Shahid Rajaee (Emtiaz) Trauma Hospital, Shiraz, Iran, Islamic Republic of; [3]Shiraz University of Medical Sciences, Shiraz, Iran, Islamic Republic of; [4]Shiraz University, Shiraz, Iran, Islamic Republic of; [5]Anesthesiology and Critical Care Research Center, Shiraz, Iran, Islamic Republic of

**Correspondence:** G. Sabetian

*Intensive Care Medicine Experimental* 2017, **5(Suppl 2):**0639

**INTRODUCTION.** A positive correlation is evident between intensive care unit (ICU) (beds) occupancy rate and mortality. reduce in likelihood of ICU admissions following high ICU congestion can be a main reason.

**OBJECTIVES.** The aim of this study was to assess the effect of increasing ICU capacity in a trauma center with unchanged admission protocol, on ICU admission rate and mortality.

**METHODS.** We conducted an observational retrospective cohort study on patients admitted in referral trauma ICU, affiliated with Shiraz university of medical sciences.

This study observed in two periods, at first period our hospital had 4 general ICUs.

And in second period had 6 general ICUs.Each ICU comprises of nine beds.

ICU Triage decision makers were emergency medicine, surgery and neurosurgery attending physicians, all were faculty of Shiraz university of medical sciences and they did not change between these two periods.

These data were extracted from patients' documents. We compared rate of admission, number of patients with less than 48 hours ICU stay, patients mortality rate, age and ICU length of stay, between First and second period.

**RESULTS.** During the study period a total of 731 patients admitted in ICUs that comprise of 149 women (20%) and 582 men(80%). Age range was between 14 and 95 years (mean 41.6 years). Admission rate in second period (n = 456) comparing to first period (n = 275) was significantly increased, ($p < 0.001$).In whole study population. The proportion of mortality in second group,54 out of 456 cases, comparing to first group, 84 out of 275, showed significant drop in mortality rate from first period, 84 deaths (30.54%), to second period,54 deaths (11.84%). ($p < 0.001$)Patients admitted in ICU for less than 48 hours were 52 out of 275 cases (18.9%) in first period and 143 out of 456 case (31.4%) in second period. Which demonstrates a significant increase in less than 48hours admission rate after expanding ICU capacity. ($p < 0.001$)Considering patients admitted for < 48 hours, we saw no significant improvement in mortality rate in second group ($p = 0.07$) A logistic regression analysis was conducted to ascertain the effects of age (being >40 or = < 40 years of age), year of admission (admitted in first or second period) and duration of ICU stay on mortality *rate*.

**CONCLUSIONS.** Increase ICU bed availability leads to growth in ICU turn over probably due to effect on triage decision making. Near half of this increase belongs to patients admitted in ICU for less than 48 hours. Increase ICU turn over reduced mortality. Decrease in mortality following ICU expansion seems to be unrelated to age and has same proportion in different age groups.

**REFERENCE(S)**

1. Kim, S.H., et al., Association Among ICU Congestion, ICU Admission Decision, and Patient Outcomes. Crit Care Med, 2016.

### 0640

#### Evaluation of a novel classification of heat illness

T. Yamamoto, Y. Oda, T. Yagi, M. Todani, T. Nakahara, M. Fujita, Y. Kawamura, K. Kaneda, R. Tsuruta

Advanced Medical Emergency and Critical Care Center, Yamaguchi University Hospital, Ube, Yamaguchi, Japan

**Correspondence:** T. Yamamoto

*Intensive Care Medicine Experimental* 2017, **5(Suppl 2):**0640

**INTRODUCTION.** The Japanese Association for Acute Medicine Committee recently proposed a new classification system for the severity of heat illness that is now used by healthcare professionals and non-medical personnel to avoid underestimating the severity of heat stroke, the most serious heat illness. It is classified simply into three stages based on symptoms and management/treatment:

Stage I is a condition that may be managed on site;

Stage II is a condition requiring immediate examination at a medical institute; and

Stage III is a condition requiring admission to hospital for at least one of the following: neurologic disorder, hepatic failure, renal dysfunction, or coagulation disorder.

Stages I, II, and III broadly correspond to heat cramp and heat syncope, heat exhaustion, and heat stroke, respectively. However, the use of the new classification for assessing the severity of heat illness has not been validated.

**OBJECTIVES.** Our objective was to examine whether this new severity classification shows similar validity to an internationally accepted classification for heat illness, such as heat stroke and heat exhaustion.

**METHODS.** A nationwide surveillance study of heat illness was conducted between June and September, 2012, at emergency hospitals in Japan. We used these data to compare the distribution of severity on admission and patient outcomes between the new classification and the existing international classification.

**RESULTS.** Of 2130 patients surveyed at 103 emergency hospitals, the severity of heat illness and the outcome were recorded for 1799 patients who were included in this study. The distribution of the severity of heat illness determined using the new classification was significantly correlated with that determined using the international classification ($\rho = 0.448$, $P < 0.001$). However, of 879 patients classified as stage I, 564 (64.2%) had heat exhaustion. Meanwhile, 564 (49.5%) of 1139 patients with heat exhaustion were classified as stage I. We found that Stage II and heat exhaustion, and Stage I and other heat illness (e.g. heat cramp and heat syncope) did not match on a one-to-one basis. In addition, Stage III may include other forms of mild to severe heat illness associated with organ dysfunction. Although the mortality rate was not significantly different between the new classification and the international classification, there were no deaths among patients classified as Stage I using the new classification.

**CONCLUSIONS.** The new classification classified the severity of heat illness consistently with the international classification. There were no deaths among patients classified as Stage I, indicating that it may avoid underestimating the patient's severity. However, Stage III included many patients with mild to moderate disorders, except for heat stroke. Future studies should evaluate sensitive indicators of organ failure associated with the outcome of heat illness.

None.

**GRANT ACKNOWLEDGMENT**

No grants received.

### 0641

#### Acceptance of a new electronic medical records system among the nursing staff in ICUs based on TAM2

A. Furmanov[1], A. Avidan[2], F. Dekeyzer Ganz[3]

[1]Hadassah Hebrew University Medical Center, Neurosurgical Intensive Care Unit, Jerusalem, Israel; [2]Hadassah Hebrew University Medical Center, Anesthesiology, Jerusalem, Israel; [3]Hadassah Hebrew University Medical Center, Henriata Szold School of Nursing, Jerusalem, Israel

**Correspondence:** A. Furmanov

*Intensive Care Medicine Experimental* 2017, **5(Suppl 2):**0641

**INTRODUCTION.** Technology is widely used in nursing field in the intensive care units (ICU); thus, nurses must be prepared to accept and work in a high technology environment. However, in many cases nursing staff have been reported to avoid technology and computing. Little is known about the acceptance of technology and computing among nursing staff in ICUs, especially in hospitals in Israel.

**OBJECTIVES.** The aim of this study was to examine factors associated with the acceptance of a new electronic medical records system among the nursing staff in ICUs in Israel based on the Technology Acceptance model (TAM) and Theory of Planned Behavior (TPB).

**METHODS.** It´s was a prospective, descriptive-correlational study. Ethical approval was received from all participating institutions. Study population included 152 nurses in five ICUs of four Israelis tertiary-care academic medical centers. A Davis TAM2 questionnaire was used to examine the perceived usefulness and ease of use, attitudes and acceptance of ICU electronic medical records system. Factors influencing attitides and acceptance of the system were compared using stepwise regression.

**RESULTS.** 123 nurses responded to the questionnaire (80.9% response rate). Subjective norms to use technology explained 60% ($R^2$) of technology acceptance. Output quality and perceived ease of use explained other 59% of the variance in perceived usefulness. Perceived ease of use had the greatest influence on perceived usefulness (correlation 0.72, $p < .001$). Perceived usefulness had the greatest influence on acceptance (correlation 0.67, $p < .001$).

**CONCLUSIONS.** The TAM2 was found to be a good predictor of acceptance of new electronic medical records system by ICU nurses. Therefore, this model can be used as a basis for designing interventions aimed at improving ICU nurse acceptance of new technologies.

**REFERENCE(S)**

Aggelidis, V. P., & Chatzoglou, P. D. (2009). Using a modified technology acceptance model in hospitals. International Journal of Medical Informatics, 78(2), 115–26. http://doi.org/10.1016/j.ijmedinf.2008.06.006

Davis, F. D. (1989). Perceived Usefulness, Perceived Ease of Use, and User Acceptance of Information Technology. MIS Quarterly, 13(3), 319–340. http://doi.org/10.2307/249008

Holden, R. J., & Karsh, B.T. (2010). The Technology Acceptance Model: Its past and its future in health care. *Journal of Biomedical Informatics*, *43*(1), 159–172. http://doi.org/10.1016/j.jbi.2009.07.002

Ketikidis P., Dimitrovski T., Lazuras L., Bath P.A., (2012). Acceptance of health information technology in health professionals: an application of the revised technology acceptance model. *Health Informatics Journal*. Jun; 18*(2)*:124–34. DOI: 10.1177/1460458211435425

Venkatesh V., Davis F.D., (2000). A Theoretical Extension of the Technology Acceptance Model: Four Longitudinal Field Studies. *Management Science* 46*(2)*:186–204. DOI: 10.1287/mnsc.46.2.186.11926

### 0642

#### Health records on ICU - is digital really the future?

A. Wong, H. Surgenor, M. Rowland

Oxford University Hospitals NHS Trust, Adult Intensive Care Unit, Oxford, United Kingdom

**Correspondence:** A. Wong

*Intensive Care Medicine Experimental* 2017, **5(Suppl 2):**0642

**INTRODUCTION.** High quality patient records are an integral part of clinical practice, as a means to document and communicate essential information regarding a patient's condition and the care they receive.

In the ICU, the sheer volume of data is overwhelming and arguably beyond the processing power of the average human. It is therefore important that this information is recorded in an accessible, complete, and organised format.

Electronic Clinical Information Systems (CIS) have been advocated as a way forward in medical record-keeping in order to appropriately manage but also analyse the large quantities of information available.

However even in high-technology environments such as ICU, the implementation of CIS alongside electronic patient records (EPR) is not universal and is subject to local variation.

**OBJECTIVES.** The aim of this study was to compare the quality of medical documentation in paper versus digital (CIS) patient records in the context of intensive care as part of a quality improvement project.

**METHODS.** A retrospective analysis of medical records was undertaken in 3 critical care areas within the same trust which utilise different ways of recording patient records (1 paper based vs. 2 different CIS platforms).

For all patients on each unit at the point of survey, records up to the first 5 days of their admission were analysed. A variety of parameters were used to assess the quality of records and a neutral party was used to adjudicate legibility.

**RESULTS.** Our findings showed that EPR was superior to traditional paper records in a number of areas e.g. legibility, dated, timed. In areas where EPR was found lacking, this was due to intrinsic software limitations which could be improved with better templating of data entry (Table 166).

**CONCLUSIONS.** By the standards set by the Royal College of Physicians on quality of medical record-keeping, our findings show that electronic CIS records were superior to traditional paper records.

These parameters may have implications for the fulfilment of treatment plans, patient safety, continuity of care and potential litigation issues.

Those areas where digital records were not superior were on account of intrinsic software limitations which could be improved with better templating of data entry. Other advantages of EPR/CIS such as built-in clinical decision support modules, improved auditing ability and notification prompts should also be considered.

The decision to move towards electronic patient records/ CIS needs to be individualised to the particular ICU taking into account work flow and logistics.

**REFERENCE(S)**
1. The Royal College of Physicians, 2011. Evidence on the quality of medical note keeping: Guidance for use at appraisal and revalidation. London
2. Friedberg, M., Crosson, FJ., Tutty, M. Physicians' Concerns About Electronic Health Records: Implications and Steps Towards Solutions. Health Affairs Blog 2014

**Table 166 (Abstract 0642).** Comparisons between healthcare records

| | Paper | CIS 1 | CIS 2 |
|---|---|---|---|
| Number of notes reviewed | 19 | 9 | 6 |
| Clearly identified home consultant | 17 | 9 | 6 |
| Pt identifiers on each entry | 11 | 9 | 6 |
| Entries filed in chronological order | 15 | 9 | 6 |
| All entries legible, timed, dated and signed | 1 | 9 | 6 |

### Caring for the ICU patient: Challenges and opportunities

#### 0643

#### The time course analyses of the most stressful symptoms and their impact on your daily practice

H. Horiuchi
Kurashiki Central Hospital, Emergency Medicine, Kurashiki, Japan
*Intensive Care Medicine Experimental* 2017, **5(Suppl 2):**0643

**INTRODUCTION.** Ideally, patients in the intensive care unit (ICU) should be comfortable at all times and assessment is required for relief of the patients' self-reported symptoms. Pain, Thirst, Anxiety, Dyspnea and Poor sleep are known as the most stressful symptoms for ICU patients. However, it is not known the time dependent change of them.
**OBJECTIVES.** In this article, we focused on five patient symptoms and assessed how they changed by lapse of time.
**METHODS.** All consecutive patient admitted to our hospital were enrolled from February 2016 to Januraly 2017. We included patients who were intubated over 24 hours, and the mental status with Richmond Agitation-Sedation Scale (RASS) of −1 or 0. Patients sustained traumatic brain injury or living with dementia were excluded. Primary outcome was patient symptoms (thirst, anxiety and dyspnea) during intubation. We evaluate these symptoms every day using 0–10 numeric rating scale (NRS). We descrived patient's data as median (interquartile range).
**RESULTS.** Of 401 patients, 160 patients were excluded. Median age was 65(IQR 42 to 74). Table 167 presents the results of the median NRS of five symptoms. A score on thirst were positively associated with the ICU length of stay until day 10 and a score on dyspnea were positively associated until day 7, while a score on pain were not changed. The degree of satisfaction of sleep was once decreased at day 7 but it substantial increase at day 10. A score on anxiety were inreased at day4 after an initial fall but it continued to fall after day6.
**CONCLUSIONS.** It must be allowing the clinicians to the better consideration and management for the tracheal intubated patient to recognize the change of the most stressful symptoms.

**REFERENCE(S)**
Intensive Care Med (2015)41:1347–1350

**Table 167 (Abstract 0644).** See text for description

| | Day 1 | Day 2 | Day 3 | Day 4 | Day 5 | Day 6 | Day 7 | Day 8 | Day 9 |
|---|---|---|---|---|---|---|---|---|---|
| Pain | 2.00 | 2.00 | 2.00 | 2.00 | 2.00 | 2.00 | 2.00 | 1.50 | 2.00 |
| Thirst | 5.00 | 5.00 | 5.00 | 5.00 | 5.00 | 5.00 | 5.50 | 6.00 | 6.50 |
| Anxety | 3.50 | 3.00 | 3.00 | 5.00 | 5.00 | 5.00 | 5.00 | 4.00 | 3.50 |
| Dyspnea | 2.00 | 2.00 | 2.00 | 2.00 | 3.00 | 3.00 | 4.00 | 2.00 | 3.00 |
| Sleep | 4.00 | 5.00 | 4.00 | 5.00 | 5.00 | 5.00 | 2.00 | 3.00 | 4.50 |

#### 0644

#### Dysphagia in acute cervical spinal cord injury-development of a screening tool through a Delphi process of expert consensus

J. McRae[1,2], C. Smith[3], S. Beeke[3], A. Emmanuel[2]
[1]Royal National Orthopaedic Hospital, Therapies Department, Stanmore, United Kingdom; [2]University College London, Division of Medicine, London, United Kingdom; [3]University College London, Psychology and Language Sciences, London, United Kingdom
**Correspondence:** J. McRae
*Intensive Care Medicine Experimental* 2017, **5(Suppl 2):**0644

**INTRODUCTION.** Dysphagia in cervical spinal cord injury (CSCI) is associated with respiratory complications, increased length of stay and mortality risks [1]. The cause is multi-factorial with subtle clinical presentation [2]. Early dysphagia identification helps to prevent complications (including silent aspiration) however a lack of national guidance and effective screening methods has led to varied clinical practices in non-specialised units resulting in poor outcomes [3].
**OBJECTIVES.** To use a Delphi technique, gathering expert consensus on risk factors for dysphagia, to develop a screening tool and clinical recommendations for critical care healthcare professionals.
**METHODS.** Following a literature review, 85 statements were generated across seven domains: co-morbid factors, definition, screening, assessment, identification, management, to form the first round of a Delphi. A multi-disciplinary international expert panel of 27 clinicians ranked the statements using a 5 point Likert scale. Statements not achieving consensus of >70% of the panel were modified and recirculated for a subsequent round until levels of agreement were static. Participants received individual feedback at the end of each round detailing their response compared to the group's response.
**RESULTS.** Following two rounds of the Delphi, 62 statements achieved consensus (round 1, 59%; round 2, 48%) with statements for dysphagia screening and assessment achieving the least consensus. Based on the results, a screening tool was developed to identify dysphagia risks with 3 domains: injury risk (brain injury, cervical surgery, spinal injury level and severity); clinical risk (intubation, tracheostomy, ventilation, and nutrition) and urgency (chest infection, pyrexia, oral care and suction). A set of clinical recommendations provided additional guidance on optimal respiratory and nutritional management.
**CONCLUSIONS.** This Delphi study achieved multi-professional consensus on factors contributing to dysphagia risk in CSCI allowing development of a screening tool. This will aid referral to Speech and Language Therapy for prompt and consistent diagnostic assessment and intervention in non-specialised units. A future multi-site pilot study of the tool is necessary to validate its utility and impact on patient outcomes.

**REFERENCES**
1.Shem, K., et al., *Dysphagia and respiratory care in individuals with tetraplegia: incidence, associated factors, and preventable complications.* Top Spinal Cord Inj Rehabil, 2012. **18**(1): p.15-22.

2.Shin, J.C., et al., *Dysphagia in cervical spinal cord injury*. Spinal Cord, 2011. **49**(9): p. 1008–13.
3.Smith, M., *Efficacy of specialist versus non-specialist management of spinal cord injury within the UK*. Spinal Cord 2002.**40**:p.11-16.

**GRANT ACKNOWLEDGMENT**
Jackie McRae was funded by a research fellowship from the National Institute of Health Research.

### 0645

#### Witnessing a paradigm shift: from curative to palliative care

F. DeKeyser Ganz[1], M. Ben Nun[2]
[1]Haddasah Hebrew University Faculty of Nursing, Jerusalem, Israel; [2]Rabin Medical Center, Petach Tikva, Israel
**Correspondence:** F. DeKeyser Ganz
*Intensive Care Medicine Experimental* 2017, **5(Suppl 2):**0645

**INTRODUCTION.** Between 10-20% of ICU patients die in the ICU or soon thereafter [1]; many after a decision to curtail curative treatment [2]. Palliative care is considered an optimal treatment paradigm when such decisions have been made [3]. However, studies have shown that there is a reluctance among critical care practitioners to embrace palliative care [4]. Efforts are being made around the world to inform physicians and nurses about the advantages of palliative care in the ICU [5]. Few studies have described the process of how such programs have changed caregivers' attitudes.
**OBJECTIVE.** To describe nurses' experiences of the process of transforming from a belief in only curative care to an integration of curative and palliative care in the ICU.
**METHODS.** Three cohorts of participants (n = 73) of a 112-hour, 6 month, Palliative Care in the ICU course were asked two questions, "What does palliative care mean to you?" and "How has palliative care been implemented on your unit?". Six Knowledge Cafes were conducted, at the start and end of each course. A Knowledge Cafe consisted of participants responding to each question in small groups and then each group sharing their responses with the entire cohort. Responses were recorded on paper for each small group and for the entire cohort, for all three cohorts. Responses were then reviewed independently by two reviewers and themes were identified.
**RESULTS.** At the beginning of the course, respondents reported what they were observing at their patients´ bedsides. They identified the need for palliative care (relief of pain, decreased suffering and dying with dignity) but were unable to deliver it. Six months later, three themes emerged related to the meaning of palliative care:

a. holistic care
b. effective and open communication within the staff and with families;
c. empowerment of participants to take an active role related to palliative care at the unit level.

At the beginning of the course, participants reported little or no palliative care delivery on their unit. Nurses recognized the need for palliative care but were unable to provide, consult or initiate such care. At the conclusion of the course, participants reported a slight change in the care environment, where nurses and physicians were more aware of palliative care; and communication with families and within the staff was greater. However, a third theme emerged, frustration, where nurses felt empowered to initiate palliative care measures but were unable due to the current healthcare environment.
**CONCLUSIONS.** Nurses can be empowered to become more active palliative care practitioners. However, constraints in the healthcare system can limit their ability to practice to the full scope of their training.

**REFERENCES**
1: INARC, 2012; Moran & Soloman, 2013
2: Curtis & Rubenfeld, 2006
3: Sprung et al., 2003
4:Cox & Curtis, 2016
5: Mosenthal et al., 2012

### 0646

#### Nursing interventions to increase sleep quality for ICU patients

A. C. van Wijk, S. Evelein, T. van Galen
VU University Medical Center, Department of Intensive Care, Amsterdam, Netherlands
**Correspondence:** A. C. van Wijk
*Intensive Care Medicine Experimental* 2017, **5(Suppl 2):**0646

**INTRODUCTION.** Although sleep is essential for physical and mental well-being, a Dutch study showed 48% of ICU patients having significant sleep problems[1]. Interrupted sleep is, in addition to pain, anxiety, and thirst, one of the most important stress factors for ICU patients[2]. For ICU patients, all stages of sleep are reduced and REM sleep is often absent. Because sleep is frequently interrupted, the patients´ sleep is superficial which can interfere with the recovery and enhance delirium onset[3].
Nightly noise contributes significantly to sleep disturbance[4.] Community Noise Guidelines[5] prescribe an average noise level of no more than 30 dB in hospitals where patients are treated. The maximum noise level may not be higher than 40 dB.
**OBJECTIVE.** How can we improve patients sleep using non-drug nursing interventions?
**METHODS.** The Pain-Agitation-Delirium (PAD) team created a nurse-nightly checklist for achieving noise reduction and improving patient comfort that can contribute to improve sleep quality. The implementation program contained increasing nurses´ awareness regarding sleep quality and potential risks, introducing the Nightly checklist and the RCSQ score.

| Patient related | Room related | Alarm/ machine related | Care related |
|---|---|---|---|
| Patient sleep habits | IV med/bags laid down | Perfusors/infusions changed earlier to reduce alarms | Quiet behind nurse station/corridor |
| Comfortable position | Sleeping signs in corridor | Turn down the volume | Strive maintain night/ day rhythm |
| Sleeping aids | Screen between patients closed | Turn alarm wide | After midnight staff only allowed in room when necessary |
| VAS score <5 | Door of room closed if savety permits, turn intercom on | | Do rounds every other hour by stable patients |
| Sleep medication | Low light, dark room | | Enter the delirium score through CAM-ICU/DOSS/RASS |
| | Move patient if necessary | | Enter the sleep score through RCSQ |

*[Nurse-Nightly checklist]*
After implementation, PAD team members did daily rounds to ensure the checklist has been done and answer questions.
**RESULTS.** For measuring noise levels the Digital Noise Meter Db Logger Noise Monitor were used. During 20 nights noise measurements were performed on fixed moments. RCSQ reporting was done by the shift nurses and doctors. The RCSQ score was evaluated daily besides regular items like PAD scores.

| | Before Nightly checklist | After Nightly checklist |
|---|---|---|
| Perfusor alarm | 76 Db (max. noise level) | 52 Db (max. noise level) |
| Ventilator alarm | 85 Db (max. noise level) | 67 Db (max. noise level) |
| Corridor noise level (23.00-06.00) | 65 Db (average) 100 Db (peak) | 48 Db (average) |
| Sleep score RCSQ (20 patients) | 4.8 (average) | 6.4 (average) |

*[Noise monitor data]*
**CONCLUSIONS.** After implementing the nightly checklist, patients reported better sleep quality, probably due to reduced noises during the night shift. Patients are less frequently disturbed by changing alarms and syringes or IV bags as anticipated on with the checklist. Nursing and other disciplines became more aware of noises during the night shift.
More investigation with concerns to improved sleep quality is needed. We want more focus on the relation between improved sleep and the effects on delirium onset and prevalence. We also need to investigate the effects of reducing ICU alarm levels on patient safety when noise level guidelines are abided.

1.(Kritiek, 12/2013, p.24-25)
2.Park, 2013 en Darbyshire, 2013
3.Verhagen, 2005 en Pugh, 2007
4.Sleep Sci. 2014 Dec; 7(4): 209–212..Environmental noise and sleep disturbances: A threat to health?Demian Halperin
5. (WHO, 1999)
6. Barr J. Clinical Practice Guidelines for the Management of Pain, Agitation, and Delirium in Adult Patients in ICU. Crit Care Med 2013; 41: 263–3067. Boelens AM, Izaks GI, Nurse Academy, jaargang 2, nummer 2, zomer 2010

### 0647

#### A pilot exploratory study to understand the prevalence, risk and fear of falling in individuals following critical illness

S. M. Parry[1], L. Denehy[2], Z. A. Puthucheary[3], P. E. Morris[4]
[1]The University of Melbourne, Department of Physiotherapy, Victoria, Australia; [2]The University of Melbourne, Department of Physiotherapy, Melbourne, Australia; [3]University College London Hospitals NHS Foundation Trust, Institute of Sports and Exercise Health, Division of Critical Care, London, United Kingdom; [4]University of Kentucky Healthcare, Division of Pulmonary, Critical Care and Sleep Medicine, Kentucky, United States
**Correspondence:** S. M. Parry
*Intensive Care Medicine Experimental* 2017, **5(Suppl 2):**0647

**INTRODUCTION.** Whilst impairments in strength and physical functioning are common for survivors of critical illness [1], no studies have examined whether these individuals have a heightened falls risk.

**OBJECTIVES.** In survivors of critical illness to explore:
1) falls risk and fear of falling over the first six months post discharge; and
2) the prevalence, time to first fall and severity of falls; and
3) the relationship between fear of falls and reported falls.

**METHODS.** Nested pilot study within a prior NIH-prospective study examining early rehabilitation [2]. Fear of falling was assessed using the Falls Efficacy Scale-International tool [3] and falls risk was assessed using the Falls Risk for Older People in the Community Setting [4]. Both measures were assessed at 2, 4, and 6-months post hospital discharge. The number of falls were monitored using a falls calendar, which were returned on a monthly basis for six months post discharge [5]. A fall was defined as an ´unexpected event in which the participant comes to rest on the ground, floor or lower level.´ [5] Further details about the nature of the fall, falls severity and medical care required were reported.

**RESULTS.** 11 participants were recruited. At 2-months post discharge, 55% (n = 6/11) had a high fear of falling and 27% (n = 3/11) had a high falls risk. At 6-months; 36% (n = 4/11) had both a high fear of falling and falls risk. In the first six months post hospital discharge, 55% (n = 6/11) of participants had a fall, with 36% (n = 4/11) falling more than twice in the six-month period. The number of falls over six months were moderately related to fear of falling at 2-months ($r = 0.68$, $p = 0.03$). Five out of six participants who fell had their first fall in the first 3 months post discharge and three had moderate severity injuries which required medical attention.

**CONCLUSIONS.** This pilot study suggests falls may be a common complication for survivors of critical illness with associated higher falls risk and fear of falling. Further research is required to understand the incidence of falls and the associated risk factors to minimise the morbidity associated with survivorship.

**REFERENCE(S)**

1. Latronico et al. (2017): The ICM research agenda on ICU-AW. ICM In Press;
2. Morris et al. (2016): Standardised rehabilitation and hospital length of stay amongst patients with acute respiratory failure: RCT JAMA 315 (24): 2694–2702; Delbaere et al. (2010): The FES-I. A comprehensive longitudinal validation study Age Ageing 39 (2): 210–216;
3. Russell et al. (2008): The reliability and predictive accuracy of the FROP-COM tool Age Ageing 37 (6): 634–639;
5. Lamb et al. (2005): Development of a common outcome data set for fall injury prevention trials: The PROFANE consensus J Am Geriatr Soci 53 (9): 1618–22.

**GRANT ACKNOWLEDGMENT**
P.M was in receipt of NIH - National Institute of Nursing Research and National Heart, Lung Blood Institute NIH 1R01NR011186-01 funding and SMP is funded by a NHMRC Early Career Fellowship.

### 0648

#### Utilization of restraints in critical care unit

R. Farshatov, V. Karan, T. Vildanov
Bashkir State Medical University, Ufa, Russian Federation
**Correspondence:** R. Farshatov
*Intensive Care Medicine Experimental* 2017, **5(Suppl 2):**0648

**INTRODUCTION.** In intensive care units, restraints are usually used either as an independent tool or in combination with sedatives to facilitate optimal environment for mandatory therapeutic procedures and diagnostic evaluations in patient care, to prevent adverse events leading to deleterious consequences such as patient removal of catheters, surgical drains and devices necessary for life support [1–3].

**OBJECTIVES.** To study the perspective of the healthcare professionals on the utilization of restraints in the intensive care unit.

**METHODS.** To implement the research objectives 116 respondents were surveyed. Respondents were divided into critical care providers (group A, n = 98) and other healthcare professionals (group B, n = 18).

**RESULTS.** Respondents considered restraints as the only way to "pacify" patients and to establish a safe environment for them(group A - 47.8% positive responses, in group B - 72.2%).When a patient is agitated or develops a delirious behaviour and interfere with the performance of procedures, 66.3% of the critical care providers prefer restraining interventions.Methods alternative to restraining therapies, are often used by 41.8% of critical care providers and 22.2% of other healthcare providers. Among the alternative methods, respondents consider the use of sedatives, analgesics, neuroleptic agents, neuromuscular blocking agents and several of other methods as beneficial..The healthcare providers surveyed are convinced that for the implementation of restraining interventions, obtaining formal informed consent of patient's legal guardian of surrogate is not required (group A- 83.7% affirmative responses, in group B - 83.3%). The healthcare professionals who considered consultation of a psychiatrist as a mandatory condition for utilization of restraining therapy (group A - 26.5%, in group B - 22.2%).Healthcare providers rarely encounter with complaints of patients or their guardians about the use of restraints (group A - 5.1%, in group B - 0%).Very few respondents believed that fixation had any adverse effects on the health of the patient (group A-9.2% of positive responses, in group B - 11.1%).

**CONCLUSIONS.** Thus, the perspective of the healthcare providers surveyed on the question about the utilization of restraining therapies was contradictory as most respondents recognize the need for restraints on ICU patients and consider it to be the only possible method.

**REFERENCE(S)**

1.Chang LY, Wang KW, Chao YF (2008) Influence of physical restraint on unplanned extubation of adult intensive care patients: a case–control study. Am J Crit Care 17:408–415.

2. Bray K, Hill K, Robson W, Leaver G, Walker N, O`Leary M, Delaney T, Walsh D, Gager M, Waterhouse C (2004) British Association of Critical Care Nurses position statement on the use o restraint in adult critical care units. Nurs Crit Care 9:199–212.

3. Martin B, Mathisen L (2005) Use of physical restraints in adult critical care: a bicultural study. Am J Crit Care 14:133–142

### 0649

#### Separator fluid volume requirements in multi-infusion settings

F. Doesburg[1], D. Middendorp[1], W. Bult[1,2], D. J. Touw[2,3], M. W. Nijsten[1]
[1]University of Groningen, University Medical Center Groningen, Department of Critical Care, Groningen, Netherlands; [2]University of Groningen, University Medical Center Groningen, Department of Clinical Pharmacy and Pharmacology, Groningen, Netherlands; [3]University of Groningen, University Medical Center Groningen, Department of Pharmacokinetics, Toxicology and Targeting, Groningen, Netherlands
**Correspondence:** F. Doesburg
*Intensive Care Medicine Experimental* 2017, **5(Suppl 2):**0649

**INTRODUCTION.** Intravenous (IV) therapy is a widely used method for the administration of medication in hospitals worldwide. ICU and surgical patients in particular often require multiple IV catheters due to incompatibility of certain drugs and the high complexity of medical therapy. This increases discomfort by painful invasive procedures, the risk of infections and costs of medication and disposable considerably. When different drugs are administered through the same lumen, it is common ICU practice to flush with a neutral fluid between the administration of two incompatible drugs in order to optimally use infusion lumens. An important constraint for delivering multiple incompatible drugs is the volume of separator fluid that is sufficient to safely separate them.

**OBJECTIVES.** In this pilot study we investigated whether the choice of separator fluid, solvent, or administration rate affects the separator volume required in a typical ICU infusion setting.

**METHODS.** A standard ICU IV line (2m, 2ml, 1mm internal diameter) was filled with methylene blue (40 mg/l) solution and flushed using an infusion pump with separator fluid. Independent variables were solvent for methylene blue (NaCl 0.9% vs. glucose 5%), separator fluid (NaCl 0.9% vs. glucose 5%), and administration rate (50, 100, or 200 ml/h). Samples were collected using a fraction collector until < 2% of the original drug concentration remained and were analyzed using spectrophotometry.

**RESULTS.** We did not find a significant effect of administration rate on separator fluid volume. However, NaCl/G5% (solvent/separator fluid) required significantly less separator fluid than NaCl/NaCl (3.6 ± 0.1 ml vs. 3.9 ± 0.1 ml, $p < 0.05$). Also, G5%/G5% required significantly less separator fluid than NaCl/NaCl (3.6 ± 0.1 ml vs. 3.9 ± 0.1 ml, $p < 0.05$). The significant decrease in required flushing volume might be due to differences in the viscosity of the solutions. However, mean differences were small and were most likely caused by human interactions with the fluid collection setup. The average required flushing volume is 3.7 ml.

**CONCLUSIONS.** The choice of separator fluid, solvent or administration rate had no impact on the required flushing volume in the experiment. Future research should take IV line length, diameter, volume and also drug solution volumes into account in order to provide a full account of variables affecting the required separator fluid volume.

**GRANT ACKNOWLEDGMENT**
This study was funded by Fresenius Kabi.

### 0650

#### Nurse driven experience of peripheral insertion central catheters (PICC) in an adult intensive care unit: prospective observational study

A. F. Forero, C. A. Santacruz Herrera, G. Garcia, J. C. Cavidades, E. Celis, M. Correal
Hospital Universitario Fundación Santa Fe de Bogota, Intensive Care, Bogota, Colombia
**Correspondence:** A. F. Forero
*Intensive Care Medicine Experimental* 2017, **5(Suppl 2):**0650

**INTRODUCTION.** Central peripheral insertion catheters (PICC) can remain from days to months allowing the administration of fluids, medications, and/or parenteral nutrition in critically ill patients. However, the best technique for PICC placement is still controversial.

**OBJECTIVE.** To describe the experience of nurse driven PICC placement in a cohort of adult ICU patients using dynamic ultrasound (D-US) guided compared to the anatomical landmark (LM) technique on the outcomes of number of attempts before successful cannulation, misplaced catheters and complication rates.

**MATERIALS AND METHODS.** 84 surgical ICU patients with PICCs inserted by ICU nurses were included from 2015 to 2017 (45 women and 39 men). Median age was 55 years [IQR 38.2 - 72]. The main indications for PICC placement was the initiation of vasoactive drugs and electrolyte replacement. The median number of days with a PICC catheter was 8 days [IQR 4–10.7]. Categorical data was reported as percentages and continuous data as median [IQR]. Comparisons between the two groups were done using generalize linear models. We used R language version 1.0.136 for all statistical analysis

**RESULTS.** The number of attempted punctures were lower using the D-US guided technique (estimate −0.8, 95% CI −1.1 to −0.5, $p < 0.001$). Fewer misplaced PICCs were found using the D-US technique (estimate −0.2, 95% CI −0.4 to −0.1; $p = 0.001$). Catheter-associated infections and peripheral vein thrombosis were significantly lower using the D-US technique (estimate −1.7, 95% CI −3.2 to −0.6, $p < 0.01$) as compared to the LM technique.

**CONCLUSION.** Dynamic US-guided PICC placement was associated with fewer attempts before successful central venous cannulation and complication rates. It may also be associated with less misplaced catheters. This study highlights the importance of maintaining a highly-trained nursing team to achieve optimal results.

### 0651

#### Alarm reduction in the ICU

W. Veugen, E. A. Icke
VU University Medical Center, ICU, Amsterdam, Netherlands
**Correspondence:** W. Veugen
*Intensive Care Medicine Experimental* 2017, **5(Suppl 2):**0651

**INTRODUCTION.** To monitor patients on an ICU, we extensively use various medical devices. These devices have the ability to generate alarms when abnormal values appear or when there is a malfunction. The majority of alarms in the ICU are false alarms[1] and are often induced by nurses during daily routine activities like arterial blood sampling or physical care. Therefore many alarms are nurse-driven and potentialy preventable. The risk of too many alarms is alarm-fatigue, which can lead to longer response times or missing important alarms[2][3][4].

**OBJECTIVES.** Can the number of alarms be reduced by promoting nurse awareness, a modified protocol and by training?

**METHODS.** Improving began with a baseline-measurement in which the average number of alarms per bed and day were measured over a 4 week period. Alarms were divided into high- or medium priority and technical alarms. The data was obtained from the Philips Central monitor database.

Introduced improvement measures consisted a modified alarm protocol with the primary goal to reduce self-induced alarms during nursing care by using the alarm-pause button more effective. The modified protocol also included redefined alarm limitations, in wich the nursing staff can ajust the alarms and when it´s necessary to consult a physician. The modified protocol was implemented by means of an educational and communication program. One month after implementation the 4 week follow-up measurement was repeated.

**RESULTS.** During the two measurement periods the occupancy rate on the 18 bed ICU department was 90 - 95% with no significant changes in the ICU casemix.

| | High priority | Medium priority | technical |
|---|---|---|---|
| Before inroduction | 20,4 | 217,9 | 5,8 |
| After introduction | 14,5 | 178,8 | 4,8 |

*[Average monitor alarms, per bed, per day]*

| High priority | Medium priority | Technical |
|---|---|---|
| 29 | 18 | 18 |

*[Alarm reduction in %]*

**CONCLUSIONS.** Introducing a modified alarm protocol and increasing the awareness regarding alarm-fatique, we achieved a substantial reduction in (false) alarms.

However we believe that there are still many false alarms and that there is room for further improvement. With these results, the ICU nursing staff can be motivated for a further reduction of alarms. Besides altering nursing workflow, exploring technical solutions[5] for preventing alarm-fatigue should be initiated by using alarm software and other technical solutions to improve ICU alarm management.

**REFERENCE(S)**

[1] Siebig S, Kuhls S, Imhoff M, et al.: Intensive care unit alarms—How many do we need? Crit Care Med 2010

[2] "Alarm Related Terms". Retrieved 18 October 2013.

[3] "The Joint Commission Sentinel Event Alert". Medical device alarm safety in
hospitals. The Patient Safety Advisory Group. Retrieved 21 October 2013.

[4] ECRI Institute. Top 10 health technology hazards for 2014.
Health Devices.

[5] Blum, JM; Tremper, KK (February 2010). "Alarms in the intensive care unit: too much of a good thing is dangerous: is it time to add some intelligence to alarms?". Critical Care Medicine.

**GRANT ACKNOWLEDGMENT**

None.

### 0652

#### Exploring the role of organizational factors to variations in incidence of delirium in ICU patients

P. Rood[1], G. Huisman-de Waal[2], H. Vermeulen[2], L. Schoonhoven[3], P. Pickkers[1], M. van den Boogaard[1]

[1]Radboud University Medical Center, Intensive Care, Nijmegen, Netherlands; [2]Radboud University Medical Center, Radboud Institute for Health Sciences, Nijmegen, Netherlands; [3]University of Southampton, Faculty of Health Sciences, Southampton, United Kingdom

**Correspondence:** P. Rood

*Intensive Care Medicine Experimental* 2017, **5(Suppl 2):**0652

**INTRODUCTION.** Delirium occurs frequently in ICU patients and is associated with numerous deleterious outcome. Early recognition of delirium is essential for treatment and improvement of ICU outcomes. Several studies on ICU delirium incidence have been published which reported a large variety on delirium incidence. Unknown is to what extend this variety exist and also it is unknown for what reason, other than patient characteristics, this wide variety can be explained.

**OBJECTIVES.** To systematically review the variety of reported ICU delirium incidences, and the role of organizational factors to delirium incidence in adult ICU's.

**METHODS.** A systematic review was undertaken following the Cochrane methodology searching for 'Critical Care', 'Delirium' and 'Incidence' and synonyms. Medline, Embase, Cinahl and Cochrane Library databases were searched till January 27$^{th}$, 2017. Studies were independently assessed for eligibility by two researchers (PR, MB), differences were settled by a third researcher (HV). Solely prospective cohort studies which primary observed delirium incidence were included. There was no language restriction. Methodological quality was assessed using the STROBE checklist. All available organisational factors were extracted from the included studies and analyzed using meta-regression analysis.

**RESULTS.** A total of 9,357 studies were found, of which nineteen studies met the inclusion criteria. All included studies reported at least ICU delirium incidence. The overall good quality (median 32/38 points [IQR 30–35]) prospective cohort studies published between 2005 and 2016, and originated from 17 countries. A total of 11,965 ICU patients were included, which originated from medical, surgical and mixed populations. In total five different delirium assessment methods were used. Incidence rates varied from 4-55%. The median ICU delirium incidence is 26% [IQR 21–41]. Location, hospital type and admission type, screening frequency and executive screener did not significantly influence the variance found. Variations in research methodology might have influenced the incidence found.

**CONCLUSIONS.** The reported ICU delirium incidence varies largely. This variety cannot be explained by the organisational factors which we could extract. It may be more plausible that differences in patients characteristics and treatment differences are responsible for this variety in reported ICU delirium incidences.

### 0653

#### Five-year survival and causes of death of children after intensive care

E. Kyösti[1], J. H. Liisanantti[1], O. Peltoniemi[2], P. Ohtonen[3], T. I. Ala-Kokko[4]

[1]Oulu University Hospital, Division of Intensive Care Medicine, Oulu, Finland; [2]Oulu University Hospital, Pediatrics, Oulu, Finland; [3]Oulu University Hospital, Division of Operative Care, Oys, Finland; [4]Oulu University Hospital, Division of Intensive Care Medicine, Oys, Finland

**Correspondence:** E. Kyösti

*Intensive Care Medicine Experimental* 2017, **5(Suppl 2):**0653

**INTRODUCTION.** Recent short term mortality in children admitted to an intensive care unit (ICU) has varied from 1.3 to 3.9%. (1, 2) Studies of long-term outcome after an admission to ICU are scarce. An earlier study comparing outcomes following ICU discharge with non-ICU admitted age matched control population found a 20-fold increase in mortality after five years follow-up. (3) Studies of long-term outcome following ICU discharge are needed to understand the effectiveness of intensive care and long-term sequelae of critical illness.

**OBJECTIVES.** To compare long-term mortality after ICU discharge and causes of deaths in children with normal population.

**METHODS.** The mortality and causes of deaths following ICU discharge in all Finnish children admitted to ICU during years 2009 and 2010 were analyzed retrospectively. The study population was followed-up to the end of year 2014. The causes of death in patients alive following 30 days after ICU discharge were compared to a cohort of one million Finnish children of the same age.

**RESULTS.** Totally 2792 children were admitted to the ICU during the study period. Of those, 53 children died during ICU stay, and 2739 were discharged alive. Thirteen children died within 30 days of discharge and 68 children between 30 days and the end of the follow-up. In the control population, there were totally 1037 deaths (0.10%) between years 2009 and 2014. Standardized mortality rate (SMR) for the ICU admitted children for five years was 53.4 (95% CI 44.7 to 63.2). One year after the discharge the SMR was 16.7 (12.1-22.6). The most common causes of death in post ICU 30 day survivors were neoplasms (35.3%), neurological (17.6%) and metabolic diseases (11.7%) when in the control group they were traumas (45.3%), neoplasms (12.7%) and neurological diseases (9.5%).

**CONCLUSIONS.** There was more than 50-fold mortality in a cohort of ICU-admitted children compared to Finnish children at the same age and the difference was nearly 20-fold one year of ICU discharge.

Medical causes of death dominated in post 30 day ICU survivors´ group and trauma deaths in control group.

**GRANT ACKNOWLEDGMENT**
Supported by Finnish Foundation for Pediatric Research.

### 0654

#### Implementation of nutrition support guidelines in children receiving ECMO

T. J. Velasquez[1], G. Mackey[2], A. Marroquin[2], P. Bastero[2], M. A. Musick[2], R. A. Orellana[2], C. Gazzaneo[2], J. Thomas[2], L. S. Shekerdemian[2], J. A. Coss-Bu[2]
[1]Texas Children's Hospital, Clinical Nutrition, Houston, United States; [2]Texas Children's Hospital / Baylor College of Medicine, Department of Pediatrics, Houston, Texas, United States
**Correspondence:** J. A. Coss-Bu
*Intensive Care Medicine Experimental* 2017, **5(Suppl 2):**0654

**INTRODUCTION.** Extracorporeal Membrane Oxygenation (ECMO) is commonly used to support critically ill children with life threatening respiratory or cardiac failure. Adequate nutrition support for children on ECMO should be based on their metabolism, metabolic reserves, and nutritional needs.

**OBJECTIVES.** The study aim was to assess nutritional support before and after implementation of nutrition support guidelines (NSG) in critically ill children receiving ECMO.

**METHODS.** Records of children on ECMO (2/11-6/16) were reviewed. Data collected: type of ECMO: venous-arterial (VA), venous-venous (VV), oxygenation index (OI) and vasoactive-inotropic score (VIS). Nutritional status was assessed by weight for age (WFA: underweight), weight for height (WFH: acute malnutrition), height for age (HFA: chronic malnutrition) z-scores and caloric (CI) and protein (PRO) intakes recorded after initiation of ECMO. Energy and protein needs estimated by Schofield and ASPEN, respectively. Nutrition support was compared before and after implementation of NSG. Values are mean ± SE.

**RESULTS.** Fifty-six patients (30 males) included; median (IQR 25$^{th}$-75$^{th}$) age was 8.1 (2–15) yrs, weight, 27 (11–45) kg, and height, 124.5 (84–155) cm. Forty-four and 12 patients received VV and VA ECMO, respectively; OI and VIS scores at time of initiation of ECMO were 37 ± 3 and 18 ± 3, respectively; ICU length of stay (LOS): 21 (7–43) days; Hospital LOS: 33 (18–71) days; ECMO duration: 9 (4–20) days; WFA, HFA, and WFH/BMI z scores of, −0.88 ± 0.36, −0.97 ± 0.38, and −0.13 ± 0.22, respectively. The prevalence of undernutrition, acute and chronic malnutrition was 45%, 29%, and 45%, respectively; albumin was 2.89 ± 0.14 mg/dl at time of initiation of ECLS. Patients with acute malnutrition (n = 16) vs. no malnutrition (n = 40) had a ICU LOS, 43 (21–54) vs. 15 (6–30) days, ($p < 0.01$); Hospital LOS, 59 (33–82) vs. 28 (9–59) days, ($p < 0.05$); and duration of ECMO; 19 (9–31) vs. 7 (3–14) days, ($p < 0.01$); average CI and PRO intake on days 1–4 of 34 ± 4 kcal/kg/d vs. 19 ± 2 kcal/kg/d ($p < 0.005$) and 2.01 ± 0.3 g/kg/d vs. 1.21 ± 0.2 g/kg/d, ($p < 0.05$), respectively. The proportion of patients receiving enteral feeds on days 1,3, 5, and 7 of ECMO were: 14.2%, 44.6%, 57.5%, and 62.5%, respectively; (Day 1 vs. all, $p < 0.005$).

| | Day 1 | | Day 3 | | Day 5 | | Day 7 | |
|---|---|---|---|---|---|---|---|---|
| | Before n = 28 | After n = 28 | Before n = 28 | After n = 28 | Before n = 28 | After n = 28 | Before n = 17 | After n = 23 |
| CI kcal/kg/day | 14 ± 3 | 18 ± 3 | 23 ± 4 | 34 ± 4a | 16 ± 3 | 31 ± 4a | 26 ± 4 | 38 ± 4 |
| Needs met (%) | 34 ± 6b | 38 ± 5b | 56 ± 7b | 72 ± 7b | 64 ± 7b | 81 ± 5a,b | 62 ± 8b | 80 ± 5a,b |
| PRO g/kg/day | 0.72 ± 0.2 | 0.98 ± 0.2a | 1.4 ± 0.2 | 2.4 ± 0.3a | 1.1 ± 0.2 | 2.1 ± 0.2a | 1.9 ± 0.3 | 2.6 ± 0.2a |
| Needs met (%) | 31 ± 8c | 37 ± 8c | 63 ± 9c | 95 ± 10a | 81 ± 8c | 106 ± 7a | 85 ± 9 | 107 ± 7 |

a $p < 0.05$ vs. before implementation of NSG, by Mann–Whitney test; b $p < 0.005$ vs. estimated caloric needs; c $p < 0.05$ vs. estimated protein needs, by paired t-test.

*[Nutrition Support]*

**CONCLUSIONS.** Almost half of the children were malnourished at time of ECMO initiation. Patients with acute malnutrition had longer PICU and hospital length of stay and duration of ECMO. Malnourished patients received better nutrition early after initiation of ECMO support and two-thirds of the patients were receiving enteral feeds by day seven. Implementation of NSG improved nutrition intake in this population of critically ill children.

**REFERENCE(S)**

1) Jaksic T, et al. J Parenter Enteral Nutr. 2010 May-Jun;34(3):247–53.2) Kyle UG, et al. J Acad Nutr Diet. 2016 May;116(5):844–851

**GRANT ACKNOWLEDGMENT**
Internal Funding Baylor College of Medicine/Texas Children´s Hospital.

### 0655

#### Total exchange transfusion in critically ill children with severe pertussis

S. Carmona-González[1], C. Ramos[1,2], J. C. Silva[3], V. Abrego-Moya[2], G. M. Vargas-Duarte[1], O. Valencia-Urrea[1], R. Treviño-Perez[1], H. Tcharmtchi[3], S. Thammasitboon[3], J. A. Coss-Bu[3]
[1]Escuela de Medicina y Ciencias de la Salud, Tecnológico de Monterrey, Departamento de Pediatría, Monterrey, Mexico; [2]Hospital Regional Materno Infantil de Alta Especialidad (HRMIAE), Departamento de Pediatría, Monterrey, Mexico; [3]Texas Children's Hospital / Baylor College of Medicine, Department of Pediatrics, Houston, Texas, United States
**Correspondence:** J. A. Coss-Bu
*Intensive Care Medicine Experimental* 2017, **5(Suppl 2):**0655

**INTRODUCTION.** Bordetella pertussis infection is a common, under recognized, and vaccine-preventable cause of critical illness with a high mortality in infants worldwide. The syndrome of hyperviscosity and arteriolar thrombosis is responsible for the development of complications including refractory hypoxemia, pulmonary hypertension and cardiocirculatory collapse. Hyperleukocytosis is a severe form of the disease with up to 80% mortality rate.

**OBJECTIVES.** This study aimed to determine clinical outcomes of exchange transfusion in infants with severe pertussis.

**METHODS.** Medical charts of patients of patients < 1 year admitted to the Pediatric Intensive Care Unit (PICU) of HRMIAE with clinical diagnosis of severe pertussis over a 5-year (01/2010-07/2015) period were reviewed.

**RESULTS.** Sixty-seven infants (M/F:31/36), age 2 (1–2) months (median, IQR), and PICU length of stay (LOS) and mechanical ventilation (MV) days of 6 (3–13) and 3 (0–7) days, respectively. White blood count (WBC) on admission was 35,890/mm3 (20,940-52,870). Forty-eight patients received supportive treatment (ST) and 19 patients had exchange transfusion (ET). Patients in ET group had higher WBC. Overall mortality was 19.4%. Admission WBC >35,000/mm3 was associated with mortality (unadjusted OR 7.4, 95%CI 1.5-36.7), and was an independent predictor of mortality (OR 8.6, 95%CI 1.4-51.4) adjusted for age, prematurity, MV and inotrope requirement. No mortality difference between ET and ST.

| | Age months | Bordetella CX (+) n(%) | WBC Count/ mm3 | Inotropic use n(%) | PICU LOS days | MV days | Mortality n (%) |
|---|---|---|---|---|---|---|---|
| ST n = 48 | 1 (1–2.0) | 24 (50) | 29,875 | 22 (45) | 5 (3–10.5) | 3 (0–6) | 8 (16) |
| ET n =19 | 1 (1–2.8) | 11 (57) | 55,200* | 14 (73) | 10 (6–19.8)* | 6 (2.3-9.8)* | 5 (26) |

Values are median (25th-75th IQR); * $p < 0.05$ by Mann–Whitney

*[Clinical Outcomes]*

**CONCLUSIONS.** Critically ill infants with severe pertussis had significant mortality and exchange transfusion had no effects on survival. Patients who received exchange transfusion had increased PICU length of stay and duration of mechanical ventilation. Admission white blood count was a strong predictor of mortality. Additional studies are required to identify exchange transfusion candidates according to admission white blood count.

**REFERENCE(S)**

1) Kuperman A, et al. Transfusion. 2014 54(6):1630–3.
2) Rowlands HE, et al. Pediatrics. 2010 126(4):e816-27.

**GRANT ACKNOWLEDGMENT**

Internal Funding Baylor College of Medicine/Texas Children´s Hospital.

### 0656

### Mobilising within the paediatric intensive care unit - a service evaluation

L. McGibbon, B. Johnstone, J. Hills, K. Sharp
National Health Service Scotland - Royal Hospital for Children Glasgow, Physiotherapy Royal Hospital for Children Glasgow, Glasgow, United Kingdom
**Correspondence:** L. McGibbon
*Intensive Care Medicine Experimental* 2017, **5(Suppl 2):**0656

**INTRODUCTION.** Early mobilisation within the adult critical care setting has been found to be beneficial. Benefits reported include; reduced length of stay, improving quality of life, decreased days on ventilator and prevention of intensive care acquired weakness[1]. In paediatric critical care, early mobilisation is in its infancy[2].

**AIMS.** The aim of this project was to ascertain current mobilisation practices within a paediatric intensive care unit (PICU) and identify potential barriers to mobilisation.

**METHODS.** An eight week prospective service evaluation was carried out. The Manchester Mobility Scale[3] was utilised following adaptation to suit our paediatric population. Data was collected by a physiotherapist, Monday - Friday at 4pm. Data collected included; ventilation status and maximum mobilisation achieved during that day. In addition, if the child did not take part in any activity, the reason was discussed with nursing staff.

**RESULTS.** 92 patient admissions (age range 1 month - 17 years mean, 3.96 years) were observed during the data collection period accounting for 529 PICU bed days.

At least one mode of mobilising (passive movements to independent mobility) was recorded during 58 (63%) of the admissions however 34 (37%) patients did not mobilise at all during their PICU stay. The three most common modes of mobilising were; Lift/hoist in to chair (33.5%), 'up for cuddles' (21.8%) and 'mat play'(16%).

Early mobilisation (during first 5 days of admission) occurred for patients intubated on 5 out of 83 days (6%) and for patients non-intubated on 44 out of 69 days (64%). Patients who were intubated mobilised on 93 of the 253 days (36.8%) and patients who were non-intubated mobilised 216 of the 276 days (79.7%).

The most common reason for not mobilising was the patient was perceived to be 'too unwell'.

**CONCLUSIONS.** Patients were mobilised during their PICU admission with some evidence of early mobility. However, intubated patients were less likely to mobilise than non-intubated patients particularly in the earlier stage of their admission. Guidelines and education for staff may help to increase opportunities for all children within PICU to experience the benefits of early mobilisation.

Further research is required within the paediatric population to develop greater knowledge in this field.

### 0657

### Unresolved problems about determination of brain arrest in pediatric patients in Japan

T. Ishihara, Y. Nagai, T. Sueishi, M. Motomura, I. Watanabe, O. Saito, T. Nitsu, N. Shimizu[1]
Tokyo Metropolitan Children's Medical Center, Emergency and Critical Care Medicine, Fuchu, Japan
**Correspondence:** T. Ishihara
*Intensive Care Medicine Experimental* 2017, **5(Suppl 2):**0657

**INTRODUCTION.** In 1985, criteria for brain arrest excluded children under 6 years of age was indicated but transplantation from the brain arrest donor was not permitted. The Japanese Organ Transplantation Law was established in 1997 and transplantation under brain arrest could be done only in adult. Although in 2010, organ transplantation has been permitted from under 15 years of age after Japanese Organ Transplantation Law was revised. Unfortunately, only 12 cases have been transplanted from brain arrest children until 2016.

**OBJECTIVES.** To identify the unresolved problems about determination of brain arrest from the survey of brain arrest at Tokyo Metropolitan Children´s Center is objective.

**METHODS.** Pediatric patients who admitted PICU of Tokyo Metropolitan Children's Center and were suspected of brain arrest due to deep coma, dilatation of pupil, loss of brain stem reflex and loss of spontaneous breathing during 2010.3 to 2016.12 were included this study. Clinical records were studied retrospectively.

**RESULTS.** During study period, 4,973 patients admitted to PICU and the number of patients who were thought to be brain arrest were thirty-one. Twenty-six cases were excluded due to various reasons. Eleven patients were dead due to collapse of circulation before determining the brain arrest. Six cases couldn't be denied for child abuse, due to no witness of out-of-hospital cardiac arrest. Three cases couldn't be assessed due to luck of system for estimation about brain arrest. Two cases were excluded because of the age, they were under 12 weeks after birth. There were another reasons such as social problem, findings of electroencephalogram, family's wish of continue for treating and severe infection. Five cases were thought to be a brain arrest if they were estimated following the law. There was no one who was diagnosed as a brain arrest by law, it was because that their families wished the palliative care or rejected transplantation from their child.

**CONCLUSIONS.** In our country, number of brain arrest pediatric patients estimated by law is very law. According to that, there are few reports about the epidemiology of brain arrest in children. It accounts for only 3% of all the transplantations from brain arrest. In our country, transplantation from the victim of child abuse is not permitted, and difficulty of denying the child abuse makes it difficult to proceed to estimation for brain arrest in children. Insufficient grief care to family about end-of-life is also the main reason for difficulty of proceeding the evaluation of brain arrest by law.

### Acute heart failure and myocardial ischemia

#### 0658

**Effect of levosimendan on the short-term clinical course of cardiomyopathic patients after cardiopulmonary bypass surgery**

M. M. Alnajjar[1], W. A. Abokhudair[2], M. F. Rajab[1], H. M. Albuti[3], M. M. Shennawy[4], A. A. Badawy[1]
[1]King Fahd Armed Forces Hospital, Jeddah, Cardiac Care Unit, Cardiac Services Department, Jeddah, Saudi Arabia; [2]King Fahd Armed Forces Hospital, Jeddah, Head of Adult Cardiac Surgery Department, Jeddah, Saudi Arabia; [3]King Fahd Armed Forces Hospital, Jeddah, Clinical Pharmacy Department, Jeddah, Saudi Arabia; [4]King Fahd Armed Forces Hospital, Jeddah, Cardiac Anaesthesia Unit, Cardiac Services Department, Jeddah, Saudi Arabia
**Correspondence:** M. M. Alnajjar
*Intensive Care Medicine Experimental* 2017, **5(Suppl 2):**0658

**INTRODUCTION.** Levosimendan is an inodilator indicated for the short-term treatment of acutely decompensated severe chronic heart failure, and in situations where conventional therapy is not considered adequate. Levosimendan improves cardiac function by a novel mechanism of action compared to currently available drugs. We hypothesized that, in patients with severely compromised ventricular function, the use of levosimendan would be associated with better postoperative cardiac function than with inotropic drugs that increase myocardial oxygen consumption.
**OBJECTIVES.** To evaluate the efficacy and safety of levosimendan, given intravenously to cardiomyopathic patients during and after cardiopulmonary bypass surgery
**METHODS.** 250 patients with a preoperative ejection fraction ≤30% scheduled for elective cardiac surgery with cardiopulmonary bypass were subjected to two different inotropic protocols: group A (levosemindan goup) levosimendan started immediately after the release of the aortic cross clamp by loading dose 6 μg / kg administered in 10 minutes then 0.1 mg /kg/ min continuous intravenous infusion in addition to our standard protocol started immediately after finishing the loading dose and maintained for 48 hours infusion. And group B (usual protocol group) received our standard protocol for these cases milrinone ± dobutamine and noradrenalin, adrenalin and dopamine according to blood pressure. The treatment was masked to the observers.
We assessed Haemodynamics of patients using Flotrac, Invasive arterial pressure monitoring, blood lactate level in ABG samples.
**RESULTS.** Cardiac Index was similar between groups initially after surgery, but it declined 12 h after surgery in the usual inotropic group but not in the levosemindan group ($P < 0.05$ between groups) despite similar filling pressures. Total dose, duration of inotropic drug administration and norepinephrine dose were lower in the levosimendan group than in the usual inotropic group ($P < 0.05$). The duration of tracheal intubation and Intensive care length of stay were shorter in the Levosemindan group compared with the group B (P < 005). Three patients in the group B died within 30 days of surgery but only one in levosemindan group.
**CONCLUSIONS.** In cardiac surgery patients with a low preoperative ejection fraction, Cardiac index and stroke volume was better maintained when adding levosimendan to our inotropic protocol. Also, ICU LOS was decreased with no increased risk of adverse Cardiovascular events.
**REFERENCE(S)**
1. Toller WG, Stranz C. Levosimendan, a new inotropic and vasodilator agent. Anesthesiology 2006;104:556–69.
2. Raja SG, Rayen BS. Levosimendan in cardiac surgery: current best available evidence. Ann Thorac Surg 2006;81:1536–46.
3. Toller W, Heringlake M, Guarracino F, et al. Preoperative and perioperative use of levosimendan in cardiac surgery: European expert opinion. International Journal of Cardiology 184 (2015) 323–336.

#### 0659

**Growth differentiation factor (GDF)-15 is associated with mortality in patients with severe acute heart failure or cardiogenic shock**

M. Lenz[1,2], K. Krychtiuk[1,2], S. Kastl[1], J. Wojta[1,2,3], G. Heinz[1], W. Speidl[1]
[1]Medical University of Vienna, Department of Internal Medicine II, Vienna, Austria; [2]Ludwig Boltzmann Cluster for Cardiovascular Research, Vienna, Austria; [3]Core Facilities, Medical University of Vienna, Vienna, Austria
**Correspondence:** M. Lenz
*Intensive Care Medicine Experimental* 2017, **5(Suppl 2):**0659

**INTRODUCTION.** Growth differentiation factor (GDF)-15 levels are associated with all-cause mortality in patients with acute coronary syndromes, however data in patients with acute heart failure are conflicting.[1]
**OBJECTIVES.** The aim of this study was to investigate the predictive value of GDF-15 in patients with severe acute heart failure (AHF) or cardiogenic shock requiring the admission to an intensive care unit.
**METHODS.** We included 90 consecutive patients with AHF or cardiogenic shock admitted to a cardiac ICU. GDF-15 was measured at admission using ELISA and patients were followed for 30 days.
**RESULTS.** Mean age of the patients was 62.1 ± 16.0 with a male to female ratio of 76.7% to 23.3%. Median NT-proBNP levels were markedly increased (median: 4986, IQR 1525–23842 pg/mL). 30-day mortality was 35.6%. In the group of non-survivors, GDF-15 was significantly increased (median: 7119.5, IQR = 3816.2 - 10168.2 ng/mL) as compared to the group of survivors (median: 2719.7, IQR 1472.9 - 7099.9 ng/mL), with a p-value < 0.001. Patients within the third tertile of GDF-15 had a 5.1-fold increased risk of mortality ($p < 0.005$), independently of demographics, NT-proBNP and vasopressor usage. Interestingly, GDF-15 and NT-proBNP showed additive prognostic value. When patients were stratified according to the median of NT-proBNP and GDF-15, those with both GDF-15 and NT-proBNP levels above the median had the highest risk of dying (HR 5.8, $p < 0.005$).
**CONCLUSIONS.** GDF-15 is a strong predictor of mortality in patients with severe acute heart failure or cardiogenic shock requiring the admission to an intensive care unit. Furthermore, it adds additional prognostic value to NT-proBNP levels.
**REFERENCE(S)**
[1] Hagström E, James SK, Bertilsson M. Growth differentiation factor-15 level predicts major bleeding and cardiovascular events in patients with acute coronary syndromes: results from the PLATO study. Eur Heart J. 2016 Apr 21;37(16)

#### 0660

**Mortality after weaning from extracorporeal emembrane oxygenation**

H. J. Lee, S. Park, E. R. Kim, Y. T. Kim
Seoul National University Hospital and Seoul National University College of Medicine, Department of Thoracic and Cardiovascular Surgery, Seoul, Korea, Republic of
**Correspondence:** H. J. Lee
*Intensive Care Medicine Experimental* 2017, **5(Suppl 2):**0660

**INTRODUCTION.** Extracorporeal membrane oxygenation (ECMO) is an important rescue method for patients with cardiac or respiratory failure regardless of underlying diseases. With the development of instruments and skills, we can apply ECMO with lower complications and a higher survival rate. However, several patients detoriate after weaning from ECMO.
**OBJECTIVES.** This study aims to evaluate the survival after weaning from ECMO.
**METHODS.** We retrospectively reviewed clinical records of patients who underwent ECMO from 2014 to 2016 in a tertiary university hospital. According to the indications, ECMO support was classified as respiratory, cardiac, and ECMO cardiopulmonary resuscitation (ECPR).

**RESULTS.** ECMO was applied to 172 adult patients. Respiratory support was 46 (26.7%), cardiac support was 70 (40.7%), and ECPR was 56 (32.6%). The mean duration of ECMO support was 7.9 ± 12.0 days. Successful weaning from ECMO occurred in 88 (51.2%) (respiratory 23 (50.0%), cardiac 43 (61.4%), and ECPR 22 (39.3%), respectively). Survived to discharge were 65 (37.8%) (respiratory 19 (41.3%), cardiac 31 (44.3%), and ECPR 15 (26.8%)). After weaning from ECMO, 23 patients (26.1%) died in hospital and 4 patients (4.5%) died after discharge from hospital. The cause of death in hospital after weaning were heart failure (n = 5), infection (n = 16, 14 patients with pneumonia, 6 with blood stream infection, and 5 from gastrointestinal origin), and neurological dysfunction (n = 2). In the multivariable logistic regression, higher age and longer duration of mechanical ventilator support were associated with mortality after ECMO weaning.

**CONCLUSIONS.** Mortality after weaning from ECMO was 30.7%. Most of patients deteriorated with infectious complications after ECMO removal rather than with primary causes of respiratory or cardiac failure.

**REFERENCE(S)**

1. Schmidt M, Bailey M, Sheldrake J, et al. Predicting survival after extracorporeal memebrane oxygenation for severe acute respiratory failure. Am J Resp Crit Care Med 2014;189:1374–82.

2. Schmidt M, Burrell A, Roberts L, et al. Predicting survival after ECMO for refractory cardiogenic shock: the survival after veno-arterial-ECMO (SAVE)-score. Eur Heart J 2015;36:2246–56.

**GRANT ACKNOWLEDGMENT**

Authors have nothing to disclose.

### 0661

#### Mini-invasive mechanical circulatory support for cardiogenic shock or refractory cardiac arrest: long-term outcomes from a prospective study

P. Ostadal, A. Kruger, D. Vondrakova, M. Janotka, J. Naar, M. Mates, P. Kmonicek, D. Doubek, P. Plasil, P. Jehlicka, P. Neuzil, I. Skalsky

Na Homolce Hospital, Prague, Czech Republic

**Correspondence:** P. Ostadal

*Intensive Care Medicine Experimental* 2017, **5(Suppl 2):**0661

**INTRODUCTION.** Mini-invasive mechanical circulatory support (MCS) systems are increasingly used in patients suffering from severe or rapidly progressing cardiogenic shock and from refractory cardiac arrest. However, current evidence on long-term outcomes of this therapeutic approach is still insufficient.

**OBJECTIVES.** The aim of our study was to assess one-year mortality in patients treated with MCS for severe circulatory collapse.

**METHODS.** We analyzed data from a group of patients treated with mini-invasive MCS in our center from March 2008 until March 2016. One-year survival data were obtained from all subjects. Mortality of patients with severe or rapidly progressing cardiogenic shock (CS group), subjects with refractory cardiac arrest treated by extracorporeal cardiopulmonary resuscitation (ECPR group) and patients with non-urgent MCS insertions for support of high-risk interventions (NU group) was compared using log-rank test.

**RESULTS.** One-hundred-and-twenty-nine individuals were enrolled into the study. TandemHeart system was used in 16 patients, PulseCath in 14 subjects, Impella 2.5 in 2 individuals and extracorporeal membrane oxygenation in veno-arterial configuration (VA-ECMO) in 111 patients. One-year mortality in the CS group (N = 87) was 40.2%, in the ECPR group (N = 33) 75.8% and in the NU group (N = 23) 8.7%, P < 0.001. In a subgroup of VA-ECMO treated patients, on-year mortality in CS individuals (N = 71) reached 43.7%, mortality of ECPR (N = 33) was 75.8% and mortality in NU subjects (N = 7) was 14.3%, P < 0.001.

**CONCLUSIONS.** Mini-invasive MCS represents often last chance to survive severe circulatory collapse. The highest survival rate was found in patients with non-urgent MCS use, followed by CS patients and the worst survival was observed in the ECPR group. Nevertheless, our data indicate that this therapeutic option offers considerable long-term survival in these patients with extremely high predicted mortality.

**GRANT ACKNOWLEDGMENT**

The study was supported by an Institutional grant MH CZ - DRO (Nemocnice Na Homolce - NNH, 00023884), IG150501

### 0662

#### Veno-arterial extracorporeal membrane oxygenation for refractory cardiogenic shock: very precocious lactate-clearance as prognostic determinant

D. F. Ottolina, T. Fossali, A. Perotti, R. Colombo, A. Corona, B. Borghi, P. Bergomi, E. Ballone, R. Rech, A. Castelli, E. Catena[1]

ASST Fatebenefratelli Sacco, Anestesia Rianimazione i Ospedale Sacco, Milano, Italy

**Correspondence:** D. F. Ottolina

*Intensive Care Medicine Experimental* 2017, **5(Suppl 2):**0662

**INTRODUCTION.** Cardiogenic shock (CS) is a life-threatening complication of several different conditions, as acute myocardial infarction, fulminant myocarditis, incessant arrhythmia, leading to cardiocirculatory shock with a insufficient peripheral perfusion and multi-organ dysfunction syndrome. In those cases veno-arterial extracorporeal membrane oxygenation (VA-ECMO) is used in order to maintain an adequate peripheral perfusion putting myocardial at rest and as a bridge-to-diagnosis/bridge-to-treatment. Several studies have examined possible factors predicting survival in patients under VA-ECMO support, and several score have been created. Until now there are not precocious prognostic determinant for non post-cardiotomy cardiogenic shock (1).

**OBJECTIVES.** To assess the presence and the trend of possible biochemical markers that correlate with survival.

**METHODS.** Among all VA-ECMO performed in an Italian ECMO-tertary single-center hospital from 2014 to April 2017, we have considered those used for support to CS, caused by different pathologies (Table 168).

VA-ECMO was performed in a standardized protocol, maintaining 36° C of internal body temperature, positioning intra-aortic balloon pump and inotropic support with epinephrine. An electronic-dedicated database was created to prospectively collect clinical and pathophysiological variables.

**RESULTS.** We have analyzed data from 17 VA-ECMO. The overall ICU-survival was 59% while 6-months survival was 50% (Table 168), with a CPC score 1–2 at 3 months of 100%. The mean VA-ECMO duration was 154 [79–336] *vs* 43 [16–165] hours (median, 25th-75th perc.) respectively in survivors and deceased patients. We observed that in survivors arterial lactate had a very fast clearance, with a significant decrease already at twelfth hour, while in non-survivors lactate had not a variation (Fig. 229). This observation was also applied in relation to weaning from VA-ECMO (lactate values: 6,6 ± 4,4 *vs* 2,9 ± 2,3 p < 0.05, at start and at twelfth hour respectively).

**CONCLUSIONS.** This preliminary study for the first time suggests that lactate clearance already 12 hours after VA-ECMO implantation could be a prognostic factor in patients with cardiogenic shock from different etiologies, highlighting the importance of early recovery of vital organs perfusion in a framework of effective systemic disfunction.

**REFERENCE(S)**

[1]Venoarterial Extracorporeal Membrane Oxygenation for Acute Fulminant Myocarditis in Adult Patients: a 5-year-multi-institutional experience. Lorusso R, Centofanti P, Gelsomino S, et al. Ann Thorac Surg 2016; 101; 919–26.

**Table 168 (Abstract 0662)**. See text for description

| | N° | ICU-Survival (%) |
|---|---|---|
| *Total Cardiogenic shock* | 17 | 59% |
| Cardiogenic shock after cardiac arrest (CA) | 6 | 50% |
| Cardiogenic shock without CA | 11 | 64% |
| *Etiologies* | | |
| Acute myocardial infarction | 7 | 43% |
| Acute myocarditis | 7 | 57% |
| Capillary leak syndrome | 1 | 100% |
| Malignant arrhythmia | 2 | 100% |

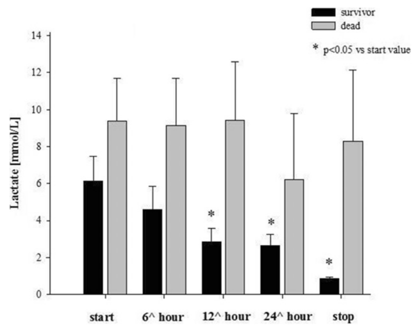

**Fig. 229 (Abstract 0662)** See text for description

### 0663

#### False positive ST elevation in a primary PCI program in ICU

O. Moreno Romero, M. Muñoz Garach, M. E. Poyatos Aguilera, A. Carranza Pinel, M. Colmenero Ruiz
Complejo Hospitalario de Granada, Granada, Spain
**Correspondence:** O. Moreno Romero
*Intensive Care Medicine Experimental* 2017, **5(Suppl 2):**0663

**INTRODUCTION.** Is well known the importance of correct and rapid diagnosis of ST-segment elevation myocardial infarction (STEMI) for the appropriate management of patients, particularly since primary percutaneous coronary intervention (pPCI) programs have been established. This is why it becomes remarkably important to minimize false positive events. In our city, Granada, a coaching and training course directed to emergency department physicians and extra-hospitalary emergency units was given before starting the program and in the first 3 months of it, to improve the electrocardiographic reading, fast diagnosis and STEMI activation code for a primary revascularization.
**OBJECTIVES.** First, to analyze the prevalence, epidemiology and etiologies of false-positive diagnosis of STEMI who underwent pPCI. Secondly, to compare extra and intra-hospital false positive activation rates in the first and second semester, after finishing the coaching program.
**METHODS.** Observational study based on spanish ARIAM (Acute Myocardial Infarct Delay Analysis) database of STEMI who underwent pPCI in our ICU (18 beds) from the beginning of the program in February 2015 until February 2016.
From the total of STEMI with pPCI as reperfusion treatment (150), 70% were male, a false-positive diagnosis of STEMI was made 18 patients (12%). 12 (66.66%) occur during the first 6 months of implementing the pPCI program (8 activated by extra-hospital units, 4 intra-hospital). And 6 (33.33%) in the second semester (50%-50% activation percentage extra and intra-hospital units). Common causes for the false-positive diagnosis were X syndrome (including coronary spasm) in 8 patients (44.44%), in these female were more frequent (65%), thoracic pain with new diagnosis of LBBB in 4 patients (22.22%), myocarditis in 3 patients (16.66%) and altered repolarization in 3 patients (16.66%).
**CONCLUSIONS.** Non ischemic causes of ST elevation are frequent. Our false positive results are in the line of other studies. As we analyzed, rates obtained during the first semester were higher than last period, probably due to the implementation of a training course to extra-hospital units as we see the same results in extra and intra-hospital units after finishing the coaching program. Causes of non ischemic ST elevation are similar to other trials.

### 0664

#### Causes and relevance of non-revascularized STEMI patients in a primary PCI program in ICU

O. Moreno Romero, A. Carranza Pinel, M. Muñoz Garach, M. Colmenero Ruiz
Complejo Hospitalario de Granada, Granada, Spain
**Correspondence:** O. Moreno Romero
*Intensive Care Medicine Experimental* 2017, **5(Suppl 2):**0664

**INTRODUCTION.** Is well known that the recent implementation of primary PCI programs around the world for acute STEMI patients has changed the type of primary revascularization, hospital time stay and outcomes of these patients.
**OBJECTIVES.** To analyze the causes of non-revascularized STEMI patients (with PCI or fibrinolysis) after the implementation of a primary PCI program (24 hours/365 days) in Granada.
**METHODS.** Observational study, before and after the implementation of a primary PCI program in January 2014 of all patients with STEMI admitted in our ICU unit of 18 beds from January 2013 to January 2015.
Main variables studied: demographic (age, sex), KILLIP, type of revascularization (fibrinolysis, primary PCI, rescue PCI), causes of non-revascularization (absolute/relative contraindication, lack of electrocardiographic criteria, delayed, presentation of limitation of therapeutic effort and others).
**RESULTS.** In the first period (Jan 2013 - Jan 2014), 150 STEMI were admitted in our ICU. 67.3% were revascularized, 22.7% with primary PCI and 44.7% with fibrinolysis. Causes of non-revascularization were: 51% non EKG criteria and 18.3% delayed STEMI.
In the second period (Jan 2014 - Jan 2015), from 170 STEMI admitted, 80.9% were revascularized, all of them underwent primary PCI. The most important reason of non-revascularized STEMI was non-EKG criteria, in the 73.9% of them.
In both periods the proportion of contraindications were similar (18.4% vs 17.4%) as well as the limitation of therapeutic effort (18,4% vs 17,4%).
**CONCLUSIONS.** The introduction of a primary PCI program (24h/365 days) has increased the global revascularization rate and modified the non-revascularized STEMI causes, mostly due to the time window widening of this treatment.

### 0665

#### A single-centre retrospective observational study to evaluate Tako-Tsubo syndrome

C. Collado Moreno, I. Valiente Aleman, I. Diaz Torres, S. Blasco Turrion, I. Lobo Torres, I. Dominguez Fernandez, I. Perez Lopez, F.J. Morales Ponce
Hospital Universitario Puerto Real, Cadiz, Spain
**Correspondence:** C. Collado Moreno
*Intensive Care Medicine Experimental* 2017, **5(Suppl 2):**0665

**INTRODUCTION.** Takotsubo syndrome (TTS) was first described in Japan in 1990 by Sato (1) and Mayo Clinic criteria were proposed in 2004 and modified in 2008 (4) facilitating the diagnosis of TTS.

**OBJECTIVES.** The aim of this study is to describe prevalence, clinical features, treatment and short-term prognosis of patients diagnosed with TTS.

**METHODS.** A restrospective observational study of patients admitted to Intensive Care Unit (ICU) of our hospital meeting the criteria for TTS was performed between March 1st 2007 and February 28 th 2017.

**RESULTS.** A total of 1602 patients with diagnosis of acute MI were registered but only 30 patients fulfilled inclussion criteria for TTS.The prevalence of TTS was 1.87% with a mean age of 59.73 ± 10.9 years. TTS was more frequent in women (83.3%). Cardiovascular risk factors were hypertension (56.6%), dyslipidemia (30%), diabetes (20%) and smoking (13.3%). Anxiety or depressive disorders were only found in 2 patients (6.6%). Preferred treatments at discharge were aspirin (96%), beta blockers (90%), ACEI/ARBs (83.3%), statins (70%) and DAPT (53.3%). One month follow-up of these patients revealed one death (3.3%) due to cardiovascular disease. This patient suffered a second episode of TTS after an elective major surgical intervention.

**CONCLUSIONS.** We found a limited number of patients, nevertheless TTS showed an important short-term mortality and prevalence between Acute Coronary Syndromes admitted to our ICU. Clinical features were similar to the International Registry (2) except anxiety or depressive disorders. Beta blockers were preferred to ACEI/ARBs.

**REFERENCE(S)**

(1) Sato HTH, Uchida T, Dote K, Ishihara M. Tako-tsubo-like left ventricular dysfunction due to multivessel coronary spasm. In Kodama K, Haze K, Hori M, eds. Clinical Aspect of Myocardial Injury: From Ischemia to Heart Failure. Tokyo, Japan: Kagakuhyoronsha Publishing Co.; 1990, pp. 56–64

(2) InterTAK Registry

(3) Takotsubo Syndrome - Stress-induced Heart Failure Syndrome European Cardiology Review, 2015; 10 (2):83-8Volume 10 Issue 2 Winter 2015 Eric B Tomich

### 0666

#### A bedside predictive model of mortality in the octuagenarian and over octuagenarian undergoing heart surgery

A. Corona, E. Maccarrone, V. Beqiri, F. Sodi, G. Spinelli, C. Di Nobile, C. Savi, E. Catena
PO Luigi Sacco, Milano, Italy
**Correspondence:** A. Corona
*Intensive Care Medicine Experimental* 2017, **5(Suppl 2):**0666

**INTRODUCTION.** Up to date octuagenarians and over-octuagenarians represents 6-10% of patients undergoing heart surgery (HS) (1). The 30th day mortality seems to be higher (3,1-21%, vs. 1,6-2,2 p < 0.001) if compared with younger patients, even if not associated with longer ICU stay.

**OBJECTIVES.** Retrospective observational cohort study to asses predictors of 30th day mortality.

**METHODS.** We considered all the patients undergoing any type of HS since January 1994 through 2015 at Luigi Sacco Hospital, Milano. Patients were divided into two group in relation if their age was ≥85 or < 85 yrs. End point variable is 30th day mortality. On all the patients we collected the following variables:

(i) demographics, chronic diseases and type of cardiac diseases;
(ii) risk score (i.e. NYHA, Euro-Score) and left ventricular ejection fraction;
(iii)intra- and post-operative variables (i.e. organ failure, vaso-active drugs, IABP, duration of mechanical ventilation).

Stata 12.1 was used for statistical analysis.

**RESULTS.** A total of respectively 889 (6.9%) over 85 yrs. and 11966 < 85 yrs patients were found.

The 30th day overall mortality was 4.3% higher in the over 85 yrs. Same difference was confirmed after stratifying patients according type of HS, particularly in case of combined and aorta surgery. A Cox´s proportional model found the following variables (out the 32 considered) as predictors of 30th day death:

(i) pre-operative chronic renal failure [HR: 1.826, (95% CI: 1.141-2.933) p = 0.012];
(ii) vasculopaty [HR: 2.088, (95%CI: 1.396 - 3.125) p < 0.0001];
(iii)aorta disease [HR: 2.512, (95% CI: 1.489 - 4.238) p = 0.001)];
(iv)pre-operative cardiogenic shock [HR: 7.654, (95% CI: 2.327 - 25.17) p = 0.001];
(v) post-operative septic shock [HR: 5.410 (95% CI: 2.459 - 11.90) p < 0.0001];
(vi)post-operative acute hepatic failure: [HR: 3.969 (95% CI: 2.194 - 7.180) p < 0.0001]. Surprisingly age ≥85 [HR 1.576, (95% CI: 0.843 - 2.915) p = 0.156] was not confirmed as a variable significantly affecting the mortality. The age interaction with the predictors was tested without finding any statistical significance or model modifications.

**CONCLUSIONS.** Despite its limitations, our study suggests that HS might be an option for octuagenarian and over since an age ≥ 85 yrs. does not seem to impact on post-operative mortality.

**REFERENCE(S)**

Bhamidipati CM, LaPar DJ, Fonner E Jr, Kern JA, Kron IL, Ailawadi G. Outcomes and cost of cardiac surgery in octogenarians is related to type of operation: a multiinstitutional analysis. Ann Thorac Surg. 2011 Feb;91(2):499–505.

**GRANT ACKNOWLEDGMENT**
None

### 0667

#### Evolution of risk factors in patients with ST-segment elevation myocardial infarction in a second level hospital and in Andalusia

A. Vallejo-Báez[1], M.-V. De la Torre-Prados[1], P. Nuevo-Ortega[1], E. Cámara-Sola[1], T. Tsvetanova-Spasova[1], A. Fernández-Porcel[1], C. Rueda-Molina[1], L. Salido-Díaz[1], M. Mateos-Rodríguez[1], I. García-Gómez[1], ARIAM Group
[1]Málaga/Ibima, Intensive Care Unit, Universitary Hospital Virgen Victoria, Málaga, Spain
**Correspondence:** P. Nuevo-Ortega
*Intensive Care Medicine Experimental* 2017, **5(Suppl 2):**0667

**INTRODUCTION.** The control of cardiovascular risk factors (CRF) is important to decrease the incidence of ischemic heart disease and mortality of acute coronary syndrome (ACS).

**OBJECTIVES.** To analyze over a period of 10 years the evolution of CRF in patients with ST-segment elevation myocardial infarction (STEMI), admitted in a 18 beds medical-surgical Intensive Care Unit (ICU), compared to other hospitals in the region (Andalusia) and its relationship to mortality.

**METHODS.** It is a prospective cohort study of patients with a diagnosis of STEMI admitted to a second level Universitary Hospital Virgen de la Victoria (UHVV) ICU, n = 2188 or, in other ICUs of Andalusia, n = 23167. The data were extracted from the Spanish record of Acute Myocardial Infarction Delay (ARIAM) between 01/01/2005 to 31/12/2014. Comparing the evolution of variables studied in three intervals of time: 2005–2007, 2008–2010 and 2011–14 and the results obtained in Andalusia. Descriptive and comparative statistical analysis was developed using SPSS version 18.0 (SPSS Inc., Chicago, IL, USA).

**RESULTS.** The percentage of each of the cardiovascular risk factors is similar in HUVV and Andalusia although, the percentage of smokers is greater in HUVV. There is no difference between the periods of time; except the obesity that increases significantly in the third period (2011–14), 8% in HUVV and 5% in Andalusia, Table 169. STEMI mortality is related significantly with women, hypertension, Mellitus Diabetes, dyslipidemia and inversely related to smoking cessation.

**CONCLUSIONS.** CRF remain stable over the decade analyzed. Control of the CRF is important to prevent ischemic heart disease and the data suggest that we should have an impact on health education to reduce the number of smokers and increase mediterranean diet and exercise habits to control body weight.

**Table 169 (Abstract 0667)**. Cardiovascular Risk Factors in HUVV and Andalusia

| Variables | UHVV 2005-07 N=534 | UHVV 2008-10 N=679 | UHVV 2011-14 N=975 | UHVV 2005-14 N=2188 | Andalusia 2005-07 N=6322 | Andalusia 2008-14 N=6727 | Andalusia 2011-14 N=10118 | Andalusia 2005-14 N=23167 |
|---|---|---|---|---|---|---|---|---|
| *Smoker | 48% | 50% | 48% | 49% | 40% | 41% | 42% | 41% |
| *M. Diabetes | 29% | 27% | 25% | 27% | 29% | 30% | 29% | 28% |
| *Hypertension | 50% | 49% | 49,3% | 49% | 50% | 48% | 49% | 49% |
| * Dyslipidemia | 37% | 34% | 37% | 36% | 38% | 39% | 38% | 39% |
| **Obesity | 5,5% | 5,6% | 13,3% | 8% | 8% | 12% | 13% | 12% |
| *Family H. | 11% | 10% | 10% | 10% | 10,5% | 12% | 12% | 12% |

*HUVV* Hospital University Virgen de la Victoria, *M. Diabetes* Mellitus Diabetes, *Family H.* Family History
(*) Chi Square p=ns; (**) Chi Square p=<0,05

**Table 170 (Abstract 0667)**. Mortality and cardiovascular risk factor

| Variables | UHVV, STEMI Survivors N=2068 | UHVV, STEMI No survivors, N=120 (5,4%) | UHVV 2005-14 N=2188 |
|---|---|---|---|
| *Smoker cessation | 17% | 10% | 17% |
| *M. Diabetes | 26% | 42% | 27% |
| *Hypertension | 49% | 60% | 49,3% |
| **Dyslipidemia | 36% | 37% | 36% |
| **Obesity | 8% | 11% | 8% |
| **Family H. | 11% | 10% | 10% |
| *Women | 21% | 41% | 22% |

*UHVV* Universitary Hospital Virgen de la Victoria, *STEMI* ST-segment elevation myocardial infarction, *M. Diabetes* Mellitus Diabetes, *Family H.* Family History
(*) Chi Square p=<0,05; (**) p=ns

### 0668

#### Acute heart failure in a Tunisian medical ICU: prognosis and risk factors

E. Ennouri[1], I. Ben Saida[1], A. Ben Abdelhafidh[1], K. Meddeb[1], M. A. Boujelbèn[1], N. Fraj[1], J. Ayachi[1], W. Zarrougui[1], H. Kallel[1], A. Khedher[1], A. Azouzi[1], I. Chouchene[1], M. Boussarsar[1,2]
[1]Farhat Hached University Hospital, Medical Intensive Care Unit, Sousse, Tunisia; [2]Ibn Al Jazzar Faculty of Medicine, Research Laboratory N° LR 14 ES 05 Interactions of the Cardio-Pulmonary System, Sousse, Tunisia
**Correspondence:** M. Boussarsar
*Intensive Care Medicine Experimental* 2017, **5(Suppl 2):**0668

**INTRODUCTION.** Acute Heart failure (AHF) is a prevalent and increasing disease and represents a frequent cause of critical care admission. It is associated with an important morbi-mortality. The outcome for such patients is poor with low survival rates. AHF is also an increasing burden on health care systems. The correct risk stratification of patients could improve clinical outcome and resources allocation.

**OBJECTIVES.** To describe the characteristics of ICU admitted patients with AHF and identify independently associated factors.

**METHODS.** It is a retrospective study of admitted AHF patients in the medical ICU of Farhat Hached teaching hospital between January 2011 and December 2016. Variables found to be statistically significant in univariate analysis were included into a multivariate regression model to identify factors independently associated to poor prognosis. For all tests, a *P* value of less than 0.05 was considered statistically significant.

**RESULTS.** 138 patients were included with an average age of 66.33 ± 15.6 years. 41.3% were women. Hypertension 74(53.6%), Diabetes mellitus 64(45.7%), and chronic obstructive pulmonary disease 23(16.7%) were the most outstanding associated pathologies. SAPSII was 38.24 ± 37. 91(65.9%) patients received vasopressors. Acute coronary syndrome 56(48.6%) was the most common cause of AHF. Mean duration of ICU stay was 4.27 ± 4.99 days. Mortality rate was 53.6%. Univariate analysis identified several factors associated to fatal outcome: physiological reserve [McCabe score ≥2 (p = 0.000), Knaus C or D (p = 0.01), OMS ≥3 (p = 0.04), NYHA ≥ 3 (p = 0.029)], severity on admission [severe acute respiratory failure (p = 0.02), vasopressors use (p = 0.00), shock (p = 0.02), SAPSII (p = 0.00), and Glasgow coma scale ≤12 (p = 0.028)] and reference from cardiology Ward (p = 0.03). A multivariate regression model identified the following factors as independently associated to mortality: OMS ≥ 3 (OR, 4.44 ; 95%CI, [1.64 -12.01] ; p < 0.003), vasopressor use (OR, 6.76 ; 95%CI, [2.23 - 20.43]; p < 0.001), SAPSII (OR, 1,05 ; 95%CI, [1.01-1.09] ; p < 0.005) and admission from cardiology ward (OR, 7.98 ; 95%CI, [1.77-35.99] ; p < 0.007).

**CONCLUSIONS.** The present study revealed a relatively poor prognosis of AHF patients admitted to the ICU. Physiological reserve (OMS), severity on admission (SAPSII and vasopressors use) and the reference from the cardiology ward were identified as independent predictors of fatal outcome.

### 0669

#### Gender and ST-segment elevation myocardial infarction in a second level hospital

M.-V. de la Torre-Prados, A. Vallejo-Báez, E. Cámara-Sola, P. Nuevo-Ortega, T. Tsvetanova-Spasova, C. Rueda-Molina, A. Fernández-Porcel, L. Salido-Díaz, M. Mateos-Rodríguez, I. García-Gómez, ARIAM Group
Málaga/Ibima, Department of Intensive Care Medicine. Universitary H Virgen de la Victoria, Málaga, Spain
**Correspondence:** M.-V. de la Torre-Prados
*Intensive Care Medicine Experimental* 2017, **5(Suppl 2):**0669

**INTRODUCTION.** Clinical differences in relation the gender would permit to adapt the messages in acute coronary syndrome (ACS) to decrease the mortality in women group.

**OBJECTIVES.** To analyze over a period of 10 years in men vs. women with ST-segment elevation myocardial infarction (STEMI) and admitted in a 18 beds medical-surgical Intensive Care Unit (ICU), the differences in clinical variables N-US; mso-fareast-language:ES;mso-bidi-language:AR-SA´ > acute coronary syndrome (ACS) to decrease the mortality in women group.

**METHODS.** It is a prospective cohort study of patients with a diagnosis of STEMI admitted to a second level University Hospital Virgen de la Victoria (UHVV) ICU, n = 2188. The data were extracted from the Spanish Record of Acute Myocardial Infarction Delay (ARIAM) between 01/01/2005 to 31/12/2014. Comparing clinical variables studied in both groups. Descriptive and comparative statistical analysis was developed using SPSS version 18.0 (SPSS Inc., Chicago, IL, USA).

**RESULTS.** STEMI mortality is significantly high in women, with more cardiovascular risk factors and delay from first symptoms to diagnosis and treatment strategies. Women are oldest, have more cardiovascular risk factors than men and significant differences in mortality. Creatinine mg/dl and hematocrite at 48 hours show significant differences in both groups: 2.7 ± 12.5 vs. 1.8 ± 12.5 and 38 ± .4.6 vs. 42.4 ± 5.6. Grace and Crusade scores in women are significant highest than men: 142 ± 36 vs. 132 ± 34 and 43 ± 13 vs. 25 ± 14. The Table 171 shows more results.

**CONCLUSIONS.** Women with STEMI arrive later to Health System and the delay continues in diagnosis and treatments, this group is oldest and have more cardiovascular risk factors than men.

**Table 171 (Abstract 0669)**. Gender and ST Segment Myocardial Infarction

| Variables | Women, n=493 | Men, n=1716 | Total, n=2209 |
|---|---|---|---|
| *Age (years), p=<0.05 | 66,55±12.5 | 60,46±12 | 62±12.4 |
| ***M. Diabetes %, p=<0.05 | 32 | 25 | 26 |
| ***Hypertension %, p=<0.05 | 65 | 45 | 50 |
| **FS-CSS, p=<0.05 | 76 [41-196] | 60 [30-160] | 65[30-162] |
| **FS->3h Fx, %, p=<0.05 | 38.2 | 27.7 | 30.1 |
| **Time FS-PPCI, P50 minutes, p=>0.05 | 290[180-572] | 225[145-553] | 230[150-476] |
| ***Killip ≥ II%, p=<0.05 | 14 | 10 | 11 |
| ***Non-survivors%, p=<0.05 | 11 | 4.6 | 6 |

*FS* First Symptoms, *CSS* Call Sanitary System, *EH* Emergency Hospital, *>3h Fx* >3 hours Fibrinolysis, *PPCI* Primary Percoutaneous Coronary Intervention (*) T Student, (**) Kruskal-Wallis, (***) Chi Square

### 0670

#### Evolution of the delays in ST-segment elevation myocardial infarction in a second level hospital and in Andalusia

A. Vallejo-Báez, M.-V. de la Torre-Prados, P. Nuevo-Ortega, E. Cámara-Sola, T. Tsvetanova-Spasova, A. Fernández-Porcel, C. Rueda-Molina, L. Salido-Díaz, I. García-Gómez, M. Mateos-Rodríguez, ARIAM Group
Málaga/Ibima, Department of Intensive Care Medicine, Universitary H Virgen de la Victoria, Málaga, Spain
**Correspondence:** P. Nuevo-Ortega
*Intensive Care Medicine Experimental* 2017, **5(Suppl 2):**0670

**INTRODUCTION.** In ST-segment elevation myocardial infarction (STEMI)) is essential to reduce delays between the symptoms, the call to health care system and the coronary reperfusion to improve the prognosis.

**OBJECTIVES.** To analyze in 10 years, the evolution of period of times between the first symptoms and reperfusion coronary interventions in patients with ST-segment elevation myocardial infarction (STEMI), admitted in a 18 beds medical-surgical Intensive Care Unit (ICU), compared to other hospitals in the region (Andalusia) and its relationship to mortality.

**METHODS.** It is a prospective cohort study of patients with a diagnosis of STEMI admitted to a second level Universitary Hospital Virgen de la Victoria (UHVV) ICU, n = 2188 or, in other ICUs of Andalusia, n = 23167. The data were extracted from the Spanish record of Acute Myocardial Infarction Delay (ARIAM) between 01/01/2005 to 31/12/2014. Comparing the evolution of the variables studied in three intervals of time: 2005–2007, 2008–2010 and 2011–14 and the results obtained in Andalusia. Descriptive and comparative statistical analysis was developed using SPSS version 18.0.

**RESULTS.** We studied 2188 STEMI patients in UHVV group with a mean age of 61.8 ± 12.2 years old (61.3 ± 12.3 vs. 61.9 ± 12.5 vs. 62.2 ± 11.69, p= > 0.05) and 23% were female (23% vs. 22% vs. 22%; p= > 0.05). In Andalusia group (n = 23167) mean age was 62.3 ± 11.7 years old (62.5 ± 11.7 vs. 62.1 ± 11.8 vs. 61.9 ± 11.9; p= > 0.05) and 23% were female (24% vs. 23% vs. 22%; p= > 0.05). We found no significant diferences in mortality in three periods, 6.8%, 5.7% and 5% in UHVV vs. 5.9%, 5.9% and 4.7% in Andalusia. We can see the results in Table 172.

**CONCLUSIONS.** There is a reduction in the delay in relation to coronary reperfusion times, fibrinolysis and primary percutaneous coronary intervention (PPCI), but there is no change in the depedent patient time how is the call to health care system after the first syntoms, that would permit to reduce the mortality.

**Table 172 (Abstract 0670)**. Delays in STEMI in UHVV and Andalusia

| Variables | UHVV 2005-07 N=534 | UHVV 2008-10 N=679 | UHVV 2011-14 N=975 | UHVV 2005-14 N=2188 | Andalusia 2005-07 N=6322 | Andalusia 2008-14 N=6727 | Andalusia 2011-14 N=10118 | Andalusia 2005-14 N=23167 |
|---|---|---|---|---|---|---|---|---|
| **FS-CSS, P50 | 59 | 60 | 58 | 60 | 60 | 60 | 50 | 54 |
| **FS-HE, P50 | 120 | 135 | 129 | 126 | 120 | 130 | 120 | 130 |
| **FS-Ext. Fx., P50 | 87 | 92 | 91.5 | 90 | 90 | 95 | 90 | 90 |
| **FS-Fx, P50 | 140 | 126 | 120 | 130 | 140 | 135 | 120 | 130 |
| ***FS-PPCI, P50 | 290 | 220 | 210 | 220 | 255 | 245 | 210 | 215 |

*UHVV* Hospital Universitary Hospital Virgen de la Victoria, *FS* First Syntoms, *CSS* Contact Sanitary System, *HE* Hospital Emergency, *Ext. Fx* Extrahospital Fibrinolysis, *PICP* Primary Percutaneous Coronary Intervention
*Values expressed as minutes and median (P50), **Kruskal-Wallis p=>0,05, ***Kruskal-Wallis p=<0,05

### 0671

#### Ten-years evaluation of patients with myocardial infarction and nonobstructive coronary arthery

C. Collado Moreno[1], I. Diaz Torres[2], I. Valiente aleman[2], I. Perez Lopez[1], M. J. Dominguez Rivas[2], S. Blasco Turrion[1], I. Dominguez Fernandez[2], P. J. Gonzalez Perez[3], F. J. Morales Ponce[1], J. C. Rodriguez Yañez[4], on behalf of the ARIAM Andalucia Registry Investigators.
[1]Hospital Universitario Puerto Real, Cardiology, Cadiz, Spain; [2]Hospital Universitario Puerto Real, Medicina Intensiva, Cadiz, Spain; [3]Universidad de Cadiz, Cardiology, Cadiz, Spain; [4]Universidad de Cadiz, Medicina Intensiva, Cadiz, Spain
**Correspondence:** J. C. Rodriguez Yañez
*Intensive Care Medicine Experimental* 2017, **5(Suppl 2):**0671

**INTRODUCTION.** Myocardial infarction (MI) with angiographically no obstructive coronary arthery (MINOCA) is an important subtype of MI where the underlying cause is not immediately apparent and its management is often suboptimal.

**OBJECTIVES.** The objectives of this study are to describe the clinical characteristics of patients with MINOCA, its prevalence, risk factors, treatment at discharge and outcome.

**METHODS.** We performed a restrospective observational study of patients admitted to a single center Intensive Care Unit with diagnosis of acute MI from the ARIAM Andalucia registry between January 1st 2007 and December 31 st 2016. All patients underwent coronary angyography.Those patients with normal result or coronary stenosis less than 50% were diagnosed as MINOCA.Follow-up data were available by merging data from the DIRAYA. Statistics: categorical variables are presented as frecuency values and compared by chi-square test and continuous variables are presented as mean ± standar deviation (SD).

**RESULTS.** We identified 54 (3.2%) patients with MINOCA among 1676 patients screened.Clinical investigations on most of these patients were suboptimal. MINOCA was more frequent in women (8.66%) than in men (2.1%) and in NSTEMI (4.2%) than in STEMI (2.7%) p < 0.001 and p < 0.05 respectively.Mean age was 60.59 ± 13.93 y. Most frequent cardiovascular risk factors among MINOCA patients were hypertension (55.6%), smoking (51.9%) and hyperlipemia(35.2%). Median risk score GRACE was 123 ± 32.68 and CRUSADE 27.4 ± 16.07. LVFE were > 50%(59.39%); 50-40% (24.1%); 40- 30% (11.1%) and < 30% (5.5%). Preferred treatments at discharge were aspirin (96%), statins (86%), ACEI/ARBs (80%), beta-bloquers (70%) and DAPT (64%). One patient had died at 3 month follow-up (1.85%).

**CONCLUSIONS.** Diagnostic workup of patients with MINOCA is often suboptimal, and can be a cause of short-term mortality, despite an adequate medical treatment. More studies on aetiology and outcomes of MINOCA are needed to improve care on these patients.

**REFERENCE(S)**

1 Medical Therapy for Secondary Prevention and Long-Term Outcome in Patients with Myocardial Infarction with Non-Obstructive Coronary Artery (MINOCA) Disease.Lindahl B, Baron T, Erlinge D,

Hadziosmanovic N, Nordenskjöld A, Gard A, Jernberg T.Circulation. 2017; doi: 10.1161/CIRCULATIONAHA.116.026336. [Epub ahead of print]
2.Systematic review of patients presenting with suspected myocardial infarction and nonobstructive coronary arteries.Pasupathy S, Air T, Dreyer RP, Tavella, Beltrame JF.
Circulation. 2015 Mar 10;131(10):861–70.
**ACKNOWLEDGMENTS AND SOURCES**
The ARIAM Andalucia registry and DIRAYA program are supported by the Servicio Andaluz de Salud.

### 0672

#### A crossover study of the hemodynamic effects of body temperature vs. room temperature fluid boluses in healthy volunteers

O. Wall[1], L. Ehrenberg[1], E. Joelsson-Alm[1], J. Mårtensson[2], R. Bellomo[2], C. Svensén[1], M. Cronhjort[1]
[1]Karolinska Institutet, Department of Clinical Science and Education, Section of Anaesthesiology and Intensive Care, Södersjukhuset, Stockholm, Sweden; [2]The University of Melbourne, Department of Intensive Care, Austin Hospital, Melbourne, Australia
**Correspondence:** O. Wall
*Intensive Care Medicine Experimental* 2017, **5(Suppl 2):**0672

**INTRODUCTION.** Fluid boluses are often administered with the aim of improving tissue perfusion in critically ill patients. The hemodynamic persistence of these boluses is poorly understood (1) and also whether the temperature of fluid has an impact on the hemodynamic response (2). This study´s aims were to look at the effects on circulation of different temperatures of infused fluids. Particularly, we aimed to assess whether the temperature of the fluid influences heart rate (HR), mean arterial pressure (MAP), systolic blood pressure (SBP), diastolic blood pressure (DBP) and cardiac index (CI)

**OBJECTIVES.** Primary endpoint: Difference in CI at 15 minutes after bolus start. The hypothesis was that CI would increase more with cold fluids.

Secondary endpoints: Change in HR, MAP, SBP, DBP, CI and temperature during 120 minutes.

**METHODS.** We performed a randomized, controlled study with a cross-over design. 21 healthy volunteers aged 18 or over were included. Exclusion criteria: ASA class of 2 or above or known pregnancy. Monitoring with pulse oximetry, ECG, non-invasive cardiac output and non-invasive blood pressure (Clearsight®) was performed. Body temperature was measured by a heat flux sensor (3M SpotOn®). The subjects were randomized to receive either 500ml of Ringer´s acetate at room temperature 22°C or heated to body temperature 38°C. Fluid was infused over 15 minutes. For two hours after infusion start HR, SpO2, MAP, SBT and DBT, temperature and CI were measured continuously and registered every 5 minutes during infusion and then every 15 minutes. The subjects would then return another day to receive the other temperature infusion.

**RESULTS.** Preliminary results showed no statistically significant difference in CI at 15 minutes or at any of the time points. There was a difference in MAP between groups, with a statistically significant increase in the cold group during infusion and at 15 minutes after bolus start.

**CONCLUSIONS.** Our preliminary analysis showed no statistically significant difference in CI between cold or warm fluid boluses in healthy volunteers.

However, we found a statistically significant increase in MAP and DBP which could be of clinical significance and merits further study in the clinical setting.

1.Bihari S, Prakash S, Bersten AD. Post resusicitation fluid boluses in severe sepsis or septic shock: prevalence and efficacy (price study). Shock. 2013;40(1):28–34.
2.Frank SM, Cattaneo CG, Wieneke-Brady MB, El-Rahmany H, Gupta N, Lima JA, et al. Threshold for adrenomedullary activation and increased cardiac work during mild core hypothermia. Clin Sci (Lond). 2002;102(1):119–25.

**GRANT ACKNOWLEDGMENT**
Olof Wall was supported by a research grant from Stockholm County Council

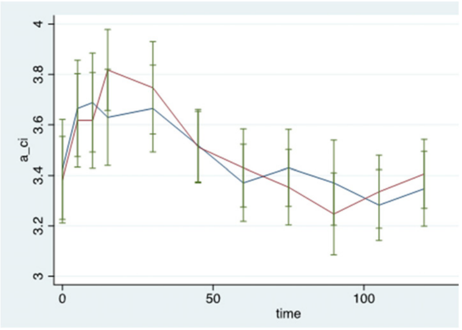

**Fig. 230 (Abstract 0672)** Mean CI (L/min/m$^2$) during 120 minutes, Blue line = Cold group, Red line = Warm group, Green bar = 1 Standard Error

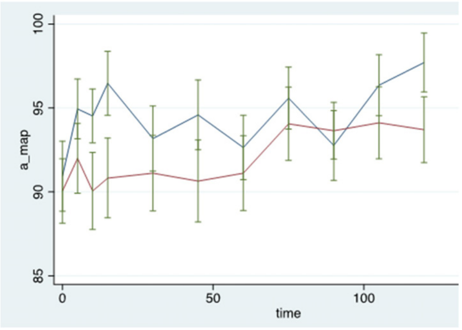

**Fig. 231 (Abstract 0672)** Mean MAP (mmHg) during 120 minutes, Blue line = Cold group, Red line = Warm group, Green bar = 1 Standard Error

### Non-invasive ventilation: Clinical studies

### 0673

#### Effect of high-flow nasal cannula oxygen therapy on thoraco-abdominal synchrony in pediatric patients after cardiac surgery

T. Itagaki[1], E. Nakataki[1], M. Onodera[1], J. Oto[2], M. Nishimura[1]
[1]Tokushima University Graduate School, Emergency and Critical Care Medicine, Tokushima, Japan; [2]Tokushima University Hospital, Emergency and Disaster Medicine, Tokushima, Japan
**Correspondence:** T. Itagaki
*Intensive Care Medicine Experimental* 2017, **5(Suppl 2):**0673

**INTRODUCTION.** High-flow nasal cannula (HFNC) oxygen therapy creates positive oropharyngeal pressure, washes out the nasopharyngeal dead space, and improves oxygenation [1]. We reported the effects of HFNC oxygen therapy on thoraco-abdominal synchrony in adults [2], however the effect has not been evaluated in pediatric patients.

**OBJECTIVES.** The objectives of this study were to:

1) clarify the effect of HFNC oxygen therapy on thoraco-abdominal synchrony in pediatric patients with mild-to-moderate respiratory failure;

2) investigate optimal flow of HFNC oxygen therapy in this population.

**METHODS.** We surveyed pediatric patients with body weight of 2 to 10 kg. Thoraco-abdominal synchrony was evaluated with respiratory inductive plethysmography (RIP). Before extubation, transducer bands were circumferentially placed around the rib cage and the abdomen. RIP was calibrated by using isovolume maneuver. After extubation, we applied oxygen via face mask for 30 minutes (CTL1) and observed respiratory status. We enrolled the patients when they showed one or more of the followings: $SpO_2 < 95\%$; breathing frequency > 50 breath/min; dyspnea, or asynchronous or paradoxical breathing pattern. Each patient received 1 or 2 L/kg/min via HFNC for 30 minutes, then another flow via HFNC for 30 minutes. After HFNC oxygen therapy, they were returned to face mask (CTL2). Rib cage and abdominal movement were converted into volumes, and two quantitative indexes: maximum compartmental amplitude/tidal volume ratio ($MCA/V_T$) and phase angle were calculated. Data were analyzed by repeated measures analysis of variance with multiple comparisons for an effect over time. $P < 0.05$ was considered statistically significant.

**RESULTS.** Ten patients were enrolled (mean age; 10 ± 8 months, mean body weight; 6.7 ± 2.4 kg). Table 173 shows measured variables during face mask and HFNC oxygen therapy. Compared with CTL1, breathing frequency, $MCA/V_T$, phase angle and minute volume significantly decreased at a flow 2 L/kg/min ($p < 0.05$ for all) but not at a flow 1 L/kg/min. $PaCO_2$ did not differ among oxygen therapies. Any measured variables did not differ between CTL1 and CTL2.

**CONCLUSIONS.** HFNC oxygen therapy at a flow 2 L/kg/min improved thoraco-abdominal synchrony and decreased breathing frequency in pediatric patients after cardiac surgery.

**GRANT ACKNOWLEDGEMENT**

None

**Table 173 (Abstract 0673)**. Measured variables

| | CTL 1 | HFNC 1 L/kg/min | HFNC 2 L/kg/min | CTL 2 |
|---|---|---|---|---|
| Breathing frequency, bpm | 37 ± 7 | 35 ± 7 | 33 ± 8* | 36 ± 10** |
| Phase angle, degrees | 32 ± 20 | 21 ± 12 | 19 ± 15* | 22 ± 13 |
| $MCA/V_T$ | 1.07 ± 0.08 | 1.03 ± 0.04 | 1.01 ± 0.02* | 1.02 ± 0.02 |
| Minute volume, mL/kg | 303 ± 130 | 245 ± 98 | 242 ± 83* | 261 ± 76 |
| $PaCO_2$, mmHg | 36 ± 5 | 35 ± 5 | 34 ± 5 | 35 ± 4 |

*CTL* control. Data are expressed as mean ± SD. * = p < 0.05 vs CTL1; ** = p < 0.05 vs 2 L/kg/min

### 0674

#### A randomized cross-over physiological study of high flow nasal oxygen cannula versus non-invasive ventilation in adult patients with cystic fibrosis: the HIFEN study

M. C. Sklar[1,2,3], M. Dres[2,3,4], N. Ritayamai[2,3,5], B. West[6], T. Pham[2,3], D.-L. Grieco[2,3], I. Telias[2,3], D. Junhasavasd[2,3], M. Rauseo[2,3], C. Campbell[2,3], D.E. Tullis[6], L. Brochard[2,3]

[1]University of Toronto, Anesthesia, Toronto, Canada; [2]University of Toronto, Interdepartmental Division of Critical Care Medicie, Toronto, Canada; [3]Keenan Research Centre, Li Ka Shing Knowledge Institute, St. Michael's Hospital, Toronto, Canada; [4]Sorbonne Universités, Neurophysiologie Respiratoire Expérimentale et Clinique, Paris, France; [5]Siriraj Hospital Respiratory Care Unit, Division of Respiratory Diseases and Tuberculosis, Department of Medicine, Faculty of Medicine, Bangkok, Thailand; [6]St. Michael's Hospital, Division of Respirology, Toronto, Canada

**Correspondence:** M. C. Sklar

*Intensive Care Medicine Experimental* 2017, **5(Suppl 2):**0674

**INTRODUCTION.** Non-invasive ventilation (NIV) is the first option for the treatment of patients with exacerbations of cystic fibrosis (CF). High flow nasal oxygen cannula (HFNC) has demonstrated benefits in terms of survival in patients with acute hypoxemic respiratory failure and in preventing postextubation failure. This technique may also have benefits in patients with hypercapneic respiratory failure including CF patients. We hypothesize that HFNC would not be inferior to NIV in terms of reducing work of breathing and improving breathing pattern in CF patients requiring ventilator support.

**OBJECTIVES.** To compare HFNC vs. NIV induced changes in inspiratory work of breathing assessed non-invasively by the thickening fraction of the diaphragm (TFdi), and breathing pattern, $CO_2$ level, hemodynamics, dyspnea and comfort.

**METHODS.** CF patients with exacerbation requiring ventilator support were stabilized and ventilated with HFNC and NIV for 30 minutes in random order. TFdi was measured using ultrasound at baseline and at 25 minutes with each device. Pulse oximetry ($SpO_2$), transcutaneous $CO_2$ ($PtcCO_2$) were continuously recorded; respiratory rate, tidal volume ($V_T$) and minute ventilation (MV) were measured by bio-impedance; hemodynamics, dyspnea (high scores associated with more shortness of breath) and comfort (high scores associated with better comfort) were assessed by visual analog scales.

**RESULTS.** 15 patients were enrolled (mean age 31.3 years, mean $FEV_1/FVC$ 49.0%, mean $FEV_1$ 28.0% predicted). TFdi was similar with the two techniques. Compared to baseline, HFNC significantly reduced the respiratory rate by 19% vs 2.8% for NIV (p < 0.01) and NIV significantly increased mean arterial pressure by 6% vs 0.6% for HFNC (p < 0.01). HFNC compared to NIV resulted in a significantly higher $V_T$ by 10.8% (p = 0.03). No differences were found for heart rate, MV, $SpO_2$, $PtcCO_2$, dyspnea or comfort (Table 174).

**CONCLUSIONS.** There was no difference between HFNC and NIV with respect to diaphragmatic work in stabilized CF patients who had an indication for ventilator support. These preliminary data suggest that HFNC may confer physiological benefits by decreasing respiratory rate, and constitute an interesting alternative or complement to NIV.

**GRANT ACKNOWLEDGMENT**

This study was supported by Cystic Fibrosis Canada and by a grant from Siriraj Hospital in Bangkok. LB's laboratory received a grant and equipment from Fisher Paykel for the study. LB is the Keenan Chair in Critical Care and Acute Respiratory Failure.

**Table 174 (Abstract 0674)**. See text for description

Median (interquartile range) from baseline after 25 minutes in diaphragmatic work, respiratory variables, hemodynamics, and level of dyspnea and comfort between high flow nasal oxygen cannula (HFNC) and non-invasive ventilation (NIV)

| | Baseline | HFNC | NIV | *P** |
|---|---|---|---|---|
| Diaphragm thickening fraction (%) | 31.9 [28.4;43.6] | 33.2 [19.8;37.5] | 35.3 [28.3;40.7] | 0.76 |
| Respiratory rate (bpm) | 21 [18;26] | 18 [14;20] | 19 [18;26] | **0.02** |
| $SpO_2$ (%) | 93 [91;94] | 94 [93;95] | 93 [92;94] | 0.10 |
| $PtcCO_2$ (mmHg) | 54 [45;58] | 54 [42;57] | 53 [41;59] | 0.53 |
| Tidal volume (mL) | 286 [224;550] | 281 [238;456] | 281 [241;433] | **0.03** |
| Minute ventilation (L/min) | 6.9 [5.9;10.1] | 4.9 [4.3;5.8] | 5.6 [5.1;7.4] | 0.08 |
| Mean arterial pressure (mmHg) | 85 [80;90] | 84 [80;92] | 91 [82;98] | **<0.01** |
| Heart rate (bpm) | 108 [91;113] | 102 [92;110] | 101 [96;113] | 0.95 |
| Dyspnea | 1 [0;2] | 1 [0;2] | 1 [0;2] | 0.20 |
| Comfort | 9 [8;10] | 6 [5;8] | 7 [6;8] | 0.36 |

*HFNC vs NIV

### 0675

#### High flow nasal cannula vs standard oxygen face mask during physiotherapy in brain injury patients: a feasible study

A. N. Cracchiolo[1], D. M. Palma[2], R. Tetamo[2]
[1]Arnas Ospedale Civico Di Cristina Benfratelli, Terapia Intensiva Polivalente con Trauma Center, Palermo, Italy; [2]Arnas Ospedale Civico Di Cristina Benfratelli, Palermo, Italy
**Correspondence:** A. N. Cracchiolo
*Intensive Care Medicine Experimental* 2017, **5(Suppl 2):**0675

**INTRODUCTION.** In severely brain injured (BI) patients many cardiorespiratory complications may be observed due to hypoventilation and inability to maintain airway clearance after extubation. Physiotherapists play an important role inside the intensive care unit (ICU) as members of multidisciplinary team in preventing cardiorespiratory complications. In the last years increasing interest about the use of High Flow Nasal Oxygen (HFNO) has been noted in patients affected by acute respiratory failure.

**OBJECTIVES.** We tested if HFNO is more effective than standard oxygen (O2) therapy administered via facial mask (FM) to keep an appropriate oxygen saturation and respiratory rate in patients affected by BI during chest physiotherapy.

**METHODS.** From July 2016 to February 2017 we treated in our multidisciplinary ICU 20 patients affected by BI due to ischemic (13) and hemorrhagic (7) stroke. Inclusion criteria were as follow: Glasgow Coma Scale (GCS) ≥ 8 at study entry, weaned from mechanical ventilation (MV) at least from 48 hours with spontaneous breathing and no need of Non Invasive Ventilation, respiratory rate (RR) ≤ 30, hearth rate (RR) ≤ 90. Patients were uniform for age (mean age 66 years old), sex (12 female), BMI (mean 26) and duration of MV. Supplementary O2 was administered with HFNO and FM at FiO2 = 0.5 and flow = 50 L/min for the former and with FiO2 = 0.5 and flow 8 L/min for the latter. We recorded for every patient oxygen saturation (SaO2), HR, RR and systolic arterial pressure (SAP) 5 minutes before start, 15 minutes after the start and 5 minutes after the finish of physiotherapy. Every physiotherapy treatment took 30 minutes.

**RESULTS.** All patients were treated twice daily until discharge from ICU. The average of ICU length of stay after MV discontinuation was 6 days (4–8). A total number of 130 treatments were given by 2 physical therapist. The first treatment was performed alternately with FM or HFNO and each patient received the same number of treatment with both methods. The average values of vital signs recorded were similar at baseline in all patients. The results are summarized in Table 175. We found that RR showed a great reduction during physiotherapy when supplementary O2 was administered with HFNO whilst it increased when oxygen was administered with FM. It is possible that positive end expiratory pressure (PEEP) related to HFNO could play a role. Substantial letup were observed also in HR while short change in SAP were seen and none in SaO2.

**CONCLUSIONS.** HFNO is feasible to use at bedside, well tolerated by patients and shows during physiotherapy great reduction in RR probably related to increased availability of O2 and a variable level of PEEP. More studies in this setting are needed to better assess these results.

**Table 175 (Abstract 0675)**. See text for description

| HFNO | BASELINE | TREATMENT | AFTER | FM | BASELINE | TREATMENT | AFTER |
|---|---|---|---|---|---|---|---|
| SAO2 | 100% | 100% | 100% | SAO2 | 100% | 100% | 100% |
| HR | 74 | 64 | 70 | HR | 72 | 81 | 70 |
| RR | 18 | 12 | 14 | RR | 18 | 29 | 20 |
| SAP | 149 | 154 | 147 | SAP | 152 | 170 | 155 |

### 0676

#### Optiflow can be safely used by the outreach team to prevent unplanned ICU admissions

M. Noureldin, F. Rubulotta[1]
Imperial College, London, United Kingdom
**Correspondence:** F. Rubulotta
*Intensive Care Medicine Experimental* 2017, **5(Suppl 2):**0676

**INTRODUCTION.** Acute respiratory failure (ARF) is worldwide the most common cause of in-hospital emergency. Optiflow is a new medical device, which allows the comfortable delivery of high flows humidified warm (37 °C, 44 mg/L) oxygen directly into the nares. Optioflow supplies also positive end-expiratory pressure (PEEP) 3 ± 1 mmHg. Our hypothesis is that early use of Optioflow by nurses working for the outreach team in hospital wards can be safe, effective and cost-effective method to prevent aggravation of ARF and unplanned transfer to the Intensive Care unit (ICU).

**OBJECTIVES.** To investigate the use of Otpiflow ventilation by our outreach nursing team in the ward in relation to the management of ARF (ref 1).

**METHODS.** Authors did include all consecutive adult patients treated between January 2014 and January 2015 with optiflow within 4 hours from outreach nurse alert due to hypoxia (PaO2/FiO2 < 100 mmHg or PiO2/FiO2 < 200mmHg and PEEP > 5 mmH2O) in the general medical, general surgical, neurosurgical and neurology wards of a large teaching hospital in London, UK. We collected demographics, reasons for admission to the hospital, time and needs to start optiflow (including differentiation between type one and type two ARF), and hospital outcome. All patients did receive the best available standard treatment (including chest physiotherapy and nebulisers) in the wards.

**RESULTS.** 223 adult (age >18y old) patients were randomized and 43 patients were included. Patients' median age was 60 year old, 62% were male, SAPS II 45 +/− 17, type one ARF in 82% of cases.
(graphic 1 shows the referring teams). Main reasons why patients were not included in this study were consent, treating physician choice, transfer to the ICU, lack of available optiflow machine. Graphic 2 shows the reason why treatment was started in the ward. Graphic 3 shows optiflow use by the outreach nurse in the ward.No complications were recorded using optiflow in surgical or medical hospital wards even with limited monitoring and staff. Optiflow was always initiated by the outreach nurse with no medical input.

**CONCLUSIONS.** Optiflow was used successfully to follow up patients in the surgical ward post extubation in the operating theater. Optiflow alone reversed ARF in 26% of patients in the general medical, general surgical, neurosurgical and neurology wards. This device can be safely used by experienced nurses (outreach team) in the ward.

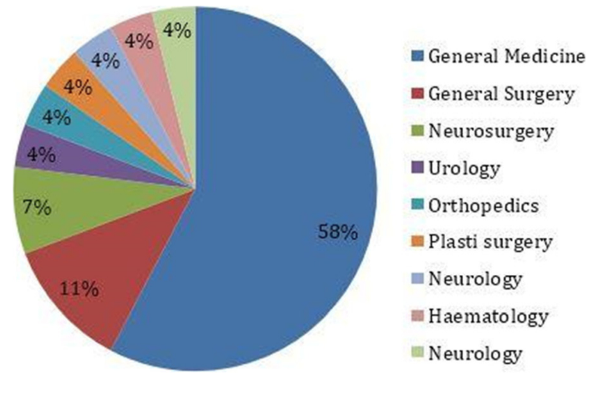

**Fig. 232 (Abstract 0676)** See text for description

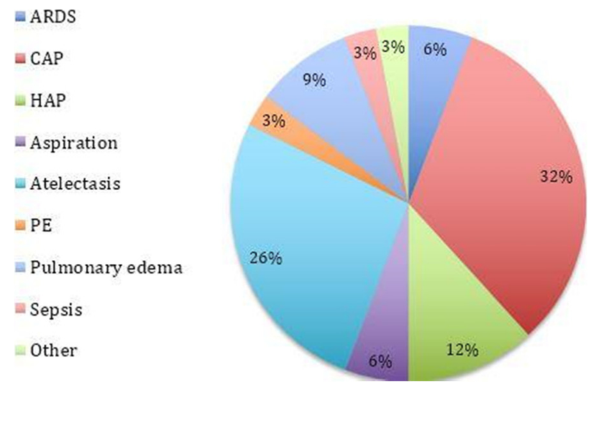

**Fig. 233 (Abstract 0676)** See text for description

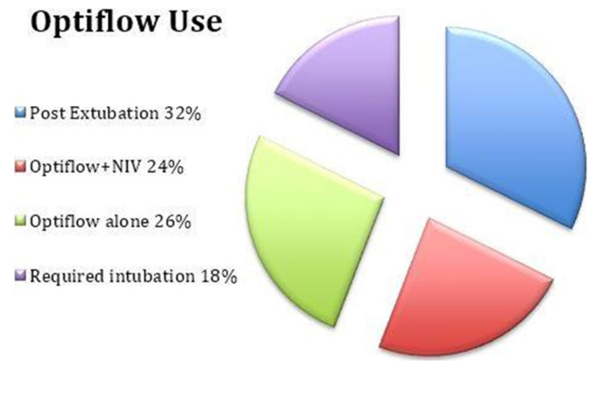

**Fig. 234 (Abstract 0676)** See text for description

### 0677

#### Early alert of failure in critical patients with high flow oxygen therapy

J. F. Martin-Lazaro, R. Despiney, M. Brys, S. Carty, J. Napier
Newham University Hospital, Intensive Care Unit, London, United Kingdom
**Correspondence:** J. F. Martin-Lazaro
*Intensive Care Medicine Experimental* 2017, **5(Suppl 2):**0677

**INTRODUCTION.** High flow oxygen therapy (HFOT) has shown to be a good technique for patients with Type 1 respiratory failure (T1RF). In Type 2 respiratory failure (T2RF) is more controversial and it is noted that there is a gap in the literature in identifying clinical parameters that highlight the high risk patients likely to fail HFOT or identify failing HFOT therapy in T2RF.

**OBJECTIVES.** To describe the outcomes with HFOT in the critical patient with respiratory failure and to establish clinical parameters that identify high risk patients likely to fail HFOT or deteriorating patients on HFOT.

**METHODS.** We conducted a retrospective observational study on the use of HFOT use (Optiflow; Fisher & Paykel Healthcare, Auckland, New Zealand) in an ICU setting at Newham University Hospital in London. We included 97 consecutive patients which were identified as having either T1RF or T2RF. We collated their vital signs, arterial blood gases before initiation of HFOT and one hour after and their outcomes (improvement, non-invasive ventilation, intubation or palliative). Statistical analysis for description of the sample and averages comparison was performed by Excel and SPSS.

**RESULTS.** Our population consisted of 97 patients (n = 97): 55 males (56.70%) and 42 females (43.30%) with an average age of 63 ± 17 years. T1RF was the reason for admission in 46 patients while 51 patients had T2RF. Patients were stratified into high risk for use of HFOT according to their co-morbidities and we found 62% of our patients were high risk. We observed an improvement in vital signs with statistical significance ($p < 0.005$) in both T1RF and T2RF. HFOT was used successfully with patient discharge from ICU in 62 patients (63.92%). Failure of HFOT was considered when intubation or NIV was required, which occurred in n = 9 (9.28%) and n = 18 (18.56%) respectively. Palliative care was applied in 8 patients (8,25%). In both types of respiratory failure it was observed that poorer outcomes were associated in patients with an acidosis prior to HFOT pH (≤7.30) and tachypnoea (RR ≥ 30). Particularly in T2RF with these clinical parameters, it was observed that 55% required NIV and 18% intubation.

**CONCLUSIONS.** In our population of critical patients, HFOT showed to be a good strategy for management of T1RF and T2RF failure. However it was observed that poorer outcomes were associated with pH ≤ 7.30 or/and RR ≥ 30. Failure of improvement in these clinical parameters suggest a high probability of therapy failure and need for treatment escalation.

**REFERENCE(S)**

Bräunlich J, Köhler M, Wirtz H. Nasal highflow improves ventilation in patients with COPD. Int J Chron Obstruct Pulmon Dis. 2016 May 25;11:1077–85.

### 0678

#### Dyspnea in patients receiving non invasive ventilation in the ICU: prevalence, risk factors and prognosis impact

L. Dangers[1], A. Demoule[2], C. Montlahuc[3], A. Kouatchet[4], F. Meziani[5], S. Jaber[6], S. Perbet[7], M. Resche-Rigon[3], E. Azoulay[3]
[1]Hôpital Pitié Salpetrière, Assistance Publique Hôpitaux de Paris, Paris, France; [2]Groupe Hospitalier Pitié-Salpêtrière, Paris, France; [3]Hôpital Saint-Louis, Assistance Publique Hôpitaux de Paris, Paris, France; [4]Centre Hospitalier Universitaire, Angers, France; [5]Nouvel Hôpital Civil, Centre Hospitalier Universitaire, Strasbourg, France; [6]Hôpital Saint Eloi, Montepellier, France, [7]Centre Hospitalier Universitaire Estaing, Clermont-Ferrand, France
**Correspondence:** A. Demoule
*Intensive Care Medicine Experimental* 2017, **5(Suppl 2):**0678

**INTRODUCTION.** Dyspnea is frequent and intense in intubated patients. It is a threatening sensation, is reported by patients as one of the worse experiences of the intensive care unit (ICU) stay and is associated with adverse outcomes. Non invasive ventilation (NIV) is increasingly used and has become the cornerstone therapy of acute respiratory failure (ARF). However, little attention has been given to dyspnea in patients receiving NIV.

**OBJECTIVES.**

1) To quantify the prevalence and intensity of dyspnea in patients receiving NIV for ARF at admission and in response to NIV,
2) To examine the factors associated with dyspnea,
3) To investigate the impact of dyspnea on NIV success or failure and on post-ICU quality of life and burden.

**METHODS.** Second analysis of a prospective observational cohort study in patients who received NIV for ARF in 54 ICUs in France and Belgium, in 2010/2011. Dyspnea measurement was assessed with a modified Borg scale at admission and in response to the first NIV session. Patients with a dyspnea intensity < 4 (defined as light to moderate) were compared to those with dyspnea intensity ≥4 defined (moderate to severe).

**RESULTS.** Among the 426 patientswho received NIV for ARF, the median dyspnea on admission was 4 [3–5] and decreased to 3 [2–4] in response to the first NIV session ($p = 0.001$). Moderate to severe dyspnea in response to NIV was independently associated with severity as assessed by the SOFA (OR 1.09, $p = 0.023$), anxiety (OR 1.84, $p = 0.009$), leaks (OR 2.15, $p = 0.002$) and poor NIV tolerance (OR 2.01, $p = 0.012$). NIV failure rate was 31% (n = 133). Four factors independently predicted NIV failure: moderate to severe dyspnea in response to NIV (OR 2.24, p = 0.001), *de novo* ARF (OR 2.41, p < 0.001), SOFA score (OR 1.35, p < 0.001) and poor tolerance to NIV (OR 1.81, p = 0.046). Finally, dyspnea was associated with higher hospital length of stay (8 [4–21] vs. 6 [3–11] days, p = 0.014), hospital mortality (29% vs. 14%, p = 0.001) and day-90 mortality (54% vs. 35%, p < 0.001) but was not associated with higher post ICU burden and altered quality of life as assessed by the HADS (hospital anxiety and depression scale), SF-36 (physical and mental health) and IES-R (impact of event scale).

**CONCLUSION.** In patients receiving NIV for ARF,

1) dyspnea is frequent, intense and linked to anxiety,

2) dyspnea exposes patients to a higher risk of NIV failure,

3) dyspnea is associated with an increased mortality and length of stay.

### 0679

#### High-flow nasal oxygen cannula in patients with chronic obstructive pulmonary disease requiring ventilator support

N. Rittayamai[1], P. Phuangchoei[1], N. Praphruetkit[2], J. Tscheikuna[1]
[1]Faculty of Medicine Siriraj Hospital, Division of Respiratory Diseases and Tuberculosis, Department of Medicine, Bangkok, Thailand; [2]Faculty of Medicine Siriraj Hospital, Department of Emergency Medicine, Bangkok, Thailand
**Correspondence:** N. Rittayamai
*Intensive Care Medicine Experimental* 2017, **5(Suppl 2):**0679

**INTRODUCTION.** Non-invasive ventilation (NIV) is the first line management in patients with chronic obstructive pulmonary disease (COPD) who develop acute hypercapneic respiratory failure. High flow nasal oxygen cannula (HFNC) is a heated humidified, high-flow oxygen delivery system that has demonstrated benefits in patients with acute hypoxemic respiratory failure and prevention of extubation failure. Current evidence of using HFNC in hypercapneic respiratory failure is still limited. However, the mechanisms of HFNC in particular reducing deadspace potentially decreases respriatory effort and it would also has a beneficial effect in COPD patients. Our study evaluated the effect of HFNC at different flow rate and NIV on patient inspiratory effort assessed by esophageal pressure (Pes) swing, respiratory and hemodynamic variables.

**OBJECTIVES.** To evaluate Pes swing, gas exchange, breathing pattern and hemodynamic variables of NIV and HFNC with different flow rate.

**METHODS.** A prospective physiological study was conducted in COPD patients who had an indication for non-invasive ventilator support. Patients were ventilated with NIV and HFNC at flow rate of 10, 20, 30, 40 and 50 LPM for 15 minutes in each step. Pes was recorded and Pes swing was calculated using AcqKnowledge data acquisition system. Respiratory rate, blood pressure, heart rate, pulse oximetry ($SpO_2$) and transcutaneous $CO_2$ ($PtcCO_2$) were also continuously recorded throughout the study.

**RESULTS.** A preliminary results from 10 patients (mean age 75 ± 9 years, mean post-bronchodilator $FEV_1$/FVC 45.9 ± 7.7% and $FEV_1$ 51.6 ± 18.0% predicted) demonstrated that patient inspiratory effort assessed by Pes swing was lowest during NIV in comparison to HFNC but not statistical significant different (P = 0.056) (Fig. 235). Higher flow rate of HFNC significantly decreased respiratory rate in comparison to HFNC at flow rate of 10 LPM (22 ± 4 vs 24 ± 4 breaths/min) but there was no significant difference when compared with NIV (22 ± 4 vs 23 ± 6 breaths/min). HFNC at optimal flow rate significantly improved oxygenation (P = 0.001). HFNC also demonstrated a trend towards decrease in $PtcCO_2$ when compared to NIV but no statistical significant difference was found (P = 0.671). There were no differences in respiratory rate, mean arterial pressure and heart rate between the two devices (Table 176).

**CONCLUSIONS.** NIV seems better than HFNC in terms of reducing patient inspiratory effort. HFNC improves oxygenation and may improve ventilation in comparison to NIV. HFNC may be an alternative method for hypercapneic COPD patients requiring ventilator support.

1.Papazian L, et al. Intensive Care Med 2016;42:1336–49.
2.Moller W, et al. J Appl Physiol 2015;118:1525–32.
3.Rittayamai N, et al. Intensive Care Medidine Experimental 2016;4(Suppl 1):A345.

**GRANT ACKNOWLEDGMENT**
This study was supported by Faculty of Medicine Siriraj Hospital, Mahidol University, Bangkok, Thailand.

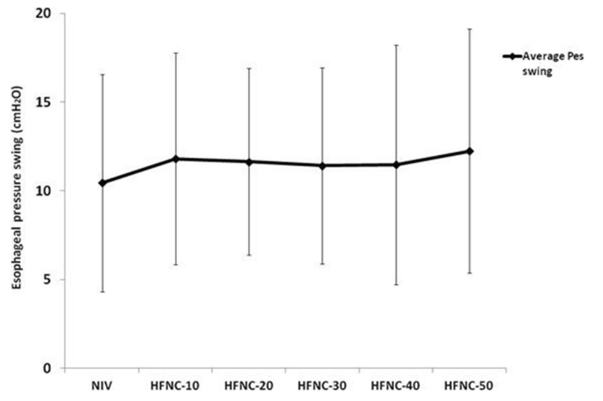

**Fig. 235 (Abstract 0679)** Esophageal pressure swing during NIV and HFNC

**Table 176 (Abstract 0679)**. Respiratory and hemodynamic variables

| | NIV | HFNC-10 | HFNC-20 | HFNC-30 | HFNC-40 | HFNC-50 | P-value |
|---|---|---|---|---|---|---|---|
| Pes swing (cmH2O) | 10.4±6.1 | 11.8±6.0 | 11.7±5.3 | 11.4±5.5 | 11.5±6.7 | 12.2±6.9 | 0.056 |
| SpO2 (%) | 95.2±2.3 | 95.0±2.5 | 96.2±2.6 | 96.8±2.3 | 97.3±2.0* | 97.6±2.1* | 0.001 |
| PtcCO2 (mmHg) | 42.2±6.8 | 41.6±7.6 | 41.4±7.6 | 41.3±7.2 | 41.1±7.0 | 40.4±6.5 | 0.671 |
| Respiratory rate (/min) | 23±6 | 24±4 | 22±5# | 22±4# | 22±4# | 22±4# | 0.042 |
| Mean arterial pressure (mmHg) | 104±21 | 96±14 | 93±9 | 93±11 | 91±12 | 94±15 | 0.051 |
| Heart rate (/min) | 94±19 | 95±18 | 91±15 | 91±17 | 92±18 | 96±19 | 0.155 |

### 0680

#### Analysis of a prediction scale for the failure of non-invasive ventilation in patiets with hypoxemic respiratory failure

A. Lopez Martinez, A. Carrillo Alcaraz, D. O. Torres Serrato, A. Burruezo, F. Sotos, P. Bayoumy, A. A. Agamez, A. Montenegro
Hospital JM Morales Meseguer, ICU, Murcia, Spain
**Correspondence:** A. Lopez Martinez
*Intensive Care Medicine Experimental* 2017, **5(Suppl 2):**0680

**INTRODUCTION.** Patients with hypoxemic respiratory failure (ARF) may be treated with noninvasive ventilation (NIV) but assuming a high percentage of failures. The prediction of NIV failure by a prediction rule would facilitate a better selection of patients with greater possibility of success through non-invasive support.

**OBJECTIVES.** To analyze the predictive capacity of NIV failure using the HACOR scale in patients with hypoxemic ARF.

**METHODS.** Retrospective study on a prospective database. All patients with hypoxemic ARF admitted consecutively to the ICU for a period of 20 years and receiving NIV were analyzed. The HACOR scale is based on easily observed variables measured at the onset of NIV: heart rate, pH value, state of consciousness using the Glasgow coma scale, oxygenation using the PaO2 / FiO2 ratio and respiratory rate. With a score greater than 5, patients with a high risk of NIV

failure, defined as those requiring intubation after the technique is used, are identified. Quantitative variables are expressed as mean ± standard deviation, median (first and third quartile), and qualitative variables as absolute and relative frequencies. The comparison between quantitative and qualitative variables is done by Student´s T test. ROC curves are constructed and the area under the curve (AUC) is calculated with its 95% confidence intervals.

**RESULTS.** During the study period, 2529 patients with hypoxemic ARF received NIV, mean age 67.3 ± 16.8 years, 1290 men (51%) and 509 (20.1%) with do not intubation order. The main reasons to start NIV were heart failure (944 patients, 37.3%) post-extubation ARF (459 patients, 18.1%) and pneumonia (412 patients, 16.3%). HARCOR scale value was a mean of 4 (2,6). Successful NIV was achieved in 1629 patients (64%) with an HACOR score of 3.5 ± 2.3 compared to the NIV failure group with a score of 7.21 ± 3.9 ($p < 0.001$). The AUC of the HACOR scale for predicting NIV failure was 0.805 (IC-95% = 0.789-0.821), whereas for failure in the first hour of NIV was 0.950 (IC-95% = 0.941-0.958) and 0.851 (IC-95% = 0.836-0.864) for the first 6 hours of NIV.

**CONCLUSIONS.** In our cohort of patients diagnosed with hypoxemic ARF and treated with NIV, the HACOR scale shows the best ability to predict the failure of the technique in the first hour of treatment and with good performance up to the first 6 hours, being slightly less effective to predict failure during the treatment.

Duan J, Han X, Bai L, Zhou L, Huang S. Assessment of heart rate, acidosis, consciousness, oxygenation, and respiratory rate to predict noninvasive ventilation failure in hypoxemic patients.

### 0681

#### Non invasive ventilation use in Severe Hypercapnic Encephalopathy

K. Meddeb[1], J. Ayachi[1], E. Ennouri[1], H. Kallel[1], M. A. Boujelbèn[1], N. Fraj[1], Y. Hamdaoui[1], W. Zarrougui[1], A. Ben Abdelhafidh[1], N. Sma[1], A. Azouzi[1], A. Khedher[1], I. Chouchene[1], I. Ben Saida[1], M. Boussarsar[1,2]
[1]Farhat Hached University Hospital, Medical Intensive Care Unit, Sousse, Tunisia; [2]Ibn Al Jazzar Faculty of Medicine, Research Laboratory N° LR14ES05 Interactions of the Cardio-pulmonary System, Sousse, Tunisia
**Correspondence:** M. Boussarsar
*Intensive Care Medicine Experimental* 2017, **5(Suppl 2):**0681

**INTRODUCTION.** Although Severe Hypercapnic Encephalopathy (SHE) is a critical situation, where intubation delays can have disastrous outcomes, a first short trial of NIV could identify a spectrum of responsive patients thus avoiding IMV and its complications, when performed by an experienced team.

**OBJECTIVES.** To assess NIV use, efficiency and outcomes in Severe Hypercapnic Encephalopathy (SHE) compared to Mild to Moderate Hypercapnic Encephalopathy (MMHE).

**METHODS.** Retrospective, observational, monocentric study conducted in an 8-bed medical Tunisian ICU from January, 2000 to December, 2015. Were included all consecutive patients admitted for acute on chronic respiratory failure (AE/CRF). Two groups were individualized according to the severity of hypercapnic encephalopathy (SHE and MMHE). Were compared patients characteristics, initial clinical presentation, SAPSII, ABG's on admission, NIV use and outcomes.

**RESULTS.** Within the study period were admitted 1245 patients for AE/CRF, 727(58%) patients presented with SHE on admission, among them only 31(4,3%) patients received a first trial of NIV. 518 patients were admitted with MMHE with 343 (68,5%) patients receiving firstline NIV ($p = 0.0001$). SHE patients had less Home Mechanical Ventilation and Long Term Oxygen therapy compared to patients who presented with MMHE, respectively, 21(2,9%) vs 33(6,6%), $p = 0.002$ ; and 127 (17,5%) vs 117(23,4%), $p = 0.011$. On admission, they had higher SAPSII score, 35,63 ± 13,44 vs 28,45 ± 10,37, $p = 0.0001$, with lower *pH* levels, 7,30 ± 0,11 vs 7,33 ± 0.08, $p = 0.0001$. First line NIV success rate of 8(25,8%) was achieved in the SHE group, vs 218(63,3%) in the MMHE group, $p = 0.0001$. Intubation delay was lower in SHE patients, 1,38 ± 1,15 days vs 2,4 ± 3,07 days, $p = 0.0004$. Intubation rate was higher in SHE group, 23(74,2%) vs 125(36,4%), $p = 0.0001$. There was no significant difference in term of overall mechanical ventilation duration, Ventilator Associated Pneumonia density incidence and length of stay between the two groups. Mortality rate for SHE patients was higher, especially those who were intubated after first line NIV, 378(52%) vs 78(26,4%) for MMHE patients, $p = 0.0001$.

**CONCLUSIONS.** Initial NIV is rarely performed for Severe Hypercapnic Encephalopathy, but when implemented, 25% rate of NIV success has been achieved. However, intubation delay is lower for these patients compared to non SHE patients, showing an adapted behavior of the attending physician in SHE.

### 0682

#### High flow nasal cannula oxygen versus non-invasive ventilation in acute respiratory failure? A systematic analysis of available literature

L. Beng Leong, N. W. Ming, L. W. Feng
Ng Teng Fong Hospital, Emergency, Singapore, Singapore
**Correspondence:** L. Beng Leong
*Intensive Care Medicine Experimental* 2017, **5(Suppl 2):**0682

**INTRODUCTION.** Non-invasive ventilation (NIV) and high flow nasal cannula (HFNC) are used in acute respiratory failure (ARF).

**OBJECTIVE.** We conducted this systematic review to examine their use.

**METHODS.** We searched several databases; including randomized trials comparing at least NIV with HFNC or NIV + HFNC with NIV. Primary outcome was mainly intubation rates. Secondary outcomes included ICU mortality and morbidities. We reported medians (IQRs), p values, odds ratios (ORs)
[95% CIs].

**RESULTS.** Five trials were included; three compared HFNC to NIV. One compared HFNC, NIV and oxygen therapy (OT) while one compared HFNC + NIV with NIV. They recruited patients with acute hypoxaemic RF largely defined as $PaO_2/FiO_2 \leq 300$ mmHg. Heterogeneity occurred in patient populations, primary outcomes and study design; preventing result pooling. Patient populations ranged from post cardiothoracic surgery to pneumonia. Two trials were done post extubation, two pre-intubation and one during intubation. Three trials reported patient-centred primary outcomes (PCPOs) [re-intubation and Day 28 intubation rates]. The other two trials reported lowest $SpO_2$ during flexible bronchoscopy (FB) and intubation. Three and two studies had superior and non-inferior design respectively. Of the trials with PCPOs, the ORs for intubation or re-intubation for the NIV vs HFNC group ranged from 0.80 (95% CIs: 0.54 - 1.19) to 1.65 (95% CIs: 0.96 - 2.84). For the trials with surrogate primary outcome, only one reported higher lowest $SpO_2$ during intubation in the NIV + HFNC vs NIV group [100 (IQR: 95–100)% vs 96 (IQR: 92–99)%; $p = 0.029$]. Important secondary outcomes were lower ICU mortality rate in HFNC vs NIV or OT group and better patient tolerability favouring HFNC over NIV.

**CONCLUSIONS.** Results were conflicting but highlighted important future research directions. These include selection of patients with acute hypercapnic RF, more severe hypoxaemia ($PaO_2/FiO_2 \leq 200$ mmHg); inclusion of an OT arm, superior trial design and PCPOs.

5. Jaber S, et al. Apnoeic oxygenation via HFNC oxygen combined with NIV preoxygenation for intubation in hypoxaemic patients in the intensive care unit: the single-centre, blinded, randomized controlled OPTINIV trial. Intensive Care Med. 2016;42:1877–87.

### 0683

#### Trends in use of NIV in acute exacerbation of chronic respiratory failure (AE/CRF) in a Tunisian medical ICU, 2000–2015

K. Meddeb[1], A. Khedher[1], Y. Hamdaoui[1], J. Ayachi[1], N. Fraj[1], M. Boujelbèn[1], J. Baroudi[1], N. Sma[1], E. Ennouri[1], W. Zarrougui[1], A. Ben Abdelhafidh[1], H. Kallel[1], I. Ben Saida[1], A. Azouzi[1], I. Chouchene[1], M. Boussarsar[1,2]
[1]Farhat Hached University Hospital, Medical Intensive Care Unit, Sousse, Tunisia; [2]Ibn Al Jazzar Faculty of Medicine, Research Laboratory N° LR14ES05 Interactions of the Cardio-pulmonary System, Sousse, Tunisia
**Correspondence:** M. Boussarsar
*Intensive Care Medicine Experimental* 2017, **5(Suppl 2):**0683

**INTRODUCTION.** NIV has become a cornerstone for the supportive therapy to manage critically ill patients with AE/CRF. It reduces the need of mechanical ventilation, complication rates and mortality. These benefits deserve more evaluation, especially in developing countries where organization difficulties may impede NIV efficiency.

OBJECTIVE. To assess trends in use of NIV in AE/CRF in a Tunisian medical ICU.

**METHODS.** Retrospective, observational, monocentric study conducted in an 8-bed Tunisian medical ICU from January, 2000 to December, 2015. Were included all consecutive patients admitted for AE/CRF. Were assessed : patient's characteristics, trends in use of NIV, success and failure rate of NIV use, length of ICU stay, ventilatory free days and mortality.

**RESULTS.** Among the 4650 patients admitted within the 16 years' study period, 1245(26,7%) presented with AE/CRF. They were 66,72 ± 12,99years aged, 913 (73,3%) were COPD, 145(11,7%) restrictive disease, and 109 (8,8%) OHS. 648 (52,1%) had mMRC ≥ 4. Mean SAPSII was 32,93 ± 13,03. 727(58,4%) patients were presented with a severe hypercapnic encephalopathy. Mean pH and PaCO2 were respectively 7,31 ± 0,11 ; 63,17 ± 22,37. Overall firstline NIV use was registered in 374(30%), 226(60,4%) patients succeeded.Length of stay (LOS), ventilator free days (VFD) and mortality were respectively 10,3 ± 9,59 ; 3,37 ± 4,7 ; 26,2%. Compared results between three predefined periods (P1, P2, P3) are displayed on the Fig. 236.

**CONCLUSIONS.** Despite a progressive increasing rate of NIV use over the course of sixteen years, this did not result in a similar progression neither of its success rate with no significant impact nor on mortality rate, probably due to ICU organizational problems, that may have interfered with NIV management and resulting in a steady gain in the learning curve.

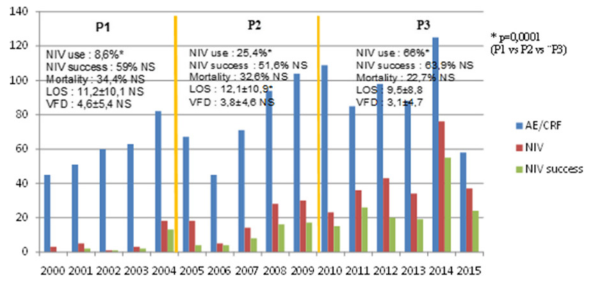

**Fig. 236 (Abstract 0683)** See text for description

### 0684

#### Factors associated with NIV failure in patients admitted for acute exacerbation of chronic respiratory failure (AE/CRF) in a Tunisian medical intensive care unit

K. Meddeb[1], J. Ayachi[1], H. Kallel[1], N. Fraj[1], M. A. Boujelbèn[1], E. Ennouri[1], N. Sma[1], W. Zarrougui[1], A. Ben Abdelhafidh[1], I. Ben Saida[1], A. Azouzi[1], I. Chouchene[1], A. Khedher[1], M. Boussarsar[1,2]
[1]Farhat Hached University Hospital, Medical Intensive Care Unit, Sousse, Tunisia; [2]Ibn Al Jazzar Faculty of Medicine, Research Laboratory N° LR14ES05 Interactions of the Cardio-pulmonary System, Sousse, Tunisia
**Correspondence:** M. Boussarsar
*Intensive Care Medicine Experimental* 2017, **5(Suppl 2):**0684

**BACKGROUND.** The use of NIV has increased over the span of two decades considering its various benefits. On the other hand, NIV failure is regarded to be associated with worse prognosis, which makes the identification of factors related to NIV failure necessary.

**OBJECTIVES.** To identify risk factors of NIV failure in AE/CRF in a Tunisian medical ICU.

**METHODS.** Retrospective observational study conducted in an 8-bed Tunisian medical ICU over a 16 years period (January 2000 to December 2015). Were included all consecutive patients admitted for acute on chronic respiratory failure for whom NIV was initiated within the ICU as first line ventilatory support. Were collected patients' characteristics, clinical presentation on admission, SAPSII, NIV success rate and outcomes. Univariate and Multivariate analyses were conducted to identify factors independently associated to NIV failure.

**RESULTS.** 1245 patients were admitted to the ICU for AE/CRF. They were 66,72 ± 12,99years aged, 913 (73,3%) were COPD, 145(11,7%) restrictive disease, and 109(8,8%) OHS. 648 (52,1%) had mMRC ≥ 3. Mean SAPSII was 32,93 ± 13,03. 727(58,4%) patients presented with a severe hypercapnic encephalopthy. Mean pH and PaCO2 were respectively 7,31 ± 0,11 ; 63,17 ± 22,37. Overall firstline NIV use was registered in 374(30%), 226(60,4%) patients succeeded. Length of stay (LOS), ventilator free days (VFD) and mortality were respectively 10,3 ± 9,59days ; 3,37 ± 4,7days and 26,2%. Univariate analysis identified that patients who failed NIV had less Home Mechanical Ventilation 24(10,6%) vs 6(4,1%), p = 0.022 ; with severe initial clinical presentation SAPSII 31,5 ± 12,3 vs 26,5 ± 8,6, p = 0.0001, severe hypercapnic encephalopathy on admission 23(15,5%) vs 8(3,5%), p = 0.0001 ; circulatory failure on admission 20(13,5%) vs 15(6,6%), p = 0.026 ; pH, 7,31 ± 0,08 vs 7,34 ± 0,08 (p = 0.0001) ; PaCO2, 67,7 ± 21,1 vs 62,7 ± 21,1 mmHg, p = 0.026. Multivariate analysis identified SAPSII (OR, 1.036 ; 95%CI [1.012-1.060] ; p = 0.003), hypercapnic encephalopathy score on admission (OR, 2.41 ; 95%CI [1.65-3.52] ; p = 0.0001) and pH ≤ 7,30 on admission (OR,1,95 ; 95%CI [1,22-3,13]; p = 0.005) as independent risk factors of NIV failure.

**CONCLUSION.** The present study showed that independent factors of NIV failure were Hypercapnic Encephalopathy score, SAPSII and pH level on admission.

### 0685

#### Compared NIV use and outcomes in Acute Exacerbations of Chronic Obstructive Pulmonary Disease (AECOPD) vs acute exacerbations of restrictive diseases

K. Meddeb[1], J. Ayachi[1], E. Ennouri[1], N. Fraj[1], M. A. Boujelbèn[1], Y. Hamdaoui[1], N. Sma[1], W. Zarrougui[1], A. Ben Abdelhafidh[1], I. Ben Saida[1], A. Azouzi[1], I. Chouchene[1], A. Khedher[1], M. Boussarsar[1,2]
[1]Farhat Hached University Hospital, Medical Intensive Care Unit, Sousse, Tunisia; [2]Ibn Al Jazzar Faculty of Medicine, Research Laboratory N° LR14ES05 Interactions of the Cardio-pulmonary System, Sousse, Tunisia
**Correspondence:** M. Boussarsar
*Intensive Care Medicine Experimental* 2017, **5(Suppl 2):**0685

**INTRODUCTION.** Acute hypercapnic respiratory failure (AHRF) due to Acute Exacerbation of Chronic Obstructive Pulmonary Disease (AECOPD) is considered the main indication to Non Invasive Ventilation (NIV). However, this seeming, better response of NIV in AE/COPD compared to other causes of AHRF, needs further investigations.

**OBJECTIVES.** To assess the differences of NIV use and outcomes when in AECOPD compared to other causes of AHRF.

**METHODS.** Retrospective observational study conducted in an 8-bed Tunisian medical ICU during 16 years from January 2000 to December 2015. Were included all consecutive patients admitted for AHRF. Two groups were formed, patients admitted for AECOPD and patients admitted for AHRF other than AECOPD. Were compared patients characteristics, SAPSII, ABG´s on admission, NIV use and its success rate, NIV and invasive mechanical ventilation (IMV) duration, Ventilator Associated Pneumonia (VAP) density incidence and mortality rate.

**RESULTS.** Among 1245 admitted for AHRF, 913(73,3%) were admitted for AECOPD. Other causes of AHRF were mainly acute on restrictive chronic respiratory failure (11,7%) and Obesity Hypoventilation Syndrome (8,6%). Sex Ratio was 8,13:1, in the AECOPD group, and 0,45:1 in the non AECOPD group (p = 0.0001). Patients in the AECOPD had a lower rate of Home Mechanical Ventilation 3,7% vs 6,3% (p = 0.048), and had less associated comorbidities such Hypertension 21,6% vs 38% (p = 0.0001), Diabetes Mellitus 13,4% vs 24,4% (p = 0.0001), and chronic heart failure 16,3% vs 8,2% (p = 0.0001). They presented more severe clinical status attested by a higher SAPSII score 33,7 ± 13,4 vs 30,8 ± 11,9 (p = 0.001). There was no significant difference between the two groups regarding firstline NIV use 28,6% vs 34% (p = 0.64) and its success rate 59,4% vs 62,8% (p = 0,5). For patients who failed NIV mean intubation delay was estimated at 2,32 ± 2,8 for COPD patients vs 2,31 ± 3,2days in non COPD patients with no significant difference. Total intubation rate combining firstline IMV and NIV failure was estimated at 758(83,1%) in COPD patients vs 261(78,6%) in non COPD patients. They presented overall higher VAP density incidence 22,75/1000VD vs 18,18/1000VD (p = 0.041) but when considering patients who had first line NIV there was no significant differences in terms of VAP density incidence, mechanical ventilation duration, length of stay or mortality rate between the two groups.

**CONCLUSIONS.** The present study did not show a better response of AECOPD to NIV compared to other causes of AHRF, this may be due to the initial severity of the COPD patients.

### 0686

#### One year outcomes of ICU survivor's patients after an acute exacerbation of a chronic respiratory disease (AE/CRD)

J. Baroudi[1], I. Ben Saida[1], K. Meddeb[1], J. Ayachi[1], N. Fraj[1], M.A. Boujelbèn[1], E. Ennouri[1], H. Kallel[1], W. Zarrougui[1], A. Ben Abdelhafidh[1], A. Azouzi[1], A. Khedher[1], I. Chouchene[1], M. Boussarsar[1,2]
[1]Farhat Hached University Hospital, Medical Intensive Care Unit, Sousse, Tunisia; [2]Ibn Al Jazzar Faculty of Medicine, Research Laboratory N° LR14ES05 Interactions of the Cardio-pulmonary System, Sousse, Tunisia
**Correspondence:** M. Boussarsar
*Intensive Care Medicine Experimental* 2017, **5(Suppl 2):**0686

**BACKGROUND/AIM.** ICU mortality of chronic respiratory patients was decreasing by the use of NIV. There are only few reports on long-term survival for chronic respiratory failure. The purpose of this study is to determine long-term outcomes of ICU survivors admitted for an acute exacerbation of chronic respiratory disease (AE/CRD).

**METHODS.** A prospective cohort study was performed in a Tunisian medical ICU between 2014 and 2016. A follow-up was performed on ICU survivors admitted for AE/CRD during 1-year using phone interviews. The study collected: clinical features at admission, acute management procedures, functional characteristics and vital parameters (BP, HR, ABG's) at ICU discharge. Outcomes during 1 year after ICU discharge (mortality, readmissions) were assessed. Survivors and died patients after discharge were compared.

**RESULTS.** We identified 325 ICU survivors with 97(30%) admitted for AE/CRD. Among these patients, 61(62%) had COPD, 26(26.8%) had chronic restrictive respiratory disease and 10(10.3%) had overlapping syndromes. Mean age was 66 ± 13 years. 68% were male. Mean SAPSII at ICU admission was 30 ± 9. Mean length of stay was 10 ± 9 days. 51(52%) patients were treated with NIV and 46 (48%) patients required first line invasive mechanical ventilation or after NIV failure initiated. Clinical features at discharge evaluated by mean value of BP, HR, pH, PCO2 and PO2 were respectively (11,7 ± 1.7mmHg; 87 ± 13 b/mn; 7.42 ± 0.05; 49 ± 12mmHg; 92 ± 45mmHg). Mean value of functional status was 3.3 ± 0.7. After ICU discharge only 79(81.4%) patients were respondent. Among them 30(38%) patients died during 1 year. Median survival was 110 days. 90-day mortality rate was 46.7%. Hospital readmission rate was 48% during 1-year of follow up and 22(27.8%) patients were ICU readmitted. Univariate analysis identified two factors associated with post ICU mortality: SAPSII was higher in died patients (33.48 ± 11.3 versus 28.9 ± 7; p = 0.03) and functional status measured at ICU discharge was also reduced among this group (4 ± 0.5 versus 3 ± 0.7; p = 0.000).

**CONCLUSION.** Like post ICU survivors, patients admitted for AE/CRD have decreased long term survival rates. Patients at risk of dying after ICU discharge were those with severe acute disease at ICU admission and poorer functional status at discharge.

### 0687

#### Nasal mask continues positive airway pressure versus high flow nasal cannula after extubation for high risk patients

M. Sakuraya, A. Takaba, K. Natsuki, T. Tsutsui, K. Yoshida, T. Kondo
Ja Hiroshima General Hospital, Hatsukaichi, Japan
**Correspondence:** M. Sakuraya
*Intensive Care Medicine Experimental* 2017, **5(Suppl 2):**0687

**INTRODUCTION.** Reintubation is associated with poor outcome. Non invasive Positive Airway Pressure Ventilation (NPPV) after extubation for high risk patients could be used to prevent reintubation. However, patients are often intolerant of NPPV. High Flow Nasal Cannula (HFNC) might increase comfort level. Tolerance of NPPV is different among interfaces. Nasal masks are better tolerated than the full-face masks.

**OBJECTIVES.** We compared nasal mask NPPV with HFNC after extubation.

**METHODS.** This retrospective cohort study was conducted in a medical and surgical ICU between April 2014 and December 2016. The patients who were underwent nasal mask continuous positive airway pressure (N-CPAP) or HFNC after extubation were included. We investigated comfort level of interface (NRS: numeric rating scale), modified borg scale, critical-care pain observation tool (CPOT), the Ratio of Partial Pressure Arterial Oxygen and Fraction of Inspired Oxygen (P/F) and rentubation.

**RESULTS.** N-CPAP group patients (n = 23) were younger and more obese than HFNC group patients (n = 30). NRS (N-CPAP: 2, 2–3 vs. HFNC: 2, 1–3), modified borg scale (2, 1.5-3 vs. 2.5, 2–5) and CPOT (1, 0–2 vs. 2, 1–3) were not significant different between two groups. Although P/F didn't change after etubation in N-CPAP group (mean P/F at SBT 252 vs. mean P/F after extubation 246), P/F decreased after extubation in HFNC group (277 vs. 217, p = 0.005). Three patients were reintubated in HFNC group, but there is no reintubation in NPPV group.

**CONCLUSIONS.** N-CPAP might be used to avoid impairment of oxygenation and as tolerated as HFNC after extubation.

**REFERENCE(S)**

Liesching T, Kwok H, Hill NS. Acute applications of noninvasive positive pressure ventilation. Chest. 2003;124:699–713.

### Infection and immunomodulation

#### 0688

**Protective effects of interleukin-33 in critically ill patients**

S. Stojkovic[1], K. Krychtiuk[1], M. Lenz[1], M. Brekalo[1], K. Huber[2], J. Wojta[1], G. Heinz[1], S. Demyanets[3], W. Speidl[1]
[1]Medical University of Vienna, Cardiology, Vienna, Austria;
[2]Wilhelminenhospital Vienna, 3rd Medical Department, Vienna, Austria;
[3]Medial University of Vienna, Department of Laboratory Medicine, Vienna, Austria
**Correspondence:** S. Stojkovic
*Intensive Care Medicine Experimental* 2017, **5(Suppl 2):**0688

**INTRODUCTION.** Patients admitted to a medical intensive care unit (ICU) are characterized by an activated immune system and exhibit a high mortality rate irrespective of the underlying cause of admission. Interleukin (IL)-33 has been shown to be protective in experimental sepsis models and it has been demonstrated that circulating levels of its "decoy" receptor soluble ST2 (sST2) are associated with outcome in critically ill patients.
**OBJECTIVES.** The aim of the present study was to investigate whether circulating IL-33 is associated with 30-day mortality in patients admitted to a medical ICU.
**METHODS.** In this prospective, observational study, both IL-33 and sST2 levels were assessed in 223 consecutive patients at ICU admission using specific enzyme-linked immunosorbent assays (ELISAs).
**RESULTS.** During the 30-day follow-up, 58 patients (26%) died. Circulating IL-33 was detectable in 166 patients and in 57 patients serum IL-33 was below the detection limit. Both detectable IL-33 and sST2 below the median were strong predictors of survival in critically ill patients independent of acute physiology and chronic health evaluation II (APACHE II) score. IL-33 and sST2 predicted risk independent from each other. Patients with both, non-detectable levels of IL-33 and sST2 levels above the median, showed a dramatically increased mortality risk (HR 6.9 95% CI 3.0-16.2; p < 0.001).
**CONCLUSIONS.** Low levels of IL-33 and increased levels of sST2 predict mortality risk in critically ill patients independent from each other and APACHE II score. Both together showed additive predictive value suggesting a pathogenic role of the IL-33/ST2 system in critically ill patients.

#### 0689

**Mortality associated to high mean platelet volume and other inflammation markers in critical patients**

A. Montaño Jimenez, A. Sanchez-Calzada, J. Franco, J. Aguirre, G. Camarena
Centro Medico ABC, Intensive Care Unit, Ciudad de Mexico, Mexico
**Correspondence:** A. Montaño Jimenez
*Intensive Care Medicine Experimental* 2017, **5(Suppl 2):**0689

**INTRODUCTION.** Sepsis is one of the main causes of admit ion to the intensive care unit (ICU), its early diagnostic and management significantly reduce mortality. Hematological anomalies are common amongst patients with inflammatory process in the ICU, sepsis physiopathology has been related with coagulation alterations, platelet activation, and a decreased platelet count. Platelet count is used for diverse severity indexes. Currently, there has been a growing interest towards the platelet function, especially regarding the mean platelet count, which when increased suggests platelet activation and production. It has been associated with thrombotic process such as ischemic cardiomyopathy and cerebrovascular events, as well as its increase has been related to inflammation and sepsis.
**OBJECTIVE.** To assess clinical prediction for mortality of the mean platelet volume (MPV) and other inflammation markers in critical patients on admission.
**METHODS.** We conducted an observational cohort with critical patients from two intensive care units in Mexico City, Mexico. Demographic, Clinical, laboratory data was collected for all patients. We estimated the following severity indexes on admission: sequential organ failure assessment (SOFA) score with acute physiology and chronic health evaluation (APACHE) II, Predisposition, Insult/Infection, Response and Organ Dysfunction (PIRO). Inflammation markers included were serum procalcitonin (PCT), ultrasensitive reactive protein (CRP) and MPV for all patients. Cutoff points were obtained for the inflammation markers and survival analysis was performed for the most accurate cutoff values for each one. Statistical analyses were done in STATA SE 11.0.
**RESULTS.** We included 202 patients with a mean age of 61(SD ± 18) years, 50% females, median and interquartile range (RIQ) for length hospital stay of 17 (11–30) days. Overall mortality of 16.83%. The median and RIQ for severity scores were for SOFA, PIRO and APACHE II of 7 (5–10), 7(4–9) and 9 (7–12), respectively. Inflammation markers with a Median serum level of CRP 6 (RIQ 2–13)mg/dL, Median PCT value of 0.07(RIQ 0.05-5)ng/dL, Median for MPV of 7.3(RIQ 6.8-8.9)fL/μm$^3$. Areas under the curve (AUC) and 95% confidence interval (CI) for PCT, CRP and MPV of 0.77(0.68-0.85), 0.52 (0.42-0.63) 0.74 (0.65-0.83). ROC analysis obtained the following cutoff values for PCT (> = 2ng/dL), CRP (> = 9mg/dL) and MPV (> = 8 fL/μm$^3$). Cox regression analysis showed that for selected cutoff points mortality risk was as follows: PCT > =2ng/dL, HR = 5.2 (IC 95% = 2.02-13.8); CRP > = 9 mg/dL, HR = 1.08 (95% CI = 0.54-2.1), p 0.8; MPV of > =8 HR 2.32 (95% CI = 1.07-5.03).
**CONCLUSION.** High MVP and PCT on admission were associated with mortality in ICU critical patients. PCT was an independent factor for mortality.

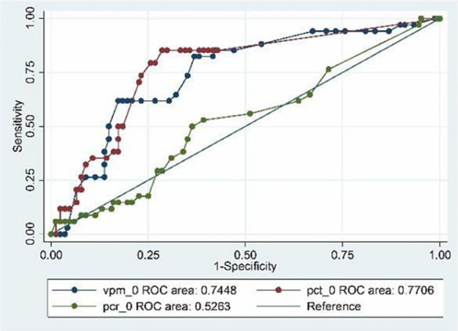

**Fig. 237 (Abstract 0689)** ROC curves

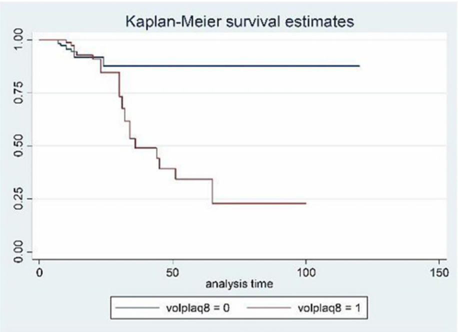

**Fig. 238 (Abstract 0689)** Survival estimates

### 0690

#### Population pharmacokinetics of continuous infusion piperacillin in critically ill patients

S. A. M. Dhaese[1], J. A. Roberts[2,3], M. Carlier[4], A. G. Verstraete[4,5], V. Stove[4,5], J. J. De Waele[1]
[1]Ghent University Hospital, Intensive Care, Ghent, Belgium; [2]University of Queensland, Burns, Trauma, and Critical Care Research Centre, Brisbane, Australia; [3]Royal Brisbane and Women's Hospital, Brisbane, Australia; [4]Ghent University Hospital, Laboratory Medicine, Ghent, Belgium; [5]Ghent University, Clinical Chemistry, Microbiology and Immunology, Ghent, Belgium
**Correspondence:** S. A. M. Dhaese
*Intensive Care Medicine Experimental* 2017, **5(Suppl 2):**0690

**INTRODUCTION.** Most population pharmacokinetic (PK) models for piperacillin have been designed based on intermittent and/or extended infusion data. To our knowledge, there are very few PK models for piperacillin that have been constructed based on continuous infusion (CI) data[1].

**OBJECTIVES.** To evaluate the population pharmacokinetics of CI piperacillin in critically ill patients.

**METHODS.** Patients admitted to the Ghent University Hospital ICU receiving piperacillin/tazobactam by CI were eligible for inclusion. Patients younger than 18 years or patients receiving extracorporeal membrane oxygenation or renal replacement therapy were excluded. Daily blood samples for piperacillin assay were analysed in the Ghent University Hospital laboratory using ultra-high performance liquid chromatography coupled to tandem mass spectrometry. Population PK modelling was undertaken using Pmetrics[2]. Probability of target attainment (PTA's) for 100% fT > 4 xMIC were compared for different CI regimens (8g, 12g, 16g, 20g and 24g per day), for varying renal clearances (30 ml/min to 300 ml/min) at the MIC breakpoint of 16 mg/L (representing a worst-case scenario). The cumulative fraction of response (CFR) was calculated using EUCAST data on the wild-type MIC distribution of *P. aeruginosa*.

**RESULTS.** 110 patients with a total of 270 samples were described using a one-compartment model. Drug clearance was best described using Cockroft-Gault creatinine clearance estimation (CLcr) as a model covariate. The estimated population PK parameters for clearance (CL) and volume of distribution (Vd) are described in Table 177.

The PTA for a standard CI piperacillin dosing regimen (4g loading dose + 16g/24h) has a probability of ≥ 90% to achieve target concentrations in patients with a CLcr ≤ 47 ml/min (Fig. 239). CI of 16g/24h piperacillin achieves target concentrations against 80% of the wild-type *P. aeruginosa* isolates in patients with a CLcr ≤75 ml/min and target concentrations against 80% of the susceptible *P. aeruginosa* isolates (MIC ≤ 16 mg/L) in patients with a Clcr ≤ 120 ml/min(Table 178).

**CONCLUSIONS.** Based on CI piperacillin data, we constructed a one-compartment model with renal clearance as a covariate. In ICU patients, the risk of target non-attainment increases with increasing renal clearance.

[1]Gonçalves-Pereira J, Póvoa P. Antibiotics in critically ill patients: a systematic review of the pharmacokinetics of β-lactams. Crit Care 2011;15:R206.

[2]Neely MN, van Guilder MG, Yamada WM, Schumitzky A, Jelliffe RW. Accurate detection of outliers and subpopulations with Pmetrics, a nonparametric and parametric pharmacometric modeling and simulation package for R. Ther Drug Monit 2012;34:467–76.

**GRANT ACKNOWLEDGMENT**

SD is funded by a NHMRC CRE grant awarded to prof Roberts. Funding for a research stay at the BTCCRC (Brisbane) was provided by the Faculty of Medicine and Health Sciences of Ghent University (Belgium).

**Table 177 (Abstract 0690)**.Piperacillin population PK parameter estimates

| PK parameter | Mean | Median | SD |
|---|---|---|---|
| Vd (L) | 25.54 | 23.53 | 9.91 |
| CL (L/h) | 8.38 | 7.68 | 3.65 |

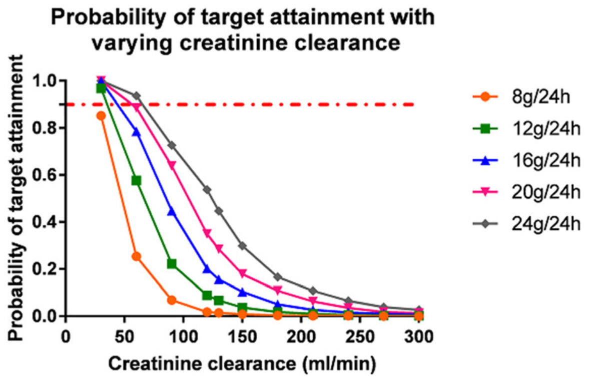

**Fig. 239 (Abstract 0690)**: PTA ~ CLcr (100% fT > 4xMIC) and MIC = 16 mg/L

**Table 178 (Abstract 0690)**. Cumulative Fraction of Response (CFR) ~ CLcr

| CLcr (Cockroft-Gault) ml/min | CFR, 100% fT>4xMIC) All P. aeruginosa isolates | CFR, 100% fT>4xMIC susceptible P. aeruginosa isolates (MIC ≤16 mg/L) |
|---|---|---|
| 30 | 0.88 | 0.99 |
| 60 | 0.84 | 0.97 |
| 90 | 0.78 | 0.91 |
| 120 | 0.70 | 0.82 |
| 150 | 0.64 | 0.75 |
| 180 | 0.56 | 0.65 |
| 210 | 0.48 | 0.56 |
| 240 | 0.44 | 0.51 |
| 270 | 0.39 | 0.45 |

### 0691

#### Adequate use of antibiotics in the Intensive Care Unit of a regional hospital

A. Ubeda[1], I. Fernández[1], R. Torcuato[1], J. Garcia[1], A. Fregosi[1], L. Alonso[2]
[1]Hospital Punta de Europa, Intensive Care Unit, Algeciras, Spain; [2]Pediatric La Lobilla, Estepona, Spain
**Correspondence:** A. Ubeda
*Intensive Care Medicine Experimental* 2017, **5(Suppl 2):**0691

**INTRODUCTION.** Inadequate antimicrobial treatment is known to be associated to worst outcome in bacteremia.

**OBJECTIVES.** To analyze the adequacy of antimicrobial use (ATB) in patients admitted to the ICU with sepsis.

**METHODS.** Descriptive retrospective analysis performed in an ICU with 12 beds during the year 2016. Adequate ATB was defined as the one with in vitro activity against the isolated microorganism. Not desescalate when indicated was considered unsuitable. Demographic variables, comorbilities, risk factors, severity scores, ICU and hospital length of stay (LOS), ATB and mortality were recorded. Statistical analysis: categorical variables (frequencies and percentages) and numerical variables (mean and standard deviation or medians and interquartile range). Comparisons: X2 test (percentages), student test (medias) and Kruskal- Wallis test (medians). Statistical significance with $p < 0.05$.

**RESULTS.** 94 patients were included: 58.8% men, 61.6 ± 14.3 years. APACHE II 17.6 ± 5.9. SOFA 7.4 ± 3.4. Not adequate ATB (61.7%), adequate (38.3%). 48.9% were medical and 35.1% surgical. The most frequent infectious focus was abdominal (44.7%) followed by respiratory (26.6%) and urinary (13.8%). ATB monotherapy (51.1%), combined treatment (28.7%) or sequential (20.2%). Reason for ATB change ($p < 0.001$): ATB not adequate (not change 58.6%, de-escalation 5.2%, escalation 34.5%), adequate ATB (not change 63.9%, de-escalation 30.6%, escalation 2.8%). There were no significant differences between groups (not adequate vs. adequate) in terms of patient type, comorbidities, risk factors, site of infection, severity scores at ICU admission, ICU and hospital LOS. The most frequent inappropriate ATBs were piperacillin-tazobactam (25.9%), ceftriaxone (13.8%) and imipenem (12.7%). The differences found in mortality were not statistically significant (not adequate 41.4% vs. adequate 27.8%, $p = 0.182$).

**CONCLUSIONS.** In our Unit, the percentage of inadequate ATB was high. Patients with adequate ATB had a higher proportion of de-escalation.

### 0692

#### Control strategies for an Acinetobacter Baumanni outbreak on a multidisciplinary icu during "zero resistance project"

O. Badallo Arevalo[1], M. del Valle Ortiz[1], E. Martinez Barrio[1], M. Gero Escapa[1], M. Martinez Barrios[1], R. Vara Arlanzon[1], D. Iglesias Posadilla[1], J.A. Fernandez Ratero[1], S. Ossa Echeverri[1], C. Carbajales Perez[1], G. Megias Lobon[2], B. Carreira Gonzalez[3], C. Diaz Tobajar[1]
[1]UCI Hospital Universitario De Burgos, Intensive Medicine, Burgos, Spain; [2]Hospital Universitario De Burgos, Microbiology, Burgos, Spain; [3]Hospital Universitario De Burgos, Preventive Medicine, Burgos, Spain
**Correspondence:** O. Badallo Arevalo
*Intensive Care Medicine Experimental* 2017, **5(Suppl 2):**0692

**INTRODUCTION.** Multidrug-resistant (MDR) pathogens have increased worldwide causing a public health crisis.Here the importance of control strategies to avoid the spread of MDR bacteria."Zero Resistance" Project consists of 10 recommendations that deal with all the factors that stimulate the apparition of MDR bacteria in critical patients.

**OBJECTIVES.** To discuss the impact and steps taken in an Acinetobacter Baumanni (AB) outbreak within an ICU.

**METHODS.** Observational prospective study of an AB outbreak in our ICU from April to September 2016 during the "Zero Resistance Project(RZ)"

Epidemological, clinical and environmental samples were collected. Classification of the strain was performed.Reinforcement of the RZ protocol was implemented, increasing patients insulation in a cohort way, maintaining in this area just exclusive staff with restricted entrance. Cleaning trolleys were replaced and a strict protocol was developed so as to perform portable X-Rays with specific staff, washing matress covers in addition to this.

Mean and typical deviation were presented for quantitative variables and rates for qualitative variables.

**RESULTS.** 34 patients were detected, 88.2% were male with an average age of 59.85(SD 14.8). Mean APACHE II 23.12(SD 12.77). Mean stay of 26.55 days(SD 19.13) and colonization period of patients in ICU with AB were 13.57 days(SD 8.31).

From those, 79,4% of them were colonized patients with AB and 20.6% developed infection. 11.8% were infected outside the ICU, including the primary case.These outside infections were 2 bacteriemia associated to catheter, however the ones got in ICU were 2 urinary infections, a pneumonia with bacteriemia, a bacteriemia secondary to surgical wound infection and a peritonitis.

655 environmental samples were collected (the epidemiological ones according to RZ protocol):infusion pumps, medication dispensing machine, cleaning trolleys, monitors, X-Ray devices, lead shields, ultrasound machines, mattresses.76 samples proved positive (11.6%).As posible reservoirs:X-Ray devices and claning trolleys.

During the outbreak, the consumption of hydroalcoholic liquid rose from 24L to 168L /1000 stays from July to September. The fulfillment of hand hygiene reached 80%.

The strain of AB found was carbapenemasa OXA-23 like producer.

Global mortality within ICU was 20.6%. Mortality directly related to AB was 5.8%.

**CONCLUSIONS.** RZ Project allowed to detect AB outbreak early with the colonized cases for most of them.Enviromental samples identified the posible reservoirs.The use of hydroalcoholic liquid was increased, an excelent adherence to hand hygiene.With all these strategies the outbreak was controlled in 3 months and the amount of infected patients was low, being also low the mortality attributable.

**REFERENCE(S)**

1. http://hws.vhebron.net/resistencia-zero/RZero.asp.
2. Estudio Nacional De Vigilancia De Infección Nosocomial De Medicina Intensiva:http://hws.vhebron.net/envin-helics/

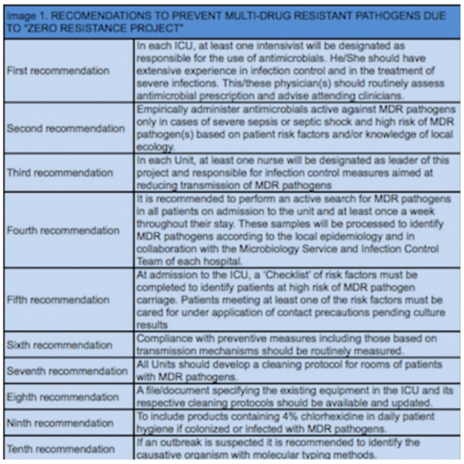

| Image 1. RECOMENDATIONS TO PREVENT MULTI-DRUG RESISTANT PATHOGENS DUE TO "ZERO RESISTANCE PROJECT" | |
|---|---|
| First recommendation | In each ICU, at least one intensivist will be designated as responsible for the use of antimicrobials. He/She should have extensive experience in infection control and in the treatment of severe infections. This/these physician(s) should routinely assess antimicrobial prescription and advise attending clinicians. |
| Second recommendation | Empirically administer antimicrobials active against MDR pathogens only in cases of severe sepsis or septic shock and high risk of MDR pathogen(s) based on patient risk factors and/or knowledge of local ecology. |
| Third recommendation | In each Unit, at least one nurse will be designated as leader of this project and responsible for infection control measures aimed at reducing transmission of MDR pathogens |
| Fourth recommendation | It is recommended to perform an active search for MDR pathogens in all patients on admission to the unit and at least once a week throughout their stay. These samples will be processed to identify MDR pathogens according to the local epidemiology and in collaboration with the Microbiology Service and Infection Control Team of each hospital. |
| Fifth recommendation | At admission to the ICU, a 'Checklist' of risk factors must be completed to identify patients at high risk of MDR pathogen carriage. Patients meeting at least one of the risk factors must be cared for under application of contact precautions pending culture results |
| Sixth recommendation | Compliance with preventive measures including those based on transmission mechanisms should be routinely measured. |
| Seventh recommendation | All Units should develop a cleaning protocol for rooms of patients with MDR pathogens. |
| Eighth recommendation | A file/document specifying the existing equipment in the ICU and its respective cleaning protocols should be available and updated. |
| Ninth recommendation | To include products containing 4% chlorhexidine in daily patient hygiene if colonized or infected with MDR pathogens. |
| Tenth recommendation | If an outbreak is suspected it is recommended to identify the causative organism with molecular typing methods. |

**Fig. 240 (Abstract 0692)** See text for description

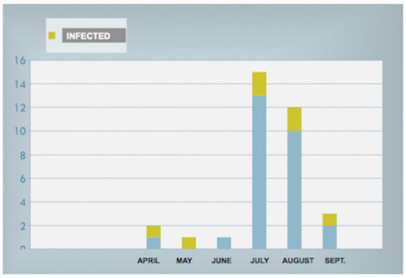

**Fig. 241 (Abstract 0692)** See text for description

### 0693

#### Outcomes of patients with severe influenza infection admitted to an intensive care unit

C.-M. Chen[1,2]
[1]Chi Mei Medical Center, Intensive Care Medicine, Tainan, Taiwan, Province of China; [2]Chia Nan University of Pharmacy and Science, Department of Recreation and Health-Care Management, Tainan, Taiwan, Province of China
*Intensive Care Medicine Experimental* 2017, **5(Suppl 2):**0693

**INTRODUCTION.** Influenza is often under-diagnosed in acute-care hospitals. It is also the greatest current pandemic disease threat to humankind and places a large burden on healthcare providers and society. In February 2016, there was a large outbreak of influenza in Taiwan, and a high percentage of patients required ICU admission for intensive care.

**OBJECTIVES.** We wanted to assess clinical manifestations and prognostic factors of critically ill patients with severe influenza admitted to the intensive care unit (ICU) in Taiwan's recent outbreak.

**METHODS.** This retrospective study was done in a tertiary referral hospital with 96 adult ICU beds. Patients admitted between January 1, 2015, and March 31, 2016, were identified and their medical records were reviewed. The primary endpoints were outcomes and predictors of in-hospital mortality.

**RESULTS.** There were 125 patients with an average Acute Physiology and Chronic Health Evaluation II (APACHE II) score (20.8), Therapeutic Intervention Scoring System (TISS) score (20.8), and Glasgow coma scale score (9.8). Hypertension (62.4%) and diabetes mellitus (40.8%) were the two most common underlying diseases. Ninety-eight (78.4%) patients had at least one organ failure: the lungs were the most common (71.2%), followed by the heart (53.6%), and metabolic acidosis (32.8%). Two of the most common symptoms of patients at ICU admission were fever (68.0%) and cough (78.4%). Ninety-two patients had comorbid cultures of gram-negative bacteria (62.4%), gram-positive bacteria (22.4%), and fungus (17.6%). Thirty-three patients (26.4%) died; most (40.9%) were middle-aged (50–65 years old). Cox regression analysis showed that multiple organ failure (MOF) (hazard ratio [HR] = 3.618; 95% confidence interval [CI] = 1.058-13.662) and negative fluid balance (HR = 0.362; 95% CI = 0.140-0.934) were significantly associated with mortality.

**CONCLUSIONS.** The mortality rate of severe influenza patients admitted to the ICU was high, especially in middle-aged adults. Mortality predictors were MOF and negative fluid balance. Additionally, comorbid bacterial and fungal cultures were common in this cohort.

**REFERENCE(S)**

1.Taylor G, Abdesselam K, Pelude L, Fernandes R, Mitchell R, McGeer A, Frenette C, Suh KN, Wong A, Katz K *et al.*: Epidemiological features of influenza in Canadian adult intensive care unit patients. *Epidemiology and infection* 2016, 144(4):741–750.

2.Shah NS, Greenberg JA, McNulty MC, Gregg KS, Riddell J, Mangino JE, Weber DM, Hebert CL, Marzec NS, Barron MA *et al.*: Severe Influenza in 33 US Hospitals, 2013–2014: Complications and Risk Factors for Death in 507 Patients. *Infection control and hospital epidemiology* 2015, 36(11):1251–1260.

**GRANT ACKNOWLEDGMENT**
None

### 0694

#### Carbapenem regimen for empirical therapy of community-acquired infections requiring intensive care unit admission

S. Rebollo Acebes[1], R. Jiménez Sánchez[1], A. Ortín Freire[1], S. Sánchez Argente del Castillo[1], M.J. Del Amor[2], M.M. Ortíz[2], M. Viqueira[2], L. Herrera Para[1], S. Moreno Aliaga[1], A. Fernández Martínez[1]
[1]Hospital General Universitario Santa Lucía, Servicio Medicina Intensiva, Cartagena, Spain, [2]Hospital General Universitario Santa Lucía, Servicio de Microbiología, Cartagena, Spain
**Correspondence:** S. Rebollo Acebes
*Intensive Care Medicine Experimental* 2017, **5(Suppl 2):**0694

**INTRODUCTION.** Infections with associated organ dysfunction require appropriate and effective empirical antimicrobial treatment as a key point. However, some broad spectrum antibiotics like carbapenems should probably be reserved for targeted therapy, given the increasing occurrence of multidrug resistant bacteria (MDR).

**OBJECTIVES.** To study if a carbapenem regimen for empirical treatment of community-acquired infections provides some benefit over narrower spectrum antibiotics.

**METHODS.** We retrospectively compared patients admitted to our ICU with community-acquired infections treated with empirical carbapenem with those who received other antibiotics. We studied demographic, clinical and outcomes data in both groups.

Groups data were compared using t-Sudent, U-Mann–Whitney, Chi-Square o Fisher test as appropriate.

**RESULTS.** A total of 335 patient with community-acquired infection were admitted and analyzed; 57 patients (17%) received carbapenem and 278 other different antibiotics. No differences in age were observed. APACHE II was 21.1 (95% IC 18.9-23.2) in carbapenem group and 19.1 (18.3-20) in non-carbapenem group (p 0.09).

Patients in carbapenem group were inmucompromised in higher proportion (19.3 vs 8.3%, p 0.016), had more bacteremia (56.1% vs 29.1%, $p < 0.001$) and presented septic shock at admission in higher rate (66.7% vs 46.8%, p 0.005) No differences were found in terms of incidence of inadequacy of the empirical treatment (antibiogram based)

Length of ICU and hospital stay (LOS) did not differ between groups (4 vs 5 and 14 vs 13 days respectively) and no differences in ICU or hospital mortality were found.

When we analyzed only patients with septic shock, carbapenem was prescribed more often in medical patients (94.7 vs 78.5, p 0.014), inmunocompromised (21.1 vs 10%, p 0.07) and bacteremic (60.5 vs 33, p 0.002) When we analyzed only patients with highest APACHE II (>25), similar results were observed. In both subgroups, no differences in LOS or mortality were found. In inmunocompromised patients, despite higher APACHE II in carbapenem group (28.6 (22.8-34.3) vs 20.2 (17.2-23.1), p 0.01), we observed no differences in outcomes.

**CONCLUSIONS.** A carbapenem regimen in community-acquired infections were used more often in inmunocompromised, septic shock and patients with bacteremia. Neither globally nor analyzing these subgroups, differences in outcomes were found when a carbapenem was used. This data should make us consider with caution to prescribe a carbapenem in community-acquired infections when the risk of MDR is low.

**REFERENCE(S)**

- Lee CH. Int J Antimicrob Agents. 2017 Mar 14.
- Tamma PD. Clin Infect Dis. 2015 May 1;60(9):1319–25.

### 0695

#### Does colistin have a role in the empirical therapy in severe sepsis/septic shock in critical care units where multi-drug-resistant Acinetobacter baumannii is endemic?

I. Bahar[1], G. Elay[2], A.U. Kılıc[3]
[1]Izmir Clerk University Atatürk Education and Research Hospital, Izmir, Turkey; [2]Ersin Aslan Educational Research Hospital, Gaziantep, Turkey; [3]Erciyes University, School of Medicine, Kayseri, Turkey
**Correspondence:** I. Bahar
*Intensive Care Medicine Experimental* 2017, **5(Suppl 2):**0695

**INTRODUCTION.** Turkey is one of the countries in which Carbapenem-resistant *Acinetobacter baumannii* (CRAB) is most commonly seen (1). Polymyxin E (colistimethate) is one of the several effective antibiotics against CRAB (2). The effect of AB infection on mortality has been found to be indefinite, and mortality has rather been attributed to the underlying disease (3). The early antibiotic administration is one of the mainstays of treatment in sepsis (4). Clinicians suspect AB to be the infectious agent in patients with sepsis in locations where CRAB is endemic; however, clinicians have question marks in their minds while initiating empiric colistin treatment.

**PURPOSE.** In our study, we aimed to investigate whether CRAB affects mortality in cases with severe sepsis/septic shock treated with colistin initiated based on culture results or initiated empirically.

**METHODS.** Our study was conducted retrospectively in the Erciyes University Faculty of Medicine Tertiary Care Medical and Surgical Intensive Care Units between January 2015 and January 2016. The patient data were obtained from the electronic recording system of the hospital and the patients' files. Patients who were administered colistin for severe sepsis/septic shock were included in the study.

**RESULTS.** A total of sixty-nine patients were included in the study. The empiric group (Group 1) consisted of 33 (47%) patients while Group 2, in whom the treatment was initiated based on antibiotic sensitivity test results, consisted of 36 (53%) patients. The median ages in the empiric and sensitivity test groups were 63.5 and 58.6 years respectively (p = 0.76). There were no differences between the groups regarding the demographics. The mean APACHE II score was higher in the empiric treatment group. There were differences between the two groups in terms of days on which antibiotics were started (p = 0.02), and the cultural breeding ground (p = 0.01).

**CONCLUSION.** We could not find any effect of empiric initiation of MDRAB treatment in patients with severe sepsis/septic shock in whom the information about the pharmacology and tissue penetration of colistin used in the treatment of AB, which has increased virulence and that the treatment has become difficult, is limited.

**GRANT**

There was *no funding* source for this study.

**ACKNOWLEDGMENTS**

The authors thank all the doctors, nurses, technicians in the study. Ethics Committee of Erciyes University Medical Faculty.

### 0696

#### Prospective observational cohort study evaluating antibiotic prescription pattern and micribiologic isolates and their correlation with hemodynamic stability in ICU patients

M. Bhattacharyya[1], A. Saha[2], T.K. Chatterjee[2], S. Todi[3]

[1]AMRI Hospitals Dhakuria, Kolkata, India; [2]Jadavpur University, Clinical Pharmacy, Kolkata, India; [3]AMRI Hospitals Dhakuria, Critical Care, Kolkata, India

**Correspondence:** M. Bhattacharyya

*Intensive Care Medicine Experimental* 2017, **5(Suppl 2):**0696

**INTRODUCTION.** Antibiotics are the most commonly prescribed drugs in ICU. In the era of antibiotic resistance it is a common and difficult problem while choosing empiric antibiotics during septic episode.The choice of antibiotics mainly depends on clinical diagnosis, culture sensitivity, and local flora. Whether severity of illness really matters is not well known. Antibiotic stewardship is the mainstay to prevent resistance.

**OBJECTIVES.** To study the antibiotic prescription pattern and whether the choice of empiric antibiotic varies according to hemodynamic stability in patients admitted in ICU.

To study the microbiological isolates and their variability according to hemodynamic stability in patients admitted in ICU.

**METHODS.** All adult patients more than age of 18 years admitted in ICU, who have received antibiotics and whose cultures have been sent were included.Patients discharged against medical advise and patients with treatment withdrawn were excluded from study.Prospective observational cohort study, carried out in ICU, AMRI Group of Hospitals, Kolkata,India. Data were collected from the patients file and nursing chart from July 2016 to March 2017. Microbiologic isolates and antibiotics advised were noted.Patients were divided into stable and unstable group according to hemodynamic parameter and usage of type of antibiotic and microbiological isolates were correlated.

**RESULTS.** 786 sepsis episodes were analysed. Mean age was 64years, male predominant and average APACHE IV score is 58(sd25). We had 444 patients in unstable group of whom 71% got discharged and 86% got discharged in stable group.More combination therapy has been used in hemodynamically unstable patients($P < 0.05$).There was no difference in usage of BLBLI, carbapenem in stable or unstable group. Pattern of drug resistance in the isolates did not reveal any signficant difference.

**CONCLUSIONS.** There is a tendency to administer combination antibiotics in more sick patient who are in shock.However,in less sick and hemodynamically stable patient there is a tendency to use higher antibiotics as empiric one though p value is not in significant range.

**REFERENCE(S)**

1. Drupad HS, Nagabushan H and Prakash GM. Prospective and observational study of antimicrobial drug utilization in medical intensive care unit in a tertiary care teaching hospital. International Journal of Pharmacological Research.2016; Volume 6 Issue 01; DOI:10.7439/ijpr; ISSN: 2277–3312.

2. Julian Mettler, Mathew Simcock, Pedram Sendi, Andreas F Widmer, Roland Bingisser, Manuel Battegay, Ursula Fluckiger and Stefano Bassetti. Empirical use of antibiotics and adjustment of *empirical antibiotic therapies in a university hospital: a prospective* observational study. BMC Infectious Diseases,7:21; **DOI:** 10.1186/1471-2334-7-21;26 March 2007.

3. Kollef MH, Fraser VJ:Antibiotic resistance in the intensive care unit. Ann Intern Med. 2001 Feb 20;134(4):298–314

**GRANT ACKNOWLEDGMENT**

None

### 0697

#### Frequency of identification and results of treatment of patients with *Pseudomonas aeruginosa (P.aeruginosa)* in a Russian cancer research center in 2011–2014

O. Obukhova, I. Kurmukov, S. Kashiya

N.N. Blokhin Russian Cancer Research Center, Moscow, Russian Federation

**Correspondence:** O. Obukhova

*Intensive Care Medicine Experimental* 2017, **5(Suppl 2):**0697

**INTRODUCTION.** The prevalence and relatively high incidence of infection, and a significant effect incidence of infection, and a significant effect on prognosis of *P.aeruginosa* bacteremia led to the need to include antibiotics with antipseudomonal activity in the program of antibiotic prophylaxis and empiric antibiotic therapy in cancer patients (pts). However, consecutive use of such programs and some other factors over time significantly change the spectrum of pathogens of hospital infection, including bloodstream infection (BSI).

**OBJECTIVES.** We determined the frequence of BSI caused by *P.aeruginosa,* the causes of its occurrence and the immediate results of treatment of this BSI in pts in a large specialized oncological clinic (950 beds, more than 22000 hospitalizations per year).

**METHODS.** In our retrospective cohort study, included pts with microbiologically documented BSI treated in our clinic from 01/01/2011 to 31/12/2014. The endpoint of clinical efficacy (primary endpoint) was 30-day mortality. The secondary endpoints were hospital length of stay (HLOS) after diagnosed BSI (for surviving pts) and the possibility of chemotherapy treatment (for pts who planned this treatment before detection of BSI).

**RESULTS.** During the study, 88217 hospitalizations of pts older than 18 years with malignancy were recorded. In 432 pts, 654 episodes of BSI were microbiologically confirmed. In 17 pts (3.9% of all pts with BSI), *P.aeruginosa* (2.8% of all episodes of septicemia) caused 18 episodes of BSI. One patient had 2 episodes of BSI with an interval of more than 10 months, during which he received chemotherapy for multiple myeloma. The median age was 57.5 years (from 22 to 74 years); male were 12 (71%). 13 pts had solid tumors, 4 had hemoblastoses. In 3 pts with solid tumors, the prevalence of malignancy was stage II, in 10 pts - stage III-IV. In 6 pts the BSI of *P.aeruginosa* was one of the complications after surgical intervention on the abdominal organs. In 14 cases, BSI was secondary, and developed in pts with multiple organ dysfunction (MOD) and was associated with generalization of infection due to pneumonia (n = 5), cholangitis (n = 4), severe enterocolitis (n = 2), peritonitis (n = 2), wound infection (n = 1). In 4 cases, BSI was a catheter-related. The 30-days mortality after detection of BSI of *P.aeruginosa* was 61%; all episodes of catheter-related BSI (n = 4), 2 of BSI due to cholangitis and 1 of secondary BSI in peritonitis were cured. The median HLOS of 7 cured pts was 9 days (4 to 15 days), with 4 pts continuing to receive outpatient antibiotics. In the study cohort, the BSI of *P.aeruginosa* in cured pts was not the reason of failure or delay of chemotherapy more than 30 days.

**CONCLUSIONS.** Gram-negative bacteria P.aeruginosa caused only 2.8% of microbiologically confirmed BSI in our clinic in 2011–2014. The overall mortality in BSI of P.aeruginosa remains high; however, it is mainly due to already severe MOD.

**GRANT ACKNOWLEDGMENT**
None

### 0698

#### High variability of Css concentrations of meropenem in critically ill patients

M. Miralbés Torner[1], S. Carvalho Brugger[1], A. Aragones Eroles[1,2], M. Vallverdú Vidal[1], S. Iglesias Moles[1], J. Schoenenberger[1], B. Balsera Garrido[1], J. Trujillano Cabello[1], M. Palomar Martinez[1]
[1]Hospital Universitari Arnau de Vilanova de Lleida, Lleida, Spain, [2]Institud de Recerca Biomédica de Lleida (IRB), Lleida, Spain
**Correspondence:** M. Miralbés Torner
*Intensive Care Medicine Experimental* 2017, **5(Suppl 2):**0698

**INTRODUCTION.** Meropenem (MER) is an extended-spectrum betalactam antibiotic (ATB) broadly used in intensive care units. It has been suggested that standard doses of MER might not be sufficient for critically ill patients.

**OBJECTIVES.** Evaluate the effectiveness of continuous infusion (CI) administration of MER in critically ill patients in order to maintain serum concentrations 4–6 times above the minimum inhibitory concentration (MIC) for 100% of the interval time of infusion.

**METHODS.** Open, prospective, single-center study. All consecutive patients in whom treatment with MER was indicated from October 2014 to March 2017 were included. A 1g MER loading dose was given followed by a 6g MER CI over 24 hours. Serum concentrations were determined by high-performance liquid chromatography (HLPC) 24 hours after the start of the infusion, determining free steady state concentrations (ƒCss). The objective was maintaining ƒCss 4–6 times above the MIC corresponding to the clinical breakpoint for *Pseudomonas aeruginosa* from our hospital database: 8 µg/ml for MER. When the target was not achieved, the dose was adjusted. Univariant, multivariant and logistic regression analysis were performed. A p value < 0.05 was considered statistically significant.

**RESULTS.** We enrolled 49 patients, 37 male (75.5%) with mean age 62 years. A total of 33 patients (67.3%) presented with septic shock and 13 (26.5%) severe sepsis. Mean APACHE-II score was 21 ± 8. In 19 patients (38.8%) bacteremia was present. In 21 (43.8%) patients, levels were below target. Those patients were hemodynamically stable, had better renal function, were not cirrhotic, did not have bacteremia and did not have hyperbilirubinemia nor coagulopathy (Table 179). In multivariate analysis we identified as risk factors for infradosification: plasmatic creatinine [OR 6.1 (14–27.2)] and not bacteriemic patients [OR 0.08 (0.1-0.6)].

15 patients (31.2%) surpass our target. Those patients received higher doses calculated by mg/Kg/day (p 0.039).

**CONCLUSIONS.** 43.8% of our patient did not achieve the goal even optimizing ATB administration. We identified as risk factors for infradosification: hemodynamic stability and renal function. We identified the dose administration as risk factor for MER supradosification.

**Table 179 (Abstract 0698)**. Comparison

| CHARACTERISTIC | INFRATARGET(N=21) | TARGET(N=12) | p |
|---|---|---|---|
| AGE(SD) | 62(15) | 62(10) | 0.996 |
| BACTEREMIA | 4(19%) | 8(81%) | 0.018 |
| APACHE-II(SD) | 20(8) | 25(9) | 0.122 |
| NO VASOACTIVE DRUGS(%) | 15(78.9) | 4(21.1) | 0.033 |
| CREATININ(SD) | 1(0.7) | 2(1) | 0.003 |
| UREA(SD) | 57(28) | 108(74) | 0.008 |
| GLOMERULAR FILTRATION(SD) | 86(31) | 49(26) | 0.001 |
| BILIRRUBIN(SD) | 1.3(2) | 3.8(3.7) | 0.039 |
| INR(SD) | 1.3(0.1) | 1.9(1) | 0.009 |

### 0699

#### Linear regression model to predict piperacilin-tazobactam steady-state concentrations in critically ill patients

M. Miralbés Torner[1], S. Iglesias Moles[1], S. Carvalho Brugger[1], A. Aragonés Eroles[2,3], M. Vallverdú Vidal[1], B. Balsera Garrido[1], J. Schonenberger[2], J. Trujillano Cabello[1], M. Palomar Martinez[1]
[1]Hospital Universitari Arnau de Vilanova de Lleida, Intensive Care, Lleida, Spain; [2]Hospital Universitari Arnau de Vilanova de Lleida, Hospital Pharmacy, Lleida, Spain; [3]Institud de Recerca Biomédica de Lleida (IRB), Lleida, Spain
**Correspondence:** M. Miralbés Torner
*Intensive Care Medicine Experimental* 2017, **5(Suppl 2):**0699

**INTRODUCTION.** Piperacilin-tazobactam (TZP) is a broad-spectrum -lactam antibiotic broadly used in ICUs. Recent studies show that normal doses may not be sufficient in critically ill patients.

**OBJECTIVES.** Identify factors to predict Piperacilin free steady state concentrations (ƒCss) in critically ill patients.

**METHODS.** Open, prospective, single-center study from October 2014 to March 2017. All consecutive patients in whom treatment with TZP was indicated were included. A 4g (TZP) loading dose was given followed by a continuous infusion (CI) of 16-24g over 24 hours. Serum concentrations were determined by high-performance liquid chromatography (HLPC) 24 hours after the start of the infusion, determining free steady state concentrations (ƒCss). The objective was maintaining ƒCss 4–6 times above the MIC corresponding to the clinical breakpoint for *Pseudomonas aeruginosa* from our hospital database: 16/4 µg/ml for TZP. When the target was not achieved, the dose was adjusted. Univariant and linear regression analysis were performed. A p value < 0.05 was considered statistically significant.

**RESULTS.** We enrolled 145 patients (mean age 64 years, 73.1% male). A total of 104 patients (71.7%) presented with septic shock at ICU admission. Mean APACHE-II was 20 ± 7.7. 29 patients (20%) presented positive blood cultures. 36 patients needed CRRT (24.8%). We classified ƒCss ranges into three groups: $ƒCss_{T< 4xMIC}$ (35 patients, 24.1%), $ƒCss_{T4\text{-}6xMIC}$ (36, 24.8%) and $ƒCss_{T>6xMIC}$ (74, 51%). Linear regression analysis was performed in order to identify factors to predict TZP Css. Results are shown in Table 180 (R-squared = 0.559).

Creatinine concentration was de most important determinant of elevated TZP Css, followed by being female and APACHE-II score.

**CONCLUSIONS.** We identified factors to predict TZP Css: age, sex, APACHE-II Score, creatinine levels, TZP dose and the need of CRRT. A high interindividual variability of Css in patients studied was shown that is important to determine Css.

**Table 180 (Abstract 0699)**.Model with linear terms and no interaction

| CHARACTERISTIC | COEFFICIEN | STANDARD ERROR | LOWER LIMIT OF 95% CI | UPPER LIMIT OF 95% CI | p |
|---|---|---|---|---|---|
| CONSTANT | −45.20 | 18.48 | | | |
| AGE | 0.53 | 0.24 | 0.06 | 1.004 | 0.029 |
| FEMALE | 30.30 | 7.19 | 16.08 | 44.53 | <0.001 |
| APACHE-II | 1.66 | 0.47 | 0.73 | 2.59 | 0.001 |
| CREATININE | 33.86 | 3.70 | 26.54 | 41.17 | <0.001 |
| 24g/day TZP | 35.85 | 7.55 | 20.92 | 50.78 | <0.001 |
| CRRT | 19.48 | 8.1 | 3.46 | 35.49 | <0.001 |

### 0700

#### Impact of carbapenem use on procalcitonin clearance in septic patients and its correlation with outcomes

R. Jiménez Sánchez[1], S. Rebollo Acebes[1], A. Ortín Freire[1], L. García de Guadiana[2], S. Sánchez Argente del Castillo[1], L. Herrera Para[1], A. Fernández Martínez[1], A. Moreno Aliaga[1], M. Galindo Martínez[1], M.D. Rodríguez Mulero[1]
[1]Hospital General Universitario Santa Lucía, Servicio Medicina Intensiva, Cartagena, Spain; [2]Hospital General Universitario Santa Lucía, Servicio de Análisis Clínico, Cartagena, Spain
**Correspondence:** R. Jiménez Sánchez
*Intensive Care Medicine Experimental* 2017, **5(Suppl 2):**0700

**INTRODUCTION.** Carbapenems are the most potent class of β-lactams and are typically used for the treatment of the most severely ill patients. Adequate procalcitonin clearance after the start of antibiotics has been associated to better prognosis in septic patients.

**OBJECTIVES.** We hypothesized that use of carbapenem as empirical treatment in patients with sepsis may lead to a higher decrease in procalcitonin levels, assuming a correlation of this clearance with outcomes and the theoretical higher potency of carbapenem.

**METHODS.** We made a retrospective analysis of a prospective database for procalcitonin kinetics. We studied procalcitonin at admission and on subsequent days and its relationship with the kind of antibiotic used as empiric therapy.

Comparison of variables was made using t-Sudent, U-Mann–Whitney, Chi-Square o Fisher test as appropriate.

**RESULTS.** We studied 190 patients. Of those, 52 (27.4%) received imipenem or meropenem as empirical antibiotic. No differences were observed in terms of age and lactate or SOFA at admission. APACHE II was higher in carbapenem group (22 (16.25-17.75) vs 19 (15–25); p .024) and CRP at admission higher in non-carbapenem group (19.7 (12.30.5) vs 26 (16.3-34.2); p .045) More patients in carbapenem group had any kind of inmunodepression (28.8 vs 13%; p .01).

Procalcitonin at admission, 48, 72 and 96 hours was similar between groups. When we analysed procalcitonin clearance (delta-PCT), we found a trend to a higher one at 96 hours in carbapenem group (86.6% vs 82.9%; p .065), without differences at earlier days. By other side, the proportion of patients reaching 80% of procalcitonin clearance at 96 hours was higher in carbapenem group (70.6% vs 51.8%), without reaching statistical significance (p .061).

No differences in mortality at 28 days was observed among patients who received carbapenem versus another antibiotic. As expected, age, severity scores and lactate were higher in non-survivors, and procalcitonin at 48, 72 and 96 hours and delta of procalcitonin at those times were lower in non-survivors.

**CONCLUSIONS.** In our patients, the use of a carbapenem as empirical antimicrobial therapy in septic patients did lead to a trend to greater decrease in procalcitonin levels. However, we could not demonstrate a relationship with patients outcomes. The small sample size may have limited these results.

**REFERENCE(S)**

Schuetz P. Crit Care Med 2017; 45:781–789

## Markers of sepsis and organ dysfunction

### 0701

#### Determination of 1,3 beta D Glucan in the peritoneal fluid for the early diagnosis of fungal peritonitis: a pilot study

E. Novy, F.-X. Laithier[1], P. Guerci[1], M.-C. Machouart[1], E. Frealle[2], E. Albuisson[1], M.-R. Losser[1]
[1]University Hospital of Nancy, Nancy, France; [2]University Hospital of Lille, Lille, France
**Correspondence:** E. Novy
*Intensive Care Medicine Experimental* 2017, **5(Suppl 2):**0701

**INTRODUCTION.** Early diagnosis of fungal peritonitis is a challenge. Yeast-positive peritoneal fluid culture remains the gold standard but suffers from a delayed time response. Decision to start an empiric antifungal treatment is based on direct examination (DE) and/or the use of predictive scores such as the Peritonitis Score (PS), with sensitivity (Se) and specificity (Sp) of 84% and 50% respectively. New diagnostic tools such as fungal PCR or serum 1.3 Beta-D-Glucan (1.3 BDG) emerged.

**OBJECTIVES.** To evaluate the usefulness of the 1.3 BDG assay in the peritoneal fluid for the early diagnosis of fungal peritonitis and to compare it with the conventional methods and with universal fungal PCR.

**METHODS.** This retrospective study was conducted at the University Hospital of Nancy between April 1st and December 31th 2016 after ethics committee approval. All peritonitis admitted in the intensive care unit were included. On the peritoneal fluid were performed: DE, yeast culture, universal fungal PCR and 1.3 BDG. Demographic data and microbiological data were collected. Yeast culture of the peritoneal fluid was used as a reference to determine the Se and Sp of the other techniques. An ROC curve was performed for 1,3BDG. Statistical analysis was performed with SPSS V22 (IBM).

**RESULTS.** This study included 33 patients with an average age of 65 years (±13.1), 54% of whom were women. Severity scores (APACHE 2 and SOFA) averaged 45 (±18.6) and 9 (±3), respectively. Sixty percent of patients were in septic shock and 27% were mechanically ventilated for more than 48 hours. Most were secondary peritonitis (97%), mainly postoperative (42%) and supra meso-colic (63%). Yeast culture was positive for seven patients (21%) with 57% of Candida *albicans*. Initial anti-fungal treatment was adequate in 85% of cases. The mortality at Day 28 was 15% and the average length of stay was 10 days (1–31). PS was ≥3 and DE was positive for 5 patients (15%). PCR was positive for 3 patients (9%). The ROC curve for the 1.3BDG identified a cut off at 320 pg/ml with an area under the curve at 0.76 (95% CI [0.6-0.92]). Considering cut-off value of 320 pg/ml for a 1.3BDG, it provided a Se/Sp of 100% and 57%, respectively, with a negative predictive value (NPV) of 100%. Se/Sp of the other diagnostic methods were 57/96% (ED), 14/84% (PS) and 42/100% (PCR), respectively.

**CONCLUSIONS.** This is the first study assessing the level of 1.3 BDG specifically in the peritoneal fluid. In our sample size, an assay of less than 320 pg/ml could eliminate a fungal peritonitis with a NPV of 100%.

### 0702

#### Presepsin usefulness in intensive care unit

S. Miori[1], K. Donadello[1], L. Gottin[2], G. Cogo[1], E. Polati[1]
[1]Department of Surgery, Dentistry, Paediatrics and Gynaecology, University of Verona, AOUI-University Hospital Integrated Trust of Verona, Anesthesia and Intensive Care B Unit, Verona, Italy; [2]Department of Surgery, Dentistry, Paediatrics and Gynaecology, University of Verona, AOUI-University Hospital Integrated Trust of Verona, Cardio-Thoracic Anesthesia and Intensive Care Unit, Verona, Italy
**Correspondence:** S. Miori
*Intensive Care Medicine Experimental* 2017, **5(Suppl 2):**0702

**INTRODUCTION.** The first cause of mortality in critically ill patients is sepsis. Various sepsis biomarkers have been studied in the past years and commonly used ones include C-Reactive Protein (CRP) and Procalcitonin (PCT). A new biomarker is sCD-14-st, known as Presepsin (P-SEP), derived from mCD14, co-receptor of the *Toll-like Receptor 4* for LPS. Its utility in critical ill patients has yet to be defined.

**OBJECTIVES.** To prospectively assess the potential role of presepsin in the diagnosis of sepsis and its prognostic value in a critical care setting.

**METHODS.** All adult patients admitted to one of the ICUs of the University Hospital Integrated Trust of Verona were prospectively screened from June 2016 to January 2017. Patients < 18 yrs, those admitted for < 48-hr observation, and those who refused to participate were excluded. Sepsis and septic shock were defined daily (patient-day) according to new standard criteria[1].

We recorded demographic characteristics, daily clinical and laboratory data, procalcitonin (PCT) and presepsin (P-SEP) plasmatic levels. Serum P-SEP levels were measured on ICU admission and then daily until ICU discharge using luminescent monoclonal antibodies ("pathfast method"). At the fifth day we used SOFA score variation (delta-SOFA score) to divide patients in favourable (delta-SOFA < 2) and unfavourable (delta-SOFA > 2) clinical course.

**RESULTS.** 97 patients (57 men) were included. Median age was 73 (62–80) yrs, APACHE II and SOFA score at admission were 14 (10–21) and 7 (5–10), respectively. Admissions were due to medical conditions (n = 66), elective surgery (n = 24) and emergency surgery (n = 7). Sepsis was diagnosed at admission in 69 patients (71.1%), whose 29 had septic shock (29.8%). Overall ICU mortality was 17.5%.

P-SEP levels in septic shock patients were significantly higher than in those classified as septic and non-septic (1966 (909–4845) vs 1226 (730–2223) vs 504 (373–856) pg/ml, respectively. PCT showed the same trend and thus similar accuracy, as showed by ROC AUC of 0.82 for both biomarkers (P-SEP best cut-off = 1036 pg/ml).

No correlation was found between P-SEP and the APACHE II score (r = 0,29). However, differently from PCT, P-SEP trend was significantly different between survivors/non-survivors and, during the first five days, between favourable/unfavourable course (p < 0.001, two way ANOVA.

**CONCLUSIONS.** In ICU patients P-SEP concentrations are increased in septic patients and show similar prognostic power than PCT. P-SEP trends may represent a promising early prognostic marker of both recovery and mortality.

**REFERENCE(S)**

[1]Singer M. et al. **JAMA**. 2016 Feb 23;315(8):801–10.

**Table 181 (Abstract 0702)**. Patient-day P-SEP levels

| Class | n° (%) | P-SEP pg/ml Median (Q1-Q3) | p-value | Class | n° (%) | P-SEP pg/ml Median (Q1-Q3) | p-value |
|---|---|---|---|---|---|---|---|
| Sepstic shock | 189 (18.8) | 1966 (909-4845) | p<0.001 | "Sepsis" | 681 (67,9) | 1330 (779 -3051) | p<0.001 |
| Sepsis | 492 (49) | 1226 (730-2323) | p<0.001 | | | | |
| Local infection | 92 (9.1) | 448 (354-727) | p>0.05 | "No sepsis" | 323 (32,1) | 504 (373- 856) | |
| No infection | 230 (22.9) | 597 (387-874) | | | | | |
| Total | 1003 | | | | 1003 | | |

**Table 182 (Abstract 0702)**. Patient-day P-SEP-Outcome

| | Survivor (pg/mL) | Non-survivor (pg/mL) | p-value |
|---|---|---|---|
| Day 1 | 825 (437 - 1395) | 1632 (453- 3870) | p<0.001 |
| Day 2 | 881 (428 - 1337) | 1825 (599- 3760) | |
| Day 3 | 765 (450 - 1320) | 1885 (826- 3654) | |
| Day 4 | 784 (451 - 1621) | 2570 (958- 4154) | |
| Day 5 | 794 (401- 1643) | 3231 (131- 5752) | |

**Table 183 (Abstract 0702)**. Patient-day P-SEP levels-Clinical Course

| | Favourable Clinica Course | Unfavorable Clinica Course | p value |
|---|---|---|---|
| Day 1 | 849 (456-1455) | 772 (127-2360) | p<0.001 |
| Day 2 | 887 (479-1591) | 1333 (458-2543) | |
| Day 3 | 807 (479-1419) | 1138 (490-2361) | |
| Day 4 | 810 (472-1539) | 1812 (826-4085) | |
| Day 5 | 837 (444-1668) | 1624 (660-3231) | |

### 0703

#### Baseline angiotensin levels and ACE effects in patients with vasodilatory shock treated with angiotensin II

R. G. Wunderink[1], T. E. Albertson[2], L. W. Busse[3], A. M. Deane[4], A. Khanna[5], M. T. McCurdy[6], M. Ostermann[7], P. J. Young[8], A. N. Chavez[9], D. R. Handisides[9], S. Chen[9], L. S. Chawla[9], G. F. Tidmarsh[9], R. Bellomo[10]
[1]Northwestern University Feinberg School of Medicine, Department of Medicine, Pulmonary and Critical Care, Chicago, United States; [2]University of California Davis School of Medicine, Department of Internal Medicine, Sacramento, United States; [3]Emory University School of Medicine, Department of Medicine, Atlanta, United States; [4]University of Melbourne, Intensive Care Unit, The Royal Melbourne Hospital, Melbourne, Australia; [5]Cleveland Clinic, Anesthesiology Institute, Center for Critical Care and Department of Outcomes Research, Cleveland, United States; [6]University of Maryland School of Medicine, Division of Pulmonary and Critical Care Medicine, Baltimore, United States; [7]King's College London, Guy's and St. Thomas' Hospital, Department of Critical Care and Nephrology, London, United Kingdom; [8]Wellington Hospital and Medical Research Institute of New Zealand, Intensive Care Unit, Wellington, New Zealand; [9]La Jolla Pharmaceutical Company, San Diego, United States; [10]The University of Melbourne, School of Medicine, Melbourne, Australia
**Correspondence:** R. G. Wunderink
*Intensive Care Medicine Experimental* 2017, **5(Suppl 2):**0703

**INTRODUCTION.** Shock affects 1/3 of the patients in the intensive care unit with survival time often measured in hours.[1,2] Vasodilatory shock (VS) is the most frequent sub-type of shock and patients with VS requiring high dose vasopressors have a high mortality.[1,3] Previous reports suggest low angiotensin II (Ang II) levels and decreased angiotensin-converting enzyme (ACE) activity predict worse outcomes in sepsis.[4] ATHOS-3, a randomized, double-blinded, phase 3 clinical trial demonstrated that administration of human Ang II significantly increased blood pressure in VS with a trend toward improved mortality.

**OBJECTIVES.** To assess if Ang I/II ratio predicts outcomes in distributive shock.

**METHODS.** Ang I and Ang II were measured at baseline prior to study treatment as part of a pre-specified analysis. Ang I, Ang II, and

Ang I/Ang II ratio were dichotomized by median value. Mortality at day 28 was summarized by Kaplan-Meier estimates and differences in survival compared by log-rank test. Proportional hazards models were conducted for multivariate analyses with covariates of age, gender, race, body mass index, baseline mean arterial pressure, baseline serum albumin, baseline APACHE II score, geographic region, recent exposure to ACE inhibitors, recent exposure to angiotensin receptor blockers, history of acute respiratory distress syndrome (history or x-ray finding), history of sepsis, baseline norepinephrine-equivalent dose, and baseline endogenous Ang I and Ang II levels.

**RESULTS.** A relatively low Ang II state, as assessed by higher Ang I/II ratios (≥1.63, median across the study population), was associated with higher mortality across the full population in multivariate analysis (HR = 1.78; 95% CI: 1.25 - 2.53, p = 0.002). The risk of death within the placebo arm was significantly associated with an elevated Ang I/II ratio, as assessed by multivariate analysis (HR = 1.77; 95% CI: 1.10-2.85, p = 0.019).

In the Ang II treated arm, the high ratio effect was attenuated in multivariate model (HR = 1.64, 95% CI:0.97-2.79, p = 0.066). This attenuation was associated with a significant treatment effect of Ang II compared to placebo on mortality for patients with high Ang I/II ratio (HR = 0.64; 95% CI: 0.41-1.00, p = 0.047).

**CONCLUSIONS.** A relatively low Ang II state, as assessed by higher Ang I/II ratios, predicted increased mortality in patients with VS, suggesting the vital importance of ACE effects. In addition, the predictive capacity of a high Ang I/II ratio was attenuated by the administration of Ang II.

**GRANT ACKNOWLEDGMENT**

Funded by La Jolla Pharmaceutical Company

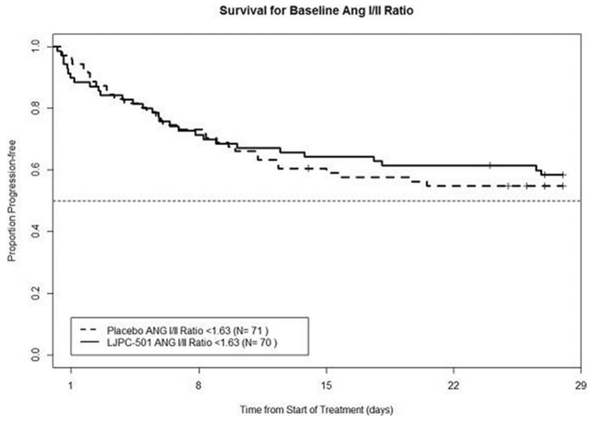

**Fig. 242 (Abstract 0703)** Survival Day 28 by Baseline Ang I/II Ratio <1.63

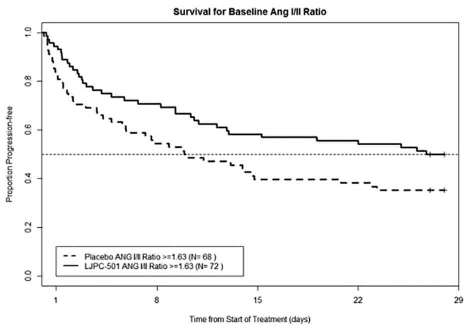

**Fig. 243 (Abstract 0703)** Survival Day 28 by Baseline Ang I/II Ratio >/=1.63

### 0704

#### Significance of stratified values of serum troponin on in-hospital mortality in patients with sepsis or septic shock

D. A. Hammond[1], C. E. Renna[2], T. L. Wiley[3], N. Raj Bhandari[1], J. T. Painter[1]
[1]University of Arkansas for Medical Sciences, Pharmacy Practice, Little Rock, United States; [2]University of Wisconsin, Pharmacy, Madison, United States; [3]University of Nebraska Medicine, Pharmacy, Lincoln, United States
**Correspondence:** D. A. Hammond
*Intensive Care Medicine Experimental* 2017, **5(Suppl 2):**0704

**INTRODUCTION.** Elevated cardiac troponin values are commonly observed in sepsis and septic shock and reflect cardiac ischemia. The impact of these elevations on patient outcomes remains controversial.

**OBJECTIVES.** The study purpose was to determine the association of serum troponin values within 24 hours of intensive care unit (ICU) admission with mortality in sepsis and septic shock.

**METHODS.** This was an IRB-approved, single-center, retrospective, observational study of patients admitted to an ICU between September 2012 and May 2015. The primary endpoint was in-hospital mortality. Patients were at least 18 years of age and possessed a diagnosis of sepsis or septic shock. Categorical data were analyzed using Chi-squared or Fisher Exact tests. Student t test was used for continuous data with equal variances, and a student t test with the Satterthwaite method was used for continuous data with unequal variances. Multivariate logistic regression analysis was performed using forward selection (p < 0.05 used for variable entry into the model) to evaluate variables associated with in-hospital mortality.

**RESULTS.** In total, 360 patients formed 2 groups: low (≤0.2 ng/mL, n = 236) and high troponin (>0.2 ng/mL, n = 124). The only differences in baseline characteristics were a greater percentage of patients with chronic kidney disease (21 vs. 10.6%) and a lower percentage with liver disease (7.3 vs. 15.7%) in the high troponin group. Average troponin values were 0.07 and 2.72 ng/mL, respectively. There was no association between troponin and in-hospital mortality (odds ratio 1.155, 95% confidence interval 0.690-1.933, p = 0.584).

**CONCLUSIONS.** This study did not find a significant increase in mortality with elevated troponin values in patients with sepsis or septic shock.

**REFERENCE(S)**
Sheyin O, Davies O, Duan W, Perez X. The prognostic significance of troponin elevation in patients with sepsis: a meta-analysis. Heart Lung. 2015;44(1):75–81.
**GRANT ACKNOWLEDGMENT**
None.

### 0705

#### Biomarkers and sepsis after cardiac surgery: C-reactive protein kinetics, is it reliable?

C. Santonocito[1], I. De Loecker[2], F. Chiarenza[3], G. Cucuzza[3], F. Sanfilippo[3], S. George[3], M. Astuto[4], J. L. Vincent[2]
[1]Oxford University Hospitals NHS Trust, Anesthesia and Intensive Care, Oxford, United Kingdom; [2]Erasme Hospital, Université Libre de Bruxelles, Department of Intensive Care, Brussels, Belgium; [3]Oxford University Hospitals NHS Trust, Oxford, United Kingdom; [4]Policlinico University Hospital Catania, Catania, Italy
**Correspondence:** C. Santonocito
*Intensive Care Medicine Experimental* 2017, **5(Suppl 2):**0705

**INTRODUCTION.** Sepsis is a leading cause of morbidity and mortality in acutely ill patients, and after major surgery the early diagnosis of sepsis is challenging [1].

**OBJECTIVES.** To analyze CRP and WBC kinetics during the first postoperative (PO) week after cardiac surgery and between groups of patients with and without postoperative infection (POI).

**METHODS.** This is a multicenter retrospective observational study conducted from Nov 2013 to Aug 2014. We screened all post cardiac surgery patients admitted to the Cardithoracic Critical Care Unit at JR Hospital in Oxford and to the Intensive Care Unit at Erasme University Hospital in Bruxelles. We included all adult (>18 yrs) patients post cardiac surgery (elective/emergency), ON/OFF CPB. We excluded: ongoing preoperative infections (including endocarditis), thoracic interventions, trauma, pregnancy, immunocompromised, cirrhotic patients. Four groups were identified: CABG (ON/OFF CPB), valve replacement (R), valve repair (r), CABG + valve r/R. For all subgroups, we separated patients who developed a PO infection from the others. EUROSCORE, SOFA score, clinical, microbiological data, CRP, WBC values, antibiotic therapies were daily recorded up to 7 POD in ICU and in the ward. Intraoperatively, as routine practice, at Erasme Hospital, a bolus of steroid was administered before starting on CPB.

**RESULTS.** A total of 511 patients were screened, and 429 included. Baseline patients characteristics are presented in
(Table 184). When compared ON-CPB vs OFF-CPB CABG, considering patients without POI, no difference was found on CRP kinetics at JRH, while data from Erasme showed CRP significant rising during POD1, 2 and 3 in the group OFF-CPB CABG (Fig. 244). No difference was observed for WBC count. In the ON-CPB CABG group, infected patients had higher CRP values than patients with no infection from POD3 to POD7 (Fig. 245), with cut off values ranging from 131 to 153 mg/dl (Table 184); and from 8 to 18.5 mg/dl when steroids were administered preCPB (Table 186). Moreover, when no steroids were given, we got higher WBC values in the patients group with POI from POD0 to POD3. In the OFF-CPB CABG and post valve surgery groups, no difference was found for CRP and WBC kinetics between patients with and without POI.

**CONCLUSIONS.** Interestingly, the ON-CPB group did not show higher CRP and WBC values than the OFF-CPB group despite the use of CPB, probably due to the high biocompatibility of the new pump circuits. After ON-CPB CABG, higher CRP values after POD3 could help in detecting PO infections. CRP got higher sensitivity and specificity from POD3 to POD7, while WBC count from POD0 to POD3. Post valve and valve + CABG surgery no WBC count difference was found between infected and not infected.

**REFERENCE**
**1. Angus DC,** van der Poll T. *Severe sepsis and septic shock.* N Engl. J Med. 2013 Aug 29; 369(9):840–51

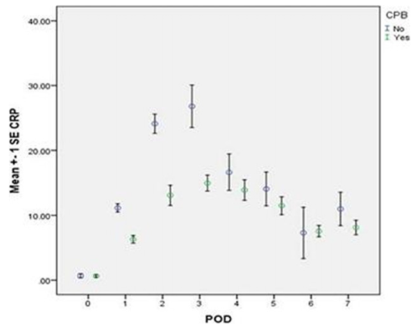

**Fig. 244 (Abstract 0705)** See text for description

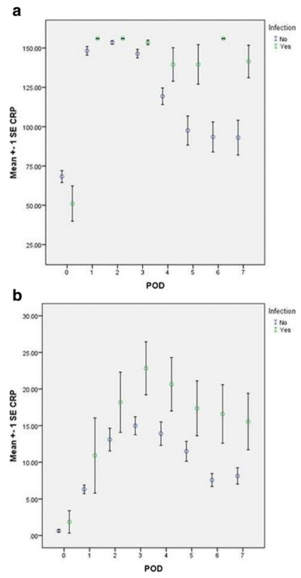

**Fig. 245 (Abstract 0705) a** CRP values (mg/dl): post ON-CPB CABG (UK). **b** CRP values (mg/dl): post ON-CPB CABG (BE)

**Table 184 (Abstract 0705)**. Baseline patients' characteristics

| Sex | Male/Female | 96/333 | 22,4%/77,6% |
|---|---|---|---|
| Age (mean±SD) | 67,9±11 | | |
| Admission type | Elective/Emergency | 299/130 | 69,7%/30,3% |
| Type of surgery | CABG/Valve replacement/Valve repair/Valve+CABG | 255/90/21/63 | 59,2%/21% 4,9% 14,7% |
| CPB | NO/YES | 86/343 | 20%/80% |
| LV function preop. | Normal/Abnormal | 348/81 | 81,1%/18,9% |
| RV function preop. | Normal/Abnormal | 428/1 | 99,8%/0,2% |
| PMH:diabetes | NO/YES | 317/112 | 73,9%/26,1% |
| PMH:smoker | No/Yes/Unknown | 117/97/215 | 27,3%/22,6%/50,1% |
| Infection | NO/YES | 384/45 | 89,5%/10,5% |

**Table 185 (Abstract 0705)**. Cut off values for CRP (mg/dl) (JRH-UK data)

| Postoperative day (POD) | Cut off (mg/dl) | Specificity | Sensitivity |
|---|---|---|---|
| 3 | 142 | 0.19 | 1 |
| 4 | 153 | 0.69 | 0.71 |
| 5 | 139 | 0.74 | 0.85 |
| 6 | 151 | 0.80 | 1 |
| 7 | 131 | 0.8 | 0.73 |

**Table 186 (Abstract 0705)**. Cut off values for CRP (mg/dl) (Erasme Hospital-BE

| Postoperative day (POD) | Cut off (mg/dl) | Specificity | Sensitivity |
|---|---|---|---|
| 3 | 13.5 | 0.44 | 1 |
| 4 | 18.5 | 0.76 | 0.66 |
| 5 | 15 | 0.72 | 0.66 |
| 6 | 9 | 0.73 | 0.83 |
| 7 | 8 | 0.68 | 1 |

### 0706

#### Predictive utilities of neutrophil gelatinase-associated lipocalin (NGAL) in severe sepsis

W. Chang, S. Zhu, C. Pan, J. Xie, S. Liu, H. Qiu, Y. Yang
Zhongda Hospital, School of Medicine, Southeast University, Department of Critical Care Medicine, Nanjing, China
**Correspondence:** W. Chang
*Intensive Care Medicine Experimental* 2017, **5(Suppl 2):**0706

**BACKGROUND.** Increases in the plasma concentration of neutrophil gelatinase-associated lipocalin (NGAL) due to increased production by renal proximal tubular cells occur relatively soon after acute kidney injury. However, plasma NGAL concentrations are also elevated in inflammatory conditions as a result of enhanced production by non-renal cells. In this study, we aim to investigate the predictive value of plasma NGAL in patients with severe sepsis.

**METHODS.** A total of 124 patients were enrolled in this prospective observational study. Peripheral blood samples were obtained at admission, on day 2, and day 7, and measurements of plasma NGAL (immunofluorescence assay) along with blood cell counts, CRP, plasma lactate, procalcitonin (PCT), and creatinine were made. Receiver operating characteristic (ROC) curves were generated to assess the utility of plasma NGAL in prediction of 28-day mortality and need for CRRT. Based on clinical variables, Cox proportional hazard regression curves were built with and without plasma NGAL for 28-day mortality prediction to determine NGAL's contributive predictive value. A possible association between plasma NGAL and PCT levels (acting as surrogate for infection) was also investigated.

**RESULTS.** Relative to surviving patients (at 28 days), plasma NGAL was significantly increased in non-survivors group on day 2 and 7.

| | Survivors (n = 82) | Non-survivors (n = 42) |
|---|---|---|
| Day 1 | 241.5 (126.8-523.5) | 352.0 (181.0-801.0) |
| Day 2 | 233.0 (106.5-469.3)$^{*}$ | 413.0 (224.5-1050.0)$^{§}$ |
| Day 7 | 161 (98.8-262.3)$^{*, †}$ | 458.5 (212.0-862.0)$^{*, §}$ |

Plasma NGAL concentration presented as median (IQR), ng/mL
$^{*}$P < 0.05 compared with day 1 within group, $^{†}$P < 0.05 compared with day 2 within group, $^{§}$ P < 0.05 compared between groups
*[Change of NGAL in survivors and non-survivors]*

At cut-off values of 240.5 and 261 ng/mL, respectively, plasma NGAL on day 2 and 7 (but not at admission) predicted 28-day mortality with AuROC values of 0.675 (95% CI 0.570-0.780) and 0.752 (95% CI 0.619-0.885), respectively.

| | AuROC (95% CI) | Cut-off value (ng/mL) | Sensitivity | Specificity | PPV | NPV |
|---|---|---|---|---|---|---|
| Day 1 | 0.584 (0.476-0.691) | 282 | 57.1% | 59.8% | 42.1% | 73.1% |
| Day 2 | 0.675 (0.570-0.780) | 240.5 | 75% | 52% | 43.3% | 80.9% |
| Day 7 | 0.752 (0.619-0.885) | 261 | 73.1% | 75.6% | 49.3% | 89.6% |

*AuROC* area under receiver operating characteristic, *PPV* positive predictive value, *NPV* negative predictive value
*[Plasma NGAL in prediction of 28-day mortality]*

Moreover, patients categorized according to the NGAL cut-off values on day 2 and 7 showed significant differences in 28-day mortality. Addition of day 2 NGAL to the clinical model resulted in a net reclassification index (NSI) increment (SE) of 0.17(0.08) for prediction of 28-day mortality. Moreover, NGAL levels at admission and day 2 (cutoff values, 294 and 107.5 ng/mL, respectively) outperformed creatinine in prediction of the need for CRRT, with AuROC of 0.726 (95% CI 0.612-0.841) and 0.692 (95% CI 0.564-0.820), respectively.

| | AuROC (95% CI) | Cut-off value | Sensitivity | Specificity | PPV | NPV |
|---|---|---|---|---|---|---|
| Plasma NGAL | | | | | | |
| Day 1 | 0.726 (0.612-0.841) | 294ng/ml | 75% | 63.0% | 32.8% | 91.28% |
| Day 2 | 0.754 (0.640-0.868) | 444.5ng/mL | 66.7% | 74.5% | 39.1% | 90.1% |
| Day 7 | 0.908 (0.832-0.983) | 363.5ng/mL | 81.3% | 88.6% | 56.5% | 96.3% |
| Plasma Cr | | | | | | |
| Day 1 | 0.692 (0.564-0.820) | 107.5μmol/L | 66.7% | 67% | 32.7% | 89.3% |
| Day 2 | 0.738 (0.609-0.866) | 113.5μg/mL | 73.9% | 72.4% | 39.7% | 91.9% |
| Day 7 | 0.815 (0.686-0.944) | 120.5μg/mL | 75% | 80.3% | 40.9% | 94.6% |

*AuROC* area under receiver operating characteristic, *PPV* positive predictive value, *NPV* negative predictive value, *Cr* creatinine
*[Plasma NGAL and Cr in prediction of CRRT]*

A direct association between plasma NGAL and PCT quintiles (ng/mL) ranging from < 0.05 (normal) to >10 (septic shock) was also observed (P < 0.05).

**CONCLUSIONS.** Plasma NGAL discriminated 28-day survivors from non-survivors on day 2 and 7. Based on ROC values, Day 2 (but not admission) plasma NGAL was a relatively robust predictor of 28-day mortality prediction - this predictive value increased on day 7. Furthermore, plasma NGAL outperformed creatinine in the prediction of need for CRRT. Finally, the direct relationship between plasma NGAL and PCT requires further evaluation.

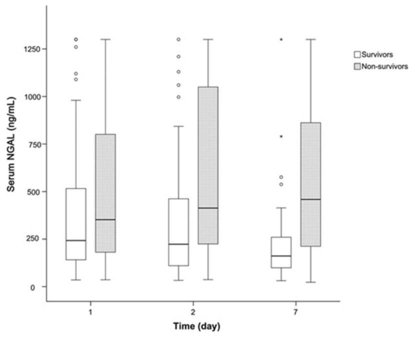

**Fig. 246 (Abstract 0706)** NGAL change in survivor and non-survivor

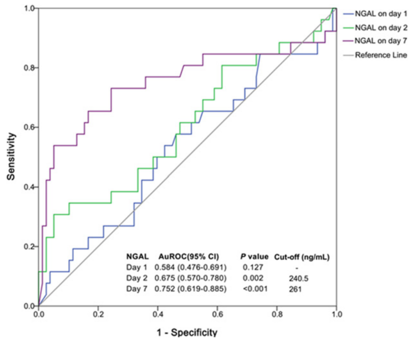

**Fig. 247 (Abstract 0706)** NGAL in 28-day mortality prediction

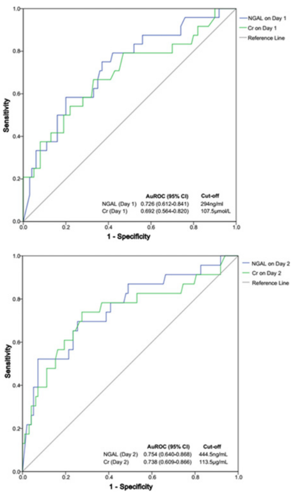

**Fig. 248 (Abstract 0706)** NGAL and Cr in CRRT prediction

### 0707

#### Neuroinflammatory markers for infectious delirium in cerebrospinal fluid

A.M. Peters van Ton[1], M. Verbeek[2,3], P. Pickkers[1], W.F. Abdo[1]
[1]Radboud University Medical Center, Department of Intensive Care Medicine, Nijmegen, Netherlands; [2]Radboud University Medical Center, Department of Neurology, Nijmegen, Netherlands, [3]Radboud University Medical Center, Department of Laboratory Medicine, Nijmegen, Netherlands
**Correspondence:** A.M. Peters van Ton
*Intensive Care Medicine Experimental* 2017, **5(Suppl 2):**0707

**INTRODUCTION.** The risk of developing long term cognitive impairment and dementia is increased after systemic infections. The greatest risk is in those patients that develop an encephalopathy (delirium) during the infection. Therefore a common pathophysiology in systemic inflammation and neurodegenerative diseases is assumed. Recently, neuroinflammation has been shown to play a role in both disease entities, however little evidence is available on which biochemical pathways are dysregulated in the brain during infections and delirium.

**OBJECTIVES.** In this observational retrospective pilot study we studied whether there are differences in protein expression in CSF between infectious patients with or without delirium. Furthermore we assessed if protein expression in CSF in infectious delirium shows similarities to patients with mild Alzheimer's disease (AD).

**METHODS.** Stored CSF samples from adult patients with a systemic non-neurological infection (with or without delirium) at the time of lumbar puncture were eligible. Samples were compared to healthy controls and to patients with mild AD which served as 'positive' neuroinflammatory controls. CSF samples were analyzed using a proximity extension assay (PEA). We compared expression levels of 133 inflammatory and brain-specific proteins between groups with unpaired t-tests and corrected for multiple comparisons with the False Discovery Rate adjustment. In order to better understand the biological significance of the differentially expressed proteins we performed an Overrepresentation Test using PANTHER Classification System with Bonferroni correction for multiple testing.

**RESULTS.** Fifteen samples from infectious patients with delirium and 30 samples from infectious patients without delirium were included and compared to 15 healthy controls and 29 patients with AD. IL-6, CXCL1, IL-8 and CXCL6 were significantly upregulated in CSF from infectious delirium patients compared to healthy controls (FDR adjusted p-value $< 0.05$ and fold change >2). 54 proteins were significantly upregulated in patients with delirium relative to patients without a delirium. Although pro-inflammatory protein expression was increased in infectious delirium relative to AD, protein expression in patients with AD and infectious delirium showed the largest overlap (Chi-square test $p < 0.0001$).

**CONCLUSIONS.** Infectious delirium was associated with increased pro-inflammatory protein expression in CSF relative to infectious patients without delirium, to healthy controls and to patients with mild AD. The largest overlap in CSF composition was between infectious patients with delirium and patients with AD. This suggests an overlapping pathophysiology and could attribute to understanding why some patients go on to develop cognitive failure after suffering from an infection.

**GRANT ACKNOWLEDGMENT**
This work was supported by a grant from The Netherlands Organisation for Health Research and Development (ZonMW Clinical Fellowship number 90715610).

### 0708

#### Pro-BNP, inflammatory cascade and organ dysfunction in sepsis

R. Hernandez-Vaquero[1], I. Granja Gomez[1], E. Rodriguez-Ruiz[1], A. Lopez Lago[1], J.L. Garcia Allut[1], A. Estany-Gestal[2], M.A. Garcia Gonzalez[3]
[1]Hospital Clínico Universitario, Intensive Care Unit, Santiago de Compostela, Spain; [2]Fundación Ramón Dominguez, Unidad de Epidemiología e Investigación Clínica, Santiago de Compostela, Spain; [3]Instituto de Investigaciones Sanitarias (IDIS), Grupo de Genética y Biología del Desarrollo de las Enfermedades Renales, Santiago de Compostela, Spain
**Correspondence:** R. Hernandez-Vaquero
*Intensive Care Medicine Experimental* 2017, **5(Suppl 2):**0708

**INTRODUCTION.** Pro-brain natriuretic peptide (Pro-BNP) levels are elevated in patients with heart failure, as well as in some patients with a variety of conditions including renal failure and sepsis. Numerous studies suggest that high Pro-BNP levels represent a reliable marker of sepsis-induced myocardial dysfunction, and are associated with increased mortality. However other findings indicate that the relationship between Pro-BNP and myocardial dysfunction is weak, and data on the prognostic impact of high Pro-BNP levels in septic patients are conflicting. The primary conditions predisposing patients to Pro-BNP elevation in sepsis are largely undetermined, and clinical studies did not find correlation between cytokine concentration and septic myocardial dysfunction.

**OBJECTIVES.** The purpose of our study was to determine the relationship between the plasma levels of Pro-BNP and different biomarkers of the septic inflammatory cascade (TNF-α, IL-1β, IL-6, IL-8, C-reactive protein). Also, our aim was to analyze the correlation between Pro-BNP and diagnosis (severe sepsis vs. septic shock), APACHE II and SOFA score, and to check its ability to predict mortality.

**METHODS.** We assessed 58 consecutive septic patients admitted to our medical-surgical ICU. Plasma levels of Pro-BNP and inflammatory mediators (TNF-α, IL-1β, IL-6, IL-8, C-reactive protein) were determined on day 1. APACHE II and SOFA score were also calculated on day 1. Spearman, Kruskal-Wallis or Mann–Whitney tests were done to study the association of Pro-BNP with the different inflammatory mediators, APACHE II, SOFA and mortality at day 28.

**RESULTS.** It was found significant association between the levels of Pro-BNP and the plasma concentrations of TNF-α ($p < 0.001$), IL-6 ($p = 0.005$) and IL-8 ($p = 0.025$). There were 18 patients with severe sepsis and 40 patients with septic shock. Pro-BNP levels were significantly elevated in septic shock patients compared with those with severe sepsis ($p = 0.006$). Also, we found significant correlation between Pro-BNP and SOFA ($p = 0.01$), dependent on its renal ($p = 0.003$) and cardiovascular ($p = 0.036$) components. Mortality at day 28 was 24.1% (44 survivors and 14 non-survivors), but Pro-BNP did not show correlation with it.

**CONCLUSION.** We found that Pro-BNP was associated to certain acute phase mediators (TNF-α, IL-6 and IL-8), linking Pro-BNP levels to the inflammatory cascade. Also, Pro-BNP was significantly higher in septic shock patients, and its levels were correlated to organ dysfunction, due to the renal and cardiovascular components of the SOFA score. However, Pro-BNP has bad prognostic performance, not showing correlation with mortality. The role of Pro-BNP in sepsis remains not fully established, and larger studies including assessment of myocardial dysfunction should be done.

### 0709

#### Evaluation of body fluid biomarkers for neuroaxonal injury in cerebrospinal fluid and plasma for diagnosis, monitoring and prognostication in patients with sepsis-associated encephalopathy

J. Ehler[1], M. Wittstock[2], A. Petzold[3], P.S. Rommer[4], G. Richter[1], G. Nöldge-Schomburg[1], M. Sauer[1]
[1]University Medical Center Rostock, Department of Anaesthesiology and Intensive Care Medicine, Rostock, Germany; [2]University Medical Center Rostock, Department of Neurology, Rostock, Germany; [3]University College London, Institute of Neurology, Department of Neuroimmunology, London, United Kingdom; [4]Medical University of Vienna, Department of Neurology, Vienna, Austria
**Correspondence:** J. Ehler
*Intensive Care Medicine Experimental* 2017, **5(Suppl 2):**0709

**INTRODUCTION.** Sepsis-associated encephalopathy (SAE) is relevant for short- and long-term outcome in patients with septic shock (1). Diagnosis and monitoring of SAE are sophisticated due to a high proportion of sedation among these patients. Body fluid biomarkers might therefore be valuable, but the majority of available studies on NSE and S100B in SAE provided contrary results (2). The potential role of neurofilament (Nf) heavy (NfH) and light chains (NfL) as neuroaxonal injury markers (3) in SAE has not been investigated.

**OBJECTIVES.** To evaluate the relevance of NfH and NfL in cerebrospinal fluid (CSF) and plasma to diagnose, monitor and predict outcome in patients with SAE.

**METHODS.** 25 intensive care unit (ICU) patients with septic shock ($n = 20$) and without sepsis ($n = 5$) were prospectively included in a single-center observational study between 2012 and 2016 (ethics board identifier: A 2012–0058, ClinicalTrials.gov: NCT02442986). Main exclusion criterion was a previously known patient history of cerebrovascular disease as ischemia, hemorrhage, tumor or dementia. All patients were clinically assessed for signs of SAE in sepsis or delirium in patients without sepsis by an interdisciplinary team consisting of intensivist and neurologists. Electroencephalography (EEG) and delirium screening methods (CAM-ICU) were used to detect and to assess the severity of SAE or delirium. CSF analysis ($n = 12$) after sepsis onset and longitudinal plasma biomarker measurements for NSE, S100B, NfH and NfL on study days 1, 3 and 7 were performed in all patients.

**RESULTS.** 18 patients with SAE showed a significant increase of NfH ($p = 0.043$) and NfL ($p < 0.001$) between study day 1 and 7 in comparison to a slight, but non-significant increase of both markers in patients without SAE or delirium ($n = 7$). NSE and S100B values did not change significantly in both groups. Non-survivors until day 100 after study inclusion had significantly higher NfL values in CSF ($p = 0.01$) as survivors, all other markers did not change significantly between the groups. Plasma levels of NfH at day 1 ($p = 0.017$) and day 7 ($p = 0.029$) and S100B values at day 3 ($p = 0.005$) were significantly higher in non-survivors until day 100. In total 6 patients with sepsis and 1 patient without sepsis died until follow-up at day 100.

**CONCLUSIONS.** In comparison to NSE and S100B both NfH and NfL might represent promising biomarkers to monitor SAE and to predict outcome in sepsis. Larger longitudinal body fluid biomarker studies are indicated.

**REFERENCE(S)**

(1) Iwashyna TJ, Ely EW, Smith DM, et al.: Long-term cognitive impairment and
functional disability among survivors of severe sepsis. JAMA 2010; 304:1787–1794
(2) Zenaide PV, Gusmao-Flores D: Biomarkers in septic encephalopathy: a
systematic review of clinical studies. Rev Bras Ter Intensiva 2013; 25:56–62
(3) Petzold A. Neurofilament phosphoforms: surrogate markers for axonal injury,
degeneration and loss. J Neurol Sci 2005;233(1–2):183–98.

**GRANT ACKNOWLEDGMENT**
None

### 0710

#### Diagnostic value of procalcitonin and presepsin as a prognostic marker for infectious complications in liver transplant recipients

M. Katsin[1], M. Hurava[1], D. Serbina[2]
[1]Republican Scientific and Practical Center for Organ and Tissue Transplantation, ICU, Minsk, Belarus; [2]Belarusian State Medical University, Minsk, Belarus
**Correspondence:** M. Katsin
*Intensive Care Medicine Experimental* 2017, **5(Suppl 2):**0710

**INTRODUCTION.** Clinically significant infections (CSIs) are life-threatening but difficult to diagnose after liver transplantation (LTx) [1].

**OBJECTIVES.** This study investigates the value of presepsin (PSEP) and procalcitonin (PCT) as a prognostic marker for CSIs in LTx recipients.

**METHODS.** The clinical course of 33 LTx recipients was retrospectively studied. Study group included patients who developed CSI postoperatively. CSIs were defined as pulmonary, bloodstream, or intra-abdominal infections with criteria proposed by the Centers for Disease Control and Prevention [2]. Control group included patients with uncomplicated course of LTx.

**RESULTS.** Groups were comparable in demographic and anthropometric parameters, as well as the level of inflammatory markers (procalcitonin, presepsin, CRP), and white blood cells count before surgery. The presepsin concentration in study group was significantly higher in POD 1 (4275 ± 3878 ng / ml vs 1916 ± 1413 ng / ml, p = 0.027), POD2 (4978.5 ± 2473.3 ng / ml vs. 2029, 5 ± 1260 ng / ml, p = 0.002) and POD 5 (5397.06 ± 3916.09 vs. 2473 ± 1140.7 ng / ml, p = 0.002) than in control group. There was no significant difference in procalcitonin concentration in POD1 (24.73 ± 17.87 ng / ml vs. 16.54 ± 21.74 ng / ml, p = 0.126), however, significant difference in the concentration of procalcitonin was observed in POD2 60.07 ± 49.26 ng / ml versus 16.38 ± 15.44 ng / ml, p = 0.0003) and POD5 (13.22 ± 8.73 ng / ml vs. 4.09 ± 7, 03 ng / ml, p = 0.003). There was no significant difference in concentration of CRP and the level of white blood cells count during the first five days after TP. According to the ROC analysis, the area under the curve (AUC) for presepsin in POD1 was 0.75, which was much, but not significantly, higher than for procalcitonin (AUC 0.51, p = 0.124). The diagnostic cut-off of presepsin in POD1 is 1693 ng / ml, sensitivity is 81.2% and specificity is 66.7%. In the POD 2 the AUC for presepsin was 0.883, for procalcitonin - 0.922 (p = 0.679).

**CONCLUSIONS.** Presepsin is the earliest laboratory marker for postoperative infectious complications and sepsis in liver transplant recipients. In our study we found the diagnostic cut-off of presepsin which was defined as 1693 ng/ml in POD1.

**REFERENCE(S)**

1. Van den Broek MA, Olde Damink SW, Winkens B, et al. Procalcitonin as a prognostic marker for infectious complications in liver transplant recipients in an intensive care unit. Liver Transpl. 2010 Mar;16(3):402–10.

2. Teresa CH, Mary A, Margaret AD. CDC/NHSN surveillance definition of health care-associated infection and criteria for specific types of infections in the acute care setting. Am J Infect Control. 2008 Nov;36(9):655.

### 0711

#### Specific neurobiomarkers NSE and S100beta are increased in patients with septic shock

K. Erikson[1], J. Liisanantti[1], J. Koskenkari[1], R. Kamakura[2], K.-H. Karl-Heinz Herzig[2], H. Syrjälä[3], T. Ala-Kokko[1]

[1]University of Oulu, Department of Anaesthesiology, Division of Intensive Care Medicine, Oulu, Finland; [2]University of Oulu, Institute of Biomedicine and Biocenter of Oulu,, Oulu, Finland; [3]University of Oulu, Department of Infection Control, Oulu, Finland

**Correspondence:** K. Erikson

*Intensive Care Medicine Experimental* 2017, **5(Suppl 2):**0711

**INTRODUCTION.** Clinical assessment of central nervous system dysfunction to detect encephalopathy in septic shock with multiple organ failure is challenging. IL6 and TNFα are known to activate brain responses during inflammatory processes, which clinical significance is unknown. (1, 2).

**OBJECTIVES.** The aim of the present study is to evaluate the NSE and S100 levels in septic shock with multiple organ failure.

**METHODS.** Neuron specific marker NSE and glial cell specific marker S100beta of 31 adult patients admitted to ICU due to sepsis were analyzed. The inflammatory response was evaluated by analyzing inflammatory markers including TNFα, IL6, PCT and CRP and the need of noradrenaline. Multiple organ failure (MOF) was determined as SOFA score of 3 or 4 in two organ systems during the blood sampling.

**RESULTS.** A total of 20 (64%) patients had NSE and 23 (74.2%) had S100beta above normal values (17ug/L and 0.11 ug/L, respectively). The median APACHE II, SOFA, PCT and CRP levels as well as the cumulative noradrenaline dose were similar between those with NSE or S100beta above or lower than normal values. TNFα was higher in those with S100beta above normal value (42.1 pg/mL [29.8-65.1] vs 20.1pg/mL [19.4-22.8], P = 0.013). There was a positive correlation between S100beta and TNFalpha as well as S100beta and IL6 (R = 0.452, P = 0.012 and R = 0.453, P = 0.012, respectively). There were no differences in the levels of either S100beta or NSE between patients with or without MOF.

**CONCLUSION.** Majority of patients with septic shock had increased levels of neurobiomarkers. Proinflammatory markers (TNFalpha, IL6) correlated with glial cell marker S100beta.

2. Heming, N, et al. [Neuroanatomy of sepsis-associated encephalopathy.] Critical Care2017 21:65

**GRANT**

Oulu University Hospital EVO-grant

**ACKNOWLEDGMENT**

We thank RN, research nurse Sinikka Sälkiö for technical assistance and helping out in data collection.

### 0712

#### A real life, in hospital, pilot study to evaluate changes in inflammatory markers & hemodynamic parameters after Extracorporeal Cytokine Adsorption Device (ECAD) therapy along with Standard Of Care (SOC) in patients of sepsis and or septic shock as per Ronco´s peak concentration hypothesis

P. Sakhavalkar[1], P. Sathe[1], T. Baronia[1], S. Chaudhary[1], D. Makwana[2], S. Yadav[1]

[1]Ruby Hall Clinic; ICU, Pune, India; [2]Ruby Hall Clinic, Pune, India

**Correspondence:** P. Sakhavalkar

*Intensive Care Medicine Experimental* 2017, **5(Suppl 2):**0712

**INTRODUCTION.** Sepsis is life threatening organ dysfunction caused by dysregulated host response to infection[1] Despite SOC mortality remains 40-60% [2].Regulating the disproportionate cytokines could reduce the end organ damage.ECAD removes key cytokines in vitro &i n vivo from cytokine storm seen in sepsis[3,4]

**OBJECTIVES.** To assess pre & post ECAD safety, efficacy,hemodynamics & outcome among septic patients.

**METHODS.** Observational,prospective,pilot study done in tertiary ICU in Pune,India during Aug 2016 to Mar 2017 after ERB approval.Inclusion criteria: confirmed diagnosis of sepsis or septic shock and hospitalized in ICU,age 18–80 yrs,evidence of at least 1 organ dysfunction during sepsis,at least 6 hours of antibiotic therapy & SOC as per Sepsis-3.Exclusion criteria: pregnant/ nursing mother, established septic shock for >48 hrs,more than 3 failed organs at entry, platelet count < 20,000/$mm^3$,uncontrolled hemorrhage within last 24 hrs, active malignancy on chemo or radiation therapy within last 60 days,CPR during current episode of sepsis,patients with immune deficiencies or on immunosuppresant therapy. Blood samples collected for cytokines IL-1,IL-6,IL-10,TNF α,CRP before and after each ECAD. APACHE II & SOFA calculated.All patients recieved SOC. Averageg duration of ECAD 12 hrs each.

**RESULTS.** Case series of 10 patients, 6 men &4 women with septic shock and or MultiOgan Failure(MOF) from the ICU treated with SOC along with ECAD as an adjuvant therapy.Total 18 ECAD used, minimum 1 and maximum 3 per patient. Average APACHE II score reduced by 18.95%. All surviving patients showed decrease in IL-6 levels except for two.[Table 187]. 6 out of 10 patients survived despite of average predicted mortality rate of 27.21% prior to ECAD therapy. Limitations of study: small sample size & no control group.

**CONCLUSIONS.** ECAD substantial reduced IL6, IL10, CRP, APACHE II & predicted mortality scores and improved MAP values along with SOC.ECAD can be a beneficial adjuvant therapy in sepsis and /or septic shock and / or MOF. It is a safe and well tolerated therapy in critically ill septic patients. Bigger, prospective, multicentric randomized control study would help to reconfirm findings of this pilot study.Sepsis is a life threatening condition that arises when the body´s respouse to infection injures it´s own tissues, hence the ways to modify such damaging immune dysregulation could be the future of Sepsis management to improve outcomes

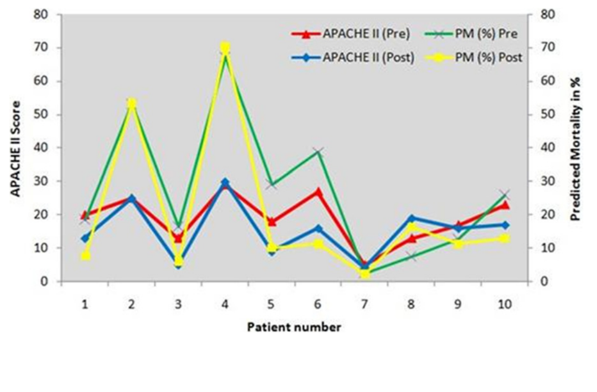

**Fig. 249 (Abstract 0712)** Pre and Post ECAD APACHE II & predicted mortality

**Table 187 (Abstract 0712).** Demography and Efficacy parameters of patients

| SERIAL NO | AGE | SEX | PRE ECAD APACHE II | POST ECAD APACHE II | PRE ECAD IL6 LEVELS | POST ECAD IL6 LEVELS | PRE ECAD MAP (mm Hg) | POST ECAD MAP (mm Hg) | OUTCOME |
|---|---|---|---|---|---|---|---|---|---|
| 1 | 73 | F | 20 | 13 | 1013 | 80.64 | 67 | 94 | SURVIVED |
| 2 | 60 | M | 25 | 25 | 2517.56 | 50.8 | 59 | 76 | DEATH |
| 3 | 25 | M | 13 | 5 | 31.24 | 15.36 | 78 | 84 | SURVIVED |
| 4 | 65 | M | 29 | 30 | 7.84 | 3.24 | 60 | 81 | SURVIVED |
| 5 | 34 | F | 18 | 9 | 51.4 | 33.27 | 57 | 82 | SURVIVED |
| 6 | 64 | F | 27 | 16 | 147.16 | 5.12 | 65 | 90 | DEATH |
| 7 | 22 | F | 5 | 4 | 4.4 | 4.4 | 92 | 87 | SURVIVED |
| 8 | 60 | M | 13 | 19 | 14.08 | 1269.2 | 108 | 120 | DEATH |
| 9 | 64 | M | 17 | 16 | 131.2 | 147.16 | 100 | 106 | DEATH |
| 10 | 66 | MALE | 23 | 17 | 15.36 | 21.12 | 112 | 72 | SURVIVED |

### 0713

#### Do Procalcitonin, CRP and WBC measure the same thing in septic critically ill?

A. Corona, V. Beqiri, D. Testa, E. Maccarrone, F. Stretti, A. Gandola, A. Veronese, C. Di Nobile

PO Luigi Sacco, Milano, Italy

**Correspondence:** A. Corona

*Intensive Care Medicine Experimental* 2017, **5(Suppl 2):**0713

**INTRODUCTION.** Procalcitonin (PCT) is a more than promising marker to be used to guide antibacterial therapy and reduce its duration in terms of reduce risk of development of antimicrobial resistance.

**OBJECTIVES.** We study the correlation betwen PCT, CRP and WBC in septic shock.

**METHODS.** Since January 2012 to December 2016, patients experiencing a septic shock and admitted with a first 24h SAPS II > 25, associated with a SOFA-score > 4 and likely ICU length of stay ≥ 72 hrs. were enrolled for the study. We studied the correlation between PCR, CRP and WBC in septic shock by linear regression and Bland Altman analyses.

**RESULTS.** During the study period, 768 (38.5%) out of 1990 patients admitted to ICU - median (IQR) age 61 (58–77), 1st 24 hrs. SAPS II 58 (47–78), SOFA-score 12 (10–14) were enrolled for the analysis. Correlation between PCR, CRP and WBC in the 142 (18.5%)/768 recorded septic shock are shown in Figs. 250 and 251, respectively linear regression and Bland Altman analyses. PCT is not correlated neither CRP nor WBC, whereas the latter two showed a similar trend and closer correlation.

**CONCLUSIONS.** PCT is not correlated neither CRP nor WBC, whereas the latter two showed a similar trend and closer correlation.

**REFERENCE(S)**

Jeong-Am Ryu, Jeong Hoon Yang, Daesang Lee, Chi-Min Park, et al. Clinical Usefulness of Procalcitonin and C-Reactive Protein as Outcome Predictors in Critically Ill Patients with Severe Sepsis and Septic Shock.PLoS One. 2015; 10(9):

**GRANT ACKNOWLEDGMENT**

None

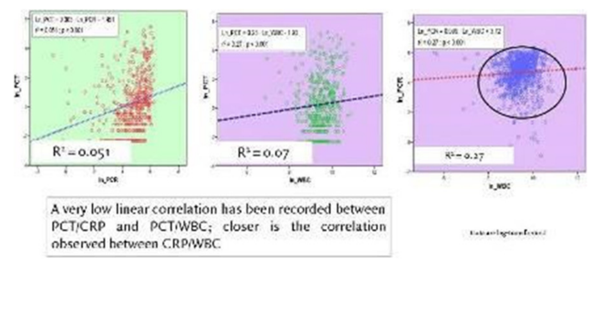

**Fig. 250 (Abstract 0713)** See text for description

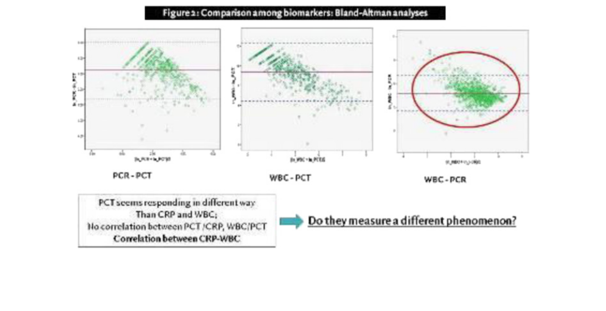

**Fig. 251 (Abstract 0713)** See text for description

### 0714

#### Leukocyte antisedimentation rate (LAR) as a sign of developing sepsis in burns

C. Loibl[1], L. Szélig[1], L. Bogár[1], P. Kovács[1], M. Matancic[2], A. Pankaczi[1], A. Miseta[3], C. Csontos[1]

[1]University of Pécs, Clinical Center, Department of Anaesthesiology and Intensive Therapy, Pécs, Hungary; [2]University of Pécs, Clinical Center, 1st Department of Internal Medicine, Pécs, Hungary; [3]University of Pécs, Clincal Center, Department of Laboratory Medicine, Pécs, Hungary

**Correspondence:** C. Loibl

*Intensive Care Medicine Experimental* 2017, **5(Suppl 2):**0714

**INTRODUCTION.** Despite improvement in care of burned patients sepsis remained the leading cause of death. The diagnosis of sepsis is difficult due to the existing systemic inflammatory response syndrome (SIRS) which can mimic the signs of sepsis in the absence of real infection too. Early detection of infections can be lifesaving in burn victims because an earlier diagnosis can lead to earlier initiation of antibiotic treatment which is associated with better outcome. On the other hand unnecessary antibiotic treatment can be avoided.

**OBJECTIVES.** Our aim was to evaluate the predictive power of leukocyte antisedimentation rate (LAR), serum C-reactive protein (CRP) and procalcitonin (PCT) levels regarding mortality risk and development of septic complications.

**METHODS.** In our prospective observational study 21 patients admitted to our intensive care unit immediately after severe burn injury were included. Blood samples were taken at admission and on the following mornings till discharge from ICU. The initial kinetics of the parameters were analysed for 5 days (T1-T5). The patients were divided into survivors and non-survivors. Sepsis was diagnosed by clinical signs according to the guideline of the American Burn Association. The kinetic of the measured parameters were analysed 3 days before (D-3-1) and 3 days after (D + 3-1) when sepsis was declared.

16 healthy volunteers with similar age and sex to the observed population served as control group.

**RESULTS.** The patients' age median was 66 (IQR: 49–80) years. Median abbreviated burn severity index was 7 (IQR: 5–8) and total burned surface area (TBSA) 30 (IQR: 25–40). Ten patients became septic,

7 patients died due to infectious complications. LAR levels in survivors were significantly higher compared to controls on T3-5 ($p < 0.001$) whereas LAR levels of non-survivors were significantly lower on T2

($p < 0.05$) and became higher on T5. Non-survivors and survivors showed significant difference on T2, T4 and T5 ($p < 0.05$) with higher levels in survivors. CRP levels were above the upper normal limit in survivors and non-survivors from T2 without significant difference between groups. PCT level were slightly elevated in non-survivors without significant difference between groups. In septic patients LAR showed a significant drop on D-1 ($p < 0.05$). CRP levels showed this decreasing tendency too, but this was not statistically significant. PCT levels failed to predict this. It became significantly elevated only since the onset of sepsis.

**CONCLUSIONS.** Patients who showed a drop in LAR levels on T2 were more likely to die. A sudden decrease in LAR levels may predict the onset of sepsis in contrast to the other studied parameters among our patients.

**REFERENCE(S)**

Lavrentieva A et al. Burns 2007; 33: 189–94.

Jeschke MG et al. Ann Surg 2008 September; 248(3): 387–401

**GRANT ACKNOWLEDGMENT**

None

#### 0715

#### Procalcitonin to predict the onset of septic shock and guide antibiotic therapy

A. Corona, F. Stretti, A. Gandola, D. Testa, A. Veronese, C. Soru, F. Cantarero, C. Di Nobile, R. Colombo, E. Catena

PO Luigi Sacco, Milano, Italy

**Correspondence:** A. Corona

*Intensive Care Medicine Experimental* 2017, **5(Suppl 2):**0715

**INTRODUCTION.** Procalcitonin (PCT) is a more than promising marker to be used to guide antibacterial therapy and reduce its duration in terms of reduce risk of development of antimicrobial resistance. We evaluated its efficacy both in predicting septic shock and in likely antibiotic duration saving.

**OBJECTIVES.** To asses the role of PCT and its trend in guiding antibiotic therapy in ICU.

**METHODS.** Since January 2012 to December 2016, patients experiencing a septic shock and admitted with a first 24h SAPS II > 25, associated with a SOFA-score > 4 and likely ICU length of stay ≥ 72 hrs. were enrolled for the study. On all the patients the following was collected: demographics, comorbidities and admission diagnosis together with severity of illness, duration of ICU supportive therapies; moreover PCR, CRP and WBC were daily collected in association with type and duration of antibiotic therapy.

**RESULTS.** During the study period, 768 (38.5%) out of 1990 patients admitted to ICU - median (IQR) age 61 (58–77), 1$^{st}$ 24 hrs. SAPS II 58 (47–78), SOFA-score 12 (10–14) were enrolled for the analysis. A total of 142 (18.5%) out of the enrolled patients experienced a septic shock and were associated with significant higher median (IQR) level of PCT if compared to those with only sepsis findings [respectively: 25 (4–37) and 1 (0.2-2), p = 0.0005]; moreover early PCT values were lower in survived patients [respectively: 4 (2–21) and 27 (5–39), p = 0.0005]; conversely we do not find a PCT cut off value considerable as a good predictor of septic shock, even though a PCT ≥ 1.85 has been found related to a specificity of 65.7% and sensitivity of 62% to predict septic shock. PCT was an helping tool in stopping antibiotic therapy and saving its costs only in survived patients and not in dead ones. Actually it decreased of ≥ 80% in day 5$^{th}$ of therapy in over 90% of patients that were discharged to the ward.

**CONCLUSIONS.** PCT may be a good performing biomarker to inform practice in antibiotic therapy stopping and stewardship.

**REFERENCE(S)**

de Jong E, van Oers JA, Beishuizen A, et al. Efficacy and safety of procalcitonin guidance in reducing the duration of antibiotic treatment in critically ill patients: a randomised, controlled, open-label trial. Lancet Infect Dis. 2016 Jul;16(7):819–27.

### Medical emergency teams

#### 0716

#### Quick alert score using simple parameters for screening high risk patients in the general ward

H.-J. Kim[1], H. Jin Won[2], Y. Koh[2], C.-M. Lim[2], S.-B. Hong[2]

[1]ASAN Medical Center, Department of Clinical Epidemiology and Biostatistics, Seoul, Korea, Republic of; [2]ASAN Medical Center, Department of Pulmonary and Critical Care Medicine, Seoul, Korea, Republic of

**Correspondence:** H.-J. Kim

*Intensive Care Medicine Experimental* 2017, **5(Suppl 2):**0716

**INTRODUCTION.** Rapid response system (RRS) screens high risk patients earlier in the general ward and performs interventions to prevent an unexpected intensive care unit (ICU) admission and cardiac arrest. Recently, scoring systems such as modified early warning score (MEWS) or national early warning score (NEWS) were used in mandatory in several hospitals.

**OBJECTIVES.** This study was done to evaluate whether new scoring system (Quick alert score, QA score) composed of vital sign and laboratory data could effectively screen high risk patients by the ward care team.

**METHODS.** All patients with which was screening by Medical Alert System (MAS) and randomly selected controls with sampling ratio of 5% were selected among adult admission during October 2013 were extracted for development of the QA score, and vital sign and laboratory data considering SBP, PR, RR, lactate, total $CO_2$ were collected. Same process was conducted among admission during December 2013 for validation. Multivariable logistic regression was used to create the QA score using bootstrapping methodology with external validation.

**RESULTS.** Of the 12,523 measures check-up, 5,700 measures were screened by MAS (45.5%), and 387 were activated by medical alert team (MET) (3.1%). As MAS screening were conducted each time of repeated measures, analysis was conducted spell-based. The QA score was developed using combination of the vital sign and laboratory test, and variables independently associated with ICU admission on logistic regression. The receiver operating characteristics curve analysis of the QA score was c = 0.82 (95% confidence interval, 0.79-0.84). External validation, performed on 13,166 measures, exhibited excellent discrimination (c = 0.76; 95% confidence interval, 0.73-0.79).

**CONCLUSIONS.** The QA score using the vital sign and lactate score could be a good scoring system for screening high risk patients in general ward.

**REFERENCE(S)**
1. Subbe CP, Kruger M, Rutherford P, et al. Validation of a modified Early Warning Score in medical admissions. QJM 2001;94:521–526.
2. Huh JW, Lim CM, Koh Y, et al. Activation of a medical emergency team using an electronic medical recording-based screening system*. Crit Care Med 2014;42:801–808.

**GRANT ACKNOWLEDGMENT**
This study was supported by a grant of the Korea Health Technology R&D Project through the Korea Health Industry Development Institute (KHIDI), funded by the Ministry of Health & Welfare, Republic of Korea (grant number: HI15C1106H).

### 0717

#### ICU's rapid response team patients triaged to remain on ward despite positive activation criteria - should the 'ICU without walls' be further expanded?

J. Tirkkonen[1,2], T. Kontula[1], S. Hoppu[1]
[1]Tampere University Hospital, Department of Intensive Care Medicine, Tampere, Finland; [2]Seinäjoki Central Hospital, Department of Anaesthesia and Intensive Care Medicine, Seinäjoki, Finland
**Correspondence:** J. Tirkkonen
*Intensive Care Medicine Experimental* 2017, **5(Suppl 2):**0717

**INTRODUCTION.** Rapid response teams (RRTs) are one of the core elements when intensive care is expanded to general wards (the critical care without walls concept).[1] The objective RRT activation criteria define vital dysfunctions, and are applied by the ward staff with the subjective 'worried criterion' to trigger RRT. Most patients are triaged to stay on ward, although some of them fulfill the activation criteria during the actual RRT review also. Outcome of these patients has not been studied, despite they likely are high risk patients that deteriorate further on.

**OBJECTIVES.** To compare those RRT patients triaged to stay on ward despite they fulfill RRT activation criteria during the actual review to those that don't. If the patients with positive activation criteria are at higher risk for adverse events, follow-up by the ICU liaison nurses could be targeted to these patients.[2]

**METHODS.** We collected prospectively data on RRT activations in Tampere University Hospital, Finland, between 1 May 2012 and 30 April 2015. Hospital's RRT activation criteria were: heart rate < 40/min or > 140/min, systolic blood pressure < 90 mmHg, peripheral arteriolar oxygen saturation < 90%, respiratory rate < 5/min or > 24/min and AVPU ≤ 3. The local Ethics Committee approved the study protocol (Approval no: R10111).

**RESULTS.** During the study period, 860 patients had their first review and were triaged to stay on ward with active treatment plan, and 564 (66%) of these patients had objective vital dysfunctions noted by the RRT during the review while 296 (34%) did not. Patients with objective vital dysfunctions during the review were of comparable age and comorbidity index as compared to the stabile patients and had as often had a preceding ICU admission or surgery. Despite the patients with altered vital signs received more often RRT interventions such as fluids and medications as compared to the stabile patients, they required a new RRT review more often and had higher in-hospital mortality rate and higher fixed mortality for up to one year (37% vs. 29%, p = 0.014). Table 188 presents the multivariate regression model of the factors that were independently associated with 30-day mortality among the 860 patients triaged to stay on ward; having altered vital signs according to the RRT was one of them (OR 1.74; 95% CI 1.12 - 2.70).

**CONCLUSIONS.** Patients triaged to stay on ward despite acknowledged vital dysfunctions by the RRT are high risk patients that could potentially benefit from routine follow-up by the ICU's liaison nurses. The 'ICU without walls' concept could be easily expanded to include these high risk patients before they deteriorate beyond salvation.

**REFERENCE(S)**
Hillman K. Critical care without walls. Curr Opin Crit Care 2002;8:594–9.
Endacott R, Chaboyer W, Edingto, J Thalib L. Impact of an ICU Liaison Nurse Service on major adverse events in patients recently discharged from ICU. Resuscitation 2010;81:198–201.

**GRANT ACKNOWLEDGMENT**
None.

**Table 188 (Abstract 0817).** See text for description

| | Multivariate analysis | | |
|---|---|---|---|
| | Odds ratio | 95% CI | *p*-value |
| Age | 1.03 | 1.02 – 1.05 | < 0.001 |
| Non-elective hospital admission | 2.74 | 1.59 – 4.74 | < 0.001 |
| Charlson comorbidity index | 1.18 | 1.08 – 1.29 | < 0.001 |
| Vital dysfunctions recorded by the RRT | 1.74 | 1.12 – 2.70 | 0.014 |
| Medical patient | 1.56 | 1.06 – 2.29 | 0.024 |
| Afferent limb failure | 1.55 | 1.05 – 2.28 | 0.026 |
| Surgery 0–24 h before the review | 0.75 | 0.41 – 1.39 | 0.360 |
| Preceding ICU admission | 0.89 | 0.53 – 1.51 | 0.668 |
| Sex (female) | 1.08 | 0.74 – 1.58 | 0.702 |
| Review during on-call time[a] | 1.00 | 0.64 – 1.57 | 0.995 |

The Hosmer-Lemeshow goodness-of-fit Chi-square (8) with p = 0.910 indicated a good fit of the model. RRT, rapid response team; CI, confidence interval; CCI, Charlson comorbidity index; ICU, intensive care unit.

### 0718

#### Arterial blood gas analysis during ICU's rapid response team review - do we need it, we have vital signs!

J. Tirkkonen[1,2], S. Hoppu[1]
[1]Tampere University Hospital, Department of Intensive Care Medicine, Tampere, Finland; [2]Seinäjoki Central Hospital, Department of Anaesthesia and Intensive Care Medicine, Seinäjoki, Finland
**Correspondence:** J. Tirkkonen
*Intensive Care Medicine Experimental* 2017, **5(Suppl 2):**0718

**INTRODUCTION.** General ward staff trigger ICUs' rapid response teams (RRTs) to attend patients with altered vital signs.[1] RRTs may conduct further diagnostics, including point-of-care (POC) arterial blood gas (ABG) analysis, interventions and escalate the level of care if deemed appropriate. However, there is no robust evidence, that advanced bed-side diagnostics *per se* improve patient outcome in comparison to, for example, simple ICU liaison nurse assessment based on patient's status during an RRT review.[1]

**OBJECTIVES.** We aimed to investigate, whether altered vital signs, defined as simple RRT activation criteria (heart rate < 40/min or > 140/min OR systolic blood pressure < 90 mmHg OR $SpO_2$ < 90% OR respiratory rate < 5/min or > 24/min OR AVPU assessment ≤ 3, detect the patients whose POC-ABG analysis reveal hypoxemia/acidosis/hyperlactatemia/decreased base excess. RRT criteria were tested both with and without the AVPU assessment.

**METHODS.** Prospectively collected data on ICU's RRT activations between 1 January 2011–30 April 2015 in Tampere University Hospital, Finland. POC-ABG analysis was conducted if deemed appropriate by the RRT physician, and vital signs measured by the RRT staff during these reviews were compared to the results of the ABG analysis. The *i-STAT*® handheld device (Abbott Point of Care Inc., NJ, USA) was used for the ABG analysis. The local Ethics Committee approved the study protocol (Approval no: R10111).

**RESULTS.** During the study period, 2652 RRT reviews were conducted. After the exclusion of no POC-ABG reviews (2203) and POC-ABG reviews with complete but unreliably data (6), 443 RRT reviews with full data on both vital signs and POC-ABG results were included. Median ($Q_1$, $Q_3$) age of the patients was 69 (58, 77), 68% (301) were male and 18% (80) had been electively admitted to the hospital. Vital signs were defined as abnormal in 76% (337) of the patients according to the RRT criteria (AVPU not included) and in 86% (381) of the patients according to the RRT criteria including the AVPU. Sensitivity of the RRT criteria including the AVPU was 89% (80% without the AVPU) for any mild deviation and 96% (91%) for any severe deviation in ABG (Fig. 252).

**CONCLUSIONS.** RRT criteria including the assessment of the level of consciousness detected patients with decompensated acidosis/deoxygenation/hyperlactatemia/decreased base excess in POC-ABG with good sensitivity. Sensitivity was excellent when RRT criteria were compared with severe disturbances in POC-ABG (and thus to rule out patients with severe ABG disturbances). We conclude, that simple vital sign measurements are sufficient to rule out patients with severe ABG disturbances during ICU's RRT reviews.

**REFERENCE(S)**

1. Jones D, DeVita M, Bellomo R. Rapid-response teams. N Engl J Med 2011;365:139–46.

**GRANT ACKNOWLEDGMENT**

None.

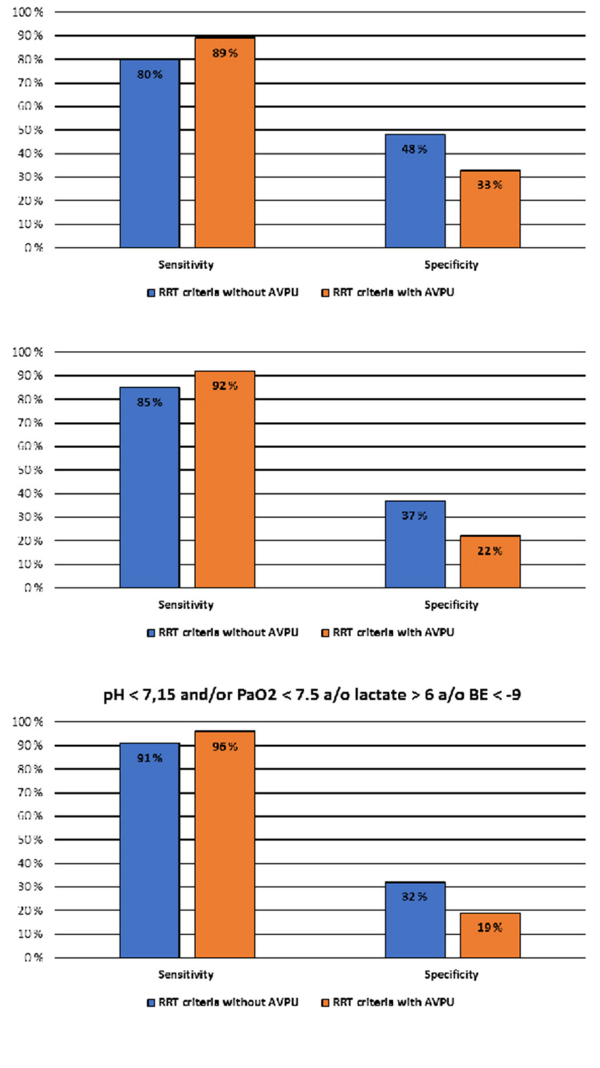

**Fig. 252 (Abstract 0718)** pH < 7,35 and/or PaO2 <10.8 a/o lactate >2 a/o BE<-3

### 0719

#### Medical emergency team - is afferent limb (METal) working?

A. Correia[1,2], I.J. Pereira[1,2,3], P. Barbosa[1], C. Granja[1,2,3]

[1]Algarve Hospital Center, Intensive Care, Faro, Portugal; [2]University of Algarve, Department of Biomedical Sciences and Medicine (DCBM), Faro, Portugal; [3]Faculty of Medicine of the University of Porto (FMUP), Center for Health Technology and Services Research (CINTESIS), Porto, Portugal

**Correspondence:** A. Correia

*Intensive Care Medicine Experimental* 2017, **5(Suppl 2):**0719

**INTRODUCTION.** Adequate monitoring, early recognition of physiological abnormalities and response trigger are fundamental cornerstones to achieve Medical Emergency Team (MET) positive outcomes[1–3].

In our hospital, a MET was implemented in Oct 2015. During the first year, 629 MET activations occurred (activation rate 30,2/1000 inpatients). Data analysis revealed a constant decrease in the total activation number and an increased percentage number of activations due to unexpected cardiac arrests. Concerns related with the contribution of the afferent component of the system to these results have been raised, justifying the present study.

**OBJECTIVES.** To compare the outcomes of patients who fulfilled activation criteria and for whom the MET was activated against those for whom there were no interventions by the MET; to determine the vital signs (VS) assessment standards in the wards and to determine if the presence of MET criteria was associated with unplanned admissions to Intensive Care Unit (ICU)/Intermediate Care Unit (IntCU), unexpected cardiac arrests (UCA), increased hospital length of stay (LOS) and death at hospital discharge.

**METHODS.** Retrospective observational single-center study. Patient clinical records during a ten day-period following admission date were reviewed against MET activation criteria. Two groups were established: *fulfill MET´criteria* patients (FMC) and *do not fulfill MET´ criteria* (NFMC) patients.

**RESULTS.** 211 patients were included, 53 of those (25,1%) fulfilled MET criteria during the study period. The MET was activated for 6 of those patients (11,3%). FMC and NFMC had similar distribution of age, sex, age-adjusted Charlson Comorbidity Index and parental unit. FMC group patients were more likely to die in the hospital (OR =28; 95% CI; 1,81-4,855). In FMC group the intervention of MET was not associated with different LOS, unplanned ICU/IntCU transfer, UCA and in-hospital mortality.

Overall, VS were more frequently assessed at least 3 times per day (n = 126/73,9%). In 75 cases (35,3%) the assessment was not directly related to medical prescription. Respiratory rate (RR) was only included in VS assessment of 2 patients (0,9%).

**CONCLUSIONS.** The number of patients evaluated by the MET was low when compared with the number of ward patients that fulfilled the MET activation criteria. We were unable to find statistical significant effects on outcomes, probably related with the low number of MET activations, although we found a clinical significant positive association between the presence of MET criteria and mortality during in-hospital stay. We found discrepancies between vital sign assessment pattern and medical prescription, more significant in surgical patients. The RR, a vital sign included in several severity scores, was seldom recorded.

1.Simmes *et al.Nethe j critic care.* 2012;16(6):195–199.

2.DeVita *et al.Resuscitation.*2010;81(4):375–382.

3.Nurmi *et al.Acta Anaesthesiol Scand.* 2005;49(5):702–706.

**GRANT ACKNOWLEDGMENT**

None

### 0720

#### The impact of introducing the early warning scoring system and protocol on clinical outcomes in tertiary referral university hospital

Y. Sutherasan[1], A. Suporn[2], P. Theerawit[1], A. Nongnuch[3], P. Phanachet[4], C. Kositchaiwat[5]

[1]Ramathibodi Hospital, Mahidol University, Division of Pulmonary and Critical Care Medicine, Department of Medicine, Faculty of Medicine, Bangkok, Thailand; [2]Ramathibodi Hospital, Mahidol University, Department of Medicine, Faculty of Medicine, Bangkok, Thailand; [3]Ramathibodi Hospital, Mahidol University, Renal Unit, Department of Medicine, Faculty of Medicine, Bangkok, Thailand; [4]Ramathibodi Hospital, Mahidol University, Division of Nutrition and Biochemical Medicine, Department of Medicine, Faculty of Medicine, Bangkok, Thailand; [5]Ramathibodi Hospital, Mahidol University, Division of Gastroenterology and Hepatology, Department of Medicine, Faculty of Medicine, Bangkok, Thailand

**Correspondence:** Y. Sutherasan

*Intensive Care Medicine Experimental* 2017, **5(Suppl 2):**0720

| | Control group | Intervention group | P value |
|---|---|---|---|
| Rate of ICU transfer No. (%) Total | 22(3.9%) | 33(5.7%) | 0.159 |
| Low risk | 9(1.8%) | 10(2.1%) | 0.684 |
| Moderate risk | 7(26.9%) | 6(8.7%) | 0.021 |
| High risk | 6(20.7%) | 17(40.5%) | 0.080 |
| In-hospital mortality No. (%) Total | 11(2%) | 15(2.6%) | 0.473 |
| Low risk | 4(0.8%) | 7(1.5%) | 0.297 |
| Moderate risk | 4(15.4%) | 2(2.9%) | 0.026 |
| High risk | 3(10.3%) | 6(14.3%) | 0.624 |

**INTRODUCTION.** National Early warning scores (NEWS) are widely used in general wards to identify the patient at risk of deterioration. The specific interventions responding to the different values of NEWS should be determined to improve the patients' outcomes.

**OBJECTIVES.** This study aimed to assess the impact of a hospital protocol responding to the deterioration of the patients stratified by NEWS on patients' outcomes regarding in-hospital mortality and the percentage of intensive care unit (ICU) transfer.

**METHODS.** We conducted a prospective observational cohort study in adult medical patients admitted to a university hospital in Bangkok. A 4-month period of pre-protocol (November 2015-Febuary 2016) was assigned as a control group and a protocol period (March 2016-June 2016) was assigned as an intervention group. On admission, the vital signs (respiratory rate, pulse rate, systolic blood pressure), oxygen saturation, the presence of oxygen supplement and neurological status were used to calculate the NEWS. Patients were categorized to low, moderate and high risk based on their NEWS. Either essential managements and/or ICU transfer were provided to patients based on the hospital protocol. The primary outcomes were compared between the control group and intervention group.

**RESULTS.** A total of 1145 patients were enrolled for analysis, 564 patients were in the control group and 581 patients were in the intervention group. The mean age was 65 ± 16.12 years and 53.3% of patients were male. The EWS of patients at admission in the control group was lower than the intervention group (1.77 ± 2.158 vs. 2.4 ± 2.4; P = 0.000).

The percentage of patients with moderate and high risk were higher in the intervention group than the control group (11.9% vs. 4.6% in moderate risk group, 7.2% vs. 5.1% in high risk group; P = 0.000). There were no significant differences in in-hospital mortality and the percentage of ICU transfer between these two groups. Among 95 patients (8.3%) with moderate risk, the in-hospital mortality and the percentage of ICU transfer in intervention group were lower than control group [2.9% vs. 15.4%; P = 0.026; relative risk(RR) of 0.188, 95% confidence interval(CI) 0.037-0.968 and 8.7% v.s. 26.9% P = 0.021; RR of 0.322, 95%CI 0.12- 0.87, respectively].

**CONCLUSIONS.** The implementation of a hospital protocol responding to the deterioration of the patients stratified by NEWS is associated with a reduction of in-hospital mortality and the percentage of ICU transfer in patients with moderate risk.

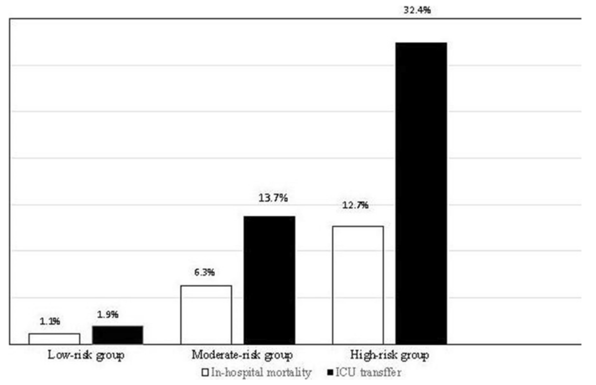

**Fig. 253 (Abstract 0720)** See text for description

### 0721

#### Epidemiology and clinical characteristics of rapid response team activations

S.W. Kim[1], H.Y. Lee[2], M.R. Han[3], Y.S. Lee[3], E.H. Kang[3], E.J. Jang[3], K.S. Jeun[3], S.C. Kim[3]

[1]Daejeon St. Mary's Hospital, College of Medicine, The Catholic University of Korea, Seoul, Department of Internal Medicine, Daejeon, Korea, Republic of; [2]Uijeongbu St. Mary's Hospital, College of Medicine, The Catholic University of Korea, Uijeongbu, Korea, Republic of; [3]Seoul St. Mary's Hospital, College of Medicine, The Catholic University of Korea, Seoul, Korea, Republic of

**Correspondence:** S.W. Kim

*Intensive Care Medicine Experimental* 2017, **5(Suppl 2):**0721

**INTRODUCTION.** To ensure patient safety and improvements in the quality of hospital care, rapid response teams (RRTs) have been implemented in many countries, including Korea. The goal of an RRT is early identification and response to clinical deterioration in patients. However, there are differences in RRT systems among hospitals and limited data are available.

**OBJECTIVES.** In this study, we retrospectively reviewed the RRT activation records of our institution to determine the clinical characteristics and predictors of survival of Korean patients who required an RRT activation.

**METHODS.** In Seoul St. Mary's Hospital, the St. Mary's Advanced Life Support Team was implemented in June 2013. We retrospectively reviewed the RRT activation records of 287 cases from June 2013 to December 2016. The RRT was activated by phone calls (74.9%) and a screening system (25.1%).

RESULTS : The median response time and median modified early warning score were 8.6 (5.6-11.6) minutes and 5.0 (4.0-7.0) points, respectively. Eighty percent of RRT activations occurred on weekdays, not including holidays. Residents (35.8%) and nurses (59.1%) were the main activators of the RRT. Of the RRT activation cases, presence of malignancy and a postoperative status were 34.8%, and 69.3%, respectively. The survival rate was 83.6% and survival was mainly associated with malignancy, Acute Physiology and Chronic Health Evaluation-II (APACHE-II) score, and the time from admission to RRT activation. RRT activation with screening showed a better outcome compared to activation via a phone call in terms of the intensive care unit admission rate and length of hospital stay after RRT activation.
**CONCLUSIONS.** Malignancy was the most important factor related to survival. In addition, RRT activation with patient screening showed a better outcome compared to activation via a phone call. Further studies are needed to determine the effective screening criteria, and improve the quality, of the RRT system.

### 0722

#### The effectiveness of intra-hospital transportation of critically ill patients by the rapid response team

W.G. Kwack[1,2], M. Yun[2], D.S. Lee[2], H. Min[2], Y.Y. Choi[2], Y. Kim[1,2], S.-H. Lee[1,2], Y.J. Lee[1,2], J.S. Park[1,2], Y.-J. Cho[1,2]
[1]Division of Pulmonary and Critical Care Medicine, Department of Internal Medicine, Seoul National University Bundang Hospital, Seongnam, Korea, Republic of; [2]Interdepartment of Critical Care Medicine, Seoul National University Bundang Hospital, Seongnam, Korea, Republic of
**Correspondence:** W.G. Kwack
*Intensive Care Medicine Experimental* 2017, **5(Suppl 2):**0722

**INTRODUCTION.** Critically ill patients could have various risks including life threatening events during intra-hospital transportation. The incidence of global adverse events was reported from 20% to 68% and cardiac arrest was 0.34 ~ 1.6% [1,2]. The level of competence of accompanying members during transportation is also considered as one of the risk factors.
**OBJECTIVE.** To elucidate whether intra-hospital transportation by the rapid response team (RRT) influenced on patient´s safety and outcome compared with transportation by general members.
**METHODS.** A single-center retrospective cohort study was done from January 2016 to February 2017. This study included a total of 135 transports in 111 adult patients admitted to the medical intensive care unit due to respiratory failure under mechanical ventilation. Forty-five transports required a portable ventilator received escort by the RRT. Other 90 transports as control group were selected by age and sex matching. Major adverse event was defined as any situation needed cardiopulmonary resuscitation. Minor adverse event was desaturation and hypotension whether requiring intervention or not.
**RESULTS.** Of all subjects, the mean age was 68.8 ± 9.6 years and male 55 (59.3%). Although there was no significant difference in APACHE II score between both groups, mean score was 35.2 ± 6.5 in the RRT group and pre-transport demand of oxygen supply and sedative agents were higher in the RRT group than control group (P < 0.01). There was no significant different frequency of cardiopulmonary resuscitation in both groups. However, transportation by the RRT had more minor adverse events than general transportation (22.2% vs. 6.7%, P < 0.01).
**CONCLUSION.** Under intra-hospital transportation supported by the RRT, no significant major clinical deterioration occurred in critically ill patients who were highly vulnerable to adverse events.
**KEYWORD.** Transportation, Critically ill, Intensive care unit
**REFERENCES**
1. Papson JP, Russell KL, Taylor DM (2007) Unexpected events during the intrahospital transport of critically ill patients. Acd Emerg Med 14:574–577
2.Fanara B, Manzon C, Barbot O, Desmettre T, Capeller G (2010) Recommendations for the intra-hospital trnasport of critically ill patients. Critical Care 14:R87

### 0723

#### Risk factors for cardiopulmonary arrest during the intra-hospital transport of critically ill patients with rapid response team

H.J. Min[1], D.S. Lee[1], Y.Y. Choi[1], M.A. Yun[1], I.A. Song[1,2], Y.J. Cho[1,3], Y.H. Jo[1,4], Y.J. Lee[1,3]
[1]Seoul National University Bundang Hospital, Critical Care Medicine, Sung Nam, Korea, Republic of; [2]Seoul National University Bundang Hospital, Anesthesiology, Sung Nam, Korea, Republic of; [3]Seoul National University Bundang Hospital, Internal Medicine, Sung Nam, Korea, Republic of; [4]Seoul National University Bundang Hospital, Emergency Medicine, Korea, Republic of
**Correspondence:** H.J. Min
*Intensive Care Medicine Experimental* 2017, **5(Suppl 2):**0723

**INTRODUCTION.** There are few studies that describe the level of activity and risk factors for cardiopulmonary arrest(CPA) during the process of intra-hospital transport(IHT) of critically ill patients with the monitoring of rapid response team(RRT).
**OBJECTIVES.** The aim of this study was to describe the activity of RRT's IHT and identify risk factors associated with CPA during IHT.
**METHODS.** In this retrospective cohort study, we reviewed and extracted data from electronic medical records of consecutive adult critically ill patients who were transported under the monitoring of RRT from September 2012 to May 2016. We analyzed the detailed activity of RRT's transport monitoring and the outcomes of interest were CPA during IHT.
**RESULTS.** A total of 540 patients were included. The mean age was 65 years, and male patients constituted 65.4% of the cohort. The mean frequency of IHT with RRT was 12 times per month. Mean Charlson comorbidity index was 5.7 point and in-hospital mortality rate was 43.1%. The portable mechanical ventilator was used in 253(46.9%) patients and 18(3.3%) were transferred with ECMO. Continuous inotropes and sedatives were used in 241(45.6%) and 172(31.9%) patients, respectively. Although all cases were accompanied and monitored by RRT members, 12(2.2%) CPA occurred during IHT. Prior ischemic heart disease and peripheral vascular disease were more prevalent in the CPA(+) group than in the CPA (−) group (25.0 vs. 5.3, p = 0.004 and 8.3 vs. 0.9%, p = 0.016, respectively). Patients with CPA showed higher in-ICU mortality (66.7% vs. 33.0%, p = 0.015). The proportions of patients who were transported with ambu bagging and with 3 or more inotropes were greater in the CPA (+) group than in the CPA (−) group (58.3 vs. 22.9, p = 0.004, 25.0 vs. 5.1%, p = 0.003, respectively). Also, a significant difference in the FiO2 was observed (91.6 vs. 70.2%, p = 0.040). Using multivariate analysis (logistic-regression), gender, age-adjusted Charlson comorbidity index, department of patient admitted, FiO2 level, ambu bagging, and use of 3 or more inotropes were assessed as predictors of CPA in critically ill patients during IHT under RRT monitoring. The transport with ambu bagging (OR 3.829, 95% CI 1.106-13.256, p = 0.034) and use of 3 or more inotropes (OR 5.730, 95% CI 1.331-24.666, p = 0.019) were found to be independent predictors of CPA during IHT with RRT.
**CONCLUSIONS.** Transport with ambu bagging and use of 3 or more inotropes were significant risk factors associated with CPA during IHT of critically ill patients under RRT monitoring. When IHT is needed, very cautious measures should be taken before and during IHT and further prospective study is needed to develop in-hospital protocols to reduce CPA during transport of critically ill patients.
**REFERENCE(S)**
Adverse Clinical Events During Intrahospital Transport by a Specialized Team: A Preliminary Report. Am J Crit Care. 2011 March ; 20(2): 153–162. doi:10.4037.
**GRANT ACKNOWLEDGMENT**
Thank you for SNUBH RRT.

### 0724

#### Non-teaching hospital rapid response team's effectiveness - 5 years' data

S. São João, J. Azevedo, G. Domingos, I. Alves, R. Ribeiro
Centro Hospitalar de Setúbal, Setúbal, Portugal
**Correspondence:** J. Azevedo
*Intensive Care Medicine Experimental* 2017, **5(Suppl 2):**0724

**INTRODUCTION.** The implementation of the Rapid response team (RRT) at our hospital started in May 2011.
The performance of the RRT improves the quality and safety of the care provided and this result in a reduction of the number of adverse events and unexpected cardiac arrests[1].

**OBJECTIVES.** After 5 years of activity of our RRT, the main objective of this study is to evaluate its effectiveness, based on selected indicators.

**METHODS.** The RRT performance data was consulted and specific indicators were selected for analysis: incidence of activation, response time, transfer rate to intensive care unit (ICU) of another hospital, unexpected cardiorespiratory arrest (CRA), hospital survival rate after Advanced Life Support (ALS), rate of inappropriate CRA alarms, average ICU delay, rate of readmissions at ICU, number of inappropriate admissions in ICU, ICU mortality rate and mortality rate after ICU. A benchmarking analysis methodology was used.

**RESULTS.** In our hospital, the incidence of RRT activation has increased progressively, in line with what was already reported in several studies[2]. The response time remained below 5 minutes. In the first year, RRT transferred to another hospital 29.8% of the total number of patients admitted at ICU. This percentage has decreased (between 5.8 and 9.6%) in the following years. The unexpected CRA rate varied between 2.4 and 4.3% hospital admissions, being under the maximum threshold. In some years, the hospital survival rate after ALS is lower than the target. This is the result of a late or inappropriate activation of the RRT. Some inappropriate CRA activations are maintained (between 3 and 15 cases, per year). The average waiting time and rate of readmissions at the ICU suffered only little variation within the period of study. Between 42 and 56 admissions to the ICU were avoided annually (patients too well or too bad). The ICU mortality rate remained constant, whereas a reduction in the mortality rate after ICU discharge was noticed over the last 2 years, agreeing with what authors had also observed[3].

**CONCLUSIONS.** The RRT performance had a remarkable impact on the reduction of inappropriate admissions at the ICU, optimizing the management of hospital beds. The ICU mortality rate seemed to be not affected by the RRT performance, but a progressive reduction of the mortality rate after ICU discharge was observed.

**REFERENCE(S)**

1 Jung B et al. Rapid response team and hospital mortality in hospitalized patients. Intensive Care Med (2016) 42:494–504

2 Buist M et al. Six year audit of cardiac arrests and medical emergency team calls in an Australian outer metropolitan teaching hospital. BMJ (2007); 335:1210–2

3 Al-Qahtani S et al. Impact of an intensivist-led multidisciplinary extended rapid response team on hospital-wide cardiopulmonary arrests and mortality. Crit Care Med. (2013) Feb;41(2):506–17

**GRANT ACKNOWLEDGMENT**

The authors gratefully acknowledge use of the casuistry of the Centro Hospitalar de Setúbal´s ICU.

### 0725

#### Rapid response team: what is the best team for the medical care? Comparative study of patient care by the intensivist physician and the emergency physician in a tertiary hospital in Guarulhos - São Paulo, Brazil

A.M. Bonatto[1], R.D.R. Rodrigues[1], P.F.G.M.M. Tierno[1], R.A.S. Becker[1], R.V.D.S. Bento[1], G.S. de Lima[1], A. Oliveira[1], M.J. Caillares[1], D.D.O. Darmochid[1], K.C.C.d. Santos[1], E.A.C. Marcos[2]

[1]Hospital Municipal Pimentas Bonsucesso, ICU, Guarulhos, Brazil; [2]Hospital Municipal Pimentas Bonsucesso, Nursing, Guarulhos, Brazil

**Correspondence:** A.M. Bonatto

*Intensive Care Medicine Experimental* 2017, **5(Suppl 2):**0725

**INTRODUCTION.** The Rapid Response Team (RRT) is an integrated system that assesses patients with signs of acute clinical deterioration or cardiorespiratory arrest in all sectors of the hospital through the activation of the blue and yellow code, aiming to reduce mortality in non-critical sectors. At Hospital Municipal Pimentas Bonsucesso in Guarulhos, a city in the metropolitan region of São Paulo, the RRT was implemented in 2014, with medical care provided by the Emergency Room (ER) team. As from March 2016, the Intensive Care Unit (ICU) team became responsible for the RRT.

**OBJECTIVES.** To compare the outcome of the patients cared by both teams.

**METHODS.** Retrospective study of RRT care from March 2015 to August 2015 provided by the ER physician, and from March 2016 to August 2016 provided by the ICU physician. All the RRT calls were recorded in a specific form and the outcomes evaluated were: Time lapse between the RRT call and the medical care, how many patients per calls were directed to the critical sectors (emergency and ICU) and hospital discharges per call.

**RESULTS.** A total of 112 RRT calls were recorded during the study period. Of the 112, twenty-five yellow-code calls happened from March to August 2015, totaling 22% of the calls and eighty-seven yellow-code calls from March to August 2016, totaling 78% of the calls. This difference between the groups was significant, as there was an underreporting of the codes by the nursing team in the ER care. It took 5.28 seconds on average to call the ER team and an averaged 4.04 seconds to call the ICU team. This demonstrates an improvement in the ICU medical team care, meeting the 5-minute target for the calls. Regarding transfers, 15 critical-care patients were referred to the critical sectors by the ER team totaling 60% of the pacients and 53 critical-care patients by the ICU team, totaling 60,9% of the pacients. Regarding hospital discharge, 20 were by the ER team, totaling 80% of the calls, and 71 by the ICU team, totaling 81.6% of the calls.

**CONCLUSIONS.** Both study teams have not produced different outcomes.The RRT calls have gradually increased after the training of the teams and this demonstrates a positive outcome in the patients´ survival.

**REFERENCE(S)**

Hillman K, Chenj, Cretikos M, et al. Introduction of the medical emergency team (MET) system: a cluster-randomised controlled trial.- Lancet 2005:365:2091–7. Daryl A.Jones, M.D., et al. Rapid Response Teams. NEJM.ORG 2011; DeVita MA, Bellomo R, Hillman K, et al.Findings of the first consensus conference on medical emergency teams. Crit Care Med 2006;34:2463–78.Winters BD, Pham J, Pronovost PJ.Rapid response team - walk, don´t run.JAMA 2006; 296:1645–7.

### 0726

#### In hospital medical emergency team activation - a single center review

R. Assis[1,2], J. Cabrita[1], A. Araújo[1], N. Lopes[1], L. Pessoa[1], T. Pereira[1], N. Catorze[1]

[1]Centro Hospitalar do Médio Tejo, Intensive Care Unit, Abrantes, Portugal; [2]Centro Hospitalar do Médio Tejo, Internal Medicine Department, Abrantes, Portugal

**Correspondence:** R. Assis

*Intensive Care Medicine Experimental* 2017, **5(Suppl 2):**0726

**INTRODUCTION.** Medical Emergency Teams (EMT) are a cornerstone of quality medical care. They are formed by doctors and nurses with advanced life support skills who respond to intra-hospital calls on patient's clinical deterioration and cardiopulmonary arrest. Initially created to respond to Cardiopulmonary Arrest (CPA) situations, their action has expanded to rapid clinical deterioration, allowing for better patient outcome, using the New Early Warning Scale (NEWS) as activation method.

**OBJECTIVES.** The study aims at characterizing the activation of the Medical Emergency Team of the researchers Hospital, evaluating the activation criteria and reasons, location of activation and the destination of patients after the EMT intervention.

**METHODS.** Observational Retrospective Study based on information retrieved from the EMT database between the 1st of January 2013 and the 31st of March 2017, based in the Intensive Care Unit.

**RESULTS.** During the period evaluated, there were a total of 272 activations of the EMT. Being a Hospital with a predominance of Medical Specialties, the vast majority from the activations came from the Medical Ward (68%), followed by the Emergency Room (18%) and the Orthopedic Surgery Ward (6%). The activation was made by fellow doctors on 50% of the cases, the rest being made by nurses. In regard to the cause of activation, CPA corresponded to 39%, followed by Respiratory Distress with 36%, Glasgow Coma Scale inferior to 8 (7%), Requirement for Definitive Airway (5%), Severe Hypotension (4%), Severe Arrhythmia (3%), Status Epilepticcus (2%) and Severe Metabolic Acidosis (3%). By the end of EMT intervention, 35% of the patients,the majority with acknowledged Limitation of Treatment Status, died; 31% remained in their respective Department, 24% were admitted to the Intensive Care Unit and 4% were transferred to another Hospital for requiring differentiated treatment not available at the institution.

**CONCLUSIONS.** The study reveals that the most frequent motive of EMT activations at the researchers' Hospital remains to be CPA. However, there are an increasing awareness to the role of EMT as the activations came equally from Doctors and Nurses and 61% of the calls did not require Advance Life Support. It is crucial to raise further awareness to all Hospital personnel in order to recognize potentially life threatening conditions and call for the EMT earlier. This is the only way to better the outcomes, as the initial recognition and measures taken before the arrival of the EMT comprises the most fragile link in the chain of survival.

**REFERENCE(S)**

Jones D, Bellomo R, DeVita MA. Effectiveness of the Medical Emergency Team: the importance of dose. Critical Care. 2009;13(5):313.

Tee A, Calzavacca P, Licari E, Goldsmith D, Bellomo R. Bench-to-bedside review: The MET syndrome - the challenges of researching and adopting medical emergency teams. Critical Care. 2008;12(1):205.

### 0727

#### Use of modified early warning score (MEWS) in patients admitted to the intensive care unit from the ward

A.S. Ribeiro, J.E.G. Rodrigues, A.P.R. Veiga, A. Etzel
Centro Universitário Lusíada, Emergency, Santos, Brazil
**Correspondence:** A.S. Ribeiro
*Intensive Care Medicine Experimental* 2017, **5(Suppl 2):**0727

**INTRODUCTION.** Patients admitted to the hospital ward who evolve into clinical deterioration, culminating with cardiac arrest and death, often present changes in physiological parameters many hours before these events. These changes are often not adequately recognized or conducted and, when those patients are transferred to the intensive care unit (ICU), such changes increase their mortality. Early identification of clinical deterioration can be performed by simple protocols based on records of vital signs. Such immediate actions as calling an experienced doctor or even the transfer to an ICU may enable an improvement in prognosis.

**OBJECTIVES.** In this study, we evaluated whether the changes in physiological parameters and a higher score in MEWS of patients admitted to the hospital ward correlated with greater severity and mortality in the ICU and, secondly, whether there was a correlation between the MEWS and the length of stay and the intensity of provided care.

**METHODS.** This one-center historical cohort was conducted in the ICU of the Hospital Guilherme Alvaro of Santos, Brazil. Patients admitted to the ICU from the hospital ward were evaluated. The calculation of MEWS was performed with the physiological parameters, measured upon the request for a place in the ICU.

**RESULTS.** Eighty one patients presented the average age of 58.3 + 16 years, predominance of females (62%) and the most common comorbidities were high blood pressure (44%), diabetes mellitus (32%), cardiopathies (24%), cancer (18%) and chronic obstructive pulmonary disease (9%). The average MEWS was 4.7 + 2.4 and the average simplified acute physiology score (SAPS 3) was 66 + 15 points, and these two parameters showed good linear relationship ($r = 0.55$; $p < 0.01$). Endotracheal intubation was performed in 52% of patients who remained an average of 7.9 (1–138) days using invasive mechanical ventilation (IMV). The use of central venous catheter was frequent (86%), besides that 55% required vasoactive drugs (VAD), 28% required transfusion of erythrocytes and 20% required hemodialysis. There was nosocomial infections by multidrug-resistant bacteria in 20% of patients. It was observed that the use of VAD was twice as likely to be risky when used in patients with high score ($p = 0.002$). Patients with high score (≥5) were 1.5 times more likely to acquire nosocomial infections by multidrug-resistant bacteria ($p = 0.08$). The chance for the need of endotracheal intubation was increased by 2.65 times in individuals at the top level of the score ($p < 0.01$). Time of IMV and ICU lenght of stay did not correlate with MEWS ($p = 0.23$ and 0.5, respectively). Patients who evolved into death in the ICU and hospital, showed to be 2.12 times ($p = 0.001$) and 2.47 ($p < 0.01$) more likely to belong to the score high level, respectively.

**CONCLUSIONS.** The MEWS correlated positively with SAPS 3 and patients with high scores showed a greater need for the use of intensive support and a higher chance of mortality.

### 0728

#### Non-teaching hospital's rapid response team's performance in cardiac arrest - 5 years' data

J. Azevedo[1], S. São João[1], A. Batista[1], G. Domingos[2], I. Alves[2], R. Ribeiro[2]
[1]Centro Hospitalar de Setúbal, Anaesthesiology, Setúbal, Portugal;
[2]Centro Hospitalar de Setúbal, Intensive Care, Setúbal, Portugal
**Correspondence:** S. São João
*Intensive Care Medicine Experimental* 2017, **5(Suppl 2):**0728

**INTRODUCTION.** Rapid response teams (RRT) have been established to manage unstable patients in general wards, with the aim of preventing further deterioration leading to cardiac arrest[1].

Our team consists of a doctor and a nurse on call for all critical events happening inside the hospital, excluding the emergency department. Our RRT may be activated for events on admitted patients, visitors or staff.

**OBJECTIVES.** The main objectives of this study were to audit our RRT performance in in-hospital cardiac arrest, to compare it to the benchmark, to identify potential pitfalls and to seek opportunities for improvement.

**METHODS.** We have retrospectively analyzed the RRT's performance of CPR during a period of 5 years (May 2011–2016). We evaluated response times, the 1$^{st}$ documented rhythm and the outcomes for return of spontaneous circulation (ROSC) and survival to discharge. Additionally, we examined the cases in which RRT decided not to attempt resuscitation (DNAR).

**RESULTS.** Among 1856 events occurred during the period of study (May 2011–2016), CPR was performed in 228 cases. The mean time to arrival on code was under 3 minutes. Most frequent first rhythm documented was asystole (62.7%), second being PEA (23.2%) and lastly VF/ pulseless VT (14.0%). ROSC was obtained in more than 40% of all cases, improving in the last 3 years. The rhythm with more ROSC was VF/ pulseless VT (nearly 1/3 of the cases). Survival to discharge on all rhythms was 16%. RRT decided DNAR in 11% of total calls.

**CONCLUSIONS.** Our data indicates a higher number of asystole cases in comparison to international data[2], which may reflect delayed recognition of arrest, although this percentage is decreasing in our hospital during the last 3 years. More than half of all cardiopulmonary arrests are preceded by deterioration in vital signs, which are often not appropriately evaluated, suggesting that many of these adverse events could be prevented by early identification and treatment[1]. Despite this, survival to discharge is comparable to literature (16% vs 18%).

DNAR implementation indicators remain below the target (5%) in relation to the DNAR rate established by the RRT. This result could be improved if the physician responsible for the patient start to ask RRT´s opinion regarding DNAR of certain patients prior to the occurrence of a potential critical event.

**REFERENCE(S)**

1. Sandroni et al. Rapid response systems: are they really effective? Critical Care (2015)

2. Morrison et al. Strategies for Improving Survival After In-Hospital Cardiac Arrest in the United States: 2013 Consensus Recommendations - A Consensus Statement From the American Heart Association. Circulation (2013)

### 0729

#### Non-teaching hospital rapid response team's casuistic - 5 years' data

J. Azevedo[1], S. São João[1], G. Domingos[2], I. Alves[2], R. Ribeiro[2]
[1]Centro Hospitalar de Setúbal, Anaesthesiology, Setúbal, Portugal;
[2]Centro Hospitalar de Setúbal, Intensive Care, Setúbal, Portugal
**Correspondence:** S. São João
*Intensive Care Medicine Experimental* 2017, **5(Suppl 2):**0729

**INTRODUCTION.** Rapid response teams (RRT) have been established to manage unstable patients in general wards, with the aim of preventing further deterioration leading to cardiac arrest[1]. Our team consists of a doctor and a nurse on call for all critical events happening inside the hospital, excluding the emergency department. Our RRT may be activated for events on admitted patients, visitors or staff.

**OBJECTIVES.** The main goal of this study was to understand, since the implementation of the RRT, the standard of its performance and effectiveness.

**METHODS.** We have retrospectively analyzed the RRT's performance during 5 years (May 2011–2016). We analyzed the available data regarding: who activates the team, in which time of the day, from where are the calls made, as well as the gender and age of our patients, which organ system triggers the activations, and finally, the effectiveness of RRT for the operation of our hospital.

**RESULTS.** Among 1856 events that occurred during the period of study, most of the activations are triggered by physicians (62%) and cardiovascular problems are the main cause (782) followed by respiratory problems (646), with little variation in the different years.

Half of the activations take place between 8 am and 4 pm. The age range of patients is very extensive, from infants up to 102 years, with a predominance above 65 years. Calls for help mostly come from Intermediate Care Units, consistently each year.

Annually, 20-30% of the patients assisted by the team are treated/stabilized using the satellite beds of the Intensive Care Unit (ICU). The number of readmissions to the ICU has not decreased (4.4%), but the number of admissions avoided has increased (40 in 2016), as well as the inappropriate admissions to the ICU has decreased (4 in 2106).

**CONCLUSIONS.** From all the patients assisted by the RRT, the use of the satellite beds allows anticipate the need for higher level of care and is a very effective way to expand the intensive care beyond the beds limit of the ICU.

The benefit obtained from the RRT´s performance is mainly due to the improvement of care to the patient outside the ICU, and this effect can be translated not only into a reduction in the number of readmissions to the ICU, but also in early readmission when clinically indicated. These results are due to the effectiveness of the team that improves ICU treatment capacity and allows to optimize the use of ICU beds.

**REFERENCE(S)**

1. Sandroni et al. Rapid response systems: are they really effective? Critical Care (2015)

### 0730

#### The National Early Warning Score (NEWS) for outcome prediction in medical patients in emergency department with medical criteria of admission in medical ward: a pilot study

J. Ruiz Izquierdo[1], L. Simón Pascua[2], L. Martínez Pujol[1], B. Sánchez González[1], G. Figueras Solé[3], G. Muñoz Gamito[3], J. Trenado Álvarez[1]
[1]Hospital Universitari Mutua Terrassa, Intensive Care Department, Terrassa, Spain; [2]Hospital Universitari Mutua Terrassa, Internal Medicine, Terrassa, Spain; [3]Hospital Universitari Mutua Terrassa, Emergency Department, Terrassa, Spain
**Correspondence:** J. Ruiz Izquierdo
*Intensive Care Medicine Experimental* 2017, **5(Suppl 2):**0730

**INTRODUCTION.** There are no widely used scores specifically designed to detect deteriorating patients, who may be at risk to develop serious adverse events (SAEs) [1] in emergency department (ED).Among different early warning score (EWS), the National Early Warning Score (NEWS) has been well established. Its purpose was to introduce a standardised trigger system to identify acutely ill patients throughout hospitalisation [2].

**OBJECTIVES.** NEWS score calculated in ED before to be transferred to medical ward would show an association with short- and long-term adverse outcomes.

**METHODS.** Prospective, observational feasibility pilot study was performed at a University Hospital. All patients ≥ 18 years old presenting to the ED during 3 weeks with criteria of admission in any medical ward were included.2 trained researchers collected data (demographic, treatment, past medical history and vital signs). Data used to calculate NEWS were collected at the moment to transfer. Outcomes registered: Serious events (urgent medical attention, ICU admission and hospital mortality) and length of stay. NEWS score classified the patients into 3categories: low risk (0–4 points), medium (5–6 points or any NEWS Individual parameter scoring = 3) and high (≥7 points).

**STATISTICS.** Qualitative variables are expressed as percentages and compared using the X2-test; Descriptive statistics were used to determine patient characteristics (presented as mean and SD or median and inter-quartile range (IQR) or mean ± SD. Student's t test was used for independent. Level of significance was placed at $p < 0.05$. The statistical analysis was performed using specific software (IBM SPSS Statistics for Windows, Version 19.0. Armonk, NY: IBM Corp).

**RESULTS.** 192 patients were included. The most frequent department of admission was internal medicine (59.4%). Aetiologies of admission were: infectious (30%), respiratory (24%) and cardiac (14.6%).

**CONCLUSIONS.** In our pilot study the NEWS measured at transfer time to a medical ward can be of additional value to detect patients at risk of presenting serious events once they are transferred from the ED department. It may be useful for clinicians receiving these patients at medical wards and so enhance the quality and safety of care. We expect to complete the study to achieve stronger conclusions.

**REFERENCE(S)**

1. Hogan H, Healey F, Neale G, Thomson R, Vincent C, Black N. Preventable deaths due to problems in care in English acute hospitals: a retrospective case record review study. BMJ Qual Saf 2012;21(9):737–45.
2. Royal College of Physicians. National Early Warning Score (NEWS): Standardising the assessment of acuteillness severity in the NHS. Report of a working party. London: RCP, 2012.

**Table 189 (Abstract 0730)**. Characteristics of the study population

| | |
|---|---|
| Sex male, n (%) | 97 (50.5) |
| Age. Median (IQR) | 74 (66-85) |
| Aggregate_NEWS:0-4, n (%) | 83 (45.6) |
| Aggregate_NEWS:5-6, n (%) | 46 (25.3) |
| Aggregate_NEWS ≥7, n (%) | 53 (29.1) |
| NEWS Individual parameter scoring 3, n (%) | 78 (42.9) |
| Any serious event, n (%) | 19 (10) |
| Hospital LOS. Median (IQR) | 8 (5-10) |
| Hospital Mortality, n (%) | 13 (6.8) |

**Table 190 (Abstract 0730)**. Factors associated with any serious event

| | Any serious event | No serious event | p |
|---|---|---|---|
| NEWS Individual parameter scoring 3. n (%) | 14 (73.7) | 64 (39.3) | 0.004 |
| Age. Mean (SD) | 78.8 (13.9) | 72 (16.6) | 0.06 |
| Hospital LOS. Mean (SD) | 8.8 (5.4) | 8 (3.9) | 0.6 |
| Aggregate_NEWS: 0-4. n (%) | 4 (21.1) | 79 (48.5) | 0.06 |
| Aggregate_NEWS: 5-6. n (%) | 7 (36.8) | 39 (23.9) | 0.06 |
| Aggregate_NEWS ≥7. n (%) | 8 (42.1) | 45 (27.6) | 0.06 |

**Table 191 (Abstract 0730)**. Factors associated with the aggregate NEW

| Aggregate_NEWS | 0-4 | 5-6 | ≥7 | p |
|---|---|---|---|---|
| Hospital LOS. Mean (SD) | 6.96 (3.9) | 8.3 (4.2) | 9.3 (3.6) | 0.04 |
| Age. Mean (SD) | 70.1 (17.7) | 75.2 (18.3) | 74.7 (11.9) | 0.15 |
| Any serious event. n (%) | 4 (21.1) | 7 (36.8) | 8 (42.1) | 0.06 |
| Hospital Mortality. n (%) | 3 (23.1) | 5 (38.5) | 5 (38.5) | 0.23 |

### Outcome 2

#### 0731

#### Perceptions of escalating organ support and prolonged intensive care admission amongst non-intensivists: the elephant in the room?

J. Gross[1], M. Dhankhar[1], L. Deterville[2]
[1]Northwick Park & St Mark's Hospitals, London, United Kingdom; [2]Imperial College Healthcare NHS Trust, Post Graduate Medical Education (Lead Provider), London, United Kingdom
**Correspondence:** J. Gross
*Intensive Care Medicine Experimental* 2017, **5(Suppl 2):**0731

**INTRODUCTION.** The decision of whether or not to escalate organ support and/or refer to ICU in the event of critical illness is complex and should take into account the pros and cons of treatment escalation and patient's wishes where possible[1]. Clinicians that do not necessarily hold an Intensive Care background are often on the frontline for making treatment escalation decisions.

**OBJECTIVES.**

1. To determine whether or not there is an existing knowledge gap amongst non-ICU physicians surrounding the short term adverse effects and longer-term physical and psychosocial morbidity relating to escalation of organ support and prolonged ICU admission
2. To determine whether or not an education session about ICU changes outlook and positively influences confidence in making treatment escalation plans (TEPs)
3. To investigate factors which might account for variation in practice when making TEPs and potential barriers for earlier implementation.

**METHODS.** An education session about ICU was delivered to non-ICU clinicians of varying grades and levels of seniority during a regional registrar training day and a hospital grand round session. All delegates were asked to complete a survey following the session.

**RESULTS.** Of the 103 clinicians who attended the sessions, the majority (79%) were medical registrar level (ST3-ST8), 46 (45%) had never had any previous intensive care training and 35 (34%) had little or no prior knowledge of the potential negative impact ICU can have on patients. Sixty nine delegates (67%) thought the session altered their outlook in how to implement TEPs, DNACPR orders and appropriate involvement of the ICU team and 92 (89%) felt they now had more confidence in discussing such issues with patients and families. Seventy four (72%) and 70 (68%) claimed that they would welcome further training and guidance in this area respectively.

Although 95 delegates (92%) felt it was important for the ICU team to be involved when making treatment escalation decisions, 47 (46%) found it difficult in practice to get the ICU team to aid with the decision making process. Ninety one (88%) felt the TEPs should be made before the onset of critical illness in select patients but time constraints and potential conflicts with patients and relatives were seen to be the greatest barriers for earlier implementation.

**CONCLUSIONS.** There is an existing knowledge gap amongst non-ICU clinicians about the negative impact ICU can have on patients and further education and training in this area may positively influence the process of how TEPs are formulated. Involvement of the ICU team may be important to aid the decision making process and support parent teams in reaching a consensus. Whilst earlier TEPs were thought to be important in certain patients, several barriers were highlighted, which need to be tackled in order to facilitate earlier formulation of TEPs in practice.

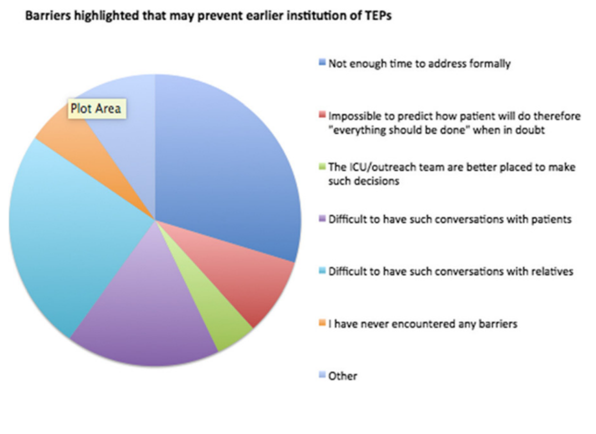

**Fig. 254 (Abstract 0731)** Barriers to earlier implementation of TEPs

#### 0732

#### The gap between expectation and reality - survey among medical laypersons on critical care topics

M. Lew[1], F. Eisner[2], P. Eller[2], M. Hoffmann[1,3], T. Pieber[1,2], K. Amrein[1,2]
[1]Medical University of Graz, Department of Internal Medicine, Clinical Division of Endocrinology and Diabetology, Graz, Austria; [2]Medical University of Graz, Department of Internal Medicine, Intensive Care Unit, Graz, Austria; [3]University Hospital of Graz, Graz, Austria
**Correspondence:** M. Lew
*Intensive Care Medicine Experimental* 2017, **5(Suppl 2):**0732

**INTRODUCTION.** Most of the financial and human resources of a hospital are needed in the ICU (intensive care unit). Despite the enormous developments in intensive care over the past years, the general public has unrealistic expectations and for example overestimates the survival rate after cardiopulmonary resuscitation. This is certainly influenced by the fact that most people obtain their medical knowledge from nonprofessional information sources like TV-series and ad-hoc internet searches.

**OBJECTIVE.** To uncover the population's level of knowledge about critical care topics.

**METHODS.** 145 women and 93 men (adult medical laypersons), from the German-speaking countries, predominantly from Austria, were surveyed in the form of a short online or paper questionnaire. The 11, partly open-ended questions, focused on various intensive care topics. At the end, the participants were asked to declare, from which sources they get their information about these topics. Then there was a statistical evaluation of the data differentiated by gender, age, level of education, and place of residence (urban or rural areas).
**RESULTS.** The most frequently mentioned diagnoses on an intensive care unit were cardiovascular problems, especially heart attack, but also fractures, serious injuries as well as strokes. A large proportion of the participants (49%) thought that 85% of all patients, hospitalized at an ICU, are still alive one year after their discharge. 45% of the participants estimated the survival rate after cardiopulmonary resuscitation at 50% and 31,5% at 85%. Sepsis was defined as blood poisoning by 47% of the interviewed and 25% didn't know the word Sepsis. Nearly all people (98%) recognized a defibrillator as an electrical device, which is used for resuscitation. The three most commonly used information sources were family and friends, the internet as well as television.
**CONCLUSIONS.** In conclusion, several misconceptions of the respondents can be detected. This lack of adequate knowledge can lead to subjective dissatisfaction and disappointment in the event of illness. Therefore, it is important to undertake further larger studies to uncover the current population's level of knowledge. In addition, it is necessary to improve the quality of the information available to laypersons and to adjust the often too optimistic expectations to reality.

### 0733

#### Relationship between mortality and number of total lymphocytes and lymphocyte sub-populations in ICU patients at admission

M.D. Arias-Verdu[1], C. López-Caler[1], M. Guerrero-Marín[2], C. Cabrera-Morales[3], A. Caballero-González[3], E. Aguilar-Alonso[4], R. Rivera-Fernández[2]
[1]Hospital Regional Universitario Carlos Haya de Málaga, Intensive Care Unit, Málaga, Spain; [2]Complejo Hospitalario de Jaen, Intensive Care Unit, Jaén, Spain; [3]Hospital Regional Universitario Carlos Haya de Málaga, Inmunology Laboratory, Málaga, Spain; [4]Hospital Infanta Margarita de Cabra, Intensive Care Unit, Córdoba, Spain
**Correspondence:** M.D. Arias-Verdu
*Intensive Care Medicine Experimental* 2017, **5(Suppl 2):**0733

**OBJECTIVE.** To evaluate the relationship between mortality and number of total lymphocytes and lymphocyte sub-populations at ICU admission.
**METHODS.** Cohort study. We exclude coronary patients and programmed surgery. Statistical analysis:
T Student, $\chi 2$ and multiple logistic regression.
**RESULTS.** N = 99 patients. Age 51.76 ± 15.89 years, APACHE II 17.91 ± 6.97, total lymphocytes at admission 1197 ± 1758. Hospital mortality 24.5%.
Patients who died had a higher APACHE II (23.86 ± 6.49 vs 15.93 ± 5.94) ($p < 0.001$) and lower total lymphocytes (900 ± 629 vs 1304 ± 1998), but the differences were not e.s. (p = 0.334).
58% of patientes had less than 1000 lymphocytes at admission and mortality was 28.6% vs 19.5% (p = 0.30). 28% of the patients had less than 600 lymphocytes with a 40.7% mortality vs 18.6% (p = 0.025). Regarding the different subpopulations, there were no differences between dead and alives.
58.6% had low CD3 (less than 690 lymphocytes) and their mortality was 26.3% vs 22% in patients with a normal number of CD3 (p = 0.62). 57.6% CD4 low (<410 lymphocytes) mortality 23.2% vs 26.2% (p = 0.73). 46.5% had low CD8 (<190 lymphocytes, mortality was 31.1% vs 18.9% (p = 0.160). 45.5% had low CD16 (<90 lymphocytes) Mortality 28.9% vs 20.8% (p = 0.351). And 23.2% had low CD19 (<90 lymphocytes) mortality was 36.4% vs 21.1% (p = 0.141).
Logistic regression showed that hospital mortality was related with total lymphocytes below 600 (OR: 3.1, CI: 1.73-8) but not with a low number of lymphocytes from the different lymphocyte subpopulations.
**CONCLUSIONS.** A high percentage of patients presented a low total lymphocytes and low lymphocytes in the various subpopulations at admission. A number of total lymphocytes less than 600 is associated with higher mortality and a low number of lymphocytes in different subpopulations does not provide additional information regarding mortality.

### 0734

#### Long-term outcome in ICU patients treated with renal replacement therapy - a 2 yr follow up study in Ireland: the impact of pre-existing CKD on survival and renal function

G.J. Fitzpatrick, A.A. Mahmoud, D. Greaney, A. Magee, M. Donnelly
Tallaght Hospital & Trinity College Dublin, Department of Anaesthesia & Critical Care, Dublin, Ireland
**Correspondence:** G.J. Fitzpatrick
*Intensive Care Medicine Experimental* 2017, **5(Suppl 2):**0734

**INTRODUCTION.** ICU patients requiring CRRT (ICU/CRRT) have a high hospital mortality and reduced long term survival (1). Renal function may be decreased in survivors. These consequences may be more pronounced in patients with pre-existing CKD.
**OBJECTIVES.** To examine the impact of pre existing CKD on long term outcome in ICU patients treated with CRRT and to assess renal outcome in 2 year survivors.
**METHODS.** We conducted a retrospective cohort study of all patients admitted to our ICU in 2013 and 2014 requiring CRRT. Data was obtained from Clinical Information and laboratory systems and entered into a database for analysis. Parameters recorded included age, gender, APACHE 11 score, SOFA score and serum creatinine prior to admission and on hospital discharge. Patients with CKD were defined as those having an abnormal creatinine 3 months prior to ICU admission. No known CKD (non CKD) was defined as no clinical history of CKD and no abnormal creatinine in the 2 years before ICU admission. Surviviors to hospital discharge were followed up to 2 yrs to determine survival, dialysis dependency and renal function for those who were dialysis independent. Categorical variables were compared using chi-square test and continuous variables using Mann–Whitney U test. The study received ethical committee waiver. Primary outcome was 2 yr. mortality. Secondary outcomes included renal function at 2 years.
**RESULTS.** During the study period there were 686 admission to ICU. 201 patients (29.3%) were treated with CRRT. Following exclusion of patients with ESRD and those dialysed for reasons other than kidney injury 181 were included for further analysis. Of these 56 (31%) had CKD prior to ICU admission. Overall 83 (46%) patients survived to hospital discharge. There was no difference in hospital mortality between CKD and non CKD patients (55% v.54%). At 2 year follow up 48% of CKD patients discharged alive had died compared with 21% of non CKD patients ($p < 0.01$). Two patients were lost to follow up. 53 non CKD patients were dialysis independent on discharge. Of these 42 (79%) survived to 2 years. The eGFR in these at 2 yrs was < 60 mL/min/1.73 $m^2$ in 14% of patients, >60 mL/min/1.73 $m^2$ in 43% and unavailable in 40%. 10 CKD patients dialysis independent at discharge survived to 2 yrs. In 4 of these (40%) serum creatinine was higher than their pre admission value (mean increase = 50.75 mmol/L, range = 22–94). In the other 60% creatinine was either unchanged or slightly less than pre admission value.
**CONCLUSIONS.** Pre-existing CKD was associated with a non significant trend towards dialysis dependency on discharge (p = 0.58) and a significantly lower 2 yr survival ($p < 0.01$). 14% of non CKD patients who survived to 2 years had moderate renal impairment.

**REFERENCE(S)**

De Corte et al. Long Term outcome in ICU patients with acute kidney injury treated with renal replacement therapy: a prospective cohort study. Critical Care 2016 20:256

### 0735

#### Evaluation of health related quality of life in trauma patients, three years after ICU hospitalization: a retrospective study in South of Iran

G. Sabetian[1], V. Rezaee[2], H. Tabei[2], F. Zand[3], M. Masjedi[3], S. Hooshmandi[2], B. Maghsoodi[3]
[1]Shiraz University of Medical Sciences, Trauma Research Center, Shiraz, Iran, Islamic Republic of; [2]Shiraz University of Medical Sciences, Shiraz, Iran, Islamic Republic of; [3]Shiraz University of Medical Sciences, Anesthesiology and Critical Care Research Center, Shiraz, Iran, Islamic Republic of

**Correspondence:** G. Sabetian
*Intensive Care Medicine Experimental* 2017, **5(Suppl 2):**0735

**INTRODUCTION.** Health-related quality of life (HRQOL) considerably decreases after trauma.

**OBJECTIVES.** This study aimed to assess and compare quality of life, three years after injury and hospital stay in trauma patients admitted to an intensive-care unit (ICU) for 24 hours at least with non-ICU trauma patients to identify potential factors associated with outcome.

**METHODS.** In this cross sectional retrospective study, data of 204 trauma patients(103 ICU and 101 non ICU patients)who admitted to a referral center in IRAN were evaluated.HRQOL was measured using the Medical Outcomes Study Short Form 36 (SF-36). Patients were called and asked to answer SF-36 questioner according to current condition.

**RESULTS.** The mean age of ICU and non-ICU patients were 34.34 and 37.68 years respectively. The majority of patients in both ICU and non-ICU groups were male (83.5%, 88.1%) and lower than 60 years old (91.3%, 92.1%). The injury severity score (ISS) was marked as critical in 66.99% of ICU patients. Except for social functioning, the mean SF-36 scores in ICU patients were lower than the similar indices in non-ICU patients in all other domains(in Physical Function-Bodily Pain-Role Emotional -Mental Health $p < 0.001$).Insignificant differences between ICU and non-ICU patients were observed for only two subscales.

**CONCLUSIONS.** Although a significant improvement was observed in the HRQOL after three years period in our sample, for most subscales of SF-36 ICU patients' scores were lower in comparison to the non-ICU population. This study emphasizes on the role of trauma specialist teams, including physical therapists, social workers and psychologists, in sufficient pain management and consideration during care. Finally, providing adequate information and long term follow-up of trauma patients can oblige health professionals by identifying patients that require additional help and support after discharge from acute care medical centers.

**REFERENCE(S)**

1- Hofhuis JG, Spronk PE. Health-related quality of life and influence of age after trauma: An overview. J Trauma Acute Care Surg. 2014;76(2):549–56.
2- Mörsdorf P, Becker S, Holstein J, Burkhardt M, Pohlemann T. [Quality of life after multiple trauma.]. Der Chirurg. 2014;85(3):210–4.
3- Sluys K, Häggmark T, Iselius L. Outcome and quality of life 5 years after major trauma. J Trauma. 2005;59(1):223–32.

**GRANT ACKNOWLEDGMENT**

This study was funded by Vice-Chancellery of Research and Technology in Shiraz University of Medical Sciences, Shiraz, Iran

### 0736

#### High rate of postoperative complications after ICU discharge in patients with free flap reconstruction due to cancer of the head and neck

S. Lahtinen[1], P. Koivunen[2], T. Ala-Kokko[3], J. Liisanantti[3]
[1]Oulu University Hospital, Department of Anesthesia, Oulu, Finland; [2]Oulu University Hospital, Department of Otorhinolaryngology, Oulu, Finland; [3]Oulu University Hospital, Department of Anesthesia, Division of Intensive Care Medicine, Oulu, Finland
**Correspondence:** S. Lahtinen
*Intensive Care Medicine Experimental* 2017, **5(Suppl 2):**0736

**INTRODUCTION.** The high rate of postoperative complications in free flap surgery due to cancer of the head and neck is a known phenomenon but previous studies have not analyzed the complications in detail or the onset of complications (1–3).

**OBJECTIVES.** The aim of this study was to evaluate the complications during and after the postoperative surgical ICU stay among the patients operated due to cancer of the head and neck with free flap repair.

**METHODS.** Retrospective analysis of 136 patients, who underwent head and neck cancer operation with free flap repair in Oulu University Hospital during 2008–2015.

**RESULTS.** The median age of patients was 65 [59–74] years. The median ICU stay was 0.9 [0.8-1.7] days and hospital stay 13 [9–17] days. 51 patients (37.5%) had medical complications and 56 patients (41.2%) had surgical complications. Both, medical and surgical complications were recorded in 20.6% (N = 28) of the patients. Those with complications had a higher rate of alcohol abuse (25.3% vs 10.5%, $p = 0.030$), were more often recorded as ASA 3–4 (63.3% vs 40.4%, $p = 0.008$), had a longer operation (566 [452–652]min vs 490 [425–556]min, $p = 0.003$) and higher intraoperative blood loss (750 [410–1150]mL vs 520 [300–950]mL, $p = 0.037$) compared to patients without complications. The median onset of medical complications was 5[3–7] days for pneumonia (N = 36), 7[5–9] days for pulmonary odema (N = 13), 3[1–8] days for myocardial infarction (N = 7) and 8[7.5-11.5] days for sepsis (N = 9). The median onset of surgical complications was 5[2–10] days for wound infection (N = 39), 6 [1–9] days for wound hematoma (N = 22) and 10 [3–14] days for flap failure (N = 15).

**CONCLUSIONS.** More than half of the patients had complications, which were related to alcohol abuse, higher preoperative risk and more complicated intraoperative course. Most of the complications were recorded after ICU discharge and infectious complications seem to occur after the fifth postoperative day.

**GRANT ACKNOWLEDGMENT**

None

### 0737

#### Description of predictive model of hospital mortality after intensive care unit discharge

C. Lopez-Caler[1], R. Vela-Colmenero[2], F. Rosa-Salas[2], D. Iglesias-Posadilla[3], R. Montoiro-Allue[4], J.M. Rodriguez-Roldan[5], J.E. Barrueco-Francioni[6], M. Redondo-Orts[2], R. Rivera-Fernandez[2]
[1]Intensive Care Unit - Hospital Universitario Carlos Haya, Malaga, Spain; [2]Intensive Care Unit - Complejo Hospitalario, Jaen, Spain; [3]Intensive Care Unit - Hospital Universitario, Burgos, Spain; [4]Intensive Care Unit - Hospital Clínico Universitario Lozano Blesa, Zaragoza, Spain; [5]Intensive Care Unit - Hospital Universitario Severo Ochoa, Madrid, Spain; [6]Intensive Care Unit - Hospital Arnau de Vilanova, Valencia, Spain
**Correspondence:** R. Vela-Colmenero
*Intensive Care Medicine Experimental* 2017, **5(Suppl 2):**0737

**INTRODUCTION.** About one third of hospital mortality in critically ill patients occurs after Intensive Care Unit (ICU) discharge[1].

**OBJECTIVES.** To perform a predictive model of mortality after ICU discharge.

**METHODS.** Multicentric prospective study in 7 hospitals in Spain. We are collected periods of two or three months during 2011, 2012 and 2015.
Data were expressed as the mean and standard deviation for quantitative variables and percentages for qualitative variables. For the comparison of two means we used the Student's t-test and the chi-squared test was used to compare proportions. Multiple logistic regression was used for multivariable analysis and area Under the ROC curve for analizing discrimination. Statistically significant differences: $p < 0.05$.

**RESULTS.** 1934 patients with a mean age of 61.22 ± 15.58 years, SAPS-3 45.26 ± 13.68 points. Predicted mortality by SAPS 3 in ICU

was 17% and observed hospital mortality was 15%. ICU mortality was 10% and mortality after ICU discharge (Ward mortality) was 5.%.
1731 patients were transferred to Ward hospitalization area, with mortality of 5.6% (97 patients). Mean age of 60.66 ± 15.62 years, SAPS 3 at ICU admission 43.06 ± 11.86 points and SOFA score at the last day of 0.91 ± 1.61 points.
Ward mortality of patients with Medical pathology (754 cases) was 6.5%, elective surgery (801 cases) 5.5% and emergency surgery (173 cases) of 2.3%(p = 0.185).
Patients who died after ICU discharge were older (65.72 ± 14.61 vs. 60.36 ± 15.63 years) (p < 0.001), higher SAPS-3 score at ICU admission (54.06 ± 13.07 vs 42.4 ± 11.43) (p < 0.001) and higher SOFA score at last day of ICU stay (2.8 ± 3.21 vs 0.9 ± 1.61) (p < 0.001).
Pre-admission functional status (by Glasgow Outcome Scale) is related with mortality after ICU discharge. Mortality pf patients with Normal status (72.6%) was 2.8%, mortality of patients limited and self-sufficient status (21.3%) was 10.8%, and mortality of patients limited and non-autosufficient status (6.1%) was 21% (p < 0.001).
Multiple logistical regression: Mortality after ICU discharge was related with SOFA score at last day of ICU stay (OR 1.39(1.19-1.46)), SAPS-3 score at ICU admission (OR 1.06(1.04-1.08)), type of pathology (medical OR 1, emergency surgery OR 1.56 (0.93-2.61) and Scheduled surgery OR 0.3(0.009-0.97)) and pre-admission functional status (normal status OR 1, limited and self-sufficient status OR 2.64(1.59-4.39), limited and non-autosufficient status OR 4.89(2.54-9.24)). Discrimination of this model evaluated by Area under the ROC curve: 0.84(0.8-0.88).

**CONCLUSIONS.** Mortality after ICU discharge is related to SOFA score at last day of ICU stay, severity illness at admission by SAPS-3 score, type of pathology and pre-admission functional status. These variables perform a predictive model with a high discrimination capacity.

### 0738

#### Factors associated with outcomes in patients with prolonged ICU length of stay

J.C. Lopez Delgado[1], I. Martínez de Lagrán Zurbano[2], M.L. Bordejé Laguna[2], J.I. Herrero Meseguer[1], R.E. Avila-Espinoza[1], G. Moreno-Gonzalez[1], M.Á. García Martínez[3]
[1]Hospital Universitari de Bellvitge, Intensive Care, Hospitalet de Llobregat, Spain; [2]Hospital Germans Trias i Pujol, Intensive Care, Badalona, Spain; [3]Hospital Universitario de Torrevieja, Intensive Care, Alicante, Spain
**Correspondence:** J.C. Lopez Delgado
*Intensive Care Medicine Experimental* 2017, **5(Suppl 2):**0738

**INTRODUCTION.** Critical illness leading to prolonged length of stay in an intensive care unit(ICU) is associated with significant mortality. A prognosis of these patients is difficult to assess due to many interrelated factors that influence outcomes.

**OBJECTIVES.** To evaluate factors associated with outcomes in patients with prolonged ICU length of stay(PICULOS).

**METHODS.** Prospective, observational study in two tertiary-level university hospital. PICULOS was defined the need of >7 days of ICU stay. Clinical and laboratory data were recorded on admission and weekly, as well as outcomes, emphasizing infections and mortality.

**RESULTS.** 115 patients were included. Mean age was 62.7 ± 14.5years; 64.3%(n = 74)were male; BMI:29.7 ± 2.1Kg·m $^{-2}$;APACHE II:19 ± 7.60.8% (n = 70) were medical patients.Hospital mortality was 21.7%(n = 25).77.4%(n = 89)were under Enteral Nutrition and 15.7%(n = 18) under Parenteral Nutrition. Nutritional support was initiated 26 ± 14h after ICU admission. Mean ICU stay was 22 ± 15days and mean hospital stay was 43 ± 29days.We showed the following differences between survivors and non-survivors:APACHE II(18 ± 7vs.23 ± 6;P < 0.001),bilirubin on admission (0.85 ± 0.7vs.1.96 ± 4.1mg/dL;P = 0.017), prealbumin on 7$^{th}$day (17.8 ± 7.6vs.12.3 ± 6.5mg/dL;P < 0.001), platelets on 7$^{th}$day(302 ± 158 vs.209 ± 111nL$^{-1}$;P = 0.004)and bilirubin on 7$^{th}$day (0.73 ± 0.66vs.2.8 ± 7.7mg/dL;P = 0.001). Higher levels of prealbumin on 7$^{th}$day (HR:0.924;95% IC:0.858-0.995;P = 0.036)were associated with lower mortality at the multivariate analysis.Patients with severe lower levels of prealbumin on 7$^{th}$day (<23mg/dL) showed worst hospital survival(87.8%vs.61%;P = 0.002).

**CONCLUSIONS.** A higher prealbumin on 7$^{th}$day was associated with better survival during hospital stay in patients with PICULOS. Factors associated with nutritional and inflammatory status may be associated with the outcome of these patients.

**REFERENCE(S)**

Williams TA et al. Effect of length of stay in intensive care unit on hospital and long-term mortality of critically ill adult patients. Br J Anaesth. 2010;104:459–64.
Gentile LF et al.Persistent inflammation and immunosuppression: A common syndrome and new horizon for surgical intensive care. J Trauma Acute Care Surg..2012;72(6):1491–501.

### 0739

#### Long-term outcome according to the occurrence of in-hospital cardiovascular events after liver transplantation

N.T.B. Scholte, M.J. Lenzen, H.J. Metselaar, C.A. den Uil
Erasmus MC University Medical Centre, Rotterdam, Netherlands
**Correspondence:** N.T.B. Scholte
*Intensive Care Medicine Experimental* 2017, **5(Suppl 2):**0739

**INTRODUCTION.** Cardiovascular events frequently occur within 30 days after liver transplantation (LT) (1). It is not known whether these events affect long-term outcome.

**OBJECTIVES.** We investigated the prevalence of in-hospital cardiovascular events after liver transplantation, and the correlation with long-term outcome.

**METHODS.** We performed a retrospective study in consecutive patients who were subjected to LT in the time period 1986–2017 at the Erasmus University Medical Centre, Rotterdam, NL. The primary endpoint was all-cause mortality at 5 years, stratified according to the presence of in-hospital cardiovascular events (CVEs; CVE+ vs. CVE-).

**RESULTS.** A total of 915 patients underwent LT. 113 out of 915 patients (12%) experienced a cardiovascular event during the in-hospital period. CVE patients were divided in subgroups; ventricular tachycardia, ventricular fibrillation or CPR (n = 30, 27%), atrial fibrillation or other supraventricular tachycardia (n = 30, 27%), bradycardia (n = 8, 7%), myocardial infarction (n = 14, 12%), venous thrombosis or thrombo-embolism (n = 13, 12%), cerebrovascular accident (n = 10, 9%), heart failure or cardiomyopathy (n = 6, 5%), and vascular injury related to surgery (n = 2, 2%). In the CVE+ group, patients were older (53 ± 11 vs. 48 ± 13, p < 0.001), had more hypertension (32% vs. 20%, p = 0.01) and had more often a history of cardiovascular disease (15% vs. 9%, p = 0.04). 30-day mortality was 26% vs. 5% (log rank p < 0.001). At 5 years, the overall survival of 30-day survivors was 81%. There was no significant difference between both CVE groups. (Figure 255).

**CONCLUSIONS.** This study demonstrates that the occurrence of in-hospital cardiovascular events do not affect long-term outcome in 30-day survivors of liver transplantation. We should therefore 1) perform better preoperative risk stratification and 2) must make urgent efforts to tide patients over the early postoperative phase so that they can have good longer term outcome.

**REFERENCE(S)**

1. Safadi A, Homsi M, Maskoun W, Lane KA, Singh I, Sawada SG, et al. Perioperative risk predictors of cardiac outcomes in patients undergoing liver transplantation surgery. Circulation. 2009;120(13):1189–94.

**GRANT ACKNOWLEDGMENT**
None

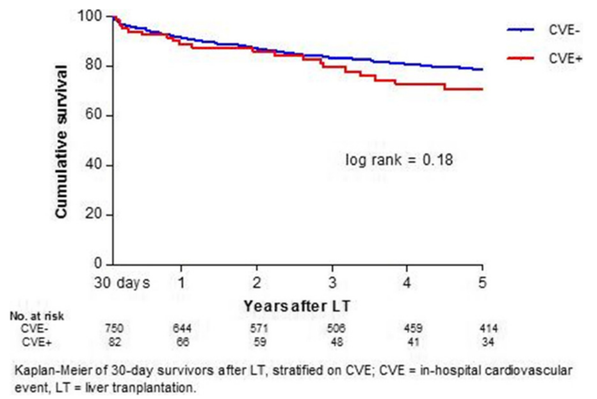

**Fig. 255 (Abstract 0739)** Kaplan-Meier 30-day survivors

### 0740

#### Characteristics of patients and predictor factors of mortality after ICU discharge. One year survey

J. Baroudi[1], K. Meddeb[1], N. Fraj[1], J. Ayachi[1], N. Sma[1], M.A. Boujelbèn[1], E. Ennouri[1], H. Kallel[1], W. Zarrougui[1], A. Ben Abdelhafidh[1], I. Ben Saida[1], I. Chouchene[1], A. Khedher[1], A. Azouzi[1], M. Boussarsar[1,2]

[1]Farhat Hached University Hospital, Medical Intensive Care Unit, Sousse, Tunisia; [2]Farhat Hached University Hospital, Research Laboratory N° LR14ES05 Interactions of the Cardio-pulmonary System, Sousse, Tunisia

**Correspondence:** M. Boussarsar

*Intensive Care Medicine Experimental* 2017, **5(Suppl 2):**0740

**BACKGROUND.** ICU survivors, have a high and ongoing risk of death after ICU discharge.

**OBJECTIVES.** To investigate the patients' profile after ICU discharge during 1 year and to determine prognostic factors in longterm mortality.

**METHODS.** An observational prospective cohort study including all survivors, after an ICU stay in medical ICU between January 2014 and December 2015. Patients were followed up via phone calls at 3, 6 and 12 months after discharge. A multivariate Cox model was used to predict 1year survival. Factors associated with longterm mortality were presented as a hazard ratio (HR). The overall survival was analyzed on the basis of the KaplanMeier curves. A Pvalue lower than 0.05 was accepted as significant.

**RESULTS.** Among 325 ICU survivors only 215 were followed. The overall cumulative rate of death in hospital survivors was 63(29%) patients after 1 year of ICU discharge. Median survival time was 30 days. Clinical features of deaths were: Mean age 58.6 ± 2.3years, mean Charlson index 2.61 ± 1.8 mostly involving chronic respiratory disease n = 108(50.6%) and chronic heart failure n = 99(46%). Physiological reserve was altered in 129(60%) before ICU admission. ICU admissions were more often for acute respiratory illness in 160 patients(74.6%) and mean SAPSII was 34.9 ± 13. 109 patients(51%) received invasive mechanical ventilation. 28(44,4%) required inotropic agents. The most common ICU adverse events were respectively acute renal failure 26(41.3%), cardiac arrhythmia 21(33.3) and nosocomial infection in 13 (20.6%). Autonomy was reduced in 86(40%) of patients. ICU length of stay was 9.3 ± 10.9 days. Multivariate Cox regression analysis shows only two independent variables associated with long term mortality: Physiological reserve status before ICU (HR, 2.02 ; 95%CI, [1.153.51] ; p = 0.013), and reduced autonomy at ICU discharge (HR, 2.01 ; 95% CI, [1.16 3.46], p = 0,012).

**CONCLUSION.** Post ICU mortality rate proves to be high. Death delay after ICU was significantly shortened by altered physiological reserve prior to ICU admission and autonomy at ICU discharge.

### 0741

#### Characteristics and outcome of patients admitted to the intensive care unit with a decompensation of obstructive sleep apnea-Hypopnea syndrome requiring mechanical ventilation

D. García Huertas[1], F. Manzano[1], M.M. Jiménez Quintana[1], A. López Fernández[1], S. Ramiro González[1], A. Fuentes Calatayud[1], A. Ruiz Perea[1], M. Colmenero[2]

[1]Hospital Universitario Virgen de las Nieves, Granada, Spain; [2]Hospital Clinico San Cecilio, Granada, Spain

**Correspondence:** D. García Huertas

*Intensive Care Medicine Experimental* 2017, **5(Suppl 2):**0741

**INTRODUCTION.** Decompensated Obstructive Sleep Apnea-Hypopnea Syndrome (OSAHS) is a condition that sometimes requires admission in an ICU to treat hypercapnic respiratory failure.

**OBJECTIVES.** This study was undertaken to determine the characteristics and outcome in patients with decompensated OSAHS admitted to the ICU and to describe the pattern of utilization of mechanical ventilation in these patients.

**METHODS.** A retrospective cohort study over a 3 year-period (2014–2016) in an ICU of a University Hospital. We described the epidemiology, treatment and outcome of OSAHS patients admitted in ICU requiring mechanical ventilation. Variables related to comorbidities, functional status (number of hospitalizations and visits to the Emergency department in the previous year), acute physiology derangement (APACHE II) of the episode and type of mechanical ventilation (invasive vs noninvasive) were recorded. A bivariate analyses (chi-square, student-t) was made to compare the results between the two groups of patients related to the type of mechanical ventilation used. Results are expressed as percentages and mean with standard deviation. P < 0,05 was considered significant.

**RESULTS.** Twenty six patients were included.The mean age was 65 ± 12 years, 53.8% were female, 34.6% were diabetic, 23.1% suffered chronic right heart failure. The APACHE II score was 19 ± 3 points. Three out of 26 patients (11.6%) had at least one hospital admission in the previous year and 13 out of 26 (50%) had a visit to the emergency department. All patients began with non invasive ventilation (NIV). Six out of 26 (27%) patients required invasive mechanical ventilation (IMV) due to progressive respiratory failure. Duration of mechanical ventilation was 3.6 ± 2.6 days (0.8 ± 1.7 days for the subgroup of invasive). Eleven out of 24 patients (45.8%) were moved to the ward with noninvasive ventilation. Overall ICU and hospital mortality was 7.7% (2 of 26) and 30.8% (8 of 26), respectively. Those who died, 5 were subjected to limitation of therapeutic effort, 2 from cardiac cause and 1 refusing treatment. Six out of 19 patients (31.6%) with NIV died versus 2 out of 7 patients (28.6%) with IMV (p = 0.88). Non-survivors were older (68 ± 15 vs 64 ± 11, p = 0.43), had higher APACHE II score (21 ± 3.5 vs 18.7 ± 2.8, p = 0.08) and greater number of hospitalizations (0.7 ± 2.1 vs 0.1 ± 0.3, p = 0.21) and visits to emergency department (2.5 ± 5.5 vs 0.8 ± 0.9, p = 0.17).

**CONCLUSIONS.** The use of non-invasive ventilation in decompensated OSAHS has a similar failure rate than in COPD, but the duration of invasive mechanical ventilation appears to be much shorter.

### 0742

#### 30 days´ post ICU mortality in a Tunisian medical intensive care unit (ICU)

J. Baroudi[1], K. Meddeb[1], M.A. Boujelbèn[1], J. Ayachi[1], N. Sma[1], N. Fraj[1], E. Ennouri[1], W. Zarrougui[1], A. Ben Abdelhafidh[1], H. Kallel[1], A. Azouzi[1], I. Ben Saida[1], I. Chouchene[1], A. Khedher[1], M. Boussarsar[1,2]

[1]Farhat Hached University Hospital, Medical Intensive Care Unit, Sousse, Tunisia; [2]Ibn Al Jazzar Faculty of Medicine, Research Laboratory N° LR14ES05 Interactions of the Cardio-pulmonary System, Sousse, Tunisia

**Correspondence:** M. Boussarsar

*Intensive Care Medicine Experimental* 2017, **5(Suppl 2):**0742

**INTRODUCTION.** Intensive care mortality has been decreasing over the last decade. However, mortality after ICU discharge and its risk factors remain not well known. Studies showed that death occurs mostly at the first month after discharge. Data are lacking for the post ICU mortality in Tunisian ICU´s patients.

**OBJECTIVES.** To analyze the short-term mortality after ICU discharge and its predictor factors.

**METHODS.** An observational prospective cohort study performed in a medical adult ICU with approximately 250 admissions per year. The study included all survivors after ICU admission. Data were collected between January 2014 and December 2015 and the mortality was assessed by telephone interviews at thirty days after ICU discharge. Factors associated with post hospital mortality are presented as odds ratios. A P value lower than 0.05 was considered significant.

**RESULTS.** Among 573 patients admitted in ICU between January 2014 and December 2015. 215 were included. A total of 63(29%) patients died after ICU discharge during the same year and 34(19%) at the first month. Physiological reserve (OR, 2.73 ; 95%CI, [0.9-7.5] ; p = 0.05), severity at ICU admission (SAPSII > 30 OR, 1.07 ; 95%CI, [1.03-1.13] ; p = 0.002), the functional status (OR,10.9 ; 95%CI, [3.8-31.1] ; p = 0.000) and tachycardia on ICU discharge (OR, 1.04 ; 95%CI, [1–1.07] ; p = 0.01) were the only independent factors of 30 days post ICU mortality.

**CONCLUSION.** 30 days´ post ICU mortality in the present study proves to be rather important. Physiological reserve and severity on ICU admission, then functional autonomy and tachycardia at ICU discharge were the only independent risk factors of 30 days´ post ICU mortality.

### 0743

#### Survival determinants in patients with ICU-tracheostomy

V. Inal, S. Efe
Trakya University Faculty of Medicine, Yogun Bakim, Edirne, Turkey
**Correspondence:** V. Inal
*Intensive Care Medicine Experimental* 2017, **5(Suppl 2):**0743

**INTRODUCTION.** Tracheostomy in ICU patients has been a matter of unsolved debate, yet.

**OBJECTIVES.** In this retrospective study, we objected to elucidate pros and cons of tracheostomy in our mixed- type ICU-population, and help to quantify literature data, for future evidence based approaches.

**METHODS.** Retrospective data of mixed-type ICU, in 2016, were analyzed in tracheostomy patients, for 90-day mortality rates and determinants of survival. Regression and survival analysis were executed at significance of $p < 0.001$ (CI: 95%); all cases were weighted for APACHE II scores.

**RESULTS.** 34 patients out of 347 (9.7%), 11 female and 23 male, at mean age of 61.5 (19–91) years, mean APACHE II scores of 19.5 ± 6.5, found mean length of stay (LOS) 50.8 (8–182) days, 17 were died included 5 after discharged (mean 48.2 (3–130) days), 14 discharged to wards, and 3 discharged to palliative-care-centers. Among 34 tracheostomy patients; 15 were from ED, 5 from outer-centers, 17 post-CPR and 10 were post-op or trauma patients. Analyses referred to patients from ED were 5.1x, from outer-centers 6.5x, and post-CPR 6.96x less likely to survive. 24 of tracheostomy were performed by intensivists in ICU and 10 by surgeons in op-theater. Tracheostomies were performed on mean 15.9 ± 8.1 days (14–18), and ICU cases were 1.1-2.0x more likely to survive. At the time of tracheostomy; severe infection, assumed by multiple antibiotics, added about a 2x risk to survive. In addition to these; tracheostomy performed before 10 and after 30 days (2x vs 6x) also significantly increased mortality risk. However, no significant correlation was shown between tracheostomy and ICU LOS, in not only between 347, but even within 34 tracheostomy patients.

**CONCLUSIONS.** Take home messages of this study are; (1) ICU-tracheostomy was not uncommon (about 10%), (2) but even a half were dead in 90-day, (3) ED admissions were more prone to die, (4) more severe the infectious condition less chance to survive at the time of tracheostomy, (5) tracheostomy performed by intensivists in ICU, and between on 14-18$^{th}$ days had better survival, (6) no correlation found between tracheostomy and ICU LOS.

### 0744

#### Correlation of gastric residual volume and illness severity based on modified sequential organ failure assessment in critically ill patients

D. Aditianingsih, M. Farina, L. Sutanto
Universitas Indonesia Ciptomangunkusumo Hospital, Anesthesiology and Intensive Care, Jakarta, Indonesia
**Correspondence:** D. Aditianingsih
*Intensive Care Medicine Experimental* 2017, **5(Suppl 2):**0744

**INTRODUCTION.** The Sequential Organ Failure Assessment (SOFA) score has been recommended for predicting in-hospital mortality in critically ill septic patients. [1] The modification sequential organ failure assessment (MSOFA) score is a simplified version of the SOFA score, that eliminates the platelet count, uses arterial oxygen saturation ($SpO_2$), and replaces serum bilirubin with scleral icterus or jaundice. [2] Sepsis may impair gastrointestinal motility, and causes increasing gastric residual volume (GRV) that often occurs in the critically ill. Patients with increasing GRV had a higher ICU mortality compared with patients with decreasing GRV. [3]

**OBJECTIVES.** This study determined the validation of MSOFA to predict mortality in sepsis, the correlation of GRV with MSOFA, and whether it could complement the parameters of MSOFA scoring system.

**METHODS.** This study was a prospective cohort study of 148 septic patients enrolled in the ICU Cipto Mangunkusumo Hospital Indonesia. MSOFA score and total GRV were measured in the first and second 24 hours of treatment.

**RESULTS.** Both MSOFA score day 1 and 2 was associated with 28-day mortality ($p < 0.001$) with AUC 0.754 and 0.846. There is a correlation between 24-hour gastric residual volume day 2 with the MSOFA day 2 ($p < 0{,}001$; r 0,52). The scoring category of 24-hour GRV day 2 was made into 5 categories (score 0–5) based on the ROC curve percentile. The addition of GRV score on the MSOFA scoring system on day 2 had a good ability to predict the mortality [AUC 0,851; CI 95% (0,788- 0,914)].

**CONCLUSIONS.** There was a correlation between GRV with disease severity scores MSOFA for septic shock patients on the second day of ICU care. The addition of the second 24-hour GRV score to MSOFA could be used to predict the critically ill patient mortality.

| VARIABLE | AUC | 95% CI |
|---|---|---|
| MSOFA score day 1 | 0,754 | 0,667-0,842 |
| MSOFA score day 2 | 0,846 | 0,783-0,909 |
| GRV day 1 | 0,674 | 0,577-0,771 |
| GRV day 2 | 0,731 | 0,638-0,823 |
| MSOFA score day 1 with GRV day 1 | 0,797 | 0,719-0,874 |
| MSOFA score day 2 with GRV day 2 | 0,851 | 0,788-0,914 |

*[MSOFA score and GRV to predict 28-day mortality]*

**REFERENCE(S)**

1. Raith EP et al. Prognostic Accuracy of the SOFA Score, SIRS Criteria, and qSOFA Score for In-Hospital Mortality Among Adults With Suspected Infection Admitted to the Intensive Care Unit. *JAMA*. 2017;317(3):290–300.

2. Halim DA et al. Comparison of Apache II, SOFA, and Modified SOFA scores in predicting mortality of surgical patients in intensive care unit at Dr. Hasan Sadikin General Hospital. Crit Care & Shock. 2009;12:157–169.

3. Elke G et al. Gastric Residual Volume in Critically Ill Patients: A Dead Marker or Still Alive? *Nutr Clin Pract*. 2015;30:59–71.

### Weaning from mechanical ventilation

#### 0745

**Low central venous oxygen saturation before a spontaneous breathing trial predicts weaning failure**

E.D. Valenzuela Espinoza[1], S. Dubó[2], A. Aquevedo[3], M. Jibaja[4], D. Berrutti[5], C. Labra[6], R. Lagos[6], M.F. García[7], V. Ramírez[7], M. Tobar[7], F. Picoita[7], C. Peláez[7], D. Carpio[6], L. Alegría[6], C. Hidalgo[8], K. Godoy[8], R. Castro[1], M. Silva[6], J. Bakker[1,9,10], G. Hernández[1]
[1]Pontificia Universidad Católica de Chile, Medicina Intensiva, Santiago, Chile; [2]Universidad de Concepción, Kinesiología, Concepción, Chile; [3]Hospital Dr. Sótero del Río, Medicina Intensiva, Santiago, Chile; [4]Hospital Eugenio Espejo, Medicina Intensiva, Quito, Ecuador; [5]Universidad de la República, Medicina Intensiva, Montevideo, Uruguay; [6]Pontificia Universidad Católica de Chile, Santiago, Chile; [7]Hospital Eugenio Espejo, Quito, Ecuador; [8]Hospital Guillermo Grant Benavente, Concepción, Chile; [9]Columbia University Medical Center, Pulmonology and Critical Care, New York, United States; [10]New York University Medical Center, Pulmonology and Critical Care, New York, United States
**Correspondence:** E.D. Valenzuela Espinoza
*Intensive Care Medicine Experimental* 2017, **5(Suppl 2):**0745

**BACKGROUND.** Abnormal central venous oxygen saturation ($ScvO_2$) reflects an disbalance between oxygen demand and oxygen supply. In addition, weaning from mechanical ventilation by a spontaneous breathing trial (SBT) results in a significant increase in oxygen demand. Therefore, we hypothesized that the presence of low $ScvO_2$ at the beginning of SBT would be a risk factor for weaning failure.

**METHODS.** In a multicenter observational study, patients mechanically ventilated for more than 24h and clinically ready for a SBT were studied. Study variables were recorded at baseline, 2min, 30min, and at the end of the SBT. Decisions to extubate where taken by the attending physician based on their usual practice without knowledge of $ScvO_2$ levels. Weaning failure was defined as a decision not to extubate at the end of the SBT, or reintubation within 48h after extubation. The baseline values of $ScvO_2$ were categorized in quartiles. One-way ANOVA test and kruskal wallis test were used to analyze parametric and non-parametric continuous variables. A Chi-square test was used for qualitative variables. Multivariate regression analysis (corrected for the presence of a history of cardiac dysfunction) was used to find independent associations between $ScvO_2$ and weaning failure.

**RESULTS.** 204 patients from 5 centers were included. Demographic and clinical characteristic are show in the Table 192. 156 (76%) patients were successfully weaned. Of the patients that failed weaning (n = 48), 37 (77%) were not extubated at the end of the SBT, and 11 (23%) were reintubated within 48h.

At baseline patients that failed to wean had lower central venous pressure, higher respiratory rate, and lower $ScvO_2$ (Table 192).

Sixty patients (29%) patients had a $ScvO_2$ less than 70%. Multivariate regression analysis revealed the 25$^{th}$ quartile ($ScvO_2$ = 63 ± 4%) to be an independent risk factor for weaning failure OR 2.78 95% CI (1.09-7.08; P = 0.03). No other variables recorded were related to weaning failure.

**CONCLUSIONS.** In a mixed group of critically ill patients ready for a SBT after at least 24h of mechanical ventilation, a low $ScvO_2$ at the start of the SBT was an independent risk factor for a failure to wean.

**Table 192 (Abstract 0745)**. Demographic and clinical characteristic

| Patients | All | Successfully Extubated | Weaning Failure |
|---|---|---|---|
| N | 204 | 156 | 48 |
| Age (years) | 53 ± 21 | 54 ± 21 | 53 ± 20 |
| Male gender (%) | 111 (54) | 86 (55) | 25 (48) |
| Obesity | 40 (20) | 31 (20) | 9 (19) |
| APACHE II score | 20 ± 8 | 20 ± 8 | 20 ± 10 |
| SOFA score | 9 ± 4 | 9 ± 4 | 8 ± 3 |
| SOFA score (day of SBT) | 4 (2,6) | 4 (2,6) | 4 (2,6) |
| ICU LOS (days) | 6 (3,10) | 6 (3,10) | 6 (4,10) |
| Mechanical Ventilation (days) | 5 (3,9) | 6 (3,9) | 5 (4,7) |
| **Clinical Variables** | | | |
| Heart rate, beats per min | 87 ± 18 | 86 ± 18 | 89 ± 19 |
| Systolic blood pressure, mmHg | 132 (123,151) | 132 (121,151) | 130 (124,150) |
| Central venous pressure, mmHg | 10 (7,12) | 10 (7,13) | 9 (6,11)* |
| Respiratory rate, breathing per min | 19 (15,22) | 18 (15,22) | 20 (17,25)* |
| Oxygen Saturation, % | 96 (95,98) | 96 (95,98) | 97 (94,98) |
| $ScvO_2$, % | 73 ± 7 | 74 ± 7 | 71 ± 8* |

Values are expressed as mean ± SD or median (interquartile range) or N (%); APACHE, Acute Physiology and Chronic Health Evaluation; SOFA, Sequential Organ Failure Assessment; ICU, intensive care unit; LOS, length of stay; $ScvO_2$, central venous oxygen saturation; *(p<0.05) comparing the groups successfully extubated and weaning failure.

#### 0746

**Evolution of diaphragm activity during spontaneous breathing trials**

S. Hemvimon, N. Rittayamai, S. Sompradeekul, N. Chierakul
Faculty of Medicine Siriraj Hospital, Division of Respiratory Diseases and Tuberculosis, Department of Medicine, Bangkok, Thailand
**Correspondence:** S. Hemvimon
*Intensive Care Medicine Experimental* 2017, **5(Suppl 2):**0746

**INTRODUCTION.** Diaphragm ultrasound is a noninvasive and realtime tool to evaluate diaphragm function and activity. Diaphragm thickening fraction (TFdi) during tidal breathing has a good correlation with patient effort determined by pressure–time product and it would be used to estimate respiratory muscle workload during spontaneous breathing trials (SBT) whereas rapid shallow breathing index (RSBI) is the most accurate index used in daily practice for predicting weaning outcome. Diaphragm dysfunction may also lead to weaning failure.

**OBJECTIVES.** The primary objective was to evaluate diaphragm activity determined by change in TFdi at tidal breathing during the course of SBT. The secondary objectives were the difference in 1) TFdi and RSBI between patients who pass and fail SBT 2) diaphragm function determined by diaphragm excursion ($DE_{max}$) and $TFdi_{max}$.

**METHODS.** A prospective cross-sectional study was done in 45 ventilated patients who were ready to be weaned. Patients who had diaphragm paralysis or neuromuscular disease were excluded. SBT was performed using flow-by technique for 30 minutes. Diaphragm thickness (Tdi) was measured at the right hemidiaphragm in the zone of apposition using a 10 MHz linear ultrasound probe. TFdi was calculated from the percentage change between inspiratory and expiratory Tdi as the following formula: TFdi (%) = (($Tdi_{insp}$ - $Tdi_{exp}$)/$Tdi_{exp}$ x 100). We recorded TFdi at tidal breathing and RSBI at 0, 5, 10, 15 and 30 min of SBT. We also evaluated diaphragm function by measuring $DE_{max}$ and $TFdi_{max}$ during maximum effort before starting SBT.

**RESULTS.** TFdi was significantly increased at the end of SBT ($TFdi_0$ = 29.8 ± 13.8% and $TFdi_{30}$ = 37.4 ± 13.0%; P < 0.001). In patients who failed SBT (13/45; 28.8%), TFdi at the onset of SBT was a significant higher in comparison to patients who passed SBT

(36.3 ± 16.4% vs 27.1 ± 11.9%; P = 0.043) representing higher inspiratory effort in failure group. There was also a significant difference in RSBI at the onset of SBT between success and failure of SBTs (57 ± 20.1 vs 80.6 ± 32.7; P = 0.005). In the failure group, $DE_{max}$ and $TFdi_{max}$ were lower than the success group but there was no statistical significant difference. In patients who were ventilated less than 7 days (n = 25), we found that there was significant higher $DE_{max}$ than the failure group (26.8 ± 9.0 vs 19.2 ± 6.4 mm; P = 0.002).

**CONCLUSIONS.** Patient efforts determine by TFdi significantly increased during the course of SBT. TFdi immediately increased and significant difference at the onset of SBT in the failure SBT group.

**REFERENCE(S)**

1. Goligher EC, et al. Evolution of diaphragm thickness during mechanical ventilation. Impact of inspiratory effort. Am J Respir Crit Care Med 2015;192(9):1080–8.

2. Vivier E, et al. Diaphargm ultrasonography to estimeate the work of breathing during non-invasive ventilation. Intensive Care Med 2012;38(5):796–803.

**GRANT ACKNOWLEDGMENT**

This study was supported by Faculty of Medicine Siriraj Hospital, Mahidol University.

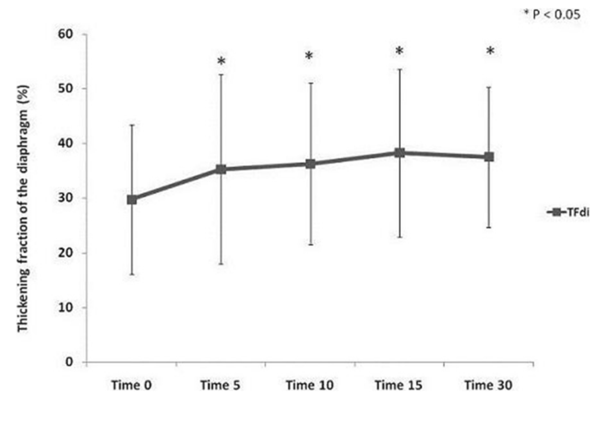

**Fig. 256 (Abstract 0746)** Evolution of TFdi during the course of SBT

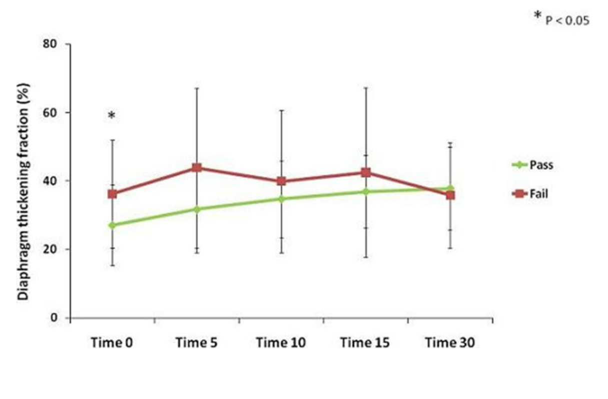

**Fig. 257 (Abstract 0746)** TFdi between success and failure of SBTs

### 0747

#### Impact of sleep quality on duration of weaning from mechanical ventilation

A.W. Thille[1,2], F. Reynaud[1,2], S. Barrau[1,2], D. Marie[1,2], L. Rousseau[1,2], C. Rault[1,3], R. Coudroy[1,2], J.-P. Frat[1,2], V. Diaz[1,3], J.-C. Meurice[1,4], R. Robert[1,2], X. Drouot[1,3]

[1]INSERM CIC 1402, Groupe ALIVE, Poitiers, France; [2]CHU de Poitiers, Service de Réanimation Médicale, Poitiers, France; [3]CHU de Poitiers, Explorations Fonctionnelles, Poitiers, France; [4]CHU de Poitiers, Pneumologie, Poitiers, France

**Correspondence:** A.W. Thille

*Intensive Care Medicine Experimental* 2017, **5(Suppl 2):**0747

**INTRODUCTION.** In difficult-to-wean ICU patients, the influence of sleep quality on weaning duration of mechanical ventilation has never been studied. We aimed to compare sleep quality between patients with a short weaning duration and those with a prolonged weaning duration.

**PATIENTS AND METHODS.** Prospective physiological study performed in a French teaching hospital. All patients intubated at least 24h and difficult-to-wean, i.e. those who experienced at least one weaning trial failure, could be included. Patients with continuous sedation, central nervous system or psychiatric disease, or underlying neuromuscular disease were excluded. A complete polysomnography was performed during the night following the first weaning trial failure. Peripheral muscle strength, maximal inspiratory pressure and delirium were measured at time of polysomnography. Weaning duration was defined as the time from polysomnography to extubation. Prolonged weaning was defined as a weaning duration at least 3 days (failure of at least 3 weaning trials) according to the international conference consensus of weaning. Altered sleep quality was defined as atypical sleep or absence of REM sleep (1).

**PRELIMINARY RESULTS.** Over a 24 month-period, 52 difficult-to-wean patients were screened and 33 patients were analyzed. Among them, 14 patients (42%) had prolonged weaning (>3 days) and 19 patients (58%) had short weaning.

Patients with prolonged weaning had shorter rapid eye movement (REM) sleep duration than patients with short weaning (0 minute [0–5] vs. 9 [0–52], $p = 0.038$). Atypical sleep was more likely to be found in patients with prolonged weaning: 79% (11 of 14 patients) vs. 32% (6 of 19 patients), $p = 0.01$. The weaning duration was longer in patients with altered sleep quality than the others (6.2 ± 5.7 days vs. 3.3 ± 2.7, $p = 0.03$ log-rank test). Absent electroencephalographic reactivity at initiation of polysomnography was more frequently observed in patients with prolonged weaning: 71% (10 of 14 patients) vs. 26% (5 of 19 patients), $p = 0.01$. The score of delirium at time of polysomnography as well the total dosis of sedation prior to polysomnography did not differ between groups. Maximal inspiratory pressure was similar in the 2 groups and the only difference was a lower peripheral muscular strength in the prolonged weaning group as indicated by a lower MRC score group (31 points [24–55] vs. 58 [46–60], $p = 0.01$).

**CONCLUSION.** Our preliminary results show that ICU patients with altered sleep quality at the beginning of weaning from mechanical ventilation, i.e. atypical sleep or absence of REM sleep, had prolonged weaning.

**KEYWORDS.** Weaning; Mechanical ventilation; Sleep; Polysomnography; Intensive care unit.

(1) Drouot X, Roche-Campo F, Thille AW, Cabello B, Galia F, Margarit L, d´Ortho M-P, Brochard L (2012) A new classification for sleep analysis in critically ill patients. Sleep medicine 13:7–14

### 0748

#### Predicting spontaneous breathing trial and extubation success: role of arterial blood samples

I. Paredes Borrachero, L. Vargas Guaman, F.J. Moran Gallego, M. Vecino Montoya, P. Carneiro Pereda, J.M. Romeu Prieto, P. Sánchez Rodríguez, G. Hernández Martínez, A. Canabal Berlanga, M.Á. Arrese Cosculluela

Virgen de la Salud University Hospital, Intensive Care, Toledo, Spain
**Correspondence:** I. Paredes Borrachero
*Intensive Care Medicine Experimental* 2017, **5(Suppl 2):**0748

**INTRODUCTION.** Current recomendations (1) for respiratory failure criteria of spontaneous breathing trials (SBT) report considering failure in patients who fulfill the following criteria:

1) PaO2 ≤ 50-60 mmHg on FiO2 ≥ 0.5 or SaO2 ≤ 90%;
2) PaCO2 > 50 mmHg or an increase in PaCO2 > 8 mmHg;
3) pH < 7.32 or a decrease in pH ≥0.07 pH units.

However, more recent data (2), confirm that patients with PaCO2 > 45 mmHg irrespective of pH value at the end of the SBT benefit with noninvasive ventilation (NIV) in term of survival rate, suggesting that the former criteria should be modified.

**OBJECTIVES.** As patients at risk for hypercapnia are mainly COPD patients, we aimed to assess whether arterial blood sample could be avoided in non-COPD patients.

**METHODS.** We performed a prospective single center study, in a ICU with a weaning protocol including preventive therapy for high-risk patients (those who present at least one high-risk factor for reintubation) with both high-flow conditioned oxygen therapy or NIV. The high-risk factors include: age > 65 years, cardiac failure, moderate-to-severe COPD, APACHE II > 12 points the extubation day, body mass index (BMI) > 30, patients at high-risk for developing laringeal edema, patients considered unable to deal with respiratory secretions (inadequate cough reflex or >2 suctioning within 8h previous to extubation), difficult or prolonged weaning, ≥2 comorbidities (Charlson index), and prolonged MV defined as MV > 7 days. Under those conditions, we performed two analysis:

1) the probability of developing hypercapnia at the end of the SBT in a non-COPD patient;
2) we compared oxymetric values from an arterial blood sample to oxymetry. Consecutive patients were enrolled since January 2016 to January 2017. Statistical analysis included ROC curves and multivariate analysis to adjust for covariates.

**RESULTS.** 200 patients were analyzed, including 35 COPD. The 12% (24/200) of patients failed the SBT, 33% were COPD patients (8/24). Respiratory related reintubation rate was 10% (21/200) and all cause reintubation rate was 12% (25/200). The 54% (19/35) of COPD patients presented with hypercapnia, while only 4 non-COPD patients presented a CO2 level at the end of the SBT above 45; all of them were surgical patients, and the reason for the hypercapnia was residual effect of sedatives. None of them required reintubation secondary to hypercapnic failure. According to the oximetric values, the ROC curve for PaO2 and SaO2 were similar (0.73 vs 0.74, respectively; p = 0.65).

**CONCLUSIONS.** Non-COPD patients do not seem to benefit with arterial blood samples in terms of improvement in diagnostic quality of SBT failure criteria.

**REFERENCE(S)**

1.- Boles JM, Bion J, Connors A, et al. TASK FORCE Weaning from mechanical ventilation. Eur Respir J 2007; 29:1033–1056.

2.- Ferrer M, Sellarés J, Valencia M, et al. Non-invasive ventilation after extubation in hypercapnic patients with chronic respiratory disorders: randomised controlled trial. Lancet 2009; 374:1082–88

### 0749

#### Manual ASV vs INTELLiVENT-ASV for the patients after cardiac surgery - are automated ventilators better for the patients?

T. Kobayashi[1,2], Y. Onodera[2], H. Suzuki[2], M. Nakane[1], K. Kawamae[2]
[1]Yamagata University Faculty of Medicine, Emergency and Critical Care Medicine, Yamagata, Japan; [2]Yamagata University Faculty of Medicine, Anaesthesiology, Yamagata, Japan
**Correspondence:** T. Kobayashi
*Intensive Care Medicine Experimental* 2017, **5(Suppl 2):**0749

**INTRODUCTION.** Automated ventilators (INTELLiVENT-ASV:I-ASV), with which physicians only need to set target $P_{ET}CO_2$ and $SpO_2$ levels, were developed to provide more appropriate ventilation to patients and reduce medical staff workloads. However, I-ASV has not been thoroughly investigated for the patients after cardiac surgery. Thus, we compared ventilator settings adjusted by I-ASV and manual ASV (M-ASV) after cardiac surgery in our ICU.

**OBJECTIVES.** To assess feasibility of I-ASV for the patients after cardiac surgery.

**METHODS.** We enrolled patients who underwent cardiac surgery from July 2013 to April 2014. After ICU admission, patients were randomized into two groups: I-ASV and M-ASV. With I-ASV, $SpO_2$ target shift was fixed to +3 and $P_{ET}CO_2$ target shift was adjusted for $PaCO_2$ of 35–45 mmHg. Ventilator settings were automatically adjusted until spontaneous breathing test. With M-ASV, PEEP, $F_IO_2$ and %minute volume (%MV) were initially decided by ICU physicians. PEEP and %MV were adjusted by physicians and $F_IO_2$ was adjusted by nursing staff based on arterial blood gas analysis. Ventilator settings (PEEP, $F_IO_2$, %MV), P/F ratio, PaO2, respiratory rate (RR) and $PaCO_2$ were recorded each hour after ICU admission for 24 hours. Statistical analysis was made by Wilcoxon rank sum test, and p-values of < 0.05 were considered significant.

**RESULTS.** Data from 48 patients were analysed (I-ASV: 28, M-ASV: 20). No safety issues occurred. All of the parameters (PEEP, $F_IO_2$, %MV, P/F ratio, PaO2,RR and PaCO2) were not detected significant difference as a trend, except for $F_IO_2$ from one to six hours after the operation. The $F_IO_2$ of I-ASV group is much lower than that of M-ASV group (I-ASV versus M-ASV: $F_IO_2$-1h 0.3(0.3 to 0.34) versus 0.4 (0.35 to 0.49), $F_IO_2$-2h 0.3 (0.3 to 0.33) versus 0.4 (0.3 to 0.4), $F_IO_2$-3h 0.3 (0.3 to 0.32) versus 0.4 (0.3 to 0.4), $F_IO_2$-4h 0.3 (0.3 to 0.33) versus 0.38 (0.3 to 0.4), and $F_IO_2$-6h 0.3 (0.3 to 0.32) versus 0.35 (0.3 to 0.4), respectively; P < 0.05).

**CONCLUSIONS.** I-ASV ventilator adjustments were not much different compared with M-ASV settings. $F_IO_2$ during early phase was set lower by I-ASV, although oxygenation was not different. Thus, INTELLiVENT-ASV use may be feasible for patients after cardiac surgery.

### 0750

#### Predictors for early weaning failure from mechanical ventilation in critically ill surgical patients

Y.T. Jung, S.H. Lee
Yonsei University College of Medicine, Department of Surgery, Seoul, Korea, Republic of
**Correspondence:** Y.T. Jung
*Intensive Care Medicine Experimental* 2017, **5(Suppl 2):**0750

**INTRODUCTION.** Prolonged mechanical ventilation (MV) is associated with high morbidity, mortality, and health care costs.

**OBJECTIVES.** The aim of this study is to evaluate predictors for failure of early weaning from MV in critically ill surgical patients.

**METHODS.** The medical records of 926 critically ill patients who underwent emergency gastrointestinal surgery for diffuse peritonitis between January 2007 and December 2015 were reviewed retrospectively. All patients who underwent MV during operation and required continuation of MV during postoperative period were included. Early weaning failure from MV was defined as the failure of extubation or the need for reintubation within the first 48 hours after surgery. Clinical and laboratory parameters before surgery and within 1 day after surgery were investigated.

**RESULTS.** This study included 315 adult patients, of whom 207 (65.7%) were successfully weaned within the first 48 hours after surgery. Patients who failed extubation within the first 48 hours after surgery showed significantly higher 30-day mortality than those who were successfully extubated (28.7% versus 6.3%, p < 0.001). Serum creatinine levels of > 1.2 mg/dL (odds ratio [OR] 2.984; 95% confidence interval [CI] 1.130-7.882; p = 0.027), platelet counts of < 140,000/μL (OR 3.866; 95% CI 1.418-10.540;
p = 0.008), delta neutrophil index of > 25% (OR 3.503; 95% CI 1.264-9.705; p = 0.016) measured immediately after surgery, failed spontaneous awakening trial (SAT; OR 3.993; 95% CI 1.052-15.157;
p = 0.042) and spontaneous breathing trial (SBT; OR 10.231; 95% CI 3.413-30.674; p < 0.001) within the first 48 hours after surgery were independent predictors of early weaning failure from MV.

**CONCLUSIONS.** Elevated serum creatinine level, low platelet counts, high delta neutrophil index immediately after surgery, failed SAT and failed SBT within the first 48 hours after surgery were independent predictors of early weaning failure from MV in critically ill surgical patients.

**REFERENCE(S)**

1. Thille AW, Harrois A, Schortgen F, et al. Outcomes of extubation failure in medical intensive care unit patients. Crit Care Med 2011; 39:2612–2618

2. Penuelas O, Frutos-Vivar F, Fernandez C, et al., for the Ventila Group. Characteristics and outcomes of ventilated patients according to time to liberation from mechanical ventilation. Am J Respir Crit Care Med 2011;184:430–437

3. Meade M, Guyatt G, Cook DJ, et al. Predicting success in weaning from mechanical ventilation. Chest 2001;120:400S-24S

### 0751

#### Model of success prediction in weaning of mechanical ventilation with clinical-objective-ultrasonographic variables in ICU

J.D. Salmerón-González, O. Zacamitzin-Contreras, J. Diaz, R. Silva, M.N. Gómez-González, R. Soriano-Orozco, X. Ramos-Ramos, P.L. González-Carrillo

Instituto Mexicano del Seguro Social, Unidad de Cuidados Intensivos, León, Mexico

**Correspondence:** J.D. Salmerón-González

*Intensive Care Medicine Experimental* 2017, **5(Suppl 2):**0751

**INTRODUCTION.** Diaphragmatic dysfunction in controlled mechanical ventilation (MV) is generated in at least 24 hours and has been associated with the development of diaphragmatic atrophy. Direct diaphragm measurement has a role as a predictor of success or failure in weaning of mechanical ventilation.

**OBJECTIVE.** The goal of this study was to determine the efficacy in weaning of mechanical ventilation from a protocol of ultrasound diaphragm measurements in association with clinical-ventilatory variables.

**MATERIAL AND METHODS.** This prospective, observational study was conducted at the Intensive Care Unit, Centro Médico Nacional del Bajío UMAE 1, in León Guanajuato, between February 2016 to January 2017. Patients were admitted over 18 years and mechanical ventilation over 24 hrs. A prospective clinical data sheet was used to collect clinical variables (GCS 13–15 points, hemodynamic stability, presence of tusigo reflex and swallowing), ventilatory variables (CROP index, SpO2 > 90% FiO2 < 60 mmHg PaO2/FiO2 > 150 PEEP < 8 cmH2O respiratory rate (RR) < 35, RR/tidal volume < 105) and diaphragmatic ultrasound measurements (diaphragmatic excursion, diaphragmatic contraction velocity).

**RESULTS.** We included 90 patients, mean age of 49.18 years, female gender 54%, male 46%; the following significant results were obtained: respect to clinical variables, the presence of tusigo reflex and swallowing with a $p = 0.001$, CROP index >13 with a $p = 0.03$, PEEP < 8 cm H2O with a $p = 0.001$, RR/tidal volume with a $p = 0.0001$; respect to diaphragmatic ultrasound measurements, diaphragmatic excursion with a $p < 0.05$.

**CONCLUSIONS.** There is a positive correlation between the diaphragmatic excursion and a success weaning of mechanical ventilation, as well as other clinical variables (presence of tusigo reflex and swallowing, CROP index, PEEP and RR/tidal volume).

### 0752

#### Respiratory measurement of volume change using Expiron® during spontaneous breathing trials in ICU

J. Kim[1], S. Na[2], H.-B. Kim[1]

[1]Yonsei University College of Medicine, Department of Anesthesiology and Pain Medicine, Seoul, Korea, Republic of; [2]Yonsei University College of Medicine, Seoul, Korea, Republic of

**Correspondence:** H.-B. Kim

*Intensive Care Medicine Experimental* 2017, **5(Suppl 2):**0752

**INTRODUCTION.** Surgical patients receiving opioids for pain control are at greater risk of respiratory depression following extubation. To decrease postoperative pulmonary complications, incentive spirometry has gained widespread use in the postoperative period by encouraging patients to achieve maximal inspiration by providing visual feedback. However, there is a limit to the use of incentive spirometry in patients who have undergone tracheotomy. Recently, impedance-based noninvasive respiratory volume monitor (RVM, ExSpiron, Respiratory Motion, Inc, Waltham, MA) became available to continuously measure minute ventilation (MV), tidal volume (TV), and respiratory rate(RR).

**OBJECTIVES.** This study was designed to determine if RVM can be used to determine respiratory depression after SBT.We also investigated whether RVM can measure respiratory changes before and after deep breathing training (DBT) after SBT.

**METHODS.** Fifty two postoperative patients completed the study. Of the 51 patients, 31% were tracheotomy patients who were unable to use inspirometry. During the spontaneous breathing trials, continuous respiratory data were recorded from an impedance-based RVM (ExSpiron, Respiratory Motion, Inc, Waltham, MA) simultaneously with ventilator data Hamilton G5 (Hamilton Medical AG; Bonaduz, Switzerland). The TV measured by the ventilator during this period was then entered into the RVM to calibrate the TV estimated by the RVM during this same period. After mechanical ventilator weaning, we have continued respiratory monitoring by RVM without any intervention. During immediately post-extubation periods, patients are continuously injected with remifentanil for pain control. After 1 hour, patients underwent DBT for 15 minutes using RVM monitoring. We did arterial blood gas analysis before and after the deep breathing exercise.

**RESULTS.** 51 subjects (28 females/23 males; age: 59.9 ± 12.8 years; BMI: 22.8 ± 3.3 kg/m2, mean ± SD) completed the study. 21 patient data were used to study the accuracy of RVM compared to ventilator in TV measurements. TVs as measured by RVM and ventilaotr during SBT were strongly correlated (R = 0.95 ± 0.1). As expected, almost all patients after extubation showed respiratory depression on RVM. On the ABGA, mean PaO2 decreased (−46.2 ± 38.1, $p < 0.001$) after extubation.

After DBT using RVM, PaO2 showed an average increase of 8.8 mmHg.(−8.8 ± 25.4, $p = 0.017$).

**CONCLUSIONS.** After mechanical ventilator weaning, most patients underwent respiratory depression. RVM can be used successfully for postoperative respiratory physiotherapy as well as accurate measurement of respiratory volume.

**REFERENCE(S)**

1. Williams GW, 2nd, George CA, Harvey BC, Freeman JE. A Comparison of Measurements of Change in Respiratory Status in Spontaneously Breathing Volunteers by the ExSpiron Noninvasive Respiratory Volume Monitor Versus the Capnostream Capnometer. Anesth Analg 2017;124:120–6

### 0753

#### Factors associated with delayed weaning from mechanical ventilation in patients after cardiac surgery

P. Kossivas[1], S. Dimopoulos[1], D. Doubou[1], S. Nanas[2], K. Marathias[1], A. Karabinis[1], C. Routsi[2]
[1]Onassis Cardiac Surgery Center, Surgical Intensive Care Unit, Athens, Greece; [2]School of Medicine, National and Kapodistrian University of Athens, Evangelismos Hospital, Department of Intensive Care, Athens, Greece
**Correspondence:** C. Routsi
*Intensive Care Medicine Experimental* 2017, **5(Suppl 2):**0753

**INTRODUCTION.** Patients undergoing cardiac surgery are generally able to wean from mechanical ventilation within the first postoperative hours. Some patients however, require mechanical ventilation for a longer period and this is associated with morbidity and costs.

**OBJECTIVES.** The aim of this study was to identify factors associated with delayed weaning from mechanical ventilation, following cardiac surgery.

**METHODS.** A prospective study was performed including consecutive, adult patients who had undergone an elective cardiac surgery and were admitted to ICU. Demographic and clinical characteristics, pre- and intraoperative parameters and type of surgical procedure were recorded. The patients underwent a weaning trial under low-level pressure support. They were divided into 2 groups: Group A included patients who weaned from mechanical ventilation within 12 hours after surgery and Group B those who were mechanically ventilated for more than 12 hours following ICU admission.

**RESULTS.** Ninety patients (mean age 67 ± 10 years, 62 males) who have undergone a cardiac surgical procedure (coronary artery bypass grafting, valve replacement, ascending aortic aneurysm repair or combined surgery) were studied. Group A included 70 patients (mean duration of mechanical ventilation 8.5 ± 1.6 hours) and Group B the remaining 20 patients (mean duration of mechanical ventilation 55.6 ± 35 hours).

Compared to patients in Group A, the Group B patients were older (mean age of 72 ± 9 vs. 65 ± 11 years, $p = 0.004$); they had increased duration of cardiopulmonary bypass and aortic cross-clamping (171 ± 28 vs. 93 ± 30 min and 123.8 ± 21.3 vs. 70.6 ± 27.6 min, respectively, $p < 0.001$) and more red blood cells (RBCs) transfusion (4.5 vs. 1.6, $p = 0.027$). On ICU admission, central venous oxygen content ($ScvO_2$) was significantly lower in Group B patients compared to Group A (64 ± 7 vs. 67 ± 5, $p < 0.001$), and serum troponin I was significantly higher (13.7 ± 2.7 ng/ml vs. 4.2 ± 2.8 ng/ml, $p < 0.05$). During the weaning trials the respiratory frequency / tidal volume was lower in Group A patients compared to Group B (32.6 ± 7.8 vs. 42.3 ± 17.9 respectively, $p = 0.038$). Also, combined surgery as compared to a single surgical procedure was associated with delayed discontinuation from the ventilator ($p = 0.043$).

**CONCLUSIONS.** Increased age, increased duration of extracorporeal circulation and aortic clamping, RBCs transfusion number, low $ScvO_2$ and high troponin I values following surgery, and combined surgery should be considered as factors that delay the weaning from mechanical ventilation in patients after cardiac surgery.

### 0754

#### Prognostic factors and outcomes of unplanned extubation

C.-M. Chen[1,2], W. Chou[2,3]
[1]Chi Mei Medical Center, Intensive Care Medicine, Tainan, Taiwan, Province of China; [2]Chia Nan University of Pharmacy and Science, Department of Recreation and Health-Care Management, Tainan, Taiwan, Province of China; [3]Chi Mei Medical Center, Physical Medicine & Rehabilitation, Tainan, Taiwan, Province of China
**Correspondence:** C.-M. Chen
*Intensive Care Medicine Experimental* 2017, **5(Suppl 2):**0754

**INTRODUCTION.** Endotracheal intubation with mechanical ventilation (MV) support is an important intervention for managing patients with respiratory failure in the intensive care unit (ICU). However, 2-16% of patients on MV undergo potentially life-threatening unplanned extubation (UE), which is defined as an accidental or a patient-induced removal of an endotracheal tube.

**OBJECTIVES.** We reviewed the outcomes of unplanned extubation (UE) in patients in a medical center's 6 intensive care units (ICUs) and calculated their mortality risk.

**METHODS.** We retrospectively reviewed the medical records of all adult patients in Chi Mei Medical Center who underwent UE between 2009 and 2015. The following data were collected: demographic and clinical variables, severity of the patient's condition, and outcomes.

**RESULTS.** During the study period, there were 305 episodes of UE in 295 ICU patients (men: 199 [67.5%]; mean age: 65.7 years; age range: 18–94 years). The mean Acute Physiology and Chronic Health Evaluation (APACHE) II score was 16.4, mean therapeutic intervention scoring system (TISS) score was 26.5, and mean Glasgow coma scale score was 10.4. One hundred thirty-six patients (46.1%) were re-intubated within 48 h. Forty-five died (mortality rate: 15.3%). Multivariate analyses showed 5 risk factors—respiratory rate, APACHE II score, uremia, liver cirrhosis, and weaning status—were independently associated with mortality.

**CONCLUSIONS.** Five risk factors including a high respiratory rate before UE, high APACHE II score, uremia, liver cirrhosis, and not being weaned from intubation—were associated with high mortality in patients who underwent UE.

**REFERENCE(S)**

1. da Silva PS, Fonseca MC. Unplanned endotracheal extubations in the intensive care unit: systematic review, critical appraisal, and evidence-based recommendations. Anesth Analg 2012; 114:1003–1014

2.CM Chen, KC Cheng, SC Hsing, AC Cheng, MY Sung, MY Su, KC Cheng. Age is an important predictor of failed unplanned extubation. International journal of gerontology 2010; 4:120–129

**GRANT ACKNOWLEDGMENT**
None

### 0755

#### Comparison of four ventilation modes for weaning mechanical ventilation

M.A. Amezcua-Gutiérez, S.E. Zamora-Gómez, L.A. Gorordo-Delsol, J.O. Montoya-Rojo, I.M. Lima-Lucero, E.S. Montesinos-Cruz, J. Garduño-López, M. Hernández-Romero, M.A. Toledo-Rivera, M.A. Carrasco-Flores, G.D. Hernández-López, J.A. Castañón-González
Hospital Juárez de México, Intensive Care Unit, México City, Mexico
**Correspondence:** M.A. Amezcua-Gutiérez
*Intensive Care Medicine Experimental* 2017, **5(Suppl 2):**0755

**INTRODUCTION.** The weaning is a key element of mechanical ventilation (MV), occupying up to 50% of its duration. It's crucial to identify the right time to extubate, since re-intubation is associated with an increased risk infections, prolonged ICU-LOS, death and substantially increased costs. Innovative modes of MV, mainly based on complex closed loop, have been developed and are available for clinical use, therefore, it is necessary to compare them with conventional modes for weaning.

**OBJECTIVES.** Determine the relationship between success in weaning and the ventilatory modes used at the ICU of a thertiary hospital Mexico City.

**METHODS.** A retrospective analysis, during last 5 months of 2016, collected patients who received MV and were ready for weaning, randomly assigned to one MV mode for weaning: Pressure Support (PSV), Mandatory Minute Volume (MMV), Proportional Assist Ventilation (PAV) and Adaptive Support Ventilation (ASV). Standardized protocols were followed for each MV mode.

**RESULTS.** We collected 51 patients (28 males). The causes of MV were: acute respiratory failure (ARF) 27%, neurocritical care 24%, abdominal surgery 16%, thoracic surgery 10%, acute heart failure 10%, and other 14%. The distribution by mode of MV was PSV = 28, ASV = 10, PAV = 7 and MMV = 6. The average time of MV was 4.72, 3.1, 5.77 and 2.73 days respectively, with an overall average time of

4,05 days. Total patients per group, successful weaning, mean and variance in Table 193.

The analysis of variance show the sum of the squares = 4.5098, 3 degrees of freedom intergroups and 47 intragroups, through F-test we obtain F = 0.2616 and a ciritical value for F = 2.8023, and p = 0.8526, so there is no variation in the 4 ventilatory modes used in for weaning.

**CONCLUSIONS.** The PSV mode was the most used, similar to international literature. There is no significant difference between the 4 modes used. Nevertheless, the study regarding modes to facilitate weaning remains of interest, large sample are needed to elucidate the role of ventilatory modes, as well as to analyze other variables that may contribute to the success or failure of the weaning protocol.

**Table 193 (Abstract 0755)**. Weaning data

| Ventilation mode | Total patients | Succesful weaning | Mean | Variance |
|---|---|---|---|---|
| ASV | 10 | 9 | 0.9000 | 0.1000 |
| PAV | 7 | 6 | 0.8571 | 0.1429 |
| PSV | 28 | 25 | 0.8929 | 0.0992 |
| MMV | 6 | 6 | 1.0000 | 0.0000 |

#### 0756

#### Anxiety and dyspnea measured before, during and after tracheostomy mask weaning trials

L. Rose[1], M. Nonoyama[2], L. Istanboulian[3], I. Fraser[3]
[1]University of Toronto, Toronto, Canada; [2]UOIT, Oshawa, Canada; [3]Michael Garron Hospital, Toronto, Canada
**Correspondence:** L. Rose
*Intensive Care Medicine Experimental* 2017, **5(Suppl 2):**0756

**INTRODUCTION.** Anxiety is a commonly reported and distressing symptom recalled by ICU survivors in association with mechanical ventilation. Repeated weaning failure may contribute to worsening of anxiety. No studies have measured anxiety and dyspnea in patients experiencing progressive tracheostomy mask (TM) trials following repeated weaning failure.

**OBJECTIVES.** To quantify anxiety and dyspnea before, during, and after daily progressive TM weaning. Secondary objectives were to examine correlations between anxiety, dyspnea, and respiratory rate and to explore anxiety over time.

**METHODS.** We included patients admitted to a specialized weaning centre in Toronto, Canada. We excluded patients unable to rate anxiety due to impaired neurological status or inability to understand English. We measured anxiety (1–10 scale; 1 = no anxiety and 10 = extreme anxiety) at 3 time-points, (T1) immediately prior to TM trial commencement; (T2) 30 mins prior to anticipated completion; and (T3) 30 mins after completion on successive weaning days. At the same time-points we measured dyspnea using the modified Borg Scale and documented respiratory rate. Measures were repeated across the number of days to wean. Due to the large range in the number of weaning days, anxiety scores were averaged across days for each time point and analyzed using a two-way repeated ANOVA, with time as the within factor and days to wean (tertiles < 7 days, 7–16 days, >16 days) as the between factor. At each time point, we calculated Pearson's correlations between mean anxiety, dyspnea, and respiratory rate.

**RESULTS.** We recruited 21 participants (mean (SD) age 63 (12.6) years; 62% male) with a mean (SD) duration of mechanical ventilation of 84 (68.8) days prior to weaning centre admission. Participants received 2 to 58 days of TM trial weaning. Anxiety decreased over time from a mean (SD) score of 4.77 (1.59) (T1), to 4.55 (1.74) (T2) and 3.92 (1.71) (T3) (f = 9.25; p = 0.001); however, the pattern of change varied according to number of days to wean (f = 3.39; p = 0.01). Those weaned in < 7 days or 7–16 days had a large (4.40 to 3.33) or small (5.62 to 5.58) decline in anxiety from T1 to T2, respectively. Those who took >16 days had increased anxiety at T2 (4.22 to 4.76). Anxiety was highest at all three time points among those who took 7–16 days to wean. Anxiety at T2 was correlated with dyspnea measured at all three time-points (r = 0.46 to 0.58; each p < 0.05); respiratory rate at T2 only (r = 0.51; p = 0.02). Anxiety at T1 was not correlated with dyspnea or respiratory rate, while anxiety at T3 only related to dyspnea at T3 (r = 0.47; p = 0.04).

**CONCLUSIONS.** Overall, anxiety was moderate before, during, and after TM trial weaning. Anxiety decreased substantially during TM trials for those experiencing the shortest weaning duration and conversely increased during the trial for those experiencing the longest durations. Anxiety during a TM trial correlates with dyspnea.

**GRANT ACKNOWLEDGMENT**
Michael Garron Hospital

### Prognostication 2

#### 0757

#### An audit of the total parenteral nutrition branch of the magnificent seven care bundle created by the intensive care operational delivery network In the East of England - are we practicing what we preach? By dr alice webb

A.K. Webb, P. Bishop
Colchester General Hospital, Intensive Care, Colchester, United Kingdom
**Correspondence:** A.K. Webb
*Intensive Care Medicine Experimental* 2017, **5(Suppl 2):**0757

**INTRODUCTION.** The Magnificent Seven bundle is designed to ensure consistent, optimal and evidence-based care throughout the region. One of it's branches is on TPN and states: 'Parenteral nutrition should not be given to adequately nourished, critically ill patients in the first seven days of an ICU stay.' This is based on evidence (1,2,3) that showed that 'In patients who are adequately nourished prior to ICU admission, parental nutrition initiated within the first seven days has been associated with harm, or at best, no benefit, in terms of survival and length of stay in ICU. These findings are true even among patients who cannot tolerate enteral nutrition'.

**OBJECTIVES.** To assess our adherence to the TPN branch of the Magnificent Seven, to investigate any failings and to look at some basic outcomes to see they are seemingly resulting in harm to patients or excess cost.

**METHODS.** Retrospective audit of the year of 2016 for ICU patients who received TPN. This included
19 patients. Case notes were used to find: how long after admission to ICU TPN was started, duration of TPN, no. of bags of TPN, duration of hospital stay and ICU stay, in hospital mortality and 90 day mortality and reason for ICU admission.

**RESULTS.** There were a total of 19 patients, 6 of these patients were started on TPN after 7 days following admission (group A), 13 patients were started on TPN before 7 days (group B), 1 of which was clearly documented as being underweight prior to admission so was in line with guidelines.10/13 patients started against guidelines were post-operative from bowel surgery. Group B had a mean 37 total hospital days/person and median 30 days. Group A had a 72.3 mean total hospital days/person and a median 45.5. Group B had a mean 8.7 ICU days/person and median value of 7. Group A, 60.6 mean ICU days/person and a median of 35.5. In group B 2/13 died in hospital and 1/11 remaining died before 90 days after discharge. In group A 4/6 died during their stay, 0/2 remaining died before 90 days following discharge. In group B there was a mean of 13.5 TPN bags/person and a mean of 21.2 TPN bags/person in group A.

**CONCLUSIONS.** We are not adhering to our guidelines, the majority of these failings are in post-operative patients, and therefore we need to work with the surgical team to make an agreement on policy. Hospital/ITU stays, mortality, number of TPN bags used and duration of TPN was longer in group A, which may indicate the severity of illness of these patients, which I would argue would suggest a more genuine need for TPN rather than poor outcomes due to TPN. I noted that documentation of nutritional status in ICU was poor and I am planning on creating a brief nutritional assessment tool. I will re-audit in 9 months.

**REFERENCE(S)**

*1) Casaer MP, Mesotten D et al. N Eng J Med. 2011; 365: 506–17*

*2) Guidelines for nutrition support therapy in the adult critically ill patient, crit care med 2009; 37: 1757–61*

*3) Buzby GP. World J Surg 1993;17: 173–7*

### 0758

#### The use of Delta neutrophil index for predicting infectious mortality in critically ill surgical patients with Acinetobacter baumannii pneumonia: a case–control study

K. Kim, J. Jang, K. Bae

Wonju Severance Christian Hospital, Trauma Center, Wonju, Korea, Republic of

**Correspondence:** K. Kim

*Intensive Care Medicine Experimental* 2017, **5(Suppl 2):**0758

**INTRODUCTION.** *Acinetobacter baumannii* (AB) has become one of the most important nosocomial pathogens. AB was previously considered to have low virulence and colonization was considered to be more common than infection. However, recent increases in the incidences of multi-drug-resistant (MDR) and carbapenem resistant (CR) AB infections and in difficulties associated with their treatments pose serious medical problems. Studies on the correlation between the DNI with severity and infectious mortality in sepsis patients due to pneumonia are limited.

**OBJECTIVES.** The aim of this study was to evaluate the usefulness of the DNI as a predictor of infectious mortality in surgical patients with AB pneumonia.

**METHODS.** The medical records of 104 surgical patients with AB pneumonia treated from March 2011 to October 2014 were analyzed retrospectively.

**RESULTS.** The mean patient age was 60.8 ± 18.8 years, and the mean APACHE II score was 15 ± 5.3.At the time of culture, 18 patients (15.4%) had renal failure, and the median DNI was 2.7 (0–39.4) %. Twenty-four patients (23.1%) died from infection. Univariate analysis indicated that several factors were associated with infectious mortality, namely age, occurrence of shock, renal failure, low platelet count and elevated DNI at the time of culture. Logistic regression analysis revealed that elevated DNI (OR 1.136, 95% CI 1.001-1.288), acute renal failure (OR 3.811, 95% CI 1.025-14.176) and decreased platelet count (OR 0.994, 95% CI 0.989-1.000) at the time of culture are independent risk factors for infectious mortality. When a receiver-operating characteristics curve was constructed to determine the optimal cut-off value to predict infectious mortality within 7 days of the bacterial culture, the area under the curve was 0.839 (95% CI 0.694-0.985) and the cut-off DNI value was 6.85%.

**CONCLUSIONS.** DNI may be an effective predictor of infectious mortality in critically ill surgical patients with AB pneumonia.

**REFERENCE(S)**

1. Liu QQ, Li WZ, Du XM, et al. Risk and Prognostic Factors for Multidrug-Resistant Acinetobacter Baumannii Complex Bacteremia: A Retrospective Study in a Tertiary Hospital of West China. *Plos One.* 2015;10(6).

2. Kim SB, Min YH, Cheong JW, et al. Incidence and risk factors for carbapenem- and multidrug-resistant Acinetobacter baumannii bacteremia in hematopoietic stem cell transplantation recipients. *Scandinavian journal of infectious diseases.* 2014;46(2):81–88.

3. Park BH, Kang YA, Park MS, et al. Delta neutrophil index as an early marker of disease severity in critically ill patients with sepsis. *BMC infectious diseases.* 2011;11.

4. Seok Y, Choi JR, Kim J, et al. Delta Neutrophil Index: A Promising Diagnostic and Prognostic Marker for Sepsis. *Shock.* 2012;37(3):242–246.

**GRANT ACKNOWLEDGMENT**

We thank the trauma team and the staff of the trauma intensive care unit Wonju Severance Christian Hospital for their enthusiasm and commitment to patient care.

### 0759

#### Treatment intensity may not predict prognosis for patients admitted in ICU with relapsed acute myeloid leukemia

M. Guillot[1], M.P. Ledoux[2], T. Braun[1], Q. Maestraggi[1], B. Michard[1], V. Castelain[1], R. Herbrecht[2], F. Schneider[1]

[1]Hôpitaux Universitaires de Strasbourg, Réanimation Médicale, Strasbourg, France; [2]Hôpitaux Universitaires de Strasbourg, Hématologie, Strasbourg, France

**Correspondence:** M. Guillot

*Intensive Care Medicine Experimental* 2017, **5(Suppl 2):**0759

**INTRODUCTION.** Admission of cancer patients with poor prgnosis like relapsed acute myeloid leukemia (AML) continue to be controversial. The ICU trial may be an alternative in this case to ICU refusal.

**OBJECTIVES.** The objective of this study was to find variables to predict prognosis in ICU: invasive mechanical ventilation (IMV), vasopressor (VAS), dialysis (RRT).

**METHODS.** Retrospective monocentric study of consecutive patients with a relapsed AML admitted to the 30 beds medical ICU of an academic hospital between 2002 and 2014.

**RESULTS.** Between 2002 and 2014, 24 patients with relapsed AML were admitted in the ICU. At admission, patients were 54 years old, IGS 2: 64 ± 24, Lactates: 4.9 mmol/L (±4.7). Eight patients underwent bone marrow transplant (BMT). Five patients had graft-versus-host disease (GVHD). BMT was signicantly associated with higher mortality [Odds ratio (OR) 13.0 (95% confidence interval (95% CI) 1.7-99, 43)—p: 0.02]. 7 BMT patients died in ICU. Neutropenia [OR 0.33 (95% CI 0.05-1.87)—p: 0.4] and GVHD (OR 2.0 [95% CI 0.07- 51.6)—p: 1.0] were not able to predict mortality in ICU. Mortality in ICU was 37%. 4 patients died from acute illness before day 3. Among the 24 patients admitted in ICU, none of the life-sustaining interventions at admission were associated with higher mortality: invasive mechanical ventilation [OR 9.14 (95% CI 0.9-92.4) - p: 0.08], vasopressor perfusion [OR 6.2 (95% CI 0.6-62.2)—p: 0.18] and renal replacement therapy [OR 4.8 (95% CI 0.3-65.8)—p: 0.27]. On day 3, life supports were not associated with higher mortality: invasive mechanical ventilation [OR 3.7 (95% CI 0.32-41.1)—p: 0.35], vasopressor perfusion [OR 2.0 (95% CI 0.27-14.7)—p: 0.64] and renal replacement therapy [OR 1.2 (95% CI 0.08-16.45)—p: 1.0].

**CONCLUSIONS.** Mortality in ICU was 37% in patients with relapsed AML. In fact, temporary full-code ICU management in patients with relapsed AML seems to be appropriate. None of the life-sustaining interventions at admission and on day 3 were able to predict survival. An ICU trial of 3 days might not be enough to appraise precisely the outcome. In case of relapsed AML with BMT, ICU management is still challenging.

**REFERENCE(S)**

Azoulay E et al. Intensive care of the cancer patient: recent achievements and remaining challenges. Ann Intensive Care. 2011;1(1):5.

### 0760

#### Could central venous pressure predict mortality in severely ill septic patients? A pilot study

Y. El Basha[1], A. Fouad[1], S. Osman[1], W. Hemimy[1], I. Rasmy[1], H. Mostafa[1], A. El-adawy[1], A. Hasanin[1,2]

[1]Cairo University, Anesthesia and Critical Care Medicine, Cairo, Egypt; [2]Elameen Hospital, Critical Care, Taif, Saudi Arabia
**Correspondence:** I. Rasmy
*Intensive Care Medicine Experimental* 2017, **5(Suppl 2):**0760

**INTRODUCTION.** Although the role of central venous pressure (CVP) in fluid management is declining, CVP measurement is still essential in evaluation of hemodynamic state and cardiac efficiency. Higher CVP was previously reported to be associated with poor outcomes in septic patients.

**OBJECTIVES.** The aim of this work is to evaluate the baseline CVP as mortality predictor in critically ill septic patients with high severity scores.

**METHODS.** A pilot study was conducted including a cohort of severely ill septic patients with Acute Physiology and Chronic Health Evaluation (APACHE) II score above 20. All patients were mechanically ventilated and on norepinephrine infusion. Severity scores (APACHE II and SAPS II), demographic data, vital signs (heart rate and arterial blood pressure), CVP, serum albumin, and in-hospital mortality were reported. Area under receiver operating characteristic (AUROC) curve was calculated for all parameters for prediction of mortality. Sensitivity, specificity, and cutoff values were also determined for variables with AUROC > 0.6.

**RESULTS.** Among 23 patients aged 48 ± 12 years, median APACHE II score was 28 with interquartile range of (22–30). There were 15(65%) male patients and 6(26%) survivors. APACHE II showed good predictive properties for in-hospital mortality with AUROC of 0.73(0.47-0.991). Baseline CVP also showed good predictive properties for in-hospital mortality with AUROC of 0.721(0.503-0.938). A CVP value of 8 $cmH_2o$ could predict mortality by sensitivity of 94% and specificity of 33%. A CVP value above 12.5 $cmH_2o$ predicted mortality by sensitivity of 41% and specificity of 100%.

**CONCLUSIONS.** High CVP on ICU admission could predict in-hospital mortality in septic patients with high severity scores. Larger studies are recommended to confirm our findings with inclusion of more survivors.

### 0761

#### Evaluation of the feasibility and performance existing Early Warning Systems to identify patients at risk for adverse outcomes in low-middle income country setting

A. Beane[1], A.P. De Silva[2], N. De Silva[3], J.A. Sujeewa[3], R.M.D. Rathnayake[3], P.C. Sigera[1,2], P.L. Athapattu[4], D.G. Mahipala[4], A. Padeniya[5,6], A. Rashan[1], S. Munasinghe[1,2], K.A.S. Jayasinghe[7], A. Dondorp[8], R. Haniffa[1,7,8]
[1]Network for Improving Critical Care Systems and Training, Colombo, Sri Lanka; [2]National Intensive Care Surveillance, Ministry of Health, Colombo, Sri Lanka; [3]DGH Monargala, Monaragala, Sri Lanka; [4]Ministry of Health, Colombo, Sri Lanka; [5]Lady Ridgeway Hospital, Colombo, Sri Lanka; [6]Government Medical Officers's Association, Colombo, Sri Lanka; [7]Faculty of Medicine, University of Colombo, Colombo, Sri Lanka; [8]Mahidol Oxford Tropical Medicine Research Unit, Bangkok, Thailand
**Correspondence:** A. Beane
*Intensive Care Medicine Experimental* 2017, **5(Suppl 2):**0761

**INTRODUCTION.** Early Warning Systems (EWS) to identify hospital patients at risk for deterioration common in high-income countries (1, 2) have not been widely evaluated in resource-poor settings.

**OBJECTIVES.** This study aimed to assess the feasibility and performance of selected aggregate weighted track and trigger systems (AWTTS) and single parameter track and trigger systems (SPTTS) to discriminate patients at risk of adverse outcomes in a low-middle income setting; Sri Lanka.

**METHODS.** Available physiological variables, adverse outcomes and survival status at hospital discharge were extracted from existing records for all patients (age >17 years) admitted to District General Hospital (DGH), Monaragala over an 8 month period, where no EWS exists. Discrimination for selected AWTTS was assessed by the AUROC. The prognostic value of admission SPTTS, using available single and paired parameters, to predict death was evaluated.

**RESULTS.** Of the 16,386 patients included, 502 (3.06%) had 1 or more had adverse outcomes; 100 (0.64%) cardiac arrests; 83 (0.51%) unplanned admission to ICU; 253 (1.54%) transfers to tertiary facilities; and 149 (0.91%) deaths. Availability of physiological parameters on admission was systolic blood pressure 86.80% (CI 86.27, 87.31), heart rate 90.97% (CI 90.52, 91.40); oxygen saturation 23.94% (23.29, 24.60) and assessment of mentation (32.89 (32.17, 33.61). Best performing AWTTS and SPTTS had equally adequate ability to predict death when applied at admission. Discrimination of AWTTS was poor (AUROC < 0.60) in predicting other adverse outcomes.

| AWTTS | AUROC (CI) admission: Death patients | AUROC (CI) 24 hours: Death patients | AUROC (CI) admission: Patients with events | AUROC (CI) 24 hours: Patients with events |
|---|---|---|---|---|
| MEWS score with missing values imputed | 0.667 (0.615, 0.720) | 0.490 (0.423, 0.557) | 0.617 (0.591, 0.642) | 0.386 (0.355,0.416) |
| NEWS score with missing values imputed | 0.677 (0.624,0.731) | 0.583 (0.525, 0.641) | 0.602 (0.574,0.629) | 0.475 (0.450,0.501) |
| SEWS score missing values imputed | 0.702 (0.656, 0.748) | 0.599 (0.547,0.651) | 0.609 (0.585,0.633) | 0.510 (0.489,0.532) |
| CART score missing values imputed | 0.781 (0.744, 0.818) | 0.744 (0.699,0.740) | 0.636 (0.611,0.630) | 0.569 (0.541,0.596) |
| ViEWS score missing values imputed. | 0.677 (0.624, 0.730) | 0.585 (0.526,0.643) | 0.601 (0.574,0.629) | 0.476 (0.450,0.502) |

*[AWTTS and outcomes]*

**CONCLUSIONS.** There was poor availability of physiological variables used by EWS to detect patient deterioration. AWTTS and SPTTS have similar ability to predict mortality when applied on admission. Awareness of the importance of bedside observations as a way of alerting clinicians to patients at risk of avoidable adverse outcomes is needed. SPTTS may have a place in this setting as a trigger system to help identify patients on admission who may benefit from increased monitoring.

The authors wish to acknowledge the support received from consultants in charge of wards, DGH Monargala: Dr AC Weerarathne, Dr K Jayasuriya, Dr KCC Gamage, Dr HKSI Premaratne, Dr SSM Kariyawasam and the team of facilitators: N Dullewe, L Pieris, CS Suraweera, ET Jagoda and Dr MF Miskin.

**REFERENCE(S)**

1. Hillman K. Introduction of the medical emergency team (MET) system: A cluster-randomised controlled trial. Lancet. 2005;365:2091–7.
2. Patterson C, Maclean F, Bell C, Mukherjee E, Bryan L, Woodcock T, et al. Early warning systems in the UK: variation in content and implementation strategy has implications for a NHS early warning system. Clin Med (Northfield Il) [Internet]. 2011 Oct 1;11(5):424–7.

**GRANT ACKNOWLEDGMENT**

This study was supported by National Science Foundation, Sri Lanka (Grant Number: RG/2015/HS/02), Mahidol Oxford Tropical Medicine Research Unit (MORU) and Ministry of Health, Sri Lanka.

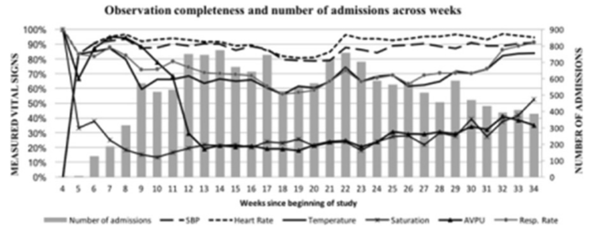

**Fig. 258 (Abstract 0761)** Availability of observations

### 0762

#### Utility of the SOFA score to predict the hospital mortality after intensive care unit discharge

R. Vela-Colmenero[1], M.I. Ruiz-Garcia[1], V. Chica-Saez[1], J.M. Rodriguez-Roldan[2], R. Montoiro-Allue[3], D. Iglesias-Posadilla[4], M. Redondo-Orts[1], S. Rodriguez-Fernandez[5], J.E. Barrueco-Francioni[6]
[1]Intensive Care Unit - Complejo Hospitalario, Jaen, Spain; [2]Intensive Care Unit - Hospital Universitario Severo Ochoa, Madrid, Spain; [3]Intensive Care Unit - Hospital Clínico Universitario Lozano Blesa, Zaragoza, Spain; [4]Intensive Care Unit - Hospital Universitario, Burgos, Spain; [5]Intensive Care Unit - Hospital Serrania, Ronda, Spain; [6]Intensive Care Unit - Hospital Arnau de Vilanova, Valencia, Spain
**Correspondence:** R. Vela-Colmenero
*Intensive Care Medicine Experimental* 2017, **5(Suppl 2):**0762

**INTRODUCTION.** About one third of hospital mortality in critically ill patients occurs after Intensive Care Unit (ICU) discharge.

**OBJECTIVES.** To evaluate the utility of SOFA score at last day of ICU stay to predict ward mortality after ICU discharge.

**METHODS.** Multicentric prospective study in 7 hospitals in Spain. We collected periods of two or three months during 2011, 2012 and 2015. Data were expressed as mean and standard deviation for quantitative variables and percentages for qualitative variables. For the comparison of two means we used the Student's t-test and the chi- squared test was used to compare proportions. Multiple logistic regression and area under the ROC curve for analizing discrimination were used.

**RESULTS.** 1934 patients with a mean age of 61.22 ± 15.58 years, SAPS-3 45.26 ± 13.68 points. Predicted hospital mortality by SAPS 3 was 17% and observed hospital mortality was 15%.ICU mortality was 10% and mortality after ICU discharge (Ward mortality) was 5.6%.

1731 patients were transferred to Ward hospitalization area, with mortality of 5.6% (97 patients). Mean age of 60.66 ± 15.62 years, SAPS 3 at ICU admission 43.06 ± 11.86 points and SOFA score at the last day of 0.91 ± 1.61 points.

Ward mortality of patients with Medical pathology (754 cases) was 6.5%, elective surgery (801 cases) 5.5% and emergency surgery (173 cases) of 2.3%(p = 0.185).

Patients who died after ICU discharge were older (65.72 ± 14.61 vs. 60.36 ± 15.63 years) (p < 0.001), higher SAPS-3 score at ICU admission (54.06 ± 13.07 vs 42.4 ± 11.43) (p < 0.001) and higher SOFA score at last day of ICU stay (2.8 ± 3.21 vs 0.9 ± 1.61) (p < 0.001).

SOFA score at the last ICU day: 1050 patients (60.8%) with 0 points and Ward mortality of 2.4%, 297 patients (17.2%) with 1 point and mortality rate of 4.4%, with 2 points (N = 159, 9.2%) and mortality of 8.2%, 96 patients (5.6%) with 3 points and mortality of 17.7%, and 124 patients (7.2%) with 4 or more points and mortality of 22.6% (p < 0.001).

Discrimination capacity evaluated by SOFA score at the last day of ICU stay was 0.74 (0.68-0.8) and by SAPS-3 at ICU admission was 0.75(0.68-0.80).

There is complementarity between both. Multivariate analysis indicates that conventional hospitalization area mortality after ICU discharge is related by SAPS-3 at admission (OR 1.06(1.04-1.08)) and SOFA score at the last day of ICU stay (OR 1.36 (1.23-1.50)). Discrimination of this model by Area under the ROC curve:0.79(0.74-0.84).

**CONCLUSIONS.** Mortality of ICU patients after ICU discharge is related by SOFA score at last day of ICU stay, severity illness at admission by SAPS-3 score. Both variables are complementary to predict conventional hospitalization area mortality after ICU discharge.

### 0763

#### Evaluation of prognostic scoring systems in patients with cirrhosis admitted to an intensive care unit

M.C. Rebelo[1], T. Alves[2], A.G. Almeida[1], T. Lamas[1], I. Gaspar[1], I. Simões[1], E. Carmo[1]
[1]Centro Hospitalar de Lisboa Ocidental- Hospital Egas Moniz, Unidade de Cuidados Intensivos Polivalente, Lisbon, Portugal; [2]Centro Hospitalar de Médio Tejo, Torres Novas, Portugal
**Correspondence:** M.C. Rebelo
*Intensive Care Medicine Experimental* 2017, **5(Suppl 2):**0763

**INTRODUCTION.** Critically ill cirrhotic patients have high mortality rates, particularly when they present with acute kidney injury (AKI) on admission. Careful and evidence-informed identification, management and prognostication of cirrhotic patients at risk of developing multiple organ dysfunction are a high priority. However, there isn't still a consensus regarding which are the best and reproducible prognostic models in critically ill cirrhotic on admission to the ICU.

**OBJECTIVES.** The aim of the present study is to assess the accuracy of specific prognostic models [Acute Physiology and Chronic Health Evaluation (APACHE) II, Simplified Acute Physiology Score (SAPS) II, Acute Kidney Injury Network (AKIN), Model for End-stage Liver Disease (MELD) and Child-Pugh-Turcotte (CPT) in predicting mortality in cirrhotic patients admitted to a medical Intensive Care Unit (ICU).

**METHODS.** We retrospectively reviewed medical records of 72 cirrhotic patients admitted to an ICU in a Tertiary Care University Hospital from January 2001 to December 2016. Demographic, clinical and laboratory data recorded on the first day of ICU admission and scoring systems applied (SAPSII, MELD, APACHEII and CPT) were collected. Some of the scores not recorded at admission (AKIN) were calculated based on the medical records. The discriminatory capacity of the different scores were evaluated using areas under the receiver operating characteristic curve (AUROC), posteriorly the areas were compared using DeLong et al. (1988) method.

**RESULTS.** In our study population, 72% were male patients, average: 60 ± 13 years. The ICU mortality was 52.8%. The main causes of cirrhosis were alcoholism (70.8%), followed by Hepatitis C (20.8%). The major cause of ICU admission was septic shock (38.9%), followed by hypovolemic shock (36.1%). In this study, 87.5% patients needed organ support at admission (renal replacement therapy: 47.2%, mechanical ventilation: 70.8% and vasoactive therapy: 73.6%).

By using AUROC, the SAPSII score had a good discriminative power (AUROC: 0.92, IC 95%: 0.83-0.97), followed by AKIN (AUROC: 0.85, IC 95%: 0,745 to 0,923), MELD (AUROC: 0.83, IC 95%: 0,72 to 0,91), all p < 0.05.

In Pairwise comparison of ROC curves, SAPSII presented the best discriminatory power when compared with CPT (p < 0.001) and APACHE (p < 0.020).

**CONCLUSIONS.** The SAPSII, AKIN, MELD and APACHE scores showed well discriminative power in predicting ICU mortality in this group of patients. The SAPSII scoring system proved to be a reproducible evaluation tool with excellent prognostic abilities for these patients. The AKIN classification is also a simple and easily applied evaluative tool. Since it is difficult to predict outcome in cirrhotic ICU patients, a combination of prognostic scores such as SAPS and AKIN could be used as complementary tools during the difficult decision-making process on whether to withdraw or pursue care for cirrhotic patients in an ICU.

### 0764

#### Simplifying cardiac surgery risk assessment

M. Pérez Cheng[1], J.M. González Santos[1], P. Pabón Osuna[1], J.L. Vicente Villardón[2], C.A. Nariño Molano[1], V. Sagrado Meneses[1]
[1]Complejo Asistencial Universitario de Salamanca, Salamanca, Spain; [2]Universidad de Salamanca, Salamanca, Spain
**Correspondence:** M. Pérez Cheng
*Intensive Care Medicine Experimental* 2017, **5(Suppl 2):**0764

**INTRODUCTION.** Cardiac surgery is the field where risk assessment has developed the most, designing increasingly complex models with different statistical techniques.

**OBJECTIVES.** This study is aimed to design a simplified cardiac surgery risk score based in the number of risk factors of each patient to evaluate operative mortality in our patients. This is defined as inhospital mortality or mortality by 30 days after the operation for patients discharged from the hospital.

**METHODS.** Data of all patients undergone heart surgery at the University Hospital of Salamanca, Spain, between 2001 and 2016 were collected. The data set was divided into two subsets: a developmental one for modeling and the other, validation subset, for model testing. Results were compared with EuroSCORE II. Accuracy was defined with a receiver-operating characteristics operator and calibration with a Hosmer-Lemeshow test. Data was analyzed with SPSS v.22 and MedCalc v.13.

**RESULTS.** In the developmental subset we included 3950 patients who had undergone cardiac surgery between 2001 and 2012, with 6.7% mortality. We identified predictors of mortality with $x^2$ and t-Student tests ($p < 0.05$) and recode the variables in "presence" or "absence" of each risk factor. We used the Youden index to determine the age and creatinine cut point. Logistic regression was used to establish the independent variables associated with mortality. These variables were female sex, age greater than 71 years, extracardiac arteriopathy, active endocarditis, NYHA class IV, left ventricle ejection fraction lower than 30%, pulmonary artery pressure greater than 55 mmHg, critical preoperative state, emergency operation, serum creatinine greater than 1.32 mg/dl, isolated CABG and three or more procedures. Only 591 patients (15%) didn't present any risk factor and none of themhad more than nine. We do not include isolated CABG since it appears as a "protector" factor in the logistic regression analysis. $x^2$ test was used to evaluate if the number of risk factor was related with mortality. Observed mortality increased with more risk factors.The validation series included 1263 patients between 2013 and 2016. Discrimination, assessed with the ROC curve, was good with our model (AUC 0.81, CI 0.76-0.86) and also with EuroSCORE II (AUC 0.80, CI 0.76-0.85), but EuroSCORE II was poor calibrated in the validation data with a significant Hosmer-Lemeshow test ($x^2$ 17.90, $p$: 0.02).

**CONCLUSIONS.** Using a model that only considers the number of risk factors without taking into account the weigh of each variable, is statistically consistent. Complex models don't assure good calibration and discrimination and are less user friendly.

**REFERENCE(S)**

Ghavidel AA, Javadikasgari H, Maleki M, Karbassi A, Omrani G, Noohi F. Two new mathematical models for prediction of early mortality risk in coronary artery bypass graft surgery. *J. Thorac. Cardiovasc. Surg*. 2014;148(4):1291–8 e1.

### 0765

#### Performance of prognostic severity scores in patients with community acquired pneumonia admitted to ICU

W.J. Jung[1], J.Y. Jung[2], M.S. Park[2], Y.S. Kim[2], K.S. Jung[2]

[1]Korea University Anam Hospital, Seoul, Korea, Republic of; [2]Yonsei University College of Medicine, Seoul, Korea, Republic of

**Correspondence:** W.J. Jung

*Intensive Care Medicine Experimental* 2017, **5(Suppl 2):**0765

**INTRODUCTION.** There are limited data on the performances of pneumonia severity scores and intensive care unit (ICU) scores to predict mortality in community-acquired pneumonia (CAP) and Healthcare-associated pneumonia (HCAP) patients hospitalized to ICU.

**OBJECTIVES.** The purpose of this study is to evaluate the performance of the pneumonia severity index (PSI), CURB-65 (confusion, urea, respiratory rate, blood pressure, age $\geq$ 65) score, and Acute Physiology and Chronic Health Evaluation (APACH) II score for the prediction of mortality in non-nosocomial pneumonia patients admitted to ICU.

**METHODS.** We retrospectively evaluated 162 patients admitted to ICU with non-nosocomial pneumonia between 1 January 2008 and 31 December 2011. The performances of PSI and CURB 65, APACHE II score were evaluated for mortality prediction.

**RESULTS.** Of total 162 patients, 76 (46.9%) patients died in hospital. Mean age of patients is 68.1 years and 112(69.1%) are men. 128 (79%) patients had ventilator care. PSI and APACHE II were significantly lower in survivors than in non-survivors (145.2 vs. 166.1, $P < 0.001$; 20.6 vs. 29.8, $P < 0.001$), but CURB-65 was not (2.4 vs. 2.7, $P = 0.075$). Receiver operating characteristic curves showed that APACHE II (area under the curve [AUC] = 0.79) performed better than PSI (AUC = 0.67, $P < 0.016$) and CURB-65 (AUC = 0.58, $P < 0.001$) to predict in-hospital mortality.

**CONCLUSIONS.** APACH II predicted mortality of non-nosocomial pneumonia patients requiring ICU admission better than PSI and CURB-65 do.

**REFERENCE(S)**

Restrepo, M. I., Mortensen, E. M., Velez, J. A., Frei, C. & Anzueto, A. A comparative study of community-acquired pneumonia patients admitted to the ward and the ICU. *Chest*. **133**, 610–7 (2008).

Mandell, L. A. *et al*. Infectious Diseases Society of America/American Thoracic Society consensus guidelines on the management of community-acquired pneumonia in adults. *Clin Infect Dis*. **44**, S27-72 (2007).

Knaus WA, Draper EA, Wagner DP, Zimmerman JE: APACHE II: A severity of disease classification system. Crit Care Med 1985, 13: 818–829

**GRANT ACKNOWLEDGMENT**

We declare that they have no conflict of interest.

### 0766

#### Hypocalcaemia is an important indicator of severity in critical

Q. Zhang

Burn Center of Ruijin Hospital, School of Medicine, Shanghai Jiao Tong University, Shanghai, China

*Intensive Care Medicine Experimental* 2017, **5(Suppl 2):**0766

**OBJECTIVES.** This research studied the importance as an indicator of hypocalcaemia in model of extensive burn patients which the capillary leakage occurred then calcium banded with albumin can leak to interstitial space. Then we try to find an pritical tool to evaluation the severity of leakage of capillary in critical ill.

**METHODS.** This is a retrospective study to study the influence factor resulted in hypocalcaemia in extensive patients in initial phase post-burn when the capillary bed emerged a typical leakage situation. The patients enrolled in this study should have a Burn Surface area was larger than 50%TBSA, time of admission < 24 hrs post-burn injury and the age of the patients were older than eighteen years old. We applied Chinese fluid resuscitation formula continuous infused fluid contained colloidal solution in initial phase post-extensive burn.

**RESULTS.** A total 142 cases treated in our burn center from January 2010 to July 2015 enrolled.

The average initial calcium of all studying case was 1.87 ± 0.20mmol/l (normal value ranged from 2.25 to 2.75). 97.2%extensive burn patients in our center suffered from hypocalcaemia and 36.6% of them were severe hypocalcaemia (lower than 1.8 or 1.75). From dynamic tendency of calcium of this study we could find: average calcium decreased in first three days then rebounded steady. The calcium change in survival group was similar to the overall performance. But the calcium in no-survival group decreased sharply. The lowest level in this group could be found in second day post-burn with respect to lowest level was three in survival group. In rebounding phase in no-survival group, we could find a twisted curve, up and down. Firstly we found the calcium between survival and no-survival group are significant different. But we did not found the difference in kaplan-meier survival curves between two groups. The factors influence the mortality is not calcium, but the age of the patients, the area of full-thickness burn wound and inhalation injury. Then we found: total burn area, full-thickness burn area and length of time from injury to admission are the risk factors result in severe hypocalcaemia. Then we compared pH value in different group of total burn area, full-thickness burn area and length of time from injury to admission. We found: in groups of larger total burn area, larger full-thickness burn area and longer length of admission groups, the initial pHs are lower than the control groups. In this study, we found that serum albumin level in larger total burn area, larger full-thickness burn area and longer length of admission groups are lower than in control groups.

As a result, the group of patients in severe hypocalcaemia had high incidence of morbidity of longer mechanical ventilation and stay in ICU.

**CONCLUSIONS.** Calcium level could be applied as a tool to evaluate the severity of leakage of capillary in critical patients.

### 0767

#### Severity scoring systems are less valid in patients with long ICU stay

A. Hasanin[1,2], H. Mostafa[1], S. Habib[1], Y. Hammad[1], A. El-Adawy[1], A. Lotfy[1], A. Kamal[1], A. Mukhtar[1]
[1]Cairo University, Anesthesia and Critical Care Medicine, Cairo, Egypt; [2]Elameen Hospital, Taif, Saudi Arabia
**Correspondence:** S. Habib
*Intensive Care Medicine Experimental* 2017, **5(Suppl 2):**0767

**INTRODUCTION.** Severity scoring systems are increasingly used for benchmarking in intensive care unit (ICU). Acute physiology and chronic health evaluation (APAHCE) scoring system and simplified acute physiology score (SAPS) are two famous systems used for benchmarking. Both APACHE and SAPS scores are calculated from first admission day data; thus, we hypothesized that the accuracy of these scores for mortality prediction would be lower in patients with longer length of stay (LOS).

**OBJECTIVES.** To evaluate the accuracy of APACHE II, APACHE IV, and SAPS scoring systems in patients with different categories of LOS.

**METHODS.** A retrospective study was conducted in surgical and trauma ICU at Cairo university hospitals including patients admitted from June 2013 till December 2016. Patients were divided into three groups: Two-day-stay group (LOS ≤ 2 days), Five-day-stay group (LOS ≤ 5 days), and Long-stay-group (LOS > 5 days). Receiver operating characteristic (ROC) curves were constructed for APACHE II, APACHE IV, and SAPS II scores as mortality predictors in the three groups.

**RESULTS.** The data of 1812 patients was available for analysis. Median length of stay was 5 days with interquartile range of (2–10) days. The incidence of mortality was 47% (860 patients). Five-day-stay group included 1028(57%) patients; whereas Two-day-stay group included 308 (17%) patients and long-stay-group included 476(26%) patients. Area under ROC (AUROC) curve for APACHE IV, APACHE II, and SAPS II scores as mortality predictors was highest in Two-day-stay group {0.883(0.854-0.911), 0.877(0.848-0.906), and 0.870(0.839-0.900)} followed by Five-day-stay group {0.745(0.702-0.787), 0.747(0.704-0.790), and 0.716(0.670-0.761)}, then Long-stay-group {0.637(0.598-0.676), 0.614(0.575-0.653), and 0.603(0.563-0.642)} (P value < 0.001).

**CONCLUSIONS.** Validity of Scoring systems decreases with longer LOS. We recommend further adjustment for these scoring systems according to LOS.

### 0768

#### Scores to predict liver graft disfunction. Which is the best?

M.E. Herrera-Gutiérrez[1], M.D. Arias-Verdu[1], J. Barrueco-Francioni[1], I. De Dios-Chacón[2], G. Quesada-García[1], G. Seller-Pérez[1]
[1]Hospital Regional Universitario Carlos Haya de Málaga, Intensive Care Unit, Málaga, Spain; [2]Hospital Son Llatzer, Intensive Care Unit, Palma de Mallorca, Spain
**Correspondence:** M.D. Arias-Verdu
*Intensive Care Medicine Experimental* 2017, **5(Suppl 2):**0768

**OBJECTIVE.** There are different diagnostic scores for assessing graft dysfunction (GD) after liver transplantation. We want to determine if there are differences between scores to assess liver GD and their ability to predict prognosis and mortality.

**METHODS.** Prospective cohort of patients who received liver transplant (LT) performed over the period 2009–2014, follow up until the end of 2016. We compared classic criteria (CC) of GD against Makowska (MK), Ardite (Ar) Nanashima (Nn), Dhillon (Gh) and MEAF. We analyzed hospital stay and mortality. Data: mean (mean error) and median (quartiles). Non-parametric tests: Chi-square, Kappa and Log-Rank for p < 0.05. Approved by Ethical committee.

**RESULTS.** N = 253. 76.3% male. Mean age 54.7 years. Ethanol etiology 45.8%, Mean MELD 16.36 (0.43), SOFA on admission 6.53 (0.19). Median days in ICU 3 (3–5) and in hospital 8 (6–13). ICU mortality 4.7%, in Ward 2.8% and at discharge 22.1%.

Severe GD was detected in 13.8% with CC, Dh 30.8%, Nn 20.6% MEAF 13.4%, MK 6.3% and Ar 0.7% and concordance with CC was low; Kappa for Ar 0.49 (0.47-0.50), Nn 0.55 (0.39-0.68), Mk 0.46 (0.29-0.64), GDh 0.44 (0, 32–0.56) and MEAF 0.41 (0.250-0.58).

Logistic regression showed that ICU stay and mortality was related with CC (OR 5.85 (4.47-56.18), MEAF (OR 11.09 (3.29-37.41), AR (OR 7.11 (2, 08–24.29), Nn (OR 8.96 (2.58-31.07), Mk (OR 9.54 (2.51-36.19) and Gh (OR 4.88 (1.43-16, 75), but after ICU discharge only MEAF was related to hospital mortality. Regarding survival, only CC (p 0.034) and MEAF (p 0.006) showed significant relationship.

**CONCLUSIONS.** Although all dysfunction scores had a goog relation with hospital mortality and therefore apparently assessed well GD, the MEAF showed the closest relationship and was related to the long-term prognosis, so its use may have an advantage over the others diagnostic scores.

### 0769

#### Usefulness of quick sepsis-related organ failure assesment to predict severity in influenza A H1N1v pneumonia patients

A. Estella[1], F. Valenzuela[1], M. Recuerda[1], J.F. Rodriguez Gutierrez[2], L. Perez Fontaiña[1]
[1]Hospital SAS Jerez, Unidad de Gestión Clínica de Medicina Intensiva Hospital del SAS de Jerez, Jerez, Spain; [2]Hospital SAS Jerez, Unidad de Gestión Clínica de Hematología Hospital del SAS de Jerez, Jerez, Spain
**Correspondence:** A. Estella
*Intensive Care Medicine Experimental* 2017, **5(Suppl 2):**0769

**INTRODUCTION.** In 2016 has been published quick Sepsis-related Organ Failure (qSOFA) like a new screening system for detection of septic patients with suspected infection outside ICU. Usefulness of this score may be to identify bedside patients and rapidly life-threatening patients.

**OBJECTIVES.** The aim of the present study is to analyze the usefulness of the application of qSOFA outside the ICU in the estimation of the risk of patients with Influenza A H1 N1 v pneumonia.

**METHODS.** Retrospective study performed in a 17-bed ICU. Inclusion criteria were patients admitted in emergency department with a microbiological confirmed pneumonia caused by Influenza A H1 N1 v. The qSOFA scale was by a retrospective analyses of clinical data at admission in the emergency room department. We analyzed clinical features, destination: ICU versus hospitalization wards and mortality. Statistical analysis was performed using SPSS, v.18 program.

**RESULTS.** 24 patients were included, five patients were admitted in medical wards and 19 in ICU requiring mechanical ventilation most of them (78.9%). Mortality was 21.1%. Only 10.5% of patients admitted to the ICU had a qSOFA with a score of 2. 57.9% had qSOFA scores of 1 and 31.6% of 0. Tachypnea of more than 22 breaths per minute was the most common clinical sign in the group of patients admitted in ICU, 68.42%. Majority of patients admitted in medical wards (80%) obtained a qSOFA score of 0, 20% had a qSOFA of 1. Only a 50% of died patients had a qSOFA score of 2 points, the remaining deceased patients obtained a qSOFA score of 0 points.

**CONCLUSIONS.** Use of qSOFA score in the emergency department may underestimate the risk of patients with influenza A H1N1 v pneumonia.

Tachypnea was the most altered qSOFA score parameter in severe patients with Influenza A pneumonia H1 N1 v.

### 0770

#### Venoarterial difference of carbon dioxide as positive predictor of mortality in patients with septic shock in comparison with other approaches

G. Merinos-Sánchez[1], M.A. Morales-Segura[2], A. Domínguez-Maza[3], C.C. Calzada-Mendoza[2], J.J. Acevedo-Mariles[4], A. López-Gómez[5], L.A. Gorordo-Delsol[6]
[1]Hospital General de México 'Dr. Eduardo Liceaga', Emergency Medicine Department, México City, Mexico; [2]Instituto Politécnico Nacional, Escuela Superior de Medicina, México City, Mexico; [3]Hospital General 'Dr. Manuel Gea González', Respiratory Critical Care Unit, México City, Mexico; [4]Hospital General 'Dr. Manuel Gea González', Emergency and Internal Medicine Division, México City, Mexico; [5]Hospital General 'Dr. Manuel Gea González', Emergency Medicine Department, México City, Mexico; [6]Hospital Juárez de México, Intensive Care Unit, México City, Mexico
**Correspondence:** G. Merinos-Sánchez
*Intensive Care Medicine Experimental* 2017, **5(Suppl 2):**0770

**INTRODUCTION.** A persistently high veno-arterial carbon dioxide ($\Delta PCO_2$) difference is related to dysoxia and increased multiple organ dysfunction in patients with septic shock, variations in $\Delta PCO_2$ have shown an independent behavior from macrocirculation variables or from products of oxygen metabolism, turning $\Delta PCO_2$ into a marker that identifies patients at high risk of death in septic shock. Although there are predictors of mortality such as SOFA, SAPS II, APACHE II and MPM II, which provide important information on severity but require multiple data (demographic, clinical and biochemical) to be calculated.

**OBJECTIVES.** To demonstrate that $\Delta PCO_2$ is a better predictor of mortality in patients with septic shock compared to APACHE II, SOFA, SAPS II, MPM II.

**METHODS.** We performed an observational, retrospective study of patients who were hospitalized in the Emergency Department of a tertiary level hospitalin México City during the first 10 months of 2014, with diagnosis of septic shock.

**RESULTS.** We reviewed 47 files, the highest mortality occurred in males (53.2%) from 49 to 58 years. The most frequent diagnosis was pulmonary sepsis (23.4%). The positive predictive value (PPV) for $\Delta PCO_2$ was 87%, with a specificity of 80%, only overcome by APACHE II and MPMII, however, there was no statistically significant difference between the use of both scales to predict mortality with respect to $\Delta PCO_2$ p = 0.052 and p = 0.56 respectively. A p = 0.006 was observed in the medians test for the $\Delta PCO_2$ and MPM II scales. Sensitivity, sp...

Kruskal-Wallis test for analysis of several scales comparing $\Delta PCO_2$ with APACHE II, shows no statistically significant (p = 0.052), with SAPS II (p = 0.70), as did SOFA (p = 0.030), and MPMII (p = 0.56).

When performing the concordance analysis between each scale and mortality, we found that by means of Kendall´s Tau-b for ΔPCO2 there is a significant association (p = 0.009), APACHE II (p = 0.001), SAPSII (p = 0.02) and SOFA (p = 0.019).

| Scale | Sensitivity | Specificity | Positive predictive value | Negative predictive value |
|---|---|---|---|---|
| ΔPCO2 | 62 | 80 | 87 | 50 |
| APACHE II | 50 | 86 | 88 | 44 |
| SOFA | 53 | 80 | 85 | 44 |
| SAPS II | 50 | 80 | 84 | 42 |
| MPM II | 71 | 86 | 92 | 59 |

*[Sensitivity, specificity and predictive values]*

**CONCLUSIONS.** The venous $\Delta PCO_2$ only obtained a better PPV compared to SOFA and SAPS II, while it behaved similarly with APACHEII and MPMII; Exceeded all scales with 92% of PPV for mortality

**GRANT ACKNOWLEDGMENT**
None

### 0771

#### Hepatitis C virus infection impact on post acute myocardial infarction mortality-a 12-year data

S.-H. Kuo[1], W.-T. Hung[1], C.-C. Cheng[1,2,3], P.-L. Tang[1], W.-C. Huang[1,2,3], S.-C. Lin[1], K.-C. Lin[1], J.-S. Yang[2], C.-H. Chiang[1], F.-Y. Kuo[1], C.-C. Hung[1], H.-C. Lin[1], Y.-S. Chen[1], G.-Y. Mar[1], H.-T. Chang[1], C.-P. Liu[1,3]
[1]Kaohsiung Vaterans General Hospital, Critical Care Center and Cardiovascular Medical Center, Kaohsiung City, Taiwan, Province of China; [2]Fooyin University, Department of Physical Therapy, Kaohsiung, Taiwan, Province of China; [3]National Yang-Ming University, School of Medicine, Taipei, Taiwan, Province of China
**Correspondence:** S.-H. Kuo
*Intensive Care Medicine Experimental* 2017, **5(Suppl 2):**0771

**INTRODUCTION.** Acute myocardial infarction is one of the leading causes of death worldwide despite advances in revascularization and medications. Therefore, assessing the risk factors of outcomes after AMI remains an important research topic. HCV infection was hypothesized as a contributing risk factor of endothelial dysfunction, atherosclerosis,[1]

and CAD,[2] with the association in between remains controversial.[3]

**OBJECTIVES.** The present study aimed to analyze the impact of HCV infection on 12-year mortality after AMI using Taiwan National Health Insurance Research Database (NHIRD).

**METHODS.** Approximately 23,000,000 cases diagnosed with AMI between January 2000 and December 2012 were identified from the NHIRD and finally leaves 186,112 cases by exclusion criteria.

Then 4,659 with HCV infection without interferon therapy were enrolled and further divided into those with liver cirrhosis (n = 107) and those without (n = 4,552). Propensity score-matching technique was applied by o*ne-to-one matching using the background* variables. The data from 4,552 AMI patients with HCV infection but without liver cirrhosis and 4,552 matched controls were included in our final analysis. For the outcome analysis, survival was defined as the time interval from the hospital admission date to the NHI coverage end date.4. RESULTSThe 12-year mortality rate was significantly higher in AMI patients with HCV and cirrhosis than HCV without cirrhosis or controls (P < .0001). The patients with HCV without cirrhosis had significantly higher long-term mortality rates than the matched controls (P < .0001).

The hazard ratio (HR) for mortality was higher in patients with HCV without cirrhosis (HR: 1.09; 95% CI: 1.04-1.15) and those with HCV and cirrhosis (HR: 2.23; 95% CI: 1.82-2.73). HCV influenced outcomes among the subgroups of whom were male (HR: 1.06), were younger (HR: 1.27), had hypertension (HR: 1.10), had dyslipidemia (HR: 1.19), or received percutaneous coronary intervention (HR: 1.20)

**CONCLUSIONS.** HCV infection was demonstrated to influence the 12-year mortality of patients after AMI in this study. Additionally, the mortality rate was higher among the patients with HCV and liver cirrhosis, and also long-term outcomes in patients after AMI among the subgroups of male patients; younger patients; those with hypertension; those with dyslipidemia; and those who underwent PCI.

without antiviral treatment: A cohort study from an endemic area, Int. J. Cardiol. 181 (2015) 27–29.
[3] R.J. Wong, F. Kanwal, Z.M. Younossi, A. Ahmed, Hepatitis C virus infection and coronary artery disease risk: a systematic review of the literature, Dig. Dis. Sci. 59 (2014) 1586–1593.

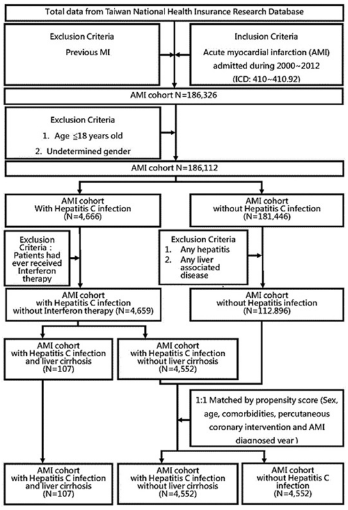

**Fig. 259 (Abstract 0771)** Inclusion–exclusion Flow Chart

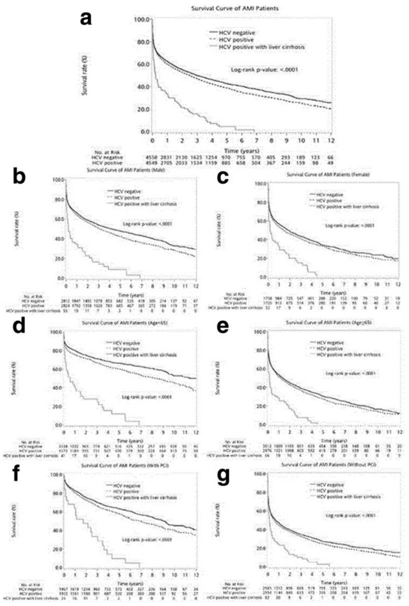

**Fig. 260 (Abstract 0771)** Kaplan-Meier Survival Curve

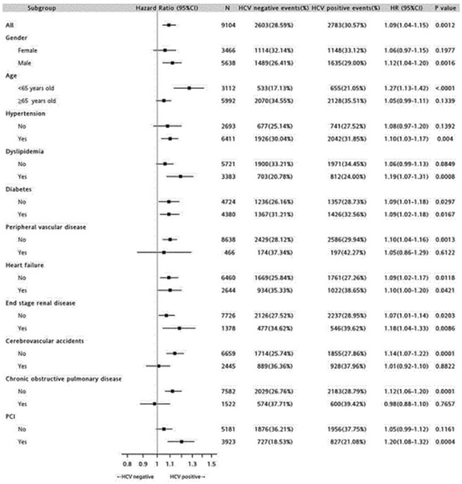

**Fig. 261 (Abstract 0771)** Forest plot evaluating the impact of HCV

### Challenges in neurointensive care

#### 0772

#### Impact of CT scan utilization on surgical management and outcome in patients with severe traumatic brain injury at a tertiary care referral hospital in Tanzania

H. Schenck[1], X. Wu[2], L.M. Gerber[2], H. Shabani[3], M.M. Santos[3], J. Ngerageza[3], P. McClelland[4], E. Kim[4], P.E. Stieg[4], R. Härtl[4], H.S. Mangat[5]
[1]Maastricht University Medical Centre+, Maastricht, Netherlands; [2]Weil Cornell University, Healthcare Policy & Research, New York, United States; [3]Muhimbili Orthopaedic Institute, Neurological Surgery, Dar-es-Salam, Tanzania, United Republic of; [4]Weil Cornell University, Neurological Surgery, New York, United States; [5]Weil Cornell University, Neurology, New York, United States
**Correspondence:** H. Schenck
*Intensive Care Medicine Experimental* 2017, **5(Suppl 2):**0772

**INTRODUCTION.** Traumatic brain injury (TBI) is a leading cause of death and disability in the developing world. Guidelines-based management is limited in low-middle income countries by resource availability, utilization and cost[1.] Computed tomography (CT) is particularly expensive, not consistently available, yet forms the core of surgical management of severe TBI patients. There is a lack of data on the pattern of utilization of CT in TBI patients in resource-limited settings in East Africa,[2] while in other developing countries it is significantly inderutilized[3]. Therefore, an impact assessment of CT use in the management of severe TBI patients is necessary to develop culturally competent evidence-based protocols.

**OBJECTIVES.** We evaluated the utilization of CT scan in the management of severe TBI and its impact on surgical treatment and 2-week patient mortality at a tertiary care referral hospital in Dar-es-Salaam, Tanzania.

**METHODS.** This is a retrospective analysis of prospectively collected data of all severe TBI patients admitted to Muhimbili Orthopaedic Institute, Dar-es-Salaam, Tanzania, 2014–2017. The Brain Trauma Foundation TBI-trac® database was implemented locally and utilized for this project. Epidemiological and treatment data was exported in an anonymized manner and analyzed.

**RESULTS.** 253 patients with severe TBI were enrolled. Average age was 33.65years, 87% were male, median initial GCS was 7, and overall mortality was 56.1%. From the 44.7% patients who had at least one brain CT scan: 92% had post-traumatic abnormalities,

35.5% underwent surgery and 46.5% died. Of the sub-group of patients who had a post-traumatic abnormality on CT, 36.6% were operated and 51.4% died. From the group of patients who did not undergo a CT scan on admission, only 12.2% underwent surgery and 66% died.

**CONCLUSIONS.** The majority of patients did not undergo brain CT after severe TBI at our tertiary care hospital with a high overall 2-week mortality. Patients who underwent a cranial CT had a higher surgical rate and lower mortality than patients that did not have a CT on admission. The surgical mortality of severe TBI patients, with or without an admission CT is still high.. We are currently exploring obstacles in routinely obtaining CT, and factors determining CT-based surgical intervention in these patients in order to help develop an evidence-based approach to lowering mortality after severe TBI.

**REFERENCE(S)**

1. Ohaegbulam et al. Surg Neurol Int 2011; 2:182
2. Ogbaide E, et al. Sahl Med J 2015;18:27–30
3. Harris OA et al. J Neurosurg 2008; 109:433–38.

### 0773

#### Cerebral blood flow dynamics during the acute course after severe subarachnoid hemorrhage studied by bedside Xenon-enhanced CT

H. Engquist[1,2], E. Rostami[1], P. Enblad[1]
[1]Uppsala University, Neuroscience, Neurosurgery, Uppsala, Sweden; [2]Uppsala University, Surgical Sciences, Anesthesia and Intensive Care, Uppsala, Sweden
**Correspondence:** H. Engquist
*Intensive Care Medicine Experimental* 2017, **5(Suppl 2):**0773

**INTRODUCTION.** The dynamics of cerebral blood flow (CBF) disturbances after subarachnoid hemorrhage (SAH) and the relation to delayed cerebral ischemia (DCI) remains poorly elucidated.

**OBJECTIVES.** The aim of the present study was to assess global and regional CBF in different phases of the acute course after severe SAH, hypothesizing that CBF disturbances are more pronounced at day 4–7 compared to day 0–3 and that there is a gradual restitution of CBF at day 8–12. The influence of HHH-therapy on CBF in patients with DCI was also addressed.

**METHODS.** Patients diagnosed with SAH and requiring mechanical ventilation due to their neurological state were prospectively enrolled in the study. Bedside measurements of regional CBF were scheduled for day 0–3, 4–7 and 8–12, using Xenon-enhanced CT (XeCT). Patients with clinical suspicion of DCI receiving HHH-therapy during their course after SAH were grouped accordingly to allow analysis of the influence of DCI and HHH on CBF. Regional CBF was characterized by the proportion of area with local blood flow below specified thresholds and by blood flow in the worst vascular territory in each patient.

**RESULTS.** During 2013–2016, a total of 216 valid XeCT-measurements were performed in 119 SAH-patients. Fifty patients had measurements at both baseline (day 0–3) *and* day 4–7; in non-DCI patients (n = 36) global cortical CBF decreased from 33.7 (IQR 27.5-45.4) to 31.7 (IQR 25.3-38.3) ml/100g/min (*P = 0.028), whereas DCI-patients receiving HHH-therapy (n = 14) showed an increase from 31.9 (IQR 23.0-35.0) to 38.8 (IQR 28.4-42.6) ml/100g/min (*P = 0.009). Regional CBF in the worst vascular territory followed a similar pattern for the respective groups. Thirty-three patients had measurements at baseline *and* day 8–12; no statistically significant differences in CBF-parameters compared to baseline could be observed in neither non-DCI patients nor DCI patients receiving HHH in this late phase.

**CONCLUSIONS.** Our study revealed different patterns in CBF-dynamics of poor grade SAH patients depending on the occurrence of DCI and subsequent HHH-therapy. In non-DCI patients there was a decrease in CBF at day 4–7 compared to baseline, whereas CBF increased significantly from a lower baseline level in DCI patients receiving HHH-therapy. At day 8–12 no significant differences in CBF parameters compared to baseline were found in either of the groups.

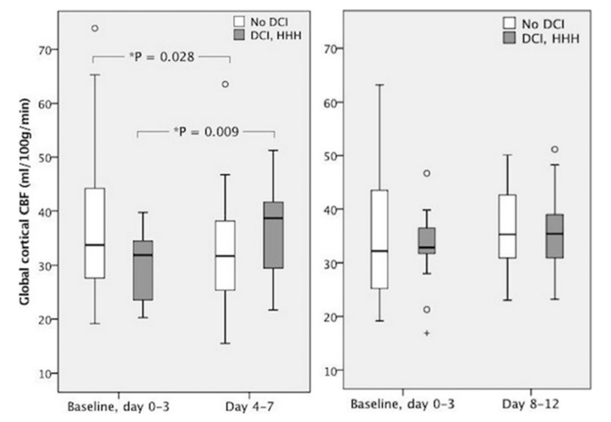

**Fig. 262 (Abstract 0773).** Global cortical CBF at day 4–7 and 8–12 after SAH

### 0774

#### One year mortality after intravenous fibrinolysis for acute ischemic stroke

R. Assis[1,2], A. Araújo[1], N. Lopes[1], L. Pessoa[1], T. Pereira[1], N. Catorze[1]
[1]Centro Hospitalar do Médio Tejo, Intensive Care Unit, Abrantes, Portugal; [2]Centro Hospitalar do Médio Tejo, Internal Medicine Department, Abrantes, Portugal
**Correspondence:** R. Assis
*Intensive Care Medicine Experimental* 2017, **5(Suppl 2):**0774

**INTRODUCTION.** Ischemic Stroke is the leading cause of death amongst Portuguese Population. The existent pre Hospital triage and quick reference to a Stroke Unit, allied to the early recognition of acute neurological signs has contributed to the increasing numbers of effective fibrinolysis in Acute Ischemic Stroke Patients, decreasing morbidity and mortality. However, there are no studies evaluating late outcomes of this population after treatment.

**OBJECTIVES.** The objective of the study is to evaluate mortality and its cause of patients at 3 months, 6 months and 12 months after treatment with Intravenous Fibrinolysis with Alteplase at the researchers' Intensive Care Unit and characterize this population.

**METHODS.** Observational retrospective study based on information retrieved from the Clinical Records of patients admitted in the Intensive Care Unit for Intravenous Fibrinolysis between the 1st of January 2010 and 31st of December 2015, and its statistical analysis.

**RESULTS.** Among the total of patients included (n = 102), 63% were male with an average of 70 years old. The average admission National Institutes of Health Stroke Scale (NIHSS) score was 14 and the average Symptoms-to-Needle time was 156 minutes. There were a total of 23 deaths occurring in the first 3 months after treatment. Of these, 4 were due to Intraparenchymal Hemorrhage after Fibrinolysis, 7 to ineffectiveness of Thrombolythic treatment, 10 to Aspiration Pneumonia, 1 to Septic Shock, and 1 patient died at home, with no reference to cause of death on Clinical Records. Between 3 months and 6 months occured two deaths, caused by Septic Shock in patients with established Limitation of Treatment. Between 6 and 12 months there were no registered deaths.

**CONCLUSIONS.** With the State of The Art Organization and Protocols, Mortality due to to Ischemic Stroke has reduced. The vast majority of deaths occurred in the first 3 months after treatment. Patients who died within 3 months had an average NIHSS stroke of 20 at 12 hours after Fibrinolysis, similar to those who died between 3 and 6 months. Patients who survived the first 12 months, had a lower NIHSS score at 12 hours (7). Although Intraparenchymal Hemorrhage and unresponsiveness to treatment are unavoidable, causes such as Aspiration Pneumonia, which occurred after hospital discharge must be avoided. The focus must shift to the optimization of post-Hospital care in order to help patients recover after stroke and minimize complications which may worsen their condition and lead to death, especially in the first trimester. This would preferably be done by multidisciplinary teams involving Neurology, Internal Medicine and Physiotherapy support and adequate infrastructures and response time.

**REFERENCE(S)**

Brown D., Al-Senani, et al.; Defining Cause of Death in Stroke Patients; Am J Epidemiol 2007;165:591–59

Vernino S., Brown R. D., et al.; Cause-Specific Mortality After First Cerebral Infarction, A Population-Based Study; Stroke. 2003; 34:1828–1832

### 0775

#### The intensive care unit experience after the implementation of a stroke system of care in the autonomous community of Madrid (SPAIN)

N. Martinez Sanz, S. Alcantara Carmona, J.M. Mateos Pérez, P. Matía Almudevar, L. Pérez Pérez, A. Pérez Lucendo, A. Naharro Abellán, J. Veganzones Ramos, P. Rodriguez Villamizar

Hospital Universitario Puerta de Hierro Majadahonda, Madrid, Spain

**Correspondence:** N. Martinez Sanz

*Intensive Care Medicine Experimental* 2017, **5(Suppl 2):**0775

**INTRODUCTION.** Although intravenous thrombolysis (IVT) remains the standard of care in acute ischemic stroke (AIS), in selected patients, endovascular therapies (ET) can improve recanalization and functional outcomes. Because both treatments are limited by a narrow window of time, stroke systems of care (SSC) are necessary. In 2013 a SSC was implemented in Madrid and Hospital Universitario Puerta de Hierro de Majadahonda (HUPH) became a primary stroke center of care, patients were admitted to the ICU for initial management.

**OBJECTIVE.** To analyze patients admitted in our ICU with the diagnosis of AIS and their evolution since the implementation of the SSC.

**METHODS.** Descriptive and retrospective study (October 2013-September 2016). Patients admitted to ICU with an AIS diagnosis who received treatment with IVT and/or ET were studied. Demographic data, severity scores [APACHE II and baseline and at ICU discharge NIHHS (National Institute of Health Stroke Scale)], vascular territory involved, arrival from other hospitals, timelines, ICU complications, ICU and hospital stays and mortalities, destination upon discharge, and overall three months mortality were recorded. Statistical analysis: STATA (v. 14.2).

**RESULTS.** 171 patients were treated for IAS in the period studied: 54 (31.6%) received treatment with IVT and 117 (68.4%) with ET. Table 194 describes the main characteristics of AIS patients. 74 (43.3%) patients came from other hospitals (5 in the ITV group and 69 in the ET group). AIS location, treatment received and timelines are on Table 195.

ICU complications involved 39 patients (23%), being the most common: infections and neurological. Median ICU length of stay was 1 day (1–2) and 9 days (4–13) in-hospital, with a mortality of 13 (7.6%) and 20 (11.7%), respectively. The patients were discharged: home 67 (39.2%), referal hospital 63 (36.8%) and chronic facilities 21 (12.3%). Overall three months mortality was 25 (14.6%).

**CONCLUSIONS.** Acute ischemic stroke was a common disease that concerned slightly more to males, usually with more than one comorbidity. Patients had a short ICU stay with low mortality and complications. In general, patients experienced a neurological improvement upon discharge from the ICU and most of them were discharged to home or referal hospital but some needed longer stays in chronic facilities.

In our experience, implementation of a stroke system of care is feasible. Nevertheless, coordination between different hospitals and healthcare networks and also primary stroke centers with endovascular experience are required to achieve good outcomes. It would be desireable to find new strategies to shorten times.

**Table 194 (Abstract 0775)**. See text for description

| Baseline demographics and clinical characteristics | |
|---|---|
| Age (years | 69 (58 - 78) |
| Sex (male) | 95 (55.6) |
| Hypertension | 107 (62.6) |
| Diabelles mellitus | 37 (21.6) |
| Hyperlipidemia | 74 (43.3) |
| Obesity | 21 (12.3) |
| Smoking | 68 (39.8) |
| Atrial fibrillation | 44 (25.7) |
| COPD | 14 (8.2) |
| Coronary artery disease | 23 (13.4) |
| Previous stroke (ischemic/ hemorrhagic) | 22 (12.9) |
| APACHE II | 11 (8 - 14) |
| NIHSS (baseline) | 16 (11 - 20) |
| NIHSS (discharge ICU) | 6.5 (2 - 14) |

Data are shown as absolute values (%) or as median (q25 – q75). COPD: Chronic obstructive Pulmonary disease. NIHHS: National Institute of Health Stroke Scale

**Table 195 (Abstract 0775)**. See text for description

| Acute isquemic stroke location, treatment and timelines | | |
|---|---|---|
| Vascular territory involved | LMCA: 77 (45.03)<br>RMCA: 57 (33.3)<br>Basilar: 8 (4.7)<br>ACA: 3 (1.8)<br>ICA: 8 (4.7)<br>Multiple location: 17 (9.9)<br>No stop: 1 (0.6) | |
| Treatment | IVT: 54 (31.6)<br>IVT + ET: 65 (38)<br>ET: 52 (30.4) | |
| Timelines (min) | Onset to hospital:<br>Onset to HUPH:<br>Symptoms to thrombolysis:<br>Arrival to puncture:<br>Puncture to reperfusion:<br>Onset to reperfusion: | 90 (65 – 125)<br>147 (85 – 240)<br>130 (110 – 165)<br>65 (45 – 115)<br>61 (40 – 94)<br>313 (273 – 390) |

Data are shown as absolute value (%) or as median (q25 – q75). LMCA: left middle cerebral artery, RMCA: right middle cerebral artery, ACA: anterior cerebral artery, ICA: internal carotid artery, IVT (intravenous thrombolysis), ET: enovascular treatment, HUPH: Hospital Universitario Puerta de Hierro.

### 0776

#### External ventricular drain-related infections in neurocritical care patients: a retrospective cohort study

M. Rojas-Lora[1], C. Cabellos-Minguez[2], I. Zabaleta-Carvajal[3], E.P. Plata-Menchaca[4], C. Lerma-Briansó[5], V. Fuentes-Mila[5], A. Martínez-Pascual[5], I. Romera-Peregrina[5], R. Contreras-Medina[5], N.D. Toapanta-Gaibor[5], J. Sabater-Riera[5], R. Máñez-Mendiluce[5], L. Corral-Ansa[5]

[1]Hospital Universitario de Bellvitge, Critical Care Medicine, L´Hospitalet de Llobregat, Spain; [2]Hospital Universitario de Bellvitge, Infectious Disases, L'Hospitalet de Llobregat, Spain; [3]Hospital Universitario de Bellvitge, Neurosurgery, L´Hopitalet de Llobregat, Spain; [4]Instituto de Investigación Biomédica de Bellvitge, Research, L'Hospitalet de Llobregat, Spain; [5]Hospital Universitario de Bellvitge, Critical Care, L'Hospitalet de Llobregat, Spain

**Correspondence:** M. Rojas-Lora

*Intensive Care Medicine Experimental* 2017, **5(Suppl 2):**0776

**INTRODUCTION.** External ventricular drain infection (EVDI) is associated with increased morbidity and mortality.

**OBJECTIVES.** The aim of this study was to describe the clinical characteristics of patients requiring placement of an external ventricular drain admitted to a third-level hospital, and to identificate risk factors for acquiring EVDI.

**METHODS.** A retrospective cohort study was performed from January 2015 to December 2016, which included 100 patients admitted to a third-level hospital in Barcelona, Spain. Patients included were those who required insertion of an external ventricular drain catheter (EVDC). EVDI was defined as a positive culture of cerebrospinal fluid with biochemical and cytology abnormalities. Clinical and laboratory data were registered. Follow-up was conducted until hospital discharge. We assessed short-term outcomes as well.

**RESULTS.** A total of 100 patients required EVDC placement during study period. Mean age of the sample was 53 years ± 14; 58% of patients were women. Admission diagnosis was subarachnoid hemorrhage in 43% of patients. The main EVDC placement indications were obstructive hydrocephaly (58%) and intraventricular hemorrhage (29%). Median duration of EVDC placement was 8.5 (4–14) days. Median of Glasgow Coma Scale (GCS) was 14 (9–15) and 10 (7–14) days at hospital and critical care unit (CCU) admission, respectively. Overall in-hospital mortality was 24%, being the admission diagnosis the most frequent cause of death. A total of 17 patients (17%) presented EVDI. The most frequent microbiological isolations were: *Enterobacter spp., Staphyloccoccus epidermidis* and *Pseudomonas aeruginosa* (23.52%, 17% and 17%, respectively). Patients who acquired an EVDI had a higher CCU and hospital length-of-stay (LOS) than non-infected EVDC patients [25 (10–44) vs 6 (1–21) days, p = 0.001; and [55 (38–98) vs 31 (16–58), p = 0.006, respectively]. A bivariate analysis was performed to analyze risk factors associated with EVDI. Previous craniotomy and EDVC-related complications (EVDC misplacement and the presence of intracranial hematoma located in EVDC path) were factors significantly associated with EVDI.

**CONCLUSIONS.** EVDI are a frequent entity in neurocritical care patients. Main factors associated with acquisition of an EVDI are previous craniotomy, EVDC misplacement and the presence of intracranial hematoma located in EVDC path. CCU and hospital LOS of EVDI patients were significantly higher than patients without EVDI.

**REFERENCE(S)**

1. Tunkel AR, Hasbun R, Bhimraj A, Byers K, Kaplan SL, Scheld WM, et al. Infectious Diseases Society of America's Clinical Practice Guidelines for Healthcare-Associated Ventriculitis and Meningitis. *CID* 2017; 00:1–32.

2. Ramanan M, Lipman J, Shorr A, Shankar A. A meta-analysis of ventriculostomy-associated cerebrospinal fluid infections. *BMC Infect Dis* 2015;15:3.

### 0777

#### Relationship between postoperative blood pressure lability and delirium in neurosurgical patients

R. Badenes[1], M.L. Gracía-Pérez[1], M.E. Del Río[1], B. Monleón[1], F.S. Taccone[2], F. Bilotta[3]

[1]Hospital Clinic Universitari de Valencia, Anesthesiology and Surgical-Trauma Intensive Care, Valencia, Spain; [2]Hôpital Erasme, Université Libre de Bruxelles., Brussels, Belgium; [3]Sapienza University of Rome, Rome, Italy

**Correspondence:** R. Badenes

*Intensive Care Medicine Experimental* 2017, **5(Suppl 2):**0777

**INTRODUCTION.** Postoperative delirium (PD) is one of the most common complications following surgery with a reported incidence ranging from 10-70% depending on the type of surgery. One possible area of intervention to prevent postoperative delirium (PD) is postoperative blood pressure management. However, the relationship between postoperative blood pressure and PD is unclear.

**OBJECTIVES.** The goal of this study was to test the hypothesis that postoperative fluctuations in blood pressure increased the occurrence of postoperative delirium in patients undergoing neurosurgery.

**METHODS.** Study subjects were undergoing neurosurgery, who were enrolled in an ongoing prospective observational study of the pathophysiology of postoperative delirium. Postoperative blood pressure was measured and predefined criteria were used to define hypotension. Delirium was measured by the Confusion Assessment Method on the first two postoperative days.

Fluctuation in a patient's blood pressure was quantified by calculating the variance of the patient's blood pressure record during ICU. Variance is a measure of the data spread. Blood pressure fluctuation was calculated according to the formula: variance = $(\text{å}_{i=1}^{n} (x_i - x_m)^2/n - 1$, where $x_i$ is a patient's blood pressure at a particular time point, $x_m$ is the mean of the patient's blood pressure, and n is the number of blood pressure measurements.

Patients were categorized based on whether they developed PD. We analysed continuous data that were normally distributed by Student's t-test and continuous data that were not normally distributed by Mann–Whitney analysis. The analysis of normal distribution was performed by the Kolmogorov-Smirnov test. Dichotomous data were compared with the Fisher exact test. We used backwards stepwise multivariate logistic regression by including those univariate variables that differed between patients with and without PD, with a P-value of < 0.1.

**RESULTS.** PD was observed in 45 (9.2%) patients. Mechanical ventilation for < 48 h [odds ratio (OR), 3.94; 95% confidence interval (CI), 1.72-9.03], postoperative blood pressure variance (OR, 3.0; 95% CI, 1.29-6.96), prior stroke (OR, 2.79; 95% CI, 1.12-6.96), and age (per year of age; OR, 1.01; 95% CI,1.01-1.07) were independently associated with PD.

It´s the first study that found that increased fluctuations in blood pressure in neurosurgical patients to be predictive of PD.

**CONCLUSIONS.** Postoperative fluctuations in blood pressure increased the occurrence of PD in patients undergoing neurosurgery. Maintaining blood pressure at a stable level, based on preoperative values, appears to help preventing PD.

### 0778

#### Diffuse axonal injury, epidemiology and functional prognosis in short and medium term

A. Touceda, M. Segura, I. Gallego, E.M. Menor, M.D. Rivas, D. Mosquera

Hospital Alvaro Cunqueiro, Intensive Care, Vigo, Spain

**Correspondence:** A. Touceda

*Intensive Care Medicine Experimental* 2017, **5(Suppl 2):**0778

**INTRODUCTION.** Diffuse axonal injury (DAI) constitutes a pathology with a low incidence, but with catastrophic results for the patient and society. There are very few studies in the literature that review its epidemiology and the prognosis of these patients.

**OBJECTIVES.** To analyze the clinical characteristics of patients with TBI and DAI confirmed in MRI, as well as their prognosis and functional assessment in the short and medium term.

**METHODS.** Retrospective, descriptive study of patients suffering from TBI, admitted in ICU, over a four-year period (November 2011-November 2015).

**RESULTS.** A total of 257 patients were included, 176 men (68.5%) and 81 women (31.5%). Of these, 108 were mild TBIs (42%), 56 moderate (21.8%) and 93 severe (36.2%). The most frequent mechanism was accidental falls (36.6%) followed by motorcycle traffic injury (16%).

DAI was suspected with the initial CT in 21 cases, rising to 26 in patients admitted with poor neurological evolution, although it could be only confirmed by MRI in 13 of them (5.1%). The majority were severe, grade III (61.5%). As in the global TBI, it was more frequent in men (69.2%), although the mean age was lower (42.3 +/– 21.8 years) and secondary to high energy injuries, mainly car crashes (30.8%). Length of stay in ICU (28.3 +/– 22.2 days) and total hospital admission (52.7 +/– 43.5 days) were also longer.

In 76.9% of the cases were severe TBI (10 patients), and were classified radiologically on a Marshal Scale of 2 (53.8%). The overall mortality with TBI was 28.1%, according to the APACHE II score at admission (mean 15.6 +/– 8.5). However, mortality in the subgroup of patients with DAI was lower, only in 7.7%, in those with radiological confirmation of the lesion, probably due to a selection bias, failing to consider those patients in whom If there was an initial suspicion and no MRI was performed (12 patients), which had a mortality rate of 66.7%.
DAI was associated with a poor functional prognosis (assessed according to the GOS scale) at medium (6 months) and long term (more than 1 year), GOS ≤ 3 in 53.8% and 50% of cases respectively, with significant statistic association regarding the global of traumatic brain injuries, which is also corroborated in a longer stay in ICU and hospitalization.
**CONCLUSIONS.** DAI is still a challenge from a diagnostic and therapeutic point of view within patients suffering from TBI, with a significant functional repercussion for the patient, severe deficits and major dependence in many cases, as well as important costs for the health system, due to prolonged stays in intensive care and hospital units, as we demonstrated in our series.
Although the mortality observed in these patients is low, we believe that it may be explained by the underdiagnosis of LAD, either because of a low suspicion in the context of TBI, or because of the initial severity of the lesion and the short-term poor prognosis that prevents radiological confirmation in many cases.

### 0779

#### Non-convulsive status epilepticus: an under-recognised cause of coma

P. Matía Almudévar[1], S. Alcántara Carmona[1], A. Naharro Abellán[1], J. Veganzones Ramos[1], A. Pérez Lucendo[1], L. Pérez Pérez[1], P. Rodríguez Villamizar[1], J. Palamidessi Domínguez[1], N. Martínez Sanz[1], B. Lobo Valbuena[2], M. Valdivia de la Fuente[1]
[1]Hospital Universitario Puerta de Hierro Majadahonda, Majadahonda, Spain; [2]Hospital Universitario del Henares, Coslada, Spain
**Correspondence:** P. Matía Almudévar
*Intensive Care Medicine Experimental* 2017, **5(Suppl 2):**0779

**INTRODUCTION.** The absence of clinical manifestations of non-convulsive status epilepticus (NCSE) makes it a challenging diagnosis amongst ICU comatose patients. For this reason NCSE is frequently poorly recognised and subsequently an under treated cause of persistent altered mental status in the ICU, which can lead to long-term impairment.
**OBJECTIVES.** To describe our experience in the recognition of NCSE together with risk factors associated to its appearance, treatment strategies used and evolution of this group of patients.
**METHODS.** Retrospective and observational study (Jan 2012 - Dec 2016) carried out in the medical ICU of a Spanish tertiary university hospital. We reviewed every EEG performed in our ICU in the period studied. Patients with anoxic encephalopathy following cardiac arrest were excluded.
**RESULTS.** During the period studied 310 patients underwent EEG monitoring, and 10 NCSE were identified, six of them during the last twelve months. Demographic data are described in Table 196.
All of our patients had at least one risk factor for developing NCSE. In six of them reason for ICU admission was a central nervous system insult and other two had suffered a previous brain injury. Upon the time of NCSE diagnosis five were being treated with beta-lactam antibiotics and three had metabolic disturbances.
Regarding NCSE treatment, the initial antiepileptic drug (AED) of choice for all patients was levetiracetam. Seven of them required the addition of a second AED for seizure control: lacosamide (5) and continuous intravenous (CIV) valproate (2). Three patients had refractory status epilepticus in spite of the use of two AED so they also received treatment with phenytoin (1), IV clonazepam (1) or CIV valproate (1). Until control with AED of NCSE was assured all patients were treated with concomitant intravenous anesthetic drugs [IAVD: propofol + remifentanil (9) or propofol + remifentanil + midazolam (1)] between EEGs.
Mean time from diagnose to complete seizure control without IVAD was 4 ± 2 days. The fact that EEG registration is only available during working hours (8 am to 3 pm; Monday to Friday) should be taken into account. Two of our patients had a relapse during AED tapering. In two cases, limitation of life sustaining treatment was decided before resolution of NCSE.
In terms of prognosis, four patients died in the ICU and two more within the next sixty days after ICU discharge.
**CONCLUSIONS.** Physicians in our ICU are starting to think more about the possibility of underlying NCSE as a cause of unexplained coma.
The appearance of NCSE in our series was related with the presence of at least one risk factor.
In our study treatment of NCSE was challenging and most patients required at least two AED.
EEG follow-up is crucial to determine treatment success.
NCSE patients had a high ICU and short-term mortality.

**Table 196 (Abstract 0779).** See text for description

| Demographic data | |
|---|---|
| Age | 67 ± 10 |
| Male | 6 |
| Mean APACHE II score | 22 ± 7 |
| Diagnose upon admission: | |
| CNS acute insult | 5 |
| Altered mental status | 3 |
| Sepsis | 2 |
| Median SOFA score at diagnose | 6 (3-11) |
| Median time from admission to NCSE(days) | 2 (0-11) |

*CNS: central nervous system; SOFA: Sequential Organ Failure Assessment; APACHE II: Acute Physiology And Chronic Health Evaluation II; NCSE: Non-convulsive Status Epilepticus*

### 0780

#### Experience in management of status epilepticus in an intensive care unit (Medical ICU) in a secondary-level university hospital

J.L. Martinez Melgar[1], E. Moreno Lopez[2], E. Sanmartin Mantiñan[1], A. Ortega Montes[1], I. Cenoz Osinaga[1], J. Bravo Doviso[1], T. Sanchez de Dios[1], A. Pais Almozara[1]
[1]Complexo Hospitalario Universitario de Pontevedra, Intensive Care Unit, Pontevedra, Spain; [2]Complexo Hospitalario Universitario de Ferrol, Anesthesiology and Postoperative Care, Ferrol, Spain
**Correspondence:** E. Moreno Lopez
*Intensive Care Medicine Experimental* 2017, **5(Suppl 2):**0780

**INTRODUCTION.** To analyze our experience in the management of patients admitted to our Unit (medical ICU- tertiary university hospital) with the diagnosis of epileptic seizures and/or epilepsy in a six-year period (2010–2015).
**METHODS.** Descriptive and retrospective study, carried out during a six-year period, of patients admitted to our Unit with the diagnosis of epileptic seizures and/or epilepsy, according to criteria for inclusion in Minimum Basic Data Set (MBDS). The following parameters were recorded: age, gender, source of admission to the ICU, past medical history, reason for admission, classification, diagnostic methods, monitoring, antiepileptic drugs (AEDs) and control of status, Patients with epileptic seizures after anoxic enchepalopathy were excluded. Statistical analysis: quantitative variables are expressed as the mean and standard deviation (SD), and qualitative variables as a percentage. The difference between qualitative variables are expressed using chi-square test and quantitative using an ANOVA analysis.

**RESULTS.** We analyzed 51 patients (36 male), mean age:67 ± 18 years old, source of ICU admission: emergency 16 patients, neurology 18, other departments 4 and another hospital 13. Past medical history: epilepsy 17, CTE 7, alcoholism 8, psychiatric pathology 6. Diagnosis: structural pathology 10, epilepsy 16 (de novo 2, recurrence 14), CTE 5, toxic-alcoholism 6, infection 5, limbic encephalitis 4, metabolic 3 and prion disease 2. Diagnostic methods: EEG 100%, bispectral index (BIS) unilateral 72,5% y bilateral 27,5%, head CT scan 100% (with contrast 66,7%, without 33,3%), brain MRI 35%, others 9%. EEG recorded. < 4 : 35%, 4–6: 12% and >6: 12%. EEG pattern: spike-and-wave:59%, polispikes:42%, mixed: 35%, others: 9%. AEDs (second line) used: phenytoin 82%, valproic 64%, levetiracetam 34%, lacosamide 18%, combination (<2 AEDs):48%, and combination (>3 AEDs:) 35%; AEDs (third line): midazolam (MDZ) 82%, propofol (PF) 18%, MDZ + PF 64%, phenobarbital 19%; AEDs (fourth line): corticosteroids 6%, immunoglobulins 9%, plasmapheresis 6%. control of status epilepticus: established (>24hours):16%, refractory(24-72hours):23%, super refractory (>72hours):12%.

**CONCLUSIONS.** In our series (data collected according to MBDS) 47% of the patients admitted to our Unit with the diagnosis of epileptic seizures and/or epilepsy needed treatment for status, being the most common causes: relapse of known epilepsy and cerebrovascular disease respectively. The most frequent EEG pattern was spike-and-wave (59%) with a mean number of EEG recorded of 4–6 in 53% of the cases. In terms of AEDs (second line) used, pheniytoin and valproic were administered in 88 and 69% of the cases compared with AEDs (third line) used MDZ and PF in 84% and 49% of the cases respectively.

### 0781

#### Development and validation of the abbreviated cognitive failure questionnaire in intensive care unit patients: a multi center cohort study

A. Wassenaar[1], J. de Reus[1], R. Donders[2], L. Schoonhoven[3,4], A. Slooter[5], D. van Dijk[5], P. Pickkers[1,6], M. van den Boogaard[1]

[1]Radboud University Medical Center, Department of Intensive Care Medicine, Nijmegen, Netherlands; [2]Radboud University Medical Center, Department for Health Evidence, Nijmegen, Netherlands; [3]University of Southampton, Faculty of Health Sciences, Southampton, United Kingdom; [4]Radboud University Medical Center, Scientific Institute for Quality of Healthcare, Nijmegen, Netherlands; [5]University Medical Centre Utrecht, Department of Intensive Care Medicine, Utrecht, Netherlands; [6]Radboud University Medical Center, Radboud Center for Infectious Diseases, Nijmegen, Netherlands

**Correspondence:** A. Wassenaar

*Intensive Care Medicine Experimental* 2017, **5(Suppl 2):**0781

**INTRODUCTION.** Intensive Care Unit (ICU) survivors often develop physical, mental, and cognitive impairments following ICU discharge described as Post Intensive Care Syndrome (PICS). In particular long-term cognitive impairment is a growing public health problem, as it occurs in up to 62% of the ICU survivors. Despite its frequent occurrence, post-ICU cognitive impairment remains inadequately characterized. Post-ICU cognitive impairment leads to everyday cognitive failures that negatively influence daily routine and HRQOL. The Cognitive Failures Questionnaire (CFQ) is the most widely used and broadest measure in terms of covered domains of failures. Although the CFQ is used in previous research to measure cognitive functioning in ICU survivors, this questionnaire has never been validated for that purpose. Given the multifaceted nature of PICS and the decreased capacity of ICU patients, developing and validating an optimal short form of the CFQ for ICU patients is desirable.

**OBJECTIVES.** To develop and validate a shortened version of the self evaluating CFQ to measure cognitive failure in ICU patients.

**METHODS.** A multicenter cohort study in two Dutch academic hospitals. Exclusion criteria were: admitted to the ICU for < 1 day; no delirium assessment during ICU stay; and an incomplete CFQ. Between 12 and 24 months after ICU discharge, patients' cognitive functioning was evaluated using the CFQ. Forward selection in a linear regression model was used in the first hospital to assess which of the 25 CFQ items could be omitted without loss of correlation. Selection criteria: a minimal number of CFQ items with a maximum R Square and a proportional coverage of the items on the dimension structure memory, distractibility, social blunders, and names. The performance of the shortened CFQ was determined in the second hospital using Pearson's correlation. A Bland Altman plot was used to examine whether the reduced-item outcome scores, the predicted scores, of the CFQ were replaceable for the 25-item CFQ outcome scores.

**RESULTS.** In total 1759 patients were included, 914 in hospital one and 1020 in hospital two (Table 197). The selection criteria were most optimal for the model with 14 CFQ items. The R Square of this model was 0.973, with a correlation of 0.987. This 14-item CFQ score predicted cognitive failure with a Pearson's correlation of 0.986 ($p < 0.0001$). The mean (SD) of the difference scores was −0.26 (2.675) and 95% of the difference scores were falling within +5 and −5.5 on a 100-point maximum score (Fig. 263). Meaning the 25-item CFQ outcome scores are replaceable by the 14-item CFQ outcome scores.

**CONCLUSIONS.** In this study we developed and validated a shortened CFQ version to examine cognitive failure in ICU patients. This abbreviated CFQ provides us with insight in the ICU patients' cognitive functioning, to ultimately better recognize the Post Intensive Care Syndrome.

**Table 197 (Abstract 0781).** Patient characteristics

| Variable | Hospital 1 N= 914 | Hospital 2 N=1020 |
|---|---|---|
| Age in years, mean (SD) | 63 (14) | 58 (16) |
| Male, N (%) | 608 (67) | 628 (62) |
| Admission type, N (%) Surgical - Medical - Trauma - Neurological/-surgical | 665 (73) - 131 (14) - 41 (5) - 77 (8) | 503 (49) - 339 (33) - 0 (0) - 178 (18) |
| Acute admission, N (%) | 388 (43) | 689 (68) |
| APACHE II score, mean (SD) | 14 (5) | 18 (7) |
| APACHE IV score, mean (SD) | 52 (20) | 62 (26) |
| CAM-ICU positive, N (%) | 171 (19) | 428 (49) |
| ICU Length of stay in days, median [IQR] | 1 [1-2] | 5 [3-9] |
| Complete 25-item CFQ, N (%) | 819 (90) | 918 (90) |

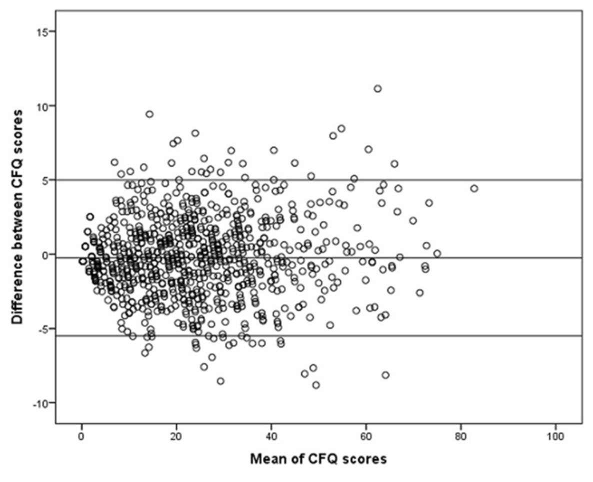

**Fig. 263 (Abstract 0781).** Bland Altman plot of the 25-item CFQ and

### 0782

#### Intracortical electroencephalography monitoring in comatose patients with acute brain injury

M.A. Hernández-Hernández[1,2], J.L. Fernández-Torre[2,3,4], D. Mato-Mañas[5], G. García-Catalán[5], E. Marco de Lucas[6], M. Martín-García[3], R. Martín-Láez[5]
[1]Marqués de Valdecilla University Hospital, Intensive Medicine, Santander, Spain; [2]Biomedical Research Institute (IDIVAL), Santander, Spain; [3]Marqués de Valdecilla University Hospital, Clinical Neurophysiology, Santander, Spain; [4]University of Cantabria, Department of Physiology and Pharmacology, Santander, Spain; [5]Marqués de Valdecilla University Hospital, Neurosurgery, Santander, Spain; [6]Marqués de Valdecilla University Hospital, Radiology, Santander, Spain
**Correspondence:** M.A. Hernández-Hernández
*Intensive Care Medicine Experimental* 2017, **5(Suppl 2):**0782

**INTRODUCTION.** Nonconvulsive seizures (NCSzs) and nonconvulsive status epilepticus (NCSE) are relatively frequent in comatose patients with acute brain injury (ABI). The use of conventional surface electroencephalography (EEG) for continuous monitoring has significant limitations in the intensive care unit (ICU) setting including poor signal-to-noise ratio, insensitivity to capture localized seizures and movement artifacts. Early experience with the use of mini-depth electrodes suggest that many of the limitations of scalp EEG recordings can be overcome

**OBJECTIVES.** We describe our experience in patients with severe ABI in whom intracortical EEG (iEEG) and multimodal neuromonitoring was carried out.

**METHODS.** Commercially available eight-contact Spencer minidepth electrodes (AD-Tech, Racine, WI) designed for clinical iEEG recording were choosen for use. Intracortical electrode location when possible, was placed next to the damage tissue. In addition, 21 needle electrodes attached with collodion placed according the International 10–20 System were included. EEG was recorded using a digital video-EEG monitoring system (XLTEK). Moreover, insertion of other monitoring devices including intracranial pressure monitor (ICP), brain tissue oxygen monitor and brain temperature sensor was performed. This protocol was approved by the local Ethics Committee.

**RESULTS.** There were 12 patients (8 men and 4 women). The mean age was 53 (range 22–73) years. The study included: 2 patients with traumatic brain injury (TBI), 6 spontaneous intracerebral hemorrhage (SIH) and 4 aneurysmal subarachnoid hemorraghe (SAH). Glasgow coma scale (GCS) mean at ICU admission was 6 ± 2. iEEG monitoring was started within 48 h after admission and the mean duration was 4.5 (range 2–8) days. No complications related with Spencer electrode insertion were observed. One patient had NCSzs and two patients had lateralized periodic discharges (LPDs), only visible on iEEG recording. One patient had LPDs 48 hours prior to developed cerebral vasospasm. Asymmetry of the background activity, rhythmic delta waves and occasional focal epileptiform discharges were some of the most important neurophysiological findings. Good outcome at hospital discharge (modified Rankin scale scores ≤ 2) was 16.6% and one patient died.

**CONCLUSIONS.** iEEG monitoring is a feasible technique that can be performed without complications in patients with severe ABI, and its use can shed light on the intrinsic pathophysiological mechanisms of NCSzs and LPDs in comatose subjects.

**REFERENCE(S)**

1. Waziri A, Claassen J, Stuart RM, et al. Intracortical electroencephalography in acute brain injury. Ann Neurol. 2009; 66(3):366–771.
2. Claassen J, Perotte A, Albers D, et al.Nonconvulsive seizures after subarachnoid hemorrhage: Multimodal detection and outcomes. Ann Neurol. 2013 Jul;74(1):53–64

### 0783

#### Cerebral autoregulation during targeted temperature management in cardiac arrest patients

I.A. Crippa, J.-L. Vincent, J. Creteur, F.S. Taccone
Université libre de Bruxelles / Hôpital Erasme, Bruxelles, Belgium
**Correspondence:** I.A. Crippa
*Intensive Care Medicine Experimental* 2017, **5(Suppl 2):**0783

**INTRODUCTION.** Survivors from cardiac arrest (CA) often experience poor outcome. Targeted temperature management (TTM) is regarded as an effective neuroprotective strategy, but ensuring adequate brain perfusion remains of paramount importance. Little is known about cerebral autoregulation during TTM.

**OBJECTIVES.** The aim of this study was to investigate the association between cerebral autoregulation and outcome in CA patients.

**METHODS.** We prospectively studied 22 adult (>18 yo) patients treated by TTM following CA (January 2015 - March 2017).Exclusion criteria were: CA due to trauma, sepsis or intoxication; irregular heart rhythm; severe hemodynamic instability or hypercapnia; cardiac mechanical support. Transcranial Doppler (TCD) was performed insonating the left middle cerebral artery (LMCA) with a 2 MHz probe (DWL, Germany) once successively during the hypothermic (HT) and normothermic (NT) phase. LMCA flow velocity and arterial pressure from peripheral arterial catheter were recorded simultaneously (Doppler Box, DWL). Patients were in steady state condition, with no concurrent stimulus or change in therapy. CAR analysis was performed using MATLAB (MathWorks, US). Mean arterial pressure and FV were first averaged on a 10 sec moving window with 50% overlap and then Pearson´s correlation coefficient was calculated. CAR was considered as altered if Mxa >0.3.

**RESULTS.** Fourteen (44%) patients survived at ICU discharge. TCDs was performed 13 [8–17] and 39 [36–47] hours after CA during HT (core temperature 33.6 [33.1-33.9]°C) and NT (37.0 [36.6-37.4]°C) phases, respectively. During HT all patients were sedated and 25 (78%) were paralysed. During NT 13 (41%) patients were sedated and 4 (12%) were paralysed. Arterial pCO2 were 38 [35–42] mmHg and 37 [34–39] mmHg at time of TCD examination during HT and NT respectively. Overall, Mxa was 0.21 [−0.05 to 0.54] and 0.43 [0.12 - 0.84] during HT and NT, respectively (p = 0.09); CAR was altered in 12/32 (37%) and 19/32 (59%) patients during HT and NT, respectively (p = 0.47). There was no significant difference in Mxa in survivors (p = 0.6), but in non-survivors Mxa increased from 0.21 [0.002 - 0.58] during HT to 0.61 [0.32 - 0.85] during NT (p = 0.04). CAR during NT was more commonly altered in non-survivors than in survivors (14/18, 78% vs 5/14, 36% p = 0.03).

| | |
|---|---|
| Age (yrs) | 65 [53–73] |
| Male | 26 (81) |
| APACHE II | 27 [24–31] |
| Time to ROSC (min) | 20 [11–36] |
| CA: witnessed | 10 (31) |
| CA: cardiac | 17 (53) |
| CA: anoxic | 7 (22) |
| Arterial hypertension | 15 (47) |
| Diabetes Mellitus | 3 (9) |
| ***Median [IQR] and n (%)*** | |

*[Characteristics of population and CA]*

**CONCLUSIONS.** Cerebral autoregulation is altered in a significant proportion of CA patients. During normothermia, non-survivors are more likely to have altered cerebral autoregulation than survivors.

### 0784

#### Apnea test completion failure determinants in suspected brain dead children

E.A. Volakli, P.-E. Mantzafleri, S. Kalamitsou, A. Violaki, E. Chochliourou, M. Svirkos, A. Kasimis, K. Papadimos, O. Vrani, M. Sdougka
Hippokration General Hospital, Pediatric Intensive Care Unit, Thessaloniki, Greece
**Correspondence:** E.A. Volakli
*Intensive Care Medicine Experimental* 2017, **5(Suppl 2):**0784

**INTRODUCTION.** Apnea testing is the cornerstone of clinical diagnosis for Brain Death (BD) deternination in suspected BD children. However, data on determinants of apnea test completion failure in children are limited.

**OBJECTIVE.** To study the feasibility and safety of apnea testing in children, and examine the factors associated with apnea test completion failure.

**METHODS.** Retrospective 17 years study of all BD patients in a 8bed multidisciplinary PICU of Northern Greece. Data collected: Demographics, Pediatric Risk of Severity Score at admission (PRISM III-24), baseline and apnea ventilation and oxygenation parameters, inotropic support, and the way of conducting and the outcome of apnea testing. Two apnea tests (A, B) per patient were aimed if possible, by a panel of three senior physicians, according to tailored by the Greek law guidelines for children. Statistical analysis: Mann–Whitney U test, $\chi^2$ Fisher's exact test; data expressed as percentages or median (IQR), as appropriate.

**RESULTS.** During the study period 48 suspected BD pts were recorded, male (52%), aged 66 (25.50-100.50) months, PRISM III-24 21 (14–26), co morbidity (52.10%), ischemic-anoxic brain injury (56.30%), severe head trauma (37.50%), inotropic support (78.50%). Apnea test were not performed in 5 patients (10.41%, 1 hypoxia, 1 shock, 3 time not permitted), 1 patient had only A and 1 only B test recorded (lack of data), given a total of 84 apnea tests for evaluation. A-apnea test was aborted 6 times
(4 hypoxia-2 shock) and B-apnea 8 times (6 hypoxia-2 shock), given a total abortion rate of 16.66%. Apnea was conducting through tracheal insufflation of oxygen (2–6 lit/min, max 12 in case of developing hypoxia) in 29/84 cases (34.52%), CPAP 5–10 cm $H_20$ in 20/84 (23.80%), lack of data in 35/84 (41.66%). No death and only 1 complication was recorded (1 pneumothorax during tracheal insufflation that led to test abortion but 2 successful tests followed). Age (p = 0.82), PRISM III-24 (p = 0.320), and baseline PA-a$O_2$ (p = 0.708), $PaO_2/FiO_2$ (p = 0.274), and PEEP (p = 0.069) were not related with apnea completion. Patients that did not manage to complete apnea test had significantly higher baseline Peak pressures (26.14 vs. 22.24 cm$H_20$, p = 0.017), higher $PCO_2$ levels (36.51 vs. 32.36 mmHg, p = 0.002), and lower ph (7.40 vs. 7.45, p = 0.04). Sex (p = 0.230), head trauma (p = 0.236) and inotropic support (p = 0.283) were not associated with the success of apnea testing. 4 pts became organ donors (8.33%).

**CONCLUSIONS.** In one out of ten suspected BD pts apnea testing could not be conducted at all, and in the remaining the abortion rate was 16.66%. The main reason for abortion was hypoxia (10/84, 11.90%) followed by shock (4/84, 4.76%). Higher baseline Peak and $PCO_2$ pressures, probably reflecting more severe lung injury, and lower ph were associated with apnea test completion failure in our cohort.

**REFERENCES**
1. Neurology (2008); 71(16):1240,
2. Neurocrit Care (2010); 12:352–355.

### 0785

#### Cerebral perfusion scintigraphy study for brain death diagnosis - 10 years case series

E. Trigo[1], M. Simoes[1], P. Martins[1], J.P. Sousa[1], A. Simoes[1], M.J. Cunha[2], J. Pimentel[1]
[1]Centro Hospitalar e Universitario de Coimbra, Servico de Medicina Intensiva, Coimbra, Portugal; [2]Centro Hospitalar e Universitário de Coimbra, Serviço de Medicina Nuclear, Coimbra, Portugal
**Correspondence:** E. Trigo
*Intensive Care Medicine Experimental* 2017, **5(Suppl 2):**0785

**INTRODUCTION.** Over the last years in Portugal, solid organ donation and transplantation have had a significant increase. The Centro Hospitalar e Universitario de Coimbra (CHUC) has played a major role at national level, as it is the main donation center in Portugal. The diagnosis of brain death is mainly clinical with very strict and well defined rules. However, in some special cases, such as the usage of some drugs (central nervous system depressants), metabolic disturbances and severe facial trauma (that impairs the clinical diagnosis), imaging techniques that evaluate brain perfusion are required to support its diagnosis. The most common exams used in this setting are angiography and brain perfusion scintigraphy, the later performed in the last 25 years in our hospital. These exams have allowed the diagnosis of brain death in some cases, otherwise not possible.

**METHODS.** Retrospective analysis of the brain perfusion scintigrams performed with the objective of brain death diagnosis over the last 10 years in CHUC. The absence of radionuclide activity of Tc-99 m-HMPAO intracranially on static SPECT images, creating a so-called "Hollow skull" appearance, is indicative of brain death.

**RESULTS.** Between 2006 and 2016 were performed 86 brain perfusion scintigrams for brain death diagnosis in 78 patients (20 female, 58 male, mean age 48 years). Most patients had severe traumatic brain injury (39,7%), but in some cases other causes were present, like stroke and aneurysms (38,5%). The main reason for usage of this imaging technique was the presence of barbiturates or benzodiazepines. 71 exams were positive for brain death (15 negative, some were repeated afterwards according to clinical suspicion). Most patients came from the Intensive Care Unit. 58 patients (74,35%) were solid organ donors.

**CONCLUSIONS.** The brain perfusion scintigraphy is a key element for diagnosis of brain death in selected cases, allowing faster diagnosis and therefore increasing solid organ donation. However, its usage is impaired by the time required for its execution and the unavailability at bedside.

### 0786

#### Predictors of organ donation among patients with brain death in ICU

M. Nakamura[1], C. Tchaicka[2], G.E. Rodio[1], E. Padilha[3], I.R. Porto[2], P.A.D. Duarte[2]
[1]Hospital Universitário do Oeste do Paraná, Cascavel, Brazil; [2]Hospital Universitário do Oeste do Paraná, General ICU, Cascavel, Brazil; [3]Hospital Universitário do Oeste do Paraná, Dept of Transplantation and Organ Donation, Cascavel, Brazil
**Correspondence:** I.R. Porto
*Intensive Care Medicine Experimental* 2017, **5(Suppl 2):**0786

**INTRODUCTION.** In developing countries, there are few data on predictive factors related to the outcome of organ donation in patients with brain death (BD) in the ICU. The objective of this study was to evaluate the clinical-demographic profile of adult patients with brain death in a general ICU, and to identify and analyze clinical and demographic predictors for organ donation.

**METHODS.** Retrospective cohort study, analyzing medical records of all patients hospitalized in the adult ICU of a Brazilian teaching hospital for a period of 1 year, with clinical diagnosis of BD (non-reactive coma, fixed/dilated pupils, absence of cough reflexes and no respiratory movements, several hours without sedatives and without other causes of diagnostic confusion). According to Brazilian Ministry of Health, in addition to the clinical diagnosis, laboratory confirmation (arteriography, EEG, transcranial doppler) is needed for the diagnosis of BD. Descriptive statistical analysis was performed. All donations were with heart-beating donors. It was made logistic regression analysis, aiming at finding predictors of success for donation.

**RESULTS.** During the study period, 625 patients were admitted to the ICU with acute neurological diseases. Of these, 85 had clinical diagnosis of BD and were included for analysis. Among donors (n = 9), all had kidney removal, 7 donated liver, and 1 donated heart; 77.7% males, age 39.6 years, and APACHE II 25.5 (compared to 69.7%, 41.2 and 26.9 among non-donors). Two thirds of the patients were victims of trauma, with a high incidence of smoking but not of alcoholism or use of illicit drugs. Almost half (47%) of BD patients were already in coma (with Glasgow score = 3) on admission at Emergency Department. Among donors, the mean time between acute neurological event (stroke / trauma) and the first confirmatory test (after clinical trials) was 237 hours, and between the event and organ withdrawal was 269 hours. In turn, the time between the first diagnosis of non-reactive coma (Glasgow score = 3) and the first confirmatory test for BD was 162 hours, and the time between Glasgow score = 3 and organ withdrawal was 208 hours. The main predictive factors (of success for donation) found by logistic regression were: lower incidence of renal insufficiency (lower maximum creatinine value), higher sodium value, lower arterial lactate, shorter time between hospital arrival and Glasgow score = 3.

**CONCLUSIONS.** In this group of patients with Brain Death in ICU, 10.6% became effective donors of vital organs (heart, liver, kidneys). Among the predictive factors of success for donation, it is noted that persistently elevated arterial lactate was a marker of not being able to reach the donation. On the other hand, patients that developed coma and evolved to BD long after hospital admission had lower chance to become an organ donor.

### Spotlight on N&AHP practice

#### 0787

#### Impact of a dedicated nurse led intravenous therapy team on catheter associated blood stream infection rates across a hospital organisation

S. Gopal[1], M. Cooper[2], A. Nevill[3], S. Rowlands[4], D. Golding[4], N.S. Karthikeyan[1]
[1]The Royal Wolverhampton NHS Trust, Critical Care Services Directorate, Wolverhampton, United Kingdom; [2]The Royal Wolverhampton NHS Trust, Microbiology Department, Wolverhampton, United Kingdom; [3]University of Wolverhampton, Faculty of Education and Wellbeing, Wolverhampton, United Kingdom; [4]The Royal Wolverhampton NHS Trust, Infection Prevention and Control, Wolverhampton, United Kingdom
**Correspondence:** S. Gopal
*Intensive Care Medicine Experimental* 2017, **5(Suppl 2):**0787

**INTRODUCTION.** Catheter Associated Blood Stream Infections (CABSI) remain a significant cause of Device Related Hospital Acquired Bacteraemia (DRHAB) and lead to increased hospital length of stay, increased cost as well as morbidity and mortality. Over the last decade, several initiatives have proven effective in reducing the incidence of CABSI including insertion and care bundles and specialised insertion teams1,2. Nurse led insertion teams have been effective within defined clinical areas including Neuro- Intensive Care Units and in Paediatrics3,4. However, no previous study has assessed the impact of a nurse led Intravenous Therapy Team on CABSI rates across an entire hospital organisation.

**OBJECTIVES.** We hypothesized that the introduction of a dedicated Nurse Led Intravenous Therapy Team across a hospital organisation would reduce the incidence of CABSI throughout the institution.

**METHODS.** An Intravenous Therapy Team consisting of trained nurses was introduced in August 2012. The purpose of the team was to establish intravenous access in any patient throughout the hospital including all wards and the intensive care unit. A doctor or nurse could refer any patient in whom intravenous access was required. The Intravenous Therapy Team would liaise with the clinical team and aim to establish either an ultrasound guided peripheral venous line or a peripherally inserted central catheter (PICC) line depending upon the clinical need. As well as establishing venous access the Intravenous Therapy Team would also take responsibility for the ongoing care of the line, including daily line inspection, dressing changes and line removal.

**RESULTS.** A database of all DRHABs has been maintained since Jan 2009. This revealed a baseline median incidence of 6 CABSI per 1000 line days across the hospital organisation. Within six months of introducing the Intravenous Therapy Team the median incidence of CABSIs had reduced to 3 CABSI per 1000 line days across the organisation. To ensure that this result was not due to the Hawthorne effect, data has been prospectively collected throughout the study. The median incidence of CABSI has remained at 3 CABSIs per 1000 line days since the inception of the team to the present with more than 3000 devices inserted.

**CONCLUSIONS.** A dedicated Nurse led Intravenous Therapy Team to establish ultrasound guided peripheral venous lines and PICC lines across an entire hospital organisation resulted in a significant reduction in the incidence of CABSI in our institution. This model is both feasible and highly effective in reducing the incidence of CABSI across a hospital organisation.

**REFERENCE(S)**

1. Pronovost P, et al. N Engl J Med. 2006
2. Berenholtz SM, et al. Crit Care Med. 2004
3. Gamulka B, et al. Pediatrics. 2005
4. DeLemos C, et al.Crit Care Nurse. 2011

#### 0788

#### Experience of 'parallel communications' training, a novel communication skills workshop, in 50 critical care nurses in a UK Hospital

S. Brown[1], M.V. Durve[2], N. Singh[3], W.-H.E. Park[2], B. Clark[2]
[1]Charing Cross Hospital, Imperial College Healthcare NHS Trust, Critical Care, London, United Kingdom; [2]Charing Cross Hospital, Imperial College Healthcare NHS Trust, Oncology/ Palliative Care, London, United Kingdom; [3]East Sussex Healthcare NHS Trust, Critical Care, Eastbourne, United Kingdom
**Correspondence:** S. Brown
*Intensive Care Medicine Experimental* 2017, **5(Suppl 2):**0788

**INTRODUCTION.** The literature consistently states that critical care patients and their families want clear and honest information regarding prognosis and treatment[1]. However the evidence also shows that families are more dissatisfied with communication than with any other aspect of care delivered on critical care units[2].

Health-care professionals(HCPs) in our institution reported that previous communication skills training(CST) had not prepared them for dealing with real-world clinical situations such as discussing escalation of care. 'Parallel Communications' is an innovative, goal orientated, simulation workshop developed to address this unmet need.

**OBJECTIVES.** The aim of this study was to investigate the workshop's impact on Critical Care nurses(CCNs) confidence to approach difficult conversations.

**METHODS.** The 2-hour workshop was delivered to by 2 facilitators to groups of 6–10 CCNs.

Anonymised data was collected regarding the CCNs' confidence on a scale of 1 (not confident) to 10 (as confident as possible) regarding different aspects of communication.

Paired statistical tests were used to assess the impact of training on the CCNs' pre/post-training scores.

**RESULTS.** Full data sets were available for 50 CCNs.

Analysis showed statistically significant improvement in CCNs confidence regarding:

- Explaining complex information to relatives(mean 1.6, $p < 0.001$)
- Managing uncertainty(mean = 2.2, $p < 0.0001$)
- Recognising distress(mean = 1.4, $p < 0.0005$)
- Responding helpfully to distress(mean = 2.1, $p < 0.005$)
- Anticipating concerns at end of life(mean = 1.8, $p < 0.005$)
- Talking about withdrawal of treatment(mean = 2.8, $p < 0.0005$)
- Discussing escalation of treatment and resuscitation status(mean = 2.8, $p < 0.0005$)
- Talking to bereaved relatives(mean = 1.98, $p < 0.0005$)
- Talking to families with children about end of life(mean = 2.1, $p < 0.0005$)

Of the CCNs that answered 39/40(97.5%) rated this workshop as very useful or useful. 28/50(56%) reported that they would alter their behaviour as a result of teaching.

**CONCLUSIONS.** This study shows that Parallel Communications a 2-hour CST workshop, improved CCNs' confidence in multiple aspects of communication. 54% reported that they would alter their behaviour.

Whilst confidence does not equate to competence, increasing CCNs' self-confidence and providing a safe environment to rehearse difficult scenarios may increase their willingness to engage in difficult conversations rather than avoiding or blocking these topics[3.]

**GRANT ACKNOWLEDGEMENT**
Macmillan, HENWL

### 0789

#### National Study exploring inter-professional shared clinical decision regarding fluid resuscitation

F. Dekeseyr-Ganz[1], J. Benbenishty[2]
[1]Haddasah Hebrew University Faculty of Nursing, Jerusalem, Israel; [2]Hadassah Medical Organization, Jerusalem, Israel
**Correspondence:** J. Benbenishty
*Intensive Care Medicine Experimental* 2017, **5(Suppl 2):**0789

**INTRODUCTION.** The ICU is a dynamic, rapidly changing environment where clinical decisions are often made under stress. The quality of these decisions and their outcomes is affected by the level of collaboration, communication and shared decision making (SDM) between physicians and nurses. A model was developed that describes ICU shared decision making and the factors that affect it.

**OBJECTIVES.** Explore ICU inter-professional shared clinical decision regarding fluid resuscitation in a national study.

**METHODS.** Prospective, multi-center survey investigating the decision making process regarding delivery of fluid resuscitation to hemodynamically compromised patients in the ICU. Inclusion criteria-all nurses and physicians working in ICUs in Israel The questionnaire, based on actual ICU scenarios, with additional demographic data.

**RESULTS.** The frequencies of the levels of shared decision making used to decide whether to administer a fluid bolus were similar in this study. Most nurses reported that they exchanged or relayed information (Level 2 of the model) (n = 73, 67%), deliberated (Level 3 of the model) (n = 69, 63%), or shared decision making (Level 4 of the model) (n = 74, 68%), either often or always. Other methods of decision making, such as administering the bolus and then calling the physician (n = 78, 72%) and only providing information that supports the nurse´s decision (n = 41, 38%) were also used.

Most nurses in the sample delivered boluses of 500 ml (n = 73, 67%). The type of fluid administered was predominantly either Full Normal Saline (n = 25, 23%), Hartmann Solution (n = 44, 40%), or Glucose/ Saline (n = 25, 23%).

Changes in arterial pressure according to the arterial line was the most common sign used to determine a clinical response to the bolus (n = 98, 90%), followed by urine output (n = 82, 75%), and Central Venous Pressure (n = 60, 55%).

The level of shared decision making was not related to any of the work or personal characteristics measured.

**CONCLUSIONS.** ICU nurses, regardless of their role or other characteristics, use different strategies to obtain medical orders for fluid bolus administration. This is in contrast to a body of literature stating that physicians often dominate clinical decision making in the ICU. These results imply that at least in situations related to fluid bolus administration, high levels of collaboration are found. It has long been shown that higher levels of physician-nurse collaboration are associated with improved patient and staff outcomes. Therefore, it would seem that outcomes related to fluid bolus administration are potentially optimized.

Nurses should be encouraged to share clinical decisions at the highest level, when clinically appropriate.

### 0790

#### Pre-operative mobility is an important predictor for determining intensive care unit admission following lung volume reduction surgery

J.L. Arnold, T.J. Scott
Royal Brompton Hospital, Rehabilitation and Therapies, London, United Kingdom
**Correspondence:** T.J. Scott
*Intensive Care Medicine Experimental* 2017, **5(Suppl 2):**0790

**INTRODUCTION.** Lung volume reduction surgery (LVRS) aims to relieve symptoms and improve function in advanced emphysema. The NETT trial (2003) validated outcomes of LVRS and a subsequent study shows durable long term effects (Ginsberg et al., 2016). Complications include admission to the Intensive Care Unit (ICU), potential intubation, increased length of stay (LOS) and persistent air leaks. Post-operative physiotherapy (PT) aims to ensure lung re-expansion, effective sputum clearance and increase exercise tolerance, with ICU patients potentially requiring more intensive PT for weaning/ rehabilitation for ICU acquired weakness (Stiller, 2013). A survey of PT provision for patients undergoing thoracic surgery at UK tertiary centres shows mobilisation and the active cycle of breathing technique were the most prevalent techniques (Agostini et al., 2013).

**OBJECTIVES.** This study aims to review outcomes of patients requiring an ICU admission post LVRS compared to ward based care and review if there are any predisposing factors that may be linked with an ICU admission. This study also aims to ascertain whether PT requirements differ in these cohorts.

**METHODS.** Data was collected retrospectively on all LVRS patients over a 6 month period (Oct '16- April '17). Data was collected pre and post-operatively and included FEV1, pre-operative mobility and oxygen ($0_2$) requirements, lobe resected, LOS, number of PT interventions and type, post-operative mobility, on-going 02 requirements and any complications on discharge. Data was statistically analysed to determine trends in patients being admitted to ICU and outcomes.

**RESULTS.** 22 data sets (n = 17 male) were gathered and analysed (Stata Version 14). All patients were reviewed by PT day 1 post LVRS as per local thoracic protocol. There was a weak correlation between pre-operative $FEV_1$ (mean 26%) and resulting post- operative mobility (p = 0.75). Of these, 5 patients were admitted to ICU. There was no link between those on pre-operative $0_2$ therapy or lobe removed and an ICU admission. Patients of increased age were more likely to have an ICU admission (not statistically significant). There was a statistically significant link using Mann–Whitney analysis between low pre-operative mobility and an ICU admission (p = 0.03). The ICU group had greater improvement in their mobility (median 6m to 60m), compared to the ward group (median 90m to 200m). Patients on ICU required more PT intervention (median n = 36, IQR 28–38) than the ward group (median n = 7, IQR 4–10), with a greater LOS (median n = 45, IQR 39–66 compared to n = 12, IQR 10–18).

**CONCLUSIONS.** Correlation was found between low pre-operative mobility levels and admission to ICU which may further the need for research into the effectiveness of pre-operative rehabilitation. PT techniques remain the same among both groups with ICU patients requiring greater input due to LOS which has financial implications. Conclusions must be interpreted with caution due to the small sample size.

### 0791

#### An observational study of light intensity in critical care

R. Greer[1], H. Durrington[2], R. Clarke[2], F. Martial[2], J. Blaikley[2], R. Lucas[2], P. Dark[2], D. Ray[2]
[1]Manchester Royal Infirmary, Department of Anaesthesia and Critical Care, Manchester, United Kingdom; [2]University of Manchester, Manchester, United Kingdom
**Correspondence:** R. Greer
*Intensive Care Medicine Experimental* 2017, **5(Suppl 2):**0791

**INTRODUCTION.** Ambient light is important in the regulation of circadian rhythms working via retinal photoreceptors. Critical care supplies specialised intervention constantly over 24 hours. Disrupted circadian rhythms have been shown to worsen outcome from critical illness. Within critical care mechanical ventilation, sedation, illness severity and ambient light and noise may disturb circadian rhythm.

**OBJECTIVES.** Light intensity was measured within the intensive care unit over during daytime and overnight.

**METHODS.** We measured light intensity in three different bed locations over 24–48 hours. A battery operated light meter was placed close to the patient at eye level. It measured light intensity every 15 seconds. The light sensor was optimised for human vision (infrared filtered). Three bed spaces were used, a typical space on the open ward, a side room with a window and a side room without a window. Bedside lights were of three kinds - ceiling lights (150–200 lux), pendant lights for invasive procedures (160,000 lux) and low level lights at the bedside for overnight activity. No change in clinical practice was implemented. Normal practice was to switch the overhead lights on at 08:00 and off at 23:00. Light intensity was measured as mean and SEM against time. Paired t-tests were used to compare means.

**RESULTS.** Overall mean illuminance during the daytime was 159 lux and at night was 10.5 lux. Light intensity was analysed by location. During daytime light intensity was significantly greater in the side room with the window compared to the other locations. During night time there was significantly lower light levels in the side room without a window compared to the other locations.

We identified patterns in the change of light intensity. On the open ward gradual changes in light intensity occurred consistent with dawn and dusk. The side room without a window demonstrated a pattern of lighting consistent with artificial light. This 'block' of light abruptly reduces when the light is switched off. In addition, overnight, bright pulses of light are observed as procedures or checks are carried out on patients.

**CONCLUSIONS.** Light intensity varies within intensive care units and is dependent on access to natural light and clinical activity. Normal sunlight can have intensity as high a 100,000 lux and an overcast day can record levels of 1000 lux. Levels of light less than 200 lux have been demonstrated to be insufficient to maintain circadian rhythms. We measured a mean levels of 159 lux during daytime. In addition we demonstrated interruptions overnight. The authors suggest that this pattern of lighting may be implicated in the disturbance of circadian rhythms. This may affect the incidence of delirium and clinical outcomes in critical care.

**REFERENCE(S)**

Gazendam JAC et al. Altered circadian rhythm in patient in the ICU Chest 144; 483–489

**GRANT ACKNOWLEDGMENT**

None

### 0792

#### Translation and preliminary validation of the Self-Competence in Death Work Scale in Spanish nurses

O. Valles Fructuoso[1], C. Alfonso Arias[2], L. Masnou Brichs[3], A. Ruz Peña[4], C. Sepúlveda Bravo[5]

[1]Hospital Universitari de Bellvitge, Intensive Care, L'Hospitalet de Llobregat, Spain; [2]Hospital General de Catalunya, Intensive Care, Sant Cugat del Vallès, Spain; [3]Hospital Comarcal de Blanes, Emergency, Blanes, Spain; [4]Hospital General de Catalunya, Pathological Anatomy, Sant Cugat del Vallès, Spain; [5]Universitat Internacional de Catalunya, Sant Cugat del Vallès, Spain

**Correspondence:** O. Valles Fructuoso

*Intensive Care Medicine Experimental* 2017, **5(Suppl 2):**0792

**INTRODUCTION.** Intensive care nurses often are confronted by death in their work. They may experience challenges to self, such as aroused emotions and queries about life´s meaningfulness. Assessing their level of "self-competence´´ in coping with these challenges is crucial in understanding their needs in death work.

**OBJECTIVES.** To translate and to adapt the Self-Competence in Death Work Scale (SC-DWS) for the context of the Spanish nursing professionals, and to determine his preliminary psychometric properties.

**METHODS.** An observational and transversal design was used. Analysis data was done with SPSS, v.22.0, calculating the descriptive indexes, exploratory factorial analysis and Pearson's correlation.

**RESULTS.** A total of 106 participants were recruited. A Cronbach's alpha coefficient of 0,72 was obtained for the 16 items of the Spanish SC-DWS form. A factorial structure of five factors were found in his set explaining 59,9% of the total variance. *Confrontation of the uncertainties*, *acceptance of the death*, *emotions before the death*, *emotions of the work* and *impotence and limitations* were identified as subscales in the five-factor structure. Correlations of the whole scale and subscales with measures of Perceived Life Significance (p < 0,01), Hospital Anxiety, and Depression (p < 0,01), Death Anxiety Inventory Revised (p < 0,05) and General Self-Efficacy (p < 0,01) provided evidence for the construct validity. Reliability analyses showed that the entire scale and subscales were internally consistent.

**CONCLUSIONS.** The results suggest that the Spanish form of SC-DWS has adequate construct validity and acceptable metric properties. This scale may facilitate helping professionals´ understanding of their self-competence in death work, so appropriate professional support and training may be obtained.

**GRANT ACKNOWLEDGMENT**

To Wallace Chi Ho Chan and Joaquín Tomás- Sábado for thei help and support.

### 0793

#### The presence, severity and duration of multiple organ dysfunction syndrome in trauma

H. Farrah

St George's University Hospital NHS Foundation Trust, General Intensive Care Unit, Tooting, United Kingdom

*Intensive Care Medicine Experimental* 2017, **5(Suppl 2):**0793

**INTRODUCTION.** Trauma admissions to Intensive Care Units often develop a dysregulated host response to injury leading to multiple organ dysfunction syndrome (MODS) (1), and consequently an extended length of stay.

**OBJECTIVES.** To evaluate the severity and duration of MODS in ICU trauma admissions and the associations with patient outcomes.

**METHODS.** This retrospective, observational study from April 2016-December 2016 reviewed 200 consecutive trauma admissions to an adult general ICU of an urban Major Trauma Centre. Patients aged under 16 years, burns, for palliation or organ donation were excluded. Further exclusions were made in cases of poor data quality, leaving a total sample size of 164 patients. Data collected included demographics, mechanism of injury, admission Glasgow Coma Score (GCS), lactate, APACHE II score, injury severity and sequential organ failure assessment (SOFA) up to day 28 in ICU. MODS was identified by calculating daily SOFA scores as a measure of organ dysfunction with a score of ≥5 defining a patient as being in MODS (2). Significance was measured between patients who developed MODS and those that did not (nMODS group). Significance was determined using a Mann Whitney U test (indicated by ~) or a Chi squared (indicated by⊞) and set at p = 0.05. The primary outcome was mortality during the in hospital phase of care, and secondary outcome was length of stay for survivors.

**RESULTS.** 164 patients were reviewed of which 104 (63%) developed MODS Table 198. Admis.... Patients who developed MODS did so within 48 hours of admission, were more severely injured and admitted with a significantly lower GCS, higher admission lactate, and SOFA and APACHE II scores (Table 198-Results shown as median values with interquartile ranges). Median SOFA scores for patients that developed MODS remained ≥5 until day 12, with the neurological and respiratory components taking until days 25 and 22, respectively, to recover. Patients without MODS required significantly less organ support, had shorter ICU and hospital length of stays, and significantly lower mortality (Table 199).

**CONCLUSIONS.** In this study, the presence of MODS after trauma significantly increased ICU length of stay due to prolonged neurological and respiratory dysfunction. The presence of ICU delirium perpetuating neurological dysfunction should be investigated.

**REFERENCE(S)**

1. Singer MD. et al. The Third International Consensus Definitions for Sepsis and Septic Shock (Sepsis-3). JAMA. 2016;315(8):801–810

2. Shepherd JM et al. Shock. 2017:;47(4):429–435.

**Table 198 (Abstract (0793).** Admission Demographics

| % and Median Values (Interquartile Range) | All Patients | MODS | nMODS | P value |
|---|---|---|---|---|
| Size | n=164 | n=104 | n=60 | |
| Age Years | 51 (33-76) | 52 (37-77) | 44 (28-76) | 0.13⊞ |
| Male (%) | 69% | 67% | 72% | 0.43⊞ |
| Blunt Trauma (%) | 92% | 91% | 87% | 0.34⊞ |
| Admission GCS | 13 (6-15) | 8 (3-14) | 15 (14-15) | <0.01~ |
| Admission Lactate | 2.0 (1.3-3.1) | 2.4 (1.6-3.6) | 1.4 (1.0-2.1) | <0.01~ |
| Admission SOFA Score | 5 (3-8) | 7 (5-9) | 2 (1-3) | <0.01~ |
| Admission APACHE II Score | 12 (7-17) | 14 (9-19) | 8 (5-13) | <0.01~ |
| Injury Severity Score | 16 (6-27) | 20 (9-29) | 9 (4-17) | <0.01~ |

**Table 199 (Abstract 0793).** Outcomes

| % and Median Values (Interquartile Range) | All Patients | MODS | nMODS | P Value |
|---|---|---|---|---|
| ICU Length of Stay Days | 2 (2-5) | 4 (2-8) | 2 (2-2) | p=<0.01~ |
| Hospital Length of Stay Survivors Days | 14 (9-27) | 19 (10-33) | 13 (8-22) | p=<0.01~ |
| Mortality | 14% | 21% | 2% | p=<0.01⊞ |

### 0794

#### The impact of nursing rounds on the practice environment and nurse satisfaction in neuroscience intensive care unit

C. Gu[1], A.P.Y. Ng[2], H.S. Lim[2]
[1]Tan Tock Seng Hospital, Nursing Service/NICU, Singapore, Singapore; [2]Tan Tock Seng Hospital, NICU, Singapore, Singapore
**Correspondence:** C. Gu
*Intensive Care Medicine Experimental* 2017, **5(Suppl 2):**0794

**INTRODUCTION.** Nursing round is an innovative learning method and has been shown to have a potential in keeping nurses abreast on current evidence and improving intra- and inter-professional relationships. Implementation of nursing rounds in intensive care unit is feasible, and it has been used in many settings as an education and practice improvement strategy. However, to date, there are no published papers on the impact of nursing rounds in intensive care in the Asian context.

**OBJECTIVES.** To determine the impact of nursing round on nurses' perception on the practice environment and their work satisfaction in the neuroscience intensive care unit (NICU).

**METHODS.** In this study, we used a pre-test and post-test quasi-experimental study design. The Practice Environment Scale of the Nursing Work Index (PES-NWI) and the Index of Work Satisfaction (IWS) scale were used to measure study outcome. An informed consent was taken and a convenient sample of 65 participants was enrolled. Nursing rounds was held once fortnightly from July to December 2016. Each session lasted forty-five minutes to an hour, during which one case study was discussed. After implementing nursing rounds for six months, the same post-intervention questionnaires were distributed to and collected from the same 65 participants. All the data were analysed using STATA Version 13.1.

**RESULTS.** The PES-NWI sub-scale of staffing and resource adequacy improved after the intervention period (pre - 11.09; post - 11.74, p = 0.0144). While there was no significant difference in the PES-NWI sub-scale of nursing foundations for quality of care, scores improved after the intervention period (pre - 29.09; post - 30.15, p = 0.0712). Upon further breakdown analysis, use of nursing diagnoses, which is under the PES-NWI sub-scale of nursing foundation for quality of care, showed a statistical difference between the pre- and post-intervention period (pre - 2.48; post - 2.74, p = 0.0296).

**CONCLUSIONS.** With reference to the results as whole, by increasing the use of appropriate nursing diagnoses, nursing rounds could have helped build nurses' confidence and equipped them in making sound clinical decisions. This may have in turn improved staff capability and efficiency in the settings of an intensive care unit, resulting in a positive impact on staffing and resource adequacy. The limitation of this study includes the time constraint being and a relatively small sample size. Hence, a longer study with a larger sample size, with the use of a control group, may give more insight into how and to what extent nursing rounds can affect nurses' perceptions of the work environment and nurses' work satisfaction.

**REFERENCE(S)**

Laibhen-Parkes, N., Brasch, J., & Gioncardi, L. (2015). Nursing grand rounds: A strategy of promoting evidence-based learning among paediatric nurses. *Journal of Pediatric Nursing, 30*, 338–345.

**GRANT ACKNOWLEDGMENT**

"I'm a Nurse Scientist" Grant Tan Tock Seng Hospital, Singapore

### 0795

**Withdrawn**

### 0796

#### Critically ill patient safety in a simulated area: nursing students

A. Aliberch[1,2], M. Llaurado-Serra[1], J. Castillo[1,3], S. Pilar[1,4], C. Alfonso[1,4], E. Rodríguez-Higueras[1]
[1]Universitat Internacional de Catalunya, Sant Cugat del Vallès, Spain; [2]Hospital Clinic, Barcelona, Spain; [3]Hospital Universitari de Bellvitge, Barcelona, Spain; [4]Hospital General de Catalunya, Sant Cugat del Vallès, Spain
**Correspondence:** M. Llaurado-Serra
*Intensive Care Medicine Experimental* 2017, **5(Suppl 2):**0796

**INTRODUCTION.** Patient safety is a key point in patient care and is considered as a critical issue in national and International policies and guidelines.

Nowadays, in the college environment, the knowledge that students acquire is based on the competences acquisition instead of just theoretical knowledge.

The learning outcomes chosen which are related to different competences are included in the Strategic plan of patient safety from the Spanish Ministry of Health, social services and equity.

**OBJECTIVES.** To investigate the acquisition level of the learning outcomes of the students related to critically ill patient safety.

**METHODS.** Observational study carried out in a Spanish University during the courses 2014–2016. Students were in their 3rd year of of Nursing Degree. In the subject evaluated nursing students have 4 ECTS where they learn different aspects related to critical care (surgery, general care in the ICU, applied pharmacology, advanced cardiopulmonary resuscitation and electrocardiography). The learning methods used are: theoretical lectures, case studies and simulation lab. The evaluation of the subject is mainly performed by an objective structured clinical examination (OSCE) and simulation lab cases.

The OSCE had 15 stations due to the amount of students. Of all the stations, 6 had an evaluator inside and in the others the students had to do a written theoretic-practical assessment. In the OSCE, students were evaluated of 7 "critical items" across the stations which were defined as those which in case they are not accomplished the patient safety could be put in danger.

The punctuation of the critical items was dichotomous (yes/no). Students had to perform correctly half of them to be able to pass the exam independently of the rest of the OSCE results.

Results are presented descriptively with absolute and relative frequencies and median and percentiles as appropriate.

**RESULTS.** One hundred forty-four students were evaluated. One hundred fourteen were female and 83.9% were younger than 26 years old. In the OSCE the critical items evaluated and their results are: differentiation of arterial and venous catheter was performed correctly by 48.9% of the students, identification of the patient's allergies (66.7%), correct practical calculation of the drug to administer (42.2%), recognition that the enteral nutrition is being administered through the central venous catheter (47.5%) and correct calculation of a theoretical drug calculation problem (63.8%).
Of the total critical items that were evaluated, students failed from none till all of them. The median of correct critical items was 3 ($p_{25}$ 2 - $p_{75}$ 5). 68 students (48.2%) performed correctly ≥4 critical items.
**CONCLUSIONS.** Our results point out several points where improvement measures have to be implemented. The simulated environment facilitates the student's training in patient safety. Training in patient safety is aligned with the national and international strategies in quality of care.

### 0797

#### Nurse led early and progressive mobilization in ICU

A. Negro[1,2], L. Miconi[1], M. Dossi[1], T. Tira[1], G. Ponzetta[1], C. Garrido Caballo[1], S. Colombo[1], L. Cabrini[1,2], A. Zangrillo[1,2]
[1]IRCCS San Raffaele Hospital, Milan, Italy; [2]Vita-Salute San Raffaele University, Milan, Italy
**Correspondence:** A. Negro
*Intensive Care Medicine Experimental* 2017, **5(Suppl 2):**0797

**INTRODUCTION.** Critically ill patients admitted to Intensive Care Units (ICU) spend long periods of time in bed, with well known physical and psychological consequences[1]. Early and progressive mobilization was introduced as a component dell´ABCDEF bundle[2], but turns out to be one of the most complex phases to implement, especially in the Italian ICUs, often characterized by the absence of physical therapists and the nurse/patient ratio different from 1: 1, seen in the other countries.
**OBJECTIVES.** To describe the implementation of the Early and Progressive Mobilization program in the general ICU of a big University Hospital in Italy, without physiotherapists.
**METHODS.** In november 2014 a plan to guide the Mobility was developed and education sessions were performed. Episodes of advanced mobilization are monitored: dangling, out of bed and walking. Patients in ICU for more than 24 hours were included.
**RESULTS.** In the first year post implementation 321 episodes of active mobilization were performed: 155 dangling, 166 out of bed and 4 walking (29,6% of patients). In the second year there were 462 episodes (35,8% of patients) :139 dangling, 277 out of bed episodes and 36 of walking.
The number of patients mobilized out of bed in VAM increased progressively, from 8 in 2015 to 21 in 2016. Adverse events were rare and never serious (hypotension, desaturation, panic, Atrial Fibrillation).
The number of personnel involved in the mobilization sessions has remained constant over time (2.1 DS 1–4).
**CONCLUSIONS.** From the obtained data it is clear the feasibility and safety of the early and progressive mobilization managed and performed exclusively by the ICU nurses.
However, it is not enough to reach the systematic and regular approach obtained in centers with dedicated staff.
**REFERENCE(S)**
1. S. Parry, Zudin A. Puthucheary *The impact of extended bed rest on the musculoskeletal system in the critical care environment* Extreme Physiology and Medicine 2015;4:16
2. Eduard E. Vasilevskis, E. Wesley Ely, Theodore Speroff, Brenda T. Pun, Leanne Boehm, Robert S. Dittus, *ICU-Acquired Delirium and Weakness—Crossing the Quality Chasm* Chest 2010;138 (5) 1224:1233

### 0798

#### A retrospective observational study of the compliance of patient pain, agitation and delirium assessment in a general intensive care unit

J. Cardoso Pereira[1], S. Leyland[2], J. Mellinghoff[2]
[1]St George's University Hospital NHS Foundation Trust, General Intensive Care Unit, London, United Kingdom; [2]St. George's University Hospitals NHS Foundation Trust, London, United Kingdom
**Correspondence:** J. Cardoso Pereira
*Intensive Care Medicine Experimental* 2017, **5(Suppl 2):**0798

**INTRODUCTION.** Pain is the most common memory patients report after their ICU stay. Critically ill patients may be unable to self-report their pain due to the use of mechanical ventilation and sedative agents that alter their level of consciousness. In addition, agitation in critically ill patients may be a result of inadequately treated pain, anxiety or delirium all associated with increased mortality, prolonged ICU stay and cognitive impairment. As such, assessment of Pain, Agitation, and Delirium (PAD) has been recognized as a pivotal part of patient care (1).
**OBJECTIVES.** This study took the format of an audit undertaken in November 2016 and February 2017 investigating the compliance of PAD assessment in terms of frequency and choice of tool applied with the intention to compare the findings with current best practice guidelines.
**METHODS.** Data collection took place during the two time periods within an 18 bedded adult general ICU. All patients were included who required either level 2 or level 3 care.
The following data points were collected by the authors:

1. Is there any documentation related to PAD assessment? What is the frequency of the assessment?
2. Was the patient sedated and did he have a sedation hold / SAT? (Sedation awake trial).
3. Which analgesic / sedative agent was administered?
4. Which pain tool was used? Was this documented?
5. Has the patient a prn analgesia prescription (yes/no)? What was the frequency in the last 24 hours?
6. Did the patient have acute/ chronic pain issues?

**RESULTS.** The results indicate that nurses collected the Richmond Agitation Sedation Score (RASS) more often than the pain score (Critical-Care Pain Observation Tool). The reason behind this may be explained by the fact that a box to document the RASS is included on the observation chart. Interestingly delirium status, assessed by CAM-ICU was documented more often than pain score.
**CONCLUSIONS.** The results indicate that there exists a lack of knowledge regarding PAD assessment in view of the assessment tools applied and their frequency of documentation.
Consequently, assessment practices during the study periods did not conform with what is regarded current best practice. We recommend the implementation of training package
to improve compliance with this important aspect of patient care followed by another audit to ascertain if any change has been achieved. In addition, the routine use of stickers.

### 0799

#### Implementation of the ICU diaries in the Italian contest

A. Negro[1,2], G. Arena[1], D.F. Manara[2]
[1]IRCCS San Raffaele Hospital, Anesthesia and Intensive Care, Milan, Italy; [2]Vita-Salute San Raffaele University, Milan, Italy
**Correspondence:** A. Negro
*Intensive Care Medicine Experimental* 2017, **5(Suppl 2):**0799

**INTRODUCTION.** Patients and family members recovering from critical illness may experience physical and psychological disorders which impair healthy recovery. Intensive care diaries have been developed as a tool to improve factual memory of time spent in intensive care, with an aim to improve recovery from critical illness. (Ullman AJ. 2015).
Diaries have been introduced as a tool for helping patients to remember or rebuild their Intensive Care Unit (ICU) stay (Petersson 2015). The use of ICU Diaries in the Italian contest hasn't yet been described.
**OBJECTIVES.** The aim of this study was to implement the use of the ICU-diaries in the IRCCS San Raffaele Hospitals's General ICU, in Milan, Italy.
**METHODS.** The diary consisted in a small notebook that it was given to the patients and their families. An educational program was conducted for the ICU team.
**RESULTS.** The sample included 19 diaries of patients hospitalized between 2 to 43 days of Intensive Care. The authors of the diaries are the patients´ family members, friends and the ICU nurses. Through the qualitative analysis of the 19 diaries were identified and classified 12 recurring themes (narrative units) according to the topics discussed. Looking at the results it appears that the diaries represented a much more complex care intervention and not only a vehicle to complete the gaps in the period in the ICU memory. The writers shared their feelings, their time and offered moral support to the patients during hospitalization.

**CONCLUSIONS.** The implementation of ICU diaries in Italy resulted feasible, an important families' participation to the project has been achieved. The diaries reppresented an activity of care for the patient, and a helpful therapeutic activity for the family to face its relative's critical ill. There is need for more research to be performed about diaries, expecially in the Italian contest.

### 0800

#### Incidence and prognostic factors for central venous catheter thrombosis in critically ill pediatric patients

P. Zuñiga[1], P. Schneider[1], A. Castillo[1], F. de Barbieri[2], V. Gonzalez[2], J. Martinez[1]

[1]Pontificia Universidad Católica de Chile, Facultad de Medicina, Departamento de Pediatría, Santiago, Chile; [2]Pontificia Universidad Católica de Chile, Facultad de Medicina, Departamento de Radiología, Santiago, Chile

**Correspondence:** P. Zuñiga

*Intensive Care Medicine Experimental* 2017, **5(Suppl 2):**0800

**INTRODUCTION.** Venous thromboembolism has increased in pediatrics and the most frequently associated risk factor is the presence of a central venous catheter. This pathology can determine morbidity, however, there are currently no pediatric protocols validated for its prevention. The aim of this study was to determine the incidence of venous thrombosis and to identify risk factors in pediatric patients with central venous catheters, as an initial approach to the creation of thromboprophylaxis protocols.

**PATIENTS AND METHODS.** Prospective cohort study. Patients younger than 15 years who required the installation of a transient central venous catheter in the pediatric intensive care unit of the Pontificia Universidad Católica de Chile between May and November 2016 were included. Data were extracted from their clinical file, antithrombin III activity was measured and during the first 48 hours after catheter removal a Doppler ultrasonography was performed to detect thrombosis.

**Results:** 31 patients were included. The incidence of VT was 45.1%, all were asymptomatic. The factors that were significantly associated with VT were: need for vasoactive drugs, patients undergoing cardiac surgery, presence of a 3-lumen catheter and jugular location compared to subclavian. There were no significant differences in the level of antithrombin III.

**CONCLUSIONS.** In this group of patients, the incidence of central venous catheter-related venous thrombosis was 45%, however, all patients were asymptomatic. Studies with a greater number of patients are required to establish risk factors and the need for thromboprophylaxis.

### 0801

#### Picking up the pieces -qualitative evaluation of follow-up consultations with patients post intensive care treatment

A.O. Glæemose[1], A.L.B. Hanifa[1], B.S. Laursen[2,3]

[1]Aalborg University Hospital, Intensive Care Unit R, Clinical Nursing Research Unit, Aalborg, Denmark; [2]Aalborg University Hospital, Clinical Nursing Research Unit, Aalborg, Denmark; [3]Aalborg University, Department of Clinical Medicine, Aalborg, Denmark

**Correspondence:** A.O. Glæemose

*Intensive Care Medicine Experimental* 2017, **5(Suppl 2):**0801

**INTRODUCTION.** National and international studies show that critically ill patients suffer from Post Intensive Care Syndrome (PICS). PICS is defined as new or worsening problems in physical, cognitive or mental health status arising after critical illness and persisting beyond acute care hospitalization(1)

In an intensive care unit in a University Hospital in Denmark patients are offered a nurse-led consultation 3 months post intensive care unit (ICU) admission, to help them cope with PICS and identify opportunities for further intervention.

**OBJECTIVES.** 1) To describe former intensive care patients' experiences of the consultation, specifically regarding content and setting. 2) To explore the benefits of the consultation with regards to the individual patient's symptoms of PICS.

**METHODS.** A two-part qualitative study: 1) an observational study of the current follow-up consultation; 2) a semi-structured interview with 10 patients, conducted approximately two weeks later, where the interview guide was based upon observations and statements arising during the initial consultation.

**RESULTS.** The study is ongoing, however, the preliminary findings suggest that the content and setting are of upmost importance. Revisiting the unit and experiencing the setting in person plays a huge role in coping after surviving critical illness. Involving relatives also proves to be of great importance. During analysis of the data regarding the benefits of the consultation in relation to the patient's symptoms of PICS, four major themes arose:1) Confronting the demons.2) Coming to terms with the reality of having been critically ill.3) Making sense of the symptoms.4) Regaining a sense of normality.The themes are closely linked, as all are related to the patient's ability to cope with the traumatic event. An important benefit for many patients turned out to be a sense of relief knowing that other patients experience the same symptoms. Many patients did not know how their symptoms were related to having been critically ill, and described themselves in condescending terms and this caused a great deal of anxiety.

**CONCLUSIONS.** The patients who participated in the study were generally pleased with the consultation. The most important part of the consultation proved to be viewing the unit, as seeing the setting and hearing the sounds initiated coping. The consultation helped the patients understand the symptoms they were suffering from, and to make sense of what had happened during their stay in ICU. The findings are of importance when developing the follow-up service in the future.

**GRANT ACKNOWLEDGMENT**

None

## Poster Corner Sessions Wednesday, 27 September 2017

### Patient safety

### 0802

#### Impact of incorporating High Fidelity Manikin (HFM) simulation in Medical Emergency Response Team (MERT) training on achieving AHA recommendations for acute cardiopulmonary events

R. Hellmann[1], D. Klein[1], P. Jadav[1], M. Krishna[2], Touro College of Osteopathic Medicine, Middletown

[1]Touro College of Osteopathic Medicine, D.O., Middletown, United States; [2]Orange Regional Medical Center, Pulmonary, Critical Care & Sleep Medicine, Middletown, United States

**Correspondence:** R. Hellmann

*Intensive Care Medicine Experimental* 2017, **5(Suppl 2):**0802

**INTRODUCTION.** The American Heart Association (AHA) published guidelines outlining a universal standard of care for patients who experience in-hospital resuscitation or receive post cardiac arrest care following an in-hospital or out-of-hospital event (1). While some studies have suggested that MERT response helps to reduce mortality from unexpected cardiac arrest and reduce the number of unexpected ICU admissions (2), there remains a paucity of data on the impact of simulation based MERT training using HFMs on achieving the recommended AHA guidelines.

**OBJECTIVES.** HFM use in simulation training has demonstrated a benefit in clinical outcomes by providing a more realistic setting to improve clinical skills and facilitate communication amongst emergency team members (3). This study aims to explore if HFM simulation use during MERT training will result in better adherence to the AHA guidelines and improve patient outcomes and decrease mortality.

**METHODS.** We performed a retrospective blinded cohort study of cardiopulmonary/cardiac arrest codes called at ORMC before and after the implementation of the HFM-MERT program.

Analysis included Student´s T test and Chi-Square of AHA metric adherence both during the code and post-resuscitation. Additionally, we compared hospital staff confidence in the MERT between groups via anonymous survey and the impact HFM-MERT training had on hospital acute care mortality rates.

**RESULTS.** Statistically significant differences were found in time to delivery of first shock for ventricular fibrillation (VF) or pulseless ventricular tachycardia (pVT). No other comparison reached statistical significance. There was a downward trend in acute care mortality rates. There was an upward trend of hospital staff confidence in the MERT, as well as willingness to call the team.

**CONCLUSIONS.** We hypothesized that HFM incorporation would significantly improve current MERT training for acute cardiopulmonary events. Though HFM-MERT training showed little improvement with respect to AHA metrics, it demonstrated the strong impact of MERT training on team dynamics. MERT training improved communication amongst team members and strengthened the confidence of hospital personnel in the MERT. When correlated with a downward trend in mortality, HFM use in MERT training demonstrates great utility.

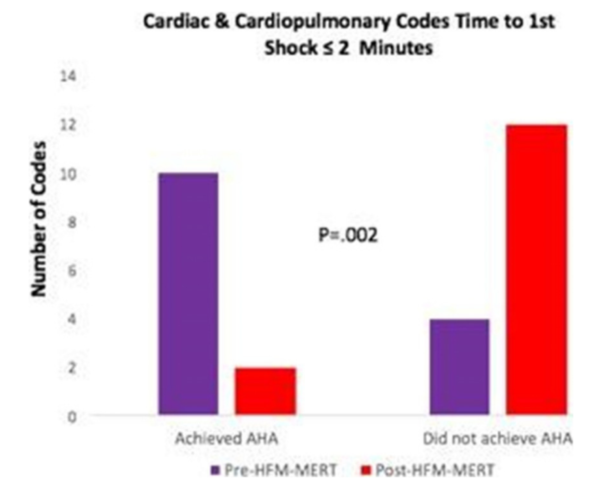

**Fig. 264 (Abstract 0802).** Cardiac & Cardiopulmonary Codes Time to 1st Shock

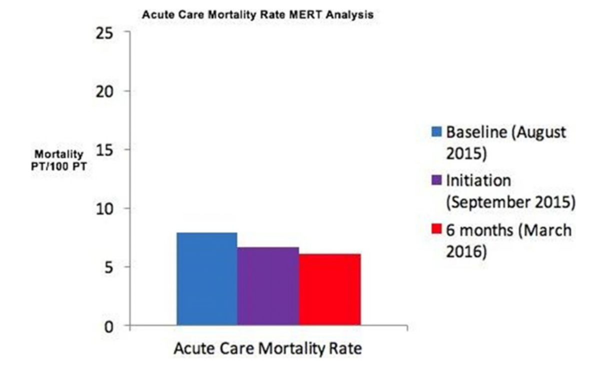

**Fig. 265 (Abstract 0802).** Acute Care Mortality Rates

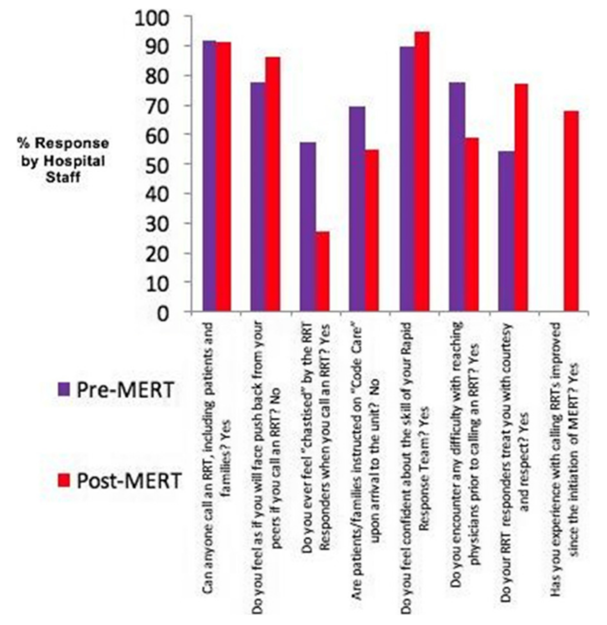

**Fig. 266 (Abstract 0802).** Hospital Staff Survey MERT

### 0803

#### Adverse events in critical care: search and active detection through the trigger tool

F. Molina[1], P. Mejía[1], A. Cardona[2], O. Monroy[3], D. Restrepo[2], D. Rodas[1], J.G. Barrientos[1]
[1]University Pontificia Bolivariana, University Clinic Bolivariana, Intensive Care, Medellín, Colombia; [2]University Pontificia Bolivariana, Medellín, Colombia; [3]University Pontificia Bolivariana, Quality, Medellín, Colombia
**Correspondence:** F. Molina
*Intensive Care Medicine Experimental* 2017, **5(Suppl 2):**0803

**INTRODUCTION.** Adverse events are defined as an injury caused by medical treatment and not by the underlying disease. There are two methods of quantification and search for adverse events: passive and active. Of the latter, the Trigger Tool looks for signs of potential unpleasant events in a sample of medical records.

**OBJECTIVE.** It intends to establish the incidence of disadvantageous events by using the Global Trigger Tool in a high complexity Intensive Care Unit.

**METHODS.** Analytical cross - sectional study between January 1 to December 31, 2016, in a uci of university character of 12 beds in the city of Medellín, Colombia. Trained reviewers conducted a retrospective examination of medical charts searching for clue events that elicit the investigation, in order to detect an unfavorable event. Information was processed through SPSS software version 21; for numerical variables, the mean was reported with standard deviation. Percentages were calculated for qualitative variables.

**RESULTS.** 244 triggers occurred; 82.4% of subjects presented with at least one, and the average was 3.37 (SD 3.47). A total of 178 adverse events took place in 48 individuals, with an incidence of 52.1%.

On average, four events per patient were recorded, and for each unfortunate event, 1.98 triggers were presented. The most frequent displeasing issues were: pressure ulcers (17.6%), followed by complications or reactions to medical devices (4.3%), lacerations or skin defects (3.7%), the least presented was delayed diagnosis or treatment (0.56%). 38.4% of mishap events caused temporary damage that required intervention; 48.9% of adverse events were preventable.

| TRIGGER | n | % |
|---|---|---|
| Skin defects or lacerations | 36 | 14,7 |
| Excitation or drowsiness of the patient | 34 | 13,9 |
| Unscheduled withdrawal of surgical catheter, probes, drains or other devices | 34 | 13,9 |
| Hypotension | 33 | 13,5 |
| Initiation of antibiotics after 48 hours of admission | 28 | 11,4 |

*[Triggers.]*

| ADVERSE EVENT | n | % |
|---|---|---|
| Pressure ulcers | 62 | 17,6 |
| Complications or reactions to medical devices | 15 | 4,3 |
| Lacerations | 13 | 3,7 |
| Drug-induced hypotension | 10 | 2,8 |
| Nosocomial pneumonia | 9 | 2,6 |

*[Adverse events]*

**CONCLUSIONS.** Almost half of the unfavorable issues were classified as avoidable, which leaves a very wide field of work in terms of preventative activities for the occurrence of such events.

**GRANT ACKNOWLEDGMENT**

University Pontificia Bolivariana

University Clinic Bolivariana

### 0804

#### Risk reduction in ICU aminoglycoside prescribing through a multidisciplinary quality improvement initiative

N. Cox, E. Ahmadnia, M. Passariello

Royal Brompton Hospital, Adult Intensive Care Unit, London, United Kingdom

**Correspondence:** N. Cox

*Intensive Care Medicine Experimental* 2017, **5(Suppl 2):**0804

**INTRODUCTION.** Aminoglycoside antibiotics are commonly used for the treatment of severe bacterial infections in the intensive care unit [1]. However these drugs require close monitoring to prevent adverse effects such as nephrotoxicity and ototoxicity [2]. This is of particular concern in critically unwell patients, in whom pharmacokinetics are less predictable [3]. Incorrect dosing can contribute to this risk, with studies of surgical patients suggesting that only 30% of patients receive the appropriate dose of gentamicin [4].

**OBJECTIVES.** To determine the burden of high trough serum aminoglycoside concentrations, and the impact of a quality improvement project on these.

**METHODS.** A quality improvement project was conducted at a large UK University adult cardiothoracic ICU from January to March 2017. During an initial 15 day audit period, the following were recorded: the incidence of high trough levels of aminoglycosides, the response to a high level (in terms of drug administration and change in prescription), as well as overall adherence with local guidelines. Preliminary interventions involved raising awareness among staff via clinical governance meetings and daily safety briefings, as well as specific departmental pharmaceutical teaching for all ICU medical and nursing staff. A second analysis of practice was conducted following these interventions.

**RESULTS.** During the first audit period there were 8 patients on intravenous aminoglycosides and 16 instances of a high trough level - subsequent doses were correctly omitted in all cases in response to these high trough levels. These omitted doses accounted for 57% of all prescribed doses (16/28), and an amendment in prescription to prevent further high levels was only made in 25% of cases (4/16). Inappropriate prescribing was identified as the chief cause for high trough levels: prescriptions were not appropriately adjusted for renal function and ideal body weight. During the second audit period (following systematic multidisciplinary departmental teaching), there were 7 patients on aminoglycoside antibiotics and only 7 incidences of a high trough level - again all doses were correctly omitted. This time only 21% of all prescribed doses were omitted (7/33) and correct adjustments to prescriptions were made in 40% of cases (2/5).

**CONCLUSIONS.** At a University cardiothoracic ICU, inappropriate aminoglycoside prescribing may have contributed to over half of all prescribed doses needing to be omitted, increasing the risk of inadvertent toxic dosing. Department-wide teaching in aminoglycoside prescribing and monitoring appeared to be effective in reducing the incidence of high trough levels and subsequent risk of toxicity.

### 0805

#### Application of the real time random safety audits (AASTRE) in an intensive care unit

P. Blanco-Schweizer, M.M. García García, E. Prol Silva, A. Prieto de Lamo, P.A. Merino, L. Tamayo Lomas, J.M. Martinez, P. Mateo, L. García, R. Vidal, J. Blanco Varela

Hospital Universitario Rio Hortega, Intensive Care, Valladolid, Spain

**Correspondence:** P. Blanco-Schweizer

*Intensive Care Medicine Experimental* 2017, **5(Suppl 2):**0805

**INTRODUCTION.** Patient´s safety in Intensive Care Units has become a real concern among care givers. Different tools have been proposed to reduce errors during everyday care. Real time random safety audits (AASTRE) is a validated checklist that has 37 safety measures which are randomly checked in ICU patients in order to detect and solve errors in many aspects of their health care.

**OBJECTIVES.** To describe the results of the application of AASTRE in a 17 bed medical and surgical intensive care unit (ICU) and to address the feasibility of it.

**METHODS.** Observational clinical study in which all patients admitted to the ICU between 01/11/2016 and 28/02/2017 were included. The AASTRE was performed 2 times a week. Patients who underwent the audits were randomly selected. 10 out of 37 validated safety measures grouped in 10 blocks were randomized to be checked in the selected patients. Data are expressed in as means, medians and proportions, and comparisons are made with Chi-squared test and Fisher´s exact test as needed.

**RESULTS.** Number of patient-days 302. Number of measures assessed 1984 of which 617 were not applicable. 30 times were these audits supposed to be made and 25 times (83.3%) were they actually performed. 3 times it was forgotten and 2 times because of lack of time. Proportion of changes carried out in patient management as a result of the application of AASTRE (IPR-AASTRE) 7.89%. Potential total changes that could have been carried out 11.56% with a difference between both of 3.67% (CI 95% : 1.97%-6.35%, p < 0.001). IPR-AASTRE and Potential total changes when occupation above the median (14 beds) Odds ratio = 0.81 (CI 95% 0.46-1.40, p > 0.05). The blocks in which IPR-AASTRE and potential changes were the highest were: mechanical ventilation, treatment, hemodynamics and techniques and tests (Table Error! Reference source not found.).

IPR-AASTRE and potential changes were compared by several groups such as physitian:patient ratio and nurse:patient ratio showing contrary results. SOFA score and length of stay do not represent major changes (Table 201).

**CONCLUSIONS**

1. The power of the real time random safety audits (AASTRE) to detect potential measures that have to be checked or modified for the everyday patient care in this unit is high. The real changes made after this detection (IPRA-AASTRE) hardly ever reach 100%.
2. A patient:physitcian ratio higher than 3 increases potential changes but reduces IPR-AASTRE while the opposite happens with patient:nurse ratio higher than 2.
3. Performance of the AASTRE rounds is feasible in an intensive care unit like the one tested with a high percentage of accomplishment.

**REFERENCE(S)**

1. M. Bodí, M. Olona, M.C. Martín, R. Alceaga, J.C. Rodríguez, E. Corral, J.M. Pérez Villares and G. Sirgo. (2015) Feasibility and utility of the use of real time random safety audits in dult ICU patients:a multicenter study. Intensive Care Med(41)1089-198.

2.G.Sirgo, M. Olona, M.C. Martín, F. Esteban, A. Pobo, M. Bodí, ART-SACC study experts. Med Intensiva(38)473-482.

**Table 200 (Abstract 0805)**. Results of the study per block of measures

| BLOCK | IPR-AASTRE (% of total) | POTENTIAL TOTAL CHANGES (% of total) | p |
|---|---|---|---|
| Mechanical ventilation | 14.29 | 23.08 | > 0.05 |
| Haemodynamics | 5.86 | 13.28 | < 0.05 |
| Renal function an CRRT | 2.63 | 2.63 | 1 |
| Sedation and analgesia | 4.21 | 11.58 | 0.06 |
| Treatment | 25.58 | 36.88 | < 0.05 |
| Techniques and tests | 14.77 | 18.18 | > 0.05 |
| Nutrition | 3.23 | 3.23 | 1 |
| Nursing care | 6.51 | 8.46 | > 0.05 |
| Structure | 5.56 | 7.15 | > 0.05 |

**Table 201 (Abstract 0805)**. Results of study per group

| | Patient:physitian ratio | | Nurse:physitian ratio | | SOFA score | | Length of stay (days) | Length of stay (days) | Length of stay (days) |
|---|---|---|---|---|---|---|---|---|---|
| | <= 3 | > 3 | <= 2 | > 2 | <= 7 | > 7 | < 7 | 7-14 | > 14 |
| Potential total changes (% of total) | 11.25 | 28.6 | 12.89 | 3.57 | 22.92 | 23.82 | 2.94 | 5.14 | 5.97 |
| IPR-AASTRE (% of total) | 7.61 | 2.17 | 7.89 | 3.57 | 13.6 | 15.42 | 7.28 | 7.85 | 7.23 |

### 0806

#### Medication safety on the ICU - a 3 year analysis and improvement process in a tertiary level ICU

A. Wong, C. Lumley, ICU Medication Safety Group

Oxford University Hospitals NHS Trust, Adult Intensive Care Unit, Oxford, United Kingdom

**Correspondence:** A. Wong

*Intensive Care Medicine Experimental* 2017, **5(Suppl 2):**0806

**INTRODUCTION.** It is estimated that 1.5 million preventable adverse drug events occur annually in the USA with the ICU being particularly at risk. It is estimated that the average ICU patient has over 150 activities per day. Although medication administration accounts for most of the errors in the acute care setting, errors in prescribing, transcribing, preparing and dispensing medications also occur. The complex nature of healthcare means that the cause of such errors are multi-factorial [1, 2]. As a result, it is difficult to pinpoint the problem areas and solutions necessary to avoid preventable medication errors in these settings. We decided to tackle this issue through a multimodal, multidisciplinary approach in a tertiary level ICU.

**OBJECTIVES.** To improve medication safety at all stages of the process: prescribing to administration and recording.

**METHODS.** A multidisciplinary medication safety group (MRG) team reflective of the workforce on ICU was convene under the leadership of a dedicated ICU pharmacists. A process was established such that every step of the administration of medication was re-examined. Error reporting was encouraged through a no blame culture. Investigation pathways were streamlined to ensure that reports and lessons were produced in a timely fashion. Innovative initiatives were rolled out through the use of named champions on the shop floor.

**RESULTS.** In the 2 years prior to the establishment of the MRG, there were 149 reported medication errors with 1 of moderate harm. In the year following the establishment of the MRG, the number of reported errors had increase to 131. Non were graded as severe or resulting in death. The majority of these reports were near misses rather than actual errors. Where errors had occurred, these were primarily during the administration phase although prescribing errors still occurred.

Initiatives introduced during this period included:

- Smart electronic prescribing software
- Red aprons to prevent nurse interruptions
- Short "At A Glance" notices highlighting key lessons
- Monthly analysis of error trends
- Staff support and reflection when errors occur
- Representation at senior trust level on medical safety initiatives

**CONCLUSIONS.** Medication errors are a considerable source of mortality and morbidity on the ICU. Increased reporting of errors and near misses are reflective of an open, honest culture within the organisation. A multidisciplinary and multimodal approach is required to address the issue. Simple, ground-led initiatives have the potential to reduce the level of harm without significant investment in resources.

**REFERENCE(S)**

1. Krahenbuhl-Melcher A, Schlienger R, Lampert M, Haschke M, Drewe J, Krahenbuhl S. (2007), "Drug-Related Problems in Hospitals", Drug Safety, 30 (5): 379–407.
2. Valentin A, Capuzzo M, Guidet B, Moreno R, Metnitz B, Bauer P, Metnitz P. (2009) "Errors in administration of parenteral drugs in intensive care units: multinational prospective study", BMJ, 338;b814.

### 0807

#### Errors in preparation and administration of medication in a Tunisian teaching hospital medical ICU: an evaluation of professional practices (EPP) study

A. Khedher[1], M.A. Boujelbèn[1], W. Zarrougui[1], J. Ayachi[1], N. Fraj[1], E. Ennouri[1], H. Kallel[1], A. Ben Abdelhafidh[1], I. Ben Saida[1], A. Azouzi[1], I. Chouchene[1], M. Boussarsar[1,2], H. Fares[3], G. Belhabib[3], O. Gloulou[3], N. Chouchen[3]
[1]Farhat Hached University Hospital, Medical Intensive Care Unit, Sousse, Tunisia; [2]Ibn Al Jazzar Faculty of Medicine, Research Laboratory N° LR14ES05 Interactions of the Cardio-pulmonary System, Sousse, Tunisia; [3]Farhat Hached University Hospital, Department of Pharmacology, Sousse, Tunisia
**Correspondence:** M. Boussarsar
*Intensive Care Medicine Experimental* 2017, **5(Suppl 2):**0807

**INTRODUCTION.** The preparation and administration of drugs are a series of complicated technical skills especially in critical care. Hence, errors are particularly prevalent during this process. The consequences of such errors vary from relatively harmless to lethal.

**OBJECTIVES.** To determine the frequency, typology and impact of medication's administration and preparation errors in a medical ICU.

**METHODS.** An EPP study was conducted in an 8-bed tertiary medical ICU. Data were collected by a trained pharmacist observer who directly accompanied nurses during intravenous (IV) or per os (P.O.) drug rounds between October and December 2015. Details of the process of preparation and administration of the selected drugs were compared to an informed checklist which was based on reference books and manufacturers´ instructions. The medication errors were classified according to the stage of the medication process and the impact categorized according to the National Coordinating Council for Medication Errors Reporting and Prevention (NCC MERP) taxonomy.

**RESULTS.** 76 patients were managed within the 3 months study period. A total of 25 drugs (20 IV and 5 P.O.) were selected for this study. Were inspected 610 preparations and administrations. Of those, 96(15.7%) errors were identified : 75(78.1%) in the preparation process and 21(21.8%) in the administration process. IV antibiotics were involved in the highest rate of error 54(54.2%) followed by the cardiovascular drugs 12(12.5%). The most common errors in the preparation process were: wrong amount of dilution for 39% of the selected IV drugs, crushing problem for 100% of the P.O. drugs and not inspecting for expiration date for 52% of all the drugs. The most frequent errors in the administration stage were wrong time (12%) and incorrect administering rhythm (27%). For respectively 64% and 16% of the screened drugs, the errors were categorized as resulting in the need for increased patient monitoring but no patient harm and as an error reaching the patient but did not cause patient harm.

**CONCLUSIONS.** This study shows that there are more medication errors in the preparation than in the administration stage. This could have important implications for critically ill patients safety. Further educational activities, epidemiological knowledge, detection of errors, and improvements in performance are needed to reduce the risk of medication errors.

### 0808

#### Use of a novel “simbulance” transfer course to better prepare staff for critical care transfers

E. Joynes, S. Gupta, D. Windsor, S. Twigg
Gloucestershire NHS Foundation Trust, Critical Care Department, Gloucester, United Kingdom
**Correspondence:** S. Gupta
*Intensive Care Medicine Experimental* 2017, **5(Suppl 2):**0808

**INTRODUCTION.** There is an increasing demand for inter-hospital transfers due to centralisation of specialist care without universal availability of retrieval teams and increasing bed shortages. Currently there is no single recommended national training course in the UK.[1] A preliminary survey of trainees in our department (n = 17) found that 65% had received formal transfer training and less than 50% were aware of national guidelines. Inadequacies in training along with human factors such as working in an unfamiliar environment and poor teamwork are known to perpetrate incidents.[2,3]

**OBJECTIVES.** A one-day, inter-professional simulation course was aimed at trainee doctors and critical care nurses. Bespoke simulation was provided by South Western Ambulance Service Network in the form of a clinical simulation vehicle “simbulance.” The model aimed to address knowledge of transfer processes and human factors such as familiarity with the non-hospital environment. Objectives aimed to improve attendees' knowledge of guidelines and confidence in carrying out transfers by defining risks, demonstrating use of equipment and stabilisation of patients, and completing documentation.

**METHODS.** A retrospective survey was carried out anonymously using a 5-point Likert questionnaire to assess whether each learning objective was met and to determine the usefulness of training. The mean for each question was calculated and written feedback was collated.

**RESULTS.** We distributed 16 questionnaires and received 15 back. There was strong evidence to demonstrate that all course objectives were met as respondents declared high confidence in these (all means were between 4.2 and 4.6). Participants agreed that they had learnt (mean 4.6) and organisation was high quality (mean 4.7). Average confidence levels increased by a point (mean before training 3.3, after training 4.4). Written feedback was positive and highlighted areas for improvement including the allocation of lead nursing roles.

**CONCLUSION.** Evidence shows simulation improves training in critical care.[4] The need to improve staff preparation for inter-hospital transfers was met through the development of a “simbulance” course. We have demonstrated improved learning and satisfaction amongst attendees and aim to roll out this course biannually to allow accessibility.

**REFERENCE(S)**

1 Droogh JM, Smit M, Absalom AR et al. (2015) Transferring the critically ill patient: are we there yet? Crit care. 19(1): 62.

2 Cook CJ, Allan C (2008) Are trainees equipped to transfer critically ill patients. JICS. 9:145–7.

3 Flabouris A, Runciman WB, Levings B (2006) Incidents during out-of-hospital patient transportation. Anaesth Intensive Care. 34:228–36.

4 Frengley RW, Weller JM, Torrie J et al. (2011) The effect of a simulation-based training intervention on the performance of established critical care unit teams. Crit Care Med. 39: 2605–11.

### 0809

#### How to convince an entire staff of the advantages of using AnaConDa supported by evidence based practice

C. Naalden, Sedation group
Akershus University Hospital, ICU, Lørenskog, Norway
**Correspondence:** C. Naalden
*Intensive Care Medicine Experimental* 2017, **5(Suppl 2):**0809

**INTRODUCTION.** Use of ICU gassedation with the help of AnaConDa is new in Norway. Our ICU experienced resistance with the implementation because we were unsure and negative because of a lack of training and knowledge.

**OBJECTIVES.** First we needed to identify the knowledge level of the ICU staff. What kind of information and training did our staff need to be confident using this form of treatment? Our ICU department has dedicated 6 workdays for competence development training per year, in cooperation with the competence development nurse. We used time at 2 of those days to ensure our staff got sufficient knowledge and confidence in using gassedation.

**METHODS.** We have our competence development days put in a system. There is an intern training, simulations, theory and practical education. After the analysis and research of the knowledge gap we could work towards a targeted training. With this evidence based practice training we have convinced the staff of the benefits. The ICU nursing staff is now interested to use the AnaConDa system.

**RESULTS.** These competence development days has resulted in an increased use of the AnaConDa device. From an insecure start to turn it into a positive implementation. We are only having 2 laggards among 90 ICU nurses. It has been proven that 2 hours theory, and then practical training with the AnaConDa, has been successful.

**CONCLUSIONS.** With the help of competence development days put in a system we can make a positive improvement by staff taking time to read up in the specific subject and teaching their colleagues. Using Evidence Based Practice thinking and training in our department, we now wish to develop training of other rarely used medical devices due to the successful implementation with the AnaConDa device.

**REFERENCE(S)**

Meiser, Andreas og Laubenthal, H (2005). *Inhalational anaesthetics in the ICU: theory and practice of inhalational sedation in the ICU, economics, risk-benefit.* Best Practice & Research Clinical Anaesthesiology (19), 523–538.

Mesnil, Malcie et al., (2011). *Long-term sedation in intensive care unit: a randomized comparison between inhaled sevoflurane and intravenous propofol or midazolam.* Intensive Care Med (37), 933–941.

Nordtvedt, Monica W., et al. (2012). *Jobb Kunnskapsbasert. En arbeidsbok.* Oslo: Akribe.

**GRANT ACKNOWLEDGMENT**

Lisbeth Grenager our compentence development nurse

### 0810

#### Critical care transfers - how is the North East North Central London network is performing?

O. Nikolic[1], F. Kovari[2]
[1]Royal Free Hospital NHS Foundation Trust, London, United Kingdom; [2]North Middlesex University Hospital Trust, London, United Kingdom
**Correspondence:** O. Nikolic
*Intensive Care Medicine Experimental* 2017, **5(Suppl 2):**0810

**INTRODUCTION.** In London Critical Care Networks are coordinating level 1–3 transfers. There are several hundreds of critical care transfers happening yearly in London area and this number is expected to be on the rise.

**OBJECTIVES.** Our aim was to audit the quality of all interhospital transfers happened in year 2015/16 in our Network (NENC-North East North Central Critical Care Network). Also we wanted to see how accurately transfers are documented in the newly introduced audit sheets hoping that we can identify incidents and develop further learning needs along with a new transfer protocol.

**METHODS.** We have collected one hundred and two audit sheets from eight different network hospitals. We audited data of transfer level, referring team, locations, diagnosis, organ support provided, comments of the teams and incidents.

**RESULTS.** We have analyzed one hundred and two audit sheets. The total number of level 3 transfers was sixty eight (67%), level 2 twenty six (25%), level 1 three (3%) and we had five (5%) patients where the data was missing.

We have identified seven (7%) incidents where one was linked to equipment failure one was indicated as incident but no recorded reason was given and in five occasions patients have deteriorated.

When we checked on the working diagnosis we found that the majority of the transfers were done due to neurology and cardiology reasons (13-13%) followed by gastroenterology and liver problems (11%) renal (8%) and respiratory (7%) problems.

The working diagnosis was not recorded 40% of the cases.

Urgent transfer was carried out in sixty eight patients ((61%) elective in twenty one (21%) non clinical transfer was done in only five occasions (5%).

**CONCLUSIONS.** Our network within London (NENC) has been reestablished only a year ago. We carried out this audit to see the compliance to the current practice and identify the areas where improvement is much needed.Of serious concern was the significant lack of data capture despite a standardized transfer form. This may be due to lack of time when managing a critically ill patient or lack of introduction to transfer processes in frequently rotating doctors.

Last year we have set up a transfer training course and wrote a new guideline on critical care transfers.We are liaising with the hospitals within the network to emphasize the need of better documentation and capturing all patients undergoing inter hospital transfers and put a lot of effort to train the frontline staff regularly.

**REFERENCE(S)**

1. Intensive Care Society. Guidelines for transport of the Critically ill adult. London: Intensive Care Society, 2011.

2. Department of Health. Comprehensive Critical Care. A review of adult critical care services. London: Department of Health, 2000

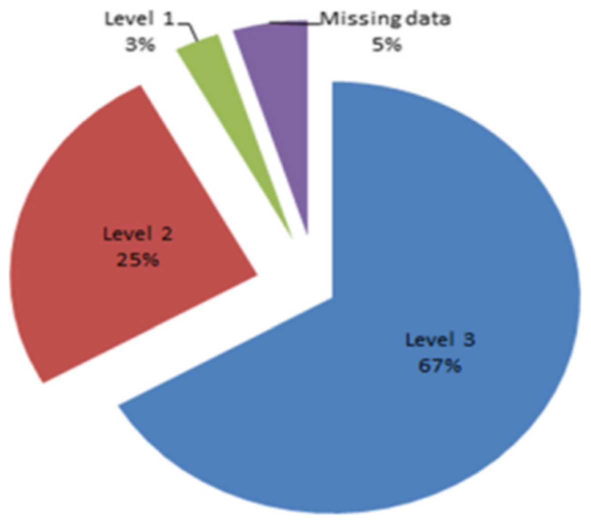

**Fig. 267 (Abstract 0810).** Level of care

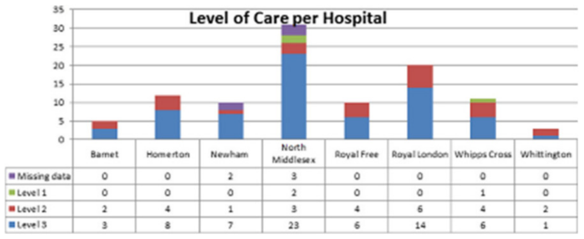

**Fig. 268 (Abstract 0810).** Level of care hospitals

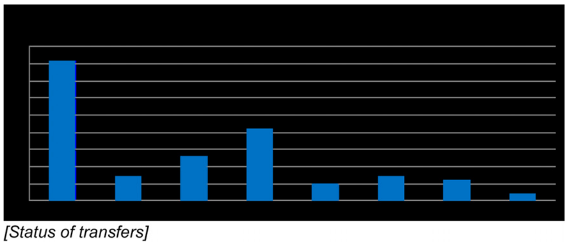

**Fig. 269 (Abstract 0810).** Status of transfers

### 0811

#### Effect of securement device for arterial catheter on catheter-related complications

Y. Tsunano, E. Nakataki, T. Itagaki, J. Oto, M. Nishimura
Tokushima University Hospital, Tokushima, Japan
**Correspondence:** Y. Tsunano
*Intensive Care Medicine Experimental* 2017, **5(Suppl 2):**0811

**INTRODUCTION.** Arterial catheter is used for continuous hemodynamic monitoring in the ICU. The catheter is often indwelled in radial artery, and motion of wrist leads to occlusion of the catheter, hematoma, bleeding, flare and loss of arterial waveform. The securement device, StatLock® (C.R. Bard, Inc. NJ), fixes the catheter at constant angle and reduces the catheter shaking by the motion of wrist.

**OBJECTIVES.** We investigated if the securement device prevented complications related to arterial catheters.

**METHODS.** This was the prospective randomized controlled study in a 10-bed medical-surgical ICU. We included patients 18 years or older who were expected to be inserted catheter in radial artery more than 2 days. They were randomly assigned to a securement device group (StatLock®) or control group. In the control group, catheter was secured by dressing tape (IV 3000 ®, Smith& Nephew). We recorded complications related to arterial catheters, bleeding, flare, and loss of arterial waveform. In addition we evaluated bacterial contamination of the catheters.

**RESULTS.** From May 2012 to October 2015, the catheters of 183 patients were included in this study. Twenty three patients were excluded due to short duration of arterial catheter insertion or poor records. Eighty samples in StatLoc group, 80 samples in control group were evaluated. Age, APACHEII score and the catheter-insertion period were not different between the groups. The incidence of complications was significantly lower in StatLoc group (26.3% vs 12.5%; p = 0.028). Bacterial contamination did not differ between the groups (7.5% vs 6.3%; p = 0.75).

**CONCLUSIONS.** We investigated if arterial catheter securement device prevented the complications at the arterial catheter insertion site. The incidence of complications significantly decreased with the device.

### 0812

#### Multidisciplinary care can protect patients from radiation exposure at percutaneous coronary intervention

W.-C. Huang[1,2,3], W.-H. Wang[1], S.-H. Kuo[1], K.-C. Wei[1], Y.-Y. Yen[1], S.-L. Wu[1], K.-J. Hsu[1], K.-C. Hsu[1], C.-C. Hung[1], C.-H. Chiang[1,2], C.-C. Cheng[1,2], F.-Y. Kuo[1], K.-H. Liu[1], P.-L. Kang[1,2], G.-Y. Mar[1], S.-C. Lin[1], K.-C. Lin[1], W.-T. Huang[1], C.-P. Liu[1,2]
[1]Kaohsiung Vaterans General Hospital, Critical Care and Cardiovascular Center, Kaohsiung City, Taiwan, Province of China; [2]National Yang-Ming University, School of Medicine, Taipei, Taiwan, Province of China; [3]Fooyin University, Department of Physical Therapy, Kaohsiung, Taiwan, Province of China
**Correspondence:** S.-H. Kuo
*Intensive Care Medicine Experimental* 2017, **5(Suppl 2):**0812

**INTRODUCTION.** The use of radiation is associated with a risk of causing skin or eye damage and inducing malignant disease. However, most interventional cardiologists neglected protection of radiation exposure to the patients or operators.

**OBJECTIVES.** This study aimed to improve protection of radiation exposure to the patients received percutaneous coronary intervention via multidisciplinary care.

**METHODS.** A multidisciplinary team, including interventional cardiologists, intensivist, dermatologist, radiation technicians and nursing staffs, was organized since Jan, 2016. The patients were divided into three groups: pre-interventional group (n = 61) from March to November 2016, Interventional group (n = 48) from December 2016 to January 2017 and post-interventional group (N = 42) from February to March 2017. The early mobilization is defined as mobilization within 48 hours after admission. The key interventions include reduce frame rate of *fluoroscopy with 15* frames/seconds instead of conventional 30 frames/seconds, reduce frame rate of *video recording with 7.5* frames/seconds instead of conventional 15 frames/seconds, guideline for radiation protection and innovative head, eye and neck protection device. A *P*-value < 0.05 was considered statistically significant.

**RESULTS.** The baseline characteristic of three groups did not differ in age, body weight, body height or fluoroscopy time. The eye radiation dose of patients improved from 4.5 ± 4.0 mSV in pre-interventional group, to 2.3 ± 2.5 in interventional group and 0.9 ± 0.7 mSV in post-interventional group (p < 0.001). The neck radiation dose of patients reduced from 17.5 ± 16.6 mSV in pre-interventional group, to 8.2 ± 8.8 in interventional group and 4.8 ± 4.2 mSV in post-interventional group (p = 0.001). The back radiation dose of patients reduced from 189.4 ± 292.1 mSV in pre-interventional group, to 48.2 ± 54.2 in interventional group and 43.6 ± 56.1 mSV in post-interventional group (p = 0.004). The radiation dose of doctors reduced from 2.07 ± 1.46 mSV in pre-interventional group, to 1.30 ± 1.29 in interventional group and 1.46 ± 0.71 mSV in post-interventional group (p = 0.036).

**CONCLUSIONS.** This study demonstrates multidisciplinary with lower radiation protocol and innovative protection device can reduce eye, neck and back radiation dose at percutaneous coronary intervention. Furthermore, the radiation dose of doctors also reduced.

### 0813

#### Multi-infusion training

R.A. Snijder[1], P.P.A. Klaver[1], A. Carter[2], P. Lucas[3], A.I. Rem[1], E. Batista[4], J. Preller[5], A.M.D.E. Timmerman[1]
[1]University Medical Center Utrecht, Utrecht, Netherlands; [2]University College London Hospital, London, United Kingdom; [3]VSL Dutch Metrology Institute, Delft, Netherlands; [4]Portuguese Institute for Quality, Lisbon, Portugal; [5]Cambridge University Hospitals NHS Foundation Trust, Cambridge, United Kingdom
**Correspondence:** A.M.D.E. Timmerman
*Intensive Care Medicine Experimental* 2017, **5(Suppl 2):**0813

**INTRODUCTION.** There is a significant risk associated with infusion technology in the critical care setting. Due to limited vascular access, clinicians typically connect multiple infusion pumps to one infusion set and catheter. This practice is called multi-infusion. Within the completed Metrology for Drug Delivery (MeDD) project[1], we have gathered ample evidence that multi-infusion is associated with dosing errors due to ambiguous physical effects (e.g. Fig. 270).[2] Despite the fact that many of these dosing errors are due to technical properties, they can be mitigated or even prevented if multi-infusion users (physicians, nurses, technicians) are given proper and comprehensive training.

**OBJECTIVES.** To develop a useful multi-infusion training e-learning course.

**METHODS.** First, we gained insights in the physical mechanisms of multi-infusion through systematic literature analysis[2], experimentation and modelling. Next, a "survey of best practices", in which healthcare professionals were surveyed, was used to list the current practices in infusion therapy and identify the potential niche where education is required. Surveying professionals is continued during the development of the e-course.

**RESULTS.** It was decided to develop a multi-infusion training e-course using the ESICM e-learning platform. From surveying clinicians, the consensus was that the course should fundamentally be presented from a clinical perspective. Consequently, our preliminary results present ten realistic critical care cases resulting in adverse events due to physical effects. Each case will consists of approximately ten multiple choice questions. The topics range from basic drug concentration and flow rates relationships, to a profound understanding of the implication of the common (dead) volume, i.e. the volume between a mixing point and the outflow into the patient. The learners will be supported by illustrative artwork and some textual theory. We plan to present the preliminary results of the e-learning course on site and at congresses. This will enable us to continuously improve the content throughout the development phase.

**CONCLUSIONS.** The preliminary results have been presented to clinicians, who stressed the importance of an e-learning course about multi-infusion. We will continue development accordingly.

**REFERENCE(S)**

1. Lucas P. MeDD - Metrology for drug delivery, EU-funded research project [Internet]. 2015 [cited 2015 Jan 1];Available from: www.drugmetrology.com

2. Snijder RA, Konings MK, Lucas P, Egberts TC, Timmerman AD. Flow variability and its physical causes in infusion technology: A systematic review of in vitro measurement and modeling studies. Biomed Tech 2015;60(4):277–300.

**GRANT ACKNOWLEDGMENT**

This project received funding from the European Metrology Programme for innovation and research (EMPIR). The EMPIR is jointly funded by the EMRP participating countries within Eurametand the European Union.

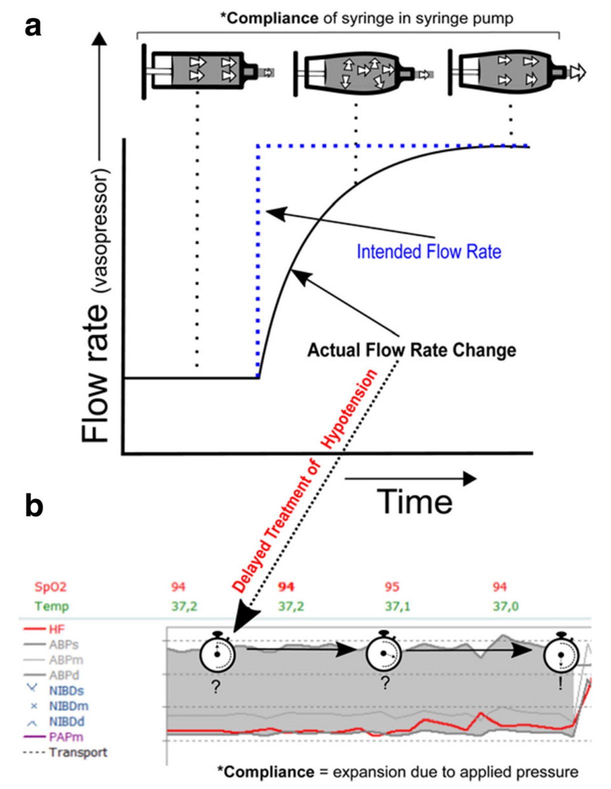

**Fig. 270 (Abstract 0813).** Physical Effect

### 0814

#### The role of early enteral nutrition in the prevention of postoperative intestinal failure

M.V. Petrova[1], A.V. Butrov[1], M.V. Vatsik[2], M.F.I. Nakade[1], R. Mohan[1], M.N. Storchai[1], G.R. Mahmutova[1]

[1]RUDN University, Anesthesiology and Intensive Care, Moscow, Russian Federation; [2]City Hospital 64, Anesthesiology and Intensive Care, Moscow, Russian Federation

**Correspondence:** M.V. Petrova

*Intensive Care Medicine Experimental* 2017, **5(Suppl 2):**0814

**INTRODUCTION.** Postoperative Intestinal Failure (IF) - frequent complication in early postoperative (PO) period after abdominal surgery. Administration of enteral nutrition (EN) in patients' undergone surgical interventions is most difficult decisions in abdominal surgery. IF is one of the cause in development of multi-organ failure & adverse outcomes.

**OBJECTIVES.** To evaluate effectiveness of various substrates for early enteral administration as prophylaxis for IF in PO patients.

**METHODS.** Study included 52 patients who underwent various emergency abdominal interventions. Patients are divided into 3 groups using single blind study method:

Group 1: 17 patients who received enterally glutamin (500 ml) on 2nd day, received EN on 3rd day.

Group 2: 17 patients, on 2nd day administration EN (semi-elemental feeding diets, 100–300 ml).

Group 3: 18 patients, on 2nd day enteral administration of saline solutions (Trisol: 500–800 ml - consist of KCl, $NaHCO_3$, NaCl), 3rd day- EN.

In PO period, following parameters were evaluated: auscultative assessment of peristalsis, measurement of intra-abdominal pressure (IAP), ultrasound visualization of intestinal structures, absorption function of GIT according to amount unabsorbed enteral volume.

**RESULTS.** IF stage in PO period was determined by classification developed during study, including clinical signs of intestinal paresis, IAP & ultrasound imaging of intestines. Suggested 1st, 2nd, 3rd degrees of IF. At stage 1 of study, when comparing severity of IF on 3rd day of PO period, patients in group 1 were found to have least signs of IF (mean value- 1.8 ± 0.68). In patients of 2nd group, IF was more pronounced (2.17 ± 0.2). Differences between groups are not significant ($p > 0.05$). Patients of group 3, IF was most pronounced (2.8 ± 0.3). Differences between 2nd & 3rd, between 1st & 3rd groups are significant ($p < 0.05$).

Manifestation of IF is characterized by several factors, such as IAP, intestinal wall thickness, intestinal lumen diameter. To determine most informative factor, a correlative analysis was performed. It has been revealed that strong & medium strength correlations found between degrees of IF & level of IAP, between degrees of IF & intestinal wall thickness. Connections are statistically significant (at the level of 0.01). Since to determine thickness of intestinal wall by ultrasonic scanning requires special skill of an anesthesiologist & ultrasound machine, which is not always possible to realize in intensive care departments, determining level of IAP acquires special significance. Level of IAP in group 1 is 13.7 ± 1.35 cm. In 2nd group, 16.7 ± 2.4 cm. In 3rd group −19.3 ± 1.6 cm ($p > 0.05$).

**CONCLUSIONS.** IAP can be used as prognostic indicator of postoperative IF & use it to determine PO nutritional therapy, which must be initiated with administration of enteral glutamine.

**REFERENCE(S)**

Loris Pironi, ESPEN endorsed recommendations./ Clinical Nutrition// V-34, 04.2015, P171-180

**GRANT ACKNOWLEDGMENT**

Not applicable

### 0815

#### Incidents without damage notification system: 5 years of experience in intensive care unit (ICU)

C. Carbajales Pérez[1], M. Gero Escapa[2], R. Vara Arlanzón[2], O. Badallo Arévalo[2], M. del Valle Ortiz[2], M.E. Martínez Barrio[2], M.E. Perea Rodríguez[2], D. Iglesias Posadilla[2], E. Pérez Cabo[2], P. de la Torre Velez[2]

[1]Hospital Universitario de Burgos, Intensive Care Unit, Burgos, Spain; [2]Hospital Universitario de Burgos, Burgos, Spain

**Correspondence:** C. Carbajales Pérez

*Intensive Care Medicine Experimental* 2017, **5(Suppl 2):**0815

**INTRODUCTION.** The critical patient care environment is extremely complex. In Intensive Care Medicine,factors such as the seriousness of the patient condition,the commu- nication barriers,the invasive therapeutic and diagnostic procedures used,and the volume of information managed all contribute to the appearance of incidents related to patient safety. Some of them can cause temporary damage requiring additional observation and care, prolonging hospital stay and-in some cases-causing permanent damage or even death.

**OBJECTIVES.** Analysis of Regional System of incidents without damage (SISNOT) registrated in ICU. Evaluate contributing factors (CF) and improvement strategies to consolidate the safety culture in our ICU.

**METHODS.** Prospective observational descriptive study from December 2012 to January 2017. We describe these variables: types of incidents without damage (IWD), day of the week and shift with greatest number, severity of potential damage: Mild (minimum damage: more observation or minor treatment), moderate (short-term damage: additional treatment or procedure) and severe (permanent or long-term damage). We describe CF in each group and the suggested improvements.

**RESULTS.** IWD were registered in this period. The most frequent ones belong to patient care (Group 1: 22.8%), drugs errors (Group2: 22%), related to equipment and devices (Group 3: 16.9%) and Diagnosis and clinical follow-up (Group 4: 10.2%). About potential damage: 16.7% without damage, 29% mild severity, 39.2% moderate and 13.7% severe. Mondays (18.4%) and Thursdays (16.9%) were days with higher risk of IWD.

In morning shift happens 52.2%. The CF grouped by type of IWD were: Groups 1,2 and 4: teamwork problems (18.2%, 12.3%, 22.3%) and lack of communication (16.7%, 33.2%, 32.5%). Group 3: factors about patients (23.5%), equipment and devices (28.8%).

Suggested improvements were: Log and plan tasks in group 1 (62.6%) and 3 (63.4%). Improve communication between professionals in group 2 (51%) and 4 (43.8%).

**CONCLUSIONS.** The most frequent IWD were: patient care and drugs. The moderate potential damage notifications showed the highest incidence. An increase of IWD was detected in the highest medical activity periods. The main CF were the teamwork problems and communication skills. Suggested improvements were based on the protocolization, task planning and professional communication skills.

**REFERENCE(S)**

- *Analysis of contributing factors associated to related patients safety incidents in Intensive Care Medicine*. Medicina Intensiva Volume 39, Issue 5, June-July 2015, Pages 263–271

- *Analysis of an incident notification system and register in a critical care unit*..Enfermeria Intensiva Volume 27, Issue 3, July-September 2016, Pages 112–119.

- *Los sistemas de registro y notificación de efectos adversos y de incidentes: una estrategia para aprender de los errores.* Revista de Calidad Asistencial Volume 20, Issue 4, June 2005, Pages 216–222.

### 0816

#### Assessing additional intravenous access in patients with indwelling peripherally inserted central catheters

P.J.P. Bird[1], A. Bhalla[2], M. Norman[2], I.D. Welters[2]

[1]University of Liverpool, Liverpool, United Kingdom; [2]Royal Liverpool University Hospital, Critical Care, Liverpool, United Kingdom

**Correspondence:** P.J.P. Bird

*Intensive Care Medicine Experimental* 2017, **5(Suppl 2):**0816

**INTRODUCTION.** Peripherally Inserted Central Catheters (PICCs) are used for long term IV access in critically ill patients[1] to deliver intravenous (IV) medications, parenteral nutrition and govern contrast administration. PICCs are associated with fewer complications than central lines and should avoid recurrent phlebotomy[2]. However, patients with PICCs frequently receive additional IV access, defying the purpose of PICCs as alternatives to cannula insertion. Peripherally inserted cannulas may be unnecessary and associated with increased infection risk[3].

**OBJECTIVES.** This audit investigates how often IV cannulas are inserted additionally in patients with PICCs.

**METHODS.** In February/March 2017 all critical care patients with PICCs were included. A data collection table was completed for each patient with new/indwelling PICCs, including identifiers, date of PICC insertion, additional IV access, and PICC line integrity. Additionally, all peripheral IV cannula access was recorded to investigate adequacy of documentation, insertion origin and time *in-situ*.

**RESULTS.** 21 PICCs were inserted in 18 patients. 23.8% of patients with PICCs required additional intravenous access, most often in the form of a cannula (19%) or a central line (4.8%). Blocked PICC lumens were reported in 30%. The most common reason for extra cannula insertion was for contrast injection (66.7%), followed by intravenous medications administration (33.3%) for which the number of PICC lumens were insufficient.

78.4% of cannulas were documented appropriately. 39% of cannulas were inserted outside critical care, most commonly in the emergency department (41%), and remained *in-situ* for a mean of 2 days (SD = 1.41).

**CONCLUSIONS.** This study suggests almost a quarter of patients with PICCs receive additional IV access, mainly for contrast injection. Careful planning of the patients' treatment needs, including contrast imaging, is required to choose the optimal PICC before insertion. Patients on numerous intravenous medications may require devices with multiple lumens. Modern PICCs with options to use high pressure injection should be chosen for patients requiring imaging with contrast agents. More attention should be paid to the correct documentation and timely removal of intravenous devices after successful insertion of PICCs to further reduce infection risks.

**REFERENCE(S)**

[1] Xu T, Kingsley L, DiNucci S, Messer G, Jeong J, Morgan B et al. Safety and utilization of peripherally inserted central catheters versus midline catheters at a large academic medical center. American Journal of Infection Control.2016;44(12):1458–1461

[2] Mattox E. Complications of Peripheral Venous Access Devices: Prevention, Detection, and Recovery Strategies. Critical Care Nurse.2017;37(2):e1-e14

[3] Ozer B, Tatman-Otkun M, Memis D, Otkun M. Nosocomial infections and risk factors in intensive care unit of a university hospital in Turkey.Open Medicine. 2010;5(2)

**GRANT ACKNOWLEDGMENT**

Bhalla A, Welters ID, Main N, Allen B.

### Sepsis: Prognostication

#### 0817

#### Diagnostic and prognostic role of repeated thromboelastography (TEG®), presepsin and copeptin in sepsis and septic shock: an observational, prospective, multicenter study

M. Ulla[1], L. Bonaccurso[1], A. Iannello[2], M.G. Bocci[3], S. Pini[4], G. De Pascale[5], P. Malacarne[4], G. Mengozzi[2], D. Pasero[1], L. Brazzi[1]

[1]AOU Città della Salute e della Scienza, Anesthesia and Intensive Care, Turin, Italy; [2]AOU Città della Salute e della Scienza, Clinical Biochemistry, Turin, Italy; [3]Policlinico Universitario A. Gemelli, Intensive Care Unit, Rome, Italy; [4]AOU Pisana, Anesthesia and Intensive Care, Pisa, Italy; [5]Policlinico Universitario A. Gemelli (policlinico, Intensive Care Unit, Rome, Italy

**Correspondence:** M. Ulla

*Intensive Care Medicine Experimental* 2017, **5(Suppl 2):**0817

**INTRODUCTION.** New sepsis definitions focus on suspected infection and organ damage (1). No point-of-care tests currently exist to precisely define infection presence and severity. Inflammation and coagulation are closely linked: thrombotic and bleeding complications often occur during sepsis and septic shock, resulting in increased mortality. Thromboelastography (TEG®) and point-of-care viscoelastic methods quickly assess the whole coagulation process and seem to be correlated with disease severity in septic patients (2,3). Surviving Sepsis Campaign reconsidered the role of biomarkers, but Presepsin and Copeptin displayed potential correlation with sepsis and its complications.

**OBJECTIVES.** To investigate the diagnostic and prognostic role of seriated TEG, Presepsin and Copeptin, in patients admitted to Intensive Care Units (ICUs) with sepsis.

**METHODS.** This is a multicenter prospective observational study conducted in 4 Italian Hospitals. Adult patients with sepsis/septic shock admitted to ICU were included. Conventional blood tests, TEG analysis and biomarkers were obtained at admission (T0), after 72 hours (T1) and after 7 days (T2). Bleeding events, transfusions requirement, clinical data and scores were recorded up to day 7. ICU/hospital length of stay and 28 and 90-days-mortality were recorded.

**RESULTS.** The study is currently ongoing, 43 patients (M/F = 29/14; mean Age = 67, 95%CI 63–72) have been enrolled so far. Preliminary results report four cases of bleeding at T0 (9%) and 6 overall (14%). Patients with lower MA values at TEG displayed more bleeding events and higher need of transfusion at T1 and T2. Patients who died within 28 days reported lower MA levels than surviving (56.2 ± 23.8 vs 81.2 ± 5.9, $p < 0.05$). A pattern of hypocoagulability (MA ≤ 51mm) seems associated with longer ICU [30 (25–42) vs 11 (9–13) days] and hospital length of stay [37 (29–39) vs 28 (20–33), $p < 0.05$]. Data regarding Copeptin and Presepsin are preliminary and currently under investigation.

**CONCLUSIONS.** According to our preliminary analysis lower MA measured by TEG seems to be associated with an increased risk of bleeding and need of transfusion in patients with sepsis/septic shock. Moreover, an hypocoagulability status among septic patients correlates with an increased mortality rate and longer ICU and hospital length of stay.

**GRANT ACKNOWLEDGMENT**

None of the authors received any financial support and declare any conflict of interest.

#### 0818

#### Validation of SOFA, qSOFA and SIRS to predict mortality and organ failures among sepsis patients admitted to medical intensive care unit in middle-income country

B. Khwannimit, R. Bhurayanontachai, V. Vattanavanit

Prince of Songkla University, Internal Medicine, Hat Yai, Thailand

**Correspondence:** B. Khwannimit

*Intensive Care Medicine Experimental* 2017, **5(Suppl 2):**0818

**INTRODUCTION.** The recent Sepsis-3 provides a change of 2 or more points in the Sequential Organ failure assessment (SOFA) score and quick SOFA (qSOFA) as the clinical criteria and risk assessment for sepsis patients instead of using the Systemic Inflammatory Response Syndrome (SIRS) by Sepsis-1.

**OBJECTIVES.** The aim of this study was to validate and compare performance of SOFA and qSOFA with SIRS to predict the outcomes in sepsis patients.

**METHODS.** A retroprospective analysis was conducted of prospectively collected data from all consecutive sepsis patients admitted to the medical intensive care unit of a tertiary university teaching hospital in Thailand from 2007 through 2016. The primary outcome was hospital mortality and the secondary outcomes were ICU mortality and organ failures.

**RESULTS.** A total of 2,234 patients were included. Mean age was 58.7 ± 20.3 years, mean APACHE II was 23.6 ± 9.8 and mean SOFA score was 8.6 ± 4.4. The sources of ICU admission were from general wards (58.4%) and the emergency department (41.6%). The most common sources of infection were respiratory (50.1%). Microorganisms were isolated from hemocultures in 670 patients (30%) and documented from any sites of infection in 1,738 patients (77.8%). The hospital and ICU mortality rate was 44.4% and 32.1%, respectively. Organ failure was documented in 2,060 patients (92.2%). The SIRS criteria were met by 2,156 patients (96.5%), 1,998

patients (89.4%) met the qSOFA criteria and 2,144 patients (96%) met SOFA criteria (Fig. 271). Overall, 1,918 patients (85.5%) had both criteria for Sepsis-1 and Sepsis-3 definitions. However, 200 patients (9.3%) and 77 patients (3.6%) met the SIRS criteria but not the qSOFA and SOFA criteria, respectively, and 39 patients (1.8%) met neither the SOFA nor qSOFA criteria. In contrast, 66 patients (3%) met Sepsis-3 criteria but not SIRS. Patient with did not met SIRS criteria had the highest hospital and ICU mortality as well as more organ failures (Fig. 272). The SOFA score presented the best discrimination with an area under the receiver operating characteristic curve (AUC) of 0.836 (95% CI 0.819-0.853). The discrimination of the SOFA score for hospital mortality was significantly higher than the qSOFA (AUC 0.799, 95%CI 0.782-0.816, P = 0.0001) and SIRS (0.585, 95%CI 0.564-0.607, P < 0.0001) (Fig. 273). The qSOFA showed better discrimination for hospital mortality than SIRS (P < 0.0001). Also, SOFA score had better performance than other scores for the secondary outcomes and all subgroups of sepsis patients according to age, sepsis severity and admission source.

**CONCLUSIONS.** The SOFA score presents better prognosis accuracy for mortality and organ failures than the qSOFA and SIRS criteria among sepsis patient admitted to ICU. Our findings support for Sepsis-3 using SOFA in ICU setting.

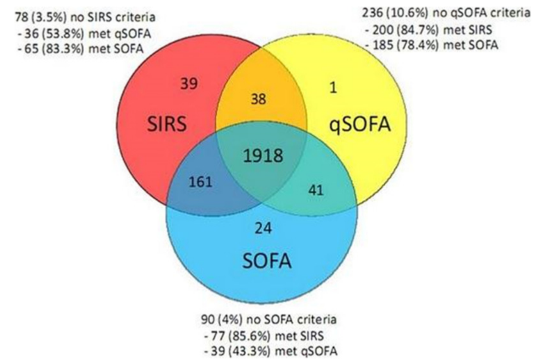

**Fig. 271 (Abstract 0818).** Diagram representing distribution of SIRS, qSOFA a

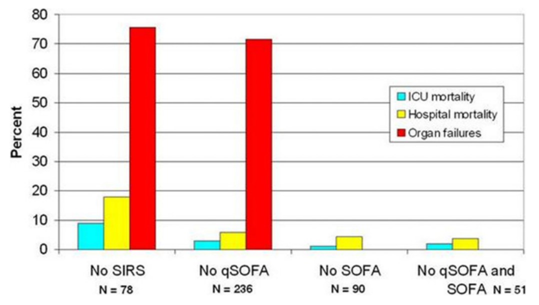

**Fig. 272 (Abstract 0818).** Mortality and organ failures in ICU sepsis patient

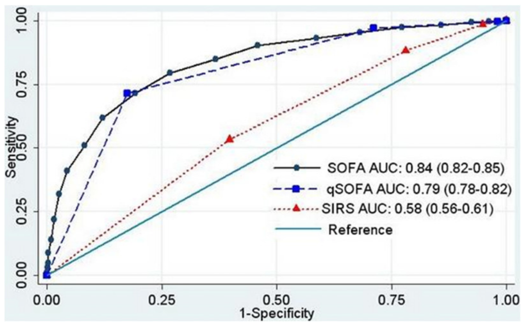

**Fig. 273 (Abstract 0818).** Comparison of ROC curves for prediction of hospita

### 0819

#### Hourly rate of lactate decrease is related to 30-day mortality in septic shock patients

P. Promsin[1,2], J. Grip[1], N. Tardif[1], O. Rooyackers[1], J. Wernerman[1]
[1]Karolinska Institutet, Department of Clinical Science Intervention and Technology (CLINTEC), Division of Anesthesia and Intensive Care, Stockholm, Sweden; [2]Faculty of Medicine Siriraj Hospital, Mahidol University, Department of Internal Medicine, Division of Critical Care, Bangkok, Thailand
**Correspondence:** P. Promsin
*Intensive Care Medicine Experimental* 2017, **5(Suppl 2):**0819

**INTRODUCTION.** A decrease in lactate levels during septic shock resuscitation is associated with favorable outcomes. Previous studies proposed different desirable rates of lactate decrease (mostly 10-30%) over a variable time period (mostly 6 hours)[1]. However, optimal change rate of lactate values over shorter time intervals are not well studied.

**OBJECTIVES.** The aim of this pilot study was to identify which percentage of lactate change in a 1-hour period best determined mortality in septic shock patients. The second aim was to examine how this hourly rate is associated to other clinical parameters in relation to mortality.

**METHODS.** This is a retrospective study of patients admitted in an intensive care unit (ICU) of a university hospital during 2015. All patient information was retrieved from ICU and hospital electronic medical record. Adult (>18 years) patients with a septic shock diagnosis were included using these criteria 1) infection-related primary diagnosis 2) hypotension receiving vasopressors 3) initial lactate at ICU ≥ 2 mmol/L. Lactate values were obtained until normalization (≤1.5 mmol/L) or last measurement within 24 hours, if no normalization occurred. Percent average hourly change rates (LAH) were calculated from all consecutive lactate values of each individual.

**RESULTS.** Of 693 ICU patients admitted, 60 patients met the inclusion criteria. The most common source of sepsis was intraabdominal infection (32%). Median age, Sequential Organ Failure Assessment (SOFA) score, baseline lactate values were 65(56–73) years, 10(7–13) and 4.0(2.7-7.4) mmol/L, respectively. ICU and 30-day mortality rates were 18% and 33%. Within 24 hours, 678 lactate measurements were used in the analysis. Receiver operating characteristic (ROC, SPSS) curves of LAH, baseline lactate values and SOFA score demonstrated that LAH was the best indicator of 30-day mortality (area under ROC curves (AUC) 0.763, 0.704 and 0.645, respectively). The best cutoff value of LAH was −2.65% with sensitivity 85% and specificity 60%. Univariate comparison of all variables revealed that older age, higher creatinine, higher baseline lactate, higher lactate at 6 hours and LAH > −2.65% (mean of lactate decrease < 2.65% per hour) were significantly associated with mortality (all, p < 0.05). After adjustment in multivariable logistic regression, older age, higher baseline lactate and LAH > −2.65% were independent factors of 30-day mortality (OR 1.139, 95%CI 1.042-1.244, p = 0.004; OR 1.381, 95%CI 1.078-1.770, p = 0.011 and OR 7.751, 95%CI 1.462-41.099, p = 0.016, respectively).

**CONCLUSIONS.** Mean lactate reduction less than 2.65% per hour during first 24 hours, higher baseline lactate values and older age were independently associated with 30-day mortality in ICU septic shock patients.

### 0820

#### A prognostic model for post-operative risk stratification of critically ill patients with faecal peritonitis

A. Tridente[1,2], J. Bion[3], G. Mills[4], A. Gordon[5], G. Clarke[6], A. Walden[7], P. Hutton[8], P. Holloway[5], J.-D. Chiche[9], F. Stuber[10], C. Garrard[8], C. Hinds[11], on behalf of The GenOSept and GAinS Investigators

[1]Whiston Hospital, Prescot, United Kingdom; [2]University of Liverpool, Liverpool, United Kingdom; [3]University of Birmingham, Birmingham, United Kingdom; [4]University of Sheffield, Sheffield, United Kingdom; [5]Imperial College, London, United Kingdom; [6]Wellcome Trust, Oxford, United Kingdom; [7]Royal Berkshire Hospital, Reading, United Kingdom; [8]John Radcliffe Hospital, Oxford, United Kingdom; [9]Hospital Cochin, Paris, United Kingdom; [10]Bern University Hospital, Bern, Switzerland; [11]Barts and the London Queen Mary School of Medicine, London, United Kingdom

**Correspondence:** A. Tridente

*Intensive Care Medicine Experimental* 2017, **5(Suppl 2):**0820

**INTRODUCTION.** Prognostic models of illness severity are useful both clinically and for research purposes. A specific tool for prognostication in critically ill patients with faecal peritonitis is not available.

**OBJECTIVES.** We aimed to develop two prognostic models, for the prediction of 6 months and 28 day mortality of post-operative critically ill patients with faecal peritonitis

**METHODS.** Patients admitted to intensive care units with faecal peritonitis and recruited to the UK GAinS or European GenOSept studies up to January 2011 were divided into a derivation and an geographic validation subset; patients subsequently recruited to GAinS were used for temporal validation. Using all 50 clinical and laboratory variables available on day 1 of critical care admission, Cox proportional hazards regression was fitted to select variables for inclusion in the prognostic model, using stepwise selection and non-parametric bootstrapping sampling techniques. Using Area under the Receiver-Operator Characteristic curve (AuROC) analysis, the performance of the models was compared to SOFA and APACHE II.

**RESULTS.** Five variables (age, SOFA score, lowest temperature, highest heart rate, haematocrit) were entered into the prognostic models. The discriminatory performance of the 6 month prognostic model yielded an AuROC 0.81 (95% Confidence Interval, CI, 0.76 - 0.86), 0.73 (95% CI 0.69 - 0.78), and 0.76 (95% CI 0.69-0.83) for the derivation, geographic and temporal external validation cohorts, respectively. The 28 day prognostic tool yielded an AuROC 0.82 (95% CI 0.77 - 0.88), 0.75 (95% CI 0.69 - 0.80) and 0.79 (95% CI 0.71-0.87) for the same cohorts. These AuROCs were superior to those obtained with the SOFA and APACHE II scores.

**CONCLUSIONS.** The two prognostic models developed for 6 month and 28 day mortality prediction in critically ill septic patients with FP, in the post-operative phase, enhanced the SOFA score´s predictive utility by adding few key variables: age, lowest recorded temperature, highest recorded heart rate and haematocrit. Before considering introduction of the scores into clinical practice to inform decision making and the design of clinical trials, it is necessary to conduct external validation in larger cohorts of their predictive capability.

**GRANT ACKNOWLEDGMENT**

GenOSept part-FP6-funded

CIBERES partial support of FP6 projects (Spanish minister of Health)

### 0821

#### Prognostic value of pulmonary vascular permeability index in patients with sepsis

Ž. Drvar, M. Pavlek, D. Tonkovic, D. Bandic-Pavlovic, M. Peric

Clinical Hospital Center Zagreb, Anesthesiology, Reanimatology and Intensive Care, Zagreb, Croatia

**Correspondence:** Ž. Drvar

*Intensive Care Medicine Experimental* 2017, **5(Suppl 2):**0821

**INTRODUCTION.** There are no early clinical signs suggesting mild acute respiratory distress syndrome (ARDS). Lately, pulmonary vascular permeability index (PVPI) and extravascular lung water index (EVLWI) are being focused on as indicators of mild ARDS.

**OBJECTIVES.** Investigating PVPI and EVLWI dynamics in development of pulmonary oedema in patients with sepsis by PiCCO monitoring.

**METHODS.** It was a 3-years-duration prospective observational study at Zagreb University Hospital Center. Examined group: 50 patients (29 male, age 55 ± 11yrs and 21 female, age 50 ± 10yrs) after urgent abdominal surgery, with mild ARDS. Control group: 50 patients (28 male, age 46 ± 9yrs and 22 female, age 45 ± 10yrs) after urgent surgery, without mild ARDS. Diagnosis of sepsis was established both clinically and by laboratory findings, mild ARDS was defined by Berlin definition. All patients were mechanically ventilated and analgosedated with midazolam and sufentanil applied via syringe pump system. PVPI and EVLWI measurements were obtained (in both study groups) three times a day in the same 8-hours interval for seven consecutive days by PiCCO monitoring (Dräger Infinity R PiCCO Smart Pod TM 2005). A 4F arterial thermodilution catheter (Pulsiocath PV2014L16N) was placed in the aorta via femoral artery using Seldinger technique. Concomitantly, oxygenation ratio ($PaO_2/FiO_2$), lung compliance and albumin levels were assessed. APACHE II score was 22.78 ± 3.62. Both measured and calculated data were statistically processed using Smirnov-Kolmogorov test and independent T-test. Values of P < 0.05 were considered statistically significant.

**RESULTS.** From examined group, 21 patients died before day 28. There was no statistical difference in PVPI and EVLWI between examined and control group during first 72 hours. The average PVPI was 2.2 ± 0.37 and EVLWI at baseline was 11 ± 4. After day 3, PVPI and EVLWI was significantly higher in non-survivors than survivors (3.93 ± 0.72 vs. 1.90 ± 0.38; P < 0.001 and 14.67 ± 4.64 vs. 6.41 ± 0.84; P < 0.001). PVPI was correlated to $PaO_2/FiO_2$ (r = −0.300; P < 0.001), lung compliance (r = −0.643; P < 0.001) and albumin levels (r = −0.375; P < 0.001).

**CONCLUSIONS.** PVPI and EVLWI in patients with sepsis demonstrated correlation with markers of mild ARDS. Dynamics of PVPI and EVLWI could be outcome indicators for patients with mild ARDS in sepsis. PVPI is more accurate than EVLWI as an indicator and correctly reveals the degree of pulmonary vascular permeability and levels of serum albumin. Reduction of PVPI and EVLWI in early treatment period was associated with better patient outcome.

**GRANT ACKNOWLEDGMENT**

None.

### 0822

#### Prognostic utility of the combined SOFA score, lactate, procalcitonin and C-reactive protein in severe sepsis

W. Chang, Y. Yang

Zhongda Hospital, School of Medicine, Southeast University, Department of Critical Care Medicine, Nanjing, China

**Correspondence:** W. Chang

*Intensive Care Medicine Experimental* 2017, **5(Suppl 2):**0822

**BACKGROUND.** We aimed to select and combine clinically feasible parameters comprehensively reflecting the pathogenesis of sepsis to predict 28-day mortality in severe sepsis, which includes SOFA score (organ functionality), serum lactate (tissue perfusion), procalcitonin (PCT) (the severity of infection) and C-reactive protein (CRP) (host immunologic response).

**METHODS.** A total of 124 patients diagnosed as severe sepsis were finally enrolled in our study. Peripheral blood specimen was obtained immediately at admission before the administration of antibiotics, and SOFA score was recorded simultaneously. Serum lactate, PCT and CRP levels were measured. All the patients were treated uniformly by our clinical work-team, and followed up to 28 day, with 28-day mortality as the primary outcome.

**RESULTS.** SOFA score, lactate and procalcitonin were significantly elevated in non-survivor group and could predict 28-day mortality.

Receiver operating characteristic (ROC) curve indicated that area under ROC (AuROC) was 0.686 (SOFA score) > 0.67 (lactate) > 0.611 (PCT) > 0.529 (CRP). Combined use of SOFA score, lactate, PCT and CRP via Cox proportional hazards model revealed the increment of AuROC from the maximum of 0.686 (SOFA score) to 0.706.

Net reclassification index (NRI) analysis showed that the combination is superior to any of single use of the parameters, and the addition of lactate to the combined model could significantly improve the predictability of the 28-day mortality in severe sepsis.

| Models | NRI (95% CI) | P value |
|---|---|---|
| SOFA vs. SOFA + Lac + PCT + CRP | 0.39 (0.02, 0.75) | 0.04 |
| Lac vs. SOFA + Lac + PCT + CRP | 0.46 (0.10, 0.82) | 0.02 |
| PCT vs. SOFA + Lac + PCT + CRP | 0.58 (0.22, 0.93) | 0.002 |
| CRP vs. SOFA + Lac + PCT + CRP | 0.55 (0.20, 0.91) | 0.004 |
| Lac + PCT + CRP vs. SOFA + Lac + PCT + CRP | 0.34 (−0.03, 0.70) | 0.08 |
| SOFA + PCT + CRP vs. SOFA + Lac + PCT + CRP | 0.43 (0.08, 0.78) | 0.02 |
| SOFA + Lac + CRP vs. SOFA + Lac + PCT + CRP | 0.10 (−0.28, 0.47) | 0.6 |
| SOFA + Lac + PCT vs. SOFA + Lac + PCT + CRP | 0.17 (−0.20, 0.54) | 0.38 |

*[NRI]*

**CONCLUSION.** The combination of SOFA score, lactate, PCT and CRP could improve the predictability of 28-day mortality in severe sepsis, and was superior to any single use of the parameters. However, the accuracy for the current biomarkers are still inadequate, and the combined use of the burgeoning novel biomarker reflecting the pathogenesis of sepsis may potentially improve the accuracy in sepsis diagnosis and stratification, which needs further elucidation.

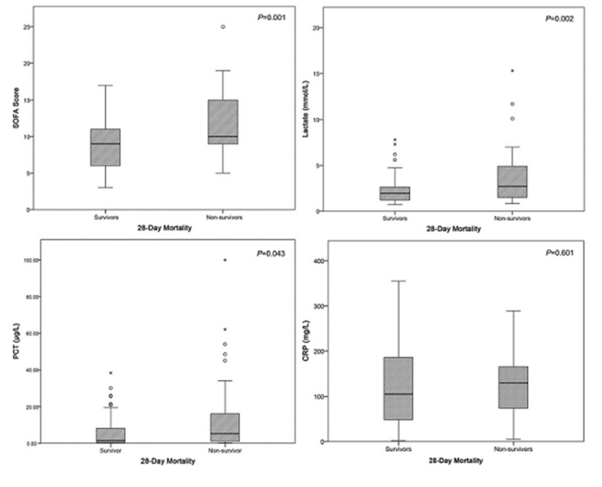

**Fig. 274 (Abstract 0822).** Levels in Severe Sepsis

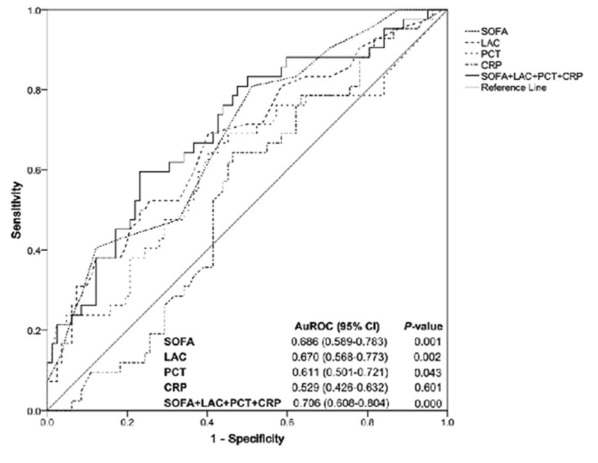

**Fig. 275 (Abstract 0822).** AuROC comparison

### 0823

#### Predictive value of hypotension duration before initiation of effective antimicrobial therapy on clinical outcome of critically ill patients

K. Aganovic

Clinical Center University of Sarajevo, Sarajevo, Bosnia and Herzegovina

*Intensive Care Medicine Experimental* 2017, **5(Suppl 2):**0823

**INTRODUCTION.** Sepsis is a clinical syndrome characterized by systemic inflammation due to infection, with a continuum of severity ranging from sepsis to septic shock. Timely and appropriate recognition and treatment can reduce in-hospital mortality and morbidity.

**OBJECTIVES.** To analyse the impact of duration of hypotension on mortality of patients with sepsis/septic shock.

**METHODS.** A prospective study performed between September 2010 and September 2012, in medical six-bed intensive care unit in Clinical Center University of Sarajevo. A total of 116 patients were admitted to the ICU during the study period. Analysis of clinical and labaratory variables (including duration of hypotension, creatinin, lactates, duration of mechanical ventilation) between survivors and nonsurvivors, was performed.

**RESULTS.** Analysis of association of procentage of survivors in relation to time intervals (hours) showed the continual declination of surviving rate depending on duration of hypotension. Survival was 86% if effective antimicrobials were administered within 60 mins of initial evidence of hypotension, 71% in the second hour and 20% in eighth hour. The area under the ROC curve, calculated only for duration of hypotension, as discriminatory variable for lethal outcome, was AUC=0.848 (p<0.0001). Duration of hypotension more than critical 5 hours showed sensitivity of 73.3%, specificity of 84.4%, positive predictive value (PPV) 88.1% and negative predictive value (NPV) 66.7% to the lethal outcome, with the + LR 4.7 and -LR 0.32. Multiple logistic regression analysis showed that basic predictive variable in this model was duration of hypotension, with age as contributing factor to the lethal outcome.

**CONCLUSIONS.** Our data strongly support current international guidelines, with accent on early validation of hypotension as first and the most important step in strategy for early recognition, diagnosis and appropriate treatment of criticaly ill patient.

**REFERENCE(S)**

(1) Kumar A, Roberts D, Wood KE Light B, Parrillo JE, Sharma S, et al. Duration of hypotension before initiation of effective antimicrobial therapy is the critical determinant of survival in human septic shock. *Crit. Care Med.* 2006 Jun; 34 (6):1589–1596.

(2) Zanon F, Caovilla JJ, Michel RS, Cabeda EV, Ceretta DF, LuvkemeyerGD, et al. Sepsis in the Intensive Care Unit : Etiologies, Prognostic Factors and Mortality. Revista Brasileira de Terapia Intensiva (RBTI). 2008; 20 (2):128–134.

(3) Labelle A, Juang P, Reichley R, Micek S, Hoffmann J, Hoban A, et al. The determinants of hospital mortality among patients with septic shock receiving appropriate initial antibiotic treatment. Crit Care Med. 2012 Jul;40(7):2016–21.

### 0824

#### Quartiles of lactate concentration in sepsis and mortality

A. García-de la Torre[1], M.-V. de la Torre-Prados[2], E. Cámara-Sola[2], P. Nuevo-Ortega[2], A. Fernández-Porcel[2], T. Tsvetanova-Spasova[2], L. Salido-Díaz[2], C. Rueda-Molina[2], I. García-Gómez[2], M. Mateos-Rodríguez[2]
[1]Málaga/Ibima, Universitary H Virgen de la Victoria, Clinical Chemistry Department, Málaga, Spain; [2]Málaga/Ibima, Universitary H Virgen de la Victoria, Department of Intensive Care Medicine, Málaga, Spain
**Correspondence:** M.-V. de la Torre-Prados
*Intensive Care Medicine Experimental* 2017, **5(Suppl 2):**0824

**INTRODUCTION.** The measurement of the lactate levels can be helpful in the diagnosis and assessment of the sepsis, as well as to measure its control.

**OBJECTIVES.** To analyze the relation of lactate as biomarker of easy measurement with sepsis scores and mortality in critically ill adults patients who have admitted in intensive care unit (ICU) with organic disfunction.

**METHODS.** It is a cohort study in critically ill adults admitted in a polivalent ICU. Demographic, clinical parameters and Lactate within 24 hours were studied from severe sepsis (SS) or septic shock (SSh) onset, defined according to the Surviving Sepsis Campaign (SSC) criteria (2012).

We tested for differences in baseline characteristics by lactate interval using a Kruskal-Wallis test for continuous data or $X^2$ test for categorical data and reported the median and interquartile ranges. SPSS version 18.0.

**RESULTS.** We analysed 780 consecutive episodes of SS (15.8%) or SSh (84.2%). The median age was 64 (inter-quartile range [IQR]:51–73) years old; male: 60.7%. The main sources of infection were: respiratory tract 36% and intra-abdomen 30.9% and 67% had medical pathology. APACHE score was 24 [20–29] and SOFA score 9 [7.25-11], mortality was 27.43%.

The median lactate levels were significantly higher in non-survivors 2.6 [1.8-3.73] vs. 4.21 [2.6-6.86], p < 0.001. AUC values was 0.75 (95% CI: 0.69-0.81) with an optimal cut-off value of 3.7 mmol/L (76.3% sensitivity and 62% specificity).

Quartiles of blood LT concentration were quartile 1 (Q1): 2 mmol/L or less, quartile 2 (Q2): 2.01-2.91 mmol/L, quartile 3 (Q3): 2.92-4.34 mmol/L, and quartile 4 (Q4): 4.35 mmol/L or greater (Table 202). The median Lactate concentrations of each quartile were 1.53 (Q1), 2.46 (Q2), 3.59 (Q3), and 6 (Q4) mmol/L (P < 0.001).

The differences between these quartiles were that the patients in Q1 had significantly lower APACHE II scores (p = 0.003) and SOFA score (p = 0.02) compared with patients in Q2, Q3, and Q4. The patients in Q4 had significantly higher APACHE II scores (p = 0.0001), SOFA score (p < 0.001) and ICU mortality (p = 0.005) compared with the others quartiles. Patient in Q1 had majority respiratory tract infection source (46.6%) and significantly less SSh patients (p = 0.01) and Q4 had significantly more SSh patients (p = 0.03) and the main sources of infection were intra-abdomen (41.9%), p = 0.042.

**CONCLUSIONS.** This study confirms the lactate as prognosis biomarker in sepsis and good relation with severity scores and mortality.

**Table 202 (Abstract 0824).** Severe Sepsis and Septic Shock and blood lactate

| Variables | Lactate < 2 N=194 | Lac= 2-2.91 N=193 | Lac= 2.92-4.34 N=193 | Lac>4.34 N=194 |
|---|---|---|---|---|
| Age (years) | 63 [46-73.25] | 65 [53.5-75] | 62 [49-72] | 65 [54-72] |
| *APACHE II | 22.5 [16-27] | 23 [19-28] | 24[20.75-29] | 27 [23-31] |
| *SOFA | 8 [7-10] | 9 [7-11] | 9 [8-11] | 11 [9-13] |
| *Lactate (mmol/L) | 1.53 [1.29-1.84] | 2.46 [2.22-2.65] | 3.59 [3.2-4] | 6 [4.99-7.72] |
| **28 day-Mortality (%) | 13.7 | 20.3 | 24.9 | 48.2 |
| SSh (%) | 75.1 | 83.9 | 89.6 | 91.2 |

*SSh* Septic Shock, *Lac* Lactate
(*) Kruskal-Wallis, p=<0,05, (**) X2 test, p=<0,05

### 0825

#### Significance of stratified values of serum lactate on in-hospital mortality in patients with sepsis or septic shock

D.A. Hammond[1], T.L. Wiley[2], C.E. Renna[3], N. Raj Bhandari[1], J.T. Painter[1]
[1]University of Arkansas for Medical Sciences, Pharmacy Practice, Little Rock, United States; [2]University of Nebraska Medicine, Pharmacy, Lincoln, United States; [3]University of Wisconsin, Pharmacy, Madison, United States
**Correspondence:** D.A. Hammond
*Intensive Care Medicine Experimental* 2017, **5(Suppl 2):**0825

**INTRODUCTION.** Lactate can represent tissue hypoperfusion in sepsis and septic shock. Early identification of patients at greater risk for a poor outcome may allow for resource allocation to manage these patients.

**OBJECTIVES.** The study purpose was to determine the association of serum lactate values within 24 hours of intensive care unit (ICU) admission with mortality in sepsis and septic shock.

**METHODS.** This was an IRB-approved, single-center, retrospective, observational study of patients admitted to an ICU between September 2012 and May 2015. The primary endpoint was in-hospital mortality. Patients were at least 18 years of age and possessed a diagnosis of sepsis or septic shock. Categorical data were analyzed using Chi-squared or Fisher Exact tests. Continuous data were analyzed with one-way ANOVA. Multivariate logistic regression analysis was performed using forward selection (p < 0.05 used for variable entry into the model) to evaluate variables associated with in-hospital mortality.

**RESULTS.** In total, 489 patients formed 3 groups: low (<2 mmol/L, n = 223), intermediate (2- < 4 mmol/L, n = 141), and high lactate (≥4 mmol/L, n = 125). Patients in the high lactate group had higher SOFA and APACHE IV scores and rate of septic shock (72.8%). Average lactate values were 1.29, 2.78, and 8.06 mmol/L, respectively. Compared to low lactate, high (odds ratio (OR) 2.151, 95% confidence interval (CI) 1.247-3.708, p = 0.0059) but not intermediate lactate (OR 1.248, 95% CI 0.732-2.129, p = 0.416) was associated with increased in-hospital mortality.

**CONCLUSIONS.** Patients with sepsis or septic shock with a high serum lactate were 2.15-times as likely to experience in-hospital mortality than those with a low serum lactate. Serum lactate monitoring is useful for risk assessment in patients admitted to the hospital with sepsis or septic shock.

**REFERENCE(S)**

Marty P, Roquilly A, Vallee F, et al., Lactate clearance for death prediction in severe sepsis or septic shock patients during the first 24 hours in Intensive Care Unit: an observational study. Ann Intensive Care. 2013;3(1):3.

**GRANT ACKNOWLEDGMENT**

None.

### 0826

#### Analysis and relation between lymphopenia and mortality in septic patients

N. Palomo-Lopez, A. Diaz-Martin, S. Escalona-Rodriguez, D. Cuenca-Apolo, J.M. López-Sanchez

Hospital Universitario Virgen del Rocío, Sevilla, Spain

**Correspondence:** N. Palomo-Lopez

*Intensive Care Medicine Experimental* 2017, **5(Suppl 2):**0826

**INTRODUCTION.** Septic shock maintains high mortality. Certain patient related factors, like lymphopenia, may be investigated in order to use it as prognostic biomarker.

**OBJECTIVES.** The objective of this study is to valuate the lymphopenia as a biomarker of unfavourable prognosis in patients with septic shock. Our aim is to establish relation between lymphopenia and mortality and the requirement of vasoactive support on the fourth day of ICU admission.

**METHODS.** Retrospective study including patients with sepsis of any source of infection that requires ICU admission during the period 2008–2014. We defined lymphopenia < 700 lymphocytes x$10^3$cells/microL in those patients with leucopenia (leucocytes < 3000x$10^3$cells/microL). The relation between the presence of lymphopenia and exits was calculated by Chi Square. To graduate severity of illness we used APACHE score.

**RESULTS.** There were included 163 patients with septic shock, with abdominal focus in 61 cases (37.4%) and respiratory focus in 47 cases (28.8%). Severity at admission in ICU was APACHE median 19 (26.06-11.97). At admission, 94 patients (54.9%) needed vasoactive support that was maintained on day 4 of admission in 49 of those patients (34.3%). The mortality rate was 69.65%, 32 cases.

In 46 episodes (28.2%) we found lymphopenia at ICU admission, 19.4% of those maintained the same situation at day 4 of admission. We found a statistical significant significance between lymphopenia at admission and mortality (p < 0.05). In addition, in those cases where lymphopenia persisted on the fourth day of admission (31 cases, 19.4%), tendency of mortality reaches 77.4% (24 the cases) (p < 0.05).

**CONCLUSIONS.** Our results suggest that septic shock patients with lymphopenia at ICU admission may have higher risk of mortality. That risk increases if lymphopenia persists on day 4 of admission

**REFERENCE(S)**

1. Drewry A, Samra N, Skupky L, Fuller B, Compton S et al. Persistent lymphopenia after diagnosis of sepsos predicts mortality. Shock. 2015; 42: 383–391

2. Moserrat J, de Pablo R, Diaz-Martín D, Rodríguez-Zapata M, de la Hera A et al. Early alterations of B cells inpatients with septic shock. Critical Care 2013 17:R105.

**GRANT ACKNOWLEDGMENT**

We gratefully appreciate the invaluable of ICU staff

### 0827

#### Related prognostic factors in elderly patients admitted in ICU with severe sepsis and septic shock

L. Sanchez[1], J.R. Martinez[1], G. Moreno[1], J.C. Lopez[2]

[1]Hospital Plato, ICU, Barcelona, Spain; [2]Hospital Universitario de Bellvitge, ICU, Barcelona, Spain

**Correspondence:** L. Sanchez

*Intensive Care Medicine Experimental* 2017, **5(Suppl 2):**0827

**OBJECTIVES.** The objective of this study was to evaluate variables related to mortality of aged patients, with severe sepsis and septic shock in the ICU of secondary level hospital.

**METHODS.** This is an observational retrospective cohort from January 2008 to June 2016, in an ICU of a secondary level Hospital in Barcelona.

Sepsis/septic shock patients of any etiology, > 70 years old, that required ICU admission were included. Patients with order DNR or limitation in vital support at admission were excluded. A descriptive analyses of the patient's demographic characteristics was performed.

The main group was divided into two groups, below and above 80 years of age.

A multi variate analyses to determinate intra hospital mortality risk factors and at 90 days of admission for each group was performed. P < 0,05 was considered as significant. Statistic SPSS 24 Statistics IBM was used.

**RESULTS.** 152 patients above seventies were included. The global mortality intra hospital was 32% and at 90 days was 44%.

The characteristics of each group were: below 80 y: mean age 74,8 +/– 3,1 with intra hospital mortality 33,7% and 46,5% at 90 days, while above 80 y were mean age 83,65 +/–3,1, with intra hospital mortality 33,3% and 42,4%, at 90 days.

The multi-variable analyses showed that for all the patients studied, the independent factors for intra hospital mortality were the worse PaO2/FIO2 (PAFI) relation, hypoalbuminemia at admission, leucopenia & diabetes mellitus; while at 90 days was hypoalbuminemia at admission.

For patients of the first group (70–80 years old), higher dose of noradrenaline, and the worse PAFI relation, were statistical significant as independent factors for intra hospital mortality, while the hypoalbuminemia at admission was the independent factor for mortality at 90 days.

For >80 years group the presence of neuropathy, disseminated intravascular coagulopathy (DIC), and the need for red blood cell transfusion, were independent mortality factors at 90 days.

**CONCLUSION.** Our studied population is fragile one, as per age as per multi pathology and have an elevated death risk compared with general population.

The mortality in our study population is high (33%), if compared with general population with sepsis/septic shock, that is around 20-30% according different series.

As we expected, the mortality was associated with severe respiratory failure, more hemodynamic instability and worse nutritional status at admission.

Limitations: One center Study, retrospective, small n, and was not possible to identify the cause of death at 90 days.

### 0828

#### Is uremia a good severity predictor in sepsis?

E. Vendrell Torra[1], E.P. Fernández Fernández[2], J. Méndez Barraza[1], E. Güell Quintilla[1], C.A. Socolich Mansilla[3], W.F. Salcedo Medina[3], M.C. de la Torre Terron[1], D. Salat Foix[4], P. Pujol Valverde[1], J.C. Yébenes Reyes[1]

[1]Hospital de Mataró, Intensive Care Unit, Mataró, Spain; [2]Hospital de Mataró, Internal Medicine, Mataró, Spain; [3]Hospital de Mataró, Emergency Department, Mataró, Spain; [4]Hospital Germans Trias i Pujol, Intensive Care Unit, Badalona, Spain

**Correspondence:** E. Vendrell Torra

*Intensive Care Medicine Experimental* 2017, **5(Suppl 2):**0828

**INTRODUCTION.** Early detection is critical for the management of sepsis. Urea is elevated in hypovolemic patients, and has been used as a biomarker of severity of several diseases, so its measurement could help in the early detection of sepsis.

**OBJECTIVES.** To evaluate the significance of elevated urea levels in the initial phase of sepsis, its usefulness as an indicator of severity and as an aid to guide early management.

**METHODS.** Prospective observational study. We collected demographic, clinical, and analytical data of patients for whom the sepsis code was activated in our center from January to December 2016. Data were described (frequency, mean and standard deviation or median and interquartílic range), and those with possible clinical significance were analyzed using the Student t-test for independent samples or the correlation coefficient.

**RESULTS.** The sepsis code was activated 65 times for 64 patients (54.6% male, age 74 ± 13.9 years). Fifty-four cases (84.4%) were community-acquired and the most frequently encountered varieties were respiratory (22; 34.4%), abdominal (16; 25%) and urinary (16; 25%). Bacteremia was documented in 38 (59.4%) cases. Lactate levels were 4.0 ± 2.3 mmol/L. SOFA score was 4 ± 2.7. Almost half of the patients were admitted to the ICU (30; 46.9%) with a mean length of stay of 5 days. The median duration of hospitalization was 14.3 ± 9.5 days. In-hospital mortality was 23.4%.

Urea level was 63.3 ± 63.9 mg/dL, and 67% of patients had an abnormal uremia (>50 mg/dL). There was a moderate correlation between these levels and the severity rating scales such as the APACHE-II (correlation coefficient [CC] 0.36) or SOFA (CC 0.334). Patients with urea >100mg/dL were more likely to need ICU admission (RR 1.34, 95%CI 0.7972 to 2.2621) and had higher intra-ICU (RR 1.09, 95%CI 0.24-4.84), in-hospital (RR 1.19; 95%CI 0.44-3.19) and 30-day (RR 1.23, 95%CI 0.59-2.56) mortality. Urea values were also correlated with a higher leukocyte count (CC for leukocyte/neutrophil 0.43/0.39, respectively) and base consumptions (CC for apH, aHCO3, aBE close to −0.4, and CC for vHCO3, vBE close to −0.3).

**CONCLUSIONS.** 67% of patients for whom the sepsis code is activated had abnormal uremia. Otherwise uremia seems to be associated to higher mortality at 30 days. This result was not statistically significant, and should be confirmed in future studies.

### 0829

#### Prognostic value of venoarterial carbon-dioxide gradient in patients with sepsis

U. Batranovic[1], S. Milic[1], V. Carapic[1], A. Andrijevic[1], D. Obradovic[2]
[1]Institute for Pulmonary Diseases of Vojvodina, Intensive Care Unit, Sremska Kamenica, Serbia; [2]Institute for Pulmonary Diseases of Vojvodina, High Dependency Unit, Sremska Kamenica, Serbia
**Correspondence:** U. Batranovic
*Intensive Care Medicine Experimental* 2017, **5(Suppl 2):**0829

**INTRODUCTION.** Central venous-arterial CO2 difference (Pv-aCO2) reflects adequacy of microcirculatory venous flow. Widening of Pv-aCO2 due to CO2-stagnant phenomenon is described in the low flow states. Pv-aCO2 was proposed as an additional resuscitation target for patients with septic shock. (1,2)

**OBJECTIVES.** The aim of this study was to examine correlation between changes in Pv-aCO2 and SOFA score as well as different blood flow indices 12 hours after onset of resuscitation in patients with sepsis or septic shock. Secondary aim was to evaluate association of delta CO2 6 hours after onset of resuscitation and patient outcomes (length of stay in the ICU, mortality).

**METHODS.** Prospective observational study included 150 patients with sepsis. Simultaneous measurements of lactate, mixed venous oxygen saturation (ScvO2) and delta PCO2 were performed at onset of resuscitation (T0) and after 6 hours (T6). Delta PCO2 was calculated as a difference between arterial PCO2 and PCO2 from mixed venous blood. Organ dysfunction was evaluated with the SOFA score at T0 and after 48 hours (T48). Mortality was assessed after 28 days. For data analysis purposes two groups were created based on delta SOFA [(1) patients with SOFA score decrease (delta SOFA < 0); (2) patients without SOFA score decrease (delta SOFA ≥ 0)] and based on Pv-aCO2 [(1) patients with high Pv-aCO2 (≥0.8 kPa); (2) patients with normal Pv-aCO2 (<0.8 kPa). Repeated measurements were analyzed with Duncan´s Multiple Range Test.

**RESULTS.** On overall, 109 patients were male (72.6%), the average age of patients was 58.7 ± 13.4 years, 75 patients (50%) met criteria for septic shock. Average length of stay in the intensive care unit was 10.9 ± 10.7 days (4–15 IQR). Average APACHE score on admission to the intensive care unit was 21.5 ± 7.1 (IQR 16–27). The total 28-day mortality was 42%. Patients with high and normal Pv-aCO2 differed only with respect to highest respiratory SOFA score (p = 0.01) Change in Pv-aCO2 between T0 and T6 was not in correlation with change in SOFA score between T0 and T48 (p = 0.12). Moderate statistically significant correlation was found between Pv-aCO2 and lactate at T6 (r = 0.2), and moderate inverse correlation between Pv-aCO2 and ScvO2 at T0 (r = −0.4) and T12 (r = −0.25) and ScvO2 and lactate at T0 (r = −0.27) and T12 (r = −0.18). Pv-aCO2 at T6 was not associated with 28-day mortality and length of stay in the ICU.

**CONCLUSIONS.** We did not observe statistically significant correlation between delta PCO2, different blood flow indices, SOFA score and patient outcomes. Further investigation is needed to examine delta PCO2 as a valid resuscitation target.

**REFERENCE(S)**

1. Lamia B, Monnet X, Teboul JL. Meaning of arterio-venous PCO2 difference in circulatory shock. Minerva Anestesiol. 2006;72(6):597–604.
2. Vallet B, Pinsky MR, Cecconi M. Resuscitation of patients with septic shock: Please "mind the gap"! Intensive Care Med. 2013;39(9):1653–5.

### 0830

#### The effect of pH enhances the prognostic value of central venous-to-arterial carbon dioxide differences in early septic shock

J. Mesquida[1], P. Saludes[1], A. Pérez-Madrigal[1], L. Proença[1,2], E. Cortes[1], L. Enseñat[1], C. Espinal[1], G. Gruartmoner[1]
[1]Hospital de Sabadell, Corporació Sanitària Universitària Parc Taulí, Universitat Autònoma de Barcelona, Critical Care Department, Sabadell, Spain; [2]Hospital Prof. Dr. Fernando Fonseca, Serviço de Medicina I, Amadora, Portugal
**Correspondence:** J. Mesquida
*Intensive Care Medicine Experimental* 2017, **5(Suppl 2):**0830

**INTRODUCTION.** Central venous-to-arterial carbon dioxide differences ($P_{cva}CO_2$) have been proposed as an additional tool in the hemodynamic resuscitation process. Correcting $P_{cva}CO_2$ by the arterial-to-venous oxygen content difference ($P_{cva}CO_2/C_{av}O_2$), as an approximation of the respiratory quotient, seems to improve the prognostic performance of $P_{cva}CO_2$ alone. However, since $PCO_2$ and the content of $CO_2$ ($CCO_2$) might be affected by several factors, such as the Haldane effect, some authors advocate for the use of $C_{cva}CO_2/C_{av}O_2$ instead of the $P_{cva}CO_2/C_{av}O_2$.

**OBJECTIVE.** To explore the factors that might intervene in the difference between $P_{cva}CO_2/C_{av}O_2$ and $C_{cva}CO_2/C_{av}O_2$, and to analyze their association with mortality.

**METHODS.** Observational study in a 30-bed mixed ICU. Fifty-two septic shock patients within the first 24 hours of ICU admission were studied. After restoration of mean arterial pressure, hemodynamic and metabolic parameters were simultaneously evaluated. A total of 110 sets of measurements were performed. *Statistical analysis*: Differences in the studied variables between survivors and non-survivors were analyzed using the t-Student test. Linear regression tests were used to explore the association of $CO_2$-derived parameters with other metabolic and hemodynamic variables.

**RESULTS.** At inclusion, the variables associated with ICU-mortality were arterial lactate, pH, $P_{cva}CO_2/C_{av}O_2$, and the difference between $P_{cva}CO_2/C_{av}O_2$ and $C_{cva}CO_2/C_{av}O_2$ (Fig. 276). Initial central venous oxygen saturation ($S_{cv}O_2$), $P_{cva}CO_2$, $C_{cva}CO_2/C_{av}O_2$, and cardiac index did not show any difference between survivors and non-survivors.

In a multiple linear regression analysis, the difference between $P_{cva}CO_2/C_{av}O_2$ and $C_{cva}CO_2/C_{av}O_2$ was independently associated with pH (B −2.73, 95% CI −3.43, −2.03; p < 0.001) (Fig. 277), $S_{cv}O_2$ (B 0.02,

95% CI 0.01, 0.03; p < 0.001), and baseline $C_{cva}CO_2/C_{av}O_2$ (B −0.28, 95% CI −0.46, −0.1; p < 0.01). A stepwise regression analysis showed that pH was the single best predictor for the magnitude of the difference between $P_{cva}CO_2/C_{av}O_2$ and $C_{cva}CO_2/C_{av}O_2$ (R Square 0.41), with very limited effect of both $S_{cv}O_2$ and $C_{cva}CO_2/C_{av}O_2$.

**CONCLUSIONS.** In a population of early septic shock patients, the $P_{cva}CO_2/C_{av}O_2$ ratio and the difference between $P_{cva}CO_2/C_{av}O_2$ and $C_{cva}CO_2/C_{av}O_2$ were associated with ICU mortality, whereas $C_{cva}CO_2/C_{av}O_2$ was not. The main determinant of the magnitude of the difference between $P_{cva}CO_2/C_{av}O_2$ and $C_{cva}CO_2/C_{av}O_2$ was pH. Therefore, the effect of pH on the $PCO_2$-$CCO_2$ relationship might amplify the prognostic performance of $CO_2$-derived parameters in early septic shock.

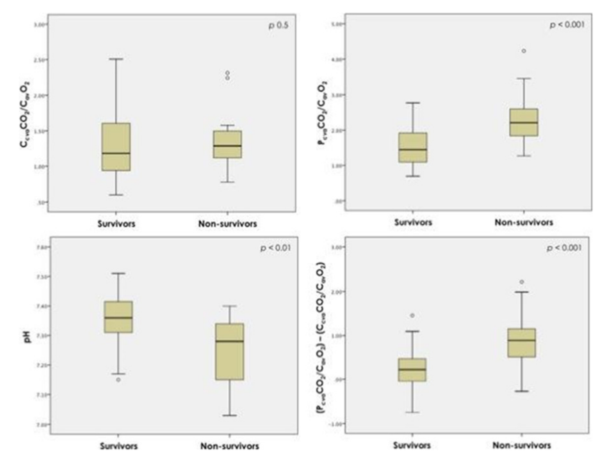

**Fig. 276 (Abstract 0830).** $CO_2$-derived parameters and pH at inclusion, according to ICU-mortality

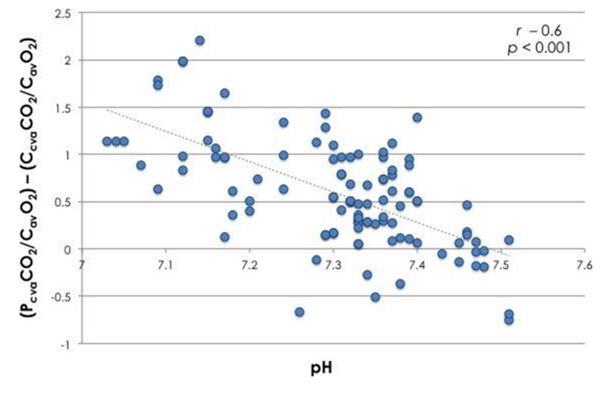

**Fig. 277 (Abstract 0830).** Relationship between arterial pH and the difference between $P_{cva}CO_2/C_{av}O_2$ and $C_{cva}CO_2/C_{av}O_2$

### 0831

#### Significance of Neutrophil Lymphocyte Ratio in outcome of sepsis

G.L. Keegan[1], I. Welters[2]
[1]University of Liverpool, School of Medicine, Liverpool, United Kingdom; [2]University of Liverpool, Liverpool, United Kingdom
**Correspondence:** G.L. Keegan
*Intensive Care Medicine Experimental* 2017, **5(Suppl 2):**0831

**INTRODUCTION.** The Neutrophil Lymphocyte Ratio (NLR) has been used to predict both long and short term outcomes in patients, with high NLR levels being an independent indicator of both in hospital, 28 day and 6-month mortality, and has consistently been linked to prognosis in critically ill patients. Since up to 50% of patients with bacteraemia can exhibit a normal White Cell Count (WCC) and CRP, further markers of sepsis are needed for early diagnosis. NLR may help predict the outcome of critically ill patients.

**OBJECTIVES.** The aims of this study were to investigate 1. The time course of NLR over a 4-day period in septic versus non-septic critically ill patients, and 2. If NLR was predictive of ICU and 28-day mortality.

**METHODS.** Patients admitted to the ICU department at the Royal Liverpool University Hospital between January 2008 and December 2012 were included. Demographic data included age, sepsis status, type of sepsis, date of sepsis/septic shock, length of stay in ICU, 28-day mortality, ICU outcome and gender. Routine blood results were recorded for the first four days of ICU admission (WCC, lymphocytes, neutrophils, platelets, lactate, albumin, CRP, creatinine, urea and haemoglobin). Acute Physiology and Chronic Health Evaluation II (APACHE II) score was also recorded on admission and Sequential Organ Failure Assessment Score (SOFA) was documented daily. Using the neutrophil and lymphocyte counts patient's NLR values were calculated for the first 4 days of intensive care admission.

**RESULTS.** A total of 522 patients were included. Mean age was 59.8 [SD ±16.4], 58.3% were male and 58.5% had sepsis on admission. 28-day mortality was 20.7%, and ICU mortality was 23.8%. Mean NLR in survivors decreased over a 4-day period, whereas mean NLR in non-survivors remained high throughout the 4-day period. In both septic and non-septic patients, the mean NLR decreased over the 4-day period, showing sepsis was not a main discriminator for outcome. The area under the receiver operating curve was highest for lymphocyte count (0.577; CI 0.517-0.637). NLR, Plt and NC resulted in AUC of 0.560, 0.539 and 0.511 respectively.

**CONCLUSIONS.** NLR measured on intensive care admission is predictive of outcome in critically ill patients and its time course over the first four days differs significantly between survivors and non-survivors. However, performance is not good enough for introduction into clinical practice.

**REFERENCE(S)**

1. Akilli NB, Yortanli M, Mutlu H, Gunaydin YK, Koylu R, Akca HS, et al. *Am J Emerg Med* 2014;32(12):1476–80.
2. Bozbay M, Uyarel H. *J Crit Care* 2015;30(4):822.
3. Salciccioli JD, Marshall DC, Pimentel MA, Santos MD et al. *Crit Care* 2015;19:13.

Antimicrobial use and infection prevention

### 0832

#### Optimization of piperacillin/tazobactam therapy in critically ill patients: a pilot study

V.D. Gumucio-Sanguino[1], A. Padullés-Zamora[2], S. Cobo-Sacristán[2], E. Esteve-Pitarch[2], E.P. Plata-Menchaca[3], M. Rojas-Lora[1], J. Sabater-Riera[1], SIRAKI Group
[1]Hospital Universitario de Bellvitge, Critical Care Department, L'Hospitalet de Llobregat, Spain; [2]Hospital Universitario de Bellvitge, Pharmacy, L' Hospitalet de Lobregat, Spain; [3]Instituto de Investigación Biomédica de Bellvitge, Research, L' Hospitalet de Lobregat, Spain
**Correspondence:** V.D. Gumucio-Sanguino
*Intensive Care Medicine Experimental* 2017, **5(Suppl 2):**0832

**INTRODUCTION.** The pharmacokinetic (PK) of β-lactam antibiotics in critically ill patients with sepsis presents high interindividual variability. There is an increasing interest in the use of continuous infusion of β-lactams to optimize antibiotic concentrations throughout the dosing interval.

**OBJECTIVES.** To evaluate whether piperacillin/tazobactam (P/T) administrated by continuous infusion achieved plasma concentrations (Cp) above the MIC. This study also sought to determine the time needed to achieve the PK/PD index.

**METHODS.** A prospective, pilot study was performed in the intensive care unit of a tertiary university hospital for adults. Patients were included if they met the following criteria: normal renal function (GFR CKD-EPI formula > 60 mL/min), sepsis and indication for P/T therapy. P/T was administered as a 4 g loading dose followed by intravenous continuous infusion of 12 g/24h. Patients who had already initiated P/T therapy in intermittent infusion (4 g/8h) were switched to continuous infusion without administration of a loading dose. P/T concentration was determined after loading dose (Cmax) and at steady state, 24h hours after the continuous infusion was started (Css). Total plasma concentrations were measured using Ultra-High Performance Liquid Chromatography-Tandem Mass Spectrometry. We used a theoretical MIC breakpoint of 16 mg/L for P/T.

**RESULTS.** Twenty-one patients were enrolled (41% women); with a median age of 47 years

(IQR: 41.8- 54.3). Mean GFR was 90 mL/min (IQR: 63–90). Sites of infection were: lung (n = 17, 81%), central nervous system (n = 2, 9.5%) and primary bloodstream infection (n = 2, 9.5%). Concentration measurement from patients was available as follows: Css (n = 12 patients), Cmax and Css (n = 5), and Cmax (n = 4). Median Cmax and Css were 93.4 mg/L (IQR: 33–143.8) and 51.4 mg/L (IQR: 32.6-73.1) respectively. Patients who received a loading dose of antibiotic (n = 9, 43%) reached a higher Css than those who did not (70 mg/L vs 46.2 mg/L). Cmax > 16 mg/L was achieved in 89% patients, 78% Cmax > 32 mg/L and 67% Cmax > 64 mg/L. All patients reached Css > 16 mg/L, 76% achieved Css > 32 mg/L and 41% achieved Cmx > 64mg/L.

**CONCLUSIONS.** In this pilot study, we found that the use of P/T by continuous infusion allowed reaching PK/PD target in almost all patients within the first 24 hours of therapy. This target was achieved immediately after a loading dose in 89% of the patients.

**REFERENCE(S)**

Felton TW, Hope WW, Lomaestro BM, Butterfield JM, Kwa AL, Drusano GL, et al. Population Pharmacokinetics of extended-infusion piperacillin-tazobactam in Hospitalized Patients with nosocomial Infections. *Antimicrobial Agents and Chemotherapy* 2012; 56: 4087–4094.

### 0833

#### Assessment of enteral paromomycin to eradicate colistin and carbepenemase resistant microrganisms in rectal colonization to prevent ICU - acquired infections

C. Sanchez Ramírez[1], M.A. Hernández Viera[1], L. Caipe Balcázar[1], S. Hípola Escalada[1], M. Cabrera Santana[1], R.E. Morales Sirgado[1], M.E. Gallardo Santos[1], C.F. Lübbe Vázquez[1], S.M. Marrero Penichet[2], F. Artiles Campelo[3], M.A. De la Cal López[4], S. Ruiz Santana[1]

[1]University Hospital of Gran Canaria Dr Negrín, Intensive Care Unit, Las Palmas de Gran Canaria, Spain; [2]University Hospital of Gran Canaria Dr Negrín, Pharmacy Department, Las Palmas de Gran Canaria, Spain; [3]University Hospital of Gran Canaria Dr Negrín, Microbiology Department, Las Palmas de Gran Canaria, Spain; [4]Getafe Hospital, Intensive Care Unit, Madrid, Spain

**Correspondence:** C. Sanchez Ramírez

*Intensive Care Medicine Experimental* 2017, **5(Suppl 2):**0833

**OBJECTIVE.** To assess the value of enteral paromomycin to decontaminate patients with rectal colistin and / or carbepenemase (CPN) resistant microorganisms colonization to prevent the development of ICU-adquired infections.

**METHODS.** All consecutive patients admitted to the ICU from October 2011 to September 2016, expected to require tracheal intubation for longer than 48 hours were given SDD with a 4-day course of intravenous cefotaxime, plus enteral colistin, tobramycin and nystatin in an oropharyngeal paste and in a digestive solution. Oropharyngeal and rectal swabs were obtained on admission and once weekly. Patients with rectal swabs colonized by colistin and / or carbepenemase resistant microorganisms were treated with enteral paromomycin 1 gr every 6 hours a day, in order to become it negative and prevent nosocomial infections. Categorical variables were summarized as frequencies and percentages and the continuous ones as medians and interquartile ranges (IQR) or means and standard deviations. Statistical significance was set at $p \leq 0.05$.

**RESULTS.** We applied paromomycin treatment to 82 colonized patients with rectal colistin resistant microorganisms. All of them but two had colonization by Extended Spectrum Beta-lactamases (ESBLs) producing *Klebsiella pneumoniae*. One patient was colonized by ESBL producing *Enterobacter spp* and the other by ESBL producing *Escherichia coli*. Demographic data and type of admission are shown in Table Error! Reference source not found.

Sixty-seven out of 82 (81.7%) negativized the rectal exudate after paromomycin. Of those negativized, 25 patients received an appropriate antibiotic during the application of paromomycin. Five out of the seven patients with CPN were decolonized. Twenty-seven patients died at ICU discharge and 15 patients died in the ICU without being negativized two of them with CPN resistant microorganisms. Only 3 patients developed infections after decolonization.

**CONCLUSION.** Our data show that enteral paromomycin is effective in treating rectal colistin and / or carbapenemase resistant microorganism colonization to prevent the development of ICU nosocomial infections.

**Table 203 (Abstract 0833).** See text for description

| | |
|---|---|
| **Patients, n** | 82 |
| **Age, years, SD** | 62,1± 14,5 |
| **Male/female, n, %** | 53(64)/ 29(35) |
| **APACHE II on admission, n(IQR)** | 22(16;27,5) |
| **SOFA on admission, n(IQR)** | 9,5(7;12) |
| **APACHE II at paromomycin treatment, n(IQR)** | 17(12,75;21,25) |
| **SOFA at paromomycin treatment, n(IQR)** | 5,5(3;9) |
| **Glasgow Coma Score, n(IQR)** | 13,5 (4,5;15) |
| **ICU stay days, n(IQR)** | 59 (30;111) |
| **Renal Replacement Therapy, n** | 35 |
| **Traumatic patients, n** | **7** |
| **Medical patients on admission, n** | **51** |
| **Parenteral nutrition, n** | **20** |
| **Diabetes mellitus, n** | **32** |
| **Neutropenic patients, n** | **2** |
| **Immunosuppression, n** | **6** |
| **Deaths, n** | **27** |
| **MV >7 days, n** | **82** |
| **Paromomycin treatment days, n(IQR)** | 14(8;26,25) |

ICU: Intensive Care Unit; n: number; MV. Mechanical Ventilation; SD: Standar Deviation; IQR:Interquartile Range; n:number

### 0834

#### Five years of selective digestive decontamination in a mixed intensive care unit at a university hospital: impact on colonization, nosocomial infection and antibiotic consumption

C. Sánchez Ramírez[1], S. Hípola Escalada[1], M. Cabrera Santana[1], M.A. Hernández Viera[1], L. Caipe Balcázar[1], R.E. Morales Sirgado[1], C.F. Lübbe Vázquez[1], S.M. Marrero Penichet[2], F. Artiles Campelo[3], P. Saavedra Santana[4], S. Ruiz Santana[1]

[1]University Hospital of Gran Canaria Dr Negrín, Intensive Care Unit, Las Palmas de Gran Canaria, Spain; [2]University Hospital of Gran Canaria Dr Negrín, Pharmacy Department, Las Palmas de Gran Canaria, Spain; [3]University Hospital of Gran Canaria Dr Negrín, Microbiology Department, Las Palmas de Gran Canaria, Spain; [4]University of Las Palmas de Gran Canaria, Mathematics and Informatic Department, Las Palmas de Gran Canaria, Spain

**Correspondence:** C. Sánchez Ramírez

*Intensive Care Medicine Experimental* 2017, **5(Suppl 2):**0834

**OBJECTIVES.** To prospectively evaluate the impact of Selective Digestive Decontamination (SDD) application on nosocomial infections and colonization rates, after 5 years in a mixed ICU.

**METHODS.** This study was conducted in a 30-bed-medical-surgical ICU. All consecutive patients admitted to the ICU from October 1,

2011 to September 30, 2016 expected to require tracheal intubation > 48 hours were given SDD (SDD study group) with a 4-day course of intravenous cefotaxime, plus enteral colistin, tobramycin, nystatin in an oropharyngeal paste and in a digestive solution. Oropharyngeal and rectal swabs were obtained on admission and once weekly. We used ENVIN nosocomial infection criteria. We compared all patients admitted to ICU with nosocomial ICU infections from October 1, 2010 to September 30, 2011 (non-SDD group) to the SDD study group. Categorical variables were summarized as frequencies and percentages and the continuous ones as means and standard deviations (SD) when the data followed the normal distribution or medians and interquartile ranges (IQR) when they did not. The percentages were compared using the test of chi-square test or Fisher exact test, means with the t-test and medians with the Wilcoxon test for independent samples. Those variables that showed statistical significance in the univariate analysis were introduced in a multivariate logistic regression analysis. For each one of the infections (catheter-related and other secondary bacteremias, pneumonia and urinary infections and antibiotic resistant bacteria (ARB) infections) the incidences per 1000 days of exposure in each cohort and the corresponding relative risks were obtained using the Poisson regression. Statistical significance was

$p \leq 0.05$.We analyzed colistin and tobramycin resistant colonization and also antibiotic consumption as Defined antibiotics Daily Doses (DDD).

**RESULTS.** Results are shown in Tables 204, 205 and 206.

There were no statistical significant differences between both groups in type of admission or demographic data. Patients with SDD had significantly less Extended Spectrum Betalactamase (ESBL), Gram Negative Bacteria Multirresistant (GNB MR) and *Acinetobacter spp* infections. We had also a significant reduction in nosocomial pneumonias, urinary infections and other secondary bacteremias and ARB infection rates, in SDD group versus non SDD. There was no infection by *Clostridium difficile*. The exogenous infections were 74,8%. Colistin resistant colonization was 17,3% and tobramycin resistant colonization was 24,6% of samples. There was a decrease on the DDD/100 ICU stays after SDD.

**CONCLUSIONS.** After 5 years applying SDD a significant reduction of infections by ESBL, GNB MR and *Acinetobacter,* was observed. A significant decrease of nosocomial pneumonia, secondary bacteremias, urinary and ARB infections rates was also shown. An antibiotic consumption reduction was found after SDD. Low rates of colistin and tobramycin resistant colonization bacteria have been observed.

**Table 204 (Abstract 0834).** Univariate analysis

| | Selective Digestive Decontamination | | | |
|---|---|---|---|---|
| | Total | No | Yes | |
| | N = 452 | N = 110 | N = 342 | P |
| Age, years | 60.8 ± 15.5 | 59.5 ± 15.8 | 61.2 ± 15.4 | .331 |
| Male / Female | 65.2 / 34.8 | 67.3 / 32.7 | 64.5 / 35.6 | .598 |
| Apache-II | 21.8 ± 7.5 | 21.2 ± 7.7 | 22.0 ± 7.5 | .326 |
| Glasgow | 15 (9 ; 15) | 15 (8 ; 15) | 15 (9 ; 15) | .105 |
| Urgent surgery | 129 (28.5) | 34 (30.9) | 95 (27.8) | .527 |
| Immunossupression | 40 (8.8) | 8 (7.3) | 32 (9.4) | .503 |
| Neutropenia | 15 (3.3) | 3 (2.7) | 12 (3.5) | .691 |
| Parenteral nutrition | 155 (34.3) | 26 (23.6) | 129 (37.7) | .007 |
| *Patient type* | | | | .199 |
| Medical | 318 (70.4) | 79 (71.8) | 239 (69.9) | |
| Scheduled surgical | 62 (13.7) | 10 (9.1) | 52 (15.2) | |
| Urgent surgical | 72 (15.9) | 21 (19.1) | 51 (14.9) | |
| Traumatic patient | 55 (12.2) | 17 (15.5) | 38 (11.1) | .230 |
| Coronary patient | 94 (20.8) | 19 (17.3) | 75 (21.9) | .295 |
| Renal Replacement Therapy | 163 (36.1) | 34 (30.9) | 129 (37.7) | .196 |
| Nosocomial pneumonia | 182 (40.3) | 59 (53.6) | 123 (36.0) | .001 |
| Urinary infection | 121 (26.8) | 29 (26.4) | 92 (26.9) | .912 |
| Catheter related Bacteremia | 169 (37.4) | 26 (23.6) | 143 (41.8) | < .001 |
| Secondary bacteremia | 102 (22.6) | 31 (28.2) | 71 (20.8) | .105 |
| Inflammatory response | | | | < .001 |
| Non sepsis | 21 (4.6) | 2 (1.8) | 19 (5.6) | |
| Sepsis | 139 (30.8) | 23 (20.9) | 116 (33.9) | |
| Severe Sepsis | 69 (15.3) | 34 (30.9) | 35 (10.2) | |
| Septic Shock | 223 (49.3) | 51 (46.4) | 172 (50.3) | |
| Renal failure | 108 (23.9) | 40 (36.4) | 68 (19.9) | < .001 |
| COPD | 62 (13.7) | 9 (8.2) | 53 (15.5) | .052 |
| Cirrhosis | 26 (5.8) | 6 (5.5) | 20 (5.8) | .893 |
| Diabetes mellitus | 142 (31.4) | 34 (30.9) | 108 (31.6) | .895 |
| MRSA infections | 10 (2.2) | 4 (3.6) | 6 (1.8) | .243 |
| Acinetobacter infections | 18 (4.0) | 13 (11.8) | 5 (1.5) | < .001 |
| ESBL infections | 119 (26.3) | 38 (34.5) | 81 (23.7) | .024 |
| Pseudomonas MR infections | 36 (8.0) | 10 (9.1) | 26 (7.6) | .616 |
| GNB MR infections | 23 (5.1) | 12 (10.9) | 11 (3.2) | .001 |
| Death | 164 (36.7) | 36 (32.7) | 128 (38.0) | .321 |

Data are means ± SD, medians (IQR) and frequencies (%); COPD: chronic obstructive pulmonary disease; MRSA: methicillin resistant Sthaphylococcus aureus; ESBL: extended spectrum betalactamase; MR: multiresistant; GNB: gram negative bacteria

### 0835

#### Effects of a novel seven-species probiotic against oropharyngeal bacterial infestation in adult trauma Intensive care unit patients: a randomized double blind clinical trial

M. Masjedi[1,2], S. Raoufi[3], G. Dabiri[1], B. Maghsoudi[4,5], G. Sabetian[1,6], F. Zand[5,7], P. Vatankhah[7], M. Yazdani[3]

[1]Shiraz University of Medical Sciences, Shiraz, Iran, Islamic Republic of; [2]Shiraz University of Medical Sciences, Anesthesiology and Critical Care Research Center, Shiraz, Iran, Islamic Republic of; [3]Lorestan University of Medical Sciences, Nursing, Khoramabad, Iran, Islamic Republic of; [4]Shiraz University of Medical Sciences, Anesthesia and Critical Care, Shiraz, Iran, Islamic Republic of; [5]Anesthesiology and Critical Care Research Center, Shiraz, Iran, Islamic Republic of; [6]Trauma Research Center, Shahid Rajaee (Emtiaz) Trauma Hospital, Shiraz, Iran, Islamic Republic of; [7]Shiraz University of Medical Sciences, Anesthesiology and Critical Care, Shiraz, Iran, Islamic Republic of

**Correspondence:** M. Masjedi

*Intensive Care Medicine Experimental* 2017, **5(Suppl 2):**0835

**INTRODUCTION.** Sepsis is still amongst the leading cause of mortality in intensive care units.Ventilator-associated pneumonia results from invasion of the lower respiratory tract and lung parenchyma by microorganisms residing mainly in oral cavity. Trauma victims are at increased risk of aquiring hospital infections specially when admitted to intensive care unit.

**OBJECTIVES.** The aim of our study was to investigate the efficacy of a new probiotic combination, containing 7 bacterial species, against oropharyngeal bacterial infestation in adult trauma intensive care unit patients.

**METHODS.** One hundred and fifty patients were placed in the two treatment groups by computerized random allocation in a 1:1 ratio and received either probiotics or placebo. Oropharyngeal cultures were taken on the 1st (before the intervention), 4th, and 6th days of admission.

**RESULTS.** The culture results of the 1st, 4th, and 6th days were comparable and no statistically significant difference was noticed in the two arms of the study.

**CONCLUSIONS.** Based on the results of our study, Administration of probiotics to alter early oropharyngeal cavity infestation with PPM in adult trauma patients admitted in Intensive Care Unit appears to be non-efficacious, even when a 7- species combination is used.

**REFERENCE(S)**

1- Chan EY, Ruest A, Meade MO, Cook DJ. Oral decontamination for prevention of pneumonia in mechanically ventilated adults: systematic review and meta-analysis. BMJ. 2007; 334(7599):889.

2- Schlundt J. Health and Nutritional Properties of Probiotics in Food including Powder Milk with Live Lactic Acid Bacteria. Report of a Joint FAO/WHO Expert Consultation on Evaluation of Health and Nutritional Properties of Probiotics in Food Including Powder Milk with Live Lactic Acid Bacteria. FAO / WHO. Retrieved 17 December 2012.

3- Bengt Klarin, Göran Molin, Bengt Jeppsson, Anders Larsson. Use of the probiotic Lactobacillus plantarum 299 to reduce pathogenic

**Table 206 (Abstract 0834).** Nosocomial infection rates

| | | SDD | | | |
|---|---|---|---|---|---|
| | | No N = 110 | Yes N = 342 | P | RR (95% CI) |
| Pneumonia/MV | Neumonias/1000 days of MV | 10.3 | 4.3 | < .001 | 0.414 (0.307 ; 0.559) |
| Urinary Infections /urinary catheter | Urinary Infections /1000 days of urinary catheter | 3.79 | 2.49 | .035 | 0.658 (0.445 ; 0.971) |
| CRB/CVC | CRB/1000 days of CVC | 3.59 | 4.02 | .595 | 1.120 (0.739 ; 1.697) |
| Secondary Bacteremias / Stay days | Secondary bacteremia./1000 stay days | 4.69 | 1.77 | < .001 | 0.378 (0.261 ; 0.546) |
| ARB infections/ stay days | ARB infections./1000 stay days | 9.59 | 2.81 | < .001 | 0.293 (0.223 ; 0.383) |

MV: mechanical ventilation; CRB: catheter related bacteremia; CVC: central venous catheter; ARB: antibiotic resistant bacteria; RR: relative risk; CI: confidence interval.

**Table 205 (Abstract 0834).** Multivariate analysis

| | P* | OR (95% CI) |
|---|---|---|
| ***Inflammatory response*** | | |
| Non sepsis | < .001 | 1 |
| Sepsis | | 0.389 (0.080 ; 1.888) |
| Severe Sepsis | | 0.081 (0.016 ; 0.400) |
| Septic Shock | | 0.363 (0.078 ; 1.691) |
| ***Parenteral nutrition*** | .004 | 2.180 (1.257 ; 3.780) |
| ***Catheter related Bacteremia*** | .001 | 2.334 (1.363 ; 3.997) |
| ***Renal failure*** | .002 | 0.422 (0.248 ; 0.719) |
| ***Acinetobacter infection*** | < .001 | 0.106 (0.034 ; 0.338) |

bacteria in the oropharynx of intubated patients: a randomised controlled open pilot study. Crit Care. 2008; 12(6): R136.

**GRANT ACKNOWLEDGMENT**

This study was financially supported by the Vice chancellery for research affairs of Shiraz University of Medical Sciences, Shiraz, Iran

### 0836

#### Probiotics; are they efficient in preventing ventilator associated pneumonia in adult trauma victims admitted in intensive care unit? A prospective clinical study

M. Masjedi[1,2], G. Dabiri[3], G. Sabetian[1,4], F. Zand[1,2], B. Maghsoudi[1,2], P. Vatankhah[1], V. Khaloo[3], B. Pourabbas[3,5], S. Bolandparvaz[4,6], H. Abbasi[4,6], S. Payedar[4,7], H. Khalili[4,8], A. Abbasian[3]

[1]Shiraz University of Medical Sciences, Anesthesia and Intensive Care, Shiraz, Iran, Islamic Republic of; [2]Anesthesiology and Critical Care Research Center, Shiraz, Iran, Islamic Republic of; [3]Shiraz University of Medical Sciences, Shiraz, Iran, Islamic Republic of; [4]Trauma Research Center, Shahid Rajaee (Emtiaz) Trauma Hospital, Surgery, Shiraz, Iran, Islamic Republic of; [5]Alborzi Clinical Microbiology Research Center, Shiraz, Iran, Islamic Republic of; [6]Shiraz University of Medical Sciences, Surgery, Shiraz, Iran, Islamic Republic of; [7]Anesthesiology and Sritical Care Research Center, Surgery, Shiraz, Iran, Islamic Republic of; [8]Shiraz University of Medical Sciences, Neurosurgery, Shiraz, Iran, Islamic Republic of

**Correspondence:** M. Masjedi

*Intensive Care Medicine Experimental* 2017, **5(Suppl 2):**0836

**INTRODUCTION.** Administration of probiotics is proposed as a novel approach in decreasing the incidence of ventilator associated pneumonia. Previous studies utilized 1–3 species probiotics. On the other hand, trauma patients are at increased risk for infection due to multiple factors.

**OBJECTIVES.** The aim of our study was to evaluate the efficacy of a new 7-species combination of probiotics on the incidence of ventilator associated pneumonia in adult trauma patients admitted in intensive care units.

**METHODS.** As a prospective, double blind, placebo-controlled study; one hundred and fifty patients were randomized into two groups of probiotics and placebo in a 1:1 ratio. Both groups received the drugs as suspensions in the oropharyngeal cavity and stomach.

**RESULTS.** No difference was noted in the incidence of early-onset ventilator-associated pneumonia between two groups. There was no adverse event due to probiotic administration.

**CONCLUSIONS.** This study suggests that administration of probiotics does not alter the incidence of early-onset ventilator-associated pneumonia in adult trauma ICU, even when a 7-species combination is applied.

**REFERENCE(S)**

1. Schultz MJ, Haas LE. Antibiotics or probiotics as preventive measures against ventilator-associated pneumonia: a literature review. Crit Care. 2011;15(1):R18.

2. Wang J, Liu KX, Ariani F, Tao LL, Zhang J, Qu JM. Probiotics for preventing ventilator-associated pneumonia: a systematic review and meta-analysis of high-quality randomized controlled trials. PLoS One. 2013; 8(12):e83934.

3. Wood GC, Boucher BA, Croce MA, Fabian TC. Lactobacillus species as a cause of ventilator-associated pneumonia in a critically ill trauma patient. Pharmacotherapy. 2002; 22(9):1180–2.

**GRANT ACKNOWLEDGMENT**

This study was financially supported by the Vice chancellery for research affairs of Shiraz University of Medical Sciences, Shiraz, Iran

### 0837

#### The effects of ceftolozane/tazobactam vs. piperacillin/tazobactam in a model of multi-resistant *Pseudomonas aeruginosa* pneumonia

G. Li Bassi[1], E. Aguilera Xiol[1], A. Motos[1], F. Pagliara[2], J. Bobi[1], T. Senussi[2], F. Idone[3], R. Amaro[1], L. Fernandez[1], M. Rigol[1], O. Ranzani[1], D. Nicolau[4], C. Sutherland[4], P. Pelosi[2], M. Antonelli[3], A. Torres[1]

[1]Hospital Clinic, Pulmonary and Critical Care Medicine, Barcelona,, Spain; [2]IRCCS AOU San Martino IST, Department of Surgical Sciences and Integrated Diagnostics (DISC), Genova, Italy; [3]Catholic University of Rome, Rome, Italy; [4]Hartford Hospital, Hartford, CT, United States

**Correspondence:** G. Li Bassi

*Intensive Care Medicine Experimental* 2017, **5(Suppl 2):**0837

**INTRODUCTION.** In patients with pneumonia, caused by multi-drug resistant *P. aeruginosa,* ceftolozane/tazobactam could be a valuable therapeutic alternative.

**OBJECTIVES.** We studied, in an animal model of severe *P. aeruginosa* pneumonia, the effects of ceftolozane/tazobactam vs. piperacillin/tazobactam on pulmonary bacterial burden.

**METHODS.** Sixteen pigs (32.9 ± 1.7 Kg) were anesthetized, mechanically ventilated for 76 hours, and challenged intrabronchially with *P.aeruginosa*, as previously reported (1)(ceftolozane/tazobactam and piperacillin/tazobactam minimal inhibitory concentration of 4 and 64 μg/mL, respectively). After 24 hours from bacterial inoculation, animals were randomized to receive: ceftolozane (50 mg/Kg) /tazobactam (C/T) every 8 hours, saline solution (Control) or piperacillin (200 mg/Kg) /tazobactam every 8 hours (TZP). These dosages were adjusted, based on preliminary studies to model appropriate humanized pulmonary epithelial lining fluid concentrations. Tracheal secretions and bronchoalveolar lavage (BAL) fluids were cultured daily. The animals were euthanized after 72 h from bacterial challenge, and pulmonary lobes biopsied for quantitative cultures.

**RESULTS.** Five animals were enrolled into the C/T and control groups; whereas, 4 animals were included into the TZP group. One animal, in the C/T and TZP group each, died of septic shock, before administration of antibiotics. In the C/T group, tracheal secretions *P.aeruginosa* concentration was 6.9 ± 0.3, 5.6 ± 0.4 and 4.8 ± 1.3 log CFU/mL, at 24, 48 and 72 hours of MV, respectively; whereas, in the TZP group it was 6.9 ± 0.9, 6.6 ± 1.1, and 6.4 ± 1.2 log CFU/mL and in the control group 7.3 ± 0.3, 7.5 ± 0.6 and 7.6 ± 0.6 log CFU/mL ($p < 0.01$). In the C/T group, BAL *P.aeruginosa* concentration was 4.6 ± 1.7, 3.7 ± 1.2 and 2.6 ± 1.2 log CFU/mL, at 24, 48 and 72 hours of MV, respectively; whereas, in the TZP group, it was 4.9 ± 0.4, 3.3 ± 1.3, and 3.2 ± 1.8, and in the control group 5.1 ± 0.8, 5.1 ± 0.7 and 4.7 ± 0.7 ($p < 0.01$). Finally, lung tissue *P.aeruginosa* burden was 3.7 ± 2.6 log CFU/gr in the C/T group, 4.0 ± 1.9 in the TZP group, and in the control group 5.2 ± 1.5 ($p = 0.06$).

**CONCLUSIONS.** In a pig model of severe multi-resistant *P.aeruginosa* pneumonia, ceftolozane/tazobactam swiftly decreases pulmonary bacterial burden.

**REFERENCE(S)**

1) Luna et al. *Chest.* 132: 523–31.

**GRANT ACKNOWLEDGMENT**

Merck Ltd, which is the manufacturer of ceftolozane/tazobactam

### 0838

#### Risk factors for extensive colonization or infection by Candida in patients hospitalized in intensive care unit

A. Margaritis[1], N. Zotos[2], M. Gerasimou[2], E. Tatsina[2], M. Bolosi[3], D. Lepida[1], O. Ygropoulou[1], G. Papadopoulos[3]

[1]General Hospital of Ioannina 'G Hatzikosta', ICU, Ioannina, Greece; [2]General Hospital of Ioannina 'G Hatzikosta', Microbiology, Ioannina, Greece; [3]Ioannina University Hospital, Anesthesiology, Ioannina, Greece

**Correspondence:** A. Margaritis

*Intensive Care Medicine Experimental* 2017, **5(Suppl 2):**0838

**INTRODUCTION.** Patients admitted to the ICU are particularly susceptible to Candida infection because of the severity of their underlying illness and the excess use of medical and surgical interventions. The frequent use of antibiotics, central venous catheters and other intravascular devices as well as poor gut motility or abdominal surgery place these patients at high risk of infection, which contributes to the morbidity and mortality of the already critically ill patient.

**OBJECTIVES.** The aim of this study was the identification of risk factors of extensive colonization or infection by Candida in patients hospitalized in ICU.

**METHODS.** The last two years, clinical specimens of 565 patients hospitalized in General Hospital ICU were examined (blood, wound, urine, ends of catheters, and bronchial secretions cultures), for the identification of Candida for colonization and cause of infection. Specimens were cultured on Sabouraud Dextrose Agar (Biomerieux,France) with chloramphenicol (0,005%) and incubated at 37° C, 72 h, and for the blood cultures, blood culture bottles Bact/Alert 3D(Biomerieux, France) were used. For the identification of Candida species, MicroScan WalkAway (Siemens) system was used. Among the risk factors checked for spread colonization or infection by Candida were: instestinal colonization by Klebsiella pneumonia (KPC-Kp) and/or Enterococcus (VRE).

**RESULTS.** Among 565 patients, 49 patients' (9%) developed at least one culture.

49 Candida species were identified in 17 blood cultures, 4 wound cultures, 10 urine cultures, 15 end of catheter cultures, and 11 bronchial secretions cultures.

26 (53%) were C. albicans,23 (47%) were non-albicans[C. Parapsilosis (12), C. glabrata (6), C. tropicalis(2), C. krusei(2), C. pseudotropicalis(1)].

In multifactorial statistical analysis, hospitalization during summer months, female sex, obesity, parenteral feeding, metronidazole intake, transplantation, bacteremia due to KPC-Kp, proved statistically significant risk factors for extensive colonization or infection with Candida. Also, the prophylactic use of fluconazole is a statistically significant inhibitory factor in the development of extensive colonization or infection by Candida.

**CONCLUSIONS.** It seems that there are several risks factors for the development of colonization and infection in ICU patients by Candida. The use of broad spectrum antibiotics for a long time in ICU patients infected with KPC-Kp, is a major factor because of the high incidence of bacteremia due to KPC-Kp in our region. The prophylactic use of fluconazole reduces the colonization and infection by Candida significantly.

### 0839

#### Evolution of Pseudomona aeruginosa in an intensive care unit (ICU) with the use of an empirical protocol of antibiotic rotation

A. Ramírez Rodríguez, H. Rodríguez Pérez, A. Casamitjana Ortega, E. Hernández Medina, Á. Villanueva Ortiz, L. Ugalde Jáuregui

Hospital Universitario Insular de GC, Las Palmas de Gran Canaria, Spain

**Correspondence:** H. Rodríguez Pérez

*Intensive Care Medicine Experimental* 2017, **5(Suppl 2):**0839

**INTRODUCTION.** The increase of multiresistant germs in the ICU is a problem of great medical, social and economic impact. The adequacy of the antibiotic treatment is essential to face this concern.

**OBJECTIVES.** To describe the evolution of Pseudomona aeruginosa resistance in our ICU, where an empirical protocol of antibiotic rotation is used.

**METHODS.** It is a prospective, observational study, from 2003 to 2016, carried out in a polyvant ICU of 24 beds of a third level hospital. An antibiotic rotation protocol for empirical coverage of Bacillus Gram Negatives (BGN) was established based on the experience of Raymon et al. University of Virginia, 1997–1999, depending on the infection focus and the time of the year.

| Period (1st protocol 2003) | Nosocomial pneumonia | Other: sepsis without focus / peritonitis |
|---|---|---|
| January, February, March | Meropenem | Cefepime |
| April, May, June | Ciprofloxacin | Piperacillin-Tazoactam |
| July, August, September | Ceftazidime | Meropenem |
| October, November, December | Piperacillin-Tazobactam | Ciprofloxacin |

*[Empirical antibiotic rotation protocol]*

**RESULTS.** The protocol has required modifications according to the characteristics of the microbiota of the moment, being an active and dynamic process. First modification in 2007: outbreak of Pseudomona aeruginosa resistant to ciprofloxacin. Action: withdrawal of ciprofloxacin. *Results*: decreased resistance to the antibiotic by the microorganism. Second modification in 2009: outbreak of Klebsiella pneumoniae ESBL. Action: change of ceftazidime by cefepime and suspension of aztreonam. *Results*: control of the outbreak. Third modification in 2013: outbreak of Klebsiella pneumoniae ESBL during summer time (period with cefepime). Action: restrict cefepime in the problematic months, increasing the use of carbapenemics. *Results*: control of the problem in the following years with even absence of outbreaks from 2013 to 2016. Consequences: increase in the resistance of Pseudomona aeruginosa due to the pressure of carbapenem use. Fourth modification in 2016: outbreak of Klebsiella pneumoniae ESBL. Action: class restriction, excluding carbapenemics from the empirical antibiotic rotation.

**CONCLUSIONS.** The availability of an empirical antibiotic rotation protocol has enabled us to control outbreaks by multiresistant germs. But we know that when the use of one antibiotic is restricted by replacing it with another, the selective pressure simply shifts, so resistance to alternative antibiotics may occur.

1.- Is crop rotation of antibiotics the solution to a resistant problem in the ICU? Niederman M.AM J Respir. Crit Care Med 1997;156:1029–31

2.- Impact of aminoglycoside cycling in sex tertiary intensive care units: prospective longitudinal interventional study.Francetic et al. Crit Med J. 2008; 49:207–14.3.

### 0840

#### Implementation of the Spanish ICU 'ZERO RESISTANCE' project in Andalusia

A. Estella[1], I. Alonso[2], J. Machado[3], J.C. Pozo[4], E. Morales[5], M.E. Yuste[6], J. Garnacho Montero[7]
[1]Hospital of Jerez, Unidad de Gestión Clínica de Medicina Intensiva Hospital del SAS de Jerez, Jerez, Spain; [2]Unidad de Gestión Clínica de Medicina Intensiva Hospital Virgen del Rocio, Sevilla, Spain; [3]Unidad de Gestión Clínica de Medicina Intensiva Jaen, Jaen, Spain; [4]Unidad de Gestión Clínica de Medicina Intensiva Hospital Reina Sofia de Córdoba, Córdoba, Spain; [5]Unidad de Gestión Clínica de Medicina Intensiva AGS Sur de Granada, Granada, Spain; [6]Unidad de Gestión Clínica de Medicina Intensiva Complejo Hospitalario de Granada (Hospital Clínico Universitario San Cecilio), Granada, Spain; [7]Unidad de Gestión Clínica de Medicina Intensiva Hospital Virgen Macarena de Sevilla, Sevilla, Spain
**Correspondence:** A. Estella
*Intensive Care Medicine Experimental* 2017, **5(Suppl 2):**0840

**INTRODUCTION.** Infections by multidrug-resistant pathogens (MDR) is a public health crisis. The Spanish Society of Intensive Care Medicine (SEMICYUC) and Spanish Society of Intensive Care Nursing (SEEIUC) have endorsed quality security projects known as "ZERO programmes". ZERO Resistance (ZR) aims to reduce the incidence of ICU acquired MDR infections.

**OBJECTIVES.** The aim of the present study is to know the degree of implementation of ZERO Resistance project recommendation.

**METHODS.** Multicenter descriptive transversal study. We conducted a written survey with 14 multiple-choice questions covering all measures addressed by the project recommendations. It was sent to all local nurse and physician ZR responsible of each ICU in Andalusia. Time of response was thirty days.

**RESULTS.** Twenty-eight medical-surgical ICU received the questionnaire: twenty-five answered responses were obtained. 68% were ICU with more than 10 beds. Majority, 80%, were university hospitals, 36% with neurosurgery department and 40% with cardiac surgery services. 88% had postgraduate medical training EIR residency program. 40% responded not to carry out ZR recommendations before its implementation and 28% partially.

In Table 207 we show degree of implementation of ZR recommendations:

**CONCLUSIONS.** In Andalusia ZR project has improved the application of measures to combat bacterial resistance. Two thirds of ICU have modified the way of working due to ZR project.

We observe a great variability in the participant ICU about the recommendation of active surveillance of MDR pathogens in time and samples performed.

**Table 207 (Abstract 0840).** Degree of implementation of ZR project

| | |
|---|---|
| Intensive care physician/nurse designed responsible of ZR project | 92%/80% |
| Empirical administration of antimicrobials active against MDR only in cases of septic shock and high risk of MDR pathogens. | 80% |
| Active search for MDR pathogens : All patients on admission and at least once a week/Patients with risk factors for MDR/ Sporadically/Never | 36%/32%/ 20%/4% |
| Microbiological Surveillance sampling: nasal, rectal and oropharyngeal swabs/Rectal swabs/Oropharyngeal swabs | 68% 16% 4% |
| Checklist of risk factors of MDR pathogens/carriage ZR project recommended checklist | 44% 32% |
| Isolation precautions close rooms Cleaning protocols for rooms of patients with MDR pathogens/A file document specifying ICU equipment and its respective cleaning updated protocols available. | 96% 88% 60% |
| Molecular typing methods to identify causative outbreak organism | 76% |

### 0841

#### Incidence and risk factors of central venous catheter associated infection in a Tunisian MICU

N. Fraj[1], M.A. Boujelbèn[1], J. Baroudi[1], N. Sma[1], E. Ennouri[1], W. Zarrougui[1], H. Kallel[1], A. Ben Abdelhafidh[1], I. Ben Saida[1], A. Azouzi[1], J. Ayachi[1], I. Chouchene[1], A. Khedher[1], M. Boussarsar[1,2]
[1]Farhat Hached University Hospital, Medical Intensive Care Unit, Sousse, Tunisia; [2]Ibn Al Jazzar Faculty of Medicine, Research Laboratory N° LR 14 ES 05 Interactions of the Cardio-Pulmonary System, Sousse, Tunisia
**Correspondence:** M. Boussarsar
*Intensive Care Medicine Experimental* 2017, **5(Suppl 2):**0841

**BACKGROUND.** The use of central venous catheters (CVCs) has greatly improved the quality of care in ICU patients, despite of its benefits, CVCs cause serious infectious complications leading to an increasing risk of Mortality.

**AIM.** To determine the incidence of CVC-AI and their risk factors among patients admitted in a Tunisian medical intensive care unit (MICU).

**METHODS.** A prospective cohort study was conducted between September15$^{th}$, 2015 and March 15$^{th}$, 2017 in an 8-bed MICU were included all patients with more than 48H of ICU stay. CVC-AI was ?A3B2 show $132#?>defined according to the CDC of ATLANTA criteria. The diagnosis of CVC-AI was based on clinical and/or laboratory signs/clues. For all subjects, age, sex, underlying diseases, SAPS II score, ICU length of stay, exposure to CVC (number of CVCsplaced, insertion site, insertion ward and insertion length) were collected. Risk factors were analyzed by conditional stepwise logistic regression. P values of less than 0.05 were regarded as statistically significant.

**RESULTS.** Among 258 eligible patients, 180(69.9%) had a CVC. 35 patients (19.44%) contracted a CVC-AI accounting for a total of 19.5/ 1000 CVC-AI per CVC-days. Among these infections, 17(60.7%) were systemic CVC-AI (with negative blood culture) and 10(35.7%) were bloodstream CVC-AI. The mean SAPS II of patients who developed CVC-AI was 32.8 ± 14.5, their mean Charlson comorbidities index was 2.1 ± 1.9, the mean overall duration of catheterization per patient was 16.5 ± 10.8 days and the mean CVC insertion-length was 5.4 ± 4 days. Gram negative bacteria were found in 53.5% of CVC-AI (n = 15) with MDR *Acinetobacter baumannii* as the commonest pathogen (n = 7). *Staphylococci* were isolated in 3 cases. Mortality rate of CVC-AI was 48.6% (n = 17). Risk factors of CVC-AI in the univariate analysis were: insertion site (p = 0.19), an insertion ward distinct than ICU (p = 0.18), a high Ramsay score (mean = 4.9 ± 1.2 ; p = 0.013) and a long insertion length (mean = 6.9 ± 4.7 ; p = 0.01). The introduction of these respective factors in a binary logistic regression model identified only a high Ramsay score (OR = 1.61, $CI_{95\%}$[1.138,2.282], p = 0.007) and a long insertion length (OR = 1.11, $CI_{95\%}$[1.03,1.20], p = 0.007) as independent risk factors of CVC-AI.

**CONCLUSION.** In a monocenter cohort study, CVC-AI had a high density and is associated with a poor outcome. A high Ramsay score and a long insertion length of CVC were the main independent risk factors associated to CVC-AI.

### 0842

#### Appropriate antibiotic therapy rates in ICU

O. Moreno Romero, M. Muñoz Garach, M.E. Yuste Ossorio, A. Carranza Pinel, M. Colmenero Ruiz
Complejo Hospitalario de Granada, Granada, Spain
**Correspondence:** O. Moreno Romero
*Intensive Care Medicine Experimental* 2017, **5(Suppl 2):**0842

**INTRODUCTION.** Is well known that innappropriate empirically administered antibiotics in critical ill patients are usually linked to extended intensive care unit stay and an increased risk of morbimortality.

**OBJECTIVES.** To describe the apropiate antibiotic therapy rates of patients admitted in an intensive care unit of 18 beds with community or nosocomial infections (intra-ICU or extra-ICU).

**METHODS.** Prospective study during 7 months (january-July 2016) of all patients with an infection and positive cultures admitted in ICU. Variables analized: demographic (age, sex), APACHE II, reason for ICU admission, infectious syndrome, adequacy of antibiotic treatment to our hospital empirical guidelines and targeted therapy based on antibiogram. An "appropriate" antibiotic treatment was considered if the microorganism was sensible to at least one antibiotic administered empirically. Analysis was performed by two physicians independently. In case of disagreement they proceeded to the evaluation by a third to tie. All rates are presented as percentages.
**RESULTS.** 612 patients. In 103, 152 infections were recorded; 65 with positive cultures (30 community infections, 20 nosocomial intra-ICU and 15 nosocomial extra-ICU or health care related). Mean age was 65.88 years (44–80), 55% males. Empirical treatment was adjusted to guidelines in all occasions and all types of infection. It was appropriate by antibiogram in all (15) nosocomial infection extra-ICU, in 28 (93.3%) comunity infections, and 18 (90%) nosocomial intra-ICU infections. Antibiotic de-escalation was performed in a high percentage (66.1%).
**CONCLUSIONS.** Our appropriate antibiotic therapy rates are susceptibly high. We only failed with empirical treatment in 10% patients with nosocomial intra-ICU and 6.7% of community infections. In the first case due to an outbreak multiresistant Acinetobacter baumanni, and in the second case due to patients without risk factors for unusual germs.

### 0843

#### Surveillance results of invasive device associated infection rate in a Turkish tertiary level intensive care unit

H. Başar[1], F.Ş. Erdinç[1], Ç. Kaymak[1], Ç.A. Hatipoğlu[1], A. Özcan[1], G.T. Ertem[1], N. Özcan[1], C. Bulut[2]
[1]University of Health Science, Ankara Training and Research Hospital, Ankara, Turkey; [2]Kırıkkale University, Kırıkkale, Turkey
**Correspondence:** Ç. Kaymak
*Intensive Care Medicine Experimental* 2017, **5(Suppl 2):**0843

**INTRODUCTION.** Intensive care units (ICU) need to be monitorized for nosocomial infections. Patients followed in ICUs can be colonized with resistant microorganisms during their ICU stay and as a result, they have an increased risk of hospital acquired infections.
**OBJECTIVES.** The aim of this study was to evaluate the central line (central venous catheter) associated bloodstream infection (CLA-BSI) rate, catheter associated urinary tract infection (CA-UTI) rate, invasive device use rate and causative microorganisms isolated in these infections, between 2007 and 2016.
**METHODS.** This study was conducted in an Anesthesiology and Reanimation Intensive Care Unit. Ten year's surveillance data was evaluated in this study. Central line associated bloodstream infection rate per 1000 central venous catheter-days, catheter associated urinary tract infection rate per 1000 urinary catheter days and device utilization ratios are retrospectively evaluated. Microbiological culture results of invasive device associated infections were also evaluated. Hospital acquired infection definitions are made according to the CDC criteria.
**RESULTS.** A total of 5406 patients were enrolled in this study. During the study period, central venous catheter usage ratio was 65.18% in the ICU for 42452 bed days and urinary catheter usage ratio was 90.19% in the ICU for 42065 bed days. The rate and number of central venous catheter associated bloodstream infection were 10.45 ± 8.36 and 258, respectively. The catheter associated urinary tract infection rate and number were 10.63 ± 3.34 and 470, respectively. Most commonly detected causative agents were *Acinetobacter baumannii* (16.58%) for central line associated bloodstream infections and *Candida* species (7.71%) for catheter associated urinary tract infections, throughout the study period.
**CONCLUSIONS.** Use of central venous catheter and urinary catheter increase the incidence of invasive device associated infection rate in ICU. The high mortality rates in patients with invasive device could be related to high infection rates and inappropriate antibiotic use. Incidence of infections can be reduced by education of health care givers and use of barrier preventive methods.
**REFERENCE**
Timsit JF., et al. BMC Infect Dis 2014;28(14): 489
**GRANT ACKNOWLEDGMENT**
None

### 0844

#### In vitro activity of antimicrobial drugs against pathogens commonly isolated in tracheal secretions of patients in the ICU

A. Margaritis[1], N. Zotos[2], M. Gerasimou[2], M. Bolosi[3], E. Tatsina[2], E. Kosma[1], D. Lepida[1], O. Ygropoulou[1]
[1]General Hospital of Ioannina 'G Hatzikosta', ICU, Ioannina, Greece; [2]General Hospital of Ioannina 'G Hatzikosta', Microbiology, Ioannina, Greece; [3]Ioannina University Hospital, Anesthesiology, Ioannina, Greece
**Correspondence:** A. Margaritis
*Intensive Care Medicine Experimental* 2017, **5(Suppl 2):**0844

**INTRODUCTION.** With mechanical ventilation widely used in intensive care unit, the ventilator associated pneumonia (VAP) has become a common and serious complication in critically ill patients. VAP can prolong the duration of mechanical ventilation and is associated with increased mortality. Culture results are used to confirm the clinical diagnosis and to adjust or sometimes withdraw antibiotic treatment. Tracheal aspirates have been shown to be useful for these purposes.
**OBJECTIVES.** To study the in vitro activity of antimicrobial drugs against multi-drug resistant pathogens isolated in samples of tracheal secretions in patients in the ICU.
**METHODS.** We studied 1004 samples of tracheal secretions of patients in the ICU of our hospital over a 6 years period. The study was retrospective.
The cultivation of the secretions was semi-quantitative and the Maki method was employed. Isolates of a growth rate of $10^{\wedge 5}$ cfu/ml were evaluated. The identification and susceptibility testing were performed by the Microscan Walkaway system (Siemens) and the evaluation of the results took place according to CLSI directions. Moreover, the highest antimicrobial sensitivity rates of the most frequently isolated pathogens were recorded.
**RESULTS.** 881 isolates were identified, 372 out of which were A. Baumanii strains, 94 were S.aureus strains and 91 were K.Pneumoniae strains. According to the antimicrobial susceptibility testing results, the highest antimicrobial sensitivity rates of the A.baumanii isolates were 100%, 40.7% and 27.9% with regard to Colistin, Ampicillin/Sulbactam and Gentamycin respectively.
As far as P.aeruginosa isolates are concerned, the antimicrobial sensitivity rates were: Colistin:100%, Piperacillin/Tazobactam 42.3%, Cefepime 26.8% and Ceftazidime 23.4%.
As for the S.aureus isolates (77.4%) the antimicrobial sensitivity rates were: Teicoplanin and Linezolid 100%, Vancomycin 100%, Trimethoprim/Sulphamethoxazole 96.8%.
Finally,the antimicrobial sensitivity rates of Klebsiella pneumoniae were: Tigecycline 100%, Colistin 100%, Gentamycin 69.2% and Cefepime 51.6%.
**CONCLUSIONS.** The results above provide us with essential information about the in vitro activity of antimicrobial agents against multi-drug resistant pathogens isolated frequently in tracheal secretions of patients in the ICU. On gram (+) bacteria there is a high in vitro sensitivity in different antibiotics. Against P. Aeruginosa and A. Baumanii the only antibiotic that is effective in vitro is Colistin. Against K. Pneumoniae are effective Colistin, Tigecycline and maybe Gentamycin. It is clear that against gram (–) bacteria we have limited antibiotics at our disposal in our ICU and in Greece in general.

### 0845

#### The link between gastrointestinal tract colonisation and infectious complications in intensive care unit patients after cardiac surgeries

G. Visockyte[1], J. Stanaitis[1], D. Ringaitiene[2,3], R. Ambrazaitiene[4], J. Sipylaite[2,3]
[1]Vilnius University, Faculty of Medicine, Vilnius, Lithuania; [2]Vilnius University Hospital Santariskiu Klinikos, Center of Anaesthesiology, Intensive Care and Pain Management, Vilnius, Lithuania; [3]Vilnius University, Faculty of Medicine, Clinic of Anaesthesiology and Intensive Care, Vilnius, Lithuania; [4]Vilnius University Hospital Santariskiu Klinikos, Centre of Laboratory Medicine, Vilnius, Lithuania
**Correspondence:** G. Visockyte
*Intensive Care Medicine Experimental* 2017, **5(Suppl 2):**0845

**INTRODUCTION.** Nosocomial infections are a major problem with high morbidity and mortality rates in intensive care units (ICU).

**OBJECTIVES.** To evaluate the link between epidemiological stool cultures, later infectious complications and clinical outcomes of patients after cardiac surgery at the Vilnius University Hospital Santariskiu Clinics (VUL SK) ICU.

**METHODS.** A retrospective analysis included 52 post-cardiosurgical patients, who were treated in the ICU and had stool cultures analysed. Epidemiological and diagnostic bacterial samples were evaluated to assess the type and multiresistance of bacteria as well as concurrence. Data on infectious complications, duration of hospitalisation and the clinical outcomes were collected. Results were analysed in patients with and without acquired infectious complications. Statistical evaluation was completed using Mann–Whitney-Wilcoxon test, F and T tests, Fisher test and Spearman correlation test. P value < 0.05 was used.

**RESULTS.** There were 52 patients (27 women, 51.9%) with average age of 66.2 ± 13.3 years included in the study. Thirty eight patients (73.1%) were diagnosed with infectious complications in the ICU (1$^{st}$ group), and 14 (26.9%) were not (2$^{nd}$ group). The mortality rate was 39.5% (n = 15) only in the 1$^{st}$ group. Multiresistant bacteria in the epidemiological stool samples were found in 86.8% and 100%, concurrence of bacteria in epidemiological and diagnostic samples - in 34.2% and 21.0% of cases in the 1$^{st}$ and 2$^{nd}$ group respectively. Treatment time before hospitalisation to ICU was medians 15,5 and 7 days (p = 0.02); duration of the operation - 342.1 ± 150.5 and 457.8 ± 165.1 min (p = 0.02); duration of artificial blood circulation - 114.9 ± 96.4 and 193.8 ± 130.1 min (p = 0.021); duration of the aortic clamping - 66.9 ± 64 and 115.1 ± 78.9 min (p = 0.028), hospitalization time in the ICU - medians 17.5 and 9 days (p = 0.014) in the 1$^{st}$ and 2$^{nd}$ group respectively. Statistically significant correlation between clinical outcomes and bacterial concurrence in epidemiological and diagnostic samples (r = 0.4, p = 0.003) same as clinical outcomes and complication occurence (r = 0.38, p = 0.005) was observed. There was no association between epidemiological and diagnostic sample concurrence, multiresistant bacterial colonisation and the occurence of infectious complications.

**CONCLUSIONS.** Multiresistant bacteria were found in 90.4% of all epidemiological stool samples. Patients, who had infectious complications in the ICU, were hospitalised significantly longer and in one third of cases epidemiological and diagnostic samples concurred. Clinical outcomes had a direct relationship with the concurrence of epidemiological and diagnostic bacterial samples and occurence of infectious complications.

**GRANT ACKNOWLEDGMENT**
N/A

### 0846

#### Significantly more de-escalation of empiric anti-infective therapy in severe sepsis and septic shock without negative impact on mortality by a goal-directed intervention including feedback of quality indicators and de-escalation rates

H. Rüddel[1,2], D. Schwarzkopf[2], D.O. Thomas-Rüddel[1,2], C.T. Mathaeus-Krämer[2], K. Reinhart[1,2], F. Bloos[1,2]
[1]Jena University Hospital, Dept. of Anesthesiology and Intensive Care Medicin, Jena, Germany; [2]Jena University Hospital, Center for Sepsis Control and Care, Jena, Germany
**Correspondence:** H. Rüddel
*Intensive Care Medicine Experimental* 2017, **5(Suppl 2):**0846

**INTRODUCTION.** In sepsis, severe sepsis and septic shock administration of broad spectrum antibiotics is a mainstay of therapy. Guidelines recommend de-escalation as early as possible following microbiological findings and clinical improvement [1, 2]. De-escalation is associated with less resistant microorganisms, fewer side effects, and lower costs [3]. Yet, de-escalation is practiced in only 10% of cases in studies of clinical practice [4].

**OBJECTIVES.** To investigate if a multi-faceted hospital-wide intervention can elevate rate of de-escalation of empiric anti-infective therapy in patients with severe sepsis and septic shock.

**METHODS.** During the sustainability phase of the MEDUSA-trial [5], all participating hospitals (40 German hospitals of all levels of health care) received feedback on quality indicators on the quality of diagnosis, treatment and outcome of patients with severe sepsis and/or septic shock. Hospitals in the intervention group (n = 21) received change management counselling focusing, amongst others, on de-escalation of anti-infective regime. Hospitals in the control group (n = 19) had received counselling without focus on de-escalation for 24 months before the analysis, hospitals in the intervention group had received only four educational seminars during the same period. Significance testing by Generalized Hierarchical Linear Models controlling for clustering, interaction effect of intervention was tested by difference-in-differences-analysis.

**RESULTS.** 6.576 patients were prospectively included between 07/2013 and 06/2015. De-escalation rose from 14.4% before to 21.7% during intervention in the intervention group (OR 1.61 [1.29, 2.00], p = < =0.001). Change in the control group was not significant (16.0% to 16.8%, OR 1.11 [0.93, 1.34], p = 0.254). Impact of intervention was significant between groups (p = 0.012).

**CONCLUSIONS.** A multi-faceted hospital-wide intervention focusing on de-escalation of anti-infective therapy can significantly raise rate of de-escalation in patients with severe sepsis and/or septic shock without negative impact on 28-day mortality.

**GRANT ACKNOWLEDGMENT**
German Federal Ministry of Education and Research (FKZ 01EO1002)

Renal replacement therapy

### 0847

#### Continuous renal replacement therapy is associated with acute myocardial injury in critically ill patients

S. Marat[1], F. Salerno[2], I. Ball[1], C. Ellis[3], C. McIntyre[2]
[1]Schulich School of Medicine & Dentistry, Western University, Division of Critical Care Medicine, Department of Medicine, London, Canada; [2]Kidney Clinical Research Unit / University of Western Ontario, Department of Medicine, London, Canada; [3]Schulich School of Medicine & Dentistry, Western University, Department of Medical Biophysics, London, Canada
**Correspondence:** S. Marat
*Intensive Care Medicine Experimental* 2017, **5(Suppl 2):**0847

**INTRODUCTION.** Intermittent renal replacement therapy is associated with dialysis-induced acute myocardial injury, in both chronic[1] and acute[2] treatments. Continuous Renal Replacement Therapy (CRRT) is often favoured in critically ill patients with acute kidney injury (AKI), hypotension or shock. Lower ultrafiltration rates, characteristic of CRRT, reduce systemic hemodynamic stress and may be cardioprotective. We assessed the impact of CRRT on the development of acute segmental myocardial injury, in critically ill patients requiring dialysis for AKI.

**OBJECTIVES.** To assess the impact of CRRT on global and segmental left-ventricular function in critical ill patients requring dialysis for AKI. Out hypothesis was that CRRT, with its charecteristically lower ultrafiltration rates, would not induce cardiac stunning (i.e. will be cardioprotective).

**METHODS.** We used 2D-echo and speckle tracking analysis software (EchoPAC, GE Healthcare) to measure global and segmental left-ventricular myocardial longitudinal strain in 12 critically ill patients presenting with AKI. Measurements were made at baseline immediately prior to and 4, 8 and 24 hours after initiation of CRRT.

**RESULTS.** Measurements were completed in 11 patients. 10/11 patients developed new regional wall motion abnormalities, with 8 developing these as early as 4 hours after CRRT start. The number of affected segments varied from 1 to 11 (out of 12). Of 11 patients, 7 (58%) died in the ICU, with 5 of those dying within 2 days of CRRT initiation.

**CONCLUSIONS.** Our results show that CRRT is associated with new regional myocardial injury. This injury was associated with high mortality. These results are concerning with respect to the presumed safety of CRRT (compared to other dialysis modalities) in critically ill patients. Further direct comparison of CRRT with other dialysis modalities is warranted to assess the potential for relative cardio-protection.

**REFERENCE(S)**

[1] McIntyre CW, Blood Purif. 2010;29(2):105–10

[2] Mahmoud et al., Intensive Care Med. 2017 (epub ahed of print)

**GRANT ACKNOWLEDGMENT**

Dr Slessarev is suported by Resident Research Career Development Fund, Schulich School of Medicine & Dentistry.

### 0848

#### Evolution of continuous renal replacement therapy prescriptions along a decade in a tertiary referral hospital: a prospective observational cohort

E. P. Plata-Menchaca[1], G. Moreno-González[2], L. Anguela-Calvet[2], G. Muñóz-del Río[2], A. Salinas-Cánovas[2], X. L. Perez-Fernandez[2], P. Rebelo-Esquibel[2], K. Maisterra-Santos[2], A. Farré-Estebe[2], I. Romera-Peregrina[2], M. González-Romero[2], J. Pérez-Sánchez[2], A. Martínez-Pascual[2], V. Fuentes-Mila[2], R. Abellan-Lencina[2], J. Ballus-Noguera[2], J. Sabater-Riera[2], SIRAKI Group

[1]Universidad de Barcelona, Facultad de Medicina, Barcelona, Spain; [2]Instituto de Investigación Biomédica de Bellvitge, Research, L'Hospitalet de Llobregat, Spain; [3]Hospital Universitario de Bellvitge, Critical Care Medicine, L'Hospitalet de Llobregat, Spain

**Correspondence:** E.P. Plata-Menchaca

*Intensive Care Medicine Experimental* 2017, **5(Suppl 2):**0848

**INTRODUCTION.** Acute kidney injury (AKI) is estimated to occur in about 7-18% of hospitalized patients, and approximately 30-50% of patients admitted to the intensive care unit (ICU). Since more than a decade, continuous renal replacement therapy (CRRT) has been settled as part of organ support for patients presenting severe AKI during ICU stay.

**OBJECTIVES.** The aim of this study was to describe the changes in CRRT prescription practices and to assess patient outcomes comparing different study periods.

**METHODS.** A prospective observational cohort study was performed from 2006 to 2016. We enrolled 446 patients receiving CRRT during ICU admission to the Bellvitge University Hospital intensive care unit (ICU), which is a tertiary referral hospital in Barcelona, Spain. Patients were divided in 4 groups according to the year in which they were admitted to the ICU (2006, 2010, 2014 and 2016), in order to assess differences in clinical characteristics, RRT prescription practices, and to analyze outcome differences in terms of survival at the end of follow-up. Comparisons between groups were performed using a two-way analysis of variance (ANOVA) statistical method.

**RESULTS.** We observed the following differences among groups: mean age 63.3 ± 14 years/F 2.48 ($p = 0.06$), days from ICU to CRRT initiation (2.5 ± 6/F 4.1, $p = 0.007$), number of days on CRRT (5.8 ± 6/F 3.15, $p = 0.025$), prescribed blood flow (177 ± 38 ml per minute/ F 33.6, $p < 0.001$) and urine output 24 hours before starting CRRT (399 ± 47 ml/F 4.43, $p = 0.005$). The majority of patients were critically ill surgical patients in the 2006 group as compared to the other groups (52% vs 36 vs 38% vs 33% of patients, $p = 0.04$). Continuous venovenous hemodialysis (CVVHD) was more frequently prescribed in the 2016 group as compared to the 2006 group (15.9 ± 8 vs 4.6 ± 7/F 14.5, $p < 0.0001$). Extracorporeal circuit lifespan was different between groups [(18.7 ± 18, 22.1 ± 21, 38.8 ± 24, and 38 ± 27 hours/F 14.7, $p < 0.0001$; in the 2006, 2010, 2014 and 2016 group, respectively]. Event-time distributions were estimated for overall survival with the use of the Kaplan-Meier method, through which no differences on survival at 28 and 90 days were found between groups (log-rank test; $p = 0.254$ and $p = 0.41$, respectively).

**CONCLUSIONS.** Differences were observed in CRRT prescription practices during the last decade. However, in terms of survival, there were no differences between groups of patients requiring CRRT during ICU stay in each study period.

**REFERENCE(S)**

Chawla LS, Bellomo R, Bihorac A, Goldstein SL, Siew ED, Bagshaw SM. Acute kidney disease and renal recovery: consensus report of the Acute Disease Quality Initiative (ADQI) 16 Workgroup. *Nat Rev Nephrol* 2017;13:241–257.

### 0849

#### Mortality in patients requiring renal replacement therapy in intensive care in St Helier Hospital, London in 2012–2013

G.P. Faria, S. Ahmed, R. Owusu-Ankomah, G. Correa, S. Glasgow, L. Mulleague, D. Makanjuola

Epsom and St Helier University Hospitals NHS Trust, General Medicine, Surrey, United Kingdom

**Correspondence:** G.P. Faria

*Intensive Care Medicine Experimental* 2017, **5(Suppl 2):**0849

**INTRODUCTION.** There has been a recent worldwide drive to recognize and treat Acute Kidney Injury (AKI) as it is one of the poor prognostic markers of patient outcomes.

**OBJECTIVES.** We studied mortality among various subgroups of patients who required Renal Replacement Therapy (RRT) in the Intensive Therapy Unit (ITU) at St Helier Hospital, London, UK in the year 2012–13, to identify at risk groups.

**METHODS.** We identified 208 patients who required renal replacement therapy in St Helier ITU between 2012–2013. These patients were admitted to the ITU from various specialties including General Medicine, General Surgery, the Renal Unit and Accident and Emergency. We then collected data from multiple sources including: patients' notes, "Ward Watcher" (software used in ITU for all admissions and interventions), intensive care notes and the renal unit haemodialysis input software (CV5). These patients were then further divided into subgroups as per their baseline kidney function (i.e.: AKI on no known background of kidney disease, Chronic Kidney Disease [CKD], End-Stage Renal Failure [ESRF] and Unknown Baseline [UKB]). We studied their mortality in the ITU, in hospital after step-down from the ITU, in the renal unit and for a further 3 months, 6 months and 1 year.

**RESULTS.** We found that the AKI group had the highest mortality when compared with the other groups (CKD, ESRF and UKB). 37% of patients in the AKI group died in ITU compared to 25% of CKD patients. The outcome of ESRF patients was slightly better than the other groups with 19.7% mortality. Similarly, the in-hospital mortality of the AKI group was highest at 44.8% when compared to the other groups. We found that one year mortality in these subgroups ranges from 33% to 52.5%.

**CONCLUSIONS.** The AKI group has the highest mortality both in the ITU and in-hospital, despite Renal Replacement Therapy. Harel Z

et al. [1] suggested outpatient review for AKI survivors can change their outcome. Through this study, we recommend that there should be a framework to identify and treat the AKI group early, as well as the involvement of a team of experts, including nephrologists, for consultation in hospital. This is in order to avoid these patients ending up requiring RRT, as the prognosis is poor at this stage.

**REFERENCE(S)**

[1] Harel Z et al.,;Nephrologist follow-up improves all-cause mortality of severe acute kidney injury survivors. Kidney Int. May 2013

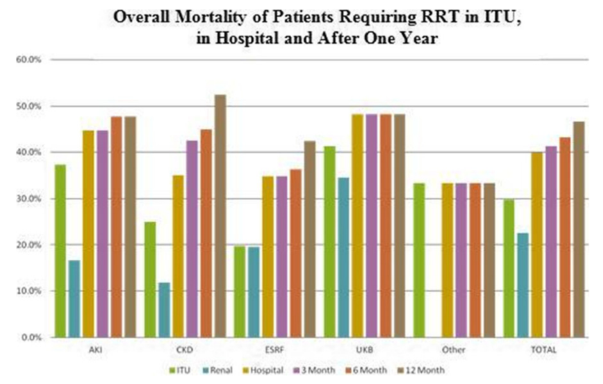

**Fig. 278 (Abstract 0849).** Overall Mortality of Patients Requiring RRT in ITU

### 0850

#### Impact of early initiation of continuous renal replacement therapy in critically ill patients with acute kidney injury

S.Y. Lim[1,2], S.-K. Cho[2], Y.J. Lee[1,3], Y.J. Cho[1,3]
[1]Seoul National University Bundang Hospital, Interdepartmental Division of Critical Care Medicine, Department of Internal Medicine, Seoul, Korea, Republic of; [2]Korea University Anam Hospital, Korea University College of Medicine, Department of Internal Medicine, Seoul, Korea, Republic of; [3]Seoul National University College of Medicine, Department of Internal Medicine, Seoul, Korea, Republic of
**Correspondence:** S.Y. Lim
*Intensive Care Medicine Experimental* 2017, **5(Suppl 2):**0850

**INTRODUCTION.** The optimal timing for initiation of continuous renal replacement therapy (CRRT) in critically ill patients with acute kidney injury (AKI) remains controversial. The generally accepted absolute indications for starting CRRT in critically ill patients include persistent hyperkalemia, severe acidosis and hypervolemia.

**OBJECTIVES.** Therefore, we compared the outcomes of patients who received CRRT without any of these indications with patients with one or more of these indications.

**METHODS.** Retrospective cohort study of patients undergoing CRRT from 2015 to 2016 in a tertiary university hospital. We defined CRRT initiated without any absolute indications as early group and CRRT initiated in the presence of any of these indications as late group. Patients with chronic kidney disease stage 5 or on dialysis were excluded. The primary clinical outcome variables were renal recovery, and 90-day mortality.

**RESULTS.** Out of 118 patients treated with CRRT, 34 (28.8%) patients initiated CRRT after meeting at least one absolute indication (late group) whereas 84 (71.2%) patients started without any indications (early group). The late group had lower renal recovery than the early group (8.8 vs. 28.9% late vs early, p = 0.019). At 90 days after CRRT, the mortality rate in the late CRRT group was 79.4% and that in the early group was 57.1% (P = 0.023). Delayed initiation of CRRT was independently associated with greater odds of mortality and renal recovery in multivariate cox regression analysis. (hazard ratio : 1.729 95% CI 1.017-2.941, P = 0.043).

**CONCLUSIONS.** Our study could suggest that initiating CRRT in critically ill patients with AKI should not be delayed until fulfillment of urgent indications.

### 0851

#### A retrospective audit of renal replacement therapy practices in intensive care units in a tertiary hospital in Singapore

R.M. Lee, Y.H. Lau, L. Li, J.J.E. Tan
TTSH, Singapore, Singapore
**Correspondence:** R.M. Lee
*Intensive Care Medicine Experimental* 2017, **5(Suppl 2):**0851

**INTRODUCTION.** Acute kidney injury (AKI) in the critically ill causes significant morbidity and mortality. There are no local studies to date about its prevalence in critically ill patients. We performed a retrospective audit of the renal replacement therapy (RRT) practices and outcomes in a single tertiary hospital.

**OBJECTIVES.** The primary objectives were the epidemiology of AKI in our hospital and its management in terms of incidence of RRT; types of RRT modalities used; dose prescribed and whether it was delivered. Secondary objectives include ICU, hospital mortality and length of stay.

**METHODS.** This is a retrospective audit of case records of all patients admitted to all intensive care units (ICUs) at a single tertiary hospital from 1 July 2013 to 30 June 2014. All patients receiving RRT for AKI were included. Critically ill patients previously on long term dialysis were also included, unless the admission to ICU was purely for scheduled dialysis to take place.

**RESULTS.** Over a 12 month period with 2353 patients admitted to the ICUs, there were 223 patients (9.8%) requiring RRT resulting in 622 episodes of RRT. The median APACHE II score of our patients was 35 (range +/− 13) and the median SOFA score 12 (range +/− 7). The majority of admissions were from general medicine (N = 136, 52.5%). 295 (47.4%) episodes of RRT were in ESRF patients while 357 (52.6%) of episodes of RRT were in patients with AKI according to RIFLE grading, with 42.8% RIFLE F at initiation of CRRT. 84% of RRT was prescribed by the renal physicians in our ICUs. Overall mortality of patients who had RRT was 42.6%. This was considerably higher than the mortality rate of 19.1% of patients who did not have RRT. Out of the 257 episodes of CRRT, the most common modality was CVVHDF (25.56%). The most common modality used amongst the non-CRRT modes of dialysis was SLED (36.5%). Most patients completed their prescribed session of RRT (60.13%). For CRRT, the median duration of uninterrupted RRT was 20 hours (10–25.5h) and the average delivered dose of RRT was 33.79ml/kg/h correlating to the prescribed dose per kg body weight.

**CONCLUSIONS.** This first audit of RRT practices in ICUs in a single Singapore hospital has provided us data for the first time on RRT practices and outcomes of patients. There exists differences in epidemiology, practices and prescription between medical and surgical ICUs even within a single institution. It is reassuring that the dose of CRRT achieved and outcomes of our patients are comparable to published centres. We will be embarking on a national intensive care audit of RRT practices by using this data to help us improve on management of RRT in the critically ill as well as plan healthcare resource allocation to improve systems of care for better patient outcomes.

### 0852

#### Causes of death in patients with acute kidney injury treated with renal replacement therapy

I. Ertugrul[1], A.C.J.M. de Pont[2], N.P. Juffermans[2]
[1]Academic Medical Center, University of Amsterdam, Department of Intensive Care, Amsterdam, Netherlands; [2]Academic Medical Center, University of Amsterdam, Amsterdam, Netherlands
**Correspondence:** I. Ertugrul
*Intensive Care Medicine Experimental* 2017, **5(Suppl 2):**0852

**INTRODUCTION.** The mortality of patients with acute kidney injury (AKI) has not decreased over the last 60 years and is even higher in patients treated with renal replacement therapy (RRT). Recent studies suggest that fluid overload, immunological impairment and the cardiorenal syndrome may all contribute to mortality among AKI-patients.
**OBJECTIVE.** To determine the most important cause of death among AKI-patients treated with RRT.
**METHODS.** We conducted a systematic review according to the PRISMA statement. We searched Cochrane, Pubmed, Embase and Web of Science for original articles describing the cause of death in AKI patients treated with RRT. Studies were analyzed according to their primary endpoint, either in-hospital mortality or mortality after hospital discharge. Two by two table analysis was used to compare incidences between groups.
**RESULTS.** Eleven studies with a total of 8804 patients were included: one randomized controlled trial and ten observational studies. The most frequently reported cause of AKI was ischemia, followed by sepsis and nephrotoxicity. Both in-hospital mortality and mortality after discharge occurred significantly more frequently among patients with Acute Kidney Injury Network (AKIN) stage 3 than among patients with AKIN stage 1 (40 vs 16% and 34 vs 16% respectively, $p < 0.00001$). Patients with AKI had a significantly worse long term survival with 1 and 10 year survival rates of 89% and 44% respectively, compared to 95% and 73% respectively for patients without AKI ($p < 0.001$).
Among the studies analyzing in-hospital mortality, the cause of death was documented in more than 90% of the cases. With respect to mortality after hospital discharge, causes of death were documented in 95% of the cases in one study and in 4% of the cases in the other. In-hospital mortality ranged from 17 to 85% and mortality after hospital discharge from 38 to 81%. Among the studies analyzing in-hospital mortality, infection was the most frequently recorded cause of death, ranging from 35 to 81%, followed by cardiac causes, ranging from 8 to 32%. Among the studies analyzing mortality after hospital discharge, cardiovascular causes of death were most frequently reported. However, the incidence of cardiovascular causes of death among AKI-patients after hospital discharge was similar to the incidence of cardiovascular deaths in the general population.
**CONCLUSION.** This systematic review demonstrates that for AKI-patients treated with RRT, the most important cause of in-hospital death was infection, which may be due to both immunological impairment and exposure to nosocomial pathogens. After hospital discharge however, the main cause of death among AKI-patients was cardiovascular, with an incidence similar to the incidence in the general population.

### 0853

#### Effectiveness of blood gas analyzers measuring ionized calcium concentration in CRRT using regional citrate anticoagulation

M.S. Satorra[1], A. Campos[1], T.M. Tomasa[1], S. Malumbres[2], O. Plans[1], Y. Rovira[1], E. Mor[1], V. Philibert[1], M. Ayala[1], P. Ricart[1]
[1]Hospital Universitari Germans Trias i Pujol, Intensive Care Unit, Badalona, Spain; [2]Hospital Universitari Germans Trias i Pujol, Laboratory Unified Metropolitan Area North, Badalona, Spain
**Correspondence:** M.S. Satorra
*Intensive Care Medicine Experimental* 2017, **5(Suppl 2):**0853

**INTRODUCTION.** Citrate has been recommended as the first-line anticoagulant for continuous renal replacement therapy (CRRT) in critically ill patients (KDIGO 2012). Citrate inhibits the coagulation cascade by lowering the ionized calcium (iCa) concentration in the filter. Regional citrate anticoagulation protocols recommend postfilter iCa monitoring to adjust citrate flow and optimize anticoagulation to ensure circuit livespam. Blood gas analyzers are often used to determine iCa concentrations. However, these instruments are optimized for physiological iCa concentrations which might make them less suitable for measuring low iCa in blood with a high concentration of citrate.
**OBJECTIVES.** To compare the measurement of very low iCa concentrations using 2 different blood gas analyzers placed in the intensive care units of our hospital (ICU1 and ICU2) compared to the general laboratory gas analyzer (Lab).
**METHODS.** Prospective observational study conducted from May 2015 to June 2016. Analyzed a total of 64 samples from 13 patients taken from the extracorporeal circuit and collected through syringes for blood gas analysis (Smiths Medical) balanced lithium heparin. Samples are processed by the central ABL800 analyzer (Radiometer Copenhagen) (Lab) and blood gas analyzers point of care GemPremier 3500 (Lab Instrumentation-IZASA) (ICU1) and GemPremier 3000 (Lab Instrumentation-IZASA) (ICU2).
The analytical method used by blood gas instruments is potentiometry (electroanalytical technique for determining the concentration of electro-active species in a solution using a reference electrode and a working electrode). Statistical analysis was performed with MedCalc software, version 9.2, using Passing-Bablok regression (nonparametric test for comparison of experimental methods and validation measuring instruments) and Cusum test used to establish a model of linearity between variables.
**RESULTS.** The correlation analysis between the analyzers ICU1 and Lab shows the presence of a constant but non-proportional error between the two methods. There are no constant or proportional errors between Lab and ICU2 nor between ICU1 and ICU2.

| Correlation analysis | Regression line | Intercept (IC 95%) | Slope (IC 95%) |
|---|---|---|---|
| Lab vs. ICU1 | y = −0.1760 + 1.2000X | −0.4030; −0.1 | 1; 1.8 |
| Lab vs. ICU2 | y = −0.1417 + 1.0556X | −0.48; 0.01 | 0.6; 2 |
| ICU1 vs. ICU2 | y = 0.0100 + 1.0000X | −0.09; 0.1 | 0.66; 1.33 |

[Results]
**CONCLUSIONS.**
1. No statistically significant correlation was observed comparing the Lab blood gas analyzer with ICU1 and ICU2.
2. Measurements obtained by the Lab analyzer are consistently higher than those obtained by the point of care ICU1 and ICU2 analyzers.
3. ICU1 and ICU2 blood gas analyzers are interchangeable with each other, so that can be used interchangeably to control low iCa concentrations in CRRT using regional citrate anticoagulation.
**REFERENCES**
1. Discrepant post filter ionized calcium concentrations by common blood gas analyzers in CRRT using regional citrate anticoagulation. Patrik Schwarzer, Sven-Olaf Kuhn, Sylvia Stracke et al. Crit.Care (2015) 19:321
2. Citrate anticoagulation for CRRT: don't always trust the postfilter iCa results! Heleen M. Oudemans-van Straaten and Marlies Ostermann. Crit. Care (2015) 19:429.

### 0854

#### Hemofiltration induces generation of microvesicles and tissue factor in sepsis

G.F. Lehner[1], U. Harler[1], C. Feistritzer[2], V.M. Haller[1], J. Hasslacher[1], R. Bellmann[1], M. Joannidis[1]
[1]Medical University Innsbruck, Division of Intensive Care and Emergency Medicine, Department of Internal Medicine, Innsbruck, Austria; [2]Medical University Innsbruck, Department of Internal Medicine V - Haematology and Oncology, Innsbruck, Austria
**Correspondence:** G.F. Lehner
*Intensive Care Medicine Experimental* 2017, **5(Suppl 2):**0854

**INTRODUCTION.** Microvesicles (MV) are extracellular vesicles known to be associated with cellular activation and inflammation. Hemofiltration is an effective blood purification technique for patients with renal failure and possibly also eliminates inflammatory mediators in the setting of sepsis. On the other hand, proinflammatory stimuli are induced by blood contacting the artificial membrane during extracorporeal blood purification.

In chronic dialysis patients a systemic increase of MV has been described.

**OBJECTIVES.** The aim of the study was to investigate if hemofilter passage of blood in CVVH alters MV composition and levels in critically ill patients with sepsis.

**METHODS.** Pre- and postfilter blood as well as ultrafiltrate samples from intensive care unit patients with severe sepsis were obtained during continuous veno-venous hemofiltration (CVVH). MV subtypes in blood were analyzed by high-sensitivity flow cytometry. Additionally, tissue factor (TF) levels and MV-associated TF-activities as well as MV-activities were quantified. All parameters were corrected for hemoconcentration applied during CVVH.

**RESULTS.** Twelve patients with severe sepsis on hemofiltration were analyzed. Significant increases of platelet-derived CD41+ MV (1.13 (1.07-2.08) fold, $p = 0.0335$) and presumably mostly leukocyte-derived CD31+/CD41- MV (1.65 (1.22-2.24) fold, $p = 0.0015$) as well as significantly higher TF-activities (1.20 (1.06-1.50) fold, $p = 0.0076$) and TF-levels (1.12 (0.99-1.15) fold, $p = 0.0376$) were detected postfilter compared to prefilter. No significant differences concerning AnnexinV+ MV or MV-Activity were detected. No MV-activity was measurable in ultrafiltrate samples. Increments of AnnexinV+, CD41+, CD42b + and CD31+/CD41- MV post- to prefilter correlated with filtration fraction (all $p < 0.01$).

**CONCLUSIONS.** Hemofiltration induces the release of MV subsets, indicating platelet and presumably leukocyte activation during hemofilter passage. Moreover, TF is generated within the hemofilter. Increases of MV subsets within the hemofilter correlate with filtration fraction. No significant clearance of MV by a single hemofilter passage during CVVH could be detected.

**GRANT ACKNOWLEDGMENT**

Supported by funds of the Oesterreichische Nationalbank (Oesterreichische Nationalbank, Anniversary Fund, project numbers 13861 and 15708

### 0855

#### A protocol for routine regional citrate anticoagulation on the Aquarius platform results in significant improvement of filter life

L. Jackson, S. Schwarze, G. Marshall, J.R. Prowle, C.J. Kirwan
Barts Health NHS Trust, Adult Critical Care, London, United Kingdom
**Correspondence:** C.J. Kirwan
*Intensive Care Medicine Experimental* 2017, **5(Suppl 2):**0855

**INTRODUCTION.** Regional citrate anticoagulation (RCA) is the optimum form of anticoagulation for continuous renal replacement therapy (CRRT). Following technical modifications to the Nikkiso Aquarius platform a post-dilution CVVHF RCA protocol was introduced demonstrating significant improvement in filter lifespan for patients contraindicated to heparin [2]. This RCA protocol has been introduced as routine care in our large ICU providing CRRT for ~250 patients a year.

**OBJECTIVES.** Assess the clinical impact on filter life of a protocol with RCA as first line anticoagulation on the Aquarius platform.

**METHODS.** A retrospective comparison of consecutive patients requiring CRRT. 41 patients using RCA as first line anticoagulation on the Aquarius (group 1) versus 24 where heparin was first line anticoagulation (group 2).

**RESULTS.**

**Group 1:** 41 patients (141 filters) were included; median age 56.5 (27–75). Of these patients, 29 were medical admissions, 9 surgical and 2 polytrauma. 18 had end stage renal failure. 61% of filters clotted, 6% were stopped due to access issues, 7% due to technical issues, 23% following a clinical decision, 6% reached end of filter life (72 hours) and 4% were had no reason for cessation recorded. Of the 141 filters, 96 used RCA (68%), 27 (19%) used heparin and 18 (13%) used no anti-coagulation (100% pre-dilution).

10 (25%) patients did not start RCA as first line anticoagulation. 6 had severe lactic acidosis (>10mmol/L) and 2 had acute on chronic liver failure which are local policy contraindications to routine RCA use, it was unclear in the other 2. There were 4 occasions where RCA was switched to heparin, twice for metabolic alkalosis, once for hypocalcaemia and once for multiple clotted filters.

**Group 2:** 24 patients (63 filters); median age 61 (27–85) who were audited immediately prior to the introduction of RCA. 15 were medical admissions, 5 surgical and 4 polytrauma of which 5 had ESRF. 42 (68%) of filters clotted and 2 (3%) reached end of filter lifespan. 36 (57%) received no anticoagulation (100% pre dilution) ($p < 0.0001$ compared to group 1) the rest received heparin infusion (aim APTTR 2–2.5).

The median filter life span (censoring for reasons other than clotting) in all patients increased from 18 to 28 hours following the introduction of a protocol using RCA as first line therapy (Fig. 279, $p = 0.016$).

**CONCLUSIONS.** RCA on the Aquarius platform is safe and effective when incorporated as standard anticoagulation for critically ill patients. It allows for significantly fewer filters to be used without anticoagulation and improves filter life span when compared to heparin or pre-dilution alone.

**REFERENCE(S)**

Kirwan et al. Implementation of a Simplified RCA Protocol… 2016 Blood purification 42(4)

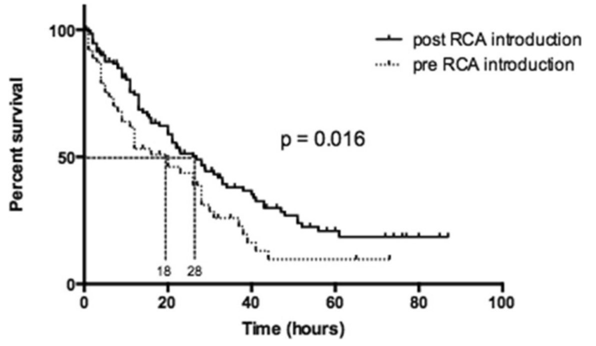

**Fig. 279 (Abstract 0855).** See text for description

### 0856

#### Line tip position has the greatest influence on filter life span for critically patients who require CRRT

L. Jackson, S. Schwarze, G. Marshall, J.R. Prowle, C.J. Kirwan
Barts Health NHS Trust, Adult Critical Care, London, United Kingdom
**Correspondence:** C.J. Kirwan
*Intensive Care Medicine Experimental* 2017, **5(Suppl 2):**0856

**INTRODUCTION.** Despite significant improvements in filter life span through the use of regional citrate anticoagulation and diligence to anticoagulation protocols, clotting remains the major cause of filter loss in critically ill patients who require CRRT. In an effort to improve local practice we looked for other factors in the process of CRRT that may influence filter life span.

**OBJECTIVES.** To analyse a cohort of patients receiving CRRT in a busy trauma heavy, non cardiac, critical care unit with a view to identifying factors that influence filter life span.

**METHODS.** A retrospective review of 41 patients using either, regional citrate anticoagulation (RCA), heparin or pre-dilution alone as an anticoagulation strategy. A Cox-Hazed Analysis for filter loss due to clotting was done to identify influences on filter life span.

**RESULTS.** 22/41 were male. Median age 56.5 (27–75) years. Reasons for admission: 6 polytrauma; 7 cardiogenic shock; 17 sepsis; 9 haemorrhagic shock, and 2 other. 18 had end stage renal failure. 141 filters were used with a median life span, censored for reasons other than clotting, of 28 hours. 96/141 (68%) filters used RCA, 27 (19%) heparin and 18 (13%) used no anti-coagulation (100% pre-dilution). 86 (61%) filters clotted. 15 patients had specific tunneled haemodialysis lines of which 2 were in a femoral vein accounting for 29 and 17 filters respectively. The line tip was positioned in the right atrium or superior vena cava for 66, the brachiocephalic vein for 26 and inferior vena cava in 49 filters respectively. Figure 280 shows filter life span related to line tip position.

A cox hazard analysis for filter loss due to clotting adjusted for anticoagulation using RA/SVC as reference demonstrates a hazard ratio for clotting of 3.37 (1.82-6.26; $p < 0.0001$) for brachiocephalic and 1.99 (1.17-3.38; $p = 0.011$) for inferior vena cava tip position. No other factor, including choice of anticoagulation influenced filter clotting.

**CONCLUSIONS.** Line tip position has an overwhelming influence on filter clotting and life span.

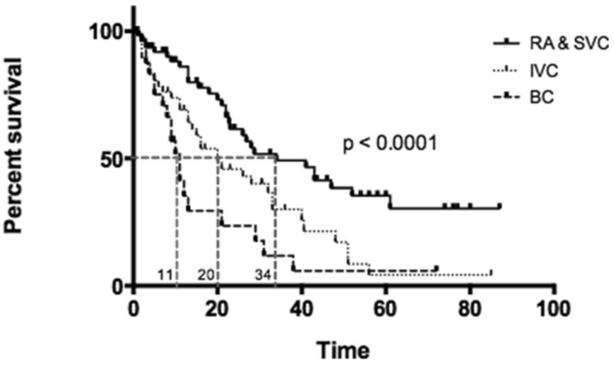

**Fig. 280 (Abstract 0856).** See text for description

### 0857

#### Regional citrate anticoagulation for continuous renal replacement therapy in the adult intensive care unit at Glenfield Hospital, Leicester, United Kingdom

A. Brookes-Elbaz, S.M. Cooke, A. Cavayas, G.K. Lau
Glenfield Hospital, Department of Critical Care, Leicester, United Kingdom
**Correspondence:** A. Brookes-Elbaz
*Intensive Care Medicine Experimental* 2017, **5(Suppl 2):**0857

**INTRODUCTION.** Regional citrate anticoagulation for Continuous Renal Replacement Therapy (CRRT) is recommended over heparin in patients who do not have contraindications for citrate[1]. This offered many potential advantages in terms of safety, efficacy and costs[2]. At the time of implementation, no other cardiac intensive care units in the United Kingdom have introduced citrate anticoagulation in this subgroup of patients.

**OBJECTIVES.** Assess the benefits, efficacy and safety of regional citrate anticoagulation in a predominantly cardiac surgical unit in the United Kingdom.

**METHODS.** We collected data before and after the introduction of regional citrate anticoagulation system on the choice of anticoagulation, reasons for the filters ceasing, filter lifespan, and blood product usage. Patients who were receiving Extra-Corporeal Membrane Oxygenation therapy (ECMO) were excluded from the analysis, as they were systemically anti-coagulated.

RESULTS.

**Lifespan of the filtration system (hrs)**

***Citrate***: (N = 132): mean (42.56), SD (26.71)
***Heparin***: (N = 148): mean (25.99), SD (22.89)
***Epoprostenol***: (N = 108): mean (15.23), SD (15.74)
***Other:*** (N = 27): mean (18.69), SD (17.78)
ANOVA: $p < 0.0005$

**Red cells transfused while on filtration (units/24 hours)**

***Citrate***: RBC (0.46), FFP (0.21), cryo (0.18), plt (0.11): ANOVA: $p < 0.019$
***Heparin***: RBC (0.32), FFP (0.04), cryo (0.09), plt (0.06): ANOVA: $p < 0.025$
***Epoprostenol***: RBC (0.92), FFP (1.85), cryo (1.38), plt (0.02): ANOVA: $p < 0.019$
***Other***: RBC (1.41), FFP (0.24), cryo (0.09), plt (0.00): ANOVA: $p < 0.579$

**CONCLUSIONS.** The lifespan of the filtration system with regional citrate anticoagulation was longer than for those using conventional anticoagulation. In addition, the transfusion of blood products was reduced when compared when other forms of anticoagulation for CRRT.

The introduction of citrate anticoagulation has dramatically improved the lifespan of our continuous haemofilters in our intensive care unit. It has been assessed as a safe method of anticoagulation with reduced downtime and overall more effective filtration delivery to our patients.

### 0858

#### Use of oXiris during continuous renal replacement therapy (CRRT) in patients with septic shock and AKI: a case series

F. Prato, M. Tengattini, G. Tasso, I.M. Berto, U. Colageo, C. Pissaia
Ospedale Degli Infermi, ASL BI, Biella, Italy
**Correspondence:** F. Prato
*Intensive Care Medicine Experimental* 2017, **5(Suppl 2):**0858

**INTRODUCTION.** Sepsis is a major causes of death in the intensive care unit (ICU) and improving its prognosis remains an important challenge for the physician. Sepsis induced Acute Kidney Injury (SAKI) is an important complication of sepsis. Immunomodulation could be beneficial and CRRT with adsorbing membranes may be useful in this clinical setting. The oXiris filter is a registered product for CRRT already safely used in routine care to adsorb endotoxin and remove cytokines.

**OBJECTIVES.** The aim of the study is to evaluate the inflammatory and hemodynamic response during CRRT with oXiris membrane.

**METHODS.** A total of 17 septic shock patients with renal failure (6 women, 11 men), with mean age 67 yr. (range 27–81), were treated between January and December 2016. Average baseline SOFA was 14.2. Seven patients suffered from intra-abdominal sepsis. Data were retrospectively analyzed.

oXiris was used as adjunctive therapy in combination with Standard of Care treatment of septic shock in order to control cytokines storm and improve hemodynamic stability. Continuous veno-venous haemodiafiltration (CVVHDF) was performed within the first 24–48 hours from ICU admission, using Prismaflex together with citrate anticoagulation.

C-reactive protein, procalcitonin and leukocytes levels, dosages of vasopressors and MAP were monitored at T00 (admission in Hospital) T0 (admission in ICU), Ti (start of CRRT), T24 (after 24h of CRRT) and Tf (end of CRRT).

**RESULTS.** Seven treated patients survived and were discharged from ICU.

No significant complications occurred during the CRRT.

Within the first 24 hours of treatment a rapid reduction in vasopressor requirement was registered in all patients: noradrenaline dose decreased from 0.40 (0.09-0.90) to 0.21 (0.0-0.69) mcg/kg/min.

Moreover there was a decrease in inflammatory markers: procalcitonin decreased from 11.01 (2.15-100) to 7.90 (0.11-23.17) ng/dl and C-reactive protein decreased from 24.19 (2,90-63.95) to 16.05 (2.92-42.21) mg/dl.

An improvement in renal function with a creatinine lowering from 3.32 (0.86-7.09) to 1.54 (0.42-3.04) mg/dl was also observed.

**CONCLUSIONS.** In our experience, a timely use of CRRT with oXiris membrane in combination with the Standard of Care therapy was clinically feasible, improved hemodynamics and had a positive effect on renal function. We did not detect any significant side effect related to citrate.

**REFERENCES**
Honore PM et al., Newly designed CRRT membranes for sepsis and SIRS-a pragmatic approach for bedside intensivists summarizing the more recent advances: a systematic structured review. ASAIO J. 2013 Mar-Apr;59(2):99–106
Rhodes, A., et al. Surviving Sepsis Campaign: International Guidelines for Management of Sepsis and Septic Shock: 2016 Intensive Care Med (2017) 43: 304.
Villa G et al. Nomenclature for renal replacement therapy and blood purification techniques in critically ill patients: practical applications. Critical Care. 2016;20:283.

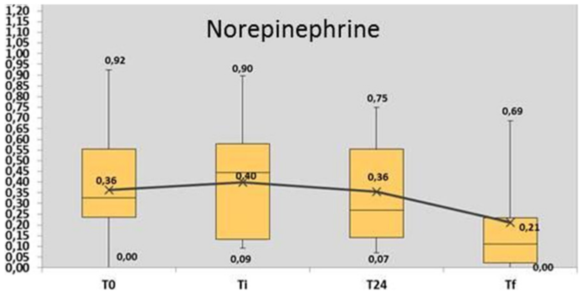

**Fig. 281 (Abstract 0858).** Norepinephrine

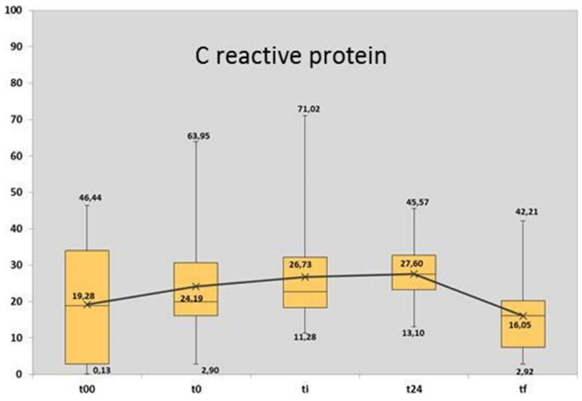

**Fig. 282 (Abstract 0858).** C reactive

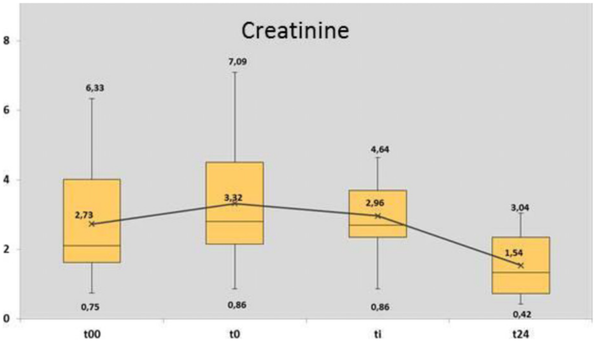

**Fig. 283 (Abstract 0858).** Creatinine

### 0859

### Hemofiltration outside ITU for persistent AKI in a cardiothoracic centre

P. Flaxman, C. Hernández Caballero, S. Marshall, J. Durrant, H. Street, E. Dunthorne
Hospital, London, United Kingdom
**Correspondence:** C. Hernández Caballero
*Intensive Care Medicine Experimental* 2017, **5(Suppl 2):**0859

**INTRODUCTION.** Persistent acute kidney injury requiring prolonged renal replacement therapies is becoming more frequent as does the complexity of patients treated in intensive care. When conventional hemodyalisis is not available in a cardiothoracic centre, a satellite hemofilration service led by the ITU team could be an alternative to a longer ITU stay.

**OBJECTIVES.** To assess the performance of our ITU led satellite hemofiltration service for persistent AKI.

The satellite hemofiltration programme in our centre is led by a highly specialised group of outreach nurses that organise the filtration regime in conjunction with the medical ITU team. Once our ITU patients are established on an alternate hemofiltration regime, they are discharged to the ward where they are followed up by the outreach team.

**METHODS.** We collected demographic data of all patients that continued to require intermittent hemofiltration after their discharge from our intensive care unit during 2016.

**RESULTS.** 54 patients required prolonged intermittent hemofiltration performed outside ITU during 2016 in our centre. The mean age of these patients was 55 years. The most frequent reasons for admission were kidney injury during their admission to hospital (16, 29.6%) and postoperative care of cardiac surgery (16, 29.6%) followed by heart transplanation (8, 14.8%) and acute heart failure (4, 7.4%).

14 patiens had chronic kidney disease in a hemodyalisis program at their admission to ITU.

55% were urgent admissions and 10% of the patients required mechanical circulatory support (ECMO, left ventricular assist devices or total artificial heart).

10 patients died in hospital before discharge.

2 patients presented haemodynamic instability during the filtration sessions that required intermitent vasopressor support and one patient sustained a cardiac arrest during hemofiltration due to severe metabolic acidosis.

The initial pattern of nocturnal hemofiltration every 48 hours could be modified (i.e. less frequent hemofiltration sessions) in 25 patients during their hospital stay due to renal function improvement.

All the14 CKD patients who were on hemodyalisis before their admission to ITU were eventually transferred back to a renal unit, plus 7 more patients that did not show renal recovery.

**CONCLUSIONS.** A satellite hemofiltration programme is an efficient and safe service development that allows the provision of medium and long term renal replacement therapies to cardiothoracic specialised centres where conventional hemodyalisis is not available.

### 0860

### Continuous renal replacement therapy in critically ill children: risk factors and outcome

T.M. Alayed, N.U.R. Siddiqui, A. Alturki, F. Aljofan, A. Eleyadhy
King Faisal Specialist Hospital and Research Centre, Department of Pediatrics Critical Care, Riyadh, Saudi Arabia
**Correspondence:** N.U.R. Siddiqui
*Intensive Care Medicine Experimental* 2017, **5(Suppl 2):**0860

**INTRODUCTION.** Continious renal replacement therapy (CRRT) has become preferred dialysis option to support children with AKI admitted to Pediatric Intensive Care Unit(PICU).

**OBJECTIVES.** To determine the use of CRRT and its outcome among critically ill children.

**METHODS.** Retrospective observational cohort study including all children admitted from 1–14 years of age in PICU of King Faisal

Specialist Hospital & Research centre, Riyadh, Saudi Arabia, who had underwent CRRT from July 2009 to June 2015.

**RESULTS.** Over all 96 patients were recruited with mean age of approximately 6 yrs. (+/− 4.37), with male preponderance. Most common diagnosis among these patients was Acute Leukemia 19.8% (19/96) followed by Renal diseases, immunodeficiency, Metabolic diseases and Lymphoma. The most common indication for CRRT was fluid overload 67.2% (65/96) followed by tumor lysis syndrome, and metabolic encephalopathy. We report median length of CRRT/patient 66 hrs (IQR, 34.57-164.2), with median average circuit life 30.86 hrs (IQR, 16.42-45). Most common CRRT catheter site was Internal jugular Vein 77.1% (74/96), followed by femoral vein and Subclavain vein with most common CRRT catheter size 11.5 Fr 38.5% (37/96) followed by 6 Fr, 8 Fr, and 10Fr. Also the most common CRRT modality used was CVVHDF 82.3% (79%) followed by CVVHD, and CVVH with heparin use of 24% (23/96). We report the mortality among critically ill children admitted in PICU requiring CRRT 50% (48/96), with 86.6% (26/30) among Stem cell transplant patients. We also report increased mortality among patients with hematological diseases (100%), and Immunodeficiency (86.6%), with least mortality 18.75% among patients with primary renal disease. We identified Patients who underwent Stem cell transplant, presented with septic shock, use of inotropic support before and during CRRT, Indication of fluid overload for initiation of CRRT, Patient in Failure as per pRIFLE criteria and in oliguric renal failure before CRRT, as the risk factors significantly associated with increased mortality.

**CONCLUSIONS.** We report CRRT to be a preferred modality of choice and better tolerated among critically ill pediatric population to treat AKI and fluid overload.

### 0861

#### Fluid removal with ultrasound driven protocol improved efficacy and safety of dehydration in critical illness

L. Wang, X. Guan, B. Ouyang
The First Affiliated Hospital of Sun Yat-sen University, Department of Critical Care Medicine, Guangzhou, China
**Correspondence:** L. Wang
*Intensive Care Medicine Experimental* 2017, **5(Suppl 2):**0861

**INTRODUCTION.** Fluid overload causes an increased morbidity and mortality in post resuscitated patients[1]. However, studies to identify the start point and end point of fluid evacuation are still lacked in current clinical practice[2, 3].

**OBJECTIVES.** Create and testify an individualized fluid removal protocol with ultrasound volume assessment in post resuscitated critical illness.

**METHODS.** A prospective controlled clinical trial was conducted. Fluid removal was performed following either the doctor's clinical experience with common monitoring (Control group), or an individualized dehydration stewardship with inferior vena cava diameter (dIVC), respiratory collapse index (IVC-CI) and lung ultrasound B-lines monitoring (Ultrasound group). The primary end points were the start point, end point and the length of fluid removal, and the complications related with fluid overload or over-dehydration during fluid removal period. The secondary endpoints were the hospital expense, the length of ICU stay, the length of hospital stay, the mortality in ICU, and the mortality in hospital.

**RESULTS.** Total 86 patients were enrolled initially and 80 patients were matched and compared finally. Fluid removal was started earlier (21.3 ± 14.6 h vs. 34.6 ± 26.3 h, Log-rank p = 0.005), and finished earlier (69.3 ± 33.3 h vs. 124.7 ± 52.8 h, Log-rank p < 0.001) in Ultrasound group than in Control. The fluid removal period was shorter in Ultrasound group than in Control (46.3 ± 29.7 h vs. 91.5 ± 41.8 h, p < 0.001). Daily negative fluid balance was more in Ultrasound group than in Control (−990.4 ± 636.1 ml vs. -672.6 ± 577.9 ml, p = 0.004) during fluid removal period. Less hypotension occurred (0/40 vs. 2/40) in Ultrasound group than in Control. Also, greater improvement of oxygen index was achieved in Ultrasound group than in Control (1.0[158.5] vs. 0[142.5], p = 0.009).

**CONCLUSIONS.** Individualized dehydration with ultrasound driven protocol improves the efficacy and safety of fluid evacuation.

**REFERENCE(S)**

1. National Heart L, Blood Institute Acute Respiratory Distress Syndrome Clinical Trials N, Wiedemann HP, Wheeler AP, Bernard GR, Thompson BT, Hayden D, deBoisblanc B, Connors AF, Jr., Hite RD, Harabin AL, (2006) Comparison of two fluid-management strategies in acute lung injury. The New England journal of medicine 354: 2564–2575
2. Vincent JL, De Backer D, (2013) Circulatory shock. The New England journal of medicine 369: 1726–1734
3. Malbrain ML, Marik PE, Witters I, Cordemans C, Kirkpatrick AW, Roberts DJ, Van Regenmortel N, (2014) Fluid overload, de-resuscitation, and outcomes in critically ill or injured patients: a systematic review with suggestions for clinical practice. Anaesthesiology intensive therapy 46: 361–380

ARDS: Clinical studies

### 0862

#### The spectrum study severe hypoxemia: prevalence treatment and outcome in 2016

D. Grimaldi, on behalf the SRLF Trial Group and the CERC
Erasme University Hospital, ICU, Bruxelles, Belgium
**Correspondence:** D. Grimaldi
*Intensive Care Medicine Experimental* 2017, **5(Suppl 2):**0862

**INTRODUCTION.** Limited information exists about the prevalence, subsequent management, and outcomes of hypoxemia among ICU patients

**OBJECTIVES.** To assess the prevalence of hypoxemia among ICU patients and to stratify them according to the severity of hypoxemia (PaO2/FIO2 between 300 and 201 in mild, between 200 and 101 in moderate, and < 101 mm Hg in severe). Management and outcomes of hypoxemic patients and the proportion of them who met the criteria for ARDS were analysed.

**METHODS.** International, multicenter, 1-day point prevalence study conducted during the spring of 2016 in 117 ICUs in 6 french-speaking countries.

All patients, hospitalized or admitted in each participating ICU during the day of the study were susceptible to be enrolled.

Hypoxemia was defined as ratio of arterial oxygen tension to inspired fraction of oxygen (PaO2/FIO2) of 300 mmHg or less.

The investigators recorded patient's characteristics (comorbidities, SAPS-2 score), physiological data during the day of the study and ICU mortality

**RESULTS.** Of 1604 patients included, 859 (54%) were hypoxemic, 440 (51%) were mildly, 345 (40%) moderately and 74 (9%) severely hypoxemic. Among hypoxemic patients, 176 (20%) fulfilled ARDS criteria (37% mild, 46% moderate and 16% severe). Characteristics of patients and cause of hypoxemia according to hypoxemia severity are reported in the Table 208. Pneumonia was the main cause of hypoxemia (53%) and of ARDS (79%). Oxygen was administrated using invasive ventilation in 525 (61%) patients, low-flow oxygen in 191 (22%), NIV in 84 (9.8%) and high-flow oxygen in 45 (5.2%). The proportion of patients under invasive ventilation and high-flow oxygen was greater in severely hypoxemic patients whereas NIV was equally used across the different severity's classes (Table 209).

Among hypoxemic patients under invasive mechanical ventilation, 77% received a tidal volume of 8 mL/kg or less of predicted body weight with a median PEEP level of 6 [5–10] cmH2O. Among ARDS patients, 145 (83%) received a tidal volume of 8 mL/kg or less with a median PEEP of 8 [6–12] cmH2O and a median plateau pressure of 23 [19–27] cmH2O.

Prone position was used in 22 patients, Veno-Venous ECMO in 21 and NO in 19 mostly in moderately and severely hypoxemic patients.

In the whole population, ICU mortality was 20%, 12% of non-hypoxemic patients and 27% of hypoxemic one (p < 0.001). ICU mortality increase with hypoxemia severity (21% in mild hypoxemic patients, 26% in moderate hypoxemic patients, and 50% in severe hypoxemic patients, P < 0.001) In multivariate analysis among hypoxemic patients, severe hypoxemia remained independently associated with ICU-mortality OR 2.7 (1.7 -4.3).

**CONCLUSIONS.** Hypoxemia is frequently present in ICU patients (more than the half), ARDS represented only a minority of these patients. The severity of hypoxaemia is associated with mortality

**Table 208 (Abstract 0862).** Patients' characteristics

| | Mild n=440 | Moderate n=345 | Severe n=74 |
|---|---|---|---|
| Age, years med (IQR) | 65 [54-74] | 65 [53-73] | 58 [47-69] |
| Female, N (%) | 142 (32.3) | 109 (31.6) | 22 (29.7) |
| Medical admission | 327 (74.5) | 281 (81.7) | 67 (90.5) |
| SAPS II | 42 [30-56] | 43 [31-57] | 45 [34-61] |
| COPD | 141 (32.1) | 127 (37.1) | 20 (27.4) |
| Pneumonia | 189 (43.0) | 204 (60.2) | 60 (82.2) |
| Acute on CRF | 73 (16.6) | 80 (23.5) | 13 (17.8) |
| CPE | 64 (14.6) | 51 (15.0) | 12 (16.4) |
| Aspiration | 30 (6.8) | 33 (9.7) | 11 (15.1) |

**Table 209 (Abstract 0862).** Ventilatory parameters according to hypoxemia seve

| | Mild n=440 | Moderate n=345 | Severe n=74 |
|---|---|---|---|
| Low flow O2 | 136 (30.9) | 55 (15.9) | 0 (0) |
| High Flow O2 | 11 (2.5) | 23 (6.7) | 11 (14.9) |
| Non invasive ventilation | 43 (9.8) | 33 (9.6) | 8 (10.8) |
| Invasive ventilation | 239 (54.3) | 231 (66.9) | 55 (74.3) |
| TV ≤8ml/kg IBW | 165 (71.7) | 178 (79.1) | 50 (92.6) |
| PEEP | 5 [5-8] | 7 [5-10] | 10 [8-12] |
| Plateau pressure* | 20 [16-24] | 23.5 [20-28] | 25.5 [23-29.5] |
| Sedation | 121 (51) | 159 (69) | 41 (76) |
| Paralysis | 11(5) | 44 (20) | 28 (52) |

### 0863

#### Prediction of respiratory effects of 16 h period of prone positioning (PP): a prospective study in patients with transpulmonary thermodilution monitoring

W. Huber, V. Vieth, T. Lahmer, U. Mayr, S. Rasch, A. Herner, R. Schmid, M. Braun
Klinikum rechts der Isar der TUM, Munich, Germany
**Correspondence:** W. Huber
*Intensive Care Medicine Experimental* 2017, **5(Suppl 2):**0863

**INTRODUCTION.** Prone positioning (PP) has been demonstrated to reduce mortality in ARDS. Although PP is considered as a beneficial and cost-effective therapy, it requires substantial personal resources and has potential side effects (catheter removal; loss of airway). Furthermore, a-priori criteria for the efficacy of PP are not clearly characterized.
**OBJECTIVES.** Therefore, this study analysed potential predictors of the efficacy of PP including baseline haemodynamics derived from transpulmonary thermodilution TPTD (PiCCO; Pulsion Medical Systems; Germany) and intra-abdominal pressure (IAP).
**METHODS.** We analyzed 54 cycles of a 16h PP-period in 26 patients (10 f; 16 m) with ARDS according to the Berlin and AECC-definitions before 1[st] cycle of PP (Berlin:14 moderate, 12 severe; AECC: 26 ARDS) and before all cycles of PP (Berlin: 29 moderate; 25 severe; AECC: 54 ARDS). Ventilator settings, blood gas analysis, TPTD-data and IAP were recorded immediately before and at the end of 16h of PP.
**RESULTS.** Mean values of $pO_2/F_iO_2$ (160 ± 55 vs. 104 ± 29 mmHg; $p < 0.001$), oxygenation index OI ((OI = mean airway pressure*$F_iO_2/pO_2$) 11.3 ± 5.3 vs. 18.8 ± 5.6 $cmH_2O$/mmHg; $p < 0.001$) and modified LIS (sum of points without X-ray; 7.7 ± 1.4 vs. 8.9 ± 1.1; $p < 0.001$) significantly improved after 16h of PP compared to baseline, while $pCO_2$ did not change (52 ± 17 vs. 55 ± 15mmHg; $p = 0.170$). Decreases in OI of ≥ 3, 5 and 10 mmHg/$cmH_2O$ were found for 42, 28 and 8 out of 54 cycles of PP, respectively. Decreases in the modified LIS-score of 1 or 2 points were found in 37 and 21 out of 54 cycles of PP. Deterioration in LIS or OI or at least one of both criteria were found in 5, 5 and 7 out of the 54 cycles of PP.
In univariate analysis improvement of the OI was associated with higher OI_base ($r = 0.535$), higher Berlin-category ($r = 0.475$), higher LIS ($r = 0.407$) and higher P_mean ($r = 0.523$; $p < 0.001$ for all correlations), but not with P_peak, PEEP, driving pressure, IAP or $PCO_2$.
Among TPTD-parameters there was a trend for an improvement in OI with higher baseline values of pulmonary vasculary permeability index PVPI ($r = 0.241$; $p = 0.080$). In a binary regression analysis including these variables only OI and $pCO_2$ at baseline were independently associated with an improvement of OI of ≥10 mmHg/$cmH_2O$.
An improvement of OI of ≥10 mmHg/$cmH_2O$ was predicted by baseline OI (AUC = 0.899; $p < 0.001$), $pO_2/F_iO_2$ (0.875; $p = 0.001$), Berlin-definition (0.815; $p = 0.005$), modified LIS (0.779; $p = 0.013$) and PVPI (0.743; $p = 0.029$). A model including OI and $pCO_2$ at baseline provided an AUC of 0.937 ($p < 0.001$).
Higher values of $pCO_2$ (≥53 mmHg) and GEDVI (≥801mL/$m^2$) were the only significant predictors of a *deterioration of OI and/or modified LIS* in univariate, multivariate and ROC-analyses.
**CONCLUSIONS.** High OI and lower $pCO_2$ before PP were the strongest predictors of successful PP. Among TPTD parameters high PVPI and a GEDVI below the upper normal range were associated with pulmonary improvement by PP.

### 0864

#### Time course of pulmonary and haemodynamic parameters during a 16h period of prone positioning (PP): a prospective study in patients with transpulmonary thermodilution (TPTD) monitoring

W. Huber, V. Vieth, T. Lahmer, U. Mayr, S. Rasch, A. Herner, R. Schmid, M. Braun
Klinikum rechts der Isar der TUM, Munich, Germany
**Correspondence:** W. Huber
*Intensive Care Medicine Experimental* 2017, **5(Suppl 2):**0864

**INTRODUCTION.** Prone positioning (PP) has been demonstrated to reduce mortality in ARDS. Nevertheless, PP carries some risks such as catheter removal, loss of airway and potential haemodynamic side effects. While the *respiratory* effects of PP have been intensely investigated, there is a lack of data on *haemodynamic* effects.
**OBJECTIVES.** This study investigated haemodynamic and pulmonary effects over time during a 16h period of PP in ARDS-patients with TPTD monitoring (PiCCO; Pulsion; Germany).
**METHODS.** We analyzed 54 cycles of 16h PP in 26 patients (10 f; 16 m) with ARDS according to the Berlin and AECC-definitions (before the 1[st] cycle of PP Berlin:14 moderate, 12 severe; AECC: 26 ARDS). Ventilator settings, blood gas analysis and TPTD-values (PiCCO) were measured immediately before PP as well as after 0.5h, 2h, 8h, 16h of PP and finally 0.5h after turning to supine position (SP) again.
**RESULTS.** Overall, a 16h period of PP resulted in significant improvement of the oxygenation index OI (OI = mean airway pressure*$F_iO_2/pO_2$) -5.5 ± 5.0 $cmH_2O$/mmHg), $pO_2/F_iO_2$ (+52 ± 49 mmHg) and modified LIS (sum of points without chest-X-ray: −1.13 ± 1.29 points). By contrast, the return to SP resulted in a deterioration of OI (+2.5 ± 3.8 $cmH_2O$/mmHg), $pO_2/F_iO_2$ (−21 ± 59 mmHg) and modified LIS (+0.49 ± 1.03 points; all $p < 0.001$).
Beneficial respiratory effects of PP compared to baseline were detectable as early as 30 min after PP with decreases of OI (−3.9 ± 5.0 (30min); −4.2 ± 4.6 (2h); −4.5 ± 4.7 (8h); −5.5 ± 5.0 $cmH_2O$/mmHg (16h)) and modified LIS (−0.52 ± 1.13 ($p = 0.002$); −0.74 ± 1.23; −0.78 ± 1.18; −1.13 ± 1.3) as well as with increases in $pO_2/F_iO_2$ (+46 ± 58; +48 ± 56; +46 ± 46; +51 ± 49 mmHg) after 30min, 2h, 8h and 16h of PP, respectively ($p < 0.001$ except as indicated).

Pulmonary vascular permeability index PVPI decreased after 8h (−0.22 ± 0.74 ($p = 0.015$) and 16h of PP (−0.35 ± 0.78; $p = 0.003$). Extravascular lungwater index EVLWI slightly increased 30 min after the start of PP (+0.72 ± 2.4; $p = 0.047$). However, on the long run EVLW decreased after 8h (−1.5 ± 3.2 ($p = 0.001$) and after 16h of PP (−1.4 ± 4.2; $p = 0.007$).
Decreases in EVLWI and PVPI after 16h of PP were significantly associated to improvement in OI ($r = 0.513$; $p < 0.001$; $r = 0.415$; $p = 0.002$) and $pO_2/F_iO_2$ ($r = -0.436$; $p = 0.001$; $r = -0.270$; $p = 0.048$). Furthermore, decreases in EVLWI were significantly associated with an improvement in modified LIS ($r = 0.317$; $p = 0.019$). Decreases in EVLWI and PVPI within 16h provided ROC-AUCs of 0.727 ($p = 0.042$) and 0.793 ($p = 0.009$) regarding an increase in OI of at least 10 points during 16h of PP. PP did not result in changes of GEDVI, CVP or cardiac index CI after 0.5, 2, 8 and 16h of PP.
CONCLUSIONS.
1.) PP effectively improved OI, $pO_2F_iO_2$ and LIS without haemodynamic impairment.
2.) PP resulted in a significant improvement of EVLWI and PVPI.
3.) Changes in EVLWI and PVPI over time were significant predictors of substantial improvement in pulmonary function.

### 0865

#### Diabetes mellitus is protective against the development of ARDS: an analysis of the LUNG SAFE database

A.J. Boyle[1,2], F. Madotto[3], J.G. Laffey[4,5], G. Bellani[3,6], T. Pham[7,8,9], A. Pesenti[10,11], B.T. Thompson[12], C.M. O'Kane[2,13], A.M. Deane[14], D.F. McAuley[2,15]
[1]Royal Adelaide Hospital, Intensive Care Unit, Adelaide, Australia; [2]Queen's University Belfast, Wellcome-Wolfson Institute for Experimental Medicine, Belfast, United Kingdom; [3]University of Milan-Bicocca, School of Medicine and Surgery, Manza, Italy; [4]St Michael's Hospital, Department of Anesthesia and Critical Care Medicine, Keenan Research Centre for Biomedical Science, Toronto, Canada; [5]University of Toronto, Departments of Anesthesia, Physiology and Interdepartmental division of Critical Care Medicine, Toronto, Canada; [6]San Gerardo Hospital, Department of Emergency and Intensive Care, Monza, Italy; [7]Groupe hospitalier des Hôpitaux Universitaires de l'Est Parisien, AP-HP, Hôpital Tenon, Unité de Réanimation médico-chirurgicale, Pôle Thorax Voies aériennes, Paris, France; [8]Université Paris Diderot, UMR 1153, Inserm, Sorbonne Paris Cité, ECSTRA Team, Paris, France; [9]Université Paris Est Créteil, UMR 915, Inserm, Créteil, France; [10]Università degli Studi di Milano, Istituto di Anestesia e Rianimazione, Milan, Italy; [11]Ospedale Maggiore, Istituto di Ricovero e Cura a Carattere Scientifico, Milan, Italy; [12]Harvard Medical School, Division of Pulmonary and Critical Care Unit, Department of Medicine, Massachusetts General Hospital, Boston, United States; [13]Belfast City Hospital, Regional Respiratory Centre, Belfast, United Kingdom; [14]The University of Melbourne, Intensive Care Unit, The Royal Melbourne Hospital, Melbourne, Australia; [15]Royal Victoria Hospital, Regional Intensive Care Unit, Belfast, United Kingdom
**Correspondence:** A.J. Boyle
*Intensive Care Medicine Experimental* 2017, **5(Suppl 2):**0865

**INTRODUCTION.** Diabetes mellitus is a prevalent disease affecting almost 400 million people worldwide, and is therefore a common co-existing disease in critically ill patients [1]. Previous studies have been conflicting whether diabetes reduces the risk of the Acute Respiratory Distress Syndrome (ARDS).
**OBJECTIVES.** To evaluate the associations between pre-existing diabetes and ARDS in critically ill patients with acute hypoxaemic respiratory failure (AHRF).
**METHODS.** An analysis of a global, multi-centre prospective observational study (LUNG SAFE) [2] was performed. All intensive care unit (ICU) patients receiving mechanical ventilation over a 4-week period meeting criteria for AHRF were included (n = 4499 patients from 459 ICUs across 50 countries). Important clinical characteristics were included in a stepwise selection approach (forward and backward selection combined with a significance level of 0.05) to identify a set of independent variables associated with having ARDS at any time, having ARDS after two days of meeting AHRF criteria, and for 90-day mortality.
**RESULTS.** Of the 4499 patients with AHRF included in this study, 3022 (67.2%) fulfilled ARDS criteria at admission or developed ARDS during their ICU stay. In patients with AHRF, diabetes mellitus was less common in patients with ARDS (n = 657 [21.7%]) than in those who never developed ARDS (n = 376 [25.5%]; $p = 0.005$). In multivariable analysis, diabetes mellitus was associated with a reduced likelihood of having ARDS (Odds Ratio 0.84 [95% CI 0.72, 0.97]; $p = 0.02$) (Table 210). There was no association between diabetes mellitus and likelihood for developing ARDS after two days of AHRF (OR 0.85 [0.60, 1.22]; $p = 0.39$). Diabetes mellitus was not associated with a difference in mortality in patients with ARDS (OR 1.19 [0.92, 1.37]; $p = 0.27$).
**CONCLUSIONS.** In a large cohort of patients with AHRF, diabetes mellitus was associated with a reduced likelihood of having ARDS but did not protect against ARDS that developed after two days. In patients with ARDS, diabetes mellitus was not associated with a difference in mortality.
**REFERENCE(S)**
1. Kar P, Jones KL, Horowitz M, Deane AM. Management of critically ill patients with type 2 diabetes: The need for personalised therapy. World J Diabetes. 2015;6:693–706.
2. Bellani G, Laffey JG, Pham T, et al. Epidemiology, patterns of care, and mortality for patients with acute respiratory distress syndrome in intensive care units in 50 countries. JAMA. 2016;315:788–800.

**Table 210 (Abstract 0865).** Multivariable analysis for having ARDS

| Variable | Odds ratio (95% CI) | p-value |
|---|---|---|
| Diabetes | 0.84 (0.72, 0.97) | 0.02 |
| Baseline PaO2:FiO2 ratio (per mmHg) | 0.995 (0.994, 0.996) | <0.0001 |
| Immunosuppression | 1.88 (1.48, 2.38) | <0.0001 |

### 0866

#### Long term hemodynamic effects of prone position in ARDS patients

M. Ruste[1], M. Aublanc[1,2], H. Yonis[1], A. Louf-Durier[1], F. Lissonde[1], Z. Riad[1,2], L. Bitker[1], C. Guérin[1], J.-C. Richard[1,2,3]
[1]Hospices Civils de Lyon, Hôpital de la Croix-Rousse - Service de Réanimation Médicale, Lyon, France; [2]Université de Lyon, Université Lyon I, Lyon, France; [3]CREATIS INSERM 1044 CNRS 5220, Villeurbanne, France
**Correspondence:** M. Ruste
*Intensive Care Medicine Experimental* 2017, **5(Suppl 2):**0866

**INTRODUCTION.** A short term beneficial effect of prone position (PP) on cardiac index has been shown in 50% of 18 ARDS patients, and was related to an increase in cardiac preload in preload responsive patients [1].
**OBJECTIVES.** The aim of this study was to evaluate the long term hemodynamic response to prone position in a larger series of ARDS patients.
**METHODS.** Single center retrospective observational study performed on ARDS patients hospitalized in a medical ICU between July 2012 and December 2016. Patients included were adults fulfilling the Berlin definition for ARDS, undergoing at least one PP session, under hemodynamic monitoring by the Picco ® device, with availability of hemodynamic measurements performed before ($T_1$), at the beginning ($T_2$), at the end ($T_3$), and after the prone position session ($T_4$). Prone position sessions were excluded if they were performed > 10 days after ARDS onset. The following variables were recorded: demographic, SAPSII, ARDS severity and risk factor, SOFA score and cumulative fluid balance at PP onset, delay between ARDS session and PP session, hemodynamic, arterial blood gas, ventilatory settings, plateau pressure, catecholamine dose and additional treatments. Statistical analyses were performed using prone position session as statistical unit and mixed models. $p < 0.05$ was chosen for statistical significance. Data are expressed as mean ± standard deviation.
**RESULTS.** 99 patients fulfilled the inclusion criteria over the study period, totalizing 186 prone position sessions (2 ± 2 sessions per patient). Patients' age was 65 ± 11 y, 67% were male, 73% fulfilled the criteria for severe ARDS, and SAPSII at ICU admission was

63 ± 17. ARDS risk factors were pneumonia in 73 (74%), aspiration pneumonia in 32 (32%), and sepsis in 9 (9%) patients. Duration of prone position sessions was 16 ± 3 h. Hemodynamic measurements were performed 3 ± 2h ($T_2$) and 13 ± 3h ($T_3$) after PP onset, and 2 ± 2h ($T_4$) after return to supine position. At session onset, SOFA score was 15 ± 4, and cumulated fluid balance was 2.3 ± 6.6 L. Vasopressor were used in 86%, inhaled nitric oxide in 22%, and neuromuscular blocking agents in 92% of the sessions. Hemodynamic and respiratory parameters before, during and after the PP sessions are reported Table 211.

**CONCLUSIONS.** Prone position is associated with a moderate but sustained increase in global end-diastolic volume, reversible after return to supine position. This effect could explain a change in cardiac index induced by postural change in preload-dependent patients.

**REFERENCE(S)**

Jozwiak M. Am J Respir Crit Care Med 2013;188:1428–33.

**Table 211 (Abstract 0866).** See text for description

| | T1 | T2 | T3 | T4 |
|---|---|---|---|---|
| Heart rate (min-1) | 97 ± 22 $^{†}$ | 98 ± 20$^{†,‡}$ | 95 ± 20 | 94 ± 20 |
| Cardiac index (L.min-1.m-2) | 3.5 ± 1.3 $^{†}$ | 3.5 ± 1.2 $^{†}$ | 3.5 ± 1.1$^{†}$ | 3.3 ± 1.1 |
| Global end-diastolic volume (mL.m-2) | 719 ± 193 | 734 ± 179 $^{*,†}$ | 751 ± 194 $^{*,†}$ | 713 ± 199 |
| Global ejection fraction (%) | 22 ± 7 | 21 ± 7 $^{*}$ | 22 ± 7 | 21 ± 7 |
| Vasopressor dose (µg.kg-1.min-1) | 0.91 ± 1.70 | 0.91 ± 2.09 | 0.83 ± 1.70 $^{*}$ | 0.88 ± 1.80 $^{*}$ |
| Dobutamine dose (µg.kg-1.min-1) | 2.5 ± 5.9 | 2.4 ± 5.6 | 2.7 ± 6.0 | 2.8 ± 6.1 |
| PEEP (cm H2O) | 10 ± 3 $^{†,‡}$ | 10 ± 3 $^{†,‡}$ | 9 ± 9 | 9 ± 3 |
| PaO2/FiO2 (mm Hg) | 112 ± 27 | - | 178 ± 63 $^{*,†}$ | 151 ± 57 $^{*}$ |
| PaCO2 (mm Hg) | 45 ± 10 | - | 44 ± 11 | 44 ± 10 |

Multivariate analysis identified as independent predictors of cardiac index: global end-diastolic volume, global ejection fraction, $PaCO_2$, $PaO_2/FiO_2$, heart rate and PEEP level. Multivariate analysis identified as independent predictors of global end-diastolic volume: body position, age and sex

$^{†}$ $p < 0.05$ vs $T_4$, $^{‡}$ $p < 0.05$ vs $T_3$, $^{*}$ $p < 0.05$ vs $T_1$

### 0867

#### Role of hypertonic saline nebulization therapy in patients with early acute respiratory distress syndrome

A.H. Dahruog, T.M. Zaytoun, H.M. Abdella

Faculty of Medicine, Alexandria University, Critical Care Medicine, Alexandria, Egypt

**Correspondence:** A.H. Dahruog

*Intensive Care Medicine Experimental* 2017, **5(Suppl 2):**0867

**INTRODUCTION.** Acute respiratory syndrome (ARDS) is a life threatening condition with high mortality rates. It is characterized by inflammation of the lung parenchyma leading to impaired gas exchange with concomitant systemic release of inflammatory mediators causing protracted inflammation, increased vascular permeability, increased permeability of alveolar epithelial cells, extravasation of plasma and leucocyte infiltration, and frequently resulting in multiple organ failure. Since inflammation is thought to contribute to the pathogenesis of ARDS, it is rational to explore modulating therapies for this inflammation, provided the adverse effect of such treatment is not excessive. [(1)]

Hypertonic saline nebulization (NaCl 3%) is a potent anti-inflammatory agent, and immunomodulator, which exert inhibitory effects in several stages of the inflammatory cascade and would seem to be a logical choice for treatment of ARDS.[(2)]

**OBJECTIVES.** to assess the effect of hypertonic saline nebulizer on patients with early ARDS as regard morbidity (using Murray lung injury score [$PaO_2$/ $FiO_2$, lung quadrant infiltration, PEEP, compliance], and follow up of lung mechanics) and mortality.

**METHODS.** This study included 60 patients according to sample size admitted to the Department of Critical Care Medicine at the Alexandria Main University Hospital meeting criteria of ARDS according to Berlin´s definition. They were categorized into two groups group I (control group) included thirty patients, and group II (study group) included thirty patients who received 4ml of hypertonic saline nebulization once daily for 7 days. All cases were subjected to history taking, clinical examination, assessment of disease severity (APACHEII), laboratory investigations, ABG, and chest X-ray, with measurement of lung mechanics (compliance, airway resistance, peak and plateau pressures, PEEP), hypoxic index, lung injury score (LIS), and SOFA score.

**RESULTS.** Hypoxic index, LIS and SOFA score were significantly improved in hypertonic saline group than control group. Also, intensive care unit (ICU) stay and mechanical ventilation days were reduced in the hypertonic saline group with statistically significant difference. Survival was significantly higher in the hypertonic saline group.

**CONCLUSIONS.** Initiation of hypertonic saline nebulization therapy for patients with early ARDS appears to be tolerable and may be beneficial with significant improving in oxygenation with trend to decrease mortality, ICU stay, and mechanical ventilation days and so may be added to protective lung strategy.

**REFERENCE(S)**

1 - Ware LB, Matthay MA. The acute respiratory distress syndrome. N Engl J Med 2000; 342:1334–49.

2 - Elkins MR, Robinson M, Rose BR, Harbour C, Moriarty CP, Marks GB, et al. A controlled trial of long-term inhaled hypertonic saline in patients with cystic fibrosis. N Engl J Med 2006;354(3):229–40.

**GRANT ACKNOWLEDGMENT**

Alexandria critical care medicine department staff members

### 0868

#### Carbon dioxide venous-arterial difference and shunts during prone position in influenza AH1N1 ARDS patients

J. Garduño López[1], S.E. Zamora Gómez[1], M.A. Amezcua Gutierrez[1], J.O. Montoya Rojo[1], I.M. Lima Lucero[1], E.S. Cruz Montesinos[1], M.A. Carrasco Flores[1], M. Hernández Romero[1], M.A. Toledo Rivera[1], G.D. Hernández López[1], C.A. Jiménez Pérez[2]

[1]Hospital Juárez de México, Intensive Care Unit, Mexico, Mexico; [2]Hospital Juárez de México, Internal Medicine, Mexico, Mexico

**Correspondence:** J. Garduño López

*Intensive Care Medicine Experimental* 2017, **5(Suppl 2):**0868

**INTRODUCTION.** ARDS due to influenza AH1N1 infection had a very harmful impact around the world but developing countries were more affected. Prone position (PP) has shown lower mortality in moderate and severe ARDS patients especially when it's applied early. Venous-arterial pCO2 difference (CO2 v-a difference) has been considered as a marker of cardiovascular system efficiency, patients with CO2 v-a difference >6 mmHg had worse outcome; in the same way, as long as shunts increase, the severity of disease increase.

**OBJECTIVES.** Analyze the clinical outcome of AH1N1 ARDS patients according to shunts and CO2 v-a difference behaviour before and after prone positioning.

**METHODS.** A retrospective analysis of severe ARDS patients secondary to Influenza AH1N1 infection treated in a tertiary hospital intensive care unit in Mexico city, from January to march 2017, recording intrapulmonary shunts by Fick method, CO2 v-a Difference before and after six hours of PP. Clinical outcome was analyzed.

**RESULTS.** Twelve patients analyzed, (10) 86.4% male and (2) 13.6% female, a survivor rate of 33.3%, 4 survivors, 8 non survivors. Mean pre prone position shunts was of 42.2%, 33% in survivors, and 46.65% in non survivors; mean pos prone position (6 hrs) shunts was of 31.39%, 22.9% in survivors, and 35.64% in non survivors. Mean CO2 v-a difference pre prone position was of 10.95 mmHg, 8.2 in survivors and 12.32 in non survivors; mean CO2 v-a difference pos prone position (6 hrs) was of 7.67 mmHg, 3.5 in survivors and 9.75 in non survivors by Pearson lineal correlation of CO2 v-a difference and shunts before prone position was 0.712 with a bilateral meaning of 0.009, this finding were not found after PP. **CONCLUSIONS.** In AH1N1

ARDS patients submitted to prone position, we found non survivors had higher shunts and CO2 v-a difference previously and posterior to prone position in contrast with survivors, reflecting the severity of disease. We found a good correlation between shunts and CO2 v-a difference behavior in pre prone position, statistically significant, however we didn't found correlation posterior to prone position. The gasometrical behavior during ARDS patients remains a topic of interest for medical research, so prospective studies with a biggest sample are needed to elucidate the use of CO2 v-a difference and shunts as a predictor of mortality in patients in prone position.

**REFERENCE(S)**

1. Mallat J. Difference in venous-arterial carbon dioxide in septic shock. Minerva Anestesiol. 2015 Apr;81(4):419–25. Epub 2013 Nov 26.
2. Kopterides P, Siempos II, Armaganidis A. Prone positioning in hypoxemic respiratory failure: meta-analysis of randomized controlled trials. Journal of Critical Care 2009; 24(1): 89–100
3. Protti A, Cressoni M, Santini A, et al. Lung stress and strain during mechanical ventilation: any safe threshold? Am J Respir Crit Care Med 2011; 183: 1354–62.

### 0869

#### Safety of prone position in patients with ARDS after abdominal surgery

L.A. Gorordo-Delsol[1,2], J.A. Castañón-González[1], M.A. Amezcua-Gutiérrez[1], J.O. Montoya-Rojo[1], J. Garduño-López[1], I.M. Lima-Lucero[1], E.S. Cruz-Montesinos[1], G.D. Hernández-López[1]
[1]Hospital Juárez de México, Intensive Care Unit, México City, Mexico; [2]Hospital de Traumatología 'Dr. Victorio de la Fuente Narváez' - IMSS, Intensive Care Unit - Shock Unit, México City, Mexico
**Correspondence:** L.A. Gorordo-Delsol
*Intensive Care Medicine Experimental* 2017, **5(Suppl 2):**0869

**BACKGROUND.** Several studies have demonstrated the benefits of prone position ventilation in patients with severe ARDS (1,2); however, controversy over the safety of this maneuver continues (3) especially in morbid obesity (4) and after abdominal surgery (5).

**METHODOLOGY.** ICU patients with a diagnosis of severe ARDS treated with prone position were prospectively enrolled from 2014 to 2016. Patients demographics, APACHE II, SOFA and Braden scores, free days of ventilation (FDOV) and lenght of stay in the ICU (LOS-ICU), vascular access dysfunction, endotracheal tube occlusion, unplanned extubations, duration of prone, pressure ulcers (PU) and complications of surgical wounds were recorded.

**RESULTS.** Eleven patients with severe extrapulmonary ARDS after abdominal surgery, were proned 57.2 (±17.2) continuous hours. APACHE II, SOFA and Braden scores at admission were 25 (±5), 12 (±3) and 10 (±2) points respectively. After 12 h of prone position PaO2/FiO2 ratio increased (83.82 ± 19.12 vs 180.14 ± 63.95, $p = 0.03$) and at 48 h pulmonary shunts decreased (46 to 23% $p = 0.03$). We recorded 16 (±3.5) mean of FDOV and 13 (±4) LOS-ICU, all survived.

Two patients had grade II PU in both labial commissures, and another 2 patients developed grade I PU in knees, no central venous catheter dysfunction was recorded. One arterial line was removed, 1 patient with a femoral Mahurkar catether had obstruction that required modifications in limb position. No orotracheal tube oclussions, unplanned extubation or surgical complications were recorded. None of them presented complications of the surgical wound (eventration, infection or need of urgent surgery).

**DISCUSSION.** Benefits of prone position are clear (1–5), some authors have proposed limits to the maneuver (3), in spite that obese (4) and patients with abdominal surgery have been reported to respond well without increased complications (5). We conclude that prone position is safe when there are protocols and trained personnel.

### 0870

#### Evolution of acute respiratory distress in third-level ICU

Á. Vidal[1], D. Robaglia[1], A. Tejero[1], V. Hortiguela[1], J.M. Milicua[1], P. Turrión[1], E. Gómez[1], M. Perez[2], C. Pérez[1]
[1]Hospital Fundación Jiménez Díaz, Madrid, Spain; [2]Hospital Universitario Rey Juan Carlos, Móstoles, Spain
**Correspondence:** Á. Vidal
*Intensive Care Medicine Experimental* 2017, **5(Suppl 2):**0870

**INTRODUCTION.** ARDS is a pathology of high incidence in intensive care units, with a high cost and high morbidity and mortality due to the difficult management of these patients, since it lacks specific treatment.(1)

**OBJECTIVES.** To study the evolution of the ventilatory and gas exchange parameters in patients with ARDS and to verify if the recommendations of the last guidelines of the protective ventilation are carried out in a third level UVI.

**METHODS.** An observational, prospective study of patients admitted in a polyvalent UVI at a tertiary hospital since 2015 until now, who present ARDS according to the Berlin criteria (2). We recorded the variables of the chosen days, collecting; gravity scales (APACHE, SOFA), respiratory mechanical variables (peak pressure, plateau, PEEP, CrsL), gas exchange variables (pafi, pCO2, ph), until the patients are released from the unit (including complications / mortality).

**RESULTS.** A sample of 34 patients with ARDS (age 59.85 ± 13.69 years, 67.6% males) has been analyzed, the severity of admission according to the APACHE II scale was 19.76 ± 8.7, The cause of the admission was abdominal in 11.8%, postoperate in 14.7%, respiratory in 52.9%, VMNI pre IOT was started in 44.1% and GAF in 14.7% with a duration of both techniques of 0.65 ± 1.45 days. The cause of ARDS was respiratory in 61.8%, abdominal in 26.5% and 8.8% by sepsis, with a percentage of extrapulmonary ARDS of 41.2%. In the Table 212 we can see the evolution of the respiratory and gasometric parameters in the different days.

Prono was performed in 44.1%, corticosteroid therapy in 41.2%, time of MV of 16.41 ± 9.9 days, need of reintubation 11.8%, tracheotomy in 29.4% and barotrauma 11.8%. With all this we obtained a stay in UVI of 19.76 ± 9.88 days, a mortality of 50% being the causes of death; respiratory rate in 32.4% and FMO in 14.7%.

**CONCLUSIONS.** The most commonly used mode is VC. No parameters are changed, the same PEEP is maintained and almost the same vt. The pressures have been adjusted to the recommendations, with peak pressure under 40 cmH2O and plateau under 30 cmH2O. Vt stands at 7 ml/kg, higher than we expected (this may be due to the increase in obesity in the population and to the low adjustment of this volume to the ideal weight).

A high mortality could be attributable to the high rate of severity of these patients, with a non insignificant association with respiratory failure.

(1)Bellani G. JAMA. 2016 Feb 23;315(8):788–800
(2) Slutsky A. JAMA. 2012;307(23):2526–2533

**Table 212 (Abstract 0870).** The respiratory and gasometric parameters

| | Patients in VM | Mode | VT (ml/kg) | Peak pressure (cmH2O) | plateau (cmH2O) | PEEP (cmH2O) | CrsL (ml/cmH2O) | PaFi | pCO2 (mmHg) |
|---|---|---|---|---|---|---|---|---|---|
| 1° Day | 100% | VC 79,4% PC 20,6% | 7±1,2 | 36,88 ±4,6 | 28,59 ±3,6 | 14,62 ±4,44 | 30,47 ±9,9 | 97,21 ±41,7 | 67,35 ±23,4 |
| 2° Day | 97,05% | VC 70,6% PC 17,6% | 7,34 ±1,31 | 36,39 ±5,0 | 28,06 ±4,35 | 14,45 ±3,28 | 32,94 ±13,25 | 119,94 ±53,5 | 60,70 ±17,10 |
| 4° Day | 91,17% | VC 50,0% PC 32,4% | 7,35 ±1,6 | 33,23 ±5,18 | 26,65 ±4,07 | 14,16 ±3,25 | 33,90 ±12,01 | 161,29 ±68 | 57,13 ±16,21 |
| 7° Day | 70,58% | VC 29,4% PC 20,6% PS 17,6% | 7,50 ±1,70 | 32,08 ±90 | 24.08 ±6,45 | 13,63 ±3,33 | 40,96 ±15,08 | 182 ±70,34 | 53,38 ±13,41 |

### 0871

#### Comparison of respiratory mechanics and gas exchange between extra and pulmonary ARDS in a polyvalent ICU

Á. Vidal[1], D. Robaglia[1], A. Tejero[1], J.M. Milicua[1], V. Hortiguela[1], P. Turrión[1], E. Gómez[1], M. Pérez[2], C. Pérez[1]
[1]Hospital Fundación Jiménez Díaz, Madrid, Spain; [2]Hospital Universitario Rey Juan Carlos, Móstoles, Spain
**Correspondence:** Á. Vidal
*Intensive Care Medicine Experimental* 2017, **5(Suppl 2):**0871

**INTRODUCTION.** The pathogenesis of acute respiratory distress syndrome (ARDS) has been explained by the presence of a direct (pulmonary) or indirect (extrapulmonary) insult to the lung parenchyma. Evidence indicates that the pathophysiology of ARDS may differ according to the type of insult. (1).

**OBJECTIVES.** To analyze the differences in ventilatory mechanics and gas exchange between ARDS of pulmonary or extrapulmonary cause in a polyvalent ICU.

**METHODS.** 34 Patients with ARDS, according to Berlin Criteria were studied consecutively since 2015 until now. Population was classified into two groups according to its cause: pulmonary (ARDSp) or extrapulmonary (ARDSe). Data were collected on days 1$^{st}$, 2$^{sd}$, 4$^{th}$ and 7$^{th}$: ventilatory parameters (PEEP, peak pressure and plateau, tidal volume and compliance), gaseous exchange (pO2/FiO2, pCO2 and pH) and rescue maneuvers (prone, systemic steroids, and neuromuscular relaxation). There are also recorded, age, gender, days of mechanical ventilation and ICU admission, SOFA score and mortality.

**RESULTS.** Both groups were homogeneous in: age, gender, severity (APACHE II scale, SOFA). The intubation was delayed less than one day from its admission in the unit in both groups. (60% ARDSe was used VMNI (p =0,04) and 60% of ARDSp was GAF (*p* =0,9)). Ventilatory and oxygenation parameters and the use of relaxants were similar in both groups.

Tidal volume was different in the 1$^{st}$ after intubation 6,79 ± 1,09 in ARDSp (compared to 7,47 ± 1,14 ml/kg with *p* =0,037). 2$^{sd}$ day pO2 was 100,94 ± 33,31 in ARDSp (compared to 75,15 ± 12,15 mmHg with *p* =0,003). 4$^{th}$ day no differences were found. Of note, at the 7$^{th}$ day PCO2 (57,06 ± 14,18 vs 44,43 ± 4,68 mmHg with p =0,014) and RR (29,88 ± 3,723 vs 25,29 ± 5,05 rpm with *p* =0,035) were higher in ARDSp comparing to ARDSe. The ph was lower in ARDSp than ARDSe 7,37 ± 0,08 vs 7,44 ± 0,056 (*p* =0,045).

Not differences were observed in the days of MV nor in the length of stay.

Rescue manoeuvres were performed more frequently in SDRAp; Prone position in 44,11% of all patients (80% vs 20% in ARDSe (*p* =0,026)), corticoids in 41,17% of all patients (71.4% vs 28,6% in ARDSe (*p* =0,211)). Mortality was higher for ARDSe with 58.8% (*p* =0,037) (main cause respiratory failure in 54.5% of cases) compared to 41.2% in ARDSp (being the main cause multiorgan failure (80%)).

**CONCLUSIONS.** In these patients, no statistically significant differences were found in the first week (exudative phase): either in ventilatory mechanics or in gas exchange. The differences were only found in the gaseous exchange of the 7$^{th}$ day when the proliferative phase is initiated. The SDRAp had higher pCO2 values and, therefore, required higher RR to maintain the pH range.

On the other hand, more rescue manoeuvres were used in ARDSp so they had less mortality, and their mortality was mostly associated with multiorgan failure rather than respiratory failure.

(1) P. Pelosi, L. Gattinoni. Eur Respir J 2003; 22: 48s56s.

### 0872

#### Acute respiratory distress syndrome: descriptive study in a Tunisian ICU

T. Merhabene, A. Laajili, A. Jamoussi, T. Khzouri, S. Ayed, J. Ben Khelil, M. Besbes
Faculty of Medicine of Tunis, Intensive Care Unit, Ariana, Tunisia
**Correspondence:** T. Merhabene
*Intensive Care Medicine Experimental* 2017, **5(Suppl 2):**0872

**INTRODUCTION.** Despite advances in critical care management of acute respiratory distress syndrome (ARDS), its prognosis reminds worse. The aim of our study was to describe clinical and paraclinical characteristics and evolving therapeutic of ARDS.

**METHODS.** It was a retrospective cohort study from 1st January 2015 to 30th of November 2016. We described epidemiology, etiology, treatment and outcomes of patients admitted to ICU during this period.

**RESULTS.** 76 ARDS were enrolled; overall incidence was 65 episodes/1000 admissions. Median age was 59 years [18–87] and sex ratio = 1.37. Co-morbidities were present in 71% of cases. The mean IGS II and APACH II scores were respectively 41 and 19. At admission, Septic shock was observed in 30% of patients, it was associated to acute renal failure in 29% and to multi organ failure in 32%. Median pH and PaO2 / FiO2 ratio was respectively 7.35 [6.9-7.5] and 116 [35–261]. ARDS was classified severe in 54%, moderate in 38% and mild in 8% of cases. Community acquired pneumonia was the most common cause (80%). Its origin was bacterial in 54% of cases, viral in 17%, parasitic in 8% and fungal in 4%. All patients underwent echocardiography. It showed an acute cor pulmonale in 17% of patients with mean PAPS of 35mmHg [15–80]. Mechanical ventilation was necessar in all cases; it was initially non-invasive in 60%. 52% received a positive end-expository pressure (PEEP) less than 12 cm H2O.The means of PEEP, respiratory rate and plateau pressure were respectively 10 cm H2O [0–18], 25 c/min [14–36] and 28 cm H2O [19–47]. Neuromuscular blockers were needed in 65% cases. Prone position was indicated in 45% (P/F less than 150). Inhaled nitric oxide was used only in two patients. Evolution was characterized by the occurrence of acute renal failure in 16% and nosocomial infection in 38% with a predominance of ventilator-associated pneumonia (65%). Means duration of mechanical ventilation was 10 days [2–46] and length of ICU stay was 11 days [2–57]. Overall mortality rate was 74%. It was 33% in mild ARDS, 75% in the moderate form and 80% in the severe one. Common cause of death was refractory hypoxemia with multi organ failure syndrome (40%). Multivariate analysis showed that independent factors predictors of mortality were age ≥ 58 years, IGS II and APACH II scores, presence of coma, PaO2/FiO2 < 150, plateau pressure ≥28 cmH2O, use of mechanical invasive ventilation, vasopressors, neuromuscular blockers and prone positioning.

**CONCLUSION.** Our study confirms that ARDS is associated with high mortality. Presence of comorbidities, multiorgan failure at admission and degree of hypoxemia were the major factors for worse prognosis.

### 0873

#### Prone positioning in severe Acute Respiratory Distress Syndrome (ARDS)

A. Laajili, T. Merhabene, A. Jamoussi, S. Ayed, J. Ben Khelil, M. Besbes
Faculty of Medicine of Tunis, Intensive Care Unit, Ariana, Tunisia
**Correspondence:** T. Merhabene
*Intensive Care Medicine Experimental* 2017, **5(Suppl 2):**0873

**BACKGROUND.** Prone positioning (PP) has been used to improve oxygenation in patients who require mechanical ventilator support for management of ARDS. Randomized controlled trials demonstrated that survival rate is significantly better with PP.

**PURPOSE.** We proposed to determine the effect of PP on prognosis of ARDS.

**METHODS.** It was a retrospective study, including 76 cases of ARDS admitted in our respiratory ICU from January 2015 to November 2016. We have defined 2 groups: GI (44 patients positioned in PP), GII (32 without PP). We compare clinical data, therapeutic, side effects and outcomes. PP was indicated in severe cases of ARDS with persistent hypoxemia despite ventilatory optimization. PP was maintained for 18 consecutive hours daily. Discontinuation of PP was decided after clinical improvement.

**RESULTS.** At admission, no significant difference was found between the 2 groups in mean age (56vs50 years, p = 0,5) and APACHEII score (20 ± 10vs17 ± 7, p = 0,09). However, sex ratio (1vs2.5; p = 0,03) and mean IGSII score (44 ± 18 vs 35 ± 13; p = 0,02) were significantly different. Cardiovascular co-morbidities were more frequent in GI (75%vs66%, p = 0,3). Septic shock was present in 36% in GI vs 22% in GII (p = 0,1). Pneumonia was the most common cause in 2 groups (73% vs 90%, p = 0,053). Patients in GI were more hypoxemic and significant differences were noted in the means of pH (7.31 ± 0.15 vs 7.42 ± 0.12; p = 0,001), PaCO2 (55 ± 23mmHg vs 43 ± 19mmHg; p = 0,019), and PaO2/FiO2 (103 ± 51 vs 135 ± 54; p = 0,01). All patients underwent echocardiography. Acute cor pulmonale was more frequently observed in GI (27% vs 3%; p = 0,01). Invasive mechanical ventilation was needed in all patients of GI, otherwise it was prescribed in only 50% in the second group(p < 0,001). Mean duration of mechanical ventilation was significantly longer in GI (12 ± 9 vs 7 ± 10 days; p = 0,04). Mean positive end-expiratory pressure (PEEP) received in GI was significantly higher than that of GII (12 ± 5 vs 8 ± 3 cmH2O; p = 0,002). 66% of patients in G1 received a PEEP more than 12 cmH2O in contrast of 6% in GII (p = 0,002). Mean plateau pressure were similar in 2 groups (29 ± 4 vs 29 cmH2O). Use of neuromuscular blockers was significantly more frequent in GI (100% vs 19%; p < 0,001). Most frequent side effects occurred in GI were pressure ulcer and hemodynamic instability. GI had significantly more nosocomial infection (48% vs 25%; p = 0,044), longer mean duration of ICU stay (12 ± 10 vs 10 ± 9 days, p = 0,3) and higher mortality rate (89% vs 56%; p = 0,001). Multivariate analysis showed that female gender (p = 0,03), presence of acute cor pulmonale (p = 0,01), invasive mechanical ventilation with use of sedative and neuromuscular blockers (p < 0,001), PEEP ≥ 10 cmH2O (p = 0,007), and nosocomial infection (p = 0,044) were independent predictor factors of mortality.

**CONCLUSION.** Our study doesn't confirm the benefit of PP in ARDS. However, patients who require this technique were more severe, have more organ dysfunction and consequently a worse prognosis.

### 0874

#### Effect of prone positioning on intraocular pressure (IOP) in patients with acute respiratory distress syndrome (ARDS)

S. Saran[1], M. Gurjar[1], V. Kanaujia[2], P. Ghosh[1], A. Azim[1], P. Mishra[3], B. Poddar[1], R.K. Singh[1], A.K. Baronia[1]
[1]Sanjay Gandhi Postgraduate Institute of Medical Sciences (SGPGIMS), Critical Care Medicine, Lucknow, India; [2]Sanjay Gandhi Postgraduate Institute of Medical Sciences (SGPGIMS), Ophthalmology, Lucknow, India; [3]Sanjay Gandhi Postgraduate Institute of Medical Sciences (SGPGIMS), Biostatistics, Lucknow, India
**Correspondence:** S. Saran
*Intensive Care Medicine Experimental* 2017, **5(Suppl 2):**0874

**INTRODUCTION.** Increase in intraocular pressure (IOP) has been reported due to prone position in awake volunteers[1] and patients undergoing surgeries in operation room.[2,3] Effect of prone positioning on IOP in critically ill ARDS patients, where it is one of the lifesaving interventions and is usually for more prolonged duration compared to operating room, has not been studied earlier.

**OBJECTIVES.** To evaluate the effect of prone positioning on IOP in moderate to severe ARDS patients.

**METHODS.** After ethical approval, this prospectivestudy was done in a 12 bedded ICU of tertiary academic hospital. All adult ARDS (moderate to severe) patients, ventilated as per ARDS net protocol and planned for prone ventilation, were considered. Patients having clinical conditions with suspected raised intracranial pressure, previous eye trauma or disease or surgery, patients with history of glaucoma and prone positioning of less than 6 hours duration, were excluded. During prone, neck was turned to one side with all protective measures. IOP was measured by hand held applanation tonometer (Tono-Pen AVIA®) in 30-$45^0$ head-end elevation position: before prone (T1), after end of the prone session at 10 (T2) and 30 min (T3). Tonopen reading displayed after 10 applanations with 95% confidence limits were recorded. Data represented as median (IQR) and Wilcoxon test applied to compare IOP at various time periods with p < 0.05 considered statistically significant.

**RESULTS.** Sixteen patients, age 61 (51–66) years, medical 15, male 11, SOFA score 12 (10–14), PaO2/FiO2 ratio of 90 (63–120), all in septic shock (on norepinephrine at 0.3 mcg/kg/min) were included. Duration of prone position was 15 (12–18) hours. Median IOP (mm Hg) before prone in right eye was 12 (9.5-16.7), which was found to increase to (with p value) 18 (0.01) and 15.5 (0.01) at 10 and 30 min respectively after end of the prone session. In left eye, IOP increased from 13 (12–17.25) to 20 (0.01) and 16 (0.01). Analysis as per eye position during prone: IOP in dependent eye increased from 13 (10.2-16.7) to 19 (0.02) and 14 (0.015) respectively [Fig. 284a and b]; whereas IOP in non-dependent eye changed from 12 (11–17) to 20 (0.001) and 16 (0.03) respectively [Fig. 285a and b].

**CONCLUSIONS.** There is significant increase in IOP due to prone positioning among ARDS patients. Also, IOP continued to rise even at 30 min, though with decreasing trends, after end of the prone session.

**REFERENCE(S)**

1. Grant GP et al. Effects of prone and reverse trendelenburgpositioning on ocular parameters. Anesthesiology 2010;112:57–65.
2. Deniz MN et al.The effect of head rotation on intraocular pressure in prone position: a randomized trial. Braz J Anesthesiol.2013;63:209–12.
3. Carey TW et al. Effect of the degree of reverse trendelenburg position on intraocular pressure during prone spine surgery: a randomized controlled trial. Spine J. 2014;14:2118–26.

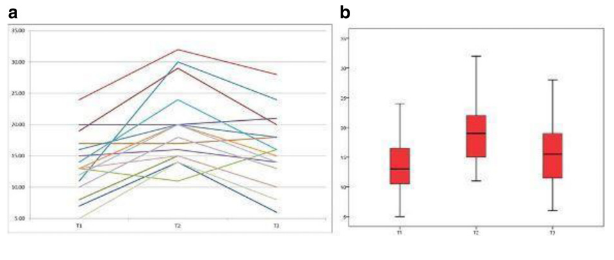

**Fig. 284 (Abstract 0874).** See text for description

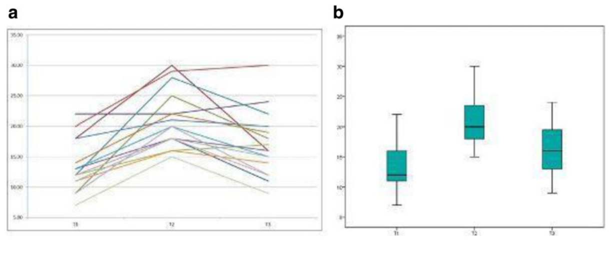

**Fig. 285 (Abstract 0874).** See text for description

### 0875

#### Airway and transpulmonary driving pressure selected by INTELLiVENT-ASV after recruitment in ARDS patients

A. Garnero[1], D. Novotni[2], J.-M. Arnal[1,2]
[1]Hôpital Sainte Musse, Réanimation Polyvalente, Toulon, France; [2]Hamilton Medical, Bonaduz, Switzerland
**Correspondence:** A. Garnero
*Intensive Care Medicine Experimental* 2017, **5(Suppl 2):**0875

**INTRODUCTION.** In acute respiratory distress syndrome (ARDS), airway driving pressure ($\Delta P_{AW}$ = Plateau pressure ($P_{PLAT}$) minus total PEEP ($PEEP_{TOT}$)) reflects the strain applied to the respiratory system. $\Delta P_{AW}$ below 15cmH$_2$O is associated with increased survival [1–2]. However, transpulmonary driving pressure ($\Delta P_L$ = end-inspiratory transpulmonary pressure ($P_{LEI}$) minus end-expiratory transpulmonary pressure ($P_{LEE}$)) is a better assessment of the strain applied to the lung. Treatment strategy leading to $\Delta P_L$ below 10 cmH$_2$O is associated with increased survival [2]. INTELLiVENT-ASV is a full closed loop ventilation mode that adjusts minute volume according to end-tidal CO2 and select tidal volume according to respiratory mechanics.
**OBJECTIVES.** This prospective physiological study assess $\Delta P_{AW}$ and $\Delta P_L$ selected by INTELLiVENT-ASV after recruitment in ARDS patients.
**METHODS.** The study was conducted in the general ICU of Hopital Sainte Musse, Toulon, France. Eligible participants were adults aged 18 or over, with early onset moderate or severe ARDS, invasively ventilated for less than 24hours. Exclusion criteria were contraindication to insert nasogastric tube, bronchopleural fistula, emphysema, pneumothorax, increased intracranial pressure, acute cor pulmonale, hemodynamic instability, and pregnancy. Patients were deeply sedated. An esophageal balloon was inserted [3]. A 10s sustained inflation recruitment maneuver was performed targeting a transpulmonary pressure at 27 cmH$_2$0 during the maneuver. Then a decremental PEEP trial was performed to determine the level of PEEP associated with $P_{TPEE}$ at 1-2cmH$_2$O. PEEP was manually set at this level. Airway and esophageal pressure ($P_{ESO}$) were measured at end-inspiration and end-expiration using 5s end-inspiratory and end-expiratory occlusions, respectively. Transpulmonary pressure was calculated as airway minus esophageal pressure at end-inspiration and end-expiration. $\Delta P_L$ was calculated as the difference between $P_{LEI}$ and $P_{LEE}$.
**RESULTS.** Nineteen patients were included between February 2016 and March 2017, 9 moderate and 10 severe ARDS, 18 were paralyzed. Sex ratio = 11/8, Age = 60 ± 18y, SAPSII = 57 ± 22, BMI = 26 ± 4, 8 patients died.
For the total respiratory system: $PEEP_{TOT}$ = 18 ± 5cmH$_2$O, $P_{PLAT}$ = 27 ± 5cmH$_2$O, $\Delta P_{AW}$ = 9 ± 2cmH$_2$O, $V_T$ = 5.8 ± 1.0mL/Kg$_{PBW}$, $C_{RS}$ = 42 ± 17ml/cmH$_2$O.
For the lungs: $P_{ESOTI}$ = 19 ± 6cmH$_2$O, $P_{ESOTE}$ = 16 ± 5cmH$_2$O, $P_{LTI}$ = 8 ± 3cmH$_2$O, $P_{LTE}$ = 2 ± 2cmH$_2$O, $\Delta P_L$ = 6 ± 3cmH$_2$O, $C_L$ = 73 ± 39ml/cmH$_2$O.
**CONCLUSION.** After a recruitment maneuver and a PEEP set to achieve positive $P_{LEE}$, INTELLiVENT-ASV selected low and safe $\Delta P_{AW}$ and $\Delta P_L$ in moderate to severe ARDS patients. This strategy seems lung protective.

**REFERENCES**
[1] Amato MB et al. N Engl J Med. 372:747–755, 2015.
[2] Laffey JG et al. Intensive Care Med. 42(12):1865–1876.
[2] Baedorf Kassis E et al. Intensive Care Med 42:1206–1213, 2016.
[3] Akoumianaki E. Am J Respir Crit Care Med. 189(5):520–531, 2014.

### 0876

#### Characteristics and outcomes in patients with adenoviral pneumonia causing severe respiratory distress syndrome

C.H.K. Lee[1], S.Y. Low[1], T.T. Tan[2], H.C. Chew[3]
[1]Singapore General Hospital, Respiratory and Critical Care Medicine, Singapore, Singapore; [2]Singapore General Hospital, Infectious Diseases, Singapore, Singapore; [3]Mount Elizabeth Novena Specialist Centre, Respiratory Medical Associates, Singapore, Singapore
**Correspondence:** C.H.K. Lee
*Intensive Care Medicine Experimental* 2017, **5(Suppl 2):**0876

**INTRODUCTION.** Human adenoviruses cause infections ranging in severity from mild symptoms to life threatening disease. Previous studies have demonstrated higher morbidity and mortality in populations including children and immunocompromised patients who were infected with the adenovirus.
**OBJECTIVES.** This study was conducted to investigate the characteristics and sequelae of patients with severe pneumonia secondary to adenovirus infections requiring intensive care support.
**METHODS.** Retrospective analysis of patients admitted in the medical Intensive Care Unit (ICU) of a tertiary hospital between January and March 2013 who had community acquired pneumonia with adenovirus isolated from the respiratory tract. Bronchoscopy and bronchoalveolar lavage were performed and samples were sent for viral cultures, antigen and polymerase chain reaction (PCR). Demographics, comorbidities, initial symptoms and travel history of the patients were collected. We recorded outcomes including mortality, ICU stay duration, use of antiviral agents, requirement for inotropic support, extra corporeal membrane oxygenation (ECMO) and renal replacement therapy.
**RESULTS.** Out of fifty patients admitted for severe pneumonia, eleven (22%) were detected to have adenovirus infection with severe acute respiratory distress syndrome (ARDS). Adenovirus serotype 7 was identified in 91% (10/11) of the patients. The mean age was 49 years and mean BMI was 28.3. 36% (4/11) of the patients were immunocompromised. Fever and cough were the commonest initial presenting symptoms accounting for 82% (9/11) of all patients. The mean maximum temperature was 38.9 degrees Celsius, although temperature did not correlate with disease severity. Other symptoms including dyspnea, sore throat and rhinorrhea were present in less than half of the patients. 36% (4/11) had rhabdomyolysis. 2 of the patients had underlying end stage renal disease. Among the remaining patients 78% (7/9) developed acute kidney injury, of whom 3 required renal replacement therapy. 36% (4/11) had disseminated intravascular coagulopathy (DIC). 91% (10/11) required inotropic support. 3 patients received ECMO, of whom 1 survived. All 5 patients who received anti-viral treatment passed away. Overall mortality was 64% (7/11) and mean ICU stay duration was 18 days.
**CONCLUSIONS.** Adenovirus serotype 7 infection is an important cause of fulminant pneumonia with systemic involvement in both immunocompetent and immunocompromised patients. Antiviral agents do not seem to alter the course of the disease. ECMO support may be considered for rescue ventilation of patients who present with worsening respiratory failure, but this is often associated with prolonged ICU stay and overall mortality remains high.
Nutrition, energy expenditure and prognosis

### 0877

#### Resting energy expenditure in elderly critically ill patients and matched healthy control cohort - a comparison of predictive equations versus indirect calorimetry

M. Kott[1], K. Rembarz[2], D. Bläser[1], M.J. Müller[2], N. Weiler[1], I. Frerichs[1], G. Elke[1]
[1]Universitätsklinikum Schleswig-Holstein, Campus Kiel, Department of Anesthesiology and Intensive Care Medicine, Kiel, Germany; [2]Christian-Albrechts-Universität Kiel, Institut für Humanernährung und Lebensmittelkunde, Kiel, Germany
**Correspondence:** M. Kott
*Intensive Care Medicine Experimental* 2017, **5(Suppl 2):**0877

**INTRODUCTION.** Elderly patients represent a growing proportion of critically ill patients with a high nutritional risk. Therefore, estimation of resting energy expenditure (REE) should be accurate to prevent over- or underfeeding.
**OBJECTIVES.**
1) To analyse REE measured by indirect calorimetry (mREE) in intensive care unit (ICU) patients aged ≥75 years as compared to REE calculated by predictive equations (cREE).
2) To compare mREE in these patients with mREE in matched healthy controls
3) To identify variables independently associated with possible mREE differences between ICU patients and healthy controls.

**METHODS.** Prospective observational matched cohort study in a tertiary medical centre. Eligible were mechanically ventilated patients aged ≥75 years with an $FiO_2$ of ≤0.6. REE was measured on the first day of ICU stay using the M-CVOX device (GE Healthcare, Helsinki, Finland). cREE was calculated using the following equations: ACCP, Ireton-Jones, Faisy-Fagon, PennState, Müller, and Harris and Benedict. Bland Altman test was used to analyse agreement between mREE and cREE in ICU patients. The clinically acceptable range of cREE was defined as mREE ± 10%. Healthy volunteers from a prospectively collected institutional database were matched to the ICU patients by age, sex, and body mass index. Measurement of REE in the control group was performed using indirect calorimetry by a ventilated canopy (CareFusion, Yorba Linda, USA). Multivariate linear regression analysis was performed to identify variables that influence differences in mREE.

**RESULTS.** We included 90 elderly ICU patients (median age: 80 years, 25%/75% percentile: 77–84 years). The median cREE differed from mREE in the equations of Ireton-Jones (Δ mREE - cREE: −109 kcal/d [25%/75% percentile: −297 kcal/d - 147 kcal/d], p = 0.085; proportion of patients within ± 10% of mREE: 36.7%; $R^2$ in ICU patients: 0.615), Faisy-Fagon (−353 kcal/d [−486 - 96], p < 0.0001; 23.3%; 0.318), PennState (+54 kcal/d [−105 - 248], p = 0.023; 48.9%; 0.211), Müller (+105 kcal/d [−82 - 334], p < 0.0001; 41.1%; 0.326), and Harris und Benedict (+139 kcal/d [23–407], p < 0.0001; 40%; 0.377), but not in ACCP (−33 kcal/d [−247 - 288], p = 0.641; 21.1%; 0.448). In the matched pair analysis (n = 58), mREE in the ICU cohort was higher compared to the healthy control (1457 kcal/d [1247–1876] vs. 1351 kcal/d [1187–1503], p = 0.008). Maximum temperature (p = 0.008), respiratory rate (p = 0.002) and $FiO_2$ (p = < 0.0001) were independently associated with differences in mREE in the matched pair cohort.

**CONCLUSIONS.** REE calculated by established equations systematically over- or underestimated measured REE, respectively in up to 80% of elderly critically ill patients. Measured REE in these patients was significantly higher compared to healthy controls matched by age, gender and body mass index. Physiological variables independently associated with a higher mREE may in part explain this difference.

None

**GRANT ACKNOWLEDGMENT**

None

### 0878

#### Bodyweight- adjusted energy expenditure deceases with age in critically ill medical patients

M. Wewalka, M. Schmid, M. Schneeweiss, B. Seeland, C. Zauner

Medical University of Vienna, Internal Medicine III, Gastroenterology and Hepatology, Vienna, Austria

**Correspondence:** M. Wewalka

*Intensive Care Medicine Experimental* 2017, **5(Suppl 2):**0878

**INTRODUCTION.** Resting energy expenditure (REE) is known to decline with age in healthy subjects. This decline is independent of the age-related decrease in lean body mass (1). Whether this holds true in critically ill medical patients has not been investigated so far.

**OBJECTIVES.** To evaluate the changes of energy metabolism and its association with age in critically ill medical patients.

**METHODS.** 200 critically ill medical patients with need for mechanical ventilation underwent indirect calorimetry within 72 hours of admission after an overnight fast to determine REE. REE was adjusted for body weight (aBW). To obtain age groups, patients were divided into quartiles (I: 18–35 years, n = 21; II: 36–52 years, n = 43; III: 53–69 years, n = 93; IV: 70–86 years, n = 43). Sex, SAPS II score, temperature at time of intervention, height, weight and BMI were assessed. Kruskal-Wallis Test was used for group comparisons. Parameters that were significant in univariate regression analysis entered the multivariate regression model.

**RESULTS.** Groups differed significantly in age (P < 0.01). SAPS II score (P < 0.01) and BMI (P = 0.01) increased with age, whereas body temperature was highest in the youngest patients (P = 0.02). REE was similar in all groups (I: 1927 (1489–2289), II: 1767 (1413–2015), III: 1625 (1348–1819), IV: 1648 (1310–1843) kcal/day; P = 0.09), yet, REEaBW significantly decreased with age (I: 27 (23.4-32), II: 21.4 (19.6-24.3), III: 20.9 (17.4-23.4), IV: 18.7 (16.7-22) kcal/kg/day, P < 0.01).

In the multivariate model with REE as the dependent variable and age, height, BW, BMI, body surface area, and temperature as the independent variables, only age (R = −4.95 (95%CI −7.91 to −1.99, P = 0.01)) and temperature (R = 108.64 (95%CI 69.03-148.24, P < 0.01)) remained independent predictors. In a multivariate model with REEaBW as the dependent variable, both age (R = −0.99 (95% CI −0.14 to −0.06), P < 0.01) and temperature (R = 1.25 (95% CI 0.64-1.85, P < 0.01) were significant independent predictors.

**CONCLUSIONS.** REEaBW decreases with age in critically ill medical patients. In multivariate regression analysis, age and temperature are independent predictors of both REE and REEaBW.

**REFERENCE(S)**

(1) Fukagawa NK, Bandini LG, and Young JB. Effect of age on body composition and resting metabolic rate.Am J Physiol Endocrinol Metab 259: E233-E238, 1990.

**GRANT ACKNOWLEDGMENT**

Nothing to declare.

### 0879

#### The obesity paradox in critically ill patients: a protective factor?

N.M. Filgueiras Filho[1,2,3], J.C. Oliveira[4,5], R.L. Silva[2,5], R.C. Menezes[5], I.B. Ferreira[1,5], A.L. Silva[2,5], C.S. Ballalai[2,6], C.V. De Lucia[2,6], A.L. Gobatto[7], GEMINI

[1]Universidade do Estado da Bahia, Medicina, Salvador, Brazil; [2]Universidade Salvador, Medicina, Salvador, Brazil; [3]Hospital da Cidade, Intensive Care Medicine, Salvador, Brazil; [4]Escola Bahiana de Medicina e Saúde Pública, Medicina, Salvador, Brazil; [5]Núcleo de Ensino e Pesquisa - Hospital da Cidade, Medicina Intensiva, Salvador, Brazil; [6]Núcleo de Pesquisa Clínica - UNIFACS, Medicina, Salvador, Brazil; [7]Hospital da Cidade, Medicina Intensiva, Salvador, Brazil

**Correspondence:** N.M. Filgueiras Filho

*Intensive Care Medicine Experimental* 2017, **5(Suppl 2):**0879

**INTRODUCTION.** Obesity is a worldwide health issue associated with increased morbidity and mortality in the general population. However, the impact of obesity on outcomes in patients admitted to the Intensive Care Unit (ICU) has not been definitively determined. A paradoxical connection between higher Body Mass Index (BMI) and lower mortality has been referred to as the obesity paradox in critically ill patients.

**OBJECTIVE.** To evaluate the impact of the body mass index (BMI) on mortality in patients admitted to the ICU of a general hospital in Northeast region of Brazil.

**METHODS.** Prospective cohort study placed in a general ICU. In the study were included all patients above age of 18 years old admitted to general ICU, from August 2015 to April 2017. BMI was calculated using the formula: BMI = body weight/height$^2$ (kg/m$^2$), and patients were grouped as underweight (<18.5 kg/m2), normal weight (18.5-24.9 kg/m2), overweight (25–29.9 kg/m2) and obese (≥30 kg/m2). To determine the hazard ratio of ICU death, we developed a multivariate Cox regression.

**RESULTS.** There were analyzed 1177 patients, 605 (51.4%) female. Mean age was: 67 ± 18 years. The mean BMI was 25.5 ± 5.5 kg/m$^2$, 42.7% had normal BMI, 7.9% were underweight, 32.3% overweight and 17.1% obese. The ICU mortality rates in the whole cohort was 18.1%. On multivariate Cox regression analysis, being obese (HR: 0.547; 95% CI, 0.338 - 0.888; p = 0.015) was independently associated with a lower mortality, while underweight (HR: 1.547; 95% CI, 1.055 - 2.269; p = 0.025) showed a higher mortality risk.

**CONCLUSION.** The results showed that being obese was associated with decreased risk of ICU mortality, while being underweight showed a higher ICU mortality risk for this sample.

### 0880

#### A new prognostic marker in the intensive care unit (ICU): the psoas muscle area index measured by abdominal computed tomography (CT) targeted on the third lumbar vertebra (L3)

E. Lascouts[1], A. Mulliez[2], G. d'Assignies[3], N. Rotovnik Kozjek[4], A.-M. Makhlouf[5], I. Ceniceros Rozalen[6], J.-C. Preiser[7], Z. Krznaric[8], K. Kupczyk[9], N. Cano[10], C. Pichard[11], R. Thibault[12,13], Phase Angle Project Investigators
[1]CHU Rennes, Rennes, France; [2]CHU Clermont-Ferrand, DRCI, Clermont-Ferrand, France; [3]CHU Rennes, Service de Radiologie, Rennes, France; [4]Institute of Oncology, Clinical Nutrition Unit, Ljubljana, Slovenia; [5]Hôpitaux Universitaires de Genève, Nutrition Unit, Geneva, Switzerland; [6]Clinic USP Palmaplanas, Department of Intensive Care, Palma de Majorque, Spain; [7]Hôpital Universitaire Erasme, Department of Intensive Care, Brussels, Belgium; [8]University Hospital Center, Departement of Internal Medicine, Zagreb, Croatia; [9]University Hospital No 1 CM NCU, Department of Anesthesiology and Intensive Care, Bydgoszcz, Poland; [10]CHU Clermont-Ferrand, CRNH, Clermont-Ferrand, France; [11]Hôpitaux Universitaires de Genève, Nutrition unit, Geneva, Switzerland; [12]CHU Rennes, Nutrition unit, Rennes, France; [13]Institut NuMeCan, INRA-INSERM-Université Rennes 1, Rennes, France
**Correspondence:** R. Thibault
*Intensive Care Medicine Experimental* 2017, **5(Suppl 2):**0880

**INTRODUCTION.** Critical illness leads to fat-free mass loss that worsens prognosis (1). Fat-free mass included muscle mass, bone tissue, and total body water. No standardized method of muscle assessment is validated for the clinical practice.

**OBJECTIVES.** To assess whether psoas muscle mass is associated with day (D) 28 mortality in the ICU.

**METHODS.** Ancillary study of the international PHASE ANGLE (PhA) PROJECT (1). Inclusion criteria: adult medical / surgical ICU, length of stay >48h, no pacemaker or defibrillator, routine abdominal CT within 9 days postadmission. Transversal (TDPM) and axial right psoas diameters were measured on a single L3 CT image by a non-expert-operator blinded of D28 mortality. Psoas area index (PAI) = TDPM*ADPM*1/height(m)$^2$. PhA measured by bioimpedance analysis (BIA) at admission. Statistics: area under the Receiver Operating Characteristic (ROC) curve (AUC) evaluating D28 mortality by PAI. Factors associated with D28 mortality: multivariable logistic regression (adjusted odd ratio (aOR)
[95% confidence interval]).

**RESULTS.** Among the 931 patients analysed in PhA PROJECT, 193 were performed abdominal CT; up to date, 154 patients were included (we plan to show the analysis for 200 patients at ESICM congress): Ljubljana (n = 49), Geneva (n = 43), Palma de Majorque (n = 31), Brussels (n = 15), Zagreb (n = 13), Bydgoszcz (n = 3); 64 ± 14 yrs, men 54%, body mass index 26.1 ± 5.1, APACHE II 19 ± 9. PAI was very easy and quick to be measured by non-expert operator. TDPM (10 ± 4 vs 14 ± 4 mm/m, p = 0.004) and PAI (245 ± 102 vs 363 ± 158 mm/m$^2$, p = 0.007) were lower in non-survivors than in survivors in men (non-significant in women). AUC ROC men: 0.75 [0.61-0.88]; women: 0.56 [0.32-0.81]. PAI thresholds associated with D28 mortality: < 337.3 (men); < 170.1 (women) mm/m$^2$. Were associated with D28 mortality: admission for pneumonia (aOR 14.84 [1.19-185.25], p = 0.04), low PAI (5.87 [1.97-17.51], p = 0.001), age (1.05 [1.01-1.10], p = 0.01), and admission PhA (0.52 [0.31-0.87], p = 0.01).

**CONCLUSIONS.** Muscle mass loss defined by psoas area index on L3-targeted CT scan is associated with D28 mortality in the ICU patients.

**REFERENCE(S)**

(1) Thibault R et al. Intensive Care Med 2016;42:1445–53

**GRANT ACKNOWLEDGMENT**
European Society for Clinical Nutrition and Metabolism (ESPEN).

### 0881

#### Effect of vitamin D levels on mortality in criticallyill patients: single center observational study

M. Sungur, G. Elay, K. Gundogan, R. Coskun, I.H. Akbudak, Z. Simsek, M. Guven
Erciyes University, School of Medicine, Intensive Care Unit, Kayseri, Turkey
**Correspondence:** M. Sungur
*Intensive Care Medicine Experimental* 2017, **5(Suppl 2):**0881

**INTRODUCTION.** Vit D insufficiency is common in hospitalized patients and especially in critically ill patients. Vit D insufficiency in critically ill patients may worsen immune and metabolic dysfunctions and may result in worse outcomes.

**OBJECTIVES.** To assess vitamin D levels during acute and chronic disease periods in critically ill patients and to investigate its effect on clinical parameters.

**METHODS.** His prospective study was conducted at medical Intensive Care Unit of Erciyes University, Medical School. The study included patients aged ≥18 years who werea dmiddet to medical intensive care unitfor > 48 hours. Vitamin D levels were measured at baseline and on the days 3, 7 and 10.

**RESULTS.** Overall, 62 patients were recruited to the study, including 30 men (48%) and 32 women (52%). Mean age was 53 ± 20 years. Median vitamin D levels at baseline and on the days 3 and 7 were12.8 (2.80-104.0) mcg/L, 8.35 (1.80-96.30) mcg/L, 9.30 (4.60-37.00) mcg/L, respectively. Low vitamin D level was detected in 84% of patients at baseline whereas in 90% of patients on the day 3. No significant correlation was detected between ICU mortality and 6-month mortality rate and vitamin D levels (p > 0.05).

**CONCLUSIONS.** Vitamin D level was found to be low at admission to ICU and during follow-up. No significant correlation was detected between vitamin D levels and mortality.

**REFERENCE(S)**

1.P. Lee, J. A. Eisman, and J. R. Center, "Vitamin D deficiency in critically ill patients,"The New England Journal ofMedicine, 360: 1912–1914, 2009.
2.P. Lee, "How deficient are vitamin D deficient critically ill patients?" Critical Care,15: 154, 2011.
3.L. R. Matthews, Y. Ahmed, K. L. Wilson, et al., "Worsening severity of vitamin D deficiency is associated with increased length of stay, surgical intensive care unit cost, and mortality rate in surgical intensive care unit patients,"American Journal of Surgery, 204: 37–43, 2012.

**GRANT ACKNOWLEDGMENT**
This project was supported by Erciyes University Scientific Research Unit.

### 0882

#### The mortality of elderly patients with severe accidental hypothermia in our intensive care unit

R. Tate
Aso Iizuka Hospital, Intensive Care Medicine, Iizuka, Japan
*Intensive Care Medicine Experimental* 2017, **5(Suppl 2):**0882

**INTRODUCTION.** Accidental hypothermia is classified into HT I~IV by Swiss staging system. Patients in HT IV are recommended for rewarming by ECMO[1]. The use of ECMO as rewarming method for patients in HT III may be considered in the situations of life-threatening arrhythmia, hypotention(SBP < 90mmHg) etc[2]. But generally elderly patients are not with no medical justification of ECMO. in Japan, aging society is proceeding and the age of accidental hypothermic patients may increase.

**OBJECTIVES.** To evaluate the mortality of elderly (older than 70 years) patients admitted with accidental hypothermia to our intensive care unit(ICU).

**METHODS.** A retrospective study was conducted in our ICU during January of 2012 to March of 2017, containing 9 patients admitted with accidental hyperthermia to our ICU by searching the electronic medical records database. Demographic variables, core temperature and Swiss staging system on arrival, rewarming method, neurological prognosis, ICU mortality and hospital mortality are collected.

**RESULTS.** The median age of our patient collection was 81.9 years(3 patients were 90 years or older), 30% were male. 2 patients were CPA on arrival, 1 patients turned to CPA after arrival. In regard to Swiss staging system, 7 patients were HT III and 2 patients were HT IV. Rewarming methods were "active external and minimally invasive rewarming" in 4 patients, "Intravascular catheter rewarming" in 1 patients and V-A ECMO in 5 patients(2 were 90 years or older).ICU mortality was 40% and hospital mortality was 50%(1 patients died of cerebral infarction). 3 of 5 patients for whom V-A ECMO was used as rewarming method were able to discharged from ICU. In regard to neurological prognosis, all of 5 patients who were discharged from hospital recovered from accidental hypothermia almost perfectly.

**CONCLUSIONS.** For only old age, we should not hesitate to implement V-A ECMO for accidental hypothermic patients. Further studies are needed to show the effect of V-A ECMO for very elderly and accidental hypothermic patients.

**REFERENCE(S)**

1. Douglas J.A. Brown, M.D., Hermann Brugger, M.D., Jeff Boyd, M.B., B.S.,and Peter Paal, M.D.
N Engl J Med 2012;367:1930–8

2. Peter Paal, Les Gordon, Giacomo Strapazzon, Monika Brodmann Maeder, Gabriel Putzer, Beat Walpoth, Michael Wanscher, Doug Brown, Michael Holzer, Gregor Broessner and Hermann Brugger
Scandinavian Journal of Trauma, Resuscitation and Emergency Medicine(2016) 24:111

### 0883

#### The clinical characteristics and risk factors of critical illness-related corticosteroid adrenal insufficiency

S.Y. Park[1], S.Y. Yu[1], J.H. Kim[1], J.H. Lee[2], S.J. Park[1], H.B. Lee[1]
[1]Chonbuk National Universiy Hospital, Internal Medicine, Jeonju, Korea, Republic of; [2]Chonbuk National Universiy Medical School, Preventive Medicine, Jeonju, Korea, Republic of
**Correspondence:** S.Y. Park
*Intensive Care Medicine Experimental* 2017, **5(Suppl 2):**0883

**INTRODUCTION.** Cortisol is necessary to survive critical illness, but the optimal level is unclear. Absolute adrenal insufficiency is rare among critically ill patients and the incidence is estimated to be less than 3%.

However, relative adrenal insufficiency (RAI) has been reported as a predictor of mortality in critical illnesses including septic patient, its effects on clinical outcome and risk factors for critically ill patients are controversial.

**OBJECTIVES.** We conducted the present study to assess the clinical characteristics and risk factors of RAI in critically ill patients.

**METHODS.** We retrospectively assessed the medical records of 237 patients who checked a random cortisol in the Medical Intensive Care Unit (MICU) at the Third University Hospital, from January 2016 to February 2017.

RAI was defined a random serum cortisol level of less than 15 μg/dL or random cortisol level of 15 to 34 μg/dL with post-cosyntropin (250 μg) stimulation cortisol levels to increase less than 9 μg/dL.

**RESULTS.** Of the 237 cases, 62 patients with a risk of RAI (random cortisol levels < 34 ug/dL) were included, and ultimately 48 patients had relative adrenal insufficiency (77.4%).

The length of MICU stay day (12.7 ± 10.2 vs. 10.3 ± 9.6, p = 0.437) and mortality rate (27/48 vs. 10/14, p = 0.308) were not statistically different between two groups (RAI + vs RAI -, respectively). There was no significant difference between the RAI positive group and the negative RAI group in baseline characteristics such as age, sex and other clinical features.

In subjects who had random cortisol levels of 15 μg/dL to 34 μg/dL (n = 33), the incidence of RAI was 19 (57.6%) and a positive result of cosyntropin stimulation test was associated with sepsis-related syndrome (16/33, 84.2%, OR 9.27, 95% CI 1.25-68.70).

**CONCLUSIONS.** In critically ill patients, RAI is relatively common but it was not associated with deteriorated outcomes in this study. However, in subjects with random cortisol levels between 15 μg/dL and 34 μg/dL, sepsis-related syndrome may be a risk factor for relative adrenal insufficiency.

### 0884

#### Artificial nutrition evaluation in the ICU: energy balance and protidemia assay contribution

T. Khzouri, M. Fatnassi, A. Ben Jazia, R. Sebaii, N. Brahmi
Toxicological Intensive Care Unit Tunis, Toxicological Department, Tunis, Tunisia
**Correspondence:** T. Khzouri
*Intensive Care Medicine Experimental* 2017, **5(Suppl 2):**0884

**INTRODUCTION.** The modalities and efficacy evaluation of parenteral nutrition among critical ill patients have been the subject of several studies which led to recommendations.

**OBJECTIVES.** We carriedout a study which aims to assess the degree of our practice adherence to these recommendations, to evaluate protidemia assay contribution in the assessment of nutritional status and its impact on morbidity and mortality.

**METHODS.** It is a prospective observational study spread over 12 months from January to December 2012 in a teaching intensive care unit including all patients admitted in our ICU for a stay longer than seven days and required artificial nutrition.

**RESULTS:** A total of 42 patients aged of 42 ± 17 were eligible; they were 26 males and 16 females, admitted for medical reason.Thegravityscoreswererespectively 46.8 ± 14.9 for SAPS II, 19.7 ± 7 for APACHE II and 7.6 ± 3.5 for SOFA.A pre-existing hypoprotidemia of 51 ± 5.7 g/l was detected in twenty seven patients (64%), 26 among them responded to nutritional depletion criteria according to French National Authority for Health score with a hypoalbuminemia mean of 20.7 ± 4.74 g/l.During week, 1$^{st}$ 24 patients (57%) had received a total parenteral nutrition, 6 patients (14%) an entirely enteral feeding and 10 (24%) benefited from mixed nutrition. A nutritional support was provided during 72 ± 19% of the whole hospital stay.

Descriptive study showed that during the three first days, prescribed and really received caloric intakes (Kcal) were widely below those required; with a caloric supply average of 8.67 ± 9.10 Kcal/kg/j. Caloric supply has steadily increased to reach 15.61 ± 8.58 at the end of the first week and to stabilize at 21.8 ± 7.74 Kcal/kg/24h at the second week.Our results concluded that enteral feeding was started from the second day, and mixed one for 24% of the whole first week with a caloric supply lower than 20 Kcal/kg/j for 29 patients (69%) who are considered as undernourished group and an adequate delivery ≥ 20 Kcal/kg/j for 13 patients (31%). Moreover, protein intakes average was of 0.52 ± 0.28 g/kg/j during the first week.

The ICU mortality was 45.2% (n = 19). The analysis of risk factors associated to morbidity and mortality showed that only initial plasma protidemia level is strongly correlated to infectious complications occurrence (p = 0.022) with no effect on mortality. No correlation was noted between plasma protidemia level and energy balance with $R^2$ respectively of 0.009 (during the 1$^{st}$ weed), 0.2 (during the 2$^{nd}$ week) 0.043 (during the 3$^{rd}$ week) and 0.12 (during the 4$^{th}$ week).

**CONCLUSIONS.** Despite adherence to the recommendations was not always respected and our patients were heterogeneous,our results were similar to those reported in literature. To build on protedemia rate to evaluate nutrition´s efficiency, can't be definitely recommended due to the protein metabolism imbalance in the initial phase of assault. However, it can be considered as a biological parameter predicting morbidity.

### 0885

#### Does admission vitamin D level reflect chronic health status and predict outcome of critical illness?

A. Khaldi, D.H. Prevedello, J.-C. Preiser
Université Libre de Bruxelles / Hôpital Erasme, Intensive Care, Brussels, Belgium
**Correspondence:** A. Khaldi
*Intensive Care Medicine Experimental* 2017, **5(Suppl 2):**0885

**INTRODUCTION.** Vitamin D deficiency is frequent in Northwestern countries. In critically ill patients, the role of vitamin D can be important.

**OBJECTIVE.** The objective was to identify if vitamin D deficiency could be associated patients' chronic co-morbidity, the severity of acute illness and ICU- and hospital-related outcomes.

**METHODS.** Consecutive patients admitted over a 2- month period in a medical/surgical 32-bed ICU were included. In these patients, demographic data, Charlson co-morbidity score, severity scores (SAPS 3 and SOFA) and 25-OH vitamin D (chemiluminescence, DiaSorin) were collected at admission. ICU and hospital length of stay (LOS) were collected. The coefficients of determination ($R^2$) between 25-OH vit D and Charlson score, SAPS 3, SOFA, ICU and hospital LOS were calculated.

**RESULTS.** 457 patients were included (59 ± 19 years, gender (257 males), category of admission (38% medical, 62% surgical) SAPS III (44 ± 20), SOFA (3,1 ± 3,7), Charlson (1,9 ± 2,1). 25-OH vit D levels was measured in 299 patients at ICU admission and was averaged 17,5 ± 10,4 ng/ml ; 61 (20%) patients were moderately deficient (level >20 - ≤ 30 ng/ml), 80 (27%) patients were deficient (>12- ≤ 20 ng/ml) and 124 (42%) patients were severely deficient (≤12 ng/ml).$R^2$ between 25-OH vit D and Charlson score, SAPS 3, SOFA, ICU and hospital LOS were 0,006; 0.01; 0,002; 0,001; 0,0004, respectively.

**CONCLUSIONS.** In the population studied, vitamin D deficiency is frequent. However, there was no correlation between vitamin D, chronic or acute morbidity and ICU- and hospital LOS.

**GRANT ANKNOWLEDGMENT**
This research received no grant from any funding agency in the public, commercial, or not-for-profit sectors.

### 0886

#### A systematic scoping review on the incidence and risk factors of hyperglycaemia in ICU adult patients

M. Miret[1], A. Danel[1], E. Olariu[2], N. Pooley[2], J.-C. Preiser[3]
[1]Nestlé Health Science, Vevey, Switzerland; [2]PHMR Ltd, London, United Kingdom; [3]Erasme University Hospital, Université Libre de Bruxelles, Department of Intensive Care, Brussels, Belgium
**Correspondence:** J.-C. Preiser
*Intensive Care Medicine Experimental* 2017, **5(Suppl 2):**0886

**INTRODUCTION.** Stress hyperglycaemia(SHG) is common in critically ill patients and is strongly associated with poor outcomes in intensive care unit(ICU) patients. Many studies have been conducted to gain an insight into the epidemiology of SHG, but a general overview of the data is lacking.

**OBJECTIVE.** To map the literature describing the epidemiology of SHG in ICU patients to identify the prevalence, the incidence of SHG and the risk factors(RF) for developing SHG.

**METHODS.** We followed the Joanna Briggs methodology on systematic scoping reviews to identify relevant publications in Medline, Embase, and The Cochrane Library from January 2000 to December 2015. Two reviewers assessed studies for eligibility. Studies were included if they reported on either the prevalence or incidence of hyperglycaemia or risk factors for hyperglycaemia in adult ICU patients. No definition for hyperglycaemia or RF was set a-priori. Data extraction was performed by one reviewer and checked by another. The results presented here are for incidence and RF(if RF were reported in more than one study).

**RESULTS.** We identified 3062 relevant records: 385 full-text articles were screened and 92 studies(8,082,774 patients) were included in the review. Of these, 10(13,773 patients) reported on the incidence of SHG in the ICU. The included studies were conducted in general ICU patients (606 patients), stroke patients (1,385 patients), trauma patients (1,350 patients), cardiac surgical patients
(1,565 patients), a mix of medical and surgical ICU patients (8,307 patients), and surgical ICU patients (560 patients). Incidence was defined in most of the studies (seven) as new cases of hyperglycaemia that developed during the ICU stay without mentioning a specific time period. Studies were mainly conducted in samples with mixed diabetic status (five studies;3,026 patients) and very few in non-diabetic patients (two studies;1,606 patients). Incidence levels varied widely, most probably due to variations in hyperglycaemia definitions (109 mg/dl to 240 mg/dl). SHG incidence ranged from 9.6% (blood glucose level [BGL] > 180 mg/dl) to 99% (BGL > 109 mg/dl). Studies reporting on the incidence of SHG in ICU patients are highly heterogeneous limiting the comparability of results across studies.
RF for SHG were reported in five studies, with diabetes being the most frequently reported RF
(two studies;3,257 patients).

**CONCLUSIONS.** Stress hyperglycaemia is frequent in critical care patients irrespective of their underlying condition. Assessing the risk factors at admission may help controlling the incidence of stress hyperglycaemia and setting up appropriate therapeutic and nutritional measures. Homogeneity in blood glucose cut off points as well as time of measurement is determinant to compare study results.

Grant Acknowledgement
This work was supported by Nestlé Health Science. AD,MM are permanent employees of Nestlé Health Science.EO,NP,JCP have received consulting fees from Nestlé Health Science.

### 0887

#### The novel use of point of care ultrasound to predict resting energy expenditure in critically ill patients: prospective observational study

M. Abdel Ghany, E. Ayman, W. Hamimy, A. Mukhtar
Cairo University, Cairo, Egypt
**Correspondence:** M. Abdel Ghany
*Intensive Care Medicine Experimental* 2017, **5(Suppl 2):**0887

**INTRODUCTION.** There is increasing interest in the use of ultrasound to assess and guide the management of critically ill patients.

**OBJECTIVES.** The objective of this study was to evaluate the novel use of point of care ultrasound to predict the resting energy expenditure (REE) measured by indirect calorimetry in critically ill patient.

**METHODS.** All consecutive patients that were admitted to intensive care unit (ICU) and required mechanical ventilation were enrolled in the study. Bed side ultrasound was used to measure muscle layer thickness (MLT) and cardiac output (CO). MLT was measured within 24 of ICU admission. Two measurements of MLT were made at two sites; mid-upper arm and thigh anteriorly using linear array transducer. MLT was determined and summed up. Stroke volume was obtained by placing pulsed wave Doppler in the middle of left ventricular outflow tract using phased array transducer. CO was calculated by multiplying SV and heart rate. REE was calculated using indirect calorimetry via metabolic module on General Electric ventilator (Engstrom Carestation and Carescape R860, GE Health care, USA). A multiple regression analysis was applied to a set of independent variables to develop the predictive equation of REE.

**RESULTS.** Forty patients were enrolled in the study. The mean (sd) REE was 1995 (414) kcal/d. Twenty five (62.5%) patients had septic shock at ICU admission. A multiple regression model using the aggregate ultrasound muscle thickness measures with estimates of CO yields an adjusted $R^2$ of 0.7 ($p$ < 0.0001). REE (kcal/d) = 128 + 159.7*CO+ 148.5* MLT. Neither age nor gender made significant contribution in predicting REE.

**CONCLUSIONS.** This study demonstrated that point of care ultrasound could be a useful tool in predicting REE of critically ill patients through measurement of both MLT and CO.

**GRANT ACKNOWLEDGMENT**
Funded by Cairo University

### 0888

#### Does the ratio between ICU admission blood glucose and chronic glycemia predict the outcome?

D.H. Prevedello, A. Khaldi, J.-C. Preiser
Université Libre de Bruxelles / Hôpital Erasme, Intensive Care, Brussels, Belgium
**Correspondence:** D.H. Prevedello
*Intensive Care Medicine Experimental* 2017, **5(Suppl 2):**0888

**INTRODUCTION.** Optimal blood glucose levels (BG) in critically ill patients are mostly unknown and could differ according to the diabetic status. In patients with diabetes, the usual BG level can be deducted from HBA1c using the formula eAG (estimated average glucose) = (AG (mg/dl) = 28.7 x A1C - 46.7, R (2) = 0.84, P < 0.0001)[1]. As HbA1c doesn't suffer influence from diseases in acute phase, it is a safe diagnostic marker of diabetes[2], and we could estimate a confiable average glucose during this period.

**OBJECTIVE.** Identify if the ratio between admission BG and estimated average glucose (eAG) was related to the patients' chronic comorbidity, severity of acute illness and ICU- and hospital-related outcomes.

**METHODS.** Observational study. Consecutive patients admitted over a 2- month period in a medical/surgical 32-bed ICU were included. In these patients, demographic data, Charlson comorbidity score, frequency of known diabetes mellitus (DM), unknown DM (HBA1c > 6.5% without prior diagnosis of DM) severity scores (SAPS 3 and SOFA), BG and HBA1c were collected at admission. As well as, ICU and hospital length of stay (LOS) were collected. Glycemia ratio (GR) was calculated as the ratio between admission BG and eAG (derived from HBA1c)[1]. The coefficients of determination (linear regression = $R^2$) between GR and Charlson score, SAPS 3, SOFA, ICU and hospital LOS were calculated.

**RESULTS.** 316 patients were included from 483 admissions (excluded < 18 years and patients without BG or HbA1c at ICU admission). The mean age was 59 years (SD 16) and 58, 23% represented by men. We analyzed the categories of admission (35, 76% as medical, 53, 2% as surgical), SAPS III average (44,8 /SD 20,07), SOFA in first-ICU day (mean 3,14/SD 3,6) and also Charlson score (mean 1,87/SD 2,02). Among included patients, known DM was in 51 patients (15, 6%), unknown DM in 8 (3, 13%). Mean HBA1c, admission BG and GR were 5,78 (SD 1,02); 151 (SD 65,32); 1,28 (SD 0,43), respectively. GR was ≥ 1 in 225 patients (71, 2%). Linear regression between GR and Charlson score, SAPS 3, SOFA, ICU and hospital LOS were 0,0007, 0,007, 0,02, 0,0004, 0,004, respectively.

**CONCLUSIONS.** In the population studied, stress hyperglycemia was frequent as indicated by the value of GR ≥1. However, there was no correlation between GR, chronic or acute morbidity and ICU- and hospital LOS.

**GRANT ANKNOWLEGMENT**
This research received no grant from any funding agency in the public, commercial, or not-for-profit sectors.

### 0889

#### Nitrogen balance in nutritional monitoring in critical patients

C.R.F. Lordani[1], R.G. Eckert[2], T. Lordani[3], I.R. Porto[4], P.A.D. Duarte[4,5], N.M.P. Valério[6]
[1]Hospital Universitário do Oeste do Paraná, Dept of Nutrition, Cascavel, Brazil; [2]Hospital do Câncer / UOPECCAN, Dept of Nutrition, Cascavel, Brazil; [3]Hospital Universitário do Oeste do Paraná, Cascavel, Brazil; [4]Hospital Universitário do Oeste do Paraná, General ICU, Cascavel, Brazil; [5]Hospital do Câncer / UOPECCAN, General ICU, Cascavel, Brazil; [6]Universidade Estadual do Oeste do Paraná, Cascavel, Brazil
**Correspondence:** I.R. Porto
*Intensive Care Medicine Experimental* 2017, **5(Suppl 2):**0889

**INTRODUCTION.** Protein catabolism is an important accompaniment to critical illness, particularly when there is an intense inflammatory process. Nitrogen Balance (NB) can be a useful tool in monitoring and individualizing the proteic nutritional delivery in critical patients. The objective of this study was to evaluate the usefulness of NB in nutritional monitoring and to identify risk factors related to the status of protein catabolism, and to evaluate the evolution of NB at different moments, seeking to correlate with outcomes in the ICU.

**METHODS.** Retrospective cohort study. Data from consecutive adult patients admitted to the ICUs of two hospitals were evaluated. NB was calculated on the 1st, 5th and 10th day, only in the ICU.

**RESULTS.** A total of 234 patients (63.7% male, 52.7 years old, 17.9% medical, 15.8% trauma, 60.7% elective surgery, APACHE 18.3) were evaluated. In the evaluation of the 1st NB, there were no patients with NB positive. The most of patients had nitrogen loss between 0 and −5 g (41.0%) or between −5 and −10 g (23.1%); 17.5% had severe hypercatabolism. This pattern didn't change in the evaluation of the 2nd and 3rd NB, although in the second evaluation there was a mild trend (not significant) to less values of severe hypercatabolism. The factors correlated with a more negative NB were the use and time of invasive MV, trauma or medical cause of admission, and COPD or cancer comorbidities. There was no correlation between NB values (at any time of hospitalization) and higher mortality. On the other hand, when comparing patients whose 1st NB had higher or lower catabolism, the variation for the 2nd NB (that is, the maintenance of catabolism or induction of anabolism) showed that, in patients with initial normal or mild catabolism, the survivors had a fall (more negative) of NB between the 1st and 2nd NB, while the patients who died had an increase of this value (becoming less negative). Among the patients with higher hypercatabolism, both groups (survivors and death) had a positive variation, but the survivors had an even more positive difference (that is, they became less negative in the 2nd NB) (Fig. 286).

**CONCLUSION.** In critically ill patients, NB evaluation showed almost universal hypercatabolism. Serial BN assessment could identify patterns of catabolism and response to therapy.

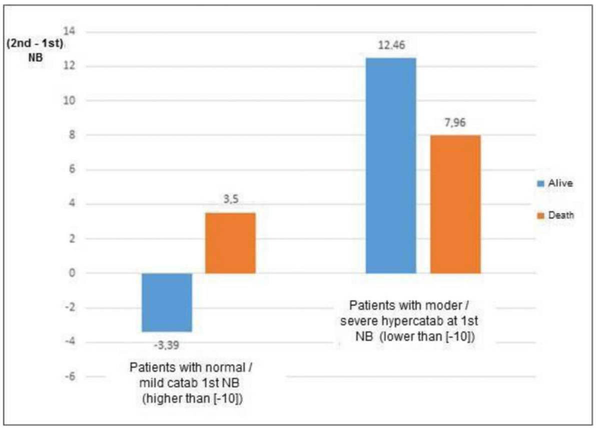

**Fig. 286 (Abstract 0889).** Variation between 1st and 2nd NB, in grams, according to the value of 1st NB. Positive values indicate that the BN value was higher in the 2nd evaluation (5th day) than in the 1st evaluation (admission)

#### 0890

#### The severity of catabolic reactions in patients with traumatic brain injury

A. Shakotko, Z. Marutyan, S. Petrikov, V. Movsisyan, A. Evdokimov, A. Kinichemova
N.V. Sklifosovsky Research Institute of Emergency Medicine of the Moscow Healthcare Department, Moscow, Russian Federation
**Correspondence:** Z. Marutyan
*Intensive Care Medicine Experimental* 2017, **5(Suppl 2):**0890

**INTRODUCTION.** Dire period of TBI is characterized by the elaboration of severe catabolic reactions. However, the level of catabolism with concomitant traumatic brain injury (SCMT) in patients is staying insufficiently studied.

**AIM.** The aim of work is to conduct a comparative analysis of the severity of catabolic reactions for patients with combined and isolated TBI.

**METHODS.** We surveyed 47 patients with serious TBI and oppressed wakefulness level upon the admitting to hospital up to 10 or less points on the GCS, 27 of the victims were had a TBI severity according to the ISS scale - 50 ± 7 points, 20 patients had isolated TBI. The average age of victims was 38 ± 11 years, the ratio male/ female – 41/6.

All victims was carried out by standard intensive care according to Russian and international recommendations. At 3–7, 8–14, 15–21 days from the moment of injury, all patients underwent the assessment of the level of urea in daily urine with the subsequent calculation of the nitrogen losses.

The obtained findings were compared between groups of patients.

**RESULTS.** The level of catabolism in victims with combined and isolated TBI was very high during the whole observation time (the loss of nitrogen and 23.7 ± 9.3 g/day (n = 125) and 24.9 ± 7,3 (n = 71), respectively). However, in patients with isolated TBI catabolism was more denominated in the first 7 days after injury (the loss of nitrogen and 26.5 ± 6.5 g/day (n = 20) compared with 22.2 ± 9 g/day (n = 27) in patients with SCMT). In further period of observation, the level of catabolism was comparable in victims of both groups: 8–14 day −28,5 ± 6.2 g/day (n = 27) in patients with SCMT and 26.5 ± 6.5 g/day (n = 20) in patients with isolated TBI; 14–21 day - to 26.6 ± 3.8 g/day (n = 25) and 25,9 ± 10.3 g/day (n = 15), respectively; more than 21 days −20,5 ± 8.9 g/day (n = 46) and 18.8 ± 4.5 g/day (n = 16), respectively.

**CONCLUSIONS.** Victims have been pointing out with isolated and concomitant TBI during the first 3 weeks after injury. The level of catabolic reactions in victims with isolated and concomitant head injury is comparable in 8–21 days from the date of injury, but in the first 7 days post-traumatic period, the catabolism is more conveyed in patients with isolated TBI.

#### 0891

#### The impact of DPP-4 inhibitors to long-term outcome among diabetes patients after first acute myocardial infarction

M.-T. Wang[1], S.-H. Kuo[1], S.-C. Lin[1], P.-L. Tang[1], W.-T. Hung[1], K.-C. Lin[1], C.-C. Cheng[1,2,3], J.-S. Yang[3], G.-Y. Mar[1], H.-T. Chang[1], C.-P. Liu[1,3], W.-C. Huang[1,2,3]
[1]Kaohsiung Vaterans General Hospital, Critical Care and Cardiovascular Center, Kaohsiung, Taiwan, Province of China; [2]National Yang-Ming University, School of Medicine, Taipei, Taiwan, Province of China; [3]Fooyin University, Department of Physical Therapy, Kaohsiung, Taiwan, Province of China
**Correspondence:** S.-H. Kuo
*Intensive Care Medicine Experimental* 2017, **5(Suppl 2):**0891

**INTRODUCTION.** Dipeptidyl-peptidase-4 (DPP-4) inhibitors are one of the classes of oral anti-hyperglycemic agents mediated through the incretin hormones. Previous studies of cardio-protective effects of DPP-4 inhibitors have not sufficient evidence of the relationship between DPP-4 inhibition and actual cardiovascular outcomes.

**OBJECTIVES.** The aim of our study is to evaluate the impact of DPP-4 inhibitors on the survival of diabetes patient with first acute myocardial infarction (AMI) through analysis of data from the Taiwan National Health Insurance Research Database.

**METHODS.** This was a nationwide, propensity score-matched case–control study of patients admitted to hospitals between January 2000 and December 2012 with primary diagnosis of first AMI. Among the 186,112 first AMI patients, 72,924 patients with diabetes were identified. A propensity score, one-to-one matching technique was used to match 2,672 controls to the DPP-4 inhibitors therapy group for analysis. Controls were matched on the following variables: sex, age, hypertension, dyslipidemia, diabetes, peripheral vascular disease, heart failure, cerebrovascular accidents, end-stage renal disease, chronic obstructive pulmonary disease, and percutaneous coronary intervention.

**RESULTS.** DPP-4 inhibitors improve the overall 3-year survival rate (log rank p value < 0.0001), whether male, female, younger or elder patients. Cox Proportional Hazard Regression showed DPP-4 inhibitor is beneficial in diabetes patients after AMI (HR = 0.86; 95%CI = 0.78-0.95), especially in those patients with hypertension (HR = 0.87; 95%CI = 0.78-0.97; P = 0.0103) and cerebrovascular disease.(HR = 0.83; 95%CI = 0.72-0.97; P = 0.018) and without dyslipidemia(HR = 0.78; 95%CI = 0.67-0.92; P = 0.0029), without peripheral vascular disease (HR = 0.86; 95%CI = 0.78-0.96; P = 0.0047), without heart failure (HR = 0.84; 95% CI = 0.73-0.96; P = 0.0035), without end stage renal disease (HR = 0.86; 95% CI = 0.77-0.95; P = 0.0035), and those without chronic obstructive pulmonary disease (HR = 0.87; 95%CI = 0.78-0.97; P = 0.0096).

**CONCLUSIONS.** This study demonstrated DPP-4 inhibitors therapy had better long-term survival in diabetes patients after first AMI, regardless of gender group.

### Cardiac surgery and pulmonary arterial hypertension

#### 0892

#### Hypotension after anesthesia-to-intensive care unit drop off in cardiac surgery patients; frequency and associated outcomes

S. Cengic[1], M. Zuberi[1], V. Bansal[1], E. Rodrigues[2], E. Festic[1]
[1]Mayo Clinic, Department of Critical Care, Jacksonville, United States; [2]Mayo Clinic, Department of Anesthesiology, Jacksonville, United States
**Correspondence:** S. Cengic
*Intensive Care Medicine Experimental* 2017, **5(Suppl 2):**0892

**INTRODUCTION.** Hypotension has been reported in up to a third of post-cardiac surgery patients upon arrival to the Intensive Care Unit (ICU). This may predispose patients to adverse clinical outcomes.

**OBJECTIVES.** We aimed to investigate the frequency of initial hypotension upon arrival to ICU after cardiac surgery, the underlying causes and associations with clinical outcomes. We hypothesized that patients who develop hypotension after anesthesia-to-ICU drop off would have longer mechanical ventilation duration (MV) and longer ICU and hospital lengths of stay (LOS).

**METHODS.** This was an IRB approved retrospective study of post-cardiac surgery patients at a tertiary academic medical center in the USA in 2016. We excluded patients undergoing transplant surgery. We abstracted baseline demographics, comorbidities and all pertinent clinical variables including vitals, laboratories, type and urgency of surgery, bypass and cross-clamp time, medications and blood products delivered during the surgery and immediately prior to transfer to ICU. The primary outcome was hypotension defined as systolic blood pressure < 90 mmHg or mean arterial pressure < 65 mmHg per arterial catheter tracing within first 30 minutes upon transfer from the operating room (OR) to ICU. The secondary outcomes were duration of MV, ICU and hospital LOS and hospital mortality.

**RESULTS.** Of 218 patients, 12 were excluded as they lacked detailed blood pressure recordings. The majority of patients were white (82%), male (69%), of median age 67 (59, 73). The most frequent surgeries were coronary artery bypass grafting and/or valvular surgery (82%). The overall mortality was 4%. A half of the patients (103) were hypotensive immediately upon transfer to the ICU, while only 26% were hypotensive before leaving the OR (53). Of 153 patients without hypotension before leaving the OR, 48% (73) became hypotensive upon arrival to the ICU. Of 53 patients with OR hypotension immediately before the transfer, 30 continued to be hypotensive on ICU arrival. One clinical variable that appeared associated with the observed discrepancies was bolus dosing of vasopressors around the transfer. About 64% (30) of patients who became newly hypotensive upon ICU arrival had received vasopressor bolus prior to the transfer, while 23% (13) of the hypotensive OR patients didn't receive vasopressor bolus on the transfer. The patients with the initial ICU hypotension, compared to those without, had trend for longer duration of MV (5 [3, 17] vs. 4 hours [2, 6]), longer ICU LOS (2 [1, 4] vs. 1 day [1, 2]), longer hospital LOS (7 [5, 11] vs. 7 days [6, 9]) and significantly higher mortality (9% versus 0%, p = 0.002).

**CONCLUSIONS.** Hypotension upon anesthesia-to-ICU drop off is frequent and may be associated with adverse clinical outcomes. Further research and quality improvement strategies directed towards use of bolus vasopressors are needed to reduce the rates of hypotension immediately post anesthesia-to-ICU drop off.

### 0893

#### Early surgery and functional outcomes of critically ill patients with *Staphylococcus aureus* infective endocarditis

S. Abid[1], G. Papin[1], B. Mourvillier[1], J. Lebut[1], R. Smonig[1], E. Magalahes[1], O. Andremont[1], C. Dupuis[1], W. Juguet[1], M. Neuville[1], E. Mariotte[2], L. Bouadma[1], J.-F. Timsit[1], R. Sonneville[1]

[1]APHP-Medical and Infectious Diseases ICU Bichat Hospital, Paris, France; [2]APHP-Medical ICU Saint Louis Hospital, Paris, France

**Correspondence:** R. Sonneville

*Intensive Care Medicine Experimental* 2017, **5(Suppl 2):**0893

**INTRODUCTION.** *Staphylococcus aureus* is the most common cause of infective endocarditis (IE) and is associated with a severe prognosis[1]. Patients frequently require ICU admission because of acute circulatory failure or neurological complications, or the need for emergent valvular surgery[2,3]. The benefit of early surgery on functional outcomes remains uncertain.

**OBJECTIVES.** We aimed to identify risk factors for poor functional outcomes in critically ill patients with *Staphylococcus aureus* IE.

**METHODS.** We conducted a retrospective analysis of consecutive patients admitted to the medical ICU with acute left-sided defined *Staphylococcus aureus* IE according to the DUKE criteria, from January 2007 to December 2016. Factors associated with a poor functional outcome, defined by a score > 3 on the modified Rankin scale (i.e., severe disability or death) at 90 days, were identified by multivariate analysis. The model was built using clinically relevant variables associated with outcome by univariate logistic regression analysis (p < 0.2). Organ failures were defined by a score >2 on each component of the SOFA score. Data are presented as median (interquartile) or numbers (percentage).

**RESULTS.** A total of 110 consecutive patients were studied (age: 62 [52–70] years, SOFA score: 9 [5–13], GCS: 15 [13–15]), of whom 78 (71%) patients underwent early valve replacement surgery 2 [1–5] days after admission. Mechanical ventilation, renal replacement therapy, catecholamines were needed in 56 (51%), 22 (20%), 59 (54%) respectively.

At 90 days, 73 (66%) patients had a poor functional outcome (i.e. 60 (55%) deaths and 13 (12%) severe disabilities) (Fig. 287). The only independent factor positively associated with a poor outcome was renal failure at ICU admission, (Odds ratio (OR) = 5.52, 95% confidence interval (95CI), 1.56-19.56). By contrast, early surgery, performed within 48 hours after admission, had a protective effect (OR = 0.08, 95CI 0.02-0.34) (Table 213).

Septic shock, neurological complications and IE characteristics were not independently associated with functional outcome.

**CONCLUSIONS.** Our study suggests that about one-third of patients admitted to the ICU for *Staphylococcus aureus* IE have a good functional outcomes at 90 days. The main factor at admission associated with poor neurological outcome is renal failure. Our study confirms the benefit of early valve replacement surgery on functional outcomes, even in the most severe patients.

**REFERENCE(S)**

1. Sonneville, R. *et al.* Neurologic complications and outcomes of infective endocarditis in critically ill patients: the ENDOcardite en REAnimation prospective multicenter study. *Crit. Care Med.* **39,** 1474–1481 (2011).

2. Mirabel, M. *et al.* Long-term outcomes and cardiac surgery in critically ill patients with infective endocarditis. *Eur. Heart J.* **35,** 1195–1204 (2014).

3. Leroy, O. et al. Infective endocarditis requiring ICU admission: epidemiology and prognosis. Ann. Intensive Care 5, 45 (2015).

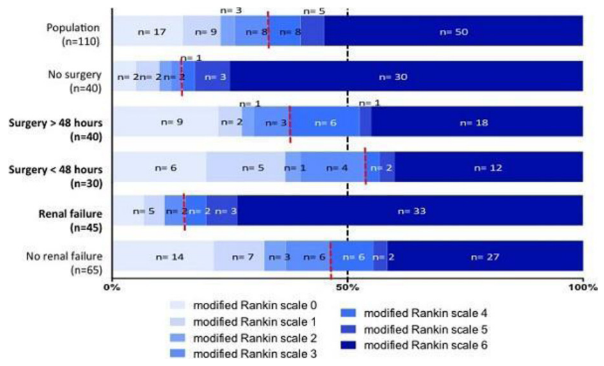

**Fig. 287 (Abstract 0893).** See text for description

**Table 213 (Abstract 0893).** See text for description

| | Population (n=110) | Good outcome* (n=37) | Poor outcome** (n=73) | OR univariate | p | OR multivariate | p |
|---|---|---|---|---|---|---|---|
| Renal failure at ICU admission | 45 (41%) | 7 (19%) | 38 (52%) | 4.65 [1.81-11.94] | <0.01 | 5.52 [1.56-19.56] | <0.01 |
| Blood lactate level >2mmol/l | 27 (25%) | 5 (14%) | 22 (30%) | 2.76 [0.95-8.0] | 0.06 | 3.38 [0.83-13.74] | 0.09 |
| No surgery | 40 (36%) | 6 (16%) | 34 (47%) | 1 | <0.01 | 1 | <0.01 |
| Surgery ≤ 48 hours | 30 (28%) | 16 (43%) | 14 (19%) | 0.15 [0.05-0.48] | | 0.08 [0.02-1.40] | |
| Surgery > 48 hours | 40 (36%) | 15 (41%) | 25 (34%) | 0.29 [0.1-0.86] | | 0.37 [0.10-1.40] | |

* Good functional outcome is defined by a modified Rankin scale 1-3; ** Poor functional outcome is defined by a modified Rankin scale 4-6; Data are presented with n (%).

### 0894

#### Monocytes CD14+ with HLA-DR+ expression counts in peripheral blood before cardiac surgery predict ICU mortality

A. Levit[1], Y. Petrishchev[2], H. Partilova[2]

[1]Ural State Medical University, Ekaterinburg, Russian Federation; [2]Regional Hospital №1, Ekaterinburg, Russian Federation

**Correspondence:** A. Levit

*Intensive Care Medicine Experimental* 2017, **5(Suppl 2):**0894

**INTRODUCTION.** A low HLA-DR antigen expression on monocytes was observed after aortic surgery according to some researchers [1] and is a marker of complicated postoperative course after aortocoronary bypass [2]. A low level of monocytes with HLA-DR+ expression is considered as a marker of immunosuppression in septic shock and is associated with the development of an unfavorable outcome [3]. The issue of the impact of preoperative level of CD14+ monocytes with HLA-DR+ expression on the outcome of cardiac surgery remains open. This circumstance was the basis for our study.

**OBJECTIVES.** We examined 40 patients undergoing elective cardiac surgery, 20 male and 20 females. No one had SIRS criteria or infection before surgery. The mean age was 62 years (26; 79); Euro SCORE II 2.35% (0.4; 13.3). The following operations were performed: CABG - 10, CABG and valves replacement - 10, valves replacement - 20. The duration of CPB was 103 min (0; 190). Length of stay in ICU was 48 hours (24; 600). ICU mortality was 5% (2 patients).

**METHODS.** We analyzed the monocyte $CD14^+$ with $HLA\text{-}DR^+$ expression counts in peripheral blood in all patients before surgery and 24 hours after by flow cytometry. The results obtained were analyzed statistically in order to identify the predictors of unfavorable outcome.

**RESULTS.** According to the results of ROC analysis, the preoperative monocyte $CD14^+$ with $HLA\text{-}DR^+$ expression counts of less than $0.32 \times 10^9$/L were a good prognostic marker for lethal outcome (sensitivity 100%, specificity 87%, AUC 0.93, and $p < 0.05$).

**CONCLUSIONS.** The preoperative characteristics of the monocyte link of innate immunity can be predictors of unfavorable outcome of cardiac surgery.

**REFERENCE(S)**

1. Differential down-regulation of HLA-DR on monocyte subpopulations during systemic inflammation. O. Y. Kim, A. Monsel, M. Bertrand, P. Coriat, J-M. Cavaillon, M. Adib-Conquy. Crit Care. 2010; 14(2): R61.
2. Blood monocyte subpopulations during uncomplicated coronary artery bypass surgery. Golovkin A.S., Matveeva V.G., Kudryavtsev I.V., Grigoriev E.V., Velikanova E.A., Bairakova Yu.V. Med. Immunol., 2012, vol. 14, N 4–5, pp 305–312.
3. Persisting low monocyte human leukocyte antigen-DR expression predicts mortality in septic shock. Monneret G1, Lepape A, Voirin N, Bohé J, Venet F, Debard AL, Thizy H, Bienvenu J, Gueyffier F, Vanhems P. Intensive Care Med. 2006 Aug;32(8):1175–83.

**GRANT ACKNOWLEDGMENT**

Our study was not granted.

### 0895

#### Mortality and therapeutic interventions in patients with pulmonary hypertension following emergency admission to critical care

D. Kerr[1], K. Bauchmuller[1], A. Raithatha[1], R. Condliffe[2], D. Kiely[2], G. Mills[1]

[1]Sheffield Teaching Hospitals NHS Foundation Trust, Department of Anaesthesia and Critical Care, Sheffield, United Kingdom; [2]National Pulmonary Hypertension Service, Pulmonary Vascular Disease Unit, Royal Hallamshire Hospital, Sheffield, United Kingdom

**Correspondence:** D. Kerr

*Intensive Care Medicine Experimental* 2017, **5(Suppl 2):**0895

**INTRODUCTION.** Management of pulmonary hypertension (PH) is challenging, particularly in the context of critical illness. Although overall prognosis is poor, recent advances in disease-specific treatments have significantly improved 5-year survival [1].

Little is known about outcomes in patients with pre-existing PH admitted to critical care, and whether the use of ICU-specific therapies has any significant impact on survival.

**OBJECTIVES.** To characterise ICU outcomes in patients with PH and assess the impact of various critical care interventions on mortality.

**METHODS.** Retrospective observational study of adult patients with PH admitted to the ICU of a UK national PH referral centre with an emergency non-surgical diagnosis between April 2000 and December 2016. Data on patient characteristics, indications for ICU admission and critical care interventions were retrieved from our electronic patient record (Metavision). Association of interventions with mortality was assessed by logistic regression analysis.

Results are expressed as median (range), mean (SD) and odds ratios (OR) with 95% confidence intervals (CI).

**RESULTS.** One hundred and thirty nine patients (64.7% female) with a median age of 53 (18–83) were included in this study.

Pulmonary arterial hypertension was the most prevalent underlying cause for PH (68.3%), followed by chronic thromboembolic disease (12.2%), multifactorial/unclear aetiology (7.2%), and PH associated with lung (6.5%) and left heart disease (5.8%).

Primary reasons for ICU admission included heart failure (38.1%), sepsis (31.6%), arrhythmia (9.4%) and acute renal failure (7.9%). The mean APACHE II score was 15.85 (SD 5.979) and median admission lactate was 1.250 mmol $l^{-1}$ (0.4-14.1).

Non-invasive and invasive ventilation, renal replacement therapy (RRT), vasopressor and inotropic support were provided in 48.9%, 5.8%, 14.4%, 28.8% and 22.3% of patients, respectively.

Critical care mortality was 30.2% and overall hospital mortality was 39.6%. Use of CPAP, invasive ventilation, vasopressor or inotropic support was associated with increased hospital mortality, whereas RRT was not (see Table 214).

**CONCLUSIONS.** Patients with PH admitted to critical care have a high hospital mortality. Ventilatory and haemodynamic support appear to be independent risk factors in this patient group, whereas RRT does not.

These observations might reflect the detrimental effects of positive pressure on the pulmonary circulation and right heart, and the strong prognostic significance of worsening acute heart failure. The use of RRT in patients with PH is an area that warrants further investigation, as this therapy may have a potential role in optimising right ventricular preload via tight regulation of fluid balance.

**Table 214 (Abstract 0895).** Association of ICU interventions and mortality

| | Univariate analysis | | | Multivariate analysis | | |
|---|---|---|---|---|---|---|
| | OR | 95% CI | p-value | OR | 95% CI | p-value |
| CPAP | 2.151 | 1.066-4.342 | 0.033 | 2.880 | 1.308-6.339 | 0.009 |
| NPPV | 1.759 | 0.597-5.178 | 0.306 | | | |
| IPPV | 12.104 | 1.445-101.369 | 0.021 | 10.021 | 1.070-93.839 | 0.043 |
| RRT | 1.298 | 0.500-3.374 | 0.592 | | | |
| Vasopressors | 4.483 | 2.054-9.786 | 0.000 | 2.978 | 1.223-7.254 | 0.016 |
| Inotropes | 4.571 | 1.942-10.753 | 0.000 | 2.746 | 1.020-7.394 | 0.046 |
| IV Iloprost | 0.947 | 0.458-1.962 | 0.884 | | | |

### 0896

#### Diagnosis and haemodynamics of pulmonary hypertension in the cardiothoracic ICU

E. Galiatsou, J. Cevallos, D. Hall, C. Hernandez Caballero

Harefield Hospital, ITU, London, United Kingdom

**Correspondence:** E. Galiatsou

*Intensive Care Medicine Experimental* 2017, **5(Suppl 2):**0896

**INTRODUCTION.** Pulmonary hypertension (PH) and right ventricular (RV) failure can be detrimental in the critically ill, post cardiac surgery and post heart/lung transplantation. The existing haemodynamic classification of pulmonary hypertension (PH) defines two groups, pre- and post capillary PH, which can be either isolated post capillary (Ipc-PH) or combined post- and pre capillary (Cpc-PH).

**OBJECTIVES.** The aim of this study was to look at the diagnosis and haemodynamic classification of pulmonary hypertension in a cardiothoracic intensive care unit (ICU) and how these affected the use of inhaled nitric oxide (iNO).

**METHODS.** This was a retrospective and observational study. Thirty nine consecutive ICU patients who had undergone pulmonary artery catheterisation (PAC) on admission were included. The following parameters were recorded from patients´ medical charts: PAC measurements and echocardiography within the first 6h following admission, and treatment with iNO. We calculated diastolic pressure gradient (DPG) as diastolic pulmonary artery pressure-pulmonary capillary wedge pressure (PCWP) and classified PH as pre-capillary (mean pulmonary artery pressure mPAP ≥ 25mmHg and PCWP ≤ 15mmHg), post-capillary (mPAP ≥ 25 and PCWP > 15mmHg), Ipc-PH (PCWP > 15mmHg and DPG < 7mmHg and/or pulmonary vascular resistance ≤3WU), and Cpc-PH (PCWP > 15mmHg, DPG ≥ 7mmHg and/or PVR > 3WU).

**RESULTS.** Thirty-nine patients (14 female/25 male, age 55.44 ± 14.42), all of them sedated and mechanically ventilated were included in the study. Thirty four were postoperative cardiothoracic admissions and five had cardiogenic shock. According to the PAC measurements PH was present in 32 patients. A full haemodynamic profile (with cardiac output, pulmonary artery pressures, PCWP measurement) was available in 21 of the 32 patients with PH. Of those 21 patients, 6 had pre-capillary PH and 15 post-capillary PH (12 Ipc-PH and 3 Cpc-PH). Eleven patients had PH that could not be classified as PCWP measurements were not available. Twenty two of the 39 (20 of the 32 with PH) had been started on iNO treatment. Fifty percent of those with Ipc-PH as well as 82.5% of those with unclassified PH (9 out of 11) were on iNO. An echocardiography scan was available in 20 of the 39 patients, with evidence of RV impairment present in 10 out of the 20. Of the 9 patients with unclassified PH who were on iNO, 3 had echocardiography scans. These 3 scans showed evidence of RV impairment.

**CONCLUSIONS.** In the acute setting, the use of PAC as a haemodynamic monitoring tool in patients at high risk for PH does not explore the method to its full potential and according to the international guidelines. When analysed, PAC data show that post-capillary PH due to left heart disease is the most common from of PH in the cardiothoracic ICU. Echocardiography as a monitoring tool is probably underused.

**REFERENCE(S)**

European Heart Journal 2016:37, 67–119.

### 0897

#### Troponin T: an indicator of adverse outcomes following lung transplantation

J.P. Scott[1], B.A. Boilson[2], S.G. Peters[1], M.E. Wylam[1], C.C. Kennedy[1], H. Sekiguchi[1], R.C. Daly[3]
[1]Mayo Clinic, Pulmonary and Critical Care, Rochester, United States; [2]Mayo Clinic, Cardiology, Rochester, United States; [3]Mayo Clinic, Cardiac Surgery, Rochester, United States
**Correspondence:** J.P. Scott
*Intensive Care Medicine Experimental* 2017, **5(Suppl 2):**0897

**INTRODUCTION.** Chronically immunosuppressed patients remain at increased risk of acute illness and of adverse outcomes as reflected by the chronic health indices of the APACHE scoring system(1).
Although cardiac conditions, including infarction are enumerated in the APACHE IV system, ischemia is explicitly considered. Evidence does exist that in ARDS patients elevated troponin T values are associated with reduced survival and the addition of such values to APACHE improves the ROC curve in ARDS(2). We have studied the effect of troponin levels in lung transplant patients, a moderately immunosuppressed patient group subject to frequent readmissions and with a high mortality rate following ICU admissions to assess the effect of such changes on patient survival.

**OBJECTIVES.** Following the description of the importance of Troponin T as a prognostic marker in kidney and liver transplantation; to evaluate the role and specificity of low level troponin T levels at evaluation for lung transplant and during episodes of illness.

**METHODS.** We have studied 90 patients mean age 54.2 (+/− 10.6) years, 42 female in whom troponin levels were routinely collected before transplantation, at two months and during acute illnesses. Analysis of data was by non-parametric testing of survival using Kaplan-Meier curves for troponin values =/< 0.01, 0.02 - 0.19 and 0.2 or above.

**RESULTS.** Results demonstrate very poor survival for elevated troponins pre-transplant, and with maximal troponin values during acute illness. For pre-transplant serum troponin levels survival falls with higher troponin group: Median survival 2780 days (Group 0), vs 2332 days (Group 1) vs 706 days (Group 2) Log-Rank, p < 0.0001; Wilcoxon, p < 0.0001. Maximum serum troponin value after transplant were also highest in patients with the highest mortality after transplantation, p = 0.0006 Wilcoxon and p = 0.0003 Log-rank tests.

**CONCLUSIONS.** Troponin levels in serum likely reflect some measure of cardiac injury, most likely ischemia induced by hypoxia in patients with end stage lung disease awaiting lung transplantation. Maximum values tend to occur during severe illnesses or prior to death when hypoxia and ischemia are likely to be present. We conclude that troponin values before as well as after lung transplantation are inversely related to patient survival and these observations are similar to those in renal and liver transplant patient populations.

**REFERENCE(S)**

(1) Zimmerman JE, Kramer AA, McNair DS, Malila FM. Acute Physiology and Chronic Health Evaluation (APACHE) IV: Hospital mortality assessment for today's critically ill patients. Crit Care Med 2006; 34:1297–1310.
(2) Rivara MB, Bajwa EK, Januzzi JL, Gong MN, Thompson BT, et al. (2012) Prognostic Significance of Elevated Cardiac Troponin-T Levels in Acute Respiratory Distress Syndrome Patients. PLoS ONE 7(7): e40515. doi:10.1371/journal.pone.0040515

### 0898

#### Can right atrial pacing suppress postoperative atrial fibrillation after atrial valve replacement, especially in patients with atrial valve stenosis?

H. Ueta[1], D. Kawakami[1], M. Suga[1], Y. Nakamori[1], J. Ito[1], T. Shimozono[1], H. Mima[1], T. Morimoto[2], Kobe Minatojima Group
[1]Kobe City Medical Center General Hospital, Anesthesiology and Critical Care, Kobe, Japan; [2]Hyogo College of Medicine, Department of Clinical Epidemiology, Nishinomiya, Japan
**Correspondence:** H. Ueta
*Intensive Care Medicine Experimental* 2017, **5(Suppl 2):**0898

**INTRODUCTION.** After cardiac surgery, atrial fibrillation (AF) occurs at a frequency of 30-60%. Various research studies have been conducted about postoperative AF (POAF). According to American College of Chest physicians the Guidelines, for the prevention of POAF, the use of β-blockers, amiodarone, and bilateral atrial pacing are recommended.However, in almost all studies, the objective was coronary aortic bypass grafting (CABG), and not aortic valve stenosis (AS).

**OBJECTIVES.** In this study, we aimed to examine whether right atrial pacing suppresses POAF, especially among patients with AS.

**METHODS.** We conducted a single-center, retrospective observational study of patients with AS, admitted to the general ICU at Kobe City Medical Center General Hospital, between 2011 and 2016. Patients who underwent aortic valve replacement were included. We defined the patients with sinus rhythm or overdrive right ventricular pacing as the control group (CG), and the patients with overdrive right atrial pacing as the atrial group (AG). Fisher's exact test was used to analyze the frequency of POAF between the two groups.A logistic regression model was used to define the risk factors, including overdrive atrial pacing with statistical software 'R'.

**RESULTS.** We included 184 patients. The atrial group consisted of 24 patients; and the control group, 160 patients. No significant differences in baseline characteristics, including preoperative and operative factors, were observed between the two groups. Among the postoperative factors, the use of β-blocker showed a statistical significant difference ($p$ = 0.039). As for the frequency of POAF, no significant difference was found (AG 45% vs. CG 45.8%, $p$ = 1.00). Even when patients were under β-blocker medication, no statistical significant difference was found (AG 42.1% vs. CG 50%, $p$ = 0.776). In the condition of the left atrial diameter of >4.0 cm, no significant difference was found (AG 60.7% vs. CG 33.3%, $p$ = 0.407). By using logistic regression models that compared AG with CG, we identified the role of the aorta cross-clamp time (odds ratio [OR] 0.99; 95% confidence interval [CI] 0.03-2.57, $p$ = 0.27), left atrial diameter (OR 1.44; 95% CI 0.92-2.38, $p$ = 0.10), and atrial pacing (OR 0.97; 95% CI 0.40-2.35, $p$ = 0.95).

**CONCLUSIONS.** For patients with atrial valve stenosis, right atrial overdrive pacing could not suppress the POAF after surgical aortic valve replacement.

**REFERENCE(S)**

▪ Arsenault KA, et al. (2013) Interventions for preventing post-operative atrial fibrillation in patients undergoing heart surgery. Cochrane Database Syst Rev. Jan 31; (1):CD003611. doi:10.1002/14651858.CD003611.pub3. Review.

▪ Hogue CW Jr, et al. (2005) Epidemiology, mechanisms, and risks: American College of Chest Physicians guidelines for the prevention and management of postoperative atrial fibrillation after cardiac surgery. Chest 128 (2 Suppl): 9S-16S

**GRANT ACKNOWLEDGMENT**

None.

### 0899

#### Mortality factors associated in patients with infective endocarditis managed with emergent cardiac surgery

N. Palomo-López, G. Rivera-Rubiales, Y. Corcia-Palomo, L. Martin-Villen, A. Herruzo-Aviles, R. Hinojosa-Perez

Hospital Universitario Virgen del Rocío, Sevilla, Spain

**Correspondence:** N. Palomo-López

*Intensive Care Medicine Experimental* 2017, **5(Suppl 2):**0899

**INTRODUCTION.** Patients with infective endocarditis has a high mortality especially if they required surgery

**OBJECTIVES.** To know the factors associated with mortality in patients with infective endocarditis (IE) admitted in the intensive care unit (ICU) and who were emergency surgically operated.

**METHODS.** Observational, prospective and cohort study. We included patients with infectious endocarditis from January 2008 to December 2016 who needed urgent surgey. We collected demographic variables, type of valvular surgery, doses of amines at admission, microbiological results, empirical and target antibiotics and outcome. Amine doses were classified into two groups: low doses 0.1-0.3 mcg/ kg/ hr and moderate doses > 0.3 mcg/ kg/ hr. Descriptive statiscal analysis was performed, presenting the qualitative variables as frequencies and percentages, and quantitative as mean (+/− SD) or median (interquartile range ICR) depending its distribution. We use chi square for bivariate analysis of qualitative variables.

**RESULTS.** We included 46 patients, 34 were men (73.9%). Mortality rate was 30.4%. The most frequent surgery was on aortic valve, in 25 patients (61%), followed by the mitral-aortic valve (8 patients, 19.5%) and the mitral valve (7 patients, 17.1%). There was no significant relation between type of surgery and mortality (p = 0.399). A total of 18 patients (39.1%) did not require amines at ICU admission, while 47.8% received low doses and 13% moderate doses. The different needs of amine were not either significantly related to higher mortality (p = 0.216). In 43 patients (93.5%), microbiological isolation was obtained, with 71.7% receiving target antibiotic therapy, significantly associated with greater survival (81.2% vs 50.0%, p = 0.03).

**CONCLUSIONS.** Patients who are urgently operated due an IE have a mortality rate in our series of 30.4%. Preoperative hemodynamic instability requiring different doses of vasoactive amines does not seem to be associated with higher mortality, nor the type of valvular surgery. Administration of target antibiotic therapy by antibiogram was significantly associated with increased survival.

**REFERENCE(S)**

1. Kung D. Timing of surgery in infective endocarditis. Heart 2015;101:1786–1791.

2. Jia L, Wang Z, Fu Q, Bu H, Wei M Could Early Surgery Get Beneficial in Adult Patients with Active Native Infective Endocarditis? A Meta-Analysis..Biomed Res Int. 2017;2017:3459468

3. Cresti A, Chiavarelli M, Scalese M, Nencioni C, Valentini S, Guerrini F, D´Aiello I, Picchi A, De Sensi F, Habib G. Epidemiological and mortality trends in infective endocarditis, a 17-year population-based prospective study. Cardiovasc Diagn Ther. 2017 Feb;7(1):27–35

**GRANT ACKNOWLEDGMENT**

We gratefully appreciate the invaluable of ICU staff.

### 0900

#### Results of percutaneous aortic prosthesis implantation in patients older than 75 years with severe symptomatic aortic stenosis and high surgical risk

M. Mancisidor Urizar, M. Cano García, L.D. Muñoz Jiménez, C. Sánchez González, J.A. Bullones Ramírez, J. Álvarez Rubiera, M. de Mora Martín, C.A. Urbano Carrillo

Hospital Regional Universitario de Málaga, Málaga, Spain

**Correspondence:** M. Mancisidor Urizar

*Intensive Care Medicine Experimental* 2017, **5(Suppl 2):**0900

**INTRODUCTION.** The implantation of a percutaneous aortic prosthesis (TAVI) is the treatment of choice for patients with severe symptomatic aortic stenosis who are not candidates for surgery or who have a high surgical risk, in most cases in elderly patients.

**OBJECTIVES.** The aim of our study was to analyze the results of TAVI implantation in our center in patients older than 75 years.

**METHODS.** Prospective and monocentric study of patients submitted to TAVI implantation at our center between June 2010 and February 2017. Clinical variables, risk scores, variables related to the procedure, complications and follow-up events were collected.

**RESULTS.** Among 140 patients in whom this procedure was performed, 116 patients (82.2%) who were ≥75 years old were analyzed. The mean age was 81.9 ± 3.3 years. The prevalence of risk factors and comorbidities was high: hypertension 99p (85.3%), dyslipemia 60p (51.7%), diabetes mellitus 51p (44.0%), smoking 34p (29.3%), atrial fibrillation 44p (37.9%), pacemaker (8.9%), COPD 23p (19.8%), obesity (BMI > 30 kg / m2) 47p (40.5%), chronic renal failure 75p (64.7%), previous neoplasia 13p (11.2%), ischemic heart disease 38p 32.8%). The mean EuroScore was 20.8 ± 12.9 and the STS score 4.7 ± 3.4. The functional grade was: grade II 23p (19.8%), grade III 85p (73.3%) and grade IV 8p (6.9%). 24.1% (28p) had some degree of ventricular dysfunction, 13.9% had significant mitral regurgitation (16p) and 25.8% (30p) had pulmonary hypertension. In addition, 31p (29.0%) had some previous conduction disorder. The immediate success rate of the procedure was 98.3%. After the intervention, pacemaker implantation was performed in 38 patients (32.8%), 6 (5.2%) patients developed renal insufficiency (5.2%), atrial fibrillation in 9p (7.8%), bleeding with need for transfusion in was observed in 10p (8.8%). The percentage of significant mitral regurgitation reduced to the half (7.7%). The median number of days of admission was 9 days. The percentage of in-hospital death was 10p (8.6%, technical complication 2p, arrhythmia 1p, heart failure 5p, other 2p. At year, of follow-up survival was 83.5% and 72.2% at 2 years.

**CONCLUSIONS.** The majority of patients undergoing TAVI were over 75 years old, with a high prevalence of risk factors and comorbidities. Both, success and survival of the procedure at follow-up in a high-risk surgical population, was high.The most frequent complication was the need for definitive pacemaker implantation.

### 0901

#### Aortic surgery with moderate hypothermia, cardiac arrest and antegrade cerebral perfusion. Results

V.A. Hortigüela[1], R. Hernández[2], D.C. Robaglia[1], A.I. Tejero[1], A. Vidal[1], H. Pérez[1], J.L. Pérez[1], V. Trasmonte[1], P. Calderon[2], C. Pérez[1], G. Aldámiz[2]

[1]Fundación Jiménez Díaz, Intensive Care Medicine, Madrid, Spain; [2]Fundación Jiménez Díaz, Cardiac Surgery, Madrid, Spain

**Correspondence:** V.A. Hortigüela

*Intensive Care Medicine Experimental* 2017, **5(Suppl 2):**0901

**INTRODUCTION.** Neurological protection in cardiac surgery for a good functional prognosis is the main concern of intensive care physicians and surgeons.

**OBJECTIVES.** Results of aortic surgery with moderate hypothermia (18°-26° C), cardiac arrest and antegrade cerebral perfusion: evaluation of mortality, neurological complications and functional prognosis at short and mid time.

**METHODS.** Observational retrospective descriptive study of 46 consecutive patients (20 emergent, 3 urgent, 23 elective) operated as described above between 2012 and 2016 at Fundación Jiménez Díaz

Hospital in Madrid. Emergent surgeries were for acute dissection. Different demographic, clinical, laboratory and intraoperative data were collected (cardiopulmonary bypass (CPB), cardiac arrest (CA) and antegrade cerebral perfusion (ACP) times were collected. We follow patients up to 6 months post-intervention (Table 215).

**RESULTS.** Eight patients died (17%): seven were emergent aortic dissection (35% mortality in this group) and one was elective surgery.

Focal neurological deficit was observed in five of the dead´s group (63%), in two of the survivors (5%) and in six of the dissections (30%).

There were three (37%) cases of abnormal awakening in the dead´s group, twelve among the survivors (32%) and ten (50%) into the dissections.

Patients with adverse neurological events had higher CPB times with statistically significant results (*T student* p 0.018, CI al 95% 6.94-59.8). The CA and ACP times were higher in this group respect the group without neurological alterations.

Patients with worst functional outcome (according to the Glasgow Outcome Scale (GOS) 1–3) were older, had higher body mass index (BMI), more cardiovascular history, worse EUROSCORE I, and longer surgical times. Emergent surgery was more common in patients with GOS 1–3 (Chi2, OR 0.058, 95% IC 0.006-0.557).

**CONCLUSIONS.** As expected, patients with emergent aortic surgery had higher mortality and more neurological complications, associated with longer CPB, ACP and CA. Patients with worse functional evolution had major preoperative risk, more incidence of emergent surgery and longer interventions.

Preliminary results of this study suggest the possibility of creating evolutionary risk scores based on these and other clinical and surgical variables.

**REFERENCE(S)**

- Preventza O. **Moderate hypothermia at warmer temperatures is safe in elective proximal and total arch surgery: Results in 665 patients.** J Thorac Cardiovasc Surg. 2016 Sep 24. pii: S0022-5223(16)31162-X. - Poon SS. **Is moderate hypothermic circulatory arrest with selective antegrade cerebral perfusion superior to deep hypothermic circulatory arrest in elective aortic arch surgery?** Interact Cardiovasc Thorac Surg. 2016 Sep;23(3):462–8.

- Gong M. M**oderate hypothermic circulatory arrest in total arch repair for acute type A aortic dissection: clinical safety and efficacy.** J Thorac Dis. 2016 May;8(5):925–33.

**GRANT ACKNOWLEDGMENT**

No.

**Table 215 (Abstract 0901).** COLLECTED DATA

| I.PRE-SURGICAL DATA | II.SURGICAL DATA | III. PERFUSION DATA | IV. INTENSIVE CARE DATA | V.HOSPITAL STAY DATA | VI.AFTER HOSPITAL DISCHARGE DATA |
|---|---|---|---|---|---|
| Age Weight Height Body mass index Arterial hypertension Mellitus diabetes Previous cerebrovascular disease EUROESCORE I | Priority Kind of surgery Surgery description Transesophageal echocardiogram (TEE) Periaortic Ateromatosis in TEE Corticoids and/or Pentothal during surgery Minimum temperature Kind of cerebral perfusion Local hypothermia Arterial cannulation Venous cannulation Extracorporeal Circulation Time (ECCt) Myocardial ischemia time (MIt) Brain perfusion time (BPt) Circulatory stopping time (CSt) | Flow machine of ECC Maximum pH during ECC Minimun pCO2 during ECC Minimun mean arterial pressure during ECC Minimun Brain perfusion pressure during ECC Minimun Hematocrit during ECC Maximun blood glucose during ECC Maximun blood Lactic during ECC Minimun right INVOS Minimun left INVOS Minimun Hematocrit during overheating Minimun pCO2 during overheating Maximun pH during overheating Maximun Lactic during overheating Trasfusión Blood products | Hematocrit at admission pH at admission pCO2 at admission Glucose at admission Lactic at admission Agitation on awakening Neurological deficit Antipsictics Brain TC Time until extubation Stay ICU time GOS at ICU discharge | Brain TC Stay hospital time Neurological deficit GOS at hospital discharge | Neurology Medical consultation Chief neurological complaint Brain TC Brain MNR GOS 6 months after surgery Surgery related death |

### 0902

### Mortality of patients with severe aortic stenosis treated with a self-expanding prosthesis

L. Olivencia[1], P. Castán[2], R. Rivera[2], M. García[2], A. Reina[2]

[1]Complejo Hospitalario Granada, Granada, Spain; [2]Complejo Hospitalario de Granada, Granada, Spain

**Correspondence:** L. Olivencia

*Intensive Care Medicine Experimental* 2017, **5(Suppl 2):**0902

**INTRODUCTION.** Transcatheter aortic valve implantation (TAVI) is a safe and effective alternative to surgical treatment in patients with severe aortic stenosis (AS) and those who are inoperable or at high surgical risk.

**OBJECTIVES.** The objective of this study was to evaluate the mortality of consecutive patients with severe AS treated with TAVI.

**METHODS.** This prospective single-center registry study from a tertiary hospital included all consecutive patients who underwent percutaneous aortic valve implantation between June of 2015 and december of 2016. Clinical follow-up was carried out during clinic visits or via telephone and lasted a minimum of 3 months and a maximum of 1,5 years. No patients were lost to clinical follow-up.

**RESULTS.** We recruited 86 patients, 74 (86%) treated with a transvascular (TV) and 12 (14%) transapical (TA) catheter-based techniques. The mean age at implantation was 78.1 ± 8.57 years, 43 (50%) were male, the mean logistic EuroSCORE was 15.24 ± 9.6% and the mean logistic EuroSCORE 2 was 3.83 ± 3.5%. The baseline clinical characteristics are shown in Table 216 and in-hospital complications are shown in Table 217. A total of 18 (15.5%) patients died during the follow-up period: 2 (2.3%) during hospitalization (one TA in ICU and one TV in hospitalization plant), and 16 (13.2%) during subsequent follow-up. The most common cardiovascular causes were heart failure and stroke.

**CONCLUSIONS.** Short-mid-term mortality in AS patients after TAVI is aceptable. The main causes of death are cardiovascular in the first year.

**Table 216 (Abstract 0902).** See text for description

| | |
|---|---|
| Diabetes mellitus | 39 (45.3%) |
| Dyslipidemia | 41 (47.7%) |
| Smokers/Ex-smokers | 2 (2.3%)/20 (23.3%) |
| Hypertension | 70 (81.4%) |
| Left ventricular ejection fraction (LVEF%) | 55.6 +-13.15 |
| Chronic obstructive pulmonary disease | 22 (25.6%) |
| Chronic ischemic heart disease | 16 (18.6%) |
| Pacemaker | 6 (7%) |
| Previous stroke | 6 (7%) |
| Previous Kidney injury | 38 (44.2%) |

**Table 217 (Abstract 0902).** See text for description

| | |
|---|---|
| In-hospital deaths | 2 (2.3%) |
| Cardiac Tamponade | 2 (2.3%) |
| Cardiogenic Shock | 3 (3.5%) |
| Septic Shock (catheter infection) | 1 (1.2%) |
| Acute myocardial infarction | 4 (4.7%) |
| New onset Atrial fibrillation | 6 (7%) |
| Permanent Pacemaker | 10 (11.6%) |
| Stroke | 1 (1.2%) |
| Major bleeding and vascular complication | 2 (2.3%) |
| Acute kidney injury/hemodialysis | 30 (34.9%)/1 (1.2%) |

### 0903

#### Inhibition of PAK-1/ROS signaling by induced pluripotent stem cell (iPSC) suppresses pulmonary arterial smooth muscle cells proliferation

M.-W. Ke[1], M.-C. Shen[1], S.-H. Kuo[2], C.-C. Cheng[2,3,4], S.-R. Wann[5], Y.-T. Chang[5], C.-W. Shu[1], W.-T. Hung[2], S.-C. Lin[2], K.-C. Lin[2], G.-Y. Mar[6], C.-P. Liu[6], W.-C. Huang[2,3,4]
[1]Kaohsiung Veterans General Hospital, Department of Medical Education and Research, Kaohsiung, Taiwan, Province of China; [2]Kaohsiung Veterans General Hospital, Intensive Care Unit, Kaohsiung City, Taiwan, Province of China; [3]National Yang-Ming University, School of Medicine, Taipei, Taiwan, Province of China; [4]Fooyin University, Department of Physical Therapy, Kaohsiung, Taiwan, Province of China; [5]Kaohsiung Veterans General Hospital, Department of Emergency, Kaohsiung City, Taiwan, Province of China; [6]Kaohsiung Vaterans General Hospital, Cardiovascular Medical Center, Kaohsiung City, Taiwan, Province of China
**Correspondence:** S.-H. Kuo
*Intensive Care Medicine Experimental* 2017, **5(Suppl 2):**0903

**INTRODUCTION.** Pulmonary arterial hypertension (PAH) is characterized by the dysregulated proliferation of vascular smooth muscle cells. In our previous study, the induced pluripotent stem cells (iPSC)-based therapy showed benefits on the improvement of hemodynamic function, and on attenuating the inflammation in lung of monocrotaline (MCT)-induced pulmonary hypertension rats. However, the underlying mechanisms are still not fully understood.
**OBJECTIVES.** In this study, we aim to explore whether iPSC-based therapy can suppress the proliferation of PASMCs in vivo and in vitro, as well as the underlying mechanisms will also be investigated.
**METHODS.** The animal model of PAH was established by subcutaneous injection of monocrotaline (60 mg/kg) into male SD rat. The iPSC-based therapy were administrated as (1) immediately intravenous injection after MCT, or (2) fourteen days after MCT injection. The types of iPSC-based therapy were further divided into the iPSCs itself (once per week) and the conditioned medium of iPSCs (once every 24 hours). The lung tissue of each group would be collected on $28^{th}$ day following the protocol. The proliferation of PASMCs will be examined by immunohistochemistry and western blot with antibodies against different proliferative cell markers. In vitro, human PASMCs were co-cultured with iPSCs to investigate the effects of iPSC treatments on the proliferation of human PASMCs. Meanwhile, the underlying signaling pathways of iPSC-based therapy on the proliferation of human PASMC will be explored.
**RESULTS.** In addition to the improvement of the hemodynamic values of RVSP, Fulton index and the severity of pulmonary vascular remodeling, iPSC-based treatments critically decreased the expression levels of Hif-1a, NF-kB and AKT molecules in the lung specimen of MCT-induced PAH rats. In vitro assay showed that either hypoxia or PDGF could enhance the proliferation and migration of human PASMCs (hPASMCs). Interestingly, iPSC-based treatments could attenuate the proliferation and migration of hPASMCs under hypoxic or PDGF stimulation. For cellular mechanism study, western blot showed that Hif-1a, NF-kB and AKT protein levels of hPASMCs were decreased following iPSC-based treatments. Furthermore, iPSC-based treatments also reduced the hypoxia and PDGF-induced increase of reactive oxygen species (ROS) level in hPASMCs, whereas western blot indicated that the decrease of ROS level in hPASMCs might be through the downregulation of PAK-1 protein in the groups of iPSC-based treatments.
**CONCLUSIONS.** In this study, evidence showed that both iPSCs and iPSC-derived conditioned medium could provide therapeutic benefits on the reversal of experimental PAH rats. Importantly, the results further indicated that the iPSC-based treatments could suppress the abnormal proliferation and migration of PASMCs via the downregulation of PAK-1/ROS/Hif-1a signaling.

### 0904

#### Enhanced expression of MMP1 and MMP10 gene by pro-inflammatory macrophage(mø) in pulmonary arterial hypertension (PAH)

W.-C. Huang[1,2,3], M.-W. Ke[4], S.-H. Kuo[1], C.-C. Cheng[1,2,3], M.-C. Shen[4], W.-T. Hung[1], S.-C. Lin[1], K.-C. Lin[1], W.-T. Huang[1], S.-R. Wann[5], C.-P. Liu[6]
[1]Kaohsiung Veterans General Hospital, Intensive Care Unit, Kaohsiung City, Taiwan, Province of China; [2]National Yang-Ming University, School of Medicine, Taipei, Taiwan, Province of China; [3]Fooyin University, Department of Physical Therapy, Kaohsiung, Taiwan, Province of China; [4]Kaohsiung Veterans General Hospital, Department of Medical Education and Research, Kaohsiung, Taiwan, Province of China; [5]Kaohsiung Veterans General Hospital, Department of Emergency, Kaohsiung City, Taiwan, Province of China; [6]Kaohsiung Vaterans General Hospital, Cardiovascular Medical Center, Kaohsiung City, Taiwan, Province of China
**Correspondence:** S.-H. Kuo
*Intensive Care Medicine Experimental* 2017, **5(Suppl 2):**0904

**INTRODUCTION.** PAH is characterized by vascular remodeling of pulmonary arteries, which is associated with abnormal proliferation of pulmonary arterial smooth muscle cells, deposition of extracellular matrix proteins and perivascular inflammation. Matrix metalloproteinases (MMPs) are critical to the maintenance of the homeostasis of extracellular environment. Several studies have discussed the role of different MMPs in the pathogenesis of PAH with the underlying mechanism still unclear.
**OBJECTIVES.** We try to explore the role of MMP1 and MMP10 in PAH, and to investigate the molecular mechanisms involved in the regulation of MMP1 and MMP10 expression.
**METHODS.** We compared the expression levels of MMP1 and MMP10 in the serum between healthy and PAH group. In vitro, human monocyte-derived mø from each group were subjected to the induction of MMP1 or MMP10 expression following LPS/IFNg stimulation. The expression of mRNA and protein of MMP1 or MMP10 was analyzed by semi-quantitative PCR and western blot. The human monocyte cell line, named THP-1, in combined with chemical inhibitors, including PD98059, LY294002, SB203580, SP600125, STAT1, and STAT3 were used to investigate the signaling pathways involved in the regulation of MMP1 and MMP10 expression. For *in vivo* study, the lung specimens from monocrotaline (MCT)-induced PAH rat were examined for the expression of MMP1 and MMP10 by semi-qPCR and immunohistochemistry(IHC), respectively.
**RESULTS.** In human serum, the level of MMP1 and MMP10 is significantly higher in PAH patients. The elevated expression MMP1 and MMP10 was confirmed by *in vitro* cultured primary human mø that robust up-regulated MMP1 and MMP10 expression was observed from mø derived from PAH patients under the stimulation of LPS/IFNg. Due to the variation between primary cultured mø, the human THP-1 cell line, which was polarized by LPS/IFNg stimulation, provided more convincible data of the up-regulation of MMP1 and MMP10 expression in pro-inflammatory mø. After treatment of inhibitors, the expression of MMP1 was significantly suppressed when the ERK signaling was interfered, whereas MMP10 expression was associated with ERK, JNK, and STAT3 signal cascades. For *in vivo* evidence, the expression of MMP1 and MMP10 was elevated in the lung tissue of MCT-induced PAH rats compared to the PBS control group. IHC staining revealed the co-localization of MMP1 or MMP10 molecule with the COX-2-positive mø, which meant the expression of MMP1 and MMP10 was highly associated with the pro-inflammatory mø phenotype.
**CONCLUSIONS.** In this study, we provided evidence that MMP1 and MMP10 were significantly up-regulated in pro-inflammatory mø *in vitro* and *in vivo* in both primary cultured human mø, THP-1 cell line and MCT-induced PAH rat model. The elevated MMP1 and MMP10 levels in the serum of PAH patients might be developed as useful biomarkers for PAH. We further indicated that blockade of JNK and ERK signaling pathways may be a novel treatment of PAH.

### 0905

#### Systematic echocardiographic monitoring after cardiac surgery is associated with prolonged inotrope infusion and favourable outcomes: results of a multicenter study

L. Valeanu, M. Stefan, S. Provenchere, D. Longrois
Bichat-Claude Bernard Hospital, Department of Anaesthesiology and Critical Care, Paris, France
**Correspondence:** L. Valeanu
*Intensive Care Medicine Experimental* 2017, **5(Suppl 2):**0905

**INTRODUCTION.** Clinical monitoring and postoperative care for adult cardiac surgery underwent continuous improvement following introduction of minimally invasive cardiac output monitoring and echocardiography in intensive care, with a decline in mortality and morbidity. However, the last multicentre study reporting the possible changes in practice was performed in France in 2001.

**OBJECTIVES.** We present data from a prospective, observational study, regarding the current clinical practice in cardiac surgery and postoperative care.

**METHODS.** After approval from the Ethics and Research Committee, 32 centres in France included patients undergoing cardiac surgery from 2 November to 20 December 2015. Patients with transcatheter aortic valve replacement, extracorporeal life support, congenital cardiac surgery, wound infections or pericardial drainage were excluded.

**RESULTS.** Tables 218 and 219 present comparative data from our centre and the whole cohort. While the populations were similar in terms of pre-operative risk and co-morbidities, there are significant differences concerning type of surgery, mean extracorporeal circulation (ECC) and cross clamping time.

Compared to other centres, our centre showed remarkable preference for echocardiography. No patient had invasive intraoperative CO monitoring, 36% of patients were monitored intraoperatively by transesophageal echocardiography (TEE), with all patients being monitored by TEE or transthoracic echocardiography (TTE) in the ICU.

Using this monitoring protocol, there was no significant difference between the percent of patients receiving vasoactive drugs after ECC, the mean lactate and ScVO2 values after ICU admission. ScVO2 measurement was performed in 8.6% of patients in our group, compared with 36.5% in the cohort.

Concerning the duration of vasoactive drugs use, no significant difference was observed for norepinephrine. However, dobutamine infusion time was significantly longer in our group, suggesting that repeated evaluation of cardiac function using echocardiography may be more accurate in guiding inotrope treatment and maintaining an adequate cardiac output when compared to other monitoring techniques.

Milrinone and levosimendan remain peripheral in clinical practice in the whole cohort and their use could not be evaluated.

There were no significant differences in postoperative complications or duration of ICU stay.

**CONCLUSIONS.** Our data shows that cardiac output evaluation and vasoactive drugs length of infusion can be safely guided using echocardiography as the main postoperative monitoring method in cardiac surgery. Advanced knowledge of echocardiography should be a must for all ICU teams involved in postoperative cardiac surgery care.

**Table 218 (Abstract 0905).** See text for description

| *p<0,05 | Bichat Number | Total Number |
|---|---|---|
| Included patients | 115 (%) | 2623 (%) |
| Mean Euroscore II | 3.89+/-2.36 | 3.75+/-0.5 |
| Mean ECC time*(minutes) | 67+/-33 | 97+/-48 |
| TEE intraoperative/ICU | 42 (36)/6 (5) | 1139 (36)/250 (8) |
| TTE ICU* | 110 (95) | 1327 (42) |
| CO monitoring intraoperative*/ICU* | 0/3 (2.6) | 387 (15)/338 (10) |
| Vasoactive drugs | | |
| Norepinephrine (NE) | 58 (50.43) | 1560 (50.32) |
| Dobutamine | 20 (17.39) | 422 (14.25) |

**Table 219 (Abstract 0905).** See text for description

| *p<0,05 | Bichat Number | Total Number |
|---|---|---|
| Postoperative measurement | | |
| ScVO2* | 10 (8.69) | 1134 (36.58) |
| ScVO2 value (mean) | 69.8+/-4.87 | 71.18+/-10.29 |
| Lactate value (mean) | 1.79+/-0.71 | 1.79+/-1.42 |
| Duration of vasoactive drugs infusion (hours) (mean) | | |
| NE | 25.62+/-56.8 | 36.69+/-79.84 |
| Dobutamine* | 94.21+/-110.49 | 59.98+/-68.78 |
| All complications confunded | 52 (45) | 1338 (43) |
| Mean ICU stay (days) | 4+/-4.8 | 4.1+/-4.8 |

### 0906

#### Red blood cells morphology monitoring as a predictor hypervolemia and pulmonary edema in cardiosurgery tetralogy of fallot

R.A. Ibadov, N.A. Strijkov, I.M. Baibekov
SC National Specialized Center for Surgery named after Academician V. Vakhidov, ICU, Tashkent, Uzbekistan
**Correspondence:** R.A. Ibadov
*Intensive Care Medicine Experimental* 2017, **5(Suppl 2):**0906

**INTRODUCTION.** Hyperfunction of subcluvian- pulmonary anastomosis in patients with tetralogy of Fallot (TOF) is known to be a common condition in the early post-operative period. Morphology of peripheral blood cells significant changes after the cardiosurgery of TOF patients.

**OBJECTIVES.** Come to know to determine morphometry of peripheral blood cells of TOF patients in the number of pathologically shaped red blood cells (PS RBCs) as a predictor of development of anastomosis hyperfunction/hypervolemia and pulmonary edema.

**METHODS.** 81 TOF patients aged 1–22 years (mean age 8.7 ± 0.9), in all the cases, the modified Blaloc-Taussig (BT) anastomosis was formed. Artificial lung ventilation was carried out to the SPA patients in the standard regimes in early post-operative period. Morphometry collate control: scanning electronic microscopy (SEM) with elaborated and patented the methods of studying the discrete structures, namely the express-method of "thick drop" (TDEM) for practical and research purposes.

**RESULTS.** Morphometry of peripheral blood cells in patients with tetralogy of Fallot demonstrated that the number of pathologically shaped RBCs has increased up to 41% after the cardiosurgery TOF, these were mainly erythrocytes with a ridge (up to 19%).

The early post-operative period after formation of subclavian-pulmonary anastomosis is characterized by the decrease of erythrocytes up to 56-57%, while the number of pathologically shaped erythrocytes in peripheral blood below 49% is the morphological predictor of anastomosis hyperfunction development.

**CONCLUSIONS.** Morphometry of peripheral blood cells of patients with tetralogy of Fallot has revealed the increase in the number of pathologically shaped red blood cells, mainly RBCs with a ridge. The anastomosis surgery leads to the predominance of the RBCs pathological shapes in peripheral blood. Progressive deterioration of RBCs morphology is a predictor of the anastomosis hyperfunction development with hypervolemia of the pulmonary circulation and pulmonary edema after the surgery.

The morphological monitoring of correlation between normal and pathological forms of erythrocytes can be the criterion of effectiveness of medical and diagnostic tactics in cardiologic resuscitation.

### Acute brain injury 2

#### 0907

#### Incidence and risk factors for the development of hyperammoniemia during treatment with valproic acid

C. De Fazio[1,2], M. Goffin[1], S. Spadaro[2], A. Brasseur[1], E. Antonucci[1], I. Romeo[1], J.-L. Vincent[1], J. Creteur[1], B. Legros[3], F.S. Taccone[1]
[1]Hopital Erasme, Dept. of Intensive Care, Brussels, Belgium; [2]Università Ferrara, Arcispedale Sant'Anna, Dept. of Morphological Surgery and Experimental Medicine, Ferrara, Italy; [3]Hopital Erasme, Dept. of Neurology, Brussels, Belgium
**Correspondence:** C. De Fazio
*Intensive Care Medicine Experimental* 2017, **5(Suppl 2):**0907

**INTRODUCTION.** Elevated blood ammonium levels (EAL) is a potential side effects of valproic acid (VPA) treatment. It has been described during long-term VPA treatment, but less in critically ill patients.

**OBJECTIVE.** Our aim was to evaluate the incidence and the factors associated with EAL in these patients.

**METHODS.** We reviewed the data of all adult patients treated in our mixed 35-bed Dept of Intensive Care over a 9-year period (2008–2016) who: a) were treated with VPA for more than 72 hours and b) had at least one measurement of ammonium and VPA levels during the ICU stay; patients with Child C liver cirrhosis were excluded. EAL wer defined as ammonium levels above 60 mcg/mL.

**RESULTS.** Of a total of 1938 patients treated with VPA, a total of 319 patients met the inclusion criteria (median age 64 years; male gender 77%). More than 75% were admitted for neurological reasons. Overall ICU mortality was 30%. Median ammonium levels were 88 [63–118] mcg/dL. EAL was found in 245 (77%) patients; including 75 (31%) with ammonium levels between 100–150 mcg/dL, 23 (9%) between 151–200 mcg/dL and 18 (7%) above 250 mcg/dL. Median time from start of VPA therapy to EAL was 3 [2–5] days. Median maximum VPA level during therapy was 76 [57–93] and was significantly higher in EAL patients than in the others (79 [63–97] mcg/mL vs. 61 [45–79] mcg/mL, $p < 0.01$). In 98/245 (40%) of EAL patients, VPA was interrupted; VPA interruption was more frequent in patients with ammonium levels > 100 mcg/dL than others ($p < 0.001$). In a multivariable analysis, high VPA concentrations, the use of mechanical ventilation and the occurrence of sepsis were independently associated with EAL during VPA therapy.

**CONCLUSIONS.** In this study, EAL was a common finding during treatment with VPA in acutely ill patients. VPA levels, sepsis and mechanical ventilation were risk factors for EAL.

#### 0908

#### Does antibiotic prophylaxis influence the incidence of EVD-related infections? A prospective multicentric study

J. Berger-Estilita[1], M. Passer[2], M. Giles[2], T. Merz[3]
[1]Kantonsspital Olten, Perioperativ Medizin, Olten, Switzerland; [2]Royal North Shore Hospital, University of Sydney, Department of Intensive Care Medicine, Sydney, Australia; [3]Inselspital, Universitätspital Bern, Intensivmedizin, Bern, Switzerland
**Correspondence:** J. Berger-Estilita
*Intensive Care Medicine Experimental* 2017, **5(Suppl 2):**0908

**INTRODUCTION.** The insertion of an external ventricular drain (EVD) is frequently a lifesaving procedure in the neurologic intensive care unit. EVDs are not devoid of complications: EVD-related infections, for example, significantly prolong hospital stay, increase costs and often negatively affect prognosis. To prevent EVD infection the use of systemic antibiotic prophylaxis is common practice.

**OBJECTIVES.** We aim to investigate if type of antibiotic prophylaxis (single shot at insertion vs. continuous) influences the incidence of EVD-related infections.

**METHODS.** This observational prospective study was conducted for 24 months at the University Hospital of Bern (Switzerland), Royal North Shore Hospital and the Alfred Hospital (Australia). Patients over 16 years of age who had an EVD placed were included. EVD-related infection was diagnosed by the treating physician based on clinical grounds and/or analysis of blood and cerebrospinal fluid samples. The study was approved by the Ethical Committee of the 3 centers.

Baseline characteristics include patient demographics, medical history, indication for placement of an EVD and type of antibiotic prophylaxis used, as well as severity scores. The statistical analysis included Chi square test for categorical and Student´s T for continuous variables. Results are presented in average ± standard deviation. Statistically significant when $p < 0{,}05$. Data were analyzed with SPSS 24.0.

**RESULTS.** We enrolled 187 patients who required insertion of an EVD. One hundred and two (54,5%) were males. The average age was 53,65 ± 17,9 and 6,5% had co-morbidities. The reasons for admission were subarachnoid haemorrhage (45.5%), intracranial haemorrhage (25,7%) and traumatic brain injury (28,9%). The mean GCS on admission was 8,83 ± 4,1. EVD-related infection was present in 31 patients (16,6%). There was no significant difference in age and LOS for the presence of EVD-related infection.

A significant difference in GCS score for EVD-related infection (M = 10,39, SD = 4,01) and no EVD-related infection (M = 8,52, SD = 4,1) was observed; $t(185) = 2{,}32$, $p = 0{,}022$, two-tailed. Peri-procedural prophylaxis was used in 66,8% of patients. There was no significant association between EVD-related infection and type of prophylaxis used, $c^2(1, n = 187) = 0.86$, $p = .35$, $phi = -.08$).

**CONCLUSIONS.** Our preliminary analysis of a large cohort of neurosurgical patients with EVD showed no association between EVD-related infection and the type of antibiotic prophylaxis used. We conclude that single shot prophylaxis is as safe as continuous administration, with evident advantages for costs and possibly less development of antibiotic resistance.

**REFERENCE(S)**

Dey, M., et al., Curr Neurol Neurosci Rep, 2012. **12**(1): p. 24–33.
Hagel, S., et al., Interdiscip Perspect Infect Dis, 2014. **2014**: p. 708531.

**GRANT ACKNOWLEDGMENT**
None.

#### 0909

#### Lacosamide use in the ICU

L. Martinez Pujol, J. Ruiz Izquierdo, R. Algarte Dolset, B. Sánchez Gonzalez, J. Trenado Álvarez
Hospital Universitari Mutua Terrassa, Intensive Care Department, Terrassa, Spain
**Correspondence:** L. Martinez Pujol
*Intensive Care Medicine Experimental* 2017, **5(Suppl 2):**0909

**INTRODUCTION.** Seizures frequently result in emergency episodes (EE) which may require the attention of critical care or may occur in Intensive Care Unit (ICU) patients. Wide variations in response are seen with first line antiepileptic drugs (AEDs) [1]. When convulsive Status epilepticus (SE) was analysed in a population based study, it was refractory in 27%, and, in a retrospective study in an ICU, 43% of the patients had seizures refractory to first-line AEDs [2]. Lacosamide (LAC) is a new AED. Recently experiences shows positive clinical experience of LAC in SE and acute repetitive seizures (ARSs) [3].

**OBJECTIVES.** The primary study objective was to describe the characteristics related to the use of LAC. Our secondary objective was to assess the effectiveness of IV LAC in EE and in patients with SE or ARSs.

**METHODS.** A retrospective, descriptive and single-centre study in our ICU was conducted. All ICU patient ≥ 18 years, with the diagnosis of seizures for which IV LAC was used (January 2014-December 2016) were analysed. Demographic data, seizure type, aetiology, number of AEDs used and order in which they are used, time from IV LAC initiation to seizure cessation, IV LAC dosage and side effects were collected. Qualitative variables are expressed as percentages and compared using the X2-test; quantitative ones are expressed as median and inter-quartile range (IQR), and analysed using Student´s t-test.The level of significance was placed at $p < 0.05$.Statistical analysis was performed using specific software (IBM SPSS Statistics for Window s, Version 19.0. Armonk, NY: IBM Corp).

**RESULTS.** 30 patients; median age 66 (55–77),sex male 46.7%, past medical history of epilepsy 26%, previous use of AEDs 81%, non-convulsive SE 16.7%. Figure 288 shows the type of seizure.

Figure 289 shows seizure aetiology.

Figure 290 shows AEDs treatment order.

Tables 220, 221 & 222 shows LAC treatment use characteristics and efficacy according with treatment order and by type of seizure respectively.

**CONCLUSIONS.** Data shows that IV LAC resulted in a fast and well tolerated cessation of different types of seizures. Our observations suggest that IV LAC may be a potentially effective alternative treatment when standard AEDs fail or are not recommended.

**REFERENCE(S)**

1. Novy J, Logroscino G, Rossetti AO. Refractory status epilepticus: a prospective observational study. Epilepsia 2010;51(2):251–6.

2. Aranda A, Foucart G, Ducasse JL, Grolleau S, McGonigal A, Valton L. Generalized convulsive status epilepticus management in adults: a cohort study with evaluation of professional practice. Epilepsia 2010;51(10):2159–67.

3. Legros B, Depondt C, Levy-Nogueira M, Ligot N, Mavroudakis N, Naeije G, et al. Intravenous lacosamide in refractory seizure clusters and status epilepticus: comparison of 200 and 400 mg loading doses. Neurocrit Care. 2014 Jun;20(3):484–8.

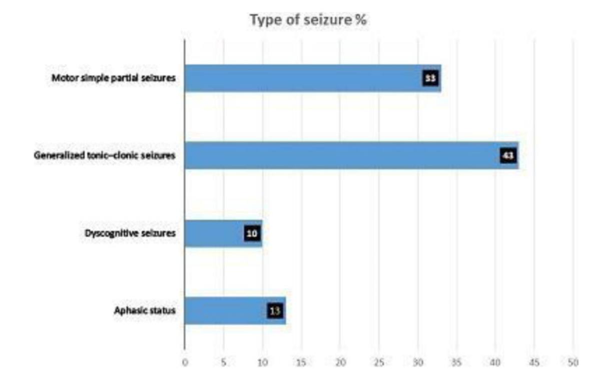

**Fig. 288 (Abstract 0909).** Type of seizure

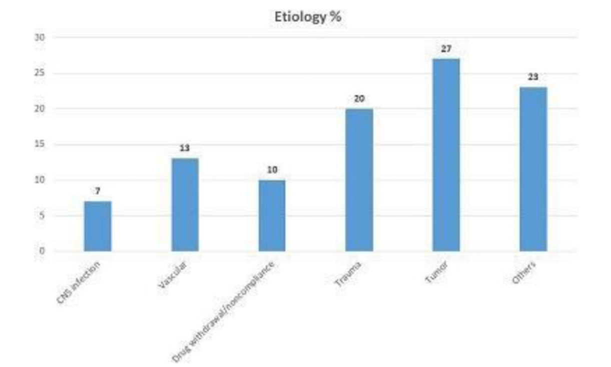

**Fig. 289 (Abstract 0909).** Aetiology

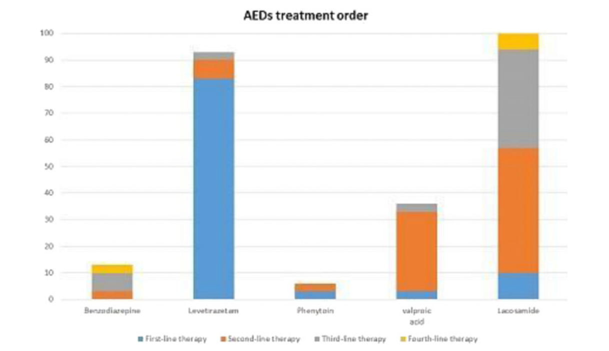

**Fig. 290 (Abstract 0909).** AEDs treatment order

**Table 220 (Abstract 0909).** LAC treatment use characteristics

| | |
|---|---|
| Cessation of seizures after IV Lacosamide, n (%) | 26 (86.7) |
| Further AED therapy needed to control seizures, n (%) | 3 (10) |
| Seizures not controlled, n (%) | 4 (13) |
| Latency time < 6 hours to seizure cessation, n (%) | 24 (80) |
| Loading Dose > 200 mg, n (%) | 22 (75) |
| Need to increase Lacosamide dose, n (%) | 4 (13) |
| Lacosamide at hospital discharge, n (%) | 12 (40) |
| Adverse events, n (%) | 1 (3) |
| ICU mortality, n (%) | 7 (23) |
| Hospital mortality, n (%) | 9 (30) |

**Table 221 (Abstract 0909).** LAC efficacy according with treatment order

| | Fisrt-Line | Second-Line | Third-Line | Fourth-Line | *p* |
|---|---|---|---|---|---|
| No Cessation of seizures, n (%) | 1 (25) | 1 (25) | 2 (50) | 0 | 0.56 |
| Cessation of seizures, n (%) | 2 (7.7) | 13 (50) | 9 (34.6) | 2 (7.7) | 0.56 |

**Table 222 (Abstract 0909).** LAC efficacy by type of seizure

| | Cessation | No cessation | *P* |
|---|---|---|---|
| Aphasic status | 0 | 4 (100) | 0.23 |
| Dyscognitive seizures | 0 | 3 (100) | 0.23 |
| Generalized tonic-clonic seizures | 1 (7) | 12 (92.3) | 0.23 |
| Motor simple partial seizures | 3 (30) | 7 (70) | 0.23 |

### 0910

### Postmortem purines in blood and cerebrospinal fluid - stroke patterns

E.V. Oreshnikov, S.F. Oreshnikova, A.V. Oreshnikov
Chuvash State University, Internal Medicine, Cheboksary, Russian Federation
**Correspondence:** E.V. Oreshnikov

*Intensive Care Medicine Experimental* 2017, **5(Suppl 2):**0910

**INTRODUCTION.** Diagnostic and prognostic value of the intravital parameters of purine metabolism in acute cerebral pathology, including stroke, is studied in detail. Meanwhile, a study of postmortem biochemical processes may provide additional scientific information on the diagnostic value of the parameters of purine metabolism in neurointensive care.

**OBJECTIVES.** Investigate the postmortem parameters purine metabolism in stroke.

**METHODS.** In 50 adult ICU stroke patients, in the first 2 hours after the fact of biological death, the samples of cerebrospinal fluid (CSF) and venous blood on the were performed spectrophotometric determination of the concentration of adenine, guanine, hypoxanthine, xanthine, uric acid, malondialdehyde as a marker of free radical oxidation.

**RESULTS.** Postmortem CSF levels of uric acid and malondialdehyde significantly higher in male patients than in female; uric acid in CSF significantly lower in the presence of intravital arterial hypertension, heart failure, pneumonia. Pneumonia is also associated with a higher postmortem blood concentration of malondialdehyde, and multiple organ failure with a higher concentration of uric acid and malondialdehyde in CSF. The ratio of the CSF concentrations of uric acid / xanthine higher in ischemic, than in hemorrhagic stroke. The ratio of the concentrations of uric acid / xanthine, xanthine / hypoxanthine, uric acid / hypoxanthine was significantly lower in the presence of pneumonia in patients with stroke.

**CONCLUSIONS.** Oxypurines significantly associated not only with gender, arterial hypertension or stroke type, but also such lifethreatening conditions as pneumonia, heart and multiple organ failure. Possibly, the mandatory inclusion of purines in the vital panel of monitored biochemical parameters will improve the results of neurointensive care.

### 0911

#### Importance of purine metabolites in preeclampsia and acute cerebral stroke

E.V. Oreshnikov[1], S.F. Oreshnikova[1], E.N. Vasiljeva[2], T.G. Denisova[2]
[1]Chuvash State University, Internal Medicine, Cheboksary, Russian Federation; [2]Chuvash State University, Obstetrics and Gynecology, Cheboksary, Russian Federation
**Correspondence:** E.V. Oreshnikov
*Intensive Care Medicine Experimental* 2017, **5(Suppl 2):**0911

**INTRODUCTION.** Along with the classic triad edema, proteinuria, hypertension, more than a quarter century, many clinicians as an indicator of preeclampsia using the high content of uric acid in blood serum hyperuricemia.

It was also found that the hypoxanthine, xanthine and uric acid (UA) are present in the brain, and their content is changed after ischemia, UA is the end product of purine degradation in the brain, xanthine oxidase is also present in the brain, it catalyzes the oxidation of hypoxanthine to xanthine, and then in UA and can be a source of free radicals, inhibition of xanthine oxidase and exogenous administration of UA accompanied by explicit antiischemic and neuroprotective effects in the experiment and clinic, while the endogenous increased its production, with the "side" synthesis of xanthine oxidase oxygen free radicals, reflects the severity of ischemic and reperfusion injury. We also know that most fatal path pathogenesis (and tanatogenesis) in preeclampsia the development of cerebral stroke.

**OBJECTIVES.** Our attention was attracted by a comparative assessment of the features of purine metabolism in women with preeclampsia and acute cerebral stroke.

**METHODS.** The study involved 33 patients with preeclampsia and 350 patients in the
acute period of cerebral stroke, in which, in addition to conventional laboratory parameters were determined in the blood and cerebrospinal fluid of guanine, hypoxanthine, adenine, xanthine and uric acid direct spectrophotometry.

**RESULTS.** It was established that between preeclampsia and cerebral stroke, there are clinical and pathobiochemical parallels, including according to the characteristics of purine metabolism. Hyperuricemia the most famous and at the same time the most pronounced adverse metabolic factor (marker, predictor) for preeclampsia, and for cerebral stroke. High value content oxypurines (hypoxanthine, xanthine and uric acid) in the cerebrospinal fluid a good sign for a stroke, and low for preeclampsia.

**CONCLUSIONS.** Liquor can be seen not only as a medium of administration of drugs for spinal anesthesia, but also and a source of valuable diagnostic (and predictive) information, including in preeclampsia. The level of uric acid and other purine both patients with preeclampsia, and cerebral stroke, it is desirable to investigate not only in serum but when possible, and in cerebrospinal fluid.

### 0912

#### Frequency and types of delirium in patients with ischemic and hemorrhagic strokes

A. Gritsan[1], N. Dovbysh[1], E. Korchagin[2], O. Chernechkiy[3]
[1]Krasnoyarsk Regional Clinical Hospital, Krasnoyarsk State Medical University, Anaesthesiology and Intensive Care, Krasnoyarsk, Russian Federation; [2]Krasnoyarsk Regional Clinical Hospital, Krasnoyarsk, Russian Federation; [3]Krasnoyarsk Regional Clinical Hospital, Anaesthesiology and Intensive Care, Krasnoyarsk, Russian Federation
**Correspondence:** A. Gritsan
*Intensive Care Medicine Experimental* 2017, **5(Suppl 2):**0912

**INTRODUCTION.** Delirium is a syndrome that can develop on the background of many diseases. The development of delirium leads to an increase in the length of the patient´s stay in the intensive care unit and the hospital, and increases the mortality. The frequency of occurrence of delirium in intensive care units reaches 32.3% according to a number of authors and in specialized intensive care units can be much higher [1].

**OBJECTIVES.** To evaluate the frequency and types of delirium in patients with ischemic and hemorrhage stroke.

**METHODS.** We conductedan observational study of 40 patients admitted to the intensive care unit for patients with acute neurologic pathology. Patients were divided into 2 groups - the 1st group (n = 17, 42.5%) patients whom delirium was diagnosed and the 2nd group (n = 23, 57.5%) - patients without delirium (Table 223).

We studied types of stroke in the groups. The presence of delirium was carried out on the scale ICDSC [2] in the first 24–48 hours from the moment of admission; also, we assessed the degree of depression of consciousness on the CGS and FOUR scales; the degree of agitation, sedation was assessed on the RASS scale. Statistical analysis was performed using the Mann–Whitney test for independent samples and the Pearson´s chi-squared test for non-parametric variables.

**RESULTS.** In 1st group, ischemic stroke (IS) was diagnosed - 11 (65%) patients, hemorrhagic stroke (HS) - 6 patients (35%), of them in the form of intracerebral hematoma (ICH) - 3 patients (17.5%) and in the form of subarachnoid hemorrhage (SAH) - 3 patients (17.5%). In the 2nd group, IS was diagnosed in 16 patients (70%), HS - in 7 patients (30%), ICH - 6 patients (25.6%) and SAH- 1 patient (4, 4%).

The degree of severity of depression on the CGS and FOUR scales, the degree of sedation / arousal on the RASS scale are presented in Table 224.

The degree of depression of consciousness by CGS and FOUR was more pronounced in group 1.

In the 1st group subsyndromal delirium was diagnosed in 12 patients (71%), depending on the ICDSC score, and delirium was diagnosed in 5 patients (29%).

Depending on the RASS score from 5 patients with delirium 2 patients had hypoactivedelirium, 1 patient -hyperactive, and 2 patients had mixed delirium.

CONCLUSIONS

1. The frequency of delirium in patients with Ischemic and Hemorrhagic Strokes was 42.5%.
2. Subsyndromal delirium developed more often (71.0% of cases) in patients with Ischemic and Hemorrhagic Strokes (ICDSC score less than 3 points); and types of delirium were hypoactive and mixed variants.

3. The type of stroke (IS or HS) did not affect the frequency developmentof delirium.

**REFERENCES**
1. Salluh JI, et al. Delirium Epidemiology in Critical Care Study Group: Delirium epidemiology in critical care (DECCA): an international study. Crit Care 2010,14(6):R210. 10.1186/cc9333
2. Bergeron N, et al. Intensive care delirium screening checklist: evaluation of a new screening tool. Intensive Care Med 2001,27(5):859–864.

**Table 223 (Abstract 0912).** Characteristic of patients

| Group | Sex, m/f | Age, year, M±σ | p |
|---|---|---|---|
| 1 group | 7 (41%)/10 (59%) | 60.2±11.0 | >0.05 |
| 2 group | 14 (61%)/9 (39%) | 64.1±15.1 | >0.05 |

**Table 224 (Abstract 0912).** See text for description

| | CGS | FOUR | RASS |
|---|---|---|---|
| 1 group, Me [25;75] | 14 [9;15] | 16 [9;16] | 0 [-3;0] |
| 2 group, Me [25;75] | 15 [14;15] | 16 [16;16] | −1 [-1;0] |
| p | <0.05 | <0.05 | <0.05 |

### 0913

#### Comparison of metabolic pathways utilizing quantitative plasma metabolomics in critically ill burn and neurosurgical patients

E.T. Van Matre[1], S.W. Mueller[1], K. Quinn[2], K. Doenges[2], M. Armstrong[2], T.H. Kiser[1], R. MacLaren[1], D.N. Fish[1], R. Reisdorph[2], N. Reisdorph[2]
[1]University of Colorado Skaggs School of Pharmacy and Pharmaceutical Sciences, Clinical Pharmacy, Denver, United States; [2]University of Colorado Skaggs School of Pharmacy and Pharmaceutical Sciences, Pharmaceutical Sciences, Denver, United States
**Correspondence:** E.T. Van Matre
*Intensive Care Medicine Experimental* 2017, **5(Suppl 2):**0913

**INTRODUCTION.** There is evidence that patients with acute cerebral nervous system injuries have temporary impairment of cellular immune function placing them at increased risk of infection. Cellular alterations leading to amplification of the inflammatory cascade and the various directs effects of T and B cells on neuronal cells have been described.

**OBJECTIVES.** Characterize and compare metabolic pathways in acute critically ill neurosurgical (NS) patients to critically ill burn patients utilizing metabolomics.

**METHODS.** Blood samples were obtained at baseline (D0) and day 14 (D14). Samples from 32.

(22 NS and 10 burn) patients were analyzed. Baseline characteristics were similar between the two groups except age was significantly higher in the NS group (56.4 ± 14.2 vs 38.5 ± 14.2). We conducted a metabolomics analysis where an organic extraction resulted in lipid and non-lipid fractions; these were analyzed by untargeted mass spectrometry. A database containing publicly available and in-house spectral data was used to annotate metabolites. Metabolites of D0 NS vs D14 NS, D0 burn vs D14 burn, and D0 NS vs D0 burn were compared by unpaired t-tests. Statistical significance was set at $p < 0.05$ with a fold change of >2.

**RESULTS.** The change between D0 vs D14 NS patients in the lipid phase untargeted metabolomics yielded 79 tentatively annotated with 6 significant pathways: glycerophospholipid (GPL) metabolism, glycosylphosphatidylinositol (GPI)-anchor biosynthesis, regulation of autophagy, phospholipid biosynthesis, alpha linolenic acid and linoleic acid metabolism, and biosynthesis of 12-, 14- and 16-membered macrolides. The change between D0 vs D14 burn patients showed 53 tentatively annotated metabolites with 6 significant pathways: GPL metabolism, GPI-anchor biosynthesis, regulation of autophagy, phospholipid biosynthesis, sphingolipid metabolism, and arachidonic acid metabolism. Finally, the comparison of D0 NS and D0 burn patients showed 89 tentatively annotated metabolites with 9 significant pathways: GPL metabolism, GPI-anchor biosynthesis, regulation of autophagy, phospholipid biosynthesis, alpha-linolenic acid metabolism, biosynthesis of 12-, 14- and 16-membered macrolides, isoquinoline alkaloid biosynthesis, arachidonic acid metabolism, and biosynthesis of alkaloids derived from shikimate pathway. Targeted analysis of lipid mediators supported a role of arachidonic metabolism and inflammation at D14 NS patients.

**CONCLUSION.** Our findings suggest there are significant alterations that occur in several lipid-rich pathways. These differences are seen over a 14 day period and baseline between NS and burn critically ill populations. These alterations may describe immunosuppression seen within acute NS patients. Untargeted aqueous fraction analysis, bioinformatics analysis, and validation of differential expressions of cytokines are in progress.

### 0914

#### Mapping spatial biochemical changes in a mouse model of traumatic brain injury with Raman imaging

A. Ercole[1], J. Surmaki[2], L. Ansel-Bollepalli[1], F. Pischiutta[3], S. Bohndiek[2], E. Zanier[3]
[1]University of Cambridge, Cambridge, United Kingdom; [2]University of Cambridge, Department of Physics, Cambridge, United Kingdom; [3]IRCCS - Istituto de Ricerche Farmacologiche Mario Negri, Department of Neuroscience, Milan, Italy
**Correspondence:** A. Ercole
*Intensive Care Medicine Experimental* 2017, **5(Suppl 2):**0914

**INTRODUCTION.** Traumatic brain injury (TBI) is a leading cause of death and disability. Ultimate tissue fate is determined not only by the initial disruptive force but also by a cascade of complex inflammatory processes that may be long-lived and long-ranged. Label-free methods for probing the biochemical signatures of injury processes that are also sensitive to structure may offer insights into TBI pathophysiology beyond conventional histochemistry.

**OBJECTIVES.** We have previously demonstrated [1] that Raman spectroscopy (RS) is sensitive to local biochemical signatures of TBI across the cortical surface of a mouse model of TBI. In particular, the technique is sensitive to protein and lipid changes which may be of particular mechanistic interest. We seek to extend this technique to cross-sectional imaging to investigate the biochemical effect of TBI on deep structures.

**METHODS.** Adult C57BL/6 mice were exposed to a controlled cortical impact model of severe TBI. Mice were sacrificed at 2 and 7 days after injury (alongside sham TBI) and perfused with PBS. Fresh frozen tissue was sectioned and imaged using a Raman microscope (WITec GmbH, Ulm, Germany) equipped with a 785nm single mode diode laser (XTRA II;Toptica Photonics Inc., USA), a 300mm triple grating imaging spectrometer (Acton SpectraPro SP-2300; Princeton Instruments Inc., USA) with 600mm$^{-1}$ grating and a thermoelectrically cooled CCD camera (DU401A-BV; Andor, Ireland).

**RESULTS.** We were able to produce false-colour images of biochemical fingerprints over time. Strong signals at 1003cm$^{-1}$ and 1560cm$^{-1}$ are seen at 2 days and persist to 7 days post injury in the pericontusional tissue. These are likely to be protein signatures either as a result of vascular injury haemorrhage or leakage, or from the presence of inflammatory processes in the region of the contusion. At the same time, we see striking reductions in other Raman peaks such as 1301cm$^{-1}$ and 1440cm$^{-1}$ that are associated with lipid components in the ipsilateral corpus callosum and internal capsule quite some distance from the lesion at 2 days. These signal partially recover by 7 days.

**CONCLUSIONS.** We have performed the first RS imaging studies in a TBI model and have demonstrated not only that RS is sensitive to temporal biochemical changes, but also that signal changes have an anatomically sensitive long-range spatial distribution. This approach is able to detect multiple chemical species simultaneously in the same sample without the need for staining.

**REFERENCES**
[1] Surmacki JM, Ansel-Bollepalli L, Pischiutta F, Zanier ER, Ercole A, Bohndiek SE. Label-free monitoring of tissue biochemistry following traumatic brain injury using Raman spectroscopy. Analyst. 2016;142(1):132–9.
**GRANT ACKNOWLEDGEMENT**
Funded by departmental resources.

### 0915

#### Ammonium tetrathiomolybdate, a novel sulphide donor, given on reperfusion, reduces cerebral infarct size and improves functional outcomes

B.P. Mendonça[1], J.S. Cardoso[1], A. Manfredini[1], M. Michels[1], A. Dyson[2], M. Singer[3], F. Dal-Pizzol[1]
[1]University of Southern Santa Catarina, Laboratory of Experimental Pathophysiology, Criciúma, Brazil; [2]University College London, Bloomsbury Institute of Intensive Care Medicine and Magnus Oxygen, London, United Kingdom; [3]University College London, Bloomsbury Institute of Intensive Care Medicine and Magnus Oxygen Ltd, London, United Kingdom
**Correspondence:** B.P. Mendonça
*Intensive Care Medicine Experimental* 2017, **5(Suppl 2):**0915

**INTRODUCTION.** Early revascularization of ischaemic organs is key to improved outcomes yet the consequent reperfusion injury may be harmful in itself. Reperfusion injury is largely attributed to excess production of reactive oxygen species by mitochondria. As sulphide inhibits mitochondria and reduces ROS production, sulfide donors can potentially confer therapeutic utility in this setting.
**OBJECTIVE.** To determine the efficacy of ammonium tetrathiomolybdate (ATTM), a novel sulphide donor, on infarct size and functional outcomes, when given at reperfusion following transient middle cerebral artery occlusion (tMCAO) in rats.
**METHODS.** tMCAO was induced by insertion of an intraluminal filament into the middle cerebral artery of isoflurane-anaesthetised rats for 90 minutes. Animals were treated with IV ATTM (10 mg/kg bolus just before reperfusion, then 10 mg/kg infusion over 60 min) or saline. Animals were then allowed to recover. Infarct size, brain interleukin (IL)-1 and IL-6, oxidative damage markers and motor function were determined at either 24 hours or 7 days. Statistical comparisons between saline and ATTM-treated animals were performed by Student's t-test.
**RESULTS.** ATTM given at reperfusion significantly ($p < 0.05$) decreased infarct size, inflammation (IL-1 and IL-6) and oxidative damage, and improved performance in the rotarod test, a surrogate of motor function, both at 24 hours and 7 days. Figure 1 shows results at 7 days post-tMCAO.
**CONCLUSION.** ATTM given at reperfusion showed both functional and histological benefit in a clinically realistic stroke model. This was associated with reductions in brain oxidative damage and inflammation.
**FUNDING.** Magnus Oxygen, MRC Newton Fund and Fundação Apoio a Pesquisa do Estado de Santa Catarina.

### 0916

#### Imaging cerebral blood flow-metabolism uncoupling and mitochondrial dysfunction after good-grade subarachnoid hemorrhage

H.S. Mangat[1], X. Mao[2], D. Shungu[2], J. Cho[2], Y. Wang[2], P.E. Stieg[3]
[1]Weil Cornell University, Neurology, New York, United States; [2]Weil Cornell University, Radiology, New York, United States; [3]Weil Cornell University, Neurological Surgery, New York, United States
**Correspondence:** H.S. Mangat
*Intensive Care Medicine Experimental* 2017, **5(Suppl 2):**0916

**INTRODUCTION.** Subarachnoid hemorrhage (SAH) causes high morbidity and mortality. 50% of patients suffer cerebral vasospasm and 30% cerebral infarction. PET studies have shown cerebral blood flow (CBF)-metabolism uncoupling after poor grade SAH.[1,2] However, PET cannot be routinely performed and this limits understanding the role of metabolic dysfunction in early brain injury.
**OBJECTIVES.** To utilize Magnetic Resonance (MR) based imaging of neuronal energy metabolism and determine if cerebral blood flow-metabolism uncoupling occurs after mild SAH.
**METHODS.** Patients with Good-grade SAH who underwent aneurysm coiling and had no clinical deficit or cerebral infarction were included. Structural MRI, Multi-slice $^1$H MRS were obtained. NAA, CSF lactate were measured and expressed as ratios of root mean square (RMS) of background and compared to controls from data library and to CBF from most proximate CT perfusion.
Separately, ASL and QSM sequences were obtained in four patients. CBF maps were created from ASL sequences, and $CMRO_2$/OEF were calculated from QSM using Minimum Local Variance (MLV) method.[3]
**RESULTS.** Average age was 58 years for MRS patients, Hunt Hess score was 2.43, modified Fisher score was 2.79. 3 patients had DCI and none had cerebral infarction. Median discharge GCS was 15. MRS was done at 9.9 days from admission. SAH patients demonstrated significantly reduced NAA/RMS in frontal lobes compared to controls (16.18 ± 4.96 vs. 20.93 ± 5.56, $p = 0.042$) but not in temporal (16.49 ± 4.37 vs. 19.37 ± 4.38, $p = 0.09$) or occipital lobes (20.62 ± 4.50 vs. 21.05 ± 4.23, $p = 0.41$). CSF lactate was significantly higher in SAH patients (7.74 ± 2.27 vs. 4.02 ± 0.76, $p = 0.001$). NAA/RMS did not correlate with CBF in pooled data ($R^2 = 0.02$, $p = 0.40$) or in frontal lobe rCBF ($R^2 = 0.001$, $p = 0.92$); nor with CSF lactate ($R^2 = 0.02$, $p = 0.53$).
4 patients also underwent ASL, QSM, $CMrO_2$ imaging. Overall grey matter (GM) CBF was 55.52 ± 1.93ml/100g/min, $CMrO_2$ was 139.25 ± 7.88μmol/100g/min and OEF was 33.5% Mean GM CBF was 55.9ml/100g/min in frontal, 57.0 in parietal, 52.7 in temporal and 56.5 in occipital lobes; GM $CMrO_2$ was 131μmol/100g/min in frontal, 138 in parietal, 150 in temporal,138 in occipital lobes; GM OEF was 32% in frontal, 32% in parietal, 37% in temporal, 33% in occipital lobes. Regional $CMrO_2$ and OEF were 10% lower compared to controls.
**CONCLUSIONS.** MR based spectroscopy and metabolic imaging shows reduced regional NAA, elevated CSF lactate as well as depressed $CMRO_2$ and OEF with normal range CBF. This implies perfusion independent mitochondrial dysfunction (decreased $CMrO_2$ and OEF) resulting in decreased NAA production and increased lactate generation.This is a novel MR based paradigm that is feasible and reveals the potential role of mitochondrial dysfunction in early brain injury after SAH.
**REFERENCE(S)**
1. Sarrafzadeh A,et al. Stroke 2004; 35:638–643.
2. Sarrafzadeh A,et al. Journal of Neurorad 2005;32.
3. Zhang Jingwei,et al. Magn Res Med 2017.

### 0917

#### Pupillometry characterisation of the ´sluggish pupil´

H.K. Kanthimathinathan[1], R. Nagaraj[1], J. Spry[1], K. Morris[1], B. Scholefield[1,2]
[1]Birmingham Children's Hospital, Paediatric Intensive Care Unit, Birmingham, United Kingdom; [2]University of Birmingham, Birmingham, United Kingdom
**Correspondence:** H.K. Kanthimathinathan
*Intensive Care Medicine Experimental* 2017, **5(Suppl 2):**0917

**INTRODUCTION.** Monitoring the pupillary light reflex is an essential part of neuromonitoring. In the neurocritical care setting, a sluggish pupillary response is considered as an early warning sign of brain injury. Although the term "sluggish pupil" has been widely used at the bedside for decades, the sluggish pupillary response has not been well characterised before. Automated infrared pupillometry is able to quantify individual components of a pupillary light reflex - latency, speed of constriction and percentage change in pupil size.

**OBJECTIVES.** Using automated infrared pupillometry to study the differences in latency, speed and magnitude of change in pupil size, between pupils reported as “sluggish” and “brisk” in a pen-torch test.

**METHODS.** Prospective observational study of children admitted to a paediatric intensive care unit (PICU) after either traumatic brain injury or cardiac arrest between November 2016 and March 2017. Pupillary light reflex was tested using an automated infrared pupillometer (Neuroptics NPi-200) once a day for the first 7 days or until PICU discharge by trained nurses or medical staff. Pupillary light reflex of each eye was assessed with a pen-torch; size of pupils (in mm) and pupillary response (brisk or sluggish) were documented before the pupillometry. Characteristics of sluggish pupillary response were compared against brisk pupillary response using univariable analysis. Microsoft Excel 2013 and R (version 3.2.2) were used in statistical analysis. Data presented as median (interquartile ranges) or percentages and non-parametric statistics were used.

**RESULTS.** 60 pupillary light reflex readings were obtained in 7 patients (median: 4; range: 2–24 readings per patient) during the study period. On pen-torch assessment 39 were recorded as sluggish and 21 as. Univariable analysis identified significant differences in size, speed and magnitude of change but not latency of the pupillary reflex to a light stimulus. (Table 225)

**CONCLUSIONS.** When a pupillary response is characterised as “sluggish”, automated infrared pupillometry commonly reveals a significantly smaller change in pupil size and a reduced constriction velocity.

**GRANT ACKNOWLEDGMENT**

Charitable research grant from Birmingham Children's Hospital Research Foundation Charity.

**Table 225 (Abstract 0917).** Univariable analysis of pupillary response

| Measurement | Brisk | Sluggish | p-value |
|---|---|---|---|
| Pupil size by pen torch (mm) | 3 (2-4) | 2 (2-3) | 0.01 |
| Pupil size by pupillometer (mm) | 3.02 (2.3-4.4) | 2.26 (2.08-2.41) | 0.002 |
| Latency (s) | 0.23 (0.2-0.23) | 0.2 (0.17-0.23) | 0.21 |
| Maximal Constriction Velocity (mm/s) | 1.9 (1.5-3.1) | 0.8 (0.4-1.1) | <0.001 |
| Percentage change in size (%) | 31 (21-33) | 8 (4-12) | <0.001 |

## Oral Sessions Wednesday, 27 September 2017

### Haemodynamic resuscitation of shock states

#### 0918

#### Pooled analysis of higher vs lower blood pressure targets for vasopressors

F. Lamontagne[1], A.G. Day[2], M.O. Meade[3], D.J. Cook[3], G.H. Guyatt[3], M. Hylands[1], P. Radermacher[4], J.-M. Chrétien[5], N. Beaudoin[1], P. Hebert[6], F. D'Aragon[1], F. Meziani[7], P. Asfar[5]

[1]Université de Sherbrooke, Sherbrooke, Canada, [2]Kingston General Hospital, Kingston, Canada, [3]McMaster University, Hamilton, Canada, [4]Ulm University Hospital, Ulm, Germany, [5]Angers University Hospital, Angers, France, [6]Université de Montréal, Montréal, Canada, [7]Centre Hospitalo-Universitaire de Strasbourg, Strasbourg, France

**Correspondence:** F. Lamontagne

*Intensive Care Medicine Experimental* 2017, **5(Suppl 2):**0918

**INTRODUCTION.** Current sepsis management guidelines recommend titration of vasopressor (VP) infusions to an initial mean arterial pressure (MAP) target of at least 65 mmHg[1]. Whether and how to individualize VP therapy for septic shock requires a better understanding of the association between patient characteristics and response to different MAP targets.

**OBJECTIVES.** We conducted an individual patient data meta-analysis of recent trials to determine if patient variables modify the effect of different MAP targets.

**METHODS.** We searched MEDLINE, EMBASE, and the Cochrane Central Register of Controlled Trials for RCTs of higher vs lower blood pressure targets for VP therapy. We excluded crossover designs, RCTs of unapproved vasopressors, and experiments < 24h. Pooling individual patient data, we used a generalized linear (logistic) mixed model with a random effect for site and fixed effects for treatment assignment and trial. Tests for subgroup effects used interaction terms between a priori selected pre-randomization variables (chronic hypertension, congestive heart failure, age, duration of vasopressor therapy before enrolment) and treatment. The main outcome was 28-day all-cause mortality.

**RESULTS.** This analysis includes 894 patients from two multicenter trials. Controlling for trial and site, the summary odds ratio for 28-day mortality for the higher vs lower MAP targets was 1.15 (95% CI, 0.87 to 1.52). There was a significant subgroup effect for duration of VP before randomization (interaction p = 0.017), but not for chronic hypertension, congestive heart failure, or age (Fig. 291). Odds of death was similar in higher and lower MAP arms among patients on VP ≤6 hours before randomization, but odds increased in higher MAP groups in patients on VP >6 hours before randomization (OR 3.00, 95%CI 1.33-6.74). The increment in VP exposure associated with higher MAP targets was more pronounced among patients enrolled >6 hours after initiation of therapy (interaction p =0.047). The ratio of geometric means of norepinephrine equivalent in the higher vs lower MAP arm was 2.1 (95%CI 1.7-2.6) among patients with ≤6 hours of VP compared to 3.7 (95%CI 2.2-6.2) among patients with >6 hours of VP before enrolment.

**CONCLUSIONS.** This individual patient data meta-analysis suggests that higher blood pressure targets may increase mortality for those patients who do not otherwise respond quickly to therapy. Lower blood pressure targets were not associated with patient-important adverse events in any subgroup, including chronically hypertensive patients.

**REFERENCE(S)**

1. Rhodes A, Evans LE, Alhazzani W, et al. Surviving Sepsis Campaign: International Guidelines for Management of Sepsis and Septic Shock: 2016. Intensive Care Med 2017

**GRANT ACKNOWLEDGMENT**

This analysis was funded by a grant from the Fonds de Recherche du Québec - Santé.

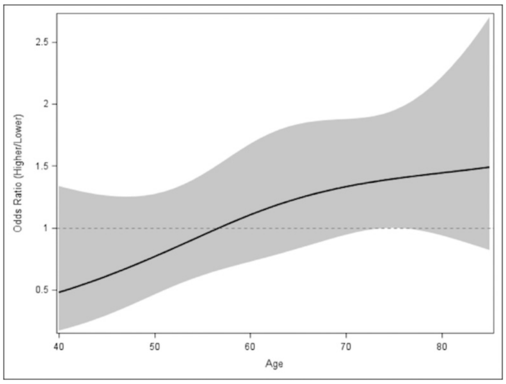

**Fig. 291 (Abstract 0918).** Odds ratio of 28-day mortality by age

#### 0919

#### Veno-arterial carbon dioxide difference/arterial-venous oxygen difference ratio but not central venous oxygen saturation or lactate predict increase in oxygen consumption in fluid responders after cardiac surgery

K. Pilarczyk[1], J. Lubarski[2], E. Demircioglu[2], B. Huschens[2], H. Jakob[2], F. Dusse[3]

[1]imland Klinik Rendsburg (managed by Sana AG), Department of Intensive Care Medicine, Rendsburg, Germany; [2]West German Heart and Vascular Center, University Hospital Essen, Department for Thoracic and Cardiovascular Surgery, Essen, Germany; [3]University Witten/Herdecke, Medical Centre Cologne-Merheim, Department of Anaesthesiology and Intensive Care Medicine, Köln, Germany

**Correspondence:** K. Pilarczyk

*Intensive Care Medicine Experimental* 2017, **5(Suppl 2):**0919

**INTRODUCTION.** Fluid responsiveness is defined as an increase of stroke volume after a fluid challenge. However, primary goal of volume resuscitation in critically ill patients is an increase in oxygen consumption ($VO_2$).
**OBJECTIVE.** To evaluate the ability of mixed venous oxygen saturation ($SvO_2$), lactate and markers of anaerobic metabolism (mixed venous-to-arterial carbon dioxide tension difference ($\Delta PCO_2$) and $\Delta PCO_2$/arteriovenous oxygen content difference ratio ($\Delta ContO_2$ ratio) to predict whether a fluid-induced increase in oxygen delivery ($DO_2$) results in an increase in oxygen consumption ($VO_2$).
**METHODS.** 45 consecutive hemodynamically stable patients after cardiac surgery were enrolled in this study. Hemodynamic variables, Lactate and $SvO_2$ were assessed with pulmonary artery catheter (PAC) before and after a crystalloid fluid challenge of 6ml/kgBW. Fluid and $VO_2$ responsiveness was defined as increase of stroke volume and $VO_2$ > 15%, respectively, after fluid challenge.
**RESULTS.** 28 out of 45 patients were fluid-responders (62.2%). In these patients, $DO_2$ increased significantly ($\Delta DO_2$ 98. ± 107.3 ml/min) whereas $DO_2$ decreased in fluid-non-responders due to a hemodilution-induced decrease in hematocrit ($\Delta DO_2$ -9.3. ± 81.4 ml/min). An increase in oxygen consumption ≥15% ($VO_2$-responders) was observed in 29% of these 28 volume-responders. Compared with $VO_2$-non-responders, $VO_2$-responders were characterized by higher $\Delta PCO_2$ (4.7 ± 1.8 vs. 3.5 ± 1.1, p = 0.04) and $\Delta PCO_2/\Delta ContO_2$ ratio (4.7 ± 1.9 vs. 0.9 ± 0.3, p > 0.001) whereas lactate (2.2 ± 1.3 vs. 1.8 ± 0.8) and $SvO_2$ (66.9 ± 7.1 vs. 65.3 ± 6.7) did not show any group difference. A fluid-induced increase in oxygen consumption greater > 15% was not predicted by baseline SvO2, Lactate and $\Delta PCO_2$ but $\Delta PCO_2/\Delta ContO_2$ ratio (AUC 0.731 ± 0.091, p = 0.03).
**CONCLUSION.** Whereas $SvO_2$ and lactate failed to predict $VO_2$ responsiveness, $\Delta PCO_2/\Delta ContO_2$ ratio might help to identify patients that will increase oxygen consumption after fluid administration. This suggests that after assessing hemodynamic fluid-responsiveness, indicators of anaerobic metabolism e.g. $\Delta PCO_2/\Delta ContO_2$ ratio should be considered to identify patients that will benefit from fluid expansion in terms of an increase in $VO_2$.

### 0920

#### The effects of passive leg raising can be detected by the plethysmographic oxygen saturation signal

A. Beurton[1,2], J.L. Teboul[1,2], V. Girotto[1,2], L. Galarza[1,2], C. Richard[1,2], X. Monnet[1,2]
[1]Hôpital Bicêtre, Hôpitaux Universitaires Paris-Sud, Assistance Publique - Hôpitaux de Paris, Le Kremlin-Bicêtre, France; [2]Inserm UMR S_999, Université Paris-Sud, Le Kremlin-Bicêtre, France
**Correspondence:** A. Beurton
*Intensive Care Medicine Experimental* 2017, **5(Suppl 2):**0920

**INTRODUCTION.** An accurate assessment of the hemodynamic effects of a passive leg raising (PLR) test and of a volume expansion (VE) require a direct measurement of cardiac output (CO). On the waveform of oxygen saturation assessed by plethysmography, the perfusion index (PI) is the ratio between the pulsatile portion, due to systolic increases caused by blood flow, and the non-pulsatile portion. We hypothesized that the PI changes could detect the CO changes during PLR test and VE.
OBJECTIVE. We hypothesized that the PI changes could detect the CO changes during PLR test and VE.
**METHODS.** In critically ill patients, we measured PI (Radical 7, Masimo) and CO (PiCCO, Pulsion Medical Systems) before and during a PLR test and, in patients in which volume expansion was decided, before and after the infusion of 500 mL saline.
**RESULTS.** We included 72 patients. Norepinephrine was administered in 52 patients (0.5 ± 0.5 μg/kg/min). Three cases were excluded due to a poor plethysmographic signal.
The PLR test was positive (increase in CO ≥10%) in 34 patients. In these patients, CO and PI increased significantly during the PLR test by 19 ± 10% and 52 ± 35%, respectively. In the 38 patients with a negative PLR test, neither CO nor PI changed significantly during PLR. Volume expansion was decided in 27 patients. It increased CO by more than 15% in 25 patients with a positive PLR test. In these patients, CO and PI significantly increased during volume expansion, by 24 ± 11% and 50 ± 52%, respectively. Considering the whole population, the correlation between the PI changes and the CO changes for all interventions was 0.67 p < 0.001.
A positive PLR test was detected by changes in PI with a good accuracy (area under the receiver operating characteristics curve: 0.93 (95% confidence interval: 0.83-0.98, p < 0.001). During the PLR, if PI increased by >9%, a positive response of CO could be diagnosed with a sensitivity of 100% (88-100%) and a specificity of 76% (59-88%).
**CONCLUSIONS.** The changes in PI of the pulse oximetry signal seem to accurately reflect changes in cardiac output during PLR test and volume expansion. This could be a reliable way to assess the hemodynamic effects of the PLR test in a totally non-invasive practice.

### 0921

#### Clinical examination for diagnosing circulatory shock: the Simple Intensive Care Studies-I

B. Hiemstra, R.J. Eck, G. Koster, A.M.G.A. de Smet, F. Keus, I.C.C. van der Horst, SICS Study Group
University of Groningen, University Medical Center Groningen, Critical Care, Groningen, Netherlands
**Correspondence:** I.C.C. van der Horst
*Intensive Care Medicine Experimental* 2017, **5(Suppl 2):**0921

**INTRODUCTION.** The latest consensus on circulatory shock advocates clinical examination and ultrasonography for diagnosing circulatory shock. Propagation of daily clinical examination contrasts with available studies, which are of limited quality and quantity so that its level of evidence is considered 'best practice'. We hypothesize that clinical estimation of circulatory shock, should be based on multiple variables including combinations of clinical, laboratory and ultrasonography variables.
**OBJECTIVES.** To evaluate the value of clinical examination, biochemical and ultrasonography variables in the critically ill, specifically for estimation which combinations of variables are associated with cardiac output.
**METHODS.** We initiated the Simple Intensive Care Studies-I (SICS-I) which was designed as a prospective cohort study to include all patients acutely admitted to the intensive care unit. Clinical examination was performed in a standardized fashion in all patients according to predefined criteria including variables of heart rate, blood pressures, central venous pressure, mental state, auscultation of heart and lungs, respiratory rate, urine output, capillary refill times, central to peripheral temperatures gradients, skin mottling; biochemical variables including lactate; and ultrasonography of heart and lungs. These clinical, biochemical, and ultrasonography variables were recorded following a published protocol (NCT02912624). Circulatory shock was defined by the requirement of vasopressors and/or inotropes and measured by cardiac output using transthoracic ultrasonography. All researchers, including medical students, underwent focused training for recording of all variables, including obtaining specific ultrasonography images. Cardiac function was clinically estimated as well before being measured by ultrasonography.
**RESULTS.** Between March 2015 and December 2016 a total of 704 out of 791 eligible patients were included. An independent Core laboratory assessed that ultrasonography images from 632 patients (90%) were of sufficient quality. Vasopressors and/or inotropes were used upon admission in 363 cases (52%). 173 patients (25%) had died at 90-day follow-up. The data of all patients included until July 1st, 2017 will be analysed to identify combinations of variables independently associated with cardiac output. At the congress, these results will be presented and these variables will inform the second phase of the on-going registry.
**CONCLUSIONS.** Standardized clinical examination and ultrasonography in critically ill patients by novices is feasible. We will be able to answer whether clinical assessment of the presence of shock can reliably be established and if so, which combinations of variables are most informative for estimation of the patients' cardiac output.

REFERENCE(S)

The protocol of this cohort study including references is registered at clinicaltrials.gov (NCT02912624).

### 0922

#### Non-invasive Cardiac output, Oxygen delivery and sub-lingual Micro-circulation as a predictive tool in acute MedICAL admission patients - COM-MICAL study

J.F. Doyle, J. McKinlay, E. Potter, E. Dhillon, M. Sukthankar, N. Doyle, K. McCullough, L. Forni

Royal Surrey County Hospital NHS Foundation Trust, Surrey Peri-Operative, Anaesthesia and Critical Care Research Group (SPACER), Guildford, United Kingdom

**Correspondence:** J.F. Doyle

*Intensive Care Medicine Experimental* 2017, **5(Suppl 2):**0922

INTRODUCTION

Early identification of patients who may need admission to an intensive care unit (ICU) is a top research priority[1]. Predicting patients that are likely to deteriorate and may need admission to ICU is extremely challenging with current monitoring and scoring systems as yet unproven in determining outcome[2]. We hypothesise that by non-invasively monitoring variables usually only utilised within a critical care environment, we may build a more comprehensive and timely picture of a patient's physiology.

OBJECTIVES

To assess feasibility of monitoring cardio-respiratory, microcirculatory and novel renal indices in acute medical patients and observe patient outcomes including discharge, intensive care unit referral and admission and renal dysfunction.

METHODS

During a 7-day sprint every 24 hours all acute medical patients were screened. Testing consisted of: Non-invasive cardiac output (CO), cardiac index (CI) and systemic vascular resistance (SVRI) measurements with LIDCO CNAP, a pulse wave power analysis. Microvascular flow was measured using sidestream dark field (SDF) imaging. Microvascular flow index (MFI) and POEM scores were performed in addition to automated measurements (sPVD and sPPV). Other variables included DO2 and AKI (Nephrocheck).

RESULTS

From 124 screened patients, 58 were included in analysis. Median age was 65 with 60.3% male vs 39.7% female. Testing occurred a median of 24hrs post-arrival. Microvascular flow was able to be assessed in 45/58 (77.6%) of patients, movement artefact being the significant barrier in those unable to be assessed. Cardiac output (CO) measurements were possible in 57/58 (98.3%) of patients. Median CO overall was 4.67l/min with CI 2.57l/min/m$^2$ and DO2I 379.4ml/min/m$^2$. Parameters were assessed in relation to 30 day mortality, whether at baseline or worse on hospital discharge and whether referred to ICU. Absolute CO, CI, DO2 or DO2I did not correlate with mortality or worse outcomes. However, patients with a DO2I of < /= 475ml/min/m$^2$ had significantly worse outcomes (p = 0.041). For microcirculation, parameters were not associated with mortality or hospital outcome status.

CONCLUSIONS

Single point non-invasive CO and micro-circulation measurements were not associated with outcome in this feasibility study, although it should be noted that sample size calculations were not performed owing to the nature of the study. DO2I cutoffs may be a useful predictive measure. Testing of the documented variables in acute medical patients was feasible and has indicated areas for more focussed study in select acute medical patient cohorts.

GRANT

Not funded.

ACKNOWLEDGEMENT

LiDCO CNAP was donated for study.

## Understanding organ failure in sepsis

### 0923

#### A new approach of sepsis heterogeneity

G. Papin[1,2], S. Bailly[1], C. Dupuis[1,2], S. Ruckly[1], M. Gainier[3], L. Argaud[4], E. Azoulay[5], C. Adrie[6], B. Souweine[7], D. Goldgran-Toledano[8], G. Marcotte[9], A. Gros[10], J. Reignier[11], B. Mourvillier[1,2], J.-M. Forel[12], R. Sonneville[2], A.-S. Dumenil[13], M. Darmon[14], M. Garrotte-Orgeas[15], C. Schwebel[16], J.-F. Timsit[1,2], OUTCOMEREA Study Group

[1]UMR 1137, IAME Team 5, INSERM/Paris Diderot University, Paris, France; [2]Hôpital Bichât Claude Bernard, Réanimation Médicale, Paris, France; [3]Hôpital Sud, Réanimation Médicale, Marseille, France; [4]Hospices Civils de Lyon, Réanimation Médicale, Lyon, France; [5]Hôpital Saint Louis, Réanimation Médicale, Paris, France; [6]Hôpital Delafontaine, Réanimation Polyvalente, Paris, France; [7]Hôpital Gabriel Montpied, Réanimation Médicale, Clermont-Ferrand, France; [8]Hôpital de Gonesse, Réanimation Médico-Chirurgicale, Gonesse, France; [9]Hospices Civils de Lyon, Réanimation Chirurgicale, Lyon, France; [10]Hôpital de Versailles, Réanimation Médico-Chirurgicale, Versailles, France; [11]Hôpital de Nantes, Réanimation Médicale, Nantes, France; [12]Hôpital Nord, Réanimation Médicale, Marseille, France; [13]Hôpital Béclère, Réanimation Médico-Chirurgicale, Clamart, France; [14]Hôpital de Saint Etienne, Réanimation Médicale, Saint Etienne, France; [15]Hôpital Saint Joseph, Réanimation Médico-Chirurgicale, Paris, France; [16]Hôpital Albert Michallon, Réanimation Médicale, Grenoble, France

**Correspondence:** S. Bailly

*Intensive Care Medicine Experimental* 2017, **5(Suppl 2):**0923

**INTRODUCTION.** The lack of effective specific therapies of sepsis could be partly explained by the heterogeneity of this syndrome.

**OBJECTIVES.** The aim of this study was to identify more homogeneous clusters of patients with sepsis and septic shock according to their initial clinical and biological characteristics.

**METHODS.** All patients admitted for sepsis or septic shock, according to the new Sepsis 3.0 definition, were included from a national prospective multicenter ICU cohort. A first test set of patients was used in an unsupervised clustering to build clusters independently of patient's outcomes. After description of main characteristics of each cluster, risk of mortality at 28 days was compared using logistic regression before and after adjustment on SOFA score at admission. A binary tree was built to assign prospectively a new patient into cluster. Performance of binary tree was evaluated on the validation set using ROC curves.

**RESULTS.** The test set included 4,050 patients (67%) with a median age 65 [53–76] year, a median SAPSII score 46 [34–60] and 28 days mortality 26% [25%-28%]. Six distinct clusters were identified: pulmonary sepsis cluster n = 1,603 (40%), meningo-encephalitis cluster n = 149 (4%), surgical sepsis cluster n = 623 (15%), immunocompromised patients cluster n = 338 (8%), COPD exacerbation cluster n = 243 (6%) and extra-respiratory chronic diseases cluster n = 1,094 (27%). Clusters[j1] were different in term of duration of stay and procedures use (p < 0.001 for all comparisons). Day 28 mortality was different between clusters (p < 0.001), higher for extra-respiratory chronic diseases cluster OR = 2.6 (IC95%: 2.1-3.0), immunocompromised patients cluster OR = 2.1 (IC95%:1.6-2.8) and surgical sepsis cluster OR = 1.5 (IC95%:1.2-1.9). The difference in cluster prognosis persisted after adjustment on SOFA score for immunocompromised patients cluster OR = 1.4 (IC95%:1.1-1.9) and for extra-respiratory chronic diseases cluster OR = 1.5 (IC95%:1.3-1.9). Pulmonary sepsis cluster was used as reference class. Binary tree accurately identified 6 discriminant variables to assign patients into clusters (Fig. 292).

**CONCLUSIONS.** Using unsupervised clustering, we identified 6 more homogeneous clusters of ICU patients admitted for sepsis. These clusters were associated with very different procedure use, duration of stay and prognosis even after adjustment on SOFA scores. Considering these clusters may improve homogeneity of patients enrolled in future clinical trials.

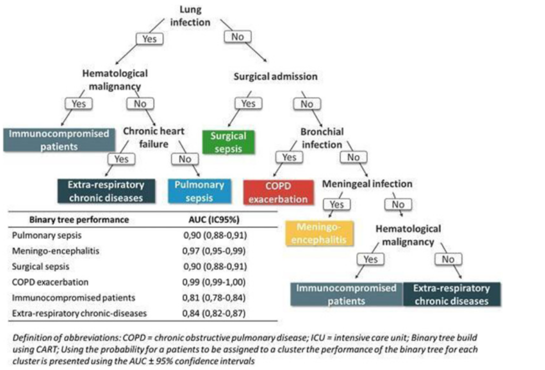

**Fig. 292 (Abstract 0923).** BT

### 0924

#### Arterial spin labeling magnetic resonance imaging evaluation of cerebral perfusion in health and under vasopressor therapy for sepsis

M.-H. Masse[1], M.A. Richard[1], M. Mayette[1], C. St-Arnaud[1], F. D'Aragon[1], N.K. Adhikari[2], W. Fraser[1], A. Carpentier[1], S. Palanchuk[1], D. Gauthier[1], L. Lanthier[1], M. Touchette[1], A. Lamontagne[1], É. Croteau[1], J. Chénard[1], S. Mehta[3], Y. Sansoucy[1], M. Lepage[1], F. Lamontagne[1]
[1]Université de Sherbrooke, Sherbrooke, Canada; [2]Sunnybrook Health Sciences Centre, Toronto, Canada; [3]University of Toronto, Toronto, Canada
**Correspondence:** F. Lamontagne
*Intensive Care Medicine Experimental* 2017, **5(Suppl 2):**0924

**INTRODUCTION.** The precise mechanisms underlying sepsis-associated encephalopathy remain unclear, but reduced cerebral blood flow (CBF) - alone or in conjunction with altered autoregulation - is proposed as a potential contributor.

**OBJECTIVES.** The objectives of this interrupted time series study were to compare CBF in
1) healthy volunteers with and without sedation;
2) septic patients receiving vasopressors to achieve higher vs lower mean arterial pressure (MAP) targets; and
3) between septic patients and healthy volunteers.

**METHODS.** We measured CBF using magnetic resonance imaging (MRI) with arterial spin labeling (ASL) conducted in a 3.0 T MRI scanner (Philips Ingenia, Philips Healthcare, Best, Netherlands). ASL detects magnetization from the nuclear spin of hydrogen atoms induced with radiofrequency pulses within water molecules. By comparing the T1-weighted contrast obtained by ASL to nonlabeled images, CBF can be calculated.

CBF was measured in healthy volunteers while awake and under moderate sedation with a continuous infusion of propofol (titrated for a Richmond Agitation-Sedation Scale level of −2 to −3). With septic patients who were sedated with propofol and already treated with vasopressors (norepinephrine), we measured CBF at a MAP of 65 and 75 mmHg. We randomized the order of allocation for sedation (in healthy subjects) and MAP targets (septic patients) and the outcome measures (calculation of CBF) were blinded.

**RESULTS.** Twelve healthy volunteers (7 with chronic hypertension) and 10 septic patients (4 with chronic hypertension) were enrolled; mean ages were 44 and 61 years respectively (p = 0.003). The mean APACHE II score of septic patients at ICU admission was 33 (SD 9.4) and the study was conducted, on average, 1.5 days following ICU admission (after the initial resuscitation). In healthy volunteers, we observed no difference in CBF measured with and without sedation (24.9 vs. 24.8 mL/100g/min; p = 0.93). Similarly, in septic patients, CBF measured at a MAP target of 65 mmHg (40.4 mL/100g/min) was not different from CBF measured at a MAP target of 75 mmHg (41.3 mL/100g/min; p = 0.65). We found no interaction between chronic hypertension and the effect of sedation or MAP targets. CBF measured in sedated septic patients was 62% higher than in sedated healthy volunteers (p = 0.001).

**CONCLUSIONS.** In the subset of septic patients who participated to this study, CBF was higher than in sedated healthy volunteers and autoregulatory capacity was preserved. Further research is required to understand the clinical significance of sepsis or vasopressor-induced cerebral hyperperfusion and to reassess the neurological effects of current protocols of vasopressor therapy in sepsis.

**GRANT ACKNOWLEDGMENT**
This study was funded by an internal grant awarded by the Centre de recherche du CHU de Sherbrooke.

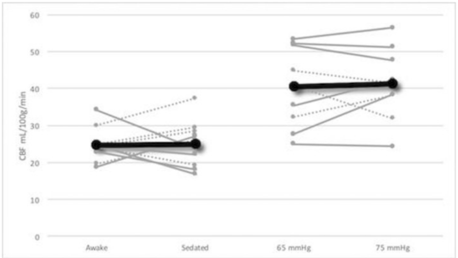

**Fig. 293 (Abstract 0924).** Cerebral blood flow in volunteers and patients

### 0925

#### Defining sepsis on the wards: results of a multi-centre point-prevalence study comparing two sepsis definitions

M. Kopczynska[1], T. Szakmany[2,3], Welsh Digital Data Collection Platform Collaborators
[1]Cardiff University, Division of Population Medicine, Department of Anaesthesia Intensive Care and Pain Medicine, Cardiff, United Kingdom; [2]Royal Gwent Hospital, Critical Care Directorate, Newport, United Kingdom; [3]Cardiff University, Anaesthesia, Intensive Care and Pain Medicine, Cardiff, United Kingdom
**Correspondence:** M. Kopczynska
*Intensive Care Medicine Experimental* 2017, **5(Suppl 2):**0925

**INTRODUCTION.** Sepsis is defined as dysregulated host response to infection, resulting in acute organ dysfunction [1]. While the condition has been thoroughly studied in the Intensive Care Unit (ICU), accurate data collection outside of this setting is less well-developed [2]. **OBJECTIVES.** Our aim was to prospectively determine the point-prevalence of sepsis in Emergency Departments and general wards according to both SEPSIS-1 and SEPSIS-3 definitions and determine predictive capabilities of these tools using our previously developed electronic data collection tool [3].

**METHODS.** Patients in the Emergency Department or in an in-patient ward in 13 hospitals with NEWS > 3 and suspected or proven infection were enrolled over a 24-hour period. Primary outcome was mortality within 30 days of recruitment.

**RESULTS.** Out of the 5422 patients screened, 431 fulfilled inclusion criteria and 380 (88%) were recruited. 212 were identified having sepsis using the SEPSIS-1 definition. Using the SEPSIS-3 definitions with SOFA ≥ 2, 272 patients, with qSOFA 50 patients were identified. For the prediction of primary outcome SEPSIS-1 criteria had a sensitivity of 67% (95%CI, 55%-77%) and specificity of 47% (95%CI, 41%-53%), SEPSIS-3 criteria had a sensitivity of 86% (95% CI, 76%-92%) and specificity of 32% (95%CI, 27%-38%). SEPSIS-3 and SEPSIS-1 definitions were associated with an HR of 2.7 (95% CI, 1.5-5.6) and HR of 1.6 (95% CI, 1.03-2.5), respectively. Scoring system discrimination was highest for SOFA (AUROC 0.70 [95% CI 0.63-0.77], p < 0.001), followed by NEWS (AUROC 0.58 [0.51-0.66], p = 0.028). SIRS (AUROC 0.55 [95%CI 0.48-0.62]) and qSOFA score (AUROC 0.56 [95%CI 0.49-

0.64]) could not statistically predict outcome in this patient population (p = 0.21 and 0.09 for SIRS and qSOFA, respectively).

**CONCLUSIONS.** Sepsis prevalence is between 4% and 5% on the wards and EDs, depending on the definition used. SEPSIS-3 definition identified patients with the highest risk. SOFA and NEWS were found to be better predictors of poor outcome than qSOFA or SIRS.

**REFERENCE(S)**

1. Singer M, Deutschman CS, Seymour CW, et al. The Third International Consensus Definitions for Sepsis and Septic Shock (Sepsis-3). *JAMA* 2016;**315**:801–10

2. Szakmany T, Lundin RM, Sharif B, et al. Sepsis Prevalence and Outcome on the General Wards and Emergency Departments in Wales: Results of a Multi-Centre, Observational, Point Prevalence Study. PLoS ONE 2016;11:e0167230

3. Sharif B, Lundin RM, Morgan P, et al. Developing a digital data collection platform to measure the prevalence of sepsis in Wales. *J Am Med Inform Assoc* 2016;**23**:1185–9

**GRANT ACKNOWLEDGMENT**

The authors would like to acknowledge the support of the Fiona Elizabeth Agnew Trust and the Welsh Intensive Care Society for the funding provided.

### 0926

#### Elevated β-lactam concentrations are associated with EEG abnormalities in patients with sepsis

B. Moro Salihovic[1], R. Rozenblum[1], M. Beumier[1], B. Legros[2], N. Gaspard[2], G.S. Casu[1], M. Hites[3], F. Cotton[4], J.-L. Vincent[1], J. Creteur[1], F. Jacobs[3], F.S. Taccone[1]

[1]Erasme Hospital, Université Libre de Bruxelles, Department of Intensive Care, Brussels, Belgium; [2]Erasme Hospital, Université Libre de Bruxelles, Department of Neurology, Brussels, Belgium; [3]Erasme Hospital, Université Libre de Bruxelles, Department of Infectious Diseases, Brussels, Belgium; [4]Erasme Hospital, Université Libre de Bruxelles, Department of Clinical Biochemistry, Brussels, Belgium

**Correspondence:** B. Moro Salihovic

*Intensive Care Medicine Experimental* 2017, **5(Suppl 2):**0926

**INTRODUCTION.** Although β-lactam concentrations have been associated with worsening neurological status, no data have been reported on electroencephalographic (EEG) findings.

**OBJECTIVES.** The aim of this study was to assess the association between b-lactam concentrations and EEG abnormalities in ICU patients with sepsis.

**METHODS.** We reviewed all ICU patients over a 4 year period (2012–2016) who were treated with meropenem (MEM), piperacillin-tazobactam (TZP), ceftazidime/cefepime (CEF) or aztreonam (AZT) and in whom at least one concomitant β-lactam trough concentration ($C_{min}$) and EEG monitoring were available. Drug levels were measured using high-performance liquid chromatography; $C_{min}$ was normalized to the clinical breakpoint of *Pseudomonas aeruginosa* (as determined by EUCAST) for each drug ($C_{min}$/minimum inhibitory concentration [MIC]). EEG abnormalities of interest were: a) ictal EEG pattern; b) generalized periodic discharges (GPDs).

**RESULTS.** We collected 391 $C_{min}$ values (168 MEM, 157 TZP, 55 CEF, 11 AZT) in 277 patients. Overall ICU mortality was 40%. There were no differences in the frequency of EEG abnormalities between antibiotics (ictal EEG pattern = 14% for MEM, 18% for TZP, 22% for CEF, 18% for AZT; GPDs = 12% for MEM, 10% for TZP, 15% for CEF, 30% for AZT). However, the incidence of ictal EEG pattern and of GPDs increased progressively with increasing $C_{min}$/MIC ranges (p = 0.14 and p = 0.18, respectively). The occurrence of at least one EEG abnormality was significantly higher for higher $C_{min}$/MIC ranges (from 19% for $C_{min}$/MIC ≤1.0 to 44% for $C_{min}$/MIC ≥7.0; p = 0.009).

**CONCLUSIONS.** There was a significant correlation between high β-lactam trough concentrations and an increased occurrence of EEG abnormalities in ICU patients with sepsis. Monitoring of β-lactam levels should be considered when neurological and/or EEG alterations occur in patients with sepsis.

### 0927

#### Dynamic evolution of SOFA-Score before Sepsis-1 onset in patients with ICU acquired sepsis

S.-O. Kuhn, C. Scheer, M. Gründling, S. Rehberg

University Hospital of Greifswald, Department of Anesthesiology, Greifswald, Germany

**Correspondence:** S.-O. Kuhn

*Intensive Care Medicine Experimental* 2017, **5(Suppl 2):**0927

**INTRODUCTION.** The new Sepsis-3 definition is based on an acute change in total Sequential Organ Failure Assessment score (SOFA) of 2 points due to infection [1]. SOFA score ≥2 points is associated with a mortality rate of 10% and is supposed to offer better and earlier risk stratification [2]. Former Sepsis-1 criteria failed to recognise SIRS-negative septic patients. However, in ICU-acquired sepsis the diagnosis is challenging because of preexisting organ dysfunction and therapy.

**OBJECTIVES.** To determine changes in organ dysfunction measured by SOFA score during 3 days before sepsis onset according to former Sepsis-1 criteria.

**METHODS.** Retrospective analysis of patient data from an ongoing quality improvement program in sepsis at the University Hospital of Greifswald, Germany. Between 2010 and 2015 all patients of a tertiary surgical ICU with ICU-acquired sepsis or septic shock according to the Sepsis-1 criteria were included. The development of organ dysfunction measured by SOFA score was determined retrospectively from the day of former sepsis-1 onset back to day −3.

**RESULTS.** 118 patients with ICU acquired sepsis were included. Because of an elevated total SOFA score 59 patients (50%) fulfilled sepsis-3 criteria already up to 3 days earlier than sepsis onset based on sepsis-1 criteria. In 8 patients (6.8%) sepsis onset was postponed by one day according to sepsis-3 (Fig. 294). Particularly, respiratory and cardiovascular failure had the highest proportion of all SOFA subscores but also an alteration in mental status appeared very frequently (Fig. 295). However, the frequency of organ dysfunction gradually increased in all SOFA subscores from day −3 to former Sepsis diagnosis.

**CONCLUSIONS.** In critical ill patients SOFA score is already higher than 2 points before sepsis onset. Accordingly, there was a gradual increase in SOFA-subscores up to 3 days but most obviously one day prior to sepsis-1 onset. As a consequence, continuous monitoring of organ dysfunction including all SOFA subscores is required for an early diagnosis of sepsis. In particular hemodynamic instability and deterioration of pulmonary function are indicative for impending sepsis. An automated scoring is recommended to handle the mass of data, but technical implementation at the bedside may be challenging for most hospitals yet.

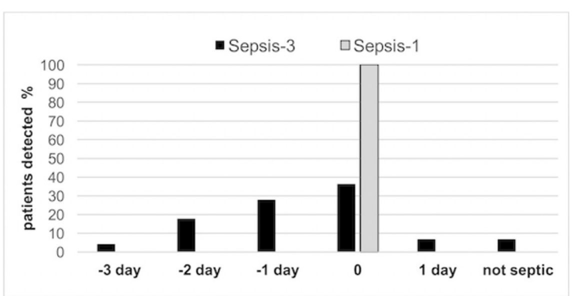

**Fig. 294 (Abstract 0927).** See text for description

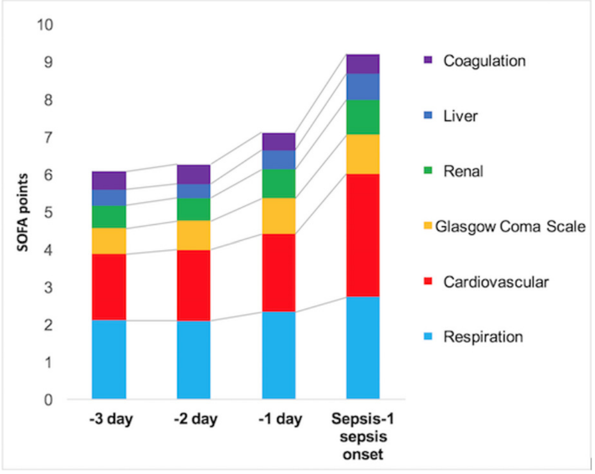

**Fig. 295 (Abstract 0927).** See text for description

### Mechanical ventilation clinical studies

#### 0928

**Acute hypoxemic respiratory failure: which patients need intubation?**

J.-P. Frat[1,2], S. Ragot[3], R. Coudroy[1], J.-M. Constantin[4], C. Girault[5], G. Prat[6], T. Boulain[7], A. Demoule[8], J.-D. Ricard[9], K. Razazi[10], J.-B. Lascarrou[11], J. Devaquet[12], J.-P. Mira[13], L. Argaud[14], J.-C. Chakarian[15], M. Fartoukh[16], S. Nseir[17], A. Mercat[18], L. Brochard[19], A.W. Thille[20], R. Robert[20], REVA Network

[1]CHU Poitiers, Medical ICU, Poitiers, France; [2]Université de Poitiers, CIC 1402, Poitiers, France; [3]INSERM CIC 1402, Biostatistics, Poitiers, France; [4]CHU Clermont-Ferrand, Pôle de Médecine péri-opératoire, Poitiers, France; [5]Normandie Univ, Department of Medical Intensive Care, Rouen, France; [6]CHU La Cavale Blanche, Service de Réanimation Médicale, Brest, France; [7]CHR Orléans, Réanimation Médico-Chirurgicale, Orléans, France; [8]Groupe Hospitalier Universitaire Pitié Salpêtrière, Service de Pneumologie et Réanimation Médicale, Paris, France; [9]Assistance Publique des Hôpitaux de Paris, Hôpital Louis Mourier, Service de Réanimation Médico-Chirurgicale, Colombes, France; [10]AP-HP, Hôpitaux Universitaires Henri Mondor, Service de Réanimation Médicale, Créteil, France; [11]Centre Hospitalier Départemental de La Roche sur Yon, Service de Réanimation Polyvalente, La Roche sur Yon, France; [12]Hôpital Foch, Réanimation Polyvalente, Suresne, France; [13]Assistance Publique - Hôpitaux de Paris, Groupe Hospitalier Universitaire de Paris Centre, Hôpital Cochin, Réanimation Médicale, Paris, France; [14]Hospices Civils de Lyon, Groupement Hospitalier Universitaire Edouard Herriot, Service de Réanimation Médicale, Lyon, France; [15]Centre Hospitalier de Roanne, Réanimation Polyvalente, Rouanne, France; [16]Assistance Publique-Hôpitaux de Paris, Hôpital Tenon, Service de Réanimation, Paris, France; [17]CHU de Lille, Centre de Réanimation, Lille, France; [18]CHU Angers, Département de Réanimation Médicale et Médecine Hyperbare, Angers, France; [19]St Michael's Hospital, Keenan Research Centre and Critical Care Department, Toronto, France; [20]Chu Poitiers, Medical ICU, Poitiers, France

**Correspondence:** J.-P. Frat

*Intensive Care Medicine Experimental* 2017, **5(Suppl 2):**0928

**INTRODUCTION.** In patients with acute hypoxemic respiratory failure, noninvasive ventilation (NIV) and high-flow nasal cannulae oxygen (HFNC) are alternative strategies to face mask oxygenation. Endotracheal intubation is frequently needed in these patients with a risk of delay and early predictors of failure may help clinicians to decide early.

**OBJECTIVES.** To identify risk factors associated with intubation in hypoxemic patients treated with different noninvasive oxygenation techniques for severe hypoxemic acute respiratory failure.

**METHODS.** This is a post-hoc analysis of a multicenter, randomized, clinical trial (1) of patients admitted to ICU for acute hypoxemic respiratory failure with a $PaO_2/FiO_2$ ratio ≤ 200 mm Hg and treated by HFNC, standard oxygen or NIV,.The criteria for intubation were predetermined including worsened or persisted respiratory failure, impairment of neurologic status and hemodynamic instability.

Clinical parameters and blood gas samples were collected at inclusion and H1 in each group of treatment: standard oxygen, HFNC and NIV.

**RESULTS.** After adjustment on treatment, two factors were associated with intubation at baseline: the presence of bilateral pulmonary infiltrates at admission (aOR 2.33, 1.10-4.94, p = 0.03) and respiratory rate (aOR 1.04, 1.0-1.09, p = 0.05). Under HFNC or standard oxygen, patients with a respiratory rate ≥ 30 breaths/min had a higher risk of intubation (aOR 2.82, 1.05-7.57, p = 0.04 and aOR 5.77, 1.78-18.65, p = 0.003, respectively). Under NIV, a tidal volume exceeding 9 ml/kg at one hour was independently associated with intubation (aOR 2.92, 1.05-8.10, p = 0.04) and 90-day mortality.

**CONCLUSIONS.** Bilateral pulmonary infiltrates upon admission predicted the need for intubation in patients with acute hypoxemic respiratory failure breathing spontaneously. The respiratory rate was a predictor of intubation under HFNC or oxygen, but not under NIV. A high tidal volume (≥9 ml/kg) under NIV was a strong predictor of intubation and remained independently associated with mortality.

**REFERENCE(S)**

1. Frat JP, Thille AW, Mercat A, et al. High-flow oxygen through nasal cannula in acute hypoxemic respiratory failure. *The New England journal of medicine* 2015; **372**(23): 2185–96.

#### 0929

**Diaphragm twitch pressure and thickening fraction are strong predictors of liberation from mechanical ventilation**

M. Dres[1], E. Goligher[2], B.-P. Dubé[1], E. Morawiec[1], D. Reuter[1], J. Mayaux[1], T. Similowski[1], A. Demoule[1]

[1]APHP, Service de Pneumologie et Réanimation Médicale, Paris, France; [2]University of Toronto, Toronto, Canada

**Correspondence:** M. Dres

*Intensive Care Medicine Experimental* 2017, **5(Suppl 2):**0929

**INTRODUCTION.** Diaphragm function is a crucial determinant of liberation from mechanical ventilation. However, the level of diaphragm function required for successful liberation is unknown.

**OBJECTIVES.** The objective of this study was to identify optimal threshold values to predict successful liberation for different measures of diaphragm function.

**METHODS.** In patients undergoing a first spontaneous breathing trial (SBT) after at least 24 hours of mechanical ventilation, diaphragm function was evaluated by twitch tracheal pressure in response to magnetic phrenic stimulation (Ptr,stim) and by diaphragm ultrasound (thickening fraction [TFdi] and excursion [Exdi]). The rapid shallow breathing index (RSBI) was also calculated. Receiver operating curves (ROC) were computed to determine the best cut-offs predicting successful liberation (defined as a successful SBT and extubation without re-institution of ventilation in the subsequent 48 hours).

**RESULTS.** 76 patients were evaluated: 43 were successfully extubated and 33 failed the SBT or required ventilatory support after extubation. The optimal cut-offs of Ptr,stim, TFdi, EXdi and RSBI to predict successful liberation were respectively 7.8 $cmH_2O$ (sensitivity 81%, specificity 82%), 29% (sensitivity 77%, specificity 94%), 0.95 cm (sensitivity 68%, specificity 65%) and 50 $breath.min^{-1}.l^{-1}$ (sensitivity 85%, specificity 64%), respectively. The areas under the ROC were similar for Ptr,stim and TFdi (0.89 and 0.88, p = 0.74, respectively) and significantly higher than that of EXdi and RSBI (0.71 and 0.76, p = 0.51, respectively).

**CONCLUSIONS.** Successful liberation can be predicted with a lower value of Ptr,stim than the value defining diaphragm dysfunction. Ptr,stim and TFdi had similar strong performances in the prediction of weaning success.

**GRANT ACKNOWLEDGMENT**

BERNHARD DRÄGER Award for advanced treatment of ARF 2015

### 0930

#### The efficacy of ultrasound assessment of diaphragmatic function in guiding weaning from mechanical ventilation in critically ill patients with abdominal sepsis

H. Eltrabily, A. Hasanin, M.S. Soliman, A. Mukhtar, W. Hamimy
Faculty of Medicine, Cairo University, Anaesthesia and ICU, Cairo, Egypt
**Correspondence:** H. Eltrabily
*Intensive Care Medicine Experimental* 2017, **5(Suppl 2):**0930

**INTRODUCTION.** Ultrasound assessment of diaphragmatic function has been developed recently providing an easy and safe method for evaluation of diaphragmatic excursion and thickening. Many parameters have been developed to aid weaning from MV such as $PO_2/FiO_2$ ratio and rapid shallow breathing index (RSBI). However, sensitivity and specificity for most variables are still variable in literature.

**OBJECTIVES.** We hypothesized that ultrasonographic measuring of diaphragmatic thickening and excursion may be useful tool to predict outcome of weaning trials in mechanically ventilated patients with abdominal sepsis.

**METHODS.** 30 mechanically ventilated patients with abdominal sepsis were included. Measurements of diaphragmatic excursion (DE) and diaphragmatic thickening fraction (DTF) were obtained during spontaneous breathing trial on both pressure support mode (PS) and T tube. Outcome of weaning was recorded. Data analysis was done after dividing patients into 2 groups, successfully weaned, failed weaning.

**RESULTS.** successful group showed higher DTF and DE compared to failed group. In successful group, DTF was 53.5 vs. 23.9 for failed group, Area under the curve of DTF was 1, sensitivity: 100%, and specificity: 100% at cutoff value of 30.In successful group DE was 17 mm vs. 8 mm for the failed group, Area under the curve of excursion on PS was 0.882(0.738-1.027), sensitivity: 94%, and specificity: 85% at cutoff value of 10.4.No difference was found between area under the curve of DE, DTF and RSBI.

**CONCLUSIONS.** DE and DTF are valuable tools in predicting outcome of weaning trial in patients with abdominal sepsis, they proved equal sensitivity and specificity to RSBI.

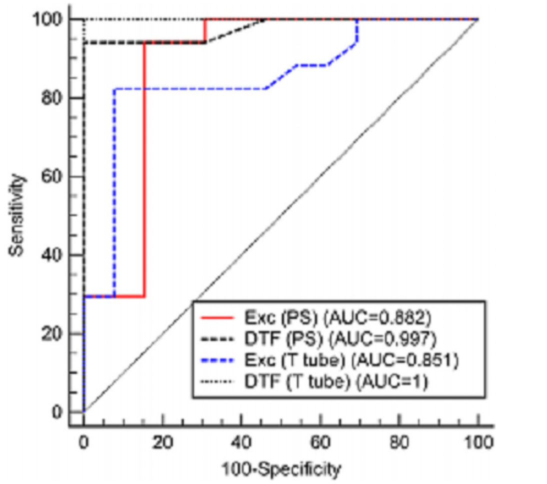

**Fig. 296 (Abstract 0930).** Area under the curve of ultrasound measurements

### 0931

#### Ventilator-associated events: prevalence, outcome and preventability

J. R. Azevedo, W.S. Montenegro, C. A. Sousa, M. M. Silva, A. L. Leitao, J. P. Maranhao, R.S. Araujo
Hospital Sao Domingos, ICU, Sao Luis, Brazil
**Correspondence:** J. R. Azevedo
*Intensive Care Medicine Experimental* 2017, **5(Suppl 2):**0931

**INTRODUCTION.** In 2013 the Centers for Disease Control and Prevention (CDC) proposed new surveillance definitions for patients receiving mechanical ventilation, changing the focus from ventilator-associated pneumonia (VAP) to all events associated with mechanical ventilation (VAE).

The new surveillance paradigms include ventilator-associated conditions (VAC), infection-related ventilator-associated conditions (IVAC) and possible VAP.

**OBJECTIVES.** To determine the prevalence of ventilator-associated events, to analyze the patients profile, morbidity and mortality compared to patients who did not develop VAE. We also analyzed the impact of each component of the ventilator bundle on the incidence of VAEs, duration of mechanical ventilation, hospital length of stay and hospital mortality.

**METHODS.** Included in the study were 438 patients admitted to the surgical (13 beds) and medical.

(32 beds) ICUs of a tertiary hospital from March 2013 to February 2016, aged above 18 years and submitted to mechanical ventilation for at least 4 days. At admission and daily the following data were collected by one of the members of the prevention of VAE team: Head of bed elevated (≥30°); daily interruption of sedation; gastric ulcer prevention; thromboembolism prophylaxis; oral care with chlorhexidine gluconate; aspiration of subglotic secretions and monitoring of endotracheal tube cuff pressure

**RESULTS.** Four hundred and thirty-eight patients were submitted to mechanical ventilation for at least 4 days and were analyzed. Ventilator-associated events developed in 60 (13.4%) patients, 11 (2.2%) of them had IVAC and 19 (4.3%) possible VAP. When patients who developed VAE were compared with those who did not, there was no difference between the two groups in demographic data and severity scores. Patients who had VAE had worse outcomes including increased duration of mechanical ventilation and higher ICU and hospital mortality (Table Error! Reference source not found.).

In Table 227 we compare patients with IVAC and VAP with those without VAE. Patients with IVAC exhibited higher ICU mortality. Duration of mechanical ventilation was expressively higher in patients with VAP ($p < 0.001$). ICU LOS also was higher in patients with VAP.

The impact of each component of the preventive bundle on the incidence of VAEs is evaluated in Table 228. The prevention of gastric ulcer was significantly protective for VAP (OR,0.84; 95 CI, 070–1.01; $p = 0.05$). When analyzing the association between components of the preventive bundle and other outcomes, oral care with chlorhexidine was associated with a significant increase in hospital mortality (OR, 9.09, 95% CI,1.11-74.25, $p = 0.03$).

**CONCLUSIONS.** VAEs are associated with significant morbidity and mortality. From all the preventive bundle measures, prophylaxis of gastric ulcer was significantly protective for VAP. On the other hand, oral care with chlorhexidine was associated with significant increase in hospital mortality.

**Table 226 (Abstract 0931)**. Comparison between VAE x Non VAE patients

| | Non VAE (n=378) | VAE (n=60) | *p value* | Abbreviations: |
|---|---|---|---|---|
| Age, Yr, Mean (SD) | 70.2 (18.30) | 66.5 (19.7) | *0.143* | VAE= Ventilator- associated events; |
| Male, n (%) | 174 (46.0) | 32 (53.3) | *0.292* | APACHE= Acute Physiology and Chronic Health Evaluation; |
| APACHE* IV score, Mean (SD) | 76.0 (25.9) | 73.4 (28.7) | *0.476* | SOFA= Sequential Organ Failure Assessment; |
| SOFA** score on admission, Mean (SD) | 5.8 (3.8) | 5.5 (3.7) | *0.577* | MV= Mechanical ventilation; |
| Diagnosis at admission, n (%) Medical/Surgical | 277 (73.3)/101 (26.70 | 52 (86.7)/ 8(13.3) | | LOS=Length of stay; |
| Duration of MV, days, median (IQR) | 9.0 (6.0 - 17.0) | 16.0 (11.0 - 30.0) | *<0.001* | IQR=Interquartile Range. |
| ICU LOS***, days, median (IQR) | 22.0 (12.0 - 39.0) | 25.5 (15.0 - 41.5) | *0.150* | |
| ICU mortality, n (%)/Hospital mortality, n (%) | 170 (45.0)/225 (59.5) | 41 (68.3)/ 46 (76.7) | *0.001/ 0.011* | |
| Adherence to preventive bundle % (SD) | 85.92 (19.1) | 86.2 (18.9) | *0.407* | |

**Table 227 (Abstract 0931).** Comparision between Non VAE, IVAC and VAP

| | Non VAE (n=378) | IVAC (n=11) | VAP (n=19) | *p value (Non VAE x IVAC)* | *p value (Non VAE x VAP)* | Abbreviations |
|---|---|---|---|---|---|---|
| Age, Yr, Mean (SD) | 70.2 (18.30) | 65.5 (21.4) | 62.8 (19.4) | *0.405* | *0.088* | VAE = Ventilation -Associated Events; |
| Male, n (%) | 174 (46.0) | 7 (63.6) | 9 (47.4) | *0.249* | *0.909* | IVAC=Infection-Related Ventilator-Associated Conditions; |
| APACHE IV score, Mean (SD) | 76.0 (25.9) | 76.9 (30.9) | 64.6 (28.5) | *0.905* | *0.064* | VAP = Ventilator-associated Pneumonia; |
| SOFA score on admission, Mean (SD) | 5.8 (3.8) | 6.1 (3.6) | 4.7 (3.5) | *0.770* | *0.255* | APACHE = Acute Physiology and Chronic Health Evaluation; |
| Diagnosis at admission, N (%) Medical/Surgical | 277 (73.3)/ 101 (26.7) | 10 (90.9)/ 1 (9.1) | 17 (89.5)/ 2 (10.5) | | | SOFA = Sequential Organ Failure Assessment; |
| Duration of MV, Days, Median (IQR) | 9.0 (6.0 - 17.0) | 15.0 (9.0 - 25.5) | 19.0 (15.0 - 30.0) | *0.046* | *<0.001* | LOS = Length of Stay; |
| ICU LOL, days, Median (IQR) | 22.0 (12 - 39.0) | 23.0 (11.5 - 42.0) | 29.0 (21.5 - 44.0) | *0.882* | *0.038* | MV = Mechanical Ventilation; |
| ICU Mortality n (%)/Hospital Mortality, n (%) | 170 (45.0)/ 225 (59.5) | 9 (81.8)/ 9 (81.8) | 11 (57.9)/ 14 (73.7) | *0.016/ 0.137* | *0.270/ 0.219* | IQR = Interquartile Range. |
| Adherence to preventive bundle % (SD) | 85.92 (19.1) | 93.5 (6.8) | 84.7 (22.7) | *0.479* | *0.447* | |

**Table 228 (Abstract 0931).** Impact of adhesion to preventive bundle on VAE´s

| | Outcome, OR (95% IC) | | | | | | |
|---|---|---|---|---|---|---|---|
| | VAE | *p value* | IVAC | *p value* | VAP | *p value* | Abbrevitions |
| BUNDLE (%) | | | | | | | VAE = Ventilator-Associeted Events; |
| Head of bed elevation | 0.97 (0.91 - 1.04) | *0.39* | 0.99 (0.87 - 1.13) | *0.89* | 1.12 (0.81 - 1.56) | *0.49* | IVAC = Infection -related Ventilator Events condictions; |
| Daily Interruption of sedation | 2.00 (0.0 - infinite) | *0.98* | 2.61 (0.0 - infinite) | *0.99* | | | Ventilator-Associated Pneumonia; |
| Stress ulcer prevention | 0.96 (0.82 - 1.13) | *0.62* | 7.26 (0.0 - infinite) | *0.99* | 0.84 (0.7 - 1.0) | *0.05* | DVT = Deep Venous Thombosis |
| Oral care | 0.97 (0.93 - 1.01) | *0.20* | 1.02 (0.9 - 1.15) | *0.68* | 0.96 (0.84 - 1.11) | *0.58* | |
| DVT prevention | 1.00 (0.96 - 1.05) | *0.86* | 1.02 (0.93 - 1.3) | *0.79* | 1.09 (0.96 - 1.24) | *0.18* | |
| Aspiration of subglotic secretions | 1.01 (0.96 - 1.07) | *0.62* | 7.28 (0.0 - infinite) | *0.95* | 1.00 (0.95 - 1.04) | *0.84* | |
| Monitoring of endotracheal cuff pressure | 0.96 (0.91 - 1.01) | *0.11* | 22.2 (0.0 - infinite) | *0.94* | 0.95 (0.86 - 1.05) | *0.33* | |

### 0932

#### Preventing reintubation: role of the different high-risk factors for reintubation in the selection of appropriate therapy. A post-hoc analysis

G. Hernandez[1], C. Vaquero[2], L. Colinas[1], R. Cuena[3], L. Vargas[1], M.L. Rodriguez[1], A. Canabal[1], O. Roca[4,5], J.R. Masclans[5,6,7], on behalf of the Spanish Multidisciplinary Group of High Flow Supportive Therapy in adults (HiSpaFlow)
[1]Virgen de la Salud University Hospital, Critical Care, Toledo, Spain; [2]Ramon y Cajal University Hospital, Critical Care, Madrid, Spain; [3]Research Unit, Medical Council, Toledo, Spain; [4]Vall d´Hebron University Hospital and Vall d´Hebron Research Institute, Critical Care, Barcelona, Spain; [5]Instituto de Salud Carlos III, Ciber Enfermedades Respiratorias (Ciberes), Madrid, Spain; [6]Del Mar University Hospital, Critical Care Department, Barcelona, Spain; [7]IMIM (Medical Research del Mar Hospital Institute), Barcelona, Spain
**Correspondence:** G. Hernandez
*Intensive Care Medicine Experimental* 2017, **5(Suppl 2):**0932

**INTRODUCTION.** A multicenter randomized trial has recently been publicated reporting that high-flow oxygen therapy (HFOT) is noninferior to noninvasive ventilation (NIV) for preventing reintubation in patients at high-risk for reintubation[1]. However, as HFOT and NIV have different physiopathological mechanisms, specific subgroups could theoretically benefit with any one of the two therapies.

**OBJECTIVES.** Our aim was to assess whether specific subgroups of high-risk patients for reintubation, classified according to the presence of any one of the 10 different high-risk factors may benefit with HFOT or NIV.

**METHODS.** The 10 different high-risk factors analyzed were: age >65 years, cardiac failure as the primary indication of mechanical ventilation, moderate-to-severe COPD, APACHE II >12 points the extubation day, body mass index (BMI) >30, airway patency problems including patients at high-risk for developing laringeal edema, patients considered unable to deal with respiratory secretions (inadequate cough reflex or >2 suctioning within 8-h previous to extubation), difficult or prolonged weaning, ≥2

comorbidities (Charlson index), and prolonged MV defined as MV >7 days. A post-hoc analysis was performed to assess the reintubation rate according to the presence of the 10 different high-risk factors, trying to classify patients in term of response to both therapies. An effect modification or interaction analysis was performed, including an effect measurement on additive and multiplicative scale. A normal linear regression analysis (RDI) and Cox model (RRI) analyses were performed to adjust for the presence of additional high-risk factors and covariates.

**RESULTS.** The effect modification or interaction analysis showed a significant benefit for NIV in patients with a BMI >30 (Table 229). No differences were obtained in any other high-risk factor. The adjusted RDI was 7.6 (95%CI 3 to 12.3; p = .001) and the adjusted RRI was 1.3 (95%CI 1.08 to 1.5; p = .005), confirming the results.

**CONCLUSIONS.** Patients with a BMI >30 could benefit with preventive use of NIV inmediately after extubation. However, a randomized trial is needed to confirm this result.

**REFERENCE(S)**

Hernandez G, Vaquero C, Colinas L, Cuena R, Gonzalez P, Canabal A, Sanchez S, Rodriguez ML, Villasclaras A, Fernandez R. Effect of postextubation high-flow nasal cannula vs noninvasive ventilation on reintubation and postextubation respiratory failure in high-risk patients. A randomized clinical trial. JAMA 2016; 316(15):1565–1574.

**GRANT ACKNOWLEDGMENT**

On behalf of the Spanish Multidisciplinary Group of High Flow Supportive Therapy in adults (HiSpaFlow).

**Table 229 (Abstract 0932)**. See text for description

| Risk factor | RDI | 95% CI (p) | RRI | 95% CI (p) |
|---|---|---|---|---|
| Prolonged MV | .06 | -.08 to .20 | 1.15 | .62 to 2.13 |
| APACHE II | .10 | -.03 to .23 | 1.46 | .77 to 2.78 |
| Weaning | .04 | -.12 to .20 | 1.03 | .56 to 1.90 |
| Age >65 y | .04 | -.10 to .17 | 1.22 | .65 to 2.28 |
| Air Way | -.20 | -.59 to .19 | .39 | .05 to 3.08 |
| Secretions | .04 | -.14 to .21 | 1.01 | .55 to 1.86 |
| BMI >30 | .19 | .03 to .35 (.02) | 2.51 | 1.10 to 5.70 (.03) |
| COPD | .16 | -.02 to .33 | 1.83 | .89 to 3.80 |
| Cardiac failure | .22 | -.05 to .50 | 2.63 | .82 to 8.36 |
| Comorbidities | .12 | -.02 to .25 | 1.78 | .87 to 3.64 |

### Trauma and other emergencies

#### 0933

#### Substance abuse as co-morbidity factor in mechanically ventilated intensive care patients

K.H. Tollisen[1,2], M. Bjerva[3], F. Heyerdahl[3], D. Jacobsen[2]

[1]University of Oslo, Institute of Clinical Medicine, Faculty of Medicine, Oslo, Norway; [2]Oslo University Hospital, Department of Acute Medicine, Oslo, Norway; [3]Oslo University Hospital, Departement of Anaesthesiology, Oslo, Norway

**Correspondence:** K.H. Tollisen

*Intensive Care Medicine Experimental* 2017, **5(Suppl 2):**0933

**INTRODUCTION.** Chronic substance abuse is an important co-morbidity factor in the critically ill. Intensive care studies addressing the combined impact of substance abuse associated with either alcohol or illicit substances are needed.

**OBJECTIVES.**

1) To identify patients with chronic abuse of alcohol, illicit substances or prescription drugs in mixed population of mechanically ventilated ICU-patients.

2) To study a) the distribution of these patients between the medical, cardiologic and surgical ICUs and b) the association of chronic abuse and length of stay, duration of mechanical ventilation and in-hospital mortality.

**METHODS.** Prospective study of mechanically ventilated ICU-patients in four ICUs at the Oslo University hospital Ullevål during 1 Year (2014–2015). Chronic substance abuse of alcohol, illicit substances or prescription drugs was evaluated based on medical records, drug screening, and the AUDIT-C questionnaire (cut off ≥9 /12 for men, and ≥ 8/12 for women).

**RESULTS.** 604 patients were included. 161/604 (**27%**) were classified with chronic substance abuse (15 (3%) "unknown"). Of these, 89/605 (15%) were admitted due to substance abuse-related diagnoses, while 72/605 (12%) had non-drug related admission diagnoses. The medical ICU had the highest proportion of patients with chronic abuse with 55/154 (36%), followed by the surgical ICUs 81/328 (25%) and the cardiologic ICU 25/122 (21%). The main agents of abuse were: alcohol in 104/ 604 (17%), illicit substances 51/604 (8%) and prescription drugs 6/604 (1%).

Chronic substance abuse was associated with younger age, median 56 vs 62 years (p = 0.03) and male gender, 132/161(82%) vs. 283/428(66%) (p < 0.01). The highest prevalence was found among male patients in the age group 30–60 in the medical ICU where 32/52 (62%) were classified with chronic abuse; 20/52 (39%) with illicit substances and 12/52 (23%) with alcohol as drug of abuse. We found no association with chronic abuse and in-hospital mortality (p = 0.4). In the surgical ICUs, chronic abuse was associated with shorter time in the ICU 7.6 vs 11 days (p = 0.01) and shorter time on mechanical ventilation, 4.6 vs 7.7 days (p < 0.01). No such differences were found in the medical or in cardiac ICUs.

**CONCLUSIONS.** Chronic substance abuse is common among mechanically ventilated patients, especially in the medical ICU, but was not associated with higher in-hospital mortality. In the surgical ICUs chronic abuse was associated with shorter time in the ICU and duration of mechanical ventilation. Overall, alcohol was the main agent of abuse, but illicit substance abuse was more frequent among male patients in the medical ICU. Future studies of substance abuse in the ICU should therefore include illicit substances in addition to alcohol.

#### 0934

#### DNA methylation patterns following major traumatic injury - a comprehensive genome wide analysis

J. Borgel[1], H. Torrance[1], M. Barnes[2], D. Watson[2], R. Longbottom[1], C. Hinds[1,3], R. Pearse[1,3], K. Brohi[4], M. O'Dwyer[1,3]

[1]Centre for Translational Medicine and Therapeutics, William Harvey Research Institute, Barts and the London School of Medicine and Dentistry, Queen Mary University of London, London, United Kingdom; [2]Centre for Translational Bioinformatics, William Harvey Research Institute, Barts and the London School of Medicine and Dentistry, Queen Mary University of London, London, United Kingdom; [3]Royal London Hospital, Barts Health NHS Trust, Adult Critical Care Unit, London, United Kingdom; [4]Centre for Trauma Sciences, The Blizard Institute, Barts and The London School of Medicine and Dentistry, Queen Mary University of London, London, United Kingdom

**Correspondence:** J. Borgel

*Intensive Care Medicine Experimental* 2017, **5(Suppl 2):**0934

**INTRODUCTION.** Prolonged ICU admission and excess mortality following major traumatic injury is consistently associated with an exaggerated inflammatory response(*1*,*2*). Upstream regulatory elements of this immune response remain elusive. DNA methylation is a key regulator of gene expression in physiologic and pathologic states(*3*) and consequently may be of importance in the regulation of genomic activity following traumatic injury.

**OBJECTIVES.** To explore alterations in patterns of DNA methylation following major traumatic injury and associations with clinical outcome measures.

**METHODS.** Consecutive adult trauma patients, requiring ICU admission following their initial resuscitation, were recruited with mixed leukocyte buffy coat samples collected within 2 hours of injury and again 72 hours later. DNA was isolated from buffy coat samples (Quick-DNA Universal Kit, Zymo) and subject to sodium bisulfite treatment (EZ DNA Methylation-Lightning Kit, Zymo) to generate methylation-specific base changes. Genome-wide DNA methylation was determined using the Illumina HumanMethylation 450k BeadChip (Illumina) which allows methylation-specific hybridization to an array of ~485,000 CpG sites spanning the entire human genome. Differentially methylated positions (DMPs) were identified using a stringent statistical analysis with multiple hypothesis testing and cell proportion correction. We set a false discovery rate (FDR) of 5%. Ontology analysis was performed using the XGR tool (eXploring Genomic Relations) on R.

**RESULTS.** 86 patients were recruited. Median age was 38 (IQR 26–57), 80% were male, median ISS was 29.5 (IQR 22–38), and admission base deficit was −4.2 (IQR −7.3–1.3). 62 (72%) of patients developed a nosocomial infection and 78 (91%) survived to hospital discharge. 152 DMPs were observed between the two time points. 113 CpG positions were hypomethylated and 39 were hypermethylated (Fig. 297).

Differences were subtle and defined by intermediate methylation (20% < methylation values < 80%). Interestingly, DMPs were preferentially found within genes or in genomic locations associated with regulatory regions such as promoters, transcription factor binding sites and DNAse hypersensitivity sites. Moreover, the 138 DMPs overlapping gene bodies or promoter regions identified genes involved in inflammatory pathways, the immune response and xenobiotics metabolism (Fig. 298). Finally, the DNA methylation changes were also detected in categories associated with apoptosis, cell movement and cell proliferation (Fig. 298).

**CONCLUSIONS.** Severe traumatic injury induces DNA methylation alterations which have the potential to regulate the post-traumatic inflammatory response and warrant further investigation as therapeutic targets.

**GRANT ACKNOWLEDGMENT**

Barts Charity

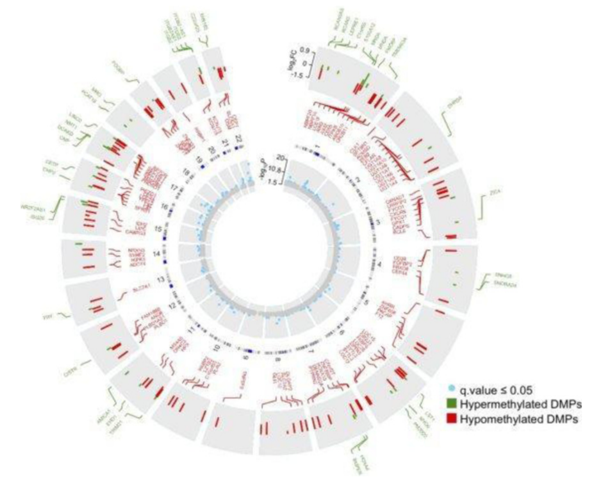

**Fig. 297 (Abstract 0934).** See text for description

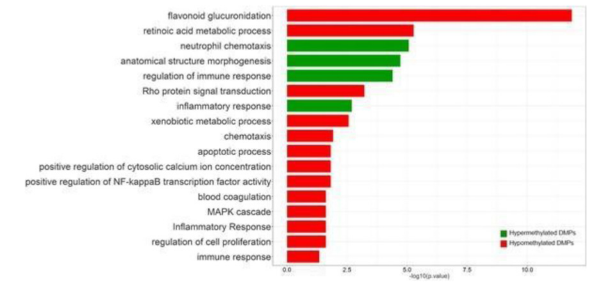

**Fig. 298 (Abstract 0934).** See text for description

### 0935

#### Microcirculatory impairment is associated with multiple organ failure following traumatic haemorrhagic shock: results of the MICROSHOCK study

S. D. Hutchings[1,2], D. Naumann[2,3], P. Hopkins[1], C. Mellis[4], S. Sartini[5], J. Mamuza[5], M. Midwinter[6], T. Harris[5], J. Wendon[1]
[1]King's College London, London, United Kingdom; [2]Royal Centre for Defence Medicine, Birmingham, United Kingdom; [3]University of Birmingham, Birmingham, United Kingdom; [4]King's College Hospital, London, United Kingdom; [5]Barts Health NHS Trust, London, United Kingdom; [6]Bundaberg Hospital, Bundaberg, Australia
**Correspondence:** S.D. Hutchings
*Intensive Care Medicine Experimental* 2017, **5(Suppl 2):**0935

**INTRODUCTION.** Patients who survive traumatic haemorrhagic shock (THS) are at risk of developing multiple organ failure (MOF). One possible mechanism is tissue hypoperfusion leading to endotheliopathy and inflammatory potentiation. Current resuscitation strategies focus on pressure based haemodynamic parameters rather than flow based measures of tissue perfusion. Microcirculatory flow dynamics can be accurately recorded using hand-held video-microscopic techniques, and may predict organ failure following traumatic haemorrhagic shock.

**OBJECTIVES.** To assess the relationship between microcirculatory dysfunction and organ failure following THS in a multi-center study.

**METHODS.** A multi-centre prospective longitudinal observational study was undertaken at three UK Major Trauma Centres. The protocol was published [1]. Injured patients who required blood products, were intubated, and had a lactate >2 mmol/l were eligible for inclusion. Patients were enrolled after initial resuscitation, within 12h of admission to intensive care (D0) with further time points at +24h and +72h. Sublingual incident dark field microscopy was performed at each time point and cardiac output assessed using oesophageal Doppler. MOF was defined as SOFA ≥6 at day 7 post injury. Microcirculatory Perfused Vessel Density (PVD) at D0 was compared between patients with and without multi-organ failure (MOF). Receiver Operator Characteristic (ROC) curves were constructed to predict the incidence of MOF at day 7 for different threshold values of PVD, lactate, cardiac index, and lowest systolic blood pressure (SBP) at D0.

**RESULTS.** 60 patients were recruited with a median age of 47 (IQR 27–52) years, ISS of 27 (IQR 20–34) and initial lactate of 4.6 (IQR 3.4-9.0) mmol/l. 6 (IQR 4–10) units of packed red blood cells were required in the first 24h. Patients who went on to develop MOF had significantly lower PVD on enrollment than those who did not. SOFA and Denver scores were significantly higher on day 4–7 in the group with below average PVD at study enrolment.

PVD was the best predictor of MOF (AUC 0.81 (0.59-1.0)) when compared to lactate (AUC 0.75 (0.5-0.99)), cardiac index (AUC 0.69 (0.41-0.97)) and lowest SBP (AUC 0.52 (0.29-0.73)) at D0.

**CONCLUSIONS.** Microcirculatory hypo-perfusion immediately following traumatic hemorrhagic shock and resuscitation is associated with increased MOF. Microcirculatory parameters are better prognostic indicators for the development of MOF than more traditional global haemodynamic parameters or biochemical biomarkers. Microcirculatory perfusion is a potential end point of resuscitation following THS.

**GRANT ACKNOWLEDGMENT**

National Institute of Academic Anaesthesia, Royal Centre for Defence Medicine, UK National Institute for Health Research.

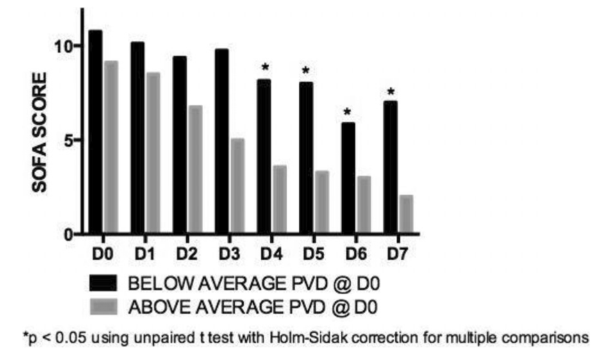

**Fig. 299 (Abstract 0935).** Daily SOFA scores & PVD

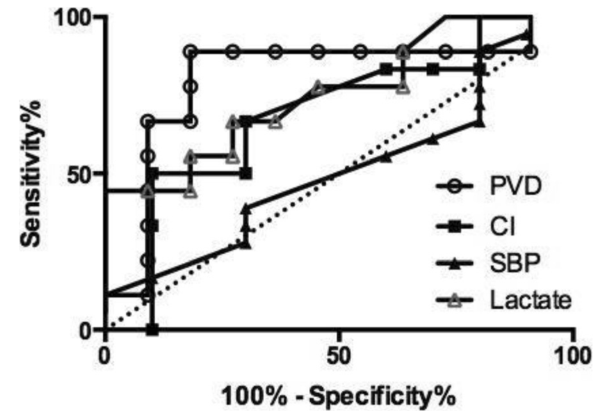

**Fig. 300 (Abstract 0935).** ROC curve to predict MOF

### 0936

#### Quality of life to one year and relation with clinical status at intensive care unit discharge in traumatic brain injury patients

R. Vela-Colmenero[1], M. Gordillo-Resina[1], J. Machado-Casas[1], E. Aguilar-Alonso[2], E. Perez-Mesa[1], M. Delange-Van Der Kroft[3], F. Fernandez-Ortega[4], M. Redondo-Orts[1], M.D. Arias-Verdu[4], E. Curiel-Balsera[4], M.A. Prieto-Palomino[4]

[1]Intensive Care Unit - Complejo Hospitalario, Jaen, Spain; [2]Intensive Care Unit - Hospital Infanta Margarita, Cabra, Spain; [3]Intensive Care Unit - Hospital Axarquia, Velez Malaga, Spain; [4]Intensive Care Unit - Hospital Universitario Carlos Haya, Malaga, Spain

**Correspondence:** R. Vela-Colmenero

*Intensive Care Medicine Experimental* 2017, **5(Suppl 2):**0936

**INTRODUCTION.** Many instruments have been developed to evaluate hospital mortality, but less attention has been paid to the long-term functional status and quality of life of traumatic brain injury patients.

**OBJECTIVES.** To analyze in traumatic brain injury critical patients admitted to intensive care the relation between the quality of life after one year and clinical status at intensive care unit discharge.

**METHODS.** Prospective cohort study of traumatic brain injury patients admitted in the University Clinical Hospital (Malaga) between 2004 to 2008. Data were expressed as the mean and standard deviation for quantitative variables and percentages for qualitative variables. We used ANOVA to compare means, Newman Keuls test, Pearson correlation and multiple lineal regression. Statistically significant differences $p < 0.05$.

**RESULTS.** 531 patients. Mean age 40.35 ± 19.75 years, APACHE-II 17.94 ± 6.97, admission GCS 7.53 ± 3.83 points. Computerized tomography (CT) on admission by Marshall score was: diffuse injury type I (10.4%), type II (28.1%), type III (24.5%), type IV (8.3%), mass evacuated (22.6%), mass not evacuated (6.2%). Hospital mortality 28.6%. 171 patients died at first year (32.2%) (Lost 6.6%) and 181 at 4 years (34.1%) (Lost: 16.2%).

The evaluation of the quality of life was performed by PAECC (Project for the Epidemiological Analysis of Critical Care Patients) QOL (Quality of Life) questionnaire (0 points: normal quality of life, 29 points: worst score). Mean score at first year follow-up 9.44 ± 8.73 points (N = 325), indicating high deterioration in quality of life.

There is an association between between quality of life after one year with age (r =0,163, p = 0,002), the depth of coma by GCS (r = −0,373, p < 0,001), APACHE II (r = 0,382, p < 0,001) and ICU stay (r = 0,401, p < 0,001).

We evaluated the clinical status at intensive care unit discharge through the analysis of oral communication, 75 patients not present problems, and their quality of life per year was 3.37 ± 5.05 points, 168 spoke with difficulty, and their quality of life was 7.82 ± 6.24 points and 82 patients not could speak and their quality of life 18.27 ± 9.06 points (p < 0.001). Statistically significant differences between three groups.

Multivariate analysis by multiple lineal regression found association between quality of life for one year with functional status by oral communication, age and ICU stay. There was no statistically significant relationship with APACHE II, admission GCS and tracheostomy.

**CONCLUSIONS.** ICU patients with Traumatic brain injury have an deteriorated quality of life after one year. Quality of life for after one year was related with functional status at ICU discharge evaluated by oral communication.

### 0937

**Withdrawn**

## Perioperative intensive care

### 0938

#### Surgical patients have favourable outcomes after admission to intensive care unit. A case-matched study using data from the Swedish Intensive Care Registry 2012–2014

M. Jawad[1], A. Baigi[2], S. Wilhelm[3], C. Mårdh[4], M. Chew[5]

[1]Central Hospital in Kristianstad, Kristianstad, Sweden; [2]Gothenburg University, Gothenburg, Sweden; [3]University Hospital in Linköping, Linköping, Sweden; [4]Swedish Intensive Care Registry, Kristianstad, Sweden; [5]Linköping university, Linköping, Sweden

**Correspondence:** M. Jawad

*Intensive Care Medicine Experimental* 2017, **5(Suppl 2):**0938

**INTRODUCTION.** The allocation of intensive care unit (ICU) beds for post-surgical care is widely debated, and there is no consensus as to whether it improves outcomes. Their availability and disposal are different between countries (1). The role of ICU in the perioperative management is widely considered to be beneficial (2) but needs evaluation due to recent findings indicating possible lack of benefit (3). In Sweden, lower admission rates to ICU do not seem to worsen perioperative outcomes (4) however there are few studies with long-term follow up.

**OBJECTIVES.** To study outcomes of patients undergoing surgical procedure prior to ICU admission (operated), comparing them to a matched population not having surgery (non-operated). The primary outcome parameters were 30-day and 1-year mortality.

**METHODS.** Quasi experimental case-matched study on data collected by the Swedish Intensive Care Registry on adult patients admitted to ICU during 2012–2014.

**RESULTS.** A total of 72,242 patients (12,880 operated patient) had mean 30-d and 1-y mortality rates of 21.0% and 31.2% respectively. After case-matching with age, sex and Simplified Acute Physiology Score III (SAPS3), operated patients (8,703) had significantly lower mortality rates than non-operated patients (33,567). Standardized mortality ratios (observed to estimated 30-day mortality rates) were 0.91 for operated and 1.07 for non-operated patients. For operated patients, factors independently associated with 30-day mortality included age (OR 1.03), SAPS3 (OR 1.08) and number of transfers to other ICUs (OR 1.22 per each transfer). For 1-year mortality, independent risk factors were age
(OR 1.03) and SAPS3 (OR 1.07). Sex, urgency of operation and planning of ICU admission were not significant risk factors. Main diagnosis was an important determinant of 30-day mortality: trauma
(OR 2.79), cardiovascular (OR 2.64), CNS (OR 2.35), gastrointestinal (OR 2.3), renal (OR 1.99), sepsis or infection (OR 1.88) and so on, but no such effect was observed for 1-year mortality, where malignancy was important (OR 1.49 and 1.81 respectively). Medical and surgical complications had paradoxical effects (OR 1.42 and 0.78 respectively). Direct admission to the ICU after surgery had a favourable effect in comparison to later admission from the ward.

**CONCLUSIONS.** Patients undergoing a surgical procedure prior to ICU admission have lower mortality rates compared to a matched population not undergoing prior surgical procedures. Short and long term mortalities were determined by age and SAPS3 scores. Main diagnosis was also an important predictor but only for short-term mortality.

### 0939

#### Fluid management in patients undergoing major abdominal surgery - influence of 0.9% saline versus an acetate buffered balanced infusate on the necessity of cardiocirculatory support: a prospective, randomized, controlled, double-blind study

C.A. Pfortmueller[1], G.-C. Funk[2], C. Reiterer[1], O. Zotti[1], B. Kabon[1], E. Fleischmann[1], G. Lindner[3], Vienna Interdisciplinary Reseach Group on Fluids, Electrolytes and Acid–base Disorders in Critical Illness
[1]Medical University of Vienna, Department of General Anesthesiology, Intensive Care and Pain Management, Vienna, Austria; [2]Otto Wagner Hospital and Ludwig-Boltzmann Institute for COPD and Respiratory Epidemiology, Department of Respiratory and Critical Care Medicine, Vienna, Austria; [3]Hirslanden Klinik Im Park, Department of General Internal Medicine & Emergency Medicine, Zurich, Switzerland
**Correspondence:** C.A. Pfortmueller
*Intensive Care Medicine Experimental* 2017, **5(Suppl 2):**0939

**INTRODUCTION.** Infusion solutions are among the most administered medications in hospitals.

We present the first randomized-controlled double-blind trial investigating whether the type of crystalloid used has an influence on cardiovascular stability in patients undergoing major abdominal surgery.

**METHODS.** Patients received either 0.9% saline or an acetate-buffered crystalloid for intraoperative volume replacement. Outcomes were primarily necessity for vasopressors, secondarily the total dose of catecholamines, total perioperative fluid and unplanned ICU admissions.

**RESULTS.** A total of 60 patients were randomized:, thirty to 0.9% saline and 30 to acetate-buffered balanced crystalloid. Significantly more patients needed vasopressors for circulatory support in the 0.9% saline group (53% vs 23%,p < 0.033). Mean dose of norepinephrine adjusted for body weight and anesthesia-duration was higher in the saline group [0.000502mcg/kg/min (standard deviation (SD) 0.001047] compared to the balanced group [0.000044mcg/kg/min (SD 0.000092)],p < 0.003. With ongoing surgery patients in the 0.9% saline group more often needed vasopressors (p < 0.0194). Cox regression revealed that a high amount of administered fluid (p < 0.001), allocation to the saline group
(p < 0.006) and a lower mean arterial blood pressure (p < 0.027) were associated with vasopressor necessity. The saline group developed hyperchloremic metabolic acidosis. There was no difference in total perioperative fluid and unplanned ICU admissions.

**CONCLUSIONS.** Use of 0.9% saline in patients undergoing major abdominal surgery leads to increased need of vasoactive substances for hemodynamic support, absolute doses of vasopressors and hyperchloremic acidosis compared to patients receiving a chloride-reduced, balanced crystalloid.

### 0940

#### The effect of using an enhanced recovery protocol for patients undergoing emergency laparotomy across 28 hospitals in England, UK

G. Aggarwal, N. Quiney
Royal Surrey County Hospital NHS Foundation Trust, Anaesthetics, Guildford, United Kingdom
**Correspondence:** G. Aggarwal
*Intensive Care Medicine Experimental* 2017, **5(Suppl 2):**0940

**INTRODUCTION.** Patients undergoing emergency laparotomy (non-traumatic) are known to have mortality rates of 15% or more in the US and the UK. Emergency laparotomy is carried out widely and significant numbers of patients die following surgery. Audits have shown standards of care vary greatly and improvements in outcome might be achieved by using an enhanced recovery model. Previous small scale studies have suggested that improving standards of care for these patients can considerably improve outcomes.

**METHODS.** A care bundle was developed using best evidence to identify key elements of care that are commonly deficient in the care of patients undergoing emergency laparotomy. 29 hospitals in the south of England (population of 8 million people) participated in a quality improvement initiative using this care bundle and the IHI Model for Improvement. This included development of regular data feedback (runcharts, SPC and CUSUM plots), coaching on quality improvement methodology and leadership and widespread inter-hospital learning and spreading of best practice ideas.

**RESULTS.** Early signs suggest a reduction of crude in-hospital mortality of 15-20% and a highly significant reduction in length of stay across all hospitals.Improvements in adherence across all elements of the care bundle have been shown in many hospitals and over 98.7% of patients have benefitted from one or more improvements of care.

**CONCLUSION.** This is the first large scale programme to demonstrate that the use of a simple care bundle, focusing on improving the quality of care for patients undergoing emergency laparotomy can result in both substantial mortality and length of stay reductions.

### 0941

#### Is there any association between metabolic syndrome and postoperative complications after coronary artery bypass surgery?

F. Ampatzidou, T. Tsermakian, L. Karagounis, A. Gogakos, T. Karaiskos, A. Madesis, G. Drossos
G.Papanikolaou General Hospital, Cardiac Surgery Department, Thessaloniki, Greece
**Correspondence:** F. Ampatzidou
*Intensive Care Medicine Experimental* 2017, **5(Suppl 2):**0941

**INTRODUCTION.** Metabolic syndrome, affecting large population especially in western countries has negative impact on outcome after cardiac and non-cardiac surgical procedures.

**OBJECTIVES.** To investigate the effects of metabolic syndrome on postoperative morbidity and mortality after coronary artery bypass surgery (CABG).

**METHODS.** A total of 1324 CABG procedures under the use of cardiopulmonary bypass were performed at our institution from June 2012 to March 2017. According to the International Diabetes Federation definition, 295(22.3%) patients- Group A- fulfill the criteria for metabolic syndrome. Our institutions' prospectively collected data were used and the following factors were compared between Group A and control Group B: acute kidney injury(AKI) defined by RIFLE criteria, need for renal replacement therapy (RRT), post-op non-invasive ventilation(NIV), re-intubation, prolonged ventilation defined as ventilation > 24 hours, postop pneumonia, red blood cells (RBC) transfusions, post-op atrial fibrillation, sternal wound infections, low cardiac output syndrome, Multiple Organ Dysfunction Syndrome and mortality. Univariate logistic regression method was used for statistical analysis.

**RESULTS.** Group A consisted of patients with metabolic syndrome mean aged 65.7 ± 8.9 vs 64.9 ± 9.8, percentage of females 16.5% vs 14.4% and prognostic Euroscore II 1.8 ± 1.8 vs 1.6 ± 1.5. From the above described 12 postoperative outcome indices, postoperative respiratory complications requiring NIV and the total RBC units transfused, were found to have statistical significant difference -defined as $p < 0.05$ -between the 2 groups.

| | Group A N = 295 | Group B N = 1029 | p value |
|---|---|---|---|
| Postop NIV (n,%) | 51(17.3%) | 64(6.2%) | <0.01 |
| RBC units (mean,SD) | 3.28 ± 3.2 | 1.87 ± 1.84 | <0.01 |

[Results]

**CONCLUSIONS.** Incidence of postoperative NIV requirement and RBC transfusions in early postoperative period are higher in patients with metabolic syndrome who undergo CABG.

**REFERENCE(S)**

Tung A. Anaesthetic considerations with the metabolic syndrome. Br J Anaesth
2010;105:Suppl 1:i24-33

### 0942

#### Can an early prediction tool appropriately stratify patients into risk groups for developing intensive care delirium?

W. Dean[1], S. Cowan[1], R. Goudie[2], J. Preller[1]
[1]Cambridge University Hospitals NHS Foundation Trust, Cambridge, United Kingdom; [2]MRC Biostatistics Unit, Cambridge, United Kingdom
**Correspondence:** W. Dean
*Intensive Care Medicine Experimental* 2017, **5(Suppl 2):**0942

Delirium is a common complication of critical illness(1). Strategies to reduce the incidence of delirium(2) may be of most benefit if aimed at patients at the highest risk. E-Pre-Deliric(3) is a tool for predicting individual risk of developing delirium in ICU using patient characteristics at admission. We have piloted the use of the E-Pre-Deliric score in a large teaching hospital ICU.

Data was collected for all admissions over a five week period. E-Pre-Deliric was calculated on admission (Age, admission type and urgency, mean arterial pressure, serum urea and presence of respiratory failure, dementia, alcohol abuse or steroid use). Delirium incidence, C-Reactive Protein (CRP) and White Cell Count (WCC) were collected retrospectively. Predictive performance of E-Pre-Deliric was assessed by calculation of the area under the ROC curve (AUROC) and logistic regression used to assess association between inflammation and delirium.

The study included 61 patients. Appropriately 88% of planned twice daily CAM-ICU scores were recorded. The AUROC of the E-Pre-Deliric score in our sample was 0.64 compared to 0.76 in the original study. Differences between our population and the E-Pre-Deliric validation cohort will be presented. As per Table 230, the delirium population had higher admission and peak CRP levels. Mean WCC was initially lower in the delirium population but on average, reached a higher peak.

Our sample contained a group of delirium positive patients admitted after surgery. These patients had relatively low E-Pre-Deliric scores (due to the elective nature of their intubation) but remained ventilated for longer than average, exposing them to the deliriogenic effects of sedation and ventilation.

Our analysis also suggests an association between the level of inflammatory response and development of delirium. An increase in CRP of 50 was strongly associated with an increase in the rate of delirium (OR 1.45, P = 0.005). An increase in WCC of 5 was more weakly associated with an increase in the rate of delirium (OR 1.39, P = 0.039). The distribution of peak WCC and CRP in patients with and without delirium is shown in Figs. 301 and 302. The burden of inflammation is not accounted for in the E-Pre-Deliric score, and in our sample may explain some of the patients with a low E-Pre-Deliric score who developed delirium (Fig. 303).

Future study will use these results as a baseline whilst we attempt to create a prediction score more closely tailored to predict delirium within our ICU population.

1. *Incidence, risk factors and consequences of ICU delirium*. Ouimet S et al. 2007, Intensive Care Med, pp. 33:66–73.
2. *A multicomponent intervention to prevent delirium in hospitalized older patients*. Inouye SK et al. 1999, N Engl J Med, pp. 340:669–676.
3. *Multinational development and validation of an early prediction model for delirium in ICU patients*. Wassenaar A et al. 2015, Intensive Care Medicine, pp. 41(6):1048–1056.

**Table 230 (Abstract 0942).** Comparison of mean CRP and WCC

| | Mean Admission WCC (109/L) | Mean Peak WCC (109/L) | Mean Admission CRP (mg/L) | Mean Peak CRP (mg/L) |
|---|---|---|---|---|
| Whole sample (n = 61) | 11.2 | 16.5 | 99.9 | 169.9 |
| Delirium Negative (n = 37) | 12.4 | 14.5 | 78.1 | 134.9 |
| Delirium Positive (n = 24) | 9.7 | 19.5 | 128.7 | 215.4 |
| Low Risk (E-Pre-Deliric <20) (n = 28) | 12.7 | 18.8 | 89.8 | 181.3 |
| Medium - High Risk (E-Pre-Deliric >20)(n = 33) | 10.0 | 14.6 | 108.5 | 160.2 |
| E-Pre-Deliric <20 and Delirium Positive (n = 10) | 9.9 | 23.5 | 121.7 | 214.8 |

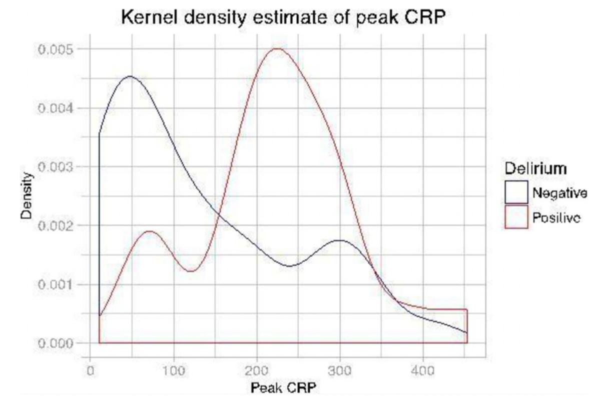

**Fig. 301 (Abstract 0942).** See text for description

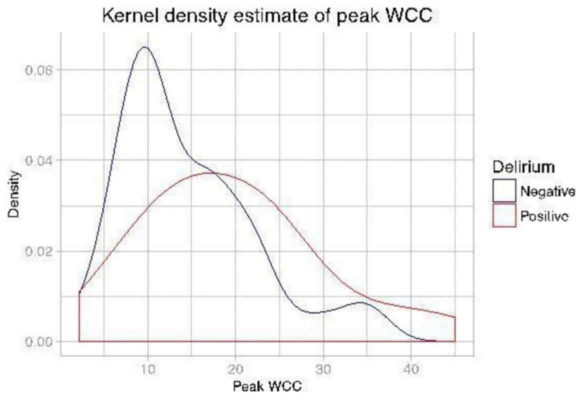

**Fig. 302 (Abstract 0942).** See text for description

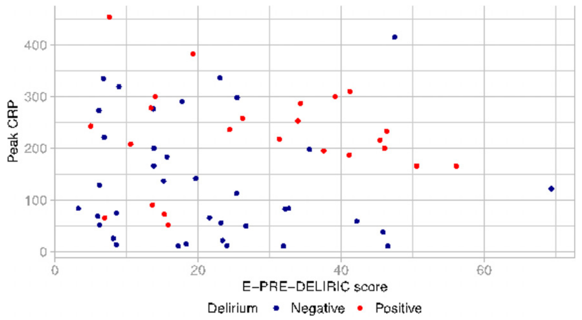

**Fig. 303 (Abstract 0942).** See text for description

### Antimicrobial use in the ICU

#### 0943

#### Plasma pharmacokinetic of a 140 mg loading dose of caspofungin in ICU patients with multiple organ failure and invasive candidiasis - the CASPOLOAD study

M.P. Lê[1,2], L. Bouadma[2,3], O. Andremont[3], M. Neuville[3], B. Mourvillier[2,3], R. Sonneville[3], E. Magalahes[3], J. Lebut[3], A. Radjou[3], R. Smonig[3], M. Lermuzeaux[3], C. Dupuis[2,3], M. Wolff[3], L. Massias[1,3], J.-F. Timsit[2,3,4], CASPOLOAD
[1]APHP Hopital Bichat, Laboratoire de Pharmacologie-Toxicologie, Paris, France; [2]INSERM, Université Paris Sorbonne Cité, Paris, France, IAME UMR 1137, Paris, France; [3]APHP Bichat, Medical and Infectious Diseases ICU, Paris, France; [4]OUTCOMEREA, Paris, France
**Correspondence:** J.-F. Timsit
*Intensive Care Medicine Experimental* 2017, **5(Suppl 2):**0943

**INTRODUCTION.** Invasive candidasis (IC) are associated with a high mortality in ICU. Indeed, treatment is often delayed and the volume of distribution of antifungal agents is increased in this population of patients. Consequently, for candins optimal concentration is difficult to obtain, particularly in sanctuary sites. .[1–3] The extensive use of antifungal agents is nowadays associated with significant trend of increase in the MICs of yeasts. For candins, an increase of MICs, especially for *C parapsilosis* and *C glabrata* has been repeatedly described and MICs >0.25mg/L became common[4–7] and recently associated with clinical failures.[8]

**OBJECTIVES.** To determine the pharmacokinetic (PK) profile of a caspofungin (CAS) loading dose of 140mg in ICU patients with multiple organ failure (MOF) and IC.

**METHODS.** Prospective, single-center, observational study [NCT#02413892]. Inclusion criterions were: adults (>18 yo) admitted in ICU with invasive mechanical ventilation, receiving epinephrine/norepinephrine for septic shock and with suspected or proven IC. Intensive PK blood sampling was performed at the end of 1h-CAS infusion and 2h, 3h, 5h, 7h and 24h post-infusion. Plasma CAS concentrations were determined using UPLC-MS/MS.[8] Pharmacokinetic parameters [estimate (RSE%)] were estimated by non-linear mixed effects modeling (population-based) approach, using Monolix software. CAS $AUC_{0-24}$ of CAS of 98mg.h/L was considered adequate. CAS PK/PD target should be $C_{max}$/MIC ratio > 10.[3] Descriptive results are presented as median (IQR).

**RESULTS.** 15 patients were included: 73% men, age 54 yo (34–65), BMI 26.5 kg/m2 (21.8-29.8), SAPS score 57 (45–78), SOFA score 14 (8–16). IC was proven in 3 cases (candidaemia), probable in 7 cases (sepsis multiple colonization and BD glucan >80pg/mL), and possible in 5 cases. Five patients were on ECMO and 9 were treated with renal replacement therapy. Among them, 14 completed PK profiles where a 2 compartments model best fitted the data (Table 231 & Fig. 304).

**CONCLUSIONS.** In our cohort of severe ICU patients with MOF, the 140mg CAS loading dose allowed to achieve plasma AUC0-24 on the first 24h higher than reported AUC0-24 at 3rd day, in ICU patients receiving standard administration of CAS (70mg then 50 mg daily).[9] High dose of CAS is known to be safe[10–11.]

Our results suggest that a higher loading dose might help reaching the PK/PD target in severe ICU patients with MOF.

**REFERENCE(S)**

1 Sinnollaredy *et al., Int J Antimicrob Agents* 2012
[2] Nguyen *et al., J Antimicrob Chemother* 2007
[3] Andes *et al., Antimicrob Agents Chemother* 2003
[4] Fournier *et al., J Antimicrob Chemother* 2011
[5] Lortholary *et al., Antimicrob Agents Chemother* 2011
[6] Dannaoui *et al., Emerg Infect Dis* 2012
[7] Alexander *et al., Clin Infect Dis* 2013
[8] Farowski *et al., Antimicrob Agents Chemother* 2010
[9] Van der elst *et al., Antimicrob Agents Chemother* 2016
[10] Betts *et al., Clin Infectious Dis* 2009
[11] Safdar *et al., Bone Marrow Transplant* 2007

**GRANT ACKNOWLEDGMENT**
Investigator grant Merck

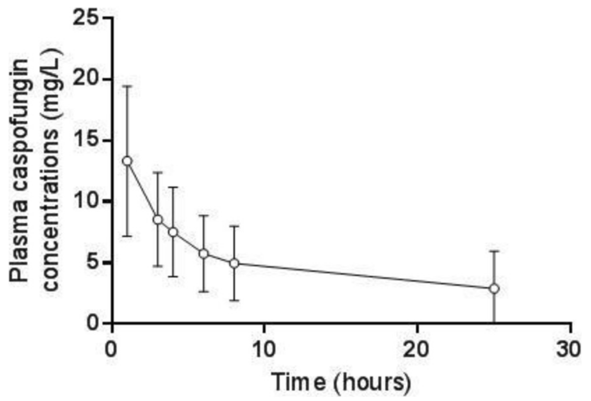

**Fig. 304 (Abstract 0943).** See text for description

**Table 231 (Abstract 0943).** See text for description

| Model parameters, *estimate (RSE%)* | |
|---|---|
| CL (L/h) | 0.218 (68%) |
| $V_1$ (L) | 8.99 (13%) |
| Q (L/h) | 1.7 (19%) |
| $V_2$ (L) | 30.1 (29%) |
| Proportionnal error (%) | 10.9 (12%) |
| Inter-individual variabilities (%) for: | |
| CL | 139% (36%) |
| $V_1$ | 46% (21%) |
| Q | 64% (22%) |
| $V_2$ | 68% (31%) |
| **Secondary pharmacokinetic parameters, *median (IQR)*** | |
| $AUC_{0-24}$ (mg.h/L) | 103.1 (88.3-140.8) |
| Nb of patients $AUC_{0-24}$ >98mg.h/L, n (%) | 8 (57%) |
| $C_{max}$ (mg/L) | 12.1 (8.1-19.8) |
| $C_{max}$/MIC ratio, MIC=0.25 mg/L | 48.2 (32.4-79.2) |
| $C_{min}$ (mg/L) | 1.8 (1.4-3.4) |

### 0944

#### Infection-related ventilator-associated complications in critically ill patients colonized with extended-spectrum beta-lactamase-producing *Enterobacteriaceae* (ESBLE): correlation with ventilator-associated pneumonia due to ESBLE and impact on carbapenem exposure

F. Barbier[1], S. Bailly[2], C. Schwebel[3], J.-M. Forel[4], E. Azoulay[5], B. Misset[6], B. Mourvillier[7], J. Reignier[8], M. Darmon[9], J.-R. Zahar[10], D. Goldgran-Toledano[11], E. de Montmollin[12], B. Souweine[13], J.-F. Timsit[14], OUTCOMEREA Study Group
[1]La Source Hospital - CHR Orléans, Medical ICU, Orléans, France; [2]Paris Diderot - Sorbonne Paris Cité University, UMR 1137 - IAME Team 5 - DeSCID Decision SCiences in Infectious Diseases, Control and Care INSERM, Paris, France; [3]Albert Michallon Hospital, Grenoble 1 University, Medical ICU, Grenoble, France; [4]North Hospital, Aix-Marseille University, Medical ICU, Marseille, France; [5]Saint-Louis Hospital, ECSTRA Team, Biostatistics and Clinical Epidemiology, Paris Diderot Sorbonne University, Medical ICU, Paris, France; [6]Charles-Nicolle Hospital, Rouen University, Medical ICU, Rouen, France; [7]Bichat-Claude Bernard Hospital, Medical ICU, Paris, France; [8]Nantes University Hospital, Medical ICU, Nantes, France; [9]North Hospital, Jacques Lisfranc Medical School, Jean Monnet University, Medical ICU, Saint-Etienne, France; [10]Avicenne Hospital, Paris-Nord University, Infection Control Unit, Bobigny, France; [11]Le Raincy - Montfermeil Hospital, Medical ICU, Le Raincy - Montfermeil, France; [12]Delafontaine Hospital, Medical ICU, Saint-Denis, France; [13]Gabriel Montpied Hospital, Clermont-Ferrand University, Medical ICU, Clermont-Ferrand, France; [14]Bichat-Claude Bernard Hospital, UMR 1137 - IAME Team 5 - DeSCID Decision SCiences in Infectious Diseases, Control and Care INSERM, Paris Diderot - Sorbonne Paris Cité University, Medical ICU, Paris, France
**Correspondence:** F. Barbier
*Intensive Care Medicine Experimental* 2017, **5(Suppl 2):**0944

**INTRODUCTION.** Infection-related ventilator-associated complications (IVAC) as defined by the Centers for Disease Control and Prevention are common events in intensive care unit (ICU) patients receiving mechanical ventilation (MV). The characteristics of IVAC have not been investigated in patients colonized with ESBLE.

**OBJECTIVES.** To appraise the correlation between IVAC and (i) ventilator-associated pneumoniae (VAP) due to ESBLE, (ii) other ICU-acquired ESBLE infections and (iii) carbapenem exposure in ESBLE carriers.

**METHODS.** Inception cohort study from the French longitudinal prospective multicenter OUTCOMEREA database (1997–2015) including all ESBLE carriers with MV duration > 2 days and ≥ 1 episode of IVAC after carriage documentation.

**RESULTS.** Among the 318 ESBLE carriers included in the study, 225 (70%) did not develop VAP, 73 (23%) developed ≥ 1 non-EBLSE VAP, and 20 (7%) developed ≥ 1 ESBLE VAP (median number of IVAC episodes, 1 [1–2], 3 [2–4] and 2 [1–5], respectively, *P* < 0.01). A total of 576 IVAC episodes were analyzed, including 361 (63%) with no identifiable infection, 124 (21%) related to non-VAP infections (ESBLE infections, n = 20), 73 (13%) related to non-ESBLE VAP (concomitant non-VAP ESBLE infections, n = 5) and only 18 (3%) related to ESBLE VAP. Overall, ESBLE infections accounted for 43 (7%) episodes of IVAC. No significant variation was observed across the 4 IVAC groups in terms of prior MV duration, requirement for invasive procedures other than MV, SOFA score values at IVAC onset, episode ranks in patients with ≥ 2 IVAC, carriage type (imported versus ICU-acquired), species distribution of ESBLE, and non-carbapenem antimicrobials use within the 3 preceding days. None of the 143 episodes of IVAC which arose under carbapenem therapy were related to an ESBLE VAP (Table 232). The empirical initiation of a carbapenem-based antimicrobial regimen was frequent in IVAC not related to an ESBLE infection, notably VAP. VAP-related IVAC were associated with a poorer outcome than non-VAP IVAC (Table 232).

**CONCLUSIONS.** The occurrence of IVAC in ESBLE carriers rarely results from an ESBLE infection, especially VAP. No risk factor for ESBLE VAP as a cause of IVAC could be identified. Rapid diagnostic tools may help restraining the empirical use of carbapenems in this situation.

**REFERENCE**
None

**GRANT ACKNOWLEDGMENT**
COMBACTE-MAGNET, OUTCOMEREA

**Table 232 (Abstract 0944).** Main characteristics of IVAC in ESBLE carriers

| Variable | No identifiable infection (n=361) | Non-VAP infections (n=124) | Non-ESBLE VAP (n=73) | ESBLE VAP (n=18) | P value, global comparison | P value, non-ESBLE VAP versus ESBLE VAP |
|---|---|---|---|---|---|---|
| Carbapenem therapy on the day preceding IVAC onset, n (%) | 103 (28) | 25 (20) | 15 (20) | 0 | 0.01 | 0.03 |
| Empirical carbapenem therapy started at IVAC onset, n (%) | 39 (9) | 19 (15) | 31 (42) | 10 (56) | <0.01 | 0.43 |
| ICU LOS after IVAC, days, median [IQR] | 7 [5-10] | 8 [5-11] | 12 [8-18] | 12 [8-16] | <0.01 | 0.61 |
| MV duration after IVAC, days, median [IQR] | 5 [2-8.5] | 7 [3-9] | 10 [7-16] | 11 [8-15] | <0.01 | 0.78 |
| Death during IVAC, n (%) | 59 (15) | 20 (19) | 19 (27) | 5 (29) | 0.05 | 1.00 |

### 0945

#### Adequacy of b-lactams therapy during extracorporeal membrane oxygenation

I. Romeo[1], C. De Fazio[2], O. Lheureux[2], M. Belliato[1], F. Mojoli[3], F. Jacobs[4], F. Cotton[4], J. Creteur[2], J.-L. Vincent[2], F.S. Taccone[2]
[1]Fondazione IRCCS Policlinico S Matteo Pavia, Università di Pavia, Pavia, Italy; [2]CUB Erasme (Université Libre de Bruxelles), Dept of Intensive Care, Brussels, Belgium; [3]Fondazione IRCCS Policlinico S Matteo Pavia, Università di Pavia, Intensive Care, Pavia, Italy; [4]CUB Erasme (Université Libre de Bruxelles), Brussels, Belgium
**Correspondence:** I. Romeo
*Intensive Care Medicine Experimental* 2017, **5(Suppl 2):**0945

**INTRODUCTION.** Infection is frequent in patients treated by extracorporeal membrane oxygenation (ECMO); however, antimicrobial concentrations can be significantly altered during ECMO when standard regimens are used. The aim of this study was to describe the adequacy of therapy during b-lactams administration in ECMO patients.

**METHODS.** We reviewed all patients in whom b-lactams (meropenem = MEM; piperacillin/tazobactam = TZP; cefepime or ceftazidime = CEF) therapeutic drug monitoring (TDM) was assessed while on ECMO support (from January 2010 to December 2016). Drug regimens were adjusted on renal function (i.e. creatinine clearance using the Cockroft-Gault formula). Daily creatinine clearance (CrCL) was also measured using 24-hr urine collection. Drug concentrations were measured 2 hours after the onset of a 30-min drug infusion and just before the next dose and were considered as "adequate" if drug levels remained between 4 and 8 times the clinical breakpoint of the minimal inhibitory concentration for Pseudomonas aeruginosa during 40% for MEM, 50% for TZP or 70% for CEF of the dose interval (i.e. "target"). Using a one-compartment model, b-lactams pharmacokinetics (PKs) and concentrations at target time-points were calculated. Drug concentrations below or above these targets were considered as "insufficient" or "excessive", respectively.

**RESULTS.** A total of 90 TDMs (MEM = 47; TZP = 36; CEF =7) were obtained in 53 patients treated with ECMO (35 veno-venous and 18 veno-arterial). The proportion of insufficient drug levels was higher for TZP (15/36, 42%) than for MEM (8/47, 17%) or CEF (0/7; p = 0.01). Excessive drug concentrations were more frequent in MEM (19/47, 40%) than TZP (8/35, 22%) or CEF (1/7, 14%; p = 0.05). TDMs in patients on continuous renal replacement therapy (CRRT, n = 39) showed less frequent insufficient drug levels than those without CRRT (3/39 vs. 20/51; p = 0.007). Among those patients without CRRT, there was a significant although weak correlation between CrCL and drug clearance (p = 0.01; $r^2$ = 0.12).

**CONCLUSIONS.** The adequacy of b-lactam therapy is highly variable among different drugs and might be influenced by CRRT or renal clearance.

### 0946

#### Risk factors of clinical failure during treatment of postoperative peritonitis: results of the DURAPOP trial

P. Montravers[1], F. Tubach[2], M. Esposito-Farèse[3], S. Lasocki[4], T. Lescot[5], B. Veber[6], P. Seguin[7], C. Paugam[8], H. Dupont[9], DURAPOP Trial Group
[1]CHU Bichat Claude Bernard, DAR, Paris, France; [2]CHU Pitié-Salpêtrière, Assistance Publique Hôpitaux de Paris, INSERM, UMR 1123, ECEVE, CIC-EC 1425, Paris, France; [3]CHU Bichat Claude Bernard, INSERM CIC-EC 1425, Paris, France; [4]CHU Angers, DAR, Angers, France; [5]CHU St Antoine, DAR, Paris, France; [6]CHU Rouen, DAR, Rouen, France; [7]CHU Rennes, DAR, Rennes, France; [8]CHU Beaujon, DAR, Clichy, France; [9]CHU Amiens, DAR, Amiens, France
**Correspondence:** P. Montravers
*Intensive Care Medicine Experimental* 2017, **5(Suppl 2):**0946

**INTRODUCTION.** Risk factors of clinical failure in the management of postoperative peritonitis (POP) have been rarely analysed.

**OBJECTIVES.** From the data collected during the prospective randomized multicenter DURAPOP study (NCT01311765) addressing the issue of duration of antibiotic therapy in POP patients (pts), the risk factors of failure have been analysed.

**METHODS.** Patients who underwent reoperation for POP, received adequate surgical and antibiotic (AB) therapies and were alive at Day-8 after surgery were analysed. Failure between D8 and D45 was defined as death, need for reoperation, percutaneous drainage and/or new AB therapy. The following data were collected: demographic characteristics, underlying diseases (Charlson score), severity at reoperation for POP (SAPS II and SOFA scores), surgical and operative characteristics, microbiologic results, empiric and targeted antibiotic therapy (tAB), presence of multidrug resistant bacteria (MDR) in clinical and hygiene samples on admission and during ICU stay. At D45, pts who had a clinical failure were compared with those having a successful outcome. Potential predictive factors which level of association to failure in univariate analysis was < 15% (p < 0.15) were analysed in multivariate logistical regression. Results are presented in median (IQR), proportions and adjusted odds ratio (OR) (95% confidence interval (CI)).

**RESULTS.** Among 236 pts included for POP, 141 (60%) had a clinical failure (death (n = 30), reoperation (n = 58), percutaneous drainage (n = 33) or new AB (n = 95)). No difference between groups was observed for the demographic parameters (age [IQR] : 66 [57:76]; male gender: 64%; no underlying disease: 56%; Charlson score : 4 [2:7]), severity at the time of surgery for POP (SAPS II score 45 [34:54], SOFA 6 [4:8]), surgical and operative characteristics or microbiologic results (data not shown). In univariate analysis, factors associated to clinical failure were renal replacement therapy on the day of surgery for POP (OR 2.90 ; 95%CI [1.0-8.10], p = 0.042), initial gastro-duodenal surgery (0.50 [0.20-0.90], p = 0.023), tAB using piperacillin/tazobactam (1.90 [1.10-3.30], p = 0.031), tAB using third generation cephalosporins (0.40 [0.20-0.90], p = 0.022), tAB using glycopeptides (2.20 [1.10-4.50], p = 0.032), MDR organisms cultured on admission in hygiene samples (2.00 [1.00-4.20], p = 0.055) and acquisition of MDR bacteria during ICU stay in hygiene samples (8.10 [1.90-35.60], p = 0.005). In multivariate analysis, the risk factors associated to failures are presented in the Table 233.

**CONCLUSIONS.** Among ICU pts reoperated for POP and receiving an adequate AB, clinical failures are frequent. The identified risk factors reflect situations where the prescribers target difficult to treat microorganisms in using broad spectrum AB. Similar observations are made with acquisition of MDR bacteria in hygiene samples during the ICU stay.

**GRANT ACKNOWLEDGMENT**
PHRC National 2009 (AOM 09024)

**Table 233 (Abstract 0946).** Risk factors associated to failure

| | Odds ratio | 95% CI | p |
|---|---|---|---|
| tAB using carbapenems | 2.52 | [1.11-5.70] | 0.026 |
| tAB using piperacillin/tazobactam | 2.45 | [1.26-4.78] | 0.008 |
| tAB using glycopeptides | 2.41 | [1.06-5.49] | 0.035 |
| MDR bacteria acquired during the ICU stay in hygiene samples | 7.86 | [1.73-35.75] | 0.007 |

### 0947

#### Pharmacokinetics of meropenem in patients with septic shock

F. Sjövall[1,2], A. S. Alobaid[3], S. C. Wallis[4], A. Perner[5], J. Lipman[4,6], J. A. Roberts[4,7]
[1]Lund University, Lund, Sweden; [2]Skane University Hospital, Intensive Care Unit, Malmö, Sweden; [3]University of Queensland Centre for Clinical Research, Brisbane, Australia; [4]University of Queensland Centre for Clinical Research, Burns Trauma and Critical Care Research Centre, Brisbane, Australia; [5]Copenhagen University Hospital, Rigshospitalet, Copenhagen, Denmark; [6]Royal Brisbane and Women's Hospital, Brisbane, Australia; [7]The University of Queensland, Centre for Translational Pharmacodynamics, Brisbane, Australia
**Correspondence:** F. Sjövall
*Intensive Care Medicine Experimental* 2017, **5(Suppl 2):**0947

**INTRODUCTION.** Early and appropriate antibiotic therapy appears to be the most effective way of improving patient outcomes in sepsis. Sepsis may induce altered pharmacodynamics due to changes in renal function, increased capillary leakage and tissue edema. Therefore, typical dosing may not achieve adequate concentrations and so the question of what is the appropriate dose and administration strategy for the chosen antibiotic is generally not well defined.

**OBJECTIVES.** The objectives of this study were to describe the population pharmacokinetics of meropenem in a large cohort of patients with septic shock and evaluate the probability of target attainment and the fractional target attainment of different dosing regimens for treatment of two challenging pathogens that pervade critical care units (*Acinetobacter baumannii* and *Pseudomonas aeruginosa*).

**METHODS.** A single centre observational pharmacokinetic study including adult patients with septic shock treated with meropenem. Seven blood samples were drawn during one administration period to create a pharmacokinetic profile. Concentrations of meropenem in plasma were measured by a validated HPLC-MS/MS method. To describe total meropenem concentrations, one and two-compartment models were developed. Monte-Carlo simulations were employed to determine the probability of target attainment (PTA) of achieving a PK/PD target of $f$T > MIC = 40%. Fractional target attainment (FTA) calculations were performed to assess the likely success of treatment for empirical or targeted therapy of *Acinetobacter baumannii* and *Pseudomonas aeruginosa.*

**RESULTS.** Fifty patients were included. A two-compartment linear model best described meropenem pharmacokinetics and including creatinine clearance (CrCL) improved the fit of the model. A continuous infusion increased the likelihood of attaining the target PTA or FTA. At the highest creatinine clearence level (CrCL 200 ml/min) 4–6000 mg was required for intermittent dosing to have a 100% likelihood of attaining the targeted PTA. This could be reduced to 2000 mg with continuous infusion. For empirical treatment of *A. baumannii*, a daily dose of 8000 mg of meropenem was required for intermittent boluses and prolonged infusion which could be reduced to a daily dose of 6000 mg with continuous infusion. For *P. aeruginosa*, a daily dose of either 2000 mg q8 (6000 mg) or 1000 mg q6 (4000 mg) achieved FTA ≥90%. At CrCL of ≤ 100 ml/min, all PTA and FTA targets could be reached with intermittent dosing of 1000 mg q3.

**CONCLUSIONS.** In conclusion, meropenem pharmacokinetics is mostly influenced by renal clearance and less so by distribution volume. In patients with septic shock and a possible augmented renal clearance doses should be increased and/or administration should be performed with prolonged or continuous infusion to increase the likelihood of achieving targeted plasma concentrations. In patients with normal to slightly reduced renal function however, standard dosing seems to be sufficient.

Outcome after intensive care.

### 0948

#### Treatment, risk of death and chance of discharge vary between weekdays and weekends in intensive care units

P. Zajic[1], P. Bauer[2], A. Rhodes[3], R. Moreno[4], T. Fellinger[2], B. Metnitz[5], F. Stavropoulou[2], M. Posch[2], P.G. Metnitz[1]

[1]Medical University of Graz, Dept. of Anaesthesiology and Intensive Care Medicine, Div. of General Anaesthesiology, Emergency- and Intensive Care Medicine, Graz, Austria; [2]Medical University of Vienna, Centre for Medical Statistics, Informatics, and Intelligent Systems, Vienna, Austria; [3]St George's University of London, St George's University Hospitals NHS Foundation Trust, London, United Kingdom; [4]Centro Hospitalar de Lisboa Central, Hospital de São José, Unidade de Cuidados Intensivos Neurocríticos, Lisboa, Portugal; [5]Austrian Centre for Documentation and Quality Assurance in Intensive Care, Vienna, Austria

**Correspondence:** P. Zajic

*Intensive Care Medicine Experimental* 2017, **5(Suppl 2):**0948

**INTRODUCTION.** Recent studies and meta analyses suggested that ICU and hospital admission on different days of the week may be associated with varying outcomes [1]. Apart from these indirect effects, little is known about the immediate effects of weekend ICU stay on treatment, risk of death and chance of discharge.

**OBJECTIVES.** To assess whether ICU stay at weekends (Saturday - Sundays) differs from weekday ICU stay (Monday - Friday) in terms of treatment, mortality risk or discharge chance.

**METHODS.** Retrospective study in the registry set up by the Austrian Centre for Documentation and Quality Assurance in Intensive Care (ASDI). All adult patients, who were admitted to one of the 119 ICUs participating in the project between 2012 and 2015, were included. ICU readmissions during the same hospital stay were excluded.

A multivariable competing risk analysis (Fine and Gray proportional subdistribution hazards model) concerning ICU mortality and ICU discharge was performed. The following variables were used in the model: weekday of admission, weekday of event (death or discharge), SAPS 3 score, year of admission, month of admission, type of admission, and centre. Intervention rates, both inside and outside the ICU, as documented by the TISS-28 score, were calculated for every weekday. All comparisons were drawn using Wednesday as the reference day.

**RESULTS.** A total of 151,268 patients was included in the analysis. Risk of death in the ICU was significantly lower at weekends than during the week (HR [95% CI] for Saturday = 0.93 [0.87-1.00] and Sunday = 0.85 [0.80-0.91]). Chance of discharge was reduced (HR [95% CI] for Saturday = 0.63 [0.62-064] and Sunday = 0.56 [0.55-0.57]). Patients received specific interventions at a significantly lower rate at weekends compared to weekdays. A single intervention was documented in 11.7% of patients at Wednesdays, 10.0% at Saturdays and 9.9% at Sundays ($p < 0.001$). Multiple interventions were performed in 8.3% of patients at Wednesdays, 7.7% at Saturdays and 7.8% at Sundays ($p < 0.001$). 15.5% of patients received interventions outside the ICU at Wednesdays, while only 11.5% and 10.6% did so at Saturdays and Sundays ($p < 0.001$).

**CONCLUSIONS.** Weekend ICU stay is associated with lower risk of death than during the week. This effect may, at least partly, be explained by a concomitantly reduced chance of ICU discharge. Risk of death may therefore be reduced due to improvements in patients' physiology. The lower rate of disease specific interventions at weekends could also be explained that way. Future research needs to investigate, whether weekend ICU discharge could be beneficial, since it is generally deemed safe [2].

### 0949

#### Safety climate, process adhesion and outcomes in critical care units: a secondary analysis of the CHECKLIST-ICU trial

F.G. Zampieri[1], L.P. Damiani[1], F.R. Machado[2], F.A. Bozza[3], P. Vendramim[4], J.I. Salluh[3], A.B. Cavalcanti[1]

[1]HCor-Hospital do Coração, Research Institute, São Paulo, Brazil; [2]Universidade Federal de São Paulo, Anesthesiology ICU, São Paulo, Brazil; [3]D'Or Institute for Research and Education, Rio de Janeiro, Brazil; [4]Hospital Samaritano, São Paulo, Brazil

**Correspondence:** F.G. Zampieri

*Intensive Care Medicine Experimental* 2017, **5(Suppl 2):**0949

**INTRODUCTION.** Safety climate has been suggested to be associated with better outcomes in critically ill patients, but evidence is sparse. It is also unclear whether safety climate is associated with adherence to best practices.

**OBJECTIVES.** To assess whether safety climate, assessed through Safety Attitudes Questionnaire - ICU (SAQ-ICU), is associated with hospital mortality and with adherence to standard-of-care practices in ICUs.

**METHODS.** We prospectively collected SAQ-ICU domains (teamwork climate, safety climate, job satisfaction, stress recognition, perception of managements and working condition) in the ICUs participating in the CHECKLIST-ICU trial. Data was grouped for the analysis considering each phase at each ICU as a distinct cluster. At least 75% of all health practitioners in each unit answered the questionnaire. The association between SAQ components and hospital mortality was assessed through mixed regression model correcting by SAPS 3 score at the patient level with each cluster as a random intercept. Model's selection was done using stepwise Deviance's Analyses. The association between SAQ components and adherence to best practices and support use was assessed through linear regression.

**RESULTS.** 236 clusters with a total of 13,638 patients and 12,933 health professionals were included; 118 centers (6,877 patients, 6,558 health professionals) on the first phase and 118 (6,761 patients, 6,375 health professionals) on the second phase of the trial. Nurse assistants were the most common responders (46.2%), followed by nurses (16.7%) and physicians (15.9%). SAQ values between phase 1 and phase 2 were correlated (lower correlation coefficient was 0.32 for stress recognition domain and higher was 0.61 for working condition). There was a high correlation between all SAQ components except for stress recognition that represented a different domain. There were only weak associations between SAQ domains and hospital mortality (Fig. 305).

In mixed effect model after stepwise regression, only SAPS3 score and SAQ safety climate domain (OR 0.987 [0.978-0.996]; $p = 0.006$) were associated with outcome. Higher levels of ICU's stress recognition were associated with higher mean unit SAPS 3 score, higher use of central venous catheter, increased use of analgesics, higher use of tidal-volumes bellow 8 mL/kg and longer length of stay but not with adherence to other practices such as thromboprophylaxis and head elevation.

**CONCLUSIONS.** A better safety climate was weakly associated with lower hospital mortality. Admission of more severely ill patients was associated with higher stress in ICU staff. It is unclear whether the association of stress with best practices may be only due to higher illness severity.

**REFERENCE(S)**
CHECKLIST-ICU Investigators. Effect of a Quality Improvement Intervention With Daily Round Checklists, Goal Setting, and Clinician Prompting on Mortality of Critically Ill Patients. JAMA 2016
**GRANT ACKNOWLEDGMENT**
Supported by PROADI-SUS

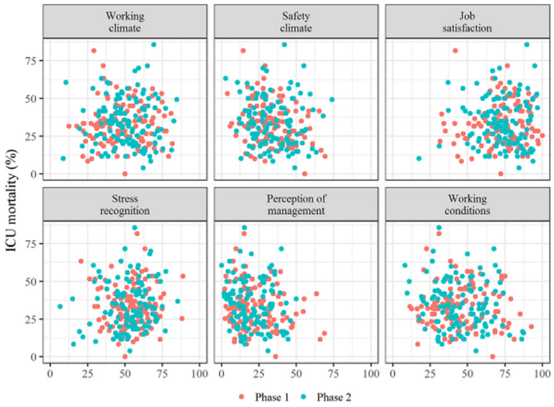

**Fig. 305 (Abstract 0949).** See text for description

### 0950

#### ARDS cases volume and ICU mortality: insights from the CUBREA database

M. Dres[1,2], P. Austin[3], P. Aegerter[4], T. Pham[2,5], B. Guidet[6], A. Demoule[1], A. Vieillard-Baron[7], L. Brochard[2,5], G. Géri[7], on behalf of the CUBREA Investigators
[1]Hôpital Pitié Salpetrière, Assistance Publique Hôpitaux de Paris, Service de Pneumologie et Réanimation Médicale, Paris, France; [2]St Michael's Hospital in Toronto, Li Ka Shing Knowledge Institute, Keenan Research Centre, Toronto, Canada; [3]University of Toronto, Institute of Health Policy, Management and Evaluation, Toronto, Canada; [4]Hôpital Ambroise Paré, Assistance Publique-Hôpitaux de Paris, Unité de Recherche Clinique (URC), Assistance Publique-Hôpitaux de Paris, Hôpital Ambroise Paré, Unité de Recherche Clinique (URC), Boulogne, France, France; [5]University of Toronto, Toronto, Canada; [6]Hôpital Saint Antoine, Assistance Publique Hôpitaux de Paris, Service de Réanimation Médicale, Paris, France; [7]Hôpital Ambroise Paré, Assistance Publique Hôpitaux de Paris, Service de Réanimation Medico-Chirurgicale, Boulogne-Billancourt, France
**Correspondence:** M. Dres
*Intensive Care Medicine Experimental* 2017, **5(Suppl 2):**0950

**INTRODUCTION.** The relationship between case volume and outcomes has been established for a number of scenario in the intensive care unit (ICU) but never in patients with acute respiratory distress syndrome (ARDS).
**OBJECTIVES.** We aimed at determining whether ICUs caring for higher volume of ARDS would or would not be associated with lower ICU mortality.
**METHODS.** ARDS cases were identified from coding system through a regional database (2004 to 2012). Volume was calculated as the cumulative annual mean of ARDS cases. Severity (SAPS2) and ICU mortality between categories of case volume were investigated. Multivariable analysis using mixed effects models was performed to adjust for severity of illness and confounding factors.
**RESULTS.** Over the study period, 8,383 ARDS patients among 31 ICUs were entered in the analysis. Overall, SAPS2 was 58 (43–74) while ICU mortality was 53.7%. SAPS2 was significantly higher in high volume ICUs (>65 ARDS per year) as compared to low (≤29 ARDS per year) and medium volume ICUs (>29-65 ARDS per year): 61 [46–77] vs. 55 [41–72] and 55.0 [40–72] respectively ($p < 0.01$). ICU mortality was similar across the three ARDS volume categories (53.6%, 54.1% and 53.3% in low, medium and high-volume categories ICUs, respectively). After adjustment for confounders, ARDS case volume was independently associated with ICU mortality (Odds ratio for log-transformed volume: 0.77 [95% confidence interval 0.62-0.96], p = 0.02).
**CONCLUSIONS.** ICUs caring for higher volume of ARDS were associated with lower ICU mortality. The underlying factors driving this relationship should be determined by further studies.

### 0951

#### Frailty and functional status as decision factors regarding admission to critical care

A. Tridente[1,2], K. Wilkinson[1], M. Marnell[1], J. Feeney[1], R. Varia[1], F. Andrews[1], A. Raithatha[3]
[1]Whiston Hospital, Prescot, United Kingdom; [2]University of Liverpool, Liverpool, United Kingdom; [3]Sheffield Teaching Hospitals NHS Foundation Trust, Sheffield, United Kingdom
**Correspondence:** A. Tridente
*Intensive Care Medicine Experimental* 2017, **5(Suppl 2):**0951

**INTRODUCTION.** Pressure on Critical Care (CC) services is significant, with a progressively more complex patient population with increasing comorbidities. Admission may be inappropriate if the patient is unlikely to benefit from it. Guidance published in the past may no longer reflect current practice [1–2]. Clinical frailty is associated with worse outcomes in critically ill patients [3].
**OBJECTIVES.** We aimed to establish the impact of frailty measures and other factors on the decision to admit critically ill patient to CC.
**METHODS.** Data on unplanned patient referrals made between November 2013 and February 2015 were collected, including patient demographics, acute physiological parameters, prior hospital length of stay (LOS), comorbidities and indices of frailty. Logistic regression analysis was used to assess factors influencing admission, using STATA 10. Results are expressed as median (interquartile range) and odds ratios (OR) for admission, with 95% confidence intervals (95%CI).
**RESULTS.** Data were collected on 943 patients referred to CC, of whom 411 (43.6%) were admitted. Median age was 65 (50–76) years, 482 (51.1%) were male and the median LOS prior to referral was 0 (0–2) days. The majority of patients were referred out of hours (496, 52.6%). The majority of patients were referred from medical specialties (384, 40.7%), the emergency department (261, 27.7%) and surgical specialties (198, 21%). Among those not admitted, 125 (13.3%) were declined as functional status was deemed too poor and 80 (8.5%) because death was deemed certain. At single variable logistic regression analysis adjusted for age and gender, predictors of admission were an increase in EWS (early warning score, an index of acute physiological derangement, OR for each point increase 1.14, 95% CI 1.04-1.26, p =0.007), shorter hospital stay (OR for each additional day inpatient stay 0.97, 95%CI 0.94-0.99, p = 0.033), lower frailty score (OR for each point increase in score 0.77, 95%CI 0.68-0.86, $p < 0.001$), being self-caring (OR 2.52, 95%CI 1.74-3.65, $p < 0.001$). Being housebound (OR 0.34, 95%CI 0.23-0.49, $p < 0.001$) or wheelchair-bound (OR 0.35, 95%CI 0.21-0.6, $p < 0.001$) were both significantly associated with admission being declined.
**CONCLUSIONS.** Frailty and functional status appear to be the major factors in decision making regarding admission to CC, after adjusting for age and gender. Quantification of frailty is a valuable tool in admission prediction and may allow risk stratification and adequate resource allocation, deserving further investigation

**REFERENCE(S)**
1 ACCCM/SCCM task force. CCM;27:633–638.1999
2 American Thoracic Society. AJRCCM;156:1282–1301.1997
3 LeMaguet P et al. Intensive Care Med. 2014 May ; 40(5):674–82.

#### 0952

**Targeted temperature Management in intensive care: an ESICM international survey of critical care practice**

A. Wong[1], G. Debaty[2], R. Helbok[3], G. Meyfroidt[4], S. Shepherd[5], E. Zanier[6], K. Donadello[7], ESICM TTM Group
[1]Oxford University Hospitals NHS Trust, Adult Intensive Care Unit, Oxford, United Kingdom; [2]University Hospital of Grenoble Alps, La Tronche, France; [3]Medical University of Innsbruck, Department of Neurology, Neurocritical Care Unit, Innsbruck, Austria; [4]University Hospitals Leuven, Department of Intensive Care Medicine, Leuven, Belgium; [5]Papworth Hospital NHS Foundation Trust, Cambridge, United Kingdom; [6]Mario Negri Institute for Pharmacological Research, Milan, Italy; [7]University of Verona, Anesthesia and Intensive Care B, Department of Surgery, Dentistry, Paediatrics and Gynaecology, Verona, Italy
**Correspondence:** A. Wong
*Intensive Care Medicine Experimental* 2017, **5(Suppl 2):**0952

**INTRODUCTION.** Targeted Temperature Management (TTM) has multiple potential applications in various ICM settings. The publication of two landmark trials showing improved neurological outcome in post cardiac arrest patients who were cooled to 33-34°C[1, 2], led to the practice becoming more widespread. The publication of the TTM trial[3] in 2013 by Nielsen *et al.* dramatically changed the landscape. Subsequently, other trials such as the Eurotherm trial[4] has led to renewed debate and discussions on its use.

**OBJECTIVES.** A European Society of Intensive Care Medicine (ESICM) taskforce was put together to survey the practice of temperature management amongst the intensive care fraternity.

**METHODS.** An online survey exploring various areas of TTM including indications, management, duration, method etc. were sent to all members of the ESICM. The survey was also promoted on the ESICM website (www.esicm.org).

**RESULTS.** Overall, 722 responses were received, with representation from all continents, with the majority from European countries (68%). A variety of hospital types were represented - although university hospitals accounted for nearly 50%). The majority of respondents (92%) applied some form of temperature management strategy for patients with post anoxic coma. The implementation of TTM for traumatic brain injuries (28%) or as a treatment for intracranial hypertension (21%) was lower. The majority of respondents (72%) had a SOP/protocol when instituting TTM which ensured a more consistent approach to the delivery of treatment in this patient group. Although intra- and endo-vascular cooling devices are available in the market and are utilized by respondents, the majority (76%) use much less invasive and simple procedures such as surface blankets/pads and ice packs to initiate and maintain temperature management. During the rewarming phase, the majority do so at a rate of 0.5oc/hr (53%).

**CONCLUSIONS.** TTM is a management strategy which is widespread amongst critical care physicians. Although there is almost universal agreement that it should be utilised in post cardiac arrest patients and that a protocol is used, other aspects are more variable. TTM is less frequently used in peri-operative and septic patients. A consensus recommendation for the use of TTM in neurocritical care and other ICU patients is urgently needed.

**GRANT**
Work carried out with support from BARD

**REFERENCE(S)**

1. The Hypothermia After Cardiac Arrest Study Group: Mild therapeutic hypothermia to improve the neurological outcome after cardiac arrest. NEJM 2002; 346: 549–556
2. Bernard SA, Gray T, Buist MD et al. Treatment of comatose survivors of out-of-hospital cardiac arrest with induced hypothermia. NEJM 2002; 346: 557–563.
3. Nielsen N, et al. Target Temperature Management 33°C vs. 36°C after Out-of Hospital Cardiac Arrest. NEJM. 2013. 369(23):2197–2206.
4. Andrews PJ, Sinclair HL, Rodriguez A et al. Hypothermia for Intracranial Hypertension after Traumatic Brain Injury. NEJM 2015; 373(25): 2403–12

### Surviving critical care

#### 0953

**Weekend ICU admission is associated with increased risk of death**

P. Zajic[1], P. Bauer[2], A. Rhodes[3], R. Moreno[4], T. Fellinger[2], B. Metnitz[5], F. Stavropoulou[2], M. Posch[2], P.G. Metnitz[2]
[1]Medical University of Graz, Dept. of Anaesthesiology and Intensive Care Medicine, Div. of General Anaesthesiology, Emergency- and Intensive Care Medicine, Graz, Austria; [2]Medical University of Vienna, Centre for Medical Statistics, Informatics, and Intelligent Systems, Vienna, Austria; [3]St George's University of London, St George's University Hospitals NHS Foundation Trust, London, United Kingdom; [4]Centro Hospitalar de Lisboa Central, Hospital de São José, Unidade de Cuidados Intensivos Neurocríticos, Lisbon, Portugal; [5]Austrian Centre for Documentation and Quality Assurance in Intensive Care, Vienna, Austria
**Correspondence:** P. Zajic
*Intensive Care Medicine Experimental* 2017, **5(Suppl 2):**0953

**INTRODUCTION.** Studies conducted in undifferentiated patient cohorts suggest that patients admitted to hospitals at weekends are at increased risk of death [1]. Results in critically ill patients, however, are contradictive [2]. This may be due to differences in severity of illness, patient disposition or regional variation in organisation and medical practice.

**OBJECTIVES.** We investigated whether the day of the week of ICU admission influences either the hazards of death in the ICU or the chances of discharge from the ICU.

**METHODS.** Retrospective study in all adult patients admitted to 119 ICUs participating in the benchmarking project of the Austrian Centre for Documentation and Quality Assurance in Intensive Care (ASDI) between 2012 and 2015. Readmissions to the ICU during the same hospital stay were excluded.

Multivariable competing risk analysis using the Fine and Gray proportional subdistribution hazards model concerning ICU mortality and ICU discharge was performed. The model was built around the following variables: weekday of admission, weekday of event (death or discharge), SAPS 3 score, year of admission, month of admission, type of admission, and centre. Wednesday was chosen as the reference value.

**RESULTS.** This study included 151,268 patients. Risk of death following weekend ICU admission was higher compared to weekdays; HR (95% CI) were 1.15 (1.08-1.23) for Saturday and 1.11 (1.03-1.18) for Sunday, respectively. Chance of alive ICU discharge after weekend ICU admission was simultaneously reduced; HR (95% CI) were 0.98 (0.96-1.01) for Saturday and 0.91 (0.88-0.94) for Sunday.

**CONCLUSIONS.** Patients admitted to intensive care units at weekends are at increased risk of death in the ICU compared to patients admitted to the ICU during the week. This effect cannot solely be attributed to severity of illness or discharge practice. Further research is needed to identify possibilities for changes in practice and structures to improve outcomes in the future.

### 0954

#### Factors associated with non-response at quality of life follow-up among survivors of septic shock. A registry-based post-hoc analysis of the TRISS randomised trial

M.-B.N. Kjaer[1], C.B. Mortensen[2], P.B. Hjortrup[3], S.L. Rygård[3], I. Andersen[4], A. Perner[3]
[1]Copenhagen University Hospital, Rigshospitalet, Intensive Care 4131, Copenhagen, Denmark; [2]Zealand University Hospital, Køge, Dept. of Intensive Care, Køge, Denmark; [3]University of Copenhagen, Intensive Care, Rigshospitalet 4131, Copenhagen, Denmark; [4]University of Copenhagen, Section of Social Medicine, Copenhagen, Denmark
**Correspondence:** M.-B.N. Kjaer
*Intensive Care Medicine Experimental* 2017, **5(Suppl 2):**0954

**INTRODUCTION.** Septic shock is associated with reduced health-related quality of life (HRQOL), which may negatively impact the survivors for years after hospital discharge [1,2]. In long-term follow-up of HRQOL, a substantial number of patients do not respond, which hamper interpretation of the results [2,3].

**OBJECTIVES.** We aimed to assess risk factors for non-response to HRQOL survey at 1-year follow-up in ICU patients with septic shock.

**METHODS.** We studied all the Danish survivors in the Transfusion-Requirements in Septic Shock (TRISS) trial patients who were sent the Short Form Health Survey (SF-36) 1-year after randomisation [4,5]. Covariates from the trial database and nation-wide registries using the unique national identification number were used to explore risk factors for not responding. Five covariates were pre-specified to be included in the primary multivariate analysis: age, number of days in hospital from randomisation to follow-up, and level of education, cohabitation, and employment status at follow-up. Survival from 1-year follow-up to the final follow-up (median 2.7 years after randomisation) was compared between non-responders and responders.

**RESULTS.** Out of 321 eligible patients; we included 308 of whom 100 (35%) were non-responders. In the primary analysis, lower age (odds ratio 1.03, 95% CI [1.01-1.05]), more admission days in hospital (1.006 [1.001-1.011]), and living alone (4.33 [2.46-7.63]) were associated with non-responding whereas level of education and employment status were not. Non-responders had a hazard ratio of 1.63 [0.97-2.72] for mortality from 1-year follow-up to final follow-up as compared to the responders.

**CONCLUSIONS.** Being younger, spending more days in hospital and living alone were all associated with non-response at 1-year HRQOL follow-up among ICU patients with septic shock.

**GRANT ACKNOWLEDGMENT**
The TRISS trial was funded by the Danish Strategic Research Council and Rigshospitalet and supported by the Ehrenrich and ACTA foundations.

**REFERENCE(S)**

1. Prescott HC, Osterholzer JJ, Langa KM, et al. (2016) Late mortality after sepsis: propensity matched cohort study. BMJ 353:i2375.
2. Iwashyna TJ, Ely EW, Smith DM, Langa KM (2010) Long-term cognitive impairment and functional disability among survivors of severe sepsis. Jama 304:1787–1794.
3. Eddleston JM, White P, Guthrie E (2000) Survival, morbidity, and quality of life after discharge from intensive care. Crit Care Med 28:2293–2299.
4. Holst LB, Haase N, Wetterslev J, et al. (2014) Lower versus Higher Hemoglobin Threshold for Transfusion in Septic Shock. N Engl J Med 371:1381–1391.
5. Rygård SL, Holst LB, Wetterslev J, et al. (2016) Long-term outcomes in patients with septic shock transfused at a lower versus a higher haemoglobin threshold: the TRISS randomised, multicentre clinical trial. Intensive Care Med.

### 0955

#### Is post-sepsis syndrome different from post-intensive care (ICU) syndrome? A cohort and propensity score matched analysis

K. Thompson, C. Taylor, S. Jan, Q. Li, N. Hammond, J. Myburgh, B. Venkatesh, S. Finfer
The George Institute for Global Health, Division of Critical Care and Trauma, Newtown, Australia
**Correspondence:** K. Thompson
*Intensive Care Medicine Experimental* 2017, **5(Suppl 2):**0955

**INTRODUCTION.** Post-Sepsis Syndrome and Post-ICU Syndrome are terms recently adopted to characterise the health-related quality of life (QoL) issues affecting patients after sepsis or after treatment in an ICU.[1,2] Whether these syndromes are different is not known.

**OBJECTIVES.** To determine QoL, late mortality, costs and healthcare resource use of ICU patients with sepsis compared to those without sepsis.

**METHODS.** After obtaining ethical committee consent we analysed data of ICU patients with sepsis recruited to a randomised controlled trial in New South Wales, Australia. We conducted a cohort and propensity score matched (PSM) analysis using baseline variables including indices of severity of illness compared to ICU patients without sepsis. We assessed QoL using the EQ-5D-3L at 6 months and mortality, costs (in $AU) and healthcare resource use at 2 years.

**RESULTS.** QoL was assessed in 4975 patients: 1320 with sepsis and 3655 without. There were no significant differences in QoL. For the analysis of mortality, costs and healthcare resource use, 3442 patients were included: 905 patients with sepsis and 2537 without. At 2 years, patients with sepsis had higher mortality: 371/905 (41%) vs 860/2537 (34%) HR1.34, 95%CI 1.18 to1.52, $p < 0.01$; increased costs: ICU: 47,206 ± 55,121 vs 34,142 ± 43,175; $p < 0.0001$; Hospital: 73,516 ± 61,100 vs 64,676 ± 56,293; $p < 0.0001$; and extended length of stay: ICU: 10.0 ± 11.9 vs 7.1 ± 9.1 days;
$p < 0.0001$; Hospital: 22.7 ± 21.6 vs 20.4 ± 19.7 days; $p = 0.003$. Patients with sepsis had fewer hospital readmissions: 506/904 (56%) vs 1597/2563 (63%); $p = 0.0002$.

1612 patients were included in the PSM analysis: 806 with sepsis and 806 without. 90/905 (10%) patients with sepsis could not be matched due to high illness severity. There were no differences in QoL, late mortality or hospital readmissions. Costs were higher for patients with sepsis: ICU 47,298 ± 53,730 vs 38,952 ± 46,778; $p = 0.009$; Hospital 74,120 ± 60,750 vs 65,806 ± 56,856; $p = 0.005$, and length of stay longer: ICU: 10.0 ± 11.9 vs 8.0 ± 9.8 days; $p < 0.0001$;
Hospital: 22.8 ± 21.2 vs 19.1 ± 19.0 days; $p = 0.0003$.

**CONCLUSIONS.** Compared to patients without sepsis, patients with sepsis report similar QoL but have increased hospital costs, length of stay and fewer hospital readmissions. Aside from cost, these differences are not significant when patients are matched for severity of illness and other baseline variables, notwithstanding the limitation of our inability to match 10% of patients in the sepsis cohort.

**REFERENCE(S)**

1. Winters BD, Eberlein M, Leung J, Needham DM, Pronovost PJ, Sevransky JE. Long- term mortality and quality of life in sepsis: a systematic review. Critical care medicine 2010;38:1276–83.
2. Elliott D, Davidson JE, Harvey MA, et al. Exploring the scope of post-intensive care syndrome therapy and care: engagement of non-critical care providers and survivors in a second stakeholders meeting. Critical care medicine 2014;42:2518–26.

**GRANT ACKNOWLEDGMENT**
N/A.

### 0956

#### Incidence of Post Intensive Care Syndrome (PICS) in a large cohort of ICU survivors: results of an ICU aftercare program

D. van Rijswijk, A. Rutten, G. van der Nat, N. van der Lely, D. Ramnarain
Elisabeth-TweeSteden Hospital Tilburg, Intensive Care, Tilburg, Netherlands
**Correspondence:** D. van Rijswijk
*Intensive Care Medicine Experimental* 2017, **5(Suppl 2):**0956

**INTRODUCTION.** ICU mortality rates have decreased dramatically. Physical complaints, cognitive dysfunction and negative emotional outcomes become more evident in patients discharged from intensive care, leading to impaired quality of live. Identifying ICU related risk factors associated with this so called PICS is therefore of great importance.

**OBJECTIVES.** The aim of this study was to examine the incidence of somatic complaints, cognitive dysfunction, depression, anxiety and post-traumatic stress disorder (PTSD) after intensive care treatment. Secondly, we searched for possible ICU related risk factors for anxiety, depression and PTSD. Also, we studied the association of somatic and cognitive dysfunction with these negative emotional outcomes.

**METHODS.** Patients treated in ICU between January 2012 and December 2016 for a minimum of 4 days were invited to the post-ICU aftercare program. Six weeks after hospital discharge, patients received an invitation letter along with three questionnaires: a health-related questionnaire for identifying somatic and cognitive complaints, the Hospital Anxiety and Depression Scale (HADS) and the Impact of Event Scale - Revised (IES-R). Patient characteristics and ICU characteristics were obtained from electronic patient records.

**RESULTS.** The population consisted of 439 patients (53,5% male), with mean age 57 years (SD = 15.8). Median Apache II and Apache IV were 17 and 55, respectively. Median ICU LOS and hospital LOS were 8 and 21 days, respectively. Sixty nine percent of the patients were mechanically ventilated with a median of 6 days. The prevalence of clinically significant anxiety and depression was 24.3%, respectively. Prevalence of PTSD was 17.3%. Most frequently reported somatic complaints were muscle weakness (50.1%), fatigue (75.8%) and pain (41.9%). Concentration problems (53.1%), memory problems (44.6%) and reduced thinking ability (54.7%) were frequently reported cognitive complaints. These somatic and cognitive complaints were significantly associated with anxiety, depression and PTSD. LOS ICU and hospital LOS were significantly associated ($p < 0.05$) with PTSD, showing less PTSD in groups with longer stay in ICU and hospital. Logistic regression revealed female sex as an adjusted independent risk factor for PTSD (OR = 1.55, 95%CI = [0.93 - 2.60]) and anxiety (OR = 1.89, 95%CI = [1.22 - 2.93]). No risk factors for depression were identified. Delirium, APACHE scores, length of mechanical ventilation and drugs administered during ICU treatment were not related to anxiety, depression or PTSD.

**CONCLUSIONS.** Incidence of PICS related symptoms was high. Patients with anxiety, depression and PTSD symptoms reported more somatic and cognitive complaints. Female sex was an independent risk factor for anxiety and PTSD. Longer ICU LOS as well as hospital LOS were not associated with increased risk of PICS.

### 0957

#### Long-term mortality after severe sepsis and septic shock in Swedish intensive care units 2005–2015

G. Strandberg[1], S. Walther[2], C. Agvald Öhman[3], M. Lipcsey[4]
[1]Uppsala University, Department of Surgical Sciences, Uppsala, Sweden; [2]Linköping University, Linköping, Sweden; [3]Huddinge University Hospital, Stockholm, Sweden; [4]Uppsala University, Uppsala, Sweden
**Correspondence:** G. Strandberg
*Intensive Care Medicine Experimental* 2017, **5(Suppl 2):**0957

**INTRODUCTION.** Several studies from large ICU databases have reported decreasing in-hospital mortality for sepsis and related syndromes, but data are scarce on outcomes after hospital discharge.

**OBJECTIVES.** To investigate the evolution of crude and adjusted mortality at 30, 90 and 365 days in patients with severe sepsis and septic shock treated in Swedish ICUs during the period 2005 to 2015, and whether age and sex distributions, Charlson Comorbidity Index (CCI), Simplified Acute Physiology Score (SAPS3), distribution of admissions between hospital types, proportions of medical and surgical admissions or selected interventions in the ICU changed during the study period and if these factors were associated with mortality.

**METHODS.** Data were retrieved for all patients with severe sepsis and septic shock defined according to the SCCP/ACCM criteria, admitted between 2005 and 2015 (ICD codes R57.2, R65.1 or A41.9) from the Swedish Intensive Care Registry, SIR, (with data from 48% vs. 92% of all Swedish ICUs in 2005 vs. 2015). Charlson Comorbidity Index (CCI) based on discharge diagnoses from year 2000–2015, and survival were captured after linking with databases from the Swedish board of Health and Welfare. Multilevel logistic regression was used to analyze survival, adjusting for patient and admission characteristics (including SAPS3, CCI, admission type and selected interventions).

**RESULTS.** 29864 unique sepsis patients were identified. The crude mortality was 31.8 vs. 29.5% at 30 days and 40.1 vs. 36.2% ($p < 0.05$) at 90 days in 2005 vs. 2015, and 48.6 vs. 44.7% ($p < 0.05$) at 365 days in 2005 vs. 2014. SAPS3 adjusted 30 day standardised mortality ratio was unchanged between 2008 and 2015 (0.88 vs 0.87, n.s.). Median CCI increased 2005 to 2015 (1 vs 2, $p < 0.01$), while SAPS3 score decreased slightly between 2010 and 2015 (68.3 vs 66.8, $p < 0.001$). Median ICU length of stay decreased from 2.9 days in 2005 to 2.3 days in 2015, $p < 0.001$). The CCI was a strong independent predictor of mortality (Odds Ratio (OR) per point increase was 1.095 at 30 days and 1.24 at 365 days). SAPS3, age, invasive ventilation, medical admission and female sex implied higher odds of death at all time points, while admission year (2008–2014) and hospital type had no significant impact on mortality in the adjusted regression model. Findings were stable in a sensitivity analysis with data from units reporting to SIR during the entire study period.

**CONCLUSIONS.** Mortality in severe sepsis and septic shock is high, with more than one in three patients not surviving three months after ICU admission, and risk-adjusted mortality has remained unchanged in Sweden 2008–2015.

Organisation issues in intensive care

### 0958

#### Improving End of Life (EOL) care in the Neurosurgical Intensive Care Unit (NICU): a pilot project in Singapore

Y.-L. Wong[1], C.H. Poi[2], H.L. Tan[1], L. Li[3]
[1]Tan Tock Seng Hospital, Anaesthesiology, Intensive Care and Pain Medicine, Singapore, Singapore; [2]Tan Tock Seng Hospital, Palliative Medicine, Singapore, Singapore; [3]Tan Tock Seng Hospital, ICU Operations, Singapore, Singapore
**Correspondence:** Y.-L. Wong
*Intensive Care Medicine Experimental* 2017, **5(Suppl 2):**0958

**INTRODUCTION.** Patients with poor prognosis in NICU are not referred early to palliative physicians to address care needs. This results in missed opportunities for exploring/establishing patient's and families' priorities and goals of care when prognosis is poor. As of 2014, only about 30% patients with poor prognosis in NICU was referred to palliative care.

Poor prognosis is defined as a) Hypoxic Ischaemic Encephalopathy, b) Severe head injury with poor neurological prognosis, c) extensive intracerebral/subarachnoid haemorrhage, d) low presenting GCS < =6.

**OBJECTIVES.** We aim to improve early referral rate from current 30% to 100% of palliative care patients with poor prognosis in NICU within 6 months.

**METHODS.** We did a literature review on this topic. Improving Palliative Care in ICU (IPAL-ICU) consensus group suggested that a well-structured palliative care initiative in the form of an integrative or consultative or a combination of both into standard ICU care can provide important benefits for patients and families. [(1)] Systemic review by Aslakson et al. suggested that proactive palliative care in ICU decreases hospital and ICU LOS. (2)

Our project is carried out by following Plan-Do-Study-Act (PDSA) tool. The team first examined the referral process and drew out the cause and effect diagram. After that, team members voted and identified the main concerns for action and brainstormed on interventions. As of a result, a new early NICU-Palliative Collaboration & Intervention Workflow was designed, along with 5 strategies planned and implemented over the 3 PDSA cycles.

**RESULTS. Run Chart or Evidence of Audit and Frequency**

Patient's data was gathered every 2 weeks, interventions implemented over 3 PSDA cycles, with introducing 1–2 new strategies each cycle every 2 wks. At the 4th PSDA, the percentage of palliative care referrals in patients who fit criteria for referral sustained at 100%.

**CONCLUSION.** Palliative care in the ICU has strengthened interdisciplinary work to improve accessibility of services to the patients. The importance of buy-in from different level and different healthcare workers is crucial to the achieved improvement. Continual engagement of multi-disciplinary teams at all levels and providing regular feedback to the referring team to ensure continual communications & feedback are other keys.

Using the same model of care, this project has spread to other ICUs in TTSH through defining referral criteria/trigger for rest of ICUs as well as continual engagement & collaboration of involved stakeholders.

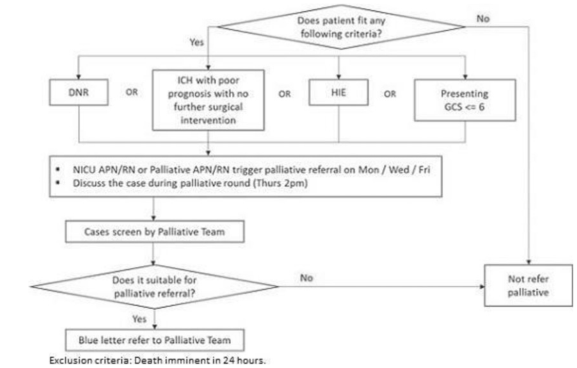

**Fig. 306 (Abstract 0958).** Early NICU-Pall Collaboration&Intervention Workflow

| Cause and Problems | Pre-Intervention Phase | Intervention I 1st cycle 16-29 March 15 | Intervention II 2nd cycle 30 March-12 April 15 | Intervention II 3rd cycle 30 March-12 April 15 |
|---|---|---|---|---|
| Inadequate Structure for referral | Consultant in ICU trigger referral | EOD screening by NICU APN & Pall Care APN | Daily screening by NICU NIC with standardised workflow | → |
| Inadequate clear & consistent referral indications | Old Referral criteria: -D3 HIE -Severe HI with poor neurological prognosis as agreed by NS and ICU team -Extensive IVH/SAH with no further surgical intervention | More comprehensive referral criteria: i) HIE ii) Severe head injury w poor neurological prognosis iii) Extensive ICH/ SAH iv) Low presenting GCS of ≤ 6 | → | |
| Inadequate standardized communications between the ICU and palliative care team | Weekly palliative physician joining NICU morning rds | | | Weekly Palliative rounds at scheduled time and day of the week 1) Palliative round to include debrief and feedbacks of patients' outcome to primary surgical team and intensivist 2) Consultants are informed of outcome of patients |
| Variable buy in from primary physicians (eg intensivists and neurosurgeons) | - | - | | |
| Low level of nursing confidence in supporting family in EOL conversations | 1) Communication workshops for all nurses 2) Upload communication skill video for maintenance of communications skills training for 3A nurses | Since Nov 15 to March 15 → | | Communication board by nurses |

**Fig. 307 (Abstract 0958).** Interventions

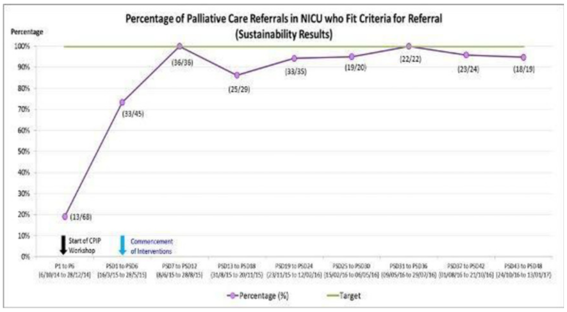

**Fig. 308 (Abstract 0958).** NICU Results

### 0959

### Crew Resource Management (CRM) training in the ICU. Is once a year enough?

U. Pietsch[1], M. Filipovic[2], W. Schuhwerk[3], G.-R. Kleger[1]

[1]Kantonsspital St. Gallen, Department of Intensive Care Medicine, St. Gallen, Switzerland; [2]Kantonsspital St. Gallen, Departement of Anesthesiology and Intensive Care Medicine, St. Gallen, Switzerland; [3]Rea2000, Resuscitation- and Simulation Training Center for Professionals and Laypersons, St. Gallen, Switzerland

**Correspondence:** U. Pietsch

*Intensive Care Medicine Experimental* 2017, **5(Suppl 2):**0959

**INTRODUCTION.** Fifteen years after the release of the Institute of Medicine landmark report [1], patient safety improved, but is still not enough. According to a recently published National Health Service -report, many medical systems are still not designed with patient safety in mind [2]. Most of these safety issues are not due to inadequate medical knowledge, but to problems in transforming that knowledge into meaningful clinical actions under real patient care conditions [3]. In complex systems like intensive care, medical decisions have to be made under uncertainty and time pressure. Working in multi-professional teams requires coordination and communication skills. Human factors are attributed with the majority of adverse events in the ICU. Creating a sound safety climate is therefore essential [1]. Studies have demonstrated that simulation and CRM training provide a valuable tool [4] to train the management of complex medical situations and may increase patient safety.

Little is currently known about the optimal frequency of simulation and CRM training.

**OBJECTIVES.** To investigate the effectiveness of simulation training to improve the CRM skills of ICU providers. A group of ICU-nurses and physiscians in training were observed over three subsequent years (2014, 2015, 2016). The providers were asked to rate her skills (1 = best, 6 = worst) with a self evaluation questionnaire before and after each training. Nonparametric tests including the Bonferroni correcture were performed. We hypothesized that a one day training once a year would be insufficient to improve CRM skills in complex ICU situations.

**RESULTS.** 65 ICU-nurses and 10 physicians completed a total of 194 questionnaires. We observed a consistent improvement in CRM relevant items between pre and post training evaluation. In contrast to this finding, the providers rated their baseline skills equal during the whole observation period.

**CONCLUSIONS.** Multidisciplinary simulation-based educational training significantly improves self-estimated competence and awareness of CRM in a medical complex ICU-setting within one session. However, the sustainability of these efforts is probably poor. We feel, that more than one training session per annum will be required to influence patient outcome and safety in daily routine. Further studies are needed to find the ideal dosage and frequency of a long lasting CRM training effect.

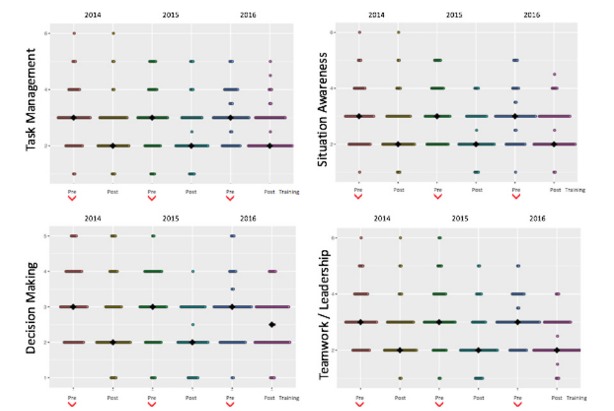

**Fig. 309 (Abstract 0959).** See text for description

### 0960

#### Virtual critical care follow up as an aid to triage patients, providing the right care, for the right patient at the right time

R. Spiritoso, M. Krupa, P. O'shea
St George's University Hospitals NHS Foundation Trust, London, United Kingdom
**Correspondence:** R. Spiritoso
*Intensive Care Medicine Experimental* 2017, **5(Suppl 2):**0960

**INTRODUCTION.** Critical illness predisposes patients to physical, psychological and cognitive complications that impact on their quality of life and ability to function independently (1). The critical care follow up service provides support through the journey of recovery (2). We currently provide an outpatient follow up clinic for this group of patients. The clinic runs one afternoon per week and aims to see 4 patients. Criteria for invite to clinic is based on length of stay (LoS) >4 days. Limited resources meant we were unable to offer a face to face appointment to all patients within a specific time frame (3 months post hospital discharge). New criteria were developed focusing on prioritising the presence of delirium, prolonged intubation and ventilation, critical illness polyneuropathy and signs/symptoms of Post Intensive Care Syndrome (PICS) rather than LoS. Virtual critical care follow up was offered at 3 months post discharge to triage patients and to reassess those who partially met follow up criteria. A senior nurse used a structured questionnaire via telephone to assess domains of PICS. Findings were then discussed with the consultant. Patients were subsequently discharged or offered a face to face appointment.

**OBJECTIVES.** To assess whether "virtual" follow up is a useful tool in identifying and prioritising at risk patients.

**METHODS.** A comparative and retrospective study.

**RESULTS.** Between December 2015 and July 2016, 53 patients met follow up criteria identified as LOS > 4 days. 66% (n = 35) attended; 34% (n = 18) DNA (did not attend). This equates to four clinic days.

Between August 2016 and March 2017, 62 patients met the new follow up criteria. Of those, 79% (n = 49) attended clinic, 21% (n = 13) DNA'd. In addition, 26 patients who partially met criteria were contacted by telephone. Of those, 69% (n = 18) were discharged, 4% (n = 1) was invited to the clinic and 4% (n = 1) declined. 23% (n = 6) were uncontactable.

Post introduction of the virtual clinic 88 patients were followed up versus the 53 of the first 8 months. In total, 25 clinic slots (6 days) were spared and face to face appointments offered to the most appropriate patients.

**CONCLUSIONS.** Intensive care admissions are life changing experience and patients need adequate support through the journey of recovery. The introduction of the virtual clinic in conjunction with meticulous selection criteria allowed us to increase the number of patients followed up and reduced the DNA rates.

**REFERENCE(S)**

(1) John A Griffiths et al. Follow-up after intensive care. Continuing Education in Anaesthesia, Critical Care & Pain | Volume 4 Number 6 2004.
(2) Richard D Griffiths and Christina Jones. Recovery from intensive care. ABC of intensive care. BMJ. 1999 Aug 14; 319(7207): 427–429.

### 0961

#### The evolution of social media in critical care conferences - improving engagement and learning beyond borders

I. Capel[1], O. Olusanya[2], A. Wong[1]
[1]Oxford University Hospitals NHS Trust, Adult Intensive Care Unit, Oxford, United Kingdom, [2]North Hampshire NHS Foundation Trust, Basingstoke, United Kingdom
**Correspondence:** I. Capel
*Intensive Care Medicine Experimental* 2017, **5(Suppl 2):**0961

**INTRODUCTION.** Social media allows for the rapid, effective dissemination of information between an individual and their followers. Twitter, an online social media platform, allows the individual to disseminate information in the form of "tweets" of 140 characters or less. In health education, this allows educators to rapidly disseminate information in public to a wide and varied audience. The use of social media has become increasingly common in medical practice leading to formation of specific committees to tackle this area in professional bodies and conferences.

**OBJECTIVES.** To analyse the trends and impact of social media at two major critical care conference over the years.

**METHODS.** Symplur analytics were used to review the impact of Twitter as a means of enhancing education at the ESICM (2014–16) and the UK Intensive Care Society (2015–16) meetings using their official hashtags. Information was obtained on number of tweets, impressions and participants, as well as data regarding the most prolific tweeting individuals. Those "tweeting" were further characterised by their professional role, be it physicians, societies, commercial companies or professional journals.

**RESULTS.** The use of twitter expanded dramatically over the specified timeframe. At the ESICM LIVES meeting, both number of tweets and impressions increased more than eightfold over the designated timeframe. Online participation also increased; mean tweets/hour and mean tweets/participant increased eightfold and fourfold respectively. This implies increased use of twitter among conference participants. Individual physicians, as opposed to societies, contributed a higher proportion of the top 10 ranked accounts by tweets, mentions and impressions in 2016, compared with 2014. The ICS conference demonstrated similar results; participants using twitter at the conference, and the official hashtag, increased from 395 in 2015 to 1107 in 2016. Volume of tweets and impressions also increased over the two years.

**CONCLUSIONS.** Twitter is an important and expanding means of intra- and interdisciplinary communication at major European conferences and has great potential in continuing professional development and medical education. The largest contribution towards this electronic conversation currently comes from individual physician accounts. An enhanced social media strategy at future conferences, in particular from intensive care societies and online journals, will increase engagement and education. Societies and professional organisation should consider the use of social media an integral part of their communication and educational ambitions.

**REFERENCE(S)**

Hillman T & Sherbino J. Social media in medical education: a new pedagogical paradigm? http://dx.doi.org/10.1136/postgradmedj-2015-133686

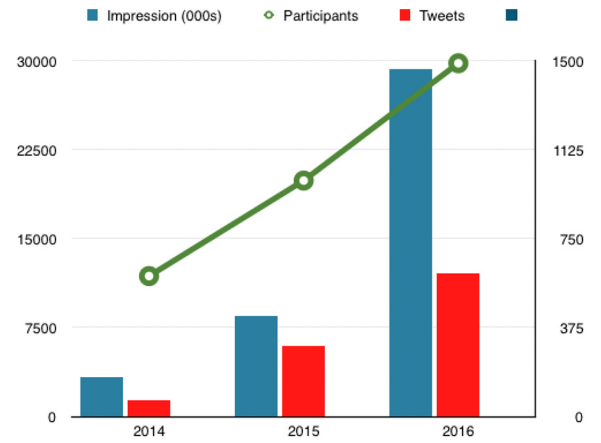

**Fig. 310 (Abstract 0961).** Trend analysis of ESICM LIVES twitter activity

### 0962

#### Extracorporeal membrane oxygenation in Korea - expenditure and impact of hospital volume on outcome: analysis of national insurance data 2009–2014

S. Kim[1], G.Y. Suh[2], C.K. Tay[3], Y.H. Cho[4], J. Park[1], J. Cho[5], H. Park[5], D. Kang[5], J.H. Yang[1], C.R. Chung[2], K. Sung[4], Korean ICU Nationwide Data (KIND) Study
[1]Samsung Medical Center, Critical Care Medicine, Seoul, Korea, Republic of; [2]Samsung Medical Center, Seoul, Korea, Republic of; [3]Singapore General Hospital, Respiratory and Critical Care Medicine, Singapore, Singapore; [4]Samsung Medical Center, Cardiothoracic Surgery, Seoul, Korea, Republic of; [5]Samsung Medical Center, Center for Clinical Epidemiology, Seoul, Korea, Republic of
**Correspondence:** S. Kim
*Intensive Care Medicine Experimental* 2017, **5(Suppl 2):**0962

**INTRODUCTION.** The use of extracorporeal membrane oxygenation (ECMO) has increased worldwide in recent years. However, characteristics of adult ECMO versus non-ECMO patients admitted to the intensive care unit (ICU) have never been studied. The impact of ECMO volume on survival has also not been investigated in any real-world cohort

**OBJECTIVES.** To investigate trend of ECMO use in a nationwide data base.

**METHODS.** Retrospective analysis using data obtained from the Korean Health Insurance Review and Assessment (HIRA) Service for adult patients with an ICU admission, and those who received ≥1 ECMO run between August 1, 2009 and July 31, 2014. Information including demographics, ICU and hospital length of stay, cost, specific treatments, and in-hospital mortality was collected.

**RESULTS.** During the 5-year study period, 1, 265, 508 patients were admitted to the ICU and 6078 (0.5%) underwent ECMO. An increasing annual national ECMO volume (218%; 768 cases in 2010 vs. 1675 in 2013) was observed. ECMO patients were younger (median age 59 vs. 64, p < 0.0001) but had more comorbidities such as diabetes mellitus, cardiovascular and chronic kidney disease than non-ECMO patients. The median expenditure and in-hospital mortality of an ECMO patient were higher (US $24,000 vs. US $5200, p < 0.0001; 63.1% vs. 12.5%, p < 0.05 respectively) than a non-ECMO patient. The number of hospitals performing ECMO also showed an increasing trend. Eighty-four such hospitals existed in 2013 compared to 42 in 2009. Using multivariable analysis, age ≥50 years, the need for continuous renal replacement therapy, and an annual hospital ECMO volume < 20 were negative prognostic factors for survival to discharge

**CONCLUSIONS.** The prevalence of ECMO among ICU patients was 0.5%. The expenditure and in-hospital mortality of an ECMO patient were four and five times higher respectively than a non-ECMO patient. Our study suggests that an annual hospital ECMO volume ≥20 may improve survival.
Is there research in ethics?

### 0963

#### Emergency Department (ED) Initiated Palliative Care (PC) in End of Life (EOL) ICU consults. A randomized clinical trial

R. Levy[1], Y. Han[1], Y. Dayan[1], M. Ganz[1], E. Gabrielle-Jocson[1], P. August[2]
[1]New York Presbyterian Hospital (Queens), Medicine, New York, United States; [2]Weil Cornell University, New York, United States
**Correspondence:** R. Levy
*Intensive Care Medicine Experimental* 2017, **5(Suppl 2):**0963

**CONTEXT:** Family caregivers of patients with end of life (EOL) illnesses experience significant emotional distress.

**OBJECTIVE.** To determine whether ED initiated Palliative Care meetings led to improved family satisfaction.

**DESIGN.** Randomized Clinical Trial conducted from March 2016 to March 2017. Patients were enrolled in the study if they were ED patients with an ICU consult and had one of the following characteristics: Dementia, Advanced cancer with metastatic disease, COPD on home 02, previous Stroke and requiring ventilator support, greater than 75 years old with 2 life-threatening co-morbidities, or nursing home patient with either peg tube or tracheostomy.

**INTERVENTIONS:** Patients in the intervention arm received a PC consult within 24–48 hours.

**MAIN OUTCOMES AND MEASURES:** The primary outcome was family satisfaction as measured by the FAMCARE survey. Results were obtained 2 weeks after discharge or 8 weeks after death. Secondary outcomes included hospital length of stay, ICU length of stay, days on mechanical ventilation, code status, and anxiety and depression symptoms at enrollment measured by HADS and Zarit Surveys.

**RESULTS.** A total of 120 patients were studied. 57 received early PC and 63 usual care (43 hours vs. 162 hours respectively) (p < .0001). Family satisfaction was significantly higher amongst the group receiving early PC compared to usual care (14.6 vs 11.0) (max score 20) (p = .001) (95% CI, 1.465-1.566).
Comparing the intervention group with usual care, mortality was not statistically significant (29.8% vs 34.9) (p = .55). Patients receiving early PC though had less days on the ventilator (7.1 vs 12.3) (P = .01) and a decreased hospital length of stay (9.29 days vs 12.54 days) (p = .02). With early PC, code status was changed sooner (3.2 days vs 7 days) (p = .03) and more frequently (56.1% vs. 36.5%)(p = .03) and patients were placed on comfort care sooner (4.75 days vs 10.6 days)(p = .01) and more often (44% vs 25%)(p = .03). ICU length of stay (4.45 days vs 5.5 days) (p = .22) was not statistically significant. Depression and anxiety scores measured by HADS and ZARIT surveys at enrollment were not significantly different either (19.9 vs 18.0) (p = .37) (23.1 vs 20.8) (p = .30).

**CONCLUSIONS.** Family members of EOL ICU patients benefit from ED initiated PC consultation. Our results show early PC compared to usual care lead to improved family satisfaction scores and less ICU interventions without shortening survival.

### 0964

#### Limitation of life-sustaining treatments in Indian ICUs: data from The Indian Intensive Care Case Mix and Practice Patterns Study (INDICAPS Study)

J.V. Divatia[1], F.N. Kapadia[2], INDICAPS Study Investigators
[1]Tata Memorial Hospital, Anaesthesiology, Critical Care and Pain, Mumbai, India; [2]PD Hinduja National Hospital, Medicine and Critical Care, Mumbai, India
**Correspondence:** J.V. Divatia
*Intensive Care Medicine Experimental* 2017, **5(Suppl 2):**0964

**INTRODUCTION.** In the past two decades, there has been a tremendous growth of intensive care medicine in India. The Indian Intensive Care Case Mix and Practice Patterns Study (INDICAPS Study) accrued data on several aspects of intensive care organization, case-mix and practices in 4038 adult patients from 120 ICUs.[1] We analysed data from this study to obtain information on limitation of life-sustaining treatment (LST) in Indian ICUs.

**OBJECTIVES.** We aimed to study the proportion of patients as well as patient and organizational characteristics associated with LIM-LST.

**METHODS.** The INDICAPS Study (clinical trials.gov NCT01384929) was an observational, 4-day point prevalence study performed between 2010 and 2011. ICU and patient characteristics, and interventions were recorded for 4038 adult patients from 120 ICUs present in the ICU on study day, and outcomes till 30 days after the study day. Any form of limitation of life-sustaining treatment (LIM-LST), including Full treatment but no cardiopulmonary resuscitation (No-CPR), withholding (WH) or withdrawal (WD) of LST, or Terminal Discharges (TDs) to a location outside the hospital, were recorded. TDs were presumed to have died, and non-survivors were patients who died in the ICU as well as TDs. Multivariate logistic regression analysis were performed to determine patient and organizational characteristics associated with LIM-LST.

**RESULTS.** 546 patients died in ICU and 183 were TDs; thus 729 /4038 patients (18.1%) were non-survivors. 453 (62%) non-survivors had no treatment limitation, while 276 non-survivors had LIM-LST. 183(25%) were TDs, 35 (4.8%) had No-CPR, 45 (6.2%) had WH and 13 (1.8%) had WD of LST. Two-thirds of all LIM-LST were TDs.

Non-survivors with LIM-LST, compared to those receiving full treatment, were older (57.8 ± 16.9 vs. 54.3 ± 18.0 yrs, p = 0.008) and had lower APACHE II scores (22.4 ± 9.2 vs. 24.4 ± 8.7). Fewer non-survivors with LIM-LST received mechanical ventilation (31.3% vs. 52.7%, p < 0.001), vasopressors (25.5% vs. 46.2%, p < 0.001) and dialysis (30.7% vs. 39.8%, p = 0.04). Results of multivariate analysis of factors associated with LIM-LST are shown in Table 234. Self-paying patients, presence of cancer and treatment in a private hospital ICU were independently associated with LIM-LST, while severity of illness, cirrhosis, severe sepsis, closed ICUs and ICUs with an ICU-training programme were not.

**CONCLUSIONS.** Terminal discharge from the ICU is widely practiced, while witholding and withdrawal of life-sustaining treatments are uncommon. Limitation of life-sustaining treatment is not related to severity of illness. Self-paying patients and treatment in private hospitals ICUs are independently associated with limitation of life-sustaining treatment. This suggests that legal and societal issues related to end-of-life care need to be resolved.

**GRANT ACKNOWLEDGMENT**

Indian Society of Critical Care Medicine

**Table 234 (Abstract 0964)**. Multivariate analysis

| | Odds Ratio | 95% Confidence Interval (Upper) | 95% Confidence Interval (Lower) | p |
|---|---|---|---|---|
| Presence of Cancer | 2.20 | 1.18 | 3.40 | 0.011 |
| Self-paying patient | 1.70 | 1.08 | 2.71 | 0.023 |
| Private Hospital (vs. Public Hospital) | 1.59 | 1.01 | 2.53 | 0.047 |
| Age | 1.01 | 1.00 | 1.02 | 0.04 |
| Receiving vasopressors/ inotropes | 0.60 | 0.47 | 0.86 | 0.005 |
| Mechanical ventilation | 0.53 | 0.31 | 0.76 | 0.001 |

### 0965

#### Sexual health in ICU survivors

J.A. Malmgren[1], S. Lundin[1], A.-C. Waldenström[2]

[1]Sahlgrenska University Hospital, Department of Anaesthesia and Intensive Care, Gothenburg, Sweden; [2]Gothenburg University, Gothenburg, Sweden

**Correspondence:** J.A. Malmgren

*Intensive Care Medicine Experimental* 2017, **5(Suppl 2):**0965

**INTRODUCTION.** Well described burdens of disease after intensive care include cognitive, physical and psychiatric dysfunction. Sexual health, classified as a human right by WHO, has not been as thoroughly evaluated after intensive care as symptoms in other domains, and has not been evaluated using patient-developed outcome measures. Furthermore, neither SF-36 nor EQ-5D contain questions regarding sexual health. Previous studies of sexual health after intensive care (1) and after trauma (2) have shown an increase in sexual dysfunction and a raised level of post-traumatic stress, but have not correlated this to effects on quality of life.

**OBJECTIVES.** The primary objective was to test the feasibility of patient-developed questions on sexual health and intimacy in an ICU-specific quality of life questionnaire. The secondary objectives were to compare responses from ICU survivors with those from healthy controls, to evaluate the prevalence of specific problems regarding sexual health, and to correlate the degree of symptoms to self-perceived quality of life.

**METHODS.** The creation of an ICU-specific quality of life follow-up questionnaire through semi-structured interviews with ICU survivors has been previously described (3). Sixteen questions on sexual health and intimacy, covering issues such as lust, sexual activity, the ability to handle closeness from loved ones, sexual counselling and impact on quality of life specifically from these issues were included.

Survivors from three mixed, adult ICUs were screened. Eligible patients were all patients with ICU LOS >72 hours, discharged from ICU six months to three years prior to study start, and with non-neurological/neurosurgical admission diagnosis. Controls matched for age and gender from the Swedish Population Register were obtained. Survivors and controls received an invitational letter followed by a phone call asking for participation. Participants were reminded by phone, had the questionnaire not been returned in two weeks.

**RESULTS.** Of 493 eligible patients, 393 has returned the questionnaire (response rate 79,7%). Response rates for questions on sexual health and intimacy were similar to those of other questions. Preliminary analysis of reported symptoms in ICU survivors shows that a substantial proportion of responders has severe problems concerning intimacy, lust and sex life, and with a considerable effect on quality of life.

Data from controls are still being collected.

**CONCLUSIONS.** The high response rate on questions on sexual health and intimacy suggests that it is feasible to include these questions and that these issues matters for ICU survivors.

Symptoms of sexual function should be asked for in post-ICU clinics as well as in primary care, and sexual counselling considered.

1 Griffiths et al.; ICM 2006;32

2 Ulvik et al.; ICM 2008;34

3 Malmgren et al.; ICMx 2016;4 (Suppl.1);A355

**GRANT ACKNOWLEDGMENT**

Sahlgrenska University Hospital, Gothenburg Medical Society, Swedish Heart and Lung Foundation

### 0966

#### Moral distress in patients´ relatives in ICU end-of-life care decisions

U.G. St Ledger[1,2], D.F. McAuley[1,2], J. Reid[3], A. Begley[4], L. Prior[5], B. Blackwood[1]

[1]Queen's University of Belfast, Centre for Experimental Research, School of Medicine, Dentistry and Biomedical Sciences, Belfast, United Kingdom; [2]Belfast Health & Social Care Trust, Critical Care, Belfast, United Kingdom; [3]Queen's University of Belfast, School of Nursing and Midwifery, Belfast, United Kingdom; [4]Virtual University of Uganda, Kampala, Uganda; [5]Queen's University of Belfast, Centre of Excellence for Public Health Research, Belfast, United Kingdom

**Correspondence:** U.G. St Ledger

*Intensive Care Medicine Experimental* 2017, **5(Suppl 2):**0966

**INTRODUCTION.** Difficult end-of-life decisions are frequently required to be made in the intensive care unit (ICU) for incapacitated patients, with potential to trigger moral distress. Moral distress is an intense emotional reaction that occurs when an individual feels constrained from taking moral action, and has known detrimental consequences (McCarthy and Deady, 2008). Prior research was focused largely on moral distress as an occupational hazard for clinicians and little was known of the existence and impact of moral distress in patients' relatives.

**OBJECTIVES.** This empirical investigation aimed to identify the triggers and constraining factors involved in the development of moral distress and of the ensuing consequences for patients' relatives in the making of end-of-life care decisions in ICU.

**METHODS.** A qualitative narrative thematic analysis of in-depth digitally recorded interviews with N = 20 ICU patients' relatives closely involved in N = 15 patient cases of non-escalation and withdrawal of therapy and organ donation following brain-stem death and circulatory death. The study was conducted in a large tertiary referral ICU in Northern Ireland (NI), from August 2012- November 2013. This abstract is part of a larger doctoral study that also included ICU physicians and nurses.

**RESULTS.** Half of relatives interviewed experienced considerable moral distress. Relatives, unreconciled with the experience, told chaotic, anguished stories of constraint and wrong-doing and of failure of familial obligation. The patient's admission to ICU generated a hope narrative and futility-based moral distress. Moral distress triggers included: breaches of communication and care giver continuity at critical junctures; the ICU environment; restricted access and autonomy; prolongation of death versus prolongation of life; failure to oversee 'the good death´; and organ donation processes. Strategies to mollify moral distress involved quests for truth and justice. Satisfaction with communication and care and a sense of the right decision, made at the right time, staved off moral distress. Moral distress impacted on relatives' health, personal relationships, working lives and grieving process.

**CONCLUSIONS.** Findings have important implications for: (a) the educational preparation of ICU clinicians to recognise, minimise and manage the ethical challenges and impact of end-of-life decisions for relatives; (b) person-centred practices that facilitate relatives' familial obligations; (c) relatives' support needs during and after the ICU episode and during and after the death; and (d) ICU design.

**REFERENCE(S)**

McCarthy, J., Deady, R. (2008) Moral distress reconsidered. Nursing Ethics 15:254–262.

**GRANT ACKNOWLEDGMENT**

Funded by the Research and Development Office, Public Health Agency, NI.

### 0967

#### Factors in the family approach process that influence organ donation consent rates: a nationwide cohort study

M. Witjes[1,2], P.E. Vorstius Kruijff[3], B.J.J.M. Haase-Kromwijk[2], J.G. van der Hoeven[1], N.E. Jansen[2], W.F. Abdo[1]

[1]Radboud University Medical Center, Intensive Care, Nijmegen, Netherlands; [2]Dutch Transplant Foundation, Leiden, Netherlands; [3]Amphia Hospital, Intensive Care, Breda, Netherlands

**Correspondence**: M. Witjes

*Intensive Care Medicine Experimental* 2017, **5(Suppl 2):**0967

**INTRODUCTION.** The high number of unregistered potential donors in the national Donor Register lead to a high refusal rate by the next of kin, which is one of the major bottlenecks in the donation process (1).

**OBJECTIVES.** To determine the influence of different factors in the family approach for organ donation on consent rates, including the training Communication about Donation (CaD). This training is offered to Dutch physicians involved in the donation process since 2012 (2). The results are used to provide tools for physicians on how to inform a grieving family about donation and make the donation request.

**METHODS.** Intensivists specialized in organ donation evaluated all organ donation requests in the Netherlands with physicians who made the request, according to a standardized questionnaire. Different aspects of the family approach were evaluated. We analyzed all questionnaires that were retrieved between January 2013 and June 2016. The consent rates were compared by univariate analysis and entered into the multivariate analysis using a stepwise binary logistic regression model.

**RESULTS.** During the 3.5-year study period, 2095 donation requests were evaluated amounting to 83% of all donation requests. After excluding the cases in which the potential donor is registered with consent or objection in the Donor Register, 1322 questionnaires were analyzed. Independent predictors of consent to donation included complete understanding of the term 'brain death' by the family (OR, 2.4; 95% CI, 1.4-4.2; p = 0.002), explicitly asking whether the family understood 'brain death' (OR, 1.7; 95% CI 1.1-2.5; p = 0.011), requesting organ donation during bad news conversation (OR, 1.8; 95% CI, 1.2-2.6; p = 0.004), and consulting a transplant coordinator prior to the donation request (OR, 3.4; 95% CI, 2.3-4.9; p < 0.001). The family consent rate was 40.8% for the family approaches performed by CaD-trained physicians in comparison to 36.3% for the family approaches performed by physicians who were not trained (p = 0.134). Training seems to have less effect on consent rates in ICU physicians (41% versus 39%, p = 0.591) than in non-ICU phyiscians (40% versus 22%, p = 0.072).

**CONCLUSIONS.** It is of importance to give the family a clear understanding of brain death and involve a transplant coordinator early in the donation process. Separating donation request from the bad news conversation is not necessary when the family accepts the loss of their loved one and is ready for the next step. Also, ICU physicians trained in CaD seem to have higher consent rates than non-ICU physicians who were not trained.

**REFERENCE(S)**

1. Annual report 2015 of the Dutch Transplant Foundation. Available at: http://www.transplantatiestichting.nl/sites/default/files/product/downloads/nts_jaarverslag_2015.pdf.

2. Jansen NE, et al. Organ donation performance in the Netherlands 2005–08; medical record review in 64 hospitals. Nephrol Dial Transplant 2010;25(6):1992–7.

**GRANT ACKNOWLEDGMENT**

None

## Poster Corner Sessions Wednesday, 27 September 2017

### Monitoring invasive and non-invasive ventilation

### 0968

#### How to assess $FiO_2$ delivered under oxygen mask in clinical practice?

R. Coudroy[1,2], A.W. Thille[1,2], X. Drouot[2,3], V. Diaz[2,3], J.-C. Meurice[2,4], R. Robert[1,2], J.-P. Frat[1,2], FLORALI study group

[1]CHU de Poitiers, Service de Réanimation Médicale, Poitiers, France; [2]INSERM CIC 1402, Poitiers, France; [3]CHU de Poitiers, Service de Neurophysiologie, Poitiers, France; [4]CHU de Poitiers, Service de Pneumologie, Poitiers, France

**Correspondence:** R. Coudroy

*Intensive Care Medicine Experimental* 2017, **5(Suppl 2):**0968

**INTRODUCTION.** The actual $FiO_2$ delivered under oxygen mask in patients with acute respiratory failure and the factors that may influence the $FiO_2$ are poorly known. In clinical practice, different methods including formula or conversion tables based on oxygen flow can be used to estimate delivered $FiO_2$.

**OBJECTIVES.** We aimed to assess first the factors influencing measured values of $FiO_2$, and second the best method to estimate measured $FiO_2$ in patients breathing under oxygen mask.

**METHODS.** We included ICU patients admitted for acute hypoxemic respiratory failure from a previous prospective trial (1) in whom $FiO_2$ was measured under oxygen mask using a portable oxygen analyzer. We collected demographic variables and respiratory parameters that may influence measured $FiO_2$. Low $FiO_2$ was defined according to median measured $FiO_2$.

For each patient, measured $FiO_2$ was compared to "Calc + 3%" formula ($FiO_2$ = oxygen flow in liters per minute x 0.03 + 0.21) to

"Calc + 4%" formula ($FiO_2$ = oxygen flow in liters per minute x 0.04 + 0.21), and to a conversion table (2). A ±10% limit of agreement for each estimation method was arbitrarily considered acceptable.

**RESULTS.** Among the 265 patients included, oxygen flow was 15 [12–15] l/min and measured $FiO_2$ was 65% [60–73]. After adjustment on oxygen flow, the three variables independently associated with low measured $FiO_2$ using multivariate analysis were patient's height, a low $PaCO_2$, and a respiratory rate greater than 30 breaths/min.

Values outside the limits of agreement accounted for 55% of cases for the Calc + 3% formula, 69% for the Calc + 4% formula, and 94% for the conversion table ($p < 0.0001$). As compared to measured $FiO_2$, an overestimation of more than 10% was observed in 18% of cases for the Calc + 3% formula, 62% for the Calc + 4% formula and 94% for the conversion table ($p < 0.0001$).

**CONCLUSIONS.** The 3 strong predictors of low $FiO_2$ delivered under mask were tallness, high respiratory rate and low $PaCO_2$. None of the tested methods estimated accurately measured $FiO_2$ in patients with acute respiratory failure breathing oxygen through a mask. The Calc + 4% formula and the conversion table overestimated measured $FiO_2$ values in most of cases, therefore artificially overestimating the severity of hypoxemia using the $PaO_2/FiO_2$ ratio.

**REFERENCE(S)**

1. Frat JP, Thille AW, Mercat A, Girault C, Ragot S, Perbet S, et al. High-Flow Oxygen Through Nasal Cannula in Acute Hypoxemic Respiratory Failure. N Engl J Med. 2015;372(23):2185–96.

2. Vincent JL, Rello J, Marshall J, Silva E, Anzueto A, Martin CD, et al. International study of the prevalence and outcomes of infection in intensive care units. JAMA. 2009 Dec 2;302(21):2323–9.

### 0969

#### Electrical activity of the diaphragm in children following extubation after cardiac surgery

N. Shioji, T. Kanazawa, T. Iwasaki, K. Shimizu, T. Suemori, K. Sugimoto, Y. Kuroe, H. Morimatsu

Okayama University Hospital, Okayama, Japan

**Correspondence:** N. Shioji

*Intensive Care Medicine Experimental* 2017, **5(Suppl 2):**0969

**INTRODUCTION.** Electrical activity of the diaphragm (Edi) can reflect respiratory drive. Edi monitoring during mechanical ventilation allows clinicians to detect patient-ventilator asynchrony and overassistance. However, interpretation of Edi values following extubation after pediatric cardiac surgery is difficult because reference values are lacking. Moreover, no information is available about the time course of Edi value following extubation in this cohort.

**OBJECTIVES.** The aim of this study was to investigate the Edi values of 24-hours time course following extubation after pediatric cardiac surgery.

**METHODS.** This was a prospective observational study. Institutional Review Board approved this study. Children less than 16 years of age who underwent cardiac surgery were included. A specific nasogastric tube (NAVA catheter; Maquet, Solna, Sweden) was inserted after surgery and adjusted the position of catheter by chest radiography and "NAVA catheter positioning screen" on the ventilator (Servo-I, Maquet, Solna, Sweden). We recorded Edi values every 1-minute from 1-hour before extubation to 24-hours after extubation period. These data included inspiratory peak Edi (Edi-peak) and expiratory minimum Edi (Edi-min). These data were stored in ventilator software. Among these data, we used Edi-peak for analysis.

**RESULTS.** Thirty-three patients were included in the study. The median age was 13.5 (IQR: 2.3-27.3) months of age. The median RACHS-1 score was 3 (2–3). A total of 25 children (75%) underwent corrective operation. Twenty-six children (78%) were extubated in ICU. Duration of mechanical ventilation was 24.5 (0.6-71.2) hours. All children received CPAP + PS mode before extubation. High-flow nasal cannula ((prophylactic use (n = 3), therapeutic use (n = 3)) was used for 6 children (18%). There was no reintubation. Dexmedetomidine was used for 23 children (70%).

The median Edi value during 24-hours course postextubation period was 7.3 (6.8-7.8) μV. This value was close to that in patients with normal pulmonary function as previously reported. No significant change was observed in median Edi value between 0 to 1 hour before extubation (6.3 (3.1-10.8) μV) and 0 to 1 hour after extubation (7.3 (2.6-14.1) μV) (P = 0.12). Against our expectations, no remarkable change of Edi value was observed throughout the 24-hours course following extubation period (The lowest median Edi value = 6.0 (3.8-14.2) μV at 8 to 9 hours, the highest median Edi value = 10.4 (6.0-14.9) μV at 23 to 24 hours following extubation). There were no correlation on Edi values with age, body weight, and RACHS-1 score.

**CONCLUSIONS.** No remarkable change of Edi value was observed throughout the 24-hours course following extubation after pediatric surgery. Median Edi value of 24-hours course of postextubation periods was 7.3 (6.8- 7.8) μV in this cohort.

### 0970

#### Value of transcutaneous carbondioxide monitorization reflecting arterial carbondioxide measurement in the ICU

N. Boyacı[1], S. Mammadova[2], N. Naurzvai[2], M. Guleryuz[2], K. Inci[1], G. Gursel[3]

[1]Gazi University School of Medicine, Department of Internal Medicine, Division of Critical Care, Ankara, Turkey; [2]Gazi University School of Medicine, Ankara, Turkey; [3]Gazi University School of Medicine, Department of Pulmonary Critical Care Medicine, Ankara, Turkey

**Correspondence:** G. Gursel

*Intensive Care Medicine Experimental* 2017, **5(Suppl 2):**0970

**INTRODUCTION.** Transcutaneous (PtcCO2) pCO2 monitoring provide the measurement of pCO2 noninvasively and continuously. The oxygen saturation can also be monitorized with these monitors. But the results of studies on how well PtcCO2 reflects the arterial CO2 pressure (PaCO2) are contradictory.To evaluate the matching of PtcCO2 measured with PaCO2; and to find out that we can reliably reduce the need to take arterial blood gases (ABG) with these monitors.

**OBJECTIVES.** To evaluate the matching of PtcCO2 measured with PaCO2; and to find out that we can reliably reduce the need to take arterial blood gases (ABG) with these monitors.

**METHODS.** Patients who were referred to the intensive care unit (ICU) were included in this study, and PtcCO2 measurement and ABG analysis were performed to determine the consistency between the results.

**RESULTS.** Thirty measurements were taken from the deltoid zone and 26 from the cheek zone of thirty-four patients. Five of 35 patients (14%) could not be measured from the deltoid region. The saturation and pulse rate were not detected at 8 of the deltoid zone measurements (26,7%). When it is analyzed that the consistency between the ABG values and the PtcCO2 measurement; correlation coefficients between PtcCO2 and PaCO2 from the deltoid and the cheek region were r:0,915 and r:0,946 (p = 0,0001). In comparison with the Bland-Altman method, the mean of the differences in the deltoid measurements was −1,38 ± 1,18 (p = 0.252) and the mean of the differences in the cheek measurements was −5,12 ± 0,92 (p = 0,0001). When the saturations were compared, no significant difference was found between the measurements of each region.

**CONCLUSIONS.** These results suggest that transcutaneous PtcCO2 and saturation measurements made from the deltoid region can be used instead of arterial blood gas analysis. With increasing patient numbers, it will be possible to determine the sensitivity of the measurement and to determine the conditions such as the weight of the disease, blood pressure, hemoglobin level, measurement area that can affect the measurement.

**GRANT ACKNOWLEDGMENT**

Study was supported by Gazi University Scientific Research Projects Foundation (Project No:01/2015-48)

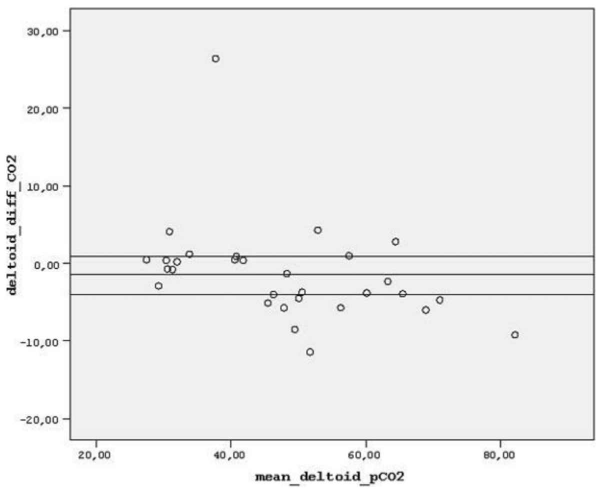

**Fig. 311 (Abstract 0970).** Graph.1

### 0971

#### Feasibility of assessing diaphragm and intercostal thickening fraction after extubation: a preliminary study

M. Dres[1,2], D. Luca Grieco[2], I. Telias[2], J. Gu[2], D. Junhasavasdiskul[2], O. Smith[2], G. Sandhu[2], E. Goligher[3], L. Brochard[2,3]
[1]Assistance Publique Hôpitaux de Paris, Pneumologie et Réanimation Médicale, Paris, France; [2]St Michael's Hospital in Toronto, Li Ka Shing Knowledge Institute, Keenan Research Centre, Toronto, Canada; [3]University of Toronto, Toronto, Canada
**Correspondence:** M. Dres
*Intensive Care Medicine Experimental* 2017, **5(Suppl 2):**0971

**INTRODUCTION.** After extubation, patients are at risk of complications that could potentially worsen their prognosis. Early identification of inappropriate diaphragm and intercostal activity may help to implement preventive strategies.

**OBJECTIVES.** To describe the level of activity displayed by the diaphragm and intercostal muscle in extubated patients.

**METHODS.** Patients who were intubated since at least 48 hours and who successfully passed a spontaneous breathing trial were enrolled in the study. Within the two hours following extubation, we used ultrasound to measure diaphragm (TFdi) and intercostal (TFic) thickening fraction as surrogates of muscle activity.

**RESULTS.** Preliminary data concerning the first 39 patients are presented. Patients were ventilated since 6 (3–8) days on the day of extubation, SOFA score was 4 (3–5) and pre-extubation rapid shallow breathing index was 50 (40–70) breath.min$^{-1}$. TFdi and TFic were obtained in 36/39 patients. TFdi was 17% (14–23) and TFic was 7% (4–9). TFdi lower than 20% (as a surrogate of diaphragm weakness (1)) was found in 22/36 (61%) patients and TFic higher than 5% (as an arbitrary surrogate of intercostal recruitment) was found in 10/36 (28%) patients.

**CONCLUSIONS.** These preliminary data confirm that diaphragm weakness is frequent during the weaning period. Intercostal muscle recruitment is present in one third of patients being extubated. Whether assessment of intercostal recruitment by ultrasound may help to identify patients at high risk of post extubation complications should be now determined.

**REFERENCE(S)**

1. McCool FD, Tzelepis GE. Dysfunction of the diaphragm. N Engl J Med. 8 mars 2012;366(10):932-42.

**GRANT ACKNOWLEDGMENT**

Bernhard Dräger Award for Advanced Treatment of Acute Respiratory Failure 2015 (Martin Dres).

### 0972

#### Topographic distribution of lung elastic energy in ventilated patients assessed by computed tomography

E.-L. Skogseid[1], M. Pellegrini[1], A. Larsson[1], G. Perchiazzi[1,2]
[1]Uppsala University, Hedenstierna Laboratory - Surgical Sciences, Uppsala, Sweden; [2]Bari University, Emergency and Organ Transplant, Bari, Italy
**Correspondence:** E.-L. Skogseid
*Intensive Care Medicine Experimental* 2017, **5(Suppl 2):**0972

**INTRODUCTION.** Excessive stress and strain during Mechanical Ventilation (MV) may induce Ventilator-Induced Lung Injury (VILI). Recent studies have asserted the importance of Driving Pressure (ΔP) and Mechanical Power (MP) when titrating MV [1,2]. However, ΔP and MP reflect the mean mechanical properties of the lung and cannot distinguish possible heterogeneity in the parenchyma. Injurious focal stress and strain levels can occur long before the mean measurements of lung mechanics deteriorate.

**OBJECTIVES.** The aim of the study was to compute the topographical distribution of the elastic energy inside lung parenchyma combining information obtained by Computer Tomography (CT) with spirometric tracings, quantify the heterogeneity of energy distribution and assess whether it had any correlation with respiratory parameters sampled at the Airway Opening (AO).

**METHODS.** We studied eleven mechanically ventilated patients (age 55–87) without pulmonary pathologies. Flow, volume and pressure were sampled at AO, together with esophageal pressure. CT-scans were performed at mid-thoracic level at nine different lung volumes during hold maneuvers: at Functional Residual Capacity and from 4 to 11 ml/Kg (in steps of 1 ml/Kg), keeping a Positive End Expiratory Pressure (PEEP) of 5 cm$H_2O$. Image analysis was performed by the Image Processing Toolbox for MatLab R2016a. Coupling information from the CT-images with ventilator data allowed to create distribution maps of compliance, strain and ultimately elastic energy. In order to assess the heterogeneity of energy distribution we used the iterative function of Quadtree Decomposition, which can identify homogeneous areas of the parenchyma. The correlation between the level of heterogeneity and different global breath parameters (ΔP, plateau pressure, transpulmonary pressure and tidal volume) was investigated as well as the correlation between the elastic energy content derived from the energy maps and the elastic energy of the respiratory system at the studied volumes measured at AO.

**RESULTS.** We obtained the energy distribution maps of the eleven patients. In the single patients we found different patterns of correlation between the heterogeneity of energy distribution and the global breath parameters mentioned before (p-value ranging between 0.004 and 0.7). No statistically significant correlation was found between the parenchymal content of energy and the mean elastic energy calculated at AO.

**CONCLUSIONS.** We found the method of computing distribution of elastic energy and quantifying parenchymal heterogeneity feasible.

The fact that correlation between parenchymal elastic energy and AO parameters can display different behaviors may indicate that at parenchymal level, elastic energy distribution may be influenced by another set of local mechanical properties that cannot be assessed at AO.

**REFERENCES**
1. Gattinoni L, et al. Intensive Care Med 2016: 42;1567–1575
2. Amato MBP, et al. N Engl J Med 2015: 372;747–755

### 0973

#### Respiratory mechanics in mechanically ventilated patients with acute respiratory failure: analysis during controlled ventilation versus partial ventilatory support

J. A. Benítez Lozano[1], P. Carmona Sánchez[2], M. Delgado Amaya[1], H. Molina Díaz[1], J. M. Serrano Simón[2]
[1]Hospital Quirón, Intensive Care Unit, Málaga, Spain; [2]Hospital Universitario Reina Sofía, Intensive Care Unit, Córdoba, Spain
**Correspondence:** J. M. Serrano Simón
*Intensive Care Medicine Experimental* 2017, **5(Suppl 2):**0973

**INTRODUCTION.** Noninvasive methods have been developed for the calculation of respiratory mechanics during pressure support ventilation (PSV), this techniques on based on resistance (Rs) and elastance (Ers) data of respiratory system obtained during relaxed ventilation using a linear regression (1), assuming constant respiratory system Rrs and Ers with linear relationship between flow, volume and pressure. However, during spontaneous ventilation the are dynamic nonlinearity relate to frequency dependence (2) and other phenomena as pendelluft, turbulences and inertial forces (3,4).

**OBJETIVES:** To evaluate the fit between the respiratory mechanics obtained during mechanical ventilation controlled (CMV) by five methods and the data obtained during PSV by a new method based on calculation of instantaneous muscle pressure (pmus), distending pressure (DP) and total pressure (TP) by the equation of motion (EM).

**METHODS.** We studied a group of mechanically ventilated patients during CMV with constant flow and relaxed, due to acute respiratory failure of different diseases and at PSV mode. Airway, esophageal pressure, and airway flow were recorded, sampling 561 Hz. Elastance (Ers) and Resistance (Rrs) of respiratory system were obtained from multiple linear regression (MLR), time constant (TC), occlusion (Occ), fast Fourier transform (fft) and new method variant of isovolume technique (iso*). This data were compare with obtained with PSV by calculation pmus by substraction of chest wall elastic load from Pes, DP, and TP using EM. Data are expressed as mean (SD) in absolute values, and as a percentage. The comparison of the data was made using one-way ANOVA, and Bonferroni post-hoc. The Bland-Altman analysis and linear regression was applied to assess the agreement.

**RESULTS.** 30 patients were studied. For all data: Ers: Iso*27.41 ± 7.85 cmH2O/L; MLR 27.49 ± 7.83 cmH2O/L; TC 24.5 ± 7.36 cmH2O/L; Occ 23.13 ± 6.32 cmH2O/L; fft 30.15 ± 8.33 cmH2O/L. PSV: 31.65 ± 11.51 cmH2O/L.Rrs: Iso* 17.2 ± 3.58 cmH2O/L/sec; MLR 16.41 ± 3.36 cmH2O/L/sec; TC 19.15 ± 3.61cmH2O/L/sec; Occ 18.53 ± 4.21cmH2O/L/sec; fft 16.67 ± 3.54cmH2O/L/sec. PSV: 15.86 ± 5.71 cmH2O/L.Ers PSV vs Tc and Occ: $p < 0.05$. The mean comparison between Rrs did not show statistical differences. The concordance analysis is shown in the Tables 235 and 236 for Ers and Rrs, respectively.

**CONCLUSIONS.** The wide dispersion and agreement make it unacceptable to superpose respiratory mechanics data obtained from controlled ventilation to pressure support ventilation, particularly in the resistances.

**REFERENCES**
1. Iotti G,et al. Chest 1995;108:208–15.
2. Romero PV, et al. J Appl Physiol 111: 420–426, 2011.
3. Yoshida T, et al. Am J Respir Crit Care Med. 2013 Dec 15;188(12):1420–7.
4. Cotton DJ, et al. Respiration Physiology. 1988;71:117–130.

**Table 235 (Abstract 0973).** The Bland-Altman analysis and R2 for Ers

| Ers PSV vs: | Means differences (SD), (cmH2O/L) | 95% Limits of agreement | % Error | R2 |
|---|---|---|---|---|
| Iso* | −4.25 (8.76) | −21.77 to 13.28 | 29.66 | 0,42 |
| MLR | −4.15 (8.72) | −21.6 to 13.29 | 29.49 | 0,43 |
| TC | −7.15(7.09) | −21.35 to 7.04 | 24.39 | 0.65 |
| Occ | −8.52(10.94) | −30.4 to 13.36 | 39.94 | 0.13 |
| fft | −1.51(7.12) | −15.75 to 12.74 | 23.05 | 0.62 |

**Table 236 (Abstract 0973).** The Bland-Altman analysis and R2 for Rrs

| Rrs PSV vs: | Means differences (SD), (cmH2O/ L/sec) | 95% Limits of agreement | % Error | R2 |
|---|---|---|---|---|
| Iso* | 1.34 (5.4) | −9.47 to 12.15 | 32.67 | 0.16 |
| MLR | 0.54 (5.59) | −10.65 to 11.24 | 34.66 | 0.11 |
| TC | 3.29 (6.31) | −9.07 to 15.65 | 36.05 | 0.02 |
| Occ | 2.67 (5.6) | −8.53 to 13.87 | 32.57 | 0.16 |
| fft | 0.81 (5.12) | −9.45 to 11.07 | 32.49 | 0.22 |

### 0974

#### Accuracy of P0.1 displayed by modern ventilators - from bench to patients

I.G. Telias[1,2,3], D. Junhasavasdikul[1,3,4], N. Rittayamai[5], L. Chen[1,3], L.G. Domenico[1,3,6], M. Dres[1,3,7], M.C. Sklar[1,3], E.C. Goligher[1,2], L.J. Brochard[1,3]
[1]University of Toronto, Interdepartmental Division of Critical Care Medicine, Toronto, Canada; [2]University Health Network and Mount Sinai Hospital, Division of Respirology, Department of Medicine, Toronto, Canada; [3]St Michael's Hospital, Keenan Research Centre for Biomedical Science, Li Ka Shing Knowledge Institute, Toronto, Canada; [4]Mahidol University, Division of Pulmonary and Critical Care, Department of Medicine, Faculty of Medicine Ramathibodi Hospital, Toronto, Canada; [5]Siriraj Hospital Mahidol University, Division of Respiratory Diseases and Tuberculosis, Department of Medicine, Faculty of Medicine, Bangkok, Thailand; [6]Catholic University of The Sacred Heart, 6Department of Anesthesia and Intensive Care Medicine, Rome, Italy; [7]Sorbonne Universités, Neurophysiologie Respiratoire Expérimentale et Clinique, Paris, France
**Correspondence:** I.G. Telias
*Intensive Care Medicine Experimental* 2017, **5(Suppl 2):**0974

**INTRODUCTION.** Airway occlusion pressure (P0.1) is a non-invasive measurement reflecting the patient's respiratory drive and estimating inspiratory effort. It could become an important tool to monitor spontaneously breathing patients under mechanical ventilation. Modern ventilators display P0.1 but their accuracy is unknown.

**OBJECTIVES.** To compare P0.1 given by 5 ventilators (P0.1vent) and P0.1 measured offline from airway pressure tracing (P0.1ref)
1) using a bench simulation study and
2) in patients breathing spontaneously under assisted ventilation.

METHODS.

**1) Simulation**

A lung simulator (ASL 5000) was connected to 5 ventilators (**Maquet SERVO-i, SERVO-u, GE Engström, Draeger Evita-XL and Puritan-Bennett 840).** Normal and obstructive lungs were used with 24 patterns of inspiratory effort. **SERVO-i** and **SERVO-u** give P0.1 for each breath (5 consecutive P0.1vent recorded and 3 additional occlusions done). Other 3 ventilators need activation of a P0.1 manoeuver to display P0.1 (3 manoeuvers activated).

**2) Patients**

Data from 2 studies on inspiratory effort during mechanical ventilation were analyzed (*STUDY1*: 13 patients, 25 recordings using **Evita-XL** and *STUDY2*: 10 patients, 20 recordings using **840**). P0.1 manoeuvers were activated randomly, with P0.1vent recorded and P0.1ref measured. Variability of P0.1 was also assessed.

Bland and Altman method was used to assess accuracy and precision.

RESULTS

**1)Simulation(Fig. 312)**

Absolute bias for **SERVO-u**, **SERVO-i**, **Engström** were similar and low (between 0.3 and 0.4cm$H_2O$). However, for **SERVO** ventilators bias was negative (ie underestimation of P0.1ref) and became more negative at high values. For **Engström** bias was positive and stable across the range of P0.1. Limits of agreement for **SERVO** ventilators and **Engström** were large (SD close to 1cm$H_2O$). Accuracy and precision of **SERVO** ventilators was improved by using a pressure trigger.

For **Evita-XL** and **840,** accuracy and precision of P0.1 was good (bias 0.3 and 0.1cm$H_2O$) with narrow limits of agreement (SD 0.3 and 0.1cm$H_2O$).

**2) Patients**

Both ventilators had low bias: −0.1 cm$H_2O$ for **Evita-XL** and 0.02cm$H_2O$ for **840**. Limits of agreement of individual comparisons were considerably larger than in the bench simulation (SD 1.0 and 1.2cm$H_2O$ respectively). When the average of 3 consecutive P0.1 was analyzed, limits of agreement were smaller (SD 0.7 and 0.6cm$H_2O$ respectively).

Coefficient of variation of P0.1ref in patients in *STUDY1* was 20% (10-80%) and in *STUDY2* 40% (10-90%).

**CONCLUSIONS.** Bias for all the ventilators was acceptably low. However, the precision of **SERVO** and **Engström** was low. **SERVO** ventilators significantly underestimated P0.1, mainly at higher values; accuracy improved with pressure trigger. Precision of **840** and **Evita-XL** was better but lower in patients than on the simulation, and variability of P0.1ref was very high. The average of at least 3 consecutive values of P0.1 should be recommended in clinical practice.

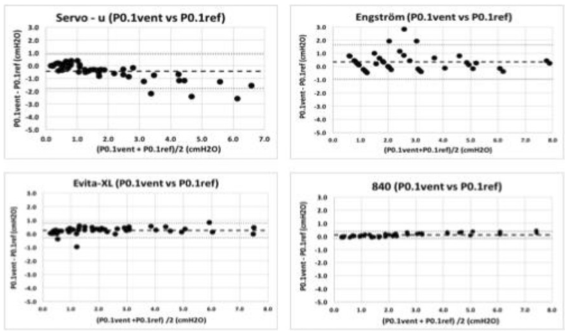

**Fig. 312 (Abstract 0974).** Bench simulation-Bland and Altman

### 0975

#### Evaluation of driving pressure ventilation during surgery

G. Tuan[1], D. Casserly[1], C.B. Groba[2]

[1]Brighton and Sussex Medical School, Brighton, United Kingdom; [2]Brighton and Sussex University Hospitals, Critical Care, Brighton, United Kingdom

**Correspondence:** C.B. Groba

*Intensive Care Medicine Experimental* 2017, **5(Suppl 2):**0975

**INTRODUCTION.** Lung protective ventilation (LPV) strategies use lower end-inspiratory ($P_{plateau}$) airway pressures, lower tidal volumes (VT) and positive end-expiratory pressures (PEEP) and have been associated with survival benefits in randomized clinical trials involving patients with the acute respiratory distress syndrome (ARDS) and in surgical populations. Individual lung mechanics is determined by lung size and lung compliance. Then, a LPV strategy should adjust ventilation parameters to the factual functional size and compliance of the lung. There are two common methods to obtain driving pressure (ΔP): the difference between $P_{IP}$ and PEEP (dynamic) and the difference between $P_{plateau}$ pressure and PEEP (static). $P_{Plateau}$ were not available, therefore dynamic measurement were used.

**OBJECTIVES.** Evaluation of ventilation settings and assessment of ΔP used during surgery.

**METHODS.** We prospectively collected self-reported initial ventilator settings, and those adjustments recorded during surgery, from 140 unselected patients undergoing a variety of surgical interventions.

**RESULTS.** We obtained 195 matching data (PIP and PEEP) points. The mean dynamic ΔP was 17.17 cmH2O (9–33). For patients ventilated with volume assisted ventilation (VAV) the mean pressure was 17.35 cm$H_2O$ and for those ventilated with pressure assisted ventilation (PAV) 17.05 cm$H_2O$ (9–28). The median tidal volume used perioperatively in both males and females was 500 ml, ranging from 450-715ml (males) and 325-575ml (females), There were no statistical significance differences among volumes delivered between the two modes of ventilation PAV, VAV or ΔP. However, 33.6% fall outside of 6-8ml/kg PBW range. PEEP was applied in 75% of cases. The level of PEEP ranged from 0 cm$H_2O$ to 11 cm$H_2O$. PEEP of 2-8cm$H_2O$ was administered in 92 cases (73.6%). The most frequently applied level of PEEP was 5cm$H_2O$ (38.4%) and zero (25%).

**CONCLUSIONS.** The results are somewhat unexpected as the driving pressure used was higher than reported for this group of surgical patients without major lung comorbidities. However, dynamic measurements are influenced by airway resistance and segmental pulmonary time constants that may have influenced these results. Delivered $V_T$ were relatively higher in females, in relation to their predicted body weight; nevertheless, only a third were greater than 8ml/kg and only 15% above 10ml/Kg. The PEEP applied to these surgical patients was relatively low, comparing with patients reported with lung injury. Surprisingly, the use of zero PEEP was relatively high (25%). In conclusion, to comply fully with a current recommendation for lung LPV strategy a further educational effort will be necessary.

**REFERENCE(S)**

Bellani G et al. JAMA 2016;315:788–800. Futier E et al. N Engl J Med 2013;369:428–37. Amato M et al. N Engl J Med 2015; 372:747–755

**GRANT ACKNOWLEDGMENT**

None.

### 0976

#### Assessment of neuro-ventilatory drive during pressure support ventilation (PSV): A 12 hours study

R. Di Mussi[1], S. Spadaro[2], D.C. Francesca[1], P. Roberta[3], A. Sara[1], C. Antonio[4], B. Francesco[1], C.A. Volta[2], G. Salvatore[1]

[1]Bari University 'Aldo Moro', Bari, Italy; [2]Ferrara University, Ferrara, Italy; [3]Pavia University 'San Matteo', Pavia, Italy; [4]Policlinico Hospital, Bari, Italy

**Correspondence:** R. Di Mussi

*Intensive Care Medicine Experimental* 2017, **5(Suppl 2):**0976

**INTRODUCTION.** Ideally, assisted ventilation should support spontaneous work of breathing and normalize the neuro-ventilatory drive. The diaphragmatic electrical activity (EAdi) can be continuously monitored at the bedside through the NAVA catheter tool (Servo i ventilator, Maquet) and is a reliable surrogate of the neuro-ventilatory drive. Pressure support ventilation (PSV) is the most widely used mode of assisted ventilation and is commonly set on clinical basis, by keeping tidal volume (VT) between 5 and 8 ml/PBW and respiratory rate (RR) between 15 and 30 b/min.

**OBJECTIVES.** To evaluate to what extent the clinical PSV setting impacts on the neuro-ventilatory drive, expressed in terms of EAdi.

**METHODS.** EAdi was continuously recorded in 10 patients during 12 hours of PSV (from 8 am to 8 pm). Patients were kept at a light sedation level (RASS score from 0 to −1). The total number of mechanical breaths was calculated. For each mechanical breath the

EAdi peak, VT and RR were also measured. The EAdi peak was classified as:
- NO: EAdi below 1 µV
- LOW: With EAdi between 1 and 5 µV
- NORMAL: with EAdi between 5 and 15 µV
- HIGH: with EAdi over 15 µV.

**RESULTS.** The results are expressed as mean ± SD. Figure 313 shows that the 48 ± 30% of the breaths presented a"NORMAL" EAdi, according to manufacturer specifications, suggesting an adequate neuro-ventilatory drive and hence an optimal PSV assistance.The LOW EAdi breaths were 28 ± 26%. The NO EAdi breaths were 9 ± 16% (range 0,24 - 50%). The High EAdi breaths were 15 ± 13%. The breathing pattern parameters (Table 237) were not significantly different in the 4 breaths cathegories.

**CONCLUSIONS.** The relevant amount of NO, LOW and HIGH Eadi breaths suggests that periods of, respectively, over-assistance and under assistance frequently occur during PSV. Of note, the breathing pattern parameters did not differ between the "NORMAL" and the "LOW, NO and HIGH" EAdi breaths and, furthermore, were in the clinical range of "adequate" PSV setting. EAdi monitoring is a promising tool to reveal the quality of assistance during PSV.

**REFERENCE(S)**

Beloncle F, Piquilloud L, Rittayamai N, Sinderby C, Rozé H, Brochard L.-A diaphragmatic electrical activity-based optimization strategy during pressure support ventilation improves synchronization but does not impact work of breathing.- Crit Care. 2017 Jan 31;21(1):21.

Liu L, Liu H, Yang Y, Huang Y, Liu S, Beck J, Slutsky AS, Sinderby C, Qiu H. -Neuroventilatory efficiency and extubation readiness in critically ill patients.- Crit Care. 2012 Jul 31;16(4):R143.

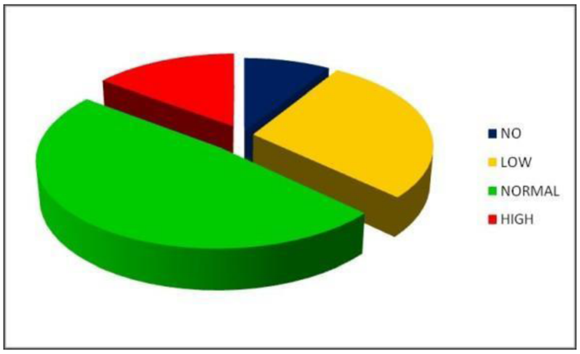

**Fig. 313 (Abstract 0976).** % of breaths in the four EAdi categories

**Table 237 (Abstract 0976).** Breathing pattern parameters

| | NO | LOW | NORMAL | HIGH |
|---|---|---|---|---|
| VT (l) | 0.42 ± 0.05 | 0.48 ± 0.13 | 0.52 ± 0.14 | 0.57 ± 0.17 |
| RR | 24.35 ± 4.53 | 22.71 ± 6.85 | 23.02 ± 7.85 | 24.37 ± 9.35 |
| Tinsp mech (seconds) | 0.91 ± 0.15 | 0.93 ± 0.18 | 0.91 ± 0.17 | 0.87 ± 0.14 |

### 0977

#### Correlation of Oxygen Index, Oxygen Saturation Index and P/F ratio in invasive mechanically ventilated adults at a tertiary care hospital in Mumbai, India

S. Vadi
GH, Mumbai, India

*Intensive Care Medicine Experimental* 2017, **5(Suppl 2):**0977

**INTRODUCTION.** Certain patients may not require invasive arterial line for blood pressure monitoring... whether there is a correlation between the P/F ratio, SpO2/FiO2 ratio, OI and OSI, and hence whether monitoring of SpO2/FiO2 and OSI (in lieu of P/F) would give equivalent information without compromising on information. The same idea hold true even for many patients who are financially non-affording, even for invasive arterial lines. To know if there is a correlation between PaO2/FiO2 ratio and oxygenation index

**OBJECTIVES.** $PaO_2/FiO_2$ (P/F) ratio is used to determine oxygenation status among mechanically ventilated adults, but requires arterial puncture and does not account for mean airway pressure (MAP). Oxygen Index (OI = MAP x $FiO_2 \times 100 \div PaO_2$) may be more representative of oxygenation, while Oxygen Saturation Index (OSI = MAP x $FiO_2 \div SpO_2$) can be continuously measured through non-invasive pulse oximetry. We evaluated the correlation among P/F ratio, OI, and OSI in adults post-invasive mechanical ventilation.

**METHODS.** Data were prospectively collected from hospital records and arterial blood gases were recorded at the time of the order for patients ≥ 18 years who were under invasive (endotracheal intubation) mechanical ventilation at medical or surgical wardsfrom December 2015 to August 2016. Only patients with reliable pulse oximetry ($SpO_2$) measurements were included. $FiO_2$, MAP and $SpO_2$ were recorded at the time of daily arterial blood gas sampling ($PaO_2$). OI and OSI were calculated based on these measures. Linear mixed effect models were used to estimate the correlation coefficients between repeated measures of P/F ratio and OI, PF/Ratio and OI, and OI and OSI using PROC MIXED in SAS 9.4.

**RESULTS.** A total of 203 measurements for 70 patients were collected over a maximum of 11 days after mechanical ventilation (day 1). Mean age was 60.4 years (standard deviation (SD) 14.0) and 62.9% (n = 44) were males. On the day 1 of mechanical ventilation, 44.3% (n = 31) and 24.3% (n = 17) of patients had a P/F ratio < 300 and < 200 respectively, and 15.7%, 20%, 18.6%, 20%, 20%, and 5.7% of patients had a SAPS II score of 0–29, 30–40, 41–52, 53–64, 65–77, and ≥78. Mean P/F ratio, OSI, and OI was 345.92 (SD 148.51), 0.061 (SD 0.042), 4.88 (SD 5.22) over 203 observations respectively. The relationships between these measures were non-linear. After natural log transformation, the correlation between P/F ratio and OI (r = −0.94) and OI and OSI (r = 0.82) were strong, but weaker between P/F ratio and OSI (r = −0.69).

**CONCLUSIONS.** There was strong correlation between P/F ratio and OI, and OI and OSI measurements among adults under invasive mechanical ventilation. Future studies are needed to evaluate whether monitoring OSI and/or OI over P/F ratio will impact treatment outcomes.

**REFERENCE(S)**

Ann. Intensive Care (2015) 5:24
Critical Care 2012;16:91
Indian Pediatrics 2016;:53;432–433
Neonatology 2015;107(3):161–166

**GRANT ACKNOWLEDGMENT**
None.

### 0978

#### Prophylaxis post-extubation failure based on peak expiratory flow

P. Carmona Sánchez, J. Muñoz Trujillo, M.V. Martínez de Pinillos, I. Ben Abdellatif, M. Bueno Bueno, R. Carmona Flores, J.M. Serrano Simón
Hospital Universitario Reina Sofía, Intensive Care Unit, Córdoba, Spain
**Correspondence:** P. Carmona Sánchez
*Intensive Care Medicine Experimental* 2017, **5(Suppl 2):**0978

**INTRODUCTION.** Failure of extubation is associated with high mortality rate. Cough strength measured with peak expiratory flow (PEF) is a strong predictor of success or failure (1). The respiratory assistance in patients with weak cough and risk of extubation failure can reduce reintubation (2).

**OBJECTIVES.** We evaluate the prognosis of prophylactic noninvasive assistance respiratory in patient with high risk (PEF < 60 L/min).

**METHODS.** Prospectively collected data from December 2014 to March 2017. PEF was measured with Cosmed Pony Graphic® spirometer v4.0 before extubation. We included patients mechanically ventilated >24h, without tracheostomy, who passed successfully a spontaneous breathing trial at least of 30 min on pressure support ventilation 5–8 cmH2O, CPAP, or T-T. Weak cough was defined by PEF < 60 L/min, the patients were then extubated regardless the PEF. We applied prophylactic noninvasive ventilation (BiPAP/CPAP) or high flow nasal cannula (HFNC) in the patients with PEF < 60 L/min, and conventional oxygen if PEF > 60 L/min. Demographic and clinical characteristics data were collected and compared between boths groups, according to the PEF. Extubation failure was defined by the need of reintubation within 48h following extubation. Continuous variables were expressed as mean ± SD or median (IRQ) and categorical variables as absolute value and percentage. Differences between groups were assessed using the Fisher exact test; Mann–Whitney U test or Student´s t -test as appropriate.

**RESULTS.** 212 Patients were studied, 140 males (66%); 59.75 ± 13.77 years. PEF < 60 L/min was identified in 63 (29.7%) patients. Patients with PEF < 60L/min presented a higher proportion of underlying COPD (26.6% vs 9.5%, p =0.04). Prophylatic assistance was effectively applied to 71% of the patients with PEF < 60 L/min, as CPAP 17,7% and BiLevel 35,5%, HFNC 46,8%. Extubation failure: Total 40 (18,9%) patients, PEF < 60L/min 18(28.6%) vs PEF >60 L/min 22(14.8%) (p = 0.02), RR = 0,4 (0.21-0.88). We found not differences between diagnosis and extubation failure. Duration of mechanical ventilation: 7.22 days (PEF < 60L/min) vs 5.44 days (PEF >60L/min), p = 0.05. Not differences in total stay in ICU between both groups, but we found significant differents in the patients that need reintubation (p = 0.00). Mortality was more higher in those patients with PEF < 60 L/min (19,4% vs 7,6%, p = 0.017).

**CONCLUSIONS.** The patients with PEF < 60/L/min had four times more extubation failure, however, the rates of reintubation are less than the data public for similar group at risk (2). We could speculate that application of prophylactic respiratory assistance in patients with weak cough could be reduce risk of extubation failure, as mortality. BiPAP mode was the assistance more effective, perhaps relate to more proportion of COPD in the group of risk.

### 0979

#### Assessment of a non-invasive respiratory volume monitor in subjects under non-invasive ventilatory support

M. Dres[1], B. Harvey[2], D.L. Grieco[1,3], I. Telias[1], M. Rauseo[1], L. Brochard[1]
[1]St. Michael's Hospital, Keenan Research Centre, University of Toronto, Toronto, Canada; [2]Respiratory Motion, Inc, Waltham, United States; [3]Catholic University of The Sacred Heart, Anesthesiology and Intensive Care Medicine, Rome, Italy
**Correspondence:** D.L. Grieco
*Intensive Care Medicine Experimental* 2017, **5(Suppl 2):**0979

**INTRODUCTION.** Non-Invasive Ventilatory Support (NIVS, e.g., CPAP, BiPAP, High-flow $O_2$) is currently used across the continuum of care. In the critical care setting, NIVS can help reduce the time patients spend on mechanical ventilation to help reduce complications. Currently, it is challenging for clinicians to both evaluate the need but also the effectiveness of NIVS. We wanted to test the ability of a recently developed non-invasive respiratory volume monitor (RVM, ExSpiron, Respiratory Motion, Waltham, MA, USA) that provides continuous measurement of minute volume (MV), tidal volume (TV), and respiratory rate (RR) to provide this monitoring in such conditions of ventilation. The RVM has previously been shown to have better than 10% accuracy for MV, TV, and RR in both non-intubated and intubated patients.[1,2]

**OBJECTIVE.** Evaluate the RVM's accuracy when monitoring healthy subjects under NIVS.

**METHODS.** Six healthy subjects completed this pilot study (3 males, BMI = 21.1 kg/m$^2$ (19.1-23.1)). MV, TV, and RR data were simultaneously recorded by the RVM and Ventilator (Engström Carestation, GE Healthcare) for 3, 5 min-long trials under different vent settings: CPAP 0 cm$H_2O$ (CPAP0), CPAP 5 cm$H_2O$ (CPAP5), and pressure support 5cm$H_2O$ with PEEP 2 cm$H_2O$ (PS5). Relative errors between RVM and Ventilator measurements of expired TV (from proximal flow sensor) were calculated over 1 min segments and bias, precision, and accuracy were calculated using Bland-Altman analyses. All data are presented as mean ± SEM. One-way ANOVAs were performed to compare RVM measurement bias, precision, and accuracy between the different ventilator modes.

**RESULTS.** Subjects maintained an average MV of 7.1 ± 0.9 L/min, with individual breath TVs ranging from 81 to 1371mL and RRs ranging from 5.0 to 35.7bpm. During spontaneous breathing (CPAP0) measurement bias in TV measurements between the RVM and the Ventilator was 3.5 ± 3.1% with a precision of 4.3 ± 1.4% and an overall accuracy of 8.3 ± 1.5%, corresponding to measurement error of 51.6 ± 9.4mL (Table 238). During NIVS trials (CPAP5 and PS5), measurement bias, precision and accuracy remained practically unchanged (p = 0.66, 0.42, and 0.54, respectively). Figure 314a displays the strong correlation between RVM and Ventilator TV measurements. Figure 314b shows that the difference between RVM and Ventilator TV measurements is similar across the range of measured TVs. Figure 315 depicts that volume traces from the RVM and Ventilator are highly correlated (R ≥ 0.95) for all three Ventilator setting trails.

**CONCLUSION.** RVM provides clinically-relevant accuracy when monitoring healthy subjects under non-invasive ventilatory support in order to evaluate its effectiveness. This study is consistent with previous studies evaluating the accuracy of the RVM compared to spirometry and mechanical ventilation.

**Table 238 (Abstract 0979).** RVM's bias, precision, and accuracy

| | CPAP 0 | | CPAP 5 | | PS 5 | | P-value |
|---|---|---|---|---|---|---|---|
| | % | ml | % | ml | % | ml | |
| BIAS | 3.5 | 20.4 | −0.2 | −7.6 | −2.3 | −14.9 | 0.66 |
| PRECISION | 4.3 | 24.1 | 2.9 | 14.2 | 5.9 | 32.7 | 0.33 |
| ACCURACY | 8.3 | 51.6 | 11.8 | 62.7 | 9.9 | 58.6 | 0.54 |

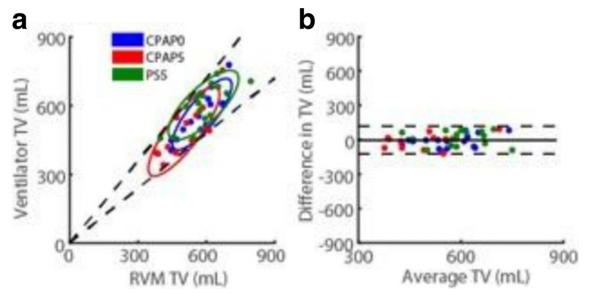

**Fig. 314 (Abstract 0979).** See text for description

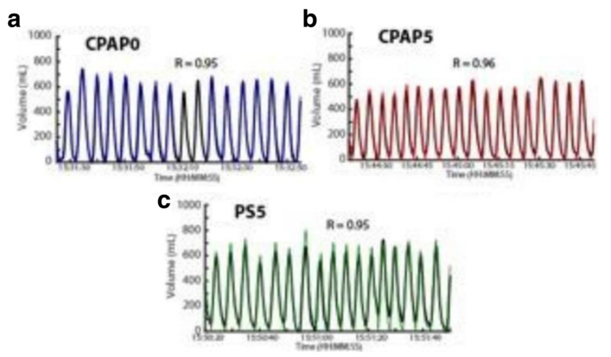

**Fig. 315 (Abstract 0979).** See text for description

### 0980

#### Evaluation of exhaled carbon dioxide measured by volumetric capnography with a respiratory model mimicking NPPV

K. Yarimizu[1], Y. Onodera[1,2], T. Kobayashi[3], M. Nakane[2,3], K. Kawamae[1]
[1]Yamagata University Faculty of Medicine, Anesthesiology, Yamagata, Japan; [2]Yamagata University Hospital, Critical Care Center, Yamagata, Japan; [3]Yamagata University Faculty of Medicine, Emergency and Critical Care Medicine, Yamagata, Japan
**Correspondence:** K. Yarimizu
*Intensive Care Medicine Experimental* 2017, **5(Suppl 2):**0980

**INTRODUCTION.** Recently, ventilators have been equipped with volumetric capnography (VC), and measuring exhaled $CO_2$ as volume ($VCO_2$) has become convenient. Because $CO_2$ is an endproduct of the body's metabolism, $VCO_2$ reflects the patient's metabolic state. To measure $VCO_2$, all exhaled gas must be collected from the patient. Therefore, studies are done in a closed circuit, such as intubated patients. The Hamilton G5 (Hamilton Medical, Switzerland) is a VC-equipped ventilator that can also apply NPPV with a double circuit, which enables simultaneous collection exhaled gas from the patient and measurement of the amount of leak. It is better to have no leaks to measure $VCO_2$, but in clinical practice, it is impossible to set the mask without a leak. The leak is also needed to reduce $CO_2$ rebreathing caused by the dead space of the interface.

Therefore, we conducted a study to evaluate the relationship between measured $VCO_2$ and leaks by using a respiratory model with known and steady $CO_2$ exhalation and to see if VC can measure $VCO_2$ in a condition mimicking NPPV.

**OBJECTIVES.** To evaluate whether VC can measure $VCO_2$ with a leak mimicking NPPV.

**METHODS.** The spontaneous breathing model (LUNGOO: Air Water Safety Service, Japan) was used as a lung model with these settings:compliance,50 ml/cmH2O; resistance, 10 cmH2O/L/s; inspiratory time,1 second; tidal volume,500 ml; and respiratory rate,15 breaths/minute. $CO_2$ was infused in this lung model to reach a $VCO_2$ of 200 ml/min, measured by the VC in the ventilator connected to the lung model. The Hamilton G5 ventilator was set on NIV mode with a PS of 5 hPa, PEEP of 5 hPa and a flow trigger of 3 L/min. An adjustable leak port was inserted between the lung model and the ventilator circuit.

The amount of leak measured by the ventilator was changed from 0 to 80%, and $VCO_2$ measured by the VC was recorded.

**RESULTS.** $VCO_2$ at a leak rate of 0% was 207 ml,a value equivalent to measured $VCO_2$ in the IPPV mode. The amount of leak and measured $VCO_2$ had a strong negative correlation ($R^2$ = 0.96). With leaks under 25%, there were differences between the actual and estimated $VCO_2$ (obtained by multiplying the collecting ratio [1-leak ratio] by the actual $VCO_2$, which was 200 ml/min in this model[dotted line in Fig. 316]). These differences were thought to be caused by excess leak compensation flow that diluted measured $CO_2$ and increased the leak during exhalation, which leads to an underestimation of $VCO_2$. Therefore, to estimate actual $VCO_2$ with NPPV using a Hamilton G5, divide the $VCO_2$ measured with a leak over 25% by the collecting ratio.

This measurement of $VCO_2$ with NPPV may allow us to distinguish patients with dyspnea, whether they have infection (high inflammatory status) or not. Further bench and clinical study is needed.

**CONCLUSIONS.** The VC in an NPPV-mimicking condition may assist in estimating actual VCO2 when it is considered with the amount of leak.

**GRANT ACKNOWLEDGMENT**
NONE.

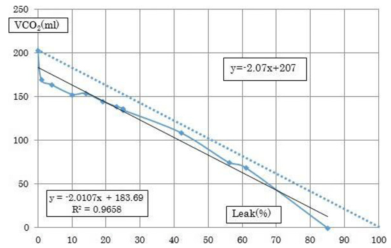

**Fig. 316 (Abstract 0980)** See text for description

### 0981

#### Ultrasound measurements of diaphragm thickening to predict NIV outcome in patients with de-novo ARF admitted to the emergency department: a pilot study

G. Mercurio[1], S. D'Arrigo[1], D. Eleuteri[1], L. Montini[1], G. Maddaloni[1], M.G. Annetta[1], G. Bello[1], M. Calabrese[2], M. Antonelli[1]
[1]Università Cattolica del Sacro Cuore-Fondazione Policlinico Universitario A. Gemelli, Department of Anaesthesiology and Intensive Care, Rome, Italy; [2]Università Cattolica del Sacro Cuore-Fondazione Policlinico Universitario A. Gemelli, Department of Cardiovascular Science, Rome, Italy
**Correspondence:** G. Mercurio
*Intensive Care Medicine Experimental* 2017, **5(Suppl 2):**0981

**INTRODUCTION.** A strict correlation between unsuccessful non-invasive ventilation (NIV) and poor outcome has been suggested, particularly in de-novo ARF patients [1].

**OBJECTIVES.** The aim of the present pilot study is to assess whether diaphragm thickening (DT) as measured by ultrasound may predict NIV outcome in patients with de-novo ARF admitted to the Emergency Department (ED) at Gemelli's Hospital, Rome-Italy.

**METHODS.** All consecutive patients with de-novo ARF requiring NIV treatment through a facial mask were included into the study. The exclusion criteria were age < 18 years, pregnancy, diaphragm paralysis, neuromuscular disorders, COPD, severe obesity (BMI > 35), palliative NIV, ineffective cough and/or inability to protect airways.

NIV success was defined as the improvement in dyspnea and gas exchange, as well as the decrease in respiratory rate (RR) ≤ 25 breaths/min within the first 96 hours of the study.

NIV failure was defined as the need of ETI and/or failure to reach an improvement in dyspnea and gas exchange, as well as the decrease in RR ≤ 25 breaths/min at any point of the study.

DT was measured as previously described [2] by 2 trained operators at baseline, at 1 hr, at 4 hours and 12 hours in the first 24 hours of ED treatment and then at any 24 hours until the next 96 hours.

Receiver operating characteristic (ROC) curve analysis was performed to assess DT ability to discriminate between patients who succeeded weaning and those who failed.

**RESULTS.** Nine of the 20 screened patients were enrolled into the study (5 M and 4 F). The median age and the SAPS II score (interquartile range) were 77 years (71–83) and 46 (42–53), respectively. The NIV treatment was success in 3 of the 9 patients (33%). We found an overall good repeatability of DT assessment, with intra-class correlation coefficients well above the 0.96 with 95% confidence interval from 0.94 to 0.98. The optimal criterion value for DT that distinguished between NIV success and failure was the cut-off > 33.3% and 37.3% for the operator 1 and 2 respectively (Figs. 317 and 318).

**CONCLUSIONS.** DT may represent a valid, feasible and non-invasive method to predict NIV outcome in acutely ill patients with de-novo ARF. However, larger studies are necessary to confirm our preliminary results.

1.Demoule A, et al. Benefits and risks of success or failure of noninvasive ventilation. Intensive Care Med 2006; 32:1756–1765.

2.Matamis D, et al. Sonographic evaluation of the diaphragm in critically ill patients. Technique and clinical applications. Intensive Care Med 2013; 39:801–810.

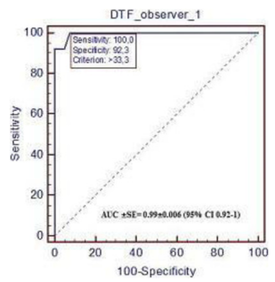

**Fig. 317 (Abstract 0981)** ROC for observer 1. AUC = area under curve

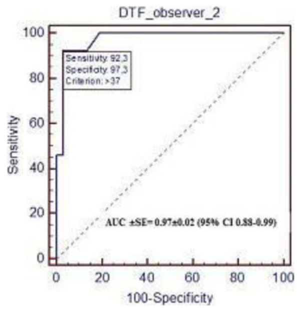

**Fig. 318 (Abstract 0981)** ROC for observer 2. AUC = area under curve

### 0982

#### A novel face mask design: effect on CO2 rebreathing during non invasive ventilation

S. Calcinati, N. Patroniti, D. Signori, D. Ferlicca, G. Bellani, G. Foti
Milano Bicocca University, Monza, Italy
**Correspondence:** N. Patroniti
*Intensive Care Medicine Experimental* 2017, **5(Suppl 2):**0982

**INTRODUCTION.** Non-invasive ventilation is commonly used to treat respiratory failure, allowing to reduce the risk of endotracheal intubation and post-extubation respiratory failure[1]. A wide range of interfaces are available, but some concerns exist about carbon dioxide (CO2) rebreathing due to increased dead space related to the internal volume of the devices.[2]

**OBJECTIVES.** Objective of this study is the evaluation of a novel mask with different access for inflow and outflow gas, in reducing CO2 rebreathing.

**METHODS.** We compared, in a bench test, a traditional mask (with a single connector with circuit) and the novel mask (DiMaxZero, Dimar, Medolla, Italy) applied to a mannequin connected to a breathing simulator (IngMar medical ASL 5000). A know CO2 flow was delivered in the mannequin's trachea.

We recorded airway pressure, airflow and CO2 concentration (and CO2 flow as the product of the two), testing all the following different combinations: CPAP (with 60–90 l/min continuous flow and Mechanical PEEP valve set at 8 cmH2O), Pressure Support of 6 and12 cmH2O (Medtronic Puritan Bennett 840) with zero and 15 l/min Flow-By), with respiratory rate 15 and 30 bpm and with $VCO_2$ of 200 and 300 ml/min, tidal volume was set at 500 ml.

**RESULTS.** Mean volume of CO2 rebreathed and minimum CO2 inspiratory concentration were significantly lower with the novel mask compared to the traditional one in all condition tested (as shown by the figure), except for two conditions.

The 15 l/min bias flow significantly decreased the CO2 rebreathing with the novel mask, whereas it had no effect with the traditional mask.

**CONCLUSIONS.** The novel mask design allows a lower CO2 rebreathing, both during CPAP and PSV, effect enhanced by the adjunct of a flow-by.

Further studies are required to establish the clinical relevance of this.

**REFERENCE(S)**

1 Nava S, Hill N. Non-invasive ventilation in acute respiratory failure. Lancet.
2009 Jul 18;374(9685):250–9
2 Taccone P, Hess D, Caironi P, et al.: Continuous positive airway pressure delivered with a “helmet”: Effects on carbon dioxide rebreathing. Crit Care Med 2004; 32:2090–2096

Multidrug resistance

### 0983

#### Risk factors for multiresistant bacteria: a Spanish multicentric study

S. Carvalho Brugger[1], M. Miralbés Torner[2], G. Jimenez Jimenez[2], C. Climent Company[3], O. Badallo Arebalo[4], A.I. Ezpeleta Galindo[5], M. Solsona Perlasia[6], F.J. González Molina[7], M. Catalan González[8], M. Ortiz Piquet[9], P.M. Olaechea Astigarraga[10], J. Trujillano Cabello[2], M. Palomar Martínez[2]
[1]Hospital Universitari Arnau de Vilanova de Lleida, ICU, Lleida, Spain; [2]Hospital Universitari Arnau de Vilanova de Lleida, Lleida, Spain; [3]Hospital del Mar, Barcelona, Spain; [4]Hospital Universitario De Burgos, Burgos, Spain; [5]Hospital Universitario Royo Vilanova, Zaragoza, Spain; [6]Consorci Sanitari del Maresme, Mataró, Spain; [7]Hospital Universitario Mutua Terrasa, Terrasa, Spain; [8]Hospital Universitario 12 de Octubre, Madrid, Spain; [9]Hospital Universitario Lucus Agusti, Lugo, Spain; [10]Hospital Galdakao, Usansolo, Spain
**Correspondence:** S. Carvalho Brugger
*Intensive Care Medicine Experimental* 2017, **5(Suppl 2):**0983

**INTRODUCTION.** Due to increased prevalence of multiresistant bacteria (MRB), one of the goals of clinicians in recent years has been to understand patient characteristics that increase the risk for infection caused by MDR pathogens.

**OBJECTIVES.** To validate the Spanish Resistencia Zero (RZ) check-list for MRB detection, as well as to find out other risk factors (RF) for MRB colonization or infection.

**METHODS.** A prospective multicentric study during 2016. Surveillance and/or diagnostic cultures were carried out in patients admitted to ICU. We analyzed RF and comorbidities. Univariable and multivariable analysis for MRB RF with binary logistic regression methodology ($p < 0{,}05$) were performed.

**RESULTS.** A total of 2270 patients from 9 Spanish ICU were included, 288 (12,7%) of whom presented MRB at admission. One or more RF

according to the RZ check-list were present in 68,1% of them, with accumulation of risk. Having a RF, increased the probability of being colonized or infected by MRB by 4,5 (IC 95% 3,5-6,0). The most common RF was previous hospitalization in the last months (28% of admitted patients).
Prior colonization by MRB was the strongest association with the presence of MRB at ICU admission. All RF analyzed reached statistical significance in the univariate analysis and 4 out of 6 did in the multivariate analysis. Some comorbidities as immunosuppression, renal failure and organ transplantation showed significance as added RF (Table 239). At admission, 71.9% of MRB detected were colonization, while 28.1% were infections. These findings could not be related with increased mortality.
**CONCLUSIONS.** Patients who meet at least 1 RF from the RZ check-list have an increased risk of being MRB carriers. However, more than 30% of MRB were detected in patients without RF, that lead us to look for other comorbidities or situations (immunosuppression, renal failure and organ transplantation) that help us to suspect and early identify MRB.

**Table 239 (Abstract 0983)**. RZ Check list for MRB

| RZ CHECK LIST | OR (IC 95%) UNIVARIATE | OR (IC 95%) MULTIVARIATE |
|---|---|---|
| Hospitalization >5 days in prior 3 months | 2,8 (2,2-3,6) | 1,5 (1,1- 2,1) |
| Institutionalized patient | 4,5 (2,6-7,9) | 3,6 (1,9- 7,0) |
| Prior MRB colonization/infection | 31,9 (20,8-48,8) | 23,7 (15,1-37,4) |
| Antibiotherapy >7 days in prior month | 4,2 (3,2-5,5) | 1,8 (1,2- 2,7) |
| Chronic kidney disease with dialysis | 3,2 (1,6-6,7) | NS |
| Colonization susceptibility (bronchiectasis, cystic fibrosis) | 2,4 (1,3-4,2) | NS |

**Table 240 (Abstract 0983)**. Comorbidities related to MRB

| COMORBIDITY | OR (IC 95%) UNIVARIATE | OR (IC 95%) MULTIVARIATE |
|---|---|---|
| Kidney failure | 2,2 (1,7-2,9) | 1,8 (1,4-2,4) |
| Immunossupression | 2,3 (1,6-3,2) | 1,8 (1,2-2,6) |
| Organ transplantation | 3,9 (2,1-7,1) | 1,3 (1,1-4,7) |
| Liver cirrhosis | 1,7 (1,1-2,7) | NS |
| Chronic obstructive pulmonary disease | 1,4 (1,1-1,9) | NS |
| Malnutrition | 1,5 (1,2-2,0) | NS |
| Diabetes mellitus | 1,6 (1,2-2,0) | NS |

### 0984

#### Successful control of ST101 colistin-resistant carbapenemase - *bla*OXA-48 producing *Klebsiella pneumoniae* in a Greek ICU

A. Mavroidi[1], M. Katsiari[2], S. Likousi[1], E. Palla[1], O. Kordanouli[1], C. Nikolaou[2], Z. Roussou[1], C. Mathas[2], E. Platsouka[1]
[1]General Hospital of Nea Ionia,"Konstantopouleio-Patission", Department of Microbiology, Athens, Greece; [2]General Hospital of Nea Ionia,"Konstantopouleio-Patission", Intensive Care Unit, Athens, Greece
**Correspondence:** A. Mavroidi
*Intensive Care Medicine Experimental* 2017, **5(Suppl 2):**0984

**INTRODUCTION.** Carbapenem-resistant *Klebsiella pneumoniae* (CR Kp) are endemic in Greece, whereas colistin resistant (COL-R) Kp are increasingly reported (Mavroidi *et al.,* 2016).
**OBJECTIVES.** The investigation of the incidence, spread and molecular characterisation of CR Kp from bloodstream infections (BSIs) in the ICU of a Greek hospital during 2014–2016.
**METHODS.** Identification of the isolates to the species level and antibiotic susceptibility testing were performed by the semi-automated system MicroScan® (Siemens Healthcare, PA, USA). The minimum inhibitory concentrations (MICs) of imipenem, meropenem and colistin were additionally determined using the MIC Test Strips (Liofilchem S.R.L), according to the interpretive criteria of the Clinical and Laboratory Standards Institute (CLSI, 2015). Phenotypic screening for carbapenemase production was performed by the boronic acid/ EDTA combined-disk test. DNA extraction was performed using the QIAcube (Qiagen, Düsseldof, Germany). The presence of carbapenemase- and ESBL- encoding genes was confirmed by polymerase chain reaction. Genotyping of the isolates was performed by multilocus-sequence typing (MLST, http://bigsdb.pasteur.fr/klebsiella/klebsiella.html). Clinical data of ICU patients with BSIs were reviewed retrospectively and statistical analysis was performed by using the Fisher exact test and the Mann-Witney U test.
**RESULTS.** CR Kp producers (n = 235) were isolated from bronchial secretions (n = 92, 39.3%), blood (n = 50, 21.2%), CVCs (n = 39, 16.7%), urine (n = 28, 12%) and other clinical specimens of ICU patients. During 2014–2016, a rise of Kp from BSIs was observed (from 9.4% to 27% of CR Kp); the majority of them were *bla*OXA-48 (n = 25, 50%) and *bla*KPC (n = 13, 26%) producers. In 2014, only 3 *bla*KPC-producers of MLST ST258 from BSIs were documented. During 2015, COL-R *bla*OXA-48-co-producing CTXM type enzymes of MLST ST101 Kp were documented (n = 12, 60% of 20 CR Kp), while they represented 48% (n = 13) of 27 CR Kp in 2016. All ICU patients affected by *bla*OXA-48 producers were put under strict contact ?A3B2 show $132#?>isolation or cohort care, along with appropriate infection control measures.The last patient affected by a *bla*OXA-48-producer was documented on October 2016; thus, we assume that the outbreak was successfully controlled. No statistical significant differences were observed for the death rates, the APACHE II score, the length of stay in the ICU and previous antibiotic treatment with carbapenems and/or colistin between the *bla*OXA-48 producing and other carbapenemase-producing Kp.
**CONCLUSIONS.** During 2014–2016, the rise in the incidence of CR Kp from BSIs was mainly due to the emergence and dissemination of COL-R *bla*OXA-48 producers of MLST ST101. The outbreak was successfully controlled by implementation of strict infection control measures.
**REFERENCE**
Mavroidi A *et al.*, 2016, Microb Drug Resist. 22:392–398.
**GRANT ACKNOWLEDGMENT**
No funding.

### 0985

#### Detection of multirresistant bacteria on ICU admission

S. Carvalho Brugger[1], M. Miralbés Torner[2], G. Jimenez Jimenez[2], C. Climent Company[3], O. Badallo Arebalo[4], A.I. Ezpeleta Galindo[5], M. Solsona Perlasia[6], F.J. González Molina[7], M. Catalan González[8], M. Ortiz Piquer[9], P.M. Olaechea Astigarraga[10], J. Trujillano Cabello[2], M. Palomar Martínez[2]
[1]Hospital Universitari Arnau de Vilanova de Lleida, ICU, Lleida, Spain; [2]Hospital Universitari Arnau de Vilanova de Lleida, Lleida, Spain; [3]Hospital del Mar, Barcelona, Spain; [4]Hospital Universitario de Burgos, Burgos, Spain; [5]Hospital Universitario Royo Vilanova, Zaragoza, Spain; [6]Consorci Sanitari del Maresme, Mataró, Spain; [7]Hospital Mutua de Terrassa, Terrasa, Spain; [8]Hospital Universitario 12 de de Octubre, Madrid, Spain; [9]Hospital Universitario Lucus Agusti, Lugo, Spain; [10]Hospital Galdakao, Usansolo, Spain
**Correspondence:** S. Carvalho Brugger
*Intensive Care Medicine Experimental* 2017, **5(Suppl 2):**0985

**INTRODUCTION.** One of the recommendations of the Spanish "Resistencia Zero" Program (RZ) is to complete a check list upon patient admission in Intensive Care Unit (ICU) to identify those patients at high risk for colonization or infection by multiresistant bacteria (MRB).
**OBJECTIVES.** To search the relation between most common MRB and specific risk factors (RF) for colonization or infection, and the samples where they are identified, assessing the cultures profitability.
**METHODS.** A prospective multicentric study, during 2016. Included patients admitted to ICU to whom surveillance and/or diagnostic cultures were carried out. It was applied a check list of RF for MRB

according to the RZ program, and applied contact precaution measures in patients at high risk for MRB. We analysed other RF and comorbidities. Univariant and multivariant analysis for MRB RF with binary logistic regression methodology (p < 0,05) were performed. The difference between groups of MRB was made by Chi-square test for qualitative variables and the Kruskal-Walls test for the continuous ones. Statistical significance was set at P < .05.

**RESULTS.** 2270 patients from 9 Spanish ICU were included; in 288 (12,7%) patients one or more MRB were detected at admission. A total of 161 patients (55,9%) were ESBLs carriers, 90 (31,2%) MRSA, 42 (14,6%) P aeruginosa, 7 (2,4%) carbapenemases, 6 (2,1%) Acinetobacter spp and 14 (4,9%) others MRB carriers. In 81 cases (28,1%) the presence of a MRB caused infection. There was a strong association between RF and the presence of P aeruginosa (OR 37,1, IC 95% 8,9-154,0), followed by Acinetobacter, ESBLs and MRSA, in this order. There were RF in 64,4% of patients with MRSA, 83,3% Acinetobacter, 68,9% ESBLs, 95,2% P aeruginosa and all (100%) carbapenemases, although this one did not reach statistical significance on RF analysis. MRSA was identified in 68,9%, 33,3% and 20% by nasal, pharyngeal and rectal swabs, respectively, and in 10% by diagnostic cultures. P aeruginosa was identified in 52,4% by rectal swab, 33% by pharyngeal swab and in 26,2% by diagnostic cultures. Rectal swab identified 66,7% of Acinetobacter. And ESBLs were identified mainly by rectal swab (81,4%), while the diagnostic cultures were positive in 21,1% of the cases these bacteria were present.

**CONCLUSIONS.** We demonstrated a predominance of ESBLs, followed by MRSA, most of them present as colonization. There was a strong association between almost all MRB analysed and the RF included in the RZ check list, and probably because of the sample size some of them did not reach statistical significance in this analysis. The surveillance cultures performed at ICU admission are important, once the diagnostic ones detected less than 30% of MRB.

J Montero et al. (Scientific Expert Committee for the "Zero Resistance" Project). Combatting resistance in intensive care: the multimodal approach of the Spanish ICU "Zero Resistance" program. Critical Care (2015) 19:114.

### 0986

#### High-dose colistin combined with continuous veno-venous haemofiltration for treatment of multidrug-resistant Gram-negative infection in critically ill patients

A. Verdoodt[1], P.M. Honoré[2], R. Jacobs[2], V. Van Gorp[2], I. Hubloue[1], H.D. Spapen[2]

[1]University Hospital, Vrije Universiteit, Brussels, Emergency Medicine, Brussels, Belgium; [2]University Hospital, Vrije Universiteit, Brussels, Intensive Care, Brussels, Belgium

**Correspondence:** A. Verdoodt

*Intensive Care Medicine Experimental* 2017, **5(Suppl 2):**0986

**INTRODUCTION.** Multidrug-resistant Gram-negative (MDR-GN) infections susceptible only to colistin (COL) are emerging in critically ill patients. Pharmacological studies suggest that a higher COL dose (i.e. a 9 MIU loading dose followed by a maintenance dose as high as 15 MIU daily) is needed to ensure microbiological and clinical cure. However, such high dose may enhance COL-related renal toxicity (1). Since COL is effectively removed from the blood by continuous veno-venous haemofiltration (CVVH), in particular when membranes with high adsorptive capacity are used, this technique may allow to administer high COL doses without inducing or enhancing nephrotoxicity.

**OBJECTIVES.** To assess safety and clinical/microbiological efficacy of high-dose COL treatment under "prophylactic" CVVH.

**METHODS.** Retrospective study of adult ICU patients with MDR-GN infections who received COL (colistimethate sodium, Colistineb™), alone or in combination with meropenem, for at least 5 days. COL was administered as a 9MIU loading dose followed by 3 x 4.5MIU daily. CVVH was performed under citrate anticoagulation at a dose of 35mL/kg/h using a highly adsorptive AN69 ST filter with 1.5m$^2$ surface area. Clinical and microbiological efficacy were assessed at the end of therapy. Clinical efficacy was defined as favourable (clinical improvement) or failure (persistent or progressive infection). Microbiological response was defined as eradication (negative cultures), presumed eradication (clinical efficacy but no microbiological data) or failure (pathogen persistence). Results were expressed as means ± SD or (range). In survivors, serum creatinine was evaluated before and at the end of therapy and at hospital discharge.

**RESULTS.** 16 patients (10 males, age 57 ± 15 years) were treated. APACHE II and SOFA score were 26 ± 10 and 8.8 ± 3.5, respectively. Pneumonia was present in 14 and urosepsis in 2 patients. Causative pathogens were *P. aeruginosa* (n = 8), *K. pneumoniae* (n = 6) and *Enterobacter* species (n = 2). COL MICs ranged from 0.03 to 3 mg/L. COL was given as monotherapy in 8 subjects. COL + CVVH was provided for 13 (6–27) days. A favourable clinical response was obtained in 14 (88%) patients. Accordingly, microbiological eradication was complete in 10, presumed in 4 and absent in 2 subjects. Length of ICU stay was 48 (13–128) days. Seven (45%) patients left the hospital alive. Serum creatinine (n = 6) at the predefined time points was 2.12 ± 1.52; 1.59 ± 0.96; and 0.91 ± 0.29 mg/dL, respectively. One patient required intermittent dialysis at ICU discharge.

**CONCLUSIONS.** In patients with MDR-GN infections, CVVH using an highly adsorptive membrane may represent a valuable option to enable safe and effective high-dose COL treatment.

(1) Vicari G, Bauer SR, Neuner EA, Lam SW. Association between colistin dose and microbiologic outcomes in patients with multidrug-resistant Gram-negative bacteremia. Clin Infect Dis 2013; 56 (3): 398–404.

**GRANT ACKNOWLEDGMENT**

None

### 0987

#### Active screening of multi-drug resistant (MDR) bacteria in patients admitted to the ICU. Results after 25 months

A.N. García Herrera, M. Barber Ansón, J. Lobo Palanco, M.P. Anguiano Baquero, M. Loinaz Bordonabe, T. Caldito Ascargorta, J.C. Moncayola Vicen, V. Mínguez Exposito, M. García Parra, E. Erice Azparren

Complejo Hospitalario de Navarra, Pamplona, Spain

**Correspondence:** A.N. García Herrera

*Intensive Care Medicine Experimental* 2017, **5(Suppl 2):**0987

**INTRODUCTION.** Active screening of MDR bacteria is one of the key points of the "Zero Resistance" project - developed by the Spanish Society of Intensive Care Medicine and Coronary Care Units (SEMICYUC) - the main objective is to reduce the cumulative incidence of patients with ICU-acquired MDR infections.

**OBJECTIVES.** To describe the incidence and most common MDR pathogens identified at admission and during stay in a medical surgical intensive care unit of a tertiary class hospital and compare the results with the national data.

**METHODS.** Prospective, descriptive study. All patients admitted to the ICU from june 2014 to june 2016 were included. Nasal, rectal, inguinal and oropharyngeal swabs (bronchial aspirates in intubated patients) were collected. Pathogens under surveillance included methicillin-resistant *Staphylococcus aureus* (MRSA), vancomycin-resistant *Enterococcus* (VRE), extended-spectrum β-lactamase-producing *Enterobacteriaceae* (ESBL-E), Carbapenemase-Producing Gram Negative Bacilli (CPGNB), Multidrug-resistant *Pseudomonas aeruginosa* (MDRP) and Carbapenem-resistant *Acinetobacter baumannii* (CRAB). Data expressed in means and percentages. Same data were obtained from the National Nosocomial Infection Surveillance Study (Estudio Nacional de Vigilancia de Infección Nosocomial, [ENVIN]) for the same period of time and both results were compared.

**RESULTS.** During the 25 months period 1,554 patients were admitted to our unit, with a total stay of 13,767 days and 5,304 days of antibiotic therapy (385 antibiotic´s days / 1000 stays). MDR bacteria were identified on admission in 144 patients (9.27%), in 115 patients

(79.9%) was colonization and in 29 (20.1%) an infection. During the stay in ICU, MDR bacteria were isolated in 48 patients (3.1%), in 32 patients (66.7%) was colonization with a colonization density of 2.39/1000 stays and in 16 patients (33.3%). Was an infection, with an infection density of 1.16/1000 stays.
The following tables show the number of patients with each species of MDR bacteria that were isolated (colonization or infection) on admission and during their stay in our ICU (Table 241) and the same data from ENVIN´s database (Table 242).
**CONCLUSIONS.** Comparing with the national results, in our ICU we do not have VRE. The highest percentage of MDR bacteria that produces infection in our ICU is MDRP. We use less antibiotics (national rate: 611 antibiotic´s days/1000 stays) and our density of MDR bacteria infection is slightly lower at admission (national density: 1.43 /1000 stays).
**REFERENCES**
1. Informe Resistencia Zero. ENVIN: http://hws.vhebron.net/envin-helics/InformesRZero/BMR.asp
2. Montero JG, Lerma FA, et al. Combatting resistance in intensive care: the multimodal approach of the Spanish ICU "Zero Resistance" program. Crit Care. 2015 Mar 16;19:114.

**Table 241 (Abstract 0987)**. ICU results. Screening of MDR bacteria

| MDR pathogens | At admittance | Colonization during ICU stay | Infection during ICU stay | Total |
|---|---|---|---|---|
| MRSA | 36 (73,5%) | 11 (22,5%) | 2 (4%) | 49 |
| MDRP | 13 (43,3%) | 9 (30%) | 8 (26,7%) | 30 |
| CRAB | 2 (50%) | 1 (25%) | 1 (25%) | 4 |
| ESBL-E | 103 (85,8%) | 13 (10,8%) | 4 (3,4%) | 120 |
| CPGNB | 1 (50%) | 0 | 1 (50%) | 2 |

**Table 242 (Abstract 0987)**. National results. Screening of MDR bacteria

| MDR pathogens | At admittance | Colonization during ICU stay | Infection during ICU stay | Total |
|---|---|---|---|---|
| MRSA | 2278 (77,1%) | 386 (13,1%) | 291 (9,8%) | 2955 |
| MDRP | 963 (42,1%) | 592 (25,9%) | 734 (32%) | 2289 |
| CRAB | 402 (32,3%) | 496 (39,8%) | 348 (27,9%) | 1246 |
| ESBL-E | 3752 (63,4%) | 1380 (23,3%) | 782 (13,3%) | 5914 |
| CPGNB | 594 (46%) | 478 (37%) | 218 (17%) | 1290 |
| VRE | 144 (56,9%) | 87 (34,4%) | 22 (8,7%) | 253 |

### 0988

#### Mucoid phenotype and severe infection by klebsiella pneumoniae (MuSIK)

T. Fauvet[1], A. Tarantola[2], J. Colot[2], A. Merlet[3], B. Marot[3], M. Série[3]
[1]Université de Limoge, Limoge, France; [2]Pasteur, Nouméa, France; [3]CHT Nouméa, Nouméa, France
**Correspondence:** T. Fauvet
*Intensive Care Medicine Experimental* 2017, **5(Suppl 2):**0988

**INTRODUCTION.** Since the 1980s, a particular capsular phenotype of Klebsielle Pneumoniae (KP) has emerged in Asia, known as hypermucoid, which is responsible for complicated bacteremia of multiple septic sites (liver, CNS, muscles). (1)
In New Caledonia, KP is a major cause of infection,about one third of the strains responsible for bacteremia have this phenotype. (2)
**OBJECTIVES.** The objective of this study was to compare the clinical severity of patients with community and nosocomial bacteraemia with KP according to their hypermucoid (KPHM) or non-KPHH character.
**METHODS.** This an observational retrospective study including successively all patients with bacteremia at KP during the period from May 2013 to March 2015 at the territorial hospital of New Caledonia. The hypermucoid character of the strains was defined by the use of the string test followed by molecular analysis to determine the capsular serotype.
After a double seizure and anonymization of the data, a bi- and multivariate analysis was carried out according to the mucoid nature or not of the KP strain and the clinico-biological characteristics of the patients during their hospitalization.
**RESULTS.** Fifty-five bacteremic patients were included in the study, 27% of the strains isolated were hyper mucoids. Infected populations are comparable and have a high incidence of diabetes in both groups (43.6%). Hypermucoid strains accounted for two thirds of community infections whereas the non-mucoid profile was predominantly found in nosocomial infections (72.5% vs 33.4%, p = 0.01). The rate of hospitalization in intensive care units is high (KPHM 46.7%, KPNH 52.5%) with an average IGS2 score (KPHM 54.1 (±20.8) KPHM 63.9 (±22.3) p = 0.28) without any difference between the two groups. There was no significant difference in mortality (KPHM 46.7% vs KPNH 15%, p = 0.07) but patients with a KPHM strain had a longer hospital stay (73.5 days versus 50.7 days, p = (OR 1.41, CI 95% 1.0-1.96, p = 0.045), a higher number of infectious metastases (OR 7.06, CI 95% 1),and a longer persistence of positive blood cultures despite the implementation of a suitable treatment,
25–39.64, p = 0.026).
Community-acquired or nosocomial KP bacteraemias represent severe infections where resuscitation is required in half the cases in patients with high gravity scores. This probably explains why there is no difference in mortality between the two groups. However, it can be assumed that the trend of excess mortality in the KPHM group is not significant due to insufficient sample size.
**CONCLUSIONS.** Due to their persistent and metastatic nature, bacteremia with KPHM should be considered severe and should be closely monitored. Prolonged antibiotic terapy should be discussed.
**REFERENCE(S)**
(1) Virulence 2013,2150-5594
(2) Int J Infect Dis 2015,120-19712

### 0989

#### Incidence of extended-spectrum beta-lactamase-producing enterobacteriaceae hospital-acquired infections as an independent factor associated with ICU-mortality: a longitudinal ICU-based surveillance study

B.K. Tan[1], E. Vivier[1], J.-R. Zahar[2,3], G. Bourdin[1], J.-B. Putegnat[1], S. Lagrange[1], F. Saintot[1], S. Rosselli[1], C. Pommier[1], L. Parmeland[4], C. Haond[5], K. Ait-Bouziad[6], M. Hocine[6], L. Témime[6]
[1]Saint Joseph-Saint Luc Hospital, Intensive Care Unit, Lyon, France; [2]Avicenne Hospital, Paris-Nord University, Bacteriology, Virology & Hygien Department, Bobigny, France; [3]Assistance Publique des Hôpitaux de Paris, Paris, France; [4]Saint Joseph-Saint Luc Hospital, Biology Department, Lyon, France; [5]Hospices Civils de Lyon, Edouard Herriot Academic Hospital, Hospital Hygien Department, Lyon, France; [6]Conservatoire National des Arts et Métiers, MESuRS Laboratory, Paris, France
**Correspondence:** B.K. Tan
*Intensive Care Medicine Experimental* 2017, **5(Suppl 2):**0989

**INTRODUCTION.** Multidrug-resistant bacteria are common in ICU. However, the incidence of extended-spectrum beta-lactamase-producing (ESBL) Enterobacteriaceae currently increases and could be a threat for the success of empiric antibiotic treatment and the survival of critically ill patients.
**OBJECTIVES.** Consequently, we design an observational study to assess the impact of ESBL-producing Enterobacteriaceae hospital-acquired infections on mortality in ICU.
**METHODS.** In a 350-bed general teaching hospital in Lyon (France), we retrospectively analyse the mortality rate and its factors independently associated in our medical and surgical 12 bed-ICU, using a 6-year longitudinal data from January 2011 to December 2016 ?A3B2 show $132#?>collected from the biological, pharmaceutical, hospital hygiene and medical information departments. Monthly incidences of hospital-acquired infections concerning ESBL Enterobacteriaceae, AmpC beta-lactamase-hyperproducing Enterobacteriaceae, methicillinresistant *Staphylococcus aureus* (MRSA) with or without bloodstream microbial identification, are analyzed using generalized estimating equations with a multivariate Poisson regression.

**RESULTS.** During the study period, 3279 adult patients are included in our ICU. Mean age is 64.7 ± 2.4 years-old. Sex ratio is 1.7 ± 0.5. Mean Simplified Acute Physiologic Score II (SAPSII) is 46.8 ± 7.2. Mean length-of-stay is 6.7 ± 2 days.

Monthly incidence of monthly ESBL-producing coliforms rectal carriage at admission in ICU is 9.8 ± 7.8 cases/1000 patients bed-days whereas monthly is 4 ± 3.8 cases of ESBL-producing hospital-acquired infections/1000 patient bed-days. Monthly incidence of deaths in ICU is 32.3 ± 11.6 cases/1000 patients bed-days.

In multivariate analysis, deaths incidence in ICU is independently predicted by incidence of ESBL-producing coliforms hospital-acquired infection (p = 0.005), SAPS II (<0.001), percentage of patients over 80 years-old (p = 0.001) and central venous catheter use (p = 0.02) whereas carbapenems consumption seems an independent protective factor (p = 0.001).

**CONCLUSIONS.** The burden of antibiotic resistance currently increases and should be considered according to the local microbial flora. In particulary, extended-spectrum beta-lactamase coliforms hospital-acquired infections could threat the survival of infected critically ill patients if first-line empiric antibiotherapy is not adequate. Consequently, improving critical care should include fight against multidrug-resitant bacteria diffusion combined to progress in availibility of new tools for fast antimicrobial resistance identification and better knowledges of trends in local antimicrobial resistance.

**REFERENCE(S)**

[1] Fabbro-Peray P et al. Infect Control Hosp Epidemiol. 2007; 28: 265–72.

[2] Cosgrove SE. Clin Infect Dis.2006; 42: S 82–9.

[3] Blot S. Clin Microbiol Infect.2008; 14: 5–13.

### 0990

#### The impact of decontamination strategies in ICU-acquired respiratory infections by multidrug resistant pathogens

C. Gonçalves[1], A. Cipriano[1], H. Castro[2], V. Lopes[3], T. Cardoso[2], I. Aragão[2]
[1]Centro Hospitalar do Porto, Infectious Diseases, Porto, Portugal; [2]Centro Hospitalar do Porto, Intensive Care Unit (UCIP), Porto, Portugal; [3]Centro Hospitalar do Porto, Microbiology, Porto, Portugal
**Correspondence:** C. Gonçalves
*Intensive Care Medicine Experimental* 2017, **5(Suppl 2):**0990

**INTRODUCTION.** Digestive decolonization strategies have been associated with lower rates of resistant pathogens colonization, less antibiotic consumption and better outcomes, probably related to lower nosocomial infection rates.

**OBJECTIVE.** To determine the impact of two decolonization strategies [oral care with chlorhexidine (CHX) 1% (oroCHX-1%) and selective oropharyngeal and digestive decontamination (SDD)] when compared to standard care (SC) in the rate of respiratory infections by multidrug resistant (MDR) pathogens.

**METHODS.** Prospective interventional cluster study in a 12-bed mixed ICU of a tertiary care university hospital, including all patients who were admitted for a minimum period of 48 hours, between ?A3B2 show $132#?>February 2015 and July 2016 (18 months). There were three study periods: **baseline** (from February to July 2015) where usual care was provided (daily bath with CHX and 3 times daily oral hygiene with CHX 0.12%), **SDD** with oral care 4 times daily with digestive and oropharyngeal application of a suspension containing colistin, tobramycin, nystatin antibiotics (in enteral suspension, no intravenous antibiotic use) (August 2015 to January 2016) and **oroCHX-1%** with 4 times daily oropharyngeal application of CHX in a 1% concentration, until extubation (February to July 2016). MDR was defined as a pathogen resistant to at least one antibiotic in three different classes to which it should be sensitive.

**RESULTS.** During the study period 458 patients were admitted into the ICU, with a mean (±SD) age of 61 ± 15 years, 291 (64%) were male. The mean (±SD) SAPS II was 49 ± 15. In 79 patients (17%) a total of 84 ICU-acquired respiratory infections were diagnosed (66 pneumonias and 18 tracheobronchitis). The number of respiratory infections was 26/143 (18%) in the baseline, 32/173 (18%) in the SDD (p = 0.943) and 26/142 (18%) in the oroCHX-1% period (p = 0.978); infections by a MDR pathogen were 4/25 (16%), 10/20 (50%) (p = 0.023) and 7/19 (37%) (p = 0.164), respectively (Table 243).

**CONCLUSION.** In our unit, decolonization strategies did not result in a significant decrease in ICU-acquired respiratory infections; in the SDD period there was a significant increase in the proportion of ICU-acquired respiratory infections by a MDR pathogen when compared to the baseline period.

**Table 243 (Abstract 0990)**. Patients characteristics in each study period

| | Standard Care(n=143) | SDD(n=173) | oroCHX-1%(n=142) |
|---|---|---|---|
| Mean Age±SD | 61±16 | 61±16 | 61±15 |
| Male, n(%) | 95(66) | 114(66) | 82(58) |
| SAPSII ±SD | 50±14 | 49±16 | 49±17 |
| Admissions from the community, n(%) | 87(61) | 106(61) | 93(66) |
| VAP rate[a] | 16.5 | 18 | 17.7 |
| VAT rate[a] | 3.9 | 8.2 | 2.3 |
| MDR, n(%) | 4(16) | 10(50) | 7(37) |
| ICU days until MDR isolation, mean(±SD) | 17±11 | 12±8 | 21±15 |
| ICU mortality, n(%) | 44(31) | 39(22) | 26(18) |

*VAP* ventilator associated pneumonia, *VAT* ventilator associated tracheobronchitis, *ICU* intensive care unit, *MDR* multidrug resistant
[a]Per 1000 of invasive ventilation

### 0991

#### Multi drug resistant bacteria in the critical elderly patients

J. Higuera, D. Cabestrero, L. Rey, A. Caballero, G. Narvaéz, M. Aroca, S. Saéz, C. Soriano, S. García, R. De Pablo
Ramon y Cajal University Hospital, Intensive Care, Madrid, Spain
**Correspondence:** J. Higuera
*Intensive Care Medicine Experimental* 2017, **5(Suppl 2):**0991

**INTRODUCTION.** Multi drug resistant bacteria (MDR) is a common problem in intensive care units. MDR infections are associated with adverse outcomes such as, increased mortality, length of hospital stay and health care related costs. Elderly patient are likely to have a higher incidence of these due to increased colonization.

**MATERIAL AND METHODS.** Our goal is to describe and to analyse the incidence of colonization by MDR (MRSA, ESBL-Producing and KPC-producing) in elderly patients. Describe if these colonization's are associated with higher mortality rates or higher length of hospital stay.

We performed a retrospective study. We included all patients with eighty years and above (+80) who entered in the ICU between April 2013 and August 2016. We collected all the carrier results and we analysed the variables (sex, mean age, severity indexes, diagnostic tabulations, and length of stays, mechanical ventilation, the use of vasoactive drugs, renal replacement therapies and mortality). Carrier's control were collected at the patient´s ICU admission and once a week during their stay.

Qualitative variables with normal distribution have been expressed as number and percentages and were analysed using chi-square tests. Quantitative variables were expressed as mean ± standard deviation (Rank) and were analysed using t-student tests and anova.

**RESULTS.** A total of 76 patients were collected. 48.7% Women (37) 51.3% Men (39). Mean age 83 ± 2.4 (80–90) years, Severity indexes: SOFA 7.5 ± 4.1 (1–18) APACHE II 19.6 ± 7.9 (7–39) SAPS II 55.1 ± 17 (30–100). Diagnostic tabulation was cardiovascular (15/76) gastrointestinal (12/76) Infectious (14/76) Neurocritical (13/76) Surgical (9/76) Respiratory (12/76) Traumatic (1/76). The mean length of stay was 4.75 ± 5.8 days. 3% of patient's required renal replacement therapy (1/76), 60.5% (46/76) required mechanical ventilation and 60.5% (46/76) required vasoactive drugs. The mortality rate of this group of patients was 34% (26/76) with limitation of the therapeutic effort in 9 cases (11.8%).

The incidence of colonization by MDR was 17.1% (13/76) (MRSA (3/76) ESBL-Producing (8/76) and KPC-producing (2/76)). There is no statistically significant differences in severity indices between the two groups. Colonization by MDR bacteria has not been associated with higher mortality rate, mechanical ventilation, vasoactive drugs or renal replacement therapy. The mean length of stay of colonized patients has been 8 ± 8 days vs 4 ± 5 days in not colonized patients. (P < 0.003).

**CONCLUSIONS.** The increasing prevalence of MDR bacteria is a common problem in our intensive care services. MDR infections are related with higher mortality and length of stay. However their presence in the carrier control in elderly patients seems to be associated with higher length of stay in elderly patients in our study.

### 0992

#### In vitro antimicrobial resistance of strains of Acinetobacet baumanii, Pseudomonas aeruginosa and Klebsiella pneumoniae isolated in the ICU of a trietary hospital during 2 years

A. Margaritis[1], N. Zotos[2], E. Tatsina[2], M. Bolosi[3], E. Kosma[1], D. Lepida[1], O. Ygropoulou[1], K. Milios[1]

[1]General Hospital of Ioannina 'G Hatzikosta', ICU, Ioannina, Greece; [2]General Hospital of Ioannina 'G Hatzikosta', Microbiology, Ioannina, Greece; [3]Ioannina University Hospital, Anesthesiology, Ioannina, Greece

**Correspondence:** A. Margaritis

*Intensive Care Medicine Experimental* 2017, **5(Suppl 2):**0992

**INTRODUCTION.** Gram (−) bacteria are the causing agents of severe infections in patients in the I.C.U. Hospital -acquired infections caused by multi-drug resistant strains are frequent and despite the precautionary measures taken, they lead to an increase in the time of hospitalization, morbidity rates,mortality rates and hospital expenses.

**OBJECTIVES.** To record and study the antimicrobial resistance of the most common drug-resistant gram(−) bacteria isolated in the ICU of our hospital during two years.

**METHODS.** We studied and recorded 34 drug-resistant strains of Acinetobacter baumanii, 32 strains of Pseudomonas aeruginosa and 9 strains of Klebsiella pneumoniae isolated in the ICU during two years. The common culture media were used for the cultivation of the specimens. The identification of the strains as well as the antimicrobial susceptibility testing were performed by the Microscan Walkaway (Siemens) system. The antimicrobial susceptibility testing was verified by the E-test method according to CLSI directions.

**RESULTS.** There was no resistance to Colistin and Tigecycline detected. High resistance levels to Imipenem (A.baumanii 69.7%-P.aeruginosa 45.2%- K.pneumoniae 55.6%) were noted. Thus,it can be seen that multi-drug resistant strains with high resistance levels to Carbapenems have emerged.

**CONCLUSIONS.** The continual monitoring of the resistance rates of multi-drug resistant strains of A.baumanii, P.aeruginosa and K.pneumoniae isolated in the ICU is inevitable, given the fact that few antibiotics can be used against these strains.

### 0993

#### Infection with MDR bacteria (Klebsiella Pneumoniae, Pseudomonas Aeroginosa, Acinetobacter Baumannii complex) in multi-specialty intensive care unit

A. Margaritis[1], N. Zotos[2], M. Bolosi[3], M. Gerasimou[2], E. Tatsina[2], O. Ygropoulou[1], O. Mousafiri[1], G. Papadopoulos[3]

[1]General Hospital of Ioannina 'G Hatzikosta', ICU, Ioannina, Greece; [2]General Hospital of Ioannina 'G Hatzikosta', Microbiology, Ioannina, Greece; [3]Ioannina University Hospital, Anesthesiology, Ioannina, Greece

**Correspondence:** A. Margaritis

*Intensive Care Medicine Experimental* 2017, **5(Suppl 2):**0993

**INTRODUCTION.** There has been observed an increasing incidence of resistant Gram-negative strains in Greece in last decade. It is important for institutions to track rates of antibiotic resistance in their ICUs and detect any potential risk factors for infection with Multi-Drug Resistance (MDR) bacteria.

**OBJECTIVES.** The study of several potential risk factors for infection with MDR bacteria in patients hospitalized in our intensive care unit.

**METHODS.** Data from a sample of 294 patients hospitalized in ICU of general hospital from 1/1/16 to 31/12/16 were studied. The factors that were studied are: age, sex, main duration of stay in ICU, hospital origin (the same or other), APACHE II score, PDR, previous MDR bacterial colonization, previous antibiotics use, duration of mechanical ventilation, immunosupresion, diabetes, hypoproteinemia and the precense of central venous catheter.

Mono-factor analysis ($\chi 2$-test, t-test, Mann–Whitney test) and multi-factor analysis (multiple logistic regression) were used for the investigation of the correlation between risk factors and the possibility of MDR infection.

**RESULTS.** Between 294 patients, 45 (15,3%) were infected with MDR bacteria.

Monofactor analysis showed statistical correlation for the following factors: duration of stay in ICU, hospital origin, APACHE II score, previous MDR bacterial colonization, previous use of carvapenems, duration of mechanical ventilation, immunosupresion, diabetes, hypoproteinemia and presence of central venous catheter.

Multi-factor analysis showed statistically important correlation for the following independent factors: previous use of carvapenems, hospital origin, presence of central venous catheter.

**CONCLUSIONS.** Between potential risk factor, previous use of carvapenems shows a very strong correlation with MDR infection. Other important factors are: the presence of central venus catheter, hospital-to-hospital patient transfer, previous colonization with MDR bacteria and the duration of mechanical ventilation. Recognition of these factors and the effort to minimize them contributes to reduce MDR, hence reduce patient morbidity and mortality hospitalized in ICU.

### 0994

#### Complications and morbidity of multidrug-resistant *Acinetobacter baumannii* infections in the critically ill patient

R. Rodríguez García, L. Forcelledo Espina, I. Astola Hidalgo, B. García Arias, L. Cofiño Castañeda, L. Viña Soria, L. López Amor, G. Muñíz Albaiceta, D. Escudero Augusto

Hospital Universitario Central de Asturias, Oviedo, Spain

**Correspondence:** R. Rodríguez García

*Intensive Care Medicine Experimental* 2017, **5(Suppl 2):**0994

**INTRODUCTION.** Intensive Care Units are the most affected by the massive use of antibiotics, which causes the appearance of multiresistant strains. Nosocomial infections caused by *Acinetobacter species* is an emerging threat. *Acinetobacter baumannii* is an opportunistic gram negative pathogen with increasing relevance in a variety of hospital acquired infections especially among critically ill patients.

**OBJECTIVES.** The aim of our study was to determinate the pattern of *Acinetobacter* infections and its association with length of stay in patients admitted to our ICU.

**METHODS.** We made a retrospective and observational study of patients admitted to the ICU during one year and who have infected by *A. baumannii*. On admission, we recorded clinical and laboratory parameters.

**RESULTS.** 40 infections were identified. The mean age was 63.75 + 13 years and 65% were men. On admission the mean value of APACHE II was 23.26 + 5.8. 57.5% came from the emergency department, 30% from the hospital ward and 12.5% from another hospital. 72.5% had co-morbidity. Neurocritical illness (32.5%) was the most frequent cause of hospitalization and the others were 30% septic, 17.5% post surgical, 12.5% politraumatized and 2.5% with cardiac arrest. 70% of patients had hemodynamic instability, 52.5% respiratory failure and renal dysfunction 47.5%. Highest rates of resistance (100%) was detected against penicillins, cephalosporins and even extended spectrum antibiotics including carbepenems and quinolones. Invasive mechanical ventilation was performed in 39 (97.5%) patients. All patients had had central venous and urinary catheters and 5% an external ventricular drainage. Pneumonia developed in 12 patients, and there were 10 tracheobronchitis, 8 bacteremias, 6 urinary infections, 3 intra-abdominal infections and 1 ventriculitis. 75% were coinfection with other microorganisms (Gram negative 35%, Gram positive 22.5%, both 17.5%). 77.5% had previously colonized, 60% tracheal, 42.5% rectal, 30% nasal, 20% cutaneous and 10% urinary. Respiratory system was most commonly involved. The most frequent infections were pneumonia (30%) and tracheobronchitis (27.5%) against bacteremia (22.5%) and urinary tract infection (15%). Overall mortality was 32.5%, significantly higher in patients with pneumonia (66.7%, p 0.001) than in all other conditions (urinary tract infections, bacteremia or tracheobronchitis). The average stay in ICU was of 64 ± 41.16 days.

**CONCLUSIONS.** Multidrug-resistant *A. baumannii* infections increase and are related with high clinical severity and with longer duration of stay in ICU. The use of invasive procedures exhibit an increasing trend. In more than 2/3 the infection appears after colonization, mainly if it is respiratory, with a higher mortality in the case of pneumonia.

### 0995

#### Antimicrobial resistance in a medical ICU, incidence and risk factors

M.A. Boujelbèn[1], N. Fraj[1], J. Baroudi[1], N. Sma[1], K. Meddeb[1], E. Ennouri[1], W. Zarrougui[1], H. Kallel[1], A. Ben Abdelhafidh[1], I. Ben Saida[1], A. Azouzi[1], A. Khedher[1], I. Chouchene[1], J. Ayachi[1], M. Boussarsar[1,2], A. Ammar[3], A. Ben Cheikh[3], H. Ghali[3], O. Ezzi[3], M. Mahjoub[3], N. Bouafia[3], M. Marzouk[4], J. Boukadida[4]

[1]Farhat Hached University Hospital, Medical Intensive Care Unit, Sousse, Tunisia; [2]Ibn Al Jazzar Faculty of Medicine, Research Laboratory N° LR14ES05 Interactions of the Cardio-Pulmonary System, Sousse, Tunisia; [3]Farhat Hached University Hospital, Hospital Hygiene Department, Sousse, Tunisia; [4]Farhat Hached University Hospital, Microbiology and Immunology Laboratory, Sousse, Tunisia

**Correspondence:** M. Boussarsar

*Intensive Care Medicine Experimental* 2017, **5(Suppl 2):**0995

**BACKGROUND.** Infections with antimicrobial-resistant bacteria (AMRB) oppose tremendous challenges in the spectrum of critically ill patients. These infections are associated with higher morbidity, mortality and healthcare costs.

**OBJECTIVES.** To assess the incidence of hospital acquired infections caused by AMRB in an 8-bed Tunisian MICU and to describe/detail the antibiotic resistance profiles of infecting pathogens.

To determine the independent risk factors of AMRB infections in the MICU.

**METHODS.** An 18 months prospective study from September 15$^{th}$,2015 to March 15$^{th}$,2017 were induced in the adult medical ICU of Farhat Hached University Hospital in Sousse-TUNISIA. All patients with more than 48H of ICU stay.All the resistance pattern were tested for all isolated bacteria. Univariate and multivariate analysis were performed to identify independent risk factors of AMRB infections. P values of less than 0.05 were considered as statistically significant.

**RESULTS.** From 258 eligible patients during the study, 40 developed 45 AMRB infections with a density incidence of 12.7/1000 patient-hospitalization-days. 24 (53%) of AMRBs were responsible for a Ventilator Acquired Pneumonia. The most frequent isolated microorganisms were: MDR *Acinetobacter baumannii* (only to colimycin and rifampicin susceptible) 33(73.33%), Extended-spectrum beta-lactamase producers 8(17.77%) represented essentially by *Klebsiella pneumoniae* and finally Methicillin resistant *Staphylococcus aureus* 4(8.88%).

Independent risk factors of AMRB acquired infections were the duration of sedation (OR = 1.15, $CI_{95\%}$ [1.05,1.27], p = 0.004) and the number of antibiotics received prior to the infection (OR = 1.6, $CI_{95\%}$ [1.01,2.52], p = 0.0046).

**CONCLUSION.** The study demonstrated the emerging trend of AMRB in our MICU with the peculiar presence of MDR *Acinetobacter baumannii.*

### 0996

#### Carbapenemase-producing enterobacteriaceae in the intesive care unit

M.I. Rubio Lopez, M. Lopez Sanchez, C.M. Blanco Huelga

Marqués de Valdecilla University Hospital, Santander, Spain

**Correspondence:** M.I. Rubio Lopez

*Intensive Care Medicine Experimental* 2017, **5(Suppl 2):**0996

**INTRODUCTION.** Carbapenemase-producing Enterobacteriaceae (CPE) are an important and increasing threat to public health worldwide.

**OBJECTIVES.** To describe intensive care unit (ICU) mortality, patient demographic data and infection type in patients with CPE isolates.

**METHODS.** Retrospective observational study between January 2016 to April 2017 in 30 beds ICU of a tertiary hospital center. Patient (p) and CPE isolates dates were collected included demographic data, underlying condition, associated infection, CPE colonization/infection, type infection, resistance to antibiotics, therapy, mortality and stay in ICU.

**RESULTS.** The first CPE isolates in our ICU was in February 2016. During study period 14 p infected and/or colonized by CPE were detected in our ICU (11 p acquired in ICU). 3 p presented rectal colonization by CPE without infection. All patients presented carbapenemase oxa-48 producing *Klebsiella pneumoniae* and 2 patients carbapenemase oxa-48 *Escherichia coli.*

Median age was 63,21 ± 12.86 years old and 71% were male. Median Charlson score was 3,93 ± 2,58 and median APACHE II was 20,30 ± 6.9. Systemic antibiotic exposure within 90 days before of isolate was documented in all patients. 85,7% of the patients were immunosuppressed. Underlying condition: liver transplant 1p, Fournier gangrene 2p, colorectal surgery 4p, hepatobiliary surgery 1p, meningoencephalitis 1p, gastroenteritis 1p, SDRA (H1N1) 2p, pneumoniae 2p. 6p presented several CPE infections. Site for infection included abdomen 6p (54,5%), lung 5p, ulcer 2p, surgical site 3p, bloodstream 2p, catheter 1p. 63,6% of patients associated fungal infection and 100% bacterial infection. All CPE was resistant to penicillin, cephalosporin, fluoroquinolones, ertapenem and gentamicin. Resistance to tigecycline was 57%, resistance to imipenem 35%, resistance to colistin 28%, resistance to meropenem 28%, resistance to cotrimoxazole 21%, resistance to amikacin 7%. Combined therapy was used in 9p and monotherapy in 1 p. Treatment was readjusted in 6p. Definitive therapy in survived patients was: ceftazidime/avibactam + colistin (1p), ceftazidime/avibactam + amikacin (1p), meropenem + amikacin (1p), meropenem + amikacin + colistin (2p). Mortality for patients with CPE infection was 54,5% (5 medical patients, 1 surgical patient). Stay in ICU was 28 days (IQR 1–75).

**CONCLUSIONS.** CPE isolates were carbapenemase oxa-48 producing *Klebsiella pneumoniae* and carbapenemase oxa-48 *Escherichia coli.* 57% patients with CPE were surgical patients. 85,7% of the patients were immunosuppressed. The most frequent CPE infections were abdominal infections. All patient with CPE isolates presented co-infections. CPE isolates showed less resistance to amikacin and ceftazidime-avibactam. CPE infections associate high mortality.

### 0997

#### Vancomycin-resistant enterococci (VRE) infections in a Tunisian medical ICU: a poor outcome

A. Jamoussi[1], H. Doghri[1], T. Merhebene[1], E. Mhiri[2], L. Slim[2], J. Ben Khelil[1], M. Besbes[1]

[1]Faculty of Medicine of Tunis, Medical ICU, Ariana, Tunisia; [2]Faculty of Medicine of Tunis, Microbiology Departement, Ariana, Tunisia

**Correspondence:** T. Merhebene

*Intensive Care Medicine Experimental* 2017, **5(Suppl 2):**0997

**INTRODUCTION.** Vancomycin-resistant enterococci (VRE) was first identified in the mid-1980s. Then it emerged as a significant cause of nosocomial infections in many intensive care units (ICU) across the world.

In our 22-bed medical ICU, cases of VRE infections were first identified in 2013.

**OBJECTIVES.** The aim of this study is to describe microbiological characteristics and outcomes of Vancomycin-Resistant Enterococci infections.

**METHODS.** Patients who developed VRE infections during their ICU stay (from January 2013 to march 2017) were included. We recorded demographic, therapeutic and evolutive outcomes.

**RESULTS.** During the study period (4 years and 3 months), 16 patients were included. The median age was of 55.5 years [26–82] with a sex-ratio of 2.2.

At admission, medium IGSII, Apache II and SOFA scores were respectively of 45, 17.68 and 6.6.

The median duration of hospitalization before VRE infection was of 17.5 days [2, 69]; the hole median of length of stay was 33 days [2,79].

The infection sites were bacteremia (n = 10), urinary tract infection (n = 5) and non-bacteremic catheter related infection (n = 1). Species isolated were E.faecium in all cases.

All patients received antibiotics before this infection episode among them 62.5% (n = 10) received 4 or more different antibiotics. The most often prescribed antibiotics were colimycine (n = 12, 75%), carbapenems (n = 11, 68.8%), amoxicillin-clavulanate (43.8%; n = 7) and glycopeptides (25%, n = 4).

Other nosocomial infections with multi-drug resistant bacteria preceding VRE infections were identified in 68.8% of patients (n = 11). These latter have all *Acinetobacter Baumanii* nosocomial infection before VRE's one.

Among all patients, 11(68.8%) received an effective antibiotherapy with Linezolid from whom only 4 patients survived. Concerning the 5 left patients, VRE identification results were received post-mortem.

In-hospital mortality was of 75% (n = 12).

**CONCLUSIONS.** Vancomycin-resistant enterococci infections are emerging and frequently occur after other nosocomial infections, especially *Acinetobacter Baumanii*.

Despite effective antibiotherapy, it is associated with high mortality.

### Poisoning, toxicology, pharmacology

#### 0998

#### Levamisole, a cocaine adulterant, impairs endothelium-dependent relaxation in the rabbit carotid artery

P. Marchio, S. Guerra-Ojeda, M. Gimeno-Raga, M.D. Mauricio, S.L. Valles, M. Aldasoro, A. Jorda, C. Aldasoro, J.M. Vila
University of Valencia, Department of Physiology, Valencia, Spain
**Correspondence:** P. Marchio
*Intensive Care Medicine Experimental* 2017, **5(Suppl 2):**0998

**INTRODUCTION.** Around 30% of emergency department visits for drug abuse are related to acute vascular complications of cocaine [1]. Levamisole, an antihelminthic drug limited to veterinary use, is currently used as a cocaine adulterant [2]. In *in vitro* studies, levamisole acts synergistically with cocaine, enhancing the sympathetic response [3], and potentiating the contractile response to endothelin-1 in rabbit carotid artery [4]. Therefore, levamisole could aggravate cocaine's actions on vascular tone. However, the effects of levamisole on relaxant response to acetylcholine (Ach) has not yet been evaluated.

**OBJECTIVES.** To evaluate the effects of levamisole on endothelium dependent and independent relaxant response in rabbit carotid artery.

**METHODS.** Rabbit carotid rings were mounted for isometric tension recording in organ baths. Concentration-response curves to Ach ($10^{-9}$ to $10^{-5}$M) were obtained in the absence and presence (30-minutes incubation) of cocaine ($10^{-5}$ and $10^{-4}$M), levamisole ($10^{-5}$ to $10^{-3}$M), and the combination of both drugs. In addition, Ach curves in the presence and absence of levamisole ($10^{-3}$M) were performed in the presence of indomethacin (Indo, $10^{-5}$M) and L-$N^G$-Nitroarginine methyl ester (L-NAME, $10^{-4}$M) to study prostanoids and NO-dependent vasorelaxation, and superoxide dismutase (SOD, 200 U/L) and ascorbic acid (AA, $10^{-3}$M) to evaluate free radicals production and scavenging. To evaluate independent vasorelaxation, curves of sodium nitroprusside (SNP, $10^{-10}$ to $3x10^{-6}$M) were performed in the presence and absence of cocaine $10^{-4}$M and levamisole $10^{-3}$M.

**RESULTS.** Neither cocaine nor levamisole $10^{-5}$M modified the relaxant response to Ach. Levamisole ($10^{-4}$ and $10^{-3}$M) significantly reduced the maximal relaxation (Emax) induced by Ach in a concentration-dependent manner (85 ± 5% for control, 71 ± 2% and 46 ± 3% for levamisole $10^{-4}$M and $10^{-3}$M, respectively). Cocaine plus levamisole did not potentiate the effects of levamisole. Reduced Ach response in the presence of levamisole ($10^{-3}$M) was not modified by Indo or L-NAME. Conversely, both SOD and AA partially prevented the effects induced by levamisole $10^{-3}$M in a similar manner (Emax 70 ± 7% for SOD and 77 ± 4% for AA, $p < 0.05$ compared to levamisole $10^{-3}$M). Neither cocaine nor levamisole modified the relaxant ?A3B2 show $132#?>response to SNP.

**CONCLUSIONS.** Levamisole impairs endothelium-dependent relaxation to acetylcholine in a concentration dependent manner. This effect is independent of prostanoids and NO, and is partially mediated by oxidative stress, which is reversed by ascorbic acid. Our results suggest that levamisole could worsen acute cardiovascular complications induced by cocaine. Moreover, ascorbic acid could be a promising adjuvant therapy in levamisole-adulterated cocaine abuse.

**REFERENCE(S)**

[1]Bachi K et al. Atherosclerosis. 2017 Mar 14.
[2]Brunt TM et al. Arch Toxicol. 2017 Mar 17.
[3] Hofmaier T et al. Neurochem Int. 2014 Jul;73:32–41.
[4] Marchio P et al. Critical Care 2017, 21(Suppl 1): P156.

#### 0999

#### 2,6-Diisopropylphenol (Propofol) promotes osteoclastic bone resorption by increasing DC-STAMP expression

E.J. Kim[1], J.Y. Yoon[1], S.W. Baik[2], S.W. Shin[2], J.U. Yoon[2], A. Cho[3], J.H. Cheon[2]
[1]Pusan National University Dental Hospital, Department of Anesthesia and Pain Medicine, Yangsan, Korea, Republic of; [2]Pusan National University Yangsan Hospital, Department of Anesthesia and Pain Medicine, Yangsan, Korea, Republic of; [3]Pusan National University Hospital, Department of Anesthesia and Pain Medicine, Pusan, Korea, Republic of
**Correspondence:** J.H. Cheon
*Intensive Care Medicine Experimental* 2017, **5(Suppl 2):**0999

**INTRODUCTION.** Bone remodeling involves the osteoclast-mediated bone resorption followed by the osteoblast-mediated bone formation. Disruption of bone remodeling can lead to pathologic state of bone structure and function. In a recent study, α-tocopherol increased osteoclastogenesis and osteoclast fusion and the molecular structure of α-tocopherol is similar to propofol.

**OBJECTIVES.** Propofol is a commonly used intravenous anesthetic and sedative agent, and the effect of propofol on bone remodeling can be critical to the patient who has pathological bone diseases. On the basis of previous studies, we investigated the effects of propofol on osteoclastogenesis and bone resorption, as well as signaling pathway of osteoclastogenic gene expression using bone marrow-derived macrophages (BMMs).

**METHODS.** BMMs were cultured with M-CSF alone or M-CSF plus RANKL in the presence of propofol (0 ~ 50 mM) for 4 days. Mature osteoclasts were stained for TRAP and the numbers of TRAP-positive multinucleated osteoclasts were measured. To examine the resorption activities of osteoclasts, bone resorption assay was conducted. To identify how propofol affect on the formation of multinucleated osteoclast, we focused on DC-STAMP, a protein essential for pre-osteoclastic cell fusion. The mRNA expression of DC-STAMP was analyzed by RT-PCR and quantitative real-time PCR.

**RESULTS.** Propofol increased the formation of TRAP-positive multinucleated osteoclasts and the number and size of osteoclasts in a dose-dependent manner.
In addition, bone resorption assay showed that propofol increased the bone resorption area on dentin discs.
The mRNA expression of DC-STAMP was upregulated most strongly in the presence of both RANKL and propofol. However, SB203580, p38 inhibitor, suppressed significantly the mRNA expression of DC-STAMP which was increased by propofol.
**CONCLUSIONS.** We have newly demonstrated that propofol enhanced osteoclast differentiation and maturation, as well as increased bone resorption subsequently. Additionally, we identified the regulatory pathway involving osteoclast cell-cell fusion, which was enhanced by propofol through p38 mediated DC-STAMP expression.

**REFERENCE(S)**

1. Karsenty G, Kronenberg HM, Settembre C. Genetic control of bone formation. Annu. Rev. Cell Dev. Biol. 2009; 25: 629–648.
2. Fujita K, Iwasaki M, Ochi H, Fukuda T, Ma C, Miyamoto T, Takitani K, Negishi-Koga T, Sunamura S, Kodama T, Takayanagi H, Tamai H, Kato S, Arai H, Shinomiya K, Itoh H, Okawa A, Takeda S. Vitamin E decreases bone mass by stimulating osteoclast fusion. Nat Med. 2012; 18(4): 589–94.
3. Yagi M, Miyamoto T, Sawatani Y, Iwamoto K, Hosogane N, Fujita N, Morita K, Ninomiya K, Suzuki T, Miyamoto K, Oike Y, Takeya M, Toyama Y, Suda T. DC-STAMP is essential for cell-cell fusion in osteoclasts and foreign body giant cells. J Exp Med 2005; 202(3): 345–51.

**GRANT ACKNOWLEDGMENT**
None

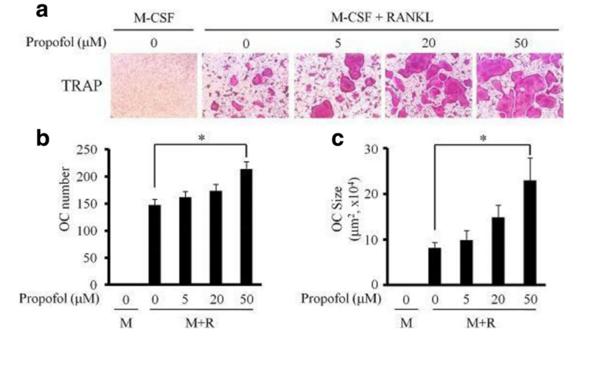

**Fig. 319 (Abstract 0999)** See text for description

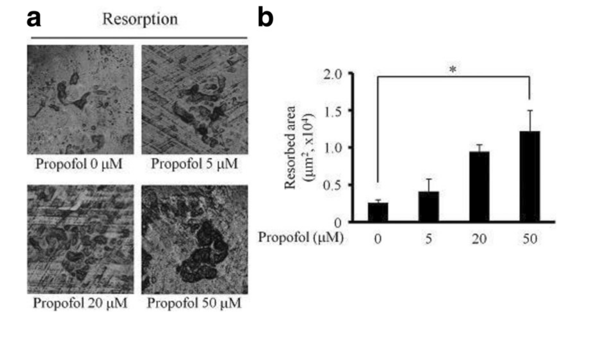

**Fig. 320 (Abstract 0999)** See text for description

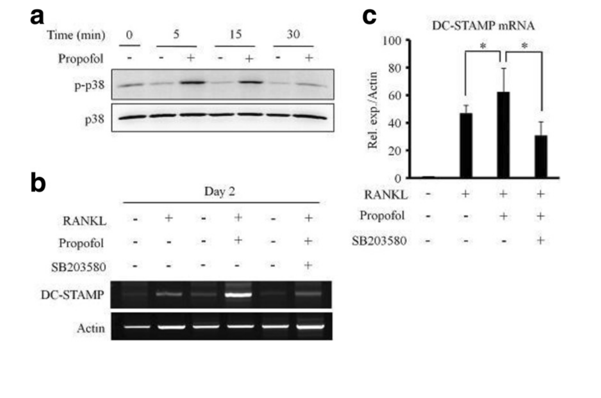

**Fig. 321 (Abstract 0999)** See text for description

### 1000

#### Metformin dose-dependently impairs excretion of the same drug in an isolated perfused porcine kidney model

R.A. Posma[1], L.H. Venema[2], D.N. van Heereveld[2], D.J. Touw[3], M.W. Nijsten[1], H.G. Leuvenink[2]
[1]University of Groningen, University Medical Center Groningen, Department of Critical Care, Groningen, Netherlands; [2]University of Groningen, University Medical Center Groningen, Department of Surgery, Groningen, Netherlands; [3]University of Groningen, University Medical Center Groningen, Department of Clinical Pharmacy & Pharmacology, Groningen, Netherlands
**Correspondence:** R.A. Posma
*Intensive Care Medicine Experimental* 2017, **5(Suppl 2):**1000

**INTRODUCTION.** Metformin is widely used as oral antihyperglycemic drug to treat patients with type 2 diabetes. It is presumed that partial inhibition of complex I of the mitochondria is causative for the pleiotropic effects of metformin.[1] The major mode of elimination is excretion of unchanged drug in urine.[2] Patients with impaired renal function are therefore at risk to accumulate metformin and develop lactic acidosis.[2] Renal elimination of metformin occurs primarily through active transport by the proximal tubules, requiring energy to function.[1] Since it is known that metformin inhibits mitochondria and a high density of mitochondria is present in the proximal tubule to transport metformin and other compounds, we hypothesize that toxic concentrations of metformin inhibit its own secretion.
**OBJECTIVES.** To determine whether addition of increasing metformin concentrations to an ex-vivo isolated perfused porcine kidney affects renal elimination of metformin.
**METHODS.** During four hours of normothermic oxygenated machine perfusion of six porcine kidneys with autologous blood, we studied whether gradually increasing the metformin concentration in the perfusion fluid affects metformin clearance. Creatinine was added to the perfusion fluid to estimate the glomerular filtration rate. Urine was collected throughout the experiment and metformin concentration was determined in both plasma and urine using liquid chromatography-tandem mass spectrometry analysis. Data are expressed as mean ± standard error of the mean.
**RESULTS.** For the first 90 minutes, metformin plasma concentration remained below 5 mg/L, which approximates the therapeutic range. Subsequently, the metformin plasma concentration increased every half hour with 38.1 ± 17.5 mg/L until a peak concentration of 271.2 ± 87.8 mg/L was reached at the end of the experiment, which is highly toxic. Creatinine clearance remained practically unchanged with a grand mean of 1.4 ± 0.2 ml/min/100g. Metformin clearance increased over time with a maximum of 20.2 ± 16.6 ml/min/100g at 90 minutes, and declined steeply thereafter (Fig. 322). Compared to six controls, no difference in fractional sodium excretion was found, suggesting that tubular function was not significantly affected. Oxygen consumption in the metformin group decreased after two hours, while oxygen consumption increased in controls within the same period.

**CONCLUSIONS.** Our experiments suggest that metformin elimination is impaired when an isolated perfused kidney is exposed to toxic concentrations of the same drug. Further studies are warranted to identify causative mechanisms behind this clinical important phenomenon.

[1]Foretz M, et al. Metformin: from mechanisms of action to therapies. Cell Metab. 2014;20(6):953–66.

[2]Lalau JD, et al. Metformin and other antidiabetic agents in renal failure patients. Kidney Int. 2015;87(2):308–22.

**GRANT ACKNOWLEDGMENT**

None.

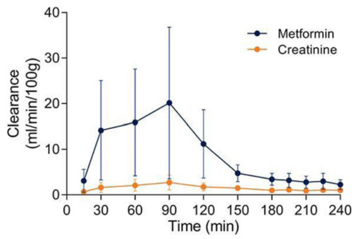

**Fig. 322 (Abstract 1000)** Metformin and creatinine clearance

### 1001

#### Exploring population pharmacokinetic models in patients treated with vancomycin during continuous venovenous haemodiafiltration (CVVHDF) on different anticoagulant modalities

M. Coyle[1], R. Munshi[2], H. O'Keeffe[3], E. Deasy[1], G.J. Fitzpatrick[4], P.J. Lavin[3], M. Donnelly[4], D.M. D'Arcy[2]

[1]Tallaght Hospital, Pharmacy Department, Dublin, Ireland; [2]Trinity College Dublin, School of Pharmacy and Pharmaceutical Sciences, Dublin, Ireland; [3]Tallaght Hospital, Trinity Health Kidney Centre, Dublin, Ireland; [4]Tallaght Hospital, Department of Anaesthesia & Critical Care, Dublin, Ireland

**Correspondence:** M. Coyle

*Intensive Care Medicine Experimental* 2017, **5(Suppl 2):**1001

**INTRODUCTION.** Uncertainty remains concerning pharmacokinetics (PK) of antimicrobials in critically ill patients due to the scarcity of data and the heterogeneity of the patient cohort.

**OBJECTIVES.** To describe the population PK estimates and the influence of patient covariates on PK of vancomycin, following intermittent infusion in critically ill patients receiving CVVHDF and on different anticoagulant modalities.

**METHODS.** Vancomycin dose and concentration data (peak, trough) were collected retrospectively from the electronic health records of 31 critically ill patients (n = 280 levels). A one-compartment model was used to describe the vancomycin concentration-time profiles, using Pmetrics software[1]. Model selection was based on the plots of observed vs. predicted concentrations, bias, imprecision, and reduction in −2*LL, AIC, BIC. For brevity, only $R^2$ values are presented here, from observed vs. population predicted concentration plots. Dosing intervals were classified according to the dialysis modality for the majority of the dosing interval i.e. 1) dialysis with citrate anticoagulation (60 levels) 2) dialysis with non-citrate anticoagulation (120 levels) or 3) not on dialysis (44 levels). Continuous covariates were: cumulative fluid balance, effluent flow rate, blood flow rate, body weight, albumin concentration and age.

**RESULTS.** An acceptable base model was produced using the Elimination Rate Constant (Ke) and Volume of Distribution (V) ($R^2$ 0.51). The mean ± SD of vancomycin PK estimates were: V 80.65 ± 22.65 L; Ke 0.03 ± 0.01 $h^{-1}$. The best population predicted models included cumulative fluid balance and albumin concentration as covariates ($R^2$ = 0.59 for both). Furthermore, the population model of the citrate anticoagulation group correlated best with the population predicted models as compared to the non-citrate dialysis and not on dialysis cohorts, with $R^2$ of 0.86, 0.58 and 0.28, respectively.

**CONCLUSION.** Cumulative fluid balance and albumin concentration data along with citrate anticoagulation status are suggested as covariates for further analysis with richer data, to optimise covariate PK modelling of vancomycin in ICU patients on CVVHDF, and support dose optimisation. The less variable PK estimates in the citrate group model suggest that vancomycin dosing while on citrate anti-coagulation might be more straightforward than in the other two categories.

1. Available for download from LAPK website at http://www.lapk.org

**GRANT ACKNOWLEDGMENT**

Faculty of pharmacy, Umm Al-Qura University, Saudi Arabia. (R Munshi)

### 1002

#### Effects of acute acetyl cholinesterase inhibition on thyroid hormonal status

C. Cobilinschi[1], R.C. Tincu[2], P.T. Neagu[3,4], I.F. Tincu[5], Z. Ghiorghiu[2], R.A. Macovei[2,6]

[1]Clinical Emergency Hospital Bucharest, Anesthesiology and Intensive Care, Bucharest, Romania; [2]Clinical Emergency Hospital Bucharest, Anesthesiology and Intensive Care II Toxicology, Bucharest, Romania; [3]Clinical Emergency Hospital Bucharest, Plastic Surgery and Microsurgery, Bucharest, Romania; [4]University of Medicine and Pharmacy Carol Davila, Plastic Surgery and Microsurgery, Bucharest, Romania; [5]Clinical Emergency Hospital for Children Grigore Alexandrescu, Pediatrics, Bucharest, Romania; [6]University of Medicine and Pharmacy Carol Davila, Clinical Pharmacology, Toxicology and Psychopharmacology, Bucharest, Romania

**Correspondence:** C. Cobilinschi

*Intensive Care Medicine Experimental* 2017, **5(Suppl 2):**1002

**INTRODUCTION.** Conditions like trauma, burns, sepsis or acute intoxications have significant effects on the endocrine status. Alterations of the pituitary-adrenal axis are long acknowledged but the hypothalamic-pituitary-thyroid axis may also be affected. The systemic response caused by an acute disease characterized by modifying the thyroid hormonal axis is known as "*sick euthyroid syndrome*". It has previously been established that there is a complex relationship between thyroid hormones and cholinergic function, thus an increase in the acetylcholine levels is expected to produce an alteration in the thyroid hormone status.

**OBJECTIVES.** The present study aims to observe the effects of acetyl cholinesterase inhibition on thyroid hormone status and gland tissue changes.

**METHODS.** We conducted a prospective experimental study on twenty adult male Wister rats. In the first step blood samples were collected in order to establish normal values for thyroid stimulating hormone(TSH), tri-iodothyronine(T3) and tetra-iodothyronine(T4). Secondly, 0.1mg/kg Clorpyrifos was administered by oral gavage to induce acetyl-cholinesterase inhibition. After developing cholinergic symptoms new blood samples were taken to determine the level of cholinesterase, TSH,T3 and T4. Hormone levels were quantitatively determined through ELISA tests and spectrophotometric method was used for cholinesterase. Thyroid gland was excised in eight rats for hystopathological examination.

**RESULTS.** The cholinesterase levels showed a major inhibition immediately after poisoning compared to standard levels in the control group, thus confirming the status of intoxication. Comparing to baseline values determined in the control group, we obtained a significant increase in T4 levels (*p = 0.01*) measured both at two hours and 48 hours after administration of the organophosphate in sample rats. Similarly, the T3 almost doubled its values 2 hours after poisoning (mean 4.2ng/ml *versus* 2.5ng/ml at baseline). Surprisingly, the TSH revealed an acute elevation with an afterward slow descending trend at 48 hours ($p = 0.1$), approaching initial control values. Beside serological significant changes in hormone levels, preliminary results observed in study rats indicate histopathological alterations of the thyroid tissue caused by organophosphate exposure.

**CONCLUSIONS.** The present study demonstrated that acetyl-cholinesterase inhibition caused major alterations in hormone levels. Inhibition of acetyl-cholinesterase is associated with alteration of thyroid response in subjects with no prior thyroid disease, causing the "*sick euthyroid syndrome*". This condition is often associated with a poor survival prognosis, despite adequate hormonal supplementation.

**REFERENCE(S)**

Warner, M. H. & Beckett, G. J. Mechanisms behind the non-thyroidal illness syndrome; Angelis, S. De *et al*. Developmental Exposure to Chlorpyrifos Induces Alterations in Thyroid and Thyroid Hormone Levels Without Other Toxicity Signs in Cd1 Mice. (2009)

### 1003

#### Interventions of pharmaceutical care unit in improving the pattern of use and decreasing the direct cost of two highly cost medications at intensive care units in a referral teaching hospital

A. Vazin[1], I. Karimzade[2], N. Hatami[2], M. Mahi Birjand[2]
[1]Shiraz University of Medical Sciences, Clinical Pharmacy, Shiraz, Iran, Islamic Republic of; [2]Shiraz University of Medical Sciences, Shiraz, Iran, Islamic Republic of
**Correspondence:** A. Vazin
*Intensive Care Medicine Experimental* 2017, **5(Suppl 2):**1003

**INTRODUCTION.** Irrational use of medicines is a global challenge at all levels of health care especially in Intensive Care Unit (ICU). Beside the high proportion of inappropriate use of human serum albumin (HSA) and intravenous (IV) pantoprazole, the elevated cost, have prompted interventions aiming to rationalize use of them.

**OBJECTIVES.** The aim of the present study was to evaluate the interventions of pharmaceutical care unit in improving the pattern of use and decreasing the direct cost of two highly cost medications at ICU wards in a referral teaching hospital in Iran.

**METHODS.** This prospective, interventional study was performed during 6 months in ICU wards in a referral teaching hospital. Indication checklist drafts of HSA and IV pantoprazole as two highly cost medications were prepared by clinical pharmacists.The head of different ICU departments were evaluated and approved these drafts. At the time of ordering these medications, the physician team was requested to fill out the checklist. The checklists were then examined by the trained pharmacists according to clinical and para-clinical conditions of the patients. The clinical pharmacist were authorized to reject the orders if there was no indication or rational for giving the studied medications. Six month time period was considered as pre-intervention phase.

**RESULTS.** Mean Acute Physiology and Chronic Health Evaluation (APACHE) III scores on ICU day 1 were not different. The implementation of guidelines resulted in reduction in HSA and IV pantoprazole use. The direct cost of HSA and IV pantoprazole decreased substantially (37.56% and 41.66% respectively). There was no significant length of ICU stay pre and post intervention.

**CONCLUSIONS.** Guideline implementation by pharmaceutical care unit associated with considerable decrease in the amount of use and direct cost of HSA and intravenous pantoprazole with no negative impact on ICU outcome in ICUs of a referral clinical setting in Iran.

**REFERENCE(S)**

1- Perner A, Junttila E, Haney M, Hreinsson K, Kvåle R, Vandvik PO, Møller MH; Scandinavian Society of Anaesthesiology and Intensive Care Medicine. Scandinavian clinical practice guideline on choice of fluid in resuscitation of critically ill patients with acute circulatory failure. Acta Anaesthesiol Scand. 2015 Mar;59(3):274–8
2- Caraceni P[1], Domenicali M, Tovoli A, Napoli L, Ricci CS, Tufoni M, Bernardi M. Clinical indications for the albumin use: still a controversial issue.Eur J Intern Med. 2013 Dec;24(8):721–8.
3- Tan B, Norman R, Litton E, Heath C, Hawkins DJ, Krishnamurthy R, Sonawane R, Anstey MH. Incidence and cost of stress ulcer prophylaxis after discharge from the intensive care unit: a retrospective study. Crit Care Resusc. 2016 Dec;18(4):270–274.

**GRANT ACKNOWLEDGMENT**
We would also like to thank staffs of pharmaceutical care unit and ICUs of Nemazee hospital for support to accomplish this study

### 1004

#### Acute tramadol intoxication in addicted patients

A. Ben Jazia, T. Khzouri, M. Fatnassi, N. Brahmi
Toxicological Intensive Care Unit Tunis, Tunis, Tunisia
**Correspondence:** A. Ben Jazia
*Intensive Care Medicine Experimental* 2017, **5(Suppl 2):**1004

**INTRODUCTION.** Tramadol is an opioid analgesic widely prescribed all over the world. Its toxicity is poorly documented in both international and local scientific litterature.

**OBJECTIVES.** We aimed to study the characteristics of tramadol poisoning in both suicidal attempt and addiction.

**METHODS.** It was a retrospective observational study spread over 84 months from January 2010 to December 2014 in a teaching toxicological center including tramadol poisoned patients.

**RESULTS.** Ninety four patients aged of 30 ± 10 years were eligible with an age range peak from 21 to 30.The prevalence of tramadol exposure has multiplied by three from2010 to 2013. In 67% of cases poisoning occured in suicidal attempt. Patients were 49 males and 45 females. 23% of them exhibited respiratory failure symptoms requiring ICU admission. 77% had moderate serotoninergic signs and were outpatients.The examination revealed that 42 patients (45%) were addicted to tramadol.In this addictive population, a history of psychiatric disease was noted in 14% of cases and addiction to another drug in 19%.Clinical exam showed drowsiness in 54%(n = 23) of cases, vomiting and nausea in 54%, epigastralgia in 24%.The main biological abnormalities were hyperglycemia(n = 3) hyperleukocytosis (n = 3) and respiratory acidosis (n = 2). Sixteen patients were admitted in ICU. Half of them (n = 8) required mechanical ventilation. Naloxone was used only in one case. All patients were discharged from ICU in a delay of 12 ± 6 hours.

**CONCLUSIONS.** Despite the favorable prognosis of tramadol poisoning unlike other opioid substances, it is imperative to maintain vigilance and take efficient measures such as awareness, shortening the prescription period and avoiding its prescription on special grounds as Health staff, psychiatric patients and other types of addiction.

### 1005

#### Clinical, toxicological and therapeutic characteristics of phenobarbital poisoning

T. Khzouri, A.Ben Jazia,M.Fatnassi,Y.Blel,N.Brahmi
Medical Intensive Care Unit, CAMU, Tunis, Tunisia
**Correspondence:** T. Khzouri
*Intensive Care Medicine Experimental* 2017, **5(Suppl 2):**1005

**INTRODUCTION.** Despite therapeutic progress in the management of the epilepsy, phenobarbital poisoning still constitute a health problem in developing countries, as a result of suicide attempt or anesthesia accident.Rarely,intoxication may occur in children or among drug addicts.

**OBJECTIVES.** We aimed to assess the frequency and the characteristics of phenobarbital poisoning.

**METHODS.** It was a descriptive retrospective study in a teaching toxicological center over a period of 295 months from January 2004 to July 2016. All patients admitted for phenobarbital poisoning were included.Diagnosis of phenobarbital poisoning was retained if supra -therapeutic phenobarbital ingestion was declared and confirmed by positive serum level.

**RESULTS.** One hundred and five patients aged of 25 years [12–68] were eligible. They were females in 65% (n = 68).A history of epilepsy was noted in 75% of cases.The frequency of phenobarbital poisoning was stable at 13% from 2008 to 2012, then decreased remarqbly to achieve 1% in 2015.The average supposed ingested dose was 1350 mg [350–5200]. The delay between ingestion and hospital arrival was 4 hours [1–48].At hospital arrival,clinical exam revealed drowsiness in 82%, agitation in 11%, coma in 6% and hemodynamic failure in 8%.Forty four patients (42%) required mechanical ventilation. Eleven patients (11%) presented inhalation pneumonia.Gastric lavage was performed in 10% of cases,activated charcoal was administrated in 23% of cases and alkaline diuresis in 9%.The mean phenobarbital serum level was of 57 ± 39.1 mg/L with a range of 2 to 214 mg/L. The average duration of mechanical ventilation was of 2,55 hours [1–30], and the hospital stay of 1.5 [0.5-18] days. All patients were discharged alive.
**CONCLUSIONS.** Through this study we demonstrate firstly that phenobarbital- poisoning must be seriously considered when it occurs. Secondary, that its frequency is decreasing in our country at the expense of the emergence of other anticonvulsivant drug poisoning.

### 1006

#### Baclofen poisoning: an epidemiological retrospective study in a Tunisian intensive care unit

A. Ben Jazia, M. Fatnassi, T. Khzouri, M. Khelfa, H. Fredj, N. Brahmi
Toxicological Intensive Care Unit Tunis, CAMU, Tunis, Tunisia
**Correspondence:** A. Ben Jazia
*Intensive Care Medicine Experimental* 2017, **5(Suppl 2):**1006

**INTRODUCTION.** Baclofen is commonly used as treatment of spasticity, and recently prescribed in treatment of alcohol withdrawal. It has spasmolytic action resulting from antagonistic activity at presynaptic GABA receptors of the spinal cord. Because it´s wide prescription, it became one of the principal agents of poisoning.
**OBJECTIVES.** We present a retrospective study that aims to report the clinical outcomes and management of twenty cases of baclofen poisoning requiring ICU admission.
**METHODS.** Retrospective review of medical observations of patients admitted to our intensive care unit for baclofen poisoning.
**RESULTS.** Between January 2013 and April 2017, 27 patients were enrolled in this study. Mean age was 27.2 ± 10 years. Sex ratio was 0.33. Mean APACHE II scale was 9 ± 4. Mean IGSII scale was 21 ± 11.Poisoning severity scale was moderate in six patients and severe in twenty one. Poisoning was deliberate in 100% of cases. Mean ingested dose was 458 ± 349 mg. The majority of patients presented to the emergency room at 4.8 ± 5 hours after ingestion. Digestive decontamination was performed in 7% (n = 2) of patients. Clinical presentation was dominated by neurological symptoms; including coma (n = 17), hypotonia (n = 5), areflexia (n = 7), agitation (n = 8), seizures (n = 3) and delirium in 1 case. Doses greater than 250 mg were predictive of coma (GCS $\leq$ 8) AUC = 0.763, with a sensibility of 80% and a specificity of 75%, Odds Ratio = 0.450 (0.150 1.349) IC 95%. All patients evolved favorably.
**CONCLUSIONS.** Baclofen overdose causes mainly neurological effects and except for bradycardia cardiovascular effects were uncommon. Doses greater than 250 mg were predictive of coma, requiring mechanical ventilation and long ICU admission. Prognosis is good if full supportive care is administered properly, except for the length of stay. Extracorporeal purification may be proposed even on patient with normal or limited glomerular filtration because baclofen pharmacological features.

### 1007

#### Drug related adverse events in activation of Inha University Hospital Rapid Response Team

J.H. Cho[1,2], J. Kim[1], J. Park[3], J.H. Jang[3], H. Cho[3]
[1]Inha University Hospital, Pulmonology and Critical Care Medicine, Incheon, Korea, Republic of; [2]Inha University, College of Medicine, Internal Medicine, Incheon, Korea, Republic of; [3]Inha University Hospital, Comprehensive Intensive Care Center, Incheon, Korea, Republic of
**Correspondence:** J.H. Cho
*Intensive Care Medicine Experimental* 2017, **5(Suppl 2):**1007

**INTRODUCTION.** Drug related adverse events are frequently encountered especially elderly patients.[1] Since 2015 October, Inha University Hospital Rapid Response Team (INHART) had been activated. During reviewing cases, INHART activation due to drugs were collected and analyzed.
**OBJECTIVES.** We analyzed drugs relate events that activated INHART and outcomes.
**METHODS.** From 2016-Jan to 2017-Apr, data were reviewed retrospectively. Demographic data, causes of activation, interventions and immediate outcomes were collected.
**RESULTS.** In those periods, 17,404 cases were activated by calling and screening. Sixteen events (0.09%) were considered as drug related adverse reactions. Male were 8, mean age was 71 (SD, ±15) years old. Ten patients were received operation such as orthopedics and two were sleeping endoscopy. Signs of INHART activation were hypoxemia (13 cases), mental change (2 cases) and respiratory arrest (1 case). Drugs were sedatives and opioids. Six patients were performed intubation and two were cardiopulmonary resuscitation. Others received oxygen therapy. Two patients with malignancy died.
**CONCLUSIONS.** Though low incidence of drug related adverse events were noticed, the sedatives and opioids should be used in caution. Monitoring oxygen saturation and mental status should be checked continuously.
**REFERENCE(S)**
[1] BMJ 2000;320:741–4
**GRANT ACKNOWLEDGMENT**
This work is supported by Inha University Research Grant.

### 1008

#### Epidemiological profile of acute poisoning during pregnancy

M. Fatnassi, T. Khzouri, A. Benjazia, I. Sedghiani, M. Trabelsi, N. Brahmi
Tunisian Medical University, CAMU, Tunis, Tunisia
**Correspondence:** M. Fatnassi
*Intensive Care Medicine Experimental* 2017, **5(Suppl 2):**1008

**INTRODUCTION.** Acute poisonings during pregnancy represent a therapeutic challenge to health care providers because of potential immediate life threat and possible involvements for both the mother and fetus.
**OBJECTIVES.** Our study aims to describe the epidemiological profile of acute poisonings in pregnant women and evaluate their short-term effects on the fetus.
**METHODS.** It is a retrospective study carried out in a teaching polyvalent and toxicological intensive care unit (ICU) including all poisoned pregnant women from January 2008 to December 2016.
**RESULTS.** During the study period, 57 patients aged of 28 ± 5 among 9618 admissions were eligible with a prevalence of 0.6%.The poisoning was accidental in 66.66%(n = 38) of the cases. Patients were married in 96% (n = 55) and unemployed in 47% (n = 27) of the cases. Carbon monoxide (CO) was incriminated in 66.66% (n = 38), medical drugs in 21% (n = 12) and pesticides in 7% (n = 7). 38.5% (n = 22) of poisoning occurred in the first trimester. The clinical status was generally good with resolutive symptoms. Two cases of pelvic pain and metrorrhagia were reported as well as 3 cases of active fetal movements change. Treatment was symptomatic; activated charcoal was administered to 2 women and gastric lavage was performed in 3 cases, 10 patients received antidotes and 11 women among 38 have benefitted from hyperbaric oxygen therapy. One case of maternal death was reported. Fetal evolution was marked by 3 fetal deaths in utero and 2 threats of abortion. The long-term evolution was marked by the occurrence of one pregnancy interruption, one death in infancy and 3 cases of health problems (sigmoid kidney, heart tumors and strabismus).
**CONCLUSIONS.** Most cases of acute poisoning during pregnancy were accidental due to the carbon monoxide exposure. In addition to the maternal risks associated with the poisoning, fetal complications may be serious.

### 1009

#### Acetaminophen inhibits complex I-related mitochondrial function in human platelets and hepatocytes

S. Piel[1,2], E. Åsander Frostner[1,2], F. Sjövall[1,3], E. Elmér[1,2], M.J. Hansson[1,2]
[1]Lund University, Mitochondrial Medicine, Lund, Sweden; [2]NeuroVive Pharmaceutical, Lund, Sweden; [3]Skåne University Hospital, Intensive Care and Perioperative Medicine, Malmö, Sweden
**Correspondence:** S. Piel
*Intensive Care Medicine Experimental* 2017, **5(Suppl 2):**1009

**INTRODUCTION.** Although safe at therapeutic levels, acetaminophen (APAP) has been associated with hepatotoxicity at high doses, and is the leading cause for acute liver failure in the Western World. Drug-induced oxidative stress exceeds the cell's antioxidant system, leaving it vulnerable for oxidative damage. The role of mitochondrial injury in this conditions remains unclear. [1] Currently, the only available treatment is N-acetylcysteine whose effectiveness depends on early administration with the aim to prevent liver damage rather than rescue. Therefore, alternative treatments, especially for late-stage presenting APAP overdose, are needed. [2]
**OBJECTIVES.** We evaluated the effect of APAP on mitochondrial function in human blood cells and hepatocytes to elucidate the role of mitochondria in APAP-induced hepatotoxicity and, potentially, find alternative treatment strategies.
**METHODS.** Human platelets were isolated from healthy donors [3] and HepG2 cells were cultured in MEM supplemented with FBS, NEAA, glutamine, penicillin and streptomycin at 37°C and 5% $CO_2$. Oxygen consumption was evaluated in the presence of APAP and different protocols were applied to study mitochondrial function. In permeabilized cells a Substrate-Uncoupler-Inhibitor-Titration (SUIT) protocol [3] was used to identify the site of mitochondrial damage. The cell-permeable succinate prodrug NV241 [4] was used as diagnostic tool to assess mitochondrial complex II function in intact cells.
**RESULTS.** APAP inhibited complex I-related oxygen consumption of intact human platelets and HepG2 cells in a dose-dependent manner. When the cell-permeable succinate prodrug NV241 was given to intact cells complex II-related oxygen consumption reached levels as those seen in controls (Fig. 323.). It was possible to exclude complex II as site of drug-induced toxicity pointing towards direct or indirect inhibition of mitochondrial complex I-related function by APAP. The SUIT protocol applied on permeabilized cells further confirmed this finding.
**CONCLUSIONS.** APAP induced mitochondrial dysfunction through complex I in both human platelets and hepatocytes. Bypassing APAP-induced complex I dysfunction using cell-permeable succinate prodrugs, such as NV241, presents an alternative treatment strategy for APAP-induced mitochondrial dysfunction and, potentially, related hepatotoxicity.

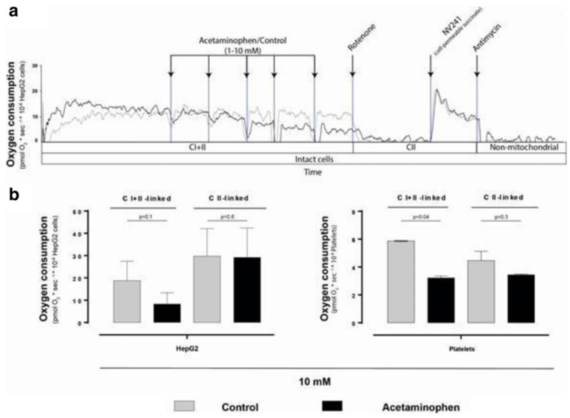

**Fig. 323 (Abstract 1009)** See text for description

### 1010

#### Metformin Associated Lactic Acidosis (MALA) - analysis of patients admitted in the Intensive care unit

N. Zarcos Palma, D. Pereira Anjos, M. da Cruz, F. Santos, R. Lopes, M.J. Fernandes, E. Lafuente
Centro Hospitalar do Tâmega e Sousa, Penafiel, Portugal
**Correspondence:** N. Zarcos Palma
*Intensive Care Medicine Experimental* 2017, **5(Suppl 2):**1010

**INTRODUCTION.** Metformin is widely used in the management of patients with type 2 diabetes. MALA is the most dangerous side effect, characterized by lactic acidosis occurring in patients chronically taking metformin or in overdose with metformin. The literature assigns an incidence of 1 to 5 per 100,000 patient/years and mortality up to 50%.
**OBJECTIVES.** To evaluate MALA's cases and to define the indicators of greater prognostic weight.
**METHODS.** Retrospective analysis of patients with MALA, between 2011 and 2017, using the PICIS computer database. In addition to the demographics and severity indicators at admission and at 24 hours, we compared the deceased and surviving patients trying to find prognostic markers and mortality markers.
**RESULTS.** Twenty-three patients were included and there was an increase in incidence per year with a predominance of females (73.9%). The average age was 68.5 ± 9.4 years, and SAPS II was 63.8 ± 21.6. Of the included patients, 82.6% were hypertensive, 65.2% dyslipidemic, 39.1% were obese and 43.4% were cardiac insufficient. The values at admission and at 24 hours were respectively: pH 7.05 ± 0.2 and 7.4 ± 0.1; lactate levels 11.6 ± 5.7 and 7.5 ± 5.8 mmol/l; creatinine 6.8 ± 4.6 and 2.3 ± 1.2 mg/dl; prothrombin time (TP) 17.8 ± 11.1 and 16.7 ± 5.6sec. The average time of hospitalization was 4.4 ± 2.7 days. Renal replacement therapy with bicarbonate was performed in 95.7% of the patients, with a mean duration of 39.3 ± 9.9 hours and an effective effluent dose of 34.5 ± 4.1 ml/kg/h. There was a verified correlation with mortality for lactate persistence and TP at 24h (odds 1.14 95% CI:ROC 0.800/odds 1.547 95% CI:ROC 0.952) respectively. Comparing deceased and survivors, we found statistical significance for age (p0.001), SAPS II (p0.04) and TP at 24h (p0.001). Mortality was 34.8%.

**CONCLUSIONS.** There was an increase of patients with MALA per year. Hemodialysis initiated on admission appears to be an effective therapy. The persistence of high TP at 24 hours showed a relation with mortality. The reduced number of the sample and the non-dosing of metformin were the main limitations.

S. Olivera-Gonz´aleza, et all: Hepatotoxicidad por metformina. Med Intensiva. 2010;34(7):483–487.

J. ORTEGA CARNICER, A. AMBRÓS CHECA, et all: Sobredosis de metformina secundaria a insuficiencia renal aguda. A propósito de 6 observaciones. Med Intensiva. 2007;31(9):521–5

**GRANT ACKNOWLEDGMENT**

Thanks to the intensive medicine service and the Hospital for the possibility of unveiling this analysis.

### 1011

#### Pharmacokinetics and pharmacodynamics of paracetamol in critical care patients: an observational study

R. Howarth, A. Herbert, J. Baldwin, S. Laha
Lancashire Teaching Hospitals NHS Foundation Trust, Preston, United Kingdom
**Correspondence:** R. Howarth
*Intensive Care Medicine Experimental* 2017, **5(Suppl 2):**1011

**INTRODUCTION.** Paracetamol is commonly used in critically ill patients for both its analgesic and antipyretic properties, however the understanding of its pharmacokinetics in this population is limited, meaning current dosing regimens may be insufficient to achieve a therapeutic effect [1,2]. In healthy patients at the end of a 15-minute infusion of 1g IV paracetamol, the maximal plasma concentration is approximately 30 mg/L. Analgesic effect commences within 5 minutes of infusion, peaks within one hour, and lasts 4–6 hours. Antipyrexial activity typically reduces fever within thirty minutes, and lasts around six hours. Plasma concentrations of paracetamol between 10-20mg/L have been shown to produce antipyretic effect but the concentrations to produce analgesia are not well defined [3].

**OBJECTIVES.** We aimed to determine whether paracetamol pharmacokinetics and pharmacodynamics in critically ill patients differs from the currently accepted mechanisms.

**METHODS.** This prospective study was performed in a mixed medical, surgical, and neurosciences critical care unit. Patients receiving the maximum dose of intravenous paracetamol per 24-hour period were recruited. Serum paracetamol levels were measured prior to administration of IV paracetamol, and then at 15 (end of infusion), 30, 60, 120, and 180 minutes post-infusion. Levels were taken again once daily on each of the following two days. Temperature, blood pressure, C-reactive protein level and neutrophil count, were also recorded, along with reason for critical care admission, APACHE II and ICNARC scores.

**RESULTS.** Following intravenous paracetamol administration in critically ill patients, therapeutic and peak levels in plasma are reached within 15 minutes of administration, but remain therapeutic only for the first hour following administration. Plasma levels of paracetamol have been shown to be sub-therapeutic by 120 minutes post-infusion in 80% of study participants.

**CONCLUSIONS.** The pharmacokinetics of IV paracetamol appear to be disturbed in critically ill patients, questioning its utility as an analgesic and antipyretic agent in this population when used in its current form. The results of this study will be used to guide further research into the understanding of pharmacokinetics and pharmacodynamics of paracetamol in critically ill patients.

### 1012

#### Design and synthesis of a Salbutamol-boron compound and the effect on smooth muscle as a possible therapeutic option in obstructive respiratory diseases

M.A. Amezcua Gutierrez[1], S.E. Zamora Gómez[1], M.A. Soriano Ursúa[2], L.A. Gorordo Delsol[1], M.E. Zuñiga Serrano[3], J.O. Montoya Rojo[1], J. Garduño López[1], E.S. Cruz Montesinos[1], I.M. Lima Lucero[1], M. Hernández Romero[1], M.A. Carrasco Flores[1], M.A. Toledo Rivera[1], G.D. Hernández López[1]
[1]Hospital Juárez de México, Intensive Care Unit, Mexico, Mexico; [2]Instituto Politécnico Nacional, Investigation Division, Mexico, Mexico; [3]Instituto Mexicano del Seguro Social, Emergency Deparment, Mexico, Mexico
**Correspondence:** M.A. Amezcua Gutierrez
*Intensive Care Medicine Experimental* 2017, **5(Suppl 2):**1012

**INTRODUCTION.** Chronic Obstructive Pulmonary Disease (CPOD) is an important cause of morbidity and mortality in critical ill, being a common reason for intensive care unit (ICU) admission. At present, there is no cure for COPD, sintomatic farmacologic treatmen is based in b2 adrenergic agonists, however, there is a loss of efficacy after frequent use due to reduced number of b2 adrenergic receptors and the induction of increased heart rate. The design of selective ligands, both agonists and antagonists to b2 adrenergic receptor (β2AR) is a topic of interest.

**OBJECTIVES.** Evaluate the interaction with adrenergic b2receptor and relaxant effect on smooth muscle of a designed and synthesized salbutamol-boron compound.

**METHODS.** We performed the design of β2AR related compounds, evaluating the effect of some substituents added to pharmacophore group. The structures were evaluated according to their affinity with three-dimensional models of β2AR. This assessment was performed using ligand-protein docking methodology, as well as to validate the estimating procedure and affinity interactions to the receptor. Three compounds were synthesized, in order to explore the effect of chemical structural changes on biological activity. M compound was obtained with adequate purity to be evaluated in its capability to relax smooth muscle in the guinea pig trachea rings.

**RESULTS.** The M compound is 32.26 times more potent than salbutamol for generating a smooth muscle relaxant effect. ICI 118,551 behaves as a competitive antagonist of the relaxant effect of the compound M, suggesting that the relaxation was mediated by interaction with the β2AR. Additionally, the acute toxicity profile of the M compound in CD1 mice, showed similar or lower toxicity than other reported arylethanolamines, and Raman spectroscopy studies showed a lower income to mice's central nervous system compared with boric acid in equimolar concentrations.

**CONCLUSIONS.** The presence of boron and exposed hydroxyls of M compounds increased their affinity with b2AR (as estimated in silico). Thus, the boron substituent allows interaction with the hydroxyl groups of the 203, 204 and 207 serines of the receptor, promoting their activation and resulting in a more potent compound than salbutamol. Further studies are necessary to determinate the pharmacodynamic and pharmacokinetic characterization of M compound, to elucidate other mechanisms that may be involved in the relaxing effect of this compound.

**REFERENCE(S)**

1. Amezcua-Gutiérrez MA, Ciprés-Flores FJ, Trujillo-Ferrara JG, Soriano-Ursúa MA. A clinical linkage of recent advance in structural biology of beta2 adrenoceptor. Current Drug Target 2012; 13(10); 1336–46.

2. Woodruff PG, Agusti A, Roche N, Singh D, Martinez FJ. Current concepts in targeting chronic obstructive pulmonary disease pharmacotherapy: making progress towards personalised management. Lancet 2015;385(9979):1789–98.

### Challenges in the immunocompromised patient

#### 1013

#### Do admission triage criteria predict 28th-day mortality in critically-ill cancer patients?

T. Heziyev[1], B. Halacli[1], O. Diker[2], E. Ortac Ersoy[1], S. Ocal[1], A. Topeli[1]
[1]Hacettepe University Faculty of Medicine, Medical Intensive Care Unit, Ankara, Turkey; [2]Hacettepe University Faculty of Medicine, Medical Oncology, Ankara, Turkey
**Correspondence:** A. Topeli
*Intensive Care Medicine Experimental* 2017, **5(Suppl 2):**1013

**INTRODUCTION.** Cancer patients requiring intensive care unit (ICU) admission are increasing. However, need for optimal use of ICU resources, mandates employement of appropriate triage criteria and use of prognostic factors especially in critically-ill cancer patients (CICP).
**OBJECTIVES.** To determine whether classification of CICP according to admission triage criteria as "full-code" (FC), "ICU-trial" (ICU-T) and "no-indication" (NI) predicts 28th-day mortality and to determine factors affecting 28th-day mortality.
**METHODS.** CICP admitted to an 8-bed oncology-ICU of a university hospital between 1.10.2013-31.3.2016 were recruited, retrospectively. Patients were classified to FC, ICU-T and NI groups together with a medical oncologist, according to time of cancer diagnosis, current disease status, current treatment plan, anticipation of complications, pallative care and so forth, as proposed by Azoulay *et al.* (reference). Comparisons were done between 3 groups and between survivors and non-survivors. Kaplan-Meier survival analysis was performed and logistic regression analysis was done to determine independent predictors of 28th-day mortality.
**RESULTS.** Out of 306 CICP, 181 (59.2%) were classified in FC, 46 (15.0%) in ICU-T and 79 (25.8%) in NI groups. There were no difference between groups in terms of age, gender, APACHE-II and SOFA score. Charlson co-morbidity and ECOG performance scores were worst in NI group (p = 0.0001 for both). More patients in the NI group had solid type of cancer, progressive disease and metastatic cancer (p = 0.0001 for all). Among patients, 28th-day mortality rate was 37.6%, 52.2% and 64.6%, respectively (p = 0.0001). Mortality rate was highest in NI group and lowest in FC group according to Kaplan-Meier survival analysis (log rank test, p = 0.049). In logistic regression analysis, NI admission criteria was an independent risk factor for 28th-day mortality as compared to FC criteria (OR (95% CI): 2.57 (1.38-4.78); p = 0.003). Other factors affecting mortality were a high admission SOFA score (for each 1 point increase OR (95% CI): 1.12 (1.03-1.21); p = 0.006), having a solid cancer as compared to hematologic cancer (2.02 (1.11-3.70); p = 0.022) and an increased admission lactate level (1.15 (1.02-1.30); p = 0.022). Hospital mortality rate was 59.1%, 76.1% and 78.5%, respectively (p = 0.004).
**CONCLUSIONS.** Critically-ill cancer patients with no-indication for admission to the ICU had the worst 28th-day mortality, having a solid cancer, a high SOFA score and high admission lactate level being other factors affecting mortality. Therefore, a simple admission triage criteria as "full-code", "ICU-trial" and "no-indication" might be used to predict short term survival in CICP.
**REFERENCE(S)**
Azoulay *et al*. Intensive care of the cancer patient: recent achievements and remaining challenges. Annals of Intensive Care 2011;1:5.

#### 1014

#### Neurologic complications of HIV/AIDS in critical care: epidemiological profile of a Colombian cohort

F.J. Molina[1], S. Penagos[2], N. Zapata[2], J.P. Villa[2], C. Rendón[1], M. González[2], J.P. Aristizábal[2], L.M. Echeverri[2], L. Durango[2], C. Galeano[2], S. Zapata[2], J. Cardona[2], S. Rivera[2], C. Agudelo[3], A. Hidrón[4], Infectious diseases in ICU
[1]University Pontificia Bolivariana, University Clinic Bolivariana, Intensive Care, Medellín, Colombia; [2]University Pontificia Bolivariana, Medellín, Colombia; [3]University Pontificia Bolivariana, Infectious Diseases, Medellín, Colombia; [4]Hospital Pablo Tobón Uribe, Infectious Diseases, Medellín, Colombia
**Correspondence:** F.J. Molina
*Intensive Care Medicine Experimental* 2017, **5(Suppl 2):**1014

**INTRODUCTION.** Neurologic complications of human immunodeficiency virus (HIV) infection remain common, despite effective antiretroviral treatment (HAART). Neurologic manifestations may be due opportunistic infection,inmune reconstitution,or the virus itself. In lower and middle-income countries, where widespread access to HAART is still an unaccomplished goal, little information exists in the literature on survival trends in neurologic complications of HIV-infected patients admitted to ICU.
**OBJECTIVES.** We describe the epidemiologic profile, outcomes and mortality predictors of HIV-infected patients admitted with neurologic complications to ICU in Medellín, Colombia.
**METHODS.** This is a substudy of a clinical, retrospective study of HIV-infected patients, ≥18 years old, admitted for more than 24 hours to any of 5 different ICUs in Medellin, Colombia from January 1, 2008 through December 31, 2014. A descriptive analysis was performed. Variables associated with mortality and those with biologic plausibility were entered in a logistic regression model constructed in order to identify independent factors associated with mortality.
**RESULTS.** A total of 409 patients, 183 (44,7%) were identified with neurologic complications. It is the second reason for admission to ICU. The main cause is respiratory failure. In bivariate analysis of neurological variables, predictors of hospital mortality were: Toxoplasmosis (p 0,039) and Cryptococcus (p 0,0162). In the multivariate analysis controlling for age, gender, CD4 count < 350 cells/μL, HAART in the last 6 months, admission for opportunistic infection and APACHE-II score at ICU admission,independent predictors of hospital mortality were:vasopressor support (RR 4,5; 95%CI 2,19-9,2) and invasive mechanical ventilation (RR 2,7; 95%CI 1,12-6,5).

| | |
|---|---|
| Age (years) median (IQR) | 37 (19–64) |
| HIV diagnosis on admission (%) | 57 (31%) |
| CD4 count (cells/ul) median | 129 |
| HAART before admission (%) | 57 (31,1%) |
| APACHE median (IQR) | 18 (3–35) |
| SDRA n (%) | 66 (36,1%) |
| mechanic ventilation n (%) | 130 (71%) |
| renal failure n (%) | 59 (32,2%) |
| Catecholamines n (%) | 93 (50,8%) |
| Mortality n (%) | 102 (55,7%) |

*[Characteristics of patients with neurologic compli]*

| | |
|---|---|
| Occupant space injury n (%) | 89 (48,6%) |
| Meningitis n (%) | 76 (41,5%) |
| Neurovascular compromiso n (%) | 10 (5,5%) |
| Associated dementia n (%) | 8 (4,4%) |

*[Etiology of central nervous system involvement]*

| Variable | RR | 95% CI |
|---|---|---|
| Catecholamines | 4,5 | 2,19 - 9,23 |
| mechanic ventilation | 2,71 | 1,12 - 6,55 |

*[Multivariate analysis of characteristics associate]*

**CONCLUSIONS.** Neurologic complications are the second reason for admission to ICU. The main causes of central nervous system involvement are occupant space injury and meningitis. Toxoplasmosis and cryptococcus are associated with mortality. Mortality in our cohort remains extremely high
**REFERENCES**
1. Matinella A, Lanzafame M, Bonometti MA, Gajofatto A, Concia E, Vento S, Monaco S, Ferrari S. Neurological complications of HIV infection in pre-HAART and HAART era: a retrospective study. J Neurol. 2015 May;262 (5):1317–27
2. Akgün KM, Pisani M, Crothers K. The Changing Epidemiology of HIV-Infected Patients in the Intensive Care Unit. J Intensive Care Med 2011;26:151–164

3. Akgün KM, Miller RF. Critical Care in Human Immunodeficiency Virus-Infected Patients. Semin Respir Crit Care Med. 2016 Apr;37(2):303–17

**GRANT ACKNOWLEDGMENT**

Pontificia University Bolivariana.

### 1015

#### Should the patients with cancer be admitted to the ICU? A results of a survey involving intensivists and oncologists

A.-S. Moreau, N. Bertrand, A. Escande, S. Javed, C. Desauw, S. Nseir

CHU de Lille, Lille, France

**Correspondence:** A.-S. Moreau

*Intensive Care Medicine Experimental* 2017, **5(Suppl 2):**1015

**INTRODUCTION.** The admission incidence of patients with cancer to the intensive care units (ICU) is increasing. New targeted anticancer therapies have changed the prognosis of many cancer patients with new outcomes but also new adverse events possibly leading to ICU admission. However oncological prognosis remains poorly known from intensivists and on the other hand, oncologists are often not fully aware of the modalities and consequences of a long stay in the ICU. Two points of view may indeed be in opposition.

To investigate this point, we performed an observational study, comparing the answer of both oncologists and intensivists to a questionary regarding different oncological and acute illness conditions.

**METHODS.** Four clinical cases of oncological patients were presented, and for each of them, two acute clinical conditions requiring an ICU admission (one reversible illness and one less reversible). Oncologist and intensivists from 4 university hospitals in France were asked if ICU admission was relevant according to the oncological status and the acute clinical condition. When ICU admission was considered, the ICU therapeutic plan was asked (full code or ICU trial, as previously published (1).

**RESULTS.** 121 physicians completed the survey, 70 intensivists and 51 oncologists. Median experience in both groups was the same (7 years [0.5-34] versus 5.3 years [0.5-35]). Apart from the first clinical case (young women with breast cancer at diagnosis with 100% of intensivists and oncologists admitting her to the ICU, whatever the acute clinical condition was), the rate of transfer to the ICU increased each time a clinical acute situation was described ($p < 0.001$), meaning the only oncological status was not enough to decide. The results are described in Table 244.

There was a no significant difference regarding the rate of transfer to the ICU between intensivists and oncologists in all but one case (clinical case 2). In this last case, the difference between oncologists and intensivists disappeared once the acute complication was described. However, even though intensivists agreed to take the patient in charge, they more often decided to introduce therapeutic limitations, than oncologists.

**CONCLUSIONS.** Our study shows that the only oncological status was not enough to make a decision to transfer or not, unless in extreme good prognosis case or in case of terminal oncological disease. The reversibility of the acute illness should be carefully evaluated by intensivist and oncologist together in order to define the better ICU care plan, particularly regarding the ICU care with therapeutic limitations. Both specialties recognize a need for training, especially in the context of new therapeutic agents.

**REFERENCE(S)**

1. Azoulay E, Soares M, Darmon M, et al. (2011) Intensive care of the cancer patient: recent achievements and remaining challenges. Ann Intensive Care

**Table 244 (Abstract 1015)**. Comparison of admission to the ICU rates according

| | Baseline, n (%) | | P | Baseline, n (%) | | P | Baseline, n (%) | | P |
|---|---|---|---|---|---|---|---|---|---|
| | Intensivist | Oncologist | | Intensivist | Oncologist | | Intensivist | Oncologist | |
| Clinical case #1 | 120 (100 %) | | | 121 (100%) | | | 121 (100%) | | |
| | 70 (100%) | 50 (100%) | NS | 70 (100%) | 51 (100%) | NS | 70 (100%) | 51 (100%) | NS |
| Clinical case #2 | 31 (26%) | | | 83 (68 %) | | <0,001 | 75 (61 %) | | 0.001 |
| | 11 (16%) | 20 (41%) | 0.003 | 47 (67%) | 36 (71%) | 0,68 | 40 (57%) | 35 (69%) | 0.2 |
| Clinical case #3 | 38 (32%) | | | 94 (78 %) | | <0,001 | 70 (58 %) | | <0.001 |
| | 21 (31%) | 17 (35 %) | 0,7 | 53 (76%) | 41 (80%) | 0,54 | 42 (60%) | 28 (55%) | 0.57 |
| Clinical case #4 | 2 (2 %) | | | 24 (20 %) | | <0,001 | 60 (50 %) | | <0.001 |
| | 1 (1 %) | 1 (2 %) | 0.38 | 11 (16%) | 13 (25%) | 0.18 | 29 (41 %) | 31 (61%) | 0,004 |

### 1016

#### Critically ill patients with hemoglobinopathies: clinical features, management and outcome

S. Zayet, A. Jamoussi, T. Merhebene, S. Ayed, J. Ben Khelil, M. Besbes

Faculty of Medicine of Tunis, Medical ICU, Ariana, Tunisia

**Correspondence:** T. Merhebene

*Intensive Care Medicine Experimental* 2017, **5(Suppl 2):**1016

**INTRODUCTION.** Epidemiologic data about hemoglobinopathies in Tunisia reported a prevalence of 4.48% reaching 12.50% in some focus regions (1). Progression of such diseases, especially sickle cell disease, could be complicated; that's how intensivists could be involved in the management of acute complications (2) considered as risk factors for early death (3).

**OBJECTIVES.** We aim to describe the clinical manifestations, management of complications and outcome of sickle cell disease and thalassemia in intensive care unit (ICU).

**METHODS.** We conducted a retrospective and descriptive study, including patients with a history of hemoglobinopathies hospitalized in a 22-bed medical ICU of a teaching hospital in Tunisia. The study duration was of 11 years and 3 months, from January 2006 to March 2017.

**RESULTS.** During this study, 32 patients were included (Sickle cell disease (n = 26),drepano-thalassemia (n = 4) and thalassemia (n = 2)). The average age was 28.9 years ± 8.46 [9–47 years] with a male predominance (sex-ratio = 2.55). The mean of APACH II score was 16.7. Hemoglobinopathies were diagnosed at the mean age of 7.9 years ± 7.46 [1–25 years] with a regular follow up in 84.4%. A family history of hemoglobinopathy was found in 10 cases. 13 patients underwent cheolcystectomy. The delay before ICU referral was of 184 hours [24–720 hours]. The reason for admission was acute respiratory failure in 30 cases (93.8%) and coma in 2 cases (6.8%). The mean LDH was 1666 U/l [11-6950U/l]. Vaso-occlusive crisis were manifested by acute chest syndrom in 15 cases (46,9%) and osteoarticular pain in 5 cases (15.6%); it was complicated with multi-organ failure in 2 cases (6.2%).The diagnosis of severe community acquired pneumonia was retained in 16 cases (40.6%) and pulmonary embolism (PE) in 6 cases (18.8%) with an average obstruction index of 27.9% .4 patients were presented with septic shock (12.5%). A symptomatic treatment based on oxygen, analgesics, hydration and heparin at preventive dose was prescribed in 62.5% case (n = 20). 18.7% of admissions required blood transfusion (n = 6). Mechanical ventilation was needed in 9 cases (average duration = 7.12 days [1–18 days]) and vasopressives in 5 cases (average duration = 4.6 days [1–16 days]). Mean length of ICU stay was 6.5 days [1–31 days]. Five patients (15.6%) died from multiorgan failure or massive PE.

**CONCLUSIONS.** Life-threatening complications in hemoglobinopathies require admission to ICU; especially acute respiratory failure that could be from various etiologies. Mortality within these patients remains considerable.

**REFERENCE(S)**

1: Hemoglobinopathies in Tunisia. An updated review of the epidemiologic and molecular data. Fattoum S. Tunis Med 2006 Nov;84(11):687–96

2: Critically ill patients with sickle cell disease. Habibi A. Réanimation 16 (2007) 310–317

3: Mortality in sickle cell disease. Life expectancy and risk factors for early death. Orah S. NEJM 330(1994):1639–44

### 1017

#### Incidence, risk factors and outomes of onco-hematological patients in ICU

C.C. Geremia[1], J.S. Seeber[2], V.C. Maraschin[3], P.A.D. Duarte[4]

[1]Universidade Estadual do Oeste do Paraná, Cascavel, Brazil; [2]Hospital do Câncer / UOPECCAN, Cascavel, Brazil; [3]Faculdade Assis Gurgacz, Cascavel, Brazil; [4]Hospital do Câncer / UOPECCAN, General ICU, Cascavel, Brazil

**Correspondence:** P.A.D. Duarte

*Intensive Care Medicine Experimental* 2017, **5(Suppl 2):**1017

**INTRODUCTION.** Despite medical advances in recent years, cancer patients have also shown an increase in the incidence of complications, with life-threatening conditions that have led to increased demand for intensive care. The present study aims to establish the epidemiological profile of onco-hematological patients, as well as to identify the risk factors associated with ICU mortality.

**METHODS.** Retrospective cohort study analyzing medical records of adult patients with hematological malignancies (leukemias, lymphomas, myeloma, and severe myeloproliferative diseases) who were admitted to the ICU of a Cancer Hospital in a 2-year period.

**RESULTS.** In the study period, there were 904 admissions to the ICU, 68 (7.52%) of patients with hematological malignancies (63.2% male, age 55.4 years). Most common diagnosis: Acute leukemia (27.9%), Lymphoma (25%), Multiple myeloma 19.1%, Chronic leukemia 7.3%. Mortality was 77.9%. 73.5% used MV. Acute respiratory failure was the main cause of ICU admission, with 45.6% of patients, followed by neurological causes, with 22.1%. By logistic regression analysis, the main predictors of death were: time of MV, time of use of vasoactive drugs (VAD), recent diagnosis of the neoplasia, and no previous chemotherapy.

**CONCLUSIONS.** The mortality of onco-hematological patients in ICU was very high, particularly with patients with recent diagnosis (still without beggining of chemotherapy).

### 1018

#### Sepsis code in patients with solid and hematological malignancies at a tertiary hospital. Risk factors for mortality

I. Romera[1], M. Santafe[1], J. Baldirà[1], L. Fox[2], C. Díaz-Lagares[1], J.C. Ruíz-Rodríguez[1], M. Pérez[1], R. Ferrer[1]

[1]Vall d´Hebron University Hospital, Critical Care Department, Barcelona, Spain; [2]Vall d´Hebron University Hospital, Hematology Department, Barcelona, Spain

**Correspondence:** I. Romera

*Intensive Care Medicine Experimental* 2017, **5(Suppl 2):**1018

**INTRODUCTION.** Patients with solid or hematological malignancies are at high risk of developing sepsis, which is related to a high mortality. So, efforts are needed in order to improve their prognostic.

**OBJECTIVES.** To analyze the characteristics and identify risk factors for mortality in oncological and hematological patients with sepsis, identified and managed in a protocolized basis (Sepsis Code).

**METHODS.** Prospective study, including adult (+18 years) patients with solid or hematological malignancies and sepsis, during the first fifteen months of activity of the Sepsis Code (15.10.2015 to 31.01.2017), at a tertiary center. Approved by Vall d´Hebron Hospital ethics committee (PR(AG)336/2016). X-Square, Fisher´s test, T-test, U Mann–Whitney and logistic regression were employed as required. Quantitative variables are reported as median (IQR) and categorical as N (%).

**RESULTS.** Sixty-seven patients were included, 43 (64%) men, with an age of 67 (52–71) years. Thirty-eight (57%) had hematological malignancies (16 (24%) acute leucemia,16 (24%) lymphoma) and 29 (43%) solid cancer (lung cancer, 8 (12%)). In 38 (57%) septic shock was diagnosed, while 24 (36%) had severe sepsis and 5 (7%) sepsis. The source of the sepsis was: respiratory in 27 (40%), intraabdominal 20 (30%), urinary tract 10 (7%). In 7 (10%), the source was not identified. The antibiotics more often employed were meropenem in 32 (48%) and piperaciline-tazobactam 29 (43%). Combination therapy with an aminoglycoside was prescribed in 22 (33%). Fourteen (21%) received specific coverage against gram positives and 8 (12%) antifungal therapy. In 51 (76%) a pathogenic microorganism was found, being the most frequently isolated, E. coli 13 (19%), K. pneumoniae 11 (16%) and P. aeruginosa 5 (8%). APACHE II score was 21 (16–27) and SOFA 6 (4–10). According to SOFA criteria, the following organ dysfunction was observed: hemodynamic 46 (71%), respiratory 33 (53%) and 27 (41%) renal. Twenty-eight (42%) were neutropenic. At sepsis code activation, lactate levels were 3 (2–5) mmol/dL, CRP 18 (12–28) mg/dL and procalcitonin 12 (2–36) ng/mL. Thirty (46%) required ICU admission, 35 (52%) vasoactive drugs, 13 (20%) mechanical ventilation and 4 (6%) renal replacement therapy. Thirty-eight (55%) died during hospital admission. The following factors were related to mortality: lung cancer and acute leukemia (17 (74%) vs 19 (45%), $p = 0.03$), APACHE II (24 vs 19, $p = 0.008$), respiratory dysfunction (26 (77%) vs 10 (32%), $p = 0.001$), mechanical ventilation (12 (92%) vs 24 (46%), $p = 0.014$) and increase of CRP after 48 hours (10 (71%) vs 6 (30%), $p = 0.021$). In the multivariate analysis, only mechanical ventilation (OR: 11.9, IC95%: 1.4-100, $p = 0.025$) and APACHE II (OR:1.1, 1.0-1.2, $p = 0.024$) reached statistical significance.

**CONCLUSIONS.** Sepsis in patients with solid and hematological neoplasms has a significant mortality, mainly in those requiring mechanical ventilation. APACHE II score is also related to mortality.

### 1019

#### Usefulness of SOFA score as prognosis factor in hematopoietic stem cell transplant recipients admitted to an intensive care unit

M. Santafe[1], I. Romera[1], L. Fox[2], C. Díaz-Lagares[1], P. Barba[2], J. Baldirà[1], M. García[1], M. Pérez[1]

[1]Vall d´Hebron University Hospital, Critical Care Department, Barcelona, Spain; [2]Vall d´Hebron University Hospital, Hematology Department, Barcelona, Spain

**Correspondence:** M. Santafe

*Intensive Care Medicine Experimental* 2017, **5(Suppl 2):**1019

**INTRODUCTION.** Hematopoietic stem cell transplant recipients (HSCT-R) are a population with a high risk of developing severe complications that require admission to an intensive care unit (ICU), which is related to a high mortality. So, efforts are needed in order to improve their prognosis.

**OBJECTIVES.** Our aim was to describe the cohort of HSCT-R admitted to a tertiary hospital ICU and analyze factors that are likely to affect their survival.

**METHODS.** Retrospective study, including adult (+18 years) patients who received an HSCT and required admission to ICU between 1st January 2010 and 28th February 2017 at a tertiary hospital. Chi-Square, Fisher exact test, Student T test, Mann–Whitney U-test, and logistic regression were employed as required. Quantitative variables are reported as median (IQR) and categorical as frequency (%).

**RESULTS.** Sixty-four patients were included, 41 (64.1%) men, with a median age of 52 (35–60) years. The underlying leading conditions were leukemia, with 27 (42%) patients, and lymphoma with 24 (38%). Allotransplant was done in 53 (83%). Fourteen (22%) had received a previous HSCT. SOFA on admission was 9 (7–11) and organ failures were distributed as follow: respiratory in 31 (48%), hemodynamic in 29 (45%), hematological in 36 (53%), renal in 10 (16%), liver in 9 (14%) and neurologic in 5 (8%). APACHE II score was 23 (17–28). The most frequent diagnosis at admission were pneumonia, that affected to 29 (45%), septic shock in another 29 (45%) and sinusoidal occlusive syndrome in 5 (8%). Neutropenia was present in 32 (50%). Forty-seven (73%) required vasoactive drugs, 33 (52%) high flow nasal cannula (HFNC), 37 (58%) mechanical ventilation (MV), 20 (31%) neuromuscular blocking agents, 9 (14%) prone positioning and 19 (30%) renal replacement therapy (RRT). Twenty-six (41%) were discharged to the ward and 16 (25%) survived at hospital discharge. SOFA score on admission was related to mortality (OR 1.29 IC 95%:1.0-1.6, p = 0.02). Patients with SOFA on admission > 11 had 100% mortality (p = 0.025). Another factors related to mortality were MV (OR = 6.6, IC95%:1.8-23.9, p = 0.04) and RRT (OR 9.07 IC95%: 1.1-74.5, p = 0.04). We did not find any differences related to the type of HSCT or to the presence or length of neutropenia. Patients on HFNC on admission who failed and required mechanical ventilation had similar mortality than patients initially on mechanical ventilacion, 90% and 87%, respectively (p = 1). In the multivariate analysis, MV (OR = 6.32, IC95%:1.62-24.63, p = 0.008), and SOFA score on admission (OR1.28 IC95%:1.02-1.61: p = 0.035) maintained statistical significance.

**CONCLUSIONS.** Recipients of HSCT admitted to an ICU have a high ICU and hospital mortality. SOFA score on admission and requirement of MV are independent predictors of hospital mortality.

### 1020

#### Impact of trombopenia and neutropenia as a prognostic factor in hematological patients in a critical unit

G.A. Narváez Chávez, J. Higuera Lucas, M.L. Aroca, L. Jaramillo Valarezo, J. Rengifo, A. Lietor Villajos, D. Cabestrero Alonso, R. De Pablo
Ramon y Cajal University Hospital, Intensive Care Unit, Madrid, Spain
**Correspondence:** G.A. Narváez Chávez
*Intensive Care Medicine Experimental* 2017, **5(Suppl 2):**1020

**INTRODUCTION.** The hematological patient are a continue challenge to the intensive care because their underlying conditions presents special characteristic which modify the complications they presents and their management.

**OBJECTIVES.** The aim of this study is to assess thrombopenia and neutropenia as a prognostic factor in oncohematologic patients (pts) who required admission to intensive care unit (ICU), either at admission or during their hospitalization, their relationship to the prognostic scales: SOFA, APACHE and SAPS II, respiratory distress (ARDS) and mortality.

**METHODS.** An observational and retrospective study was conducted, we reviewed all pts with hematology malignances that were admitted to the ICU between May 2013 to February 2016.

We analyzed the following variables: age, sex, type of hematological disease, organ failure, SOFA, APACHE II y SAPS, thrombopenia and neutropenia at admission and minimum values during their stay, presence of ARDS and ICU mortality.

We used the $X^2$ and the results are shown using the median values and their range.

**RESULTS.** A total of 141 pts were admitted, with a mean age of 55.1 ± 14.9 years. Men were 57.1% and 42.1% were women.

The underlying hematological condition was leukemia (53%), lymphoma (24%), myeloma (15%) and a solid tumor (8%). The main reason for admission was hemodynamic failure 77.1% and respiratory failure 71.4%. 26 pts (18.4%) were diagnosed with ARDS.

The mean SOFA severity score was 9.3 ± 4.3 (2–20); APACHE II 22.64 ± 13.15 (3–65) and SAPS II: 50.27 ± 20.82 (2–101). Mortality rate was 38.6% (54 patients).

Thrombopenia (<50000 platelets), occurred in 59 pts (41.8%) on admission and during their stay were developed in 100 patients (70.92%). Neutropenia (<1000 neutrophils) was recorded on admission in 43 pts (30.49%) and 59 pts (41.84%) were developed during hospitalization.

Using the $X^2$ test we found that thrombopenia, both at admission and its minimum value, was related to the mortality (p 0.016 and p 0.003); as well as SOFA (p 0.0001; p 0.001) but not with APACHE II or SAPS II.

Using the same test ($X^2$), there was no statistically significant relationship between neutropenia at admission and the minimum value during stay with mortality or prognostic scales: SOFA, APACHE II and SAPS II.

Statistically significant relationship was found between neutropenia at admission and the presence of ARDS (p 0.007). Applying X2 showed a relationship between ARDS and mortality (p 0.0001).

**CONCLUSIONS.** Thrombopenia is related to mortality in oncohematologic patients in our setting, regardless of the underlying disease and the reason for admission.

Thrombopenia is only related to the SOFA,

In our study the neutropenia can't be considered an independent factor of mortality in oncohematologic patients. A larger study could better assess these results.

There is a relationship between the presence of ARDS and the presence of neutropenia at admission.

### 1021

#### Hemophagocytic lymphohistiocytosis in intensive care unit

F. Frantzeskaki[1], E. Paramythiotou[1], M. Theodorakopoulou[1], P. Tsirigotis[2], A. Armaganidis[1]
[1]Attikon University Hospital, 2nd Critical Care Department, Athens, Greece; [2]Attikon University Hospital, 2nd Department of Internal Medicine, Athens, Greece
**Correspondence:** F. Frantzeskaki
*Intensive Care Medicine Experimental* 2017, **5(Suppl 2):**1021

**INTRODUCTION.** Hemophagocytic lymphohistiocytosis (HLH) is an uncontrolled hyperinflammatory state caused by impaired natural-killer (NK) and cytotoxic-T-cell function, which can lead to multiorgan failure and death. Triggering factors are infections (EBV, CMV, HIV), connective tissue diseases, lymphoid malignancy and drugs.

**OBJECTIVE.** The aim of the present study was to retrospectively investigate the prevalence of HLH in critically ill patients. We also studied the possible triggering factors and the subsequent morbidity and mortality associated with the syndrome.

**PATIENTS AND METHODS.** Mechanically ventilated patients, treated in ICU during five years, were included in this retrospective observational study. Disease severity was estimated by Acute Physiology and Chronic Health Evaluation (APACHE II) score and the degree of organ dysfunction was quantified by the Sequential Organ Failure Assessment (SOFA) score. Clinical and laboratory data were recorded. HLH diagnosis was based on five out of the following eight HLH criteria: persistent fever, splenomegaly, bicytopenia (hemoglobin < 9g/dL, neutrophils < $1x10^9$ /L, platelets < $100x10^9$ /L), hypofibrinogenemia (<150mg/dL) and/or hypertriglyceridemia (>265mg/dL), hemophagocytosis, low NK cells activity and high concentration of sCD25 (soluble receptor for interleukin 2). The presence of immunosuppression, the sepsis stage, ferritin levels, specific therapeutic interventions and morbidity and mortality data were also recorded. Multivariated analysis was used, in order to evaluate the risk factors associated with mortality.

**RESULTS.** During the study period, twelve patients fulfilled the HLH diagnostic criteria. The mean (±SD) APACHE II and SOFA scores were 20 ± 7 and 10 ± 3 respectively. Septic shock was present in five patients (41%). Regarding the possible triggering factors, lymphoid malignancy was present in four patients (30%), while CMV infection was observed in one patient and systemic lupus erythematosus in one patient as well. Hemophagocytosis on bone marrow aspirates was found in seven patients (58%). The mean time from ICU admission and HLH diagnosis was 7 ± 5 days. Regarding the therapeutic interventions, intravenous immunoglobulins were administered in eleven patients, while one patient received dexamethasone and etoposide. The mean (±SD) ICU stay was 20 ± 18 days, while the observed ICU mortality was 58%. ICU mortality was significantly associated with SOFA score (p = 0.01) and advanced age (p = 0.03).

**CONCLUSIONS.** HLH is a life threatening, usually underdiagnosed syndrome in critically ill patients. Malignancies, connective tissue diseases and several infections might trigger the syndrome. The intensivist should be aware of this possible diagnosis and timely proceed to the appropriate treatment, in order to limit the HLH-related mortality.

### 1022

#### Haematological malignancy on the ICU: retrospective review of outcomes in a single UK centre 2012–2016

A. Skorko, M. Thomas, T. Gould
Bristol Royal Infirmary, Bristol, United Kingdom
**Correspondence:** A. Skorko

*Intensive Care Medicine Experimental* 2017, **5(Suppl 2):**1022

**INTRODUCTION.** Admission to ICU with a haematological malignancy continues to be a contentious decision. Previously published data shows that survival has improved over the last 30 years. We examine the outcomes of patients admitted with a haematological malignancy into our ICU over the last 5 years, to understand how survival has continued to change.

**METHODS.** We retrospectively examined data for 139 patients coded with a haematological malignancy who were admitted to our ICU between 2012 and 2016, who between them had 161 ICU episodes (22 patients had multiple admissions). Data was analysed in stata.

**RESULTS.** Overall hospital and 6 month mortality during the study period were 46.6% and 64.4% respectively. Of those discharged from ICU, 7.6% died before hospital discharge.

Median ICU LOS was 3.5 days (IQR 1.8 to 7.9 days). Median hospital LOS after ICU was 19.3 days (IQR 6.75 to 27 days) before death or discharge.

Figure 324 lists the demographics of patients, divided by haematological diagnosis. This shows that patients with acute leukaemia tended to be younger, had more exposure to chemotherapy around their ICU admission, and more likely to be bone marrow recipients. Despite this their ICU survival was much better than other diagnoses.

Virtually all patients received at least one organ support, 88% requiring at least one advanced level organ support, 23% required renal replacement therapy. As the number of organs requiring support increased, so mortality increased (p < 0.001) (Fig. 325).

Overall 71% of all admissions were associated with chemotherapy treatment. Of those receiving chemotherapy; 49% died, versus 44% in those not (p = 0.52). Chemotherapy did not increase risk of death (OR 1.25 CI 0.6-2.5, p =0.41).

72 (45%) admissions were associated with bone marrow transplantation(BMT), 27 during the index admission, 45 historically. Hospital mortality in those receiving BMT during the current admission was 48%, versus 46% in those not (p = 0.83). BMT was not associated with increased risk of death (OR 1.2 (CI 0.6-2.2, p = 0.54).

Of those admitted, 23 (14%) had a new diagnosis of malignancy. Mortality was not correlated with a new diagnosis, 39% vs 47.7% (p = 0.446), OR of death 0.7 (CI 0.28-1.7, p = 0.44).

**CONCLUSIONS.** Mortality amongst patients with haematological malignancy admitted to ICU is higher than general ICU mortality but is lower than historical data.

Chemotherapy, a new diagnosis of malignancy, or a history of BMT were not correlated to hospital outcome in our patient cohort. Age and number of organs requiring support were correlated with outcome.

Taccone at al**. Characteristics and outcomes of cancer patients in European ICUs.** *Critical Care* 2009, **13:**R15

Bird et al. **Outcomes and prognostic factors in patients with haematological malignancy admitted to a specialist cancer intensive care unit: a 5 yr study**. British Journal of Anaesthesia 2012; 108 (3): 452–9

| | Acute leukaemia (n =74) | Lymphoma (n = 51) | Chronic leukaemia, myeloma and other myelodysplastic disorders (n= 36) |
|---|---|---|---|
| Mean age (years) | 46 | 56 | 61.6 |
| Sex M:F | 38:36 | 34:17 | 24:12 |
| ICNARC score on admission (mean) | 22.4 | 21.2 | 21.7 |
| ICU admission as part of first presentation of disease | 21% | 12% | 2.9% |
| Bone marrow transplantation (BMT) during admission | 24.6% | 12% | 12% |
| Chemotherapy as part of admission | 81% | 74% | 51% |
| Bone marrow transplant prevalence | 50% | 29% | 21% |
| Hospital mortality | 37.8% | 55% | 52.8% |
| 6 month mortality | 60.8% | 65% | 71.4% |

**Fig. 324 (Abstract 1022)** Patient specifics, by diagnosis

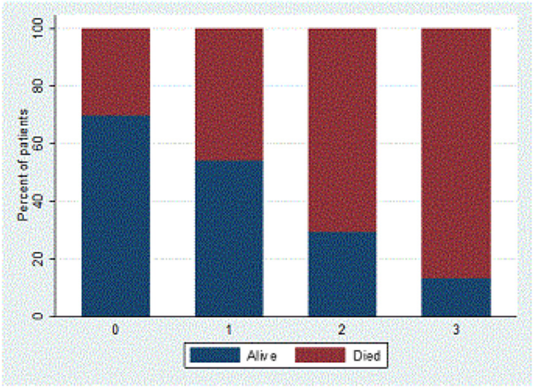

**Fig. 325 (Abstract 1022)** Number of organs supported

### 1023

#### The haematological malignancy patient: can we improve the selection for ICU admission?

U. Azram, N.M. Robin, S.R. Singh
Countess of Chester Hospital, Anaesthesia & Critical Care, Chester, United Kingdom
**Correspondence:** U. Azram

*Intensive Care Medicine Experimental* 2017, **5(Suppl 2):**1023

**INTRODUCTION.** ICU patients with a haematological malignancy have a higher ICU, hospital and 3 month mortality (46.4%, 59%, 77%) compared with general ICU patients (22%, 30%,37%) [1]. Despite advances in both haematological and ICU treatments, mortality remains high. Selection for ICU treatment in this group of patient is important to improving outcomes. Known negative prognostic factors include underlying diagnosis (acute leukaemia), second line treatments (chemotherapy or bone marrow transplantation), invasive ventilation [1] and multiple organ failure (SOFA score > 11) [2]

**OBJECTIVES.** The aim of the study was to identify factors associated with survival and construct pre-ICU strategies and selection for admission to ICU guidelines.

**METHODS.** We carried out a retrospective analysis of patients admitted to the adult ICU at the Countess of Chester hospital, UK with haematological malignancy over a 5 year period (2010 to 2014) looking at factors that may correlate with mortality.

**RESULTS.** In total we had n = 28 patients in over a 5 year period. The data was analysed using Chi squared test with Yates´ correction.

| Factor | Survived | Died | P value | Significance |
|---|---|---|---|---|
| Haematology consultant referral | 14/15 (93.3%) | 7/13 (53.8%) | 0.028 | Yes |
| Invasive ventilation | 6/15 (40%) | 12/13 (92.3%) | 0.006 | Yes |

*[Factors affecting ICU mortality]*

| Haematological diagnosis | N | APACHE 2 predicted mortality (%) | ICU mortality (%) | Hospital mortality (%) | 3 month mortality (%) |
|---|---|---|---|---|---|
| Acute leukaemia/AML | 5 | 72.5 | 80 | 80 | 100 |
| Lymphoma/ NHL | 14 | 59 | 50 | 64 | 86 |
| Myeloma | 8 | 58.7 | 12.5 | 12.5 | 50 |
| CML | 1 | 97.1 | 100 | 100 | 100 |

*[Outcome by haematological diagnosis]*

**CONCLUSIONS.** This study showed that factors associated significantly with a favourable outcome includes consultant to consultant referral and a diagnosis of myeloma. Single organ failure not requiring invasive ventilation had lower mortalities. The results also show that 53.8% of patients admitted to the ICU had sepsis. Of the 28.5% of patients admitted with respiratory failure a significant proportion of these had pneumonia. Sepsis and respiratory distress/ pneumonia make up the majority of reasons for ICU admission. Hospitalised haematology patients should undergo even more rigorous screening for sepsis using the qSOFA criteria. The threshold for referral should be lower in this group of patients as we know that prolonged pre-ICU stay confers poor prognosis [1]. In the UK this may be a role for critical care outreach. The referral to ICU should be consultant to consultant to factor in haematological diagnosis, prognosis, relapse, secondary treatments and recognise futility. In addition to a clinical assessment, SOFA scoring should be included in the decision to select for ICU admission.

**REFERENCE(S)**

[1] Hampshire PA et al. Admission factors associated with hospital mortality in patients with haematological malignancy: a secondary analysis ICNARC CMPD. Critical Care 2009, 13:R137

[2] Silfvast T et al. Multiple organ failure and outcome of critically ill patients with haematological malignancy. Acta Anaesthesiol Scand 2003; 47: 301–306

**GRANT ACKNOWLEDGMENT**

No conflicts of interests to declare

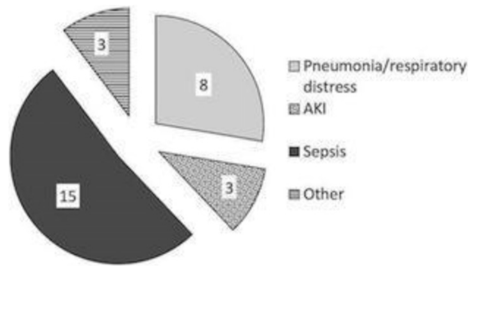

**Fig. 326 (Abstract 1023)** Admission diagnosis

### 1024

**Levosimendan in critically ill cancer patients with chemotherapy associated acute heart failure**

A. Turki[1], R. Hamacher[2], N.K. Steckel[1], P. Wohlfarth[1], D.W. Beelen[1], T. Liebregts[1]

[1]University of Duisburg-Essen, University Hospital Essen, Department of Bone Marrow Transplantation, Essen, Germany; [2]University of Duisburg-Essen, University Hospital Essen, Department of Medical Oncology, Essen, Germany

**Correspondence:** T. Liebregts

*Intensive Care Medicine Experimental* 2017, **5(Suppl 2):**1024

**INTRODUCTION.** Cardiovascular complications are one of the most common complications associated with anticancer therapy. Although treatment- induced cardiotoxicity may occur in up to one-third of cancer patients, there are very limited data on the management of chemotherapy induced acute heart failure necessitating ICU admission. Levosimendan, a calcium sensitizing inotropic agent, has been shown to improve hemodynamic function in patients with decompensated heart failure but data in cancer patients are lacking.

**OBJECTIVES.** We therefore aimed to characterize the effect of levosimendan on hemodynamic performance in patients with anticancer therapy associated acute low-output heart failure.

**METHODS.** We conducted a retrospective analysis of thirty cancer patients admitted to ICU with acute heart failure attributable to chemotherapy induced cardiotoxicity. All patients received levosimendan as continuous infusion of $0.1 \mu g kg^{-1} min^{-1}$ for 24h. Hemodynamic measurements were performed at baseline and at 6, 12 and 24h after start of levosimendan using a Swan-Ganz thermodilution catheter. Additional dobutamin was allowed in stable doses and a mean arterial pressure was maintained at 65mmHg by noradrenaline if necessary.

Changes in hemodynamic variables over time were analyzed by Wilcoxon rank test.

**RESULTS.** Causes for acute heart failure were toxicity due to anthracyclines (30%), myeloablative conditioning regimen (40%), tyrosine kinase inhibitors (10%), protein kinase inhibitors (10%) and carboplatin (10%). Patient demographics were; Median age 61years, male/female 45% vs.55%, BMI 23.9, allogeneic HSCT recipients 45% and autologous HSCT recipients 5%. Invasive mechanical ventilation was initiated in 55%, non-invasive mechanical ventilation in 10%, CRRT in 50%, VA-ECMO in 20%.

The median ICU severity scores were; APACHE II 20, SAPS II 45. Overall ICU mortality was 35%.

During the levosimendan infusion there was a significant ($p < 0.001$) increase in cardiac index from 1.6 ± 0.3 to 2.0 ± 0.5 (6h), 2.2 ± 0.5 (12h) and 2.6 ± 0.5 L*min$^{-1}$*m$^{-2}$ (24h). Patients showed an increase ($p < 0.001$) in stroke volume from 29.8 ± 11.6 to 36.6 ± 15.9 (6h), 41.0 ± 17.3 (12h) and 47.8 ± 15.5 mL/beat (24h). Pulmonary-capillary wedge pressure dropped from 17.2 ± 2.7 to 15.4 ± 2.5 (6h; p = 0.02), 13.9 ± 2.0 (12h; $p < 0.001$) and 10.9 ± 3.1 mmHg (24h; p = 0.002). Systemic vascular resistance index decreased from 3182.7 ± 902.7 to 2894.5 ± 783.1 (6h; p = 0.07 ns), 2637.7 ± 768 (12h; p = 0.04) and 2315.1 ± 773.3 dyn*sec*cm$^{-5}$ (24h; p = 0.001). Brain natriuretic peptide declined from 3465 ± 1565 to 3162 ± 1476 (6h; p = 0.07 ns), 2135 ± 1364 (12h; p = 0.002) and 806 ± 622 pg/ml (24h; p = 0.008).

**CONCLUSIONS.** Levosimendan substantially improves hemodynamic performance in patients with severe low-output heart failure associated with anticancer therapy.

### 1025

#### Study of total lymphocytes and lymphocyte subpopulations in ICU at admission

M. Guerrero-Marín[1], M.D. Arias-Verdú[2], C. Cabrera-Morales[3], E. Aguilar-Alonso[4], A. Caballero-González[5]

[1]Complejo Hospitalario de Jaen, Intensive Care Unit, Jaen, Spain; [2]Hospital Regional Universitario Carlos Haya de Málaga, Intensive Care Unit, Málaga, Spain; [3]Hospital Regional Universitario Carlos Haya de Málaga, Immunology Laboratory, Málaga, Spain; [4]Hospital Infanta Margarita de Cabra, Intensive Care Unit, Cordoba, Spain; [5]Hospital Regional Universitario Carlos Haya de Málaga, Inmnology Laboratory, Málaga, Spain

**Correspondence:** M.D. Arias-Verdú

*Intensive Care Medicine Experimental* 2017, **5(Suppl 2):**1025

**OBJECTIVE.** To evaluate the incidence of lymphopenia and the frequency of values lower than normal in lymphocyte subpopulations in ICU patients at admission.

**METHODS.** Cohort study, evaluating the number of total lymphocytes as well as the different lymphocyte subpopulations the first day at ICU admission. We excluded coronary and programmed surgery patients. Data as mean + standard deviation and absolute and relative frequencies. Satistical Analysis: Pearson correlation. $p < 0.05$ as ss.

**RESULTS.** N = 99 patients. Mean age 51.76. Mean APACHE I 17.91

Total number of lymphocytes1197 ± 1758. For the different subpopulations the values were CD3: 726 ± 635, CD4: 434 ± 355, CD8: 238 ± 230, CD16: 131 ± 125, CD19: 206 ± 187.

Regarding the number of total lymphocytes, 80% have less than 1,500 lymphocytes on the day of admission, 58% less than 1000, 44% less than 800, 28% less than 600 and 25% less than 500.

Among the lymphocyte subpopulations, 58.6% had low CD3 lymphocytes (less than 690), 57.6% low CD4 (<410), 46.5% low CD8(<190), 45.5% low CD16 (<90) and 23.2% low CD19 (<90).

There was no ss between the number of total lymphocytes and the severity assessed with APACHE II ($r = -0.135$, p = 0.203), neither between APACHE II and the number of lymphocytes from different subpopulations.

**CONCLUSION.** A high percentage of patients presented a low total lymphocytes and low lymphocytes in the various subpopulations at admission. Total lymphocytes less than 600 is associated with higher mortality and low number of lymphocytes in different subpopulations does not provide additional information regarding mortality. We found no statistically signification between the degree of lymphopenia and APACHE II.

### 1026

#### Adverse outcomes including sepsis are associated with thrombocytopenia following liver transplantation

A. Strachan, L. Abeysundara, S. Mallett

Royal Free Hospital, Perioperative Research Department, London, United Kingdom

**Correspondence:** A. Strachan

*Intensive Care Medicine Experimental* 2017, **5(Suppl 2):**1026

**INTRODUCTION.** Platelets are innate inflammatory cells and, in addition to their traditional role within haemostasis, are newly considered to have a key role within immunity performing a range of adaptive responses to an antigen challenge. In disease, their immune role is dysregulated and this has been associated with adverse surgical outcomes. Studies have demonstrated a correlation between sepsis and platelet nadir with a platelet count < 60x10$^9$/L predicative for surgical complications following liver transplantation (LT).

**OBJECTIVES.** To explore the relationship between platelets, sepsis and outcome measures within our liver transplant cohort.

**METHODS.** A case note review of 97 LTs taking place between 2015–16 within our centre. Data collected included perioperative transfusion, indices of infection (positive cultures, antibiotic use, CRP and WBC) on Day 0–7, 14, 21 and 28. Platelet count at the same time points was collected. Outcome data included acute kidney injury (AKI) and length of ICU admission.

**RESULTS.** 13% of patients had a platelet drop from baseline (median reduction 18% [range 14–34]). The incidence of platelet rise was 87%; median increase was 38% [25–54]. A significantly higher incidence of AKI (p = 0.032) and longer ITU admission (p = 0.002) was found when the platelet count dropped below 60x10$^9$/L post-operatively. Platelet counts on post-operative days (POD) 5, 6 and 7 were significantly lower in patients who developed systemic infection ("sepsis" group) compared to patients who had no evidence of sepsis ("no sepsis" group): POD5 p = 0.021, POD6 p = 0.006, POD7 p = 0.007. Median time to initiation of antimicrobial therapy was POD 9 (IQR 4–15). Comparison of demographic data, baseline platelet, white cell counts and creatinine, intra- and post-operative transfusion were comparable between groups. While the reduction in platelet count was comparable between both groups, the sepsis group had a slower recovery of platelet count and a lower peak upon recovery (Graph 1).

**CONCLUSION.** Low platelet count is independently associated with worse outcomes, namely increased incidence of post-operative infection, AKI and ICU admission. As with previous research, our results suggest a high suspicion for sepsis is required in patients who are thrombocytopenic following liver transplantation and in these patients a lower threshold for commencing antimicrobial therapy is likely necessary.

1.Pamecha V, Mahansaria SS, Kumar S et al. Association of thrombocytopenia with outcome following adult living donor liver transplantation. Transpl Int. 2016;29:1126–35

2.McCaughan GW, Herkes R, Powers B et al. Thrombocytopenia post liver transplantation. Correlations with pre-operative platelet count, blood transfusion requirements, allograft function and outcome. J Hepatol. 1992;16:16–22.

3.Lesurtel M, Raptis DA, Melloul E et al. Low platelet counts after liver transplantation predict early posttransplant survival: the 60–5 criterion. Liver Transpl. 2014;20:147–55

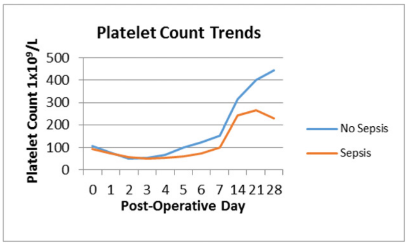

**Fig. 327 (Abstract 1026)** Postoperative platelet count in each group

### 1027

#### Characteristics of immunocompromised patients with influenza A virus admitted to the intensive care unit

C. León Moya[1], A. Gutiérrez Pizarraya[1], M.L. Cantón Bulnes[1], Á. Arenzana Seisdedos[1], A. Rodríguez[2], J. Garnacho-Montero[1]
[1]Hospital Universitario Virgen Macarena, Sevilla, Spain; [2]Hospital Universitari Joan XXIII, Tarragona, Spain
**Correspondence:** C. León Moya
*Intensive Care Medicine Experimental* 2017, **5(Suppl 2):**1027

**INTRODUCTION.** Information about immunocompromised patients infected with influenza A virus requiring admission to the ICU is lacking. The objective of this study was to know the clinical characteristics of these patients and to identify variables associated with mortality.

**METHODS.** A prospective multicenter observational cohort study was based on data from the GETGAG/SEMICYUC registry (2009–2015) collected by 148 Spanish ICUs. All patients admitted to the ICU with the diagnosis of influenza A virus infection were included. Immunosuppression was defined as any primary immunodeficiency or immunodeficiency secondary to human immunodeficiency virus (HIV) infection, active malignancy, immunodeficiency secondary to radiation treatment or use of cytotoxic drugs or corticosteroids (daily dose >40 mg of prednisolone or the equivalent for >2 weeks), immunological disease, solid organ transplant, and haematological disease. Factors associated with mortality in immunocompromised patients were assessed by logistic regression analysis.

**RESULTS.** Of 2315 patients with influenza A infection, 289 (12.5%) were classified as immunocompromised. Mortality was significantly higher in immunocompromised patients (47.1% vs 20.1%; p < 0.001). Severity of illness and the rates of health-care infection, early use of oseltamivir and administration of corticosteroids were significantly greater in immunocompromised subjects. In this group of patients, factor independently associated with mortality were: APACHE II score (OR =1.09, 95% CI = 1.04-1.13, p < 0.001), stage 3 of acute renal insufficiency (OR = 2.33, 95% CI = 1.69-4.63, p < 0.05), use of vasopressor drugs (OR = 4.69, 95% CI = 2.44-9.03, p < 0.001) and the use of corticosteroid (OR = 1.89, 95% CI = 1.06-3.37, p < 0.05).

**CONCLUSION.** Immunocompromised individuals with influenza A admitted to the ICU have a poorer outcome than patients without immunocompromise. In this population, we should avoid the use of corticosteroids because it is independently associated with a higher mortality.

### Liver failure: Nutrition and metabolism

### 1028

#### Development of a multivariate prediction model for mortality in a cohort of 1177 critically ill patients with hypoxic hepatitis

A. Van den broecke[1], L. Van Coile[1], A. Decruyenaere[2], H. Van Vlierberghe[3], K. Colpaert[4], J. Decruyenaere[4]
[1]Ghent University, Faculty of Medicine and Health Sciences, Ghent, Belgium; [2]Ghent University Hospital, Department of Internal Medicine, Ghent, Belgium; [3]Ghent University Hospital, Department of Hepatology and Gastroenterology, Ghent, Belgium; [4]Ghent University Hospital, Department of Intensive Care Medicine, Ghent, Belgium
**Correspondence:** A. Van den broecke
*Intensive Care Medicine Experimental* 2017, **5(Suppl 2):**1028

**INTRODUCTION.** Hypoxic hepatitis (HH) ("ischemic hepatitis" or "shock liver") is a common cause of hepatic dysfunction in an ICU, is histologically characterized by centrilobular liver cell necrosis and is caused by an insufficient oxygen delivery to hepatocytes. Typical for HH is the sudden, significant rise (>5x ULN) of aspartate aminotransferase (AST) in response to hemodynamic failure.

**OBJECTIVES.** The aim of this study was to identify mortality risk factors of HH using a multivariate prediction model and to assess the predictive accuracy of 2 existing models (Fuhrmann et al. [2] and Raurich et al. [3]).

**METHODS.** The study cohort consisted of 1177 adults with a first episode of HH (AST >5x ULN) out of 29874 patients admitted to the ICU of the Ghent University Hospital (UZG) between January 1st, 2007 and September 21st, 2015. The incidence of HH was 4.2% and the hospital mortality 42.8%. Further detailed study results are described in another report [1]. 29 parameters were retrospectively collected as potential risk factors based on clinical importance. The association with mortality was studied using logistic regression at the time of maximal AST value. Predictive accuracy of the models was assessed by the area under the receiver operating characteristic curve (AUC), the calibration curve and the Hosmer-Lemeshow (HL) test. As the 2 existing models were developed in patients with AST >20x ULN, only this subgroup (n = 460) was used for their validation.

**RESULTS.** Resp. 8 and 2 risk factors are excluded based on missing values and correlation. The remaining 19 variables have 1153 complete cases. After a backward elimination procedure, the multivariate UZG model indicates that age (OR 1.03 per year), cardiac failure (OR 8.55), septic shock (OR 12.25), hypovolemic shock (OR 7.99), lung embolism (OR 14.04), acute respiratory failure (OR 2.38), acute-on-chronic respiratory failure (OR 6.68) and mechanical ventilation (OR 2.24) are most significantly (all P < 0.001) associated with mortality. Additionally, INR (OR 1.27 per unit, P = 0.002), hyperthermia (OR 14.58, P = 0.019) and AST (OR 1.01 per 100U/L, P = 0.05) are significantly associated. pH (OR 0.56 per 0.1) and trombocytes (OR 0.98 per $10^4$/ml) are inversely associated (both P < 0.001). The UZG model has a good discrimination (AUC 0.79) and shows a good fit between the observed and expected mortality (HL: P = 0.67). On the contrary, the models of Raurich et al. and Fuhrmann et al. have a weak discriminative capacity (AUC 0.59 and 0.67, resp.).

**CONCLUSIONS.** This is by far the largest single-centre cohort study of HH described in literature. 7 mortality risk factors associated with HH are identified. Unlike the 2 existing models, the UZG model has a good discrimination and calibration.

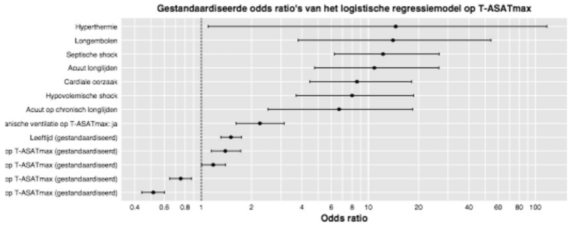

**Fig. 328 (Abstract 1028)** Logistic regression

### 1029

#### Epidemiology, cause, evolution and outcome in a cohort of 1177 critically ill patients with hypoxic hepatitis

L. Van Coile[1], A. Van den broecke[1], A. Decruyenaere[2], H. Van Vlierberghe[3], K. Colpaert[4], J. Decruyenaere[4]
[1]Ghent University, Faculty of Medicine and Health Sciences, Ghent, Belgium; [2]Ghent University Hospital, Department of Internal Medicine, Ghent, Belgium; [3]Ghent University Hospital, Department of Hepatology and Gastroenterology, Ghent, Belgium; [4]Ghent University Hospital, Department of Intensive Care Medicine, Ghent, Belgium
**Correspondence:** L. Van Coile
*Intensive Care Medicine Experimental* 2017, **5(Suppl 2):**1029

**INTRODUCTION.** Hypoxic hepatitis (HH) ("ischemic hepatitis" or "shock liver") is histologically characterized by centrilobular liver cell necrosis and is caused by an insufficient oxygen delivery to hepatocytes. Typical for this type of liver cell necrosis is the sudden, significant rise (>5x ULN) of aspartate aminotransferase (AST) in response to cardiac, circulatory or respiratory failure. Other causes of AST rise should be excluded before diagnosing HH. Treatment of HH relies on the prompt correction of the underlying dysfunction.

**OBJECTIVES.** The aim of this study is to describe the epidemiology, cause, evolution and outcome of critically ill patients with HH. More insight may raise more awareness and could result in a faster diagnosis, which may contribute to a better prognosis.

**METHODS.** The screened population consists of all adults (n = 29874) admitted to the ICU at the Ghent University Hospital between January 1st, 2007 and September 21st, 2015. 4012 patients had at least one episode of AST-levels above 5x ULN. After exclusion of 2835 patients with another cause of elevated AST-levels (e.g. liver surgery, toxic hepatitis, cholangitis,…), a cohort of 1177 first episodes of patients with HH were identified. These patients are classified by underlying cause of HH. The most important outcome variable is ICU and hospital mortality.

**RESULTS.** The incidence of HH is 4.2%. The male/female ratio is 1.5 and the median age is 66 years. The causes of HH are cardiac failure (40.2%), septic shock (28.3%), post-anesthesia without overt evidence of an acute cardiac or respiratory event (11.6%), hypovolemic shock (8.9%), acute respiratory failure (6.0%), acute-on-chronic respiratory failure (3.1%), pulmonary embolism (1.4%) and hyperthermia (0.5%). The hospital mortality associated with HH is 42.8%, of which 90.4% occurred during ICU stay. Pulmonary embolism and septic shock have the highest (56.2% and 52.9%, resp.) and fastest (median survival 5.4 and 12.3 days, resp.) mortality. Contrary to previous studies, patients with a limited rise of AST (5xULN < AST < 10xULN) were also included. This subgroup contains 439 patients and has a mortality of 26.2%. Another subgroup of 136 patients appeared to develop HH after major surgery with general anesthesia despite having no problem during surgery. This subgroup, with still a considerable mortality of 7.4%, was not described in previous studies.

**CONCLUSIONS.** This is by far the largest single-centre cohort study of HH described in literature. With an incidence of 4.2%, HH is a frequent cause of hepatic dysfunction in critically ill patients and is associated with a high hospital mortality of 42.8%. The principal causes are acute cardiac disease and septic shock, which include more than 2/3 of all episodes. Clinicians should search actively for an undetected underlying hemodynamic or respiratory problem even in patients with moderately elevated AST values or with AST elevations after major uncomplicated surgery.

### 1030

#### Thromboelastometry - relation to the severity of liver cirrhosis in patients considered for liver transplantation

G. Dumitrescu[1], A. Januszkiewicz[1], A. Ågren[2], M. Magnusson[3], S. Wahlin[4], J. Wernerman[1]

[1]Karolinska Institutet, Anestesia and Intensive Care, Stockholm, Sweden; [2]Karolinska Institutet, Department of Medicine, Division of Hematology, Coagulation Unit, Stockholm, Sweden; [3]Karolinska Institutet, CLINTEC, MMK, Clinical Chemistry and Blood Coagulation Research, Stockholm, Sweden; [4]Karolinska Institutet, Division of Hepatology, Centre for Digestive Diseases, Stockholm, Sweden

**Correspondence:** G. Dumitrescu

*Intensive Care Medicine Experimental* 2017, **5(Suppl 2):**1030

**INTRODUCTION.** The severity of liver disease is assessed by scoring systems which include the conventional coagulation test PT-INR. However PT-INR is not predictive of bleeding in liver disease and thromboelastometry has been suggested to give a better overview of the coagulation system in these patients. It has now been suggested that coagulation as reflected by tromboelastomety, particularly maximum clot firmness (MCF), may also be used for prognostic purposes in patients with stable chronic liver disease.

**OBJECTIVES.** The objective of our study was to investigate if thrombelastometry may discriminate the degree of liver insufficiency according to the scoring systems Child Pugh and Model for End-stage Liver Disease (MELD).

**METHODS.** Forty adult patients with stable chronic liver disease of different etiologies and stages were included in this observational cross-sectional study. The severity of liver disease was evaluated using the Child-Pugh score and the MELD score, and blood samples for biochemistry, conventional coagulation tests and thromboelastometry were collected at the time of the final assessment for liver transplantation. Statistical comparisons for the studied parameters with scores of severity were made using Spearman´s correlation test and receiver operating characteristic (ROC) curves.

| | Child-Pugh score A + B | Child-Pugh score C |
|---|---|---|
| No. | 22 | 18 |
| Child-Pugh score | 8(6–9) | 11(10–13) |
| | MELD ≤16 | MELD >17 |
| No. | 21 | 19 |
| MELD score | 13(7–16) | 20(17–27) |

*[Number of patients (No). Scores as median (range).]*

**RESULTS.** Spearman's correlation coefficients indicated that the thromboelastometric parameters did not correlate with Child-Pugh or MELD scores. The ROC curves of the thromboelastometric parameters could not differentiate advanced stages from early stages of liver cirrhosis.

**CONCLUSIONS.** standard thromboelastometry cannot discriminate the stage of chronic liver disease in patients with severe chronic liver disease.

**REFERENCE(S)**

1. Tripodi A, Primignani M, Chantarangkul V, et al. The coagulopathy of cirrhosis assessed by thromboelastometry and its correlation with conventional coagulation parameters. Thrombosis research. 2009;124(1):132–136.

2. Wiesner R, Edwards E, Freeman R, et al. Model for end-stage liver disease (MELD) and allocation of donor livers. Gastroenterology. 2003;124(1):91–96.

3. Lentschener C, Flaujac C, Ibrahim F, et al. Assessment of haemostasis in patients with cirrhosis: Relevance of the ROTEM tests?: A prospective, cross-sectional study. Eur J Anaesthesiol. 2016;33(2):126–133.

**GRANT ACKNOWLEDGMENT**

The study was supported by grants to Jan Wernerman from the Swedish Medical Research Council (project 04210) and the County Council of Stockholm (project 502033).

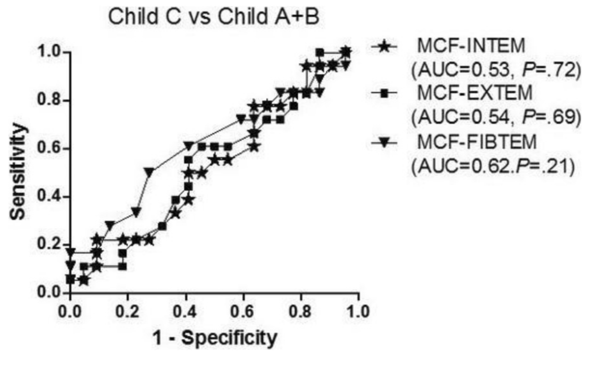

**Fig. 329 (Abstract 1030)** ROC curves of MCF for Child-Pugh groups

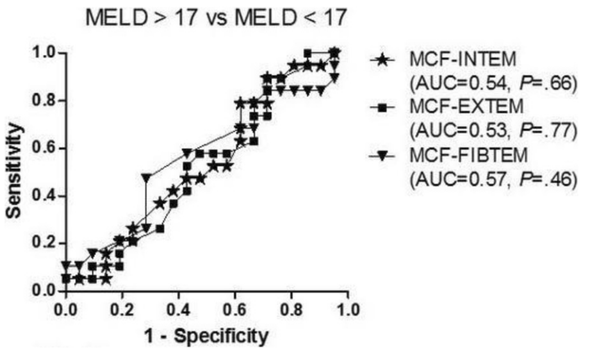

**Fig. 330 (Abstract 1030)** ROC curves of MCF for MELD groups

### 1031

#### Cirrhotic patients colonized by candida. Incidence and prognostic

J. Higuera, G. Narvaéz, L. Rey, A. Blandino, L. Jaramillo, Á. Caballero, D. Cabestrero, S. García, C. Soriano, R. De Pablo
Ramon y Cajal University Hospital, Intensive Care, Madrid, Spain
**Correspondence:** J. Higuera
*Intensive Care Medicine Experimental* 2017, **5(Suppl 2):**1031

**INTRODUCTION.** Candidemia is associated with signficant mortality in cirrhotic patients admitted to intensive care unit. There is a strong trend towards higher prevalence of colonization's by candida albicans and other species of candida in cirrhotic patients. Our objective is to describe the incidence of candida colonization in this patients and if their presence is associated with higher mortality or length of stay in the absence of candidemia.

**MATERIAL AND METHODS.** A retrospective study has been performed with all patients admitted to the Intensive Care Unit for hepatic decompensation between April 2013 and May 2016. The following variables were collected: Mean age, APACHE II, SAPS II, SOFA, MELD, cause and stage of cirrhosis, use of vasoactive drugs, mechanical ventilation and mortality. Presence or absence of candida (type) in carrier control. Carrier's control has been collected at the patient´s ICU admission and once a week during their stay. Patients with candidemia were excluded.

Qualitative variables with normal distribution have been expressed as number and percentages and were analysed using chi-square tests. Quantitative variables were expressed as mean ± standard deviation (Rank) and were analysed using t-student tests and anova.

**RESULTS.** 110 patients have been included. 80 men, 30 women. The mean age has been 57.5 ± 10.35 (34–85) years. APACHE 21 ± 9.4 (5–50); SAPSII 51 ± 19.3 (10–116); SOFA 10.2 ± 6.9 (1–65); MELD at admission 18.7 ± 9.27 (6–47) MELD maximum 23.64 ± 11.8 (6–55); Length of stay has been 8.67 ± 8.7 (1–48) days. The etiology of liver disease was alcohol in 41 of the patients and viral (HCV) in 40 cases. 77 patients required mechanical ventilation (70%); 75 patients required vasoactive drugs (68%) 45 patients were diagnosed as septic shock (41%). Candida colonization has been observed in 44 patients (40%). Albicans: 26; Krusei: 2; Bracarensis: 2; Glabrata: 4; Guillermondi: 1; Lusitaniae: 1; Parapsilosis: 4; Trompicalis: 4. The mortality rate of the study has been 30/110 (27.3%). None of our patients had invasive candidiasis.

There is no statistical significant difference between APACHE II, SAPS II, SOFA, MELD and length of stay between patients colonized by candida and non-colonized patients. Patients colonized by candida do not present higher mortality rates, mechanical ventilation, and use of vasopressors or septic shock. When performing the same analysis in patient whose cause of liver disease was alcoholic or HCV, we did not objectify differences. There is statistical difference in length of stay in patients which the cause of hepatic decompensation was HCV and were colonized by candida and non-colonized patients. 10.6 vs 5.83. (P < 0.05).

**CONCLUSIONS.** The incidence of cirrhotic patients colonized by candida is high. Candidemia is associated with signficant mortality in cirrhotic patients, however, colonization does not seem to be associated with higher mortality rate or length of stay during admission in to ICU.

### 1032

#### Non-selective beta blockers ameliorate outcome in patients with cirrhosis at the ICU

T. Horvatits[1], K. Rutter[1], A. Drolz[1], K. Roedl[1], J. Kluwe[2], S. Kluge[1], V. Fuhrmann[1]
[1]University Medical Center Hamburg-Eppendorf, Dep. of Intensive Care Medicine, Hamburg, Germany; [2]University Medical Center Hamburg-Eppendorf, Dep. of Medicine, Hamburg, Germany
**Correspondence:** T. Horvatits
*Intensive Care Medicine Experimental* 2017, **5(Suppl 2):**1032

**INTRODUCTION.** Non-selective betablockers (NSBBs) represent a central therapeutic principle in management of portal hypertension by lowering hepatic portal pressure. Deleterious effects of NSBBs in patients with refractory ascites and spontaneous bacterial peritonitis have been reported. However, controversial findings regarding beneficial effects in end-stage liver disease set in motion a debate on when to discontinue betablocker (BB) therapy.

**OBJECTIVES.** We aimed to assess the impact of BB treatment on outcome and clinical implications such as course of organ failure in patients with cirrhosis at the ICU.

**METHODS.** This retrospective study was performed at the Medical University Centre Hamburg-Eppendorf. Patients with liver cirrhosis being admitted to ICU were included in this study. Data on prescription of NSBB medication as well as clinical and laboratory findings were analysed in all patients.

**RESULTS.** 411 patients with liver cirrhosis were enrolled in this study. The most common cause of cirrhosis was alcoholic liver disease (71%) followed by hepatitis C virus infection (18%), and others. 92 patients (22%) had NSBBs and 44 (11%) had selective BBs (SBBs) in their current medication when being admitted to ICU. Taking discontinuation and new prescription into account, 82 patients (20%) received at any time during their stay at ICU NSBBs and 72 (18%) SBBs. Overall 63% of patients did not receive any BBs after being admitted to the ICU.

Patients on NSBBs had a lower MELD score than others (p < 0.05). However, rate of ACLF did not differ between groups NSBB (68%), SBB (69%), without BBs (66%) (p = n.s.). Administration of NSBBs was associated with improvement of ACLF during first 48-hours (25% vs.16%).

In patients on NSBBs, improvement of 28-day survival was observed compared to SBB group and those without BBs (22% vs. 39% vs. 41%, p < 0.05), as shown in Fig. 331. In multivariate model NSBB treatment was significantly associated with improved 28-day survival independently of age, sex and severity of ACLF (grade) (HR 0.49, 95%CI 0.3-0.82; p < 0.05).

**CONCLUSION.** We observed that NSBB treatment was linked to improvement of ACLF during course of time. Furthermore NSBBs were associated with improved short-term survival in liver cirrhotic patients at the ICU.

NSBBs seem to represent an important therapeutic principle in patients with ACLF. Early termination of NSBB treatment may be associated with worse outcome.

**GRANT ACKNOWLEDGMENT**
This research received no specific grant from any funding agency in the public, commercial, or not-for-profit sectors.

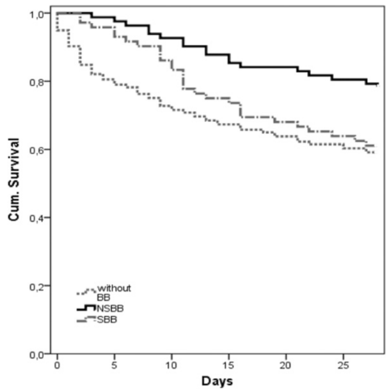

**Fig. 331 (Abstract 1032)** See text for description

**CONCLUSIONS.** Cirrhosis and especially ACLF is associated with metabolic acidosis and acidaemia owing to lactate and unmeasured anions. Acidosis and acidaemia, respectively, are associated with increased 28-day mortality in liver cirrhosis. Lactate and unmeasured anions are main contributors to metabolic imbalance in cirrhosis and ACLF.

### 1033

#### Acid–base status and its clinical implications in critically ill patients with liver cirrhosis and acute-on-chronic liver failure

A. Drolz[1,2], T. Horvatits[1,2], K. Roedl[1,2], K. Rutter[1,2], R. Brunner[2], C. Zauner[2], P. Schellongowski[3], G. Heinz[4], G.-C. Funk[2], M. Trauner[2], B. Schneeweiß[2], V. Fuhrmann[1,2]
[1]University Medical Center Hamburg Eppendorf, Intensive Care Medicine, Hamburg, Germany; [2]Medical University of Vienna, Internal Medicine III, Vienna, Austria; [3]Medical University of Vienna, Internal Medicine I, Vienna, Austria; [4]Medical University of Vienna, Internal Medicine II, Vienna, Austria
**Correspondence:** A. Drolz
*Intensive Care Medicine Experimental* 2017, **5(Suppl 2):**1033

**INTRODUCTION.** Acid–base disturbances are frequently observed in critically ill patients at the intensive care unit (ICU). To our knowledge, the acid–base profile of critically ill cirrhotic patients has not been evaluated with respect to acute-on-chronic liver failure (ACLF), and compared to critically ill patients without acute or chronic liver disease.
**OBJECTIVES.** Assessment of acid–base status and its clinical implications in critically ill patients with liver cirrhosis.
**METHODS.** One hundred seventy-eight critically ill patients with liver cirrhosis admitted to the ICU at the Medical University of Vienna compared to 178 matched controls. Arterial blood samples were collected on ICU admission. Quantitative physical-chemical analysis was performed to assess acid–base status.
**RESULTS.** Median SOFA score on admission was 13 (IQR 10–16) in cirrhotic and 12 (IQR 8–16) in matched controls without liver disease ($p = 0.105$). Patients with and without liver cirrhosis showed hyperchloremic acidosis, antagonized by hypoalbuminemic alkalosis. Cirrhotic patients, especially those with ACLF, showed a marked net metabolic acidosis owing to increased lactate and unmeasured anions. This metabolic acidosis was partly compensated by respiratory alkalosis; yet the mechanism of compensatory respiratory alkalosis became ineffective with progression to ACLF. Accordingly, acidaemia was present in 62% of patients with ACLF grade III compared to 19% in cirrhosis patients without ACLF. Acidaemia and metabolic acidosis were associated with 28-day mortality in cirrhosis. Patients with pH values < 7.1 showed a 100% mortality rate. Acidosis attributable to lactate and unmeasured anions was independently associated with mortality in liver cirrhosis.

### 1034

#### Critical liver cirrhotic patient who is admitted to an ICU, what can we expect?

A. Agrifoglio, L. Cachafeiro, E. Herrero, M.J. Asensio, M. Sánchez, J.M. Añón, A. García de Lorenzo
Hospital Universitario la Paz, Critical Care Medicine Service, Madrid, Spain
**Correspondence:** A. Agrifoglio
*Intensive Care Medicine Experimental* 2017, **5(Suppl 2):**1034

**INTRODUCTION.** Cirrhotic patients who need critical care support show high morbidity and mortality rates compared with other critically ill patients. Their prognosis is, in fact, influenced by both the severity of the underlying hepatic disease and the worsening of extrahepatic organ function. Because Intensivists require objective measurements of prognosis when making decisions, such as triage or treatment limitations, we performed a retrospective study over a period of 5 years to reassess the prognosis of Intensive Care Unit (ICU) admitted cirrhotic patients.
**OBJECTIVES.** To evaluate the hospital and one year mortality of cirrhotic patients entering our ICU, as well as the development of organic failure during their admission.
**METHODS.** We retrospectively collected all patients with clinical and/or histological diagnosis of hepatic cirrhosis admitted to the ICU between January 2011 and December 2015. We excluded liver transplants and collected: demographic and epidemiological data, reason for admission to the ICU, developed organic failure, need of organic supports (Mechanical Ventilation MV, Continuous Renal Replacement Therapy CRRT, and vasoactive drugs) and ICU and one year mortality.
**RESULTS.** During this period 4880 patients were admitted to the ICU. APACHE II mean of 18 points. Of these, 198 patients diagnosed with hepatic cirrhosis on admission, APACHE II mean: 17. Mean age was 52.5 ± 11 years, 68% were men. The mean stay of these patients was 8.1 days. Reasons for admission: 12% related to complications directly associated with liver disease; 88% due to habitual causes of ICU admission: community and nosocomial sepsis, polytrauma, post-surgical patients, neurocritical, exacerbated COPD, intoxications, acute pulmonary edema, cardiorespiratory arrest, etc. Cirrhotic patients, in relation to the total number of patients admitted to the ICU, required MV in 38% vs 39%, CRRT 18% vs 8% and shock with need for vasoactive drugs 75% vs 33%. The mortality of cirrhotic patients was higher than expected for their APACHE II (40% vs 17-27%) and in relation to subgroup mortality was 99% if they required CRRT; 83% with vasoactive drugs and if they needed MV >48h, 70%.
**CONCLUSIONS.** The cirrhotic condition in our patients resulted in a high ICU mortality, higher than expected for their APACHE II, although the reason for admission was 88% due to extrahepatic causes. The prognosis proved disastrous if the patient developed organic failure on admission. Of the patients who survived their ICU admission, 48% had died at one year.
**REFERENCES**
- Gines P, Fernández J, Durans F, Saliba F. *Management of critically-ill cirrhotic patients*. J Hepatol. 2012;56 Suppl 1:S13-24.
- Das V, Boelle PY, Galbois A, Guidet B, Maury E, Carbonell N, Moreau R, Offenstadt G. *Cirrhotic patients in the medical intensive care unit: early prognosis and long-term survival. Crit Care Med.* 2010 Nov;38(11):2108–16.

### 1035

#### Coagulogramm vs thromboelastogramm tests in patients underwent liver transplantation

T. Kuandykov, V. Mutagirov
National Scientific Center of Surgery, Anesthesiology and Intensive Care, Almaty, Kazakhstan
**Correspondence:** T. Kuandykov
*Intensive Care Medicine Experimental* 2017, **5(Suppl 2):**1035

**INTRODUCTION.** Optimal coagulation management remains one of the greatest challenges in orthotopic liver transplantation. Using coagulation tests and thrombelastography (TEG) may beneficial in determining the optimal coagulation management (1).

**OBJECTIVES.** To evaluate coagulation state after liver transplantation in patients who was not admitted to transfuse FFP.

**METHODS.** After local Ethic Committee approval and informed consent we studied 30 patients with liver cirrhosis, aged 41.3 ± 11.8 (24–56) y.o., weight 58.6 ± 6.9 (48–69) kg. MELD 16.2 ± 2.9. We studied activated partial prothrombine time (APPT), international normalized ratio (INR), fibrinogen A (FGA), platelets count (PLT), thrombelastography (K; R; α angle; MA; LY30), ICU stay and in-hospital stay duration.

**RESULTS.** The coagulation tests showed significant impairment of coagulogramm tests: prolonged of APPT, increased of INR and decreased of FGA level through 10–12 days after liver transplantation. While thromboelastography tests showed slight hypocoagulation status in patients only through 1–7 postoperative days (Table 245). There was no any bleeding in postoperative period in all patients.

ICU stay duration was 16.4 ± 5.7 days. In-hospital stay duration was 42.7 ± 14.3 days.

**CONCLUSIONS.** In this trial TEG tests showed a sufficient potential of haemostasisin in patients after liver transplantation on $4^{th}$ postoperative day in contrast to coagulogramm tests. Despite of impared coagulogramm tests coagulation status in patients after liver transplantation presents not critical disorders which does not require active management.

**Table 245 (Abstract 1035)**. Coagulation and TEG tests after LTx

| | Day 1 | Day 3 | Day 7 | Day 12 |
|---|---|---|---|---|
| APPT (sec) | 58.1±18.3 | 39.3±9.2 | 26.3±6.4 | 32.9±3.7 |
| INR | 2.7±0.7 | 2.2±0.3 | 2.1±0.7 | 1.5±0.7 |
| PT (%) | 34.8±19.1 | 36.4±8.3 | 49.2±5.5 | 59.0±9.9 |
| FGA (g/l) | 0.7±1.3 | 1.9±0.8 | 1.9±0.7 | 2.6±0.5 |
| PLT ($10^*9$/ul) | 55.7±26.3 | 49.3±22.7 | 53.5±18.2 | 106.0±48.2 |
| R (min) | 8.1±1.9 | 4.2±2.6 | 4.2±0.7 | 5.3±0.7 |
| K (min) | 4.9±3.8 | 4.2±2.0 | 2.9±1.9 | 2.2±1.1 |
| Alpha angle | 51.6±19.4 | 56.1±12.4 | 67.5±9.9 | 70.3±10.2 |
| MA (mm) | 39.7±19.6 | 53.3±21.3 | 60.1±15.8 | 66.9±18.8 |

### 1036

#### Cholestasis in patients after cardiac arrest

K. Roedl[1], C. Wallmüller[2], A. Drolz[1], T. Horvatits[1], K. Rutter[1], H. Herkner[2], F. Sterz[2], V. Fuhrmann[1]
[1]University Medical Center Hamburg-Eppendorf, Department of Intensive Care Medicine, Hamburg, Germany; [2]Medical University of Vienna, Department of Emergency Medicine, Vienna, Austria
**Correspondence:** K. Roedl
*Intensive Care Medicine Experimental* 2017, **5(Suppl 2):**1036

**INTRODUCTION.** Sudden cardiac arrest (CA) is one of the leading causes of death in adults in many parts of the world. Every year estimated 350.000 to 700.000 people in Europe are suffering CA and receive cardiopulmonary resuscitation (CPR). Cholestasis can be found in up to 20% of critically ill patients. To date, data occurrence and outcome of cholestasis after CA and CPR is scarce.

**OBJECTIVES.** Aim of the study was to determine occurrence and outcome of cholestasis in patients after in- and out-of-hospital CA.

**METHODS.** Assessment of occurrence of cholestasis was performed in a cohort of 1068 consecutive patients with CA and successful CPR that were treated at the Medical University Vienna, 266 (25%) patients with cholestasis could be identified. Patient characteristics, admission diagnosis, severity of disease, course of the disease and 28d mortality were assessed. Cholestasis was defined as rise of serum bilirubin over 2 mg/dl.

**RESULTS.** Overall, 266 (25%) patients developed cholestasis after successful CPR. Of these 213 (80%) were male with a median age of 59,5 (47–70) years. CA was witnessed in 236 (89%) cases. 213 (80%) of the patients suffered the CA out-of-hospital. Cardiac events leading to CA were observed in 176 (66%) of patients. Initial rhythm was shockable (VT/VF) in 150 (56%), non-shockable (PEA/Asystole) in 107 (40%) and unknown in 9 (4%) patients. Time to ROSC was 18 (9–28) minutes.

133 (50%) of patients with cholestasis were dead or had bad neurological outcome (CPC III/IV) within 28 days following CA.

**CONCLUSIONS.** Cholestasis is a frequent finding in patients following successful CPR. Cause of cardiac arrest is often cardiac.

### 1037

#### What does it mean the elevation of procalcitonin (PCT) after a liver transplant?

G. Seller-Pérez[1], J. Barrueco-Francioni[1], M.D. Arias-Verdu[1], M. Diez De Los Ríos[2], R. Lozano-Sáez[1], M.E. Herrera-Gutiérrez[1]
[1]Hospital Regional Universitario Carlos Haya de Málaga, Intensive Care Unit, Málaga, Spain; [2]Hospital Regional Universitario Carlos Haya de Málaga, Clinic Laboratory, Málaga, Spain
**Correspondence:** M.D. Arias-Verdu
*Intensive Care Medicine Experimental* 2017, **5(Suppl 2):**1037

**OBJECTIVE.** Procalcitonin (PCT) is considered a marker of sepsis although there are publications that claim there are increases not related to infections. We want to analyze the behavior of PCT after a liver transplant which causes are related to its alteration.

**METHODS.** Prospective cohort (n = 158) of patients who received liver transplant (LT) performed over the period 2009–2014, follow up until the end of 2016.

We determine PCT (reference 0.5 ngr/ml) and graft liver function during the first three days. Data as average (mean error). Outcome variables: graft dysfunction (GD) according to MEAF score, renal disfunction according to AKIN and hospital mortality. Analysis: non-parametric test and Chi-square test for $p < 0.05$. Approval by Ethical Committee.

**RESULTS.** PCT levels were increase in our patients after liver transplant, with levels of 8.38 ± 0.93 ng/dL on day 1 (range 0.05-68.26), 13.34 ± 1.28 the second day (range 0.18-68.26) and 9.62 ± 1.23 on the third (0.19-38.35). Only 18 patients (11.4%) had a PCT below 0.5 ng/dL.

The distribution by percentiles was: Median 3.17, quartiles 1.23,8.3, 95, 27.66 ng / mL.

We analyzed the relationship between renal dysfunction and PCT levels and although PCT levels were higher according to the degree of renal dysfunction, this relationship was not statistically significant. AKIN 0: 11.17 ± 1.47, AKIN I: 12.65 ± 1.12 ; AKIN 2: 19.15 ± 1.17; and AKIN 3: 21.50 ± 3.24 (p 0.91).

We also found no relation s.s. between graft dysfunction and PCT levels: 12.42 ± 1.3 in patients without graft dysfunction vs 23.46 ± 7.35 with dysfunction (p 0.25).

We found relation ss between PCT and hospital mortality :12.26 ± 1.13 ng / dl in vivo vs 23.46 ± 7.35 in deaths (p 0.025).

**CONCLUSION.** PCT levels are elevated after liver transplant without infection, which decrease its usefulness as a marker of infection and makes it necessary to define a new threshold of normality for this scenario. Although we can not explain this behavior, this elevation does not seem to be related to the function of the graft neither to the presence of acute renal dysfucntion.

### 1038

#### Acute liver failure due to Amanita phalloides poisoning: single centre experiences

E. Kieslichová[1], M. Protuš[1], S. Fraňková[2], D. Merta[1], J. Froněk[3], J. Šperl[2]
[1]Institute for Clinical and Experimental Medicine, Anaesthesiology, Resuscitation and Intensive Care, Prague, Czech Republic; [2]Institute for Clinical and Experimental Medicine, Hepatogastroenterology Department, Prague, Czech Republic; [3]Institute for Clinical and Experimental Medicine, Transplant Surgery Department, Prague, Czech Republic
**Correspondence:** M. Protuš
*Intensive Care Medicine Experimental* 2017, **5(Suppl 2):**1038

**INTRODUCTION.** Amanita phalloides poisoning is an uncommon, but potentially fatal cause of acute liver failure (ALF). In the Czech Republic Amanita phalloides intoxication occurs in 10–20 patients per year with 25% lethality. The management of amatoxin poisoning includes: preliminary medical care, supportive measures and specific treatments including extracorporeal liver support systems. In selected patients liver transplantation (LT) is the only successful modality of treatment.

**OBJECTIVES.** The aim of the study was to describe the characteristics of the patients with ALF due to amanita poisoning, analyse the results of treatment and evaluate their outcome.

**METHODS.** 24 consecutive patients with ALF due to Amanita phalloides poisoning admitted to ICU in the Institute for Clinical and Experimental Medicine (IKEM) in Czech Republic between July 2007 and April 2016 were studied retrospectively. Diagnosis of Amanita poisoning was based on the recent ingestion of mushrooms with gastrointestinal symptoms, laboratory markers of ALF and on the mycological examination. Demographic data, clinical data, laboratory parameters and therapeutic approach were collected. Standard medical treatment included oral decontamination with activated charcoal, intravenous rehydration with balanced crystalloid solutions, N-acetylcysteine and silibinin, continuous veno-venous hemodialysis in case of acute kidney injury or hyperammonaemia. In selected patients fractionated plasma separation and adsorption (FPSA, Prometheus, Fresenius Medical Care) was performed. During the study period, the decision for urgent LT was based on King's College criteria.

**RESULTS.** The cohort consisted of 13 males and 11 females (age 48.6 ± 17.4). All patients were already referred to ICU IKEM from other hospitals, 2.5 ± 1.3 days after mushroom ingestion. Mean time from ingestion to onset of gastrointestinal symptoms was 12.2 ± 9.7 hours and time to hospital admission was 28.5 ± 22.9 hours. At admission to IKEM, encephalopathy was present in 9 patients, 2 patients required vasopressoric support and 1 was mechanically ventilated. In total, 8 patients with poor prognosis were listed for urgent LT, 7 of them were listed within 2 days after admission. Six patients underwent FPSA procedure prior LT. One patient died before LT due to intracranial hypertension. Seven patients underwent successful LT; in 4 cases AB0 incompatible and in 2 cases, a split LT was performed. One patient died 2 months after LT due to mycotic sepsis, one patient underwent re-LT. Seven LT patients and all patients who had not meet King's College criteria and therefore were not listed for LT are alive.

**CONCLUSIONS.** Our results show that early and complex supportive therapy including extracorporeal liver support and availability of urgent LT is successful in treatment of patients with ALF induced by Amanita poisoning. King's College Criteria seem to be suitable for predicting prognosis and deciding in favour of LT in these patients.

### 1039

#### Retrospective review of patients with acute on chronic liver failure admitted to 2 British district general hospital intensive care units. Presentation, management and one year mortality

N. Singh, A. Trimmings
East Sussex Healthcare NHS Trust, Intensive Care, Eastbourne, United Kingdom
**Correspondence:** N. Singh
*Intensive Care Medicine Experimental* 2017, **5(Suppl 2):**1039

**INTRODUCTION.** Acute on chronic liver failure (ACLF) is a recently defined syndrome involving acutely decompensated cirrhosis and organ failure. Patients with ACLF who require admission to intensive care units (ICU) have poor outcomes. O'Brian et al. found mortality for patients admitted to UK ICUs had a mortality between 65 and 90%[1]. More recently Baja et al. found the mortality of patients admitted to North American Liver units with ACLF and sepsis to be between 28 and 77%[2].

**OBJECTIVES.** To determine if patients with ACLF admitted to our ICUs had a mortality similar to the published data. To determine if the commonly used markers/scores of physiological derangement in these patients were associated with mortality.

**METHODS.** Hospital electronic information systems were searched to find patients with cirrhosis and organ failure who were admitted to 2 British ICUs with a combined total of 19 beds in 2014/15. Data was collected on the cause of cirrhosis, Child Pugh score and Model for End Stage Liver Disease - Sodium (MELD Na) score on admission, levels of organ support, 30 day mortality and one year mortality. The unpaired t-test was used to look for association between the variables recorded and mortality.

**RESULTS.** 42 patients were admitted with ACLF. 29 (69%) were male, 32 (76%) had alcoholic liver disease and the mean age was 56. The median MELD Na score was 28 and the interquartile range was 22 to 33.75. 35 (83%) were Child Pugh grade C and 7 were B. 23 (54%) patients had an acute kidney injury.

28 (66.6%) patients died within 30 days and a further 4 died within a year. In total 32 (76%) died within 12 months. There was a statistically significant association with MELD Na scores ($p = 0.027$) and serum creatinine values ($p = 0.027$) on admission with one year mortality. There was no statistical association with Child Pugh score, serum bilirubin and serum creatinine. 11 (26%) patients had a MELD Na score greater than or equal to 34 and none of them survived.

**CONCLUSIONS.** Patients admitted to our ICU's with ACLF had a high mortality similar to the published data. We found a statistically significant association between mortality and on admission MELD Na scores and serum creatinine levels.

**REFERENCE(S)**

1. Prevalence and outcome of cirrhosis patients admitted to UK intensive care: a comparison against dialysis-dependent chronic renal failure patients. O´Brien AJ, Welch CA, Singer M, Harrison DA.. Intensive Care Med. 2012 June;38(6):991–1000

2. Survival in infection-related acute-on-chronic liver failure is defined by extrahepatic organ failures. Bajaj JS1, O´Leary JG, Reddy KR, Wong F, Biggins SW, Patton H, Fallon MB, Garcia-Tsao G, Maliakkal B, Malik R, Subramanian RM, Thacker LR, Kamath PS Hepatology. 2014 July;60(1):250–6.

**GRANT ACKNOWLEDGMENT**
None

### 1040

#### Secondary sclerosing cholangitis in patients with traumatic and non-traumatic brain injury

J. Schäper[1], L. Berger[1], B. Kitze[2], C. von der Brelie[3], H. Schwörer[4], V. Ellenrieder[4], M. Quintel[1], T. Randall[5]
[1]University Medical Center Göttingen, Anaesthesiology, Göttingen, Germany; [2]University Medical Center Göttingen, Neurology, Göttingen, Germany; [3]University Medical Center Göttingen, Neurosurgery, Göttingen, Germany; [4]University Medical Center Göttingen, Gastroenterology, Göttingen, Germany; [5]Asklepios Kliniken Schildautal Seesen, Neurology, Seesen, Germany
**Correspondence:** J. Schäper
*Intensive Care Medicine Experimental* 2017, **5(Suppl 2):**1040

**INTRODUCTION.** There has been an increasing number of reports on critically ill patients with secondary sclerosing cholangitis (SC-CIP) during or after an ICU stay. The development of casts in the intrahepatic bile ducts, and pronounced cholestasis are pathognomonic in SC-CIP. In the further course liver cirrhosis with indication for liver transplantation may develop.

**OBJECTIVES.** To test the hypothesis that in our institution SC-CIP has disproportionately often occurred in patients with brain injury during the last years, and to identify influencing factors.

**METHODS.** Retrospective data analysis (01/2010 to 11/2015) of clinical variables from the patient database management system IntelliSpace Critical Care & Anesthesia (ICCA™). An IT-based database search with illness specific keywords resulted in 239 patients, 30 thereof fulfilling the SC-CIP criteria.

Recording of clinical variables from the beginning of ICU stay until the beginning of cholestasis (definition: bilirubin ≥ 2.4 mg/dl and/or γGT ≥ 1000 U/l). Validation of diagnosis through ERCP/MRCP. Statistics: Spearman correlation. Statistical significance was assumed at $p < 0.05$.

**RESULTS.** 30 out of 35 SC-CIP patients showed traumatic or non-traumatic brain injury. 70% thereof were male. 100% were mechanically ventilated, developed SIRS or sepsis, and 87% continuously received high doses of ketamine (≥3 mg x kg body weight$^{-1}$ x h$^{-1}$). There was a significant correlation between the beginning of cholestasis and days on mechanical ventilation (R = 0.948) as well as the days on anti-infectives (R = 0.793).

**CONCLUSIONS.** This is the so far largest report on patients with brain injury developing SC-CIP. Nevertheless, the number of cases did not allow statistical evaluation of risk factors. However, formerly postulated SC-CIP triggers, such as MAP < 65 mmHg, number of transfused erythrocyte concentrates, obesity, prolonged prone position, PEEP > 10 mbar and high doses of catecholamines could be ruled out for this cohort. SIRS/Sepsis and a low oxygenation index have been confirmed as potential relevant factors. In addition, neuroprotective sedation with high doses of ketamine appears to be a contributing factor to the development of SC-CIP and should be re-evaluated for this use.

Leonhardt et al. Critical Care (2015) 19:131; Weig et al. European Journal of Medical Research (2012), 17:30.

### 1041

#### Efficiensy of plasmadsorbtion in patients with mechanical jaundice complicated with liver falure

A. Fomin, G. Titova
M.F.Vladimirskiy Moscow Regional Research Clinical Institute, Moscow Region, Moscow, Russian Federation
**Correspondence:** G. Titova
*Intensive Care Medicine Experimental* 2017, **5(Suppl 2):**1041

New methods of extracorporal blood correction and detoxification in condition of liver failure of patients with mechanical jaundice need an assessment of their efficiency and safety, especially against initial hypocoagulation and system introduction of heparin.

**RESEARCH OBJECTIVE.** Assessment of efficiency of plasma sorption on Plasorba BR-350 and its influence on the condition of blood coagulation system of patients with mechanical jaundice.

**MATERIALS AND METHODS.** The research is conducted for 18 patients aged 47–67 with the mechanical jaundice which developed as a result of biliary obstruction (choledocholithiasis). Initial level of general bilirubin was from 285 μmol/l to 589 μmol/l. The plasmadsorbtion procedures were carried out on the Octa Nova device, manufactured by Asahi Kasei Medical, Japan, with use of Plasorba BR-350 sorbent developed on the basis of anion-exchange resin for plasma sorption. Each patient had three procedures with processing of two volume of plasma circulation per procedure. One procedure was carried out just before operation (one day before), and another two - in the postoperative period. The duration of procedure averaged 4 hrs 30 min. The blood flow rate was 130–160 ml/min. The plasma flow rate was 25–30 ml/min. Anticoagulation was ensured by washing the bloodlines and a column with a sorbent with normal saline with heparin - 4,000 units of activity per 1 L. 5,000 units of activity of heparin were injected intravenously at the beginning of procedure. The biochemical values and coagulogram test results of patients were studied prior to procedure, during procedure and upon termination of procedure.

**RESULTS.** As a result of selective plasmadsorbtion after carrying out procedures with two circulation plasma volumes, by the end of the procedure, there was a significant decrease in total bilirubin level by 68.6 +/− 3.8%, conjugated bilirubin by 64.6 +/− 6.4%, unconjugated bilirubin by 66, 9 +/− 9.8%, and bile acids by 51,4 ± 2,8%. Other blood chemistry parameters did not show any changes. The study throughout the treatment did not show the decrease in dynamics of hemoglobin and platelets. There was no negative dynamics in change of INR (international normalized ratio), APTT (activated partial thromboplastin time), level of fibrinogen, prothrombin, anti-thrombin III. No bleeding complication occurred in any patient during the procedure of plasma sorption. Other biochemical values did not change significantly.

**CONCLUSIONS.** The researches showed that Liver Support plasma sorption is effective in condition of mechanical jaundice as a detoxication method. Based on the lack of bleeding complications in patients during the procedure and lack of changes in coagulogram after the procedure, this liver supporting method can be recommended for patients with high level of bilirubin in condition of mechanical jaundice at the stages of preparation for operational treatment of the bile ducts obstruction and in the postoperative period.

### 1042

#### Biomarker of inflammation or dysfunction after graft liver transplant. What c-reactive protein (CRP) means?

G. Seller-Pérez, M.D. Arias-Verdú, J. Barrueco-Francioni, M.M. Arrebola-Ramírez, R. Lozano-Sáez, M.E. Herrera-Gutiérrez
Hospital Regional Universitario Carlos Haya de Málaga, Intensive Care Unit, Málaga, Spain
**Correspondence:** M.D. Arias-Verdú
*Intensive Care Medicine Experimental* 2017, **5(Suppl 2):**1042

**OBJECTIVE.** After liver transplant, CRP has a controversial paper, because can be elevated (after inflamation) or normal (after disfunction). We want to analyzed the value of CRP after liver transplant and his relation with graft disfunction (GD).

**METHODS.** Prospective cohort (n = 183) of patients who received live rtransplant (LT) performed over the period 2009–2014, follow up until the end of 2016.

We determine CRP (reference 5 mgr/l) and graft function during the first three days. Data as average (mean error); Outcome variables: GD according to MEAF score and mortality. Analysis: non-parametric and Chi-square test for $p < 0.05$; ROC curves, area under the curve (AbC) and Youden index (best cut point). Approval by CEI.

**RESULTS.** Mean PCR levels was 57.46 (2.96) mgr/l on day 1, 86.99 (4.06) on day 2 and 64.03 (5.34) on day 3.Lower register PCR was 51.91(2.80) mgr/l.

These levels were no trelated to the confounding variables analyzed (including renal function by AKIN),but we found relation statisticaly significant with noradrenaline [81,55 (4,10) mgr/l without NA vs 103,17(10,22) with NA, p0.02].
However, CRP was negatively related to mortality [alive 53.94 (2.89) vs 24.45 (6.72) in deaths, p 0.018; AbC 0.75 (0.60-0.91) p 0.007] and also with GD [alive 54.43 (3.15) vs 36.68 (4.02) in deceased, p0.026; AbC 0.61 (0.51-0.71), p 0.078].
The cut-off point detected for GD was less than 68mgr/l, sensitivity 96.15% and specificity 34.4%, Youden index 0.31.
**CONCLUSION.** Although CRP levels are elevated after liver transplant, a less elevation (below 68 mgr/l) is associated with graft dysfunction. However, this marker does not seem to add information to other scores to detect liver dysfunction.

### Rehabilitation and recovery

#### 1043

#### The association between family-centered nurse communication and acute stress in the ICU

F. DeKeyser Ganz[1], N. Bekman[2]
[1]Haddasah Hebrew University Faculty of Nursing, School of Nursing, Jerusalem, Israel; [2]Hadassah Hebrew University Medical Center, Jerusalem, Israel
**Correspondence:** F. DeKeyser Ganz
*Intensive Care Medicine Experimental* 2017, **5(Suppl 2):**1043

**INTRODUCTION.** Patient/family centered care in the ICU has been receiving increasing attention over the past few years. One cornerstone of this approach is patient/family centered communication [1]. Family members of patients in the ICU have been shown to suffer from high levels of post-traumatic stress long after discharge from the ICU [2]. One possible method to decrease this stress is to improve staff communication with family members while the patient is still in the ICU. Few have investigated whether high quality family centered communication was associated with decreased family member stress levels while the patient was still in the ICU. Finding such an association and eliciting what forms of communication are most associated with decreased acute stress can be a first step in designing interventions to decrease family member short and long term stress.
**OBJECTIVES.** To determine whether there is an association between family member perceived acute stress and family centered communication during ICU hospitalization.
**METHODS.** Three questionnaires were distributed to family members while their loved one was hospitalized in the ICU (a demographic questionnaire, the Perceived Stress Scale[3] and the Family Centered Communication Questionnaire, developed by one of the authors) in a surgical and medical ICU.
**RESULTS.** The sample consisted of 100 family members who were found to be mild/moderately stressed ($M$ = 1.97/4, $SD$ = 0.63) and were satisfied with their communication with the nurses ($M$ = 4.29/5, $SD$ = 16.8). The majority of respondents were children (n = 54, 54%) or spouses (n = 31, 31%) of the patient with a mean age of 48.4 years. Average patient stay was 6.7 days ($SD$ = 5.2). Most respondents classified their loved ones´ condition as stable (n = 53, 53%).
Significant negative correlations were found between mean total acute stress scores and communication (r = −.36, p = .003) and with the communication sub-scale scores of responding to emotions (r = −.32, p = .005), managing care (r = −.33, p = .004) and developing and maintaining relationships (r = −.37, p = .001), but not for decision-making or exchange of information.
**CONCLUSIONS.** Good communication is associated with decreased acute stress levels among family members, especially related to emotional and relationship issues. It is therefore recommended that efforts be made to improve communication with family members, especially in these areas.

**REFERENCES**
1: Davidson JE et al. (2017). *Crit Care Med*, 45(1): 103–128
2: Petrinec AB, Daly BJ (2016). *West J Nurs Res*, 38(1): 57–78
3: Cohen S, Williamson G (1988). *The Social Psychology of Health*. Newbury Park, CA

#### 1044

#### Development of a nurse led ICU follow Up clinic using limited resource

S. Galt, T. Scott, K. Massie, L. Allen
NHS Grampian, Aberdeen Royal Infirmary, Aberdeen, United Kingdom
**Correspondence:** S. Galt
*Intensive Care Medicine Experimental* 2017, **5(Suppl 2):**1044

**INTRODUCTION.** Any critically ill person is predisposed to extended physical and psychological ill health[1] and should be offered the support of a specialised follow up clinic[2]. Research shows that patients who survive critical illness have complex physical and mental problems causing persistently poor quality of life, referred to as 'Post Intensive Care Syndrome'[3].
**OBJECTIVES.** We proposed to pilot a nurse led out-patient follow up clinic into the role of the current ICU in-patient follow up team.
**METHODS.** Patients who were ventilated for >3 days and lived locally were offered an appointment 12 weeks after hospital discharge. Discretion was also used to invite any patient who may benefit from follow up. Appointments lasted 1 hour with time to visit to the ICU. Telephone consultations were also offered. Due to limited resources we ran a nurse led clinic with ICU consultant support as required. Physiotherapists were on hand, and clinic space away from the ICU was used. Clinic format was based on quality of life assessment tools, and audited through evaluation forms returned to the clinical effectiveness department for external review.
**RESULTS.** The number of patients invited was lower than expected. Out of a possible 54 patients, 31 attended clinic between September 2015 and March 2017. Feedback was received from 18 patients, and was invariably positive.
Referrals were made to Neuropsychology, Dietetics and Cardiology. The Follow Up nurse, physiotherapist or Consultant dealt with most other issues at the time of consultation. A letter was sent to each patient's GP summarising the appointment and recommendations. Contacting patients was often difficult so an appointment letter was sent out. Some clinics had to be re-scheduled due to other work demands. Common reasons for patients being unable to attend clinic were noted.
**CONCLUSION.** The pilot was planned to run for 1 year, but numbers were lower than anticipated and so we aim to review data after 50 patients. Feedback so far suggests that the clinic is beneficial. Once all data is available consideration will be made to source funding towards staff resources.
Other expansion ideas include patient support groups. We are currently exploring the possibility of utilising university resources for rehabilitation needs. Ongoing qualitative auditing of patient benefit will be essential to justify the service.

**REFERENCES**
1. Griffiths J A, Barber V S, Cuthbertson B H, Young J D (2006) A national survey of intensive care follow-up clinics. Anaesthesia Vol 61 pp950-955
2. National Institute for Health and Clinical Excellence (2009) Rehabilitation after Critical Illness Guidance.nice.org.uk/cg83
3. Needham D M, Davidson J, Cohen H, et al. Improving long-term outcomes after discharge from intensive care unit: report from a stakeholders' conference. Crit care med 2012;40:502–9

**GRANT ACKNOWLEDGMENT**
Not applicable.

**Table 246 (Abstract 1044)**. Patient Feedback

| Patient Feedback Comments from Follow-up Clinics |
|---|
| "Amazed to be given the appointment" |
| "The nurse was excellent at the clinic and made talking about my ICU experience easy as she was very compassionate, understanding and empathetic" |
| "Think that it is a good service to provide. Allows you to focus on your progress since leaving hospital and let you know what you are going through is normal or as expected" |
| "I found the session most reassuring and put a lot of my anxieties to rest, real and imagined!" |
| "The session put my mind at rest because I was very distressed at the memory loss, the inability to count and writing which was illegible. Thought I had a stroke or dementia. Very worrying until I visited the clinic nurse. " |
| "I am a lot clearer about what was done to me and have a better idea of what happened to me" |

**Table 247 (Abstract 1044)**. Reasons for Not Attending Clinic

Patients contacted and reasons not seen at clinic

| Preferred a telephone consultation | Message left no return call | Patient didn't feel they required an appointment | Cancelled clinic appointment | Ongoing hospital treatment or palliation | Moved away or lost to follow up | Died | Unable to get Transport |
|---|---|---|---|---|---|---|---|
| 5 | 10 | 2 | 2 | 6 | 4 | 6 | 2 |

### 1045

#### Introducing early and structured rehabilitation in critical care: a quality improvement project

D. McWilliams[1], C. Snelson[2], H. Goddard[3], B. Attwood[3]
[1]Queen Elizabeth Hospital NHS FT, Birmingham, United Kingdom; [2]Queen Elizabeth Hospital NHS FT, Critical Care, Birmingham, United Kingdom; [3]South Warwickshire NHS Foundation Trust, Warwick, United Kingdom
**Correspondence:** D. McWilliams
*Intensive Care Medicine Experimental* 2017, **5(Suppl 2):**1045

**INTRODUCTION.** Early and structured rehabilitation in critical care has demonstrated significant improvement in physical and clinical outcomes [1]. Despite this increasing evidence base, uptake and delivery of these interventions remain low [2].

**OBJECTIVES.** We aimed to assess the potential impact of introducing an already established and effective programme of rehabilitation into an external critical care unit.

**METHODS.** All patients admitted to critical care for ≥ 4 days and discharged to the ward were included in the trial. Baseline data was collected for a period of 3 months from 01/12/2016 until 28/02/2017 to confirm current practice. After this period an expert physiotherapist in critical care rehabilitation worked with the home team for a training period of 1 month. This training period included an analysis of potential local barriers to early rehabilitation practice and the introduction of a structured approach to rehabilitation according to a previously published protocol. To assess any changes in rehabilitation practice outcome measures included time to first mobilise and highest level of mobility achieved within critical care.

**RESULTS.** Baseline date was obtained for 31 patients over a 3 month period and compared to 9 patients admitted for ≥ 4 days during the training period of the study (see Table 248). Time to 1st mobilisation was reduced by 2.2 days for patients with a critical care length of stay of ≥ 4 days, with the greatest reduction seen for those patients ventilated for 4 days or more (11.6 vs 5.8 days). This was associated with a slight increase in Manchester Mobility Scores (MMS) score at critical care discharge and reductions in critical care and hospital length of stay.

**CONCLUSIONS.** Early results from our quality improvement project demonstrate a potentially positive impact of introducing a programme of early and structured rehabilitation in critical care. On going data collection will now occur for a period of 3 months to help confirm these findings. If successful this could provide a framework for introducing similar programmes to other critical care units nationally.

**REFERENCE(S)**

1. Kayambu G, Boots R, Paratz J. (2013) Physical therapy for the critically ill in the ICU. Crit Care Med. 41(6):1543–54.
2. Bakhru RN, Mcwilliams DJ, Wiebe DJ, et al. (2016). ICU structure variation and implications for early mobilization practices: an international survey. Ann Am Thorac Soc. 13(9):1527–37.

**Table 248 (Abstract 1045)**. Patients admitted to critical care for ≥ 4 days

| | Baseline | Intervention |
|---|---|---|
| n | 31 | 9 |
| Age (years) | 65.7 | 69.2 |
| Mobilised within critical care | 23/31 (74%) | 9/9 (100%) |
| Time to 1st Mobilise (days) | 6.2 | 4.0 |
| Mean MMS at Critical Care Discharge | 3.7 | 4.6 |
| Critical Care length of stay (days) | 9.9 | 8.7 |
| Ventilation days | 4.2 | 4.7 |
| Ward length of stay (days) | 13 | 9.8 |
| Total hospital length of stay (days) | 24 | 18.5 |

**Table 249 (Abstract 1045)**.Data for patients ventilated for ≥ 4 days

| | Baseline | Intervention |
|---|---|---|
| n | 12 | 4 |
| Age (years) | 64.0 | 66.3 |
| Mobilised within critical care | 5/12 (42%) | 4/4 (100%) |
| Time to first mobilise (days) | 11.6 | 5.8 |
| Mean MMS at critical care discharge | 3.5 | 4.0 |
| Critical Care length of stay (days) | 17.4 | 13.5 |
| Ventilation days | 11.3 | 10 |
| Ward length of stay (days) | 17 | 10.3 |
| Total hospital length of stay (days) | 34.4 | 23.8 |

### 1046

#### Timing and feasibility of early mobilization in critically ill patients: a retrospective observational study

B. Broens, M.C. de Waard, S. Hania, S. Stapel, H.M. Oudemans- van Straaten
VU University Medical Center, Adult Intensive Care Medicine, Amsterdam, Netherlands
**Correspondence:** B. Broens
*Intensive Care Medicine Experimental* 2017, **5(Suppl 2):**1046

**INTRODUCTION.** Early mobilization is an important intervention to prevent or limit Intensive Care Unit (ICU)-acquired weakness,[1,2] while delaying mobilization can adversely influence outcome.[3] Nevertheless, an appropriate timing of initiation is not defined. In addition, patient-related factors can be barriers for the initiation or continuation of a mobilization intervention.[4]

**OBJECTIVES.** To identify factors that influence the timing and feasibility of early mobilization in critically ill patients.

**METHODS.** In this retrospective observational study, data was derived from electronic patient and mobilization files during a four-month period. Inclusion criteria were: age > 18 years, BMI < 35 and ICU admission ≥ 3 weekdays. Timing was investigated with a survival analysis for the ability to mobilize passively or actively, stratified by various patient characteristics. If necessary, these were divided by the 33$^{th}$ and 66$^{th}$ percentiles. The ability to mobilize was based on cooperation, physical stability and muscle strength using a mobilization protocol. Contraindications, indicating physical instability, included hemodynamic instability, poor oxygenation, elevated intracranial pressure and recent seizures. Feasibility was presented as numbers and percentages, for exercise therapy and cycle ergometry separately.

**RESULTS.** During the study period 50 patients [36M, 14F] were included, with a median age of 66 [52–74] and a median Sepsis-related Organ Failure Assessment (SOFA) score of 10 [8–12]. After 5 weekdays, 90% and 32% of the patients were able to mobilize passively and actively, respectively ($p < 0.0001$). Patients with a SOFA score > 12 were less able to mobilize than patients with a SOFA score ≤ 12 ($p < 0.05$). The most common barriers to initiate an exercise therapy or cycle ergometry session were contra-indications (36% and 31%, respectively) and no physiotherapy visit (23% and 20%, respectively), while fatigue was the most common reason for the discontinuation of a physiotherapy session (33% and 47%, respectively).

**CONCLUSIONS.** Most critically ill patients were able to mobilize passively even in the first days of ICU admission. Failure to mobilize was predominantly associated with severe disease (SOFA > 12). Feasibility can mainly improve with a more fitting visitation strategy and the application of passive physiotherapy in case of fatigue. These findings may contribute to the enhanced implementation of early mobilization in critically ill patients.

**REFERENCE(S)**

[1] PMID: 20711065
[2] PMID: 18596631
[3] PMID: 26655865
[4] PMID: 22807652

**GRANT ACKNOWLEDGMENT**
None.

### 1047

#### Using an intervention mapping approach to reduce the emotional impact of the transmission from an intensive care unit to the follow-up ward for patients and their relatives

M. van Mol[1], E. Kompanje[1], D. Gommers[1], M. Nijkamp[2]
[1]Erasmus MC University Hospital, Intensive Care Unit, Rotterdam, Netherlands; [2]Open University of the Netherlands, Faculty of Psychology and Educational Sciences, Heerlen, Netherlands
**Correspondence:** M. van Mol
*Intensive Care Medicine Experimental* 2017, **5(Suppl 2):**1047

**INTRODUCTION.** It seems clear that three basic human needs (i.e., autonomy, competence and relatedness), are comprised in patients that are discharged from an intensive care unit (ICU) to the follow-up ward. This means; having to deal with loss of control (inflicting autonomy), not having one´s health outcomes in one´s own hands (decreasing competence) and feeling alone in recovery from severe health threats (negatively affecting relatedness). Therefore, it is of utmost importance to support ICU patients and their relatives to help them regain their feelings of control, their self-efficacy and social contacts.

**OBJECTIVES.** Aiming to develop an intervention reducing the emotional impact of the discharge from the ICU that is robust, effective and useful in practice because it is grounded on theoretical and empirical evidence.

**METHODS.** Intervention Mapping (IM), a six-step theory- and evidence-based approach, was used to guide the development process. The first step, a problem analysis, comprised a literature review, semi-structured telephone interviews with former ICU-patients and their relatives, and qualitative roundtable meetings for all eligible nurses (i.e., 135 specialized and 105 general ward nurses). Performance and change objectives were formulated in step 2. In step 3, theory-based methods and practical applications were selected and directed at the desired behaviors and the identified barriers. Step 4 designed a revised discharge protocol taking into account existing interventions. In step 5, a training for nurses addressing the emotional impact of admission into the ICU was executed. The knowledge gained was expressed by change scores before and after the training on a scale from 1 to 10. The evaluation of the new discharge protocol (IM step 6) is in progress and not included in this study.

**RESULTS.** Four former ICU patients and two relatives underlined the importance of the need for effective discharge information and supportive written material. They also reported a lack of knowledge regarding the consequences of ICU admission. The intervention was designed to target knowledge, attitudes, self-efficacy, and perceived social influence. Building upon IM steps one to three, a discharge protocol was developed that is relevant and feasible within current daily practice. The knowledge raised significantly from 5.7 (±1.6) to 6.7 (±1.5) in ICU nurses and from 4.8 (±1.6) to 7.2 (±1.3) in general ward nurses.

**CONCLUSIONS.** Intervention mapping provided a comprehensive framework to improve ICU discharge by guiding the development of a theory- and empirically-based discharge protocol that is robust and useful in practice. In this way, the evidence based renewed practice enables to respond better to the instrumental and affective needs of patients and their relatives.

**GRANT ACKNOWLEDGMENT**
This work was supported by a grant from Erasmus MC, “Evidence Based Care by Nurses”, project 2015–15404.

### 1048

#### A service development initiative of extended hours working for physiotherapy services in critical care

O.D. Gustafson, J.A. Grant
Oxford University Hospitals NHS Foundation Trust, Adult Intensive Care Unit, Oxford, United Kingdom
**Correspondence:** O.D. Gustafson
*Intensive Care Medicine Experimental* 2017, **5(Suppl 2):**1048

**INTRODUCTION.** Physiotherapy services in critical care units are commonly delivered during traditional daytime working hours (08:00–16:00). Provision outside of these hours is delivered by a variety of physiotherapists that may not be specialists in critical care as part of an overnight on-call service. The demands on this service are increasing annually with more patients being treated by non-critical care specialist physiotherapists. Altered working patterns for critical care physiotherapists have previously demonstrated positive effects on clinical care with significant additional investment[1], however it is unclear if this is possible without investment.

**OBJECTIVES.** To assess the feasibility of providing an extended hours physiotherapy service to ICU with no additional investment.

To assess the impact of extended hours physiotherapy on the frequency of overnight on-call physiotherapy interventions.

To assess the impact of extended hours physiotherapy on the delivery of rehabilitation interventions.

**METHODS.** An adjustment to the ICU physiotherapy team working patterns at a UK general tertiary adult ICU from May 2016 to March 2017, ensuring one critical care physiotherapist working until 20:00, Monday to Friday. The feasibility of the change in working patterns was evaluated by recording the number of uncovered extended hours shifts. The impact on overnight on-call activity was evaluated by recording the number of physiotherapy interventions during the extended hours period (16:00–20:00) that would have otherwise been covered by the on-call physiotherapist. Information was also recorded on the number of rehabilitation interventions completed during the extended hours period that would have previously been missed.

**RESULTS.** Extended hours working was provided on 84% of eligible days, with an average of 4.4 patient interventions per extended hours period. There were 136 referrals to the on-call service over the 11 months, with 205 acute physiotherapy interventions provided during the extended hours period that would have otherwise resulted in a referral to the on-call service. This translates into a cost saving of £5,920 over the 11 month period. There were on average 10 ?A3B2 show $132#?>additional rehabilitation sessions completed per month during the extended hours period.

**CONCLUSIONS.** This service development initiative was feasible to deliver with no additional investment. It resulted in a 60% reduction in referrals to the on-call physiotherapy service and an additional 110 rehabilitation interventions.

### 1049

#### A benchmark survey of compliance with National Institute of Clinical Excellence (NICE) clinical guideline 83-rehabilitation after critical illness in adults, in intensive care units within Great Britain

L.A. Mylott, M. McKeever, K. Morris
Guy's and St Thomas' NHS Foundation Trust, Physiotherapy, London, United Kingdom
**Correspondence:** L.A. Mylott
*Intensive Care Medicine Experimental* 2017, **5(Suppl 2):**1049

**INTRODUCTION.** The initiation of rehabilitation in Intensive Care Units (ICUs) is widely accepted and is supported by NICE guideline (CG83) published in 2009 [1]. The processes and systems present in ICUs to ensure compliance with the guideline vary despite an increasing evidence base. Existing literature tends to focus on the act of rehabilitation itself rather than how it is achieved. CG83 advocates a multi-disciplinary team (MDT) approach, the setting of goals and regular assessment alongside the involvement of the patient and their family. This project sought to understand how ICUs ensure they are complying with these recommendations.

**OBJECTIVES.** To determine the compliance of ICUs in Great Britain (GB) with CG83 [1] with reference to evidence of goal setting, use of outcome measures (OMs), MDT, patient and family involvement and the documentation of these activities.

To recognise areas for improvement, identify best practice and disseminate findings.

**METHODS.** Survey questions were developed through an iterative process. These were emailed to 75 eligible centres in 2016, identified through the interactive Chartered Society of Physiotherapy forum. Sole paediatric centres were excluded. The need for informed consent was waived by the local research and ethics committee.

**RESULTS.** A demographic representative sample of 48 surveys was returned giving a response rate of 64.5%. Documentation of goal setting occurred in 38 (78%) units. Goal setting meetings took place in 13 (27%) units with 8 (17%) including more than two disciplines from the MDT. Six (13%) units involved the patient and/or family. Documentation of goals varied from inclusion in individual notes to the use of rehabilitation pathways and boards in a patient's bed space. Utilisation of OMs to aid goal setting occurred in 33 units (69%) with 29 (60%) using the Chelsea Critical Care Assessment Tool. Issues with consistency, communication and time were described as barriers to compliance to CG83. Proposed changes or current improvement work was reported in 11 (29%) units.

**CONCLUSIONS.** This small benchmark study suggests that compliance with CG83 differs between units and that there is a great variation in the processes surrounding rehabilitation after critical illness. The result of this study is timely with regards to the current consultation on CG83 and is an opportunity to highlight the importance of considering the procedures in place to ensure compliance. A few novel practices were discovered through this project, however, the authors also suggest collaboration with other areas, such as stroke and older persons, who have reputable and established rehabilitation care pathways. Review of current practice will empower staff to drive change and produce more robust systems that will result in the best care for patients.

### 1050

#### Impact of implementation of protocol of early mobilization in a spanish intensive care unit: a pilot study

M. Magret, P. Perelló, J. Mariné, Z. Ramos, A. Piquer, A. Arasa, M.T. Cabas, A. Rojas, R.M. Sansegundo, M. Bodí
Hospital Joan XXIII, Tarragona, Spain
**Correspondence:** M. Magret
*Intensive Care Medicine Experimental* 2017, **5(Suppl 2):**1050

**INTRODUCTION.** Early mobilization (EM) can benefit ventilated patients admitted to intensive care unit (ICU) through prevention or attenuation of ICU acquired weakness. Literature shows that EM is feasible and safe.

**OBJECTIVES.** To determine the feasibility and safety of the implementation of EM protocol in our ICU and to assess the impact on days of mechanical ventilation (MV), length of ICU and hospital stay and hospital discharge destination.

**METHODS.** Retrospective cohort study in a general ICU. Adult patients who had been on MV for more than 48 h and Barthel Index > 70 were included in the protocol of EM during 6 month. These were compared with a historical control who received standard physical therapy. Three active mobilization events were defined: sit sitting chair, standing and walking. We defined six activity-related adverse events as fall to knees, tube removal, systolic blood pressure >200mmHg, systolic blood pressure < 90mmHg, oxygen desaturation < 80% and extubation. The characteristics were analyzed using median, interquartile range, mean and standard deviation (SD) for continuous and ordinal variables, and absolute frequency and percentage for categorical variables. Characteristics on the length of stay (LOS) in ICU and in hospital, duration of MV and discharge destination of both groups were compared using Pearson´s T-Student and $\chi 2$.

**RESULTS.** During the study period we included 24 patients in the EM group and these were compared with 18 historical control patients. We conducted a total of 82 activity events in the EM group. These included 31(37.8%) sit in chair, 33(40.2%) stand up and 18(22%) ambulate. In 44.4% of the ambulation the patients were on MV. The median distance ambulated was 75.7 meters. Adverse events were infrequent, occurring in 1 of 82(1.2%) activity events. There were no differences in baseline characteristics between the groups. The mean hospital LOS was 32.2(SD26.6) vs 39.2(SD 21.2), p = 0.35, the mean ICU LOS was 21.7(SD 20.6) vs 22.4(SD12.2), p = 0.8 and the mean duration of MV was 15.6(SD18.7) vs 13.6(SD 9.13), p = 0.65. The majority of patients included in the EM were discharged at home compared with control group (75%vs45%,p = 0.04).

**CONCLUSIONS.** The implementation of EM protocol is safe. Although no statistical differences were found regarding the hospital and ICU LOS and duration of MV, patients included in the EM had less hospital length of stay and a higher percentage was discharged at home. The functional improvement of the patients, besides having clinical significance as the rest of variables, had statistical significance despite the population limitation.

**GRANT ACKNOWLEDGMENT**
No

### 1051

#### A short structured skills training course for critical care physiotherapists in a lower-middle income country

T.S. Tunpattu[1], V. Newey[2], P.C. Sigera[3], A.P. De Silva[3], A. Goonarathna[1], I. Aluthge[4], P. Thambavita[1], R. Perera[4], A. Meegahawatte[3], I. Isaam[3], A.M. Dondorp[5], R. Haniffa[3]

[1]National Hospital of Sri Lanka, Colombo, Sri Lanka; [2]Barts Health NHS Trust, London, United Kingdom; [3]Network for Improving Critical Care Systems and Training, Colombo, Sri Lanka; [4]School of Physiotherapy and Occupational Therapy, Colombo, Sri Lanka; [5]Mahidol Oxford Tropical Medicine Research Unit, Bangkok, Thailand

**Correspondence:** R. Haniffa

*Intensive Care Medicine Experimental* 2017, **5(Suppl 2):**1051

**INTRODUCTION.** Lack of regulated and systematic postgraduate training or continuous professional development (CPD) for the physiotherapists in Sri Lanka was identified as the greatest barrier to the growth of critical care physiotherapy in an island wide survey involving all state physiotherapists working in critical care
(Sigera et al. 2016).

**OBJECTIVES.** The aim is to describe the delivery and acceptability of a short, structured training course for critical care physiotherapy and its effects on the knowledge and skills of the participants in Sri Lanka, a lower-middle income country.

**METHODS.** The 2-day program combining short didactic sessions with small group workshops and skills stations was developed and delivered by local facilitators in partnership with an overseas specialist physiotherapist trainer.

The impact was assessed using pre/post-course self-assessment, pre/post-course multiple-choice-question (MCQ) papers and an end-of-course feedback questionnaire.

**RESULTS.** Fifty-six physiotherapists (26% of critical care physiotherapists in Sri Lanka) participated. Overall confidence in common critical care physiotherapy skills improved from 11.6% to 59.2% in pre/post-training self-assessments respectively. Post-course MCQ scores (mean score = 63.2) and percentage of passes (92.5%) were higher than pre-course scores (mean score = 36.6, percentage of passes = 13%).

| Responses (n = 56) | Rating: 8–10 (High) | Rating:4–7 (Moderate) | Rating:1–3 (Low) | Missing |
|---|---|---|---|---|
| Overall, the course was enjoyable | 41(73.2%) | 8(14.3%) | - | 7(12.5%) |
| Overall the course was worthwhile | 39(69.6%) | 7(12.5%) | - | 10(17.9%) |
| This course has increased my critical care knowledge | 42(75%) | 6(10.7%) | - | 8(14.3%) |
| This course has increased my critical care skills | 34(60.7%) | 13(23.2%) | 1(1.8%) | 8(14.3%) |
| The quality of the teaching was of a high standard | 42(75%) | 7(12.5%) | - | 7(12.5%) |
| The quality of the educational materials provided was of a high standard | 22(39.3%) | 26(46.4%) | 1(1.8%) | 7(12.5%) |
| There was sufficient time allocated in the course to cover all the material appropriately | 14(25%) | 30(53.6%) | 4(7.1%) | 8(14.3%) |
| I would recommend this course to a colleague working in critical care units | 42(75%) | 7(12.5%) | - | 7(12.5%) |

*[Feedback from participants]*

Overall feedback was very positive with 75% and 61% of the participants were highly satisfied with the course's contribution to improved critical care knowledge and skills respectively.

**CONCLUSIONS.** In this setting, with the lack of a formal CPD, there was limited opportunity for physiotherapists involved in critical care to improve their knowledge and skills. The positive feedback and the significant increase in self-perceived confidence to perform skills associated with critical care physiotherapy treatments, suggests this method of locally up-skilled faculty delivering practical training may be of value in this setting and may be of benefit for filling the theory to practice gap for clinicians where opportunity for more formal specialist training is limited.

We wish to acknowledge the Government Physiotherapists' Association, all the physiotherapists who participated in the study and the staff of National Intensive Care Surveillance for their kind assistance. We are grateful to Ms. Abi Beane for the assistance given when preparing this manuscript.

**REFERENCE(S)**

Sigera, P.C. et al., 2016. National Profile of Physiotherapists in Critical Care Units of Sri Lanka: A Low-Middle Income Country. *Physical therapy*, p.ptj.20150363.

**GRANT ACKNOWLEDGMENT**

The training was sponsored by the School of Physiotherapy and Occupational Therapy, Ministry of Health and by the Mahidol Oxford Tropical Medicine Research Unit.

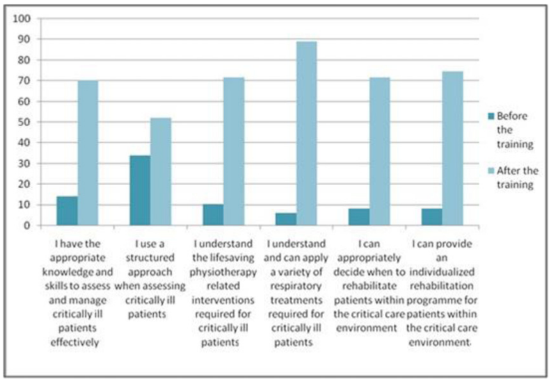

**Fig. 332 (Abstract 1051)** Overall confidence and skills

### 1052

#### Does surgical rib fixation affect the rate of pulmonary complications following major trauma?

T.J. Lea, D. McWilliams

Queen Elizabeth Hospital NHS FT, Birmingham, United Kingdom

**Correspondence:** T.J. Lea

*Intensive Care Medicine Experimental* 2017, **5(Suppl 2):**1052

**INTRODUCTION.** Flail chest injuries cause significant morbidity and are associated with prolonged periods of mechanical ventilation and post traumatic pneumonia[1,2]. Recent evidence of surgical rib stabilization has been shown to be associated with reduced ventilator days, lower incidence of pneumonia, and reduced medical costs[3]. Surgical rib stabilisation was introduced at our hospital in July 2015.

**OBJECTIVES.** To evaluate the impact of surgical fixation of flail chest on development of post injury pulmonary complications.

**METHODS.** Data was collected from all patients admitted to a large West Midlands multi trauma centre with flail chest injury between November 2015 and August 2016. Primary outcome was development of pulmonary complications, assessed using the Brooks Brunn tool. Secondary outcomes included incidence of mechanical ventilation and tracheostomy rates. Data was analysed using the Fisher exact test.

**RESULTS.** A total of 36 patients were admitted with flail chest injuries during the trial period, of which 10 underwent surgical fixation (see Table 250). Pulmonary complication rates were significantly higher in the fixation group (90% vs 35%, $p < 0.01$), with higher rates of mechanical ventilation also seen (100% vs 35%, $p < 0.001$). No significant differences were seen in terms of ISS or APACHE II scores between groups at baseline.

**CONCLUSIONS.** Patients admitted with flail chest injury undergoing surgical fixation were more likely to suffer with pulmonary complications and require invasive ventilation. A number of patients in the non-fixation group did meet the criteria for surgical fixation and it is not clear why surgery was not performed. It may be that only those already developing complications were surgically fixed hence the higher rates seen.

**REFERENCE(S)**
1. Campbell DB. Trauma to the chest wall, lung, and major airways. *Semin Thorac Cardiovasc Surg*. 1992;4:234–240.
2. Fitzpatrick D C, Denard P J, Phelan D, Long B W, and Madey S M, Bottlang M Operative stabilization of flail chest injuries: review of literature and fixation options. *J Trauma*.2010
3. Tanaka H, Yukioka T, Yamaguti Y, Shimizu S, Goto H, Matsuda,H, Shimazaki S. Surgical stabilization of internal pneumatic stabilization? A prospective randomized study of management of severe flail chest patients. *J Trauma*. 2002;52(4):727–32.

**Table 250 (Abstract 1052)**. Results

| | Surgical stabilization (n=10) | Conservative Management (n=26) | p |
|---|---|---|---|
| Age (years) | 65.1 | 57.9 | |
| Male n(%) | 9 (90%) | 19 (73%) | |
| ISS | 26.6 | 24 | 0.409 |
| APACHE II | 13.9 | 12.5 | 0.570 |
| PPC | 9 (90%) | 9 (35%) | <0.01 |
| Mechanically Ventilated | 10 (100%) | 9 (35%) | <0.001 |
| Ventilator Days | 11.9 | 12.8 | 0.958 |
| Tracheostomy | 4 (40%) | 8 (31%) | 0.701 |

### 1053

#### A review of mobility practices with orally intubated patients in a UK, general ICU

J. Grant, O. Gustafson
Oxford University Hospitals NHS Foundation Trust, Oxford, United Kingdom
**Correspondence:** J. Grant
*Intensive Care Medicine Experimental* 2017, **5(Suppl 2):**1053

**INTRODUCTION.** It has been over a decade since studies were published demonstrating that mobilisation whilst orally intubated is both safe and feasible. Despite this the incidence of mobilisation while intubated is variable, with point prevalence studies reporting rates of 1-8% in patients ventilated for more than 24 hours[1,2]. A more recent study has also demonstrated that mobilisation activities make up only 7% of physiotherapy interventions provided to orally intubated patients[3].

**OBJECTIVES.** To evaluate current mobility practices for orally intubated patients comparing to previously published data.

**METHODS.** A retrospective review of consecutive patients admitted to a single general tertiary ICU between January and March 2017 that required mechanical ventilation. Information was collected on the number of patients mobilised whilst orally intubated, the type of mobilisation activity and any adverse events that occurred during mobilisation.

**RESULTS.** Information was collected on 69 patients, 10 (14%) of whom were mobilised whilst the endotracheal tube was still in situ. The incidence of mobilising whilst orally intubated increased as the number of ventilated days increased - 27% of the 37 patients ventilated for three days or greater and 31% of the 26 patients ventilated for greater than 5 days. The mobilisation sessions consisted of two activities - sitting on the edge of the bed with or without assistance, or a passive transfer to a chair (eg. lateral transfer or hoist). They each made up 53% and 47% of mobility sessions respectively. In those patients ventilated for more than 5 days, mobilisation activities made up 12.3% of all interventions delivered to patients whilst they were intubated. There were no adverse events during any mobility session.

**CONCLUSIONS.** The proportion of patients who were mobilised whilst orally intubated was almost double those previously reported. Mobilisation activities comprised a greater proportion of the intervention provided by physiotherapy. The prevalence of mobilising orally intubated patients increased as the duration of intubation progressed to over five days.

**REFERENCE(S)**
[1]Nydahl P, Ruhl P, Baryoszek G, Dubb R, Filipovic S, Flohr H, Kaltwasser A, Mende H, Rothand O, Schuchhardt D, Schiabbauer N, Needham D (2014) Early mobilisation of mechanically ventilated patients: a 1 day point-prevalence study in Germany, *Critical Care Medicine*, 42 (5)1179-86
[2]Berney S, Harold M, Webb S, Seppelt I, Patman S, Thomas P, Denehy L (2013) Intensive care unit mobility practices in Australia and New Zealand: a point prevalence study, *Critical Care Resuscitation*, 15 (4) 260–65
[3]McWilliams D, Duffy L and Nelson C (2017) Current rehabilitation practices for patients admitted to critical care in the UK: a 5 day point prevalence survey of 12 adult general intensive care units, *Journal of the association of chartered physiotherapists in respiratory care*, 48 5–14

### 1054

#### Use of ergometric bicycle in ICU cancer patients

A. Pereira[1], C.A. Luchesa[1], D. Becker[2], P.A.D. Duarte[1]
[1]Hospital do Câncer / UOPECCAN, General ICU, Cascavel, Brazil; [2]Hospital do Câncer / UOPECCAN, Cascavel, Brazil
**Correspondence:** P.A.D. Duarte
*Intensive Care Medicine Experimental* 2017, **5(Suppl 2):**1054

**INTRODUCTION.** Early mobilization is a strategy that aims to reduce the time of weaning from ventilation and help in the functional recovery in ICU patients. It can be performed through therapeutic activities such as bed motor exercises, transfer to the chair and sit at the edge of the bed, with progression to orthostatism and ambulation. The objective of this study was to evaluate the safety of the use of the ergometric bicycle in patients hospitalized in the ICU of a Cancer Hospital.

**METHODS.** Pilot interventional study, non-randomized, non-controlled. Interventions of active lower limb exercises were performed in the cycle ergometer, the duration determined by the subjective perception of effort and sign of fatigue of the patient. Hemodynamic and respiratory variables were evaluated at three different moments: before, during and after exercise.

**RESULTS.** 48 patients (72.9% male, age 57.1 years; APACHE 12.8; 87.5% solid tumours; 43.7% of the patients were using MV at the beggining of the procedure) participated in the study. Comparing the initial and final values of the analyzed variables, there was a non significant increase in the blood pressure after exercise, although the procedure proved to be safe, without significant hemodynamic or respiratory changes. No patient needed to discontinue the procedure.

**CONCLUSION.** The use of the cycloergometer in cancer ICU patients was safe (including patients in MV), provided that the activity was performed with adequate monitoring.

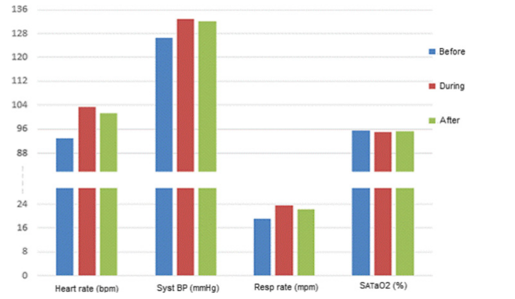

**Fig. 333 (Abstract 1054)** Respiratory and hemodynamic monitoring during and after the procedure (n = 48)

### 1055

#### The feasibility of using the Sarah Combilizer ® tilt table in early mobilization in the intensive care unit: an observational study

K.O. Poulsen[1], M.B. Borup[2], T. Stroem[1], E. Laerkner[1]
[1]Odense University Hospital, Department for Anaesthesiology and Intensive Care, Odense C, Denmark; [2]Odense University Hospital, Department for Rehabilitation, Odense C, Denmark
**Correspondence:** K.O. Poulsen
*Intensive Care Medicine Experimental* 2017, **5(Suppl 2):**1055

**INTRODUCTION.** Early mobilization in the ICU is associated with several positive outcomes, such as reductions in ICU and hospital length of stay and shorter duration of delirium.
Early mobilization can be defined as the use of physical activity during the first 5 days of critical illness (1).
The Sara Combilizer® is a modern tilt-table that can lift the patient to standing position even if the patient is currently not able to stand by himself.
**OBJECTIVES.** This study aimed to test, whether the Sara Combilizer was feasible to use in early mobilization. The objective was also to describe characteristics of the studied group.
**METHODS.** Adult patients admitted to a 27-bed general ICU in Odense University Hospital during a 3-month period was enrolled. Mechanical ventilation was an inclusion criteria. Patients permanently without the ability to stand and with contraindications to mobilization were excluded.
When enrolled, baseline data was collected. The patient was daily assessed regarding the possibility and appropriateness to use the Sara Combilizer. If not, the reason was explored.
**RESULTS.** 214 patients were admitted during the period. 28 met inclusion criteria. Loss to follow up: 8. 20 were analyzed. 10 of these did use the Sara Combilizer.
The mean age was 68,9 years. 6 were female.
SOFA scores at admission was 7,6 (SD 3,1) at 1st mobilization 5,9 (SD 2,4) and at 1st mobilization with Sara Combilizer 7,1 (SD 3,2)
APACHE II score was 26,3 (SD 6,0)
Main admission diagnosis was Sepsis (N = 6, 30%), respiratory failure (N = 5, 25%), Neuological (N = 5, 25%) and Surgical (N = 4, 20%)
Time from admission to first mobilization was 37,5 hours (95% CI 21,4-53,5 h.)
The primary method to mobilization was hoisting to chair (n = 18). Sitting on edge of bed (n = 1) and Sara Combilizer (n = 1) was also used. Patients were hoisted to chair before physical training was feasible.
The Sara Combilizer was not used on the day of admission, and the use was increasing from day 2 to 5. Most patients used the Sara Combilizer only once, since they were able to train standing position next day.
The reasons for not mobilizing the patient with the Sara Combilizer was in this study primarily related to patient conditions, but also to the level of knowledge about the Sara Combilizer.
**CONCLUSIONS.** The use of the Sara Combilizer is feasible in the first five days of critical illness for the studied group of adult patients, undergoing mechanical ventilation. Baseline data for the group was described. Mobilization to chair took place before the patient began physical training with the Sara Combilizer.

**REFERENCE(S)**

1 Schweickert WD, Pohlman MC, Pohlman AS, Nigos C, Pawlik AJ, Esbrook CL, et al. Early physical and occupational therapy in mechanically ventilated, critically ill patients: a randomised controlled trial. Lancet. 2009;373(9678):1874–82.

**GRANT ACKNOWLEDGMENT**

None

### 1056

#### Predictive value of cerebral oximetry for acute outcomes after pediatric cardiac surgery: a prospective observational study

M. Flechet, F. Güiza, D. Vlasselaers, L. Desmet, S. Lamote, H. Delrue, M. Beckers, M.P. Casaer, P. Wouters, G. Van den Berghe, G. Meyfroidt
KU Leuven, Laboratory of Intensive Care Medicine, Leuven, Belgium
**Correspondence:** M. Flechet
*Intensive Care Medicine Experimental* 2017, **5(Suppl 2):**1056

**INTRODUCTION.** Near-infrared spectroscopy (NIRS) has been proposed as a technique to monitor cerebral tissue oxygen saturation (SctO2) non-invasively and continuously. NIRS-based monitors are increasingly used in perioperative monitoring of children undergoing surgery for congenital heart disease (1). However, the usefulness of postoperative NIRS-based monitoring remains unclear.
**OBJECTIVES.** To assess whether SctO2 is predictive for prolonged intensive care unit (ICU) stay and for prolonged duration of invasive mechanical ventilation in critically ill children after pediatric cardiac surgery.
**METHODS.** Single-center prospective, observational study (Clinical Trials.gov, NCT01706497) of 177 critically ill children and infants with congenital heart disease, younger than 12 years old, admitted to the pediatric ICU between October 2012 and November 2015. Children were monitored with the FORESIGHT cerebral oximeter from ICU admission until they were weaned off mechanical ventilation. Using the first 24 hours of SctO2, we defined a SctO2 desaturation score, using the percentage of area below 50%, and a low frequency variability, using the standard deviation of a smoothed signal created by taking the median of a rolling 20-sample window of the initial SctO2 signal.
**RESULTS.** Outcome was predictive value of postoperative SctO2 for duration of ICU stay (Table 251, median (95% confidence interval), 4 days (3–8)) and duration of mechanical ventilation (median (95% confidence interval), 111.3 hours (69.3-190.4)). In a multivariable bootstrap analysis adjusted for age, weight, gender, PIM2, cyanogenic cardiopathy and time prior to SctO2 monitoring, risk factors associated with worse outcomes included increased SctO2 variability and elevated SctO2 desaturation score below 50%.
Patients with cyanogenic cardiopathy had lower SctO2 ($P < 0.001$), stayed longer in the ICU ($P = 0.002$) and were longer mechanically ventilated (Table 251, $P = 0.002$). In these patients, only the SctO2 low frequency variability was a significant predictor of adverse outcomes. On the contrary, in patients without cyanogenic cardiopathy, the variability was not predictive for adverse outcome but the mean SctO2 and the desaturation score below 50% were predictive.
**CONCLUSIONS.** Increased SctO2 variability and elevated SctO2 desaturation score are associated with longer ICU stay and with longer duration of mechanical ventilation after pediatric cardiac surgery. Further research is required to investigate how these measures can be used to drive therapeutic interventions and to investigate additional possible clinical applications.

**REFERENCE(S)**

1. Murkin JM, Arango M: Near-infrared spectroscopy as an index of brain and tissue oxygenation. *Br J Anaesth* 2009; 103:i3-i13

**GRANT ACKNOWLEDGMENT**

MF and GM receive funding from the FWO (11Y1116N, 1846113N). MC receives funding from the UZLeuven KOF. GVdB receives long-term research financing via the Flemish government Methusalem-program.

**Table 251 (Abstract 1056)**. Baseline and outcomes characteristics

| | All patients | Cyanogenic | Non cyanogenic | P-value |
|---|---|---|---|---|
| n | 177 | 70 | 107 | - |
| Age, month, median (IQR) | 4 (1-14) | 4.5 (1-11) | 4 (1.5-17) | 0.15 |
| Male gender, n (%) | 107 (60.5) | 42 (60.0) | 65 (60.7) | 0.92 |
| Weight, kg, median (IQR) | 5.2 (3.8-8.0) | 5.3 (3.6-7.9) | 5.2 (3.8-9.3) | 0.13 |
| ECMO during ICU stay, n (%) | 8 (4.5) | 6 (8.6) | 2 (1.9) | 0.06 |
| SctO2 during first 24 hours, mean (SD) | 70.2 (8.0) | 66.1 (8.4) | 73.0 (6.4) | <0.0001 |
| Duration of SctO2 monitoring during first 24 hours, median (IQR) | 19.5 (6.7-23.4) | 21.8 (12.3-23.6) | 16.6 (7.0-23.0) | 0.03 |
| ICU LOS, day, median (IQR) | 4.0 (3.0-8.0) | 6 (4-14) | 4 (2-6.5) | 0.002 |
| Duration of invasive mechanical ventilation, hour, median (IQR) | 111.3 (69.3-190.4) | 143.0 (92.2-338.8) | 93.9 (49.9-153.6) | 0.002 |

**Table 252 (Abstract 1056)**. Association between SctO2 and outcomes

| | Outcomes | Coefficient | R-squared | P-value |
|---|---|---|---|---|
| SctO2 predictors | ICU LOS | | | |
| Low frequency variability | | 3.26 (3.16-3.35) | 0.382 (0.379-0.385) | 0.04 (0.03-0.04) |
| Desaturation score | | 21.21 (21.01-21.70) | 0.382 (0.379-0.385) | <0.0001 (<0.0001-<0.0001) |
| SctO2 predictors | Duration of mechanical ventilation | | | |
| Low frequency variability | | 75.60 (73.23-77.36) | 0.385 (0.382-0.389) | 0.04 (0.04-0.05) |
| Desaturation score | | 503.98 (496.42-511.37) | 0.385 (0.382-0.389) | <0.0001 (<0.0001-<0.0001) |

#### 1057

#### Paediatric In-bed cycling: a safety and feasibility evaluation in intensive care

S. Sargent, K. Sharp, J. Hills, B. Johnstone
Royal Hospital for Children, Glasgow, NHS Greater Glasgow and Clyde, Physiotherapy, Glasgow, United Kingdom
**Correspondence:** S. Sargent
*Intensive Care Medicine Experimental* 2017, **5(Suppl 2):**1057

**INTRODUCTION.** Early mobility in intensive care (ICU) is important to optimise functional recovery whether mechanically or spontaneously ventilated. Early mobilisation is crucial in the prevention of ICU acquired weakness and can improve pulmonary function and decrease ICU length of stay[1]. However, opportunities to mobilise can be limited. In-bed cycling (IBC) can enable activity of patients when mobilising out of bed is restricted. The use of IBC has been found safe and feasible within adult and paediatric ICU (PICU)[2, 3] with further research ongoing in Canada for children in critical care[4].

**OBJECTIVES.** The aim of this project was to determine if the use of IBC was safe and feasible in PICU within Royal Hospital for Children, Glasgow.

**METHODS.** An evaluation of the benefit of the RT-300 Paediatric Moto-Med Bike was completed over 4 months. Eligible patients were cardiovascularly stable with calf length greater than ten inches, intact skin integrity and no contraindications to exercise. Active or passive cycling sessions were completed dependent on patient's ability. Outcomes measures were heart rate (HR), blood pressure (BP), respiratory rate (RR), oxygen saturations and tidal volumes ($V_T$). These were measured before, during and after treatments.

**RESULTS.** Six patients participated in the evaluation, age range ten to eighteen years old. Treatments varied from five to ten minutes with 83% of sessions including active pedalling and a mean of five sessions per patient. All patients remained clinically stable during the sessions with no adverse events. All outcome measures remained within safe parameters according to each child's age and baseline. 87.5% of sessions increased their $V_T$ with a mean change of 12.5% and over 70% of sessions maintained or increased their oxygen saturations during and after treatments. Participants and families reported the pedals were enjoyable, motivating and enhanced their care and experience in PICU.

**CONCLUSION.** The use of IBC was found to be safe, feasible and enjoyable in a PICU setting. No adverse events occurred and the bike was found to be a useful adjunct to increasing physical activity and early rehabilitation within PICU. Further research is required to determine if IBC has a benefit on respiratory and physical function. This may facilitate early weaning of mechanical ventilation or impact on PICU length of stay.

### ECMO

#### 1058

#### Association between higher PEEP and improved pulmonary vascular dysfunction in severe ARDS patients on ECMO

E. Spinelli[1], T. Mauri[1], N. Bottino[1], S. Crotti[1], D. Tubiolo[1], S. Caminada[2], F. Bichi[2], G. Grasselli[1], A. Pesenti[1]
[1]Fondazione IRCCS Ca' Granda Ospedale Maggiore Policlinico, Università degli Studi di Milano, Milano, Italy; [2]Università degli Studi di Milano, Milano, Italy
**Correspondence:** E. Spinelli
*Intensive Care Medicine Experimental* 2017, **5(Suppl 2):**1058

**INTRODUCTION.** Decreased pulmonary arterial compliance (Cpa) is a sign of pulmonary vascular dysfunction that predicts worse outcome in patients with acute respiratory distress syndrome (ARDS), even in presence of normal pulmonary vascular resistance (PVR). Extracorporeal membrane oxygenation (ECMO) could potentially improve pulmonary haemodynamics by facilitating the application of protective ventilation and decreasing hypoxic vasoconstriction.

**OBJECTIVES.** To describe pulmonary haemodynamics and their correlation with respiratory mechanics and ventilator settings in severe ARDS patients on ECMO.

**METHODS.** Hemodynamic measurements obtained from pulmonary artery catheter (systolic and diastolic pulmonary arterial pressure = PAPs and PAPd, pulmonary arterial occlusion pressure = PAOP, cardiac output = CO), arterial and mixed venous blood gas analyses, ventilator settings and respiratory mechanics (positive end-expiratory pressure = PEEP, Plateau pressure = Pplat, tidal volume = TV, driving pressure = ΔP) measured during the first 24 hours of veno-venous ECMO were prospectively collected in 51 consecutive severe ARDS patients treated between 2009 and 2016 at our center. PVR (= PAPm-PAOP/CO), Cpa [(CO/heart rate)/(PAPs-PAPd)], intrapulmonary shunt (Qs/Qt) and respiratory system compliance ($C_{rs}$ = TV/ΔP) were calculated. In-hospital mortality was recorded.

**RESULTS.** All patients received protective mechanical ventilation (PEEP 18 [15–22] $cmH_2O$, TV 4.3 [2.9-5.3] ml/kg IBW, Pplat 28 [26–30]$cmH_2O$,) and ECMO support (blood flow 3 [2.7-3.6] L/min, gas flow 4 [3–6.4] L/min) to achieve adequate gas exchange (pH 7.39 [7.36-7.42], $PaCO_2$ 43 [38–48] mmHg, $PaO_2$ 84 [69–96] mmHg) despite severe lung injury ($C_{rs}$ 28 [18–37] ml/$cmH_2O$, Qs/Qt 50 [42–62]%).

Mortality was 33%. With a $SvO_2$ of 84 [84–87]%, PAPs was 41 [33–47] mmHg and PAPd 25 [19–31] mmHg with a PAOP of 18 [14–21] mmHg, resulting in low PVR of 159 [122–194] dyn*s*cm-$^{5}$, but impaired Cpa of 5.38 [3.28-7.46] ml/mmHg. In the whole population, Cpa was positively correlated with PEEP (r 0.31, p 0.026). Patients with PEEP higher than the median value (18 cm$H_2O$, "high PEEP" group) received ventilation with lower ΔP and TV and showed a greater Qs/Qt as compared to "low PEEP". Interestingly, Cpa showed a trend toward improvement in the "high PEEP" group (Table 253).

When we restricted our analysis to the patients with $C_{rs}$ lower than the median value (i.e. those who might benefit more from higher PEEP), Cpa was more significantly correlated with PEEP (r 0.5, p 0.007) and was significantly higher in the "high PEEP" group (Table 254), despite a lower $PaO_2$ (65 [57–75]mmHg in "high PEEP" vs 86 [71–105] mmHg in "low PEEP") with no difference in $PaCO_2$ and pH.

**CONCLUSIONS.** In this population of severe ARDS patients on ECMO, PVR was not elevated, but Cpa was reduced. Application of higher PEEP with reduced ΔP and TV seems to be associated with improved arterial pulmonary compliance in the subgroup of patients with more severe lung injury.

**Table 253 (Abstract 1058)**. All patients (N=51)

| | LOW PEEP (n = 25) | HIGH PEEP (N =26) | p |
|---|---|---|---|
| PEEP (cmH2O) | 15 [14-18] | 21 [18-22] | < 0.001 |
| Pplateau (cmH2O) | 27 [25-29] | 28 [27-31] | 0.007 |
| ΔP (cmH2O) | 11 [10-13] | 8 [7-8] | < 0.001 |
| TV (ml/kg) | 4.8 [4.2-6.3] | 3.2 [2.3-4.3] | 0.001 |
| Crs (ml/cmH2O) | 28 [20-36] | 29 [18-38] | 0.94 |
| Qs/Qt (%) | 47 [39-57] | 58 [45-67] | 0.03 |
| PAOP (mmHg) | 17 [13-21] | 18 [16-20] | 0.63 |
| PVR (dyn*s*cm-5) | 162 [118-189] | 159 [127-231] | 0.57 |
| Cpa (ml/mmHg) | 4.9 [3.2-6.7] | 6.3 [4.4-7.8] | 0.15 |

**Table 254 (Abstract 1058)**. Patients with lower Crs (n=26)

| | LOW PEEP (n = 13) | HIGH PEEP (n = 13) | p |
|---|---|---|---|
| PEEP (cmH2O) | 15 [14-16] | 22 [20-23] | < 0.001 |
| Pplateau (cmH2O) | 27 [26-29] | 30 [28-32] | 0.004 |
| ΔP (cmH2O) | 12 [11-14] | 8 [8-10] | <0.001 |
| TV (ml/kg) | 4.2 [3.1-5] | 2.3 [1.6-2.8] | < 0.001 |
| Crs (ml/cmH2O) | 18 [16-22] | 18 [14-26] | 0.63 |
| Qs/Qt (%) | 50 [41-61] | 67 [57-80] | 0.006 |
| PAOP (mmHg) | 16 [13-21] | 18 [16-21] | 0.34 |
| PVR (dyn*s*cm-5) | 183 [123-226] | 173 [116-208] | 0.59 |
| Cpa (ml/mmHg) | 4.9 [3.3-6.4] | 7.4 [5.4-9.1] | 0.026 |

### 1059

#### Predictors of outcome in veno-venous extracorporeal membrane oxygenation patients

B. Halacli[1,2], H. Xu[1], S. Ledot[1], S. Singh[1,3], S. Price[1], B. Patel[1,3]
[1]Royal Brompton and Harefield NHS Trust, Adult Intensive Care Unit, London, United Kingdom; [2]Hacettepe University Faculty of Medicine, Medical Intensive Care Unit, Ankara, Turkey; [3]Imperial College London, Section of Anaesthetics, Intensive Care and Pain Medicine, London, United Kingdom
**Correspondence:** B. Halacli
*Intensive Care Medicine Experimental* 2017, **5(Suppl 2):**1059

**INTRODUCTION.** Severe acute respiratory failure (SARF) is the most common and lethal single organ failure in the intensive care unit (ICU) and has a mortality rate of 45% to 55%. Extracorporeal membrane oxygenation (ECMO) is an effective rescue support therapy in those patients who fail conservative management strategies. According to the database of Extracorporeal Life Support Organization (ELSO) registry from "The Respiratory Extracorporeal Membrane Oxygenation Survival Prediction (RESP) Score" study, mortality rate of VV-ECMO for SARF in adults was around 40% (Reference).

**OBJECTIVE.** To investigate pre-ECMO/referral factors predicting the outcome of patients who underwent VV-ECMO in a tertiary referral center.

**METHOD.** A retrospective evaluation of referral characteristics of SARF patients admitted to one of the five commissioned ECMO centers in United Kingdom (UK) who received VV-ECMO treatment between 1$^{st}$January 2010-1$^{st}$ January 2017. Patients' demographics, co-morbidities, aetiology and other available related pre-ECMO variables were noted from referral forms. Patients were separated into survival and non-survival groups in terms of ECMO run.

**RESULTS.** Among 861 referred patients, 228 patients were retrieved. After exclusion, 183 patients VV-ECMO were evaluated. Median age was 45 years old (IQR; 35–55). 102 patients (55,7%) were male. Mean BMI was 29,9 ± 8,8. Median Charlson Co-morbidity Index (CCI) was 1 (0–3). Aetiology of SARF was mainly bacterial (53,4%) and viral (27,3%) pneumonia. ICU mortality was 26,8% (n = 49). Non-survivor patients were older (p = 0,014) and most were >65 years old (p = 0,039). Patients in the survivor group tended to have a higher body mass index (BMI) (p = 0,052) whereas low BMI was associated with the non survivor group (p = 0,014). The presence of septic shock (p = 0,008) and nitric oxide (NO) usage (p = 0,01) before ECMO implementation were seen significantly more in non-survivors. In contrast to this, neuromuscular blockage (NMB) agents usage (p = 0,045) was high in survivors (Table). After logistic regression analysis; the presence of septic shock (p = 0,033, OR: 3,712; 95% CI: 1,115-12,356) and usage of NO (p = 0,021, OR: 4,989; 95% CI: 1,279-19,462) pre-ECMO were independently associated with ECMO outcome.

**CONCLUSION.** We found several variables associated with outcome in our cohort of SARF patients receiving VV-ECMO at a tertiary referral centre and mortality was evidently lower compared to ELSO registry. Further comparative study of such variations in larger databases is important to determine appropriate benchmarking and selection criteria for successful ECMO outcomes.

### 1060

#### Evaluation of almitrine infusion trial during veno venous extracorporeal membrane oxygenation for severe acute respiratory distress syndrome in adults

C. Guervilly[1], P. Esnault[2], J. Bordes[2], S. Hraiech[1], R. Rambaud[1], S. Lehingue[1], M. Adda[1], J.M. Forel[1], L. Papazian[1]
[1]Medical Intensive Care Unit, Respiratory Distress and Severe Infections, North Hospital, URMITE CNRS-UMR 6236, Aix-Marseille University, Assistance Publique Hôpitaux de Marseille, Marseille, France; [2]Intensive Care Unit, Sainte Anne Military Hospital, Toulon, France
**Correspondence:** C. Guervilly
*Intensive Care Medicine Experimental* 2017, **5(Suppl 2):**1060

**INTRODUCTION.** Veno venous extracorporeal membrane oxygenation (vvECMO) can be proposed in the most severe forms of acute respiratory distress syndrome unresponsive to prone position. vvECMO.allows the perfusion into the right atrium of oxygenated blood with high partial pressure of O2 (PvO2).Therefore, the hypoxic pulmonary vasoconstriction (HPV) is mainly abolished during vvECMO. However, vvECMO weaning may be challenging in some patients or severe hypoxemia may occur. In those situations, infusion of almitrine, a selective pulmonary vasoconstrictor, could be of interest by restoration of HPV, by reducing intrapulmonary shunt and thereafore increasing systemic arterial oxygenation.

**OBJECTIVES.** Main objectives of this study were to describe the physiological effects and the safety of an almitrine infusion trial in patients treated by vvECMO. Second objective was to identity associated factors with response to almitrine infusion.

**METHODS.** We conducted a retrospective, study at the medical ICU of the North Hospital (Marseille, France). Between January 2013 and June 2016, all patients admitted with severe ARDS requiring support with vvECMOwere screened for inclusion. Almitrine infusion trial was considered in patients with persistent hypoxemia or after more than 10 days of vvECMO without success for weaning. Contraindications of the trial were: acute cor pulmonale (ACP), hemodynamic instability with hyperlactacidemia, and liver failure. Trial consisted of a 30 minutes infusion of 0.5 mg/kg almitrine.

A positive test was defined by an increase in PaO2 to FiO2 ratio > 20%.

**RESULTS.** During the study period, an almitrine infusion trial was performed in 25 patients. Five patients received 2 tests, and one, 3 tests. The almitrine infusion trial was attempted after a median of 11 days on vvECMO. Twenty trials (62.5%) were considered as positive. After a positive trial, the median increase of the $PaO_2/FiO_2$ ratio was 35%. After multivariate logistic regression analysis, an inhomogeneous ARDS was the only independent factor associated with a positive trial (OR = 10.3; IC95% [1.3 - 84.2]). Eighteen patients (72%) were considered as responders. The characteristics between the responder and non-responder patients were not different. More patients were rapidly weaned definitely from vvECMO

(into 72 hours after the trial) in the responder group: 45% *vs.* 0% ($p$ = 0.049). There were no reported complications after the almitrine infusion trial. However, 4 patients (22%) had adverse events probably linked to prolonged continuous almitrine infusion, including 2 patients with acute cor pulmonale.

**CONCLUSIONS.** During vvECMO, a majority of patients are responders to almitrine infusion trial.

A significant number of patients was rapidly weaned from vvECMOafter a positive trial. Due to the rate of adverse events, the clinician must put in balance the benefit-risk ratio before the prescription of a prolonged continuous infusion of almitrine.

**GRANT ACKNOWLEDGMENT**

None

### 1061

#### Incidence of right ventricle failure in patients with acute severe respiratory failure requiring extracorporeal membrane oxygenation

A. Fabbri, A. Falvo, J. Powell-Tuck, N. Desai, G. Glover, C. Langrish, D. Wyncoll, A. Retter, C. Meadows, N. Barrett, S. Tricklebank, N. Ioannou, L. Camporota

Guy's and St Thomas' NHS Foundation Trust, London, United Kingdom

**Correspondence:** A. Fabbri

*Intensive Care Medicine Experimental* 2017, **5(Suppl 2):**1061

**INTRODUCTION.** The incidence of acute cor pulmonale (ACP) in patients with acute respiratory distress syndrome (ARDS) is around 22% and is associated with a higher mortality[1]. Little is known about the incidence of ACP in adult patients receiving extracorporeal membrane oxygenation (ECMO). The gold standard for the diagnosis of ACP is echocardiography, although there is growing interest in the assessment of ACP using CT scans.

**OBJECTIVES.** We studied the incidence of ACP in patients with severe ARDS requiring ECMO. ACP was assessed using echocardiography and CT imaging.

**METHODS.** We included all patients with severe ARDS admitted to our Severe Respiratory Failure Centre between January 2011 and March 2015 who received a CT scan and an echocardiogram after being established on ECMO. ACP was defined on CT as a right ventricular to left ventricular diameter ratio (RVd/LVd) of either >0.9 or >1 [2]. Echocardiogram criteria used to define ACP were RVd/LVd and the presence of a D-shaped left-ventricle (paradoxical septal movement). Right ventricular failure (RVF) was diagnosed on visual assessment or tricuspid annular plane systolic excursion (TAPSE) < 17mm. [3].

**RESULTS.** we included 107 patients. The prevalence of ACP with echocardiography was 13.7%. RVF was 37.1% using TAPSE < 17 and 30.8% on visual assessment.

The prevalence of ACP with CT was 54% using a RVd/LVd ratio > 0.9 and 33% with a ratio > 1. Bland-Altman analysis on RVd/LVd ratio with CT scan and echocardiography showed a Bias −0.13, LOA −0.35 to 0.22. We also compared the ratio between the areas of the RV and LV (RVa/LVa) using CT scan and echocardiography as a further marker of ACP. Prevalence of RVa/LVa ratio on CT scan >0.9 was 17.8%. The RVa/LVa shows the greatest agreement (Bias = 0.0079, LOA - 0.416 to 0.431) compared to other measurements.

**CONCLUSIONS.** The incidence of ACP in a large population of patients with ARDS on ECMO support is lower than in mechanically ventilated ARDS without ECMO. Moreover, CT appears to have great potential in the indirect assessment of RV function. Integration of the two investigations could be useful in the detection of RVF, particularly as many patients with ARDS have CT scans. Further studies should assess the temporal association between ECMO and resolution of RVF.

**GRANT ACKNOWLEDGMENT**

None.

### 1062

#### Risk factors and outcome of right ventricle failure in patients with acute severe respiratory failure on extracorporeal membrane oxygenation

A. Fabbri, A. Falvo, J. Powell-Tuck, N. Desai, G. Glover, C. Langrish, D. Wyncoll, A. Retter, C. Meadows, N. Barrett, S. Tricklebank, N. Ioannou, L. Camporota

Guy's and St Thomas' NHS Foundation Trust, London, United Kingdom

**Correspondence:** A. Fabbri

*Intensive Care Medicine Experimental* 2017, **5(Suppl 2):**1062

**INTRODUCTION.** Right ventricle failure (RVF) in patients with acute respiratory distress syndrome (ARDS) is associated with a higher mortality. In these patients, the combination of higher airway pressures, a severe deficit in gas exchange, and the low lung volume, may increase their risk of acute cor pulmonale (ACP) and its associated morbidity and mortality [1]. The most severe form of ARDS with refractory hypoxaemia and hypercapnia may require extracorporeal membrane oxygenation (ECMO) support. ECMO may lead to a reduction of the ventilatory pressure that, in association with near normal gas exchange, may decrease right ventricle (RV) afterload and therefore RVF [2].

**OBJECTIVES.** we studied the risk factors for RVF and the outcome of RVF in a large population of patients with ARDS on ECMO support.

**METHODS.** This is a retrospective single-center cohort study of patients admitted to the ICU of Guy's and St Thomas' hospital for ARDS on ECMO support. We collected demographic data, ventilatory settings just before starting ECMO, arterial blood gases pre-ECMO; presence of comorbidities, therapy with vasopressors before ECMO, laboratory results, length to stay (LOS) on ECMO and survival. RVF was assessed echocardiographically using the tricuspid annular plane systolic excursion (TAPSE) and the visual assessment. [3]

**RESULTS.** Of all factors included in a logistic regression model only body mass index (BMI) was independently associated with lower risk of RVF with an OR 0.85 (0.73-0.99); p = 0.039.
There was no difference in the median value of the RVF and LOS on ECMO between survivors and non-ICU survivors.
One of the other possible determinants of pulmonary vascular resistance and therefore RVF, is represented by the lung volume. Interestingly, we found that patients that displayed greater increase in lung volume after increasing airway pressure from 5 to 45 $cmH_2O$ (recruitability), showed a trend towards higher likelihood of RVF visual: OR 1.29 (0.98 to 1.68); p = 0.06. Similarly, in patients with low potential for lung recruitment, TAPSE was higher compared to patients with high potential for lung recruitment TAPSE median (SD) 2 mm vs 1.7 mm, but statistically not different.
**CONCLUSIONS.** In this study we show that BMI - probably because of the effect of the chest wall elastance - protects from RVF and ECMO may protect the right heart by allowing reduction of pressure, particularly in patients with higher recruitability. In order to demonstrate the possible protective role of ECMO against RVF further studies and comparisons with adult ARDS patients treated with conventional ventilation are required.

**REFERENCES**
[1] Mekontso Dessap, *Intensive Care Medicine*, 2016, *42*(5), 862–70.
[2] Fan, Intensive *Care Medicine*, 2016, *42*(5), 712–724.
[3] Lang, *European Heart Journal Cardiovascular Imaging*, 2015, *16*(3), 233–70.

**GRANT ACKNOWLEDGMENT**
None.

### 1063

#### The RESP score for prediction of survival in patients with acute respiratory failure on extracorporeal membrane oxygenation: Korean multicenter study

M.S. Baek[1], H.J. Kim[2], C.R. Chung[3], S.H. Park[4], B.J. Kang[5], J.Y. Oh[6], S.Y. Park[7], W.H. Cho[8], Y.S. Sim[9], Y.J. Lee[10], S.H. Park[11], J.-H. Kim[12], S.-B. Hong[1]
[1]Asan Medical Center, Department of Pulmonary and Critical Care Medicine, Seoul, Korea, Republic of; [2]Asan Medical Center, Department of Clinical Epidemiology and Biostatistics, Seoul, Korea, Republic of; [3]Samsung Medical Center, Department of Critical Care Medicine, Seoul, Korea, Republic of; [4]Kyung Hee University Hospital at Gangdong, Department of Pulmonary and Critical Care Medicine, Seoul, Korea, Republic of; [5]Ulsan University Hospital, Division of Pulmonology, Department of Internal Medicine, Ulsan, Korea, Republic of; [6]Dongguk University, Ilsan Hospital, Divisions of Pulmonology and Critical Care Medicine, Department of Internal Medicine, Goyang-si, Korea, Republic of; [7]Chonbuk National University Hospital, Department of Internal Medicine, Jeonju-si, Korea, Republic of; [8]Pusan National University Yangsan Hospital, Department of Internal Medicine, Yangsan-si, Korea, Republic of; [9]Hallym University Kangnam Sacred Heart Hospital, Division of Pulmonary and Critical Care Medicine, Department of Medicine, Seoul, Korea, Republic of; [10]Seoul National University Bundang Hospital, Division of Pulmonary and Critical Care Medicine, Department of Internal Medicine, Seongnam-si, Korea, Republic of; [11]Hallym University Sacred Heart Hospital, Division of Pulmonary and Critical Care Medicine, Department of Medicine, Anyang-si, Korea, Republic of; [12]Bundang CHA Hospital, Division of Pulmonary and Critical Care Medicine, Department of Medicine, Seongnam-si, Korea, Republic of
**Correspondence:** M.S. Baek
*Intensive Care Medicine Experimental* 2017, **5(Suppl 2):**1063

**INTRODUCTION.** Use of extracorporeal membrane oxygenation (ECMO) for acute respiratory failure (ARF) has been substantially increased worldwide since the 2009 A (H1N1) influenza pandemic. However, because the ECMO therapy is associated with higher mortality, enormous financial burden and the significant complications such as bleeding, appropriate selection of the patients who could benefit from ECMO therapy is important. Recently, several outcome prediction models for ECMO treatment such as the ECMOnet score, the PRESERVE score and the RESP score have been developed.
**OBJECTIVES.** The aim of this study was to evaluate the outcomes of the ECMO patients and to validate the previous outcome prediction models in Korean cohort.
**METHODS.** This was a retrospective multicenter study for ARF patients who did not respond to conventional treatment at 11 hospitals. From January 2014 to December 2015, patients over the age of 19 years who underwent ECMO support were included.
**RESULTS.** During the study period, 209 ARF patients were received ECMO therapy. Successful weaning rate was 147 (70.3%) and 96 patients (45.9%) were alive at hospital discharge. Venovenous ECMO mode was used for 72.3% of patients, and median duration of ECMO support was 7 (3–14) days. In all patients, the median age was 58 (45–65) years and 138 patients (66.0%) were male. Acute physiology and chronic health evaluation (APACHE) II score was 20 (14–27) and sequential organ failure assessment (SOFA) score was 8 (5–12). Before ECMO initiation rescue therapies were following; 98 prone positioning (48.8%), 134 neuromuscular blocking agents (64.1%), 50 nitric oxide (23.9%) and 31 steroid use (14.8%).
The receiver operating characteristics curve analysis of the each outcome prediction score was performed: the RESP score, AUC = 0.66 (95% confidence interval [CI], 0.58-0.74); PRESERVE score, AUC = 0.63 (95% CI, 0.55-0.72); APACHE II score, AUC = 0.57 (95% CI, 0.49-0.65); SOFA score, AUC = 0.52 (95% CI, 0.44-0.60).
**CONCLUSIONS.** Our study suggests that the proposed PRESERVE and RESP score did not predict properly outcome for patients treated with ECMO for severe ARF.

**GRANT ACKNOWLEDGMENT**
This study was supported by a grant of the Korea Health Technology R & D Project through the Korea Health industry Development Institute funded by the Ministry of Health & Welfare, Republic of Korea (HC15C1507).

### 1064

#### Low flow veno-venous extracorporeal carbon dioxide removal ($ECCO_2R$) in a cardiothoracic transplant centre

V.P. Laxton, A. Rosenberg, C. Walker
Harefield Hospital, Royal Brompton and Harefield NHS Foundation Trust, Anaesthesia and Critical Care, London, United Kingdom
**Correspondence:** V.P. Laxton
*Intensive Care Medicine Experimental* 2017, **5(Suppl 2):**1064

**INTRODUCTION.** Low flow veno-venous extracorporeal carbon dioxide ($CO_2$) removal ($ECCO_2R$) is a safe and effective treatment for hypercapnic respiratory failure that has been successfully used to enable lung protective ventilation in acute respiratory distress syndrome (ARDS)[1], to avoid tracheal intubation in exacerbation of chronic obstructive pulmonary disease (COPD)[2,3] and as an adjunct therapy in life threatening acute asthma.[4]
Our centre is a quaternary referral centre specialising in heart and lung transplantation and cardiothoracic surgery with ready access to many different modalities of extra-corporeal and mechanical circulatory support and thus presents a different demographic to previous published case series.[5]
**OBJECTIVES.** To evaluate the use of $ECCO_2R$ at a quaternary referral centre for cardio-thoracic surgery, heart lung transplant and extra corporeal life support (ECLS).
**METHODS.** Case notes of all patients receiving $ECCO_2R$ at this centre between February 2014 and March 2017 were reviewed. Demographic, physiological and outcome data were examined.
**RESULTS.** 16 adult patients were commenced on $ECCO_2R$ using the Hemolung® (ALung Inc, Pittsburgh, PA) for ARDS, pneumonia, and to facilitate weaning from extracorporeal membrane oxygenation systems (ECMO). 9 patients (56%) had previous bilateral lung transplants.
Median age was 38 years and at commencement of therapy median SOFA score was 17 points. At instigation of $ECCO_2R$ median blood gas analysis values were pH 7.17, $PaCO_2$ 11.47 kPa and $PaO_2$ 16.09 kPa.

At 24 hours lung protective ventilation was achieved in 100% of patients. The median pH was 7.36 and median $PaCO_2$ 6.23 kPa. The mean time to normal pH, in patients not being weaned from ECMO, was 14.3 hours.

Median duration of $ECCO_2R$ was 121 hours and this was weaned successfully in eight (50%) patients, withdrawn for futility in six (37.5%), upgraded to ECMO in one (6.25%) and was ceased for device failure due to major air entrainment in one patient (6.25%).

No serious adverse outcome resulted from device failure. Median mechanical ventilation time was 16.5 days and intensive care unit (ICU) length of stay (LOS) was 23.5 days. All patients successfully weaned from $ECCO_2R$ survived to discharge from ICU.

**CONCLUSIONS.** $ECCO_2R$ has proved a useful addition to the armamentarium for management of severe respiratory failure in our centre. The treatment achieved the therapeutic goals of normalising blood pH (with lowered $PaCO_2$) and attaining lung protective ventilation in our population of lung transplant recipients and cardio-thoracic surgical patients.

**REFERENCE(S)**

1. Bein T et al. *Intensive Care Med* (2013) 39: 847–856.
2. Cole S et al. *J Intens Care Soc* (2014) 15: 2–4.
3. Abrams DC et al. *Ann Am Thor Soc* (2013) 10: 307–314.
4. Brenner K et al. *Perfusion* (2013) 29: 26–28.
5. Moss et al. ASAIO J. (2016) 62: 458–62

**GRANT ACKNOWLEDGMENT**

None

### 1065

#### Liver function before ECMO initiation in lung transplantation and outcome

M. López Sánchez[1], M.A. González-Gay[2], F.J. LLorca Díaz[3], M.I. Rubio López[1]

[1]Hospital Universitario Marqués de Valdecilla, Intensive Care Medicine, Santander, Spain; [2]Hospital Universitario Marqués de Valdecilla, Rheumathology Service, Santander, Spain; [3]Cantabria University, Preventive Medicine and Public Health, Santander, Spain

**Correspondence:** M. López Sánchez

*Intensive Care Medicine Experimental* 2017, **5(Suppl 2):**1065

**INTRODUCTION.** Indications for Extracorporeal Membrane Oxygenation (ECMO) use in lung transplantation (LT) are: bridge to transplantation, intraoperative extracorporeal respiratory and/or circulatory support and treatment of primary graft dysfunction (PGD) in postoperative period. Several mortality risk factors pre-ECMO initiation has been investigated. Hepatic dysfunction represents an independent risk factor for poor prognosis in critically ill patients, especially in those with heart failure. In ECMO patients with LT this risk parameter has not been studied.

**OBJECTIVES.** To estimate if liver function parameters and the presence of liver disease at the beginning of ECMO were associated with ICU mortality.

**METHODS.** Retrospective observational study between January 2009 to December 2016 in 12 beds intensive care unit (ICU). Inclusion criteria for ECMO entry: bridge to PT, intraoperative support and in PGD. ECMO systems: centrifugal pump and polymethylpentene membrane oxygenation with Bioline® coated circuits and cannulas. Demographic data, liver disease and liver function parameters were collected at the time of ECMO initiation. ECMO indications, type of ECMO support and ICU mortality were also recorded. Liver disease and liver function parameters were compared between survivors and non-survivors. Continuous variables, reported as mean ± standard deviation (SD) were compared using the Student t-test. Categorical variables were compared using the $X^2$ test.

**RESULTS.** 35 patients (p), 23 male (65.71%) were included with a median age of 49.57 ± 13.14 years. Median APACHE II was 19.85 ± 7.73 and median SOFA was 7.11 ± 3.02. VA ECMO was used in 22 patients (62.85%) and VV ECMO in 13 patients (37.14%). Intraoperative support was used in 54.28% (19 p); bridge to LT in 20% (7 p) and PGD was the indication in 25.71% (9 p). Median Cardiac Output (CO) was 4.74 ± 1.95. Interstitial lung diseases disease (ILD) were the principal indication for LT in 13 p (37.14%), and idiopathic pulmonary fibrosis was the most frequent disease. Liver disease was present in 11 patients and more frequent in survivors than in non-survivors (45% vs 13.33%; p = 0.046). Liver diseases were: hepatic steatosis (2 p), Gilbert disease (2 p), hepatitis B (1 p), hepatitis C (1p), liver transplant (1 p), alpha 1-antitrypsin deficiency (1 p), liver stasis (1 p), hepatotoxicity attributed to atorvastatin (1 p), hepatic hemangioma (1 p). Total Bilirubin (mg/dl) was higher in survivors than in non-survivors (1.03 ± 0.73 vs 0.60 ± 0.45; p = 0.05) and no significant statistical differences between survivors and non-survivors was observed in liver enzymes. ICU mortality was 42.85% (15 patients).

**CONCLUSIONS.** In this review with ECMO patients and LT, total bilirubin before ECMO initiation was higher in survivors than in non-survivors (p = 0.05). Liver disease was more common in survivors than in non-survivors with statistical significance (p = 0.046). ICU mortality was 42.85%.

### 1066

#### The efficacy of veno-venous extracorporeal membrane oxygenation for patients with acute respiratory failure

K. Tokuda[1], J. Maki[1], T. Fujiyoshi[1], N. Kaku[2], S. Mizuguchi[2], T. Akahoshi[2], S. Hoka[3], Y. Maehara[1,2]

[1]Kyushu University Hospital, Intensive Care Unit, Fukuoka, Japan; [2]Kyushu University Hospital, Emergency and Critical Care Center, Fukuoka, Japan; [3]Kyushu University Hospital, Department of Anesthesiology and Critical Care Medicine, Fukuoka, Japan

**Correspondence:** K. Tokuda

*Intensive Care Medicine Experimental* 2017, **5(Suppl 2):**1066

**INTRODUCTION.** Respiratory management using veno-venous extracorporeal membrane oxygenation (VV-ECMO) has been widely used to rescue patients with acute respiratory failure (ARF), such as acute respiratory distress syndrome. We investigated the management of ARF patients using VV-ECMO at our institute.

**METHODS.** Patients who received VV-ECMO for the management of ARF between 2013 and 2015 were reviewed in this retrospective observational study. We collected data regarding patient demographic characteristics, duration of VV-ECMO management, and the outcome. We divided the patients into two groups (survivors and non-survivors) according to the outcome and compared the groups to determine the prognostic factors.

**RESULTS.** A total of 11 patients (8 men and 3 women) were enrolled. The median age was 35 years (range, 1–71 years). The median duration of VV-ECMO management was 10 days (range, 4–71 days), with 0–10 replacements of the ECMO circuit. Five patients could be weaned from VV-ECMO (survivor group). Although there was no significant difference in the duration of VV-ECMO management between the groups (median 8 and 16 days, respectively, in survivors and non-survivors; p = 0.12), the duration tended to be longer in non-survivors than in survivors. The causes of death in the non-survivor group were VV-ECMO withdrawal because of no pulmonary improvement (n = 4), bleeding tendency that prevented the use of VV-ECMO (n = 1), and cardiac arrest due to multiple organ failure (n = 1).

**DISCUSSIONS.** VV-ECMO may have induced “lung rest” during mechanical ventilation until the primary lung condition improved, thereby preventing irreversible lung injury and allowing weaning from VV-ECMO in the survivor group. In contrast, in the non-survivor group, VV-ECMO was withdrawn in some cases because of a worsening general condition due to emerging co-morbidities, such as infections and uncontrolled primary lung disease. Long-term VV-ECMO was continued for two deceased patients, as the decision to discontinue VV-ECMO could not be made, despite the unlikely prospect of effective treatment of the primary disease.

**CONCLUSIONS.** Although VV-ECMO may be an effective intervention for patients with ARF, we must also bear in mind that VV-ECMO should be discontinued when treatment of the primary disease is unlikely to be successful.

| | Survivors (N = 5) | Non-survivors (N = 6) | P-value |
|---|---|---|---|
| Male: Female | 4:1 | 4:2 | 1.00 |
| Age (years) | 27.0 (1–60) | 42.5 (14–71) | 0.51 |
| MV before VV-ECMO (days) | 2 (0–5) | 4 (0–5) | 0.74 |
| SOFA score at VV-ECMO induction | 9 (5–13) | 10.2 (6–13) | 0.70 |
| P/F ratio at VV-ECMO induction | 53 (48–115) | 64 (27–76) | 0.63 |
| Duration of VV-ECMO (days) | 8 (5–12) | 16 (4–71) | 0.12 |
| ECMO circuit replacement (times) | 1 (0–3) | 2.5 (0–10) | 0.14 |

MV mechanical ventilaton, SOFA sequential organ failure assessment
Data are expressed as median (minimum - maximum)
*[Patient Demographics]*

### 1067

#### Determinants of respiratory drive, effort and driving transpulmonary pressure in early severe ARDS patients undergoing ECMO

T. Mauri[1], G. Grasselli[1], A. Zanella[1], T. Langer[1], E. Spinelli[1], M. Albanese[2], M. Lazzeri[2], G. Florio[1], F. Bichi[1], M. Panigada[1], A. Pesenti[1]
[1]Fondazione IRCCS Ca' Granda Ospedale Maggiore Policlinico, Milano, Italy; [2]University of Ferrara, Ferrara, Italy
**Correspondence:** T. Mauri
*Intensive Care Medicine Experimental* 2017, **5(Suppl 2):**1067

**INTRODUCTION.** $CO_2$ removal by ExtraCorporeal Membrane Oxygenation (ECMO) might control the respiratory drive and effort of patients with the Acute Respiratory Distress Syndrome (ARDS) and it could even induce apnea.

**OBJECTIVES.** We report preliminary data of a study aimed at assessing effects of extremely elevated $CO_2$ removal by ECMO (i.e. ≥90%) on respiratory rate, effort, transpulmonary pressure and work of breathing of early severe ARDS patients.

**MATERIAL AND METHODS.** We enrolled 5 patients with severe ARDS on veno-venous ECMO since ≤7 days. Esophageal pressure ($P_{es}$) tracings were continuously recorded with airway pressure and flow. Richmond agitation sedation scale value of −2 to 0 and inspiratory trigger were obtained by lowering sedation. Then, patients were switched to Pressure Support Ventilation (PSV) set to obtain tidal volume of 4–6 ml/kg while on clinical positive end-expiratory pressure (PEEP) and ECMO settings. Clinical $FiO_2$ and ECMO blood flow (BF) were left unchanged. ECMO gas flow (GF), instead, was increased from clinical baseline to the one removing ≥90% of total $CO_2$ production, as testified by ECMO $CO_2$ extraction ($VCO_2$-ECMO, obtained by analysis of gas passing through the expiratory outlet of membrane lung) divided by total $CO_2$ production ($VCO_2tot$ = natural lung $VCO_2$, assessed by volumetric capnography, + $VCO_2$ECMO). Then, by offline analysis, we measured respiratory rate (RR), $P_{es}$ swings ($\Delta P_{es}$), inspiratory muscular pressure ($P_{mus}$), tidal volume ($V_t$), driving transpulmonary pressure ($\Delta P_L$) and the pressure time product ($PTP_{es}$).

**RESULTS.** Patients were 53 [48–60] yo and undergoing ECMO since 5 [5–7] days. On the morning of the study: ECMO BF was 3.28 [3.21-3.29] l/min and GF 6 [4–7] l/min, PEEP was 15 [10–18] $cmH_2O$, respiratory system compliance ($C_{rs}$) 28 [18–39] ml/$cmH_2O$ and intrapulmonary shunt 41 [34–53] %.

To reach target $CO_2$ extraction, ECMO GF was increased to 15.0 [12.0-17.5] L/min, obtaining $VCO_2$-ML/$VCO_2$tot of 98 [91–100] %. With these settings, blood gas analysis showed $PaO_2$ 77 [60–93] mmHg, $PaCO_2$ 42 [41–46] mmHg, pH 7.45 [7.43-7.46] ; RR was 23 [20–31] bpm, minute ventilation 3.4 [3–5.7] l/min, $\Delta P_{es}$ 2 [2–4] $cmH_2O$, $P_{mus}$ 4.3 [4.0-5.5] $cmH_2O$, $\Delta P_L$ 10 [8–11] $cmH_2O$ and $PTP_{es}$ 165.2 [89.7-221.3] $cmH_2O*s*min^{-1}$.

Apnea (defined as absence of inspiratory effort for ≥10 seconds) was detected in only 1 patient.

$\Delta P_{es}$, $P_{mus}$ and $\Delta P_L$ don't seem to correlate with extracorporeal support but rather with more severe lung injury (i.e., lower $C_{rs}$). RR and $PTP_{es}$, instead, decreased with increasing $CO_2$ extraction and weren't correlate with $C_{rs}$.

| | Crs | VCO2-ML/VCO2tot |
|---|---|---|
| RR | r: 0.159 P-value: 0.798 | r: −0.796 P-value: 0.107 |
| ΔPes | r: −0.690 P-value: 0.198 | r: −0.101 P-value: 0.871 |
| ΔPL | r: −0.850 P-value: 0.068 | r: 0.293 P-value: 0.632 |
| Pmus | r: −0.701 P-value: 0.187 | r: 0.048 P-value: 0.938 |
| PTPes | r: 0.189 P-value: 0.811 | r: −0.968 P-value: 0.032 |

*[Pearson's Correlation]*

**CONCLUSIONS.** In early severe ARDS patients undergoing ECMO, extremely high levels of extracorporeal support couldn't induce apnea and didn't seem to correlate with effort and transpulmonary pressure.

### 1068

#### Pulmonary arterial hypertension (PAH) in ARDS patients undergoing veno-venous extracorporeal membrane oxygenation

C. Valsecchi[1], V. Punzi[1], S. Spina[1], A. Asta[1], M. Bombino[2], A. Pesenti[3], N. Patroniti[1,2]
[1]Università degli Studi Milano Bicocca, School of Medicine and Surgery, Milano, Italy; [2]San Gerardo Hospital, Anesthesiology and Critical Care, Monza, Italy; [3]Università degli Studi di Milano, Department of Pathophysiology and Transplantation, Milano, Italy
**Correspondence:** C. Valsecchi
*Intensive Care Medicine Experimental* 2017, **5(Suppl 2):**1068

**INTRODUCTION.** Veno-venous extracorporeal membrane oxygenation (VV-ECMO) is a well-established therapy in patients with acute respiratory distress syndrome (ARDS) unresponsive to conventional therapy. Only few data are available on pulmonary arterial pressure and incidence of PAH in ARDS patients undergoing VV-ECMO.

**OBJECTIVES.** To describe the effect of VV-ECMO on mean pulmonary arterial pressure (PAPm), and to assess association of PAPm with the severity of native lung dysfunction throughout the ECMO treatment

**METHODS.** A retrospective analysis of prospectively collected data was performed in all consecutive ARDS patients undergoing VV-ECMO from January 2003 to August 2015. All patients were monitored with a pulmonary artery catheter (PAC). In 34 (36%) patients PAC was available before ECMO starting. In all other patients PAC was inserted within 12 hours from ECMO starting. PAPm and other respiratory and hemodynamic variables were collected before ECMO institution and every day up to disconnection from ECMO. Use of pulmonary artery vasodilators and dobutamine was recorded. For the purpose of the study pulmonary arterial hypertension (PAH) was defined as PAPm higher than 30 mmHg.

**RESULTS.** The cohort is based on 94 patients (65% male, 45.7 ± 13.2 years old, overall mortality 34%).

Before ECMO PAPm was 32.6 ± 7.6 (IQR 24–32, 56% with PAH); vasodilators, dobutamine, or both were present in 4, 15, and 1 patients respectively. PAPm was correlated with PaCO2, arterial pH (pHa), and pulmonary artery occlusion pressure (PAOP).

Starting of ECMO was associated with a significant decrease in PAPm. Changes in PAPm were correlated with changes in PAOP, PaCO2, and pHa.

During ECMO treatment highest PAPm was 36.6 ± 7.9 (IQR 31–40). Patients were classified in three groups according to highest PAPm: PAPm < 31 mmHg (19, 20%), between 31 and 40 mmHg (53, 56%) and > 40 (22, 24%). Patients of the high PAPm group had a lower respiratory system compliance (Crs) and higher intrapulmonary shunt (Va/Q).

Mortality was 16%, 28%, and 59% in the low, medium, and high PAPm group respectively.
Pulmonary vasodilators were administered in 26 patients (22 sildenafil, 12 NO, 9 prostacyclin; 12 patients received 2 or more pulmonary vasodilator).

**CONCLUSIONS.** Occurrence of pulmonary arterial hypertension was common both before ECMO institution and during ECMO treatment. Before ECMO starting the main cause of PAH was left ventricular failure with increase in PAOP and/or respiratory acidosis with hypercapnia. Starting of ECMO determined a decrease in PAPm mainly due to the reduction in PAOP and relief of respiratory acidosis.
Patients with PAPm higher than 40 mmHg showed higher mortality, had a lower Crs and higher Va/Q. These data suggest that ventilatory management of the native lung may have a great effect on the occurrence of APH and mortality.

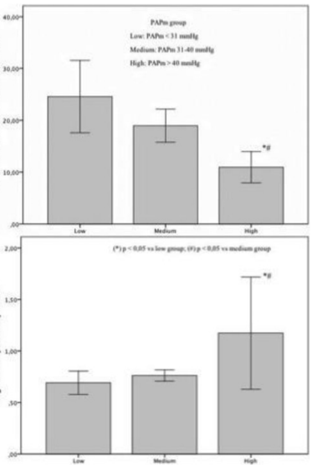

**Fig. 334 (Abstract 1068)** See text for description

### 1069

#### Assisted spontaneous breathing trial during veno-venous extracorporeal membrane oxygenation

S. Spina[1], V. Punzi[1], A. Asta[1], C. Valsecchi[1], M. Bombino[2], G. Foti[1,2], A. Pesenti[3], N. Patroniti[1,2]
[1]Università degli Studi Milano Bicocca, School of Medicine and Surgery, Milano, Italy; [2]San Gerardo Hospital, Anesthesiology and Critical Care, Monza, Italy; [3]Università degli Studi di Milano, Department of Pathophysiology and Transplantation, Milano, Italy
**Correspondence:** S. Spina
*Intensive Care Medicine Experimental* 2017, **5(Suppl 2):**1069

**INTRODUCTION.** Protective mechanical ventilation (MV, low tidal volume and plateau pressure) is the cornerstone treatment of acute respiratory distress syndrome (ARDS). In this context, veno-venous extracorporeal membrane oxygenation (VV-ECMO) has become an important tool. After the acute phase and while still on ECMO, patients may be switched from controlled to assisted MV with possible improvement of respiratory muscles function, decrease of diaphragm dysfunction and reduction of sedation needs.

**OBJECTIVES.** To assess patients during spontaneous assisted breathing trial undergoing VV-ECMO to determine
factors predicting success in switching to assisted MV and the effects on main respiratory and hemodynamic parameters.

**METHODS.** A retrospective analysis of prospectively collected data was performed. All consecutive ARDS patients from 2003 to 2014 undergone spontaneous assisted ventilation during ECMO were included. Data are reported as median (interquartile range) or mean ± SD. Two-way repeated mea- sure ANOVA was used to assess differences between days (prior to and after assisted ventilation trial) and between patients who failed and succeed the trial. A logistic regression was used to identify independent factors predicting spontaneous assisted breathing success.

**RESULTS.** During January 2003 and December 2014, 75 among 100 patients receiving VV-ECMO for ARDS, underwent at least one trial of switching from controlled to assisted MV while on ECMO (60% male, 44 ± 13.4 years old, BMI 26.5 ± 6.7). A total of 89 trials were performed. Time of trial from starting ECMO was 10 (3.5-16) days; 64, 8 and 3 patients underwent 1, 2 and 3 trials, respectively.
show main respiratory results. Success patients had a significant lower inspired fraction of oxygen (FiO2), before the trial. After switching to assisted ventilation, success patients showed a significant increase ($p < 0.05$) in compliance of respiratory system (Crs),
tidal volume and partial pressure of oxygen in the arterial blood (PaO2). Crs and FiO2 showed to be independent prognostic factors to predict trial success (OR 1.054 (95% CI 1.003-1.107) and OR 0.008 (95% CI 0–0.414), $p < 0.05$, respectively). No significant changes on hemodynamics were recorded.

**CONCLUSIONS.** Patients succeeding an assisted MV trial during VV-ECMO show an improvement in respiratory mechanics with increase in Crs, tidal volume and PaO2. A higher Crs and lower FiO2 independently predict success of a spontaneous assisted breathing trial in patients with ARDS during VV-ECMO.

**Table 255 (Abstract 1069)**. See text for description

| Crs (#, §) | Success | Failure |
|---|---|---|
| Before | 32.03 ± 12.84 (*) | 26.77 ± 9.23 |
| After | 36.75 ± 15.15 (#) | 26.77 ± 9.23 |
| Vt (§) | | |
| Before | 299.42 ± 90.91 | 295.92 ± 87.02 |
| After | 337.65 ± 118.97 (*) | 285.31 ± 94.57 |
| RR (*) | | |
| Before | 11.55 ± 5.40 | 12.23 ± 5.63 |
| After | 16.60 ± 7.51 | 19.27 ± 6.62 |

*Crs* Compliance of respiratory system, *Vt* tidal volume, *RR* respiratory rate
(*) p< 0.05 before vs. after; (#) p< 0.05 success vs. failure; (§) p< 0.05 interaction

**Table 256 (Abstract 1069)**. See text for description

| FiO2 (#) | Success | Failure |
|---|---|---|
| Before | 0.52 ± 0.14 | 0.62 ± 0.20 |
| After | 0.50 ± 0.14 | 0.63 ± 0.22 |
| PaO2 (§) | | |
| Before | 86.12 ± 17.91 | 82.51 ± 18.87 |
| After | 88.43 ±17.66 (#) | 77.72 ± 14.06 |
| PaCO2 (*) | | |
| Before | 48.72 ± 6.95 | 47.94 ± 7.32 |
| After | 46.69 ± 6.29 | 45.50 ± 7.09 |

*FiO2* inspired fraction of oxygen, *PaO2* partial pressure of oxygen in the arterial blood, *PaCO2* partial pressure of carbon dioxide in the arterial blood
(*) p< 0.05 before vs. after; (#) p< 0.05 success vs. failure; (§) p< 0.05 interaction

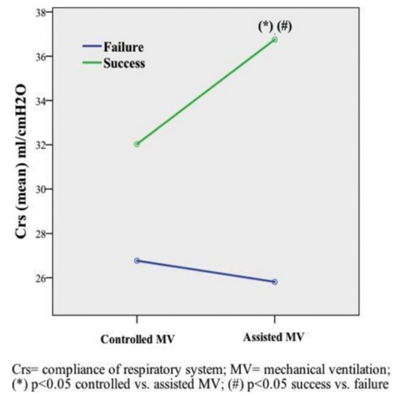

**Fig. 335 (Abstract 1069)** See text for description

### 1070

#### Accuracy of very low tidal volume delivery from ICU ventilators. A bench study with implications for ventilator setting in ARDS patients under ECMO

B. Moro[1], L. Baboi[1], H. Yonis[1], M. Aublanc[1], F. Lissonde[1], A. Louf-Durier[1], Z. Riad[1], J.-C. Richard[1], C. Guérin[1,2], M. Mezidi[1]
[1]Hopital de la Croix Rousse, Lyon, France; [2]INSERM 955, Créteil, France
**Correspondence:** M. Mezidi
*Intensive Care Medicine Experimental* 2017, **5(Suppl 2):**1070

**INTRODUCTION.** Once ARDS patients are under ECMO it has been claimed that the primary goal is resting the lung. THis can be done by lowering tidal volume (VT).A 1–4 ml/kg predicted body weight VT range has been reported in the literature in this condition. We were wondering whether such low VT can reliably be delivered by ICU ventilators, an issue which has never been investigated on the bench. Our hypothesis was that the common 10% accuracy in VT delivery was not reached by most of them.

**OBJECTIVES.** To compare VT actually delivered across ICU ventilators when set at low VT.

**METHODS.** A pneumatic test lung (TTL, Michigan inc. Grand Rapids, USA) was set at 20 ml/cmH2O compliance and 20 cmH2O/L/s resistance. Five ICU ventilators V 500 (Drager), Carescape R 860 (GE Healthcare), Servo U (Maquet), PB980 (Covidien) and G5 (Hamilton) equipped with Heated humidifier (Fisher-Paykel MR 850) set off and adult ventilator circuit (RT 380 EVAQUA Fisher Paykel) were tested. Each ICU ventilator was set in BTPS condition, at PEEP 12 cmH2O and FIO2 0.21. Airway pressure and airflow (Hans-Rudolph pneumotachograph) were measured (Biopac M150) proximal to the lung model. For each ventilator a 20 ml stepped series of VT ranging from 100 to 280 ml was delivered for 10 breaths each, at 30 then at 15 breaths/min respiratory frequency (f). VT was measured by numerical integration of the flow signal. Accuracy was assessed as VT error (=(VT measured-VT set)/VT set*100). VT error was compared across ICU ventilators and set VTs by using two-factor ANOVA at each respiratory rate with Tukey post-hoc comparisons.

**RESULTS.** There was a significant effect of ICU ventilator, nominal VT and their interaction on VT error. Figure 336. shows Box and whisker plots of VT error across ventilators and nominal VT at f 15 breaths/min. The results were basically the same at f 30. Symbols correspond to pairwise **not significant** differences between ICU ventilators at each nominal VT. Set VT was under delivered with every ventilator. The 10% limit of accuracy (horizontal broken lines) segregate ICU ventilators into two groups at every nominal set VT: two ventilators were within and three were out the 10% accuracy limit.

**CONCLUSIONS.** The very low VT values investigated were not properly delivered by common ICU ventilators with marked differences between them. The inaccuracy was consistent across VT and f. This result has potential clinical implications and should be confirmed in the clinical setting.

**GRANT ACKNOWLEDGMENT**
None

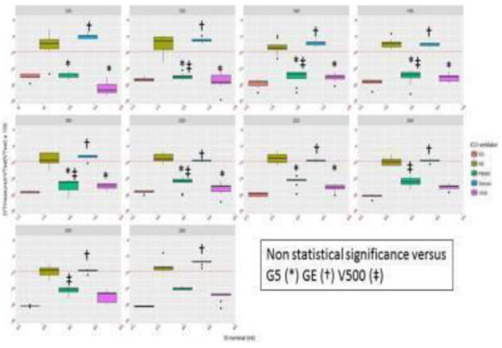

**Fig. 336 (Abstract 1070)** See text for description

## Sepsis: Miscellaneous issues

### 1071

**Withdrawn**

### 1072

#### Postoperative lymphopenia: an independent risk factor for postoperative pneumonia after lung cancer surgery

G. Dupont[1], L. Flory[1], A.-C. Lukaszewicz[2], J. Morel[1], G. Monneret[3], S. Molliex[1]
[1]Centre Hospitalier Universitaire de Saint Etienne, Anesthésie Réanimation, Saint Etienne, France; [2]Hospices Civils de Lyon, Anesthésie Réanimation Hôpital Pierre Wertheimer, Lyon, France; [3]Hospices Civils de Lyon, Laboratoire d'Immunologie, Hôpital Edouard Herriot, Lyon, France
**Correspondence:** L. Flory
*Intensive Care Medicine Experimental* 2017, **5(Suppl 2):**1072

**INTRODUCTION.** Postoperative pneumonia (POP) is one of the most common complications after lung cancer surgery with a reported incidence ranging from 9 to 25%. POP are associated with worsen outcome. Identifying risk factors are necessary to prevent POP. Postoperative lymphopenia (POL) is described for more than thirty years. Because lymphocytes cells are a major component of infection control, POL have been proposed as a risk factor for postoperative infections but was never identified as such in a multivariate analysis.

**OBJECTIVES.** We aimed to identify postoperative lymphopenia as a risk factor for postoperative pneumonia after lung cancer surgery.

**METHODS.** We have performed a retrospective single center study, conducted in Saint Etienne University hospital, France, from January 2013 to May 2015. We have included patients admitted for lung cancer surgery (lobectomy, bi-lobectomy, pneumonectomy) aged ≥ 18 years old and with no history of immunosuppressive state. Lymphocyte blood counts were collected the day before the surgery and at postoperative day 1, day 3, day 7. Patients with POP must met two majors criteria (fever > 38°C, abnormal radiographic finding) plus one minor criteria (leukopenia < 4.0 $.10^9$ cells.$l^{-1}$ or leukocytosis ≥ 12.0 $.10^9$ cells.$l^{-1}$, a new rise in the C-reactive protein value, an increase and modification of the expectorate with purulent aspect) or one major criteria plus three minors criteria. A logistic regression model adjusted on risk factors for POP currently described was used to explain postoperative pneumonia.

**RESULTS.** Two hundred patients were included. Forty-three patients (21.5%) developed a POP. The median time to the occurrence of POP was 2.5 ± 1.5 days. Preoperative lymphocyte count was 1.8 ± 0.6 $.10^9$

cells.$l^{-1}$ and 2.0 ± 0.7 .$10^9$ cells.$l^{-1}$ ($p = 0.09$) for the patients with and without POP respectively. In the two groups of patients, we have observed a decline of lymphocyte cells at postoperative day 1 corresponding to the nadir. In multivariate analysis adjusted on ASA score ≥ 3, sex, age > 65 years old, COPD, active smokers and chronic heart failure, postoperative lymphopenia at day 1 was significantly associated with an increased risk for POP (odd ratio 2.63 CI 95% [1.03 - 5.40]). Using the receiver operating characteristics curve, we have identified lymphocyte count ≤ 1.19 .$10^9$ cells.$l^{-1}$ at postoperative day 1 as the best risk threshold to develop a POP (sensitivity = 74%, specificity = 49%, predictive positive value = 29%, predictive negative value = 88%). POP was more often seen at postoperative day 7 when patients had a lymphocyte count below the threshold at postoperative day 1($p = 0.003$).

**CONCLUSIONS.** Our study showed that lymphopenia occurred at postoperative day 1 is associated with postoperative pneumonia in lung cancer surgery.

### 1073

#### New diagnostic strategy of sepsis induced disseminated intravascular coagulation (SEDIC): a validation study

T. Kitamura, H. Ishikura, Y. Irie, K. Hoshino, K. Muranishi, Y. Nakamura, Y. Kawano
Fukuoka University Hospital, Department of Emergency & Critical Care Medicine, Fukuoka, Japan
**Correspondence:** T. Kitamura
*Intensive Care Medicine Experimental* 2017, **5(Suppl 2):**1073

**INTRODUCTION.** Sepsis remains the commonest cause of emergency admission to ICU globally. However sepsis is still a lethal clinical syndrome with a high mortality rate worldwide. The majority sepsis patients have coagulation abnormalities. In the pathogenesis of sepsis, inflammation and coagulation play a pivotal role. Evidence of an extensive cross-talk between these two systems is increased recently. However, there are different diagnostic criteria in sepsis and disseminated intravascular coagulation (DIC). Therefore we defined the new diagnostic criteria of sepsis induced DIC (SEDIC) as follow; Presepsin (PSEP) level ≥ 900 pg/ml and Protein C (PC) activity ≤ 45% [1].

**OBJECTIVES.** In this study, we attempted to validate this scoring system.

**METHODS.** A single center, retrospective, observational study was carried out. Patients who were diagnosed with sepsis were included in this study. The definition of sepsis was performed according to the new sepsis criteria named Sepsis-3. The blood samples were collected at the time of admission. The patients were classified into the following three groups according to PSEP level and PC activity. (1) SEDIC: PSEP ≥ 900 pg/mL and PC ≤ 45%. (2) non-SEDIC: PSEP < 650 pg/mL and PC > 45%, or 650 < PSEP < 900 and PC > 55%. (3) pre-SEDIC: the range out of SEDIC and non-SEDIC. The scoring system for Japanese Association for Acute Medicine (JAAM) DIC was used for diagnosis of DIC. We examined the severity of illness of the patients was evaluated according the APACHE II score and organ failure was assessed by the SOFA score. All patients were followed up for 28 days after enrollment in the study, and 28-day all-cause mortality was assessed.

**RESULTS.** Three hundred twenty seven patients were enrolled for this study from July 2011 to April 2016. Ninety seven patients (29.7%; 97/327) were SEDIC, 136 were pre-SEDIC (41.6%; 136/327) and 94 were non-SEDIC (28.7%; 94/327). SEDIC scoring system significantly reflected the positive rate of JAAM DIC (SEDIC 72.2%, pre-SEDIC 42.6%, non-SEDIC 22.3%; $p < .05$), JAAM DIC score (SEDIC 4.7 ± 2.0, pre-SEDIC 3.7 ± 2.1, non-SEDIC 2.5 ± 1.6; $p < 0.05$), and SOFA score (SEDIC 9.8 ± 3.8, pre-SEDIC 8.2 ± 3.3, non-SEDIC 6.3 ± 3.1; $p < .05$). The 28-day mortality rate was significantly worsened, depending on the cutoff points in the respective criteria (SEDIC 27.8%, pre-SEDIC 22.1%, non-SEDIC 6.4%; $p < .05$).

**CONCLUSIONS.** From these results, we strongly believed that the SEDIC scoring system has an acceptable property for the diagnosis of sepsis induced DIC even if we use Sepsis-3 for the definition of sepsis. We can measure PSEP and PC with very simply and quickly within thirty minutes. So, we strongly suggest that this scoring system can be useful for early treatment in a critical care setting.

### 1074

#### Time course of septic shock in immunocompromised and non-immunocompromised patients

M. Jamme, F. Daviaud, J. Charpentier, N. Marin, M. Thy, Y. Hourmant, J.P. Mira, F. Pène
Cochin University Hospital, Réanimation Médicale, Paris, France
**Correspondence:** M. Jamme
*Intensive Care Medicine Experimental* 2017, **5(Suppl 2):**1074

**INTRODUCTION.** Septic shock affects a wide range of patients with non-immune and immune comorbidities. Its prognosis commonly relies on the sole vital status, whereas it is also associated with the development of severe infectious and non-infectious complications.

**OBJECTIVES.** We addressed the impact of underlying immune conditions on the course of septic shock with respect to both mortality and the development of acute complications.

**METHODS.** This was an 8-year (2008–2015) monocenter retrospective study. Patients diagnosed for septic shock within the first 48 h of ?A3B2 show $132#?>intensive care unit (ICU) admission were included. Patients were classified in four subgroups with respect to their immune status: non-immunocompromised and immunocompromised distributed into hematological or solid malignancies and non-malignant immunosuppression. Outcomes were in-hospital death and the development of ICU-acquired infectious, hemorrhagic and ischemic complications. The determinants of death and complications were addressed by multivariate competing risk analysis.

**RESULTS.** Eight hundred and one patients were included. Among them, 305 (38%) were immunocompromised, distributed into solid tumors (122), hematological malignancies (106) and non-malignant immunosuppression (77). The overall 3-day, in-ICU and in-hospital mortality rates were 14.1%, 37.3% and 41.3%, respectively. Patients with solid tumors displayed increased in-hospital mortality (cause-specific hazard (CSH) 2.16 [95% confidence interval 1.49-3.11], $p < .001$). ICU-acquired infections occurred in 211 (33%) of the 3-day survivors. In addition, 95 (11.8%) and 70 (8.7%) patients exhibited severe ischemic or hemorrhagic complications during the ICU stay. There was no association between the immune status and the occurrence of ICU-acquired infection. Non-malignant immunosuppression and hematological malignancies were independently associated with increased risks of severe ischemic events (CSH 1.83 [1.07-3.23], $p = .03$) and hemorrhage
(CSH 2.12 [1.06-4.25], $p = 0.03$), respectively.

**CONCLUSIONS.** The underlying immune status impacts on the course of septic shock and the further susceptibility to complications. This emphasizes the complexity of sepsis syndromes in relation with comorbid conditions, and raises the question of the relevant endpoints in clinical studies.

**REFERENCE(S)**

Daviaud F, Grimaldi D, Dechartres A et al. Timing and causes of death in septic shock. Ann Intensive Care. déc 2015;5(1):16
Tolsma V, Schwebel C, Azoulay E et al. Sepsis severe or septic shock: outcome according to immune status and immunodeficiency profile. Chest. nov 2014;146(5):1205-13
Azoulay E, Mokart D, Pène F et al. Outcomes of critically ill patients with hematologic malignancies: prospective multicenter data from France and Belgium–a groupe de recherche respiratoire en réanimation onco-hématologique study. J Clin Oncol. août 2013;31(22):2810-8.

**GRANT ACKNOWLEDGMENT**
The authors declare have not conflict of interest

### 1075

#### Impact of chronic hypertension on time to goal mean arterial pressure and clinical outcomes in critically ill patients with septic shock requiring vasopressors

Q.M. Yeo[1,2], C. Li[1], K.M. Olsen[1], D.A. Hammond[1]
[1]University of Arkansas for Medical Sciences, College of Pharmacy, Little Rock, United States; [2]Changi General Hospital, Department of Pharmacy, Singapore, Singapore
**Correspondence:** Q.M. Yeo
*Intensive Care Medicine Experimental* 2017, **5(Suppl 2):**1075

**INTRODUCTION.** The mean arterial pressure (MAP) impacts microvascular blood flow and reflects adequacy of tissue perfusion. The 2016 Surviving Sepsis Campaign recommends vasopressors target MAP ≥65 mmHg in patients with septic shock. It is unclear if patients with chronic hypertension (HTN) achieve target MAP faster and experience different outcomes.

**OBJECTIVES.** To evaluate the impact of chronic HTN on outcomes in patients with and without chronic HTN who developed septic shock requiring vasopressors.

**METHODS.** A retrospective cohort study was conducted for critically ill patients admitted to medical and surgical intensive care units (ICU) at our institution between May 2014 and July 2016. Patients were included if they were ≥18 years old, admitted to an ICU for ≥48 hours, and diagnosed with septic shock requiring vasopressors. The primary outcome was time to goal MAP (≥65 mmHg). Secondary outcomes included duration of vasopressor use, days free of vasopressor support, mortality, ICU and hospital length of stay. Differences between groups were compared using t or Wilcoxon Rank Sum test for continuous variables and Pearson´s Chi-square or Fisher's exact test for categorical variables. To adjust for imbalances in baseline characteristics, inverse probability of treatment weighting (IPTW) procedure was performed, where probabilities to be in the HTN group were estimated based on baseline demographic and clinical characteristics of patients using logistic regression.

**RESULTS.** In total, 133 patients were included, of which 75 (56.4%) had HTN. Baseline demographics were similar between groups except patients with HTN were older (62.8 y vs. 56.2 y; p = 0.011) and more frequently had ischemic heart disease (28.0% vs. 3.5%; p < 0.001) and diabetes
(42.7% vs. 8.6%; p < 0.001). Time to goal MAP (HTN vs. no HTN: 12.6 h ± 16.5 vs. 14.2 h ± 29.6; p = 0.692), duration of vasopressor use (70.7 h ± 65.7 vs. 84.7 h ± 97.0; p = 0.326) and days free of vasopressor support (14.6 d ± 19.6 vs. 20.4 d ± 24.5; p = 0.131) were similar between groups. Patients with HTN had higher in-hospital (49.3% vs. 31.0%; p = 0.034) and 28-day mortality (53.3% vs. 31.0%; p = 0.010). Duration of ICU stay was shorter (6.6 d ± 6.1 vs. 9.9 d ± 10.0; p = 0.033) in patients with HTN, but hospital lengths of stay were similar (17.5 d ± 20.2 vs. 23.9 d ± 26.3; p = 0.115). After IPTW, duration of ICU stay was no longer significantly different but in-hospital (50.2% vs. 27.3%, p = 0.004) and 28-day mortality (52.7% vs. 27.3%, p = 0.001) remained higher in patients with HTN.

**CONCLUSION.** The time to goal MAP and vasopressor free days were similar regardless of HTN diagnosis. Chronic HTN was associated with a higher mortality rate.

**GRANT ACKNOWLEDGMENT**
None.

### 1076

#### Thyroid function in septic patients and their prognosis

A. García-de la Torre[1], M.-V. de la Torre-Prados[2], T. Tsvetanova-Spasova[2], P. Nuevo-Ortega[2], E. Cámara-Sola[2], A. Fernández-Porcel[2], C. Rueda-Molina[2], L. Salido-Díaz[2], M. Mateos-Rodríguez[2], I. García-Gómez[2]
[1]Malaga/Ibima, Universitary H Virgen de la Victoria, Clinical Chemistry Department, Malaga, Spain; [2]Málaga/Ibima, Universitary H Virgen de la Victoria, Department of Intensive Care Medicine, Málaga, Spain
**Correspondence:** A. García-de la Torre
*Intensive Care Medicine Experimental* 2017, **5(Suppl 2):**1076

**INTRODUCTION.** Sepsis caused serious disturbances in pituitary-thyroid axis functions, with an abnormal thyroid hormones releasing, causing a no thyroidal illness syndrome, also known as low T3 syndrome or euthyroid sick syndrome.

**OBJECTIVE.** To analyze the thyroid function of septic patients and its relation with outcome.

**METHODS.** Prospective study in a cohort of 150 consecutive adults with severe sepsis (SS) Septic Shock (SSh) according to Clinical Guidelines for Surviving Sepsis Campaign (SSC, 2008) during 20 months in a polyvalent Intensive Care Unit (ICU). We studied thyrotropin (TSH), free triiodothyronine fraction (fT3) and free thyroxin fraction (fT4) serum levels, APACHE II and SOFA score. Statistical analysis was performed using SPSS 18.0 for Windows (SPSS Inc. Chicago, IL, USA).

**RESULTS.** We analyzed 150 episodes of SS (16%) or SSh (84%), the median age of the patients was 64 (inter-quartile range, 48.7-71) years; the main sources of infection were: respiratory tract (38%) and intra-abdomen (45%); 70.7% had medical diseases. APACHE II score was 25 [21–30], SOFA score was 10 [7.75-11] and 28-day mortality was 22.7%. Our data shown 18.3% with low levels of TSH (<0.2uUI/mL), 20.3% had low levels of fT4 (<0.75 ng/dL) and 91.4% low levels of fT3 (<2 pg/mL). The profile of death patients were men (64.7%, n =22), with significantly higher average age (63 vs. 57 years; p = 0,049), as well as clinical severity scores, APACHE II (29.8 vs. 24.1; p < 0.001) and SOFA (12.1 vs. 8.9; p < 0,001) and major dysfunction organs number (4.6 vs. 3.6; p < 0,001). Non-survivors had significantly lower TSH 0.85 vs. 1.4 UI/mL; p = 0.042, and fT3 1.2 vs. 1.39 pg/mL, p = 0.031. However fT4 did not show statistical significance 0.42 vs. 0.58ng/dL, p = ns.

**CONCLUSIONS.** Most of our septic patients present an altered thyroid function. Our data indicate that low TSH and fT3 serum levels could be a significant prognostic factor in patients with SS and SSh.

### 1077

#### Influence on outcome of microbiogical documentation of sepsis patients detected by a sepsis team in a teaching hospital

A. Lartategi, R. Zaragoza, S. Sancho, V. Ramirez, C. Ibañez, R. González, B. Bonet, C. Martínez, J. Camarena
Hospital Universitario Dr. Peset, Sepsis Unit, ICU, Valencia, Spain
**Correspondence:** R. Zaragoza
*Intensive Care Medicine Experimental* 2017, **5(Suppl 2):**1077

**INTRODUCTION.** Sepsis is a time-dependent process that must be early detected and treated. Automatic alerts may play an important role in prompt detection but no data about the role of microbiological documentation has been recently provided.

**OBJECTIVES.** The aim of this study were to asses the principal "microbiological" features of the patients with severe sepsis and septic shock treated by a Sepsis unit in a teaching hospital, to describe their updated prognosis and specially to analyze the influence on global mortality of microbiological documentation (MD) of these septic patients.

**METHODS.** During a four years and four months period (October-2012-January 2017), 2322 severe sepsis and septic shock patients detected by a sepsis unit form ICU using an electronic automatic alert in a teaching hospital were prospectively evaluated. Clinical and microbiological variables were recorded. Any patient with a confirmed sepsis was potentially eligible through the whole hospital. Several multivariate analysis was performed to describe independently factors associated to global mortality in these patients using SPSS package (16.0).

**RESULTS.** Among 2322 electronic activations 631 of them corresponded to septic shock (27.1%) and the rest to severe sepsis (62.9%). Only 27,9% of patients were admitted to ICU. Their mean APACHE II and SOFA score were 18.05 ± 6.06 and 5.21 ± 3.3 respectively. The most frequent sources of infections were the respiratory focus (40,9%), urinary (26,1%) and abdominal (18,6%). Global mortality was 20.7%. The majority of episodes were community adquired (57.6%). Associated bacteremia was present in 33,5% of episodes.MD was achieved in 51.3%. The most frequently microorganism isolated were: *Escherichia coli* (19.1%), *Staphylococcus aureus* (4,6%), *Streptococcus pneumoniae* (3.9%) and *Pseudomonas aeruginosa* (3.8%).In univariate analysis MD was associated to lower mortality (18.4% vs. 23.1%; $p = 0.05$). However multivariate analysis confirmed APACHE II (OR 1,12; p = 0,0001), age (OR 1,02; p = 0,0001) and origin of infection (OR 32; p = 0,0001) as independently factors associated with global mortality but no MD (OR 1,15; p = 0,33).

**CONCLUSIONS.** MD was achieved in a half of patients with sepsis detected by a sepsis team in a teaching hospital, but had no any influence on global mortality.

### 1078

#### Endotoxin and cytokine elimination in patients with septic shock: an observation study

M. Kiselevskiy[1], E. Gromova[2], L. Kuznetsova[3], Y. Dolzhikova[1], I. Chikileva[1], R. Vlasenko[1], S. Lodinov[4], N. Krotenko[4]
[1]Russian NN Blokhin Cancer Research Center, Cell Immunity, Moscow, Russian Federation; [2]Russian NN Blokhin Cancer Research Center, Intensive Care Unit, Moscow, Russian Federation; [3]Russian NN Blokhin Cancer ResearchCenter, Intensive Care Unit, Moscow, Russian Federation; [4]SP Botkin Hospital, Intensive Care Unit, Moscow, Russian Federation
**Correspondence:** M. Kiselevskiy
*Intensive Care Medicine Experimental* 2017, **5(Suppl 2):**1078

**INTRODUCTION.** Lipopolysaccharide (LPS) of Gram-negative bacteria and inflammatory mediators play a key role in sepsis pathogenesis. LPS-adsorbers are applied to eliminate LPS. However, there are few studies of their clinical efficacy, besides they were conducted in small cohorts of patients.

**OBJECTIVES.** The aim of the study was to evaluate clinical efficacy and endotoxin and cytokines elimination by Alteco LPS Adsorber in patients with septic shock.

The study included 20 patients with confirmed Gram-negative sepsis. Endotoxin elimination was performed using hemoperfusion with the Alteco LPS Adsorber (Alteco Medical AB, Lund, Sweden).

Methods. Blood serum samples were taken before and immediately after the procedure, column washouts were taken as well. Samples were stored at - 70 °C. The values of IL- 4, IL- 6, IL-8, IL-10, IL-18 in serum and washout from LPS-adsorber were measured by ELISA. Concentrations of LPS were measured by LAL-test.

**RESULTS.** 28-day survival rate in patients with septic shock after the hemoperfusion comprised 25%. All the surviving patients leaved hospital in satisfactory condition. Normalization of hemodynamic parameters was noted in the group of the surviving patients after the hemoperfusion, which allowed significantly lowering vasopressor load. Moreover, the procedure resulted in decrease of leukocytosis and numbers of young lymphocyte forms. PCT level also declined on the average from 14.29 to 8.6 ng/ml. The SOFA score dropped from 14 to 11. Similar but less prominent trend was observed in the group of nonsurviving patients. Patients diagnosed with septic shock and with a suspected Gram-negative infection did not always have a high endotoxin level. High or extremely high LPS level was observed in 50% of the patients. LPS concentration in blood usually decreased 2–10 folds after the hemoperfusion. Stable LPS decrease required from 2 to 6 hemoperfusion procedures. We did not observe significant changes of endotoxin level after the hemoperfusion in group of the patients with low initial LPS level. Drop of IL-6 and IL-8 levels was observed if their initial concentrations exceeded 100 pg/ml.

**CONCLUSION.** Alteco LPS Adsorber allows effective elimination of LPS from the blood stream even at its extremely high concentrations. Stable decrease of LPS level requires more than 2 hemoperfusion procedures. LPS decrease was accompanied with normalization of clinical and laboratory parameters (patients' condition). The decrease in the SOFA score indicated significant improvement in organ function.

### 1079

#### Effect of two levels of mean arterial pressure on microcirculatory reserve in septic shock patients

M. Jozwiak[1,2], M. Chambaz[1,2], P. Sentenac[1,2], C. Richard[1,2], X. Monnet[1,2], J.-L. Teboul[1,2]
[1]Service de Réanimation Médicale, Hôpital de Bicêtre, Hôpitaux Universitaires Paris-Sud, Assistance Publique - Hôpitaux de Paris, Le Kremlin Bicêtre, France; [2]Inserm UMR S_999, Univ Paris-Sud, Le Kremlin Bicêtre, France
**Correspondence:** M. Jozwiak
*Intensive Care Medicine Experimental* 2017, **5(Suppl 2):**1079

**INTRODUCTION.** There are currently some contrasting findings concerning the level of mean arterial pressure (MAP) to target in septic shock patients in terms of microcirculation.

**OBJECTIVES.** To compare the microcirculatory reserve at two levels of MAP in septic shock patients.

**METHODS.** We measured the cardiac index by transpulmonary thermodilution and microcirculation parameters by using near-infrared spectroscopy in 22 septic shock patients receiving norepinephrine, within the first six hours of resuscitation and with a MAP > 75 mmHg. Both variables were measured at MAP > 75 mmHg (so-called "high MAP") and at MAP 65–70 mmHg (so-called "low MAP") after decreasing the norepinephrine dosage. The muscle tissue oxygen saturation ($StO_2$) was measured at the thenar eminence and the microcirculatory reserve was measured as the $StO_2$ recovery slope obtained after a vascular occlusion test.

**RESULTS.** The MAP decreased from 81 ± 3 to 67 ± 3 mmHg and this was associated with a decrease in mean $StO_2$ recovery slope (3.00 ± 1.40 *vs.* 2.61 ± 1.46 units/sec, respectively, $p < 0.05$) without any change in mean $StO_2$ (81 ± 8 *vs.* 81 ± 9%, respectively) and in cardiac index (3.26 ± 1.09 vs. 3.12 ± 1.05 L/min/m$^2$, respectively). There was a large interindividual variability in terms of relative changes in $StO_2$ and in $StO_2$ recovery slope. A significant relative change in $StO_2$ was observed in 19 patients: $StO_2$ decreased in 11 patients, increased in 8 patients and exhibited no change in three patients. All patients except one exhibited a significant relative change in $StO_2$ recovery slope: it decreased in 16 patients and it increased in 5 patients. Patients with significant relative changes in $StO_2$ or in $StO_2$ recovery slope had a similar baseline cardiac index. Changes in microcirculation parameters were not different in hypertensive patients (n = 17) and in non-hypertensive patients (n = 5).

**CONCLUSIONS.** The mean $StO_2$ recovery slope decreased when MAP dropped from "high" to "low" values in septic shock patients, without any change in mean $StO_2$. Nevertheless, there was a large interindividual variability with two opposite behaviours: the vast majority of patients had a higher $StO_2$ recovery slope at "high MAP" whereas some other had a higher $StO_2$ recovery slope at "low MAP". Thus, monitoring the microvascular reserve in septic shock patients makes sense in order to individualize the MAP target.

### 1080

#### Inflammatory mediators and coagulation in heat stroke

A. Vázquez-Sánchez[1], J. Marín-Corral[2], C. Vilà-Vilardell[2], C. Climent-Company[2], I. Dot-Jordana[2], M.P. Gracia-Arnillas[3], J.R. Masclans-Enviz[1], J. Nolla-Salas[1]
[1]UPF-Hospital del Mar, ICU, Barcelona, Spain; [2]Hospital del Mar, ICU, Barcelona, Spain; [3]UAB-Hospital del Mar, ICU, Barcelona, Spain
**Correspondence:** A. Vázquez-Sánchez
*Intensive Care Medicine Experimental* 2017, **5(Suppl 2):**1080

**INTRODUCTION.** Heat stroke is a life-threatening illness characterized by central nervous system disfunction and severe hyperthermia; some evidence suggests a relationship between heat cytotoxicity, coagulation and multiple organ damage.

**OBJECTIVES.** To assess differences in inflammatory mediators and coagulation among a group of heat stroke patients (HS) compared to a group of healthy volunteers (HV) and a group of patients with hyperthermia by other causes (HOC).

**METHODS.** Descriptive and prospective study including all patients admitted to the hospital with HS diagnosis since July 2009 until September 2016, and a group of HOC admitted in the same period and a group of HV. We recorded demographic and clinical data: age, sex, vital constants at admission, risc factors, APACHE II, daily SOFA, need for respiratory support, renal replacement therapy, vasoactive drugs, cooling method, length of stay and evolution. Laboratory data were recorded at 0,12 and 24 hours. We determined baseline inflammatory mediators levels (IL6, IL8, IL10 & TNFα) and FXa-activity in HV, and basal,12 and 24 hours in HOC and HS patients. The study was approved by the Hospital Ethic Commitee. Differences between groups were assessed using chi-square for categorical variables and Student´s t-test or Mann–Whitney test for continuous variables, considering $p < 0{,}05$ to be significant.

**RESULTS.** Six HV, 40 HS and 28 HOC patients were included, similars in age, sex and APACHE II. HOC patients had more comorbidities (75% vs 50%; $p < 0.05$), need for ICU admission (85,7% vs 37,5%; $p < 0.001$), vasoactive drugs (53,6% vs 20%; $p < 0.01$), mechanical ventilation (67,9% vs 25%; $p < 0.001$), higher SOFA at admission [8(6–11) vs 4(2–8); $p < 0.01$], 24 [7(4–9) vs 2(0–4); $p < 0.001$] and 48 hours [5(3–9) vs 1(0–3); $p < 0.01$], length of stay [17 (5–41) vs 3(1–10); $p < 0.001$] and mortality (35,7% vs 7,5%; $p < 0.01$). They received more antithermic drugs (92,9% vs 70%; $p < 0.05$) while HS patients received more physical cooling measures (67,5% vs 14,3%;$p < 0.01$). We found no differences in any of the recorded clinical data at admission. Liver and kidney function were similar in both groups. No differences were found in Procalcitonin levels on admission, 12 and 24 hours. Significant differences in other laboratory data and inflammatory mediators and FXa-activity are shown in Tables 257 and 258.

**CONCLUSIONS.** HS and HOC patients have high levels of IL6, IL8, IL10 and TNFα. Only TNFα is higher in HS than in HOC. FXa-activity decreases in HS and HOC in similar levels, werheas other coagulation data are worse in HS.

**Table 257 (Abstract 1080)**. Laboratory data HOC vs HS

| | HOC | HS | |
|---|---|---|---|
| Protein C Reactive(mg/dl) 0h 12h 24h | 10 (2-19) 13 (4-22) 11 (5-26) | 0,3 (0,1-2,9) 1,8 (0,3-7,5) 1,5 (0,3-5,7) | ** *** *** |
| Hemoglobin (g/dl) 0h 12h 24h | 14,3 (1,7) 12,5 (1,7) 12 (1,5) | 12 (2,3) 10 (2,4) 10,3 (1,9) | *** ** *** |
| Lactate (mmol/L) 0h | 2,1 (1,6-2,9) | 2,9 (2,1-4,8) | * |
| Platelets ($x10^3$/uL 12h 24h | 200 (134-334) 195 (128-319) | 150 (112-197) 134 (104-180) | * * |
| Fibrinogen (mg/dl) 0h 12h 24h | 477 (179) 503 (177) 507 (130 | 359 (133) 317 (140) 349 (152) | * *** * |
| Protrombine time (%) 24h | 71 (18) | 57 (18) | ** |

* p<0,05; ** p<0,01; *** p<0,001

**Table 258 (Abstract 1080)**. Inflammatory mediators & FXa-activity

| | HV | HOC | HS |
|---|---|---|---|
| IL-6 0H IL-6 12H IL-6 24H | 1,00 (0,71 - 2,98) | 148,79 (17,69 - 589,32) ### 96,39 (5,93 - 236,42) 66,67 (20,23 - 161,25) | 95,01 (21,03 - 350,43) ¶¶¶ 38,68 (18,15 - 136,34) 23,55 (7,15 - 32,77) |
| IL-8 0H IL-8 12H IL-8 24H | 2,55 (1,88 - 6,45) | 79,93 (10,00 - 174,87) ### 45,58 (4,26 - 82,98) 14,55 (1,94 - 46,82) | 29,94 (5,13 - 144,69) ¶¶ 22,71 (3,10 - 147,70) 10,66 (6,69 - 36,36) |
| IL-10 0H IL-10 12H IL-10 24H | 4,31 (3,42 - 5,96) | 17,93 (8,58 - 95,85) ### 22,45 (7,28 - 95,95) 11,12 (3,37 - 38,73) | 81,75 (13,54 - 224,77) ¶¶¶ 14,48 (4,46 - 34,00) 5,18 (0,48 - 19,47) |
| TNFα 0H TNFα 12H TNFα 24H | 59,33 (30,66 - 73,66) | 217,00 (132,66 - 394,41) ## 175,33 (68,66 - 285,00) 189,16 (164,08 - 343,66) | 290,83 (226,16 - 398,75) ¶¶¶ 294,41 (236,00 - 368,62)* 296,16 (214,33 - 406,75)* |
| FXa 0H Activity FXa 12H Activity FXa 24H Activity | 110,04 (11,45) | 88,38 (24,45) ## 87,10 (27,53) 86,54 (13,67) | 87,96 (18,19) ¶¶ 77,70 (21,44) 77,54 (18,70) |

• * p < 0,05 between HOC & HS • ¶¶ p < 0,01 between HS & HV; ¶¶¶ p < 0,001 between HS & HV • ## p < 0,01 between HOC & HV; ### p < 0,001 between HOC & HV

### 1081

#### The relationship between serum zinc level and coagulation system in sepsis-induced coagulopathy

Y. Irie, H. Ishikura, R. Hokama, M. Koie, K. Muranishi, K. Hoshino, R. Yuge, T. Kitamura, Y. Kawano
Faculty of Medicine, Fukuoka University, Fukuoka, Japan
**Correspondence:** Y. Irie
*Intensive Care Medicine Experimental* 2017, **5(Suppl 2):**1081

**INTRODUCTION.** Recently, it was clarified that $Zn^{2+}$ plays a pivotal role of inflammation. According to the several reports that $Zn^{2+}$ led to suppression of inflammation and its deficiency resulted in further deterioration of severe organ dysfunction including coagulopathy in patients with sepsis. However, there have been no reports that the relationship between the serum zinc level and sepsis-induced coagulopathy.

**OBJECTIVE.** The purpose of this study was to investigate the relationships between serum zinc levels and coagulopathy and the prognosis of the patients with sepsis.
**METHODS.** This study was conducted a single-center retrospective study from June 2016 to March 2017. Blood samples for measuring the markers were collected on admission. And serum zine level and platelet counts, PT-INR, FDP, D-Dimer, PIC, TAT, PAI-I, antithrombin (AT) were measured. The definition of sepsis was performed according to the Sepsis-3.
**RESULTS.** Of the 128 patients, 50 patients were sepsis and 78 patients were non-sepsis. The serum zinc level was significant lower of sepsis group than that of non-sepsis group (33.4 ± 16.4 vs 62.8 ± 17.8μg/dL, p < 0.01) at the ICU admission. In 50 sepsis patients, the number of JAAM DIC positive patients (DIC+) and negative patients (DIC-) was 26 and 24, respectively. In sepsis patients, there was no significant difference in the serum zinc level between survivor and non-survivor on day 14 of ICU stay (33.9 ± 16.9 vs 30.8 ± 13.9μg/dl). Similarly, there was no significant correlation between serum zinc level and JAAM DIC score, APACHE II score and SOFA score in the sepsis patients. There was also no significance in the serum zinc level between DIC+ and DIC- (35.4 ± 14.9 vs 31.5 ± 17.9μg/dl). About the relationship between the serum zinc level and the levels of coagulation molecular markers, we found the significant positive correlations to platelet counts (r = 0.46, p = 0.0007), AT activity (r = 0.524, p = 0.001), and the negative correlations to PAI-1 (r = −0.580, P = 0.001).
**DISCUSSION.** From these results, we confirmed that the serum zinc level decreased under sepsis condition. Previous study reported that $Zn^{2+}$ was involved in the activation of platelets[1)]. Furthermore, $Zn^{2+}$ was an essential element for constructing the cross-linked structure of fibrin and platelets during platelet aggregation[2)]. As these results, zinc will be consumed under the sepsis-induced coagulopathy. From these reports, we suggest that zinc may be involved considerably in the coagulation system under the sepsis condition.
**CONCLUSIONS.** Serum zinc level decreased in sepsis patients. And this reduction in zinc may be associated with sepsis-induced coagulopathy.
**REFERENCE**

1) Metallomics. 2016;8:91–100.
2) Thrombosis and Haemostasis. 2016;115:533–42.

### 1082

#### Effects of different vasoactive drugs on global and intestinal perfusion during endotoxic shock in rabbits

N.E. de Oliveira[1], J.V. Gandolfi[2], L.M. Contrim[2], R.M. Pereira[2], L.O. Fernandes[2], F.G. Soriano[1], S.M. Lobo[2]
[1]Faculdade de Medicina de São José do Rio Preto - FAMERP, Intensive Care, São José do Rio Preto, Brazil; [2]Faculdade de Medicina de São José do Rio Preto - FAMERP, São José do Rio Preto, Brazil
**Correspondence:** N.E. de Oliveira
*Intensive Care Medicine Experimental* 2017, **5(Suppl 2):**1082

**OBJECTIVE.** To evaluate the effect of different vasoactive drugs on global perfusion, mesenteric flow and gut injury.
**METHODS.** Experimental study of endotoxic shock in New Zealand rabbits treated with different vasoactive drugs and distributed in 5 groups (6 rabbits in each group): A (control), B (noradrenaline), C (noradrenaline + dobutamine), D (vasopressine) and E (vasopressine + dobutamine). All animals received adequate fluid resuscitation. Mean arterial pressure (MAP), aortic blood flow ($Q_{ao}$) and superior mesenteric artery flow ($Q_{SMA}$) and lactate were measured at baseline, 1, 2, 3 and 4 hours after endotoxin injection (LPS - *E. coli*-055:B5, SIGMA-ALDRICH, 1 mg/kg). In addition, enterocyte damage was evaluated by measurements of citruline and Intestinal Fatty Acid-binding Protein (I-FABP) at baseline and after 4 hours.
**RESULTS.** All groups remained with adequate MAP during the whole experiment. Increased serum lactate was more pronounced in group D (2,0 ± 0,4 mmol/l to 4,6 ± 0,7 mmol/l; p = 0.002). The largest reduction of $Q_{ao}$ occurred in group D (64 ± 17,3 ml/min to 38 ± 7,5ml/min; p = 0.04). $Q_{SMA}$ declined more significantly in groups D and E and remained lower than in the other groups during four hours (group D: baseline: 65 ± 31 ml/min; 1h: 37 ± 10 ml/min; 2h: 38 ± 10 ml/min; 3h: 46 ± 26 ml/min and 4h: 48 ± 15 ml/min p < 0,005; group E: baseline: 73 ± 14 ml/min; 1h: 28 ± 4.0 ml/min; 2h:37 ± 6.4 ml/min; 3h: 40 ± 11 ml/min; 4h: 48 ± 11 ml/min, p < 0,005). Citrulline significantly decreased in groups D (p = 0.014) and E (p = 0.019) in comparison to group A.
**CONCLUSION.** Vasopressin is effective in maintaining MAP during endotoxic shock in rabbits but may induce intestinal injury, despite adequate fluid resuscitation.

### 1083

#### Real-time qSOFA predicts critical care admission and hospital mortality in sepsis

A.N. Ahmad[1], E.J. Bates[2], P. Khan[2], R. Loveridge[2]
[1]King's College London, Faculty of Life Sciences and Medicine, London, United Kingdom; [2]King's College Hospital, Critical Care, London, United Kingdom
**Correspondence:** A.N. Ahmad
*Intensive Care Medicine Experimental* 2017, **5(Suppl 2):**1083

**INTRODUCTION.** In 2016, the Third International Consensus Definitions for Sepsis and Septic Shock (Sepsis-3) were published, in which the quick Sepsis-related Organ Failure Assessment (qSOFA) score was proposed (1). A positive qSOFA score is defined as ≥2 of: respiratory rate ≥22/min, altered mentation, systolic blood pressure ≤100mmHg.
**OBJECTIVES.** To demonstrate the place of the qSOFA score in risk stratifying patients on the ward in need of critical care, irrespective of the meaning behind the score, and to compare it to older established scoring systems.
**METHODS.** King's College Hospital inpatients first referred to our iMobile critical care outreach team (CCOT) between May 2016 and January 2017 were identified on outreach records, the data was prospectively collected. Systemic inflammatory response syndrome (SIRS) criteria (positive if ≥2), qSOFA (positive if ≥2) and National Early Warning Score (NEWS; positive if ≥5) scores were calculated. We recorded evidence of likely infection, and whether the referring nurse or doctor was worried about the patient.
**RESULTS.** A total of 544 case notes were analysed. The mortality rate was 21.7%. qSOFA and NEWS scores performed well for identifying patients with a higher mortality and rate of critical care admission, whereas SIRS was a poor predictor.
Patients had a significantly higher mortality when qSOFA score positive [Age adjusted odds ratio (OR), 3.651; 95% confidence intervals (CI), 2.168-6.148; p < 0.001] or NEWS score positive (OR, 2.743; 95% CI, 1.209-6.227; p = 0.016) compared with SIRS criteria (OR, 0.583; 95% CI, 0.299-1.136; p = 0.113).
Patients who were positive for either NEWS, qSOFA or both were significantly more likely to be admitted to critical care (OR, 2.992; 95% CI, 0.024-1.155; p = 0.024). NEWS ≥5 alone did not discriminate for ICU admission (p = 0.338), and neither did SIRS (p = 0.396). However, qSOFA alone was highly significant

(OR, 2.007; 95% CI, 1.411-2.856; p < 0.001).

We analysed the effect of individual variables on mortality, finding that altered mental state (p < 0.001), lactate >2mmol/L (p = 0.004), and a worried referrer (OR, 3.366; 95% CI, 1.41-8.039; p = 0.006) were all significant predictors.

Our results show clearly the benefits of including whether the referring clinician is concerned when referring to CCOT, a point promoted by the Sepsis Trust (2) but not included on current scoring systems.

**CONCLUSIONS.** For patients with suspected infection, qSOFA performed better than NEWS, and could be used as a trigger for referral to CCOT. SIRS is not sufficiently sensitive or specific. The worried referrer should be a red flag criterion for any deteriorating patient.

**REFERENCE(S)**

1. Singer M, Deutschman CS, Seymour CW, et al. The Third International Consensus Definitions for Sepsis and Septic Shock (Sepsis-3). JAMA. 2016;315(8):801–810

2. www.sepsistrust.org

**GRANT ACKNOWLEDGMENT**

Not applicable

### 1084

#### Metabolic acidosis in patients with sepsis

I. Vasileiadis[1], M. Kompoti[2], E.-S. Tripodaki[3], N. Rovina[1], K. Pontikis[1], E. Ntouka[4], E. Stavrou[4], A. Koutsoukou[1]

[1]ICU, First Department of Respiratory Medicine, Medical School, National and Kapodistrian University of Athens, Sotiria Hospital, Athens, Greece; [2]Thriassion General Hospital of Eleusis, Athens, Greece; [3]First Department of Internal Medicine, Agios Savvas Regional Cancer Hospital, Athens, Greece; [4]Emergency Department Laboratory, Sotiria Hospital, Athens, Greece

**Correspondence:** I. Vasileiadis

*Intensive Care Medicine Experimental* 2017, **5(Suppl 2):**1084

**OBJECTIVE.** To compare increased lactate concentration (Lac) and decreased strong ion difference (SID) as main causes of metabolic acidosis in septic patients upon their ICU admission.

**METHODS.** 94 (35 Female) septic patients were enrolled in the study. Arterial blood samples were analysed in the blood gas analyser on the day of admission. Patients were categorized in three groups based on the base excess (BE) value: < −2 mEq/L (metabolic acidosis), −2 mEq/L ≤ BE ≤ + 2 mEq/L (normal), > +2 mEq/L (metabolic alkalosis). Other recorded parameters were: Lac and the chloride to sodium concentration ratio ([Cl]/[Na]), indicating decreased SID acidosis when >0.75. We also measured albumin in plasma (Alb), to assess hypoalbuminemia, which has an alkalinizing effect that can hide an underlying acidosis when conventional markers as the pH or BE are used.

**RESULTS.** Table 259 compares the three forementioned patient groups in respect to markers indicating the acidotic disorder [Lac > 2 mmol/L, [Cl]/[Na] > 0.75] and also in respect to Alb < 3.5 g/dl. It depicts the absolute number of patients and the percentage relative to the total number of patients in each group. Cl levels differed significantly in the three groups (107.43 ± 5.88 vs 105.77 ± 5.74 vs 102.63 ± 5.41 mEq/L, p = 0.016), being higher in Group 1 patients while Na levels (137.25 ± 4.63 vs 139.71 ± 4.24 vs 140.05 ± 6.10 mEq/L, p = 0.01) and SID (33.92 ± 4.01 vs 38.16 ± 3.69 vs 41.37 ± 3.20 mEq/L, p < 0.001) were lower in Group 1. Lac showed no significant difference in the three groups (1.90 ± 1.40 vs 1.64 ± 1.12 vs 1.58 ± 0.67 mmol/L, p = 0.81).

**CONCLUSION.** Lactate levels did not differ in patients with and without metabolic acidosis. A decreased SID was mainly noted in septic patients with metabolic acidosis.

**Table 259 (Abstract 1084)**. See text for description

| | SBE<-2mEq/L | −2mEq/L≤SBE≤+2mEq/L | SBE>+2mEq/L | Total | p value |
|---|---|---|---|---|---|
| Cl/Na>0.75 | 28 (82.4%) | 11 (50%) | 2 (9.1%) | 41 (52.6%) | p<0.001 |
| Lac>2mmol/L | 11 (32.4%) | 5 (20%) | 6 (26.1%) | 22 (26.8%) | p=0.569 |
| Alb<3.5g/dl | 34 (97.1%) | 26 (100%) | 24 (100%) | 84 (98.8%) | p=0.485 |

### 1085

#### Mean platelet volume and its ratio to platelet count as a prognostic marker in septic patients

A. Ortín Freire[1], R. Jiménez Sánchez[1], L. García de Guadiana[2], S. Rebollo Acebes[1], L. Herrera Para[1], A. Fernández Martínez[1], M. Galindo Martínez[1], S. Sánchez Argente del Castillo[1], S. Moreno Aliaga[1]

[1]Hospital General Universitario Santa Lucía, Servicio Medicina Intensiva, Cartagena, Spain; [2]Hospital General Universitario Santa Lucía, Servicio de Análisis Clínico, Cartagena, Spain

**Correspondence:** A. Ortín Freire

*Intensive Care Medicine Experimental* 2017, **5(Suppl 2):**1085

**INTRODUCTION.** Sepsis involves a dysregulatory process with an intense systemic inflamatory response. Platelets have a crucial role in inflammation and immunomodulation and mean platelet volumen (MPV) represents a measure of platelet size and reactivity.

**OBJECTIVES.** To study the value of several platelet indices for predicting mortality in patients with sepsis and septic shock.

**METHODS.** We prospectively enrolled patients admitted with sepsis/septic shock to our Intensive Care Unit during a two-year period (2015–2016) We measured different demographic, clinical and laboratory data at admission, including platelet indices, and we studied differences between survivors and non-survivors.

Comparison between survivors and non-survivors was made using t-Sudent, U-Mann–Whitney, Chi-Square o Fisher test as appropriate. Receiver operating charasteristic (ROC) curves were used to assess diagnostic accuracy.

**RESULTS.** A total of 219 patients were studied. Of them, 47 (21.5%) died within 28 days after ICU admission. Non-survivors were older (70 (IC 95% 66.1-73.8) vs 64.1 (61.8-66.4), p 0.023), had higher APACHE II (27.3 (25.5-29.1) vs 18.5 (17.6-19.4), p < .001) and higher lactate (4.6 (3.4-5.89) vs 2.6 (2.3-3), p < .001) and SOFA (9.5 (7–12.3) vs 7 (5–9); p < 0.001) at admission.

No differences were observed in platelets count (164 x $10^3$ (103–253) vs 152 x $10^3$ (90–259)) and mean platelet volume (11.1 fL (10.8-11.5) vs 11 (10.8-11.2))at admission between survivors and non-survivors. The ratio MPV to platelet count was higher in non-survivors (11.2 (IC 95% 7.2-15.3) vs 8.4 (7.2-9.6)) but it did not reach statistical significance.

ROC curves for predicting mortality at 28 days showed an area under the curve (AUC) for MPV and MPV/platelet count of 0.525 and 0.547 respectively, while AUC for APACHE II score was 0.829 (IC 95% 0.765-0.893, p 0.033) and for SOFA score of 0.715 (0.633-0.797, p 0.042).

**CONCLUSIONS.** In our patients with sepsis/septic shock, mean platelet volumen and MPV to platelet count ratio measured at admission, did not predict 28-days mortality.

**REFERENCE(S)**

- Oh GH. Shock. 2017 Mar;47(3):323–330
- Zampieri et al. Annals of Intensive Care 2014, 4:20

**Publisher's Note**

Download to read this full article text.

Publisher’s Note

Springer Nature remains neutral with regard to jurisdictional claims in published maps and institutional affiliations.

